# The Host R‐M Systems Change the Host Range of *Staphylococcus* Phage EBHT

**DOI:** 10.1002/mbo3.70165

**Published:** 2025-11-27

**Authors:** Henni Tuomala, Julia Holtel, Melina Markkanen, Sheetal Patpatia, Katariina Kaansalo, Clara Rolland, Oliver W. Bayfield, Kira Ranta, Mikael Skurnik, Johannes Wittmann, Saija Kiljunen

**Affiliations:** ^1^ Research Program Unit, Faculty of Medicine University of Helsinki Helsinki Finland; ^2^ Institute for Functional Gene Analytics Bonn‐Rhein‐Sieg University of Applied Sciences Rheinbach Germany; ^3^ Department of Microbiology, Faculty of Agriculture and Forestry University of Helsinki Helsinki Finland; ^4^ Viral Zoonosis Research Unit, Faculty of Medicine University of Helsinki Helsinki Finland; ^5^ Leibniz Institute DSMZ‐German Collection of Microorganisms and Cell Cultures GmbH Braunschweig Germany; ^6^ York Structural Biology Laboratory, Department of Chemistry University of York York England; ^7^ HUS Diagnostic Center, Clinical Microbiology University of Helsinki and Helsinki University Hospital Helsinki Finland

**Keywords:** phage resistance mechanisms, phage–host interactions, phage–host specificity, *Staphylococcus aureus*

## Abstract

Therapeutically utilized phages should optimally be produced in defined bacterial strains that are free of prophages and virulence factors. However, phage–host interactions in these production strains may be very different from clinical strains. Here, we characterized a lytic *Staphylococcus aureus*–specific phage vB_SauP_EBHT (EBHT), which had a dramatic change in its host specificity when produced in alternative host 19A2 compared with the original isolation host DSM 104437, even though there were no changes in the phage genome, proteome, structure, or adsorption efficiency. The reason for the altered host range was revealed to be based on different methylation patterns of the EBHT genome by host restriction–modification (R‐M) systems in the two hosts. Even though the alternative host 19A2 produced a higher burst size, the host range of the produced phages was narrower. Together, these results illustrate that the most efficient production host may not necessarily be the most optimal one and that bacterial R‐M systems should be considered when selecting the optimal phage‐production host.

## Graphical abstract

The study shows how Staphylococcal restriction–modification (R‐M) systems methylate the phage genomic DNA and change the phage host range. The host R‐M systems should be considered when selecting phage manufacturing hosts, as the manufacturing host with a higher yield can result in a narrower phage host range.

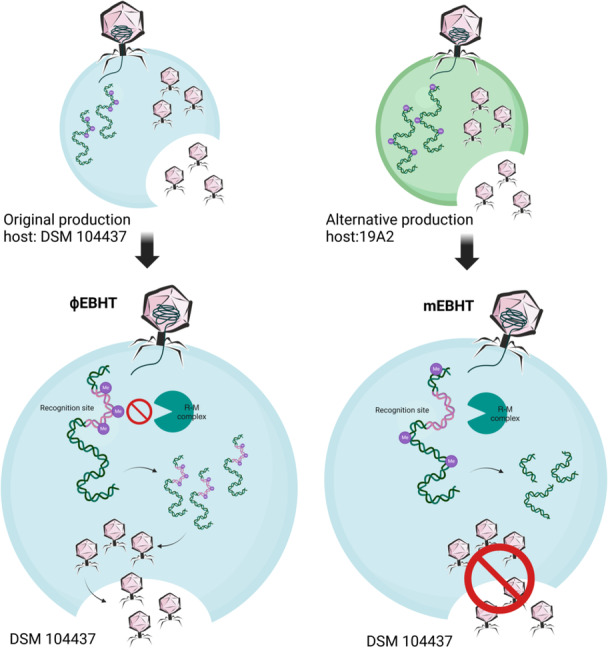

## 1 Introduction

*Staphylococcus aureus* is a major nosocomial pathogen that develops resistance at an alarming rate against its main treatments, semisynthetic penicillins (e.g., methicillin and oxacillin) and the last‐resort antibiotics (e.g., vancomycin, daptomycin, and linezolid) (Esposito et al. 2023). Infections caused by methicillin‐resistant *S. aureus* (MRSA) are increasingly difficult and costly to treat and have higher mortality rates than methicillin‐susceptible *S. aureus* (MSSA) strains. *S. aureus* adapts efficiently to changing environments due to intrinsic resistance to many antibiotics and a plastic genome that allows efficient genetic exchange through mobile genetic elements (MGEs) and high diversity of plasmids, which increases the clinical significance of *S. aureus* (Mccarthy et al. 2012; Bosi et al. 2016; Lindsay and Holden 2004). For example, MGEs participate in the transfer of methicillin resistance‐containing gene cassette, Staphylococcal chromosomal cassette mec (SCCmec), to new *S. aureus* cells (Ballhausen et al. 2014; Weterings et al. 2017). While *S. aureus* is highly adaptable, its core genome remains stable and enables strain classification into clonal complexes (CCs), which then help monitor *S. aureus* strains during outbreaks (Murray et al. 2022; de Kraker et al. 2016; Mork et al. 2020; O'Neill 2014; Pirnay et al. 2022). In 2024, the World Health Organization (WHO) listed MRSA as a high‐priority target for research and development of new treatments (WHO 2024). As a response to the upsurging antimicrobial resistance (AMR), phage therapy faces renewed interest, and MRSA is among the most extensively studied target pathogens for phage therapy applications (Wittebole et al. 2014; Uyttebroek et al. 2022; Kaźmierczak et al. 2014).

Lytic *S. aureus*–specific phages having myoviru*s* or podovirus morphotypes are well suited for phage therapy (Azam and Tanji 2019; Cui et al. 2017). Myoviruses are known for their broad host ranges that usually include several *S. aureus* strains and, at times, even the close relatives of *S. aureus*. They recognize and attach to unmodified wall‐teichoic acids (WTAs) on the host surface to initiate infection (O'Flaherty et al. 2004; Xia et al. 2011). Similar to myoviruses, podoviruses also have broad host ranges, are well suited for treating MRSA infections, and use WTAs as receptors. For example, Podoviruses ɸ44AHJD, ɸ66, and ɸP68, considered to be suitable for the treatment of MRSA infections, require *O*‐linked β‐*N*‐acetylglucosamine (β‐*O*‐GlcNAcylation) of the WTAs by the TarS glycosyltransferase for infection. Interestingly, the TarS‐catalyzed β‐*O*‐GlcNAcylation is also necessary for methicillin resistance. Deletion of the *tarS* gene resulted in susceptibility to methicillin and resistance to phages ɸ44AHJD, ɸ66, and ɸP68 (X. Li et al. 2015; Moller et al. 2019; Jurado et al. 2022).

Most known *S. aureus*–specific phages that have siphovirus morphology are temperate. Multiple prophage genomes reside in almost all *S. aureus* strains, and they play a significant role in driving *S. aureus* evolution forward by enhancing host fitness and facilitating horizontal gene transfer (HGT) and lateral transduction. For example, prophages can supply the host cell with virulence factors, aid in immune evasion, and promote the mobilization of MGEs. The features that improve bacterial fitness can jeopardize antibiotic and phage treatment by providing resistance mechanisms against both treatments. Prophages hinder antibiotic treatment by transferring AMR genes to new host cells and mediate phage defense through, for example, abortive infection and superinfection exclusion (Kuntová et al. 2023; M. Li et al. 2012; Moller et al. 2022; Humphrey et al. 2021; Chen et al. 2018).

The phage resistance mechanisms of *S. aureus* target the adsorption, biosynthesis, and assembly stages of the phage life cycle (Moller et al. 2019). For *S. aureus*, the Type I restriction–modification (R‐M) and clustered regularly interspaced short palindromic repeats (CRISPR)‐Cas (CRISPR‐associated) nuclease systems constitute intrinsic phage resistance mechanisms. These systems are widespread within *S. aureus* strains and other prokaryotes, and approximately 80% and 40% of prokaryotes carry R‐M and CRISPR‐Cas systems, respectively (Shaw et al. 2023; Xiao et al. 2024). The R‐M systems process incoming DNA by distinguishing between self and foreign DNA. The system is composed of three subunits, HsdS, HsdM, and HsdR. While HsdS recognizes the target sequences, HsdM methylates the hemimethylated sites, and HsdR cleaves the unmethylated sequences. Furthermore, the Type I R‐M systems control HGT and allow the exchange of genetic material between *S. aureus* strains that belong to the same CCs at a higher frequency than between different CCs. This tolerance toward genetic material from related strains contributes to the evolution of the distinct *S. aureus* lineages (Moller et al. 2019; Monk et al. 2015; Cooper et al. 2017; Goerke et al. 2009; Zschach et al. 2018; Shaw et al. 2023b). The CRISPR‐Cas system is part of the adaptive immune system of bacteria. It consists of conserved spacer sequences that separate short and unique nucleotide sequences. These short nucleotide sequences of foreign DNA are incorporated into the CRISPR complex. After incorporation, they act as templates for the recognition of foreign DNA, which are then cleaved by the Cas‐complex (Hille et al. 2018).

In July 2023, the European Pharmacopoeia Commission (EPC) published in the Pharmaeuropa the general framework for phage therapeutic applications and quality control (Eur 2023). The European Medicines Agency (EMA) has also published a guideline for phage therapy in veterinary medicine (CVMP 2022). Both publications state that only lytic phages are acceptable for treatment, and neither phages nor their production hosts should contain detrimental genetic factors that include known AMR genes, virulence factors, and prophages (Guideline on quality 2023; European Directorate for the Quality of Medicines & HealthCare 2023). Often, the isolation hosts for new phages are wild‐type bacterial strains resistant to phages from existing collections. They tend to be more virulent than what is accepted for production hosts, and thus, the production hosts of phages can differ from their isolation hosts (Fong et al. 2021; Bae et al. 2006). Understanding the impact of the host strain on phage characteristics during the manufacturing process and treatment period can improve the design and outcome of phage therapy (Moller et al. 2019, 2022).

In this study, we have analyzed the properties of *S. aureus*–specific phage vB_SauP_EBHT (EBHT) to determine its suitability for phage therapy and the interactions between EBHT and its two production hosts DSM 104437 and 19A2 that were isolated from human and pig, respectively. EBHT was originally isolated against DSM 104437 but was after that produced in the alternative host 19A2 as part of an in vivo phage treatment trial (Tuomala et al. 2021). The aim of this study was to understand how phage‐production host affects phage infection efficacy and host range and the impact the production host may have on the treatment product and outcomes of phage therapy.

## 2 Results

### 2.1 vB_SauP_EBHT Is a Lytic Phage Isolated From a Pig Snout Sample

**Figure 1:**
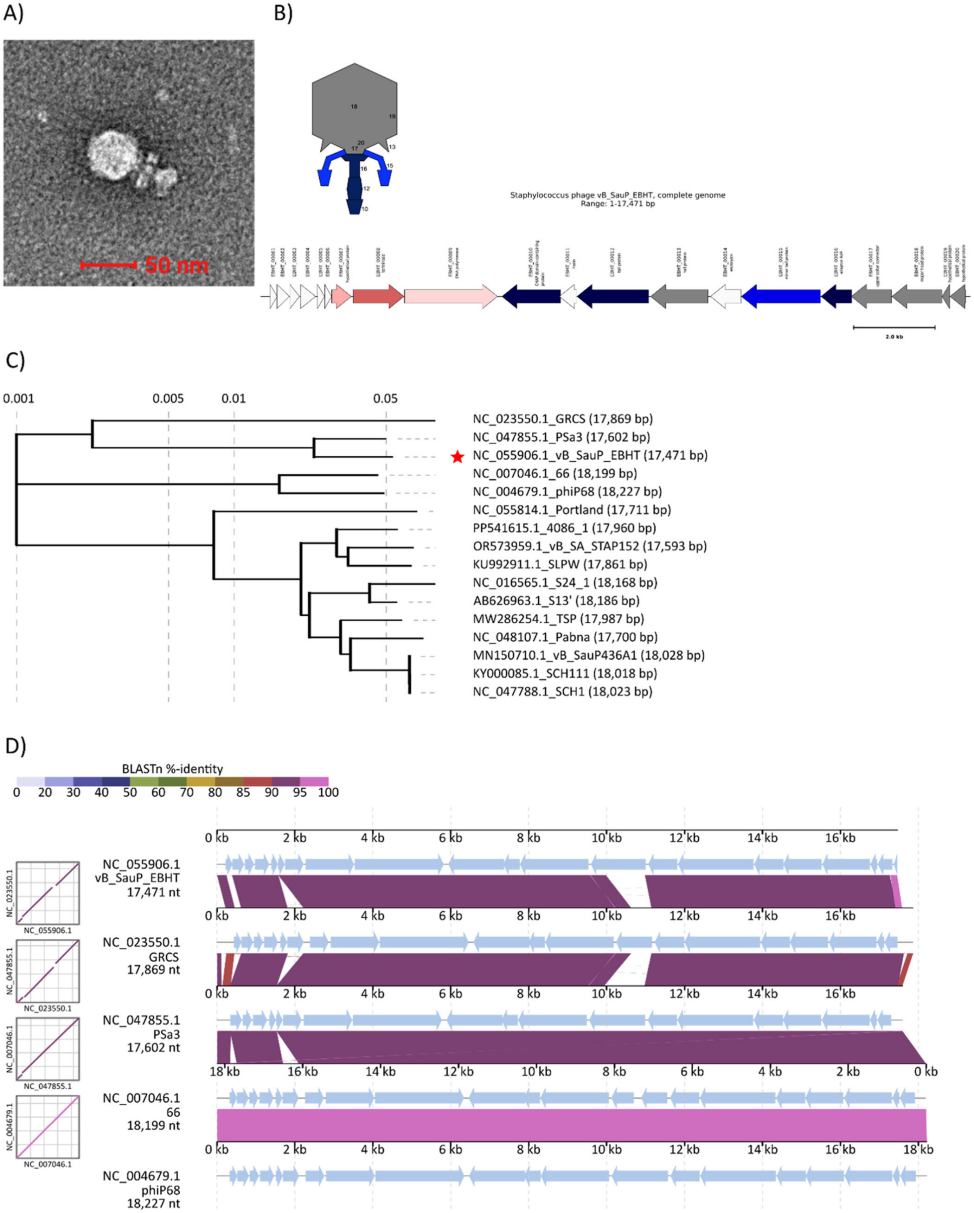
Morphological, genomic, and phylogenic characterization of vB_SauP_EBHT (EBHT). (A) EBHT was imaged with Transmission Electron Microscopy. The scale bar equals 50 nm. (B) Organization of the EBHT genome and prediction of the location of structural proteins in the EBHT particle. Annotations are derived from the EBHT sequence in GenBank (NC_055906.1). (C) Whole‐genome phylogenetic analysis of EBHT and phage genomes with 93% query coverage and 90% nucleotide identity in BLASTN search. The phylogenetic tree was generated with the Viral Proteomic Tree server (ViPTree, version 4.0). (D) Alignment of EBHT, GRCS, PSa3, 66, and ɸP68 genomes with the DiGAlign (version 2.0) online tool.

EBHT was isolated from a pig snout sample against a clinical isolate DSM 104437, and this is the first study that reports its isolation and sequencing. The negatively stained micrographs of EBHT showed a typical podovirus morphology with a small isometric head and a short noncontractile tail (Figure 1A,B). The length of the phage capsid was 42.4 ± 1.5 nm, while the tail length was 29.6 ± 5.8 nm. The sequencing and genome analysis results revealed that EBHT is a phage with a 17,471‐kb‐sized genome having a GC content of 29.6% and 20 predicted protein‐coding open reading frames. The functions of the predicted gene products included DNA replication (DNA polymerase), lysis, packaging, and structural proteins (Figure 1B). None of the gene products was related to proteins involved in the lysogenic life cycle, antibiotic resistance, or bacterial toxins.

For whole‐genome phylogenetic analysis, 14 phage genomes having at least 94% genome coverage and 90% nucleotide identity in BLASTN search against the GenBank nucleotide database were selected. The phylogeny analysis suggested that EBHT belongs to the genus Rosenblumvirus with *Staphylococcus* phagePSa3 and GRCS (Figures 1C and S1). BLASTN pairwise comparison of EBHT and PSa3 revealed genome coverage of 98% and nucleotide identity of 94.68%. The genome coverage and identity between EBHT and GRCS were 91% and 94.95%, respectively. The alignment of EBHT and the four most closely related phages, PSa3, GRCS, 66, and ɸP68, showed over 90% similarity between the phages (Figure 1D).

### 2.2 The EBHT Host Range Depends on the Production Host

**Figure 2:**
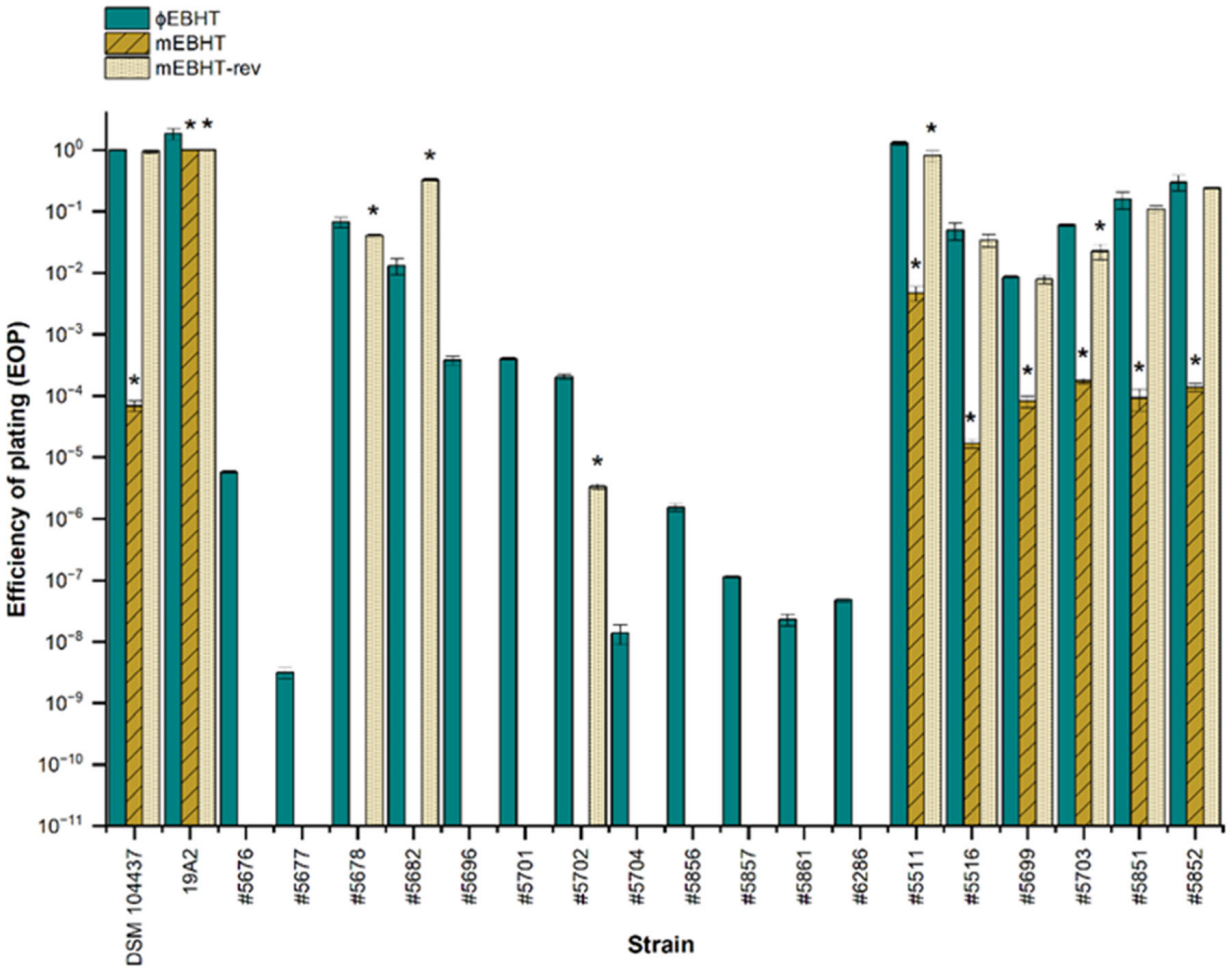
Efficiency of plating (EOP) determination for ɸEBHT, mEBHT, and mEBHT‐rev phage variants. The EOP values were calculated as the relation between the titer of the phage variant in its original host (DSM 104437 for ɸEBHT and 19A2 for mEBHT and mEBHT‐rev) and the test strain. The EOP in the original host strain was given the value 1. The EOP values were determined from the mean and standard deviations of triplicate test results. The *y*‐axis shows the EOP value on a logarithmic scale, and the test strains are presented on the *x*‐axis. The absence of the bar indicates that the strain is resistant to the phage variant. One‐way analysis of variance was used for statistical analysis with a 0.05 confidence level, and a significant difference in the EOP of ɸEBHT is indicated with the *symbol. EBHT, vB_SauP_EBHT.

Bacteriophage EBHT was produced in two host strains; the phage variant produced in the original isolation host DSM 104437 is denoted as ɸEBHT, and the phage variant produced in strain 19A2 is denoted as mEBHT. Interestingly, the mEBHT variant infected host DSM 104437 with a 6.8 ∗ 10^5^‐fold lower efficiency than strain 19A2 (Figure 2). After five rounds of plaque purification, ɸEBHT and mEBHT variants were re‐sequenced, and their genomes were found to be 100% identical. Raw sequencing reads of ɸEBHT and mEBHT variants were submitted to the Sequence Reads Archive project PRJNA1160023 with sample codes SAMN50554998 and SAMN50554999, respectively.

The host ranges and the host range differences of ɸEBHT and mEBHT variants were tested with 162 *Staphylococcus* isolates. The results showed an extensive difference between ɸEBHT and mEBHT host ranges; ɸEBHT infected 89 (54.9%) strains, in contrast to mEBHT, which infected 32 (19.8%) strains (Tables 1 and S1). MRSA, MSSA, and coagulase‐negative *Staphylococcus* strains included in the host range screening originated from Finnish and German hospitals and Finnish pigs. The host range differences of EBHT variants extended to all groups of *S. aureus* isolates, in which ɸEBHT infected more strains than mEBHT. ɸEBHT infected most efficiently clinical MRSA strains, while mEBHT infected most efficiently pig MRSA strains. The host ranges of the phage variants were most similar within the pig isolates, with only one differing strain. Neither variant infected the coagulase‐negative *Staphylococcus* isolates.

**Table 1:** The host ranges of ɸEBHT and mEBHT phage variants within different groups of *Staphylococcus* strains.

Bacterial host	Total number	Infected with ɸEBHT (*n*)	%	Infected with mEBHT (*n*)	%
All *Staphylococci*	162	89	54.9	32	19.8
Clinical *Staphylococci*	108	59	54.6	18	16.7
*Staphylococcus aureus*	130	89	68.5	32	24.6
Clinical *S. aureus*	86	59	68.6	18	20.9
All MRSA	63	54	85.7	20	31.7
Clinical MRSA	45	41	91.1	6	13.3
All MSSA	67	35	52.2	12	17.9
Clinical MSSA	41	19	46.3	11	26.8
Pig *S. aureus*	22	13	59.1	12	54.5
Pig MRSA	18	13	72.2	12	66.7
Other *Staphylococci*	32	0	0	0	0

Abbreviations: EBHT, vB_SauP_EBHT; MRSA, methicillin‐resistant *S. aureus*; MSSA, methicillin‐susceptible *S. aureus*.

The reversibility of the mEBHT host range was studied by determining the efficiency of plating (EOP) of a mEBHT plaque (denoted as mEBHT‐rev) picked from a lawn of host DSM 104437 bacteria and comparing the EOP of mEBHT‐rev to the EOPs of ɸEBHT and mEBHT. The infection efficacy of mEBHT‐rev in host DSM 104437 returned to the same level as the infection efficacy of the original ɸEBHT variant, and its EOP in host 19A2 remained similar to the EOP of mEBHT (Figure 2). EBHT host range reversibility was tested in an additional 18 strains that were either resistant to mEBHT or were infected more efficiently with ɸEBHT than mEBHT. mEBHT‐rev infected nine of the isolates. All of the strains intermediately sensitive to mEBHT were infected with mEBHT (*n* = 6), but only three of the 12 mEBHT‐resistant isolates were infected with mEBHT‐rev. Thus, the host specificity of mEBHT‐rev did not recover fully, but it resembled more the host specificity of ɸEBHT than mEBHT.

### 2.3 The Production Host Does not Affect the Proteome or Adsorption of EBHT

The influence of the production host on the EBHT phage proteome was evaluated by comparing proteomics data of purified ɸEBHT and mEBHT phage particles. The total number of proteins with a read coverage above the cutoff value of 4.0 identified in LC/MS–MS analysis was 13 and 12 for ɸEBHT and mEBHT particles, respectively (Table S2). ɸEBHT and mEBHT particles included eight proteins originating from the EBHT genome, and the remaining proteins in the samples originated from the propagation host. LC/MS–MS analysis identified structural proteins from both EBHT variants: tail fiber protein, tail protein, major capsid protein, and putative upper collar protein. Additionally, the CHAP domain protein and three hypothetical proteins encoded by the phage genome were present in ɸEBHT and mEBHT particles. All phage‐originating proteins were identical between the two phage variants, while differences were found in the host‐originating proteins. The mEBHT particle was also imaged with Transmission Electron Microscopy (TEM), and the structure was identical to the structure of ɸEBHT particle (not shown).

ɸEBHT sample contained three unique host‐originating proteins: Aconitate hydratase, 6,7‐dimethyl‐8‐ribityllumazine synthase, and Inosine‐5′‐monophosphate dehydrogenase (Table S2). These proteins participate in the metabolism of strain DSM 104437. mEBHT sample contained two unique host‐originating proteins, bacterial nonheme ferritin and Glutamine synthetase type I, which are also part of the metabolism of 19A2. Both purified phage samples included two host‐originating peptidases: deblocking aminopeptidase and M42 metallopeptidase. Peptidases from the ɸEBHT sample had different amino acid sequences than the peptidases in the mEBHT sample. The identity between the deblocking aminopeptidases was 99%, and the identity between the M24 metallopeptidases was 98%. It is possible that the host‐originating proteins were not part of the actual phage particles but were remnants from the phage samples during the purification process, which relied on protein separation based on size and the strength of the negative charge of the target particle.

**Figure 3:**
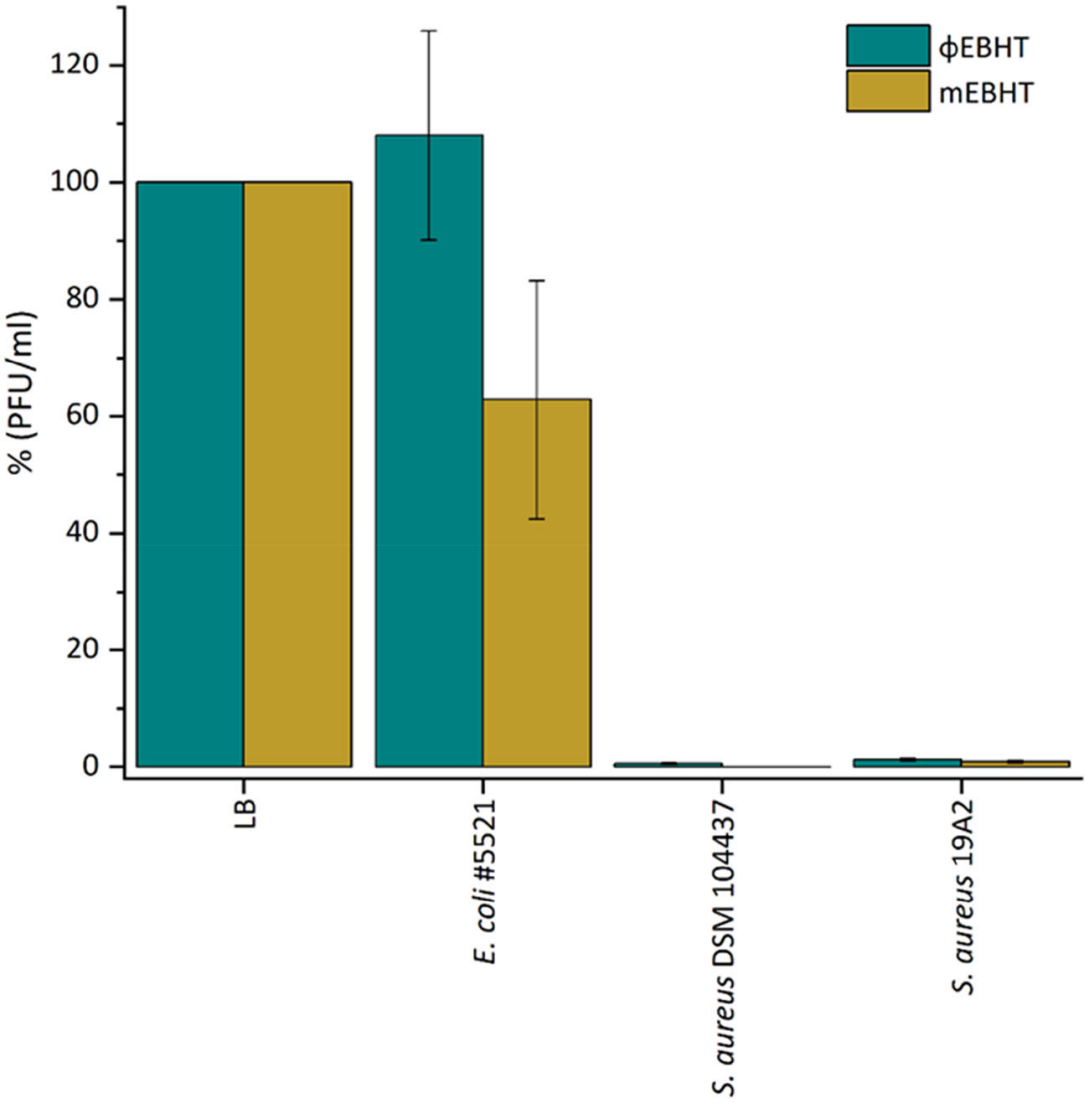
EBHT adsorption on host strains. The adsorption of ɸEBHT and mEBHT phage variants was determined in opposite host strains (ɸEBHT in 19A2 and mEBHT in DSM 104437) and the original host as a positive control. LB broth was used as a negative control, and unspecific adsorption was determined in *Escherichia coli* strain #5521. All platings were performed as triplicates, and the mean and standard deviations were used to determine the adsorption rates. The phage quantity in LB represents a 100% residual PFU/mL value. EBHT, vB_SauP_EBHT; LB, Luria–Bertani; PFU, plaque‐forming units.

The adsorption assay was used to test if the host‐originating aminopeptidases affected EBHT receptor binding by modifying receptor protein structures through their protein‐degrading function. Phage adsorption was tested in host strains DSM 104437 and 19A2 (Figure 3). Over 99% of both phage variants adsorbed on both bacterial strains. The mEBHT adsorption was not inhibited on host DSM 104437, as 99.4 ± 0.1% of ɸEBHT particles and 99.9 ± 0.01% of mEBHT particles adsorbed on the host DSM 104437. Some unspecific adsorption was detected for the mEBHT variant in the control strain *Escherichia coli* #5521, even though this result may come from the high standard deviations of both EBHT variants in this strain. The results show that the change in EBHT host specificity was not a result of a reduction in the adsorption efficacy.

### 2.4 The Infection of mEBHT in Host DSM 104437 Does not Proceed after the Initial Adsorption Step

**Figure 4:**
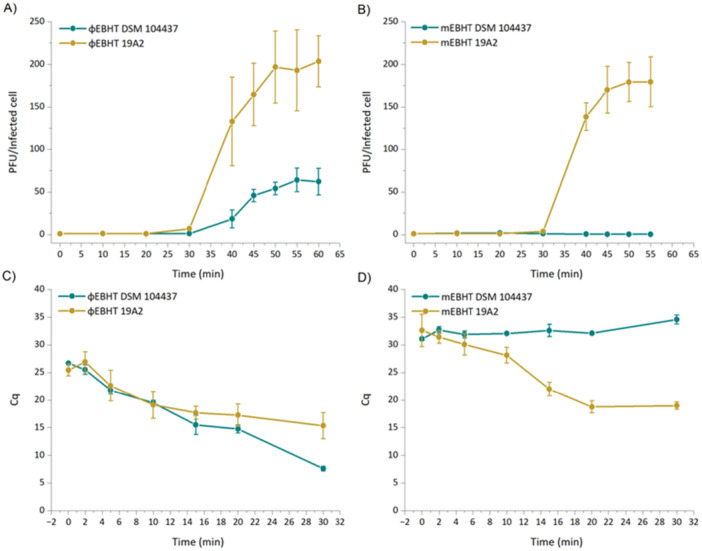
One‐step growth curves and phage DNA replication assay results of ɸEBHT and mEBHT phage variants. The one‐step growth curve experiments were performed for ɸEBHT and mEBHT to study the differences between their life cycles in their own and opposite host strains. The experiment was performed three times for each phage–host pair, and the means and standard deviations were used to calculate the plaque‐forming units/infected cell (PFU/infected cell). The PFU/infected cell result at 0 min was given the value 1, and the burst sizes were determined at 55 or 50 min. (A) ɸEBHT growth curves in both host strains. (B) mEBHT growth curves in both host strains. The proliferation of the EBHT genome in both host strains was analyzed with a qPCR assay, using the original hosts as positive controls. Bacterial cells were infected with the phage and sampled at time points: 0, 2, 5, 10, 15, 20, and 30 min. The bacterial DNA was isolated with a Nucleo‐Spin Microbial DNA‐kit, and EBHT‐specific primers were used to quantify the EBHT genome at different time points. All samples and controls were analyzed as triplicates. (C) ɸEBHT genome quantities in hosts DSM 104437 and 19A2. (D) mEBHT genome replication in hosts DSM 104437 and 19A2. The *y*‐axis shows the Cq values of the qPCR assay, and the *x*‐axis shows time as minutes. EBHT, vB_SauP_EBHT; PFU, plaque‐forming units; qPCR, quantitative polymerase chain reaction.

As EBHT adsorption to both host strains was similar and therefore did not affect its host specificity, a one‐step growth assay was performed to explore differences in ɸEBHT and mEBHT life cycles in the host strains DSM 104437 and 19A2. The length of the ɸEBHT and mEBHT infection cycles differed; ɸEBHT infection took 60 min to progress from initial infection to full lysis in both host strains, while the mEBHT infection cycle took 55 min in host 19A2 (Figure 4A,B). At timepoint 60 min, the mEBHT infection in 19A2 entered already the second cycle (not shown). The latent period was 30 min for both phage variants in their respective host strains and ɸEBHT in host 19A2. Both phage variants had considerably bigger burst sizes in host 19A2 than in host DSM 104437. The average burst sizes of ɸEBHT and mEBHT in 19A2 were 193 ± 48 PFU/infected cell (55 min timepoint) and 179 ± 23 PFU/infected (50 min timepoint), respectively. In host DSM 104437, the ɸEBHT burst size was only 64 ± 14 PFU/infected cell (55 min timepoint). No new mEBHT particles were produced in host DSM 104437 during the 55 min assay, indicating that the phage infection cycle was considerably slower or totally inhibited in this host.

To study EBHT genome injection and replication after infection, the quantities of ɸEBHT and mEBHT genomes were measured in both host strains with a quantitative polymerase chain reaction (qPCR) assay for 30 min. The ɸEBHT genome replicated in both host strains, although the replication was slightly more efficient in host DSM 104437 (Figure 4C). As expected, the mEBHT genome replicated well in host 19A2, but there was no visible reduction of Cq value in host DSM 104437 (Figure 4D). On the contrary, the Cq value showed a slight increase in this strain, indicating possible degradation of the phage DNA during the 30‐min assay time. Interestingly, the quantity of mEBHT DNA seemed to plateau in both host strains after 20 min, whereas the amount of ɸEBHT DNA still increased between time points 20 and 30 min. The plateau at 20‐min time point coincided with the lysis of host bacteria that started 30 min after infection.

A standard curve of qPCR performed with a dilution series of phage lysate as template showed that a 5‐unit difference in Cq value corresponded to approximately 10‐fold change in phage titer (PFU/mL) (Figure S2). This indicates that the genome copy numbers of ɸEBHT and mEBHT in host 19A2 increased by approximately 100‐ and 900‐fold, respectively. The copy number of ɸEBHT in host DSM 104437 increased even more, approximately 10,000‐fold. This implies that the genome copy numbers were not linear to the number of the newly formed infectious phage particles and that a substantial excess of genome copies was formed for mEBHT in host 19A2 and ɸEBHT in host DSM 104437. One has to keep in mind, though, that qPCR reactions using templates as different as DNA isolated from infected bacterial cells and dilution series of phage lysate may result in different Cq values, making the standard curve and the estimation of genome copy numbers rough estimates.

### 2.5 Host Strains DSM 104437 and 19A2 Contain Different Numbers of Type I R‐M Subunit‐Encoding Genes

Genomic DNA of the host strains DSM 104437 and 19A2 was sequenced to characterize the strains and study their potential role in the host range variation of EBHT. MLST typing classified the host strain DSM 104437 to the CC22, while Verkola et al. showed earlier that the host strain 19A2 belonged to the CC398 (Verkola et al. 2022). Phage Search Tool Enhanced Release (PHASTER) tool detected one intact prophage (NC_011344) and one incomplete and questionable prophage genome (NC_048657 (22)) in the genome of DSM 104437, whereas 19A2 contained two prophage genomes, one intact (NC_022758) and one incomplete (NC_007045). DefenceFinder identified complete R‐M Type I and IIG, Abi2, Pycsar, and ShosTA phage defense systems in the DSM 104437 genome and complete RosmerTA, R‐M Type II, Theoris I, and RloC phage defense systems in the 19A2 genome (Table S3). Additionally, DefenceFinder identified several genes involved in incomplete phage defense systems (Table S4). Phage defense mechanisms identified with the PADLOCK tool partially differed from the ones identified with DefenceFinder (Table S3). Both host genomes contained CRISPR/Cas systems, yet their protospacer sequences did not contain sequences from the EBHT genome.

Further analysis of the R‐M systems showed that the different analysis tools classified the R‐M systems into different R‐M types. RAST classified all identified R‐M systems as Type I in contrast to DefenceFinder and PADLOCK, which associated the subunits from host DSM 104437 with the Type I, Type II, Type IIG, and Type IV R‐M systems. The subunits from the host 19A2 genome were associated with Type I and Type II R‐M systems in the 19A2 genome (Table S5). Additionally, PADLOCK classified some of the R‐M subunit‐encoding genes to belong to an unspecified group of proteins related to DNA modification systems. The number of genes related to R‐M systems depended on the analysis tool, and each tool identified at least one unique R‐M‐related gene in DSM 104437 and 19A2. As an example, RAST analysis showed that the number of genes encoding for HsdR, HsdM, and HsdS subunits also differed between the host strains; the DSM 104437 genome contained three HsdR, four HsdM, and three HsdS subunit‐encoding genes, in contrast to the 19A2 genome that contained two HsdR, three HsdM, and one HsdS subunit‐encoding genes. A screening of the HsdM subunits against REBASE and NCBI Conserved domain databases indicated that the HsdM subunits from the two host strains cause different types of modifications. The methyltransferases (MTases) from host DSM 104437 were recognized as N6 adenine‐specific MTase when analyzed with the NCBI conserved domain database and/or REBASE. HsdM subunits from host 19A2 (scaffolds 0 and 2, Table S5) were also recognized as N6 adenine‐specific MTases, at least by one of the tools. HsdM subunit from 19A2 (scaffold 4, Table S5), on the other hand, was detected with both tools as a C5‐type methylase (Supporting Information File 2).

Possible target recognition sequences of HsdS subunits from the host strains were studied with REBASE and conserved domain databases. REBASE detected at least one target sequence for three HsdS subunits from host DSM 104437 (scaffolds 4, 9, and 10, Table S6B) and one target sequence for the HsdS subunit from host 19A2 (scaffold 2, Table S6B). Target sequences of HsdS subunits of DSM 104437 (scaffolds 4 and 9, Table S5B) were found in the EBHT genome from three and four sites, respectively. The HsdS subunit target sequence from host DSM 104437 (scaffold 10, Table S5B) and 19A2 (scaffold 2, Table S6B) were not found in the EBHT genome. Comparison to the NCBI conserved domain database also identified that the HsdS subunit in DSM 104437 (scaffold 10, Table S6A) resembled three HsdS subunits with known target sequences. Two of these target sequences were present in the EBHT genome in three and two sites. The HsdS subunit from host 19A2 resembled one HsdS subunit with a known target sequence, which was not present in the EBHT genome. HsdS subunits from host DSM 104437 (scaffold 4, Table S6A) and 19A2 (scaffold 2, Table S6A) resembled HsdS subunit S.Sau13435ORF2165P of *S. aureus* strain NCTC 13435 according to the NCBI conserved domain database. Yet, REBASE showed that these two HsdS subunits had different target recognition sequences. The different tools for analyzing host phage‐resistance mechanisms and the host R‐M systems gave differing results. Altogether, the analysis shows that the DSM 104437 genome contained more R‐M systems than the 19A2.

### 2.6 ɸEBHT and mEBHT Harbored Distinct Methylation Profiles

**Figure 5:**
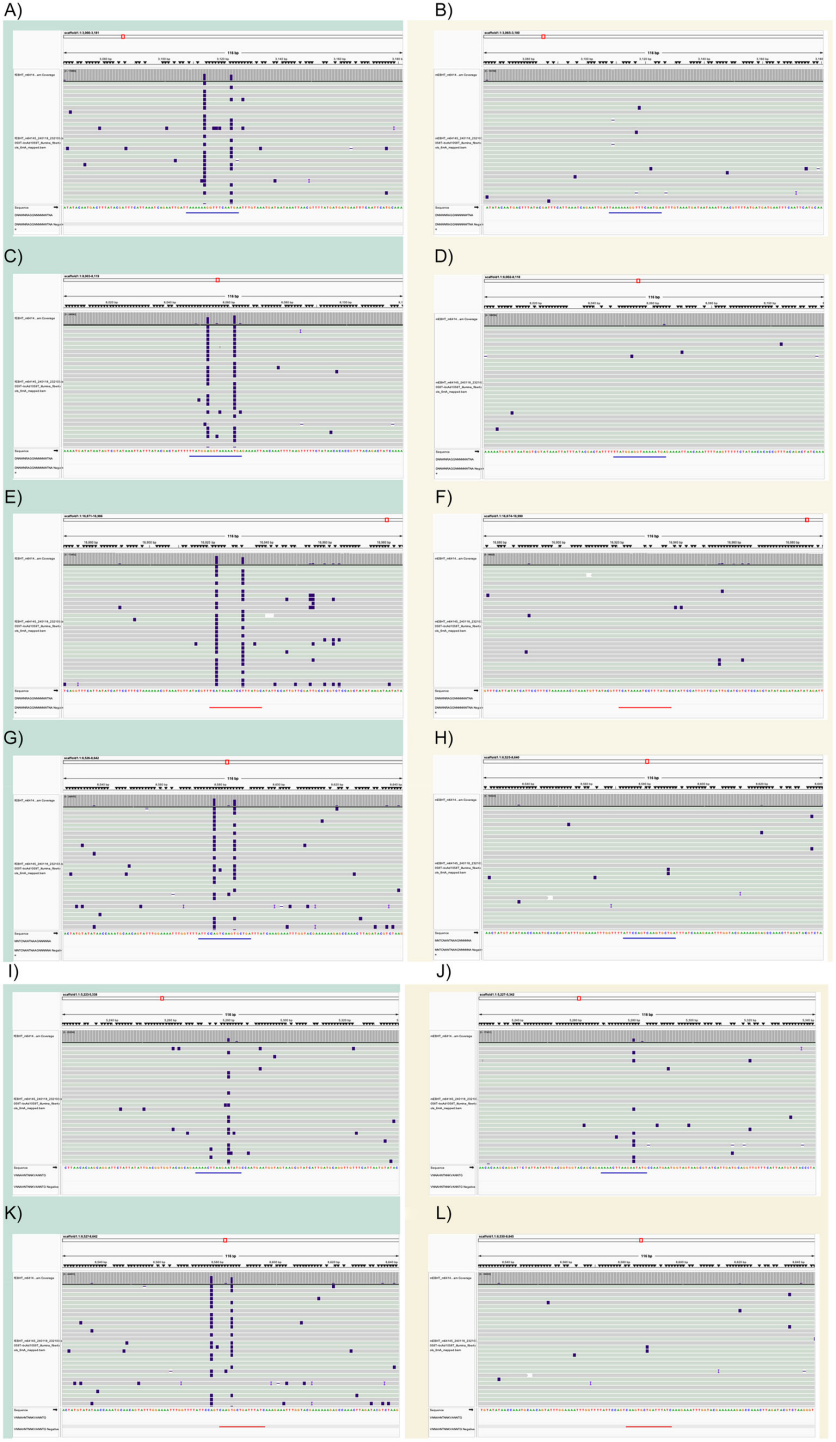
Methylated sites in ɸEBHT and mEBHT genomes. (A–L) The genomic views of ɸEBHT and mEBHT visualized in Integrative Genomics Viewer (IGV) (Robisson et al. 2011), where the HiFi reads mapping to the genome sequences are shown as rows and the nucleotide bases as columns. Modified bases of type m6A are highlighted in dark color. Views for ɸEBHT are on the left‐hand side with a pale turquoise‐shaded background (A, C, E, G, I, and K), while mEBHT views are on the right‐hand side with a pale‐yellow background shading (B, D, F, H, J, and L). Each view is a snapshot of approximately 30 visible individual reads. Information on all reads' base modifications is gathered into Table S7. Each view pair (ɸEBHT and mEBHT) shows the same sequence locations in both variants in all six methylated sites, indicating the differences in the methylated bases at these loci between the variants. The views and their corresponding sequences are (A, B) TAAAAAAGGTTTCAATGA, (C, D) TTATGGAGGTAAAAATGA, (E, F) GCATAAAGGATTTTATGA, (G, H) ATTCCAGTCAAGTGCTGA, (I, J) AAAACTTAAGAATATG, and (K, L) GATAAATCAGCACTTG. These sequences were derived from the preliminary screen by MultiMotifMaker (Beaulaurier et al. 2019), which resulted in three predicted methylated sites with ambiguous sequences (DNNWNRAGGNNNNNWTNA, MNTCNANTNAAGNNNNNA, and VNNAHNTNNKVANNTG) (see Table S7). The locations of these methylated sites are denoted as annotations below the mapped reads in blue, or in the case of reverse orientation, in red. Sites (E, F) GCATAAAGGATTTTATGA and (K, L) GATAAATCAGCACTTG were in reversed orientation. All six distinct sites were detected in the genome sequences once, and in the case of ɸEBHT, all sites were moderately or strongly methylated (methylated fraction ranging from 0.304 to 0.656, Table S7), while for mEBHT, only one of the sites (J) AAAACTTAAGAATATG) was counted as methylated (methylated fraction 0.293, Table S7). Sites were considered methylated if their methylated fraction exceeded a threshold of 0.2. EBHT, vB_SauP_EBHT.

PacBio sequencing was used to detect methylated bases from the ɸEBHT and mEBHT genomes to clarify the role of host R‐M systems in defining the EBHT host specificity. Methylated bases were detected based on delayed kinetic events. The number of total detected base modifications was slightly higher for mEBHT than for ɸEBHT (1339 and 1328, respectively), while for solely m6A modifications, it was the opposite (19 and 32, respectively). No m4C were identified from ɸEBHT or mEBHT genomes. For further analysis, we chose to focus on m6A modifications due to their significance for phages as they are the most prevalent modification type in prokaryotes (Beaulaurier et al. 2019). The preliminary de novo methylation motif search with MultiMotifMaker identified in total of five putative ambiguous motif sequences, which have the possibility of being methylated (T. Li et al. 2020). More detailed investigation revealed that these ambiguous motif sequences matched six distinct specific motif sequences present in the EBHT genome. All six methylation motifs were present once in the genomic sequence, and their methylated fraction exceeded the cutoff value 0.2 in the ɸEBHT genome, indicating that they were all methylated. However, only one of the motifs seemed to be methylated in mEBHT, and it was at a lower level compared with ɸEBHT. The methylated fractions—as in the ratio between the modified bases and all adenine bases at the motif site—for all six motifs were relatively high in ɸEBHT, ranging from 0.304 to 0.656 (Table S7 and Figure 5). For mEBHT, the highest methylated fraction was 0.293, while the remaining five motifs possessed values below 0.025 and were thus considered unmethylated (Table S7 and Figure 5).

## 3 Discussion

Phage therapy is one of the urgently needed alternatives for treating MRSA infections. Successful implementation of phage therapy requires a broad collection of well‐characterized lytic phages that fulfill the criteria for therapeutic use and a thorough understanding of phage biology and phage–host interactions (Leptihn and Loh 2022). The present research aimed to characterize the *S. aureus*–specific phage EBHT to establish its suitability for phage therapy and to explore interactions between EBHT and its two production hosts, DSM 104437 and 19A2. EBHT is a typical representative of the genus Rosemblumviruses, which are lytic phages with a podovirus morphology and genome sizes < 20 kb. A previous study outlines the structure of *S. aureus* phage P68, a close relative of EBHT. The structural analysis reveals that P68 employs tail and head fibers for host recognition and attachment, which facilitates the cleavage of the host surface with enzymes located on the tail spike. This mechanism enhances the attachment of the phage particle to the host cell, allowing the tail tip to penetrate the cytoplasmic membrane and trigger the release of the P68 genome (Hrebík et al. 2019). Given the genomic similarities between P68 and EBHT, it is probable that EBHT employs a similar approach for host cell recognition and infection. The structural features of P68 and EBHT are consistent with those of other podoviruses (Hrebík et al. 2019). The characterization of EBHT revealed that it indeed is suitable for phage therapy as it does not encode any gene products associated with the lysogenic life cycle or virulence factors that would compromise treatment outcomes. This result is consistent with our earlier findings, where EBHT was part of a phage cocktail that caused no adverse effects for pigs during an animal trial that assessed the efficacy of phage treatment in the eradication of MRSA from healthy carrier pigs (Tuomala et al. 2021). Previous animal studies, individual case studies in humans, and clinical trials reported similar results on the safety of bacteriophages (Wittebole et al. 2014; Concept paper on quality 2022; Guideline on quality 2023; European Directorate for the Quality of Medicines & HealthCare 2023). Meanwhile, the efficacy of phage therapy remains inconclusive. Some published clinical trials on phage therapy have failed to establish its efficacy, in contrast to individual case studies that report more successful results. Individual case studies show that 50%–80% of patients receiving phage therapy experienced clinical improvement, bacterial eradication, or both (Uyttebroek et al. 2022; Pirnay et al. 2024). Often, this difference is associated with the use of predesigned phage products during clinical trials, while individual case studies often use personalized phage products during treatment. However, these results require cautious interpretation as case studies do not include controls and there may be a publication bias toward successful treatment cases.

The EBHT host bacteria, DSM 104437 and 19A2, contained AMR genes and prophages in their genomes and therefore are not optimal phage‐production hosts for clinical use (Guideline on quality 2023). The host range screening showed that EBHT infected both human and pig isolates from the Finnish and German strain collections and would therefore be suitable for the treatment of infections caused by *S. aureus* strains of human and pig origin. Furthermore, the EBHT host range was not limited to MRSA strains, but it infected MSSA strains as well. The ability of EBHT to infect zoonotic *S. aureu*s strains is valuable for the One Health application of phage treatment, as human health is inseparable from the health of animals and the environment (Verkola et al. 2022).

Our study revealed a drastic difference between EBHT host specificity when the phage was produced in human isolate DSM 104437 (ɸEBHT) and pig isolate 19A2 (mEBHT). We established that these host specificity differences were not due to mutations in the phage genome or inhibition of phage adsorption on the host cell surface. Instead, mEBHT adsorbs efficiently to both host cells, but its genome is replicated only in its production host, 19A2, and not in host DSM 104437, where the mEBHT infection cycle is disrupted at the replication stage. No EBHT‐specific CRISPR spacer sequences were present in either host bacterial genome, but they carried different R‐M systems. The CRISPR/Cas system was thus excluded as the cause of disrupted mEBHT infection in DSM 104437, and these findings suggested that host R‐M systems determined the EBHT host specificity. PacBio sequencing of ɸEBHT and mEBHT genomes provided further evidence for this discovery; the phage genomes had different methylation patterns that were acquired during propagation since the EBHT genome did not encode any Mtases. Hence, the host‐specific methylation patterns allowed the bacterial strain to recognize the EBHT genome as “self” or “non‐self” DNA. The detection of six methylated m6A motifs in ɸEBHT—of which only one was shared in mEBHT—likely reflected the host‐specificity, which was lost in mEBHT, leading to significantly lower host infection efficacy and diversity. Most likely, the methylation patterns in the mEBHT genome differed from the ones that would protect it in human isolates, leaving mEBHT more sensitive against the bacterial R‐M systems. This would also explain the similarity of ɸEBHT and mEBHT host specificities within the pig isolates. Given the limitations related to de novo detecting methylation motifs in phage genomes, we acknowledge the possibility that some methylation information may have been overlooked, for instance, in mEBHT, in which only a few indications of methylation were detected now. However, we were able to indicate clear differences in methylomes of these two genomes with the available tools, and these results provide strong evidence for the importance of methylation for host infectivity and diversity.

We also studied if the mEBHT host range was revertible back to the host range of ɸEBHT. To this end, we picked a mEBHT plaque (mEBHT‐rev) from the DSM 104437 lawn and carried out a host range analysis with a selected subset of the strains. We observed that the behavior of mEBHT‐rev resembled ɸEBHT more than mEBHT but did not revert completely. We hypothesize that one round of plaque purification in DSM 104437 was not enough to turn the methylation pattern back to that of ɸEBHT but may have resulted in a mixed pattern. However, we did not analyze the methylation pattern itself or study whether further infection rounds would revert the host range completely back to the original one. Thus, this hypothesis remains speculative.

A recent study utilized a multistage infection system to show that the *H. pylori* phage KHP30T infected its most recent host strain more efficiently than other hosts. The improved infection efficacy was attributed to the methylation pattern obtained from its most recent host. The KHP30T study gave further support to the hypothesis that host‐originating methylations provide phages an adaptive mechanism to evade R‐M systems and enhance their infectivity toward a specific host, supporting the findings from this study (Takahashi et al. 2025). Several other earlier studies show similar results and have clarified the pivotal role of R‐M systems and the HsdM MTase subunits for genetic exchange and phage infection. For example, Type I R‐M systems regulate plasmid transmission between *Staphylococcus* cells by blocking HGT between *S. aureus* strains of different CCs (Mccarthy et al. 2012; Monk et al. 2015). A study by Waldron and Lindsay showed that the Type I R‐M system prevented plasmid acquisition and phage infection when the infecting phage was produced in a *S. aureus* host strain belonging to a different CC; five phages produced in an *S. aureus* strain belonging to CC51 infected a CC8 *S. aureus* strain RN4220 that has a truncated HsdR subunit but not its parental strain 8325‐4 with a functional HsdR subunit or the RNA4220 strain harboring a plasmid with a functional HsdR subunit (Waldron and Lindsay 2006). Another study by Xu et al. presented a Type IV 5mC modification‐dependent restriction enzyme SauUSI in *S. aureus* that controls plasmid transmission in *S. aureus* subspecies USA300 strains (Xu et al. 2011). The same SauUSI restriction enzyme, when cloned into an *E. coli* host, rendered the strain susceptible to Dcm^−^ λ phages while preserving the host resistance to Dcm^+^ λ phages (Xu et al. 2011). Other known, complete phage defense systems relying on methylation, such as Bacteriophage exclusion (BREX) or DISARM, were not identified from either DSM 104437 or 19A2 genomes analyzed in this study (Drobiazko et al. 2025; Ofir et al. 2018).

Previous studies focused on the R‐M subunits of bacterial hosts or the MTases present in phage genomes. The recent advances in third‐generation sequencing technologies, for example, PacBio and Oxford Nanopore, have enabled phage genome methylome analysis. A study focusing on three *Lactococcus* phages used PacBio sequencing to detect methylated sites attributed to phage‐encoded MTases and highlighted their role as an adaptive antirestriction mechanism and as a driver for phage evolution (Waldron and Lindsay 2006; Espinosa et al. 2024; Murphy et al. 2014). These MTases in *Lactococcus* phage genomes have a similar role in defining the host specificity of the studied phages as the MTases of DSM 104437 and 19A2 have on EBHT host specificity. Host‐dependent host specificity of phages complicates the selection criteria of production hosts since it is not enough to consider only the virulence factors of host bacteria and phage infection efficacy. EBHT demonstrates how phage infection efficacy varies depending on the production host and how good infection efficacy may not be beneficial for manufacturing therapeutic phage products. One‐step growth curve assay and EOP analysis showed that both EBHT variants infected host 19A2 more efficiently, making 19A2 the obvious but not optimal choice as an EBHT production host. Implications of methylation patterns of phage genomes extend to all phage‐production methods. For example, cell‐free phage production is gaining attention as it provides high yields and purity of final products (Emslander et al. 2022). Without the R‐M systems of bacterial host, phage genomes lack base modifications, which can lead to unintended qualities of the final product and render the phage noninfectious toward clinical strains. A comprehensive understanding of host‐dependent phage host‐specificity is a necessity to design efficient therapeutic phage products and enhance the outcomes of phage treatment.

## 4 Methods

### 4.1 Bacterial Strains, Media, and Basic Methods

The bacterial strains used in this study for phage enrichment and host specificity experiments are described in Table S1. Clinical MRSA strains were obtained from Hannover Medical School (MHH), Germany, and the Hospital District of Helsinki and Uusimaa Diagnostic Center, Finland. MSSA and coagulase‐negative *Staphylococcus* strains were obtained from the German Collection of Microorganisms and Cell Cultures (DSMZ) and the Hospital District of Helsinki and Uusimaa Diagnostic Center. Livestock‐associated MRSA and MSSA strains were provided by Annamari Heikinheimo and were isolated from Finnish pig farms (Verkola et al. 2022). All bacteria were cultured at 37°C in Luria–Bertani (LB) medium (Sambrook and Russel 2001). LB soft agar was supplemented with 0.4% (w/v) agar, and LB plates were solidified with 1.5% (w/v) agar. EBHT lysates were produced with the standard liquid method described elsewhere (Leskinen et al. 2017). The lysates were centrifuged at 5000–8000*g* for 10–20 min at +4°C and filtered through 0.22 µm syringe filters (Sartorius). Phage titers were determined with the standard double‐layer method in either DSM 104437 or 19A2 (Sambrook and Russel 2001).

### 4.2 Isolation of EBHT

A freshly filtered wastewater sample was mixed 1:1 with LB medium and an overnight culture of strain DSM 104437. The enrichment was incubated for 24 h at 37°C with shaking, the cells were centrifuged for 20 min at 8000 rpm, and the supernatant was filtered through a 0.22‐µm filter (Millipore). Plaque assays were performed according to the double agar layer method using DSM 104437 as the host strain. The obtained plaques were purified 10 times.

### 4.3 Phage DNA Isolation, Sequencing, and Sequence Analysis

For Illumina sequencing, large stocks of 200 mL of EBHT were further purified and concentrated in Centricon Plus‐70 centrifugal units (Millipore) according to the manufacturer's instructions. The Centricon‐purified phages were further purified by CsCl gradient ultracentrifugation at 35,000 rpm for 2 h. Fractions of 200 µL were sampled from the top of the CsCl gradient and dialyzed in 400 mL sodium‐magnesium (SM) buffer at room temperature using a dialysis membrane (14 kDa cutoff, Carl Roth GmbH). After 2 h of dialysis, the dialysis buffer was exchanged, and the dialysis step was repeated once again. The titers of the obtained fractions were determined, and the fraction with the highest titer was eventually used for electron microscopy and genome DNA extraction. The phage DNA was extracted using an innuPREP Virus DNA kit (Analytik Jena AG, Germany). The EBHT genome was sequenced using the MiSeq platform (Illumina Inc.) in a 75‐bp paired‐end double‐indexed run. De novo genome assembly and follow‐up analysis were performed using the Geneious software (Biomatters Inc.) applying default parameters, and the phage termini were determined with PhageTerm (Garneau et al. 2017). The EBHT genome was annotated automatically with the rapid prokaryotic genome annotation tool (Prokka) (Seemann 2014). The automatic gene predictions of the EBHT genome were verified manually with BLASTP and HHpred (Gabler et al. 2020). The Viral Proteomic Tree server (ViPTree, version 4.0) was used for whole‐genome phylogenetic analysis, and the Virus Intergenomic Distance Calculator (VIRIDIC) was used to calculate intergenomic distance and similarities between aligned phage genomes (Moraru et al. 2020; Nishimura et al. 2017). Phage genomes with at least 93% query coverage and 90% nucleotide identity in BLASTN search were selected for alignment (Priyam et al. 2019). The EBHT genome was submitted to GenBank with accession number MT926124.1 and was later used to create reference sequence NC_055906.1.

### 4.4 Transmission Electron Microscopy

ɸEBHT was concentrated and prepurified for imaging and proteomics analysis, and these steps were performed with Vivaspin 20 ultrafiltration units with a 100‐kDa cutoff filter membrane (Sartorius). For prepurification, the phage lysates were concentrated into 1/10 of the starting volume and washed twice with 1 Vol of SM buffer (100 mM NaCl, 10 mM MgSO_4_, 50 mM Tris–HCl, and pH 7.5). The final volume was adjusted to be between 1.0 and 1.3 mL. Prepurified phage particles were further purified with ion exchange chromatography (IEX) using an ÄKTA Purifier (GE Healthcare, Chicago, IL, USA) HPLC system with an anion exchange CIMmultus QA column (*V*, 1 mL) (Sartorius). A salt step gradient program was designed according to a method described by Hietala et al. (2019). In brief, prepurified phage was injected into the HPLC system, and unbound proteins were washed with buffer A (20 mM Tris–Cl and pH 7.5). The lightly bound proteins were washed with buffer A supplemented with 150 mM NaCl, and the phage particles were eluted with buffer A supplemented with 250 mM NaCl. The purified phage samples were changed from elution buffer to a final volume of 300 µL SM buffer with Vivaspin 6 ultrafiltration units with a 100‐kDa cutoff filter membrane (Sartorius). The phage titer in the final product was determined with the standard double‐layer method

Carbon‐formvar‐coated 400 mesh copper grids (Agar Scientific Ltd., Stansted, UK) were plasma cleaned in a PELCO easiGlow for 60 s at 0.38 mbar (air) and 20 mA. The sample, 5 µL, was applied to a grid for 60 s and then removed by wicking with filter paper. The grid was washed with 5 µL of deionized water and stained with 5 µL of 2% w/v uranyl acetate (Agar Scientific Ltd.). Grids were imaged using an FEI Tecnai 12 G2 BioTWIN microscope with a tungsten filament operating at 120 kV.

### 4.5 Host Range Determination and Reversibility Test

The host specificity determination of EBHT was performed with 162 *Staphylococcus* strains using a standard spot assay with three phage dilutions for Finnish *Staphylococcus* isolates (Sambrook and Russel 2001). The German *Staphylococcus* isolates were tested with a liquid phage susceptibility test as described elsewhere (Patpatia et al. 2022). EBHT variants in their production hosts were used as positive controls, and LB broth was used as a negative control for the assays. For EOP determinations, 10‐fold dilutions of phage samples were titrated with the double‐layer method as triplicates, and the EOP was calculated by comparing the ratio of titer in the test strain divided by the titer in the host strain (DSM 104437 for ɸEBHT and 19A2 for mEBHT).

The reversibility of mEBHT infection in host DSM 104437 was tested by plating mEBHT against the host DSM 104437 with a double‐layer method. A single plaque was picked from a low‐EOP culture of host DSM 104437 (denoted as mEBHT‐rev) and resuspended in 500 µL SMG buffer (100 mM NaCl, 10 mM MgSO_4_, 50 mM Tris–HCl, pH 7.5, and gelatine). The remaining bacteria were removed with 100 µL of chloroform for 15 min at RT and centrifuged at 11,000*g*, 5 min, RT. The EOPs of ɸEBHT, mEBHT, and mEBHT‐rev were determined in a set of 20 *S. aureus* strains, which were not infected or infected with reduced efficiency by mEBHT. Host strains DSM 104437 and 19A2 were included as controls for the EOP determination. One‐way analysis of variance with a 0.05 confidence level was used for statistical analysis. The data plots were visualized in OriginPro 2024 (OriginLab, Northampton, Massachusetts, USA).

### 4.6 Proteomics Analysis and Phage Adsorption Assay

For proteomics analysis, EBHT was purified with IEX as described previously for TEM imaging. The protein concentration of purified phage was measured using QubitTM Fluorometer and QubitTM Protein Assay Kit (Invitrogen by Thermo Fisher Scientific) according to the manufacturer's instructions (Sambrook and Russel 2001). Purified phage particles were subjected to liquid chromatography–mass spectrometry–mass spectrometry (LC/MS–MS) analysis at the Proteomics Unit, Institute of Biotechnology, University of Helsinki. For the LC/MS–MS analysis, the phage particles were reduced with tris(2‐carboxyethyl)phosphine and alkylated with iodoacetamide before digestion with trypsin. The proteomic analysis was performed, and data were collected as described in Leskinen et al. (2017). The amino acid sequences of ɸEBHT and mEBHT were annotated against the annotated sequence of vB_SauP_EBHT (GenBank accession number MT926124) and the annotated sequences of host strains DSM 104437 and 19A2.

To study phage adsorption on host cells, in total 500 µL (2.0 ∗ 10^9^ CFU/mL) of host DSM 104437 or 19A2 was mixed with 2.0 ∗ 10^8^ PFU of ɸEBHT or mEBHT in 100 µL and incubated for 5 min at RT. The cells were separated by centrifugation at 13,000*g*, 3 min, RT, and the remaining bacteria were removed with 300 µL chloroform at RT for 15 min. The double‐layer method was used to determine the phage quantity, and all titrations were performed as triplicates. The adsorption efficiency of ɸEBHT and mEBHT particles was tested in both host strains using the original host as a positive control. For the negative control, the phage was mixed with Luria broth, and *E. coli* strain #5521 was used to determine the unspecific adsorption of ɸEBHT and mEBHT particles. The data plots were visualized in OriginPro 2024 (OriginLab, Northampton, Massachusetts, USA).

### 4.7 One‐Step Growth Curve

The one‐step growth curve assay was performed according to the protocol described in Pajunen et al. (2000) and T. Li et al. (2020). In short, a 10‐mL host culture was grown until OD_600_ = 0.45, and the cells were infected with 0.0005 multiplicity of infection of phage. The mixture was incubated for 3 min at 37°C, after which the cells were collected by centrifugation at 5000*g*, 15°C for 5 min. The supernatant was removed, and the cells were re‐suspended in 10 mL of fresh growth media. A 150‐µL sample was taken at time points 0, 10, 20, 30, 40, 45, 50, and 55, and a dilution series of each sample was plated according to the standard double‐layer method. For each phage–host pair, the experiment was repeated three times. The mean values and standard deviations were calculated from the three replicates, and the growth curves were generated by assigning the value 1 for the PFU/mL of the sample taken at the 0 min time point. The growth curves were visualized in OriginPro 2024 (OriginLab, Northampton, Massachusetts, USA).

### 4.8 Phage DNA Proliferation Assay

The quantification of phage DNA inside the host cells was performed by infecting a host culture (*V*, 30 mL) grown until OD_600_ = 0.8 with 2 MOI of phage (9 ∗ 10^10^ PFU in 225 µL for ɸEBHT and 8.4 ∗ 10^10^ PFU in 210 µL for mEBHT). Samples (*V*, 2 mL) were collected immediately after infection and 2, 5, 10, 15, 20, and 30 min postinfection. The bacterial cells were pelleted by centrifugation at 16,900*g*, 2 min, +4 C, and washed three times with 1 mL of ice‐cold 10 mM Tris–Cl, pH 7.4 buffer solution. The DNA was isolated using a NucleoSpin Microbial DNA‐kit (Macherey‐Nagel, Germany), and the total DNA was quantified using a QubitTM Fluorometer and QubitTM Broad Range DNA assay kit (Invitrogen by Thermo Fisher Scientific, USA). The quantity of EBHT DNA was determined with a qPCR device (C1000 Touch Thermal Cycler with CFX96 Real‐Time System, Bio‐Rad, USA), using 10 ng total DNA as a template. A standard curve was created by using 1 µL of a 10‐fold dilution series of ɸEBHT lysate (2.8 ∗ 10^5^–2.8 ∗ 10^9^ PFU/mL) as template. The primers used for qPCR were EBHT‐tp‐for (5′‐AGCGTGATTTCGGGTCGCTA‐3′) and EBHT‐tp‐rev (5′‐AGTGGCATGACGCACAAGG‐3′), which recognize a 1075 bp region from the EBHT tail fiber gene. The total volume of each qPCR reaction was 20 µL and contained 10 µL of Maxima SYBR Green/ROX qPCR Master Mix (2X) (Thermo Scientific, USA), 10 μm of each primer, and 1 μL of the standard, the sample, or sterile mQ‐H2O for control. Triplicates were used from each standard, sample, and control. The qPCR conditions used for the experiment were 10 min at 95°C, followed by a 15 s denaturation step at 95°C and a 1 min annealing and extension step at 63°C. The Cq values were blotted against assay time in OriginPro 2024 (OriginLab, Northampton, Massachusetts, USA).

### 4.9 DNA Isolation, Sequencing, and Sequence Analysis of Host Strains

The genomic DNA of host DSM 104437 was isolated using a Nucleo‐Spin Microbial DNA kit (Macherey‐Nagel, Germany). The DNA concentration was determined using a QubitTM Fluorometer and QubitTM Broad Range DNA assay kit (Invitrogen by Thermo Fisher Scientific, USA) according to the manufacturer's instructions. The DNA isolation, sequencing, and characterization of the host 19A2 genome (accession number ERS6643341) are described elsewhere (Verkola et al. 2022). DSM 104437 sequencing and library preparations were completed at Novogene (UK) using the Illumina platform. The sequences were assembled with the A5 pipeline, and the raw reads were submitted to Sequence Reads Archive with BioProject ID: PRJNA1160023 (Tritt et al. 2012). The CC of strain DSM 104437 was determined with PubMLST (Jolley et al. 2018). Prophages present in the host strains DSM 104437 and 19A2 were identified with PHASTER provided by Wishart Lab, University of Alberta (Arndt et al. 2016). DSM 104437 and 19A2 genomes were screened for the presence of CRISPR/Cas systems with CRISPR/Cas Finder version 1.1.0, and the spacer sequences were compared with the EBHT genome (Couvin et al. 2018). The phage defense mechanisms in the bacterial host genomes were identified with DefenceFinder and PADLOCK online tools (Tesson et al. 2022; Payne et al. 2022). R‐M systems and genes encoding for the R‐M HsdR, HsdM, and HsdS subunits in the host genomes were identified using RAST, BLASTN, DefenceFinder, and PADLOCK (Aziz et al. 2008). R‐M subunit‐encoding genes that were identified with two or more tools were included in the study. HsdS subunits from hosts DSM 104437 and 19A2 were analyzed against REBASE and NCBI Conserved domain databases to identify target recognition sequences (Wang et al. 2023; Roberts et al. 2010).

### 4.10 PacBio Sequencing and Methylation Analysis of EBHT Genomes

PacBio sequencing was used to study the methylomes of the ɸEBHT and mEBHT genomes. For DNA isolation, EBHT lysates (10^10^ PFU/mL) were precipitated with PEG‐8000 as described by Sambrook and Russel (2001), and the pellets were dissolved in a 3.5 mL Tris‐magnesium buffer (50 mM Tris, pH 7.5–10 mM MgSO_4_) followed by removal of polyethylene glycol (PEG) with repeated chloroform washes. The PEG‐treated samples were further concentrated and purified through ultracentrifugation with glycerol step gradient using Optima L‐80 XP Ultracentrifuge (Beckman Coulter, USA) with SW 55 Ti rotor (Beckman Coulter, USA) at 40,000*g*, 4°C for 3.5 h. EBHT pellets were dissolved in 1.2 mL of SMG buffer, supplemented with 8% sucrose. Host DNA and RNA were removed with 1 µg/mL DNaseI and RNaseA treatment at +37°C for 30 min, and the phage capsids were degraded with Proteinase K (20 mg/mL), 10% sodium dodecyl sulfate, and 0.5 M ethylenediaminetetraacetic acid (pH 7.5) treatment at 56°C for 1.5 h. Phage DNA was extracted using a Maxwell device and Viral TNA–DNA isolation kit (Promega, USA) according to the manufacturer's instructions.

To allow the base modification detection in the downstream analyses, kinetics tags were attached to the HiFi reads running the “ccs‐kinetics‐bystrandify” program of the SMRT Link tool package (version 1.1.0, http://www.pacb.com/support/software-downloads/) in the command line. Genome assemblies of ɸEBHT and mEBHT were indexed using “pbmm2 index” (pbmm2 version 1.9.0, https://github.com/PacificBiosciences/pbmm2). The HiFi reads with kinetics tags were aligned against the indexed genomes using the “pbmm2 align” program (pbmm2 version 1.9.0, https://github.com/PacificBiosciences/pbmm2) with the option “—preset CCS.” The mapped reads were indexed using “pbindex” (version 1.0.0) and the assembly files with “samtools faidx” (version 1.16.1) for the next steps (H. Li et al. 2009). To predict the IPD (interpulse duration) scores and their positions in the fEBHT and mEBHT genomes, analysis with the “ipdSummary” (version 3.0) program was performed with the option “—identify m6A,m4C” to include bacteria‐related base modification types. All the above programs were run within an in‐house Snakemake workflow (version 7.17.1) (Mölder et al. 2021). Finally, for de novo methylated motif search program MultiMotifMaker (https://github.com/bioinfomaticsCSU/MultiMotifMaker) (T. Li et al. 2020) was run with the command “java‐jar artifacts/MultiMotifMaker.jar find” with kinetics results obtained from the ipdSummary program as the input. The visualization of de novo predicted methylated sites was done in R Studio (version 4.4.0) (R Core Team 2024) using the package “pheatmap” (version 1.0.12) (Kolde 2019).

## Author Contributions

**Henni Tuomala:** data Curation, formal analysis, investigation, methodology, visualization and funding acquisition and writing – original draft (lead). **Julia Holtel:** investigation and writing – original draft (supporting). **Melina Markkanen:** software, visualization and writing – original draft (supporting). **Sheetal Patpatia:** investigation and writing – review and editing. **Katariina Kaansalo:** investigation and writing – review and editing. **Clara Rolland:** investigation and writing – original draft (supporting). **Oliver W. Bayfield:** investigation, visualization, instrumentation, data curation and writing – review and editing. **Kira Ranta:** investigation and writing – review and editing. **Mikael Skurnik:** funding acquisition, project administration (supporting) and writing – review and editing. **Johannes Wittmann:** supervision, data curation and writing – original draft (supporting). **Saija Kiljunen:** conceptualization, supervision, resources, project administration (lead), funding acquisition, writing – review and editing.

## Ethics Statement

The authors have nothing to report.

## Conflicts of Interest

Saija Kiljunen is a cofounder and shareholder in PrecisionPhage Ltd. Other authors do not have competing interests. Funders had no role in designing the work, analyzing the data, or writing the manuscript.

## Supporting information


**Supplementary Figure S1:** Intergenomic genome similarity heatmap of EBHT generated from the same phage genomes included in the whole‐genome phylogenetic analysis (Figure 1.) and was generated with Virus Intergenomic Distance Calculator online server (VIRIDIC). **Supplementary Figure S2:** qPCR standard curve prepared from a dilution series of ΦEBHT lysate (2.8*105 PFU/ml –2.8*109 PFU/ml). In average, 10‐fold change in PFU/ml equals to Cq‐value change of 5 units. **Supplementary Table S1:** Bacterial strains and EBHT host range test results. **Supplementary Table S2:** Proteomics analysis. **Supplementary Table S3:** Complete phage defense mechanisms of EBHT host strains DSM 104437 and 19A2. **Supplementary Table S4:** HMMERS from DSM 104437 and 19A2 genomes that were associated with phage defense systems using DefenceFinder –tool. **Supplementary Table S5:** Restriction‐modification systems identified from DSM 104337 and 19A2 genomes with DefenceFinder, PADLOCK, RAST and BlastP. **Supplementary Table S6:** Restriction‐modification specificity (HsdS) subunits from DSM 104437 and 19A2 genomes compared to NCBI Conserved domain database (A) and REBASE database (B). **Supplementary Table S7:** The table lists the detected methylated sites and their methylated fractions in ɸEBHT and mEBHT genomes. All six m6A methylated sites were methylated in ɸEBHT, while only one of them showed lower‐level methylation in mEBHT. Methylated fraction refers to the ratio between the number of detected methylations on the site of the methylated motif and the total number of methylated motif sites.

Supplementary_file_2.

## Data Availability

The data that support the findings of this study are openly available in GenBank at https://www.ncbi.nlm.nih.gov/genbank/ with accession numbers MT926124.1 and NC_055906.1 (EBHT genome), the European Nucleotide Archive, https://www.ebi.ac.uk/ena/browser/home, with accession number ERS6643341 (*S. aureus* 19A2), and Sequence Reads Archive, https://www.ncbi.nlm.nih.gov/sra, BioProject ID PRJNA1160023 BioSamples SAMN43589536 (*S. aureus* DSM 104437), SAMN50554998 (ΦEBHT), and SAMN50554999 (mEBHT).
