# Supplementary material for: The Host R‐M Systems Change the Host Range of Staphylococcus Phage EBHT
Source: Microbiologyopen. 2025 Nov 27;14(6):e70165. doi: 10.1002/mbo3.70165 (PMC12660161; doi:10.1002/mbo3.70165)
Supplement: Supplementary file 1 — Supplementary Figure S1: Intergenomic genome similarity heatmap of EBHT generated from the same phage genomes included in the whole‐genome phylogenetic analysis (Figure 1.) and was generated with Virus Intergenomic Distance Calculator online server (VIRIDIC). Supplementary Figure S2: qPCR standard curve prepared from a dilution series of ΦEBHT lysate (2.8*105 PFU/ml –2.8*109 PFU/ml). In average, 10‐fold change in PFU/ml equals to Cq‐value change of 5 units. Supplementary Table S1: Bacterial strains and EBHT host range test results. Supplementary Table S2: Proteomics analysis. Supplementary Table S3: Complete phage defense mechanisms of EBHT host strains DSM 104437 and 19A2. Supplementary Table S4: HMMERS from DSM 104437 and 19A2 genomes that were associated with phage defense systems using DefenceFinder –tool. Supplementary Table S5: Restriction‐modification systems identified from DSM 104337 and 19A2 genomes with DefenceFinder, PADLOCK, RAST and BlastP. Supplementary Table S6: Restriction‐modification specificity (HsdS) subunits from DSM 104437 and 19A2 genomes compared to NCBI Conserved domain database (A) and REBASE database (B). Supplementary Table S7: The table lists the detected methylated sites and their methylated fractions in ɸEBHT and mEBHT genomes. All six m6A methylated sites were methylated in ɸEBHT, while only one of them showed lower‐level methylation in mEBHT. Methylated fraction refers to the ratio between the number of detected methylations on the site of the methylated motif and the total number of methylated motif sites. [file MBO3-14-e70165-s002.docx]

**The host R-M systems change the host range of *Staphylococcus* phage EBHT**

Henni Tuomala, Julia Holtel, Melina Markkanen, Sheetal Patpatia, Katariina Kaansalo, Clara Rolland, Oliver W Bayfield, Kira Ranta, Mikael Skurnik, Johannes Wittmann, Saija Kiljunen


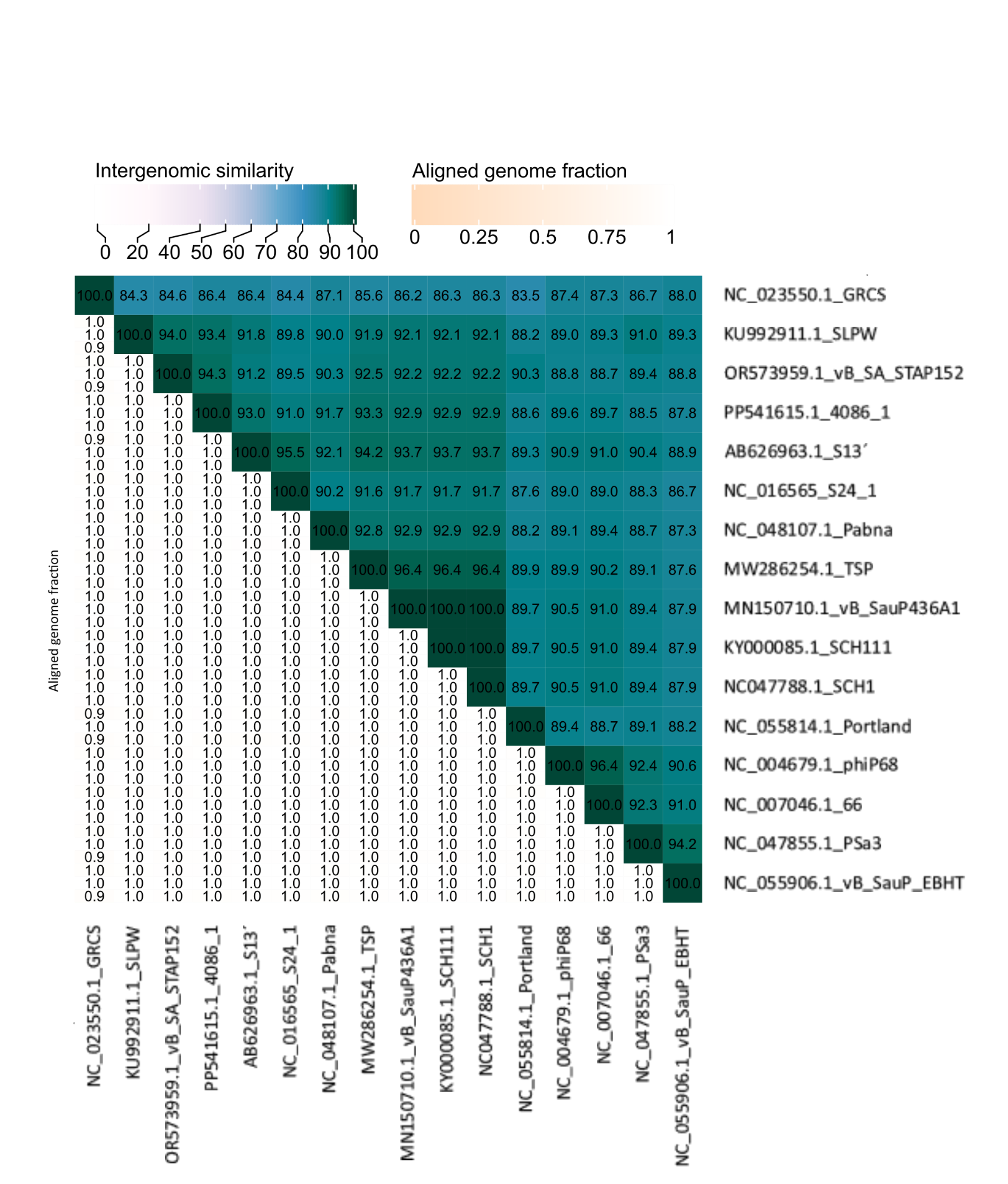


**Supplementary Figure S1.** Intergenomic genome similarity heatmap of EBHT generated from the same phage genomes included in the whole-genome phylogenetic analysis (Fig 1.) and was generated with Virus Intergenomic Distance Calculator online server (VIRIDIC).

**Supplementary Table S1**. Bacterial strains and EBHT host range test results

| Species | Strain |  | Origin | Publication | ɸEBHT | mEBHT |
| --- | --- | --- | --- | --- | --- | --- |
| *S. aureus* | DSM 104437 | MRSA | Hannover Medical School | Not yet published | + | + |
| *S. aureus* | 19A2 | MRSA | Pig | Heikinheimo et al. 2016 | + | + |
| *S. aureus* | #6472 |  | HUSLAB | Leskinen et al. 2017 | + | + |
| *S. aureus* | #6469 |  | HUSLAB | Leskinen et al. 2017 | - | - |
| *S. aureus* | #6466 |  | HUSLAB | Leskinen et al. 2017 | - | - |
| *S. aureus* | #6465 |  | HUSLAB | Leskinen et al. 2017 | + | + |
| *S. aureus* | #6462 |  | HUSLAB | Leskinen et al. 2017 | - | - |
| *S. aureus* | #6457 |  | HUSLAB | Leskinen et al. 2017 | - | - |
| *S. aureus* | #5849 | MRSA | HUSLAB | Leskinen et al. 2017 | + | + |
| *S. aureus* | #5850 | MRSA | HUSLAB | Leskinen et al. 2017 | + | + |
| *S. aureus* | #5851 | MRSA | HUSLAB | Leskinen et al. 2017 | + | + |
| *S. aureus* | #5852 | MRSA | HUSLAB | Leskinen et al. 2017 | + | + |
| *S. aureus* | #5853 |  | HUSLAB | Leskinen et al. 2017 | + | - |
| *S. aureus* | #5854 |  | HUSLAB | Leskinen et al. 2017 | - | - |
| *S. aureus* | #5855 |  | HUSLAB | Leskinen et al. 2017 | - | - |
| *S. aureus* | #5856 |  | HUSLAB | Leskinen et al. 2017 | + | - |
| *S. aureus* | #5857 |  | HUSLAB | Leskinen et al. 2017 | + | - |
| *S. aureus* | #5858 |  | HUSLAB | Leskinen et al. 2017 | - | - |
| *S. aureus* | #5859 |  | HUSLAB | Leskinen et al. 2017 | + | + |
| *S. aureus* | #5860 |  | HUSLAB | Leskinen et al. 2017 | - | - |
| *S. aureus* | #5861 |  | HUSLAB | Leskinen et al. 2017 | + | - |
| *S. aureus* | #5676 |  | HUSLAB | Leskinen et al. 2017 | + | - |
| *S. aureus* | #5677 |  | HUSLAB | Leskinen et al. 2017 | + | - |
| *S. aureus* | #5678 |  | HUSLAB | Leskinen et al. 2017 | + | - |
| *S. aureus* | #5679 |  | HUSLAB | Leskinen et al. 2017 | - | - |
| *S. aureus* | #5680 |  | HUSLAB | Leskinen et al. 2017 | - | - |
| *S. aureus* | #5681 |  | HUSLAB | Leskinen et al. 2017 | - | - |
| *S. aureus* | #5682 |  | HUSLAB | Leskinen et al. 2017 | + | - |
| *S. aureus* | #5683 |  | HUSLAB | Leskinen et al. 2017 | - | - |
| *S. aureus* | #5684 |  | HUSLAB | Leskinen et al. 2017 | + | + |
| *S. aureus* | #5685 |  | HUSLAB | Leskinen et al. 2017 | - | - |
| *S. aureus* | #5686 |  | HUSLAB | Leskinen et al. 2017 | + | + |
| *S. aureus* | #5687 |  | HUSLAB | Leskinen et al. 2017 | - | - |
| *S. aureus* | #5688 |  | HUSLAB | Leskinen et al. 2017 | + | + |
| *S. aureus* | #5689 |  | HUSLAB | Leskinen et al. 2017 | - | - |
| *S. aureus* | #5690 |  | HUSLAB | Leskinen et al. 2017 | - | - |
| *S. aureus* | #5691 |  | HUSLAB | Leskinen et al. 2017 | + | + |
| *S. aureus* | #5692 |  | HUSLAB | Leskinen et al. 2017 | + | + |
| *S. aureus* | #5693 |  | HUSLAB | Leskinen et al. 2017 | + | + |
| *S. aureus* | #5694 |  | HUSLAB | Leskinen et al. 2017 | + | + |
| *S. aureus* | #5695 |  | HUSLAB | Leskinen et al. 2017 | - | - |
| *S. aureus* | #5696 | MRSA | HUSLAB | Leskinen et al. 2017 | + | - |
| *S. aureus* | #5697 | MRSA | HUSLAB | Leskinen et al. 2017 | - | - |
| *S. aureus* | #5698 | MRSA | HUSLAB | Leskinen et al. 2017 | - | - |
| *S. aureus* | #5699 | MRSA | HUSLAB | Leskinen et al. 2017 | + | + |
| *S. aureus* | #5700 | MRSA | HUSLAB | Leskinen et al. 2017 | - | - |
| *S. aureus* | #5701 | MRSA | HUSLAB | Leskinen et al. 2017 | + | - |
| *S. aureus* | #5702 | MRSA | HUSLAB | Leskinen et al. 2017 | + | - |
| *S. aureus* | #5703 | MRSA | HUSLAB | Leskinen et al. 2017 | + | + |
| *S. aureus* | #5704 | MRSA | HUSLAB | Leskinen et al. 2017 | + | - |
| *S. aureus* | #5705 | MRSA | HUSLAB | Leskinen et al. 2017 | - | - |
| *S. aureus* | #5530 |  | HUSLAB | Leskinen et al. 2017 | - | - |
| *S. aureus* | #5531 |  | HUSLAB | Leskinen et al. 2017 | - | - |
| *S. aureus* | #5526 |  | HUSLAB | Leskinen et al. 2017 | - | - |
| *S. aureus* | #5527 |  | HUSLAB | Leskinen et al. 2017 | - | - |
| *S. aureus* | #5528 |  | HUSLAB | Leskinen et al. 2017 | - | - |
| *S. aureus* | #5515 |  | HUSLAB | Leskinen et al. 2017 | + | - |
| *S. aureus* | #5516 |  | HUSLAB | Leskinen et al. 2017 | + | + |
| *S. aureus* | #5511 |  | HUSLAB | Leskinen et al. 2017 | + | + |
| *S. aureus* | 7065_6_10P |  | Pig | Heikinheimo et al. 2016 | - | - |
| *S. aureus* | 7936_6_10 | MRSA | Pig | Heikinheimo et al. 2016 | + | + |
| *S. aureus* | 7936_16_20 | MRSA | Pig | Heikinheimo et al. 2016 | - | - |
| *S. aureus* | 1333_6_10 |  | Pig | Heikinheimo et al. 2016 | - | - |
| *S. aureus* | 1057_11_15 | MRSA | Pig | Heikinheimo et al. 2016 | - | - |
| *S. aureus* | 7502_1_5P |  | Pig | Heikinheimo et al. 2016 | - | - |
| *S. aureus* | 6161_1_5 | MRSA | Pig | Heikinheimo et al. 2016 | + | + |
| *S. aureus* | 6161_6_10P |  | Pig | Heikinheimo et al. 2016 | - | - |
| *S. aureus* | 3582_11_15 | MRSA | Pig | Heikinheimo et al. 2016 | + | + |
| *S. aureus* | 0812_1_5 | MRSA | Pig | Heikinheimo et al. 2016 | + | + |
| *S. aureus* | 0812_6_10 | MRSA | Pig | Heikinheimo et al. 2016 | + | + |
| *S. aureus* | 0812_11_15 | MRSA | Pig | Heikinheimo et al. 2016 | + | + |
| *S. aureus* | 0812_16_20 | MRSA | Pig | Heikinheimo et al. 2016 | + | + |
| *S. aureus* | 0250_1_5 | MRSA | Pig | Heikinheimo et al. 2016 | - | - |
| *S. aureus* | 0250_6_10 | MRSA | Pig | Heikinheimo et al. 2016 | + | - |
| *S. aureus* | 0250_11_15 | MRSA | Pig | Heikinheimo et al. 2016 | - | - |
| *S. aureus* | 0250_16_20 | MRSA | Pig | Heikinheimo et al. 2016 | - | - |
| *S. aureus* | 0186_11_15 | MRSA | Pig | Heikinheimo et al. 2016 | + | + |
| *S. aureus* | 6672_1_5 | MRSA | Pig | Heikinheimo et al. 2016 | + | + |
| *S. aureus* | 6672_6_10 | MRSA | Pig | Heikinheimo et al. 2016 | + | + |
| *S. aureus* | 6672_11_15 | MRSA | Pig | Heikinheimo et al. 2016 | + | + |
| *S. aureus* | DSM 104801 | MRSA | Hannover Medical School |  | + | - |
| *S. aureus* | DSM 104790 | MRSA | Hannover Medical School |  | + | - |
| *S. aureus* | DSM 104802 |  | DSMZ |  | + | - |
| *S. aureus* | DSM 104791 | MRSA | Hannover Medical School |  | + | - |
| *S. aureus* | DSM 104805 | MRSA | Hannover Medical School |  | + | - |
| *S. aureus* | DSM 104792 | MRSA | Hannover Medical School |  | + | - |
| *S. aureus* | DSM 104808 | MRSA | Hannover Medical School |  | + | - |
| *S. aureus* | DSM 104793 | MRSA | Hannover Medical School |  | + | - |
| *S. aureus* | DSM 104809 | MRSA | Hannover Medical School |  | + | - |
| *S. aureus* | DSM 104797 | MRSA | Hannover Medical School |  | + | - |
| *S. aureus* | DSM 104811 | MRSA | Hannover Medical School |  | + | - |
| *S. aureus* | DSM 104798 | MRSA | Hannover Medical School |  | + | - |
| *S. aureus* | DSM 104814 | MRSA | Hannover Medical School |  | + | - |
| *S. aureus* | DSM 104800 | MRSA | Hannover Medical School |  | + | - |
| *S. aureus* | DSM 104783 | MRSA | Hannover Medical School |  | + | - |
| *S. aureus* | DSM 104866 |  | DSMZ |  | +/- | - |
| *S. aureus* | DSM 104785 | MRSA | Hannover Medical School |  | + | - |
| *S. aureus* | DSM 104869 | MRSA | Hannover Medical School |  | + | - |
| *S. aureus* | DSM 104787 | MRSA | Hannover Medical School |  | + | - |
| *S. aureus* | DSM 104870 |  | DSMZ |  | + | - |
| *S. aureus* | DSM 104788 | MRSA | Hannover Medical School |  | + | - |
| *S. aureus* | DSM 104875 |  | DSMZ |  | + | - |
| *S. aureus* | DSM 104789 | MRSA | Hannover Medical School |  | + | - |
| *S. aureus* | DSM 104926 |  | DSMZ |  | + | - |
| *S. aureus* | DSM 104857 |  | DSMZ |  | + | - |
| *S. aureus* | DSM 104930 |  | DSMZ |  | + | - |
| *S. aureus* | DSM 104859 |  | DSMZ |  | + | - |
| *S. aureus* | DSM 104931 |  | DSMZ |  | + | - |
| *S. aureus* | DSM 104865 |  | DSMZ |  | + | +/- |
| *S. aureus* | DSM 104437 |  | DSMZ |  | + | - |
| *S. aureus* | DSM 104827 | MRSA | Hannover Medical School |  | + | - |
| *S. aureus* | DSM 104806 | MRSA | Hannover Medical School |  | + | - |
| *S. aureus* | DSM 104829 | MRSA | Hannover Medical School |  | + | - |
| *S. aureus* | DSM 104807 | MRSA | Hannover Medical School |  | + | - |
| *S. aureus* | DSM 104817 | MRSA | Hannover Medical School |  | +/- | - |
| *S. aureus* | DSM 6732 |  | DSMZ |  | + | - |
| *S. aureus* | DSM 104818 | MRSA | Hannover Medical School |  | + | - |
| *S. aureus* | DSM 13661 |  | DSMZ |  | + | - |
| *S. aureus* | DSM 104819 | MRSA | Hannover Medical School |  | + | - |
| *S. aureus* | DSM 26309 |  | DSMZ |  | + | - |
| *S. aureus* | DSM 104823 | MRSA | Hannover Medical School |  | + | - |
| *S. aureus* | DSM 28763 |  | DSMZ |  | + | - |
| *S. aureus* | DSM 104824 | MRSA | Hannover Medical School |  | + | - |
| *S. aureus* | DSM 102264 |  | DSMZ |  | + | - |
| *S. aureus* | DSM 104863 |  | DSMZ |  | - | - |
| *S. aureus* | DSM 346 |  | DSMZ |  | - | - |
| *S. aureus* | DSM 799 |  | DSMZ |  | - | - |
| *S. aureus* | DSM 3463 |  | DSMZ |  | - | - |
| *S. aureus* | DSM 6148 |  | DSMZ |  | - | - |
| *S. aureus* | DSM 17091 |  | DSMZ |  | - | - |
| *S. aureus* | Newman |  |  | Duthie and Lorenz 1952 | - | - |
| *S. aureus* | TB4 |  |  | Bae et al. 2006 | - | - |
| *S. intermedius* | #6209 |  | HUSLAB | Leskinen et al. 2017 | - | - |
| *S. intermedius* | #6210 |  | HUSLAB | Leskinen et al. 2017 | - | - |
| *S. intermedius* | #6211 |  | HUSLAB | Leskinen et al. 2017 | - | - |
| *S. intermedius* | #6212 |  | HUSLAB | Leskinen et al. 2017 | - | - |
| *S. intermedius* | #6213 |  | HUSLAB | Leskinen et al. 2017 | - | - |
| *S. lugdunensis* | #6214 |  | HUSLAB | Leskinen et al. 2017 | - | - |
| *S. lugdunensis* | #6215 |  | HUSLAB | Leskinen et al. 2017 | - | - |
| *S. lugdunensis* | #6216 |  | HUSLAB | Leskinen et al. 2017 | - | - |
| *S. lugdunensis* | #6217 |  | HUSLAB | Leskinen et al. 2017 | - | - |
| *S. lugdunensis* | #6218 |  | HUSLAB | Leskinen et al. 2017 | - | - |
| *S. epidermidis* | #6219 |  | HUSLAB | Leskinen et al. 2017 | - | - |
| *S. epidermidis* | #6220 |  | HUSLAB | Leskinen et al. 2017 | - | - |
| *S. epidermidis* | #6221 |  | HUSLAB | Leskinen et al. 2017 | - | - |
| *S. epidermidis* | #6222 |  | HUSLAB | Leskinen et al. 2017 | - | - |
| *S. epidermidis* | #6223 |  | HUSLAB | Leskinen et al. 2017 | - | - |
| *S. haemolyticus* | #6224 |  | HUSLAB | Leskinen et al. 2017 | - | - |
| *S. haemolyticus* | #6225 |  | HUSLAB | Leskinen et al. 2017 | - | - |
| *S. haemolyticus* | #6226 |  | HUSLAB | Leskinen et al. 2017 | - | - |
| *S. haemolyticus* | #6227 |  | HUSLAB | Leskinen et al. 2017 | - | - |
| *S. haemolyticus* | #6228 |  | HUSLAB | Leskinen et al. 2017 | - | - |
| *S. saphrophyticus* | #6229 |  | HUSLAB | Leskinen et al. 2017 | - | - |
| *S. saphrophyticus* | #6230 |  | HUSLAB | Leskinen et al. 2017 | - | - |
| *S. saphrophyticus* | #6231 |  | HUSLAB | Leskinen et al. 2017 | - | - |
| *S. saphrophyticus* | #6232 |  | HUSLAB | Leskinen et al. 2017 | - | - |
| *S. saphrophyticus* | #6233 |  | HUSLAB | Leskinen et al. 2017 | - | - |
| *S. pseudointer* | #6234 |  | HUSLAB | Leskinen et al. 2017 | - | - |
| *S. pseudointer* | #6235 |  | HUSLAB | Leskinen et al. 2017 | - | - |
| *S. pseudointer* | #6236 |  | HUSLAB | Leskinen et al. 2017 | - | - |
| *S. pseudointer* | #6237 |  | HUSLAB | Leskinen et al. 2017 | - | - |
| *S. pseudointer* | #6238 |  | HUSLAB | Leskinen et al. 2017 | - | - |
| *S. saphrophyticus* | DSM 20229 |  | DSMZ |  | - | - |
| *S. gallinarum* | DSM 20610 |  | DSMZ |  | - | - |

**Supplementary Table S2.** Proteomics analysis.

| Proteins originating from EBHT phage | | Peptide coverage | |
| --- | --- | --- | --- |
| Protein name | **Function** | **ɸEBHT** | **mEBHT** |
| EBHT_rev_-_CHAP_domain_protein_CDS_translation | Putative peptidoglycan cleavage | 65.48 | 27.69 |
| EBHT_rev_-_tail_fibre_protein | Host recognition and attachment | 103.54 | 157.30 |
| EBHT_rev_-_hypothetical_protein_CDS_translation_pI_3 | Unknown | 104.37 | 52.17 |
| EBHT_rev_-_hypothetical_protein_CDS_translation_pI_4 | Unknown | 48.23 | 43.97 |
| EBHT_rev_-_hypothetical_protein_CDS_translation_pI_6 | Unknown | 97.75 | 108.82 |
| EBHT_rev_-_major_capsid_protein_CDS_translation | Structural protein | 102.12 | 146.15 |
| EBHT_rev_-_putative_upper_collar_protein_CDS_translation | Putative Structural protein | 101.98 | 81.98 |
| EBHT_rev_-_tail_protein_CDS_translation | Structural protein | 22.56 | 22.79 |

| Proteins originating from the host | | Peptide coverage | |
| --- | --- | --- | --- |
| **Protein name** | **Function** | **ɸEBHT** | **mEBHT** |
| Aconitate hydratase (EC 4.2.1.3) | Citric acid cycle | 41.77 * | NA |
| Deblocking aminopeptidase (EC 3.4.11.-) | Amino acid metabolism | 22.02 * | NA |
| Deblocking aminopeptidase (EC 3.4.11.-) | Amino acid metabolism | NA | 4.29 * |
| Bacterial non-heme ferritin (EC 1.16.3.2) | Oxidoreductase | NA | 14.46 * |
| 6,7-dimethyl-8-ribityllumazine synthase (EC 2.5.1.78) | Riboflavin synthesis | 29.34 * | NA |
| M42 glutamyl aminopeptidase, cellulase | Amino acid metabolism | NA | 19.69 * |
| M42 glutamyl aminopeptidase, cellulase | Amino acid metabolism | 19.28 * | NA |
| Glutamine synthetase type I (EC 6.3.1.2) | Nitrogen metabolism | NA | 54.81 * |
| Inosine-5'-monophosphate dehydrogenase (EC 1.1.1.205) | Purine metabolism | 15.71 * | NA |

* Protein is found from only one of the purified phage variant particles.

**Supplementary Table S3.** Complete phage defense mechanisms of EBHT host strains DSM 104437 and 19A2

| DSM 104437 |  |  |  |
| --- | --- | --- | --- |
| **Tool** | **System** | **Gene product** | **Scaffold** |
| DefenceFinder | RM_Type_IIG^1^ | RM_Type_IIG_Type_IIG | Scaffold 9 |
|  | RM_Type_I^1^ | RM_Type_I_Mtases | Scaffold 10 |
|  | RM_Type_I^1^ | RM_Type_I_S | Scaffold 10 |
|  | RM_Type_I^1^ | RM_Type_I_Reases | Scaffold 10 |
|  | Abi2 | Abi2_Abi_2 | Scaffold 4 |
|  | Pycsar^1^ | Pycsar_AG_cyclase | Scaffold 10 |
|  | Pycsar^1^ | CBASS_2TM_5 | Scaffold 10 |
|  | ShosTA^1^ | ShosTA_ShosT | Scaffold 28 |
|  | ShosTA^1^ | ShosTA_ShosA | Scaffold 28 |
| PADLOCK | SoFic | SoFic | Scaffold 1 |
|  | pycsar_effector^1^ | PycC | Scaffold 10 |
|  | pycsar_effector^1^ | PycTM | Scaffold 10 |
|  | RM_type_I^1^ | MTase_I | Scaffold 10 |
|  | RM_type_I^1^ | Specificity_I | Scaffold 10 |
|  | RM_type_I^1^ | REase_I | Scaffold 10 |
|  | Mokosh_TypeII | MkoC | Scaffold 10 |
|  | ShosTA^1^ | ShosT | Scaffold 28 |
|  | ShosTA^1^ | ShosA | Scaffold 28 |
|  | RM_type_IV | mREase_IV | Scaffold 29 |
|  | DMS_other | MTase_I | Scaffold 4 |
|  | DMS_other | Specificity_I | Scaffold 4 |
|  | RM_Type_IIG^1^ | REase_MTase_IIG | Scaffold 9 |
|  | DMS_other | Specificity_II | Scaffold 9 |
| RAST | RM_type_I | Type I r-m, restriction subunit R (EC 3.1.21.3) | Scaffold 3 |
|  | RM_type_I | Type I r-m, methyltransferase subunit M (EC 2.1.1.72) | Scaffold 4 |
|  | RM_type_I | Type I r-m, specificity subunit S | Scaffold 4 |
|  | RM_type_I | Type I r-m system, restriction subunit R (EC 3.1.21.3) | Scaffold 10 |
|  | RM_type_I | Type I r-m, methyltransferase subunit M (EC 2.1.1.72) | Scaffold 10 |
|  | RM_type_I | Type I r-m system, specificity subunit S | Scaffold 10 |
|  | RM_type_I | Type I r-m system, restriction subunit R (EC 3.1.21.3) | Scaffold 10 |
|  | RM_type_I | Type I r-m, specificity subunit S | Scaffold 28 |
| **19A2** |  |  |  |
| **Tool** | **System** | **Gene product** | **Scaffold** |
| DefenceFinder | RosmerTA^1^ | RosmerTA__RmrT_2603008502 | Scaffold 2 |
|  | RosmerTA^1^ | RosmerTA__RmrA_2603008502 | Scaffold 2 |
|  | RM_Type_II^1^ | RM_Type_II__Type_II_REases | Scaffold 4 |
|  | RM_Type_II^1^ | RM_Type_II__Type_II_MTases | Scaffold 4 |
|  | Thoeris_I^1^ | Thoeris_I__ThsA_new_grand | Scaffold 20 |
|  | Thoeris_I^1^ | Thoeris__ThsB_Global | Scaffold 20 |
|  | Thoeris_I^1^ | Thoeris__ThsB_Global | Scaffold 20 |
|  | RloC | RloC__RloC | Scaffold 22 |
| PADLOCK | SoFic | SoFic | Scaffold 0 |
|  | RosmerTA | RmrT | Scaffold 2 |
|  | RosmerTA | RmrA | Scaffold 2 |
|  | DMS_other | MTase_I | Scaffold 2 |
|  | DMS_other | Specificity_I | Scaffold 2 |
|  | thoeris_type_I^1^ | ThsA1 | Scaffold 20 |
|  | thoeris_type_I^1^ | ThsB1 | Scaffold 20 |
|  | thoeris_type_I^1^ | ThsB1 | Scaffold 20 |
|  | RM_type_II^1^ | REase_II | Scaffold 4 |
|  | RM_type_II^1^ | MTase_II | Scaffold 4 |
| RAST | RM_Type_I | Type I r-m, specificity subunit S | Scaffold 3 |
|  | RM_Type_I | Type I r-m, methyltransferase subunit M (EC 2.1.1.72) | Scaffold 3 |
|  | RM_Type_I | Type I r-m, restriction subunit R (EC 3.1.21.3) | Scaffold 5 |
|  | RM_Type_I | Type I r-m, restriction subunit R (EC 3.1.21.3) | Scaffold 9 |

^1^ System identified with DefenceFinder and PADLOCK

**Supplementary Table S4.** HMMERS from DSM 104437 and 19A2 genomes that were associated with phage defense systems using DefenceFinder -tool

| DSM 104437 | | | | | |
| --- | --- | --- | --- | --- | --- |
| **Contig** | **Hit Identifier** | **Gene name** | **I e-value** | **Profile coverage** | **Sequence coverage** |
| scaffold_0 | scaffold_0_21 | Dodola__DolB | 1,80E-24 | 0.44 | 0.205 |
|  | scaffold_0_29 | Gabija__GajB_2 | 3,90E-66 | 0.749 | 0.745 |
|  | scaffold_0_80 | Dnd__DndA | 1,70E-44 | 0.867 | 0.829 |
|  | scaffold_0_148 | ShosTA__ShosT | 2,20E-08 | 0.608 | 0.702 |
|  | scaffold_0_179 | PsyrTA__PsyrT | 1,80E-112 | 0.514 | 0.581 |
|  | scaffold_0_187 | Dpd__QueC | 3,70E-42 | 0.927 | 0.915 |
|  | scaffold_0_188 | Dpd__QueD | 6,30E-19 | 0.95 | 0.843 |
|  | scaffold_0_189 | Dpd__QueE | 1,10E-13 | 0.606 | 0.517 |
|  | scaffold_0_372 | Dodola__DolB | 8,30E-26 | 0.659 | 0.327 |
| scaffold_1 | scaffold_1_36 | Retron_I_A__ATPase_TypeIA | 2,10E-10 | 0.485 | 0.491 |
|  | scaffold_1_77 | UG6__DnaG | 1,80E-37 | 0.896 | 0.517 |
|  | scaffold_1_132 | recD_IV-D_1 | 7,10E-81 | 0.845 | 0.775 |
|  | scaffold_1_135 | Dnd__DndA | 4,50E-115 | 0.995 | 0.976 |
|  | scaffold_1_141 | CBASS__AG_E1_ThiF | 4,20E-34 | 0.627 | 0.601 |
|  | scaffold_1_192 | UG9__DNA_pol | 7,20E-31 | 0.715 | 0.448 |
|  | scaffold_1_213 | RM_Type_II__Type_II_MTases_FAM_29 | 2,80E-06 | 0.42 | 0.728 |
|  | scaffold_1_213 | RM_Type_II__Type_II_MTases_FAM_13 | 5,20E-09 | 0.412 | 0.718 |
|  | scaffold_1_217 | Dnd__DndA | 2,70E-92 | 0.973 | 0.933 |
| scaffold_2 | scaffold_2_118 | WYL_I_II_III_IV_V_VI_1 | 3,20E-50 | 0.735 | 0.983 |
|  | scaffold_2_154 | CBASS__AG_E1_ThiF | 3,30E-48 | 0.992 | 0.699 |
| scaffold_3 | scaffold_3_95 | Rst_gop_beta_cll__cll | 2,90E-22 | 0.732 | 0.701 |
|  | scaffold_3_96 | RM__Type_I_REases_FAM_2.einsi_trimmed | 7,40E-28 | 0.779 | 0.8 |
|  | scaffold_3_96 | RM__Type_I_REases_FAM_0.einsi_trimmed | 2,50E-193 | 0.9 | 0.968 |
| scaffold_4 | scaffold_4_25 | DarTG__DarG | 1,10E-65 | 0.756 | 0.693 |
|  | scaffold_4_68 | Abi2__Abi_2 | 2,90E-23 | 0.683 | 0.578 |
|  | scaffold_4_106 | RM_Type_II__Type_II_MTases_FAM_28 | 8,50E-16 | 0.989 | 0.507 |
|  | scaffold_4_106 | RM_Type_II__Type_II_MTases_FAM_14 | 1,30E-18 | 0.479 | 0.487 |
|  | scaffold_4_106 | RM_Type_II__Type_II_MTases_FAM_13 | 2,30E-23 | 0.446 | 0.495 |
|  | scaffold_4_106 | RM_Type_II__Type_II_MTases_FAM_6 | 2,10E-23 | 0.448 | 0.486 |
|  | scaffold_4_106 | RM_Type_II__Type_II_MTases_FAM_33 | 6,70E-41 | 0.463 | 0.497 |
|  | scaffold_4_106 | RM__Type_I_MTases_FAM_3 | 1,10E-49 | 0.501 | 0.85 |
|  | scaffold_4_106 | RM__Type_I_MTases_FAM_1 | 1,40E-56 | 0.686 | 0.934 |
|  | scaffold_4_106 | PrrC__EcoprrI | 9,60E-158 | 0.961 | 0.987 |
|  | scaffold_4_106 | RM__Type_I_MTases_FAM_0 | 1,40E-181 | 0.956 | 0.979 |
|  | scaffold_4_107 | RM__Type_I_S_03 | 2,60E-23 | 0.992 | 0.952 |
|  | scaffold_4_107 | RM__Type_I_S_02 | 3,10E-31 | 0.819 | 0.995 |
|  | scaffold_4_107 | RM__Type_I_S_51 | 4,30E-32 | 0.886 | 0.514 |
|  | scaffold_4_107 | RM__Type_I_S_52 | 3,30E-36 | 0.44 | 0.902 |
|  | scaffold_4_107 | RM__Type_I_S_04 | 7,60E-38 | 0.623 | 0.95 |
| scaffold_5 | scaffold_5_1 | RT_I_II_III_IV_V_VI_1 | 4,90E-87 | 0.731 | 0.625 |
|  | scaffold_5_39 | Lamassu-Fam__LmuB_SMC_Mrr | 5,20E-07 | 0.906 | 0.232 |
|  | scaffold_5_39 | Lamassu-Fam__LmuB_SMC_Cap4_nuclease_II | 6,40E-11 | 0.411 | 0.499 |
|  | scaffold_5_39 | Lamassu-Fam__LmuB_SMC_Hydrolase_protease | 2,10E-14 | 0.769 | 0.78 |
| scaffold_7 | scaffold_7_7 | Lamassu-Fam__LmuA_effector_Protease | 3,00E-15 | 0.813 | 0.607 |
|  | scaffold_7_54 | Dodola__DolB | 3,10E-31 | 0.546 | 0.399 |
| scaffold_8 | scaffold_8_77 | Retron_I_A__ATPase_TypeIA | 7,20E-08 | 0.47 | 0.227 |
|  | scaffold_8_77 | Lamassu-Fam__LmuB_SMC_Mrr | 3,00E-08 | 0.697 | 0.339 |
|  | scaffold_8_77 | Wadjet__JetC_I | 1,70E-11 | 0.473 | 0.469 |
|  | scaffold_8_77 | Lamassu-Fam__LmuB_SMC_Hydrolase_protease | 1,30E-13 | 0.491 | 0.413 |
|  | scaffold_8_77 | Lamassu-Fam__LmuB_SMC_Cap4_nuclease_II | 2,60E-17 | 0.789 | 0.408 |
|  | scaffold_8_92 | PsyrTA__PsyrA | 1,40E-24 | 0.523 | 0.722 |
|  | scaffold_8_92 | ShosTA__ShosA | 7,40E-61 | 0.723 | 0.759 |
|  | scaffold_8_107 | DEDDh_I_II_III_IV_V_VI_1 | 1,40E-48 | 0.994 | 0.108 |
| scaffold_9 | scaffold_9_2 | RM_Type_II__Type_II_MTases_FAM_6 | 1,20E-08 | 0.4 | 0.337 |
|  | scaffold_9_2 | RM_Type_II__Type_II_MTases_FAM_28 | 1,80E-10 | 0.412 | 0.361 |
|  | scaffold_9_2 | RM_Type_II__Type_II_MTases_FAM_33 | 1,10E-28 | 0.473 | 0.373 |
|  | scaffold_9_2 | RM_Type_IIG__Type_IIG_1 | 5,00E-218 | 0.797 | 0.978 |
|  | scaffold_9_12 | PsyrTA__PsyrT | 5,60E-84 | 0.502 | 0.728 |
|  | scaffold_9_38 | csf4_IV-A_1 | 8,00E-36 | 0.916 | 0.692 |
|  | scaffold_9_38 | DEDDh_I_II_III_IV_V_VI_1 | 2,70E-42 | 0.988 | 0.173 |
|  | scaffold_9_58 | Wadjet__JetC_III | 4,80E-06 | 0.642 | 0.094 |
| scaffold_10 | scaffold_10_55 | CBASS__2TM_5 | 1,50E-37 | 0.96 | 0.981 |
|  | scaffold_10_56 | RM_Type_II__Type_II_MTases_FAM_6 | 1,90E-12 | 0.476 | 0.527 |
|  | scaffold_10_56 | RM_Type_II__Type_II_MTases_FAM_29 | 1,70E-13 | 0.402 | 0.453 |
|  | scaffold_10_56 | RM_Type_II__Type_II_MTases_FAM_14 | 6,10E-15 | 0.409 | 0.434 |
|  | scaffold_10_56 | RM__Type_I_MTases_FAM_1 | 4,60E-50 | 0.714 | 0.711 |
|  | scaffold_10_56 | RM_Type_II__Type_II_MTases_FAM_33 | 4,70E-51 | 0.525 | 0.545 |
|  | scaffold_10_56 | RM__Type_I_MTases_FAM_3 | 1,40E-55 | 0.687 | 0.859 |
|  | scaffold_10_56 | PrrC__EcoprrI | 1,20E-64 | 0.942 | 0.98 |
|  | scaffold_10_56 | RM__Type_I_MTases_FAM_0 | 9,20E-182 | 0.979 | 0.984 |
|  | scaffold_10_57 | RM__Type_I_S_03 | 3,30E-18 | 0.502 | 0.645 |
|  | scaffold_10_57 | RM__Type_I_S_01 | 3,90E-27 | 0.5 | 0.844 |
|  | scaffold_10_57 | RM__Type_I_S_02 | 5,20E-31 | 0.42 | 0.455 |
|  | scaffold_10_57 | RM__Type_I_S_52 | 3,00E-31 | 0.663 | 0.959 |
|  | scaffold_10_57 | RM__Type_I_S_04 | 3,60E-36 | 0.733 | 0.951 |
|  | scaffold_10_57 | RM__Type_I_S_51 | 1,40E-39 | 0.826 | 0.978 |
|  | scaffold_10_57 | RM__Type_I_S_06 | 8,40E-57 | 0.739 | 0.968 |
|  | scaffold_10_58 | RM__Type_I_REases_FAM_2.einsi_trimmed | 4,20E-14 | 0.861 | 0.827 |
|  | scaffold_10_58 | RM__Type_I_REases_FAM_0.einsi_trimmed | 1,10E-253 | 0.993 | 0.976 |
|  | scaffold_10_68 | gcu233__gcu233 | 3,20E-49 | 0.977 | 0.957 |
|  | scaffold_10_74 | Mokosh_type_I__MkoA_D | 1,10E-63 | 0.676 | 0.768 |
| scaffold_12 | scaffold_12_7 | MazEF__MazF | 2,00E-09 | 0.856 | 0.835 |
|  | scaffold_12_26 | DISARM__drmC | 6,10E-14 | 0.491 | 0.251 |
|  | scaffold_12_53 | Veles__VlsC2 | 3,30E-26 | 0.919 | 0.936 |
|  | scaffold_12_53 | Veles__VlsC1 | 1,80E-26 | 0.926 | 0.95 |
| scaffold_17 | scaffold_17_48 | Lamassu-Fam__LmuB_SMC_Cap4_nuclease_II | 3,80E-05 | 0.439 | 0.128 |
| scaffold_18 | scaffold_18_7 | Gao_Iet__IetA | 1,70E-30 | 0.563 | 0.324 |
|  | scaffold_18_27 | Gao_Qat__QatD | 2,80E-12 | 0.953 | 0.977 |
| scaffold_21 | scaffold_21_43 | Lamassu-Fam__LmuB_SMC_Cap4_nuclease_II | 2,70E-08 | 0.777 | 0.784 |
|  | scaffold_21_43 | Lamassu-Fam__LmuB_SMC_Mrr | 4,20E-09 | 0.746 | 0.79 |
| scaffold_26 | scaffold_26_22 | Veles__VlsC1 | 1,20E-28 | 0.953 | 0.923 |
|  | scaffold_26_22 | Veles__VlsC2 | 5,10E-30 | 0.966 | 0.929 |
| scaffold_27 | scaffold_27_5 | DEDDh_I_II_III_IV_V_VI_1 | 1,10E-36 | 0.988 | 0.822 |
|  | scaffold_27_16 | Gabija__GajB_3 | 5,90E-16 | 0.66 | 0.386 |
|  | scaffold_27_16 | Gabija__GajB_1 | 3,90E-44 | 0.927 | 0.826 |
|  | scaffold_27_16 | UG35__UvrD | 3,00E-103 | 0.535 | 0.596 |
|  | scaffold_27_16 | Gabija__GajB_2 | 2,10E-115 | 0.777 | 0.893 |
| scaffold_28 | scaffold_28_27 | ShosTA__ShosT* | 3,80E-52 | 0.945 | 0.774 |
|  | scaffold_28_28 | PsyrTA__PsyrA | 7,70E-28 | 0.445 | 0.628 |
|  | scaffold_28_28 | ShosTA__ShosA* | 7,00E-77 | 0.861 | 0.926 |
| scaffold_32 | scaffold_32_5 | Veles__VlsC1 | 5,40E-17 | 0.939 | 0.955 |
|  | scaffold_32_5 | Veles__VlsC2 | 7,00E-20 | 0.933 | 0.939 |
| **19A2** | | | | | |
| **Contig** | **Hit Identifier** | **Gene name** | **I e-value** | **Profile coverage** | **Sequence coverage** |
| scaffold_0 | scaffold_0_70 | csf4_IV-A_1 | 6,90E-36 | 0.916 | 0.692 |
|  | scaffold_0_70 | DEDDh_I_II_III_IV_V_VI_1 | 7,30E-42 | 0.988 | 0.173 |
|  | scaffold_0_95 | PsyrTA__PsyrT | 3,10E-84 | 0.504 | 0.73 |
|  | scaffold_0_132 | Retron_I_A__ATPase_TypeIA | 1,60E-10 | 0.485 | 0.491 |
|  | scaffold_0_173 | UG6__DnaG | 1,50E-37 | 0.896 | 0.522 |
|  | scaffold_0_228 | recD_IV-D_1 | 1,40E-80 | 0.844 | 0.774 |
|  | scaffold_0_231 | Dnd__DndA | 3,80E-115 | 0.995 | 0.976 |
|  | scaffold_0_237 | CBASS__AG_E1_ThiF | 1,20E-34 | 0.668 | 0.64 |
|  | scaffold_0_294 | Dodola__DolB | 9,50E-04 | 0.624 | 0.726 |
|  | scaffold_0_300 | UG9__DNA_pol | 4,20E-31 | 0.717 | 0.45 |
|  | scaffold_0_321 | RM_Type_II__Type_II_MTases_FAM_29 | 7,40E-06 | 0.42 | 0.728 |
|  | scaffold_0_321 | RM_Type_II__Type_II_MTases_FAM_13 | 1,10E-08 | 0.412 | 0.718 |
|  | scaffold_0_325 | Dnd__DndA | 2,34E-93 | 0.981 | 0.941 |
| scaffold_1 | scaffold_1_6 | Dodola__DolB | 1,40E-24 | 0.44 | 0.205 |
|  | scaffold_1_263 | Lamassu-Fam__LmuB_SMC_hypothetical | 1,20E-06 | 0.755 | 0.415 |
|  | scaffold_1_263 | Lamassu-Fam__LmuB_SMC_Mrr | 3,90E-08 | 0.697 | 0.339 |
|  | scaffold_1_263 | Retron_I_A__ATPase_TypeIA | 5,00E-08 | 0.472 | 0.228 |
|  | scaffold_1_263 | Wadjet__JetC_I | 6,10E-11 | 0.487 | 0.502 |
|  | scaffold_1_263 | Lamassu-Fam__LmuB_SMC_Hydrolase_protease | 1,50E-13 | 0.491 | 0.413 |
|  | scaffold_1_263 | Lamassu-Fam__LmuB_SMC_Cap4_nuclease_II | 9,60E-18 | 0.777 | 0.396 |
|  | scaffold_1_280 | PsyrTA__PsyrA | 1,80E-24 | 0.523 | 0.722 |
|  | scaffold_1_280 | ShosTA__ShosA | 8,50E-61 | 0.726 | 0.749 |
|  | scaffold_1_295 | DEDDh_I_II_III_IV_V_VI_1 | 1,20E-48 | 0.994 | 0.108 |
| scaffold_2 | scaffold_2_9 | RosmerTA__RmrT_2603008502 | 7,40E-83 | 0.967 | 0.961 |
|  | scaffold_2_10 | RosmerTA__RmrA_2676476075 | 8,50E-26 | 0.871 | 0.875 |
|  | scaffold_2_10 | RosmerTA__RmrA_2641389401 | 6,00E-34 | 0.97 | 0.964 |
|  | scaffold_2_10 | RosmerTA__RmrA_2734955840 | 2,70E-35 | 0.792 | 0.811 |
|  | scaffold_2_10 | RosmerTA__RmrA_2617826694 | 4,50E-36 | 0.552 | 0.523 |
|  | scaffold_2_10 | RosmerTA__RmrA_2585209417 | 1,40E-40 | 0.957 | 0.964 |
|  | scaffold_2_10 | RosmerTA__RmrA_2753529055 | 3,30E-46 | 0.887 | 0.941 |
|  | scaffold_2_10 | RosmerTA__RmrA_2634932349 | 1,40E-49 | 0.915 | 0.934 |
|  | scaffold_2_10 | RosmerTA__RmrA_2662548665 | 1,20E-58 | 0.902 | 0.964 |
|  | scaffold_2_10 | RosmerTA__RmrA_2664250653 | 5,00E-63 | 0.934 | 0.972 |
|  | scaffold_2_10 | RosmerTA__RmrA_2634882613 | 5,60E-65 | 0.919 | 0.964 |
|  | scaffold_2_10 | RosmerTA__RmrA_2677172838 | 6,70E-66 | 0.926 | 0.934 |
|  | scaffold_2_10 | RosmerTA__RmrA_2600853143 | 5,90E-67 | 0.951 | 0.972 |
|  | scaffold_2_10 | RosmerTA__RmrA_2623274509 | 2,00E-67 | 0.927 | 0.944 |
|  | scaffold_2_10 | RosmerTA__RmrA_2603008502 | 4,00E-173 | 0.982 | 0.995 |
|  | scaffold_2_60 | RM__Type_I_REases_FAM_2.einsi_trimmed | 1,80E-27 | 0.779 | 0.8 |
|  | scaffold_2_60 | RM__Type_I_REases_FAM_0.einsi_trimmed | 3,20E-193 | 0.927 | 0.946 |
|  | scaffold_2_61 | Rst_gop_beta_cll__cll | 2,80E-22 | 0.736 | 0.688 |
|  | scaffold_2_237 | Lamassu-Fam__LmuB_SMC_Cap4_nuclease_II | 3,30E-05 | 0.439 | 0.128 |
|  | scaffold_2_252 | DarTG__DarG | 1,30E-65 | 0.756 | 0.693 |
|  | scaffold_2_331 | FS_Sma__Sma | 2,50E-157 | 1 | 0.996 |
|  | scaffold_2_344 | RM_Type_II__Type_II_MTases_FAM_28 | 7,10E-16 | 0.466 | 0.493 |
|  | scaffold_2_344 | RM_Type_II__Type_II_MTases_FAM_14 | 1,30E-18 | 0.449 | 0.474 |
|  | scaffold_2_344 | RM_Type_II__Type_II_MTases_FAM_13 | 3,10E-23 | 0.439 | 0.486 |
|  | scaffold_2_344 | RM_Type_II__Type_II_MTases_FAM_6 | 1,00E-23 | 0.465 | 0.487 |
|  | scaffold_2_344 | RM_Type_II__Type_II_MTases_FAM_33 | 1,10E-40 | 0.499 | 0.495 |
|  | scaffold_2_344 | RM__Type_I_MTases_FAM_3 | 5,40E-50 | 0.686 | 0.85 |
|  | scaffold_2_344 | RM__Type_I_MTases_FAM_1 | 2,30E-56 | 0.961 | 0.934 |
|  | scaffold_2_344 | PrrC__EcoprrI | 6,50E-158 | 0.956 | 0.987 |
|  | scaffold_2_344 | RM__Type_I_MTases_FAM_0 | 1,40E-181 | 0.992 | 0.979 |
|  | scaffold_2_345 | RM__Type_I_S_02 | 1,40E-24 | 0.886 | 0.995 |
|  | scaffold_2_345 | RM__Type_I_S_52 | 6,40E-26 | 0.658 | 0.947 |
|  | scaffold_2_345 | RM__Type_I_S_51 | 7,50E-28 | 0.436 | 0.542 |
| scaffold_3 | scaffold_3_147 | Dodola__DolB | 1,90E-31 | 0.685 | 0.403 |
| scaffold_4 | scaffold_4_123 | RM_Type_II__Type_II_REase23 | 7,30E-36 | 0.889 | 0.987 |
|  | scaffold_4_124 | RM_Type_II__Type_II_MTases_FAM_21 | 1,40E-23 | 0.415 | 0.44 |
|  | scaffold_4_124 | RM_Type_II__Type_II_MTases_FAM_16 | 1,80E-26 | 0.541 | 0.452 |
|  | scaffold_4_124 | RM_Type_II__Type_II_MTases_FAM_22 | 3,20E-31 | 0.451 | 0.412 |
|  | scaffold_4_124 | RM_Type_II__Type_II_MTases_FAM_0 | 2,30E-47 | 0.555 | 0.51 |
|  | scaffold_4_124 | DISARM_2__drmMII | 2,80E-48 | 0.499 | 0.452 |
|  | scaffold_4_124 | Druantia_II__DruM | 1,90E-68 | 0.848 | 0.781 |
|  | scaffold_4_124 | RM_Type_II__Type_II_MTases_FAM_2 | 2,30E-130 | 0.987 | 0.781 |
| scaffold_5 | scaffold_5_76 | Gabija__GajB_3 | 5,10E-16 | 0.66 | 0.386 |
|  | scaffold_5_76 | Gabija__GajB_1 | 3,30E-44 | 0.927 | 0.826 |
|  | scaffold_5_76 | UG35__UvrD | 2,60E-103 | 0.535 | 0.596 |
|  | scaffold_5_76 | Gabija__GajB_2 | 1,80E-115 | 0.777 | 0.893 |
|  | scaffold_5_87 | DEDDh_I_II_III_IV_V_VI_1 | 9,60E-37 | 0.988 | 0.822 |
|  | scaffold_5_100 | Veles__VlsC1 | 1,00E-28 | 0.953 | 0.923 |
|  | scaffold_5_100 | Veles__VlsC2 | 4,40E-30 | 0.966 | 0.929 |
| scaffold_6 | scaffold_6_38 | Dpd__QueE | 1,20E-13 | 0.606 | 0.517 |
|  | scaffold_6_39 | Dpd__QueD | 5,40E-19 | 0.95 | 0.843 |
|  | scaffold_6_40 | Dpd__QueC | 3,20E-42 | 0.927 | 0.915 |
|  | scaffold_6_48 | PsyrTA__PsyrT | 1,70E-112 | 0.517 | 0.585 |
|  | scaffold_6_78 | ShosTA__ShosT | 5,60E-08 | 0.608 | 0.702 |
|  | scaffold_6_147 | Dnd__DndA | 1,20E-44 | 0.867 | 0.829 |
|  | scaffold_6_199 | Gabija__GajB_2 | 5,80E-67 | 0.749 | 0.749 |
| scaffold_7 | scaffold_7_60 | CBASS__AG_E1_ThiF | 3,60E-48 | 0.992 | 0.699 |
|  | scaffold_7_96 | WYL_I_II_III_IV_V_VI_1 | 2,70E-50 | 0.735 | 0.983 |
| scaffold_8 | scaffold_8_32 | Lamassu-Fam__LmuB_SMC_Mrr | 8,50E-07 | 0.403 | 0.234 |
|  | scaffold_8_32 | Lamassu-Fam__LmuB_SMC_Cap4_nuclease_II | 1,30E-13 | 0.771 | 0.5 |
|  | scaffold_8_32 | Lamassu-Fam__LmuB_SMC_Hydrolase_protease | 1,30E-14 | 0.824 | 0.791 |
| scaffold_9 | scaffold_9_70 | gcu233__gcu233 | 4,90E-59 | 0.971 | 0.983 |
| scaffold_10 | scaffold_10_8 | UG9__DNA_pol | 3,90E-29 | 0.647 | 0.88 |
| scaffold_11 | scaffold_11_45 | gcu233__gcu233 | 1,70E-49 | 0.977 | 0.957 |
| scaffold_12 | scaffold_12_8 | Dodola__DolB | 7,10E-26 | 0.659 | 0.327 |
| scaffold_13 | scaffold_13_23 | Veles__VlsC2 | 5,30E-26 | 0.919 | 0.936 |
|  | scaffold_13_23 | Veles__VlsC1 | 3,60E-26 | 0.926 | 0.95 |
| scaffold_15 | scaffold_15_7 | Gao_Iet__IetA | 1,50E-30 | 0.563 | 0.324 |
|  | scaffold_15_27 | Gao_Qat__QatD | 2,40E-12 | 0.953 | 0.977 |
| scaffold_18 | scaffold_18_7 | MazEF__MazF | 1,70E-09 | 0.856 | 0.835 |
|  | scaffold_18_27 | DISARM__drmC | 4,00E-14 | 0.494 | 0.253 |
| scaffold_20 | scaffold_20_5 | TniQ_I-F_1 | 6,70E-17 | 1 | 0.253 |
|  | scaffold_20_10 | Lamassu-Fam__LmuA_effector_Sir2 | 1,10E-17 | 0.619 | 0.556 |
|  | scaffold_20_10 | Thoeris_II__ThsA_new_petit | 2,70E-41 | 0.861 | 0.457 |
|  | scaffold_20_10 | Thoeris_I__ThsA_new_grand | 1,50E-171 | 0.987 | 0.983 |
|  | scaffold_20_11 | Thoeris__ThsB_Global | 7,30E-35 | 0.58 | 0.395 |
|  | scaffold_20_12 | Thoeris__ThsB_Global | 9,40E-20 | 0.829 | 0.97 |
| scaffold_22 | scaffold_22_19 | Lamassu-Fam__LmuB_SMC_Cap4_nuclease_II | 9,60E-10 | 0.465 | 0.648 |
|  | scaffold_22_19 | RloC__RloC | 1,10E-98 | 0.678 | 0.97 |
| scaffold_23 | scaffold_23_7 | UG9__DNA_pol | 2,60E-27 | 0.653 | 0.87 |
|  | scaffold_23_10 | WYL_I_II_III_IV_V_VI_4 | 2,10E-11 | 0.977 | 0.532 |
|  | scaffold_23_10 | WYL_I_II_III_IV_V_VI_1 | 4,60E-32 | 0.984 | 0.997 |

**Supplementary Table S5.** Restriction-modification systems identified from DSM 104337 and 19A2 genomes with DefenceFinder, PADLOCK, RAST and BlastP

| DSM 104437 | | | | |  |
| --- | --- | --- | --- | --- | --- |
| **Scaffold** | **Start Position** | **DefenceFinder** | **PADLOCK** | **RAST** | **BlastP** |
| Scaffold 1 | 219,964 | RM_Type_II__Type_II_Mtases | n/a | Adenine-specific methyltransferase | class I SAM-dependent methyltransferase |
| Scaffold 3 | 115,401 | RM__Type_I_Reases | n/a | hypothetical protein | sce7726 family protein |
| Scaffold 3 | 118,390 | n/a | n/a | Type I r-m system, restriction subunit R | type I restriction endonuclease subunit R |
| Scaffold 4 | 105,213 | RM__Type_I_Mtases/RM_Type_II__Type_II_Mtases ^3^ | DMS_other_MTase_I^6^ | Type I r-m system, DNA-methyltransferase subunit M | type I restriction-modification system subunit M |
| Scaffold 4 | 106,762 | RM__Type_I_S | DMS_other_Specificity_I^6^ | Type I r-m system, specificity subunit S | restriction endonuclease subunit S |
| Scaffold 9 | 560 | RM_Type_IIG__Type_IIG/RM_Type_II__Type_II_Mtases ^*^ | RM_Type_IIG_Rase_MTase_IIG | hypothetical protein | N-6 DNA methylase |
| Scaffold 9 | 2,451 | n/a | DMS_other_Specificity_II | hypothetical protein | restriction endonuclease subunit S |
| Scaffold 10 | 64,453 | RM__Type_I_Mtases/RM_Type_II__Type_II_Mtases ^3,*^ | RM_Type_I_Mtases | Type I r-m system, DNA-methyltransferase subunit M | class I SAM-dependent DNA methyltransferase |
| Scaffold 10 | 65,957 | RM__Type_I_S ^*^ | RM_Type_I_S | Type I r-m system, specificity subunit S | restriction endonuclease subunit S |
| Scaffold 10 | 67,173 | RM__Type_I_Reases ^*^ | RM_Type_I_Reases | Type I r-m system, restriction subunit R | type I restriction-modification system endonuclease |
| Scaffold 28 | 23,486 | n/a | n/a | Type I r-m system, specificity subunit S | restriction endonuclease subunit S/(hypothetical protein) |
| **19A2** | | | | |  |
| **Scaffold** | **Start Position** | **DefenceFinder** | **PADLOCK** | **RAST** | **BlastP** |
| scaffold_0 | 368,677 | RM_Type_II__Type_II_MTases | n/a | Adenine-specific methyltransferase | class I SAM-dependent methyltransferase |
| scaffold_2 | 71,656 | RM__Type_I_REases | n/a | Type I r-m system, restriction subunit R | type I restriction endonuclease subunit R |
| scaffold_2 | 353,187 | RM__Type_I_Mtases^3^ | DMS_other_MTase_I^6^ | Type I r-m system, DNA-methyltransferase subunit M | type I restriction-modification system subunit M |
| scaffold_2 | 354,736 | RM__Type_I_S | DMS_other_Specificity_I^6^ | Type I r-m system, specificity subunit S | restriction endonuclease subunit S |
| scaffold_4 | 127,785 | RM_Type_II__Type_II_REases^*^ | RM_type_II_REase_II | hypothetical protein | type II restriction endonuclease |
| scaffold_4 | 128,810 | RM_Type_II__Type_II_Mtases^4,5,*^ | RM_type_II_MTase_II | DNA-cytosine methyltransferase | DNA (cytosine-5-)-methyltransferase |
| scaffold_20 | 6,214 | n/a | n/a | Type I r-m system, restriction subunit R | type I restriction enzyme endonuclease domain-containing protein |

^1^ N6A methylation according to REBASE

^2^ C5 methylation according to REBASE

^3^ Annotated also as PrrC__EcoprrI in DefenceFinder

^4^ Annotated also as Druantia_II__DruM in DefenceFinder

^5^ Annotated also as DISARM_2__drmMII in DefenceFinder

^6^ Group of DNA-modification systems (DMS) that captures various DNA-modification system proteins

*Recognized as a complete system in DefenceFinder

^n/a^ Not recognized as a gene associated with r-m systems

**Supplementary Table S6**. Restriction-modification specificity (HsdS) subunits from DSM 104437 and 19A2 genomes compared to NCBI Conserved domain database (A) and REBASE database (B).

| A. | | | | | | | | | |
| --- | --- | --- | --- | --- | --- | --- | --- | --- | --- |
| **DSM 104437 HsdS subunits** | | | | | | | | | |
| **Scaffold** | **Start** | | **Similarity to Conserved domain database^1^** | | | **Target sequence** | | **n (sites)** | **location** |
| 4 | 106,762 | | *Shewanella oneidensis* MR-1 (S.SonII) TRD2-CR2 | | | TCTANNNNNNRTTC | | 0 | n/a |
|  |  |  | *S. aureus* S.sau13435ORF2165P (NCTC 13435) TRD1 – CR1 | | | Unknown | | n/a | n/a |
|  |  |  | S.SauL3067ORFAP, TRD1 – CR1 | | | Unknown | | n/a | n/a |
| 9 | 2,451 | | MgeORF438P-TRD-CR_like | | | Unknown | | n/a | n/a |
|  |  |  |  |  |  |  |  |  |  |
| 10 | 65,957 | | *E. coli* str. K-12 substr. MG1655 S subunit (S.EcoKI) TRD2-CR2 | | | AACNNNNNNGTGC | | 0 | n/a |
|  |  |  | *Salmonella enterica* Potsdam S subunit (S.StySPI) | | | AACNNNNNNGTRC | | 3 | 11,549 (+)  13,973 (+)  13,982 (-) |
|  |  |  | *S. aureus* MSHR1132 S subunit (S.Sau1132ORF3780P) TRD2-CR2 | | | CAAGNNNNNRTC | | 2 | 9,130 (-)  16,436 (-) |
| 28 | 23,486 | | n/a | | | n/a | | n/a | n/a |
| **19A2 HsdS subunits** | | | | | | | | |  |
| **Scaffold** | **Start** | | **Conserved domain database similarity** | | | **Target sequence** | | **n (sites)** | **location** |
| 2 | 354,736 | | *S. aureus* NCTC 13435 S subunit (S.Sau13435ORF2165P) | | | TCTANNNNNNRTTC | | 0 | n/a |
|  |  | | *S. aureus* 3067 S.Sau3067ORFAP S subunit  *Photorhabdus luminescens* S subunit (S.PluTORF4319P) | | | Unknown  Unknown | | n/a | n/a |
| **B.** | | | | | | | | | |
| **DSM 104437 HsdS subunits** | | | | | | | | | |
| **Scaffold** | | **Start** | **REBASE identity** | | **Target sequence** | | **n (sites)** | | **location** |
| 4^2^ | | 106,762 | S.SauNRS271I | 100% | AGGNNNNNNTGAR | | 3 | | 3,114 (+) |
|  |  |  | S.Sau5464I | 100% |  |  |  |  | 8,053 (+) |
|  |  |  | S.Sau5096I | 100% |  |  |  |  | 16,920 (-) |
| 9 | | 2,451 | S.Sau13616III | 100% | YACNNNNNTGG^2^ | | 4 | | 3,800 (+) |
|  |  |  | S.Sau13142III | 100% |  |  |  |  | 6,496 (+) |
|  |  |  | S.Sau11963II | 100% |  |  |  |  | 8,576 (-) |
|  |  |  |  |  |  |  |  |  | 10,465 (-) |
|  |  |  | S.Sau12035III | 92% | CCANNNNNGTR | | 4 | | 3,800 (+) |
|  |  |  |  |  |  |  |  |  | 6,496 (+) |
|  |  |  |  |  |  |  |  |  | 8,576 (-) |
|  |  |  |  |  |  |  |  |  | 10,465 (-) |
| 10^2^ | | 65,957 | S.SauNRS271II | 100% | GAAGNNNNNTAC | | 0 | | n/a |
|  |  |  | S.Sau5464II | 100% |  |  |  |  |  |
|  |  |  | S.Sau13142I | 100% |  |  |  |  |  |
| 28 | | 23,486 | n/a | | n/a | | n/a | | n/a |
| **19A2 HsdS subunits** | | | | | | | | | |
| **Scaffold** | | **Start** | **REBASE similarity** | | **Target sequence** | | **n (sites)** | | **location** |
| 2^2^ | | 354,736 | S.SauSTI | 100% | ACCNNNNNRTGA | | n/a | | n/a |
|  |  |  | S.Sau64038I | 100% |  |  |  |  |  |
|  |  |  | S.Sau8726I | 100% |  |  |  |  |  |

^1^ Subunit were assumed similar if conserved domain database e-values between query and reference sequence were equal to or smaller than 7.1*10^-6^

^2^ REBASE detected a larger number of identical HsdS subunits. and only three are shown in the table.


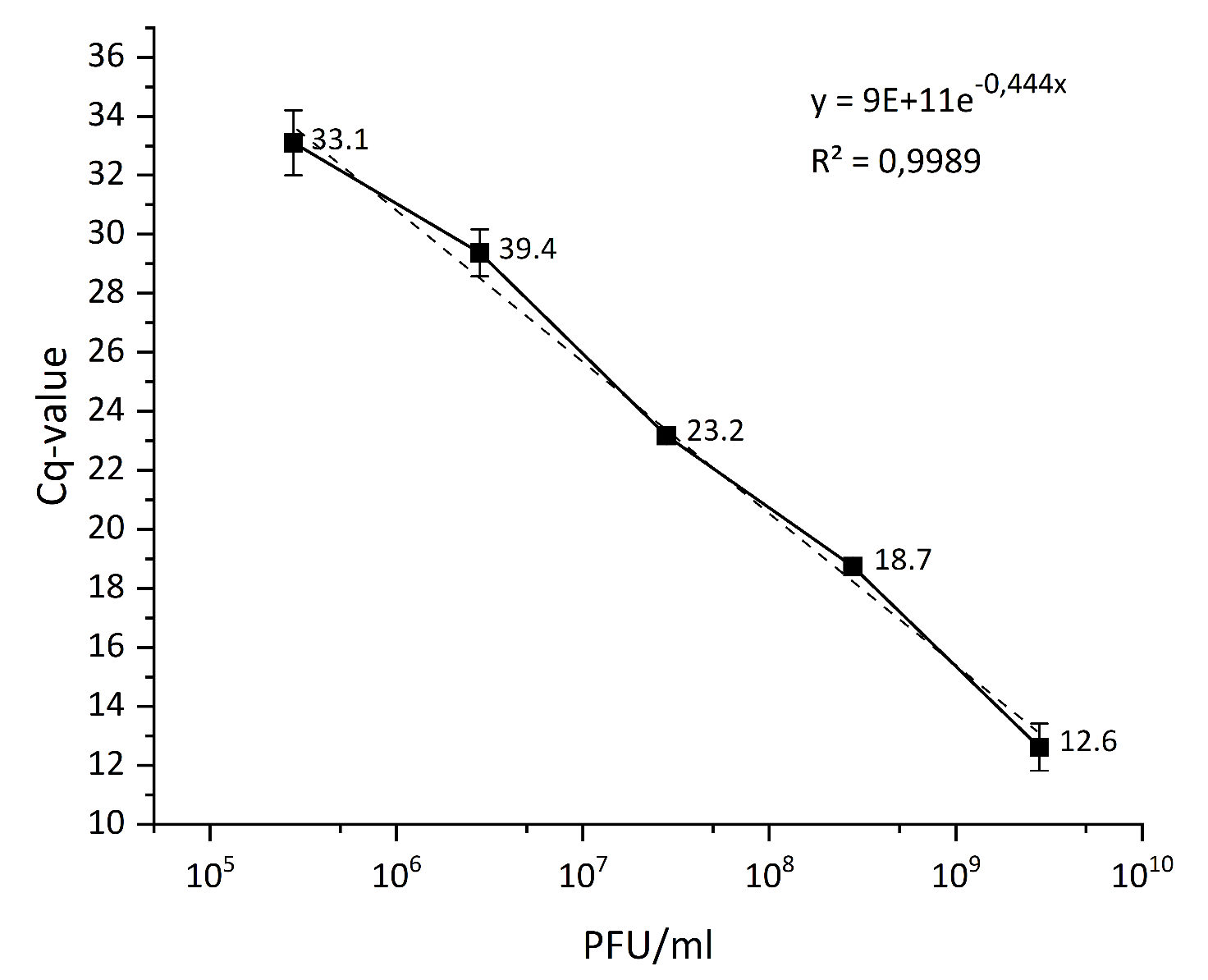


**Supplementary Figure S2.** qPCR standard curve prepared from a dilution series of ΦEBHT lysate (2.8*10^5^ PFU/ml – 2.8*10^9^ PFU/ml). In average, 10-fold change in PFU/ml equals to Cq-value change of 5 units.

**Supplementary Table S7.** The table lists the detected methylated sites and their methylated fractions in ɸEBHT and mEBHT genomes. All six m6A methylated sites were methylated in ɸEBHT, while only one of them showed lower-level methylation in mEBHT. Methylated fraction refers to the ratio between the number of detected methylations on the site of the methylated motif and the total number of methylated motif sites.

|  |  |  | **ɸEBHT** | | | | | **mEBHT** | | | | |
| --- | --- | --- | --- | --- | --- | --- | --- | --- | --- | --- | --- | --- |
| **Ambiguous Motif Sequence** | **Motif Sequence** | **Position (bp)** | **6mA (A+) Count** | **6mA (T-) Count** | **Average Likelihood (%)** | **Total A+ or T- bases** | **Methylated Fraction** | **6mA (A+) Count** | **6mA (T-) Count** | **Average Likelihood (%)** | **Total A+ or T- bases** | **Methylated Fraction** |
| **DNNWNR_A_GGNNNNNWTNA** | TAAAAA_A_GGTTTCAATGA | 3,114 | 45,957 | 57,293 | 98 | 174,436 | 0.592 | 1,361 | 1,545 | 97 | 143,480 | 0.020 |
| **DNNWNR_A_GGNNNNNWTNA** | TTATGG_A_GGTAAAAATGA | 8,053 | 67,417 | 78,236 | 98 | 245,664 | 0.593 | 1,848 | 2,348 | 97 | 187,860 | 0.022 |
| **DNNWNR_A_GGNNNNNWTNA** | GCATAA_A_GGATTTTATGA (TCATAAAA_T_CCTTTATGC) | 16,932 (negative strand) | 29,272 | 39,709 | 98 | 109,049 | 0.633 | 103 | 129 | 97.5 | 86,190 | 0.003 |
| **MNTCN_A_NTNAAGNNNNNA** | ATTCC_A_GTCAAGTGCTGA | 8,578 | 79,278 | 81,203 | 99 | 244,805 | 0.656 | 667 | 684 | 97 | 185,114 | 0.007 |
| **VNNAHNTNNKV_A_NNTG** | AAAACTTAAGA_A_TATG | 5,280 | 25,514 | 41,708 | 97 | 220,828 | 0.304 | 19,616 | 31,040 | 97 | 172,743 | 0.293 |
| **VNNAHNTNNKV_A_NNTG** | GATAAATCAGC_A_CTTG (CAAG_T_GCTGATTTATC) | 8,585 (negative strand) | 69,856 | 70011 | 98 | 244,819 | 0.571 | 581 | 625 | 97 | 185,050 | 0.007 |
