# Supplementary material for: The Host R‐M Systems Change the Host Range of Staphylococcus Phage EBHT
Source: Microbiologyopen. 2025 Nov 27;14(6):e70165. doi: 10.1002/mbo3.70165 (PMC12660161; doi:10.1002/mbo3.70165)
Supplement: Supplementary file 2 — Supplementary_file_2. [file MBO3-14-e70165-s001.pdf]

>DSM104437_scaffold_0
GTCATGTTATAATTTTTATACATAAGGCACCTCGTTAATTTAGTTTAGTGGTATTTATTAAATTATACGAAGG
GACCCAACACAGAAAATTCATTTTATTGAATTTTACATTTATGTGCAAGTTGGGCAAAGTGTCTTATTTTTTT
AAGTATTTAAAAGTAAAATTACATGTTAATACGTAGTATTAATGGCGAGACTCCTGAGGGGGCAGTGCCAGTC
GAAGACCGAGGCTGAGACGGCACCCTAGGAAGGGACCCATCATCAAAAATTCTATTTATAGAATTTTACAGTA
ATGTGCCAGATGGGCAAAGCGAAGCCATTCAATACGAAGTATTGTATAAATAGAGAACAGCAGTAAGATATTT
TCTAATTGAAAATTATCTTACTGCTGTTTTTTAGGGATTTATGTCCCAGCCTGTTTATTATTTAACGTCTACC
TCTAGGATTAAAAGCATCTTTAAGCCCTTCACCTACAAAGTTTATACTTAATATAGTTAATGTAATCATTATA
GCAGGTGGCATCCATATCCACGGTTTACCACTTATAACATCCCCTTCTTGAGCATCACTCAACATATTACCCC
AAGATGGGATTGCTTTACTAATTCCGAATCCTAAAAAACTCAATCCTGATTCCACTACAATCATACCGGCAAA
TAACAATGTTGCTTGTACGATAACTACACTTAATATATTCGGCAAAAGATGTTTCAAAATAATTTTATATGTG
GGTGTACCAATTGATTTTGCTGCCAAAAAGTATTCATTTTCTTTTTCTTGAAGTACTTTACCACGAACAAGTC
TTGCAATACCTCCCCAACTTAATACGATAATAACTAGAACAAGAATTATGGCAGATCCATAAGGATTTTTAAT
TTTATCTCCAAGTGCAGCATTTAATACAATTGCAAATATTAAAAATGGAAATAACATAACAAATTCGGTTATT
CTCATTAATAATGTATCAACAATACTTCCAAAATAACCAGAAATCACTCCAACTGTAATTCCAATCAAAAGCA
TTCCTATTGTAGATGTAATTCCAATGGATAATGAAATACGACCTGCATATAACAAACGACTAAAGTTATCCCT
ACCACCGGAGTCTGTACCAAGAATGTTTTGTGCTGTCATTTCACCTTTTATATTTAATAAATCTTGTTGGTTC
ACTGGAAAAGGAGCTATTAATGGCGCTATAATTGATACAATAGTTATGATTAATAAAAAAATAATCGATAACA
TTGCAATTTTATTATGAATAAATTTAGACAATGCAATTTTAAAAGGCGATTTTGACTTATTTTGCATTCCAAT
CCCCCCTAGTTACTTCTAATTCTTGGATCTATAAATCCATAAGTAATATCAGAAATCAAATTACCCAGTAAAC
CTAAAAATGAGAAAAACAACGTTAATGCCATCATTAATGGATAGTCTTGACCTATTACCGATTCTAAAAATAA
TTTACCGATACCGTTATATGAAAAGATAGTCTCAGTAATCACAGCTCCACCTAAAATACTTACAATATCAGCA
CCTAAAAATGTAATAATAGGTATTAAAGAATTTCTCAATATATGTTTATTATAAATTTTATTCATAGATAATC
CTTTTGACCTTGCCGTCAATACATAATCTTTTTTAGAATTTTCAATAATATCATTACGTAAATATTGAATATA
ACCAGCAGTAGATAATAATCCTAAAGTTAATGCAGGCAAAAATGTATGATAAATTTTACTCATATAATATTCA
AAAGTACCTTCTTTAAGGTTAATATCAACAGAACCTTGAAACGGAAACCATTGTAATTCAAAAGAAAAAATAA
AAATTGCAAATACTCCCGCAATAAAAGATGGAATAGCTAACATCAAATAATTAAATATTTGCACAGTATAATC
AGTCAAACTGTAAGAATATCTACCTGACGTTATTCCTAAAGCAAATGAGATAATATAAGTAATAATTAATGAC
ATAGCACCGAGTAATATTGTATTTGGAATTCTTTCCTCAATAACATCAATTACCGGCCTTTTATATTTGATTG
ACTCCCCTAAATCACCATGTATAACATGATTGACCCATCTAATGTATTGATGTGATATAGAGTCGTTGAGGCC
TAACTTTTCCCTTTGTGCATTTATCGCTTCTTGTTTTATTTTTCCGTTTTGTAAATCTGAAAATGGATCTCCT
GGTTGAATGATAGCTAATGAAAATACAACAATAGAAATTAAAATTAGTAACGGAATCATTAAACCTAATCTCT
TTAATATTAATTTAACCATTTAAATCACCTACTTTATTTACATAAAAACAAGCAACTTGATGTTCATCACCAA
TGTCCTTTAATTGTGGTATATTTTCTTTACATTTATCTTTTGCAATCGGACATCTTGTATGAAAAGGACAACC
TTGCGGCGGATTACTTGGCGAAGGTAATTCTCCTTTTAATATAATTCTATTGTTATTATGTTTATCAATTTGT
GGTATTGATGAAATCAACGCTTTTGTATATGGATGTTTGGGATTTTCATAAATTTCTTTATCAGATGCGATTT
CAACTATATGACCTAAATACATAACTCCAATGACATCACTTATATGTTTTACTACACTTAAATCATGTGCGAT
AAATAAATAGCTTAAGTTAAATTGTTCTTGTAAATCTTTTAATAAATTCAGTACTTGAGATTGAACAGATACA
TCTAATGCACTTACAGGCTCATCAGCAACAATTAAACTCGGACGCAAAGCCAATGCTCTTGCAATTCCCACTC
TTTGTCTCTGTCCACCTGAAAATTCATGTGCATATTTATAATATGCATCTTCACTTAGGCCAACACATTTTAA
TAAATATAGTACTTCTTTTTTTATTTCTTCTTTTGGCAATTTTTTATAATTTAAAATAGGTTCTGAAATGATA
TCTCCAACCATTTGCATCGGATTCAATGATGCATACGGATCTTGAAATATCATCTGATATTGTTGTCGTGATT
TTCTGAGTTTTTTACCTTGTAATCTTGTTATATCTTCACCATTAACAATTATTGAGCCTGAAGTTGCATCTTC
AAGCCTGATAATCACTTTACCTAACGTTGACTTACCACAACCCGATTCTCCTACTAATCCAAAAGTTTGTCCT
TTTTTTATTGAAAACGATACGTCATCAACAGCTTTAACGTACTGAGTTTTTTGTAATAATCCAGTTTTAATTT
TGTAGTATTTTTTTATTTGGTTGACTTCTAAAATATTTTCCATTCTTATATCTCCTTATATTCATTTTTGTAC
AAATGACAACGTACAATGACGTCTTCACAAACATGCATTTTAGGAGATTGATTATTACAAATATCCATTTTTT
TGTTACATCTATTTGCAAATCTGCACTTATTAACGTGAAATGCTTGGATTGATGGCACAATTCCTTCTATCGT
TTCAAGTCGTTTCTGCTCTTCTTTAAGTTTTGGAATTGTTGATAATAATTTTTGGGTATAAGGATGTTTCGGA
TTGTGTAGTATTTCTTTTATGCCTCCAAATTCTACAATTTGACCTGCATACATAACTAAGACTTTATCGCAAA
ACTCAGCAACTACACTCAAATCATGTGTAATCATCATAATTGCCATTTGCGTTTCCTTTTGCAATTCTTTTAA
TAAGTCTAATATTTGCGCTTGAATCGTGACATCCAATGCTGTTGTAGGTTCATCAGCAATTAATAATTTAGGA
GAACATGAAATGGCCATTGCAATCATTACTCTTTGTCTCATACCACCAGATAATTGATGAGGATAGCTATTTA
ATATTTCATCTTGTCTAGCAATTCCAACTTTTTTTAGTAAATCTTTTGCTAAGTTATTTGCTTCTTTTTTAGA
AATTTTTTTATGTGATTTTATAGATTCCACAAGTTGATTTTTTATGGTAAATACAGGATTTAAAGCAGTCATA
GGTTCTTGAAAAATCATAGCAATATCATTTCCTCGAATATCTAGCAATTGCTTCTCATTGAGCGTATCGATTC
GTTTACCATCAAATATAACTTCTCCACTACTTAGTTTAGATATCTTTTCTGGTAATAATTTAATAATTGACTT
ACTTAATATGGACTTACCGCAACCAGATTCCCCTACTATACCCAAAACTTCTCCTTTAGATAATTCAAAAGAA

ATATTTTCAACAGTTGCTAACCATTCATTATTTATTTTTAATGATGTTTCTAAATCTTTAACCTCTAACAATA
CATTATTCATAACATCCCCCCATATAAGTAAACCTCGACAAAATCGTCGAGGTTACATTACTAAGCTTCTTTA
GTTAAATTATATAAACTTTGATCAGTTCCAATTTCAATGTCTAAGTTTCGTACTTTATCGTTAACAATAGTTA
TCGATTTACCTTGGAACATTGGTAATCCAGGTAACTCATCATTCATATATTTTTGCCATTTAACATAAATATC
TTTACGTTTCTTTTCATCAATGCCTACTTTTTCGAAGTCTAATGCATCATCTAAATATTGATCTGATTTTGGT
AAAACTGTTCTCATTTCATTTTGAGGTCTATCAGTGTGGTATAAATCTGATGGATCTGGATCTGTACCTCCTG
CCCATGATCTAAAGTACACTTCCATATCTTTAGATGCATTTGCTAAGTCTTCATTATATTTACCGAATTCTAC
TAACTTCACATTTGTTTTCAAGCCAACTTTTTCCCAGAAATCTTTTATCGCAGCAGTTCTTGGTTCAAAAGTA
GGATTTGAACCTGAATAATGTTTAAAGTTAATCTCAAATTTATTACCTTTAGGATCTTCTCTAAATCCATCAC
CATCTCTATCTTTATAACCTAACTTATCTAAGATTTTTTTAGCTTTTTCAGGATCATATTTATAATCGTTTAG
TTCCTTAGGATCAGCAGCTATCCAATGCATTGATGGTACAAAACTATTTATTTCACTAGCGTAACCATTGAAA
AATGCTTTGATCCATTTTTCTCTATCAATTGCATAAAGCATTGCTTTACGTAATTCTTTGTCTTCATATTTTG
GTCTCACTTTACCAGTTTTATTAGATTTTTTATCGTAATCATGAGATACGAATCCTATTAAACCGTAGTCTAA
GCTTGGCGCAGATAATACCTTGAGACCAGCATTAGATGACTTAGCATCTTTTGCCATTGCACCGGTAGCATCA
TTCGCAACATCAATATCGCCTTTTTCCATTGCCTTAATAATTTGAGCTTGATCAATAACTTTTAAATTGATTT
TGTCTAGTGCAGGCTTACCTTGCCAATAATCATCAAATTTAACGAGTTGAACAGCCTCACCTGGAACGATTTT
CTTAACTTTATACGGTCCAATACCAATAGGATATTTTCGGATTTTATCTGATTTCGCTAAATCTTTAATTGGT
ACATCTGATAAATATTTTTTACTTAATAAAGGTCCAGTAATAAATCCTGTTAAGTAATTTTCTTGTTTTTTAT
CAAATGTAACCTGCATAGTGTAGTCATCTATTTTCTTCAATCCGCTAATATGATCAGTTTTTCCTTCATGATA
ATCTTTCGCACCTTGGATATTTTCTACACTTGGATAATAAGCACCTTCGTAGTCCTTGTTAGCTAAGACTTCA
ATTGAATAAATCCAATCATCAATTTTCAATTCATTACCATCATGCCATTTAATACCTTTTTTAATTTTAAATT
CTATTTTCTTAGCCGGCTCGATGTCCTTCCATGAAGCAATATATGCCTTAGGACGTATTTTTTTATCAGTTGC
TAATAAGCTTTCGTTAAAATATCCCTCAACTACAGCATCCGCATGTGTACTATTTAATAACGAAGAATAAACA
CCACTTGGAGGTGCTGTTAATGCTACATTTAAGGTACCACCTTTATGTTTTGAGGTTTCTGTTTTAGTAGCAT
CTTTTACTCCTTCGTCTTTATTACTGCTTTTTCCGCAAGCACTCAACACTAAAACGATAACAATAAGTATTGA
AATATATTTAATTAGCTTCCCCATTTATATGCCTCCTACTTTCAATCTAATTTTTCTTTTTAGTTTACTCATA
GAAAAAATTATTACAAGTTTATTTTTAGAATATTCAGTTATTTATGCATTTAATCAATTTCAGTAATTTAAAA
AACGACTTATTTTAAATTTTTATTCCCAAAGTTTCTATCCAACACAAAAAGACTGGAGATATGTCTCCAGCCT
AATTTACAAAGCATATTATTTTTTTCTTCTTACCTGTTTCTTTATCTATCGATTTATCAATATAGACATGTTTA
AGTGAGTTATTTGGACCAAATTTATGGTAAATTAATCCTTTTACTTGAGGATTTGTTAAATGTGCAACACCTT
TTTGATAAATTGGTGCTACCGGTGCATCTCCTAGGAACATTTCTTCTGCTTTTTTCAAGTTTTCATATCGTTC
GTTCGGTTGAAGTGCCAATTTGGTTCTTGCTACTTTAAGTAATTGATCATATTCTTTATTACCCCAGTCTGTA
TTATTTTGTGCGCTACCTGTGGTCATTGTTTCTAAATAAGCCATAGGATCAGGGTAATCTGCAGACCAACCAC
TAAGTGATGCTTCAAAATTGTTACTTAACTCTAATGAAACTCTTTGCTTAAATGGCAATTGCTTAATTTTTAA
AGTAACACCTGGTAAATTTTTCTCAACTTGCGATTTGATATATTCTGCAGATATTTTTGCATCTGGCGTATCT
TCTGTGTTCATAGAAAATGTCACTTCATTTTTACCTAACTCTTTTTTAGCTTTTTCCCAATGTGCTCTTGCTT
CTTTAGGATTATATTTTAACGGTGAATTCATGGTACTCGCATAATCTTTGCCATTCGGTGCTTTCGCAATTCC
TTTGGCTGTTAGTGTATCGGAAGGAATTGAGCCATTGTTTTTCACTGAATCAACGTATCCTTTTTTATCTATT
GCTTGTGCGATAGCCAGTCTTAAATTTTTGTTTTTAAAATCAGGATATTGTTTTTCATTCATTTTTACAAAAA
ATGTCCCAGTCGTTAACACAAAGTTTAATCCTTTGTTATCTTTATATTTATTTACTTGATCTGCAGTAATAAC
TGCGTCATCTACTGATTCAGTATCATACAATGATGCACCGGCTTGTAAGTCTTTAATAACTTTATAATTCACT
TTATCTAATTTTACATTCTTTTTATCCCAATAATACTGATTTTTAGATAGTAAGGTTTTATCTTCTTGTTTCC
AATCATCAACTTTAAATGGACCATTGTATACCGCTCTATCAGCTGCCGTACCGTAATTTTTACCATATTTTTT
GGCAACTTTTTCATTTTGAGGTGCAAATGTATTAAGTGCTAATAATTGATTAATATATGGAACCGGCTTTTCT
AATTCAATTTGTAATGTTTCATCATTTAATGCTTTGATACCTAATTGTTCTACAGGTTTCTTACCAGTACTAA
TATCACTCGCATTTTTAATGTCCCCCATAATGTATGCAAATTCAGAACCTGTTTTAGGGTCAACTGTTTTTCT
CCAAGCATAAACAAAGTCTTGTGCAGTCACTTTGTCACCATTGCTCCATTTAGCATCGCTTCTTAATTTAACC
TTTATAGTTTTACCATCTTTACTCTTTTCAGGAAAAGCTTTCGCAACACCTAACACCGGTTTGTCACCTTTTC
CTAATGTGTATAAACCTTCGAATGTTTGCGCAGTCACTTCAGAAGATATTTCATCCGTTATTAATGATGTATC
AAGGGATGTTAAGTCTGATGACAAAATTTTTCTGAATACTTGACCTTTATCTGAATAAATACCATCATCATTA
GCACAACCACTTAATGCAATTGTAGCAATCAAAATTAAAATAAGTGTTCTAAATTTTCTCGTCATTGCATTTC
CCCCTTCCCTTAATTTTGTGTCACCAATTTATTTTCTCGTAGTTGTGCCGCTTCTTCTTCAGTACTAAAGACA
AAATGCTGTGGTCTTATTTCATGTAATTGTCTTAAATGATTATTTGCTTCATCATCAATATAACTAAATCGTT
TGCGACTACGTTCCGATTCAGGATCCGGTTGAGGAATTGCCGACAATAAAGACTTAGTATAATCATGTAATGG
ATATTGATAAATATCGTCTGCTGGTCCGATTTCTACAATTTTCCCAAAATGCATGACTGCAATTCGATCAGAG
ATATACTTCACCATTGATAAATCATGTGCAATAAATAGGAATGTAATACCTTTTTCACGCTGTAATTTTAATA
ACAAATTGACTACTTGAGCTTGAATCGAAACATCCAATGCTGAAATAGGTTCATCTGCAATAATAAATTCTGG
TTCAACGGCTAATGCACGGGCAATTCCAATACGTTGGCGTTGTCCGCCTGAAAATTCATGAGGATAACGATTG

GCATGTTCTTTACTTAATCCAACAGTTTCAAGTAAATCATAGACACGTTTTTTACGGTCACGTTTATCAGTTG
CTAAATGATGGATATCAATACCTTCAGCTACTATATCCATTACTTTTAACCTAGGATTTAAAGACTCATATGG
GTCTTGAAAAATCATCTGTATCTTTTTATTAAATTTAAGCAAATCTTTGCGTTTACGAATCTTTTGTATATCA
ATACCCTCATACAAAATTTCTCCACTTGTAATATCATTAAGTTTGATAATTGATTTACCAGTTGTAGATTTAC
CACACCCCGATTCTCCTACTAAACCTAATGTTTCCCCTTTGTATAATTCAAACGAAATATTTTCAATCGCTCT
CACTTCGTTTTTCTTTCCTGCGTTAAAATATTGCTTTAAATTTTAATAGATAATAGCACTTCATCATTTTTC
ATTGAACGACACCCTTTCTACCTTGAGTGGTTTTTCATAATTATTAGGCATCGGTTTCATACATTGTTTTACC
AGCTCGGGTAGTTCAACTTTTGGTGCACGTGCGTCTAATAACCAAGATTTCACAAAATGTGTCGGTGAAACTT
TAAACCACGGTGGTTCTACTTTAAAATCAATATCTAATGCATATTGACTACGTCTCGCAAATGCATCACCTTT
AGGTGGGTGTAATAAATCAGGTGGCGCTCCAGGAATCGCTAGTAATGGTGTGTCATTTGTTGTTGATAAATCA
GGCATTGACGATAATAATCCCCATGTATATGGATGCTTTGGATCATAAAATATTTCGTTAACATCTCCTGTTT
CAACCATTTGACCACCATACATAACTGCCACTCTATCAGCAATATTCGCAACAACCCCTAAATCATGCGTTAT
AAAAATAATTGCTGTATCGATTTTTTGTTGTAGTTCTTTCATTAAATCTAAAATTTGTGCCTGCATCGTTACG
TCTAATGCAGTCGTTGGTTCATCAGCAATGAGCACTTTAGGTTCACAAGCTAATGCGGTTGCAATAACAATTC
TTTGCCTTTGTCCACCTGAAAATTGATGTGGATATGCTTTAAATCTTTTTTCTGCATTTGGTAAACCTACAAG
ATTTAGTATTTCCAATGCGCGCTTTTTAGCTTGTGCTTTACTATAATTTTTGTGCTTAATTAATGGTTCCATG
ACTTGTTTACCAATTTGCATCGTTGGGTTTAAAGATGTCATTGGATCTTGAAAGATCATTGAAATATCTTTGC
CACGTAATTTAATTAACTCATTTTCAGGTTTTTTTGCTAAATCTTCCTCTAAAAATAAAATTTCTCCCTTTTT
AATTCTTCCTGTGTCCCCTTGGAATAATTTTGTAATTGCTTTTGTTGTTACAGATTTACCTGAACCTGATTCA
CCAACAATTGCCAATGTTTCCCCTTTGTTTAAATAAAAATCTACACCTCTCACTGCCTGCACTTCCCCTGCTG
TAATATCAAAGGAAACATGCAAATCATTTACTTCTAATATTCTTTCAGTCATATGCTATGCCCCCTTTTATTT
ACGCATTTTCGGATCAAATGCATCACGTAATCCATCACTAAATAAGTAAAAGAATAGAATTAATAAACTTAAA
ATCATTGCTGGTATAAATAATTCATGTGGATAAATTAATAACATTGCGCGCCCATCATTTACTAATGACCCTA
ACGATGTTTGAGGTGCGGGTACACCTATACCAATGAAACTTAAAAATGCTTCGAAGAAAATAGCACTAGGTAC
TGTAAACATTGATGTAACCACGATAGCACCTAATGTATTAGGTAAAATATGCTTAAATATCAATTTGAATTTT
GAAGCCCCCAATGTTTTCGAAGCCATGACAAACTCTTGATTTTTTAATTTTAAAAATTCTCCACGTACAACTC
TGCTCATGCCTAACCAGCCTGTGATAGACATAGCCAATATAATTGTCCAAATGGATGGTTCAAAAATTAATAC
AAATAAAATTACGACAATTAAATTCGGAATAGATGCTATGACTTCAAGTATACGTTGCATAATCGTATCGACA
CGTCCACCGAAGAATCCAGAAATCGCACCATATACAACGCCAATAAAAATATCTAACATCGCTGCAACAACAC
CGATAAACAATGAAATTTGAGCACCTTTCCATGTTCTTGTCCATAAATCTCGACCCAACTGATCAGTACCAAA
CCAATAATTTTCTTTAGCATTTGCTGCTTTATAAGCATCCTTGCCATCTGCATCTTTACCATCAAAAGGTAAA
AATGGAACTTTGTCTAATACAGGTATTTTTGCCGGAAGATTTCTATGTTGTACATTTTGTTCAGCATAATCAT
GTTTATTTATAACTGGACCGATAAAAGCAAATATTACAATGATAATTAAACCTATCATACCGACAACAGCTAA
CTTATTTCGTTTTAATTGAGCCCAAGCATCTTGCCAAAAGTTTTTGCTTTCTCGTTGCATTTCAGGTTCCTGA
TTAAAATCTAATTCTCTAATAATAAAATCAGATGATGCGATAGCGTCAGAGGTATGCGTCATAGCTGCATTAG
AATGATCTTCGTTAATCGACAAATTGTTTTTATTTTCAGCCATTATTTTTTACCTCCTTGAACACGAATTCGT
GGATCTATCACACCGTACAAAATATCTACAATAAAAATAGAAACGATAAACAGTGTGCTAAATAATAGTGTGA
TTGCCATGATTACTGAAAAATCATTTGTTGTAATTGAACGTACGAATTGATCCCCTAATCCAGGAACCCCAAA
AATATTTTCAATAGTTAAAGTGCCTGTTAAAATACTTGCTAACATGGGAACGATAATTGTAATAATTGGAATT
AAAGCATTTCTAAGTGCATGTCCAAAAAGTACACGCATTGTCGAATTACCTTTAGCTCTCGCTAATAAAATGT
AGTCTGAACTTAATACCTCAATCATCTCTGCTCTTATGTATCTGGAGACAGTTGCTAAAACAGCTGCAGATAA
TGCAAGTGACGGTAATACCGCGGTCGAAAAACCTTCCCATCCAGCTACTGGGAACCATCTTAATTTAACTGCA
AATACATATTGTAAAAGTACAGCAAGTACAAAAGATGGTACAGATACTGCAATAACTGAAATAACTGTAGTTG
TATAGTCAACCCAAGAATTTTGTTTAGTAGCTGCTGCAACACCTAAAATAAGTCCCAGTATCACACCGATGAA
CATTGCTGTAAGACCCATTTCAAAAGAAGGTAGTAGTCTCGGTTTAATCAAATCCCACACAGGTTGATTATGA
TACTGGAATGAATTACCAAAATCGCCTGTAACAACATTTTTTAAATAATGTAAATACTGCGTAGCTACAGGAT
CATTTAATCCATATTTTTCATTTAAAATTTCTTTTTGTTCAGCATTTAATTTAGCATCGTTAAATGGCGAACC
TGGCATTAATTTCATTAAGAAAAATGTAATTGTAATAATAATAAATAAAGAAATAAGCATATAAATAAATCGT
TTGAAAATATATTTCCCCATCTAATCCCCTCCTCTAACAAATTCCGATTTCTAAATTTCCATTCCCTTCAAAA
TTTTCTGAAATCTTAACTAATATTATATATACAATATTTACTATATATCAATATTTTTTTGCTTTAAACTAAT
GAAGAATGATTGATTTTAGAATTTCAATAAAGCTGAAGAAATTAAAAAATCATTCCTCAATGTCTCATTTTAA
TGTAAACTATGCGTAAGAGTTTTCTATAAGTGGGAGTGATCAAATGTTGAAAAAATGGCTTGGAATGGCACTA
ATAACGCCAATATTAACGTTTATTATATGGGTTTTTAATAGCCATACTATCATTACATATTTAAATATATTGT
TTTATGTTTCATTAATTATTTTTATTAGCATATTTTTGATTTTACTTGTACAAGAAGGCATTTTTGATGCGAC
AAGTTATGGTTTTAGAAGATTAAAGTATCAAATGTCATCTTCGAAAAAGAAGAAGTCTATCTCTGATGATCCA
TTTTTCAACCCACAAGAGGTAAAAAAAGAGCATTACTTTGTTTCTACATGGATAATCCCCTTATTATTAATAA
ATATTCTATATTTTATTATGACGATTGTGCTGTCGCTCATCTTAGTATAAAGAAACAGAAAAAAATCTTCAGTA
ACTAAAACAACGTTACTGAAGATTTTTATAATTATGCTTCAAATTTCTTGAATACTAATACTGCGTTATGTCC

ACCGAATCCTAAGCTATTACTCATTGCATAAGTAATATCAAGGTCTTGCGCTTCATTTGGAACAATATCCAAA
TCACATTCTGGGTCTGGTGTTACCGCATGAATTGTCGGTGCGACTTTAGAGTCTTTAATTGAAAGCGCTGAGA
AGATTGCTTCAATTCCACCTGTTGCACCAAGTAAGTGACCAGTCATTGATTTTGTTGAGCTAACTTTTAAGTG
TTTAGCTGCTTCACCAAATGTATTTTTAATAGCTTTAACTTCATTTAAGTCACCAACAGGAGTACTTGTACCA
TGGGCATTTAAGTATTGTACATCTTTAGGTTCAATACCAGCATCATCCATAGCTGCTTGCATTGCTCTAGAAC
CGCCTTCACCTTCTGGAGCTGGCGCTGTAATATGATAAGCATCACCTGTAGTACCATAGCCAACTATCTCAGC
ATAAATATTGGCACCTCGAGCTTGTGCTGATTCTAAAGATTCGATTACTAAAATACCAGCACCTTCACCCATA
ACAAAACCGTCTCTACCTTCTTGGAATGGACGACATGCTGTTTCAATGTCATCATTTGTAGAAAGCGCTCGAC
TTGCACTGAAACCAGCAATTGCCATATGAGTAATTGGTGCTTCTGTACCACCAGTAATCATTGCATCTGCATC
ACCGCGTTGCACAATTTTAAATGCTTCTCCGATTGAATTTGTACCTGTTGCACATGCTGTAACTGTTGCACCA
TTTGGTCCTTTTGCACATAAGTCAATTGATACTTGCCCAGTTGCCATATCAGGAATTAACATTGGTACGAAAA
ATGGACTCACACGTCTTGGGCCTTTATCCATTAATTGTTTATGTGCAATTTCAAATGTTTCCATACCACCGAT
ACCAGAACCAATCCATACACCGATTCGATCTGCAGTATTTTCATTGATATCTAATTGCGCATCTTTAACAGCC
TCTCTAGCTGCTACAATTGCATATTGAGTAAATCTATCCATACGACGCGCTTCTTTTTTGTCGATATGATCTT
CAATATTAAAGTTTTTAAGTTCTCCTGCTAAGTGAACGCTATAAGGTTCAGTATCGATACGTGTAATTTTATC
GATACCATTTACGCCTTTTAGAGCATTCTCCCATGTTGTTTTGACATCATTACCGATTGGAGAAAGGGCTCCC
ATACCTGTAATAACTACTCTTTTATTTTGACTCATTCGTTATCCTCCTATTTTCCCCATTTTATTGTCATTGC
GCCCCAAGTTAGGCCGCCACCGAATCCGACAAGAACAATTGTATCATCATCTTTGAGTTTACCATTTTTTAAT
TCTTGATCGATACTTAAAGGTATTGACGCAGCTGAAGTATTTCCATATTTATTTACAGAAACACTCATTTTGT
CTTTTGAAATACCTAAGCGTTCTCTAGCTGATTCCATAATTCTAATATTAGCTTGATGAGGAATAAATAAATC
TATATCATCTGATGTTAAATTCGCTTTTTCAACTACACGTGTTGATGCATCACCCATAATTCTAACAGCAAAT
TTAAATACTTCTCGACCATTCATTTTCAGTTTACCAGTATCTTTATCTAAATATAAATGTTTACCACCAGTGC
CATCAGAACCCATTTCATAACTTATAATACCTCTGCCTTCTGAAACTTCACCGATAATAACCGCACCTGCACC
ATCTCCAAATAGAACTGCAGTAGAACGGTCAGTTAAATCTGTTATTTTAGATAATTTATCTGCACCGACAACT
AAAATATTATGATAATCTCCAGATTGAACATATTGTTTAGCTGTAATCATTGAATACATAAATCCTGAACATG
CTGCAAGTTGATCCATAGAGGCAACTTTGCCCGTCCCTAAACGTTCTTGCAACATATTTGCGACAGTTGGAAA
TGGCATATCTCCAGTTGCTGTGGCAACAATTATCATATCTATATCTTCGGGCTGAATACCAGCGTCAGCGATT
GCTTTTACACTTGCTTCATATGCTAAATCTGAAGTATCTTGATCGTCATCTGCCCAATGTCTTTCTTTAATTC
CAGTCATCTTAGAAATCCATTCATCAGATGTATCTAAAAATTGCTCAAAATAGGCATTGTCAATAATCTTTTC
TGGTGCATATGCACCAAAACCTTTAATACCCACGTTCATAGTTGAACACCTTCTTAATAAAATTTTTAATACC
AGGTATTAATTTAACATAGATTACTTTTTAAACTCAAGTCAGAGGTAGGAATATTGACTAGTTAATAAAATTT
TAATGCTTTATTATCGTCAAATTCCAGTATGAAATTTGTTTTAATGTGCTTAAATTAGAATAGCCATTCAGAT
AAAATCATTTATTTAAACTGTATAATATGGTCAAAATATATGAGATGTTACTATACAAATTTCTAACTTTCAC
TTAAAATAAAATATATATAATTAAAGTGGAGGTAGAATGACATGAAACACCTCGTAACATTCTTTTGGGCATT
ATTATTAATGCAAATGGTTAACTTTGTACTTAATAGCCTAGTAGGTGGAGACAGCTTAAATTTAGTGAATCCA
ATTATCATGGCAGTATTGTTCACGATTTTCACAGCAATTTTTGCTGCAGTAATTAAACCGCCGAAAGATTCAT
CACAATAACTATTTAGTATAAAATGAAATATGTCTTTCTTATTATTGAAGCTGCCCAGATAAAGGCGGCTTTT
ATTTTTGGCCATTAATGTTTTGTATTTTGAATATTTTAAAGCTCAATGACCTCTAACCCATCACTTTTAAATT
TAGAAAAAGCCTTAATACAAAGATGTCATTTACGAGTAATCTTTGTATCAAGGCAATTATATTATAATCATCA
CTGTAAAATTAAATGCTTCATGTTGAATTTGATAATTTTAAGTGTTTATTTAGTTTTTACATTAATATCAATT
TGTTTAATTGAATTTGAATCAAACAAGTTTGCTGTGTAGCTATCTTTCTTAAGATCCACTTCTTTTTTACTAC
TATCTTTCCAAGTAATCGTGTAAACGGCAGATTTTGCATTTCGAATCTTCTCAGGTGTTACACCGCGTTCATC
ATATAATAAAGCTTTAACTTTATTTCCTAAATCTAAATATGATAATTGGCTATCTTTTGGTAAATTTAAGTAA
GATTGATTGAAAGCCATAATGCCATCTACTGCAATTGTATATGGAACATTTTGCATTTCTTTAGCTTTTTGAG
TTGCTGGTTGTTTGTCCTTTTCTGCTGCAGATACCTCATTACTTGCCACAGTAGCGCCAGTCGTTGCGATCAT
GCCCAGTGCCAAAGTTACAGTTATAAATGATTTTAATTTCATATAATCTCTCTCCTTTGTGAGTTAAAGTATC
TTACAAAATCAATATAACACAAATTGAAAAGTTTCAAAAATTATAAGTATGTAAATTGTTTACTTGTTTTTAA
AAATAAATTTTAATTAATGTAAAAGCTATAATTGTTAATACTTTCTATTTTTTCAAATAAATTCACATTACAA
TAACTAAGTTTACATTTCTTAAATTTAATTACTTCTTTGCATTATAGCGAACGCCGTACCTAATATATAAATT
AAAACCTTTGTTTCTTTGTACTTTTAATTGAACAATTTTATATTGATGAGATATTTAGTATTAAATTTTTCTT
CCTATAAGGTTAATACTTGTTTGATCAATTTTATGTTGATTTACTTCTTTTAAAAATTCATTCAAGTAGAACC
CATTCTTCAAATTTAAAGAATGATCTAACGCATAAACTGTTAATTCATATTGATGATCTTGATCAGGTGGTGT
AGGTCCTACATAGTGATTTTCTATTTCTGAAAAATCATCAGCTAACAAACCACTAGTGAAGCTATTTTTACCT
TGTACATAGTCGCCCTTTGTTCTTGATAAATCAGGTTTAATTGAAATTGAATCACGAGTGACACTTACATTAG
CAACGCTCCAATGAATCCATGCAAATCCGCAAACTGGTATTGCATCATAATCTATCAGAGACCATGCAAGATA
TTTAGTATCTTTTGGAAGTTCTGTTATTTCAAACGGGAATGATTTAATTGGATTGCCATTTATTAAATCACTT
ATATCTGCGCCTGCCGCATATTCATATGGTATATTATTTCCTTTAAATTCTGTGTTGATTTTCATGTTGATCT
CTCCTTAAATAAAATAATGTTAGTAAATATATAAACGTCAATAATGACATAAATAACATTAAATATTGAATAG

TTCCAATTGAATATAGGTAACCACCAATAAGTAAAAATGCTATATTAAAAATAAAGTACAGTGTTTCCGCGTA
ATTAAGTATAGCTGCTAAGTTACTACTATTTGAAAGTTCATTAATTGTCTGAGGACGAGTTAAGCCTGAAGAA
ATCCCTAAAAATAAAATAGCGATTGTAATTGCAATAATATTTTGGAATGTATATATTATTAAAAATATTACTA
AACAAATTTCAGATAGAAATTTATAATTCAAATATCTAGTCAAGTAGCGGGAAGAAATGTAACCCATTATTGT
ATAACTAGTTAATACAGATATAAATTGAACGTTATTCAGTTTTAAATCAATATACAAATTAATTGGAATAAAT
CCTACAAAAAACCCTAATATTAAAGCTCTTAAAAAAGAATAATGTAATACCCAAAATTTCTCATCAGCCAATA
ATTTTTTTCTCTTTCTCTTAGATTCCGATTTTATATTACAATCAACGTTTTCAACCAATGTGAGTAGAATAAC
TAAAATTGTTAAAATCGAAAATAAACCTGTCATATAAATCGGCCATTTTATATTAATTCCATAAAGGTAACTC
CCAATAATTCCTGAAATTAATAATGATAAAAACATATAACCATTAGATTTATTTTGTATAAGTTTATGATTTT
TTATATTCATTTTAATAATAGAAGTATCTACTCCAGCCATTATTGAATAACTCAAACCTAATAACAGTTGGGC
TACTAATAAAATATAAAAATGGCTTGGATACAATAATAATATTAAGCCTATTATTTTAAATACCTCACTCACA
ATTAATTTATAAGCATCTTTTATCTGATAAATTCTAAATATTTTCTCTTTATATAAACCGAACAAAAAAGCTG
CAACTCCATATGTCGCCATTAGTATTTCTAACTGCATTATAGAATAGTTTTGTGCTAAAAAATAAATTAATAA
AAATGGCAAATAGAAGTAAGCTCTTGACAAAATTCGATATAAATAAATTCCAAGTATTAATTTATTCATATTG
AATACCTGAACCTAGTTTTGCTTTTATTTCTTCGAATTTTATATCCCCTTCTCTTAAGAGTTCAATTTTATTC
CCCGTCACTTTAATAATCGTACTTGATGATTTATATTTACTTTTATTTTTACTCCTAAGCATATAATTAACAT
TATTCCCCATATGTTTAATTGCTTCATTTTCAGTTATCAATATATCATCAGCAGTTCCTGAAATATTCGCTGA
AGTGATAGCAATTGGTGATTGAATGTAGGAAATTAGATTTCTCATTGTTTTATTTTGAACACATCCAATCGCA
ATACTATCTGAATTATTAAGCATGTAGTTATAATTCGTTTTATTTTTCAAAATAATATTTAATGGACCTGGCC
AAAATATATTAACCAGTTTATCTACTAATTCTACATTTTTATTTTCTCCATATAATTTCCAGTCTTCTGGTTC
ATCAATAAATAAAGATAACGGTTTGTCTTTTGAACGCTTTTTATATTTAAATATTCTATCAATGCAAATTTTA
TTGTTAGGCAAGCTGCATAAATTATAATTTGTATCCGTGGGAACAATTATGGGGTAACTATTTTGTAGTTGTA
AGTATAATTTTTCAAAGCTTTCAACAGATTCATTTAAAATTTCCATCTTTACTCCTCCCTTTTATTACCTGAA
GTAAGTCTGCTGTATCTTCAGGTACAACCTCAGGTATGAATTTGTTTATGAGTTTATAAAATGATATGTTTTC
CTCAATATCTTTAGGATGCACAATTGATTTAAATTTATTTTTCAAAATTTTCTTACTCGAAATATTACTTATT
GGAAATTGCACTGCTTTTGAATTTTTTAATCCTAAATCACTAGGTAATATATACTGGTACATACTTATATTTT
TATTAGTGCCATCTTGATGAATGCCTGATTCATGTTTAAATATATTTTTTCCGATAACAGGTTTATTTTCAGC
TAATCGAATATCAAGTATTTTACTTATATTCATGCTAGTTTTATAAATATCTGGTAAATTCAATGAAGTCATT
ACTATTTGCTTTTTTGTTAAAATTGCTATTATCTCTTCAATTGAGGCATTACCTGCTCTTTCACCAATTCCTA
AAAAAGTTGTCTCGATTTGCTGTGCACCATTTAAAATAGCAGCAAATGTGTTGGCAGTAGCCAATCCAAGATC
ATTATGACAATGAGCAGAAAAAGTTATATTTGGATATTTTTTTACAAATTCCTTAAAAATATTTCCATATTCA
AATGGAGTTGAACATCCAACAGTATCAGCAAAAGTAACTGTTCTTATTGGAAATTTTGAAATCATTTCCATAT
ATTCACATAATTTTTCTAATGAACATCTTGTAGCATCTTCAAAACAAATATCAATTGCTACTTGATTTACCTT
TATCATATTTAAACACTTATTAATTCGTTCATTGTAATACTCACTTGTGAAGTTAAGTTTTTCTTTTATGTGT
AAATCAGATATTGGTAAAAGTATTTTTACTGCTAGGTTATGTATTTTTAAATTTAGAATTTTATTAATTTCAT
TTTCATCTAGCCGAGTTAAGACTACTATTTCTTTATCATTATTTAAAATTTCTCGGTATTGATGAATATTATA
TTCATCCTCAAAAGTTGTGCACATGCCAACTTCCACAGAATTTATGCTTATCTTATTTATTTCTTGTAACACT
TCTTTTTTTACAATAATGCTTTTACGAACATTACTTTGTTGCATACCATCCCTTATTGTATTATCTTGAATTC
GAATCATATTACTTCACCCCCTCGGTAACCTCTGATATAATATTAATATTATTTTTATGCAATAAAAATTAAA
TATTATCTAATGAATTATTAACTTTTTTGAAAGAGGTAAGTTCATGAATTTGGATTGGTACTATACATTTGTC
ATTTTAGCGAAGACGTTAAACTATCGATTAGCTAGTGAAGAAATTAATCTCACTATACCTTCTATACATAAGC
AAATTAAAAATTTAGAACAGCATCTCAATGTGAAACTATTTGAAACTTATAAAAATCAAATTATCTTGACTGA
AGATGGTCATACATTTCTTCCAATTGCGCAAAGTTTTATTGAACAATATGAAAGTGGCATCAAGCATATCCAA
CTTAAAAAGACGATGTTTCAATCGAAATTAAATGTTGTCGTGTCTTCATATATCGCGACATTCATTATGCCCA
AATTTTTAAAATCTTTCTTTAACGAGCATCCGTTTATTGATGTTTCTCTCCATGTCAAAAATGAAAATATTGA
AAAAGATATTAATAATCATACGTATGACATCGGGATTAGCAGAAATCAACCTAAACTAAGAGAAGTTCATTCT
GAAAAAGTATGCGAAGGTAAAATTGTATTGATTGCTCCCAATAAAGAGAATAATCATCTTCTAACCGAGGCAT
CTTTATTTGAAAAATACAAAATAATAAGTGATAACCATCCAGAATATTGGTCATCACTTAAAAATAATATTTT
AAATATTTATGAAAAAGCACAATTTCTATCTATCAATGATGTGCATACTTCAATTAAATTAATCGAAATGAAT
CAAGGTATATCATTCCTGCCTATTTATATAACAACAGATAGTGATTACAATATTTCGGTTATTAACACGAAAA
TTTTACAAGCACCTATTTCATTTACATATATATACAGCAAAAAAGAAAGTCCAGAAATATTGGCGTTTATTAG
ATCATTTAAAAAGTATATTGCCAATGAACAATTATAATAAATTTCAAATCTAAAAAACCAAGAATGCGATTAA
TCATCACATTCTTGGTTCAATTTTATTCATGAATTTTTTCAACATTAAACGTTAAGTTATTGTCTGAATTTAA
ATTAACTTTAATCGTTGTACCTTCTGGGAATCCCTCTTTAATCATCATACGTGCTAATGGTGTTTCAATTTGG
CGTTGTACAAAACGTTTTAATGGTCTTGCACCGTATTGAGGTTCATAAGCCTCTTGACCTAGCCAAGCTTTAG
CATCATCAGAAACTTCAATTGAGATTCGTTGTTCTAGTAATCTTATATTTAATTGCGTTAAGATTTTATCTAC
AATCATACTCATGTCATCAATAGATAATGGTTTAAATAATACGATATCATCCATACGATTCAAAATTTCTGGT
TTGAAATATGCATTTAAACTTGTCATAACAGCTTTTTCTGTTGATTCTGTAATTTCACCAGTCTCTTTTACGT

TTTCTAATAAAACTTGAGATCCAATATTACTTGTCATAATAATAATAGTATTTTTAAAATCAACGCTACGTCC
TTTAGAATCAGTTAAACGACCTTCATCTAAAATTTGCAATAATACATTAAAGACGTCAGTATGCGCTTTTTCA
ACCTCATCTAATAAAATAACTGAGTATGGATTACGACGAACCGCTTCAGTTAATTGACCCCCTTCATCATGTC
CAATATATCCTGGAGGTGCCCCTATCAATCTTGATACTGCATGTTTTTCCATATATTCACTCATATCAATACG
AATCATATGTTTTTCAGAATCAAATAATGATGCAGCTAATGATTTAGCTAATTCAGTTTTACCTACTCCAGTT
GGACCTAGGAATAAGAAACTACCAATAGGTCTGTTTGGATCTTTAATACCTGCTCTTGCTCTAACTACTGCAT
CTGAAACCAGGTCAACCGCTTTATCTTGACCTACAACACGTTTATGCAAGATGTCACTTAAGTGAAGTAATTT
TTCACGTTCTGTTTCAACTAATTTTGAAACTGGTATGCCTGTCCATTGGCTGACAATATCGCCAATTTCTTCG
TCTGTTACAACTTCACGAATCATTCGATCTGTATCTTCACCTTGCTCATCTTGGAAATTATCCTCTAATTCTC
TAAGTTCTTTTTCCAATTGAGGAATTGTTCCATATTGTAGTTCAGCAGCTTTTTCTAAATTGTTATTTGTTTG
TGCATCTTCCAACGCTTGTCTACTTTCATCTAGTTGCGCACGTTTTTCTTGTAAATTTGCTATTTTTTCTTTT
TCTGATTCTACACGAGATTGAAGTGCTGCTTGTTTCTCTTTTTCATTGGCAAGCTCTTCTTGTAGTTCTTGTA
ATCTCTGTTTGCTCGCATTGTCAGATTCATTTTTAAGTGCGCTTTCTTCAATTTCTAATTGCATGACACGTCT
ATTAACTTGATCCAATTCAGTTGGATTTGATCCCATTTCCGTACGAATTGTTGCACATGCTTGGTCAACTAAA
TCAATCGCTTTATCTGGTAAAAAACGATCAGTGATGTAACGATCAGACAATTCAGCGGCAGCAACTAAGGCTC
TATCTTGAATACGCACACCATGATACACTTCATATCGTTCTTTTAAACCACGTAAAATTGAAATTGTATCTTC
AACATCAGGCTCACTAACTGCTACTTTTTGGAAACGACGCTCTAATGCCGAATCTTTTTCAATATATTCTCGA
TATTCATTTAAAGTTGTTGCACCAATACAATGTAACTCTCCTCGTGCTAACATTGGTTTTAGCATGTTGCCTG
CATCCATGGCACCATCTGTTTTACCAGCACCAACAAGCATATGGATTTCATCAATAAATAATATAATTCTACC
ATCAGACTCTTTAACTTCTTTTAGGACTGCTTTTAATCTCTCTTCAAATTCACCACGATATTTAGCGCCCGCT
ACTAATGCGCTTAAATCTAACTCAAAAACAGTTTTATCTAATAATGATTCTGGCACATCTTTCTTAACTATAC
GCTGCGCTAATCCTTCAACAATTGCAGTTTTACCAACACCTGGTTCACCAATGAGCACAGGGTTGTTTTTAGT
TTTACGACTTAAAATACGAATCGTATTTCGAATTTCTTCATCTCTTCCTATAACAGGATCCATTTTACCTTGT
CTAACTTCTTCTACTAAGTCGCGGCCATATTTAGCTAATGCTTCGTAATTAACTTCTGGATTTTGTGATGTCA
CGTGATTTCCCCCTCTTACTTTTTTAATAATTTCTTTGATAACTTCTACTTTATTATTTATATAATGTTTTGT
TGTTTGATCAATGTCCATTGCCGAACGTAAAATATGCTCCATTGAAATATATTCATCTTCATATTCTTTCATG
TATGATTCAGCCTTAGTTATCAATTGGTTTGCTTGTTGGCTAATATATTGACCATATTGTATATTGTCACCTT
CTACAGATGCATACGTGTTTAGTTTGTCTTCATAAGCTTTGTTTAATTGATCTACCTCAATATTTGCTCGTTC
TAAAATACTTTTATATAAGCTTTCACTTTCATTTAAGGCAGCGCTTAAAATTGCCTCAATTTCTATATTTTGT
AATTTATGTTGCTGACTCAGTTCAACTGCTTGTTGTAAAGCATTTTGAACAGCATATGTCATTTTATTTATAT
CCATATAATTTCACCTCTAATTTGTAGAGTTGGTCAAGTTGCACTTTTGACTTTGACTTTCTTTGACCTTAAT
TATATTATAGTCCCAATATAATCATTTATCAACTCTTTTACACTTAAATTTCCTAATATTTGTAAATAATTTT
TTGAATTAAAAAAATCTAGCTAATGTTACGTTACACATTAACTAGACTTATTAAACATATGTTATTTCTTTTT
AAGTGCTTTTAATATTTCATCTTTTAAAGCTAGGACTCCTTTGTACTCTAAATGTACACCGTCTGGTGCAAAA
TATTCACTATGTCCTTGTGATCGCTTATACCAATCAATTAATGTGACATTGGACTTTCGTTTCGCTGCGTCAG
CTAATAATCGATTTACATTTGCCTCATAAATTCTTGGAACACGTGTATTAACTAAATAAATCTTGGCTTTTCC
AAATTGATTTAGTAAATCGTCGAGCTGTTTGACAGTAAAGTCGCCATTTGTACCTAATTCTAATACGACTTGA
TCAGATGATTTTTTATATTGTGAATAATTCGCTTTAACTAAAGGTAAGGTTTGATACAATTGACGCCCTACTT
TTCCATCAATTCTAGACTTAGGAACTGATGACTTAAATGACTCACCGATATCAACCATTACTGAATCACCGAT
TAAAAGAGGTTTAATATTATTATATACGTCGGAAGATTCCTTTTTCTTCTCACTTACTAAGCCATCAATGTGT
ATGTTATCAATTGGTGCTATCCGCATTAAATAATTATCTTCAATAGTGGTATCAAAGCTATTCGCTTTTTCTC
CAATAATGTCTTTACCATATTTATTGAATGCACCTACTAAAATCAACATAAATGGAATTAACAAGGTTACTAC
AATTGCCATTCTTATAAATTGTGGTATATAAGAAGGTCGCCAATTTAAAGCTTTAATACCTTCTTTTCTAAAT
GGAGTTTCTATAAAGCGATATGATAGCTCTGCAAATATAATTGTTAAACTTATATCTATAAAGTACACATATA
CAGGTATCTGTCCGTCTACATAGTAACTATGTACGAAACTAATTACTGCAAAATGCCATAAATATAAACTATA
AGACCTTTTCCCGATAAATACTAACACTGGATTTGAAAATATCTTCGCTATCCATGTAGATGGATGAACGACA
CTGGCAATAATAAATAACGTTAATATGGATATTAAATAGAAACCACCATCATATATCCAATTCGTCTCATCAT
TAATAATGAAAAATAATAATATAAGTATTATAAATGATAAACTACCTATGCTATCAATAACATATTTTACAAC
TTTAGGTGGATCATTTTTCAATTTAAACGGTGGCCATAAAAAAGCTAAAATAACACCCAGTAACAATGTCTGT
AATCTAGTATCTGTACCAAAATACACTCGTGAATGATCCCCATTAATACTGTAGATAAACATCATTAACCCTA
AAGAAATTATTGATACTCCCCAAAAAATAAATCCTATTTTGTATCGCTTTTTAATTGTTAACAATAATGTAAC
CAAAATAACTGGGAAAAATATGTAAAACTGTTCTTCAATTGCTAAAGACCATAAATGCTTTAATGGCATAAAT
GAAAATTGCTCAAAATAATTAACATCTTTTGCTATATACCACCAGTTTGATACATAAAATATCGCAGCAATAA
TATCATGTTTAACCCTAATGATATTATCTGATTTTAATAATAAGGTTGCTGTCCCTACAACCATTAATAAAAC
TATGACTGCTGGTAATAAACGTTTTAAACGACGTATCCAAAAGCTTTTCAATTTAATGATACCTGTGTCATCA
TACTCTTTTAGTAATAAGCTCGTAATTAAATAACCAGAGATCACAAAAAAATGTATCCACACCTAAAAAGCCAC
CTGTCAACCATTGCTTATTTAAGTGGTAAATAATAATTCCTAGAACAGCGATTGCCCTCAAACCATCGAGCCC
TGGCATATATCTCATTTTCTTATACTTTGTAAAACCCTTTGTTTTGTTCATTTTTGCATTCTTCCCTTTTAAA

ACTGTTCTTCTTAGATGCTTAATTAAATTTAGTTATGCTGTTTAAAAGAATATTGAAATGCATATGTATATTA
TTGAATTACGACATCATCAAAATCATATTGACTAAAATACTGTTAAATTAAAAAAATTACCAATGATGATTCT
TACTTCGAAATCCAATTTGTAATGCAACTCGGCAACTTAAAAACTATGAAGTATTATGTATTGTAATATAACT
GTAATATAAATTCAATTTATTATAAAAATTTTCAAGAAAATATTCAACTAGAAAATGAATTGTGCGCTCTTAG
AAGTGAGAAACACTGTCTTAATTCATATTTTTTGAAACAAGTTAGATATAAATTTTCAAAATAAAATCAGAAC
CTAGAACATAAATAAGGCTCCCTTCAAAATTTTCATTTTTCAATGTCTACTTTGAAGGGAGCTTATTCACAAT
GAATTATACTCTACAAGGTTATATTGACTGCGGGGCCCAAACACAGAGAATTTCAAAAAGAAATTCTACAAGC
AATGCGAGTTGGGGAGCGGGCCCAAACACAGAGAATTTCGAAAAGAAATTCTACAAGCAATGCAAGTTGGGAG
CGGATCCCCAATTGGATTTCCAATTTCTACAGGCAATGCACGTTTATGTTAGCTCACACCAAGTGCAATCTTA
GCGTAACGTGACATCATATCTTTTGTCCAAGGTGGACTCCATACGATATTCACTTCAGTATCCTGAATTTCAG
GAATCTCTGCTAATACTGTTTTAACTTGATCAATAATTTGAGGTCCCATTGGACATCCCATTGATGTTAAAGT
CATATCAACTGTACATACGCCTTCATCATCAACATTCACTTTGTATACTAAACCTAAATTAACGATATCAATT
CCTAATTCAGGGTCAATTACCATTTCTAATGCACCTAAGATACTATCTTTCAATGCCTCTTCCATCCATATCA
CCTCTTTAATGTCATATTATTCATAATATATCAAATATCCGACAAAACGCCAATAAAATGCTATGATGTATCT
ATATGAACTAAGCAACTTATGAGGAGAGAGATATGCAACCACATTTAATATGTCTAGACTTAGACGGAACATT
ATTAAACGATAACAAAGAAATTTCATCATATACTAAACAAGTATTAAATGAATTACAACAACGTGGACACCAA
ATTATGATTGCGACTGGCAGACCTTATCGTGCAAGTCAAATGTATTATCATGAATTAAATTTAACGACACCAA
TTGTTAATTTTAATGGCGCTTACGTACATCACCCTAAAGATAAAAACTTCAAAACTTGCCATGAAATTTTAGA
TTTAGGCATCGCACAAAACATTATTCAAGGATTACAACAATATCAAGTATCGAATATTATAGCAGAAGTGAAA
GATTATGTTTTCATTAACAATCATGATCCAAGATTATTTGAAGGTTTTTCAATGGGTAATCCAAGAATTCAAA
CTGGTAATTTACTTGTCCACTTGAAAGAATCCCCTACCTCAATTTTAATTGAAGCCGAAGAAAGTAAAATACC
TGAAATCAAAAATATGCTTACTCATTTTTATGCCGATCATATTGAGCATCGACGCTGGGGCGCACCATTCCCT
GTCATTGAAATTGTAAAACTTGGTATTAATAAAGCAAGAGGCATTGAGCAAGTTAGACAATTTTTAAATATTG
ACCGAAATAATATTATTGCATTCGGTGATGAAGATAATGATATTGAAATGATTGAGTACGCCCGCCATGGTGT
TGCTATGGAAAATGGTTTGCAAGAACTTAAAGATGTAGCGAATAATATTACATTCAACAATAATGAAGATGGC
ATTGGTCGATATTTGAATGATTTCTTTAATTTAAATATTAGATATTACTGTTAATTTATAACTAATCATTTTA
TAATATTTTAAAACAATAGGAGGTAAGTTACGATGCCCAAAATAGTCGTAGTCGGAGCGGTCGCTGGCGGTGC
AACATGTGCCAGCCAAATTCGACGTTTAGATAAAGAAAGTGACATTATTATTTTTGAAAAAGATCGTGATATG
AGCTTTGCTAATTGTGCATTGCCTTATGTCATTGGCGAAGTTGTTGAAGATAGAAAATATGCTTTAGCGTATA
CACCTGAAAAATTTTATGATAGAAAGCAAATCACAGTAAAAACTTATCATGAAGTTATTGCAATCAATGATGA
AAGACAAACTGTAACTGTATTAAATAGAAAGACCAACGAACAATTTGAAGAATCTTACGATAAACTCATTTTA
AGCCCTGGTGCAAGTGCAAATAGCCTTGGCTTTGAGAGTGATATTACATTTACACTTAGAAATTTAGAAGACA
CTGATGCTATCGATCAATTCATCAAAGCAAATCAAGTTGATAAAGTATTGGTTGTAGGTGCAGGGTATGTTTC
ATTAGAAGTTCTTGAAAATCTTTATGAACGTGGTTTACACCCTACTTTAATTCATCGATCTGATAAGATAAAT
AAATTAATGGATACCGACATGAATCAACCTATACTTGATGAATTAGATAAGCGGGAGATTCCATACCGTTTAA
ATGAGGAAATTGATGCTATCAATGGAAATGAAATTACATTTAAATCAGGAAAAGTTGAACATTACGATATGAT
TATTGAAGGTGTCGGTACTCACCCCAATTCAAAATTTATCGAAAGTTCAAATATCAAACTTGATCGAAAAGGC
TTCATACCGGTAAACGATAAATTTGAAACAAATGTTCCAAACATTTATGCAATAGGAGATATTGCAACATCAC
ATTATCGACATGTCGATCTAACGGCTAGTGTTCCTTTAGCTTGGGGCGCTCACCGTGCAGCAAGTATTGTTGC
CGAACAAATTGCTGGAAATGACACTATTGAATTCAAAGGCTTCTTAGGCAACAATATTGTGAAGTTCTTTGAT
TATACATTTGCGAGTGTCGGCGTTAAACCAAACGAACTGAAGCAATTTGACTATAAAATGGTGGAAGTCACTC
AAGGTGCACACGCGAATTATTACCCAGGAAATTCCCCTTTACACTTAAGAGTATATTATGACACTTCAAACCG
TCAGATTTTAAGAGCAGCTGCAGTAGGAAAAGAAGGTGCAGATAAACGTATTGATGTACTATCGATGGCAATG
ATGAACCAGCTAACTGTAGATGAGTTAACTGAGTTTGAAGTGGCTTATGCACCACCATATAGCCACCCTAAAG
ATTTAATCAATATGATTGGTTACAAAGCTAAATAAATGAATCATGCGATTCAATCTAATCTTAATTGTCTAGG
TTAGAATTATGTTGGACTGGTACTACTATCCAGTCCATTTTTTATGTTTAACATTTTTAAAATCAAAAAAGAC
ATAAGGTCTTGGACTAATAATTGTCCATGCCTTATGTCATAAACTATATGTCTTATCAATTAGCCAATACCGA
ATAATTTTGATATAGGCCCTAACGGTAGAATGACACCTAATACCATTGTGATGATAATTAATGCAATTGTTAT
CCAAAACATTGTGTGACTTTGTTCATGTCTCTTTCTTTTAGCAATCGACACTTCCATCAATCCAACTACTGCA
ACACCACACAGCATTTTCAATGTAAGCAACATATGATTTGCCCCGCCATTCATAAATGACTGAATTAATATCC
AAAATCCTGAAATTAACGTCAACAGCATAAATAAGCGTAAAATCATGTGCAACGGTTTGAAAAATGGTGATCC
GCCTTGATTTTTTGAAATGTTTAAGTATGTAGCGATAAATAAAATAATCGCTAATACCCAACTTAATATATGT
AAATGTAACATACTGATTCCCCCCACTTTAATAATTTATATTATTAAATTAAAGCTTCTTGGGATTAATACCC
ACTTGCTTGTAATTTAATCATGATTTGATTATACACGAATATATATTCTACCACACTTCTATATTTGAGAGGA
AGAACATGACATTTTATTCCTTATTAGAATATTGTGCATCTGCTGTAAAATAATCAACTACTTCTTAATATCA
ATATTTCACTTTCATCTCAAAATGGTAACATTATAAACAATGATCTTTAACACCTTATTAGAAAGCAAAAAA
CTAACCAATCTATATAAAGACTGGTTAGCTTTTTAAATGATAATTATTTAGCGATATAAGTTGTCAGCGTTCC
AATATTATCAATAGTCACTTTAACTTCATCACCTGGTTGTAAGAATTTAGGTGGTTGCATACCTGCACCAACG

CCTGCTGGTGTACCAGTTGCAATAATATCTCCCGGATGTAGTGCAACATATTTTGAAATTTCTTCTATTAATT
CATCAATTTTAAGAATCATTTCGCCAGTGTTACCATCTTGTCTAATTTCATTGTTAACTTTTGTAACAATATT
TACATTTTCAGGTAATGGTAGTTCGTCTTTAGTAACGATATAAGGACCCATTGGGCAACCGCCAGTTAAACTT
TTTGATAAAAATGCTTGATCTTGTTCACTTTGTGCTTTGCGATCAGTGATATCGTTAATAATTGTATAGCCGT
AAACATAATCTAAAGCTAATGCTTTTGGAATCTTTTCACCAGACTTACCAATAACAATACCTAATTCACCTTC
ATAATCTAATTGATCAGTAATATCTTTATGATTTGGAATTGTTGCATTATCTCCTGTTAAAGATGACGCTGCT
TTTGTAAATACATATAATCTTTCTACTTCATGATTTAATTCGTTCGCATGATCTTTGTAATTTCTACCAAAAG
CAATCACATTATTCGGAGGTGTTACTGGTGGTAAGAATTCAATGTCATTAAATGAAATTTTATAGTCTTCAGC
TTTGCCGCTATCTTCTGCTGCTACAACTGCTTTACGTACTTGTTCTTGAAAATCTAAAGTATGATTTTGTTGT
AAACCAGCTAACAATGTTTTAGGATGGAAATCTCCTTCTGCAAAGTCAGCAAATACTTGTGTTAAATCCCATA
CAGCATCTTCGCGTTTTACTTTAACGCCATATGAAGTTTTGTCATTATACTTGAATGATAAGAATTTCATTCA
TTCTCAACTCCTCATCTTTATCTTAATTCACATTATAACTTTTTTCGTTATCAAATAACAAATAAATAAGTAA
GACAATTTTGAAAATGAGTTGTGTTCATTCTGCTACAAGGGCTTTGCACTTAATCGAAATTTTTTTATTCTTT
TGAAAATCAAAATACTACAGTTGCAATGTACCAAATTTGAAGAAGTATAAATAACCTTTAACTTCTTTATTAA
GAATCGTTTGAAGCGTATTTTGATAATATTTCATCTGTATCTTATATTTATTTTTTAATTGTGTACCAATTTC
TTCATCTGTCATCCCACGGCGACGATTAAATGCATCGGTTTTATAGTCTACAAAATAATGCACACCATCTTTA
ACAAAGATTAAGTCAATCATACCTTGAATAATTGAGACGTCTTCGTCTCCTTGTGGCAATTGGTCAACTAATG
CTTGGTTAACTACAAACGGTAATTCACGATAAACTTGCTCTGCTTCAGCAATAATCGAATATAACTCACTATT
GATAAATGTCATTATTTCATCCATACGGATATCTTTTTTCGCATCTGCTTCGATAATATGTTTATCGATTAAT
CCATCGATATACTGATGTAACTCAACTTCAGATATGCGTTCTTTTTTGAATGGTAAATGTTGCATCACTGTAT
GCATTAACGTACCAATTTCATTCGCTTTTCGTTTACCTTGTTCACTTAGAAATTTAGGTCGTTCATACGTTGA
AAAACCGATACGATATTGTCTTACTCGTTCGTAACTTGTGCCACTTTCTTCTGTTTCATATTGCCTTTTCAAT
TCAGAAACAGATTGTTTTGATGGCTTTTTAGTATCATTTACATATGGATATTGATAATCAAGTTGGTGTTTAA
TTTGTGCTTTAACATCCTCATTACCATTTTGCATAGTTTCTAATTGATTAACCGAACGATATTCATTATTATC
TAAAATGGTTTCTGTAGACACATCTTCAAAGTATATAATTGAAATATTTACATTCGGACGACTACTATCTTCA
ACTTGTGCTATATCTTTTTCAAATTTTAAATCATCTGGAATTGACGCAGATTGATGTTTAGATAAAATACTAT
AAATAAGATGGAACGGATTTGGTGAAGTTAATCGTTCATTGACAGCAATATGCTCACCAGAAATAGACAATTG
CTCTAGTTCTAGTAACGATTTATCATTTTTCACTCTACCAATTAAATAAAGTTGTTCTTTCGCTCTTGTTAAT
GCTACATAGACTAATCGCATTTCTTCTGACACAAGTTCTTTTTCGGCAACAGCTTTATATGCAACCGAAGCTA
AAGATGGAAATGCCATTTCTTTATCCACATCAAAATAATCCATTCCGAGACCAAATTGCTGATTTAAAATAAC
TGGTTGTTTCAAATCACGTTTATTAAAATCTTTTGACAATCCAGAATAAATGACAAATGGAAACTCTAGACCT
TTACTACTATGAATTGTCATCATTCTAACGACATTATCGTTTGGACCAACTACATTTTCCTCACCAAAATCTT
TGCCTCTTTCAATCAATTCATCGATAAAACGAATAAATTGATATAAACCTCTAAAACTTGAATTCTCAAACTC
GATAGCTTTATTAAATAAACCATAAAGGTTTGCACGTCGTCCACGTCCACCAATAAGTCCACTAAAGTATTGA
ATAACATAATGATCATTATAAAATTTATCAATTAACTGATACACCGGATGATCTTTACTATATTGTTGGTAAC
TTTGAATATCTGATAAAAACATCTTTAATTTATCAACTAAAATAGCATCTGCTACTTCGTCATTAATGTAATT
TACAATCGATTGATAGAAGTAGTCATCATTTGGACTCAATATTCTAATTTGAGCTAATTCGTCTTCTTTGAAC
TGATATATAACGGAGCGCATTAACCCAACTAAATAAATATCTTGTAATGGATTATCTATCGCTCTTAAAAATG
ATAATACTAAGCGGACTTCTGTTTGTTCAAAGTAACCTTCACGACTATTCACATGGAATGGAATATCTTCATT
TTTAAAGGCTTGTTGTAAATTGCGAGCTTGTCCAAAGCTGCGTTCTAGAATAACGATATCTTTGTATGTCGCA
CTTCTATAGCTTCCTGTTTTCATATCATAAACTTTTTGATGTTCTAAGATATCTTTAACTTGTTCTACTATAA
AATGCGCTTCTTGTTCACTACCAGTTAAATCACTATGTTCTTGATCCGCTTCAACAAGCACTTTTAAGTTTAC
TGGATGGTCCGATTCATCATATGGTGCACCATAATACAATTGTGCTGATTCATCATAATTTACTTCACCGACT
TGTTCATCCATCATATGTTTGAATATATAGTTAGTCGTTGACAGTACTTCTTTTCGAGAACGGAAGTTTTGCG
ACAAATCAATTCGACGTCCAGTGCCATCTCCATTTATAGTAAAGCGTTGATACTTTTCAATAAATAAACTTGG
ATCAGCTTGTCTAAATTTATAAATGGATTGCTTAACATCTCCAACCATAAATAAATTACCATTATGTTCTGCA
CCCGTTTTTATGCAAGATAGTATTTTCTCTTGAACTCGGTTCGTATCTTGATACTCATCGACCAATATTTCTT
GGAAGTGTTGACGGTATGATTCGGCAATTTCTGAAGGCGAACCATCCTCATTAGTTAAAATTTGTAATGCAAA
ATGTTCATAATCAGAAAAATCTAAAATATTTTTGCTACGCTTTTTTCGATTGAATTCTGACATAACATCTTTC
ACAATACGCGCAAGGTACTTTACTCGTGGCGCCAATTGTTGCATATCAGCTTTCAAATCATCAGCTTCTCTTG
AAAAGTAATCACTCTTCACTTTATCAATTAATGATTTATATTTTTTATACTGAAGTTTAGCATCTTCTAAGGC
ATCGACCATCATTTCATTTGCTTCTTTAATTTTCGCTGTTACATTAGGCAAACGCGCGCCAAATTCGTGACCA
GTTAAATAAGGTATATCAATAAAGCCACCTTCTAAAACACGCCCCATTAAACGTCGTTCATCTTCTATAACAG
CTAAATGTTTATCGACGCCATCCATCATACTAAACAAATCATTCGCCTTATTTAAAGCATCATAGGCAGCTGT
GATAAATACTTTAGATAAGTCTGTTAGTAGTTGAATAAGTTGCGCTTGTTGTGCTTCTTCTTCGTATGGTGTC
ACCAATTGATCCAACCAATTTGTAGGATTTGGATTTGCAACGCTAAAGAAATACAATTGTTTAATAATCATTC
GAAACTGATCATCACTTCTATCTGAAGACAATTGCTCTGTTAATTCAATAAAAGCAGGATCAAGGATATCGTA
ATGTTGTTCTATGACCTCATCTATCGTTTGTTCTAATAATAAAATATTTTCAGCTTCACTGCTTGTTCTAAAG

TTCGGGTCAATATTTAATACATCATAATGCTGTTGAATTAATTTCAAGCAAAAACTATGAAGTGTAGATATTT
GTGCTTGATGAATTTTGATACGTTGGTTTTTCAAGTGTGCATTTGCAGGATCAGCAATCGATGCCTCTTGAAT
ACGTTGGTCTACACGATGCTTCATTTCACGTGCGCTTAAGTTTGTAAACGTTACGACTAAAAGTCGATCGACA
TCAATGCCATCACGTAAAATCTTTTGGATAATACGCTCAACTAGTACAGCTGTTTTACCTGAACCTGCCGCGG
CTGCAACAAGTACATCTTGTCCAGTTGCGTAAATACTTTGCCATTGCGCGTCAGTCCAAATCACGCCTTGTGG
TTTCTCTGGAATTGTCATCTATTGCTCACCCCCAAATTCATCATTAATGTTAATATTTTGAATTGCTTCAATT
GGATTTATTGTTTCATCTACAGTTCGATATCGCTTACTATCAATCATGCCATCTACATGACATACCGATTGAT
AACTACAAAAAGCACATGGCAATTTTTGTTTGTACTTTAATGGTGCAACTTCAGTATGTCCATCCATAATATT
TGAAGCGGTTTCTATAAAATTCTCTTTGTTATGTTGGATGAATTTATAAATCGTTGCTTCATCTGCAACTTGA
CTGCCTCGTTTACTCAAAGAGCCATCTTTATTCAAACCAACTGGTACAATATCTGAAGTGAATTTAGGTTCTA
AACGAATATCCAATGCATCAATAACAGTTTGGTCTGCATTCACTAAACCACTCAGCTTAAACTTTTTAATTAA
ATCTTGTTCTAATTTATCTTCATCAATATCTGCCCAAGATTTAAATTTAATTCTAGGTTCATGTACATGGAAG
TATAATAATCCACCTGGCTTCACAATATCTGTTAATCCAAGGCGTTGTTTATTTTGTAAAACGATATCCATGT
ATGTCATCATTTGCATTTGCATACCATAATATACTTTCGTTAAATCAAGTGTCGCACTACCTTCAGAGGATTT
ATAGTCAATGATATTAACAAAACTTGTATCATTCTTTGTATACGTATCGATACGGTCAATTTGCCCTCTAATA
TTAATTGGAATACCTTGAGTTGTCGTTAATGTTTGTGCAATTAATTCGTCATTTGTTCTTGGTTTCCTTCTAA
AACTTGTCTCAAAATGTTTTGGCATAAACTTTGAATACGTGCCTTGATATTTTAATGCGCTTAGTGTTGTTTC
TACAATAGCGCCAATGCGTCTTGATAAATAACGATAGTAAGCTGAAGAATTTAATAAATTAAACTGAACTTTA
GGTAAAATTTGTTCCAATGCTTCATTCGTTAATTGTCTTATTTTTTTCAGGTCTAATTGTTTAAAATCGCCAT
TAATACGTTCAGATATATATTTTAAAACGGAATGGAAAATATCACCTAAATCAAAGTTTTGAAGTTCATACTT
CGTTCGCTCATTTAGTTTAAGACCATGTGAGGCATAGTGTTTAAATGGGCATTGTTGATAACCTTCAAATCGA
GATACACTGGCATTGATTTCCTTACCATATAAATCTTTAGACAACGTTTCACCTAATTTTACAGTTTCATTGT
CAAACGTTAATGCTGACATCAAATAATCTAAACCTTGATTTAAATGATCGCTATCTCTAATTACTTGATAAGC
ATCTAACCAACTATCAGCCACAATTTCATCATCTAACCAAGCACGCAATGCTTCAAATAATGTAATTTTGGTT
TGCTTAGCATGTTGCATTAGTGACAATGGGTTAACTTCATGGTATTGAGGAATGTTAGTAATTTCCAATTGGT
TGAACAATGATTGAATTTGATTTAAAAATGGGCTGATCTCCTTATCATCACCACTTGATCCCATTAGACTGTA
AGAAAATGTAACATCTCCCTTAGCTCTAGTCATAGCAACATAGCAAACAAATGCTTCATCCATCTGTAAAATA
TCTGATGTAGGACTCAACTCTACATTTGCTTGTTGTTCAAAATATTTCTTTTCTTCATCAGTAATTAAACTTG
ATGCAGTTACTGGTTGTGGCATGGTGCCGTCGTTCATTCCAACTAAGTAAACATGTTGCTTATTGTCGACTTT
AGCCAAATCCATCGTACCAATACTAACTTGATCTAATGTTTGTGGAATCATGACAAATTCTAATTGTTCTAAA
CCAATATCAAATACTTCTAAGAAACGTTCCATCGACATTGGTTCATCTCCAAATACTAGAACTAAGTCATCAA
GGATTTGAATTAAGCCATTCCATATTTGATCAATTTCCTCCGCCTTTTCATGATTACCATTTAAATCAAGTTC
ATCTCGCTCTGTCATCAATTGATTTGGCAGTTCGAAATATTCCATACTTTCATAAAAAGCAGTTGCAAAGTCT
TTTACAGTTTCCGCTTGTGACATTTGCTTTTCAAAATGTAAAATTTTATCAATGACATCTTTCTTTAACTTAA
CGACTTGTTTAAATGTGTTACGTTCATCTTCGGTCAGTTTATGCGCTTTACGCCCCATTTTGCTAAAATGTTC
GACATTAAATAGCTCATCATCTAACCAACGTTTACCGTATATACCACGTTCAAGTACAAAATTTTCAAGTAAA
TCAACTAAGTATGCACTTTTTAGATATGATGCCGTTAACACATCAGTCTTCAATAAGCGTAGCATTGGATTCA
CTTGCCAATTAGATTGAATAACTTCAATCAATGAACGAATCATTTCCATGACCGGATGATGTGTCATCGAACG
CTTTGTATCAATATTATAAGGAATATTATAAAGCGGTAATATGGAATCAAATAAATAAGCATAAGATTCATCA
CGATATAAAATAGCAATATCTTGATATCGTAATTGCTTATCACGAATATCAACGATGATACGTCGCGCAATTT
CATTTATTTCCTCTCTCATAGTCGCAGATTCTAAAATATTGATATGACCTTGACATGCCACTCGATTGATTTG
AAGTGCATCAAATTCTTGTTCAAGATGCTTTAAATCTTGATTATTGAAGCGATATAATTGCTTGAAATATTGA
CGTTCAATAGAAATATTGAGTTCATTTGCTATTTCTTCAATATGTCGTAACACTTCCGATGGTTTTCTAAATA
AACTAAATTGATCGTGGTTACCATCTGTCGTCAATATAATTGTGACACTCTTCGCATATTTAATTAATCCTTT
GATTATTAAATACTCAATCGTTGAAAAGTTGTGAAAACCATCAATATATATATCAGCACGTTTTAGCCACTCT
GATTTCGGCATACAATCAATAAAATATTGTAATGAATCCTCACCCGTAATAAACTCGTTTTGAATGCGTTGTT
CGAACTCACGGTATATTAAAGCAATATCCTCTAACTTATTTTTAGTTCGAGTTTGCATATTTTTATCAGCAAT
AAAGTGTTCTAAATGTTCAGGCGTTACTGCATATTTTTTAAAATCTTGAATTTGTTCTGTTAATTTTTCACTA
AAACCATAATATTTTGCTTGTGATTGATAAAGTTTTAAATACTTTTGTTGTTCTTGAACAATGTTATAAATCA
TCATTTCCGTTGCAGCTTTAGATAACTTTTGTTCGCTATAACTACCAACTTCTTGGAAAATACGATGACTTAA
TCGTTCAAAATGCAACACTTCTGTTCTTAAACTACCATTTAATTCCGGATCATTGACAAAGGCTTGTTCTAAT
TGAAATGTACTTTGAGTTGGCGCAATTAAAATGATTGGATCTCCAAGCGGATCCTGTTTCATTTTTTGTTTTA
TTTCGGTTAACATTTTCGTAGACTTACCTGTTCCTGCTCTTCCTAAATAAGCATGTAATGTCATGACAAACCC
TCACTTCCTTTGACACATATTTTAACACAAAGTGATTGTGATTGATTTATTATAAGTTCATTTCGAACTTAGC
TTTTATTGTGGTACTAACAAAATAAAACATCGTTTATCAGTATTAAAACCGACAAACGATGTTTTATTTGTTT
CATATTAATTTTTAGTGTTTTCAGGATTGAAATTATGTTTAAATTCACTAAATGGCCAGAATCTAAATGAAAC
TTTGCCAACAATTTGGTCTTCATCAATGAGGCCAAACGCACGGCTATCTTTACTTACTTCACGATTATCTCCA
AGCACTAAATATTTACCTTTTGGAATGACATTTGATTTAGGATTCGCATTCGGTAAATCTTTAACTTGGAAAG

TCCCAGTAATATAATCACCTTGTTTATGTTTTAAATTGTAGTTTAAATATGGTTCATCTTGTTTTTTACCATT
GACATATAATGTATCATTTTTATATTCTACTTTATCACCAGGAACACCGATAACACGTTTAACATAGTCATCA
TTTTTGTTTGCATGGAAGACAACTACATTACCTTTTTCCAAACCACCTGTTTTATATCCAATAATGTTTACAG
CTACTCGCTCACCATCTTTCAAAGTTGGATCCATTGATTCACCTTTAATCATATATGGCGTAACAATAAATTT
ACCTACTATAAATAAAATGACAAAAGCGACTGCAATTGAAATAATCCATTCCAATAATTCTTTTTTCAATTTT
GACACCTCTTTTTAAGATTTGAACTGAATCGTCCATTTTGAAAAAGGATAATATCTCAAACTTATATTACCAA
TAATATCCTTTTTATCAATTAAACCAAATTGTCTAGAATCATGCTGATTGTTATCATAATCATTTAGCACAAC
AAAATTGTTAGGCGGTATAATATCTCCATCTAATTCTTTAAAATTGCGCAAACTAAAATCTTTAATTTTTCTG
TTCTTGGCATAAGATGCGTCAACCGGTCGGTCATCACGGTATAATTGTCCCTGACGAAACGCCATTGATTGAC
CAGGTTTGGCAATAATCCGACTAGTATATATCTCGTTACCACGCCTATATGTAATGATATCACCATTATTCAA
TTGATTAAATGTAACTTTAATTTTATTTACAATAACACGATCCCCTTTGTTAAGGGTTGGTGACATATCATTA
TTCGGAATGACATGACCAACTATTACAAAAGTTTGTACGAACAGTACAATGATAATAGCAAGTATCAATGAAA
TCAAATATTTTACAACTTTTTTCACGATGTCACTCCTTTTTCGACCCCATAAAATGCTGTTCTAACTTTTTTC
CAACAATCCAAAATACAACCAACACAACTAACATTAATATCCCTCTTAAAGGTTGCGTTAAAATTGTAGTAAT
TTCTTTTCCTAAATAACCTAAAATAATTGTTGAAACTAACTTTGACGATGCCAAAACAATGAAATAATATTTA
GGTCTAATATGAGATAGACTCGCTACAAAATTTATTAATGTATTTGGCGTAAAAGGAAAACAAAGTAAAATAA
ACAATGGGATTAATCCTTGGCGATCAATAAAACTAATCAAGCGTTGAACAGCAGTACGTTGTTTAATTCGCTG
CATCCTCTCAGTGTTCACCCATCGTTTACAGATCAAATAGACTGTAAATGTTCCAGAAATTAATCCAAGCCAA
CTAATCAATATACCTAAAATAGGTCCATAAGCTTGAATGTTAATTAAAATATAGAGTGCTAAAGGAAATACTG
GAATTATCGCTCTAATATATAACAATATAAATCCAGGTAAATAACCAAACTGTCGAAATATCTCAAACCATTC
TTCTACTTGATGAAACGACAAATCATCAATCCCTTTCTTTGGCTGAAGATAATTATTCTTACATTATAAAGTT
AATTACTAAGTAAAGAAACATTAACATGTAATCATGCTAATAAATCAGTCTCTAGTAGTATAACAATATTATT
GTATGACAGATGTTATTTTTTCTTTAATTTTCTCTTAACATCTTGTCACATTAGCTAGTAACCTAGTTCTTGC
ATATAAATAAAAACGAGGTTGTCATTACATTTTTACATTTGTAATGACAACCTCGAAAATATGCTTTAAATAG
TTAACGTCTAGTTCTATCTTTTCAAACTTCACTAATCTTTGAAGTAATGTATTTTATAAACGTTCTTCTAATT
CTTTTTTCAAGTCTTCAAAACCAGGTTTACCTAATAATGCGAACATGTTTTGTTTATATGCTTCTACACCAGG
TTGGTTAAATGGATTTACGCCTAATTGGTATCCACTCATTGCACAAGCAAGTTCGAAGAAGTATACGACATAA
CCGAAAGTTTCTTCATCTAATTGTGGAATGTTCACTACCATGTTAGGAACACCACCATCAGTATGTGCTAATA
ATGTACCTTCGAATGCTTTTGTGTTAACTTCGTCGATTGTTTTACCAGCTAAATAATTTAATCCGTCTAGATC
ATCACTATCTTTTTCAATAGTAATATCATATTTAGGATGATTTACTTTTACAACTGTTTCGAATAAGAAACGA
CGTCCTTCTTGTACATATTGACCTAAAGAATGTAAATCAGTTGTGTAGTTGGCACTTGAAGGATAGATACCTT
TGAAGTCTTTACCTTCTGATTCACCAAATAATTGTTTCCACCATTCATTAAAGTATTGCATAGATGGTTCATA
GTTAATCAACATTTCTGTTGTATAACCTTTTGCATATAAAATGTTTCGAATTGTCGCATATTGATATGCAATG
TTTTCTTCTAATTTATCTGAAGATAATTCTTCACGTGCTTTTGCAGCACCAATCATCATAGCTTCGATGTTAA
TTCCAGCTGTTGCAATTGGTAATAATCCTACTGCTGTTAAAAAAGAATATCTTCCACCTACATCATCAGGTAC
GATAAACGTTTCATAACCTTCGTTTGTAGCCAACTGTTTTAAAGCACCTTTTTCTTTATCCGTTGTTGCAAAT
ATACGTTTTTGTGCTTCTTCTTTACCGTATCTTTCTTCAACTAATTGTTTGAACAATCTAAATGCAACTGCTG
GTTCTGTAGTTGTACCAGATTTAGAAATAACGTTTACAGAGAAATCTTTGTCTGCTAAATAATCAACTAACTC
TTTCGTATATGTTGATGACAAGTGATTACCAACAAATACAATTTCAGGGTATTCATTGCTGTTTCTAAATGAT
GACGTTAACATTTCGATTGCTGCACGTGCACCTAAGTAAGAACCACCAATACCGATGACTACTAAAACATCAG
AATTTTCTTTAATGCGTTTTGATGCTTCAACAATTCTTGAAAATTCTTCTTTGTCGTAATCAACTGGTAAATC
AACCCAGCCTAAGAAGTCACTACCTGCACCAGTACCTTCATGAATTGTTTTGTGAATTGATTTAACAATTTCT
TGTTGTTGTTTTAATTCGTGTTCACCGAAAAATTCTAACGTTTTACTAAAATCTAATTGAATATGAGTCATCT
ATAAAAGCCTCCTGTTTCATTTAATATCTTAATTTTACTAAGTTTACGTTTGCGATTCAATTCACAAGTTTGT
ACTTAATCAATAAAATTGATATTTATGTGACGTTTCACACCTATTTTTCAACAAACATTTCAAACTGGCGTCA
AACACTCAAACATTTTTTATACAATTTGCATGTTTTTTAAAATACTTTTAAACATTGATAAATAAGCATTTAT
ACTTACAACTCTAACTATCTCTATCGCATTTTATGAATAAATATGTAAAATAATGGTTTTATTTTCATTAAAA
GTTTGCTATCATGTGTTCATACGAAATATTCAGATAACTACAATGAGGTGAATTTTATGAAAGAGAAAATTGT
TTTAGCATATTCAGGAGGACTAGATACAAGTGTGGCCGTTCAATGGCTTATCGACAAAGGATACGATGTTGTA
GCTTGTTGCCTAGATGTTGGTGAAGGTAAAGATTTAGACATCGTTTATAAAAAAGCTTTAGATATGGGAGCAG
TTGAATGTCATATTATTGATGCAACAAAAGAATTTAGTGATGAGTATGTAAGTTATGCAATCAAAGGAAATTT
AATGTATGAAAATGCATATCCATTAGTTTCAGCTTTATCAAGACCATTAATCGCTAAAAAATTAGTAGAGATT
GCTGAGAAAACAAATTCAGTAGGTATTGCACATGGTTGTACTGGTAAAGGGAATGACCAAGTACGTTTCGAAG
TTGCCATTAAAGCATTAAACCCATCATTGAAAGCATTCGCACCTGTACGTGAGTGGGCATGGAGTCGTGAAGA
AGAAATCGATTATGCAATTAAACATAATATCCCTGTATCAATCAACCATGATTCACCTTATTCTATCGATCAA
AATCTATGGGGCAGAGCGAATGAATGTGGTATTTTAGAAGATCCTTATGCTGCGCCACCAGAGGATGCGTTCG
ATCTAACAAATGCTTTAGAAGAAACACCAGATACTGCTGATGAAATCATTTTAACGTTTGATAAAGGCATCCC
AGTTCAAATTGATGGCAAAACATATGAATTAGACGATTTAATTTTAACGTTGAATGCATTAGCTGGTAAGCAT

GGTATCGGAAGAATTGACCATGTAGAAAATAGACTTGTAGGTATCAAATCAAGAGAAATTTATGAAGCACCTG
CTGCAGAAGTTATTTTAAAAGCGCATAAAGCATTAGAAACGATTACGTTAACGAAAGATGTCGCACACTTTAA
ACCAATCATCGAGAAGCAATTTGCTGAACAACTATACAATGGACTTTGGTTCTCACCTTTAACTGATAGCTTA
AAATTATTTATTGATAGTACTCAGCAATACGTAAGTGGTGATGTCAGAATTAAATTATTCAAAGGTAATACCA
TCGTAAATGGTAGAAAATCACCTTACACATTATATGATGAAAAATTAGCAACTTATACAAAAGAAGATGCATT
TAATCAAGACGCTGCTGTTGGCTTTATCGATATCTATGGTTTACCTACTCAAGTAAATGCGATGTTGCATGGA
GGCTATAGCAATGAGCAATAAAGCTTGGGGCGGTAGATTTGAAGTACAACCTGAAGAGTGGGTTGACGACTTT
AACGCATCCATTACTTTTGATCAAACGCTCATAGATCAAGATATCGAAGGCAGCATTGCACATGCAACTATGC
TTGCGAATCAAGGCATTATTAGTCAACAAGACAGCGAACAAATTATACAAGGACTAAAATCTATTCAACATGA
TTATCATCAAGATCAAATTCAATTTAGTGCATCATTAGAAGATATTCATTTAAATATTGAACATGAATTAATT
AAACGTATTGGTGATGCTGGTGGTAAGTTGCATACTGGACGCAGTAGAAACGATCAAGTTGCAACAGATATGC
ACTTGTACACTAAGAAACAAGTGCAAGATATCATCGCATTAATTAAGTCGTTACAAAGTGTAATTGTAGACAT
CGCTTCCAATAATGTTGATACAATTATGCCTGGTTATACTCATTTACAGCGTGCACAGCCAATTTCATTTGCA
CATAATATTATGACTTATTTTTGGATGTTACAACGAGACCAACAACGATTTGAAGATAGTTTAAAACGAATCG
ATATTAATCCTTTAGGTGCAGCAGCCTTAAGTGGTACCACATACCCTATCGATAGACACGAGACAACAGCATT
GTTGAACTTTGGCAGTCTCTATGAGAATAGCCTAGATGCTGTTAGTGACAGAGACTATATTATTGAAACATTG
CATAATATTTCTTTAACGATGGTTCACTTATCACGCTTTGCAGAGGAAATTATTTTCTGGTCCACAGACGAAG
CTAAATTCATTACATTATCAGATGCATTTTCAACTGGCTCATCTATTATGCCACAAAAGAAAAATCCTGATAT
GGCAGAATTAATTAGAGGTAAAGTTGGTCGAACGACTGGTCATTTAATGAGCATGCTTATGACTTTAAAAGGA
TTACCTCTAGCATATAACAAAGATATGCAGGAAGATAAAGAAGGTTTATTCGATGCTGTCCATACAATTAAAG
GTTCTTTACGTATTTTCGAAGGTATGATTCAAACGATGACAATTAATAAAGAACAACTCAATCAAACTGTTAA
AGAAGATTTTTCAAATGCAACGGAACTAGCAGATTATTTAGTAACTAAAAATATTCCATTTAGAACTGCACAT
GAAATTGTAGGAAAAATTGTCTTAGAATGTATACAACAAGGTCATTATTTATTAGATGTTCCTTTAGCAACAT
ATCAACAACATCATTCTAGTATTGATGCCGATATTTACGATTATTTGCAGCCTGAAAATTGTTTAAAACGACG
TCAAAGTTACGGTTCAACAGGTCAATCATCGGTCAAACAACAACTTGATGTTGCTAAACAATTACTATCACAA
TAAATACGTTAATCTACCTACCCACAATGTCTATTAAAATTACATTGTGGGTATTTTAATGCTCTCTTCGTCT
TGTTGAAAATCACATTTTTAAGATTCCTAAAATGTTTGATAATTCTTTTAAATTTATATTACAGAAATGTTAT
AAATTGTAAAAGAAATGTGTAAAGCGTTTTCACAAGCAGGTTTTTGTAGTATTTTAAAATTGTTAGACTACAA
ATAAAGAGATGAAAGGATAAAGACTATGACTAACTCTTCGAAAAGCTTCACTAAATTTATGGCTGCTTCTGCT
GTTTTTACTATGGGATTTTTATCAGTACCTACTGCTGGCGCTGAACAAACAAATCAAATTGCAAATAAACCTC
AGGCTATTCAATGGCATACAAATTTAACGAATGAGCGATTCACTACTATCGCACATCGTGGCGCAAGTGGCTA
TGCACCCGAGCATACGTTTCAAGCATATGATAAGAGTCATAATGAGTTAAAAGCATCTTATATCGAAATTGAT
TTACAACGTACCAAAGATGGCCATTTAGTTGCTATGCATGATGAAACTGTTAACCGTACAACAAATGGACACG
GTAAAGTTGAGGATTATACCCTTGATGAATTAAAACAGTTAGATGCAGGAAGTTGGTTTAATAAAAAATATCC
AAAATACGCAAGAGCAAGTTATAAAAATGCTAAAGTACCCACTTTAGATGAAATTTTAGAACGTTATGGCCCG
AATGCAAACTATTATATTGAAACAAAGTCACCTGATGTATACCCAGGAATGGAAGAACAATTATTAGCTTCAT
TGAAAAAGCATCACCTTTTAAATAACAATAAATTAAAAAATGGACATGTAATGATTCAATCATTTTCTGACGA
AAGTTTAAAGAAAATTCATCGTCAAAATAAACATGTGCCATTAGTAAAATTAGTTGATAAAGGTGAACTACAA
CAATTTAACGACCAACGCTTAAAAGAGATACGCTCTTATGCGATTGGATTAGGTCCTGATTATACAGATTTAA
CTGAACAAAATACCCATCATTTAAAAGACTTAGGTTTTATAGTACATCCTTATACAGTAAATGAAAAAGCTGA
TATGTTACGATTAAATAAATACGGCGTTGATGGTGTCTTTACAAATTTCGCTGACAAATATAAAGAAGTCATT
AAGTAGTATTGTTAAACTAGAAAACATAAATACAAAAATATAGCTATTACTAAAAAAGACAGCAGTAAGATAT
TTCCGAATAGAAAATATCTTACTGCTGTCTTTTTAATCAACTATATATAGTTGACCGTTTAATTAGAAATTCT
CTGTGTTCGGGGCCCCGGCTATTTTTGAAATAAGCTTGTTACAAGTGTATTTTTATTCAGTCAACTACTGCTA
AAATAAAATTAACGAGCTTAGGGCTTTGTTTTCTGTCCCAAGCTCGTTAAATCACATATGATAATTAATTATG
CCCAACCACGATATCTAGCTGCTTCTGCTGTACGTTTAATACCTATGATATATGCTGCAAGTCTCATATCTAT
TTTTCGATTTTGAGACAATTCGTAAATCGTATCAAATGCCGCTTCTAATTTTTCACGTAGCTTTTCATTAACT
TCTTCTTCAGACCAATAATAACCTTGATTATTTTGTACCCATTCGAAGTAAGAAACCGTTACACCACCAGCAC
TTGCTAATACGTCTGGAACTAATAATATACCACGTTCAGTTAAAATACGTGTTGCTTCTGGTGTTGTAGGTCC
ATTAGCAGCTTCAACAACGATGCTAGCTTTAATATCATGTGCATTGTCTTCTGTAATTTGGTTTGAAATAGCC
GCTGGTACTAAAATGTCACAATCTAATTCAAACAATTCTTTATTTGAGATTGTTTCTTCAAATAAATTTGTTA
CCGTACCAAAACTATCACGACGGTCTAATAAATAATCTATATCTAAGCCATTTGGATCGTGTAATGCACCGTA
AGCATCAGAGATACCTACAATTTTTGCACCTAAATCATATAAGAATTTAGCTAAGAAACTTCCGGCATTACCG
AAACCTTGAATAACAACCTTGGCACCTTCAATTTGCATATTACGACGTTTTGCAGCTTGTTCAATTGCAATAA
CTACACCTAGTGCAGTTGATCTGTCGCGTCCATGAGAACCACCCAATACAATTGGTTTACCTGTGATGAAACC
TGGTGAATTAAATTTATCTAATGCACTATATTCATCCATCATCCAAGCCATAATTTGTGAGTTTGTAAATACA
TCTGGTGCTGGAATATCTTTGTTCGGACCTACGAATTGTGAAATTGCTCTTACATATCCGCGTGATAAACGTT
CAACTTCATGAATGCTCATTTGACGTGGATCACAAACGATACCACCCTTACCACCACCGTATGGTAAGTTTAC

AATGCCACATTTCAAAGTCATCCACATTGATAATGCTTTTACTTCTTCTTCATCAACATCTGGGTGGAAACGC
ACGCCCCCTTTTGTTGGTCCAACAGCATCATTATGTTGCGCACGGTAACCTGTGAATGTTTTAACAGTACCAT
CATCCATTCGTACAGGGATACGCACTTGTAACATTCTTAAAGGTTCTTTAATTAAATCGTACATTCCTTCGTC
AAATCCCAATTTATGCAATGCTTCTTTAATAATTCCTTGAGTAGAAGTTACTAAATTATTGTTCTCAGTCATG
ATCCTTTTCGCCTCTTCTTTACCTAATGATTTCGCTTTCAAACATATTGTAACATAACGTATTCCTTTTTAAA
GCCCTTACAAACTGATTGTTACAACTTTTTGACATTATTGAAATACGTGTCTTATTTTTTCAAGTGCAAGGTC
CAATTCTTCTTTAGTAATAATTAATGGTGGTGCAAAACGAATGACAGTATCATGCGTTTCTTTACATAATAAG
CCTTCTTCTTTCAAAGCTTCACAATATGGTCTAGCACTTTCATTAAGTTCCACACCTATAAACAAACCACGTC
CACGGACTTCTTTAATTGATGGATGATCAATTTGCTTTAATTGTTCTTTAAAATAATCTCCTAATTCTAAAGA
GCGCCCTGGTAAATCCTCATCAACGATAACATCTAATGCAGCAATTGATGCAGCACAAGCAAGTGGATTACCA
CCAAATGTTGAACCATGTGAGCCAGGTGTAAAGACATCTAATACTTCTTTATCTGCTAATACAACAGAAATTG
GGAAGACACCACCACCTAGTGCTTTACCTAAAATATAGACATCAGGTTTTACATTATCCCAATCCGTAGCAAA
TAATTTACCCGAACGACCTAATCCTGCTTGGATTTCGTCAGCAATAAATAAGACATTATGTTCATCACATAAT
TCTCTAATTGCTTTCAAATATCCTTCTGGCGGTATATTTATACCCGCTTCACCTTGAATTGGTTCTACTAAAA
CTGCTGCAGTATTTTCATTAATTGCAGCTTTCAATGCATCTACATCTCCAAAATCAACTTTTCTAAATCCATC
TAATAACGGACCATAACCACGTTGGTATTCTGCTTCTGAAGATAATGAAACTGGCGCCATTGTTCGACCATGG
AAGTTACCATTAAATGCAATGATTTCTGCTTTATTTGGCTCAATTCCTTTAACATCGTATGCCCAGCGTCGTG
CTGCTTTCAAAGCTGTTTCTACTGCTTCAGCACCTGTATTCATTGGTAAAGCTTTATCTTTACCTGCCAGTTT
ACAAATTTTTTCGTACCATTCACCTAAGTTATCACTATGAAAAGCACGTGAAACTAGAGTCACTTTATCAGCT
TGATCTTTTAATGCTTGAATAATTTTCGGATGTCTATGACCTTGGTTAACAGCGGAATATGCAGATAACATAT
CCATATATTTATTGCCTTCAGGATCTTTAACCCATACCCCTTCAGCTTCTGAAATGACAATTGGCAATGGTAA
ATAATTATGTGCTCCGTAATGATTTGTTAACTCAATAATTTTTTCAGATTTAGTCATCATATCTCCCCTTTTC
ATCATTTATAACTATTATACATGAAACATTATCCAAATAATTACATTAGTTTTCAAAGCAGATACTTTTCCAC
CAAAAAAGATGAAATAATCACTAAGTTTCATTAATTTTGTCTATTTTGAAAACCCTTACATTTATAATGACAT
AATTACTTAAATGATTACAAGCAAAAGAATTGATAATTTTACACTTAATCAAAAGTATATTTTACTAAGAATA
TTTTTATTTATAAATATTGAAAACCACTAACAAATTGTATACACAATATCATTAGTGGTAACAGTTAAACACT
TATTTATCTTTACGTGGTAATGGATAGAACCCTTCATTAAATTGATGCCATAAATTAGGAGTTAAGTGATGAT
TATCTTCTCTCTCTGGATCAAAGTAATTAATAATTTCATCTTCGCGTCCATCTTTAATTTTCTCAATAAATTG
ATAATCCAGTAGTAGCTCTCTACCAATAGCTACTAAGTCAACACCAACATTTTCAACTGCATCTAAAGCTTCG
TCAGCTGTGAAAATTGAACCAATACCGATAAGTGGCATACGACCATTTATCCATTTGTGAATTAAAGGCAGTC
TTTCTTGTCCAGCGTATTTACCTTCACGTGTCGTTGCATGCGTATCCATCATTGAAACATGAATATAGTCGAT
TGGCATATGGCTAATTTTATTAACGAGTTCCTCTGTAATTTCCATTGTGATTCCTGGAGACTCCGCTTCTTCC
GGAGACAATCTGTATCCAACTATAAAGTCTTTATTGCCATACGCTTCTTTCGCTTTAAGCACTTCTTCAATCA
CAGCGACCGGGAATTTATATTGATTTGCCCATACATCATTTCTACTATTATAGTATGGTGATACAAATTGATG
AATTAAGTAATGATTCGCACCATGTATTTCAACACCATCAAACCCTGCTTCAATTGCACGTCGCGTTGCTTCA
CCAAAATCCTTGATTGCTTGTTCAATCTCTTCATTCGTCATTTCTCTAGCACTATGTTCTTGTTTCTGACCGA
AACTTTTTAAAGAAATTGGACTTGGTGCTACGACGTCTCCACCAGGTGTTAATTCAGGCAATGCTTGTGCACC
GCCATGATGTATTTGTACGAGTGCTTTGGCACCGTTTTTCTTCATTGCTGTAGCTAATCGTTTTAGTCCTTCA
ATATCACTGTCATGCGCGATTGATGGCTGTCCTGGAAATGCTTTTCCGACATCACTCACATTACTCGCAGCAT
TAATTGTAATACCAACATCTTGTGAACGCTTTTCAATATAAGGAAGTTCTACATCTGAAATAGTACCATCATC
ATTTGAAGAAATATGTGTTAAAGGGGCTAACACAAATCGATTTCTCAACTCTACTCCATTTGGTAATTCTACT
TTATCAAACAATGGTTCGTATTTACTTTTCATTACTTAATTCCTCCTTAAAATTATTGAGTTGACTATAACAT
GTAATTTTTTCATTCTCCAATTATCTGTTTATATAGTTGCAATTCGTTTAATACTTTAAGTTGTAGTTCTCAT
TTCTAAAATAAATTAAAAAAACAAATCCACATACAATATTGCAAATCAACGATTCTATCTATCAAAGATAATG
GATATGTCACCTTAAACCATTTGTACGTGTAAATAGAATCAATCATATTACGTTATCGTAGACAAAAAGAGAT
TAGTATCTAATAAAATTTAATAGATACTAATCTCTTTTATAGTCTAAGAAACTTTCATTTCAGACCCTTTGTA
TGATTTTCGGTACGATTTATCTGTTCCTTTAGTCGTTTCGAATTGCTTGCTTTGACTGTTTTATCCAGATTGG
TAAGCGTTCTTTAAGTGTTTGAAACCCATATTTTTCTGTTTCTCTGATTTCATCTAATACTGATTGTTTTTCC
TTCTTACGCTCATAATTTTTGAAGTAATCATTATCCTTTAATGATAGATTAAGCTTTCCTTCATCATCTATAG
ACAAAATTTTAGCTTTAACAATTTGGCCTTCTGATAGAAATTTCTTCAAATTATGAACGTAGTCATCCATAAT
TTCTGATATATGAATCAGTCCTTCAGTATGATTAGGGGTCTCAACAAACGCACCGTATGGTTGAATACCAGTT
ACACGCACCTTGATATGTTGGCCAATTTTGTAGTTATTCAACTTAATAACCCCTTATTTCGTTTACTTAACAC
TATTATCATAGCACAAATCGAAATGGTGGACTAATTAAAACATCAAATCATACGAATATTATTTTATAAATGA
ATAAGAATACTACAAACTTGGATTTACAAATAAAATCAGCAACAAATTCCTATCTAAATCAATATGATATCCA
GCTACTTTGTTTTCAATTTATTAACTATTTGAATAGCAATTCTCTGTAAAAACATAAAAAGCAACCTCAACTA
TTATCTTTAGAATACACATCTATTTAAAAACTGTGCTTTCTTGATAAATATATTGAGATTGCTCATCATGTTA
GTTTATTTGTTTTCGTTGGAATATATACTAATCCATTCATAAATTAGCTTTATCCGAGTTTAAAATGTTGGTA
GTTAATTATGTTTAGATTTTATTCTTCAACATCAATAGATTCAATTACAACATCATGAAGTGGTTTATCTTGT

GGTCCCACTTTTGTATTTGCAATATCTTCTAATGTAGTTTCACCATCAATGATTTGACCGAATACAGTATGTT
TTTGATCTAACCATGGTGTACCACCCTTCTCGCCATATGCATCAACGATTGGTTGTGGCCAACCACCATCTGC
AAGTTGACTTAACATATTTTGAGGTACTTCTTTCATTTGAACAATGAAAAATTGTGAACCATTTGTATTAGGT
CCTGAGTTAGCCATTGATAATGCGCCATATAAGTTAAATGCATTTAATGAAAATTCATCTTCAAAAGCACCGC
CATAAATACTTTCGCCACCCATACCAGTAGCTGTTGGATCGCCACCTTGAATCATGAAGTCATTAATGACACG
GTGGAATGTTATTCCATCATAATAACCATTTTTTGCATGTGTCACAAAATTTTCAACTGTTTTTGGTGCAATA
TTTGGAAATAATTTGAATGTCATGTCACCTTTATTTGTGTGCATAACCACTTTAATTTCACCTTGTTGTACTT
CTTTGTTTAACTGTGGATAGTTAGCCATTTATATTCTCCATTCATGTTATGATACATATATATTAACATACTT
TTAAGGGAGGAAAATAGCATGAAGTTATATCGATTTTCACACAAAACAGGTAGCTATGGTGTCACGATTGTTG
AAGAAAATGGAGACGATGTCCTTGTTAAAGTTGAACAAGTTATCAAACACCCGAAACAAGGTGATTTACATAA
CCCTACTGAAACTGAAGGTGTATTTTTCCATGAACGCAAAGCATTAAGCCATTTTGAAAAACGCTATACGAAA
CGTTCACAATTACGCGACTTCAATGTTGATGTATTGCGCTATGAAGATTCACTTCAACAAGCAATTACAGATT
TAGAAACTAAACTTAAAGCTCAGCAAACAAAACATGCTGAAATGTCTTTAGCTAGTTTAGCACGCTTAAAAGA
AGATTATTCACTTCAATACAAAGAAAATTTTAAATAAAAAATGACGTGTTTCTTCTTATTTAAATACTTCTGA
AAATAAAGCACAACAAGGAGTGTGTACGTTTTTCAGAACTATATCATTCATGTATAATGAATAACATAAATAT
TTTTTAAAGGAGGACAACATAGTGAGTTTGTTACATATTGCGGTGATATTACCGTTAATATTCGCACTCATTA
TTCCTATCCTGTATAGATTTTTTAAACGTATACATTTAGGATGGTTCGTATTACCTGTCCCTATCGTCATATT
TATTTATATGCTTACATTAATTAAAACGACGATGTCAGGTAATACTGTAATGAAAACCTTAAATTGGATGCCT
CATTTTGGTATGAACTTTGATTTATATCTTGATGGCTTAGGTTTACTCTTTAGTTTGCTTATTTCAGGAATCG
GCAGTTTAGTAGTATTATATTCAATCGGCTACTTAAGTAAGTCAGAACAACTAGGGAATTTTTATTGTTATTT
ATTACTATTTATGGGTGCAATGTTAGGTGTCGTACTTTCAGATAATGTTATTATTCTATATTTATTCTGGGAG
CTAACGTCATTTTCTAGTTTTTTACTTATTTCATTCTGGCGCGAACGACAAGCATCTATATATGGTGCTCAAA
AATCGCTCATAATCACAGTATTTGGTGGTCTATCATTATTAGGTGGTATCATCTTACTCGCTATACCAACACA
ATCATTTAGCATTCAGTACATGATTCAACATGCTTCTGAAATTCAAAATAGCCCATTCTTCATTTTTGCGATG
ATTTTAATCATGATTGGTGCTTTTACAAAATCTGCTCAGTTTCCATTTTATATTTGGCTACCAGATGCAATGG
AAGCACCTACACCAGTGAGTGCTTATCTTCATTCAGCAACAATGGTTAAAGCAGGGTTATATTTAATCGCTCG
AATGACACCAATCTTCGCAGCATCGCAAGGTTGGATATGGACTGTCACACTCGTTGGTTTAATTACGTTATTC
TGGGCTTCGCTAAACGCAACAAAACAACAAGACTTGAAAGGTATACTGGCATTTTCAACAGTATCCCAATTAG
GTATGATTATGGCGATGCTTGGAATCGGTGCCATTAGTTATCACTATCAAGGCGATGATAGCAAAATATATGC
TGCTGCATTTACGGCTGCAATATTCCATTTAATCAACCATGCAACATTTAAAGGTGCCCTATTTATGATTACA
GGTGCAGTGGATCATTCTACTGGTACACGTGATGTTAAGAAATTAGGTGGCTTACTAACAATTATGCCTATTT
CATTTACAATCACTGTTATTACAGCATTAAGTATGGCTGGTGTACCACCGTTCAATGGTTTCTTATCAAAAGA
ATCATTTTTAGAAACAACATTTACAGCAAGCCAAGCAAATTTATTCAGTGTTGATACATTAGGCTACCTATTC
CCAATCATTGGTATAGTGGGCAGTGTCTTCACATTTGTATATTCAATTAAATTTATAATGCATATATTTTTCG
GACAATATAAACCTGAACAATTACCTAAAAAAGCACACGAAGTATCAATATTAATGCTTTTATCACCAGCTAT
TTTAGCTACATTAGTAATTGTATTCGGTTTATTCCCTGGCATTTTAACTAATTCGATTATTGAACCTGCTACA
TCTTCGATTAATCATACGGTAATTGATGATGTTGAATTCCATATGTTCCATGGATTAACGCCTGCCTTTTTAT
CAACATTAGTGATTTATATTTTAGGTATCTTACTAATTGTGACATTTAGTTATTGGGTGAAATTGTTGCAACG
ACAACCTGGTAAACTAACATTCAATTATTGGTACAACAGAAGTGCAAATGTTATTCCAAACTACTCTGAAAAG
ATGACGAATAGCTATGTTACCGATTATTCACGTAATAATTTAGTTATCATTTTTGGTACATTAATTTTATTAA
CATTTGTCACTATATTCTCAGTACCTTTCAACATTAATTTTAAAGACGTTAGTCCGATTAGAATTTTCGAAGT
TTGTATCGTAATTTTATTATTAAGTGCTGCCTTTTTAATTCTTTTCGCTAAATCAAGATTATTTAGCATTATT
ATGTTAAGTGCAGTTGGATATGCTGTGTCTGTATTGTTTATATTCTTTAAAGCACCCGACTTAGCATTGACAC
AGTTTGTCGTTGAATCTATTTCAACAGCATTGTTCTTATTATGCTTCTACCACTTACCAAATTTAAATCGTTA
TAATGAAAAGCGCTCATTCCAGCTTACTAATGCTTTAATTGCTGGTGGAGTTGGTTTGTCCGTCATTATTATC
GGTTTAATTGCATACGGAAATAGACATTTTGAATCGATATCTAAGTTTTATCAAGAGCACGTTTATGATTTAG
CTCACGGTAAAAACATGGTAAACGTCATACTTGTTGATTTCCGTGGTATGGATACTTTATTCGAGTCATCTGT
ACTTGGTATCGCAGGATTAGCTGTATATACAATGATTAAATTACGTAAAAAGCGTCAAACGCAAGGTAACGAG
GTGAAAAATCATGAATAGACAACAAAATGATCTAATTTTGCAATTTGCTGCAGTTATTATCTTTTTCATGGTA
ATGGTCTTTGGTTTTTCACTTTTCTTAGCAGGACATTATACACCTGGTGGCGGTTTCGTCGGCGGGTTATTAT
TCGCAAGTTCACTTGTTATCATTACGATTGCATTTGATATAGAAACAATGCGTAAAATTTTCCCGTTAGATTT
TAAAATCTTGATAGGTATCGGATTAGTTTTTTGTATTGCGACGCCTATCGCAAGTTGGTTTTTAGGCAAAAAT
TTCTTTACGCATGTCACATTTGATATACCGTTATTTATATTAGAACCTGTTCATATGACAACAGCAGTATTCT
TTGATTTCGGTGTTTTATGTGCAGTAGTTGGTACTGTCATGACAATTATTATTTCGATTGGAGAGAACGAATA
GTGGAAATTATTATGATTTTTGTTAGTGGTATTCTCACAGCAATTAGTGTCTATCTCGTTTTGTCTAAAAGTC
TGATACGAATTGTTATGGGAACTACACTATTAACACATGCAGCAAATTTATTTTTAATAACTATGGGCGGACT
TAAACATGGTACTGTTCCAATTTATGAAGCGAACGTAAAAAGCTATGTTGATCCTATCCCGCAAGCACTTATT
TTAACAGCAATCGTTATCGCCTTTGCGACAACAGCCTTTTTCTTAGTATTAGCATTTAGAACATATAAAGAAT

TAGGCACAGATAACGTTGAGAGTATGAAAGGAGTTCCAGAAGATGATTGAAAGTAATATGCTTGTGTTAACGC
TAGTTATTCCAGTAATTACAGCCATATTACTTGTCTTTATTGGAAAGCGTCCTATTATAAAGCGTTATGTCGC
ACTAGGCGGTACATTATTAACCTTGGTCGCGGCAATTATCAATTTAGCAAATGTTGTTAAACATGGACCAATA
CGCGTAGAGCTAGGTTCATGGAAAGCACCATACAGTATTGTCTTTGTATTAGACATATTCAGTGCCCTACTAA
TAATTACTAGTATTATTATTACAGCTATTGTGATTTTATATTCATACCAAACAATTGGTATTGAACGTGAAAG
ATATTATTATTATTTTTCAGTACTATTTATGTTAATAGGTATCATTGGTGCATTCACAACTGGTGACATTTTC
AACCTATTCGTATTTTTCGAAGTATTTTTAATGTCATCTTATTTCTTATTAGTAATTGGTTCTACAAAAATTC
AACTACAGGAAACAATAAAATATGTTCTTGTTAACGTTGTATCGTCATCCTTCTTCGTAATGGGTGTAGCGAT
TTTATATTCAGTAGTAGGAACATTGAATCTAGCAGATATAAGCAATAAATTAGCTAACCTTTCAGCGCATGAC
AGTGGCTTAGTCAATATCGTTTTCATACTATTTATCTTTGTTTTTGCTACAAAAGCTGGCGTATTTCCAATGT
TTGTTTGGTTACCTAGTGCATACTATGCACCACCAATTCCAATCATTGCATTCTTTGGTGCTTTGTTAACAAA
AGTTGGTGTTTATGCAATCGCTAGAACATTAAGCCTATTCTTTAGTGATAATGTAAGCTTTTCACATTATGTC
ATTTTATTCTTAGCACTATTAACGATCATCTTTGGTTGTGTCGGTGCTGTTGCATATGCCAACATTAAAAAAA
TCATCCTATACAATGTCATGATTGCTGTAGGTGTGATCTTAGTCGGCGTTGCAATGATGACAGAGTCAGGTAT
GATTGGGGCTATTTACTATACATTGCACGACATGTTAGTAAAACTTGCATTATTCTTGCTTATCGGAATTATG
ATTAAAATTACAGGCACTGCTGATTTAAGACAATTTGGTGGCTTAATTAAACGATATCCTGTACTTGGTTGGT
CATTCTTTATCGCAGCATTAAGTTTAGCTGGCATTCCCCCACTCAGTGGCTTTTATGGAAAATTTTTCATTGT
CCAATCAACATTTGAAAGAGGATTTTATTTAAGTGGTGTCATCGTCTTATTATCAAGTTTAGTCGTATTATAT
TCTGTCATTCGCATATTCCTACAAGGCTTTTTCGGTCAACCAAAAGGATATGATCTTAATAACAAAGTCGACG
TCAAATATTTAACTACGATTGCAATCGTTGCAGTTGTGATAACTGTTTTATATGGATTGTCAGCAGATTACTT
ATATCCAATGGTAAAAGCTGGTGCAGAAACATTCTACAATCCATCAACCTACGTTAAAGCTGTATTAGGAGGT
AAATAACATGGCTGTACAATTAGTATTAAACTTTATTATCGCAGTGTTTTGGTTGTTTGTAACAAATAGTTAC
ACAACAAATAACTTTGTGTTAGGTTTCATCTTTGGCCTAGTTCTTGTTTACCTCTTACACAGAGTGTTACCAG
GTAGATTTTATGTCATCACGCTATATCGTATTATTAAATTAGTTATTATTTTCTTAATCGAACTGATTAAAGC
TAATTTTGATGTTTTGAAAATTATTATTAAACCTTCGATTAAAAATGAACCAGGATTCTTTGTTTATCACACA
GATTTAAAAAAAGATTGGCAAATTGTCTTGCTGTCTAATTTAATTACATTAACACCTGGTACTGTAGTGCTTG
GTGTAAGTGACGATCGTACAAAAATTTATATTCATGCCATAGATTTTTCAACTAAAGAGCAAGAAGTAGAAAG
TATTAAAACATCATTAGAGAAAATTGTAAGAGAGGTGGGCGAAATATAATGAATCATAATGTTATTATCGTTA
TTGCATTAATCATAGTTGTCATTTCTATGTTAGCTATGCTCATTCGCGTTGTGCTAGGCCCATCACTTGCCGA
TCGTGTTGTCGCATTAGATGCGATTGGTCTTCAATTAATGGCAGTTATAGCATTATTCAGTATTTTATTAAAT
ATTAAATACATGATTGTCGTTATTATGATGATTGGTATATTAGCTTTTTTAGGTACTGCAGTATTCTCTAAAT
TTATGGACAAAGGTAAGGTGATTGAACATGATCAAAATCATACTGATTAGTCTTGCACTTATCTTTGTTATCA
TCGGTGCTTTAATTAGCGCCCTAGCAGCTATAGGATTATTGAGACTTGAAGATGTATATTCACGTGCACATGC
TGCCGGAAAAGCATCAACATTAGGTGCAATGTCATTACTATTTGGTACGTTTTTATATTTTATTGCTACTCAA
GGTTTTGTAAATATGCAATTAATCGTTGCGATTATCTTTGTATTAATTACAGGTCCTCTTTCAAGTCATATGA
TTATGAAAGCAGCATATAATATTAAAACGCCTTATACTAAAAAGACTAAAGTCGATGAAATTTCGGAAGACTT
AAAAGACACAAAATTGTAGATTATATAACAAAATCATGAATATAAATCAACAACAAACAGCAGTAAGATGATT
CCAAATTAGGAATGATTTTACTGCTGTTTTCTTTTGACATTGTTACCTCTTTTTCAATGGTTTTTTCTTTGAC
TACAGCTTCGCCCTATCTACATATATCTCTTTAATTTAATTGCCTTTCATGTCGTTATGTATTATGATAATAA
TAATTATAAATCGTAACGATTACGCTTTAAAAAGAGAGAGGTTTTATTATGCATTGGACAATTATCGGCGGTG
GCATACAGGGAACTGCAATCGCACAAAAACTATTATCAAGCGGATTAACAACAGACCGATTAACAATCATTGA
CCCACACGAAACTTTTTGCCAAAGGTTTAACTCATTTACAAATCGAATAGAAATGCCTTATTTAAGATCACCG
ATTGTACATCACGTACATCCACAACCGTTCCATCTAAAACAATTCGCTAAACAGCACCAATATACAAATGCTT
TTTATGGTCCTTATCAACGACCTGAATTGACAATGTTTATGGATCATATTGCACATGCTTCTAAACAATATCA
ATTAGAGGATTGCTTGGTTCAAGGTTTAGTTCAAACTTTAGATAAACAAGAAGACAAATGGCATATCAAGTTA
GAAGATGGACAAATTATCACTACAGATTGCGTCGTTATTGCAATAGGTAGTACAAATATTCCGTTTATGCCTG
ACATTTTAAAAGACAAACAGAATGTAAATCATATCTTCGAGAAAGAACATGATCAAGTAGTATATGATAAGAC
CGATCATATCGTTGGTAGTGGCATTACGGCTGCACATCTTGCACTTAAATTGTTAAATCATGATAACGATAAA
AAGATTCATTTATGGCTAAATAAAGATATTGAAATACATGACTTTGATGCTGATCCTGGTTGGTTAGGTCCGA
AGAATATGTCTACATTTTTAAGTACTAAAAGCATGCCTGAAAGAAATGCCATTGTACAACGTGAACGTCATAA
AGGATCAATGCCTCACGAACTGTACTTACGCCTTAAAAAACATATTAAAAATGGTCGTATAAATGTGCATAAA
ACACCTATCACTCAAATTAGTGATGGTTTAATTAACACTGAAAATGATTCTGTTCCATATCAACAGATTATGG
TTGCAACTGGTTTTGAACAAGATTTTATGTCACAACCACTTATTAAGCAATTAATACAAAATTATGATGCACC
TATTAACGAATGTAATTACCCTGTTATTTCCGAAAAATTAGAATGGATACCAAATCTATTTGTCGCAGGATGC
TTTGCAGACTTAGAATTAGGACCATTTGGTAGAAATGTTATGGGTGGCCGTAAAGCTGCCGAACGCATTGAAC
AAGCATTTCTAAAACTACAACAATATAGCGCATAAAAAAACTGCTAGCCGTAGTTATGAATATCTCATAACAT
TTCAGCTAGCAGTTCTTTTATTTTAAAGGTTTAATAGCAACTGTACCACGCATAACTGTAATTAATTGTTCTT
TGTCATTCTTAATTTTTATATCCCATACATGTGTCGACTTACCTTGATGAATAATTTCAGCTGTCGCAGTAAC

ACGACCATCTTTAGCAGAATGAATATGATTGGCATTCATCTCTAAACCTAATGGAATAAATTTGGTTGTATCA
ATTAGATTAGCAGATCCTAATGAACATGCTGTTTCACCTAAAGCAATCGAAGCACCACCATGTAAATATCCAA
ATGGTTGTTTTACTTTATCAGTAACAGGCATAGAAATAACAACTAAACCATCTTCCTGGTGATCTATTGACAT
CTCAAATGTCTCTAATAAATGCGTCATATTTTCACCTCTTTATGCTTATAGTACCATAGCAGCAATAGTACCG
AAAATCATTAAAGGAATATTATAAAACAAGAAGTTTGGTACACATGTATCGCGTATATGATCATGTTGGCCAT
CAACATTTAATCCCGCAGTTGGTCCTAATGTTGAATCACTTGCAGGTGAACCTGAGTCACCTAATGCACTCGC
TGTTCCAATCAATGCGATTAATGCCATTGTATCTAGTCCAATTGACGCTCCAAAAGGAATGAATAATGATGCG
ATAATAGGAATGGTTGCAAATGATGATCCAATACCTAAAGTGACAATTAAACCTATCACATACATCACGATAA
TGCTAAATAATTTATTATCACCAGTAATACTTGTTAAAGTTTTAACTAATTCATCTATATCACCAGTAGCATT
CATTACACCAGCAAATCCATTTGCTGTTAAAATAACTACACCAATATAAGCCATAATTTTAATACCTTCAACA
AACTTAGCATCTAATTCATACCAATTATATGCACGTGAAATAAAGAATACGAGTACCCCGGCCAGTGCACCAA
AAATCATTGAATCTGTAAATGTTTGTACTAAAAATGTAGCTAGTATTGCTACAATTGTTACTATTAAGATATA
TGGTTTTAACTCTGTAACATTGTCACTATCTGAAATTTTACGTGTTTCATATTCACGTGGTTTACGATATACA
TATAAACCGATAAGTAAGCCAACAATATACCCCATTGAAGGAATAAGCATTGCTTTCCAAATCATATTAAACT
CAATTGGGTGATTCGCCTTTGCAAAGCCACTTTGAATAATTTGCTGGAAAATTTGACCGAATCCATAAGGTAA
TAACACATACGGGAAACATAAACCAAAACCGATAATCAAACCGATTAAACGTCTATCTATTTTTAAGTCATTA
AACAGACTTAACAATGGTGGGATGACAATTGGAATGAATGCAATATGTACAGGAATTAAGTTTTGACTCATGA
TACTCATAGCTAATAATGCAATAATTATTGTCACTTTTACTTTAACTCTTGACCATCGACTATTTTCAGCGTG
TATTGCATTTATAATTTTTCCTACTAAATAGTCTGTGATACCACTGTATGAAATTAATGCTGCAAATCCACCT
AATAAGGCATAGCTTAATGCTACCTCAGCACCATCGACTATATTTTTTCCGAATACATTAATTACTTTTTCAA
TGCTCATACCTGATACTACGCCACCAACTAGCGCACTGATAAATAAGCTTATAACTACGTTTAATCGACATAA
ACATAGCACAATCATTAAAATTACTGCTATTACTACTGCATTTATCATATGACAACCATCCTTTATCACTCTA
TCGAACTAAAGCGTTTATAGACTACATAGTATCAGTAGCTTTAACACCTGTCAATAGCAAACTATACCAATAA
TCTTTATTTTTTAGTTAATTGTCTTTCTTAAAATAATTTTATCTTTCATTAAATTAAACAATTTTACAAGCTT
GGAACACCAATCAAAATACTAAGTTCTAAAATGTAATATTAGTAGTCGTTGACTGAATGAACATATGCTTATA
ATATTTTTTTGCAATGCTAGTCAAGTTGATTTATGCTCACAAGGATATGCGATTTATATTTTCTTACAACAAT
GAAAATGCCTGATACAATGCGATCCTTTATACTTTCAAGAGTTAAATTAGAAATGTATATCAAGACACAAGTT
GTATCAGGCAAATAATTAAATTATTGTTGTTTTAACCATTGTACTATCGTCGGAATCATAAATCCTGTTGGCC
CTTTTGGACCATTATATGAAGCTTTATTAGTCGTTGCTGGACCTGCAATATCGAAATGAATGTGAGGTGTTTG
ACCACTAAAATGTGTTACAAAACTTGCCGCAAATAGCGCTTTACCTTGTCCATTCGTATGGTTAACTAAATCA
GCGATATCACTGTGTTTAATACTTGCACGTTCGGTTGCAGTAATCGGTAATTCAAATACCATTTCATCGACTT
TAGAACTTATTTGTAATATATCGTTTAATATCACTTTACTATTCGATTCAAATGCAGCAGCTTTATCATCGCC
TAGTGCAACAATTGCTGCACCCGTTAATGTAGCAAAGTCCATAATCACACTAGGCTGATATTGATTTGCATAA
TACACAGCATCTGCAAGGACTAATCTACCTTCAGCGTCTGTATTCATTACTTCTACAGTTTCACCACTTAATG
CTGTAAATACATCATCTGGCTTCATTGATGCTTCATTTATCATATTTTCAGCACAAGCAAGCACTCCGACAAT
ATTTACAGGCAGTTGTAAACGACTAGCCGCTTCAATGATACCAACGACATTCGCAGCGCCACACATGTCAAAC
TTCATTGTAGCCATGCCATTCTTCGTTTTAATACTATAACCACCAGAATCATACGTTATACCTTTACCAACTA
AGGCAATTGGTGCTTCATCTTTGTCTTTGCCATTATATGTGATGGTTACTAATCTCGGTTTATGCTTACTACC
TTTACCTACTGCTTGTAAAAGTCCGAATCCTTCAGAAACTAAAGTGTCATAATCTTTAACATCTACTTTGACC
TTTGTATTTTTAAAATGATTAACAATATCTTCTGCAAATGTTTGTGGTGTTAATACATTCGGTGGCATATTAC
TAAAGTCTCTTGCCAAATTAATGGATTGGCCAATACTGATACCCTCATGAATGAAATCTAATTCAATTAATGA
TTCACTAATAAGGTTTAAATTCGTCTTAAACGGTGCCTTCTTACTTGATTTATAATGATCGAATTCATATGTT
GCACGCTCACTTTGAATACCGCATGCCATTAATACATCTGATAACTGATCATATTTTGAAATAAATGAATCCA
TAAGTAAATAAGTATCTTCAATGTGTTCTGACTTTATGTATTGGAAAAGATGTCCCCATATTTTCAACATATC
TTGATAACTACGTGCTTTTAAGTTTCCTAAACCAACTGTAATTAAACGATATGTTTGATCTTGTACATCAAAT
GCAGTTGTATAAATCTTCCCAACTTTACTACCAATAATATGTTGATGTTTTAGTCTTTCAAGTGATTCTGTAA
TATCGATATGATTAAAACTAATGCGCTCTAACTGATTTAAATGTTCTGGTATACCAATAATCAATGTATTTAT
TTCGTTGCTTAGTGTGTTATTTAATTTAAAATTCATAATGTACCTCCTTAGATTTGATATGTTACTCACTTCA
AATTGTACAACAATAAAGCCCTTCAGTGACACTGAAGGACTTTATAAATGAATAATTTAATTTTATGTGTTTA
GCTGAAACTTTATTTAAAGCCTAGAATTTACCTTTTTTGAATGCTAAACCGATACCACCGATTTTGAATACCG
CACGTGTATCAATCACTTTTTTCATGAATGCTGCTTTTTTACCAGCGATAGGTTTACCAAATACCATACCTAC
ACCGTCATGTGAACCTAAAGAACAAACAGTTCCACGATCAACGTATTCGAATTCTTCAGTTGATTCACCGTTT
AAGATGCGTTTAATGTTTTTAGCAACACTTTCACCTTGTTGCATTGCAATTTGTGCTGTAGTTGGTAATGGAC
GTTCTTCTCCAGCTGGGATAAACGCTGAACAGTCACCAATAACAAAAATGTTGTCGTAACCATTGATTGTTAA
ATCTTGCTTTGTAACGATACGTCCACGTTTAACGCCTTCAAATGATTCTTCCATTAATTTACTACCACGTACA
CCAGCTGCCCATACTGAAGTACCTGCATTTAATTGTTGTTTTTCACCATCTACTTCAACTACAAAACCTTTTT
CGTTACAAGCAACGATTGGTGTAGCAATTTTAAATTCAACACCGCGGTCTTCTAAGTAGCTAACTGCGTGGTT
AACTAATTCTTCTGAGAACATTGGTAACATTTTAGGTGCTGCTTCAACACAAGTGATTTTAACTTTATTTTGA

TCCACACCATATTTGCTACATAATTCAGGAATTCTGTCTGTTAATTCACCTAAGAATTCAACACCAGTGAATC
CAGCACCACCAACTAAGATAGATAAATCGTTATCATCTTTTTCTTTTGATGCTGCATAGTTAGCAAATTTGTC
TTCGATATGACGTGATAATTCACGTGCTGTGATAACATTTTCAATTTGGAAAGCATGATCTTTCATACCTTCG
ATGCCGAATGTTTCACTAACGAAACCTAATGCTACTACTAAAATATCAAAGTCATAAATACCTTGATTTGTTT
CTACCTTTTTAGCATCACGGTCAATTTTTGTTACTTCTGCTTGAACAAAGTTCACTTTGTCTTTCTTCAAGAC
ACTTTCCACAGGATATAATACATCTTCATAGTTTAGTGTACCTGCTGATGCTTCATGTAACCATGTTGCTTCA
TAGTGATATTCATTTTTATTAATAAGCGTAATTTCTGCTTCTTCTGTTGATATCGCTTTTTGCAATTTAGTTA
CAGTTTGTAAACCTGCATAACCAGCACCAAGTACAAGTACTTTTTTACGATCTTGAGCCATTTAATTTCACCT
AAGCTTTCATATTTTTTTAACCAAATGCTGATAATTACTAGTAACAACTGGTCATTACTACTTTTACCACAAT
GTATACTAGTTTATCATCATTTTGTTATTTAAGTCTTTTAAATTATGTAAAAACCTGTGATGTTCACAAAAAA
GACATTTTTAATCCAATACTAATTCTATAGCTTTTATGTTATATTTTCAAGCAAGATTCAGTCAGATTGTGAA
AAAATTAATTAATTTTTTCGTAGCCTGATTTCGATTAAAAATGTATCCGTTATCACGATCATTTAAAATACTA
TTTATAGTCAGATTCTTAATTTTCAATAAGCTTTTAATAAAACAAAATATCTTTACGCAAAAATAGGACATA
ATCAAATATGTCCCTAATCATAATTGATATCAATTTGTATCCCAAAAATTGGGTTAACCATTAAAAAGTTATT
TTATTAGCAGTTTTCAGGATTACCTGCAACTTTTGCAGTTCTAAATGAACTACCACAGCCACATGAAGCAATT
GCATTAGGATTGTCGATTTGGAAACCGCCACCCATTAATGATTGCTTAAAATCAATAGTCGTACCATTTAATA
CGGGTGCATCTTTTTTTGTCTACTAAAACTTTTAATCCAAAGTATTCTAAGATTTCATCATTTTCACCAGGCGC
TTCTTCTGCACCCATACCGTATGTTAAACCAGTGCACCCGCCACCATTCACTTTAATTTTTAAATAGCCATCT
GGCATTTCATTTGCTTTAAGCATATCTTTTACTTCGTAAGCAGCTGCTTCTGTTAATATAACTGTTGGCATGA
TAACTCCTCCTTAAAAAATCCAAGTTTCTTTTATATGTGCATATATATTTTGTAATAATTCTTCCGGCGAATC
ACCTTCAACAATATCACCATTTACTAAAGCATACAACCCGGCTGAACATATACCACAATGTGTCAGGCAACCA
TACTCTAACACATCGACATCTGGGTCATTTTCCAGTTGATTAAAAACATAATCTCCACCTTTTGCCATGTTAG
AGAGACAAAATTCTACGATCGGATTCATACTTCACCTTCTTATTTCATTTGTTACAATATTATAGCATTTTAA
AACTGGTATTTTAACATGATGTGCTCAATTACAACAACTGATGTTTCTTATCCCAGTTATGTAATAGTGCCTT
AGTTAGTACAAATATTTATTTGTAGTCATTTTCAAAAAAATATATAATAAGCATATAAATACATATTTACTTT
TTTAGGGGTTATTGATGAATCAAAGACACTTGCATTTTATAACTGTTCATTAACAAATATATCTATGCACTTT
GATCTTTTCAAAATTAAAGTAAATATTAACATATAAAAGGGGTACTTCATTATGAAAAACTTAGTTTTGTTAG
GCGGCGGATATGGTAATATGCGTATCATGTCACGCATTTTAACTACTTCTTTACCACAAGATTATACAGTCAC
ATTAGTTGATCGTATGCCATTTCATGGATTGAAACCAGAATTTTATGCTTTAGCTGCGGGCACGAAATCAGAT
AAAGATGTTCGCATGAAATTCCCTAATCATCCACAAGTGAATACAGTTTATGGTGAAATTAACGACATAGATT
TAGATGCTCAAATTGTCTCAGTCGGTAACTCTAAAATTGATTATGATGAGCTAATCATTGGTTTAGGATGTGA
AGATAAATATCATAACGTTCCAGGAGCCGAAGAATATACACATAGTATTCAAACACTCTCAAAGGCTCGGGAT
ACTTTCCATAGTATTAGTGAACTACCAGAAGGTGCTAAAGTTGGTATCGTTGGTGCTGGATTAAGCGGCATAG
AACTTGCCAGCGAATTAAGAGAAAGTAGATCAGACTTGGAAATATATCTTTATGACCGTGGGCCGCGAATTTT
AAGAAATTTTCCAGAAAAAATTAAGTAAGTATGTTGCGAAATGGTTCGCCAAAAAATAATGTTACCGTTGTTCCA
AATTCAAATATTAATAAAGTTGAACCTGGTAAAATATATAACTGTGATGAACCTAAAGATATTGATTTAGTTG
TATGGACAGCAGGAATTCAACCTGTTGAAGTTGTTCGTAACTTGCCGATTGATATAAATAGTAATGGACGCGT
GATAGTTAACCAGTATCATCAAGTACCAACATATCGTAACGTCTATGTAGTTGGTGATTGTGCTGATTTACCA
CATGCGCCAAGTGCTCAGTTAGCCGAAGTTCAAGGTGATCAAATTGCCGATGTGCTTAAAAAGCAATGGCTAA
ATGAACCATTACCTGACAAAATGCCGGAACTAAAGGTACAAGGTATCGTTGGGTCATTAGGAGATAAACAAGG
TTTTGCATATATCATGGATCGCACTGTAACAGGACGACTCGCTTCTATTTTAAAATCCGGCGTACTTTGGTTA
TATAAATATCATAATGGTTAAAATTAAAAAGCAATGTCAGTTCACACTATTACTGACATTGCTTTTTGTTATG
TGTATATTATTCATTCACAAGCTTTTGGTCTATAAATCGAGTGATTTGCTTTGTTTGTATATAACCATCTGCT
ACATATTCATCGTTCATTGTAATTAATGGATAAAATAGTTCATCTTGTTCTATTCTTTCAATAAATTGTAAAT
CATGATCTGTTAAGTTGTCATTATCTTTTGTAATATCTATATACGTATATTTAAATGATATATTTGGGTATTT
TCTTTTTAATAGCGGCTGTAGCCAGTCATAAATATCTTTCGATGTTGGCGCATTAACGCAACTTGCACATATA
ACATCTGCCCCATATACTACCACACTCACGTGCTCCATATATTTATCCCCCATTGGTTTGATAGATTTTTATT
ACACTATCTATTATAATATACTAATAAAGATTATCAAATTCATCTCTCGAAAGGAGACTTGCCTGATGCCTAC
TGAAGATACAACGATGTTTGATCAAGTAGCAGAAGTTATTGAACGTCTTCGCCCATTTTTATTACGTGATGGT
GGCGACTGCTCATTGATTGACGTGGAAGACGGTATTGTTAAATTACAATTACATGGTGCATGTGGTACATGCC
CAAGTTCTACAATCACTCTTAAAGCTGGTATTGAGCGTGCATTACACGAAGAAGTGCCTGGTGTAATAGAAGT
AGAACAAGTATTCTAATATAAAGATATTAAATTAACAGAACATATTATGAATAACAACAAAGACAGGAATACT
GAATTATTTATAATCAGTTATTACCTGTCTTTTTTCGCTGTCAAATTGGACTGATGGGAAAGTTATTTTATAC
TATTTATCTGATTAATTATTTTATTATTGCCACATATCCAAGCGCATATTACTATTCTATGAAATTTAAAATA
TAAAAATAGCACATACGCTCAATCAAAATCGTTGAGTTATGTGCTATTTGTATTTTAATTTTTAGGTTTATCT
ACTTCAGGTTGTGGTTCACCTTTCATATGTTTCGCAATTTGCTCATCCATATAAACCCAACCTTTCCAACCGA
TGTGTACGGCATCACTGATAACATATTTTTCATAATCTTTATCAGTCATATCGTAAATTTTACCACCATTATC
TACAACAGTAGAATGGATTTTTTTATACACTGCTTGACGACGTTCTTTATCGATACCTATGTGGTCATACCAT

ACACCGTTTGATGGAATACTTACATATTGAACATCTGCACCAGCAGCACGCATTGTTTTTACAAGTAATTCTA
AATCTTGGAATTCTGGAGAATTAACATTGAATTCGTAGTCACGTCTAACTTTACGCTTACTTTCTTGAATTAA
TTTCCAGTATTGATCTCTAATACCAAATTTATTCGATGTAGTATCAGCTTTACCAATTTCAACTGCTTTTTGT
TTCATCTCATCCCAAGAAGCGTCTGGTTTTGTAGCAGGTTTAACATGTTCTAATGGAGATTTATCCATTGCAA
ACAATGATTTAATCGCTTCTATTTTAATCAATTGATTCTCTTTAAAACCAGAAATATAACTATCTTTAGTTTC
TTTAGGGTTTTTAGCATAAGATTTCAAGTATTCTTTATTGTGTACATGTGGAAACTGTAATAAACGTTGTGCA
TAACGACGTTTTAATTCAGTAGACATGTTTTTCTGCTGGAACATTTGATTAATTTGAGTTTGAGACATACGAG
CATCAAAGTTTTGATTCGTTAAACCATGGTTTGTAAACCATTGTGGTGAAATAATAAATGTTAATTTCTTACC
TTTTAATTTATCATACTGTGATGCAAGTTCAACTGCGTTAATTAAGTCTGTAGAACCACCAGCACCTAATAAG
AATGCTTTTTTGTTGGCGTTATGCTTATTTAATGCAATTGCAGGATTAAATGGGTCATCTTTACCTAATTCAC
TAGAGCCGTATATAGGATAATACTTGTTTGATTCGTATAACTTATCTTGAATGAGTGTGCCTTTTAGTACTTG
ATCTGTCAATGTAGTTCTATTATCTTCTACAGTCTTTTCATTTACTAATCCTGTAAACCAACTAGCAGGTAAT
AATAGAAATACAATGAATACCGCTCCACTAATTAAAATGGGTAAAAAAGGTTTTAATTTCATCGTAACTCTTC
TAATGCTTCAACGATTTTATTTGGTGTTGCCCACTCATCTCTATCAAAGTCCATAATAGATACTTCGATATCA
AGTTTATTTTGAATCTCTAATAATAATCCAACTGTTTGGAAAGAATCAATAATACCTTCTTCAAAAATTTCTA
CGTCTGGATTTTCTTTTACAATATCGTTTTCTGCTACTTCTGCTAATAAATTTAATACTTGTTCTCTAAATTC
CATAATTAAATTCTCCTTTATTATATAAGTTTACCTGAGAAGATTAAAAAGCCAAATGTTACAAAGTGGAATG
TGATCACAATGCTAAGTGCTGTTGTGAAACCATTTTGCCAACGTGGCGGATGTTTCTTGCGCCAACGTTCATA
ATAGCCATAACCTATAAACAATGCTGCATGGTATAAACCATAAACAATGTAATACACTTCGATACCATGCCAA
ATTCCCATTATGAAGAAGTTGATTAAGAATGCCACGTTAGACATTGCAAATTGACTCTTCAATAATTTTTTAC
GAGACATGTAGAATAAAGATCTCATGTAAATACAATCTCTGAACCAGAATGATAATGTCATATGCCATCTATT
CCAGAAATCTTTAATATTTTTCGCTTTGAAAGGTTTATCGAAGTTTGGTGGTGTTTTAATACCGAATAAATAA
CTAAATGCTATCGCAAATAAACTATAACCTGCAAAGTCAAAGAATAAATATAAGCTGTATGCGTACATATATA
ACCACAAATTGACAAAACCATGTAAGTCTAATTGTAACGGCATGATTGCATATGTGTTAATAAAGTAAGCAAC
AATATATTTATACAAGAAACCAAGCATAATCATGTGAATTGCTTTTAATACTAATTCACGATATTCATTGCCT
GTTGGTACTTTTTTATCGTCTTTAACGAAACGTTTGTAACGGTCGATTGGTCCAGATGAAATCGTTGGGAAGA
ATGAAATAAATTGAATTAATTTCCATACTTTAATTTCTTTGATAGAACCATCACGAATTTCCATAATTAACTG
CACACTTTTGAATGTAACATAAGAAATTCCTAAGAAACCAACAAATTCAATTAATTTGCTTTCATGGAAGTGA
ATCTGATGCCCACCTAACCATGTACTTTGTAACACTTTCACAAGTGCTAATGGCAATATTGATAAAACCATTA
CAGTTACAAATTTTGAAAATGAATTATTTTTTGGTTTTGAATGATAATAAAACATTATCAATAAAACTTGCCA
TACGACGTAAATAATAAAACTAATTAATTGAACACTTAAATACTTTTGGTCAAACAGATTATGTTTATCAGAA
GAAAAGATTAACACAATCATAAATGCTGTAACTACGCCATTATAAATGTAACTTCGCTTACCTAAAAATCCAA
GTATAATGACTGGTAATAATGCAATTAAAGCAATTAAGAAGAATGTAAAATCACCATATGGAATCATCCGTTA
ATTACCTCTGCAATTTTCTTTCTGTCAATTTTACCATTTGAAGTCAATGGTAATTGTTCCATCCATTCAAACT
TTCTAGGAATCATATACTCTGGTAAGCGTGATTTCAAGTCATTTTTAATATTTTTAGTCATTTCTGAATTGTC
CGTAACTTCAGTCGTTGGCACAATTGCACCAATTAAATGAATAACTTTATCATTTTTATATACAGGTACAACA
ATCGCTTCTTTTACGAACTCAGACTGGCGTAATTGTGTTTCAATTTCTTCTAATTCCATTCTGTAGCCATTCA
ATTTGATTTGGAAATCAATACGACCTTGAATGAACCATTGACCATTTTCAAACTTCGCTTTATCACCAGTGTG
ATAAGTACGAATACCGTCATCGAAATTAAATACTTCAGCTGTTTTTTGGTCATTTTTTAAGTATCCTAAACTT
ACACTTTGACCTTCGATAACAAGTTCACCTTCATCTGTAGTAGATAATTTTGCGCCTGGTCTTTCAACACCAA
CAGGTAATGTCGGATATTGATCTAAGATTTCTTGTGTAATTTGAATACTTGTAACTGCTACCGTAGCTTCAGT
TGGACCATATGTGTTGTAAATCGTCGCACTTGGGAAACGGCTTACTAACGCTTTTGCTGCTCTGTGAGGTAGA
ATTTCACCACAGAAGAAGAATTCGTTAAGACTACTATACTGTTCTTCATTAAGCGTTGGTAATAATAAACACA
TTTCCATAAATGATGGTGTTGATACCCAAATGTTAATCGGTGTTGCTGTTAGCATTTCATTTAATAATTTAGG
TTTATTAATCATGTTTTTATCTACAAGATTTAATGTACCGCCTGATGCTAAACATGGATAAATAGCCATTACA
GATAAGTCAAATGAAAATGGCGCTTGGTTAAGCCATTGTTGTTCATTTCCTGATTTATTAAGTTCTAACATCC
ACTCAGTAAATTGAACTAAACTTGCATATTCAATTTGAACACCTTTAGGTTCCCCAGTAGAACCAGATGTAAA
GATTGTGTATACTGTGTCGTTATCTTTAATCTGACTATCAAAAATTACTGGGTCTTGAGATGTTTTAATATCT
TCTATTGTAAATACTTCGCCTGCTAAACTTTCAAATGATTCATCAGTCGTATTAAATACAAACTCTGGTTGAA
CCTTGTTAATAATCATTTTAATACGGTCTTCAGGAATTGAAGTGTCTACAGGTACATATCCACATCCTGCTTT
AATGGCACCTATCATCCCAACAATCATATATGGTGACATGTGACCGAATAAAATCATCGGTTTCTTACTACCT
TGTAATCGATGTGCTAATTTACTAGACTCATCCATTAACTGTTGATAAGTTAATTCATCAGTTGTGTGTCTAA
CAGCAATGCTTTGTGGATTTGCATCCGCAAACGCTTGCAGCTTATTAATAATATCTGTCATATTAAGTCTCCC
TCATTAGAACTCATTATAAATGAAGTTATTGTGTGTGTCGCCACTGCCATAAATTAAATATAAAGTAATAAAT
ATTGCCAAATACAATAGTGTTAATAAATATGGTTTGAATGCTTCAACATATTTATTAGGTGGCTGTTTACTTT
TAGATTTCATATTGCACCTCTTAAAGTTCTTAGTAAAAACGCCTTTATAAAGACCGTTCAATATAAAATACGT
TTTAAAATTTGTTTTTTACAATTCATTATATCGATATTCATAATGAAATTCAATTTTAATTTTATAGATTCAA
CATAGTAATTGGTTGTCATCACTCAATTATTGTAAATGATACACTATGTTGAGAACATCATTCATTTTAAAGG

TTATTTAAACAATAAACAATTACAGTCTATATAACAATTTTGTTATATACGTCAAAAATCAAATAAACTCATC
ACATTAATATGACGAGTTTATAATGTTATTGAATTATCATCAGCGCAAATATATACATTCGCAAGTCAAGCGT
AACATATTTAACAATTGCTTTGCTTGTTTTACCAATGATTAAAAACCATACTTATTTTCAATTTACTGGAGTA
TGTGGTACCTGATTTGTCATAACCGCTTCTATATTATTAATACATAATTGAATCATATTGTCCCGTGTTGTTA
CTGATGCACTACCAATGTGTGGTGTAATCAGAACATTATCACGTCCCATTAATGGATGTGTATGATCAATCGG
TTCATTTGCTAATACATCTAAACCACATGCTAAAATTTCTTTATTGTCTAGTGCATCGATTAATGCTGTTTCA
TCTACAATTTGCCCTCTACCGATATTAATAAAAATTGCATCATTTTTCATTTGTTCAAATGCTTCAGCATTAA
ATTTATGATGTGTTTCTTTTGTAAGTGGCGCTGTACAGATGATAAAATCACTTTCTGCTAACAGCTTTTCAAA
AGAAACATATGTTGCATTAAAGTCCGCCTCTGCATCTTTATGTCTTGATCGATTATGATAAAGAATATTAGTA
TTAAACCCTTGCAACCTTCTTGCAAAAGCTTTACCAATATCTCCCATACCATATATTCCAATAGTTGAATTGA
AAACATCTTTACCTGACAATAAATAAGGACCCCAGCTTTGCCATGCATCTGCTTCTACATATTTTTCAGCTTC
TACAATACGGCGTGCTATAGCAAGCATTAATGTAAATCCTAATTCTGCAGTTGTTTCAGTAAGTACATTTGGT
GTATTCGTGACAACCACGTTATTCGCTGTTGCACTTTCAACATCGATGTTGTCATATCCTACAGCCATATTCG
CAATTACTTTTAAATTTGGTGATTGTGCTAAAATTTCTGCATCGATCTGTTCACTTAATGTGATAAAACAAGC
ATCTGCGTCACGAAGTGCTGTCGCAAATTGATCTTTAGGCATAGGCACTAATGATTTTTGCCACATAACAACG
TCACCAAGTTTACTTAATTGTTGATAAAATTTATCTGGAATTTTCCTCGAAACAACTATTTTTACCAAATCAA
TTCACCTACTTTATAAAATGACTACATATATTTTTCTAATTCAGCTATTGCTTCATTTAAATCTTTAAAAGAA
TACGTTGGTGGCACATTTTTATTTTGCACATCTTCTAACGTAGATACACCTGTTTGTACATGAATCGTATCCA
TACCTACGTTAATACCAGACATAATATCGGTATCGTACAAATCGCCTACCATAGCAACTTCGGATTTATCTAA
TCCTAAAATTTCTAATGCTTTAACCATAATGATTGGTTCTGGTTTACCAATAAATTGTGGCGATACGCCTGTC
GATACACTTACAACACTTGTAATAGCACCATTCCCAGGTAAAAATCCTCGTTCTTTTGGAATTGACACATCTG
GATTTGTAGAAATAAATGTTGCTCCATTTCTTACACCTAACGTTGCAATCGCAAGCTTTTCATATGTAACTTG
TTCGTCAAGTCCAATAACTACATAATCAACATGCTCGTCATTTTTAATGACAAGTCCCGCTTCGGTTAACGCA
GTATTTAAACCACTACCACCTAACATATATACTGATGCTCCTGGTGATTGTTCTGAAATATAATCAGCAGTGG
CTAACGCTGACGTTACAACCTCTTCTGGTTTAGCATCAATGTGCATTTCACGTAATTTTTCAGTTACTTGCTC
AGGTGTTTTCGTTGAATTATTAGTTACGTATAAATGAGGAATGCCTTTTACATTTAAATAATCGATGAATTGT
TTTGCTCCATCAATCTCATCTGTTCCCATATACATTGTGCCATCTAAATCGATTAAATACGCTTTATACTGTT
TCATTATTGATTTTCTCCTTTACCAAAAGCTGTTACTGGTACATTTTCTTGATGTAAAAATGTCGTTACTTCT
GTAATAAATTGTTTGTAATAAGGTAATGCGTCATCAAACATTGGCATGATAACCTTAATATCTAAGGCTGTGT
AATCATATGTAAATTGTTTACGAATATCGACAGTTTTATTAATCGCCTGCTGTGTTTCTTTAGTAATAACATT
TTCTAGTTCTAATATATCAATCACATCTTTATAATTACCAGGATCCCTTAAAATAAATGCATCGATAATCATA
TTCCCTATATCTACCGATGATTCTATCAACATTTGAGCAATGCGTTCAAATGCATAATGATTGTGCTTGCTCT
CATGGTAATCATCAGTTAATGCTTGTAAATAGGCTAATTTCTGAGTTAGTTTATCTTTGTCTACAAAATACAT
TCCTTGTCACCTCTCGCTTATATATTATCATAATTTTGCAGTATACTTCATTTGTCGTTATACTAACTTTACT
ACTTTTAAAAGGAGTCGCTACAAAATGATTGATATGTATTTATATGATGACAACGAAGAGAGTCAAGTTCAGT
TTGTTGGTTTTGTTGGCGAGCATAGTCGATATGATTTAATGTTAGTCCATACAAATAGGCATTATGGTAAGAC
ACTCGTACTTAATATGCAAACAAATAAATTCGGTATTATCGGTACTGATGATTTAAAAGAAGAAGGCTATATC
GCTCATATTTTAGGTGTAAATGCTGAAGAAGGCGATGAAATTACTGAGTATTTAAATGAAGTCATTCATTAAA
GATAAAAACCCCTAACTAGATTACAGGTCCATGCTTGAATTGCATGATTCCCATTTTCAATCTAGCTAAGGGT
TTTATTAATGTTCAGTTTCTTGAATGGTTGGCTTAATCTTCACATCTTGAAGTTTATTGTCGTCATCTATCAC
ATCAGACGGATGAACTTCTTCTATTAACTTTTTAACCTCTTGTTTAGACAAATGTCTTAATACAAAGTAAGGA
CAACCAAAGTTACAATATTCAAATATGTAATCTTGAATATTTGAAAAACGTTTACTCATCTCTGCTTTTTTAT
TAGAATCTTTGTAAAAGCCTTTTAATCGTAATTGATCGTAACCATAGTCACCAACTATGTAATCATATTTATC
TAAAATATCTGAATACCTAGCAATAAATTGTTCTTCATTAAAACATTCGCGATAATTTTCTATTAATTCAAAG
TAATGTTGATCTACTTTTATCAAATTCTTCACCTATCTATGACAATGAAAGGCTCAGTTTATACTTTTAGTGT
TTCATAAAAATAATGATTACTTCTATGACACGTTCGTTTTGTACAAACTAAGCCTTTATGCCTTATTAATGGT
TAAATATTAACTATTTAACTGTGCCTCACCTTGGCGTTGCTTTTCTTTAGCAGCTTCATTTACTTGCTCATCC
GCATGATAAGAACTACGTACTAAAGGTCCAGCTTGGCAATGTTTAAAACCTTTATCCATTGCCACTTTTCTTA
ATTTACCAAACTCTAAAGGCGTGTAATATTTTTGAACCTTTAAATGTTTACGTGAAGGTTGTAAATATTGACC
AATCGTTAAAATATCTACATCATTCGCACGTAAATCATCCATCGTTTCATAAATTTCTTCTATAGTTTCACCT
AATCCAACCATAATACTTGATTTAGTTGGGATATCCGGTTGTAATTCTTTTGAACGACGTAAAAACTCTAATG
TTCTGTCGTAAGTCGCACGCGCACGAACTCTCGGTGTTAAGCGACGAACAGTTTCAATATTATGGTTTAAAAT
GTCAGGTCTTGACGCCATTAATGTTTCTAACGCATCATAGTCCCCGCCCATATCTGATGGTAAAATTTCAATC
GTTGTAAATGGATTTCTTTCTCTAACTTTACGTACTGTCTCAGCATAAACATTTGAACCAGCATCTCTTAAAT
CATCACGCGCAACAGCAGTGATAACAACGTGTTTCAAATTCATTAATTCAACTGATTCAGCTACACGTTCAGG
CTCATTTAAATCAAGTTCATTAGGTAAACCTGTCTTAACCGCACAAAAACGACAAGCTCTTGTACATACGGCA
CCTAAAATCATAAATGTCGCTGTACGACGTGCACCCCAACATTCATGTATATTAGGACATTTAGCTTCTTCAC
ATACAGTATTAAGATTTTTTTCCCTCATCATCTTCTTAAGTCCTGTATAGTTTTCGTTGGTATTTAATTTTAT

TTTCAACCAATCCGGTTTACGTAATATTTCCTCGTTTTTTGTCGCCATAACAACACATACCCTCCTGTTAAGT
GTATCTTTAAACATTATATCGAAATTCATGACAAATGAAAATCCAATAATCTTACTTTTTAATATTCCAATAA
TTTTTCTTTAAATATATCTCTTAAAAACTCTGGCATTAAATACTCTTTTGGTAATTCTTTCAATATTGGGAAA
TAACGACCGAACGTATACATATCTATCGTACCTAATACATATTCTTTGTCGTCTTCGATTTCTTCTCCATTCA
GATAGTAACGCCCTGTAGAATGAATGTACCCTAAATTATAAAGAATATATCCTCCAAATATATTCCCTCTGAA
ACCCAAACCTTGTGCATGTTCATACATATATTCTTGTTTATTACTTTTAGCTATAATCTCTTTTAATTTCACA
CCAGAAAGTCTAACCCTTACCATATTAATCGGATGCGGTAACATTTGATGAATGTCATATTCTGTCACTTCAT
CTTTTACAATATCTTTAACGAGTAAGCCGGCATTGATGATGGCACATTGTGCATGTGTATACTCACAAACACT
TTGAGCTAATAAGTATGCAGCTTCAGTTATGTGATTCATACTACGCTTAAGCACTACTGGATGTTGAATTACT
GAATTGGACATTAACGTTTTTCCTTCTTCTTCAAATGAAGTTTCAACTTCAGGTAATGTTTCTAAAGGAATAA
TCTTTGCAGTTTTATGTACTACTTTATGTGCCTCAAAAGTTAAATTAACCTCTCCAAGATAATTACCATACTT
TCCAGCTGCCGCCATCAGTACACCATTATTAATTTCACCATGTTCAAAATAATGATGCGTATGACTACCAAAA
ATGACATCAATTTCAGGCAATTCTTGGCATAATGTTTCATCGAAGAAAATGCCACAATGACTTAGCACGATTA
ATACATCAAATTTACCTCGTTGAAGTTCAATTTCTTCTTTTATAGATTCAAGTGGATCGGTAACAATCCAATC
TAGCGCACGATAAAATGGGGTAAAAGGTGCTGTCGCTGCAACGAATAGTATTTTCACACCGTCTATATCCTTA
ATATAAGAAGAAACGATATTATTTGGTAAATGACCTGATTCATCTATAACATTGCTACATGTCACTATAAATT
TTGCTTCGTCGTAAAGGTGATTTAAAGCTTCGTGTGAAATGGTCATCCCTTCATTATTACCGATTGTTGCAAC
ATCACATTTTGCTTCATTTAGTAATGCCACATTCTTTTTACCTAAAGTTGCTTCAGTTATAGGTGCGGATAAA
TCTACATGATCACCTAGATCAACATATAAAGAAGGATGGTTAAGTCGTGGCCGATGTTCTGCCATATATGCTT
TAATGCGTTCGTATTCATGTAAATGACTATGAATATCGTTCGTATGATAAATTGTAAGCCTCAAATTGACGTC
CTCCTTTTAAAGTTGTATTAAATCAACGCACGAATAATTAAATATATACCCATTAACAACATTACTGTTCTCA
ATAATGTTACTACCGTATCTGACTTAATTGATTGATTCACTTTGACACCGATTTTCGCACCAAAATAACTAGA
AATAATTAAAATGATTGCATAACCCCAAGCTACGTGACCTTGAGCAATGTGCCCTATAGAACTCATGACACTT
GAAAAGAAAATCATCATCATACTTGTTCCAACAGCTACATGAGGTGGAAATCTAAATACAATAAGCATTAGTG
GCGTCATAAGTGCGCCACCTCCAATACCAAATAAACCTGTCAAAATACCAATTAAAAATGTAGTAACAAAAGC
GAATAAAGGCGGTACACTATAATGATATGTTTTACCTTTAGCATCTACATAAGTTTTTTCATACTTAGGTTTA
TCAAATATTTTAAACGGTTTAATCTTATTTCTTACCATTAATAAAATGGCTACGAAAATTAAAAAGATTCCAA
AATATAAGTTAAATGACTCAAATGTTAAATATCTACTAATGAATGACCCAAGCAATGAACCTGGTAATAATCC
AAATAAAAAGATGGAACCATTTTTAATATCAACTTGTTTTGTTTTTAAATATCCAAGTGATGAAGAAAGTCCT
GTCACAATTAGAATGACTGAAGACGTCCCTATCGCTACTTGTGTTGTAATATTATGTAGTAATCCATGTTCAA
CACCGAGGTAAACCATTGTTGGAACGATAATAATACCGCCTCCAATGCCTACGATTGACCCTATAATCGCTGA
CAAACCTCCGATTAAAACTAATAATGTAATTGTTAATAACATAGTCGTCGCCTCTTAAAATAATTTTAATTGT
TGTGGTGCTAACCCTTCATATTCTATATTCAATAATCGCTGATATGTTTTGGCATTATTTGCTGCATGACCAC
CAGAATTATTGTTAAATATGACGTAAACTTTCTTAGCTTTTTGTGCTAATATTTGTGCCTTTTGTGCCAAGTC
TATTAATTCTTGCTCATTATAATCATATAAATAGCGTACATCTCGCCATTCTTGATCTGACATATCTTTCTTA
GTCCAACCGTAATGATTACGTCCATGATAACGTACAAACGCAATTTCATTTGTGATTCGATTGACTAAAGGTA
CACTGCCATCTTTCACTTGTGGTTCATCAACTACTGCATGAATGATTTGATGTTCTGTTAAAAATGCCAATGT
TTGTTCTTTAAATGCATCACTAAACCATGATTGATGCCTAAATTCTACACACATTGGAAATGCTTGTAATTGC
TGTCTTACATACAAAATATATTTGATATTTTGTGCATTGCAGTCAAACCATGGCGGAAATTGAACTAATACCA
TTGCTAATTTTTTCTGTGTATGTAAGGGCTCTAACATATTCTTAAATTGATCAAATAGTTCTTGCCTTGTATC
TGCAAATGTTTTGTAGTCTGCATGCAATGTGAGTGCTTGATGAATTTTGACCACAAATTCAAATGTATCAGGC
GTTTCTTTTATCCATTTCAATATATTTCTTTCCGGTTGTATCGCATAGTATGTCGCATCTAATTCGACAACCG
GAAAATGTCCAGCATATGTTTTAAGTTTATCGGTTTGGCGTTCTAAATCTTCATATAATGAATAGTGATCACC
CCAACCTGTTAATCCGATGTTTATCATATATATCACCAATGTCATCATACCATATAACTTTTATCATAATCAT
TTCAGCGAACTTTCGTTTGATGTTTTTGCTTGATTAAAATCTTTCGGCGTTCTTCAGCTTGATGTTTTCGTTT
GATTATAATCATTTCAGTGAACTTTCGTATGATGATTTCGTTTGATCATAATCATTTCAGTGAACTTTCGTTT
GATGATTTCGTTTAATCATAATCATTTCAGCGAACTTTAGCTCGATAATTTCGTTTAATTATAATCATTTCGG
TGACCTTCAGCTCGATAATTTCGTTTGATTAGATTGGTAAAATAGAAACGCAATCCACAAAAATGGCAAGCAC
TAAAATAATGTTTGGGGGTGCTTGTGCTTTTGTGGATTGCGGTCGATTATTTATATTGCATGATTTGATTAAT
TTGATTGATTATATTGGACATGATGGTGTTGGCGGGATGCGTTGTTGCTAGTCGCGGGCTTTGTCCACTCCAC
ATATGTGTTAACTCTTTGTCGCCGATGTTTGCTGCGGCTTTTCTTATGCTACTTGTTAGCTCATTTTGTATTG
GATAATCTGGGATGTCGCCTTCGTATTGGGACATTTCTTCGATAAACCTATTGTTGATACCGCGTGCAAGCTT
TCCACTAAACGCTTTTGTAACGACTGTATCTGTTTCTTTACTATTTATAATTGCGTCTCGCAGTAGTTCTGAT
GCATTACTATCTTGTGATGTTAAAAATGCGGTGCCCATTTGTACCCCTTCTGCACCTAAGACAATACTTGCCA
AAACTCCTCTACCGTCCATAATTCCACCAGCGGCAATGACCGGAATTGAAACGACATCTACAATTTGTGGCAC
TAAAGATATTGTTCCAATCATAGGTAATTGATTTTTAGGTTTAAAAAATGAACCACGATGTCCACCTGCTTCA
CTACCTTGAGCAACGATAGCATCCATACCCGCTTTTTCATTCGCAATAGCTTCATCAACACTTGTTGCTGTAC
CTATTAGCTTAATGTTTGCTTCTTTCAATCTTTTTATAATAGATTCATTTGGAATTCCAAAAGTAAAACAACA

TACAGTCACTTGCTTTTTAATTATCGTATCAATATGACACTTAAATTGTTGTTCTTCGGTAATTTTTACAACC
GGCTCTTCTAAATGTAATGCGCGTCGATAAGGTTTTAACCATGCATTCATATTTTCAATTTGACTACTGGTAT
ATGATTGTTGACTTGGTACAAAGACATTTACGCCAAAAGAATTTGACGTTAATTGGCGTACATAATCTATTTC
GTCTTCTAATTGCTGCGTATTAAAGTAACCTGCGCCTATTGTGCCTAACCCACCACTGTTACTTACTGATGCA
ACTAATTTCGGTGTCGTACTTCCTGCCATACCTGCTTGTATAATTGGATATTCAATACTTAACATTTGAGTAA
GTCGATTCTTATTCCACATAGCTGTTCGCTCCTTATATAGATACGTTGCGATTTTTCCGTTGTTGAAATTGAA
TTTGCTGTTGAGAAAGTTTTTCTTTTTCCTTTTTATCCATCTCATCTTCAATTTCCATACCTAATAATTCTTC
AATTAAGTCTTCATGTGACACTATCGCTTCGGTACCACCAAATTCGTCCAACACAATTGCTAAATGTTTTCTA
GAAATAGTCATCTTACGTAATACCCATTCAGCTTTATTGTGTTCATTCACAAATAATGGCTTAGCTGAATAGT
TTGTAATTTGATCTTCTTTTTTATTACTCCAAGCCAACAGATATTTAGAATGAAACACCCCAATAATGTTATC
AATATCTCCCTCATATACTGGATATCTAGTGTATGGCTTATTCATAACCGTTTCATAAACTTCTTCGTATGTC
GCATTTGAAGCAAATGCCGTCACATTAATTCTAGGTGTTGTATCTACATCTTTTACTTTTAAATTTTCAAAAT
TAATGACACCTTCCAACCTACTCGTCTCAATTTCATTTAAAGCACCTTCATGTCCAGCAATTGCTAACATTGT
TTTAAATTCTTCTTTTGAAAATTGATGTTCTTGAGGTTGGCCCTTAGATAAACTTCGATTAATACTGTCCGTC
AACTTATTTAAAAGTAATGTGATAGGGCGAAACACAATGACACAAATATTAATAATTGGATATACAAGCCTTG
TTATTTTATCTGGAAATGTTGCAGCGACAGACTTGGGAATCACTTCGGAAATCAAAATGATAACAACTGTTAA
AACAGCTGATGCAATACCAACGCTAATCCCCCAACGTAAAGCCATAATTGTAACAAGTGTTGGTAATAAAATA
TTCGCGACATTATTCCCAATTAGAATCGTTGTAATAAACTCGCTTGGTTTTTCAAGTAACTTTACAATGCCTT
TTGCTTTTTTATCACCTTTATCAGCTTCAGTTTTAAATTTTGTTTTATTGGCAGCCGTTAATGCCGTCTCGCT
TCCTGAAAAGAAAAACGAAATAAATATCAATATAATTATGGCAATGATCACGCGTGTAGTCTCCTTATTGTCA
TATCTTATTTTTATTGTAGTTACTTAATTCCCGACGTTCTCGTTTAATAAACCAGTTAAAATTCATTATATAG
AATAGTCTTAATAATGATAATTAATATGCATTTTTAGTCATAGTTACTTTAATGCGTTCATTAGTAAAATGAT
GCTATAGTTCGACCTGATTGTTACGAATAAATTTACACAATAAAAAAATTTTAACAAATTCAGTTTATGACAT
AGTTATAATGATTTGAAACCGCTATAATGATAACAACAAGTTTCTCATAAATTTATTCTAATTATCTTTTAAA
TAACTTTAGACAAGTACTTGTTTATATCCAAATATAGAAAGAAGGATTAACATGTATTTTGTTTTAGCTATAT
TTACAATCATTAGTGCCAGTATAAGTTTAGGTTATTCAATTCAAGCATGTGCATCTAGCCATAATATAAATGC
ATATTATGCACTTAGTCGAAGCTTACCTTTATTTTAATTAGCTATTTTTTCTTTAGTCATTCATAGTGCTATA
TTTTTGATAACTATATCCATTGCAATGATTTTAGTTCAATTTTTAGATGCGATTGTTGGTTATAAAAGTAAAG
ATGTCTTTAAAACTTACGGTCCATTAGCAACATCTGTAGTGAACTTAATATTATTAATAGTTTTCTTATTTTA
ACTCACTTATACAACGAATCTTAATCGAACTAATATCGAATTATCTTTAAAGAATATTTGATGTAGTTTTCAA
TTAATTTAATAAAGACCAGCACTCTAATGCCACAATCATGTTGTATTTGTGTTGTCGCTTTATCCACCATCAA
TGATTATTTTTTACACCAATCAAAAAATCGGATTTATAAATACGCACAAAGCTTATCTATCAATCCGATTTAG
TTATAAAACCAAAAAAAGCCACATTAATGTGGCTTTTTGTTATATTCAGTATCGAAATGGTATCAATACCTAT
TTCCGGAAGTTAAGAATAGCTTAACAACGCGGTTTAAAGCTAGCCAATACTACCTTCCATTTCGAACTTGATT
AAACGGTTCATTTCGACCGCGTATTCCATTGGAAGTTCTTTTGTAAATGGTTCGATGAATCCCATAACAATCA
TTTCTGTCGCTTCTTCTTCAGAAATACCACGACTCATTAGATAGAATAATTGTTCTTCAGAAACTTTTGAAAC
CTTGGCTTCATGTTCTAATGATATTTGATCGTTGAATACTTCGTTATATGGAATTGTATCTGATGTTGATTCG
TTATCTAAGATTAATGTATCACATTCAATATTTGAACGAGCGCCTTTTGCTTTACGTCCAAAATGTACAATAC
CACGATAAATAACTTTACCACCATTTTTAGAAATAGATTTAGAAACAATCGTTGAAGATGTATTTGGCGCTTT
ATGAATCATTTTAGCACCGGCATCTTGAACTTGTCCTTTACCAGCAAATGCAATAGATAATGTACTACCTTTT
GCACCTTCACCTAAAAGAACACAGTTTGGATATTTCATCGTTAACTTAGAACCTAAGTTACCATCTACCCATT
CCATATTTCCGTTTTCATAAACAAAAGTACGTTTTGTAACTAAATTGTATACATTGTTCGCCCAGTTTTGAAT
CGTAGTATAACGAACGTGCGCATCTTTATGCACAATGATTTCCACAACAGCAGAGTGTAAAGAACTAGTTGTA
TAAACTGGTGCAGTACAACCTTCTACGTAATGTACAGAAGCACCTTCATCAGCAATGATTAATGTACGTTCAA
ATTGACCCATGTTCTCAGAGTTAATACGGAAATAAGCTTGTAGTGGCGTATCTAGTTTGATATTTTTAGGTAC
ATAAATGAACGAACCACCTGACCATACCGCTGAGTTTAACGCCGCAAATTTGTTATCTGCTGCAGGTACTACA
GAAGCAAAGTATTTTTTGAATAATTCTTCATTTTCTTGTAAAGCACTATCTGTATCTTTAAAGATAATACCTT
TTTCTTCAAGTTCTTTTTCCATATTATGGTAAACAACTTCAGATTCATATTGAGCAGAAACACCAGCTAAATA
TTTTTGTTCAGCTTCAGGAATTCCTAATTTATCGAAAGTTCTTTTGATTTCTTCTGGCACTTCATCCCATGAA
CGTTCAGCTTGTTCTGAAGGCTTTACATAGTAAGTAATGTCATCGAAATTCAATTCTGATAAGTCGCCACCCC
ATTGAGGCATTGGCATTTTATAAAACAATTTTAATGATTTAAGACGGAAATCTAACATCCATTCCGGCTCATT
TTTCATGTTAGAAATTTCTCTAACGATATTCTCAGTTAAACCACGTTCTGATCTGAAAATGGACACATCATCG
TCGTGGAATCCATATTTATAATCCCCAACATCAGGTGCTTTTTTAGCCATTTCAATCACTCCTTAAAAATATA
TTTACAACGGCGTGCGTAAGTTTTTAATTCATGATGTAAACCATATTATAACAATGACATGACATCTTATAAA
AATTTTTATACTTTTATATGTCTAATATCAAAATCATCTATGATTAACAGCATTCTATTCTTCTTCAGTCGTA
CCTTCTGCTTTACCTTCTTTAGCAACAGTACCTTTTTCCAATGCTTTCCAAGCTAATGTGGCACATTTAATAC
GAGCTGGGAATTGAGATACACCTTGCAATGCTTCAATATCTCCCATTTCTTCTGTAATCACATAGTCTTCACC
AAGCATCATTTTCGTAAATTCTTGGCTCATTTGCATTGCTTCTCCAAGTGAATGACCTTTAACAGCTTGTGTC

ATCATCGATGCACTTGCCATTGAAATCGAACAACCTTCACCTTCAAACTTAGCATCTTTTATAATGCCGTCTT
CTATATCAAATGTTAGTCGTATACGGTCACCGCATGTCGGGTTATTCATATCTACTGTCATAGACCCGTTATC
TAATACACCTTTATTTCTAGGATTTTTATAATGATCCATAATGACAGATCTATATAATTGATCTAGATTATTA
AAATTCATAAGAGAAAAACTCCTTCGTTTGTTTCAAGGCATTTATTAACTGATCAATGTCTTCTTTCGTGTTG
TATATATAAAAACTCGCTCTAGCTGTTGAAGACACATTTAACCATTTCATTAACGGTTGCGCACAATGATGCC
CAGCTCTAACCGCTACACCTTCTGTATCTACGGCTGTAGCAACATCGTGTGGATGTACATCTTGTAAATTAAA
CGTTATTACACCTGCACGACGATCCTTTGGCGGGCCATAAATTTCAATTCCTTCAATTGCAGACATTTGCTCA
TAAGCATATATCGTTAATTCTTGTTCATATTTATGAATTGCATCAAAACCTATGCGTTCTAAATAGCGAATAG
CTTCTGCAAGCCCAATTGCTTGTGCAATTAATGGAGTACCCGCCTCAAATTTAGTAGGTAAATCAGCCCATGT
TGCATCATACTTACTTACAAAATCAATCATGTCGCCACCGAACTCAATCGGTTCCATTTTTTGTAGTAACTCA
CGTTTACCAAATAATACGCCAATACCTGTTGGTCCAAGCATTTTATGACCACTAAAACTATAAAAATCAGCAT
TCATTTCTTGCATATCAAGTTTCATATGTGGTGCTGCTTGCGCCCCATCAACACTGATAATTGCGCCATGTTG
ATGAGCTATTTCTGCAATGGTTTTAACATCATTAATTGTACCGAGTACATTAGAAATATGTGCAATAGCAACG
ATCTTTGTTTTATCATTAATCGTTTGCTTAATATCTTCGATGTTTAATTCACCGTCAGCTGTCATTGGTATAA
ATTTCAATGTCGCATTTTTACGCTTTGCTAACTGTTGCCAAGGAACAATATTGGCATGATGTTCCATTTCAGT
GACAACAATTTCATCGCCCTCTTCAACATTTGCATCACCATAGCTATGTGCTACAAGGTTAATCGACGCAGTT
GTTCCGCGTGTGAAAATGATTTCTTCAAAATACTTCGCATTAATAAAACGACGAACGGTTTCACGGGCATTTT
CATAACCATCAGTTGCCAATGATCCTAATGTATGAACACCACGATGAACGTTTGAATTATAACGCTTGTAGTA
ATCCTCTAAAACATTTAACACTTGCACAGGCGTTTGACTTGTCGCTGTTGAATCAAGATATGCTAAACGTTTG
CCATTGACTTTTTGATCTAATATCGGAAAATCCTTGATTACTTCATTAACGTCAAATGAGTGTTCGGCCACTT
CATTCACGACCTTTCTTAAATAAAAATCCTAATCATTTAAATACTGACGTTGTATTAGTCTTATACCAATATC
GACAGTCTATATCTATTACAAACTTTTATTTTCAAAATATTATTTAGAAACTTTGCGTTCAATTACTTCTCTC
AATTGACGTTTAACGTCTTCGATAGGTAATTCACGTACTACTGGATCTAAGAAACCATGTATAACAAGACGTT
CCGCTTCTCTTTGAGAAATACCACGACTCATTAAATAGTAAAGTTGATCTGGATCAACACGACCTACTGATGC
AGCATGACCAGCTTGTACATCATCTTCATCAATTAATAAAATAGGATTCGCGTCACCACGAGCATGTTCAGAT
AACATTAATACACGTGATTCCTGATTAGCAATTGATTTAGTTCCACCATGCTTAATGTAGCCGATACCATTAA
ATACAGATGATGCATGTTCTTTCATAACACCATGCTTAAGGATATAACCATCTGTTTCTTTACCATATTGTAC
GATTTTAGATGTTAGATTAATTTTTTGTTCGCCTGTACCTACAACTACTGATTTAAGTGAACTTGTTGAACGA
TCACCAAATAAATTTGTTGTATTATCAATAATTTGGCTACCCTCATTCATTAAACCTAATGCCCAATTAATTG
AGGCATCCGCTTCAGTAATACCACGTCGAATGATATGACCTGTAAAGCCTTTATCCATATAGTCCACTGAGCC
ATATGTGATATTGGAGTTTGCGCCAGCAATCACTTCAGAAATAATATTTAATTGATTTCCTTCACCAGATGCA
TTTGATAAGTAATTTTCAACATATGTGACTTCGGCGCTTTCTTCAGTAACGATGATAACATGGTTATAAAAGC
TTGCATTTTCGTCGTCGTGCAACACAACGTATTGTACTGGATGTTCTACAACCACATTTTTAGGAACATAAAC
AAATACGCCACCATTAACTAATGCCGTGTGTAGCGCTGTGATACGATGTTCATCTACTGTTACTGCATCTTTC
ATAAAGTACTTTTGTACTAAATCACTATGGTTCATAAGAGCGTCTGCTAAACCTTCAACGATAACGCCATCTT
TCGATGCATTATCATCAACTTGTGTGTACGCAATCGTATTATTATGTTGAATTACTAAGTTTTTAGAATGATC
TACGTCAATAATTTCTCTTACTGACTCAGGTAATTGTGATAAAGATTGATAAACATCACCTTTTACATCGTGT
TGTTTAAAAGAATCAAAATCCCATTTTCTTAATTTTGTTTTATCAGGTTTTGGCATTTCTAAAGTTTCTGTTA
ATTTCAAAGCTTTTTTACGTAATTCTGTCATCCAAGAAGGTTCATTGTGGGCTTTAGAATAATCAACAAGTTG
TTCTTCAGAAATGTTCAAAATATCAGTTGTCATAACTTTTTCCTCCTACTTGATGAAATTTACTATTTCGTAT
AAATGTTCATTTTCATCATATATAGATGAACATCTCATGGATACTGTATTAATAAGATTATTCAGCTGAACCG
AACTCTTCTTTAACCCATTCATATCCTTCTTCTTCAAGACGTTTTGCTAATTCTGGACCACCAGATTTAACGA
CTTTACCAGCATACATTACATGTACTTTATCAGGAGTAATGTAATTTAATAATCGTTGATAGTGTGTAATCAT
TAATGCACCAAAGTTTTCCCCACGCATTTGGTTAATACCTTTAGATACAACTTTTAATGCATCGATGTCTAAC
CCTGAATCGATTTCATCTAAGATTGCAAACTTAGGTTCTAACATCATTAATTGTAAGATTTCGTTACGTTTCT
TCTCTCCACCTGAGAAACCTTCATTTAAATAACGTTGTGCCATGTCTTTATCTATGTCTAGAAAATCCATATT
TTTATCTAATTTCTTAATAAATTGCATTAAGTTGATTTCTTGTCCTTCTTCACGTTTCGCATTGATTGCTGAA
CGCATGAAATCAGCATTTGTAACACCTGTAATTTCTGATGGATATTGCATTGCCAAGAATAATCCTGCTTTTG
CTCTTTCATCAACTTCTAATTCTAAAATATTTACACCGTCTAAAAGTACTTCTCCTTTAGTTACTTCATAGCT
TGGGTGTCCCATAATTGCAGATGATAAAGTTGATTTACCTGTCCCGTTTGGTCCCATAATCGCATGTATTTCA
TCAGTGTTAATTGTCAAGTTAACACCTTTTAAGATTTCTTTATCCTCAATAGACACATGTAGGTCTTTGATTT
CTAATGTTGATGCCATTTTATATTCCCTCCGTAAAATATAAAGTTTTCTTAACTAGTTTATAATAATTTTAAT
TTGTAGTCAAAAAGACTTTGTAATAATGCGTTCAGTTAATTATAACTTACTTATACCTTAATATAAACAACTT
AAACCCTTTTTATTATTTTTAATAACTCTAAAGTACAACTCTAATCCGCTCTCTTTAAAAATATAAATGATAA
TAAGTGCACATAATTTCTCAATGGATTTTATGAATTTAAAATATGTTATCATTTCACTAGGACATTTGTAATA
TGGTATGATGCTATTTATGATTTTATTCCAATCGTTTGAAAGGAGTTATTCATGCAACAATTTAAATGGATTA
ATATTCTAAAAGGTTTTGCTATGGGAACGAGTGACTTGGTGCCTGGTGTTAGCGGTGGGACTATTGCATTATT
GTTAGGTATATACAATCAATTTATCGCTTCAATTAGCGGCATATTCTCACGACGTTTTTGGCCAAGTTTTACA

TTTTTAATCCCCATTATAATTGGAATGTTACTGGCAATGGGATCACTAAGTAATCTTTTTAATTATTTATTAA
GCCAACACCATATTCCAACTATGTTTTTCTTTGGTGGATTAATCATTGGTATCGTTCCATATTTATTGAAAAT
TTCTAACTATAAAACGTCATTTACAACTAAACATTACATGATGGTTATAGCTGGTATCGCTATCTTAATTGTT
ATTACATTAATGAATAATGGCGATAAACATGCTGGAGAAACACTTACTCTATCAACTGGTCTTATTATTAAAT
ACTTTATTGCAGGTATGTGTGCATCTAGTGCTATGTTACTCCCAGGTATTTCAGGTTCATTTATGTTATTAGT
ATTTGGCGTCTATGGCACAGTCATGCTAGCAATATCTGAAGTTGTTAAACTTAACTTTACAGGTCTTCCTATA
TTACTTGCAGTCGGATTTGGCGTGCTTGCTGGATTCATTATTTCAAGCAAAATTATCCAGTATTTCCTAACAC
ATCATAAACTAATGACTTTTGCATTAATCATTGGTTTTGTTGTTGGCTCCCTCTTTGCAGTATTTCCAGGTTT
ACCAACTAATATCGTGATGTGGTTTGTTTCATTAGTAGTATTTATCATCGGTTTTATTGTAAGTTTGACTTTA
GGTCGAATTACCGCTGAAAATGAATAGTCATTTTAGGGGTGATAAAATATTTTCAGTATCTCGTTAATCATAT
GCAATATATGTCTCGTTTTGTATACATATTATTGGATATCATTAGCGAGTTTTTATTTAGTTTTTTTCTAACA
TTAAAATAATTATTAATAAATTTTGGTATCCACTTTTTTAATACAAAAACCACCTAGCCGATGGTTATACCGA
CTAAGTGGCATGTGCAGTGTATTAGGAATAAAAAAGTAAAATTACTCGCCTTTGTTACCTTTTACTTTATCAA
TAAAATCAGTTGCTTTTTCTTTTGCATTTTCAACGAATTCTTTCGCTTTACCAGAAGCTTTATCTTCTTTACC
TTCGTTTTCTAAATTTTTATTATCAGTAACATTACCTACTGTTTCTTTAACATTACCTTTTGCTTGTTCGAAT
TTACTTTCGTCTGCCATAATAAATGCCTCCTCGGAATAATTAAATGTTATATATAATACTTACCCACTGAAAA
ATTATCTAAACATTTTACTTAAATAATTTTTGTTATTGATTTGACATCATTTTATAACTAGCGAAATAGATTC
ATCATTAACTTGAGGGAGTGGGACAGAAATGATAAAGAATCACTAATGATTTATGATGTAGTAGTCACTATCC
ATGTGAAATTAAAAATAGGAATAAATGAGTAGAACGCATGCATATAGGATTTTACTTTATCCGTAATAGCATC
TCATTCCTAAATATCATATAAATACCTGTTTAAATTAAAAAGCCCAGCAACATCACGTTACTGAGCCATTAAT
AATTATTTAGCAGGAATAACTGCACCATTGTATTTTTCATTAATGAAGTCTTGAATATCTTTAGATTGTAATA
CTTCAATTAATGCTTTGATTTTCTTATCATCTTGATTTCCTTCTTTAACAGCAATTAAGTTTGCATAAGGATT
ATCTTTCGCACTTTCTACAGCAATAGAATCTTTTTTAGGATTTAGTTTTTGTTCGATTGCAAAGTTTGAATTA
ATGATAACAGCATCCGCGTCTTCATTTTGATATATTTTAGGTAAGAATTCTGCTGATTGTTTATTATTAAACT
TAATATCTTTTTTATTCTCTGTAATATCACTAAACTTAGCATCTTCAATTTTTACGCCTTTTTTGATCTTAAT
TAAACCTGCATCAACGAAGAATTTTAAGAAGCGTCCTTGTTCAGCTGGATTATTAGACACATAAACTGTTGCA
CCTTTTGGTAATTCTTTTAAACTTTTATACTTTTTAGAGTATACAGCCATAGGTTCTAAGTGAACATCACCGG
CACTTACGATTTTGTAACCTTTATCCTTTTTCTCTGTGTTTAAATATGGTGTATGTTGGAAATAGTTTGCGTC
AATTTCACCTTTGTCTAGTAATTTATTAGGCGTAGTGTAATCGTTAATTGTTTTAATATCTAGTTCATAACCT
TTTTTCTCTAATAATGGTTTTGCTTTTTCTAAAATTTCAGCATGTGGTGCTGGTGAAGCACCTACTGTTATTT
TCTTGTCGTCACTGCCACTTTTGTTTCCATTACCGCAAGCTGCTAATACAACTGCAAATGTTAATACTAAAAT
AAGACCAAATAATTTTTTCATAAAATGAAACCCCCAATTTATCGTTTATCAAGTTTATTTGTAAGCCAATCCC
CAATGAATTGGATTATAAATACAATAATTAAAATAAAAACTGTTGATACTAAAATGACATCATTTTGATTTCG
AGTGAAACCTGTTAAGTATGCTAAATTTCCTAAACCACCGGCACCAATTACACCTGCAACTGCTGTTGAACCA
ACTAAAGCGATTGCTGTAACTGTAATGCCAGACACTAGCGCTGGCATGGCTTCAGGTAAAAGGACTTTACGAA
TTACTGTCCAAGTATTAGCACCCATTGACCAAGCCGCTTCGATGACGCCTTTATCAATTTCTTTAAAAGCAAT
TTCTACGAGCCTTGCATAAAACGGTGCTGCGCCAATGATCAAGGCTGGTAACGCACCTGTCGGACCACTTATC
GTTCCAAGTATCAAACTTGTAAATGGAATTAATAATAAAATTAAAATAATAAATGGTATCGCTCTAAATAAGT
TAACAATGAAAGAAACGATAGAATAAAATAACCTTGCACCGATAGACTTACCTTTAGCAGACAAGAATAATAA
CACACCTAAAATAAGACCAAGTATAAATGCAAATATAGTTGAGACGACTGTCATGTATAGTGTTTCGACTATT
GCAGTCCAAACTTCTGGCCACTGAATATTAGGCATTGTAATCATTTCATTTATAATTTCACTAAATGATTTAC
CCATGTCTTAACACCTCCATTTTAACTTGTCGCTCAATTAACTCTTTTTCGAATTTTCCGAAATCTACACTTG
AAATATATGGAATATGCAGAACTAAAAAGCCGACTGTTCCATTTTTTGTATTTTTAATATTTGCTTCTAAAAT
ATTAATTTTAATATCATAGGCAGTTGATAGGCTCGATACAATAGGCTCGGTTGTTGTTGAACCAGCGAAAACT
AATCTAACGATATAAGCATCTTTTTCTAATGGCTCTAATTCTGTTAAAGATGTTTCGAAATCATCATTTAAAT
CGTCTTTCACAAATCGTTTTGTCACAGTGTGTTGCGGATTTTCAAAAACCTGTGTCACCGGTCCTTGTTCTAT
CACTTTACCACTTTCCATAACTGCAACTTCATCACAAATACGACGAATGACATGCATTTCATGCGTAATTAGT
ACAATTGTTAAATTTTGTTGTTCTCTAATTTTTAGTAGTAGATCTAAAATTTCATCTGTTGTTTGCGGATCAA
GTGCACTTGTTGCCTCATCACAAAGCAAGACCGTTGGATCATTAGCTAACGCTCGTGCAATCCCAACACGTTG
CTTTTGTCCACCTGATAACTCTGATGGATAAGCTTTTTCTCTACCTTTTAAACCGACAAGTTCGACAAGTTCT
AATGCTTTTTGCTTAGCTCTCCTTCTAGGGACACCTGCAATTTCAAGCGGAAACATAATATTTTTTAACACAG
TCCTTGACCATAATAAATTAAAATGTTGGAAGATCATACTTACTTTTTGTCTTTTTGCTCTTAATCCATTTTT
GGACAATTGACCTATATGGTCCCCATCTATAATAACTTCACCTGATGTAGGCGCTTCTAAATGATTAAACATT
CGAATCAAAGTACTTTTTCCTGCTCCAGAAAAACCAATGACGCCATAAATCGATCCTGCTCGAATCGATAAAT
TAACGTGATCTACAGCAAGGACTTCTTTATTTTTAGTCCGATATTCTTTAACAACTTCTTTTAATTCAATCAC
GTTGATTCCTCCCTGTGTTGCTTAATAAAATAAAAAAATGCTTTCTCAATATCGATAGAAAAATTGAGAAAGC
AATAGTAGTATTGTTTCTCTCATCTTCAAAAGTTAAAACTTTATGTGAATTGGCACCATTTCTATATAAGACG
GTTGCCGGGCTTCGTAGGGCACATCCCTCCACCACTCTCGATAAGAGTTTACGCATCATTTAATTTGTATTAA

TCCTAACACCTTAGTAAAATTTCGTCAATAACTATTTTAAATTTTCTAACAAATCAGTCACCGATTTAAATGC
ATAAATTCGTTTTACTTCTTTATCTTTATTCATCAACAATAAAATCGGCGTAGACATGATTTGCATATCTTTA
CAAAACTGAGGATAAAAGTTTAAATCTATTTTCAATAATGGCAACTGCAATATTTCATTAGCAATGTCTAACA
TTCTTTCTGAAACCTTACAAGTACCACACGTTGGTGTATAACCAAAGATTAAATGTTTGTCTTCCTCATAAAA
TGTAGTTACATCTTTGATGTCTAATGAATTATTCATTTACTAAAACTAACCTTTCATTATTTATATTCGGTAA
AAGAGGTGTTTCTTTCTTACAAGTAAAGCCATGTTTTGAAAGTACATGCGCCAAATATTGTTTGGGGCAATTC
GCAACTTGACAGTAAGTTTTATCAATAAATATATGTTCACTTTCACTCAAATAACGTTTAAACCAATTTCTAA
TTCGATCTCCTTCGTCATCAGAATCGGCTAATACAAAAACTTGTTTATCATACAGTGATTCTATCATATCATC
AAGCTTATCTATACTCATTGTTCCATGAGTACAAATAATATTGACTGGTTCTGCAATAACCTGTTGCACCCTT
TTTTTATCAGATTTTCCTTCAACAATTATCACTTTATTTACAATAGCCATCATCATCACCCTTTAAAATCAAT
AAACATCTGTCACTGTATCATTTCACAAAATTGGTATGAATAAAACATAAATCACAAAAAATTTAAACTAGCT
TAATATAATAATTACAAAATCAATGTTTGACTAGCTGGGACATTTAACATAAGCAGACAAAGGCTAAGTCAAA
AATCAACATCCTAAAATCTACAATGTTATATTACCAATAGTTAACCAAAAGAAAATACACCTATAACAAACTT
TTCAATTATAGCGGGGCCCCAACATAAAGAAATACTTTTTCTTTAGAAATTAGTATTTCTTATGCATGAGTTT
TACTCATGTATTCCTATTTTTAAGTACACATTAGCTGTGGCTTATGAAAAAGAACCACTACATTATAAATCAC
TAGTGGTTCTTTTTCATTTCTGTCCCACTCCCTAGATTGATCTATAGATACTACACTTATTAAAGTAATATAT
TTTCATGATTCTCTTAGCTGCAATCCCATGAATACATGTAATCATCAAACTTCATAGCCTCAAGGTCAGTAGA
TTTCACTAAGATATTGGCAATACCTAAATCACCCCATATAATCGAAGAATTACCATCTCTTGTAGAATCAATT
TGCAGTAAGACTCTATCATACATTTTCAGTTCTTGATCGTTCGTTCTTGGATCTATTTGGTCAAAATATGGAT
AACCACCAAGTTGATTAAATGGTTCCTTAGCAGTAAATGTGTTACACACAGGTTCCATCAATTCATCATAACC
ACCAAACATTGATTTTAGCGCGAAGGATGGATTGACTTTATTCCACAATGCAAGCACGTCCTCATCGTACTTA
TAATCAGTACATGTAATAGTTTGTTTCGACAATCTAAATTCTATAGAAAATGCTCCACTGAACGGGATATCCT
CATTATCAGCATCTAATGACTTCAAATGCGCTTGAATATTTTCGAGTGATAAATCTGTAATTGGCTCTTTTAT
ATAAACAAGCCTTGAGTTTATATTGTTTCCCTTTAAATTTTCAGACATTCCATACATTTGATCTTCTGAACTA
ATCCAAAACTGCAATATCCCTTCTTTTACAGGAAATAATTCTTGTTGTTCTGGAAGATCATCCAAATTAATCT
GCGCTAACAATACCATTGGTACATTATTTTCATCTAATGGTATATCTTTAAAAGATTTTAAAAATGGCATTCC
ACCAATCTTACTCTGGTTTAATGTTTCTTCTTTCATCTCTTTTAGATCCATTTCAATACATGGTAGTACTGGT
AAATTACCGTTTTCTACAGATTCTACGAAACGGTTATATGCTTTTTGCTCCTTTTTAGAAAGATGACTAACGT
CTTGAATGTTTAGCATATTAACTTCCCCCAATTTTTATTTTTAAAATAGCTAAATTCATTATTTTCATCATTC
TTGAATAATAAAATGTAGCAATACTCCAAATAATAGTACCTAATAAATTTTCTTTCATATTATTTACTTACTC
AGACTAAAAATAAACTCCTCAATCTTAGATTAAGGAGTTACACGGTGATTATTCACCAATCATTTCTGAATAT
TTTTCAGCTGTTAATAAAGCTTCAAGCTGACTTTCATCACTAATTTCTACTTTTACCATCCATGCTTTTTCGT
ATGGAGATTCATTTACAAATTCGGGACTATCTTCTAGTTCTTCGTTGACTTCAACTACTTTACCAGAGATTGG
TGCATATAATTCTGATACAGTTTTAACTGATTCTACGCTACCAAACGTATCCCCTTCATTAATTTCATCATCT
GTTTCCGGTAATTCAACGAAAACAATATCACCTAACTCGCTTTGTGCGTATTCTGTGATTCCAATTGTTGCTA
CATTACCTTCAACTTTAACCCATTCATGCTCTTTTGAATATTTCAATTCATTTGGTACTGCCAAGAGAATCCC
CTCCTAATTAATCTTTACATCATTAATGATGCCATAAGGCTAGATGCTATTCAACTATATTTATAAAAATTAA
CAATTGTATAGCTTATTTCATTTTACATGTAATGAATCTAAATATCGAGATATTTAGTAAACGTTTACATTTC
ACTTACGCTAACCAAGTCTCTTTATATTGATCTTCTTTAAATCCTAATGTTATCTTATCGCCCATTACTGCTA
GAGGACGCTTTACTAACATACCATCAGATGATAACAACTCTAACTTTTCATCTTCTGATAAGGTTTGTAATTT
ATTTTTCAAATCAAGCTCACGATATTTCGCGCCGTGTGTATTAAACAATTTATTAATTTCTACGCCTGTATTT
GCAATTATTGTTTTAAATTCATTTATTGTAGGTGTATGTTGAACGATATCAATTGGTTCATAACTTACGCCAT
ATTCATCTAAAAACTTTGCTGCCTTTTTACAAGTTGTACAATTCTTATATTGGTAAAATTTAATCATACGACT
GCAACCTCCTTTATGTATTTAAAATAAATATATCATTTTCCATAGCAAAAGGCTTTCATTTTGAAGCTAAAAT
TTTTTATTATGTTATAAATTTAGCTATAATAAAAATTATTAAACTATCTAGAGGAGTTGTTACAATGCAAGCA
ATCAAAAGTAATGAATCATTTAAATCTGTAATTAATAGCGATACACCTGTAATTGTTAAATTTGAGGCAGGAT
GGTGCCCAGACTGTCGTGCTATGGATTTATGGATTGACCCAATCGTAGAACAATATAATGATTACCAATGGTA
TACTGTTAATCGTGATGAATTAGAAGATGTAGTTGTTGAAAATGAAGTTATGGGTATCCCTAGCTTGCTAGTA
TTTAAAAACGGCGACAAAATTGCACACCTTCATTCAGCAAATGCTAAGTCACCTGAACAAGTTGAATCATTTT
TAGCAGAAACTTTTAAATAAATACTAAAATAAAAATGTGTATATACTGCACAATAAAAAGCTGAGACTGTGAT
TGTACCGACTCTAGTAAGTTAGATTAAATCTCTAACTCGATAGATGTCCATCAATTCATGACTCAGCTTTTAT
TAATGTTTCTATAATTATACATATTAATATAATGTAATTAAGTTTCTATTTTTACGTGGTGCTTTAGGCATAT
CTTCTTCTAAATCCGTTAAATATAAGAATCCAGCAAGAACTTCATCTTTCTTTATACCAAGTGTTTTTCTTAC
TTTAGGATCATAGATATATAATGGCGATTTCCAACATGTACCTATTCCCGCTTCATAAAGTAACAACATAAGA
TTTTGTGCATATGCACCAAATGCAAAGTAGTTTTCATCATTTTGACGTTGTCTTGGATCTGTTTTTAAAATAA
GCAATAACATGCCACCAAGTTTCGTTACTGCATCATAATGACATTGTTGCTTATCTAATTCATTAGGAAATGC
AAATTTAGAAATATCCTTACTCATATCTCCTAATCTGTCTTTCGGAACATGCACAACTCTCCATGGTTCCCTC
ATTCCGTGATTTGGAGCATCAGCAGCTTTCTCAATTGCTTGATATAGCAATGCGTCATCTATGTGCATATCTC

TTTTAAATTTTTTCACACTTCTTCTATTAGCTATTGCTTGTTGTAATTCCATCTAAAATCGCTCCTCCACGAA
CAATGATAATACTTCTCAATTATAAGACAGCACTTACACAAAAGCAACCTAATTCATTTTATTAGTATAAAGT
CACTTGTGCTTTTAAATCAGTAACATCAATCTGTCCTGGAGCTTGTGCTTCTCCGATACAACCATAAGTCAAC
GCACCACCAAAAACGCCTTGAGCCGTTCTACTTATTAGTCCAAGTTTAGACATTGAAATACCAACAACTTTGC
AGTCCATAGTATCTGAAAATGTAGACATTGCCTGCAATAAATTTAACACATCATTTTTATTATGTGGCATTAC
TGCTAATTTAACGTATTCTGGGTTGAATTTTTGCATTTTAAAAAATATAAATTGCAATTCATCTAATGGAGGC
GTACTTTCGAAATTATGATGTGATATGACCACCTCTTTATTATACTGTTGCAAATGTGTAATGATTCTTTGAT
GTTTTTCAATGTCAATATCTTCTCGCCATTCTATATCAATCATATCTATGCCATTGATATTTGCTAAGTCTGA
TATTAAATTAAGATACAAGTCATTTGTAAATTGCCCATAGCCACCTTGTAACTTTGTACGATACGTAACCAAT
AATTTGAATGAATCTTGCATAACCTTCAGCTTTGTAATCATTTCTGCCACTTGATCAACTGTGACATTTTCAA
TTTGATCAATTCGTAATTCTAATACGTCTATTGCATCAATACGATGATTAATTTTTTGAATTAAAGTTTCTTC
GATAGATAATTGTGGCGCGATAGTCGCTACTACTTCCACATGTGTCATAGTAAATCCCTCATTTCAGATACTC
ATTATTTTTATTGTAAACAACAACTTTTTATCCTTCAATCAAACAATGCTATAAATGAAAATGATTAAGGTAT
TTTCGAATGGGTAAAGCATAAATGTATTTTAAATTAGGAGGTTATAAGTATGGCAATACATTATGAAACTAAA
GCGACGAATGTTGGCGGACGTAAAGGACATGTTTATACTGATGATCGAGCATTAGATATTGATATCGTTCCGC
CTGCTCAAGCAGATGGTAAAGCTACTAACCCCGAACAATTATTTGCAGCAGGTTATGCATCTTGCTTCAACGG
TGCCTTCGACCTAATTTTAAAGCAAAACAAAGTGCGTGATGCTCATCCAGAAGTAACACTAACAGTGAGACTA
GAAGATGATCCAGACTCAGAAAGTCCTAAATTAAGTGTTTCAATTGATGCGACAATTAAAAATGTTATATCTC
AAGAAGAAGCTGAAAAATATTTACAAATGGCTCATGAATTTTGTCCATATTCAAAAGCGACTCAAGGAAATAT
TAATGTCGATTTAAATGTAAATGTTGTAGATTAGCATTAACTTAAAGAGATTATTCAACGTTATTAATAAAAT
TCACATAAAATTCAAATTTGCCAACCAAAAATTTTTGGTTGGTTATTTTTTCTATTCGTGATTGAAATTTCTG
GCAATATTTAACTGAAAATGATTGTCCCTTAGTCATCATAAATGTGACCGGTTCCAATACTGGCTTGACTTCT
TCGCATACCGTCTACAAATAAAAGTCCAGTGTCGACCAATAACCAATTCCATAATTTCCTGAACTTAAAAATA
AATATAATTAAATTGATAATCCATTTACAAGAATGTGCATATAAATCAATGATTGCAAAATTTTAATAAACAT
TTTAATATGTAATTAAGAAATTTTTTTAGTACAAAATCTTTCTTAATAATGATTAAGGAGGTCAGTAATGATT
AAAATTAAAACATTAACGAATAATGATTTTAATGAGTATAAGAGACTTGTTTCGACAGTCAATGAAGAATTCA
CTCAAGATTCACATTATAGTCAAACAATGACTGACACCTTAATACATGACATTTTAAATCAAGGTTCACCGAA
ATGTATTGTATTTGGCTGTTATGAAAACGAAACACTTATCGCAACAGCTGCCTTAGAACAAATTCGATACGTT
GGAAAAGAACATAAATCATTAATTAAATACAACTTTGTTACTAATAACGATAAATCGATTAATAGCGAGCTCA
TTAATTTCATTATTAATTATGCACGGCAGAACAATTACGAATCTTTACTTACATCAATTGTGTCAAACAACAT
AGGTGCTAAAGTTTTCTATAGTGCACTAGGATTCGACATTCTTGGTTTTGAGAAAAATGCAATTAAAATCGGA
AACACCTATTTCGATGAACATTGGCTTTTTTATGATTTGATTAATAAGTAATACAGTTTTATATATTCTACAT
TTCTCATAAATTCAAATCATATAGGTTCTATTTTTCATACAACTACTCTACTATTGAGTAGTTGTTTTTTTTG
GTATGATATCAATAAGTTTTTTCAGAATATTCTATGTTTTAGGGGTGTCAAAACTTAATGGTAACCGCAATTA
TTCATGGATTTATCTTAGCGATTGGTCTAATTTTACCTTTAGGTGCACAAAATGTATTTATTTTTAACCAAGG
AGCTAATCAACCAAAATATAGATATGTATTGCCTGCAATAATTACAGCCGGGTTGTCAGACAGCTTACTTATC
ATTATTGCAGTGGTAGGAGTATCTATCATTATTATGTCTTTACCTGTACTTCAAGCAATTATTTATATAGTTG
GTTTAATTTTCTTGATGTATATGGCTTGGACCATTTGGCATGATAAACCCTCAACAGATGGAGAAGCTCAAAT
TATGTCTCCAATGACACAAGTAAGTTTTGCTTTATCAGTTTCATTACTCAATCCACATGCTATTTTAGATACA
ATTGGTGTAATTGGTAGTAGTGCTGCATTATATAGTGGCAGCAATAAAATTGCGTTTACAATCGCTTGTATTA
GCGTTTCATGGTTATGGTTCTTTTTACTCGCAATTTTAGGAAAAATGGTTGGATCAATTGATAAAACCGGAAA
ATTATTAACAATAATCAATAAAATATCAAGCATAATAATTATTATTGTTGCTCTGATGATATTACAAAAACTT
ATTCAATTAATATTCTAAAGCGTTATATAGTTGTATTTTAAAAGCGTTATACAAAAGACATACTTGTGTCTAT
GTATAACGCTTTATTTATTATAAATTTGGATCAATTAACTCAACAAATTTAAATGTTCCATTGTCATATTCAA
ATTTCAGGATATTACAATTACCGATATTATGCTTTAATGCTTCATCTAGTTTAAGGCAATATCTTAAAAATAC
TCCCATTATTGTTCCATGACTCACGACTAATGCATTATTCTTTGTTTGAGACATAATGTCATGTAAAGTTTTC
ACAATGCGATCTTCAACTTGTTGCCTTGCCTCTCCTTTGAAAGGAACAATTCGATCTCCGAATAAGTCCTCAG
GCTTGTATAGATTATCAAATAGATAGACTGACTCACCTTCAAATAATCCAAAATGCCATTCCTTCAGTCCCTT
AAAACGTTGATACGATTGGTTAGGTGCAACATTTTCAAGTGTGTCACTTGCGCGTTCTTGCGTTGATGATGCA
TATAAATCGAAGTTTATCCCCTTAGTTTCATAATAACTACGTGCCTTTTGAGCTTGAGCAATTCCAAGTTCTG
TTAGCGGCGAATCTCCAAATCCCTGAATTAGTCCCTTAAAATTAAACAAAGTTTGTCCGTGGCGCATTAAATA
TAACGTTTTAGCCAACAATAACACCTCTTCAATAAAAAGTATTGATAGCTCAAAATGTATTCTCTTTTTATTA
AAATGAGTACCAAATATATTAAACCATTATTTAAACTTTTTCGGTAGCAAAAACCTTTGCTTTTGATGAACAT
TTTTTTCATAAGTAATGATGATTTTAGTTTAAAAGATAATGTGACGGGGTAAAACGCAATGAAGAGTACAGAA
TTTTAAAGGAGTGAGTGTTTATGGATAATCATAATCAATGTAATTATGTGAATCCACAAAATGTTTCACTTGA
CTGGGAATGTTTTATAATTAGTAAAAGTGAAATGTTGTTAGATGGTGTACCAAATGAACTTATCAACACTTGG
TTAGATAAAGACATTATTACACCATTTTCGATAAGAAATGATGAAATAAACTTTAAAACAAAAGATATTTGGG
ATGCACTAATACATCATAATTGGTACTACTCAAATTAATTTGCCATATATAAGCTAATAGCTCTATTTGTAGT

TTCAATTAATTGATGATCTGTGTAATTAGACATCACATATGCTCTAATTACTTGGTGTCTTTCTTCTAATCGA
GGGAATGCTTTATCTTCACTAACGTGTTCGGCCAATATGCCAAATGGTGTGTTATCATTTTGAAAACCCATGA
TGAAATTGTAAAACGTCATAAGACTTACCCCCTTCATTTATTAAGCGCTATTTAAAGTGTTATTATTAATCTT
ATCAGAAAACTAGTATTTGTAAATGTGCAAAAGTGATTTTGCACATTTTTTTGTTATCTGATTCTAAAAATAT
TAACGAATTATACAATTTTAAAGGTTTTTAGTTTGTAAATATTCTTTTCATATTAATACTACACACCTCTATA
TATGCTCACTTGTTTGAATTAATAAACTTTAGCATTCTACAATAATATTTTAAAAATAACTATACTTTTTATT
TACCTATTGGAAAATAGTGTTATCATATTTATTAAGAAGCTATTTTTATACTAGTAGGTGTCAGTCATGAATA
ACATTTTGTTAAATGCTATCAATATAGTTATTACTACCACTTTTGTTATCTTTAATATTTTAATCACATATAA
TAAAGATTTAGATGATTTATGTTGGCTCCTGCCTGGTATTATCATTTGTGGTGTGATACTCATCGTATCCTTT
ACCATTGCAATGATAACTAAAAACTGGTTAAGTGAAATATTATTTTTTATAAATATCGTACTCGTTCTCTATT
ACATTTATCCTATTTTTTATAGTTTTATAGGTTAATAATTACGGTATATAAGGAGGGTTTCACATGTCTTTAC
ATTTTGCAATTCTGTTTTGGCTAGCATTAATTTTCTTAGTTGCCGCTACGTTTATACTCGTATTAATGAAAAA
AACTGGCAAAGAATCTAAAAAAGAGTCCTATTTAAGTTTTACTGTCATTCTCTATATTTTTGGATTCGCTATA
TTAATATACACATTTATATTTGGTGTGCTATAAATGAAAATCATTTTATATCCAGTCTGAAACGTTCCAATGA
TGTTTCAGACTGTTTTTTATTCTTAATAGACATTTCACACTCTCATACTTTAATATTTAAGATATGTTTTAGT
CTTAAATGCTTATTAAAGATTACAATTCTAATTTAAGACTATTCACATATATTTCTTCAGCAAGGAGCTTTTA
AGTGAACTATATTAAACGAACGATAATTTTACTTATATTATTCGTTGTCGTATCCCCTATAAACTCACCAAAA
ACAATTGCAGATAATAAGTATTCAGAAATTCAAGATGACAAATTCCAATTAAAACCTGGTGATATTATTGTTA
CAAAAGGCCCTGTCATGTGGGGATTTTTTGGTCATTGTAGTATCGCGATTGATGATAAAACGATTTTACAAAT
TGAGGGGCCTGGCGACAAACCAACTACACAATCCTTCGAATCTTTTAAATATAATTATGCAAGTGGCAAAAAT
GATTGGATGAAAGTTTATCGTTGCAGCTATCCTGGTGCAGGTAAAAAAGCGGCTGAATGGGTTAAGAAAAACT
ATGAAAATACAAATCATCGTTATCTAGTTACATTAAATTTGAACAGCAAAAAATTTACGTATTGTACTAAAAT
TATCTACCAAGCTTATAAATTTGGTGTAAGTGAAAAATCAGTTAAAAGTTACGGACTACATATTATTTCACCC
TATGCAATTAAAGATAATTTTATAGATCCATACAAACTAAGGCTTGTTAAAGCTTATTAACACAGTTTCATCA
AATAGTTAAATTAGTTTGTGTTTTGCAATAAATTGGGTATAGATTACAAATGATAATTCAGGAGGCCCAATAT
TTTGAATGAACTAACTAAAGAACAAAAGTATACAATAGCTAAATTTTACAAATTATATATTGAACGTTCGAAT
AAGGGAGAAACAGAAACAGTCGCTAACTTTTTTGGCGATGCTAAAGATGCACGTGAAAACTATTTTTGCGATC
GTGATTATCAAGACTTCTTAACTAACTGCCAAATTTTAATTCAAAACAAATACTTAACTGGTGAAGTTTTGGA
CGATAACATTTACAATATTTCTATTTTAAACAAGACATTTATTGAATTTGAACAAAATTTTGGTTGAGCATGA
ATGTCTCAACTATAAACTTTTTAACCCTATATTAAAGCAGGAGTGAAAAACATACATGAGTGAATGGCATATT
AATAATCAATCTACAAAACCTTTTTTAATACAACAAGCGGAGGAGCAATTGTCTATTGTTAAACCATTGCCTA
ATGGTAGCTTTCAAATACTCACAGAAATAGATTTGGAGAATGGCAATGTTAATAACATCGATCATTCTCTACT
TGTTTCTGTAGACCTCAAAGCATTAGAAATAAGTATATTTGACGCTGTAAATTAAACGCAATATTTTTATTTA
ATTATTTTAATGTTCCGCCTTTTAAATGAAAACAAGATTACTGAATTATTATGAGATTTTAAAAACAATAAAT
ATTCTCAATAATTAATCAGTCTTAATAAAAAATTAACAAAAAGTTGATTAGTTAAATTATTAATTAAGTAGTA
AGATATTATGCGATGTGGTATCCTATTATTGTTAAACATTTTGAGTGTTTAACATTACTTCTTGTTATCGCCA
TATACAGACTTTAATTCTCGTCATTATTAAAGTTCTGTATTGGCGCTTTTTTTTATTCAAAATCAAAAGTCGG
ACAGATGAAAAGTAAAAAACTTTTCCATCAGTTCGATTTATTTTAGAACCATTATATATTGTTAGATCTCCAC
CTATATTTATTGGACAAAAAAGTAACAGCTAGTGCTTTTACCACACCAACTGTTATCGATTTAAAGCATAAAA
AAACAGCCCTATACTAAGGACTGTTTGAAAAAGTTAAAATTACATTTTAACTACGTTTGCAGCTTGCTCGCCA
CGGTCGCCTTCAACGATGTCGAATTCAACTTTTTGGCCTTCTTCTAATGATTTGTATCCATCTTCAGCGATTG
CTGAGAAGTGTACGAATACGTCGCTACCATCTTCTCTTTCGATGAAACCAAAACCTTTTTCTGCATTAAACCA
TTTAACTGTACCGTTATTCATATTGAATACCTCCGTCGTGCTTTTGCACTAAATATTTTGTAACAAATTCATA
AATGAAAAGGAGAATATTCTATATAAATAACTCACATTTTTCTTCACGCTCTTTATTACTTAACTTTCATTAT
ACACTTTTAAATATAGAAGTAAAGCATTATTTTATTTTCTGTGATAATACACATTGAAATATATAGTAATATT
CCATTTTAATACTAAATTTAAAATTTTTATTTTCTAATATTAAATACACTTGCTTTTGATACTATTTACTTTT
TTAATTCTGAATTGATAATAAATCATATCGTGATCTAAAAATTATAAAAGTGCCACTAGCAGCAGTGACACTT
TTACAATGAGCATTATTGACCTGAATCAGCGTTGTCTTCGCTCCAAATATTTAATTTCTCTTTTTTCGCTTGT
GCTTCACTTTTTCTTAAAAGTTGTTCATGTGTATTGTTAGGTTTATAAACATAAGCAACTTTAGCCAAGCCTT
GACGAACTAAAGCTTCGTTTACCATTTTTCCATCAGCATAAATATACGCTAAGCCACGTCCATATTTATCAGT
TCTTTGACCTTTGTCAAACTCGACTTCAATTTTCTTTGCATTTTCTACCATTTTTTTCGTAAATGCACTTGCT
TCAGGGCCATATTTCTCTACACCTTTTTTAGGATGCTTTGTTTCAGGTGTATCAACTAATAATAGTCTGAATG
TCATTGGTTGACCTTTGTACATTAATTTAACCGTATCACCATCAATCGCTTTAATTAATGTCGCAGGTTCTTT
ATGTAATTTTTTAGTTGAAGTTGCACTATATACTGTTGAATCTTCAGAACCAATTCTATTTACGCCGTTATCT
GTTTGTGATGCATTTGCTGAGCTACTTAGACTTGAAACTACAACTAAAGTTAACACTAAGCAACTAGTAGCGA
AAAAGAAAAACCTCTTTGCGTATTGCCCTTTCGAAACATTACTGATAGCCATCCCTATAAGTAATATTGAAAC
AATTGCCATACATATGCCAGCACTTAATAAGTATTCTGTCATAACTAACACCTCTTTCTTTTTAGTTAATTTT
AATATTAAACGTTAATTAATTATAATTCTACATTTTTACAATTAATTAATAAAAATTTAATAATTATAATAAA

ATAACTATACTTACTTTTATGTATTTTCTATATTTTAGGTGTCAGAACTTATATATTTAGCCTTATTTGTAAA
ATTAAAAAGCCTACAACATGAATCGTTGCAGACTATCATATCGAGACAAAGAGGTTTATTAGTGTCTCTATAT
CTATGTGGAATTTGAAAAGATTATTTATAATATTTACGGGGATAAATTGTCATTTAAAAACGAGATGTATAAC
TTATAATTTACTTTGGATTAGCGTTTTTCAGTTTTCTCTATCTTATTATTCACTTTATCAATTTGTTTATTCA
AATAATTTTGATGAGACTGACTAAGTGTACTTACTTTTGCAGTTGCCTTAGCGAGATCTTCATAACTTTGTGT
ATCTTTATAATCGTTAATTAAGCTTTGTGCTTCTTTAACATGATCACGTCTAATAGCGCGTTTGTCACAGTCT
GTGAAATGTGATGAGTCAATTTCACTATAGCCATATAACCAACCTTTTTTAATGCCTGTTTGATTAGTATGTA
AATCATATTCTATGTAGTTGTCATTAGATAGTGATTTAGTGTATTTAATTGGCTCTTTTCTACTATTGAAAA
TTTCTCCTCAGTAGTAATTTCAGTATGGGTTTGTAAAATATTATTATTACCCGAATCTTTTACAACAAAATTA
GTTTCAACACTCTCTGTCACTAATGCGTTTGCGTGATTAGAATGCCATAATTGCGTCGCACAAAGTACGCCCA
TTGTTAATACTAGTACTTTCCTTTTCATTTTTTACTCCTTATTTGCATTGATTGTAAAACCGACTTTTTCACC
CTGTTTTCTTCGCCATCGAAATTATATACCAATTTTTTAGAAATAATAATTAACTTGCGATGAACAAACTATT
AACAATAATCTTGAGTCTTATATTTATCTTAATTAATAATATATTTATTTAAGATTATGTTACTTCCAACTTT
GAAAGTAGAAAAACGGTATAATTTGTTGATGGGTGTTGATTGCTAAATTGCAAAAAACATAGCCTACAACCTC
ATAGATTGCAGACTATATTTAAAATAATAGGTATTTATCATATCTCGTAAACTTAGTGCCAATTTTTATACTC
GTGGTGCTGGTAAAATGCCCTTAAATTCAGGAACAACGTGTGTAGGGCTATAACTTGGAACAGCATATTGATA
ATTTACATTTTTGATATTTAATGCTGGTTTACCAGTTTTAAAACCATTAGATTGTGAAAATGAGAAATATTTT
TTCACACCTTTAACTACTTTATAAGAATAGTAGTATTTATAGTCATATGCTTTATTTACTTTAGCATTTTGAT
GTGTTGCTGTTGTGTTATTTTGGAAACTTGGTACATGCATACGGTGAGAATTGTGACCATATGGTGGAATTAC
CTTGAAACTATTTATTTGTGGCACAACACAAAAGTGATTAATTTTAATGCTAGCATGCCCTGGTGTAACAAAT
TTATGCGCGTGATATCCAGGAACTGCAAAATGATGCTTGATAATTAAAGATTGAGATGGATGTGTATATCTAG
GCGATTCTGATGGTTTAACAATAAAGTGTTTATTAATAGAATCCTTTGCATGATTTACATGTTTATGTACATG
TGTTGATTTGTATGAAGTAATAACTTTCTTGTAGTGGGTTTGCGTAGTAATGAAGTGGTGGTTTACTTTGTTT
TGCGTAATAAATCTATTTACTTCTGGGCCTTTAGCAACAAAATGCTTATCAACTTGTTGTGAAGCTGGATTGT
TTGTTGCTACATTCACGCGATTATTGATTTCTTTGTACTCTGGCACAATGTTTCCTAACTTTGATTCTGGTAC
GACAAAGTTTTTATCTACAATTTTACTTGCTGCTGAAGGATCTGAAATCTTATTCAATTTGCCTTCAGAAACT
ACAAAATTTTTATTGTCAGTTAAATTACTAACATTTACTTTTGGTGCACTCTTAAATTCATCGTAAGCTGGAA
TAATTTTATTAATACCTGATTCTGGAACTACAAATTTTTTGTCAACACTCTCTGTCACTGATGCTTTAGCGTG
ATTTGAATTCATAATTTGTGTAGCAAATAGCGTGCTCATAGTTAAAACTAATAATTTCTTTTTCATCTGTTAT
TTCTCCTTTATATAGACTCAATATTATAACCAATTTAATTTTCCTGTTATATTCACTAACAGCATTATATACC
AGAATTTTCAGTATAATAATTAACTTGAAGTAAACGTTGTCTTAACATTTTTATTGTTTTTCAGCTTAAAATT
AATTATTGATATTGATAGTTAAGCATAATAATTTTTTCGTAATATAAAGTGAAAAAAGTAATAGTCCACACCT
GTTTAGAATGTGGACTATACTAGATTGCATCATTGAATTGATGACTTTGATATTATTTATTGCTAGTTTAAAA
TGTTGTGATTTTTACGACAAACTCATTATTTGCTTTGCAGCCATGCATTAATATTATTTGCCATTGTATACTT
TATTGATTTGTTTATTAAAACGTTTTTGATGACCTTCACTTAAAGTATTTACTTTTTGTTGTGCATTTAATCG
GTCTTTATAACTATGTGTATCTTTATAATTATCAACTAACTTTTGAGCTTCTCTAACGTGTTCACGCTTAATT
ACACGCTTCTCACGTTCAGTTAGATGAGAAGAATCAATTTGACTTATACCAACCAGTGATCCTGATTTTCTAC
CAGGTAAAGCAGACGTTTCTTCAAATTCAATAAGATGATTATGCGTATGATTTGAAGTTGTTGGTGATTGATG
CGTTGTTGTGAAATCATGACTTTCACCATTTAATCCAGCCAATTGTTTTGGTGCATTATAAATAATTTGTTGC
TGAGTTTGACGCTCAACTGTAGGCATAGGTACCTGTGTCGTTGTTTCAGTATATGTAGGCGGTGTTGTTGGTT
TCTTTGATGGCTCAGAAACCACAAGTTTCTTGTCGTTTTCGTTGTCGTCTTCGTCATATTCTAACGATACAAC
TGGTGTGTTATTTTTATTTTTTTGTTTAGCTAAAAATCGTTCTTTTATTTCTTTAAATTTCTTTTCATAATTA
GCTTTTTGTTCTTCAGTTACTTCGTTTATTTTAGATTTGTGATTTTGAGTATTTAAACTTCTTTTACTTCTTT
TAGATGTATTCGCTTTAGCTTCTTCTGTATCTGCTTTTAGTTTTTTTGCCATTACTATATTTCTTCATTACT
TTCAATTAACGCCGGTATATTCAACGGCCTATTTTCATTGATTTCAACAAAAAAATTCATCAATAATTGATTCT
AAATCTTCAACTCGTTCTTTAGATAACCTATCATTTTGAGGATGCCTTTCTTCTCTCTCATCATCACTACGGC
CAACAATCAAATCTAATTTATTATAAAGATTCTCTCTTGCTTCATGTTTATTTGGAAAAGCGTGTCCAAGCAT
TAATACTTTATTTTCTAGTTCATTTAATTTTACTCTAACTTCGTAATCTTTATCTCTATCAAATTTTTTCAAA
TCAGGATTTCTACTTTCGATATCTTGTGTCTTTCTTTCAAACTCATTTTTATTTTCCTTTATTGCTTCATGAA
TAGCTAAATACCTTTGATGTGTTAAGCCCAATACACCATTTGCAACTTGGTTGTTATTTTTTTTACTACTTTC
TATTTTTCTCTCTTCATCTAAAAACTTGTTAAGCGCATCTAATTCAGCAAATAATTTTTTTCGGTAAGTATCC
ATCGCCTCTTTATATTCAGGTTCATCATATCCATCATTTTTATTAATTTCTGATGTGCACATAACTGTTCGAA
GACTATCTTTATATTGACCTAAATTCCATGAATTTGTCTTTTGACCTTTAATTGATAAAGCTTTTGATACATA
TGGATTCTTCTCCCCAGAAACCACTGCACTCGCATGATTACTTTCCCAAATTTGTGATACACATAATGCTCCC
AATGATAAAACTAGCAATTTATTTTTCAACTGTATTTTCTCCTTAATTTTCCCTAATTAACAACCTACCTTAC
ACCCTATTTTTTCGCCAAGCCAAATTATATAACATCTACCTTTTTAAAATAATTAACTGTTGATGAATTAATT
ATTAATTATATTCAAACAATATATTTGGAAAATCCAAGTAAAAAAGCCACCTTTTAAGGTGGCTTCTTCATGA
ATCATATTATTAATTTAATATCATTATTACTTATTTCTTATCTTTATTTCTTTTTTTCTTCTGAAAAGTAGT

AATGAACCTAATGATGCTAATAATCCCCAAATTAGTGACGTATTCGCTTCATCTTCAGAACCTGTATCTGGTA
ATGGCTCTTTACTATCTTTAGCCTCATTTTTATTAGAAGCATTAGTACCATTTTTAGGTGAATTAGGCGGCAC
TACATTATTGTTAGAACCTGAGTCGGAGTCACTATCTGATTCTGAATCGCTATCTGAATCTGAGTCGCTGTCT
GAGTCGGAGTCACTATCTGATTCTGAATCGCTATCCGAATCTGAGTCACTGTCTGAGTCGGAATCGCTGTCTG
AGTCTGAGTCACTGTCGGAATCTGAATCGCTGTCTGAGTCTGAATCACTGTCTGAGTTGGAATCGCTATCTGA
ATCCGAGTCACTGTCTGAGTCTGAATCGCTATCTGAATCCGAGTCACTGTCTGAGTCTGAATCGCTATCTGAA
TCCGAATCACTGTCTGAGTCTGAGTCACTGTCGGAATCTGAATCGCTGTCGGAATCTGAGTCACTGTCGGAAT
CTGAATCGCTATCTGAATCTGAGTCGCTATCTGAATCTGAGTCACTATCTGAATCCGAATCGCTATCTGAATC
TGAATCACTATCTGAATCTGAATCGCTGTCTGAGTCGGAGTCACTATCGGAATCTGAGTCGCTATCTGAGTCT
GAGTCACTATCGGAATCTGAGTCACTGTCTGAGTCTGAATCGCTGTCTGAATCTGAATCACTCGCTGAATCTG
AATCGCTCGCTGAATCTGAATCACTTGCTGAGTCTGAATCACTTGCTGAATCTGAATCACTTGCTGAGTCTGA
ATCACTTGCTGAATCTGAATCACTTGCTGAATCTGAATCACTTGCTGAGTCTGAATCACTTGCTGAATCTGAA
TCACTTGCTGAATCTGAATCGCTTGCTGAATCTGAACCACTATCTGATGTAGAATCACTACCCGAATCTGAAC
CGCTATCTGAATTAGAATCGCTGCCAGAATCTGAACCTGGGTCAGAATCTGAATCCTCTGGAATTGGTTCAAT
TTCACCAGGCTCATCAGGTTGTTCAGGAACAACTGGTTTATCGATACCGTCACCAGAGCCTGATCCGTTATTA
AATGCTACTTCGTTGTCCCATGACATAGATCTCCATATAAAATTAGAATCATAACCATAAAATGTCGAACGTA
GTGCTAAATCACCTGTACTAGCAGGATCAATATGGCCATTAACAACTACAATATACGGTGTTGTAATTTGGTC
ATCGTCCGTAGGAAATTCTACTTTGTATTGATTAGCATTTGGAAATGAAATTCTAACTTGATTAGTTACATCT
TCAAAATCGCTAGGATTCACATAATAACTTTCAGATAAATCATTAGCATTATCGACTCTATAAACTTTAATAT
CAGTGTTTTTTGCATCTATTAACGCATTACTCTTTGTATTAGGAATTAAATTACCTGTTAAGGCAGGTAACAC
AACGTTATCTCCGCTTGGATTGACATAAATTGTTTGGCGATACGTATTATTTGTTTTATCGATTTGATCAATC
GTACCTTTAATTGATAAATTATGGAATTGTCCATATTTCTCATAGTCGATTAATACTGTCTTACTAGCAGTAT
TGGTTCCTATGCCAGTTGTCAATGTCACATTACCTGTCTTTGTAACATTTTCAGGGTCAATATAAGCTGGCAT
AGTAATATTAGCTGTTACATTTTCTTTATTATCAACATAGTCTGTAAATGTATAAATAACATTACCATCACTA
TCGATTACACCATTTGCCAATACTTGATCTCCGGCCATAATTGGTGGCACCTTAGCAGTTGAAGTTACACCAT
TTAAGTTTAATTCTTTAGGTACAGTTATTTTGAATGTGTCACCTTTAACAGCAGAATTAGGGACTGAAAAACC
ATAATTCAGTTTGACATAACCTGCTTGGTGCGGATACACAGTCGTACCAGAGTCAATAGTAACTTTCACATCT
GTCAACTGATTCGTAATATCTGTGCCAGCAGCCGGTGCATCTGCAGCTACTGCCGCTAAACTAAATGCTCTCA
TTCTAGGCGCACTTGTATTAACCGCTTGATTAACTACATCTTTATTACTTGCATCTGTACTCTGTGGAGCTGA
TTCATTGTTTGAAGGTGTTGCTTCAGTTGAAGTATCTTGCGTTGTTGAAACATTTTCCGCATTTGTAGAATTT
TGAGGTGAATTTACAGATGATACTGTATTAGTATCATTAGAAGTCGTTTCATTATTTGTTTGATTCACTAATT
CCTCCGCATTTGTATTGCTTGATTGAGTTGTTGCCGGTGTATTAGCTTGATTCGTTGCCGTAGTAGTAGCTTC
ACCAGTTGCCGGAGTTTCTTCCGTAGTTGCATTTGTTGATGCTGATTGTGTCGTTTCCTGTTGTGCCGGATTT
TGCGCCACACTTGTTTCGCCACTATTAGTGTTTGACGTTGTTTTAGTATCACTCACGTTTGTGTCGACTGTTT
TAGGTGCAGCACTAACGCTACTTGAATCATTACTTTTGCTTTCGTTACTTGCGCTATCGGATTGCGTAACACT
ATTTTCACTTGCATCTGCTTCTTTACTGCTGAGTAGTCCAAAACCGATTAACGTACCTACAAGCACTGAAGCC
ACGCCAATCGATTTTTTCCGAATTGCGTGTTTTTCTTTTTTCTTCATATTCATTTTATTCCCTCTTTTTAAAA
AGTCATTTTATATTAACTGTATACCCTTTAAAGATATATTTAATCTCCATTAATGCAATTATACACTGAAATT
GCATTATAGCAATTAATTTGTATCGATTTTTTATTATCCACAATAATACTTTCCTAACAAACATTTTATTTAT
TGCTATTTTATAAATTGCAAACGACAACGTGCGATTTCATTGCGAACAATTTGTATTATTAATATGAACTCTG
CCTAATGTAATCCTATCTTGAAATCATATTTTTTCAAAAGCAGATGTGTAATTTATGGTACCTGTTTTTCCCG
CTAAACTGTTCACTTTTAATTCTTTAATTAAAAACGCTTCGTCTGGTATACCATCATATGGTGGATAAATGTC
GTACATACTTGCGCGTTGATATCCTAGATTGGCATAATACGTCGGCCATCCTAATACACTGATAAAATTATAT
CCTTTTAATATTGCTTCTCGTTCTAATGCTTGAATCAATCGCTTCCCTATACCTTTATTTTGATGATGAATAT
CAACAGATACAGGTGCTAACACTAATCCAATTTCCCGTTGTGCTCCGTTATCAAGATAAACTTCACTTAGTAA
ACCGTGCCCTACAACTTCGTTTTGAAGAACAGCTACTATTTCTAAGTTATTGTCATACGTATCACTTAGACGA
ATTTGGTCTACTAGCTCTGATTCATTACCATAACCATGTTCACTATTTTCAAATGCCGTTCTAATTAACTGGT
CAACTTGAGCAAAGTCATGTTGATGTATTTGTCTAATTTGCATCTTTGCCCCTCCTTTTAGTTCTATCTTATA
GAAAAATGCTTTACAACATAAAGTAATATTTTATAGTAATGACGCCATTATTTCATTAAAATTGAATGATATT
TTTCGCAATAAACAACTACCCTCTTAAATCAATTTTTAGATAAACTGCAAATCATAAATTTCTTTATTATTAT
AAGTATTAAAACGGTCGATTTGACCTTTTAATATAACTTCCTGTTGTTTGCGACTTACATTATGATAATAAGT
CATACGTACATTCAAATCAGCTTCATAACCATTGTTCTCAATCGTTCTTTCTAATTCTTCGTACACATTTTTA
TTAATTGGTAACATGTACCCTTCTTTACTAGGTAAAATATTCACGTATTTTTTCTGAATGACTTTATCATTAC
CCAATAAAACCTCTATTGCAATTTTTTTAGCAGCACCGCCACCTAAATTATATAGTTTAATATAATCTTTATG
ATGATGCTCTTCTGTTGCCGAATTCATTATATTAAGTTGATCACCCTCCCTTTCTAATAAAATTTGGTTAAAA
CCTAAAGCTGGCAGAAAGCTAATTTTCATTTGATAAAGTTGAACTGAAACTGATACAAAATAAAATAAAGCCA
TAATAAATGTCCCAATAGATCCTATAGCTGAAATAATATTAATCATTAAGATATCAACCTTCCCATAAGAAAT
TTATATGATTAATTACAAACTGAAAAAGCATTTTACTTAATCGTTTATATCCAAAAACAATCATTTTAAACGT

TATATTCGACTACTCATATAAAAAGTTTTGCTTTAAAGCCTTAAGTAATTTTTTAACCATCAAGTACGATTAT
TATTACGGCAATCTATTTGCATTGACTAAAGAATACAATAACGCTTTTGAGAGCTTCCATCTTTGACCAGTAT
TTTCCAAATTAATAACACGTCTTCTTCAATACATATTCATCTCATACAAATAAGTTGATTTTTTTATTTTTAT
AAACGCAATTTCTTAACAAATCAAAATTTTACATTTAAATCTTTCGATATATTACAAGTTTTTTAAAAAAGTA
ATATATTTCTATCAATAAAAGTAATATAATATTAGTAATTATAGTTTCATTAATTTATTGCAACAATACATAG
AATTATTATTACAAGAATTATTTAATATCATCTTAGAATCTCACAAAATATCAACTTTGTTTAATTACTCAAA
ACGATAAGTCGTTTAGAGTTTTTAAAATAAATTTTTGCGAAATAAAGGAGACATGTTAAATGAAAAAAGTAAT
GGGGATATTATTAGCAAGTACACTTATCTTAGGTGCTTGTGGACATCATCAAGATAGTGCAAAAAAAGAGAGC
ACTAGTCACAAAAAGAAAGAAAATGACAATGAAGAATTAAATGAAGAACTTAAAGAATTTAAAAGCAAAAAAA
ATATGGATATAAAAATTAAAGGCGATACTATTGTTAGTGACAAATTTGAAGCTAAAATAAAAGAACCGTTTAT
CATCAATGAAAAAGATGAGAAAAAGAAATATATCGCTTTTAAAATGGAAATTACTGCTAAAAAAGACGATAAA
GATTTAAATCCATCTTCTATTTCTCATGACTATATTAATATCACTCAAGATGATAAAAATACAGTAAATAAAT
TAAGAGATGGTTATCTTTTAAGTGATAAAAAAATATAAAGATTGGACAGAACATAACCAAGATCAAATTAAAAA
AGGCAAAACTGCACAAGCCATGTTTATCTATGAATTAAGAGGTGATGGAAATATTAATTTAAATGTCCATAAA
TACTCAGAAGATAAAACAGTTGATTCTAAATCATTCAAATTTAGTAAACTTAAAACCGAAGATTTTTCTCATA
GAGCGGAAACAAGAGAAGAAGTAGAAAAGAAAGAAAAAGAATTTGAAGAAGAGTACAAAAAAGAACAAGAACG
AGAGAAAGAAAAAGAAAAGCATAAAGATGACGACCACAGTGGTTTAGATGAAGTATAAATTTATGAATGCTCG
CATTCAACGTTATAATCTTTACTGTTATTATTGTCATTATAGATTAGCTTACTTACACTCATAATGACATACT
AATTTAATTGTTAAATATAATAAAAAAGAGTAGCTCGCCTACTCTTTTTTGTTGTTTTGAATGAAGAATTATC
TTATATAAAGAATTTTTTTTATGAACAATATTAAAACTCAAAGCTCAACTTTTTTGACACTTATTTGACACAT
ATTCTTTCAACCCTTAACGCAAAATCTTATATGTACACAACTATTAATTTTTCATTCATGAAAATTTGAACCT
AAGCAGATACTATCATATCAAAGTACACAGCTAATTTAACCTTCCTCACAAGAAAAGATAAAAAACATAAAAA
AGAGCAAGGTCAACGTGTCAGAAAATTATTTTCTTGACACATATTTGACACTTGCTAGATTAAAACCCCATTA
AATCAAGGTTTCCAAGCATATTTTTATGGAGACGGCGGGATTTGAACCCGCGTCCAGAGGTCCTGATACACAT
CTTTCTACGTGTGTAGTTTATCGAAAGGTTTCGCATCATGAAAAGTGATAAACAACCAACATGATGCTAGATT
GATTAAGTTTCTTCTAAACAGACTTCAAACGGCAGTGTTTAGCATATCCTATTAAGGTTGAATCGCGTTAACA
GCACATAGGAAATGCTGTTAGGCGATGCAGAGTGCGATTAGGCAGCTACTGCGAAATTATTGTTTGATTTGCC
AGTTATTATAAACTGTGTGTGTTGATGACGAAGACAACCCTCCGACACGCTTAATGAGCTCGGGGGACCCCTG
TCGAATCCATGAACGTCCCCGAAATAATAAATCAAACAAATCATACTTTTGTAATTTACAAAAGCACATATAA
CTATATCAAATATAGCCCGATTAAGCAACTAAATCGCTTAATAACGGGCTTTCATATCGCGCGCAACATCTCG
TTTGACTGCTTTTTCTTTCAAAGCTTGACGTTTATCATATTTTTTCTTACCTCGTGCAACACCAAGTAATACT
TTACAATGTCCATGCTTCAAATAAAGCTTTAACGGCACAATCGAATAACCAATCTCACGTGTTTGATCACCCA
ATTTAATGATTTCACGCTTGTGCAATAATAATTTTCGAGAACGAAGAGGATCGTGATTAAAACGATTCCCTTC
TTCGTATGGTGCTATATGCATATTATTCAAATACATTTCACCGTTTTTAACTTGCGCATAACTATCTTTAAGG
TTAGCACTACCTCGGCGAATTGATTTTATTTCTGTGCCTTGCAATACAATTCCCGCTTCAATCGTGTCTTCAA
TATTATAATCATGTCTAGCTTTACGATTTTCCGCTAATGTACCTGGTGATTTCTTCTTAGCCATTCATACTCA
CCTCATTGCTGCTTATTTTTTCTTACGACGTGCTTTCTTTTTCACACTTTTATCTTTATAAAATGGCTTATGT
TTACTGTTTCCTGATTTGTCATTTTTACGTTCATTTTTACCTTTACGTTGCTTACCTTTTTTCTTATGACCTT
TATCATCAGATTTCGATTTATCTAATGATTTACCACGCGTTTTAGCTTGAATTGTCTTACCTCGCGCTGGGCG
CTGTGATCGATCATTTTTCGGTAAAGGCATACCTACAATTTGAAAATCAATTAATCGTTCATCTACATCAACA
TGCGTCACTTTAACCTTAATTGTGTCACCAATTCTAAATACTTTAGCTTGACGCTCACCAATTAATGCCATTT
GACGCTCTTCAAAACGGTAATAATCATCAGTCATATTCGCAATATGAACCATACCTTCTATCGTATTTGGCAA
TTCAATGAACATACCGAAGTTAGCTACTGAGCTGACAATACCTTCAAATTCATCACCAATATGTTGAATCATA
TATTCTGCTTTTTTCAATTCATCAGTATCACGTTCTGCCTCAATAGCACGACGTTCACGTTTAGAAGTATGTT
CAGCTAACTCAGGCAATTTGTCTTCCCAACGCTTCACTTCTTTGTTATCCATTGATTTCTCAATTAAATACTT
ACGGATTAAACGATGAACTGTTAAATCAGGATAACGTCTAATTGGTGATGTAAAATGCGTATAATATTCAGCT
GATAAGCCAAAATGTCCCAAGTTCACATCATCATAATGCGCTTGTTGCATTGAACGTAACATCATTGTTGAAA
TGACCATTTGTTCAGGTCGACCTTCTACTTCTTCTTGAACCTTTTGAAGTGTTGTTGGATGAATATCTTCGCC
AGTACCCTTAATCATGATGCCAAAGTTTGTAATAAAATCAAAGAATTGTCTTAAGCGATCTGATTTAGGTTGC
TCATGCACTCGGTAAATAAAAGGTACATCTAACTTACTAAAATGTTCAGCAACTGTTTCATTTGCAATTAACA
TAAATGATTCAATTAGACGTTCACCCTCGCCACGTTGTCTTAATTGAACATCTGTAGGTATACCATCTTCGTT
AACTAATACTTTTGCTTCACTAATATCAAAATCGATTTCACCACGTCGTTTTCTCATTTGAATCAAACGATTA
GATAAATCTTGTGCTAAATCTAGCATAGGCGTAATTTCATTATACTGCTCTCGAATCGCAGGATCCTTTTCAG
TAATAATCTGATTTACCGCATCATACGTCATTCGATAATCAGAATGTATAACACTATCAAAAATTTCATGTTT
AACAACGCGACCACTAGCATCAATTTCCATGCGACAGCTTAACGTTAAACGATCAACGTGAGGATTTAATGAA
CAAATACCATTGCTTAATCGATGTGGAATCATCGGGATCACACGGTCAACAAGATATACACTTGTCGCTCTAT
CATAAGCTTCTTTATCTAAAGCTGAATCTTCAGTTACATAATAACTGACATCAGCAATACTTACAGTTAATTG
CGTATTACCGTTCGCTAACTTTTTAACACTAATTGCGTCATCTAAGTCTTTAGCATCAGCACCATCAATTGTG

ATTGTCAATTCATCACGTAAATCATGACGGCCTTTAATTTCAGTATTCTCAATATGATCAGGTACTGCTTCAG
CTTCTTGTAACACTTCATCAGGAAATTCAATTTCTATGCCATGTTGATAGATAATAGATAAAATATCTACGCC
AGGATCATTTTTATGTCCTAAAATAGCAGAAATATGTCCTTCTGGATTATCTGAACCATCAGCATATTTAGTA
ATTTGAACAAGTACCTTATGCCCATCTACTGCACCTAAACTTTGTCCTTTAGGAATGAAAATATCTTGCATAA
TACGTTTATCATCTGGAATAACAAATCCGAAATGTCTAGCTTCACTATACGTACCAACAACTTGAGTTACTGA
ATGCTTCTCAATCGACTTAACTTCCCCTTCAATTTTACCTTTATGTTCACCTTTAGATTGATGTATTTCTACA
ATTACAGTATCTCCATCTAAGGCACGATTAATTTTTGTTGGGGGAATAAATATATCTTCCATATCCTCATCTT
CAGGTCTTAAGAATGCAAAGCCTTTTTTATTTTGACTTAACGTTCCCTTTATCAATTTTGAATGACCTTTTGA
ACTATGCTTTTTTTGATATCTGTCTGTTTTTGTACGTTCAATTAATCCTGATTGTTCTAATTCTACGAGCACC
TTAATTAAATCTCTAAATGAGTCGGCACTACTTAAACCTAATGCATCTTGAAAATCTGACACTGACATAGGTT
CATATTCAGGTTGATTAATAATCTCTTCTATTGATTGCTTTAAATTCATTATGCCCCTCCTTTCTAATGTTAA
AATCTCTTTTTATTCTGACCAATCTAATGACTCTAAAAATTGATAAATATCTTCAAATACTTGTTCTTTTTCT
TTATCAATCGTAATAACATGTCCAGATTCACTGTACCACTTGATATTTTTATCGTCAGAGTCTACATGTTCAT
ATATATAATTTGCGGATTGTGGATCAATCATATTGTCGTTTTCTGCTTGAATCACTAAAATTGGGTCAAACAC
CTCATCAACTTGATCTTTAATCGTATCTAATGCTTCACTTAATTCTTTTAAAGTTTCAGTTGGTTTAAAATGA
TGCATTTCGTTGTCAATCGTTTCTTGATCTTTACCTTCATACTTTTTAAAATTGCGTGCATATTCTAAAAAGC
CTTCATAAATGGCACCTTCAGTTTTGCCACCCATAGGTGCACACATCGTTACAATACCCTTTACATCTCTATT
TAAGCTTAATTTTAATGCAAAATCGCCACCTAATGACAAGCCAGCAACAACGATTTCATCATAACCTTGTTCA
ACAAGGTAGTCATAGCCATCTAAAGCATCTTTAAACCAAACAAAAGGACTTGATTGAAGTATCTCTTCTGGTG
GTGCTGCATGACCTTCATATTGCGGTGCATACGAAGTATATCCCTTTTTTTGTAAAAATCGACCTAATTGACG
TACATCAGACGAATTGCCTGTAAATCCATGTAATAATAACACGGCACGTTTGCCTTCTTCAAAAAAGAAAGGT
TTTGGTAATTTTATCTGCATAAATCTAACGCTCCCTTTGCATACTTAACTAGGCTATATATTTTACATAGCTT
ATTGTTAAACTATTACATTCTTAATTATAAATAAAAAAGCCCGACATTTACATCGCCGGACCTTACATACCAA
GATAACTTATGCAAATCATAAGTACAAAAAATAATATTGATAAAATAATTGTTAATCTATTTAAGAATAAATC
GACGCCACGTTGTTTTTGTTTACCGAATAACTGCTCAGCACCACCACTGATGGCACCTGAAAGTCCACTGCTT
TTACCTTCTTGGAGTAGTACAACAGTTATTAATGCAATACAATCAATGATTAATAATACGATTAAAAATGTAT
GCATAAATTGTCCTCCGTTCCTTATATACGACAAGTAGTATATCATATTTTTATTGTTAAAATCAATTTTCTA
GCGATTAAATGTACTTTTACGCTTAATTGAAACAACAAGCATATAAATCGCTAATGCAACCGAACCTATAATA
ACAATTAAAATTGGCAATGTTAAGTACGCACCGTGTGCAGCTTTTATTGCTTGAATTGTGATTGATAAATTAA
TCAACGTAAACAAACTATTTGATACATAAACTGCAAACATAAACCACCACACATAAGTATGTCGATTCCAAAA
AGTGATGAAGCCTGCTAGATAAGCCAAAATATACATAATACCAGTGAATCTAAAGCTGTTTAAAATGTAATCA
ATCGCTTTATCTGAAGTGTTTTGATTCGTTAAATTAAGTAATGCTTTGGCAGTCGCATTATCCAACGAAACAA
AGTAATGAATAATCAATAATATTGAGAATACGATTGATGCAGTTGCAATAAAGCGTCCTGTTTTAATTTCTTT
ATTCGTTCTATCAGCCATTTGTTTACTCCTCATATATTTAAATACGTTCTATAAAACCTTTATTTAATTTTAC
CTCTTAATAAAAGTGAATACCCTGTCATACAATATTCATTTCAACATAAATTTATTATACCAGTTTAAGAATA
CAAGTTAAATATGTGTTATTAGTTACCTCATGATTTATCAAGCCCCTTATAAATCATGATTTGAAGCCATCCA
AAATAAAAAACCTACCACAGTCTCAACTCTGTGGCAGGTTATAAATACAAGCGTGAACTTTTTAAGCGTGAAC
TCAAAAACTTTATTCAATTTATACCGACATAATAATCCTCAACTAAAATTATGGCAGTACTTAATGTTTAAAA
TGATTATTTATCTAAGTTGTAGAATGATTTGATACCGTCATATTTAGCAGTTTCAAATAATTCATCTTCGATA
CGTAATAATTGATTGTATTTAGCAATACGGTCAGTACGTGATAATGAACCAGTTTTAATTTGACCAGCGTTTG
TAGCAACAGCGATATCAGCAATTGTTGTATCTTCTGTTTCACCTGAACGGTGAGAAACTACTGCTGTGTAACC
AGCTTTTTGAGCCATTTCGATTGCATCAAATGTTTCAGTTAATGTACCGATTTGGTTAACTTTAATTAAGATT
GAGTTACCAATTCCGTTTTCAATACCTTTTGCTAAAATTTCAGTGTTTGTTACGAATAAATCGTCACCCACTA
ATTGTACACGGTCACCGATACGTTCTGTAAGTTGTTTCCAACCATCCCAGTCGTTTTCGTCCATACCGTCTTC
AATTGTAATGATAGGATATTTGTCTACTAATTGTTCTAAGTAGTCAACTTGTTCTGCAGCTGTACGTTTTGCA
CCGTGTTCGCCTTCGAACTTACTGTAGTCATATACACCATTTTCATAGAATTCTGATGATGCACAGTCAAATC
CTAAGAATACTTCTTCACCTGGTTTGTAACCAGCTGCTTCGATTGCTTGGATAATTGTTTCAACAGCATCTTC
AGTACCTTCAAATTTAGGAGCGAAACCACCTTCGTCACCTACTGCAGTTTCTAAACCACGTTTGCTTAAAATT
GATTTTAAGTTGTGGAAAATTTCAGTACCCCAACGTAATGATTCTTTGAACGTTGTAGCACCTACAGGTAAAA
TCATGAATTCTTGGAATGCAATTGGAGCATCTGAGTGAGAACCACCATTAACGATGTTCATCATTGGTACTGG
TAACTGCTTACCATTAAATCCACCTAAATATTTGTAAAGTGGTTGACCTAATAAGTCAGCTGCTGCACGTGCT
ACTGCGATAGATACACCTAAAATAGCATTTGCACCTAATTTACCTTTGTTTGGAGTACCGTCTAATGCGATCA
TCATTTTATCAATAGATACTTGATCTAATACTGAAAATTCACCTTCAATAATTTCTGGTGCGATGATTTCATT
AACGTTTTCAACTGCTTTAGTAACACCTTTACCTAAATAACGTGATTTGTCTCCATCACGTAATTCAACAGCT
TCGTGTTCACCAGTTGAAGCACCTGATGGTACTAATGCACGACCAAATGCGCCACTTTCAGTTAATACTTCTA
CTTCAACAGTTGGGTTACCACGAGAGTCTAAGACTTCGCGAGCGTAAACATCTGTAATAATTGGCATGTTTAT
AATCTCCTTTAAATAGTTATTCTATAATTATTATAGCTTTATTCTTTCATTTAACAAAATGATATTACTATTA
TTTTCAAAAAATAAGTGTCATTAAGCGTTTACTTAACATCTTTTACAATATTAGTGTTTAATTAAAGATTCAC

CTGTCATGTCTTCAGGTTGTTCTACATTTAATAAATCTAATAATGTAGGTGCTAAGTCACCTAAGCGACCAGT
TTCTCGAAGTGTAACGCCTTCTTTTGTTACAATCACTGGTACTGGGTTCGTTGTATGCGTAGTCATTGGTTGA
TCATCATCCGTCAATACTTGATCAGAGTTACCATGGTCAGCAGTAATAATTGCATAACCGTCCATGTCTAAAA
TCTTATCAACGACTTCACCCAAACATTCATCAACCGCTTCGATTGCTTTGATTGTCGGCTCAAGCATACCACT
ATGTCCAACCATATCAGGGTTAGCAAAGTTTAAAATAATTAAGTCCAAGTCACCTTTATTTAACTCTTCTAAT
AATGCATCTTTAACTTCATAAGCACTCATTTCTGGTTTCAAGTCATACGTTGCAACTTTAGGTGAATCAATTA
AACGACGGCGTTCACCTTTAAATTCCTCGTTACGTCCACCACTCATAAAGTAAGTAACGTGAGGATATTTTTC
AGTTTCTGCAATACGTAATTGTGTTAAATTGTTATTTTGTGCAATTTCACCAATTGTATTATTTAAATCAACT
TTTTCGAAAACGATAGCCGCATCGATATTGTCGTTATACTTAGTGAATGTTGCATAGAATAAGTCTTTAACTT
GTTCAACTTTAAAGCCTTCGAATGCTCTGTTCGCAAAAATTTCCGATAATTGTGCTGCTCTATCAGGTCGGAA
ATTATAGAAGATCACTGCATCTCCATCATTAACACCGTCATTTTGATTCTCAACGATGAATGGTACTACGAAT
TCGTCAGTTAAGCCCTCATTATAGCTTGCTTCTACACCTTCTTTGGCAGTTGCATAAGTTGGGGCATCAAAAT
TACGAATAGCATTGTAAGCTTTTTCTTCACGTTCCCAACGTTTGTCACGGTCCATTGCATAATAACGACCAGA
CACAGATGCAAATTGACCAATGCCTAATTCATTGAATTTAGCTTCAGTCTCTTCGATGTATTTCAAAGCGGAT
TTTTGATCTACGTCACGACCATCTAAAAATGCGTGTACGTAAACTTTTTCAACACCTTGTTTTTTAGCAAGTT
CTAACAAAGCAAATAAATGTTTGTAATGACTGTGCACACCACCGTCAGACAATAAACCAAAGATGTGTAACGC
TGAATCATGTGAATTTACGTGTGCAATTGCATTATTTAAAACATCATTTTCAAAGAAATCACCGTCTTCAATT
GATTTATTGATTCGAGTTAAACTTTGATAAACGATACGTCCTGCACCGATATTCATATGACCAACTTCTGAGT
TACCCATTTGTCCTTCAGGTAGTCCAACATCTAAGCCACTCGCTTCGATTTGAGTCGTTGGATATTTGTTGTA
ATAACGATCAAAATTAGGCTTGTTTGCTAATTTTACCGCATTACCATGTTCGCTTTCGCGGTTCGCAAAACCA
TCTAAAATAATTAACGCAGTTGGTTTCTTAGCCATGATTATTTTGCACCTTCTAACAATTGTACGAAATCTTC
AACTTTAAGTGATGCGCCACCTACTAATGCCCCATCAATATCAGTTTGTGCCATGTATTCTTTAATGTTGTTA
GGTTTAACACTACCACCATATTGAATACGAGTTGCTTCTGATACTTCTTTGCTTGATAAGTCAGCAATAGTTT
GACGTACAAATGCACACATTTCATTCGCATCTTCAGATGTTGATGATTTACCAGTTCCGATTGCCCAGATTGG
TTCATAAGCAATTACAACTGATTTTAGTTGATTTTCAGATAAACCTGCAACAGCTTTCTTAACTTGCTCACCT
ACAACATCGTTAGCTTTACCACTTTCACGCTCTTCGTCTGTTTCACCAACACATATAATTGGAGTCATTCCAT
GTTTGAAAATAGCGTGCGCTTTTTTGTTAATTTCTTTATCTGTTTCGTGGAATAATTCACGACGTTCAGAATG
ACCGATAACAACGTATTTAACGCCTAAATCTGCTAATGCAACTGGAGACGTTTCACCTGTGAACGCACCATTA
TCTTCGAAATACGTATTTTGAGCACCGATTTCTAAACCTTGTGCTTTTCCTTCTTTAACTGCAGTAGTTAATG
CATCTAATTGAATTGCTGGTGCACAAATTACTGATTCTACTTCTTTTGAATCTGGTAATGTTGGCAATGCATT
GACGAAGTCTTTTGCTTCTTGTACTGTTTTGTTCATTTTCCAGTTACCAGCTATAATTGGTGTTCTCATTAAA
GACACTCCTTGTTTTGTAAATATTTTTGAAAAGTGATGAAACACGATGTCATCTTGTGACTGTTTTCCCGTAA
CAATGTTAAACAAACATGCCACATCACTTTAAACTATCACTTTATTATTTATTATCGATTGCTTTGATACCAG
GCAATTCTTTACCTTCTAGGTACTCTAATGACGCACCGCCACCAGTTGAAATATGAGTGAAGTCATTTTCAAA
ACCTAAAGAGATTGCTGCTGCAGCTGAATCACCGCCACCGATAATCGTAATTGCATCTTTAAGGTTTGCAATT
GCTTTACATACACCAATTGTACCTTGTGCAAAGTTACTGAACTCGAATACACCCATAGGTCCATTCCATACAA
CAGTGTGCGCACCTTCTAATTCATCTGCAAATAATTTTACAGTGTTTGGTCCAATATCCATACCTTCTTGGTC
TGCTGGAATTGAATCAGATGGTACTACAGTAATTTTGGCATCATTAGAAAATTCTTTAGCAACTTTAGTGTCT
ACTGGTAATACAATTTTATCACCATGTTTTTCTAATAAATCTTTTGCGAAGTCGATTTTATCTTCTTCTAATA
ATGAAATACCAATTTCTTTACCTTGCGCTTTTAAGAAAGTATAAGCCATACCTCCGCCGATGATAATTTTATC
AGCTATGTTAACTAAGTTTTTGATGACATTAATTTTGTCAGATACTTTTGCTCCACCTAAAATAGCAACAACT
GGTTTATGTGGATCGTTAACTACGCCACCGATAAACTTAATTTCTTTATCCATTAAGAATCCAGCTGCAGTTT
CTAAATGTGTAGAAATACCAACATTAGATGCATGCTCACGATGCGCAGTACCAAAAGCATCATTTACAAACAC
ATCACCTAAAGATGCCCAGTATTTACCTAATTCTGGATCATTTTTAGATTCTTTTTTACCGTCTAAATCTTCA
TAACGTGTATTTTCAACTAATAATACGTCGCCTTCTTTAAGGTCTTTAATAGCAGCTTCAAGTTTTTCGCCGC
GTGTTTCTGGTACGAAAACAACTTCTTTATCTAATTTCTTAGATAAGTCTTCAGCAACTGGGCGTAAAGTTAA
TTTTGCTTTATCACTTTCTTCTTTTACTTTACCTAAATGTGAAAATAGTACGATTTTACCACCTTGTTCGATG
ATGTATTGAATTGTAGGTAAAGCTTGAACGATACGGTTGTCATTAGTAATTTCACCGTCTTTTAAAGGTACGT
TAAAATCAGCACGTACTAGGACTGTTTTACCTTTAAGATCTAAATCAGAAACAATTTTTTTAGCCATGGTGTT
TCCTCCTTGTAATTAGGAAATTTTATATAAAAATAAGCGGAGAAGCGTTTGTGCTCCCCGCTTACTCATAAGC
TTTAATTAATGCTAAATAGTTCTGAGCACTAGCGTATTTGAATAAAAACTATACTAAAATTATTTAGAAAGTT
CAGCTAAGTATGCTAATGTACGAACTAATTGTGCAGTATATGACATTTCGTTATCATACCAAGCTGCAACTTT
AACTAATTGACGGTCGCCAACTGACATTACACGAGTTTGTGTAGCGTCGAATAATGAACCGTAAGTCATACCT
ACAACGTCTGAAGAAACGATTTCGTCTTCAGTGTAACCGAATGATTCGTTTGAAGCATTTTTCATAGCTTCGT
TAACTTGTTCAACTGTTACGTCTTGTTTTTCTAATACTACTGTTAATTCAGTTAATGAACCTGTAGCTACAGG
AACACGTTGTGCACCACCATCTAATTTACCATCGATTTCAGGAATAACTTTACCGATAGCTTTAGCAGCACCT
GTTGAGTTAGGGATGATGTTTTCTGCCGCTGCACGAGCACGACGTTTGTCACCTTTTCTGTGAGGTGCGTCTT
GTGTATTTTGATCACCTGTGTAAGCGTGAATTGTAGTCATTAAACCTTCAACTAAACCAAAGTCATCGTTTAA

AACTTTAGCAACTGGTGCTAATGAGTTTGTAGTACATGAAGCACCTGAAACAACTGTTTCAGAACCGTCTAAC
TCTTGGTGGTTAGTGTTGAATACGATTGTTTTTAAGTCACCAGTAGCTGGTGCTGAGATTAATACTTTTTTAG
CGCCTGCTTCAATATGAGCTTGTGCTTTATCTTTATCAGTGTAGAAACCAGTACATTCTAATACTACATCGAT
ATTTAAGTCTTTCCAAGGTAATTTGCTTGCATCTGGTTCACTGAATGATTTAACTTCTTTACCATTTACGCGG
AAACCACCATCAACTACCTCTACTTCACCTGTGAAACGACCTTGCATAGTGTCATATTTTAATAAATGCGCTA
ACATGTCGTCATCTGTTAAGTCGTTTACTGCTACAACTTCAAGACCTTCTACTTCTTGAATTCTTCTGAATGC
TAAACGACCAATTCTACCAAAACCATTAATTGCTACTTTTACTGCCATTATAATGGCCTCCTTTAAAATGATA
TTTAAAAAGTATTAAACTTTTTATCTCTTATTCAAGTATTATCTTTGCTGCGGCTTCATCAGTGATTAACACT
GTATTCTTGGGTGCAATCGTCAAGTATGCTTTAATTGCTTCACCTTTCGATTTGCCTCCTGCAACTGCAAAAA
TAAAGTCTTTTGATTCAAGGTCTTCTAATTGAAGTCCAATTGTTTTAACCTTATGGACAATTTGACCTTGTGT
ATCAAAATAATAACCAAATGCCTCTCCGACAGCTTGATGATGTTGAAGTTGTTCAATGACCTTTTCAGGTGAT
TGACGTCGATGCGCCATCTTCAGCGCATCACCAATGCCGTGTAATATAACGTTTGCTTGTTTAATTTTGTCTA
AAGTGTTTATGACTGATGGCTCTAACAACAATGTGTTATATGTTGTTTCACTGACATTATCAGGTACATACAT
CGTCGTATAATAACCGCCAGCTTGTTGTGCCATACTGGCTGCAATTGTGTTTGCCTGAAAGACAACATTTTCG
CCTAGTCCACCTCTGGCTGGTACGAAGAATACATTATATGGTAATAAATGAATTGCTTCACTAACACATGCCA
TCGTGGATCCGCCAGTTACAGCAACTATCGCGTCTTCTTGTAATATGCCTTCAAGTAATTGACCTGCTTGTCT
ACCTAATTCTGTTTTAACAGATTGACTATTATCAGCATCACCAGGAACAACATGAACTTCCTTAATTTGAAAT
TTATTCTTAATACCTTCTGACAGACGATTATCATCTGCATAGATATCAAAGTAACCTTTCAATTGCGAAATCA
GTTGCTCACCTTCAGCTGTAATTTCCATTCCGGTAGGCTTAACTTTTATCAAATCTTGTTTCTTAAGCATATC
TGTTTCAGAACGCAGTACACGTTCAGTCATATCCATATGTTCGCTTAAACTTCGACGTCCGACAGGCTGATTT
TTTGAGATAGTAGTAAGAATAGAAAAACGTTTATACATTTTATCTATGAGATCCGGTATAAGCTTTTGCTGTG
CTTGCAATAAGTCTTTCACTACTACCTCCTCCTTATATTTATAAATGTAAATAAGGATATGTCACAACCAGTG
GTTGCCTTTTTAAGTCCCGCGTGGGACAAAAATTAACCTCTTTACACTTGCAATAGTACTATTCTATTTCAAA
AATTGCAAGCATAAAACTTAAATTTTCTAAATAATTTGATAAAGCGCTTGCAATACATTTTCGATTTGTTATT
CAACAAAACGTTTTATTTTATTCGCTGTAGTCAAATGTATACCCTTATACTGACTCGCTAATCATTTGCTTAT
TATTTGAACTAAGACATAAATCAATGCTCTAGAATCCAAAAAGTTACATTAGTAGTAGTTGACCGAACGAAAA
TGCACTTGTAACAAGCATTTTTCAATTAAAGTCAGGGGCCCCAACATAGAAGCTGGCGGAAAGTCAGCTTACA
ATAATGTGCAAGTTGGGGTGGGCCCCAACATAGAGAAATTGGATTTCCAATTTCTACAGGCAATGGAAGTTGG
GGTGGGACGACGAAATAAATTTTGCGAAAATATCATTTCTGTCCCACCTCACTCAAAATAAATTTCACTATCT
TCAAAATAAACAACTAACTATTTAAGTATCATCTCTCCCCTAATTAACAATTATAAAAATAAGAACCAATATT
CGAGACAACCTAATTAATTGTGTTGTCTGTCATACTGGTTCATTTTTGTTTGACTAAATACTACTTATCATTA
GCATTATTTTGTTTATTTGTTGTTGATTGGCTATTTTGTTGTGGTGCTTGTGGTTTTGCTTGTTGATTTGATT
GATTGTTTTGTTGATTATTATTTGGCTTTGGTGTTGGTTGTTGCGGCTGTGGTTTAGGCTGAGCTGGTTGTTT
ATTATCATCAGTTTTCTTCTGATTATCATCTGATTTATCTTGAGATGCTTTGTCATCCTTATCTTTCGACTCA
TCCTTTGATGCTTTATCATCATTCTCTTCTTTAGCAGGACGTTGCTTTGTAGGTGCTTGTTGCTGAGGTGGCA
CATATTGATATTGATTCTGCTGTGGCAATTGCTGATATTGGTTTGCTTGTGAATCTAGCTCATTCTGCTCTTT
TTCCTCTTGTTGCTTTTTCTTTCTCTCTTTTTCTGCATTTTCTTTTTGACGCTTTTCTTTTTGTTCCTGCTTT
TGTTCGTTCAACATACGTTCTTTAGCTTTATTCGAATGATCTACATATGCAAAAATTGCAAATACTAATCCTC
CGAGTAATAGCAATACCGTCGCAATGCTAACTACTTTCGCTGTTGTACTCATTTTCTTCTTACTTCCATCTTC
ATTTTTATTGTAGTCGTAATTTTGATTCGACATACTCTCCCTCACTTCAAACTAATTTAAATATAGAAATTAT
CACGCAACCATTAACATTTTCACAACTAATTTGTTTACCTAGTTTCTACAAATATTAATAAACATTTACTCTT
TTTGCCTTCTTCAACTTAAAACTGCCTTCACTATTACTCACTCGTAAATCGGATTCATTTTTACAAAGAAATA
GTAGTCTTATATATCTTAACATTTAATAACTAAATCAAACATATTTTGAGCCTTTTTTTGAAAATTAACATAT
TAACCTTTTTGATTTTACAACAAATTATAGCTACGTATTGAAAATTAAAGCATTAGTTTAAGCGTTGGGTTAA
AAGTTTTTATGTTTAGATTGTTATATTACTATGTATTCTTAAATTTGTTTAAAAAATTAATGCTTTAAATTGA
TGTATAATGGGAATAAGAAATAAATAAAAACGACCCGCACGATAAACGTACGGGTCCACTACTAAAGGGAGTC
AAATTTTTACCTTCGTTTGTATCATGCAGCGTTTTACACATACTTTTAAGAGATGTTTATTCGTTATCGAAGG
TACACCTTTATTATAACTTATATCATTTTTATTAAAATACAATTCAAAAAAAGTTATTGACATATTATTGTCT
AAATAATGTATAACCTTTGAACAACAATAATGATTATTAAATAGTAACTAAATACAAAATTACATGGGGTGAA
TGATAATGAAACAATACTTAATTACTGGTGGGACTGGTATGGTTGGATCTCAATTAGTTAATGAAATTAAAAA
ATCAGATTCACATATCACGATATTAACGCGACACGACCAAATTTCAAATGATAAGAAAATTTCATATGTCAAC
TGGGCTAAATCTGGGTGGGAACACAAAGTTCCTCAAAATATCGATGTGGTCATCAACTTAGCAGGTGCTACAT
TGAATAAACGATGGACACCGGAATATAAACAAACGCTAATGCTAAGTAGAATTCAATCTACGCAAGCTTTATA
TGAATTGTTTAAATCACGTAATAAAGCACCTAAAGTTTTATTTAATGCTAGTGCCACTGGTTACTATCCTCCT
GATTTATTTATGAGTTACACAGAAGTTTATAAAACTTTACCTTTTGATTTCTTATCAGATATTGTGTATCAAT
GGGAACGTTTCGCACAACAATTTGAACAATTAGGTACACGAGTTGTCATCGGTCGTTTCGGTATAATTTTATC
GAATGAAGGCGGTGCGTTACAAACAATGAAACTGCCATACGAATATTACATTGGTGGTAAATTAGGTTCTGGT
CAACAATGGTATTCATGGATTCATATCAATGATTTAATTCAAGCTATTTTATTTTTAATAAATAACGAGTCAG

CTAGTGGTCCGTTTAATTTAACTGCACCTATACCTGAGCGTCAAAATTTATTTGGCTACACTTTAGCAAGAGC
TATGCATAAGCCTCATGAAACTTGGGCACCAAGTCTTGCAATGCGTCTCATACTTGGTCAAATGTCAACAGTA
GTATTGGATACTCAAAAAGTATTACCTAATAAAATTCAAGCATTGGGATTCCAATTTAAATATAGTAATTTAA
AAATGGCACTTGAAGATTTAATTAGTAAGTGAGTGGGACATTGATCTCTCTCCTACTCCACTTCAAGCAAAAA
GTCTAGGAGATAATTCGTTAAATCTCTCTAGACTTTTCTATTTGCTGATATTACATATAGATATAACTTTAAA
GTCACTTATGAATATTGGTTGATTAATTTTAAAGTAAATACGAAAAGGCCACTTGAAACAAAATATTCAAATG
ACCTTTTACTACTTTATAAAATTAAATGTATGATTTGTATTCAGGATACCAAATTTCAGCTTCAATTGCTTTG
TCAATATCATCAATAGTTTCAGCGTTTAATCCTTGATCAATGACACTTTGTGCAACCGCTTTGGCTATGGCAC
GAGAACATTGATGTATTTTCTCGACAGGTGGTAAGACTGCAGCACCTGGTTCATCTGGATTGACCATGCCACC
AAGTGCATGACTCGCAGCTGATAGAATCTGTTGATTTACTTTCTTCGCTTTTGACGCAATAAGTCCCAAACCT
AAACCAGGATACATCAATGCATTATTTGCTTGACCAATACTGTAATTCACACCATTGTATTCAATGTCATTAT
ATGGGATACCAGTTCCAATTAATGCTTTTCCGTTAGTCCATTTTAATAAATCTTGTGCAGTTGCTTCTGCTAA
TTTTGTAGGATTAGACAATGGGAAAATCATTGGACGTTCTGTATGTTTTAACATCTCTTTTACTGCATCTTCT
GTAAATGCACCTGTCTGAGTTGATGTACCGATTAAAACAGTAGGCTTAATATTTTCAATTACTTCTTTTAAAT
TTTTCCAGTTTACTGTATTTGAAAATTCATTCCTATTTTTAGCAAATACTTGTTGCGCTTCAGTTAAATCATC
CATATCATCAAACAACAATCCCTGTTTATCCATAATGAAGAAATGCTGATTTGCTTTTTCTTCTGAAACACCC
TGTTTAATTAGTTCATCTTTTAAAATATTGGTAATACCCATACCAGCTGTACCACCACCATAAACTAAAAACG
TCTGATTCGTATAATCTACTTTAGAAATATTTAATGCACCTAGTACGCCAGCTAATGTAACTATACCTGTACC
TTGAATATCATCATTAAAAGTTGGTAACGTATCTTCATATTTATCTAAAATATTTTTGGCATGCCCTCTACCA
AAATCTTCCCAATGTAATAGTGCATTAGGATATTCTTTACGCACTGCACTCACAAATTTATCTACAAATGCGT
AATATGTCTCATCATCGACACGTTTATGACGATTTCCTAAATAAAGGTCATCCTTTAGTAATTGTTCATTATT
AGTACCTACATCTAAAGATACAGGTAGTACTTGTGAAGGATTTATTCCAGCTGCTGCTGTATATACTATTAAT
TTTCCTATCGCAATATCTACACCATCAACACCCCAATCGCCGATACCTAATATACCTTCCGCATCTGTCACTA
CAATTAATCGAATATCTCTGCCATTTGCAACGTTGCGCAATTCTTGTTCAATTTGGTCTTGTCTATCAACAGA
TAAAAATACTGCATCTTGGGGTCTGCTGAAGTTCTCATTATATTGTTCAATCGATTGAGCAATAACAGGGTCA
TAAATAATTGGCATAAATTCAACTAAATGCTTTGAAGTCAATTTGTAAAACAATGTACGATTACGATTAAAAA
TTGCCATTAAAAATAAACGTTTCTCAAAATCGGTTTGTTTCGCCTTGAATTGTTCATAACATTGTTCTGCTTG
TTGTTCTAAAGTTCGAACATTCGCAGGTAATAAACCTTCAAGCCCTAACTGTTTTCTTTCTTCGTTAGTAAAA
GCTGTACCTTTGTTTAAAAATGGATTGTTAAGTAATTCAATGCCTTTCATAAAATCATCCTTTATATTTGTTA
GATTGTAGAACCGCAGAAACAACGTGTCTAATTAAATTCTCACAAATATTTTTACCCGTTATTTTTTGCTTTT
ACTCAAAATATTTTAAACAATTTATTGATACCTTGGTTTACTCATACAAAAAGAGCAGACTGCTTAGTCTACT
CTTTACTTTGAATTATTTTGTTTCAGGTACCATCACTTCATCAATTAAGCCATATTCTTTAGCTTCTTCTGCA
GTTAAGAAGTTATCACGATCTGTGTCTTTTTGTATTTTTTCAATACTTTGACCAGTACGCTCTGATAAAATGC
GGTTTAATTTTTCACGTGTTTTTAAAATGTGATTTGCAGCAATTTCGATTTCAGTTGCTTGTCCTTGAGCACC
ACCTAATGGTTGGTGAATCATTACTTCTGCATTTGGTAACGCGAAACGTTTACCTTTTGCACCAGCTGCTAAT
AAGAATGATCCCATTGATGCAGCCATACCGATACAAATTGTTTGAACATCAGGTTTAATGTGTTGAATTGTAT
CATAAATCGCAAAACCAGCTGTTACACTTCCACCTGGTGAATTAATGTATAAATAAATATCTTTCTCTGAGTC
TTGCGCTTGTAAGAATAATAACTGTGATACGATTGAATTTGCTACGTTGTCATCAATTTGTGAACCTAACATA
ATAATACGGTCTTTTAATAAACGTGAGTATATATCATATGCACGTTCACCGCGGTTTGTTGTTTCAATAACTG
TAGGAATTAAATTCATCTATATTTCCTCCTTGTAATAACTGTTACTCATTATTTTACATATTAAGTCAAATTT
GGTCAAACACTTTGACTTTAAATAGTTACATTGTTTGACGCTTTTCTCTTTCCTTTGTAAACTGATAATATCT
ATTTTAGATTGTACTTATTTTACTTCTCATAGTACAGAGATATGATTACATATTTGCTCCTTGTGGTGTAATG
GATAACACGTAAGATTCCGGTTCTTAAGATAGGGGTTCAATTCCCTTCAAGGAGGCTGTTTTAATAAAATACA
ATACTTTTCAATACTGAGAATCCCGCAATGTCGGGATTCTTATTTATGCTGATTTTGTTTTTGTCTATGATCA
GGGACTCAATTAATTATCACATTCATATTTTCTTATGACCAAATTGATACATATGATGCTATTTAATGAGAAA
ATTAGGCATCACTTGGTTATTGAATTTCCTTCATTAAACTTTCCAGCTCAATTTAATAGTTAGTCGACTATTA
TTCATTAAACACTTTTTAATCATAAAAAAGTGTTTTTGATAATTCACTACCAAAAACACCTTCTTACTTATAA
TTCTATTTGTTCACCATTTCTAATCTTATCGGCTAAATCATTAAGTTTTCTTAATCGGTGATTTACACCTGAT
TTTGAAATTGGACCAGTTGATACCATTTCTCCAAGCTCTTTCAACGAAATTTCTTGATGTTCTACTCGAATTC
TAGCAATCTCTCTCAACCTGTCTGGTAAATTTTCAATACCAATTTCTTTATCAATCAATTTAATGCTCTCAAC
TTGTTTCATCGCAGCACTAACTGTTTTATTTAGATTTGCCGTTTCACAATTAACGAGTCGGTTAACAGAATTA
CGCATATCTCTTACAATACGTACGTCTTCAAATTTTAATAACGCTTGATAGCCACCTATCAAACTAAGAAAAT
CCGAAATCTTTTCCGCTTCTTTTAAATACGTAATACTTCCTTTTTTTCGCTCTAAATGTTTGGCATTCAACTC
ATAACTATTCATTAGTTTCGTTAAGCCTTCTGCATGACTCTCATTTTGAGAAAAAATTTCCAAATGGTACGAA
GATGTTTCAGGGTTATTCACTGAGCCACCTGCCAGAAAAGCTCCTCTCAAGTAACTGCGTCTCATTTCATCAT
CTTGAATCATTGAATGATCAATTTCATGCGTAAAAATGCCGTCTTTTAAAATTCCTAATTCATCAAGAATTTC
TTTCGCTTTCATCTTTGTACGACAAATATAAATATTATTTTTTTTAAGCTTCATTTTTTTACGGACTAATATT
TCAACTTCCACATTAAAGACACGTTTAATCAACGAATAAATACGTCTTGCCGTTGTTGCATTTTCCGTTTGAA

CATTTATAACAAATTGTTGATTTGAAAGACTAAGTGCACCATTCATTCGAATCAGTGCACTGAGCTCTGCTTT
TGCATTCATTTCATCGACGTCTATTCTAGTTAATTCATTTTTCATTTCTGATGCAAAGCTCATCGTACAGTCA
TTCCTTTCTTATTTAAAACATGATTCACCTTAGAACCACTGTCTATTTTCATTTTTTTACACAGCTCTATTAT
CATATCATAATATGATTACGTTCTATATTATTTACGTTTATCACTTGGTACGAAAGGAATAGTACTAATTAAT
TCTAAAGCTATATCATAAATCATTGTCGATAACACTTTAGTATTATGTCTTACTAAATGATTTTCAGAAATTT
CAACTAAATTTGAAGATGTTTTTACATTTATGCTTTCTTTTTCAAGTTCAGCCTTATTAACTTCAACTGGTTT
AGAATGTTTTTCTTCATATTTTTTCAAAACTTGAGCATTGAAAGTTTGTGTACTACAAATGACATAATCAATA
AACGGTTGTCCAGCTTGTCTATGAATCGCATCGATATGATCTTTCACGCTATAACCATCTGTTTCCCCAGGTT
GCGTCATCACATTAGAAACATATAGCTTAGGCGCATCAGAATGAATTAACGCATCTGAAATACCATTGACACA
TAAGTTAGAAATAACGCTCGTATATAATGACCCTGGTCCAAGAACGATTAAATCTGCTTCCCTTAAAGCATCG
ATTGCTTCTTCCATTGGTTGCACATCGTTAGGTTCTAAAAACACACGATCAATTTTTTTATGTTTTTTAGGAA
TATTTGTTTCTCCAAAAACAATTTCTCCATCTTCCATAACAGCATTTAATTGCACACTTGTATTTGTAGATGG
AATGACTCTACCTTTAATATTTAAAATTTTACTTAATGCTTTAATGGCATGTCCGAAATCATTCGTAATATTA
GTCATACCTGCGATTAATAAATTACCTAATGAGTGACCGCTAATTTGATTTTCTTCAAAGCGATACTGAAAAA
GTTGGCTTAAAACTGACTCAGAATCACTTAAAGCTGCAATCACATTTCTGATGTCTCCTGGTGCTGGTATATC
CATTTCATCTCTGATTTTCCCTGTACTCCCACCATTATCAGCAACTGTTACAATCGCCGTAATATCAATTGGG
AATTCTCTTAATCCCCTAGCCATAACTGATAAGCCAGTGCCACCACCGATAAGTACAACTTTTATTTGTCTCA
TTTTTTCTCGCCACTTTCAATATGTGCGTCCCTATGATGCACATAAACATTATATTCAAATACTTCATTTAGA
TAATTACCTAGTCGTTCTGCTAATGCTACAGATCGATGTTGTCCACCCGTACAACCGATGGCAATTACTAATT
GAGATTTCCCTTCTTTTTTATACCCAGGTATCATAAAATCTAACAAATCAGTTAATTTTTCAAAGAAAATCTC
CGTCTCTTTCCATTTCATAACATAATTATAAACGTCTTTATCTAATCCTGTTAAAGGTCTTAAATCTACTACA
TAATATGGATTTGGTAAAAATCGTACATCAAATACTAAATCTGCATCCATCTGAATTCCATGTTTAAAACCGA
AACTTGTGACATTAATTGTAAAAGTTTCAAACTCTTCATCTTCATAGTATCGACGAATGCGTTCTTTTAATTC
TTTAGGTGATAACTTTGTAGTATCTATAACAAAATTAGCTATACTTCTAATTTGAGACAAATGCTCTCGCTCA
TCATTAATTGCATTGATTAAGGATCTTTTACCTTGTTCCATCAAAGGATGTGCACGACGCGTTTCCTTATATC
TTGAAATTAATTTTTCAGTACTTGCTTCTAAAAACATAACATCAATGATGACGTCACTTTCACTTTTAACTTT
ATCCACTACTGCAACTAATGAATTAAATAGTTCCTTACCTCTTAAATCAATTGCAATTGCCACTTTTCTTAAG
GATGGATTTCCTTGTTCCATCAACTCTACAAATTTAGGCAATAACACTGGTGGTAGATTATCTACACAAAAAT
ATCCCATGTCTTCTAAACATTGAATAACCAAAGATTTACCTGCGCCAGATAAACCTGTTACAACTAATAGTTC
ACTTTTACTTTTTTCTTTTTCATTATTATCCATTATTTTTTCACCGCCAAAACGAATTTCATGATGTTAATTT
AAATGTTCTATGACAAAATTAAGCAACGATGTTTTATATTTATTTTTAACTATGCCTAGATTAAATTTCTAAA
AATATACATCATTTAAAAGGAGATTGGGACATAAATCAATGTCCTAGGGTCTATAATATTATATTGCTAGTAG
TTGACTGAATGAAAACGCGCTTGCAACAAGCTTTTTTCAACTCTAGTCAGGGGCCCCAACACAGAGAAATTGG
ATTTCCAATTTCTACAGACAATGCAAGTTGGGGTGTGGCCCCAACACAGAGAATTTCGAAAAGAAATTCTAC
AGGCAATGCGAGTTGGGGTGGGACAACGATAAAGAAATACTTTTTCTATAGAAATTAGTATTTCTTATGCATG
AGTTTTACTCATGTATTCCTATTTTTAAGTACACATTAGCTGGGCTAATGTGTAAGAACCACTACATAATAAA
TCATTAGTGGCTCTTTATCATTTCTGTCCTACTCCCATAAAGTTATAAGTTTAATATAACATATAAAAAAACT
AGAACTCATTGAAACGCACAAATGATACGTACTAAGTTCTAGTTTTCTATATAATTTGCTTTAATTTAAAAGT
AAGACATTATTTTAACAATGTGATGACACTATTAAAATAAATATCTAAATAATTTACAACTCAACATCTTAAT
TCGACTTCGAATTAAGCTTGATCGTTTAAATGTTCAATATATTCCGCTGCACTTTGCGCTGCAATACTACCAT
CGCCGGTAGCAGTGACAATTTGGCGTAAACCTTTGTCGCGAACATCTCCTGCTGCAAAAATACCTGGTACTGA
TGTTGTCATATCATCTTTTGTTACAATATAACCAACATCATTTGTAATACCTAAGTCTTTAAATGGCGCTGTT
AATGGTTTCATACCAATATAGATGAATACACCATCAGCCTCGTGTGTTTCTTCTGAACCATCTTTTGTAGACG
TTAATGTCACAGAACCCACTTTGCCGTCTTTTTCATTAATTGATTTCAAAGTATGACTCCAAATAAAGTCGAT
TTTATCATTTTTGAATGCTCTATCTTGTAAAATACGTTGTGCACGTAACTCATCACGACGGTGAACAATTGTT
ACTTTGTCAGCAAATTTAGTTAAGAATGTTCCCTCTTCTACTGCTGAGTCACCACCACCGATAACGAATAGGC
GTTTATTTTTAAAGAATGCACCATCACATACTGCACAATAACTTACACCGCGTCCACCAAGTTCTTGTTCACC
CGGAACACCAATTTTCTTGTATTCTGCACCTGTAGCAATAATGACCGCTTTAGCTGTTAATTCTTTGTTACCA
AAGTTAATCACTTTATATTCGCCTTTATCTTCTACAGATTTAATATCTCCATATTGATAAACTGCACCAAACT
TTTTAGCGTGTTCAAACATTTTTGTAGATAAATCTGGACCTGTAATCATTTCGAAACCAGGGAAGTTCTCTAC
TTCTTCTGTATTAGCCATTTGACCGCCTGGAATACCTCTTTCAATCATAACTGTTTTTAAATTAGCACGTGAT
GCGTATACTGCAGCAGTCATACCAGCTGGACCTGCACCGATAATTGCTATATCAAAATCTATTTCAGTCATTT
TATTAACGCCTCCTCATTATTAATCATTATGCGCATTATATAATAAATCTAACTTTTCATAAATCTATATGCT
CAAGAGAAATTCAATCATTTTGTTCAGTTTATATTGTGTTATGCCTAACCATGTTGTAATTTGGTTCTTTGTA
ACATTTCGAGGTTGATTTTTAAAATACAAATAAATAAACGCACCGATATATGGCTCAACATCAGTTAAATCTA
CTTTTTCAGCAATTATGAGTTCACCTTGATTAATCCATGCAACCATTACATCATTTTCACTCACAAATAATTC
ATTATGGTAAAGCGTTAACAAGCCGCGATGAATGAAGTCTAATTTATTGAGCGTTAAACCTTGAACTAAATAC
GTTAAATACAATTTCTCATAATTATTTAGATTTTCCAGAACCTGCCAAATACTTTCCGTCATCACAATTTCTT

TACCATTTAATTGATCCAATAAAAAAATACCATATAAACGATAATGACTGTCATCACTTTGAAGTAATGGCAA
AATATGTTGGTCGAAAACTTCTTTGCTATTTTCAATTACCCAAGGCGCATGTCCAATTTCCACTTTAGAAATT
TGCTTCAATTTATCCCAGTAGTAATGACTTTCGTCTTCTTCACCTAAATAATAATAATTATATGCTAAAGCAT
TGTACATTTGAATTGATAAAAATTTCCCTTTTTTATATAAAGGGTACAACAATTGTTGTGATGCACGATACTG
CTTTAAATAACTTAAAACAATACCTAATTTAAAACTTTCATCGTCATTCATAGGTACAACTTTATTTAATATT
TTTAAATATTTTTGGTATTGTTCATTTTCCTTAGTGTTATAAAGTAGCAAAGTATAATGGCATAATGCATATA
TATCTGTACTATCCTCTTGTAATAGTGCTTCGTACAATGCTTTAGCCGTGTCATATTCGCCGAGATATAAATA
ACACATTGCCAATAAATTACGTACGACACGATGATCTTGAACTTCTTGTGGTTGATGTAAGACATAATTCTTT
GCATCTTTTAATCGACCTTGAGAAAAAAGATATTGGAATAGTATTTGAACTACAAACAATTGAGCTTCAAGTT
CAATTTGTTCTTCACTTTGATATTTCACATCAAACATTTGATCTAATTCATCTCTATAATCTTGGTCGTCGCT
AACAATAACATAATTAATACCAAACAAGAATGCCTTGTTTGGTTCGTTAACTTCAATGTTGAGCTGACTCAAT
TCATAAAAGCTATCTTCTAGATGCCTATTATAAATAATATTGTCATAAAATAAATGTTCTGCTTTTTTAGCAA
TACCAAGTTGCACTAGACATTGTGCATAATCAATTTGAATTTCCAGATCATCAGGTGACAATTCTAACACTTT
TTCAAAGTATTCAGCAGCTCGTTTATATTCTCTTTGTTTAAACTTCTGATTAGCCATTTTACGATAAAATGCA
TCATCAAAAGTCATTGGAATTACGTTATTATTCTTTTGCGCCATAAAATTGTCACCTCCTAATCATTTTTATA
TACATAGGGTTGCCATATGCAAATCCATTATCCGGTATATCTTTTGAAACAACCGTACCAGCTGCAACTTTTA
CATTGTCACCAATCGTTATACCGGGTAAAATGGTAGCATTTGCACCAATCAAAGTGTTAGATCCTATCGTCAC
TGGTCCATAACGAAATTCATCAACTAATGCTTCATGCGTCAAAATTGTTACATTGTAACCAATAACACTGTTA
CTACCAATCGTAATCAGTTCTGGGTAAAAAATATCTAACATTACTTTGTAAGCTATCGACGATTGATTACCGA
TATTAATATTTAAAAGTTGTTTATATATATGTCTTTTCAGTACCATACTTGGAATAAATTTCGAAAATTCAAT
GATAATTACATTCTTAAAAACTTTCGAAAATTTAACAAGACGGTATACACGCCATAAAGGGTTTGTATGATGA
TGTGTTTTTGATAAAAATTTTCTCAAAATATCACTACTTCACTTTTTTTGTTGGCCATGGAAGCGCCCCAACT
TTGCTATACAACGGTGGATTATACTTAATCCTTCTATATACAATTAAACTTATACTTATTAAAATTAAAAGAA
TTGATACTAATTGTGCAACTCTAATATTACTTGTGAGCATTAAGCTATCTGTACGTAATCCTTCTATAAAGAA
TCGACCAATTGAATACCAAGTTAAATATAAAAAGAATGTTTCTCCTAATTTTAAATGTTTACGAATATTAACT
AAGATAATAAATCCAGCGACATCCCAAATGGATTCATATAAGAATGTTGGATGATAATATTGGCCGTTGATAT
ACATATTTTCTATTATAAAATTAGGCAAATGTAATTGTTCTAAAAAAGCGCGTGACACAGGTCCACCATGTGC
CTCGTGATTCATAAAGTTACCCCAGCGTCCAATTCCTTGCGCTAAAATTATACTTGGCGCAACGATATCACCA
ATTTGAAATGGGTTTAAATTTTTCACTTTACATACAATAACACCAGCAATAAAGCCACCTATTAAACCACCAT
GTATTGCTATACCACCATGCCATATTTTAATAATTTCACCTGGATTTTCCGCGTAATATGGCCATTGGAAAAT
CACGAAATAGATTCGTGCCGCGATAAATCCAAATAGTGCACTATAAAAAATAATATCTACTAAAGTATCTTTA
TGTAATCCTGCTTTAACTAGTGCACGTTGTGCAACAAAGTAACCAAGTAGTATTCCGACAGCAATGATAATTC
CATACCATCGTACACTCAGTGGTCCTAAGTTAAATGCCACAGGATCTATATAGTTAAATACAATACCCATTCA
ACCTACTCCTCACTCTTATGACTGTTCTTGATAATTTCTTCATTTAATCTTTCACTAAATTCTTCGGCAGTGT
TAATGCCCATGATATTTAATCGATAGTTCATTGCAGCGACCTCAATAATTACCGCAACATTTCTACCAGGTCT
TACAGGTATTGTTTTTTTAGTTATTTCAGTATCTAAAATACTTAACGTCTCTTCATTAAGACCTACGCGGTCA
TATAACTTTTGCTTGTTCCAGTTTTCCAAATTAATATTTAATCTAATTCGTTTTTCAGTTAATATTGAACCCG
CGCCAAATAAAGTCATAACATTGATAATACCTAGTCCACGTATTTCTAATAGATGTTCTATTAACTTTGGTGG
TTTCCCTATTAGTTCATCTTTATTAATTTGACGTATTTCTACATTATCATCTGCTACTAGTCTATGCCCACGC
TTCACTAATTCTAACGCAGTCTCACTTTTACCAATACCAGAATCACCGGTAATTAATACGCCTACACCGTAAA
CATCCACTAAAACACCATGTAAAGATGTCGTCTTTGCAAGTGCATGCTCTAAAAACGTTGTTAATCGACTCAT
TAAACTTGTAGTTGCATCTTTAGCAACTATAAGTGGCGTATTTAATTCTTTTGCAGCTTCAACTAATTCTTCT
GGTGGCTGCAATCCACGTGTCACAATAATTGCAGGCGTTTCTGGTCTGCATAGTTTACGCATACGACCTGCGC
GATCCTTATCTGGTAATAAATTGTAAAACGATAGTTCCGTTGTTCCTAATAGTTGTATTCTATCTGACGCATA
ATGTGAAAAATAACCTGCCATCTCTAAGCCCGGTCTTGATATATCAGCATTTTTAATTGGCTTCGATAGTCCT
TCTTCACCAGCGATTAAATCTAACTTTAATGTTTCAACTAGTTTTTCTGTCGTTAACATGGGTTCACCTCAAT
TGTATTTATCCCTACTCTTACATCTCTTCTTATCATATCAAAAATATAACACCAATCTACATTGAAAAGCTAA
AATAAATATTAATTGTTCATTATTGTTATTATTTTACAAGTCAATATCAATCATTAATTTATTGAATGCTTAA
TTTACATGGTTCATATCGACATAAAAGTCCAAGCCATAAATCAATGACCTAAACTCTAAAATGATATATTGGA
ATTAGTTGGCGGGGCCCCAACACAGAGAAATTGGATTCCCAATTTCTACAGACAATGCTAGTTGGGGTGGGAC
GACGAAATAAATTTTGCGAAAATATCATTTCTGTCCCACTCCCAGTTCGATTTATGTTTAGCAACTCTAGATT
GACCCTATCTTAACATAATTCTTTTTACGCTTCTTTTCTTTCTACTATTCATTACTAAATTAATAAAAAGAAG
TGAAGGATAAATTTACAACATCCTTCACTTCTTTTAATCTTATTTATCTTCAGTATTTTGTTTATCTCGTTCA
AGTACTTCTTTTAAATACTTTCCTGTATAGGATGACTTTGTCTGAGCAATATCTTCGGGTGTGCCAGTCGCAA
CAATAGTACCACCGCCACTACCACCTTCAGGACCTAAGTCGATAATATAGTCTGCTGTTTTGATAACATCTAG
GTTATGTTCAATAATTACAACAGTATCACCATTTTCAACTAATCGGTTTAATACTTTTAATAATCTGCTAATA
TCGTCAACATGTAACCCTGTTGTTGGTTCGTCTAGGATATAAATAGATTTACCAGTTGAACGTTTATGAAGTT
CAGATGCAAGTTTCACACGTTGAGCCTCACCACCTGATAACGTTGTAGCTTGTTGACCTAATGTGACGTATCC

AAGACCAACATCAACTAGTGTTTGTAACTTGCGCTTAATCTTAGGAATATTTTCAAAAAATTGTGTTGCTTCT
TCAACAGTCATTTCTAAAATGTCAGCAATATTTTTACCTTTGTAAGTAACCTCTAGTGTCTCACGATTATATC
GTTTACCATCACACACTTCACAAGGAACATAAACATCAGGTAAAAAATGCATTTCAATTTTAATAATACCGTC
ACCTTTACAAGCTTCACAGCGTCCACCTTTTACATTAAAACTAAAACGCCCTTTTTGATATCCTCGGATTTTA
GCTTCATTTGTTTGCGCAAACACATCACGTATATCATCAAACACACCAGTATATGTTGCTGGATTAGAGCGTG
GCGTTCTACCTATCGGTGATTGATCAATATCAATAATTTTATCAAGTTGATCAATACCTTCAATCTTATCGTA
CAATCCTGGCTTTACTTTAGATTTATTGATTTTTTGAGCTAATGATTTGTATAATACTTCATTTACTAATGAG
CTTTTACCAGAACCTGATACACCTGTAACGACCGTCATGATTGATAGTGGTATGTCCACATCAACGCCTTTAA
GGTTGTTACTTCTAGCTCCACGTATAGAAATTTTACGATCTGAAGCCGGTCTGCGATATTCAGGTACGTCAAT
ACGTTTCTTACCACTCAAGTATTGTCCTGTTAAAGATTTTTTATCTTTCATTACCTTTTGAGGTGTCCCACTA
GACACAATTTGTCCTCCATGTTCACCAGCACCAGGCCCTATATCCACTAAGTAATCAGCCGCACGCATTGTAT
CATCATCGTGTTCAACTACAATTAAAGTATTTCCTAAATCTCTCATTTCTTTAAGTGTATTAATTAATCGATC
ATTATCTCTTTGATGCAGTCCAATTGATGGCTCATCTAATACATATAAGACACCAGTCAAACGCGACCCAATT
TGCGTTGCCAATCGAATACGTTGTGCTTCACCACCTGAAAGTGTACCTGAAGCTCTGTTTAGCGTTAAATATT
CAAGTCCGACATTATTTAAAAAAGTGAGTCGGGAAATAATTTCTTTCAATATTTGATTTGCAATCGCTTGATC
TTGTTCTGACAAATCAATGTTTTTATAATAGTTCAGCGCTTGACTGATTGAGTATTCGACTACTTCACCAATA
TTTAAACCACCTACATAAACAGATAACGCTTCACGACTCAATCGCTTTCCATGACAAGTTTCACAAGGTAGTT
CAGTCATATATTTACTCATCATTTCACGTGTATATTCTGAAGGAGATTCATGGAATCGTCTACTTATATTAGG
AACTACACCCTCGAAAACCATTGTTCGTTTTCTAGTACCACCTTGACGTTGTGTAAATGTAAATTCAATTTCT
TTGTCACCCGAACCATACAATAAAATATCACGTTGACGTTCTGTTAACTTTTTAAAAGGTTTATCCATATTGA
TTTTATAAACTTCACAAACACGTTTTAACAATGTTGGATAAAAATCAGAACTCGTCGGTATCCAAGGTTCTAT
TGCACCTTCGTTTAGCGTCTTATCTTTGTCTGGAACAACCAAGTCTACATCGACTGTTAACTTTTGGCCTAAG
CCATCACATGTTGGACAAGCACCAAAAGGACTATTAAAGCTAAACATTCTTGGTTCTAATTCTCCGATTGAAA
ATCCACATATAGGACAAGCATGGCTTTCTGAAAACTTAAGGTCTTCCCCGTCAATGACATCAACTGTTAATTG
TCCTTCTGAAAGCTCTAACGCAGTTTCTATAGAGTCAGCTAGTCGTGTTTCAATTCCATCTTTAACAACTAAT
CGGTCTACAACAACTTCTATTGTATGATTCTTGTTCTTATCTAATGTAGGTACATCATTTACATCAACAATTT
CGCCATCGATTCTTAAACGTACATAACCTTTTTTACCAATATCTTCGATTAGCTTTTCATGACTACCTTTACG
ATGAGCGATGACAGGTGCTAATAATTGAATCTTTGTACGTGCCTCTAACTCCATAATGCGGTCAACCATTTGT
TGTACTGTTTGTGATTCAATTTCTATATTGTGATTTGGACAGTAAGGTTTACCAACACGTGCATATAACAAAC
GTATATAATCATATATTTCTGTTACTGTTGCAACAGTTGATCTTGGATTTTTACTTGTTGTTTTTTGATCTAT
TGAAATTGCTGGCGATAATCCTTCGATTGTATCAACATCTGGTTTGTCCATTTGGCCTAAAAATTGACGCGCA
TAGGCACTTAATGATTCAACATAACGTCGTTGTCCTTCAGCATATATAGTATCGAATGCTAATGACGATTTAC
CTGACCCAGATAATCCTGTCATAACAATTAATTTATTTTTAGGTAGTTCGATATCAATATCTTTCAAGTTATG
CGCACGAGCACCTTTTACTACTATGGATGGTTCTTTCATTTACTTGTCACCCTTCTGCTTTTAATTCAAATAA
CATATCTCTTAATTCTGTAGCTTTCTCGAAATCTAAATCTTTCGCTGCTTGTTTCATTTCTTTTTCTATATTG
TCGATTGTCTTTTGACGTTCTTTTTTCGTCATCTTCTTAGGTATCACAGTTTGTGCTTTGTCATTATTTTCGT
CATTTTCAACAGTAGCACTAATTAAATCATGTATTTTTTTATTAATTGTTTTAGGTGTAATACCATGTTTTTC
ATTATGTTTCATCTGTATTTCTCGACGACGTTGTGTCTCATCAATTGCATACTTCATCGAATCAGTCATTTTA
TCGGCATACATAATGACTTCACCTTTATCGTTACGCGCAGCTCTACCTATTGTTTGAATTAAAGAGCGGTTAG
AACGTAAAAATCCTTCTTTATCTGCATCTAATATGACAACTAGAGAAACTTCTGGTATATCAATACCCTCTCT
TAATAAATTAATACCTACGATAACATCATATGTACCCATTCGTAAGTCTCTAATTATTTCGATTCGTTCTAAT
GTCTTGATTTCTGAATGCAGATAATTAACTTTAATACCCGCTTCTTTCATGTATGTGGTTAAATCTTCACTCA
TCTTTTTAGTGAGCGTTGTAACAAGTACGCGTTCATTACGTTCAACCCTTGTTTGAATTTCACTTAATAAATC
GTCAATTTGATTTTCAGTAGGTCTAACCTCAATCTTAGGATCCAGTAAACCAGTAGGACGAATAATTTGTTCA
ACCATCTTATCCGTATGTTCAATTTCGTATGGTCCAGGCGTTGCAGATACATACACAAGTTGTTTTGTCTTTT
CTTCAAATTCTTCAAATTTAAGAGGACGGTTATCTAATGCACTCGGTAATCTAAATCCATGATCCACCAAAAC
TTGTTTACGCGCTCTGTCTCCGTTATACATGCCTCGAACTTGCGGTAATGTCACATGTGATTCATCAATCATT
ACTAACCAATCATCGCCAAAGTAATCCAATAAAGTATATGGTGTCGAACCCAGTGGTCGCAAAGTTAAATGTA
CGGAATAGTTTTCAATTCCTGAACAGAATCCCATCTCTCGCATCATTTCTAAATCATAGTTGGTACGCTGTTC
TAACCTTTGCGCTTCTAGTAATTTATTCTCATTTCGTAATTCTTTCAATCGTTCTTCCAATTCTTTTTCAATA
CGTTCAATCGCAACTTTCAACTTTTCTTCACGTGTTACGAAGTGAGAAGCTGGGAATATCGCAAAATGTTCTC
TTTCTTTCAACACTTCACCTGTTAGGTAGTTAACTTCTCGGATACGGTCAATCTCATCGCCGAAAAACTCAAC
CCTTATACAAAGTTCTTCTTTAGAGGCTGGGAATATTTCAACTACATCACCACGCACTCGAAACGTTCCTCGT
TGGAAATCGATGTCATTTCGTGTATATTGCACATCTACAAGTTTTCTAAGTAATTCACTTCTATCCATTTCCA
TACCAACTCGAACACTTACTACTAAATCTTTATATTCTTCAGGATTACCTAAACCATATATACAACTTACACT
AGCAATAATAATTACATCATCGCGTTCAAATAATGCACTTGTAGCAGAATGTCGTAGTTGATCAATTTCATCA
TTGATTGAGGCATCTTTTTCAATAAAAGTGTCAGTAGACGGTACGTATGCCTCAGGTTGATAATAATCATAGT
AACTTACAAAGTATTCCACCCTGTTTTCAGGAAAAAATTCTTTAAACTCACTATATAATTGTCCTGCTAATGT

TTTGTTATGCGCGATAATTAACGTTGGTTTCCCAACTTCTTTAATAACATTACTCATCGTAAATGTTTTCCCT
GTGCCAGTTGCACCTAATAAAGTTTGATGTCTTTTCCCCGCTTTAATACCTTCCACGATTTCCTTAATTGCTT
GCGGTTGGTCACCTTGAGGCTCAAAATCAGAATGTATTTTAAAAGGATAATGTTCAACCATTGTCACATATAC
CTCCTCTTTCAACGTATTATTTATATTTCCGCTCAACTCTTCGCTTTCTCATTACATATTTTAGCAAAGTAGT
CACACAAAAAGCAAACGTTTGTTCGTAAAAATATCGAACAAAGAAAAACAAACATAGTTGTGCTAACTATGCT
TGCCTTAAATCAAATCGTTTATCTTGTTATTTTAGTATGTAAATACACATTATATTAATCATCATTTTGTTT
CAACAAATTTGTTTGAAACATTATTTTAAAGTTAATCTTAGCGATCTTCATCTTGATGTTTATGAAATTCGAG
TTGATCTATAATTAAATAACCAGCTAATAATGACACTACATCAATAAAAATAATCCACTCGTTATGGAAATAC
TCTTTATAGATTGAGGCACCAATTAAAATTAATGTCAGAATAGTACCGACCCATTTACTTCTTGTTATTACAC
TAAATAATACTACCAAGACACATGGAAAGAATGCTGCGCTAAAATACCATATCATTCATTTTCCTCTTTTCTT
TTATTTAAAATGTTCATGGTTGTTTCTCTTAATTCTGTTCTAGGTATAAAGTTTTCAGTCAACATTTCTGGAA
TGATATTATTAATAAAATCTTGTACAGATGCTAAATGGTCGAATTGAATAATTGTTTCTAGACTCATTTCATA
AATTTCGAAAAATAATTCTTCAGGATTACGTTTTTGTATTTCTCCAAATGTTTCATAAAGCAAATCAATTTTA
TCAGCAACTGAAAGTATTTGGCCTTCTAATGAATCATCTTTACCTTCTTGCAGTCGTTGCTTATAAACATCTC
TATATTGTAATGGAATTTCTTCTTCAATAAAGGTCTCTACCATTTCTTCTTCAACTTGCGAAAATAATTTTTT
TAATTCACTACTCGCATATTTAACAGGTGTTTTTATATCACCAGTAAACACTTCAGCGAAATCATGATTTAAT
GCTTTTTCATATAAGCTTTTCCAATTAATCTTTCGTCCATGATATTCTTCAACTGTTGCTAGATATTGAGCAA
TTTTAGTTACTTTAAAGGAGTGTGCTGCAACATTGTGTTCAAAATATTTAAATTTTCCAGGTAATCTTATAAG
TCTTTCCATATCTGATAATCTTTTAAAATATTGATGTACACCCATTTCAATTACCTCCTCCATTAATTAATCA
TAAATTATACTTTCTTTTTACATATCAATCAATTAAATATCATTTAAATATCTTCTTTATATAACTCTGATTA
AATGATACCAAAAAATCCTCTCAACCTGTTACTTAAACAGGCTAAGAGGATAGTCTTGTCTTGATATATTACT
TAGTGGATGTAATTATATTTTCCTGGATTTAAAATTGTTCTTGAAGATTTAACATTAAATCCAGCATAGTTCA
TTTCAGAAACAGTAATTGTTCCATTAGGGTTTACAGATTCAACAACACCAACATGTCCATATGGGCCAGCAGC
TGTTTGGAAAATAGCGCCAACTTCTGGTGTTTTATCTACTTTAAATCCTGCAACTTTTGCTGCGTAATTCCAG
TTATTTGCATTGCCCCATAAACTTCCTATACTTCTACCTAATTGTGCACGACGATCGAAAGCATAATATGTGC
AGTTTCCATAAGCATATAAGTTTCCTCTGTTAGCAACTGATTTATTGTAGTTATGTGCAACAGGTACAGTTGG
AACTGATTTTTGTACTTGAGCAGGTTTGTATGCTACATTAACTGTCTTAGCTACTGCTTGCTTAGGTGCTTGC
TTAACTACTACTTTTTTAGACGCTTGTTGTACAGGTTGTTGTACTACCTTTTTAGCTTGGCTTGCTTTTCTTA
CTGGTGATTTAACCGCTTTAGTTTGTTTCACTTTATTTTGAGGCACAAGTGAAATCACGTCACCAGGAAAAAT
TAAAGGTGTTACACCAGGATTGTATGAATATAATTGATTCAACGTTAAGTGATGCTCTAATGCAATCTTATAT
AATGAATCGCCAGCAACTACTGTATAAGTTGTCGGTGATTGCGTTTGTGCTTGAACATTTGATACATAATTAT
GTTGAACAGGTGTTTTTACTTGTGTGCCATGTTGTTGTGCATGTGCTGCATTATTTAAAGCTAAAAAAGCTAA
CACTGACGAAACCGTCACTGTAAGAGATTTTTTCATCTTGCTGTCATTCCTTTGCTGTTAGAATTTTAAGTAT
GCAAATACTATAGCACAATACATTTTGTCAAAAGCTATTGTTATAACGATGTAATCAAATGGTTAACAATATA
AAAAGAATACAACCTTTTATCATAGTGTAAAATGTATTCATACCATGTAATTGAGAACGTTTTCAACGATTAA
TTCAATACCTCGAAAATCGCCATAGGTAATATTACTAAATGCACACTGCATATGTTGTTTTAACAAACACAAC
TTTTAAAAAATATATTCTAACCCTATCTACCCAATTGTACTTAAATATTCATAAACAAATCATATTCCAAAAT
CTAATTTACAATTTATTTAGCTACCTATAAAAAACCAAAAACCGACGCCCTTTTAGAGCCTCGGTTTTAAAAT
ATATATTAATCGTGTGACATTGTCTGTCTTAAATATGATTCGATAAATGGTCCAATATCTCCATCCATCACTG
CATCAACCTTACCTGTTTCTTCGTTCGTACGATGATCTTTCACCATTGAGTATGGATGGAAAACATATGATCT
AATTTGGCTTCCCCATCCGATTTCTTTTTGTTCGCCACGAATTTCAGCCATTTCACGTGCCTGCTCTTCCAAT
TTTAATTGATATAATTTAGACTTTAACATTTTCATAGCTGCTTCACGGTTTTTAATTTGAGAACGTTCATTTT
GGTTATTAACAACTATACCTGAGGGGTGGTGGGTAATTCGTATTGCCGATTCAGTTTTGTTAATATGCTGACC
ACCTGCACCAGAAGCTCTGAATGTATCAACTGTAATATCATCCGGATTGATTTCAATCTCTATTTCATCATTA
TTAAAATCTGGAATCACGTCACATGATGCAAATGATGTATGACGACGTCCTGATGAATCAAATGGAGAAATTC
GTACTAGTCGGTGTACACCTTTTTCAGCTTTTAAATAACCATAAGCATTATGCCCTTTGATGAGCAATGTTAC
ACTTTTAATCCCTGCTTCATCTCCAGGTAAATAATCAACAGTTTCAACTTTAAAACCTTTCTTCTCACAATAA
CGTTGATACATTCTAAATAGCATATTAGCCCAATCTTGAGACTCCGTGCCACCTGCACCAGGATGTAACTCTA
GAATTGCATTATTGGCATCGTGAGGCCCATCTAATAATAATTGCAATTCGTATTCATCCACTTTAGCCTTAAA
ATAAATGACCTCTTGCTCTAAGTCTTCTTTCATTTCTTCATCAAATTCTTCTTGTAATAAATCCCAAGTAGCA
TCCATGTCATCTACTTCTGCTTGTAGTGTTTTATAACCATTAACTATTGCTTTTAACGCATTATTTTTATCTA
TAATATCTTGCGCTTTCGTTTGGTTATCCCAAAAATTAGGTTCTGCCATCATTTCTTCATATTCTTGAATATT
AGTTTCTTTGTTCTCTAAGTCAAAGAGACCCCCTAATTTGTGTTAAATCTTGATTATACTTATCTATATTTCG
TTTGATTTCTGATAATTCCATAGCCTTCGCTCCTATTTATAATTCAATTCAAGTCATTGATTTGTATCATTTA
TAATGCTAAATTTTAACATAATTTTGTTAAATAACAATGTTAAGAAATATAAGCACACTGACAATCAGTTTAT
GCTTTTATTGTTAAAAATCAGTACATTTATCATCGATATATGCCTAAACCTATTTTTTAAAACTAAGTACATA
ACAACGTTTAACAACTTCTTCACATTTTTTAAAGTATTTAACGCTTGTAAAATAAAAAGACTCCTCCCATAAC
ACAAACTATAGGTGTTTAATTGGAAGGAGTTATTTTATATCATTTATTTTCCATGGCAATTTTTGAATTTTTT

ACCACTACCACATGGACAATCATCGTTACGACCAACTTGATCGCCTTTAACGATTGGTTTCGGTTTCACTTTT
TCTTTACCATCTTCAGCTGAAACGTGCTTCGCTTCACCAAACTCTGTTGTTTTTTCACGTTCAATATTGTCTT
CAACTTGTACTACAGATTTTAAAATGAATTTACAAGTATCTTCTTCGATATTTTGCATCATGATATCAAATAA
TTCATGACCTTCATTTTGATAGTCACGTAATGGATTTTGCTGTGCATAAGAACGTAAGTGAATACCTTGACGT
AATTGATCCATTGTGTCGATATGATCAGTCCAATGGCTATCAATAGAACGAAGTAAAATCATACGCTCAAACT
CATTCATTTGTTCTTCTAAGATATCTTTTTGACTTTGATATGCTGCTTCAATCTTAGCCCAAACGACTTCGAA
AATATCTTCAGCATCTTTACCTTTGATATCATCCTCTGTAATGTCACCTTCTTGTAAGAAAATGTCATTGATG
TAGTCGATGAATGGTTGATATTCAGGCTCGTCATCTGCTGTATTAATATAGTAATTGATACTACGTTGTAACG
TTGAACGTAGCATTGCATCTACAACTTGAGAGCTGTCTTCTTCATCAATAATACTATTTCTTTCGTTATAGAT
AATTTCACGTTGTTTACGTAATACTTCATCGTATTCTAAGATACGTTTACGCGCGTCGAAGTTATTACCTTCT
ACACGTTTTTGTGCTGATTCTACAGCTCTTGATACCATTTTTGATTCAATTGGTGTAGAGTCATCTAAACCTA
GTCGGCTCATCATTTTCTGTAAACGTTCAGAACCAAAACGAATCATTAATTCATCTTGTAATGATAAATAGAA
GCGACTATCCCCTTTATCACCTTGACGTCCAGAACGACCACGTAACTGGTCATCAATACGACGAGATTCATGT
CGTTCTGTACCTATTACTGCTAAACCGCCTAATTCCTCTACGCCTTCACCTAATTTGATATCTGTACCACGAC
CAGCCATGTTAGTGGCAATAGTAACGGCACCTTTTTGTCCAGCGCCTGCAACAATTTCAGCTTCACGTTCATG
ATTTTTCGCATTTAACACATCATGACGGATACCACGTTTTTTTAGTAAATTTGAAATATATTCAGAAGTCTCA
ACTGCAACAGTACCTAATAGCACTGGTTGCCCTGCCTTGTGTTTTTCAACAACATCTTCTACTACTGCATCAA
ATTTACCTTTTTGGCTAATGTAAATTAAATCAGACTTATCGTTACGTTGCACAGGTTTATTTGTCGGAATTTG
AGTTACTGTCATGTTATAAATATTTCTAAATTCTTCTTCTTCAGTTTTAGCTGTACCTGTCATACCCGCAAGT
TTATTGTACATTCTGAAATAGTTTTGGAATGTAATAGACGCCATAGTTTTAGATTCATTTTGAATTTGAACGC
CTTCCTTCGCTTCAATAGCTTGGTGTAAACCTTCCGAGAAACGACGGCCTGGCATTGTACGTCCTGTAAATTG
GTCGACAATTAATACTTCGCCATCAACAACCATATAGTCTACGTCACGTTGCAATGTAACGTGCGCACGTAAA
GCTGTGTTGATATGACTAATAACATCAACATTTTGTACATCATATAAGTTTTCAACTTTGAACATACGTTCAG
CTTTATCCGCACCTTGTTCTGTTAAATGTACTGCTTTTGTTTTTTCATCATATTTATAATCTTCGTCCTGCTT
TAACATTTTCGCAAAAACATTTGCTTGTGTATAAAGTGACGTTGACTTTTCAGCTTCACCAGAAATAATTAAT
GGCGTACGTGCCTCATCAATTAAAATTGAGTCAACCTCATCAATGATTGCGAAATGTAATGGACGCATTACTC
TATCTTCAGAATAATTCACCATGTTATCTCTTAAATAGTCAAAACCTAACTCATTGTTAGTACTGTAAGTAAT
GTCTTGCGCATATGCTTCACGTTTTTCTTCAGTCGTCTTACTGTTTAAGTTTAATCCGACAGTCAAACCTAAG
AAGTTATATAACTCAGCCATTTCTTCACTTTGAACACTTGATAAATATTCATTGACTGTAATAACGTGAACAC
CTCTACCAGCTAATGCATTTAAGTATGTTGGCATTGTCGCTGTTAACGTTTTACCTTCACCTGTTCTCATCTC
AGCGATATCACCTTTATGAATTGCAATACCACCCATAATTTGAACTTTATATGGTGTCATATTGAATACACGT
TTAGAGCCTTCTCTAACAAGTGCATATGCTTCTGGTAAAATTTTATCTAAATAATCATTTTGCTTTTTGACAT
TATCAATGTCAGCTAATTCTGTTTGGAATTGTTTCGTTTTATTACGAATTTCTTCATCAGTTAAAATTGCCGT
TTTTTCTTCTAAAGCGATTACTTTATCAGCAAGTTTACCTAACTGTTTAATTTCTTTATTATTGCCATCAAGA
ATTTTTGATAAAAATCCCATTTCGTTCGCTCCTTTAGCTAAAAAACTGTTTGGCCTACAACAATATATCTTAT
CATTTATAGTTAGAAAATTATACTTATTTACTCATTTGTAGAATCAATATAAATATATTTATGACATACTTCA
TTCACATTCTGTTGACAACAAGTTTATCACTAATAAATATATTCTCAATACGCAATTATACTTTTTAATAAAT
TATATTATAAATATTTTACGATTTTTGACTTAGACTATACAATAGACTGACATACTATTATTAACTTAACATT
CAAATATATACATCCATTAACATTAGCATAGTCACTATGTTTCATTCAACAAATTGCACAAACGAACTGTGAA
ATAGTTAGAAATTGCTTTTGAAGTCTAAAAAAAGCACTTGTGCAAAAACACAAGTGCTTTAAACTTAATTTAT
TGTTCACTAGTTTGAATCAAGCCATATTTACCGTCTTTACGGCGGTAAACGATACTTGTTCCATCAGTTTCTC
TGTCTGTGAATACAAAGAAATCATGACCTAATAGATTCATTTGTAATACCGCTTCTTCTGAATCCATTGGTTT
TAAGCTGAATTCTTTTGAACGAATAATTTCTATCTCGTTATCATCGTAAGCGTCATTATCAACTTGTGTTTCT
TGCATTTCTTGTAATTCGGCAACAAACACTTCTTGATCTCCTCGATCACGGCTCTTACGATTAATACGTGTTT
TATATTTTCGAACTTGTCTTTCAAGTTTATTATTAATTAAATCAATACCTGCGTATAAATCATCGTTTCGCTC
TTCAGCTCTTAACGTAACATTTTTCAATGGAATTGTTACTTCAATTTTAGTAGCTGAATTTGAATAAGTTTTA
ACTTTAACATGCGCCACTGCATTTGGTACGTCATTAAAATAACGTTCCAACTTACCAATTTTTTCCTCAATAT
AGTTGCGAATAGCATCTGTGATAGTGAGGTTATCTCCATGAATTTCAAATCTAATCATAGTAATCTCTCCTTA
AACCTCTTTATTGGTAACTCTTTATTATATTTTAACATTTTTACGCTATCGTGCAAACGCAAACACTTTGAAT
TTTCTGATATTTTTAGCATATAATTTACACCCTGCACGATGAATTGTTAATCCAGTTGTATAAATATCGTCAA
CGAGTAATATTTCCTTGCCGTTCAAATCTAATTCCGTATCAATAATAAATGGATTTTCATCTGCCAGACGCTC
TTTCTTAGTTAAATGAGACTGTTTTGGTCTATTTGACATCTTTAAAATCTTATCAAAGCGAATCCCTTTAGCT
TTTAGTACTGCTTCTACCGGGTTAAATGTTCTAGATAAATCATGTGCCGGCGAAGAAGGAATTGGCACAATAT
AGTCATAAGATGTTTGTGGTATTTCAATCAAATGTGCCAATAATTCACATAAATAATAGTCTTTCAAAAATTT
ATACTGATGTATCATCTCTTTCATTAAACCATCATATTGAAATTGACAATATAATTGTTCCATTAAATTAAAG
TGTGCCGATAGAAACTTGCAGTCTAAACAATACGCTTCATTTTGATTTAAGTATTTTAAGCATCTTGAACATC
GCCTTGCTTTAATATCAAGTTTAATATTGTCCCAATTCTCTTTGCATCTGTCACATAATCTATTAGGTTTCTT
GAACAAATTATAAATGGTTATATTTTCATATAACTTAGCACCACAACTCAAACAATTATTCATCAATCCAACC

TCTTTTTAATGCTAATTTGTTCATCTTTTGAATCTCTTTTTTAGCTTGAATCATATTCATACTTACTCCTTCA
TGAAAAAACAATACTTTTCCAGTAGGACATTCTAATTTTCGTCCAACACGTCCAGCAATTTGTATTAAAGCCT
CTTGAGTATATTGATGCGCATCGATAACAACCACATCCAAATTTGCCATTGTAAATCCACGTTCTAATATTGT
CGTAGTAAAAATGACATCAAATTGTCCATTCCTTAATTGTTCAACTTTTTCAAAGCGAAAAACATCCTCGCTA
TGGACATATGTTAATTTAGTAATTTTCTGCTTATAAACCGAAAATGTTTTAATCATTGTTTCTATATTGTTAA
AAAACACCAGTGTATAACGTTGTTTATTAATTTGATCTTGTAAAATTCGATATAACATTTTCTGAATCTTATT
ATTATTGAGTTTGAAATAGCGATATTTAGGAACTGGAAGTGATTTTTTATGAAAGCGAGCTGGCAATTTAATT
ACATTTTCGTGGGGAATCTCTGACAGAAGTTGTTTCGGTGGTGTTGCTGTCATATAAATTGTTGCATGTTCAA
CTTTAGAAGATGACTTCAATGCTTGTTGTAAACTTTTATCCATTGATAAAGGAAAGGCATCGACTTCATCAAT
AAAAATAGTATCAAAGTGCTGTTTGAATCGGTAAAGTTGATGCACTGTGCATACAACAAAATGCCCTTCAAAT
TGTTGACTTGATTGCTGGTGTAGTATGTCTATATCTTCATTAAGAAATGCGTCTTTAATACGTTTACTAATTT
CTACAACAACATCTACACGTGGTGACACAATAGCTATATTATCTCCCTGTCGTCTTGCATATTGAATGCCTTG
AAACATCATTTCTGTCTTACCTGCACCTGTTACTGCATATAACAAAATCGTTTGTCTCATTCTAATGGCTCGA
ACAATATGTTCTGAGGCATAAGACTGCTGTTCCGACAATTCAAATGAGAGATGATAATAGGCTTGTGATGAAC
TTTGCGAACTCTCTGTTATTTTATATCTTGTTACATTATCCATTCGACCCAGTGATATACAATTTCTGCAATA
TACAATTTTTTTGGAATGTACTGAGGAATCATACTGATAAAAATGCTTTGGATTTGTACTTTCACATTGAACA
CAATAATAGTTTGAATTTTGTTTGATGACACCTGTTACTTCTTTTTTAATTTTTTCATTAGTAAGTGAAGTAT
GAGGATGAATTAATTTCCCGTAATGTTTAATGCTTAAAACCTCATTTCAACGTAAAAAAGAGTCTAATATTAT
AATGATTTTCTTATATCTTTAGCATTTTGACATTCAAATGATGAACATTTAAAGCCATGTATGCAAGAGATAA
TTTCCAATTACAATATAGACTCTTTTATTAAAATTTTATAATTATGTTAATCTTATTTTTCTGCCAACATAGC
CTAAACCTAATCCACCAGAACCTAAATGTGCTGCAACAACTGGACCAAACTCAGAGTATGCTACTTGATAACC
TGAAGGACAATCTTCTTGTAACTTTTTGTATAACGCTTGACCATCTTCAAAATGATCTCCATTTATGACAAAT
AAAGTTACTTCTTCAAAGTCTTTTACAATGTCTAACACTTTCTTTTCTAATGTTTGAATGGCACGCTTTTTAG
TACGAACTTTTTCTTCTGGTATAATCTTGCCATCTTCAAACTTAAGAACTGGCTTCATTTTTAATAATGTTCC
AACCCATGCTTGAGCACCAGTAATTCGACCACTTTTTTGTAAATTCTTTAAGTCATCAACAATTAAATATGCA
CCAGTGTGTTCACGCATATTAGTTAAATCATCAATAATTTGCTGTGGTTCGTATCCTTCTTCAACCATTTCAA
TAGCACGTAATACATAACAACCTTCAATCATTGCTGCAAGCTTACTATCAAATGCATGTACATTAACACCTTC
AACCATTTCCCCTGCTTGATAACTAGATTGATAACTTCCACTAATTCCACTTGATAAGCAAATGACAATGATA
TCTGTGTAACCTTGATCTCTTAGCATTTCATATTTAGTAATCCATTCGCCAATTGCTGGTTGGCTTGTTGTTG
GAATCGTTTGAGACGATGCCATTTTATTATAAAATTCTTCAATTGCTATTTCATTACTTTCTGTAAAGTTCTT
CCCATCATCAAAAGTCACACTTAATGGCGCTATTTGAATATTATATTTATCGATTAAGTCCTGCGACAGATAA
CTTGTAGAATCGGTCATCACAGCAATTTTCATAGTCGTCCTCCTAAAATATACTCATAACAATGATACAATAT
TTCAGTTAAAAAGAAAATGGCAATCAAACGATTTATAGTCATGTCATTTTCAGTGTATAATGAAGATTAAATA
ATCTTAAGCTTATGAATTAAGGAGTTATTATGACACAAAATATCATTACTGTTAAAAAAGAACACATTATTGA
AAATGTGATTAGTAAATCAAAATTCATTGCTCATATCAAACCTGTTCAAAATGAAGATGAAGCTAAAGCATTT
ATTGCTGCAATAAAAAAAGAACATAAAGATGCCACTCATAATTGTTCTGCATATACTATTGGACCTGAAATGA
ATATTCAAAAAGCGAATGATGATGGTGAACCGACAGGAACTGCCGGTGTCCCAATGTTAGACATATTAAAAAA
GCTCGATGTTCATAATGCGTGTGTCGTAGTAACAAGATATTTTGGTGGTATTAAATTAGGTGGCGGCGGTTTA
ATTCGCGCCTATAGCGGTGCTGTTAGAGACGTCATTTATGATGTCGGACGTGTCGAACTACGAGAAGCAGTAC
CATGTATAGTAACACTGAATTATGATCAAACTGGTAAATTTGAATATGAATTAGCTTCAACTCATTTTATGTT
AAGAAACCAAACATACACTGATAAGGTAAGCTACTATATCGATGTCGTTAAAAGTGACTATGAAGCGTTTATT
AACTTTTTAAATCAAATGACTGCTGGAAATTTCGATTTGACTGAAGAAACTCCAAAGCAGTTACCTTTCGATA
TACCAACTGAATAAAAATAAAAACACTAGCTTAGGATGGCTAACTCTAATTGCGTATTGTTACTTTGCAATTT
TGAAAGCACGTTCCATGCTAGTGTTTTTTGTGAGTATGCCTTCCTTTATAACAACATAAAAGTACAGACATCA
TTTTTTATTTTATTACTTACCGTTTTAAAATCTAATCTTCTTTGTGTGACGATTTCCGACTAATTAAATTTAA
TATTGGTCGGTAGTTGTTATCTATTAATCCTGTAAATTCAACAATTAATTCAATGCTAAATATGATTAATATA
AACATTAATACAACACCTAATGGTGGCGATACATACAAGATAATGCTCGAAAGACTAAATAAAATAGAGATTG
AATAGATTAATAATACTGTTTGTCTATGTGTGTAGCCTAATGCTAATAGTTTATGATGCAAATGCGATTTATC
AGCTTGCATTATATGCTGCCCTTTTTTCACACGTCGAATCATTGCGAACAAAGTATCAATGAATGGCACTGCT
AGGATAACAATTGGGAAGAACAATGCAATAATTGTAATATTTTTGAATCCGAGCAAAGAAAGGAATCCGATGA
TAAATCCAATCATTAAAGCCCCACTATCACCTAAAAATATTTTGGCAGGATGGAAATTGTAAAATAAAAACCC
AATTAAAGAGCCTAATAAAACACAACAAATCATCGTTATGAAAATATTAGCTTGTAAAATTGCAATGAATCCT
ATTGTAATGAGTCCTATTGCAGAAACACCCGACGCCAAACCATCGAGTCCATCAATTAAGTTAATTGCATTTG
TAATAGCAACAATCCAAATCACAGTAATTGGAATACTAAGAAATCCAAAATGAATCGTTGTACCCATTGGCAA
CGAAATAAAATCAATAGTCACACCATAAAAAGCAACTACTAAGGCAGCGGCAATTTGACCAGCCAATTTTATA
TACGGTTTCAAATCGTAGATATCATCTACAAGCCCAAGTACGTACATAATAATCGCACCAATAATAAGTGGTT
TGATTTCTGTTTCAATAGGATGACCAATCCAAATACCTATTAAAAATGAAAAGAGAATCACTGTACCACCCAT
AACTGAAATAGGTTTAGTGTGTACTTTTCTAAAATTTGGTTTATCAACTAAATTTAATCTTTTCGATATTGCA

ATAACAATTGGTGTTATCGTCAAACTGACAATCATTGTTACTGCAACTAGTAATAATGTAACCATTTATTCAC
CTTCATCGATATTAATTGTTTTTAACGTTATATTTATTTAATATTGTCTTTATAGAATGATATATCTATTTTC
TTCGTATATCTTATAGCTTTATTATAAATTAATTCGCTCAATTAATGATATCATATTTACAACTATACGATTC
ATTTCTTATATATCGCCTATTATTCATTTTTTCATCATATCATTTAAGGCAACATTTTAACATCAAAGTCTAT
ATCATACATTAATTTTATATGTAAACTAATGACAAATTGTAAGTTTTCACTTACAATAGCTAGTGGTCAATTA
AAGGAGTCGATTCAGACATGTTCGAAGCATTTATATACAATATATCTGTTATAGTCGCTGGAATCTATTTATT
CCATCGCTTACAGTATTCAGAAAACAAACGTATGGTTTTTCTAAGGCTTATGTCACTGTTTAATGACAATC
GTATCATTATTATTATCTGTCTACCCTATCCCTTATCGTGAAGATTATTTAATTCACTTAACATTCGTTCCTT
TATTGTTTTTAGGTAGATTTACAAATATGGTCTATACATTATCTGCCACAGTAATTGTAGCAATTGTTGAAAT
TGTAGTATTTAACAACTCGATTATGTACGGTGTTACGTTGATTGTAATTGCCGCAGTAACAAGTGCTATTGGA
CCATTTTTAAAACAAAATGATGTGCTATCATTATTAATACTTAATGTTGTTACAATAATCATTTTATTTGGTG
TCGCATTAGTGAGTCCTATTTACACACTATCGGAAGTTATCATTTTAATACCAATTTCATTAATTATCACTTT
AGCTTCTGCGATAACATTTGTCGATATTTGGCATTTCTTCTCACTCGTAAATCGTTATGAAAACGAAGATAAA
TATGATTACTTAACAGGCTTAGGCAATGTAAAAGAATTTGATAGACATTTAAATGAAATTTCACGAAAAGCTG
AAAAAGAACATCAAAGTATCGCATTATTATTAATCGATATCGATGGATTTAAAGATGTCAATGATACCTACTC
ACACAAATCAGGTGATGCTGTATTAAAACAAATGTCTCAATTACTTAAAAACTATGTGCCGAATCAATTTAAA
ATTTTTAGAAATGGTGGCGAAGAGTTCTCAGTTGTCATTCACAATTATTCACTTGATCAAAGTGTTAAATTAG
CTGAAAATATTCGTTCGGGTGTTGAAAAATCATCATTCCATTTACCAAACAAAGAAGTTATTAAATTGTCAGT
ATCTATCGGTGTTGGTTATTTAACAGATGACGATCCAAAATCACAGCGTAAAGTGTTTAAAGATGCAGACGAT
ATGGTGCATGTAGCCAAAAATCAAGGGCGAAACAAAGTAATGTTTAACCCTATTATCAATTTATAACAACGAC
AAAACTGTTAATCCTTTGCTATTATTAAATCATAGTATAATAGCAAAGATTAACAGTTTTTCTTATTTAAATT
TGAACCATACAAGTTCATCATACTAACATAAAGTCTCACTATTGAAAGTTGCAACAACAAGTTGTAAACTTCA
ATTGTTCAAATAAATAACACATATCATTGGAGGTGAATTGATATGCCAGATTCAATCACAATTATAGATGAAA
ACAAAGTGATTGATGTTGTATTAATTGCAGGTAGAATTTTACTTGAATCAGGTGCTGAAACATATCGAGTTGA
AGATACAATGAACCGTATCGCACATAGTTATGGTCTTCATAATACATATAGTTTTGTCAGTTCAACTGCAATT
ATTTTTTCATTAAACGATCGAACAAGTACAAGATTAATTCGTGTACAAGAGCGTACAACAGATTTAGAGAAAA
TCGCTTTAACGAATAGTCTTTCACGTAAAATATCAAATAAAGAACTCACAATTGATGAAGCTAAATCTGAATT
TATTCATTTACAGCATGCATCATTACAATATTCTTTTTTAACGAATTTCTTTGCAGCTGCCATTGCATGTGGC
TTCTTCCTATTTATGTTTGGAGGTGTCGCATCAGATTGTTGGATTGCAGTCATTGCTGGTGGATCCGCATTTT
TAACATTCAGCTTTGTGCAACGCTATATACAAATTAAATTTTTCTCAGAGTTTGTTGCAGCCGCTGTGGTCAT
ATCAATTGCAGCTACATTTACTAAATTAGGTATTGCAACCAATCAAGATATCATTACTATAGCCAGTGTCATG
CCACTTGTCCCTGGTATATTAATTACCAATGCCATTCGAGATTTATTAGCGGGTGAATTACTTGCAGGTATGT
CTCGCGGTGTTGAAGCTGCATTAACAGCATTCGCAATCGGTGCTGGTGTCGCTATCGTTTTATTAATCATTTA
AGGAAGGACTGTTAAAGCATGCTATTTTATTTATTTCATTTTACAATCAGCTTTATATCAACAGTACTTTTCT
CTATCATTTTCAATGCACCCAAACGTCTCTTAGTAGCATGTGGATTTGTAGGTGCCATTGCATGGACGATTTA
CCAATTAACGGTAGATTTAGAGTTTGGAAAAGTTGGCGCTTCATTTTTGGGAAGCTTAATTTTAGGCTTAATG
AGTCATACTATGAGTCGCAGATATAAACGACCGGTAATTATATTCATAGTGCCAGGCATTATACCATTAGTAC
CTGGTGGTGCAGCTTATCAAGCGACTCGTTTTTTAGTATCGAATGATTATACAAGTGCTGTAAATACATTTTT
AGAAGTTACACTGATTTCAGGTGCGATTGCTTTCGGTATATTAGTTTCTGAAATTCTATATTACCTATACACA
CGTATCAAACAACTGTATGGTAAAATCAAAGGTAAGACATATAAAAAATCTTACAACATGAATAATAGAGTTT
AAGGGGTCGAATATAATGAAGAACCAATTGATAGATAGATTAACAAGATATACGACAATTGATACGCAATCTG
ATCCAAAATCTACAACAACACCTTCAACTGAAAAGCAATGGGATTTGTTACATTTATTAGAAAAAGAATTACA
ACAATTAGGATTGCCAACTGATTTAGATGAAAATGGCTACTTATTTGCTACATTAGAAAGTAACATCGATGTC
GACGTCCCAACAGTTGGATTTTTGGCGCATGTTGATACATCACCTGATTTCAATGCTTCAAATGTTAAACTGC
AAATTATTGAAAACTATGATGGTAAGCCATACAAACTAGGCAATACTAAACGTGTCTTAGATCCTAAAGTATT
TCCAGAACTTAACAGTTTAGTTGGTCATACATTAATGGTAACTGATGGTACATCGTTACTAGGTGCAGACGAT
AAAGCGGGTATTGTGGAAATTATGGAAGCTATTTGTTATTTACAAGAACATCCAGAAATTAAACATGGTACCA
TTCGCATTGGATTTACACCAGACGAAGAAATCGGTCGTGGTCCACATAAATTTGATGTTGACCGCTTCAATGC
CGATTTTGCTTATACTATGGATGGTAGTCAATATGGAGAATTACAATATGAAAGCTTTAACGCTGCTGAAGCG
ATTATTACATGCCACGGTGTGAATGTACATCCTGGTTCAGCTAAAAATGCAATGGTAAACGCAATACGTTTAG
GTGAACAATTCGATAGTTTGCTACCTGATAGTGAAGTTCCGGAGCGAACAGAAGGATACGAAGGCTTTTATCA
CTTAATGAACTTTGAAGGAACTGTTGAAAAAGCAACTTTGCAATACATTATTCGTGATCATGATAAAAAACAA
TTCGAATTGCGTAAGAAACGTATTTTAGAAATACGTGACGATATCAATGCCCATTTTGAAAATTATCCAGTTA
AAGTTGATATATCGGATCAATATTTCAATATGGCAGAAAAAATATTACCATTGCCTCATATTATTGATATACC
TAAACGTGTCTTTGCCAAATTAGATATTCCAGCAAACACTGAACCTATTCGCGGTGGTACAGATGGTTCACAA
TTGTCATTTATGGGGTTACCAACTCCAAACATCTTTACAGGTTGCGGTAACTTCCACGGCCCATATGAATATG
CATCCATTGATGTCATGGAAAAAGCAGTACAAGTAATCATTGGAATTGTAGAAGATATCGCTGAAAATCACTA
AAAACAAACGATTATATACTAGTTCAACTCAAGCTTAATTAATATAAAAAATCACGCTAACATTCAGTGCAAT

GATAGCGTGATTTTTCATATTTTTTACGTATACTTTCGATCTTCTAATTCGTTTTGCTAACCATCATAAATGT
TATTAACCATCATGACAGCATCACCAATTTAGTCCTTCTTTACTAAACCTCAACATTAAAATGTGTTTTCAAC
AGTTTTAAAGACTGAACAGTATAATGCCTAATTTGTAATCCCATTTTAAAGTCAGTATAACTTTCCGGCATAT
CGATAAAGGTATTAAACATTGCTGTAACCCCTTGTGATTCAAATAAATCAAACTTTTCAGCAGTTGCACAAAT
TGCAATGGCAACCTTTTGATGTTTATGACAAAGTTCTGCAATACGCAATGTTGTCGTTTCTAGCAACTGATCA
TTTTCGTTTAATCCTTCTCCAAAAATAATTAAATCCGCTTGTTCAACTAAATTTTCTAAATGTGTTAGTTGGT
CTACTAATGCATGACTGGTTAATATTTCAGCTTGATACAGTCCATTCAAGACTGCTGCAATTCCACCACCAGC
ACCACCACGTTCAACTGGACCAATTGCAATTTTCAATTCACTTTTAAATAACTCACTAAAATACCAAATTAAA
TTATCGATTTCTGCTGCTTGATTATGATTCAACTGATGCGCATCATAAGTTTGCATGATTTCACTTTGCTTAC
CATATAATCGACTTGAAAAATCCGACATTACTTGAATTCTTGCTGTTTCCATTTTAGGGTGTAAGTTTGACAT
ATCCATACGACGAATATATTTAATTACACCAGCACCTTGTCTCATATCTACGACACGCCCTTCGTCATCATAG
AATTGAGCACCTAATGCTTGTAACATACCTGCACCAGCATCAAAACTATCAATCCCACCTAGTGAAATTACAA
CATGTTTTGCGTCGTTATCTAATGCATGTTTAATCATTTCTCCTAAACCATAACTTGTTCGTTCAACAATTGG
TTTTTTACCTTTTAAAAATAAATTCCCCTCGATAACGGTCATCCCTGTATCAGTTTGTCCGTAAACACCTTCA
ACTTCATTCATATCTGCATCATGTACTGGTATACGATACTTTTGCCCAGATTGCCATAAAAATACAGAATCTA
ATAATTCATGTCTTCCATTAAACAATGGTACTTGAACTACATCTGCAGTTTCAATTTGGCTTGCAACTGCCTC
TTCAACATATCTATTAGCTTGATAACTCGAAATAATTCCATGAAACTCATCCATGGCTACTAATACTTTCATA
ACCTTGACCACCTTACTTTATTATTTGTTGTGATACGTTTTTTATTCTTAAAAGATTAATCTTAAATTTTAGA
TTTTATCTCGTGATTATATGCGAACATTTTACATGACATGATTATTAATTATTCCAGTAAATAACAGTATGTT
TCTTCTGGCTTAGCATGTATTTCCTATTAAACTAACTTACAAATCATGTTTAAATTTTCTTATTGGATTCGTC
GTTTTCCAAAATATTTTCAATAAAAATACTCGAATACATTATACATATGTTTGTAAAAAAGTACTAACTTAAC
TACTTCTATAAGTTACAAAAAATAAAAGGTCAGAACATCATTATAGCCGAACTACCCTGATTCACACGAATTT
AAGTTGTGCTATTCACGTCTAGTAGTGTAAGGCAAAATGTTCCAACCCTATATTTTCATTATTCTTCTGCTTG
TGCTAATGACGACACATTAATATCACCGTGGTCTCGATTCCAAACCATTTCACCATTTACAAAATAAAATGCT
TGAGGTGATTCATGTTTAACATTCGTTTTTTTAGCAATATAATCTGACAAATCGCGTTCTTGTTGGACAATCA
AATAATAACCGTCCATATCGCGTTCATATAAAAATTTATTAAATTGATCGTACGCATTTGCCGATATTGGACA
AGTTTCACTATGTTTTAATACAAAAACATATTTATTTTCCTCAATAACCTGTTCAAATTGGTCAATTGAACTT
AGCTTTATAGCCACTCTATTCACCTCTAAAAATATTTATCACATTTAAATAGCATCTGCGTGACATTTTTATA
CATATAGTTAATATCAATGTTTACCATTATACACATAGTAAGTTGACATGTTAACCATAAATTCAAAATTTTT
AACTTTGAAAAATGATTATAGTTATTTTTCTTCTAATTTTTCAAGCTTTTTTTCAAAGGCTTTATCGCCGTGA
GTTATATCTATACCCTTTTTCGGCAACTTATCTACATCGACTTTTGATAACTTCTCACTAACCTTTATTGTTT
CAATACGTGTATTATAATAGTTCTGCAGACTGTAATACATTTCTATTGCGTTTTTCCTTGCATTATTAACATG
CTTATCACTAATATTAGTTTGGTTAATATCGGTAATTTGTTTTTCAATCATTGGCATAATGTGATTTTTAATC
GCGCCTTTTACTTCATTTTCTTTACTATCATCTAGGTTCTTCTTCGCAGTATCTCTTAACGCTTTATTATTAT
GTTCTAATTTTGTCGTAAGATTAGTTGCTTCACTTTTATTATCAGCTAATTTAATTTCTGATTCAACTTTGTA
TCTATTTTTTTCAAATTGTTTCGTATAATCTAAAATGTCTTCGTTATACTTGATAGATTGATTACATAAACCT
ATGAATTTTTTTAATTGATATATCGCATCCTTCTTCTCATTTGCAAGCGTCATATATTCTTTTTTTAATTTCT
TAACTTTCATTGTATCATCAGGCAAATTTTTTGCTGACTTATAATATGCTTCAAATTTAGGTATGAGATGGTT
TTTAACATCTTCTTGTAGTGCCTTAAATTCTTTACTATTTTTATCAGTTGTATCAGTTTTACTTAGATGATCA
ATTTCTTTCAAATGTATATTATCCATGACATGTTGTAATTCTTTTTGTTTTTGCTCAACATCTTTAAATTGCT
TAGTAAATTGATGTTGTGCCTCTTTTTCTTTATTACCACAAGCACTTATTACTACAAATAAAATCGCTAAAAC
AGCGATAATAACAATTTTTTTCATGATTGCACTCCTTAACAGATTCATTATACAAAAACATGTTTATTATGAA
AATGAATTATGTTATAGTATTCCAGACTTTATCGTGATTAAATTAATTTTCAATATCAAAGGAGTTTATCATG
TATTCAAATGTTCAACCATTCATTAATAGTGATTTAACTAATTGTTTTCAATCACAATACACCGAAATTGAAT
CATTATTATTCAACCTCTTAGACAGCCCAGTAAAATATACAAAACATATTGGTGGTACGCATCATTTTAAATA
TTCAACAGAGCCAATTTTAGATATTTTAGTAGGTGTAGAAAATTTGCATGATATTACATCCTTAGATGAAAAA
CGTTTAAATTATGTTGGGTTTTATCGCTTGCATCATAAATATAATAAGAAAGTTATGATGGCGAAGTTTAATA
ATTTTCATGAACTAAAACAACAGGTGCGTCTACATATCATTCAAATGAATACCCAAACATTTGAATCTTATCT
TGCACTTGATTATCTCTTATCGACTAATGATGAGATTGCTTTACAATTCAATACTAAAAAGCATCAAATATTG
CAACATAGCTTTACAATTAGACAATATGAGATGCAAAAGCAAAAATACTTTGAAAAACTATGTAAAAAATACT
TATAACGACAATAGTGCCGTATTTTATAATTATACTAATTCTAATACTTTCTTTTCAATTTTCGCAAATGAAT
TTTAAAATTGGTATAATACTATATGATATTAAAGACACGAGAAAGGATGTACTGAGAAGTGATAAATAAAGAC
ATCTATCAAGCTTTACAACAACTTATCCCAAATGAAAAAATTAAAGTTGACGAACCTTTAAAACGATACACTT
ATACTAAGACAGGTGGTAATGCCGACTTTTATATTACCCCTACTAAAAATGAAGAAGTACAAGCAGTTGTTAA
ATATGCCTATCAAAATGAGATTCCTGTTACATATTTAGGCAATGGCTCAAATATTATTATCCGTGAAGGTGGT
ATTCGTGGCATTGTAATTAGTTTATTATCACTAGATCATATCGAAGTATCTGATGATGCGATAATAGCCGGTA
GCGGCGCTGCAATTATTGATGTCTCACGTGTTGCTCGTGATTACGCACTTACTGGCCTTGAATTTGCATGTGG
TATTCCAGGTTCAATTGGTGGTGCTGTGTATATGAATGCTGGCGCTTATGGTGGCGAAGTTAAAGATTGTATA

GACTATGCGCTTTGCGTAAACGAACAAGGCTCATTAATTAAACTTACAACAAAAGAATTAGAGTTAGATTATC
GTAATAGCATTATTCAAAAAGAACACTTAGTTGTATTAGAAGCTGCATTTACTTTAGCTCCTGGTAAAATGAC
TGAAATACAAGCTAAAATGGATGATTTAACAGAACGTAGAGAATCTAAACAACCTTTAGAGTATCCTTCATGT
GGTAGTGTATTCCAAAGACCGCCTGGTCATTTTGCAGGTAAATTGATACAAGATTCTAATTTGCAAGGTCACC
GTATTGGAGGCGTTGAAGTTTCAACTAAACACGCTGGTTTTATGGTAAATGTAGACAATGGAACTGCTACAGA
TTACGAAAACCTTATTCATTATGTACAAAAGACCGTCAAAGAAAAATTTGGCATTGAATTAAATCGTGAAGTT
CGCATTATTGGTGAACATCCAAAGGAATCGTAAGTTAAGGAGCTTTGTCTATGCCTATAGTTTATGGTTCATT
AATCGATACTGAAACACGCTGTCGCCATTATTTTACCGAAGAAGATATTATTGCTATTAAATTTAAATGTTGT
AATAAATACTATCCATGCTATAAGTGCCATAATGAGTTTGAAAAGCACGCCATTAAGCGTTGGTCTGAGCCTT
CATTTAACGAAAAAGCAATCTTATGCGGTGTATGCAAACACGAATTAACAATCAATGAATATATGATGGTTGA
ACGTTGTCCAAATTGTCAATCTCGATTTAACAATCGCTGCAAATATCATTATCATATTTATTTCGAAATTTAA
ACAACATAGCCAAAACATTATAGGGAGTGGGACAGACATGATAAAGCACCACTTCTATAGAATTCCTTTTTGA
TATTCTCTATGTTTGGGCCCCGCCAACTTGCATTGTCAGTAGAAATTGGAAATCAAATTTCTCTATGTTGGGA
GCCCACTGGTATTGAAATGAACTTGTTACAAGTACATGTTCATTCAACCAACGACAAACAACAGAACATTTTA
GAGTCTAGGACATTGATTAACGTCCCAGATTCCTTTTTAAAAGAAAAGTGGGGAACAATGATTAAGACCTTT
AACCATTGTTCCCCACTTTTAAAATTATTTTACAACTTTTTCAAGTTCCTCAATTTGTTTAATTGTAGTTGTA
GTTGATCCAGCTGCAAAGTACCATAATTTAGGATCTAAATTATAAACTTTGTCTTCTTTAATTGCTTTAACAT
TTTTTAATACAGGATTATTTAATGCTTGTTTCGCAGTTGATTTACCACTTACAGCCTGTCCTCTATCCATTGC
TAAAATAACATCTGGATTTTCTTTATTAATGTATTCGTTAGAGACATTTTGTCCGTGGTTACTATTGCTTACT
TTTTTATCAACCGCGTTGAAACCTAATGTATCGTAAACTAATCCACCGAAACGACCTTTAGGTCCATAAGTAG
ATAATTCACCTTCATTAACTAATAAATACATAACTGATTTGTTGAACTTCTTAGTTTTATCTTTCATTGAGGC
AATTTTGTTATCTAAATCTTTATTTAATTCTTTAGCTTTATCTTCTTTGTCGTAAATTTTACCGATATTTTCA
GTGTTTTGTTTCATTGAACCAATTAAGTTCTTTTCATCTGCACCAACATAAACAATTTTTGCTTTTGGTGCAG
CTTTTTTGAATTCATCTAAATTCTTTTGATTAGCTGTACGGCCTGAGATAAAGATTACTTCTGGTTTTGTTGC
TGCAATTTTATCGAAATTAACTTCTTTCAAGTTACCAACATTTGTATACTTGTCATCTTTAAATGACTCTAAG
AAGTTCGGTAATGACTTGCCGCCTTCCCCTTTAGGTAATGCTTTTACTTTGTCTGATAAGCCCATTTCTTTCA
TTACATCTAATGCACCATAATCTAATACAACTGCATTTTTAGGATTTTTTGGTACTTCAACAGTTTCTTTAAC
TTTTTTAGCATCGCTACCATCTTTTTTCTCGCCACGCATTTTATAGTTGTTTTCAATTTTCACTGTATCTTTA
GAACCTTTAGTTTCTGATTTTGATTGTTCTTTATCAGAATTGTTACCACATGCTGCTAATAAAAACATTACTG
CTACTACTAAATATAAGACTGTTTTCTTCATAATATAATTTTCTCCTCTTTTGGCTTAATCTGTGTGCTTGAT
GTGAAAAATAAATAAATGATTTATAGTTAAACTTTCTTTAATTGGTGCATCACCCCTTTAAAGTGTTTCCGAA
TTATCAATCCTTAAAATATAAACAAATCCTTTGATTTTTTATATGTTCAATTTGAATATCCATGTCATATAAA
TCACTTAAAACACTACTTTGAATGACATTATCTTTCAAATCTGATTTAACTAGTTGTCCGTTTTTCAGTGCTA
CAATTTGATCTGAATAACAGGACGCAAAGTTAATATCATGTAACACAATGACAATCGCTTTATTCATTTTATG
TGCTAACATTTTTAACGTTTGCATAATTTGAACAGCATGCTTCATATCTAAATTATTTAACGGTTCATCTAAA
AGAATATATTCAGTATCTTGTGCAATGGTCATCGCTATATAGGCACGTTGACGTTGTCCACCAGACAATGATT
TTATATTACGCTTTCTAATATCTTGTAATTGCAACAAATCTAAAGCATCATTAACAATTTCATGATCTTCTTT
CGTTAAACGTCCTTTAGAATAAGGGAATCGTCCGAAATTAACTAATTGTTCTACAGTAATGTTCATTTCTGTA
TGGTTCGTTTGCTTTAAAATCGATATCTTTTTCGACAAATCATTGTTTTTATAATCAGACATAAGCTGTCCAT
CTATTTTTACTTCCCCTTCATCAAACCGAATTAAGCGACATATCGCTGAAAGTAACGTACTCTTCCCTGCTCC
ATTAGGTCCAATTAAAGAAGTCAACTTACCTTTTTCAATTTCTATGCTAATATCGTCCAATATCATTTGATTA
TTTATAGTTTTAGTTAAATTTCCAACTTGAATCATTGCGCATTTCTCCTTCTAACTAATAGATAAATGAAATA
ACTTCCACCAATTAAATCAATAAGTATACTCATTTCTGTCGTAGCCTCAAACACATTTTCAACTACCCATTGC
GCACTAAATAAACTAATCCAACTCAAGCAAATTGTCGCGATTAATATATATTTATGTTCATACGTCTTCATTA
GTTCATGCGCTAGATTTACAGTCAATAAGCCTAAAAATGTAATTGGTCCTACTAATGCAGTTGACACAGAAAC
TAAGACAGAAACTATTACAAGTAGAATCCGCGTTAATTTTTCATACGATATACCAAGATTAATTGCTTCAGCA
CGACCTAAAAGCAATACATCTAAATACGGCAATAGAAAAATTGTAATGACTAATAAGATTACTAATAATACTG
CTGAAAAAGAAACTAAATTCGAATTAGAGGCATTAAAATTAGCAAACATACTACTTTGTATTGCTAAAAATGA
TTCAGGATCCATAATCAATTGAATAAAACCTGTAATGCTTCTAAAAAATGTTCCTAAAAGGACACCAATAAGT
AAGATAAAATAAATTGAAAAATGTCCAATTTTAAAGATACCTTGGAATAGGATTAGTGCGAATAACACCATCG
TTATAAGTGTTAATATAAAATTCAAATATACATTAGTAACCCACACCGATTGAATTCCAAATAAAAAGACTGG
CAATACTTTGATAAATAAATAAACTGCATCTAATCCCATTATTGATGGTGTCAATAAACGATTATTTGTAATC
GCTTGGAAAATAACAACGGATGTTGCAATGGCAGCACCTACTAAAATAATTAATATGAACTTTCTTAAACGAT
TTGAAAATTGATATTCAAATATTTCAAAATCAATACCAACAAATAAGTACAGCATACTAATTAAGAACGTGAC
TACAATTAAAAGCATTATTTTTTTATTATTGTTGCGCATAATTTTTCCTACCTTTCATAAGTAAGATAAGGAA
AATGATTGTTCCAAATACACCTATTGTTAAACCAATATTAATTTCATAAGGATAAACTACAATTCTGCCAACT
ATATCAGAAAACAATACAAAGATGGCACCTAACATCATCGTATGAGGGATAGCATTTTTTAAATGATCGCCTC
TATAAATCGAAATAATATTTGGTATTACTAGTCCTAAGAACGGTAATGTTCCAACAGTCACCACTACCAATGC

TGTAATAGTTGCAGTAATAAACAATGCGATGTTAATTAATTTTTCGTAACTCAAACCTAAATTATTGGTAAAG
TCTTTACCCATTCCTACAATTGTGAAATGATTCGCAAAAAGATATGTCAATGCTAAAAGAGGAATACTTAAAT
ACAAGATTTCATAACGTCCACTTGTGATAATGGCAAAGTTCCCGTTAAGCCAGTTACCAATGCTTTGAACTGC
GTTCGTACGTAATGAGATAAACGTTGTAAAACTAGCAACAATGCCACCCATCATAATCCCTAATAGTGGTACA
AAGATGACATCTTTCACTTTGATAAAATCAATAATTTTAACAAATAAAAATGTTCCGCCGATGCTGCAAATAA
CAGCAAATACTAATTTTAATAAAATATGACCAGTTGGAAAGAATAATAAGGCAATTAAGATACCTAATTTTGC
CCACTCCATCGTCCCAGCTGTAGTTGGACTAACAAACTTATTTTGCATCATTTGTTGCATGATTAGGCCAGCT
AAAGCTAATGAACTACCAGCAATTAAAATACTCATCGTTCTCGGTATTCGGCTTGAGAACAATATATTTTGCT
GTGAGTCTGATAAATGAAAGATATCTTTAATAGACAGTTCACTGACACCTATAAACAACGAAATGATTGTTAG
CAATATAAGTAATAGAAGCAAAATTTTTCCCTTTAATAAATACTTCATTACAAAATTTTCTCCCTTTAATTTA
ATGATAATTATTATCATTGATAACATTTCTCATTAATGGTATCGTTTTTTGTCACTCATTTCAATAGCCTTTC
AAATAATTTATTGTCGATTTTATTGATATTTGCAATTAAAATAGAAATCAATTTTTGTAAATGTGTATTTTAG
ATGTATATAGTAAAATAGCACACCCTTTTGTAATGCAATATCTTGTATACTTTCTAGCCTCATTGACATCATT
AAATGCAATTTTTACTTTTTCAAAATGTGCATACGTTTTTAAGCATAGAGAAAAGACCTTCAACATGTCGGAA
GCGAATGTCATCGCTTTGCCCTTTCATGTGAAGGTCTTTTTAATTTTAATTATTATTTGTTGTCAAATACAAA
GTCATCATCTTGTAATGCTTCTACATTTAATGCTAATACATAACCATCACCTTTTACAGAGAAGAAGTCATGG
TTTTTAGTTGTTGTATCTAAGGCATTCTCAATGATTGGGTTAAATTCACGTTCCTCAAAATAAGGTTCAAAAC
CTAAATTTGAAAGTGCTTTATTTCCATTATATTTAACATAGTTTAACACATCTTCAGTGATTCCAAGATCATC
GTATAACATTTTTGTGTATGACTCTTCATTTAAATACAAGTCATTTAGCAATTTATACATTTCTTGATCAGCT
TTTTGCTTCTCACTTTCAGATAGTTCATTTCGTAAATGCTGTGCATCTAAACCAGTAAACACACCATGAATAG
ATTCATCTAAAAGAATTTTACGAATGATTTCTCCTGATGTCGTCATTTTCCCTTGACCAGCTAAATATAGCGG
ATAATAGAAACCAGAGAAGAATAAGAATGTTTCTAAAAATACACTCGTAACTCTGGCCATATATTGGTCATAT
ATGGAAGCTTCTTTACCCCAAAGTTTGTGGTAATTAGCAACAATTTTATCTGATTTATATTTTAAATGTGGTT
CTTCTAAAACCCATTCATCTAATAAATAGTTTGTTTCACTAGATGGTAATAACGTTGTGAAAATATGCGAATA
ACTTTTCGCGTGTATTTGCTCCATCATCGCCATAAATGAATAAACTGCTTTTTTTCTTAAGTCAGTCGTATGT
AGCATGACTAAAGGCATACCATCATCTGCTTGATGTGTATCTAAGCCTGTTAAACCAGCTAATGCTTTTTTAA
ATGTGTCTTGTTCAGCTTCTGACAAAGTCTTCCAACTTGCAATGTCTTTTGATACTTTAAATTCTGTTTCAAC
CCACATTTGAGATATATTTTGTCTCCAAAACATATTCGTCATATCTTCTTGTGTGTTCCAATTAACAGCTATC
ATGAATATAATTTCCTCCTGTAATGTTACTCTCAATAATTGACAAATATAGACATGGCAGACTAATCATCTGT
TATTTTAAACGAGATGATTAGCTGTTTGTCATTTTCAACATTTAATTGTTAGATAGAACAACTTGTACATTCT
TCTACACTTAATAATTTATTTCTAGTATAGTAAAGTGATTTTAATCCTTTATAGTGCGCATATACATATAATC
TTGCTAACTCACGTGTAGAAATTTCAGAATTAACATAAAGTATCGTTGAGATACCTTGGTCAATATGCGTTTG
AATTGTCGCAATTAAATCGATTAATTTCATCTGATCAGTATTGAATGCTGATTTGTAGTACCACATTGTTTGT
GGTGATAAGAATGGCATAGGGTAAAATGTTTCCGCATTACCATAAGTACGACGTTCAATTTGATCAACGATTG
GCATTACAGAACTTGTAGCATTTTGAACATAAGAAATACTTTGTGTTGGAGCAATAGCTAATCTATATGCATG
ATATAAACCATATTGTTCAACATCTTGTTGTAGTTTCTTCCAATCCTCAGAAGTAGGAATAACCATACCGTCG
AATAATTCACGTACTTTTTCGAATTGAGGTTCAAATTCTTGAGTTGTATAGAACTCGAAATATTTGCCATTAG
CATAATCAGACTTTTCAAAGTCTTGATATTTGATACCACGCTCTTTAGCGATTTCCATTGAACGTTCGATTGA
GTAGAAATTCATCATCATAAAGAAGATATTTGCAAAATCTTTTGCTTCTTCTGACTCATAACCAATTTTATTT
TTTGCTAGGTAACCGTGTAAATTCATGACACCAAGACCAACTGAATGTAATTCACTATTAGCTTTTCTAACTC
CTGGAGCATTTTGAATATTTGCTACATCACTTACAACAGTTAATGCGTCCATACCAGAGTGAACTGAATCTCT
GAATTTTCCGCTTTCCATTACATTTACAATGTTTAATGAACCTAAGTTACAAGAAATATCACGTTTAATTTCG
TCTTCAATACCATAATCGTTAATAATTGAAGTTTCTTGTAATTGGAAAATTTCAGTACATAAGTTACTCATTT
TAATTTGTCCAATATTTGAATTCGGATGCACTCTATTAGCATTATCTTTAAACATTAAATATGGATAGCCTGA
TTGTAATTGTGTTTGCGCAATTAAATTCAACATTTCACGTGCATTCTTTTTCTTTTTCTCAACATTTGGATTT
GCAACCATATCATCATAATATTTTTCTAAATCAATATCGTCTAATGTAACACCGTATTCTTCTTTAACTGTAT
GAGGTGCAAACATATAAAAGTCTTTACCTTCTTTAGCTAAATCGAAGAATTTAGATGGAACAATTAAACCAGT
TGATATTGTAGATAAACGTAAATCTTCATCCGCATTTACTTTTTTAGTATCTAAAAATTCTTCTACATCATAA
TGGAAGATATTTAAGTACACAGCACCAGCACCAGGGCGTTGACCTAGTTGATCTGCATAGCTAAAGCCACCTT
CAAGTGACTTAGCAATAGGTAAAACACCTTTCGCTACGCCTTTAATTCCTTTAATCGCTTCACCACGTGCACG
CAATTTAGATAAGTTAATTGCTACGCCGCCACCAATTTTGCTTAATTGTTTTGCAGTTGAATCAATAAAGTTA
ATTGAATTTAAGCTGTCATCCACTTCTAATAAGAAACATGACACTAGCTCACCACGACGCACACGGCCTGCGT
TTAAAAATGTTGGTGTCGCTGGTTGATATCTTTGTTCAACCATAGCAGAAATAAATTGCTTAGCTTGTGCTTT
ATTACCATTTGCTAGGTATAAAGCAACAATTGCAACGTGTTGATTATAGTCTTCTAAATATTGACTTTTATCA
TTTGTTTTCAAAGCGTAATCTTTGAAAAATTTACTAGCTGACATATAACTTGCAAAATTAAACGGGATTGATT
TTGCATAATCAGTGATTTCAATTAGATCCGCTTCACTATAAATATCAAACACATTGAAATAAAAATCGTTGTC
TACTAAATAACGTAAACGTTCGATTTCAGTGTCGAAGAAGATTGTTTTATCTTTTACTTCTTCTAAATAAGCT
ACTAAAGCTTCTTGGTCTTTTTCTAAACTAAAGAATCCATCTTCTCTTCGTTTAGTGACCTCATTATTTAATT

CAATATGATTGTACTTTTTCTCTTCCATGGTTTTCATTAAAATTACCCACCTTGTTCTTAAATTCAATAACGT
CTTTGTTTTTTCCATGTAACTCAAACTTCATTAATAAAGGGACATTATACTCTTCTGATATCGTGCGACCCGC
TTTTGCGAAATTTAGTCCCCAATTTCGATTACCGCTAGCTGCCACACCTCTGATGTATTGATGATTAACTTCT
AAAAAAGATTGAACGGGTTCTGGTACTTCTCCAAATCCAATAGTGCCAGTAACGATAATAAACGGTTCATGAA
CTGGTTCCATACAATTTTCTGCTGTAATCTCAAGCGTATTTTCAAGTTCTGTTCTCTTAATGAAACGACGGAC
ATTTCCAGTAAATGAAAAATATATTATTTTCATTGGATCACCTTCTTTCTTCAACCCTTTTAAATAAAGTAGT
CATTACAATTAAAAAATGCTTTAACAACATGGTTACTATGGTTATCTCAGCATTGTATTAACTATAATGAAGC
TACTTATTTTCTCAAATGTAAACCAATTGCATAGATTTATATTTGAGAAAATAGTAGCTTATATGGGTTGAAT
GTATAAAAATCACTATATGTAGTGTTTACATCATTGGATATCTTCCATTTGCTCAGTACATTTTCTAAAAATA
TGACTATTAATTGATAATGACTATTAAATTTCTGTGTTAAATTACTTTTAAATGTAATTTCTTCATAAATAAA
GCATTTGCATACAAAACACAAGATATTGTGTCTTTTTTGTTTCACCAACACTATATTATGTGTTTCATTATAC
ATAAGACACACTATGATTTAAAGTGCCATTTTCTAATCGAATGTTCGTAAAACGTTCCAACTAGGCACCAATA
CTTAATTAGAAAAACTTGAACAAATTAAAAAAATTTTTTCAGTAGATATCACTTGATTTTTTAAAATCTGCGT
CAATCTCAATTGTATCAACAGAAATTAAGAAAAAGCTTATTAGCTTGATACTGAATTGATATTTGTTTTATGT
AATAACACAAACATTCATAAAAGTGTAATGCCCTATTATTCCGACAAATAAACACAGAACGTTTGTTCGGTAC
AGTCTATTATAGGCTTTTTATAGTAAATTTCAACTATTTATGCACAACATGTTGTTTTATGCTAATATAAATA
CGTTAATTTTATATAAAAGAGGCGATAAAATTGAATCCTAAAGTAAAAGGTATCATTGCCATTTTAATTTCGG
CTATTGGTTTCAGTTTTATGTCAGTGTTTTTCAGACTTGCAGGTGATCTTCCTGTTTTTCAAAAATCATTAGC
ACGAAACTTAGTAGCAATGTTTATTCCATTATTTTTTATATATAAATATCATCAACCAATGTTTGGTAAACTC
TCAAGCCAACCATTATTATTAACACGTTCGACACTTGGACTTATAGGTGTATTATTAAATATTTACGCCATTG
ACCATATGGTACTTAGTGATGCCGATTCATTAATGAAACTCAATCCTTTCTGGACGATTTTATTAAGTATTGT
TTTCTTACATGAAAAGGTAAGGAAGTATCAAATAACAGCAATGATAATTGCTATTTTAGGTATGCTTTTAATA
GTGAAACCTGAATTTTCATCATCTATGATTCCATCACTAGCTGGACTATTTTCAGGTATTTTTGCAGCATCGG
CATATACATGTGTTCGCGCACTTAGTACTAGAGAAGCACCATACACCATTGTATTTTACTTCTCATTATTTTC
AGTCATCGTATTAATACCATTTACTGCATATACATATGAACCAATGAGTCAGATGCAAATACTTTATCTATTA
GGGGCTGGCTTAGCTGCCGCTGTGGGTCAAATTGGTGTTACATTGGCATATAGTTTTGCTGCTGCTAAAGATA
TTTCTATTTTCACGTATGCATCTATAATTTTTACAGCAATACTTGGATTTATTCTATTTGGAGAATCTCCAGA
TTTCTATGCAACATTAGGTTACGTCGTTATTATCGGTGCAAGTTATTACATGTTTGAAAAAGCACGCCGAGAC
GCCAAAATAATAAAAAAATAAATTTAAAGGAGCGCTTTATATGGCACATGGCCGTCAACAAGATGAATTACAA
GATATAACTTTATTAGGAAATCAAGACAATACTTACAATTTTGACTATCGTCCTGATGTTTTAGAATCATTCG
ATAATAAACATCAAGGTCGCGACTATTTCGTAAAATTCAACTGTCCAGAATTCACATCATTATGTCCGATTAC
TGGGCAACCAGATTTTGCAACTATCTACATTTCTTATATTCCAAATGTTAAAATGGTTGAATCAAAATCGTTG
AAACTTTACTTATTTAGTTTCAGAAATCACGGTGATTTCCACGAAGATTGTATGAATATTATTATGAATGACT
TGATAGAGCTTATGGATCCACATTATATTGAAGTTTGGGGCAAGTTCACACCACGTGGTGGAATTTCTATTGA
CCCTTATACAAACTATGGACGTCCAAATTCTAAATATGAAAAAATGGCTGAGCATCGTCTAATGAATCATGAT
TTATATCCAGAAAAAATAGATAACCGTTAATTTGTTTCCCTTTTCAAAGCACATCAGTATGTTGGTTACTACC
AACTTGTTGATGTGCTTTTTTAATATATATTGTAAATTAACTTCAATCATTAAATCCCAAAGTACACCAAAAG
AATATTAATACACTAATTAAACAACATTTTTATAGAAACCTATTGCACTTTAACGTCAATAAGTATATTTTTA
TATTATCTCTAATTAATTGTGCGCGCTTAATAACAGAATATTCTCAATATTTTTATTTTTTTGTGATTTATTG
GAATATTTAGTTGATAAGGTACAATCAAATTTACTTAAACTATTGTATTAGGGGAAGAAAGGATGGGATGTAT
AAATGACACAACAAAACTCCCATGGAAATCAAATTCAAGACATACCTCAAACAGGATTTTTCGGGCATCCTCG
AGGACTAGGTGTACTCTTCTTTGTAGAGTTCTGGGAAAGGTTTAGTTATTATGGCATGCGTGCCCTACTCATT
TTCTACATGTACTTTGCCGTAACAGATAATGGCCTTGGAATTGATAAAACAACAGCTATGTCAATTATGTCAG
TTTATGGTTCATTAATCTATATGACATCAATACCAGGCGGATGGATTGCTGACAGAATTACAGGTACTAGAGG
CGCTACTTTATTAGGTGCAGTCTTTATTATTATCGGACATATTTGTTTAAGCTTACCATTTGCATTAATCGGC
TTATTCACATCAATGTTCTTCATCATTATTGGTTCAGGTTTAATGAAGCCAAATATTTCAAATATCGTTGGCC
GTTTATATCCTGAAAATGATAGACGTATGGATGCAGGTTTTGTTATTTTCTATATGTCAGTTAATATGGGTGC
ATTATTATCACCTATTATTTTGCAACACTTTGTTAATGTTAAAAACTTCCACGGCGGATTCTTGATTGCAGCA
GTTGGTATGGCATTAGGTTTAGTATGGTATGTACTTTTCAACCGCAAAAACTTAGGTAGCGTTGGTATGAAAC
CTACTAACCCATTGACACCAGCTGAAAAGAAAAAGTATGGTCTTATTATCGGAAGTGTTGTCTTAGCAATTGT
ATTAATTATCGTTATTGGTGCATTAACTAACTCATTATCATTTAACTTAGTTAGTAATACTGTTTTAGTATTA
GGTATTGCATTACCAATCATTTACTTCACTTTAATTATTAGAAGTAAAGATGTCACTGATACTGAACGTTCTC
GTGTTAAAGCATTTATTCCATTATTTATTCTTGGAATGGTGTTCTGGGCTATTCAAGAACAAGGGTCTAATGT
ATTAAACATTTATGGAATTGAACATTCAGATATGAAATTAAACTTATTTGGTTGGAAAACAAACTTTGGTGAA
GCGATCTTCCAATCAATTAACCCATTATTTATTTTATTATTAGCTCCAATTATTTCACTTTTATGGCAAAAGC
TTGGAACTAAACAACCTAGCCTGCCAGTAAAATTTGCAATTGGTACGTTCTTAGCAGGTGCGTCATACATACT
AATTGGTATTGTAGGTTATGCATCAGGTTCATCAAACTTCTCAGTTAACTGGGTTATTTTATCGTACATTATT
TGTGTTATCGGTGAGCTATGCTTATCACCAACGGGTAATAGTGCTGCTGTTAAGTTAGCACCTAAAGCATTTA

ATGCCCAAATGATGAGTATTTGGTACTTAACTAACGCTTCTGCACAAGCAATCAATGGTACATTAGTTAAGTT
AATAGAACCACTAGGTCAAACAAATTACTTTATTTTCTTAGGTGTTGTTGCAATTATTGTTACAACAATTGTA
TTAGCATTCTCACCTTTAATTATTAAAGCAATGAAAGGTATTCGTTAATATTGTTGACCTAAATTAAAAAACA
GTAAGTCATTTAAATGGCTTACTGTTTTTTTATAGGTTTCCATTAATTAAATTCAAGTTATCAGTATAAATAA
AAACTTAATATGCTCATTATAGACAGCTATAACTATATTTTCTCGTCCCACTCTATAGAGATTAATTTCATTT
ATTACCATTTTCTCTATTTAAAAACATATTTTTGGTATTAGCAATTAAAAGTGGGTATATATATTTAATGAAC
CAATTTTTAGAAAGTAGGCTAAACTATGGAAAACAAATATACACATGGTGTCCTCTTTTACCATGAACACAGT
GGATTAAAAAATATTAATCAAGGTATTGGGGAAGTTACAACAGCATTAAGTTCAATTTGTAAGCATCTTTCTA
TTCAATTGAGTGAAAGCGAAGGTGACATTATTAAATATTGCCAAGAAATCAAAACTAAAAACTATGCAAAAGA
TGTAGATATATTATTTATTCTTGGCGGTGATGGCACGGTTAACGAACTTATAAATGGTGTTATGGCACATAAT
CTTCAACTCCCTATCGGCATTTTACCAGGCGGTACTTTTAATGATTTTACAAAAACGCTAAATATTGCACCTA
ATCATAAACAAGCTAGTGAACAAATGATTTCTGCACAAGTTGGTACATACGATGTAATAAAAGTAAATAATCA
ATATGCACTCAACTTTGTTGGCTTAGGTCTAATTGTTCAAAACGCTGAAAACGTACAAGATGGGTCAAAAGAT
ATATTTGGTAAATTAAGTTATATTGGATCAACTGTTAAAACACTGCTAAATCCAACACAATTTAATTATCAAT
TATCAATTGATGATAAGACATATTCTGGCGAAACGACCATGATACTTACAGCAAATGGTCCATTCATTGGTGG
TAGTCGTATACCATTGACTGATTTGTCTCCTCAAGATGGTGAACTGAACACTTTCATTTTTAATGAACAAAGT
TTCAGTATTTTAAATGATATTTTTAAAAAGCGTGATAGCATGAATTGGAATGAAATTACTCAAGGTATTGAGC
ATATACCTGGTAAAAAGATTTCACTTACAACAGATCCAGCAATGAAAGTTGATATTGATGGCGAAATTTCATT
AGAAACACCTATTGATATTGAGGTTATACCAAATGCCATTCAGTTACTTACTGTCAATGATTTGTAAGCATCC
GGAATGATCTTAACGGGATATTATATTAGGTTTAGAGTGTAATCATAAGATTCAACGCACCTTATATTCGTTT
TTATTAGCTTTAAGCTATTACCCTGTTTCAAAAACAGACATATAATTTTGGCGCATGCCACTAAAATGATTGA
TGTTGTATTTTAAATCATAGTGGTTTATGCGCCTTTTTCAAATCCTATAAAAAATCGGATGACGTGTAATCTG
CCATAGATTTACACATTCATCCGATTTATAATAATGAGATAGACTAACATTTATTGAGAGCGGGACGGAAATG
ATATTTTCGCAAAATTTATTTCGTCGTCCAGTCCCAACTTCATAATAGTATATCCATCATCTTTGCTACATAA
AATGCTTCAAGTGATATATTTTTATTTCTCAATATTATCTAAAAAATACTGTTCTACATCTTTCCAACCATTT
ACGCGTTCAAATCGATCATCATTAATATTATGCACTGCTGTAAACATAATCGGTGTACCAGTAAAAATTTCAA
GCTGTCTAGGATTGTCATCTATTAAATAATCAGCTTTAACAATGTTTTTTCTACCACAAAAAACAAAATGCTG
TGGATCTAAGAATGGAAAGAACTCTAGTAACCATTCATATTTATCACTAAATGATGTTGGTACATCCATTGCT
GCTGTAGCAATATATACATCATAATGTTCAGTTAATTTTTTTACAACTTCTTGTGCATGCGGCATCACTTTAA
GATGTCTGAAGAAGCCTGGTTCTCTCAATACTTCTGTAATTAATCCATCATGTTCAGGAATAACATGTTTAAG
TTTTTGACCATTCAAAGCTTCCATTTTAATACCTAAATCCGCTCTAAAATTGACAGCATCAATGATTTCTCCT
AATGTATCTGCCAATACTTCATCCATATCAATCGCGATTGATTTACGGGTCATTTTAATCTTCTCCTCAAAAT
ATCAGTATTAATTCAGTATAGCAAAAAAGCAATTGATACAAAACAATATCATCTATTGTTGCTTTTTACAATA
AATGCTCTCACCTCATAATTCACACTGCATTTTGGCACATATTCATACCATGTGGTGCCCTTTTCTATTAGTA
TTTAAAGTTTGATAAAACTTCTAGCATTTTATCATTTTGTTCTTTAAAACCAATTGTAATTCTAACACCAGTT
GGAAATGGTCGCGTAATACACCCTACATTTAAAAGCGCTTCATAAAGTTCATTTACCCGCTTTGTTTTTACAA
ATATAAAATTCGTTTGACTTGGTAAGAAATGCTCACTTTGAGGTAATTGATAAAATCTTTCGCGTTCAACACT
ATTTTTATGTGTTACCTCTTTTAAATATTGTTGATCTTCAAGTGCTGCAACTGCTGCGTATTCAGATATACGT
GTCACATTAAATGGTGGTCTAATGATGTTCCATTTTTCAATCGCATGTTCACTTGCTACCACATATCCTACAC
GTAAACCCGCTAATCCATACGCCTTTGAAAATGTACGTAATAAGAAAGCATTGTCATATTTTTGTTGCAAAGC
AAGTGTATCCGGGTAGTCCTCTGCTGTCACAAATTCAAAATAAGCTTCATCTATAATTACTGGTACATGTGGA
GGTACTTGAGATAAAAACGAATCTAAGCTCTCATGATTAAAATATGTACCTGTAGGATTATTTGGATTACATA
ACCATACCAATGACGTATCTTCATTAACTTCTTTTAAAATACCTTCTAAATCGAAGCCACCATCTTTTAAAGG
TACTTGTATCACATTAGCTGATTCAACAATCGCATTGTGATAATATTGACCGAATGTCGCTTCACTTGTAACA
ATAGTATCCCCTGGCGTTAATACAGCTCTAGAAATCATTAATATAACTTCATCTAATCCCGCACCAAATAAAA
TGCGTGATTGATCTACATTTAAATGTTTACTAATCGCCGCTTTTAATGTCGGTGATCCTGTTTCAGGATAATA
ATATAACTCATCTAAGTGTGCTGATATCGCTTCTTTAACTTTTGGCGATGGTCCATACAAATTTTCATTTGAT
GCAAGTTTATATAAATCTCCTTCAATGCCATACTTTTCTTTCAATGCCCTTGGAGATAAACCAGGCTGATATG
CTGATAGTTGATTAAGTTGTTCTTTCATAATAAAAACAACCTCCTTAGAATTAACTGAAATTTTAAAAAACTT
ATATTCATTATATCGCTCTATGTTTCTAAAATGCAATGCTACTATTTTCATACATTACTAATGTAAATAGTAA
TTAGCATAACAAATTCAATTATTAATATTGTATGTTTAAATTGTACATAAACATAGATGTAAATATAGCAACG
CCACATAAGATATAATGGCGTGCCCCTCATCTTATGTGGCGTTGGTCGTTATTTGATTAACCCTTTTGCTTTT
AAATAATCTTTCGCAACTGTATATGGGTCTTCATTTTTAACTGTTACTTTATAGTTCATCACTTGCATATCTT
CATCAGATATTTTGTTTTCTAATTTGTTTAACGGTTTCTTAATTTCTGGATGTTTCTTTAAAAAGCTTTCTTT
AAATAATGGTGCTCCTTGATATGGTGGAAATACGTGCTTATCATCTTTTAACACAACCATATCATATTGTTTT
AATTCAGCGTCAGTTGAATATGCATCTATTAAATTAATATTACCTTTATTGATCGCTTGATATCTCAACTTAG
GTTCCATTGTACGTATGTTATCTAAATTTAAATTATAAGCCTTTTGAACAGCTTTGTAACCATCTGGACGATC
ATTAAATTCCAATGTAAATCCTGGTTTAAGTTGATCTTTAACCTTATTTAAATCACCAATTGTACGTATATTA

TGTTGTTTAGCAAAATCACGTTTTACAGCTAAAGCATACGTATTGTTATACTTCATTGGTTTTAACATAGTCA
TTTGATATTTCTTTTCAAGACTTTGCTTAGCTTGTTCATAAACTTTTTTCTCTTCTTTTGACTTCAATGGTTC
TTTTGTTAATTCACCTAAAACTGTTCCAGTAAACTCTAAATACCCATCTATATCATCAGATTTTAAAGCATTA
AATAAAAATGCTGTTTTCCCCATACCATCTTTCACTTCTACAGTATTTTTGGTCTCTTCTTCTATTAAAATTT
TATACATATTTGTAATAATCGAAGGCTCGGAGCCAAGCTTTCCAGCTAACGTAATTTTATCACCTTTTTGTGC
AAACATAGGAATAGCGATAGCCAGTATAATAATCATCACTATAACACCTAACGTCATCAATAACTTTTTATAA
GATAATTTAGCCATAAATCTTAAAATTAAATCAAATATAATTGCCAATAAGGCTGCTGGAATTGCACCTAATA
ATATCAATGATGCATTGTTACGGTCTATACCTAATAAAATTAAATCTCCTAGTCCGCCTGCACCAATTAATGC
TGCTAGTGTTGCTGTACCTATAATTAATACCATAGCCGTTCTTACACCAGCCATTATAACAGGCATTGCTATC
GGAAGTTCGACTTTAGTTAAACGTCTAAATGGTTTCATACCTATACCTTTAGCCGCTTCAATGAGTGATGGAT
CAACTTCTTTAATTCCAGTATACGTATTCCTTAAAATTGGTAACAACGCATACACTACAAGTGCGATAATTGC
TGGCACACGACCGATACCAAATAAAGGAATCATTAAACCTAATAATGCCAACGATGGTATGGTTTGAAGAATT
GCCGCAATATTCATTACGATTTCAGATATCGTTTTAGTCTTCGTTAATAAAATACCTAATGGTACCGCAATAG
CAGTTGCAATCAATAATGCGATAAATGATATTTGAATATGTTCTATCATTGTCGAAAAGAGTTGTCCCTTACG
TTCACTCAATATGTCGAAAAAGTTAGTCATGTTGAGCTACCTCCTTTTTCTGGGACAAATATTTGAAGATATC
TTTCCTATCAATAACATATTGACCTACGCTATCTTCTTGCATGACAATGACACGCTCGCTCTCTGATAAAAGT
TGATACAATACTTCAATTGGTTGATTGTCATAAACAATTGGATAAGCGCTCATAGATGTAACCTCATCGATTG
GTTTCATAATATCCAAGTCACGGATAATTGCGTTCTCTTCAACACATGGCGCATCATCTTCTAAATGACTACC
CATAAATTGTTTAACAAATTCACTTTGAGGATTATTTTTAAATCCTTCTGGTGTGTCAATTTGTTCAATATGC
CCTTCATTCAAAAGACAAATCTTATCACCAAGTTTCATCGCCTCTTGAATATCATGTGTAACAAATATGATTG
TCTTCTTAATTTTAGTTTGTAATTCAATTAAATCATCTTGAAGTTTTTCTCGGCTGATTGGGTCTAATGCACT
AAACGGTTCATCCATTAAAATAACTGGTGGATCAGCTGCTAACGCACGTATAACTCCTACACGTTGTCGTTGC
CCCCCTGACAATTCATCAGGTTTTCTGTTTTTATATTTTTCAGGTTCTAATCCAACCATTTCAAGTAATTCAT
CTACTCTTTTATCTATATCTTTTTCTTTCCACTTTTTCATTTGTGGCACTTGTGCAATATTTTCTTTGATTGT
CATATGTGGGAATAATGCAATCTGCTGCAATACGTATCCAATATCCCAACGCATTTCGTATACTGGATAATCA
CTTATTGGTTTATCTTTAAAGTAAATATAACCTTCACTTAAGTGAATGAGTCGATTAATCATTTTTAATGTCG
TAGTTTTTCCACAACCTGAAGGTCCAATGAGCACAAAAAATTCACCCTCATTAATATTGAAACTAATGTTATC
GACAGCAACATGTTTGCCATAACGCTTAGTTACATTTTTAAACTTAATCACTTTGCCACCTCTTTTTTTCTCA
TAGCATAAAACCGAGATTATATGTATGTATTCCCTATTTAACCACGTTTATTACAATTTTCAAATTTAAATGA
TTTATCCTTGAACTTTTTTAACAAAATAATGAATAATAGGTAATCTCCAGTTAAGAAATAGTGTTATTTTACT
TTGAATTCAAAAAACACCCAGTAAAACAAGGAATGTTTACTAGGTGCCTTCACTATACTTTGGCTTTATAATT
TTGAATCGTTTCTAAAAATGCTGGACAATAATGTTTTAATTTGTAACTACCTACGCCATCAATATTAATCATA
TCTTGTTTCGAAGCAGGCTTACGTTTAGCAAATTCCTCTAGCGTGTAATCAGAAAATATACTTACAGGTGCTA
TCGTTAATTTGTCACTTAACTTTTTACGAACTTCTACCAACTGACTGAATAATGCTCGGTCAACCCCTTCAAC
CGTATTTATAAATACTTTTTCAGTCGCTTTTTGCTTAAATGGTGTTGTGAATACTTCTACTTCATTACTGAGT
AATTTTTTAATTGAAGTATCACACATTAATATTTCATCATTTTCATTTAAGAACCCTTTGAATCTTAATTCAT
CTATTAAGTGACTTAATTCTGATGTTGTGTAACCTTTCATTAAACCATGGGTTGAAATTTGGTCATAACCTTT
ATACTTAATATAATCTGTTGACTCTCCTCTTAATACTTGGATGATAACACTATAACTCTCTTGTTGTTTCATA
CGAGCGATGCAACTAATAATCATCTTAGCTTCTTGTGTCATATTATATGATTTATCTTGTTGAACACAATTAC
TACATTGTTCACATTCTTCTAATTTTTCATTCGGTTCAAAATAATGGACAATTGTTGCTTCTAAACATTTTTT
TGTTTTTGTATATTGAATCATTTTAGTTAACTTTTCACCCATTTTATCTTTATAGTCATCATCAGCTTGAGAG
ACTGTTATAAAATACTCGTGTAAATTGATATCGCGTTCGCTAAATAACAAAATACATTCACTTTTCAACCCGT
CACGACCTGCACGACCCGCTTCTTGATAATAAGATTCTAAATCTCCAGGCATATTATAATGAATAACAAAGCG
TACATTGGATTTATCAATACCCATACCAAAAGCATTTGTAGCAACGACTACTTTAACACGATCAAATAAGAAA
TCATTCTGCGCTTCTTCTCTTTCTTTATTGCTCAAACCTGCATGATATATAACACTTTCAATGTTCTGGCTTT
CTAAGGCTTCTTGAAGCTCTTCAACTTGCTTACGTGTAGAACAATAAATAATACCTGCATCTTCATCGTGTGT
TTTAATATAATCCAATATAAATTTTTGACGTTGATAAGTAGGATTTACTTTAAAAATTAAGTTTCTACGCTTA
GTACTCGTTTTAATTTGATCAGTTTGAGCGATATTTAACTTTTCTCTAATATCTTGCTGTACTTCAACTGTTG
CAGTTGCTGTCAATGCAATAATTGTAAAATCTTGTGGTAACATAAACACTTTTGAAATAACATTTTGGTAACT
CGGCCTGAAATCATGTCCCCATTTAGATATACAATGCGCTTCATCAAACGCCACTAAATGAATCTTGATACGT
TGAAGCAAATTTAAAAAATATCGATTTTCAAATCGTTCTGGTGCAACATATAAAAATTGAATATTCCCACTTG
TTAATTCTTTTTCAATATGTTGTTGTTCTTTTTGAGTCAAACTACTGTTTAAAAAAGCAGCTTGAATCCCCAT
TGCCTTTAATTGATCCACTTGATCTTTCATTAATGAAATTAGTGGACTTATAACAATCGTTGTACCACCTAAC
AATAAACCTGGTACTTGATAGCATATAGACTTACCCCCACCAGTTGGTAAGACACCAAGCACATTACGATGGT
CTAATACTTTGCTAATAATTTCTTCTTGTCCTGGTCGAAACGTTTCATACCCAAAGTAATGCGATAATGTTTG
TTGCATCATAAATTGACCCCTTATTGTTGTTCTTTAATTTCTTCTAACTCACTCCATCTTGTGATGTCTAAAT
CATATTGAATTTCAAGTTGTTCTTTTTCTTCGTTTAATTCTTTAATTTTCCCATAATCTGCACTTGCCTCAAT
CATGAGCACATCAATTTCTTCCATTCTTACTTCCGATTGTTCTATACGTTTCATCAATTGTTCATATTCTAAT

TTTTCTTTATATGATAACCCATTTTTCTTACGTACAGTTGTAGAAGATTTAGATTGTTGCTTCAATGTGGATT
TATTTTTATCTAATGATTTTTTATAACTTTCATAATCTTCAAAAGTTCCGATAATCTTTTCCATCTGACCATC
ATGAATAAACCAATATGACTGTGCAACTTTATTTAAGAAGTAACGATCATGGCTTACGGTAATCACTGTACCA
CCGAAAGTATGAATATAATCTTCAAGTATTGTTAAAGTCTCGGTATCTAAATCATTTGTCGGTTCATCTAACA
ACAGAACATTTGGCTGGTGTACGAGTAGACGTAATAAATACAAACGCTTTTGCTCTCCACCAGATAATTTATA
AACTTTTTTACCATGAGTTGCACTTGGAAATAAAAATCGTTCAAGAAGTTGTGTAATAGATACCGAGGTTCCA
TCTTTTTCTTTTGCAATCTCACTTTCTTCTCTTAAATAATCTATCATACGAATATCTCTATCCAAGGTCTCAT
CTGTTTGCTTAAAATAAGCTATTTTAACCGTCTGCCCAGTCTTCAATTTACCTTCGAATTGTTGGTCTTCTCC
ACTCAAAATATTTAAGAGTGTTGTTTTACCAGCTCCATTTGGACCAACAACACCAATACGCTCACCTTTTTGA
ATAATTTCCGTTAGATGTTCAAATAATACTTTATCATTAATAGCCTTTGATAAGTCTTCTAATTCGAACACTT
GCTTACCTAATCTTGAATAAGCAAGATTCAATTCACCTTTATCGTCTTTATATTGCTGGTTAACTTCATTTTC
TAGGTCATTAAATCGATTAATTCTAGCTTGTTGCTTTGTAGTACGAGCCTTAGCTCCAGCCCTCATCCAAGCA
AGTTCTTCCTTATATAAAGCTCGTTGCTTTTGTTGTTGCTTTTGAAGTGTTACTTCTCTTTCAGCGCGCATTT
CAATATAAGATTCATAGTTACCAGGATATGACGCTAACTTACCTCTGTTTAGTTCAATAATTCTAGTGGAAAC
TTCATTTAAAAAATATCGATCATGGGTTACGAATAAAACTGTATGAGGATATTGCTTCACATAATTGATCAAC
CAGCTGATTGATTCGAAGTCTAAATGGTTCGTAGGTTCATCTAATAACAATAAATCTGGTTGTTCTATTAATG
TTTTAGCAAGTACAACACGTTTTTGTTGTCCGCCGGATAATTCTTTAATGTATTTAGTAGTATCATGTATACC
TAGTTTTGAGAGGATTGTTTTAATTTCAGCGTTATAGTCCCAAGCATCATGTTGATCCATAGCATCTTGCGCA
TCCATCATTCGCTTGAACAATTTATCACTTTGGTCATCCGCATAAGCTTGTACTGCCTGCTCATATTGCTTGA
TGATGCGTAAAGTTGTTGTATCAGAACTTAATACTGCATCAAAAACAGTCATATCTTCATTAAGATCCTGTTT
CTGAGACGAATAACGAATTCGATATTGATTTGGATGCATAACATTGGCTGTAAAATCATCATCAATACCACCA
ATTACTTTTAACAATGTACTTTTCCCTGTACCATTTATGCCTACTAAACCTATTTTTTCACCTTCTGAAATTG
ATAAATCTAGATTATCGAATATAATCTTATCGGCATAAGATTTATTTAAATGTTCAATTTTATATGCTTCCAT
ACCCATCTTCCTTCTTCTGTTGAAATCTCTACGTACTTAAATCAGTTCTATACATTATACACCTTTTTGAAAT
CAAAAGAAATCGATACTGTTAAGTGTCATAATTTAATAAATTGTTGCTTCAACTATAAATAAAATTTGATAAG
GCTTTGATGACTATGCTAATGATCTTTATAATATACCTTTTTATCAACACATATAATGTAAAACATAATATAA
AAGAACATAATAAAAACCCGGATTGATATTTTATCAATCCGAGTTCGTGTTTAAATATTATTTTTTAGAGTTT
GCTTTAGGTCCTGTTTCATATTTATACTTCGAAGGATTTACCTTTTTGAAGTCTGGATTTTTGTAGAATCTAA
ACAAATCACCATTAAGCACGTTGTCACTCATTTCGAGATCCTTTTCAACTTGCTTTTTATTCTTTTCGAAATC
AGCTGGTTGAGTAGTTATGAGTTCATTATTTTTATTAGAATAAATCTTACCATTAACATATTTATAATCTTTT
GTTATAAAGTCACCATTTCTGAATGGAACTACTTGATTATGACCTTTAGAGAATAAATCAGTACCGAACATTA
AATAATTCTTCGTATCTATACCAGCCAAATGTAAAATTGTTGGCATTACATCGACTTGACCAGCATATTCATT
ATTGATACCACCAGATTTACCAGGGATTTTAATCCAGAAACCAGTTCTGTTTAAATCTGTAAATTTAGCTGGT
GTGATTTTTTCACCTAATAGTTTTTCCATGGCATTGTTATGGTTTTCAGAGATACCATAGTGGTCACCATAAA
TCATAATCACTGAATTGTCATATAATCCTTTTTTCTTCAAGTCATTAATATATTCTTCTAATGCTTCGTCTAA
ATAACGTGCTGTTTGAATATAACCATCAACTGTTGCATCACCTGTGTTTGACTTCTCAATAGTTGCATCCTTT
TCATCTAATGTGAATGGATAGTGGTTAGTCAATGTAATTAAATGAGAATAGAATGGTGATTTCATCTTAGCTT
GATAATTAGCAGAATCTTTAAAGAAGATTTTGTCTTTCAAGCCTAAGTTTACAACGTTTTTATCAGACATGTC
ATAGTATGTTGCATCATAGAATTTATCGATACCAAAGTGTTTATATACTTGGTCTCTGTTCCAGAATGTTTTA
TAGTCACCGTGCATAACATCAGATTTGTAACCTTGCTTTTGATCTAAAATTGCTGGTAATGACTGATACGTAT
TATCTCCTTTTAATGAAAAGGCAGAACCTTGCGGTAAGCCGTATAAACTGTTATCCATTGTAAATTCAGAGTC
AGATGTTTTACCTTGACCTGTTTGATGGAAAAAGTTAGGGAAATATGTGAATTGCTCTTTCCCACTTGATAAT
TTGTTTAAAAACGGTGTTACTTCTTTACCATTAACCTTTTTATTAATTAAGAAGGTTTGGAAACTTTCTAAAT
GAATCTTAATAATATTTTTCTTCTTTGCCACCCCATAATATTCTGGGTTAGGCTCTGTTTGACGTTGCTTCGT
ATAATTTAATACTTTTGTTAAGTCATCTTCAGATGCTAACGCCTTTTGTTGATTATTTTCGATAGTTTTAACA
CCATCGTAAACTGTAAAGTTATAAGGCCCTAAATATTTCACTAAATATTTATGGTCAAATGTACGTGTTAATA
ATTCTGGTCTGTCAGTTTCAGCAAAAGCTAAGTTTAAGAAGAATAATGCTACTGAAGCCGCCATTACGACAGG
AACAAATTTCTTACTAAATGCTTTTGTGTCTAACCATTTTGTTTTAAATATTAAAATGAATAAGTAAACTAAC
GTATCAATGAAATAAACAAAGTCATACCATTTGAATGATGCACTAACCGCACCACCCATAGATTCTACGTTAC
CTACTTGGTTTAAAGTACTAAACGTTAAAAAATCAGAGAAGAATCTAAAGTACACAACATTGGCATATAATAG
GAACGTCAATAAGAAGCCGCCTATGAACATGAACCAAAATGCTTTTTTGCCTTTAAAGAATAGGAACACACTT
AAAACCAGTGCTACTAAACTATAAGGATTCATCAATAATATTAAGTTTTGTACTAAACCTTTAACACCTAAAG
AAAAATCAACATAATAAGAAAAATACGTCTTCAAGGTAATCGTTATTACGGTTAATAAGAAGAACGCAAAAAG
ACTAATTTTCTTTTTTTGTGAACTCATGATTCTTTCCCCCGTTATTTAGATAATAAATCTTGCGAAAGTACTT
GAATAAATTAGATATTAACATTATGTAGTTAATTAATAAGTGCGTTTAAAAGTTAACATTGTCATAATTATAG
TAATTACTTTCAGATTCAAGTTTATAAATTTCATTATTTGTAGCTTTCAACTTTGATTTAACAAGTATTTTGG
GCTACAACTCATATTTGCTTGTGTAAGTTTCTTTATTTAATATTATCATTCAATTAACAAATTTAAAATTTGT
AATCAAATTGTAATAATAAGCATAGCTCAATTAATAAATATAGAGTAATTTTAGTCAAAATTCAAGTAAAAAC

ACACTTTATCTTTTCATTTTACTGTGAAACGAATTTGAATATGATACATTTCGCAGATAAAACACAGTATTAT
TTCATTATACATTAGGAGGAAATTATTTGCATCGGACTCGAGTATGAGCTACGTACCAAATATTATTTCATCA
TTTCTAATTGCATTCTATATTCTATTCCATGGAATAATTGATTTAACCAGTTACTATACTTAACAAGGTTCTT
TTCAGCCGTTAAAATATCTATAAATTGAAATTTCAAACGTGATCCTTGCGGTTTTTGTGCTAATTTCGTTAAA
TGATAACTTGCGATTGTACCGATTTGCGGATAGCTACCTAGCGTATAATGATCATTTAATAAGATAATAGGTG
TGCCATCACGCTTAATTTGTATCGTTCCTTTTTTGACAGTCTGATACGGTGGCATATCTTCATAAAAGGCTTT
GATTTTTTCACCTTCAAGCATCATCCCCATGCGATTTGCTTTGCTCGTTACTTTATAATCATGTTTCACAAAT
CTTTGAATGGCATCTTCTTTAAAATCTTCCGTACCTTTATTTTTAATGACATGAAATACATCAGACATATAAT
TAAATGACAAGGCGTATCCATCAATACCCCAATCTGTTTGTTTCGTGTGAGCAAGGTTTTCAAATAACTTATG
ATGACGAGCTGTATAATCTCTCTTAAGCTTTATTTCATCGCCATCTTGTAATGTTCTTCCTTTAAAACCACCA
ATTTTTACATTAAAGTCGGTTGAATTAGATCCTAACCATGCATCTAATTCAAATCCGCCTCCCACAGCTAAAT
ACACTCGCGATGTATAACTTGTTTCTCTAAACTTTAAAACATCGCCTTTATCTAACAAATACAATTTATATGG
AGATATAGTCATATGCTCAGTGTATGCTTTGACATTACCCCCTGCTAATGCAATCAGCGTAGGTTCTGTAAAA
CGAATCTTTGCCATTTTATTAGTCATTTCCAAAGTTGCTTCATTCTTGTCATTAGCAACTAATCGATTAGCAA
TTTCATGTGCTAAAGTATCAAGTGCACCACATGGAATTACACCATCATGTTCATATCCCCTTCTACCAAAGTC
CTGAAAGCTACTGAATAAGCCACTTTTTTCAATTATGATTGACATCTTTAAACGCTCCTACTTCTAATTCATT
TTCATTGATAGGTCTAAATTTAATATTATCGCCTAACTTTAAGCGTGCGAAATCTGATTCTTTCGGATTAAAA
AGTGATAATGGTGTATAACCAATAACTAACCAATCATTAATTGTATCCGTTGTTACAATACCGCATTTTTTCC
CTTCAAGTACTACAGAACCAGCTGGAATGAATTTTTTCTGTTTACTCGTGTGATTAATATACAATTTCTTATT
TAATCCAGTTAAATAAGGAAACCCAGGTGAATATCCCATCATCGAAACAAAATATTGCGCCTTAGAATGTAAT
TCAATAAAAGTTTCTAGCTTGATTTTGTAATGTTTTAAAAGTGATTCTAAATCCGGACCATATTTCGAACCAT
ACACAATTGGTATTTCAATCAAATCTTGCTGGTTAAAATCATGTTTTATGTTTAAATGAATCGATTCTATTAG
TGCTTTCATGTATAAAAAAGGTGATTGTATATTATGGTGTTTAATCATATCCCTTGCGTCATAGACAATCATC
ATGTCTGATTCTGATGGCACAATTTCTATAATAAATGGATAATTTTGTTCAATTAAATATGAGCGAAGTGTTA
ATAAATCTTCGGTTAAACTTTTAGATACTTCTTTTTCAATTGCGACTACAATGGCTTGGTCACCTTGACTATA
TATTTTCATTTGTACACCTCTTCAATATTTGGTTACTTTACTTTCATTTTTAATTAATAGAATCGATTGAATA
GTTCATTTTATCTACTCAACTAAATTTACTGATGATACATTAAATCCATTGCTCGCACAACAAAAGGCATGAG
TTCGTGAATTAATGCATATACAAGGATAAAGCTTATAACAGTAGTAATTGTTGCTATCAAACGAACAACATAT
ATTCTATTTTCAGATAGCAATCGATTCACTAATCGATAATTTACGTATACAAGAATTAACAACAGCACAATAT
AAACAATAATCATATTTGGCCCCATTTAATTTTTAAATTTGTTTTCACTTCGTTTTCTACATTATTATCATAC
TAATTTTAAAGGCAAAGGTGGACATCGGCAACCTCTCGTAAACTATTTATCAAAAATAAACGTATCTCATTGT
TATGATATTTATAAATCAATTCGTTTTTATTAAAGTCTTTTTCAACAAGTTTGTCACTATCTATTAAATAATC
ACGCATGCATCCTTTTAAGAAATCATCTTTATAACTTGGTGTGTACCATTTTCCATCCTCTTCAATGACAATG
TTGCCAATATCAAATTCAAGGACCTTGCCGTCCTCTGAAGTTAGCAATATTAAATCTGTTTCATGATTGTGTG
CTAAATGCTTTCGTTCTGTCGTTTTATTATTTATAAAAGTTGGATTAACTGCTTTGGGCAACACTTGAAATTT
TGCTGTAAAACTACTTTTCTCAGGTAAAGGTGACACTATCGTTTCAAATTCGCTTTCAGCATTTAATACGATT
TTAAGTCTATATTGGCCACTGTGATACTTTGTTGTTACATCGTTTAATTCATTTCGCCATGCATGTTCATCAA
ATTTAAAGTTTAATCGCTCAGAAGAACATTTTATGCGATTAGTATGATAAGTAAGTCTAGGGATATGTCCATT
ATCAATTTTCATTGTTTCAAATAATTGCATTATAACATCTCCAAAATCTTGGTTTTTGCGTAAAATTCATTTA
CTTCATCTTTTGGCTTAGAATCAATTGTAATACCTGCTCCGACTCCATAAATCGCTTGTCCATATTTATACTC
AATAGTACGAATTGGAATATTAAAAATCATTTTATCATCTTCAGTTGGAAGTAATAGTCCAATTGCTCCGCAG
TATATACCACGAGGTGAACTTTCTAATTGTTTAATATATTTCATTGTATTCAGTTTCGGTGCACCTGTTATCG
AACCACAAGGAAATAACGATGTTAAAATTTGAGTTAAGTCTGTATTATTTTTTAAAGTTCCACTTACCATCGA
AGTCATTTGAAATACAGTTTTATATGCCTCAATAAAAAATAGTTTATACACCTTAATTGAGCCACTCTGTGAT
ATTCTCCCTATATCGTTTCTTAGTAAATCAACAATCATGACATTTTCTGCACGATCTTTCGAAGAAGTTTGCA
ATGTTTTATAATACTGTTGATCTTCAGCTTCCGTTTTTCCTCTAGGCATTGTCCCTTTCATCGGTTTGCTTAT
GATAACGTTATCGACATTGTTAAATTGTCCTTTTTGAAAAAATAATTCTGGTGAGATAGACGCTACTTGGATT
TCATCTGTTTGTAATAACGCAGTATAATTACCATTACTAAATTGAGTTAATCGTTCATATAAAGTACTAATAG
GATAATAAATGTTATCTGTTAAGCGCGCCGTATAGTTCACTTGATACGTTTCGCCTTCGACAATTGCTTGTTG
GACACGTTTAATATTAGTCATCATAACCTCAGAAGATTCAACAAATGAAAAATGATGCTTTGATACATAAGAA
GTTTGATGTTCATATGTTGAATTTATGCTTTCCGCTTTTTCAAAACTATAAGCTACTGCATAAATATCATCTT
TAGCTAATGAATGTGTACACATAACATGATTAAAATACTTTGCCGCTTCGTAACTTAAATATAACGAGACATA
TCTACCTTGTCGTTGCTGCGCTTGTGCAAAGTGTATCACTTCTCCCACATCAGCCAACTTAGTAGCAACATAC
TTCTTTATAAATCCCTTTAATTGAATATGGTATTGCTTATATTCATTTTCAGTTAAATAGTAGCGATAATTAT
ATTCTATTCTCACAGTAATCACCTACCTTCGATAAAAATAATTCAACTTGTCGATAACCGTATTCACTCAAAA
TAGATTCAGGATGATATTGCACACCAAAAACCGGAAATCTAATATGCTCAAATGCCATAATAATCGCTGCATC
GTTTTTTGCTGTAATCTTTAAGCAATTTGGAAAAGTCGCTCCGTCAGCAATTAATGAATGATAACGCATTACA
TTGAAATTTTGAGGCAGTCCTTGAAAAATACCTTCATTGGTATGGCGTAACTGTGTAGTATGTCCGTGTACAG

GATGATAGCCGTGAATGATATTTCCACCAAAATAAGACACGATACATTGAAATCCTAAACATACACCTAGTAT
AGGTACACGCTGATAAAATTGTTCTAACACTTCATTCAAGATAGGATAATCATCCGGATTACCCGGCCCAGGT
GAAATAACAATTGCTTTTGGCTTCATATTAATGACGTCTTCTATCAGCAGATTATCAATACCAACAACTTGAA
CTGTTAGTTTCGTTTGAGTCTTAATATAGTCTATTAAATTATATGTAAATGAATCATTATTATCTATGACTAG
AATCATTGTATACTCCGTTCTAAATGTGTTTTATTTTTATAATATGTATTGGATGTAGCTAAAACTTTAAAAG
CATTGTCATTATCCTGACCTTGATTTTAACTAATATATGGTATATTCTATTCATCGTACATAAATGAATATCA
GAGGTTCCTAGCTGAAACCCTCTATAAAAAACTAGACATTGAAATTTCAAACATTCGTTGGTAAACCTTTAAT
ATATATTATAGGAGCAACCCATTTCTTTCGTCTATCTTTTATGGAGATAGGCTTTTTTTATTTTATAAATTAA
AAACGGGCTTATCAATGCCATTAACCTCTTTATTAATATTATTAATGATAGGAGCAATTACAAAATGGAAAGT
GTATTAAATAATGAAAAAGCCATCGTTGTCTTTAGTGGTGGTCAAGACAGTACAACATGTCTCTTTTATGCAA
AAAAACATTTCAAAGAAGTTGAACTCGTAACGTTTAATTATGGCCAAAGACATGATACTGAAATTGAAGTCGC
AAAACAAATTGCACAAGATCAAGGAATGAAACATCACGTTTTAGATATGTCATTATTATCACAACTTACTCCA
AACGCATTAACACAACATGATATGGAAATTACTAATAATGAAGATGGTATACCTAATACATTTGTTCCAGCTA
GAAATTTACTTTTCTTGTCGTTTGCAGGCGCTCTAGCTTATCAAATTGGGGCTAAGCATATTATTACAGGCGT
ATGTGAAACAGACTTTTCAGGCTACCCAGACTGTCGCGATAGTTTTATAAAATCAATGAACGTAACATTAAGC
CTAGCTATGGACAAAGATTTTGTCATTCATACTCCTTTAATGTGGTTAAACAAAGCAGAAACGTGGAAATTAA
GTGATGAACTCGAAGTTTTAGATTATATTCGTACAAAAACATTAACATGCTATAACGGTATCATTGGGGATGG
CTGTGGTGAATGTCCAGCTTGTCATTTACGTCAACGTGGACTAAATCAATATCTTGAAAGTAAAGGAGCGCTT
TAAAATGTTACAACAAATCTATCCTAGTACAACGCATCCATATCAATTCGAATTAAATAAAGATTTTAATTTT
TCAGCTGCACATCACATTCCATGTGAAGAAGCAGGTATTTGTCAAAATGTCCATGGTCATACTTACTTTGTTA
ATTTAACAATTGTCGGTGATAAACTAGATGACACTGGCTTCTTAGTGAACTTTAGCCATTTGAAAAAGATGAT
ACACGGTAAATTTGACCATCAACTGTTAAATAACTTACCTGCTTTTAAAAACAAAATCCCTTCAACTGAAATC
GTAGCGGAAACAATTTATCAAATTGTTAAAGAAAATTTGGCATCGCTCGAACACCAACCAAAATGTATTCAAG
TATTTGTAAGAGAAACACCAACAAGTTATGTCGTATTTAGACCAAAGGAACAGGTGTAACTAATGGCTAAAAT
TCCTGTACTCGAAATATTCGGCCCAACCATTCAGGGTGAAGGTCGAGTAATCGGTAGAAAAACGATGTTTGTG
AGAACAGCTGGATGTGATTATCGCTGTAGCTGGTGTGATTCTGCATTTACTTGGGATGGTAGTGCTAAAGGCG
ATATAAAACTCATGACCGCTGAAGAAATTTATGATGAATTAAAACGTATTGGTGGCGATTTATTTAACCACGT
TACAATTTCTGGTGGTAACCCAGCATTAATTAAAGGTATCCAAGAGTTAGTTAACTTATTTCAAGATAAAGGT
ATTTTCAGCGCACTGGAGACACAAGGCAGTAAATTCCAACCTTGGATGACACAAATTGATGATTTAACGATTA
GTCCAAAACCACCAAGTTCAACTATGACACCAGATTTAAAAAAGTTAGATGAAGTGATTACACAATGTGTACC
TTCCTCATTAAACTTAAAAGTAGTTGTATTCGATGATAAAGATTATGATTTTGCCAAAATGATACACCACCGT
TATCCAAATATTCCCTTCTATTTACAAGTTGGTAACCCATATTTATCAGACAGCGTAGATAATCATACCGAAA
AGTTGTTAGAACGCTACGAGCAGTTGGTGGATTTAGTTATGCAAAGTAATGATATGAATCACGTTTATGTGTT
ACCACAACTTCATACATTACTTTGGAGTAATAAAAAAGGTGTATAAAGGATAACTTATTTTACTTTTTACAAG
TCATATCTAATGAATAACAATATATTAATTGATATAATAGTTGATTGAAGTATAACGAACAGGAGTCCATCAT
GATTGTATATATATTAATTAATATTGCTATCGTCATACTTATAACTGGATTCAATTTATATAGGCATCAAATG
CAACATTTATCATTAAGTGCAATGTTGTTATCTATTACGATAAACGCCTTTATAAATACATTCATAATTGATA
AATATAACTTTATTACTCTGTGTACTATAACAATGTTTATCATTTGGACGATACTACAGTTTTACATTGATAA
AAAATTAAAACCTGTATACATTACAGACCAAAAATTTATTGCTATCATATTAACGATTGTTGTCAGTTTAACA
CAACGTGTTACCGACTTTTCAAGTACACAATCAATATATATGTCTATTCCATTTTTAGCCCCTGCCATTTTCA
TCATTGGTGGTATTATGTTGTTTATTTCAACATTTAATAACTTAGATGAAACTGCTGAAAATAATAACAAAAT
AAAGAAACTAATGATTAAAGGACTTATCATTATTAACATTTCATTTATCGTTATGATGGTTTTAACACCATAT
TGGTACTTGTATTTAATCGTCTATCTTATTTTCTTGTTGTTTTTATTGTGGCAAAAGGTTTATAAATTTTAAT
ACCAAAACTATAAAACACTTCTGATATTCTTAGTTCGATATATCAGAAGTGTTTTATAGTGATAACTAGTTTA
GATAAATATTTTCTTACTTAAAAAACCGCCCTCTTCTTATTTTGCCTCCTATTTATTTAAATCAGACAATTAT
TTTCATTTTCAAATTATTCTTTCTTCAATATTAGTTAAGCGATATTTAAACGAAGTTAAGAATTAGTTAATGG
CATATTATTTGCCTTCATTTTAAACTTAACTTATCAAATTGAAGAAATGAGGAGTTAGCATGAATACAAAATA
TTTTTTAGCAGTTGGTGCTGTTGCCGCTGTATTAACTTTAGGCGCTTGTGGTAATTCTAATTCACAAGATCAA
GGTAACAAAACTGAACAAAAAACAAAGTCAGAAGACAGCAATGTTAAAACTGATAAAACAAAACACCTAACAG
GTACATTCAGTTCTAAAAACGGTGAAACTGTTGAAGGTAAAGCTGAGATTAAAAATGGTAAATTAATGCTTAC
TAACTACAAATCATCAAAAGGTCCAGATTTATACGTCTACCTAACAAAAAATGGCGACATTAAAAACGGTAAA
GAAATCGCAATGGTTGACTACGATAAAGAAAAACAAACATTTGATCTTAAAAATGTAGATTTAAGCAAATATG
ATGAAGTAACAATCTATTGTAAAAAAGCTCACGTCATCTTCGGCGGCGCTAAATTAAAATAATCAATTTCTGA
GTTAAACTTTTATTTACAACATACTATTACTATACATTACAAATTTTAAAAATATACATTACACTCATTACTC
AATGGAAAGCGTATGATTTCACAGCCCCCCCTAGCTTGTAGAAATCATACTTTCCTTTTTTCAATATATATAC
AACTATTAAATCCCATAAGATTGTAGAGCACATAAGTAAATATTTTTAGAGCTTGAGGTTTGTTTAGCTTAAG
CAGCCCATGAGCTCAAACACTTCCTGTTCACATAACACTACAAATCGCATTATGTTGCTTAATCTTATGTTTA
TATAAATTACACACAATAAATAGAAAGAATGTGAACATCATGAATAAATTATTGCTACTCGTTACATTTATCA

TTCGTGTGGGTTCAGGTATTGTTATGTTAATGCAAGGCTACGAAAAATTAACGGGCGGATTTACGCTGAAAGG
TTTAGTCCCAGTCATAGCTAACAATACTGATTCACCAGAGTGGTATAAGTGGTTTTTCGCAAATATAGTTGCA
CATACGACGTCATTATTTGATATTATTGTTCCACTTGGAGAGATTGCAATTGGATTAGGTTTAATTTTTGGAG
TTTTTGCATATGCTGCTAGTTTCTTTGGAGCCTTTGTTATGATAAATTATATCTTAGCAGATATGATATTTAC
GTATCCTCTTCAATTAACTTTCTTTATCCTTTTACTAATGAGTCACTCATTGTTAAAACAGATTTCACTTAAA
GAAATCATTAATTACTTTAGAGGTCGTAAGAACAGAGGTGAAAAAATAGATGACCCACTTACTGATCGTGGAT
GATGAACAAGACATTGTAGACATTTGTCAAACCTATTTTGAATATGAAGGTTACAAAGTAACAACGACAACTA
GCGGTAAAGAAGCAATTTCTTTACTATCAAATGATATTGATATCATGGTACTTGATATCATGATGCCAGAAGT
TAATGGTTACGACATTGTCAAAGAAATGAAAAGGCAAAAATTAGATATCCCCTTTATCTATTTAACTGCCAAA
ACACAAGAACATGATACCATTTACGCCTTAACTTTAGGTGCAGATGACTATGTCAAAAAACCATTTAGTCCAA
GGGAACTCGTTTTACGTATTAATAATTTACTTACAAGAATGAAGAAATACCATCATCAACCAGTTGAACAACT
GTCGTTTGATGAATTAACACTTATTAACTTAAGTAAAGTTGTGACTGTAAATGGTCACGAAGTCCCTATGCGT
ATTAAGGAATTTGAGTTATTGTGGTATTTAGCTTCTAGAGAAAATGAAGTTATTTCTAAATCAGAATTACTTG
AAAAAGTTTGGGGATATGACTATTACGAAGATGCTAATACCGTGAATGTCCATATACACCGTATTAGAGAAAA
ATTAGAAAAAGAGAGCTTTACAACATATACCATCACAACTGTATGGGGATTAGGATATAAATTTGAAAGGAGC
CGATAATGGTGTTATCAATTAGAAGTCAAATCATTATTGGCGTCGTATCGAGTATACTATTAACTTCAACTAT
TTTAGCAATTGCATATATTTTAATGTGGTTTAACGGCCATATGACACTAACTTTGACCTTAACGACAATAATT
ACAAGCTGTTTAACCTTATTAATATGTAGTATTTTTATTAATCCACTTATACAAAAAATTAAGCAGTTTAATA
TAAAAACTAAGCAATTTGCTAACGGAAATTACGCAAGCAATGATAAAACGTTTAATTCACCAAAAGAAATTTA
TGAATTAAATCAATCTTTTAATAAAATGGCTTCTGAAATTACGCAACAAATGAATCAAATTAAATCCGAACAA
CAAGAAAAAACAGAACTGATTCAAAACTTAGCCCATGATTTAAAAACACCTTTAGCAAGCATTATTTCATATT
CTGAAGGACTACGTGATGGTATAATCACAAAGGATCATGAGATTAAAGAGTCATACGACATATTAATTAAACA
AGCAAACAGATTATCAACATTATTTGATGATATGACTCATATTATCACTTTAAATACAGGTAAAACATATCCC
CCAGAATTAATACAACTAGACCAATTACTTGTATCAATATTGCAACCATATGAGCAACGTATCAAACATGAAA
ACCGCACATTAGAAGTGAATTTCTGTAGCGAAATTGATGCATTTTATCAATATCGAACGCCACTTGAGCGTAT
TTTAACAAACTTACTTGATAATGCGCTAAAATTTTCAAATGTTGGTAGTCGCATTGATATTAATATTAGTGAA
AACAAAGATCAAGATACTATCGACATTGCTATTAGCGATGAAGGTATTGGCATTATACCAGAACTACAAGAAC
GTATATTCGAACGTACATTCAGAGTAGAAAACTCTCGTAATACAAAAACGGGTGGTTCTGGATTAGGCTTATA
TATAGCTAATGAACTCGCGCAACAAAATAACGCAAAAATCAGTGTAAGCAGTGATATAGATGTAGGAACTACG
ATAACTGTGACATTACACAAATTAGACATTACGTCATAATCCGATTTATTTATAAAATAAAATGCAAAGACTA
AAAAGAAGCTCCCATTAATGAGGGCTTCTTTTTTTGTTTATTTAGAATAAACTTTATGGGTATCCTTCTCAGC
ATTTTCAATACTTGAAAGATTTGTAGCTTGAATAATATATTTAGGACGTGCCTTAACTTCATAATATATCCTG
CCAATATATTCACCTACAACACCAATTGAAATTAACTGTATGCCGCCTAATAATAAAATAGCTGCAATCGTTG
AAAAATATCCCGGAATATTAACACCAGATATCATAATATTGATGAATAAATAGATAATATATAGAACGCTTAT
TGAAAAGATAAATAAACCAAGATAAATCATCATTCTCAAAGGTTTACTATTAAAGGAAATCAATCCATCAATA
CCATAATTAAATAACTTTTTAAATGACCATTTAGATTCACCTTTTTGTCTCTCAACATTTTGATACGTAAACA
CTTTAGTATTATAGCCTATCCATTCAAATAACCCTTTTGAAAATCGATTATATTCTTCAAGTGATGCAATGGA
TTTAACAGCTCTTTGGCTTAAAAGTCTAAAATCACCAACACCATCATCAAATTGTACTTCTTCTACAAAGCAA
TTAACTAACTTATAATACAAATGGCTTAATGTTTTGCGACTAAAATTTTCACCACTTCTATCACGCTTTGCAA
TCACTTGATCATAGCCTTCCATAAAACCTTCAACCATTTTCGGAATATATTCAGGTGGATGTTGCAAATCACA
ATCTATCATGATGACTGCATCAAATTCAGTACTATGCTGGTAACCTGCAATCATAGCTGCTTCTTTTCCAAAA
TTTCTACTAAAAGAAATATAGCTGACATGTCTATCTATTGTGGCAAGATGTTGCATTTCATCTATAGTGGTAT
CCGTACTACCATCATCTATGAAAAGCATATTATAATCATAGCCTTTCACACTACTATCTTGTGAAAGTATTTC
AGTTAATTGTTGATGTGTTTGTGTAATGACTTCCCCTTCATTAAAACAAGGAATGACGACTCTAATTTTCATC
TATGTCCATCCTTTTTATTTAAAAGATTTTGAATGTACCTGAGTATTGTTTTATCAAATAGTAAAAACCAACT
CATTTATTTTAATGTACACATTAAAAGGCATTCATAAACGTAAAATTTAACATCCAGTTATTTTAAATCCCCA
ATTTTCACATCATCTGGATCTTTACCTTGTCTTGCATTTCTATTTAAAGCATCAATTTCAGCTACTTCTGTTA
ATTCTAAATTAAAATCTAAAATATCTATATTTTCTTGAATGCGTTTAGGTGTCTGAGATTTTGGAATAATAAT
TCTATTGTGTGCTAAATGCCAACGCAATACAACTTGTGCTGGTGTTTTATGATATTTTTCAGCAATTTTAACA
ATGACAGGGTCGTCTAGTAGTCCTCTATTTCTCATCAAAGGCATCCATGCAGTCACTTTAATATCGTGACGAT
CACAAAATTCTTGTAATTCTTGTTGGTTAAAATATGGATGTACCTCAATTTGATTCACCATTGGTTTGATACT
TGATTGAGCCATTAATTTTTCTAGATGATGAACATTAAAATTACATACACCTATTGCTTTTACCTTACCTTGC
TCGTAAAGTTCTTCCATAGCTTTATATGTTTCTAAAAATAGACCATCTGCTTCACAAGGCCAATGTATTAGAA
ATAAATCAAGATAATTAGTTTGTAAATTTTCAATCGATTTGTTGAAATATTCGAATGTTTTCTCATAACCTTG
ATAGTCATTCCATAACTTCGTTGTTATAAACAAATCTTCTCTATCGACGCCATTATCCTTTAATGCTCGACCT
AGTGAAGCCTCATTATCATAAAAGTATGCAGTATCAAACGCTCTATAGCCTGCGTCAATTGCAGCATTTACAA
CTTTAGTCATATCTTCGTCAGAGATTTTATAAACACCTAATCCAACTGAAGGCATCGGGTATCCATTATTTAA
TATTTGTATCTCATTCAACATCTTTATATCTCTCCAATCTATGTATCTTTTATATCTTTACATTACCCTAATT

TTTTCAACAAACTCAATTAATACGAATTATCGCTTTCAATAAAAAATATTCATTAAATCATTAAAGATATTAA
GTTCCAATACTATTTTCACTTTTCATTAATTTTAGTCAAAAAAATAACCAACCAAAAATGAATTAAATCATTC
TTAGTTGGTTTATATATTATTATCTATTCTGATTTTTCATCTTCATCAGACTGTCCGATAGTAGGTCTCGCTT
CATTAAACTCATAGTTTAACATCACCCAAATAATTTGGTGGTTATCGATTTCTGAAACAACCCAGCGATCATA
AGTTGTATCCACGTAATCATCTTTTTGTAAGTTGGTATTACGAGATTGTAACCATCCACCTATCGTATCAATA
TCCTCAGAGTCATCAAATTCTATACCGAACTCTTCAGTTAAATCATCCAATAGTACTCTACCATTTACTTGGA
ATGTCTTATTATCAATTTTAACGATATCATTCACTTCATCATCATCAAATTCATCACGAATTTCCCCAACGAT
TTCTTCTAAAATATCTTCCATCGTTAAAATACCTGCCGTTCCACCATATTCATCTATAATAAGACTCATATGT
ACATGTTCACGTTGCATTCTAATTAATGCATCACTGATACGTGTTGTCTCTGAAATCATTGGCAACTCATGTA
TATAGTTTGCTATTTTAATAGTTTTTCCAGAAGCATATTCAGTTAAAAATTCTTTGACGTTAATAAATCCTTT
AATGTGGTCTTTATCACCATCATCAGTAATTGGATAACGCGTAAATTGATGTTCTTTTATTGTTTCTAGTAAT
TCGTCTACATTAAAAGGTTCATTTAGTGTAATCATTTGAGTTCTAGGTACCATTATATCTTTTGCATGTCTTT
CATCGAATGAAAAGATATTTTGCATATACGCCAATTCAGTTTGGTTGATTTCTCCACCATTATAACTATTGTT
AATAATAATTTTGATTTCTTCTTCTGACATTGCATCGGTTTGGGCATCAGGATTTACACCAAACATTCTAATA
ATAACACGTGCAGAACCATTCATCAGCCAAATCAATGGTTTCATAATGTTACCGAAATAGAACAATGGTCTTG
CATATACTAAAGCAAGCTTTTCAGTATGTTGAATCGCTATAGATTTAGGCGCTAATTCACCAAGTACTACATG
CAAATACGTAACGATTATAAATGATACTGCAAACGAAATCGTCGTCGTTAATGCAGTTGGTAAATTGATTGCT
TCAAATATCGGGTGTAATAGCTTTTCAAACGTTGGTTCACCAAGCCAACCTAACCCTAAAGATGTTACTGTTA
TACCTAACTGACAAGCAGAAAGATAATAATCTAGATTAGCAATCATCTTTTTTACTATTTTAGCAGGTTTATT
TCCTTCATCTGCTAGCTGTTCAATTCTTGTTGCTCTAATTTTTACTAATGCAAATTCTGAACCAACAAATACA
GTGGTTAATGCAATTAATAGAATAAATATAATCAAACTAATTATGGTCGAAGTTTCCAATTAATTCCCTATTT
CTAGGGATTCACCTCCATGTTATATGTGTCACTCATGGGTAACACGCATTCAAATTTATCACTATGACTTAAA
TTACAACTACATGTTATCGCCTTCCCATTTAGACACCCCCAGAAAAAAATGTTATTGCTTCTATTTTATCATA
ATATAAAGTGCTTATGTTAACAGATTAAATCTATTGCATACATTTAATTATGATTATCTATTAGCTAAATGTG
TGAACCTTACCTTGTTTTATTGTAGATTTTACATTCATGTCATCATCTAGAATAACAAGGTCTGCATCCTTAT
TTACTTTAATACTACCTTTTCTATCATCGATACCTAATGCAATGGCTTGATTTAAACTTGTTACACGCCATAA
ATGATCTAATGTATCACCTGTAAATGATATTAAGTTACGTAACCCATGATTCATTTTTAAAATACTACCAGCA
AGCGCACCATTTGCAAGACGTGCTTGTTGTGATTGAACAGTTACTTTTTGTCCACCCAAATCATATTCTCCTT
CAGGCATACCTTTTGCACGCATTGCATCTGTAATTAAATAAAAACGTTCATTACCTTTCATACGGTAAGCAAT
TGCAACCGATGCCGGATGAGAATGTGTGCCATCAACAATCATTTCGGTATGTAGAGCATCATTCAACCATGCT
ACTCCAAAAACACCTGGTTCTCTATGTTGGAATGGCGTCGCTGCATTATATAAATGCGTGACATGTTTAGCTC
CTCGCTCAACAGCTTCAACTGCTTCTTCGTATGTTGCCACTGTATGACCAATTGAAAAAATAATGTCATCTTT
ATACGTTTCAAGCGCTTCTTTTGCACCTTCAACTTCAGGTGCAAACGTCATAATCTTTATTAATCCGTTAGCA
GTCTCTTGAAAATGTTTAATTTTATCGATAAATGGACGTACAACGTATTGCGGATGTTGAGCACCAACTTTAT
TTTCAGATATAAATGGCCCTTCCAAATGTATACCTACAATTTCCGCTGCATTGTGAACATCTTGCTCCGCTTC
ATATTTAGCAATATTTATAAGTGCATTATCTATTTTATCAGTAGATTGCGTCATTGTAGTGGCCAAGTATGAT
GTCGTCCCTTCAGACAACAAATTTTCGGATAGATATTTTAAGCCATCGTATGACCCATCCATTGCATCTTGAC
CATAACCACCATGAATATGTATATCAATAAAACCTGGTAATACATGATGACCTTTAGCATCAATCACTTGAAT
TTTATTTGTCGTATCATTATCAATTGCTGCTTTATCATTTACTTCTCCAATTGCAACAATCTGTCCATCTTTC
ACATGAATGTAACCATTATCGATTTTGCCATCTTCAGTATAAACTTTGCCGTTATATATAATTAATTCTGACA
CGTATAAACCCTTCTTTATTTATAAATTTTCACTTTAAGACACTCATATTTTACTATATCTGCTTCGTTTGGC
ATAACTTTTTGCAAAATATTAAAAAAGTCTATACATTTGTGGTGTCTATTTCTTCATTCATTTATCTAAAAAT
AAAAACACCTTCATATCAATTCATAAAAACTGAATTAATAGAAAGTGTTTTTACAGCTTTATATAAACTACCG
TTAACACGATAAATAAAACTAACATATATTCATTGAACATACAACTTAATATGAAGCTCTTTGCTAACAATCA
TTTTAAATCATTAAAACTATTCGTCCATTGATTTTGAAGCTTCGATTTCTGTTTCAGTTAACTTTGGTTTAAT
AAGTCCATAAATTAGCGCTGAAACTAATGTACCTACTACAAGTGCAATAAGTGTTTGCAGTAAATGTGCACCA
TCCGTACCGACAATTACGATAATACCACCGTGTGGCGCAGTAATTCGAGAACCTAAACCTAAAGCGATTGCGC
CACCTATACCTGACCCAATCATCATTGAAGGGATAACACGTAATGGATCGGCAGCCGCGAATGGGATCGCACC
TTCTGTAATAAAGGACATACCCATCACATAGTTAGGGATAATTGAACCACGTTGTTCTTTTGTAAATTTACGT
CTGAAAATTAACATCGCTGTCGCAATTGCTAACGGTGGAATCATACCACCAATCATTGCAGCTGTAATTGGTG
CTGCATTACCTTCAATCAACGCACCTGTTGCAAAAACATATGCCGCTTTGTTGAATGGACCACCCATATCAAT
CGCCATCATAGCGCCAATAACTAAACCTAATAATACAATATTAGAACCTGATAAATTGTTTAATCCATCTAAT
AACAAATGATTTAACCAAGATGCTGGTGGATTAAAGACATAAATCATCAATAATCCTGTAGCTGTCACTGTTA
ATAGTGGGTAAATTAATGTCGGCTTTAATCCCTCTAATGCTTGTGGTAACTTACGTGTCATGGCTTTAACACC
TTGTGTTAAGTAACCTGCTAAGAAACCTGCAATAATACCACCAATAAATCCTGAACCACCTGAAATTGCTAAC
ATACCACCTACAAGACCTGACGCGAATCCAGGTTTATCTGCAATACTACGTGCAATGAAACCAGATAAAATTG
GGATAATTAATGCGAATGCACTTTTACTACCGATATTCCAAAGTTGTTCTGCAAACGCGTTATAATCTGAACT
TTTCGGATCAAATGAGTTTGCACCAAATAAAAACACGATTGCCATTAAAATACCACCAGATATTACAAGTGGT

AGCATGTTAGAAACACCGTTCATTAAGTGTTTATAGAATGCTTTACCTGGGCTTAATTTTTCTTGGTTGTCAT
TTGAATGACCTTTTCTGTTACCATCACGAGCAACAAAAGGTTTACGACTTGTATCTAATGCTTTATTAATTAA
TTCTTCTGGTCGTTTAATACCGTCTGCGACTGGTACTTCTACGACATTTTTACCATCAAATCGATCCGTCTCA
ACATGAACATCTGCAGCAACAATGATACCTGTTGCATTTTCAATATCTTGTTCAGTTAAATGGTTTTTAATAC
CGCTTGAACCATTCGTTTCTACTTTAATTTTAATACCCATCTTATCCGCTTGCTTTTTCAATGCATCACGTGC
CATATATGTGTGTGCAATACCTGTTGGACAAGCAGTTACAGCTAATACATATGGCTCATTAGATTGTTTAGAT
GATTGTTCTGCAGCTGCAACTTGTTGTGCTTCAGCTTCTGCCTCTTCTTCTTTTGTCGCTTCATCATCAGCAT
GATCAATAATTGCTAACACTTCTTCTGGAGACGATGCATGTAATAATTTCTCACGAACATTTTCATCCATTAA
AATACCAGATAGTTTAGCTAAAGCATCTAAATGTGTTTGGGCACCACCTTCTGACGCTGCAATCATAAAGAAT
AAGTGTGCTGGTTGCATATCCAAACTTTGATAATCTACGCCTGCTTTAGATTTACCAAATGCAATAGCTGGTG
ACTTAACTGCTGCCACTTTGGCATGTGGAATAGCAATACCTTCACCGATACCAGTTGTACTTTGTGATTCTCG
ATTGTGAATCGCTTCCTTAAATGACGCGACATCACTTAATTTACCTGCTTTGTCTAATTGATTTACTAACTCA
TCAATAACACCATTTTTGTCATTTGCCATTAAATCCATTGCTATCGTATCTTTTGTTAATAACTCTGTTACTC
TCATTATTTTCACTCCCCATCAAGTACGCTAATCGTAACTTGTGATTTTATTTTTTCTATAGCGTCCCGTGTT
GCTAAGTCCTCATCAAATGCCGTGGCAGTACCGCATGCAACTGCTTGTTGGAATGCTTTTTCAATCGTTAAAC
CTGAAGCAATTCCAGCCACCATGCCTGCAACTGTACTATCACCAGAGCCAACTGTATTAACCACTTTCCCTTG
TGGATTAACTGCTTTAATACTGATTTCTTTATCAATATAAATAGCACCATCACCGCCAAGTGAGACAATAACA
GATTGCGCACCTTTATCAACTAACAAACGACCATATTTAATAACATCTGCGTCTGAGTTCACTGTTGTATTAA
ACATCACTTCTAATTCATCTTTATTAGGTTTAATAAATAGTGGATGATATGGTAAAACGCTTTCAGCCAATTC
TTTTTCAGCGTCGACTACTAATTTAGCACCTGTCTGTGCTGTAATTTGTGCAATTTGCGCATACGCATCGCTT
GGAATACTACTTGGTACACTTCCAGCAACAATAACTATATCTTCGCTTGTTGTATTTTTAATTTGTTGTAACA
GTTGTTCAAATTGTGTTGACGTTATATGAGGACCAGGTGCATTGATTTCTGTTTCTTGTCCTGTTTTTAATTT
CACATTAATACGTGTATCTTCATCAACTTCAATAAAATTCGATTGAATTGCACTGTTATTTAATGTATCTATA
ATGAATTTCCCAGGAAATCCACCTGCAAATCCCAAGGCAGTTGACTCAACATCCAATGTCTTTAAAACACGTG
AGACATTAATACCTTTTCCCCCAGCGAATTTATATGTTGCTGTTGCTCTATTCAAACCATCAATTTTAAAATC
ATTCGTAAAAATGACATAGTCAATTGAAGGATTGAAAGTCACTGTATAAATCATAAAGTCCCTCCTATAAAGT
GATACTTTTGTTGGTATTCTTTTAACGATTCTTGATTTAATGCTTTTTCAGATGTGATGACGGTCGTACTTTC
TAGCAAAGGTACACGAGCAAAATATACTTTATTAAACTTAGAATGATCTATAAGTACAAATGATTGATTGGCT
AATGACATTGCTGTTTGTTTAACTAATGCCTCTTGCTCATCGGGAGTAGTTAATCCAAGTTCAATATCTAATC
CATTCATCCCGATAAAAGCTTTATCGAAACAATATCGTCTTAATATCTCCATGGCACTTGAACCAATCGTAGC
AAGTGTATTTTCTTTAACTTGACCACCTAGCATAATTGTTTTAATACCTTTTTTAAGTAAAGCTTCTACATGT
GTTAAACCATTGGTTACCACAATGATATCTTTCGCTTGAATATATTTAATTAGCTCCAATGTAGATGAACCAG
CATCGATAAATAAGCATTCATTATCGTTGATTTGATTAGCTGCTATTTTAGCAATCATTTTCTTTTCATCGAG
ATTAGTTGCTAATTTTTCAGTTAAATTCGCCTCAACCATACGATTTTCTTTTAACATTGCACCACCATGCACA
CGTTGCAATTTCCCTAATTGTTGTAGTTTAGATAAATCTCTTCGTATTGTTGAAGCACTGCAGCCAGTTCGAT
CTATTAATTCTTGTAAAGTCAAAAAATCTTTGTGCGAAAGTTCTTCTAATATTAACTCGTGTCTTTTTTCTGT
AATTATCATCATCACCCCATCTCACTCTGTATTATAATGAGTACGCTTTCACTTTTCAATCATAAATTACCAA
AAACAATCAAAAACAACCAAATTGTGACGATAAAATTACATTTTTATATTCAAATACTCATTTGTAATGACTG
TTTATGATCGTAATATGCTAATTCAATCAAAAAGGAGTGTGGCAGAAATGAATTTTATAAAAATTTCATCTCT
GTCACACTCCTAATCTTGCCCCATACTTTTTGTTGTTATTAATCATTCATGGATAACTTCTCCAATTTTGCCT
TTAGTTATTGCAATCAAATCCTCAACAGCTATTGTGATTTGCATTGTTCGAAGCCCACCACTCACACTGATAT
GACTATAATTTTCACAGGATTTGTCAACGACTGTTGGAAACAATGTTTTCATACCAACAGGCGAACACCCACC
ACGAATGTATCCCGTTACATTTTTCAAATTATCTAAAGGCATAAGCTGCAATTTCTTCTCTCCAACCAAAGCA
GCTGCCTTTTTCATATCTAAAGTTTCACTTACTGGGATAACAAATACAAAATGTTCATGTTTTGTATTTTCTA
GAACAAGCGTTTTAAATACTGTTTTAACATTTGCTTGTATGAGTTCTGCGACTTCTACACCCGATAAATGTTC
TTCTGGTACCTCAAAGCTATGAACATCGTATTTAATTTTTGCACGATCAAGCATACGCATCGCGTTCGTTTTC
TTATTTTTAGCCATTTCAACACCTCATTTATACTTCTTCAGAACTATATTAACATTTTAAAATACATTACGGA
TTGGTTGATTTTGACATAACATTTACATCATTTAATAAGTTGAGTTCACCTTTTCAAATTAAATTATTGCAAA
ATAAAAACAGTATATCCAATTAGAATACACTGTTCCATCTATTATTTCGTTATTATGCCTTGATTTGAAATAC
TTACATGTTGCTTACTAGTTGAATTATCATTGGAATACACATAATTATATCCATGATTTGTCTTAGAAATTCT
TTCTAAACCATAATTATCATTGATATGCTTATCTGCATAACGATCAGCAATAACTTGGCACTCTTGTTCGTTA
AAAGAATTTGATTTATTTGCATTCACACGATGCTTAAAATGATTTTGCTTTTTCACATTTTCATTCACTTCAG
ATGCTTTAGCAGTATGAATATTGAGAATATCCATTTTCTTTTTATAAGTATCTTCAGTTGATACATTTATCGC
GTTAAGTGTATTAATCGTTTTATGTACCTTAGCAAGCGCATCTTCATTTGTTGCTAATATTAATAAACCAATT
AAACAAACGGTTAATATTACAGCAACAAAGCCTAAAAATAAATTTTTTACTGTCATCCCCTTACCCACATTTC
CTTACTCATTTTTTATCATCTTAACACATTAATGTTAATGAATTAGGTATGTGGATTGCAATTTAAAAAGAAT
GTAATATTTGTAACATGTTTATTACTCAAAATCATTTTTTGTTACATATAACATTTAAATATAAAGACTAAAA
CATTTAAAAGTTTTTAATCTTTATAAATGTAAAGCATCGACTTTTCTTCACTATAATTGTAAGAAAATCGATG

CTTATTTTGAAATGCTTTAGTTGTCGAAATACATTAATCGAATGACAACCATGTGCGATATTACAAATCTTGT
TGTTTTAACTTAGCTCTATGTTGCTTTTCAATTAAAACAATAACAACACCTGCTAATGACACACCTATAGCCA
TATAAATTGGTGCTTCAATGTGTACATCAAATAACGCACCTGCGATTAAAGGACCTATGAAATTACCCATACT
AGTGAATGTCGAGTTTAACCCACCTGCAAATCCCTGTCTATCACCAGCAATATTTGAGAAATAATTTGTAATA
GCAGGTCTTATCATATCGAACCCGATAAAGACAACAAAGCTAATTACCATAATTGTCCAGTAACCATCGGCAA
TAACTAATAGCACTAATACAATAACTGAATATATTAATGACCATGCAATAAATGTTAACTCAGAGAAGTATTT
CATAAATTTATCAAAGAAATAAATTTGGAAAAGTGCACCAAAGATACCGCCACCTGTAATTGCAATCGAAATA
TCTTTAGGTGAATAATTTACTTTATACGATGTATATAAAGAATACAGTGTTTCAAATGCCGATAAACCGAACG
CTAAGACGAGCGTTAAAATTGCTGGCGTAATAAAGACTTTCCAATCAATTTTCGTTAACAATTGGGGCTCAAG
TTTTTGGAAACCACTTGTCGTAGACTTTTTCGGATCATGAATCAATACAACTGACATTATGAAAGCTAAAATG
CCTAATGCACCTGCAAAATAAAATGGCATACGATGTGAAACTTCTGCCATAAATCCACCAATCCCTGGTCCTA
AAATGAATCCAGAATTGATAATCGCTGACATGTAGCCAAAGTTTTTTGCTTTTTGATGGCTTGGTGAAACATC
TGCAATCAAACCTGTCACACCAGGCATTACCATACCAGCACTCATACCACCAATCACTCTCGATAACATCAAT
ACCGAAAAATTGTGGCCAACTGCAAACATAAATTCTGACACTGAGAATAAAATTAACCCTATACATATAATTA
ATTTCTTCCCTAATTTGTCAGCTAGCGTACCACCAAACGGCGATATAATCATTTGAGATAACGCAAAAGCAGC
AACTAGTAATCCTAAATCACTACCAGTTAATCCCAAATCTTTTAAATAAACAGGCAAGACTGGTATTACTAAA
CCGATACCTAAAAAAATCAAGAAAATATTAAAATATAAGACAAAAATCTGTTTATTCATATGCTCACCTCTTT
TTTCTCCATGTCATGCTTAAAGCTGTTATTCTTCTTTTCCACGACAGATTGCAATTCATGCTAAATATTCGTA
AAAAGTTTCTATATTGTATTACAACATTGTATTACAACATTGCTACATTTGACAATATTTTTTCTTTGTAATT
ATCACTATTTTCCATTTAATTGTATAATTAATGACATATTAAAATAAAGGGGATTGATCATATGAAATTGCAA
AAAAGTAATCATACTATACTACTAGTGTTAGAAAAAGGCGAAGATATCGTTGAATGTATAACAAACTTTGCAG
ATGACCAAGACTTAACATTTACATCTGTCAGCGGCATTGGTGCGTGTGATGACGTCGTTTTAAAATTTTTCAA
TTTAACAACGAAACAATACGAAGAAAAACATATTACTGAGCCACTGGAATTGACGAGCTTATTAGGTAATATT
TCTCGTCTAGAAAATGGTCATTTTGCACATTTGCATGCTACTTTCGGTACTCAATCATATGAAACTTTTAGCG
GACATCTCGCTAAAGCAATTGTTTCTGCAACTGCTGAAATCATTCTTACTGTAACTGATTTGGACATTCAACG
TTCATTCAAAGATGCAGTCGGTTTAAATTTACTTGATCCTCAATAGCACTTTACTTTTTAGTTGAATCAAATT
TTATTACAAGTTATCATAAGTGCTTAATCATAAATGCATTCTCGTTTATTAATATACTTCAACGAGTTAAATT
AAATTTTTAATTAGGAATTGAATGCGTTTATAACTATCACATTTGAATGAATAACTTTTTCATTAGAAAGTAG
TGTTTATTATTGATGCAATCTCGATACATCGCTAGAATTTACTTTTTAATTTTATTTATCGTCTCCCTTTTTG
AGACTAAAATTTTTCAATTTATGACTTTAAATTTGTTTTTAGCATATATACCTTTTGAGCTTAGTCTATTATT
ACGATTATTCAAACCGAATAAAAAGTATGAATGGCCACTATTTATCATATATAGTTTTATATTTGTGTTACTT
TTACCAAATACATTTTATATGGTAACTGATTTAATCCATTTGAACCAATTTCAATTCAACTTTTACGCAGGAC
TAAATTTAAGTGAGTGGAAATACTTTACCTATTTACTGCTTGGTGTCTTCTTAGCGGTTTATGTGATGATTTT
AATCTATTTTGAAATACTAACATTGACTTCTCACCGTTGGCTTAATAGATTAATTGTTGTCGTGCTAATGTTT
TTAAATGGATTCGGAATTTATATTGGTCGTTTCTTACGCTTACATTCCGTTTATTTCTTTAATGAGCCCTTAC
ATGTTCTAAATCACATTCTAGAAAGTCTAACGCTTAAAACTGCTATGTTTGTATGTTTTATGGTTGTCATGCA
AGCAGCAATTCTATTATTTGGAAAAGGAGTGCGTTTGAACAAGTGACAATAAATATAATCGTTTTAATAGTAT
CTATCATTGTCTTTCAATTGATTATTGGACACATATGGCATGATATTGGTTTAAGTTATTTAAGAAGTATCTT
ACTCATGATGCTGCCATTTGGACTAGGTGTATTTATACAACAAGTGTCGTATTACGAACGACAATATCCTAAA
TGGCAAGTGCCTCAAAACATAAAAGTTAGATTGAAATATATTTATCTCGCTACGTTTTTAGAGTATGTCGTAC
TATACCTCACTTTATTTACAGACATACTAAGATAACATCAAATTAATAAGAAAACTAAATACAACTTATATAT
TTGAGGAAAGCCAATTTCTATCACATCAATAATAGAAATTGGCTTATTTATATTGCTTAAGAACTTTATATTC
TGCTCCAGATTTTACTCATGCCAGATCTATCAATTGAGCCGAATTAGTCTAGCGCTTTAAATGTAGCTAAAAC
TTGTGTACGTTTTTCTTGATGATCTACCATTTGTCTTGGATAATGACTACCTAATTCAATCCCCTGTTCAAAA
AGGTTGGACTCATTGCGTTGTGTATCATGCAAATATTTTGCATCAATTTGATTAAAAATCGGAAGATATGTTT
TGATATACAAAGCTTTAGCATCAAAGCGTTCACTCTGTCTTATTGGATTAAACATTCTAAAATACGGCACTGC
ATCCGTACCTGTAGAAGCAGACCATTGCCATCCATGAATATTTGATGCTGCATCATAGTCAATAAGGTGCTTT
CTAAAGAATTTTTCTCCCCATGTCCAATCTATAAATAAATCTTTGGTTAAAAATTGCGACACAACCATTCTCA
TTCGATTATGCATAAAACCAGTTTGTGTCAATTCCATTATTGCTGCATCAATGATTGGAAATCCTGTTTGCCC
TTCGCACCATGCATTAAAATTCGCTTCATTTTGCGACCATTTTATCTGTCGATATTTAGGTTTGAAAGCTTGG
TATGAGGTTTCAGGATACTGTGTCATTAACACATAATAAAATTCTCTAAAAATGAGTTCACGTATAAATGCTT
CATAGTTTGCCTCATCTTCATCATAACGCGCCAATAAATCATTAAAAATTTCAATAATATCTAATAATCCATA
TGCTAAAGCAACACTTAGCTGACTTGTTAACACTTCAGGTAAATAGTCTCTTCCGCTTTCGTAATTCTGTATA
TCTTGATCTAAAAAAATGTTGCCATTTATTCTGTTCAATTATTTGTGAGGATCCAAATGTTATGTCATCAAACA
CTAAATCATCAGGTGATGCAATGACGAAGTCTTCGAATGATTTTAAATCATAGTGATATACGTCTCTATGCCT
CAAGTAAGGTCGCCATTTTTTATAGAAACTAGTAAAAACTTTGTATGGCTCCCCTTGTTGATTCATTGTTTTA
CTCGCTTTAAAGTAATGATTCCCACGTATTAAAGTAACGGCAATACGTGCTTCATTGAATAAAGAACGTTGAT
GTAAAATATCATATTCTTCAAGATGATAACTCATAATATCACCCGCAATCACTACTTCAGATATATTGTTTTC

TTTACAAAATGAAATGATATCGTCATAAGACATGACATAAGGTGTGAGCTTTTCTGCGTGCAACGATTGGTAA
AAGCGCTGCAAAGTACCGCGATAAAACTGTGCTTTCAAATCAGAAGCTGAAGACATGTCCTCTTCCGGAATGA
TAAAATAACATGCATCAATGTCTTCTTTTTGTTGATAAATATATTCAAATAATGGATTGTGTTCCATTCGAAA
CATTCGATTTAATAACACAGCAATTGCCATAAACAAAACACCCTTCAATTATCTTTTTATTTTATTAAGCTTT
TTGTCACTTTAAAATAACCTAGATTCTTAATTATATTTTTAATTAAAGCAAACCTAACCATTTCCAATAAGTC
ATACTAAAAACTATCACTAGAATATACCCTACAATTGTCAATGGTATACCTGCCTTCAAGAAATCTTTAACCG
TAAAAGTACCAGTGCCATAAGCCAACATATTTTGAGGTGCACTAACCGGTAATAAGAACCCAAAGCTAATAAC
AAATTGTTGAATTAAAACAAATCCTATAGACTGGTCTCCTAAGTGTAACGTAGAGGTTAGCGAAATAAAAACA
GGTATTAAGGCTGATGATAAACTTGTTGCACTCGCAAAGCCCAAATGAATCAATATATTAAAAAGCGTGATAA
GTGCAATTGTCGCGATAATAGGTAAATGTTTTAAACCTAAAACACCAAAAGTTTGATCACTTAACCATTGAGC
TGCACCTGTTTTCAAAAGAACGTTACCTAATGAAATACCTACACCAAACACGATAATTGTTCCCCATGGTATT
TTATTTTCAACATGTTTCCATGTCATGACACCAATTTTCGGCATTAACATAACACCTAAAGCAATAATAGTAA
TGGATGCAGAGTCAATCGGATGTAATACTTTTTCAGTTGACCAAAACAGTAATAATAACATGGATATAACAAT
TAAACGCCATTCACGTGCGCTAACGGGGCCAAGTTTATGCAATTCTTCTTTTATTAAATCTTTGCCACCTTCT
ATTGTATTGATTTCTGGAGGCATCACTTTAATCATGATGAAATATAAAGCTATGGACATAACTATGGACCAAG
GCGCTGCATATAAGAACCACTCGCCCCATGAAACATCAAATCCTAATTGATGGTTTATAAAATTAATCGCTAC
GATATTTTGTGCTGCCGCCGTTTTGATACCAATATTCCAAATTGACACAGCTTGTACTGAGGTAATTATTAAT
AAAGACGCTAACTTGCTATCTTTGGAAACTTTAAATGCCGCAATCATACCCAGCAAGATTGGTACAACTGCCC
CTGCTCTAGCTGTTGCAGAAGGAACGAAAAATGCAAGTACAATTGAAACAATAATTGCTCCAATAACTATATT
TCTAGTTTTATTACCAACAATTGATAACACTAAAAGAGCTAGTCTTTTATGCAAATTCGTTTCTTGCATAGCA
GCAGCCAAAAATAATGCGGCTGCAACGAGAGCTACAGCTGAAGTTGCAAATCCACTAAACGCTAATGTTAATG
CATGATTAGTTCCTAGAAGGTCACTTCCAGCTAAAACAGCACTGCCACTTTTCGGATTACCTAGCTTCTCCCC
TAAATTTTGAACCGGGCTAAATCCTAAAAGTAATATCATTAAGCCAATAATTAAAGTTGCTGACACCGGATAT
GATACAGCTTCCGTTACCCACATAATAACTGCAAAAGCTAAAATAGCTAGTACTGCCTTAGCCATTATTGGCA
GACTTGAAGGTGCCGGGAGTAATAGTACAACTATAAGTATTATAAAACTCATGACAATCCAAAATGGCTTGTA
TTGCTTTTTTGTCTTTTCTTTCTTTTGTTGTTCATGTTCCACTACATGTTTTTCTTCTGACATAACAGTCACC
CCTTTGTTTAATCTTAATTTAATTATAACAATTTTTTAAAACATACATTTTTAATTTATGCAAAATACACAAT
TTTATCGAAATATTTTGTAAATAATATTGCTAGGTGATGAGTTATCTCGGTTAATTACAAAACGATATCTAAG
TTTAAATATTAGATCATAAAAAAACTATCTACTGAATATCCTCATAGCATCATTTTTCTAAGCAAAAGTTAAA
CTTAGTTAGATAAAATGCAAATGAGATTATTGTAGATAGTCTCTTTTTAATATTTAAAAATGATTAATGCCAT
AAACATAAATAATTCCAAAGAATGCCGCGCCACTAAGCACTGTTAATATAAGACTTAGTAACAATGTGCGTTT
ATAATGTTTAATAAATGCTATGAACAAAATGACTACATTATACGCAAAGAAAAGTCCGAATAATGTCATTGTT
ATTTTCAAATCAGAATAGAAAATATTTAATAGTAATACAATGACTGCATAAATTAAATACGGAATAACTATAA
ATTTGCGTTCAAATCTTAATTGTTCGAAACGTTTATCTTCGTTTGTCATGATATGCTCCTTTATGATTTAAAA
GTAAATAAGTTTACGGAATATCTTGTCCGATAATAGCGGTGTAAATGTCAAACCACGACTGATCATCTAAATT
AAGTTGTAGCCCTTCGATTGCTTGATCAATACGCTTTAACTGACTTGTTCCAAGTATCGGCATGATACGATGC
GGTATTTTTACTAACCATGCTATCATCACAGCTGTGTCACTCACACCATATTTGTCAGCTATTGATTGAACAA
CTTTCATAATACGTTGCGCTTTAATATCTTCCTTGTCGAAAATTTTACCGCCTGCAAAAGGACTCCAAGCCAT
AATTTGAACATGGTTTTGATACATTGAATCCATTGTTCCATCTTGTAAACTATCAACGTGATATGGCGATAAT
TCTAATTGATTGATGCTAATATGTAGTCTTTCTTTCATAATATATTGATTTAACAATTGGTATTGTGAATGAT
TAAAATTCGACACCCCGAATGACTTCAACTTACCTTGTTTAACAAGTTTAGTTAATGCATCAGCAACTTGTTC
TGGATCCATCAATGGTGAAGGACGATGAATGAGTAGACTATCTAAATAATCTACATTCAAATTGATTAATGAC
TGTTCAACAGATTTCACGATGTGCTTACTACTCAAATCATAACGATGTCCATTTGTAAAATCAAATTGCTTAG
AAGGCAAAATGATACCACATTTCGTAACAATTTGAATTTTATTTCTTAATTCGGGTGATAAATCCAAAGCATT
ACCAAACAGTGATTCACATTGATAATCCCCATAAATATCAGCATGATCCATCGTGGTAATCCCACGTTCAACT
AATTCATTTATAAAATAATTTAACTCTTTCGCAGTCATCTTCCATTCATTTGCACGCCAAAAACCTTGTACAA
GCCTAGAAAAATGAACATAGTGATTAATCATTATTTGTTCCATATCTCATCCACTCCATCCCTCAATGTCACA
AAATATTCACAATAAAAGTATTAAGAAATTAATGTGCTATTTCCGTTAAATTGAAACAACACATTTTCTGATT
TACGTAAATTGTAGCATATCCAATTGATTACGTATGTGATTTGTATCTAAAGTTTCACCAATAATAACAATCG
TTAATGGTAAGTGCATGCCAATTATTCCATAGTCTGGTAAACCATATGCATATTGAAATTCATAAATTGCATT
TGGTTGATCTCTAAATGATACATAACCTTTCAAACGTAGAACAGATTCCGGTAATTTCATTATAAATTGATAA
AACAATTGACGATCAATCGGACCTGTAAATGTGTAAGTCATACTTTTAATCCCATGATGATGGTGATGATGAG
ACGATATCTCTCTGTCTTTACCAGGTAAGTCCAATTCTTCCAACGAAACTTGTCCATAGGTTGTTTTATAAGT
TGGAATACTTGCACAAATTATACTTAATTGCTTATCAATTTTTTCAAGACTGTCATCAGTTACAAGATCAATT
TTATTAATAATAATCATGTCACTTAGTTTCAACTGATCTTCCATCAGCGAAACTGTATTTTCGGTATATTGAT
GACGTTCCAAAAATCGAGTCGCATCTAATACACCATAAATAATTGGTTTTTCAAAGAAATTAACGATTTGCGG
ATCTTGACATGCAACTAGTAATTCCAAAGAATGCGCAATGCCTGTCGCTTCTATGATGACATGATTAACGTCC
CCTTTTAAAGCAATGGCTTTTAGTTCATAGACAAGTTCTTGTTTAAGATCGCAACAAACACAACCATTAATCA

ATGAATGGACTTCAATTTCATTTGAAATACTATTGCTATCAATATCAAAAGTACCGAATTCATTCATGATGAT
TTTAATTTTCTCATCATTTTTTAATAATTCACTAATATAATGTGTCAGTAACGTGGTTTTACCACTACCCAAA
AATCCGTTAATTATGGATATTCTTATTTTTTCATCTTTATTATTTTTCATTTTATATTCTCTTTTCTATTACA
GTTATTTTTAAAAATGATAACATATATATTAAATTATCTTTTTGCTTGATTTAATCAAAAATATCTTCTAAAC
TTTAAACATACTTTAATTTTAGCATGGCATTGAATCAAAGAAAGTTGTGAATTAATAAACAATCAACTTTTTA
ATGATTACCAATCCGAAAAGAGAAAAAAACGGATAGTATGTCTGGAGGAGAACTTTAATGTCTGATCAACATA
ATTTAAAAGAACAGCTATGCTTTAGTTTGTACAATGCTCAAAGACAAGTTAATCGCTACTACTCTAACAAAGT
TTTTAAGAAGTACAATCTAACATACCCACAATTTCTTGTCTTAACAATTTTATGGGATGAATCTCCTGTAAAC
GTCAAGAAAGTCGTAACTGAATTAGCACTCGATACTGGTACAGTATCACCATTATTAAAACGAATGGAACAAG
TAGACTTAATTAAGCGTGAACGTTCCGAAGTCGATCAACGTGAAGTATTTATTCACTTGACTGACAAAAGTGA
AACTATTAGACCAGAATTAAGTAATGCATCTGACAAAGTCGCTTCAGCTTCTTCTTTATCTCAAGATGAAGTT
AAAGAACTTAATCGCTTATTAGGTAAAGTCATTCATGCATTTGATGAAACAAAGGAAAAATAATTAACTTTTG
TCATGACAATTAAAGTAATGTTTAGAATTTATTAAGAATAGAAAAACAATTAGCACGCGTAAGCTTGTTAGTT
AAAAAACTGCTTGAAAGGTTTCTTAGCCTATCAAGCAGTTTTTTTATGCATTATATTGAATCATATTCCATAA
AGCACCTTGATTAGCGAGTAATTGTTGATAGTTTCCCTTTTCAACTATTTTACCATTTATCATCACAATGATG
GTCTCGAAACGTGACAATAAAGTTAAATCGTGTGTAGCAACAATTAATGTTTCTGCATGTGCTTCAATTAAAT
CCATAACTTTTAAACTATTTTGTTTATCTAATGCAGTTGTTGGTTCATCTAAAATCCATGTTGATACAGTATC
TTTTAATAACATCCTCGCAATCGCTAAACGCTGAATTTCTCCGCCAGATAATGTATGACCATCTAAGTCAATT
TGACGTTCTAGTGCCAAATGTTCTAAATCTAATTGCTTAAATATTGCTTGCACCGCTTCATCTTTTTCATCGG
TAAATAAATTTTGACGTATTGTACCATCAAATAATTGTTGAGATTGTAGCAAGACATTTAACGATTCAAACTT
ATCTTTGTCATCTATTTCAAACATATCCATATTTTCGAAACGAATAGAGCCACTATCTAATTGATATAACCCA
GCCATAATTTGTAGTAATGTACTTTTTCCTGAACCAGAAGGACCCACAATCGCAATCTTTTCGCCTCTATTAA
CATTAAAATTAATATCCGACAACACATACGTTTGCTGGTTCCAATACTTAAAACTAGCGTCCTTAACTTCATA
AATGTTCGTTGCATCATACTTATCATTAAGACGGTTTTTTCCATTAGTAGAAGGTACAGATATCACTTCATTA
ATATCATGCAATGCTTGGTCAGTATCCGCTTTATAATACGCGACATTTGTCATTGGTACAGCTTGTTCAAATA
AAGTTAAGACCATTAAAACTATACTCGTCATATAAATAATATTTAGTTGGCCTGCATTAATTTGAATTAATCC
TAGAACTAAACTACCAAAAATCGAAAGCATAGCAATAATATTTAATATAAAATCATAAATCGTTAAAAAGCGT
TGCTCTTTAAGTTGTAATTTATCAAAATGATTTAATTTAGCCATCAAATTATCTCGATAATGATTAATTTGAT
TAAATCGACGTAGTTCATCCATACCAGCTTTATAATCATAAAAATGATTTAAAAAACGGGCCTGTTCATTAGC
TGCATGTTTCTTTAAAGTACGTGCTTTTTTTGAGCTTAACCAAGGAACAACGAGTAAAGTAAACAACATGCTA
ACCATAATCAATAGCGCATGGCTGATTGAAATGAACGCCAAAACTATTACTGTAACTAGCGCTGTCAAACCGA
TAACGACTGGTGGATAATAAACACGTAAATATATATTTTGTAATGCCTCAACACGACTAATCATACGTGAAAT
TAAATCACTAGAACTCAGTTTACGGTAAACATTAGGAATGACATTTACTAATTTACCGAAAAACTGTACCCGA
ATATCACGTAGCATTGTAAATGTAGCTTTATGAGAAATAAGACGCTCTACGTATCGAGTAATAGCTCTTAAAA
ACCCAAACAATTTTACTGTTACGACTAAAATCATCAGAGCGTATAGTGGCGCACCAAGTGCACTTTGTGTCAC
CATATAACCACTTAAGAAAAACATGGCGAGCGCAACTAAACTTCCACAAACACCAACAACTATAGCTAACAAT
AAATCCTTATCTACTTGAAATTTTAGTCGTGTTTTCATAAGTCATCACCATTTTCTGTTATATTTACTGAAAT
ATTGCGATCATCAGCAATCAGTCTTCCATTTTCTATATACAAACGTCGTTGTAAATGGCGAATTGTATTATCT
CTATGTGCAATGACAATCATCGTTGTATCTTTAAAATGTTGAAACAGAACGTTCTGAATCATGTGTTCTGTTT
GAATATCTAAACCAGTTGCAGGCTCATCAAATATAACGAGATCTGGCTTCATAACTAAAAGACGGCAAAGTTC
TATGCGTCTCATCTGTCCACCAGATAACATTTCGCCTCCTTCACCTATTATTGTGTTAATGCCTTGTGTGAAA
GATTGCACTTTGTCTAATAAACCTACTTCGTCTAGCACTTCTTCAATAGTATTATTTTCTATATCTTTAAACA
TCGTAATATTCTCTTTTATAGAAGCACTGAAAATATATGGCTGTTGACTAAGTATTCCTATATTTAAATCACG
CTTGTTTGTACTTATAGTACCTATTGTTGGCTGATAAACACCTGCAATAAGATGTGTCAAAGTGGATTTACCT
GCCCCGCTTGGACCTACAAGTGCAATTTGATCACCTTTAAATATTTCCAAATTTAAATCATTTAATACCAATC
TATCAGAATCATCATATCGAAATGATATGTCTGTTAACTGAATAAATGGCTTTTGATTTTCCTCATACTTTAA
TAGAAATTCATTATTATTGTTCGGTTGTTCTAAAAACTCAAACACAACGTCACTGGCACCTTCACTTTGTTTT
CCAGTATGGAACGCTTGCCCTAAGTCCTTAATTGCATTATAAAATTCAGGCGCTAAAATAATCGCAATTGCCG
CAGTTTTAAAATCAATATTATGAAATACTACTAAGCTTAGCGTTGCTTCCAATGCAACCAATCCAATACCTAA
CATACTTATAAATTCGAGCATTAATCCCGATAAAAAAGCACTGCGTAAAATGCGCATTGTTAAAGTTCTAAAC
TGAGTACTATCGTCGTAAATATGCTTCTCTGTTTGCTCTGTACGATTAAATAGCTTTAACGTCACTAAACCTT
TAGCAATATTTAAAAACCGTTGACTAAATTGATTCAAATAAGTCATTTGATCTTTTGACTCATCTCGCGTTTT
CAAACCGAAAATAATATAAAACAAAGGAATAAATGGTGCAGTTATTAACATAATTAATGCGGTATTGAAATGG
ATGAAAAACATTGCAATGATTATGATGAGCGGAACCATCATTGATTTGAACACTTGAGGCAAATAACTCTTAT
AAAAAGGTGCTAGACCATCAATGTTTTCTGTGAGTATAGTCATTTGTTCACCGATTGGATGACCATTATTTTT
ATAAATAACCCGCTGTCTAAGCATATGCTTAACTTTAAATGCTAATGTATCACCTAACCATTGATTTAGAAAT
TGCACAGTTGCTCTTAAAAGTAAAACACCTAATAAAATAAATAATACAATCCATAAACCTTGAAATTGATGTC
TTATAATTTTAGCTAAAAAATCTGCTATTAAAATATTTTGCGTTATAACGAGTATGCCGAGACCAGTACTGAC

CATGAACATGAGTACCGGAAAAATTTTATATTGAAACAGTATTGTTGTTAATTTTTTCACAATTATATCACCT
AAACCTATTATAAAAGGTTTTCCTTAAAATCAAATTAAATAGCTCAATAAAATATTGTAGTTTTACATGTAAA
AGAAAATGAATATATACATCTTTAGTCTAGTATTATAACTATAACTAGCATGTCTTTAAATGAATATGCTATC
ATGATATGGTAATTTTGATATTTAAATAAATTAAATTGAGTAATGAAAGTGGGTACATTATGTTTATCATTGA
ATTAATAAAAGGTATTATCTTAGGGGTCGTCGAAGGATTAACAGAATTTGCACCTGTTTCCTCTACTGGACAT
ATGATTCTAGTTGATGATATGTGGTTAAAATCATCTGAATTTTTAGGTTCTCAATCAGCATTTACATTTAAAA
TCGTCATCCAATTAGGATCCGTCTTTGCAGCAGCATGGGTATTCCGCGAACGCTTCTTAGAGATTTTACATAT
TGGTAAACACAAACATGTTGAAGGAGAAAACGATCAACAAAGACGTTCAAAGCCAAGACGTTTAAATTTATTA
CATGTATTAGTGGGTATGGTGCCAGCAGGTATTTTAGGCTTACTATTTGATGATTTCATCGAAGAACATTTAT
TTAGTGTGCCAACTGTTATGATTGGCTTATTCGTAGGTGCTATCTATATGATTATTGCTGATAAATACTCAGC
TAAAGTTAAAAACCCACAAACAGTAGATCAAATCAATTATTTCCAAGCGTTTGTAATTGGTATCTCTCAAGCA
GTAGCTATGTGGCCTGGTTTCAGCCGTTCAGGCTCAACAATTTCAACTGGGGTATTAATGAAATTAAATCATA
AAGCTGCATCAGACTTCACATTTATTATGGCTGTTCCAATTATGTTAGCAGCTAGTGGTTTATCTTTATTAAA
ACATTATCAAGATATTCAGATTGCTGATATACCTTTTTATATTTTAGGCTTTTTAGCTGCATTTACTGTTGGA
CTAATTGCAATCAAAACATTCTTGCATCTAATTAACAAAATCAAACTAATTCCATTTGCCATTTACAGAATTG
TATTAGTAATCTTTATTGCTATACTTTACTTCGGATTTGGCATTGGTAAAGGTATTTAATTTCCTTTTAAAAT
ATAGACTGTCAATTAAGGAACCACTTGATTGGCAGTTTTTATTGTCTTCTTTTAAAAAACATCTGAAATACTT
AATAGTGATTCACTACTATCATCGTTTATATTGATGTATCATAGGGTATGTAAAATTAATAAAGTAATTATGG
AGGCAAGATCTATGGATAAGAAAAAAGTCATCAAATTTATGATTAATGTATTACCAATTGTATTGGTACCGTT
AATTGTTGAACGTAAACGTATCAAACAACATCCGGACGTACAAAAAGTTACAGATGCTACAAGTAAAGTTGCT
TCAAAAACATCTGCAGCAATCAGTAACACAGCGAGTGATGTTAAAGAATATGTCGGCGATAAAAAACAAGATT
TTGAAAATAAGCGTGAACTTAAAAAGTTTGCTAGAGAACATGATCCTGCCTATATTGAGAAAAAAGGCGAAAA
ATTAGCTAAACAAAATCGTAAAGACGCTGATAAAATGAATAAAATACTTCAAAAAAATATCGAAAAGCGTCAT
AAAGAAGAGCAAAAAGCCCGCGAAAAGAATGAAATACAACGTGTTAAAGATATGAAAAAATCACAAAAATACG
AAGAAAAAGCAGGCTTAACACCTAATAAATTAGATGAGAAAACTGAGAAAAAAGGCGATAAACTAGCTGAAAA
AAATCGCAAAGAAATCGCTAAAATGAATAAAAAGTTACAAAAAAATATTGAAAAACGACACAAAGAAGAACAA
AAACGCCAACAAGAAGCTGATAAAGCACGCATCAAGTCATTTAAAAAATATAAAGATTATGTTGCCAAAAGCG
CCTCTCAACAAAATAAAGAAAACAATACTGAGGCATAATTGTGACACATATTATTATTGATGGAGATGCTTGT
CCTGTTGTTGATTCTATTATAGATTTAACAACTGAGACAGGCATTTTTGTGACAATTATTCGGAGCTTCAGCC
ATTTTTCGAACCAATTATATCCCCCGCATGTATCAACATTATATGTTGATGATGGACCAGATGCAGTTGATTA
CAAAATTGTTCAATTATCAACGAAAGATGATATCGTAGTCACTCAAGATTATGGACTCGCAAGTTTATTAGTC
GACAAAGTCTTAATTGTCATGCATCATAATGGGAAGATATACAATTCCAAAAATATTCAACAACTATTAGATA
AACGATATATGAATGCACAAATTAGAAAACAAGGTGGTCGCCACAAAGGTCCCCCACCTTTTACAAAGCAAGA
TCAAAAAGTGTTTGAACAATCACTATTAAAAGTCATTCATCGAATTAAGGAGCTGGATTAACATGAAACGAAT
CGCAGTTTATTGTGGTGCAAGCAAAGGTCATGACCCTTCATATGTACAAAAAGCATACGATTTAGGTAAATAT
TTTGCTGAACAAGGTTATGAATTAGTATTTGGTGCAGGGTCAATTGGTATTATGGGTGCCATTCAAGATGGTG
TATTAGATCATGGCGGCAAAGCTATTGGCGTCATGCCTAAAATGTTAGATGAACATGAAATCACAAGCCAACG
TTTAACTGAATTAATTCTAGTTGATTCTATGCATGAACGTAAAAATAAAATGGCAGAACTTGCAGATGCATTT
GTCATGGCTCCCGGCGGCGCAGGTTCACTAGAAGAATTTTTCGAAATGTATAGTTGGGCTCAAATTGGTATCC
ATGAAAAGCCTATTGCCATTTATAATATTAATGGCTTTTTCAATCCACTACAAACAATGATTGACCATATGAT
TGAGGAAGGTTTTATTGATCCTAAGTATCGTGCACTTGCACCGTTATGTGACACTAAGGAATCATTAATAGAA
TCTATATTGAATTTCAAACCTCTAGGTACTCGTTCATACGATTAATCATTCTAAGGTTAAAAAATAAACAAAC
ACCCCTAATATTGTTATCATCAGCTAAACAATATTAGGGGTGTTTGTTACGCTTATGTATAAATTTTTCACTT
TCGAAGATTAATGTAATAGCGCACTTGCCTTTTTAATAAATGATACTTACTTAGATTGGGTAATTCGAAATGA
TCATACACTGTCATGCCAATTTTTTCCATTACACGTTGTGAAGCTTTATTTGCTTCTGCTGTAAAACTATAGA
CATCGTATATTTGATGTTGTTCTGCTAACTTCAACGCTACCTTTGCGCCTTCAGTTGCTAATCCTTTTCCCCA
AAATTCTGGCAACAAGCGCCAACCTATTTCATAAAGCGGTAATTCTTTAAATGGATAATCGCTTGTTTCTGGA
ATATAATTCAAACCTATAAAGCCTATCCATTGATGGGACTCTTTATCTTCTACAGCAAATAATCCAATGCCAT
AATCTTTAATAACCGCATCCATAGTTCTCATATCTAATTCTGAACGACGATAACTCAATAAACTTGGAAAATA
TTTACGTACGTCATAATTCGCATTCATTTTTTGAAAAGGTAACAGATCATCTTCATGCCAGTCTCTTAATATT
AAACGCTCTGTTTCACAATAAATCATCGATTGACACTCCTATATAATTTCTCAATAAAAAACACAAAATCGAT
ATTTTGATCAACTTTGCGTTTTAGATAGCAGTTTATTTTGATTTAGAAATATAACTTATAAGTTCAACATGAA
CTGGCACTTCTTTTAAACGTGCTCTATCTGATTTCAGCGCAGTAAAAGTAGCATCTATAAAATCTTGTTGATG
GTTTAAACGATATGTAGCAACAAAAGTATCTGTATCGGACTCAAAGTTTGGTAAATGTGCAATTACACGCTTA
ATTTCGCCAGTAGAATCAGCAATCGTGCGTCCTGTAATATTTTCTAATTCTCTACCTAATTCTGGCAAAGTAA
TATCTTGACCCTCTAAAATTCTAAAATCTTGTTCATGCATGCCGTTTCTCTCCTTTATTAATTACGTTGTCTA
TTTTTACATTCTGTTTCAATCATGACCACATTTTAATTAATACATACCCTTAAAATAAGGTTTAAAACGCTGT
ACCTTTCATCTATAACAAGAAAGCAATTCAATTATGATTGGTGTTTGTCATTAGCTTTTTGATCTTTATCTGT

TGATGTAGGCTTTTTGTGATTATCTTTATCCGATGACTTAGTTTGATCTTTTGATTGCTTATCTTGTGATGCC
TCTTTATCTTTATCTTTATTTTTGTCTTCGTCTTTTTTATCATCCTTGTTATCTTCTTTTGAAGGTTTTAAAT
CTTTCACTTCTTTTTCAAGGTCTTTTTGCTTATCTGCTAATTTTTCTTTTTCTTGTTGAAGCTTTTCTTTTTT
ATCTTTTAATTCTTTATTTTCTTTTTGTAAATTATCGATGTCCTTTTCCAGAGTGGCTTTCTCTTGGCTTTTA
CCACAACCTGTTAAAAATAGCATGACCGCCATAATACTGATGATTAATTTCTTCATATAAAACTCCTTAACGT
CATTAAATCTTTTTTAATTTTAACATGAATTAGCAACATAATCCTAATTCTAATCAATATCTTTATACATTTT
CACATGACTCACTTGATAACCTAAATCTTTATTCAAAGATATCATTGGTAAATTACTTTTATGAATTGTACTG
CTTATTCGCTTTGCATTCATAGTTTTTGCCCATTTTTCAAGCGCAATCTTCAGTTGCGTAGCTATTCCCAGTT
TGCGAAATTGTGGTTCAACATATAGCAGTTCAGTGTTAACCATACTTTTTTCATTACTAAAATGTCCCCAAAT
AAACGCTATTAATTGACCTTCATTTTCATATATATAAATTTTATCATTTGTATGTTCTAATCTTGAACATATC
ATCTCGTACCTAAGAGCAATACTCAGTTTAGTACATTTATAGTTGGATTCTACTTGCGACAATAATGTCTCAT
GTATATTAGCTATTTGCTTGATATAATTATGTTCATCTTTATTAAGTGTCCTCATTTTCCTCTCCAATATTAA
TCGTTATATTTATGCGTTTAATATGATATGATTGACCTTATTAATACTATAAAATCATTTCCATTTTAATTTG
GAATTAAACATTTTTAAAGGAGCGCTTAATTAACAATGAAAAGTACTGCGCAATTAACAAAAGAAAATAATGT
TAAATCATTAAGATTAAGCAATACTGATAGAGAAATCTTCGAGAATTATATGACTTATATGCGCTCTGACTTT
CGCGTCAATCCTCATGATACAGAGTTAATTATTAACCGTATACTTAAACAATTATTAAGCGCAGAACAACATG
GTTTATTAGCCTTAGATTTTTTCAATCACGATCCAAAAGCACATGCAATAAAAGAACTAAAAGCGATGCCTAA
TGAAACATTTAAAAATATTTTTAAATATATATATCAGCACATCGTTCTATTAATTGGGATTGTTAGTTTTCTC
AAAGGATTTTTAGGATTTTTCATGGAAAAAAGTGGAAGTAATTTATATTTTGTTTCGTTCCCGTTTTCAGTAG
TCGTCGGATTCTTTATTGTCTTTTTATTTATATGGTTTAGTTTTAAAACAATACAATTGCAGTGTTTTAACAA
TTCAAATTGGATATGGATATTCACCTATTTAGCAATCATTCTACTAATTGTAGGATTTTTCTATGTATTCTTT
ATTCCACAATCATTACTTGCCTTTGGACCATATATTCAGGTAAGCAATTGGGTATTTATTATATTTTCATTTA
TTGTTATGCCTATTGGATTACGCATTGAAAGAAATATTTCGAAAAAACATTCTCATACATTTTTATAAACTAA
AAGCTTATTACATCATATGCGAATTTGGTACTGTTCATGATAAAATCACAAAATTACAGTTACCGAAAAACAT
ATTTTGCAATAAGCTTTTTATTAATTAATCAATAAAGCATCTATAAAGAACCATACAATCATTATAAACATAA
TGATTGCTTGAGCTATACTACTTGTTAAAAATGCTACGATTGAACCTATACTTACTTTAACTGCTCTTTCAAA
TGAATAGCCTTGTATTAATTCAACTATGAATACCAAAATAAAAGGTATAATAATAATTCCAAATGGCGGTAAA
ACAAAACATCCAATAACCACACCAATTAAAGCTGCATACTCTCCAAACTTAGAACCACCGAAGCGATTCACAA
AATATTTATTAGCTAAAAAGTCCGCACATAAAATTAATATTGTTAGCAATGCCATAGATACATAAAACACCCA
TGATAAATGCTGATTATGAAAGCCAAATTGATAGATTAGAAAGCCAACCCATAATACTAAAACAGAAGGAATA
ATCGGCTTAATCAACCCAACAAATGCTAACATGAAGGCAGCAATGATAAGTAGCCATAAAATAATTGTCATGT
TGATATCACATCCTCTTTTGTAATTTTTTGATTTTTAGTAAAGAATATAAGTATCATACCTAACATGATTGAT
GCTGCTGATACAAAAAATACATTTTCCAAACCTACCCAGTGACTCATAGCACCACCTAATAAGTTACCGCCTA
ACTGTCCAATAACCATTGCATTAGAAAATAGTGTTGAGGCGTATCCTGGGAAATCTGGTAAGATATCTTGGAA
ATAACTAATACCAATTCCTAAAAGAACCGCTAAGAAAATAGCTAAAAACACCTGTCCTGCTAACATCATATAG
AAGTTTTTAAATACCCCAATGCTGAAGTAGAATAAACCACCAAATATCGCTCCATAAATCAACAATGTTCGAG
TCTGTAATCTAGATGATAAAACGCCAAGAATGATCATAAATGGCACTTCTAAACCAGCACATAAACTAGCTAA
ATAACCGACATGTTGCTCATTTTCTTTTAAATAATCAGTAACAAATAAAGGCATATTCATCGTATACATCCAT
TGTCCAATGTGTAATAAAATAAATGCAATAAATGGTAATAAAAGTGTTTTGTCTTTAAACATATTAGGAGCAA
TTTTTTCAACATGTTGTTGCGTACTAATAGGGTGTTTAATGTTTAAATCCTTATAGAAAAACACTTGAAGTAC
TAAAGTAAATAAAATGATACTTATTGTTCCACCAAACAATCCAGCATAGCCTTTTAATCCGATTAATTGGGCA
CCAATAAATGGACCAAATAAAAAGCCCAATGAGAACATTGAACGTAATACTGTGTTGGCAAATTGAGCTCTAT
CTTTAGAGCTTGAAACATTGATAGATTCTCTAGCAGATGCGTAAAGTTGGGGCATTGCTGGTGCAAATAATCC
TTGGAAAATCGCATATAGTAATATGAATAACCAGATTGTATCTACAAAAAAGTATATTGAAAAACCAAGCGCA
CCCATTAATAATGCGAGAATAATAATAATTTTTCTATTAAAGTGATGCGTATCCGAAAATCTAGCAATAATTG
AATTTACTGTAAACTGGCTAATCGCTGCAGATGCTAGAAGTAATCCATACTGATTTGTTGTCATACCTAAATC
TTTAGTTGCAAAAAGAACAAGATATGGGACCGTAACCGCAATACCCATACCTAGTAGAAACATATTAGCAACA
AAGAGTTTATAATTCTTTATTTGTAATAACGCTGCAAACATATCCATAACCCCACTTCTTAGAGCCCCTTTTA
ATTTATTAATTAGGGGCTCTTATGCAGTTGGTGCGTTAGCAATCAACTGTAATCCTTTATCCCCTTTTAATTT
ATTAATTAGGGGCTCTTTTGCTGTTGGTGCGTTAGCAATCAACTGTAATCCTTTATCCCCTTTTAATTTATTA
ATTAGGGGCTCTTTTGCTGTTGGTGCGTTAGCAATCAACTGTAATCCTTCGTCCCCTTTTAATTTATTAATTA
GGGGCTCTTATGCAGTTGGTGCATTAGCAACCAACTGTATTCCTTTGTCCCCTTTTAATTTATTAATTAGGGG
CTCTTTTGCTGTTGGTGCATTAGCAAACAACTGCGTTCAACTTAACCGAATAGTTTAAAATTAAATACTAACC
TTAAATTAGTTTAAGACTACGCCTTTGGTTGTTCAACAAAGCTCGCCATGAGATTTACAAACGAATCAACTTG
TGGCAATTGCAACATGCTCGGATCATAACTCATAAATGTCGAACGAATCAGCGGTTCATTATCAATTTCTACT
TTTTCAAACTCAAATTGTTCTTTGCTGATATTTTTCATCATAATTTCTGGCAAGATTGTAACACCTACACCAC
TAATCAACATTTCTTTGCAAGTTGCTACTTGATCCACTGTAATAGTTGCATGGTAATCTTGTTCTAAATTATC
GTTATACCATTGTTTTATTTGATTTATATAAATCGGGTCAGCTTGAAACTCTATAAATGGTAACTTTGTAACA

TCATCTCGTCTATTTTTTGGAAAAATAAAATAATGATCATCATTAAATAAGTGTGTGTTAGCTAAATTCATTA
CCTTATTTCCACGAGTTATCATAACATGATAATCTCTATGATTTGCTTTAATTTGTTCAGTTGAACCAACTTG
CACTTGTATTTCAACATTAGGAAATTGGGCATTATATAGGCTCAAAACTTCAGGAAGTAAGGTTTGTCCAATC
AAAGAAGAACACCCGATTGATATTGTTCCATTCACTTCACCAATATGTGCCTGCATTTTGTCAAAAAATAATC
GCTCTCTTTTCAACATGTCACGGGCATGCTCAATAATCATTGTTCCTTCAGTTGTTGTAATCAATTGTTTTTT
TGTTCTGATAAAAATATCTACTCCAAAAGCATTTTCAATAGCTTTTAGTCTTTGTGTAACAGCAGGTTGAGAT
ATATATAAAATTTCAGCCGCTTTACGTAACGTTTTCGTTTCGTCTAATGTTATTAGTAAACGATAGTCTTCAA
TCTTCATAATTTCCCCCCCATAAATTATTCAATTATTGAACTTTCATGACTACGAGCATTCAAGAGTTCATTA
CTAACGAATAATTTTCACCAATTTTATTGGTATGGCTGCAGCTTGAATTACTTAGTTTTCTTTTGTTGTTGGT
GATTTTTAGTTTGATTATATTGCTTAGGTTTTATTTGTTTGCTTTTTTCAAAATTAGTTTTATTTTGTGGCTT
TTGATGATTTTTTTGAGCCTTTGCATTAATTTTATTAAAGCAGTACATGATTTTCTTTTGGAATCCTTTAAAA
TCATTTTCTAACTCTGCCATAATTTGATGTGCAATCATATATGCTTCATGAAATTGCTTTTTTGTAATTTGCT
CACTTTCTAATGCAAACATTAAATCATCTTCATCTACCAACTCATATTCACCACTTGGTAATGCTAAAACATC
TAAACATAAATCTACTGTACGCGCATTGCCTTTTTGCGTTATATTTTTGATATTTATATCAAAATAATATTCT
AATGGATTGCCTTTATTATCAAACATTACAGTTATACTATAACGTTTCTTTTCAGGTAGTATTTGCAACCATT
TATAATTGTCATCTGCGACAATGATATTTTGTCCAACTACCGTCACCTCTAAAGGATCACGAACCTTTTTCAT
CGTGACTAAGCCTATAATACCCTTAAACCTATTATTATTAACTTTTACCTCTGTGTATTCCCTATCAATTAGG
CGACGCCAGTGACGTTTATCTATATACTTTACTTTCACAGTCACCAACTCCTTGTCATTATTATATAAAACTA
CATAAATGATGTCATGTCATGATACAGTTTTATAGTTTTTGTTAATCATATGAAAATATTTATTATTATTTTA
CTACAACTCGCTTCAATTTACTTAAAATAGACAATATTAATTAGATAGTACACACATTTCTTCATAAAAGTGA
TTTTTCAAAAATATAAATAACACACTCTTATCGTTTTCAAAATCATTTAATGCTATTTTCATTAAAAACATCT
GAAGCATCAAATCTATTCTGATTCAATCAAGAATACATATAAAAATGAAGTGACTCAAAGGTTTATTAACAAC
CTTCAAACCACTTCATTGATTCTTTTTTATTTTAAAGCATATTTTTCGATTACTGATTTAAGATGCGGATATT
GATCAAATAATTCAGATTGCTTAAACAATTCAAATTGCTTAAACTCAAATGCCGGTTGACACATACAACCTAC
TAAACTAAAAGTATTTGAATTTTCAATTGAAGAAGCAAAAATTGTTCCTTTAGGCACTACATATTGCAATACA
TCTCCATTTTGGATATCAGTACCCAATGTTGCAGTCATATATTCTCCATCCGGATTTATCATATGAATTGTTA
GAGAATCACCGGCATGATAGTACCATACTTCATCAGCATCAATTCGATGAAAATGCGAAATATTGTCATCTGT
AAGTAAAAAATAAATACTGCTAAACGGCGCTCTGTGTCCATCTTTTAATACTTCTCGGATTGTCTCTCTATAA
AAACCACCTTCAGGATGCGATTCGAGTTGTAATTCATTAATCCATTGTTCTGCTGTTTTCATTATTTCAAATC
CACATTATGGAATACGTTTTGTACATCTTCTAAATCTTCTAATGCATCGATTAATTTTTCAAATGTTACTTGG
TCCGCTTCAGAAAGCTCAATATCTGTTTGAGGTAACATTTCAAATTCTGCTACTTTAAATTCTTCTACACCAG
CTGCACGAAGCGCATCTTGAACAACCGCAAATTGATCTGGTTCAGCATAGACTATTGTCAATCCATTGTCGTC
AATCACATCATTTACATCTACATCTTGTTCCATTAATGTTTCAAGTATATCGTCAACAGATTTACCTTCAATA
CCAAACGTTGCCACATGGTCAAACATATAAGCAACTGATCCAGATACACCCATATTACCGCCGTTTTTACCAA
AAGCAGCACGCACATCAGATGCAGTACGATTTACATTGTTTGTTAACGCATCAACAATAAGCATTGATCCGCT
TGGGCCAAATCCTTCATATCTTAGGTGATCAAAGTTTTCGTCTCCAGCACCCTTAGCTTTATCTATTGCTTTT
TCAATAATATGATTCGGTACTGAATATGTCTTAGCGCGTTCAAGTACCAATCTTAAAGCTTGGTTAGATTCTG
GGTTTGGTTCACCAGATTTTGCTGCAACATAAATCTCCTTACCAAATTTCGCATATATTCTACTTGTATTTTT
ATCCTTTTGGGCCTTTTTTTCTTTAATGTTATTCCATTTACGTCCCATAATGTCATCTCGCTTTCGAACTGTT
TAATTAACAACATATATTATACATGATTTTTTTATGATTATATACATTTTCACAATTAACATTATTGAACTAC
GATTTCTTCAATCTGTTACTTAGTTTTAAAATATGTATAAATTAAAAATGTGCCGATAAGCAATTTCTATGCT
TTCGACACTCAATTTCAATTAAATATTTAAAAATTGTTCTACATCTTCAAATAAAGCATTAAATTTAGATATA
TCATCATCATTTAAATAAATATAAATTTTACGCTCATCTTTAGATGATCTAACTTTACTAAGTCTTTCTTTTT
CAATTAGTCTTCTTATAGAAACTAATAATTTTGTCCTACTTAAATCTAGCTCATCCATTGCAGTTTTTAAAAA
TGATTGCATAAGTACTTTTTCATCTTTGCAATACTTCATCGTTAAATCTAAGACTGCTAAATCATTTAGCGAC
AACTTATATTTTTTATCAATATATTCAGATAATGCTTTATATTGTTTATAGAAGCCAAGCAATGTTTCTAATT
TCTCAGTATTCAAAATGTTGCACCCCCAAATATATAAAGACAATGCTTCTGTCATTATTATCTTAAATTTTTC
TGCCATTGTCTTTCTCATTTGTTTTTAATACGTTGTCAAAATTACCAACAAATGCAAATTAAATTTTGCAATA
CATATCAAGGTGTTATTTTTTTCCTATTTTAATTATGTATAACGCATTAAAAGTAACGTTAATGTTTAAGGAA
TTATTACTAAAGTTATGCTCTTTAATCTCGATTGCTGGTTTAAGGTAGTTGTCAATTTTAGAGAGTAAACTGG
GGTTCCATTCATATTTATCTCTTAATTCTTTAGAAATCAAATGATTAATGTTCCCACTATTTTCATCTAACGT
TTCTATTACAATTAGATATTTATCGTTTAAAACATTATTTTTGAAATTTATATAAACTTGACTATCCTCAAAA
TTGCTCATAATTGTCTCGCTCTCAATCACTCGCCAATCATAAATAACCAGGCAATGATAATTCTCTATATCAA
AAATAGAATAATAACTGTTTTCGTATTTTGGTTTCGAGACATATTCGATAAACTTTTCTAATATAAGACCTAG
TGTTGTTTTAAATCCATTTTTATCATATAGATGCATCGCATTAAATGTTTGATGATTATCAAAAATGTTTAAT
CCAAAACCATCAAAGTTATGATACAGCGCACTTAATGTCTTATAAATTAATGGAGCACTATTACTTAAATCTC
GTTCTTTATTATTAAGCAGTTTATGATTTTCAACATTTAGAAAAATCAGCTTTTCTTGTTCTATATCCAATTC
GTTAATAACTTTTTTTAAATTTTTAAAATGCAAAATATTTTTCAATAGTAACTCATCACTTTCCCCCATCAAA

TAAGGGCTACTTAAATTTGCATTATCTATTATTAACGAATTGAATTTAAGTCGCTTTATTTGTGATACTAGTG
ATTTAAAAACTGAAGGTTTATCAAATAAACAGGTAATATTCAATCCTAATTTCACATTTAACATGATGTTATG
TAGTTTTAAAATCATACGGTAAATTGTCCTAATTTCCATCAATGATAAATCAAAAACAAAATGCACTTCGTAA
TTATTATGATGATTTATTTCGTTTGGAGATAATTGTCTAATAACATTCATAAAAGCTTCTTCAACATAATAAG
TCGTTTCAATATCATTTATAAAAGCTTCTTCAACATAATAAGTCGTTTCAATATCATTTATATTAAATGCTAT
CTTTAAATCACTTTCAATTATGGTTTTAATGATTGTAAACTTTTCTTCTTCGGACATGGTATTTTTACTTTTT
AAAATCGATGGCATGATGAAAAAGACAACTTGGCTGTTCTTGTTCAATAAGGTTTTAAAAATTCCTTCTAAGA
AAACAAGTTTCATTTCTGTATAAGTATTAATTTGAACAACCGTTTGAAATAACTTAGGTATTTCAAATTTGTC
TTCGTCAATATGTATAACATCAGTTAGGTGATTTTTATAATGATCAATATAATTCAATATGACTTCTTTTACT
TCTTGGGCCATACCGCCTACAAGTGGCTCTGGATTAAGCATTATACATTTATTAAATTTTGATAAATTACGAT
ATTCATTCGGTGTTATATCCATATAACTTTTAAACATTTTAGAGTACGTTGAGCTGCTGCTAAACCCTAAATG
TTCACTTATATTGCTAATAGTACTATCTGTAGTAAGTAGAATCTCTATGGATTTACCAATTTTCAAAGTATCA
ATATATTTTTTAAAACCCATGCCCATAAGTAAGTGGAATTGCGAAGACAGATTTGACTTTGAAACAAATAATT
TATCAGCGACACTTTTTAATGTCAATCGTTGATTTACATTTTGATAAATATATTCGAGCTCATCTCGCAAATC
ACCATAATAAGCATAACGATATTGATTTTGCGCAATCTTTTTATCCATACTTGCTTCTTTTGCAATGATTTTA
ATAATATCCTGTAATTTTCTAATATCAGCGTCATTAAGAGGCTGATTATCAAGGTATCGATATGTCAATTTTA
ATAATAAGCATTTCAAATTATAGGATGATTTAATTAACTTTGCTGTATAGTGATAATTAAAGAAAGTAAAGCC
TAATTCCGCAAACCAATCGCTACTCAAATACAACATGATCGTTTTTTTATTATTCTTAATACTAAATACATCC
AGATGATTAATGATATATATATCATCTTCAATTATTTTACTTTTACGAAATTTTTGTAGTTCGATTTCCCCGT
CAATTGGCCAAAATAAGACTATGCCATCTTGGCAACGCGTTGTCGCATATTCATTATTAGTAAGTATATGCAA
GCATGATTTTGCCATAACTTTACCTCTTTCTATATTTAAATACCACTTTTATCATTCCCTATAATATTTAACT
TATTTAATTAAACGTAAATTTAACACAATACGAATACCAAATCAAAAATAAATAAAACTATATAATGGTTATT
TTTTAAACTTATATATCAATCATTTAAACATTACAAAAATAGATGTAGTCAGTTTAATTAAACTATCTAATTG
AAAACGCACTACTTTTTAGCATTTTCAAAATAATTTTAAATGACCACATCTACAACGTATTACTATTATCTTT
TGTTAGCCTTACATTCATTTTCAATTTAAAAATAAACACTTATTTAAATCACATTCTACTTTTGAGATGAGCT
CAAACTAACATTAAATTGTTTATATTATCTATACTTATTCCTATTAATATTTCGATTTTAAAATTAATACGTA
GCTATACTTTACTCTTCTAATAAAAGTGTAATTAGAAAGGCAAGTATACCTGTTTAAAGTTTATCTTCTAAGA
TTTGTTTACTTTAGAACTATAGCAGTTAATCATTTCAATGATTAATAATATCTTTACAATTAAGGTAATATAT
TTAAATTACCCTATCTAAATTTCTAATTTATGACAATCGCTCCAATTTAAGGTTGATATTCATTGTTGATTAT
TCCAAAACTTTATACAAATTTCTTTATTAATTTAAATGTTTATCTTCTGAACATATTAGCAAGATAAAGTATA
TCCTTAATAATATTCAACAAATTAATAAATAGATTAAATCCCATTTCTCTTGGTGAATAGTCACCTCTTTTTA
AACGATTAAAATCATACAAAGTATAAAGTAGAAATAACAACAGACTAACGACGGTAATAACAGTATAAAAAAT
AGGATTATGCAGAAAAATACCAATTAGACTCGCAATAATTAAAGTTATTAATGTAACGAATAAATACTTACCT
ATACTTGATGCATCTCCAACGAAGAAATAACCAATAATACCAAATGCTATAAATGCAAAAATTGCTAATGCGA
TATTTTTATAGAAAATATCTGGTCCTAAATTTTGTAAATACGTGGTAAACGTTGCGTATGACAATAAGCCAAT
TACTACAGCGTAAATATGTGAAATGATTAAACCGAACCGTCTCGCTCTCTCAAATACAAGTGTTGCTAAAATA
ATAATCAGTAATCCAAATGACAAGGGTTGTCGCCAACTTAATGGTAAAAACTGACCTAGATAGCATCCTATGC
CAAATATAATCCAGTAATACATAAAATAAAGCCACACTTTACTTATTGATTGAGCATGCTGGTTTTGCTGAAA
ATATGAATGATTTGTATTTTGCGACAAAATAATTCCTCCTTCTACTGTCGTTTTATGCGTCAAATTTTACAGT
AAATTTTGCTTCATCAAAAGAAATAACCTTTAACAAGTACAGTAATTTTACATTTTACAATGTTACAAAATAT
AAATTTTTATAATTAGTTAAAATCACTAAAACGCTTTTATACACTATCAAATCAGCATTTATAAAAATATGAA
CCGATATCCTAAAATGTTAATAATATTACAAGATAATAACAAACCACACAAAGCTACTTATTTTTGATAATAT
GGAAAGCGTAATATAAAACGAAAACTTAATTTACTATATAAATTGTCTTAATAATTTTTAAAAGTAGTAAATC
TTAATTTTAAGGAGGAGTCCCTTTGAAAAAATTAGCATTTGCAATAACAGCAACATCTGGTGCAGCTGCATTT
TTAACGCATCATGATGCACAAGCTTCTACACAACATACAGTACAATCTGGTGAATCATTATGGAGTATTGCTC
AAAAATACAACACTTCAGTAGAGAGTATTAAACAAAATAACCAATTAGATAACAACTTGGTATTCCCTGGTCA
AGTTATCTCAGTAGGTGGAAGTGATGCACAAAATACGTCAAACACTTCTCCACAAGCTGGTTCAGCATCATCT
CATACTGTACAAGCTGGTGAATCATTAAATATCATTGCTAGCAGATATGGTGTTTCAGTTGATCAATTAATGG
CAGCCAATAACTTACGTGGTTATTTAATTATGCCTAACCAAACATTACAAATTCCTAATGGTGGATCAGGTGG
TACAACACCAACAGCTACAACAGGTAGCAATGGCAATGCATCATCTTTTAATCACCAAAATTTATACACTGCT
GGTCAATGTACATGGTACGTGTTTGACCGTCGTGCTCAAGCTGGTAGTCCAATTAGCACATATTGGTCAGACG
CTAAGTATTGGGCTGGTAACGCAGCTAATGATGGTTACCAAGTAAACAACACACCATCAGTTGGTTCAATTAT
GCAAAGCACACCTGGCCCATATGGTCATGTTGCTTATGTTGAACGTGTCAATGGTGATGGTAGTATCTTGATT
TCTGAAATGAATTACACATATGGTCCATACAATATGAACTACCGTACAATTCCAGCTTCAGAAGTTTCTAGCT
ATGCATTCATCCATTAATTAAATAAATTGTACTGATATATACTAGCAATTCACATCATGTGAGATTGCTAGTT
TTTTATTTTTGAAAAAAATTTTCATTTTGGTACAAAAAATTATCTCACCCTTCCCTATCATACTTATTTATAT
TTTGTATGAATGGTAGTTAGGTAAAAAATAATAACCTACCTATTTGATTAAATTAAATGCATAATATATTAAA
AATCAAAATAATGCAACTAAGTTGACTATATAATGATTTAATTTGTTATACCAGTATTTTACGCTTTTTCGTC

TACATATACAAATTTATATTAAATAAAGTCCAATACAATTTAGGTTAATCAAACAAGTTGATAGCTATTTAAT
TATTCATTCATTGCAGAATATAAACTATTAAATCATTATTTTACGCTTACTTATATTTTAATGACCTAACTGT
TTATGTTCCATGGAATACATTTATAACTTAGCCCCTAATTAAAATGCTTTGTCTTGGTCATTCTATGTAAATT
CTATAAAATATGTTATCTATTTACATAAAAAACTGAACTTCAATACCACCACATTTTTGTGATACTGAAGTTC
AGTTTAGTTTTATTTTCAATTAGAAAAATAAGTTAAGTATATAGAATAGTAAACCTGCTAACAATGCTGAAAT
AGGTAATGTAATCACCCATGTAATGATCATTCGTTGCGCAGTACTCCATTTTACACCTTTAGCTCGGTTAGAA
GCACCAACACCTAAGATTGATGATGACACAACGTGAGTTGTTGATAATGGGAAATGTAGCGATGATGCAACAA
AAATTGTTAATGCAGATGATAAATCGGCCGCAGCACCATTTGCTGGACGTATTTTCATAATATTACCACCTAC
AGTTTTGATAATTTTCCAGCCACCAATTGCAGTACCAAGCCCCATTGCTGTCGCACAGGCAAATTTTACCCAT
AACTGTGGTTCAACGCTGCCATCATTCTGTACATTAGCAACAATCAATGCCAACGTAATAATACCCATTGATT
TTTGCGCATCATTCGTACCGTGAGAGAATGATTGTAACGCTGCTGTGAAAATTTGGAAAAATCTAAAGTTACG
ATTCGCTCTTGTTAAATTTGCATTTTTAAAGATAACTTTAAAAATTGAATACATCAAGAAACCAACACAAAAT
GCGATAATCGGTGAAACGATTAATACAATAATAATTTTTGTGAAACCTTGGTAATGTAACACTCCAAATGAGC
CTTCAGATGCGATTGCTGCTCCCGCAATTGAACCTATAAGTGCATGTGAAGACGAACTTGGAATTCCGTAAAA
CCAAGTAGCTAAATTCCAAACAATAGCCGCAAGTATTGCAGCTAACACAACAACTAAGCCATTTTCCAATTTA
AATGGATCGACAATGTCTTTAGTAATGGTGCCTGCAACGCCCGTAAATGTTAAAGCACCTATAAAGTTCATCA
CTGCTGCCATTAAAATTGCCGTTTTAGGCGTTAACGCTCTAGTAGATACAGCAGTAGCTACTGCATTGGCTGT
ATCATGGAATCCATTGATAAAGTCAAATATCAGCGAGAAAATAACTACAGCTATAGTGACGATGATTATATAT
GACATAAATATAAACTCCCCTTAGCTATTTTTCATAATAATAGTTTCAAAATTATTTGCTACGATTTGACATT
TATCAGCGATTTCTTCCATGCTTTCATAAATATCTTTTATTTTAATTAAAGTGATTGGATCTGTTTCGCTATT
GAAAATATGTTTAATTGACTGTCTTAAAATACCATCACAGTTTGTTTCAAATTCTTTAATATTAATTGAATGA
ATACGCATATGTGATAATTTTTTATCGACTAATAAGCCGACAGCAAGTTTCATTTCTGCAACTGCTTTTTGAA
TGTTATCAACAAACTCAGCCATATATTCATCTGTGTATTCGATTGAATACATTTCAAACATAGCTGCCGTTTC
TTCAATTGCATCTAAAACATCATCAATTGCATCACATAATGATAAAATATCTTCACGTTCAATTGGTGTGATA
AATGTTTGATTTAAATCAGTAATTACTTGATGTACTAATTCGTCACCATGTGACTCATAAGTTTTAATGTTGT
CTGAGTATGCTTTTAAATCTAAATGAGTATTGAAATCCATTTTACCGAATTCAATAGCAGCACGATCCAGATT
GAAAACCATCTCTTCTAATTGAACCATAAACTTATCTTTTTTCTTACTAAACATTTAAAATCCTCCATTTAAG
CGATTGTCACCAATCACATTCAGTTATAATTTGTTTCAAATTAAGACAAGTGAATTTACAAACTAATGATACA
AATTTGTTATTATCAATCGTCAGTATAATTTAAGTGTACTGATATTAATTTCAAAAATGCCTCACAGTAAACA
ATTTACTGTATTTGCCCTTATATGAAAATAGCACAAAATTTACAATCCCACAATTCAATTTACAAATTTTTAA
CAATTCCGAAACTTAACTTTTACATAAAATTTAAATAGTGGTAGGCATTTGTTTTGAAAAACTTGATATATAG
GCAATTTGTTATCTACAAAACTCTAATAATTGTAAATGGACATAGTTGGAAATTAAACAACGATGCAATATTT
ATTACATATCTTTTACATATTAAAACTCACTTATCTTTCTTCTAACTATTCTTATTTCATATTTCTTTCAATG
CCTATGGCTCTATTTTCTCACATTTAATACAATTTACAAAGTTGAAACATATACATTAAAACTGTCGTGCCCG
GATTTATAACTATCCAAAAATAAAAGCTTTCAGTATAGCTCATGAATCTTAATTCACTAGCCTACTGAAAGCT
GCTATATATATTAAATTGAGTGTCTGATTGTACGCTTAGAATGATTATAAGCTGTTATTGCAAAAATGGCATA
CATACAAATATATAATCCCATTACTATAAATATCGGTATTTGATTCGTTGTCCCCATCAATTTCATATATGCT
AATGATGTAAAATATGCATGTGAAAGTGCAATGACTAATGGCAATCCAAAATTAAACGCAATTTTAAATTTTA
ATCCTTTTGCCATATCTTTTTGTGTAAATCCAAGTTTTCTAAGAATACTATAATTTTCTAACTCGTCTTCAGT
TTCATCTATTTGTTTAATGTATATGATACAACATACGGCAATTAGGAAACTAATTCCTAAAAATGAGGTTACA
AATAACAATATTCCCGTTAAACTCGATATCGAACTTATGGCTTCACTTCTTGTTTCAATAGATTTGTCAACTT
TATTTGTTGCTTTTTCCAAAGCTAACATATCTTTTTTATGTTTTAAATCAAATCCAAATTGAGAAACAATGTG
TTTTGGCTTAGTATACTTTCTTATCTCTTGATAATCTTGATCGTTTAACACAAATGTCGGTCCACCTAAGTCA
ACATCAGTCATAAAGTAAATTTTATTGATATCTTTACGTAATTTAATATCCACATGGTGCTTTTTCGTTCCTA
TCACTGCCTTACCATGTTTCTTATGCTTCACTAAATCTTTAATAGAGCCTTCCGCTACAAATAAATCAGCCTG
ACCACGTTTCAAATCCGTATTAGGAATATATTTATCACTAGTAATTGTTACATTATATGGTTCCTTTGCTTTT
ATATCAAATAAGTCATCCTTATAAAGTTTTGTATGAATCACTTCTTTATAATTATAAAAATGAGGAATTTTTT
GGTTGTTCAATTCACTTGCTAACTGATTCGCCTTCTGTTGATCTTTAATCGTAACATCATGAGGTGACGTGTA
CTTAATTTCACTTGATAAAGTTGCTCTACTGATAGCAGCAAAACAAAGTACTGATAAAGTAATAGCTGAAATA
ATTGCCATAACAGTAAGTGAAAATGCATTTTTCTGAATACGATACATAATAGATGAAGAAAACATGACATCAT
TTACACTTATTACACCTTTTTTAAACCTTTTAATAATTTTAAATACCAGGGATACTGAACTTTTAAAAAATAA
ATACGCCCCAATTACTGTCGCAAACAATATAAACATAAGGACGCCAAATGCATCATAGTAATGTACAATGCGT
AAAGAGTAGTAATACCCACTGATGATTAAAATAATACCTAGAATTCCTAGTATAACTTCTTCAAATGTAATTT
TATTATGATTCATTTGCTTAGCCTGATTATGCTTTGATAGTTCTGAAATAGATTGTTTCAATACTAAGATAAG
ATTTTGAGCTGCAGTTAGGAAATAAGCAACGCCAATTAATAATAGCGTCTCAAACACTGCTTTAAAGCTGAAA
ATAATTGGAACCTTTTCTTTGATACCTAATAAAGTAAACACTATCATTAATAAAATTTTAGAACCAAAGATAC
CAATCGTAATGCCCAATATAGCTGTTATTGAAAATATTAATAATTGTTCAAACATCAATATATATATATAATGTT
GTATTTGGATAAACCTAATGTTTGATATAATGCAAGTTCAGAACTACGACGTTTAAGAAATAGTAAATTGGCA

TATAGCAAAAAGGCAATAATGATAAAAAATAGAAAGTAACTACCTACTTGCGATCCTTCTTTAATAATGGGAT
ATGTCTCAGTTATGTTAATGCTATGAGTATATTTTAGTGCTACGAAGCTAAAAAACAAAACTACACTTGTAAT
TAGCGAAAAAAGATAAATGGCATAATGTGATAAATTTTGGCGGAAATTTTTAAATATAATCTCATTAAAACTC
ATAATTAATGCCACCTAAAACACTTTGTACACGTATGATTTCTTTAAAGAAAGTATGTTTGTCATCATCACCT
TGATATAACTCCGTGAATATTTGCCCATCTTTAAGCATCACTACTCGATTTGCATAGCTAGCTGCAACCGGAT
CATGTGTCACCATAATAATCGTCGTCTTAATCTCTTCATTCATTTTCATTAATCGCTTTAATAAATCTTGTGT
ACTTTTCGAATCGAGTGCACCTGTAGGTTCATCGGCAAATATAATAGAAGGCAATGTTATAAATGCTCTGGCA
GCTGATGTTCGTTGTCTTTGACCACCAGACAATTCGGATGGATATTTATCACTGATGTCTAATATATTTAATG
CTTCTGCCACTTTTTCATAACGATCTAACATTGTGTCCTTATCTAATTTCTGAACCGTTAATGGTAACATGAT
ATTTTCTTTAACAGTCAATGTATGCAGTAAATTATATTCTTGAAAAATAAAACCAATATCATGCTTTCGAATA
TCAGATAATGCTTTGTTTGAAAGCTTTTCAAGTTTGTGTCCTTTTAGTGTGATGGAACCTTGCGAAATATAAT
CAATTGAACTCAAAACATTTAATAAGGTCGTTTTACCAGATCCTGAAGGTCCCATAATAGCAATAAATTCACC
TTCTTCAACAGACAGATTGATATCTCGCAGAACTTCTTGTGCCATTTTCTTATTGCCATATATTTTTGTTAAT
TGTTTTACTTCTAATATTGTCACTTTAACACTCCTATATTTATCTTAACTTCATTTCCTTTATATTCAATTTG
GCACGCTTACCTTCAATTTTGTATTTAATTAACATCTATTTGATTATATCGTTGAAGTGGTCATGATGTATCA
ATTCTAAGTAACAAAACGCATGTTTAAAATGACAGATTTGTCACTTCTGACATGCGCTCAACAATTTCATTTT
GTAATGGGAAGATCAATTTAACCGTTGTTCCTTTTCCAATAGTCGAAGTGACTTGTAATTGTATTCCTAATTG
ATCTTTCACACTATCCACTAAGTATAGACCCATGCCGGAGGATGTCGTTTCATTTCTGTTAGCCGTTGACGTA
AACCCACGTTCAAATATTCGAGGCATATCTTTTTTACTAATACCTCTTCCGTGATCCTTGATGATTAACGCAA
CATGTTGATCAATTAATTCCGTTGAAATATCAACATTATAATTCTCACTATATTTCAAAGCATTTGATAAAAT
CTGTCTAATAATCATACGACACCATTTTATATCCGTGTACACATGATTATCCACTTTAAAGTCAACATCAAAA
CCAATACCTTTAACCTGACTAATATGTCTTGTTAATTGTATTTCATCAATGACCATGCGTTTAAGTGACACGT
AATCAAAATACATATCTTTGCGTTGAGATTCTAATCTCGTAATATACAATTGCGTATCTAACATCGAGTTAAT
ACGTGACCATTCAAATAATAGCGCTTGTTTTCTTTCTTGATTCTCTTCTTGGTCAATTAATAATTTCATGGCT
GTCACAGGTGTTTTTATGTCGTGCACAAATTCTGTAATGGTTTGTTCATGCATGTTCAATTGTAACTGTTGGT
CGACCACTTTATCTTTATGCGCTAAAATTTGACGATATAAATAATCAACTGTATGACGTTGAAATGGCGTTTC
CGCTAAATCTTTATGTTTAATTTCTTCTATTTCTTTATCTTTGTCAAAATGCTTATATAATTTTACTTCTTTA
AAATATGTCAATATAAGAAAAATCATTGTTAAACTTAAATTCAAAGAAACAATATAAAATAAACTGTCTATTG
GAAAATCATAATCGATAAGACTAATGCCTAACATAAGCAAGTTTAAAAACAATATCCAAAATATCCAGTTCAT
GCGAGATTTCAAAAAATAAGCTACCCATTTCAAATTATTCATGAGCCATATATCCTTTTCCTACTTTTGTTTC
GATTGCACTATCCATACTAATTTCAGATAATTTTTTTCGTAAACGATTCACATTTACTGTTAACGTATTATCA
CTAACAAATGCTTCATCATCCCATAATGCAGTGATAATTGTATCTCTCGAAACGATTTGATTTTTTTTGGTAA
TAAGAATTTCTAAAATAATCATTTCTGTTTTAGACAGGAAAATCGTCTGATCACCTTTTTGTATACTGTCTTT
TGATAGATCAACGACAGCATCTTGCCAAGTCAATGTACGTTTTTCTTCAGCTGTAAACTCATAGACACGACGA
TAAATCGCTTGTAATTTAGCAATTAATACATTTGTATAAAAAGGCTTTTGCATATAATCATCTGCGCCAAGTT
CCATACTCATCACTTGATCCATCGGGTTGTCACGAGATGATAAGAATAATATTGGCACATTGGAAACTTCTCT
CATTTTTCTGCACCAATAAAACCCATCATATTTAGGTAATTGAACATCCAATATAACAATTTCAGGATTAAAA
CTTTCAAATGTATCCATTACTTTGCCGAAATCTTCAATACCAGCAACATTAAAATCCCATTGTTCTAATTCTT
TTTTCAATTCTTGAAACAAAGTATTGTCATCTTCTACTAGTAGTATTTGCATCCATATCACCCAATATCATTT
AGTATATTTCATATTTTCTCCTGATTTTAATCGACTTTGAAAATCTTTAATCCGGCAGTCAACTTCAAAGCCA
TGAATCATCATTTTTTTGCATTGGTGCTTGTATAAAGTAATAAATCGGCCAAAATAATCGAGGGATATAATCGT
ATAGATGTATATAAACGACTGCCGACTCTTTGATTAATCTAAATTCTAACTTCCCTTGATTAACTGTATATTT
TTTCACTAAACTTCCACTCAATAAAATTAAAGTTATTATTCCATCAGCTGTCTCTTCTATTTTAAATACTGCG
AGCGGTCGCACCTTATTCTTAATATATATCTTAAATTGATCATGTGATTTTTCTGTTTTCACAAAAGTTCCTT
TAGTATAACCTATCCATGCAATAAAATGGTTTACAACGTTCTTTAATGTCCATCCCTTTGGTAAAACTACCTT
CATCGTTGATCTAACATCATCATACTTTGAAACTTGTAATTCTACATTAACTAAAGAACGTTTAAAAACTAAA
TTTGTTGTTTCTACAGTTGTACCATATGCACCTAGGCGTTCAATTGTTTCATTATCATAACGACTCCCAGGTA
TGTAGATTACTTTTTTTACTTGATTGATAGCAGCTGCTCGACCAAAATTATCTGCTGCGATTAATGTTAAATC
TCTTGCTGATGATTGTGTTATTTTGGCAGAGTTCTTTGTTGGGTCGATAAAGAATACAGCAATATCTACTTGA
TTCATTGCTGCAACAACCTGTTCGTAATGAAATATATCACACTGAATCCAAGTCATTTCAACATCATCTGTTT
TTTTATTGTCTGGATATTTTGATATAGCAAAAAGTTCCGCATCATTTTCAATCACTTCACTTAAATACTTACC
AATATACCCTGTTCCACCTGCTAATAAAACTTTAGGTTTCATCTAAAATACTCCTTTAAACTGTAACCAAAAA
ACATATTACTCTACCTTTTGGTTAAATATATATTATAATAGTAGCAAATGTTTTAAAATTTCAAAATACTGGA
GGCTTTTTATGGCCCATATTATACGTAGAGTTAGTATCAAAGATGTAGAAAATTTCATTTCAATGTTAGCGAA
AATATACGACGAATCTCCGTATATGTTTTACACACCAGGAGAATATGATCCTAGCGTCACATCGGCTAGTAAA
CAATTAGAAGAATATATCACTTCTCCACATAAAGTCATCTTTGTTGCTGAAAGTGATGAACAACTCGTTGGCT
TTGCCTTTGTTAATACGACACCATTTCAACGCATTAAACATGTTGCTAAAATTGATTTAGGTGTAAAGAAATT
ATATCAACATCGTGGAATTGGCCAAGCACTTCTTGATGCCATTATGGCTTGGTGTTTAAACAATCAAATACAC

CGAATTGAAGCAAATGTACCACTCAATAACCAACCTGCCCTCGAGCTTTTTAAAAGTGCCGATTTTCAAATCG
AAGGCGTTTTAAAAGATAAGTTATTTATCGATGGTAAATATTACGATGACTATATGATGGCTAAAATTCTTAA
TTAAAGCTATTTTATCATAATCTTGTATCAGAATCGCATAACAACGAATTTAATGGTTACCTAATACATTACT
CATCCTTATCAATGTCATCTAATTTTAAATGAATGCGTATACTCTTATTCATTTATCAAATTTAAATTCAAAA
TAAAACACCACTAATGTGTAATTGATTAACTATCAACTACGGTTAGTGGTGCTTTATATATGTTGTTTAGTTT
TCCTTACTATAAAAATCTTCGAGTCTAAAATCTATATCCGGTAATTTTTCATATATCTTATCCTGATACTGAT
TGTTTTGATTGTAAGCTTCTTCAGTAATTAACCTACCTTTACGCTTAATTCCATAATCTTGTACGAATAAATT
ATTATTTCTATTTATTTTGTAATACGGTACAACATTCGCACTAAATTTTACTCGTTGATGAATAAGTTCCATT
GCCAATGCATAGTATTTATTTGTCTCATATAACCATAAATGATCTATTGATACACCTGAAACAAATACATCTT
TTAATAATATTCTTACATTCGGCTTAAATGTGCTATGTCTATCTAAATAATTTTTAAACAAAACACGTTGTAT
ACGTCCAGTAACCATAACGCGTTTATTTTTAAATGGTTTTAATGCTGACCTTGTCATTCCCTTTCACAACCTT
ATTTCAAATCACTTCGTGCTCAATCTATATTTTAATTTTTCTTTTGAATTCTTTTCAATTTATAAATTTATTA
TATCATGTACACACTGTTTTTCGACTATTTAGTTGTAAAAAGTTATTTTCAGGTTTAAAACAAATTAGTTTGT
AAAATAAATGTCACATCATAATAGAGTGAGATAGAAATGATAAAGAACCGCTAACAATTTATTATATCTCAGA
CTCTTACACATTAGCCCCACATGTTTTAAATTCTTCGTTTTCAATTTGAAGGTCTTAAATGTTGTTTAAGTAA
TAAATTACTACTGTTTATTATTAGTTTAAAGGATTTAACGGTAATTCATTACTCGGATTTTCTATTTGTGGTT
TCTCTTCAGTTTGAATACCACTAGAAGATGTATTACGACTTAAGAACAGTAACACTTTTTTGATATTATCTAT
CGATTCAGGTTTATTAAGGTGAAATTGATACTGATGATGTAAATGATGCGTACCATCATAAAACAAAGTATCT
ACTGGTACATTCAATTCTTGTAATTTCTTACTGAATTCTATATTTTGACTTTCGAATGGATCGCTATCTCCAA
CAGATAAAAATGTTGGTGGATAATTTTTTGTCGATTGTTTTACTGTCGACATTTGTGAAATGTTTTTAAAACT
CTTTTCCCAATCTTCTTCGCCAGTATAACTTTTCATAAATAACTGTATTCTTGGAAACTCAGTTTCTCTAACT
GTTTGCATATTATAAAAACCACCAAATAGTATAGCACCTTTAATTTGCGATGGTTTGAATGACTGATCAAATT
TCATGGCTTTTCTTAAGCGATCATTCGTCTGTATTGCCGTAAATTGACTAGCCAGTTGTGCGCCAGCAGAATC
ACCGCCAATAACTACTTGATTAAAATCAATAGGTAAATTCATTTTATTTTCTTTAATGAATTGAGTTGCTTGA
TTCATTTGAATTAATGGTGTGGGATATTTATATTGTGGCGCCAATGCATAATTTACATTCACAACAATGTACC
CTTGTTCAGCAATTTTCGCTAATAATGGGTTTTTATACTGCTTATCACCCGCAATATAACCACCACCGTGCAT
CCAAAAAATAACTGGCAGTTTGGCATTAGAAGACATATCAACAGGTGTAATAATATCTAATTTACTGTTAGGG
AAAACTCTATTGTATGTAAGATTTTGATAAATGCGCACATTTTTATTTTTAATATTAATTTTGTCTTTATTAG
CATGGTTCTCATTTGTAGTTTGGTCATAATGTTGTTTCAGATGAAATGCTAACAATACACTGATTACAGTTAT
AAAAAGCGCAGTTATCATCGTCCATTTATTATCTCTATTCATTACATCGTACCTCTCTATTTGTTAGACGCTC
ATATTAATAGACTGAATAAATATAAAAAGCAATTCGCCCAATAAGAGTAGAGACGAATTGCTTTTAAGGTTAA
AACAAAGTGATTCAATTGTAAAGTTTCGGATATCCATCATATGATATTAAGCAATATGTTTTTTATCCGCATT
TTTACGTTTGAAATATTTAATAATTACAGCTACTACAATAAATAAGCCTACATATCCCATAATTGTATATAAC
TTATTAATTAATTCAGCAAATCCTACGAAACTTAATAAATAACCTGCTACCATCATTATAATAATAAAGATAT
GATAATTTTTACTGTATGGTTCTGTAAAACGTGCTGCAAATGAATACATTAGTCCTAGAATAGTATTATACAT
AACCGCAAGCATAATAACAGATAACACGGTTGCTATTAACGGATGAATGTTATTGGCTAATGTTAATGTTGGA
ATTGATGCATTTTTAATTGTAGGATATTCACTTTGTAAAGCAAAGTTGATCAATGCTAGTAATACAGTATAGA
TAATACCACCATACATCGCGCCTGCACCTGACACTGTACGTTTTTCAGTATCCCCACCGATTGCTACGATGGT
ACTAAAACCTACAGAAAAAGCTAATCCACCATATATTGTACCAAACCAGATTCCCTTCCAGATACTTGCTTCA
GGCACTACTTGGTTAACTTCTGCTAATGAAACATGACCTTTAAATAAATAAACGCCAGCGATTAATACAACTA
AAACAATTAAAAATGGTGTAACAACACCAAGGGCACGTACTATCTTATTAAAGTCTAATAGCAATGTTACATA
AATTGCTAATGTCATAATTAATGCACCTAGCCATGTAGGTATGTTATAACTTTCTTCAAATGTTGCGCCTGCA
CCAGCTATCATGGTTACAGCTATACCAAATAAGAAGAAGATTAAAATATAATCAAAGACTTTACTAAACTTTT
CACCGAATACGTATTGTAATGTTGATTCATGGTTTGTCGCTTCAAAGGCAGTACCAATTTTTGCTACTTGGCG
CCCAATAAAAGCTAAAATAAAACCAGATATAATCACACCCAAATAAGCCCACAAGCCATATTTAGTGAAAAAT
TGCATAACTTCTTGTCCTGTTGAAAATCCTGCGCCCACTACAATGCCGACGTAGGCAAATCCAATTTTTGTTG
CTTCTTTATTCATCATCATGTGAATAATAGACCTCCTTAATTTTGCACAGTGTGCATTATAACGTACTGAAAA
TTAATTGCAAGTCACATAGGTTATTTTTCACTTTTTCAAAATTGCTCAATCCATTCACATTATCACGTTCAAT
GACATTTAAAAAAATTATAAATTTATGCTTTTATAATGCAGTCATACGAACCTTTGTATAGGAAAAATGATTA
ATAATTGTGCTTCTTAACTTACGTATTTTTAGTTAAATTTCGAAAAATACATATCAAAATCTCTCAGTTAATA
AGGTTTAAACCATCTATCGTATGTTCTTTATTAAAATGTATAAATTTATAAAATGTTGTATAAAAACCATTTG
TGCACACAAGTACACTTCGTTAGTTTTTAAGTATAAAAAAAGGCTGGAACATATGTCCCAACCCAACCAAATG
TTAAAACTATTATTAAGCTAATATAATTGAAATGTAATTAACGTGCATTACGTTGGTGTAATTCATTTATATC
AACAACAACGTTGTCCATACGTTTGGCAGTGTCTTTTAAGACGTTACGAGCAACATTATTATTAGGATCTAAT
TTTAAAATTTGCTTGGCGATATCAAAAGCCTTCTTATCTGAAATAACAGGTGTTGGTATCGCATGCAATAATA
ACATTTGTAACAAACTTTTAACTTCTTTGCCGTCAGTAAGTTTTATGGAAGCGTCTGCATGATAATAAGCAGA
ATCCAAAGCACCTTCATATTCACTTAGTGGATACACAAGCAATAAAAATGCTAAGTCATGCATTTCAGATGAT
TCTTCGTACTTAATCATATCTAAAATACATGTATAAAACATCATACTTTCTACTTCATGCGCACTTGAAATAT

ATGCTTCTTCAAATTCCATAAAATCTGTTTCGGACATCAATTGTCGTACTAAATCAAACTCGCCATTCAATAC
ATGTTTTTTTATAAGGTGTTGCATGGCAATGTCCTCCTATTTCTCCAGATTTTATTAAACATTTACATATATA
CTATCATATTTTTTATAAAGTTTCATTTGAAAAAGGTTTACATTTTTCATTATTAGTAAATTTTAACTAAAAT
GATTGTTTGATTTCTGCTAATGCATCATCAATTGAACCGCCGATTGACAGCTTTACTGCTGCGATAAAACTGC
CTTCAACAATTGGTGCATCAACTTTTAACACACGATGATTACCATCATACATTTCAATTGCCATATCTACATT
CATTTCTGAAGATCCAATATCGTAAAAACACATTGCATCATCATCTAATTCAGTTAAAGCTTCTTGAATGATA
TCAAATGAAGTTCCAATTGAACCATCTGGTAATCCCCCGATTGGTATAATATCAACGTCACCAGCCATTTGCT
TTAACAAAGATTTTGTACCATTTGCAATTTCTTTACTATGGCTAACAAGTATAATTTTAGGCATTATCTTCAT
CTCCTATCAAAGCGTTTAAAATATAAACCATACTTTGAGCACCTGGATCTATATAACCTTTTGATTCTTCTCC
AAAATATGCAGCTCTACCTTTCGTTGCTACCATATCTTTTGTATTATCTGCTAATTGTTGTAAATCATTAAAT
GTTAAAGTTTCACCATTTTTAAGCTTCTCTGCTGCTCGTGCTACTACATCATACATTGTCTTTTCATTTAAAG
TAACTTTCCCACGTGATTCGACCGCTTCGGCAAATGCTTGAATTAGTGTAGTGAAATCTTGATTATCCATATC
ATCTTTAGTAACTGCAGACATTTTAACAAAGCTAAAGCCATACAGTGGTCCTGATGCACCGCCAACATTTGAC
ATCAATGCCATACCAGTCGATTTGAACAATGATTGCATTGAGCTATCATCAAGTTTTTCTTTAAGACTACTAA
ACCCTCGAACCATGTTAACCCCGTGGTCACCATCACCAATTGCTCGATCTAATTCAGTTAATTCAGATTCATG
TTTTTTAAACGTTTCTTCTAAATTTAATAAACGCGCTTTCATATCATTCACTTTCATTTGTGCCACCTCATAA
ATGTATATTTATTCATATTCACTGCTTATTTAAAGTATTGACTTGTTGTTGGTGCTAAAAATGCTTCCAGATA
TTCTGGTTTATTAGGCACGATAGTTATAGAAAAACCTTGCATGTCTAAAGATGTCATATAATCACCAACAAAC
CATTTAACAACATTAACCGTTCTTGCAGTTAAATTTTGTTGAATATATTTAGTTACGATATTTAATTCAGATA
ACGGCGTACCTCCCATTCCATTTACCATTACTATGACCTCATTGGCAGTAACTTCTTTATACAATTCGTCTAA
TAATGTTCTAACAATATGATCAATATCCTTTACTTCTTCCCTATGAATACCTTTTTCACCATGTATACCAATG
CCAATTTCCATTTTGTCGTCTTCAATATCAAAGCCATATTTTCCAGTAGTTGGAACAAGCGGTGGCTCAATTG
CCATACCAATACTTTTAATTTCAGGTAACAACGCTTCTACACGCGATTTAATCTCGCCTAATGCGAGCCCTTT
TTCTGAAAGATAACCGGCAAGCTTATGAACAAACACTGTTCCTGCAACACCACGACGTTGTGCTTCATTTGTC
ACAGCAATGTCGTCACGAACAATAACTGTTTCAACATTTATACCTTCCATTTCTGCAAGCTCTTGTGCCATTT
CAAAATTCATCACGTCACCTGCATAGTTTTTCACAACTAGTAATACACCATCACCTGTATCTACTGCTTTAAT
AGCTTCTAATATTTTATCTGGTGTAGGTGAAGTAAATACCTCGCCACAAACCGCTGCATCTAGCATACCTTCT
GCAACAAAACCGGCATGCGCAGGTTCATGTCCGCTTCCACCTCCAGAGACTATTGCTACACCATGTTCTTTCT
TATCTTTTTTTACAATAACTGTATTAGCAATCAGATCTAACTCTGGGTGCGCAATTAACAATCCTTCAAGCAT
ATCAGTTAAAAATGTTTCTTTTTTATTGATTAACTTTTTCATCATGTTGTACCTCCTTGGTATTATCAGTCAT
AGTATAAAACAGAATAAATGAATGCGCTATCATAAAGGGTTAACCTGATACCGTTATCAAATATATTTTTGAT
AAGATCCTCTTGATAACTTAATGGTTCATTATTGAAAAAGTAAATAGTCTTGAGTGACTCATTAAATAAAAAA
TGCTATGAAAAGCCTTATAATAAAGTGCCTTTCATAGCAATATGTTGTGTCCTTTGACACTATACGTTTTTCG
TTTTGTACATTAAATATAAGAAATACGGTGCACCAATAATTGCTACGACAATACCTGCTGGAACCCCACCTGG
TTGTAATACAATTTTGCCAATTGTATCAGCTATAACAAGTAAACATGCCCCTACTAAAATGGCAATTGGTAAA
AACAACTGGTGACGTGGTCCAACGATACGTTTGGCAATATGCGGGCCCATTAATCCGATAAACGAAATTGAAC
CTGCTACTGCTACAGCAGCAGATGATAACATCACTGCGATAAAGAATAATATTAAACGTTCTCTGCTTAACCT
TACACCTAGACCTCGTGCAATATTATCACCCGTATGAATAATATTTAGTGTATTCGATTTAAATAGTAAGTAA
GGAATAATAATCAACACCCACGGTAAAAATGCAATGACAAATGGCCATTCGTCACCCCAAATATTACCTGCAA
ACCAAGCAGCGATGAAATCAGATTGCTTATCATCAAATTTTGACATAATTGTAATTGAGCCACCATACAATGC
TGTTTGTAAACCTACACCTATTAATACCATACTCGCAGGTGTAACACCTTCATTTTTATTAAAACTGAAAATA
AAAATAATCAATGCAGCGGTGATACCACCTAGTATACTTATTAACGGCAGTACATAAACAAAGTTGTCAGCAT
TAATTTTACCAATTGCAATAAATAATGCGATTGCAAATCCGCCGCCTGCATTAATACCTAATATACCGGGTTC
AGCTATTGGATTTTTTGTGACACTTTGCACTATTGCACCACTAATACTAAGCGCTGCGCCTGCCAAAATAGTA
ATCATCATCCGAGGTAACCTGAAATCTAATAAGATTAGCTCATCAATGGCATCACCTTGTCCAATTAAAGTTT
TGAAAAATCTTTCAACTGGTATGTTGTATTCACCTGAGGTGATACTCCAAGTACAACCTAGAAATAGTAGAAT
GCTAAAAACAGCCAGTGCAATCCATCGTCTGCGTTTATTATTTGAACTAATCATATTGAGCGTCCTCCTTTTT
TAACTAAATATAAAAAGTAAGGAACACCGATAAATGAAATGATTGCACCAACAGGCGCTTCTCCTAAATATCG
TGCTATCACATCGGCAACAAGCACGAGTATCCCACCCAACAAGGCTGTTAATGGTAGAATTTTAGCATAATCA
GTTCCAATTAAAAATCTTGCTATATGAGGTACCATCAAACCTACAAATGCAACTTGTCCAGCGATAGCAACTG
CTATACCTGCTAGAATCATAGCAATAATTAAACATATGCCTCTGATCATTGTTACATTTTGACCTAAACCTTT
AGCTAATGATTCACCAAGATTTAAAATGGTAAGTTGTTTACTAATTGTTAATATAATGAATAACGCAATTCCA
ATTAATGGAATTGCCCACTTAAGGTGTGACCATGTTGTGCCTGAAACGCCTCCAGCAGTCCAAAATGTTACTG
TTTGATTTAGTCTAAAAGCTAATGCAATACCTTGACTTAGCGCTGTTAACATAGCACTTACTGCTGCACCCGC
TAAAATAATACGCATCGGATTAAATCCATCACGTCTAGATCGGCCTATCATTAATACAATAGCCCCTCCTAGA
ATAGCACCTAAAAATCCAGCAAACATCAATATTAAAAATGAAGTGTTTGGTAAAACTGCATATGTTAATGCTA
AAGCAAATGAAGCACCTGAATTTAAACCTATGAGCGCCGGATCAGCAAGACCATTACGAGTAACACCTTGTAT
AATCGCACCAGAAACTGCAAGCGCCATACCCACAATTACTGCTGCTATATTTCTGGAATCCTAATCTCATTG

ATGATGTTTTGCTGCTGATTGCTAGGATTATAATTAAAAATAGCCTCTATAATTGTAGAGGCTTGAATTTTGG
CGTCACCTATTAATGTAGAAATAAATAGTATGATTAGTAGTATCATACTTAAACCTATAATATAGTATAAAAA
CTTCAATGGCGTTGGATTCTCTCTATTTGTCATGTTAATTGTCCTTTTTATCATATTAACTTACTTAATTAAG
AATAAGCTCTGCGACATAAGTCATAAGTTACCAGTAAAGGTTTTCCAGTTTTAGGATCTTTACTTAAAACAAC
ATCAATATTAAAAACTTTTTCTAAAATTTCCTGTGTTAATACGTCTTCTGTTGAACCTGTAGCGATGATATCG
CCTTCTTTCATCGCAATAAGATGATCTGAGAAACGAATCGCTTGGTTAATATCATGAAGAACCATGACAATTG
TACAACCTTGTTCCTGATTTAACTTCTGAACTAATTCTAGTATTTCTAATTGATGACAGATATCTAAATATGT
TGTTGGTTCGTCTAAAAAGATAATATCAGTTCTTTGTGCTAATGCCATTGCAATCCAAACACGTTGTCTTTGA
CCACCACTTAAATCATTGATTGAACGGTGTCGAAATGTATCAGTTCCTGTAACTTCCATTGCCCAATCAATTT
CTTTCTTATCCTCAGCAGTTAATCTACCAAATCCTTTTTGATGTGGAAAACGACCATATGAAACTAATTCTCC
AACAGTTAAGCCATCTGCTACTTCAGGTGATTGAGGTAAAATAGCTATTTTTTTTGCAATCTCTTTCGTAGAT
TGTGTATGAATATTTTCATCATCTAAAAATACTTCACCTTCTTTAACTGCCAATAAACGTGACAATGCCTTTA
GCAAAGTAGATTTCCCGCAGCCGTTAGGACCAATGATTGACGTCACTTTGCCATCTGGTATTTCAACATCTAA
TTTATTTATAATCGTGTTATCCCCGTAACCAATTTTAACTTGTTGTCCATGCAAACGATTCATAATTTCCCTA
CTTTCATTAAATTCTTTCTGTTTATAAAAAATAATTTCTATTTTTAAATTATCAATTTTCAAAGGCACCCCAA
TTGATAATGATTATCATGAACATCATTATAACATTTTTCAATCTTATTGACTAACATTACTTTTTAAATTGGA
TAGCTCGATTTGTCATGTCTTGTATATTACTTTTATAAAATAAAAAACGCCCACAGATAAGTCTTCATAGTTC
AAAAACTTGTCCGTGGACTTCTATTTAAGTATGTGTGCTCATACCATTTATTTATTCATCTGCAAGAAAGCCA
TTACCATAGACATCTCTTACATCATGAATTACAAGGAATGCATCTTTATCGATTTGTTTAATTAATCGCTTTG
CTTTTGAAACTTGTGTTTTAGAAATAACAACGTATAAGACATCTTTTTCTTCACGCGTATAATAGCCATGTCC
GTTTAAAATGGTTAAACCTCTTCCAATTTGCTCGTCTATTGCTTTGGCAAGTTTGTCGGGATTAGTTGAAATA
ATCGTCATAGCTTTTTTAGTGTTTAAACCTTCTATGACATATTCCATCACTTTTGTTCCTATATAAAGTGATA
TTACTGTTACTAATACTTTATCAAGTGGAATAACTGTAAGTGAAATTGCAACAACGATCATATCGAAGAAAAG
CAAAGCATATGGCGTGCTTACATCGAGGTATTTCGTTGCAATTCTCGCCAAAATTGTTGTACCTGCTGTTGTA
CCGCCTGCAAGGATAATTACTCCGATTCCTAGTCCAACGCTTACACCACCAAAAATGGCATTCACAATGCTGT
TTCCAGTTTCAACTTGCCATGATTCTGTTAAACTCAAAAATATTGAAATAAGAATTGTTACAAGAATAGTTAA
GTACATACTTCTCTTACTCAAAAATTTATAACCTATGGCAATCAATACTGCGTTGACCAAGAAGTTAGTGATG
GCTGGTGAAATATGAAACGCATAATATAAAATAATTGCTAAACCTGTAACCCCGCCTTCACCTAAGTTACCAG
AAATAATAAATGCATTTACACCTGCAGCAAAGATAAATGAACCTAAGACAACTAGTATTAAATCTTTAACCGT
TTTATTCACGAAACCATCCCCTTTATATATTTATTAGACTATAAAAATAGTAGCACAACAAAAATGCTGTCAC
AACTCTATGGAAAGAGTTCTAACAAAGATTTACTATTTAACTAATTTATATGAAAAATAGCTTTCCCTTATAA
ATATCACTCACAATATAATAATTAAAAATATTCTGAATTTTTAATTTACTTTCATTTCATTGTCTGCTATAAT
GTTTTTAACTTCTTCAATAGAGAAGTTATCTCCTTTGTGTTGTTTATAATAACAAAACAAATTTTGCTCGAAG
TACTATGACTGTACCTAGTCCTTACTGTCATATGTACAATAATATTCATTTCCCATAAAAGCCAAGTGTATTA
CCATGTGCACTTGGTTTTTATAATGCTTCAATTTCGGTAACTTTAAAAATATTTAAATATCTTTATATCAATA
ATAAAAAAGTCCAAGCATCTCACCTATATCAATAGACGACATGCCTGAACATATTTATTAGTTAAAATGTATT
ATCGAAGTCACACTTCAACATATTACCATGCAAATAAACCAACGAAAGCTGCTGTTAATAATGAAACTAGGAT
ACCTGATAATAGCATCATAGGCACATATTTAGATACAAAGTCTGATGTCTTTTTATCAACAATGCCTTTCAAT
GTACCGATAATCATACCAATCGTTGAGAAGTTTGCAAATGAAATTAAGAATGTTGTAATAACCGCACGATGGT
GTGGTGTATACGATGCAATATCTTTAGAAATTTCACCCATAACAACAAATTCATTTGTAACAATTTTCTTAGC
CATTTGTTGTGCTACCAACCACGCTTCATCATAAGGTAAACCGAGTAATAGCGCAAATGGATACATAAACACA
CCTAAAATTTGGTTTAAACCGAAACTACCTTTTATGCCTATCCATCCTGCTATCAATCCTGTAATCAAATTGA
TAAAACGATCAAATAGATCAGCTAACGCTACAAAACTAATAACAAATGCGATGATGATTAATACTAATTTACC
TGCTGCTAATACAGAATCTCCAAGGAATGAGAAGAATGGTTGACGTTCAACTTCATTGTTTTTAAGACTGTAA
ATAATATCTTCTTTCTCTTCAACACTTACTGGATTCAACAAGCATGACACAATAATCGCGTTAACGATATTTA
GTGGAATTGCCGTTAGTACCAGTTCTCCTGGTACCATTTGTACATACGCACCTACAATAGCTCCCGATACAGA
GCTCATTGACATCATTGCGATTGTTAATACACGCATTTCATTCATACGTTTTAGTTGCTCACTTGATACGGCT
AATGCTTCAGTATTTCCTAAGAACATCATTTCTATCCCAAAGAATGACTCGAATTTAGGTTGTCTTGTTACTT
TAGCTAGTAACCAACCAATACCTCCAATAATTTTCGGTAAAATATTAAAGTACATTAAGATATCAAATAATGG
CACTATTAATAATATTGGGAATAAGGCTGCAACAGCCATATCCATCATTTTAACATTTGTCAAACTTGCAAAT
GCAAAACCTGTACCAGCATGCGCTGACTGAACTACCCAAGCGATACCATTGGCTGCTCCTCTTACTGCTTTTT
GACCCCAATCAAAATAAATAAAGAACCATGCTAAAAACAGGTTTAAAACAACTAAGATCCCAATTGATTGCCA
TTGGATATTTTTGCGATCTCTTGAAAATAATACCGCAATACCAAGAAATACAATTAGACCAATAATGTTGATT
AATAAAAACATTTGGCACCCACCTATGATTTAATTTTTTAAAAATTTAGAAACATTGAAATATCAAACTTCTG
TACCGTGGACTCTAAAATCAGTTAAATTGCTATGTAAAATATGAAGAATTATGACTCGTGGCTACAGTATTTA
ATATATGTATGTTTGCTTATAACTTGTATTATACCATGAGCATTTTTTTCTTAAAATATCGATTTCATTAATT
TAAGAATTATTATGTCGTAACTTCTCAAATGATTTAAAATAGACAAAAGCTTGAAGCAATTTATTAACATCTA
TATCCATCAATGTGCCACGCTTTAATTTCCTCGATGGTATATCTGTACTTGCTCCAAGCTTACTTATATATAT

AAAATTAATCTGTTAATTTTTGAGACACTACAAAGTTTTTATACTTCGCATGTGATGCCATTAGTTCTGAATG
CGTACCTTTACCTGTTACCTGTCCTTTGTCTAAGAATATAATTTGACCGGCTTTTTTAATTGTAGACAAACGA
TGCGCAATGACAATCGTTGTTCTACCTTCCATCAATGTTTCTAAAGCTTCTTGAATTTTCAATTCACTTTCAC
TATCGAGATTAGCTGTTGCTTCATCAAGTAACAAAATATCGGGATTTTTAACAAAACTTCTAGCAATATCAAT
ACGTTGACGTTGTCCGCCAGACAGTTTCAATCCTCGTTCACCTACAAGCGTGTCATATCCTTCATCAAATTGC
ATGATAAAATCATGACAGTTCGCTAATTTAGCATAATTAATAAGTTCTTCATCTGAAACATGACGATTAATTC
CGTATAAAATATTGTCTCTAATTGTACCACTCATCATCGAATTTGATTGCATAACATATCCAATTTTGCGTCG
CCACTTAGATAACGGGATATCATAGACACTTTCAAGGCCATATTTAATATCACCTGACTCAATTTCATACATA
CGTTCTATCAGATTAAATATCGTACTTTTACCAGATCCAGAAGGACCTACAAAAGCACTCACTTGCCCTTGTG
GGATTTGGAACGACACATCATCTAATATTTTCTTCACATCATATTTAAAGTCTACATGTTCAAATGACAACAC
ACCGTCATCAATTAATACATTTTCAGAATCTTCAAGAGCTTCTGTCGGTTCAATAGGTTCTTGCATGATTTCG
TATATTCTACTACTTGCACCGACTGCCTTTTTATAGTCTGTAACTAACGTTGAAAGATTGATTAAAGGCATCG
ATAATTGAATAACATAAAATATCATTGCAATTAATGTACCTGCAGTGATTGCACCAGTCGCAATTTCTAATGC
ACCAAAACCTAAAATAATTGCAATTGTTAGCAACATAACAATACCTGAAATTGGTTGTACAACTGCCGCAATT
TTAGCCTGTTTCAAACCTAATTTATATATTTCATTCAAATTTTTATGTGCATTATCTAATTCAAGTCGCTCTG
TATTTGATATTTTAACAAGACGCATTTCAGTTAGGACACGCCCTAACAAACCACTAAAGTTTGCAATTTCAGA
TTGTGTATTTGTCGATATTTTTTGCATAATACGTCCTAGAGGAATCATAATTAAAACAAATATCGGTATCGTT
ATAAATGTTAATAATGTCATTTTCCAATCTAAAATAAATAACATGATTAGTGACCCAACTAATGTAACGATTG
ATGGTAATAAGTTGGGTAGCTTTTGTGAAATAAATTCATTTATCACTTTCGTATCGTCAGTTAATCGACTCAT
TAATTGACCACTTTCATTTTTATCAAAGAATGGCATTTTTAATTGTATGATATGCTCCCATAAAACTGAGCGT
ATCGCGTAAATAATTTTTTCACCAATTTTACTTAATAAATATAAACCTAATCCGCTTAATAAAGCATTGATGA
CAAAGATACCACCAAATAATGCGATTAGATTCCAATTGATATGGCTCACGGAAAATTTATCTACAATTCGTCC
AGTAAACAGTGGCACTAATAGTCCACTTAAGCTCCCTAGTGATGAAATAGTGATAGCTGCAACGATAAGACCC
ACTGGCCATGATAGTTTTTTAAATAAGAAAAACAATGGATTTTCTCGTTTCATAGTTTTACCTCTTTTGTTTG
AAATTTATAGTTAATTCATTTAGATAGATACATGCATTTCTATCATATACTTTCTCCCAACAAAACTTTAAAA
ATCAAATAAAGTATTGCCCCAAAATACCGTTCATAACATTTATATGATAGAATATTTCTATTGCATTTTTTGT
AATAACTTCTCAAAGTTTTTATATTAAAACATGATAATTAATCGCTTTTGAGTAAGTTTGCTCTTCGTACAAA
CATTATAACACCTTTAGCTACACACGTAATATATTTGCATTATTTTTCAAATAAACCGCAAGAAACTTAATTT
TCAACAATTTAATTAAGGTGAAGTGAATTAGCAATGATTTCCGTAATTAGGAATGACCATTGCTTTTAGGGAA
TATTTAAGAATTGGAAAAGGGAAGATTAACGCTTTATGAAAAATTTAATATCTATTATCATCATTTTTATGTTT
AACATTAAGTATTATGACACCATATGCACAAGCTGCTAACAGTGACGTAACCCCTGTACAAGCAGCAAATCAA
TATGGTTATGCAGGTTTGTCGGCTGCATACGAACCGACGAGTGCTGTTAATGTTAGTCAAACTGGACAATTAC
TGTATCAATACAATATCGATACTAAGTGGAATCCAGCGTCTATGACTAAATTAATGACAATGTACTTAACATT
GGAAGCTGTAAATAAGGGGCAGCTTTCACTTGATGAAACTGTCACAATGACGAACAAAGAATATATTATGTCT
ACACTACCTGAGTTGAGTAATACGAAACTATATCCTGGACAAGTATGGACAATCGCAGACCTATTACAAATTA
CAGTATCTAATTCTAGTAATGCCGCGGCATTAATTTTAGCTAAGAAGGTATCAAAAAACACCAGCGATTTCGT
TGATTTAATGAATAACAAAGCTAAAGCTATCGGAATGAAAAATACACATTTCGTCAATCCAACGGGTGCTGAA
AATTCAAGATTACGTACATTTGCACCAACAAAGTATAAAGACCAAGAACGTACTGTAACGACTGCTAGAGACT
ATGCCATTTTAGATTTACACGTGATTAAAGAGACACCTAAAATATTAGACTTTACAAAGCAGTTAGCACCAAC
AACGCATGCAGTTACGTATTACACATTCAACTTTTCATTGGAAGGTGCAAAAATGAGTTTGCCTGGTACAGAT
GGTTTAAAAACTGGATCAAGTGATACAGCAAATTACAACCATACGATTACTACCAAACGAGGTAAATTTAGAA
TTAATCAAGTTATCATGGGTGCGGGAGACTATAAAAACCTTGGTGGCGAGAAGCAACGTAATATGATGGGGAA
TGCATTAATGGAACGCTCATTTGATCAGTATAAATATGTAAAAATATTGTCTAAAGGTGAGCAAAGGATAAAT
GGTAAGAAATATTATGTTGAAAACGATCTTTACGATGTTTTACCAAGTGATTTTAGTAAAAAAGATTATAAAC
TTGTAGTCGAAGATGGTAAAGTACACGCGGACTATCCAAGAGAATTTATTAATAAAGATTATGGACCTCCAAC
TGTAGAAGTTCATCAGCCAATTATTCAAAAGGCAAATACTGTTGCTAAAAGTATGTGGGCAGAACATCCATTA
TTCACTATCATTGGTGGTGCATGCCTAGTCGCTGGATTAGCACTAATTGTTCATATGATAATCAATCGTTTAT
TTAGAAAAAGAAAATAAAACATACTAAAAACGGACAAGTTGCACATTATAAAGCTGCGAAACTTGTCCGTTTT
ATATTTATTTTATAATAACCTACGTTAATTTATCGTTTAGTATCGATAGTTCTATATAATTTATTTAGCATCT
TTACCATATAATTCTTGTTTGATTTTAGTCGTCGAAATGCCTTCTGTACGTTTTAAATAAATGACTTCACATT
TATCCTTTAAGAAGTCGAATTCACCTTCCCAGTCATGTCCCATAACAAAAACATCTACATCAAATTTTTCGAC
ATCGTCTTCTTTTTGTCCCCAGCCCTTTTCTGGAATGACTAAATCGACATAGCGTATTGATTCAAGCATCATT
TTTCGTTGTTCATAATCATAATAAGATTTTTTATGTTTAATTTGATTAAATTCATCTGTTGATAATGCTACTA
TTAAATAATCGCCCATCTCTCTTGCACGACGAAGCAATTCGATATGACCATAGTGAAGTAAGTCATATGTGCC
ATATGTTATTACACGTTTCATTAAGTTATCCTCCTCATATTGTCACATCATAGGATTGCAGTTTATGGTCATC
AATGTACACGTTATCGTTTTAACATTGATTTTGTTCGATGTGCAAACTTTCTCCCTTTAAGTTGTATCAAGAA
TTTTTTATAAGTCGGCCTATTAATGCCCACTTGAATTAATGTTTGTCGCCACAATTCGTATATTTCTCTATCC
CAATTCTCACCAATAAATTCAACTGTATTTATAATGTCAAACATGTTTTGCGATACATAATCAGAATGCTGCA

TAGACATAATATAGTCTTTTACGAGTTGAATTGTCGTGTGCGTTACTGCTGGGTGTTCATTCATATAATATTT
TAATGAATTATTGAATAAAATACGATCAACCATATCACTATATAATAGCCTTTGTTCAGGTAATAGTAACATT
TCCATGACTCGTTGTCTTATTTTCATAATTTCTGTGATATATCTATTAAAATCTTCATCGTTACTTGTAGCAT
GTGTATCTATATCGTTATCTCCGACAATCATCTGTGAAACTAAATGTATATCCGTAGCTTTTTGCATCGCCTT
GACAAGTATTGCGTGATTGTATGTGTTAGCTAAAGTTTCGTCACACTGTAATTCAGCAAATTTAGCACTGAAT
AACTTTCCGTCAAAAGACATCATTGATAACAAATCTGGTTGTTCCATTAATGTAACGATTCGATTGTCTTTAA
TAAACTCATCAGCACTTTTCAATTTCCATTCACCATGGTAATTGTAAGCTCGCTGCCCTATTACCATATCAAT
ATTATCATTATTTTTGAAAGCTGCAGTTAGACTATCTAATTTACCTGGCAATACGATTGATGTTGGATTTATG
ACCATAAATGCGTCACTATCATGTGGATCTGTCATTTTGATATCCATCAAACCTTTATTAATACACGTAGCTA
TCGAAGCATTTTGTAATTGTATTAATGAAATATGCTTGAGTTCTCTTTGTAACCGTTCAATCATAGGAATTGT
TTGATCGGTAGAACCTCCATCAATACAAAGGATTCTATAGTGTTCTTTACTCTCAATAGATATTAACAATTGT
CGAATTGTTGCCTCATTATTACATGTAGGTATGATTATCGTAAGCCTCATTTTGTCACCATCTTATCTATATA
TTCTGTGAGCTGATGTAAACTTTTATCAGTATTATACTTATGCCAATCTTTAAATAACGGACTTAATAGATGT
TCTTTTTCTTGTATCGTCATTATTAAATCTTCTTCAGTATACACTTTGTAGCTATCCGGTATTGCTTTGTAAA
ATTGATTCAGGCCTCTCACTTTATCATATGCCTCTTCATCATACACATAAAATATAGTTGGAATATTTAACAA
GCTAGCTTCTATTGGCAGCGAACTATAGTCGCTAATAATTATATCTGACATTAGCATTAATGTAGACGTGTCG
ATTGAAGATACGTCATCAATGTCTGAATGTTCAATTGATGGATGTAATTTATTAATCAGTGTATATCCTGGTA
AACAGTTTTCAAAATAAGCTTTATCAATAGCCCTATTATCTGCTTTATCTTCTCTATATGTTGGTACATATAA
TGCCAACTTATTTGTAATTCCATATTTATGCTTTAACTCTGCCTTAACCGTTGCTCTATCAGCTGTGTAATAT
TTATTAATTCTCGGAAGCCCAAAATACAACATTTGCTCTTCTGTTGCACCTAAAGACTGTTTAAAACATTGTG
ACATTTGTTCACAACCCACTAAGTAAAAATCCGTCGCTTGATAAACTTTACGGTACTGCTGAACCATTCCCTT
GTCAGACACATCGACTTGATGATCTGTTAATCCAAAGTTTTTAATGCACCACTTGCATGCCACGTTTGAACA
ATGTGTTGATTAGAAGTCTTATTATATCCACCTAGTAATAGGTAATAATTATCGATAATAATCATCTGCGCGC
TTTTCAAAGCCTTAATTTGTTTTACCAATGTTCGATTAGTCATTTCTATCACATCAACATCGTCGCTAAGTTC
AGATAAATAAGGCGCTTGTTTTGGTGTTGTTAAAACAGTTTTCTGATACGACGAATTATTTAATGCTTTGATG
ATAGGCTTAATATCTTCTGGAAAAGTCATCATAAATACGATATGCGGTTTATCAATCACTTGAGGCGTAATCA
TTTTAGAAAGTATTCGAACTACCAAATGATAAAATTTCTTTATTAAAACGTTCATAATACACCAACTTAATAT
GTTATTTACTTAAATTATAAACAAAAATGAACCCCACTTCCATTTATTAATGGTTAGCGGGGTTTCGTCATAT
AAATATATTACAAGAAATCTGCAAATTGATCTCTATATTTCATGTGTAAGTACGCACCAATTGCAAAGAAAAT
GGCAACAATACCGAAATTGTATAACATTAATTTCCAATGATCCATGAAATACCATTCGTGATATAAAATTGCT
GCACGGTATGATTCAGCAATAAAGTAAACTGGATTATATTTCATCATTTCATGAATTAAACCACTGATACCAT
GGTTCTTTGGTAGCCACAAAATTGGTGAAAAGTAAAATAATATTCTTAATATTGCTTGCATTAACATTTGTGT
ATCTCTAACTAACACACCGAGTGTTGATGTTAATAACGTCACCGAGGCAGTTAAGAAAAAACAAAACGGTACA
TATATCAATAATTGAATGATATGTATTGATGGATAAATACCAGTAAACATACATGCAATTATCACAAGTAAAA
GTAAGCCTAAATGTCCATAAAATCTACTTGTCACAATATATGTCGGTATTATCGATAACGGGAAGTTCATTTT
CGATACTTGATTAAACTTTTGTGTAATTGCTTTAGTACCTTCTAAAATACCTTGGTTGATGAAGAACCACATA
CTGATACCAACCAATAACCAATAAACAAAAGGTACACCATGAATTGGTGCATTACTTCTTATTCCTAATCCAA
AAACCATCCAGTAAACCATAATTTGCATAACAGGGTTAATTAATTCCCAAGCCACACCTAAATAGTTACTATG
ATTAATAATTTTAACTTGAAACTGAGCCAGTCTTTGAATTAAATAAAAGTTCTTTACATGTTCTTTAAAAACT
GTTCCTATTGCTGACATTCCATTAAACCACACTTTCAAATGTTTAACTATTTCTCTACTTAACTAAATAGTAT
TATAATAATTGTTGTAAATACTATCACTATACATGGATGCTATCAAAATTATTGTCTAGTTCTTTAAAATATT
AGTTTATTACAAATACATTATAGTATACAATCATGTAAGTTGAAATAAGTTTAGTTTTTAAATATCATTGTTA
TCATTGATGATTAACATTTTGTGTCAAAACACCAACTCTGATAATAACAAAATCTTCTATACACTTTACAACA
GGTTTTAAAATTTAACAACTGTTGAGTAGTATATTATAATCTAGATAAATGTGAATAAGGAAGGTCTACAAAT
GAACGTTTCGGTAAACATTAAAAATGTAACAAAAGAATATCGTATTTATCGTACAAATAAAGAACGTATGAAA
GATGCGCTCATTCCCAAACATAAAAACAAAACATTTTTCGCTTTAGATGACATTAGTTTAAAAGCATATGAAG
GTGACGTCATAGGGCTTGTTGGCATCAATGGTTCCGGCAAATCAACGTTGAGCAATATCATTGGCGGTTCTTT
GTCGCCTACTGTTGGCAAAGTGGATCGTAATGGTGAAGTCAGCGTTATCGCAATTAGTGCTGGCTTGAGTGGA
CAACTTACAGGGATTGAAAATATCGAATTTAAAATGTTATGTATGGGCTTTAAGCGAAAAGAAATTAAAGCGA
TGACACCTAAGATTATTGAATTTAGTGAACTTGGTGAGTTTATTTATCAACCAGTTAAAAAGTATTCAAGTGG
TATGCGTGCAAAACTTGGTTTTTCAATTAATATCACAGTTAATCCAGATATCTTAGTCATTGACGAAGCTTTA
TCTGTAGGTGACCAAACTTTTGCACAAAAAATGTTTAGATAAAATTTACGAGTTTAAAGAGCAAAACAAAACCA
TCTTTTTCGTTAGTCATAACTTAGGCCAAGTGAGACAATTTTGTACTAAGATTGCTTGGATTGAAGGCGGAAA
GTTAAAAGATTACGGTGAACTTGATGATGTATTACCTAAATATGAAGCTTTCCTAAACGATTTTAAAAAGAAA
TCCAAAGCCGAACAAAAAGAATTTAGAAACAAACTCGATGAGTCCCGCTTCGTTATTAAATAAACCGAAAAAA
CCGAGAATCTCCATTTAAGGATTTCCTCGGTTTTATTTTTGTCATCATGATTATTTCGCCTTTTTTATTTTTC
TTTTTGCTTTGGCTATTTTATACATAAATATAGGAATACTCTTTAATCTACCAATACGTTTCCAATCTATTAA
TGCTCTATATATCCATTCAATGTTTAATTTTCTAAAGATATAGGGCGCTCTCTTTTTAGCCCCAGCAAATACT

TCAAGAGAACCACCTACGCCCATCATCACTGTAGATTCAAATTGGTTTTCATGTGTCATAATCCATTCTTCTT
GTTTAGGGAATCCCATACCTACAAATATGTAATCAGGTTTAAACAGTTTAATTCGTTTCACTACTGTCTCATC
TTCCAAATCAATATAACCGTGATGATGTGCAAACGATATGTTTGGATATCTTTGTTGTAATGCATATTGTGCC
GCTTCTACAACTTCATTAGTTGCCCCTAGCAAAAATACTTTTTGATGATTTACATGAGCAATTTTCAAACATT
CATCCATCAACTCAATACCAGGTATACGATGCGCTAGAGGTTGCTTTAAACGATGCGAAGCTTTGACTACTCC
TGTCCCATCAGCAACAATATAGCTCGCTTGATTTATTAACTCTAAATACGCTTGATATGTCGTCGCGTAATTC
ACTATTTCAGGGTTGGCTGTTACTATAAAAAGATTATTCGTTGATTGATTTGCAAAAAAGGTTTTAATATTTT
CAACCATTTGCAACATTGTTGTATTATCAAAATCGACCCCTAAAATGTCAACTTTGGCTGTATTGGATCTTTC
TTCAACAGTCATAACAGGAATCTCCTATCTTATTTCATTATTATGTTAGTGTAATCAATATTAACAGTATTTA
TATTTATCCTATTTATATCGAAATGATTTTGCTTTATTTTAACACATATTCACCTTTTCAAAAATTAAAATTA
GTTTAACTAAGGTAAGTCTAAAGTCTAACGCTATTAATGCCAATATCATTTATAATAAGTGATAAGCAAACTT
TAAATTAATATCTTATATGAGGTTAACAATGAATTATTTATCAAACTTTTTTAATACGACAATTCAACTTAAT
ATATACATTATCGTTATTGTTGCAGCTTGTTATATTGCTATTCATCAATATAGACATAAGGCTGTTTTGAACT
ATTTAGATGTCATTTTAAACTATATCCCTGTTTTAACACATGAATTTGGTCATGTATTGTTTAATAAACTCGC
TGGTGGCCGTGCAAAAGATTTAGTCATTGTAACAAGTCCACGAGAGCGACAACAGACATTACAACAAGGATTC
GCAATTACACAATCTAGACATCTAGCAGGCCAATGGCTTACAACGATTGGTGGTTATTTTATGCCACCTATTA
TGTTATTGATAGGTTTAGCGTCGAGCCATTATCAGATACCTAGTTTCTTTATATTTACCTACTTATTGATATT
TATTTATTTTTTAATTTTAACATCTAGAAAAGGCTCACCCATTGTTGTTATAACTTTAATTTCTATCATGTTG
TATTTTATATTAAAAGATGAAAATATAGTAGAAATTCAACTACTTGTCACAATGAGTTATCAATATATTTTAG
GTGTATTATTAGGCGAAGTTCTACAATCTAGTTGGACGATTGCTAAATTGACTTTCCAGCGTCCGAGTCCACA
GTGGGATGGTAGTGCACTTAAAGAACTCAGTCATGTACCTATCTTCATTTATAGTACAATCTGGATTATCTTC
AATCTTTATGCAGTGAATATTTTACTCAAATATACTCACTTGATAAATTAACTAATAAAAAAGAAGCCTAAAG
CAATTATCCATATGTCTTTATCAGTACAATCATGGATATCTTTATGGCTTCTTTATTTTATTTTTCAGCAAAC
ATGTTCATTGCATTTTCGTAACTGACAATAATTACATTATCATTACGTTTAATAATAATTACTTTATTCATTT
CATCTTTCGATACAATTTCCACTTCATTACCAATAGAAATATCTTTACTAGACAAATATATTAGCAAATCGGT
CTTATCTCTCACACGTTTGATTGTAACGATATCACCAGGTTCATAATTCAAAATCGTTGTTATATATTTCTCT
TTATATTCATTATTTCTAGGAATCACACCGCCGTGCGGGCAAGTTTCTGGGAAATTTAACAGGCTATCCAGCC
TTTCAACAAATAAATCTGAAATTCGATGTTCTAAAATTTCTGCTTCTTGATGTACTTCTTCCCAATTATATTT
CAATATTTCTATTAAAAATAATTCTAATAGTCGATGTCTCTTAATGATATCAAGCGTATGCGTTAAACCATCC
TCTGTTAATCTAACACCTTTGTATGGTTTTGTTTCAACATAGCCTGCTTTTTCAAGACGTCCTACCATTTCAC
TTACAGATGGAGGCTTAATATTTAAAAATTGAGATAAGATTTTATTTGTCACAAAGTTTTTATCGCCATTATT
CGTAAGGATTGCCTTTAAATAGTCCTCTTTTTCTTCAGTTAACATACTTTCACCTCACATACATTGTCTATAT
TATATCACGAATTTACTTGACATGATAAATATTCTCAGTTTATTATACAATTAATTAGGTTAGCCTAAACTTT
TAATTAGGAGGTATAAACGTTTGTTAGAAACAAAAGATTTAAATCTGTTTTTAGGTAATAAGCATGTACTTAA
AAACATTTCCTTATCGATACCAGTACGCGGCGAAATAATTGGTATCATGGGCCCGAATGGTGCTGGTAAATCT
TCCCTTATCAAGTCTTTAATTGGTGAATTTAATGCTACCGGTACTAAATTGTTATATAACAAACCTATACAAC
AACAACTGCAACATATTACATATATTCCACAAAAAGCACATATTGATTTAGATTTTCCTATAAGTGTGGAACA
AGTGATTTTATCAGGTTGCTACAAAGAAATTGGATGGTTTAGACGACCTAATAAATCAGCAAGGGATAAACTC
AAACAGTTATTAAGCGATTTAGAATTAGAATCTTTACGTCATCGACAAATTTCAGAATTAAGTGGTGGACAAT
TACAACGTGTGCTAGTAGCAAGAGCATTGATGTCCGAAAGTGAAGTTTATTTTCTAGATGAGCCGTTTGTCGG
AATTGATTTTAGTAGCGAAAAATTAATCATGACAAAAATCGAGAACTTAAAACAACAAGGAAAACTTATTCTT
ATCATCCACCATGATCTATCAAAAGCAAAGCAATACTTTGATCGCATTATTCTATTAAATCAAACATTACGAT
ACTTCGGTGATAGTGAAGAGGCTATGAGTGTCACTCGCTTAAACGAAACATTTATGAGTAGCACTGACTGTAG
TGACCCTAGTCAAAGGAGCAATATAACATGTTAGAGTTTGTCGAACATTTATTTACATATCAATTCTTGAATC
GAGCATTGATAACTTCAATTATTGTAGGGATAGTTTGTGGTACAGTTGGTAGTTTAATTGTATTACGTGGTCT
TTCATTAATGGGAGATGCAATGAGTCACGCAGTATTACCTGGTGTTGCCCTATCATTCTTATTGGTATTCCA
ATGTTTGTAGGCGCACTTATAACTGGTATGATCGCAAGTATTTTTATCGGTTATATCACATCTAGTAGTAAAA
CGAAACCTGACGCCGCTATTGGAATTAGTTTTACCGCTTTCTTAGCTTCGGGGATTATTATTATTAGCTTAAT
AAACACTACGACAGATTTGTACCATATTCTGTTTGGTAATTTACTGGCAATCACAAATAGTGCATTTTTAACA
ACTATTGTGATTGGTTCAATCGTCCTTATTCTAATCATTATTTTCTATCGTCCATTAATGATTTCTACATTCG
ATCCAACGTTTAGTAGAATGAGTGGTCTAAATACGACGTTATTACATTACTTTGTGATGTTGTTACTCTCATT
AGTAACAGTAGCAAGTATTCAAACGGTAGGTATTATTCTTGTAGTTGCATTATTAATTACACCTGCATCTACA
GCATTTTTAATTAGTAAAAAACTATACTCAATGATGATTATAGCAAGTTTAATAAGTGTCATAAGTTCAATTG
TCGGACTTTATTACAGCTATATTTACAACATTCCTAGTGGTGCGACAATCGTACTTTGTACGTTTGTAATTTA
TATCATCACATTATTTTTCACAAAATTTACGAATAGAAAGAAACGAGGAAGTTTAACATGAAAAAATTAGTAC
CTTTATTATTAGCCTTATTACTTCTAGTTGCTGCATGTGGTACTGGTGGTAAACAAAGCAGTGATAAGTCAAA
TGGCAAATTAAAAGTAGTAACGACGAATTCAATTTTATATGATATGGCTAAAAATGTTGGTGGAGACAACGTC
GATATTCATAGTATTGTACCTGTTGGTCAAGATCCTCATGAATATGAAGTTAAACCTAAAGATATTAAAAAGT

TAACTGACGCTGACGTTATTTTATACAACGGATTAAATTTAGAGACTGGTAACGGTTGGTTTGAAAAAGCCTT
AGAACAGGCTGGTAAATCATTAAAAGATAAAAAAGTTATCGCAGTATCAAAAGATGTTAAACCTATCTATTTA
AACGGTGAAGAAGGCAACAAAGATAAACAAGATCCACACGCATGGTTAAGTTTAGATAACGGTATTAAATACG
TAAAAACAATTCAACAAACATTTATCGATAACGACAAAAAACATAAAGCAGATTATGAAAAGCAAGGTAACAA
ATACATTGCTCAATTGGAAAAATTAAATAATGACAGTAAAGACAAATTTAATGACATTCCAAAAGAACAACGT
GCCATGATTACAAGTGAAGGTGCCTTCAAGTACTTCTCAAAACAATACGGTATTACACCAGGTTATATTTGGG
AAATTAACACTGAAAAACAAGGTACACCTGAACAAATGAGACAAGCTATTGAGTTTGTTAAAAAGCACAAATT
AAAACACTTATTAGTAGAAACAAGTGTTGATAAGAAAGCAATGGAAAGTTTATCTGAAGAAACGAAGAAAGAT
ATCTTTGGTGAAGTGTACACAGATTCAATCGGTAAAGAAGGCACTAAAGGTGACTCTTACTACAAAATGATGA
AATCAAATATTGATACTGTACACGGAAGCATGAAATAACACGCTGTGTTTTAATGAAGTAAGATGAATTGATG
TTGATGCAACCTAAAATATTGGTATCTCCAATATTTTAGGCCACACATCAACATAACAAAGTCGAAGGCTAAT
AGTCCCATATCGTGCGTTAAATATATATTACCCTCCTATTAATATATATACCGTTCCCGATCGCACGATATGG
TGGTATTAGAACTTCTCTTTGAACGAAAGAGAAAAGCTAGAAGTTCTTATGCAGTTTTAATTAAACTGTCGTT
CCCTTCATCTCTTTTAACCACAGAGATGCGTTAGAAGTTCTTCTAATACAATTTATACAACGCCATTCCCTAC
ACACTCTTATAAAAGAGATTCACGCGCGTCAATAAATTGTATTACATACTAACTAAAAAGCTTTTCTTAATCG
TACTAACGAAGTTAGAGGTTCTTATGTGATTGGACAACTGTTCTCCCTACTAGACTTTTAGCATAGCTGGTTA
AAAGTACTATTACAGATTGTTCAATTGTATGCCCCTTTCCTTTGAACGGAATATTAAAAGCTATGTGCAGTAA
CTTTTAATAGGCATACCACTTTTGCATCAAGCTATAAATAATCGCATACTTTGATATTTAATCTGGCCTATCA
AAGTAAACATCTATGGTTTCCTCCCCCCATAGATCCTTAAGCCACACTCATCCGATGTCATATGAGTGTGGCT
ATTTTTTGTGAATGCACGTATCTTCTTAATTTAAATGACTTCGATTAATCTATCTCCTCCAATTTTTAAAACT
TATTTCAATTAATTCTCAAATATTAATAATTCGTATAGTTTAAAACTATCTATTCAATTCCCCTAAAATAAAA
GGGATTGATGAAAAGCCATTCATTTTTTCATCAATCCCTTTTAGCATAATTACGAACTACGTCATACTATCTA
CAATAACCATTTCATCATAATTAATGGCCTCTCTTAGTTTTAACGCTGTACCTTCTGATACTTCATCTGTATC
AATCAACTCACGTAAAATGCGACGTTGTTCTCTCAATGCATTTAATTTAATTTTAGTAATCGTATCTTCACTT
GCTGAGTTGAAGAAATTAGATGGTGTTAAGTTTTCAATTCGCATTAAATAACCGTCACAAACCATTCCAACTT
CAAGTGTATTATCTTTTGTTGTTTCCTTAGACAATCGACTAACCACATTATAATGGACTAATTTATTAATTTT
ATTTAATTCCATTAAGTTATCCGTAACACTAAGTGAAGATGCCGAGTTCACTCTCGTTTGTACTCTTCGTTTT
AATAAAGCACCTCGTAATAATACAATCATTCTTCTTATTAATGATGCTTGTCTATATACTTGTGTTCTTTCAG
CATAACGCATATAGTTTTCAAGTACACTATTCGTTATTTGTCCTTCATCTACTAAAGACTCTAATGTTTTTGT
TTCTACATTAAAAGCAATTTTTTGTAGACGTTCTAATTCTTTAGAGTTTTCATCATCTTTCTCTACAGTTTTT
AAAAATGCTAATTTATCATGATATTCTTTAATCACGTTACCATATTTAAAACTTGTTTCGAAAGTAGATTTTT
GATTTAGATAATCAATAACTTGTTCTAATATATAAATTCTAGCAACTTTAAACGACATATTGCCAATTACTGT
TTTAGGTGCAGGTTTCGTTAATAGTGGCAATAATACTTGCGCAACTACCAAACTAATAATAACCATACCAGAT
GCAATAAATAATAAGTCGTTTCTATACGTAAAAGCATGATGCCCTGCTAAAAAATACGGTAACGTTAATGCGA
TTGCTAAAGAAATTGTTCCATGCACACCACATAACGTCATAATTAAAGCGTATAAACTTCGCTTTGGTGGTTT
CTCAGTCGTTGGATTATCATCATCATTTTTAGTCATCATTTTTTGGAATGGACTGATGGCTAAATAAAAATAA
GGATATAAGACATAAACCCAAACAAATCTAAATAGATAGACAGCTAAAGCAACAACAATAGTGATGCCTATTA
AAAAGATTAAATTGTGCGGTTCTGTTTTGATAATTTTAATAATAACTTCAGGTACTAAAAATCCTAATATTGA
AAAAACAAAGCCATTTAAAACATAACCTAGTATATTCCATGTATGATTGTAACTCATTTGTAGTTGTGTACGT
ACTTGCATAATTCTGTCACGTTCGAAACCATGTACAAGTCCTGCAACTACTGCTGCAATGATTCCTGATGCGT
GAAACAATTCAGCAATTAAATACGTAACAAATGGTGTTAACAATTGAATAATTGTAAACATATTAATGTTTTC
ATATCCTCGACGCATCAATGTTAATCGGAACCTTACTAATGCCATACCTATAAGTAAACCAACCACTGCGCCA
CCAATTGATGCAATTAAAAACAACTGAACAGCATCAACAAGTGAAAAAGCACCTGTAACTAATACTCCAACAG
CTATTTTAAATGAAATAATACCAGCAGCATCATTCAATAATGACTCACCTTCAAGAATTGTCATTGCTCCTTT
TGGCAAGACCTTTCCTTTAGTGATTGCTTGCACTGCTACTGCATCAGTAGGGCAAAGAATGGCAGCAATTGCA
AATGCTGCTCCAATAGGTAAATCTGGCCAAATCCAATGAATAAATAAACCTACACCTATCACAGTAGTAATGA
CTAATCCTAATGCCATCATCATCACTGGCTTAATATATTTTCTTAAATGGACTCTAGAAACATTAACACCTTC
TACAAATAACAAAGGCGCAATCATTGTTACCATAAACAATTCAGAATCAAAATTAAATTGAACAGGGATTGGG
GTAATAAATAGTAACATGCCCAAGAAAATTTGTATAAATGCTAGGGGTACTTTAGGTATGAAAGTATGGACAA
ACGAACTTAGTATCACAACAGCTATAAATATAAGAATTGTTTCAAATATTTCCAAACTTTCACCTCTCTAAAA
AGTATTGTTTAATTGAAAATTAAGTATCACATCTCGTTGTAATTATACTTTAGAGGATAAATTGAGTTAGCGA
CCACAAAAGCACTTTAATATAGATATATGTCTACGATTGCAGTACTTAAATTTGCAATTATTTAATTTTATTT
TATCACTAATTGTTTGTATAAATAAACAACTTGCTTTCACATAACAACATTAACTTATAATACAAAAAATGAG
CACCTTAAAATCGACTAACCAATTTCAAAGTACTCTTTTAATGATTAATTTTGAAAACAAATCTTCAAAAGCA
TCGTTATGCTTAACAATTTAGCCAACACTTCAATCGTTTTGATACCATTTCTTACGATGCTCTTCTCGTTTTT
TAGCACGTAATTGTAATGCTTCTGTAGAGTTTTGTTCATTTGAACTTAATAATATTGATGCATGTGTGTGATC
ATCATTTTTTCGATACATATAAGCGCCGTTGCGATAAGCAGCGCGAGCGACTAAGTGCATGCCGACGGGTGAA
GTTAAATTAATAAAAACAAGTGACAGTAATAAACGCACACTGAAAAATCCTGTATTCACAATAAAATAAATTA

ATACACCGATTAAAGTTAATAACACTGATAAAGTTGAACTTTTTGTCGCAGCGTGACTTCTTAAGAAAACATC
TTGGAATTTCACGATACCTATTGCACTAATAAGAGCAATAAAACTACCTAACAACAACATCACAGCAGCAATA
AGACTAAAGATTTCTTTTGTTATTTCCATTAAACACATGCCCCCCACCAATAAAGCGTGATATTGAAACAGAA
CTTACAAAAGATATAATGGCAATGAGCATGATTGAATCTAAGAAAGAAACGGTGCCCATAAGTACACTTAACA
CACCCACAATTGACATTACGACAGCACTTGTTGTATCAAATGTAACGACACGATCTGCTGTTGTAGGTCCCTT
GATTAATCTAAATAAACAGATGATTAATGCAATTCCAAAAATAATGAGTGAACTAATAATCATAATATGTGTT
ATTGTTTGTATCATCGCGACACCTCCAATATTAAGTCTTCATAATGCTTAATACTTCTTAACAAACTATCTTT
TTCTTTTTCTGACACGTCGATACTATGAATAAAAAACTTTTTAGAGTCTTGAGAAATTCGTATTACTGTAGAC
CCTGGAGTTATAATAATTAAAATTGTTAAAAATGTTATTGCCCAGTCACTTGTTAGTCTTGTTTCATATGAAA
GTAATCCAGGGTTCATATCTTTTGTTTTAAAAAGAATATAATTAATCGTGCTAATGCTAGATGTTATTAATTG
ATATAAATAAACACCTAAAAATTTAATAGCTACCCAAATTTTTCTAACATAAAAATCATCGCTGAAAAACCTG
TGTAATATATAAATGACAATTAGACCAATTAGATATCCAGAAAAGAAAGTCGAGAATTTAAAATGATCTTCAT
CTTGAAATAATACCCATAAGAATGCAATGATAATATTTAAAACTATTTGATTCATTTAGTCCTCTCCTTTCAA
ATGCGGATTTACAAGTTTTTGATATAATTGATCACTCGTGTTCAACTCAGTTGCATCACTTGTAACATTTAAC
ACTACAGGCGCAGCAATTCCGATTGCGATAACCACAACTACTAAAATACTTAAAATTCTTTTTCGATATAGTG
GGATTTTCTTAAAATTAACTTCCTCCCCATCTTTATCTCCAAAATACATATAAAAAAGTATCCTAAATAAACT
GTACATTGCAATTAGACTAGTAATAATCATTAACGCTAGTCCAATATAATTGCCATTTTGCAATGCGCCTTGG
AAAATAAGTACTTTCCCCGGAAAGCCACTAAATGGGGGCACGCCGCCAATAGCAAAAATCATTATAATAAACG
CAACTCCAAATAAAGGTTCTTTTTTAGCTAAGCCATTCAAATATTGATATTGTCGATAGCCTGTAATGTAAAC
TAAACTACCAATAATAAAGAATAGCAATGTTTTTACAACAATGTCATTTACCAAATAAAATATTGCACCATTA
ATACCTGCAAACGTGTTTGTTCCCAAACCTAAAATGATAAATCCTATTGAGATTATGACTTGGTAAGCTGCAA
TCTTTTTAATATCTTTATAAGCAATGACACCTATAGCGCCGATGACCATAGTTATAGCAGCCATAGTTGCTAG
CAATGGATGTATGAGATCATTATGTTGATCGAATAGTAAAGTGAAGAATCGAATTAATGCATAGGCCCCTACT
TTGGTCATTAACGCTGCAAATAATGCTGCAAGCTCAGTATTTAACACTGCGTATGCTTTGGGTAGCCACATAA
AAAGAACCAGCGCTGCTTTCGCACTAAATGCGACTAAGAAGATTAATGAAATCATTGTTACAGTGCGATTATC
TCCCATATCATTCAAACGCATTGCAATATGTGAAAAGTTTAATGTACCTACTGTTTTATAAAGTAAACCTATA
CCTAATAAGAATAGCCATGAACCAATAATATTCAAGACAACATAAATAATTGCGGCACGTAATTGTTCAACAG
ATTGTCCAAGTGTAATGAGTACAAATGACGCAAGTAACATAATTTCAAACATGACGTATAAATTAAATAAATC
TGATGTTAGAAAAGAGCCTATCACGCCAACGCTTAAAAATAATATGAACGATGGCAAGTGATAACGATTTGCT
TTATGTTCGCCACGCCCAAATCCGTATGCCATAATTAAAGTAATCACAAATGAAGCGGTTGTAACCATAATTA
AACTTAAAGAATCTCCTAAAAACTGTATACCAAAGGGCGCTGACCATCCTCCAAAGTCTAGCGTAATTGGACG
GTGACGCTGAACATAAATTAATAACATTAATGAAATAATTGTGGTGATAGTCATTGTACCTAAGTATAAATAT
TTAGAAATACGATCATTATTTTTTAAAAATACAAGGATTAAGGCACAAAGGAATGGTAATAACATTGGTAAAA
TCAATAAGTTACTTAGCATCATCTTCCCCCCTTAGGCCTTCAATTTCATCTTCTTTTGTTACTTTATAAGTTC
TATAAACAAGTACAAGTAAAAACGCAGTCATCCCAAACCCTATAACTATTGCAGTTAGTACAATAGCTTGTAA
CAAGGGATCAACAAACAATTGGTTTCCACCAGTTATTAATGGTTCTGATCTACTAGAACCATACGTTCCCATA
CTCATAATAATGAGATTACCAGCATGAGTATATATTGAAATTCCAATTACAATACGAATTAAATTGATTGATA
AAATCATATATGTTCCTATAAACACTAAAAATCCTATAACTAGTAATAATATTAAATTCATGATCGACCTCCG
CTAAGCGACAACATCACTGTGACAATAACACCAACAACTGAGAATAAAATACCTAATTCAAAAAGTGTTATTG
TACTTACATGAATTTGCCCTAAAATTGGAAGTAACCAAGTTGTTTCATATTGAGACAAAAATGGTTTTCCAAA
AAACATAGGTATTATCGCAGTAATAGATGATACCAATGCTCCAATAATCATTAAAATTCTAAAATCAATCGGT
AAACTTTCTAAAACCTCTTCAACATTAAAAGCCAGAAACATTAAAATAAACGCTGAACTAAATATTAAACCAC
CAATAAAGCCACCACCAGGATTATTATGACCTGCGAAGAAGACATAGAATCCGAAAGTCAATAAAATAAATAC
AACAAGTTTCGTGACCGTTCTTAACACGACATCATTCTCTTTCATCTTGTCCCCTCCGATCTTGATAATTTAA
TAATGTGTAAATACCTAGCCCAGTAATAATTAACACTAATCCTTCAAATAATGTATCTAATGCTCTAAAGTCA
CCAAGTATCGCATTTACAATATTTTTACCACCTGTTAGTTTGTCAGCTTTTAAATAAAAGTCTGATATTGATG
ATAAACCATCTGTTTGTTGTGTAATAAAAATTAATGATACAACAATAAGTGCCATCAAGAGTGATACAGAAAT
TTTAATTATTTCTCTTTTTTTTGTTAGCGTTAGATCTTGGCACGTTTGGTAATCTTGAAAAACTAACAATAAAT
AGTATCGTCGTTATTGTTTCAACTACTAGCTGAGTCAATGCTAAATCAGGGGCTTTCATTGCTATAAAGAATA
AGGTCACAACAAATCCGATGACTCCATTTAAAATGACCATTGTCAGTCGTTGACGTATAAAAATTAAAGAAAT
ACCAATTGTGACAGTTACGATTGCTAATATAATTTCTAATGCCCCAAATTCAGAAACATGTAACTGATGTACT
TTTGGAAGTCCAATTCGAATATAACCATATCCAATGATAATCATAAATATGCCTAAGGTCATAATAATGTATT
GGTTTAAACGATCTTGCATAACACGTTTAAATCGCTTCGTAGCAAACTTTTCAAAATGTCGATATACCATCTC
ATAGCTTTTTGAAACTGAAATCTGTCTAATTTTACCTGTGAACACTTTTTTCCAATCTACTTTGATTGCTAGT
ACACTACCCAATAAAATAATGATGATGGTTAAAAGAAGCGGTATGTTAAATCCATGCCATTGCGAAACATGTG
GTGCCAATTGATCAATTTGATGATTACCACCTGATACAGCTCTTAATGCTGGAACGATAATCCCCTTCCCAAA
TATATTTGGTACAAAAAAGATTACAGGTACTAGCACCATTAATATAAGAGATGGTAAACTAAACAACCATGGT
TCGTGGATATTTTTTTTAGTAAAAACCTTAGAATCATATTTTGTCCAAAATACTTCTTTTACCATGTATAATG

CATATGTGAATGTAAAAACACTCGCAATAACACCAACAAACACAATACTTATCATTGAAATCAAACTAAATTG
GGATAATTGTCCAGTTTGTGTTAATGCATCTAAGAACATTTCTTTACTTAAAAATCCATTTAAAAATGGTACT
CCAGCCATAGATAGAGCCGCTATCGTCATGACTAGATTCATTTTAGGAAATAGTTGACGCATTCCACTTAAAA
TTCGTATATCCCTTGAACCTGCTTCATGATCTAAAATACCTACTCCCATGAAAAGCGCACATTTAAAGATGGC
ATGATTCATTAGATGAAATAGCGCACCAAATAATACAAATACATAAATAGATGCTATTGCGTTTTGTTGGTGT
TGAGCATATCCACCACCGATACCGACCATTGCCATAATCATTCCTAGTTGACTGATTGTTGAATATGCTAGGA
TTCCTTTTAAATCCCATTGTTTTAAAGCTGTGATTGAACCAAATAACATTGTTATTAACCCGACAAAGGTTAC
GATATAAATATACATATTGCTTAGACCTAACAAAGGTGTAAATCTAAGTAATAAAAAGATACCAGCTTTAACC
ATAGTGGCTGAATGTAAATATGCACTTACAGGTGTAGGCGCTGCCATTGCCCTTGGTAACCAAATATGAAATG
GGAATTGTGCAGACTTTGTAAATGCACCTAATAAAAACATGATAATCATAGGGATAAATAAAGCATGGTTCTT
AATATGATCTGCTTGTCCTAATATCTCTGTGATGTTATTCGTTCCTGTCATGATATACAACATGATAAAACCA
ACTAATAATGCCAAACCACCAAATACTGTAATCATAAATGATTGCATCGCACCAAATTGACTATCTCCGTTGT
TATACCAATATGAAATTAATAGAAATGATGATACGCTAGTTAGTTCCCAAAACACATACATTAATATTGTATT
ATCAGCTAATACAATACCGAGCATGCTAAACATAAATAGCGTTAAGTATAAGTAAAACCTTGGTAAATTATCT
TTACGAGAGGATAAATATTGTGTTGCATAGAAGAATACAGCAATTCCAATAAGTGAAATAATAAGAGAAAACA
TTAAACTCAAACCATCTAAACGTAAATCTACATTAATATCTAATGAGGTCATCCACGGAACGGAAACAGACAA
ATAATGCTGTTTAATTACTGATGGAACTTGTAACAAAAAATAAATAGATGCTATTACAGGTGCAGTCAGTGCT
ATGTATCCTGCATATTTAGCCATTGCTCTACGTTTAGATGTTAATAATATCATCGCCATAATTACTAGTACGG
CAATTAATAGATAAACCAAACTCATTATTAGCCTCCTTTGTCGTTATAATTGTAATGAAATATAAATACTGTG
TTCACATTCATTTTCTAAACCGATAAAATTTAGTGTTTCAATAGCAGATTGATGCCCTAAATACTTTTGAATG
ACAGGAATAGGTATGCCCTTTTGATATGCATGATACGCAAATGTCTTACGCAATGTCGTTAGTCCTACATTCT
CTATGCCAGCCTTAATTGCTGCTTGATGAATAATACGATAGGCTTGTTGCCTAGATAATACTTGATTAGTTCT
CAATGATTGAAATAATACATCTTCATTTGACAGACTTTTGTTTTCAATATAGTGAAGTAACTCATTAGATAAT
GTTTCTGGCAATCTAATTTTAATCAATGAGTGATAATGGGCTTTACACAATTCGCTTAGTGTCCCCTTTTCAA
TCAGTCGTTTAGTATCTTTAACAGTAATTGTTAATAATTCTGTAATTTTCATACCACTATGTATAGCTAATTT
AAAAAACAAATAATCCCTATCAGATCGAGCTTTGAGTGCGTCATACATTTTATTAATATCATCATTGCATTTA
ATTACTTCTACCTTGTTCATATGTCCCACCTCGCTTTAAGTGTCTTAATCATAAACATTAAGTATTTAAGTCA
CATATAATTATAACCTTTTTTATAGAATTTAAACAAGAATAATAAAATTAACCCAATTATTTTTTTCTTTCGA
TATATAGTGTGAAAGGAGGTTTTAAAATATGAGTAAAACAAATCACATTACAATTGTATTATCATTTACAAAA
CTAGATACAAATGGTAAACAAACAGAATTTAAGCGTCGATTCGCCAATATTAATCCTGAAGTATCAAACGACC
AAATTAAAACATTTAGCAAACTTATCGAACGACTTACTGGAGAAACATACAACAACATCGAAGTCATCAAATC
TGTATCAATTTAATCAGGAGGACACTTTCATGACCAAAACACTTGAATTAAGTTTTAAATCTTCACTAGACAA
ACCTGTCAAATTGCAATTACCACAATTAAACAACATCGTAACCGAGCAAGTTGCACGTGATAGTATGAATGCA
TTAGTAGATTTAAATATTATAAAGTCAAATAGTGGCGCTATCACCAAAGTACATTCTGCTCAAATTATTGATA
AAACGACGACCGTTTTATTTGAAGCAAAGAAATAGATATAAAGAAAGCGCAATTAATTTAGTATAAATACTAA
CACCCTAGACTTTCATGATGATTGTCTAGGGTGTTTTTGTTGTGTTAATGCCCTGTTTTTTTCAACATTATTG
TAAATGAGTTGTATATTTTCTCTATAATGATAATGTATACATATGATTTTTATTATTAACTTTGGAAAGTGAG
ACTTTATTCATATGCTTCTTTTCTATATCATTGGTGTCCTAGTTGGAATGCTTATTCCTATCCAAACATCTGT
CAATTCAAGACTATCACTGTATACTAAATCGCCTTTCTATACTTCATTCATTTCATTTTCAGTTGGAACAATT
TGTTTAATTATTTTAAATATCATTATCAATCCAGAAGTATTTACTATTCACTTTTACAATAATCAATCATTTA
ACAATACATGGATAGTCGGTGGTTTGCTTGGGGTTAGCTTTTTAACTGGCAATTTATTGTTATTGCCAAAATT
AGGTGCAACATTAACTGTAATTGCAACAGTTGCGGGTCAAATTATTATGGGTGTCATAATTGATACATTTGGC
TTATTTGGTGCTACAATTCATGATTTTAATTTAATTAAAGCAATTGGTGTATTGTTACTCATTATCGGTATCG
TCATAATGAATCAATTTAACAAGAATAATTTATTACTAACTGATCAAAAGTATTTACTGTTTTGGCTTCTATT
AGGATTTATTTTTGGTTTCTTTCCACCTATTCAAACGACAATTAATAGTGCTTTAGCAAGTCATACTCATTCA
CCAGCCTTTGCATCATTAGTATCATTTACAATTGGGTCAATAACGCTATTGATTTTAACCGCTATTTTTAATC
GTTCTTTAAAACTAAATACATATCATTTGAAATTCGGCAAACTAAAACCTATCTATTTACTGGCGGTATACT
AGGCATGGCTTTTGTAACAGCTAACATTATCTTAATGCCTCATATGGGTGCAGCATTAACAACACTTATTGGG
ATGTTTGGTCAGATTCTAATGGGCATATTGATAGATCACTTTGGATTATTTGGTTCACCTAAAATAGCAATGA
CATCCAGAAAAACTATTGGTCTATTATGTATTTTGACAGGCATTATACTTTTAAGATTATTTTAAATTAACTT
TTAGCTTATCATTTTAACTTGTAATTATTTTTAAAAGTGATAAGCTATTTTTTTGTGGTCTAAATTAGTTTTA
ATAAGTACTAATATGGGTAGTATGCTTTGACACAACAAATTTTAATTTAGCAAATTCGATAGTCAACTCATTC
TTAAGACCTAAATTAATGTTGTTTTTTAATAATTTATACCAAATTAATAGCAAAAATTATGTTATTCGTGCTA
ATATTTCATAGTTGGTTATTCAATTAATTAAAAATAAGTCAAAATGCACAACTTTAATTCATTCATTGAGTCG
AGTTTGAAAAATAAAGGTGCTATAACGTATGATCAATTATCGTACTTTCTATTATTTGTTACCCGTTATCAAT
CGGAATAACGTATAGACACTTTAACGTGCTATAGATTGGTTTTAATCACTAAATTAGTGTGTTTTCCTTATCA
TTAAAACTGCACTGGGAATTACTAAATTAAAAAAATTATAAAAATTTTTCTTTTTTAGTGATAAAAGTTCGAA
AAATGGGTATAAATATTAGAAGAAGTTAACTTGAAAGCGTTAAGCTATGACAATGAATCACTTTAGATACACA

TTGAATATTGAAACATTTAATTGCGCTAAATCGTTTCATAAAATAAATTACCTTATACTGACGATTAAATTAA
GGTAAATTATAAAAAATGCTGATATTTTTGACTAAACCAAATGCTAACCCAGAAATACAATCACTGTGTCTAA
TGAATAATTTGTTTTATAAACACTTTTTTGTTTACTTCTCATTTTTAATTAGTTATAATTAACTAAATAATAG
AGCATTAAATATATTTAATAAAACTTATTTAATGCAAAATTATGACTAACATATCTATAATAAATAAAGATTA
GATATCAATATATTATCGGGCAAATGTATCGAGCAAGATGCATCAAATAGGGAGGTTTTAAACATGGCAATTA
CAAAAATCAATGATTGCTTTGAGTTGTTATCAATGGTCACTTATGCTGACAAATTAAAAAGTTTAATTAAAAA
GGAATTTTCAATTAGCTTTGAAGAATTCGCTGTATTGACATACATCAGCGAAAACAAAGAGAAAGAATACTAT
CTTAAAGATATTATTAATCATTTAAACTACAAACAACCACAAGTTGTTAAAGCAGTTAAAATTTTATCTCAAG
AAGATTACTTCGATAAAAAACGTAATGAGCATGATGAAAGAACTGTATTAATTCTTGTTAATGCACAACAACG
TAAAAAAATCGAATCATTATTGAGTCGAGTAAATAAACGAATCACTGAAGCAAACAACGAAATTGAACTATAA
TTTTGTTTAGCGCAATTTGGTGAAGTTTGATAGATGATACATTCTATTAAACTTCCTTTTTTTATGCTCTTTT
TACCTAATTGTTAAGAGGTTTTGCACTAATGGCACTTAATATAAGGAAGATAGCCGCATAACGAGCAGTATAT
ATCAAATACATTTCAAAAAAAGCTGATTTCTATCAAATAATGAATAGTAATCAGCTTTTTCACATTGCCTAAG
GACTTTATGCCCTAGGTTTATATATCACATATTATGATACGCTTTTTAAAAAATTTAAATAATAATTCATAAA
TGCTTCTGGTTCTTCAATATGCGGTGCATGTCCTGAATGTTCGAAGATTTTTTTCGTAACATTTAAAAAATGC
GCTTCCAATTTAAAATGATTTTTATCTTTAATCAAAGGATCATGTTCCCCATTAATTAACAAAGTAGGTACAC
ATGTACGTTTTTTTAAATACATAGGTTCATCTTTATGCAAAATTAATGAACGAATTGCCACCGCACTATCATC
CTCAGACTTAGTGCTATATATTCTTTGTTTTTGATACCATTTCATCGTCTTCTCTTGATTTCTAAACAATTTA
GAAAATAAAAGAATATACGCTTCAGACTTATTAAAACCCGCTATATCATCTTGATATTGTATTAACAATTGAG
TAAAGCTGTGTATTAAGTCATCTTTCTTCGATGCAATGGTAGTTAATGATTTTACACGATAAGCATATTTATC
TGTAAATAACTTAGCAACTACCCCACCTAAATCATGTCCTAGAATATGTGCCTCATGAATATTTAACTTCTCC
ATTAGAATTTTTAAATCCTCAACGTGATCGTTTAAATCGTATGATTCACTTTTAGAAGACTTGCCATGACCTC
TTAAATCGTAAGTAAGTACATTATATGATGATGCTAGTTGATGTTGCAAATCTTCAAATCCAGCTAAATTACC
ATCCAATCCATGAAGTAATATTATAGGATCCCCTTTACCTTCAATTTGATAAGTTAATTGGATTTGAGGATTA
ATTGTGACTTTATTCATTGTGACACCTCCCTCAATAAACTATATACCCAACCTTCATTCTATATACGCTTCCA
TTTTATAAACAATTTTTTTAAATTATTTATAAATTACCAAAAGTTATTCCAACAATAAAAAAACTATTCATCT
CAATTAATACAATTAATCAAGTGAATAGTTTTATCATATTTATAATCTTGTACGTGAAGATAATCTAATTGTG
AAATATATAACTAGAACTACTAATAATATCCATATAATTAATGAAAAAATCTGGGCTGATTGTGGATTATGAA
TCATTGCATCGACGGTTTTTTGACAAAATAATAAGCCGATTCCCGAAAACTCAAGTCGCTTATAAAATATGCC
GGCAATTACTAAAATAATACCAATGAAAAAGAAAAATTGATGTCCAAAATTTGTAATTGGTATAACGCCCCAA
GTAATATTAAATGCTTGTAATATAATTAATAACAAGGATATGATAGCGATAATTGTACCCATAATCATTTCTA
TTTTATTACTACGCAAGTAATTCTTTTGAGACACTCTTAGGTATACAACAATAGTCATTGGAAAAATCACTAA
ACAATAAAATAGACTCATTCCAGTAATAGCTTGAAACTGAACATACTGTTTATCATATAAATAGGATATGAGA
ATGAAATTTATAATAAAGAAAATAACTGGATTCATTTGTAAAGCAAACAAATTCCTTTGAATCATTAATATGA
TTTGTGCTGGAGATTTTCTTTTATATTCAATATAATCTTGATCTTTCTTTTCAGAATTTTTTATATCAGTAAT
TACTTTCTCTTTAATATCATCAATCAAATGCGGTGATAAACCTTTATGATTAAAGTAATCTTCTATATTTTCT
AGTAAAATTTTATCGTTAATACGCATTGGTAGTACTCCTTTAAAATGAAACATCTTTATTTATTGTATTGGTT
TAATATCCCCTTCGCAAATCATAAAAATTAATATTCACTGTATTATGCCATGTAATAAAATAACCGACAAGTA
TTGCGATTTAACTTGTCGGTTAAGCACGATTAATTTAATTATTATTTTTCCAAATCAATACGATATAATTTTA
TGTTTTTATCTTTTGGTGCTGAGGCAAATTGATAAGTCGTTTTGCCTTTTTCAATATATCCAAAATTACCAGC
AACTTTGCTATATTCCGTATGTGTTACTGCTTTGCCAACATAATTTTTAACAGCTTGATATGTTGGACCATTA
GCATCATTATACATCATTGAAATATGAGTTACTTTACCATTGTTTTTCTTACCATCAGTAGTTACTAATAACA
TACCTTTTTTAGTATGGAATTCGTAATAATGTTCAGTTCCATCTTCATTGTAAGAATACATCGGATTTTTGTA
CTTAGTTAAAACATCTTTAATAGATTCGCCAATTTTCACATTTTCTAATGACTGATCACCCTTAATTAATTGT
TTAACAGTATCAATAGAGTTGCCACTTGCTGCTTCGGCACTAGTGTTCACTAAGCCTACTCCCATTACAACAG
AACATGCAATTATACTTGCCGTTAGTAGCTTTTTCATAAGCACTCTCTCCTTTTATTTATATCGTCTTGTGCT
CACAACCATTATACAACAATCATTTCTAAAACAACAGTCATATTACAATTATATTACAAATAATAATTACTAC
TTTTAAATTTCACTTATCACTAAAAATTAAACATGCTTTTCATCGTGATGTTTGTTGATAAATGAAGCTGTAT
CTTCGACGAATCTTTCTTGCTCTTCAACAAATGGGAATGCACTTGATTCTTGATATACTTCAAAGTCCGCATT
TTGGATTAAATCAGCTACTTCTTTAGCTTCTAATCTTGTTGTTCTTTCACCGAATTCTCCTGCAATAATTTTT
GTCGGTACTACGACATTTCTATAAGTTTGAGAAATATCAGCGTTTTTGAATACCTCTTTAACTGCTTGAATTT
CTTCTTTAGTTGATATTTCATTTGTATCTACGACATGTTTGAGGAATCGATTCATTTTTCTCGGACGATAATA
CTTACGCTTATTTAAAAATTTATCTTGTTTTTCAGGATCCCAGTTTCGAATAATATGGGCATATTTTCTAAAT
AAACGTTCTTCCGGTAATTCACCTTCAATAGATGTTGGATTTACCAACGTAAGTGATGATGTAAATTCAGGAT
AACGTACTGAAATATCCGCACCAATGATTCCACCCATTTCATGGCATACAAATGCAACTTCTTCAATATATAA
ATATTTAAGTAATTCAACAATGTCATCAGAAAAATCTTTTATTTCAATGTGACGAGGTTTATCAGAATAGCCA
TGTCCACGTAAATCAATTAACACAACTTGAAATGATTTTGCTAATTCTGCTGCTAATTTATTAAACACAGAAT
AATTATCAAGTACAGTATGAATCAATACGATAGGATATCCTTCACCTAATGTACTGTAATGTATCGATGTTCC

ATCTTTTCTAGTAAATAGATCCATAATTTTCTCCTTTATTATCATTTTTTATTGGTAAAATGGTTAATAATTC
TTCCACTGTTGTAATTGTAAAATCAACTTCTTCTTCGAGTGGTTCAATTTCAGCATCTTCTTGTTTATACCAT
ACACTAACCATGCCCATAGCTCGTGCTGGAGCTACATCATTTAACGCATCATCGCCAACATACATAATTTGCT
CAGGTAATACCCCTAGTTGATCAATCATATCTTCAAAAATTTTAGGATGTGGTTTACGAAAACCAACTGTTTC
TGATGTTGATAAATAATTAATAACATGCATCAAACCAAGTGAATGTAATCGAAATTGTTTAATCTTAGATTTA
CCATTTGCGATAACACCAATTTGAAAGCCTTTTTGCGATAGCTTTTCTAGTGTATACAAAGTATCGTAATAAG
GAAATACATAACGATAAAAATGCATTTCAAAATCATTAAATAAGTCTTTCCAAGTTAACCTATCTACATGGAA
ACGTTTAATGATTTCTTTATATAAATCAGGTTTATCATTATCTTCATCGTCATCTAGCTCAATAAATGCTTTT
TTAAAATCTGCCAGTTGAACATGAATTAAGTAGTCATGAAATCGTTCATATTGCTCTTCGATAAATTTCTCTC
GAGATTTAACTCTGTCCAACAACGTACCTTCTAAATCAAATACAACTGCTTTAATTTGATTCAAATCCATCGC
TTATGCCCTCTTTATGTTTATAGTTATTTTTATTTTAGCAAATGTCATGTGCTAGTGTAACCGTTTCGACTGA
GATATTAGCATGTAAAATAACACTGGTATACCAATTAATGCAATAATAATGCTTGCCGGTATTTGTACTGGTG
ACAGTATATGGCTACCAATAAAATCTGCCATAACCATTATTTGTCCTCCAATTACAATATTCAAAGCCAATTG
TCTTCCTAATGATTGTTTCCACTGCCATTTTCTAATGAGTTGTGGTATCACCATACCAATGAATCCAATGATG
CCAACATATGCGACATTTATCGCCGTTATCATAGAAGCTATACATAACGCGATATATGTAATCAATTGAACAT
TTAAGCCTAGTGACTGACTTTTTAGTTCTCCTAATTGCAATAACTTTAGTTGATTAAGAATGATAAATATACC
AAACAGTGCAATGATAAATGTGATTGCTATTATGGATACATTTGAGTATTCTGCATCACCAAAACCACCAAAC
AGATAATTGGCAATTGTATTTAATTTGCGAGGTTTTAATAAAATCAAAAAATATAGAAGTGAATTGAATAACG
CACCAATCATTAAACCACTTAATATTAAGATTCTAACTGGATAGCCTTGGCTCAATACCGACGTAATGACTAA
TACAGTTATTAATGTTATCAAACTAAATGTTATTGAAAATACAGGAATCCATAACGTTGTTAAACCTAAAAAT
AATGCTAATCCTGAACCAAATGTAGCGCCGCTTGCCAATCCTAATGTAAAGCTATCTGCCAATGCATTATTTA
AAACTGTTTGAAATATAAGGCCTGCAACAGTTAATATCATACCTGTCAACAATGCTTCTAATAATCTCGGTAA
TCTAACATTGATTAGAATAGACTGGTTAAATACATCATTGATATCGCCCAACTGCCAAAATAGATATATGCTA
GTTGTTATAATCAATATGACTATCCAGCTCAATAATACTTTATTAAATGTCATAGCATTCTCTTTTCTTTCAT
CGATAATTACATTCATTATAGCAATCACATACTCGCGTTGCTATCATTCACTAAATATATTTAAAAACCCCT
TATCTCAAACATGTATTTTTTTACATGTAGCAATAAGGGGCATAATTAGAATGGTTTATTTTCTATAAATTGC
ATCTCTTAATTCTTTTAATCCTTCATCAATACGTGGACCTGGTCTTGATACTTCATCACCATTTACAACTTCA
ATACGTGTATTCTTGACAGCATTAATTTTATTGAATCCACCTCTTTTTTTGATGATATCCATATAATCTGATC
TTGTCTTAGCTTCCGTCGAAATTAATATATCTGGGTTCTTTTTAATAATACTTTCCTTCGTTACAGGGTTCCA
ACCATTAATGTCACTATACACATTTTGGGCTTCTAATTTTTCTAACATATCATTGAAAAATGTATGCTTCCCT
GCTGTATATATTTCAGGCTTTGATGAAACCTCAATAAATACTTTTGATTTTTTATGATGAGCAGGAATTGAAT
CTATGACTTTATCGATATTATCTTTAGTTTCCTCAACAAGTTGTTCAGCCTGCTTATCATGATGCGTTAATTT
CCCAATTTGCTTAAATGTGTTGTAAGTTTCATCAATTGATTGTGCATCTTTAACATATACTACTTTGATGCCT
TGTTTCTCTAATGATGACAATACTTTATTAGCAGTTGCCTTTTGCGACTCATGCGCAAGAATTAGATCTGGCT
TTGCCTTTAAAAGTTCCTCTTTATTTAGATTCAAAGCATCAAATTGTTTCTTACCCTTTTTCACATCTTTTGG
ATAATCATCAACCGTTGAAACACCAACTATGTATTTACCTAATCCTAATTCATATAAAATTTCAGTATTACTA
GGCATTAACGAAACAATTCTATGGTACGGCGATTTAGACGAGCCATTATCATCGCTACCTTGTTTATGATTAT
CTTTCATACCACAGCCACTCAGGACAAGCATAAAAATCAAAATAAAAGCAATTAACGATTTCTTCACATTATC
ACATCTCCTTTTTAATCCCACTTTTCGCTTTAGCGATTAGAGGGATTTATGCAGTTGTGCGGTAGCATCCAAC
TGCGTTCCTCTTCATCTTTAGCGATTAGAGGCTTTTATGCAGTTGTGCCTTAGCATCCAACTGCGTTCCTCTT
CACCTTTAGTGATTAGAGGCTTTTATGCAGTTGCGCGGTAGCATCCAACTGCGTTCCTCTTCACCTTTAGCGA
TTAGAGGGATTTATGCAGTTGTGCCCAAGCATCCAACTGTTTTTCACTTTATATGCTTCTATTTATTATATAA
GTCCACATACTAAAAGAAAATAGTAAATTATTTTTTATATTTCATATAAACTCATCTATCTAACTTCACAGCG
TTTACTTTTTAAAGTAAGGTTCTAAAAAATCATAATTCTTTATATCTTTGTCTCTAAAGTAATGTTTACCAAA
TACATCTAACAGAGCATGAAATTCATTAGCATCTTGATTTGTAAAATGATTTGGTAATGTGACTACTTTTTTT
AATTGATCATAACTTTTAGTGTTTTCATATCCTAACTTATCATATATTTTTCTTGTATAGCTATCTGGAATAA
ATACAATGCGTCCGAATATATAAACAAGTAAGACATCTGCTGTTTCACTTCCAATACCTTTCAAAGATAATAA
TTCTTTTCTTAATTCACCTTTATATCGCTCATTAATCTCTTGATAATTGAAATGATGTCGTGCTAACCATGTT
AATAATGTTTTAATCGTCAGTGATTTACTTTTATAAAAACCACTTGAATGTATCAATGATTGTAACGTTTCAA
TAGATAGTTCTAATATATGATTTGGATTAAAATGCGTGTGTTCTTTAATCTGATTCAATGCAATTTCTGCATT
TCGCCATCTAGTATTTTGAACTAAAATTGCACCTAACATCATTTCAATGTCATTATCAGCAGGCCACCAATTT
TGCGGTCCCATATGCCTATATAACAATTTATACAATTCATCAGTTCCTAACATTTACATCCTCCTTTTAGAGG
GAGTGGGACAGAAATGATATTATCGTAAAATTTATTTCGTCGTCCCACCCCGGCAAGGTTGACTAGAATTGAA
AAAAGCTTGTTACAAGCGAATTTTCGTTCAGTCAACTACTGCCATTATAACTTTGTAGAGCATAGAACATTAA
TTTATGTCTCAGGCTCTCTTATGCAGTTGTGCGCAAGCAAGCCAACTGCGTTCTCTTAACCTTATTCACATAC
TAAGCATTCCCCAATATGTCACATACCTAATTAAAAACACTCCAACACAAAGTATCTTTCGAGAAACTCTACG
TTGGAGTGTATATAAATGTTCTTACATTGATTCAGGTGCGCTTACACCGACCATTGCCAATGCATTTTTCAAT
GTAATTCTAACCGCTTCAATCATAGCAACATGTGCTTTTGTTTTTTCAATATCATCTGTTAACACTTTTTCAG

CATTATAGAATTTATGGAAATGAGAAGCTAAATCTTGAATATAATTAGTAATTCTATGTGCCGATCTATGCTC
AGCAGCACTTTCAATTGTAGGTTCGAAATCAGCTACTTTTTTCAACAATTCAATCGCTTTTTCATTAGTAATC
GTTGTAAAATCATTCGCAGCAGTCACTTCAATACCTTGCTCTTTTGCTTGTTTTAAAATTGAACAAATACGTG
CGTGAGCATATTGAGCATAATAAACTGGGTTATCTTGAGATTGCTCTTTCGCTAATTCCATATCAAAATCAAA
GTGACTATCAGGACTACGCATAGTTAAGAAATAACGTGCAGCGTCAACGCCAACTTCGTCCATAATTTCTCTT
AATGTAATGGCATTACCAGTACGTTTACTCATCTTCACTTCTTTACCATTTTCCATTAAACGAACCATTTGCA
TGATTTGAATTTCTAAACGATTACTATCTACACCAAACGTTTCAAGAGATGCTTTCAAACGATTAATATAACC
ATGATGATCAGCACCAAATAAATCGATTAAAATGTCATTACCACGTTTAACTTTATCGAAGTGGTACGCAATA
TCTGGTAAGAAATACGTATATGTACCGTCATTTTTAATTAATACTCTGTCTTTGTCGTCTTTAAAATCAGTTG
TACGTAACCATGTAGCGCCATCAGCTTCATACGTATAACCTAATTCTTTCATTTTTGCTAAAACTTCAAGAAT
TTCGCCTTTTTCATATAAAGATGTTTCACTAAACCAATTATCAAAATGCGTATTGAACTCTGCTAAATCATTT
TTCAATTTAGCCATTTCGTATTCTACGCCTAATTTTCTAAATTCTTTCAAACGTGCTTCTTCAGAATAATCTT
TAATTTCAGGGTGTTTCTCTGCTAAATCTTTACCTATTTCAATAATATCTTTTCCATTATAGCCATCTTCTGG
CATTGAATAACTATTGTCACCTAAAGCTTCAAAGAAACGTGTTTCAATCGAACGCGCTAAGTTAGTAATTTGA
TTACCAGCATCATTAATATAATATTCACGTGTTACATTATAGCCAGCTGCAGTTAAAATATTAGCTAAAGCAT
CACCAACTGCTGCATTTCTAGCATGACCAATATGTAAATCTCCTGTAGGGTTAGCTGAAACATACTCAAGCAA
TACATTTTGCCCTTTTGATTCATTTACATGTCCAAATTGATCACCTTTTTCAATTGCTTCAGGAATAATTGCT
GTTAAATACTGATTATCTAAGTAAAAATTAATGAATCCTGGACCAGCAATGTCAATTTGTTTTACATGTGCTT
TTTCAGTATCTAAGTTATCAACAATCGCTTGAGCAATTTCACGAGGATTACGCTTTGCAATCTTAGTCAGTAC
CATCGCAATATTAGTAGCATAATCTCCATTTTTTGTATCTTTAGGAACTTCAATTTTAATATCAGGAATCTCA
TCTGCTAATCCTGCTTTGTTAATACTTGCTGCAATTTCTTCTACTAATGTTTGTTTCACTTGATCAATAATAT
TCATTCGCCTATCTCCTTATAGTTAATTTCATATTGATAAGAACCCATTTTTTCATTATCTTGATATAATTCA
TAATGAATCTTTAGCTTGCCACCGTCTCCACTTACGAAATGTAAAATGCGTAATGTTTTAACTTCTAGTGGAA
TTCGTCCAGCCGATATATCGTAAAAAGTTGTTGTCGTTTGTCCTTCAACAAAATGCAAATTCATATTAATGTC
GCCTTTACGAATCAACTTAACGCTATCATCATCCACTTTTATTGTAACATTAACTGTTGCATCCTCAATTTGT
TCTACATACCGAATAAAGTTAGATTGCCTTTGTTGCCAAGTTCCTTCAGTAGTAAATTCAAATTTTTCTTTTT
CGTTGTGCTGTTTCAACACTTGCTTTGTTTGAATACTTACTTTTTTATCCAATTTACTACTCACCTTAATCTT
CGCATAATAGAAATATTATAGCATAAAATAAGGTCATCTTGTTATGCCTTCTTAAAGCTAAATAGGTTCAATT
AATTAACAATTCCATACTTAATCAACGCTTCATATATGTAAGCCATTTTATAAAATACAAACAACACCTCAAA
ACCAAAAAACACGAGATTTTCACTATCTTTCATGAAAACCTCGTGTTTGATTATAATGATAGTGTTCAGAGCA
CTACCTTCTTCATCTATATTTAATCAATTAATATTTTTAAAATTTAATAACCATTCTACCAGTTATAGTACCA
TTTTCCATTTCTTCAAAAATATCATTGATTTCTTCTAATTTTCTTAATTGAACTTTAGGTGTTACTTTATTTT
CAGCAGCAAATTCAAACGCTTCACGTAAGTCTTGTCTTGTACCAACAAGTGAACCTACTACTTCAATACCGTC
AAGCACTAATCTTGGGATATCTAAGTTCATTTTATCAACAGGTAAACCAACAGCAACAACTCTTGCACCAGCT
TTTACAACATCAACCGCTTGGTTAAATGGCGTCTTAGCAACTGAAGTTACCACTGTTGCATCTAATCCTTTGT
TATCAGTTAATTTCATAACTTCTGCAACTGGATCAACATCTTTAGAATTAATAATAGCATCAGCACCTAATTC
TTTCGCGAATGCTAATTTATCATCATTGATGTCGAAGGCAACAATTTTAGCCCCCATAACGTTTTTAGCATAT
TGTAAAGCTAGGTTACCTAAACCACCTATACCAAAAACACCTAACCATTGTCCAGGTTTTACATTACTTACTT
TTACAGCTTTATAAGTTGTCACACCTGCGCATGTAATAGAAGACGCTGCTGCTGGATCTAATTTTTCAGGTAC
TTTCACAGCATAGTCTGCAGTAACAATAACTTGTTCAGCCATTGCACCATCTACTGTATAACCAGCATTTTTC
ACACTACGGCAAAGTGTTTCACGACCTGTTGTACAATATTCACATCTTCCACAGCTTTCGAACATCCAAGCGA
TAGACACACGGTCTCCAATTTTTAATGATTCTACATCTTCCGCAACTTCGATGACTTTACCAATACCTTCATG
ACCTAAAGTAACGCCTGTAACATCACCAAAATCAGCATTCTTAACATGTAAATCGGTATGACAAACGCCACAA
TATTCCGTTTGTACCAACGCTTCACCAGGTTTTAAAGCTCTTATCTTTTTGTCCTCAATACTTACTTTGTGAT
CTTTCGTTACAACTGCTGCTCTCATAATAATGTCCTCCTTATAGTCACTCCAGTATGTATGATATTAATTGGC
TATTACTTTCAAAAAAGAAAACGAATAATTATAGATGTAATTTTTATTTGTAATAGACGCCAGATTAAAACAT
ACTACTGTGACCTTGTTTTTAGTTAGATATCAACCGTTAACTTTTAACGTTTCCCTAGTTAATATGTTAAAAG
AAATGACATAATCGATATGCTAACGTGTAATTTTTACTTCACATATATTTATTTAAAGATAATTTATAATACT
CTTATCTTCACTCTTATTATAATGTGAATTAATTCACAAAGTATATAGCTATGATCAAAAAAATGTGTCAGTT
ATTCAAATTTTTTATTAACTACAGATTAATTTAATTTCAAAAAAATTAAAAAAACTCCCAATCAATCTAAGAC
TTTATTTTCACCTCAAGATTTACGTCGTCATTGAGAAATGAATCATATCTTTATAGATTAATTGGGAAATTTT
AGTTTCTAATATTTAATTAAATGCTTGATGTAAAGCATCAGAAGAATGCTTCCACATACCTTCATCATGGTTC
ATTAAAAAGTCTTTTAAAATAGCTTTATTCTCGTCACCAATTTGATCAATGATAATATGATGTTTCATTGATT
TATCCATCATATTAACATGTTCAGGCATACTTTTATAACCACGACGAATTGCTTTATTCACTAATAAATAGCA
CGCTGTTAAACCAGCATAATATGGTCCTTGTTCATTACGTTCTGTTGTTACCCAACACAACCAATATTCTTTA
GCTTCATCGCCTTCAACAGCAGCTTTATCAGTAATCCATTTCACGCCTTTTTCAACTTTGGCGCGAGCATGCA
TGCCTCCAATATCAATAAATGCTTCTTTGTTAGCAACATCGATAAAAACTGGTGCAATATTGTCTAAGCTAAT
TGAACCAATATTTGTACCTTTATGACCATCTAGAGGGTCATTTTTTATGATATTAAAATTAAAGCCTTTTTTC

TCAGACAATGATATAACCTCCATATTTAACGATAACAATTTTAGCCCTTTTTAATTAAGCTCATTAATCAAAT
TTTCAATAGCTTCTCTAATTGTCTTATCTTTAATCTTTGCAACCATTTCTCTAGGATAATATAATTTATAGTC
TTGGCGATTAATGCCTGTAATACTATGAATGACTAAAGATTGATTGCTTATTTCTCTAATCGTACCATCTTGT
CTTAATAAATGAATCGGTTTGCGATTTGACCCAGGTCGATCATAGTCATATGGCAAATCCGAAAATGCTTCAC
TCACAAAATAATAATCTGGATTAATACCACCTGCTTCAAACAGTTCTTGCAGTTCTGATATTGTAATAATTGA
GCCATCAAATGGAATATATTTAAATAAGTCTCGATTAATAAATCGACTTGCTAAATCACTTAAAATAGCATCA
TCTTCTTTTGTCCATTTTTCCAAATAATACGTAACTACCGCTTCATCGAGTTCAACATATTGTTCAATCGTAA
CTGTCTCTTCAAAAAATGGAATAAAATCATGTGGATGCAACTTAAATTCATAGCCTTCATTATAAAGCTGTTT
TGCGCGTTTCAAACAATTATTAAGCAGCACTTCTCCACCACGACTAACTGGGTGGAAATAAATTTGCCAATAC
ATTTGATAACGACTCATAATAAAGTTTTCAACTGCATGCATACCACTTTCTTTAATTAGTACTTCATCTTTAG
AAGGTCGCATTAATCTTAAAATACGTTCCATATCAAAAGCACCATATGATACACCTGTGAAATACGCATCACG
TTGTAAATAATCCATTCTATCCGCATCGATTTGTGAAGAAATCATCGAAATGACCAATTTATTATGATGCGTT
TTATTAATTACTTCCGCAACTTCTCTTGGAAACTCAGGCGCCACTTTACGTAATACAGCATTCACCTCAGTAT
CTCCAGTAATAATCGCTTGTGTGTATGCTTCATGGTCTGTATTAAATATTTTTTCAAAACTATGTGAAAATGG
ACCATGTCCTAAATCATGTAATAATGCAGCACATAATGCCAACGGTCTATCTTTATTGTCCCATGCATCATGA
CCAATAAATGACTCATCAATTAATCGTCTAACTATTTCATACACACCTAAAGAATGTCCAAAGCGACTATGTT
CTGCTGTGTGAAAAGATAGGTACAGTGTTCCTAGTTGTCTAATTCGACGTAACCTTTGGAATTCCTTAGTTTT
AATTAAATCCCATATCAATTGATCTTCAACATGAATATATCGATGTATCGGGTCTTTAAAAACTTTTTCTTCT
ACTAATTTTAAATCTACATATGCGTTAGTCATTATTCCCCTCCTTTTCGTTTAATATAATATTTAATTTACTT
AAAATGCTTTGTACATAAGTGCTAAGTCTAACTTTTCGCCATACATTTCTGGCTCATAAGAGCGTAAGATTGT
AAAACCTTGCTCTTTATAGTAAGCTACTGCTTCTTCATTTTTATTATCTACTTCTAAGTAAACACCTTCAAAT
TTATCTTCAAAACGTGATAATCCTTCATTTAACAATGCTGTACCATAACCTGTATGTTGCGATTCTGGTTTAA
CATAATGAGCTGATAAATATAATTCTTCACCGTAAATAAAGTTAGCAAAGCCAACGATGTCATTACCTTCTTC
AACGACTAAGAATAATTGTTCTTGAAGTCTTTTCTTTAAATGATGTTCATTATATGAAGCTTCTAACAAGTGA
TTAACTGTTGTCGCAGCGTATATATTTAAGTATGTATTAAACCAAGCTTTAGTTGCGACATCTCTAATTTGAA
CAACATCTTTTTCAGTTGCTTGTCTTACCTTGAACATGACTTTCTCCCCTTATTAACAAGTTTTAATAACGGC
ATTATACCACAACTTGCTCAATACTTAATAAACAATGATTGTCTATTCAATTTATATATTTATATTTTCCGTT
AAAATTAAAAATAAAAAATAACGAAGCAAAAAATCACTTCGTTTAGTATGAGGTATGTCTTATTGCAATATAC
TATTCCACTCAGTTGCACGTGCTAAGGCATAGTTGTCTTTCATGATGTCACCAGGCTTTTCAGCAGTTCCAAT
AATATAACCATTTAAAGTGGCACCTAAAAAGTCTAAACTATATTTCATTTGCGTAATTGCTGGCTTCGCTTTT
ATTTTTGGACAATCTCCACCAACTAAAATAACTCTAAAATCCTTTTCGGCCATTTGTGCCTTAAAATTAGGAT
ATCGTTTATCTTGTAATGTTTCTGACCAATGTTCGATAAATGCTTTCAATGGTGCTGAAATGCTATACCAATA
CACTGGTGATGCAAAAATAATTGTATCACTAGCCAATATTTTATCTAGAATCGGCAAATAGTCATCGTCATAT
GAAGTAATAGTCTCTGCTGTATGTCTCACGTCACGTATCGGTTTAAACTGATGTTGTGTCACGTCAATCCATT
GATACTCTAAATCTTGCAAAGCGAATTTTGTTAATTGTGCAGTATTACCGTTTGGTCTACTCCCACCAAACAA
AACAGTAATCATTTTAGCCTAACCTCACTTTTGATTAATAAATATCTGTGTTTTTCGTTACCTAATTATACTA
TCATAAGCTTTGCCTACCGAATAGTAAAACGCTTACAACTTTTATATAAATTTGACGAAATTTCGTCATCCTT
ATATAACGTCGTTTGTGATGGGAGTGGGACAGAAATGATATTTTCGCAAAATTTATTTCGTCGTCCCCCCCAA
CTTGCACATTATTGTAAGCTGTCTTTTCGCCAGCTTCTATGTTGGGGCCCGCCAACTTACATTGCCTGTAGAA
TTTCTTTTTGAAATTCTCTGTGTTGGGGCCCACACCCCAACTTGCATTGCCTGTAGAATTTCTTTTCGAAATT
CTCTGTGTTGGGGCCCCTGACTAGGATTGAAAAAAGCTTGTTACAAGCGCATTTTCGTTCAGTCAACTACTGC
CAATATAACTTCGTAGAGCATAGAACATTGATTTATGTCCCAGCCTGATATCACCATTAAATACAATTCATTT
AGTCTTCAATCGGAAACAGTTCATCGATATATTGAATCTCATCATCTGATAAAACGATATCTGCAGCTTTAAT
ATTTTCAACGACTTGTTCTGCAAGTTTTGCACCAGGAATAATCACATCGATAGCTGGTCTCGTTAAATAAAAT
GCTAATACAATGTTCGCAATTGAAGTTTGATGTGCTGCAGCTATGCTTTCCAAAGCTTTTACGCGACGCACAT
TTTCTTCAAATACACCTGGTTTAAAATCACGACGTGTAGTACGATGGTCACTAAATTTAGTGTTCTCATCATA
TTTTCCAGCTAAAATACCGGATGCTAATGGGAAATATGGAATAAATGTGATTTGGTGATCAACACAATATTGT
AATACTGCCTCATTTTCGCGATGCAATAAATTATATTCTAACTGTACAACATCAACGTAACCATCTTTATTTG
CTTCTTTAAGTTGATCTAATGTGAAATTTGATACACCAATGGCTTTAATCTTCCCTTGTTCCTTAAGCTCTTG
TAATGCTGCAACTGCTTGATCTTTCGGAGTGTTGTCATCCGGAAAATGAATATAATATAAATCGATATAATCA
GTTTGTAGACGTTTCAAACTATTCTCAACTTGTTGTTTTAAATATTCAGGTTGATTGTTCTGATGTACTTCTT
GATTTTCATCAAATTCATGAGACCCTTTTGTAGCAATTTTAATTTGCTCTCGCGGATATTCTTTAACAACTTC
TCCAACCAATTCTTCTGATCGTTCTGGCCCATAAATATATGCCGTATCTAATAAATTAATACCATGATTAATG
GCTTGACGAACAACATCTTTTCCTTGTTCTTCATCTAAGTTCGGATATAAATTATGCCCACCTACTGCGTTCG
TCCCAAGTGCGATTGGAAACACTTCAACATCAGATTTACCTAAGTTTACAAATTGCTTCATTAGACCCAGCTC
CTTATCTTTATAACTTTTCCACTATCACACTACCATGATTTAATACAACTTCCAAATGTTAGAATTAGAGCTT
GAATTTAAGCGGTGTAATAGGAAAACGTTATATAAAACTCAGTTAAAATAAGTATTGCTTAAATTGATATGCA
TATAGCGCGATTGCTCGTTGACCTTTAAATTACATGTACTTTTGAATCAGAAATTGAAAAACATTTAAGAACG

ACAACATTAAAGCCATTACAAATTTAATTAGAAATACTATAATGTTTTGATTTGCATAACAAAGAAATTAAAA
CTGTTAATATTAACATGGGATATCCATTATAAAATATTCATCTTTGTAGTTGATAATTCTATATTTCGGATTT
TTATTCATTCTATCTATGAGTCAAATTTTTATATTAAATTTAATAATTTTAGATTTAAAAAACTCATTTATTA
TTCAGCTTTTTTAAGAAGTATTACATGAGCTTTTTTAATGCCAATGGGAACAATGTTTATAAAAAAGAAAAAC
ATAGTTACTGCACCCCAACAGATAAATATAATTATATTTTGAACATCTATAGTTATCAGGGCATATAAAGTGA
AAAATGAAAAACCACCGAAATATAAATATGCAAATATTATAAAGAAAAAAATGTTTTAAATTAGGTAGCAATAT
CATTTTCTGACTTTTTTCTTTATTCATAGTAAAAACACTTAATGCTAGCCTACGATTTAAATTTAGGTAGAAA
AATAAAATACCAAGAAATACAACAAAAAATATGGCTATTACTATTGTTTTTTCCAATCTAACATTAAATAAAG
GCATATACTTTCTAAAAGTCACACTAAATAAAACTGAACTACCTAATGCTGGCCAAAATGCATTGTTTTTAGC
AGGTTTGAAAGTATTTAAACTTTCTAATTCTTCTTGACTGATCTTGATGTATCTTTTTGGTGTCAACCAATTA
AGCAATGGAATGAATAAAACTAACCAATTATTTATTATATTCACCATCAAATATTCATCATTATATTTAATAA
TTCTATATTTGGGATTTTTGTATATAGCTATTACCTCGCAAAGCAATGTCTCCACATCCCTTTAATTTTATGT
GTAATACATTTTTCGATACTTCAAAAGACATTAAAATATTATCAACTTACTATCATTAAAAATTTTATTCATT
GTTATTTGCATATCTTTAATCTGAATTTATTACTACCACTTAATTTTTAAGTATGACATGAATGTTTTTATCT
ATAATTAAAGCTATATTCACTAGAAAAAAACCCATTATTGCTGTAATCCAAGCAAAATATACTATTATGTTTT
GATAACTCATTGATAATAGAGCATCTAGGAATAGCATAGTCAATCCGCCAAATAAAATATATCCAAATAATGT
GAAACAAACATTTTTTAGTGTAGGTATTAAAAAAATTTTTGACCTTTTATTTTTAGTATTATAAATATTTAAA
GATGATTTTTTAATTAGGCGTACATAAAATATTAAAATCCCTATAAATGTGATGCAACATATGGCAATAACTA
ATTTTTTATCAAGTTGAACGTCAAGTAAATGTGTATATTTTCTTAATGCAACTCCCAGCAAAGCAGAGCTACC
TGCAACTGGCCAAAAAACATTCTTTTTAGCAGGTTTAACAATATTTAAATTTTCAAACTCTTCTCTACTAATT
TTGACGTATCTTTTGGTGATGAACCAATTAATAAAAGGAAAAAACAAACTAATCCAAGTGCTTATTATATCGA
TCATTAAGTACTCATCATTATATTTAATAATTCTATATTTAGGATTTTTATTGATGATTTTAGATTCGCACAG
CAATGTTTACACCCCCTAATTAGCAATTGAAAACCAACTAAATTTGTAACTATTTATTAGACTTTAAAAATAT
ATGTACTTTTTTATCTATAATTGAATGCATATTCATTAACATAAATAGCATAGTCATGAAAAGCCAAGAAATA
TATAAGATTAAATTTTGTACATTTTCAAACACCAACAAGTAAAATGCCCCATAAGACATGAAACCAGTAAAGA
GGTAATAAAAAATTGTGAAACACATATTTTTAAAGGAGGGTAATAATCTTAATTTAAGTTCATTATTTTTATT
TTCCTTATAAACATTTAATGTTAATTTCTTATTTAGATAACAATAAAAAAATATAACCCCTAAAAAACTAATA
AAACAGCTGCTCCAAACAATTAGTTTTTCTAATTGAAAATCAAATAAATAAGTATTTCTTCTTACAGTACCAC
CAAGTAACACAATACCCGCGAATATAGTCCATCCTATCGATTTATTTTTAACTGGTTTGACAATATTTAATTT
TTCATAATCATTCTTAGTGATTTTGACATATGTTTTCGGTATGAGCCAGTTAATAAATGGAAAGAAGAAAACA
ATCCAATTACTTGCTAAATCAATCATTAAGTATTCACTTTCGTATTTGATTATTCGATATTTAGGATTTTTAT
TAATAACTTTAGATTCGCAAAGCAATATTTACCAACTCCATTTCATAGGATTAATATGATTACTAATACTTTT
TGTGGCATCACCAATGGTATTACCAACACTATCTACAGCTTTTTCTGCAGTTTTGTTTACAGCATCAATACCC
TCGTCTATTTTATGTCCTGCCCAGTCCACTTTATCTTTTATGCCAAAATAATTATTTTGATAAAGATAATCAG
AAACAATAGATCCAATAATACCTGCTGTTAAAATACCTACCACAGCCCAACCGACTGGATTACTTACTAAAGC
CGCTACACCAAATGCTGCAACAGATCCAGCTGCAAGCGTCATACCATTATGCGACAACGCCTCCCCAAATGTT
TTATCATCATTTGCAAGGTCATCATACATACCTATACCAAATCCTACACCTGCAACACCTTTACCTAGATATT
TTCCACCTTTAGCAAATTTACTACCATTTTCTATAAACACATTACCTGATGTACGTTTGACTTCCACAAATGA
ATTTGGACCGGCTGGGCCTTTCACTCCACCTGCTGTATTGATAAATACACCGAATTTACTTGCATTTATACCG
CCTTGCTCTAAAAGTGTTGACGTAATATCTAATCCTATATCTCGTTTAATACTATCTTTATTGTCATTTATAT
ATTTCATTATACTTTTCGGGATATCGTCTTCTGGATGTTCTTTGGCATATGCCTTTATAACAGCAAAGTCTGC
TTTATTTAAAGTTTCTTTCTCTGCTTTATGTTCAATTTTCCCCATAGCAACTTTCAAATATTTTTCATGACTT
GCTTTGGCCCAATCAAGTTCTTTACCTGAAGGAATGTTAAATTGATTTGTTGAAAAGTTCCAAAAAATTTTGCG
CTTGGGTAAGTCCTTGTTGGACAATTTTTTGAAATTCTTCAACTTCTTTAAATATTTCTGGTGATTTTTGATT
AAACTCACGCAATTTGCGTAGCTTCTCTTCTAATTCATGTTTTTGTTGACCTAATGTTTGTATTATTTGTTGG
TTCGATGAAATGGCTTGCTGATTATCGGAAGCATGCTTTTTCAAATTGTTAGTTAAATTTTCATATCGCGTAA
TTTGTTGACTTAATGATCTGATATCTTCTTCAAGCTCTGATTCTTTTAAAGATATGCTATCAACCTCACTCGT
ATAACGTGACACAAAATTATCGCAAGCTTGCTTCGTTAAATCACTCAATGTTTTCATACTTGTTGATAATGGA
ATTAACACCGTACTAAAAAATTGTTTAGCTGACGTATACGCTTTACCTTTAAGCGCATCATCATTGATAAATT
GAGTAATTGCTTTTTCCAACGCATCATAATTTGAATTCATTGTTTGACTCAAATTACCCACACTTGAAGCTTG
GTTTCGAGATCTGTCTAAATACATGTCAATACTCATCGGCATGCTCCTTTTTCAAAAATATATGATTTTCAAA
CTATTTAAAATTAAATGCTTTTTACATCTATAAAGTTGTAAAATTTTAAAACTCAGCGATGATTATTTCTTAT
GTAAAGGGGTCTAGATGCAGGTAAATTGAGATAACATGTCGCCTTTTTTCTTATTTTAGCATATGGATATAAT
GGTGTCTTTGTATATTCACAATTAATCAATAAAAATTATCTTTCAATATTTTAATTTTATTGCGACAACATCC
TTAATCTAATATATATTAATATCTCAAAATATATTTACGATTTAAATATGTCACCAGTTGTTGAAAATACTTC
CTCATCATGCGAAATATCAAAAAATATCTAAAATACGAATAAGTTTACAAAATCACACAACATCATCATTCAA
AATTTTATTGCCCTCATTTCAATATTCATTTCATATTTAACAGTTCATACATAACTAAAATTTCAATTGTGCA
ATCTCACCGTTAATGCAACAACTTTCAAACAATAAATCATCACAATAACCACACCTAATTCAACACTTTTCAA

ACATAAGTATTGACACATTGAGCAAAATGATTTTTAATTGTAACTAATACAGTTACAATTATGAGGTGAGAAA
CATTGAATTTAGAATTTAACATTGCCGTGCATGTATTAGCTTTTTTAACTAAGCATCATTCAGAAAAATTCAA
TAGTAGTTCATTAGCTGAATTAACTTGTTTAAATCCTGTTCAATTACGACGCGTGACGACTCAACTTGTCGAT
TTAAAAATGATTAACACAATACGGGGTAAAGATGGTGGATATTTAGCAAATGATCAAAGTGCTGATGTCTCTC
TAGCAACATTATATAAACATTTTGTCTTAGAGAAAAAACACCACACACGTCTATTTACTGGCGACGAAGGCAG
TCACTGTCAAATTGCTCGTAATATTGCAACTACCATGTCACATTATCAGCAAGACGAACAGAATATCATTATT
AATTTTTATAATGGAAAAACAATCAAAGATGTCATTGAAGACATTCAAAAGGAGGATTTATGTCATGAAAACA
TATGATTTAATTGTAATAGGATTTGGGAAAGCTGGTAAAACTTTAGCGAAATACGCTGCATCAACAGGTCAAC
ACGTCGCAGTTATCGAACAATCTTCGAAAATGTATGGAGGCACTTGTATCAATATAGGATGTATACCTTCGAA
GACACTTGTACATGATGGATTAGAAGGCAAATCCTTTGAAGCAAGTTATAATCGTAAAAACGATGTTGTCAAT
GCGCTAAACAATAAAAATTACCACTTATTAGCAGACGATAACAACATTGATGTACTGGATTTTAAAGCGCAGT
TTAAATCTAATACTGAAGTGAATTTATTAGATCAGCATGGCGATATCGCCTATAGTATTACTGCACCTCATAT
CATTATTAATACCGGTGCTACCTCTGTCATTCCTAACATAAAAGACCTTGATCAAGCTAAACATGTCTTCGAT
TCAACAGGTTTATTAAACATTAACTATCAACCTAAGCACCTCGTTATTGTAGGTGGCGGTTATATCGCCTTAG
AATTTGCTTCAATGTTTGCGAATTTAGGTAGTAAGGTAACAGTATTAGAACGTGGTGAATCATTTATGCCACG
CGAAGATCAAGATGTCGTTGCACATGCTATTACTGACTTAGAAAATAAAGGCATTGCATTGCATACAAATGTT
GAAACGACTGAATTGTCATCTGACGATCATCATACAACAGTCCGTACTAACGTTGGCAACTTTGAGGCTGATG
CAGTACTTTTGGCTATCGGACGCAAACCGAATACAGATTTAGCTTTAGAAAATACTGATATCGAATTAGGCGA
CAGAGGCGAAATTAAAGTCAATGCTCATCTTCAAACAACTGTGCCGCATATTTATGCTGCAGGTGATGTTAAA
GGCGGACTTCAATTTACGTATATATCTTTAGATGATTATCGAATTATCAAATCAGCGTTATATGGTAATCAGT
CACGTACGACTGACAATAGAGGCAGCGTGCCTTATACAGTATTTATAGATCCACCATTATCACGTGTTGGATT
AACTAGTAAAGAAGCAGCCGCTCAACATTATGATTACACTGAACACCAACTTTTAGTAAGTGCAATACCTCGT
CACAAAATTAACAATGATCCAAGGGGTTTATTTAAAGTAGTCATTAATAATGAAAATAATATGATTTTAGGTG
CTACATTATATGGTAAGCAATCTGAAGAATTAATTAATATAATTAAACTTGCGATTGATCAAAACATTCCATA
TACCGTATTACGAGATAATATTTATACACATCCTACGATGGCCGAATCATTTAATGATTTATTTAATTTCTAG
ACAAAACATAAAAACCTGGTGGCACGCATTGAATGATGCTGCCATCAGGTTTTATTGTTGTGCTTTTCGCTTT
TCTAATTTTTCTTTAAGCTTTCTATCTTGTTCTTCTTTACGACGTTTACGTTCTTCATGTCGTTTTCTTAAAC
GCTCTTCTTCTTCAGAATCACGTGGTTTCTTTAATTGTTGAGAAACTTTTTCGATTAATTCTTCTTCAGTAAG
CGCAGCCAGTGGGCGGTTATTAACAAAAGTGAATGTTTTTCGGCGTCCAGGTCCACAATAAGATTGACAACCT
ATCACGATTTCAGCATCGGGATCTAATTTTTCCAACTTCTTTTGTAACGTTCTTATATTGACTGCCTGACATT
CATCACAAATAAGGAATGTATTTTTCATATTGCTACCCACCTTTCTTTATCATATCTATATCGTCGATTTCAT
TAATTTTTTCGTTAACTCTATCTATTTTACTCTTTTTAATATTTTTTTCAAGATACGTAACACGGCTGGCAAT
AAAAAATGGAGCATTTATCTTCTAATTAAATTAGATGATTGCTCCCCTATCAAATCATTTATTGCCCATGATA
AATATTAAATTTTAATGGTTTAATACCATGTTTTGTCCATTCATCATAAATTTTTTCTTTATCTACATCTTTA
TTGATAATTGTAATACCACAGTCTCCACCACCAGCGCCTGATGTTTTAGATGCGCCGTGATACTTTTCAGCAA
TATCACACAAATATTTTAGCTTTTCAGTTTCTATATCAACTGTAGCTTCTTTATCCATACGTTGAATAATTGT
ACGATTCTGACGCACCATCTTTTGCACACCTTTAATGTTATTTGTTTTAAAAGCATGAATAAGCTTTTCAACA
CAACGATGTGAATCTTCTAAGAAGTCACCGTAAAATGAAGGATCTGATTTCAAACGTTTCACTTCGCTAACAA
AGTGTGGTGATGACGCCGGTGAGCCAGTCCAACCGATAAGTACTTCCATATTTTCAGGTGCTTGTAATGGTTC
GATGTGCAATCCAGGCCAGTTTTTGATTAAAACTTCTTCAACCGTAGTATCTTCAATTTGATGCTTAACCCAT
TCATGATCAAAAGTACTATACGCTAGCCATCCACTATATACACTCACAGCAATATCTCCGCATGAACTTAAAC
TTTGTAACTTCATATTTGCAATCACTGCTAGTTTATAAATGTATAAATTAGATAACTTCATATCATAAAATTC
ATTTAATACTTTTATAACTGACACAAGTACTGCTGCACTTGAACCTAATCCATATTTATGACCATTTGAATCA
TCTAAATTACTATCAATAGTCAGATGAAAATGCTTCATCGCTATATCGCAACTTTTCGCGTATTGTTCAAATA
TTTCAATAGCTGTGACCACATAATTTAATTGTTTTGCTGCATGTGGATCTGAAATGACAATACTATCTTCATC
TCTACTAAATGTAACTGGGTTATGATGTAATGCTTTTGAATGAATGGTACCTTTATATTGGTCTGCTTCTTCA
ATAGTAGCAGTTACAAAACGATCTAACGCAATAAGTACAGATTTATATCCTGGTTCTGTTACAGCATATTCTC
CAGCAATATAAAGTTTTCCGGGTGCTTTGACCTGAATCATTTTATCTCTTCCTTACTCAATTATTTCAATTCC
TGTGGCAATAATGTCACTATCAATAATTTGGTTATTATCAAACTGTGTTAATAATTTATCTATAATCTGTTGC
TTGTTTTTCTTTTCTACAAGTATTTTCACATTAGGACCCGCATCCATCGTAAAATAACACGGATATCCCGCTT
CTCGGCATTCGTGAACGAGCGCCATTACATCATAACTTTCTTGCACAAGATATGTGAACGGCGGTGTTGATCC
TAGATTCGTGGCATGCATACGCAAACCATTTTCTTCAATTACTTCACCAAGACGTTTAAAATCTTTGTCTTGA
ATCGCTGCTTTTGCTTCAGCTAAATCTTCATCAATATGATCTAACCAATATTGATAAAACCTTGATGTGTTTC
GTGTCAATGACATACCATATCGACTAGGTACCTTTTTAGAATGTTGATTAATCACAACAAATATCATGGCAAG
GTCATCTTCAAAATGATTCGATTCAAGTGGAACGGCATATGACGTCTCATCACTATACCCTTTTTCCCATTCT
GCAAATCCACCATAAATACTACGCGACGCAGAACCCGAACCAATTCGCGCCAATCTCGATAAATCCTTATCTG
ACAGCTGCATGTCTAGCGCTTGATTACAAGCTGCTGCTAAAGCTGCATATGCGCTTGCCGATGAAGCCAACCC
TGCTGCTGTTGGTACAAAATTGTCGCTTTCAATTTCTGCATACCAATCGATGCCAGCTCTATTTCTGACAATA

TCCATATATTTTGAAATTTTCTCTAATTCTTTGCCACTAACCTTTTCACCATTCAACCAAAATTGATCCTGTG
TTAACTGGTCGTTAAAAGTGACTTTCGTTTCAGTGTAAAATTTTTCTAATGTAACAGATATGCTATTATTCAT
TGGAATGATTAGTGCTTCATCTTTTTTACCCCAATATTTTATAAGTGCAATATTCGTATGTGCACGTGCTTTG
CCACTTTTAATCAACGCATTAACCTCCTAAATTCTCAATCCAAGTATGTGCTGCACCAGCTTTTTCTACAGCT
TTTACAATATTTTTCGCTGTTGGTAAATCTTTGGCAAGCAATAACATACTTCCGCCACGACCAGCACCAGTAA
GTTTTCCAGCAATCGCACCATTTTCTTTACCAATTTTCATTAATTGTTCTATTTTATCATGACTAACTGTCAA
CGCCTTTAAATCCGCATGACATTCATTAAAAATATCCGCTAAGGCTTCAAAGTTATGATGTTCAATCACATCA
CTCGCACGTAAAACTAACTTACCGATATGTTTTACATGTGACATGTACTGAGGATCCTCACAAAGTTTATGAA
CATCTTCTACCGCTTGTCTTGTTGAACCTTTCACACCAGTATCAATAACAACCATATAGCCGTCTAAACTTAA
CGTTTTCAATGTTTCAGCATGACCTTTTTGGAACCAAACTGGTTTGCCTGATACAATCGTTTGCGTATCAATA
CCACTTGGTTTACCATGTGCAATTTGCTCTGCCCAATTAGCCTTTTCAATGAGTTCTTCTTTCGTTAATGATT
TCCCTAAAAAATCATAACTTGCACGAACAAAAGCAACCGCGACAGCTGCACTCGATCCTAATCCACGTGATGG
TGGTAAATTCGTTTGGATCGTTACTGCTAGCGGCTCTGTAATATTATTTAATTCTACAAAACGGTTCACCAAA
GACTTAAGATGGTCAGGCGCATCATATAACATACCATCGTAAACATCGCTTTTAATAGACGAATAGTTCCCGC
TCTCTAAGGCTTCTATTAAAACTTTGATTTTACCTGCGTTAAACGGTACTGCAATAGCAGGCTCTCCAAATGT
AACAGCATGTTCTCCTATTAAAATAATCTTACCTGTCGATTCCCCATATCCTTTTCTTGTCATGTCAATATCA
CCTTTTATATTTATCCTATACTTGATTCATTATTTTTATTTATTAGTAAAAGACATCATATTCTAAGTTGCAT
ACGCATTCGCGTCAAATTTCATTGCAGTCTTTATCTCACATTATTCATATTATGTATAATCTTTATTTTGAAT
TTATATTTGACTTAACTTGATTAGTATAAAACTAACTTTCGTTTACTTCAAAGTTTAAATCTTATCGAGTGAT
ATTTCAGATTCTTTATCTTTTTATAAAATAGCCCTACAATTTATAATTTTCCACCCTAACTATAATACTACAA
ATAATAATTGGAATATATAGATTTACTACTAAAGTATTAGAACATTTCAATAGAAGGCCGTTTCTTTCATAGT
CATACGCATTATATATACCCTATTCTCAATCTATTTAATACGTAAAACATGAAATTTTCTTATTAAATTTATT
ATTTCAATCATATCATTACTTTTAATTTAATGATGTTCAATTTAAATATTAGGCCAATAACATATTTATGCTA
TTTATGGATACTTTCAAAAATAACAGCCCCAAACGATAACTTGAAAGGGGCTGTTAAATATTTAACTATTGCA
TTTGGTCTATCATTTTCTTGTTTCTTTCAATCATTTTATCAAAATACGTATCGTATCTTTGCCATTCTTCTTG
AGTAATGGGCGTCATATTTAATACACCGCCAAGATCTTTGATTGCATATAATAGTAAAAACATTACATCTTGA
ACAGTAATCGTTTTGTTAAGCAATGTCTCTAATGAGGCCATACAAGATGGTTCAATTTCAGGATATTTAAATT
TAGTCACTTCACCTTTTAAAGCATGTTCATAAAATGTTTGCATCATCAATGCACGTTCTGAACCAGAGCCTTC
AACACAAAGATAAATTTGTACAGCAATACCGCCTCTAACTCTTCGTTGCGATATGCCTGCAAATTTTTTACCA
TCGATACTTAAGTCAAATTTTCCTGGGCAATAAGAATGTTCAATTTCCATCGTATCAATATCAACATTCTCAT
CTTCGAACATTTTGCTAATTAAAAGATACATCACAGTAAACGCTTCATCAATCGTTGTTTCTGTTTGTCCTTT
GAACATCAGCGATATATTTAATACACCTTGATCTAGAACGACACCTAAGCCACCAGAATTTCTAACAATGGCA
TTATAACCAATCTCATTCGTTAAATAATCAATGCCATCTTTTAAAAACGGCAATCTTGAATCATGAATACCAA
GAATAACAGTATGTTGATGAATCCAAGTACGCACAACATTATCTGATATATCTTTGCCCACACTTTCACAAAA
TGTATCATCGAATGCGAAAGATTGCATAGGCTCTAATCCAGAAGAATGATCGATATATCGCCAGTTGACGCCA
TTAAAATATTTACTCGCTAAATCCATCGTTATTGTAAGGCTTGCGCTGCTGTGATAATTGAAAGATTGTATAC
ATCTTCAATTGAACAGCCACGTGATAAGTCATTTACTGGAGAATTTAAACCTTGTAATACTGGACCAACTGCA
TCATATCCACCTAAACGTTGTGCAATTTTGTAACCAATATTACCAGCTTCTAAACTTGGGAATACAAAGACAT
TTGCATCACCTTGTAATTTAGCACCTGGCGCTTTTTTCTCAGCAACACCTGGTACAATCGCAGCATCAAATTG
GAATTCTCCATCAATGATTGCTTCTAATTTTTCTTCTTCAGCTTTTTGTTGTGCTAATTTGACAGCTTCTTGA
ACTTTTGTCACGTCGTCTGATTTAGCAGACCCTTTTGTTGAAAAGCTTAACATTGCAACTTTTGGATCCATGC
CAAAGCTTAATGCTGATTTTGCACTTTCTACTGCAATTTCTGCAAGTCCTTGTGAATCAAGTTCTGGATTGAT
TGCACAATCACCAAAGATGTATTGTTCATCACCTTTAATCATAAAGAAGATACCTGATGTTCTTGATACACCT
GGTTTCGTTTTGATGATTTGTAAAGCTGGACGCACAGTGTCTCCTGTTGAATGTGCTGCACCACTAACTAAAC
CATCTGCTTTACCAGCATAAACAAGCATTGTACCGAAGTAGTTCACATTGTTTAATAATTCTTGCGCTTGTTC
TTCAGTCGCTTTACCTTTACGTCGTTCAACAAATGATTGGACTAATTCAGCTTTAAATTCACTTGTCGCAGGA
TTAATTAATTCAATATTAGAAATATCAAGATTAAGTTTTTGCGCTAAAGATTGAACCTTAGTCTCATCACCTA
ACACGATTGGTGTAACATAATCTGTTGCTTGTAATTGTGTTGCAGCTGTTAGAACACGCTCGTCCTCTCCTTC
AGGTAATACGATTTTAACGTTTTTACCAGAAAGTTTGTCTTTTAATACATTTAATAAATCAGCCATAATGTCC
TCCTGTAATATAAATCTTATTAATCATTCACGGTATAATTATACGCCATTTTATTTCATAATGCCACGTCACC
AATTTGTAATCATAATTTTATCGTTACCCCTTTGCTTTATGATAAAATTTATAAAGAACTGATGATTTTTGAA
AAGGAGCGATAAACATGAGTCAAGCAGCCGAAACATTAGATGGTTGGTATAGTCTACATTTATTTTATGCAGT
TGATTGGGCATCATTACGTCTAGTTCCAAAGAACGAACGCGATGCACTTGTCACTGAATTTCAATCATTTTTA
GAAAATACAGCAACTGTAAGATCATCAAAATCTGGTGATCAAGCTATTTATAATATAACTGGTCAAAAAGCAG
ATTTGTTATTATGGTTCTTACGTCCTGAAATGAAGTCTTTAAATCATATTGAAAATGAATTTAACAAATTGCG
CATTGCTGACTTCCTAATCCCTACATATTCATATGTTTCAGTCATTGAATTGAGCAATTATTTAGCTGGTAAA
TCTGATGAAGATCCTTATGAGAACCCTCATATCAAAGCAAGATTATACCCAGAATTACCACATTCTGATTATA
TTTGTTTCTATCCAATGAACAAACGTCGTAATGAAACTTATAACTGGTACATGTTAACTATGGAAGAACGCCA

AAAATTAATGTATGACCATGGTATGATTGGTAGAAAATATGCTGGCAAAATCAAACAATTTATTACTGGTTCT
GTAGGGTTTGATGATTTCGAATGGGGCGTAACATTGTTCTCAGATGACGTATTACAATTCAAAAAAATTGTAT
ACGAAATGCGCTTTGATGAAACAACAGCACGATACGGTGAATTCGGTAGTTTCTTTGTAGGACATATTATTAA
CACAAACGAATTCGATCAATTCTTTGCGGTTTCTTAATACATTGGTACGTTTATAAATTAATAAAAAATTCCA
AGCTTATCGGTTTAAGCTTGGAATTTTTCGTTTATCTTCAGTATATTCCCGTATACATAAGACGTGATTTGGT
AAATAGTTGAAATCTGTATGTTTAAACTTATATATATGTGCTAATGTATTATCAATAACAAAGTACACTTTGC
TCATAGCAAAGTGTACCCGAGTAGTCTTCCTTGGGAGAACTTTAACTACTATCACTACATATAAACGTTAACC
TCAATAGAAATTATACAGTCGCTACTCTATACAATTTTTGTAATGGTTAACTAATATTATTTTAACCTATTTG
AAATATTTGAAACATATTTTTGTCGAATTTTTTTCAATAATTTTTCCTTTTTATACTTCAAGAGAATTTTAAC
TACTAAAAATTCCGATGATTATTATTACAATAGTATCAAATATTAGTTTTTTAAAATCAATAACAACTTATCA
AAAAGCTCATGTGGTTATTTTATAGTGTATAAACTATAATGAGTATTAAATTCTTATAAACAATGGTGATGAA
ATGGACATAAATTCAGAAGAATACAAACAAGAGGTACTTATCAAAGACGTTGTCATGCTTGCTGCTCGCATAC
TATTAGAATCTGGTGCAGAAGGTACGCGTGTAGAAGATACCATGACACGTATTGCAAAAAAACTTGGTTACAG
TGAAAGTAACAGCTTTGTTACAAACACTGTCATCCAGTTTACGTTACATTCGGAATCGTTTCCTAGAATATTT
AGAATTACCTCTCGAGATACAAACTTAATAAAAATTTCTCAAGCTAATAAAATTTCGCGTCAAATTACAAACA
ATGAAATTTCTTTAGCCGAAGCAAAAACGCAACTTGAAAAAATATATGTTGCTAAGCGTGACAGCAGTCTTCC
CTTTAAAGGTTTTGCTGCAGCAATGATTGCAATGAGTTTCTTATATTTACAAGGTGGTAGATTGATTGATGTT
TTAACTGCGATATTAGCAGGTAGTCTAGGATACCTAGTCACTGAGATTTTAGATCGCAAGTTACATGCACAGT
TTATCCCAGAATTCATTGGTTCATTAGTTATTGGGATTATCGCCGTTATTGGACATACACTTATCCCAACAGG
TGACTTGGCAACTATTATCATTGCGGCAGTCATGCCTATTGTTCCTGGTGTATTAATAACAAACGCAATACAA
GATTTATTTGGTGGACACATGTTGATGTTCACAACGAAATCATTAGAAGCATTGGTTACTGCGTTTGGCATCG
GTGCTGGCGTTGGTAGCGTATTAATTTTAGTATAGGAGTATCAGACTATGTTTTGGATCTTAAACTTTATCTT
TAGCTTTTTAGCTTCAATGTTCTTCTGCGTCATTTTTGATGCACCTAGAAAATTATATCTATCATGTGGTTTC
GTTGGTACATGTGGATGGATGGTTTACACCTTATTCTTCAACGGCTTTAATGTGCACACTATATACTCTAGTT
TCTTTGGTAGTTTAGCATTAGGCTTGTTAAGTCATTATATGGCTCGTAAACAAAAAGAACCTGCCATCATTTT
TATGGTAACGGGTATCATTCCATTAGTACCTGGTGGATTAGCTTACGATGCAACGAAAAATTTAGTCTTATTA
AATTTCAGCACAGCAATAAATACAATGTTAGAAGTAACACTTATTGCAGGCGCCATCGCATTAGGTTTATTAT
TCGCCGACCAAATTTCCAAATTAATTGTTTCTGGGTTCGTGAAATCTTTTAAACGATTATAAATATTGTTAGA
AATGGCTCAGCCCAAACTCTTAGCAATTATATATAAGGTTCAGCTGCCTCGTCTTAAATGTCAATTTATAATA
CAAAAAATCGCACGATACGCCTCCGAATGAAGAAGGTATATTGTGCGATTCTTTTATTATTTAGTTTTTTCTT
TTTCAGGTTCTTCAATCATATCATCAACTTCATTTTCACTTCTATTTAAAATCAAGCGACTTAATTCATCATT
ATCCATATTTTTCAATTTAGGATATCGAATCACAAAGAATATTAAACCGATGATTAACCATCCAAGTAATGCA
ATATAAGACGGTGCAGTCAGTGCTGCAGGAGAACCTGGCACTAATAACAACGCTAAGAAAATGAATGATACAA
ATGAGCCGATAATAGCAAACGTTTTGTAAACCGGTGCATACGTATTACTTTGTTTGTTATAACTGAATAATTT
CGCTGCAGACAAACATGTAATAAAGTAGGCAATGGATACACCTGTAGATGACATATCTACAATCCAAGTCAAT
GCAGTTCTTCCTAGCCAAGGTGCAATTAACGACACACCTACTAGGAATATGATTGCGACATATGGTGTTTTGT
ATTTACTATGTAATTTACTAAACATTGTTGGCATAATACCTGAACGTCCCATAGAAAATAACAAGCGACTTGA
ACTCATCAAGAATCCATTTAAACCAGTAAATATACCCATCATAATTGCAATTGCTAATACACCTAATCCAATA
TAACCAAATGCTGTTTGTGTAACAGCACCTGTTAACCACAACTGCCCATTTAAACTTTGATGACTTGTTGATA
ACCAACCAGTGTATAAAATCATGACAACATAAGTTAATGATGCTGCTAATAAACTGTACACGATAAGCTTAAA
TGTCTTGTTTGGTGCAAAGTTAAACTCTTCTGCTGTTTGTGGAATATTATCAAATCCAACATATGCCCATGGT
GCCACGGATACAATAACCACAATAGACACTAACCATCCTTTGCTAGGTTCAGCTAACGGTTGTAAATTTTCAA
GTGCAAAATTATTACCAAAGAATGAACCAAAGAACATCAATAATACGACGATTACCATCGCCACACAGAAATA
ATATTGTAATGATCCAGATACACTTGCGCCACGAATCGTTACTAGCATGAATACAAGTAGTAATACGGTCGCA
ATAATGATTTCCGTAATATAAACGTCCCAGCCCGCAATGGTGTATAGTTTCCCATTATTTAAGACATCTGGCA
ATAAGAATTTAACTAGTAAACTGAATGCGGTCGCATTTAAAGCAACGACACAGACATAACCAAAAGTTAAAAA
CCATGATGAGAAGAAACTCACATATCTGCCGAAACTTAAGAAACTAAAGGCAAACGCGCCCCCTGATACTGGA
AATCTCTCTACTAATGCGCCATAACTAACCGCAATTAATATCATTAATAATGCACCAATAACAATACCAATTG
ATGCTGCAATCGGACCTGACTGCTTAATCCAGTCTCCTGGTAAGATGAATGCGCCCCATCCGATACATGAACC
ATATGCAATCGCCCATACAAACTTTTCAGATAGGTTTTGTTTTAAATCGCCTCTATCTATTTGCTTATTCTTT
TTTTCCATAAATAAAAACTCACCTCGAAGGTATTCTATACCCAAAAGATGAGTTTGTAACTCTTTTTCTTGAT
TATTTTTATTAAAAATTTAATGTTTAGTTACATAGGTAATCTAAAGTTTTAAAATTTAACCAGCAAATTTGAA
TGTCGCAATGATTAACATTATCCATCCAATGATGAACAATACGCCACCAATTGGCGTAATCGCACCTAAAACT
TTAATTTGAGTTAATACTAAAATATATAATGATCCACTAAAGAAAATAATACCAGCAAATATTAACCAGCCAG
CCCAGTTAACATTGATTGAAGTTGTACCACTAATTACACCTATAATTAATAATGCTAAGCCATGGTACATTTG
ATACGTCGTTGCTTTTTCCCATACTGATAAATAGTGATCACTTATTTTTCCTTGTAAACCATGCGCACCAAAT
GCACCTGTTCCGACAGCCATCATCGCGTTTAATGCACCTAAAATAATAAATAATTTCATTGCTACCTCTCCTT
ATATAATTCAGTATTAATAAATGTTTAAATCCGAACACTCGAATATAAGTATATGTGTCTTGATTATTAACCT

ACATACTTAACAAGCTATTAAGTAATTATGTTTCATTGTTGCTTTTTAAAAATCAAATATGGATTCACCATTG
CCAATGTCATCATCTGTCGCTAATCTTTGTTGTTGGTTTGAAGGTTGTGAATATGACTTATTATGATGATGTG
CTGAATGCGTATTTACTTTACCACCCATCGCTTCAATTTCAGCAGCTGTCACTTGATGAGTTGGCTGCGTTAT
TTGTGATTGTGGCATTTGATTATGGTTAGCAATACGACGGTTCATTTGTTGTTCACCAATATGTGGTGTATTT
GATGTTGAAGCATATAAAGATGTTAATATATGTATGGCATACATATGTTTTTCAAAGTCATGGTCATGCTGCG
CCTGCTCTGCTTGTACTAATTCTTGCTCAATTCGTGCTATTAAAGTTTCTCTATTCACCATCTATGCCTCACT
TTCACACCAATTAATTGGTGATTTTCCTACTGACTCTAAATAGGCATTCGCTTTGGAATACGGTTTTGATCCA
AAGAATCCTCTATATGCAGACAGTGGACTAGGATGCACTGATTTTATAATACAATGTTTAGATGTATCGATAA
GCTTTATTTTTTGCTGTGCAGGTTTCCCCCACAAAATAAAGACAACATGTTCTTTATAATCAGACACTGCTTT
AATAATTTCATCAGTAAATGTTTCCCAACCAATATCACGATGAGAATTTGCTTCACCCTGTCTTACGGTTAAA
ACTGTATTCAATAACAAGACGCCTTCTCTTGCCCAATCTTGTAAATGCGGTGTTTGTCTAACACATCCAATAT
CATCTGCTAATTCTTTATACATATTACGTAAAGATGGAGGGAATTTTGCGTTAGGTTGCACTGAAAATGCTAA
TCCATGTGCTTGGTTTGGACCATGATACGGGTCTTGTCCTAATATAACAACTTTGATATTTTCAAACGGTGTT
AAATCAAACGCTTGATATATATTTTCCCTATCAGGGTATACGATTGCAGTCGAATATTCTTTTTCTAAAAAAT
CATGCATAACTTTAAAGTCATGTTTCGTTGTTATGTCATGAAAAAATTTGCGACCATTCCATTCAATCTCACCT
CAGGACATATTTTAACATATCACACTTATCTAAAGTGAATGACATTTTCAAAAGTTACAATAGTATGAATGAT
TCATTTCAACAATCATCAAATATTGAATGCTTAATATTTTTCAATTAGGTTAAAAATGATATGATGAATACAA
TATTATTATGTAGGAGTGAAGCTCAATGGCTTTAAAGAAAGTTTTAACAATTGCCGGTTCTGACACAAGTGCT
GGCGCAGGTATGCAGGCAGATTTAAAAACATTTCAAGAATTAGATACGTATGGTATGGTCGCTTTAACTGCCA
TCGTTACTATGGATAAAGATACATGGTCACACGATGTCACACCATTACCTATGGATGTATTTGAAAAGCAACT
TGAAACGGCTTTATCAATCGGACCTGATGCTATTAAAACAGGTATGTTAGGTACTGAGGAGATAATCAAACGT
GCTGGAGAAGTATATGAGGCATCTAATGCACAATATTTTGTAGTAGATCCTGTCATGGTTTGTAAAGGCGAAG
ATGAAGTGCTTAATCCTGGAAATACTGAGGCGATGATTAAATATTTACTACCTAAAGCAACGGTAGTGACGCC
AAACTTATTTGAAGCGGGTCAATTATCAGGATTAGGTAAATTAAATTCAATTGAAGATATGAAAAAGGCTGCA
ACAATTATCTTTGATAAAGGCGCACAGCATGTCATCATTAAAGGTGGCAAAGCTTTAGATCAAGATAAATCGT
ATGACTTATACTATGATGGTCAAACATTTTATCAACTAACAACAGACATGTTCCAACAAAGTTATAACCATGG
TGCTGGTTGTACATTTGCTGCGGCAACAACTGCATATTTAGCTAACGGTAAGTCACCGAAAGAAGCTGTGATT
AGCGCGAAAGCTTTCGTCGCTTCTGCTATTAAAAACGGTTGGAAAATGAATGATTTTGTTGGTCCTGTGGATC
ACGGTGCATACAACCGTATTGAACATATCGATGTTGAAGTAACAGAGGTTTAACGATTAGGCGGTTCATCTAT
AAATCAACATTATTAGCCACACCACTTAAATTGGGTATGAACTCAATTTATGTGATGTGGTTATTTTTTTATA
ATCATCATGCTCTGTATATAATTTGAATTTGAAAGTTACATATAAGCGTTATTAGCTTTTATTGAAAATTTCA
TACATATCGAACAATACATACAAATATTTCCTATACATCTACATCTATGACTAAATAACCCTTTATTTTCAAG
ATGAACTTAAACTCATCTATATGCTCAATACCTCTATCATTATCATTCTTTTGCGATAGATGCCTCTCTAATC
ATATATGTTATATTTCCCAAATGACTGCCAGGCGACTCATTAAATATATATTTATTTAGAACTTTTATACATA
AATAGTCTTAGTAAATCATACGTTCTATGTCTGATAAAAAGTTGATACTTAAGGTTGATGTCACACATAAAAA
TGTTATTTATAATATAACCATAGAGTAGCAAAGGAGGTAATATAGGTTATGATTATTTATCGACAGTATCACC
ATGAAGGCGCACCAGTTTATGAAATTATAACCAAAACGTTTCAGCATGTTTCAATTAAATGTGACGATTCATT
TAGTGATACTGAAATATTCAAATTGCTCTCTTTATTACAAGACGATATAGATCATATGAAAGTTAGTTAAATT
GACTAATCGTTCCAATAATCCAGAATTTATTAAACATTGATATAGTTGTTATTATTATTTTAGATTAATGCAT
CCCCGTGCAAAGAAAGAATCGTAACCTATATTCATCTCTGTTCCCAATAATAATAACAATACATAACTGATAT
TAGCTGTCCTTTCATCTCCTAATTAATATGCGTTCATGCAGCTAATATCGGTTATTTTTTTGTGCTCAAAGAA
ATTTTTATTTTAATCTCTCCATCGTCATTAATTGCTATAATTTCTTGCTCGTGTTGTTCAATGAATTCGATAT
TTTCATTTATTAAAATAGGTTTAACGTAGTTAATTTGACACTGGTTTGTCTCTAATGACATAGCCATCAGCAT
CATTTGACTCACTAATTGTCCTGGCACAATCTCATTATGTATTGGATTATCATCATTTACGAGCGCCAAATAT
TCATTTATCCATATCTCATTAAACTTCATCTCTACTTCATCGCCTTAATAAAAGTTTGTGTAACCGTTATGAC
ATGTTGATCATTTTTAGTTAATGTTAAAGCCATTGTATAACGTGTGATATTGCGAATCTGTCGGCATTCAATA
TCTTCTAAACGTCCTACATATATTGTGTCTACTTCTATCTTTTGAGTTTGATTAATTGCTGATTTTGTTAAAA
TCAGTTCGCTATTTGCATATTTTTTAAATAAATCAAATTTTGACCATAATGACGCACACATTAATGGTGGTAC
TTCTTTTCTATAAGGTTGGTTAAAACACTGATAATATGCAGCCACACTATCCTTAGAAAATGACACATTTAAT
CGCTGGTTAGAATCGAGTGAATAATGCTGCATTTCCTAGACCTCCCCCAATCCCCATAGATGCAATCATAGTC
TCTTTGTCAAACATATAAAATAATCGCGTCACTAATTGGGCACCGCTTGCACCGTATGGATGACCTGATGCTA
ATGCACCACCCCATATATTTAATTGCGTATTTGAAATATTTAAAGCTTGTTGGCAGGCAACCACCTGTGCACT
GAACGCTTCGTTAATTTCAATGACTTCAATATTTTCTATCGTTAATTGATTTCTTTTTAGTAAGTTGGAAATG
GCTGGTACTGGACCAATGCCAGGAAAATTAGAATCAACACCTACCGTAACACCATCTTTAAATAATAAACCAT
GCTCGAAACCTAATTCGTATGCCATATCTTTTTCCATAATAAGCAATAAAACTGCACCATCATTTTTCATACA
ACTATTCGCAGCGGTAACGGTCCCACCTTTGATCACGGGCTTAAATCGGCCAAAGTTATCTTTCGGAATATGT
GATTTTAGACTTTCATCAGTGTTGAATATTTCTCCTTTAACGGTTATAGGTAATATTTCCTGAGAAATATTTC
CATTCTTTACATTTTCCGCTGTCAATTGATGACTTCGATAAGCAAATTCATCTTGTAATTCTCTTGAAACATC

ATACATCTTGGCCACATTTTCAGCACCTTGAATCATTGATGGGTCGCTCATTTCAGGTGCAAATGATGCACGC
TCATAAAACTCAGGTAATGCTGTTTCGTACACAGAATGCGGTCGTTTGATTTTCCAAGGTGCTCGACTTGTAC
TTTCAACACCACCTGCAATATATACCTTGCCAGCTCCGGCTTGGATCATGCGACATGCATATTGAACACTTTC
AAGTCCAGACCCACATTGCCGATCGATTGTGACGCCAGGTATTGAATCTTTAAGCCCCGCTTCAAGCAATGCT
TTTCTTGCAATATTGCCACCATTCCCAACAACATTACCTAAAACTACATCATCTATTTTAGATATTACCTCTG
GATACTTCTCTTTAAAATGTTGGAATAAAGGTTTAAGCAATTGTTCTGGCTCTAAATGTTTTAAAGTGCCACC
ATATTTCCCAAATGCAGTTCGTTTAGCTGCAACTATGACTGCTTGATTCATATTATAATTCACCTCTCAAATA
CATCGATATCATTTTTTCTCTAGCAATTTTACCACTTGCAGTGTAATACATCTTTTCTACATGATGAATCATC
GATGGAATTTCATAGCGTTTCACTTTCTTTTTTAAAAATTGTTTTACATTTTTATGTGTAAGTGTCACATCAC
TAGAATAAAGCAATACGCCTATTTGACCAAAACGCTCATTTGGAATACCGATGATAATTGCTTCATCAATGCT
CGACGATTGCGTTAAAAGGCGTTCAACATGTGCTGGATATATATTTTGACCACCAATAATTAACATATCCTGT
TGACGTCCCGTTAAATATAAATACTGCTCTTTTACATAGCCATTATCATTAGTAACAAACCATTCATCATTAT
TTATACATTGTTCACTTACATAGCCACTAAACATCATATTACTTTTTATACAAATAGTTCCTATACCATTGTG
ATCGTGATCCGTTGTTTTCAATTCCACATTTGGAAATAGCACACCTACTGATTCAACTGGTGCTTGCTGATTC
AAGTTATAGCTGATAAAACTGGTTTCCGATGTACCAAAAAATTCAATCAAATTTATGTCATTTGCTTGATTTT
TTATCTTTTTAAAAATAGAAGAATGCAGCTTATCTCCACTGCTAAAAAATGATTGGATTGTATGTTCATTGTT
GTAAACTAACAATAATGATTTAATCATCGTTGGAACAAGAAACATAGCAACTTTGTATGATGATATTTTACGA
CATTGATTAAGTAACTTTTCAGGATGAAAAGTGGTCTGTCCTATAAAAGTACGACCGGAACTTAAAGCAAACA
ATAACGCATATAATGTTAACGAGTGCGATAGTGGTCCAGGGGCTGCTATTGCATTTTCATTTTTTAACATCAA
CATTTCATTAACTTCAAAAGAAGCCAACCATGAATCTTCATCACGATAATATGCTTTTGGCAGTCCAGTTGTC
CCTGAAGTAAAACCAATATGTAATAAATTGGGATAGTGCTGTAATTGCGTTGAATCAATAAACATCGGTGAAT
CTATACTCTGCATGAGACCTGTATCTCCAATTACATCTTTAATACCATATTTTTCAATCAATTGACGATGTAT
AGTACTTTGCCATTTACCTTCCATCATCATAGGCACTTTATGTAAACGATGCAAAGCTAAATAACAAATAATA
CTTTGCACCGGTGATTTCATATTAATAGCAACGACAGGATTAAGTGAATATTTCTGCAAAGATTCAACTGCAC
TAGTGATGCGGGCATTTAGTTGACTATATGTAATTGTTTCATCATCGATATGTAATGCTATGTCATTAGGTTT
ATTTTGAGTATGTGTTTTCAACTGTTCTAAAATTACGTTCATCAATATCCCTCACTCAATGTAAACTTTAAAA
TACATCTTGTTAACATTATATAATGAATAAGTATTTTTATCAGGTAAACCTGCCTATTATAAAAATCAAACTT
AAGCCCTAGATAAATTTGATACTGTAGTACCATGTTTTTTATTAGATAAGACGTTACTATATGTATATAGAGA
AAAGAAGTTATCGCAATCGCAATAACTGACACAAATAAGTCACCCAATCTATTTAAGTTTGTGTTCTCTATAA
TAATATTGAAGGTTAGAGTCTCAATATTAATTGCGTTGGACAAAATACTCTAAGACATATTTACATCCTAACA
TTTCAATAGCAAGAGAATTTTAAGTATTCAAAGCTAAACATATGCTTTAAGTCCCCTCTTTCCCCCATTGCGA
TAATCATTGCAGTGGGGGTTTATGTATATACAAAAACTTTATTACGGCTAATTACAGTTCTCAATTTAAAAAG
CACTTTAAAGTTAGCAGAACCTACTAGCTTTAAAGTGCTTATATACGATTGATTTATACTTTTTATTAAAATC
TCTAATTCTTACGTTCTTTAACCCACCATAATGCTTCTTTTGGGTGTTCTGCATAATAAGCTCTATCTTGCTC
GTTATCTACATTTGGATACGAACCTTTTAGAGATTTTCCTGCTGTACTTAAATGTAAGAATGTAATAACTAAA
AAGCCAATAACACTGATTGCTGTTAAATAATACGCAGGTGCTAATGGATCTCCAGTTTTCGTAACTAACCATG
TTGCAACTAATGGCGTCGTACCACCAAATATCGAAACAGAGATATTAAATGTTACTGATAAAGTTCGATATCT
TATATGACTGTAAAACATCGTTGGTAACGACCCTGGCATTGTCGCTTCGTAAGTTGATAAGAAAAATCCTAAT
ATAAATATACCGATTACTATTACAACAAATGATTGTGAATGTAATAACATAAATGCGATGATACTAAATAATG
TTAGCCCACCAGTACCAATTAGAAATACTTTCTTTTCACCTATTTTATCCGCTAACTTACCAAACATTAATGC
TAATGGAATCATTATTGCCATGACACAAGTAATTAATACACTTGTTGTCGTTGCATCTAATTTAATAACTTGT
TCTAAATAGGTTGGTAAATATGCAGTTACCATATAGTTCGTAACATTGAAGAATACAACAGCTACAAAACACA
CAAATATATCTTTGTAATAAAATCTGATGATTTGTAAAAAGTTAATGTTGTCTCGTTCTGGTTGTGTTGCAAC
ATCATTTTCGAAAACTGGTGATTCTTCAAGCTTACGACGTAAATATAAGCCGAATAATCCTAGGAATAAACCA
AGTAAGAATGGGATTCTCCAACCAAATGATGCCATTTGTTCATCTGTTAAAAAGAATGTTAATACAGCAATCA
TAATTGAAGCAGCTATGTAACCTGATAATGTCCCAATTTCTAGTCCACTACCTAATGAGTTACGAAGCTTATC
TGGAGATGATTCGGCAACATATGTCATTGCCCCCGCATACTCTCCACCTGTTGAAAATCCTTGCAGTACTCTT
GCAAGCAATAATAGTATTGGTGCCCAAAGTCCAATTTGATCATAGCTTGGCAATAATCCAATGGTTAATGTTG
AAAATGCCATTAAAATAATTGTAGATGTTAATACAACTTTACGTCCATATTTGTCACCAATAATACCAAATAC
GACACCACCAATTGGTCTTAATAAAAACGCAATGGCTAATGCTGCGAAAGTCAACATTTGTCGAATGTCTGCA
TTCTCTACTGGAGAGAAGAAGTTCGCTCCAATGTACGCAGTTGTATATGCATAGACACCAAAATCGAACCATT
CCATTGCATTACCGATACCGGTTGCAACAACGGTTTTTTTAGCTTTCTTTGCATCCACCATGTTAATATTCTC
TTTATTAAAATCCATGAATTCATACACTCCCTCTTGTATGTTTCATAATTATACGGAGTTAATTAGTTTTGTC
AAATTTATAAGAATTTAAACTTTTATATAAATACAAAAGTATGTTTGTTCAAAAATTTCACTTTATCGAATTA
AATTCAATGATTTTATACCCTCATACATTTGAATTAAACAATAATAATTCTTAAATCATCGAACGATTAACTT
TTTGGAATTATAACTAAAAATAGCGATAGCACACAACTTCATGTAAGTCATATGCTATCGCCATTTTCGACAT
CATATTTAAATGTTTATATTCGATTTACTTCAATAACTTTGTCTTTACTTTAATTTATTTGTACAAAAATAAA
TGATCAACGAATCAGACATCACTCTTTAAAAATTCATGATGTTTAATAAATATTTATTTTAAATTCCTAATCT

AAAGCGAAATATTTCTATGTATAATCTAATATTTATGAATATACATTTTAACCATGGTATTCTCTGTTTTAAC
AAATTTTATAATACTGTATTAATTTCTGATGGATCATTGATTATAAAATCTGGATTTGAATGAAGTAATGACC
TTGCATCATGTGCTCCCCATGTTACTGCTGCAGATGGTACACCAGCATGTTGCGCCATCTCAACATCAAATGT
TGAATCACCAATATAAACCGTTTGTTGGCTATTTAAATTGTAGCGTTGCACAATTGTGTGTATGCCTTCAGGG
TTTGGTTTATATGCACTTACTTGATCGGATCCAACAGCTTCGGTAATCAAGTGATTTAATCCAATAGCCGATA
AATTTCTTTCTAATACATCACTCTTCTTACTAGACACCACGAAAAGTTTTTTCCCTTGGTTATACAAACTTGT
AATGGCTTCAGTTATACCCGCAAATTCATAAATATAGTCCTTTTCAATAGATTGATATGTATGTCTAAATGTA
TCGATTAACTTTGCTAATGCTACTTCATCTAATGGTCGGTCTGCTAATTTTAAAAATGATTCTTCAATAGGTA
TTCCCATATAATGCGTTATTTCTTTAGATGATGGTTCCGTTAAGCCACATGCTTTAAATGCACTTTGTGTTGC
TACTTCACCACATTTTTTCGTGTCTGCCAACGTACCATCAAAATCAAAAATATAATTGTCAAACTTCAATATT
AACACCCCTATTCGTTTATTATTGATATTATACTATAATTTATTATTTAAATAAAAAAATAATCGATAATATT
TCTGCCTAGGCCTCATATTATCGACTATCTTTGTTTTATTCTAAATTAGCATGATTTTGTTGCATCGTTTGTT
CCGTTACATTCATTTCAGTCATCAATCCCATTACAACACTATCTAAAAATAATTGAGATGCTTGTTCAAACAA
ACTTCCTAATGGTTGTGCCGAGCCTTGTTCATCATATTTTGTACCTGCAGGCAAAACGATGTTCGTCCCAGCT
AGATTGCCTATTGCAGAATCTTTATTTGTAGTAATTAATACGATGTCAGCACCTACTGATTTTGCTTTGTCTG
CTAATAATCTTAAATGTTCCGTGGAACCTGAACCAGAGATAATTACAAATACATCATTCGACTTAATCGCAGG
TGTCGTTGATTCTCCAACAACATGTGCCTGTTTGCCGAGCTGATTTAAGCGCATTGCAAAGCTATTCGCCACG
AATCCTGAACGTCCTTTGCCAGCTACAAATATATGTTTAGCATGTAGTATTTTGGACGCAAACGTTGAAAATT
CATCCGCTTCAACATGTGACAAAGTCATCTTTAATTCATCTAGAATTAATTGATAGTCACTAAATTCAGCCAT
ATTACTTACCTTCGATTGCAGCGCGACATTGTTTTGCAGCTTCTACTGGATCATCTGCATTTGCGATTCCGCC
ACCAACAATAACAAGATCAGGACTTTCAGCGACAATTTCTTTAATTGTATCTGGTTTAATTCCACCTGCTACT
GCAACTTTAGAATTTTTAATAACAGATTTAACGGTTCTTAAACTTTCTAATGGTGATTGCCCTTCTGCTTGTA
AATCATAACCAGTGTGTACTGCAATATAATCCGCACCCATTTCATCTAGTTCTTTTGCACGTTTTTCTAAATC
TTGAACAGCAATCATATCAACTAGTAATTGTTTATTATTTTTATGAGCTTCTTCAATAGCTGCTTTAATTGAT
GCATCTTCTGCAACACCTAGTATTGTAATTACATCCGCGCCAAATTTAATTGCTTGGCTAACTTCATAATCAG
CTGCATCCATAATTTTCATGTCTGCTAATACTTTTACATTACTAATGTTGTCTGCCATATGTTTAACTGCTGG
TAAACCTTCGTTGTAAATGATTGGCGTACCGATTTCTACGATATCTACATAATCTTTTACTTTATTTGCTAAC
TCAGCCGCGTCTTCTTTGTTTAATAAATCAATTGCTAATTGTAATTCCACTAAAAATCATCCTTCCAATGCTT
TTTATCTTTCTATTGGAACCTTTCCCGAGGCATTTCTTAATTAAACATTGCAATCAATCCATTTCATCGACAT
GAATTTTCGCAACATTTTTCGGGCAAGCTTCGTCGTCTAAATATATCTCAACATTCGGGTGTTTGTGAAGTAA
TGTGGCTGGAACATCAACAGAAATTTCCTGATTTAATAAATGTGTAATAGCAGCACGTTTCTTTTCACCAAAT
GCGAGTAAAATGATACGTTTGGCTTGAAGAATATTAGCAAGTCCCATCGAAATGGCTTGCTTTGGAACATCAT
CTTCGTTTTTAAAATATCGACTATTAGCCTTAATAGTACTTTCAGTCAAATCAACGATATGAGTAACGCTATC
AAACGGCGTACCAGGTTCATTAAATCCAATATGACCATTTTCTCCAATACCTAAAATTTGAATATCACGTTGA
CCTTGTTGTTCTAAAACGTCATTATATTTTGACGCTTCCGCATTCATATCATCGGCATCTCCATTTGGAATAT
GAATGTTCTTTCTATTAAAATAAGGATATTGTTTGAAAAGCATGTCATCCATATAATAGTGATAACTTTGCGG
ATGTGATGCGGTTAAACCTACATATTCGTCTAAATTAAACGTGGATACATTATCTACGTTTAACTGATTTTTA
TTTAATAACTTAACAAGTTGTTCATACAAATCTGTCATTGTACCACCAGTTGCTAAACCTAGTTTACAGTGCT
GATTAAATGCCATCTCTTTATATAACTCACATGCAACATAGAATGATGCTTGTTTTTTCGATCCTAAGTTTAA
TACTTTCATTGTTTACATGCCTCCACAAATTTAATACATTTTAAAAACTTATGAATTCATTCATACACCATTA
ATATAGCATCATTTTTAATGTTTAACACTTTTCAAATTCCAACAAATTTTTGATTTTTAAAAGTAAAAAACGA
ATGCTGTATGATTACGTAATCCTGAGTAATTACCCTTCATGAAAATTAATTTATAAATAATTGAGCAATCGTA
TACAATTATCGAATAGTTTTTAGTAAAATAGAAAGATAAACACATTGATAGTGTAAGGAGTGAACGTGTTGGA
ACCAATTAAAGAACAAGAAGTTATAGATTTACTTGCTTCTTTTGAACATAAACCTGTGTATCTGCATGTTGAA
ACGACAAATGGGGCCTATGCGAACCATTTCGATCAACGTGTTTTTAATGCTGGTACTTTTTTAAGAAACATAC
AAATAACATATACGCATGCACAACTAAAAGGTGGTAATAAAGAACCTTATCGCATTGGTCTTAAACTATCAAA
TGGTGGTTGGGTTTATGTACAAGGTCTAACGCATTTTGAGGTCAATGAACATGATGAATTTTTAATTGCAGGT
TTTAATTACGAAGGTCAACTCGCTGCAGCACTTCAAATTAGTGAGCGACCATTTAATTTATAGAGGAGGCATC
AACATGACTGACGAAAGACATGTATTAGTAATATTTCCACATCCTGATGATGAAACCTTCTCATCTGCAGGCA
CATTAGCAAGTTATATCCAAAAAGGCATACCTGTAACATATGCATGTTTAACCCTAGGACAAATGGGCAGAAA
TTTAGGTAATCCCCCATTTGCAACACGCGAATCATTACCAAGTATTCGTGAGCGTGAACTAGAAGAAGCTTGT
AAAGTTATTGGTATTACTGATTTAAGAAAAATGGGTTTACGTGACAAAACTGTCGAATTTGAACCCTACGAAC
ATATAGATGGTATGATTAAATCTTTAATCGATGATACAAATCCATCATTGATTATTTCATTTTATCCTGGATA
TGCAGTACATCCTGATCACGAAGCAACAGCAGACGCTGTAATTCGAACGGTTGAACGCATGCCTAAAGAAGAA
CGCCCTCGTTTAACTTTAGTTGCGTTCAGTAATGATGCAACCGAAGCTCTTGGAGAACCTGATATTCAAAATG
ATATTACAGATTTTAAAGAACTTAAAATTAAGGCATTTGAAGCACATGCTTCTCAAACAGGTCCATTTTTAAA
ACAATTAGCAAGTCCTGAAATCGATGGTAAACAACACAGCTTTTTAACAGTCGAACCATATTGGACATATCAC
TTTAAATCTTAACGGAGGAAAAAAATGACTGAATTTGATTTATCGACACGAGAAGGTCGTTGGAAACACTTTG

GTTCCGTAGATCCTATTGAAGGCACGAAACCTACTACTAAGAACGAAATGACAGACTTACAAAGTACACATAA
AGATTTTTTATTTGAAATAGAAGAAGTTGGTATTAAAAACTTAGTGTACCCTGTACTTGTTGACCAGTATCAA
ACAGCTGGCACATTTAGTTTTTCAACAAGTTTAACAAAAGACGAAAAAGGTATTAATATGAGCCGTATAATTG
AAAGTGTTGAAAAGCACTATGATAATGGTATTGAACTTGAATTTAATACACTTTATCAGGTGTTACGCACTTT
ACAAACGAATATGAAACAAAATGCGGCAGGCGTTGATGTTTCTGGGAAATGGTTCTTTGATCGCTATAGTCCA
ACCACAAATATTAAAGCAGTTGGTAATGCCGATGTCACTTATGGATTAGCAATTGACGGTGATAAAGTTACAC
GTAAAGAACTTACTATTGAAGCAACTGTCACAACGCTATGCCCTTGTTCAAAAGAAATAAGTGAGTATTCAGC
GCACAACCAACGTGGTGTTGTCACTGTTAAAACATATATTAATAAAGATCAAGACATAGTTGATGACTATAAA
AATAAAATTTTAGATGCAATGGAAGCTAATGCAAGTTCAATTCTATACCCTATTTTAAAACGTCCTGATGAAA
AACGTGTGACAGAGCGTGCATATGAAAATCCACGTTTTGTCGAAGACTTAATTCGTTTAATTGCCGCGGACTT
AGTTGAATTTGATTGGTTAGACGGTTTCGATATCGAATGTCGTAATGAAGAGTCTATCCATCAACATGATGCT
TTCGCTAAATTAAAGTATCGCAAATAATATCATATGATTAATTCAAACGCGTAACAGTAGTTGCGCGTTTTTA
TTATACCTTCTAGAGTTGTATATCTCTTTTATAAATCTTCTCATTTGATGTGCCTGAGTCATAAATCAATTTC
CTAAACTCTACAATGATATATTGGTCGTAGTTGACTGAATAACTACTACCATTATAAATATCAAAACTTGTTG
TTTTATAAATAAACACATCTTCCAGTTAAAAGAAGTAATTAAATATTATGTGATATAATTATAAAAAAATAGAT
ATAAGTGGTGTTGTAAATGAATTATTTTGTAGGTAATAGTTTAGGTGTTAATTTAACTGGGATAGAGAAAGCA
ATTATTAATCGTTTAAATTTATTTAAAGAAATGGGAAGACCAGCGCAATGTGTATTTCTGTCATGGAATAGAT
ATTTATATAGAAATGCCCAAAATTATATTACTAGTTCTGACTATATTAATATGTATGATTTCTTCCAAGAAGC
AACATATTTAGAACGCAATGAACCATTTGACTGGTTGTCGTATTGGACTGATGAATGTCATTATACTTTAAAA
CATGTTGAAAATAGTCATGACTTTAGAATATATGATCAAGAGCGTTTCTTAATGTATGCCCATTTTCAAGATC
CTAAATATCGTATATTAGATTATGTGAATCATTTTGATAGTCAAAGACGAAAAGTAAAACGTGATTTTTATGA
TGTGCGTGGCTTTTTAAGTTGTAGTAGGATTCTTGTTGATAAACAACAAACATTATGTGAATTTTTCTATAAT
CCAGAAGGTGACACAAAATTAGAAAAGTATTTTTCTTACAAAGATGGCAAACCAGAAGTACAAAAAAATTATTG
TTTACTATGCTAACAAACAATATTTCTTTAATAATGAAACGGAATTAGGCGCATTTTTCATTAAACAACTATA
TCAACATGGGGATTTATTTTTCAGTGACCGTAATGTGTACACAGCACCTATTTTCAACTTAACGCCTGAGTCA
ATCCCAGTAGTAGCTGTGCTTCACAGTACACACATTAAAAATATTGATGCATTAGATTCATCGCCATTTAAAA
ATGTATATAAAGCGATGTTTGAAAATTTATCTCGATATCGTGCAATTATCGTTTCTACTGAGCAGCAAAAATT
AGATGTTGAAAAACGTATTAACCGTACTATTCCAGTTGTTAATATTCCTGTCGGCTATAGTGAAACAATTGAT
ACACCAGTCCAAACATTAGATCAGCGCAGTGTTAAATTAATTTCCGTTGCTCGTTATTCACCTGAAAAACAAT
TACATCAACAAATTGAATTAATTAAACGCTTAGTATCGTATGTCCCAAAAATAGAGCTTCATATGTATGGTTT
TGGTTCTGAAAGTAAAAAATTAAATGAATTGATACAAAAATATGGATTGGAAAATCATGTTTACTTACGCGGC
TTTTTAAGCAATCTAGACCAAGAATATAGCGATGCCTATTTGAGTTTAATAACAAGTAACATGGAAGGTTTCT
CTTTAGCGCTACTTGAATCTTTAGCACATGGTGTTCCAGTTATTAGTTATGATATTAAATATGGACCTAACGA
ATTAATTACGCCTGATTTCAATGGTTATTTAATTACTAAAAATGATGAAGATGCCCTATTTGATAAAGTAAAA
TATGTGATTGATCATCCTGAAGTACAGCAACGACTGTCAAAAGGTAGTTTGGCAAAAGCGCAACAATATAGTA
AAGCGTCGTTAATTAAACAATGGGACCAATTTGTTCGTCTTATATAAATGTACTTCACCTTTATTTTTATAAA
AAAAAAGCGTTTTCCTATGTCATTTAACTAAACATGTAAGTTCGTATGAACGTTGTTTGTTAAATAGATGATT
CTAGGAAAATGCTTTTTTCTTTTGACTTAGTTTAAAATATTTTGCCACTTTGTACTGATAGTAGTTGCATTGT
ACTGTTGTGCAGATTCTATGCTATTAATCGAAAACTGTTGCAATTTTTGAGTATTATTTAGTAATTGGTCGAC
CTTTTCAACCATTTGATTGATGTCACCTTGAGGTACTAAATAGCCATTAAATCCATCTTGAATCAGTTCTGAT
GGACCATAATCTACATCATAACTGATCACTGGTGTACCTACTGAAAGCGACTCTAAAATTGCTAAGCCAAAAC
CTTCCATTTTACTTGTCGATAACATCAGTTCTGCTTTAGCAATCTCTTCATTAATATGCGTCTTAAAACCATG
AAATTTTACATGTTCCGATAAATGATAATCTTCTACAAGTTGTCGATATTCTGACAAACCATTTCCATGTCCA
TAAATATTTAATTGAATATTGGGATGTTTTGTTACTAATTGCTTGATCACTTCAACTTGATGTTTAATTTGTT
TATTTTCAACGAGGCGAGCAATTGATATGATATGATTTTTCTCCTTTTGATTGATATCAAATTGATACTTTAA
ATTTGCCACGTAGCCAACCGGAATATTGATAACTGGTATTTTATTTTCAATATATTGTGAAATATCTTGGCAT
TGCTGTTCTGTTGATACAACAATCGCTTTATAACGTGTTAAATTATTAAACACTGTTTTATAAAAACTTTTTA
TACCATTACCGGTGTCGGCTAAATGTGTACTGTGGAGCACAACAATAACTGGAATACTTTGATTTAATCCCGC
TATAACATTTCCTAATTCATGAGGACGATCTAATATGATTTGATCATTATTTTTACATAATTGATGGAGAAAA
TATTGAACTAATTCATCTTCTGTATCAAAAAATTGTTGATGCTGATCTTCATTTAAGATAACCTTTGTGAGCG
TGTTTTTCCCTTTTATATCGTCGAAATATTTTTGGATGACGATTTCCCCATTAGGTGTATAGTAATTTTCGAG
TACAATCCGTTGTCCTTCACCTAAAATTCGAGAACAACTTAAAAAGCCTCTTCCATCATACAATTCGCGTTTT
ACTTTTCTTCTTTTATGATCAAAATAATTCACATAGTTTAAATGATGATACTGTTTATCTAAAAAATGTGCAT
ACATTACAAATTGCTCTTCATCATATATTCTGACATCATTTGAATTTTCCACAAATTTCAATGTGTACCTACA
TGACTTTTCCCAATACTGTATCCAGTTAACTTGCTTTGTCTTTTTATAATTGATTGCTTTTTGAAAATAGTCA
TACATTGTAAATACATCATTTTCAATCTGATGTTGCTTCGCATATGTGTATGAATAAGGATTCCATTTAACAT
ATACACATTTTGAAGATATGCCGTGTTGTTTGAACAACTTCAATCTATTTATTTGCGCTTTTTCTACACCTGT
AATTTTACTTTCTAAAATTGTTCCTAAAATGTAATTCATATTATCGCCTCATATAAGTTTTATTCCGTATCTT

TATTGTTTATTTTATATGAAAAATACATCTATTGCATGTGTAATTATAAAAAAACCAGGCCACAAGGACCTGG
GTCATATTGTATTATTTGTTTTGTTTTTTGCGACGACCGAATAACAATAATGAACCTAATGCTGCAAATAATC
CACCAAATAACGTTGCGTTATTTGAGCCGTTATTTTCACTACCTGTTTCTGGTAATGCTTTTGCTTTATTGTG
ATGGTCTTTAGTAGTACTCATTGGTTTAACAGGTGTATGTTTTCCTGCATCCGAGTCCGAATCGCTATCTGAG
TCTGAGTCGCTGTCCGAATCTGAGTCGCTATCTGAGTCTGAGTCGCTGTCCGAATCTGAGTCGCTGTCTGAAT
CTGAGTCGCTATCTGAGTCTGAATCACTGTCCGAATCTGAGTCGCTATCTGAGTCTGAGTCGCTATCTGAATC
TGAATCGCTGTCTGAGTCTGAGTCACTGTCTGAGTCTGAGTCGCTATCTGAGTCTGAGTCACTGTCTGAATCT
GAGTCGCTATCTGAGTCTGAATCGCTGTCTGAATCTGAGTCGCTATCTGAGTCTGAATCGCTGTCCGAATCTG
AGTCGCTATCTGAGTCTGAGTCGCTATCTGAATCTGAATCACTATCTGAGTCTGAGTCGCTGTCTGAATCTGA
ATCGCTGTCTGAGTCTGAATCGCTATCTGAGTCTGAATCGCTATCTGAGTCTGAATCGCTGTCTGAGTCTGAA
TCACTGTCTGATGTATCTTCTTCGAAGTATCCGTTATCAAGTGTGAAATCATCATGATCCGTAATTGTTACGT
CAACTTCGCCACCATCTGCATCTTTATCATCTTCAGTTGTATTTGTAACTGTTTGTGTTAAGCCAGCAGGCTT
TTCAAAAATAACTTTGTATTTACCGCTATCTAAATTATCGAAACGATATTTACCATTTTCATCTGTTTTAGTT
GTTCCAATTACTTCGCCTTTTTCGTTTTGCAATGTAACTGTCACATCTTTAATACCTTTTTCAGTTGAGTCTT
GTTTGCCGTCTTTGTTACTGTCGTACCAAACATAATCACCTAAACTGTATTTTGGTGTTTTATAGAAACCACT
GTCTAATGTCATATTATCTGCATCTTTAATGACACCTGTTGTTGTTAAACCATTAGAATCTTTATCTTCAGCT
GTAGTATTTTTAACCGTTGGCGTGTAACCTGCTGGTGTTTCAAATTCTACTGTATAGTTACCATTATCTAAAT
CTGTAAATTTATATTTACCCGAGGCATCAGTAGTAGTTGTACCAATAACTTTTCCAGTACTATCTTTCAATGT
AACCTTGACATCTTTGATACCAGGTTCATTAGCATCTTGGATACCATCTTTATTTGTATCTTCCCATACATAG
TCACCAAGATTATATTTAGGTTCTTTATAAAAACCAGTGTCTAAAGACATATCATCTTTACCATTAATTTTAA
CAGTTACAGAACTACCATTTGAGTCTTTTTCACCATCAGTTGTTCCATTTTCTTTTGTTGGAAGGTATCCAGC
TGGCGTTTCGAATTTAACTGTATAAGTTTCTCCGTCTTTCAAACCACCGAATTCATAATGACCATTCGCATCT
GTTCTTACTGATTTTGTAGTACCGTCCGGGTAAGTTAATGTAACTAAAACGTTTGCCATTGGCTTTTCTTTTG
AATCTGTACCTTGAACACCGTCTTTATCAACGTCTTCCCATACATAGTCACCAATTTTGTATAACTTTTCTTC
AGGTTTAACAGTACCGTCACCACCGCCAGAGTCATTAGAAGTTACGATGAAGTTTGAATATCCTGCATAATTA
TAATAACCATATTTATCAGTTGTTCTCATACTAGTACCTTGGGCAATATCTAGTTCGCCATCTGATGTAGGTG
TATATTTACTAACAACTTTGATAATATAGGCTGAATTAATATCACCAAAATCCAATGTTGCTACATTATTACT
AAATGATTTTTTATTATCAAATTGACTTGTTACATCTTCGTATTGACTAAAATCATAGATTCTATTACTTTGA
GGCAATTGTTGATCAGAGTTAACTTTATAAACCTTTATTTCTGTATTTTGGTCAATAATGGTACTACCATTTC
CTAGTTTAATATTTCCATAATCATCTACTTGACTACCAGCTATATCAACTTTAGTGTTGGTTGCTGATTTTTT
CAATGGGTTAACATAAATTTGTTGTTCAATAGTTTGCTTATCTTCATCTAATTTTGTAAAGATAGATTGAATG
TTTGAATCACCATGGACCATTGGATCTTGATAATCAACAGTGACATTTTGGCTTGTTTCTTTACCTGCTGTAG
CAAATGTTAAATTCAAACTTGTCTCATTTGGAACTGTTTTTTTATCAATATACGAATATAGAGTTAAGCGTGA
TTTTATATCTTCATATTTATCTACATAGTCTGTAAATGTATATGTGATTTGCTTAGTTGCTTTATCAAATGTT
CCTTTAGCAATGACCTCTCCTGATGGATCAGTAATATCGATAGGATCATTTTTATCTGTTAAATCCGAAGGAA
TTACATTCTTATCATAATTAATCGTCATTGTATCGCCTTTTTTGACTTTATTGTCAATTGTAAACTCTGTATC
ATATTCAATATCTTTACCGTCATGCGCTGCTGCCACATTATCTTTACCATCGCCAACTTTGATTGTTTGCTTC
GTCACTTTAATTAAATCATTTACATTGTTTGAAGCAACTGCTGCTGGTTGTGCAACTGCAAAGCGCATTTTTG
CGTTTACACGTTTTGGTGCAACACTTGTTGGAGCTGTAGCAACTGGTTTAGTTGAATGATCTGTATTGCTATC
ATTTCTAACCAATTCTTTTAATTTCTCAGGATTATTTTTAAGTTCTTCTTTTGACACATTTACTGGTTCTTTT
GGATTAGTTGCATCTGTAACTTGATTGTCTACTTTTGAAGGCGTTGGTTGCGGTTGTGAATTTTCAATATTTG
TAGATTCTTGAGGTGTAGTTGGTTTTTTTTGAATTTCACTTGTAGATGGTTTTGTAGTTACATCGTTATTTGT
ATTATTTGGCGCTTTCTTCTCTTCTAAAATAACAGATGTATCTTCTGTCGCGGTTTTATCAGTTGAAGGTTTA
ACATTTTCTTTTTCAATGTTTTGAGGCTTAGTTTCAGTTGTAGCTGTAACGTTATTTTGCTGTTGTTGAGTAC
TTGATGTAGTTGATTCTTCTTTAGCTTCTGGTTGTGAATCAGTATTTGTTTCTTTCTTAATTGGATTTGTTGA
ATCATTTTCTGTTGTCGAATTATTTTCAGCTTCTGACACTACTTCTTTATTATCACTAGTCGTTGCATCATCT
TGTTTTGCATTTTCTGTACTAGTGTTTTCAGCAGCTTTAGCTTCTTGGTTCCCTAGACCAAAAATCAATGTCG
TACCTACTAAAATCGATGCAGTTCCTACAGTATACTTTCTAATCGAAAATTTGTTTAAGCGATTTGAAATCAT
ACCCTTTTTTGTTATTGCCTTTTTATTATCCCTGTTAATCATATAAATCTCCTTTAAAATATCAATATTTGCA
AAAAATTAATATTTTTACATATAAAAATTTGTATTCCCATCGTAATACATTTTTAGATAAAGCACAATACTTT
AAATAAATTTATTTATCTAAGACTGTTATTTTACACTTTGGTTTGAATAAATTAATTCTAGTAATTTTTAATT
ATTCGTTTGTTTTTTATATCAATTGTTTTAAATGGTTTGATACTGTGTTTAATTGCTTTTCTTATTCGTTTGT
ACTTTTAATGTAAAGATGAATTAAATTTATGTTGATTTATGTTATATTTTTTAAAAAATTGCACTATTTAATC
TCAAACATAAGATACATTTCATAAATTTTGAAATAAAAAACCAGGCCTCACGGACCTGGTATAAGCTGTATTA
TTTATTTTGTTTTTTACGACGACCGAATAACAATAATGATCCTAATGCTGCGAATAATCCGCCAAATAACGTT
GCGTTATTTGAGCCGCTATTTTCATTACCTGTTTCTGGTAATGCTTTTGCTTTATTGTGATGGTCTTTAGTAG
TACTCATTGGTTTAACAGGTGTGTGCTTACCTGCATCTGAGTCTGAATCACTGTCTGAATCTGAGTCGCTATC
TGAGTCTGAGTCGCTATCTGAATCTGAATCACTGTCTGAGTCTGAGTCGCTGTCCGAATCTGAGTCGCTGTCT

GAATCTGAATCGCTATCTGAGTCTGAGTCTGAATCGCTATCTGAATCTGAGTCTGAATCGCTATCTGAATCTG
AGTCGCTGTCTGAATCTGAATCACTGTCTGAATCTGAATCGCTGTCTGAATCTGAGTCGCTATCTGAGTCTGA
GTCGCTATCTGAGTCTGAATCGCTGTCTGAGTCTGAGTCGCTATCTGAGTCTGAATCGCTGTCCGAATCTGAG
TCGCTATCTGAGTCTGAGTCGCTGTCTGAATCTGAATCGCTGTCTGAGTCTGAATCGCTATCTGAATCTGAAT
CGCTATCTGAGTCTGAGTCGCTATCCGAATCTGAATCGCTGTCTGAGTCCGAATCGCTATCTGAATCTGAGTC
GCTGTCTGAATCTGAGTCGCTATCTGAATCTGAGTCGCTGTCTGAGTCTGAGTCGCTATCTGATTCTGAATCG
CTATCTGAATCTGAGTCGCTATCTGATGTATCTTCTTCGAAGTATCCGTTATCAAGTGTGAAATCATCATGAT
CCGTAATTGTTACGTCAACTTCGCCACCATCTGCATCTTTATCATCTTCAGTTGTATTTGTACCTGTTTGTGT
TAAGCCAGCAGGCTTTTCAAAAATAACTTTGTATTTACCGCTATCTAAATTATCGAAACGATATTTACCATTT
TCATCTGTTGTAGTTGTTCCAATTACTTCGCCTTTTTCATTTAATAAAGTAACTTTAACATCTTTGATACCTT
TTTCAGTTGAATCTTGTTTGCCGTCTTTATTACTGTCGTACCAAACATAATCACCTAAACTATATTTTGGTGT
TTTATAGAAACCACTGTCTAATGTCATGTTATCTGCATCTTTAATGACACCTGTTGTTGTTAAACCATTAGAA
TCTTTTTCAGTATCATTTCCAGAAGTTACTGAAGTTGGTGTATAACCTGATGGTGTCTCGAATTCAACTTTAT
AAGTTCCATTGTTTAAATCAGTGAATTGATATTTACCATTTTCATCTGTTGTAACTGTTTTTAAAACTTTGTC
GTTTTCATCTTTTAACGTAACTGTTACACCTGAAATACCCTTTTCATCTTTATCTTGAACACCGTTTTTATTT
GTATCTTCCCATACATAGTCACCTAAGTTGTAAGTCGGTTTGTAGAAACCAGAGTCAATAGTATCGTTATCTT
TATCTTTAATGACACCTGTTGTTGATGTACCATTTGAATCTATACCTTCATCAGTTCCTGAACCTACTTGTGT
TGGTGTGTAACCTGATGGTGTTTCGAATTCAACTTTATAAGTTCCATTTTCTAATCCAGTAAATTGATATTTA
CCATCTTTATCTGTTTTAGTTGTTTGTAAAACTTCACCGTTTTCATTTTTCAATGTAACTGTTACGCCTGAAA
TACCTTTTTCAGTTGAATCCTGCTTACCATCTTTATTTGTATCTTCCCATACATAATTACCTAAATTATATTT
TGGTGTTTTGTAAAATCCACTATCTAATGTCATGTTATCAGCACCATTAATAACACCTGTTGTTGTTAAACCA
TTAGAGTCTTTTTCAATGTCGCTACCAGATGTTACTGTAGTCGGTGTATAGCCTTCTGGTGTAGTAAATTCAA
CTTTATAATTACCATTACCTAAATCAGTAAATTTATATTTGCCATCAGCGTCTGTTGTAACTGTTTTTAACAC
GTTACCGTTTTCATCTTTTAATGTTACCGTTACGCCAGATATACCTTTTTCATCTTGATCTTGAATACCATTT
TTATTTGTATCTTCCCATACATAGTCACCTAAGTTGTATTTAGGTTTGTAAATACCTAAGTCTGCAGATAAGT
TATCTTTGCCATTAACTGTAATAACTGAAGATAAGCCGTTTGAATCTAATTCTTCGTTATTACCTTGTTTTGA
AGGGGTTACTTCATAACCTTTTGGTAAGTTTGAAAATTCTACACGGTAATCTCCATTAGGTAAGTTTGGAATC
AAGTACCCTCCTTTTTCATCAGTAATCGTTCTTCCAACTTCTTTATTAGTTTTATTGTCATATGCAACCACTG
TTACACCTTTTACTCCAACTTCCCCTAATTCTTGAATACCATTTTTATTTGTATCTTCCCAAACGTAGTTACC
AATTTTATAGACTTCTTGACCAGCACCACCACTTTGGTTATTAGTAAATCCTAAAGCATTGCCAGTAGAAACG
GATCTGTTTCCATCAGAAGTCAAAGTTGCCATTTGAACAAGTGTTGGACTTTCACTAGTTGTAAATTCAAATT
TTGTATCCACCATAACTACATAAGAATTGTTAATGCTACCAAAATCAACATTTACACTGTCATTAGTTCCGTA
TGTAATCTTATCTTTAAATTGTTCAGTCACATCTATAAGTTGATTTGTATTAACATCATAACCTTTGTTTAAA
ACATAGTCTTTTGGTGCTTGATAAATTTTAATTTTAGTTACATTTTTATTTATTTGACCTACATTGTCAGGAT
AATCCTTATGATAAGCTTCAACTTTTAATTTAGCATTTGTTAAAGTTTTTTCCGATGGATTTACATAAACAGT
CTGTATATAGTAACCCGGGTCTTCTGCATTACCTGCATGTGAAACAGTTTCAGTAAATGCTGAACCTATAGAA
TTATTATCTCTAGAAACATAATCTGGATATTGAATGTTAGCAGTTGTTTTTGTTGTAGTATTACCTATCGTAA
CATTAAACTCAACATCGTTTTTACTAACAGGAATTGTATCAGCATCCATATAAATTGAATAATTAATTCCCAT
TTGTACAGAATTAAATCTATCTACATAATCTGTAAATGTATATGTAATTAAATTATTTGCAGTATCATGTTTT
GCAGTCGCAATTGTTTCACCATTATTTGGATCTTTAATATCTCCAATATTTTTAATATCTTCCGGATTCAATC
CATATACTTGTACTGTATCTGAGTATTTTATTGTGAAATAATCACCTGATTTAACTTTGTCATCAACTTTAAT
TTGTGATTTTAATGCTAGATAATCTTGTGCTGGTACGATTTTATTGTTTTTATCTGCATCAACGACAGTTAAT
GTTGTATTTGATGTGATTAAATCATTAACATTTTTAGCCTCTGTTGATGATGGCTGTACTGCTGCTATACGCA
TTCTTGTATTCAAACGTTTAGGTGCTGTACTTTTTGGCAAAATGATATCTGCATTATTTTCATTATTTGAATT
ACTATTGTTATCAACAAGAGTTTCATCATTACTCTTGATAGCATCACTTTTAACATTTAATGTAGTTGATTCA
GTTTTGGCATCTACCTTTTTGTTTTCCTCATTAGTTGGTTGAACATTTACCACTGATTTATTCTCTTGCAAAT
CAGGTTGTAACGCTTCTTGATTACTTATAGTTTGTTTAGTGTTTAAATCTTCATTCGTAGATTTTGGTGAAGC
TTGCTCATCTGATTTGGCAGTTGAAACTTCAACTTTATTTCCAGTGGTAGATTGTACACTTTCTTTTTCTATT
AATTTATTCCCATTTGAAGTCGTTTCATTACCTTGAGATGATACCATTTCTTTTTGATTATCATTTTTAGTAT
TGTCTTCTTGATTTAGTTGCTGCATATCAACTTTATCACTCGATTGATTATCACTTGCTGAAGTTGTCGCTTC
GTTCAATTCTTTATTAGTACTTTCTGCAGCCTTTGCTTCTTGGTTCCCCAGACCAAAAATTAATGTTGTACCT
ACTAAAATTGATGCTGTTCCCACTGTGTACTTTCTAATCGAAAATTTATTTAATCGATTGGATACCATGCCTT
TTCTTGTTATTGCCGTTTTATTTTCTCTGTTTAGCATTAGATTACTCCTAATTCATCAAATTTTTAAATAATA
CAATTGTTTTAAATACAAAAATGTATATCAATATAGTATTACATTTTTAGATAAAGCACAATACTTTAATTAT
TTTTCTTTATCGTAAAACGTTATTTAACATTCGTGTTTAAATGAAAATTTTTATGAGTTTTGTAATCTTTATT
TAATTATCATAAAAAATAGTATTATTTGCCCTTAAAATTAATATCTTAGCTTTTCTAATTCATAGACAATTAC
ATTTCTGTAACAAATTAAATTGTATCTATTCCTTAAAGATTTTTTGTTTTATATCTGGGAATTTCTAAACAGA
AAAAACCAGGCCACATGGACCTGGTTAAGTTAATCATATTATTTATTTTGTTTTTTACGACGACCGAATAACA

ATAATGATCCTAATGCCGCGAATAATCCACCGAATAATGTGCCATTATTTGAATTATTATTTTCACTACCTGT
TTCTGGTAATGCTTTAGCTGTTTTATGCTGATCTTTAACCGTACTCATTGGTTTAGCCGGAGTATGTTTACCT
GCATCTGAATCTGAATCGTTGTCTGAGTCCGAATCGCTATCTGAATCTGAGTCGCTGTCTGAATCTGAGTCGC
TGTCTGAATCTGAGTCGCTGTCTGAATCTGAATCGCTATCCGAGTCTGAGTCGCTATCTGAGTCTGAGTCGCT
ATCTGAATCTGAATCTCTGTCTGAATCTGAATCTCTGTCTGAGTCTGAGTCGCTATCTGAATCTGAGTCGCTG
TCTGAATCTGAATCGCTATCTGAATCTGAGTCGCTATCTGAATCTGAATCGCTATCTGAATCTGAGTCGCTAT
CTGAATCTGAATCACTGTCTGAGTCTGAGTCGCTGTCTGAATCTGAATCGCTATCTGAATCTGAGTCGCTGTC
TGAATCTGAGTCGCTATCTGAGTATGAGTCGCTATCTGAGTATGAATCGCTGTCCGAATCTGAGTCGCTATCT
GAGTCTGAGTCGCTATCTGAGTCTGAATCGCTGTCTGAATCTGAGTCGCTATCTGAGTATGAATCGCTGTCCG
AATCTGAGTCGCTATCTGAGTTTGAGTCGCTGTCCGAATCTGAGTCGCTATCTGAGTCGCTATCTGATGTTTC
TTCTTCGTAGTAACCATTATCAAGTGTGAAATCATCATGATCCGTAATTGTTACATCAACTTCGCCACCATCG
GCATCTTTATCATCTTCAGTTGTATTTGTACCTGTTTGAGTTAAGCCAGCAGGTTTTTCAAAGATAACTTTGT
ATTTACCACTATCTAAATTATCAAAGCGGTATTTACCATTTTCATCTGTTTCAGTTGTACCAATTACTTCGCC
TTTTTCGTTTTGCAAAGTAACTTTAACACCTTTAATTCCTTTTTCAGTCGAATCTTGTTTACCATCTTTATTA
CTGTCGTACCAAACATAATCACCTAAACTATATTTTGGCGTTTTGTAGAATCCACTATCTAATGTCATGTTGT
CAGCGTCTTTAATGACACCTGTTGTAGTTAGTCCATCAGAATCTACAGCATCATCTGTACCTGCATTTGCAGT
TGTCGGTGTATAACCGGCTGGTGTTGAAAACTCTACACTATAAGTTCCATTGCTTAAACCAGTGAACTGATAT
TTACCATTTTCATCTGTTGTCGTACGATCTAATTCTTTACCGTTACTATCTTTAAGAATGACATAAACACCTT
TAATCCCTTTTTCATTGGCATCTTGTTTACCATCTTTATTTGTATCTTCCCATACATAGTCACCTAGATTATA
TTTCTTTTGGTCGCCATTAGCAGTTGATGAGCCATTCACATTTGAATAACTGTTTGACCAACTACTTTTTCCA
TTTTGTGTTTCTAAAGTATAATCAATTTTCCCATTATCTGTTGAACTATTATCTGGATAAGCAACTTGTTGAA
TGATGTATTGTTTATCACTACTAGATTGACCATTCAATAAATCTACTGTCGCCGTCTTATTATCATTACTGTA
TGTAATTTTAAATTTATCAGTGACATCTGTAAGTTTTGAAGTATCTGGGGTGAAACTATCCACAAACTGATTT
TGGTTAGTTACTTCATAAATTTTAAAGTTTTTAGCATCTGGATTAAATTTATAACCAGTTAAATTTGTTACTA
ATGTTTCTTTTGTATATTTATTTTTAGGTTGGTTTACATAAACAGTCATATTACGTGATAAATCTTCATTGTT
AATATAGTTTGTACTTGAAATGAGTTGTTGTCCTTTTTTATTACCATAATCGACAATGACGTCTTTACTATAT
TTATCGTTTCCTAATGTAACTTCCATTTTATAAGCAGTTTTATCAGTTGTTGCATTTTCACGTTTGGCGAATG
AAACTTGTTCAAAGCTACCGCTAACATTTGTATATTGATCTACATAATTCGTAAAAGTATACGTTGTTGTATT
TGTTTTACTATCGTAAATACCTTTTGCAATAATATTACCTTGGGCATTATATAAATTTTGATTTTGTGCAGGT
AATCTTACAGAACCTGGACGGAAATATTGACCATATTTAAAAGTAAATGTATCGCCCTCTTTAACAGAATCAT
CGATTGTGTAATTCGCTTTTAATTTTAAAACATCACTTGAAGTTGCCCAAAATTCAGTTTTGCCAGTATCTTT
ATTTAAGTGTCCTTTATCAATAGCAATATCAATATTTGAAAAATGAACTTTATCATTAACATTTGTACCTTGT
TGTGGTGCTGCTACTGTGTTCACAGCCATACGGTTTAATGTTCTTGGTTTAATAGTCGTTGTTTTAGGAGTAG
CTGAAACATCTTTAGCCTGTGTTAAATTACTTTTATCAGTTTCATTACTATATGTAGTTGATGATTTATCATT
TGTTGTTACATTGCTAGTTTGTGTAGTAGATTGATTAGCTGTAGCGTTTTGTGGTGATTGCATGTTACTACTA
GTTTCTTTAACTGTTGCACTATCACTCATTGTCACTTTAGGCTGATCTGCAGTTGCAGTTTGCGTATTGTCTT
TTAGTTGACGACTATCAACTTTTTCAGTTGTTTTATTCTCACTTGTGCCTGTCGTTTCATTTTTTGATTGATT
TAATTCTCCATTCGTATGTTCTGCCGCTTTAGCTTCATGACCACTTAACCCAAAAATCAATGTTGTCCCTACT
AAAATTGAAGCAGTACCTACAGAATACTTTCTTATCGAAAATTTGTTTAATCGATTTGGTATCATGCCTTTTC
TATTTGTTGCTGTCTTTTTATTATTCATTTAATAATACTCCTTTAAAATATCAAAATTTGATAAATATAATAT
TTTGCATATATAAATTTCTGCATTACTATCGTAATATAAATTTCAATAAAGTGCAATACTTTAATTATTTTTC
TTTATTTTACAATGTTATTTGCTACAAATATTAATATGAATTCCAGTTTAAGAATTGCAATTTTTAATACTTT
TTTTCTTTAATTGCTTTAAATTATTTATCTATATTTTTAAAACTCACTTTTAATCTAAGCTTGTTTTCAATTC
GCTATTACATCTTATTTTAATTAATAACATTGCTCACAACAGTAATCTCAAAGGCTTACTATTGGTGCATTAG
AACAAAAAATAGGCATGTGACTTAAAAGCATTCTCAATGAAATACCTTAAGCTACATACCTATTTTTAATGTA
TATTTACATATTTTTTAATATTATTTTAAATGTTCATATGGACTATTATTCACAAAAGATACAATTTGATCGA
CAAATAATCTTGCTCGTAATTGGAATTCACTATCATTTAAGTAATATGAACCATCTTCATTTTTAGCAAAATC
AGAATCATGTGTTGCTATTTGAGTTGGTACAGCAATGCCTAGTAAACTTCTGACGATTACTCTTAAATGTGAC
AATGGCTCTGAACTAACAATACCACCACTATTTCCTATTAAGCCTACAGGTTTCATTTTAAAATAATCCATAT
TTAGATGATCTAATGCATTTTTCAATATTCCAGAATATGAACCATGATAGTTTGGCGTTCCTAATATTAAAAA
GTCCGCCGCCATTGCTTTCTCTTTTAAATCTTTCATATTTTGTTTGATTTCATCAATAGACGGTGTTGTTCCT
GAAAAATCTAATTGATTTAACGGTTTTTCTGCTAAATCGAATATTTCCGCTTCAATATCATGTGTTTTAAAAT
GCTCAGTTAAGTATCTTGCTAGTGCACTTGTATGTGAATTCACTTGTGCACTGCCAATAATAATTAATCCTTT
CATATTTGACCCCTCTTTTTTTATTAAACTTGTTGTTCACGTAATAATTTCATAATAGCTTTTCCAACACCAC
TATTTTCATTCGTATCTGTGACATATTTCGCTATTTTTTTAACTTCTTCTGCACCATTTTCCATCGCAACTGG
ATAGCCAACTTTCTCTAACATTGATAAGTCATTTAAATTGTCACCTATTGCCATGACATCTTTCATTTCAATC
CCTAATCTTTCGGCAATTGTTTCTAGCGCAATACCTTTTTGTGCATCTGAATGCGTTATTTCTATATTTCCTC
TCGAAGATGATGATATAGCTAAATTCGGAGATTCAGCTAAAATTTTACTAGCTTTGTCAATTTTTTCTAAATT

TCCATCAAATGCTAATATTTTCATAATTAATTCACCAGGTATGTTTTCAATAGCATCATAATTATCAACAACT
TTCAACGTACCATTATCTATGCGTCTTTGAATACCATTTTTAATACGCTCAACGTTTGCATGTTGACCTGCAC
GCTCAGCAATATCTATGTAAATGTCTAAATCTCTTTGTGGATCTTCAGTATAAATCGCACGACTCGTGTATAC
TTGATAATAAATACCTGCATCTTTTAAAACATTTGTAATTTTGTGTACTAACGATTTATTAAGGTGTGAAGTG
CTCATTACATTGAAAGTTTCATCACGTACTTCAGCACCATTCAAACAAATATATGGTACTGTTAAATCTGTGT
CAGCAACTGGTGCTTGTGCTTCATAAAATGCTCGACCTGTCGCGATAACAACCGTTATCCCTTGTTCTTGAGC
GTATTTAATCGCATCAATATTAGGTTGAGAAATTTCATGTGCTGCATTAAGTAGCGTACCATCCATATCAGTG
GCTATTAGTTTTATCATTATGTTACCTCGTTTCGTAAATTTAAAATCTTGTTCTTAAATAAGAATATATACTC
AGCGCACATACTTTTCTATTAACATTTATATTGTCATTAATTTATCATATAATGTAATTCTAACAAATTTTAA
TTAGTATGTACTATCGTCTAATTGGTGGATTTCTTATTGGCTCTTAAGTTTTTAAAAAAATGTTGTTAATAATG
TGCTACATGCTTCTTTAAGTACACCTTTATCAACAATTGCACGATGATTAAAATTAGATTGTTGCAATAAATT
CATTAAACTGCCACTACAACCACCTTTAGGATCATCTGCGCCATAGACGACTCTTGGAATGCGACTCATTACA
ATTGTTCCTGCGCACATGACACATGGTTCTAAGGTTACATATAATGTGCAACCTTCTAAACGCCAACTACCTA
ACACTTTGGCTGCACGTTCAATTGCAATATGTTCAGCATGCGCCGTTGGTTGTTGTAGTGTTTCTCTTAAATT
ATGTGCTCTAGCGATAACTTCATCATCTTTAGTGATGATAGCACCTATAGGTACTTCGCCTAGTTGAGCTGCT
TTTTTAGCTTCTTCAATCGCTAATGTCATAAAATATATATCATTTGTCATTTATGTCCAGATACCTCACTTAT
GGTACAATACTCAATGTCTATTTTAACATTTGGAGATGTGAATATGAACAAACCTTTTATAGCAATTGAAGGT
CCTATTGGCGTAGGTAAATCTTCACTTGCACACAAATTAAGTCAAACTTTAGATTTTTATGAAGAAAAAGAAA
TCATCACAGAAAATCCATTTTTATCAGACTTTTATGAAGATATCTCTAAATGGAGTTTTCAAACTGAAATGTT
CTTTTTATGCAATAGATATAAGCAATTTCAAGATGTAACACAACTAAATCAAGGTGTAGTTAGTGATTATCAT
ATACATAAAAATAAGATATTTGCTAAAAATACTTTGAGTTCTGTTGAATTTCAGAAATTCAGTAAAATTTATG
ATATTTTAACTGAAGATATGATTATGCCGAATATGATTATCTTTTTAGATGCAGACCTTGATGTGTTAAAATC
TAGAATTGCTAAACGTAACCGTAGTTTTGAGCATCAAATAGAAGATGAATATCTGTTAAAGTTAAAAAAAGAT
TATCGTGAGTATTATGAGTCCTTACAAAGTAATGGTTCAAATGTAGTTTTAATCGATACAACTTCTATTGATT
TTCTTAAAAATGAACAAGATTACGAAGATATATTACATATTATATTACCTATGATAGGAGATATTACCAATGA
ATAATTACGGTATTCCACAAAATGCCATTATAACCATTGCAGGTACAGTTGGTGTTGGAAAATCAACACTAAC
GCAAGCACTTGCAGATAAATTAAACTTTAAAACGTCTTTTGAAAATGTCGAACATAATCCATATTTAGATAAA
TTTTACAGCGATTTTGAACGATGGAGTTTCCATTTGCAAATTTACTTCTTAGCTGAACGTTTTAAAGAACAAA
AGCGTATGTTTGAATATGGTGGTGGCTTTGTCCAAGATCGATCAATTTATGAAGATGTTGATATTTTTGCAAA
AATGCATGAAGAAGAAGGCACAATGAGTAAAGAAGATTTCAAAACATATTCAGACTTATTTAATGCCATGGTC
ATGACACCTTATTTTCCTAAACCTGATGTAATGATTTATTTAGAATGTAACTATGATGAGGTCATTGATCGTA
TTATTGAACGTGGTCGCGAAATGGAAATTAATACAGACCCTGAATACTGGAAAAAGCTATTTAAACGCTATGA
CGATTGGATTAATAGCTTTAATGCATGTCCAGTTGTACGTATCAATATTAATGAATATGATATCCATAAGGAC
CCCGAATCTTTAAATCCTATGATAGACAAAATTGCTCGAATTATTCAAACATATCGACAAGTAGATACACGAT
AAAAGACTAAAGACATAGCGTATATGTTTATATTCAATGTATATTCCATAGATATCATCGATTACATTATCAA
TTCTATCGAATACATTAATTCACATATACACTATGTCTTTCTTTTTAATTTAAAGCTTCTAAAATATCTGCCG
CACTATTTAAAATAATATCAGCTTCATGTAATTCTTCTTTTGTTGCAATACCTGTTAATACACCTATTGCCAT
ACCTAAATTTGCATTACTTGCTGTCTTCATATCATTAGCAGTGTCTCCTACTATAGCTACTTTCTGAGGATCT
ACATTATATTGCTCAAATAAAGGCGATAATACTTTAGGATTTGGCTTCTCATAGGCATCCGCTTCGGTAGAAA
TGATCAAATCGAACAACGAGATAGCATTGGTATGTGCTAAAAATTGTTCTACACCTTTTTTAGTATCACTCGT
AACAATACCAAGTTGATAGCCTTTTGCTTTCAAATCGATAAGTGCTTCTTTAACACCTTCTACCCAATTAATT
TCAGGAATACGTTCATCTACCAGCTTTTGACTTGTTGACTTGGACCAGTCGGTTGTATCTTGTCCCGTCACAT
CATTAAATGCCTGGATAATTTGTTGTAAAGATCCTGAACCCATCACTGATTTTGGATCAATAGATTCTTTAAT
GACACCGAGTTGTCTTAAAGCAGCTTCTTTATTATGTACTGGGAAAGTCTCAAGCAATGATTGTACAAATCGT
ACCCCTATTTTTTCCCAACTTCTATCAAATTCAATTAACGTACCATCTTTATCAAATAATATCCATTCCATTG
ATATCAATACTCCTATTTATTTATTTCGTATTATGCTGATTCTATGATATTCGTTATCCCCTGAAAATGAACT
CGTAGTATTGTTCTATTTAAATATTGAATTAAATATAATAATAAGTGAAATCCCCTTCAATACTTAACAATAA
ACATTGTAAACTTAATTTATTACCATGCTTCGCTTCATTGAAAGGGATTTTAGTCATGATTAACTTTTACATA
TTGTTTTCATGATTATATTCAATTTTTATTAATATTTTGGTACAACGACTCTCCAACCATTTTTATCTTCTAA
AGTACCATTTTGAATACCAGTATAGACGTCGTATAATTTTTGAGTAATTTCACCAGTCTCATTATTATTAATA
ACGATTTCACGATCTTCGTATCTCAATGTACCCACAGGTGAAATAACTGCTGCAGTACCACTACCAAATACTT
CTGTTAACTCACCTTTATCATATGATTCGAATAATTCATCGATTGAAACGCGGCGCTCTTCGACTTCATATCC
TAAGTTTTTAGCTAATTCGATAATAGATTTACGTGTAATACCAGGTAAAATACTGCCATTCAACTCTGGTGTA
ATTACTTTGCCATTTTCAACGAAGAAAATGTTCATGCTACCAACTTCTTCGATATATTTCTGTTCAACACCAT
CAAGCCATAATACTTGGTCATAACCTAATTTATTTGCATTAGTTTGTGCTAATAAACTTGCCGCATAGTTACC
TGCAACTTTTGCAAAGCCTACACCGCCACGAACAGCACGTACATATTCATCTTCTACATAGATTTTAGTTGGT
TTTAAAGTCTCACCACCATAATATGCACCTGAAGGAGATAAAATAATTAATAATTTATACTGATGTGATGCAC
CAACGCCAAGTGCCCCTTCTGTTGCAAAAACAAATGGACGAATATATAATGATTGACCTTCCCCTTCAGGAAT

CCAATCTCTTTCTATATCAACTAATTGTTTTAGCCCCTCTAACAATTCTGCTTCGTCTACTTGAGGCATTTCT
AATCGTGCTAACGAGTTATTAAGACGCTTAAAATTTTCTTCAGGACGGAAAAGTGCAACTTCCCCATCTTTTT
TATATGCTTTTAATCCTTCGAATACCGATTGACCATAATGAACACCTTGTGCAGCAGGTGAAATTTCAATAGG
ACCATAAGGTACTATCTTCAAATCATGCCATCCTTTTTCTGCATCATAATCATAACTCAACATATAATCAGTA
AAATATTTACCAAAACCTAGTTGAGATGTATTTGGTTTTTGTTTTAATGTTTCTCGTCGTTCAACTTTAACTG
CTTGTGACATGGTGATTGCCTCCTAATAATATTGTATAAGAATTTGTTTAACTTAAATTATAACAATCCATAT
TTTGCTGTTCAACAAATTTTCTAAAAATTCAAAATTAATTAACAGATTTCTAGAAAGACTATATCTTTTAGTA
TAAACGTATTAATTTCACAGAGACAAGTAATCTGTGTTTTACTAATATACTTTACATACAAAAAACTCTTTAC
TTTAAAATGAACTAAGCTCGCGAATTCAATAAGTATAATGAATAATATTAGAATTCATGCACTAGTTTATTAA
AATAAAGAGTAATTTAAAATATCATTCCGTGTATTAAAGTGAATGGAAATGATTAGTTATTATTTTTAACAGT
ATCTTTTTGTTCAATAGCTTCTAACATTAATTTAGTCATGCTCGCTAAATCATATTTAGGATCAAATCCCCAT
TCGCCACGTGAACAGCTTGTATCAATAGAATCCGGCCAACTATTAGCGATACCTTGTCTAATAGGATCAACAT
CGTAATCTAATGTAAAATTTGGATAGTATTCTTGAATTGCTTCTTTTACCATCTCTGGATCAAAACTCATTGC
GCTCAAATTATAACCGTTTCTAGTTTCTAATTTAGCGTCGTCTGCTTCCATAAGTTTAATAATTGCTTCAATT
GCATCATCCATATACATCATATCCATATACGTGCCTTTATCTATGAAGCTTGTATAATGACCCTCTCTTACTG
CTTTGAAGTATATTTCAACAGCATAGTCTGTAGTACCGCCACCTGGCTCTTTAACATGCGAGATTAAACCTGG
GAATCTAACACTTCTTGTATCTACACCAAAACGTTTGAAATAGTATTGACACAATAATTCTCCAGCTACTTTA
TTTACACCATACATTGTCGTAGGTTGCTGAATCGTTACTTGTGGCGTATTAACTTTAGGAGTTGAGTCTCCAA
ATGCACCAATTGAACTTGGTGTGAAAAAGTGCAAATTATAAGTTCTTGCAGCTTCTAATGCATTCATTAATCC
ACCCATATTTAAATCCCAAGCTAGAATTGGATTTTTCTCAGCAGTTGCTGATAATAATGCTGCCATATGCATT
AGACTATCCGCTTCAAAGTCCCTAACTAACTCAAACATACGGTCACGATCTGTTACGTCTAAGATTTCAAATG
GTCCATTTTGTACAGGTGAGTCTGCTTCAGGTTCCCTAATATCTGTAGCAAGAACATTATCTGTCCCATAAAT
TTCTCTGCACTTAACAACTAATTCTGTACCAATTTGTCCTAATGCACCAGTAATCATAATTTTTTTCATAGAA
ATATCTCCTTTGTCTCTTCTATATAGCTATAGTCCATCACAAGCGGACATAATATTCATTTTCATAATAATTA
TAATATAAAAGCGCTTTCTTGTATATATGACATGTACATGTTGCTGATATTTCTGTAAATGGAAATTCTAGTT
GTATTAATTGATTTTAGTAATTTATAGCGTTTATTATTGCTAATTACTGTCAAATTAAATTTTTTATCCCTCA
ACTCTTAAACTCTGGATATCTTTCATTATATTAGCTTTTTTATAACCATGGATATCATGTAAAGCCTTATAAG
CTTTAAATAATGTTTCATACCTTTGTACTTTTTCCGCTTCTGGATTAATTTCGTATAAAATTGGCTCTTTCAT
AGCATCAACTGCATCATTAATTGAATTATGTGCGCCACCACTGACTGCACCTAATATTGCTGCGCCTATTGCT
GGTGCATACTCACTATCCATAACAATTAATTTTTTATTCAGTACATTCGCATAGATATCCATCATAACAGCAT
TCTTTTTAGGTATACCGCCACATGCAAATACCTTTTCTACTTCCATATGCCAATCTTGATACTGTTGCATAAT
CATCTTAGTACCAAATGCTGTAGCTTCTAAATATGCTCTATAAATATCCTCATGCTTAGTTTGTAAAGTTAAT
CCAAAGATACAACCTGTTAAATTGCTATCACTTAATACACTTCGATTTCCATTATGCCAATCAAGAGCAATGA
GCCCGCTTTCACCTGGCATTTGATGTTTTATCTTTTCATTCATTAATTCAAATACAGTCATATTTCTATTTGC
TGCTTCATCTACATATGACTTTGGTGCTTGCTTAGCGACATACTCAAACAAATCACCTACTGCTGATTGTCCC
GCTTCATAAGCAAATAATTCTGGAATAATTGCTCCTTTTACAGAACCTGATATACCTGGCACTTGATGTTGCT
TTTCATTTAACATAAGATGGCATGTGCTTGTTCCCATCACCATAGTCATTTCTTTATCTTTTTCAGACCCAAT
ACCTAATAAACTAGCATGGGCATCAATAATAAAAGGACTTACCATAGTCTCTTTTGATAATCCTAATTTCTGT
GCCATTTTATCGTCCAGCTTCCCTACTACTTCACCAATATTAACAACCGGTGCAGATACTTTATCTTGAATTA
CTTTTGATAATTTGGGGTCTATTTTATCAAATAAATCATAATGAAACCCTGTTTCTTCTTCCCAAAATGCTTT
GAAACCTAATCCACAATTCGAGCGTACATTTTTATTAGTTAATTTATTTACAATCCAATCGCCCGCTTCCATA
ATATAAGCCGTTTTTTCCATAATTTCTGGTGCTCGATTCATGACCTCCATTATTTTGGGAATCATCCATTCAC
TACTAACATTATATCCATAATGGCCTAACCACTTATTATTATTTTCAATAGCAGTTTGATATAATTTCTCTGC
CTCTTTATATGCACCATGATGTTTCCAAAGTTTCACATACGCATGTGGATTGTTTTTAAATTGTTTTAAATTA
TGTACCGGGTTAAGGTTTTCGTCAGTAAAAATAATAGTAGATGAAGTAAAGTCTATACCAATACCTACTATAT
TGACTGGATCTATTTTTGATTCACGTACTATATATGATATTCCTTCTTCCATAATTTCTAAATAATCATTACT
ATTTTGAAGTGCATATGTATATGGTATTTTCAAACCATTTAATTCACTCTCAATGACACCATGTGTATATGGT
TTCACAAATTTTGATACTACTTGACCGTTAGTTGTATTAATTAAAAACACACGGCCTGAAGCTGTTCCATAAT
CAATTCCAATGCTATAAGACATCATGTTTCCTCCATATCGCTAGTTGCGGTTATTAGTTTTGCAAGCGTTTTC
TTAAATCATACTCATAATAGGTTCTATTTTACAAGTATCACTTAAATTTTGCCAAAAAAAGAGCCCTCATACA
TTTACATATGAGGCTCCTCTAATTAATATTTAATTATTTATTTTGTATTGCATTTAACATTTCATTAACTGCT
AATTTTCCTAACTCATTTGAAGCTAAAGGACTGTCACCTGTTAATAATTTACGATCTTTTAACGTTCTGCCTG
TCATATCGTCGTTAACTACTTTTAATCCTTGTTTAGTTAGTAAATCAGCAACTAACCATTTCAAGCGTCCAGG
TAAATAACCTATTTCAATATTTGCACCTTCATCTAATGAGTCAGGGAAGACACAAACAGAATATCCTTCTAAT
GGAGATTTTTCTCTGTTGAGCCCTGCTGAAAGTAGTGCTGCTGGTCCATGACATAATGTAACTATAAAGCGGT
CATTGTCTAATGCCCAATCTAATGTTTGTTGAACGTCCTCACTTTCAGAAATACCAACAACTGCAGCATGTCC
GCCTGGGATAAAGACAGATAAATAGTCTGAATCAGGTCCTAATTCATTTTTAATCACATCTGCTAATTTTTTT
GGCTGTTTTAATTTTTCTTTCAATTTATTATAAGTACTAATAACTGCCTCGTCTTCAGTTGGCATAGCCCATA

ATTCTAATTTAACTGGATAACCAGATAATGTCGCAACATCAACGTCAAAACCTGCTTCCATTAAATGATGTAA
AGGTAATAACATTTCAACAGGATGATTACCCGTAGAGAACATTTTTCCATTTTCCAATAATACATATCTCTCT
TCAGCAGCAATCATCAATACTTTCCATTTACCATCTTTATAGGCACCTTTGTGTTCAACACCATCAAAATCTG
TTTTAGTTGCTGTATATTGACTAAGGGAATATGGTGATGGGAAAAATGCGTTATCTTCTGCTTTATCTGGCGT
TGGTTGCTTACTTAATTCATTTACATCTTGTGACATAGTTTATTCCTACTTTCTCTATATAGTTACTACTAGT
ATAGACATATACTACACACTATAAACATGTATTACTATTAAAATGCTCATTATCAAAAATAATCAAAATGAAT
TTCTTTGTAAAAAATACAACTAAATTCTACAAGATGCTTTTAAAATTCAAACTTTAATTTTTGGGGAGCACTA
AACATTGTTATAAGAACTTACTGTTAATAAACTAAACATTAAAAAAAGCCCACGACAATATTTGCCGTGGGAA
TAAATAAATATTAAATCAACTTCATTTCTTTTCCTACTTTTTCATAAGCCGCAATTGCTTCATCTAACATGTC
TTTTGTATGTGCAGCTGTAGGCATATTTCTTACACGTCCTGTACCTCTTGGTACTGTTGGGAAAACGATAGAT
TTCACATAGACACCTTCGTCTTTTAAACGCTTACTAAATTCTTGAGTTGTTTTTTCATCACCAATAATTACTG
GTGTAATTGGAGTTTCTGACTCACCTGTATCATATCCTAATTTTGACAATCCATTTTTTAAATATTGTGCATT
GTCCCATAATTTATCATGTAATTCAGTTGAATCCATTAACTTTTTAACTGCTTCAGTTATTGCTTTGGTATCC
CCAGGTGCTAATGATGTAGAGAATAAGAATGGTCGTGATTGTGCTTTTAACCAATCTATTAACTCTTTTGTAC
CTGCTACATAACCGCCAACGACACCAATTGCTTTAGAAAGCGTACCTATTTGGAAATCGATTTTATCTTGTAA
ACCAAAATGTTTAACCGTACCAGCGCCTTTACCCATAACACCTGAACCATGAGCGTCGTCAACATAAGTTAAT
AAACCAAATTCTTCTGCAATTTCTACAATTTCAGGTAATTTAGCCACATCACCATCCATACTAAAAACGCCAT
CAGTGATATACATCACTTTATTGTATTGACCTGATTCAACTGCTTCTTTTGCTTTCGCACGTAAATCATCCAT
GTCTGAATGGTTAACTCGAATAATTTTAGCTTTAGATAAGCGACATCCATCAATAATTGATGCATGATTAAGC
TCATCTGATAAAATAGCATCATTTTTATTCATCACAGCCGAAATAGCAGCCATATTACAATTAAATCCTGATT
GATAAGCTATTGCAGCTTCTGTTCCTTTAAATTTTGCTAGTGTTTCTTCTAATTCGTCGTGTAAATCTAATGT
ACCATTGATTGTACGAACAGCGCCTGCACCTACACCATGTGTATCAATAGCTGCTTTTGCAGCTGATTTCAAA
TCTTCATTTGTTGCTAGTCCTAAATAATTATTTGAAGATAAGTTAATGTATGATTTCCCATTGATTTTGATTT
CTGGTCCGTTTGCACCTTCAATTGTATCTATTTCATTATACAAACCATTTTCTTTTAGATAATTTATATTTTC
CTCTAAAAACTCATGTAATGATTGAACCACAGTATATAATCCCCCTTAAGTATTGTTTGGTAATACCTTTATC
ATACAGTAATTCTACATAACTTGAAAGCCTTATCATTCATAGATAAAATATCTCAAAGCAATCATTTAGTACG
ATAATTTCATAAAATGTTATACTATAAACATTATTTTAGAAAGTAGTGATAAGGGTGTTAGATTGGTTCCAAC
TAGCAAATAATAAAGAAAATAAAACAATTCAACTCAGACGTTATTTACACCAATATCCTGAGTTATCTTTTGA
AGAATTTCAAACACATGATTATATTGTTAACCAATTAAGCCAGTTATCTTGTGATATTGAAACTCCTATCGGC
CGCAATGGTATCAAAGCCACATTTAAAGGCTTGGGCACAGGACCTACCATTGCATTAAGAGCTGACTTTGATG
CGTTACCTGTCGAGGAATTAAACGATGTGCCTTATAAGTCTAAAAATCCAGGATGTATGCATGCGTGTGGGCA
TGATGGTCATACAGCAATTTTACTTACTGTAGCAGAGATTTTAGATGAACATAAACATTTATTAGAAGGTAAT
GTTGTATTAATATTCCAGTACGGTGAAGAAATCATGCCAGGTGGTTCTCAAGAAATGATAGATGCTGGATGTC
TGGAAAATGTAGATAGAATTTACGGTACACATTTATGGAGTGGTTATCCTACTGGAACTATTCATTCACGTGC
GGGGGCAATCATGGCCTCTCCAGATGAATTTAGTGTCACAATTAAAGGTCGTGGCGGTCATGGTGCCAAACCT
CATGAAACCATTGACCCAATAGTAATCATGGCAGAGTTCATTTTAAGTGCACAAAAAATAATTTCACGTACGA
TCGATCCAGTTAAACAAGCTGTACTTTCATTTGGAATGATTCAAGCAGGCACCACAGATAGCGTTATTCCTGA
TCAAGCATTTTGTAAAGGTACAGTTAGAACTTTCGATTCAGATATACAGAATCATGTAATGGACAAAATGGAT
AAGCTTTTACAAGGTTTAGCAATAGCAAATGATATTAACTATGATTTGAATTATATTAAAGGGTATTTGCCTG
TCCATAATAACGAGAAAGCATATCAAGTTATTAAAGAAGCAACAAATGACTTACATGTACGCTTTAATGAATC
AGATTTAATGATGATTGGAGAAGATTTTTCACATTATTTGAAAGTACGACCAGGTGCATTCTTTCTGACTGGT
TGTGGTAATGAAAGTAAAGGTATTACAGCGCCACACCATAATCCCAAATTTGATATTGATGAAAAATCATTAA
AATACGCTGTAGCTGTATTTTTTAAAAATCATTGAATTAGAGCAAGTATTTAAAACAAATTAAGCATTTAAATT
AATTGAAAGATATGAATCTGAGACATAAACTAATGAAATGCTCCCTTCAAAGTAGACATTGAAAAAATGAAAA
CTTTGAAGGGAGCATTTTACAATGTCCAAGACTCAACAATTTTATTTAAACAGTATTATCAGTTATAAGTTTT
ATTAGTCAGTCTTTCATCAATTATTGTTCTTCTAAATCGAATAGCCATTACAAAATTTACACATTAAAAAAAG
ACCCCTCGATTGAGGGATCTTCTTTTTATATAAAATCTAAGAAATTAGAAATTATTTAATGATTTCAGTAACA
ACGCCTGATCCTACAGTACGTCCACCTTCACGGATTGAGAAACGAGTACCGTCTTCAATCGCGATTGGAGCGA
TTAATTCTACTGTCATTTCAACGTTATCACCAGGCATTACCATTTCAGTACCTTCTGGTAAGTGAACAACACC
AGTTACGTCAGTAGTACGGAAATAGAATTGTGGACGATAGTTTGAGAAGAATGGAGTGTGACGTCCACCTTCG
TCTTTTGATAATACGTATACTTCTGCTTTGAATTCAGTATGTGGTGTAATTGAACCAGGAGCAGCTAATACTT
GACCACGTTGTACGTCTTCACGAGCAACACCACGTAATAATGCACCAATGTTGTCACCAGCTTCAGCGTAGTC
TAATAATTTACGGAACATTTCAACACCTGTAACAGTTGTTTTAGATGTGTCATGTAAACCGATGATTTCAACT
TCTTCACCAACTTTGATTTGACCACGTTCAACACGGCCTGTAGCAACAGTACCACGACCAGTGATTGAGAATA
CGTCCTCAACTGGCATCATGAATGGTTTGTCAGAATCACGTTCTGGAGTTGGAATGTAAGTATCTACAGCTTC
CATTAATTCTAAGATTTTTTCTTCGTATTGAGCATCGCCTTCTAAAGCTTTTAATGCTGAACCAGCGATTACA
GGTACATCGTCACCTGGGAAGTCATATTCGCTTAATAAGTCACGAACTTCCATTTCTACTAATTCTAATAATT
CTTCATCGTCAACCATGTCAACTTTGTTTAAGAATACTACTAATGCTGGTACACCAACGTTACGTGATAAAAG

AATGTGTTCACGAGTTTGTGGCATTGGACCGTCAGCAGCAGATACTACTAAGATACCGCCGTCCATTTGAGCA
GCACCAGTGATCATGTTTTTAACGTAGTCAGCGTGTCCTGGGCAGTCAACGTGAGCGTAGTGACGTTTGTCAG
TTTGGTACTCAATGTGAGAAGTATTGATTGTGATACCACGTTCTTTTTCTTCTGGAGCGTTGTCAATCATGTC
ATATGATTGTGCAACTGAGTCACCATTTTTTGCTAATACAGTAGCGATTGCTGCTGTTAATGTTGTTTTACCA
TGGTCAACGTGACCGATAGTACCGATATTGGCATGTTCTTTAGAACGATCGAATTTTTCTTTTGCCATTATAA
AATCTCTCCTATTCTAATTAAAAATAATATTTAAATTTATCTCTCATGATAGTTTCTCACCATCATGAGAAAT
TATTTATAGCGATGCAAAAAGAATTTTACAATTCTTTTATAATGCATTCACCCCAAAAAATCAAACTATAAAG
ATGATAGCTTACATTTAAGCTCACCTTGTATTTTAACCTAGGCTAGCTAGTCAAAACAAGTTATATTATTCAC
CTTTATTTTTCTTGATAATATCTTCAGCGATTGATTTTGGAACTTCAGCATAGTGATCGAAGTACATAGTGTA
AGTACCGCGACCTTGAGTGTTTGAACGTAATGATGTTGCATAACCGAACATTTCTGAAAGTGGTACATAAGCA
TTAACAACTTGTGCATTACCACGAGGTTCCATACCATCAACACGTCCACGACGAGATGTTACGTCACCCATGA
TATCACCCATGTACTCTTCAGGCATTTCAATAGTTACTTTCATCATTGGTTCTAAGATTACAGGATCACATTT
TTTAGCAGCTTCTTTAAGTGCTAATGATGCAGCAATTTTGAAGGCCATTTCAGATGAATCGACATCATGGTAT
GAACCATCATATAATTTAGCTTTAACATCAATTAAAGGATAACCTGCTAAGACACCATTTTCCATAGCATCTT
TAAGACCAGCTTCTACTGATGGAATGTATTCACGAGGAACTACACCACCAACGATAGCGTTTTCGAATTCGAA
ACCTGCGCCTGTTTCGTTTGGTGTGAATTCAATGTGAACATCACCGTATTGACCACGACCACCAGATTGACGA
GAGAATTTACCTTGAACTTGTGCAGATGATTTGAATGTTTCACGATATGAAACCATTGGAGCACCTACGTTAC
ATTCAACGTTGAATTCTTTCTTCATACGGTCTACTAAGATGTCTAAGTGAAGCTCACCCATACCACCGATGAT
AACTTGTCCAGTTTCTTCGTCAGTGTGTGCATGGAATGTTGGGTCTTCTTCTTGTAATTTAACTAAAGCTTGA
GTCATTTTATCTTGGTCAGCTTTAGATTTTGGCTCTACTGATAAGTGAATAACTGGCTCTGGGAATTCCATTG
ATTCCAAGATAATGTCATTTTTCTCACCACATAAAGTATCACCAGTACCTGTATCTTTAAGACCTACCGCAGC
AGCGATATCTCCAGAGTATACAGTATCGATTTCTTGACGTGAGTTAGCGTGCATTTGTAATAAACGACCTACA
CGTTCACGTTTACCTTTAGTAGAGTTCTTAACGTATGAACCAGATGTCATTGTACCTGAATACACACGGAAGA
ATGTCAATTTACCAACATAAGGGTCAGTCATAACTTTGAACGCTAATGCAGCGAATTCAGCTGAATCGTCTGC
TTTCGCGATTACTTCTTCTTCAGGGTTGCTAGCACGGTGACCAATAATTGGTTTAACGTCTAGTGGTGAAGGT
AAGTAATCAATTACAGCGTCAAGCATTAATTGAACACCTTTGTTTTTGAAAGCTGTACCACAAAGTACTGGGT
AGAATTCTACGTTAGTAGTAGCTTGGCGGATAGCTTCTTTTAATTCAGAAACTGAAATTTCTTCGTCACCAAG
ATATTTTTCCATTAATTCGTCGCTAGTTTCTGCAACTGCTTCGATTAAGCTAGCACGAGCTTCTTCAGCTCTA
TCTAAGTGGTCTTCAGGAATTTCAATTTCTTCAATTTCAGTACCTAAATCATTTGTATATTTGAAACATTTCA
TTTCAACTAAGTCAATGATTGCTTCGAATTCGTCTTCCGCACCAATTGGTAATTGGATTGGAGCAGCGTTAGC
TTGTAAACGATCATGTAATGTACTTACAGAGTATTCGAAGTTAGCACCTAATTTGTCCATTTTGTTTACAAAT
ACGATACGTGGAACACCATAAGTTGTAGCCTGACGCCAAACTGTTTCAGTTTGAGGTTCAACACCTGATTGTG
CATCAAGTACTGTAACTGCTCCGTCAAGTACACGTAATGAACGTTCAACTTCTACAGTGAAGTCTACGTGTCC
AGGTGTATCGATAATGTTTACACGGTGACCTTCCCAAGCTGCTGTTGTTGCAGCAGATGTGATAGTAATACCA
CGGTCTTGTTCTTGCTCCATCCAGTCCATTTGTGAAGCACCTTCGTGTGTTTCACCAATTTTGTGGATACGGC
CAGTGTAATAAAGAATACGTTCAGTCGTAGTCGTTTTACCAGCATCAATGTGAGCCATGATACCGATATTACG
AGTTTTTTCTAATGAAAATTCTCTAGCCATGTATTTTTTCTCCTTCCAGTTTATATTACTGAATAAATACGAT
AGATATGGCGATGCCCTAAGCATGAACGCTTAATGAAAATTCATTAATATAGAACACACTCAGGGTAAAAGCT
TTTATCTTACCAACGGTAGTGAGCAAATGCTTTGTTTGCTTCAGCCATTTTGTGAGTGTCCTCACGTTTCTTA
ACGGCACCACCTGTATTATTTGCTGCATCTAAAATTTCGTTAGCTAAACGATCTTCCATCGTTTTTTCACCAC
GAAGACGTGCATAGTTAACTAACCAACGTAAACCTAAAGTAGTACGACGCTCTGGACGAACTTCTACTGGTAC
TTGATAGTTAGAACCACCTACACGACGAGCTTTAACTTCTAATACTGGCATAATGTTGTTGATTGCTTCTTCG
AATACTTCTAATGCATCACGACCACTGCGTTGTTCAACTAGGTCGAATGCTGAATAAAGAATTCTTTGTGCTG
TTCCACGTTTACCATCTAACATAATTTTGTTAATTAATTTAGTTACTAACTTAGAGTTATGAATTGGATCTGG
TAATACGTCTCTTTTAGGTACTGATCCTTTACGAGGCATAATGTAAATCCTCCCTTCCTTATATTAAATATTT
TAAGTTTAAGATTTAATTAAAAACTAAATTCTTAGTTTTTAGGTTTCTTAGTTCCGTATAATGAACGACCTTG
TCTACGTCCGTCAACACCTGAAGTATCAAGTGCTCCACGTACAATATGGTAACGCACACCTGGTAAGTCTTTT
ACACGTCCACCACGTACAAGTACAACACTGTGTTCTTGTAAGTTATGGCCGATACCAGGGATGTATGCGTTAA
TTTCGATGTTGTTTGATAAACGCACACGTGCATATTTACGTAACGCTGAATTAGGTTTTTTAGGTGTCATTGT
ACCTACACGAGTACATACACCACGTTTTTGTGGTGAGTTTAAGTCAGTAAATTTTTTCTTTTTACTGTTGAAA
CCTTTATTTAAAGCTGGAGAATCTGATTTTTTGATTTTGCTTTGTCTTGGTTTACGTACTAATTGGTTAATAG
TTGGCATGTGATATGTCCTCCTCTCTTCACTTTTTAATCCCACACATCCAGGTGGTTCATTTTTAGGTTAAAT
AAAATTTAGTAAGTAAAACACTTACTAATTCTCATTTAATCAATGCTACTATTGTCGCATTGACGTTAATACC
TACATGTTTACCCAAAGCATGTTTGCTTTTGAAAAAAGATACAGGTATATTTTTCTGATTGATTTGGCTTAAC
ACGCGAGTCATTAAATATACTTCAACGTCTTCAGCAATAATCAAAGATGTAACTTGATCTTTCTTTAACGCTT
TAAGCGTTTCTTTAAGACCAACTACAAAATGTTGTTTGTTAAAGCGTGCAACTTTTTCCTTAGACAATAATTT
ATCCTCCAAAGTTCTGCTTGCATCAACCTTTAATATATTAACATTGAACAAGAGAATTCGTCAACATTAAATC
AAATAATTTACATAAAAATAACAAGAGGCTAAAGCATTAGCCTCTGTTATATACTTGTTATTCCGTTACTTCA

GTTTGAGATTCAACTTCTGCAACTGGTTTAGCTGTTTTTTCGTATTTTACGTCGCTATAACGTCTCATACCAG
TACCAGCTGGAATTAACTTACCAATAATTACGTTTTCTTTAAGACCTAATAAGTCATCACGCTTACCTTTAAT
TGCTGCATCTGTAAGAACTCTTGTTGTTTCTTGGAATGATGCTGCAGATAAGAAACTTTCTGTTTCAAGTGAT
GCTTTAGTAATACCAAGTAATACTGGTTTAGCTGTTGCAGGACGCTTACGGTGTTTAAATGCTTCTCTATTTG
CATCTGTAAAGTTATGAATATCAACTAATGAACCTGGTAATAACTTCGTATCACCTGCTTCGATAATTCTAAC
TTTACGTAACATTTGTCGAACCATAACCTCAACGTGTTTATCGTCGATTTCTACACCTTGCATACGGTAAACT
TTTTGTACTTCTTTTAATAAGTAGCTTTCAGTCGCGTTTAATCCAGCAACAGATAAGTAATTCTTAGGTTCAA
TAGAACCTTCAGTTAATACTTCACCACGTTGAACTGGTTGACCGATTTCTACAATAATTCTTGAAGTACCTGA
AGCAAGGTATGATCTTGTTTCATTAGCACCTTTAACAACAATTTCTTGTTGTCTATCTTTTGCTAATTTAATA
TCTTCTACGACACCTTCGATTTCCGTAATTACCGCTTGACCTTTAGGGTTACGTGCTTCGAAAATCTCTTGAA
TACGAGGAAGACCTTGTGTGATATCGCTACCTGCTACCCCACCTGTATGGAATGTACGCATTGTAAGCTGTGT
ACCTGGTTCACCGATAGATTGGGCTGCAATTGTACCAACTGCTTCACCAACTTCAACTTTTTCACCAGTAGCA
AGGTTTTTACCGTAACATTTTTCACAAACACCATGTCGTGCGTTACAAGTAAATGCTGAGCGAATATACATTT
GTTCAATACCAGCATCTGTAATTTTCTTAGCAATTTCAGGTGTAATTAATTCATCAGGACGAATGATTATTTC
ATCAGTTTCAGGATGACGAATTGTTTCTTTAGAATAACGACCTTCAATACGTTCGATAAATGGTTCAATCATT
TCTGTACCTTCTTTAATATCAGAAACTAATAAACCTCTATCAGTACCACAGTCTTCTTCACGAACAATAACAT
CTTGTGCCACGTCAACAAGACGACGAGTAAGATATCCTGAGTCAGCTGTTTTAAGTGCTGTATCGGCAAGACC
TTTACGTGCACCGTGAGTTGAGATGAAGTATTCAAGTACTGTTAAACCTTCACGGAATGAAGATGTGATTGGT
AATTCGATAATCTTACCAGATGGTGCGGCCATCAATCCACGCATACCTGCTAACTGTGTAAAGTTAGATGCGT
TACCACGGGCACCTGAATCACTCATCATGAAGATTGGGTTAGTTTTATCAAGTGATTGCATCAATTCACCTTG
AATTTGATCTTTTGCATCTGTCCAAATTTCAACAACTGCATTATATCTTTCTTCTTCAGTGATTAAACCACGG
TTGAATTGTTTTGTAATTCTGTCGACTAATTTTTCATGCTCATCAAGTATTTGTTGCTTATCAGGTAATACTA
CGATATCAGCAACACCTACTGTAATACCAGCTTTAGATGAGAATTTGAATCCTAAGTCTTTCATACGGTCTAA
CATCATTGATGTATCAGTGATGCTAAATCTGTTGAATACTTCTGCAATAATATTACCTAAGAATTTTTTGTTG
AAAGGTTCAATTAATTCTTCATTTTCAAAGTATTCTTTTAATCCACCTTCACCTAAAGTTGTAGGATCGATGA
AATATCTGTTTGGTGTCTTTCTTTCTAAGTTTTCTTGCGTAGGTTCATTAATATAAGCAAATGAATCTGGAAT
GATTTCATTGAATATAATTTTACCTACTGACGTAGCAAGAATCTTTTTGTTTTGTTCTTCAGTAAATGTTGGA
TTATTGAATGAACTTGCATGTACACCAATTCTAGTGTGTAAATGTACAAAGCCATTTGCATATGCTTTTAATA
CTTCATTTGTATTATTAAAGATTGCGCCTGTATTTACTGCATCTTTTCTTTCTAAAGTAAGGTAATAGTTACC
AAGTACCATATCTTGTGATGGTGTAACTACAGGTTTACCATCTTTAGGGTTCAAGATGTTTTGTGCTGCTAAC
ATCAACATTCTTGCTTCAGCTTGTGCCTCTTTTGATAAAGGAACGTGAACCGCCATTTGGTCACCGTCAAAGT
CAGCGTTATAAGCTGTTGTTACAAGTGGATGTAGACGAATCGCACGACCTTCAACTAAAGTTGGTTCAAATGC
TTGAATACCAAGTCTATGAAGTGTTGGTGCACGGTTAAGTAATACAGGATGTTCTCTAATTACTTCTTCCAAT
ACGTCCCAAACTTCATCATCCATACGTTCGATTTACTCTTCGCATTTTTAATGTTAGTTGCAATTTCACGTT
GAACTAATTCTTTCATTACGAATGGTTTAAATAGTTCAAGTGCCATTTCTTTTGGTAAACCACATTGGTACAT
TTTCAAGCTTGGACCTACTGCAATAACTGAACGTCCTGAATAGTCAACACGTTTACCAAGTAAGTTTTGACGG
AAACGACCTTGTTTACCTTTTAACATATGAGATAAAGATTTTAATGGACGGTTACCTGGGCCAGTAACTGGAC
GACCACGACGACCATTATCAATTAAAGCGTCAACGGCTTCTTGTAACATACGTTTTTCGTTTTGAACGATGAT
ACCAGGTGCACCTAAATCTAATAAACGTTTCAAACGATTATTTCGATTAATTACACGACGGTATAAGTCGTTT
AAGTCACTTGTTGCAAATCGTCCACCATCTAATTGAACCATTGGACGAATTTCTGGTGGGATGATTGGAAGTA
CATCTAAAATCATCCATGAAGGTTTGTTACCTGAATTACGGAATGATTCAACAACTTCTAAACGTTTAATTGC
ACGAGTAAGTCTTTGACCAGTAGCTGATTCCAACTCATCGCGTAACAATTTAAGTTCTTCGTCAAGATCAATC
TCTTCAAGTAAATCTTTAATACCTTCTGCACCCATTTTTGCAACGAATTGACCTGGGTATTTATCATAATAAT
CTCTGAATTCAGCTTCAGATAATAAAGTTTTCTTTTCTAAACCAGTTGGACCTGGATCTACAACAACATAAGA
AGCAAAGTAAATAACTTCTTCTAATGCTCTTGGTGACATGTCAAGTAATAATCCCATACGGCTTGGTATACCT
TTGAAATACCAAATGTGAGAAACTGGAGCAGCAAGTTCAATGTGACCCATTCTTTCACGACGTACTTTAGATT
TAGTTACTTCAACTCCACATCTGTCACAGACCATGCCTTTGTAGCGAACACGTTTGTATTTACCACAACTACA
TTCCCAGTCTTTTGTAGGTCCGAAAATTCTTTCACAGAATAGACCATCTTTTTCAGGTTTTAATGTACGGTAG
TTGATTGTTTCAGGTTTTTTAACTTCACCAAAAGACCAAGAACGGATTTTTTCAGGTGAAGCCAATCCTATTT
TCATATAATGGAAATTATTTACATCAATCAAGGAGCCTACCTCCTTCAATTTAGATTAGCTGTGCTATTTGAT
TGATTTGCATTTATTTAAAATGGCTTAAAGTACTAAACTGAATCATCAATAAAATTCAGCTTAGTATCTTTTT
GCCTGTTTTGTAAATTGCGTATTAATCAGTAACTTCTTTTTGTGTTTCAGGAGCATCATTTTGTTGTAAATCT
ACTTTGCGTTCTACAACATCATCGTCATCAACGTCTGTCATTTCGATTTCATTATCTTGCTCATCCATAACTT
TTACATCTAAACCTAAACTTTGTAATTCTTTCATCAATACTCGGAATGATTCTGGAACACTTGGTCTAGAGAT
GTTTTCACCTTTAACAATAGCCTCGTATGTTTTCACACGTCCTACTGTATCATCGGATTTGTAAGTTAAGATT
TCTTGTAATGTGTATGCAGCACCATATGCTTCAAGTGCCCATACCTCCATCTCACCGAAACGTTGTCCACCGA
ATTGCGCTTTACCGCCAAGTGGTTGTTGTGTAACAAGTGAATATGGTCCTGTTGAACGCGCATGTAATTTATC
ATCAACCATGTGCGCAAGTTTCAACATGTACATTACACCTACTGAAATACGGTTATCGAATGGTTCACCTGTA

CGTCCATCATAAAGTACAGTTTTACCATCACGAGCCATACCAGCTTCTTCAATTGTTGACCATACATCGTCAT
CGTTTGCACCGTCAAATACAGGTGATGCAACGTGAATACCAAGATTTTTAGCAGCCATACCTAAGTGTAGCTC
TAATACTTGTCCGATGTTCATACGAGATGGTACACCAAGAGGGTTTAACATGATATCGATCGGACGTCCGTCT
GGTAAGTAAGGCATATCTTCTTCAGGAACAATCTTAGAAATGACACCTTTGTTACCATGTCGACCACACATCT
TATCACCAACATGAATTTTACGTTTTTGAACGATGTATACACGTACTAATTGGTTTACACCAGGTGATAATGT
ATCGTCGCCTTCTTCACGATTGAATACTTTTACATCAAGAACGATACCGCCAGCGCCGTGAGGTACACGTAAT
GAAGTATCTCTAACTTCACGTGCTTTTTCACCAAAGATTGCATGTAACAATCTTTCTTCGGCAGTTAACTCAG
TTACACCTTTAGGCGTTACTTTACCAACTAAAATATCTCCATCTTTTACTTCTGCACCAATATAAACGATACC
ACGATCGTCTAAGTTCTTAAGTGCACTTTCAGAAACATTAGGAATATCTCTTGTGATTTCTTCAGGTCCTAAC
TTAGTATCACGTGCTTCTGATTCATACTCTTCAATATGAATAGAAGTATACACGTCATCTTTCACAAGTCTTT
CACTCATGATAACGGCATCCTCATAGTTGTAACCGTCCCAAGTCATGAAACCAACTACTACGTTTCTACCTAA
TGCCATTTCTCCTAATTCCATAGATGGTCCGTCTGCTAAAATCTCGTTATACTCAACAACATCTCCAACTGCA
ACGATTGGACGTTGGTTATAACATGTACCTGAGTTTGAACGTTTAAATTTAGCTAATGGATAGCGATCTAATT
CACCTTCATGCTCAACGCCGTTCTCTTCAACTAGACGACGTACAAGAATTTCATTAGATTCAACATGTTCAAC
ACGACCTCTGTGCTTAGCTGTAATAGCCGCACCAGAATCACGTGCTGCAACGTGTTCCATACCTGTACCAACA
AATGGTGCTTCTGGATTCATCAAAGGCACTGCTTGACGTTGCATGTTCGCACCCATCAATGCACGGTTTGAGT
CATCATTTTCTAAGAATGGAATACATGCTGTCGCTGCTGAAACAACTTGCTTCGGCGATACATCCATATAATC
CATTTTTTCTTTAGCCATAACTGTGTTGTTACCACGGAAACGACATACAACTTCATCATCCATGAAACGACCA
TTTTCATCTAATTTAGAGTTTGCTTGTGCTACAACATAGCTATCTTCTTCGTCAGCTGTTAAATAGTCAATTT
GATCAGTGATAGCGTGTGTATCTAAATCAACTTTACGATATGGTGTTTCAATAAAGCCGAATTCATTTACACG
TGCATAACTTGATAATGAGTTAATCAATCCAATGTTTGGTCCCTCAGGTGTTTCAATTGGACACATACGGCCA
TAGTGAGAGTAGTGAACGTCACGTACTTCCATTTGAGCACGTTCACGTGTTAAACCACCAGGTCCTAATGCTG
ATAGACGACGTTTATGCGTTAACTCAGCTAATGGGTTTGCTTGGTCCATGAATTGTGATAATTGAGAGCTACC
AAAGAATTCTTTAATAGATGCAATAACAGGTCGAATATTAATTAATTGTTGAGGTGTGATAGACTCAGTATCT
TGAATTGACATTCTTTCACGTACAACTCTTTCCATTCTTGATAAACCGATACGGAATTGGTTTTGTAGTAATT
CACCTACAGAACGTAAACGACGGTTACCTAAATGGTCAATATCATCTGTATATCCAATACCGCTTAATAAGTT
AAAGAAGTAACTCATTGAAGCAATGATATCTGCTGGTGTAATGCATTTAACTTCTGAGTCAGGGAAAGCATTA
CCAATTACAGTTGTCGTACGACCTTCATCATCGTTAGGAACATATACTTTAATTGATTGAATTTCTACTGGCT
CGTCTATAACGCTACCATGCAATTCAAACACTTCGCTGTTTGCATTTGATTCAAGTACATCCATGATTTCGTC
GATTTTACGACGATCAAGCACTGTACCTTCTTCAACTACAATTTCACCAGTTTCAGTATTTACAATTGGCTCA
GCTAATTTTTGATTAAATAAACGATGTTTTAAATGTAATTTTTTGTTTGTTTTATAACGACCCACGCTTGCTA
AGTCATAGCGTTTTGGATCAAAGAAACGTGAATACAATAGACTTTTAGCATTTTCAACAGTTGGTGGTTCACC
TGGACGTAAACGTTCATAGATTTCTAATAACGCTTGTTCAGTGTTTTCAGTGCCGTCTTTCTCTAAAGTATTA
CGTAAATATTCATTGTCACCTAAAAGGTCAACAATTTCTTGGTCGCTTGAGAAACCTAATGCACGTAACAATA
CTGTTAATGGTAGTTTACGTGTTCTATCAATACGCACGTATACAACATCTTTAGCATCTGTTTCATATTCTAA
CCATGCACCACGGTTTGGAATAATTGTTGCATCATAGTTTTCACGACCATTTTTGTCGATTTTTTCATTGAAA
TAAACGGATGGTGAACGAACTAATTGAGATACGATTACACGTTCTGCACCATTGATAACGAACGTACCTGTAT
CAGTCATTAATGGGAAATCACCCATAAAGACTTCTTGTTCTTTAACTTCTCCTGTTTCTTTAATGATTAGACG
CACTTTTACACGAAGAGGTGCAGCATAAGTAGCGTCACGGTTTTTAGATTCTTCTAAATCATATTTTGGTTCT
CCTAAACGGTAATCCACAAACTCTAATGACAAATTACCAGTAAAATCTTCAATTGGAGAAATGTCTCTAAACA
TTTCGATTAAACCTTCTCTTAGGAACCACTCGTAAGATTTAGTTTGAATTTCTATTAAGTTTGGTAATTCTAA
TACTTCTGAAATTCTCGCGTAGTTTCTACGTTTACGATGTCTTCCATATTGGACAACTTGACCTGCCAAACAG
ATTCACCCCTCAAAAATTATGTGATCCGCTTTTAGCTAAATACCATAAGCATATTAATAAAAAGACAAAAAGA
AAACGGCAACATTAATGATGACACCATTTTCGATTCTATATTTATTCTTAATTAACTTATCTATTTAACGTAA
AAGTACACACTTATTGAACATAAATTTTTACATTGTATAACTATAACAGAATATCCATTTCATTTCAAGCTTT
TATACTTCTCAGAATGTAATATCCTTTATCTTTATTTACCACTTCTACATTTCCAAAAAGTTCATTCATTCTT
TTCTTTGCAGATGGCATACCTTGCTTCTTCTGAATTACAACGAATAGTTCACCGTTCGAGTCTAATCTATGTA
ATGCTTGCTCGAATATACGATGCACGGTTTCTTTCCCTGCTCTTATTGGTGGATTGGTTAAAATAAAATCAAA
ACTTTTGTCTTCCACAGCAGACAAAGCATCACTTTCCTTTACGATCGCATTATCAATACCATTTATTTTTTTG
TTTTTTTCAACTAAGGCTAGCGCTCTGTGATTAACATCTAGCATTGTAATTGAATGATGTGGTGATACTTTAG
CAATCATCAAACCAATTGGTCCGTAACCACAACCAACATCGGCAATTCGCTTACTTGGACCAGGTGGATGCGC
TTTTAAAAAAGTTTGAACAAGAACATCTGAACCATAATCTACTTTATCTTTCGAAAACACTCCATTATCAGTT
ATTAAATCAATTTTATGATGGTTTAATTGATATTGAATACGTTGTTCATTGCTAATTACACTTGGATCTTCAT
CGTAATAATGACTCATTATTCCACCTCATCTTCACTTTGTAACTCGTCATTATAATTATAACATTTCACTCAG
TCTTTAAATGAATAAAACCCCGTTATCAAAATAACGGGGTTTTAACTTCTCAAAGAATCATTATATTATAGCA
ATACTTGCTATAAAACTTTGATTATTAAGTTTAAGATACTTGAATTATTTTAATTCTACAGTAGCTCCAACTT
CTTCTAATTGTTCTTTAAGTTTTTCAGCTTCTTCTTTAGGTAAAGCTTCTTTGATTACTTTAGGAGCTCCGTC
TACTAATTCTTTAGCATCTTTTAATCCTAAACCAGTTGCTTCTTTAACAGCTTTAACAACTTTGATTTTAGAT

GAACCAGCTGAAGTTAACTCAACGTCAAATTCAGTTTTTTCTGCTGCAGCGTCAGCGCCACCAGCTGCACCTG
CTACTGCTACTGGAGCAGCTGCAGTTACACCAAATTCTTCTTCAATTGCTTTTACTAAGTCGTTTAATTCTAA
TACTGACATTTCTTTAATCGCTTCAATGATTTGTTCATGATTAGCCATTTTATAATTCCTCCATTATTTTTAA
TTTTTTTACGCTAAAAATTATTCAGCGCTTTCTTCTTTTTGTTCTCCAATAGCTTTAACCGCATAAGCGAAGT
TGCGTACAGGAGCTTGTAATACTGATAAAAGCATAGATACAAGACCATCGTGTGAAGGTAATGAACCAACAGT
TTTAACTTCTTCTGCTGTAATAACATTGCCTTCCATAACGCCTGATTTAATTTCTAATGCTTCATGATCTTTA
GCAAATCCAGAAATTACTTTCGCTGCAGCTACAGCATCTTCACTTGAAGTTGCAATAGCAGTAGGACCTGTTA
AGAATTCATCTAAGCCTTCGATACCAGCTTTTTCAGCTGCACGACGTACCATAGTGTTTTTGTATACTTTATA
CTCAACACCAGCTTCACGTAATTGTGAACGTAAGTCAGTAACTTCAGCTACTGTTAATCCACGGTAGTCAACG
ATTACTGTTGAAACTGAATTTGATAGTACCTCAGCAATTTCATCAACTAGTTGTTTTTTAGCTTCAATGATAG
CAGACATTCAGACACCTCCATTTAAATTTTTGGTGCTTTTTTTAAAATTAATAAAAAAAGCACTTTCTACCCA
CGGCAAAAAGTGCTTGAAAGATCATAGATCATCACGTTCAAGTCAATTTTAGCCTCGGCAGGATAAATTTTAA
GTTATAAATAACTCCTACTGTCTTAGGTAAAATAATCACAATAAGTAATATAACTTGGAAATTACTTATTGTC
AATATATTTTTACAGACTGAAAGATATTTCTTTCAGCCTGTAATTGTTTATATTATTTATTATTTGAAACTTG
CAGTATCAATTTTAACTCCAGGACCCATTGTTGTAGTTACAGCAACAGATTTGAAGTATGTACCTTTAGCAGA
TGATGGTTTAGCTTTAGCTAATACATCTTGTAAAGTATTGAAGTTTTCAATTAATTGTTCATCAGTAAATGAA
ACTTTACCAATTGATGCATGTACGATACCAGCTTTTTCAGCACGATATTCTACTTTACCAGCTTTGATTTCTT
CAACAGCTTTTTTAACATCCATTGTTACAGTTCCAGTTTTAGGGTTTGGCATTAAACCTTTTGGTCCTAATAC
ACGACCTAATTTACCAACTTCACCCATCATGTCTGGTGTAGCAACTACTACATCGAAGTCGAACCAACCTTGT
TGGATTTTTTGAACGTATTCTGCTTCACCTACATAGTCAGCACCTGCTGCTTCAGCTTCAGCAATTTTGTCAC
CTTTAGCGAATACTAATACACTTTGTGATTTACCAGTTCCGTTTGGTAATACAACTGCACCACGGATTTGTTG
GTCATTTTTACGTGTATCAATTCCTAAACGGAATGCAACTTCAACAGAAGCGTCAAAGTTAGCAATGCTTGTT
TCTTTAGCTAATTTAATTGCTTCTTCAACACTGTAGTGCTGAGTACGGTCAACTTTACTAGCTGCTTCTTGAT
ACTTTTTACCTTTTTTAGCCATTTATAGTTCCTCCTTTAGTGGTTTTAGCGGAATTTCCTCCCACATTACTTG
CTATATGCAAGTTAAGAGCAGACAACAGAAGAATTAATCTTATGAATAAATCAATCATTAATCTAGTCATTCA
GATTATATTTATAATTCATCATTATTATCTCTGTTATCTGCTTCATCGTTTAAATCATGTAATCAAATTTAAT
ATTCGTAAAATTATTCTACAACGATACCCATACTACGTGCAGTACCTTCGATAATACGCATAGCTGCTTCTTC
GTCAGCAGCGTTTAAGTCTTGCATTTTGCTGTTAGCAATTTCGCGTACTTGATCTTTAGTTACTGTACCAACT
TTAGTTTTGTTTGGTTCGCCTGAACCTTTTTCAATACCAGCTGCTTTTTTAAGTAATACTGGAGCCGGTGGAG
TTTTTGTAATAAATGTAAATGAACGATCTTCATAAACACTGATTTCTACCGGAATAATTAAACCTGCTTGATC
TTGAGTACGTGCATTGAACTCTTTACAGAATCCCATGATGTTCACACCTGCTTGACCTAATGCTGGACCAACT
GGTGGTGCTGGATTCGCTTTACCTGCAGGAATTTGTAATTTAACAACTTTATCTACTTTTTTAGCCACGATGT
GCACCTCCTTGATATCGTGATGTGGTCACAGGGCTCAGTTTTGCCCTCCCACTCTTAACATTTCGTGACGAAA
TGGGCGCTCTAAAAGCGCGACCACATAATTTTAACACTTTTCTAATTAAAAAATCAATACTCTTTTTTATCTT
TTGGTTAGTTTAATAACTTTAATTGTTAATTAAAGCTTTTCTATTTGATCGAATTCAACTTCTACTGGTGTTT
CTCGGCCAAACATATCTACTAATACTGTTAGCTTAAACTTATCTGTTTCAATTTCTTGAACTTCACCAACTTG
ATTCGCAAATGGACCTGATTTAATACGAACTTGCTCGCCAACTTCGAGTTCAACATCGATAGTCTTTTCTTTA
AGACCCATTTGTTTTAAGATGAAGCGAACTTCTTCTGGTAACAATGGATTTGGCTTAGACCCTGCACCTGCAG
AACCTACAAAACCAGTAACACCTGGTGTATTTCTTACCACATACCATGATTCATCTGTCATGATTAATTCCAC
TAAGACATATCCAGGGAATGTTTTTTTAACAGTCGTTTTAGCTTTGCCATCTTTTACTTGATTTTCTTCTTCT
TCCGGTATGACTACTCTAAAGATTTGTTCAGTCATATTCATAGATTCTACTCTTTTTTCTAAATTCTTTTTAA
CTTTATTTTCATATCCAGAATATGTATGCACTGCATACCAACGCTTTGCGCCAACTTCTTCAGACATGTCTTC
ACTCCTCTAACCAAACAATAAATTTTTCAATGCTGTAATTCCTAAATCTAAGGCATAGAAAAAGACTAAGAAG
AATATAACAGCAGAAACTACAATTACAGTATATTTAAATAGCTCTTCTTTCGTCGGCCAACTTGTTTTTTCCA
TTTCAGACTTAACGCCTTTAAAGAAACTTTCTTTTTTAGCCATCTGCTCAACCTCCAAATCCTTTGAACATAT
TATCAAAGTATTATTTCGAATGTATTTATTTCGATTCTTTATGAATTGTGTGCGCGTTACATTTTGGACAATA
TTTCTTTAAGGTTAATCTTGTTGCCGAGCCTTCTTGCTTAGGAACATTATAATTTCTATTGCCACAAGCTTCA
CAATTTAAAGGTATTTTTCTCACAATAATTCACCTTTATTCATTATAATACCAGTATACTATACATAAAATTT
ATAACAAATGTCAAACCATCAGTAAGTAACACGTTATAATTCAATTCACGTCCTAAGGCGCTTTTCAAATCAT
TTTGAAATAACGTTTTATTTTATTTTTACATCGTTGTATGGCATTATAAATCACTTTCTCTTTTACATGCATC
AATTGAGCAATTTCTCTCGGCTTATATTGTTCACACATTAAATACATGACATCTTTTTCAAATGCACTCAAAT
ACTTAAACTGACAAATCAACTCTTTAATTGCTAATTTATTCAAATAATTGTTTTCAACATCAAAACGCTTTAT
TGCACATGGATACGTAACTCTATATTCATTAATCAAATTATCCATTCTTTTATTTGTAGCTAAATATTTTCTC
AAATAATCATACTTTACAGACGTAATTAAACATTGAACATAATTTGTGAAAGGCTGCTCTCCACTAAAATCAA
ATGTTTGGACCGACCTATACATCCGTATAAGTATTTCTTGATATAAGTCTTCTAAATCATAATGATTAAATCC
AAATGTTTTGATGCGTCGAATAATTAATGGTTGTAGATCCATTACTAACTTTTCGAAGTCTTTATCATTTATT
GCATTGTTACGTTTGATTGTACTGTCTTGAGTTGTCAAATCATATTTCAAGTATTAAACTAACCCCTTCTATC
TAAAATTTAAGGTTAGTTTAATATTGTTACATTCAAAATTTCAAGATGACGGAAATGTCATTTCTTATGATGT

CCTCTTCGTATTTTTTCAAATTCTGCAAGGATTTCAGAAGATAACGGAATTCGAGTTCTTGGCTTGTTTTCAC
TTATATCATCTAATGATTTACTCACATCAATTTCATTTTCTTTTAAATCTCTCCACATTTCGCGAGATGATAT
TCTATATGCACCTGATCCAAAGATAGCATGTTGCTCACTCATATCACTTGTTACAACTGTAATATGCTTAGTA
TGCTTGTCATAAAGTTCATAAACATAACGTTCTATGAAACTATCAGCTGTTTCTTTTTCCTTGGTAAAAATCG
TTTTAACGCCATGATACATGTATTCTCTTTCAACACCCGATTGGTCATAAGCATCGAAAACACAAATAATTTC
ATCTGAAATAACTGCATTATAATTTGCAATTGCATCTATTAATTGTATTCTAGCTTCTTCTAAATTCTCTTTT
GCAATGGCGCTTAGCGTTGGTGATTGTCCTATCATATTGTATCCATCAATGATTAAGTAACGTTCTTTCATTA
TATTTCTCCAACATCATGTCTTTTTCGAAATACTTCGTACATCATTAAACTTGCTGCAACCGAAGCATTCAAA
CTGTTTACATGTCCAACCATTGGAATCTTAATATAAAAATCGCATTTATCACTTACTAGGCGACTCATACCCT
GTCCTTCGCTACCAATTACAATAGCCAATGACATGTCCGCTTCTAGATTTCTATAATCTGTTGCATTATTAGC
TTCAGTGCCAGCTACCCAAAAGCCATTATCTTTTAGTTCATCGATAGTTTTAGCTAAATTTGTCACTCGAATA
ACTGGTACATGTTCAATTGCACCTGTTGAGGCTTTTGCAACTGTTTGCGTTAGTGTAACTGAACGACGTTTAG
GAATAATAACACCATCAACTCCCGTTGCATCGGCTGTTCTTAAAATTGATCCCAAGTTATGCGGGTCTTCTAA
GCCGTCTAATATAAGTACTGTCGATAAACCTTCTTTTTCTTTTTGCTGTTTTAAAAATTGATCGAAGTCAGCA
TATTCATATGGTGCAATAAGCGCTGCAACACCCTGATGTGGTGCATTTGCTAAAAAATCTAATTTAGATTTTG
GTACAGTTTGAACAATGATTTTTTGATCTTTTGCATTTTTTAAAATTTCATTAATTTGTTGCTTTTTAATACC
TTCTTGAATCAATATCTTATTTATCGGATGCCCAGTAATAATCGCTTCTCTAACAGCATGCCTACCAACAATA
ACCGTATCTTCCACATCGTCACTACCTTTCGTTTACTATTGTTATTATTTTATTTAATAATGCCTCTAATCGT
TCTTCTCTTTTTTCTAAATAAAGAAAACCTATCACTGCTTCTATCGCTGAACTTTTACGATATGTTTGAACAT
CAGTGTTTTTAGCTTTAGTATGACTTTTCGCGTTACGCCCTCGCTTCAAAATATCCATTTCTTCGTCTGTAAA
CCATTCTTGCTCCATTAAATATTCTAACGTTTGCGCCTGACTTTTGGCAGATACATATTTTTTAGACATTTGA
TGTAGTTTATTAGGCTTACTTTTAAGCTTTAAAACGATATAGGTACGTACATATTGATCTAAGACTGCGTCTC
CCATATATGCTAAGGTCAATGGATTCAATAATTTAATGTGATTATCTTGTTGATTATCCACGTTTAAATCTAA
CCCCTTGAGGTGTGTCTTCTAATATAATGTTTTGTGATTTCAGCATGTCTCGAATTTCATCTGCTCGTGCAAA
ATCTTTGTTTTTCCTTGCTTCATTACGCTCTTCGATTAATTTTTCAACATCTTCATCCAATAATTCATCTGCA
TTTTTAGATTTTAACGGTACACCTAAAACATCGCTGAAAATTTGATAAACTGCTTTAAATTTATCAATTACTT
CTGTTGATGTTGTATTCTCTAGTACATATTTATTCGCAAGTTTTGCTAAATCATACCAAGCTGTAATTGCATT
AGCTGTATTAAAATCATCATTCATAACTGTTTCAAAACGATTTAAAATCGCATCAATTTGATCAATATATGTC
TGTTGATTTTCAATATTAGTAGCAATTTGTGCGCGCTCTTCAATTAATTGATAACTATTGCGAATACGCTCTA
GTCCACTACGTGCTGATTCTACCAATTCTAGATTATAGTTAATTGGGCTTCTATAATGTACGCTAATCATAAA
GAATCTTAGTACATCTGGATCAACTTCTTTAATAATATCGTGAACTAAAATAAAGTTGCCTAGTGATTTACTC
ATTTTTTCATTATCAATATTAATGAAACCATTATGCATCCAATAATTAGCAAATGGCGCATGATTATGTGCTT
CTGATTGTGCTATTTCATTTTCATGATGTGGAAATTGTAAATCTGAACCACCCGCATGTATATCAATTGTAGG
TCCTAGCTCATGAAATGCCATTACAGAACATTCTATATGCCATCCTGGTCTACCTTCACCAAATGGGCTATCC
CAACTAATCTCGCCAGGCTTCGCTTTTTTCCACAATGTAAAATCAAGTGCATCTTCTTTATGCTCTCCTGCAT
CTATACGAGCACCCACTTTTAAGTCATCTATGGATTGATGACTTAATTTACCATAACCTTCAAATTTACGTGT
TCTAAAGTAAACATCGCCACCACTTTCATATGCATAACCTTGATCCACCAAATCTTTAATAAATTGAATAATG
TCATCCATATGGTCCATTACCCTTGGATTTGAAGTCGCTTTTCTAACATTTAACGCACCAACATCTTCATGAA
AAGCAGCGATATATTTTTCTGCAATTTCGGGAACAGACTGATTTAATTCTTGAGAACGTTTAATTAATTTATC
ATCTACGTCTGTAAAATTTGATACATATTCTACATTATATCCTTGGTATTCAAAGTAACGTCTCACTACGTCA
TAATTAATTGCTGGTCTTGCGTTACCAATATGAATGTAGTTATATACAGTAGGACCACATACATACATTTTTA
CTTTCCCTGGTTCTATAGGCTTGAACACTTCTTTTTGACGTGTAAGCGTATTATATAATGTAATCATCTTGAA
TCTCTCCATTCCTAGTCTTTTCAAGTTGTCGTTCTAAATGCTTAATTTGTTCATAAATTGGATCAGGTAGATG
GCGATGATCAAATGTTTTTCCAACTCGAACACCATCTTGCTTAACAATATGTCCTGGTATACCAACAACCGTT
GAATAACTTGGAACTGATTGTAAAACAACTGAATTTGCACCAATATTTACATTTGAATTTATTTTAATATTTC
CTAAAACTTTCGCACCGGCTGCTATTAAAACATTGTCTCCTATATCTGGGTGTCTTTTCCCTCTTTCTTTCCC
TGTCCCACCAAGTGTCACGCCTTGATAGATTGTCACATTATCACCAATTGTACATGTTTCTCCTATTACAACG
CCCATACCATGATCTATAAATAGACGCTTTCCAATTTTAGCACCTGGATGGATTTCTATACCTGTGAAAAATC
TTGAAATTTGAGATATCGCGCGTGCTGCAACATATTTTTTTTGGTTGTATAACTTATGTGCAATCAAATGACT
CCAAACTGCATGTAAACCTGCATACGTTGTAATGACTTCTAATGTTGAACGTGCCGCTGGATCCTGCTCAAAT
ACCATTTTTATATCGTCTCTCATTCTTTTTAACAAGATCATTTCCTCCTCAATGATTGAACTACGTAAATACA
TAATTGAAGTACCTGCGAAATTAAATATCAAAAAAGCACCACTAACATACAAATTGTATTGTTAGAGGCGCTT
CCGCACGGTTCCACTCTGAATTTAGCGAATAACATTAATAATATTGCGGGCGCTTCCAAATTATCAAGGAAAC
TAAGTCAACTTAATGCTCATCACTCTCATTATATATTTAATTCATTTTACGAAGGTGCATTCATTAATTTCTA
CGTTGTACTCACAGCAACCGTACACTCTCTGCATCGTATAAATTTAATTACTAATCCTTCGTTTTATATAAAA
TACTTTATTCATATTGTTGTAATTTGTTAATACAACTTTATCATATGTGGTTACGTTATAAAATTCAAGTATA
TACTACCTTGATCTTGTCTATTTCATTACTTATATTGTTTTAAACGGTTTAGCACTTTTTCTTTACCAAGTAC
TTCAATTGTATTTGGTAATTCAGGACCATGCATTTGGCCTGTTACAGCAACACGAATAGGCATAAATAATTGC

TTGCCTTTTATTCCTGTTTCTTTTTGAACTTCTTTAATTGTCTTTTTAATTTCAGCCGCTTCAAATGGTTCAA
GTACTTCTAATTTACTGAATAAGTGCGTCATTAACTCTGGTACTTGCTCTCCATTAATCACTTGTTGTTCTTC
TTCACCAAGAGCTGGCATTTCTTTAAAGAACATTTCTGATAAAGGTACAATTTCACCGGCATAACTCATTTCT
TTTTGATAAAGCGCAATTAATTTGCGTCCCCAAGATAAATCCTCTTCTGACGGCACCTCAGGAATCAAATTTG
CTTTAATTAAATGAGGTAATGCTAATTGGAATACTGTTTCAGTATCTTTTTGTTTCATATATTGGTTATTAAC
CCATGCTAATTTTTGCTTATCGAAAAATGCTGGTGATTTTGACAAACGCTTTTCATCAAAGATTTTGATAAAT
TCTTCTTTAGAAAAGATTTCTTCTTCACCTTCAGGAGACCAACCTAATAACGCAATAAAATTAAATAACGCTT
CAGGTAAATAACCTAAGTCACGATATTGCTCAATAAATTGTAAAATTTGCCCATCACGTTTACTTAACTTTTT
ACGTTCTTCATTAACAATTAATGACATATGACCAAAACGAGGTGGCTCCCAGCCAAATGCTTCATAAATCATA
ATTTGTTTAGGCGTGTTTGAAATATGATCATCACCACGAATTACATCTGAAATTTGCATGTAATGATCATCTA
TAGCTACTGCAAAATTGTACGTTGGAATGCCATCTTTTTTTACGATAACCCAGTCACCAATACCATTTGAATC
AAATGAAATATTTCCTTTTACCATATCATCAAATGAATACGTTTGGTTTTGAGGTACTCGGAAACGAATTGAT
GGTTGGCGTCCTTCTGCTTCAAATTGTTGACGTTGTTCTTCAGTCAAATGCGCATGTTGACCACCATAGCGAG
GCATTTCACCACGAGCGATTTGCGCTTCACGTTCAGCTTCTAATTCTTCTTCTGTCATATAGCATTTATATGC
TTTATCTTCTGCTAGTAACTGATCTATTAATGGTTGGTAGATATGTTGACGTTCAGATTGACGATATGGTCCG
TAGCCATTGTCTTTATCTACAGACTCATCCCAATCTAATCCTAACCATTTAAGATTATCAAATTGTGATGTTT
CTCCATCTTCTAAATTACGTTTTTTATCAGTATCTTCAATTCGAATCACAAAATCTCCGTTGTAATGTTTAGC
ATACAAGTAATTGAATAATGCTGTTCTTGCATTACCAATATGAAGATAACCAGTTGGACTTGGTGCATATCTT
ACTCTTATACGATCGCTCATTTTTTTCACTCCTAAATTAAATATCAGATTTTCAAGTTAGTTCATATAAATTG
TTCATTTGCTATCTTCGACCGTCATAACAAATGTCTAACTCGTCTTATTGTTAAAACGAAACAATGCTTTTTA
ACATGACCTTAAAATAATTTCATTGTTCAATCATAACATAATTCCCTGGGTAATATGCTTAAATTTTAAATAG
AAAGCAGTTGTTTTTTCAACACTTTAAAAAAGCTATCCCTAAGAATCAAGACTGCACTACATAAACATTTCAA
CGACTTAAAAATGAATATTATGTACTGTGAAATTAAACTTCAAATTAACTATTCAAATACGTTAAAATTGATT
CTAATTTTGTATGTCTTGATTGCTATAAGAATAACTTTATTAATATCTAAAATTTAACACTTAATGAACTTGT
TTCAATGATATATTAGCACTATTTGTATTTTTTGATAACTATTATGTTTTGCATTTATTTATAGTTATACTTC
AAATTACAAACTTCGCCATTTCATATACCTTTTAATATCTATTTTGTTTTCGTCAACTACAGTTTTTATAATG
ATACTGTATCTTCGATTTTTTTAGCAAAAACAATTCTTCCTGAAGATGTTTGCAATAAGCTGACTACTTCTAA
ATTGACATGACTGCCAATAAGATTTTTAGCATTATCAACAACTACCATCGTACCATCATCTAGATATCCTACT
GCCTGACCAGGCTCTTTACCCATTTTTGTCAGTAAAATATGCAGTTGATCACCTTGATGTACATTAGGTTTGA
TTGCTTCTGATAAATCATTAACATTTAATGCTTTGATACCATGTACATGACAAACTTTATTTAGGTTGAAATC
TGTCGTAATAATACTTGCATGATATTGTTTAGCTAGCTTTAATAACATTGTATCAATATCACTATGTGTTTTA
GTTGGATGTATGACTTTTGTAGGATAATCTAAATCATACAATTCATTTAAAATATCTAAGCCTCTTTTACCCT
TTTCACGTTTAACACTGTCATTTGAATCTGCAACAATTTGTAATTCATTAATAACACCTTGTGGAATTAAAAT
ATTGCCATCGATAAAACCGCAACGAATGACTTCTAAAATACGACCATCAATAATTGCGCTTGTGTCGATAATT
TTTGGCGTAGCACTTTTAGTATGTTGTGACATGGAACGCGCTATATTCTCAGGTAAAAACATTAACATTTCAT
CTCGTTTTTTAAGGCCAAATTGGAAACCGAAATAACATAGTAATATCGTAATTATGACAGGAATGAAATGATT
AAAAATAGAGTTGCCAATTGATTCTAATATAAACGACACCATAACAGAAATAAGTAATCCGATTATTAAACCT
ATTGTTGCAAATAGTATTTCAACAGCACTTCTACGCATAATAAAATGTTCTAAACCTTTTATAGCGTTAGTAA
CTCGTCTAATAAATACACCAAAAATTAAGAACATAAAAATACTACCGATAATGCCATCTACATAGTGATTTTT
TAAAAAGCTGGAGTTTTGTAATCCAAGATCATTTGCAATTTCAGGAATAATAATTATTCCTAATGCGCTCCCA
ATAATTAAGTAAATAATAATAACCATTAGTTTAACGATATTCACACAATGTCCTCCTTTCTTGATGTTTTATG
AATGAAGAGCAAATGACAATGCTTCATGTACAGTAGTTACACCTATTACTTGTATACCTTCAGGATATGTCCA
TCCGCCTATATTATTTTTAGGAATAATTACACGTTTGAAACCTAGTTTTGCAGCCTCTTGCACGCGTTGTTCT
ATCCGAGATACACGACGTACCTCACCCGTTAAACCAACTTCTCCAATATAGCAATCTAATCCGTCGACAGCTT
CATCTTTAAAGCTAGACGCAGTTGCTACAATTACACTTAAATCAACTGCTGGCTCCGTTAACTTTACACCGCC
AGCTACTTTGATATAAGCATCTTGTTGTTGTAATAGATAATTTTCTTTCTTTTCCAAAACAGCCATCAACAAA
CTTAATCGATTATGATCAATCCCTGTTGCCATTCGTCTCGGATTGTTAAAAGTCGTTGGAGTTACCAGCGCTT
GAACTTCTATTAAAAGTGGTCTGGTTCCCTCCATGGTTGCAACAATTGTTGAACCTGGAACATTTGTTGAACG
TTCTTCTAAAAACATTTCAGATGGATTATTTACACCTTTTAATCCACTTTGCTTCATTTCGAAGATTCCCATT
TCATTCGTTGAACCAAAACGGTTTTTAACAGCTCGCAAAATTCGATATGCGTGGTGTTCATCGCCTTCAAAAT
AAAGCACAGTATCAACCATGTGTTCTAGCAATCTTGGGCCAGCAATTTGACCTTCTTTCGTTACATGACCCAC
TATAAAAGTTGCAATGTTCATTTGTTTAGCAATATTCATTAAACTTTGTGTACTTTCACGAACTTGTGAAACA
GAACCTGGCGCAGAGCTGATTTCAGGATGATATATTGTTTGAATCGAATCCACTACTAGTAAATCAGGTTGTT
CTTCTTTTACTGTTTGATAAATAACTTCAAGATCTGTTTCAGCTAATACTTGCAATTCACTTGAATCTTCATC
TAATCGCTCTGCACGTAATTTAGTCTGACTAAGCGATTCTTCTCCAGTAATATATAGTACTTTTTTCTTTTGA
GATAACGATGCACAAATTTGTAAAAGTAACGTTGACTTACCAATACCTGGATCCCCACCAATAAGTACTAACG
ATCCGCTCACAATACCTCCACCTAATACGCGGTTGAATTCTGCTGAATCTGTTAACACTCTCGGCGTTGTTTC
ATGTTTAATACTATTTAATTTTTGTACTTTACCTGCTAATTCCTTGGTTTTCACTCCATGTTTAGGATTGGCT

GCTTTTTCAACAATTTCCTCCATTTGATTCCAAGCGCCACAATTAGGACATTTCCCCATCCATTTAGGAGATT
GATAACCACAAGCCATACATTCAAAAATCACTTTTTTCTTGGCCAAAATTGCACCTCCACTTTCCTTTGAACA
AATCTATTTTAAACGCACATTCCCATTATGACAAATTAAATGTGAGTTACATTTGTTTGTATTTTAACATGAC
TACTAACGCAAACTGTTTTATCAGATTTATTTTTTATGAATATTATAAAATAAACTTACATCATGTTGATAGT
CGGTATCTTTCAAAGATAAAATCTTAATAATTTCTTAGTAAACTCTTTTCTCTAGATTTATCACAATATTATA
TAGACCTATTTTATTTTGACGTAAGTTGCTAGTATCTTCAAACAAAAAACCTTTATAAAAATTCATACCTTTA
TGCTATCGCTGTAGGCTCATTAACTTGTTTCATATAATTCTTAACTATCCTTTGATGATTATTTTATTAGATT
GTTTCGTTGATGGATACTTTCACGAATTTCTATAGTTCAATGCTACTAAAAAAACAGCCCAAAACTTTAATTT
GTTTTGGACTGTTTTATAATTATGCTTGCGATGGTGTTTTAGTTTCTGAAGTTTGTTCAGCAATGTCATATTT
AAACTCTTTACCATCATGATCTACTGTAACTTTCTTACCTTCAATTTGATTACCATCTAATATTAATTCACTT
AAATTATCTTCGATAGTTTTTTGTATCGCTCTAATTAATGGTCTTGCACCATATTCTGGATCATATCCTTCTT
CTGCGATTTTGTCTTTCGCTTTATCAGTAACAATAATATTTATGTTTTGTTCAGATAATCGATTTGTTAATTT
ATTAACCATCATTGTTACAATTTCTTTTAATTCTTCTTTTGTTAGTTTATGGAATACAATGATATCATCTACA
CGGTTTAAAAATTCTGGACGGAATGAATTTTTTAATTCTTTTAACATCGTTTTTCGAATTGTTCATAATCTT
GTCCATCACTTGAACCACCGAATCCAGCAAATCGTTGATCTTGTAATTCTTGTGCTCCAACGTTTGATGTCAT
TATGATAATTGTATTTCTGAAATCAACTGTACGTCCTTTTGTATCTGTCAAATGTCCGTCATCTAAAACTTGT
AATAGAATATTAAATACATCTGGATGTGCTTTTTCAATTTCATCAAATAAAATTACAGAATACGGTTTACGTC
TAACTTTTTCAGTTAATTGTCCACCATCATCATGACCAACATATCCTGGAGGAGCACCAACTAATCGGCTCAC
TGCGTGTTTTTCCATAAATTCACTCATGTCTACACGGATCATCGCATCATCATCGCCAAACATTGATTCAGCT
AAAGCTCTAGCTAATTCAGTTTTACCAACACCAGTTGGTCCAAGGAAGATAAAGCTACCAATTGGTCGTTTAG
GATCTTTTAACCCTGCACGGGCACGTCTAACCGCTTTACTGATTGAATTAACAGCATCTTTTTGCCCAATAAC
TCTCTCATGTAATGTATCTTCTAGACTAAGAAGTTTTTCAGATTCTGTTTCATTGATTTTAGTTAATGGGATA
CCTGTCCATCCTGCAATAACTTCAGCAATATCTTCTTCTGACAATGAAGTTGACATGCCATTTTGTGCATTCT
TCCATTCATTTTTAGCTTCTTCATATTGCTTTTCAAGTTTTGTTTGTTTATCACGCAGGTTAGCAGCATTTTC
AAACTCTTGAGCATGTACTGCGGCATCTTTTTCATTTTTAACTTTTTCAATTTCTTGTTCAATTTCTTTTAAA
TTATTAGGTGTCGTATGACTCTTAAGTCTTACTTTAGAACTTGCTTCATCAATTAAATCAATTGCTTTATCTG
GTAAGAAACGATCTGAAACGTATCTGTTACTTAATTTAACAGCTGCTTCAATAGCTTCGTCTGAAATATTAAT
ACGATGGTGTGCTTCGTAACGATCTCTTAATCCTTTTAAAATAGCAACTGTATCTACTACTGAAGGTTCATCA
ACTTGTACAGGTTGGAAACGACGTTCTAAAGCCGCGTCTTTTTCAATATTTTTGCGATATTCATCTAATGTAG
TAGCACCAATACATTGTAATTCACCACGTGCTAATGCCGGCTTCAAAATATTCGAAGCATCGATAGCACCTTC
AGCACCACCAGCACCAACTAAAGTATGCAACTCATCAATAAATAGGATGACATTACCTGCTTGTTGGATTTCT
TCCATAACCTTTTTCAGACGCTCTTCAAATTCACCACGATATTTAGTACCTGCAACTACTGTTCCCATATCTA
AAGACATAACACGTTTATCTTTTAATGTCTCTGGTACCTCATTATTCACTATGGCTTGCGCTAAACCTTCAGC
AATAGCAGTTTTACCAACACCTGGCTCTCCAATAAGCACAGGATTGTTTTTCGTACGTCTACTTAATACTTCA
ATTACACGTGTAATTTCTTTATCACGTCCTATAACAGGATCTAATGTACCGTCTTTGGCAATGACTGTTAAGT
CACGAGCTAAACTATCTAAAGTTGGAGTATTATTTGACTTACTAGCTTGTGCATTTTTATTACTCATTTCAGG
GTTTCCTAAAGCTTTCACAACTTGTGCACGTGCTTTAGTAATATTTAAATCTAGATTTGCAAAAACTCTTGCT
GCAACACCTTCATTTTCACGAATCAAGCCTAATAAAATATGTTCCGTTCCAACAAAATTGTGATGTAATTTTC
TAGCTTCATCCATCGATAATTCAATGACTTTTTTAGCTCTAGGTGTATAATGCAATGTACCAACATGATCTTG
ACCATGTCCGATTAATTTTTCAACTTCTTCAATTACTTTATCTTCAGTGATATTAAAACTTTCTAATACTTTT
GCAGCAATTCCTTCAGGTTCTTTCATTAATCCCAATAATAGGTGTTCTGTTCCTATATTTGAATGATTTAAAC
GAATTGCTTCTTCTTGTGCATGTGCTAATACGCGCTGTGCACGCTCAGTTAATCTACCAAATAACATAAATAA
TGACCTCCTACTTTATATGTTCTCTTAGTATATCTGCTCGTTTTTCTTTTACAGATTTGTCATCTTCTTCATC
TAATAAAAATGGTGACTGTATAGCTACCATCAATTCATTAAATTTAAAGTTTTGTAATTCAATGTAATTTAAA
TCTATACCAAGTTTAACTTCGCTTAATCTATAAGAAGCCTCTTCCATAGTTATCATTCTACAGTTTTGTAAAA
TACCTAGCGAGCGAAAAACACGGTCTTGTGTTTCTAATTGATTATAAGTGTCTAACTTTTGTCGTATTTGTTT
TTCTTCATGAATGATTTGATTAACAACTTCTGTTAATGTTTCTATGATTTCTAACTCAGATTTACCAAGTGTA
AGTTGGTTGGATACTTGATAAGTATGTCCATAAACTTGCGAACCTTCACCGTAAATACCTCTGATTGTATATC
CAAAACGATTAATGGTTTGAGCAATCCGTGTCATTCTTTTCATAATAGATAGACCTGGTAAATGTAGCATCAC
GCTTGCTCTCATACCAGTACCTATATTGGTAGGACATGTAGTTAAATAACCAAGTTGTTCATCATAACTTATA
TCAAGGCTTTTATCTAATTCATCATCAATTGATGAAGCTTGATTATATAAAGCCTGTAATGTCGTGTCAGTTC
CCATAGCTTGAATACGAATATGGTCCTCTTCATTTATCATGACACTTAAAGATTCATCATCATTCACTAATAC
TGCGGCTGCTGGTTGTTTTATTAGTTCAGGACTAATCAAATGCTTTGCAACCATCTTCATTTACTTTGTTGA
TCCATTTGATCAAGACGCATTAATTCAAAATTTGGCAAGGCATCTTGTACCTCATTTATAACTCTAAATCCAT
CATTTTCAGTAGCATACATTAGTGGATGCACATGATTTTCTAAATTACGCGCTAACCGAATTCTAGAAGACAT
AACAATGGGTGTTTCTTCATTACTTTTCATCCATTGGCTGATATTATCATGAATATTATGCGTCATCATGTTG
CACCTCACTCTCAGCTTTTAGTGCTTTAATTTCATCTCTAACAATGGCTGCTTCCTCAAAATCTTGGATTTCA
ATAAGTTTTTTCAAATATTCATTCTTTTCTTCGATTTTTCGCTTTAAAGCTATCTTTTTATGTGAAGAATGTG

GTGTCTTTCCAACGTGCTCAAATTGTCCACCTTGAACTCTGCGGACGATATCAATGATGTCATCTTTAAATGT
TGCATAACAATTAGCACACCCAAATTTACCAACATGTGCAATATCTTTCAAAGTCATATGACATGATGGGCAA
CGTTTTTCTTCTTTAAACGCTACTTCTTGAAAATTAATGCCATGTTTCGTAGCTAAATGTTGTAAAATTTGCT
TAACAACAAATGCTTCTTCGAAATTATCTTGATGTTCTTGATATTCAGGTTGTTCATTAGCTTGATTCCACGG
ATGGTGCCCCTCAGCACAAGTTTGACACACCATTTTTTCTTCTGTTTTATTTTTACTTGTAACTTTAACTTTT
AATTCCGCTTCATTAAGTTGACAATTTTCACAAAGCACTTCAACACCTCATTTCATTTAGTAATAATTTATAA
CTGGTAACAAACGTTTTAAAATATTTGCTCTAATAATATCTCTAGAAACCATATCCATTGATAGCGTTTCTCT
ATCAATAACTGCTTGAATCATTTTAGCTTCACGTTCATTTATTAACATTTTATCTAAAAGCCCATCAATAATA
TAATAAGCTTGTTGTTGAGAAATAGAAGGTCCAATCAGCTGAAGCAAATGATTAATATAACCTGTTGCATCTT
TATTTTCAATTTTAGTGATTCGGATGTAACCACCACCACCACGTTTACTTTCGATTTCATAACCATGTTCATT
AGTGAATCGTGTTTTGATTACATAATTTAATTGTGATGGTACACAATCAAAGCGCTGTGCGATATTCGCTCTC
TGAATTTCAACGACATCTTCATTCGACTCTTCAAATAAACGTTTGATGTATTGTTCTATGATGTCAGACATAT
TGTGCATGTATATCACCCCTTTTTGACCTTCTTTGACTATATTATATGACCTATTTTGACCTTTTTCAATTAA
TTTGTTTTTATATTTTCAAAAATTTATGATGTTAGCCCTTCCATTTTATTGTATGATAGATTGTAGAAAAAAC
TAAAGGAATGAGAAATATATGCATATTCTAATTGGGATTATCGGAATTATCTTCTTTTTAGCACTTGCGTTTC
TATTTAGCTCAGACAGGAAAAATATTCGCTGGAAATATGTAGGAATTTTACTTGCAATTCAGCTTATCTTTGC
ATTTATCTTGCTTAAAACTACAATGGGAATCAAGGTTATTGGTGGTATTTCAAACGGTTTTAACTACTTATTA
TTAAAAGCAGCAGATGGTGTTAACTTTGTATTCGGTGGTATTCAATATATCGATCCTAAGCAACCACCATTCT
TCTTCAGTGTATTATTACCTATCGTATTTATTTCAGCGATTATCGGTATCCTTCAATACACAAAGATTTTACC
TTTAATCATTAATGTTTTAGGATTCTTAATTTCTAAAATTAATGGTATGGGACGTTTAGAATCATACAATGCA
GTTGCTGCAGCAATTTTGGGACAATCTGAAGTATTTATTTCAATTAAAAAACAATTACCATACATACCTAAGC
AACGTTTATACACATTAACAGCTTCAGCAATGTCGACTGTTTCTGCTTCAATTATCGGTGCATACTTTACCTT
AATTGAACCTAAATATGTTGTAACGGCTGTTGTCTTAAACTTATTTGGTGGTTTCATCATCGCTTCAATTATT
AACCCTTATAAAGTTAATGAAGAAGATGACAAATTATTAGTTGAAGAAACTGAAGAAAGACAACAATCATTCT
TCGAAATGCTAGGAGAATATATTTTAGATGGATTTAAAGTAGCTGTCATTGTAGGCGCAATGTTAATCGGATA
CATTGCTATTATCGCTCTATTAAATGGAATTGTAAGCAATATCTTTAGTACAGTTTCAGGTGGCGCTATTTCT
TGGGATTTCCAAACATTAATTGGTTTTGTCTTTGCACCATTTGCATTCTTAGTCGGTGTGCCATGGCAAGATG
CAGTTCAAGCCGGTTCAGTTATGGCGACAAAACTATTATCTAACGAATTTGTAGCAATGCAAGCACTCGGTAA
ACTTAGTGATTTATCTGAACACGCAAAAGGTGTTACATCTGTATTCTTAGTATCATTTGCTAATTTTAGCTCT
ATTGGTATTATTTCAGGTGCGATTAAATCTCTTAATGACAAAAAAGGAGATACAGTTGCCCGCTTTGGTTTGA
AATTATTATTCGGTGCAACACTAGTATCTTTCATTTCAGCTGCAATCGCTGGATTCTTTATTTAACAAATACA
TCAACATCGAATTAACCCCTTCAAAATTTTGACTATTCTCAGTGTCTTTGAAGGGGTTTTTTCTTATGTATAT
CTTTATTTTTTATCATTTTTAATTTTGACATAAAAAACAACTCCTCCAAAGCTAAGTTTGAAGGAGATATTGT
CTTCTCAACAAATCTATAAATATCAATAATTCGTTGATTTAGCATACATTAAGCTATGCTTTTTTTACAATAT
GATTAATAAAGTAATCAGTTACTCTATAGTCATCTGTTAATTCAGGATGGAATGATACGCCTAAATATTTACC
TTGCTGGACAGCTACAATTTTCTCATTAACCTTACATAGGATATTTACGCCTTGACCTACTTTTTCAATGTGT
GGGGCTCTTATAAAGACACCTTCAATATCTGTAGCGATACCTTTAATATCTAATTCTGTTTCAAAGCTGTCAA
CTTGTCTACCGAATGAGTTTCGTTGTACAGTAATATTCAACTTGTTAAGGTATCCTTCTTCACCAACTATATC
TTGCGCTAGAACTATTAATCCTGCGCATGTACCAAACATAGGTAAAGTTGAATTTTGTAAAGCCTCTTTAAAT
CCATATAAATTCATTAATCGACGTAACGTTGTAGACTCGCCACCAGGTAATATTAAGCCCTCGATTTCTTCTA
ATTGTTCAACTTTTTTAACTGCAATACCTTCATGACCACTTAATTCAATATGTCTAATATGTTCACGTACTGC
ACCTTGTAATGCTAATACACCTATTTTCATATCTTACCAACCACGCTCTTGCATACGTTCTTCTAATGATAAT
TGATTGATATCTAAACCTTTCATAGCAGTGCCAAGTTCACTTGCTAATCTTCCAATTAGTTCATAGTCTTGGT
AATGTGTTGTTGCTTGAACAATTGCTTTAGCAAATTTTTCTGGATCTTCTGATTTAAAAATACCTGATCCAAC
GAATACACCATCGGCACCTAATTCCATCATTAAAGCAGCATCTTGAGGAGTCGCAACGCCACCAGCTGCAAAG
TTAACTACCGGTAAACGACCATTGTCTTTAATTTGTTTTAAAATTTCATAAGGCGCACCGATATCTTTCGCGA
AAGTCATAATCTCATCATCATTCATTACAGTCAATCGACTAACTTCTGAATTAACTTGTCTCATATGTCTTAC
AGCTTCAACAATATTACCTGTACCTGGTTCACCTTTAGTACGTAACATAGCAGCACCTTCACCAATTCTACGC
GCAGCTTCACCTAAATTACGACATCCACATACAAATGGTACTGTAAATTGATCTTTTCTTAAGTGATATTCCT
CATCTGCTGGTGTTAACACTTCTGATTCATCAATATAGTCAACACCCATCGCCTCTAATACTCTTGCTTCAGT
GATATGACCAATACGTGCTTTAGCCATGACTGGAATAGAAACAGCATTCATTACTTCTTCTACAATTTTAGGG
TTTGCCATACGTGCAACGCCACCAGCAGCTCTAATATCAGAAGGTACTCGTTCTAATGCCATAACTGCTACCG
CGCCAGCTTCTTCTGCAATTCTTGCTTGCTCAGCATTAACGACATCCATAATAACGCCGCCTTTTTGCATTTC
AGCCATACCTCTTTTGACTCTGTCTGATCCAATAATTTTACTCATTATACATAAAACCCCTTTTCAAGTTGAT
TAAATACATTGTAATGTATAAACTGATATTTAAAAAGGTACAAAATAAGACAAATTTAGGAGGTCAGTTATAA
AATTGAAAAACACTTTATACCATCAACTTTATGAAAAATTAAAAAAACAAATAATTGAAGGTCAATTTAAAGA
AGGTGATAAATTTTATTCTAAAAGACAATTAAGCAAACATTTATCTATCAGCCAAACAACCGTAGAACATGCA
TATCAACTTCTATTAGATGAAGGTTACATTTATTCAAGACCTCGCTCTGGATATTTTGTGTCCGAAATTGAAT

CTTTAACAATTTTAAACAATCAACCTATCCCCTCTCTTTTTGATGATGATTCATATAAACCAAAAGCGAGTGA
TGAAGCTTATGATTATGCTTTTAATTTGGATGAAATAGATACGAAACATTTCCCTATAGAACTTTTCAGAAAG
TATTCCAAAGATCTTTATGATACAAATCATTTGAACCAATTACGTAGAGGACATTTTCAAGGAGAATTACATT
TAAGATTTCAACTGGCATTTTATTTATTTACTAATCGTGGTGTAATTTGTGATCCTAATCAAATTATAATTGG
CTCATCTACCGAACAATTAGTAAATCAATTAGTCGATTTACTTTATACTTCAACGTTTATTATCGAAAAACCT
AGTTATCCACCAATCAAAAATATTTTAGATAAAAAACAAGTTGAATATGAACAAATTGAAGTTGAAGATAATG
GAATAAACGTAGATGAAGTTATTAAATCACAAAAAAATATAGTATATATAACGCCTTCCCATCAATTTCCAAC
TGGATATGTGATGGACTTAAAAAAGCGAACTCAATTAATACAATGGGCTCAAGAAAAAGAAGAACGATTTATT
ATAGAAGATGATTATGATTCTGAGTTTAGATATTTCGGTAAACCCATTCCAGCAATACAAGGTCTATATTCAA
GAGGAGAAAAAGTGATTTATATCAGTACATTTTCAAAGTCTATTTTTCCTAGCTGTCGTGTTGCATATATGGT
GTTACCCTATTCTATAATGAAAAAATATCATTCTCAAAATCACATTGAAGGCAACACTGTACCTGTTCATATG
CAAAACCTTATTGCAACGTTTATATCTTCCGGTGGTTTTGAAAGGCATTTAAATAAAATGAGACGAATTTATC
GTAGAAAACTAACCTATATTTTAAAAAGATTGAAGCCATACAAAGAGCAACTTGATATTCAAGGTGCTGAAAC
AGGTATGCATTTTACAATTACAGTAAAAAATGGATTAACATTACAAGAGTGTCTTGATAGAGCAAATAAAGTA
AAGTTAAAATTACAAGTTTATAATTTTGATGATGACCAAGACTATAAGAAAACCCCTAAATTTATATTAGGCT
TTGGCGGTATAGACGATGATGCTTTAAAACCGCATGTAGACGCTCTAATAAAAACATTAATTATAAAGTAACA
CTTCTTTGATAAAAGTTAGTGTTACTTATGCAATAGCTTGTCTATTGTATAATAATTAATTTCCTTTTTGTAT
TTCAATAAAGTATATAAAAGAGCAGTAAGATCATTTTCAATTAGATACCGTCTTACTGCTCTTCTCTATTTAT
ACAATATTTTGTTTTCGATGATATCATTTTAAATTTATATAAACACAAAATTAAAGTTGTACATACTTAGACA
ATCAAAAGTGCACATTATTAAATTATCATTTCCACCATGGGTTTTATAATATTTATACTTAGGATACAAAGTA
TTGACATTAATATCTAATTGTAATGTATCGATTAGGTATTGAAACGACACGCTTTTTTCATATTCTTGAAACA
TATATAGCAGTACATCTATGTCTACTTTGAAGGGAGCATTTCAATGCGGCTCATCGCATCCATTTTTTGCCTG
GCAACGTTCTACTCTAGCGGAACGTAAGTTGGCTACCATCCTCGCTAAGAACCTTTCTTGACTTGTGACAATC
GCTTGCTTCTTTCCTCTTCTTCGGCTCTCGCTTACTCATTTAGCTCTACTAAACTCGTTGCGCTCTTTTCTCG
TTTCGTCAGATTCAAACGTTTTCACTTCGCCAAGCCATTTTTCTTTGTGTTAAGAGACCTTACGGTCTCAATG
CGGCTCATCGCATCCATTTTTGCCTGGCAACGTTCTACTCTAGCGGAACGTAAGTTCGACTACCATCGACGCT
AAGGAGCTTAACTTCTGTGTTCGGCATGGGAACAGGTGTGACCTCCTTGCTATAGTCACCAGACATAYGAATG
TAATTTATACATTCAAAACTAGATAGTAAGTAAAAGTGATTTTGCTTCGCAAAACATTTAAATTTGATTAAGT
CTTCGATCGATTAGTATTCGTCAGCTCCACATGTCACCATGCTTCCACCTCGAACCTATTAACCTCATCA
>DSM104437_scaffold_1
GTCTATTTCAGCAGTGTTTGAAAGGAAGTTTATAATGAAAATAACTAATTGCAAAATAAAAAAAGAAACTATA
GTATATGAAGTTTTAACTAGTGGTAATCAACCATTCACTTATGAGTTACCTAAAGATTTATCGTCACATAATG
CGCGTAAATACTTGGAATTTATTTCACAAAAAATAGATGGAGATAAGTTAACCAAAGAAGATTCATTATGATT
TTACTAAATAAAAAAACGCCTACTAGTGTAGACGTTGAATGGTGGTGAGAATTTTATGGTAGATAAAAACAAA
AAACAAGAAACTACTCGTAGTAACCCATTAAACAAAAGTTTTGAAAAGTCAGGCGCCAGCGAAAAATTAAAAA
GCACTTTATCAGAAAAAGCTAAGAAAAAAGATTAGTATTCATTCATTAAATATAAATCCAATTTAATTTGTTG
TTTAAGGTCTACAAGCGTATGTTTAATATACAATTCATCGTTTGACGCTAAATCAGATACTTTGAAATCTTGT
CGCTCAACTTCTAGTAAATCGAAATCGCTACCAGCTGAATTATAGGTTTTAAGTTCACCCTCTTCAATGATTC
TGTTTTCAAAGTCTTTTATAACTATAAATACTGGTTTACCGTTGTTATTAAACAACTTGTCTCTTTTGTCTAA
TAAGCTTATACAATCCAATTTCATAAACTTTCTGGTTATATTAATTAACCAGATAATAAATTTAACAATTAAA
GGATTAAATACAAACACTGTTAAAACAAAAATAAATAGAAACAAAATATTTGCTTTTAGACCTGTAAGCAACT
GAATTAAATTCAAATTTTTTAAATCAACATTATTAAAAATTATAAAACTATAAAACCATATCAAACATGTTTC
AATAGAAAAAATCAATAATACAGGAGTATTGATAACCTTGTTTTTTTCACTAACTAAACCTATCATTGTTAGA
TATTTATATGGTATGTAACCTAAAACTCCTGTAAGAAGAAGCGCCCCTAGAAATTGAGTCATCTTATCACCTA
CTTTTTATTTTATTATAACACATTTAGTACCTAGTACTAAATTACGGGTAGCCCGACTACCCTTATTATTTTT
TAATATTTTATAGAACATACGTTCTTGCAGGAGGTATAAACATGTGGATTGAAAAATTTAAAAACAAAAATAA
CGAAACTAAATACAGATACTACGAGAAGTATAAAGATCCATACACAGATAAATGGAAGCGCGTAAGTGTTGTG
TTGAACAAGAATACAAAACAATCTCAAAAAGAAGCAATGTTTCGTTTAGAAGAAAAAATAAAAGAAAAACTGA
ACAACAAGTCGTCAAGCGAATTAAAAACTTTGACTTTTCACGCGCTATTAGATGAATGGCTTGAATATCATAT
AAAAACATCAGGTTCAAAGTTGACTACTCTTAATAATATAAAAATAAGAATTAGAAACATTAAACGATACAGC
TCTGAGAACTTGCTTTTAAACAAACTAGATACAAAATATATGCAGATATTTATTAATAAATTATCAGATATCT
ATTCTCAAAATCAAGTAACCCGTCAACTCGGAGATATGAAAGGAGCTATTAAATATGCAGTTAAATTTTACAA
TTATCCAAATGAATATTTGTTAACTAATGTCAAAATTCCTAAAAGAAGAAAAACAATAGAGGATATCGAAAAA
GATGAATCTAAAATGTACAACTATTTAGAAATGAACCAAGTCCTACAGATACGTGATCATATACTAAATGATA
ATAAGTTACACAAGCGAAATCGCATTTTAATTGCCAGCATCTTAGAAGTACAGGCTTTAACTGGTATGCGCAT
AGGAGAACTACAAGCACTGCAGGAAAAAGATATAGATTTATTAAACAAAACTATCAATATAACAGGTACAATT
CACCGCATTAAATACGAGGAAGGATTCGGATACAAAGACACTACAAAGACTATAAGTTCAAAAAGAAGTATCA
GCATCAATTCTAGAACCGTAGAAATTTTTAAAAAGATAATACTGGAAAACAAAATGTTGAAAAGATGGAATTC

GAGCTATGTTGACAGAGGGTTCATATTCACAACAAAAAAAGGGAATCCTTTATGTAATAATCAAATCGCCGGT
GTGCTTAAGAAAACTACAAAAGCTTTAAATATGAATAAGAAAGTTACCACGCACACATTTAGACATACACACA
TAACTTTATTAGTAGAAATGAATGTTTCTTTAAAAGCAATTATGAAAAGGGTAGGACATGTAGATGAAAAAAC
AACCATTCGCATATATACTCATGTAACTGAAAAAATGGATAGAGAACTAACTCAAAAACTCGAAAACATTCCA
AGTTAGCTTAAATCCGCCCTTTTTTTGCCCTTATATTTTTTACAAGCTTTATAAAACGCTTGAGAACACTGGC
GTTAAAGCTTTTCTTGAAATAAACATATCATCATAATGAGATGGTTCAAAAATCTGATTAACAATTAACGGTT
TCATATTTCTAACTATATCATCTAAGTGATTTTCTAAAATCGGCGAAGCTATTTTTAAATTATTTAAAAATTC
GTCTAAGTTTTTAAGTTTATTTTTTAAAACTTCAAGCATTTTATCAATTTCGTCAACATTTATTCCCATTGCC
TTTATCTTTATTTTCATTCCTGATTTGTATATTAAACTGCTTATTTCATAAATATGTCTCACAGCTGTTTCTA
CCGAATGTATAACTTCAAGAATGTAGTTTAATACTGGTTCAAAATAATTATTGTAAGTTTCTTGAACATAATC
ACTAATTTTGTTAACTGCATTATCAATATGGTTTTTTAAAATTGTCATTTTTCTGGTTAAATAATTAGAAGAT
TGTACTTGACCCGAAATCATATGTTCACCGCTATAATTAATAGATAAGTTTCCATCCATTACCGTTGCATCTT
GATGAGACATTATATTTTTAATATCGGTTATCTGCTCTCCATATACATCATTTTGCTTTTTCACAAAATTTAT
ATTATTATTCACAACATTTAAATGCTTCATCATTTCCTCTGTCACGCCATCTTCAAAATCATGATCTAAATTT
TTAAACATTTCTTTAATTACGTTATCAAGTGAATCATGTAAACTTTCTATGCCACGTTGTAATTTTTTATCTA
AATCTTCAAGAATTTCTTCAATAGTATTTAATTCAAGAATAAGGACTCCAGCTGCTGGAGCTATTAAGATTAT
TACAACAAGATTTTTTTTGACATCATCAATTGCGCTAAATACATCCTGTTTAAAAGAAGTTAATTCCCTACTC
ATTCCTTCAAGGATATCAACTTCGACAATCTTTTTAAAATCTTGTTTAAGTTTATGTTTTCTTTCTCCAAAAT
TAGAATTTTCAACCTGTGAAATATTATAGCTCTCATTCAAATAATTTACACAATCGGTTAACATATTACTTGT
TTCACCAGTTACCAAATTACTTAATGCACTAAGATTTTCTAGATTTAATTTTATAGGAGTCCCTTTACCCGTT
CTCGCTATTGAATCTCCTGTCCACACATTAATTGGAATTCTATCATCCATATTTATATCGATTTCAATAGTTT
TTTTGACTTTTTTACCATTTTTAATCATAGTATCTTTAACTGATTTAATTTTATAAACTGCTAAATCTTTATA
AGTATTATATCTTGGATTTAATTTTCTTTTATAACCTAAATGACTATTTACTAATCCATCCATCGTTGGCACA
CCAGTTTCTAAATTCACTCGTTTTCCTAGTGTCGGAGTGGCAAATGGATCCTGCAGCCAAGATAAAATATCAA
ACTTACTATGATAATTAATCATATTTTCAAAGTTTTGAGATTTGAAATTTTCCCATATTGACTTTGGTAACAT
GGCAGGGTTTGTAGAAACTATATTTTTATTCGGGTTAATCAACCCTTGGTGGTTAGCACTTGCTCCCCCTTGA
GAATTGCCTCCATCAGACTTAATAGTTTTGTTTTTATACGTAGACGGATTTTGCTTATATTTCCTTAAAAAAT
CAGAGCTTTTAAGTGTATCTGCATCTTTTAGTTTCTGTTCATACGATTTAATATAAGCATTATTTTGTTCTAA
TAAAGAAGTTGACTTATTTTTATCATTCATTAACTTTATATTTTCAATCCAATCATCAGCAACCTTCCCACGA
AAAGGATTGCTTTTCACATCACTCTTTTGGTTATCTGTACCTTGAAATAATAATACTTGTTTGCCTGTAGGTG
TTTCATTTTCATCCAATAGTTCATAAACTTTTATATCTGAACTATTATTTTTATTAGCTACTTTCGTTATCTC
TTCGACTTGTTTGAATTTTTTATCGTTAACTCTAAATTTACTACCATCTTTAATATCTTGGTAAACCCAATAA
CCACTTAACTCAGTTAAATCTCTATCGTTTATATTGTTCATTTTCACTCAACCTCAATCTTGATAGGTTTGTT
ATCACTATAATTTACTTGTTTAGTATTAATGGTAGATTTCGTTAGTTGTAAAAATATTTTCGTGTTTTCTAAA
TTAAGGTTTAACTTTTTCACGCTTTCGGATAAATCTATAGCATCATCTAATTTCTTGTCCTTTGAAAAGTTTC
TACTTTTCGAAAACAGTGTTGTATATAATTCGATTTCAGCTTTGTAGCCTACTCCTCTTCTAGCATCTTTCAT
ACCTTTTTTAAAATCTAGATTATTTTTTTAATCAGAGGTTCGTAATATTTTCTATATTCTTGAAGTGTTGGTA
TATTTGCAACGATATAAAAATATTCATTTTCATATCCACTATTTTGTGTCTTGTTAATTGCCTCTTTTGTAAA
TCCTGTATATTGGTATTTCTCTTCGTTATCTTTGAAAAATTTATATAGGTTATCATACTTTTCTTTTTGTGCT
CGATATTCAAATCCACTTAGTACTGTACCAACTAAAGTACTCATATCATCGCCCTTATCCTCACTTCTTAATG
AGCTATCACTGTCAATAATTGATTTATCAAATGGAATACTCGCATTAAATACGATATCGTGGTCATCGCAATG
CACATAAACTTCTACACCGTCGCCACTACCTACAACATTGGTAGCTTTAACTTTTAGACCGAAGTTATCCATA
AAAAATTGTTCGCCTCGTTTGGCAATTTTATCTTTATGCTTCTTCGCAAATTCAATCGCATCTTTTTCTGCAG
GTGGTTGGAAGCCTTGTCCTACATATTTTGAAGCTTCCATTTCTTCTGGTACTGATTTTGTTTCTGTATTCGT
GTCTTTACTTGATTCATTTTCCATCATGGAACATCCCCCTAAAATTAATGTCGTGGTTAAAACTGATCCAATG
AACTTTTTCATATGACACCTCGAATAATTTAATTATTACAATATATCTATATTTTACCCTATTTTTATAATCA
AATAAATAACTTTATCAAACTTTATATTTTTGTATAAATTAGTAATATATTTAATAGTTGTTATTCGCAAAAA
TCATAAATCCTATTTCCTAATTCACTTACAAAATTCCCCACTTCAATCAAAAAACGCATGCTATAAATTAGAA
TCTCTAATCAATAACATGCGTTCTTAAATAACTATATTCAGTTTTATTCTGGTTTTGGTAGTTTAATAATAAA
TGTTGTGCCTTTTCCTAATTCGCTTTTAACATCAATGGAACCACCATGCTCTTCGATAATCATTTTACAAATG
AACAAACCTAAACCGGTACCTTGTTTACCTCGCGTTCTCGCTGCATCAACTTTATAAAAACGATCAAATACTT
GTTGTAAATGTTCTGGTGCAATGCCCGTGCCTGTATCTTTAATGTATAAAATATCTTCGCTTTTATTTTCATC
ACAAGTAATTGCAATTTCATCTCCAGGTTTCGTATAACGTGATGCATTATCAATTAAGTTCGTTAGTACTTGG
TCCATGCGATCCATATCATAACTCCAAACACGCTTCTTACAATAATTAAAAGTCATATTTAGACCTAAATCAT
CAGCTTGTTGGCGATACTTAATTTTCATCTTATCTAGTAACGCTGCAATAGGCTGAACTTCTTTATTTACGGA
TAACCCTTCAGCATCCATGCGAGCAACATTTAACAATTCATTAACTAAACGATTTAAACGTTTCGATTCATCA
AGGACAATGGCAAGCGATTCTTTTATTTCATCCGGTTCTGTAACAATACCATCTACAATTGATTCAGTATAAC
CTTGAAGTAATGATATCGGTGTACGTAATTCATGTGATACATTAGCAATGAAATCTTTCTTCATTTGATCTAG

ATTGTGCTCATTAGTCATATCACGAACTGTCACAACAACACCACTTTTACCTCCCTGTTCAATCTTGTCGATA
TAGCTTGTAGTCACAACAAAGAATCGTGCATTCATTTCTAAATCGCGCATTTCAGTTTGTTTTGATTTAAAAG
TATCTTCTATTTGTCTTAATAAGAAAGCTTTAGCATCTTCATCAATATTGTCCATAATATCATTCGCCATCTT
ATTAGATAAGATAATTTGTCGACTCTCATTAATACCTAGGACGCCTTCTACCATAGAGTTAATTAAGCTGTCT
CTAATACTTTTAGATGTGGATAATGCGTCGACATGCTCTTCGATTTCTGTACTCATCTGATTAAATGCCTGCG
ATAATTGACCAATTTCATCTTTCGTTGTGACAGAAGGTTTATAAGAGTAATCCCCTTCAGATACACGTGTAGC
TTGGTCTCTTAAACGTCTTAAAGGTTTTGTAATTCTTGACGATAAGAAAAACGCAAAGACTGTTGTAATTGTT
AAGAAAATAACAGCCGTAATTATAGTGATAATCGTAATAGCATTATTTGTATCTTCGATTGATTTCAAGTCTT
TATATATAAATACTCCACTATATTTGCTATGACTATTCTTCTGTGCTTTTGTTGGATAGCCTAACAAAATATA
AGTTTGAGATGAACCCTTTTCTTTAATCGTTACATTTCGAGTAACAGATTTACCTTTATCAAACACATCGTCA
AAATGGTCGTTGTTGACTACTTCATTCAACATTTGCTTTTTAATATTAGAAAGTGAAGCCGTTGATTGACGAT
GTTTATTGTTTATAATCATCAACCCACCAGGATTTTCAATTAATGTTTGACTATATTTTATTGCTTCTTCTTT
ATTATGTGATTGTTCGACCAGTGAACTTATACGTCTAGCATCTTCTCTTATGGCATTTTCAGTTTCTTGTGTG
AAATAGTATTGCATAAAGGTAATTAAAGCAATACTTAATAAAATTAAAACTGTCGTCACTATTAAAATAATAG
TTAACCACAGTTTAATTACGACACTATTTAGCCGGCTCATCATTAGATTTAACCTCAAATTTATACCCAACGC
CCCAGACTGTTTGAATCATATGCGCAGCTTCGCTAGACACACGATTTAACTTTTCTCTAAGTCGTTTAACATG
AGTATCAACTGTTCTTAAATCACCATAGAATTCATAATGCCAAACTTCTTTTAATAATTGTTCACGGTCAAAT
ACTTTATTTGGTGTTTTAGCTAAATATATTAATAATTCGTACTCTTTAGGAGTCAAATTAACTTCTTGATTAT
CAGCAAGTACGCGATGTGCATCATTATCTATTTCTAAATGTTTAAATTCAATCACATCACGTGCGTGAGGTTC
GCTTTGTTCTACAGTTGTAGATTGCGTTCTTCTTAGAAGTGCTTTAACTCTTAAGACTACTTCTCTTGGTGAA
AATGGTTTGACGATATAATCATCTGCACCAGATTCAAAACCTTCAACACGGTTTGTTTCTTCACCTTTAGCAG
TCAACATAATAATCGGTGTTTGTTTATGTTCACGCAATTTAGTTGCCACCTGGATACCATCCATTTCAGGCAA
CATTAAATCTAGTAGTATGCAAGCATAATTATTCTCCATTGCAAGTTCATAAGCCTCTTGGCCATTACTTGCT
TCATGGATTTCAAAAGATTCTCTTTCTAAATACATTTTAAGTAATCTTCTGATTCTATCCTCATCATCTACGA
TAAGTATTTCGTTCGACATACAGGTCATACCTCCCACACATGCTTTTCTTTACAAAAGTATTATATCACAGGA
AAGCGCATACAGTTATTAAAAACCTAAAATTTTGTGAAAAAATTCTCAAACTTTGTGATAAATACATATTTTA
TTTAATTATTTCCCATGTTCTGCTAAATGACGCATCACTTTTACTTCATGAGGCGTCAATACACGTCCTTCAC
CAGCATTCAAACCGACAACATTTAAAGGCCCATATTCAATACGAGACAGTTTCGTCACTTGATGACCAAAATG
TTCGAACATTCTTCTGACTTGGCGATTACGACCTTCTGTAATTGTAATTTCAACCAATGTTGTGTTTTTATCT
TTATCTTGTTTCTTAACTTTCACTTCAGCCGGTTGCGTCATACCATCTTCTAATTCAATACCTTTTTCTAGGG
CTTTCACTTCTTCTCTCATTAAGTAACCTTTTAATTTTGCTACATATTTCTTCTTAATTTGATATCTTGGATG
TGTCATTAAATTAGTAAATTCACCATCATTTGTAAGTAATAACAATCCAGAAGTATCATAGTCTAAACGACCA
ACAGGATAAATACGTGCTTCTATATCTTTAAAGTAATCCGTAACCACTGTACGTCCTCTATCATCTGATACAC
TTGTTATCACTTGAGTTGGTTTATGGAATAAAATGTAAATTTTGTCTTCTAGTTCTATTTTAATACCTTCAAC
TTCAATCGTATCTGATGGCTTTACTTTTGTTCCTAATTCAGTGACAGTCGTACCATTCACTTTCACTTTTCCT
TCAGAAATTAAAGTTTCTGCCTTACGTCTTGAAGTATAACCGCTATTAGCTATACGTTTTTGTAATCGTTCTA
ATTCTTTAGTCATTATTTTCTCCTTTTTGATTGACTAGATTACTGAAGAAAGCATCCATTTCTTCATCGTCTT
CTTCAGTTGTCGGCAAATCTTCTATATTGGAAATACCAAATACATTTAAAAATAAATCAGTAGTAATTAACTG
TTGGCTACGCTGTTCATTAACCACTTTAGCCTCAACTAGTCCTTTGGCAATCAAAGTCTTGACCGCACCATCT
GAATTGATACTACGAATTAATTCAATATCACTTCTTGATAATGGCTGGTTATAAGCAATAATTGATAGTACTT
CCATTGCTGCTTGTGATAATTTCATTTGTGACTTTTGTTCAATTAATTGTTCAATATACGTTGCCGCTTCTTT
TTTAGTCGTTAAAACATACGTCGTTCCAAATCGTTGTATCATTAATCCATGTGATGAATAATTTTCAATTAAT
TCAACGAGTTGGTCTTTCGACATATCTAATATTTCTAATAGTTGTTTTTCATCTAAACCTTCATCGCCAGCTG
TAAATAAAAGCGACTCTAATATACCATGATTATCCAAAATGGTAGTTCACTCCTCTAATAATGTTAATATCTT
CAAAATTACGTTGTTGCTCAATATTAATTATTCCTGCTTTTGACATCTCTAAAATAGCTAAAAAGTGAGTGAC
TACTTGTTCAATTGGCTCAGAAAACGTAAACAGACTAAAGAAGTTAAAATGATCTTTATCTTTCAATCTCGAT
GTCACTTGTTCTGTAGCTTGTTGAATGGTAAATGTCTCTTTTCGGATTTCAACAGATTTAGGTGTATTTAACT
CAACTCTATTTTTAACTCTTTGATAAGCTACAATTAATTCAGTTAAATCAATCGTATGATTTGGATCCCAAGA
TTCATCTGTTTCCAAATGAGATAAATCTGTCGGTCTTTTTGTAAAATAAAAATCTCTTTCTTCTTTCATGTCA
TTTAAAATAGCAGTATATTCTTTATAATTTTGATATTCTATTAAACGCCCAACTAAATCTTCCCGTGGGTCAT
CATCAACATCCATATCTGATGTTGATTGTGGTAATAGCATCTTACTTTTAATCATTAAGAGTTCTGACGCTAA
TACTAGGTATTCACTTGCAATATTAATTTCAAGCTGTTTCATTGCATGAACGTACTGCATATACTGCTCTGTT
AATGCTTGCATAGGAATATCATAAATATCTATTTCAAATTTTTGGATAAGATGCAGCAATAAATCTAATGGTC
CATTGAAAGCATCTAATTTAACTTCATACATAATATCTACCTCGTATTGCGTCATATGTGCATAATTCTCTAA
AATATTATAGCATGAAAATAACTTACTTTTAAATTGGGGTGATAACAAAATGCAACAGGCACTGATAAATTTC
TATTATCAATTTCATACTAAGCAACATTATTTCCTATGTCATGACATTTTAGAAGATGCATGGAAAGCTGAAA
ATAACTACAGTAAGCAAGATGCAGTTGTTAGTTTAATCTTGTTTGCAACTGCTTGTTACCATTATCGCCGCAA
TAATTTAAAAGGCGCCTATAAGTCTTTTAATAAATCTAAAGAAATAATACAAAATGCTAAAGACAGAGATGCG

TTATACCTTAATTTAAATGATTATCAGTTGTTAATTGAGCAACAGATTGCAAAATTGAATGCAGCAAAGCCTT
TTTCATCTGTTATATTACCAATAACTCCAGTCTTTGAACGCATCATCAAGGCCAACTATCCAGACTATGATTA
TAATCAGGAAACAGCAACAGATCCTTTTATTGTAGACCATCACATGCGCCGTGATCGTTCAGAAGTGATTGCA
GCAAAAGAAGAAGCGATTCAATTAAGAAAACATAGACGTAATTAATCAACCAATTTAAAAAAGCCACAATAGC
ACAGACATTTTTAGTCATATGTATTGTGACTTTTGAGTTATTGTTCACTTTATGCTCTAGGATGAAATTGGTT
ATACATTTTTCTAATTTGAGATTTCGAAACATGTGTATAGAGTTGGGTAGTAGATATGTCAGAGTGACCTAAC
ATCTCTTGCACTGCTCTTAAATCTGCGCCATTTTCCAATAAATGTGTCGCAAAAGAGTGGCGTAACGTATGTG
GCGTTAACGTCTTTTTAATGTTTGCCTTTACACCATTTTGTTTAATCATTTTCCATATTGCTTGTCGCGATAA
AGGTTTACCATGCATATTTAAAAATAAGACTTCGGTAATAGTCTTTTTTAAAAGTTGCGGTCTAATCGTTTCA
ATATAAGTAGTTAAGTACTCAATGACTGCGTCGCCTAATGGTACAATTCTTTCTTTATCGCCTTTACCAAATA
CGCGTACAAATCCCATTATTAAGTTCACGTTCTCTAACTCTAAATGTATCAATTCAGATACACGCATTCCCGT
TGCGTACAGAAGTTCTAACATCGTACGATCACGATAACCATTAATTTTATTTAGATCCGGCGTTTCTAATAAA
GCCAACACTTCGTCAACATTTAAAACGTCAGGCAATTTTTTGTCATATTTTGGTGAATCTAATAATACCGTTG
GATCTTTCGCCGCATATTTTTCTCTTATAGCAAATTGATGAAAACTACGGATTGTTGAAATAAATCGCGCAAT
AGATTTAGCAGATTGCCCTTGGTCTATTAAATGCCCCAAACACTCTTGAATTAATTGTCGATCTATAAAATCA
ATATGCGAGATATGATGTTCAGTCATATAATCTTGATACTTTTTCAAATCACGTCTATAAGCACCAATTGTGT
TAGAACTTAGTCCTTTTTCAATTTGTATAAAACGCAAATATTCTTCAATAATTGTTTCCATAACCACACCTCT
AAAAAAAGCGAAGGTTAATTTAACCTACGCAATTTACTATCCTTTACCTTTAGCTTGGCACGTTTCACACACA
CCATGGAAAGTTAAACGATGATCTAAAATTTTAAAATTGAACTCATTTTCAACTCGATTTTCAACTTCTGGTA
ACAAATCTTCATCGATTTCATCTACACGACCACATTCCATACATACTAAATGATGGTGGAAATGTTTTGCGCC
TTCTTTTCTTAAATCAAAACGAGCGACGCCATCACCAAAGTTAATTTTGTCGACAACTTTTAGTTCAGCTAAC
AACTCTAACGTTCTGTATACTGTCGCCAAGCCAATTTCAGGCGCTTTATCTTTTACTTTCAGATATACGTCTT
CAGCACTTAGATGATCTTTTTCATTTTCAATTAGAACTCTAACAGTAGCTTCGCGTTGTGGCGTTAGCTTATA
TGATGATTGTTGTAATTGTTGCTTAACGCGATTTAATCGTTCTTCCAACGATGTCCACTCCCCTACTAATAAT
TAAAATCATTACAAATTATTTCAAACTTCACAATTAAAACTAACAGTTTTCTCAATAAAATGCAAGCTTTTCT
CATTTGTTATTTAGAATGATTATAATTTAATAATAGGTGTTGCAATGCAATGATAGTCTTCGCATCTTCTATT
TCTTTATTCATTAGCATCGATTTAACATTCTCAATCGGAACTTTAATGACTTCGACAAATTCATCTTCATCTA
AGTGAACTGTACCTTCTTCTAAATTATCCGTAAAATATATTGATAATTGTTCATCGCAAAAACCTGGTGAACC
ATACATATCTACAACATGTGTTAACTCTTTGGCAATATATCCTGTTTCTTCTTCTAATTCACGTTTTGCCGCT
TCGACTCTATCTTCATCATCTTCTAATTTACCAGCTGGAATTTCTAGCAATGGTTTTTCTACTGGTTTACGAT
ACTGTTTCACTAATACGACTTCTTTTTTAGGTGTCACTGCACAAACAGCAACTGCACCATTATGATAAACTAA
TTCTCTTGTTGACGTTTCACCGTTTGGTAATGTCACTGTATGAATTTCTACATCTACAATTTTGCCATTATAA
ATAACTGTTCGATCAATTGTTTTTTCATTTAAATCCATTATAATCACGTTCCTTTAAATTCATATTATATATT
GATACACTATGCTTGTTAGCTAAAGTGTATCGAAAGGAGAACAGACATGCAAAAAAATATATTAAAAAGTGGT
ATTTCATTATCTGAATTAGGTCTAGGTTGCATGAGTTTAGGTACAGATTTAAAAAAAGCCGAACAAATTATAG
ATTGTGCTGTTGAAAATGGTATCACTTATTTTGATACAGCAGATATGTACGATAAAGGTATAAATGAATCAGT
TGTTGGTAAGGCACTTCTTAAATATCAACAACGCGATGATATTGTCATTGGTACAAAAGTAGGCAATCGTTTA
ACAAAAGATGGCAGTACAACATGGGATCCGAGTAAATCCTATATTAAAGAGGCAGTTAAAGGTTCACTAAAGC
GTTTAGGTATCGATCATATCGATTTATATCAACTTCATGGCGGAACCATTGATGACCCATTAGACGAAACAAT
AAGCGCATTTGATGAATTGAAACAAGAAGGAATTATACGTGCTTACGGTATTTCTTCTATTCGCCCAAATGTA
ATTGATTATTATTTAAAACATAGTCAAATCGAAACGATAATGTCTCAATTCAATTTGATTGATAATCGTCCAG
AATCATTATTAGATGCAATTCACAACAATGATGTTAAAGTATTGGCAAGAGGACCTGTGTCTAAAGGATTATT
AACTTCAAACAGTGTTAATGTGCTAGACAATAAATTTAAAGATGGTATTTTTGATTATTCTCATGATGAATTG
GGTGAAACAATAGCCTCTATTAAAGAAATTGAAAGTAATTTATCTGCATTGACATTTAGTTATTTAACATCAC
ATGACGTGCTTGGTTCCATCATTGTAGGTGCAAGTAGCGTCGACCAATTAAAAGAAAATATTGAAAACTATCA
TACTAAAGTTAGTTTAGATCAGATTAAAACAGCAAGAGCTCGTGTAAAGGATTTGGAATATACCAATCATTTA
GTGTAGAAGTCATTTTCAGTAATAAAAACAGCAGCATGAGGCGTTTCATTATAAAAATGCCTTACTGCTGTTG
TTTATGTACAATTCGCTATAATTTATGATTAAGATTACTCACTTATGATAGAAATTAAAGCGTTGTCCTCACG
CATCAGTATTTAGTAATTTCGCCCTGCGGCAGTGCCTTAAGCAAACTTCTCCCACTTCATCTTTTAATAATTT
TATTAAAACATCTTTCTATATTTCACTTCGCATGTTGATTCATCATTATTAGTTATTATTTGTACACCCAGCA
CATTTCCTTGCAACACAAGTAGTTTGAATTTTTCACAAGTATAATATAATGTACTGTCTGAAATTTGGTCTAC
AGAAATATCGCCTAAAATATCCAGCACTGTAAATTCTTCAAACACTGATAGTTGTTCCGCATATCGTACACAA
AGTCTTACCACACTCTCCGATTGACAGTTCATTGCCATCCCACCTATTTATGCTTTATTTTTAAATAATTTAG
GAAAACATCGTTCAAAAAATCTAGGCGCAATTTGATACATTTTCAACGCATGGTGCATCCATTTAGGGCGATT
AATTTCCAATTGTTTTGTTTTAATGCCATAAATGATATCTTCTGCAAGCTGATTAGCATCAAGCATAATTTTC
CCCATCTTTTTAGCATACTTCATTGATGGATCGGCTTTTTGATGAAAAGGTGTATCAATCGGGCCAACATTAA
CTGTCATGATATGTATGTTTGGTGACTCTAGTCTTAAAGCATTCATTAATGCATAAAACCCTGCTTTCGATGC
CCCATAATGTGCAGCATTTGCTTGAGTGGAAAATGCAGCTTGACTTGAAATACCTACAATATGTGCGTTAGAT

GTTAAATATGGTCTCAACACAGTATATAAAACATTAAAACTAATTAAATTAAGCTGATACGTTTCAATCATTT
CTGAAAAACTATGGTCTGAAATAGATTTGAAATAACCTAAACCTGCACTATAAATGAATCCATCGAATGATGT
ATTGTCTTCAAATTGCAGTGCCTGTATCGACTTCAAATCATTTAAGTCACAAGGAATAACATTTATAGTTTTC
CCCAATTCTTGTTCAAAGATTCTAGTTGCTTTATCAACATCACGCACCAACAACGTTACATGCACTTTATTTT
CAAGTAACTTTCGGACAATCGATAAACCTAAACCACTCGTACCACCAGTCACTATAAAATGTTGTCCTTTCAT
CAATTAACCTCCCTTTTCAATTATATAGAATGCAATTTATCAACTTTACATAATTGAGACAAGTTGATTATCT
TTCCTAATATATATACAATAATAAGAAAATATAACATACAAATCAAAAACTAAAGGATGTGACGTTAATGAA
ACTCGTATTTTATGGAGCTGGTAATATGGCACAAGCTATATTTACAGGAATTATTAACTCAAGCAACTTAGAT
GCCAATGATATATATTTAACAAATAAATCTAATGAACAAGCTTTAAAAGCATTCGCTGAAAAACTAGGTGTTA
ACTATAGTTATGATGATGCGACATTATTAAAAGATGCAGATTATGTTTTTTTAGGTACCAAACCGCATGACTT
TGATGCTCTAGCAACACGCATCAAACCACATATCACAAAAGACAATTGCTTCATTTCAATTATGGCAGGTATT
CCGATTGATTATATTAAACAACAATTAGAATGCCAAAATCCAGTTGCTAGAATTATGCCAAACACAAATGCGC
AAGTCGGACACTCTGTTACTGGCATTAGTTTTTCAAACAACTTTGACCCTAAATCTAAAGATGAAATTAACGA
TTTAGTTAAAGCATTTGGTTCTGTAATTGAAGTATCAGAAGATCATTTACATCAAGTAACAGCTATCACCGGA
AGCGGCCCAGCATTTTTATATCATGTATTCGAGCAATACGTTAAAGCTGGTACGAAACTTGGTCTAGAAAAAG
AACAAGTTGAAGAATCTATACGCAACCTTATTATAGGTACAAGTAAGATGATTGAACGTTCAGATTTGAGCAT
GGCTCAATTAAGAAAAAATATTACCTCTAAAGGTGGTACGACACAAGCTGGCCTTGATACATTGTCACAATAT
GATTTAGTATCTATTTTCGAAGATTGTCTAAACGCTGCCGTCGACCGTAGTATTGAACTTTCTAATATAGAAG
ACCAATAAAAACAAACCCGCCAACACATGTATGCATCATCGCATACATAGTTGGACGGGTTATTTTTATAAAT
TATTACTATTTGGCAAGCATTGTTTATTACTTTGTCATTAGACTTTAAAACTATCAAAATCTTTTACAAAATT
AAAATTAGGTGTATCTTCATTTTGTATCAATGTTTGATAAATTTCATTTATATCTTCTGTATTATAGCGATTG
CTCAAATGTGTAATCAACGTACGTTTAACATTGGCTTCTTTTATCAATGCAAATACGTCTTCAATATGGCTAT
GATGATAATTGTTGGCTAAATGCTTTTCACCATCTATATAGGTCGCTTCATGTACCATCACATCAGCATCTCT
AGAAATCACACGTTCATTAGAACATGGTTTTGTATCACCAAAAATTGCTACAACTGGACCCTGTTTGGACTCA
CCTCTAAAATCTTTTGATTGATAAACTTGACCATTATGTTCAAATGTATCATGAGATTTTACTTCTTGATATT
TAGGACCTGGTTCAAGACCAATGTTTTTTAACGCTTCAACATTGATTGTACCTGTAGTTTCAGGTGCCATTAC
TCTATATCCATATGATGGAATACCATGATTAAGTAAATGCGCCTCTACAGTAAAACCATCATGATGATATGTC
AGATGATCATCGATTTCAATATATGTAATTGGATAGTTTAAATGTGACTCTGATAAATTCATAGACATTTCCA
CATATGCTTTAATTCCTTTTGGTCCAACCAATGTAAGCGGCTTCTGTTCACCGCCCTGAAAAGAACGACTAGA
AAGTAATCCTGGCAAACCAAAAATATGATCGCCATGCATATGAGTAATAAATATATGTGTCACTTTTCCTAAT
TTAATTGCATGATGTAAAATTTGGTGCTGTGTACCTTCACCAACGTCGAAAAGCCATATGGAATTGGAATATG
GTTCTAAATTTAAGGCGATTGCTTGTGTATTTCTCTCTTTTGTAGGCAAACCTGCACTCGTTCCAAAAAATGT
AACTTCCATATATGCCCCTCCTTTTCTTCAATTCATTTTATCATAAAATTTGTATCAATTTAGTTTTAATTTA
TACTTGAATCAAAATCTGTTAAAAATTCGTCTTTGCTAGTTAATTTTTCTAACTTTAACGTAGACAAAACTAT
ATAAATTTTGATAATTACGTTATACTTATCATTAATAAGTATCACATTAAACATGATACATGAATCGATATTT
CATTTAAGACACTGCATACTGTCGAGCATATTGAATGACCTGCTGAATGGATTATCTTATAATAATAAATCAT
ATATCTAATTAAGAATTGAGGTTTTAATCTTGAGTACTAAAAACAAACACATCCCTTGTTTAATCACAATCTT
TGGTGCAACTGGTGACTTAAGCCATCGTAAGTTGTTTCCATCAATATTCCATCTCTACCAACAAGACAATTTA
GATGAACATATTGCCATCATCGGTATTGGACGTCGTGACATTACTAATGATGATTTCCGTAATCAAGTAAAAT
CATCAATTCAAAAGCACGTAAAAGATACAAACAAAATTGACGCGTTTATGGAACATGTCTTCTATCATAGACA
TGATGTTAGTAATGAAGAAAGCTATCAAGAATTATTAGATTTTAGTAATGAATTAGACAGCCAATTTGAATTA
AAAGGTAATCGACTATTCTATTTAGCAATGGCACCACAATTCTTTGGCGTTATTTCTGATTATCTAAAATCTT
CTGGTCTTACTGATACAAAAGGATTTAAACGCCTTGTTATCGAAAAACCATTCGGTAGTGATTTAAAATCAGC
CGAAGCATTAAACAATCAAATTCGTAAATCATTTAAAGAAGAAGAAATTTATCGTATTGACCACTATTTAGGA
AAAGACATGGTTCAAAATATCGAGGTATTACGTTTTGCGAATGCGATGTTTGAACCATTATGGAATAACAAAT
ATATTTCAAACATCCAAGTTACATCTTCTGAAATACTAGGTGTTGAAGATCGTGGTGGTTATTATGAATCAAG
TGGCGCGCTAAAAGATATGGTGCAAAACCACATGTTACAAATGGTTGCATTATTAGCTATGGAAGCACCTATT
AGTTTAAATAGTGAAGATATCCGTGCTGAGAAAGTAAAAGTACTTAAATCACTGCGTCATTTCCAATCTGAAG
ATGTTAAAAAGAACTTTGTTCGTGGTCAATATGGCGAAGGCTATATCGATGGTAAACAAGTTAAAGCATACCG
TGATGAAGATCGCGTTGCAGATGACTCTAACACACCTACCTTTGTTTCAGGTAAATTAACAATTGATAACTTT
AGATGGGCTGGTGTACCATTCTATATTCGTACTGGTAAACGTATGAAATCTAAAACAATTCAAGTTGTCGTTG
AATTTAAAGAAGTACCAATGAACTTATACTATGAAACTGATAAACTATTAGATTCAAACCTATTAGTAATCAA
TATCCAACCTAATGAAGGTGTATCTTTACATCTAAATGCTAAGAAAAATACACAAGGTATCGAAACTGAACCT
GTTCAATTGTCTTATTCAATGAGCGCTCAAGATAAAATGAATACTGTAGATGCATATGAAAATCTATTATTCG
ATTGTCTTAAAGGTGATGCCACTAACTTCACGCACTGGGAAGAATTAAAATCAACATGGAAATTTGTTGATGC
AATTCAAGATGAATGGAATATGGTTGATCCAGAATTCCCTAACTATGAATCAGGTACTAATGGTCCATTAGAA
AGTGATTTACTACTTGCTCGTGATGGTAACCATTGGTGGGACGATATTCAATAATTGAATTAAAACGCACATG
TTAAACAAAAATAAATGAGCGAATGACAAGGATTGATTACGAATCAAGTAAATCAATTGTTCTATCATTCGCT

TTTTTTCTAGACTATATTATGAAATTATATTTTACAATGCCCAAAACTATTTTAATAATCATTGAACAAATGG
GTGTATAATTTATAGAAATAATGTAGAATAAAAATAAATGATTGAATTAATTGGAGTGAAAGTTTTGGACGTT
ATCAAGCAAATACAACAGGCAATTGTTTATATTGAAGATCGTTTATTAGAGCCTTTCAATTTGCAAGAATTAA
GTGATTACGTTGGTCTTTCGCCATACCATCTTGATCAATCATTTAAAATGATTGTCGGCTTATCTCCAGAAGC
TTATGCACGCGCGCGTAAAATGACACTCGCTGCAAATGATGTGATTAATGGTGCTACACGACTTGTAGATATC
GCTAAAAAATATCACTATGCAAATTCAAATGATTTTGCAAATGATTTTAGTGATTTTCACGGCGTATCACCTA
TTCAAGCTTCTACTAAAAAAGATGAATTACAAATTCAAGAGCGATTATATATCAAATTATCAACTACTGAGAG
AGCACCCTATCCATACAGATTAGAAGAGACAGATGATATTTCATTGGTTGGATATGCACGATTTATAGACACT
AAGTATTTGTCACATCCTTTTAATGTTCCGGATTTTTTAGAAGACTTGCTCATTGATGGTAAAATTAAAGAGT
TACGACGATATAATGACGTTAGTCCATTTGAACTATTTGTTATTAGTTGTCCTCTTGAAAATGGTTTAGAAAT
ATTTGTAGGTGTACCAAGTGAACGTTATCCTGCACACTTAGAAAGCCGATTTTTACCTGGCAAACATTGTGCG
AAATTCAATTTACAAGGTGAAATTGATTATGCAACTAATGAAGCTTGGTACTATATTGAATCAAGTTTGCAGT
TAACATTGCCATATGAACGAAATGATTTATATGTTGAAGTGTACCCTCTCGATATTTCATTTAATGACCCATT
CACTAAAATTCAGCTTTGGATTCCTGTTAAACAGAGTCCTTATGACGAAGATTAAAAATTAAAAACAAAGAAG
CCCCCTAATATATCTATAGGTCTACAAATGGCCTTAGATTCTATTAGGGGGCATATTAATATGTTAATTTAGT
TCGATAACACATGCTTCATATGGACGTAACTGTTTTAAATTAACTTTGGCATCATAATTAAATAGCTTTACTT
CTCCATGGCTTAAATCAAATGGTACAGTTAATTCTGCTTCGTGGTTAGTAAGATTACCTACAATAAGAACTTG
CTTTTCATTTAATGTTCTCGTGTACGCAAAAACTTGTGAATTTTCAGCATCTACTAAATCAAATTGACCATAT
ACGTATACATCATTAGACTTTCTTAATTGAATTAAATCTTTATAAAATTGTAATACTGAATGCTCATCTTCTA
ATTGTTGTGCAACATTGATAGTTTTATAATTCGGATTCACTGGGAACCACGGTTCACCATTTGTAAATCCTCC
ATTTAACGTATCATCCCATTGCATTGGTGTGCGAGAATTATCTCGGTTCTCATCTTTATATTTCGCAAGTAAA
GCGTCTACATCTCCACCTTGAGCTTTCACAATTTGATAGTCATTTTTAACAGCAACATCGTTAAACGTTTCAA
TACTTTCAAATGGATAATTCGTCATACCAATTTCTTGACCTTGATAAATGAATGGCGTACCTTGTTGCAAGAA
ATAAACAGCTGCATGACTTGTTGCTGATTCATACCAATACTTGTCATCGTCACCCCACGTCGATACACGTCGT
GGTTGGTCGTGATTTTCAATAAACAACGCATTCCAACCTTTATTTCAAGTTGTTTTTGCCATCTATTTAATA
CAGATTTATACGAATTTACATCAAAGTGAGAATCACCACTATTCCACAGTCCCAAATGTTCAAATTGGAATAT
CATATTAAATTTACCATTTTCTTCCCCGACCCAGTCATCAGCATCATCAGGGCTTACACCATTCGCTTCACCA
ACAGTCATAATGTCATACTTACTTAATGAGCGATCTTTCATCTCTTGTAACCAAGTTTGTATACCTGGCTGAT
TCATATCTACATCAAATGCTGGGGCATATGTTTTATCCTCAGGTACAGGTAAGTCACCCGCTTCAAACGTCTT
TTTAATATGCGTAATTGCATCTACTCTAAATCCATCAATGCCTTTATCAAACCACCAGTTCATCATTTCAAAT
ACAGCATCTCTAACTTCTGGATTAGCCCAATTCAAATCAGGTTGTTTTTTACTGAATAAATGGAAATAATATT
GCTCAGTATTAGCATCATATTCCCATGTAGATCCATTAAAGATACTTTCCCAGTTGTTAGGTTCAGAGCCATC
TGGCTTTGGATCTTGCCAAATATACCAATCACGTTTGGGATTGTCTTTACTAGATTTAGATTCTATAAACCAA
GGATGTTCATCAGATGTATGATTTACAACTAAATCTAAAATAAGCTTCATGCCTCTATCATGAACACCTTTTA
ATAAACGATCAAAGTCTTCCATCGTTCCAAATTCATCCATAATTTCTTGATAGTCACTAATATCATAACCATT
GTCATCATTAGGTGATTTAAACATTGGACTGAGCCAAATGACATCGATACCTAAATCTTTTAAGTAGTCCAAT
TTATCAATCATTCCAGGTAAATCCCCAATACCATCGTGATTACTATCATTAAAACTTCTTGGATATACTTGAT
ATGCTACTGCTTCTTTCCACCATTGCTTATTCATTTTAAAACTCCTTTGCTATCGCTGTGTTGATTTTCTTAT
TTTTAATTCTGTATCTATAATGACGAGTTCAATAACATCCTGTGCTTTATTTTTTAATATATTTAAAATTGCT
GCACCAGCCTGTTGACCTAACATTCGAGGCTCGATGTCAATACTGGTTTGAGGTGGTGACGCAATTTCGGTTA
AATAAGAATCATTGAACGTTGCTGTCATTACATCTTTCGGAATTTCAATATTAAGTTCATATAGGACACTTAA
AATCGCTAAATGTAACACAGCATCTAACGAAATGATTGCCTGTTTAATATTTGGGTCCTTCAAACGCGTATGT
AGATTTTGCATGTAATTAAAAATAACTTCTCTTTCATTACTAGTCTCAATAATTTGATAATTAATTTTATTTT
GAGAAGCTATCGTTTCAAATCCTTTTATTCTATCTTTTGAAACTTCAAAATTTCCTTTTTCTGTAATAAATAT
TAATTCATCTACACCTTGTTCAATAACATGTCGTGTCAAATTTTCAGAAGCTAATATATTATCATTATCTATG
TGTGTAAATTGATGATCTATATCCGATGTAGGCTTACCAATCACAATAAATGGCATGCTTTCATCAATTAACA
TTTGTTTAATCGGATCATTTTCTTTTGAATAGAGCAGTATAAACGCATCAACCATTCGTTGTTTAATCATTTT
ATAAACTTCATCCATTAAATCATTCAAATTATTTGAGACTGTCGTTTGTGTACCATAGCCATGCTGGTTACAC
GTTTCAGAAATTCCTAGCAATACATTGATGTAGAATGGATTCAGTCGAATAGGCTCCTCAGACCCTTTTAACA
CTAAACCAATTTTATATGTTTGGTTAGTAATTAAAGTTCTAGCAGCAGTATTAGGAAAATAATTCAATTCTTC
CATAACTTTCTTCACTTTTGAAATTGTCGCTTCGCTAATACGTTGATTTCCTTTTATAACCCTTGAAACTGTC
GAAGGAGAAACACCGGCTTTTAGTGCAACATCTTTAATCGTAACCATTTAATCACCTCCTGTTAATCACTGCG
CTGGAAAACGCTTCCAACCATTGTATAATACCAGTTTAGTCACACTTTCTAAAAAAGTCAAAAGATTTGTGCA
AACGATTGCATAAAACGATAAAAATAAAACCTTCATACTGAAATTCAATCCGAAAATCAATATAAAGGTTTGT
ATAAATATTAAAATCGATTGTTTAGTCACTAACTGCAAAATAGTTACCTTGGCCATCTTGGAAATTAAATGCA
CGTTGACCATTCATTTCTACTATATCATGCCCAGTTAAACCTAAATCATTTAATTTTGAGTATAATGCATCAA
AGTTTTTCTCTTTAAACATTAAAGATGGTGTTCCTAGGTTCACCTCTGGACTATACTTTTCAATAAATTCTTT
TGCCATAATCGTCAACGACGTTTCAGCATCTTTCGTAGGTGATACTTCAACTGCAACATAGTCCTCAGCTAAC

GGTGTTTCACTTACAACAACAAATTCTAAAGTTTCTGTCCAAAATGCTTTCGCTTTGTCAACATCATCAACAT
ATAACATAACTTGATTTAACTTTTCCATAAGTTAATACCTCTATTTCTCTATAGTACATGCTATCATAACACA
GTAAATATTTTATTACTTCACAAAATGCTTAAAAATATGTCGGGATGCTTTTAAGGTCAAGGATAATACTTGT
GTAATTTTTTATAGGTTGTAGATACTCTATCACACTCTGTTTTATATTTATCAAAAGATATAAAAAAGGATAG
TATCTTTCAACTATCCTTTAATCAATATTATTCTTCAATCCATTGTGTATGGAATACGCCTTCTTTATCTTTT
CTTTCGTACGTGTGAGCACCGAAGTAGTCACGTTGTGCTTGAATTAAGTTTGCAGGTAAATCAGCTGCACGGT
AACTATCATAGTAATTAATACTTGAAGAGAAACCAGGTGTTGGTACACCATTTTGAACACCAGTTGCGACAAC
ATCACGTAGCGCATCTTGATATTCAGTAACGATGTTTTTAAAGTAAGGATCTAGCAATAAGTTTTGTAATCCT
GGATTATTATCGTAAGCATCTTTGATCTTTTGTAAGAATTGTGCACGGATAATGCAACCTTCTCTCCAAATCA
TAGCTAAATCACCAAGTTTTAAATTCCATTCATTATCTTCACTTGCTTTACGCATTTGCGCGAAACCTTGTGC
ATAAGAACAAATTTTACTCATATATAATGCTTTACGAATTTTTTCTAAAAAGTCTTTCTTGTCACCATCAAAT
GATGCTTTTGGACCATTTAATTCTTTAGAAGCATTTACGCGCTCTTCTTTGATTGAAGAGATAAAACGTGCAA
ATACAGATTCAGTAATGATTGTTAATGGAATACCTAATTCTAATGCGTTAATTGAAGTCCATTTTCCTGTACC
TTTTTGACCTGCAGTATCAAGAATTTTTTCAACTAATGCTTCTTTATTTTCATCTAATTTCATGAAAATATCA
CCAGTGATTTCAATTAAATAACTTTCTAATTCACCAGCATTCCAGTCTTTGAACGTTTGAGCAATGTCTTCAT
GAGACATGCCTAATAATTCTTTCATCATAGCATAACTTTCTGCAATTAATTGCATGTCAGCATATTCGATACC
ATTGTGTACCATTTTCACATAGTGTCCAGCACCATTTGGACCAATATAAGTAACACATGAAGCACCGTCTTTT
GCCTTAGCAGCAATTGCATCAAGAATATCTGCAACTTTGTTATAAGCTTCTTCTTGTCCACCCGGCATTAATG
ACGGACCAGTTAACGCTCCAATTTCACCACCAGAAACGCCCATACCAATAAAGTTGATTGCACTTTGTGCTAA
TGCTTTATTACGTCTGATAGTATCTTGATAGTTTGTATTACCACCATCAATTAAAATATCTCCATCATCTAAT
AAAGGTAACAAACTATCAATCGTTGCATCCGTAGCTTTACCTGCTTGAACCATTAATAAAATTTTACGTGGTT
TTTCTAAAGAATTAACAAATTCTTCCAATGAATACGTTGGATGAATATTTTTCCCTTTTGATTCTTCAACCAT
TAAATCAGTTTTTTCACTTGAGCGGTTAAATACAGATACACTATATCCGCGTGATTCAATATTCCAAGCTAGG
TTTTTACCCATAACGGCTAAACCAATAACTCCAATTTGTTGTGTCATATTACTTACCTCACTTGTTGATTTTT
CATTAGTATTGTATCACAAAATAGACATACACTACACTAAATCATTTCGATTGTCGCGCAACTATTTTGATTA
TTTCTAACACTTGACTTGCAAGCAAGTTCAATGATTTAATCGGCATTCTCTCATTTGTTGTATGGATTTTTTC
ATAACCCACTCCTAAAATGACTGAAGGAATACCAAATGTATTAATAATACTGCCGTCTGAACCGCCACCAGAA
ATAATTGTATTTGCAGATAATCCTAAATTACGAGCACTTTCTTGTGCAATTTTAACAACCGCTTCATTATCAT
TAATTTTAAATCCTGGATAACTTTGCTCCACTGTAACTTCTGCTTTCCCACCTAATTCTGATGCAGTAGTTTC
AAACACATCAGTCATATGTTTGACTTGTGTTTTTATTCTTTCTGGATCGTGAGAACGTGCCTCTGCTTCTAAA
ATGACTTCATCTGCAACAATATTCGTAGCTGAACCGCCATGAAACTTACCAATATTGGCAGTAGTTATTTCAT
CAACTTGTCCTAATTTCATTCGACTAATTGCTTTCGCCGCAATATTAATAGCACTAACACCCTCTTTTGGCGT
ACTTGCATGAGCTGTTTTGCCAATAATTTTAGCTGAAATTAACATTTGCGTCGGTGCACCTACAACCGTAGTG
CCGACATCAGCACTTGCATCAATAGCATAACCAAAGTCCGCGTCCAACAACTCTGAATTTAATTCTTTAGCAC
CAATTAAACCTGATTCTTCTCCAACAGTAATCACAAATTGAATTTGTCCATGTGGGATTTGTTGTTCCTTAAT
CACTTGCAAAACTTCAAGCATCGCTGCTAATCCTGCTTTATCATCTGCACCTAGAATAGTCGTACCATCAGAG
TATATGTAGCCGTCTTCTTTTACAATTGCCTTTACATTAATTGCGGGTACAACAGTATCCATATGGCTCGTCA
AATATAATTTAGGTACTTCGCCTTCTTCGATAGTACTATTCATTGTACACACTAGATTATTGGCACCTAATTT
AGGATGTTTAGCCGCTTCATCTTCTTTAACATCTAACCCTAATGCTATGAATTTTTCTTTTAAAATAGGTTGG
ATTGTTGATTCATTCCCTGTCTCAGAATCGATTTGTACAAGTTCTAAAAACGTATTAAGTAATCTTTGCTCAT
TAATCATAATGGAACCCCTTTTATCAAAATTTTCAAGTACGCCAAACGGTAAAAATTGATGAAATAAAATGTT
ACAGTAATTGACGTTACACAGATTTATCAGGCTTGTAAATTGTGTCATATTATTTTCAATTTAATATATATAT
TTATTGTAACTCAAACTAAGCTTTGTCAAAAATATATTGATTGATTTTTCAATGATATCGTATAATGAGGAAA
ATGACATAAGCAAACTTACTCATGTTTTTTATTATATTCCTTTATGATGATTGCTAGTTATATCGTCTCAAGT
TAAAAGTTTTATATCTTATGTCGTAATTATTAATACAAAGGTTATTCATTTGGAGGCACACAAAATGCAAAAT
AAAGTTTTAAGAATTATCATTATCGTTATGCTTGTATCAGTTGTATTAGCATTGTTATTAACGAGTATCATTC
CAATTTTATAAACTATATCTCAACTACCTATACAAAATCATACAATTAAAAATCCATCCGTTATAAACGCATG
TATTAATAAGTTATCGTATTGCAACGATTACTTTCATACATGGGTCATACGGATGGATTATTTTTTAAGCTAC
TTCACTATGCATTTTCAATGAACCAAATTGCGATTTGATTTGTAAATATTCTTCTAATTCATTTAATATTTGA
ATAATACTTGCTCTCGAGTTAAGCGCTTTGTGCGTTGTTGGCAATGGCAGTTCATCCAATTTCAAACGCGTCT
CATACAAATTGTGTAAACGCATTGCTGTATAGTCATTACTATTCACATTTAGACCAATTTCTTTCAGCAGTGA
CGCAACATCATTTAAAAGTGGATCTTTATGACAGATACTTTCGATGAGCGGTTTCATTCTCATTAACAATTCC
ACTTGCTCTTCTCGCATATCAAAATAATGATAGTATGAATTTTCGTTTCTAACAAAATGATTTTTAACATCTC
GGAACGCGATAGACTTCGCCTTTTTAATATTTAAAAGTAACGCTTCAAATTCAATCGCAATGGTATCTTCATA
TTTTTCACAAATATAACTATATTTACTAAAAATATCAGCAATTTGTTGCTCAATTTTACATTTGTATTCGTCT
AGTTGTTTGTCTAAACTTGGCATCATTAAATTCATTGTAAATGCAATGCTTAGTCCAATTAACAGTAATAATG
TTTCATTAACAATTAAATGTACATCAATTGATTTTGCATTAAAAACATGAAGTAATATAACGCAACTCGTAAT
GACACCTTCTTGTACTTTTAATACGACAGTTAATGGTATAAATAACAATACGATAATACCGAGTACAATTGGA

CTCTGGCCTAATAAACTAAATATTGCTGAACCTAAAAACAATACTAAAAAACATGATACTAATCTTGAAATAA
TCGCTTGTAGCGAATGTACTTTTGTATGTTTAATACATAATACGACTAATATGGCGCTTGAAGCATAATTATC
TAAACCTAACAGCTTACTAATAATTACACCTAAAGTCATACCCACTGCTGTTTTTATTGTTCTAAATCCAATC
TTGTAAGGATTTAACTTTAACATGGGTTAGCGCCTCTTATCTTTCTTCACAATATTTATTGAATAATGTTTGT
AATTGATTAATTACGTTCATCACATCATGACCTTCGATTTGATGTCTTTCAATCATTTCTGTAATCTTTCCAT
CTTTTACTAATGCAAATGACGGACTTGAAGGCGCATAACCTTCGAAGTATTCACGCGCTCTTTGTGTCGCTTC
TTTATCTTGTCCAGCAAATACTGTCACTAGACGATCAGGTAATACGTCATAATGTAAAGCATGTGATGCTGCT
GGTCTTGCGATACCACCTGCACATCCACATACAGAATTGATCATAACTAGTGTTGTACCATCTTGTTTAAGAA
CTTTGTCAACATCTTCTGCAGTAGTTAATTGCTCATATCCCGCAGATTCAATTTCATTCCTTGCTTGTTCTAC
AACACCGTTCATGTATAAATCGAAATTCATGTCCATAAGTTCAATCACCTATCCCTTTATATTTAAACTATCC
TCATTCTACTAATTAATAACATATTGTTCAATAAACTAATCTGAATCACACCTATATTTAGACACAATTTTAA
CAATATACCAAACATTATTGTGCTTAAAATCATGGAAACTAATTTGTTCACCTGTTTTCATTAATATGTTTCA
AGTATGATGTCTTATTTTGACTTTACTGCAAAAATTCATTCAACCATGTTGATTATTGTTCTTTATCTTTTTT
TGAATATATTGCACATATTTTATTGCCCAAAAAAATACATCCATCGACAAGAACAAGATAAAACAAGTTGTCG
ATAGATGCATCTATGTTATCACTAATATATATTTGTATTTTCTAAAGTATACTGTTCGATACGCTGTTTAATA
TGATTCATAAATTTACCTGTTTGTAAACCATCTAAAATACGATGATCAATTGAAATACATAAATTAACCATGT
TACGAATTGCAATCATATCATTAATTACTACTGGCTTTTTAACGATTGATTCTACTTGTAAAATCGCTGCTTG
AGGGTGATTTATAATACCCATTGATGATACAGAACCAAACGTACCAGTATTATTTACCGTAAATGTACCGCCC
TGCATATCTTCAGCTGTCAATTGCTTATTACGCGCTTTTGTTGCTAAAGTATTAATTTCTCTAGCTATACCTT
TGATTGACTTTTCGTCTGCATGCTTAATCACAGGTACGTATAATTTATTTTCATCAGCAACAGCAATTGAAAT
ATTAATGTCTTTATGTAAGACAATTTCATTTCCTTGCCAGCTACTATTTAATAAAGGATATGCTTTTAAAGCA
TCTGCTACAGCTTTTACAAAGAAAGCAAAAAACGTTAGATTATATCCTTCTTTATTTTTAAAGCTGTTTTTAT
AATGATTTCTCGTATTCACAAGATTTGTAGCATCTACTTCAATCATCATCCATGCATGTGGAATCTCTGTTAC
ACTATTAACCATATTTTGCGCAATTGCTTTACGCACACCATTTACTGGTATTGTGCTGTTTTCACTATTGTCT
TCAGATGATTGGTTACTTGATGTATCTACTGATGTTGATTGTGTTTGAACTTGTTTGTCAGATTGAGCTGTGG
TACCACCATTTTCAATAACTGACATTATATCCTTCTTAGTTACACGACCTTCAAATCCACTACCTACAACTTG
TGATAAATCAATGTCATGCTCTGAAGCGAGTTTAAATACAACAGGTGAAAAGCGACCATTATTACGTGGTTGA
TTTTGTTTAGCAGTAGATGTCTGTTCCACTGTTGCACTAGCTTTTTTAGTAGATTTCTGAGTATGCTCATCCA
CATTTGCTTGTATCTCTTCAGTTGTTTCATTTGTCTTTTCATCAGCAGTTTCAATTTTACAGATAATTGTATC
AATAGCTACTGTCTGCCCCGCTTCAACTAAAATTTCTGTAATTGTTCCTGATATCGTGGAAGGGACTTCAGCT
GTCACTTTATCTGTAATAACTTCACATAATGGTTCATATTCATCAATATGATCACCAACAGAAACTAACCATT
GTTCAATGGTGCCTTCATGAACACTCTCACCTAACTTAGGCATTGTTATTTCCATGACTTTCCCTCCCTAGAA
TTCTGCTAATTCACGCATTTTATTTAAGATTTTTTCTGGATTCATCATAATTTCATTTTCTAATACAGGAGAA
AATGGCATAGATGGTACATCTGGAGCAGCTAAACGCATGATTGGTGCATCTAAATCGAACAAGCAATGCTCTG
CAATAATCGCTGACACTTCTGACATAATGCTACCTTCTAAATTATCTTCAGTTACAAGTAAAACTTTACCTGT
ATGTTTAGCACGATCAATAATTGTTTCTTTATCTAATGGATAAACAGTTCGTAAATCTACGACTTCAACATTG
ATACCGTCTGCAGCTAAAATATCAGCGGCTTGTAAGCAATAATTGACCATTAATCCATAACAAAATACTGTTA
AATCTTCACCTTCACGTTTAACATCTGCTTTTCCTAAAGGTACAGTGTAATATTCTTCTGGCACTTCTTCCTT
TAAGAAACGATAAGCTTTTTTATGCTCAAAGTATAAAACAGGATCATTTGATTCAATTGAAGATAATAATAGA
CCTTTAGCATCATACGGTGTGGAAGGAATAACAATTGTTAAACCTGGTGATGAAGCAAATATACTTTCAATAC
TTTGTGAATGATATAGTCCTCCGTGAACACCGCCACCAAATGGTGCACGAATCGTTAATGGGCATTGCCAATC
ATTATTTGAACGATAACGCATTTTCGCAGCTTCACTAATAATTTGATTTGTCGCAGGTAAAATAAAATCTGCA
AATTGAATTTCTGCAATTGGTCTTTTACCTACCATAGCTGCACCAATGGCAGTTCCAACAATATTTGACTCAG
CTAATGGCGTATCGATAACTCTGTCTTCACCATATTTTTGTTGCAGTCCTTGAGTAGTACCAAATACGCCACC
TTTTCTACCAACATCTTCACCAAGAATAAACACATCTTTATTTTGTTGTAATGCTAAGTCTTGTGCCTGGCGT
ATCGCCTCTAAATAAGATAATTTAGCCATTAGTTAAGACTCCCTTCTTCGTACACAAATGCATAGGCTTCTTC
GACACTTGGATATGGCGCGTCTTCAGCAGCCTTTGTCGCTTTATTGATGATGTCTTTATGCTCCGCTTCTATT
TCTGCCAACCAAGCATCATCGATAATGCCAGCTGAAAGCAACTCTTTTTTGAACTTTTCATTGCAGTCTGCTT
TTTTAAGCGCTTCACGCTCTTCTTTCGTACGATATTGGTCGTCATCATCTGATGAATGAGCTGTCATACGACT
TGTTACTGCTTCAATCAAAGTTGAACCTTGACCAGAAATAGCTCGATCTCTTGCTTCTTTCATCGCTTTATAC
ATTGCTAATGGATCATTACCATCTACTTGTTCACCATGTATACCGTAACCAAGTGCTCTATCCGATAATTTTT
CAGCTGCGTATTGTAATGAATCAGGTACTGAAATTGCATATTTATTATTTATAATGACACATACAAAAGGAAG
TTTGTGTACACCCGCGAAGTTTAAACCTTCATGGAAGTCACCTTGGTTTGAGCTACCTTCACCAACAGTTGCT
GTTGCAATTTTCTTCTTACCATCCATTTTTATAGCTAAAGCAGCACCAACAGCATGGGGTATTTGAGTTGCTA
CCGGTGAACTTTGTGACAAAATATTCTTAGCTCTACTACTAAAGTGTGATGGCATTTGTTTTCCACCAGAGTT
AACATCGTCTTTCTTTCCAAACGCTGATAAAAACGTATCATACGCTGATATACCCATATAAGTAACGAAAGCT
AGATCTCTATAATAAGGCGCAGTAATATCACCTTCTTCTAATGCGTATGCCATCCCAATCTGAGTTGCTTCTT
GTCCTTGACCACTTACAACAAATGGAATTTTACCTGCACGGTTCAATAACCACAGTCTTTCATCTATTTTTCT

ACCTAAATCCATCCATTTATATATTACTTTTAGGTCTTCTTCGCTAAGGCCTAATGATTTATAATCAATCATG
TTAAATCCTCCTATTTATACGTGAATAGCTCTACTTTCTGCTTTCAATCCTAATTCCATCAACACTTCAGAGA
TGGAAGGATGTGCGTGTGTTGTTAGTCCTAATTCTAATGCCGAGCCATTCATGAACTGTAACAGTGATGCCTC
ATTAATCAATTCTGTTACATGTGGACCAATCATATTAATACCCACAATTTCTTCAGTTGATTGATCAATCACC
ATTTCGCTATACCCTTCGTTTGCATCATGGCTATCAATCACTGCTTTACCAATTGCTTTAAATGGTACTTTAA
AACTTTTAACTTTCATTCCCTCTGCCTTTGCTTGCTCAATGTTTAAACCGATAGAAGCAATTTCAGGTTGTGA
ATAAATACACTTAGGCATCATGTTATAGTTTACTGGGATTGGGTTCCCTTCAAACATATGATCGACGGCCACA
ACACCTTCTTTTGATCCAACATGTGCCAATTGTAATTTTCCAATACAATCACCAGCTGCATAAATATGTTTAT
CTTCAGTTTGTTGAAATTCGTTCGTTAAAATATGTCCTGATATTGAAAGTTTTATTTTAGTGTTGTTTAAACC
AATATCTGATGTGTTAGGTTTTCTACCAATCGATAGCAACACTTTATCTACTTTAATTATGTCTGAGGAAATT
TCAAATGTAACACCATCTTCGTTAACATTTATATCATTTTCAGAAAGTTTTATTCCCTCATAGAATTTAACAC
CACGTGCTGATAATGATTTTTTTAATAGTTGTGAAGCTTGTTTACTTTCAGTTGGTAAAATTCTTTCACCTGC
TTCTATAACTGTTACGTCAACACCTAAATCTATCATCAATGATGCAAATTCCATTCCGATAACACCACCACCA
ATAATACCAATACTTGATGGTAACGTCTTTAATGATAATATATCATCGCTAGATAAAATTTTATCATGATCAA
ATGATAAAAATGGCAACTCTGCAGGCGAAGAACCAGTTGCAATTAATACAAATTGGTTGGGTAATAAGTCTGA
TTCACCATCTTCATATTCGACAGAAATTGTGCCACTTTGAGGTGAAAATATAGATGTACCTAGAATACGTCCC
GTGCCATTATAAATGTCAATGTGATTGTGTTGCATTAAATGCTTTACACCTTGATACATTTGATTAATAATGT
CTTCTTTTCGTGCCAACATATTTTCAAAATTAACATTAGCATCTTTGACATCAACGCCAAACATTGCTGCCTG
TTTTACTGTTTGAAATACTTCTGCTGACTTAAGCAGTGATTTAGTAGGAATACAACCTTTATGGAGACAAGTA
CCTCCTAATAGTTGTCGTTCTACTATTGCCACTTTCTTACCTAATTGAGACGCACGTATCGCAGCAACATATC
CTGCAGTACCTCCACCGAGAACGACTAAATCATATTGTTTCTCTGACATGTTCTTACTCCTAACTAATGATAT
ATATCCATTGAAAATTTATTAATACATAGTTTTCATGTCCATTAATTACCTATTTTACATGATTGTCTATTTA
GTTTGAATGCACATAAATAAGTCCACAAATGAGTATTCAACACACTTTGGAAATTATCTACGTCTTTGATTGT
GTTGTATCATTTCTCTTGCATTTTCTCGAGTTAAATCAGTAACACTTGCACCTGAAATCATTCGTGCAATTTC
ATCTACTTTATCATCGCCAATTAACTCTTGAACTTGTGTTGTTGTACGATCATCTTTTGATGATTTCGAAATT
AATAAATGATGGTCGCTCATCGATGCAACTTGTGGCAAGTGAGAAATACAAATAACTTGTATATATTCTGCTA
TATCTCGCATTTTCTCTGCCATTTTTTGTGCAGCTTGTCCAGATACACCTGAGTCAACCTCATCGAATAAAAT
TGCAGTTTGACCTCTCGATTTAACAAAAATACTTTTTAACGCTAACATAATTCTAGAAAGTTCTCCACCTGAC
GCAATTTTATTTAAACTTTTTAATGGTTCCCCTTTATTAGGACTGATTAAAAATTCTACAAATTCGATTCCAT
CAATATTCGGTTCTTCTAATTTTTTAAATGAAATCTCAAGATTTGCATCTTTCATTTGTAAGTTTTGAATTTC
CGATACAATGTGGTCTCTTAACTCCCTAGCGACTATACGACGCTGCTTTGATAGCGCTTGTCCAACCTCTATA
ACTTGATTATACAATGCATTTATTTCTTCTCGTAACTGCGATGTACTTTGTTCATAGTTTTCAATTTTATTGA
TTTCATTATTAAGTTTTTCTTGATATGAGATTAATTCTGAAATATCTTTTCCATATTTGCGTTTTAAATTATT
CAGCAAATTCATACGAGATTCATACTCGTTTAACACTTGTTCGTCGAATTCTGTATTAGCCATTTCATCATAT
AACTCATGTTTTGCATCTTCTAAAATGTAATAAAATTGATCAATATCTTCTTTTAATTTGTCATATTTGTTTG
GAACTATATCGTTTATTGTTAACAAATGGTTGCTTAGTTCATACAAACGATCAGTGATAGCATTTTCATCCGT
TAATGTCATATGTGCGTTATTAAGTGCTAAGCTTAATTTTTCAGAGTTTTGAATGCGTTTAATATCAATTTCA
AGTTGCTCTATTTCACCTTCTTTTAGGTGTGCTTCAGACAATTCTTCTAATTGAAATTTCATTAAATCTAAAC
GCTGCAGCAATGCTTGGTCTGCTGATTCTAAATCTTCTAACTCTTGCTTTTTGGCTTTATAATTTTGAAAAGT
TTGGTGATATTTATCCAACAAATCTTGATAACGTGATTCTGCGTAATTATCCAATAATGTTAAATGGTATTTT
TGTTTCAACAAAGACTGCGTTTCATGTTGGCCATGAATATCTAATAATTCTTGCATAACTTTTCGTAAATCTT
GTAAAGTAACTGTTTGATTATTAATTTTACAAAGACTTTTACCAGAGCTAAAAATTTCCCGTTTAACTAATAA
AAAATCTTCATCTACATCAATATCCATATTTTTCAATATATGTATAGCATCTTTACTCTCGTCAATATCAAAT
ATACCTTCGATGACAGCCTTTTTTTCACCATGTCTTACAAAATCAGATGAAGCTCTCATTCCAATTAATTGTC
CAATTGCATCTATAATAATTGACTTACCTGAACCCGTTTCACCACTTAAAACAGTTAAACCATCAGAAAATTG
AATTTCTAACTCTTCAATAATAGCAAATTGCTTGATTGATAAGGTTTGTAACATAAACTCATCGCATCCTTAT
AACAAATTGAAAATTCTTGACTTGATTTCATCACTTGCCTCTTTGCTTCGACAAATAATTAAACAAGTATCAT
CACCACAAATTGTGCCTAGTACTTCTTCCCAATTGATTTGGTCTAATATAGCTCCAATAGATTGTGCATTACC
AGGTAATGTTTTTAGAACAAGTAAATTATCAGTACCATCTATATTAACAAAGGAATCCATTAAATAACGTCCC
AATTTTTCTAAAGGATGGAATTTTCTATCATTTGGTAAACTATAAACATATTGACCTGAAGGTATAGGTACTT
TAATAAGTTGTAGTTCTTTAATATCACGAGAAACAGTTGCTTGAGTGACATTTAAATCATAATCGTTTAATCG
TTTAACTAATTCATCTTGTGTCTCTATCTGTTCATTTGAAATAATTTCTCTAATTTTTATATGCCTAACCGAT
TTTTTGGGCACAGTAAACACCTCTGATTACGAATATTTATACATTTATTTTAACACATGCACTGATTTACGAC
TACTAAACACCTTTACGTAAAAAGGGTAAACATGGTTTATCTATCTTGGTTATCTATTTATAAATATTTTTCA
TATTACGCATAACAATTGCTTAAAATATGTATAAAAATGAATATATGTGTAATAAACTTGCTAATTATTAGAT
TTAATAAGCGTCAATTGTTTGAACATATTTAATTAAAATCACATTGATATCACAGATGCGAATATTGTCGTAT
AGAAATTGAAAATTCTATTTTTAAATGAAAGACTTCAACATAATTTTAAGTTTCAACATGAGAAAAATCGATT
AACAAACAACGTCAGTTGAATATGCCTTTTGAGACATTTCAACTTTACAATTGTTGCTAATCGATATATTTG

CTTTTAGTGATCCCTGCTATAAAATAAATCAACGATTTCTAATAAGTGTTTTGTATTGAATTGTTCATCAATT
TGCGTTAGTTCATACACTGCTGCGTCTCTATGATAAGTCAATTTATCTTCTGCGCCATCTTTTCCTAATAAAC
TCACGTACGTACTTTTATTATTTTCAAGATCGCTGCCCACTTTTTTACCTAACTTCGCTTCATCACCATAGCA
GTCTAATAAATCATCTTTAATCTGAAACATCATACCTAAATGATAACTATAACTTTCTAAATGTTCTTTAGTT
GCATCATCGACATTAGCGATATCTGCTGCACTCATAACCGCAAAAGTTAATAGTGCACCTGTTTTTGTTTTGT
GTATCATTTCCAAAGTTTCAAGATCAATTGGTTGGCCTTCGCTTTGCATATCTAACATTTGTCCACCGACCAT
TCCAACATGACCACTTGCTATTGACAGCCGTTGTAGAACTTTTATTTTTACTTCATCAGTTAATCTATCATCA
CTTGAAATAAGTTCAAATGCTTTAGTTAATAAAGCATCACCTGCTAATATCGCAGTCCACTCACCATATACTT
TATGATTTGTTAATTTCCCTCGTCGATAATCATCATTATCCATCGCTGGTAGGTCATCATGAATAAGTGAATA
TGTATGAATCATTTCTAGTGCAATTGCGCTCTTCATACCTAACTCATACTCGGTATTTAGTGAATCTAAAGTG
AGTAATAACAGAACTGGTCGGATGCGTTTACCTCCAGCATTTAATGAATACAACATACTTTCTTCTAGCTGAG
TATCCATTACTGATTTATTTATCGCAACCGATAATTCATTATTGACTTCATCTATTAATTTATTCATCGGTAG
ATTCGTCATTTTTTACATCCTCAGCTTCTTCTTTTATTAAGTCATTCACCTTTTTTTCGGCATTTTTTAAAGT
TGTGTCACAAGCTGCTGATAGTTTCATACCACGTTGGTATAAATCTAATGATTCCTCTAAAGATACTGTTTCA
TTATCTAATTTTTGAACAATTTGCTCTAATTCTTGCATCATTTCTTCAAAACTTTGCGTTTCTTTAGTCATTA
TTACACCTTACTTTCGTAACTTTTGCATCTACTAAGCCATCTTTCATTGTTAACGTCAATTGATCATTTTCTG
TTAAATCTTTAGTACTCGTAATGACTTCGTCTTTTTATTAACAATTGCATATCCACGCAACATTGTATTAGT
TGGACTTAAATTGTTTAAGTTTTCTACTTTATTTTTCAAATCATTTTTATAACTTAATATCTTAGAATTCAAT
AATTTAACAAGTTGGTTTGTCAATTGAAGATTATTTTGTTGTTCTTGATTAACACTACTTAGTAACGCTTTTA
AATTATAGCGTTGTTGCAACAGCATTAATCGATGTCTCTGTTGTTCAAAAGTTGCCTGAATTTGTTGTTTCAG
TCTCTTTTCTAAATCATCTCGACGTTGTATCTGTTGATCATACAATAAAGTTGGTTGTTTAAACTTGTAATAC
GATGACAAATGTTCAACATGTTTACGTTGTTGTTCTAAATGTTTCTTGATGAAACGAGTCAATGTAAACTGAT
ATTGCTGTATTTGTTGCAGCAATTCATATTGGTCTGGTGTTGCAATAACAGCAGCTTGAGTTGGAGTCGCAGC
TCTGATGTCTGCAGCAAAATCACTTAATGTAAAGTCTGTTTCATGACCTACTGCTGATATAATCGGTGTCTTA
CAATTATATATTGCACGGACGACAGCTTCTTCGTTAAAATTCCATAAATCTTCTATGGATCCACCGCCTCGAC
CTACAATAATGGTATCTACACCTAAACTATCTGCATATTCAATTTTTTCAATAATGTCGTCCTTTGCTTTTTC
ACCTTGAACCAAAGTACTAATTTGTATTTGTTCAGCTAATGGGAAACGACTATTTATCGTTGAATGGATATCT
CGAATTGCGGCACCTGTACTCGCTGTTAAAACTGCAATTTTTTTTGGAAACTTAGGTATTGATTTCTTATTCG
CTTTATCAAAACAACCTTCTTCAGTTAATTTTTTCTTTAATGCTTCTAATTTTTGATATAAGTTCCCTATACC
ATCTAATTGCATTTTATTTACATAAATTTGATAGTTTCCACGACGTTCAAAAACAGAAACACGTGCTTCTAAT
AAGACTTCATCTCCTTCTTTAGGTTCGAAGTTTAATTTAGAAGCACTACCTTTGAACATCATGGCACTTATAA
CGCTTTCTTTATCTTTCACATTAAAGTATAAATGACCACTTGAATGCTTTTTGAAATTTGAAAGCTCACCTTT
AATCAATACAGATTGGAGATGTGGATCTTGATCAAATTTATATTTAATATATTTCGTTAAAGCTGAAACACTT
AAATAATCTGACATATAACATCACTCAATTTTATTTTTTTATATTACTCAATACACCATTTATAAATTTATAA
TGATCATCATCACTGAATTGTTTTGTTAATTCAACTGCTTCATTCATTACAACTTTAGCAGGTGTATCACTGT
GTAATATTTCATATGTTGCCATTCTTAAAATAATACGATCCGTTTTTAATAAACGTGCAATAGTCCAATCTTT
TAAATAAGGACTAATTGTCTCGTCTAATACAGGTTCGTGATCTTTAACGCCAGAAACTAGCCAATGAATAAAT
TCGAAGTCTAAATCTGGATTATCGTCTTTAATAAAGCTTATCGCTTCATTTATCGTTAAATCACTGTCCTTCA
TTTCTAATTGAAATAAAGTTTGAAAAGCTTGCACTCGGGATTCTTTACGACTCATTTTTAACTCCTTCAAACG
TTTGTATTTTTCTTTATTTAATTACTGAATTAGGTATGACATTACTTTTCAATAACGATTTGTGTAATGTGAA
TATTAATTTGCTTAGGTTCTATCGCTGTCATATTAGAAATTGAATTAAAAATTGACGTTTGAATTTTGTTTGC
AGTTTTTGAAATATTAACACCATGTTTTAATGCACAATATACATCTATATATATGCCATCTTCTTTACTCTCG
ATTTTTAAATCACGGCTTAAATTTTTACGACTAACTTTTTCTAAATTTGTTTCTTTTAATTCAGCAAAATGGC
CAGTGATGCCTTCGACTTCCGAAGTAGCTATACTTGCAATAACAGATAGCACTTCTGGCGCTATTTCTACTTT
ACCTAATTTTGAATTTGAATAATCAGTTACTTTGACCATGGATTGACCTCCTATTAACCTTCATCATTCATAA
TGCTATTTTGCTCTAAAAAGTTTGTATTAAATTTACCGCTTCTAAATATATCGTTATTCAATAATTTAATATG
GAATGGAATAGTTGTATCAATACCAAGAACCACAAATTCACTTAATGCACGAATGCCAGCCATAATCGCTTCA
TCTCGTGTCGGTTCATGTATGATTAATTTCGCTACCATCGAATCATAATATGGCGGTATCGTATAATTAGTAT
AACATGCTGACTCTATTCGAACACCATATCCACCTGGTGCAAGATATTGCTCAATTTTACCTGGCGATGGCAT
AAAGTTCTTGTAAGGATTTTCAGCATTAATTCTAAATTCAATTGCGTGTCCTGTTAATTTAATATCTTCTTGT
TTATATGGTAACACTTCACCCATAGCAACTTGTAATTGTAATTTAACTAAATCAATTCCTGTTACCATTTCAG
TTACAGGATGTTCTACTTGAATACGTGTATTCATTTCCATAAAATAAAATTTATTATCATTTAAATCATATAT
AAACTCAATTGTTCCCGCATTTTCATAATTTACAGCTTTCGCTGCACGCACTGCGGCATTTCCCATTTCACGA
CGTGTTTCATCATCTAAAATTGGGGAAGGTGCTTCTTCCACTAATTTCTGCATACGTCTTTGAATTGTACAAT
CACGTTCTCCTAAATGAATTACATTACCATAGCTGTCCCCAACAATTTGGATTTCAATATGGCGGAAGTTTTC
GATGAATTTCTCCATATAAAGTCCACCATTACCAAATGCAGTTTGAGCTTCTTGTTCTGTCATTCGGAAGCCA
GTTTCAAGTTCTTTTTCATCACGAGCAACACGGATACCTTTTCCGCCACCGCCAGCAGTAGCTTTAATGATGA
CCGGATAGCCAATTTTTTGGGCGATTTTCTTAGCTTCTGAGACGTCTTTCATTAAACCGTCACTACCAGGAAC

AACTGGAACATTGGCTTTGATCATTTCTGCCTTAGCAACATCTTTGATACCCATTTTTTGGATAGATTGATAA
CTTGGTCCAATGAACTTCAATTGGCATGCTTCGCATAATTCTGCAAAGTCAGCATTTTCAGCTAAAAAGCCAT
AACCCGGATGAACGCCATCACAACCTGTAGAAGTTGCAATAGATAAGATGTTCGGAATATTTAAATATGAATC
TTTAGACAAAGTGGGACCTACGCAATATGCTTCATCAGCAATTTGAGTATGTAGCGCATCTTTATCCCCTTCA
GAATAGATTGCAACAGTTTGGATGCCTAAATCACGACAAGCGCGAATAATCCTAACTGCGATTTCACCGCGGT
TTGCAATTAAAACCTTTTTCATTATTTCACCTTAAATAACGGTTGGCCATACTCTACCATTTGTCCGTCTTCT
ACTAAGATTTCAACAATTTCACCTGAAATTTCTGCTTGAATTTCATTAAATAGTTTCATTGCCTCTAAAATAC
ACACTGTTGTTTCATTTGAAACAGTGTCCCCAACTTGCACATATGCTTCTTCGTCTGGAGAAGGCGATTTGTA
AAATGTACCTACCATAGGTGCATTAATTGTTTTGTGATTATCTGAAGTTGGCTTTGGAGCTTCAGTTTTATTG
CTATCAGTTGATTGTACTTGAGGCATAGGCATTGCCGCAGCTTCAACTGGCATTTGTGAAATTTGTGGCGTGA
TAATCTCAGTTTCTTTTTCTTTCTTAAGCGTCACTTTGCCTTTAGTATCTTCAATATTGATTTCCGTTAAAGT
TGATTTATCCAGAATTTCAATTAATTCTTTGATTTCTTTAAAGTTCATTATTACTGACTCCTTCAGTTTGTTT
TCATCTACCCGTCTATTTTACTTGAGACAACTCTTCAATTCAAGCATGTTCATATTGCTGGCGACATTATAAG
TCTATCCCAAAGTTATAATAAAAACACATTTTAAATTAAAAACACTTGTGTATTTATTACTTAACATTGACTC
ATCTTAACTCTTGATTAAAAATTTTATGCATTCAGTTAAACATATCAATGTTATTTTTATTAGCTATTATGTT
TAATCCTCAACAATATGATATGTAGATATATACCTTTACTCATGTCTATGTTTTTTCACTACCAATTAGACGA
TTCGTTTTATATTTTAAAAATTATTTTAAAAATAAAAAGCCTCATATCAGCATATAACCTATAATCATTTTAG
GCAATAGCTTTATGAGACTTATTTGGAATATCTTACGTTAAATCAGTATAGTGTATACAAGCTATTTGTTAAC
AAATTAGAGATTATCCTCTTGAAATGTAGCTTCCATCACCAGTGTTGATAATTAAAACGTCACCTTCGTTTAC
AAATAAAGGTACATTTAATGTATAACCTGTTTCAACAGTTGCCGATTTAGTAGCACCAGTTGCAGTATCACCT
TTAATACCAGGTTCTGTTTCAGTTACTGTTAATTCAACAGTTTTAGGTAATTCAACACCGATAGTTTCACCTT
CGTATGTTTGAATTTGTACTTCCATACCTTCTTTTAAGTAATTCAATTCTTCTTTTAAGTAATCACTTGAAAG
TTCTGTTTGTTCAAAGCTTTCATTATCCATAAATACATGATTATCTCCGTCAGCATATAAATATTGCATGCGA
CGATTTTCAATCATTGCTGGTTCAACTTTTTCACCAGCTCTAAACGTTTTCTCTTGAATTGCACCAGTTCTTA
AATTACGTAATTTTGAACGAACGAATGCTGAACCTTTACCAGGCTTTACATGTTGGAAGTCTATAACTTTCCA
AATAGCGTTATCAACAGAAATTGTTAAACCTGTTTTAAAATCATTAACCGAAATCATTCAGTTTCCTCCTCAT
TTTACACGCTTATGTTAAAACTATAAGGTCTTTTGTGCATTTAGTAAAGACTTGACAACCATTTTCTGTAATT
AAAATATCATCTTCTATTCTTATACCGCCCAAACCTTCTATATAAACACCAGGTTCTACTGTAACACAGTTGT
TAACTTGAAGTTTATCTTGTATCGTACGAGCCAGCATTGGCCCTTCATGGATTTCTAAACCAATACCATGTCC
TAGTGAATGTCCAAATTCTTTTCCATACCCTTTTGACTCTAAATAGTTTCTTGAAATGGCATCAGCTTCTGCA
CCAGTCATGCCAGGTCTAATCTCATTAATTGCTTTCATTTGAGATTCAAGTACTATTTGATATATTTCTTTCA
GTTTAGGATCTGGTTCTCCAATAGCAAATGTTCTAGTAATATCTGAACAATAGCCGTTATAATACGCGCCAAA
ATCTAATGTAATCATGTCGCCTTTTTCAATAATTTTATCACTTGCAACACCATGTGGTAATGCACCTCTATGA
CCAGATGCTACAATCGTATCGAATGATGGTCCATCTGCTCCTAATTCTAGCATTTTACTTTCTAATATTGCCT
TTAATTCTTTTTCAGTCATACCTGCTTTTACAACAGTTAAAATATATTCATATGTTTCATCAACAATATTAGC
TGCTTTTTGAATTAAAGCAATTTCGTCAGCATCTTTGACGTCTCTAATTTTATCTACAGTATTAGAAATGCTT
ATTAATGATATACGGCTTTTATTTAATTCAAGGTATGTATCATAACTTACATGGTGCCCCTCAAAACCTACAT
TTTCAAAATTTTCTTGGTGTAGCAATTCTTTAATCTCACCAATAATAGTAGATTTACGATTAATAATTTCATA
ATTTGGCGCCTGCTTAGTTGCTTGATCAATATATCTAAAGTCTGTTATCAAATATTGTTTATCTTTAGATATG
ATTAGCGCACCGCTAGTCCCAGTAAATCCTGACAAATATCTTCTATTGTAATCTGAAAGTATGATTATTGCAT
CTAAATGTTTTTGTTCTAAAATACGATGTACTTGTGTTATTCTGCTCATCTTATGACCTCCTAGTGAAACGTT
TGTGCATTTCAACTTTATACATTAAAATAATATCATAATAAGGATAAAAAATAATAGATATTGATTTTAGGGA
GATAGTAATGAAAAAATTGGTTTCAATTGTTGGCGCAACATTATTGTTAGCTGGATGTGGATCACAAAATTTA
GCACCATTAGAAGAAAAAACAACAGATTTAAGAGAAGATAATCATCAACTCAAACTAGATATTCAAGAACTTA
ATCAACAAATTAGTGATTCTAAATCTAAAATTAAAGGGCTTGAAAAGGATAAAGAAAACAGTAAAAAAACTGC
ATCTAATAATACGAAAATTAAATTGATGAATGTTACATCAACATACTACGACAAAGTTGCTAAAGCTTTGAAA
TCCTATAACGATATTGAGAAAGATGTAAGTAAAAACAAAGGCGATAAGAATGTTCAATCGAAATTAAATCAAA
TTTCTAATGATATTCAAAGTGCTCACACTTCATACAAAGATGCTATCGATGGTTTATCACTTAGTGATGATGA
TAAAAAAACGTCTAAAAATATCGATAAATTAAACTCTGATTTGAATCATGCATTTGATGATATTAAAAATGGC
TATCAAAATAAAGATAAAAAACAACTTACAAAAGGACAACAAGCGTTGTCAAAATTAAACTTAAATGCAAAAT
CATGATAGGAGTCTGTTAATGCGTAATATAATATTTTATCTTGTACTTATTATTGCTGCGATTGGATTAGTAA
TGAATCTAGATGCCTTTATTTTTTCAATCGTCAGAATGTTAGTCAGCTTTGCTGTAATAGCTGGTATTATTTA
TCTTATATATTATTTCTTCATTTTAACTGAAGATCAACGTAAATACCGTAAAGCAATGCGTAAGTATAAAAGA
AATCAAAGAAGAAAATAGATAAAAAAATGGAAGCACTTGTAGGTAAAATAGTCTACGTGCTTCCATTTTTCAT
TCTAAAAACTACTTTCTAAACATCCATTCATCTGAACGATATTTTTCAGTTAATTCTTCCACTTCTGCCAATT
GAGCTTCTGTTAATTCAAGTGGCTTTAATTCTATATTTAAACCTTTCTTAAAACCTTTCTCGAAAGCTTCTTC
CATTTGACTAATAGTAATGTGTTTATCTGAAATATCATTGATGGCAACTGCTTTTTCAACGAATGCCTCTTTC
ATTTTTAATTTTAATCTTTCATTTTTATAAATAAACATATCAAACAGTTCATCAATATCAATATCTTGTAAAA

TCGAACCGTGTTGGAGGATTACGCCCTTTTGTCTCGTTTGAGCACTCCCAGCAATCTTACGGCCTTCAACAAC
TAGCTCATACCAACTTGGTGCATCAAAACACACTGAACTTCGAGGTTGTTTTAATTTTTGACGCTCTTCAGGC
GTTTTAGGTACCGCAAAATAAGTATCAAATCCTAAGTTTTTAAATCCTTCTAATAATCCTTGTGAAATCACTC
TGTACGCTTCTGTAACTGTAGAAGGCATATTCGGATGCGATTCAGGCACAATCACACTGTAAGTTAACTCTTT
ATCATGTAGCACCCCACGGCCACCAGTTTGACGCCTTACGAGACCAAAACCTTTCTCTTTAACCTTATCTATA
TCAATTTCCTTTTGTAACCTTTGGAAATACCCTATTGATAATGTTGCAGGATTCCATGTGTAAAAACGTATAA
CTGGATCAATTTCACCTCTAGAGACAAAATTTAATAACGCTTCATCCATTGCCATATTATAATATGGGTCTTT
ACTTCCTGTATTAATAAAATTCCAAGTTTCAGTCATATTCAAGAACTCCTATCAGCAAAATGTATAATATTTG
ATTCGCTAATTAATCAATTTAACTAAATGAATAATAATTGCAATTCTTTAGTGAAATATTTTGATAATTTGAC
CTAACAGTCTTATAATTATATTATCGTTTAATTAGGGAGGATGCAAGATGAGTGCTAGTTTGTACATCGCAAT
AATTTTAGTTATAGCAATTATTGCTTATATGATTGTTCAACAAATTCTTAACAAGCGAGCTGTTAAAGAATTA
GATCAAAATGAATTCCATAATGGGATTAGAAAAGCTCAAGTCATCGATGTTAGAGAGAAAGTTGACTATGACT
ACGGTCACATTAATGGGTCTCGCAATATTCCTATGACAATGTTCAGGCAACGATTCCAAGGATTAAGAAAAGA
CCAACCGGTATACTTATGTGATGCCAATGGGATTGCTAGCTATAGAGCCGCTCGTATTTTGAAAAAGAATGGA
TATACAGATATCTATATGTTAAAAGGCGGCTATAAAAAATGGACTGGAAAAATAAAGTCTAAAAAATAGTTTT
TGTAAATTTAATATACGATTTAATAAAATCTGAGTGTTAATTGATCATCAATAACAATACTCAGATTTTAATT
TTTTAACAAAGTCTGTTACTATATTTCTCTAGCTTCACTGATCATTAAACTTAGTTTCAGCATAATAAAGAAA
GTTCAGCTCATTTTCAATACGATTCAATTACCGCAATCTAAAAAATGAAAAGACAATTTCTATGAAAGAATAA
TACCAAACCCTAAGAGTTATTACTTCGGTTTAGTTTCTTGTTCAAATAGAAATTGTCTTTTTCAATTGATTT
TGAAACCATTATCCTTAAATCTTCATACAAAGTTAGAATAATAATTCTCGGAATATGTGTTTAATACTTTATT
TTTCCTGTTTAAGATTTTCAAATTTTAATATTGGTTTACGAGCAGCTGTAGCTTCGTCTAATCGATCAATCAC
AGTTGTATGTGGTGCTTCTAACACTTTATCAGGATCATTTTTAGCTTCTTCAGCAATACTAATTAATGTATCG
ATAAAATAATCAAGTGTTTCTTTAGACTCTGTCTCAGTCGGTTCAATCATCATACCTTCTTCAACATTTAATG
GGAAGTAGATTGTTGGTGGATGTACACCGAAATCTAATAATCGCTTAGCCATGTCTAAAGTACGTACACCAAA
TTCTTTTTGACGCACACCACTTAACACAAACTCGTGTTTACAATATTGTTTATAAGGTATTTCAAAGTGTTCA
GATAAACGTGCTTTAATATAATTCGCATTAAGAACCGCTGCTTCAGAAACCTCTTTAAGTCCAGTTGCTCCCA
TAGTTCTAATATACGTATAAGCTCTTAAGTAAATACCAAAGTTACCATAAAATGGTTTTACACGTCCGATAGA
ATTTTTAATGTCATTATCATATTTAAATTTGTTGCCATCTTTAATAACCATTGGCTTTGGTAAGTAACTTGCT
AGTTCTTTTACTACACCGACTGGACCTGAACCAGGACCGCCACCACCATGTGGACCAGTAAATGTTTTATGCA
AGTTTAAATGAACAGCATCAAATCCCATATCTCCTGGGCGAACTTTGTCCATAATAGCGTTTAAATTCGCACC
ATCATAATATAATAGACCACCAGCATTATGGACGATTTCACGGATTTCCATAATATTTTTTTCGAAAATACCT
AAAGTGTTTGGATTAGTTAACATAATAGCTGCTGTATTTTCATTTACAACACGTTTCAAGTCATCAATATCAA
CTTCGCCACGTTCGTTTGATTTTACAGTAACTGATTTAAATCCTGCAAATGAAGCTGAGGCTGGATTCGTACC
ATGCGCAGAATCTGGCACAATGACTTCATCACGATGACCTTCACCATTATTCTCATGATAAGCTTTAAATATC
ATCAATGCAGTCCATTCACCATGTGCACCAGCAGCTGGTTGTAATGTTACCTCATCCATACCAGTAATTTCTT
TTAATTCTTCTTGCAAACTATAAATAATTTCTAATGAACCTTGTACTTGGTCTTCATCTTGTAATGGATGTGA
TTCACTGAATCCTGGTATTCTAGCAACCTTTTCATTAATTTTAGGGTTATACTTCATCGTACATGAACCCAAT
GGATAAAATCCGTTATCTACACCGAAATTTTTATTTGAAAGTTCAGTATAATGACGTACTAAGTCTAGTTCAG
CAACTTCTGGAAACTCCGCTTTGTTTTTACGAATAAATTTATCATCTAACAATGACTCAACAGAATTTGTTTT
AATATCACTTTTTGGTAATGAATATGCATATCTGCCTTCACGAGATCTTTCAAAAATTAATGGACTTGATTTA
CTAGTCATTTAACTCACCAGCCTTTTCTACAAATGTATCGATTTCATCTTTTGTTCTTAATTCAGTTACAGCT
ATTAACATATGATTTTTAAAGTCGTCTGAAACAACACCTAAATCAAAACCACCGATAATATTGTACTTCACTA
ATTCCTCGTTAACTTGTTGAATTGGTTTGTCAAATTTGACTACAAACTCATTGAAAGATGTACCATCTAATAC
TTCAAAACCTTTTTTAATAAATTGTTGTTTAGCATAGTTAGCATGTTCTATATTTTGAACTGCAATATCATAG
ATACCTTGTCTACCAAGTGCTGACATTGCAATTGATGACGCTAGAGCATTTAATGCTTGGTTAGAACAAATAT
TAGATGTCGCTTTATCGCGTCGAATATGTTGTTCACGTGCTTGTAGTGTTAATACAAAGCCACGATTACCTTC
ATCATCTTGTGTTTGACCGACTAATCTACCTGGCACTTTACGCATTAACTTTTTCGTCGTTGCAAAATACCCA
CAATGTGGCCCACCGAATTGAGCAGGAATTCCGAATGGCTGAGTATCACCTACAACAATATCTGCACCAAATG
AACCTGGAGGTGTAAGTAATCCCAATGCTAATGGATTTGCATATACGATAAATAATGCTTTTTTATCTTCAAT
AAAGCTATGAATCTTTTCAAGATCTTCAATTGAACCGTAGAAGTTTGGATATTGTACTGCAACAGCTGCTGTT
TCATCATCCACTGCTGCTTCTAATTTTTTTAAATCTGTAACAGTGCCATCTAAATCGATTTCCACTACTTCGA
ATTCCTTGCGTGTCTTAGCATAAGTATGAAGTACTTGTAATGCTTGATAATGTAAACCTTTTGAGACTACAAT
TTTATTTTTCTTTGTTTGACTAAATGCTAAAATACATGCTTCAGCAAAGCTAGTCATCCCATCATACATAGAA
GAATTTGCTACATCCATATCTGTTAATTCACAAATTAAAGTTTGGAACTCAAAAATGGCTTGTAATTCACCTT
GAGAAATTTCCGGTTGATATGGCGTATATGCTGTGTAAAATTCTGATCTTGAAATCATAGCATCCACAACTGA
TGGCGCGTAATGATCATAAACACCAGCACCCAAAAATGATGTATGCGTTTCTTTAGTGATATTCTTGCTTGCA
ATGCGATTTAATCTTCTAAGTAACGTTGTTTCCGCTTCGCCTTCAGCAATATTTAAATCTCTATTTAATAAAA
TGTCACTTGGTACATCACCGAATAATTCTCCTATAGATTTTGCACCAATTGTTTGTAACATTTCTTGCTTGTC

TTTTTCAGTTAAAGGTATATAACGATGACTCACAATGCACACCCCTTTTTAATTATTTATCAATTTGATTTTT
CTTAACAATTTTCGCTTTTAATTGACGCTTACGAACTTGAACAAGCAACTCTTTACCCATTTCAAACTCATCT
CTTTTTATCATTGCAAGTGCAATTGATTTTCCTGATGATGGAGACTGTGTTCCTGAAGTTACTTCTCCAATAA
TATTTCCATCTAAATCCATAACTTCATAACCAGTTCTTGCAATTCCTTTTTCAAGTAATTCTAATCCCACTGT
TCTTCTTGGTGCACCATTTTCTTTTTGATCTTTTAATACAGATTTACCAATAAAATCAGCATCAATTAATGGT
TTACTTGCAAAAGCGATACCACCTTCATATGGTGTAATTGATTCAGTTAAATCTTGACCATGTAATGGCAATC
CAGCCTCTAATCTTAATGTATCACGAGCGCCTAATCCACATGGCATAACATTATACTCTAATAAACCATCCCA
AATTTTTTCAGTATCATCGATATTACAATAAATTTCAAACCCATCTTCACCTGTGTAACCTGACTGAGATAAA
ATGACGTTTGCTCCAAATAATTTGACACCCTGTTTAAATTCAAACATTTTCATTTCAGTTACATCTTCATCAA
CTAATTGATTAATTAAATCTCTAGCTTTTGGTCCTTGTATTGCTAATTGACCATATTGGTTTGATACATTTTG
TACTTCAACATCAAATTTCTCTTTGTGTTTTAAAATCCAATTAAAATCTTTTTCAGTATTAGCAGCATTAACA
ACTAATAAATAATTGTCGTCAGCTAATTGATATATTACTAAATCATCAATAATACCGCCTTCTTCATTACATA
AAGCAGTATATAATGCTTTTGAAGTAGTTAAATTATCAGTATCATTTGATAATAAATATTGCACAAACTGACT
AGCATCTTTACCTGTTACTTCAATTTCACCCATATGACTAACATCAAACAGGCCTATTTCGTATCGAACAGCA
TTATGTTCCTCTTTAATACTAGAAAATTGAACAGGCATCGCCCATCCTCCGAATTCCACAATTTTTGCACCTC
TATCAACATAATTTTGATATAAAGGTGTTTGTTTTAAATCACTTGACATTGAAATACCCCCTTAAAATTATAA
AAACAGGATAGCAATCGCTACCCTGTTTATAAATCACCAATTCAGGAACGCGTTACAGTATTATCCTTTTGCC
TGAGAGTTTCGTAATAAATTACTTGCACCTTCGGCGATAGAGTCTTAACGCTCTATTCTCTCTAATACTGATC
ACTCGCTTTTATTAAATTTAGCAATTCATAATATATTTCTGAAATTGATAGCAAATGACTATCAAATTTCTTG
AATGCGATTTCATTATATCTTAAAATCCGCGAGCAATACAAGTCATTTAACTGCATGATTGTCTTATTATTTG
CATTAGGTCGATGTGGGTCATCATTGATTCGACTATATATAATATCAATATTACAATCTAACCAAATAATGTT
TTTTTGATTTTTCAAAAAATTAAATGCCTCTTCACTTTCAATAATACCACCACCAGTAGCAATTATATCTGCA
GTGTTAATACATTCTTGCAAACATGTGAACTCTAAATTCCTGAAATATTGTTCACCATGTTTACTAAATATTT
CTGGTATTGTTAACTTATACTTTTCTTCGATATATGAATCTATATCAATAAATGATAAATTTTGCTCATCTGC
AACGTATTTACCAATCGTAGATTTACCGGTACCCATGAAACCAATTAAAATTATTGGTGATTTATCATGATTC
ATGCGTTTTCCCTTCTTTCAATAACAAAACAATCTTATAGTTATAGTCAGTTTCAATATTATATAATGTCTTT
AATTTTAAACTATATTGACTGATATAAAAAGTGAAAATGGAAAAATAAATTGCTGTTAGCATCAATAAGAACG
GCGTAACATATCCTTTTTCTTTATATAAATCAAAATTAATAAATTATTTCACATCCTTTTTAATAGTACTCAT
TTAAATTCTAGTAAGTTATACATAAATAGTTTTAGTCTGCACATTTGTACCGACTTTAACTGTTATCGTTATT
TTAATAATAGATTTATAGTAGATATTTGCAGTAAATGCTGTAACATTATTAATCATTGTTATATTTCCTCTGT
CATTTACAACTTTAATAATTTTATTATTTATTAATTTGTATTCGATCATTTCTTCACCTTTATGTAAAATGAT
ACGCTGTTGCCTAATTTCAATATCGTTTCTATCTACACCTCTAAAATCCTCTAGAATATCTCTTGAGAAAAAT
TCGAAATCTACAGTTGTTAATTCCCTACTTTCAATTAGAAAAGTTTTGCTAAGTCTAATTAAGTCTGGAACAA
TTAGTAAAGTTATACTTATAACCATCATCGCTACTAACATTTCAATAAGCGAAAAAGCTTTGACATTAATACT
GTATACATGTCTTTTGATAAGAAGTGGTATTTTTTGAAATAGCACAAATTTGTTGGTCACTTTGCTTAATATC
ATATTTCCCTATAGTTACCCCCTTCTTAAGTTCTTCTTTATTAATTTTAGATACAGTCGTCAAAATTACTTTA
GAAGCATCAATTGTTTGTAGTTTATGGTTTATACTCGCTTGCATTTGATTCATCATTGGTATCAATAGTAATG
TAATCAATCCAATTAGCAAAAATCCAGCCATACTATCTATTAAGAATGATCCTTTACACTTATAGCTTTTCAT
AACGAATTCTTCCTTTTTCAATATGGAATATTATTCTATAAATTGAATTGTTATTGTCAATTGTTATGCTACC
AAATTTATTGATATTCCCTTTTTTATCAAAGGCAATAATATCAACTTTTGCAACATTAATTATTTTGCCTACT
TTTAATTTTAGAAATCGTATTTTATTATTCTCTATTACTTTAATAGTGTCACTGTTTTCATAAAATCTAACAT
TGATATATCCTTGATTTGCTATAGCTTGCGATTTAATATAATTCAATTCAGTAATAAGAGAAATGATATTTGC
CTCGTCATCTATTACTCTAAGATTGCTTAATCCTTTAGATGTCATTGTCAAAAGTAAAAATATACTGATTAAC
ATCATTACCACAAGTATCTCAATCATAGTAAATGCTGACTGCTTTCTAATTTGCAACTGCTTCTCCATTACTA
ATTGTGATTGTCTCTCCTGATTTACATGTCTTTTGTGCTTCTTTTATAAAACCATCTGCAATTAAGTCTTCAA
TAGACGATGGATTTCTATTATGTTTCAATGCATACGCTTCAATTTGACTATTAACCATTTTTACCTGTGCATT
ACAACCTGTTGATTGTATGTGAGCAGTTTGTTTAGCAATATTTGGAATGATTAAAATTAATAATAAACTGATG
ATTAATAACACTAATAGCATCTCTATCAATGTAAACGCTTGAGTTTTCTTAAGAAATTTAAACATATATTAAC
CTCCATTATTTTATACTTTGCATCATTTGAAACATTGGTAACATAATTACCAAATAAATTGCGACAATAAATA
AACCTAAAATCAAAAATAAAATAGGCTGTAAAAACTGAGTCTGTTTTATCGCTTTATCTTCTATTTGTTTTAC
TAATATTTGCGAATATAACTTTAGTTCTACTTCTAGCTTTCCTCTCTTTTCACCTTGTAGCACAAACTTAATT
AATTGAGGCTTAAAGCATTTTAGTTTTTCTAAAATTTGAGGTAAACCATATCCCATTTCTGAATAAGTTAATA
AGTATTTACCTAGAAACTGTCTAAATGGATCACTACTATGGTTAATATAAACGTCTACTATTGATTGAAGTGT
AATACCATTTTTATAAAATAACACTAATTCATTAGTTACAAAATAAGTTTTAAATAATTGGAAATAGCCCGAT
ATTAGCGGTAGTTTCATCACAAAGTTTATCTTATTGAGCATATTTAATTTGTTATAAATTAATTTCATAATAA
TAGCCAACATAGATACTATTATGAGCATTACTACAATTATAGTAGGTAAGCTGGTAATGAAAAAAGACAATGT
TTTTTGAAAAGAAGATAGTTGAATATTCATAGAAGTATATAATTGTTGAAACTGTGGAATTACTGTGAGGTTT
AATATAATAATCATAGCAATAAAAATCGAAACTAGTATTAAGGGGTATTGCAGTGTCTTTAACAATCGTTGTT

CTGACTTTCTATTCACTTTCATATAATTTACGGTTTCTTCTAGAACGTCTATGATATTGCCAAATCTTTCTGC
CAAATATACTTGCATGACGATAGTATCGCTATAACCTATCAGTGATAATATCTGGTTGCATGGTGCACCATTT
GAAATTTCACTTAGAATGGTGGTGCCTAATTGCTTATTTTTATATGTCATTTGAAGATTTAAAAATTGAAAAC
TTTGATACAGAGTGAAACCATATTTCAACAAATTACATAAATTTAAAAGTAAATCGATTTGTTGGGCCTTACT
TAATTGTCGCTTCTTAGAATGTAGTTTAAATGTATTTATCCATTGTAGTTTCACAAATGACACCTGCTTTTGT
CATATCATCAAGTTTATCTTCTAAGTTCTTAAATGATGATGGTAATGAATGATTATGGGAAAAGAAATATCGG
AGTTGTTGCTGAGATAGAATTTCACATACTAATTGTCGCTGTTGCTTAATAGTAGTTACAAGTCGTTGGTTTA
TAATTAAGTTAGTTGCCTGTATCAATTCTTGTACAGAAACACCCATTTCTAATAGCCTTAAAATAGCACCTTT
ACAATCAGTTGCATGCAATGTAGTCAGAACAAGGTGACCACTTAAACTAGCCTGTATAACACACTTGGCAACA
TCTTTATCTCTGATTTCACCTATTAAAATAACATCGGGATCACATCTTAAAATAGCTTTAAACGAATTTACAT
AGTTAATGCCAGCTTTATCATTCACATTAATTTGAACAATACCAGGAATTTGCATCTCTACAGGATCCTCTAT
AGAAATTACATTTAAATTCAAGGCTTTATTCGCGTATGAGACCATTTGATACATTAATGTACTCTTTCCTGAA
CCAGTTGGTCCACTAAACAATAGTAATCCTTGTTTCTTGTTCATGAGGTGTTTAAAATCATTGAATTTATAAG
TTGATTTCTGTTGTTGAAAAAAATTGAGGTACAATTCTGATAACACAACTTTCTTGGCCAAGTGACAATGGTAA
TGTTGATATTCTCAAAAAATAAATTTTATTGAAATGGTAACTATATCGACCGCTCTGTGCGACTTGCTGTGTA
GAAACATCAAGCCCAGCTTGAAACTTCATATAAACTAATAACTTTTGATAAATGCCATTCCCAATTTGTTCAT
ACTGCTCCAAGTTATCATTAATTCTAAATTTAATACTTACTTCATTTTTAACTGGAATAAAATGTACATCACT
CGCTTTCATTTCTATCGCTTTATTAATTATTTCTTGAAATAGAATCTTCAAAAAAACACCTCCTACATATAAT
CACGTAGGAGGTGTTTTTATTACTTCAATTTAACCATGTAAAAATGGATTTAATTGTTCATCATCAACCGTCG
TATATGGACCATGTCCAGGGAATAAAGGTAAATCGCCTTCTAATTCAAATATTTTATCTAGAATAGAATCAAC
TAGAGTTTCATAATCACCTTTATATAAATCTGTACGTCCAATTCCATTATTAAATAATGTATCTCCAACAACT
GCGAATTCATCGAACACATATGTTAAACTTCCTGGTGAATGTCCAGGTGTGTGTAACACATTAAACTTAAATC
CTTCTATTTCTGTGCTACCTTCGTTTAACTTTTCAGGAGTTACCTTACTTGTAATAATTGGTAATCCATATTG
CTTAAATTTATCTGCCCCATTTTTAACGGGATCTTTTAGAAAATCAAACTCTGCTTCATGCATATAAACCGGG
ACATCGAATCGATCAACTATATCATCGACTGCTCCGATATGATCAAAGTGTGCATGTGTTAATAAAATAGCTT
TTAACGGTTTATTTATTTGGTTTAATTTTTTAATAATTTTTTCACTTTCACCTGAAGGGTCAATCAGAATAAC
AGCTTTGTCATTTTCGATGAAATACGTATTAGTATCAACTAAGCCTAAAGTTAAGCTTGAAATCCTCATAGGT
TTTCACCACTATTTTCTAAATGTTTTTGTACTGATGTTAGTTTATCATTCATTTTTCTAGATTTGTCTCGTCG
GTCATCAATACGGATATTTGTACAAACTCTACTTAAACCTTTATCAAAAGGTAATTCATGTATCACTTGCACA
ACTTCTAATACATCGCTTAATTCACCTTCAATTAGAGTATTCATTGGTGTTAATTGAAAGTCAATTTTACCCA
TCGCTTTATATTCTTGAAGTTTTTTCTGAATATCTGCAATATATTTACTAACACTCGGACCTTCCGTTCCAAC
TGGAATAACAACCACATCAACAATAGCCATTATTTTACCCCCTCTTTATCTAATACATAGGTCTTGATTAATC
CTGCTGCTCCTGTAATACCTGCATCATTACCTAATTTCGCTTGAACAATTTCAGTTTCAAATTGAGCAGGTGT
AAATGTTAAATTATGATATTCTGTTTTAATATTTTCAATTAAAATAGGTCCTGCAGTAGACATTCCTCCACCT
AAAACGATATATTTCGGATTACTTGTAACACTAATAATACTACATAAATATCCAATATAGTTTGCAACCTTTT
CAGTAATGAAAATACAGAATTGGTCACCAGCTTTTGCCGCATCAAAAACAGCTTTTGCTGTAACCTTATTTTC
TTTAATCAATTCTAATATAGAAGATCTAAACGTCAACTTCGGATAGTAGAAGTTAACTAAGTTAACAACGCCT
GTCGCTGAAGCAACTGTTTCAATACATCCAGAACGACCACAATTACATTTAAATCGTTGATCGAAGTCTGCTC
TAAAATGACCTATTTCTGCGCCAGAGCCATTATGACCATGTACGATTTCACCATTTGAAATAATTCCTCCACC
TAGACCTGTACCAAGTGTGATGGCAACAACATCATCGGCACCTTCACCAGCACCTTTGTGTTTCTCCCCTAAA
GCAGCTATGTTAGCATCATTATCTACATACACTGGACAATCAACGAATTGTTCAAAAATCTCACGTACATTAA
CTTTTTCTGGCCAATATAAGTTTACTGCTCCATTTACTGTACCTTTTTCAAAGTCAACAGGACCTGGAACACC
AATACCTACGCCAAGTACATTTGAAAAATTATAATTATTTTCATTTACTTTTTCAACAAACGAATCATAAATT
CCTTTCAAAAGTGTATATCCTGTACTATCAGATGTATCAGTGTGAATAGACCATTTATGTAATTGTTCTAATT
CAGGTGTGAAAATACCTAATTTACAAGTCGTCCCGCCTACATCAGCTGCTAAAATAATTTTTGTCATTTCTGT
TCATTCCTTCTCTGATTAATTATCAACGTACATTTCAAATATTCTTCCTTAGATAACAGTTCATATTGATACA
ATGATGAAATCTCCTGTTGAATCATTTCGTACATATCTTCTGGATTTTTAAAATATATTAGAAAACCGTAACT
TTTCAATAACTGTTGTACATCATAAAAGTTATTTATTTTTCGACTCATTTGATTTAGTCAACTCTTTTTCTAA
GTTAATATAATCTGTATTTTTAGGGTCTGTATTTAATGCTTTACGCACATATTTTAATGCTTTTTCATCATCA
TTTAATGAACGATTTGCTATCGCTAACTCAAAATTTAATAAACCTGATTTAGGAAACATTCTAAGTCCTCGCT
CCCATTCTGTCATACCTTCAGACTTAGAATTGATAGTAGCCATAATCATACCACTTAAATAATATGTTTGATC
ATCGGCATAATTTTTATTTATTGTCTGCTTCACAATATTTTGAGCATTATCATAATTACCACTAGTCATATCA
TCTTTGATCAATTTATTATAAATATTATCTTCTTTAATTGTAAAAATTCTAATTTGAAGTGCAATAAATATAA
CAAGCATACCAATTAGTAAAATCCAAAAAATATTACGATTCACTTTATAGTAATAGCCAATTAAAGTTATTAA
TAAACCACCAATGAATCCTCCAATATGCGCCACAATATTTATATTTGACATAAACAGAGAAACACCAACTAAT
ATCACTAATGCAATTAATAACTGCCCTAACATTTTTTTGTTAAATGTTTTTGAAACATACATCATCGCAAAAA
TTGATCCAATCAGACCAAATATAGCACCACTAGCCCCAACTGAAATTGTAGTCGTATTAAATGATAGTGATAC
AAAGTTTCCAAACAACCCTGCAATAAAGTATACAGTTAACATCCGCCATGAACCAATAATTGCTTCGACTATT

TTACCAAAAATAAATAATGAAAGCATATTCATAAGTATATGTTCAAAACTAAAATGTAAAAACATCGATGTAA
CAATTCGATACCATTCACCATGTACGACATTAAAATGCACTAACCCGCCAACATCTAATAATTTTACATCCGA
AAAATTATTTAAATATAAAATCATACATAACCATATTAAGACATTTACAAATATTATTGTGTATGTTGCCGGT
GAAAATTTCTGCATATATTTATCTAAAAAGTTATCGGTTAATACTTTGCGTTTATAGAACATATATGTCTTTT
TATCATTATCTTCTGAAATAAGTCTAGCCAAAAATATATTCGGCATATGCTTTACCAAATCTTTTGTGTGTCT
TATAACATTGAATTTAATTCTAATTGGCGAAACTTCATTTAATTGTTCTTCTGAAAATTCCGATTCTGTAAAA
TAATAAAATTCAAAACTTTGTGGTTCGAAAGAAATAAAATTTGCTATTTCATCTTTGTGTTCAAGCACTTTCG
CTTTGTCAAAACGTATTTCTTGGGTAGACTTGATATGTTGTTTAAAAATGACTACTTGTTTTTTTCTTTTATG
GGCTAACCAAATTTCTTGGTCATCTTTTTCCCTACTGACAATATCAAAATTCAAGTACCTAATCCAATAATAT
ATTGTTTTCCAAAATTGTTTGTCTATGTTCATTTAGTCCTCCACTATGCTGATTGATATATAATCAATTTATC
GACTGGTTGATCGAATGATTCCGGTTCAAATGATGTTATTTGAAAATCGTATAATAAGCTTATTGTCTTTGTC
TGATAATTAGCTAAAAACCTGTCGTAATAGCCACCACCATACCCAATTCTATATCCATCGTCTTGAAATCCAA
CACCAGGAACAACAATTAAATCTAGGTTATTCGTTGTTTCACCTTTTGAAGTTGGATAGTAAACCCCCTTATT
ATCGACATCAATATCTTTGAGATTAAATATCTCTTTAAAAGTCATTTGATGATTTAAATAATCCATTTTCGGT
ACAAAAATACGTTTATGATCCATTAAGGCTTGTTCAATAATAGAAAAAGTATCTACTTCATGATTAAAAGAAA
GAACTAGCGCAATTGCGTTTGCTTCTTTATATTCTTCAGTTGCAAAAAATTGATTTCTTAACCATGTGTCTGC
TTTTCGCTTTTCAGCCTTATTAAAATTCTTCATTTTATGTAAAATGTATTTTCTAATCTCATTTTTAGTCACA
TTATCACCCACTTTATTCAATTCTATTATATCGTTAATAACAATACATGTCATTATGTTGTCATGATGAATTA
TGCGACAATTCTAAAATTTATTATTTCAATTGAAAATCTTTTATCATTTCATACGTGTGCACAATTAAAAAGG
TGAAACCTCTTATTTTGAGATTTCACCTTTTTTATTGTTAATTTAAATTTAATTTTAATGATTATTTTGTTTC
ACGGTGTAAAGTTTGTTTGTTTTCACGTGAACAGAATTTCTTCATTTCAACACGTTCTGGATTATTTCTTTTG
TTCTTAGTTGTAATGTAGTTTCTGTCACCACATTCCGTACAAGCTAAAGTTACGTTTACGCGCATGGTTATTC
CTCCTTATCATTTCTAAATACGACTTTATTATTATACCAACATCTATAATATTTGCAAAGTTAAAATTTTACA
ACCATGCGCTACACAATCGTCAGGTTAAATTTTATTTATCTTTATCTTTATTTTTATCATCTTTACCTAACTG
CTTAAAGTAGTAGTTAATTACATCTCTACCTAAGTCTCCACCTGTTAACCATGGTGGTGGTACAGGCTGATTT
GTATATACAATTGAAAACGCTAATTTTGGATCATCAATTGGCGCGTATCCTATATAAGTAGAGTTAACTCTTG
GCTCTCCGTTTTGGAACACTTCAGCGGTACCAGTTTTACCAGCAGTAGGCACTACTGTATCTTTAAAACTAAC
ATATCCAGTACCATCTTTATCATTAAACGCCATTTTGAACCCTTCTTGAATTTGTTTGATTTCATTTTCAGTA
TTATTAACTTTGTTCAAGACGGTGCCATTAATTTTCTTCTTGAGTGGACCAACCTCATCTTTATTAGTTGATT
CATGAATCGTTAATCCAATGTGTGGTTGTATTCTATAACCATCATTCGCTATAGTTGAAACATATTGTGATAA
TTGTAATGGTGTATAGGTATCATATTGACCAATTGATAAATCTAGATAATTACCTGGATTATTTGTTAATGGT
TCGATTTGACCTTTTGTTTCATTTGGTAAATCTATACCTGTTTTCACACCTAAGCCTACTTGATTCAATCCTC
TTCTTAGCTTTTGGGCAGGTGAACTTATGTCTGATGGTAAAGCCATACCAGAATAATAAGGGTCTCCCGCTAA
TTTTAATGCTGTTTTAAACATATATACGTTTGATGAATGCATCAAAGCTTGCTTATCATTAATAGATACATGC
CCGTTTTTATTAAAGTATGATCGTTTTGTCAAACCACCTTGGAAATGTAATGGTTCATCGACCATTGTTTCTC
CAACTTTGATAGCTTTATTCTGATAACCAGCTAATAATGTTCCACCTTTTACAGAAGATCCAACTGCAAATTG
AGAAGTAAACGTACCAATGTCATAATCAGTCATTTTACCACTCTTATTAATCTGCTTTCCGGCAAGCGCAAGA
ATGTCTCCATTTTTAGGATTTTGTACAACCATCATAGCATTATCCATATCTTTTGCACCTTGGCTGCGAAGCT
TCTTAATTTGTTTATCTAATAATGCTTCTACTTCTTTTTGAAGATCTATATCGATCGTTAATTTCAAATCTTG
ACCGCGAGCGCCAGGATTTAACACTTCTGAAGATGTAACTTTACCAGATTTGTCCGTTGTGTATTTCATTTCT
TTCTTCTTACCACGCAATACATCTTCATATTGATATTCTAGGTAAGATTTTCCAACACGATCATTGCGTGAAT
ATCCTTTGGATAAGTAATGTTCTGTCAATTCTTTTGGAATACCTTCAGCAGGTGTCGATACATCTCCGAATAT
ACCTCTTAATGTGTCGCCATATGGATATTTTCTATCCCAATCCATAGACGTGTTAACACCTGGTAATTTGGAA
AGTTGCTGAGAAACTGCTGCATACTCTTTTTCACTGACATCTTCATTTTTTATCATTTGTGGATCTAAAACTG
TTCCTGCATTCATCTCTCGAAAAATGGCTAAAACTTGTAAATCTTTAGAAGACAATTCATCTAATTGTGATTT
TCCGATTTTCGATAACAGTTGTTTATCATATTGATCTTGTTTAATACTTCCATCTGCTAACATAGCTTGTTCT
TTTGTCATCATTGCTTTTGCTTTTTTAGGATGCAACTGAATCCAGAAATCTTTTTTATCACGTTCTGTAATCT
TCTTAGTATCCATCTTGATTAGCTTTGATAACTTTTCAGCCGTATCCAACATTTCCGATTGTGTTGTTTTTCG
ACCCCTAGTATATGTAATAGCCATTTTAGAAGCATTATCAACTAAAACTTTCCCATTTCTGTCTAAAATACGA
CCTCTTGGCACAGACTCATTCACTGTAATGTTTTCATCATTTTTTATAATTTGTTTATAATGTGAGCCTTGTG
CGATTTGTAAATAACCTAAACGTAGTACTAGTACTGCAAAAATAAATACAATCACACCAAATATAAAGTTAAT
TCTCTTGTTAATTGTATTTTGAACGATTTCATCATTTGATTTTTCTTTTAGTCTTTTTAACAAAACTACCTAC
CTCTATTCAAAGTCTTTCACTTTAAATCATATATGAATTTAGAAATTATTTCTATCTTTTTGACAAAAAAATA
ACGGTCTCATTTAAGAGACCGAACAAGTAATCATACTTTATTTTGTTGCATTATATAATTCGTCAACTTTTTC
CCAGTTAACTACATTCCAAAATGCGCCAATGTAGTCAGGGCGTTTGTTTTGATATTTTAGGTAATAAGCGTGT
TCCCATACGTCTAAACCTAAAATAGGTGTTTTACCCTCAGTTAATGGATTATCTTGGTTTGGTGTAGTCACAA
TTTCTAACTGGCCATTGTTTACGACTAACCAAGCCCAACCTGAACCAAAGCGTGCAGCTGCTTTGTCAGCAAA
TTCTTTTTTAAATTCTTCTAAAGAACCCCATTGTTCTTTAATTTTTTCTACAACAGTACCTTTTTCTTCTGAG

TTTGGTGAAAGTAACTCCCAGAATAATGAATGGTTTAAATGTCCACCGCCATTATTACGTACAGCAGTTTGGA
TGTTAGCTGGTACACTGTCTAAATTAGCAACAATTTCTTCAATAGATTTAGATTCTAAATCTGTACCTTCTAC
TGCAGCATTTAATTTCGTAACATAAGTGTTATGATGTCTGTCATGGTGAATTTCCATAGTTTCTTTGTCAAAA
TGTGGTTCTAATGCATCAAATGCGTATGGTAATTTTGGTAATTCAAAAGCCATAAATAATCATCCTCCTAAAA
TGTCTGTAAGTAAATAATAACAAGCAGTGACTGGTTCAACAAAGAATTTGCTTAAATACTACTACTTATTATT
TTCTCTACTCATTTAATATAACTCAAATCAAAAATAATTAAACATTTTGTATATAAAAAGTTAACAGATTTGC
CATAAAATCATACGAACGGAGTATGAAATGAACCTTTATCTTCTATAATTTAAAAAATGAGATTTATGCATAC
ATCTGACCTAATGTGTATAAATCTCATTTCTTTATATTAATCTTGGCAAGACTCACATACACCATAAACTTCA
AGTTTGTGTTTGTGAATATTAACACCAGGTAGTGATAATTTTATCTGATCTATTGGACAATAATCTATTACCT
TTGTATCTCCACACTTTTCACAGATAAAATGATGATGATGATGGTTTGTACAAGCGATTCTAAATTTCATTTC
ACCATCAAGTTCTGTATTTTCAATAATCCCTAAATCTTTAAATAAGTGCAGGTTTCTATATATTGTGTCGAAT
GAAATTCCAGGATAATTTTCATCCATAACTTGTTGTATATACTTTGCGTTTATATACTTATCTTCTTCGACAA
AAATATCTAACATATCTTTACGTTTATCTGTATATTTTAAACCGTTCTCTTTTAAAATTTTAATAGCATCATT
TGTATTCATTGATATTAGCTCCCTTTTTAAACTTCATTCGCATTTTCTGATAAGCCATTGTAATCATAAGTAA
AATAACCAGTAGAACTACAATTACACCACCCGGAGAAATGTCCATATAGAAAGCTAGGACTAAGCCTAATATT
ACTGATAATTCACCTAAAAATACACTTAGTAATATCAATTGCTTAAAACTTTTTGTTATTCGCATACTTATTG
CAATTGGTAACGTGATTAACGCACTTACTAACAGTATCCCTACAACACGCATTGAGGCAGAAATAACCATCGC
TACAATAACAATAAATAAAAATTGAATCCATTTAGGAATGCCAATGACTTTACTATATTCCTCATCAAATGAC
AATATAAATAATTCTTTATAAAACAATGTAATAAACAGAACAACTATGATGGCAATGACAATAATCGTTGTTA
AATCACTTATATTCACTGCGCTTATTGAGCCAAATAGCAATCCAACAATTTCTTGATTGAACCCATCAGCTAA
TGAAATGAAGATTGCACTCAAGGCGATACCAGCACTCATTATAATTGGAATAGCAATTTCTTGGTAAGCAGTG
TATGACGTTCTTAATTTTTCAATTAGAAGCGCACCTACTATTGCGAATAAGATTCCAAACCACATTGGATTAA
TAAATACTAGTGTTGGCATAATAGTAAGTAAAAACATACCGAAGGATATACCACCTAAAGTTACATGACTTAG
AGCATCAGCTATAAGTGATAGTCGTCTAACAACGATAAAAGCACCGATTAGAGGCGCAATAAAACCTATCAAG
ATACCACTAATTAAAGAGTACCTCATAAAATCAAAATTCAATAATGCATCTATCAATTGTGACACGCCTTTCC
ATTTTATATAAACTCAAATCTTTATTAATTACAACATTCTCGATTATGCTGATGATCGACAAAACGTACAGGA
TGTCCATAAATTTTTGAAATTTCAACTTCATCAAGTGATTTAAACTCATCAGTTGTACCATGGAAATGCAAAT
GCTTATTTAAACATGCTACTTCAGTAGCAGTATCTGCTACAACACCAATATCATGAGTAACTAAGATAATAGT
GATACCTTCTTGTTTTAATTGATCTAAAGTATTATAAAATTCACTTACATGTTTTGCATCAATACCATTCGTC
GGTTCATCAAGTACTAATACTGCAGGTTCTGAAATCAATGCTCGAGCAATCATTACACGTTGTTGTTGACCAC
CTGATAATTCTGCTATATTTTTATGAATTAAATCACTTATATTCAGTCTTTCTAGTACTTTAATCACTTTTTC
ATTATCTTTGCTATTAAATGTTTGGAAAAGACGTTTTGTCTTTGTTAATCCGCTTAAAACAACTTCTTTAACA
CTTGCTGGGAAACCTGAATTAAAGGCATTTGCTTTTTGTGATACATAGCTTAATTTAATTGATGTTTTCTTAT
TTTTAAAATCAATACCTTCAACAAAAATCTCACCACTTTGTAAAGGTAATAATCCTAGAATCAACTTCAATAA
TGTTGATTTACCAGCACCATTTGGTCCAACAATTGCTAAAAATTCACCTTTATTTATTTTAATGTTTATATTT
TCTAACACTTTTTTATGATCATAGTAGTAATTGACATTCTTCAATTCAAAGACTGGTGTCATCGTATTCTCAC
CTCGCATTCAACTATACAACTCCTAGTAACATATGTAAACAGTAATGTTTACGACTCAAAATAAGACAAAATA
AAGAGATATGCCCCCTTCAAGTTTTATTTATCGCATTTCTTGAAGAGAGCATTATCATTTTATTGTTGCATAA
CCTTATTTTTTAATTCTGGGTCAAATTGCTGTTGTTTCAACATTTCAATTTCAAGTTTATATGGCGGTTTTTT
ATTTTTCTTATCTTCACCAACATAAGGTGTTTCTAAGATTTTCGGAATATCTTTAAAACTATCATGATGCACA
ATGTAATTTAATGCATCAAAACCAATGTAACCGAAGCCAATATTTTCATGTCGGTCTTTTTGAGCGCCACGGT
CATTTTTAGAATCATTGACATGAACAACTTTGATTCTGTCGACTCCAATGATTTTATCAAATTCATTTAATAC
GCCATCAAAGTCCTCTTTCACATTATATCCAGCATCATGCGTATGACATGTATCAAAACATACTGATAAACGT
TCGTTATTATGAACTCCATCAATAATACGTGCTAACTCTTCAAACGAGCGACCAATCTCAGTACCTTTACCTG
CCATCGTTTCAAGCGCAATACGTACATTATTGTCATTCGTTAAAACTTCATTTAATCCTTCAATAATCTTATT
AATTCCGGCATCAACACCAGCTCCAACATGCGCACCTGGATGTAATACAATATCTTTAGCGCCTATAGCTTGC
GTTCTTTCAATTTCTTGTTGCAAGAAATCTACACCAAGATTAAACGTTTCTGGTTTGGTTGTATTTGCAATAT
TAATGATGTATGGTGCATGAACAACAATATTAGATAAGCCATATTTTTCCATCACTTCATGACCTTTAGTTAT
ATTTAAATCTTCAATACTTTTACGGCGCGTATTTTGAGGTGCACCAGTATAAATCATAAATGTTGTTTCACCA
TATTCATGCGCTTCTATAGCAGAACCTTCTAACATCTTTTTACCACTCATTGAAACATGTGATCCTAATAACA
TAAAACACACCTAACCTTTTTTGTTTTGCTTACGTTTTTGTCTATTTTGCTGCTTACTAAATTGTTTACGCTC
TTGACGTTTCATTTTTTCAACTTCTTGCTTAAATTTCTTCTTATAACCTGGTTTAACTTTGTTTTTAATTTTA
CTTCGAACTTTGTTCTTCACTTGATTAGTTAAATGGTCATCTTTGCGCATTCTTGCTTGACGCTGATTGTGCG
CTTTAACTTCTTTTAACTCACCATCTTTAATATCAACAGTATTGAAAACAAAACCGCGATTTTCTATTAATGA
AATATTGTGTTCTTCATCAGGACTATAAAGCGTTATTGCTACACCTTTATAATTCCCACGACCAGTTCGTCCG
ACTCTATGTGTAAAGAAGTCAATATCATTTGGCACATCAAAATTGATGACATGACTAACACCTTCAATATCAA
TACCACGAGATGCTAAATCGCTGGCAATAACGTATTGGAATTCTAAATTACGTATACGTTTCATTTGTTGTTT
ACGTTCACGTGGCGTTAAACCACCATGAATCATACCAACTTTAATACCAGCTTCATTTAGTGAACGTGCTAAA

TCATTTGCATTATCTCTACTATTACAGAAAATAATACATAAGTATGGATTTAGTATATCAATTAAATTTAAAG
TCTTTTCAACTTTAGCTGCACCTTTAGTAGGTATTAAAAAGAATTCGATGTTCTTTTTATTTTGTTTTTTACT
GTCGACAGCTACATATTCTGGATGACTTAAATATTTATTTAAAAATGGTTGTAACTGTTGTGGAATTGTAGCA
CTAAACACCGCAATATTTGCATTATCTTCCAATCTTGCAGCAATGTAATCTACATCTTCAATTAATCCTAAGT
CAATCATAAGATCCGCTTCATCAATAACTAAATATGATGCTAAGTGCACATGTAAATGTCCCGTTTTAGCTAA
GTCATTAATTCTAGTAGGGGTGCCTATAATCAATTGTGGTTGTGCATTACAACGTTGTCTATCTTTCTCTATA
TCTGTACCACCAATAAAAACTTTAACTGAAACGCCAGCTTTAAATTGGCTTAAATGGTTCGCTGCATCGTATA
GTTGTTGTGCAAGTTCTCTTGTTGGTGCAACTACGATTGCTTGTGGTTCTTTTATTTCACTATCAATTAACTG
CATTAATGGTAATAAAAATGCATGAGATTTCCCTGTACCCGTTTGAGATTGACCAATTAAATTTGTTCTCTTT
AGTATTCTTGGAATAATTCGATTCTGAATTTCAGTTGGTTTTTCAAAATTAAGGTCTTTCACAGCGTCAATTA
AACTAGATTCTAGATTAAATTGTTCGAATGGATGTTTTGCCATGATTTGGCCTCCTTATATGTTCATCTTTAC
TATTATAAAGTATAAATGTGCTATTTTAAATAAAAATATTAAAACCTCGAAATTTCAAAATTTAGAAATCCCG
AGATTTTAGTTTAAATATATTGAAATGGATCTGTATTAATTGTAGATGCTTCAACATCTATATTTATTTTTTC
TGTATTGAACCAATTCATTAGTAACGTTTTTAAACCTTCTTTCATCACATATTCGCTGTAATGATTAATATCA
ATTAAGTTCACACCATGAATTTTAGCATCTAAGGCATCATGATGTTTAATATCACCTGTAACAAAGACATCTG
CGCCTTGTTGGACAGCTTGATATTCATATCCAATACCTGAACCACCAATAATTGCAATACGTTTAATTTTCTG
ATTAGACTTACCAACAAAACGGACACTTGGGATATTTAATTTAGATTTAATATCAGCTGCGAAATCTTCCAAT
GTCATTTGATTATCCACTTCTGCCATAACGCCAAGTCCATAAAGGGATGTTTGTTTTATCTCAATAAAATCAA
ATACCGGTGTTTCATATGGATGGTATTGTTTAATTAATTGCTCAGCCCTTGACTTTTGATATGCATCTATCAT
AAATTCAATTTTAACTTCATCTACATATTCAATTTTATCAATTTGTCCTATTGTTGGATTAGCTTCACCAACT
GGTTTGAATTGCCCTCTTCCTTCACTTTCAAAGAAACAATATTCATAATTACCTTCTTGTGCTAATCCATTTT
CACTAAGCTTATCTTTAAATGGTCCAACATTATCCTTAGGTATATATGTTTGAACTTTATAGTATACACCTTG
TTGATTATTGATTATTGAAATGTTCTTCAAACCCATCGCCTTCGCCAACATCATATTGACACCATACGGATTT
ACATCTAAATTTGTATGCATCGCTATTAAATTAATGTCATGTTGAATTAGTTTTCTAATAATCAAACCATAAC
CATTAGCTTTTAATGATGTTACGCCTTTAAAGATTAGAGGATGATGACTAATAATAGTATTATAACCTTTTTC
GATTGCTTCATTTACTACTTCCAACGTACAGTCTAATGCTGTTAAAACACCAGTAACTTCAACATCTTCATCA
CCTATTAACAATCCTACATTATCCCAAGATTCAGCAGTACTAAATGGCACATGATGATCTAACAATGTCATTA
AATCAGCTATTTTCATTACTATAACACCCTTTCAATTACAGCAATTTCGTCATTAATTTGAGCTAAACGTTGA
TGATGTTGTTCAGTATTGAGTTTCGATTTAATATGATAAAGTGCTTCTAACTCTCTTTGCCATTTTTTTATAA
AATATTCATTTTTGTTGTTCAGCAATTTTGGTCCGAATTTCAATTCATCAGATGATAGCTCTATTAATTGTGT
AGAATATTCTGCTACAACAATTTCATAAATATGGCCTTTTTCTTCCATTATTATTTCATCAATTATTTCATAA
TTCAATTGTTGTAATGTTTGTCTTAAATTTTCAGTTTGGATATTACTTTGTAAAATCAACCTTGGATGTTGAC
TTAACTTATCTTGCCCATCTTTTAAAATTTTAGCAATAAGTGGTCCGCCCATACCACAAATTGTGATATTATC
GATTACGTCCTCGGGATGAATAACACTTAAGCCATCCCCTAAACGTACATCAATTCTATCTTCTAATTGATTT
GCAGCTACATTTTTCACTGCAGCTTGAAAGGGGCCTTGAATAACTTCTCCTGCAATACCGCATTCGCATAAAT
GGTTTTGAATTGCATAGATTGGCAAATAAGCATGATCTGAGCCAATATCCGCGATTGTACCTTGTTTTAAAAA
TCGACTTACCGTCGTTAATCGGTTATTTAACGAAATCATTTTATTCTCCTTTATGAAAAAAGAGGTTGAACAT
TATTCAATGTACCAACCTCACTCAAGTTAAATCTTAATCTAAAAATAAATAAGCGCTTATTCATGTCTTGGTA
TCAATCATTAAAATTGATATAAATTAATCCATAAAGTCTTTCAAACGTTTACTACGACTTGGATGTCTTAATT
TTCTAAGTGCTTTTGCTTCAATTTGTCGAATACGTTCACGTGTAACACCGAAAACTTTACCAACTTCTTCAAG
TGTTCTTGTTCTGCCGTCATCAAGACCAAATCTTAATCGTAATACATTTTCTTCTCTATCAGTTAATGTATCA
AGCACATCTTCTAATTGCTCTTTTAATAATTCATAAGCAGCATGATCTGAAGGACTTTGTGCTTCCTGATCCT
CAATAAAGTCTCCTAAATGACTATCATCTTCTTCACCAATTGGTGTTTCTAATGAAACAGGTTCTTGCGCAAT
TTTTAAAATTTCACGAACTTTTTCTGCTGGTAAATCCATTTCTTCACCAATTTCTTCTGGTGCTGGATCTCGA
CCTAAGTCCTGTAATAATTGACGTTGAACACGAATTAATTTATTAATTGTTTCTACCATATGCACAGGGATAC
GAATCGTACGTGCTTGGTCAGCAATTGCACGAGTGATTGCTTGTCTAATCCACCATGTTGCATATGTTGAAAA
CTTAAATCCTTTGTTAAAGTCAAATTTTTCAACAGCTTTAATAAGACCCATATTACCTTCTTGGATTAAATCA
AGGAATAACATACCACGACCTACGTATCTTTTAGCAATACTTACAACTAAACGTAAGTTCGCTTCTGCAAGTC
TTGATTTTGCTACTTCATCACCTTGTTCAATACGTTTGGCTAATTCGATTTCTTCTTGTGCACTTAATAAGTT
AACACGCCCAATTTCTTTAAGGTACATACGAACTGGGTCATTTATTTTAACACCTGGAGGGGCACTAAGATCA
CTTGGATTCAGTTTCTCGTCAGTATCTGAACTATCTTTTTCATTAACTAGTGAAATATCATTATCATTTAATT
GATCAAAGAAATCATCCATTTGATCAGAGTCGATATCAAAATTCTGAAGTTTTTCAGCAATTTCTTCATGACT
TAAATGACCCTCTTTTTTACCTTTTTCAATTAATTGCTTCTTAACATCTTCTAATGTTAATGTCGGATCAATT
GTTTGTTTTTTAATTTTAACTGTGTTATCAGACATGAAACGGCCTCCCGATTTTAAATATGAACATTCGAAAT
TTATTCAATATTGCTATTTTAAACTAAATTCTTAATTAATTCATCCATATTTTTAATTTTATTTTACAAATTG
GAACTAATCCCAATATTTATTTTTCAATAGTGTTGTTTAAATGCGGTAACTATCATTTACCCTTATACATTTT
GACATAATCATTATTCGTATTATTCTTTAAAATCACATGCTACATGCGTTCTTTATTCTTAGCAACAATTTGC
TGTAAATAGTATTTTTGTAATTCTACATCGCCAATCCTTGTAGCTTCCCTTAATTTATGATTCAATGACTCAA

TTGTTTCTTGTCCTTTTTCATTAATAACATTGACATAATCATCAATTTCATTTTCATATGGTTCGTCATTCAA
ATTATATTGTTCTAAGCTAATTAGTGTTTCTCTCAACTCATTTGAATTAACATACTGTACAGCATCACTGATA
TTATATTGATCATTTTCCGCATAAAAATCATGTAAGACTTCGAATACATATTTAAAATGCTGATTTGTGAAGT
TATCCTTATCAACACTTTCATAATAATTTAAAAATGTATCTTTATCTCTCATTAAATGTTTTAAAAATGCTCG
CTCCGCTTTTTCTTGACGGCTCAAATTGTCAAATTGTGCCATACCAATTGGCTCAGGTTCAATGTAACCTCCA
TACTCATCATAACCGCCATACTCATCTTCTGGATAATAATTGGCTGGTGCTTGATTGAATTGTATTTCGTTAG
CTAACTGCTCAGGACTAACATTGAAAAATGGCGCAACATCATTTATAGCCTTTTGTTGCAGAATTGATGACTT
CATAAGTGAAATGTCATGACTCAGTTCTTTCAAATAACGTTCATATGAAAGGTCATTATGTGCAATTTCATCT
TTTAATATACTTACTTTATAATGTGCAAATGACTTTTTGTCATTTTTCACAAAAGTAGTAAATGCGTCGTTGC
CATACTTACCAATGTATTCATCCGGATCCATGCCAGATGGCAATTGTATAACAAATACATTTAGCCCTTGCTG
TAACAAATGTTGACCTGTTTTAAGTGTTGCTTCACTACCCGCAAAATCCCCATCAAACATTAATGTTATATTT
GATGTTAACTTTCGTATAAAGGTGATATGTTCATCTGACAACTGTGTACCCATTGTTGCAACAACGTTTTTCA
AGCCAGCAGTATCAGATTTTATAACATCCATAAAACCTTCTAGTAATACAATTTCATCTAATTTTCTAATTGA
TTTACGTGCTTTATCTAAGTTATATAACAACTTTCTTTTTTGAAAGATAGGCGTTTCAGGACTATTTAAGTAT
TTTGGTTCTTGACCGGTATATGTTCGACCTGAATATCCAACAATTCTTCCTTGCGCATTTTTCAAAGGAAACA
TAATTCGATTTCGAAATCTATCGTAATAACTGAAATTTTCTTCGTTACGTGATAATAATCCGGCTTCATATGC
TAATTCAATATCGTAACCCTTTTTTTGAAGAAAATCATGACAAAAATGTGAGCTGTCGGGTGCAAAGCCAATG
CCTCGCTCTTTAATAAGCGCATCTGTAAAACCACGTTCTTGTAAGTATGTTAATGCTTGTTCGCCTTCGACTG
TCTTTGTTAAAGCGTAATAATAAAATTCTTGTATTAACTCATGCATTTCAATCATTTGTAAATCATCAGAAGC
AATTTGAACATTTGAGTTAGATTGTGTTGCCTCAATATCTACAGCAACATTAACTCTATCACCTAATTCTTTA
ACCGCTTCAACAAATGATATGTCTTTAATTTCTTGAGTAAATTGAAAAACATTGCCACCTTTTTTACAACCAA
AACAATGACAAATTTGTTTATCTTCAGAAACTGTAAATGAAGGTGTCTTTTCATCATGAAAAGGACACAAACC
TATATAATTGCGTCCTCTCTTTTCTAATTTTACATATTCACTTACCAAGTCTAAAATGTCGGTTTTATCTTTT
ATTTCATTAATGATCGATTGATCTATTCGCAAATTAAATCACCTATTAAGCATTAGTTATACTATTATACCTA
ATTTTCGACAAAAATGAAATCATTTCGATTTATTTTGTTCAATATAATGGATTATATCGTTTGCTGTTTCTTC
GATTGCTTTTTGAGAAACATCAATGACAGGACATCCAATTTCACTTACGATTTCTTCAAAGTAATTCAATTCT
TCTTGAATTCGTGCTTCTGTTGCATATCGAGCTGTGTCACCTAGTCCTAATTGTTTTAGTCGCTCTTTTCTAA
TGCGATTTAATTTTTCTTCACTTATTTAAGTGCGATACATTTCTTTGGATCAATATCATATAAGCCATCTGG
CGGTGTCACTTCTGGTACAATCGGTACATTCATAACTTTGTAACTCTTATGCGCTAAATACTGAGATAATGGT
GTCTTTGAAGTTCTCGAAATACCAAGTAAAACAATATCAGCTTTAGGTAATCCTTTAGGATCTTTACCATCAT
CATATTTAACTGCAAACTCTATCGCATCAATTTTCTTGAAATATGCATCATCTAATCTATGAACGATACCTGG
CTCATTATAAGGTTTTTCTTCAACCGAAGCTGATAATAAATCCATTAATGGCCCCATGATATCGACAGACTTC
AATTGGAATTCTGCTACTTTCTCACTCATATATTGCTTCATTTCAGGTTTAATAAGTGTATAAACAATGATAG
CATTTGTATCTTTTGCAACTTGAATCACTTCATCAACATCTTCAAAAGATTCAATATATGGATATCTTAATAA
TTCATTTTTACATTGCTTAGGATTGAATTGTGAAATACCTGCCCTAGCAACTAACTCTGCCGTTTCACCTATA
GAATCTGAAGCTACGATAATTTTAATTTTTTCCATTACTTCTCACCTATTCTTTATATAATGCTACTAATAAC
TTAGCTATTGTTGTTTTGGAAATTCTTCCAATTACTTCATACTTTTGATTATCTTTTTTTCTTACAATTGGAA
TCGAATCAATTTCTTTTTCAATCATTCTATCTGCTGCGTATATGACTAATTCGCTTTCCTCTAAATAAGTGAC
ATTAGGCATACGTGTCATATTTACACTGATAGGTACTGTATGAATATCTGCTCCAATCATTGAAGCTCTTAAT
AAATCTTTTCTTGAACACACACCAACAAAATCGTTATCTTCATTAATAATAAATAATGTACTTACATCTTCTA
AAAAAATTGTACAAATAGCGTCATAAACTGTTGTATTCTCTCTTAGCACAACAGGTTGAGACATATAGTCCTT
AACCTCAAATTGTCGAAGTTTTTCATTAAAAAATTTACCTTTTGATTTACCTGAATAATAATATCCAACTCGG
GGACGCGCTTCTAAAAAACCTGACATTGTTAATATCGCTAAATCTGGTCTAAGCGTTGCTCTTGTTAAATTCA
ACTTATCTGCTATTTGTTCACCAGTAATGGGTCCTTTAGTTTTAACAATTTCGATGATTCGTTCTTGTCTTTG
ACTGAGTTCTATAGGTCTTCACCCCTTTTTTATTCACTTATTATTATACCGATATTTCTTTCATGTCACAAAC
ACATTAATTTTACTTGCCTTTAAATAATCTATCAATTAGAATAAACGTACAGCGAGTTAAGGATAGTGTAAGC
TTAACAATAAGATTGGCGCAACGAATCATTTTAAAATAAAAGCGAGTGACTACACTAATTTGGGTGGAACCGC
GGGTTAAATACGTAACTTATTTTGTATTATATACAATTTAAAATATTAACTCGTCCCATGGAAGTAAAGCATA
TTTACTATGCTGTTATTTTCCATGGGGCGTTTTCATGTATGAGGAGAGGTAATTATGGCAAAAGATATGGATA
CAATTGTTTCATTAGCAAAACACAGAGGTTTTGTGTTCCCTGGTAGTGATATTTACGGTGGTTTATCAAACAC
ATGGGATTATGGTCCTTTAGGTGTTGAATTAAAGAATAATGTTAAAAAAGCTTGGTGGCAAAAAATTCATTACA
CAATCACCGTTTAACGTTGGTATCGATGCTGCAATCTTAATGAATCCAAAAGTATGGGAAGCTTCAGGACACT
TAAACAACTTCAACGACCCAATGATTGATAATAAAGATAGTAAAATTCGATATCGCGCTGATAAATTAATTGA
AGATTATATGCAGGATGTTAAAGGTGATGAAAACTTCATTGCCGATGGTTTAAGTTTTGAACAAATGAAAAAA
ATTATTGACGATGAAGGTATTGTTTGTCCTGTAAGTAAAACTGCTAACTGGACTGAAATTCGCCAATTCAATT
TAATGTTTAAAACATTCCAAGGTGTAACTGAAGATTCTACAAATGAAATTTTCTTACGTCCTGAAACAGCACA
AGGTATTTTTGTAAACTATAAAAACGTGCAACGTTCAATGCGTAAAAAATTACCATTTGGTATCGGTCAAATT
GGTAAATCATTCCGTAATGAAATCACTCCAGGTAACTTCATTTTCAGAACAAGAGAATTTGAACAAATGGAAC

TTGAATTCTTCTGTAAACCTGGAGAAGAAATCGAATGGCAAAATTATTGGAAAACTTTTGCAAGTGACTGGTT
AACAAGCTTAAATATGAGCAGTGAAAATATGCGTTTACGTGATCATGATGAAGATGAATTATCTCATTACTCA
AATGCAACAACTGATATTGAATATAAATTCCCATTTGGTTGGGGTGAGTTATGGGGTATCGCAAGTCGTACAG
ACTTCGACTTACGTAAACATGCTGAACACTCTGGTGAAGATTTCAGATACCATGATCCAGAAACGAATGAAAA
ATATATTCCATATTGTATCGAGCCATCACTTGGTGCAGATCGTGTAACATTAGCTTTCTTATGTGATGCATAT
GATGAAGAAGGCGTTGAAGGTAGTAAAGATGCACGTACAGTTTTACACTTCCATCCTGCATTAGCACCATATA
AAGCAGCGATTTTACCTTTAAGTAAGAAATTATCTGGCGAAGCGATTAAGATTTTTGAGCAATTAAGTTCTAA
ATTCTCAATCGATTTCGATGAATCACAATCTATCGGTAAAAGATACCGTCGTCAAGATGAAATCGGTACACCT
TATTGTGTAACATTCGACTTTGATTCATTAGAAGATAATCAAGTTACAGTACGTGACAGAGATTCAATGGAAC
AAGTTCGTATGCCAATCTCAGAGTTAGAAGCTTTCTTAACTGAAAAAACAAAATTCTAATTAAAAAGACTCTC
CACCATATGTGGTGGCTATATAATTTATAAAAAGAGATACTGGTTAGTAAAATTGCCAGTATCTCTTTTCTAT
TCGAAAAAGGTATTCAAATAAACACATCTATGTGGCTACTTGAATACCTTTTATTATTTTATGTTATTGTTCC
AATCTTTTTAATTGGTTGATTAGTTTCTGACTTTTAAAAAACATACCTGCATATTCACGATATAACATTAAAA
TGATATCTGACATTTCATCAATAATTTCTTGATGGATATTCAATGAATTCATTTTATCTATTGGTAATTTTTG
TAATACATCTAATAAATATAGTGTTTTATTCGATAATATAACTGCATGTACATCTTTAGAAGCCTCTTGCCTT
GAAATCGCACCGTCAAACTTAAAACTATAACCTATTAAATCTGCTTGTGTGTCATTACCACTCACAGCACAGC
GATTAAATGATGCAGTAAAACCAAATCGTTTCATACACTTTAACATAACTACGACTGACATTAACTGTGCAGA
TGTACCTGATTCTATTTTTTCAAGAACAAATTGTAATAATTGATAGTTATATGGTGCAATGTCACCTTCATCC
ATTGAGCGCTCAATAGTTTCAGCTGCCAGAGAGGCATAACTGCTTACGTAAAGGTCCATTTGTAATTTATAAT
GTTGACTAATAACATCTACAGAATTTAACGTTCCCATACCTCGCCACTGATTGTAAATAAACAAACCATAAAC
AAACAATTGCGTTTGCGCTTGTAAACCCGTCTTAACTTTTTTAGCACGCCTTGCCATAAGTGGTACTTTTGCA
CCATGCTCATTTAAAATCGTGATAATTTTATCAGATTCACCATAATCAACTGCTTTGATGATAATCCCTTTTT
GGCGCATTAACAATTATCTTCACCACTTTTAAGATTAATCTTGGTCTTCAACATAACCAATTTGGCGAATAAA
GTTAACTTTGTTTCGCCAGTCTCTTTGAACTTTGACCCATAATTCTAAGTAAACTTTAGAGCCTAGAAGCATT
TCTATATCACGTCTCGCACGTTTTCCTACTTCTTTTAACTTTTTACCGCCTTTTCCAATGACAATTCCTTTTT
GCGAATCTCTTTCAACATATATAGTTGCTTCGATATGAACGCGATCTTCGCTTTCTTTAACCATACGGTCCAC
ATTAACACCAATCGCATGAGGGATTTCTTCACTTGTAAGATGAAGGATTTTTTCACGAATGATTTCACCCACT
ACAAATTGTTCAGGATGGTCTGAAATTTGATCATCTGGATAATATTTAGGTCCTTCGGGTAAATACGTCTTTA
AAACATCAATAAAATGATCGACATTTAGCCCTTCTAATGCTGAAATAGGTACAATCTCTGTAAAGTCCATATA
ACTTTGATATTCTTCAATCTTTGGCATTAATTCATCTGGATGCACTAAATCTATTTTATTTAATACTAAAAAT
ACTGGTGTCTTAACATTTTTCAACATTTCTATAATATATTCATCGCCTCGTCCAATTTCCTCATTGGCATTAA
CCATAAACATGATTGCATCTATCTCAGATAATGTATTTTTAGCGACTTTCATCATATAGTCACCTAATTTGTG
TTTAGGTTTATGAATACCTGGCGTATCAATGAATATAATTTGCGCGTCATCTCTTGTCATAACACCTTGAATT
TTATTTCTAGTTGTTTGAGCTTTATCGGACATGATTGCTATTTTATGGCCGATCACTCTATTAACAAATGTTG
ACTTTCCTACATTTGGTCTACCTATAATTGAAACAAATCCTGATTTATGTTCTGTCATTTATTCTAAATCCTT
TCCTGAAAATCCAAATGGTAGTAACTCTGCGACTGTCATCATAACCATATCTCCTTTATGATTTGTCATATAC
ACAGGCATATCATCATCACATAATTCCTTCAAAACTTGACGACATGCACCACAAGGTGATGACGGTTTATCTG
CATCTACGGTTACAGTTATTGATTCAAAATCACCTGGTCTGTATCCTTGAGAAATTGCCGATACCAAACTAGC
TCGTTCAGCACATATCGATAATGGATAAGAAGCATTTTCTACATTGGTACCATAAAAAGTTCTACCGTCTTTC
GTTTTTAAATAAGCCCCTACTTTAAATTGACTGTATGGCGAATATGATTCTTGTTGTGCTTTTCTAACTTCTT
GAAAATAATGAGGTTGATAACTCATATATGCCTCCCTAAAATAACGCTATAAAATGTGGTAAAAATACTATTA
AACCTATAATAAATGCTAATATTGAAACTATAAGTACACTAAAAGCCGCAATATCTTTAGCGTATTTAGCTAA
ATCATGATATTCAACGGTCACTAAATCGACAACATATTCAATAGCAGTGTTTAAAGCTTCAACAGTGAGAACT
AATGCAATAGCAATGAGTATAAATATCCACTCAATCCGATTAATATTTAGTACGAGACCAAAGACAATAGCAA
CAATCATTGCAAACACATGTAAAAGAAATTTATAGTCTTTTTGAATTAAGATTTTCAGCCCATCAAGTGCATA
TTTAAACCTTTTCATAATTAGTCTCGTGTTAATCCATATGCGTTTAATATTGTATCTTGTCGACCAAACATTT
CCTTTTCATCCGCTTCAGTCATATGATCATAACCTAATAGATGCAAAAATCCATGTAATGCTAAAAATCCTAA
TTCTCGTTCAAAAGAATGTCCGTAATTGTTTGCTTGTTCTTGCGCTACATCCGTACAGATAATTATATCCCCT
AAAACACGTGGTATATCAAGATCACTAAAATCAATCTCTGGCTCATCTTCTTCTAAAGCAAATGAGATTACAT
CTGTAACTTTATCTTTATCTCTATATGTTCGATTAATTTCTTGTATTTCTTGTTTATCTACAAATGTAACAGA
AAGCTCAGCATCGTCTTCTATATGCTCTTCTTTTTTAGCAAATTCTAATAAATCTTCAATTTGTTTATACCAA
GCATCTTTAACTAAGCCTGTGTGATCGCTAAAATCTATCGTAAACATTTAATTCTCTCCTTCATAATGTTCAA
TGATCTTACTTACCAATGGATGTCTCACTACATCGCTCTGATCTAATTTCAATATACTTATACCTTTAACGTT
GTGTAACCTACTGACCGCTTCTTTAAGTCCACTTTTAACACCTTTAGGTAAATCGATTTGAGTTTGGTCACCA
GTAACTACCATTTTTGAGCCAAAACCTAGTCTTGTTAAAAACATTTTCATTTGCGCATGTGTCGTATTCTGCG
CCTCATCAAGAATTACAAATGCATCTTCTAATGTTCGTCCGCGCATATATGCAAGTGGCGCTATTTCGATAAT
GCCTCTTTCAATAAATCGCTCTGTTTGTTCACGCCCAAGAACAGTATAGAGACCATCATATAAAGGTCTTAAA
TATGGATCTACCTTTTCTTTCAAATCTCCTGGTAAAAATCCAAGTGACTCTCCTGCTTCAACAGCAGGTCTTG

TTAATACAATACGTTTAACAGCACCTTTACGGAGTTGCTTTGCCGCATAAACTACAGCTAAGAATGTCTTACC
TGTACCAGCAGGACCTATACCAAATACTAAATCATTATTTTTCATGGCATTAACATATATACGTTGCCCCATC
GTTTTCGCACGAATCGTTTTACCAAATGCATCTTTAGTTATCTCTTCATCATATAAATCTAACAGATGTTGAA
TTGTGTTATTATGCGCCATTTTAATAGCTGCTTCAACATCTTTAATTGTAATATTATTACCTAAATCAATAAC
CTTCAGCAAATTGATTAATACTGATTCCGCTTTTTCTACGTTTTCTATTTTTGTACCTTTAACGGCAACTTCT
TGTCCTCTTGCATGGATGACAACATCGAAACTCTCTTCAATTGCTTTTAAATGTTCATCATTATTTCCAATTA
AAGCTTGAGATTGGTTCATATCGTCTATTTGTATAATTCCAGGCATACACGCGCTCCTTTTCATATATCAGTT
ATATTCATTCTATGATGCTTTAATATCTACTTAATTATATCATGCAATTGATTGATTTAAACATCTTTGCATT
GACTTACTCATTTTTCTTTAATTTAAAATAATCATTTATACTTTTCACCTTGTCATAAAAGCAGATTCAATAT
TAGACTAATCAATTTTTTGTTGTAATTTAAATAACTAATATTTCTCGAAAGATTCTCTTTGTTTTTCAATTTT
TCATAACGTAAAAAAACAAGCAACTACCATTACGATAGTGCTTGTTTTCAAAAAATTTTATAATTGTTTTGGT
TTAGCTAAAATTTCTGACCATATCATACCATTGATTACTTCATCTTTATCAAATTGGAACGCTGATTTAGTCA
AATCTTTTTCGACATTTTGAGAATTAAGCAACTGCTTTAATTTCAAACGTTTTGTACGTTCAGATAAGTATTT
ATCCTCAATAATATCTCTAGCACGTTTTTCCATTTTAGCAATTTGCTTTTCTTTTTCTCTATCAATGTCAGTA
CGAATCGTTTTAATATCATCTCTAAGTGATTTTTCTAATTGACGTCTAATTTCGTCAGAATTATCTTGTCTAG
AAGGTCTATGTTTCTGCTCCACCGGTTCTGCTTTAGGCACTGTAATTGGTTTCTCTGTCATCGGTTTTGTTTC
TGGTTGTTTTTTAGGTGCCATAGGTTCTACAACAGGTTTCGATTTAGGCTCTTCCTTTGGAAGTTCATCGAAT
AAAGGTGGTAACGTATCATCATATTTTCGTTTCGATGATTTCTTTTCTTCTTCATTTAATTCTTCACTTATTT
CTTTAAACGTTCGCTCAATTTCTTCAAAAAAGCCACCTTTTTTTGGTTCATTATCGGTAGATGTTTTTTGAGG
TGGCTTTTGATTTTGTCTATCTTTATGACTATTTTCGCGCATAGTAGTAATGATAGAAATGATCACTGATATG
ACAAAAATTAGAATACCGACACTCATTTAATCACCTCTCGACTTAATGTTCAGGTGACTCATCATCACTTTGA
TCAGTTCGTTTATTAATTGCATTTCTCATGCCTGTATCAGCTTCGATATTTTTCAAATTATAATAATCTTTAA
CACTGATATTGCCTGAGCGTAATGCTTCAGCCATAGCTAATGGCACTTCAGATTCGGCTTCAACTACTTTAGC
ATGCATTTCTTGTACACGCGCTTTCATTTCTTGCTCAGTTGCTACAGCCATAGCTCTACGTTCTTCAGCTTTT
GCTTGTGCAATATTTTTGTCTGCTAATGCTTGTTCAGTTTGTAAGTCTGCACCAATATTTTTACTAATATCAA
CGTCAGCAATATCAATTGATAAAATTTCAAATGCAGTACCTGAATCTAAACCTTTGCTTAATACTGTTTTAGA
AATATTATCTGGGTTTTCAAGTACTTCTGTATGATGCTTACTAGAACCAATTGTTGAAACGATACCTTCACCA
ACACGTGCGATGATTGTTTCTTCACCAGCACCACCAACAAGTCGAGCAATATTAGCTCTAACTGTGATACGAG
CTTTGGCTTTCACTTCAATACCGTTCATTGCTACACCTGCGATAAATGGTGTTTCAATGACTTTAGGATTAAC
AGACATTTGAACCGCTTCTAATACGTCACGTCCTGCAAGGTCAATTGCAGCAGCACGTTCGAAAGGAAGATCA
ATGTCAGCACGTTGTGCAGCAATATTAGCGTCAACAACTCTGTCAACATTTCCTCCTGCTAGATAATGCGATT
CTAATTGATTTGTTGTTAATGCTAGTCCTGCTTTATGCGCTTTAATTAATGGCGCTATAACTTTTCTTGGAGA
TACACGACGTAAACGCATACCAACCAATGTACCTATACCAACATGAACGCCAGCTGCTAACGCTGAAATCCAT
AAACCAATCGGTACAAATGAGAATAAAATAAGTAATGCAACTACTATAATAACTGCTATTACGATAAAACTTA
AACTAAACATGGTATCGCTCCTTTTAATTAATCTACTTCCCTCACAACTACTCTTGTTCCTTCAACTTCAAGG
ATTTTTACCGTTTTATTGCGTAAAATAAAGTTGCCATCTGAAACAGCATCAATACGTTCATTTTCACAAAAAA
TAATCCCTGCAGGTCGAAGATCTGTAACTGTTTGAGCAGTCTTTCCTACGAGGTGCGAGCGGTTATCATGAGA
ATTGTAACCTGACTCAGAATTAGTTGAATCTTTTAAGATAACTTTATCCAAAAACGGAATCTTTCTGTTGAAA
ATCTTCACTAATATCACCCATTCTACAATCGTTAAAATCAAGGCAACGATAACATTCGCAAGCATAAATAGCA
AATTATCACCGAGCGTTGTTATGCTTATAGTTATCAGTATCATGCCAATAATACCAATTACTGCACCAACTAC
AAATAATTCAATTACAACTAATATAACGCCAATTGAGAATATTAAGATAGAATGCATATTGACATTTCCTTGG
ATTAGAAATCCCAAAAATAAAATAAGTAATGATAATGTGGCGATAATACCAGCTGCATTGATTTTTTTAGAGT
AAAGTTGATATACAAATCCTAAGAATGTCAAACAGGTTAATATTAACGTAAAAATAGGTTGAACAATTATATT
ACTAACTTGTTCAACCCAAGTATCTCCAGCCGTTGATTCCAAGATAGTTGTCATTTGTAAAAAATTATTATAA
CTCACAATTTCACCTCGCCCTCACATTAATTATACATGAAATTGAATATACTTTATAGTTATAAGAACCTATA
GAATATCAATTTGATTATTTATAAACTATATAACTTTTTTTAATTAAAAATTGACAACAAAAAAACATCACAT
ATTAGTGTCAGACACTAACTTGTGATGTTTGTTTTATATAATTAATATTCGTGTTGAGGGAGTCAACAGAGGT
ATTAATTATTTGAATTTACGTTTACGTGCAGCTTCTGATTTCTTTTTACGTTTTACGCTTGGTTTTTCGTAAA
ATTCACGTTTACGTACTTCTTGGATTGTTCCACTTTTAGAAACTGAACGTTTAAATCTACGTAACGCATCTTC
AAGTGATTCATTTTTACGTACTACTGTTTTAGACATCTGTATTTCCCTCCCTCCAAATATCAACGGAACTTTA
TAGTCAATTGCACAGTAAAACTATGCATTTATAAGTATAATATATCTGTAATTTATGGTCAATTAGTAAATTG
TTTTTTATTTGAAACATATTTCACATCAAAATCACAAAGACTTTTAGATTTTGTTCTAAAAATCTCTTAATAA
TTTATTTAATGAGAAGAGTTGCTTATATAGTAAATTGTGAAGCCGTTAAAACAACGTTACAAAACCTATATCT
TTAATACGGAACCTTATGGTATGAATCAAGGAATACTTAAACTAAAACTTCTCTATCAGATTTATTTGTTGCA
AAATCAACAACTTTAATTGCTTGCCCTTCATTTAATGGATAATTTGCTTGCGTAATTTTAACTTTTACAATTT
GACCTATGAGTGATTCGTCACCTTCAAATTGTACTTTCATATAATTATCTGCATATCCAACTAATGTACCTTC
TGTGTCACCCTGTTCCTCAGGAATTACTTCAAGCACATCTTGATCAAATTTAGACGCATATAACTTTCCGAGT
TGATTGCTTAGCGTAATTAACTTATGCACGCGTTCATTTTTAATTTCTTCATCAATTTGGTCATCCATTCTTG

CAGCTGGCGTGCCAATTCTAGGAGAATAAGGGAAAACATGCAGTTCAGAGAACTTATGCTTTACGATAAAATC
ATATGTTTCTTGGAACTCTGCTTCAGTTTCACCTGGGAAACCAACAATTACATCACTCGTAACTGCCAAGTCT
GGTAAAGCTTTATGCAATTTTGTTAATCGTTCTGAAAATCTATCCATAGTATACTTACGTCTCATACGTTTTA
ATACTGTATCTGAACCAGATTGTAATGGAATATGCAAATGACGCACAACTTTTGTTGAACGTTCTAAAACGTC
AATTACTTCATCTGTAAGTTGACTTGCTTCAATTGAAGAAATTCGAATTCGTTCTAATCCATTAATCGTTTCA
AGATCACGTAATAATTGGGCCAAGTTATAATCTTTTAAATCTTGACCATATCCACCTGTATGAATTCCCGTCA
ATACAATTTCCTTATATCCTGAATTCACTAGTTGCGTCGCTTGTTCAACTACTTTTTCCGGATCTCTTGAACG
CATTAAGCCACGAGCCCATGGAATAATACAGAATGTGCAGAAGTTGTTACAACCTTCTTGAATTTTTAATGAC
GCACGTGTTCTATCTGTAAAATATGGGACATCTAATTCTTCATATTTACGATTTTTCATGATATTTCCAACAC
CATTAATTGGTTGGCGTTCTTTACGGAATTCATCAATGTAACCTAATAGTTTATGTCTATCTTGTGTACCAAC
TACTACATCGACACCAGGAATTTCCATAATTTCAGCTGATGAAGTTTGCGCATAACAACCTGTTACACAAATT
ACAGCATCAGGATTTTGTCTTATTGCACGTCTAATAATTTGACGACTTTTTTTATCACCTGTATTCGTTACTG
TACAAGTATTAATAACAAATACATCAGCATTCGCTTCAAAGTCAACGCGCTCATAGTTTGCTTCTTTAAATAA
TTGCCAGATTGCTTCAGTTTCATAATGGTTTACTTTACAACCTAATGTGTGAAACGCAACTGTTGACATAAAT
ATTCACCCCATTAATTCATTTTCATAACTTATTGCACTTAACGCATACAATGGTGCAGTTTCTGCCCGTAAAA
TTCTCGGTCCAAGACCAACAACTGTACTAGTATTACTAAATAATGAAATTTCATTTTCTGACAAACCACCCTC
AGGACCAAAAATCATCAACACTTTATCCTGAGCCTTGAATTGTTGTAAAGTTTGCTTGAAATTGCTTAACTCA
CCATCTTTTGCTTCCTCTTCATATGCAATAAGAATATAGTCATAATTATCAATAGTATCACAAATTAATTTTA
AATTCGACTCGAATTGAATAGATGGAATCACTAAACGATAGCTTTGTTCAGCAGCTTCTTTAATTATTTTTTG
CCAACGCTCTATCTTTTTGGCAACTTTCGCCTCGTTTAATTTAACAATTGAGCGTTCCATGCTCACAGCTATA
AATGATGAAGCACCCAATTCAGTAGCTTTTTGTAGCAACCACTCATATTTGTCAGCTTTAATTAGTCCACTGC
AAATCGTAACATCAACTGGCAATTCTGTATTAATATTTTGTTTTTCTTTTAAATCAACTGCAATTTTTATCACT
TGTTATATCAGCAATTTCACATAAATAAACTGTTTGATCATTAAAAGTTAAAATAATTTTACTACCAACATCA
TATCTCATTACATTTGTTATATGATGAATATCTTCTTTTTTTGTAATAAAAAAACGCTGACTTACATCAGCGT
TTTGGTCTATGAAATAACGTTGCACATTATTCACTCACTTTCTGGCCAACAAGACAAACCCAACCGTTGTCAT
GTTGTTCTGAAATAATTTTAAAACCTACACGCTCCATATGTGACTGTATACCTTCATACTTCTCTTTTATAAT
ACCAGAAGTAATAAAATAACCGCCTTCATTTAGAGTATTATAAGCATCTTCAATCATTTCATCAATAATATGC
GCTAAAATATTTGCTATTACAATATCAAATTTTTCTGTTTCGTCTTTCAATAAGTTACCTGGAACAGCTTCAA
TTAACGTTTCACAATGATTTCTTCTGAAGTTTTCTTTAGCTACACTCACTGCCATTTCATCAATATCCAACGC
TTTAATTCGTTTTACACCGATTAGATGACTTGCAATACTTAATATACCTGAGCCAGTACCAACATCAATTACT
GAATGCTGTGGCAATACATATGTTTCTATTGCCTTCAAACACATACTTGTAGTCGGATGATCGCCTGTTCCAA
AAGCCATACCTGGGTCGAGCTCAATGCAAAGCTCTTCATCCGCTTCTTTAGCATATGTTTCCCAACTAGGAAC
TATTGTGAACTTCTTCGACGCTCGGAATGGATGGAAATAGTTTTTCCATTCATTTTCCCAATCCGTCTCTGCA
ATAATTTGCTCACTGAATTGAACGTTATGTTGATCAAGTTCATCTAAATTTAATAACTCATCTTTAATTTGCT
GTCGCAACTTATCATCATAAGTCATTTCATTAAAATAGGCTTTCAATCTTACTCCCTTATCTGGATAATCCTC
TTTTTTCAAAGCGTAAATTTCACCGTATTTATCTTCTGGTTGGTTAATTAAATCATCTGAATCTTCTATCACG
ACACCATTTGATCCATGATTTTCAAGTATATTGGTAGCCAATTCTACTGCTTCATGATTAATAATAATTGAAA
GCTCTGTCCAGTTCATACTTTATTCTCCCTTAAAGAATCTTTTTGCTCTATCTTTAAAATTCGAAGGTTGTTC
ATTAATTTCTTCACCATTTAATTGGGCAAATTCTTTCATTAGTTCTTTTTGTCTATCTGTTAATTTAGTAGGC
GTTACTACTTTAATATCAACATATAAATCTCCGTATCCATAGCCATGAACATTTTTTATACCCTTTTCTTTTA
AGCGGAATTGCTTACCTGTTTGTGTACCAGCAGGGATTGTTAACATAACTTCATTATTTAATGTTGGTATTTT
TATTTCATCGCCTAAAGCTGCTTGTGGGAAGCTAACATTTAATTTGTAATAAATATCATCACCATCACGTTTA
AATGTTTCAGATGGTTTAACTCTAAATACTACGTATAAATCACCAGCAGGTCCTCCATTCACGCCTGGAGAGC
CTTCACCAGCTAATCTAATTTGTTGTTCATTGTCGACACCTTCAGGTACTTTCACTTCTAATTTAACTGTTTT
ATTTTCAGTACCTTTTCCGTGACATGTTGGACAAGCTTCTTCAAATTCTTGACCACTTCCATTACATTTAGGA
CAAACTTGTTCAGTACGAACTCTACCTAAAATTGTATTTTGTTCAACAGCTACATGACCAGCGCCATTACAGT
AACTACAAGTCTTTTTACTTGTTCCAGGCTTTGCACCATCACCATGACATGTTTCGCATGTTACATCTTTACG
GATTGAAATTTCTTTTGTTGTACCAAATACCGCTTCTTCAAATGTCAATGTCATTGTATACTGAAGATCATCA
CCTTTTTGCGGTGCATTTGGATCTCTTTGTCTGCCGCCACCGAAGAAAGAGCTAAAGATATCTTCAAAACCGC
CGCCACCGAAGCCACTAAAACCACCAAAGTCAGAGCCATTGAATCCTTGTCCGCCAAAACCTTGCGGACCGTC
ATGTCCAAATTGATCATAGCTTGCGCGTTTATTATCATCACTTAAAACTTCATATGCTTCAGAAATTTCTTTA
AACTTTTCATCTGCACCTTCTTCTTTGTTAATATCTGGATGATATTTTTTCGAAAGCTTTCGATACGCTTTTT
TGATTTCATCTTTTGAAGCATCTTTACTAATGCCTAAAACTTCATAATAGTCTCTTTTGGCCACAGCTATCTC
TCCTTTTCTTAATTAACTCATATAGTTTAACGTAATATGTCATACTATCCAAATAAAAAGCCAAAGCCAATGT
TCTATTGACTTTGACTTTTCAGATCATGACAACATTCTAATTGTATTGTTTAATTATTTTTTGTCGTCGTCTT
TTACTTCTTTAAATTCAGCATCTTCTACAGTGCTATCGTTGTTTTGACCACCATTAGCACCTTGTGCTTGTTG
TTGCTGTTGAGCCGCTTGCTCATATACTTTTGCTGATAATTCTTGAATCACTTTTTCAAGTTCTTCTTTTTTA
GATTTAATATCTTCTATATCTTGACCTTCTAAAGCAGTTTTAAGAGCGTCTTTTTTCTCTTCAGCAGATTTTT

TATCTTCTTCACCGATATTTTCGCCTAAATCAGTTAAAGTTTTTTCAACTTGGAATACTAGGCTGTCAGCTTC
GTTTCTTAAGTCTACTTCTTCACGACGTTTTTTATCTGCTTCAGCGTTAACTTCAGCATCTTTTACCATACGG
TCGATTTCTTCGTCTGATAATGAAGAACTTGATTGAATTGTAATTCTTTGTTCTTTATTTGTACCTAAGTCTT
TTGCAGTTACATTTACAATACCGTTTTTATCGATATCAAACGTTACTTCAATTTGAGGTTTACCACGTTCAGC
TGGTGGAATATCAGTCAATTGGAATCTACCAAGTGTTTTATTATCCGCAGCCATTGGACGTTCACCTTGTAAT
ACGTGTACATCTACTGATGGTTGATTATCTACTGCTGTTGAATAGATTTGAGATTTAGATGTAGGAATCGTAG
TGTTACGTTCAATTAACGTATTCATACGTCCACCTAAAATTTCAATACCTAAAGATAGTGGTGTTACGTCTAA
TAATACTACGTCTTTAACGTCACCTGTGATAACGCCACCTTGGATTGCAGCTCCCATTGCCACTACTTCGTCT
GGGTTTACACCTTTGTTAGGTTCTTTACCGATTTCTTTTTTGACAGCTTCTTGTACTGCTGGAATACGAGTTG
ACCCACCAACTAAGATAACTTCATCGATATCTGAGTTTGTTAAGCCAGCGTCTTTCATTGCTTGGCGTGTAGG
TTCCATTGTTCTTCTAATTAATGAATCTGATAATTCCTCAAATTTAGAACGAGTTAAGTTTACTTCTAAGTGT
AATGGACCGTTTTCACCAGCTGAGATAAATGGTAATGAGATTTGAGTTTGTGATACACCTGATAAGTCTTTTT
TAGCTTTTTCAGCAGCATCTTTCAAACGTTGTAATGCCATTTTATCTTGAGATAAGTCTACGCCATTTTCTTT
TTTGAATTCTGCAACTAGGTAGTCAATAATTACTTGGTCAAAATCATCACCGCCAAGTTTGTTGTCACCGGCT
GTTGATAGTACTTCGAATACACCGTCACCTAATTCTAGGATAGATACGTCAAATGTACCGCCACCTAAGTCAA
AAACAAGAACTTTTTCATCTTTATCAGTTTTGTCTAAACCATATGCTAATGCTGCAGCTGTTGGTTCATTAAT
GATACGCTCAACTTCTAAACCAGCAATTTTACCAGCATCTTTAGTTGCTTGACGTTCAGCATCGTTAAAGTAT
GCAGGTACTGTAATTACAGCTTTGTCAACTTTCTCACCTAAATAGCTTTCAGCTGTATTTTTTAAGTTTTGTA
AAATCATAGCTGAGATTTCTTGTGGTGTGTATGATTTACCTTCAATATCTACTTTATAATCAGTACCCATATG
ACGTTTAATAGATTGAACAGTGTTTGGATTTGTAATAGCTTGACGTTTAGCTACTTCACCAACTTGAGTTTCT
CCATTTTTGAAAGCTACAACAGATGGTGTTGTACGTGAACCTTCAGGGTTTTGAATTACTTTTGGCTCATCGC
CTTCTAATACTGTTACACATGAATTTGTTGTACCTAAGTCTATACCAATAATTTTACTCATAATAAAATTCCT
CCATTTAATCATTAAATTAATTTAATTTTAAACAATGTCTTTTCGCCAAATTTAAGTTATTGGTTTACTTTGA
CCATTGATGGTCTTAATACTCTATCTTTAAGCTTGTATCCTTTTTGTAGTTCTTGAGTGATTTCGCCAGATTC
AAAATCAGGGTTATCATCTTGAACTACAGCTTGGTGAATATTTGGATCAAATGCTTCACCTTCAGTTTTAATA
ACTTCAAGACCATTATCTTTTAGTGCGTTAATCAAACTTTCATGCACCATTTGTACACCTTTTTGAAGAGATT
TAAAAGTCTCATCATCACCTTCAATTTGAAGTGCACGTTCTATATTGTCTATTGCTGGTAAAATATCTGTTAA
CACACGTTGTGCTTGATATGTTTTGTTTATTTCATTTTCTTTTTGAATTCTACGCTTATAATTTTCAAACTCA
GCGTAGAGCCTTAAATATTTCTCTTCGTTTTCATCTGCTAATTGTTGAAGTTCATTAATTTTTTGATCTTTTG
GATCTATTTCTTCAATAACATTCTCGTCAGACGTTTCTTCTATTGCTTCATCTTGTAAATGACCTTTACTTTC
TTCAGCTTGTTCAACTGAATCATCAATATTTTGTTTGACGTTTGTTTCTTCAACTGTTGATTCAGTGTTTTTT
TCAACTGATTCGTCTTTATTTGTCATTTTCTGTCCTCCAATACTTTCTAATCCATCATTACCAAATTCTATTT
AATAATTGAATGACATTTTGATAATGCATAGCTGTAGGTCCAATCACAGCGATTTGACCTTTTAACGTTTCAT
CAAAATGATATTGACTTGTTACAATTGAAATATCACTTAAGCTGTCATCAATTTCGTTACCAATTTTTACATT
AATATTTGGTGAAGATATATCTTGTAATAATTCTGCAATTCTATTTGATTCTATATATTGTAGAATGGGCTGA
ATTGAAGATACATTACTTTCATTCAATGCATCAATAAGTTTAACCTTTCCACCCATATAAATGCTATTACTTT
GATTAGAAATATGATTATTCATCGTATTTAACAATTGATTGATAAATATTTCTTCCTGCTCTGATTGAACAAA
AGAGACAATATCATCTTGTAAATTCTGATTAAACTCAGTTAGTTTGTTTGTAACAAAATTTGATATTGTATTT
AGTTTGTCGTTATTAAACGGTATGTCTGAAGCAAGATGTACATGCTCTACATGACCTGATGAAAATACGATAA
CCATTATAACTAAATTAGGATTAGCACGAATCAAGTGTACATTATTGATAATATCTTGTTTATGATTAGGATG
AACAACTAAAGTTGTATATTGAGATATATTTGATAATTCATCTGCAAAATATGTCAATGCTGATGATACATCA
TATTGATTCTCAACTAACAATTGATTTAATCGTCTTAATTTATTTGTTTTTTGATGAGATGTTTGTTCAAGTA
AACGATTGACATAATACCTAAAACCTAATTGTGATGGCGAACGCCCTGAAGAACTATGTGTCTTTTCGATATA
GTTTAAATCTTCAAGCTGTTTCATCTCATTTCTAATTGTAGCAGGACTAACATTCAAGTTATGTCGCTCAATT
AGTGTTTTAGAACCAACGGGTTGTCCAAAATCAACATAATCCTCAACAATTGCGTTTAATATACTCAATTGCC
TATCTGTAATCATGTTTTCACCTCATTAGCACTCACTTATCTCAAGTGCTAATTATAATTTATCAAATTGGTC
AAAGTAAGTCAATGTTAAAGACTCGAAATTTCAATTTTTTTTAATCATTTATTAGGAAAGCTTCAAAAACCTC
ATTACCGATGACTTTCCCTCGCTTTGTTAGTGCAATCGCATCGTTCTTTTCTAAAATTAATTCCTTCTCTTTT
AAATTATTTATTGTTTGACCAAAGACACTTTCAATAGATTGGTCAAACTTCTTTTGGAACCTACTACTACTCA
CACCTTCATTTAAACGCAACCCAAGAAACATTTCTTCTTCCATTCTCTCAGTCAAAGAAGGTTTATTTGATAC
TAAAATTGCTTTACTTTCTTTATTTATAGCTTTGATATAATGTTTCACTGGATTGATATTCGTATAACGCACA
CCATCTACATAACCACTTGCACCTGCTCCAAATCCATAATATTCCTCATTAAACCAGTAAACCTTATTATGTT
CTGATTCATGGCCATCTAATGCAAAATTAGATATTTCGTATTGATGGAAAGGAGATTGTTCTATCTTAGACAT
CAGCAACTGATACATGTCAGCACCTAAATCCTCATTAGGAAGTTTGAGCAACCCTTTTCTATACATATTATAA
AATTGGGTTTTAGGTTCAAGTATTAAGCCGTAACTCGAAATATGTTGAATATCCATATCTAAAGCTAGATCTA
AACTTTGTTCAAAATCTTCAATCGTCTGTTTCGGTAAATGATACATTAAATCTAAACTGATTGATTTAATACC
TGCGTTTTTAGCATTTAACACCGAAGTGTAAATATCTTCAGTATTGTGCGTTCTACCTAAAACAGACAATAAC
TCCGGCTTGAATGTTTGAACGCCCATTGAAATTCTGTTTACTCCATATTTCTCTAATAGTTGGACTTTCTCTT

TAGTTAACTCATCAGGATTGGCTTCAAATGTATACTCGCCTGTGATTGTAAACGTATCACGTATTGCTTTAAG
TAATCTTTCCAACTGATTAATAGAAAGGGCCGTTGGTGTGCCACCACCTACATACATGGTCATTAAGTTCCTA
TATTTGGCTGTAGACATTTCTGTTATTAGTGCATCTAAGTACTCATCTACAGGTTGATTCTGTATAAAATATT
TATTGAAATCACAATATGTACATATTCTTACACAAAATGGAATATGTATATATGCACTTTGTACCGTCATTTT
AATGCCCCGCTTCTCAGACTGTTTATCCAATCGGCTACATCCTGTTGGACTATCTGCTATTATTCTTACTTTT
AACTTTAACGTTTAATCAATGATTGTCTAATATATCGATTCATTTTAATATTTATAAATGTTAAGAGGGAGTG
AGATAGAAATAATAAAGAACCACTAATGATTTATTATGTAGTGGCTCTTACACATTAGCCAGAGGTAATATGT
ACTTAAAAATAGAATTACATGAGTTAAACTCATGCATAAGGGAGTGGGACAGAAATGATATTTTCACAAAATT
TATTTCGTCGTCCCACCCCAACTTGCACATTATTGTAAGCTGACTTTTCGTCAGCTTCTTTGTTGGGGCCCCG
CCAACCTGCATTGCCTGTAGAATTTCTTTTCGAAATTCTCTGTGTTGGGGCCCCACCCCAACTTGCACATTAT
TGTAAGCTGACTTTTCGTCAGCTTCTATGTTGGGGCCCCATTGTTATAAGCTCATTTTAACTCTGTCGATGGC
TGCCAATATAACATTGTAAAGCCCAGGACGTTAATTTATCCTAGGCTCCCTCTTTTTAGTTATTAAACTTTAT
TCATTGTAAATTGTTAATAGATTTTTTAAAAACTTATTCGTCATCCATTTTCAACACAGCCAAGAAAGCATCC
TGTGGAATTTCAACATTACCAACTGCTTTCATCTTAGCTTTACCTGCTTTTTGTTTTTCAAGTAATTTACGTT
TACGGCTTATGTCACCGCCATAACATTTAGCTAAAACGTTTTTACCCATTGATTTAATATTTGTACGCGCTAC
AATTTTTTGTCCTATTGCAGCCTGTACAGGTACTTCAAATTGCTGTCTTGGAATTAACGTTTTAAGTTTTTCA
ACTAATGCTTTACCACGTTCATACGCAAAATCTCTATGAACTATGAAGCTTAGCGCATCCACTTTATCACCAT
TTAATAAAATATCCATCTTGACTAAATTACTTTCTTTATTTTCGATGAATTCATAATCAAATGATGCATATCC
TTTAGTATTAGATTTAAGTTGATCGAAGAAATCAAATACAACTTCAGCTAAAGGTAATTCATAAACAATATTT
ACACGAATATCATCTAAATAGTCCATATTTATAAATTGTCCACGTTTACGTTGACATAATTCCATTACTGCAC
CGACATAGTCATTTGGAACCATCATAGTTGCTCGAACATATGGCTCAAATATTTTATCAATTTTATCACGATC
TGGCATTTGTGCTGGGTTATCAACCGTCACTTCTGAACCGTCCCTTAAAATACATTGATAAATTACAGATGGT
GCGGTTGCAATTAATTCAATACCAAATTCTCTTTCAATTCTTTCTTGAATTATTTCCATGTGTAACATACCTA
AGAAACCAGTTCTATAACCAAAACCTAATGCTTGTGATGATTCAGGCTCAAATTCTAATGATGCATCATTCAA
TTGTAATTTTTCTAATGCTTCTCTTAAATCATTATAATTTTTGTTATCTATTGGGAACAGTCCGCAATATACC
ATTGGATTCATTTTCTTATAACCTTGCAATGGTTCTGATGCAGGTCTACTAGCTAATGTGATTGTGTCACCAA
CCCTAGAATCATCAACATTTTTAATACTTGCAATAATATAACCAACATCACCAACTGTTAATTCATCAACTGG
AAGCTGCTTAGGTGTATTAATTCCAACTTCTGTTACTTCGAACTCTTTACCAGTGGCCATCATTCGAATTTTA
TCCCCGGCTTTAACAACACCGTCTACAATTCTTATCGATGAAATTACCCCTCTATATGGATCATACTCAGAAT
CAAATATTAACGCTTTTAGTGGTGCTTCTGGGTCACCATCTGGAGCTGGCACAACTTCAACTATTTTCTCTAG
TATCTCTTCAATTCCAATGTTAGATTTAGCACTTGCTAAAACAACATCGTCTTGGTCTAAACCTATCATATCC
TCAATTTCTTGTTTCACGCGTTCAGGTTCTGCAGCAGGTAAATCAATTTTGTTAATAACAGGCAATAACTCTA
ACTCATTATCTAATGCCAAATAAACATTTGCTAATGTTTGTGCTTCGATACCTTGAGCCGCATCTACTACTAA
AATCGCACCCTCACAAGCTGCCAAAGAACGTGACACTTCATATGTAAAATCGACGTGTCCAGGCGTATCGATT
AAATGGAATGTATAAGTATTTCCATCTTTAGCTTCGTACTTTAAACGTACTGCGTTTAATTTGATTGTAATAC
CACGTTCTCTTTCTAAATCCATTGAATCTAGTAACTGATCTTGCATATCTCTTGTTTCAACTGATTTGGTATT
TTCTAAAATTCTATCAGCCAATGTAGATTTTCCGTGGTCAATATGTGCTATAATCGAGAAATTCCTTATATTC
TCTCTTCTTTTTAAGCGTTGCTCATTATCCATTTTATCCTTCTCACTTTCATAAAATAAACCCGTATTATCTT
TCGCATCTTTGATATAATAACGTTTTTAATAGGTAAATGCAAACGCCATAATCTTGGATGAACTTTGAAATTA
TAACAATCTTTATTTAAATACATACTTGCGATATGTTAATATGTTAATTACGTACTTATAATTATAAAACGTA
TTGATTGTGTTTCCAACACAATTTATTAAATCTGCAAAATTTTAAGCTAACCCCATCAAATAAATGATTGCAC
AACGGTTAGACTTTTGTTAAAATATTTCTTGTTGTAATCAAATAAAATTTTGATAAGATGAACTCACTTTTAG
GAGGTGACAGAAATGGCAAATATCAAATCTGCAATTAAACGCGTAAAAACAACTGAAAAAGCTGAAGCACGCA
ACATTTCACAAAAGAGTGCAATGCGTACAGCAGTTAAAAACGCTAAAACAGCTGTTTCAAATAACGCTGATAA
TAAAAATGAATTAGTAAGCTTAGCAGTTAAGTTAGTAGACAAAGCTGCTCAAAGTAATTTAATACATTCAAAC
AAAGCTGACCGTATTAAATCACAATTAATGACTGCAAATAAATAATCTTTTTAAATAAAAGTTCAAGCGCATG
CTTGAACTTTTATTTTTTATAAAGATAGAATGAATAATTCCAGTATTAACTGTTTATCCATATATGATGATTT
AAGTTTATAATCAGTTTCCGCACAAGCATCTATAATATTCAATAATTCATCAAGTTGATAATGTCTTACTTGT
CCTAACGCTAATTTTACTCTGTATGGATGTACGCCTATTGTTTTAGCAATTTGCTGTCCACTATATCCTTTTT
GACTCAAAATCTTACATTGATAAAATAATCGGTAATTACTTGTAATTAGTGCAAGTAATTTAATAGGTTCTTC
TTTCATAGTTATTAAATCTTTTACTAAATGAATTGCTTGTTCTTTCTTTCTTTTCTGAATGTATTCAGTCAGT
AAAAATACATTTTGTTCTAAACTTCTATTAATAATTTGGTTAACATCCTGCTTATTAATTGTTGGTCTATCGC
CTAAAAATAAAATCAACTTTTCTATCTCTTGTGAGACAATATTAAAGTTAATACCTGTCAACTCAATAAATAA
ATCTAATGCATCTCTTTTGATATCTTTGAAATTCTCATTTAATTTACTTTGAATCCATTTTTTTATTTCTTCT
TCAGACATTTGCTCTATTTTTTTAAGCCTTGCATGCTTTTTTAGAGTTTTAGTTAACTTTTTTCTTTCATCAA
GTTTATTTTGATATATCTCAAAGACAATCAAATTTTCGCCATCATATTTTTCAATAAATTCTATTAATTGATC
TACATTATGAGCCATATCTTTTGGCGCTTTTTCACCTGTAAATATATATGCATTTTTAACCAAAATTGCTTTT
TTATCTGAAAAGAAAGGCAATGTTAATGTTTCTTCAACAATTGGTGCAATCTCTGTTTCGTATAAATTATATT

TCACAAAGTTAAAGTCATCTCTATCACTTTTCAAAAATTGTGATATGATTTCTGCACTTTGTTTTTCAACCAA
TTCAGGCACATCTCCATAAATAGCTACAATATTGTCGCTCATTCATTACACCCTTTCATTCGTTTGACACATC
AATAGATTATATCATGTTTCTGTATCTATAAACCACTTGCATTTCCATAAGAGTTTGAATCAACTTTTAAATT
ATCATCTAAGTCAATTGTAACTTGACCGTTTTGTTGACTATTGTAAATGCGACTGCGAATCCTTTGCAATCGT
TTAACAACTTCTATATTAGGAAGATGATACATATTGTTCTTCCCAGAAGAAATCAAACTTATTTTAGGCTTAA
TCATCTCTATAAATTCTTTAGAACTACTTGTCTTGCTCCCATGATGTCCTACTTTTAAAATATCAATCTCCGG
CAAGTTATATTTTTTTAGTAGTAAAGATTCATTATTTTTACTAGCATCGCCCATTAATAAAACTTTTTTATTT
TGATATGTAATCATAGTAATAATAGAATATTCATTTTTATCTCGGCTATTTGGAATAAAACTATCAAAAAATA
GAAAACTACTATCTCCAAGTTTAAAACTACTAACTTGTCTTACATCCATAAGTTTAATGTTATACTTATGGCT
TAATTTCGATAATAACATCAATGTATTACTACTATATCCCTTATTGTATATCACTATATGTTTAATTTTAATA
TGACTAATAATATATTCCACTTCACCAATATGGTCATTGTGTGGATGTGTTAAAATTAGATACTCTAATTCAT
TTATCCCTCTTTCATTTAGCGTTGGTAAAATATGATATTTAGAAATTGAATAACTAGGTTGTTTAGTATCATC
AATCACTTTCCCACCTGTATCAATCAAGACATTTTGGTTCTTACCACCTTCATATAAAATACTGTCTCCCTGC
CCCACATTTAACATTGTAATTTTGTGATGTGAATTTGTTGGAAACGTTATTAATAATGTCAGAATAATTATAG
TCCAAACCGTAACCAATATATATTTACGTTTAGCCAATAACCAAAATATGTAATAAACAGAAATTATAAATAT
TATAAATATCCAATCATTAAACTTGGGAACAGAAAAATGTGATTGCTTGATTCTTGTGAATAGGTCTAGTAAC
CAGTCATGAAAATTAAAACTTAGGTCAACCAAGTAATTTAGCGGCGTTAATCCCACAATAAAATGACTTGTAA
TAAAGAATAAAATAGATAGCGGAAACAATATAATCGAATAGTACGGTACAAAAATCAAATTAGATAAAAATCC
CACCCATTGAAGTTGATGAAAGTTTGGAATGGCAACGATAAATGAAGCTAATTGTGCAATAAACGTAATTATG
AATAATGATTGTAACTTTGACAATTGCTGTAAAAAAGGAAAAAGTAGCATAATAAAAAATGAAATGATGAATG
AAAATTGAAATCCAATATCATAAACAACTAGTGGATTTAAAATAAACATAATTATAAATGCAAATGCTAATAG
CTGAATACCCTTTATTTTAATTTGCTTAGTAATAAGCAGTACAAGAGTTGTCATTATTATAGCTCTTACAGCA
CTAGGTGCATAATTTGTGTATTGAGCAAATAAAGCTAATACAATGATTGTAATTCCTTTAATGACAAATAAAG
GTAAATTTAATCGTTTTAAAGGTTGGTAAATTAAGAATACAATTGCAGCTATATGCGAGCCACTAACTGCCAG
CAAATGATATATACCTATCTCTTTAACACGTTCCTTAAATTGCTCATTAATTTCTTTTACGTCACCAGTAATC
AATGCCATAATACGATCCGGAAACTTAATACCCGAATCATAAATTCGATTCATTATAAACTGTTTATGTTTCT
CAATTAAATTAGACCGGTTCGATTCTAGGCAGCTTTGTACAACTATACTTTGTAATTTAAGAGTTACAAACTT
ATTGTCATTAACTTTGAATTGTCCTTTAACAATACAATTACGACTTTCAATTTTCTTTAAATCAAAATTCTTT
TTATTTGTTAAAAAGAAAGGATATATTTCATTTTTTAAACTCAACCTACCTTTATATGTGTCACTACCTTGAC
GTTGAATTTGGATTACTTGAGCACGCTCATTAAACTGAGAATTACGTTCAATATAATTGATATAATTAAATAT
TGCTTGTTGTGAATAATGTAAATACCATGAGGAGAAAATGATTAAAAAGAGAGAAATAGGGGCATAAACGATT
TTATTTTTACGATAAGTAATATACAAAAGTAAAATGAAAAGAAATGTAGAGAGCACTTTGCTAGAATTCCAAA
GCACTCCTACAATCATTGATAACGCGACATACAGCAAAACATTAACCTTTAGTCAGATATTTAGCAACATATT
CTGGTGAAAATGGAATTTTTTTAAATTCAATACCAGATTGTTTGAGTAATTTAGTTGCATATTCATGGTTATG
ATAATCTTCTGCATAGTAGATACGCTTTATACCTGCTTGAATAATTGACTTTGTACAATTTAGGCATGGAAAA
TGAGTAACATAGATTGTTGCACCTTCAGTAGATACACCTTGTTTTGCACATTGTAATAAAGCATTCATTTCTG
CATGTATCGTTCTGATACAATGTCCATCTTCAATTAAACATCCTTCATCTATACAATGCACCTCGCCAGCTAC
AGAGCCATTATAACCACCAGCAATAATACGATTATCCTTAACAATCGTTGCACCTACAGATAATCTTTGACAA
GTTGAACGTAATGCTAGCAAATGACTTTGTGCCATAAAATATTCTTCCCATTTGATTCTTTCCAAGATGTTCA
CCTTCCATACTTAAAATTTAGTAACATTTTCTAATAATATAAGATTAATCACAAAAAATAAAATTTGCAATTA
AAATAATCCATTATATCGTGAAATAAGATTTCAGTTTATCAAAAGTTTTACTTCCAAAACCTTTTACTTTTTT
CAAATCGTCAATTTCTTGAAATGCACCTTGTTGGTTGCGATATTCAACAATTGCATTAGCTTTAGCTTGCCCT
ACTCCAGGAACAGACATCAATTCTGATACAGATGCGGTATTTAAATTTACTTTAGTATTATTTGTGTTCCCAT
TTTTTACATGCACACTGTTTACTCCAATTTGTGGTTCAACATTCTTTTGTCCTTTATGAGGTATGAAAATCAT
TTTTTGATCTGTTAATTTTTCAGACAAATTAATTTGACTTACATCTGCATCCTCCAATAATTGTGCTTTATCA
AGTAAATCAACTACTCTATCCTTAGATGTCATTTTATAAACATTAGGATGTTTAACAGCACCTTTTACATCGA
CATATACAGGACCTTTATTTTTGGAATTATCTCCATCTTTGACCTGGACATCTTCTAATTTGGACAAACTACT
ATTTTCACTAGTGCTTTGTTTCAGAGCAGTATCTTTATTTTCAAAATTTCTTGAAGTATAATCATCTTGTCTC
CAGAATATAAAACCAATTAATACCATAATTAAAACAACAGCACTTATAATATATAACTTCCACTGAGTTAAAA
AATCTTTATAGCGTAATAAAAATTGATACAATAAAACCACTCCTCAACTTTATACACGTCTGAGCGAGTGGTT
TTACTTTTATTTTTTCGCAATGAAAAACAATCTTTCTGCATCTTCATTATGTTCATCTATATTAAAATCAGTA
AATGTTTCAACTTGTTTAAAACCAACTTGAGCTAACCAAGACAAATATGTCTTTTCATCAAATGTTCTCTGAA
AATGAGACTCATCAAACCTTGAATATGTTTCATCTTCATGTCGAATGAAAAATGTCATATCATGATAAACACT
TAAAGGTAAATCTCCTTGCACAGCATCCCATGCTAAAAAAATGTCCCCTTTATCATCAATATAACTTTGATTA
TTAAACAAATTCATCATTTTATAAACAGTATGTACATCAAAAATAAATACACCTGAATCAGTCAGATGATGAT
AAACATTGATGAATGTTTCAATCACTGCAGTTTCATCTTGCAAATAATTTAGAGAGTCACAAAAAATAGTGAT
GATATCAAATTGTTGTTGCAAATCAAAAGATGTCATATCTCCTTCAAGCCAATTTACATTTGCTGATTTTTGA
GCTGCAACAGTCAACATATCAACACTTAAATCCATACCAGTAACATTACCTAAAGCTTCTAATTGAACTGTTA

AACTACCAGTACCGCATCCAATATCTAAAATATTTGATTCATCTTTGCAGTGATTTTTTACAATTTCAAACCA
TTTTTCATATGGTTGATCTTGAGTCAATTGATCGTACACTAGGCTCATTTCTGCATATTGCGACATAATTAAT
ACGCAACCTGACTATATGATTCTAATGGTGCATCTTGATATAACTTTTCAATATTATAATAATTTCTTTCGTC
TTTATGGAAAACATGTACCACAACATCAGCTAAGTCAATTAATATCCAACGCGCTTCATTGTATCCTTCCATA
CGTTTTACTTCTATATTTTGTTCATTGGCTACTTCTTTCACCGCTCTAGCAATCGCTTGAACTTGTCGTTCAT
TATTTCCGTGCGTTACAACAAAATAATCTGTCATATCGCTGATACCTTTCATTTCTAAAGAAATCGTATCTTC
GCCTTTTTTATTGTCAATTGCATCCACAGCAATTGCTAATAATTCTTGTGAATTCATTTAATCATCCTTTATT
CTTTCGTCACTATAGTTATAATAATTTAAACAGTCAATCGTCTTATTATATACCGTAATATCTTTCTGTATTA
AAAATAGTACTGTGCGTTTAGAAATTTCATAAATTGTCTTATCTAAACTACCTTGATTGTATGCCATATCTCG
AATATCATCAACTCCTGGGATTGTTCTTCCAGGTTCGATGTAATCTGCAATAAAAATCAGTTTTTCAGTTTTT
GTCATTTGTTGACGTCCAGTAGTATGGTATTTGATAGCCATTAATACTTCCTCATCATTGATACCATATTCAT
GTTCCATGATTGCTGCACACACAGGGCCATGCAATATTTCACTACCATAACTCAGTAGGTCATTACCTAATTC
GTATTGTCGAACAATTTGATACATTTTACCTAAATCATCATACTTACAGAAATCATGTAATACACCTGCTAAT
TCTACTTTACTAGTGTCTCCATCATAAATTTCTGCCAATTTAATAGCTGTTTCTGCAACTCTTAAAGAATGAT
TATAACGTTTCTCTGGCAGTTTCTCTTTTGCAAGCCGTTTTGCTTTTTCAATGTTCATATAATCCTTCCCCCT
TAATATAGTTTTCAACGGATTTAGGAACAAGAACTTGGATAGATTTCCCTTCACTAACTCTTTGTCGAATCAT
TGTCGAACTTATATCTACCCTAGGTATCTGAATTGCAATCATAGCATTTTCAACATTTTGACTATTTTTGTCT
CGATTTACAACTACAAAAGTAACCATTTCTTTTAAGTATTCAATTTGATACCATTTCTCTAGTTGGTTATACT
GATCCGTCCCAATAACAAAGTACAACTCACTGTCTTTGTGTTGCTCCTTGAATGCCTTGATCGTGTCATAGGT
ATAACTCTGACCACCACGTTTAATTTCATCGTCGCAAATATCTCCAAAACCAAGCTCGTCGATAATCATCTGT
ATCATTGTTAATCTGTGCTGAACATCTATAAAATTATTGTGCTTTTTCAATGGAGACATAAAACTAGGTAAAA
AATAAAATTCATCTGGCTGTAATTCATGAAATACTTCGCTAGCTACTATCATATGTGCAGTATGGATAGGGTT
AAACTGACCGCCGTAAAGTACTATCTTTTTCATTATTATGGCAATTCAATTTCTTTATTTTCTTTAGATTCTC
TATAAATCACTATCATAGATCCAATCACTTGCACTAATTCACTACGCGTAGCTTCGCTTAATGTTTCAGCTAA
TTCTTTTTTATCATCAAAGTTATTTTGTAGTACATGCACTTTAATCAATTCTCTGTTTTCTAACGTATCATCT
ATTTGTTTAATCATATTTTCGTTGATACCGCCTTTTCCAATTTGAAAAATCGGATCAATATTGTGTGCTAAAC
TTCTTAAGTATCTTTTTTGTTTGCCAGTAAGCATATGTTATTCTCCTTTTAATTGTTGTAAAACTGCTGTTTT
CATAGAATTAATATCAGCATCTTTATTAGTCCAAATTTTAAAGCTTTCCGCACCCTGGTAAACAAACATATCT
AAGCCATTATAAATATGGTTTCCCTTGCGCTCTGCTTCCTCTAAAATAGGTGTTTTATACGGTATATAAACAA
TATCACTCATTAAAGTATTGGGAGAAAGATGCTTTAAATTAATAATACTTTCGTTATTTCCAGCCATACCCGC
TGGTGTTGTATTAATAACGATATCGAATTCAGCTAAATACTTTTCAGCATCTGCTATTGAAATTTGGTTTATA
TTTAAATTCCAAGATTCAAAACGAGCCATCGTTCTATTCGCAACAGTTAATTTGGGCTTTACAAATTTTGCTA
ATTCATAAGCAATACCTTTACTTGCACCACCTGCGCCCAAAATTAAAATGTATGCATTTTCTAAATCTGGATA
AACGCTGTGCAATCCTTTAACATAACCAATACCATCTGTATTATACCCTATCCACTTGCCATCTTTTATCAAA
ACAGTGTTAACTGCACCTGCATTAATCGCTTGTTCATCAACATGATCTAAATACGGTATGATACGCTCTTTAT
GAGGAATTGTGATATTAAAGCCATCTAATTCTTTTTTCGAAATAATTTCTTTAATTAAATGAAAATCTTCAAT
TGGAATATTTAAAGCTTCATAAGTATCATCTAATCCTAAAGAATTAAAATTTGCTCTATGCATAACGGGCGAC
AAGGAATGTGAAATAGGATTTCCGATAACTGCAAATTTCATTTTTTAAATCACCTTATAAAATAGAATTTCTT
AATACAACATCAACATTTTTAGGAACACGAACGATTACTTTAGCTCCTGGTCCTATAGTTATAAAGCCTAGAC
CAGAGATCATAACATCGCGTTTCTCTTTGCCTGTTTCAAGTCTAACAGCCTTTACCTCATTAAGATCAAAATT
TTGTGGATTTCCAGGTGGCGTTAATAAATCGCCAAGTTGATTACGCCATAAATCATTAGCCTTCTCCGTTTTA
GTACGATGTATATTCAAGTCATTAGAAAAGAAACAAACTAACGGACGTTTACCACCTGATACATAATCTATGC
GCGCTAGACCGCCGAAGAATAATGTTTGCGCCTCATTTAATTGATATACGCGTTGTTTTATTTCTTTCTTAGG
CATAATAATTTTCAATTCTTTTTCACTAACTAAATGCGTCATTTGATGATCTTGAATAATACCTGGTGTATCA
TACATAAATGATGTTTCATCTAAAGGAATATCTATCATATCTAAAGTTGTTCCAGGAAATCTTGAAGTTGTTA
CTACATCTTTTTCACCAACACTAGCTTCAATCAGTTTATTAATCAATGTAGATTTCCCAACATTCGTTGTCCC
TACAATATACACATCTTCATTTTCTCGAATATTCGCAATTGATGATAATAAGTCGTCTATGCCCCAGCCTTTT
TCAGCTGAAATTAATACGACATCGTCAGCTTCCAAACCATATTTTCTTGCTGTTCGTTTTAACCATTCTTTAA
CTCGACGTTTATTAATTTGTTTCGGCAATAAATCCAATTTATTTGCTGCTAAAATGATTTTTTTGTTTCCGAC
AATACGTTTAACTGCATTAATAAATGATCCTTCAAAGTCAAATACATCCACGACATTGACGACAATACCCTTT
TTATCCGCAAGTCCTGATAATAATTTTAAAAAGTCTTCACTTTCTAATCCTACATCTTGAACTTCATTATAAT
TTTTCAAGCGGAAACAACGTCTGCAAATCACGTCATCACGAAACATATTATGCTCTGGTACAAAACCAGGTTT
ATTTTTATCTTCAGATTGAAGTGGCGCACCACAACCGATACATTTTAAAATGTCAGACAATCAATTTTCCTCC
CATGTGATATAACCTTTTTTACTGAAATGACGTAATAATCGTCTTTCAATTAACCTATTAAACTTAGTAATAA
AGCCATCAGTTCGTTTAACTGGAACGACCATAATTGTATATAAACCTCGACGATTACCACCAAATACATCAGT
AAGCATTTGGTCACCTATAACAACAGTTTGATCTGGTCTGATATTCATCTTAGTTATTGCTTTATCAAACGCT
TTCCCCATTGGCTTTCTCGCTTTAAAAATAAAATCGATGTCTAAATGCTGACTAAAACTAGCAACACGAGACT
CATTATTATTAGACACGATTGTAATAGTGATTCCTTTTTCATTAGCTTCCTTAAACCATGCTTTAACACGTTC

TGTAGGTTCTTTAACATCCCAACCTACTAGCGTATTATCTAAATCTGTAATAATACCTTTAACGCCTTTGTCC
ACTAACTTGTCTAAATCAATTTGAAATATTGATTGAACATATGAATTCGGCATAAAAAACTTGCGAACTAAAC
CCATTTAACTCACCTTTACCTTTTTATAATTGAGACACTAATGCTTCAACAGTTTGACTTGATGATACAGCTG
CTTTTTCTAAAAATGCTTCGAAGCTCATTTCCGCTTCTCCATTTGCTAAGTCTGAAACTGCACGAACTACAAC
AAATGGTACATTAAATTGATAACATGTTTGTGCAATTGCAGTTGCTTCCATTTCAACCGCCATCGCATTTGGA
AATGCTTTTTTAATTTTTTGGCGTTGTTCAACACTACCGATAAAGCTATCACCACTTACAATTAAGCCTACTT
TAGCTGTTAATTGTTGTTGTTGTACAACTTGAGATACTTTATCTATTAAAGGTTTACTTGATTGAAATGCTAC
CGGCATCTGTGGTACTTGTCCATATTCATAACCAAATGCTGTTGCATCTGCATCATGATATTTTACATCATCA
CTTATAAGAACGTCACCTACATTTAAACTTTCATCTAAAGCTCCAGCAGAACCTGTATTAATAATGATGTCCG
GTTTAAACTTATTAATTAATAATGTCGTAGAAATTGCAGCATTAACTTTTCCAATGCCGCTTTGGGTAATCAC
TACTTCTCTATCTTTTAAAATGCCAGTATAAAATTTAACATGTGCAACTGAAATTTCGCTTAATTGTGTTAAT
TTATTTTTTAATATTGTTACTTCTTCTTCCATGGCACCAATTATACCAATCATCCTTTGATTCACCTCTTTA
AAAAATCCTAGCATTTGTTATTTTATCACATTTTAATCACTACAACGACAAGTATCATGATTTTCGTTACTGT
ATAAAACTTTTGCTTTATTTTTTAAAAAAACATTCACAATAACGTGGTATCATAAATAATTATTTATGAACTC
ATAACAAGGAAATCGATGCTTCTAAATTGTCTATTTTTCAAAAACTAATCAAATCAATTACATCAGCTAATTG
TGTCAAGAAACTTTATTAAATAACAGGAAAATGATATGTTTAAAATAATAATGATGAAATAAGAGGATTCTAC
AATGGCTGAAAATAATCAAAATAGTCTCGTAACAAAGATTGCTACATACGGCAGTTTTATTGCAATTGCGTCG
TTTGTCATTTTATTTATAAGTATATTTTTAAAGTTTTCATTTAATATCGAGTTTACAGCAACAATTATGAACA
TATGTCGCTACACTCTAATTCTAGGATTTATACTTATGTCCTTACCTGATGTTGTTGATAAAAACATCAAGAA
AATTATATTCGATGCATTTATTATCATCGTCTTTATCTTCTTTTTGCTATAGTAGTTTTATTTTGTAATTACA
CAGTTAATCATATTTGTTTCGTATCGAATTCCTGTGTGCATTAAAGGTAATTCTAATAGAAAACAGCAGTAAG
ACTTTTCTGAGTGATAGCCTCCTTACTGCTGTTTTTATTTGTAACAATAAAATGACAGGTACTTTATATTTCA
CTGTCAAAGATCATTAATGATTAACTTACTTAAATTATGATTTGAATAAATAGATATCTAAATGCAATTTGTC
ACTATTGATAGTTTTATTATCAGCATACATTTTTAATACCTCATGACCATACTCACCATTTAAATTATAAAAA
TCATATTCTACATTTTTTTCATCATCATTATGATATTTAACATACCCTTTAACAATGTCTCCACCTAGTTCGT
ACAACTTATACTTCTCATTCAAATAATTTCTTAATTGAACATCTAATTCTTGAATGGTAACAATTTTCTTTTT
CGTTTTTACTGCAGTTAGTTCTATTCCTGTTTGATGTCCGTCAATCCATAAATTACAAGGTATATTCTTATAA
TCATCGTATTTATTATTGTCACTTAAAGTAACACCACCATAACTACATAGCGTTTTATTGGTTTCTCCCCCAT
GACATCCATATCCATAATTTCCAGCAAATATATCGATATCCTTATTTACAAATTTCTTTGAAAGTGCCTCATT
TTCGAATTCAACTTTAAAATCTTTTTTCCAAGCATCATTTACGAACATGTCATGAAATAACAAATCATGCTTC
AACAGTCTATCGTTTGTTTTTTTATTTTCAAGCTGAGTTGGATTAGCAAATGACACCTTAGCATTATATAGAC
GCTTGGAATCAAGTTCACTTTTATGATGTAAGTCTGAATATTCGATTGCACTTGCTGAATTTGTAAAAATTCC
TTCTAACAATAATATTAAAATAAATCCTGTTAAATATTTAACTTCAAACTCCCCCAAAACAATTACTAATTTA
CATTTGAGAGCGAGACATAAATGATAAGGGAGTGGGACAGAAATGATATTTTCGAAAAATTTATTTCGTCGTC
CCACCCCAACTTGCACATTATTGTAAGCTGTCTTTTCGCCAGCTTCTATGTTGGGGCCCCGCCGGCAAGGTTG
ACTAGAATTGAAAAAAGCTTGTTACAAGCGTATTTTCGTTCAGTCAACTACTGCCAATATAACTTTGTAGAGC
ACTGAACATTGATTTATGTCTCAGGTTCTTTAGCCTACAATTTTGATTATGTATCACGCTCATAATACTTTCC
AATTTGAAATACCCTGTTCGCAATTATTAGTTGAGTTATATCAACCAATACAGTGTATACTTAAAAATTCCAA
ACCGTTTAACAGTATATCAGAAAAAATGATAATTATTTCAATTATTTTTATTAAATTTCATAATGATTCATAT
AATATTCGCATTAAAACATCACAATACTGATACACAACAAAAAGAGCAGTACATTTGTCATGCACCACTCTCA
ACAAATATATTTTTTTAATGAAGCAATTACGCAATCTTACAAATACAATAAGTATAGCTGAATCACATATTAC
ATTTATTTTATAATCGAATTTAGAAATTCATCACTTATCTCATAAATTGTTGGTCTACTTTTAATTTTAACTA
AGTAGTTACCAAATTTTTTAATTGTATTATTAATTTTTACTCTTCCTACTCCCAATATGTTTTCTAAATCAAT
TAATGTTAATCTATTTCTTTTATTTCCAAACAATTTATCCATTGAAAGGAGATACAAAAATTCATAATCTATC
TGTTTATAATGACTAGTTAAATAAAGCGTTAATTTTTCAGTCGCATCCATTTTTGGTATTAATTCATCAAGTA
TTCTTTCTTGTCCTGCTATCAATAACTCAAGCATTGTGTCAATAAATTCCGTCAAATCACCGCAATTCAAATG
ATTTGAAGCAGTCATAAACGCCTTGTAATATTTCGATTTATTCCTATTTATAACATATGAAAATGTCAAAGCA
GTATAATTATCATAATAATCACTCAAGAGCTTGGCAATTATAAATCTTCCAACTCTACCATTGCCATCATAAA
AAGGATGTATATATTCAAACAGATAATGACTAGCCATGATTTTGAACGGCTGAGGCGCATCAAAATATTTTAA
AAATGTTAGCATTTCACCTATATATTCAACAATTTTGGTTTCAGGTTGTAACCCAACATGTATATATTTATTC
GTTGACCCATCATGTACACCGACAAAATTTTTACGAAATAGCTCTCCATCTAACTTATCTTGTTCGTTAATTT
CATTTGAAACTAATTTATCATAAATCGCTCTAATGTCTCTTACATTATCAACTTTAATTTTTTTATTAAGTTC
TATCTCTTTATATTGATCCACGAGGCCTCTGAACTTAAGAAATTCTGATGCTTGGTTATTTAACGCATGTGCA
ATCTCTTGTTTAGTACTAAATACATTTTCAATTTCATTAGTACTTTGTAATTCATCGATTAATAAATCATTAA
AATATTGTTCTCTAGCTGCATAAGGCAAAGAATTAAGTGCTCTATCAATTTTTCTACTATTAATTGATATTAA
TTCTTGTTTTTTTGATAGATTTTTAGTCACCATAAAGAATAGAGGATATTCCAAATCATTAACTTTTTTTCCA
TTTTCCATGGGTATGATATTAATATTAGTATTGAAAGATGCTAAAGAATTAAATCTTTTAGTATACTCGTCTT
TCATTTTACTTTCGTTATGCTCATGAAAAATACTTTTTAAAGTTCTGTAACCCATTTGCAGCCTCCTCTACTC

ATTTAAAATGTATTTTTTGTCCAATAAATCCATTTTCATTAGATTAGCTAAATATTATCACTTTTTTTCTTTA
AAAACTATATAAATAACTATTTTCTCGATCAATAGAAAAAGATTTTTCATCATAACAAAAAAGAGCAGTACAT
TTATCATGCACCACTCTCTTTCTTAAAATATATTCAATTATGCTTTATAAATGAATCACCGGAAATCAAAATA
CATCTTGCCTAATACTAACAACATTTACAGTGATACTATTATTCGAAATGTCTTTTAAAATAACTGAAGTACT
TCTTTGAATGAGAATATACCTGTCAATATTGTAACAAGTACTGCAACGATACCAAAGATGAACATCCAATTTG
GGTGTTTATAATCACCAACAATTGATTTCTTTTTACTTGCAATCAAAATTGCACCTAACGTAATAGGTAAAAT
CCATCCATTTATCGCGCCTGCTATAATTAAAAGGCTGATTGGTTTTCCAATAAACAAGAAAATCATTGTTGAA
ATAACGATAAACACAATGACAATTAAATTACTTCTTTCGTCAAGTGATTTATGAAGTGTTTTTAAAAATGTTG
CGCTTGTGTATGCTGAGCCAATTACTGATGACATAGCTGCAGCAAATAACACAATACCAAAAATATTCTTTCC
AATTGGTCCAATTGCGTGTTCAAAAACTGACGCTGGTGGATTTTCAGAACTTAGTGTCACACCTGTTACAACA
ACTCCTAATACCGCTAGGAATAGTAACGTTCTCATAATACCTGTAGTTAAAATACCAGCAATTGCTGATTGAT
TTACAAATGGTAAATATTGCTTACCTTTAATGCCAGAGTCTAAAATACGATGTGCACCTGCAAAGGTAATATA
ACCACCTACAGTTCCACCAACTAACGTAATGATTGGCAAGACTAATTTCATTGGATGTTCTGGCGCAAATGTA
TGCACAAAAGCATCACCATAAGGTGGATTAGAAACAAACATCACATATGCCACAACTAAAATCATCACAATAC
CAAGAATCATTGAAACAACGTCCATAATTTTTTGGCCACTTTTACTTACAAAGATTAATATTGCAAAGATTGC
AGTAATAGCTGCGCCCCATTTTACATCTAATCCAAAAATTGCATTTAAACCTAAACCGGCACCAGCAATATTA
CCAATATTAAAAGCTAGACCTCCAAATGCAATCAATATTGAGATAACAGTACCAAGCCCAGGAACAACTTTAT
TTGATATTTCTTGACCTCTTAAACCAGTGACAACCAATATGCGCCATATATTAATTTGTGCACCAATATCAAT
AATGATTGATAGTAAAATTGCGAATGCAAAACTCGCCAAAAATTGTGATGTAAATACTGCTGTTTGCGTTAAA
AATGCTGGCCCAATTGCAGAAGTTGCCATCAGAAATACAGAACCTAACAATAATCTCTTATGGTTTTTAGTAA
ATTCAAAATCACTTTCACGATGTTTAAGCTTTAAATTTCTCCCCATTTTTTAGCCCCCTATAAGGATTGAATA
TCAATGCCTTCTTTCATTAAAATTTCTCTAATTTTCGAAACAAATAATAATGCATGTTCTCCATCACCATGCA
CACAAATTGTATCTGCTTGTAACGTTACTTCCTTATTGTTTTTTGAAATAACTTTATTTTCCTTCACCATCTT
TAAAACCTGCTTAAGTGCTTCGTCAGTATCAGTAATCACAGCATCACTTTCTTTTCTACTAACGAGCTGCCCA
TCATCTTCGTATCGTCTATCAGCAAACACTTCAGAAGCTGTAATTAATCCGACATTCTTTGCTTCTGAAATTA
GATATGAATTTGCTAATCCTACTAACACTAGTGATGGATCAAAGTCATAAACAGCTTGTGCTATAACGCTTGC
TATTTCTCTGTCTTTTGCACCCATCTGATACAATGCACCATGCGGTTTAACATGATTAATTTTAACTTGATGA
ATGCGACAAAACCCTTGTAATGCACCTAATTGATAAATCATCAAATTATAAATCTCGTCGTTAGAGATATCTA
TATTTCGTCTGCCAAAGCCTTTCAAATCAGGTAAACCAGGATGTGCACCTACTGCAACATTATGTGCTTTGGC
AAGTTTTACCGTTTCATTCATTACATTTTCATCACCAGCGTGAAAACCACAAGCAACATTCGCACTTGTAATT
AACGGAATAATTTGATGATCACCACCAAAGGAATAATTTCCAAATGCTTCGCCTAAATCACAATTCAAATCAA
CTCGCATTATAATTCCACCCCTTTAACAATTTGATGTTTTTCTAAAAATTTAATATCAACATCTTTTGCATCT
CCATCACGATATAGTGGATAATTTAAAACTGCATATAAAAAATCGGCAGTTGTAGAAAATCCATCTATCACCA
TTTCATCTAAGGTGGCTTTCAACTTATCAATTGCTGAAGCTCTATCATGAGATTTTACAATTACTTTAGCTAC
TAAAGAATCATAATATGGTGAAACTTGATAACCGTGATATAGTAAAGAATCGACTCGCACATTAAAGCCTTGA
GGTAAATGTAACGCTGTCACTTTACCTGGTGTTGGTTGAAATTTCTTTTCAGGATTTTCGGCATTTATTCTCG
CTTCTATCACATGACCATTAAATTGAATATCGCTTTGTGAGAAAGGTAAATGATTATGTTCCAATAAATACAG
TTGTGCTGCAACCAAATCACGTTCTGCTCGCATCTCTGTAACAGTATGTTCAACTTGAATTCGAGCATTCATT
TCAATAAAGTAATGTGCGGTATCAGTTACTAAAAATTCAATCGTACCTGCACTTCTATAATTTGCTGCACGTG
CAACTTTAACAGCATCATTACATATTTGTTGTCGCCTTTCTTCAGTTAATGCTGCACAAGGAGATTCTTCGAT
TAATTTTTGATTTTTACGTTGTACAGAACAATCACGTTCCCCTAAATGTACATAATTATCCTGCCCATCCCCC
ATAACTTGAACTTCAACATGTTTTGCAACAGGTATAAAAGCCTCAACATAAACACGATCATCATCAAAGTATT
TTTTTCCTTCACTTTTAGCTTCTTTAAATGCCTTTTCTAAATCTTCAGCTTTCTTTACAATACGTATACCTTT
ACCACCACCGCCACTGGCAGCTTTGATAACAACTGGATAACCGATGTCTTTGGCAAGCTTCTCAATTTCAGAC
ACATGATTCACAGCACCATTTGATCCTGGAATCACAGGAACACCTGCATGATGAACTGTTTGTCTTGCTGTTA
TTTTATCCCCCATCATTTCCATCGTTTTTTTAGTAGGCCCTATAAACGCTATGCCTTGTTCCTCAACGGTTTG
AGCAAATTTTGTTGATTCTGATAAAAATCCATATCCTGGGTGAATTGCATTAGCACCAGTGATTTGTGCAGCA
GATATGATGCGGTCAATATTTAAATAACTATCTAAAGCATTAGCTTCCCCAATACATATAGCTTGATCTGCTA
AATGTACATGCAAGCTTTGCTCGTCCCGTTTTGCATAAACTGCTACAGTTTCAATCCCATATTCTCTGCAAGC
TCTTATAATCCTTACAGCAATTTCACCTCTGTTCGCAATTAAACAACGAAGCATTTACTTACCCCCTTTACTT
AATACGTACCAAAACTTGGTCGTATTCAACATTTGTGCCGTGATCAGCTACTATTTCAGTAATTTCTCCAGCA
ACATCTGTTGTTACCTCGTTTAATACTTTCATCGCTTCAACATATCCTATAATATCTCCCTTGTTAACTTTGT
CACCGACTTTCACAATTGGTTCAGTTAATTCTTTACTATCTTGTAAAAAGAATGTACCTACCATTGGTGATTT
AATGTCATGATAATCATTTGTCGAAACATCGGAGTTATCATTCGCTTTTGAAGCTGTCAAATCATTATTGTTC
ATACTTTGATTTGATTGATTACTGTGTGCAGCCAAATGATTCGAGTCAGTGAAGTTAATTTCTATTTCATCTT
CAAAATTTTTATATTTAAATTTCTTAACATCATTTTCCTTCACTAATTTGATTATTTGTTCGATTTTTTCAAT
ATTCATTTTACAAATCCCCTTTTAAAATTGTTGCTAATTTTTTCGAAGTATGTCGCAAGCTAGATGTATCAAA
AATTGGAGTCTTTTGATGACTCTTAAGAATTTCATTAAACAGAGACATTTGTTCCCGATTCTTATCTACAGCT

TCTTGGAATGATATCCATTTAAATTGAATTGTATCTTGTGGTTTCATCTGTGCTAACTTTGGCAGATCAAATT
TGCATACAGTTGCAATTTTGGTATAACCACCTATCGTTTGTTTATCATTAAGCAGAATAATAGGTTGACCATC
ATTTGGTACTTGAACACTGCCAAGAGCAACCGGTTCAGAAATGATATCTGCTTGATTAATTGGTGCAACGCTG
TCACCTTCCAAACGATACCCCATACGGTCTGATTGTTCAGTTATTAAATATGGATAATTTACAATTTTCGCTC
TAGCCTCTTCAGAAAATGCCTCGAATTGAGGTCCTTGAAGAATGTGTATAATATTATTTTCTGGTAATAAATC
GTCCTGTAAATGAATAGTCTTTCCAATGTTTTCTTTAAAGTCATTATTTATTTTCACTGTTATTACGTCATTA
GCTAATAACTTTCTACCTTTGAATCCTCCTATACTGCTTCGGGTATGTGTTGCATAACTTTCAGCAATAGAAG
GTACGTTGATAGAATGACCAAAAGTAAGATAACCGCGTGCACCTTTGGTTATAGCACCTATTTTTAAAATGTC
ACCTTTCTCAGCTAATATGACAGAATTCATTGATATAGTTTTATTATTTAGCGAGGCATTAACACTACCACCG
GTTATAACAAATGTATTTTGCGTATTAAATTGAATGGTAGGACCAATCAAAGTATATTCAATCGCTGGACCAT
CATTGTTAATTAATGACTGCGCAACCTTAAAACTAAATTGATCCATGGCACCTGAGCCTGAAAATCCAATATG
TTCATAACCTTTTCTTCCTAGATCTTGTACCGTTGAAAAGAGACCTGGTTGTAAAATCTTAATTGACATTTTC
AATCACCACCCAGTCATCAACATTAAAGTTGCCATCTGATATATCTCTTTCGATTTGTATAAATTCCTGTTCA
TCTATTGCATAAAATTGTATCCATTCTCCTGCTTCGTACATTGACATTGGTTCACGCTCGCTGCTAAATACTT
TTAACGGTGTGCGTCCAATAATTTGCCATCCGCCAGGAGAATCTGATGGATATAGTCCTGTTTGATTATTCGC
AATACCTACAGAACCTGCATGAATTTTTAACCTTGGCTGATTACGTCTAGGTGTATGTAGTTGTTGATCAAGT
CCGCCTAAGTATGGAAATCCAGGCATAAATCCTAGCATATATATTAAATAAGGTTTACTTGTATGTTTTTCAA
TAACTTGCTCAACAGTTATTCGATTATGCTTTGCTACTTCCTCAATATCAGGTCCATATGTACCACCATATTG
AACAGGTATTTTAATAATTCGATTGGTTTGATTCACAGCATGAACATTTTTTTCATTAAATTTGTTAAGTTCT
AAATTTTCAATTAATTTAGAAGATGTTATAGCTTGTTCATCAAAATATATTAGAACTGCTCGATACGAAGGGA
CAATGTCTTGAATTTCTAATATTTCTTTTTCTCGTATCCACCGTACCATTGCTGTGACATTTCGATATGTCTC
TTCGGATATATTATTTTCAAAATAAATCATAATTGTCTGCTCGTTAATAAATCTTACATCCACTCTAAATCCC
CCTTTGTATTGCAATAAACCAGTATTGAATACCTTTTCATTGTATCATTGAGAAGCACAAGTTGTTTAATAAG
TAATTCAAATCGCATACAATAACAAATTATAATCAATTTGTTTTCTTGGAAATATTTTGCTTTTTTTAAAATG
GAGTAGTTAATATTGAAACTCTTACACTAATTTAGGTTCTGCTATCAATCGGTCTGATGGAAAATTTTTACTT
TTCATCTGTCCGATTTTTTGATTTTGAATATAAAAAAGCACGACCGAAGTATCATTAACACACTTCAATCGCG
CAATTAAACAATCTATTTGATCATTTATTGGATATTAACAATTTTTACGTTCATTTCGCCACCATTAGGTAGT
GGAACACGAACTTCATCATCTAAACCTTTACCAATTAACGCTTTAGCCATTGGTGATTCATTTGAAATCTTAC
CATTAAATGCATCTGATTCAGCTGAACCAACGATTTGATAACTTTCCTCTTCATCACCTGGTAATTCTACAAA
CGTTACTGTTTTACCAATTTTAACAACGTTGTTATCTCCAGTATCTTCAATGATTAATGCATTTCTTAACATA
TGCTCAATTCTTTGAATATCTTGTTCGATGAATCCTTGTTCATCTTTTGCTGCATCATACTCAGAGTTCTCTG
ATAAGTCACCAAATGAACGTGCAACTTTAATTTTCTCTACAACTTCAGGACGCTTAACTGTTTTTAATTCTTC
AAGTTCACGCTCTAATTTTTCAAAACCTTCTTGAGTCATTGGATATTGCTTTTGATTTTCCATATTGTCATCT
TCCTTTACTGAATTATACTATTGCTTGCTAACTAAAGACTGAATTTTTGTTGTCATAATATCTATTGCAACTT
TATTGCTCCCACCTTCAGGTATAATTATATCAGCATATTTCTTAGTCGGTTCAATAAATTGGTCATGCATAGG
TCTAACAACACTTAAATATTGATTGATAACAGAGTCCATTGAACGCCCACGCTCTTTAGTATCTCGTGTTAAA
CGGCGTAATATTCTTAAGTCTGCATCTGTATCAACATATATTTTAACATCCATCATATCACGTAATACCTTAT
TTTCTAAAGCGAAAATACCTTCTACGATAATAACATCTTTAGGTTTAAAATCAATGGTAATATCACTTCTTGT
ATGACTAGCATAATCATATGTCGGTACTTCTACTGCTTTACCATTTTTCAAGTCTTTAAGATTTTCAATTAAT
AAATCATTATCAAATGCAAATGGATGGTCATAATTGGTTTCTAGGCGCTCGTCGAAAGTCAAGTGCTTTTGAT
CTTTATAATAGTAATCTTGAGCAAGTAAAGCGACACTATGACCTTCTAAGTTTTTCATAATTTCGTTAGTTAC
AGTTGTTTTTCCTGAGCCAGATCCACCAGCTATGCCAATGATTGTAGTAGCTTTCATTAGCCAATTTCCTTTC
TCATCATGTTGTTTGGATATATCGGGCGATCCACTTTAATTTGAACGATTTGTAATGGATGGCGCGCCGCGTC
TAAGCTGTTACCTTCTTCATCATAAATTGCTTCTACTACTTGTGTAAATGTTTCAATTTCTGGACCAAAGAAT
TCTATTTCTTGACCTGGTTTAAAGTTATTTCGTTGTTGAATAGTCGCAATTTTTGTATCTTCATTATAATCTA
ATACCAAACCACAAAAATCAAATGGTGATTTTTTAGATTGTTGTTGACCAAACATCTGTTCTTCATAACCAGG
TGTACCCTCAAAGAATGCTGGTGCAGTGTCTCTATTTGCACATTTATCTAACTCAATTAACCATTCCGGATTA
ATCTTAAAGTTGTCAGGATCTGCCGCATACGCATCAATGACTTTACGATATACTGAGACAACTGTTGCAATAT
AATGAATTGACTTCATACGTCCTTCAATTTTTAATGAGTCCACACCAATATCCATCATTTGAGGAATTGATTC
GATTAATTTTAAATCTTTAGGACTCATCGCAAACGGTGTAACTTCACCTTGATTATAAAATACATCAAGTTCA
CCATTATCATCAACTTCTAATAATTCATAATCCCAACGGCAACTTTGACAGCAACCGCCTCTGTTGGAATCCC
TTGCAGTCATATGATTACTTAATGTACATCTACCTGAATAGGCGATACACATAGCACCATGAATAAATGCTTC
GATTTCAATATCTACTTTTTCCTTCATTTCACGCATTTCCATCGCGCCGGTCTCACGTGCTAATACAACACGA
TCCAATCCTTCTTCTTTCCAATATTCTACAGCTTTGTAATTAGAAAGTGATTGTTGAGTAGATAAATGAATTT
CAAGTTTTGGCGCAACTTCTTTACATGTTTCGATAATTAAAGGATCTGCAACAATGATACCTGTCGCACCAGT
CTTTTCCAAATTACGCAAATATGATTCTAGACCTTCAATATTCTCATCATGTGCAATAATATTTGTCGTAACA
TAAATTTTGGCACCGTAACGGTTCGCAAATTCAACACCTTCAGCTATTTCTTCCATCGTGAAATTATCAGCAT
TTGAACGTAATCCATATTCTTGACCACCTAAAAATACGGCATCAGCGCCATAATGTACTGCTATTTTTAACTT

TTCTAAGTTTCCAGCAGGTGCTAATAATTCTGGTTTCTTCATAACTGTTTTAGGAGTTGATTTAATCTCTTCT
ATTGTCTTCATGATTAACCTCCTTAATATACCGTTTGTTTATATAAGAAACCTTCGTCAAATGGTCGATGATC
AGGTTGAATTTCTTCAATTGGGTCCATCAACATAAATTTCTCATCTTCATAGATTTCAGGATCTTCATTGTAC
AAATCTATCGCTTGACGATACTGTTCCGTTACCACATTAATATATTCTTCCGTTTGTAGAATACCATCGATTT
TAAATGAATCTATACCCGCCTCAAAAAATGGTGCTAATTCTTCAATTAAACAAATGTCGTTTGGTGACATAAT
GTGCGTACCATTGTAATCTTCGTAAACTGGGTAATTATTTTGTCTTTCTTCATCATAAAGTAATAAAGATTGT
TCATCATTGCGACGTTCAATTTTCATTTGGCGATCTTGGAACGTATAATAATTGCCTAGTAGCATACGCTTTG
ATTGGAACATACAAGTCATTCCTTGAACTTGCACCTCAATTTCCACATTTGAATTTTCTTTTATATTAATAAT
TTCATCCAAATTCAGCTCACGTGCTAAGACAGCTCTTGATGCGCCTCTTTTCCCCCAGTAATTACATTGAAAA
TGATTAGTTACTAACGTCTCTGCATTCCAATGAAGTGGTATTGGATTCTCTTGCGCCTTCACATACATTACTA
CTGCTGGATCCCCGAAAATAATTCTGTCAACTCGTATTTCATGTAAAAAATTAATATAATCTTCTACAGCATC
TAAATGATAATTATGAAATAATCCATTCACCGCCGCATATACTTTTTTATCGTTTTTGTGAGCTAATGCGACA
GCCTCTGTCATTTGTTGTCTATTGAATTCCCCTGGAAGTCTTAAACCAAACTTTTGCTCGCCAATTACAAAAG
CATCTGCACCTAAATCAATAAGTGTTTCCATATGGCTTAATGACTTGGGTGTGACAAGTAATTCTGTCATAGT
CATTCTCCTTTAATTGAAATCGCTAATCCATCGTCTATATTTAAAAAATTCGTTGTATATCCTGGTTGCTTTA
TTAACCACTCATTATAATCTTGAACCTTTTTAACCATTTGTCTTACATTTCTCGATCTAACAATCCCAATATC
CGATACAAAACCGTGATATAAAACATTATCTGTAATTACGAGACCTTGGTGCTTTAAAAGTGGTGTATATATT
TCAAAAAATTTCTTTGATTGCGCTTTTGCTGCATCAATAAATATCATATCATAAACTTTGTCATTTACATTTT
CAAATTGCTCTAAAGCATTACCTTCAATAATTCGAACCTGGTTTTCAAAATGATAAGTAGCTAAATTTTGTTT
AGCATATTGAATCATCGTTTCATTACGCTCTATCGTTGTGACATGAATGTCATCAGATATAGAAGCGAATTGC
ATAGAACTATAGCCGATTGCTGTACCAATTTCTAAAATATTTTTAACATTATTCATACGAATTAATTGCTTAA
TTAAATCTAATGTTAAACGATCTACGATTGGCACTTCATTTACTTCGGCAAATTCACGCAAAACTTCGATTGA
ACTATTTTGATGTTGATGTAAATCTATTAAATATTTTTTATTTAGGTCATCCATGTTTTAAACTTCCTTTATG
TAAAATAAGTCAATATGATTATGACAATAAAATAAATCAGCCTTCACAATTGATTATGATTTTGCCAACCAAT
TAAATGACTGATTTCGTGTTAGACGCAAAGCTATTTTATTTATAGAAGCGAATCATTCATATAAAATTTAACT
TTAGATATTTTACCATATTTTCAATAAAATTATAAGCGTTAATTATTTATACATTGCTTGACTTAAAAAAATA
CTCTTGCCTCCCCATCTTAAGGTTAGCAAGAGTAAAATCTTTTTAATTATTCTTCCATTTCAGTATTTACAAC
TTCTTCAATCATGTCCCATTCTTCATCAGTTTCGATTGGTACTAACTTACCACCGTCACCTGACTCATCTGGT
TCATTGATCATTGGTACAAGCTCAATCATATCGTCTTCATCTGATTGAGCACCTTCTTCAGCTAAGATAACAT
ACTCTTTTTTGAATTCAGGATGATAAAATTCTAAAACTTTTCGGTATAAAACTTCATTTCCCTCTTCATCGAA
TAAAGTTAATAATTCTTCTTCGTTATTAATTTCTAGTTGTGAATCATGATTATGTTCAGTCATAGTAAAATCT
CCTTTTAATGTAGTGAATCTAAATAGCCTTGTAAAATAAATACCGCTGCCATTTTATCAATCACTTGTTTTCT
TTTTTGTCTTGAAACATCTGCTTCTAATAATGATCGTTCAGCAGCCATTGTGCTTAATCTTTCATCCCACATC
ACAATCTCAATAGAAGGATAAGCTTCTAATAATTTTTCTTTATATGTTAACGAAGCTTCGCCTCGAAATCCTA
TTGAATTATTCATGTTTTTAGGTAGTCCTATTACGACTGTACCCACATTATGTTTTTTAATAATGTCTACTAA
TTGGTCAATACCTAATTCATTATTTTCTTCATTGATTCGGAGTGTGTCTAATCCTTGTGCCGTCCAACCCATT
ATATCACTAATTGCAATTCCTACCGTTCTACTACCGACATCGAGTCCTAAAATTTTATGTTGTAACATAAATT
ATTTATTTTGCTCTTTTAAATAGTAAGAAACAAGCTCTTCCATAATAACATCTCTATCAATATGACGAATTTG
ATTTCTTGCTTCATTTTGGCGTGGAATATACGCAGGGTCACCTGATAATAAATAACCTACAATTTGGTTTACG
GCATTATATCCTCGTTCATCTAATGTTCGATAAACATTATTTAAAACATCTCTTACATCTTGTGTTGGAAGTT
CTTCATAGTCGAATTTCATTGTTTTATCAAAGTTTTCCATTTGCGACACTCCTTTAATTACAAATATAACTCA
CTATCATCATACAATATTATGGCTATAAATTATAGATTTTTAATGTAATCTTTAATAAAGCTTAATGATTTTG
AGATATTTTCAGGTTGTGTACCGCCACCTTGAGCCATATCTGGACGACCGCCACCTTTACCACCAACGATTGG
TGCCATTTGTTTGATAAGATCACCGGCTTTAACGTTATTTGTTAAAGATTTAGGGACAGTTGCAACCATCGAT
ACTTTATCATCAACATTACTTGCAAGAATGATAATTGTATCTTGTAGTTTAGATTTAAAATCGTCCATTGTCG
AGCGAATTGCTTTCGCATTTGGTACATCCACTTCAGTAACCAATACTTTATAGCCATTGATTTCTTCAACTTG
ATCTTCAATATTACCCATTTTAAGTGATGTAATTTCTTTGTCACGTTGCTCTAATTGTTTTAATAATGCTTTT
TCTTCATCTTGTAATTGTGTTAACTTATCGACTACTTGATCATCAGATTTCACTTTCAGCTGTGATTTCATCG
TATTAAATTTCTCTTGAATATCTTCTAAATATAAGAAAGCTGCTTTACCTGTTAATGCTTCAATACGACGCAC
ACCAGCTCCTGTACCTGACTCACTTACTATTTTGAATAAGCCAATTTCAGAAGTATTGCGGACATGAATACCA
CCACATAATTCAATTGAAAATGGTGCCATATTTACTACACGCACAACATCACCATATTTTTCACCGAATAATG
CCATTGCGCCCATTTCTTTAGCTGAAGCAATATCCATTTCTTGAATGTTAACGTCAATACCTTTCCAAATTTC
TTCATTTACTAAGCGTTCAACTTGATCAATTTCATCCTTAGTCATTGGACCAAAATGAGAGAAATCAAAACGT
AAACGATCTGCTTCTACTAGTGAACCAGCTTGGTTAACATGATCACCCAGTACTGATTTCAACGCTGCATGTA
ATAAATGTGTTGCACTATGGTTCTTTTGAATGTCACGTCGATCATTTTGGTTCACTTCAGCAGACACTGTAGC
GCCAACATTTACTTGGCCAAATTGTACTACTCCTTTATGCAAGTTTTGACCATTTGGTGCTTTGGTTACTTCA
CTAACAGCAATTTCAAAATTGCCATTATAAACAATACCTGTATCCGCAACTTGTCCACCACTGACTGCATAAA
ATGGTGTTTCCGTTAACATGAAGTATACTGTTTCACCCGCTTCAACTTGTGAAACTTCTTCGCCATTGTATAT

CAAGTGTGTTAGTGTTGTTTGAGCTGTCGCAGTATCATAACCAACAAAAGTACTTGCAGATGTAATATTTTTC
AATACTTCACTTTGAACTTGCATTGATTGAGAATTTTGACGTGCTTGACGTGCACGATCACGTTGTTGTTGCA
TTTCTGACTCGAATGTTGTCATATCAACTTTCAATCCTGCTTGCACTGCTATTTCTTCAGTTAATTCAATTGG
GAACCCGTACGTATCATACAATTTAAATGCATCTTTCCCATTAATTTCATTTGTTGTCGCTTTAGCTTTTTTA
ATTAATTCATTTAAAATCGCTAAGCCATCTTCTAATGTTTCATGGAATCGTTCTTCTTCAGACTTTATAACAC
GCTTAATGAAATCTGCTTTTTCCTTAACATTTGGATAATATGGTTCCATAATGTCTGCAACAATATCAACAAG
TTTGTACATAAATGGCTCATTGATTCCTAACGTTTGACTAAAACGAACGGCACGACGTAACAATCGACGTAAT
ACATATCCTCTACCTTCATTGGCAGGTAATGCACCATCAGAAATTGCAAATGCAATCGTACGAATGTGGTCAG
CAATTACTTTAAATGCCACATCCTGTTCGCTGTTTACTAAATATTGTTTACCTGATACTTTTTCGATTTCATT
CATTATAGGCATAAATAAATCTGTTTCATAGTTAGTACGTACATTTTGAGAAACTGAGGCCATACGCTCAAGC
CCCATGCCAGTATCAATGTTTTTATTAGGTAATGGTGTGTAACTATGATCTTTATTATGATTGAATTCACTAA
ATACTAAGTTCCATACTTCAAGATAGCGTTCATTTTCTCCACCTGGATACATTTCTTCTGCCGGATCGTCTTG
TCCATATGCTTCTCCGCGATCATAGAAAATCTCAGTGTTCGGTCCTGAAGGCCCTTCACCAATATCCCAGAAG
TTACCTTCAATGCGAATAATACGACTTTCTTCAAGCCCAATATCTTTATGCCAAATGTTGTATGCTTCCATAT
CTTCCGGATGAATCGTAACGTACAATTTATCTGGCTCCATTCCCATCCATTTATCACTCGTTAAAAATTCCCA
AGCAAATTCAATCGCTTCTTGTTTAAAATAATCACCAATTGAGAAGTTACCTAACATTTCAAAGAATGTATGG
TGACGCGCTGTGAAACCAACATTTTCAATATCATTTGTACGAATAGCTTTTTGAGAGTTTACAATTCTTGGCT
TTTTAGGTGTTTCACGTCCATCAAAATATTTCTTTAATGTTGCTACACCTGAATTAATCCATAATAATGTATC
ATCATCAATTGGCACTAATGGTGCAGAAGGTTCAACCATATGTCCTTTTTCAACAAAGAAATCTAGATATTTT
TGTCTAATTTCACTCGCTTTTAACTTTTTCATCATTTACACATCCTATTTACTGTTTTTAAATTACCATTCCA
TAAAAATTGATGACACAGATAGTCGATTTGAAAAACTAGTATAAATCAATATCATTTTTTATTATTAAAAAAT
AAAAAACGCCCATCCTCAAAAGGGACGAACGTTATCGCGGTACCACCCTAGTTATAAATGCAATTCAACACAT
TTATCACTTTAAATCGACTATACAGTTGTGCATAAAGTAGCGTTCACTAATGTTTGTTGTACTTTTCACCAAC
CAGTACATCTCTGATAAACAAATCATTAACTACTCATCTTTATACGAATTTAATTCTATTTTAGTTACATTTA
CGCTTGTTGTCAACGTTCTATAAAGTCATACGGCGTGATTTCTCCCATATTAATCATTGGGTCAATTTTAAAC
ATTGTAGCTTCCGTTAATACATTTGTATCTGTTTTTGTTGAATCAGACGTAACTTCTTCACTATCATTCGATG
GCATTGGCGCTTCTACTTGATCATCTATTGTCGTTTGTGAAGCTCCTGTATCATTAGTTGCTGTGTTTTCTAG
CATTTCTTCATCTTCTGAATTAAAATAATTTTTCAACAATGTACATAATTGTGTTAAACGCGCTTGACCATTT
GTTTTCAATCCAATATCAAATGCTTCTGGATCACCAAGTAAAACTAAACTCGTTTTCGCTCTAGTTAAACCAG
TATATAATATCGGTCTTTGTAACATTCTAAAATACTGTTTAACAATAGGCATGATAACAATAGGAAATTCTGA
ACCTTGTGATTTATGGATTGATGTACAATAAGCATGCGTTAATTCCATCATATCTTGTTTCGTAAATGTAATT
TCATTACCTTCGAAATCCACAACAAGTACATCTTTATTAAGGGCATTTTCTTTCGCCCAAAAAATACCAACAA
TAACTCCTATGTCACCATTGAATATGTTATCATTTGGCCTATTAACAAGTTGTAATACTTTGTCACCTTTTCT
AAAGACTACATCACCAAACTCAATTTCTCGTGTGTCTTTCTTTTTAGGGTTTAAAATATCTTGTAAAACTTGA
TTTAAACGTTTAATACCGGCATTTCCTTTATACATTGGTGCAAGCACTTGAATATCAGCCATAGTATACCCTT
TATTAACAGCACTAGTAACTACCTTCTCAACAACTGTTGGTATTTGGTTTGCCTGACAGTTAATAAAACTTCT
ATCATGAAAACGCTGTGTAATATCAATTTTCTGACCCAATTTCATTCGATGTGCTAATTCTATAATGCTTGAA
CCATCTTGTTGACGATATACTTCAGTCAGATTAACTCGTGGTATAGCTTTCGATTCAATTAAATCTTTAAATA
CTTGACCAGGACCTACAGAAGGCAATTGGTCCTCATCACCTACAAATATCAATTGTGCATCTAAAGGAACTGC
ACTTAAAAATTGGTGAAACAACCAAGTATCTACCATAGACATCTCATCAATGATTATGAGTCGTGCGTTTATT
TCATTTTCTAATATATCCTCTGGCTTTGTGTCTTGATTCCAACCTATTAAACGATGAATCGTCATAGCTTCTA
ATCCAGTTGACTCTTGTAGTCTCTTGGACGCTCTTCCTGTTGGCGCTGCTAATACAACTGGATAATCATCATT
GACATAATCATCATAATCTAATGATAAGCCATGAATCTCAGCATATAATTCTACAATGCCTTTAATTACTGTC
GTTTTTCCAGTTCCCGGTCCACCGGTTAATAGCATCACCTTAGAATTGATAGCCGTTTGCAAAGCTTCTTTTT
GTGAAGCTGCATAGTTCACTTGATTCGCATCTTCTATTTCACCAATATGCATTTGTAAATCTGACTGTTCAAT
TTCTGTAAGTTTATTTGTATGCGTCTTTATTCTGAATAAGTTTTGAACACTTTTGATTTCAGAATAATACAAA
CTTGGAATTGCAACTTGTTCATTGTCAATAATTAGTCGTTTTTCCTCATTTAAGTATTGCAACATTTCGTCTA
ATTTTTCAGGTTCGATGACCTCTTCATCTTGATAATTTAATACATCAACCGTTAAATCTATAACAACATTGAT
AGGCAAATATGTATGTCCCTGTTTAATACATTCTTCTTCTAACGTATAGAGCAACGCAGCTTTTAATCGTTCA
TTATCGTTATAAGCGATACCAATATTTCTAGCAAGTTGATCTGCTTTATTAAAACCAATACCTTTAATATCAT
AAATCAATTGATATGGATTTTGGTCTAAAATAGTCAGTGTATCACCGAGATAAAACTGATAAATTGCCATTGA
AAGTTTAGGGCCAAACCCTAAATCATGTAAACGAATCATTATTTTTTCAGATTCTTGGTTTGCTGAAATTTGT
TCTGCAATTTGTTTCTGTTTCTTTTTAGATAATCCCGATACTTTTTCTAGCACTGAATGGTCATCTAAGATAT
CATTTATCGCATTGTCACCTAATGCATTAACAATATTTTGAGCTGTCTTTTTACCTACACCTTTAAACAAATC
ACTTGATAAATAACTTATAATTGCTTCTTTCGTTTGTGGCATTTCTTTTTCAAAAGTCTCTGCTTTTAATTGT
TTACCATAACGTGGATGATCAACAACTTGCCCTTTAAATGTGTAGACATCGCCTTCAACAATATTCGGAAGAA
ACCCTACAACAGTTGGCATTGTATCAAAGTCTTCATTTGTTTCAATAGTATCTACTTTAAGCACTGTATAAAA
ATTATCACTGTTTTGAAACAATATCGCTTCAACAGTACCTTTGATCATTGAATAATCAAATAGTGTAGGGTCT

GACATGCTATTCCTCCTCTTTCATTTTAGTGAATGTTTTCAGCGCATGCTGACTTAATAAGTGTTTAGGGTCG
ATAGTCACAGCTTCTTTAAAATGAGTTATGGCTTCATCAATATCTTCATTTTTCATAAATAACGCTAAGCCCA
AATTGTATCTTGCATCAACATGATTTTTATCAATCGTTAATACATGTTTAAGTTGAGTTATGGCTTCATTAAA
CATTTCTAATTGACATAATACAAGACCATATTGAAATTGAACTTCTGCATCTTTGTCTTTATCTAGTTCCGCA
GCAGTCATTAAATACGGCAATGCCAGCTTAAATGATTCTAACTGATTAAACGCCATACCGATCATATAATTAC
AATCAACTTGTTCAATCTCTGTTTGTAATGCTTGTTGATATAATTTAATAGCTTCTTGATAACGCTGCTGATT
ATAATATACATTTGCTAGATTATAAAATACTACGCCATTCTTCGGATCTATTGTTAAAGCTTTTTGGAAAAAG
CGCTCTGCCTTTTCAATCTCATTCGCATCAGCAAGTACGATACCGGCATTAATATAATTTTCAATAATTGTAG
GATCTTCTTCGATATTTCCGAACAATGCTTGTAACGCTTCTTCTATTTTTCCATTTTGTATGTATTGATAAAT
TGTTTGTTGATCTATCATTTACGAACCTCATTTCTCATCAATTATAACATCTTGATAAATTGTATGTCTCGAA
TCACTTAACAACGAATAAAATATAATCTAATATCATCTTCATTCATGAAAAAGCGGGAATGGAATAGAAATGC
TAAAGAACCATTAACGGTTTATTATGTAATGGTTCTTCCACATTAGCCACCACTATTATGTACTTAAAAATAG
GAATACATAATTAGACTCATGCATAGGGAGTGGACAGAAATGATATTTTTACAAAATTAATTTGTTTACCCC
AACTTCCATTGCCGGTTGAATTTCTTTACGAAATTCTCTATGTTGGGGCCCCGCCAACTTCCATTGCCTGTTG
AAATTGGGGTTCCAATTTCTCTGTGTTGGGGCCCCGCCAATATAACATTGTAGAGCCTAGGACATTGTGATGT
CCCAGACTCTATCATCATGAATTATTCTCATCAAAAACTGTCTTTCGTCATTTCCAACGCTGAAACTTCAAAT
AAGTAATTTATTGTTGCCATTGTTTATACAACATAATTTAATTGACCTTCATTTTTGAACACATCGTCAATTG
TTGCTCCACCAAGACACACATCACCTTGATAAAAAACAACTGCTTGTCCAGGTGTGATTGCTCTTACTGGCTC
AGCAAAAGTAACACGTAGTGCATGGTCGTTTTCACGTTTCACAAAAACTTTCGTATCTTTTTGGCGATATCTA
AATTTAGCTGTACATTCAAAACCTTGATCTAAGTCATTATCTTCTGGATTTACAAATGAATAGTCTGAAGCAA
TTAAGTAATCACTGTATAATGCATCGTGATGGAATCCTTGTTCTACATATAAAACATTATCTTTTAGATTTTT
ACCGACAACAAACCAAGGATCGCCATCTCCACCTATACCTAAACCATGTCTTTGTCCTATTGTGTAATACATC
AACCCACTATGTTTACCCATTTTCTTACCATCAAGTGTTATCATATCACCCGGTTGCGCAGGTAAATATTGAG
ATAAAAATGTTTTAAAGTTTTTTTCGCCGATAAAACAAATACCTGTAGAATCTTTTTTCTTAGCAGTAACTAG
TCCTTGTTCTTCAGCAATTCGACGCACTTCACTCTTTTCGATGTCGCCAATTGGGAACATCACTTTTGAAAGT
TGTTGTTGGGATAATTGATTCAAGAAGTATGTTTGATCTTTATTATTATCTACACCACGTAACATTTCAACAT
GACCATCTTCATGACGATGTATGCGTGCGTAATGCCCTGTTGCTACATAATCTGCACCTAAATTCATCGCATG
ATCTAAAAAGGCTTTAAACTTAATTTCTTTATTACACATAACGTCTGGGTTTGGAGTACGACCTTTTTTATAT
TCATCTAAGAAATACGTAAAGACTTTATCCCAATATTCTTTTTCAAAATTAACAGCGTAATATGGAATGCCAA
TTTGATTACACACTTCAATAACATCGTTGTAATCTTCAGTTGCAGTACATACGCCATTTTCGTCAGTGTCATC
CCAGTTTTTCATAAATATGCCAATGACATCATAACCTTGTTCTTTTAAGACGTGAGCTGTTACAGAACTATCT
ACACCGCCTGACATACCAACGACAACACGTATATCTTTATTTGACAATTATGACTCCTCCTTAAATTTAAAAT
ATATTTTATGAATTTCAGCTACAATTGCATTAATTTCATTTTCAGTAGTCAATTCGTTAAAACTAAATCGAAT
CGAATGATTTGATCGCTCTTCATCTTCGAACATTGCATCTAAAACATGCGACGGTTGTGTAGAGCCTGCTGTA
CATGCAGATCCAGACGACACATAGATTTGTGCCATATCCAACAATGTTAACATCGTTTCAACTTCAACAAACG
GAAAATATAGATTTACAATATGGCCTGTAGCATCCGTCATTGAACCATTTAATTCAAATGGAATCGCTCTTTC
TTGTAATTTAACTAAAAATTGTTCTTTTAAATTCATTAAATGAATATTGTTATCGTCTCGATTCTTTTCTGCT
AATTGTAATGCTTTAGCCATCCCGACAATTTGTGCAAGATTTTCAGTACCTGCACGGCGTTTCAATTCTTGTT
CACCGCCAAGTTGAGGATAATCTAATGTGACATGGTCTTTAACTAATAATGCACCGACACCTTTTGGTCCGCC
AAACTTATGAGCAGTAATACTCATTGCGTCGATCTCAAATTCGTCAAACTTAACATCAAGATGTCCAATTGCT
TGAACCGCATCAACATGGAAATATGCATTTGTCTCAGCAATAATATCTTGAATATCATAAATTTGTTGCACTG
TGCCAACTTCATTATTTACAAACATAATAGATACTAAAATCGTCTTATCTGTAATTGTTTCTTCAAGTTGATC
TAAATCAATAGCACCTGTATCATCAACATCTAGATATGTTACATCAAAACCTTCTCGCTCTAATTGTTCAAAA
ACATGTAACACAGAATGATGTTCAATCTTCGATGTGATAATGTGATTACCCAATTGTTCATTTGCTTTTACTA
TGCCTTTAATTGCCGTATTATTCGATTCTGTTGCACCACTCGTAAATATAATTTCATGTGTATCTGCACCAAG
TAATTGTGCAATTTGACGTCTTGACTCATCTAAATATTTACGCGCATCTCTTCCCTTAGCATGTATTGATGAT
GGATTACCATAATGCGAATTGTAAATCGTCATCATCGCATCTACTACTTCAGGTTTTACTGGTGTGGTCGCAG
CATAATCTGCATAAATTTCCATGTTTGGACACTCCTCACAATTTTATCAATGTTCCAATAATAGCACCTTACA
TACTATTTTTCTACTTTTCTGTTTAACTTTATTTATAATGTTTTTAATTATATTTTACCATTTTCTACACATG
CTTTTCGATAGGCTTTTTTAAGTTTATCGCTTTATTCTTGTCTTTTTTATAAATTTTAGTATTTGCAGATATT
TTTTCATTCGTAAAATGTAACGTACTATTATTTTGGTTATGAGCAATTTAATATTTATCTGGTTATTCGATTG
GTATACTTCTTATATCATACAAAAGGAAGGACGATACAAAAATGGCGATTAAATATTCAGCATTAAACCTTGT
CCCAATTCGAGAAGGTGAAGATGAACGAACAGCAATTAATGATATGGTTAAGCTCGCACAACATTTAGACGAA
TTATCATATGAAAGATATTGGATTGCTGAACACCATAACGCTCCCAACCTAGTAAGTTCAGCGACTGCTTTAT
TGATTCAACATACGTTAGAACATACGAAACACATACGTGTAGGTTCTGGAGGAATCATGTTACCTAACCATGC
TCCATTAATCGTTGCGGAACAATTTGGCACGATGGCAACATTATTTCCAAATCGTGTCGATTTAGGATTAGGA
CGTGCACCTGGAACAGATATGATGACCGCAAGTGCATTAAGACGAGATCAACATGATGGTGTTTATAAATTTC
CGGAAGAAGTTTCATTATTACAACAATATTTCGGTCCCGCTCACCAACAAGCATATGTTCGTGCTTATCCAGC

AGTAGGTAAAAATGTGCCTTTATATATTCTTGGTTCTTCAACAGATTCTGCACATTTAGCTGCTCGTAAAGGG
CTTCCATATGTGTTCGCTGGACATTTTGCACCTCAACAAATGAAAGAAGCTATCGAAATTTACAAAACGTTAT
TTGAACCTTCTGATGTATTAGACGAACCTTATGTTATTGTATGTTTAAACACAATCGTTGCTGAAAATGATGA
AGAAGCACAATATTTAGCTTCATCTATGGCACAAGTTATGGTTAGTATCACTCGTGGCAGAATGCAGCCCGTT
CAACCGCCAACACATGATCTACAAAATATATTAACGCCGAGAGAATATGCGATGGCTATGGAAAGACAGAAAA
TATCATTAATAGGTTCAGAAAATACTGTTCAACAAAAAATTCAAGATTTTATGGAAACTTATGGTGAAGTCAA
CGAAATTATGGCAATAAGTTATATTTATGATAAAGATATGCAATTAGACTCTTATCGTCGGTTCAAGAATGTT
ATAAATCAGATAAATGAAAAAAACACTTTATAATGTGATAAATAAACTAAGTGAAAGTATGTATCCATAATAT
TAATAAAAATAAACAGTAACAGCATTTTGAATAAAAGATGTCTTTATTGTTCAATCATTTATTTTAGTAATGA
TTCAAATTCACTTAAAATTCTAATGCAAATATGAAAGCGCCCCTTCAACTTTACACTGTGTTAGTGTTTATTT
GATGGGGCGCTTTCAAAATATTGAAAAGCATATCCAAAATTTAAAGAAATTTATTTCTCTTTATCTTCATTTT
CTTTTTTCTCTTCGTTATTCGATCCTGTATATTCATTTATCTTATCTTTTACATTTTTAACTTGTTCATTATC
GCTATTTTTAAATTTTTCTACTGCGTCTTTAGCTTTATCCATAAAACTCATATTAATCGCTCCTCTTATATTT
AATTAGTTTAATTGAACTTATTTTTTAAGTTTATCAATTGCATCAGTTATTTTGTTTTTAGCATTTTCAACAA
CTTCTTTTGCTTTACCAGTCGCTTTATCTTGCTGACCTTCTTTTTCTAATTCTTTGTTATCAGTAACGTTACC
TACTGTTTCTTTAACATTTCCTTTAAATTGATCGAACTTACTTTCGTCTGCCATAGTGAAACCTCCTTGGATG
TATATATTTATATACCACTAAGGAGGTTCGCTAAACATTATAATATGAAGTTTTTATGTTATAGTATAGTATT
TATACGATTAAATATAAAACATGTATCCGTCTAAATCTTCGCTTGTATCTACATATTCCGCTAAATATTTCAA
TGTTGTATTATCTAAAACATCTCTCACTGCATCTCTCATGCGAATCCATAGTTGTTTTTGCGCAGGTGGTTCT
GATTCAATACTTTCAACAAATGTAATTGGACCTTCCAACAGTCTTATAATATCCCCTGCTGAGATTTCTTCCG
CTGGCACTCTTAATTGGTATCCACCTTTAGCACCGCGTACACTTCGAATTAACCCCGCATTTCTTAAAGGACC
TACAAGCTGTTCTAAATATAAATCACTCAAATTATTTTCTTCAGCAATTGACTTTAATGATATACATCCTTGC
CCCTCTTTTTTAGCAAGAGAAATCATCAATGTAAGTCCATATCTCCCTTTAGTAGAAATTTTCATTGTATAAC
CTCACTTAATTCGAATATTGATATTCCCATTTTAGCATTTTTTGAGTTAAGATAGTATAAGAAAGGTGTGACA
AATGTGAGTACAGAACCATTAGCATCGAGAATGCGCCCAAAAAATATAGATGAAATCATTTCCCAACAACATT
TAGTTGGACCAAGAGGCATTATCAGAAGAATGGTTGATACAAAAAAATTAACTTCAATGATTTTTTATGGTCC
ACCTGGTATAGGCAAAACAAGTATTGCCAAAGCAATTTCGGGCAGTACGCAATATAAATTCAGACAATTGAAT
GCTGTAACTAACACTAAAAAAGATATGCAACTTGTTGTTGAAGAAGCTAAAATGTCTGGTCAAGTTATCTTGT
TATTAGATGAAATACATCGACTAGATAAAGCTAAACAAGACTTTTTATTACCTCATTTAGAAAATGGCAAAAT
CGTCTTGATCGGTGCTACAACTTCAAATCCTTATCATGCTATCAATCCAGCGATTCGTTCAAGAGCGCAAATT
TTCGAGTTATATCCTTTAAATGACGAAGATGTGCGCCAAGCGTTAACTCGTGCAATAGAAGATGATGAGAATG
GTTTGAAAACATATCAACCCAAAATTGATGAAGATGCCATGACCTACTTTTCTACACAAAGCCAAGGTGATGT
TCGTAGTGCGTTAAATGCATTGGAATTAGCTGTATTAAGCGCAGATAATGACAAAGACGGTTATCGACATGTT
ACATTGCAAGATGCTAAAGACTGTTTACAAAAAGGTGCATTTGTAAGTGATAAGGATGGTGACATGCATTACG
ATGTTATGAGCGCTTTCCAAAAAATCTATCCGTGGTAGCGACGTCAATGCCGCTTTACATTATTTAGCACGATT
AATTGAAGCTGGAGATTTACCTACAATAGTTCGACGATTACTTGTAATTAGCTATGAGGATATAGGCTTAGCC
TCACCTAATGCTGGTCAGAGAACACTTGCTGCTATTGAATCAGCAGAACGTCTAGGTTTACCAGAAGCTAGAA
TTCCACTAAGCCAAGCAGTAATCGAACTATGCTTATCACCTAAGTCAAATTCAGCAATGAGTGCCATTGATAG
TGCATTGTCCGATATTAGAAACGGTCATGTGGGCCAAATTCCAAACCATTTAAAAGATGGACATTATCAAGGT
GCTAAAGATCTAGGCCGATCTATTGGTTACAAATATCCACACCAATATGTTAATGGCTATGTTTCACAGCAAT
ATTTACCTGATAAACTTAAAAACAAAATTTATTATGAACCAAAAACGACATCTAAAAGTGAACAACAACTTAA
AGAAATATATAACAACTTACTTAAACAAAGGCCGTAAAAATAAGATTAAATCGATTACCTTTGATATTTTAGC
TATAATCAAATCAAAACTGACTAGTACACGAGGCATATTCTACTATGCTTGCGAACTAGTCAGTTTTAGTTTT
ATGGTGCGGTTACACTTGCCTTACGTTGAAAATTATGACTTTCAAAATTAATTATTGCCCTTTGTCTTTAATG
CGACGAACTGGAATATCTTTTAATACGTCATTCACCACATAACTTGCACAAATTAATCCAACAACACTTGGCA
CAAAGGCATTTGAAGAAGGTGGCATTTGTCCTTTTCGATTGATAGCATTTTTATCTCCAACTATATCTTTTAC
ATCTTCTCTTATGACAATTGGACTTTCATCTGAAAATACAACTGGAATCCCTTTACGAATTCCTAGTTTTTTC
AATTTTTGACGAATAATTTTGGCCATTGGATCGGTATGTGTTTTAGAGATATCTGAAATTGTAAAACGTGTTG
GATCTGTTTTATTTGCAGCACCCATACTGGAAATCACTTTAATCCCTCGGTCAAGACACTCTTTCATTAAGTG
TACTTTGTACATTATTGTATCACTTGCATCTACAAAATAATCTATATCGTAATTATCGAAAATTTCTTCATAT
GTCTCTTCTGTATAAAACATATGTAAAGGTGTGACTTTACAATCTGGATTAATTAATTTAATACGTTCTTCCA
TCAAAGAAACTTTACTTTGTCCTACCGTTGTAGTTAAAGCATGTAATTGTCTGTTTACATTTGTAATATCAAC
ATCATCTTTATCAATTAATATAATATGACCAATATTCGTTCTTGCTAATGCTTCAGCAGCAAATGAACCAACA
CCTCCAACGCCAAGTATGACAACAGTTTGTTGCTTCAATAAATCTAAACCTTGTTGTCCAATCGCTAGTTCAT
TTCTTGAAAATTGATGTTTCATTATTTTACCTCTTTCACTGATTTATACATAAGTACATAGTAACTTAAAATT
TTATATTTAGCATTATTGCTTTGATTATTTTTCAAAAATTCAACGAAGAAACATTTATTAAACGCTATTAAAC
CCAACTAATTCTTTATTAAAAACTTAAAGAAACGCATAAAAATACGCAAGACAAAGTCTTGCGTATCGATAGA
GTCCGTATTGCCGTAGTTATAATAGCTTGATCATTCGGCCTGTTATATACAGGTGGGTGCCCTGTTTCTTGTT

TTGTACGTCCTTCATATAAGGCGTGTACGCTGCAAGAAAACCCATTGGGCTCCCTTGATCAAAGAGTGTTAGG
CCCAAATTAAAAAGCAAACTTACGAACAACTCAGATGACTATCTTATGATGTTATATTACCACATAATTAAAA
TTAATGAAATTATAACAAACCAAAGTTTATTGATTTTTTAAAATTTAGTGACGAATTCGCAAAGAAAGTTCTT
CTAATTGTTTATCAGAAACTTCACCAGGCGCATTCGTTAATAAACATGTAGCAGATGCTGTTTTAGGGAATGC
GATTGTATCTCTCAAGTTTGTTCTATTAGTCAATAACATGACTAATCGGTCTAATCCTAATGCAATACCGCCA
TGTGGTGGTGCACCATATTTAAATGCATCTAGTAAGAAGCCGAACTGTTCTTGTGCTTGTTCTTTAGTAAATC
CAAGAACTTCGAACATTTTTTCTTGTAACTCACCATCATGAATTCTGATTGAACCGCCACCTAATTCATAACC
ATTTAATACTATGTCATAAGCATTTGCCTCAGCTTCTTCTGGCGCAGTGCCAAGCTTAGCAATATCAGCTTCT
TTTGGAGATGTAAATGGATGATGTGCTGCAACGTAACGTTTCGCATCTTCATCATATTCTAATAATGGCCAAT
CTGTCACCCATAAGAAGTTTAATTTTGTTTCATCGATTAAACCTAATTCTTTAGCTAATTTGACACGTAATGC
ACCTAAACTTTGTGCAACGACATTTGGTTTGTCTGCAACAAACATTACTAAGTCACCAGCTTCAGCACCAGTT
AATGTAAGTAATGTTTCAACATTTTCTGTTTCAAAGAAACGTCCAATTGGACCTGTCAAACCATCTTCCACAA
CTTTAACCCACGCTAATCCTTTAGCACCATAGATGTTTACAAATTCTGTTAAAGCATCCATATCTTTACGAGT
ATATTGTTCAGCCGCACCTTTAGCGACAATTGCTTTAATTTCACCATCATTTTCAACAGTATCTTTAAATACT
TTAAAGTCCATATCACGTCCTAATTGAGAAACGTCAATTAATTCCATTTCAAAACGCGTATCTGGTTTATCAG
AACCATAGCGACGCATCGCTTCTTTATATGTCATGCGTGGGAAAGCGCCATTAATTTCAACGCCTTTAACTTC
TTTAACAACTTTTTTAAGCATTTCTTCACCCATTTGCATCACATCTTCTTGGTCTACAAAACTCATTTCAATA
TCGACTTGTGTAAATTCAGGTTGACGATCTGCACGTAAATCTTCGTCACGGAAGCATTTTACGATTTGGTAGT
ATTTGTCAAATCCACTAATCATCAATAATTGCTTAAATAATTGTGGTGATTGTGGTAATGCATAAAATTCACC
ATCATGAACACGAGATGGTACTAAATAGTCACGTGCACCCTCAGGTGTTGACTTCGTTAGTACTGGTGTTTCG
ATGTCAAAGAAACCTTCATCATCCAAATATTGACGAATAGAACGTGTAATTTGATGTCTCATTTTAAATGTTT
GCGCTAACTCTTGACGACGTAAATCTAAATAACGGTATTTTAATCGAATATTTTCATCAACGTTAACATTTTC
TTCATTTATAGAAAATGGTGGTGTCTCAGATTTATTAATCACTTTAATATTTGTAACTTGTACTTCAACTTGG
CCAGTTTTAATTTTAGGATTAACTGTTTCAGGGTCACGCTTCGTAACTGTACCTTGAACTTCTACAACATATT
CAGAACGTACTGTTTCAGCAATTTTCAATGCCTCTTCTGAAAATGCAGGATTAAACACGACTTGTACAATTCC
TTCTCTATCTCTTAAATCAACGAAAATCAATCCACCTAGGTCACGACGATTGTTAACCCATCCTTTTAATGTA
ATTTCTTGTCCTAAAAATGCTTCAGTAACTAATCCACAATAAGTTGTTCTCTTACTCATATTTTAACGCCCTC
TCTACTTCTTAAAATATTCGACTAATGCGTCTAATTCAATTGTTTCAGATTCACCAGTTGTCATATTTTTAAC
ATCGATTTTATTATTTTCTAATTCTTGATCACCAATAACGATTGTAAACTTGGCACCTAAACGGTCTGCTTGT
TTCATTTGTCCTTTAATTTTACGCTGTAAGTAGTCTTTATCTGCTTTAATACCATTATGTCTCAAATGATTTA
ATAACTTCACAGCATATCGATCTGCTTGATCACCCATTGTAACAATGAATAAATCTAAGTTTTCTTCAATATC
TAATTCGATACCTTCTTCTTCAAGTGCAAGCAATAATCGTTCTATACTTAGCGCAAAACCAATACCTGTTTCA
CTTGGACCATCTAGCAATTCTAATAAACCATTATAACGGCCACCACCACAAAGCGTTGTAATGGCACCATCAT
AGTTAGGGTTATCCATCATTAATTCAAATGCTGTATGTGTATAATAATCCAATCCACGAACTAAGTTAGGATC
TTCAATATATGGAATACCTAAATCATCTAAATAAGCTTTTACTTGTTCATAATATGCCTTAGATTCCTCATTT
AAGAAATCAGTGATTCTAGGTGCAGTCTTAATCGCTTCTTTATCACGGTCAACTTTACAATCCAAAATTCGCA
TCGGATTTGTATGCAAACGTGATTGACAATCTGAACAAAATTCATGAATTACTGGTTCAAAGTGTTTCACTAA
CGCTTCGTTATATTCTTTTCGAGACGCCATATCCCCTACACTATTAATAACAAGCTTTAAATGTTTTAATCCA
AATGATTGATAAATATGCATAACCATAGCTAATACTTCTGCATCTACGCTAGGATTTTCAGCACCAATAGCTT
CTACACCAAATTGATTAAATTGACGATAGCGCCCTTTTTGCTTACGTTCATATCTAAACATCGGTCCATTGTA
ATAAAGTTTAATTGGTTGGTTTGGATTACCTTGCATTTTATGTTCAATATATGAACGTACAACTGCAGCTGTT
CCTTCAGGTCTTAATGTAATACTTCTATCGCCTTTATCTTTAAATGTATACATTTCTTTTTGTACGACATCGG
TTGAATCACCAACACCTCTTGCAAAAAGATCTGTACTTTCAAAAATTGGTGTTCTTATTTCTTTATAATTATA
AAATGTCATTAATTCATCTAATTGATTTTCAATGTAACGCCATTTCTTTGAATCTTCAGGTAAAATATCCTGC
GTCCCTCTAGGTATTTTAATCATTTTGACATCTCCCTCGGCAAACTATTTTATTTAATTACCTTTTTAAAACT
AAAACTATATACGATCACTTAACTTTGCATGATAAATCATAATTTTAAAAGCAAAAGAAAAAGCCCTTGCACA
GCCTGAAACTGCACAAGGGACGAATATCCGTGTTGCCACCCTCATTGGTTTAAAAAACATGCCTAATTGTAAT
CATCATTACTTTTTATAAGCGTGCATTAAATAAACCCACTCAAAACGTTTAACGTACGTTTAACGGCTTTACT
TTTCATAAAGCACCTCTTCTTGTGTTCAATGTATATAAACTACTCAGCATAATCTTTCAGTCTATGGATTATA
TTCCTACACAATCTTAGTTAGATTGCTACCTATTTCATTTACCATTTCAAGAATACTAACGGTTTTAGTTATA
AAATTCAATATCAATGATAACTACTTTCACATATTTTTGCAAGCCCTACGCAGAAAAATAAATTTTAAGGCCA
TCAACAATTGCTTGTTCTAAAATTTGTCTATGTAATTGATCTTTAATCATCGTTTCATCAGTTGGGTTACTAA
TATAACCTAATTCTAATAAAACAGCAGGAACTTTTGTTTGTCTTAACACTTGATAATTTTCTTGTCTTGAACC
GCGATTAGAAAGTAGACCTTTCTTCTGAATCGTAGCGTCTAACGTATCTGCTAAAGCTCTTTGATTATCATGA
TACCAATAAACTGTCATTCCATTTGCATTAGATGATTCTAATGCATCATTATGTATACTCAAATAGGCATCGC
CTTTGATATCACGATTTTCTAGTGAAACATATGTATCGTCTGTTCTTGTCATCTTAACAGTTGCGCCTTCTTT
TTCTAAAGTACGCTGCAATTCTTTTGCTGTTTTCAACGTATAGTCTTTTTCTAAACTTTTATATTTAGTATTG
CTTGAAGCACCCTGGTCACTACCTCCATGACCAGGATCAAGCACTATTGTTTTACCTTGCAAAGGATTTTTCT

CCTTCGTATTATCCGCGACAATATCTAAATTTGTGTGCCATCCAGCTATCCAACCTTTTTCATTACTGGATGT
ATCTTCAACTTCAATCCATTTACCTACTTTACCAATCTTTTTAAAATGGTCACCTTTCTCAACTTTATATATG
ACTGGATACGCAGCGTTTGGACCTGTACGTAATTCAGCATTTTCAGTTATCGTGATGTTCCCACTATCTTCAC
TATTGCTATTCAGCAATAAAAATAAAAAGATGATAAATAAGACAAAGGCAATCACTACTATTAGAGTACGTTT
ATTTTTAAGACCCTTTTTAGATAACCATGCCTCTATTTTTTTCATTGAATTTTGCCGTCCTGACTTTCATAAA
TAATAGTGACTGGACCATCATTATTTATGCTAACATTCATGTGTGTTCCAAATTCACCTGTTTTCACAGTAAG
ACCATACGCTCGTAGCGCATCATTAAAATACTCATAAATTTTTACCGCTTGATCAGGATTTTTAGAATTTGAG
AAACCTGGACGGTTACCTTTTTTACATCTGCATAGAGAGTAAATTGTGAAACTGATAGTATTTCACCATTCAT
TTGTTGGATATTAAAGTTTAATTTATTATTGTCATCTTCAAATAATCTTGCATTAGCAATTTTCTTTGCAATT
ACATCTGCATCTTGCTCTGTAGAGTTCTGACCGATACCGACTAATAAACAATATCCTTTTTTGATTTGATTAT
TTAATGTATCATTCGTCACCGATGCTTCTTTAACTCTTTGTACAACTACTTTCATATTTTGCACCTCTAGTTC
CAAACTCTTGTTACTGTATAAACATCACCAAGTTGTTTGATCTTTTCTACCACACGATAAACATCATTCACGT
TTTTCACCATGACACTAATATTTATTATTGCATTTTTATCAATATCTGAACGTCCTGAAACTTTAATTAAATT
GCCGGCTGTCGAGCTAACAGCTTGTAGTACTTCATTCAACAAGCCATTTCGGTCATACGCAGTTACCTCTAAA
TCAACCTGATATTTTTGAGTTGCGTCTTTTGATTTTACCCATTCAACATTAATTAGTCGTTCAGTTTCGTTCT
TAATATTTGGGCAATCAGTGCGATGTACTTTAATACCGTGACCTTTGGTGATATAACCTACAATATCATCACC
TGGTATAGGATTACAACATTTTGACAACTTGATAAGTACATTTTCTAAACCTTCTACATAGACACCACTATCA
GTAATGATGTTGTCTTTAATAGGCAATGATTTCGTAACTTCTTGTGCTTCATTTAAAGCACGTTGTTTATCTA
AAATACGTTGTCTTTCAGTTAATTTATTAACAATCTGTAGGGATGTCACGCCGCCAAATCCTACAGCTGCGAA
TAAATCATCTTCATTTGCAAAGTTATATTTTTCATTAACAACCTGAATATTTTTCTCTGTCAAAATATCTTCG
ACTCTAAATCCTTGCTCTTTTATTTCAGCTTCAACCATCATTCGGCCTTTTTCAATATTAGATGAACGATCTT
GTTTTTTGAAGAAACTTTTAATTTTACCTTTGGCACTAGACGATTTAACAATTTTCAACCAATCACGACTTGG
TCCATATGAATGTTTACTAGTACGTATTTCAACAATATCGCCTGTTTGTAAAATATAGTCAATTGGTACAATT
TTGCCATTCACCTTGGCACCAATCATCTTATTACCTACTTCACTGTGAATCGCATAAGCAAAATCAATCGGCA
CAGCACCATATGGCAACTCAATAACATCACTCGCTGGGGTAAATGCGTATACTTTGTCACTCTGTAAGTCATA
TTTTAAGGTTTCCATAAATTCTTGAGCGTCAGACGATGTATGATCCGCTTCAGCTAATTCTTTTAACCAATTT
AACTTATTTTGATAAGTTTGATCTTTTTCACTTACTTTTTTACCTTCTTTGTAAGCCCAGTGTGCTGCAACAC
CATGCTCAGCAATTTCGTGCATATCAAACGTTCGTATTTGGATTTCGAGCGGGTCTCCATTTGGACCTACTAC
TGTAGTATGCAATGACTGATACAAATTTTGTTTAGGCATTGCAATATAATCTTTAAAACGTCCTGGCATCGGT
TTCCATAACGTATGCACCAACCCAAGTATCGCATAACAATCATTAATAGAATTGACAATAACACGTATCGCCA
ACAAATCAAAAATTTGATCAAATTGTTTTTTCTGCTTCATCATTTTCCGATAAATACTGTAAATATGTTTAGG
TCTACCATTTATATCGCCTTCGATATTCATTCGGTCCATTTCAGTACGTATTCTATCAATAGCCGTTTCGATA
TACGCTTCACGTTCACTACGTTTCTTCTTCATTAAATTGACTATTCTAAAATATTGCACATTATCAATATAAC
GAAGAGCTGTATCTTCTAGTTCCCATTTAATTGTATTAATACCAAGACGATGTGCTAAAGGTGCATAAATTTC
TAATGTTTCTCGAGAAATTCTAATTTGTTTTTCACGCGGCATGGCTTTCAAGGTACGCATATTATGTAATCTG
TCTGCTAATTTCACCAAAATTACGCGTACATCTTTGGCAATCGCAATAAATAACTTGCGATGATTTTCAGCTT
GTTGTTCTTCTTTTGAGCGGTATTTTACTTTTTTAAGCTTCGTCACACCATCAACAATTCGAGCAACTTCTTC
ATTGAACATTTCTTTTACATCTTCAAATGTATACGGTGTATCTTCAATTACATCATGCAAAAAACCTGCGACA
ATCGTCGGTCCGTCTAATCGCATTTCTGTTAAAATACCTGCAACTTGTATAGGATGCATAATGTATGGTAATC
CGTTTTTTCGGAACTGACCTTTATGTGCTTCATAAGCAATATGATAGCTTTTTAAAACATACTCATATTCATC
TGCTGACAAATATGATTTTGCTTTGTGAAGAACTTCATCTGCACTATATGGATATTCGTTGTTCATTATATGA
TACACCCCATTCATATTTATTACTTCGCCTTAAACAATGATTTAGGTACTCTTGTTGAATAGTATTTGTCCCA
CACCAATCATACGTCCGTCGACGATAAATATTTATCCTGTCGTCATTAATCGTAATATTAATTTTACTTGAGC
GAGTTTAATTTGTATACTATTCCTACTTTTAAAACTTTTACAAAAATTCGACCTAAATCTACTGTTTCATTTT
TTAAATATTAGTTCTATGATACTACAATTTATGAAATAAATAAACGATGTTATTAAGGTATAATGCTCAATCA
TCTAACATTTTCAGTAAATAAAAAATCCTAACATCTCATGTTAAGAAAACTTAAACAACTTTTTTAATTAAGT
CATTGGTTCTTATACATTTGATGAAGGATTTCATTTGATAAAATTATATTATTTATTATTCGTCGTATGAGAT
TAAACTCATAACATCGTAATCTTTAATTTTTTCAATACCATTTAAATATTTCAATTCAATTATAAATGCAATA
CCTACTACGATACCGCCTAATTTTTCAACTAATTTTATTGCTGCTTCAATCGTACCACCAGTAGCTAATAAAT
CATCTGTAATTAACACACGTTGACCTGGTTTAATTGCATCTTTGTGCATTGTTAAAACATTTGTACCATATTC
TAGGTCATACTCATAACGAATGACTTCACGAGGTAATTTCCCTTCTTTTCTAACAGGTGCAAAGCCAATCCCC
ATTGAATAAGCTACAGGACAGCCAATGATAAAGCCACGCGCTTCAGGTCCTACAACGATATCAACATCTCTGT
CTTTTGCGTATTCTACAATTTTATCTGTTGCATAGCCATATGCTTCACCATTATCCATAATTGTAGTAATATC
CTTGAAACTAACACCTGGTTTCGGCCAATCTTGAACTTCTGATACGTATTGCTTTAAATCCATTAATATTTCC
TCCTAAATTGCTCACGACAATTGTGACTTTATCCAATTTTTTATTTCTGAAAAATCTTGATATAATAATTGCT
TTTCAACATCCATACGTTGTTGTCTTAATTGATATACTTTGCTGGAATCAATCGATCTTTTATCAGGTTGTTG
ATTGATTCGAATTAAACCATCTTCTTGTGTTACAAATTTTAAATCTAAGAAAACTTTCAACATGAATTTAAGT
GTATCTGGTTTCACACTTAAATGTTGACACAATAACATACCCTCTTTCTGGATATTTGTTTCTTGTTTAGTTA

TTAATGCTTTATAACACTTTTTAAAAATATCCATATTAGGTATACCATCGAAGTAAATCGAATGATTATGTTG
CAAAACTATATAAAGTTGAGAAAATTGCAGTTGTTGCAAGGAATTAGACAAGTCTTCCATTGACGTTGGTAAA
TCTCTTAATACTACTTTATCAGTTTGATGTTTAATTTCTTCACCATAATAATATTCATTCGCATTTACTTTAT
CACTTTTAGGATGAATAAGCACGACAATATTTTCATCATTTTCTATAAAAGGTAAACTTTTTCGCTTACTTCT
ATAATCTAATATTTGCTGTTCATTCATCGCAATATCTTGAATAATTAATTGCGGTGATTGATTACCATTCCAT
TCGTTGATTTGAACAGATCCTAATATATTAATTGGCTGTTCATCTTGTAACTCAGGTTCTAAGTGACCATTTT
GCCAAAATAGCGCGGCGATATTACTTTCACCAAGTGTCAATTTTAGATGATTTTTTTGTTGACCGATCGCCTT
AACTGAAGAAACTGACAAATCATCCATTTCAAAAATAGGTCTAGAAAAAATCTGTTCCGAAGGGTCTTAAACGA
TTCATATCACGAATATTTTTAATCGTTATATCATTTTCTGTTAATAATACGTCTACTGGCTTTACGGGATCTA
ACGAAGTTGTTTTAGATAATTCTTTCATCCATTTATTTAAACCTTCAGCTAACGATTCTATATTTTCAATATC
CATCGTCATACCTGCAGCCATATGATGGCCGCCAAATTTAGCGATTAACTCTTGATGTGCTGATAGTATTTCA
AACATCGACACTTGATCAATTGATCTGGCGGAACCTTTTGCATGATTTTGCTCCCTATCAATATTTAAAATTA
ATGTTGGCAAAGCAAATGTTTCGACAATTTTCGAAGCAACAATACCTAAGACACCTTCATGCCAATTTTCTTT
TGCTAAAAGTAAAAATAAATCTCCCTTTTTAACTTTCGTTTCTGCCATAGCCATTGCTTCTTCTGTGATAGTT
GCTACAATATCTTTTCTTTCACGGTTAAAATGTTCAACTTGTTCTGCTAAAAATGCAGCTTCTTCTTCGTCGT
CAGTCATCAACAATTCGCAAGCTAATGATGCGTCATCTAAACGACCTACAGCATTAAGTCTAGGTCCAATAAT
AAAACCAATTGTTTCTTCATCAATATTGTCATTGTATCCCGCTTCTTTTAGCAACGCTTTAACAGAGGTCGGA
CATTGATCATTTAAGACTTTTAATCCTTGTTTCACTAATGATCGATTTTCATCAGTTAAGGATACTAAATCCG
CAATGGTACCTATCGCAACTAATGCTTTAAAATAATCAGGTACATTTTCAATCAATGCTTGTGCTAATTTGTA
CGCAACACCTGCACCACACAATTGTTGGAACGGATAATTAAACGATGGATGCATTGGATGTACGATTGCATAT
GCTTCTGGTAATGTACTACCAATTTCATGATGATCAGTTACAATGACATCAACTCCTAAATCTTGAACCATTT
TAATTTCATTATGACCTTGTATGCCATTATCAACAGTTATGATTAATGTTATGCCTTCATCATGAGCATTTCT
AAATGCTAGTTCGTTTGGTCCATATCCTTCGGTAAAGCGGTTAGGAATATGCCATCCTACTTGTGCACCTAAA
AGTTGTAATGTTGTCACTAAAATTGTAGTTGAGGTAACACCGTCGGCATCGTAATCACCATAAACTAGGATTT
TCTCATCATTCGCTATCGCTCTTTTAATTCTTTCAATAGTCTTAGTCATATCGCTCAATTGCAGTGCATCATG
ATTGATATCTGTATCTGAGATGATGGCTTCTATTGTTTGTTCATCAATAATCGATTTACTTTCTAATATTTTT
TTTACGATTGGCGTTAACTTTAATTTTGATGTTAATTCATCACTTATGTATTCAGCTGGTTTAGTTAATTTCC
ACTTATACTTCGGTTTAATCATATATATTTACGCCTCATTTCCAAAAGACATTATATCTAAAATTACTTTAAA
TAAAAAGCACTTATCTAAATAATCAAGACTTTAATTATACCATTTTTTAAAAATGAATGCGGGAACTTATTTA
TATATATTTTGTTAAATTTAAACAAAGTAAAAAGCGGTATGTGAACTCTTTATTGTTTCACATACCGCTTAAT
TCATTTTAAACTAAAATCTTTTCATCGTTCGATTTCTTTTCTTTATATACAACTAATTTGTGTTTCGGCGATT
TTTTCAGCTGACGTTTTTTCATTATTCCCCATAGCGGAACGGCAATGAAGATTGAAGAGAATACACCAGAAAT
CAATCCGATAAATAATGCTAAAGTAAAGTTGAATATCGTAGGAGCACCGAAGAATAGTATAGCAACTACTACT
ACAATAACTGTTAATACTGTATTAATTGAACGTGTCATTGTCTGTCTAATTGATCTATTAACGATATCATCAA
TTTGTTCTGTTGTCGTAATCACTTTAACCTTTTGTAAGTTTTCACGTACACGGTCAAACGTTACGATTGTATC
ATTAATTGAATAACCGACAATTGTTAATACAGCGGCGATAAATGTTAAATCTACTTCAATTCTAAATAAACTG
AAAATCGCTACTATAATGAATACATCATGTAATAATGCCAATACAGATGAAAGACCCATGCGCCATTCAAATC
GTAATGATACATAGATGATGATACCTATCGATGCATAGATTAATGCAAGCATTGCATTTTTTGCTAATTCCTG
TCCAATAATTGGTGATACAGTATTAATTTGAGGTGTGTCACCGAATTTCGATTTAATATTATCACTCAATTTA
TTATCTTGAGCACGCGTTAAATCGTCTTTAAATTGAACAGTTGCTACTTTATTATCTTTACCATTGATTTGAA
TTTGATCCGCTTTAAGTCCACTATCTTTTACAACTTGCTCAACCTTTTGTTGAGTAATTGCTTGTTTAGATTG
GAAATCTACACGTGTACCACTTGAGAAATCAATTCCTAAGTTTAACTTGAAGATATAAAGAATAACTAAACCG
ACAACTACAATTAAAATACTTACTCCAATTAATGGCTTAGCTAATTTAACAAAATTCCATTTCTCGAATGAAG
TTTTAAGGTCATGAACATCTACACCTTCATTAATATCATGTCGTTTATTCTTTTTAACACCAAATAACCAAAA
TTGATTTTTGAATATATTTGATGAAACAAGTAATGATAATAAGAATCTTGATAAGAACACGGCTGTAACAAAG
ATCATTAGAATACCTAATAATAACATTGTCGCGAAACCTTTAACTGAACTTTCACCGAAGAAGAATAATACTG
CTGCGGCGATAACTGTTGTTAAGTTAGAATCAAAAATTGTTAGGAACGAACTTTTGTTTGCTTTAGAAAAGGC
TTGCTTTATCGTTCTACCTATTCGAAGTTCATCCTTAATACGCTCATACATGATAATATTGGCATCTACAGCC
ATACCTACACCTAATACCAACGCCGCTAATCCTGGTAAAGTTAGAACCCCGGAAATGAAATTAAATGCTACTA
ACGTTAGATAGATATAAGTTGTCAATGCAATAATCGCTACTAAACCAGGTAATCGGTAGAATCCAAGCATGAA
TAAATAAATTAATGCTACACCAATAAACGATGCAAACACAGTTTTATCTAATGCATCTTGACCAAATTGTGCA
CCTACTGAGTTTGAATAAATTTCTTTCAAGTCAACTGGTAAAGAACCTGCATTTAACAATTCGGCGATTTGTT
TTGCTTTTTTAACGCCTTCTTGTCCTTTAAATCCACCAGAGATTTCTACGCTATCAGAATTGATTGGTTGATC
AACACTTGCTGCAGAAATAAATTTAGGGTTTTTCTTTTGTGCTTCTTTTTTATAGCTGTCACCTTTTTTGAAA
TCTAACCAAACAACCATGACATTATCACGTTTCTTAGAGATTTCTTCCGTTACTTTTTTAAATTTGTTTTTGT
CTTTTACTTTAAAAGTAACTGTAGGCTGGTTTGTTTCCTGTTTAAATTCTTGTTTGGCAGATCCCTGTTTAAT
ATCAGAACCGCTTAATTTTACTTTATCTTCTGCATCGCGAATTGTTAAATTAGCCTGAGAAGATAAAATTTTA
CGTGCTTCATTCTGGTCTGTTACACCAGCAAGTTGAACTCTAATTCTATTAGGTTCTTCAACTTGAATTTTAG

GTTCGGAAACACCTAAAACGTTAACACGATTTTCTAATGTTTGCGCTGTTGATTGTAAGGCTTTTTTATCTAT
TTTGTCGCCTTTATTTAAAGGATCGACTTGATAAAGCACCTCAAATCCACCTTGCAAATCAAGTCCTAAATTG
ACATTCTTTATAACACTTTTATAAGTTGCAGCCATTCCGGCAAACAACAATACGACTAAAAGCAAGAACGCAA
TTATTCTACTACTTTTCTTCACATGAACACCTCATTATTTACGTATGTATTTAGAATACTTGAATACTATTTT
ATAACGCAAGTTAAATCTTTCTTACAAAATTTATTAGCCTTATACATATTAACATACTAGAGCAGGGAGTAAA
AATAGTCATAGATATATTTAATTTAAATTTAACAATTATTATGTATCACAATTTCTTTTTTTGGAATAAAAAT
AAGCTATTCTACATACTACTTATAACTTAAAAAGGATGAAACGCTATTCGTTTCATCCTTTTTAATACCACTT
CTCGTTATGAAGGGTCAACTTGTTTAATAGCAGGTTTTTCGAAAGTTAATTCAGTACCATGACCATTAACTGT
AATAACAACAGTTGTTTCATCTACTGCTTTAACAGTACCTTTAATACCACCAATAGTTGTAATTCTTTGACCA
GATTGAATGTTATTAATCAACTCACGATGCTGTTTCGCACGTTTTTGTTGTGGTCTAATCATCAAGAAATACA
TAACCGCAAAAATTACGACTATATATATTAGTAATGAAAATTGCATTTTATACGCTCCTCTTGCTTAAAAGTT
TTTTGGGTTCTCAACATTTAATCCATATTGCTCGAAGAATTCTTCTTTGAAATCTAAAAGACGATCTTCTCGA
ATGGCTTGTCTTATATCTTCCATTAATTTTAGCAGAAAATGTAAATTATGAATAGTAGTAAGACGAATACCAA
AAGTTTCCTCTGCCTTGATTAAATGACGTATATACGCTCTTGAATAGTTTTGACATGTATAACAGTCACAATT
CTCATCTAACGGTCTTAAATCATCTGCAAATTTTGCATTTTTAATAACTAAACGACCTTGCGATGTCATACAA
GTACCATTTCTGGCAATACGTGTCGGTAAGACACAATCAAACATATCCATGCCGCGAATACTACATTCGATTA
ACGCATCTGGAGATCCTACACCCATTAAATATCTTGGTTTATCTTTAGGCATAAACTGCTCTGTATGTTCAAC
CATTTTATACATAACCGGTTTAGGTTCACCAACTGACAAACCGCCGATTGCATAACCAGGAAAATCTAATTCT
ACTAAATCCTTTGCACTTTGTTCTCTTAAATCTTCATATTCGCCACCTTGTATAATGCCGAACAATGCTTGAT
CTTCAGGTCTTTGGTGTGCATCTAGACATCTTTTCGCCCAACGTGTTGTACGTTCAATAGATTTTTTTACATA
ATCATATTCAGCAGGCATTGGTGGACATTCATCAAATGCCATCATAATATCAGAACCTAAATCATTTTGAATT
TGCATTGATTTCTCAGGACTCAAAAATAATTTAGACCCATTAGTATGATGTCTAAATTCCACGCCTTCTTCTG
TAATTTTACGTAAATTACTTAAACTAAACACTTGGAAACCGCCTGAATCTGTTAGAATCGGACCATCCCAATT
CATGAATTTATGTAATCCCCCAGCGTGTTTGATAATATCATTTCCGGGTTGTAACCACAAATGATATGTGTTG
CCCAAAATGATTTTTGCTTCAATTTGTCTTAACTCTTCTGGACTCATTGTTTTAACGGTTGCTTTAGTACCAA
CTGGCATAAACATAGGTGTTTCAAATGAACCGTGTGGTGTGTGCACGATACCTAAACGCGCACCTGATTGTTT
ACAAGTTTTAATGTGTTCGTATGTTACTGCAGGCATATTCAAAATCCTCACATTCTTTTTATATAATTAACAT
TGCATCGCCAAAACTAAAGAATCTATATTCTAAATTTACTGCCGTTTTATAAGCATTCAGAACATTTTCACGA
GTACTAAACGCTGATACTAGCATAACTAATGTTGATTTTGGTAAATGAAAATTAGTAATCTGGCCATCAATTG
CTTTAAAATCAAATCCTGGATAAATAAATATATTAGTCCAGCCACTCGTTTCAACAAATTTATCATGATCGCG
TCGAATTGTTTCAAGTGTACGTGTTGAAGTTGTACCAACTGATATAATGCGATGTCCTTTGGACTCAGTATCA
TTTAATAAATCAGCTGTTTCTTGTGTCATTTGATAATATTCACTATGCATTTCGTGGTCATTCACATCATCGA
CGCTCACCGGTCTAAACGTACCTAACCCAACATGTAATGTAACAAATGCGATATTAACACCTTTATTTTTAAT
TTCAGTTAATAACTCATCAGTAAAATGTAATCCTGCTGTTGGTGCTGCCGCTGAACCACTTTCTTTAGCGTAA
ACTGTTTGATAACGATCTGGATCATCTAAACGTTCTTTGATGTATGGTGGCAGTGGCATTTCCCCTAATTCAT
CTAATCTTTCTTGTAAAATACCTTCATAATGTAAACGCATGATGCGTCCACCTTGATCCATTTCTTTTATGCA
TTCAGCTATAATTTTGCCATTACCAAAATTCAATTTATTACCAACTTTAATACGCTTAGCTGGTTTCAGTAAG
ACTTCCCAATCATTACCTTCAATTTGAGTTAACATTAACATTTCAACTTTTGCACCAGTTTCTTCTTTTAAAC
CAAAAAGTCTAGCTGGCATTACTCGCGTATCGTTAAGCACTAATGTATCACCAGGTCTAAAATACTCAATGAT
ATCTTTGAAATGTAAATGTTTCATTTCACCAGTTTCTCTATCCATGACTAATAAACGACTATGATCACGATCT
TTTAAAGGCGTTTGAGCAATTAATGATTCTGGTAAGTCATAGTCAAATTCTTCAAAATTCACGTTATTCTCTC
TCCTCATTCGACTTTGCAAAATGTTCATAAGCTAATGGTGTTGCTTTTCTGCCACGTGGCGTACGTTCTAAAA
AGCCTTTCTGAATAAGAAATGGCTCATAAACGTCCTCAATTGTAATACGTTCTTCACCAATTGTTACGGCAAT
CGTATCTAAACCAACAGGTCCACCATTATACTGCTTAATAATACAGTTCATCATTTTATGATCAATGTAATCT
AGTCCGTGTTGATCAACTTGAAGTAAACCTAATGCGTGCTTCGTTGTTTCAATGTATATTTGTTCATCTTCAT
TCACTTGCTGGAAGTCTCTTACCCGCTTCAATAGTCGATTTGCTACTCTTGGAGTCCCTCTAGAACGTTTAGC
AAGTTCAATGGCACTTTCTTCATCAATACCTGTGCCTAAAACCTCAGCTGTTCTAATAATGATTTCTTTTAAA
TCTGATTCATTATAATATTCTAATCTTAAGTGCACACCAAATCGATCCCTTAGTGGACCTGTTAAGCTGCCAG
CTCGCGTTGTTGCACCTACCAAAGTGAATGGAGGTAAGTCGATACGGATACTTCTAGCCTCATCGCCTTTACC
AATGATAATATCTAAAAAGAAATCTTCCATTGCAGGGTATAACACTTCTTCAACAACACTACTCAGTCTGTGT
ATTTCATCAATAAACAAAACATCTCCAGGTTGAAGTCCTGATAAAATTGCAGCCAAATCACCAGGTCTTTCTA
ATGAAGGCCCTGATACTGTACGTATATTAACTTCCATTTCATTGGCAATGATATTAGATAATGTTGTCTTACC
TAATCCAGGGGGGCCAAAAAGCAATACATGATCTAATGGTTCATGACGAAGTTTAGCCGCTTTAATAAATACT
TCTAAATTACTTTTTATTGAATTTTGACCAATATATTGTCGTAATCTCGTAGGTCTAAGCGACAATTCGAAAT
CAGTTTCTTCACTATGCATTGATTGATCAACCATACGCTCATTCATGACAACACTTCCCCTATTAATCTATTT
AAAATTAAGATACAACTAATTGAAGACCTGCCTTAACAGCTTCATCAACTGAGTCATATTTATTTTTATTTAA
CGTTTTCTCAACTTTTGCAAGCTCTCGTTTAGAATAACCTAATGCTTCTAACGCTAACATTGCTTCTTGCACG
AATTGATCTTGCACCGTCGAAGTAGCGTCTACTTGTAATAATGAATCGCTATCTTCTTCAGTGATTTTCACTT

TACCTTTTAAATCTAAGACAATCTGTCTTGCCGTTTTCTTACCAATTCCTGGGAATTTAGTTAAATACGTATC
ATTTTCATTTTCAATGGCACGTTTTACTTCATTAGGCGTACTTGTCGCTAAAATAGCTAAAGCTGATTTCGGA
CCAATACCAGTAACTTTAATTAAACTCAAGAACATATCTTTCTCTTCTTCACTACTAAATCCATACAATAATT
GTGCATCTTCACGAACAATTAAAGATGTATGAATTAAAACTTCATGATCTAGTTGCTTTTGAAAACGATAAGA
ATTTGGTGTTTGAATTTCATAACCAACACCAGCAGTTTCAACAACTACGTGTGTAGGATATAAATGTGTTAAC
TTACCTTTGACATACGCGTACATTATAGGCACATCCTTACATACTCATACTAATTAATTCTACTTTTGATACA
TAATCTAAATTTCTCAAAGCGCCAATAACATCTTCTACTGCAGTTTCTTTAGATTTAGCATTCAGTGATAATG
TTATTGTTGCTTTTTCTTCCATTGGAATACTTTGATGAATCGTTAATACAGATAGTTCTAACTTTGATATAAC
ATCTAGTACACGTGCCAACATACCCACAATATCTGTTACATATAAAATTAATGTAAATTCTCGATGGTCAAGC
ATTTTATCGTCTACTGGAAATATCGTTTCTCTATATTTATAAAAAGCACTTCTAGATAGATCAAACTGTTTAA
CGGCATCATAAATGGACAATGTCGGATCACTTTTTAAGGCATCTTTAATCTTCAATGTTTTAACCACGGATTC
AGGCAAGACATCTTCTCTAATTAAATAAAACTTTTTATAATCTTTATTGTCCATCATGTTACGCTCCTATTCA
ACGAATTCAAATTCTCCGCCAAGAATTCTAACGATATCACCATTTTTACAACCACGTTCTCTAAGTGCATCAT
CAATACCCATCGAACGCATTTGACGAGCAAATCGACGTACTGCTGGATCACTGTTAAAGTCAGTCATTTTAAA
CATTCTTTCAATAGCATTACCACTTACCACATAAGCACCATCATCATCTCTTGAAATTGTAAATTTATCTTGT
GACGGTGTATGTTTATATAATACTCGGTTAATGCCAACTGACTCCTCTTCTTCAACTGTGAAGTCAACATCTT
TATATTCTTCTAATTTATCTGCTATTGCATATAATAATTGATCAATATTATCACGCGTTATTGTTGAAACTGG
AATAACTGGCACATCTTCGCCAATTTCTTCTTTAAACAAGTTTAAATTATCTTGTGATTCAGGTAAATCCATC
TTGTTAGCTACTACGATTTGAGGTCTATCTTCTAAACGTTGCTCGTACGCAGCTAATTCTTGATTAATGACTT
TATAATCTTCAATAGGTTCTCTACCTTCAGAACCGCTCATATCAATCATGTGAACAATAACTTTTGTTCTCTC
TACATGTCTTAAAAATTGATGTCCTAATCCAACGCCATCAGATGCACCTTCAATTAAACCTGGTAAATCTGCC
ATAACAAAACTACGTTGATCAGGTGTTGAAACAACACCTAGATTTGGTTTAATCGTTGTAAAATGATATGCCC
CAATTTTAGGCTTAGCTTTTGAAACGATAGATAATAAAGTCGATTTACCCACACTAGGGAAACCTACTAATCC
TACATCAGCTAATAATTTCAATTCTAAAGATACATCTAATTCCTCGCCTGGTTCACCTTTTTCACTGAAGTCA
GGTGCAGGGTTTCTAGGTGTTGCAAAACGTGAATTACCTCGGCCACCTCGACCGCCCTTCGCTACTACAGCTC
TTTGACCATCTTCAACAAGATCTGCTAACACTTCGTCTGTTTCAACATTTTTAATAATTGTACCAGGTGGAAC
TTTTAATACTAAATCTTCCGCATTTTTACCATGCATATTACTACTTTGGCCATTTTCACCTTTGCTTGCTTTA
AAATGACGTTGATATCTAAAATCTAATAACGTTCTTAAACCTTCATCCACTTCAAATACGACTGAAGCACCTT
TACCACCGTCACCGCCAGCTGGTCCACCAAATGGTACATATTTTTCTCTTCTGTATGCGGTAATACCATTACC
ACCATCACCGGCTTTAAGAGATATTTTGACTTGATCGACAAACATATATCTCACCTCTTTTACATATAATTTT
CATTTTCCCAATAATTATAGCATATTAGAGCCCCTTATAGTTTAGCGATTAGGGGCTCTTATGCAGTCGCACA
TTTAGTATAACGTCTAAAATTCCCATTTTTTAATAACAAAATGAAAATAATAATGAAAAACGTTTTATAAAAA
GTAAAAAACACCAGAAGATTCACTTCTGGTGTTAACTAGACAAAATTATTCAGCTACTGCATATACAGAAACT
TGTTTTTTGTCGCGACCTTTACGTTCGAATTTAACAACGCCGTCGATTTTAGCGAATAATGTATCATCGCCAC
CACGACCTACATTTTCACCAGGGTAAATTTTAGTACCACGTTGGCGATATAAAATTGAACCACCTGTTACGAA
TTGACCGTCAGCACGTTTAGCACCTAAGCGTTTTGATTCAGAGTCACGTCCGTTTTTTGTAGAACTTACCCCT
TTTTTAGATGCGAAGAATTGTAAGTTTAATTTTAACATCGGAATGCACCTCACTTATAATTTAATCTAATATT
CTCATTATATTCTTCTTCAATAGTTTGTAAAGACACAAGCATTGTTTGAAGAATTAGTTGCGCTTCATCGTTA
TTTGTATCAACGCTTCTTATATGAAAATGACCACCATTGTCGTCATAATTGATATCTGGTCTCTCAGATGTCA
ATCCTATAATCGCATTAACACTACCAAACAATACAGCTGAAGCTCCAGCACAAACGATATCATGACCATATTC
ACCATGGTCAGCATGGCCATCCATAATAACGTCTGTTACTTTGCCTTCATCATTAACTGTAATATCAACAGTA
ATCATAATAATTACGCGTTGATTTTATCGATTGTTAATTTAGTGTATGGTTGACGATGGCCTTTTTTACGTTT
TGAATTTTTACGACGTTTGTATGTGAATACAGTAATTTTTTTACCGCGACCTTGTTTATTAACAGTAGCAGTA
ACTGTTGCACCTTCAACTGTTGGCGCTCCAACTTTAACTGAATCTCCACCTACAAATAATACTTTATCAAATG
TAAAAGTATCTCCTTCGTTTACGTCTAATTTTTCAACGAAGATTTCTTGACCTTCTTCTACTTTGATTTGTTT
TCCACCTGTTTCAATAATAGCAAACATACTTTGCACCTCCTGTATAATAAGTCACGCCATACATAGGTGACAT
TTCGTTATTGAGTCAAGCGTTTGCACTCTACGTTGTTTCGGTCGTAACTCGAGTCATTTATAAAGATAAACTC
TTCTCGTTTCTTCCTTCAACGCCTTGATTGAAAATCGCTTTCTTCATTAAAAATGTTCTTGAACCTATATTTG
AGCGGTTGTATGTAGCTACTTATAACTACTCAACTATTGCATCTTACCATTGACGACGTTTCATGTCAATGTC
ATTGTTTGTTTTTTTAAGGAACTTTATAATCAACGGATAAAGCATAATCAACAGTACAAAATTCATAATTAAT
GTTGGCAATAATCTAAAGACTACAAAATGAATAATATCAAATTGAATGAATCCTAACATACCGTATATTAATG
CCACATAGACTTCTAATAATAAGGTGCTGGCTAATATAATAATGAATAACATCGAATGATCTTTGTAAAAAAT
TTTAAAGAATCGATCTATAAGTGCTAAAAACAATATATAGCCAAATAAGTACACTCCATAAATACTACCAAAG
TATACATCAGTCATTACGCCTAAAAATATGCTGAGCAATAATGATACGCCAAAGCCACGATACACTACCATCA
TTAAAATATACATAAATGTAAGGTGTGGTACAAATACAAGTTCAAACTTACCTATGTTCATTGGAATAAGAAG
CCCAATTGCAGTATCTATATAAAATAGTAAAATACCTATCAAAAAATAATACAGTGTACGCATTATTTATCCC
TGCTTTCATCATCAGGAATTGTTTTAGGATCTCTTTTTGCAACATAAACATGACTCAAATCTGTTAAGTCTGC
ACCAGTCTTAACCCTAACTTCTTTAGCTAAGCCATATTGATCATTTTGAACCTTAGTCACTTCTCCTATATAT

AAATCACTTGGTAATTGATCAGCTAATCCACTTGTAACGACTTTATCACCTTTTGAGATATTATCTCTATTAT
TAATATCACTAATTACAAGTTCTGAGTTCTTTTCATCATAACGATCAATTAAGCCAAATATATTTTTAGAGCC
GTGTTGTATATTTACAGATAATTTACCCGCACGTGTATTAGTTGAGATTAAATCAACTTGTGAAGAAAATTTA
TTAACTTTAGTAACTCTTCCAACAAAACCTTTTGATGTCATCACAGCCATATTTGAAGTTATACCTGATTTAG
ATCCCTTATCAATAACAATTGTATTCATCCACTGATCCGGATTTCTTGCTAAAACCGTAGTAGAAATAGGATC
AAATTTTGAAATATCTTTTAAATCAAGCTCTTTTTTTAATTTTTCATTTTCCGCTTCTAATTGTTGGTTCTTA
GATTCTAACTGGCTAATCTTATTTTTAGATTCTTTAGAATCTCCTTTTTTAAAAAAGTCCCCAATCGTACCAG
CAACAAAATTAACTGGATAACTCACAACTCGTTGTCCAAAAGACACAGAATCACCTATATATTGTTCAGGAGG
TGATTGAGATTGTGAACGTATGGACAGCCCAATTAATGCAATAAAAACGATAATTGCACATAAAACAACAATT
AATTTGTTATTTTTAAAAAACTTAAGCACCCAGAACACCTCTATTATGTCAAAATATTGTATATCCTTTTCTA
TTTTATATTACTCCCATTATGAAATAAAGCACTAACTACGAATTAGTTTGAAAATGAACAACTCCGTAAAAAA
TTTGTGGCATTTTAAAAGCAAAAAAACTACTAGCAAACATTCCAGTCACTAGTAGTTTAAATAATCGATTTAA
ATTTTAAAATTATTTGTTTGTATCGTTCGAGGATTCACTGTTCGAATTCTGAAGTTGTCTCCAACGTGCGAAT
AAGTTCTGGGCCTTTTGTTGTTCATTAGCTTGTTTCTGTTTTGATTGTTCTGCCATTTGAACTACACCAACCT
TTTAAATTTATTCTAGTGACAGGATAACTAAAATATATTTCTTATGCAATAATTTCCTATTGATAAGCATTTT
CGGGTTTTAGTTGTAAATTTTGCCCTAATTCATTTAAGTCTTGTTGCATTTCAAATTCAGTATTGTAAACACG
CATTGACTCATCTCCAAATTTATAAAGAATAAATTCGTCTCCTCTTTGACCTATAATATATTGATCATTATAA
GCCATGCGATTCATTCCAGACACAGCCATAAACTCTTGTTTATCTATCATTTTAAATACATTTTTAATTTGGC
TTAATGGTACATTCTTTATTAAATCATTTTCTTTTAATGAATATTTCTCACCACTTACATAAACATGTTGGGC
ATCATTAGTGGGTTGCGTAACTTGATTCATTTGATCATTATCGTGATTTACAGGATTCCAATCTTGTTCTTTT
AAAAAAGGTGCGTATTTCAAGGCGATATAAACAATTATCACAATTGCTATTACTGCTATAATGTTTTTAATCA
TACTAAATATCAATCTCATAGTTCGCCTCCAATGTCTTCATCTAATTATATATTTTATTATGTATATTTTCCT
CAGTCTTCAGCCTTAATCATGATATATCTTAGGACTGATGCACGTTCATTTAAAATAGCTTATAATTTTGTTA
TGATTTAAAAGCATATGAGGAGGTTTGTAGCATTGAGTATACTAACAATTATTTTAATAGCACTATTAGTCAT
CCTCCTTTTTAGAGTTGGACTTTCTATTTTGCGATTTTTAATTTATGTCGGACTTGTTTTATTATGTATTTAC
TTAGGATATCAAGGTTTAATATGGTTGCTCGATTTTTTTCAAATAAATAGTGGGTTTTTACCACACTTTCAAT
TTAACAATTAAATTTATTCTGCATAACAAAACTCCTAATGTACTAGTTTAGAATTTTTTAACTAGTTCCACAT
TAGGAGTTTTAATTTGTTTTCAAGCACGTATACTACTTGATGAACAGATTACTATTACTTACGCCCAAATGCG
TTAGATAAAAATCGTATCAGCGCATAGCTACCGCTTAGTAAAAGGCCCCAAACGATTCCTGTAAAAATTGAGC
CTATTGCTACACTTAATAAAGGAAAAGCTTGTTTTGTAACAATCACACCTGCTAATGCCGTTACTAAAATACC
TAACGTAGATAAAGCTAATGTTATTAATAGATTGCGCTTTGTTACAGGTTCATTTTTAATATTTAACTGCATG
GTATAAACCATTGGCACGAGAACCCCGATAATAATAAATAGAGTCACTATTTTTCCTCCAATGAAAGTACTTC
GTAGGTATTATACACAAGATACGTCATTAATTCTATCAACTTCAATCATTTTCATCAAAGTAACCCGCTTCTA
CAAGACTCGTAAATCTATTATCACCGATTATAATATGATCCAATAAATCTATCCCTAAAATCAAACCACACTC
CTTCAACCTCATTGTTGTTATGATATCTTCTTGTGAGGGCGTTACATCACCGGATGGATGATTATGAACTGCG
ATGATTGCATTGGCATTTTCTCTCACCGCAATACTAAAAATTTCACGTGGATGTACAATCGAACTATTTAATG
TACCTTTAAAAACACAGGTTTCTTTAATCACTACATTTTTTGAATTTAACAATAAAATGACAAAATGTTCTTG
TGTTAAATCTTTCATTGTTGGAATCATATAATCAGCAACATCACTTGGTTGCGTTATTTTTATACGATTATTT
TCAGCTCTTCTCCCCATCCTTTCCCCTAGCTCAAATGCTGCTTTTAAAGTAATTGCTTTTTGTAATCCAATCC
CTTTAACTTGTATCAAATCGTTAATTGAAGATTTTTTCAATTCATTCAGATTCGAAGCAGATTTAAGCAGTTC
ATTACTAATGTCTATGCTCGAGAATCCTTTTCTTCCGGTGTTAATTAATATAGCTAATAATTCTGTATTCGAA
AGACTTTTTGCACCATGGCTTAACAAACGTTCTCTTGGCATTTCTGAAGTTACCATTTCTTTAATTTTCAAAA
ATATACGCCTCCTAAAAATTGATGGATATCATTATAAAAAAGTGAATTGATAAAAAAATGAAATAAATATAAAT
GGAACAAGGGGTAATAGTTTAATCGGCTTAAATATCATGGTAATTAAAGCAACTAAACCAGCAATGACAAATG
TAAATAAAATGACATAAATAGTGAATTGGAGAGGGAAAAACAAAGAAAGTGCAGATATTAGTAAAACGTCACC
ATAACCAATATATGCCCGAAATAAAAAGTAGAATATATGCGTGGTCATACTAATAATGATAAAAGCTACTGGA
TAAATCATACTTAACGAGAGAGAAACGATACAATAAATTATAATTAAGCGACAATCTAACATTAAAGAAGTGA
TATCGGTCATAGTAAAAATAAGCAGAAAAACATATGTAGTTATAAATAGCGTAGCATTTACGTATGTGAAATC
ATACTTAATAAAGACGATAGGTATTATAGCAAAGGTTTCCCCTAAGAAATGTGTTAGGGAAATACGCTTTCGA
CAGTTTCGACATCGCCCTTTTAATAATAAAAAACTAATAATCGGCATTAATTCATACCATTTGAGTGATGAAT
TACAATAATCACATTTCGATCTTCTATGTAAATAATCAAATGACGTTTCTTCTATAGATATAAATTGATATAG
AAAACTAAAGATACAACTGCAACTATAAGATAACAATACTACCAAATGACAACCTCCTTATGTAAATTATAGT
TAGTTATTATCAAAATGTAAATATACACTATTTTTCAAGAATTGAATCGCTTTTTCATTTAAATTTTTCAATA
TTGCTAAGCATAATTGATGGATACTTTAACAGCCCATTACTGCTCGGCAAAATTAATAATGGCAAGAAATTGA
ACCTTATAAACACATACGATTTAGAGCATAAAAAATAACCATGAAGCTCTACCTATTGATTAAATAGATTCTT
CATGGCTATTTTAGTTTTAGTTTTATAATGCTTCAAAGTCTAATTTTGATTTAACTTCACTTATGAAATACAG
ACTACCGGTAATTACTAATGTATCACCTTGATAATTTTTTATAAATTCAACGTAGTCATCTACTAATTGTATT
TCATCATTTTCAATACTACCTACAATTTCTTCTTTGCGTAACGCTTTCGGAAAATCAAATTCAGTTGCATAAA

ACGTATGCGCAATTAAACTTAAATGTTTGACCATCTCGTTAATCGGTTTTCCGTTTATTGCTGAGAACAAAAT
ATCTACTTTTTCTTTATCATGGTACTGTTTAATTGTATCAATTAGAGCATCTATACTCTCTGAATTATGTGCG
CCATCCAAAATGATTAAAGGTTTGTCATGCACCTGCTCAATACGTCCAGTCCAACGAACTGATTCAATACCGT
CTATCATCTTATTGAAATCTAATTCAATTAATCCTTGTTCATTTAATTCAATAAGAGCTGTTATGGCTAATGC
AGCATTTTGTTTCTGATGTTCACCTAACATACTTAAAATGATTGTTTCTAATTCATAATCTTTATAACGGTAA
GTAAATTCATCATTTTGCGATACAACAACAATTTCTCTATCTAATTCAATTGGCTTTGCATGTTGTTCAATTG
CGCGTTCACGAACATATTTTAATGCATCTTCATTTTTAACAGCATATATCACTGGAACGTTAGGCTTTATAAT
CGCGCCTTTATCCCTAGCAATATCTAGATACGTACCACCTAAAATATCTGTATGGTCTAGACCGATACTAGTT
AAGATTGATAAAACCGGTGTAAAGACATTTGTCGAATCGTTCTTTATACCCAATCCAGCCTCAACAATGACAA
AATCAACAGGATGTATTTCACCAAAATATAAAAACATCATCGCTGTGATTATTTCGAATTCAGTTGCAACACC
TAAATCTGTTTCACGTTCCATCATTTCACTTACTGGTTTAATACGTGATACTAATTCTACAATAGCGTCATTT
GATATTGGCACACCATTTAGACTAATTCGTTCATTAAATGTTTCAATAAACGGCGACGTAAATGTACCTACTT
CATAACCATTTTCAACTAAAGCTGTTCTAAGGTAAGCAACTGTAGATCCTTTACCATTTGTGCCACCTACATG
AATACCCTTAATGTTATTTTGAGGATTATTAAATTGTGCTAGCATCCATTCCATACGTTTAACACCTGGTTTG
ATGCCAAATTTAGTTCTTTCGTGTATCCAATACAAGCTCTCTAGGTAATTCATTGTTACTAACTCCTATGCTT
TTAATTGTTCAATTCTTGCCTTCACACCATCATATTTTTCTTGATAATCTTGTTTTTTACGTTTTTCTTCATT
TATAACCTTTTCAGGTGCTTTACTTACAAAGTTTTCATTAGAGAGCTTTTTATCTACTCTATCTAATTCGCTT
TGAAGTTTAGCTAATTCTTTTTCCAAACGGCTGATTTCCTTATCCATATCAATTAGCCCTTCTAATGGTAATA
CCACTTTACCTGCAATTACAACTGATGTCATTGCTTTCTCAGGAATTTCCACGTCAGTGCTAATATTTAAGGT
ACTAGGATTACAGAATTTGATTAAATAATCTTTGTTTTGTGATAAAGTTGTTTCAATTTCTTTATCTTTAGCT
TGAATTAAAATAGGTATTTCTTTAGACAATGGCGTATTTACTTCTACACGTGATTGTCTTACAGATTTAATGA
TTTCAACAAGTTGTTGCATTGTTTGTTTACTTTCTTCAAAAATCAATGATTCACGCACTTCTGGCCATGAAGC
TTTAACAATTGTGTCACCTTCATGTGGTAAACTTTGCCATATTTTCTCTGTTACAAATGGCATGAATGGATGT
AGCATTCTCATAATATTGTCTAAAGTATAACTCAATACTGAACGTGTAACTTGTTTTTGTTCTTCATCATTAC
CATTCATTGGAATTTTACTCATTTCAATGTACCAATCACAGAAATCATCCCAAATGAAATTATATAATGCACG
TCCAACTTCGCCGAATTCATATTTGTCACTTAGATCAGTAACTGTTGCAATCGTTTCATTTAAACGTGTTAGA
ATCCATTTATCTGCTAATGATAAGTTACCACTTAAATCGATATCTTCAACTTTAAAGTCTTCACCGATATTCA
TTAAACTGAAACGTGCCCCATTCCAGATTTTATTGATAAAGTTCCACACTGACTCAACTTTTTCAGTTGAGTA
TCTTAAATCATGTCCTGGAGATGAACCCGTTGCTAAGAAGTAACGCAAGCTATCAGCACCGTATTCGTCAATA
ACATCCATTGGATCCACACCATTACCTAATGATTTACTCATCTTACGCCCGTCTTCAGCACGAACTAAACCGT
GTAATAATACATCATTAAATGGACGACGATCTGTAAATTCTAAGCCTTGGAATATCATGCGTGCTACCCAGAA
GAAGATAATATCGTAACCTGTAACTAAGGCATTTGTTGGGTAGTATCGTTTAAAGTCTTCACTTTCTAAATCA
GGCCAACCTAACGTAGAGAAAGGCCATAAAGCACTTGAGAACCACGTATCTAATACATCTTCATCTTGTTGCC
AATTTTCAATATCAGTTGGCGCTTCTTCTCCAACATATATTTCGCCTGTTTCTTTATGATACCAAGCCGGAAT
TTGATGACCCCACCATAATTGTCTTGAAATCGTCCAGTCTCTAATATTTTCCATCCATTGGTTAAATGTATGT
TCGAAACGTTGCGGATAAAAATCAATACGATCATCTGTTTTTTGGTTATCTAATGAACGTTTCGCTAAGTCTT
CCATGCGCACAAACCATTGTGTTGATAAATATGGTTCAACAACAGCGCCAGATCGTTCTGAATGACCTACAGA
ATGAACATGATCTTCAATCTTGATAACTAAATCTTGTTCTTTTAAATCTTCAACTAGCTGTTTACGACAATCA
AAACGGTCCATACCTTCGTATTTACCAGCTTTGTCGTTCATTTTACCATTTTCATCCATAACGATAATATTTT
CTAATTGATGTCTTTGACCAATTTCAAAATCATTAGGGTCATGTGCTGGTGTCACTTTCATAGCACCAGAACC
GAAGTCTATATCAACATACTCATCTGCTAAAATAGGCAGTTCGCGTCCTACGATTGGTAATATAACAGTTTTA
CCGATTACATCTTTGTATCGTTCGTCATTAGGGTTAACAACAATCGCTGTATCACCTAACATCGTTTCTGGTC
TTGTTGTTGCAATTTCAATAAAACCTTCACCATCAGCGTAAGGATATTTAAAATGATAAAACGCACCTTGAAC
ATCTTCATGTATTACTTCAATATCAGATAAAGCTGTACGTGCTTTAGGATCCCAATTTATAATACGTTCGCCA
CGATAAATAATTCCTTTATTGTATAAATCAACAAAAACTTTTTTAACTGCTTTACTTAAACCTTCATCTAAAG
TAAAACGTTCTCTACTATAATCTAAACCTAGACCTAATTTAGCCCATTGCGCACGAATAAATGACGCATACTC
TTCTTTCCAATCCCATGCCTGTTCTAAAAACTTTTCACGACCAAGATCATATCTAGTTATTCCTTGTTCATTT
AATTTAGCTTCTACCTTTGCCTGTGTCGCAATACCAGCATGATCCATACCTGGTAAGTATAACGTATCGTACC
CTTGCATACGTTTCATACGTGTAATGATATCTTGTAAAGTTGTATCCCATGCATGTCCTAAATGTAATTTACC
AGTTACATTTGGTGGCGGGATAACGATTGTATATGTTTCTTTTGATTTATCTTCTGACGGTTTAAAATAACCA
TTCTTTACCCATTCTTCATAACGTCCCGCTTCAACTTCACGAGGATCATATTTTGGTTTCATTTCCATTTGCT
ATACCTCCTAAAAAAATAAAAAATATCCATCCTATATACAAATAGGACGGATATTCCGTGGTACCACCTATAT
TCAAGAAGGATGATTAATATCAAATTCACTCTTTTAACATAATTGGAATAATCATACCAATACTATCATCGTG
AAATTTGAAATGCTTCATCTCTTCAAGCACTCTAGATTATGATTAACGCTCAAACACGTCTTAGCCTACTATT
AATCACGTTCAGCTAAGATACTCTGTGGGCTACCTTCAGTAAAAATCATTTACATACTCACACCAAATCATAT
GCTCTCTTTAAAATAATTTGAACTTACTCTTCCCAAATCATATATTAAACTCTTAACTTATAGTATAATGATT
GACAAAATAAGTCAATGTATAGGTGGGAATAAAATGAATGATTGCGCATTTGGAACTAAAGATCCAGTCTACT
TAGACTATCATGATTATGTATGGGGACAACCGCTCTATGATAGCAAAGCATTGTTTAAACTTTTAGCATTAGA

ATCACAACATGCTGGGCTATCTTGGTTAACTATTTTAAAAAAGAAAGAAGCCTATGAAGAAGCATTTTATGAT
TTCGAACCAGAAAAGGTAGCGCAAATGACCGCTCAAGATATCGACCGCTTAATGACTTTTCCAAATATCGTTC
ATCATCGTAAAAAATTAGAAGCAATTGTTAATCAAGCTCAAGGGTATTTAAAAATTGAACAAGCATATGGTAG
TTTTAGTAAATTTTTATGGTCATATGTAAATGGTAAGCCTAAAGATTTGCAGTATGAACATGCTTCTGATCGT
ATCACAGTTGATGATACTGCAACACAACTATCTAAAGATTTAAAACAATACGGGTTTAAATTTTTAGGTCCAG
TAACAGTATTTTCGTTTTTAGAAGCAGCCGGTTTATATGATGCACATTTAAAAGATTGTCCATCAAAGCCTAA
ACACAATTAATTTGCTATAGCATCATAAAATAAACGAGACATCGTCTATTTTTAACTAATAGACTGTCTCGTT
TTTTGATGTGAAAATTTATTCAAATCATAAGTTAAAGTTCCAAGTTAATGCACTGCGAATATTAGATATCTTA
ATCTAAATATAGATGTACAACCTTTGCTTAAACCTTAATAATGAATTCTATCCTAAGCATAATTCAACTTAAA
TGGATGATAGTTTCTAATCTTCACGCTTATTCGAACGATATTTTAAAAAGTAATTAATTCCTGGTGCAATAAT
AAATAGGATGAAAAAGATTCGGAAAATATGATAACTCGTAATCATAGCAACATCGGCACCAGTAGCTAATGCA
ACTAAAACTATCTGATTAACCCCTCCTGGTGCTGCACCAAGAAACAATTCATTAATAGGATTATTATCAAAGA
AATGTATAACATAAACCATGATTAGCGCACCAATTATCAACATAATATTTTGAATTGTAATTGCGATTGCTAG
TCTACCTTTTAAATCTGACAATAAATGCGCAATTTGAACTCCAATTCTAATCATATATATTAGTTGTGCCATG
TTCAACAACCAATGATCTAGTGTAAATGTTAAATCTGTAGAAAAATTCCAAACAATTAATACAATGAGTGGTG
CTAATAATTGAAATGTTGGAAACTTTATTTTAGACATAATTAGATAAACTATAAAGATAGCTATCGCTAAAAT
AACTATTTGCCCTATGTTTAATACTTGTGATAAAGGCAAGACTTTTGTTAACTTTCCATTCGCATGCATGTTA
CCATCATGAAAAAAATATGAAATGAACGGTACTAAAACAACAACAAATATAATTCGTGATGTTTGCGTTAAGC
TAACAACTAACAAATTAGCACGTTTGTCTTGTTCAGCCATGACCAGCATTTGTGTTAGTGCTCCTGGTATAAC
ACTTAAAATAGCTGTTTCTGTATTAATACGTGCAATTTTTTTAAAAATAAATGCCATTACTAATGCAATTAAT
AATATCGAAATAGATACAACAATAATCGAAAGCCAATTGTCTTTAATATCCATAACGACATTTTTCGTAAACG
TTGATCCGATTTGCACACCTAATAGTACAATACCTAATTCACTAAGTAAGAATGGCCATTTAATATCAAGTTT
GAAAACTTTCACACAAATGATTGATGCGATAATAGGACCAAACATAAATGGGAGTAATACGTGCGATGAATAC
AATATAATACTAATAAAAAATGATAAAACGAACACAATGAAATTATTTCTATATATCATTGTCATGTTTTCCA
CTTCTTTCAATAAAAAATAAAATGACTAAATTGCTGCTTGAGCTTCACGTTTGTTAAGATAACAATATCCGCT
AGCAGTTTTGACTACAAAGCATATATGGACTTTCACTATCAAGTCGCCGCCCATGTCTGATATACATTTAAAA
AGAGCCTGAACAAAGTTCAGGCTCTCAATTTGTCCGTATATTTATTTTACAATACGACTTAAAGCCGTATCAA
ATGCTTGAATCGTTTTTTCAATATCTTCTTTCGTGTGTGCCGTAGATAAGAATGTACCTTCAAATTGAGATGG
TGGTAAAAACACACCTTCTTTTGCCATTTCTCGATACATTTCTGCAAATAATTTCAAATCACTTTTATTCGCT
TGTTCAAAATTAGTTACAGGTCCTTCATTTAAGAAATAACCAATCATTGAACCTGCTCTATTTACAGTTATTG
GTACATTGTGTTTAGCAAATACACGCTTTAAACCGTCTTCAAGTATATCGCCTAACATATTAAAATACTCATA
TGTCTCTGGCGTTAATTGGCTTAACGTTTCATAACCACTTGTCATTGCAAGAGGATTTCCTGATAACGTACCC
GCTTGATAAATATTTCCTAATGGTGCTATATGATCCATGATTTCTTTTTTACCACCAAAAGCACCTACAGGTA
GTCCTCCACCGATAACTTTTCCTAAGCAAGTTAAATCTGGTGTCACACCAAAGTGACCTTGTGCACAATGATA
ACCGACTCTGAAACCAGTCATTACTTCATCGAAAATTAGCAATGCTCCGTATTCAGTCGTAATATCTCTTAAT
CCCTGTAAAAAACCTTCAATCGGCGGTACGACACCCATATTACCAGCAACAGGTTCTACGATTACCCCAGCAA
TATCGTCTCCAAATTTTTCGAAAGCGATTTTAAGTGCATCTAAATCATTGTATGGAACTGTAATTGTATTTTT
AGCAATACCTTCAGGCACACCAGGAGAATCCGGCAATCCTAATGTTGCCACCCCAGAACCAGCTTTGATTAAT
AACGAATCACTATGACCATGATAGCAACCTTCAAATTTCACAATTTTATTTCTGCCAGTATAACCACGTGCTA
ATCTTAAAGTATCCAATGTAGCTTCTGTACCAGATGACACCATACGCACTTTTTCTATTGAAGGTACTCGGTC
AATAACGAGCTGCGCCAATTTATTTTCAAGTAATGTTGATGCACCAAAACTTGTACCTTTATCAATTGCTTCA
TGTAAATGACTAATAACTTGAGGGTCTCTATGTCCTAAAATAAGTGGTCCCCAACTTAGTACATAGTCGATAT
ACTCGTTACCATCGATATCATAAATTTTTGAACCTTTACCGTGATCCATAAAAATTGCTGGTGTATCTACTGA
TTTAAATGCGCGTACTGGACTATTTACACCACCAGGCATTAAAGTTTCAGCAACCTTCATTGCTTCTTCTGAT
TTCGTATATCTCATAAAGTAAATTCCTCCTATAGTTTATGGAAAATCATACATATAAAACCTTATTTATCTAA
ATAGCGACAAATGTCCTTTGCAAAATACGTAATAATCATATCAGCACCTGCACGTTTCATTGAAACCATTTGT
TCCATAACGACACGTTCTTCATCTATCCAACCATTTTGTGCCGCCGCTTTAGTCATACTATATTCTCCACTCA
CATTATATGCAACAACTGGAACATTCGTATGATTTTTAACATCTCGAACTATGTCTAAATAACTTAGAGCAGG
TTTAACAATCATCATGTCGCACCCTTCTTTTAAATCACTTTCTAATTCACGAAGTGCTTCCAAACGGTTAGCA
GGGTCCATCTGATACGTTTTTCTATCCCCAAATGATGGCGCTGAATCTGCTGCATCTCTAAAAGGTCCAAAGA
AACTTGATGCATACTTGACACCATAACTCATTATAGGAATATTGTAATAGCCGGCTTCATCTAATCCACGACG
AATTTCAGCAACAAAACCATCCATCATATTACTTGGCGCAATAATATCAGCACCAGCTTCCACTTGAGAAATT
GCTGTTTTAACAAGTAGTGGCAATGATTTATCATTGTCAACGTCATGTGTATGGTCATCAATCACACCACAAT
GACCATGATCAGTATATTCACATAAACAAGTGTCTGCAACAATTAATAAGTCATCATACATTTTTTTAGCAAT
ACGTGTCGCCTGTTGAATAACACCATCGTGAATGTATGCACCAGTACCTATATCATCTTTTGAGTTTGGAACA
CCGAAAAACATAATGGCACGTATGCCTAAGTCATAAGCTTCTTTTAATTCACTTTCAAGTAAATTCAAACTGA
TTTGGTATACACCTGGCAATGACTTAATTTCTTTTTTCACATCGTCTTTTTCAACTACAAAAATTGGATATAT
TAAATCTTCTTTTCTTACATGATTCTCTCTAACCATATCTCTCATTGTCGCTGATGATCTCAATCTTCTATGT

CTATCAAATTTCATTTTTAGCCCCTACTTTCTAAAATCTTTTCAATTAGTGATTCGAGTGTTTGAATTTCTGC
AATTGTTACTGGTTGTTGATATGATTTAATGGTCCGTGCTGTTTGTTCTCCAATAGCAAAATACGACTTGAAT
TTTGGTACAAATCCTTCATTAAAATAATAACGTACTGCCGACGAACTTGAAAATGTTAATGCATCGATTTGTT
GATGTTCTATCATTTCTTTAACATCTTGTATATTTTGTTTGTTAGGCACTGAAGTATATAAATCTATTTTAAC
AACTTCATTATTTTTAGATAACGCTGCTAATAACAATGGTCTCGCCAATTCACTCGAAGGCAAAAGTATTTTT
TGGTTAGTTTGATTAAATGATTTTAAAAATCCTTCTTGAGAAAAGTCGTTTGGCATAAAATCAACTCGAATGC
CAAGTGATTCACAATATTGCGCTGTCTTACTTCCTATCACAGCAATGTTATCAACATTAATTCCTTTTAGATA
TTTATAAAAGAATTTCACAGCATTTTTAGATGAAAAAACAAGCCAGTCATAGCGTTGATTTAACAAATGAATA
TCAAAATTTAGTGGCTTTATATCAATAAAGGGTTTGTGAATAATTGATACTAAATCACTTTGCATGTCATTTG
TTTGTGTCATAACTACAACTGGCTTCATATTTAAACGTCACTCCATTATTTAATGTTGTTCATTTAAGCGTTT
TATAATTTCATAAGCACCTTGCTCTTTTAATTTGTTACTCACTGTTTTGCCTAACTCAACCGGATCTGTTCCG
TTCATTGTATATTCAAATCGTTCTTTACCATCTGGGGTCATAATTAAACCTGTAAATTCGATTTCTTTTTGAT
CTGAGATTGTAGCATATCCTGCAATTGGCACCTGACAACTACCATCCATTTCTGCTAAAAACGTTCGTTCAGC
AGTCACACATTTTGCAACTTCATCATTATGTACTTTGCTTAATAATGTTAATAGTTCTTCATCGTCACTACGA
CATTCTATCCCTAAAGCACCTTGTCCGATTGCAGGTAACAATGTATCTCTATCAAGATAAGATGTTACAATAT
CATCTGACCAGCCCATTCTTCTTAAACCAGCTGCAGCTAAAATAATCGCATCATAATCTTCAGTTTGTAACTT
TTCTAATCGTGTATCTATATTACCTCTAATCCATTTAATCTCTAAATTAGGATACTTAGATAATATTTGTGCA
CCACGACGTAATGAACTAGTACCAATAATACTGCCTTCTGGCAATTGGGATAGTGGTGTATGTGTTTTAGAAA
TATACGCATCAAAAGGTAATTCTCTATCAGGGATACAACCTAATGTTAAACCTTCCGGAATTACACTTGGTAC
GTCTTTAAGTGAGTGTATTGCCATATCGATATTTTTTTCAAAAAGTTCATGTTGTATTTCTTTAACAAATAAG
CCTTTGCCTCCGACTTTAGACAATTGTTTATCTACTATACGATCGCCTTTCGTGACAATTTCTTTAATTTCAA
TTTCTAGATTTGGCTCGACAGCTTTTAATTTATCAATAAATTGCTGGCTTTGTGTTAAAGCTAATTTACTTCT
TCTGGAGCCAACGACTAATTTACGCATGTTCAATTCCTCCTAGGAACGGATTGCTCTAGATTATTTTCTCAAT
TCACAAAATGTGTTGCAAAAAATAAATTAATCATATTTAAGCAAAATAAAATAATGTTATAGTATATTAAATA
TCTTGAATTCAACCATTTGTTGATTCTAAGTAAAATATAACTTCCATATAATACTGTAATAATTGAAGAGAGT
ATTACCTTCGGGTCAATGAATATACGTTCACCAACTGAAATTACACCCCACTGTGTACCTAAAATAATACTAA
ATATGAGAATTATCCACCCACTTAACGTTGAGTAAAACACAATTGATTCAAGTGTAGCAACGCTACCTATTCT
AAAGTATTTTTGATCAAAACGCTTTTCCTTCAAATTACGGTATTGCATGATATACAGTAATGCATTGACAAAA
GCTAAGGCAAAGAAGACATAACTTAACACAGCTAGACCGATATGGACTAACAGTAACTCGTCTACAACAGCAA
TTTTCTGAACCTTATTAGTATAATGTGTCGGTTGAAATGTATTCATCCCTAAAAGTGTTAGCCCTATTAAATT
CAAGAAAAACACTGAGAAATTCAAAACTTTGATAAGGTTAAGAATTAAAGAGATTGAAATAATCAGCCAACTT
AAAGTATAGAATACATCTGAAATAGACCCTAATGGAATATGTCTAGTTTGTATAATAAAAATAGATAAAGAGA
TTGTTTGTAAAACCCAAACAATCCCCAATAAATATATGCCTAAACTTCTAATCTTATGACTTTTTTGGACAAA
ATCATAAAAATAGCAAATGATACTGATTAAGTATATTAATAATATAATTTCATTGAATCGAATAAACAGGTTT
TCTTGCATATTATCACCATATTAATATGCTTATTCAAAACTAAAGATACGTCGTGCTGAAATTTCCTTGACCT
TGCTTTCTTTTTGTTGCTTCGCTTGCTCATGAGGACATTCAGCTTCGATATCAAATATATTTTGAAATAGCTC
TAATTTTTCATTACTTTTCTTATCACTACTTAATTCTTTGGCCTGTTTAATAGGATCTTTCAACATTTGATTG
ATGATACTTTTTGTATGTTTTGATATAATTTTACGTTCTCTTTCGCTTAACCCTGGCAATTTACGATCAATAC
TGTCCATCGTTTCTGCTTGAATTGTCATAGCTTTTTCACGTAAAGCTCTAATCACTGGAACAACACCCAACAT
ACTAATCCACTCATTGTGTGCATGTATTTCTGCAGGAATTTGTTCCGAAATTGTTGCAGCCGCTAATTGTCGC
TCACGTAAGTTTGCATCAACTAAACCTTTTAAGTCATCAACATCATAATTAAAGATGTTTGTGATGGCACTAA
TACCAGGTTCAATATCTCGAGGAACTGCAATATCAATCAATACTAGTGAATCTTGCTTTCTATTTTCTGCAAT
TCTTTCTATCATTTCATTTGTAATGATATAAGATTGTGCACTCGTTGAACTAATCACAATATCTGCACTTTCA
AGTAAATTTGGTAATGATGATAGTTCATCATATTTCACTTGATGCTTTGCTGCTAATTTCATAGCATTTTCAA
TTGTTCTATTTACTACTGTAATATCAGTAATTCCAGAACCAAGAAGATTTAATAGTGATAATTCACTCATTTC
CCCTGCACCAATAATGATAGCTTGCTTACTTTTCAATTTGCCAAATACTTTTTTCGCCAACTCGACCGCAGCA
TAAGACACACTTACAGCATTATCAGCTATATCTGTTTCATTATGTGCTCTTTTTGCAAAAGTAATTGCCTGTT
TAAATAGATGATTAAAAATTATTCCTGTCGTACCTGTGCTTTGCGCTAAGAAAAATGCATCTCTTATTTGACC
TAAAATTTGAGTTTCTCCAAGTACGATTGAATCTAAACCAGAAGTGACACGCAATAAATGTTCTACTGCTTCG
TCCCCCACTTTTACTTCTGACATTGCTTTAATATCATCTACTTCAAATCCAAATGCACGAGCTAGAAATCGTT
GAATATAGTAACGACCTGTGTGAATTTGATCAACAACAGCATATACTTCAGTTCGATTACATGTTGATAATAT
GACATTTTCTAAAATAGATTTAGTTTCATATAAATCTTCATGGGCAATTCGTAAGGCATCATCTCTAAAAGCA
ACTTGCTCTCTTAGTGCAACATCAGCTGTGCGATGATTTATACTAATTGCAATAAAATGCATTTGAAACGCCC
CCATATATACTAACAATACAAATATTATAACATAATTTAAAATCCAATTAAGTACGATGCTTAAATAAATTAT
AATCTTTCTAATTTACTTTTGAGTTTTTGACAAAATGATGAACGTTGAAAAATGTTATCAATTGTGTACTAAT
TAAAAGTTTCGTGCTTACATTATTTTTAAAATTTTTGTATAAGTTTTGTCGTACAAAAACTATGAAATATACG
GTTCAATTAAATTCCATATTTGTTGTTGTTTATTATTTTTAATTGATGAATAACTTACAATTGTATCGTCTGG
GTCCATATCTAATTGTGTCTTAATATTTTTAATATGCTTTTGAACTTTACCTTTTGGAATTTTGTCTTCTTTA

GTGCATATAACTAAAGTAGGAATATCAAAATGCTTCAAATAATTGTACATTAAGATATCATCTTGTGTTGGAT
CATGTCTTAAATCAACTAATTGAATAACTAATTGCAAATTCTCTCTCTTAGTTATATATTCCTCAATCATTTT
CCCAAATTTTTCACGTTGTGTTTTACTTACTTTAGCATATCCATACCCTGGAACATCCACAAAAATAAGTTGT
TCATCTATATTATAAAAATTTAACGTTTGCGTTTTGCCGGGTTGCTGTGATGTACGTGCCATATTTTTTCTGC
CAATCATACTATTGATAAATGTAGACTTACCTACATTAGATCGACCGCTCAGTGCAACTTCAGACAATTCTGT
TTCTGGATATTGTTCTTCTTTTACTGCACTAATGATTAATTCTATATTATTAGGATTAACTTTCATTTTATAT
CCTCACTTTTTTATATTCTCTTTCAACTCTACCATTATATAATATTTGAACCTTTCGGTATACATTATAAAAA
AGCTCCGATCAAAGTTAAACTATTTGAGCGTCAACTTTGATTGGAGCTTTTCACTTTTATAACACATCAATGA
TTAAGCTGATGTTTTACTATTATTAATTAAATTACCTTCTGCGTCGTATAGTTCTGGTTCAGTTTCTTCATTA
ATTGTTTGTGCTGTAATAACTACCTTCGTTACATTTTCGTTAGAAGGCACATCAAACATAATATCGATTAACG
ATTCTTCTATGATTGAACGTAAACCACGCGCACCTGTTTTTCTTTCAATTGCTTTTTCACTAATTGCTGATAA
AGCTTCTTCAGTGAACTCTAAATCCACATCATCTAATTCCAGCATTTTAGTATATTGTTTCACAAGTGCATTT
TTAGGTTGCGTTAAGATGTTTTTCAACGCAGTTACATCTAATGTTTCTAAATTAGCTACAATTGGCACACGTC
CGATAAATTCAGGAATCAAACCATAGGCTTGCAAATCTTCTGGGCGAATTTGTGCTAATAATGCTTGTTCGTC
ATATTTATCAGCTTCATTGCTTGAGAAACCAATAACTTTTTCACCAAGACGGCGCTTAATCACTTCTTCAATA
CCATCAAAGGCACCACCAAGAATAAATAAGATATTTGTTGTATCAATTTGAATCATTTCTTGGTTTGGATGTT
TGCGTCCACCTTGTGGCGGAACACTCGCAGTTGTGCCTTCTAAGATTTTAAGCAATGCTTGTTGAACACCTTC
ACCTGAAACGTCACGTGTTATAGATGTGTTTTCAGATTTACGTGCAATTTTATCAATTTCATCTACATAAATA
ATACCTTTTTCGGCTTTATCAATGTCAAAGTCAGCTGCTTGAATTAATCTCAACAAGATATTTTCAACATCAT
CGCCTACATAGCCAGCTTCAGTTAAACTTGTCGCATCTGCAATTGCAAATGGTACATTCAACGTCTTGGCTAA
GGTTTGAGCTAATAATGTTTTACCACTACCTGTTGGCCCAATTAAGGCAATGTTACTTTTTTGTAACTCAACA
TCATCTTCTTTTGGTCCTAATTGTTGAATACGCTTATAGTGGTTATAAACAGCCACAGCTAAAGATTTTTTAG
CTTTTTCTTGACCAATAACATATTCGTTTAAATGATCCATAATTTCTTTAGGAGTAGGTAATTCTGTAATCGC
TTCAGAAGTATTTTGAGCTAATTCTTCTTCGACGATTTCTGAGCATAATTCAATACACTCATTACAAATATAT
ACACCACTTCCTGCTACAAGTTTTTTTACTTGATCTTGGTCTTTTCCGCAGAAAGAGCATTTCAAATTTTCTT
CATCTTCATTGAATTTAAACATTCTTTTTACACCCCTATTCGTTAAAGACTATACTAGATTGGATATTACAAT
GCAACATATTAACATACAAACTTTTTGCTTAAAGAATAGTAGCAGATACATAAGCTATGCATCTGCTACTCAT
TTTTGTAATTTAATATTTAATGAAGATTAATCTTCTTTAGTTCCTTCAACGAACTTTGCGTTATCTCTTAATA
AATCGATAACTTTTTGGATACGAACATCATTTTTAATGACATCAGTATTACCTAAAGTATTTTTGATATCTTC
AACTGAGATATTAAATTGTTTACTCATTTTTTTCTAATTCTTTATCGATATCTTCATCAGTAGCTTCGATTTTT
TCAGCTTCAGCGATCGCAGTTAAAGTTAAGTTAGTTTTAACACGTTGTTCTGCATCGTCTTTCATTTGCTCTC
TTAATTGAGATTCATCTTGACCTGAGATTTGGAAGTACGTTTGTAAATCTAAACCTTGTTGTTGAATTCTTTG
TGCAAATTCAGACACCATACGATCTAATTCAGTATTAACCATTGCTTCAGGAATATCGATTGTTGTATTATCA
GTAGCTTTTGTAATCGCTTCTTCTTTTTCAACATTTTCAGCATCTGTAGCTTTTTGTTCAGCTAAACGTTTAC
GTAAGTTTTCTTTATACTCGTCTACTGTATTTGCTTCCGCATCTAATTCATTAGCAATTTCATCTGTTAATTC
TGGAACTTCTTTAAATTTAATTTCGTTAACTTTTGTTTTAAAAGTTGCTTCTTTACCAGCTAATTCTTCAGCA
TGGTATTCTTCTGGGAATGTTACGACAACATCTTTTTCTTCGTCAACTTTCATACCTTCTAATTGCTCTTCGA
AACCAGGAATGAATGAACCTGAACCGATTTCTAAATCGTAACCTTCAGCTTGTCCACCTTCGAATTCTTCTCC
GTCAACTGAACCACTAAAGTCGATGTTAACTGTGTCGCCATTTTCAACAACACCATCTTCTTTAACTACCATT
TCAGCTAAATGTCCTAAGCTGTGGTCAATCGCTTCTTGTAACTCATCATCAGATAATTCAGTTTCTTGTTTTT
CAATTTCAAGACCTTTATAGTCTCCTAATTTAACTTCTGGCTCAACTGTAACTGTTGCTTCAAAAATAAAATC
TTTACCTTTTTCAATTTGAGTAACACTTACTTCTGGTTGTGCAACTGGTTTAATATCAGTTTCGTCAATTGCT
TCACCATAAGCATCTGGTAATAAAATGTCGATAGCATCTTGATATAATGCTTCTACACCAAAGCGTTGTTCGA
AAATTGGACGTGGTACTTTACCTTTACGGAATCCAGGTACGTTAATTTGTTTAACCACTTTTTTGAATGCTTG
ATCTAACGCTTTGTTTACTTTTTCTGCAGGAACAGTAACAGTTAATAAACCTTCGTTACCTTCCTTTTTTTCC
CAAGTTGCTGTCATGTATATATACCTCCATGATTAACTAATTTATTTTTTCAACTTCCCTATTATATCATAGG
TCTATTCCCTATACAAACATTGAAATCACAACGTTTATATATTTGTAAATCAACTTTTTTCGTCAAAACTATA
TTAAAATGCTGAGTATTATTTTGCAACAATTTAACTATTATTATTCAAATCTAATTGCTGAATAAAATTAAAT
GCATCGATACTTTGTAATTCACATTTAAGTCCTAACATATTTTTGAAATAACATTCGTATGCATTAATCCATT
TATTCGTTTCAAACAAAGTTTCAATATCAATTGGATAAATCATGATAGAGTGATTATTCATAATATGGTGTGC
TTCTTCAGCGATATGTAAAGCACCATCATTTAATGTTTCCATAACGTTAGGTATAACTTTTTCTTTCAGCGTT
GTATGTTCTAGCCCATTTAAATTAGCTGGTACAAAAGTTACATCCATACCATACTTTTCAATTGTCAGTTCTT
GTGTACAATTTGCAAACCTTAAATACTCAATCATTAAACTAATTAGGTTATAACTGTACGTATTAGATTTTAA
TATATATAATACCGTTTCTTGAAATTGAAAATGACCAATGTCTATTAACTTCAAAATCAAGTGCGTCTGTTCC
CTCATTGATAACGTATCAAAATCAGTTAATGACTGAGTCAATCGTTTTTCATCTTCAATTAATTTTGACTTAG
CAAATTCTTTAAGAGGATGTAATGCCATTCTTGTTTTGTGATCTTTGACTTCATCAATAATTTGATGAATTAC
TTCTACCGCTTCAAAATATTGTCCCAAACCAATCAAACTTTTCACGTAATAAATCATCAATGCATCATATTGT
TGTATGCCAGTTTTCAATAAGACAATTGTTTCTTCTCTTAATTCAAGAAATGACCCCGTCTCATATAACATAC

GGCATTTCATCATTGCTATTTCTTCAGTGAGTTCAAATTGACGTTCATATTGCACAATATATTCATACATCAG
GTCATAGTTTTGGTCTTGTTCAGCACGTTTTATATCTTTATATAACTTTATCGAAGAGTTAGGAAACTGAATA
ATATCTGACATATATGTCACCTCGTTTGATTATAAATCTTGAAGGATTAATGCGCTCCACGTTTCGAAATCAT
CATATGACTCTATGTTGCCTTTTTCAACCCATTTTATTCGTAAAGTATCTGTTTCTTTAGCTTCTACATCATT
CGTACTTACAGTGATTTTAAATACAACACCAATATGTACCTTGCCCACTTCATTATTATCGTCATTAATAAAA
CCGATATACTCCATATTTTGTGAATCTTGCTCACTTAAACCTACTTCTTCTTCTAATTCTCTCTGAGCATTAA
CTCTCAATACTTCGTTAATAGATTCTGCTCCTGGAACATCATTCATATGACCGCCTACACCTATTGAAGATTG
TCCATGCAATCGAGCTTCTCCACCGCCAGATAATCGTTCATACACTAATATCTCGCCATGCTCATTTTCAAGT
AAACAATAAGAAATGAGTTGTTTATATGATGGATCCTCTTCCATATCGCCGCGTCGCTTAACTTCATATTGAC
TTAGCGCATCAAAAATATTTTGACCTTCTGGTTTATTCTTATTTAAAAACCCATTAAAAGTATTCTTTTCATT
ATTAAAAATAATTTCTCTAGGTACTACAATGATTTGTTCATCAAATTTAGACATCGTATAAAACTCCTCACTT
TAATGTTTGTTCTCTTAAATGCATTTTAGCAAATATGTCATATTTTAGCGATTAAATGTGGCATTTTGCTATT
TAAAAAATAAAAAAGAGATGACAATGCCATCTCTTTTTTATAAGTGTTATTATTTTAAAGCATCTTTAGCTTT
AGTTACTAATTGAGCAAATGCTTTTTCATCAGAAATTGCAATTTCTGATAACATTTTACGGTTAATGTCGATA
CCAGCTTTTTTCAAACCGTTCATTAAACGTGAGTAGCTCATTTCATGTTGACGAGCTGCTGCGTTGATACGTG
TAATCCATAATTTACGGAAGTCACGTTTACGTTGACGACGGTCACGGAAAGCGTATTGACCTGATTTCATTAC
TTGTTGCTTAGCTACTTTGTATAATGTATGTTTTGAACCGAAGTAACCTTTAGCTAATTTAATCGTTTTTTTA
CGACGCGCTCTTGTTACTGTTCCACCTTTAACTCGTGGCATAAAAAAATTCCTCCTTAGGTAATTCCTACCGAT
TTCTGTATTTGTTCTTATTTTTTGTATGCTAATAATTGTTTTACACGTTTCATGTCGCTCTTAGACACTAATC
TAGCTTTACGTAATTGACGTTTTTGTTTAGTGCTCTTGTTTGCGAATAAGTGAGATGTGAAAGCTCTTGAACG
TTTTAATTGACCTGAAGCAGTTCTTTTAACACGTTTAGCTGCTCCGCGGTGAGTTTTCATTTTTGGCATAATA
TAAGTCCTCCTGTAATAGCTTTAATTATTTTTCAGCTGTTGGCGCTAACATGATAAACATTTGACGCCCGTCC
ATTTTAGGTTTTTGTTCAACTGTTGCTATATCTTTGCATTCATCTGCATATTTTTCTAGCACACGTTGACCAA
TTTCCTTATGCGTAATGGCACGCCCTCTGAAACGAATAGATACTTTACATTTATCGCCTTTAGTTAAGAATTT
ACGTCCGTTTTTCAACTTAGTTTGGAAATCATGTTCCTCAATTGTTGGACTTAAACGAATTTCTTTAACATTG
ATAATTTTTTGTTTCTTTTTCATTTCTTTTTCTTTTTTCTGTTGTTCGAATTTGAATTTACCGTAATCCATAA
TTCTTGCAACTGGTGGTTTCGCATTCGGTGCAACGACCACTAAGTCTAAATCTACACGTTCAGCCATTTCTAA
AGCTTCTCGCTTTGATTTAACACCAATTTGTTCACCATCTTGACCGATTAAACGTAATTCTTTTGCACGAATT
TTGTCATTGATTTGAGTTTGATCTTTTGCTATGGTTGACACCTCCAAAATTTTTACGAAATTTGCACCAAGCA
AAAAGGAAGAGCAGGTATAAAATACCCGCTCTTCCTTATACACAGATATGTGTAATGTGATTAACCTGCCAAC
TGCTTTATGCGTCGCTACAGGTGAGAAGCGGGTGCTTCTACTTGGTTCGTTTCGTATTCAACGTTGTTAATCA
TATCAACAATTCACATTTAAGTCAACACTATAACTGTAATTATTTTTATTTTAACCTTTTATTTCATCCATTG
ACACGTCTTGACGTAAATCTACTTGTTCTAATGGAATTTTTTTCGTTTTATATCGAAGCTTATGATAAATAAA
GAATGCTAAAAATACTGGAATTCCCATATACGTAATTAAGAAGCGACTAAAATTAAAATCTCCTGTTTTAATA
AAGTCAACATCTTGACCAATAATTACTACAATACATAAAAAGCCAGCAAATAATGGTCCGAATGGGAATAATT
TAGCAGTATATTTTAATTTAGATTTGTCATAATTTTGTTTATCAAATGCTCTTCTAAATCGATAATGACTTAC
TGCTATACCTACCCAAGCAATAAAACCAGTTAAACCACTTGCTGCAACGATATATTCGTATGCACCTTTTGTA
AGGCTTTGTAATACGAAAATAATCACTACAATGATTGCTGTAACGAGTAACGACATATATGGCACACCGTTTT
TATTTGTTTTACCAAATGCTTCAAACGCTAATTTATCTTTACTCATTGAATATAGCATTCGAGTTGAAGCATA
CATACCTGAGTTACCTGCTGATAACACAGACGTTAAAATGACTGCATTCATAAATGATGCTGCAAACGCAAAT
CCAGCATTTTTAAACACTAATGTGAATGGAGACGTTGCTACATTATCACTACCACCCATTAATGCACTACTAT
CATAAGGAATTAACATACCGATAACAAAAATGGCTAAAATGTAAAATAATAAAATTCTCCAGAATACTTGTTT
AATTGCTTTCGGCACAGCACGTTCAGGATTTTCTGATTCACCAGCCGTAATACCAATTAACTCAGTACCTTGG
AATGAGAAACCAGCGATTAAGAATACACCTAGAATTGATAACAAACTTCCGCCTAGATTGCCACCAAGAATAG
GACCTTCACCTTTATTAAATATTTCGAATCCTACAACATGACCACCCATGATTCCGACAATCGTTAATAAACC
AATTGCAATGAAAACAATAACTGTAACCACTTTTATCAATGCCAACCAGTATTCACTTTCACCATAGACGCGA
ACTGATAACGAATTCAGACTAAAAATTATAACTAAGAACAACGCACTCCATGCCCAAGCGGGTATGCCTTGCA
ATGGTGTCCAATATTGAATGACTTGTGCTGCAATCGTAATATCTGCTGCTACAGTTACTACCCAGTTAAACCA
ATAGTTCCAGCCAAGCGCAAATCCTAAAGATGGATCAACAAATCTTGTAGCATATGTACTAAATGAACCTGAT
ACTGGCAAATACGTAGCCATTTCGCCAAGTGACGTCATTAAGAAAAATACCATTATTCCGATAATTGCGTATC
CTATTAATGCACCCAAAGCACCTGCATCATGAATTGCTCCACCAGAAGTTACAAATAAACCTGTACCAATACA
ACCCCCAATCGCAATCATAGAAATATGACGATCTTTAAGTCCCCTTTTGACAACATTGTTACTTTCATTTTGA
ACTTTTGACATATAAGCTCCCTTTCCATGTTTTAGAGGCCAAGAAATAAATAACCTTTATTCACTTTCATACC
CTTTTTTACAGAACATAGTGATATTAAAAGTAAAAAAGTGGCTACATACCTTTAAGATATGCAACCACTTTGG
CAATTATCTATATAAGGTAGCACATCACATTCTGTGACAGTACAGTTCCTATTCGAAAACTGTCCCAATAGAT
ATAATTTATGGTTTATATCTATTTCGGCATCTTTACCTTTCACTTGTTCAACTTATGTACCATAAATACTTCT
GACAAGTTACTAATTAAACATGCAACCTCTAACTCAATTTAATATTTTAACTAACTTGTAATATACAGGATTC
ATCACGCATAATCAACCCTGTGAAACTTGATACGCAATAAAAATTTCAAAGCATTTTATTGCGACAACTGTCT

ATCTATGTTTTTTTAAACGAATTTCATCAACTAGATTCCAGATAAATTCATCTTTTTCAACTGTTTCTTGGTC
TTGCGATCCATATTGACGCACATTCACTTGATTATTTTCAACTTCCTTATCCCCAACTACGATTTGATAAGGT
ATTTTTTGCATTTGAGCTTCTCTAATTTTATAACCCATTTTTTCATTACGGTCATCAATACTTACACGAACGC
CTTGAGATTTCAATTCATCTTGTAATTGGCGCGCATAATCATAATGTAAATCAACGTTAACTGGAATGATTTG
AACTTGTTTTGGCGCTAACCAAGTTGGGAATGCACCTTTAGTTTCTTCAGTTAAAAATGCTACAAATCGTTCC
ATTGTTGATACAACACCACGATGAATAACAACTGGACGATGATGTTCACCATCTTGACCAATATAAGTTAAAT
CAAAACGTTCTGGTAATAAGAAATCAAGTTGTGCTGTTGATAATGTCTCTTCTTTACCCATCGCTGTTTTAAC
TTGAACATCTAGTTTCGGACCATAGAATGCCGCTTCACCAATCGCTTCTTCGTACGATAAGCCAAGCTCATCC
GCTGCCTCTTTAAGCATATTTTCAGCTTTATTCCACATATCATCATCATCAAAGTACTTTTCTTTATCTTCAG
GGTCTCTATAACTTAATCTAAAGCTATAATCCTCGAAACCAAAGTCTTTATACACATCAATAATCATGTTTAC
AACGCGTTTGAATTCTTCTTTAATTTGATCAGGTCGAACAAAGATATGTGAATCATTTAAAGTCATACCACGA
ACACGTTGTAATCCTGATACAGCACCACTTGCTTCATATCTATGCATCGTTCCTAGCTCAGCGATACGGATAG
GTAATTCACGATATGAATGTGGTTTATTCGCATAAATCATCATATGATGTGGACAGTTCATTGGACGTAATAC
CATAGATTCTGTTTCATCTAACTGCATTGGTGGGAACATATCTTCTTGATAGTGATCCCAGTGACCAGATGTT
TTGTATAAATCAACATTAGCAAGTACTGGTGTATAAACGTGGTCATATCCCATGCTAACTTCTTTATCAACAA
TGTAACGTTCAATTTCACGTCTAATTGTTGCACCGTTAGGTAACCATAATGGCAAACCAGCACCAACTAATTG
GCTATTTGTGAATAGTTCTAACTCTTTACCAATTTTACGATGATCACGTTCTTTACGCTCTTCTAACATTTGT
AAATGTGCTTTTAATTCTTTTTTATCAAAGAAAGCAGTACCGTATATACGTTGTAACATTTTGTTGTTACTAT
CTCCACGCCAGTATGCACCTGCTGTAGATAATAGTTTAAACTCTTTAATTTTAGCTGTTGATGGAACGTGAAC
GCCACGACATAAATCAGTAAAATCACCTTGACTATATAATGTTACATTTTCATCTTCAGGAATCGCGTCGATT
AATTCTAATTTGTATTCATCATTGCTGAATAACTCTTTCGCTTCATCTCGTGAAACCACTTTTCGTTCGATTT
TCATATTTTCGTTAACGATTTGTTTCATTGTTTTTTCAATTTGTTCAAAGTCATCAGATGAGATGTTTTGGTC
AATGTCGAAGTCATAGTAGAATCCACCTTCTATTACAGGACCTACACCAAATTTAACATTACCATATAACCTT
TTAATCGCGTGTGCCATTAAATGTGCAGTAGAATGACGTAATACCTCTAACGCTTCTTCACTACCTGGTGTCA
CAATTTCAATTGATCCATCAGTTTCAAGCGGTTTAGTTAAATCTACAAGTTGCCCGTTAAATTTGCCGGCAAC
AGCTTTTTTACGTAATCCAGGACTAATTGATTGTGCTATATCTTCAGTAGTAGTACCTTTATCAAACGCCTTT
TTATTACCATCTGGAAATTGAATATTAATTTGTTCCATGTTAAACCCTCCTATTTGCTTTATAAATACTTTTA
TCTGATCTCGTTAAAAAAATTGAACAACAATCATCGTTTTAACATTAAACCTATTAACAATAAACAGGGAATTT
ATGAAACCAACGAACATTTTTGTCTGAAAAAAACAAAAAATCCCGCCCCTATACAAGGGACGAGATCGTCGTG
GTACCACCCTAGTTATTTAATACAATTTATGTATTAAATATCTCTGCTTAAGATAACGGTCTTGATCCGGGTA
TTCATTACAAATACCATAAATGAAGTAGTAATCACCTAATTTATTAACCATATTCTCATCAAACAATGGCTTT
CTGTGTAATATTGATTAGATCATCTTGTCTTCAATCATTTAAACGATTTAGATTTGTATTCATTATACACCAA
TCATTTTAAAATTCAATTGTTTCTGAAATTTTCTCCTGATAAAAAGTATGGTGTTGACAAAGATTTGACACGT
TCAATAATACGTGCTGCTTTAGTCTTCTCTTCACCATCACGAGTCATCGCTAAATGATGTTCCAATTCACTAT
AGTCAAAATTAGAACTAAAGAATGTTGGTAATTCATGAACCATTCGATAATGTAGCAAAGGTCCAATTACCTC
ATCTCTCACCCATGGAGTCACTTCTTCAGCCCCAATATCATCAAGCATTAAAATGTTTGCTTCTCTTACGCGA
TGTAATTTCTTTTCAAAAGAACCATCTTTAAAGCCACCTTTTAATGTTCTAATAAATTCCGGTAAATAAATAA
TTGTCGAACGTACCTTCTTAGATTTGAGCTGATTCGCAATTGCACCTAGAATAAAAGATTTACCTGTCCCAAA
TGGACCATAAAGGTAAAGGCCTTTCACTTGTTCCCCATTAGTTATTGCTGTACAAATATCATCTGCTGCCATA
GCTACATCAAGACGGTCTCGATGATTCATATAAATATCTTTCAATTTGGCATTTAAAGTATCTCGTTGCATAT
GATGAGATGTAATTAGCTCAGCTTCAAAGCGTTCTTCGTCGTACTTGATTTTACATGGGCATTGTAAATAGCG
TATTTTAATTCGGTTATTATCAACATATAACTCAGGCACATGCCCCTTTACGAAATTTGGACAATCAGCAAAT
TTATGACCGTCATAATGTTTTTGTTGATCTTTATACTCTTGTAACACATTTAAGTCTTCATCAATCATAGCAT
TCGTTAATTCAGCTCGATGCGCTTCCAAAAATTGCTTAACATCTGGGTCATTGATTACTTCTTTTTTTATCTT
TTCTATTCTTTTTTCAAAGTCCTGCGACGTGTTAATTATACTTTTAAATTGCTTCATTATTGACTGTCCTCCT
CCCATTTTTTAGATAATTTATCTAGAAATGCTTGTCGATCTTGCTCTAATTGTTGATCATCTACGCTATTATC
TTTAGCCGAATCTTCTTCACTAGGTTTATCTCTATTTTCTAACCATTTAGGTGTTTTTTCTTTTGAAATACGA
TTACGCTGCCCATAGTATGAACCACGCTTTTGGTAATTTCCGCTAGAACCCTCATTTTTAGGTTGATTAACTT
TTTTAGCGTAATTATATGCTTCTTTAGCTGTCTTAATACCTTTTTTCTTCCAATTTGATGCTATTTCCAAAAT
ATACGCTTTAGGAAGTTTCATATCTTCTTTTAACATGACAAATTGCAACAAAATATTAATGACGCCAAAAGAC
ATTTTTTCACGTTCAATTAATTCTTCAACCATTGTCTTTTGCGATATAGTTGGTTCTGATTCAGACCAAGAAG
CTAACATATCAATTGGACTCGTTTGTTCAAGTAACTCAAACCATTCATCACTTTGTGGCTTTGGATTCACTTC
TGAAGATTTGCCCGTCGAAGATGATGTAACAGGAGATTTCACCTGTAATTTAGGCATTTGATTTTCGTGTTCC
ATTAAGTAATACGAGCGTGCTTGTTTACGCATTTCTTCAAAGGATAACTGTTGTCCACTTGTAATTGAATTTA
AAATAACATGCTTCATGCCATCTGCTGTTAAACCATATAAAGTCGCGAGTTGTGTAATTAAACGCTTTGCATC
TTTGGTAACAATGTCTTGACTAATAAAATGTTTACCTAACATTTGTCTCAACATTTCAAAGTCAAAAGATTCA
TTTGATAAATCGATACCTTGGTACGGTTCATTAATCGGAATATCACTTGTATCGATATCTATTTTTGTAGACG
GCACTTTAAAAACATCAGTAAATTGTCTTGTTACCTGTTTAAATTCACTCAAATCAATTTGTTGATACTCAAA

GTATTTCTTCAACTCATGAAATCGACGATGCTCGACTTCACTATATAAAAAGATTGACAACATTGGATCATTA
AAAAATAAATGTGCTGAAGGCGGTTGAATTAATTGGTAAACAAATTGTGTTTCTTGTTCATCATGTTTGACAA
ACGCCTTTAACAATCCAATCGCTTCAAGTAAGTCCATTTGTTGTCTAAACTCTAGTAAATTAATTTTAAGTTC
ATTCATAAAAATATAATGAGAAAGAATCAATGTTTCATTATGACTTTCTTTAACGAATTGTGTCATAAAATGG
TATAAACCCACTGCTTGTGTTCCAATTAGCGGTGTATACAGTCGATTCAATACCTCTAAATGATTCGTATTTA
AATCAAAGTGTTGCATAACTTTGAATTGATCCTTTGGTCTTAAGCCGAATTCGAAGGCTTGTCGTCCCATTTA
AGCATCACTCCGTTTGTTTTCGCTTAAAATCCCTTGCATCGATGCTAACAATTGATCAACATCTTTAAATTCT
TTATAGACTGATGCAAATCTAACATATGAAACTTGATCAACATGCATTAACAAGTTCATAACGTGTTCACCTA
TATCTCGTGAAGACACTTCCGTATGACCTTCATCTCGTAATTGCCATTCAACCTTGTTAGTTATGTCTTCAAG
TTGTTGATATCTAACTGGTCGTTTCTCACAAGAACGCACAAGTCCATTAAGTATCTTTTCTCTTGAAAACTGC
TCTCTTGTGCCATCTTTTTTCACAACTATAAGCTGACTAACTTCGATATGTTCAAATGTAGTGAAACGTGTTC
CACAATTTTCACATTCTCTTCGTCTTCGAATGGCATTTAATTCATCGGCATGCCTTGAATCTACAACTTTAGA
TTGTGTAGAATTACATTTCGGGCATTTCATTACATCACCCTCTTTATTTTGATTATGCCTAATTATACTATAA
ATCTAGAGATGAAAAAAGAATCCCTCAATTTAATTCATTTAACCAAATAATGAAACAATAAAAAACATTATAT
CGTTACTTATTAAGTAATATGCATGACAATATTATTGTATTAAAAATAAAAAACCTAACTCCGAAGTCAGAGT
CAGGCTATTAAATAATTGTATTAACTTGCACTTACAGTTTCTTTTGATGTCAAAAGTGCTCCAATTTGCTCAG
CAACATCTACAACTCTATTTGAATAACCCCATTCATTATCATACCAAGCAATAACTTTTACTTTATTCCCTGA
CATGACCATTGTTGATTTTGCATCAATAATAGCTGAATGTGGATTAGTATTGAAATCAACAGACACTAGTGGT
TGATGTTCGACTTCTATGATACCTTCTAAACCAGCATTTTCAAAAGCTTGGTTTACTTCTTCTGCCGTTACTT
CTTTTTCTAAATCAACAACTAAATCAACGAGCGATACATTCTTTGTTGGTACACGTAATGCCATGCCGTGTAA
TTTACCTTCTAATTCTGGCAATACTTCTTTTAAAGCTTTCGCCGCACCAGTAGAAGTAGGAATAATGCTTTCA
TTACATGAGCGTGCACGTCTTAAATCTTTATGTGGATTATCAATATTTTTTTGGTCATTTGTAATAGCGTGAA
CAGTAGTCATTAAACCATTAACTATTCCAAACTGATTATTTAAAACTTTTGCAACTGGACCAATGCAATTAGT
AGTACATGAAGCATTACTAAAAATGTCAAATGCTTCTATATCTAATTGGTCATCATTTACGCCTTTAACTACC
ATTTGAACATGTCCACCTTTTGAAGGACCAGTTAACAATACTTTTTTGGCACCTGCTTTAATATGTGCGATGG
CTTTATCACCATGATTAAATTTACCAGTTGCATCTATAGCAATATCGATATCTAATTCTTTCCATGGCAAGTT
TTCAGGATTGCGATCAGCAACCAATTTAATTTTATGATCACCAACTTGCAATCCGTTTTCAATTGGTTCTACT
TTTAGATTATATTTTCCATGTGTTGTATCGTAATTGATTAAATGTGCAATTGTTTCGGGTGGATAACTAGCAT
TAATCGCTACTACATTTAAATTTTTATTTTGTAATGCAATACGTAATACCATTCTTCCAATTCTACCCATACC
ATTAATTGCAATATTCGTTGACATTAAAAGCACCCCTTGATTTTTATGTGTTATACTTTATGGAATTAGTATA
GCATATTATATGAAATTTGAAAGCGTTTTCCGTCGATTTATTTATCTTTTTTATAAAAAATAGTCATTACTTA
CATACATCAACCCGTACGTAAAGTAATGACTTATTTATAGTGTAATATTAATCTTCTTCTCCGTAATTCGGCT
TTTCAATATAACCTTCTTCTTCTAACAATCTCTCAAGGTTTTGTTTTAATTCAAGTTTATCCCCTAAATTATC
GATAACATGATCGGCCATTCGGCTTTTTTTATCAATAGAAATTTGGCTATAGACACGAGCTTTCGCATCTTCT
AATGACAAATTATTACGTTGCATTAAACGATCCATTTGTATACTTTCAGAAGTGTATACAACCCACACTTCGT
CTACTGTATTTTCCAATTCATTTTCAAATAATAATGGAATATCCATGATTACATTATATCCTTGTTTTAAATA
TTCTTGCTTTTCTTCTTCCATAATATCTCGCACGATAGGATGTATGATAGCATTTAATTCTAAGCGTTTTTCT
GGATGGTTAAACACTAAATCACCCATATAACGACGATTCATCTCGCCATTTTCATCAATTGCTTCATCACCAA
AGACTTCTCGTACTTGAGCTAAACCTTTACTCCCCTTTTTAACAGCTTCCCTGGCTGCTTTATCAGCATCTAC
TACTTTAAAACCGAATACGGATAAGAGTTCTGATACTGTTGATTTTCCTGAGGCGATTCCACCTGTTAGACCA
ATAACTTTCGGCATAATTTCACTCTTTCTTTATTTTTGACATACTGGACAATAATGACTATTTCTTGTCGCGA
TGATTTTTGTTTCAATTTGACTTCCACACACTTTGCATACCGGCTGCTTATATACATTAAGATGCAATTGCAT
CTCACCAGTTTTTCCATCAGCATGACGATAATCTGAAATACTTGTACCGCCATATTTAATACCTTCTTCTAGT
ACTTCTCTAACATAATAAAAAACCATTTCTTGTTGTTGGTGTGTTAAGTCTTTTACTTTTTTATCTGGTAAAA
CACCTGCACGAAACAACGCTTCACATGCGTAAATATTTCCACAACCTGCGATTACTTTATGATCCAAAATCAC
TTGTTTGATTGGTTTATTCTTATTAGACTGTTGATGAATTCGATTTAAATAATACGTCAATGCTTCATTTGAA
AAAGGTTCAGGCGCTATTTCTAAAAATGAAGGGTAAGATGCTACAGACGCAACATTTCTAATTTCTCCAAAAC
GCCGTATATCTGAATAAATTAACTTTTTGTCATTTGACAACTCAAAAATAACATGCCAATGCTTACGATAATT
AGGTATCATAATATCTTCAAGTTCATCTACAATGAAAAAACCACCCGCCATACCTAAATGACTAATTAATGTA
CGTTGTTCTCGTTTATTATCTAGCTGAAAAACGATATATTTACTTCTTCGTTCTACATTTGTAATGGTATAGC
CTTCCGATAAAGTTTTAAAAGTATCTAATTCAATTCCTTTTATAATTGTTTCCTTGCCTTGAGCTTTACCTTC
AATTACTTTATCCGAAAATTTAACGTGTTCAATTTTTTGATTTATAACGTAGGGTTCAATTCCTCTTTTTACA
TGTTCTACTTCTGGTAATTCGGGCATACCATTAACCTCACTTTATTTTGCATCATACCAAGTTGCACCATAAC
TTGAGTCTACTTTTAATGGAACATCTAATTGCAATGCATTTTCCATTATCTCTTCTACAAATTCACTAAATGA
ATCTACTTCTGACTTAGGTACTTCAAAAATTAATTCATCGTGTACTTGTAATAATAGTTTAGCTTGATATGTT
GTCTCTTTCATTTTTTGTGCAAATTTAACCATTGCCAGTTTAATGATATCTGCAGCACTGCCTTGTATTGGCG
TATTCATAGCAGTACGTTCAGCAAAGCCGCGTAAATTAAAGTTACGACTCGTAATATCAGGAATATAGCGTCG
ACGATGTAGCAATGTTTCCACATAACCTAAAGCTTTGGCATCTTTTACAATATCAGACATATATTGTTTTACA

CCTGGGAAACTAGCTAAATAATCATCAATGAATGCTTTTGCTTTTTTACGAGTAATACCTAAACTTTGACTTA
AACCATAATCACTTATCCCATAAACAATTCCAAAGTTAACCGCTTTTGCTTGACGACGCATTAAACTATCGAC
TTGATCAGCTTCTACACCAAATACTTTCATAGCAGTTGCTGTATGAATATCATCGCCGTTGATAAATGCTTCT
TTCATACTCTCATCTTGTGTAATGTGTGCTAATACACGCAATTCAATTTGAGAATAATCTGCTGATAATATAA
CGCTATCTTTTGAAGTTGGTTTAAAGGCTTTTCTTATTTTACGCCCTTCTTCAAGTCTAACCGGAATATTTTG
TAAATTAGGATCTACACTTGATAAACGTCCAGTTTGCGCTAAAGTTTGGTTAAAACGTGTATGGATACGTTGA
TCATCACTAATTACCTTTTGCAATCCTTCAACATAAGTAGACTGTAACTTAGATAATTGACGATATTCTAAAA
TATAATCAATGATAGGATGTTCACCTTGCAATTGCTCTAAGACATCTACAGCAGTAGAATATCCTGTTTTCGT
CTTTTTAATAACAGGTAATTGTAATGTCTCAAACAATACAACACCTAATTGCTTAGGAGAATTTATATTAAAA
TCTTCACCAGCTGCATCATGGATATTTCGAATCAAGACGTCTAATTTTTCTTGAATTTCTTTTTCCATTTCTT
CTAAATCATGAACATCTGTAAATATACCAATTTCTTCCATTTCACTTAAAATCTTAGCTAGCGGTAGCTCTAA
ATCAGCTAAGAGTTCTACCTGATTGTATTCTTCTAATTGTTTATCCATATTTGGTTTCGCAAAGTAAATTGCA
TCAGTAATAGAAGCAACATATGGATTTAAAACATCATCTTCAGGTACCTTAAATTTCTTACCTTTTCCATATA
TACTCACATCGTCTTTCACAAAACTTTGACCGTACAATGAAACAACTGATTGAACATCACTAATCGTACGAGA
TGGATCAATTATATAACTGGCCAACATAATATCGAAAGAAATATTTTGAATATCAATTCCCAATCTATGTGAT
GCTACATATGTTTTTTTAGCATCATATACGACTTTTTTCGTATTCGGATTTTCTAACCATGAAACTAGTTCGG
CATAATTATTTATGTCATCCGCATTAATTACAATATGTTTCTCACCTGTAAATAAAGAGAATTTTAAAATATT
ATTTCGCAAATAGTTACCACCGTCTAATTCGAAATGGATGGCCGCTTCTTTCAATGAAGTAAAATCAACATTA
TCAAAAGACGTTTCAATTTCAATTGTCTTTTCTATTGCATCTTCAACGCTTGCTGATTGATCAATGTCAGCCA
ACAATTGTTTGAATTCTAACTTCTTAAACAATTCGATTTTTTCTTGTTGTTCATCTTGATGAGTCATTAACGT
ATCTTCAAGTTTTACTTCAATCGGACTATCTACATTAATCGTTGCTAATTCTTTACTCATTAATGCATCTTCT
TTGCTATTTTGAAGTTTCTCTTTTAACTTTTTACCTGAAATTTCATCTAAATGTTCATAGACACCTTCTACTG
TGTCAAATTGGTTTAGCAATTTTATTGCTGTTTTCTCTCCAACACCTGCAACACCTGGTATATTATCAGAAGT
ATCTCCCATTAATCCTTTCATATCAATAATTTGATTAGGTGTTAACCCGTTGTATTTTTCCGCAATAAAGTCA
GGTGTATAATGATCAACATCAGTAACACCTTTTTTAGTGTAATAAATGGTTACATTATCCGTTGCAAGTTGTG
TTAAATCTCGGTCTCCCGTAATAATAATTGTCTGAAAGCCCGCTTTATCTGCTTCTTTACTTAAAGTTCCGAT
AATATCATCTGCCTCATAGTTATCTAATTCATAACGTTTAATATGATATGCATCTAATAATTGGCGTATATAA
GGAAATTGCTCACTTAGTTCAGGCGGCGTTTTCTGGCGTCCACCTTTATATTCACTATATTTTTCATGTCTGA
AAGTCGTTTTACCTGCATCAAACGCTACTAAAAAATGATTTGGCTTTTCTTCTTTTAAAATCTTCTCTAGTAA
CATTGCAAAACCATATACTGCATTGGTATGAATGCCTGCTTTGTTTGATAACAAAGGTAATGCATAAAAAGCT
CTAAAACTTAAGCTATTACCATCGATTAATACTAATTTATTCACAATTTTAACCTCCAGAACTAATTTATATA
TATTTTAGCATATTCGACTGTGATGTCGTAATTAAAGAAACTATCTCATAGAGTATTTATACACGACCTAATT
TCAAATAAAACAAGTGACTATATAGACTAAGCTAAAAAATAAAAATGACATAATCATGATTGTTCTTGATTAG
CACTCCATAAAGTCAGGTTAAAAGTCCAACTTTTGAGATGCACATTATCATTTCATGATTATGTCTACTTTTT
AATATTTTTATGCTGCTATTTTAAATTTTTATAGGTAAGAATAAGTTATGAATCGTCAGAACCAAATGGAATA
TATTTAACTTTGGTCATTTTCGGTAATTCTTCATAATTATTAAACCACTCTTGGTAATTTTTATTGATTGGTG
ACGGATGATGCATATAATGTTCAAACGGCAATGATTCCACTGTATAAACCTCTTGTCGTGGATGATCTTTAAT
TTGTTGTTTTAATAACTGAACTCTTTTTTCATGTTCATAATGAACATAAATAAATGCACTAAGATATATCATA
GCTAAAACTAGTGATGCAACTTTAATAAAATTAACATTGATTGACTTATATTTCCGAAATTCTTTTAATAAAA
TAATTAAAATGATTACATGTATCGTATAAACAATCAAAAAATTACCTGGTTCTATTGGAGTAACAATGACTAG
TGTCGACGCCGAAACACATATTGCAATAAGTAAGGAATATAAAGTGATTTGTGTTTTACGATCATTAATAGAC
AAATAAATACCTACAAATATCGAAAACGCAAAGTAACCACATACAATTACGTTCACAAAACCAACCAATCCAA
TATCTGTATTTTTATTTAATAAAAACTGATTGTAAAATAGTAAATAATAAAGCGGTAAAGTGATAAATCCTAT
CATAATGATACGACGCTTTAATATTGTTAAATGTACATACCGATCACTTCTAAGCAGTAGATAAATAATAAGC
GCTGCAATCATACTTAAAATTATAATCTGACTGAAAATGACACCATATGGAAGTGACGTTGATATCATTTCTG
CAAATTTTGAAAAGATACCTTGATTATTTGAAACTTGTTGATATTCTGATCCTTCAAATAAAATTTTGCGATA
ATTTGGATTGGAAAACATAATGATTGTACCTATAGTAGCAATCATAAATGAAAACAATAATTTATAATTAAGT
GTTCGATTTGCAGTAAATTCATACAGAAAAGCTAAAATTAAAATGATACAGTGGAATAATGTCACATTCTCCA
TAAATAATTGACCGAAGAAGCATAAAACATAGAATAGTACAGTAACACTTACCGGCTGCTTTTCTTTATATAT
AATTGCATTAATACAATAATAAATAATAAAGAGTGAAATTAGTGTTGATGTCGCATAATTATAAAATCCTGCA
AACCAGCCATATGTATCTGCATAAATAGCACTTGGTAAAATTAACATTAAAGAAAATGCTAACAAATAATAGC
TTGTCCACGCTTTACAACGTGTAATGTGCATAATCATCCAAATGATGCCCATACTAATGAGACCATATGAAAG
CCAACGTAACCAGCTTACATGTACAGCTATAATTTCAAAGATATTTCCGATATAGCGACCATTAAGTGATGCA
AATCCTACTTTTAAAATATCAGTATTATAATTGCTAAACCATTGTAAATCATCGTGCATGAGTGGTAGTAAGA
TACCCATAAAAGTATAAAACAATAATATCGCAATTAATATCAAAGTTGTCTTGTGTAATTGAATTGTTTTCAC
TTTGCTAATCCTCAAATCTAGTTAAATTTTCCTCAACTTGTAGGTCGAAAAATTAATTCAATATTTTTAATGT
ATTTCTAATTTTCACCTATGCATGTTTCCTCAATCAAATTAGATAAACAAGGTATTTAATATTACTTTCAACA
ATTTATCTAAATCGCCCCTCGTCTTTTTCTATGACGAATGATTACACTTGTCTATTCAAGTGTTTAAATTAAG

ATTTATTTCCGTTCAAATAAAACTTATTACTTTTTAATTAAGCATCTATTTTAAGCGCGTTACTAAAATTTCT
TTATTATAATAAATATATTATAAAATTGCTTGATTCACAACAATCTATTACTTATAACTTACAAATCGACATA
TGCAAATGACAAAATTGACAACCGAATTTATTCCACTTCATTAACTCTAATTTAATACCAATAGAGATTTGAA
CGAAATAAAAAAGCAGCAACAACTTCGTGAAAAATACACGCGCTGTTACCACTTTAATTTTTATTCTTTATAA
TCTTTTAGAATAACTTTGAACGTTGAGCCTTTGCCAACTTGACTATTCACTTCAATATTGCCTTGGTGGGCCT
CTACTATATGCTTTGTAATTGATAATCCAAGACCTGTCCCACCAGAATCTCTACTTCGTGCTTTATCTACTCT
ATAAAATCTTTCAAAAATACGTTGTTGGTCTTCTAATTTAATACCTATACCAAAATCTTGTACTTCGAAAATG
ACACGAAAGTCATCTCGATACACACGAACGTTAATATCTCCATCTTCATAAGAATAGTTAATTGCATTCGTTA
ACAAATTCGTGATAACTTGAGCAATTTTACTTTCTTGTGCTTTAACAATGACATCTTTTTCAATATCAGTATG
AATGGAAATATTTTTTTGATTGGCTTGAGTCATCATATTATCAATAATACGCCGCGTTAAATCTGATAAATTC
ATATAGTCTGTATCTAACTCTGTATGTTGCTCAATATGCGATAAATCTAACAGATCTGTTACTAAAGATTCTA
TTCGATTTGATTCTTTTAAAATTATGTTTAAAAACATGTCTAATGATTCCGCATCATTTTTTGCACCATCAAT
AAGCGTTTCAGCAAACCCCTTAATTGAAGTAATAGGTGTTTTTAATTCATGTGAAACATTTGCTACAAATTCA
CGTCTTAGATTTTCAAGTTGTTTCAGATTTGTTATGTCATGCATCACAACTAAAATCCCTTGCAAACTTTTTT
GAGACCTAGTTAAAATCGGAACGCATGAAATATCAAAGTACTTGGCATGGACTTGGTTTATTGCAACTTCCAA
TTGTTCATAAATAGGTTTTTCAACTTTAAAACTTTCTAAAATTAATTGCTCAATTTCAGTATTAACATAACCG
TGATAGCCTACTTGTTCAATATTATGCGAGATGTTGAACTGTTCATAATACGCTTTATTTGCAACAACGATTT
TTCCATTTCGATCTATCATTAAAATAGCACTTGGAATATTTTCAATCGTTGTTTTTAAACGATTGGATTGCAT
TTTTTGCTCATTATTAAGCTTTTGAAGGCGTCGTGCTAGATCATTGGTAGACACAAAAAGCGCTTTAGTTTCT
ATAACATTACTTTCAGGTACACGTATGTGATAATAACCATTTGCCAACAATTGTGTTGCATAAGTAACTTCTT
GAATGGGACGGATTAATGTACGCTTAAAACTACGGCTTGCAAAATACAGACAAATGAGTACAACTAAACATGT
CAAAATAAGATATTTCCACAACGTCCAATGCATTTCTGTAATATCGTTATTGTAACCTTTAATCCATACATGA
TAACCGTTAACCTTCTTATTAAAAATAAAAACGTCCTTTTTTTGAATATAGCTATCACTATTTTGAGTGGCTT
TCAGTTTGCCACTACTAAACACCTTTTTATCATTTTTAGTTATCAATAAATCTATATTTTGCTGTTTTACAAT
TTCTTTAACTTTATCAATCTCATTATCTTGGACTAAATAAATATATGATCTTGCATCTGTTGCTAGAGCTTGT
TCGTGTTTTTCTGATAACACATATGTGATGGAAGCGTGAATAATAATGCCTAATGTAACAAAACTGATAATTA
ATATACTGCTTATCAATAACATTAAGCGGTGGTGAAACTTCATCATTGTTCTTTAGGTCTTTCCAATTTATAG
CCTAAGCCACGCACAGTTTTAATAAGTTGTGGCTTCTTAGGATTATCTTCTAATTTATCTCTTAAATGACTGA
TATGTACATCAACAATTCTTGAGTCTCCTGCAAATTCATAATTCCATACCGTATTTAACATATGCTCTCTCGT
AATGACTCTGCCTTGTCTTTCTATCAAATAAAGCAACAGTTCAAATTCTTTCGGTGTTAGCTCCAATAGCTCA
TTATGCTTATATACTTCAAAATAATCGGGTCTAATACGTATCGATCCGATGGTAATATCATCGTCAATCTCTT
CTACTTCTTTTACAAATTGAGAACGTCTTAAAATGGCTTTCACACGGGCAACAACTTCTCTAGGTGAAAAAGG
CTTAGTCATATAGTCATCGGCACCTAATTCTAAACCTAATACCCGATCAAATTCATCATTTTTCGCCGTTAAC
ATTAATATAGGGACTAAATTTTTATTTGTTCTTACAGTCTTACATACGTCAATGCCATCTTTTTTAGGTAGCA
TAACATCTAAAATAATTAAATCTGGCTGTTCATTTTCCACCTTTTCTAAAGCCTCATCACCATCAAATGCGAC
AACAACTTCATAACCAGCTGTTTCTAAGTTATATTTAAGTAATGTTACGATTGAATGTTCGTCATCTACTACC
AACACTTTTTGCGACATGGTATGCCTCCCTAACTTATAATTATATTTACATTATAACCGAACTATTTATAAAA
ATAACATCCTACACATTATCTTTACACATTTTTTACATTACTTTACACATAAATAAAATACTTCTTATATTTC
CTTCTATAATTTACATGACTTACTCTGGGACAACGAAATAAACTTTGTGAAAATATCATTTCTATGCCACTAC
CTATGCATGAGTTTTACTCATTTATTCCTAAACTTATGTACACAATCGCTTTGTCTAATGTGTAAGAAACACT
ATATAATCAATCATTAGTGACTCTTTATTATTTCTGTCCTGCTGCCAACTTCAATTCATTTAAAAAGGCGAAC
CTAGCAATTAAAGTAGCTAGACTCACCTAAATAATTAGTATTTATTTTAAATTTTTAATCAATTCATCTGCAA
ATGCTGATGTAGAAACTTCTTCAGCACCATCCATTAAACGGGCAAAGTCATAAGTAACAACTTTTGAAGCAAT
TGTATCTTCAATTGAATCTGTAATCTTATCTGCCGCTTCTTGCCATCCTAAATGTTCTAACATTAATACAGAA
CTTAAAATTACTGAAGATGGATTCACTTTATTTAAACCTGCATATTTTGGAGCTGTACCATGTGTTGCTTCAA
AAATAGCATGACCTGTTTCATAATTAATGTTTGCACCTGGCGCAATACCAATACCACCAACTTGTGCAGCTAA
AGCATCTGAAATATAGTCACCATTCAAGTTCATAGTTGCTACAACATCATGCTCAGCTGGACGAGTTAAAATT
TGTTGTAAGAAAATGTCAGCAATAGAATCTTTAATGATAATCTTGCCTTCTTTTTCAGCTTTTTCTTGTGCAG
AATTAGCAGCATCTCTGCCTTCTTTTTCAACAATTTCGTCATATTGTTGCCAAGTAAATACTTGATCACCAAA
TTCAGTTAATGCTAAATCGTAACCCCACTGCTTAAATGAGCCTTCTGTAAATTTCATAATATTACCTTTATGA
ACTAAAGTAACTGATTTACGGTTATTATCGATAGCATATTGAATAGCTGCTCTAACTAATCGCTCAGTACCTT
CTTTAGAAACTGGTTTAATACCAATACCTGAAGTTTCTGGGAATCGAATGTTTGTCGCACCCATTTCGTTTTG
TAAGAAGTCAATTACCTTTTTAACTTCTGTTGTACCTTCTTTAAATTCAATACCAGCATAAATGTCTTCAGTA
TTTTCACGGAAAATAACCATATCAACATCTTGTGGACGTTTAACAGGTGATGGTACTCCTTTAAACCAACGTA
CCGGTCTTAAACAAGTAAATAAATCTAATTCTTGGCGTAAAGCCACATTTAATGATCTAATACCACCACCAAT
TGGTGTTGTTAAAGGTCCTTTAACAGCAATTAAATATTCTTTAATTGTATCAAGTGTTTCTTGAGGTAACCAT
TCGCCAGTTGTATCAAATGCTTTTTGGCCAGCTAGCACTTCTTTCCATTCAATGCGTTTTTCGCCATTATAAG
CTTTCTCAACAGCAGCATCTATCACTCGGCTTGCTGCCTTCCAAATATCCGGTCCAATTCCATCACCGATAAT

AAATGGGATAATTGGTTCATTAGGTACGTTTAATCCTTCAGTTCCTTGAGTAATTTTTTCTGCAGTCATAGTT
ATTTTACCTCCAAATTTTACATCTTCATTTTTAATTTGTATCGATTATTTTCTTTCTTCAAGCGGAATATACT
TACGATTCGTTTCGCCAATATATTTTGCTCTAGGACGCATAATTCTATTATCTTTATATTGTTCTAAAATATG
AGCAATCCATCCTGCAGAACGACTTACAGCAAAGATTGGCGTGAATAAATCATGAGGTATTTCCATACAGTGA
TAAACACTCGCACTATAAAAATCAACATTAGGAATTAATCCTTTTTCTTCTGCCATACGTTTTTCCATTTTCA
CTGACATTTCAAATAATTCTTCACGACCAGCGTCTTTCGTAATTTGACGGCTCATTTCTCTTAAATATTTCGC
TCTAGGATCACCATCTTTATATACACGATGACCGAAGCCCATTACTTTATCTTTATTCGCAAATTTTTCATCT
AAGTAAGCATCAACATTTTCAATTGACCCAATCTCAGATAACATCGTCATAACTTGTTCGTTTGCACCACCAT
GTAATGGCCCTTTCAGAGAACCTACAGCTGCTACAATACCTGAGTACATATCTGACAATGATGATACCGCACA
ACGTGCTGTAAATGCAGATGCGTTCAACTCATGATCAGCGTGTAAAATAAGTGCTTTATTGAAGGCTTCTACT
TCTATATCTGTTGGTAATTCCCCACGTAACATATATAGGAAGTTTGCCGCATAACTTAAGTCAGGATTAGGCT
TAAGTGGTTCTTTATCTTGTCTTACTCGAGCAAACGCTGTAACTAATGATGCTACTTTAGCCTGTATACGCAT
TGCTCTTTCATAACGATTTTCATCTGATTCATTTTCTGCATCAGGATCGAAATGTGCAATATATGATAATGAC
GTACGTAATGCTGTCATTGGATGCACGTGATCTGTAACATACTCCTCAAAATGTGTATACACACGAGGATTTA
ATGTCATGTATTGATTTAATTTCCCCTTTAAATGAGCTAGCTCTTCTTCATTTGGCAATCTGTAATTCCATAA
TAGGAAAATGACTTCTTCAAATTGCGCGTTTTCAGCTAGATCATCAATATCATAGCCGGCATATGTTAATTGA
CTTTCAATAATTGAACTTATCTTAGTCTCCGCTGCGATAACCCCTTCTAAACCTCTTTGTAATTCTGCCATGA
TAAATTTCCCCTTTACTGTTTCTTTATGAAATGGCTTTCAACTAATATTATAGCTAAACGTTATTCGTTTGTG
AAATATAAAAACTGGCATTATATGTGCAACAATTATTTTGGAATTCTATGTAAATGTCACAGAATAGCCACAA
ATAGCTCTATTAAAGCTTTTTCAAGATTATATTCATCACCAACTTTTAGCATAGAATAAAATTTTCAATTGGT
TATTATATATGATTATAAAATCCAAAGTATATTAACATGGAATTTTACACATTTTTACCGAAAATAATTGTAT
ATATCCTAATATAAGTGCATAATAAACATATCTATATTTAAGGGAGATGTATTATGGCTGAAAAATTACAAAG
GGAACTGAGCAATCGCCACATTCAATTAATTGCTATTGGTGGCGCAATTGGTACAGGCTTATTCTTAGGCGCT
GGTCAAACGATTGCATTAACTGGCCCTTCTATATTACTAACATATATCATTATTGGATTTATGTTATTTATGT
TTATGCGAGGTTTAGGTGAAATCATCATTCAAAATACTGAATTTAAATCTTTCGCAGATGTAACCAATACATA
CATTGGGCCTTTTGCAGGATTCGTAACCGGTTGGACATACTGGTTCTGTTGGATTATTACAGGTATGGCTGAA
GTAACAGCTGTAGCAAAGTATGTAAGCTTCTGGTTCCCAGAAATTCCAAACTGGATTAGTGCACTTTTTTGCG
TATTGTTATTAATGTCTTTTAACTTATTGAGTGCGAGACTATTTGGAGAGTTAGAATTTTGGTTCTCTATCAT
TAAAATAGCAACAATCATTGGTTTAATTGTCGTAGGTTTCGTTATGATTCTATTTGCATATAAAACTCAATTT
GGACATGCGAGTTTCACAAACCTATACGAGCACGGTGTATTCCCTAAAGGAGCATCTGGATTCTTTATGTCAT
TCCAAATGGCCTTATTCTCATTCGTGGGAATCGAAATGATTGGTGTTACGGCAGGTGAAACGAAAAATCCTGT
TAAAACAATACCAAAAGCTATTAATAGTGTTCCTATTAGAATTTTAATATTTTATGTCGGTGCTCTAGCGGTT
ATCATGTCGATTATTCCTTGGCAACAAGTTGATCCTGATAACAGTCCATTCGTAAAATTGTTCGCATTAATTG
GAATTCCATTTGCTGCAGGCTTGATTAATTTTGTAGTACTAACTGCTGCTGCTTCATCATGTAACAGTGGTAT
TTTCTCGAATAGTCGTATGCTGTTCGGTTTATCAAGTCAACAACAAGCACCACCGAATTTCTCTAAGACGAAT
AAGTATGGCGTTCCACATGTAGCAATCTTTGCTTCATCGGCATTGTTACTCGTGGCAGCATTATTAAACTATA
TTTTCCCTGATGCTACTAAGGTATTTACGTATGTGACAACAATCTCTACAGTATTATTCTTAGTTGTTTGGGG
ACTGATTATCATCGCTTATATCAATTATAGTCGTAAAAATCCAGACCTTCATAAAAAGGCTACGTATAAATTA
TTTGGCGGAAAATATATGGGCTATCTCATTTTTGTATTCTTTATTTTTGTGTTTGGCCTATTGTTTATCAATG
TTGAAACAAGACGTGCGATATACTTTATTCCCATTTGGTTTATACTATTAGGATTTATGTACTTAAGATATAA
ACGTATCGCTGCTAAATCAAATAACTAATAACAAGTTTTACCCCTGTTGAATATATTAAAGTTCAACAGGGGT
TTTTATATGCAACTTATATATTAAATTGCGAACTTGGCTCAATACTTTTACTTTCTCATCTATTTAATAATGT
ATCATTTCAGAAATACATCCATATTTCTATTTTATAATAACTTTCCAAAGTAAAATGAGTGAAAGTTTAAAGC
TGATAATGTACATGTATAAAAGATATAAATATTTATATAGATTGCTATTCATACATTATCTTTATCAAATAAC
CTATTAATTGAGTCATAAAATACCAGATAAACCAAAAAAACGTCTTTCCATAGTTGATAAATGGAAAGACGTC
TTTATTTATAAATTATAGTACGTTTGCATATCCTTCAAAGATTTTACCTTGAGCAGCATCAATCGTAACTAAC
ATGTTATTGCTTATGTTTTTAACAGCTTTTTCTACACCTACAACTGTTGGAATACCTTTTTCTAAACCAACAA
TTGCACTTGGTGATGTAATACCATTTTCTTCTGTAATTAAGCCTAAAGCTTTTTCTACATAAGGTACAAAAGT
TTCATCGATTGAGTTAGTAACGATAACTTTGTCAGATAAATCTTTACCTTCTAAATCTTTAACAGTTTCAGCA
ACTAACGTAGTTCCAACAACTGATCCACGTCCAATACCTTGACCATTAGCAATTTCGTCACCAACTAGGTGGA
TTTTCATCATATTAGTAGTTCCAGTTTCACCAGTTGGTACACCAGCAGTAATAATGATTAAATCACCATTAGT
TACTCTACCAGTTTCAACAGCTGTTGCAACTGCATTGTTTAACAATGCATCTGTACTCTTACGTCCTTTTTTA
ACTACAGGTTGAACACCCCAAACAATTGAACATTGACGTGCAGTTTCTTCACTTGGAGTCACTGCAATAATGT
CTGAATGCGGACGATATTTAGAGATAGTACGTGCCGTTGAACCACTTTCAGTAGCAGCTACAATTGCTTTAAC
ATTTAAGTTTAAAGCTGTATGTGCAACCGAAATACCGATTGCATTCACTAATGAAGTTTCAACTAATTTAGTA
CGATCTGACAATAACTTTTTGTAATCTTGGGCTGCTTCAGCTGATACAGCAATATTTCTCATTGTTTTAACAG
CTTCTTCAGGATATAAACCAGCAGCAGTTTCACCAGATAACATTACTGCATCTGTACCATCATAGATTGCGTT
GGCAACGTCACTAGCTTCTGCACGTGTAGCACGTGGGTTACGTTGCATAGAATCTAACATTTGTGTAGCTGTA

ATAACTGGTTTACCTAATTTGTTACATTGTCTGATTAAATTTTTTTGAACCATTGGTACTTTTTCAGGTGGAA
TTTCAACACCCATGTCACCACGTGCAACCATTAAACCATCAGACACTTCAAGAATTTCCGCAATATTATCAAT
ACCTTCTTGGTTTTCAATTTTAGGGAATACTGAAATGTTAGCTTTTTGTTCTTCTAAAATTTCACGAATTTCT
AAAACATCACTAGGACGACGTACGAAACTTGCTGCAATGAAGTCAACATTTTCTTTAATACCGAAACGGATAT
CTTCAGCATCTTTTTCTGTAATACCAGGTAAACTTACTCTTACACCAGGTAAGTTAACACCTTTTTTGTTTTT
AAGCTCACCAGAGTTTAAAATATCACATTTAACTTCTTTTTAGCATGGTCAATATCTTTAACTTGTAATTCA
ATTAAGCCATCATCAAGTAAAATGTATGAACCTACTTGAACATCGTTAATTAAGTTTTCATATGTTACTGAGA
ACTTTTCCGGTGTTCCTTCAACTTCATTCATGCTAACAATAACTTCGTTACCACGTTCAAGTTCAATGATACC
GTCTTTCATATTATGCGTACGAATTTCTGGACCTTTCGTATCTAATAAAATTGCTACAATTTTGTCTAATCTT
TTAGCTACTTTACGAATTGTATCAATTCTACCTTTATGCTCTTCATGACTACCATGTGAAAAGTTTAATCGTG
CAACGTTCATACCAGCATTGATTAATTTCTCAATCATTTCTTCTGATTCTGAAGCTGGTCCAATTGTACATAC
AATTTTAGTTTTTCTCATTTTATAATTCCTCCTGAAATCTTATATAGATAACTTGTTAGCAAGTTCATATAGA
CTATAATCAAATTTATGATCTTTACCATCAAAAATTTCATCAAAAGATGTTGCTACAATTTTATTGTTCTTAA
TTCCAACACCCTTAGCTGTTTCACCTTGCATTAATAAGTCTACCGCATATCCACCTAAACGTGATGCTAAAAC
TCTATCCGCACCTGTTGGGCTACCACCACGTTGAACGTGACCTAACACAGACACTCTATTATCAACATTGATG
TATTGTGATAATTCTTTTTGACAATCTTGCGCAGTCATGCAACCTTCTGCTACAAGAACGATTGAGTGTTTCT
TACCACGTTTAATACCTTGTTCAATTTTATCAGCTATTTCTTTAATATCTGTTTTCACTTCTGGTACTACAAT
TGTCTCAGCACCAACTGATAATCCAGCCCATAATGCTAGATCTCCACAATCACGGCCCATTGCTTCAATGATA
AATGTTCGTGCGTGACTTGACGCAGTATCTCTAATTTTGTCGACTAAGCCAATAATCGTATTTAATGCTGTGT
CAAATCCAATTGTAAAATCAGTACCATTGATATCATTGTCAATCGTACCAGGAATACCGATAGTTTGAATTTC
TTTACATTCCTCACTGATGCGTTGTGCACCGCGATAACTACCGTCACCACCAATAACTACAAGGCCCTCAATC
CCTCTTTTACGTAAGTTTTCGATTGCAACTTTACGTACTTCTTGCTCCTTAAACTCTGGACATCTTGCTGAAT
ACAAGAATGTACCTCCACGCTGAATCGTATCCCCAACTGATCCTAATTCAAGTTTATGAATATCATCATTTAA
CAATCCTTGGTAACCATGATACACACCATAAACTTCAATTTCATTGTAAATTGCTGTACGAACAACTGCTCTT
ACCGCAGCATTCATTCCAGGTGAATCTCCACCACTAGTTAAAACTGCAATTTTCTTCATGACGACATACCTTT
CTATAACTGATTTATCTTTAACTCTAAATTACCACAAATTGATATGTCATTCCATCAAAACTAATGTATTGAA
TTGATGTAAACGTTTTAAATATTGAATTTTATTTTAACTTAATTAAAAAAAGATTAGTAGCCACAATTTTGAC
TACTAATCTTAGTTAAAATTCAGTATCTAAATATTAAATTATTGTTCTAATGTACTTCAATTTATGGTAAAAC
CCAGTATCGATTTAACATAAAAATGCTCAAGTTGATTATTCTATATAAGAACCGATATTTCTGAATTTTTCAA
ACCGATCATTAGCAATTTCATCACGTGATAATGACTCAAGTGAATCTAACTGTTCAACAAACGCTGATTTAAT
AGCTAAAGCTTGCTGTTCAATATCTTTATGTGCACCGCCAAGTGGTTCAGAAATGACATCATCTATAATACCT
AATTGCTTAATATCATGGGCAGTAATTTTCATTGTTTCAGCTGCAATTTTAGCCAAATTACTGTCTTTCCATA
ATAATGCCGCTGCACCTTCAGGAGATATAACAGAGTAAGTACTATTCTCTAACATCAATACTTTATTGGCAAT
ACCAATACCTAGAGCACCTCCACTGCCACCTTCACCAATGACAATCGCAATAACTGGTACTTTTAGTGAAGCC
ATCTCAATCAAATTTGTTGCGATAGATTCACTTTGTCCACGTTCTTCCGCAGCTTTACCAGGATATGCACCTT
TTGTATCTATAAATGTAAAGATAGGACGATTGAACTTTTCAGCTTGTTTCATTAAACGTAATGCTTTTCGATA
ACCTTCTGGATGCGACATACCAAAATTTCGATAAATATTATCTTTTGTATCTTTTCCGCGTTGTTGTCCAATA
ACCGTAACAGCGCGACCATTTAAAAAGCCAATGCCACCAATCATTGCTGGATCATCTCTAAAATTACGATCAC
CATGAAGTTCCATAAACGAATCAAAGATATATGGAATATAATCTAGGGTCGTAGGTCTTTCTTGCAAACGCGC
AATTTGCACACGATCCCATGGTTTAGATTTGTATATATTTTTTTAGTTTCTCGTTCCAATGACGCTTCAAGC
ATGTCAATTTCTTCTTGTAAATCCACATCATTTTTATCTTGAGATTCTTTCAAAGATTCAATTTTATTTCGAA
TTTCAAAAAGTGGTTTTTCAAAATCTAACATTATTTAGTCACCTCTTGATGGATTTTTAGAATTTCAGACAAT
GTTTGACGCATATCATTACGATGTACAACTTTGTCCAATTGTCCATGCTCTAATAAAAATTCTGCAGTTTGGA
AATCATCTGGCAATTTTTCGTTTATTGTCTGTTCAATAACTCGACGACCTGCAAAACCTATCAACGCTTTTGG
CTCACTTAAATTTATATCACCAACTGATGCAAAGCTTGCAGATACACCACCAGTAGTTGGATGTGTTAAATAT
GATATATATAATAGTCCAGCGTCAGAATGACGTTTTAAAGATACACTGGTTTTACCCATTTGCATCAAGGAAA
TAATACCTTCTTGCATACGTGCACCACCACTTGCAGAGAAAAGAATAAATGGTAAACGGTTCTCAGTGCAGTA
ATCAATGATGCGACATATCTTTTCACCGATAACCGATCCCATACTTCCCATTCTAAAACGTGAATCCATGACA
GCAACGCCAAATTTCATACCATCTAGTTGCGCTGTACCAGTCACAACTGCTTCTTTAAGACCTGTCTTTTGTT
GGTCCTTTTCAATTTTTTCTAAATAACTTGGAAAATCTAATGGATTCGCAGAGGTCATTCCCTTATCGAATTC
TGTAAATGATCCTTCATCAGAAATTGCTTCTATACGTTTATACGCAGTTAAAGCAATATGATGATCACAATTA
AAGCACACATTTAAATTTTCAGCTAATTCTTTTGTGTACATAATTTTCTTACACTTTGGACACTTAGTCATAA
TACCTGCAGGCACATCATTATTTTTAGAGTCTTGTACTGTAAGATATTTCTTTTTCTTTGTTCGATTAAAAAA
ATCTTTAAACATAGGAAAACCTCCAAATAACCTCTGTTCTAACCATTTAGAGCCTTAAACTGAACTTGACCAG
TTAAAAACGGGTTTAAATTTATTTGATTTATATTCGTTTGACTGTTGAATGTTTATCATAAACTTTCATTTTC
ATCATTTTAATGAATCTTTATTGCTAACTTTTAAGCAATTTAATTAAACGTTAATTATTTTTATTGTAAATCT
GTAAGTTTCATTGTTTTATCATACACATCTTGCGGATCAACTTCAATCCTAGCTACTCCAGATTCCATTGCCG
CTTTAGCAACATTACGAGCAACTGATGGCGCTACACGTTTATCAAACGGTCCTGGGATACAGTAGTCTTCATT

TAATTCAGAACTATCGATTAAATCAGCAATCGCTTCTACAGCTGCTTTTTTCATTTCTTCATTTATATGTGTA
GCTTCAACCTCTAATGCACCTCTAAAAATACCAGGGAAAGCTAATACATTATTAATTTGGTTAGGATAATCTG
AACGTCCAGTACCAACAACGCGTGCACCTGCCGCTTTGGCATCATCAGGTATTATTTCAGGATTTGGATTAGC
CATTGCAAATATAATTGGATTATCTGCCATACTCTTAACCATATCTTGTGACAGCGCATTAGCTACAGAAACT
CCGATAAATACATCTGCGTCTTTTACGACTTCTTCTAAAGACCCTTCAATCTTATCTTTATTTGTCCATTTAG
CTACAACATCTTTCGTAGGATTCATACCATATGAACGTCCTTCAAAAATTGCGCCTCTTGAGTCACACATAAC
CATATTTCTTACACCATACGCGTATAGTAATTTAACAATGGCTATTCCTGCTGCACCAGCACCATTTAGTACA
ACTTTTATTTTAGCAATATCTTTGTTAACAACTCTCAATGCATTTACCAAACCTGCCATTGTTACAATTGCTG
TACCATGTTGATCGTCATGGAATACCGGAATATTAGTTTCTTTTTTCAATCGTTCTTCAATTTCAAAACAACG
TGGTGCCGAAATATCCTCTAAATTAATACCACCATAATTAGGTTCTAACAACTTAACTGTTTTAATGATTTCT
TCGGTATCAGTTGTATTTAACGCAATAGGCATCCCATTGATACCAGCGAAGCTTTTGAATAATACTGCTTTAC
CTTCCATTACAGGAATACTTGCTTCAGGTCCAATGTTACCTAAACCTAATACCGCTGTTCCATCAGTAATAAC
TGCAACTGTATTCCCTTTGATTGTGTAATCATACACTTTTCTTTTATCTTCATATATATCTTTACACGGTTCA
GCAACGCCAGGTGAATATGCTAAACTTAATTCCTCTTTATTAGTAACTTTTACATTTGGTTTAACTTCTAATT
TACCTTGATTACGTTTGTGCATTTCCAATGCTTCATCTCTTAATGACATGAAATCAGCCCCTAATTCAATATT
TATTTTTAAAAAAATAACTTGGATAAAACGCATTACATTATAAAAGTAAAAATATTGGGTAAATCTGAATGAG
TAAGAATTTATGGTTTTAATTATGTAACACCAATAGCGATAAACGATAATAAAATAATATATATAAAGATACA
TTAAACCATACTATCTAAAGATATACCTTTAATTATTATAATGGATAGCAAAAACCAATATATCAAAAAGTTA
TTATTTTTTCGCACGATATATCGACAAAATTCTTTACTCAATTTATGTATACTGCTTTTTGTGCTAATTATTC
TTATGGATTAATCAATAATGTAAAGTGAAACTCATAAAAATAATAAGCATAAAAAACTAATATAAACGCAAAC
TGATGGTTAAAAAATATCTAACCATCAGTTTACTATATCATAATTTATTAGTTGATAAAAGTTATATAAGCCT
AATATCACTAGGGTTAAAGGATTGTATAAAATTATTAAACATACTATCTTTTTGATTAATATAGCCTAAAGTA
GTCATTTGTTTAATCGTTTCATCATAAAAGGATAACACAACATCATTAGCATTCTCTTTCGTAGCTTTAATCG
CCTCTTCAAACGCATCTATATGTGATTTATTTCTAATTATAATTTGTTTGGCAAATGCTAATTTTTGTTCTTC
AAAAGTGGCTAATGTCTGAATCTCATTTATAATTAATTGACGTTGTTGCTTTCTAAGGTCAAATTTCCCGCTA
ACTATAAACAAGTCATTATGTGATAACAACTCTTCGTACTTTTTAAACTGATTAGGGAAAATCACACCATCTA
AAGTTTCGATGCCATCATTTAATGTGACGAATGCCATATTTTGGCCATTTTTAGTTCGGATTTGTTTAACTTT
ATCAAACTGAACTAATATAGGCTTATTATTCTGTGCGTTACTCAATTTAAATATCGTTAAATATTGTTTGGCA
ACAAACTTTTTATCTACTGGGTGTTGCGAAACATAAAATCCTAAATATTCTTTTTCGTACTGACTAATAAGTG
CATCAGGCAATTCTTCTTTATCTTCATACATCTGTTTTGGCGTTAAAATATCAAATAAAAAACCATCTTGTTC
AATGTTTAAATCGCCATCCAACACTTGATCAATAGCTTGCAACAACGTTGAACGTGTTTTACCAAAAGCATCA
AACGCTCCCACTAAAATCAGTGCTTCAAGTAACTTTCTCGTTTTGACTCGCTTTGGTATACGTCTAGCAAAAT
CAAAGAAATCTTTAAATTTGCCGTTCTGATAACGTTCATCAACAATCACTTTCACACTTTGATAACCAACACC
TTTAATTGTACCAATTGATAAATAAATGCCTTCTTGGGAAGGTTTATAAAAACAATGACTTTCGTTAATGTTC
GGTGGCAATATAGTGATACCTTGTTTTTTTGCTTCTTCTATCATTTGAGCAGTTTTCTTCTCACTTCCAATAA
CATTACTTAAAATGTTTGCGTAAAAATAATTTGGATAACGGACTTTTAAATAGCTCATAATGTATGCAATTTT
AGAATAGCTGACAGCATGTGCTCTAGGAAAACCATAATCAGCAAATTTCAGAATCAAATCAAATATTTGCTTA
CTAATGTCTTCGTGATAACCATTTTGCTTTGCACCTTCTATAAAATGTTGACGCTCACTTTCAAGAACAGCTC
TATTTTTTTTACTCATTGCTCTTCTTAAAATATCCGCTTCACCATAACTGAAGTTTGCAAATGTGCTCGCTAT
TTGCATAATTTGCTCTTGATAAATAATAACACCGTAAGTATTTTTTAATATAGGTTCTAAATGTGGATGTAAA
TATTGAACTTTGCTTGGATCATGTCTTCTTGTAATGTAAGTTGGAATTTCTTCCATTGGACCTGGTCTATACA
AAGAAGTTACAGCCACAATATCTTCAAAGTGTTCCGGCTTTAATTTTTTTAATACACTTCTTACACCGTCAGA
CTCTAATTGGAATATGCCAGTTGTATCTCCTTGCGACAACAATTCAAACACTTTTTGATCATCGAACGGAATA
TTTTCGATATCAATATTAATACCTAAATCTTTTTTGACTTGTGTTAAGATTTGATGAATAATCGATAAGTTTC
TCAACCCTAGAAAATCTATTTTTAATAACCCAATTCGTTCGGCTTCAGTCATTGTCCATTGCGTTAATAATCC
TGTATCCCCTTTCGTTAAAGGGGCATATTCATATAATGGGTGGTCATTAATAATAATTCCTGCCGCATGAGTA
GATGTATGTCTTGGTAAACCTTCTAACTTTTTACAAATACTGAACCAGCGTTCATGTCGATGGTTTCGATGTA
CAAACTTTTTAAAATCGTCAATTTGATATGCTTCATCAAGTGTAATTCCTAATTTATGTGGGATTAAACTTGA
AATTTCATTTAATGTAACTTCATCAAACCCCATAATTCGTCCAACATCTCTAGCAACCGCTCTTGCAAGCAGA
TGACCGAAAGTCACAATTCCAGATACATGTAGCTCGCCATATTTTTCTTGGACGTACTGAATGACCCTTTCTC
GGCGTGTATCTTCAAAGTCAATATCAATATCAGGCATTGTTACACGTTCTGGGTTTAAAAAGCGTTCAAATAA
TAGATTGAATTTAATAGGATCAATCGTTGTAATTCCCAATAAATAACTGACCAGTGAGCCAGCTGAAGAACCA
CGACCTGGACCTACCATCACATCATGTGTCTTCGCATAATGGATTAAGTCACTTACTATTAAGAAATAATCTT
CAAAACCCATATTAGTAATAACTTTATACTCATATTTCAATCGCTCTAAATAGACGTCATAATTAAGTTCTAA
TTTTTTTAATTGTGTAACTAAGACACGCCACAAATATTTTTTAGCTGATTCATCATTAGGTGTCTGATATTGA
GGAAGTAGAGATTGATGATATTTTAATTCTGCATTACACTTTTGAGCTATAACATCAACCTGCGTTAAATATT
CTTGATTCACATCAAGTTGCTGAATCTCATTTTCAGTTAAAAAATGCGCACCAAAATCTTCTTGATCATGAAT
TAAGTCTAATTTTGTATTGTCTCTTATCGCTGCGAGTGCAGAAATTGTATCTGCATCTTGACGTGTTTGGTAA

CAAACATTTTGAATCCATACGTGCTTTATACCCTGAACTAAAACACTTCGATGATCTAAATACGTATCTTCAT
GCGAATCAAAAACTTGAACAATGTCACGATGTTCGTCTGCAACATTTTTAAAAATGATAATCATATTGTTAGA
AAATCGTTTTAATAATTCAAACGACACATGTTCTAATGCATTCATTTTTATTTCCGATGATAGTTGATACAAA
TCTTTTAACCCATCATTATTTTTAGCTAGAACAACTGTTTCGACTGTATTTAATCCATTTGTCACGTATATTG
TCATACCAAAAATCGGCTTTATATTATTGGCAATACATGTATCATAAAATTTAGGAAAGCCATACAATACATT
GGTGTCAGTTATGGCAAGTGCATCAACATTTTCAGACACAGCAAGTCTTACGGCATCTTCTATTTTTAAGCTT
GAATTTAACAAATCATAAGCCGTATGAATATTTAAATATGCCACCATGATTGAATGGCTCCTTTCTATTAGTT
AAGTTTTGTGCGTAAAGCTGTAGCAAGTTGCTCAAATTCATCCCAGCTGTCCACTGAAACTCCTGACGCATTC
GGATGACCACCGCCACCAAAATCTTGCGCAATATCATTAATAATCAATTGCCCTTTAGAGCGTAAGCGACATC
TTATTTCACTACCCTCATCAACTGCAAACACCCATATTTTCAAGCCTTTGATGTCAGCAATTGTATTAACAAA
CTGAGATGCTTCATTTGGCTGAATACCGAATTGCTCCAATACATCTTCAGTTATTTTAACTTGGCAGAATCCA
TCACCCATAAGTTCGAAATGTTGTAAAACATAACCTTGAAACGGCAACATTTTTGGATCCTTCTCCATCATTT
TATTTAAAAGCGCATTATGATCAATATCATGCCCAATTAACTTTCCAGCAATTTCCATAGTATGTTCTGAGGT
ATTGTTAAAAAGGAATCGCCCAGTATCACCGACGATACCAAGATATAAAACGCTCGCGATATCTTTATTAACA
ATTGCTTCATCATTAAAATGTGAGATTAAATCGTAAATGATTTCACTTGTAGATGACGCGTTCGTATTAACTA
AATTAATATCACCATACTGATCAACTGCAGGATGATGATCTATTTTAATAAGTTTACGACCTGTACTATAACG
TTCATCGTCAATTCGTGGAGCATTGGCAGTATCACATACAATTACAAGCGCATCTTGATATGTTTTATCATCA
ATGTTATCTAACTCTCCAATAAAACTTAATGATGATTCCGCTTCACCCACTGCAAATACTTGCTTTTGCGGAA
ATTTCTGCTGAATATAGTATTTTAAACCAAGTTGTGAACCATATGCATCAGGATCTGGTCTAACATGTCTGTG
TATAATAATTGTATCGTTGTCTTCGATACATTTCATAATTTCATTCAAAGTACTAATCATTTTCATACTCCCT
TTTTTAGAAAAGTTGCTTAATTTAAGCATTAGTCTATATCAAAATATCTAAATTATAAAAATTGTTACTACCA
TATTAAACTATTTGCCCGTTTTAATTATTTAGATATATATTTTCATACTATTTAGTTCAGGGGCCCCAACATA
GAGAATTTCGAAAAGAAATTCTACAAACAATGCAAGTTGGGATGGGCCACAACACAGAAGCTGACGTAATGTC
AGCTTACTATAATGTGCAAGTTGGGCTCCAACGTTTGTGCGAAATCTATCTTATGCCTATTTTCTCTGCTAAG
TTCCTATACTTCGTCAAACATTTGGCATATCACGAGAGCGCTCGCTACTTTGTCGTTTTGACTATGCATGTTC
ACTTCTATTTTGGCGAAGTTTCTTCCGACGTCTAGTATGCCAAAGCGCACTGTTATATGTGATTCAATAGGTA
CTGTTTTAATATACACGATATTTAAGTTCTCTATCATGACATTACCTTTTTTAAATTTACGCATTTCATATTG
TATTGTTTCTTCTATAATACTTACAAATGCCGCTTTACTTACTGTTCCGTAATGATTGATTAAAAGTGGTGAA
ACTTCTACTGTAATTCCATCTTGATTCATTGTTATATATTTGGCGATTTGATCGTTAATTGTTTCACCCATCT
GAGGCTGTCTTCCTAAAAGTTGCATAGACTTTAAAACATCTTGTCTATTAATCACACCCACTGTCTTTTTATT
ACTCGAAACGACAGGAATCAATTCAATACCTTCCCAAATCATCATATGCGCACAACTTGCTACTGTACTCATA
GCATTTACATAAATAGGATTTCGCGTCATCACTTTATCTATTTCGTCGTCGTCCTTTGTATTAATCATCTCTC
GACTTGTTACAATACCTACTAATTTATAAGACTCATTGACTACCGGAAATCTTGTATGGCCAGTTCGATTCGC
CATACGCTTATAATCTGCTATTTTCATCGTATCAAACAGCACAGATAAATCATCTAATGGCGTCATTATATCT
TGAACTATTAAGATATCTTTTCGTATTTTCTGATTAAAAAGTGCTTTGTTGATAATATTTGCAACTAGGAATG
TATCATAACTTGATGATAGAACAGGTAAATCATGTTCATTCGCAAAATTAATAACTTTATTAGATGGCTTAAA
TCCACCAGTAATTAATATAGCCGTACCTCTTTTTAAAGCTTCAATCTGCACATCTTCGCGATTTCCGACAATC
AATAATGTCTTTGGACCAATATATTTTAAAATATCTTTGAGTTCCATTGCTCCAATTGCAAATTTAGATACCA
TCTTAGTGATACCTTTATTGCCACCTAACACTTGGCCATCAATAATATTGACAATTTCATTAAAAGTTAAATG
TTCAATTTCATTACGATTACGTTTTTCGATTCGAACCGTACCAACACGATCTATCGTTGCGACCATGCCCATT
TTATCAGCATCTTTAATTGCACGATATGCTGTCCCTTCAGATACGTTTAAAAATTTAGCGATTTTACGCACCG
AAATTTTAGAGCCTATAGATAACGATTCAATATAATCTAAAATTTGTTCATGTTTTGTCATTCTTTACCTCTT
CTTTTCGAACAGTATTAACTACATTATAACTTTATTTGGATAAAAAGCATTGAAGTGAAATGAAATAATGAT
CGTTTCACCTATTTTATTTTTTGAAAATATACAACAAACACAAAGATCACAAAATCTTTAATTTTAAATGGAA
AAATCCATTATTATTTATTAGAATGTAAGTGAGGAGGGATGTACTAATGTATAAAAATATATTACTTGGTGTA
GACACTCAGTTAAAAAATGAAAAAGCACTAAAAGAAGTGTCTAAATTAGCTGGCGAAGGTACAGTCGTAACAG
TTTTAAACGCAATCAGCGAACAAGATGCTCAAGCATCAATTAAAGCAGGTGTTCATTTAAACAAACTTACTGA
AGAACGAAGCAAGCGATTGGAAAAAAACACGCAAAGCTTTAGAAGATTATGGTATTGATTATGACCAAATAATT
GTTCGTGGTAATGCAAAAGAAGAACTATTAAAACATGCTAATAGCGGTAAATACGAAATTGTTGTTTTAAGTA
ACCGTAAAGCAGAAGACAAAAAGAAATTTGTACTTGGAAGTGTCAGCCACAAAGTAGCAAAACGTGCGACTAT
CCCTGTATTAATCGTTAAATAAAATTTTTATCCAGAATCACAAATAATCTTTCAATCATGATGCAGTCTCAAA
CGACTAAGTAAACACAAGAAATGATTATGACTGTGGTTCTGGATTTTTTATATCGTAGTAAATTTATAATCAA
TGTCTAATTGTATAAAACTAAAATTACGAGAGTAGGTCAGAAATGATAAAGAACCACTGATGTCCCCCGTCCA
CGTCGTAACTGAATTAGTAGAATATAAAAACACCCACTAAAAATATGCAGACGATAACTTCCACATAGATTAG
CGAGGTGTTTTTTAGTGTAAAATCTATATTCTATTTAAAACTGAACAGATTCACCTGGTTTTAAAATTTGCAC
GTCCCCTACATTAACAGCATCTTTAAATTGTTGTGGATCTTGTTCGATTAATGGGAATGTATCATAATGAATC
GGTACAGAAATTTTTGGTTTAATAAATTCATTAATAGCATAACTTGCATCATCAATACCCATCGTAAAATTAT
CTCCAATTGGTACAAAACATACATCAACTGGATGACGTTTCGCAATAAGTGACATGTCACTAAACAGACCTGT

ATCACCAGTATGATAAATTGTTTTCCCTTCAACTTCAAACACGATACCCATTGGCATACCTAAATAAACTGGA
ATACCATTTTCATGTGTAAAACTTGAACTATGAAATGCTTGAACAAATTTAACGCTTCCGAAATCAAAGTTTG
CTTTACCACCAATATTCATACCATGAACATTTTCAACACCGTGATATGAAGAAAGATAGTCAGCCATTTCTGC
ACTTCCAATTACTGTTGCTCCTGTTTTCTTTGCTAGTTCCACAACATCACCAAAGTGATCAAAATGACCGTGC
GTTAAAACGATATAGTCTACCTGCACTGTTTCAATATTCAAATCACACTTAGGGTTATTTGAAATAAACGGAT
CTACGATAACTTTTTTGTTGTTCCCTTCTAAATAAATCGTTGATTGACCATGAAATGATAACTTCATTTGAGC
ATCCTCCTATCAATTGCTATATAAATTTAGTACCCTTTTGCCTCTTAATTATAACAAATTCTCAAATTTTAAA
AATTGAAAATCTAGTTAATGAATTAGCTTGATTTTGAAATCTAATAATAATTGGCATAAAATGGAAGTAATAT
TATGTTGAGGAGTGTTTATAAAATGACAAAAATATCAAAAATAATAGACGAACTGAACAATCGACAAGCTGAT
GCAGCATGGATTACAACACCGTTGAATGTATATTACTTTACTGGATACCGTAGCGAACCCCATGAAAGATTAT
TTGCATTATTGATTAAGAAAGATGGGAAACAAGTACTATTTTGTCCAAAAATGGAAGTCGAAGAAGTCAAAGC
ATCACCTTTCACAGGTGAAATCGTTGGATATTTAGACACTGAAAATCCTTTTTCACTTTATCCTCAAACAATC
AATAAATTACTAATTGAAAGCGAGCACTTAACAGTAGCACGCCAAAAACAATTAATCTCTGGTTTCAATGTCA
ATTCATTCGGAGATGTTGATTTAACAATCAAACAATTGAGAAATATTAAATCCGAAGATGAAATTAGTAAAAT
ACGTAAAGCTGCTGAGTTAGCAGATAAGTGTATCGAAATAGGTGTTTCTTATTTAAAAGAAGGTGTGACTGAA
CGTGAAGTAGTCAACCATATTGAGCAAACAATCAAACAATACGGCGTCAATGAAATGAGTTTTGATACGATGG
TTTTATTTGGAGATCATGCCGCATCACCTCATGGCACACCAGGAGATCGCAGATTAAAAAGCAATGAATATGT
ACTATTTGATTTAGGTGTAATTTATGAGCATTATTGTAGCGATATGACACGTACCATTAAATTTGGTGAACCT
AGCAAAGAAACACAAGAAATTTATAATATTGTATTAGAAGCAGAAACATCTGCAATCCAAGCAATTAAACCTG
GAATACCTTTAAAAGATATCGATCATATCGCTAGAAATATTATTTCAGAAAAAGGTTATGGTGAATATTTCCC
TCATCGCTTAGGTCATGGTCTAGGATTACAAGAACATGAATATCAAGATGTTTCAAGTACTAATTCTAATTTG
TTAGAAGCTGGCATGGTCATTACAATCGAACCAGGTATTTATGTACCAGGTGTTGCAGGTGTAAGAATTGAAG
ATGACATACTTGTCACTAATGAAGGATATGAAGTATTAACACATTACGAAAAATAAGGAGTGAGACAAAAATG
AAAAGCTTGTTACAAGCGCATTCTCATTCAGTCAAACACTGCCAATATAACATTGTAGCGCCTAGGACATAAA
TTTTTGTCCAAGTCTAAATGCAATATGTAACAAACAAGCTAGAAACACATATGCAGGTATGTTCATCAGTAAC
ATGTAATGAATCAAATCAATATCATTCATGTTCGATGATTTCTTCGCATTGTTTCTAGCTTTAATTTATCATT
ATTTAATTTTAATAACCAAGGAGATGATAACGTCATTCTTTAGTACGCTGTAATCCATTCCCTTTTCATTAAA
TTCAAATTATAATTGTAATGCTTCTTCTACAGATTTATATTCCATTTCAAATGCCTCTGCAACGCCTTTATTG
GTTACGTGACCTTTGTAAGTATTTAAACCTAATGATAATGGTTGATTTGATTTAAATGCTTCTCTATACCCTT
TATTAGCTAGCATGAGCGCATAAGGTAGCGTAGCATTATTTAAAGCTAACGTCGAAGTACGCGGTACTGCACC
TGGCATATTTGCAACTGCATAATGAACCACACCATGCTTAATATATGTAGGATCATCATGTGTCGTAATTTTA
TCAGTTGTTTCAAAAATACCGCCTTGATCAATAGCAATGTCAATAATAACTGACCCATTTTTCATTTGTTTAA
TCATGTCTTCTGTTACAAGTCTTGGCGCTTTAGCACCTGGAATTAAAACTGCACCTATTACTAAATCACTTTG
TTTAACATACAACTCAATATTCAACGGATTTGACATAATTGTATGTACACGTCCACCGAATAAATCATCTAAT
TGTTGTAAACGCTTTGGATTAACATCTAAAATCGTAACATCTGCACCTAGTCCTAGTGCAATTTTAGCTGCAT
TTGTTCCTGCTTGACCACCACCGATAATAGTTACTTTACCCTTAGGTACTCCTGGGACACCACCTAGTAGAAT
TCCCATACCACCATTAAGTTTTTGTAGGAACTCTGCGCCAACTTGAGCTGACATTCTTCCTGCTACCTCACTC
ATTGGTGATAACAATGGTAAAGATCGGTCTGGTAACTGCACAGTCTCATATGCAATGCTAATTACTTTTCTAT
CTATCAAAGCTTGTGTTAATTTTTCTTCATTTGCTAAATGAAGATAAGTGAATAATACAAGCCCTTCTTTAAA
ATATGGATATTCAGATTCAAGTGGTTCTTTAACTTTAATAACCATATCCACATCCCAAACTTTTGCTTGTTCA
GCAACAATCTCAGCACCTGCTTCTTTGTAATCTACATCTTCAAAGAACGATCCTGAACCCGCATTTGTTTCCA
CTAAAACAGTATGCCCACTTTCTACTAAAGCGTGCACACCACTTGGTGATAAACCAACACGATTTTCATTATT
TTTAATCTCCCTTGGTATACCAATTTTCATACCATCCACCTCCATAACCATCTTAACGCGACCATTTTGAAAG
CGCAATCAAAAATCCACAAAATTGTAAAGGTTATTACACTGACTTTTCCGAAAATTGTGGTAAAATATAATTA
AGAAAGAACAAGGAGGCACTTACTATGATTACTTACAAAAATATTTTAATCGCAGTTGACGGTTCACATGAAG
CGGAATGGGCATTTAACAGAGCAGTTGGTGTTGCTAAACGTAACGATGCGAAGTTAACAATTGTGAATGTAAT
TGATTCAAGAACATATTCTTCTTATGAAGTTTATGATGCTCAATTTACTGAAAAATCTAAGCATTTTGCAGAA
GAATTATTAAATGGTTATAAAGAAGTAGCTACTAACGCTGGTGTTAAAGATGTAGAAACGCGTCTAGAGTTTG
GCTCTCCTAAATCTATCATTCCTAAAAAGCTTGCACATGAAATTAATGCAGACTTGATTATGAGTGGTACATC
AGGCTTAAATGCCGTGGAAAGATTTATTGTTGGTTCTGTATCAGAATCTATCGTTCGTCATGCGCCATGTGAC
GTGTTAGTTGTTCGTACTGAAGAGTTACCAGCAGACTTCCAACCACAAGTTGCAACAACTCAATTACGTGAAA
AATATCAAAATTAAATATAGATGATCATTAGATAACTACTTGATCATTATGAACCAAGTGCAACTTTCTGCAC
TTGGTTTTTTATTGTTTATAAATAAAACTCACTTAATAATGTTTTCATAATCTTCTTCGACTACTTAATTCTT
TAAGATATTCGTGAAAAGAGACATTACACTAGTTAATTTTCAAACAATACAAAAAGCGTCTACCTCCTACATA
TAATTGTAGCGGAGATAGACGCTTAATATTTATTTAAAAATTATTTTAAACCACCGAATGTCATAACATCACG
GGCAATCATACTTTCTTCATCAGTTGGAATAACGACAACTTTAACTGGTGAATGAGGATAGTTAATAAATCCT
TCTTTACCACGTAGTAAGTTTTCATTTTTCTTAGGATCCCAGTAAACACCCATAAATTCTAAGCCTTCAAGAA
CTTTCGCACGAATTTCTACTGAGTTTTCACCGATACCTGCTGTAAATACGATAACATCAACACCATGCATTCT

CGCAGCATATGATCCAATATATTTGTGAATTTTAGAAGCAAATACATCTAAAGCCATTTGTGAACGTGCTTTA
CCTGATTCAGCTTCTTCTGATAAGTCACGTAAATCACTAGATGTACCTGATAATCCTAATAAACCTGATTCTT
TGTTTAAGATTTCCAATACTTGTTCAGCAGTTTTACCTGTTTTTTCCATAATAAATGGAATTAAAGCAGGGTC
AATATTACCAGAACGAGTACCCATTGTTACACCAGCAAGTGGTGTGAAGCCCATTGATGTATCAATAGATTTA
CCGCCATCGATAGCTGCAATTGATGCTCCATTACCAATGTGACATGAAATAATACGTAAATCTTCAATTGGCT
TATCTAACATTTCTGCCGCTCTTTGTGATACAAATTTATGGCTTGTACCATGGAAACCATACTTACGAATGCC
ATAATCTTTATAATAATGATATGGCAAGCTATATAGATATGCTTTTTCAGGCATTGTTTGATGGAATGCTGTA
TCAAAAATTGCCACATGAGGGATATTTGGTAATAATTTACGGAAAGCACGAATACCCATCAAGTTAGCTGGGT
TGTGAAGCGGTGCTAATTCGCTTAATTCTTCAATTTCCTTTTCAACCTCATCAGTAATAGCTACTGATTCAGG
GAATTTTTCACCACCATGTACAACACGGTGACCTGTTCCATCGATATCGTTAATATCATTAATAATATTATGC
GCTTTAAAAGCATCCAACATGATATCAACTGCCTCAACGTGATCCTTGATATCTTGTACTGTTTTAACTTTTT
CCCCGTTGACTTCAATTGTAAAAATTGAATCCTTCAATCCGATTCTTTCTATTAAACCTTTTGTTACTAATTC
CTCTTCAGGCATTCTAATTAATTGAAATTTTAATGATGAACTACCAGCATTGATAGCCAAGATTAATTTTGAC
ATAAGTAATGATCCTCCATCGTTTGCTTTTTATACTATTTCATTTCTCATTTTATCATTCATACAGTTTTATG
ACTATACTATTACAGATTATTTTTTAGGACGATTTGTGTCCATCCACTGATTTAACTCTGTCATAAATCCTTG
AAATTGTGAAGGATTTTTGAAATCAGGAATATTTGCCAATAATACTTCAACTGGCTTTGTTTCACCCGATTTT
TTCTTTTGTAAAATTAATATAGATTTTCGCGCTTTTTCATTTTTAAATAAAGTTGGTGGTAAATTTAAAAATG
CTTGCATCTCTGTCTCTGTTGCAATATATTTTTCAAGCTGTTTTACATGTTCACCTGTAAAAATATTACTTGG
TACCACTAGAAAGGCATATCCAGCATCTTTTAATGCATTTATTGCTTGTTCTATTAATAAATAATGTGAATAA
CTATGTCCTTCTTCAAAACCTAGCTTAAACTCCTTACTTCTTTCATCAATTGGATAATAGCCTACTGGAAAAT
CACCAATAACGATATCTGCTTCTTCTAATGGTAGTGGCATGATGGCATCTTGAGGATACACATCGAAAGGAAT
TTCTAAGAAGTTTGCTAAATGTACACTAACACGTGATAAAACTGGATCAACTTCAATTAAATGATGCATAACC
GCAATTTCAGGTAACACTTCTTTTACAGTAGCACTTAAATGACCGGCACCACTTGCAATATCAACAATATGTA
ATTCTTCTTGGTTGTTCATAAAACGCTCAACTAAAAATCCTAGTATCAATCCAATTGAATCTGGTGTAATTTG
ATGATTTGCTTGTATCTTTTCTTCCTGCATTAAACTTAAATATGCAAATTGGAATGCTTTACGTCGATCTTGT
AACGTCGATTGTTCTAACAATCCTCTTTCATTGGTATATACTTGTTCCATTGCTAGCCCAAGATTTTCAATAA
AACTTTGGCCATTTTCATTATTTAATGTTTTAGCTTTTTCATCTAATGTATGAAACAAGCGTTCCATAATTGT
TTGTTGTTCTGCCATATTCGTCCCTCTCCAATAAAAAGTGCCCATAGGTCTTAGCAATCATAGAAAACAATTC
TATTTTAATGCTGTTGACAACGATGGACACGATGTATACATTTTAAGATTTATCTCTTTAATGATTAAATAAT
TTTGCATGCAGCTAAAGCAGCATCAAAATCTGGGAAATCAGTACCTTCACTAACGATTTCTTTATAAACAACT
TTATTATCTGCATCTAATACAAATACTGCACGAGCTAATAAGCGAAGTTCTTCCATAACAACGCCATAGTTTT
CACCAAATGATAAGTCACGGTGGTCACTTAATGTAATGACATTGTCTAAACCTGCTGAAGCGCACCATCTTTT
TTGTGCGAATGGTAAGTCTGCTGAAATTGTAAGCACAATCCCCTCTTCTTTAGAAGCTTCAGAGTTGAATTTG
CGAGTCTGCTGATCACAAACACCTGTATCAATTGATGGTACCACACTAATTAATTTCTTTTTACCAGCATAAT
CTGCTAATGTTACTTGATTTAAGTCATTATCTAACACTGTAAAATCAGGTGCAAAATCACCTTCATTAATTTG
TTGACCTTTTAAGTGGATTGGTCCACCTTTGAATGTTATTTCAGTCATATACTTGACCTCCTGCATTTTAAAA
TTCTAACTACATCATACGACAATTTATGGTTTGATTACAATTTTCATGCTTTAATCAAAAGCAAATTTAATTT
TATACAAATTGTTGAATATAATCAAAAGCATTTTTTAAAATTAAAATAGCAGTAATAATAATAAATAATACTT
TTACATAGGCAACACCTTGTTTGATAGCAAAATGTGCTCCAGTATATGACCCAGCAATCATGCTTGTAGCCAT
AATTAAACCTATTACATAATCTACTTGTCCTAATACCATAAATAATACAAGCGCACCTATATTAGAAGCAAAG
TTCAAAACTTTAGCATTTCCTGCAGCACTTAAAAAATCAAAACCAAAGATTAACAATACAAAAAGCATAAATG
AACCTGTTCCCCCACCTACAAATCCATCATAAAAGCCGATTAATATAAAAAGTGCTGCAAATATTATGGCTTT
CTTAAATGTAAATTGAGTAAACGTACGTGTATTGCCCCAATCTTTTTTAAGTAATGTGAATATAAACACCGAC
GAAAGTGCAATGATGATTAAAGGTTTCAATATTTGTGACGGAACCATCGTTGCAATATATGCGCCACATGCAG
ATGCCAAAAATACAAAACCAAATAATTTGGCAACAACAAATAAGTCCACTTTACCGGACCTTATAAACTTTAT
CGTACTAGTTAAAGAACCAAATGAACTTGCCAATTTATTTGTACCTAAAGCCACAGATGGTGGTAGACCGATT
GCTAATAATGCTGGCGTAGAAATTAAACCGCCACCCCCTACAACCGAATCTATAAACGCCGCGATAAAACCAA
ATAAAATTATGATTATAATCATCGTTAAGTTCAAATCCATAGTTTAACATCCCCTTATGTTTAAATTTACTCT
AGGATTTTATTTATAAAAAGTCGTTTATTAATTGGTTTGTTTGCTGTTCTTTAATAGTTTTATAATCACTAGT
TATTTCAAGTGTTTCAATATTTTCAACAGCACGATTAATCATCTCTTCAAAATCAAAGACGCTTTCATATTGA
ACTACCTTATCAAAGTTTGGTTCGGTTACTGGATTTTTAGGGGTGAAAATTGTACAACAATCTTCAAATGGTT
GAATAGATGTTTCAAATGTGCCAATTTCTTTCGATTTAATAATAATTTCTTCTTTATCGTAAGTTAATAAAGG
ACGTAATACAGGAGTAGAAGTTACATTATTAATTGCATACATGCTATGAAGTGTTTGACTGGCTACCTGCCCT
AGGTTTTCACCATTTACAATAGCTAAAGCCCCTATTTGATGTACTAATTTATCAGCAACACGCATCATCATAC
GTCTCGTTGAAGTCATTGTATATCTTGGATGTACAACTTTATTTACCTGTTTTTGCAATTCTGTAAATGGTAC
AATATGCAATTTAATTGGTCCAACACGTTCAGCTAAAATACGTGTCAATTCAATAACTTTTTCTTTTGCTTGA
TCACTTGTAAATGGTGGACTATGGAAATGAATCGCTTCAATTGTTACGCCACGTCTCATCACTTCCATTCCAG
CAACTGGTGAGTCTATACCGCCTGAAAGCATTAGTAACGTCTTACCACCAGTACCAACTGGTAATCCGCCTGA

ACCCGGAACAACTTCTTCATACATATAAATTGCATCTAATCTAACTTCCACTCGAATTTCATGATCTGGACGT
TTGACATTAACAGAAACATTTTCAATTTGTTTCAATACGGTACCACCCAATTCACGTTGTAATTCATACGTAT
CCATTGGGAAATTTTTATCGGCACGCTTCACATCAATTTTAAATGTGCTGTTTTCTTCAAATTGCTGCGCAAA
TTTAATTGCCGCTGCACTCATTGCCTCTATTGTTTTTTCTACTTTTAATACTGGACTAATAGATTTAATACCG
AAAATTTTTGATAATCGATATGTTATTTCATTTATATCTGCATGGTCTTCAAGTTCAATATACATACGATCTC
GTTTGCCTTTAACGACAAACCCATCAAGTCCTTTTAATGACTTATTTACATTATTTCTTAATTGATTTACAAA
TTTCTTTCTATTTGAACCCTTTAATGTTAACTCCCCGTATCTAACAAGCAAGTGATCATACTTCATTATTTTA
GCAACTCCTTAATTTCCTCATAAATGATGATAAATATTTCTTTAAACCTTGCTATATCTTCTTTAGTTGTAGT
AGCCCCAAATGATAATCTTATACTACCTTCAATAGATTTGTCTGATAATCCCATTGCAGCCAATACTTCATTT
AATTTATTACGTTTAGATGAACAAGCACTCGTCGTAGATATCATAATGTCATATTTTGAAAAAGCATTAACTA
ATACTTCACCTTTTACTCCAGGAAAACTAATATTTAAAACGAATGGTGAACCTGAAGTTGAAGAGTTAATATA
AACTCCATGATATTTATTTAAAAATTGACGGACGTCATTATTTAACTCAGTAACAAATGCATTCAATGCTTCA
AAGTTTTCATTAGCTATCTTCATCGCTTTAACCATTGCAATATCATTTGGCAAATTAACTGTTCCACTTCTAA
CGCCATATTCTTGACCACCACCATGGACAGTTGGTTCAACATTTTGAATGTGATTTACAAGTAAGACGCCTTG
GCCTTTTAAACCATTAAACTTGTGTCCACTTAAACTAATACTATCTACGTTATTGAAATCCATTGAAATTTTG
CCGAATGCTTGAACCGCATCTACATGAAAATGTGCCTTAGGATAATTTTTTATAACTTCAGCCATTTGTGGAA
TAGGCTGTATTTGTCCTGTTACATTATTTACATACATACATGTTACTAAACCGACTTTGTCTGACATTAATTC
TTTGAAGTGTTCTAAGTTAATACTGCCATCTTTCTTTACATCAACATAATTAACTTTAAATCCTTCGTGCGCT
TCCAAATATCTTACAACCTCTAATACGGATGGATGCTCTAACACGGATGTAATTATTTCCTTAGCTGTATCAA
ATTTACGATAGGCAATACCTTTTAAAGCAAGATTATTGGATTCAGTTGCACCACTCGTGAAAACAACATTATA
ATTTGTTTTTGAATTAATCATTGCATTAATTTGGGCTTTTGCTTGCTGTAGTAATTGATTTGCCTGCAAACCA
GCTTTATGCGGACTATTCGGATTATAATACATTGATTGATTTACTTTTAAATAAGTATCTAACACTTCTTCAA
ATGCTTTCGTCGTTGCCGCATTATCTAGATATATCAAAATAAACACTTCTCTTTCATTAATCATCAATCGTTA
AAATTGTTTTACTCAAGCATTATACTATTATCCGACAAAAAATAAAGGTTATAGCGCAAAATAATCTTATTTC
TACTTATTAAATAAGAATTAGACTATTTCACTCTATATTAAACAGCTGTGTGATTATGCTTTTTTCACTACTT
ATATTAATTAAAACAACACTTTTGATTACGTTTATCTTTAAACAATTGCTATTTATATTTATTGTCCCTTTCA
TATATCCATATAAAAAAAGAAGCTAGACAACATACAGTTGGCTAACTTCAATTATTAAACTCATCAGTCCAAT
TTGACAGAGAGCTCATATTATTTAAGTATAACTACATACTAGTTTCTATTGCTTAATAACTTCTTCTTCAATA
TGTTTAGTGACACCTGGCTCAACACTTTCAAGAGCTTGCTCTGCAATTTCAATCGCACGCTTATAGCGATTAT
TTTTAAATAATCGTTCAGCTTCATTTAAGCTCTTATCAACATTGCTATAGTCCTTACGATATCTATTTCCATA
TTGAATTAATTTCTCTGCATAAACAGCATTAACAAGAACATCATTTGCTTCATCTTCAAATGTATTCATTTGA
ATCACAATTTTAGACACTTTATCTTTTAACTGTTTAACGTGTATTGGACGTTCACTAAATTGTTCGTTAACAT
CACGAACTTCATGATCAATTTCATTTTTCATGATGATAAATCTTTCAGGAACACTTGTTAAGTTAGAAGCAAG
TAATCGACGATACACTTCTTCTTTCTTCGATTGTACTCGTAACAGATTGTCTTCTGCTTCTGCTTCATCTTCA
CGTAATTGAATCAGATGATTTTGTAGCTTTTCTTGTTTGTCATTAATGACTGTGACATGATCTTCTAAATATT
GTAAATTATCCTGAACCTCGCTATATCTAACAGCAGATTTAGACATTTCTTTTAAAATATCATCATATACAGA
AATTAAACTTTGAATTTCATTTTCAAATTGACGAACACTTTGAGCATCAGATTCATTTATATAGTAGTTTTCA
CGTACATATTCAATTTCTGTTTGCAATGTATAATTCATGTCTTTAGCTTTGAATAAGTTATCCGTAATGATAT
CTTTTGTTTCTTCGACATCATTTTTAGCTTTAACTTCATGTTCAATTAAATCATACATGTCATCTAACTTATC
ATTGATATTAGCTAGTTTATCATTAGCTTCTTCTAATTCTAAGCGACTAATTAATGGTTCAACGAAACTAAGC
TCTGTTTTTAAGCTTTGTAATGTACTGTCTACTTTTACGTGATCCAGATCATACCCTTCAACTTTAAGATCAC
GGCAACCATATTTTAAATCTTGGAATTGACCAGGTAATTCTTTTTGAGTTTCTCTAATTAATTCTGGTATTTC
TTCCATATAAGATCTTAGCTGTTTCATTTGTTCATTCAAGGCAGCTATATGGTTGTGCGCTTGTACATAATTA
CCATCAGCTTTTAGTACTTCATATTGCTCTAACCTTGGCTCAAATTTTTCAATTTCAGTTTCAAGTAGACTTG
CTGCCTCACCAAATTGATGACGATTTGCTAAAACATCACGTTTCATTTCACGATAATCAACCTTACATTTGTC
ATATAATTCATCATTATCTTTGTATAACGCAATAATTTCATTTACATCTTCTAATTGTTGCTGATAGCTTTGT
TCGTAACTATCCATCAACTCATTTGCATCATCAATTTCACTTTGAGCTGCGTTGAAACTAAATTTATCTAATA
AAGCCTCAGCATTATGGATTTTTTCTTCCACAGGAGCTAGATACTTATTTGTACTTTCTACGTTGTCCTTTTT
CATTGCATCGTATTTCGTTTTTGTTTCACCTTTTAAATTCAACTTAGATAATTGTGCAAGGTTTTGATCAAAA
GGTAACGTCTCAATTTCATTTTTACGTTCGATTGCTTTTTCAATAATTTGTCGTTTATTTGAACGTAAATAGA
ATAATACACCTACAGCAATCAATATAATCACAATTATTGCCAAAATGATATATAACACCATATGCTTCTCCTC
CTAATTTATCATTACTATTATAACGTAATTTTGCTTAAGGATAAATTATAAAATGAATTTATTGCCATAATAT
TTTCGAAATTCTATACTATTATAAAATCTATGAAAATAAGAGGTTTTAAATATGACAACAATTAACCCAACAA
ACTATGCATTATTAAAGAAACAAGCAGCAAGCCTTATTGAAGATGAACATCACATGATTGCTATTTTAAGTAA
CATGTCTGCCTTATTAAATGATAATCTGGATCAAATTAATTGGGTCGGCTTTTACTTATTGGAACAAAATGAA
CTTATACTTGGACCTTTCCAAGGACACCCCGCTTGTGTCCACATTCCAATTGGAAAAGGTGTATGTGGTACAG
CCGTTTCAGAACGTCGTACACAAATTGTAGCTGATGTTCATCAATTCGAAGGACATATCGCTTGTGATGCTAA
TAGTAAGTCTGAGATTGTCGTTCCAATTTTCAAAGATGATAAAATTATCGGCGTCTTAGATATCGATGCCCCT

ATAACGGATCGATTTGATGACAATGACAAAGAACATCTTGAAGCAATTGTTAAAATTATTGAAAAGCAACTCG
CATAAAAGGACATCAGCATTTTCAATAAAGTGTTGACAGTTAGCAGGAAAATGTTACAATAATCTTTGTGTGA
ATTAACGAAAGTAGCAGTTGTATATTATTGAGCGCTATGTTGTTCCCAATGCGGACGTGTCACGTAACTGTCG
CTATAAGGTGAAGACACATAAAACAATATATCTTAGTAAGCATGCAACACTCTTTTTTGTTTATTCATAACAA
CAAAAAAGAATTAAAGGAGGAGTCTTATTATGGCTCGATTCAGAGGTTCAAACTGGAAAAAATCTCGTCGTTT
AGGTATCTCTTTAAGCGGTACTGGTAAAGAATTAGAAAAACGTCCTTACGCACCAGGACAACATGGTCCAAAC
CAACGTAAAAAATTATCAGAATATGGTTTACAATTACGTGAAAAACAAAAATTACGTTACTTATATGGAATGA
CTGAAAGACAATTCCGTAACACATTTGACATCGCTGGTAAAAAATTCGGTGTACACGGTGAAAACTTCATGAT
CTTATTAGCAAGTCGTTTAGACGCTGTTGTTTATTCATTAGGTTTAGCTCGTACTCGTCGTCAAGCACGTCAA
TTAGTTAACCACGGTCATATCTTAGTAGATGGTAAACGTGTTGATATTCCATCTTATTCTGTAAAACCTGGTC
AAACAATTTCAGTTCGTGAAAAATCTCAAAAATTAAACATCATCGTTGAATCAGTTGAAATCAACAATTTCGT
ACCTGAGTACTTAAACTTTGATGCTGACAGCTTAACTGGTACTTTCGTACGTTTACCAGAACGTAGCGAATTA
CCTGCTGAAATTAACGAACAATTAATCGTTGAGTACTACTCAAGATAATACCGTTCAATACAAACACCCACAA
TTGTGGGTGTTTTTTTTATTAATTTAAAAATGATAGATTCAAGATTATATCGAGCTACCCTATTTTACAATCA
ACTTAGAATTTACAAGTCTTATATGTTGAATTATCTATAATATTTCCTCATTAGTTTTTAAAATTATCAATTG
CGATATACGAATCCTCATCCCGCTATCAAATAACCATTCATCGTCTCACTTAATAAATAATTTCGGGTTATCT
GTAATTAATCCATCTACACCCATTTGTCTTAATTTTTCTCCAGTTTTCAATTTATTAACTGTATATGGCATAA
CCTGTAATTGATGATGATGTGCTTCATCAACAAATTTTCTGGTCACTAATGCATAATTAGGATTAACATAACT
AGCAATTTGAGAAATTCTTGAAAAGTTTGGCTTTTTATACCAATACTTACGTTTACTACAAAGCACACCTAAT
TCATATATACTGCCCAATGTGTTTAACTTTTCGATGCATTCGATATCAAAGGATTGTATAACTACTTGTGTCG
CATCGACTTTCTTTTCTTCCAAAAATGCCAGCAATTTACATTCTATTTCTGGATATAAATTCGGACTTTTCAA
TTCAATCAATAGCTTTTTATCATACTTTAAGCATAATGAAAGTACTTCATCTAACGTGGGTATTCGTTCTCCC
TTAAAAGCAACATCTTTATAACTACCAAAATCAAATGATTTTAATTGCGATAATGTGTAATCAGCAATACGCC
CCTTACCATCCGATGTTCTATCAATTGTTTCATCATGTATCACAACAAAATGTTGGTCTTTGGTCAAATGAAC
ATCTATTTCTAACATAGCAACATTGAGCCCCATTACCTCTCGATAACCGACCATTGTATTTTCAGGAAAATCA
CTCGGTAATCCACGGTGCGAAACAATTTGTAATTCATCTTTCAGTTTATTCAGAGTCATTGATAAGTCACCTT
TCTGTTAAACTTTGATAAACTATTCTAAAATTAAACTATCACAATTATATTGGAGGTTAAAGCAATTGCATCA
ACATGACTTTAAAGTCCAAACTTCTTGGCAAGGTGGTCGTAACAATGTCGGAAACGTTCAAGGCGACATACTT
TCAGAGAATATTTCAATACCTGCTTCTTTAGGTGGTGTTGGTATAGGAACAAATCCCGATGAAATGTTAGTAT
CAGCCGCTTCATCATGTTATATCATCTCATTAGCAGCTACTCTTGAACGTGCAAAGTTCACAGATATTTCAAT
TGAACAACAATCGATTGGAACAGCTTGTTTAAATAATGGAAAATTCAGTATGTCAAAAATTGTGCACCATCCT
CACATTGAAATTCCAAGTGATCAAATAGCACAATTAGAAAAGCGATTACCAAAATTGATAACAATTGCAGATA
ATAATTGCATGATTTCAAATGCTGTAAGAAATAATGTAGACATAAAAATCTATCCCATCATTCAGGCCAAATA
AAATCGTTACGTGATAACGTTTATTCGACAATATAGCGTTATCACTTTTTTTAGGCTTTTAAATTTTCAGAAA
ATTTGCTAAAATAGTGAAATCTTATTTATAGGAGGCATATAAATGTATTATCATCAACCGTTGTTATTAACAC
CTGGCCCAACCCCTGTACCTGATGCCATTATGAGAGAAATTCAAGCACCTATGGTTGGTCATCGTTCTAAAGA
TTTTGAAGACATCGCACAACAAGCATTTCAAGGTCTAAAGCCAATATTTGGGAGTCAAAATGATGTACTTATT
TTAACATCTAGCGGTACAAGCGTCTTAGAAGCTAGTATGTTGAACATTGTAAACCCTGAAGATCACTTCGTTG
TCATTGTTTCAGGTGCCTTTGGTAACCGATTTAAACAAATTGCACAAACTTATTACAAAAATGTGCACATTTA
TGACGTAACATGGGGAGAAGCTGTAGATGTTAAAGATTTCATCAATTACCTTTCAACTTTAGATGTTGAAGTT
AAAGCAGTATTTAGTCAATATTGCGAAACATCTACGACAGTGCTACACCCTATTCACGAGTTAGGAAATGCCA
TCAATCAATTTAATAGTAATATTTATTTTGTAGTTGACGGCGTAAGTTGCATTGGGGCTGTTGATGTTGACAT
TAACAAAGATAAAATTGATGTCCTTGTTTCTGGTAGTCAAAAAGCAATTATGTTACCTCCAGGATTAGCTTTT
GTAGCTTATAGCCACCGCGCAAAAGAACGTTTCAAAGAAGTAACTACACCAAAATTTTATCTAGACTTAAATA
AATACATTTCGTCACAAGCTGACAATTCTACACCGTTCACACCAAATGTGTCTTTATTTAGAGGTGTAAATGC
ATACGTTGAAACCGTAAAAGCAGAAGGTTTCAATCACGTAATAGCACGACACTATGCAATTAGAAATGCATTA
AGAAGCGCCTTAAAAGCATTAGATTTAACTTTATTAGCCAATGATAAAGATGCATCTCCAACGGTTACAGCAT
TCAAACCTAATACAAATGATGAAGTGAAAATAATCAAAGATGAACTTAAAAATCGCTTTAAAATAACAATTGC
TGGTGGTCAAGGCCATCTTAAAGGTCAAATTTTAAGAATTGGTCATATGGGGAAAATTAGTCCTTTCGATATT
TTATCGGTAGTATCTGCTTTAGAAATTATTTTAACTGAACACCGTAAAGTTAACTATATCGGTAAAGGTATAT
CAAAATATATGGAGGTTATTCATGAAGCAATTTAATGTACTCGTTGCAGATCCCATATCAAAAGATGGTATCA
AAGCATTATTAGATCACGAAAAATTTAATGTAGATATTCAAACTGGCTTGTCCGAAGAAGCATTAATCAAAAT
TATACCTTCATACCATGCTTTAATCGTTCGTAGTCAAACTACGGTCACTGAAAATATCATAAATGCTGCTGAT
TCTTTAAAAGTAATCGCACGCGCCGGTGTTGGTGTAGATAATATTAATATTGATGCTGCAACATTAAAAGGTA
TTTTAGTTATTAATGCCCCAGATGGTAATACGATTTCAGCTACTGAACATTCACTGGCAATGTTATTATCAAT
GGCTCGAAATATTCCGCAAGCACACCAATCACTTACAAATAAAGAATGGAATCGAAATGCATTTAAAGGTACT
GAGCTTTATCATAAAACATTAGGTGTCATTGGTGCTGGTAGAATTGGTTTAGGTGTTGCTAAACGTGCGCAAA
GTTTCGGAATGAAAATACTAGCTTTTGACCCTTACTTAACGGATGAAAAAGCAAAATCTTTAAGCATTACGAA

GGCAACAGTTGATGAGATTGCCCAACATTCTGATTTCGTTACATTACATACACCACTAACACCTAAAACAAAA
GGCTTAATTAATGCTGACTTTTTTGCCAAAGCAAAACCTAGTTTGCAAATAATCAATGTGGCACGTGGTGGTA
TTATTGATGAAAAGGCGCTAATAAAAGCATTAGACGAAGGACAAATTAGTCGGGCAGCTATCGATGTGTTTGA
ACATGAACCTGCAACTGATTCGCCTCTTGTTGCACATGATAAAATTATTGTTACACCTCATTTGGGTGCTTCA
ACAGTCGAAGCTCAAGAAAAAGTGGCAATTTCTGTTTCAAATGAAATCATCGAAATTTTAATTGATGGTACTG
TAACGCATGCAGTGAATGCACCTAAAATGGACTTAAGCAATATAGATGATACTGTAAAATCATTCATCAATTT
AAGCCAAACAGTTGGTGAATTAGCTATTCAATTAATGTACAATGCACCAAGCTCTATTAAAATTACGTACGGT
GGCGACTTAGCCTCTATTGATAGTAGTTTATTAACACGTACAATTATTACTCATATTTTAAAAGATGATCTTG
GTCCTGAAGTCAATATTATCAATGCTCTAATGTTGTTAAACCAACAACAAGTGACATTAAATATTGAAAATAA
TAAAGCAGAGACAGGTTTTAGTAACTACTTAGAGGTAGAACTATCAAACGATAGCGATTCCGTTAAAGTTGGC
GCTTCTGTCTTTACAGGTTTCGGTCAAAGAATTGTTAGAATTAATAATTTTTCTGTAGACTTAAAGCCAAATC
AATATCAAATTGTGTCATATCATAATGATACTCCAGGTATGGTAGGAAAAACTGGCGCATTGTTAGGTAAATA
CAATATCAACATTGCATCTATGACTTTAGGTAGAACTGAAGCGGGCGGAGATGCGCTAATGATTTTATCCGTT
GATCAACCTGTTTCAAACAATATAATTGATGAACTTAAACAAGTTGGTGAATACAATCAAATTTTCACAACTG
AATTGACGGTACAGTCATAAACAATTTATGTAAATTCAAAATGCCAAGAAGACATTGGATTACTTCCATTACT
TCTTGGCATTTTTTCATTATTAATTTCATTGTAACTCAATATGAAACCTTTGGTTCAATCATTCACTTCAAGT
TGTGCTGTCAAACAACAATCAAATTACAAATTATCTAGTACACCTCTAAGTTCACCTAAATGATTAATAACAT
AGTCGGCATGATGCGCTTCTAACTCACCTGCAGCATCCTTACCTTTTAAACCTGTTAATGTTCCAATAAACGT
TGCACCTATTTTTTGAGCACTTAATAAGTCAGCTAACGAATCGCCTACTATAAATACGTCATCTTTATTTACA
ATGTTATCTTGCTTATTGATATAAGATTCATATTTATCGCGATTATTACCATATAAAGCTGCGATATAACTAA
AAGGATTCGGCTTTCCTAATGGTCGTGCTTGCGGATACATATTCTCTGCTTCTAAAACATCACTTGCAGTTGC
AATAAAATCAGCTTCAAAATATGGTAACAATCCTAAATTTTCAAATGGCACAACAGTCTCAGTATAAGGACGA
CCTGTTGCAATACCTAATTCGAAACCAGCACCTTTTAAATCATTCAGAAGTACCTTAACTTCATCTACTGGTC
TCAAAATAATTTCTTGATAAATATAACCTGTCTTAAAAGTAGTTCGTGCTATTTTCTTTTCAACATCTTCATA
CAACTTCGATCCTAAATACCATTCTTGATAAACTTCTTGCGCTAACGTCCATAATGCACCCTTCAAACTAAAT
AAAGTAGCATCCGAAACATGTAACTCTGTTGTTGCAAACTCTTCCAAAGCAGCATAAATATTATTTTTACCAA
CTTTTACATTATCCAAAAATTGTAAAGGTAGTTGTTCATTTAAATTAAAACAGTCTGCTAAGTTTGTGCTTAT
ATTTTGCAACTTCAATTCGACAGGCTCATCTTGATACATGAATGCCTCAATTTCATCATGTGATAACTTTTTC
AAAATATCAATTAAATGAATACTAAAAACGATAAATAACATATCCCAATTTGAATTTAGCCCTAGCGATTTTA
ATTTGTTTAAAATCTTATCTTTTTGAAAAATTCGATTCCTAATGTCTTGTATATCGTTATCAGTCAAAGTTTC
CCAATCTATATGTGAATGAAGACCTAAATAACACTTATCCATTAATAATTCATATACCGTTAATGCAGAGACA
TCGAAACAACGTTCTTCACTTAAAAAAACGCCATCAACATCAAATAAAATTTCTTCACAATCCCCACTCCAT
TTCTGAAAATTCAGATATAAATCATTCTACTATTTGACTAAAAAAAGCGCAAACCCTATTGAAGTTGGATTTG
CGCTTTAGCTGTTTAAATTTTATAAATGTTTTTCAATTTCATCAGCAACCTGCTGTACGTGTGTACCGACAAT
AACTTGAGTTGAATGTTTACCATTAACAGTAACACCAACTGCACCGGCGTTTTTAATCTTCTGTTGATCAATA
ATAGATGTGTCTTTTAACTCTAGACGCAACCTTGTTGCACAATTAGTTAAATTAACAATATTCTCTTGACCAC
CTAAACCTTCTAATATTTGTATAGCATGTTGATGATATTTACTTTGTTTAATATCATTTTCACCAGGAGCAAT
ATTATCTTTTACAACTGTTGGATCAACTAATTCATTTTCACCTCTACCAATCGTATTCAAGTTAAATACTTGG
ATTACTACACGGAAAATCACATAGTATAAGATGAAAAATACAACACCTTGAACAAGCAACATCAATGGATGAT
TTGATACTGGATTAATTAGTGATAACACATAATCTATCAAACCTGCACTAAATGAAAATCCAGCTGTCCAATG
GAATGTAGCTGCGATAAATAAAGATAATCCTGTTAATAACGCATGAACAACATATAAGATTGGCGCAACAAAC
ATAAATGCAAACTCAATCGGCTCTGTAACACCAACGAAAAATGCTGCAACTGAACTCGCTAGGAACCAACCGT
AAACTTGTTTTTTCTGAGTCGTTTTAGCTGTATGATACATTGCTAACGCAGCCGCTGGAATACCGAACATCAT
GATTGGGAAGAATCCCGCTTGATAGCGTCCTGTAATACCTTTTATAGCATCTTTGCCACTTTGGAATTTACCA
ATATCATTAATACCAATCGTATCAAACCAGAACACACTATTCAGTGCATGATGTAATCCTGTAGGAATTAATA
ATCTATTGGCAACACCATATATGAAAGCTCCAAACGATCCTAAACCGACTATAGATTCACCAAATTTTACAAT
CCATGAATAAAGTAGTGGCCATAAGAATAACAATATGACAACTAAAAATGTACAGTAAAATGCAGTCATAATT
GGAACTAGACGTTTACCACTAAAAAATGATAATGCTAATGGTAATTCTGTTTCACTAAACTTATTGTATGCAT
AAGCTGCTATTAAACCTATTACAATACCAACAAAGACATTGCCATTATTCATCTTTTCAAAAGCTGAATTTAT
TTCCGAAGCTTTCATTCCTAATAAAGGCGCTAATTTCATTGGTGATAATACAACTGTAACTAAAAAATATCCT
AACGTAGCTGCAAGCGCGACTGCACCATCATTTTTCTTTGCCATTCCTATAGCTACACCAATTGCAAATAAAA
TACCTAATTGCTCTAAAATCGTAGTACCTACCGTAGTAAAGAACATTGCGATTTTCGGCGTCGCATGAAGTGC
ATTTAACGTATTACCAATTCCGGCAATAATTGCTGCAGCCGGTAAAATGGCAACTGGTAACATTAACGAACGC
CCTAAATTTTGGAAAAATTTATACATTGAATGTCATCCTTCTTAAAATAAGGTAGAAATATAAAGATTACTAA
TGTAACTAGAATAACTACTTCGATACTCCGTTATAGTCACCTAGGCTTACTAACCAGCTATATTTCTACCTCA
AGTTATTTTATAAACTTTTTACAATTTCATGCAATTCTTGTTGTAACTTTGCTGTTCGTGTTTCAATCTCTTT
TGTAATATAATCGATACGCTCGTTTCGTTTTAAATCTTTAGGTAAATCGTTAATATCGATTGGTTTACCAATA
TTTATGTATGCTTGTCCTGTTAAAAGACCGTGAATCTTAGTAGGACCAACATAAGCAACAGGTAATATTGGTG

ACTTACTTAACATTGCAATTGTTGAAGCACCACGTTTCAAAGGTGCACCTTCTTGCGATGTGCGAGAACCTGT
TGGGAAGATACCAACTGTCTTATTATCTTTCAACAAATTGATTGGGCGTTTTAAAGTACTAGGTCCTGGATTT
TCACGATCTACAGGAAATGCATTTAAAGACGTTAAAAATTTACCAATCCATTTATTTTTGAATAATTCTTTTT
TAGCCATATAATGAATTTGATTAGGATATAATGCCATACCTAGCATAATGACTTCGTTATAACTTTCATGCGT
ACAAGTTACGACATATTTACTATCCTTAGGAATATTATCTTTACCGATTACGTATAATGATTTTGACATTTTA
ACTAAAATGAAATTCAAAATCTTACTAATCACTGAATACATTGTGCCACCTACTTAACTTATTTGTTTATAAC
AACTAGATTTTATCACTATATTTTTACTAGAACAAGATAGTCTGAAAATACATCTAAAGTTGTATAGTGTATT
ACTAGCAATAATTATAAAAAAAAGACATAATTAAAGCATATATAAATCATATAATGGAGGTTAAGTATGTCAG
ATTTTAATCATACAGATCATTCTACAACAAACCATAGCCAAACACCTAGATACAGAAGACCTAAATTTCCATG
GTTTAAAACAGTCATCGTTGCATTGATTGCTGGAATTATTGGTGCACTTCTAGTACTTGGTATAGGCAAAGTA
TTAAATAGTACAATTTTAAATAAAGATGGTTCAACTGTCCAGACAACAAATAATAAAGGTGGCAATCAATTAG
ACGGTCAAAGCAAGAAATTCGGTACCGTTCATGAAATGATAAAATCTGTCTCCCCTACAATTGTTGGAGTTAT
TAACATGCAAAAAGCATCAAGTGTAGACGACTTATTAAAAGGCAAATCATCTAAACCATCTGAAGCTGGAGTA
GGTTCAGGTGTTATCTATCAAATAAACAACAATTCAGCTTATATCGTTACAAACAATCATGTTATTGATGGCG
CAAATGAAATTAGAGTCCAATTACATAATAAAAAACAAGTTAAAGCGAAATTAGTTGGTAAAGATGCAGTAAC
TGATATTGCTGTACTTAAAATTGAAAATACAAAAGGTATTAAAGCGATTCAATTTGCCAACTCTTCAAAAGTA
CAAACTGGCGATAGCGTATTCGCAATGGGTAACCCATTAGGATTACAATTTGCTAACTCTGTAACATCTGGTA
TCATTTCAGCAAGCGAACGTACGATTGACGCTGAGACAACTGGTGGCAATACAAAAGTCAGCGTTCTTCAAAC
AGATGCTGCTATTAACCCAGGTAACTCAGGTGGCGCATTAGTAGATATTAATGGTAATTTAGTTGGTATTAAC
TCAATGAAAATTGCTGCGACACAAGTTGAAGGTATCGGGTTTGCTATTCCAAGTAATGAAGTTAAAGTAACAA
TTGAACAACTTGTAAAACATGGTAAAATTGACCGCCCTTCGATTGGTATTGGTTTAATTAATTTGAAAGATAT
TCCTGAAGAAGAGCGCGAGCAACTTCATACTGATAGAGAAGACGGTATTTATGTCGCCAAAGCTGATAGTGAT
ATTGATCTTAAAAAAGGTGATATTATTACAGAAATTGATGGCAAGAAAATTAAAGATGATGTTGATTTAAGAA
GCTATTTATATGAAAATAAAAAACCTGGTGAATCAGTCACTGTTACCGTTATCCGTGATGGTAAAACAAAAGA
AGTTAAAGTGAAATTAAAACAACAAAAAGAACAACCAAAACGTCAAAGCCGATCAGAACGTCAATCACCTGGC
CAAGGCGATAGAGATTTCTTTAGATAATTTACAGACAGTAAAGTACATTAAGAGCCTACTCATATTCATTCCT
AAGAATGTAATGAGTAGGCTCTAATTTATTTAGCTATGCAATTATATTTATTGATAGTTAACCATGAAGTATT
TTTTCTTACCGCGACGAATAATCGTAAATTCGCCATCAATTTTATCTTCTGGTGCTAAAGCATAATTAACATC
TTGTTGTCTCTCACCATTAATATAAATCGCACCATTGTTAACATCTTCACGTGCTTGTCGTTTAGAAGGAGAA
ATGCCTGTTTCAATAAGGACTTCAACGATATTTGTTGTGTCATTTGATAATGTCACTTGAGGCACATCTTTAA
ATCCATCTTTTAATTCTTTCGCTGATAATGATTTTAAATCACCACTAAATAATGCTTGTGAAATACGGATTGC
ATCATTTAATGCATCTTCACCATGAATAAATTTAGTTACTTCTTCAGCTAATGTTTTTTGAGCTTCACGTAAA
TGCGGTGCTTCATTTTTAGATTGTTCTAAGCGATCAACTTCTTCTTTTCCTAAGAAAGTAAAGTATTTTAAGA
ATTTAATTACATCTTCGTCTGATTGATTAATCCAGAATTGATAAAATTCATAAGGACTTGTTTTTTCAGCATC
TAACCAAACAGCACCTGACTCAGACTTACCAAATTTCTTACCATCTGATTTAGTTACAAGCGGAATAGTTAAA
CCGTATGCGTCTGTTTGACCATACATACGACGCATTAATTCAATACCACTTGTGATATTACCCCATTGATCTG
ATCCACCTACTTGAATCTTACAATTCAATTCTCTATTCAAATGACCGAAATCAATAGCTTGTAAAATCGTGTA
TGTGAATTCTGTATATGAAATACCATGTTCTAAACGACTTTGGATTGAATCTTTACCTAACATGTAATTAACG
CCGACGTGTTTACCATAGTCACGTAAAAAGCTAATTAATGAGATTTGTCCTAACCAGTCTCTATTATTAACAA
GCACTGCACCATGGTCTGTTCCAAATTCAAAAATATTGTGCATTTGCTTACTAATACCTTCGATATTTTTATC
TACTTGTTCTTCTGTTTGTAGCACACGTTCTTCTGATTTACCTGATGGATCACCAATCATACCTGTACCACCG
CCAATTAAAACGATAGGACGATGTCCATGTTCTTGAAATCTTCTTAATGTTAAAAATGGTAATAAGTGACCAA
TGTGTAAGCTATCTGCTGTTGGATCGGCACCACAGTATAACGTCACTTGTTCTTTATTTAATAAATCTTCAAT
ACCTTGTTCATCAGTTTGTTGATAAATAAGACCTCTCCATTTTAAATCTTCAATTAATACATTCGTCATTATT
ATTTCCTCCTATTTATAATTATTTATTGAATACTTGTTAAAATACTTTAAAGCTTTTTGAACGTAAAAAAACC
CTTACAACAAATATGTAAGGGCGCGATTGCACGTTACCACCAAACTTAAACATAATCATAAGATAATGTTCAC
TCTATTAATGATACGTTCATTAATAAACGTAGGACATGTTAGTTATAAAGGTGTATTCATATTATTAAAAACA
CTAGTTCACAGCGACCACTAGCTCTCTGATGATTTCAAATAATATTACTTGTCCTTTTATCCTATTCTTTATG
CATTTTCCCTAATTATAATTAACGTTAAAATAAAAGTCAAATTGCTTAAATATGGTATACTATACGTAATTTA
GGAGGTTAAAGATGACGAATCAAGACAACAATCATCAATTGAATCATCGTATATATCATTTTGAAAAGATATA
TAAAGCTATCAAACATGTCATTGTTTTTATATTTATGATTTTCATTGCCATCGTTGCTATCGCTGTGATTGCG
ATGTCTTTATATTTTCATCATTTAACTAAAACGTCCGACTCATTATCAGATGATGCTTTAATAAAAAAAGTTC
GACAAATACCTGGCGATGAATTATTAGATCATAATAACAAAAATTTATTATATGAGTATAACCATTCTCAAAA
CTCACTCATTATAGGCCCTAAAACATCAAGTCCAAATGTCATTAAAGCATTAACGTCATCTGAAGACACTTTA
TTTTATAAACATGATGGCATCTTACCAAAGGCGATTTTAAGAGCAATGATACAAGATATTTTTAATACTGATC
AAAGTTCAGGTGGTAGCACAATTACACAACAACTTGTTAAAAATCAAGTTCTTACCAACGAAAAAACATATAG
TAGAAAAGCAAATGAACTTCGCCTAGCAATTAGATTAGAACACCTACTCTCAAAAGATGAAATTATATATACA
TATTTAAATATAGTTCCCTTCGGTAGAGATTATAATGGCGCTAATATTTCCGGAATTGCATCCGCTTCATATA

GTTTGTTTGGTATTCCACCAAAAGATTTATCAATTGCACAATCTGCATACCTTATCGGTTTGTTGCAAAGCCC
TTATGGCTATACACCCTACGAAAAAGATGGAACGTTAAAATCGGATAAAGATTTGAAATATAGTATTCAAAGA
CAACATTATGTATTAAAGCGTATGTTAATCGAAGATCAAATCACTGAAAAAGAATACAACGACGCATTAAAAT
ATGATATTAAATCACATTTGTTAAATCGAAAAAAGCGTTAATTGATGCTCACTTTTTAAAGTAACCACAACAA
TGAATCCAAATATTAAAAACAGCAGTAAGATTATTTTCAATTAGAAAATTTCTCACTGCTGTTCTTTTTATTT
ACTCTAAAGGAAAGCCATTAAACCAATCAATAGTTTATAAATTAATTGCTTTCACATTGTCTACAAAGTTAAT
CATACATATGCTATATCCAGTTAATTATTTAGATTCTTTTCTGAATTTAGGAATGAATAAAGCTAACATACCT
AATAACGCATATAAGCCCCACCATGATTGGCTTGAAGTTGTTTCTCCAGTTTTTGGTAGCATTTTAGATTGAC
TAAGTTTGTTTTCTTTCGATGGTGTGCCGGCCTTTTCTTTTGTTTTCACTGTTTTATCTACAGTTTTATGAAT
ATCATTCGGCAGATGTTCAGTTGTTTTTTTGTCAGTAACTTTGTCTGTATTATTATAATTTTGTTTCACTACG
TCAAGCTTTGCTGCTTTTCCAGTATGATTATTTTTATCGGCAATATGATTCAGCTGTATGACTTTGTCTTTAT
TTTTATTAGAGTCTTTATCAGTATCGACATTCGATGACATACCAACGCTATTATCGGCATCTTTATCCACATT
TCTATCAGTATCTTTGGCAATTTGAGTATCCATTTCTTTTAAATCATACTTATCGAAGTGATTATTATCCGAC
ATATCGGATAAATGATCAACATCATGTTGCACATCTTTATCAATATTATTATCAGCATCTTTAACCACGTCAG
ACTCTGGTTCTATCACATCTGCTTTATCAGACGCATCTTTAGGATTTGTTGCAGTGCTGCTATTTTCAGCTAC
ATCTGTATCAGCAACCTTACCTTCTTGTCCTGTTTGTACATTTAGCGGTTCACTCGTGTTATTTTGTGATGTA
GCATCATCTTTTGTCTTGATATCCTGATTTATAATTCTGACATGATATTGACCTTCATAACCAATGTTTGCCA
CAACGACTTTAACAATCGCATTGTAAACTGCTTTGTCAGGTATATATGGGAAAATCAGCGTTCTAGAATTATT
TTTAGGATCTTTAGAAACAGTAGTGACACGTTTACCTTCTACAATTAAATCTTTCCAGTAACTGTCATCCTTT
GTTTTCATCACTACATATTTTTGACCATTTAAAGTTGCTGTATAGAATGGATGTTCAACAAAGCCGTCCATAA
CTGACTCACTATTTTCTTCACTTTCAAAAACAACAAAATGCGCTTCTTGTAAATCTGTTAATTGATCATTTGT
AGGTGTAACATTTTCAAATTCCGTCACTGCTGATTTAACCTGATCAGCTAACTCTACTCTAGTTTGATCTAAT
TTCTTTTTATATTCCGCCTTATATTTTTCCGGCAATTTCTCTTGTAATTTTTCTAATTCATAAACTTGTCTTT
CTAACGTTTTAGCTTTGTGATACGGAGCTAATAATTTTTGTAAATTGTATGTTTCCTCATCCACATAGTCGTC
TGGGTTATTAGTAATAGGCTGTGCAAAGACCATTAGCGTATAATCATAGTCTTCATGGATGTTCTCACCATAT
TCAATAGATGACACAATTTTAACTTCTCTCGTACCATTAGATACTGGGAAACGAATATATGCATAATCTTTAT
CAGAATCATATGATACTAATTCGACTGGTAACTTTTTGTCACCTTCATAAACTTCAAATTTCTTCCATGTTGA
AGCTGTCTTTAAACCTAATTCAATTATTGGTCCTGTTTTTGTAAAAATGACAGTTGCTGGTTCAACAGTACTA
GCATAATGATAGAACTGTCTTTCACCTTTATCATTTTTCATTTGAAAATCAATTGGTCGCCAATTATCAGCTG
TATGTTCTTTATCGATGATAGCCGGGTTTTTAATTGCATCTTGTAGTGATTCATCTGCTGGTGGATACTGTTG
ATTAACATCCGACTGATTACTTGACTCATTCGTTTTATCGTTAGTATTACCATTAGAATTTGTTTCATGAGCT
GGTTGACTTGATGCTTGATCTGCATTGGCTGACGATACTGGTTGTGCAGGTTGACTCATATTGGTCGTTGCGT
ACGGTTGATCACTAGTATTGTTCGTCGTTGATGTATTTTGATTAGATGTATTCGTGTTTGTTTGATTACTTGC
GTCTGAACTTGATTGATCATTCGTTACTACTGCATCATTTTTATCTGATTTTACAAGTGAAGGATCGTTATAA
ATAGGTTTAGCAAATACTAATTTAGTATAATCATAATTTGTTTCTTCTCCATCATCAATTTGAGTCGAAGAAA
CAATTTTCAATTCTTGTGTGCCATCTGAAACTGGGAATCGAATATAGGCATGGTCTTCTGGTACAGGACTATA
TGATACAAGTCTCACTGGCAATTTTTGATTGTTTTCATAGACTTCAAACTTTTTCCATGTTGAAGCAGTATTG
ATGTCTAATTCAACTTCTGCTTTCTTTTTAGTGTAATACACATCTGCTGGATCTTTGATGCTGAAAAAGTGAT
AGTACTGCGTTTCATTGTTTTTATCTAATAACTGGAAATTGACTTGTTCTCTTGGACCTATATCATGTTCTTT
ATTTTCTAATGCAGGATCTTTAATTGCATCTTTAAGTGATTCATCCGCTGCAGGATAGTTTTTCGCAGTGTTT
GCTGGTTGCGTAGCAGGTTGTGTTTGATTTGTATCCTTTGGTGGCTGAGTTGTAGTTGCATTATTATTTTGAT
TTTCCGAGATTTTATCTGAAGTATTTGTATTTTCTGCTGCTTGCGCTTGATGTTGAGAAGTAATTAAAAATAG
TGTACTGACAATGACCGATGCAACGCCTAGAGTTGATTTCTAATAGAATAGAAAGACCTTAATTTTGGGTGA
TGTTTGTTCATGTTGTTAACAACTCCTATAAGATTATATTTAATTGATAATAATTATCAATATCAATTATTAG
TGTTAATTGTAGCGCATCCCAATTAAATATTGCAACCTATTTTATTAAAATTTTTTAATATGAATATTCATTG
CATCGCTTTCCTATTCTTCAAGCCCACTTTTTATCTTCATATACATTAATTAACCACCTTCAAACATTGTCGT
TAGATTCGCCAAATGGAATCTATTTTTAGCACAGCAAAAAACCGAACTGCTGAAATAATGCATCTCCAACAGT
CCGGTCTATTAAACTATTTACTTATCTTTATTAAACAATTGACATGATTTATTAGAATAACCCAATTGCATGA
CCATCATCAGTAACATCCATGTTTAATGCTGCTGGTTTTTTAGGTAAACCAGGCATAGTCATGATTGCACCTG
TCAACGCTACGATAAATCCTGCACCTGTTTTCGCTTCTAATTCACGAATTGTAATTTCAAATCCTGATGGTGC
ACCTAACAACGTTTGATCATCTGAGAATGAATATTGTGTTTTCGCCATACATACTGGGTAATTATCCCAACCA
TTTTCTTTAAATTGTTTTAATTGTTTATGCGCTTTACTGCTAAACGTTACTTTTGAACCGCCATAGATTTCAG
TCACAATCTTTTCAATCTTTTGCTCTAATGGTAATTCTAATTCATATAAAGGTTTAAATGAATTAGGTTGATC
AATGACTTCTAATACTTCATTTGCTAAGTCAACGCCACCTTTACCACCTTTTTCCCAAACTTCAGTTAAGGCA
ATTCGTACGTTATTTTCTTTAGCCCAAGATTTTACATATTCTACTTCTGCATCGGTATCATGTATAAATGCAT
TAATTGCAACAACCGGTTCTACACCGAATTTTTTAATATTATTAACATGACGCTCTAAATTAACAATTCCTGC
TTTTACTGCTTCTACATTTTCTTCTTTTAAATTATCTTTCGCTACACCACCATGCATTTTTAATGCACGAATT
GTCGCAACAACAACGACAGCTGCCGGATCAAATCCTGCTTCACGCGCTTTAATATCCATGAATTTTTCAGCGC

CTAAGTCTGAACCAAATCCAGCTTCCGTTACAACGATATCAGCTAAATCACGTGCTGTTTCAGTTGCTAAAAT
TGAGTTACAACCGTGTGCGATATTCGCAAATGGTCCACCATGAACTAATGCTGGTGTCCCTTCAATTGATTGT
ACTAAGTTTGGTTTAATTGCATCTTTTAAAATCATTGCAAGTGCACCTTGCACTTTTAAATCTGCAACTGTAA
CTGGCTTGCGATTTCTAGTGTAACCAATAGTAATACGACTAATTTTATCTTTTAAGTCTTTAATACTTCTACT
TAAACATAAAATCGCCATAATTTCAGACGCTACTGTAATATTAAAGCCATCTTCACGTGGTACACCATTTGTA
GGTCCACCTAACCCAACGTTTACATGTCTAAGTGCACGATCATTCATATCTAATACACGTTTCCATTCAATAC
GTCTTTGATCGATTCCTAATTCATTACCTTGGTGAATATGATTATCGATAAACGCAGACAATGCATTATTTGC
AGTTGTAATCGCATGGAAATCTCCGTTGAAATGTAAGTTGATATCTTCCATAGGTAAGACTTGCGCATAACCA
CCACCAGTCGCACCACCTTTGATACCAAATGTTGGTCCTAAAGCAGGCTCTCTTAATGCAACCATAACGTTTT
TATTTAACTCATGGAATGCATCAGCTAAACCAACTGTAACCGTTGATTTACCTTCACCAGCTGGTGTTGGGCT
CATCGCAGTTACTAAAACAACTCTCCCTTTGTTTCTCTTGGCGTAATTTTATTAATGTCGATTTTAGCTTTG
TAATGACCATAAGGTTCTAATGCATCCTCTGAAATACCTACTGATGCAGCAATATCCTTAATTGGTTGTAGTG
TTGATTGATTCGCAATATCTAAATCTGATAAATGAGTCAACTTTTTTCGCCCCTTTTTGTTTAATTTATGCAT
TTATAATATGTAAGTTGTAGCTAAGAAATTTTTACTTACATTCATATAATAACGAACTGTTAAAAACTTGTCG
ACTAAATCGGGTTAGCGCTTTCATTTTTGCAATAAATTCACTTATTAAAGTTTTGTATTATACAACTTTTAGC
GCTTGATACTATTGATAACTATATTTTTCTATTATTTAAAATAGTCATCTTCCAAAGATCATATTAAAAATAA
GAAGCAATATAAAGATATGCCATTATTGTTATGTCATCATTTTCAAATGCATTCTACGAAATGATATGACACA
AGTTAATAGCTCTTTATATTGCTTATCATAAAGTAAACTAATATTTAAACATTGACATGAAATATGTTGTCAC
CCTTTATTCTTAGAAAGAAATCTTCTATAACATTCATGTCATTATTCCATTGTACTTAAATCCCCTGCATCTA
AATTTAATTCCCAAGCTTTTAATACACGTCTCATAATTTTACCTGAACGTGTTTTAGGTAATTTATCTTTAAA
TTCGATTTCACGTGGTGCTGCATGCGCCGACAAACCTTCTTTAACAAATAAACGAATTTCTTCTTTTAATTCG
TCTGTTGGTTCATATCCTTTCCTCAGTGCAACAAACGCCTTAATTATTTCACCGCGAACCGGATCAGGTTTAC
CAATAATTCCTGCTTCGGCAACTGCTTCGTGTTCAACCAATTTAGACTCAACCTCAAATGGTCCAACTCGTTC
GCCAGCTGTCATAATTACATCATCAACACGTCCTTGGAACCAGAAATAACCATCTTCATCTTTATATGCCGAG
TCACCAGATACATACCAGTCTCCAATAAAATACGACTTATATTTTTCTGGATTCTTCCAGATACGATACATCA
TTGATGGCCAGCCTTTTTTTATAGCAAGATTGCCCATTCGATTTGGTGGTAATTCATTACCTGCATCATCGAT
AATTGCAGCTTGAATACCAGGTAATGGTTTGCCCATTGAGCCAAGCTTAACGTCCATCGTTGGATAGTTAACA
ATCATATGTCCACCTGTTTCTGTCATCCACCAAGTATCTAACACCGTTAAACCGTATACTTTTTTCGCCCATT
TTATAACTTCAGGATTTAAAGGCTCACCTACTGACAGAATCGAACGTAACGATGACAAGTCATATTTCTCAAC
AATATCGTCACCAGCACTCATTAACATTCTTAAAGCTGTTGGTGCCGTATACCAAATCGTCACTTTAAAATCT
TCAATCATACTATACCACTGTTCTGGTGAAAAGCGACCACCAGCTATACAATTTGTAGCGCCATTTAACCATG
GTGCAAAAATACCATAAGATGTTCCTGTAACCCAACCTGGATCTGCTGTACACCAATAAACATCATCTTCTTG
TAAATCTAATACATATTTTCCAGAAATATAGTGCACTAACATCGCTTGTTGAACATGCAATACACCTTTAGGT
TGCCCAGTAGAACCTGATGTATAATGTAAAATCAAACCATCATCCGACTTTAACCATTCAATGTCAAATTCAT
CGCTAGCAGTTTCCATCAAACTAATGAAGTCTATGTAATTGTCTTCTACATCCTCATCTACGACAACAATTTT
TTTCAAGTTCGGTAATTTATCTACAGGTACTCGAGGTAACAATGCCTTATTAGTAATTAACACTTTAGCTTCA
CTGTTCTCTAATCTATCCACAACTGCCTTTTCCATAAATGCTTCAAATAACGGACCAACAATTGCACCAATTT
TTAAAACACCTAACAACGCAAAATATAGTTCAGGTGTACGCGACATAAATATAAATACTCTGTCACCTTTGTC
AACTTCTGCATGTTCAGACAAAACATTCGCTGCTTTATTAGATAACCGTTGCATATCCTTATAAGTATACGAT
TCTTTTCTGTGCTCATCTTTGTAATTTAATGCTATTTTATCCCCTATTCCTTGATCTACATGGCGATCTATAC
ATTCATATGCCATGTTCATTTTTCCAGTTTCACTCCAAGAAAATGCTTGTTCTACGTCTTTCCAATTAAAAGT
ATTATATGTTTCTTCATAATCTTTAAGGTTATGTTTGCCTTGCGCTCCTTTATAAACTTCGACTTTCATTTGA
AACTCCCCCTTTGTTATGTGAAAACGCTTTCTTTCTCAATATTATACATAACATTTCTAATTATTCAAAATTT
TTACTTTTATCCTTTTTCTAGTATTAATTATTGTAAATTTTCTATATAATGAATTTAATACAATCACGACGTC
GATTGAGGAGTTGAAAATATGAATCATTTAAAGACGTATCAATCCGAAGATTATTACATTCATGACAAGCAAT
TTGTTATTGAAGGACCTTTAACATACGAAGATTTGAAAGCGCTTACTTTCGATGCGCATTTAACCGCATTTAG
AGATGCTGAAGATCAGTATGAAGCTTTGTTAGAAATTACAACATTACCAGAAGGTAGAATTTATGTTGCTCGC
CAAGATCAACTCATTGTGGGTTATGTCACTTTCCACTATCCTGATGAAATTGAGCGCTGGTCTACAGGTAACC
TTCCATATTTAATCGAATTGGGGGCAATTGAAGTCAGCATCAATTTTAGGCAATTACAACTTGCAGAAAAGCT
GATACAACTTAGCCTTTCTACACCAGAATTCGAGGATTATATCGTTATAACTACAGAATATTACTGGCATTGG
GATTTAAAAAATTCAAAGTTAGATGTATTTGACTATAAAAAATTAATGCAGCGGTTAATGGCAACTGGTGGAC
TTGAAATATTCGCTACAGATGATCCAGAAATAACAAGTCATCCAGCTAATTGTTTAATGGCAAGAATTGGCAA
AAACATTACATTAGAACAGCAACAAGCGTTTGATGATATTCGTTATATGAATCGGTTTTTCTTTTAATTTTTG
GAATATCGGAGGGATTATATGCAACAACATTCATCAAAAACTGCATATGTTTATTCAGATAAGTTATTACAAT
ATCGATTTCATGACCAACATCCCTTCAATCAAATGCGTTTAAAATTAACAACAGAGCTACTTTTGAATGCAAA
TTTATTGTCTCCTGAACAAATAGTACAACCTAGAATTGCAACAGATGACGAATTAATGTTAATTCATAAATAT
GATTACGTCGAAGCTATTAAGCATGCTTCACATGGCATTATCAGTGAAGATGAGGCTAAGAAATATGGATTAA
ATGATGAAGAGAATGGTCAATTTAAGCATATGCACCGCCATAGCGCCACAATTGTTGGAGGCGCTTTAACTTT

AGCAGATCTTATTATGTCAGGCAAAGTATTAAATGGTTGTCACTTAGGTGGTGGATTGCATCACGCTCAACCT
GGTCGAGCAAGTGGTTTTTGTATATACAATGATATTGCAATTACCGCACAATACTTAGCTAAAGAATACAATC
AACGCGTTTTAATCATAGATACCGATGCACATCATGGAGATGGTACACAATGGAGTTTCTATGCCGATAACCA
TGTTACTACTTATTCTATCCATGAAACTGGAAAATTTCTTTTCCCAGGCTCTGGTCACTATACTGAGCGCGGT
GAAGATATCGGCTATGGACACACTGTAAATGTCCCACTTGAACCGTATACAGAAGATGCATCATTTTTGGAGT
GTTTTAAATTAACAGTTGAGCCTGTCATAAAGAGTTTTAAACCTGATATTATTCTAAGCGTAAATGGTGTCGA
TATACATTATCGTGATCCACTAACTCATCTAAATTGTACGTTACATTCATTATATGAAATTCCATATTTTGTA
AAATATTTAGCTGATTCTTATACGAATGGAAAGGTAATTATGTTTGGTGGCGGAGGCTACAATATTTGGAGAG
TCGTACCACGTGCATGGAGTCATGTATTCTTAAGTTTAATTGATCAACCAATTCAAAGTGGTTATTTACCGTT
AGAATGGATTAATAAATGGAAACATTATTCATCTGAATTATTACCTAAAAGATGGGAAGATCGTTTAAATGAT
TATACCTATGTCCCTCGCACAAAAGAAATTAGTGAAAAAAATAAAAAATTAGCTTTACATATAGCGAGTTGGT
ACGAATCTACTCGTCAATAAGTAACCTAATCTGCAACGATTTTAAAAGATAAATAAAAGGCATTCTTCCAACA
CCCATATAATTCGGGTCGTTAGATGAATGCCTAATTTTGTGAATTTATTTTGTAGTTCCTCGGTATTCAATTC
TGTGAGGTAAAACTACATTTGGTTCTTCTATCTTTTCATCATTCATATATTTTGTTAATAAGCGCATCCCTAC
TGCACCGATATCATATAATGGTTGAATAACACTAGAAAGTTGTGGTCTAACCATCTCAACTAAACGTGTATTA
TTGAAACTAATAATTTGTAATTCCTCTGGAACTTTAATACCAGCATCCATTGCACTATGCATAATACCAATAG
CTTCTTCGTCACTAATACATAATATAGCATCTGGAAGATTACCTTTCATTTTGGCAAAAGCTTTTACGCCTTC
TTTATAACTTTCAGCACCAGAACAATTCAATGTATCACCTAATTGAAGACCATTTTTATTTAACACTTCAGTT
AAACCTTCTAAAACATCTTCTTGAGCTTTTTTAGAATGTTCTCCACCTACTAAAGCAAATGATTTAGCGCCTT
TTTCAATTAATTCTCCCGTAATTTCTTTCGCAGCTTCAGTAAAATCAATATTAACTGATGCTATATGTGCATC
CTTACCATTTGTTCCTGATACTACTACAGGTACAGATGATTGATTTATCAATTCTTTCATTTCTTCAGTAATT
GTACCACCAAGGAAAATAATACCATCAACTTGTTTACTTAATAAGTTATTAAAAATTTCTTTTTCCTTTTCAG
GATCGTTATCTGAATTTGAAATAATTGAGTGATATTTATACATTGTTGCAATATCTTCAAGTCCACGAGCAAG
TTGTGAATAATAGATATTAGATATATCTGGAATGATCACACCTACTGTTGTTGTCTTTTTACTAGCTAAACCT
CTAGCAACAGCATTTGGACGATAATTCAAACGCTTAATGACTTCGTTAACTTTATTTTTAGTTTCTGCTTTAA
CATTTTGGTTCCCATTAACAACACGCGACACTGTGGCCATAGAGACACGCGCTTCTCTTGCTACATCATATAT
AGTAACTGTCATAATTTCCTCCTTGTAAACGTTTTATTCATTATAATAACATGTTTTCATTTCATTTTCATAG
TTTACCATATTTTAATTTACAGTAACATATTTTTGTTTGGAAAAATTTAGCGAAAAGCTTGAAATAAAAACAA
ACGCTAAATGATAACGAACGATATATTTTAATTTTATATAAACTTTTTTCATTTTTAATTGTAATATTTTGAG
AAAGCCTTTTCAAATACCAATTGCATTTGTTGAAAAATGTTCACATTTCAGTAACAAATGTGTGAATCTATTA
TATTTACAGATAATTCATAGTTATAATTTTAATTACTGCAATCAGTCATAATCTTTAATAATTGTGAAATTGA
ACAAGTGTTGAAGTATTATAACAATCGTTAAGTGCAGTACACCTTTATTTCTATACAAAAAATAATAGCCACT
CTAAAAGTAATATACTCTTAGAATGGCTACAAGTTTAAAATTGTCGATTCAAAGATAATATTCTACTTCAACA
TGACATATTAGTTAAGTAGTCTATAGTTTCCTTGGAATATTATTTTAACTTTTTAGCGTTATATAAATCAGAC
AAAGGCTTTAATTCATCATAAAATGCTTGGAATTCATCTAAATCCATTTGTTGACCCGCATCACTAAGTGCAA
CAGATGGATCTGGATGCACCTCAGCCATAACGCCATCAGCACCTACTGCTAATGCTGCTTTCGCAGTTGGTAA
CATGATATCTTTACGACCTGTACTATGCGTAACATCTACCATGACTGGTAAGTGCGTACCTTGTTTTAAAATT
GGTACTGCTGAAATATCTAAAGTGTTACGTGTAGCCTTTTCATAAGTTCGGATTCCACGTTCACATAAAATAA
TGTTTTGATTACCTTGTGAAGCAATGTATTCAGCTGCATAAACAAACTCTTCGATTGTAGCAGATAAACCACG
TTTTAATAGAATAGGCTTTTTCGTACGGCCAGCTTCTTTTAATAACTCGAAGTTTTGCATATTACGTGCACCA
ATTTGGAATACGTCTAAATACTCATCAGCCACTTCAAAATCATTTGGATTTACGATTTCACTGACAACATTTA
AATCATATTTATCTTTAATTTGTTTAAGTATTTTAAGACCTTCAACACCTAAACCTTGGAAATCATATGGTGA
TGTACGTGGTTTAAATGCACCGCCACGAATAAATTTTTCACCTTTAGCATGTAAGTTTTTAGCAACAGCTTCA
ACTTGTTCAAATGATTCAACTGAACATGGCCCAAATACAAATGATTTATTGCCGTCTCCAATAATGCCCCCAT
TATCAAATGTTACAATCGTATCTTCAGGTTTCAACTTACGTGATACATATAAATGTTTTTCATTTTCAGATTT
TTGTAAATCTGTAGAGGCTTTGAAAATTTCTTTAAATAATTGCTTAATAGTATTATCGTTGAATGGTCCTTTG
TTACTATCGATTAAGTCGTTAAGCATTTCTTTTTCACGTTGTGGATCATAGATACGTGTACCTTGTTTTAATT
TTTCTTCCCCAATTTTTTGTGCTAGTTCACCACGTTTAGATAATAAGTCTAAAATTTGATGATTCAGTGAGAC
AATCTCACTTCTGTATGATTCTAATTTATTACTCATCCTTACTCACCTCTACGAATTATTTCAAGTATATTCC
ACTTTAAAGCAATAAAATATAATGTTAAAAAATATTCTAACAATATCATATGTATAGTTCAACCCTAAATTGA
CAGAAAATAATTAATTTTCTAACTATATGATGCTTGACTGACATTTCGATTTTCTCAGTCATTGTTTGAAAGT
TATATTCTATATTGATATTTATTAGCACATGTTAATCAATTAAATAAGTGTCATGATTATAGCTTATTTTGAG
TCAACATTACTGCTTTTAAGAAAAATTTTGGTTTCTATCTTCTATTATAAATCTAAAATTCTCATACATTCAA
CCTAACGCTTAATAAAATTTAACAACCTTTGTATATTAATATAAATCTATAGTGAACATCACTCACCAACATA
TTCAGCTATATTTGAATTAGTGAACGTCATTGTAATTACTAAGATTCATTGTTTATTTGATTATCGAATATTT
TTCCATCTTCAGCTTAAAATGTGCAATATCACTATTGTAATCTAACGATGTTACAAACAAAATAAATACATGG
AATGATCGCGTTGCCTTTCAAAATAAACTGATGATATCAAAAAAGGTAAGCACCTGAATGCCTACCTTTTATT
TTGAAATTCAATTTTTATAAATATAATACATAGCTTACTGCTATGAATGATTATTAAACGATTTTAGTATGCT

TAGTCATTAAATGTACGCTTCTCTATTTTACTTTTAGCTTTTTCAACTTTTGCATTTGATTTTTTTGATGCAT
TTGATGATTTAGCATTTGTTGCTTTTGAATTCTTATTACTAGTTTGATTATTCTTTTGAGTTGACTTACTTGA
ACTACTTTGTTTCTTAGCACCTTGTGATGATTTCTTATTATGTTGCTTTTTCTGACCTGAAGTTTTATTTGTA
GATGCTTTTGATGCATTTCGTTTATTTGAAGGTGTTGTTTTTTTAGATTGTTTACCTGATTTCTTGTCAACAT
CTGTATTATTAGTTGTTTTTTCATCAGCTTTACGTGTAATAACACCATTTTCAAATTTAGCTTCTTTTTGTTT
TACAGTGTCTTTATTATCTGTATTAACTGTTTCTGATACATCATTCTTTTTAGTAATTAATTGAGGAATTTCT
TTCAAGTCATTTTTAGCAACTGGTTTTTCTGCAAATAACGCTTCAGTTAATTGACTCTCTTTTGAACCTGGTG
TTAATTTAGCTTGCTTCTGTTTTGCAGCGTTTGCTAATCTTTCAGCTTGTGACTCTTCTTTCGATACCTTTAA
TGCCACTGCTTTTGATTCTTCATTTGATACAGCTTCACTTTTATCTGTTTCTGCTTGTGCTTCTTTTTTAGCT
TCTTGAATTTCTTGTGCCTCTTGTGATGTATCACTCAAATTATTTGCACTTGCTTCTTCTTTTATTGCTGCTT
GTTGTGCTTTTAATGCCACTGCTTTTGATTCTTCATTTGATACAGCTGCACTTTTATCTGTTTCTGCTTGTGC
TTCTTTTTTAGCTTCTTGAATCTCTTGTGCCTCTTGTGATGTATCACTTAAATTATTTACACTTGCTTCTTCT
TTTATTGCTGCTTGTTGTGCTTTTAATGCCACTGCTTTTGATTCTTTATTTGATACTTCTGCACTTTTATCCG
TTTCTGCTTGTGCTTCTTTTTTAGCTTCTTGAATCTCTTGTGCCTCTTGTGATGTATCACTTAAATTATTTGC
ACTTGCTTCTTCTTTTATTGCTGCTTGTTGTGCTTTTAATGCCGCTTGCTCATTTTTAGATTTGTTTAAAAAT
CCTTCAACACGTTCTTTTGTAAAGGCAACCGTTTCTTCAAGTTGCGTTTTTCTTTCTTCAAACTTTTGCGACA
GTTCTTGTTCTTTGACTTTTAAATCATCTGCTTTTTGATAAACTTTATTTTTAAAATACAAGCCTAAAGCTGA
ACCAACAAGCGCGCCAGTTATAAAACTAACAACAAAATCTTTACGGTTCGGTAGTAGTTCATTATGATAAGTG
TGTTTATTTTTTAATGTTCTTTTATTATTTTGTTGCTGCGTCATTTGTATATCCTCCTATAACAAATGCACTT
AAATAAGTAACAACCTCATTTAAGTGCATTTGTTTAATTATTAATTATTTATCTACTCGAGAAGTATAGCTAT
GATTTGCATCAGTTGCTACATTATTAGCTTTGTAATTTGCACTTCCACGACGGTAGTGTCTATTTTGCCATTT
GTCTGCAATTTCCATTGCAACATTTGACCATTGAACAACTTGTGAGATTTTATCTTCATTTTGAGAAATATTA
TGTGTAATTGAATTTGTTACACGATCTACAGAGCTGTTTAACGTTTGTACTGAGTCACCGATACCTTTAACAG
CATCTACAACTGAGTTTAAACGATCTACTTTACCTTGGATATCCTCAGTTAAACGGTTTACTTTATGAAGTAA
ATCTGTTGTTTCACGAGTAATACCTTGAACTTGACCTTCTACACCGTCAAGTGTTTTTGCAACATAATCTAAG
TTTTTCTTAACAGAATTTAATACAGCTACGATACCGATACATAAAATTAAGAATGCAATCGCAGCGATAATTC
CAGCAATTGGTAAAATCCAATCCATTAAAAACGCCTCCTAATTAACATGTAATAATGTCATTAATAATAAATA
CCCATACTACTCTATTATAAACATATTAAAACGCATTTTTCATGCCTAATTTATCTAAATATGCATTTTGTAA
TTTTTGAATATCACCTGCACCCATAAATAAAATAACAGCATTATCAAATTGTTCTAATACATTAATAGAATCT
TCATTAATTAACGATGCACCTTCAATTTTATCAATTAAATCTTGTATCGTTAATGCACCAGTATTTTCTCTAA
TTGATCCAAAAATTTCACATAAGAATACACGATCTGCTTTACTTAAACTTTCTGCAAATTCATTTAAAAATGC
TTGTGTTCTAGAGAAAGTGTGTGGTTGAAATACTGCAACAACTTCTTTATGTGGATATTTTTTTCGTGCCGTT
TCAATTGTAGCACTAATTTCTCTTGGATGGTGTGCATAATCATCTACAATAACTTGATTTGCAATTGTAGTTT
CATTGAAACGACGTTTAACACCACCAAACGTTTCTAATGCTTCTTTAATATTTGTAACATCTAGCTTCTCTAA
ATAACTAATCGCAATTACAGCTAATGCATTCAAAACTGTATGGTCACCATATTGTGGAGACAGGAAGTGATCA
TAAAACTCACCATCCACATACACATCAAAAGCAGTACCTTTATCCGTAATTTGAATATTTTGAGCATAAATGT
CATCCGAATCTTTAAATCCATAGTAATAAATTGGAACATCTGCTTCAATTTTACGTAAATGTTCATCATCACC
CCAAGCAATAATACCTTTTTTAACATTATGTGCCATTTCTTGGAATGCATCAAAAACATCATTAATATCTTTA
AAATAATCAGGATGATCGAAATCAATATTTGTCATAATTGCGTAATCAGGTTTATAACTTAAAAGTGACGTC
TATATTCACATGCCTCAAAAGCGAAATAATCACTTTCAGGCAATCCCATACCTGTGCCATCACCAATTAAAAA
TGAAGTCTTTTTATCACCATTCATAACATGTGATAATAAACCTGTTGTAGAAGTTTTACCATGTGCACCAGTT
ACAGCTACTGAAGTATATTGATCAATAATCTGTCCTAAAAAATCATTATAACTTACAACATCTATTTTCAATT
GATGTGCACGTACTATTTCTTCATGGCTACTCGCGAATGCATTACCTTGTATAACTACCATATCTTCTTTTAT
GTTATTAGCATCAAATGGTAATATTTTTATCCCCTTATTTCTAAGAGCAACTTCTGTAAATACGTAGTTCTCA
ATATCCGATCCTTGAACTTCATGTCCTAAATCATGCATGATTTGTGCTAATGAACTCATGCCAGAACCTTTAA
TTCCGACAAAATGATAGTGTGTCATTATATAAAACTCCTTACTCATAATTATTCTTTATTTAAATCTGCTTCC
GTAACATAAACATCCCTTGGTTTTGAACCATTAGCACTCGAAACATAACCGAGTTGCTCTAATTGATCGATAA
TTCTTGCTGCTCTATTATAGCCAATTTGGAAATGTCTTTGGATTAATGATGTTGAAATATGTCCTTCATTAAC
CATAAATGCACAAACATCATCAAATAATTCATCTTGTGATTGTGTTTGTGTTTTTTTCAACAATTCTTTTTCT
TCAAATAGATAGTCCGGTTCTCTTTGTTGTTTGATAAAATCAACAACATCATCAATTTCGTCATCAGAAACAA
ATGTACCTTGAACTCTAATCGGTTTATTCATACCGCTACCAAGATATAACATATCGCCATATCCTAACAAGCG
TTCTGCTCCACCACTGTCTAATATTGTTCTCGAATCTACACTTGATGATACCATAAATGCAATTCTTGTTGGT
ATGTTGGCTTTAATTAAACCTGTAATTACATTGACAGATGGTCTTTGCGTAGCTACTAACATATGAATACCAC
ATGCTCTCGCTTTTTGAGCAATTCTAGCAATAGACTGCTCAACTTCTTGCGGAGCCATCATCATTAAATCAGC
CAACTCATCAATTACAATGACAATTTTTGGCATTCTTTCATCATATGGTGCTTTTTTGTTAAATGCTGTTATA
TTGCGTACATGGTAATGTGCAAATAACTTATAACGTCGTTCCATTTCTTCTACGGCCCATTTTAAACTCTGTG
TAGCTGCTTTGACATCTGTAATTACCGGTGCAACTAAATGTGGCAAACCATTATAAGGAGCTAATTCAACCAT
TTTTGGATCGATAAGTAATAATCTTAATTCCTCAGGGTGATTTTTATATAGTAAAGACATCAAAATACTATTG

ATACAAACTGATTTCCCTGATCCAGTTGCACCTGCAATTAGTGCGTGTGGCGTTTTAGCAATATCCATAAGTA
ATGGTTCATTATTAATTCTATACCCCATCGCAACTGTTAATTTAGATTCAGCATTTTTAAAACTTGGAGATTC
AATAATAGAACGTAAGTTGACCGTCGTTGGATTTTGGTTCGGAACTTCAATACCAACACGACTAGTTCCTGGA
ATAGGCGCTTCTATACGAATATCTTTCGCTGCCAATGCCATTTTAATGTCATCTTGTAATGCCGTAATTCTTG
AAACTTTAACACCTTTTTCAACTGATAATTCAAATCTTGTAACACTTGGACCTTCAGTTACATCTTGTACTTC
TGCAGGTACATTAAAGTAAAATAATGCGTCATTCAGTTCTTTCTTTTTATCTGTAATCCAGTCCTCGTCCGGC
TCAATAACTTGTGGTTCTTCTAGTAATGAAACACTTGGCAATTTAATATTTGGGCCTTTACGAATCATCGGCT
TAGATGTTTGTACAGCTTGATTTGTTGCAACATCTTGTTGGTTCGTCCGAGGTCTATTTTCATTAGCTGTATC
TTCATTTTGATTTGAAAATGAAGGCTGTAAATCTTTTTGTTGATCATCATTATCTTGTTGTCCACTAGTATTG
TTTGGATGTGTTGTTTCTTCGCTTTCTTCAGTTATGTCGCTTACTTCTGAAACTGAAGACGAAGTTGATTGAT
CAACTTGCTCATTTTCCAATTGATTATCATCTTGGCTTTCTTCAAATATTTCCGTTTGAGTTGAATCAGCAGA
AGGTTTCTGCTCTGAATATTGTTGTGCATTTTGATAATCATTTTCTGTTTCTGCATGACCAATAAGTTGATTG
TTCTCAACATTGTTTGATGTCATATTATTTGTTTTATATGCATTTGTGTTTGACTCTTGTGAATCAGAGCTTG
ATGATGGTGCGGCTTGACTCGCAGGCTTGCTTTCACTCACAGCTTGTTTACTTTGTACAGGCTTTAATTCAGA
CACATTGACTTTTGAATGCTTTTTACGATCCATCATACGCTTTTTATCAGATGGCGTCATGACAACATTAAAT
GGTCTTTTACTTAAAGTTGAAACCTTTTCAGTCTGTTTCTTTGGATTTACGTTTTCAATAGTTTTCTCTTCGA
CATTTTGTTCACTTTCAGCTGCTTCGTTTTGCACATTTTCTGTAATTTCTGCGTTATGCTTTTCTATTTCATT
CATGCTTGAATCTTCCACAACATGACCATCATTCACATCATTCGTTTTCAATGAGTTGACACTTAAGTTTGTT
TGGTCATCTTTTTTATGACGCGTTGCTAATTCAATTTCATTATCAACCGTTTTATCGATATTATTAACTTGAG
ATTCAGTTACTGCGTGCTCTTGCGTTTTAGGAGCGTCTACATGCATTTCAGACGCGTTTCTATTGGTATTATC
TTCGATATTTGAAGTAGTATCCTCATCTGCTTCTGTATCATTTAATTCATAAAATTCTGCATCATCGATTTGA
CTTCCTGAATATTCTTCTTTATTCGTTGCTCCATCTTCTTCTGAATCAGCAATTAATCTGGATTGATTTTTTA
GTTCATCTTTATCATTTACTTCAACGTTATGTTGTAGTGCTGATTGATGTTGAGACGTTACATTCGAAACCGT
AACTTCATCATCTGACAATTGTTTTGTTGTCGATACTTGATTCAAACTAACTTCCTCATAATTGTATGGACTT
TCATCTACATGAGTGTCATTAGTGTCTTCATTTTGTATTGACGCTTGTTTATTTTCATCATTTGATACAGTTT
CATTCTCTATGCCATTTGTATGAAGCTGACTAGCATCTGTACTATTATCTATTAAGTCACTATCATCATTTAA
TGAACTATCACCAACATAGCGTTCCGCTTGTTTAGCATACATTTCATCAATTGCACGTTGTATTGCATCTTGC
TCTTCGTTTTTATGTTCTTCACGCTTTTGTTGCAACGCTTTTTTAAATCGACGCTTTTGAAGCACTTTACGTT
CTCGTTCACGTCTAATTTCTTCAACAATTTGTGAAGCATAAATATTTTCAATTTTGATAGTATTATCAACTTT
TGAATAATTAGGCATTGATTTTGTTGTTGATGATACATTTTCATCAGATGATTTAGATGCAGTTCCTTGTTTG
ACAACACTATCATTTTGTTTTTCTTGTTCTAATTGTTTATTTTGAGACGTTTGCGTCTTAGCTACATATTTAT
CATATTTTTGTTTATCTGACTCAACTTTTTCTGAAGGTTTACTTACAGGGATACGACCATTTTCTAACTTTTT
AGGTTTCATTGTGCCAAAAATAGCTGATGGTACCTCTGAAGTCTTGAAACTTTCTTTGTGATAATCTGGAGTT
GAATCTTTAGCACGATGATTTGTATTATTAGAATACATATTATGTGTTTTTGACTTGTGCGTTTCCTTCTCAA
TACCATTAATTGCAGAAACATATGTACCTGGCTTATTCGTATGGTAATGTGAATGATCTTTATATTTTATACT
TTGCTGTGATATTTTAGAATTCCCACGTTGTTCACTATAATTTTGTTCTTCAGTTGTTTGATTTCTTCTACGG
CGATGTCGTTTTTGTGAACGAGAATCGTGGCTTTGTTTGCGATAGTCTCGATGGTATTGTTCTTTTTCATCTG
AAATAGTATCTGCAGATTGTTCAACATTTTCATTTTCATAAGCTACGCTCATAGGAAAACGGAATTTTCCCCT
CGGACGACTATAAATATCATTATTTTGAGGCAGTAATGAGTCATGATCGTTATCTATATTTTGTGATTCTTGA
CGTCTTTTTTTCTTTCTATGAATCAAGTCATCATTTGAATCATTATCTTCGCCGAATAATTTATCAAACCAGC
TCATATTTTCACTACCTTCCTTAATTATTCAAAAAATGCTTGTCCAATTTCATAGCTGTCATTTAATACCATA
ATACCTTTTTCTTCAGGTGCATTAGGTAAATTCAATTCTTTCATTGAACAAATCATACCGCTTGAGGCAACAC
CACGTAATTCAGCATCTTTAATTACCATACCGCTAGGCATCACTGCACCTACTTTAGCAACAACAACTTTCTG
TCCAGCTTCAACGTTAGGCGCGCCACATACAATTTGTAATGTGTCATTTCCAACGTTTACATTTAGTACACTT
AATTTATCTGCATCAGGATGTTTGTCTTTAGTTTCAACGTAGCCAACTACAAATTTCGGTGATAGATCAGCAT
TTAATTTATAATCAAAACCAGCTTCTGAAATACGCTTTTGGAATACATTTACAAGTTCATCAGTTAATTTAAT
ATGACCTTTTTCTTCAATTGTTATATCTTTTGAAATTTCAAAAATATTATAACCTACAACATTACCTTCATTA
GTAATTTCAACAACATTACCTTTTTTATTGTAGTTTAATTCACCTTCAACTGGTTCAATTTGTAAAAATGCGA
CATCTCCTACATATTTAGGATTGTAAAATAAATTCATTATGACAAAACTCCTTATCCAATTATTTCTATTTAT
CTTTATTAAATTTACGACGATTTGCTTCTAAACGCTGAATCACGTTTGGATCTCTTTTTTGTTTATTATTTTT
ACCTAAAATAAATATCGGTTCAAGATGACCCTGTTTATATCCAAAGGATAATGATGTAATTGGAACTAGACCT
TTAGTGAAAAATTCCATTGTTAAATGTGCCATCACATCATATCCTGTTTTATTGCGTATATCTGCAATAATTA
ACACATCTTGGTGTGGCACTGCTACGAGCATTTCGCCTTGACATTGTGCCTCAATTTCATTTAAAAATGCAGT
ATTTAGTATCCTACTTGCATCATACCCGTCATTTGAGTTAATAAAATAAAAAATATTACCTTTTACTTCATCA
GTCGTATATGAATTTGACAATTTTCTAACATTAAACAGAGACATTTCTCTTATTTGTTGTTCAGTTAACTTCA
AATCCTCTAACATGCTTTCGTCAATTAGACGATATGATTTCCCTAAATCGACTGCATAATAAACTGCTGTTTC
TGCAGTATGCTCATCATAGATAAAAGGAACACCTTGTTTAGTTTTTTTATCAAAGCTAGTCGCTCTAATGACA
GGCATAATTTGACTAGATGATATACTCTCAAGGGTTTTATCTGCCATTTGTGCAATAGCTTCATCAACGTAAT

AAACAATTTCATCTACAATTTTTTCTTTTTTATCTTCATATTTTGCGACTATAGCGTTAAGTTTAATCGTGAT
ACCTTTGTTATTATCTGTTCGATAAATACGCAAAGTTTCTTCTTCACGATTAAATTTAAAATCAACGTCTAAA
TGGCTTAAACGTTCCTTTAATTTATCTCTCATTTGAAAGGTATTCATATGTAACACTCCTAATTTCGCACCTT
ATAATAGTTTACAATAAATCAACACATAGTAACAGAGAAAAACGAGTATTACCCCGTTTTTCTCTAGTCTAAA
TTACACGTATTGAGCTAAAAATGCATCTATCTGTTCAATTGATTTTCGTTCTTTTCCAATATAACTTCCAAGC
AGTTCACCATTTTTATATACTAGAAAACTTGGAATCCCCATAATACCATTTTCAATACAAATATCCATAAATT
TGTCACGGTCTACTGATACGAAGTCAAACATAGGATATCTCGCTTCTAATTCCGGTAAATCTGGTTCTATCAC
TCTACAATCTGGACACCAGCCTGCAGTGAATTCAAACACTGTAGCACCTTGTTTTAAAGATTCAAATTGTTGT
TCTGATTCAAGTTGTTTCATTTTTATGTCTCCTTAATTAATGATTATATATAACTATTTAGAGATAATAGTTG
TTATTACCCGATTATTTATATTGTAATGTTTCTATTTGATTATTATCTAAATTACAAATGGCTTCTGAAAGTA
AAGATCTAGCTGCAAAATAGTCTCTTATATCAAATACTGAGTCTGTACTATGAATATATCGTGCACATACACC
AATAACTGCAGTCGGAATACCAATATTAGCTTTATGAATTTCTCCACCATCTGTTCCACCTGGTGACATATAG
TATTGATGTTCAATGTCATGTGCTTCTACTAACTTTAATAAATAATCTCTAAATACAGGCTTTAAAATCATTG
TACCGTCTTTTATGCGAATTAACGTCCCTTTACCAAGTTCACCAGATAATGGTTGGTTTCCTTTAACGTCATT
GGCAGGTGAACAATCGACTACAAATGCAACGTCTGGGTCTATCATCTCTGCAGATGCTTTCGCACCTCGTAAT
CCAACCTCTTCTTGAACATTTGCGCCAACATACAAGTCTACATCTAATTCTATATCTTTTAATAATTCTAGTA
TTTCAATTGCCAAGACACAACCATAACGATTATCCCATGCTTTAGCACTATATCGATGTTCAGATAACTGTGT
GAATGGCGTGTGAGGTACAATTGTATCTCCTATATCTATTCCGCGCTCACGCACCTCATCTTCATTTTGAGCA
CCTATATCTAATGTTAAATCTTTAATTTCCGGTGCACCTTCACTACCAGTACGAAAATGTTTAGGTATATTAG
AAACAACACCGATAATTTTATCGCCATTTCTATTTTTAATTACTAAGCGTTGTCCTTGCCAAATATCATTTGC
AACACCACCTAAATTTGTGAATTGAATCATTCCATTTTTAGTGATATTTGTAATCATAAATCCGATTTCATCC
ATATGTGCTGCAATCATTACACGTTTTGCATTTGGATTTTTAGATTTTTTCACACCAAAAAATCCACCCATAC
GATTTTCAATAAATTCATCTACGTACGGCGCCATTTGCTGAGTCATATAATTTTTTACTTCTTCTTCAAAACC
TGGTGCCCCATGAAGCTCAGTTAAAGTTTGAATTCGTTGTAATGTTACTTTTTTATTTATGTTCATAAAAGTT
CTCACTCCTTATAAGTTATTATATCATCTTCAATATGGTAAACTAATGATATGAATTTGAAATCAAGGGAGCT
TAAATGATGACTAAACTGAAATATATAATTCCAACAATAATTGCAGTAGCCATTGTAATTATTTCTACCATTT
CAATCATCCAATTTATTAATCGTAAACGCTATAATCCCGTTAAAGTACTTAATGAAGTAAAATCATATTTTAT
GAATGTCAAAGGCTCATATATCGTTTATGAACCATTCGTTCATCCTGAAACTGATAAATACCGTTTAGTTTAT
CAAGGTGGAATTACAACTATTAAAAATGGTCAAAATATTCATTATGATTTTTATGCAGATGCATATACTGGTG
AAGTCATTAACATTGTAGAGCGTTAATACGAATTTTATCCATATTTGATTTGGATCGTAAAACTTAAAAATGA
GCCACACCCTAATTAGTAAGATTGGATGTGGCTCATTTTTTTATTTTTGAAAATTTGTTTAGTAATACATCTT
TATTTTAGTTATTATCAACTAATGTTTCACGTAAAATATATGCATCTATGTTTTCACCATCATCGCTGTATTT
TACAGCAAAGTAGTTTTCATCATGATAAAACAAGAACCAATATTGTTGCTGAATAAAATATGGTATCATGCGT
TCTTTTTCACGAATCGATTGCATAGGATAATCATCATATGCCGTTACCCATAGAGGATTTTTATGTGCAGTAG
TTGGGAATATATCACCCATATGAACTGCTTTATCTCCTTGACTTTCAATCGTAATAATCGTGTGGCCAAAGCT
ATGACCTCCACTATGTTGCATCTTGATACCCGGAACCGGTTCAAAATGTTTTTCGAATAAAATCAACTTGTTA
CTATAATCGCCTTTATTCTTATCCCAGTAAGTTGATTTACTTCTTATATTAGGTGCAATAAACTCATGCCACT
CATCTTGTTGCACAACATGAATCGCATTTTCAAAAATTGCATGTCCCGCTTGATCAGTCAAACCGGCAGCATG
ATCAAAATGCATATGTGTCATTAGCACATAATCAATATCCTTTGGCGTTAAATTATAATTTGCCAAATCAGCA
ATTATATGACTTTCTTCATCTACTCCAAAATTACGTAATTGCTTTTCAGATAATTTACCATTACCAATACCCG
CATCTATAATCAAATTATATTGAGCCGTTTGAATCAAAATTGGATGTGTCGGTAAATTGATTTGATTTCGTTC
ATTTGCATTGTATTGCTTTGACCACAACGGCTTCGGAACAACACCAAACATTGCACCGCCATCCATTTTTGTA
TTGCCACCATTTAGATAATGAATAGATATATCCCCGATCTTCATAACATCACCTATTCTTTCTGTATTCGTTA
TTACAATTAGCATGTGTTTATTACATCATATCACTATTCATAAATGCGAACAAAAACGAAACGCCCCACACTA
ATACACTTCCAACTACTTATAAAGATTAATGCAATCATATTATATGTAAATACATCATAACCATCGGACTGTA
ATTTTAAATTAATAATTAAAATTTCCAAGTAAAATGCAAGCTTTATTCTTTTAAATCAAAGGTATTTAGTATA
ACATTGTTCATAGGCGCAATTGTCTCCTTTAGTTTAGGTTTGGTCACTTTAAATTACAGAGGCAATTGCGCTT
TTTTATATTCAAAATCAAAAAATCGGACAGATGAAAAGTAAAATACTTTTTCTATCAGTCCGATTTATTATAG
AACCATTTTACTATAGAACCATTATAGTACGAAAAAACACTTATGATTGTATGTGTACATACCCTCATAAGTG
CTTTATTTTTGTGAATGGAATTTCGCTTCCATACGATAAATACGTGACCCTTTATTCGAAAATTTCTTTTCAT
ATTCTGTTAGAATATTACTGCCATCGTCTTCTTGATGTAAATTTAGATTTATTTTTGTAAAATACATTCCAAA
TTGAGACATACTTTCTAAACTGTAGGCAAATAGTCCTCTGTTATCAGTTTTAAAATGTAAATCTCCTTCATCA
TTTAAGATTTGTTGATACAACGCTAAAAACGTATGATACGTTAAACGTCGTTTTGCATGACGATTTTTTGGCC
ATGGATCTGAAAAGTTCAAATAAATACGCGAAACTTCGCCGTCTTTAAAATATTCATTTAATTCAATGGCATC
ATTACAAATAATCTTTAAATTTGTTAAACTCATCTCTTTAACTTTATCCAATACTTTATAAACGATACTTTTC
TCACGTTCCATTGAAATATAGTTAATATGAGGATTTTGAGCAGCTAATGTTGTAATAAACTGCCCCATACCCG
AACCAATTTCAATGTGTATCGGTTGCGTTTTATCAAACCATTCAGTCATTTTCCCTGCATGTTGACCGTCCAT
GTCAACCAATTCAGGATGATCTTTTAAATAATCTTCAGCCCATGGTTTGTATCGAACTCTCATATTTTATTCT

CCTCTTAAATAAACATGTTACTATTCATAACTTCATTTAGGAATTTAAGCCAAGTGTTCATATCCTTATATCT
TTTTTGCTCTTCATACCATTGCACAAGACCTATAGATTGAATTACCGTATACCATTTCATACGTTTATTTAAA
TTCAAGCTCTCTTGAACACCATATGTTTCAAGCCATTCAGACCATTGTTGTTGTGGAACATAGTTGTAAAGCA
GCATTCCGATATCAATTGCCGGGTCTGCGATCATTGCACCTTCCCAATCGACTAAAAATAGTTCATCTCGATC
AGATAATAACCAATTATTATGATTCACATCGCCATGTACAACAGTGAAAAAACGTGAATCTAAACTCGGTATA
TGCTCTTCTAAATAGGTTAATGATTTTCTCACAATATGATGTGTTAAAACTTCTCTTGATAAAGAGGCATTAA
TTTTATTAAGCATAATCTCAGGAGTAATAGGTTCCATTTCCATACGCTTTAACATACTTAATAAAGGTCTAGA
ATTGTGTATCTTCTTTAATAAATGTGCAACTCTTGTTTGCTTCATTTCGTTTGAAGATAGTTCACGGCCATTT
TTCCAATGTTGTGCTGTAACAACCTCGCCTGTTTCTATGCGCTTCGTCCATACTAATTTGGGCACAATACCTT
CTGCTGATAATGCCGCAATAAATGGATTTGAATTTCGTTTTAAAAACAACTTTTGTCCATCTTGTTCAGCCAT
ATATGCTTCACCAGATGCACCACCTGCTGAATCAAGTGTCCACCCTAATTGATAAAACTGCTCCAACTCGTCC
ACCTCACTTTCAATTAGAAAATGGCTCTAGAAATAGGTTTTTCAAGAGCCATATATTCTAATTTATAACACCA
TACTGGTACAAATTTTATGACCAGATAATTATTTTAAATCCTCAACCAATGCCTACATTACACGACTAAATTT
AAATCGTATTGTCTTTCATTGACACCATACATTCTATAGTCACTTACTTGACATAAAATGTTACCGTGTCTAA
AACTACATGTTTTTGTAACTTTTGTAGGCGATAAACTCTAGTTTTCAAAATAATTGCTATCCCATTTTCATGG
TTAGCATAAATTTATGAACTGTAACATTTACGTACTTAGTAAAATATGATGCACATCATATTTGTAACTCATA
GAAAATTTTATAATTTTTATCATTATATTTCAACTGAAAATGAGAAACAAAATGGCACTTTTTACTAATATGT
GTTTTCTAAACAACACTTTTAAGCTTCGTTTTAAATTATAACATAATTCACTTACGAAAGTTGATAAATTTAA
GTAATTTAATCTAAAAATATGATGAAAGAATTTTAAATACTGTGTGACTCTATATACTTTTCAAATCCTTCTT
GTAGTTGACGTGTAATTGGGCCAACTTTACCATCATTAACTGGTTCACCATCTAATTTAATAACAGGTGTAAC
CTCAGCTGAAGTACTTGAAACAATAACTTCATCTGCATTTCTCAAGAAATCTACAGTAAACGTTTCTTCTTTA
AATGGGATGTTATAGTCTTCGGCAATTTTTTTAATTACAATTCGTGTAATACCATTAAGAATATAGTTGTTAA
TCGGATGTGTATAAATCACACCGTCTTTAATTGCATAAGCATTACTTGAAGATCCTTCAGTGACAGTTTCGCC
TCGATGTTGGATTGCTTCAACTGCATTATATTTCACAGCATATTCTTTCGCTAAAACATTACCTAATAAATTT
AAGCTTTTAATATCGCAACGTAACCAACGGATATCTTCAACGGTAACACCATTCACACCATTTTCTAAATGAT
CATAAGGACGATCATAACTCTTTGTATAAGCAACAATTGCTGGTTCTACTTCAGGTGTCGGGAAGCTATGATT
TCTTTCAGCTACACCACGCGTTGCTTGAATATAAATTGCCCCAGTTTCAATTTGATTCATATCAACTAATTTA
CGAGATAGTTCAATTAATTCTTCTACAGAATAATTTAAATCTAAACCAATCTCATTGGCACTACGTAAAAATC
TTTCATAATGTTCTGTTACTGTAAATAACTTACCATTATATACTCGAATGTATTCATAAATACCATCGCCAAA
TACGTATCCTCTGTCGTTGTATGAAACCTTTGCTTCACTTGGACTTACAAACTCACCATTTAAAAAAATTTTT
TCCATATATTATTCCTCCACACATAATGAATAAATTGCTTCTAAATAAATACTAGTTGCATTAAATAACTGTT
TTTTAGTGATATATTCATTTTTTTGATGCATTAAATCTTCAGAATCACTAAACATTGCGCCAAATGCTACACC
CTTGTCTAAGTTTCTCGCATAAGTACCGCCACCTATAGTATAAGGTTCAGTCATATCATTTGTTTGATTTCTA
TATGCAGTAACTAACTTTTGTACAAAAGGATCATTTTTATCAACATAATGTGGTGGTTGGACTTTACCTAATT
TCACTTCAAAGCCATATTGTTGAATCTCATTTGCAAAACGATCCATAGCTTTTTCAAATTCAAATCCTTCGGG
ATATCTTAAATTGATACCGAAAAGACCTGCATTTTCATTATCATATGTAATAACACCAATGTTAGTTGTCACA
TCACCCATGACATCTGTATGGAATTTCATTCCCATCTTTTCACCAAAATCTGAATTAAATAAGTAGCGATTAC
TAAATGCTACAAACGCTTGTGCATTATTATCAAGATTTAATGATGCTAAGAATTTTAGTAAGTAAAGACCCGC
ATTCACACCGATAGATGGATCCATACCATGAACCGCTTTACCTTCAACTGTTAAAACTAGAATGCCACTATCA
ACAGTACTATCACCTTGTAAATGATTTTGTTCTAAAAAGTACTCAAAGTCTTGAATAACATCTGTCATATTTT
CTTTAACAAGCACTCTTGCTTCTGCATGATCAGGTACCATGTTGTAACGTTCACCAGATTTGAAAGTTATTAA
TTCATAATCAGGTTCATCTTGATCTTCAGTAAGTTTATTTTGAACTAAATCAAATGTTGTAATGCCTTTTTCA
CCATGAATACACGGAAATTCTGCATCTGGTGCAAAACCTAATGTTGGCATTTCTTCTGTTTTAAAATAGCGAT
CCGTACATTTCCAATCAGATTCTTCATCCGTACCAATAATCATATGAATACGTTTCTTCCAATCCACATTCAT
ATCTTCTAATATCTTAATTGCATAATAAGCAGCAATTGTTGGACCTTTGTCATCAAGTGTACCTCTAGCTATG
ATAGCATCTTCTGTTACAACCGGCTCAAACGGATTACTATCCCATCCATCACCAGCAGGAACAACGTCAACAT
GACATAAGATACCTAATACGTCATTTCCTTTACCTGCCTCAATTCTTCCTGCAATATGATCCACATCATGTGT
TGTAAATCCATCTCTATGTGCAATTTCATACATGTAGTCTAATGCCTTACGAGGACCTGGACCAACTGGTGCG
TCTTCTGATGCTTTCGCATCATCTCTCACACTTTCAATTGCTAATAATCCTTTTAAGTCATTAATGATTTGAT
CTTCGTATTGTTGAACTTTTTCTTTCCACATTCGAAATCGACTTCCTTTTTTCTATAAGTTAAATTCTATTTT
ACATGAAATGATATAAAAACTACAATAAGATGTCAGAAAATAATAAAAAGGAACAAAACGATGCTATCGATAT
GACACAAATCATTAATAGCTGCTTTGTTCCTTTTTTAATTTATATATTTAATATGCCAGTTCAATACCAACTT
ATTAATTGTATGCCTTATTCTTTATCAGATAATGCATTTTTATTCTTTTTTAAGTCTTCTTCAGTGACGATAC
GTAAATTATTATTTGGTGCTACGCCACCTTCATCATCAAATTTACCTTTTTCAATACTTTCGTCAGTCTTATT
GTCATATTCGGTAAATTTTGATTTTTCTTCTTCGAAAAATGCTTTTGGATTATTTTTTAATCTATTAGCATAT
TCTTTCGGATTTGTTTTTACTTCTTTAATTGTTTCATTAGCAATTGTTCCTAATTGCGTCGCTTTATCCTTAG
CATTATCTTTATAGCTTTGAGGATCTTGTTTATATTTATTATATTCTGCTTTCAGCTTGTCACGACTATCTTT
ACGTGTAACAAGTACAGCTGCTACAGCGCCACCTATACCTAAAATCGCTTTAAATAAATTACCTTTTGCCATA

TCAATCGTCTCCCTTTTATTTATAATTTAATTTGTCAAAATCATTTTCAGTTAATAAACGATATTCTCCTGAA
TCTAGATTGCTGTCCAATTCTAAATCAGCAATTTTGATACGTCTTAAATGTAATACCTCATTTTGAATGCTAT
GAAACATTCGTTTAACTTGATGATATTTTCCTTCATAAATTGTTACGTGTGACGTTTGATTATCAATATAAGT
TAAAATTGCAGGCTTAACCTTGCCATCAGACAGTGTTATACCCTCTTTAAAAGCTTGAATGTCGTCTTCAGTG
ATAGGATTTGCTGAAATAACTTCATATTTTTTAGAAACATGTTTGTTTGGACTCATTAATTCATGATTAAAAT
CACCATCATTCGTTATCAATAATAGCCCTTCTGTATCTTTATCAAGACGACCAACCGGAAAAATATTTAGATG
TTGGTATTCAGGTATTAAATCAATAACGGTTTTTGAATGATGATCTTCAGTTGCCGATATATAACCTTTTGGC
TTATTTAACATAATATAGACGTTTTCAACGTATTCTATTAATTCTCCACGAACTGATATCTTATCGTTTTCTG
GTTCTATATGTGTTTTTGCTGATTTAATTACTTGTTCGTTGACATTTACAAAGCCTTTTTTAAGTAACTGTTT
GACCTCATTACGTGTACCGACGCCCATATTTGCTAAAAATTTATCTATTCTCATCGTAAAAACCTAACTCTAC
GTCTTAATTTTTCAGGAATTTCACCTAAGAATTCGTCCGCAAGACGCGTTTTAATAGTGATTGTACCGTAAAT
TAGAATACCTACTGTAACACCTAAAATAATAATGATTAAGTAACCAAGTTTAGTAGGTTCTAAGAATAGATTT
GCAAGGAAGAATACTAATTCTACACCTAGCATCATAATAAATGAATACAAGAATATTTTTGCAAAATGAATCC
AACTATAGCTGAATTTAAACTTCGCATATTTTTTAAGAATATAGAAATTACATCCAATTGCAAATAATAATGC
GATACTAGTACTTAAAATTGCACCAGGTGTATGGAATAACATAATTAATGGATAGTTTAACGCTAACTTGATA
ACTACAGAAGCTAAAATAACATAAACTGTTAATTTCTGTTTATCTATACCTTGTAACATTGACGCCGTTACAC
TTAATAGTGAAATTAGTATTGCTACAGGCGCATAATAGAATAATAATCGACTGCCATCATGGTTAGGGTCATG
ACCTAAAACAATTGGATCGTAACCATAGAAAACTGTGAATAATGGTTGCGCCAAGGCCATAATTCCAATACTA
GCTGGAACAGTTATAAACATTAATACTCCAATTGACGTTCTAATTTGATGATGCATTTCATGTAAGCGACCTT
CTGCAAATGTTTTTGTAATATAAGGAATTAAACTCACCGCAAAACCAGCACTTAATGATGTTGGAATCATAAC
AATTTTATTAGTTGACATATTTAGCATATTAAAGAATATATCTTGTAACTGTGAAGGTATACCAACTAAAGAT
AAAGCACCGTTATGTGTAAATTGATCTACTAAGTTAAATAATGGATAATTCAAACTAACAATAACGAATGGAA
TACTATAAGCAATGATTTCTTTATACATCTTGCCATATGACACATCTATATCTGTGTAATCAGATTCGACCAT
ACGATCAATATTATGCTTACGCTTTCTCCAGTAATACCAGAGTGTGAATATACCAATAATCGCACCAACTGCT
GCTGCAAAAGTAGCAATACCATTGGCTAATAAAATAGAGCCATCAAAGACATTTAGTACTAAATAACTTCCGA
TTAATATGAAAATCACGCGTGCAATTTGCTCAGTTACTTCTGACACTGCTGTTGGCCCCATAGATTTATAACC
TTGGAATATACCTCTCCATGTCGCTAATACAGGAATAAAGATAACAACCATACTAATGATTCTTATAATCCAA
GTAATATCATCGACTGACCAACCGTTTTTATCATGTACGTTTCTAGCTAATGTTAATTCAGAAATATAAGGTG
CTAGGAAATACAGTACCAAGAAACCTAAAACACCGGTAATACTCATTACAATAAAACTCGATTTATAAAATTT
CTGACTGACTTTATATGCCCCAATAGCATTATATTTCGCAACATATTTCGAAGCTGCTAAAGGTACACCTGCT
GTCGCAACTGCAATTGCAATATTATATGGTGCATAAGCGTATGTGAACGGCGCCATATTTTCTTGTCCACCAA
TTAAATAGTTGAATGGAATGATAAAAAGTACGCCCAATACCTTGGTAATTAATATACTAATGGTAATTAAAAA
GGTTCCACGCACCATTTCTTTACTTTCACTCATTACGAATCTCCCTATCTCATGTTTATTAAAGTTTTGTAAA
CTAAAAGCTATTTCTCTGTAAAATCATTTTTCATTATTATGAATATATCACAAAACTTTATTTCATTGTCGTA
TATTCAATGAATTATCATAACAAAATTATCAACGCATTGTCATTGAATACTCGATTTTGATTAGAATATTACA
AAATTTCATATGAACATTATACTACTATTTGAGATGAACATCGCATAACAGTAGAAAAATCATTCTTATCATA
CACATACATCTTCATTTTTATGAAGTTCACATTATAAATATATTCAACATAATTGTCATCTCATAACACAAGA
GATATAGCAAAGTTTAAAAAAGTACTATAAAATAGCAATTGAATGTCCAGTAACAAATTTGGAGGAAGCGTAT
ATGTATCAAACAATTATTATCGGAGGCGGACCTAGCGGCTTAATGGCGGCAGTAGCTGCAAGCGAACAAAGTA
GCAATGTGTTACTCATTGAAAAAAAGAAAGGTCTAGGTCGTAAACTCAAAATATCTGGTGGCGGTAGATGTAA
CGTAACTAATCGATTACCATATGCTGAAATTATTAAGAACATTCCTGGAAATGGAAAATTTTTATATAGTTCC
TTTTCAATTTTTGATAATGAATCCATCATAGATTTTTTTGAGTCTAGGGGTGTTAAATTAAAAGAAGAAGATC
ACGGGCGTATGTTTCCAGTCTCCAACAAAGCACAAGACGTAGTTGATACATTAGTGACAACTATCGAACGCCA
ACATGTAACGATTAAAGAAGAAGAAGCTGTTAGTAGAATCGAAGTAAAAGAAGATCAAACTTTCACTGTACAT
ACTCAAAATAATAGTTATGAAAGCCATTCGCTAGTGATTGCTTCAGGTGGAACAAGTGTCCCTCAAACAGGTT
CAACTGGTGATGGTTATAAGTTCGCACAAGATTTAGGTCATACCATTACGGAGTTATTCCCGACCGAAGTTCC
AATTACATCAGCTGAACCTTTCATCAAATCCAATCGTCTAAAAGGTTTAAGTTTAAAAGATGTTGAATTGTCA
GTACTTAAGAAAAATGCTAAAAAACGCATTAGTCATCAAATGGATATGTTATTCACTCATTTTGGTATCAGTG
GTCCAGCTGCATTAAGATGTAGTCAGTTTGTTTATAAAGAACAAAAAAATCAAAAGACACAGCATATTTCTAT
GGCAATCGATGCATTTCCTGAATTAAACCATGAACAATTAAAACAACACATCACATCATTATTATCGGACACA
CCAGATAAAATCATTAAAAACAGCTTGCATGGACTAATTGAAGAGCGCTACTTACTGTTCATGCTGGAACAAG
CTGGAATCGATGAAAATACCACATCACATCACTTATCAAATCAACAATTGAACGACTTAGTAAATATGTTTAA
AGGGTTTGTATTTAAGGTAAACGGGACATTACCTATAGATAAGGCATTTGTCACAGGTGGTGGTGTGTCACTT
AAAGAAATTCAACCTAAAACAATGATGTCTAAATTAGTTCCGGGATTATTTTTATGTGGTGAAGTATTAGATA
TACATGGTTATACTGGTGGTTATAATATTACAAGTGCACTCGTAACAGGACATGTCGCTGGATTATATGCCGG
TCATTACGCACATGCATCAATGGAATAATAGTATAAAATTTGGTTCGATTCTCTTTAATAGATCAACTTTTTC
ATTTAATTAAAAATGACCCTAATATAACTGAGTCACTAAAAAGTGTCGTTATATTAAGGTCATTTCGTTAATT
ATGATTCTTTTTCATTTTTAGTACGTCTTCTAGCTAATAATGCTGCGCCTGTAATTAGCGCTAATTCTTTTAA

TGGTAAATCCATTTCTTCAGAACCAGTATTTGGAAGTTCTTTTTCAACTTTGTGCACTTCTTGTTTATCTTCT
TTTTTAATAGGCGTACAAACTTTTGGAGCAGGTTGCCCCACTTTTGGTGATACATTCGTCACTTCAGCTGGCA
ATTTAATTGCTAAAATTTCATCAACAATGAATTGCGTATGTTGTTTGATGTCATTTAATGTCGCATCTTCATC
AATCATTCTATTGCCATCTGCAACATATTGATCAATTAATGCTTTTACTTTAGCTAATTGTTCTGGTGTTGCG
ATTGATTTGAATTTCGCATATGTTTGTTGAGCAATGTTATCAATGCGCAGTAAGCTATTTTCTTTTTCAGTAA
TTACTGCTTCTATATCGCCTAATGCAACTTTAAATCGTTTTAAAACTGCATCAACATCAGCATTAGTGCGTGC
TATTTTTAATTCTTCATCCATTTGTAATTTTAATGCAGTTATAGCTTTTAATGCATCAGCCTTATTACGATCA
CTTACTTTTCGATAATTTTGCACTAAATGTGTAACACGTGCAAGATCATCATTAATCGTTTTTTCAGCATCTG
GCTTTTTAATAGGATGTACATCTAAATCATGTATTGTTTGTAGATTTAATGATGCTGTTTTATCAACTTGTGC
ATTGCTACGATCTTGATCAATTTGTCCAATAGCAGTGTCATAAATGTTTTGTAACTGCGCTAAAATACTATTT
CTTTCTTCTACCGTTGCTTGAACATTCGCTTCAATTGCTTGTTTTTTATCATTGAGCAATGTTGTTAATTGTT
CTCGCGCAGTTGATTTTCTATTAATAACAGGTTCAATTTCACGAATTTCATTTTTACCATCATGCAATAGATA
TGCTACATCAGCATTCGTCACTGCACTTGCAATTTGTTGTTTCGCTTTTATTAATGCTTGTTCTACTTGTGCT
ATTGCAGCATTTTGTTCTTCATCTGTCGCTTCATTATTTGCTTTAATTAAATTTATTTTATTGTAGCGATAT
TTTGAATTTGTTGTAGTGCTGTTGTTTTAACTGTTGTCGCTGGTTTAATTTGTGAAATTATCTTTTGAGCATC
GATACTATTTTGATTAACTTGTGCAGTCTTATCAGCATGATTAATCTGATCAATAGCCTGATTAAGTGCTTGA
TCTACTAAATGTTTAGCAGCTAATCTTTCTTCTTCGGTAGATAAATCACTTTGATCAATAAGTGCATTTTGAG
CTTCAGCTTTTGCGCTAACAGAATGACGCGCTGCTGGTTTAACTTGAACTTTAGGTAAAATCACTTTGATGTT
ATTGTTACCATCCGCCTCAGTTTGATCCACTTCTGCATTCGTTTTGTTTTGCGCAATGTCATTTTTGATAGTA
TTTACAATTTTATTTAACTCAGCAATAGCAATATTTCGTTCATCTTGCGTAGTATCTAGCGTATTTCGGATTG
CATCGAGTTGGTTATTATTACTTTCATCAATGTTATCCAACGCTGCACGTTTGCGTCTAACTTTCGGTTTAAT
TTGTTCGATTGCCTTAATCGTTTGATTTCTAACATCATTTACAGCTACATCTTGTGTTGTATTGTCTATATCT
TGTTGACCTTGTTTAAGTGTGTCGTTTATTTGTTGGTTTGCTTCATCTAATTCATCAGTCGTAGCATGTGGTG
TATCTTTTATAGATTCTACTTGATTATCTGCGCTCGCTTTTATGGCTTGTTGAGCTTCAGGTTTAACCACAAT
GTGCGGTTCTACGCCTTTTAACGTAGCAATACCATTTGATTCAACACGTTTCACATCATTATTCGCTATTGCT
TGATCGATGTTTTGTAATGCACGTTTTTCATTATTCGCTAATTGATTTAAAGCAACTTGTTTTTCTTCATCAG
TCGCATGTTCCGCTTGCTCTATTTCTTGCTTTTTAGCCTCATATTGTTGCTTTACTGCATCTCTAGCAGCTGC
TCTAATAATATGGTCAGGCGTCACTAATGCAATGTCTCCAAGCGCTTGATTTGTTGAGTCGTTAACTTGCTGA
TCTGTTCTATCATTCGTGATATTTGTCATAGCTTGAGTAACTAAATCATTGATTTTATCTAACGCCGCTTGTC
TTTCTTCTGCTGTCGCTTCTTTATCTTGATTGATTTGCGCACGTAATTCATTCGCTTTTTGATTGATTTTTTC
ACGTGCTGCTGGTTTAACTCGAGTTTCAGGTTGAATAATTTTTATCGCAGTTATACCATCTGTTGCCGCTTGA
TTCACTTGCTCGTTCGTTTGAGCTTGTTCAATTGCTACAAGGGCTTTTTCTTTTTCTCTAGCTAATGCTTGTG
TAGCAACTTCTTTCTCATTATCTGTTGAATCAATACTATTATCAATTTGCTGTTGCTTTTCTTTAACAGCTTT
TTCAATATCTGCAATTGCCTTTGGTTTAATTACTACTTCAGCTTCAACATTGTCTATAGCATTTATCGCTTGA
TTTGTAGTTGCGTCTACCTGATCATTTGTTTGGTTTTGATTAATTTGGTTAAATGCTTGATCTTTAAGTTGAT
TGATTTGATTAACAGCAGCCTGCTTTTCTTCATCAGTTGCATTAGGTGTTTGTTTAACCGCTTCAATACGTTT
CGCCACTTCAGCAGTGATTTTATCTCGTGCTGCTTGCTTTTTCACTACGTCAACTTGAACAGCATCAATATTA
TTTTCTGCTGTTATCGCAGCTTGGTCTACTTCATTATTGGTATTCGCTTGATTAAGACTTTCAATAGCTTGTT
GTCTGTCTTGATTCAACGTATTAATTGCAGCATTTTTCTCATCATCTGTTGCATCTGGTGTAGCATTGATTTC
AGCTAATTTAGCATTATAATGCTGATTGATTTGTGCTAATGCTGCAGGTTTTTTTACAATATTTGGCTGAATC
GCATTAATTGCTTGTGCACCTAATTGTTGCGCTTGATCTACTTCCGCGTTTGTATCAGCTTGATTTATATTAT
TAAGTGCAGCTGCTAATTCTTGGTCTACTTGATTTAATGCTATTTGCTTTTCTTCAGTTGTTGCATTTGTATT
TTGATTAATTTCTTGCTTTTTAGCAGTTGCTAAATCGTTTAATACACCTGTAGCTGTTTGCTTCTTAGTTACA
TGCGGTTGAACAGCACCGATTTGATTGACTGCATCGTCTCTAATGCTATTGACCATCGCAGTAGTAGACGTCA
CACCGATATCAGTTAATGCTCTATTTTTAAGTGCATTGACACGATCTATCGCTTCTTGTTTCTCTTCTCGAGT
CGCACCAGGTGTAGCATTGATATTTGTTATCACCTCACGTGCTTTTTCATTTACAGTATTACGAGCATCCGTT
TTAACTTGTGTCGCTGGTTGAATCACAACTATATTTTGCATACCTTGATCTTTTGCTTGTGCAACTTCTTGAT
TCGTATCTGCTGCATTAATATTTTGCTTCGCTGTGGCTACAGCTTGATCAAGCAATTGTTGTGCTGCTTGTTG
CTCTTCTAAGGTCGCATCATTATTATTAAATATCGCATTATGTTGCGTTTCAGCACTTTGATCAATAGCATTT
TTAGCATCTGTTTTAACCTTTGTAGCTGGTTCAATGGCTTGTATACCTTGAATTGCATTTGTCTTCACTTGCT
CCACATCTGCATTGGTATTAGCATTTAAAATGTTAGCATTCGCAGCAGCTACAGCAGCATTTACTTTGTCAAT
CGCTGCCTGTCTTTCTTCTTGAGTCGCATCAGGATTAGCATTGATCTCAGCGATATGTTGTTGTGCATCATGT
GATACGGCATCTCTTGCTGCCTGCTTGACAACAGTTACAGGCGCAATAGGATTAATGGCATTCAGACCATCAC
CTTTGGCTGTATCCACATCATCATTGGTTGTCGCTTGATTGATTTGCTCTAAAGCGTGGTTCGTAGCTTGCGT
TAATTCATTCAATGCTGCATTTTTCTCTTCTTGTGTTGCTTCTCTATTACTATTTATCTGTTGTCTTTTTGTT
GCTGTTGCTTGGTTAATTGCATCTCTTGCTGCTTGTTTGTGTGTTACTTGAGGGACAACAGCTCCAATAGTAT
TAATACCATTATTTTTAGCTGTTTCAACATCAGCATTGGTAGTAGCATTCGTAACATTATCAATAGCATCTGT
TTCATCCGTTGTTAATTGATTTAATGCATCTTGAATTTCATCTTGAGTAGCATCTGGTGTGTTATTGATAATT

TCTCTTTGTTTCGCAGCTTTATCACGTATTGCTTGTTTAGCATTTGGTTTAACAACTGGTGTTGCAGTGTCTC
TACTTAAAGTCTGTATACCTTGATCTTTAATTCTAGTAACACCATCATCAGTCGTTTGGTCACCAATATTTCC
AATAATTTCATTTTTATGTTCCTCGATGACTTGAATCGCTGCTTGTTTTTCTTCATCTGTCAGTTCATCATTT
TGGTTAACTAAATCTTCCATGTCATCAACTTTTCTATTAACGGCATTTTCAGCGTCAACACTTCGAATTAACG
TATGTTGCATTTGATTTGCTAATGAATCGATATCTGCCTGAGAAACTCTTTTGTTTAAAGGTACATTGTTACG
GTTTTCATCTAAAATTGTTTGTGCGCGTCTTTTAAGATCATTAAAAATATCTAATGATGCAAATGTATAATCC
GCTTGTTGAATACGTCTATCAACTTCAGCTTGTAGTGCGTCTTTATTCATGATGATATCGATTGTATACGGTT
CAGTGCTTACTGTATGACTTTCTGCAGGTGAATTAATAAAATCTTGTGAAAATGTTTTATACGTTAACGTATC
GTTAAATGTTACTGTTCTTGGTGTTGGCACATTGTTCACACGTAGCTTATACTTCAAATCCAATATTTTATCA
GGCATAAGTCGTGCCGGTGAATTCCCTGAACCTCCACCAGTACTTTTAATTGTAATAATTCGATTTGCTGCGT
CATACGTCACATTAAAATCATTGATATCAACACCTGAATTACCGCTCTGGAAATCTTTAGTCAATGAATTATT
TACATATTCAACACCTTCAGGTAATTGAATTTTATATACAAAATCATCTGTATTAAATGAAGCGCCAAAGTTA
CCATTATTCTTTAATGACGTTGTAACTGAAAATTCTTTGTTATTTGTTGCTGTTGGCTCCATTGTACGTCTTT
CAACATAGACATGCGACCCAGAATGTAGACCGATAGAGTCAACAAAGTCATAGTATTTATAACCATCACGTAA
TTGATAAATGCCACGCGGATCTGTTATTGCGTCATTTTTAGGAACAAATTGTATTTTTAAATGACTAACATTT
TCAGGTACTTTAAATAAGCGCCAAATCGGACCATAAGCGACTACTTTTTCACCAATAACTGCATCATTATCAG
CATTTTTAATGACTAAATTTGTCATACCTTGATAGTTTTTAGTAGTCATTGTATTAAATTCAAAGATTAATTC
AGAATTCGGATTTACTGTTAATGTTTTCTCGATACCATTAAAATCGCCATGGTCATTTACATCATTACCTCTA
ATACGACCTAACGCAATTACATTGCCTTGTGCTTGATAGTTCTTAGCATCTCCTGAATCAAACATACTCGTTC
TTACCATCGCATGACTAAATACCCCTACTTTACCACCATTAATTAATGTAAAACCTGGTAAATTATCAACGAC
GGTTACTGTTGGCACAGAGCGGTTCGTACTTGGTCTAATACCGTTATCATCAAATGACAGCACTTCGTTAGGT
GCATTTTGTCCTATATTATTGGCATTGGGATCAGTTGTTGGCGTGTATGGCGCTGTAATTGCAACTGGTGCAC
TACCGTTTCCTGCCGCTGGGTCTGCTGGTGTAGCCGTTGGATCTGCTGGTGTTGCATTAGGATCTGCTGGCGC
AGCACGTCTGCTTCTTTTTTTCGGTCTATTAGATGCTTCTTCAGCAATCGCAACTAACTCTGGCTTATCTGAA
GAATGACGAACATCTTCTTGAATTTCTTTTAAAGTCAGATGACGCGCTGAATCATTGTTATCTGTATTATTAG
GCACGTTTAATGTATTGTTATTTGATGATAAATTATTATTTGTATTCGCCGCTGACTCAGTTGCTGTTGACTT
AGCATCAGAATGATTATTGTTTGATGCAGATGACGTACTAGGCTGTACTGGCATTGGCACAACTTGATTTGAC
TCACCAACGTTATGATGATTAGCCAAAGCTTGGTTTGTTGATGCATTTGCCGTCGTATGTTGATTAGGTGTAT
TTTGCGAACTATTTGTAATATCTCTACTAGTATCGACATTTGTATTATGTGAGTTTCCAGGTAATGCTTGATT
TGACTCACCTTGAACATTTTGATCCGTAGTTAAGGCTTGTGCACCATTTGGGTTTGAAAGTAATAAAACTGTT
CCGATTAAAGTAGAGAATATGCCTACTTTATACTTCCTAATACTATATTTATTTTTCTTTAACAAATTCATTT
CATTTTCCTCCTGGTTGTCTGTTCTCTGCTGTGTTGTTAAGAATTATGCTAAAAATATACGCTCCCTCATATT
ATGTTTATAGCTATATTATTTTGTACAATATTTTAAGTTCACTCAATTTTATCATCATCATTTTATAAAATAA
AGTTTTATTTTATTACAATTTAGTTTCCGTTTTGTAATTAAAATCTTAATAAAACATAACAAGAACGCTTTAG
AATGTTTTGTAACAGAAAGTAATTCATACCAAAAAACACCAAGCAACCGAAACGTTACTTGGTGTCATTACAA
ATGTAATATTATTTTAAATTATGTCACTACTTTAAATACTTTTTATTTCCAAACCTTCATCGCCCCATGCGTG
CATGCCGCCTTCGACATTTACGGCATCAATGCCATTTGCCTCTAAATATTCTACAACTTTAGCGCTTCGAACT
CCACCAGCACATACAATATAATATATTTCATTTTTATTAAATGAATTTAAATTATCCGGAATGGTATCCATTG
GAATCAACTTTGCATTAGGAATATATCCCATTGCTGTTTCTTCGTCAGTACGAACATCAACAATTTGAACTGG
TTTAGATTCTAAAAGTTTGTTTTTTAATTCATCTGTAGTAATTGACTTCATTTCAAAGTCCTCCTTAAAACAT
TATTTAGCTACAATATTGACTAATTTTTGAGGAACAGCGATGACTTTCATGATGTCTTTACCTTCAATACTCG
CTTTAACATTGTCATTAGATAAGGCAATTTCTTGCATTTCTTCTTTTGATGTATCTTTAGCAATTTTAATTTT
AGCTCTCAATTTACCATTTACTTGAACAACGATTTCTACTTCATCATCTACAAGTAGTGCTTCGTCAAAAGTT
GGCCAAGGTTGGTACGTAATAGACTCTTCATGTCCTAATTTTGACCATAATTCTTCACCGATATGTGGTGCAA
TAGGTGCTAACATTTTAACGAAGCCTTCAATGTAAGGTTTATAAACTTCATCAACTTTATAACACTCATTAAT
AAATACCATTAATTGACTAATAGCAGTATTAAATCCTAATGTTTCAAAGTCTTCTGTTACCTTTTTAACAGTT
TGGTTATAAACTTTATCTAAAGATTTATTATTTGTAGTTACAATTTTTGAACTCAATGTTCCATCTTCATTTA
CCATTAAACGCCATACGCGATCTAAGAATCGACGAGACCCATCTAATCCTTTTTCACTCCATGCAATTGCAGC
ATCTAAAGGTCCCATAAACATTTCGTAAAGACGTAAAGTATCTGCACCATGAGACTGTACTATATCATCAGGA
TTGATTACATTTCCTTTAGATTTACTCATCTTCTCATTACCTTCTCCTAAAATCATACCTTGGTTAAATAATT
TTTGGAAAGGTTCTTTAGTAGGTACGATACCCAAATCATAAAGGACTTTATGCCAAAATCTTGCATATAATAA
GTGAAGAACCGCATGTTCTACTCCACCGATATATAAATCAACAGGTAACCAATGTTTTAATTTTTCAGGATCT
GCTAACATATTTTCATTTTTAGGATCGATGTAACGTAAATAATACCAACAACTACCTGCCCATTGTGGCATTG
TATTTGTTTCACGACGTCCTTTCATACCTGTTTTTTCATCTACAACATTTACAAATGAATCAATATTAGCTAG
TGGAGACTCACCAGTCCCTGATGGCTTGATTTCATCTGTTTCAGGTAACAACAATGGTAGCTCTTCTTCAGGA
ACAGTTGTCATTGTTCCATCTTCCCAATGAATGACAGGAATTGGTTCGCCCCAATAACGCTGACGACTGAATA
ACCAATCTCTTAATTTGTAATTAACTTTCTTTTCGCCAGCACCTTTTTGCTCTAATAATTGAATAGCTTTAGT
AATTGCCGCTTCATTTTCTAAACCATCAAGTTCACCAGAATTAATATGTTTACCTTCACCAGTGTATGCTGCT

TCTTCAACATTTCCACCTTCGATGACTTCAATGATTGGCAAATCAAACTTTTTAGCAAATTCATAATCTCTGT
CATCATGCGCTGGTACTGCCATAATTGCTCCAGTACCATATGTTGATAATACATAATCAGCAATCCAAATTTG
TACTTTTTCACCAGATAAAGGATTAATTGCATATGCACCAGTAAACACACCTGATTTATCTTTTGCTAAATCT
GTACGTTCTAAATCTGACTTTTTAGAAGCTTCTGTTTGATAAGCTTTTACTTTTTCTTTATATTCATCAGTAG
TAATTGAATTAACTAATGCATGTTCAGGACTTAAGACTAAGAATGATGCACCATAGATTGTATCTGGTCTAGT
CGTAAATACTTCTACTTTTCCTTCCGTATTATCTACATCAAATGAAACTTTGGCCCCTTCAGAACGTCCAATC
CAATTGCGCTGCATATCTTTTAAAGACTCAGGCCAATCTAAATCATCTAAATCTGCTAATAATTGATCTGCAT
ATTCTGTGATTTTAAGTACCCATTGTTTCATCGGCTTACGATAAACTGGATGTCCACCACGTTCAGAGACACC
ATCAATCACTTCTTCGTTAGATAAAACAGTGCCTAATGCTGGACACCAGTTAACTGCAACTTCATCAACGTAT
GCTAAACCTTTGTTATATAACTGTATGAAAATCCACTGTGTCCATTTATAGTATTCTGGATCTGTTGTATTAA
CTTCACGATCCCAATCATAACTGAACCCTAATTCTTTAATTTGTCGTTTAAAAGTTTGGATATTTTTCTTTGT
AAATTCACGTGGGTCGTTGCCAGTGTCTAAAGCATATTGCTCTGCTGGTAATCCGAATGCATCCCACCCCATC
GGATGTAATACATTATATCCTTGCATTCTTTTATATCTTGAAATGATATCTGTTGCTGTATAGCCCTCAGGAT
GTCCAACATGTAAACCAGCACCTGATGGATATGGAAACATGTCTAAAGCATAAAATTTCTTTTGACCTAAGTT
ATCATTTGTTTTAAATGTTTTATTTTCGTCCCAATAGTCTTGCCATTTCTTTTCAATTTGATTGTGGTTGTAA
TTCAACACATTTCCTCCTATTCAATAAAAAAGACACCTCTAACTATTCTATTTACATAGGGACGACATTTGCC
GCGTTACCACCCTATTTCACAAAAATTGTGCACTCATTTTAATAGTAAGAGATGTCATTCATTCAATTTAAAA
TTAATACGATTTACATTTAGTCCTTAACACTAGCTCTCAGCATCACTAGCTTTCTGTAGAAAAGCTCCAAACG
AAATTAAATTATACAAATTAATATTATGTTATAACGACATTTATTTCAAGTCTATAACGTTAATTCTATTTAC
TCATTATGCATCTATTTTTTTAGGTTGCGTATTATTCTCCTTGAAAACCATTAATAATATTAATGCAAATACA
AGTAGTAGCATCATACCGATAAACATCATGCGCATATTAAACGCATCAACTAATACACCACCAAGAAATGGAC
CAAATGCTTTTCCTACTGTAGCAGCTGAATTCACAAAACCTTGGTACTGTCCTTGCTTACCATCTGGCGCTAA
CTGATTGGCTATAGTTGGAACTGCTGGCCATACAAACATTTCTCCAAAAGTTAAAATAATCATACCGACAACA
AATATTGTAAAGTTTTCGGCAAAACTCGTGACAAAGAACGACAACATAAAAATGATGATGCCGACAAACATTT
GCTTCTTTAAGTTTCCTTTTAACAGATAGAGAACCGGTTTAATTAATGGTTGTGCTACTAAAATCATTATTCC
GTTAATTGTCCATAAAACACTATATTGTGCCATTGAAATATTAATAGATTGTGTAAATGATGCGATTGTAGAC
TCCCATTGAATATACGCAACCCAACAAATTGCAAACATTGCACAAATTAGTACTAATGAAATAAATCTTGCTT
TATTCTTTTTACCAGTAATATCTAAATGAGTTGGATATTTAACTTTCGCATTAATTTCAATATTAAATTGCGT
TACCGCGACAAGCGCAAACACAACATACATAATAAGATTGGCTAAAAAGATATAGTTAAAGCTAAATTCTGCG
ACAAAGCCGCCCATTGCAGCACCGACAGCCACACCAATATTTTGCGCTAAGTATATCGCATTAAACGTTTGTC
TTCCGCCATTTGGCCACACTGCTCCAGCCATAGCGTATATCGCAGGAATAATCATTCCGCCACCAAACCCTAA
CATTACAAGCCATACAGCATACCAAGGCCACCCGTGAAAGAAATTAAGTAGCGTTGTACTACAAAGACAAGTG
AAAGTTCCAATTAAAATCGTCTTGTATCCACCTAATTTATCAAATAGTGAACCACCTAATAAGTTTCCAATAA
CCATGCCAAATGAATTTATCATTAGCACTAAACCAGCAACAGTTAAACTTTTTCCAAGTTCTTGTTTCATATA
AATTGTATTTAAAGGCCACAAAAAACTGGAACCAGTAATATTTAACGCCATGCCAATTACTAGCCACCAGACT
GATTTAGGTATATTCATATTTCATAGTTCCTCTCTCTTTTTTGTCAAACTATAATAAACACTATAACATTAAA
ATATGTTAAAATCTGTACTTAGAACTCTTTTTTTTATAGAAAGGAAAATGACATATGGGCAATCATTTCCAAT
ACGCTTTTGAAAACAAACGTTATCACACGTGGAATTACCATTTAAAAAATAAATTTGGACAAAAAATATTTAA
AGTTGCATTGGATGGCGGGTTTGACTGTCCTAACCGCGATGGCACTGTAGCACATGGTGGATGTACATTTTGT
TCTGCTGCAGGTAGCGGAGACTTTGCAGGTAATCGTGCAGATTCAATCGCAGTACAATTTAAAGAAATTAAGG
AAAAGATGCATGAGAAATGGCACGAAGGAAAATATATTGCTTATTTTCAGGCATTTACAAATACACATGCACC
AGTTGAAGTATTAAAAGAAAAATTCGAACCTGTACTTAAAGAACCGGGTGTTGTGGGATTATCTATTGGTACG
CGTCCTGACTGTCTACCAGACGATGTTGTCGAATATTTAGCAGATTTGAATCAACGAACATACTTATGGGTTG
AATTAGGACTACAAACAATCCATCAATCAACATCTGATTTAATCAATCGTGCCCATGATATGAAAACTTATTA
TGATGGTGTGGCAAAATTACGTAAGCATAATATCAATGTATGTACACACATCATTAATGGCTTACCTGGCGAA
GACTATGACATGATGATGGCTACTGCCAAAGAAGTTGCACAAATGGATGTACAAGGTATTAAAATTCATTTAC
TTCATTTGTTAAAAGGTACACCGATGGTAAAACAATACGATAAAGGTTTATTAACTTTTATGACTCAAGAAGA
ATACACAAACCTAGTTGTGGATCAATTAGAAGTGATTCCCCCTGAAATGATCGTTCACCGAATTACCGGTGAT
GGTCCAATAGATATCATGGTCGGTCCAATGTGGAGTGTTAATAAATGGGAAGTATTAAATGGCATCGATGCTG
AATTAGCACGTAGAAATTCTTATCAAGGCTTGCGTTACAAGTCTAAGGTGAAGCAATGAAATTAGAACGTATA
CTCCCTTTTTCAAAAACACTTATTAAACAACATATAACACCAGAAAGTATTGTTGTAGACGCAACTTGCGGTA
ACGGCAATGACACTTTATTTTTAGCCGAACAAGTACCAGAAGGACATGTTTATGGTTTCGACATTCAAGATTT
AGCTTTGGAAAATACACGTGATAAAGTTAAGGATTTCAATCATGTTTCTTTAATAAAAGATGGACATGAAAAT
ATTGAACATCATATAAATGATACACATAAAGGTCATATTGATGCAGCCATCTTTAACCTAGGTTATTTGCCTA
AAGGTGATAAATCTATCGTGACAAAGCCTGACACGACAATCCAAGCTATTAATTCATTGCTATCATTAATGTC
AATTGAAGGTATTATTGTACTTGTTATATATCATGGTCATAGCGAAGGACAAATTGAGAAGCATGCATTGCTT
GATTACTTGAGTACTTTGGATCAAAAGCATGCGCAAGTTTTGCAATATCAATTTTTAAACCAACGTAATCATG
CTCCATTCATTTGTGCCATAGAAAAAATTTCTTAAGCATTTGCTGCGCTATACTATTTATAATTTAGGATTGA

GTAGTCAACATTTGAAAAATTAATACCAACAACAAAAAGAGGTTTTCAACTTAATGAAAACCTCTTTTTATGC
TATTAAACTTACACAGCAATAATTGCGTTTAAACTATTTTGCATTGCTGTTGCTCTACTTGCAATCGCATCAC
TGATGAAATTCAACAACTCTACTTTCTCTTGTTGTAACTTTTCATTGAAATGAATAATAACTGTTCTTTCATC
GTCAACAGGACGCTCTTTGTAAATCCATTCTAATTCAACTAAATTATTATACGTTCTCGTACGCTTATACGGT
TTAACTTCAACAAATCTGTCCATTTCTTTAAGCGTCATAGAACCTTTTTGCCATAAAGTTAGTAAAATTAAAA
TTTCTTCTCTAGACATTTTGTATTCGCTTTCAATCTCGCTGAAAATTGAGTTAATGTCACCCAAAAGTGTTTC
TAATTGCAAAATCCCAAATACAGTGTCGTTATTTACTTTTTTCATGCTAAACATCTCCCAATTAATAATCACA
TATTTAGCTACCATACAAGTATTAACAAACATCCCAACAATCCCAAAACTTGTATGTGCTAACTTATGCATAA
AACTACAAGTGTAAATAAACTTGCTTTCTATTCAATTGTCAAAGTTGAATAAAATTAAATAAGTATAAAAACC
AATAATCAATAGAGTTAATATTTAGAAATAAGATAATAGTACATAAAACAAATCACTCTATTACATATTAATT
TTATAATTTATATACAAAGAAAATATATCAATATTACCATTAAATTGCAACAATTCTACATATATTTCTTCAA
AAAAGATACGCATTGAATTTTTATTACATATAACTCTCATGAAAATTGATTAAACTAAATTAATTAGTTAGCC
TTAAATTTAACAAATTAAATCAAACCGAATGAGGCTAAATTGGTTATTTTTCAAGATATGATTTGTATAAATC
AAAACATTTCTTACAAATCAAATGTGTATAAAATGTGATATACATTCTCAACCTTAAAAAAGTTCTAAAAAAA
AGATGAGCATCTACTGTCTCATCTTTCAGTTCTTTTTTACGGGTCTGTTTTCTAATTTGAGCACAATCTTCGA
TTTCTTTATCTAAATGACTACCAATTAAATCTATTTCTTCTATTGTTAAATCGCTATCTCCATCTTCTTTTAT
CACTGGTATTATTTTTTTCTTCAACTAAGTCACGATATAATGCTTTTGAATTTTCGTTCAATTTCGATTCGTGA
TTTTGAATACTTTTCTTCCACACAAATGTATATCTATTGGCATTAGCTTCTACTTTTGTACCATCAATRAAAA
TTGA
>DSM104437_scaffold_2
CAACTTAAGCCAGGATACAATTTACAAATAGCGACAAATTCTCAATTTGTTTTATCCTATGACCTATTTCAAA
ACCCGACAGATACTAGAACTTTAATTCCATTTTTAACAATGATTCAAAATACCTTCGGTTATTTACCGGAGTA
TAAATCCCCTCACTAGGGCAATAGTGAGGGGATTTGGTGTATTTAGATGTTCAATTTATTATGTAACTAATTA
TATCATTGATGAACAAGGCGCTCAACGTAATATTACAATTAATGGATTGTTTCACAACATGGTAAAGCTAGGA
TGTCTTTTTATTAAGAAATAATGCAATTATTAATTTTAACTGACATATTTTATCGAATCGCTGTTTATATCTG
CCCGAATTCTGTAAGAAGCCTTAGTCATTTCAATAGCAAGGTCAAAATTTTGCCAAAATTTATAGTTATACTG
AGAAGTTTAAATTCATGTTTTCTTGAAAAACATGCGCCTTAAATGTAAACTTATTAATTATAAAAGTTTACAT
TCGGATTGAGGTGCTTATTTTTTATGAGGATTTTTATTACAAGTACGAATACTGATGTAGGCAAAACCTATGT
TACAAAGCATTTATACCATGCTTTGAAAACACGTGGTCATCGTGTTTGTGTTTTTAAACCATTTCAAACTGAG
GAACGCCAAGACGGGACATTTCCAGATTTAGAAGTATTTAAAAATGAATGTGATTTAAGCTATGACATAACGT
CACTTTATACTTTTAAGCAACCTGTATCACCACACCTTGCATTTAAAATGGCAGATCAAATTTTTCTAAATAA
GCAGCGTGTATTAGATAAGGTAAAAGTTTTAGATAAGGAATTTGATTTTATCTTAATTGAGGGGGCTGGGGGA
ATTGCCGTACCAATATATGAAGGTACAGATGATTTCTACATGACTAAAGATCTAATCAATGATTGTGCAGATT
GTGTCATCAGTGTGTTGCCATCAAAATTAGGTGCTATTAGCGATGCCATTGTTCACCAAGATTATGTTAATCA
GAATGTATCAGCGAGTAATTTTTTAATAATGAATCGCTATACAGACAGCTATATTGAAAAAGACAATCAAATG
ACGATTGGAAAATTAACAAATAAAACAGTCTATACATTTGAAGAACATGCCACGTATGAAAATTTCTCAGAAG
CATTTTTAAAACAATTAATAGGAGTTAAAAATGAATTACACACAACAACTTAAACAAAAAGACTCAGAATATG
TTTGGCATCCATTTACACAAATGGGTGTATATAGCAAAGAAGAAGCAATCATCATTGAAAAAGGAAAGGGTAG
CTACCTTTACGATACGAATGGCAATAAATATTTAGATGGTTATGCATCGTTGTGGGTCAATGTGCATGGTCAT
AATAATAAATACTTAAATAAGGTAATTAAAAAGCAACTCAATAAAATTGCCCATTCTACGCTGCTAGGATCAT
CAAATATTCCGTCAATAGAACTTGCGGAAAAATTAATCGAAATCACGCCAAGTAATCTAAGAAAAGTATTTTA
TTCTGATACAGGCAGTGCGTCTGTTGAAATCGCAATAAAGATGGCATATCAGTATTGGAAAAATATTGATAGA
GAAAAATATGCCAAGAAAAACAAGTTTATAACGCTAAATTACGGTTATCATGGGGATACGATTGGTGCGGTAA
GTGTTGGTGGTATCAAGACCTTTCATAAAATATTTAAAGACTTAATATTTGAGAATATTCAAGTAGAAAGCCC
ATCTTTCTATCGCAGTAATTACGATACTGAAAATGAAATGATGACAGCTATTTTAACGAATATAGAGCAAATT
CTAATTGAAAGAAATGATGAAATCGCAGGGTTTATATTGGAACCGTTGATTCAAGGTGCGACAGGCTTGTTTG
TTCATCCTAAAGGCTTTTTGAAAGAAGTCGAGAAATTGTGCAAAAAATACGATGTCTTATTAATTTGTGATGA
GGTAGCTGTTGGTTTTGGGAGAACTGGAAAGATGTTTGCATGTAATCATGAAGATGTTCAACCGGATATTATG
TGTTTAGGTAAGGCGATTACAGGTGGCTACTTACCACTTGCAGTTACATTGACATCTCAAAAAATATACAATG
CATTTTTAAGTGATTCGCATGGTGTGAATACCTTTTTCCATGGTCATACATACACCGGAAATCAAATCGTTTG
TACGGTTGCATTAGAAAATATAAGACTTTATGAAAAACGTAAGTTATTGTCACATATTGAAACGACATCATCA
ACACTTGAGAAACAGTTACATGCGCTGAAGCGTCATCGAAATGTTGGTGATGTAAGAGGACGAGGCTTAATGT
TTGGAGTTGAATTGGTTACAGATAAAGATAGTAAAACGCCGTTAGAAATTGAAAAAGTTGAACGTATTGTACG
TAATTGTAAAGAAAATGGGCTAATGATTAGAAATTTAGAAAATGTCATTACGTTTGTGCCAGTGTTAAGTATG
TCAAATAAAGAAGTGAAAACGATGGTACGTATTTTTAAAAAGGCAGTACATAACATTTTAGATAGGAAGTGTT
AATATGAATTTGGCTAAACGCATATTACAAGGGGAACAATTAACAAAAGAGACTGTATTGAAAATTTATGAGG
ATACTAATATTGATACCTTAGATTTATTAAATGAGGCGTACATTTTAAGAAAACATTATTTCGGTAAAAAAGT
AAAATTAAATATGATTTTAAATGCTAAAAGTGGTATATGTCCTGAGAATTGTGGGTACTGTGGACAATCACGA

GATATTAAACAAAAACAGCGATATGCTTTAATTCCAGAGGAACAAATTATCGATGGAGCAAAGGTAGCACATG
ATAATCATATTGGAACATATTGTATTGTTATGAGTGGTAGAGGGCCGAGCGATAAAGAAGTTGATCATATTAG
TAACACTGTAAGAACGATTAAATCTCAACACCCGCAACTAAAAATATGTGCATGTTTAGGATTAACGAATGAC
GAACAAGCTAAGAAACTTAAGTCTGCTGGTGTAGACAGATATAACCACAATATTAATACAAGTGAAAATTACC
ATGATAACGTCGTGACAACGCATAGTTATAAAGATAGAACAGATACGATAGAACTAATGAAAGCGAATAATAT
ATCACCATGTTCTGGCGTGATTTGTGGTATGGGAGAATCTAATCAAGATATTGTTGATATGGCATTTGCTTTG
AAAGAAATGGATGCCGACAGTATTCCGATTAATTTTTACATCCAATTAAAGGTACAAAGTTCGGTAGCATGG
ATGATTTAACACCAATGAAATGTTTAAGAATCGTAGCATTATTCCGATTAATCAATCCTACGAAAGAAATTCG
TATTGCTGGAGGAAGAGAGGTCAACTTACGTTCGTTACAGCCATTAGCATTAAAAGCGGCGAATTCAATATTT
GTCGGTGATTATCTAATTACTGGTGGGCAACCGAACCAATTAGACTACGATATGATTAATGATTTGGGCTTTG
AAATTGATTATGACACTTGTGAAAATAAGGAAAACAAGAATGATGTTTCAAGAGCAAATTGATTTATTACAAC
AGAAAGGGTTATATCGGTCGCTTAAATCGGTAGGGTATATAGATAGACAGTATATTGAAGTAGAAAATAAGCG
ATGTACGAACTATACATCTAATGATTATTTAGGATTAGGACAAATAGCGTTTGATAAGGATGATTTCGAAAGA
TTTATGCGGAAGTATAGTTATCACTTATCAAGTTCAAGATTAATTAGTGGAAGTTCGACAGCTTATGAAGAGA
TTGAAACAATGTTAGCAGGTTGGCTCGGATATAATGCATGTACTATCTTAAATAGTGGTTATGATGCTAATTT
GGCGTTATTTAATATTTTCAAAAATACAAATTGTGTCGTGTTTTCAGATCAAGAAAATCATGCGAGTATTATT
GACGGTATTAAGTTAAGTGGTTTAGAAAAAGTGATATATAAGCATCTAGATATTGCTGATTTAGAAAAAAGGT
TAGCGGAATACCCTAATCAAAATATACAAAAAATAATCATATCTGATAGTGTATTTTCAACGAATGGCGACGT
TGTGGACATTGGTCAATTAGTCTCATTAAAGCATAAATATAACGCAACACTGATACTCGATGTTTCACATAGT
TTTGGAATAGAGAATTACTCGAATTATCAAGGCATAGATATACTCACTTCTAGTTTATCTAAAGCATGTGGTG
CATACGGGGGTGTGATTTTAAGTTCAAATGATGTGAAGGATATGTTAATTAATCACGGTAGACCACTCATCTA
CTCAAGTAGTTTGCCAATTTATAATTTGTATTTTATAAAAAGAAATATTGAAAAGTTAATAAATGCTGATGAT
AGACGCACTAAATTAAATAGTTTGATTAAATATTTTAACCAAAAGTTAAAAACACTCAATGTTAATTATAATA
GTTCAAACTCGCCGATTAAGTTTATTGAGTTTGATGACATAGAAGCGGCTAAGAACATTCATCAAACATTATT
AAAGCATCATGTGTTTACAAGTTATTTAAGGTATCCAACTGTGACTAAGCCAATGTTAAGAATATCATTGTCG
TATTTTCATACTGAACAAGATGTAGATAGGTTGTTTGAAATTTTGCATCAAGAAGATTGAGGTGATCGTCATG
TATAGTATAAAAATGCGTTCTAGCAATCAAGATGTTCATATTAGTGGTGCTGAAACGATTTGTGAGTTTGACA
AAATAGAACAGACGGTACGAAGGTTTTATAATAAAGGTTTTTTCATGAAAATGGGCAACCGGATTTTCTCAA
TATAAAAATACAAAAAATCATGGAACCGATTCAACAAATAAAAGCATTACAAATTACTGAGGATGATAAAGCA
AATCTCCAACATTTAACACAAGAATGTGGTGTCACTGAACAAGCGCTAAATCAAGGGATGACATATATTAAAA
ATGAAACGGTTTATACAGGAGCTATTATTCTATCTGCTATATCTGGTAAACGTTTAGATTCATTTGGACAAAG
AGGGATTAGGGCAACGCATTTTTCGTTTGAAGATATAAATAATAAAGGTGATCTTAATGAAAGAGTGACTGAT
GCCTTAGCAATTGCTAGTTGTATCAATGCGCATCCATATGTCAAAGGAGAACTTTGTGTGTCCGATGACTTAA
CGTATACGACAGGTTATTTTGCCTCTGCTAAAATTGGTTACCATCGATTATTTGATATTAAACCAGTTAATAC
GAGATATGGAGGCAGAATAATATTTGTGGACGATCGTATTGATTTAAATCATTACATATCATTTTTAGAAAGC
ACACCGAAGCAAGTTGTTTATGAAACGGTATAGGGGTTTTAGTATGACATCAAAAGATATTACTCAAATTAGT
GTCATTGCTGCGATTTTAACCATTTTGGCAGTTTTGAAAATACCATCCATTATACCAGGTTTAGATTTTCAAT
TATCTGCACCGGCAGCATTATTGATATTAGCTTTCTTTGGAATTAAAAAGTACTTTTTAGGTGGATTATTATC
TAGCCTATTATTACTAGTATTTGGTGTATTTAATCCAATTAATGTGATTATCTCTATTATATTTAGAGTTATA
GCTATTGCAGTTGTTTATTTATTGAAAATAAATGTACTATCATTAGTTTTAGCAAGTGTATTAGGCAGTTTGG
TATCTAGGCTACTATTATCTATTATTTTAAATTTACCTGTGTGGGTAGTGTTATTAAACGCGATTCCAGGCGT
AATATTCACTTTAATTGTAGCTATTCCTTTATATCTCACATTGAGAAAAAGAATGGCAGTCTTACTAAGATAA
TAAATCAAAACACGGTCGTCACAATTATTGTTGGCGACCGTGTTTTACTAGCTATTTATTGTTTTCAGTTTCT
TTTGTACCTAACAATTTCACTTTGTGATTTTCCCAATCAATTTCATATGTTGATTTAAATGTTCTAGTTTTAA
AGTTTTTATAATTTGCGCCTGCCCAGTAGAACCCATTCCAACGAATTTGGTATAAATCCATTTCACGTTGATA
AGTTACTGTAATTTTAGATTTTTTAGCGCCATCTTGTCTGTGTGATAGTACGCTTAAAAATTCTGGATTGAAG
TTACTTCTAGATAATAATGGCATTTGGTGTTGCGCTATGAAGTTTTGGCCTGCATATGAACTGCTTTGTCTAC
CAGCTAAGAAGATTTCATTGCCATATGTTGGATGGAAGCTATCTCGGCCATAGGGGCCCCAACCATTATTCAT
AATTTTATGTGCTTCTACACCCCAACCGACATTTTTATAATTTGTGTTGCGACTTAAAGTTGTTCTGTAACTT
TCTTGTTTGTAGTTAATTGTTTCAGAAAAAGCAGTGTTTCCGTTAAGTCCACCAGATAAACCATTAGAGATAC
TGATGTCACCACCAAATGTGTATCCTAAAGTGTTTTGCACTTGAAATTCTTCATTTTGATTTTTAGGTGCATA
GTCTACTACATTAACCGAATCATTAGATTGTGAACTGATAGACACATTGTATTTTGCACCCCAATATATTTTT
GAAAAGTCATAGTCTTTAGGGTTTGGTCTTTCATAACCTGAGTTAATATTACCTGCAGCTTTAAGTACTAAAG
TATCTTTATCATAACTTTTATCTTTGATGAAATTAAATGTTAAAATCTGTGAAATTTTGAATTTATCTGAATC
TGCTGTAGCTGTAGTTTTGTATAAAGTAACTTTATCATCTACTTTTTTGACGCTAACTGGTGTGATTTTGCCT
TCAGCATTAGCAGTACCAGAAAGTAATAATAATGCCATAGATGTAGCAACGGATGATTTGACTAATTTATTCA
TGTTCATATCAATTCTGTCCTTTCACTTTGATTTCGTGTGTCTTCCAATTGACTTCATATTTCACAGTGTAGT
TTCTATTTTTAAATGCATTATGGACTCTGTGACCATCTAAATAACTGTTGCCATAATGTGTTGATCTTTTAAT

GGCATGAGTGACATCCATATTTCTTCCATAAGTGATTTCAAATTCGCTCGTGTCGCCTGAACCTTTTTCATGA
GATACTGTTGCGATAAATGAAGGGTTAAATCCACTTTGCACAAGAGGTGGTAATTCGCTGTCTGGAACAAAAT
AATCTCTTGGATCTTTGCTATTTGGTTTGTATCCTACGAATAAATCACTATCAAATGCTGACTTTTGACCTGA
TGCAGTGACGAATGAATTCGCTTTGACACCCCATAAAACACTTTTTGAGTTTTGTTGTTCCACTTCACTGACA
TAATTTTGTTGTGTATAGCTAATCGATTTAGAGTAGTTAAAGGATCCATTACCACCGAGTGATGGGGCTGATT
GGAAATTACCGCCGATATTGTATCCTAATGTCTGGCTCACATTTGTAGATTCAATTTTATTTTTCGGTAAATA
ATTGATTAAAGAAACATTTGGATCATTTGTTTTTAACCCAATATTATATTGGAAGGGCCAACGCATAGATTTA
ATATGATTTGTATTTTTATAGTTATAATATGTTGTTCGAGAGCTAATGAATCCTTGCATCTTTAGGATCAAAG
CATCTTTGTTATATTTTTTATCCTTCACAAAGTCGAATTGGATATTTTGAGTCACTCCCCATTTATTACTTGT
TTTATCTTCTGTCCTTTTGATAATTTCTACATCGTTTCCTTTACCGATGTCTTCAGTATCATTGGCAGCTTTA
GCATTTTCTAATAACGGATTGGCAAGAGGGGCAAGTAAGCTTACAGATATAGTTGTAGCTAATATTTTATTTT
TAAGCATAAGTTTCACTTTCTTTCTATAATTTTATTTAACTTAATTAAATAATAATATATTTGAACGTTATT
ATAATTAACTGTTCATTAACAATTGACCATTATCAACTTTTTTTACAATGGCTTTAAAAATGTCATAATTCCG
TTCGATTTAACCTTTTTTGTTTGGAATGCTAAAAGCTAATTTGTGAGAAATATAGTTAATAAAACCTTGGTA
TTGTTGACAGTTGTAAACAATTTAAGGTAACCGATTGCACGAATGTTTTGCTTTCATTTTTAATTAATGTTTG
TAAAATACAAATTGTGCTTTTTTACGATTTTTGATGAATCCATCTAAAAGTTAATTGTTTATTAACTTGAGAA
TATTAATTTAAGCTTTCAACTTAAATTGCTTCATTTGTATTATTTGTGTATCTACTATGGTGCGATACTTTGT
TCTTATAAAAATCAGTTGTTATTATTAAATTTAATGTACGATAAAATAATTTAATGTTATGAGGCTGATTCAA
TATGTTAAAAAATCCTGAGACAAGTATGAAGTGTCCCAGGATTTTTTAAAATGATTGAACTGTTTACTTAGGT
GTGATGCTTTTAATTTTTACTTCATGTGTTTTCCAGTTCACTTCATATTTAACTGTAACGTTTCGGTTTTTAA
AAGCATCATGTTTTCTATCAACGGCTAAACGATGTCTTGTCACGTAAGCATATGTAGCATCCATGTTTCTGCC
GTAAGTGATTTCAAACTCGCTTTTATCACCTTTACCTTTTTCGTGTGACAATGTTGTAATAAATGATGGATTA
AAGCCACTTTGAATTAAAGGAGGTAGTTGATTATCTGGGACGAAATAGTCTCTTGCTGCTGGACCAGTTGGGT
CTTGGGCAAATAAGTATTGATCATATGCAGATACTTGACCATTCGGTGTAACGAATGAATTTGCTTTCACTCC
CCATTTAACACCTTTAGAGTTCTGACTTTCTACTTCAGTAACATAGTTTTTTTGATTATAACTAATTGTTTTA
GAGTAGTTGAATGAGCCACTGCCTCCGATTGATGGCGCTGATTGGAAGTTTCCGCCGATATTATAGCCTAATT
TTTGACTAACATCTGCTGAATCAATTTTATTTTTAGGAAGATAATTGATTAAATCAACATTAGAGTCTTTCGT
TTTCAAACTGATATTATATTGAAATGGCCATATCATTCTTTTAATATATGGATATTTTTTTAAGTCTGAATAT
GTTGTTCTAGAGCTGATGAAGCCTTGCATCTTAACAACTAGGGCATCTTTGTTATATTTTTTATCTTTTACAA
AATCAAATTGAATGTTTTGAGTTATAGCTAATCGTTTGCTAGTAATGTCTTGTGTTCTTTTGATGATTTCTGC
ACCTTGCCCGATATCTTCTATCTTATTTCTGCTTTAGAAATTTCTATAAATGGATTAGCTAAAGGGGCGATT
AAACCAACTGCTAAAGTTGCTGTTAATATTTTATTTTTAATCATAGAAATCACTTTCTTTCTATTTAATTTTA
AGTTCATATATATAATAATCTATTTGTAAGCGTTTTCGAATTAACTGTTCGTTAAAAGTTAATTACTATCAAC
ATGTATTGAAGAGACATTTTTAGCATTGTTAAATGATGTTTTTGCATTATGTTATGTTTAGAAGATTTATTGG
TCGCAGGCGTTTATATAGCGACATAACTATGTTTTGCAAGTCTTATTCTATTGAATTAATGTTTAATTAAGGT
GCTTGAAGTGGAAGGGGAATGAATTGCGCAATAGTTTGTGAAAATTTGAAGTTAATAAAGTTGAGCTGATTCA
ACTAAATGATGAACTATTCGTTAACGATGTAAGGATTATTTAACAAGTTGCAGGTAAAAAAGTGGGCTGACAA
GTAGCGAAGAAACATTTAATTGATGGAAAATGAAAAAAGAACCGTAGATTCGATTGAGAATCAACGGTTCTTT
TTAATCACGGTTTATTACGAAAATTATTTAAAAAATGATGTAAAACTAGAGAAGATATTTTTGATTGATGTTG
CTGTTTCAGCTGTAGTTATAGCAGCTTTAACTACTTTTGGATTTTGATAAAATGCATATAATACTTTACCTGT
GTTTAAAGTTTCAGTTACATAGTTTCTTACTTGTGCATATGATTTAGATACATAATTTTTTTGATCTTCAACT
TTTAATGGTTGGATGTATGATTTGTATCCATTACCAAAAACTGATAGTACAGTTTTATCTATTGTACCTTTAT
ATTTAAAATATTTATCAGTGTAGTACTTTGCTGTATCATATTTTTCTTTATAGCTTTTATAAGTATCTGTTAA
ATACTTGTAACCATAGTTAAATGAATCTTTTAATGATTGGTAGTAACCTAATAATTTAGATTGAGGAACCTCA
ACTTTTGGCGATTGAATTTGAGGGACTTCAACTTTTGGTGATTCTGCTTTAGGTTTTTCAATTTGTGGTGATT
GAATTTGAGGAGCCTCAACTTTTGGCGCCACTTTCTTTTCAGCATCTTTTTGAGCTACTAATGCGTCTAATTG
TTTCTGTAAGTGCTTTTGTACATCCATAGGTGCTTTATTAACTTCACGTTGTGCTAAACGTCTGTTTTCAATT
GAATCATTTTTTTGTAATTTAGTAATGGCATCGTTTGCTGATTTCACACGCTCATCATGACGAACGATCGCTT
TTTCAATTGAAACTTTTTCTACAAAGCGGTTATTTTTATCACGTAAATCTTTAGGTAATTTTGAGTTAGCTTC
ATATGCTGCTGATTTATCGTGTGGTGCTTTGTTATCTTGGAAATCTTTAATTGCTTTATCCGCATTTTCAATA
TTTTGACGTTCTTTAGCTTTAGAAGATTGTACTGATTCTACCCAAACTTGTTGGCTTCTATCAGGGTTTTCTT
TAATTTGTGCAATGTAATTATTTTTTTCTTGTTCAGTTAAGTTATCATTTTTAAGAACATTGTAAAAAGCGTT
TTGTTGTGCAACACGTCGGTCTGGGTTCTTGCTGTCTTTAAGTGATTCAGAGAATACTTCTTGTGCACGTTCT
GGGTGTTCGCGTAATGTTTTGATGTATTGGTTACGTTGTTCTTCTGTGATACCTTTTAGGTGTAATACTTGAT
AAAAAGCTTTTTGTTGATCTGTTACGTAGTTGTTTTGAGTTGTTTGGTGCTTAGTTGAAGTTTGTTGCGTGTT
TTCACTCGCTTTTGCTTCCCCATTTGAAATCATTGTAGCTAAAGTAATTGTTGCTGCCCCAACTAGCAACTTC
GAGATATATTTATTTTTCATGTGTATTCCCTTTCTTTTTACATATTAAATTTATTGTATGCTTTTAGAATTTA
TCACATGAGAATACAAATGGAAATTAACTAATTATTAACTAATGTTAACTAATAGATTAACTAGTGTTAACTA

TGTAAAAAAGCGTTTAATTCAGAACATTCAGCGTATTTTAAGTAAGGAAGTACTATATGGAGAAATGCAATAG
TTTGCTAATAAATAGAGGAGAAAATTGTGTTTCGAAGAGAGTTTGGTAGAGTAGAAATCTCATTTGATTTGTC
ACTGATGCACTTTTTCTTCTATATAATTAGGTCACGACATTAGATAGCTTCGCAACTAATGCATAGGGTCCCA
ACATAGCAACTTTCGAGGCGAAAGTTCACAAACAGGGAGAGTTGGGCGCCTTTCTAACAAATAAATTTGAAGA
AGACGAAGGAACGCAGTTGGACGTAAAACGTCAACTGCATAAGCCTTTCTAGCTCATAAAATTCGCAGAAAAT
AAAGGAACGCATTAGATAGCTTCGCTACTAATGCATAAGCCTTTCTAGCTCATAAAATTCGCAGAAAGGCTAA
AAAATATTTTTAGGGGTTGCGAATGAATAAATTAGTTTTATTTTATAAAAATTTATAAAAATTAAAAACGTTG
TAAAATTAAAATAAATCATTCATAATATTGTTACTATACTAATTCAAGGTGGTTCAACGCATGTCTCATAATG
AAAATCTTAAATTGGCGCAACGCGGTGCTTATTTGAGTTTGATTGTATATATAATCTTATCTATTGTTAAGTA
TGTCACAGGTTTTGTCTTTAACTCTGCAGCTGTAAGAGCCGACGCATTAAACAATATGACAGATATTATCGTT
TCTTTAGCTGTTATTATTGGATTGAAAATTTCAATTAAACCTGCCGATCGAAATCATCCTTATGGCCATTTGA
AGTCTGAAAATATTTCTTCATTATTGGTGTCATTTGTCATTATGTTTGTAGGTATCCAAGTAGTTATTCAAAA
TGCACCTCGTTTGTTCAAAGAAGATGACGTTGTACCTAACGCAATAACAATTATCGTCAGCTTAATCAGTGGT
CTTGTAATGTTGATTGTATTTGCAGTCAATCAAAGACTAGCGAAAAGAACGAAAAGTAGTTCTTTAAATTCTG
CCGCCAAAGATAATTTATCAGATAGTTTAGTCAGCATTGGAACGGCGATAGGATTAATTTTTACGCAAATCGG
TTTCCCAATTGTCGATATTATTTTAGCAACATTGCTAGGCTTACTAATCGTTTATACTGGTTTTGGTATTTTT
AAAGAAGCAATTTTTATGTTGAGTGATGGTTTCAATGAAACTGAATTAGAAGCGTATCGCAATGATATTTTAG
AAGTTGATGAAGTACAAGAAGTTAAAAGTATTAAAGGGCGTTACCATGGAAGTAGTGTGTTTATTGATGTCAC
AATTGTTGTAGATGCTAACCTTTCCTTAGTAGCAGCGCATCAAATTTGTGACAATGTCGAACATCATTTGCAC
AAAAAAGGTATTTCCTCAGTATACGTTCACCCAGAACCAGACCATCTATAATGTATTTAAACGTCTTCATAGA
TATATAAATATCTGAGGAGGCGTTTTTATTACATTTATTTTTAGGTGCACCTAAAAATATTTTTTAAACCTTA
GAAAAATGGCGAACAAAGGTGTATAATATGATTTGTTATTAGGTGGTTATTGATTCATTTATGAATTAAAAAA
TGAAAATGGGGAATAGGATTTATGTATTTAATGTTTAGTGATAATTGATTTAATCATTGAATTTTAAAACATA
TTGAAAAGAAAATTGCTAAGATTAAATCATCTTAAAATATCCCTGAAATAACGTCCTAAAGATTAAAGGAAAG
AGGTTATAAGTTATGCCAAAATTAATTTTATGTCGTCATGGACAAAGCGAGTGGAATGCTAAAAACTTATTTA
CTGGATGGGAAGATGTTAATTTATCTGAACAAGGTATTAATGAAGCGACTAGAGCAGGTGAAAAAGTAAGAGA
AAATAACATTGCCATCGATGTAGCTTTTACATCGTTATTAACACGTGCTTTAGATACAACGCATTATATTTTA
ACTGAATCTAAACAACAATGGATTCCTGTATATAAAAGCTGGCGTTTAAATGAACGCCACTATGGTGGATTGC
AAGGCTTAAATAAAGATGATGCTAGAAAAGAATTTGGAGAAGAACAAGTACATATTTGGCGTCGTTCTTATGA
TGTGAAACCGCCTGCTGAAACCGAAGAACAACGTGAAGCTTACTTAGCTGATCGTCGATATAATCATTTAGAT
AAACGTATGATGCCTTATTCTGAAAGTCTGAAAGATACTTTAGTTCGAGTGATACCATTTTGGACAGATCATA
TTTCACAATATTTGCTAGATGGTCAAACGGTATTAGTTTCTGCACACGGAAATTCAATTCGAGCATTGATTAA
ATATCTTGAAGATGTGTCAGATGAAGATATCATTAATTATGAAATTAAAACAGGTGCACCGCTTGTTTATGAA
TTAACCGATGATTTAGAAGTTATAGATAAATACTACTTATAAAAAAAGAGCTGCATGTACACAAGGAGTGAGT
GTATATGCAGCTCTTAAATATGTGAAGTAATGTAAGGAAATAGTTAAGTATAGAGTTTATATTAACGAGCTAG
GGATACTCGAAAATATAGTTAGACATACAATATAGTCAAATTAAAACAATTATTTCGCTCTTTTGTGTTGCTT
AATAATCTTTAAAGCACGCTTTCTTGTTTTAATGTTAGGGCTATTTAAATTACGACGAGCAGTCTGTAAATCT
AATTTCATCTCTATCCCTCCTTGTAAATATATTATGACCGATAACTACTCATATGTAAATAGTAATGATTACG
TTTTAAAGAAATTGTAATAAAGTCGTGCTAATTTTTTTGAAAATGGGTATAATTACCGGATATCTAAAAATGT
GTGTCGTTTTTTAGATGGTGAGGGGGAAGCTTTAAATGTCGAAGAAACAAAAATTAACGATGATTATTACTAT
GCTGATGGGTGGATTTTTTGGATTATTAAATGAAACACTATTAGTGACGGCTTTACCAAGTATTATGAAAGAT
TTTGAAATTTCATATACACAAGTTCAATGGCTGACAACAGCTTTTTTATTGACTAATGGGATTGTTATTCCTT
TGTCCGCGCTTGTTATACAACGTTATACAACAAGACAAGTGTTTTTAGTGGGTATTTCTATCTTTTTCTTAGG
TACATTACTCGGCGGTTTGAGTCCACACTTTGCAACATTATTAGTTGCTAGAATTATTCAGGCGTTAGGTGCT
GGAATTATGATGCCTTTAATGATGACAACGATTTTAGATATTTTTCAACCGCATGAGCGTGGTAAATATATGG
GCATATTTGGCCTCGTTATTGGTTTAGCACCAGCTATTGGACCTACTCTTTCAGGTTACCTTGTTGAATATTT
TAACTGGAGATCGCTTTTCCATGTTGTCGCTCCAATTGCAGCTGTGACATTTTTAATTGGATTTAAAACGATA
AAAAATGTTGGAACTACAATTAAAGTACCTATTGATTTTATTTCTGTCATTTTTTCTGTACTAGGTTTCGGCG
GGTTATTGTATGGAACGAGTTCAATTTCGGAAAAAGGTTTTGATAATCCTATCGTATTAGTTTCGATGATTGG
AGGCGTTGTTTTAGTCGCATTATTTGTATTACGTCAATATCGGCTATCAACACCATTATTAAATTTTGCAGTA
TTTAAAAATAAACAATTTACAGTTGGTATCATTATTATGGGTGTCACAATGGTATCAATGATTGGTTCGGAAA
CGATTTTACCAATTTTTGTGCAAAATTTATTGCATCGTTCAGCTTTAGATTCTGGGTTAACTTTATTACCAGG
AGCAATTGTTATGGCATTTATGTCGATGACTTCAGGTGCCTTATATGAAAAGTTCGGTCCAAGAAAATTAGCT
TTAGTCGGTATGGCAATCGTTATTATTACCACTGGTTACTTTGTTGTAATGGATGAACAGACATCGACAATTA
TGTTGGCAACGGTTTATGCGATTCGAATGGTTGGTATCGCCTTAGGTTTAATTCCTGTTATGACGCATACAAT
GAATCAATTAAAACCTGAAATGAACGCACACGGTTCATCTATGACAAACACAGTACAACAAATTGCTGGTTCT
ATCGGAACAGCTGCATTAATTACTATTTTATCTCATGCAAGCAAAAACTTTTCTCCAACTATGTCAGACTATA
ATGGCATGAACAAAATTGAGATGATGAATCAAATTAAAGTTGATACGATGTTACATGGTTATCACGCTGGATT

TTTATTTGCACTATTGATTACAGTGATTAGTTTCTTTTGTTCATTTATGTTACAAGGAAAGAAAAAAGAAGTT
GATTCACATCAATAAATATAAGTTGCTAGCTATATAAAGCTTTTTAGCAAAAGTTCAACATATTGACTTATCC
GGCATTTCAGATTAAAATATTTTTATTCCGATTAGAATAATAAGAATAAGGAGATATATTCTATGAAAAGACT
TTTATTTGTGGTGATAGCTTTCGTTTTCATATTAGCTGCATGTGGGAACAATTCGTCTAAAGACAAGGAAGCT
AGTAAAGATAGCAAGACAATTAATGTTGGTACTGAGGGGACTTATGCACCATTTAGTTTCCATGATAAAGATG
GTAAATTAACTGGTTACGATATTGATGTTATTAAAGCAGTAGCTAAAGAAGAAGGTTTAAAACTTAAATTTAA
TGAAACTTCTTGGGATTCTATGTTTGCAGGTTTAGACGCAGGGCGTTTTGATGTAATCGCGAACCAAGTGGGT
ATTAATCCTGATAGAGAAAAGAAATATAAATTTTCTAAACCTTACACATTCTCAAGTGCTGTTTTAGTTATTC
GTGAAAATGAAAAAGATATTAAAGATTTTGATGATGTTAAAGGTAAGAAGTTAGCACAAACATTCACATCTAA
TTATGGTAAATTAGCTAAGGATAAAGGTGCTGATATTACAAAAGTTGATGGCTTTAACCAATCAATGGATTTA
TTATTGTCTAAGCGTGTTGATGGTACATTTAATGACAGTCTGTCATACTTAGATTATAAAAAACAAAAACCTA
ATGCTAAGATCAAGGCAATCAAAGGTAACGCTGAACAAAGTAAATCTGCATTTGCATTTTCTAAAAAAGTGGA
TGACGAAACAGTTCAAAAATTCAATGATGGCTTGAAAAAAATCGAGGAAAACGGTGAATTAGCTAAAATAGGT
AAGAAATGGTTTGGTCAAGATGTTTCTAAATCTAAATAGTGAACAACTACATGCACTTGATGCCGCAAAACAA
GCTTTTGGGCCTATGCTCGAAGGTCTGGTGAAATATTCAATTCCGATTACCCTCGTAACATTTGTATTAGGGT
TAATCATTGCATTATTTACAGCATTGATGCGCATTTCAACGAGTAAAGTTTTAAGAAGTATTGCACGTGTCTA
TGTATCTATTATTCGTGGGACACCTATGATAGTACAATTATTTATCATCTTTTATGGTATTCCAGAATTAGGT
AGATTATTAACTAATGATGCAGATAATCAATGGACATTGGCGCCTGTGGTCGCTGCCATTATTGGTTTGTCAT
TAAATGTAGGTGCATATGCTTCAGAAATTATTCGTGGCGGTATTATTTCTATACCAAAAGGACAAACAGAAGC
TGCATATTCTATCGGTATGACATATCGTCAAACGATACAGCGCATCATTTTACCGCAGGCAATTCGAGTATCT
ATACCTGCACTAGGTAATACATTTTTAAGTTTAATCAAAGATACATCATTACTAGGATTTATTTTAGTGGCTG
AAATGTTTAGAAAAGCTCAAGAAGTTGCGTCTACAACATATGAATATTTAACAATTTATGTGTTAGTTGCGCT
AATGTACTGGGTGGTATGCTTTATTATTTCAATAATCCAAGGTATCTACGAATCTTATATTGAAAGAGGGTAT
CGCTCATGATTCAATTGAACAATATCCATAAATCATTTAATGATGTTGAAGTCATCAAAGGTATTGATTTATC
TGTTGACAAAGGTGAGGTTGTAACCTTAATTGGCCGATCTGGGTCAGGTAAAACAACGTTGTTACGAATGATT
AATGCGTTAGAAATTCCAACTGAAGGTACAGTATTTGTTAACGGCAAAACATATACATCTAAAGATAAAAAAT
CACAAATAGAAGTTCGTAAACAATCTGGTATGGTATTTCAAAGCTATAATCTTTTTCCGCATAAGACGGCATT
AGAAAATGTAATGGAAGGTCTTATTACAGTTAAAAAGTTGAAAAAGGCTGAAGCTCGTGAGAAATCTCTAGAA
TTACTTGAGAAAGTTGGTTTGACACATGTCAAAGATCAACGACCACATGCATTATCAGGTGGTCAACAGCAAC
GTGTTGCCATTGCGAGAGCATTAGCTATGAATCCTAAAGTGATGTTGTTTGATGAACCAACATCTGCACTTGA
TCCTGAACTTGTGAATGATGTGTTAAAGGTGATTAAAGATTTAGCGAATGAGGGCATGACAATGGTCATTGTG
ACACATGAAATGCGTTTTGCTAGAGAAGTATCTAATAACATTGTATTCATTCATGAAGGTGTAATTGGAGAAC
AAGGGGCACCTGAAGAGATTTTTAACAATCCAAAAACTGAAGAACTAAAACGTTTTTTAAATGTTATTAATGA
AGAATAAACAAATAGAACCACGAATCTTGTTTGATTACGGCGAAGATGCCACTAGCAAGATTCGTGGTTTTTG
ATGTTACAGCATAAACTGTTAACGGCGTTTATGTTTTAAGTATGTTTGTAATTTGTAAAGCATAGGTTTGATT
GGTTTAATAAAATCACCAATATATTCTTCGACGTGTGCGTTAAATCCTTTTTTGAATTGTTGTACGCCATAAT
CTTCTGATGACTCTGAAAAGTCACCGGTAATACCATAGAAATTATAGCGATCAATATGATGCGCTTTAGCAAA
CTTAATCATTTCCCACTGCAAATGGTAGGCACCCATATAAGCATTATATTTAGGGTTTGAACCACTAGATAAG
TAATAAACTTCATGCTCATTGTAGATAAACAAAGCAGAAGCTAAGTTCAACACTGCACCATCTTGTTCAATTT
GTTCTATTGTATTATCAATTTTTCGCTTATTACTGCTTAACTGTTGTTCTAACTGTGTTCGTTTCGTTTTATT
TTTCTTTGAATTCGGACTTTCTTCCAATGCCTCTTCAACACCTGAGAGCTCAGCAGCTAATTGCTGTTGTTTC
AATTGTAATGTTTTCAAATAATCATTTAAATCGATATAAGCTAACTTTAACATGGCGTGATCACCATATAGTT
TTTGCATTTCTTCAAAGTATGGTAACTCACGGAATTTGAAACCGTGCTTTTCCTCAGCCATATGGAATAAGTC
GAAAAAGGTTTGCGTTTCATCAATCGTTAACGTTTTAGTTTTGACACCAATATCATATGTTTTTTTAATATTA
CGTCTCGTTTGATAATCCATTTCTTTTAAAAGTTGGTCTTCAGTCTTATCTTTTAAATCTAACACTGACAGCC
AACGGATTTGGCTCATTGAATCATAACCTACAGGGAAACCTTGGTGTTTATAACCTAATTTATCCATTGTTCT
AACAAATGCTCGGTTATCATAAGACTTAACAATTTCACCGTCTGCATTGCGTAAATTTTCAATTAAATATGGA
TCTACAAGGACATATAAACAATTTTGTTTCTTTAAATATGACGTTAATGCTTTAAAGAAAAATGCTACTAATG
ATTGATTTGTATAATCCATCACTGGCCCGCGATGTGTATAAAAATATTTGAAAAATTTAAGTGTGCGTGCTTC
TGTCAATAAGCATCCTGCAATCACTTGACCATTGTCATCTTTAACCCCTACAAGATGCACATCGCCTTTTAAA
TCAACTCTATGATTGTAATGAATAGCTGATTGTGTGTAATGTGAAAAATGCTTTGTTGTAAACGCATTGAACT
CATCCGAAGTCAACGTTACAAAGTTCATTTTATCCCATCCCTCTCTGAATTGAACTGTAATTTTTGAATTTAT
TTATAATGCTATTTTTTTATTTTATCAAAAACTGATTGTAAATACACATAGAAAATGAAATATATTTTTCTTT
GACTATACATAACTGTTATTTCTTTGGATATTTATATTAAATGCATTGGTAGTAGTTGTAGCGCATTGATGTT
TGACGGTGTATAGTATTAATTATATTGAAAGTTAGTTGGAAGTTGATAGTAGGAGTGGGAGCATTGAAGCGAT
TTGTGGCGACGGTATTATTATTACTAGTCTTTATATCAGGATGTGGTAATGATAAATATGTGAAAGAAATAGA
TGAAGCAGTTAAAATTCAAAATCAAAAACAAGAACAACTTGCCAAAAAAGGCAACGGTGATCGTGTTGATCAT
TTTGAACGCAAAGATGCTAATATTTATGTCTATGATAAGGATAAAATTATCATTTTAGCTTATAAACCTTTGA

GTAATGATGATGAAGTGCATTATTATGCATATGATTTTAGTGATAAACGTGTATCATATAAGCAAGATTTTGA
TTCGAGACGATATTATCAACAACATGATGCGGATTATCATGAAGAAAATATGACGAACTAGATATGAACAGGA
GTTATTATGACTAAAAAATTACTAACATTATTTATAGTGAGCATGTTAATTTTAACAGCTTGCGGTAAAAAAG
AATCAGCAACGACATCTTCGAAAAACGGCAAACCATTAGTTGTCATATATGGCGACTATAAATGTCCTTATTG
TAAAGAATTAGATGAAAAAGTCATGCCAAAGTTGCGTAAAAATTATATAGATAATCACAAAGTGGAATACCAA
TTTGTCAATTTAGCTTTCTTAGGTAAAGACTCAATTGTTGGTTCGCGTGCGAGTCATGCAGTATTGATGTATG
CACCTAAATCATTTTTAGATTTTCAAAAGCAATTATTTGCTGCCCAGCAAGATGAAAATAAAGAATGGTTAAC
AAAAGAACTATTAGATAAACATATTAAACAACTGCATTTAGATAAAGAGACGGAAAATAAAATTATAAAAGAT
TACAAGACAAAAGATAGCAAGTCTTGGAAAGCTGCAGAGAAAGATAAAAAAATAGCTAAAGATAATCATATAA
AAACGACACCAACTGCATTTATTAATGGCGAGAAAGTTGAAGATCCATATGATTATGAAAGTTATGAGAAGTT
ATTAAAAGATAAAATCAAATAGTCTTAATTGTTGAGATAAGTAAGTGATACCAATTTTTTAGGACAATATGAA
AAGGCAATTTCTATTAGCAATATGATAGAGATTGTCTTTTTAATGTATGAAGTCTAAATGGGTGGAAATTATC
ATGTAAACATTTTTTAGTAGGGAATCAGTACAGGATAGGTATTGATTCTTCCCCGTGCAAAATCTTGATTAGT
AATTTTTTTGTGTCGCTTGTTTTATTGTACAGATATTTGTACAATTGAGGTGTGTTAAAAGGAGGCGATAAAT
ATGATTATTAAAAATTATTCATACGCTCGACAGAATTTAAAGGCACTTATGACAAAAGTAAATGATGATAGTG
ATATGGTAACTGTAACATCTACTGATGATAAAAACGTAGTAATCATGTCAGAATCAGATTATAACTCCATGAT
GGAAACACTTTACCTCCAACAGAACCCAAATAATGCTGAGCACTTAGCTCAATCAATTGCAGATCTAGAACGT
GGGAAAACTATAACGAAAGATATAGATGTATAATGGCTAGGTTAAATATTACGTTTTCGCCTCAAGCCTTTGA
AGATTATAAGTATTTTCAGCAGAACGATAAAAAAATGGTGAAGAAGATTAATGAGTTACTTAAAAGTATTGAC
AGAAATGGTGCATTGGAAGGTATAGGTAAGCCTGAAAAGTTAAAATCGAATCTGACTGGGTATTATAGTAGAC
GTATCAATCACGAACATAGATTGGTTTATACAGTAGATGACAAGCATATAAAAATAGCATCATGTAAATACCA
TTATTAAATCTAAAAGTCACACTATAAAAATAGGGTAAGTTATTTTAGGACAATATGAAAAGGCAATTTCTAT
TAGCAATATGATAGAGATTGTCTTTTTTCTTATATTATGACGTTCGTAAATACTATAAAGTTTTCTGTATCAT
ATTTGTACAATAGTCATGTATTAGATAAAACGAAAAGTGGTAAATACCTAAAGTATGATTCGACTTTATATTA
TTTATAAAAACAGTGCTTGTATTTTAAGTAGCATGAGTATATAATTTTAAAACGTAAAGATTACTATTTAGGA
GGGTGACTATGAAAAAGAAATTAGGTATGTTACTTCTTGTACCAGCCGTAACTTTATCATTAGCCGCATGTGG
GAATGATGATGGAAAAGATAAAGATGGCAAGGTAACAATTAAAACGACAGTTTATCCATTGCAATCATTTGCA
GAGCAAATTGGTGGAAAACACGTGAAGGTATCATCAATCTATCCAGCAGGGACAGATTTACATAGCTATGAAC
CAACACAAAAAGATATATTAAGTGCAAGCAAGTCAGACTTGTTTATGTATACAGGGGATAATTTAGATCCGGT
TGCTAAGAAAGTTGCATCTACTATCAAAGATAAAGATAAAAAACTGTCTTTAGAGGATAAATTAGATAAAGCA
AAGCTTTTAACTGATCAACACGAGCATGGTGAAGAGCATGAACATGAGGGACATGATCATGAGAAAGAAGAAC
ATCATCATCATCATGGTGGATATGATCCACACGTATGGTTAGATCCTAAAATTAACCAAACTTTCGCTAAAGA
AATTAAAGATGAATTAGTGAAAAAAGATCTAAAACATAAAGATGACTATGAGAAAAACTACAAAAAATTAAAC
GACGATCTTAAGAAAATTGATAACGATATGAAGCAAGTTACAAAAGATAAGCAAGGTAATGCAGTATTCATTT
CACATGAATCAATTGGATACTTAGCTGATCGTTATGGTTTTGTTCAAAAAGGTATTCAAAACATGAATGCTGA
AGATCCATCACAAAAAGAATTAACTAAAATTGTTAAAGAAATTAGAGATAGCAATGCTAAATATATTCTTTAC
GAAGATAATGTTGCGAATAAAGTGACTGAAACAATTCGTAAAGAAACAGATGCGAAGCCTTTAAAATTCTACA
ACATGGAGTCTTTAAATAAAGAACAACAGAAAAAAGATAATATTACGTATCAATCATTAATGAAATCGAATAT
TGAAAATATCGGTAAAGCTTTAGACAGTGGTGTTAAAGTGAAAGACGACAAAGCTGAAAGTAAACACGATAAA
GCAATTTCTGATGGGTATTTTAAAGATGAGCAAGTTAAAGACCGTGAATTAAGCGATTATGCTGGTGAATGGC
AATCTGTTTACCCTTACTTAAAAGACGGTACGCTTGATGAAGTGATGGAACATAAAGCTGAAAATGATCCGAA
GAAATCTGCTAAAGATTTAAAAGCTTACTATGACAAAGGATATAAAACTGATATTACTAACATTGATATAAAA
GGAAATGAAATTACATTTACTAAAGATGGTAAGAAACACACTGGTAAATATGAATACAATGGTAAGAAAACAT
TGAAATATCCTAAAGGTAACCGTGGCGTGAGATTTATGTTTAAATTGGTCGATGGTAATGATAAAGACTTACC
GAAATTCATCCAATTTAGCGATCACAACATTGCACCTAAAAAGGCAGAACACTTCCATATCTTTATGGGTAAT
GATAATGACGCGTTATTAAAAGAAATGGATAACTGGCCAACATATTATCCTTCAAAATTAAATAAAGACCAAA
TCAAAGAAGAAATGTTAGCGCATTAATTCAACAGGTTTGCACACTCTACTCAACTGTTAATAGCTGAATAGAG
TGTGTTTTTTATTTCGTAGTTATCAATTTCAACAGAGGGAATGATTAATATTACTATAGTTTATAACAAATGA
TTTGGTAACATATGTTACTCAATGCATGTTAAGATGAGTAAATATAAAAGATAGGGAGTGGGAAGATGACGAT
TAAAGTTGAAGATAATAAAATTATTTTTTCAAGAACGATAGAAGCACCGATTGAAAAGGTGTTTGATGCCTAT
ACGACAAAAGCATTATTTGAAAAATGGTTTCATCCAAAAGACGCTAGTACCAAAGTGTTTCGCTTTAATGCTG
TTTCAGGCGGTGATGCATTTTATGCGATTAAAACACCTACAATGACCAGCTATACATTAGCAGAATATAAAAC
GGTTAAGCGTCCATATTTAATTGAATATATTGACTCATTTGCGACACCTCAAGGTGCAAAAGATACGAAAATG
CCAAGCATGAAAATTACTTTGTCATTTTCCAAGAGCAATACAACGAAAACGACAGTTACATCAACATCGGTAT
TTCCAACAAAAGAAGCCGCTCAACAAGTCGTAAACATGGGTGTTGAGCAAGGGATGAATCAAACATTAGATCA
ACTTGATGCTTTATTGAAATAAAGTTGTTGAAAATATGTAAAAATTTTTATATGCCATTCAGTTTATAATAAT
AACGTAATATTATTAATTGAATGAAGGATGATTCGATGGTACAACGTTACATTAAAGTATTAATATTATATAT
TTTTACTACACTTTTATCTTCAATTACTGTTACTTCGAAATGTGTACCAAATAAAGTTGTTAGATTTATTTTG

CGTACAGTCGTAGGCTACGGTATATTTGCATATGGTTTACATTACTTTTCAAATTTAAAAAAGAATAAGTAAT
ACTTACATAAAGCGAGATGAGCGCAATTAAAATGATACCAATTGTTTTATGTCGCGATATCTATCTCGCTATT
TTTATGAGTAAATATTAGTTTTGAATTGGTAAAAAGAATTTTCTGAATTTTAATCTATGAATTACATCTAAAT
ACTAATATAATTAATAGGTAAAATTTATTTGATAATAGTAAATGGGGGAAGTGCTGTGATAAAAAATAATAAA
ACAGTTGAAGATACGTATTCAACTAAAGCAATCGTAGACTCAATATCATCATCTGTTCAAATGAAACAAGTGA
TGGTACAGCAAACACCAGGACGATACATGCTAAAATCTATGATGGCAGGTTTCTTATTGTCCATTGTTACTGT
TTTTATGTTTGGTATTAAAACACAATTCGCAAGTACACATGTTGATGGTCTCATTAATTTAATGGGAGCAATT
GCTTTCAGCTTAGGATTGGTCTTAGTTGTACTAACGAATTCTGAATTATTAACAAGTAATTTTATGTATTTTA
CAGTTGGCTGGTATTATAAAGTTGTTTCTCTTAATAAGATGATGTGGATTTTATTATATTGTTTCCTTGGAAA
TATTTTAGGTGGTTTTGTATTATTCTTCTTAATGAAATATGCACATGTTATGACACCAGAAATGACACAAGCT
TTAACTGCCTTAGTTCAGAAAAAAACAGTCGATTCGACTTGGTTGAATATTTTTACAAAAGGTATTTTCTGTA
ACTTCTTTATTAATATCGGTATTTTTATTTCTATGCAATTCAAAGGCGGATTGACAAAAGCATTCTTCATTGC
TTGTGGCGTTATAGTCTTTGTATACATGGGATATGAGCACGTTGTATTCAATGCTGGTTTATATGCAGGTATG
GTGTTCTTCAACCTTGATGCAGTTTCATGGTTACACGTTTTAAAAAATATCGCTTTTGCTTTCTTAGGTAACT
TTGTCGGTGGCGGTATTTTTGTAGGTCTCGTTTATGCTTTCTTAAATGGTAAGCGTAATCGTTTAGAGCAACA
ATAATTAAGATGTAGTGATTAAATAGCGTAACAATGAATCATTCTTCCAAACCATTCATGAATTTGAATGGTT
TTTTTACGTTGAGAGAGTAATTTTTAATAAGTTTCATGAGAGTATTGGTGTATTCAGATGAAGCATACTTTCT
GACATGTGATAAATTTATTGATACACTTTGATGGATATTTATTTTAAAAATAAAATATGGTTAAATCATTTAA
AAAAGAGTGGGGATTAATATGACAAAAACGATGATTCGACTCGCAAGTGAACAAGATGCAGAAAAAATTGCAAC
AACTCATGCATGAAGCATTTACGCCTCTAAGAGAACTTGGCATAGACTGGCCTTCAGTAAATGCAAATTTAGA
TGCTGTGAAAGAAAATATAGATAAAAATACTACATTTGTCATGACAATTGATGATGAAATTATATCTACGATT
ACGGTCAGATATCCTTGGGGAAGTGTGAAAAGCATTTCGGGCTATCCTTTTGTCTGGTGGTTTGCGACAAATC
CTGAGTATGAAGGGAAAGGCTATGGTAGCCAATTACTTACTTATGTTGAAGAAACATTTTTGCGAGATACGTT
AAAGTCAGCGGCAGTTACTTTAGGTACATCTGCAAGATTGCATCCGTGGTTACTTAAAATTTATGAAAAAAGA
GGTTATGAAATATACAGCGAACATGAAAATGATGATGGCGATTTAGGTGTAATCATGCGTAAAGTATTAATAC
CTGAACGTTTTGAAGAAACGATTCTAGGCCAACCACCATTTTAAGATAATAATATACACTACAAGCGACCGAT
ATATTGATGCTTGCTCAAATGAATGAATGTTGAGTAGGGATTAACATATTGGTCGCTTTTTTGATTTAACAAA
AAGGGAAAATTTATAAGGAATATGCATGACTACAGGCATCAGGGAATTCCCTGATTTTACATTATAATGTGAT
TCTTTTCACAAAAACGAAATGATTTGGTATACAATACGTGTGAAAGGGTTGGTAAATGTGAATGGGAATATCA
TTGTTGCACATGGCATGAGGCACGGACGACAGAATCAAGCGTTAGAAGCATTTATTTCTGAATTAGTAAAAGA
TGATATACATCACTATGATATTGCATTTTTAGAAAGTGAGCATCAGGATTTAGAAACAGTAATGACGACGTTG
ATTAAAAGTGGCGTTAAAAAGCTTAAGATTGTACCTTTGCTTCTCTTTAGTGCCATGCACTATCTCAAGGATA
TACCGAATATCGTTCATGAAATGACACAGCGTTATCCAGACATAACGGTAGAAGTAAGCGAGCCACTTGGTAC
ACATCCTTTAATGAGACGATTAATTGAACAACGTATTTGTGACGCATTAGCAGGTGACGAACAACAAGTTGGA
GTTATGGTTGTTGCACATGGCAATATTAATGGCAAGTTTACAAAAGCACATGAAGAGTTACAGACATGCGTTC
AACATTTGCAGATTAATAAACCTACTTATGCGAGAACGCTATATGGTGAAATCAGTTTTATACATGATTTAGA
AGATATTGCGAGACAGTATCAAAAATTGATTGTCGTACCTTTATTTTTATATGACGGAAGACTTGTTAACAAA
GTGAAGCAGCAAATGAATGACATGGTTATTAATACAGATATACACATCACACCTTCAATTAACTTCGACCCAA
TTTTAAAGCAAATCATTAAAGATAGATTAGAAAGCTTAATGATTCCAATGAAAATATAAAAGCACTAACGAAA
AATTAAACCTTTTTTTGAAATAAATATTATGTAGTAAAGGGGCATATTTTATGGCAAAGCAAAAACTAGTAAT
GATTGGTAACGGTATGGCGGGCATTCGAACAATCGAAGAAATATTAGAGCGTGCCAATGATTTATATGATATT
ACAGTAATTGGTAAAGAACCTTATCCTAATTATAATCGCATCATGCTTTCAAATATTTTACAAAATAAAATGA
CAGTTGAAGAAACAATTATGAATCCATATGAGTGGTATGAGGAACATGGTATCGAGTTAATTACAAATGATCC
TGTAATTGAAGTAGATAGAGCAAATCAAAATGTAACGACTGCAAATGGTATTGAAGTAGCATACGATAAATTA
ATATTTGCGACAGGATCTAAAGCATTTGTCATTCCAGTGCCAGGTTCGACCTTACCAAGTGTTATTGGCTGGA
GAACAATTGATGACACTGAAAAAATGATGGACATTGCTAAGACTAAAAAGAAAGCGATTGTTATCGGTGGGGG
CTTATTAGGTTTAGAATGTGCACGTGGTTTATTAGATCAAGGTATGGAAGTAACAGTGTTGCATTTGGCTGAG
TGGTTGATGGAAATGCAGTTAGACCGTAAAGCTGGAAATATGCTTAAAGCAGATCTAGAAAAGCAAGGTATGA
AGTTTGAAATGCAAGCCAACACAACTGAAATCTTAGGAGAAGATGATGTTGAAGGTGTTAAATTGGCTGATGG
ACGCGAGATTCCGGCAGACTTAGTTGTTATGGCAGTAGGTATTCGACCATACACAGAAGTAGCCAAAGAATCA
GGTCTAGATGTTAATCGTGGTATTGTTGTCAATGATGTGATGCAAACAAGTGATAGCAATGTATATGCAGTCG
GTGAATGTGCAGAACATAACGGTAAAGTTTATGGTTTAGTTGCACCTTTATATGAACAAGGTAAAGTATTGGC
AGATCATTTAACGAATAAAGAAACAGATGGATACAAAGGTTCCACTACATTTACATCATTAAAAGTTTCTGGT
TGTGAGCTATATAGTGCTGGTCAAATTGTTGAAAATGCTGAAATCAAAGGTATTGAAATATTTAATAGTGTTG
ATAATAACTATAAAAAAGTATTTTTAAAAGATGGAAATGTGGTCGGTGCAGTCTTATATGGTGATATCGATGA
TGGTTCACGCTTTTATAACATGATGAAAAAAGGTGAATCTACTGAAGATTACACACTGGTATCATTGCTTACT
AAAGGTGGAGAAGAGGCATCGCTATCAATTGCTGATATGGCTGATGATGAAACAATTTGTGGTTGTAATGGTG
TTGATAAAGGTACTATAGTAAATGCTATTACGGAAAATGGCTTTACAACAGTTGAAGAAGTAACGGCTAAAAC

AAAAGCGGGGAATTCATGTGGTAAATGTAAACCGCAAATTGCTCAAATATTGCAGCACACCTTAGGAGATGAC
TTTGTTGCCGCAAAACCTGCTGGTATATGTGGTTGTACTGATTTGACACGCGATCAAATTGTAACGCAAATAA
GAGCAAAAGGTTTAAAAACATCTAAAGAAGTTCGACATGTTTTAAACTTTAAAAATAAAGGTGGATGTCCAAA
ATGTCGACCAGCAATCAACTATTATTTAAACATGGTTTATCCACATGATCATGAAGATGAAAGAGAATCAAGA
TTTGCTAACGAACGTTACCATGCGAATATTCAAAATGATGGTACATTTTCTGTTATACCTCAAATGCGTGGAG
GTGTTACAGATGCAGACCAACTGATTCGTCTAGGAGAAGTGGCTAAGAAATATCATGTGCCACTAGTTAAAGT
GACAGGTTCGCAACGTGTTGGTTTGTATGGAGTTAAAAAAGAAGAATTACCAAATATATGGGAAGACTTAGGT
ATGCGTTCAGCATCAGCTTATGGTAAGAAAACACGCTCAGTTAAAAGCTGTGTCGGCAAAGAGTTTTGTCGAT
TTGGTACGCAATACACGACACGACTTGGCATTCGTTTAGAAAAAACATTTGAATACATCGATACACCTCATAA
ATTCAAAATGGGTGTATCAGGCTGCCCAAGAAGTTGTGTTGAATCAGGTGTTAAAGATTTTGGTATTATCTCA
GTTGAAAATGGATTCCAAATCTATATCGGTGGTAATGGTGGTACAGAAGTTGAAAAAGCTGAATTTTTAACAA
CTGTAGAAACAGAAGATGAAGTAATCAAATTATGTGGTGCTTTGATGCAATATTATCGCGAGACAGGTATTTA
TGCTGAAAGAACAGCACCGTGGTTAAGAAGACTAGGGTTTGAAAATGTTAAAGAAGTGTTGCTTGATCCAGAA
AGACAGAATGAATTATTTGAACGTATTATGGATGCTAAAAAAGCAGTCGAAGCTGAACCTTGGGAAGCAATCA
CTAGTAATGCGCAAGCTCGAAAAATCTTTGAAGTGGAGAAGGTGTAATACATGGAAACGAAAGAGAAAATCAA
AGTGACAACTTTAGATGAATTGACACCCTTAATTGGTAAAAAGGTTATTGTCAAAGGCAAAGAAATAGGATTG
TTTTTAACTGAAAGTGGTACAATTCACGCGATTCACAATATTTGTCCGCATAAACAAGGGCCATTGTCAGAGG
GAACAGTGAGTGGTGAATATGTATTCTGTCCACTCCATGATCAAAAGATTGATTTAAATACTGGGATTGTACA
AGAACCTGATGAAGGTTGTGTAGATGTTTATGAGGTAGAAGTTACAGACGGGAACGTATATATATGTCTGTAG
ATGAATATGGCAAGGTTTACTTGATAGGTGCGGGACCAGGTAATCCGAATTATTTGACTAAAAAGGCTGAGCG
TTTAATACGTGAAGCGGAAGTTATACTATATGATCGATTAGTGAATCCGTTGATTTTACAATATGCGAATTTG
ACTACTGAAATTATCGATGTTGGTAAAAAGCCGTATGCAAAACACATTCAGCAAGAAAAAATAAATGACTGTA
TCGTGGAAGCAGCACGTCGATATAACAAGGTTGTGAGGCTTAAAGGTGGCGACCCAGCAATATTTGGACGAGT
TCAAGAAGAAGTTGACACACTAAACAACTTTAATATTGCGTTTGAAATTGTACCTGGTGTGACATCTGCGAGT
GCTGCAGTTGCTACTTTGCAGACAGGTTTGACAATGCGTACAGTTGCTAAAAGTGTGACATTTTCTACAGGTC
ACTTTAAAGATTCAGAAGAAAATGAAGTGGATGTCAATTCCTTAGTGAATGGTGGTACGTTAGCGATTTATAT
GGGGGTAAAACGTTTAGGGAAAATTATCACACAGATACAACAGTACACAGATATTGATTATCCGATAGCGATT
GTCTTTCAAGCATCGTGTTTTAACGAGTTTGTTGTTAAGGGGCATTTAAGTAATATTATTTCAAAATTGCAAC
ACTATTCAATTGAGGCGAAACCAGGTATATGTATTATTGGGGAAGTTGTTGGTTATACTGAAAACACTCCTAA
ATCATATGATCCTACGAAGCAATTTTATGTAGTAAGTGGTGCTAAACATGACGCCCTTATCCTCTGTGAACAT
TTATATGACGAAGGTTATGGCTGTTTGCTAAACCCAAATGATTCATCGAATGGCACATATCATTCGTCGCAAT
ATGATTACTATGATGCGTTTATTAAGCAGCAAGAAAATGTAACATATATTTCAACCGATCGTGCAGATGCTAA
AACAGTGTTATGTCACTAATTTATAAAAAATGAATAAGTAAGGTTTCAACCGAGAGAATATATTCGTGTTGAA
GCCTTATTTGTCGTTGTGCCAAATTTGAACGATTTAGATTGGCAATAAACATGGTCATATGTGATTGTGTAAA
ATTTCAATAAAATCCAATTATTGATAGGGAAAATCCTTAAGTAATTAGGTAATTCCCTAATATTTTATATTTG
TGAAAAAATGTACAATCTAATTAAAGCAATAGTCTTGGGCATTTTTAGTATTTAAAAACACGACATCTACACA
TCGTATTTTTATACGTAGGAGGATATAAATATGGGAAAATTTGGATTGAATTTCTTTAAGCCAACAGAAAAAT
TTAATGGGAATTGGTCGATCCTAGAAAGTAAAAGTAGAGAATGGGAAAAAAATGTACAGAGAACGTTGGAGCCA
TGATAAAGAAGTAAGAACAACGCATGGTGTTAACTGTACTGGATCATGTTCATGGAAAGTCTTTGTGAAAAAT
GGTGTGATTACCTGGGAAAATCAACAAACTGACTATCCTAGTTGTGGTCCGGATATGCCTGAATATGAACCGA
GAGGCTGTCCTCGTGGTGCATCATTCTCTTGGTATGAATACAGCCCACTTCGAATTAAATATCCATATATTCG
TGGGAAGCTTTGGGATTTATGGACTGAAGCATTAGAAGAAAACAATGGCAATCGTGTGGCTGCATGGGCTTCT
ATTGTTGAAAATGAAGACAAAGCCAAACAGTATAAGCAAGCCCGTGGTATGGGTGGACATGTGCGTTCAAATT
GGAAAGATGTTACGGATATTATCGCAGCACAATTACTATATACAATTAAAAAATATGGTCCAGATCGAATCGC
AGGATTTACACCTATTCCAGCGATGTCGATGATTAGTTATGCAGCAGGTGCTCGATTCATCAATTTGCTTGGT
GGTGAAATGCTTAGTTTTTATGACTGGTATGCAGATTTACCACCTGCCTCTCCACAAATTTGGGGAGAGCAAA
CAGATGTGCCTGAATCAAGTGACTGGTATAACGCATCATACATTATTATGTGGGGCTCTAATGTACCACTAAC
GCGTACGCCAGACGCACATTTTATGACAGAAGTTCGCTATAAAGGAACAAAAGTTATTTCAGTAGCACCTGAT
TATGCAGAAAATGTGAAGTTTGCTGATAACTGGTTAGCCCCGAATCCTGGTTCAGATGCTGCAATTGCTCAAG
CGATGACACATGTTATTTTACAAGAGCATTATGTTAATCAACCGAATGAACGCTTTATTAATTATGCGAAACA
ATATACAGACATGCCATTTATCATCATGTTAGATGAAGATGAAAATGGATATAAAGCGGGTAGATTTTTAAGA
GCAAGTGACTTAGGACAAACGACAGAGCAAAGTGAATGGAAACCGGTAATCCACGACACAATCAGTGACAGTT
TAGTGGTACCTAATGGCACAATGGGGCAACGTTGGGAAGAAGGTAAGAAGTGGAACTTAAAACTAGAAACAGA
AGATGGTTCTAAAATTAACCCTACATTATCAATGACAGAAGGTGGATATGAATTAGAAACAATTCAATTCCCA
TACTTTGATAGTGATGGAGATGGGATATTCAATCGTCCAATTCCAACTCGACAAGTCACTTTAGCAAATGGTG
ACAAAGTCCGTATTGCTACAATTTTTGACTTAATGGCAAGTCAATATGGCGTGCGTCGTTTTGATCATAAATT
AGAATCAAAAGGATACGACGATGCAGAATCAAAATATACACCTGCTTGGCAAGAAGGCATTTCAGGCGTAAAA
CAAAGTGTTGTCATTCAAGTAGCGAAAGAATTTGCGCAAAACGCTATCGATACTGAAGGGCGTTCAATGATTA

TCATGGGTGCGGGTATTAACCATTGGTTTAACTCAGATACGATTTATCGTTCAATCTTAAACTTAGTTATGTT
ATGTGGCTGTCAAGGTGTGAATGGTGGCGGTTGGGCTCACTATGTGGGACAAGAAAAATGTCGCCCAATTGAA
GGATGGAGTACTGTCGCATTTGCGAAAGACTGGCAAGGACCACCACGTTTGCAAAACGGAACAAGTTGGTTTT
ATTTTGCTACAGACCAATGGAAATATGAAGAGTCAAATGTAGATCGTTTAAAATCTCCATTAGCTAAAACAGA
GGAGTTAAAACATCAACATCCAGCTGATTATAATGTGTTAGCAGCTAGACTTGGTTGGTTACCATCATATCCA
CAATTTAATAAAAATAGCTTGTTGTTTGCAGAAGAAGCTAAAGATGAAGGTATAGATTCAAATGAAGCGATTT
TGCAACGTGCAATTGATGAAATGAAATCAAAACAAACACAATTTGCAATAGAAGATCCTGATTTGAAAAAGAA
TCATCCAAAATCATTATTTATATGGCGTTCAAACTTAATTTCAAGTTCTGCAAAAGGTCAAGAATACTTTATG
AAGCATTTACTTGGCACAAAATCAGGGTTATTAGCTACACCAAATGAAGATGAAAAGCCAGAAGAAATTACGT
GGCGTGAAGAAACAACAGGTAAGTTAGATTTAGTGGTCTCTTTAGACTTCAGAATGACGGCAACACCGTTATA
TTCTGACATTGTTTTACCGGCAGCAACTTGGTATGAAAAACATGATTTATCATCAACGGATATGCATCCATAT
GTACATCCTTTCAATCCTGCCATTGATCCATTATGGGAATCACGTTCAGACTGGGATATTTATAAAACATTGG
CAAAAGCATTTTCAGAAATGGCAAAAGACTATTTACCTGGAACGTTTAAAGATGTTGTGACAACTCCACTTAG
TCATGATACAAAGCAAGAAATTTCAACACCATACGGCGTAGTGAAAGATTGGTCGAAGGGTGAAATTGAAGCG
GTACCTGGACGTACAATGCCTAACTTTGCAATTGTAGAACGCGACTACACTAAAATTTACGACAAATATGTCA
CGCTTGGTCCTGTACTTGAAAAAGGGAAAGTTGGAGCACATGGTGTAAGTTTCGGTGTCAGTGAGCAGTACGA
AGAACTAAAAAGTATGTTAGGCACGTGGAGTGACACAAATGAAGATTCTGTCAGAACGAATCGTCCACGTATT
GATACAGCACGTAATGTCGCTGATGCCATACTTAATATTTCATCAGCTACGAATGGAAAGTTATCACAGAAAT
CATATGAGGATCTTGAACAACAAACTGGTATGCCATTGAAAGATATTTCTAGTGAGCGTGCTGCTGAAAAAAT
TTCGTTTTTAAATATAACATCACAACCACGTGAAGTTATACCAACAGCAGTATTCCCGGGATCTAATAAGCAA
GGCCGACGTTACTCACCATTTACAACGAATGTAGAACGTTTAGTGCCTTTTAGAACATTAACAGGACGTCAAA
GTTATTATGTGGATCACGAAGTTTTCCAACAGTTTGGTGAAAGCTTACCAGTATATAAACCAACGTTACCACC
AATGGTATTTGGTGATAGAGATAAGAAAATTAAAGGTGGTACAGATGCTTTGGTACTGCGTTATTTAACGCCT
CATGGAAAATGGAATATACACTCAATGTATCAAGATAATAAGCATATGTTGACACTATTTAGAGGTGGTCCAA
CGGTTTGGATATCAAATGAAGATGCTGAAAAACACGATATCCAAGATAATGATTGGCTAGAAGTGTATAACCG
TAATGGTGTTGTAACGGCAAGAGCAGTTATTTCGCATCGTATGCCTAAAGGTACAATGTTTATGTATCATGCA
CAAGATAAACATATTCAAACGCCTGGGTCAGAAATTACAGATACACGTGGTGGTTCACACAACGCGCCGACTA
GAATCCATTTGAAACCAACACAACTAGTCGGAGGATATGCACAAATTAGTTATCACTTTAATTATTATGGACC
AATTGGGAACCAAAGGGATTTATATGTAGCAGTTAGAAAGATGAAGGAGGTTAATTGGCTTGAAGATTAAAGC
GCAAGTTGCGATGGTATTAAATTTAGATAAATGCATAGGATGCCATACGTGTAGTGTGACATGTAAAAACACT
TGGACAAATCGTCCAGGTGCTGAGTACATGTGGTTCAACAACGTAGAGACTAAGCCAGGTGTAGGGTATCCGA
AACGTTGGGAAGACCAAGAACACTACAAAGGTGGTTGGGTACTAAATCGTAAAGGGAAACTTGAATTAAAATC
TGGAAGTAGAATTTCAAAAATTGCATTAGGGAAAATATTCTATAATCCAGATATGCCACTAATTAAAGATTAT
TACGAGCCATGGAACTATAATTATGAACATTTAACAACTGCGAAATCAGGTAATCATTCTCCAGTTGCTAGAG
CGTATTCAGAAATTACGGGGGATAACATTGAAATTGAATGGGGACCTAACTGGGAAGATGACTTAGCAGGTGG
TCATGTTACAGGTCCAAAAGATCCTAACATCCAAAAAATAGAAGAAGATATTAAATTCCAATTTGACGAAACA
TTTATGATGTACTTACCACGTTTGTGTGAACATTGCTTGAATCCAAGTTGCGTTGCATCGTGTCCTTCAGGTG
CTATGTATAAACGTGATGAAGATGGCATCGTGCTAGTTGACCAAGATGCTTGTCGTGGTTGGCGTTACTGTAT
GACAGGTTGTCCATATAAAAAAGTATACTTCAACTGGAAAACGAACAAAGCAGAAAAATGTACTTTCTGTTTC
CCGAGAATTGAAGCGGGGATGCCTACAGTGTGTTCTGAAACTTGTACAGGACGTATGCGTTATTTAGGTGTTT
TATTATATGATGCAGATCGAGTTCATGAAGCAGCTTCAGCAGTAGACGAGAAAGATTTATACGAAAAACAATT
AGATATATTCTTAAATCCATTTGACGAAGAAGTCATTGCACAAGCTGAAAAAGATGGCATTAGTTATGATTGG
ATTGAAGCTGCACAAAACTCACCAATTTATAAGTTAGCGATTGAATATAAACTAGCATTTCCATTACATCCTG
AATTTAGAACGATGCCGATGGTTTGGTATTGCCCACCACTTAGCCCAATTATGAGCTATTTCGAAGGTAAAAA
TACAAATCAAAATCCGGATGCGATTTTCCCAGCCATTGAAGAAATGCGCTTGCCGATTGAATATTTAGCGAAT
ATTTTCACGGCAGGAGATACAAAACCAGTAAAAGAAGCATTGCAGCGTATGGCAATGATGAGAAGTTACATGC
GTTCCCAAGTTACACAACAACCATTTGATACATCACGTTTAGAGCGATTAGGTTTAACTGAACGACAAACTAA
AGATATGTATCGCTTATTAGGACTAGCTAAATATGAAGATCGATTCGTTATTCCAACGTCACATAAGGAAACA
TATTTAGATACGTATCATGCTCAAGGTAGTACGGGATATAACTATGGTGGCGAACACTTTGGTGATAACTGTG
AAGGTTGTGGTGTTGCAGTAGGTTCAGGAAAAACTGGTCAAGAAATTTATAATGAGAATTTCTATGGAGGGAT
TTTCCGTGATTAATTTCGATAATTTTAAAAAGTATCAAGAAAGTTTTGGATATATAGCGCAACAGCTATGCTT
TCCGGAAAAATTAACATTTCATCCAAAGACTTTTGAAGAAACAATTTCGAAAGATCATCCAGGCTATGACGAT
TTAATAGCATTTAGAAATGTAATGATGACATTTTCTTTATCAGAAATCAAAGCGATTTATACAGATACATTTG
ATTTTACGAAAAAAGCACCATTATATATGACGTATAACAAGTTTGATACACAAAAAGAACGTGGGCAAATGTT
AGCGAAACTAAAGGTATTGTACGAGATGTTTGGACTAGAAATGGTAGACAATGAACTTTCTGATTATCTACCG
TTGATGTTGCAATTTTTACAAGTGGCAGAATGGAGAAATGATCCAAGAGCTGAAGAGAATATTCAATTAATCA
TTATGATTATTGAGGATGGAACATATGTGATGGCGAATGCACTTGGTGAAGATAACAATCCATATGCCTATGT
GATTCAAGCGCTTAGAAAAACATTGAAATTATGTTTGACATCACCAAAAGGAGTGAAACATCATGCTTAATCA

GTTTTTATGGGTTATATTTCCGTATTTATGTTTAGTTGTCTTTGTCGCAGGTCATATTGCACGTTATCGTTAT
GATAAATTTTCGTGGACAGCAAAATCTAGTGAATTAATTGAAAGAAAAAGATTGATGTGGGGTAGTTTATTAT
TTCACTTAGGTATTATTCCGGTATTTTTTGGACATGTTGTAGGATTATTAATACCTAAGTCGTGGATGGATGC
TGCCGGTGTGTCTGAGCATTTATATCATATTGGTGCTGTTTATATAGGGAGTATCTTTGGTATAATTACATTG
GTAGGGATGTTTTTATTAACAGCGAGACGTGTTACTACGAAAAGTATTCGCAGATTAAGTTCAGCGTCCGATA
TCTTTGTTAACTTCTTATTATTACTTATTGTATTTATGGGATGTTATGCAACATTAGTAACAAATATACAGCA
TCCAGATTTTAACTATAGAACTTCATTATCAATTTGGTTTAGACAATTATTTATGTTGAAACCAGATGCATCA
TTAATGAGTGGTGTACCAATTGCATTTAAAATTCATATATTATTAGGTTTCACTATTATGGCTTGTTGGCCAT
TCACACGTTTAGTACATGTTTGGAGTGTACCTTTGACATATATAAATCGCAGATATATTATTTACCGTAAGAA
TAAAGTGTAAGTATAAGAGGTGAAAGGATTTGACACCAGAAGCAATTATTGAAGATCGTAGATTTCAAGAGAC
ATTAGATAAAATACGCAAAGAAGAAGGTTATGATTTTGCGGCAATCGCTTTCTATGAATCTAACAAACCATCA
TCTCCAATAAAATGGCACTATGTTTCAGGTAATAAAAATAATCGGTTTAAATTAATTATTTTAAGAAAAGGCC
GTGGATTAGCTGGAACTGTTATGAAAACAGGGAAGCGTATGATTATAGCTAATGTCGGTTTAGCATTGGGACC
AGAAGAAAAGATTGACTATCCAATTTTATTAAGCGAATCATTAACAGCAGTTTTAGCAGTGCCGCTTTGGTAT
AAGAATCAAGTGTATGGCGTACTATTATTTGGTCAAAGAGATGGACGTCCTTTACCAAAAATATTTGATAACG
ATGATATTCAACGTAAGTTTGGTATTTTCAATGATGATAAATAATGAGATGAATTAGGGTGTAGGTCATGATT
AATGAGGACAGTATACAATTAGAAGCTTTATTAAAAAAATACTATGAACATTCAATCGAAAAAATAGTATTTG
CCGACGATAGTGGTAAAATTATTGCAATGAATGATGCTGCTAAAGATATATTATCTGAAGAAGATAATTACAG
TGCTGTGGCGAATGCGATTTGTCACAGATGTGAAGGGTATACAAATGCCTATGATGTACAATCATGTAAAGAT
TGTTTCTTAGAGTCCATGCAAGTACAAGCTACGAATTTTCAAGTGTTTATGAAGACGAAAGATCAAAAAGTGA
TGCCGTTTACTGCAACTTATCAGCTTATTGATCAGGATAGAGGGATTCATGCATTTACGCTTCAAAATGTCTC
ATCACAGATTGAACAACAAGAAAAATTGCATCAACAACGTATGATGCGTAAAACGATTTCCGCACAAGAAAAT
GAGCGAAAACGAATTTCTAGGGAATTACATGATAGTGTGATTCAAGAAATGTTAAATGTAGATGTTCAATTGC
GATTGTTGAAATATCAAGAAGATACAGCAAAGCTTTTGGAAGACGCAGAAAATATTGAATATATTGTCGCTAA
ATTAATTGATGATATACGTAATATGTCGGTTGAATTAAGACCCGCTTCTTTGGATGATCTAGGCCTTGAGGCA
GCTTTTAAATCATATTTTAAACAGTTTGAAGAAAATTATGGAATAAAAATCATATATACATCAAATATTAAAA
ATACCCGCTTTGATAGTGATATTGAAACGGTTGTTTATCGAGTAGTGCAAGAAGCTATTCTAAATGCGTTGAA
ATACGCTGATGTAAATGAAATAAATGTAGGTATCCGTCAAACTGGGCGACATTTGGTTGCTGAAGTGATAGAT
GCGGGAAATGGTTTTGATCCAAGTTCTAAGCCTAAAGGTTCTGGACTCGGTTTATATGGAATGAATGAGCGAG
CTGAATTAGTCAGTGGTAGCGTCAACATCGAAACGAAAATTGGTGAGGGTACCAATGTTACATTGAACATTCC
AATTTGAAACATACATTGGGGGAATAAAATTGAAAATAGTCATTGCCGATGATCACGCTGTTGTCCGTACGGG
GTTCTCTATGATTTTAAATTATCAAAATGATATGGAAGTTGTTGCAACGGCTGCAGATGGCGTCGAAGCTTAC
CAAAAAGTAATGGAATATAAACCTGATGTGTTACTAATGGATTTAAGTATGCCACCAGGTGAGTCAGGTCTTA
TCGCTACGAGTAAAATTGCTGACAGTTTTCCTGAAACTAAAATACTAATATTAACAATGTTTGATGATGAGGA
GTATTTGTTCCATGTGTTGCGTAATGGTGCGAAAGGTTACATATTGAAAAATGCACCTGATGAACAATTATTG
TTAGCTATTCGAACTGTATATAAAGGTGAAACATATGTAGATATGAAACTTACAACATCTTTAGTGAATGAAT
TTGTATCTAATTCAAATCAGGACACTGCAAACACATCAGATCCTTTTAAAATATTATCAAAACGAGAACTAGA
AATATTACCACTTATTGCCAAAGGTTACGGGAATAAAGAAATTGCAGAGAAATTATTTGTTTCTGTAAAGACA
GTAGAGGCACATAAGACGCATATTATGACAAAGCTTGGCTTGAAAAGTAAACCAGAGCTCGTTGAATATGCAT
TGAAAAAGAAATTATTAGAGTTTTAATGTTTGAAAATAATACCATACAAAGCGCCAGAGGAAACGGGGAATCT
TCTGGCGCTTTTGTATGTTTCGAAATATATTTTTTTTTATAAATCGAATGGAACCTAATCCTTTCTCCTTTCG
ATAACCTATACTAATTCGCTTTGCAAAGGTACAACTTTTAATATAGTTATTGATTTAGAGAATAAATGATTAG
TTTTCTACTTTGAACAATTATTTTAGTTGGTGTATTCAGTGTTGTAGCATCTGTATGAGGATTAAAAATTGTC
GCTCTTATTCACATTGCTCAAAAAATAATTAACAGCTTCTGAAATGTATGATGCTAAATTTTGATTATCATAT
GAATTAAAGTAATCTTTGAAACGCGTATCATCTTCATATGTTTTAGCAATACAGCATAATGTTTCATCATCAA
AATCAGCGATAGTATTCATAAAAGATTTCCATTCTGTCACGATATCATTACAGTCTTGGATTGGATAATGACT
CAAAGACATATGATTAAATTTATTGAAGATTGTTGACAATTTCTGTCTGATATCCTGTGATTGTAAGTTATCT
TTACGTCTAATAAATGATTGATATGCTTTCGTATGACCATATTTTATAGATGCTTCTTTATCATATTGTGACT
GTAGTGTCTTGTTTAAAATGCTGGTATCTATTAGAGAATGTCCTTTAATAAATTCACATACAGAGCGTCTAAT
TAATTGTAAGTCAGAAATCTTTTTATCTAACTTGCTTATTTGCTCTGATAATAACTTTCGCAATTGTTCATTA
TCATATGAAATGTATTGTTTTATTTTAGCGATATCAAAATCAAAAGACTTGAGTATTAAAATCTTTTGTAATT
TTTCTAAGTCTTGCTGTTTATAAACGCGATAATTTTTATCATTTTTATCTGGAACTAATAATCCTATTTCATC
GTAATAATGTAAAGTTCTTTTAGTTACACCTGTAATTTCAATAATATCTTTTAAAGTGTATGTGTATGCCAAA
ATTCAGTCACCTCATTTATTATTTTTTGTGCTGGAAGTGTTGTGGCAACTTCAATAGGTATGTTTGATTTTTG
TAAAAAGTATTTCACCAAGTGATAGCCACATGCATAACCGGCAGCATGAGATAAGCCAACGATAGGCCTACCT
TGAAGTTTATTAATATCATCACCATAGAGATAAGGCATCGATTCAAATATATGCTTTAAATGTAAATGATTAT
AAATAGTATTTTTAATTTTTACGTTATCACGACTCCAATTAGTATTTGTTACCCACGGTCCAATATGCGCTTT
ACCATACAATGATTCTACATAGTTTTCAGCTAATCCTTCTGCAATAATCAATTCAATCAAACTTCCGCCATCC

CAATCAATATATTGATAGCGCATATTATGATTTACTTCATGTGCAATTAAGCTTTTCATAGAATTAATTGTAC
TTGTACTCGGCACTAAATAAATCATAATATAACCTGGTATGCCACCATCACCACCACGATTTTTATTTAGATA
CATAAGTGGTTTTTGGCTATCTCCAAGTAATACTGTGAAATGATAGTTAGAAACTTGAGATGAGATACTATAA
TTTGCAAATTGATCTATAGCAACTTTTAAATATTTTTCACAATTAATCCAGAAATCATTATTCAGGTATTGTA
TTGACATTTGGTCAGTTTCAACCAGTTCTTCGGGAGACTTATGAAATACATCTAAAAGTTGAATCGCACTCAT
TGTCTGCTTGTCATCATTATGAATAGGAATCTGCTGCACCTCAAATTTCTTTTTAAAAGGTACTAATATTTCT
TTAGTGAAAATTGAATCACGTTCGTTTAATGGTGCCTTGAGTATTTTCTTATAGACGGAATCTGATCTAATAA
TATTGATTTTATACATGATAAACCTCCTTACGCTGTCAGCATAAAGGGTAACGTAACGTGATTTTCAAGCAGT
AATTGTAACTAATTGAAAAAAATTAAGAAAAGTATGTGAGCGTTCCTTAATAATATGATTAAAATGATGACAA
AATAGTGTCTAAATGCATCGTAAAGGGACATTGTATAGGGGAAATCACACCATAAATAAGGGAAAATCCTTAT
GTTCACTTTTTCACAATCATAATAAAATATATATGTAGTCAATACTTTGTCTATATTGAATGTTTTCATATAA
ATGAAAGCATTTTTAAATAACATTGACCTCTAACATATAGGCAGAGTATTGATATCTATTAAAAAATAAATGA
TTTTGGTGAAGGTGAAACGTATGTACAAAACAAAAGGTGGCTTTCAACTTACATTACAAACATTAAGTTTAGT
GGTTGGATTTATGGCTTGGAGTATTATTGCTCCTTTAATGCCTTTTATTAAACAGGATGTCAATGTAACTGAA
GGTCAAATATCAATCATTTAGCGATACCAGTTATTTTGGGATCAGTGCTCCGTGTGCCATTTGGTTATTTAA
CAAATATTGTTGGTGCTAAATGGGTATTCTTTACAAGTTTTATCGTATTGTTATTCCCGATATTTTTCTTGAG
CCAAGCACAAACGCCGGGGATGTTAATGGCATCAGGATTTTTCCTTGGCGTAGGTGGCGCAATTTTCTCTGTT
GGTGTTACATCAGTTCCTAAATATTTCCCTAAAGAAAAAGTAGGTCTAGCAAATGGTATTTATGGTATGGGGA
ATATCGGTACAGCAGTTTCTTCATTTTTAGCACCACCGATAGCGGGTATTATTGGTTGGCAAACAACAGTTAG
AAGTTACTTAATTATTATCGCTTTATTTGCATTAATTATGTTCATTTTTGGTGACACACAAGAACGTAAAATT
AAAGTGCCATTAATGGCTCAAATGAAAACATTATCTAAAAACTACAAATTATATTACTTAAGTTATTGGTATT
TCATTACTTTTGGTGCTTTTGTTGCATTTGGTATTTTCTTACCTAACTACTTAGTAAATCATTTTGGAATTGA
CAAAGTAGATGCTGGTATTCGTTCAGGTGTATTCATTGCGCTGGCAACATTCTTGAGACCAATAGGTGGTATA
CTAGGTGATAAATTTAATGCAGTTAAAGTACTGATGATTGATTTTGTTATTATGATTATCGGTGCCGTTATTT
TAGGTATTTCAGACCATATCGCATTATTCACTGTAGGCTGTTTAACAATAAGTATTTGTGCAGGTATTGGTAA
CGGCTTAATTTTTAAATTAGTACCATCATACTTCTCAAATGAAGCAGGATCAGCAAATGGTATCGTATCAATG
ATGGGTGGTTTAGGAGGATTCTTCCCACCGTTGGTCATCACATACGTAGCTAATTTAACAGGTTCAAGCCATT
TAGCATTTATTTTCTTAGCGGTATTCGGCTGCATTGCATTATTTACAATGCGCCATTTATATCAAAAAGAATA
TGGTTCATTGAAACACAGTTGATATGTAATACGAAGCATTCTTAAATTTAAATGACAAAGCCTTAATATCATG
CGCAATATTCGTAGCATGATATTAAGGCTTTAGTAGTGTATTCATTTTAAATTTAACTATGAAATTGGAATAG
ATGTTGATGAAGTAGTGTCATTGCTTGGTTTGATTTTAGGCAGGGTAATAGTTAATAGCCCATTTTCAAAACT
AGCAGTAATATGTTGCTTATCAACAGCTTCAAAATCAAATTGACGCATTAATGATTCGAAGTTACGCTCATCT
AAAATGAGTTGTTCAGATTTGTGTTTTGCGTTTCTAGTAGCTTGAATAGTGAGCGTATTATTATTGAAATCGA
TACTAATATCTTCTTTATTTACACCTGCTAGTTCAGCTTCTAAGTAATATAATTCGTCTGTTTCATATATATT
CGTTACAAATGAAGGTGTTGAAAAGTAATTAAATACTTGTTTACCTAAATCTTTAAATGTATCACTTGGATTA
CCGTTAAAAAAGTTTTGATTCTCGAATTGATTGAAGTTCAATATCATCACTTCCATTATATAAATTTGTATTA
CAAAGCCATTATATGAGTAGTATTAAAAAAGATAAAGTAAAAGGGGAGATAAAAAGTTATAAATGGGTAGATT
TGTGCCTGATAAGGAATCAGGGCAAAAGAGCTGAGCATCGAGATTTATACAAGCAATAAAGATAATGAATAAA
CATAGCGATGCATACTTCTGCCCATCACCTTATGATAGGGAGTTACTTTTTGTCGGAATTGTCGAAATTTTTC
GAAACAAAATTCCGCAAATTATTAACAATGTCTGATGCTTCTCGTTCTGAAATGTTGAATTCATTAAATACTT
TAACAGAAATTTCAGCAAGAGGACTTTTTATTGCTTTACCTTGCTCTGTTAGAGAAATTTGTAGGTTTCTTTC
ATCTTTCTCTTCACGTGTTCGAACAACGTAATCTTTCTTTTCTAATTTCTTTAGTAATGGTGTCAGTGTTCCA
GAATCTAAGAATACACGTTCACCTAATTTTTTGATGTTAAGTTTTTCATCATTTTCAATCGCCATTAAAACAA
TGTAACCAGTATAGGTTAAATCATAATCCTTAAGATAGTTTGTGTATTTTTTAATTATTTCTTTTGAAGAAAC
ATAAAATAAAAAACACAACTGTTTGCTAAGATAGCTGTTTTCTACATACATCCAATCACTCCTTGTTAAAATA
AACAATATTATGAAGTGTAATAGTTGTCAAGTACTGATTTCATCGCAATTATCGAATTGTCTATAATTGAATT
TAAATGTTAAATAATGCAAAACATATCAATATCTTAGTATTTTCATCGGTAATGACTGATATGACTGATTTTT
TAAATTGAAAAAAATGGAATTGTATCATTCTTAATTGTTAAAAGTAGTCAATTATTTTGATAGATAGTAAAAT
GAAATTTGTTATTAAATTTTTTCAAAGCATCGTGTATGTAATTCGATTTCTATAATAATATTGAGAAGAAAAA
AATTGTTACAATCTGGTAAAAACTCTTTATTAAATAAATTGATTCGTTCATACTGAATAGATAAGCAAAAAGG
AGTTACATAATGAAGAAGAAAAAAAACGTGATTAAAGTTATCATTATATTATTTATTTCATTAATTGTTGCTG
TAGCATTTTTCTTTGGTCTGAAAACATATCAAGGCCATAAGAATATTCAACTTATTGACAGTTACTTGGAAGA
GAAAAGTCTAAAAGATAAAATTAAATCAGAAAAAACAGAGTATAGTGCTAAAAAAGGTCTCTTCTATAAGGAA
GTTACATTTAAAGATGAACCTGGTGTAACTTATGTTGTGCAACCTATCAGTACTAATAAAGGATTATTTGTTG
AAGGCTTTGATACAGAAACTAAGAAAAGTTTGAAAACTGCAAAGCATAAATATTTCAATCAAAATTATAAACC
ATCAAAATAGTGAAGCGTATAACTGGTATAACAGTTTAGTGCCTTCAATTTTAATTATTTGAGGTAAACAGTT
TTTGTATGGAAATTAGTGATTTTTGACATCTATAAAAGATAAATATTTTAATTTTTTATAGATAATTATCAAT
TTTCTGATATAATTGAGTGATGTCAGATGAAGGGGAGTTTGTACTATGGCTCTATTCAAGAGAAAAATTAGCC

TACCTATGCAAGTTGTTATTGCATTAGTGTTAGGTGTTGTCGTAGGACTTTTATTATATGGACAAGAAAACGT
AGCAAATTACATTAAACCATTTGGTGACGTATTTTTAAATTTAATTAAAATGATCGTTATACCAGTTGTATTT
TGCTCGCTAGCGCTTTCTATTTCGAACGTTGGGGAATCGAAAACTGTAGGGCGTTATGGCTGGAAAACAATTT
TATACTTTGAAATTATTACAACAATCGCAATAGGTTTAGGGATTATCTTCGGTAACCTATTTAAACCAGGTGC
TGGATTAGACCCAACAAAATTACCTAAAGGTGATATTTCTAAATATCAATCAACTGCACATGCAGCAGAACAA
TCTACATATGGAAATCATTTTATTGATACCATTGTACATATTATTCCGACAAACTTTTTTGAAGCTTTAAATA
AGGGTGAATTATTACCTATTATCTTCTTCGCAGTATTCTTTGGATTAGGATTAGCTGCAGTAGGTAAAAAAGC
AGAACCAGTTAAAGAATTTTTAAGCGGATCGCTTGAAGCTGTGTTCTGGATGATTAATAAAATTTTAAAATTA
GCACCACTTGGAGTGTTTGCATTCATTTGTACTACAATTATTACATTTGGTGCATCCGCATTATTACCACTAT
TAAAATTAGTATTAGTTGTTGTCTTTGCAATGGTGTTCTTTGTATTCGCTATACTAGGACTAGTTGCATGGAT
GTGTGGTATTAATATCATGAATATTATTAGAATCTTGAAAAGTGAATTGCTTTTAGCATTTTCTACATCAAGT
TCGGAAGCTGTACTTCCTGTAATGATGAAGAAAATGGAAAACTTCGGTTCTCCAAAAGAAATTACTTCTTTTG
TTATACCAATTGGTTATACGTTTAACTTAGACGGATCAGCACTTTATCAATCTATTGCAGCATTATTCGTTGC
ACAGATGTATGGAATGCACTTAACATTATCAGAGCAAATTGTGTTGATGTTAACATTAATGATTACGTCTAAA
GGTATGGCGGCAGTACCAGGAACTTCAATCGTTGTTTTATTAACAACATTAGGTGCCATGGGCTTACCGGCAC
AAGGTTTAGCATTAATTATTGGTGTTGACCGTATCTTAGATATGGTACGTACATGTGTAAACGTTATTGGTAA
CGCATTATCAACAATCGTTATAGCTAAATGGGAAAACGTATATGACAAAGCAAAAGGTCAAGAATATTTAAAA
TCAATTTAAAAAATACTATCTGACATTTAAAGCCCCTTACAACCTTTGGTTGTTAGGGGCTTTTATGCATGCG
CTAAAGCGCAGGCGAACCGAAACGAAGTGACCTCAGGTTGTTAGGGGCTTTTATGCATGCGCAAAGCGCAGGC
GAACCGAAATGAAGGCAACCTCAGGTTGTTAGGGGCTTTTATGCATGCGCAAAGCGCAGGCGAACCGAAATGA
AGGCAACCTCAGGTTGTTAGGGGCTTTTATGCATGCGCTAAAGCGCAGGCGAACCGAAACGAAGTGACCTCAG
GTTGTTAGGGGCTTTTATGCATGCGCTAAAGCGCAGGCGAACCGAAGCGCAGTGACCTCAGGTTGTTAGGGGC
TTTTATGCATGCGCAAAGCGCAGGCGAACCGAAACGAAGTGACCTCTGGTTGTTAGGGGCTTTTATGCATGCG
CAAAGCGCAGGCGAACCGGAAATAGACTGACACAGTAATTCTTAAAAAGCAGCAGTAAGATAATTTTCAATTA
GAAAACATCTTACTGCTGCTTTTAATATCTTATATATTATTCAAAATTAAAGCTTAATTAGTGTTTCATGTGC
ATTTTGCCCATGTCATGAATTTTCATCATTAAACCATGTGGTACATCGTCATGATCAACTTTTTTTACTTTAA
CAATTTTATCAGAATCTGCTAATTTATAAACAATTACAAAATCACCTTTTTTAACTGAAACGTCTGCATCTGA
AGGTAATTTAACGTTCTTCTCAATGCTTTCATCTTTTTCACTAATCATTTTTTCAATTGTAGCATTGTCTTTC
ATAATTCCATAATATGTATCTTTCTTTCCACCAGTCATCATCATGATTACTAGTACAATACATACAATCAACA
TGATGGCAATTAAACTAATACCAAGACCAAACCCTTTTTTATTTTTCACGGTTCATCAATCTCCTTAAATGTA
GTTAATTCTGTTATACCTAAAAGTACAACAGTTTGCAAATGCAGTATTTATATTACATAAAATTAATTTAAAA
AGACACACCTTAATCACAATAAATGTGAATAAAATGTGTCTAAAATATTGTTATATAATAAAAATATAGTTAA
AAGTATTAAACGCTTTCAAAATCGAAATATATATAAATAGGATACTGTTGAAATTTATAGCTAACATGTTCAA
TTGCTGGCTAATTTTAAAACTAAAAACTTACATATTATAAATCAATTTTAATAGGTGTATCTAATGATTTATC
ATTCATTAGCTTAATGGATTGCGGAACTACTCTTAAAACACATAACTCTGGGTCTTCTTTTGAACTGAAAAAG
CTTTTGTCTTGTGTTTCCCATAGCCAATCAATCACTTCTTGTTCTGTAACGATTTCGATGGTCGCTTCCATCT
CAACGAAACTACGATTAGTTGTGTCGTTATAGCCTAACAAAACATATGCTGCTGGATTATCTTTAATTTCTTT
GACCTTAGCAGAATGGATATTCGTTTTAGTATATAAAGTAAGACCATCATTATAAAAGACCATATATCTACTA
TTAGGTTTGTTATTATATGCAGTTGATAATACACCAACTTTTGACGTTGCTAACACGTTTTCAATTGCTTGAA
TTGCTTGTGAGTTACTCATCCTACATCATCTCCTTATGATATAAATAGCCTTTAACGTAAGAAACTAAACACA
AGAGGACTGTTAGTATTTAGCTTGGTTTAAAGCAAATATATTTAACCTCGTCGTGATTTAACTTGAGTTCAAA
TGTACGACCAGTTTCTTCTTGAACTGCAAGCTCCGTTTTAGGGTAATTCGTTTTGTCTAATTCATCTAAAATT
TCTTTTTCTATATAAACAAGTTGATCGCTTATTGGAAGCAAATGTTGAATTGAACCATGTTCATGATTCAATG
TTTTAATAATCATCAAAGAGTCTTGAGGTAATTCATTATGGAAAATGAAATCTTGTTTTACGTCTGACATATA
TCGATCATTAATTTTATTGAATAATAAGAAATGGTAGTTATTATCTTTACGACTCAATACATAATTATTTGTA
ATTTCAATGTCTTCGTTAACAAAAGGTCTAAACATTTTATATAAATGCATTAATGGTAGGGGGCTACCATATT
TGTTGTACAAGGAAATATAATTTGAATGGTCATCTAATAATTGATAGCTAATGCCACCGCCATATCGTAATAA
TGCGATTAAGTGATACACTATGTCAGATAATTGAATTGGATTCTTACCATCATTAGTATATTTAAACCCGCTT
ACAGACAGGTTATTATATACAAATTGTGTTGATGATATACCTGAATCTAAAATAAATTTCTTATACTGTTCAA
AATGTGTGTCAGTTTGATGATAGTGTTGATTTTTAGTGATAAGATGTGTTGCAGTTTTTGAATTTTCAATATC
AATAAAGTAATAATGGAATTTAAGACGCTGCATGACATCATAAACTTGTTCCACGGTTGAATAAGTTTCTAAA
AAGCCATCGACTGTCACACTATATTTAATTTCTTTATTTTTGTTTTTAATTTTAAGATGACATAAATGAATTT
CTTCTGCTGTCATAGAATTAGAGCAAAATACGAGCATAAATTTAACTAATTTCTTGCTTGTCTTATAGTCTTG
ATTGCCTTGTAAAAATGTTAGTATTAATTGCTTCATCGTTTCGAATTGTTGTGTTGATTTAATAGCGATTGCT
AAACCTATATTTTTTTCGAAAAGCTTTTCAAAACATCTATTTAACAGGTTGTAATTCGTTTGGCTAATCTCAA
TATCGTGAATATCGTCAATGAAAACGACAGGTTTTGGTAAGTGCTCAAAGTTAATATCGTAATATTCATTGAA
AATAAATTGAAATAGTTCATTGAAATTACTGAATCGAACAAATACTTTGGCACGCTGACTTCGATCTTTCGGA
TTAAAATCATCTATATGAATATCTGTTGTATTTGTAGCCAACTGAGCTGTTAAATCAATTGTTGAAATTAACT

CAGTGAATTGTGATACGTTATCTTGTTGGAAGTGTATTTGTGGTGGTTCAGTAATCTTGGAAACGAGTGAACG
GAACTGTTTAGGACTAAAATGCAAGTAATTCTTAAATTGATTTGCAAAATTTGTATGACTGTTAAAACCAGCC
AATTCAGAAACAGTTGTAATAGAATGTTTTGTAGAAAGTAATAAATTGATGGCGTTAACAAGTTTAATACTTG
TAAAATAATCTTTAAAATTCATACTCAAATATCGAACAAAAAGATTAGAACAATATGATTCAGAGATGTTGCA
ATGCATCGCTATATCTTTTAAAGACAAGTGCGCATCAATATTGTCATGAATATAGTTTACACAATCTATAAAC
ACTGGATTGCTTAAAGCAATATTTGGTAAATATTCATGATCTATTCTTATAAAAGCTTCTTTAAGTAAAGTGT
CTATAATACTTTGACCTATATTTTTTCCCATTAAAGAAGAACTTGTTGGTGTACTGATTAAATCGGCAATTAA
TGACTTAATATAATTACTTGATTGTAATAAATGACGATCTAAATAACCATTAAAGAAATCATCATCTTGTTGA
TGAAAATAAAAAACGGGTATCATTAATTCGATTATATTTTCGCCGTGGTTAATATTATAAATATCACCATGGT
TAATTAAGATAATATAATTACTTACGTCTTTGGTTTCACCATTAATCGTAATATTGAGTTTACTTGTAAGTGA
AAATAGCACCATAATATTATTAATATTACGACTTGGTATTGTCGATAATGTATGATGTATTTTAAGTTGGCAT
GTCATATTTCTCTCTCCTGCCTAATATATATTGAAATCTATCATTTAAATGTAAAACATGGAAATTTTTAAAA
TCATGGATAGTATACCTATTATAGTTGAATATTTTTGAAAAGGTAATAGTGAAAAATAAAAGAAATTAATGAA
AACTCCTAGGAAAACGCTTGTTTTTATGCATTGTGCACAATATAAAAGTAAAATTAGAAAGATAGTAAGCAAA
ATTTGGAAATTTTCGATAGAGTCTAATTAAATTATTTGGTGCATAATTAATATAGATTTCAAACCTATGTATT
TCAAAAAACAATACATAGCCATTTTGAACTTACTTGCTAATCTTTTATTGATATTCAACTATGTTATAAAAAA
AGCAAATTGACCAAAATGGAAGTTTGAATCAAAAAACATTAAATGTACCCATGACTTAAATTTTATCGCCATT
ATTTAAGTTTGGACATACACTTTTGTCATCAAACGCTGAAAATTAGTACGATTGAAATCACTTCTTGTTATAA
AAATTCTTAGTAGCCCTGTTACAAAATATTCGCTACAAATTATTCAAATCAACAACAATAACCATCAACTAAG
AAATTTTAAACAATCTACTGAAAAATAAAGCGTTTAAAAGTTCTGTTTAGAAAACATCAAGATAAGTATAAAC
ATCTAGAAAAGTGAACATCTAAGAAAATCCATCTAGTTATTGCCGTAACAATTAGAATAATCATTTGAAAAAG
TGCATTAGACAACGAAATTCACCATCAAGATAATGAAGCAACAAGAAACAGCACACATCAATGAATTAACACC
TTAGATTCATCATGAGAAAATGCATGGCACAACTAGATTTACCATCAAGATAATTCTTAAGCAGCGAAGAATT
AACATCTAGAAATGCATAAACATCTTAGATTTTACATCAAGAGATGCGATAACTATAAACAACTAGAAACGTC
ACCAAGATAGTGCAACAACAAAGAATCAGCATCTTGAAATCGCACTAACATCTAAGAATCAACATCAAGTATT
ATATTAACATCATAGATAACTTCATCAAGACAGGCATCCACATCTAAGAAACTCATCTAGAATAGCTAACACC
TAACATCAAAGAATATTCATCAAGAACAGTATCTAACAGAACTTTTTGACTATATATGAATTAATTATGTTAC
TAGCTAAATCATCTATTATTAACTTTCACACTCAATTTCTACATCTAATATCAATTTATTAACAATAGAATTA
GCAGCAACAAGATATTACTTAAAATTACTCCCCCGAATTTAAAATATAAACAATGTAATAATTAAGGGTATAA
ATTGATTTATGCTGTAACTAATTAAATGTGTATGGTATCTTCTAACATCATGAAACGCATTTTAATATAGATA
TGTACATTTAAACCTTGAGAGATAGATTATAGTAAACTTTAGTACAAAGTATGAATTGCTGAACTCCAATGAC
TACTATTTGTCCGTTACAACTAAAGTTTCAATCTATCTCTTTCTTTTTGTGTTTAAAACACGACTTTGTAGTA
AATATTGAACATCATATTTATTAGCAAGTTCTGCTAAAAAATCTAATAAAGTTTGGGTGTCGATTTTACCATT
AATAAAATCAGCTTTAAGTAGTTTTGATTGTTGTCTTTCTTCTTCATTCGCACATTTTTTAAAATATCCCCAC
ATATGATCAAAAGCATTACATACACTTCCTATACTTGGTTCAATTAACAAAGCATCATCAATCATTTGTTGGA
CTTGATGAAGTGATAAATCCGTTTTTAAAGCATTCCTTATCATTTGGTAAGACTGTTGACTGTGTAACAATAC
ATGATATTTTTCCTCACGCCAAAGCTGTTCGATATGGCCACGTTCTTTCATAAAATTACCTCCGATAATTAAT
GTTAATATACATGCATATAGTATAGCAATAAAAATGAATGCGCTATCAAAAATGGTGTAGGTGTTTATATAAA
ATGATAATAGAGTGTTGCAAATATTTTTGGAACCGGTTACAATAAAGTGGAATAAATGGAACCGGTTCTAATT
AGGAGGCTAAATATATGAATTATAAGCAATCCGCAGAAGATATTTTGAACGCGATAGGCGGAGAAGAGAATTT
AGATGCAATGGCGCATTGTGCAACGAGACTACGATTAGTTTTAAATGATGAAAGTTTAGTAAATGAAGAGGCG
CTAAACAATATGGATGTAGTTAAAGGGACGTTTTCTACTGGGGGACAATACCAAATTATTATTGGGTCTGGTA
CAGTCAATAAAGTATTTAGTGAACTGGAAAAATTAACTGGAAAAGAAGCATCAACCACTTCGGAAGTCAAAGC
ACAATCTGCTAAAAATATGAATCCGTTACAGCGATTTGTAAAAATGCTTTCAGATATCTTTGTTCCGATTATA
CCAGCCATCGTTGCTGGTGGTTTATTAATGGGGTTAAATAACATTTTGACTGCGAAAGATTTATTCTTTTCAG
GTAAATCATTGATAGATGTCTATAGTCAATTTGCTGGATTAGCTGAAATGATAAATGTTTTTGCGAATGCACC
ATTTACATTATTACCAATTTTAATTGGATTTAGTGCAGCAAAACGTTTTGGTGGCAATCCGTTTTTAGGTGCT
GCATTAGGTATGATATTGGTTCATCCATCGCTAATGAGCGCATACGATTTCCCAAAAGCAGTTGAAGCAGGTA
AGGCTATTCCATATTGGGATGTATTTGGTTTGCATATTAATCAAGTAGGTTATCAAGGACAAGTGTTACCTAT
GCTTGTAGCAGCTTATATCTTAGCTTCAATTGAAAAAGGGTTACGCAAAGTTATTCCAACGGTGTTAGATAAT
TTGTTAACACCATTGTTATCTATTTTTATAACAGCATTTCTAACATTTTCATTTGTAGGTCCAATCACTCGAC
AATTAGGTTACTGGTTATCAGATGGTTTAACATGGCTTTATGAATTTGGTGGTGCAATTGGTGGATTAATATT
CGGATTATTGTATGCTCCGATTGTTATTACAGGTATGCATCATAGCTTTATAGCTGTAGAAACGACATTAATT
GCAGATGCCACAAAAACGGGTGGATCATTTATATTCCCGATTGCGACAATGTCTAATGTTGCACAAGGTGGTG
CAGCAATTGCAGCGTTCTTTATTATTAAACAAAATAAGAAGTTAAAAGGTGTGGCATCTGCCGCAGGTATTTC
AGCATTACTTGGTATTACAGAACCGGCTATGTTTGGTGTTAACTTAAAACTAAGATATCCTTTTATTGGCGCT
ATCGTTGGATCAGGTATTGGTTCAGCATATATTGCTTTCTTCAAGGTTAAAGCAATCGCATTAGGAACTGCTG
GATTGCCTGGATTTATCTCAATCAATCCAGTACATGCAGGATGGTTACACTACTTTGTTGGTATGACAATATC

ATTCATCGTTGCCATAACAGTTACTTTAATTTTATCTAAAAGAAAAGCAAATAAAGAAGTTGTAGAATAAAAA
AAGAGGTATGACAGTAATCAAAGTATCAGTCATTGCAAATGCAATAGGGTGAAACTTTAAGCTGTCATACCTT
TTAATTTAGTTATAATTTCTGTTTTCGCCATAAGAAAATACTAACAATTACGACAAGAATTAATGAAATACCT
ACCACAATAATCCAAGATATATAACTATGGTCATCAATTGGCAACGGCACATTCATACCGAAGAAACTAAATA
CGAGCGTTGGTAACGTTAATAGTACCGTGAAAAGTGTTAATGTCTTCATAATCGTATTCATATCATTAGATAA
TAAGGAAGCGTATGATGTTGTGATACTTTCTAAGATACGTTGATGTAATTCTGTTGTTTCGATGGCTTGGTTA
TTTTCAATGATTAAATCTTCTAGTAATTCTTCATCTTCTTCAAAACGTTTAATTGCAGGTAAACGGAATAACT
TTTTAATAATTGTATCGTTACCTTTTAAGGCAGCTAAAAAGTATACTAAACTTTTTTCAACTTCCATTAATTT
GAAAAGTTGTTTGTTAGTAATGTTATTTTTTAGTTCTTTCTCAATACGAATTCTACTTTTATTAAGTAATCGT
AAGTTACGATTATAATGGTCGGCAATAGTAGTTAAAATTTCTAATGCAAATCTGCTGTGAAATTTTAAATTAA
TATCACGCTTAGGTAAATTTTCTAAAAATTCATTTTCAGCGTCACAAACTGTTACGATAATACCGTTGCCAAT
AATAATGCCTAATGGAATCGTTACAAAAGATAGAACGCTACGATTAGTTGAATTGACGATAGGTAAATCAATA
ATGATTAATGAGTAACCAGTATCTTCGTCATATTCAATACGGGAACTTTCTTCTGAGTCCAAAGGGTCACGTA
TAAAGTCTTCAGGTATATTATATTGTTCCATAAGATTTTCAATTTCTTCTCGGTCTGGTTCTACTACATTAAT
CCATGACGCTGTATGATCTAATGGTGTTTTTATAATATGTTTATCTATATCTTCAGAATGTCTGAATGAAGTA
ATCATGATATATAGTTCCTTTCTAAAGGATTTAGGAAAAGCGAGTATTACTTAAGTTAGTGAATCAAGGTTTG
ATTTATGCAAACGGTTTTCTGCCATTATTCTTAATGGACCATTAAAAACAATATTATGCACATGTTTAGCTAA
TTCATCAACTGAAATGGGCTGTTTGTCCATTACCCAGTATTTAATAATTGAAATTGTTGCTCCGGAAACGTAA
CTGTAAAAATACATCTCTGGAATGCCTCCGATAGAATGATTGATACTAATATAACGTTGCATATTATTTTTAA
TATGTTCGTTGATTTTCAATTCTAATTGACTCGTGCGTTCCAATTGTAAAATAGTATGATAAAACTCTAAATT
ATCATGAATATGCTGGAAAATATTTTTGAGTATATCATTTACAAAAGTATTCGCAATGTCTTCAATTGAAGAA
CCGCGTAACAAATCAAATTGAGTATATGTCGTTAGTTCTGAAATATACTCATCTTCCATATCTGCGAGTAACA
TATATTTATCCTCGTAATGTAAGTAAAATGTCCCTCTATTGATATCAGCGCGTGTTGTTATGTCGCGAATGGT
AATCTTTTCTAAATCTTTTTCTTGTAGTAATTTAGTGAACGCTTGTTTAATGGAGGACTTTGTCTTTCTAATT
CGCCTATCTTCTTTCAAAAATAAATCAACCTCCTTGTGTTATTGAACAATGTGGGTGTATTTGTCTATTAATA
AACGTATTGATGAACATTGTGTATTGTTTATTCCCACTTACATATTATAATAGACAACATGCGAAATATTCAA
CACATTGTCTAATAAGGAGGTAATTAATAAAATGAATATATTTCAAAATAAATTATTATGGATTGCACCAATA
GCAACTATGATTATCTTGGTAATCTTTTCTTTAGCTTTTTATCCTGCATATAATCCTAAACCGAAAGATTTAC
CAATTGGTATATTAAACGAGGACAAAGGTACAACGATTCAAGATAAAAATGTTAACATTGGTAAAAAATTAGA
GGATAAATTATTAGATAGTGATTCTAATAAAATTAAATGGGTTAAGGTTGATAGTGAAAAAGACCTTGAAAAA
GATTTGAAAGATCAAAAAATCTTTGGAGTAGCTATTATTGATAAAGACTTTTCAAAAGATGCTATGAGTAAAA
CACAAAAAGTAGTTATGGATAGTAAAAAAGAAGAAATGCAACAAAAAGTTGCTTCAGGTGAAATTCCGCCACA
AGTGGTTCAACAAATGAAACAAAAAATGGGGAATCAACAAGTAGAGGTTAAGCAGGCTAAATTTAAAACGATT
GTAAGTGAAGGATCAAGCTTACAAGGTTCACAAATTGCATCAGCTGTGTTAACTGGTATGGGTGATAATATTA
ATGCTCAAATTACGAAGCAAAGTTTGGAAACATTAACGAGTCAAAATGTTAAAGTCAATGCCGCGGACATCAA
TGGTTTGACGAATCCAGTAAAAGTGGATAATGAAAAACTTAATAAAGTTAAAGATCACCAAGCAGGTGGTAAT
GCACCATTCCTAATGTTTATGCCAATTTGGATAGGTTCAATCGTAACGTCTATCTTATTGTTCTTTGCATTTA
GAACTAGTAACAATATCGTCGTGCAACATCGTATCATTGCTTCAATTGGACAAATGATATTTGCAGTTGTTGC
AGCATTTGCAGGTAGCTTCGTTTATATTTATTTCATGCAAGGCGTTCAAGGATTTGATTTTGACCATCCAAAT
CGTATCGCAATTTTTGTAGCACTTGCGATTCTTGGTTTCGTGGGTCTTATTTTAGGTGTTATGGTATGGCTAG
GTATGAAGTCAATTCCAATTTTCTTCATTTTAATGTTCTTTAGTATGCAACTTGTAACGTTACCTAAACAAAT
GTTGCCCGAAAGTTATCAAAAATATGTATATGATTGGAATCCATTCACACACTATGCAACAAGTGTAAGAGAA
CTATTATACTTGAATCATCAAATTGAATTAAATAGTACAATGTGGATGTTTATAGGGTTTATGATTTTTGGCG
CTGTATCAAGTTTAGTTTCAGCTATTGTTAGAAAACATAGCACAAAACGTACTGAAGTGCCGTCATAATTTAA
TAAATAGGAATATAGCTATACAAGACGTTTGTAGCGAGCGTCATGGTATAGCTATTTTATTTGTATAGTACTT
GATGTAAAGTTATTTTAGCTGTCATACTATGTGCTAAACCAAATTCAGTTGTGTCATATCTTCGCTTTTTTGT
CACTGTTATAATAGGTATAATCGGATATAATGAAAAGGTGAGGGAGGATTCAAAATGCGTAGGTTATTATATT
CATTTCTTTTTTATATGATCATAGGTTTATTTAGTGGATTTTTCTATAGAGAGCTAACAAAAGCATATGATTT
TACTGGTACAACACAATTATCACTTGTACACACACATACACTTATTTTAGGTATGTTTATGTTTTTAATATTA
TTACCATTGGAGAAGTTATTTAAATTATCAAGTTACTACTTATTTAACTGGTTCTTCTATGTATATAACATAG
GTGTTATCGTTACTATAGGTATGATGGTGACAAAAGGATTCTTCCAAGTCACTGGTAAATCATACTCTCCAGA
AGCATTTGCAGGCTTTGCAGGTATTGGGCACACAGGTATGCTTGCAGGTTTATTGTTGCTATTTTTCCTTTTA
AGACAAGCAATTCTTAAAGAACCACGTGATGAGTCTTTTAAAAAATAATATAACTAGTACTAAAAAATATACA
CATCTTTCAAAGGAACATTGTTGTTTCGTACTTTGAAAGGTGTGTTTTTATATTGTGATGGGATAGTTATTGA
TAATTCATGGCTAACTATGCGATTTATTTTAACAATATAAATATTAATAGTATAATGTCTTTAAATGATAATA
ATTATCAATTTGAGCGCGTTAATGAAAGAAATGAATATAGAGAGTCATAGGAGGCACATAATGAAAGACGTTA
CAATCATTGGTGGCGGTCCGTCTGGTTTGTATGCAAGCTTTTATGCTGGATTGCGAGATATGTCTGTAAGATT
AATAGATGTTCAATCTGAATTAGGGGGTAAGATGAGAATTTACCCTGAAAAAATTATTTGGGATATTGGCGGC

ATTGCCCCAAAACCTTGTCATGAAATTTTAAAAGATACAATTAAGCAAGGATTATATTTTAAACCGGAAGTTC
ATTTGAATGAACGTGTGGTAGATATTAGGAAAAAGGCAGAACGTCATTTCGAAGTTGAAACAGAAGCGGGAGA
AATATATACATCGAAAGCAGTTATCATTGCAATTGGTGCGGGTATTATTAATCCAAAACAATTAGATGTTAAA
GGTGTAGAGAGATATCAATTAACTAATTTACATTATGTTGTACAAAGTTACAGACGTTTTAAAGATAAAGATG
TATTAATATCAGGTGGAGGAAATACAGCATTAGATTGGGCGCATGATATTGCCAAGATTGCTAAAAGCGTGAC
AGTTGTTTATCGAAAAGAAGATGTAAGTGGTCATGAAGCGATGAAAACACTGGTGACAGATTTAAATGTGAAA
CTATGCCCGAAAACACGTATTAAATACTTGGTCGGCAATGATGATGAAACGCATATAAGTGAAGTTGTTTTGG
AACATGTTGAAAGTGGTGATACGCATACAGTTAAATTTGACGATGTCATTATTAGTCATGGTTTTGATCGTTG
TAATACATTATTGAGTGAAACATCTTCAAAGCTAGATATGCATGATGATTGTCGTGTTAAAGGATTTGGTAAT
ACGACAACTAGTATACCTGGTATCTATGCGTGTGGAGATATTGTTTATCATGATGCAAAATCACATCTAATTG
CAAGTGCATTTAGTGATGGTGCAAATGCAGCGAACCTTGCTAAAACATATATCCAACCAGATGCGAATGCAGA
GGGTTATGTTTCAAGTCATCATGAAGTGTTTAAAGAAGCGAATAAGACTATTGTAAATAAACATTTATACTAG
TTTGATATATTGTGTGATGAAAAGTGGGGTAAATAATGGTTAAAGTGACATATGATATTCCGACTTGCGAGGA
TTATTGCGCATTAAGGATTAATGCAGGTATGAGTCCAAAGACGCGCGAAGCAGCTGAAAAAGGATTACCTAAT
GCCTTATTTACAGTAACCTTGTATGATGAAGATCGGTTAATTGGTATGGGTAGAGTGATTGGCGATGGAGGAA
CTGTTTTTCAAATTGTTGATATTGCAGTTTTGAAAAGTTACCAAGGTCAAGGTTACGGCAGTCAAATTATGGA
ACATATTATGAAGTATATTAAAAATGTATCTGTCGAAAGTGCATACGTTAGTCTGATCGCAGACTACCCAGCG
GATAAATTATATACAAAATTTGGATTTATGCCTACCGAACCAGATTCAGGCGGTATGTATATCAAATTTTAAA
AATGAAAAATCGTACCGATAAACATACTGAGTTTTCTACACAGCTTAACTTAGTATGTTTTTGTTTTTCTAAG
TAATGATATTAAAAAAATGTGTGTTTTATGGTAAAGTTCAAGTTGATATGATATAGAATTTTCGACTTTGTAA
CTAAGGGAGTTAGAACAATGAAAATCGATACATTAAGACAAGCTATAATATATTATTTTGTTGCACTTGTGTT
ATCAGCTTTAATGATCTATTTATTTTTAACAAATATTGAGAGAGTTATTTTGTATAGTTGTGCATTCACAGTC
GTTTATTGGGCAATATATGCCTTTGATGTATATAGAAAGAGAAATTAAATATGAATTGTAAACGAATCAACAT
TTATTTTGCTTAATGTGTAGGTATGATAGGTGCTGAAAATACATGGCTTTCCAAAATACACTAAAGTAAAATG
TTAATTGATGAAGTATCGGAAATTTAATAATCCACAACAAATAAAAAGACATCTCGCATAACGGGATGTCTTT
TTCATGAATACTATTTATCTACACCGAAATCGATAACAATGCGGCCTTTATTTTCATGTTTAATTACTTTGTT
AAGTTGTTCTGGAAGTTCATCAAATGTAATAACTTGCTTGATCTCATGTAAATTTTCAGGCATTAAATCTTTT
GCGAGACGACGCCAAACGCGCTGTCTTAATTTCATAGCAGTAAATACCGAGTCAATTCCTAAAATGTTTACAC
CTCTTAAAATGTGAGGGAATACAGAATTAGTATAAGTATTACCGGCAGTCATACCAATAACTGCAATTGACCC
ACTATGATTTAAACGCTTTGTAACATAATTAATACCTTCGCCACCAACAGGGTCTACACAAGCTTGCCAAGTT
GATGATGCGAGTGGCTTTTTATGATCATCTTCAACAGGAAGTCGATCGATAACTTCTTTGGCACCAAGTTCAA
GTAATTGATCGCTAACATCTTGTTTACCTGTACTTGCGATAACTTTATAACCAAGTTCATTAAGCATGAGTAC
TGCTAAAGTACCGACACCACCTGAAGCACCGCGAACGAGTACAGGACCATCTTCAATATTCATTCCAACTTTT
TCAAGTATTTCAATTGCTAAACCGGCAGTATAACCAGCTGTGCCATATATCATTGATTCTTCTAATGTTAAAG
TATCAGGAAGCTTGATAATCCATTCTGATTTTACACGCGCATATTCACTAAAACCGCCATAATGGCTAACACC
TAGGTCATAACTCGTTACAATTACTTGTTCTCCTTTTTCAAATCCTGGTGCTTCGGATTCAACAATTGTTCCA
GCTAAATCTATTCCTGGAATCATAGGATACGATTTTACGACTGCATTATGATCTTGAGTCGCTAAAGCATCTT
TATAATTTATACCAGAGTAATGTACTTTAATCAGCACATCTCCTTTAGGTAAATCAGTAGGCGATAATTGTTT
GAAAGTTGGTGTCACTTTACCGCTCTCATCTTTATCAATTACAAACGCTTTAAATGTTTCTATCACAACGATA
CATCCTCTCTTTTTCTCTTTTGAAAATACGGCCATTTTACTGGAATAAATGTATCATTTATTGATGTTACACA
CAATCAATACAATTTGTAATTGATTTGTGTTAATGTATTTTTAAATAGTTTTGGCTGAAAAATAACATAAGTA
ATATTTTGATTCGATCACTGTTTATTGGAGGATTTATATGGCTGGGATAATCAAAGAAATTTCAAAACAAGAT
GCTGGTGAATTAGTTGAATTAGCAACTAGAACATTTTATGACACGTTTGGTTCTTACTATGATGACAAAGATT
TTGATCAATTTTTTAAAGACAATTATACTGTAGAAAAATTTACACAAGAGATTAACCATGTAGATTCATTTCA
TTATTTTTATCAAGAAGATGGTGCGAATGTTGGTTATATAAAGATGAATATTAATAGTGCTCAAACTGAAGAA
ATGGGGGAAACCTATTTAGAAGTACAGCGCATATATTTTTTGAAAGACTTTCAAGGTGGCGGAAGAGGTTCAC
AATTGATAGAATTGGCCGAAAAAATTGCACAAGAACATAATAAACATAAAATTTGGCTAGGAGTTTGGGAGCA
TAATCCTCGTGCTCAAGCATTTTATAAACGTCATGGATTTAAAGTGGTTGGTGAACATCATTTTCAAACAGGT
GATGTAACGGATACAGATTTAATTATGGAAAAAGAGTTATAATGATTGTTTTTGCAGCAGATAAAATGAAATC
ATAGTAATAAATATTGTGTATGCCTTGAAATGCTAAATGCGTAATGAATTGTTGAAGTAAAATAATTAAATTT
ATAGAAAACATAAAATTCACAAAAATTAAATAATAATGAACAAAAATTAGGGTATAATAAAAAGAACCTTAGA
TTGTAGTTATGGATATTGTGATAATTCAGATCATATTAATGTTGACGTCTTTGGTAACAAGCCATGAAAAGAT
ACACATGGTAGATATGTATTTCAAGCTTATTCAATGAATATCGAATTATAGGAGGAGATATGTATGAAAAGAT
TAGTTACAGGGTTACTAGCATTATCATTATTTTTAGCTGCATGTGGTCAAGATAGTGACCAACAAAAAGACAG
TAATAAAGAAAAAGATGATAAAGCGAAAACTGAACAACAAGATAAAAAAACAAATGATTCATCTAAAGATAAG
AAAGACAATAAAGATGATAGTAATGACGTAAACAAAGATAATAAAGATAATAGTGCAAACGATAACCAGCAAC
AATCTAATTCAAATGCAACAAACAATGACCAAAACCAAACAAATAATAACCAATCAAGTAATAATCAAGCGAA
TAATAATCAAAAATCAAGTTACGTTGCACCATATTATGGACAAAATGCCGCGCCGGTTGCACGTCAAATTTAT

CCGTTTAATGGAAATAAAACTCAAGCTTTACAGCAATTGCCAAATTTCCAAACAGCTTTAAATGCGGCTAATA
ATGAAGCAAATAAATTTGGTAGTAATAATAAAGTGTATAATGACTACTCTATTGAAGAACATAATGGTAATTA
TAAGTATGTGTTTAGTTTTAAAGACCCAAATGCAAATGGAAAATATTCAATTGTAACGGTTGATTATACTGGA
CAAGCAATGGTTACTGATCCAAACTACCAACAATAATGTTAATGTAATTATGATGCAAGTTAAAAAATTAAAA
CGGTAAACTCTATAATATGAATTAGGGTTTACCGTTTTTTTTTGCGTATTTTAAAGTATAAAAGTTTACGTTT
TATAGTACTTTTAAACCTCATTCACAGCCACTGATGAATTTAAACACTATTGATTAGATGTCATCTTAGACAA
TATAAGTTGTGAAATAGATTCTAATGGTTGTTCTTTATGTTGTATTTGTGGATATTTAATCACTGGTATTGCC
TGGGAAATTAAATTATTAATTAATAATTGTCGGTCTGTATACCAAATTTTTGATAGATGACGCCAAATATCTT
CGTTAACACCTTGAGACTCAATATATTGACTGTGATTTGGAGTGTATAATGCTACTGATTTGTCTATAGCCAT
CGCATCAAATATTATATTTGAGTAATCAGTGATAACGACATCAGACATTAGAATTAAGTCTTGAGCACTAAGA
TATTTTGGTGCAACAATCGCCTCTTCTGGTAACATCGTTTCATCAATACCCTGAACCACAACATGATAGGCTT
TGAATAAAGCATCTGATAGTGGTAATTGTTGCGCACTCACTAATCCAATAGGTGCGTATAATAAAACTGGTTT
TTGATCATTTATTTTAAACGATTTCTTATACTGTTGTTGAGTAGTACTTTCGTTACGCTTTTGTAGTAAATAT
TGGTGCTTAGGATTACCGTAAGCCAATACATTTGCTTGGTGACTAGGAAAAGCCGTTTGATAAAAGTGGCTAA
TATCATCAGCAGAATGTATAACATAATCGAATTGCAACCAGCGATTATACCTTTTAGCACGATAAAAAGGTGT
TTCTATATTTTTAATAGGCTCTTTACTATCTAAAAATAATTGCATTATTGGTGTGCCTTGATGTAATTGAATT
AAAGTACCGTTTGGTTGTAACGTTTCAGGTATATCATTTTCAACTAAAACTACGCGTGCGCTGTTAATCAATT
CGTCAGCTTTTTCAGACTTAGGTGATATGAAATGTGGTCCGCGACGATCGTCAGTAACAAAATAACAGGCAGT
CATTGGATTATGTTTAACGAAGTAAGTAAATAAATATTTTGAATTACCTATATATTGATGATGATCACCGATA
AAAACGATGTGATCTTTTAAAACGTTTGGTTGCTTCGTATACAAGCCGAAGTTTTGTAATTGTTTGTTATCAG
CACGTTGCTTTGCTATTTTGTATAACCATTTAGGTAGCATAAACTTGGTAGAATACAACCAATTATCTGGCGA
ATTAAGTTGTTCTGAATCCTCGATAAAAAGGTTGCGAAAAGGTATAGGATGATGATCAATATAAGCCATTTTA
TTCAAATAAAACTGAGACATCATATCAAAATCTTCAATAGTTTTAATAGTTTCCCATATTGAAGCACTTGTTG
CTAAAGGGGCTTTAAAATCAAAAACGACATGACCATCGATTGATAAATCACCTTTCAGAAGTGTCAATAAAGC
TAAATCGAAAATCGTTTCTACAGAGTAATAATGTATTAAATCAAGAGTCTGTTGAATCGCATATATCAGGTTA
GGATAGTAATTATTGTTTTGAGCCCAATCGGCAATGTTGATCGTTGATTGACCGAAATAGTGACAATCATTTT
TGTATTGTCCATCCACAGTATAATCAACCACGATAGACTTAGGATATAATTGAACCGCATCTAACATAGATTG
ATAAATCATTATCTCATTGGCATATGGAACGACATGTGTATAACCTTCAGACTGAGCTTTCATTAATTGAGAT
TCAAACTTTTGCATATTAGAAACATTTATCTGTTTAATCATTGCTATAACTTCCATTTCTCATCGTAGTATCT
TCCTTATTTTAGCACGGTCTTATGTAAAATTCATGAATAGTATAAGTTACTTCATTACATTAAGTACAATTCA
TTTTGATGAACAGATTTAAAATGAAAAATAAAACAATTAAATAGGAAAGTGATATTGTTAATAGTCTGAAAAA
ATGGTATATTTTAATTGTGAAAAAAATCACATTAATCTCACTGTTCTGTAAAATGTTATCGATAACAAAATGC
TATAGCCAGAGCATGATTTTAATGAAGTAATTACACTACTACTTTATGGAAAGAAGAGTCAGCCTATGTTAGT
AAATACGTTTAACCCATTTGATAATTTATTATTATCGTCTTTAATTGCAGCAATACCGATTGTACTGTTTTTA
CTATGTTTAACTGTTTTTAAAATGAAAGGTATTTATGCAGCCATTACAACACTTGTTGTAACATTACTAATTG
CAATACCATTTTTCAAATTGCCAGTTGGTATCGCTTCTGGTGCAGTAGTCGAAGGTTTCTTCCAAGGTATCAT
TCCGATTGGCTATATCGTTATGATGGCAGTATTGTTATACAAAATTACTGTTGAATCTGGACAATTTTTAACA
ATTCAAGATAGTATTACAAATATTTCACAAGACCAACGTATTCAAGTTTTACTTATTGGATTTGCATTCAACG
CATTTTTAGAAGGTGCAGCAGGATTTGGTGTACCAATTGCAATTTGTGCACTTTTATTAACACAATTAGGATT
TAATCCATTAAAAGCTGCGATGTTATGTTTAGTCGCAAATGCAGCGTCTGGTGCTTTTGGTGCGATTGGTATC
CCTGTAGGTGTTGTAGAAACGTTGAAATTACCTGGAGATGTTTCAGTATTAGGTGTTTCTCAATCAGCAACTT
TAACATTGGCAATCATAAATTTCATTATTCCATTCTTACTTATCTTTATTATTGATGGTTTTAGAGGTGTTAA
AGAAACATTACCAGCAATTTTAGTAGTTTCAATCACTTATACACTTACTCAAGGATTATTAACAGTATTCAGT
GGACCTGAATTAGCAGATATTATTCCACCGTTATTAACAATGTTAGCATTAGCAGTATTTTCTAAAAAATTCC
AACCAAAACACATTTATCGTGTTAATAAAGATGAAGAAATTGAACCTGCAAAAGCACATTCTGCAAAAGCAGT
ATTACATGCATGGAGTCCATTCATTGTATTAACAGTCATTGTAATGATTTGGAGTGCGCCATTCTTTAAAAAC
TTATTCTTACCAAATGGTGCTTTATCATCATTAGTATTTAAATACAACTTACCTGGAACAGTCAGTGAAGTTA
CACATAAACCATTAGTTTTGACTTTAAATATTATTGGACAAACAGGTACAGCTATTTTATTAACTATTATTAT
TACAATTTTAATGTCTAAAAAGGTTAACTTTAAAGATGCAGGTAGATTATTCGGTGTTACATTTAAAGAATTA
TGGTTACCAGTCCTTACAATTTGTTTCATCTTAGCAATTTCTAAAATCACAACTTATGGTGGTTTAAGTGCAG
CAATGGGTCAAGGTATTGCTAAAGCAGGTAATGTCTTCCCAGTTCTATCACCAATCTTAGGTTGGATAGGTGT
GTTTATGACAGGATCAGTTGTAAATAACAACTCATTATTTGCACCAATTCAAGCTTCTGTTGCACAACAAATT
GGAACAAGTGGTTCACTTCTTGTATCTGCTAATACAGTTGGTGGTGTAGCGGCAAAATTGATTTCACCACAAT
CAATTGCAATTGCAACTGCAGCAGTAAAACAAGTTGGTAAGGAATCAGAATTATTAAAAATGACATTGAAATA
CAGTGTATGTTTACTAATATTCATCTGTATTTGGACTTTCATCTTGTCATTACTATAAAAAAACGTATTTCAA
AATATAAATATACAGAGGGTGAGATGTTTTCTAACATCTCATCTTTTTTTAATGGATTATTAATGAAAGAAGT
TTGACATTATAATAATGGTAGAGCTTTATGTTAAAATGAAGAGTGAGTAATCAGCAATCAAATTAAATTGGTT
GATAGCTGTTAAGGTTTGTGGTTTTGTCTTTGTGCTATCGCACATAAAGTATATAATTAAAGTAGTTTCGTTA

TTATAAAATATTAATATACATAGTAGATAGTAATAGAGCATCACTATGGGAGCCTATTGAGACACTTATTGAT
TCAAAGTGGTATTAATATGTCGTATTTGTCGAACGTTCCATTATTCATTTTAAAAAGGGGGACTGTATTTGTT
ATGACAACACAACATAGCAAAACAGATGTCATCTTAATTGGTGGCGGTATTATGAGTGCAACATTAGGAACAT
TACTTAAAGAATTATCACCTGAGAAAAATATTAAAGTGTTTGAAAAATTAGCACAACCTGGCGAAGAGAGTTC
AAATGTATGGAATAATGCCGGTACAGGGCATTCAGCACTTTGCGAGTTGAACTATACAAAAGAAGGTAAGGAT
GGCACAGTTGATTGTAGTAAAGCAATTAAGATAAATGAGCAGTACCAAATTTCAAAGCAGTTTTGGGCATATT
TAGTTAAAACAGGACAATTAGATAACCCAGATCGCTTTATTCAAGCGGTGCCACACATGAGTTTTGTCATTGG
CGAAGATAATGTAGCTTTTATAAAAAGTCGTGTTGCAACGTTAAAGAAAAGTGTTTTATTCGAAAAAATGAAA
TTATCGCAAGATGAAGAAGAAATGAAATCTTGGGTACCGTTAATGATTGAAGGTCGTAAGTCTGATGAACCAA
TTGCTTTAACTTATGATGAAACTGGTACAGATGTTAACTTTGGTGCGTTAACTGCAAAGTTATTTGATAATTT
AGAACAACGTGGTGTGGAAATTCAATATAAGCAGAATGTATTAGACATCAAGAAACAGAAATCAGGGGCATGG
CTAGTTAAAGTTAAAGATTTAGAAACTAACGAAACGACAACATATGAATCTGATTTTGTATTTATTGGTGCTG
GCGGTGCGAGTTTACCATTACTCCAAAAGACTGGAATTAAACAATCAAAACATATTGGTGGTTTCCCGGTAAG
TGGATTATTCCTGCGCTGTACAAATCAAGAAGTGATTGATCGTCATCATGCTAAAGTGTACGGAAAAGCAGCA
GTGGGTGCGCCACCAATGTCAGTGCCGCACTTAGATACACGTTTTGTAGACGGCAAGCGTTCATTGTTATTTG
GTCCATTTGCAGGTTTCTCACCTAAATTTTTAAAAACAGGTTCACATATGGATTTAATTAAATCGGTTAAACC
AAATAATATCGTGACGATGTTATCTGCAGGTATCAAAGAAATGAGTCTTACGAAGTATTTAGTGTCACAATTG
ATGTTATCTAATGATGAGCGTATGGATGATTTAAGAGTCTTTTTCCCAAATGCTAAAAATGAAGATTGGGAAG
TGATTACAGCAGGGCAACGTGTCCAAGTAATCAAGGATACTGAGGATTCTAAAGGTAACTTACAATTTGGTAC
CGAAGTCATTACGTCAGATGATGGCACATTAGCTGCATTACTTGGTGCATCACCTGGTGCGTCAACAGCTGTA
GATATTATGTTTGATGTTTTACAGCGATGCTATCGTGATGAATTCAAAGGATGGGAACCAAAGATTAAAGAAA
TGGTGCCGTCATTTGGTTATCGCTTAACAGATCATGAGGATTTATATCACAAAATTAATGAAGAAGTAACTAA
GTATTTACAAGTTAAATAATAAACGAAACGGTAATGTCTTTTTTAATGCGATAGACATTACCGTTTTTTAGTG
GTTGTTTTTATTATCAGTGCTTTATATTTAGAAGCTTATCTTGATTGAAGTCATTTAAAAGTTTTGTAGCATT
TATACCTGTCTGTATCGTTTTAAACCAACAATATTTAAGATAATAAAGACTATGATAAAAATCATTAGAATTA
GTAAATCGAACCATATATTACTCCAACCTTGCCCCTTTATCATAATATTAGTTAAAGCATCACCAGCATATCG
AAGAGGGAAGAGATAGCCGATGTTACCTAGCCAATCAGGCATATTTTCAAGTGGGAAAATTCCTGAAAAGAAT
ACCTGAGGTACAGCAACTAGTGGAATAAATTGAATCATTTGAAACTCAGAATTAGCAAACGTTGAAATGAAAA
TCCCCATAGATAATGCTGTAATAGCTAAACAAATATTAATCAAAATTACATACCATAAGCTACCTGCTAAATT
AATGTTCAATAAATATATTGAAAATAGGACTATGATTAATGTTTGAATGATTGCGAATATACCATAGCCAATC
AGATAACCAAGGACGATTTCACTTCGACGAATTGAAGTTGATAATACACGTTCTAACGTACCTGTTGTACGTT
CACGTAATAAAGCTATACCTGAAATTAGAAATACAAATAAAAATACAAAGAAGCCCATTAGTATTGGAAACAT
TTTATCGAAATAAGTACTATCAGAATTACCGTATAAATAGGAATGCGAAATCTTAATATCTTGTTTGTTTGGT
GCTTTCATGGTCGGAGGTGCTTTTTCAATTAGATGCTTTATACTGCTTATTTTATCTTGTTGTATAGCTTTGC
CAATCATTTGTTTGATCATATTAGATTTGTTAGGGTCTTCATTTGTATAAATGACATGTAATGTTTGATTTTC
TTGGTAAATATAACCATCAAGATGATTATTCTCGATTTTAGATCTGATATTTTCATGAGAATGGAACGTTTGA
GTTTTTATGTCGGATGATGGCAATTGTTTTGTTATATTTGATGAAACAGATGTATCGATACCGATTTTCAAAT
CGTTTTCATCACCGTTAAAAATAAAATACATAAGCGTCAATATTAAAATTGGTGCTAGAAACATCAGCGCAAG
AGTACGTTTATCACGTAACATTTCTTTTAATACCCGTTTAACAATAGCTAAAATTTTCATGATTGACGAACCT
CCTCAGCTTTAATAAATACCGCTTCAATTGTATCTACATTAAATCTTTGTTTTAAATTGGTTGGCGTATCTAT
AGCAAATATTCTTCCGTCGACAATAAGTCCAACTTTATCGCAACGTTCCGCCTCATCCATAACATGTGTTGTG
ATGACGACACTCTTTCCGTCTTTGGTCATTTGCTTGAATTGTTGCCATATTTGTTGGCGCAATTTAGGGTCAA
TGCCTACAGTTGGTTCATCTAATATGATTAATTCTGGTTCGGCCAACAATGTTATTGCTAAAGATAATCGGCG
TTTCATGCCTCCTGAAAACTGTGCAACTTTTTTATTTAACGCTTGCTCTAAATTCACTAGAACGATATTTTTT
TCAATATTATCTTTTAATACATTTCCTTTCAGTCCTGACAAACGACCGTAAAACTCTAGGTTTTCATAAGCAG
TTAAGTTTTCGTATAATGCATCAGTTTGCCCCATATAGCCTAATCGATTTAAAGCTTTGCGATTAGGCATCTT
ATGAGCGAAAATTTCTGCAGATCCCGAATCTACTTTTTCCATACCTAGTAAACACTTTATTGTTGTTGTTTTA
CCAGAACCACTAGGACCTATTAACCCTAGCAGTTCACCTTTATTGATTGACATACTAATTTCGTTTAATACGT
GATGCTTGTTATACGTTTTTACAACTTTATCCAACTTCGCTATAGTTGCGTCCATGACAACCCCTCCTAAATT
GAGATACATTTCACTTTATTTATATATCATCTTAAGCGTATATTAAGATTGCGAATAAAATATTAACAGATAG
TGAACATATTTGCTTTTAGTGTTCAGAAAAAAGGTGGGGCATATGGATCAAACAGCTTTTACGGATAGTCGTG
TAACGAAAACGATAAATCGCATACATGATGGAATGGTAAATCTATTAAAAATGAAGGCATATAATGATATAAC
TGTTAAAGATATATGTGAAAAGAGTCAAATAAGTCGGACAACCTTTTATACACATTACAAAACTAAAGATGAT
TTTATATACAGCTATCTTAATACATTGTTAAAAGTTGCTAAAAAGAAGTTCATTAAAGAGAGGCCTATGACAC
AAGCAGTATTTTTTAAAAGGATGATTTATTATTGGCTAAGTGAAGGACAGCTCATATTGATGTTATTAGGTGA
TGAGAGTGCTTATAAATTACATCAAGTCATAAAAAAATCACTACAACAACGTATCGAAATTAATGTTGTACCA
GTATTAAATACAAAAATGTTAACGACGAAGGAAAAGTATTTTTTACTTATTTTTATGAGTAATGCAATCATTG
GTGTGTTACAAGATTGGGTCAAACGAGGATATAAAGAGAGTCCTAAAGAAGTAGCAGAAATTATGAATAAAAT

ATTTGAAAATGCATTTCGTTAGGTTTTTATCATGATAAGTTGATGTTGTAAAGGACAGTTGCAGTTACAATTT
AATTTGAAAATAGTAAAAACATATTATTCAGTTCATGCGTATTTAAAATAAATACAACATAAAGCTATACATT
GCAGAAGAGACTACTTTAATGCAATGTATAGCTTTTATAATTTTAATAAAAATCATTTTAATTGTTTCAGTTG
CTCGTTAATAGTGTCTACGTAGTTCTTGTTTTTAAAGAATTGAATAATCCAAATCAATGTGTAAACCACAATA
AAGATAATTGTAAATATGATTAGGGAATTTATTGTTAGTGGAAACCAACCGGCAAGTATTGCTAAAGGTAAGA
ATCCGACATACGTTGTTATGAAATGCACTATAGTTGCTTTAGTAATGCCCCAATCTGTGTATTTAAAGATAAA
ATCTCCAAGGAAAAAGACGACGCCTATGAGTAACCATAAAATGATAGAAATCAACATTACGGTAGTTTCTGTG
AAATGATTATAATACAATATGCCAATAGTTGATTGTGGGTTCAGTGGATAATATTTGCCGTCTGCAAATAACA
TACTAAAGAACAGTGAAAGGGACAAACCAATGATTAAGCTAATAAATAATGAGTTTTTCAAATTTTTCATATT
GATAAGCGCTCCTTTATAGATTTTAAATAACGTCTAGAAGAATAGGTGTAGTGTGCATCTTTAAGATACATAC
GTATAAGTCCATTTGGCTCTAATAATAATTTTTCAATGTAATACTTGTTGACGATTTCTGATTTGGAAATACG
AATGAAATGTTGTGGTAACTGTTTTTCTAGTTCATAAAGTCGTAATTTTAGTTTGAATTTTTGATTGCTTGTA
ATGGCTGTTACATTTTTATTAATAACTTGGAATGTTATAACATCTGATACATTAATGATATGGATTTCTTTGT
CTATGTATCCGACTAATGTATGTGATTTGTCTAAATCATTGACTGCATTAATAATACTTTGAACGTGATCATT
CATTTTAGGTGCATGTATATCAATATAAGATTCCGTCTCAGTTGCATTGATAAATAAATTGAGTTTCATCATA
GGTTAATGCCTCCTTCAAAATTATTAAACCATAAATGACCATCGATATATTTAAATTTTGTTGAATGGTAGAA
ATTAAATGTTAAGTGGCTAGAAAGCGTTAATTAATATAAAAGATACCTCCTGTAATAAAAACAGAAATGTTTT
TTCAGGAGGTAGAGATTAATGTGAATTATTTGGCAGTGTAATAGTAAAGGTGGTTACATACTCGTTACTTTGT
GTGAATTGGATTGTACCATGATGCAATTCAATGATGGATTTTGTAATTGCAAGACCTAAACCATTGCTATTAT
CATGTTTGCTCACTTTATAAAAACGTTCAAATAAACGTGCTTCAGCTTGTGGACTAATTGGTGAACCATCATT
ACTTATTGTGAAAATGATGTTGTTGTGACTATGTTGCAAAGCGATGTCAATGGCACCACCAACATCTGTATAC
TTAATAGCATTTATTAATAAATTACTTAATGCTTGATGTAACAAACGTTGATTTCCTAGGAAATCGATTGATT
CTAGGTCAGCTAAAATGATTAACGACTTTTCATCAGCAGAAAATTGTTCATGTCGAATAATATCTTTAATGAG
TTGATCGACTTGAATCTTATCATCGAATAATAAATGTTGGTGATTATCTAATTCGGATAAGAGCAACAATTCA
GTTGTTAATCCGCTCAGTTGCGTTGTAATGGTATAAATATCATTTATATATTGTTGTCGTAATGTTTTATCAG
AAGTTTGTTGTAACTCGCTTAATAAATGATGAATATGTGTTAATGGTGTTTTGATTTCATGAGATACATTTTG
TACAAAATGCTGCCTCATTTGATCAACTTGTCTCAATGACTCTCTCATCTTATTAAAGTGATACTGTAATGTT
CCAATTTCATCTTTGCGTGTTTGTTTGATAGGTGTTTCAAAATCACCATCAATTAAGCGTTCGGTCGCTAGCT
TTAACTTTTTTACTGGACGAATGATTGAATAGGTTGATGCGATAACTAATGAAATCGAGATAAATAATAACAA
CATTAACAAGACGGCTAAGAATGTTCTAAACTCGCTAAATGTTTCTCCAATATCTGGGCGCATGAATACTGCT
ATTGAACCGTCTTTTGTCTTAAAGTTAATACCAACAGTATTATCTGTTACATTGTCGAAAAATCCAGTTACGA
ATAATGCGAATGGTTTATCTTTAATACCATGGTAATCTTTATTATTTAATACGTTGTTAATAGCATTTTGTGA
TAAAGTATCTTCTCTAAACGGCTCCCCATAAAATGTCTTATGACCTTTTTGATCAACGGTCATTATTTGGTAA
TTCATTTGCCCTAAATGTTTGAAATATTGTTGAATGTGCGTTGGTTTAGCTGATTGTTCATATTGTCTTGCTT
CTTTAAGCGTCTTCATGATTTTCGCGTCATTAGATGCTTTTAAATTATAATGATAGTAAACATTTGTTAATAC
AAAACTTATTAATGCACTAAATAAAATAACCGTAATGGAATAAATCGCAATTCTAGCATAGAGTGTTTTAAAC
ATGATTCTCCACCTTATAGCCTTGTCCTCTTACTGTTTCAATTGTAAGTGTGGCATTTAATTTTTTTAATCTT
TGGCGTAGTCGCTTAATATGAACGTCAACTGTTCGTTCATCTCCTTCATAATCATAGCCCCAAATTTTTTCTA
TTATCTGTTCACGAGTAAAGATTTGCTTAGGACGTGCTGCAAGCATAAATAATAATTGAAATTCCTTGTTCGG
TAACGTCATCGTTTTATTAGATACTTGGAGTTCCAAATAGGATTGGTTTAGCGTTAAGTTGCCAATGGTCATT
TCTGAATTTGAATTGATATTATATCGACGTAATACCGCACGAATTCTAAAAATAAGTTCCTTAACCTCAAAGG
GTTTGGTTACATAATCGTCAGTACCACTTATAAACGCACGCTCTTTATCACTAAGTGCATCCCGCGCTGTTAA
CATAATAACTGGTATATCATAATCATTTTTTAATGTATTACATAATTGAAAGCCGTCCATACCATCCATCATA
ATATCTACCACTGCAATATCGACACGCTGTTTTTCTAAAAGTTTTAATGCAGCTTCACCACTTGGTTGTGTGT
ATGCATCAATGTGCTCTGTTTGTAAATGGCTAGCTATATAATTTAGGATACGAGGATCGTCATCGACAACAAG
ACATTGCACCATAGCTATAAACTCCCTTATCTTTTTCATTTATTATACATGTAAAATATTTTTGCGTAAAAAA
ACAATTGTTCATATTGAGTTCATATTTCAACCTTATACTGACGCTAAAGAAGAAATAGGGAGAAGTGAATCGA
TATGAAATTAGCGATAAAAGAGATTATGTTTTACAAATTTCGTTATATTTTAATCACATTAATCATTCTTTTA
TTAAGTATTATGGTGTTATTTATTAGTGGTTTAGCTCAAGGGCTTGGTAGGGAGAATATTTCGTTATTTGAAC
ACTTTGATAATGATGAATATGTTGTTCAAAAAATGAAAGAGCCGCAAATTGAGAAATCGCAACTTAGTGATAC
GCAACAGAATCAAATTAAAAAGGTAATTCATCAAGAACCATATAAAATGAATATTCAAACATTGAAATTATCT
AATAAAGAACAAGATGTGATTACAATGAATGACGTTAAACAACAACGCATACAACTTAAAAAAGGTGATTATC
CTAAAAATGCTCATGAGGTCGCTATTAATGATAAGTTAGCTGCAGACAACATTAGAGTCGGGGATAGATTACA
TTTTAAAAATAATTCAACTAGTTATAGAGTTTCTGGTATTTTAAACGACACAATGTATGCGCATAGTTCCATT
GTGCTATTGAACGATAACGGATTTAATGCATTGAATAAGGTTAATACGGCATTTTATCCAGTGAAAAATTTAA
CGCAACAACAACGTGATGAGCTTAATAAAATAAATGACGTTCAAGTTGTGAGTGAAAAAGATTTAACAGGTAA
TATTGCGAGTTATCAAGCAGAGCAAGCACCGTTAAATATGATGATTGTTAGTTTGTTTGCTATTACAGCAATC
GTTCTAAGTGCATTTTTCTATGTTATGACGATTCAAAAAATATCACAAATCGGCATTTTGAAAGCAATTGGTA

TTAAGACAAGACATTTATTGAGTGCGTTAGTTTTACAAATTTTAACACTAACAATAATTGGGGTAGGTATTGC
TGTGATCATCATAGTAGGACTATCATTTATGATGCCTGTAACGATGCCTTTTTATTTAACAACGCAAAATATT
TTATTGATGGTGGGGATATTTATATTAGTAGCGATTTTAGGTGCCTCACTATCATTTGTCAAATTATTTAAAG
TGGATCCTATCGAAGCAATTGGAGGTGCAGAATAATGGCATTAGTTGTTAAAGATATCGTCAAAAATTTCGGA
GAAGGTTTGTCTGAAACAAAAGTTTTAAAAGGTATTAATTTTGAAGTGGAACAAGGGGAATTTGTCATTTTAA
ATGGTGCCTCTGGTTCTGGGAAAACAACATTGCTAACGATATTAGGTGGATTGTTAAGTCAAACGAGTGGTAC
AGTGCTTTACAATGATGCGCCATTATTTGATAAACAGCATCGTCCTAGTGATTTACGATTGGAAGATATTGGG
TTTATTTTTCAATCTTCACATTTAGTTCCTTATTTAAAAGTGATAGAGCAATTGACGCTCGTAGGCCAAGAAG
CGGGAATGACCAAACAACAAAGTTCAACAAGAGCAATACAACTTTTGGAAAATATTGGTTTAGAAGATCGCTT
GAATGTATATCCGCATCAGTTATCTGGCGGTGAAAAGCAACGTGTTGCGATTATGAGAGCATTTATGAATAAT
CCGAAAATCATTTTAGCAGATGAGCCCACAGCAAGTTTAGATGCCGATAGAGCAACAAAAGTTGTTGAGATGA
TACGTCAACAAATTAAAGAACAACAAATGATTGGTATTATGATTACACACGATCGAAGATTATTTGAATATGC
TGATCGAGTGATTGAATTAGAAGATGGCAAAATAACTGATTAGTGGCTCGTAAAGACGCTAAATGTTAATGAT
TTAAGACATAGTAGTATAAAAGTTAGATAACAGAATACGATTTGGGTTTACAAAAAAGCACGTCACTATTATA
TAAAATGATGACGTGCTTTAGTTTATTTATTTTCAATATAACAATTGTCTAATCAAGGATTAACGCATATTTA
AAGATAGTTTGACGCAATATTAGAAACAACCTATAATAATAGTTTATTTGTGGGTTAACTATTATAAATAAAA
GCAGCGTATAGACATATAAACCAGCTACTTGAACAATATAACGTTAATAACAATCTATACTGATACATTACGC
CTAGATAATCTTTAATGAGCACATGTAAGAAAAAGTGATATGGTGTATGACTTCCGACACCATCGATAGATAA
ACCTAATTTTTGGGCTAGTCGTAAGGCGCGTAATACATGAAACTGACTTGTTACACAAACAATTTTAACTGCT
TCATGATACAAATTGTTGATGATTTGTTTAGAATATAAAAAGTTTGTGTATGTATTTATAGAGTGAGATTCCA
TTAGTATATCTGTTTTATCAACACCATGTGCAATCAAATAGCGTTGCATAGCTAAAGCTTCAGGAATTGGTTC
GTCTGGTCCTTGTCCGCCAGATACAATAATCTTTGTTGCTGATACTTGTTGTTGATAGATATCAAGTGCACGG
TCTAAACGCGCTGCAAGCATTGGTGTGACAAATTCGGTAAAAATACCAGCACCTAACACAATTATGATATCAA
CTTCTTTGTTGTATGATCTATGTCTATATGATACTGTCCAAACGAGATAACAAATAAAGGTTAGTAACAAGGA
AAGGCATAATATAGCTAACCACATAGACAAACCTTTCACAATAGGTGACTGAATCGTACTTATAAATAGAAGT
GCTGATGTGTAGAGTACAAATTTATATGAAAAAGATAATAATTTTTTAATAAATAAGCGACTAGAAGTATGAG
AAAATAAATATCTATGTTTGAATAGCATGATAATACTGATTATTATAAATGTTATAAACATAGACCAAGGGAA
AGTATAGGTCATGATGCTATAGATGAGTGACAAAAATATCGATATGACAACTAAGATGTAGCATGTTAAATTT
AACGTCAGAGTATAGTTGAAAATTAACGGACAAATAACGATAAGTATAAATAATAATAATAAATTCAATAACA
TACTGACACCTCGCTTATAATAAATATTAAGTATAAATGTAGATGATTTAATTTATTAAAGCAAGGAGAACGT
AGCAACATGTAAGTCTTAATTTGTTATATTATATATGGGTCAATATTTTTGTGTTTTTTAGATGTGCGATACA
ATGATTGCTGAGGATACTTTATAAAAATAGTAATTAGGGAGAAATAATTATGGTAAAACATTTACAAGACCAT
ATTCAATTTTTAGAGCAGTTTATAAATAACGTTAACGCATTAACTGCAAAAATGTTGAAAGATTTACAAAATG
AATATGAAATTTCATTAGAGCAGTCTAACGTATTAGGTATGTTAAATAAAGAACCTTTGACAATTAGTGAAAT
CACGCAAAGACAAGGTGTAAACAAGGCCGCAGTAAGCCGACGAATTAAAAAGTTAATCGATGCTAAATTAGTT
AAGTTAGATAAACCAAATTTAAATATTGATCAACGTTTGAAATTCATAACCTTAACTGACAAAGGTAGAGCAT
ATTTGAAAGAACGTAATGCGATTATGACAGATATTGCGCAAGATATTACTAATGATTTAAATTCTGAAGATAT
TGAAAATGTAAGGCAAGTATTAGAAGTCATCAATCATCGCATTAAAACATATAGCAATCATAAATAGGTTTTT
GAAGGTGTTCAAATATAAGACTAAATATGTATGTATTGAACACCTTCTTTTTAGTTATAATTGAGAGAGTACT
TTCATAAATAATGTAATAAGGTACAATTCTAAAGTAAAGTTAATCATTTCTTGAATTGTATTCTTGGGATAAT
ATTTGTAAAATAATAATGGTAAAATAATGAAATAATTTTTGTGATATACATATAACAATTGAAGAGGAACAGA
ATTGAGGTGAAAGTATGAAATCTTGCCCGAAGTGCGGACAACAAGCACAAGATGATGTACAGATATGTACACA
ATGTGGACATAAATTTGATAGTCGTCAAGCATTATATAGAAAGTCAACCGATGAAGATATACAAACTAATAAC
ATCAAGATGAGAAAAATGGTGCCTTGGGCAATTGGTTTTTTCATTTTAATCTTAATTATTATATTGTTCTTTT
TACTAAGAAACTTCAATTCACCTGAAGCGCAAACTAAAATATTAGTGAATGCGATTGAGAATAATGATAAACA
AAAAGTAGCAACATTATTAAGTACTAAAGATAATAAAGTAGATTCTGAAGAAGCGAAAGTATACATTAACTAT
ATCAAAGATGAAGTTGGGCTTAAGCAATTTGTCAGCGACCTTAAAAATACGGTACATAAATTGAATAAGAGTA
AGACCAGCGTAGCTTCTTATATTCAAACCAGATCTGGTCAAAATATATTACGTGTAAGTAAAAATGGCACTCG
TTATATCTTTTTCGATAATATGAGCTTTACTGCACCTACCAAGCAACCAATTGTTAAACCGAAAGAAAAAACA
AAATATGAGTTTAAATCTGGTGGTAAGAAAAAAATGGTTATAGCTGAAGCAAATAAAGTGACGCCAATAGGTA
ATTTTATACCGGGGACATATAGAATTCCAGCTATGAAATCAACTGAGAACGGTGATTTTGCAGGCCATTTAAA
ATTTGATTTTAGACAAAGTAATTCTGAAACGGTAGATGTTACTGAAGATTTTGAAGAAGCAAATATAACTGTA
ACTTTAAAAGGCGATACAAAATTAAATGATAGTTCTAAAAAAGTAACTATAAATGACCGTGAAATGGCATTTT
CAAGTTTCAAAACGTATGGTCCATATCCACAAAATAAAGATATTACCATTTCAGCTTCTGGTAAAGCGAAAGA
TAAAACATTTACAACACAGACGAAGACGATTAAAGCTAGCGATTTAAAATACAATACAGAGATAACTTTGAAT
TTTGACAGTGAAGATATCGAAGACTATGTTGAAAAGAAAGAAAAAGAAGAAAACAGCTTGAAGAACAAATTGA
TAGAATTCTTTGCTGGATATTCTTTAGCGAATAATGCTGCGTTTAATCAGTCGGATTTTGATTTTGTATCGTC
ATATATAAAAAAAGGATCATCTTTTTATGATGATGTAAAGAAGCGTGTATCTAAAGGAAGTTTAATGATGATT

AGTTCACCACAAATTATAGATGCCGAAAAACATGGTGATAAGATTACCGCAACCGTAAGATTAATAAATGAAA
ACGGTAAGCAAGTAGATAAAGAATATGAGCTTGAACAAGGTTCGCAAGACCGCTTGCAATTAATCAAGACATC
AGAAAAATAGAGTGTAAAACTTAAATAAGATGCATGTGTCATGGAATACAAATAACATCCTTTCAAAGTATTT
ACAATTAGTGTGAATTACTTTGAAGGGATTGTTTTTGTGAAAAAATTTATGGAGTGCTGAGGTTTATATCATT
TTATACCTTTTATAAGTTGTAATGATGTTTATGAAACTTGTGAGTAATGCTAATATGTAAAAGAGCATATTTA
AATTTAGTTTCTGTTCGTGATAGTTATAAACTAATCATTATATAAAGGAGGAAATATAGTGAATGATTTAAAG
CGTTTTAATAAGCGTTCACCGATATTTATTATTATTTTAGGATCTTTGACTGCCATTGGAGCATTATCAATAG
ATATGTTTTTGCCTGGATTACCAGATATTAGACATGATTTTCAAACAACGACGTCTAACGCACAATTAACATT
ATCGATGTTTATGATTGGTTTAGCGTTTGGTAATTTGTTTGCTGGTCCAATTTCAGATTCAAATGGACGCAGA
AAACCGCTTATTATAGCTATGATTATTTTTACATTAGCAAGTTTAGGTATTGTTTTTGTACATAATATTTGGC
TGATGATTGCATTAAGATTTCTTCAAGGAGTGACAGGTGGAGCAGCAGCAGTTATTTCAAGAGCTATAGCAAG
TGATATGTATAGTGGTAATGAACTAACTAAGTTTATGGCATTATTGATGCTTGTTAATGGTATTGCACCAGTT
GTTGCGCCAACAATAGGAGGCATAATTTTAAATTATTCTGTCTGGCGTATGGTCTTTGTCATTTTGACTATCT
TTGGCTTCGTTATGGTCATAGGTTCTTTATTAAAAGTACCTGAATCATTAACAGTGACAAATCGTGAATCAAG
TAGTGGATTAAAGACAATGTTTAAAAACTTCAAAATATTATTGAAGACGCCACGTTTTGTATTACCAATGCTC
ATTCAAGGTATGACATTTGTAATATTATTTACTTATATTTCTGCGTCTCCGTTTATTATTCAAAAAATTTATG
GCATGACTGCAATACAATTTAGTTGGATGTTTGCAGGAATTGGTATTACGCTCATTATATCAAGTCAACTTAC
TGGCTACCTTGTTGATTTTATAGACTCACAAAAATTGATGAGAGTCATGACGATGATTCAGATTATCGGTGTA
ATTTTAGTGACGATTGTATTGCTTAATCATTGGAACTTTTGGATTTTAGCGATAGGATTTATCATTTTAATAG
CACCAGTGACAGGTGTTGCGACACTTGGTTTTACAATAGCTATGGATGAAAGTAGTAGTGGTAGAGGGAGTTC
ATCAAGTTTATTAGGTTTAGTGCAGTTTTTATTTGGTGGTATTGCATCGCCATTAGTGGGGGTAAAAGGTGAA
GATAATCCTATACCTTACATAATTATTATTATAGCTACAGCAGTTATATTAATTATTTTACAGATTTATAATA
TGAAGGTATTTAAAACAAATCGATGATAAACATAGTTTCTTCTATTATATTGAATTGAAAAATTAAAAAACAA
AACTTGAGCTATTCATAGACCTATGAATTTAGCATCAAAAATAAAAATGCCGAGATTATTATGTGGAAATCTC
GGCATTTTTATATTGATAGCACCGTTACATATGTACTATTATCTGTCCATTTGGTTTAACTTTATCTAGTTGT
TTTATAGCATTTCTTTATTAGAATGAAACCGTATTATTGGTTTTGAAGTTTAATCGTATTAGCCAATAAAATG
TCAGCAACTTCTTCAGGTGATTCATTACAACCATTCGAAAGCCAATTAATAAACACCCCAGCTTGACCGCCTA
TGATATATGTAACATAAAGCTGTTTATTGGTTACTGTAGTACTATAGTCATTCATAATTTGTTGATAGTAATC
TCTCGCTAAATTTATATAGTCTATGAATAATGTTTTCATTGGATAGGTAACTAAAATGTCTTTGAAAAACTGC
TCATTGCGCTTGATATACTTAGCAATAATAATGAAGAATTTATAAAGTTTAAGCTTATCTTGTTTAATATTTT
CGAAATTATGATGTAGCGCACTTAGAAGAAATTGGAATTTTTCTTTATGCTTTGAGTTGATGGTATCCAATAA
ATCAAATTTATCTTTGTAATAATCATAAAAAGTAGAACGATTAATATTACAATACGCGCAAATCATTTTAGTG
GATATTTCTTGAAAAGGATATTCAGCCATCAAATCAACAAGTGCGTTTTGAATTTCTATTTTGGCTTGTCGTT
TCATATTGTTACACCTTTGTGAAATTTAACCAACACTCTTATAAAAATTGTTGGTGTTTTTTTAGTTATCATG
TCTCTATAATAAATAAAGTGAATTTTAAAAGCAAATAGGAGGAAATGTTTCATGAAAAAGATGATATTAATTA
ATGTCATTACGGTTGTTGTACTGCTAGCAATTGGGATTGCTGGTTTTTATTTCTGGAATAAAACAACAAGTTA
TATCACAACTGATAATGCTAAAGTAAATGGCGATCAAATTAAAATAGCAAGTCCAGCTTCTGGGCAAATCAAA
TCACTAAACGTTAAGCAAGGTGACAAACTTGATAAAGGTGATAAAGTAGCAACAGTTACTGTTCAAGGTCAAG
ATGGCGAAACTAAAGATATGGAACTTAAAATGCCACAAAAAGGTACAATTGCTAAATTAGATGGCATGGAAGG
TTCTATGGTACAAGCAGGCAATCCAATTGCTTATGCTTACAATTTAGATGATTTATATGTAACTGCAAATATT
GACGAAAAAGATATTAAAGATGTTGAAGTTGGTAAAGATGTTGATGTGACTATTGACGGTCAAAAAGCTTCTG
TTAAAGGTAAAGTTGATAGTATTGGAAAAGCTACTGCTGCAAGCTTTTCATTAATGCCATCATCAAATAGTGA
TGGTAACTATACTAAAGTATCTCAAGTTATACCTGTTAAAATTACGTTAGATTCAGAACCGTCTAAACAAGTT
GTTCCTGGAATGAACGCTGAAGTAAAAATTCATAAAAATTAAGGGGGTCATTAAATGACTACGACCTTCATTA
TTAGCTACATTGTTTTAGCGCTCATTATTGTTGGGGTTATCAATTTATTGTTAATAAGATCAAGAAGAAAAGC
AAAACGCCAACAAAAGGAACAACATTTTGCGACACGTCAATCAAATCAATCTAAATTTAAAGCAAGTGATTTA
GACAGAACAACTGATCAATCGTCACAACATACGGCGCGTGAAGAGGCACGTATTGACAATCAAGATAATCAGA
ATCAAGTTAGTCTAAACAAACAGACAGAGGGATCTGAACAAAATCAAGCATCATTCACTGATAATAATGAATC
TGATGAAGAAGTATATGCTGCTAAAAAATCCTAAATCAGAAGAATATAAAGTAAATGAAAAAATAAAAAAAGAA
CATAAAAACTTTATTTTTGGTGAAGGTGTTTCACGCGGTAAAATATTAGCGGCATTATTATTCGGTATGTTTA
TTGCGATTTTAAACCAAACATTATTAAATGTTGCTTTGCCAAAAATTAATACGGAATTTAATATTTCTGCGTC
AACAGGACAATGGTTGATGACGGGATTCATGTTAGTAAACGGTATTTTAATACCAATTACGGCGTATCTATTT
AATAAATATTCATATCGAAAATTATTTTTAGTTGCGTTAGTACTCTTTACAATTGGTTCATTAATATGTGCGA
TTTCAATGAATTTCCCAATCATGATGGTTGGACGTGTATTACAAGCCATTGGTGCAGGTGTACTAATGCCATT
AGGTTCAATTGTTATTATTACAATTTATCCACCTGAAAAACGTGGTGCTGCCATGGGTACAATGGGTATCGCA
ATGATTTTAGCACCTGCAATCGGTCCAACATTATCAGGTTATATTGTACAAAATTACCATTGGAATGTGATGT
TTTACGGTATGTTCATTATCGGAATTATTGCAATCTTAGTAGGTTTTGTATGGTTTAAGTTATATCAATATAC
TACAAACCCTAAAGCTGATATCCCAGGTATCATTTTTAGCACGATTGGTTTCGGTGCTTTGTTATACGGCTTT

TCAGAAGCGGGGAACAAAGGCTGGGGTTCTGTTGAGATAGAAACAATGTTTGCGATTGGTATTATCTTTATTA
TTCTATTTGTAATAAGAGAATTAAGAATGAAAGCGCCAATGTTGAATTTAGAAGTATTGAAATTTCCAACATT
TACATTAACAACAGTAATTAATATGGTTGTAATGATGAGTTTGTATGGTGGTATGATTTTATTACCAATGTAC
TTACAAAATTTACGTGGTTTCTCAGCATTAGATTCCGGATTGTTATTATTACCTGGTTCTCTAATTATGGGTC
TACTAGGACCATTTGCTGGTAAGTTGTTAGACACGATTGGTTTAAAACCTTTAGCCATTTTTGGTATTGCTGT
CATGACTTACGCAACGTGGGAATTAACTAAATTGAATATGGATACGCCATATATGACAATCATGGGCATCTAT
GTTCTACGTTCATTTGGTATGGCATTTATTATGATGCCAATGGTAACAGCAGCTATTAATGCGTTGCCCGGAC
GACTTGCCTCCCATGGTAATGCCTTCTTAAATACGATGCGTCAACTAGCTGGTTCTATAGGAACAGCAATCTT
GGTTACTGTAATGACAACACAAACTACACAACATTTATCAGCTTTTGGGGAAGAGTTAGATAAAACGAATCCT
GTTGTACAAGATCATATGCGTGAATTAGCATCGCAATATGGTGGTCAAGAAGGAGCAATGAAAGTGTTATTAC
AATTTGTAAATAAACTATCAACAGTTGAAGGAATTAATGATGCATTTATAGTTGCAACGATATTTAGCGTCAT
CGCCTTGATTTTATGTTTATTTTTACAAAGCAACAAAAAAGCCAAAGCTACGGCTCAAAAGATAGATGCAGAT
AATAGTATCAATCATGAATAAATAAAATAAATTAATTGAAGTGTGACTAATCAAAAATTATGTTGTGGGGACA
TGATTTTTAAAGTATCGGTGCCAATATATGGTTATCGATACTTTTTTTATTTGTTGATTTGTAGAATGTTAGA
GGAATTATATTAAAATTTGGCATTGACGTAGTAGGTCATTAATAAAGAAAAAGCAGGAAGTGGGTCAACGAAA
TGAATTTTGTGAAAATAACATTTCTGTCCCAATCCCTACTGTATATCATTATTTTAAACGAGGCATGCGATTA
CGGAAGAATAAGCTTATAACAAGTAAACCGATGCTACAGCCCAGTAAAATGATGCCGTTATGAATAGCGTCAG
CTTCTGTTATCACTTGATCTGGTGGTACATTTACATAATATTTTTTAAAAACATCTGCAATTTCTTGTGCGAG
TGCTTTAATAATATAACTGAAATACAATGTAATGACTAACATAATTGTTAACACTAAAATATTTACTACTTTT
AGAACTGTGCGATTAAATACAATTGTCAGTATTGTTATGACTAGGCTAACGATACATAAAATAAAGAAACCGT
TAAAGACTAAAATTAAGCGATCGATTCCTTGTTGTAAATCATTGATATTACCCAAATTAATATTTAATTGATG
CATTTTTCCAATTGTCTCGTTAAAATTTTTAATGCCATCAAAATCTACAGATTCTTTGGTGAAGACAATATTA
AAAATAGGTTGTTTCCACATGCTGTATCCAGCGATTGCGGTTAATATAACAACTATGATTTGAATAATCATGC
TTACCAATGAACGTTTTTTCTTACCATATCGCTTACCACGATGAATATTCGGTTGTTGAAATGTCTCATCAAA
TGGTTCTAATCGTTGATTTGGATTTGTCATGCAAGGCACCTCCGTATAAAGTTCTTTTCTTTACTTATCAATA
TGTCTAGTTGTCTGTTATAATGATGAAAAATCACTGTTCAATTGTAGTCTTAAATTTGAAAGTGAAAAAGAAA
ATATCTTCTATATTATATACTTTTTTTGGCGAAGAATGTAGGGGATAGACAATCATTGGATACATAAAATATA
TAAAGGTAGGTTAAGACATTGATTAGAAACCGTGTTATGAATAGCGTTGTCAATAAATATTTGCTTCACAATC
GCTCGATTATGTTTAAAAATGATCAAGATGTTGAAAGATTTTTTTATAAAAGAAAAATTGAAAATAGAAAAAA
ACATAAGCAGCCTTCAACATTAAATGTTAAAGCAAATTTAGAAAAGTTATCATTAGATGATATGCAAGTCTTT
CGCTTTAATTTCAGACATCAAATTGATAAGAAAATTTTATATATTCACGGTGGATTCAATGCACTACAACCAT
CACCGTTCCATTGGAGATTGTTGGATAAAATCACTTTAAGTACATTATATGAGGTTGTACTGCCTATCTATCC
AAAGACACCAGAGTTTCATATTGACGATACTTTCCAAGCGATACAACGTGTTTATGATCAATTAGTATCTGAA
GTAGGACATCAAAATGTCGTAGTCATGGGTGATGGTTCAGGTGGTGCACTGGCATTATCCTTTGTACAATCTC
TTTTAGATAATCAACAGCCATTACCGAATAAATTGTATTTAATCTCACCAATTTTAGATGCAACACTATCTAA
TAAAGATATTTCGGACGCCTTAATCGAACAAGATGCGGTTCTAAGTCAGTTTGGTGTCAATGAGATTATGAAA
AAATGGGCGAATGGCCTACCATTAACAGATAAGCGCATATCGCCAATTAATGGCACAATAGAAGGATTGCCAC
CAGTTTATATGTTTGGTGGTGGACGTGAAATGACACATCCTGATATGAAGCTATTTGAACAAATGATGTTGCA
ACATCATCAATACATTGAGTTTTATGATTATCCTAAGATGGTACATGATTTTCCAATTTATCCAATTAGACAA
TCACATAAAGCTATTAAACAAATTGCCAAATCGATAGATGAGGATGTAACACAAAATAACTAATAACATACAA
TTGATCCAGTTACGATTTCATGCAAGATGATATTTGACATGAAAACGTAATTGGTTTTTTTTGAAAATATAGC
AAGGGTTATATAGACTAATTGTTAATGAGCTTCTGAGTGAAAATCACCAATAAGTGTATTGATATGTGTGATG
ATAAAGTATTGCCCTTTAATTTGATAAAATTGATCTAAAAAATTATCGAATTGACGTGGAATATCACTAAGTT
CTGCAAAGTTAGCTGTTTTATCATAATTGCGATGAGCAACCTCATTTTGAAATTTATCATAATGTTCTAGGAA
ATGTTGATCTTCAAGCTGCTGTTCCACAATAGTCATAATATTATCGATGGCTTGCTTTTGTCCTTGAACATAA
GCCATATGCACGCCACGCTTAAGTAGCTGATTTGCTGGTCCGTTAATGTATAACCCTTTTTGTGCATTTAATT
CTGTTAATTCAAACTTAACATCATCTAATAAATCTAGTAATTCGATATGAATCTTTTGTTGTAATGCATCTGT
CATTTTAATGCTCCTTTGTGTGGAATTGCTATTTTATGCCCTAATTGTTCAGGCCAAGTGATAGTTGGTTGAT
TTTTATAATAGTGTGCTATTTGTGTTGAAAATGATTCAGGAAAAGCATCAGAACGACGTGTGTGCTGATTAAT
TCCCATCATCATAACTTCATTTGTTGATGCTAGTGTACCATCTTCTTTAGTTAATGTTAAAAATAAATGCAGC
CGCTTATAATCGTAATCATAAATATATAAAGTAACAGTAAATACATCGCCAAGAGACAATTCTGAGAGGTATG
TCGTATGTTCTTCTAGTGTAAATAGCGTATATGCTAAATTTTCGCGTTCTTTTAAAGAAAGACCGTGGCTGTA
ATTAAAACGATTCACGACGTCACTAAAAATGATATTATAATTTGCATCATGCATATGATTGTTATGGTCAATG
AATTCAGATGTTACGGTTTGAGTATGTGTAAAAAGTTGTTTGATAATAACCATCTCCTTAGTGACTATCATAC
TAAAATAAATTAAGACATACACGTTATTTAATTCATGATAAAATAATAGAGAATTCGTTATTTAGGGGGAGTA
TCGATGGCTTTAACGATACGGTATCAAACTACATATGAACCGTTTAAAGTAGTTGATGATATCAAAGAGATAC
CAAAAGATGCGACTATTGTTTGGTATGATTTTGATGAACCGAATGAACAAGAAAATGAATGGTTTAAAGCACA
TTTTAATTTTAATGATTTAGAAGTGGATGATGCAATTAACGGTATGCCACGAGCTAAATATAAATCTTATAAA

GATTATCAATACTTAGTATTTCATAGTATTATGGGGTCAAACTATTCACCAATAGCACTAAATATTTTTATAC
AAGACAATGTATTAGTAACATATCATCATCAAACGTTGGAATCATTAAATAAAGTAGTCTATAAGTATATGAA
TACATTAGATGCAGAATTAGATTGTGCAGATGTCGTCATATTAATTTTAGATATGATGGTTGATAAATATTTC
AACTTTGTATATGCACTTGAAGATAGTGTTTATCATTTTGAAGACCGCCATGTAGATGATCGCTTTAATAAAA
TGGTCATGGATAGTGTCTTTAAGTTACGTTCGGATTTGATTAAAGTAAAACGTGTACTGTTTCCTATGCAAGA
GCTAATTGATACAATGAAACAAAATGGTGATTTGATTATAGATAATAAACATTCGCTATACATTCAACATATC
GATGACCACTTGATTAAGCAACGTAATATCATTCGCACTGCTCAAGAAATGACAAACGAAATACGAGAAAATT
TTGAGTCATACACGTCATTTAGAATGAATAGTATTATGCAAGTACTGACATTAGTATCAGTTATATTTTCACC
GTTAACATTCATAGCTGGGATTTATGGTATGAACTTTGTAAATATGCCGGCATTACATTTACATTATGGATAT
TATATTTGCTTAGCTGTAATGTTTGTTATAGCTGTCGTATTAATCATCTTTTTCAGACGTAAAAAATGGTTTT
AAATTAGAAATGACGAAAAGGTAGGTTTAATCATGAGTGATTTTCAAAGAGAACAGAGAAAAAATGAACATGT
TGAAATAGCAATGGCTCAATCTGACGCAATGCATTCAGATTTTGATAAGATGCGTTTTGTGCATCATTCTATC
CCATCAATTAATGTAAATGATATCGATTTGACATCACAGACGCCTGACTTAACGATGGCATATCCGGTTTATA
TTAATGCAATGACGGGTGGTAGCGAGTGGACGAAAAACATCAATGAAAAGCTAGCTGTAGTTGCAAGAGAAAC
TGGCTTAGCGATGGCAGTTGGATCAACACATGCAGCATTGAGAAATCCACGCATGGCTGAGACGTTTACGATT
GCGCGAAAAATGAATCCTGAAGGCATGATTTTTAGCAATGTTGGTGCGGACGTACCAGTAGAAAAGGCTTTGG
AAGCAGTTGAATTACTTGAGGCACAAGCGTTACAAATCCATGTTAATTCTCCTCAAGAATTAGTTATGCCTGA
AGGGAATCGTGAATTTGTGACTTGGTTAGATAATATAGCGTCGATTGTATCACGAGTGTCTGTTCCAGTCATT
ATAAAAGAAGTTGGATTTGGTATGAGCAAAGAATTAATGCATGACTTACAACAAATAGGCGTCAAGTATGTCG
ATGTTAGTGGCAAAGGTGGTACTAACTTTGTAGATATTGAAAATGAACGTCGTGCAAATAAAGATATGGATTA
CTTATCATCATGGGGACAGTCTACAGTTGAGTCATTACTTGAAACAACGGCTTATCAAAGCGAAATTTCAGTT
TTCGCGAGTGGTGGTTTACGTACACCACTCGATGCAATTAAAAGTTTAGCACTTGGTGCAAAGGCAACTGGTA
TGTCACGTCCGTTTTTAAATCAAGTTGAAAATAATGGCATTGCACATACAGTAGCTTATGTAGAATCATTTAT
TGAACACATGAAATCAATAATGACGATGTTAGATGCTAAAAATATTGACGACTTAACACAAAAACAAATCGTA
TTTAGTCCGGAAATATTGTCATGGATAGAACAACGTAACTTGAATATACATCGAGGATAAATAAAAGAGGAGG
CGAAGGCCTCCTCTTTTATTTATGAGTTTGGTGTTTAACTAAACCATTGAATGAATCCCATAATGACTATAAC
ACCAATTAAATCGATTAAGAATGCACCAACAATAGGTACAACTAAATATGCTTTAGGTGAGTTTCCATATTTT
TTAGTAATAACATCTAAATTTGCCATGGCATTTGGCGTTGCACCAAGCCCATGCCCGATAAAACCACCTACCA
TTACTGCAGCATCATAATCTTTTCCTAAACCTCTAAATAAAATTAAAACAGCAAATAAAATCATAACGACAAC
TTGAACTAAAACGATAATAATAAGAGGTATAGCAAGTTTATAAATTTCGATTAATTGAATGCTCATTAGCGCA
AGAGATAAGAAAATACCTAATGCGACATCACCAATTTGATTAGTAATTTTTAAATCTACAATATTAAAATTAA
AACTTTCAGAGATATTTCGGACAATAACAGCTACAAATAATGAGCCAACGTAAATTGGAACATTAATCCCTGT
TTGAGATGTAAACAAATGACTGAAATAACTTCCAACTGCCATACAGAATACAACGATTGTAAATTGAATGAAG
AATACTTCAGTGGCATTAAATTTACTATGTAAATGTTCGTTATATGCTACTTGGCTATAATCTTTAAATGTAT
CGTCACTATGTTGAGGCTTTAAGTTATAACGTTTGATTAAAAATTTTACAACAGGACCACCGATAAGCCCTCC
AAATACAAGACCTAAAGTTGCAGCTGCAAGAGCCGCTGTCAGTGCTGAGTCAATGCCTAAATCTTGAATTGTC
TTACCATAAGCAGCAGCATTACCATGACCGCCTTCCATAGACATGGAACCTGCTGTTAATCCTAACAAAGGTT
TAATATTTAATACTTTTGCTAGTGATACACCAACTATGTTTTGAATGACTGAAATGATAGCACAAAACATAAA
GTATAATAGCAAGACTTTGCCACCTAATTTAAATAATTTCAATGATGCACCAAGACCGATTGTCGTAAAGAAT
GCTAACATGAAGAAATCTTGAATGAATGAAGCATCTAATTTAATCTTAACCATGCCAAATGAATCCAAAGTCG
CAACTAAAATAGCAAAGATTAAGCCGCCAATCACAGGTGCTGGTATACAAATGCGTTTTAAAAAATTAACGTG
ATTAACGATAGCTTTACCAAGTAAGTAAAGTATACAGGCTAAACATAATGTTGTAATTGCATTAAGTTCTATC
ATTTTCAAATCCCCCTTGTTGAATATCCCTCTCCCTAAATAAGCTTTTATATGTAAGCGTTATAAAAGGGCAC
TTTTAACATTATACATGCAACAAATCAATATTTCTAGTTTATGAAAATAATATCAATAATATTAATGCATCTA
AATTTAATAATTATAAATAATTCTATTGTTATATTTTATAATTAAGAACAAGTTCAAAAAGAGGAGATGAATC
AAAAAATATGATGGACAAAAAGAAAAACAATCAACCCATCCGCATGTTGACGGTACGATTGATTGACTTATAA
GGAGATAGATTTCATTTTCATGATAGTAATCAATGAAAGATGGCATTTATTTCCAAGTATCTTCGGGAAACAT
ACAATCTGATTTACGCATGCGAGACACAAATGGATTACCTTTCACTGTGTAACGTAAAGATTTATGTGTCCAA
TCACCTTTATTTGGAATACCGATTCGTGGACTAGCAATAATATCTTTAGGATATTTACGATTCTTAGTATCAA
TAGACAATCTACAGTCATTTAACGTAGCGCCATCGATAGCCCGTGGAATGTTAAATGCCTTAGTCCATTTTCC
TGGGCCATTCGTTACCTCGTAGCCTTTCTTACCTCTGTTACGGAACATAGCGGATAAACCTTCTTCTGGTTCA
ATTGCGCGGATAAGTACGCCTTCAGGTATACCTTCAGATCTTGTTACAAAATTAATGAGTAAATGCGTATGCA
TGACATGTGCATAAATTGTACCACCACGTTTATATAATGACGTAACTTTAGGTGTTATTTTACCGCCATAGCC
ATGAGCCGCACGATCATTCAAACCTAAGTATGCTTCCGTTTCCACGATGTAGCCTGTATACGTTTGAGTGGTA
TCCTGATAAATCACTTTGACACCTAATAAGTTTTTAGCAATTTGTCTTGTATCATTATTAACGAAATCCATAA
TATTCTCCTTGCAATTATATGATTAATTTAAATAAAAATGAAAAAGATATTTTTATTGTGGTAAATGTTTGAT
AAAATAGTATTGTTAAGAATTATAAAAATGTAAAGTATATACTTTGTTATGGAATATTTTATTTGCAGAGATT
GAGTCTCTTTTGAACGCGAACATTGCAGTGTGACATATTTTTAGTATAGTTAACGAAGCGTTGACTTTCAAAT

GAGTCTCCATATAGATGCGTACTTGTAGCGGGTTTTCATTTAACTAGTGATGAAATATAAAGTTCATTTTCCA
AAGTGAATGACGATTGAAATGAAGTGGCTTGATAATGAATCTTGTAAATTATAACGGTAATAATAATTAAAAA
TATAAATATGATTAACGAAAGGGAGATTACAATGGAACAATTACCTCAAGTAGGTTTTATGAAATCATTCGTA
TTATTTTGGAAAAATTATGTTAATTTTAAGGGGAGATCAAGACGTAGTGAATATTGGTATATGATGTTGTGGC
ATTTAATATTTATGGTTCCAGCTGTGTGTGTGCTAATAATAAGCCTATTTCTATTCTTTATTTCTATAGCGGC
AGATAACGAAACATTAATAGTTATGAGTATATTGATGATTATCCTTACATCTGTGTATACTTTTGTCTATAGT
TTGGCAACGTTTATTCCAAATTTAGCGTTGTGCGTTCGAAGATTTCACGATATATCAAGAACAATGTTATTTC
CAATGATTATGACAGCATTCAGTATTATATTTTATATAGTATTACAGATTATCGATAGTTATTATGACAGTGA
TTTTACCTTAATGCCGATGGGTCTTAATATTTTGTTAGTATTTCTTTATTTTGTTTATTTAAGCCTAACAATT
GTTATGATAGTATTCCTATGCTTTGATAGTAAGCCGGCAAATAAATATGGCGAAAGCCCAAAATACCCATCTT
CAATTAAAAATCAACCAGTGACATCTGAAACACCGAAAACACCGGGAGAAACAGATAATCAATTGTAATGATA
TAAATATCTCGGCACAATGATGCTGGGATATTTTTTTACCAATAAGGCAATAAAGGCATGGAAAAAAGCATAT
CCAACACTAATCATACTTAACATGCAAAGATGGTACTATTTAAATTTTTCGATTTCAGAAATTTTAAAAATAA
ATTCACTTTAAAAATATTGCATTTATAGAGAATAGAAATGTTATATTAAGATAAATACATATTGTTTTAGGAG
GCGAAGTATGGGACTTGTAATTGAACATGTTACGAAACGTTTTGGCAAGATGACGGCAGTAAATGATATTTCA
TTAGAGTTAGAATCAGGCAAAATGTTAGGTTTTCTAGGAAGAAATGGTGCAGGTAAAACAACGACATTTCGTA
TGATTTTAGGCTTAAGTGAACCAACTGAAGGACACATTACGTATAACGGTAAAAAACTAGATAAAACAATGTA
CAATCGTATTGGTTATTTACCGGAAGAACGCGGTTTACATGGGAAATTGACAGTTGAAGAAGAATTGAAATAT
TTGGCAACTTTAAAGGGAATGTCAAAAACAGAAATACAACAGCAAATATCGTATTGGCTTGAGCGTTTTGATA
TTACTGAAAACCGCAAAAAACGAATTGATAGTCTATCAAAAGGGAATCAACAAAAAATTCAGCTGTTAGCAAG
TATGTTACATAAACCAGAATTACTAATATTAGATGAGCCTTTCAGTGGTCTAGACCCAGTTAATGTTGAGTTA
TTAAAAGAAGCTGTTAAAGACTTAAATGATTGGGGCAGTACAATCGTTTATAGTTCTCACCGTATGGAGCATG
TTGAAGAATTATGTGATGATGTTTGTATTTTAGATAAAGGTCAACTTGTTGTTTCTGGTGATATCAATCATGT
ACGTGCATCTAATGGTAACAAGAAAGTTGTCATTGAATCAGAAACAACACTACCAGATTTAACAAACATTAGA
GGCATCATTCATAGTGAAAACATGAAGCAAGGTCTTCAGTTGACGATTGAGAATGAAGACGTAGCCAAAGATA
TTTATCAAGTTGTGGCACATCAAGGTTATGTTAAACGATTCCAAGTTGTAGAACCATCATTACAAGATATATT
CATTGAGAAGGTTGGTGGCAAGGATGCATAAGTTTTGGGCGACATTTACTTTGACATATATCAAAAAAAATTAA
AGCTAAATCATTTGTAATTTTTATGATTATTATTGCTGCCTTAATGATTGGCCTATCTAACATTGATAAGATT
ATTAACATGTTTGATGATGGTCCAGATAAAATTGGTGTAGCAGCACCGAATGAACAAATCTATAAGGTTTTCA
AGCAACAGGCGAATACATTTCATAGTGATGCTAAGTTTACTAAAGTTTCTATAGAAGATGCTGAAAAAGAAGT
AAAAAAACATAAACTTGATAAAGCTTATATTATCAAGGTGAATCAAAATCGCACGTTACAAGGGACAATCATT
AGTGAGAAGCGAGTAAGTCATGAAGATTCACAAAAAGTACAAGCACTGCTTACAGCTATTCAAACAAATATGG
TAGCCGGAGAATTAAATATCAACAAAGAAGACTTACAAAAATTACAAGCACAAAGTAAAGTTGATAATAAGGT
GATTTCTAATGATGAAGTCGATAAAGTATCAGAAGGACAAAAGATTTTCAACTATGCTTTAGCTTACGGCATT
ATCTTTCTAATGTTCTTTATTGTTTTAAATTATGCATCTCAAATAGCTATGGAAATTGCTAGTGAGAAAACAT
CGCGTGTCATTGAAATGATTATTACAAGTATTTCACCAATCCAACATATTTTTGCTAAAATTTTAGGCGTTAT
AGCAGTTGCCGTGACTCAGATTATTTTAATTGTATTGATGGCAGTTATATGCATTTTTGCCTTTGACTTAAAA
GACTTGCTACAAGGTTTTAACGTGGAAATGAATCAATTGTCTTGGCAAATTATTATTGTCGGAATTATTAGTA
GTATTGTTGGAATTATGGCGTATGTATTACTTGCAGCCATTCTCGGATCATTAACATCTAGAATAGAAGATTT
AAATCAATCTTTGATGCCACTGACATTACTTGGTATGATTGCATTTTATATCGCGATATTCACGATAAATAAT
CCTGATGGGCAACTTGCTTTAATCACAAGTTATATTCCGTTTTTAGCGCCATTTCAATTAGTTGTACGCGCTC
AAACTTCAGGGCTTCAAATACATGAGGTCATCTTAAGTTCATTAATCTCAATTGTAATGGTAGCTGTCTTGAT
ATGGATTGCAATTAAAACATATAAAGATTCAGTACTTACATTTGAACGTGGCTTATTTAATTCAATGAAACGT
GTATTTAAAAAATAAAATTGATGAGAGTGACACACATTTATCATATAAAATGATGATGAAAGCGTGTTACTCT
TATTTTTTTATATTTAGCTATAGAATAATGTCTAAAAAAGTGTACAATATGAATGTGAAAACGAACTTTTTAA
AATGGGAATGTGAAGTAAGAAGAAATGTAAACAAATGATATGTAGCCGATAGATTATTGGTAGCAAATGTGTG
ATGGAGTGATTTCATGTTATATCCGATTTTTATATTTATATTAGCTGGCTTATGTGAAATAGGTGGGGGATAC
CTGATTTGGCTGTGGCTTAGGGAAGGACAGTCTTCACTTGTTGGGCTAATAGGCGGTGCGATACTCATGTTAT
ATGGTGTCATTGCGACATTTCAATCATTTCCATCATTCGGAAGAGTATATGCAGCATATGGTGGCGTATTTAT
CATCATGAGTTTGATTTTTGCGATGGTCGTAGATAAACAAATGCCAGATAAATATGATGTTATCGGTGCAATC
ATTTGTATTGTAGGTGTTTTAGTAATGTTGCTACCCAGCAGAGCTTGAACACTTGTGGTTAGAAGGGAGAAGT
AAAAATGATAGTTGAAGTTGAGCACCAAAGACAAGGTGTAGATTTAATCAAAATTGATAATGATGAAACTAAA
ATTATTTTTACGAATTATGGAGCAAGAGTAGTGTCTTGGAAATATCATGATAATAATATTGTTCTTGGAAATG
TTGTTGAAGCGGATGAATTTTATCATAGTAATCCTTTTAAATTTGGTGCTTCTATAGGAAGATATAGCGGTAG
AATTGATAATGCTAAATTTAAGTTGAAAGGTAAAGAATATCAGTTAGAAAAAAACAACGGCGAACATCATTTA
CATGGTGGATGTCATGGTTTGGACAATAAGTTATTTGATTATGAAATTAGAAATGAAATTGCTCAAATTAAAG
TGATATTTAAGACGGTGTTGAAATCAGCTGATGATCATTTCCCGGGTGATATCGATGTGACGATTACACATAT
TTATGATGCGGATCACCAATGGTCGATTGAATACGAAGCAGTTGCATCAGAAGACACATTGTTTAGTCCTACG

AATCATGTGTACTTCAATTTGAATCGAGATAATAATGTCGTTGATAATCATCGCATTTCAAGTAATCAATTAG
ATATGTATGTATTAGATGAGCGAAATATTGTTACAGGTGATATTCTAGATTTACATGAAGTGTTTGAAGATAA
CAAGATAAAATTGAGTGATATTTTTACATCACAACATGCGCAATTAAGCCAACAAATGATACGTTTTGGTGGT
CTCGATCACCCATTTACAGTTGGTGAACATAAAATGTATGTTGAAAATCATGAATTTATGTTAGAGGTCGATA
CAGATATGCCTCATGTTGTATTCTTTACTTTTAATCAGCCTGATGAGTGGGATAGTCCATTTAATATTTATAA
ACCACATTCTGGCTTTACATTAGAAACACAATTTTTACCAAATGATATTAATTTATATGGTGACAAAGCACCG
TCAATTTTAAAAGCTGGTGAAGTGTTCAATTCGAAAACAAGTTTCCGTATCGAAGAAAAAGTAGAAAATGATT
AAAAGTATCGGCATTTATATAATATATATGTTGTTTGGCGGAATTTTGAATAGGTTATCAAGAATCGAATCAA
ATGAAGTGACATCATTAATATACAGTTTGAAATAAAAAAATATATAACATAAAAAAGGAATCAATAGCATCAG
TAGTTATAGATAAGACTATTATACAAACGATGTGTAAGTTGATTCCTTTTGTTTTGAAAAGTTCATTTAACAC
TTATAATGAAAGGTAATTATTTCAATGTTCTGTTATACATTTTTTGAAGTTTATCGCGTCTTGTTGGTGACAG
AAATGGATTGTTTAGTGCATATCGTAACAGATCTTGATTTTGTCTATCATTATAATAAAGGTCATTAAGCTGT
TGTACAGATAAACGATGTTCGATTGGTGCTTCTTGTATTAAGTAAAGCTGATCATTTGGAGATACATAGCCTT
GTTTTAATACCCGGAAATAGAAACCTGTTTTACCAGATGTAGACATGCGCTTCACTAAATCAGGAATATTATA
TTTTGCTTGAATTTTCCAGCATGGTTCTCTAATTTCAGAAACCTCAATTAACGCATCACCTAAACGATATTGA
TTACCAAAGTAAACATCAGATTCGTCTAAATAATCAAATGTTAAATTCTCTCCAAACATCGCATGAGTAGGTA
GTGAGGGTAAATCATCTTGATACAGTGCATAGTTGTGTTTACTAAACCCACAAACTGCTTTATGTGGCCCACC
ATGATCTTTATAAGCCTGTTCATCACCAGTGAAACCAGTTGAAGATAACCATGTTGTTTGCGAAATCTTAGTT
TTATCTAAAGCAGAGCGCATCGGTCTTTTCGAGCTATAAGGCAAATCTTGGATTTTTCCTGTCGAAATCGCGT
GAATTGATATCATATCATTCTCCTTAACGAGTATTATTTAAAATATATTGTACTATAAAACATTTTTAGAATT
GTAAATTCTGATTGATATGTTTAAAAAAATGTTAGAAATAAGGGTATGTTAATTTATAAGAGTCATGTAAAAT
ATGAATTGAATAGATTACATAAATATGGGAGGCTACCTTCATGAAAGATGTCAAAGCACTCAAGTTAATGACA
TTAAATGATGTTTTAAGTCAAATCAATGGTGACATGACGCTGGGAATTGGTACAGGAAGTACAATGGAATTAC
TCTTACCTCAAATGGCGCAACTAATTAAAGAACGTGGTTACAATATTACAGGTGTATGCACTTCTAATAAAAT
TGCATTTTTAGCAAAAGAATTAGGTATTAAAATATGTGAAATCAATGATGTTGATCATATCGACTTAGCAATT
GATGGTGCTGATGAAGTAGATCCATCTTTAAATATAATTAAAGGTGGCGGTGGTGCGCTGTTCAGAGAGAAAG
TTATAGATGAAATGGCATCGCGTTTTGTTGTGGTTGTTGATGAAACGAAAATGGTTCAATACTTAGGTGAGAT
GTTTAAGTTACCAGTAGAGGTGGATAAATTTAACTGGTATCACATATTACGTAAAATTGAATCATACGCTGAT
ATAAAAGTAGAACGCCGTGTAAACGAAGATGTCGCATTTATAACTGATAATGGCAATTATATTTTAGATTGTA
AGTTACCAAAAGGAATTGACCCATATAAGTTCCATGAGTATTTAATTCATCTGACAGGTGTGTTTGAGACAGG
ATACTTCTTAGATATGGCAGATCAAGTTATTGTAGGCACTCAAGAGGGTGTTAAAATTTTAGAAAAAATAATAT
ATAGTATAGAAAAGCGCTTGCTAGCATCATCTTTTTAAGAGGTACTAGCAAGCGCTTTGTTATTATTTGTTTT
TATCTGAAGATTGTTCTTCAGTTTTTTCGATATGTTTTTCTGCATCGATTACAACTGATGAACGTCTGTCTTC
TTTATAAGTCGCAGGTTCTTTTAATGATTGTTTTTCATCTTCATTTGAATCGTTTGTTAAATCGTCACTTTGT
GGTGCATGTTTAACTGGTTCTGCTGATTCTACTGACTCCGAAGTGATGTCATGATCTTCATCATGATTAGATT
CAGTTTGATTATCTATGTGATGTTGAGCTTCTGTTTTTTCAGTACCTTCATGAATGCTTGGTTCGTCAGCCAC
TGTATTTTCAGTATCATTTTTAGCAATACCGACCGTTGATGCAGTAGCTACACCTGTTTTTTCAGCATTTTTA
ATATTTGAAGAAGATTTTTCAGCCTCAGTATGATTAGTTTCTTTATCGTCATTGACATGGTCTAAATAATTAT
TAGGCTCAAGTTCTTCAAGGTTTCGTCTTGTTGTTAAATATGCAATACCTCGGATAATTAAGCTTAAACCAAT
GTATCCTACTGCAACGATTGCTGTTGAAATGGCGATGACTTTGATTGAAAATAGATCGCCGATTTCTTCGCTA
AACAAGAAAATAAGTCCGAATGTCATTGAAGCATGAAATGTCGTTGCAATATAAATTGTACGTCCTTTAGTCG
CTCTAATTAATTCACCAAGGATCATAGAGAATGAGAATGTATAAAGGAAGTTATAAGCAGCAAATTCTGTACC
ATAAGTTGTATTTGCTGAGAAAACTGAATACATCAAACCAACAACAATACTTGCAAAGAACGTGTTCATTTTC
GTTTCAACAATATTTTGTAAGTATGAACGGAATCCGAATTCTACTACGAAAGCCATTAGAATATGTCCAATCA
GAATGTGTGTAATAGGTACTGATAAGCCTGTTGATTGTAATAAAATAAAGCTATCTGCAAATGTATTAAAGCT
GTACATACCAATAATTAGAATAACTAAAGGTAAAATTAAAGCTAGCAATAGACGTTCAATTACTTTCAGGCTG
ATTGAGAATTTTAAACCTGCAAGTTGGACCTTTTTATATTTGAAAACGAGTATACAAATGATTGCAGCAATTA
ATGGTGCTAGATCTGTAACTTCAAATATAAAGTGTTTGACACCAATTATAGACTGGAAATCTCTGAGCATAAT
GGATAACGCCATTGTAATGACAAAGAAGACGAAAATCGTCATTGCCCATTGAAAACCAGAAATTTTATTGTTC
TTCATTATATGTAACCTCCATTAGGTAACAAGCAAAATATCTTTTAGTAACAATTATACATATTACAAGCCGA
GAGATAAATTGTCTATGTCAATATTAATGAAAATGTAATAGTCTTGACGTATTTTATTAACATAAATCAGCTA
AGTATTCGTTATTACATAGAAAATAGGGGTAAAAGTAATGTAGATAAGTATTTTTAAGATACGGATTTAACTA
TGAATTGAATTCAAAATTTCCTATACGTTATACTTTAATTGTTAATAAGCCACATAAATAAGAGGGGGAAATG
CTGTGTACAAGCAAGGTGAACCAAATTTATGGACTGGAAGGTTAGATAGTGAAACAGACCCGAAAAAATTTAG
ACATTTTCAAACAGTAACATTTGAAGATTTGTCTAAGCTGGAAAAGAGTAGTATGCCATCAGGGGTCGGTATA
TTAGGCTATGCTGTTGACAAAGGTGTTGCTTTAAACAAGGGGCGCATTGGTGCAAAAGAAGGACCAGATGCGA
TTAAACAAGCATTTGCAGGTTTGCCGGATTTGAATCAATGTGAAACTTTAGTCGATTACGGAAATGTTTATCA
TGATCATGAGGAATTAATTGATACTCAAAAAGAATTTGCTATGCTTGCAGCGAAGTCAATTGCTAATCATAGA

CAAACATTTTTATTAGGTGGTGGACATGATATTGCGTATGCTCAATATTTAGCAACACGTAAAGTCTATCCAA
CACAATCTATTGGTGTGATAAATATTGATGCGCATTTTGATACACGTGCTGAACAACAATCTACATCTGGAAC
GAGCTTTAGACAAATTTTGGAAGAAGATGAAAACACAGATTATTTAGTGCTTGGTATTGCTCAAGGTGGTAAT
ACGCAAAGTTTATTTGATTATGCTAAAGAGAAAAAGATTGATTATGTCTTTGCAGATGAATTATTGAGTCACG
TATCACCAACAATTAAAGATATGATTGAACGTTTTGTACATGAACATGATGTCATTATGTTTACGATTTGTAT
GGATGTCATTGATAGTGCGTTTGCACCTGGGGTAAGTGCGCCGGCAGTGTTAGGTTTGTATCCACATACTGTT
CTTGAATTGGCAAAACGTATTATTCCAAGTGATAAGGTGTCTTCAGTTAGTATTGCTGAGATGAATCCAACAT
ATGACGCTGACAATAGAACTGCGAAGCTCGTTGCTAATTTAGTGCATCATTTTTTAAAATAATTAAAAATATT
TATATGTGACGAAATTTGCATTGCAGGGCAGAATGTGAGTATTTCGTAATAGAAGTTATATTAACGTTGATAT
ATTTGAAAAATAATTGCAGTAAGAAGACTCTTAAGTTGTAGTCATCTTACTGCTTATTTATGTTCGTTGGTCT
GAATGTAATGATTTATACATGGAAATCATAAAATTGGTTGTGAAGTTGTTTTACAGTTGACTACGCAACTGCA
TAAGAGGCTCTAATAACTTAAGTTAAAGTCAAAAGGTATACAGTTGACTACGCAACTGCATAAGAGGCACTAA
TAACTTAAGTTAAAGGCAAAAGGTATACAGTTGACTGAGCAACTGCATAAGAGGCACTAATAACTTAAGTTAA
AGGCAAAAGGTATACAGTTGACTGAGCAACTGCATAAGAGGCTCTAATAACTTAAGTTAAAGTCAAAAGGTAT
ACAGTTGACTGCGCAACTGCATAAGAGGCTCTAATAACTTAAGTTAAAGTCAATGGTGTACAGTTGACTGCGC
AACTGCATAAGAGGCTTTAATAACTTAAGTTAAAGTCAAAAGGTATACAGTTGACTGCGCAACTGCATAAGAG
GCTCTAATAACTTAAGTTAAAGTCAAAAGGTATACAGTTGACTGAGCAACTGCATAAGAGGCTCTAATAACTT
AAGTTAAAGTCAAAAGGTATACAGTTGACTGAGCAACTGCATAAGAGGCTCTAATAACTTAAGTTAAAGTCAA
AAGGTATACAGTTGACTGAGCAACTGCATAAGAGGCACTAATTACTTAAGTTAAAGGCAAAAGGTATACAGTT
GACTGAGCAACTGCATAAGAGGCTCTAATTACTTAAGTTAAAGTCAAAAGGTATACAGTTGACTGAGCAACTG
CATAAGAGGCTCTAATAACTTAAGTTAAAGAGCCTCTAAAAGTCCATATAAATAATCTTGGCATTGTTGTACG
AATGTATGTACTTGTTCGGAGTGTTTACGCTTTTTATGATAATTGATATAAATTTTTCGGCCTAAGTTTGGTC
GAATTTTTTTATATTCTAAATTGGACGTGTGAAATGATTGGTAATAAAATCTCGGAATGATAGCGTAACCTAA
GCCGAGATGAACAAATCCAACTGCTGATTCGAATCGATCTGTTTCTACAACGACATTTGGACGAATATTTCTT
CTATTAAAATAGTCATCTAAGTGTTTGCGCACTTGAGAATTTTTGTTTGGCAATATGAGTGGCAAATTTTCAA
CATCTACCCAATTTTGATTTTTAAATGTTTCTTTAGGTGCTAATAAAATGTAAGATTCCTCATATAAAGGAAT
GGATCTTATATCTTCGTGGGTTATTTTTTCATTTGTTATAGCTAAATGAATATTAAAATTCAGTAATTGCTCT
ATAGATTGATGTTTGTCATGTATTTCATATAAACGATATTGCTGTTCAGGGTAGTCGGAATGGTGCTTTCGAA
TTAAATTCGCAATCCATTGATTCGTAGATTCAAGAGTCCCAATTTTTATCCTTGGTTCTGATGTAACGCTTAA
ATCATACATTTTTTCCATCGTGGATCGATATTGTTGAACTAATTCGCTCGCATAACGATAAAACTGTATTCCT
TTTTCGGTAATCTTGATGTCTTTTGTTGAACGTGTAAATAAGTCATAACCTAAATCTGCTTCCATTTTTTTAA
TCGTAGCAGTTAAAGATGGCTGGCTAATATGTAAAAATTGTGCAGCTTTAGTAAAACTATTATATTTCACGAT
AGCCAAGAAATATTCTAACTGAATAATTTTCATGTGTTTTGCTCCTTTTATTTATAGATTCAAGCTATGGATA
ATTAGTTTATTTATATTTGTTGGCTATATTTTAACATACTATAATGAATATTGAAGATAATAATGTAAGCGTT
TTAAATTTAGTGGAATTAACTTCTAAAAAGAACAGGAGCGAATTTTATGAGAAAAATTCAAGCAAAAAAAGGT
TTAAGTATTGAGTGTAAAGGTTGGGAACAAGAAGCAGTACTTAGAATGTTATACAACAATTTAGACCCAGAAG
TTGCTGAAAGACCAGAAGATTTAGTTGTTTATGGTGGTATTGGAAAAGCAGCACGTAATTGGGAAGCTTTTGA
AGCAATAGAGAAAACGTTACGAGAATTAGAATCAGATGAGACAATGTTAGTACAATCAGGAAAGCCAGTTGCT
GTATTTAAAACACATGAAGAAGCGCCACGTGTATTAATTTCAAATTCAGTATTAGTGCCTGAATGGGCAAACT
GGGATCATTTTAATGAATTAGATAAAAAAGGTTTAATCATGTATGGACAGATGACAGCCGGTAGTTGGATATA
TATTGGTTCTCAAGGTATTGTGCAAGGTACTTATGAGACATTTGCCGAGCTAGGTAATCAACACTTTAATGGA
GATTTAGCAGGCACTGTTACACTGACAGCAGGTTTAGGTGGTATGGGTGGTGCGCAACCGTTAGCAATTACTA
TGAATCATGGGGTAGCAATTTGCGTGGATGTTGATGAAACACGTGTTGATAAGCGAATTGATACGAAATACTG
TGATGTTAAAACAGCTGATTTAGATGAAGCATTAAAATTAGCAGAAGAGGCGAAAGAACGTGGAGAAGGATTA
TCAATCGGGTTAGTTGGAAATGCTGTAGACATCCATCAAGCGATTCTAGAAAAAGGATTTAAAATTGACATTA
TTACTGACCAAACAAGTGCACATGATCCGCTAAATGGATATGTGCCACAAGGATACTCTGTAGAAGAAGCGAA
AGTATTGCGTGAAAAAGATCCGAAAAAATATGTTGAACTGTCACAAGCTTCAATGGCAAAGCATGTTGAATTA
ATGCTTGAATTCCAAAAACGTGGCGCTGTAGCATTTGATTATGGTAACAATATTCGTCAAGTAGCCTTCAATA
ACGGAGTGAAAAATGCTTTTGACTTCCCAGGCTTTGTACCAGCTTACATTAGACCATTATTCTGTGAAGGTAA
AGGGCCATTCCGCTTTGCTGCGTTGAGTGGTGATCCAAAAGATATCGAGCGTGCGGATGAAGAAATGCGCAAA
CTTTTCCCAGAAAATGAAAAGTTATTAAGATGGCTTGATTTAGCTGAAGAAAAAATTTCATATCAAGGACTAC
CATCACGTATTGCTTGGTTAGGCTATGGAGAACGAGCGAAAATGGGCTTAGCTTTAAATCGTCTTGTACGTGA
CGGTGAAATTTCAGCACCAATTGTTATTGGACGAGATCATTTAGATGCTGGTTCAGTTGCTAGTCCTAACCGT
GAAACAGAAAGTATGAAAGATGGTAGTGACGCGGTTGGTGATTGGGCTGTATTGAATGCACTCATTAACACAG
CTGCAGGTGGTTCATGGATTTCATTCCATCACGGTGGCGGTGTTGGCATGGGATATTCACTTCATGCGGGTAT
GGTTGTTGTAGCAGATGGATCAGAGCGTGCTGAAAGAAGATTGGAACGCGTATTGACGACTGATCCAGGTATG
GGTGTTGCCCGACATGTTGATGCTGGCTATGACATCGCTATTCAAACAGCTAAAGAAAAAGGTATTCATATTC
CAATGATTGATAAAGCAGGTGATAAGTAATGAATGATTTAATAATTAATCATATAGCAGAATTAATTTTACCG

AAATCAACAGATAAACCTTTGAAGGGTAAGGAATTAGATGAATTAAATGTTGTGAAAAATGGTACAGTTGTCA
TTAAAGATGGCAAAATTGTATATGCTGGAACACATACAGATGACTACGATGCGACTGAGACGATTGATGCTAG
TGGGAAGGTAGTGTCCCCAGCATTAGTAGATGCACATACACATTTAACATTTGGTGGATCTCGAGAACATGAG
ATGTCATTAAAACGTCAAGGTAAGTCTTACCTTGAAATATTAGAAATGGGTGGTGGCATATTATCTACCGTTA
ATGCTACTAGAGAAACATCCGAAGATGACTTGTTTAAAAAAGCAGAGCATGATTTGCTTACTATGATTAAACA
CGGCGTGCTTGCAGTTGAAAGTAAGAGTGGTTATGGATTAGATAGAGAAAATGAACTGAAGCAATTGAAAGTG
TCTAATCGTTTAGCTGAGAAATATGATTTAGACATGAAACATACTTTCCTAGGGCCTCATGCTGTACCTAAAG
AGGCAAGTTCAAATGAGGCATTTTTAGAAGAAATGATTGCGTTACTTCCGGAAGTAAAACAATATGCAGACTT
TGCGGATATTTTCTGTGAAACAGGTGTATTTACAATAGAACAATCGCAACATTATATGCAAAAAGCCAAAGAA
GCAGGTTTTAAAGTGAAAATACATGCGGATGAAATTGATCCGTTAGGCGGACTGGAATTAGCAATTGATGAAC
AAGCAATATCAGCAGACCACTTAGTAGCTTCTAGTGATAAAGGAAAAGAAAAGTTGCGAAATAGTGATACTGT
AGCTGTCCTCTTACCTGCAACGACGTTCTATTTAGGTAAAGAAGATTATGCAGATGCAAGAGGCATGCTTGAC
AATAACGGTGCGATTGCATTAGCAACTGATTATAACCCTGGTAGTAGTGTCACAAACAACTTACAACTTGTTA
TGGCGATTGCAGCCTTGAAATTAAAGCTATCACCTAATGAAGTTTGGAATGCTGTTACGGTCAATGCTGCTAA
AGCAATAGACATTAACGCGGGTACAATTAACACAGGTGATAAGGCTAATTTAGTAATTTGGGATGCACCAAAT
CATGAATATATTCCATATCATTTTGGTATCAATCACGCAGAAAAAGTTATTAAAAATGGTAAGGTCATCGTTG
ATAACACATTATCTTTTAAAGCATAAAGTAATTAAAGCTCTAAGCGTTGGTTTACACTAATGTTTAGAGCTTT
TTGTGATTTGTAAAAACAAGTTAATTCGAGAGCACGTTTTTGTATAGGTTCTTAATTATAAAAGTTAGAATAG
TTTGACAATAGTGTTACGATATTATTAAACAATTGTCTATCATTAGCGAAAGTAATTAACAAGAGTACATTGT
TGTGAAATCATCGTTTAATAGTTAAAGATGATCATCGTAGGGTAGACAACATATCATTGACTTAAGGAAGGGG
AATGACCGATGGGATTAAAAGATCAAGCTGTTGCCTGGAGAAGGTATTTTCATCAATTTCCAGAACTATCCGA
TAAAGAATTTAAGACAACGCAAAAAATTAAAGATATTTTAACAGAACATCATATTAGAATATTAGACTTACCG
CTTGCGACTGGGCTTGTTGCTGAAGTGGGACAAGGACCGAGTTGTATAGCTGTTCGGGCTGACATTGATGCTT
TACCGATTCAAGAGCTTGTTGAACAGGATTTTAAATCTAAAAATGAAGGTGTGATGCATGCGTGTGGTCATGA
TATTCACATGGCTAGTATTTTGGCTACAGCTGTAAAGTTAAAAGAAATTGAGGGCACTCTTACTGGGCGTGTT
AAATTCATTTTTCAACCAGCAGAAGAACTAGGACATGGCGCTTTTAAAATTATTGAAACACATGCATTAAAAG
ATGTACAAGCAGTATTGGGTTTTCATAATGACCCATCGCGTTCGGTAGGTACATTTGCAATCAAAACAGGGGC
AATTACATCAGCAGTAGATCGTTTTGAGTTTCATATTAAAGGCGTGGGTGGTCATGCTGCAAAGCCAGAACAA
TGCAACGATCCAGTTATTGTGTTGGCGCAATTGATTAATAGTATTCAATCCATAGTTAGTAGAAATCTATCTG
CGTTTGATGAAGCGGTAGTAACCATTGGGCAAATATCATGTGGTAATACTTGGAATGTCATTGCTGACCATGC
ATATGTACAAGGCACAGTACGTAGTTTTGATCCTGTGGTGCGAAAGCTTGTAGAAACACGATTACAAAATATT
GCGGACGGGTTAGCTCAAGCATATAATATGAAAATTAATTTGAATTATACACATTTACCTGGTGCTGTGATGA
ATGATGAGGTATTGACGCATAAAGCAATAGCAGTAGCCCAACATGTTGGATATAAGATGGAAATGATGGAACA
GCCGCTTACGATTGGAGAAGATTTTTCAGGTTATAGTCAACATTTTCCTAGTGTATTTGCGTTAATTGGTTCT
CATAGTGAATATGATTTACATCATCCTCAATATAAACCGGATGAAAGGATTCTGGAAAAAGTACCTGAGTATT
TTGTTGAATTTGTAAAGAGACTATTGCATGAGTAATTCGAGCTTGTTTTTTAAAGAAGAAAGTAATTTTTAGC
GTAATCATGATTAAGTTCCTGGGAAGTTTGGGTAAATAATAACCATTAAGCGAATAAGGAGATGTTAAAAATG
GCAGCTCAAGATCCTAGAACAAAATTTAAAACAACAGATTATGAAAAACAAGAACAAGAGGTACCGGGTTTAC
AATCTGAAATGACGCCAGCACCTGATTGTGGCGAAACGTCTTACCAAGGTCATCAACGCTTAGAAGGCTATAA
AATGTTAGTTACGGGTGGTGACTCAGCAATAGGGCGTGCAGCAGCGATTGCGTATGCTAAAGAAGGTGCAGAT
GTAGCGATTAACTATCTTCCGAGTGAAGAACAAGATGCACAAGAAGTACGCCAAGTGATTGAAGAAAGTGGTC
AAAAGGCAGTGTTAATTCCTGGTGATATAAGAGATGAGCAGTTCAACTATGACCTTGTTGAACATGCATATCA
ACAATTAGGTGGCTTAGATAATGTTACTTTAGTTGCTGGACATCAACAGTATCATGATGATATTCATGGATTT
ACAACTGAGGCATTTACAGAAACATTTGAAACGAATGTCTATCCGCTATTTTGGACAGTTCAAAAAGCATTGG
AATATTTAAAACCGGGTGCGTCAATTACAACGACATCATCTGTACAAGGCTATAATCCTAGTCCTATTCTTCA
TGATTATGCCGCTTCAAAAGCAGCAATTATTTCATTAACTAAAAGTTTTTCAGAAGAATTAGGACCAAAAGGA
ATTAGAGTGAACTGTGTAGCGCCTGGTCCATTCTGGTCACCATTACAAATTTCTGGTGGACAACCTCAAAGTA
AAATCCCAACATTTGGTCAAAAAACACCTTTAGGTCGTGCAGGTCAACCTGTTGAGTTATGTGGAACTTATGT
GCTATTAGCGTCAGAAGAATCAAGTTATACAACTGGACAAGTGTTCGGTGTTAGCGGTGGAGTGCAAATAGAT
TAGGTTTGTATGAAGCGTTTAATTGAACAAAATAATATTAATTTAGCTACTTCAATCAAGTAAATTAAAATGA
ATTAGTGAAGTGTATTTTAAATAGTCGGTAAAGGTGTGTATCTTTATCGGCTTATTTTAATACCCTGTCATAA
AGAGCGTTTGCTCAGGGTGTGGATTATAAGTTTTAAGCAGAATAAACTTTCTTAATGAGGATGTCAGGACAAC
AACGAAAAAGTTAAAAACTGTTGCGGGATTGGGTTTAGGGACGGTTAGTGGAAGAAAGTTAAATTTATTAACA
ATTGAAATGCCTAAGGTTAGATATTAAACGTCAGAACGGACAAAGAATAATAGGATATGTTTTCAATTATATA
GCGAAGTATTTATATGTTAAGATGATGTGAATTACATAGAAACGCTGCCCGTAGAAGTACTGATCATAGAAAT
ACTGATAAACGTAAATTTGCAAACTCGATATTAAACAAAGGGGTTGTTATTATGGCATTAATAGTATCTAGAG
CTATATTATTTTTGATTTTAGTATTAGCTTTTGGAATAGCGACTGTTTTTCCTGAACAAAAGTTGCCAGTTTA
TATATTATTCACACTGTTTGTAGTGGTTGCGATTGTTTCGAATGTTTGGCTTACACATCAAAACGTTAAAAAG

CATATCATTAAGTTTGACAAACGTGTTGCTTACTTTTTGGGTGTGCTCTCAGGAGTATTCTACATTGGAATCG
TTATTTATGGATCAACTATTATAGGTTACTATAATTTTATATCATTTATTATATTTGCAATAAGTATCGTATT
GGTTCAAATATTAGAAAATATACTGGGTATTAATTTTAAAAATACGCTTCCATCAAACAAACAGTTGAAAGTC
ATCTCCAAGCAACGTGTTCAGCAATTTAAAGTCATTTCATTAATATTAGTAAGTGTGTCTATCGTGTTCATTT
TTATTGCATTAATGCTTAAATGGTGGCTTTTACTTGTTATTAGCATTATTGTTTTGATTATTATTACAGTAGC
AATGATTGTTTATGAAAAATATTATACGACTCCTGATTAATTTATGATTGACGTACTAAGGATTATCTTGTAA
CGTAAACGTAAATAAGCATCACTTATCTAGAGAGGTGGAGGGACTGGCCCTATGAAGCCTCGGCAACATCTCG
AATGTGCCAATTCCAGTAACCGTAATGGTTTGAAGATAAGCAGGTAAAGCACATGAAAACCTCTTTCTTCATC
GTTTGTGAGAAAGAGGTATTTTTAATTGGAAAGCAGGTAAAAAGGATGGAAGTACATAAAAAGAGCAATGCTT
GGGCATTATTCCCCTTGTTATTATTTGTGGCGTTGTTTTTAGGCGTAGGTATTATCACAGGTGATTTTACTTC
AATGCCATTAAATGTTGCAATTACGATAACGGTAATTGTGGCGTTATTAATGAATCGAAAAGAATCATTTGCT
AAAAAAGTTGAGGTCTTCACGAAAGGTGCAGGCCATTCAAACATTATTTTAATGATGTTGATTTTTATTTTAG
CAGGTGCATTTTCAAATACAGCTGAAAAAATGGGTGGCGTAAAGTCGACAGTTAATTTAGGACTATCATTAAT
CCCGGAAAATTTAATTATTGTAGGATTGTTTGTGATTTGTATGTTTGTTTCTATATCTATGGGAACGTCAGTA
GGGACTGTTGCTGCAATAGCACCTGTTGGTTACGGATTTGCACAAGCGACAGATGTACCAACTGCGCTAGCAA
TGGCTACAGTTGTTGGAGGTGCAATGTTCGGCGATAATCTATCAATGATTTCAGATACAACAATTGCTGCGGT
TAGAACACAACATACGAAAATGAAAGATAAATTCAGAGTAAACTTTAAAATTGTATTGCCAGGTGCTATTTTA
ACGATTATTATTCTGTATTTCTTAACGAATGGCATTTCTTTAAATCATGCTAAAAACTATGATTATAATCTAA
TTAAAGTTGTACCGTATATTTTAGTATTGGTATTAGCATTATTAGGTGTAAATGTCATTATCGTATTGATTGG
CGGAACATTATTAGCCGGAATCATTGGTCTCATAGATGGTTCATTCGGTTGGATGGGGCTACTAGATGCAGTG
TCCAAAGGTATTATAAGCATGGAAGATATTGCTATGATTGCGTTGCTAATTGGTGGTTTAGTGGGTATTATTC
AACACAATGGTGGTATTGAATGGTTGTTGCAATTTGTTCGCTCTAAAGTGAAATCAAAGCGTGGCGCAGAATT
AGGAATTGCTAGTTTGGTAAGTGTCGCAGATATTGCCACGGCAAATAACACCATCTCTATTATTATGTCAGGT
CCTTTAGCTAAAAATATTGCAGATGAATATGATGTAGATTCAAGGAAATCAGCAAGTATTCTTGATATATTTG
GAGGCTGCTTCCAAGGTTTATTACCATATAGCCCGCAAGTCATTTCAGCGGCTGGTGTAGCTGGTATTTCACC
GTTAATGTTATTCCCATACAGCATTTATCCTATATTATTAGGTGTATGCGGACTTGTTGCGATTTTATTTAAT
TTTCCAAAGCTAAAAAAGAACACTAGTCAAGAAATGAAACATTAATGCTTAACAATATATCGATGTAGCCAAG
TAAGGTGGCTTGTTATATTAACAGATCATGTTGTACTCACCGTATAACGCTAATCTTTTAAAAATGATGATCA
TCTATGTTTGGAACTGAGCAAAAATACGTTAAAATAAAAGTAAGAGCGAAAGTGGGTGGACAACAGTGGCAAA
ATTTAATGTAGAGAATGAACATGTCGAAGTTGAAATTGAAAAGCTTTATAAATTTTCTCCAGAGTTAGTATAC
GAAGCTTGGACAAAGAAAGATTTGTTGAAACAATGGTTTATGACATCAGCCAGAACGAATAAAGAGATTGAAG
CGGACGTTAAAGAAGGCGGTAAATATCGTATTGTAGATCAACAACGTAATGGCAAAGTAAATGTGATTGAAGG
CATTTATGAATCTTTAGTTATGGATGAATACGTTAAGATGACTATCGGTATGCCTGGACTAAGTGAAACACAA
GATGTCATTGAGGTAGAATTTTTTGAACGTGAAACAGGTGGCACCCAAATGCTATTTTATTATCGTTCATTAG
TTGAAAAAGAAAGACGATTTACTAATTTAGAATACAAGCAAAAGAAAAAAGAATATCATGATGCCATGGTGCA
TGGTTTCGAATTGATGTTTGATAAGATGTACCACGTTATCGAAACATCAACACAACAATAAAATGATAAAAGG
AAGTTATGATAGGCTCAAGGCCATTAAATCATAACTTCCTTTTTTCTAATTTGAAATATCATTATGTAGTAAA
TACTCATTATATTTTCTTACAAGTGTTTCAAAGAATATATGATAACTGATGAAGCTAGAATAGTCTGTACCAT
CAGTTAAGTAGAATGTGTTACTGGATACATAGACGTTGTAATTTGCTTTTTGAGCAAGGGTATTATCTTTCAT
TGTAGTAACAGAAATAAAGTATTTTTGTCTAAGTTGAAGCAAGTTTGTTACTTCTAATAATTCATGTGTTTCA
CCTGATAATGAAATAATAAAGAATAAATCATTAGGTTCACTCTTATTCAAAATCATTTTAAGTTCATGTTCAT
CATGAAGTACAATGACATGATTATGTAACGTTAACAATATACGCTGTGCCTCTGAAGCAACGTTTAACTGTGC
ACGCCCTGTACCATACAAATATATTGTTGAAGCTGTATGCATTTTTTCAGTAAGAAGCTGGTAATCAACGCGT
TCTAAATAACTGAATGTACTATCGATTTCTTGTTTGAAACTATCGATAGAGTTAGATGGTAATTGGTGTGTCT
TATTACTTTCAAATTTAAGGTACGATTTAAAATCGCTATAACCGTCAAAACCTAATTTGCGTGTAAATCTGTG
AATGGTTGCATTCGAGGCATGTGTGAATTGCGCTAAATCTTGTATTTTCATATTTTTACATTCATCTATATGT
GTATTGATAAAGTGAGCGATATGTAAATCATTGTCATTAAGTTTATCAAAGTTTCGATTAATGTGTTCGTCTA
AAAACATATTCAACCTCCTTGGAAATAAATTTGTGATATATGGTTTTTACATTAAAAGGTGAAATTTTTAAAA
TTTAATTCTTGATTGTATATAAGGTTATAAATGGCTAAAGTACTTACATATTTTAAAATCAAGCATATTCTTG
ACATGCTTGTGAAAAAATTTTCACTATATGAAAAGATAATCTGATTATACAAAATACTTTCAAAATACGTCAA
TGTCGTTGTAGGTGTGTAAGCGTTTACTATATATTATATTTAAATAGTAAGACATCTATTTATAAATGAATCT
AATTAGATGTATTGTTAGGAAAGATGATATGGAGGGAATAGAAGATGAATGCGATAAAGCGTTTCGGTAGTGC
AATGATTGTACCAGTATTAATGTTCGCTTTCTTTGGGATAGTGCTTGGCTTTGCGACATTATTTAAAAATCCT
ACAATCATGGGGAGTTTAGCAGATCAACATACGTTTTGGTTTAAATTTTGGTCAGTGATAGAATCTGGTGGCT
GGGTTATTTTTACGCATATGGAAGTTGTATTTGTTGTAGGTTTACCACTTTCTTTAGCTAAAAAAGCACCAGG
TCATGCAGCACTTGCAGCATTGATGGGTTACTTAATGTTTAATACATTTATAAATGCTATTTTAACGCAATGG
CCACATACGTTTGGCGCAAATTTAGAAAAAGGCGTTGAAAATGTACCTGGTTTAAAATCAATTGCAGGTATTG
CCACATTGGATACGAATATTCTAGGTGGTATTATTATTTCAGCTATTATTACTTGGATTCACAATAGATACTA

TAGTAAACGATTACCAGAAATGGTTGGTGTATTCCAAGGTTTAACATTTGTGGTTACAATTTCATTTTTTGTG
ATGTTACCATTAGCAGCAATTACTTGTGTTATTTGGCCAACTGTTCAGCACGGTATTGGTTCAATGCAACACT
TTATCATTGCTTCAGGCTATATCGGTGTATGGTTGTATCACTTCTTAGAACGTGTATTAATACCAACAGGATT
ACATCACTTTATTTATGCACCAATTGAAGTTGGACCAGTAGTAGTCAATCACGGCTTGAAAGCTGAATGGTTA
CAACATTTAAATGAGTTTGCTAAGAGTACTAAGCCATTAAAAGAGCAATTCCCTTATGGCTTTATGCTTCAAG
GAAATGGGAAAGTATTTGGATGTTTAGGTATTGCTTTAGCAATGTATGCTACGACACCTAAAGAAAATCGTAA
AAAAGTAGCAGCATTACTTATTCCTGCAACATTAACAGCAGTCGTTGTAGGTATTACTGAGCCATTAGAATTT
ACATTCTTATTCATCGCACCATACTTATTTGTTTTACACGCAGTTTTAGCAGCATCAATGGATACTTTAATGT
ACGCATTTGGTGTTGTAGGTAACATGGGTGGTGGCTTGTTAGACTTTATTTCAACAAACTGGTTGCCATTAGG
TAAAGAACATTGGGGTACTTATGTTGCACAAGTTATTATCGGTCTGATATTTGTAGCGATTTACTTCTTCTTA
TTCAGATTCTTAATTTTGAAATTTGATATTCCATTACCTGGACGTAAGAAAACAGAAGAGGAAGTTAAATTAT
TTTCTAAGCAAGATTACAAAAATAAAAAAGGTGATAGCGTAGATTCTAAACGTGCTTCAACAGGTAATGAATA
CGAAGACAAAGCTGCGTATTACCTCGACGGTTTAGGCGGCAAAGAAAATATCAAAGATGTTACTAACTGTACA
ACAAGATTACGCTTAACTGTTTATGATGAAAGTAAAGTTGCGGATACGGAATATTTCACGCATCAACAAATGG
CACACGGATTAGTAAAGAGTGGTAAGAGTATCCAAGTTGTAGTAGGTATGACAGTACCACAAGTTCGTGAAGC
ATTTGAACAAATGGTTGAGGATCAATCGTCCGAAGATAAATAATATAAAAAAAGACACATCACTATACGCAAT
CATTGTAAATGATCATCGTATAGATGTGTCTTTTGTTATGATATACGAGCTTTGCTCATAGATGACGATCTAT
AATTGGCAGATAGAATGTCGATATTTCTAGTTGCAATCGTATGTAACAATTTAGCAAATATTTCTGGCGTACA
TATAATATGTGCATGAGACTTATTCAACGTATAAGTGATATCAGTGTGATTTTGGAACATATTCATAATATCG
ATGACTTTGTCTTTAATGAAAGCATCTTGCAAGCCATCAATATTGATATCGAAACGCTTTGCAGATTGATATA
AATTATCAGAATCAAGTAATTCATTTATTTTCTTTGGTGAAAGTACTTGGAAAATACTATACTGATGAAAACT
TAAAAATGAAATGAAGTATGGAAGTTGTTGCTTTGGTAAGCTAATTTCTAATACATACTGATTAAACTGTTTG
TTTATTGAGAATTGCAACGCATCACAAAATTGTACACTTTTGCATAACTTACTTAATTTTTTTCTAGTAGTAC
GGTCATTTAAACCGGCGATGTTGATGATGTATGTTTCGAATTTATCAATTAACATGTGAGGACCTCCCGAGGA
ATACATGGCATTAAATACACGTTTAATATTTATAAAGGTGACTTAATTTTGTTCAAGTTGATTTTACCACGCT
TTTTTTCTTTATTCACTAAGACTTTTGAATGAAGTTTAAAATAATTGTTTATCAGTGATAAAATATTTGCAAT
AAGAAGAGAATGGCTAAATAATCTTAATTTTCAGAAAAGTAATTGTAACCTTACTGGTCTTATGGTAATATTT
TTCAATATTATCGACGAGGATGTGTTAACAATGTTTCAAAAATTGAGTATATTTGCCACGAAGAGTTTTTTGG
TATGGATGTTAGTAGCAGCTGTTATTGGATTTATTTTCCCACAACATGTTGCGACATTAGGTAAATGGGTACC
TTATTTACTTGGTATTGTTATGTTAGGTATGGGATTAACAATTACACCTAATGATTTCAAAATGGTCTTTAAA
GCACCTAGAGCAGTAATTATTGGTGTCTGTCTACAATTCAGTATTATGCCCACATTAGCATTTATAATTGCAA
AGTCTTTTCATTTACCACCTGATATTGCTGTTGGCGTAATATTAGTTGGATGTTGTCCGGGTGGGACATCAAG
TAATGTAATGAGTTATTTAGCCAAAGCTAACGTAGCACTTTCTGTTTCTATTACGACGGTCTCTACGTTGCTA
GCGCCATTCGTTACACCTGCGTTAATATATCTATTTGCAAATGAATGGTTGGAAGTATCTTTCCTGAGTATGT
TGTGGTCAGTTGTTCAAGTTGTATTAATTCCAATTGCTTTAGGTATTGTTTTGCAAATTATTAATCGTAAAAT
TGCTGAAAAAGCTTCTACAGCTTTGCCGATTATATCAGTTGTTGCTATTTCATTAATTTTAGCAATAGTTGTA
GGTGGCAGTAAGCACCAAATCTTAACTACAGGATTATTAATATTTTTAGTAGTTATTTTACATAACGTATTAG
GGTATACGATTGGATATTGGTTAGCTCGTCTTTTAAAATTAGATCGACAAGATCAAAAAGCAGTCAGTATTGA
AGTTGGAATGCAGAACTCTGGTTTAGCTGTGTCATTAGCAGCATTGCATTTTAATCCAATTGCAGCAGTACCA
GGCGCAGTGTTTAGTTTCATTCATAATATAACAGGGCCTATTTTAGCAAAGTATTGGTCAAAAAAGTTATAAT
TGCACTAATAGAATGAAGTGGTCATCGGACTATGTTAAGCTTTGATAAAGAGAAAAAATAGAGGAGTAAATAT
ATGTATAGAGCAGTTATATTTGATTTCGATGGAACAATAATAGATACGGAACAACATTTATTTAATGTTATTA
ATAAACATTTAGAGATGCATAATGCCGATCCTATAAGCATTGATTTTTATCGTTCTTCTATTGGAGGAGCAGC
TACAGATTTGCATGACCATTTAATTAAAGCGATTGGTTCGGAAAATAAAGATAAACTTTATGAAGAACATCAT
CTTACTAGTACAACATTACCGATGATTGATACGATTAAATCATTGATGGCATTTTTAAAGCAACGTCACATTC
CTATGGCGATTGCCACAAGTAGTGTGAAAGCGGAAATAATGCCTACCTTTAAAGCATTAGGTCTAGACGATTA
TATAGAGGTAGTTGTTGGTAGAGAAGATGTTGAACAAGTTAAACCTGACCCTGAATTATATTTATCTGCAGTA
CAACAATTAAATTATATGCCGACACAATGTTTGGCTATTGAAGATTCTGTAAATGGTGCAACAGCCGCGATTG
CAGCTGGATTAGATGTTATTGTTAATACGAATAAAATGACAAGCGCACAGGACTTTTCTAATGTAGATTATGT
AGCAAAAGATATTGATTACGATCAAATTGTAGCGCGTTTCTTTACGAAATAGGAGGCGTATCATGATGGGTTA
CATTATATTGTTTTTTCTAGCTGGTCCAGTAATTTTAGGCGTTGGAAATTTGGTGATTGGTCCTATATTTAAC
AAACAGACACCATTTCACGTGCAAGTAAGATCTTTTGTTGTTGGTTCAATGATTTACTTAATACTCGCAACAA
TTGACTATTTTTTACTATTACAAGGTAAACTTTAACGAGAAAACCACCTTACCTCATTAAATGGACGACCATA
TGTATGTGAAATGGTAGAACGTTCATGTTTATGTATGAGATAGGGTGGTTTAAATAGTTACATATATTTTAAT
AATAACGTCACGATGATAAGTACAATTAAGATAATATCTATGCCTACCATAATTGTAGCTCTTGTTGCATTAC
TTCCTTGTTCTTTTGCTGATTTCATAGCACGGTAGTTTGGCACAAAGCTAATAATTAGTAAGATTAATACAAT
TACACCAATTAATGCTGTTGTCATGATGAACGACCTCCTTTATTTTTTTCAATCAATTCCCAAATAAACGTAG
CAATCACACCGATGATTAACCCAGTGATGACACCGATTTGTACCGTTAAAAATATGGCGCCGAAAATGATACC

AATAATTAATGCAATCGGTAAAGTCGTACCGAGTTTAATCTTGCGCTCTGGAGAATTAATAATAGTAAATAAT
GTAAGACAAATGAGTATGAAAACAAGTGTTGCAATAATAGTTCTTCCAACTAAATATAGGATGTCAGGTTTTT
CCATACCGATATAATTTATGATGAAAAATGCTACAGCAAAGAGTATCGATATTTTTGTAGCACGTGGCAGTAT
TTGTTTTAACATTGATATACTCCTTTGTAATATTATTAAAATTATATCATAATTACCGAGAATAGCTGAAGTT
GTATGTGACTTAATAGTTTTCGAACAACTTTTTTATTTTTTTAGAAAAATCACAAAATAATTGTTTGCAAAGT
TGCAAAAGCCTGCTATAGTAGTTCTGTAAACGATTGCATGGTATGCAAATATTAATGTACCAAAATCGGTAAT
TTATAGTATAATTACGGCAATAAGTTTTTTTATGGATTTATTTAGTATCAATCAGAGATGGGGTAAGAAGTTA
TGGAGAACAATGAACTACAAAGGGGATTGAGTGCCCGTCAAATTCAAATGATTGCACTTGGTGGTACGATTGG
CGTGGGGCTTTTCATGGGTGCTACAAGTACAATTAAATGGACAGGCCCATCAGTTATCCTTGCATATTTAATT
GCGGGTATCTTTTTATTTTTAATCATGAGAGCAATGGGGGAAATGATTTATTTAAACCCTACAACAGGATCAT
TTGCAACATTTGCAAGTGATTATATACATCCTGCAGCAGGTTATATGACAGCATGGAGTAATATATTCCAATG
GATTGTTGTTGGTATGAGTGAGGTCATCGCAGTAGGAGAATATATGAAGTTTTGGTTCCCGGAATTGCCAACC
TGGATTCCTGGTATTATTGCTATTTTATTATTGATGGCAGCGAATTTATTCTCGGTAAAAGCGTTTGGAGAAT
TCGAATTTTGGTTTGCTTTAATTAAAGTTGTAACAATTATTTTAATGATTATTGCTGGTTTTGGTCTTATTTT
CTTTGGTTTTGGAAATGGTGGCCATGCGGTAGGTATTTCTAATCTATGGACAAATGGCGGATTTATGCCAAAT
GGTATTGTTGGTTTCTTCTTTGCATTATCAATTGTAATTGGTTCATACCAAGGTGTGGAACTGATAGGTATTA
CTGCAGGTGAAACTAAAGATCCTCAGAAAAATATCGTTAAAGCAGTGAATGGTGTTATCTGGAGAATTTTAAT
TTTCTATTTAGGTGCTATTTTTGTTATTGTTTCAGTGTATCCTTGGAATCAATTAGGAGACATTGGAAGTCCG
TTTGTTGCAACATTTGCAAAAATCGGTATTACATTTGCAGCTGGATTAATTAACTTTGTTGTATTAACTGCAG
CAATGTCAGGATGTAACTCAGGTATTTTCAGTGCGAGTCGTATGATTTATACACTTGCTCATAAAGGTGAAAT
GCCGAAAATATTTACTAAAATCATGAGAAATGGTGTGCCATTGTACACTGTTGTTGCAGTATCTCTTGGTATT
TTAATTGGTGCTTTATTAAACGTAATTTTACCTTTATATATCGATGGAGCGAAGAGTATTTTCGTATACGTTT
ATAGTGCGTCTATTTTACCAGGTATGATTCCTTGGTTTATGATTTTATTCAGTCACTTACGTTTTAGAAAATT
ACACCCTGAAGAAGTGGAAGGTCACCCATTCAAAATGCCTGGTGGCGCAGTAACTAACTATTTAACAATCTTA
TTTTTAATATTAGTATTGGTTGGTATGGTATTTAATGTTGAAACTAGAATATCAGTGCTTATTGGTGTTATCT
TCTTAACGATTGTAACGATTTATTATTTTATTAGATATAATAAAAATAATGTAAAAGCAAAATAGTATGATTA
TAATGTGCGCTTATTTTATGACAAACTTGGCCCTAGCTTATTAAAGTTAAATAGCCAAAGAAATGTTATGAGT
AGGCGCATTTTTTTATAAATATAGCTTAAATCAAAGATGTAGTATGAATTTACTTATAAGTGGCAATACTATC
GTTAGTTTTTACTGTAGGCTTTAAAATAAAAAATAGCTTTATTAAAATAACAAACGCAATTGATATATGGAAT
GAATCCGTTAATCAACTGCGTTTGTTTTTAATATAAGAAATTGAATGGCCTTATCAAATCATTGGTCATATGC
GTATGTTATACAAGATAAAAGACATGTAAGCTTTGAAAATGATGCATAAATTATTATATGACATGATTAAAGA
ATTTATATAAGACTGTTAATGTTTAAAGTTAATATTTGATAAATGTTTACGATAGTTATCTAAAGCCATTTTA
GATTGTGTTATGAAATCTAATGATGCGTGATAATTTAATGCAACATAACGATAATATAAAATATCAATAGTGA
ACATTTGAGCAAATAATGAAGTTGTTGCTCCCATATGCATCTCATTTTCATCAGTTTTCCCATAAGTTAAAAC
AATATTTGATGCCTGTGCTACGGGATTATCCCTTGTACTAGTAATTGTAATTATAGGTATATGGTAGTCATCA
ATAACTTTAACCATTGATTGCATTTCACTTTGCGTACCATTGTTAGTAATAAGAATAACACTGTCATTCGAAT
TGTGAGTTGCTAATAATGTTGCAAAAATATGTGTTTCTTGAACAAGTTGAATATTAAGACCTATTCTTGATAA
CTTTTGGTATAAGTCGGTAGCAACTACAAAAGATGCGCCAAAACCATATATAAAAATCGTCTCAGAACGTTTT
AAACAATGACATATTTGATCAATAGTTTTATCATTTAATTCGTTATTTGCATGATTAAGTGCGCGTGTAGTAC
GTGTATGGAGTTTAGTTCTTAAAGATTCTGTGCTTTCGTTATTCATTAATTCAACATTGTAAATTGATGATGC
TTTAGGAACATATTTAGATATATTTATTTTCAAGTCGTGAAAACCGCCATCAGTAATTTTCTTGCTAAATCGA
ATGACTGATGATGTACTAATATCTAATAAGCTTGCTAAGTCTTGAGACCTCATTTTAATCACCTTTTGAGGTG
AATTTAGTATAAATTTAGCTATCTTTTTTTCATTTTTAGTCATATATGGATATTGACTATCTATTTCTGTTAG
TACGTTTGACATATTAATCACTCCACTTTAACGCAATATATTTTATTAGCGTTGGCTATTGTTGTTTAACGCG
ATGGTATTTACAACTTGTAACGTTAGAGAATGAAAAATAAGCTGAATATGTTGTTTGTTTAATGATATTGAAT
TATAAAACTGTAAATTTTTTTAAGATGTATAGATAAAAAATAGCATACTCCCATGTGCAAATAATATATCAAT
ATATTGATATATTACCGTAAGTCATACTGTATATTCCCAATTTATACATTATGTACTCATCTATTTAAATCTT
AGTAAATGTAGTTATATAAGACTAAATCTACCATCTTAATCATTATGATTATAACTCGATTAATGCATCTTTA
CAATTTGAAGAAACTTTTTAATTGTATAATTTATGTAATAAAGTGAATTTTAATAAATAACTTGTTTAAATGA
CATTTTATGAAAAATAGGTTAATGAGCAAATTTAATTGCAAATAATGCGTTAGATAGTTCTGTATGATAAAAA
TTAAGATATTGAATTTTGTTACCATAGTCGACTTGTCGGAGGGGACGTTTATTAAGTAGTGTTATAATTTCAT
AATAGAAAACTTGCTTTTCGTTTATTATATATGATGTTTCAAAGCCTTTGAACTTATTCAATTCATTAGTAAC
TATTTGGTTAAATTTATAAATATGCGTTTGAATGTTCCAATTTGTCATTAAAGTATTATATTTAATCGTATCA
ATGATTTGAATCAAATTAGTAATTTGTTGTCTCATATACTTAGTTTCGTTTATCATGTAGAAGTACCTCCAAA
AATCAATTATTAAACAGGAAAGGATATAACAGTGGAAAATGAAAAAAAGAAATACACGTTTAAAGATATAGCA
TGGAGAGATTTGCTTATAATTCCAATTATAGGTGTTATATTTTATGCACTCACAATTGCAGCTTACATAGGAC
CAATTTTGTTTGGTTATTATAAAATCGATAGTAATCTGATATTTGTAGCGACTATCACACAAGCTTTAGCATA
TACGATTGGGATTGGCGCATTTTATTTATTTCATTTAAAAGTGATGCCATCAAGGCTTCGAGCTGGTTTTGCG

TATATCAAAAAATATTGGCTTAGATTGTCAATAACGTATATTATTGCTCTCATCTTAATTTATACATATGAAT
ATATGACACAGTTTCTACCTAAACATTTGCAATATAGTGAAACAGCCAATGAACTTGAGCTAAATAAAATGTT
TGAAGTGCCTGCATTTTTACCTGTAGCATTCTTATTAATTGTGATTGTAGGTCCAATAGTTGAAGAAATCGTA
TTTAGACATATTTTAATTGGAGAATTAGGTAAGAAGTTTAATTTTATAGCTATGAGTATTGTTTCGGTGTTCT
TATTTGCATTTATTCATGTGACTGATGCAAAGTCGCCATTTGAGTTTGGTCCATATCTCATACTATCCATCAT
ATTAGTTTTCACTTATATAAAATCTGGAAGAAACCTAGGTTCAACAATTGCATTACATATAGCAAATAATTTT
GTTTCTTTCGTTATATCAGTAATAAGTATTTATGCGTAGATAAAACCCCATCGACTCCATAGTTTAGGAATCG
ATGGGGTTTAAAAATTGATGATAGTTATCAATGGATATGTCTCTGGTTTGAATCGTCTTACCGAATGAATACG
AAATATCTATAATATTTTCAATTTCGAGTTCGGCAGTTTTGTCTTGAGCAATATCATAAATGACTGCATGCCA
ATGTCCATGTTTACACAAAAGGCCAATAAATATAACGTTTTCGGCTTTAATATTATCATCAACCCTATGATCT
ACTAACATCACATTTCGTTCTGTACAATAAATTAAAATGTCGGAAAACAACATTGGGAGTGTCATATGTTGTG
TGTCATCAAATTTAATATAAAAGTCCATGCGCTTAAGCAACTTTCTTAAGCGTGTTTGTGGAAGACTTAAACA
TTGTTTTATAATTGCGTTGATTTCGCTTTTATATGGTAATGTCGAGTACGACTGACTTTCATTAAGCACTAAA
AATAAGGCCGATAATTGTGTTTCAGATAAGTTTAATGCAATTTTAGATTGTGCTTGTTGTATTTGGTAACCAC
CATTTTTCCCATAATGCGCATAAATTTTAACACCTTGATTTTCTAAATCATCAATATCTCTTAAAATTGTGCG
TTTGGATACGTTGCAATATTTAGCTAATTCTAAAGCGGTCATTTTTTTATTTTGTTGAATTGCAGTAATTATT
AAATTTTGCCTTTCAGCTTTATTCATGACAGCCACCTCCTAAAAGATGATATATTACACAAAGTATAACATAA
GAGTGACAATGAGGACATTTTCAATATCTTTTTAAAAAAATCGGAAAAATTTTGTAGAAAACGCTTACATTTT
AAAAAAGATTGTGCTATGATATTACTTGTAACAGGGGGAGCGATTAAACAAAGGGGTAGAGCTATATAACAGA
TAGCTTTTAATCGTTCAAGTTACATCACAACATCTTCAATATTTATTACTTACTTTCCTTTCTATTTGTCGGC
TAGCACGTGACTAGCCGATTTTTTTATGCACAAAAAGAAGAAGTCTTCAGTTGTATTGTTGATTCTTTTAATA
TAGGGTAATAGTAAAATGATAATGATATGAGAAGTGGTGGACGAAGTGACAGATAAAACATTACAGCAAATAG
ATAAGTTAATATGTTCGTGGTTGAAACAAATAGACAATGTTATTCCACAATTAATTATGGAGATGACTACAGA
AACCAAGCGTCATCGATTTGATTTAGTTACAAATGTAGATAAACAGATTCAACAACAATTTCAACAATTTTTA
GCAACGCATTTTCCGGAACACCAACTATTAGCGGAAGAAAAAAGTAATAAAATGATTACGAATGAAATTAATC
ATTTATGGATTATGGATCCCATTGATGGAACTGCCAATTTAGTAAAACAGCAAGAAGAGTATTGTATTATATT
GGCGTATTTTTATGAAGGTAAACCAATGTTATCGTATGTATATGATTATCCGCATAAAAAGCTTTATAAAGCA
CTACGAGGAGAAGGTGCTTTTTGTAATGGAATTAAGATGGAAGAACCACCATCGTTAAAATTGGAAGACGCTA
TTATATCGTTTAATGCACAAGTGATGAATCTGGATACGGTGCAAGATTTATTTGACGCCTCATTTAGTTATCG
TTTAGTTGGTGCTTGCGGATTAGATTCTATGCGAGTGGCAAAAGGTCAATTTGGGGCACATATTAATACAAAT
CCTAAACCTTGGGACATTGCTGCGCAGTTTTTATTTGCAGAACTATTGAATCTAAAAATGACAACTTTAGATG
GTAAGGCAATTGATCATTTAAAGGGTGCACCTTTTATTATAAGTAATCAAGCATGTCATGAAACGGTACTTAA
AATTTTAAATGCAAATGGTGGTTATCAAAAATACAGATAGATATTCTATAGTGATAAAAAATGATAAAAAAATA
GTTATACGTCTACAGAAGTATAGTAAAAAAATTATTTTACTATATAATAAATAGAGAAAACAGACAAAGTATG
TAAGGAATAGTAAAGGAGTGGTGGGATTGAAACGTTCGTCTAAAAGTAAAATGAGCCTACCGAAAAAAATATT
TTTATGGGTATTCGGTATTTTAGTAATCTTAGCGATTGTAGTTGTTGTGTATGTAGCTGCTAAAATTTTTATT
ACTGGTAATAAGATACACAATCCGTTAGATCGCAATCATTCAGAATTAAGAGATAAAAAAGTGAGTTTAAACG
ATGGTGATCCATTCACAATCGCTTTATTTGGTGTCGATTCAGATGCTGATCGTAAGAAAAAGGGTGGCGGAGA
GCGTAGTGATAGTATTATGATTTTATCTATCAACCCTAAAACGAAGAAAACTGAAATAGTAAGTATACCGCGA
GATACAAGAGCAGAAATTGTTGGCCGTGGTACTACTGAAAAAATTGCACATGCCTATGCATATGGTGGACCAA
ATATGGCTGTTAAATCACTTGAAAAATTAATGAATGTGCCAATTGATCATTATGCGACAATCGATATGGATGG
TTTGCATAATATGATAGATAGCATCGGCGGTGTTGATGTAGTAAGTAATGATACATTCACTGTTGATGGTGTT
CGTTTTACAAAAGGACAGCAGACACATGTAAATGGTGATCAAGCATTGAAGTTTATTAGAAGTCGTAAAGAAG
AAGGTGCTGGTGGTGACTTTGGTCGACAACAACGTCAGCAAATTGTGCTTGAAGCGATGGCCAATAAGATTGC
AAGCCCATCATCGATTACACATTTTAATAGTCTAATGAATGAAATTCAAAATAATGTTAAAACAGATTTAACA
TTAGGTGATTTGAATACGATTAGAAGCAATTATAAAGATGCTAATGACACGATTAATAAACATCAATTGAGTG
GTCAAGGCGGCATTCAAAGTGACGGATTGTACTACTTCATTCCTAGTGAGCAGTCAAAAGCTGAAAGTACTAA
GTTATTAAAAGATAATCTAGAGTAGTAAACTAAGGGAAAGTAAGTTATCAAAATTGTACACAAACTAATTAGA
TAGAATGAGCTGTAAAAAGAATAACGAATAGAATTAATAGCCCGTAAATAGAAGGTGATAACACTTTTTATTT
ATGGGTTATTTTATTTAATGCCTGATATGCGGTTTTGTAAATTAACCCATTACAAATTCAATTTAAAAATAAA
TCATACCTTCTAAATGACAGTAGATATTTATCAGACTTGGGTTATTAATTTTCAGAAAAAGGGTATTTATAAA
TAGTATAACGATTTTTAGGTGAGCTACTTATTAAGTATTGTCATTAATAAGGTTGTGAAAGATATGGCAAAAA
AGCGTGAAACAGATAATTGGAAAGCTAATAATGATAATTATTTTCAATTAAACAAAAAGATGATTTAATTTCG
CGCAAAATATTTCAAATAAAAATGATTGCAAAAATAAAAAATACAAAAATACGCTTAACATTATACACTTAAA
GTAATACAATTATATTGATTCTATCTGAAAGATGTGTGGGGCATCGTTATTTTAGGTGGATATGAGCAATTTA
TTAAAAGTCATTTACGGAAAAATATATAGATGGGGTGAATAGTATGCAAGAACATTTGGTGGTCACGCTTGAT
GGTAAAGATTATCTTGTAGAACCAGGTACAAATTTACTTGAATTCATTAAATCGCAAGATACTTTTGTGCCTT
CAATTTGTTATAACGAATCAATGGGACCAATTCAAACGTGTGATACATGTACTGTTGAGATTGATGGTAAAAT

TGAACGTTCATGTAGTACAGTGATTGATCGTCCAATGACAGTAAATACAGTGAATAATGATGTGAAAGATGCT
CAAAAAGAAGCGCTTGATCGAATTTTAGAAAAGCATATGCTGTATTGTACAGTATGTGATTATAATAATGGTG
ATTGTGAAATTCATAATACGATGGATGCATGGGGGCTTCAGCATCAAACGTATGAATATAAAGAGAAGCCATA
CGAAAAAGATTACGGCCCATTTTATCGTTATGATCCAAATCAATGTATCTTATGTGGGCGTTGTGTAGAAGCA
TGTCAAGATATTGAAGTAAATGAAACTATTAGAATTGATTGGGATCGTGAACATCCACGTGTTATTTGGGATA
ATGATGTACCGATTAATGAATCTTCATGTGTATCTTGTGGTCAATGCGCAACTGTATGTCCATGTAATGCCAT
GATGGAAGTGAATATGGAAGGTAATGCGGGTTATATGACTGATACGGAACCTGGTTCATTAGCGGCAATGATT
GATTTAACTAAAAAAGCAGAACCTGGTTATGGTCCACTATTTGCAATCTCAGATTCAGAAGCAGAAATGCGTA
AAGAGCGTATTAAGAAAACTAAAACAGTGTGTACATATTGTGGTGTGGGCTGTTCATTTGAAGTTTGGACTAA
AGATAGAGAAATTTTAAAAGTACAACCATCACATGATTCTCCAGCAAATAAAATTGCGACTTGTGTTAAAGGT
AAGTTTTCATGGGGACATATTAATTCAGATCAACGCTTAACGAAACCATTAGTAAGGAAAAATGGTGAGTTCC
ATGAAGTAGAATGGGATGAAGCATTAAATGTCATTGCAGATAATTTTACATCTATTAAAGAAAAGTATGGCCC
AGACGCACTATCATTCATTTCTTCTTCTAAAGCGACGAATGAAGAATCGTATTTAATGCAAAAATTAGCAAGA
CAAGTTATTGGCACTAATAACGTTGATAACTGTTCAAGATATTGCCAAGCACCTGCAACAAAAGGCTTATTTA
GAACGGTTGGACACGGCGGTGACTCAGGTAGTATTGAAGATTTAGAAAAAGCAGCAATGTCTGTATTGATTGG
TACGAATACAGCCGAAGCACACCCGGTTATTGCATCACGCATGAAACGTGCTCAAAAATTATTTGGACAAAAA
ATACATGTGTTTGATATTAGAAAACATGAAATGGCTGAACGTGCGGATCGTTTCTATCAACCGAAACCAGGTA
CGGATTTAGCGTGGTTAAGTGCAGTGACGAAATATATCATTGATCATGACTTACACGATAAAGCCTTTATCGA
AGAGTGGGTTGAAGATTTTGATGAATATTACAAGTCATTAGAAACATTTACAATGGCATTCGCTGAAGAGGCT
ACTGGTATTCCAGAAGCAGAGTTGATTAAGTTCGCTGAAGAATGTGCCAAAGCTGAGTCCGTTGTCATTTGTT
GGGCAATGGGTATCACACAACAAGACATTGGAAGTGATTCAAGTACAGCAATTTCAAACTTATTATTAGTTAC
TGGAAATTATCGTCGACCTGGTACAGGCGCGTATCCATTACGTGGACATAATAATGTTCAAGGTTGTAGTGAT
ATGGGTAGTATGCCTGATAAAATTACTGGCTATCAAAGTATTGAAGCGGACGATATTCGCGCTAAATTCGAAA
AAGAATATGGCGTTAAATTGAATCCTAAAGTTGGTAAAGATAATCATGAAATGGTTGAAGGTGTCCATGATGG
GGACATTCATTCATTATATCTATATGGAGAAGATACTGGTATTGTAGATTCAAATATTAATTTTGTACAAGCT
GCTTTTGAAAAATTAGATTTCATGGTAGTCCAAGATGAATTTTTAACATTTACAGCTACATTTGCAGATGTTG
TCTTACCAGCAAGTCCTTCATTAGAAAAAGATGGTACTTTTACGAATACTGAGCGACGTATTCAACGTTTATA
CCAAGCATTGAAACCATTAGGTGATTCTAAACCTGACTGGAAAATATTCCAAGCAATTGCTAATAAATTAGGT
TTCGATTGGAATTACAAACATCCAAGCGAAATTATGGATGAAATTGCTAGACTAACACCTCTATATGCTGGGG
TAAGTTATGATCGTTTAGAAGGATTCAATAGTTTACAATGGCCTGTGCATCCAGATGGTACAGATGAACCAAT
ACTTTATTTAGAAGGATTTAATTTCGATAATGGTAAAGCAAAATTATTCCCATTATCATTTGATAATTACTTT
AAGCAAGATGAAGTTTATGATATTCATGTAAACAATGGTCGATTGTTAGAGCACTTCCACGAAGGTAACATGA
CATATCAAACACCAATGCTTAAATACAAAGTGCCACGTGCATTTGTTGAAATTTCTCCAGAGCTTGCTGAAGA
TAGAGGCATTCATGAAGGTGCAGAAGTTAAGTTGATTTCTGAAACAGGAGAAGCGGTATTACAAGTTCACGTG
ACAGATCGTGTTAAAGGTAAAGAAATCTATATTCCATTAAATAATGATGCGATGGAAAATGGAGACCTAGGTG
CAATTAACTTATTAACTAATAGTGATGTTGATCAATACACGGATACACCATCTTATAAACGTACAAGCTGTCG
TTTGGAGGTAATTACGAAACGTGGTAAGTCACCGTTGAACCCAAATAATTTCCGTGTCAATAAAAAACGTCAT
CCGCAGTACAGTGTTCAAGTACAGAAAAAATGGGAACGTCCAGATTATGTTTTCCCAGGAAATCAGGTGGATA
AATAATGGCTGAGAGAATATCTTCTAAAATTAGACGCTTAGAAAAATCAGAAGAACAAATTAAGTTAGAAAGT
TTAAATGAAGTTACAGAGGCAATTGCAGCGAATAAAGATAGTATATTAAAAGCAATCAAACTTATTAAAGTGT
TAGATGATGCTAAAATATTAGATGCTTTAAATGGCGCAGTTAGAGGACGTCAAGTGATTATTAATAAATTTGC
TGTTGAGCTCAACAAAGATATCTATACTGGTCTATTAACAAATATGGCATCAATGGTATTTTTATTAGGTGAA
TTAAATGTATCAGATTTAAGTGACTTCCTTAATAAAGTTAATAAAGGATTACATGTTGCAAATCAAGCGAGTC
CTAATGCGAAGACATCTGTAAGAAGTCTGTTAGGCGTTTTAAAAGATGACGACATGAACCGTAGTTTAACATA
TATGCTTAATATGCTAAAAGGTATGTCTCGAGAAGATTAACATTACAATGAAAAAGTGTTTCTACATGCAAGC
AGTGTAGGAGCACTTTTTTAATTTTTTTAAAAATTAAAAAGGGTGTTTTTGATGGAATAAATGAATAACATTG
AAAAACAAAAAAATCACGTGCTATTATGAATCAATCATCGACAGGCCTATGAAACATCATGTATTATTATGAT
TATGTAAGTTATTTAAATAAAAAGGGTGAGTGCATATTAATTCGAGAATGTTAAAAACGTCAGCTGTAATTTC
TAATACATTATTAGTCATAGGTTTAGTCTGTCTATTTATGATGAAATTAGTGTTAGCTATTACTTTTTTTGCT
GTTTCATTATCAATTAGTCTAGTTGTCTTTAATATGATGTTCAGAGATAGAACAACGATGAAAGTAATCGTTA
ACGCTTCTTTTTTAATCGTAATATTAGCGATTGTAGTGGCATATTTTTTATTGTCTAAGTAAGAAAAATGAGG
TTTTGTCTATATGAAGAAGAATTTCAAGTTACGCATTTCAACGCTACTATTGATAGTTATTTTAGTTGTTTTT
GCTGTATTACTCATCGTGAATGAAACTAAATTGTTTAAAAATGATGTGAATTACTCTTTTGATGAGGCTGTTT
CAATGCAACAAGGGAAAGGTATTGTACAGACAAAAGAAGAGGATGGTAAATTTGTTGAAGCAAATAATAATGA
AATTGCTAAAGCAATGACTATTTCACATAAAGACAATGATATGAAGTATATGGATATCACAGAAAAAGTGCCA
ATGTCGGAATCTGAAGTTAACCAATTGCTAAAAGGTAAGGGGATTTTAGAAAATCGAGGGAAAGTTTTTCTAG
AAGCTCAAGAAAAAATATGAGGTTAATGTCATTTATCTTGTTAGCCATGCATTAGTAGAAACAGGTAACGGCAA
ATCAGAATTAGCAAAAGGCATTAAAGATGGAAAAAAACGCTATTACAACTTTTTTGGTATAGGAGCATTCGAT

AGTAGTGCTGTTCGTAGTGGAAAAAGTTATGCTGAAAAGGAACAATGGACATCACCAGATAAGGCGATTATTG
GTGGTGCAAAGTTCATTCGTAATGAATATTTTGAAAACAATCAACTGAATTTATATCAAATGCGATGGAATCC
AGAAAATCCTGCGCAACATCAATATGCGAGTGACATTCGCTGGGCAGATAAAATTGCCCAATTAATGGATAAA
TGCTATAAGCAGTTTGGTATAAAGAAAGATGATATTAGACAAATATATTATAAATAAGACATCAGTGCTTAAA
GGAGCTGGAACAATTTATTGTTTCGAGCTCCTTTAGCGCATTCTGAGTGTGTTAGTTAAATGGATTTTAACCT
AACAAAAAACGCTATATAGCATCAAATATGCTATATCCCACATCATTGTTACAAATGTACATTATGTAAATGA
ATATTGCTGTCTAAATGTGCATGTAATATACAATGGTGCAGATAATACACTTAAGTCTTTAAAAATGAAACGT
TAGTTCCAAGAGTCATTTTTAAACAATAGTGCATGTGATAAAATAGAAAAGAATGAAAAATATAGAGGTGACA
ATATGAAGATAGCAATTATAGGTGCAGGCATCGGTGGATTAACAGCTGCTGCATTATTACAAGAACAAGGTCA
TACTATTAAAGTCTTTGAAAAAAAATGAGTCAGTTAAAGAAATTGGCGCTGGGATTGGTATCGGAGATAATGTG
CTTAAAAAACTAGGTAATCATGACTTAGCTAAAGGTATTAAAAATGCTGGGCAAATCTTATCTACAATGACAG
TGTTAGATGACAAAGATCGCCTGTTAACTACTGTTAAATTAAAAAGTAATACATTGAATGTGACGTTACCACG
CCAAACATTAATTGACATTATTAAATCTTATGTAAAAGATGATACAATATTTACAAATCATGAAGTCACGCAT
ATAGATAATGAGACAGATAAAGTTACCATACATTTCGCGGAACAAGAGAGTGAAGCATTTGATTTATGTATTG
GTGCTGATGGAATTCATTCTAAAGTGAGACAATCTGTAAATGCTGACAGTAAAGTATTATATCAAGGGTATAC
ATGCTTTAGAGGTTTAATTGATGATATTGATTTAAAGCATCCGGATTGTGCAAAAGAATACTGGGGAAGAAAA
GGAAGAGTAGGTATTGTTCCGTTATTAAATAATCAAGCATATTGGTTCATTACAATCAACTCGAAGGAAAACA
ATCATAAATATAGTTCGTTTGGTAAACCTCATTTGCAAGCATACTTTAATCACTATCCAAATGAAGTTAGAGA
GATCTTAGACAAACAAAGTGAAACAGGTATCTTATTGCATAATATTTATGATTTGAAACCACTCAAATCTTTT
GTTTATGGTCGTACTATTTTACTAGGAGATGCAGCACATGCGACAACGCCTAATATGGGGCAAGGTGCTGGAC
AAGCAATGGAAGATGCTATCGTATTAGTAAATTGTTTTAACGCATACGACTTTGAAAAAGCATTACAGCGTTA
TGATAAAATACGTGTCAAACATACTGCAAAAGTAATTAAGCGTTCTAGAAAAATCGGTAAAATTGCCCAATAT
CGTAGTCGTTTAGTTGTTGCAGTTAGAAATCGTATTATGAAAATGATGCCAAATGCATTAGCAGCTGGACAAA
CTAAATTCTTATATAAATCGAAAGAGAAATAATACAACAATATGAAAACCCCCGTATGTTGAAATGAGAGCTC
AACATATGGGGGTTCTTGTTTTTATAATGTCATTATAATAAATTCAATTATTAGTTAACGACAAATTGTGGTT
TCTCACCTTGAACGGCACTAATTGCAGCATTAGCAACAATTTTAGACATCATGTCACGTGCTTCAAATGTAGC
ATTACCAATATGCGGTGTTAATACTACATTATTAAGTGATTTTAAGTCATCGGTAATATCTGGTTCAAATTCA
TATACATCAAGTGCAGCACCTTCAATTTCATTATCTTTCAATGCTTGCACTAGTGCTTGTTCGTGCACGATTG
GACCACGAGAGGCATTGATTAAATACGCCGTAGATTTCATCATTTTAAATTGTTCTGTATCAATTAAATGATG
CATTTTAGGATTATAAGCAGCGTTGATAGTGATAAAATCTGCATTCTTTAATAGTGTATCTAAATCTACATAT
TTTGCACCGATTTCTCGTTCTTTTTCTTCTTTGCGATTAGGTCCTGTGTATAGTACATCCATGTCAAATGCTC
TTGCACGACGCGCTACTGCACTACCAATTTCACCTAAACCGATAATGCCGATTGTTTTCCCAGATACTTCTCT
ACCTCTGAAAAATAAAGGTGCCCATCCGTCAAATCCAGTTATACGTGATAATTGGTCGCCTTCAACAATACGA
CGCGCTACTGCAAGTACTAATCCAATTGTTAAATCAGCAGTCTCGTTTGTTGATGCTTTAGGTGTGTTTGTAA
CATCTATACTTTTTTCTCGAGCATACTCGATATCAATATTATTAAAACCAGCACCATAGTTGGCAATGATTTT
TAAGTCTTTACCAGCATCGATAACATCTTTATCAACGTTTGTAGATAATAAACTAATTAAGGCAGTCGCATCT
TTAACACCTTTAATTAAAGTGTCTTTATCGACTAATCCTTTACCTTCATACATTTCAACTTCAAAATGTTCTT
GTAAAAGTTTTAAACCTACTTCTGGTATTGCACCAGCAACATAAACTTTTTCCATAAAAGATCACTCCTTTTA
TCTTAGTATAGTAGAAGATTAGACAGTATACAACTATGTCATGATGTCTTGTGTATCAATGATGTAAGCGTGT
ACTTTTGATGGAGGTGATATAACTTAGGCACTGTAGAACTATGAATATTGTAATGTGGAAAAACTGGATCAAT
TAAATTAGATAACGTAGTTTTAAAGTTAAATAGTATTGAAAAAATTAATATTATGAATATGGGGGGGAAATAT
AAATAAGTAGGTGGCAACGAAAAATAGCAAAAAAAGAGCTTCTCCTATAAAGGAAAGCTCAAAGTTTTTTGAT
GACATATGTACTAGAATTAAGTTTCAAGACAATATGTATCATCGTGTTTATATTAAATATGGATGTAGTTGTA
GTTACCTGCTTCGCTTGCAGAAATAGTTCTAGAACTTACTGAGAAAGGTCCGCCACTATAATTCATTTCTGAA
ATTGTAACTGAACCATCACTGTTTACACTTTCTACATATGCAACGTGACCAAATGGTCCTTCAGAAGATTGTA
GGATAGCGCCTTTAGAAGGTGTATGGTTTACTGTGAATCCAGCACCTTGTGCAGCAGCAGCCCAATTATTAGC
ATTTCCCCAAGTAGAACCGATTTCTCCGCCTACTTTATCATATACATACCAAGTACATTGTCCTGCAGTGTAT
AAGTTACCAGAATGTGAAATTGATGATGTAGTTGTCGTAGTTGTTGTAGTCGTTGTAGTTTGAGTCGTATTAT
AGTTATAGTTGTTATAATTTGTATAATTTTCAGCAGCGTCTGCATGTTGTGCATGACCTACTAATGCTGTACC
AATTCCTGCTGTTAACGTAGTTGCTGTTACTAATTTTTTCATGAATAAAGTCCTCCAAAGTTCTATATCTTTT
TTTATAAATAAAACGTAGCGACTGTTTTATTCTCACATCTCGAATTGATGACAATAGTTACTTTAACAAAATT
AATGCTTCTTGTGGGGAATGTTATTGATTTGTAAAAGAATAAAAAAACTTTGACTAATTTTGTAATAAAAATT
AGTCAAAGTTACAATGAGATTAACAGATAATTAATAGGAAATATTTATTTGTAATATGTTTAAATAAATCGAA
TTGTTAAAGGTATTACATATTCTTGGCCATTATAATATTTGACACACGCAATAATTGTGAATACAAAAGATAA
TATTGAGAAAGCGAATATGGATAAAATACCGATAAACGTAATGATGAAACCTATAATGATAATGAAATCAATA
TCGGTAGCAATTAGGAAAACGCCAATAAAAGTGATAACGACTAAAATGATAGACCAAATAATATAAGAAATCG
TATAGTTAAGATAATTTTTTCCAGCACGATCAACTAGTTTTGATTCATCTTTTTTCAATAACCATATAATCAG
TGGACCAATAATAGATGTGAATAAACTTAATAAATAGATAAGCATCGCCATAATGTTCTCATCATTGGATTTG

CGATTCGGTTGATGATTTGTTACGTCGTTCATTTCAGTTGTCATATTAGACACTCCTTTGAAAATTGTAATAT
TATCTTTAACTATAACAAAATATAATCAAAAATAAACATGTTTATTAAACAATTATTAAAAATAAAAATAATT
GGTGGACGTCGGCGTTTAAATAGGTTAATTTAAGGTTATATATACTTAACATTTATAATGATGCGTAATGAAT
TCGCATCATTTTTATATTGTCTTACGTATAATTTGTTTTTAATTTTAACCAAAGATAGAAAGAGGGTTGTTTA
TGAAAATAGCAATTGTAGGATCAGGAAATGGCGCAGTTACGGCAGCAGTAGATATGGTGAGCAAAGGCCACGA
TGTTAAATTATATTGTCGTAATCAATCTATAAGTAAGTTTCAAAACGCAATCGAAAAGGGCGGATTTGATTTT
AATAATGAAGGTGATGAACGTTTCGTAAAATTCACTGATATTAGTGATGATATGGAATATGTTTTAAAAGATG
CTGAAATTGTTCAAGTGATTATTCCATCTTCATACATAGAGTATTATGCTGATGTAATGGCAGAGCATGTAAC
TGATAATCAGTTGATATTCTTCAACATGGCTGCAGCAATGGGGTCAATTCGTTTTATGAATGTTTTAGAAGAT
AGACATATTGAAACAAAACCACAATTAGCGGAAGCTAATACGTTGACGTATGGTACGCGTGTCGATTTTGAAA
ATGCAGCAGTTGATTTATCTCTAAATGTACGTCGTATCTTCTTTTCAACATATGATAGAAGCTGTCTAAATGA
TTGTTATGACAAAGTTTCAAGTATTTATGATCATTTAGTAAAAGAGGAAAGCTTAATTAAAACAAATCTTGAA
AATGGTAATCCTGAAGTGCATCCAGGGCCAACATTATTGAATGTCGGTCGCATTGATTATGCTGGTGAGTTCG
CTTTATATAAAGAAGGAATTACTAAACATACAGTTAGATTACTTCATGCAATCGAATTAGAACGTTTAAATTT
AGGCCGTAGATTAGGTTTTGAATTATCAACAGCTAAAGAATCACGTATTGAACGTGGTTATTTAGAACGTGAT
AAAGAAGATGAACCATTAAATCGTTTGTTTAATACAAGCCCAGTATTTTCACAAATTCCAGGACCAAATCATG
TAGAAAGCAGATATTTAACTGAAGATATTGCATATGGTTTAGTATTATGGTCAAGCTTAGGTCGTGTTATTGA
TGTACCGACACCAAATATAGATGCAGTAATTGTCATTGCATCGACAATCTTAGAAAGAGACTTCTTTGAGGAA
GGCTTAACAGTTGAATCCATTGGCTTAGATAAGCTTGATTTAGAAAAATATTTAAAATAAATGATGGCTTGAA
GATAGAAAAGGATATAGCATTATGTAAAAGCAATAAATTGAAGAAAAGAGGTTTCTCATCAATAAGCGAAGGG
GACGATAGATGATGACAAGAAAACCAACCTTTTTAGAATCAATTTCGACAATGATTGTAATGGTTATTGTTGT
TGTAACAGGCTTTGTGTTTTTTGATATTCCAATTCAAGTATTATTAATTATTGCCTCAGCATATGCCACATGG
ATTGCAAAACGTGTCGGCTTAACATGGCAAGATTTAGAAAAAGGCATTGCAGAACGTTTAAATACTGCAATGC
CTGCAATTTTAATTATACTAGCGGTAGGAATTATAGTAGGCAGTTGGATGTTTTCTGGCACAGTGCCAGCGTT
GATTTATTATGGCTTAGATTTATTAAATCCAAGCTATTTTTTAATATCAGCCTTTTTTATAAGTGCTGTTACA
TCTGTAGCAACTGGTACAGCATGGGGCTCTGCATCAACTGCAGGGATTGCACTTATTTCTATTGGTAATCAAT
TGGGGATTCCTCCAGGAATGGCTGCGGGTGCTATTATAGCAGGCGCTGTATTTGGAGATAAAATGTCACCATT
ATCAGATACAACTAATTTAGCGGCACTTGTTACTAAAGTTAATATATTTAAACATATACATTCGATGATGTGG
ACGACGATACCTGCGTCAATCATAGGTTTATTAGTATGGTTTATTGCTGGATTTCAATTTAAAGGGCATTCAA
ATGATAAACAGATTCAAACTTTGTTATCAGAGCTTGCACAGATTTATCAAATTAACATATGGGTCTGGGTTCC
CTTAATTGTGATCATTGTTTGTTTGCTATTTAAAATGGCTACAGTGCCAGCTATGCTAATATCAAGCTTTTCT
GCCATTATAGTGGGTACTTTTAATCATCATTTCAAAATGACAGATGGTTTCAAAGCAACATTTAGTGGCTTTA
ACGACTCAATGATACATCAGTCTCATATTTCATCCAGTGTGAAAAGCTTGTTAGAGCAGGGTGGTATGATGAG
TATGACTCAAATATTAGTAACGATATTTTGCGGATATGCATTTGCAGGTATTGTAGAAAAAGCAGGATGTTTA
GAAGTCTTATTAACTACTATTTCTAAAGGCATCCATTCTGTAGGAAGTTTAATATGTGTTACTGTTATTTGTT
GTATTGCGCTTGTATTCGCTGCAGGTGTTGCTTCGATTGTAATTATTATGGTCGGTGTGTTAATGAAAGATTT
GTTCGAAAAATACCAAGTCTCTCGTTCCGTTTTATCAAGAACACTTGAAGACTCAAGTACGATGGTCTTACCT
TTAATACCATGGGGAACATCAGGTATTTACTATACGAATCAACTTCATGTCTCTGTTGGAGAATTTTTCATAT
GGACAGTACCATGTTATTTATGCGCAATTATAGCAATTATCTATGGTTTTACAGGGATAGGTATTAAAAAGTC
ATCGAATTCACGTTTAACTTAATGTGAGCGTGGAATATATATAATATGTTGAAACACTTTAATCATTTATAAT
TGTAGCGGTTATAATTTGAAAAGGTTTTAACTTAGAATAAATATCCTCTATACATATACTGAATATGTTTTGT
AGCGGAACATGTTGATATATGTAATGTAAGTTTTATGTCATGATTTGTAATGACTAAATTAATTGAGAATTTG
AAGGCAAGTATATTTGTAAGTACTTTAACTAAAAATTTATCAATGTATAGCCGATTTGACGTGCCTAAATTTG
GGTGTGTCAATGGCTGTATGTTGTTTATTCTTTATTACAGAGTGAATCGGATTGGTGAAAATCGAAATTTTGA
GATTTTTACCAATTCGATTTTTTTCATAGAAATTAAAAAAGCCAACAAGGCTCTTGAAACCTTGTTGGCGTAA
AACTTAGTCATCACTAATTAGTGAATGAAGTTATAACCAGCAGCTTGGCTAGCTGAGATTGTACGTGAAGTTA
CAACACCTGGGCCATAACCATAGTTCATTTCTGAAACTCTTACTGAACCATTGCTGTTAACACTTTCAACGTA
TGCAACGTGACCGTATGCACCTTGAGTTGTTTGCATAATTGCACCAGCTTTTGGTGTATTGTTCACTGTGTAA
CCAGCTCTTGCAGCTGCGTTAGCCCAGTTACTTGCATTGCCCCAAGTTGAACCGATTTTACCACCTACACGAT
CAAATACGTAGTATGTACATTGACCAGAAGTGTATAAGTTACGTCCTGAAGTATAACCACTTGAGATTGAACG
GCCATTTGATGATGGAGCCATAGTTGTAGTTACTTGAACATTGTTGCTTGAAGTGCTGTAGCTTGCACCTAAA
CCACCAGTACGGTAGCTGTTTGTGTTGTAACTATTATAGTTATTGTAGTTATATGATTGATTATTATTTGAGT
AGTTGTTGTAACGGCTGTAGTTATTGTAGCTATAACCGTTGTTGTAATTGTTATAGTTATAGTTATTGTAACC
ATTGTAGTAGTAATAGCTGTAGTAGCCATTATCTTGGTTTAATTGACTTGGATGCCAGTTACCTTTCCATGTG
TAATGGTAGTTACCTTGTGCATCAATAGTGTAAGTATAGCTATATGATGTTGGGTCATTTGGATTATAACCGT
AGTTATCTTGCTCAGAAGCATGAGCTTGATTTCCTGATGCAATTGCGATTGTAGCGAATCCTGCAGTTGCGAT
AGTAGCTGTAGCGATTTTCTTCATTTTAAAAATATCCTCCTAAAAATTTAAATCTAAAATATTTTCGTAATG
TCCGTGTGACAAAATTAATGTTATAAGTTATCTCTCGTAATTAAACGACAAGAAAGACTATAACAGAAATTAG

CGTCCTTGTGTGCTTTGTTAACGTTTTGTAATTTTTTGCTAATATCTTGACACAATAGAATTTTAAAAGTATA
GAAATTTGCATTTTGCAAAACTTATAACTACGGCATTCTTTGTGAAAACTGAATGTTTCGAAAATAAGTCTGT
TACAAATTTGTAATATTACTGAAAATTCTAAATGTATATTTTGTGCATAATATAGGACTTTTAATCAGAAAAT
GCATAAAAAATTCGACACGAACGATCAAGTTTCTTTGATTATGAAAGCTTTGGTATTTGGATAAAAATGTATAT
TTAATGGATAAAATATCCTAATTTAGCATAAAAAAATGTTTTAATAAAAGTATTATTTGATATAATCGATTTA
TGTTTTGTTACTGCTAAAAAACATGTGGCGATATAATTGGTAGTGAATATTAGATGTTGACAATTGATTTGAG
AAAGACAAACAGGAGTAAATCAATATATTTGTATATGTGAGGATGTAAGATGACAGATAATTATTCACTACAT
ATTTTTAAAAGTATGAAAATGAGTAGTGTGAAGGAAGCAAACTTAAAAATTATATATTGGCTACAAGGTACAG
GTGTAGTTTCAATCAATCTACAACAATATGATGTAAAAAGAAATGATGTCACCGTTATTATGTTGAATGATTT
GTATCAGATTGATGGTCATGATGATAGTGTATGTTGTGTTGTTGAAGTAAGTGCAAAGACATTTTTGAAGTTT
ATGAATGCAAACTATATGATGCGTGGCAAGATATTAACAAATGAGGTTGCTAGCACTTTTAGAATCTTGATTA
AGTATTTAATTTACAGCAAAATTAAACAACAGCCTTATAATGCTAATGATAAGATTATTAATTACATGTGTGT
TGAATTAATGTCATTAAATACATACGATGGTAGTCGTCATTTAGTAGCCGAAGAAGTGCACGATTATTTAACA
AACAACCACCATAAAAAGATAAATAGGAAAGATGTCATTAATCAAGTTAGTATTACAAACAAAGCTTTAAGTG
ACATGTTTAAAGCGACACCTTATAGTAATTTTATTCAATATTTAAATCATATTAGACTGGAGTATTGCTTAAT
TGATATTTTAACTTCTAAGAAGCCCATTGAAGAAATCGCAAGTAGTCATGGTTTTAATCATTATTCTCGTTTT
ATACACCTCTTTAAAGAAACGTATGGTGACACTCCTAAATTAATTAGAAAAAGATATAGTCCAACATCTCATT
CAATTTACCATTCCCAATTAGTTGAAATTGATAAAAATATAATTGAATTATTTGATGATACTAATCAAAGTTC
TTCAAGTATGCGCGAGATTGATATACCTTTTGAGCCTACCAAAAGGAGTCAAATGTATCAACCTAAAAACATC
TATATTAAAGGAAGTAATTTTTATTGGATGGATTATTATACTATTAAACAAATCATTCAAAGATTAAGTGTTA
ATCCTGAAAACTTGCATATAATTGTTACAATCGACACAAGTAATGAGCTATCGATTGAACAAATCACTTTATT
ACTTCAAAAGATAGTGAATTACAATGTAAATGTAGTATTTCGTATTAAACATGAATATAAGGAATTGTTAACG
AGTACTGAAAGAGGCAAGATAGAAAAGCTAGTAGCTACAATTTTTAATATGACTTTTGAGAGTAATAGATGTA
AGATTGGATTTTTAATTAACGACTTAAATACTACTGCAATTCAAAAACTGAAACGTTTAATAGATAATTATAT
AGGAGTATTTGAATTAATATATCATCTGGAACCAAATGAGTTGAATGTGCGCTATTATAAAGAAATTGATCAT
CTAATAGACTATTTTATCTTACCGCTAAGACAAATAGAACAGGTGTCTATTGCACGTTCTAAAATTATATGTG
ATGTAGATTTGATTAATGAGAATGATGACTTAAAAGAAAAAATCACAATGTCTGATATTAATAAGATTACTGA
ACTTTTGACAAATTATACAAAATTTCGAGGGCTTATTATGCACATGTCAATTATAAGTGAAGCGAGCAAAGAA
ATAAATTTGACTAGATTATCAATATTCATTTTTATCATAAATATACTTAATCAATTACGAGGGATTGTTATTT
ATCAAAATGACACGATGATTGTAACGAAGTTCAAACATGAAGTACAATGTGTCATTTGTTTGCCAGCCGCATT
GTTAAATGAGAATTCAGACGCTATACGTTTATCGTGTAAGAGTCTTCAACAATTTAGCCATGCTAAAACTAAA
CAAATTGTTTTAAGTATAGATAAAGATAATCATACTTCCGAAAATGGTGTACTTGTGAATGATATTATGCAAC
AACACAGTTATTGGAAAGCTAATTTTATAGAAAGAAATATTTATGATCATTCATTAATGGTCTCAAGCAATAG
TATTGTTCATTTGAAATATCATCTTGAGATATAGGCATGGTTTAAGAGAGGTTTTAAAGATGTCTAAATTTAA
AGTTAAGCGTTTAACTGCACACATACTAATGTTGAAAGTCATTAATGATATGTTGAAATATTCATTTCATCTC
ACTTTGACTCAAGTGAAACTATTAAATCTATTAGTTAAATATGATAATTCTGATTCCAATAGCGCTTCAATAG
ATAGGTTATTAAAAATGAAAGAAATCCAATCTAAGATGGCACTTCTTCAAATGTTAAGTTATTTAAACAATCA
TCAATGGCTATTGAAAGGAAGAGATCAAAGGGATCAACGTAAATTGACTATTTCGATTAATAAAATGCATATA
GATAAAATAGAATATATGAATAATGAGCTTAGTGACTATATTGAACGTTATTTTGGTCAATATACATTTGATT
TTAGTTGTGACTATGTATCGTATTTGTTGTCGTCACAAGAATTATTTCGAAATCTAAAGTGTTATTTAAATAT
GTGTCAATTATCATTAGAAGAGTTATACGTTTAGGAATATTAAATCTGCATAAAGGTCAATTGACGGTTAAA
GAATTACAGGGTGAATTCCATCACCCGATATTTGCGGTTAGTCCGATTTTAAAGTGTTTAATTTTGAAAGGAT
TAGTTAAAAAAGAGAGATGTGAATTAGATGAAAGGCGTGTTATTGTAACAATCAAGAGAGAAAAGTTTTCTAA
AGTCACTATGCTTATTCAAGCATGTTATAATTATCTTGAAAAGGGAATACAAGTTAAGCTTAATAATAAATAG
TTAATCACTTGTATTTCATATACCTATGAAGAATAGTTTATAGTTTTAAGTGGGAGGGATATTATGATTTTAG
ATAATGTGAATCCAAATGATTTATTTCCAACTGAAAAAAAGGGTCCGTCAGTGCTAGGAATAATTGAGTATCA
AGTTCAAGGTGAGAATGAATTTGAAGGGGCATTTATAGCAACAAATGAAAGATTAATTATGAATGTAGATATG
AATGGCCAATTTTATTATAGAAGTATTAGTTATAATGAAATAGAGAAGATAGATTATGATGGTCAAACTATAA
TGTTCAAATTTAATATTGGTAATGTCCCAATGCATGATATTAAAAGTGATAACGTTGAAATGTTTGTTGAATA
TGTAAAACAACATATGATAGTATAATGTCAATGTATGTAGGTTGATAAGAAGAGTTTGAATGATAACTCATTG
CATTTAAAGATAAATGGCATGCGTTATGATTTAAAGCTCTTCTTTTTTTATATTTTATAAATTTCGTGAGCAA
GCCATCAAATTAATATAAAAAATATCAAATCAGACACGTTGATATAGATTAAAATTTTAAAAGTTTACATATC
AGTTAAGATACAAAATATTCAGACTAATAATTTTCAATTTGGCAAAATATCTTAAACATCAAATTATTGTAAG
AAATAAATGTATTTAACCATATTCTAGTAATAAAAATATTGAATTTTTATACTTATTTGTTTAGAATGAACTT
TATAACATAGTTGGATAGAGTTTCGATTTAATAAATTACATGTGAACCTTGCTACAACAAGATGTGCATCAGA
GGAGTGGTTTAATAATGAGTAAAATTAATGACATTAATGATTTAGTCAACGCAACATTTCAAGTTAAGAAGTT
TTTCAGAGATACAAAAAAGAAGTTCAATTTGAACTATGAAGAAATTTATATTTAAATCATATTTTAAGAAGT
GAGTCTAACGAAATCTCATCTAAAGAGATTGCTAAGTGCTCAGAGTTCAAACCTTACTATTTAACTAAAGCTT

TACAAAAGCTAAAAGATTTAAAATTGTTATCAAAGAAAAGAAGTTTACAAGACGAAAGAACAGTTATTGTTTA
TGTTACAGATACACAAAAAGCAAATATTCAAAAACTGATTTCAGAATTAGAAGAATACATTAAAAATTAAATC
AAGGTTAATTGCGTTTAATAACATTGAACGATAACAATTTATTAAAACGAAGTTATTTATTCAGCATTGGGAC
ATAAAATTAACTTAAAATTTAAATATTGAAGATGCTTTAATTAAAGTTAAAGACTAGCCATACCTTATTTCAG
CTTATTAAGCTTGAAACAAGGTACACTAGTCTTTTTATTTTAATATTTTCTTAGAAAATCAAGTTTACGATCA
TAAATATTTTCTGCGATATAGCTTTGGATGGTTCCAAGTATTTTCTCTATAATTTGTGTGCGATAAGCAAAAA
TTCTAACTGCAAAACCATGTGTAGGCAATTGAGAAATAGCAACACGACAATCGAATGTATTGCTATAAGAACT
AATGGTTTCATAAACTGAATCGATTAGTTTTTGGTTAACATCTTCATGTATGAAGTAAGCAGAGCCATAATGA
GAGTAATGTTCCATATAACCTATTTCATTGATCGATTGTTTATTAGGATTCAATAATAAATTATCATATGTGA
CTAACTCATCTTCAATATAAATTTCATTTATTAAATGCATATATTGATACTTGAAGGCTTCGCCAGTTTTTGA
ATAACCAGGAGTTAAAATATCAGTATAAAATAATGAACTAGAATTATTGAGATTGAACGTATTGTGTTGATAA
AATTTAGCATTTTCATATGCGATGATTGGATCAGCGACATATTCTAAATATGCGTTATCTTTCAAATTAAAAG
TTTGATACTGCTCAACATGATTGGAAGGTGTCTTGTATATTTTTGTTGCACCTTGAGATGTCAATGTCACTTT
AGCGTTATCTTCGACATTCACATTCATACGGTAACGATCTCCATCTAAATAGCCACCACCTACATTTACTATA
TAAAATGTAGGAATGGTAGATTGATTTAGATAAACTGGACGTATCACTTTAAGTGCTTTTTCAAAGAAAATAT
CTCTTGATACTGATCGATTGCCATCGAAAAACACTGTTAAATCAAGTTGCCCAGTCCATTGTTGTTCATCCAT
TATGATAATCCTTTGAGTAAAGTGTCGCGTTCAATCCAATCGATAACTTCATCCAAACCTTCGTCAGTTTTTA
AGTTAGTAAAAGTAAATGGACGTTTACCACGAAATACTTTAGTATCTTCAGCCATTTGTTCTAATGATGCACC
TACATAGGGAGCTAAATCAGTTTTGTTAATTACAAAGAAATCTGACTTAATCATACCTTGACCACCTTTACGT
GGAATCTTTTCACCTTGAGCAACATCGATAATATAAATTGAAAAGTCAACAAGTTCTGGACTAAATGTTGCTG
CTAAGTTATCGCCACCAGATTCTATGAAAATTAGTTCTATATCGTCATGACGTTCTAATAATTCGTCTATTGC
TGCAAAGTTCATAGATGCATCTTCACGAATCGCAGTATGAGGACATCCACCAGTTTCAACACCAATGATACGA
CTTTCAGGTAGAACTCCTGAATTTACTAATATCTTTTCGTCTTCTTTTGTATATATATCATTTGTAATAACGC
CGATACTCATTTCTTTTGAAAGACGTTTTACAACTTTTTCAATTAATTGTGTTTTACCTGCACCTACAGGACC
ACCAATACCAATTTTAATCGGGTTTGCCACAATTATAACCTCCTATGATATGAAAATTCTAACATTGACGTTC
TCATGCGCCATTTGATTTAGTTCTAAACCAGGCGCTGTCATGCCAAAATCTGCTTCTTTTAATTCGAAAATCT
GCTTTCTTGTTTCTTCTATATAAGGAATCATGTGAGTAACTATCTTTTGACCAGCAGTTTGTCCAAGTGGAAT
AGCACGAACAGCATTTTGAGTTAAACTTGAAACATTTTGATATAAATAGTAATCAATAATCGTTTCAATATCT
ACACCTAAATAATGGCCTAGCATAGTAAAACAAATAGCTGGATTTAACTTTGCTTTCTTATCTTGCATTTGTT
GATGATACCAAGCAATCCATGGGCTATTATAAAGTTCTAAAGCCAATTTAACCATGCGAGTCCCCATTTGTTT
TGCACCAACACGTGTTTCTTTAGGTAAGTTTTGAACAAACATCAGCTTATCTATGTGTAATACTTTTTGTGTA
TCATCATTTTCCAATGCATCATAAACTAAACGCATGGCTAAACCATCAGAATAGGTAAGTTGCTCTTGTAAAA
ACATTTTTAACCAAGCAATAAAAGTATGATCATCATGAATTATATTTCGTTGAATATATGTTTCAAGACCAAA
TGAATGACTGAAAGCACCTGTTGGAAACTGTGAATCACAGAACTGAAATAATCTTAAGTGTGTATGATCAATC
ATGAGAATGGCCTATATGTCTGAAAGCCTTATTAACTTTACGGTCTTCTCGAACATATGGGATGCCTAAACTT
TTTAATAAATCTTCAACTAAATAATCATATTGTACTAGCATTTCAGTCTCTGTAAATTGTGCTGGCAAATGAC
GATTTCCTAATTGATGGGCTATATCTCCCATTTCTTGCAATGTTCTTGGTTGAATCACTAAAAGATCTTCTGA
ATTAACATCCACAATAATCATATTATGGTCATCTGCGTATAAAATATCTCCATATTGTAAGTCAATAGGTTGT
TTTAAACGAATGCCTATTTCAGTGCCATGGTCTGTAACGACTCTTTGAATACGTTTAACGAGATCTGAATTTT
CAAGGTATACTTTTTCGACGTGCTTTTGTTTTTCTGAATTTGACAAATTGGCAATATTGCCTTGGATTTCTTC
AACAATCATTCTATGTTCCTCCTAGAATAAGAAGTATCTTTGAGTTAATGGTAACTCAGTTGCTGCATTACTT
GTAATTTTTTCTCCATCTACATATACTTCATATGTTTGTGGATCAACGTCTAATTTAGGTGTTGCGCTGTTAT
TTTTCATATCTGCTTTAGATAATTGTCTAATATTTTTAACTGGACGCACCATGCGTTTTAAATTTAATGCACG
ATTGATACCATTTTCATAAGCAGTTTTAGACACGAATGTCATTGACGTACTTGTAAGGTTTCCGCCGTATTGA
CCATACATTTTACGGTACTTCATTGGTTCAGATGTAGGGATAGAACCATTTGCATCGCCATTTACGGCAGAGT
TAATTAATCCGCCCTTTACAACTAATTCAGGTTTAACACCAAAGAAAATGGGATCCCATAAGACAATGTCAGC
TAGTTTGCCCGGCTCGATAGATCCTACATATTCAGAAATACCATGTGTAATTGCTGGGTTAATTGTATATTTA
GCGATATAACGTTTGATGCGATTATTATCATTATGTTCAAAATCACCATCTAAAGGACCACGTTGTTCTTTCA
TGCGATGTGCTACTTGCCATGTTCGTGTAATTACTTCACCTACACGGCCCATTGCCTGTGAATCGGAACTAAT
CATACTGAATACACCCATATCTTGCAGGACATCTTCTGCTGCAATCGTTTCTTTACGAATACGTGAATCTGCG
AATGCGATATCTTCAGGAATAGCCGCATTTAAATGGTGAGTAATCATTACCATATCTAAATGTTCATCTACAG
TATTATGTGTATATGGCAATGTTGGATTTGTAGATGAAGGTAAAATATTTGAAAATGCAGCGGATTTAATTAA
ATCAGGCGCATGACCGCCACCAGCACCTTCAGTATGGTACATATGAAGTACACGGTCTTTAACAGCAGCCATT
GTGTCTTCCATAAATCCTGCTTCATTTAAAGTATCTGCATGTAATGCAATTTGAATATCAAATTCATCAGCAA
CATCTAATGCATGACTCAAAGCAGATGGTGTTGCACCCCAGTCTTCATGTACTTTTAATCCAATTGCTCCGGC
ATTGATTTGTTCAATGAGTGCAGTTGGATTTGTTGCTTGTCCTTTACCTGTAAAACCGACATTAATCGGTAAA
CCTTCGGCAGCTTCTAACATTCTATGAATATGCCATGGACCTGGAGTTACAGTTGTTGCTTTAGAACCTTCTG
AAGCACCAGTACCACCACCAATATGAGTCGTAATACCACTTTCTAATGCGACCTCTGCTTGTTCAGGATTAAT

AAAATGAACATGAGTATCAATACCACCAGCAGTGACGATTTTACCTTCAGCGGCAATGATATCTGTTGTTGAT
CCTATAATAATGTCGACATTATCCATTATATCCGGGTTGCCGGCATTTCCAATGGCGAAAATATAACCATTTT
TAATACCTATATCGGCTTTAACCACTTTATCGTAATCGATAATAACGGCATTAGAAATAACAAGGTCTGCAAC
GTTCACGTCATCACGTGTTACACGAGGATTTTGCGCCATACCGTCTCTAATAGATTTTCCACCGCCAAAGGTA
GCTTCTTCACCATAAACTGCATAGTCTTTTTCTATTTGAGCAAATAGGTTCGTATCACCTAACGAATAGAATC
TCCAACAGTAGGACCGTATAAGCTCGTATATTGATTTTGCGTCATTTTAAAGCTCATGATCTTTTTCCTCCTT
TTTTATTCACGTTTTCAGCACCGTTATCTCCGAATACACCTGCATATTCATCATTTTCATCAGTCGGGCGATA
GACACGTGACTCATCGATAGGACCATTGACCATACCACGAAAACCAAAAATTTTACGTTTGCCAGCATATTCA
ACTAATTGAACTTCTTTTTTATCCCCAGGTTCAAATCGAACAGCTGCTCCAGCTGGAATATCTAAATGTTTTC
CATATGCCATTTCACGTTCGAAATCTAATGCTGCATTTGCTTCATAAAAAATGAAAATGTGAGCCCACTTGAAT
TGGTCGATCTCCTGTATTTTCAACTTCGATAACTGTTTCAGGATGATGGTTATTAATTTCAATCTCTGTACTT
TTTGTAATAATTTCTCCTGGTATCATTTGACTGCCTCCTTTAAACAATAGGGTGATGTACTGTGATTAACTTA
GTACCATCGGGGAACGTAGCCTCGATTTCGATATCTGTAATCATGTGTTCGACACCATCCATGACATCTTCTT
TGTTTAGAATTTGTCTACCATAACTCATTAACTCTGCAACGGTCTTACCATCGCGTGCACCTTCTAATAATTC
ATCGCTGATTAAAGCTAATGCCTCAGGATGATTTAGTTTCAAACCACGTGCTTTACGACGACGTGCAACTTCC
GCTGCCACTACAATCATTAATTTGTCTTGCTCTCGTTGTGTAAAATGCAAATTAAAACCCCCAATTTCATATT
AGATACAATTTACAAAATTTATATTAATCCTAATTGTTGTGATAAATAAGTAATATACAAAGTTTAATGCGTA
ACAGTAAAAAATTAATTTACTATTTCGTATTTAAAAATATATGATTACAATTACAACAAATAGCAAAAGATAT
TTAGGATTTAGAAAATAGAGTTTTTAATATAACTTTTTGTAAAATGGTAGTATATTAATGAGTAAACATTCAA
GAAAAAGGGTGGGCATTTTGAAAATGATAGATGTACTTTTGAAAAATATTTCTCAGGTTGTGTTAATTAGTAA
TAAATGGACAGGATTATTTATCTTAATAGGATTATTTGTTGCTGATTGGACAATTGGATTAGCGGCTATTGTA
GGTAGCATCATCGCCTATACTTTTGCGCGTTTTATAAATTATAGTGAAGCAGAGATTAATGATGGGTTAGCCG
GATTTAATCCAGTGTTAACTGCCATTGCGTTAACAATCTTTTTAGATAAGTCAGGATTAGATATTGTTATAAC
AATGATAGCAACTTTATTAACGTTACCAGTCGCTGCTGCAGTGAGAGAAGTTTTAAGACCATATAAAGTTCCG
ATGCTGACGATGCCTTTTGTCATTGTGACTTGGTTTACAATTTTACTATCAGGACAGGTTAAATTTGTAGATA
CATCTTTAAAGTTAATGCCTCAAAACATTGAAACGGTTAATTTTAGCAACAATGATAGAATACATTTCATTCA
GTCATTATTTGAAGGATTCAGTCAAGTATTTATCGAAGCGAGTGTAATTGGTGGCGTATGTATTTTAATCGGC
ATATTGATAGCATCAAGAAAAGCAGCACTCTTAGCTGTTATAGCTAGTTTGTTAAGCTTTATCATTGTAGCTC
TATTAGGTGGTAATTATGATGATATTAATCAGGGGTTATTCGGTTATAACTTTGTATTAATGGCAATCGCACT
AGGATTTACATTTAAAACAGAGATTAATCCTTATATTTCGACTTTTTTAGGTGTGTTATTAACAGTAGTGGTG
CAACTAGGTACAACAACATTGCTTGAACCGTTTGGCTTACCTGCATTAACATTGCCATTTATTATTGTGACTT
GGATTTTATTATTTGCTGGTATTAAACATGACAAAGTAGATGCTTGAAAGTTAAATCAAACCTAGTATTGTTT
GAATATCACCTTAAACCATACAGCGAGTTGTATAGTTTAAGGTGTATTTTTATGGAGAAAATTAATTGGATAC
TTAATCGAATTATCAACTTTCTTTTAATTGTTCACAGCACGTTCCTTTTTATTTTTAAAGTCGAGTGTTAAAC
TAGGAATAAATAATTTATATTGTGTGTTGTGTGGGGTGACTAATATGAATGATATGGATAATTCCTTTTTAAT
AACAACGGAAATTCAAAGAAAATGGATTGAAAAATTCAAAGTAATTGGAGATACATTTAAGGCTAAAGCTGAA
TATAATGATCAACATAGCCAATTTCCATATAAAAAATATTGAATGGTTAATTAAAGAAGGTTATGGAAAATTAA
CGTTACCAAAAGAATATGGTGGTGAAGGTGCGACCATAGAAGACATGGTTATTTTGCAATCATTTTTAGGCGA
ACTTGATGGTGCCACAGCATTATCTATTGGTTGGCATGTGAGTGTCGTAGGACAAATTTATGAACAGAAATTA
TGGTCTCAAGATATGTTGGAGCAATTTGCTGTTGAAATTAATAATGGTGCATTAGTTAATAGAGCAGTTAGTG
AAGCTGAAATGGGTAGTCCAACAAGAGGGGGAAGACCAAGTACACATGCTGTTAAAGCTGATGACGGGTATAT
TTTAAATGGTGTGAAGACATATACATCAATGAGTAAAGCACTAACACATATTATTGTTGCTGCTTATATAGAA
GAATTAGAAAGTGTTGGTTTTTTCTTAGTGGACAGGAATTTGCCTGGTGTTGAAATTGCCGATAATTGGGACG
TATTAGGTATGCGAGCGACAGAAAGTCATGATTTAATATTAAATGATGTAAAAGTACCATTAAAACATTTAGT
GGAAACAGAGAAAAGTAAAGCGCCAAATGGTTGGATATTGCATATACCAAGCTGTTATTTAGGTATAGCACAA
GCTGCTAGAAATTATGCTGTAGACTTTGCAATCCAACATAGCCCTAATAGTATTGAAGGAACGATTGCAACAT
TGCCAACTGTGCAACAAAATTTAGGGAAAATGGAAACGCTGTTGTTATGTGCGAGACAATTTTTATGGAGTAC
AGCAAAAGGCTATCAACAATATACAGACGAAAGTCAGATTAGAAATGCGACGAGTGCGAGTAAAGTAATGGTA
ATGAACCAAGGCCTTGAAGTGATTGATTTAGCGATGCGCATTGTTGGTGCTAAAAGCTTAGAAATGAATCGAC
CATTACAACGTTACTATCGTGACATGCGTGCAGGATTACACAATCCACCTATGGAAGATGCAGCATATACAAA
TATTGCTAAGAGTATTACAGATACATTTTAAATTTAAAAAGGTAAATCAATCGTTGAATGGCATAAAGAGATG
AAATTTGACGTTCGGATTTACCTTTTTTGTGGGAATTTATAGGTATATTAAGATATTTTGCTAAATTTTTTGC
GAAAATAAAAAAATAAATAACACTAGCTATTGTAAATGTATATGCAATCTTGTATAATTTTGGAATGATTGAT
AATCATTTTCAATAGGAGGAAATTATGAAAAAACTATTATTACCATTAATAATTATGTTATTAGTGTTAGCTG
CGTGTGGGAACCAAGGAGAAAAAAATAACAAAGCTGAAACTAAATCTTATAAAATGGACGATGGCAAAACGGT
AGATATTCCGAAAGACCCTAAACGCATTGCAGTAGTTGCGCCAACATATGCTGGTGGACTTAAAAAATTAGGT
GCAAACATTGTAGCTGTAAATCAACAAGTCGATCAAAGCAAAGTATTAAAAGATAAATTTAAAGGTGTTACAA
AAATTGGTGATGGCGATGTAGAAAAAGTTGCTAAAGAAAAGCCAGATTTAATTATTGTATACTCTACTGACAA

AGACATTAAAAAATATCAAAAAGTAGCACCAACAGTAGTTGTTGACTATAATAAGCATAAATATTTAGAACAA
CAAGAAATGTTAGGGAAAATTGTTGGTAAAGAAGATAAAGTAAAAGCTTGGAAGAAAGATTGGGAAGAAACAA
CTGCTAAAGACGGTAAAGAAATTAAAAAAGCAATTGGACAAGATGCAACAGTGTCATTGTTTGATGAATTTGA
TAAAAAATTATACACTTACGGCGATAACTGGGGTCGTGGTGGAGAAGTATTATATCAAGCATTTGGTTTAAAA
ATGCAACCAGAACAACAAAAGTTAACTGCAAAAGCAGGTTGGGCTGAAGTGAAACAAGAAGAAATTGAAAAAT
ATGCTGGTGATTACATTGTGAGTACAAGTGAAGGTAAACCTACACCAGGATATGAATCAACAAACATGTGGAA
GAATTTGAAAGCTACTAAAGAAGGACATATTGTTAAAGTTGATGCTGGTACATACTGGTACAACGATCCTTAT
ACATTAGATTTCATGCGTAAAGATTTAAAAGAAAAATTAATTAAAGCTGCAAAATAATTCAACTATATAAGTT
AGTGAAATGAGAGTCTGAAACATATCAATCTTTTGATATTGTATTAGGCTCTTGTTTTTATAGCTAGAAAGTT
AGATATTTGTATTTTTTAAAATAATAAGTGCCGTTGTTATCATTCAATTTAATTAATGATAGATTAGTATTAT
TACAGCTAAAGTAGTATACCTGAGAAATAGCTCAATGTATCTCTTCATTAATAAGTTATATCATAATTATTTT
AGTGCATACTTTATGGAAGGCATATCAGGGAATGATTATCAATTAAAGAAGAGGTTTAAAAGGATTACAACAG
AATGTTATGATTTTGTAGAAAGATATATAACAACGTTTTATAAAAACATAATATTGTTAATGGAAAATGAAAT
GTAAGGGGGATTTCGAGTGACTAAGAAAGTTTATTTTAACCATGATGGTGGTGTAGATGATTTAGTATCTCTA
TTTTTATTATTACAAATGGAAAACGTTCAATTGATAGGGGTCAGTACAATTGGTGCTGATTGTTATTTAGAGC
CATCTTTGAGCGCATCAGTAAAAATTATTAACCGTTTTTCAAATGAAGATATTCAAGTTTCCCCATCATATGA
ACGAGAGAAAAATCCATTTCCTAAAGAATGGCGTATGCATGCCTTTTTTATGGACGCATTGCCAATTTTAAAT
GAGCCAGTCAAACACGTTGCTTCAAATGTGAGCGACAAAGAAGCCTTTGAAGACATTATTCAAACTTTAAAGA
GACAATCAGAAAAAGTAACATTATTATTTACAGGCCCGCTTACAGATTTAGCAAAAGCACTACAAAAAGATTC
ATCTATCGTTCAGTATATAGAAAAATTAGTTTGGATGGGTGGTACCTTTTTACCAAAAGGAAATGTTGAAGAA
CCTGAGCATGATGGTTCTGCAGAATGGAATGCATATTGGGATCCAGAAGCGGTTAAAATTGTTTTTGATAGCG
ATATAGAGATTGATATGGTTGCTTTAGAAAGTACGAATCAAGTGCCGCTAACGTTAGATGTTAGACAAAGATG
GGCGAATGAACGCCAATATACGGGCATTGATTTTTTAGGAGTAAGTTATGCAGCGGTACCACCATTAACACAC
TTTATAACAAATTCTACTTACTTTTTATGGGATGTTTTAACGACTGCTTATATTGGTAACAAGGACTTAGTTC
ATTCAATTGAGAAAAAAGTGGATGTAATAAGTTATGGACCAAGTCAAGGTAAGACATTTGAGTGTAAAGATGG
GCGCAAAATTAATGTCATAAATCATGTAGATAACAACGCATTTTTTGATTATATAACTGCACTTGCTAAAAAA
GTAAATTAACAGCTGTGTAGAATAATTAAGATTTTAATTTATATAGAACAGCTTATTGTAAACTTTTCGTTTC
TTAAAGTTTACAATGGTGCTATAATAATGGTCATGAAATACGAAAGGAAGTAAAAAATGACAACAAAACAGTT
AGTATATACAGCTTTAATGACAGCGATTATCGCTATTTTAGGATTGGTACCAGTAATTCCACTACCATTTTCT
TCAGTACCAATTGTACTTCAAAACATTGGTATTTTCTTAGCAGGTGTGATTTTAGGACGTAAATATGGCACAT
TAAGTGTTATCGTCTTTTTATTATTAGTAGTTGCTGGCTTGCCACTTTTATCAGGCGGTCGCGGTGGCATTGG
TGTATTCGCAGGTCCTTCAGCAGGATTTTTACTATTATATCCAGTTGTAGCATTCATGATTGGTGCGATTCGT
GATAGATTCATCAATGAAATTAATTTCTGGATTTTATTCGTGGGTATTTTAGTTTTTGGTGTTATAGCATTAG
ATGTTATTGGTACATTGATTATGGGCATGATTATTAACATACCATTTACGAAAGCTATTTCAATTTCATTAGC
TTATTTGCCTGGTGATATATTAAAAGCAATTGTAGCAAGTTTGATTGGTACAGCTTTACTTAATCACTCGCAG
TTTCGTCAAATTATGGGATTAAAAAAATCATATTTAAGATAGTAAAGTAATTGAATAAGTTGCTTTGAAATTT
ATAAAAGTGAAAGGAGTAGGTGTCAATGGCTAGTATAAGTATGTCAGATATATATTGTAACGGCACTATATTT
GAAAATGACGATGAGCAGTTGATTTATTTAACACCTTCTTTTCCACAACGATATACAAGCAACACATGGATAT
ATAAAAAGATGCCTACCGAAGAGCGATGGCTGAAAGACTTAGAACGTCAACATCAATTACATACAAATCAAGG
TTCAAATCATTATGCGTTTAGTTTCCCAGAAAATGAACAACTTGATAATCATTGGATGGCTATGTTTAAAGAT
ATGAATTTTGAACTAGGTATTATGGAATTGTATGCCATAGAAAGTGATGCCCTTGCCAATTTGCCGCGTAACG
CTGACGTTGAAATTGCCATCGTTGACGAGTCGCATATAGATGCCTATTTAAAAGTTGCATATCAATTTAGTTT
GCCATTTGGAAAAGACTATGCAGATGCACATGAAAAAATGGTAAGGGAACATTATCAAAAAGATGTGATTAAA
CGCTTAGTAGCTTATTTAAACAATGAACCTATTGGCGTTGTAGATGTCATTGAAAGTGAAAATTACATTGAAT
TAGATGGATTTGGTGTATTAGAACAATTTCGACACCAAGGAATCGGTTCTACCATGCAGTCATTAGTAGGTGA
ATATGCTATATCTAGAGATCATAAACCGGTTATATTGGTGGCAGATGGTGAAGATACAGCAAAAGATATGTAT
GCAAAGCAAGGTTATGTCTATCAATCGTTTTGTTATCAAATATTAAAAGAAGATATTGGAAATTAACGATTGA
TGGTAGGTTTGTATTAGAATAAATGAAAAATATAAAAAGCAGTAAGCTGGTTTCGAGTAGAAATCGACTTACT
GCTTTTTAAGTTGTGATAAGTATATTAAACAATGTTGTTTTAAGGTTGTTGATTCATGTAGAATATCGTTCAT
TATGACACACTATAATAAATATGTTATTGTTCAGAATCAATGATACGTTCTGGATGACTGTATATATTAAAGC
CACCATTTCGAATAAATCCAACTGACGTAATGTTTAGGTCATTAGCTAAAGTTACAGCAAGCGTTGTTGGAGC
CGATTTAGATAAAATGACGCCAACACCAATTTTCGCGGCTTTAATTAAAATTTCTGATGAAATACGTCCACTG
AAAATTAATACTTTATCTCGGACAGTAATATGTCGCTGAATACAAAATCCATATAATTTATCTAGAGCGTTAT
GTCTACCAATGTCTTGTCGATGTACAAAAAATGTCAAACCATCGCTTATAGCAGCATTATGCAGGCCACCTGT
TTCTTGGTAAATATGACTTGCACTTTGTAATCGAGTCATCATGTTAATAATTTGTATTGGAGTTAAAGTGATT
TTAGACATAGATGTTTTAGCGATAGCAGCATCATTTTGAAAATAAAACTCACGACTCTTTCCACAACAAGATG
CAATCATTCGTTTTGTGGAATATTGAAAGCGATCGCCTAAATCTTTATTAAGTTCAACATGGGCAAATCCTTT
ACTGTCATCAATTAGTACAGATTTTAATTCATCTCGCTTTAAAATGGCACCTTCCGAAGCCAGAAATCCAATG

ACTAACTCCTCAAGGTTTGTTGGACTGCATATAACAGTCGCAAATTCTTCACCATTCACCATAATTGTAAGTG
GAAATTCTGTCACATATTGATCTGTCTTATTGAATAATTTTCCATCTTCATATCTAACAATTGGTTGACCTAA
AGATACATCTTTGTTCATTATCTAACCCCTTTAATTAGCTTGAACTTTATTTTAAAGTAATTTGCTTAAAATT
TTAACATATTTGCTTAAGTTTGAAATTTGATTGATAAAAATTAATAGCGAGCAATTATTTAGAATTTGATTGG
ATTTAGAAAAAGGCACACTAGCAGCTCAATTAATAAAAAATAATTTTTAGAAAGTGTGACAATTATTTGAATC
ATTATTTAAAGAGAAAACGCTTCTTTTCAAAAATATTGTTAAGTGTATAATGAAAATGTAAATATTTAATAAT
TGGGGGTATTTCGTAATGAAAATGAAACGTTTTATAGCTATTGTAATGGCATTATTTTTAGTATTAGCTGGTT
GTTCTAATTCTAATGATAATCAAGACAACAAAAAAGATGCAACAGACAATGGAAAAAAACAAGAAATTCAAGT
TGCAGCGGCAGCAAGTTTAACAGATGTAACCAAGAAATTAGCTTCAGAATTTAAAAAAGAGCATAAAAATGCT
GATATTAAATTTAACTATGGTGGATCAGGGGCATTAAGAAAACAAATTGAATCAGGCGCACCTGTTGACGTAT
TTATGTCTGCAAATACTAAAGATGTAGATGCATTAAAAGACAAGAATAAAGCACATAATACATATAAATATGC
GAAAAATAGTCTAGTACTAATTGGTGATAAAGATTCAAATTACACTTCAGTAAAAGACTTAAAAGGCAATGAT
AAATTAGCATTAGGTGAAGTGAAAACTGTACCAGCAGGAAAATATGCGAAACAGTATTTAGATAACAATAACT
TATTTAAAGAAGTTGAAAGTAAAATCGTTTATGCTAAAGATGTAAAACAAGTATTAAATTATGTTGAAAAGGG
TAATGCGAAACAAGGTTTTGTATATAAAACTGACTTATATAAACAAAACAAAAAAATTGATACTGTAAAAGTA
ATTAAAGAAGTAGAACTTAAGAAACCAATCACATACGAAGCTGGTGCTACATCGGATAGTAAATTAGCAAAAG
AGTGGATGGAATTCTTAAAATCAGATAAAGCTAAAGAAATATTAAAAGAATACCACTTTGCAGCATAAGGAGT
TGTAATCCATGCCTGACTTAACACCTTTTTGGATATCCATACGAGTTGCTGTAATCAGTACGATTATTGTAAC
GGTTTTAGGTATTTTTATATCTAAATGGTTGTATCGTCGTAAGGGTTCGTGGGTTAAAGTATTGGAAAGTTTA
TTGATATTACCTATTGTTTTGCCGCCAACGGTATTAGGTTTTATTCTATTAATCATCTTCTCGCCAAGAGGAC
CAATCGGTCAATTCTTTGCGAATGTACTACATTTACCTGTAGTGTTCACTTTGACAGGTGCTGTGATAGCATC
TGTCATTGTTAGTTTTCCACTAATGTATCAACATACTGTGCAAGGCTTCAGAGGTATAGACACGAAAATGATT
AATACAGCTAGAACAATGGGAGCAAGTGAAACGAAAATTTTCCTCAAATTAATTTTACCATTAGCTAAACGCT
CTATTTTAGCAGGTATAATGATGAGTTTTGCTCGTGCATTAGGTGAGTTTGGTGCTACATTAATGGTTGCAGG
ATATATTCCAAATAAAACGAATACACTACCTTTAGAAATATACTTTTTAGTGGAACAAGGTAGAGAAAATGAA
GCGTGGTTATGGGTATTAGTGCTAGTCGCATTCTCTATTGTGGTTATATCTACAATTAATTTATTGAATAAAG
ATAAATATAAGGAGGTCGACTAGATGCTTAAAATCAATGTGAAATATCAATTAAAGAACACTTTAATTCGCAT
CAATATAGATGATACTGAACCAAAAATTTATGCAGTTCGTGGTCCGTCTGGCATTGGTAAAACTACTGTTTTA
AATATGATTGCCGGATTACGTAAAGCAGATGAAGCTATTATCGAAGTGAATGGGCAATTGCTTACTGATACGG
CAAAAAACGTGAATGTTAAAATTCAACAACGACGTATTGGATATCTGTTTCAAGACTACCAATTGTTTCCTAA
TATGACGGTCTATAAAAATATTACTTTTATGGCTGAACCATCTGAACACATCGATCAATTAATCCAAACTTTA
AACATTGATCATTTGATGAAACAATATCCTATGACATTGTCAGGTGGAGAGGCACAACGTGTAGCACTTGCAC
GTGCACTTAGCACGAAACCAGATTTAATTTTATTAGATGAACCTTTTTCTAGTTTGGATGATACTACAAAAGA
TGAGAGTATTACATTAGTTAAACGTATTTTCAACGAATGGCAAATACCAATCATATTTGTGACACATTCAAAC
TATGAAGCAGAACAAATGGCTCATGAAATTATTACAATTGGGTAATCATTTATTTGCCATTAAAGAGTTTAGA
ACGTATTTAAAATTGTAGAAGTGAATGCTTCTATCAGCATTTTAATGATGTTTTAAACTCTTTTTTAGGGGCA
GTTTTTTGAGAGACATTGACGCGCGTCATATAATGAAAGTAATGATAAAAAGAAAGGATAACTTAATGTGAGT
CAAGAACGTTATTCAAGGCAAATTTTATTTAAACAAATAGGTGAAATAGGTCAAAGCAAAATAAATCAAAAAT
GTGCGTTGATTATTGGTATGGGCGCATTAGGTACACATGTGGCCGAAGGACTTGTTAGAGCAGGCATTGCTAA
ACTAATCATTGTTGATAGAGATTATATTGAATTTAGTAATTTACAAAGACAAACATTGTTTACTGAAGAAGAT
GCTTTGAAAATGATGCCTAAAGTGGTTGCAGCTAAAAAGCATTTGCTAGCGTTACGTAGTGATGTTGATATTG
ATGATTATATTGCCCATGTGGATTATTATTTTTTGGAAACACATGGACAGGACGTTGACGTTATTATTGATGC
AACCGATAACTTTGAAACACGACAACTGATTAATGATTTTGCATATAAACATCGTATACCTTGGATTTATGGT
GGTGTTGTACAGAGTACATATACAGAAGCTGCATTTATACCTGGTAAAACACCTTGCTTTAACTGTTTGGTAC
CACAATTGCCAGCATTAAATTTAACATGTGATACAGTAGGGGTCATTCAACCTGCCGTGACGATGGCAACAAG
TTTACAATTAAGAGATGCGATGAAAGTATTAACGGAACAACCAATTGACACAAAAATAACTTATGGCGATATT
TGGGAAGGTAGTCATTATTCATTTGGTTTCAGTAAAATGCAACGTTCAGACTGTACAACTTGTGGAGATGTAC
CAAGTTATCCGTATTTAAACAAGAATGAACAACGTTATGCAACATTGTGTGGTAGAGACACTGTACAGTATGA
AAATGCATCAATTACACACGACATTCTTGTTCAATTTTTAAAACAACATCAACTAAATTATCGCAGTAATTCG
TATATGGTTATGTTTGAATTTAAAGGACACCGCATTGTTGCTTTTAAAGGTGGAAGGTTTTTAATACATGGCA
TGACACGCACATCAGATGCCACACATCTAATGAATTTATTGTTTGGATAAAAAAAGATAAGACAAAAGGAGTG
TAATATTATGGGCGAACATCAAAACGTTAAATTGAATCGTACAGTTAAAGCAGCCGTACTAACGGTATCAGAT
ACTAGAGACATTGATACAGATAAAGGTGGTCAATGCGTGCGCCAACTATTACAAGCAGATGACGTTGAAGTGA
GTGACGCACATTATACAATTGTGAAAGATGAAAAAGTAGCCATCACGACGCAGGTAAAGAAGTGGTTAGAAGA
AGATATTGATGTCATCATTACGACTGGTGGAACAGGTATTGCACAACGTGATGTGACGATTGAAGCAGTAAAA
CCACTTTTAACTAAAGAGATAGAAGGCTTTGGGGAATTGTTTAGATATTTGAGTTATGTTGAAGATGTTGGCA
CGCGTGCATTATTGTCTCGTGCTGTAGCAGGTACAGTTAATAATAAATTGATATTTTCGATTCCAGGATCAAC
AGGCGCAGTTAAATTAGCATTAGAAAAGCTCATTAAACCAGAATTAAATCATCTGATTCATGAGCTTACAAAA

TAATTTATTGATTTGATTGGCGTTGAAAATCTCCAGATTTACCGCCAGACTTGCTTTCAAGGTAGGTTTCGCC
AATAATCATACCTTTATCAACTGCTTTCGTCATGTCGTAAATGGTTAAAGCCGTTGCTGATGCAGCGGTTAAA
GCTTCCATTTCAACACCGGTTTTGCCAGTTGTAGAGACAGTTGTTTGAATGTTTAAAGTATAAAGGGGTGCAT
TTGTTTCATCCCAGCTGAAGTGAACATCTATGCCAGTCAATGGCAATGGATGGCACATCGGAATAATTGTTGA
TGTATTTTTAGCAGCCATAATACCAGCGATTTGAGCAGTGTTCAATACATTGCCTTTGGTATTGGTGTTATTA
GAAATCTGCTTAAAAATAGTTTCATTCACAGTAATGCTTGAATGAGCGACAGCAGTTCTTTTTGTAATTTGTT
TGTCTGATACATCGACCATTTTGGCGTGGCCTTGTTGATTAATATGAGTAAACTCAGTCATTTTACCCCTCCT
AGTGCATCTAGTATATCATGAAAAAATAAAAGTTTTGGAGATGATTTTTAATGGTAGTAGAAAAAAGAAACCC
AATCCCAGTTAAAGAAGCAATTCAACGTATCGTTAATCAGCAGAGTTCAATGCCGGCAATTACGGTAGCACTT
GAAAAAAGTCTAAATCATATCTTAGCAGAAGATATTGTAGCTACTTATGATATACCAAGGTTTGATAAATCAC
CTTATGATGGTTTTGCAATTCGCAGTGTTGATTCACAAGGGGCAAGTGGTCAGAATCGCATTGAGTTTAAAGT
GATTGATCATATTGGTGCAGGTTCAGTTTCTGATAAATTAGTTGGGGATCACGAAGCGGTGCGTATTATGACT
GGAGCACAAATACCTAATGGCGCAGATGCTGTTGTTATGTTTGAACAAACGATTGAACTAGAAGATACATTTA
CAATTCGTAAACCATTTTCAAAAAATGAAAATATATCTTTAAAAGGTGAAGAAACAACGACAGGCGATGTTGT
TCTAAAAAAAGGACAAGTAATTAATCCAGGGGCTATTGCGGTCCTTGCAACATATGGCTATGCAGAGGTTAAA
GTTATTAAGCAACCGAGCGTCGCTGTTATTGCAACAGGAAGCGAATTATTAGATGTTAATGATGTATTAGAAG
ATGGAAAAATTCGTAACTCTAATGGTCCAATGATTCGTGCCTTAGCAGAAAAATTAGGTCTTGAAGTTGGTAT
TTACAAAACACAACAAGATGATTTAGATAGTGGTATACAAGTCGTTAAAGAAGCTATGGAAAAACATGATATA
GTTATTACAACGGGCGGAGTTTCTGTCGGAGACTTTGACTATTTACCTGAGATTTATAAGGCTGTAAAGGCGG
AAGTGTTATTTAATAAAGTAGCAATGCGTCCTGGTAGCGTAACAACGGTTGCATTTGCAGATGGAAAATATTT
GTTTGGATTATCTGGAAATCCATCAGCTTGTTTTACAGGATTTGAACTATTTGTGAAACCAGCTGTTAAACAT
ATGTGTGGCGCACTAGAAGTCTTCCCGCAAATAATTAAAGCAACATTAATGGAAGATTTTACCAAGGCAAACC
CATTCACACGATTTATACGTGCTAAAGCAACATTAACAAGTGCTGGAGCTACTGTAGTACCTTCAGGATTCAA
TAAATCAGGTGCGGTTGTAGCGATTGCACATGCTAACTGTATGGTCATGTTACCAGGAGGGTCACGTGGTTTT
AAAGCGGGGCATACAGTAGATATTATATTGACTGAATCTGACGCTGCTGAAGAGGAACTTCTTTTATGATTTT
ACAAATTGTAGGTTACAAAAAGTCTGGTAAGACAACATTGATGAGGCATATTGTCTCTTTCTTAAAGTCACAT
GGTTATACAGTTGCTACTATTAAACATCATGGGCATGGTAAGAAAGATATTCAATTACAGGATTCAGACGTCG
ATCACATGAAGCATTTTGAAGCGGGGGCAGATCAAAGTATTGTACAAGGTTTTCAATATCAGCAAACTGTAAC
ACGTGTAGATAATCAAAATCTTACTCAAATTATTGAAAAATCTGTTACAATTGACACCAATATCGTATTAGTT
GAAGGCTTTAAAAATGCTGATTTTGAAAAAGTCATAGTCTATCGAAATGAAGAAGAGTTGCAAGTATTACAAC
AATTGTCGAATGTTTGTTATAGCATTAATGTTAGGGAGCATGAAGATTTTACAGCATTTGAGCAATGGTTATT
AAATAAAATTAAAAATGATTGTGATACACAATTAACATAGAGGATTGAAATGAATGAAACAATTTGAAATCGT
GACAGAACCGATACAAACAGAACAATATCGTGAATTCACTATAAATGAATATCAAGGTGCAGTAGTTGTTTTT
ACCGGTCATGTTCGCGAATGGACTAAAGGCGTCAAAACGGAATATTTAGAATATGAAGCGTATATTCCAATGG
CTGAAAAGAAATTGGCACAAATTGGAGATGAAATAAATGAAAAATGGCCTGGAACGATAACGAGTATTGTTCA
TAGAATAGGGCCATTACAAATTTCAGATATCGCTGTATTAATTGCAATTTCTTCACCGCATCGTAAAGATGCC
TATCGAGCAAATGAATATGCAATTGAGCGTATAAAAGAAATTGTTCCGATTTGGAAAAAAGAAATTTGGGAAG
ATGGTTCAAAATGGCAAGGGCATCAAAAAGGGAATTATGAAGAAGCGAAGAGGGAGGAATAAGAGAGATGAAG
GTACTTTACTTCGCAGAAATTAAAGATATATTACAAAAAGCACAGGAAGATATTGTGCTTGAACAAGCATTGA
CTGTACAACAATTTGAAGATTTATTGTTTGAACGTTATCCGCAAATCAATAATAAAAAGTTTCAAGTTGCTGT
AAATGAGGAATTTGTACAAAAAATCAGATTTCATTCAACCTAATGATACTGTTGCATTAATTCCACCGGTTAGT
GGAGGTTAAGCGAGCATGAAAGCAATAATTCTTGCAGGTGGTCATTCAGTACGATTTGGTAAGCCCAAAGCTT
TTGCGGAAGTGAACGGTGAGACCTTTTATAGTAGAGTAATTAAGACATTAGAATCAACAAATATGTTCAATGA
AATTATTATTAGTACAAATGCGCAATTGGCAACGCAATTTCAATATCCAAATGTTGTTATAGATGATGAGAAT
CATAATGATAAAGGTCCATTAGCAGGAATTTATACAATCATGAAGCAACATCCTGAAGAAGAATTGTTTTTTG
TCGTTTCTGTTGATACACCAATGATTACTGGTAAAGCTGTAAGCACGTTGTATCAGTTTTTAGTTTCTCATCT
TATTGAAAATCATTTAGATGTCGCAGCTTTTAAAGAAGATGGACGTTTTATTCCAACAATTGCATTTTATAGT
CCGAATGCATTAGGCGCTATAACTAAAGCACTACATTCTGATAATTACAGTTTTAAAAATGTATATCATGAAT
TATCAACGGATTATTTGGATGTCAGGGATGTAGATGCGCCCTCATATTGGTACAAAAATATAAATTATCAGCA
TGATTTGGACGCTTTAATTCAAAAATTGTAAGCCGTTAGGAGGTCCACAAATGGTAGAACAAATAAAAGATAA
ACTAGGACGTCCCATCCGTGACTTACGGTTATCTGTGACAGATCGGTGTAACTTTAGGTGTGATTATTGCATG
CCTAAAGAGGTATTTGGAGATGATTTCGTATTTTTACCTAAAAATGAACTGTTAACGTTTGATGAAATGGCTA
GAATCGCTAAGGTATATGCAGAATTAGGTGTAAAAAAAAATACGCATTACAGGTGGAGAACCATTGATGCGACG
CGATTTAGATGTACTTATAGCTAAATTAAATCAAATCGATGGTATTGAAGATATTGGTTTGACTACAAATGGT
TTGTTATTAAAAAAGCATGGACAAAAGTTATATGATGCTGGGCTACGCAGAATTAATGTCAGTTTGGATGCTA
TTGATGATACGCTATTTCAATCAATCAATAATCGTAATATTAAAGCGACTACGATTTTAGAACAAATTGATTA
CGCGACGTCTATTGGTTTGAATGTAAAAGTAAATGTTGTTATACAAAAAGGTATTAACGATGATCAAATTATA
CCAATGCTTGAATATTTTAAAGATAAACATATAGAGATTCGATTTATAGAATTTATGGATGTTGGTAATGATA

ATGGATGGGATTTCAGCAAAGTTGTAACTAAAGATGAAATGCTTACTATGATAGAGCAGCACTTTGAAATCGA
TCGTGTAGAACCAAAATATTTTGGGGAAGTAGCAAAATATTATCGCCATAAGGATAATGGTGTTCAATTTGGT
TTGATTACAAGTGTTTCACAATCATTTTGTTCTACATGTACACGGGCAAGGCTGTCATCAGATGGGAAGTTTT
ACGGATGTTTATTTGCAACTGTCGATGGATTTAACGTTAAAGCGTTTATTCGTTCTGGCGTGACCGACGAAGA
GTTAAAAGAACAATTTAAAGCTCTATGGCAAATAAGAGATGATCGATATTCAGATGAGAGAACTGCTCAAACA
GTTGACAATCGCCAACGTAAAAAGATAAACATGAATTATATTGGTGGTTAATGTGTAGGAACCACTATATATT
ATATCATTAGAGATTCTTTAATATTTCTGTCTTACTCCCTAAAATACAATATTATTTATTAAAGTAAAAACGG
TCATATCTATGCCAGATTTAATAGAAATGATCGTTTTTAATGTTTTTACAAATTGGCGGGGCCCCAACACAGA
AGCAGACGAAAAGTCAGCTTACAATAATGTGCAAGTTGGGGAACGGGGCCCCAACATAGAGAATTTCAAAAAG
AAATTCTACAGACAATGCAGGTTGGGGAACAACGAAATAAATTTTATGAAAATATCATTTCTATCCCACTCTT
AAGAATCACTACATAATAAATCTTTAGTGGTTCTTTAACATTGATGTCACACCCCATGTCATTGAGTTGTAAT
ATATCTTTTTTAGGTATAAATGTTGTCGAATAAACAACAAGTTGTCCAAAATATATAAATCTAAACAAGATAT
ATCCAGCAATTTAATATTTGTAATAGATAAAATGCTAAGTTTGATATATAATAAATTTAAGTAATTGTATAAT
AATATGAATTACAAACATCTAAGAAGAAACATAGGAGGCATCATATTATGAGTAATAAAGTTCAACGTTTTAT
AGAAGCAGAAAGGGAGTTAAGTCAGTTAAAGCACTGGTTAAAAACAACACATAAGATTTCAATTGAAGAATTT
GTAGTACTTTTTAAAGTGTATGAAGCTGAAAAGATTAGCGGTAAAGAATTGAGGGATACATTACATTTTGAAA
TGCTATGGGATACAAGTAAAATCGATTTGATTATCCGTAAAATCTATAAAAAAGAGCTTATTTCTAAATTGCG
TTCTGAAACGGATGAAAGACAAGTATTCTATTTCTATAGTACTTCTCAAAAGAAATTGTTAGATAAAATTACT
AAAGAAATAGAAGTGTTAAGCGTTACAAACTAAAAACTTAAAAAGCATGCCAATCAGTACATCATAATTGCGT
CTTGGGGACAGACAAATGATGAATAGAGATTGGCATGCTTTTTATTTTTGAATATAAATATTTAGTTCATGGC
ATTTCTAGTTACATGACGTCCATGAATTAAGAAGTAAACAAGCATAGTAATGATTGCTAAAGCGGCCATAAAG
CCGAAGATTTCACTATATGAAAACATATGAGTAAATAACCCAAGGAATGATGGACCGAAGCCGACACCTGCAT
CTAGACCAACGTAAAAAGTAGATGTCGCGATACCATATTTAATCGGAGGTGAGACTTTTATCGCAATAGATTG
CATTGCAGATGATAAATTTCCATACCCTAAACCTAGGCAAGCTCCAGCAAGTAATATTAACCAGCTTTGATAG
CTTGAAATTAAGCACACAAATGAAAGGAAAAGCATGATAAATGCTGGGTAGACAATAATATTTTCATTTTTAT
CATCCATCAATCTACCAGCAATAGGTCTAGTAATTAACGATGCTATAGCATAGCAAATAAAGAAATAGCTTGC
TGCAGTTACTAGGTGTCGCTCTAAAGCAAATGCTTGTAAATAAGTTAGGATGGACGCATAGGTAACGCCAATT
AAAAGCATAATTACAGCAACAGGAATGGCCTCTTTTGCAATAAATTGATGAATACTAAATCTTGGTTTATCAA
TGACATTAGTTTCAGTTTTATTATTTGTTACTTCAAAATCAACTTTTATAAATAATGAGATAATGAGTCCGAG
TATGCCTAATATGACACAAATAATAAACAGTAAATCAATTGCGTATTTTGTAATAAGTAACATGCCTAGAAAT
GGGCCAATCGCTGTACCTAATACTAAACTTAAAGAAAATAAACTGATGCCTTCACTTTTTCTATTAACAGGGG
TAACGTATGCCGCAATAGTACCTGTTGCAGTTGTCACAACTGCAGTTGCGATACCGTTTATGAGACGTACAAA
GATTAAAAAAGCTAAAGATCCATCAATAAAATAAAGTAATTGCGTGATAATTAAAGCAATTAAACCAATAAAT
AATAATCGTTTAGGTCCGATTTGATTTACAAATTTACCTGTAGCAAATCGACCAATTAAAGACCCGACAATAA
ATAGTCCTACAACTAATCCGGCTAATGAATCGGAAGCGTCGAAAGCTTGCTTACTATAGCCAGCAATAACTAC
AAGTAACAAATACATGCATAGGTATACGATAAAGTTGACAAAAAAGTTGATTGTAAAACTTTTGGTGAAAATA
GGTGATTTATTTAATGAGCCTTGATTAGTCATTGTGTGCCTCCTTTAATAATATTTCATGAATTTGATTCATC
ACATTTATGGTTTCTTGCATTTGATCTTGCGAAATGTTTGTTTTGGCAACTACTGCTTGTTGGAGCTTTTGTA
CATCAAGACAAACAATCTCATATAATTCATGCCCTTTAGGTGTTAAAGTTAAAAACTTTTGTCGTTTATCCTC
TAAACTGTTTTCTACTGTTATAAGGTCATTTTCTATTAGAGCTTTTAAAGTTTTTCTTGCAGTAGGCTTTTCA
ATTGCACGTCTATGTGAAATTTCAATGAGAGTAGTGGGCTGATGTTTAGCGATATCGCGTAAGATTAACCATT
GGCCATAATATATATTGTGTTTTTCTAAAATCGGCTCGGCTAATTTTAAATAGGGGCGATATATTGTTATAAA
ACTATTGAAAAATTCTTGTGATAGCATAGTGACATCTCCTAAGATAAAATAGTTAGCTTAGCTAACCTTTTTT
GCAACAATAGTAATTATAAAACGGGAGCAATTAGAAATCAATATATAATTATTAAGAGCAAAAATAATTATAC
TTTGTTAAAATAAGCGTAATTACATGTAAATAGGGGGATACTAATGATATTGAAATTTGATCACATCATTCAT
TATATAGATCAGTTAGATCGGTTTAGTTTCCAGGAGATGTTATAAAATTACATTCAGGTGGGTATCATCATA
AATATGGAACATTCAATAAATTAGGTTATATCAATGAAAATTATATTGAGCTACTAGATGTAGAAAATAATGA
AAAGTTGAAAAAGATGGCAAAAACGATAGAAGGTGGAGTCGCTTTTGCTACTCAAATTGTTCAAGAGAAGTAT
GAGCAAGGCTTTAAAAATATTTGTTTGCGTACAAATGATATAGAGGCAGTTAAAAATAAACTACAAAGTGAGC
AGGTTGAAGTAGTAGGGCCGATTCAAATGGAAAGAGATACACATAAAGATGGTAAGGTAAAGTGGCAATTGCT
TTATATAATGAATCAGGATGATGATGAAATTAAGCCACCATTTTTTATTCAATGGGAAGAAAGTGATTCCATG
CGTACTAAAAAATTGCAAAAATATATTCAAAAACAATTTTCAATTGAAACTGTTATTGTGAAAAGTAAAAACC
GATCACAAACAGTATCGAATTGGTTGAAATGGTTTGATATGGACATTGTAGAAGAGAATGACCATTACACAGA
TTTGATTTTAAAAAATGATGATATTTATTTTAGAATTGAAGATGGTAAAGTTTCAAAATATCATTCGGTTATC
ATAAAAGACGCACAAGCAACTTCACCATATTCAATTTTTATCAGAGGTGCTATTTATCGCTTTGAACCATTAG
TATAAATATACGTAAGTGCTATGAGCGAGAATGCCCATATGAATAATGGCAAGCACAATGGAAAGAATCGTTA
ATATATTATTTAATCGTGATGACTTAATTAAAATGAAAAAGATTGATAATATAAATGTGAAAAAGATAAGTAT
AACCCGTAAACTAAAGTAATTCACGGTGAGAGGTTGACTCAATGTCATAATGATTGCAACGATGTTCATAATT

ATAAATAGACTTAAAATAATTGTTCTCATATCAAACACCTCATTGTTAGATTATTGACATTATAACAGGGGTA
TTTGTATATGAACATTAATGTGGTTGCTTGAGGAAAAATTTATTCATTGAAGTCAAGTTGGTTCATTTTAGAA
ATGAATATCGTGTTAGATGATGAAAGTATATTGAAGTATAGGTAACTAGTTGAAAAGTATTAATTGTACGATA
ACATTAAATTTAACACGAAACATAGATATAAAATGATTTACAATTAAAATGGGTAAATTTGAACTTGCTAAAC
TATTAATTGGAGCATGGACATTTCAAAAATAAGAGTTCAAATCTTACACAAGCTCTGAATCGACACTATAAGA
TACAAACTGTATAATTAAAGGTATTGTTAAATAGAAGGAGATATCATAAATCATGGAAAAGATGCATATCACT
AATCAGGAACATGACGCATTTGTTAAATCTCACCCAAATGGAGATTTATTACAATTAACGAAATGGCAGAAA
CAAAGAAATTAACTGGATGGTACGCGCGAAGAATCGCTGTAGGTCGTGACGGTGAAGTTCAGGGTGTTGCGCA
ATTACTTTTTAAAAAAGTACCTAAATTACCTTATACGCTATGTTATATTTCACGTGGTTTTGTTGTTGATTAT
AGTAATAAAGAGGCGTTAAATGCATTGTTAGACAGTGCAAAAGAAATTGCTAAAGCTGAGAAAGCGTATGCAA
TTAAAATCGATCCTGATGTTGAAGTTGATAAAGGTACAGATGCTTTGCAAAATTTGAAAGCGCTTGGTTTTAA
ACATAAAGGATTTAAAGAAGGTTTATCAAAAGACTACATCCAACCACGTATGACTATGATTACACCAATTGAT
AAAAATGATGATGAGTTATTAAATAGTTTTGAACGCCGAAATCGTTCAAAAGTGCGCTTGGCTTTAAAGCGAG
GTACGACAGTGGAACGATCTGATAGAGAAGGTTTAAAAACATTTGCTGAATTAATGAAAATCACTGGGGAACG
CGATGGCTTCTTAACGCGTGATATTAGTTACTTTGAAAATATTTATGATGCGTTGCATGAAGATGGAGATGCT
GAACTATTTTTAGTAAAGTTGGACCCAAAAGAAAATATAGCGAAAGTAAATCAAGAATTGAATGAACTTCATG
CCGAAATAGCTAAATGGCAGCAGAAGATGGAAACATCTGAAAAGCAAGCTAAAAAAGCGCAAAATATGATTAA
TGATGCGCAAAATAAAATTGCTAAAAATGAAGATTTAAAACGAGACCTAGAAGCTTTAGAAAAGGAACATCCT
GAAGGTATTTATCTTTCTGGTGCACTATTAATGTTTGCTGGCTCAAAATCATATTACTTATATGGTGCGTCTT
CTAATGAATTTAGAGATTTTTTACCAAATCACCATATGCAGTATACGATGATGAAGTATGCACGTGAACATGG
TGCAACAACTTACGATTTCGGTGGTACAGATAATGATCCAGATAAAGACTCAGAACATTATGGCTTATGGGCA
TTTAAAAAAGTGTGGGGTACATACTTAAGTGAAAAGATTGGTGAATTTGATTATGTATTGAATCAGCCATTGT
ACCAATTAATTGAGCAAGTTAAACCTCGTTTAACAAAAGCTAAAATTAAAATATCTCGTAAATTAAAACGAAA
ATAGATTAAAGACTGAAATCTGAACGCTCATAAGACTGTCATTTGCGTTCAGATTTTTTTACACAATATAGAA
TGGTTGAGTAAAATATTTTTGAATATAGTGAAAGAGGGGGAAGTACTGTGATAAAAAAGCTATTACAATTTTC
TTTGGGGAATAAGTTTGCTATCTTTTTAATGGTTGTTTTAGTTGTCTTGGGCGGTGTATACGCGAGTGCTAAA
TTGAAATTAGAATTACTACCAAATGTACAAAATCCAGTTATTTCAGTTACAACAACAATGCCGGGTGCAACGC
CACAAAGTACCCAAGATGAAATAAGTAGTAAAATTGACAACCAAGTAAGATCGTTGGCATATGTGAAAAATGT
TAAAACGCAATCCATACAAAATGCTTCAATTGTAACAGTTGAATATGAAAATAATACAGATATGGATAAAGCA
GAAGAACAGCTTAAAAAAGAAATCGATAAAATTAAATTTAAAGATGAAGTTGGTCAACCAGAATTAAGACGTA
ATTCGATGGACGCTTTTCCGGTTTTAGCATATTCATTTTCAAATAAAGAGAATGACTTGAAAAAAGTAACGAA
AGTACTGAATGAACAATTAATACCAAAATTACAAACGGTAGATGGTGTGCAAAATGCGCAATTAAACGGGCAG
ACGAACCGTGAAATCACCCTTAAATTTAAGCAAAATGAACTTGAAAAATATGGGTTGACTGCTGATGATGTAG
AAAACTATCTAAAAACGGCAACAAGAACAACACCACTTGGATTGTTCCAATTTGGTGATAAAGATAAATCAAT
TGTTGTTGATGGTCAATATCAATCTGTTGATGCTTTTAAAAACATAAATATTCCATTAACGTTGGCAGGAGGA
CAAGGGCAATCTCAATCTCAAAGTGACAATAAACAAAATTCAGCCATGTCAGACGTTAATCAGGCATCACCAC
AGCAAAATTCAAAAGCGTCATCATCAAATAATATAAGTGGTGTGCCGACAGCGAAACTAGGAGATTTAGCTGA
TATTACAGTTGGTGATGTGCGTACTTCTATTTCTAAAACGAATGGCAAGGATGCGGTTAATTTACAAATAACT
AAAGCTCAAGATGCCAATACAGTTCAAGTAGCCAAAGATGTACAACGTAAAATTGATACATTCGTTGATGAAA
ATAAAGATTTTAATGTCACAAAAACAATGGATACTGCAAAGCCTGTTGAGAAATCACTTTATACGATGGTTGA
AAAAGCATCATTAGGTACAATCGTGGCAATTATAGTTATTTTGCTGTTTTTAAGAAACATTCGTACGACGGCA
ATTTCTATTATATCCATTCCGTTATCACTTCTTATGGCGCTTATTGCTCTGAAATTGAGTGATGTTTCATTGA
ATATACTAACGTTAGGTGCATTAACAGTAGCGATTGGACGTGTGATAGACGATTCGATTGTAGTTGTTGAAAA
TATTTATCGACGCTTAACAGATTCAGAAGAACAACTAAAAGGTGAAAATTTAATTATCAGTGCGACAACTGAA
GTATTTAAACCAATAATGTCATCGACACTAGTTACTATTATCGTCTTCTTACCACTTGTGTTTGTATCAGGTT
CAGTAGGCGAAATGTTTAGACCTTTTGCATTGGCTATTGCATTTAGTTTATTAGCATCGTTATTAGTGTCAAT
TACACTCGTTCCAGCGTTGGCAGCAACACTATTTAAAAAAGGCGTTAAACGTCGTAATAAACAACATCAAGAA
GGATTAGGTGTTGTTAGTACAACTTATAAAAAAGTATTGCATTGGTCACTAAATCATAAGTGGATTGTAATTA
TATTAAGTACATTAATTTTGGTTGTAACTATTGTATTTGGAGGACCGAGACTAGGCACTAGCTTTATTTCAGC
AGGTGACGATAAATTTTTAGCTATTACTTATACACCGAAGCCTGGTGAAACGGAGCAAGCAGTGTTGAATCAT
GCGAAAGATGTTGAAAAATATTTAAAACAGAAAAAGCATGTAAAAACAATTCAATACTCAGTTGGTGGTAGTA
GTCCAGTAGATCCAACGGGTAGTACAAATAGTATGGCAATCATGGTTGAATATGATAATGACACGCCTAATTT
TGATGTAGAAGCGGATAAGGTTATTAAACATGCAGATGGCTTTAAACATCCTGGAGAGTGGAAAAATCAAGAT
TTAGGAACAGGTGCAGGTAATAAATCTGTAGAGGTTACTGTGAAAGGCCTATCAATGGATGCCATAAAATCAA
CTGTAAAAGATATTGAACAGAAAATGAAACAGGTTAAAGGACTAGCCAATGTCAAATCTGATTTATCGCAAAC
ATATGATCAGTATGAAATTAAAGTTGATCAAAATAAAGCGGCAGAAAATGGTATTTCTGCAAGTCAACTTGCA
ATGCACTTGAATGAAAACTTACCAGAAAAAACAGTTACGACTGTTAAAGAAAATGGTAAAACTGTTGATGTTA
AAGTCAAACAAAATAAGCAAACAGACTGGTCAGAAGATAAGTTGAATAATATAACTTTGAAAAAGCCGACTGG

TGGCACGATTAAATTGGGAGATATCGCTACGTTAGTTAAAACAACGACACCAAGTAAATTGACGCAAGAACAA
GGTGATTATGCAACGACGGTATCTGCTGAAGTAACAAATAAAGATGTGGGTGGCACAACACGACAAGTGATGT
CTAAAATAAATAATTTGGACAAACCGAATAATGTAAAGGTTAATATCGGTGGTGCATCAGATGATATTAACAA
TGCAATGACACAATTAGCCTTTGCAATGTTAGCTGCGATCATTATCGTATATTTAATCCTAGTTATTACATTT
AAAGGTGGCTTAGCACCATTTACAATTCTATTCTCTTTACCATTTACAGTTATCGGTGTAATTATTGCACTAT
TAATCACAGGAGAAACAATATCAGTACCAAGTTTAATTGGTATGCTAATGTTAATTGGAATCGTAGTAACAAA
TGCCATTGTGTTAATAGACCGTGTCATTAATAATGAGCAGCAGGGCATGGAGATGAAAGAAGCATTAATCGAA
GCAGGCGGTACTAGAATTAGACCGATATTAATGACGGCGATTGCAACAATTGGTGCATTAGTTCCTTTGTTAT
TTGGTCAAGATAGCTCGATTCTTATTTCGAAAGGATTAGCTGCTACAGTTATTGGTGGTTTAATTTCATCAAC
TTTATTAACACTTGTAGTTGTACCAGTAATATATGAAATTCTCTTTACTTTAAAAAAACGATTCACTAAACGA
TAGATAATATAAAAGCCATAAAAGCGGTCATGGTATTGACTACTTTTATGGCTTTTTAAAATTTGAAATTTTA
GACCTTATTTCACGTGCGATTTAATCGATGTGTTAATAAAACAATACTGGCATTCAACATGTAATCCGATAAT
AAAATAAGATTGCAATTATGTTATTGCATGTTTTGTTTATTACTTAGAATAATACGATGGTTAAGATGAAATA
TATGACAAAGATAAATAATGACATTAAATTCAAGATGTTTACGCCTTTTAGATCTTTTTTAAGCATTTTAAAT
CCTGTCACTTCGACATTACCGACTAAGAGTAATAAGACGATAAATACCCACCAATTCTCTTTTGGAAAACTCA
TCGTCGTTAATGCTAAGACGAATAATAACAACACACCCATAATAATTCTCAAAATGCGAGTAAATACAACTTC
CATTTTAAAAATACTAATAAAACTAACAATTAGATAAAGTATTGCTAGTAAAATCAGATAAAATTGCATATGA
TAACTTCCTCTCCGTTTCAAAGGGTTCATGTAGTACAGCAGCGTAGAAAACATAAATAGCAGTGTTGGGATAG
ATATGCGCGTATGTTATTAATGAGACACTAAATCATAACACGACAACACCTTGTGATTCTAGATGTAATTGAG
CGAAAAATAAAGCAATTCATCAATATAACGTATTCAGAGCAATGCTAGTCAAATTTATGTAACTAAAAGTATT
TATTGCCTGAATGATAGATTATTAATCTTGCATTTGAGCACGAACATTTCTAATTTCATTTTCAATATCTTTA
AGTTGACGAACAAGTGCGGAAGTATCTAAATCCAAAGATTCCCGTTTTTCTAAGTCGCCTTGAAGACGAACGT
AATCATATTTAAGTTCGGCTAGTTGGTTTTCTAAAGACATACTATAACACTCCTTTGAAATATAATTAAGTGC
ATTTAATGTAGATTATAGCATGAAACAGTAGACTTCTAAAATATCTGTGGAGTATGTTAAAGTTATAAATTAT
GAAGATTATTGAAAGAAGGGATAACAGTGACAGAACAATTTACTATGATTATTTTATTAATTGCGCTTGGTTA
CTTTTTGAAGCGCATTAATTTTATAAAGGCAACTGATAGCCAAGTCATTGCTACATTAGTTCTTAATGTCACA
CTCCCATCTTTAGTTATTGTGAATTTAAACAGTGCAGAATTAAAATTGTCATTCTCCATTTTACCAATTTTAA
TGATTATCTATGGTATTGTGGCGAAAATGATTGCTGTCTGGTTTTTTAGAAAATATGATAATCACATGCGTGG
ATCAGTAGGTATGATGACTGGTGCTATGAATATTGGATTGTTTGCATATCCACTAATTGAAGTGATATGGCCT
AAAACGGGGCTTATTTATTTTGGTATGGCTGATATAGGTGGTGCCTTTGTGATGTTTGGAATTACTTACTTTG
TAGGTAGCTATTTTAGTGAAGGTAGTGATCAATTCGATTTTAAATATTTAGGTAAGAAATTATTACAATCAGT
GCCACTCGTTACATACATTGTAATGTTTATATTGAATATAAGTCACATCCATTTACCACATGTGGCAATCGAT
TTCTTTAGCATACTTTCCCATGCAAATATGCCGTTATCAATGATTTTGCTAGGTGTCATGTTGAATTTTACTC
TGGAAAGAAAGTATATACCTGCTACTATTAAATATTTATGTTTGCATTATGGATTAGCATTAGTTGGGGGATT
GTTAGTACATGTGTTGTTACCGGTATCAGATGATATGATTAAGACGACGTTGTTGGTCACTTGGATGTTCCCA
GTTGGTGTCGCAATTATTTCTTATGGTATTCAGTTTAAATATCGAACACTGTCATTTATAGGTATGACGACCA
ATTTGACAATCATTATTAGTATCATTATTTTGTACGTGTATCAGGCAGTGTTTGTCTAGTTTACACATTTGAA
AATAAAATCCATTTTAATAGTCGAAGAAAATGAGAGCCCCCTTTACCACAACATGTAAATGTATATTGTGAAA
AGGGGGCTTTTATTTATGACATAGTTGTTCAACAGGCTGGGACATAAATCAATATTCTATGCTCTACGAAGTT
ATATTGGCAGTAGTTGACTGAATGAAAATGCGCTTGTAACAAGCTTTTTTCAATTCTAGTCAGGGGCCCCAAC
AAAGAGAAATTGGATTCCCAATTTCTACAGACAATGCAAGTTGGGGTGTGGGCCCCAACACAGAGAATTTCGA
AAAGAAATTCTACAGGCAATGCGAG
>DSM104437_scaffold_3
TTCAAAAGTATTTAAAATTACTGGAGAATCATATCGATTAAAAGATTATAAAACCGAAAAATCATTAAATATA
CGACAATCTTAAACCGCTAAAAAACGACATTTTCAAACCGCCATAAAACAGCATTTTTAAACCGCTATTGACA
ACCATTACATTTTTATATTTTAATATTGAAAAAATCACATTTTACATTCATTGTCATTATCTTTTTATCTTCA
AATTGCTTGAAGTCATTTAGATTCAACTACATCTATTATGTATAAATCATTAGGACGGATTTCTTTTAAACCC
GTCCCATGTTAAATTAATATATTCTATTGCTTTAATCTTGATTGCTGTATTTTATAAACGAATACTGTACCGA
TTGTAAATAAAATGATGCCAATAATGAATAATGGTATTCCAAACATATTAAAAGGTTCACCGTAAAGTTTTAT
TCCAATTTCCATAAACCTAGCTTTCATTACGAAGTAAAATAAGATAGACATAAATATAAATACTGCATATGTC
CCAATGATTGATAAAATTATCGTTGGTTTTGATATAGATTCAAGTGCTTTAGAATTTTTATGCGGTGTATAAA
CAAATAGCCACATGACATTCAGTGCCAACATTCCAAAGCTACCTAAACATAAAATAAATCCATAAACACTTGT
TAAACTTAAAATCCCTGCAATTGTTAAAAGTATCCACCAAGTATATTTCATGCTATGACACTTCTTTTCCTAT
TTTGTTATATCTTAAAACAGAAATCCCACTTATTAACCAAGCAATAAATGTTAATAAATTGGCGCTGATTACT
GCACCAATAATATACAAAATGCCACTCGCTCTATTATTCACAAACAACTTTTTCATAGCTATAATGCCTAATA
AATCTGAAGCTAAAGAAACAACAAATAATATTGCAAATATTGTCACGAATGTTGCTTGAGCAGTTTCTGGACT
TTGTATAGTTTGTTCACCATTAACAGTTGTGGTCAATTCTGGCTGTATAATCCCTGTTGCTGATAAAATTAAA
AATATTAAAAATAACAATGTTAAAAAACTTTGAATAATAATTCCAATTAAAGACGTCACTTTGATTAATTTTT

CCATTTTCCTACTCCTTAAAATTATTTTACTTTAATATCTTTATAATCTGATTGGTGTTCTTTCACGTATGTT
TTAGGATTAATATCTCTCTTATAATAAATTTTGTCGTTTTTAAACTCGTAAGTCGCGTAAAACATTTGATCTT
TATCTTTAAATAACTTATAGCTGATGATAATTAATTTGCCATCATCAAATACATAAGTATTTACTTTATCCTT
ATTAAATTCAGGATTAACTTCATTACTCTTTTTAACCATCTCTTTATGAGTTTCTTGTTGAATTTTCATTACT
TTATTAATTTGACTTTGATATTTATTTTCATTTTGCTCATTACCACAACCATAATTGATTATACTCATCATTA
TAATTAACACTCCTATAATTAATCTTTTCATTGGTTACTCCTTAATTATCTATTTTTAATAAAAAAGTCTTTC
AACTCTATCGGTTTTCGATTAGGTTTTATAAAAAATTCCCAAAAGTGGTAAGCAGGCTGTCCAGATTTTTTAT
TTTCTCTAAATGAGCGAACATGTATAAATCTAAAATAGCATGCAATCGCATATAATCCGGGAATCATATACAA
AATCGACAGTACTTTTGTAACTTTCACTCTATTAGCCAATTCAATATTATTTTGATCATATTGATAGTTTTTC
AATAAAATAAAGTAAAAAATAATTTCTAACAAAATGGTAATTGCATATGCAACCATAATTGAAATATAACAAT
AAAAGCTTACTCTATCGCCAAAATCCCTACCATACTGCAAACGATTATTAATTATACCTGCAATAACTGGTAG
AAACGCCAGTAAAATCATCATTGCAAATGGAATAACTTCAAATGATAAAAATATTTTGTTTATTCTCTTTATT
TTTTCCATTTCTACTCCTTTAATTAGTTAATTTTTCAATAGCAGCAGCTATTGAATTTATTGCATTTTCTAGT
ACAGATAATGTACTACCCAATGAATTAACTTCATTTTGCATCTCGGTAATTTTATCGATGAGTCTATCGATAT
TATCATCAATATTTTTTATACAATTTGTTATTGAACCACTTATGAGTTCACTCTCACTTTTCTCTTCCCATTT
CGAATTATTTTCTCCCTTCCAATAGGAATATTCTTTGAAAATATTCTTTGAATTTGTTCTATGCTCTTTCACT
TCCTTTTTTAAATCATTTAATTTATTCTTAATTCGTGTTGCTCTTTCTATTTTATCATTTAATTCTGCTATTT
TGGTTTTAGTTTCTGCTGACTGTTGAAGTTTATAATTCTTTTCCAATATTAAAGATGATTTCGACATTTTATT
CTTCCTTTACCATGTAATTTTAATTTTTCCTCCAAGTATAAATTTAGCATCAACTTCAATTCCTTTTTTGCCA
ACATTTACACCTCCCCCAGCTTGAGCCCCAATACTCGCGCCAAAATCAAAATTTAATCCACTAAGTGCAAAAC
CACCTTCAACCCCTGTATTAGCTCCGACTGCTTCAGCTTTCGCTTCTACTCCATCAGCACCTAAAGATCCACT
CGCACTTGCTGACAAAACTTTTGCATCTCCTTTTACATGCAAATTATTATCTTCTGATCCAAATCTTAATTGT
GCTTCCGCATCAATTAATTGCGCTTCTGCAATAGCAGATATATGCGAGTTACTAATTTCAGCTGAACCTTTTA
CACTCCCTACTTCGAGTCCTGCCTTACCTTCAACATTCTCGTACTTTACCTTACCATCTAATCCAGCTACTTT
AGCATCTCCTTTAACCCCCATCGAATCGTCGCTTCGATATGCTATTCCACTAACTTTACCTATTTCAGCTGTA
CCAACTATATGTCCATCTTTAGAAATTGCTTGAGTTCTACCTATTGTCTCATCTCCTTCTGCATAAGCAACAG
GATGATCCGCTTTCATTTTTTTAAATTCACCCTTGTTAGTTTCATTTTTATACCAATTTTCTTTTGTAATGGA
GTTTATCTTTGATACGATAATTCCATTTTTATCATGATATTCATTATTCAATTTATTTATTTGAGTTATTAAT
CCTCTTACATCATTGCTTAGACTTTCTAGTGATTGCTTACCTGTTTGATCCATAGTCTTAAGTTTTTCTTTAG
TTCTATCTATTTTTTTGGAACTTTCATTGTAATCACTTTCTACATTATCTTTCCCTAAAGACACAGTATCAAT
AAATTCTGATGCTTCAGATAGAATTGCTGGGGAATTACTTGTAAGATCTATGAATTCCTTCTTTTTTGAGACC
ACACCTTTTTTAGTTTCATTCAAAGTTTGTAAACCAATTTTGGCATTACTACTTTTATCGCATTTTTCTCCAA
GTTCTTTTAAGTGCTTTACTGATGCTTCCAACTTATCCATTAAATTAATAGCTCTAGAAATTGTGCTTATATG
TGCTGATTCAACATATTTCTTTATACTATCCGCTGTTTTTCCTTTTATACTACCAGTTTTAGATAAAAAAATAA
CATTGAGTGTAATGATTATATAATACTTCATTTAATTTCGTTTTCACATCATTTGTTTTATTAAAATATTCTT
CTAACTCTGAATTAACTAATCTAGGCATATTTTCCCCTCCCTAATTCCTATTTGCAATTTTATTTGCATTATC
TTTATCATTCCGTTGCATTGTTGATGCTATTTTATCCATTGCTTCTTTATCGCTGCCAATCATCTTAGAATAC
GTTTTCATCGCTTTCGATAAGTCGTCAATTATTTTTTCTAGTTCAACTATACTTTCTAATATAATTTCATTTT
TAGCATCTTTTATACTAGAAATTGAAACTTCACTAATCTCACTTGATCCTTTTTCAAATTTCTCTAATACATT
TTGAAATTCAATTGGATCTAAATAAATTTCTTTCCCCATACTTAAACCTCCTCAAAGTTAATTTTAAAATTAA
CAAAACTAGAACTCTTAATAACTTTTATAACATCTCCTACGGTTAAAGTTTTCATATATTTTATTATTTCATT
CAAATCCAACACATCACCATTCAGTTTAGATAGCAATTCGTAAAATTCTTTTCTATCTTTTTCATATAATCGC
CTAATACTTTCCATGATATTATTTTTAAAATCTTCAAATTTTCCAACGTTGTAATTTTCTATTTTTTTTATAT
TATTTATACATTCATCTTCCCTACCTAAAATACCTATTTCTTTTCTAGACTTGTGCACTTCAAAATTATCGCC
AAATAAGTAATATCCAGTAGCCAAAATTACTGCATAATCATAATGTTTATTTTTATTATCAAACTCTTTAAAT
AAGTATCCAATAAATGAAGAGTTCCTACGTGCTTTTTCAACTATATAATTATGACCGTTAAACTTTGGTATAT
ATAAATAATTAAATTTTTCACCTTTATTTTTAAAATTCTTTAATTTTTCTTTATTGTCAATTTTTTCACTTAT
ATTAATCAAACAATTTTCGTAATTTAGCATATATTTTTTAAATACTTCCAAATTCTTTTGATCAAAAATCCAT
AAATCAGAAATCAACTTTTTAAAAGAAAATGTATAGTCACTAAATAATTCCATGAATTTCATAGTAGCTCTGT
ACTCACTAATTTTAAAATTATAATTCATGTCTTTCTTGGCTAACTCTTTAGCTTCTAAAACCGATGCCTCATT
TAAATCTTCTTCACTCATCACTTTAATAATTTCATTGTATTTTTCAATATACATATCCTTATATCTATATTTT
TTATCATGCGCAAATGAAATAACAGTGCTATTAAAATAATTATTAATATCTACAATTATATTTCTCTTTTTCA
ATTCAATTTTCAAATATTGTGCGAAGATAATTGGCGTTGCATTAGAAAAATTAATTTCTTTCCCGTTATCAAA
TATAACTTTAGAATGTCCAACTCCAGTACCTATAATTATATTTGTATAAATATTATTGTCAGAAATCTCACCA
TTTTTCGCATTAAAATCAAATCTCATAATACTCTCCTTTTATTCAAATAATTTAATTTTTTTATATTCATAGT
TATATATATAATACATTTCATTATTCTTAAGGTTTTTTTCATTTACTTTATAACGAACTTTTATTAATTCTTG
TTCGCTAATACTAATTCCTACAAAAGCGCTACTGACTATTTCCTTAAGTAACTTTATTGAGAACTAAATCCT
CCAATTGTTTCTTGATACAATCCGTAAATAATTGGAAAGACATTATTCTTTGGTCCGTCTAAAAATATAGTTT

TTATTTCATCATTATTTGGTGATGCAATATTTATAAATTCTTTAAAATCCGATATAACTACTATCCATTTTTT
TCGTGGTTCTACATTTTTTATAAAACTCACTAATTCATTTTTAAAACTAATAATTTCTTTCTTATCTCCACAG
TACAAATAATCTTCATACCCTGTATTTTTTAATATGCCTTTTGTGTCTAAAGTTGCAATTTCATATTCATTTG
AAATTATATTTAGCTGTTTCTCTAATAAAGTTAAACCATTATCTATATCAACAGGTTTAACTGAAGTGAAAAT
ATTTGTTTGACTTAAGTCGAAACCAACTAGTTCCACATCCTCAAAGTCTAATCCTAATGGTAATTTGTGTTCT
TCATGAATGATTTTTTCCAAATCATATGTTTCTTGAATATCATCCATTAATACCTTATCTGGCATCATTGGGA
TATGTTTTGGATAATCTCCTACATAGAATTCTTTCATTTGTGCTACTTCATTTTTAATGCGCTCGTTATATGT
TTGTCCTTCTGCTAATTCAAATGGTTGCGCAATTTGGAACTGTGTGAAGTTATCATCATTTATTGCAGCTCTA
CCTTTAACATCTTTCACACCTTTTTTATAAGAACCAATAATATTTGTTAATTCATTATTTTCAAAGAGGTATA
GTGCAACTCTTGTTTTAATGTTAGTAAATATAGCTGATTTTATTGCACTTGATCTTGATCCTGAAAGAATGAT
GTATACCCCTAATGCTAAACCTTCACGTGTTACCTTAGACATCATTTCTTCATATTCTTCCATAAATGGTGAT
TCTTTCACCGTGTCGTAGTTATCGATAATTAAGAAAATATTTGGAATTGAATTTCCAGTTTCTTTATTATATT
GCTCTATATTAACTACTCTCTCTTGACTTAATAGTCTTTTTCGCTCAGAAATAATATCATGTATCTTACGTAT
CGCCTTCGCAATCTTGTCTTCTTGATCTACTGTAAAATAATCAGCAACATGTGGTATGTCTGTGACTGGCATC
AAACCATTAGTTCCGAAATCGAACAAGTACATGTGTGCTTGATCAGGACGATGGTGTCTTGCAACATCGAAAA
TAATGTTGTGTAAGAACGTTGTTCTACCATATCCTGGACTTCCGATTAACGCGATGTGACCAGCTTTTTTCAA
TTGTAATACCATCGGCCCTTGATATTGTTCTTCTGGTACGTCTTTAAGTCCTAATGTTAATTCCACTTCTTTT
GCATCATCTGACCATAATTTTCTGAAATCTGTTTCTACTAAATCTTCTTGATATACATTTTCTGGTAATGGTG
GTAACCATGGACGCTTAACTTCTTCAATTTCTAATCGTGTTGTAATAGATTCGATATGATCGATAACCGCTTC
TAACTCAGTTTGATTTTCTTTCGTTTCTTCATCTTCAAGTCCACTCAAGTCTTTGTTGATTGCTTGAAGTTGA
CCATAGTCATTAATCATGTAAATCGTCTTATCTTCAACTTCTAATTTATCGCCTTCGATGTTATATGTTGCTC
CACTCCATGCAGATTGGAACAATTCATAAATTTCATTATTACCAACTTGTAAATACGCGCGACCTGGTAATGT
AATGTCTGCTGCATCTGGTGTTTTTAAAATTTCATTACTGTCTTGTCTATCTTGTACTTTTAATGCCAATTTA
AATTTAGAGTTAGACCAAATTTGGTCATCAACAACACCCGATGGTTTTTGTGTCGCAAGTATTAAATGAATAC
CTAACGAACGTCCAATACGTGCCGTTGATACAAGTTCTTTCATAAAATCAGGTTGTTCTGATTTTAATTCGGC
AAATTCATCGGAAATAATGAATAAATGTGGCATTGGTTCTGTCGCAATACCTTCTTTAAATAACTTATGGTAT
TGATTAATATGGTTAACATCATGCTCTCCGAATAAACGTTGACGTTTTCTCAATTCGGCTTTGATTGATGTTA
AGGCACGCATCGCTTCATCGCCATCTAAGTTTGTAATCGTACCAACTAAATGGACTAAATCTTTAAATAAGTT
CGCCATACCCCCACCTTTATAGTCAATCAATAGGAATGCAACTTCATGCGGGTGAAAATTAATAGCTAAAGAT
AAAATGTATGATTGGATAATCTCAGATTTCCCTGAACCAGTGGTACCAGCAACTAAACCATGTGGTCCGTGTG
CTTTTTCATGTAAGTTCAATGATAAAATGTCATCTTTACCTCTTACACCTAAAGGTACTGCCATCGTTTTGTA
TGTTTCGTTTTGTCTCCATCGATTAACCACATCAAGCTGATCTACTTCTTTCACGTTATACATTTCTAAAAAT
GTAATACTATCAGGAATTGCATTTTTCAAATGTTCGACGTGTATCAAATTCGCCAAACGTCGCGCGATATATT
CTTTATCGACGTTATCAATATTTTCAGGTGTAAATTTCAATTGAACTAATTCTTTTTCTTTCGTAATCAGTTC
GCCTTCAGTACGAGACTTGATATCAATAATGGTATCTACATGCTCTGGCAAACTTTCAATCACATCTTCAACA
AAGATTAATGAAATACCATATTCTGATAAATCTTGGTTTACATATTCTAAAATGACGTGATCAATAATTAATG
ACATATCTGTAATGACAAACACTAATTGCGGTGTGAAAATAATTTGCTCATTACTTCTGCTGCGTTCACGCAC
AGCTTGGATACGTTCTTTAATCATGCTATAAATTGACGTTAAAATTTGGTCACGTGTTCGTTGATTGTAAACA
AACCCTCTAATGTTTTGACCTCTCAATGTCATATGTGGCAACCAACGTGCCCATTTCAATGTTTCAACTTCAT
CTTCACGTGTCACAAATAGAAACTCTAAATCATGATAACTATGGAACGTTGACAATTGGATTAGCATTTTCTC
CAATTCTTCTAAAATGAGATGTCGTGCACCAATATATGCAATTGGCCCATGATTTAAATCATTGATTAATGGT
GCTTGTTCTACATCTGTGTAAAATTCATACAATTCTTTAGCATCGTCGAATAGTTCATCACGACGTTGGTTAA
ATTCTTCTTCTTGGTAATCTAATTTGAATGACTTTTCTACATTCGCAATACCTAACTTATAATATAAGAAATC
GTGATGATGCGATGTTTTTTCATATATTCTTGGTGCTTTCGTTTCAACGATATCTTTAATTTCAGCAACCGTT
GGATAATGATAATTCAAACTAAAACGTTGTGCTTTAATCGCTTTATTAATTTCTTTAGATTTATTATCCAAAT
AAGCTTTGTAATCTTTCTCTCGTTTTTCAACATCTTTGTTATACTTTTTCTTTTCAGAGAAATACGTTGTAAT
ACCAAATACTATCGTTACTGTACTCATACCAATCATCATTAAAATATAAATACCAATTGGTCTCACTAAAAAG
ATGACAACAGTTAAAGCAATCATTACTAATGGCGGTATAATGGAACGCCATATCACTGTATTGTTCTTCTGTA
TTGGCTGTGGTGGTCTTTCAATCTTAATATCATCGGTCGGTTCACGGTGAATAATCCTTGGCGAACGATGGTA
CGTATTGTAATCATCTGCCTGTGCATGTGGCATCTCTTGTGTTAAGCGAATTAATGACGATGCCACTGTATTC
TGACTCAATACATTTAAACCATCAGCTTGTACTTCGAGCCATATCCCTTCAACATAAATATGATCACCAATGT
AAGCTTTGTTTGTTAATTGCTCTTGTAGTTCATAGTTAATATACACATCTGTATTTTTATCGTGCACAATGCG
TACGTATTGTGATTCTTGTATTGATTGAAAATCTTTAATAATGATGGCATTCATCAAGCTTTGAATAACCATA
TCATCATACGCATTTGGCCCAATTGTCATCGTATCTTGAATTGAAGGATAAGCAAATGATGCATAATCAGCTT
CTGTATAAAGTTGTAATGTAATGTCATCAAGGTCACCTTTTCTAACAAGCACCTTATTAATAGAAGTATGATT
CGCTTGCCAAGTACCTTGATTATTTTGTTCTAAATGAATGACTTCGCCTAACGATTTCAACGTAATATCTGCA
CGCTCGTCTTCGCTAATAGTATATGTCTTACCATCTCGCAAATTGAGCATCTTCAATTGTTTGTTATATTTTA
TAATCAATTTATGCATTGTCTTTGCCTCAGTCCTATACTATTTTTTTCTTTCAGCTTCTTGACGTTTTTCTTT

ATCTTTTTGTGCTTGTTCTTTTTGTTTCTTTTCGTTCTCTTCTTGTTGCTTTAATTTCTCATCTTTCGCTTTT
GCTTTCTCTTCTTCAGATTTTGCTTTTTCATCTTTAACTTGCTTTTCTTTATCTAAAATATCTTGCAATTTAT
CGTTATATTTTTTAGTTTCTTCAGAACGTTTATCATTCGATAAATCACCATTATTTTTAATCTCATTTAATTT
ATTAATCAACGCTAATTTCGTAATATCGTTATCATCTAAATATGTTGCAATGTCAATCGCTTCATCAAGATGT
CCTTGTCCTAATTCCATCCAATATAATAAGTAATCTTTGTTCGAATTTGGCGTCACATTATTAAGCAAGTTTT
CTTTCTTATCTTTTTCTAAACCTTGTTTGTTCGTTTGGATATAGCTTTTGGCATAAATGTAAAGCGCTTCTTT
GTCTAATTTTTTACCATCTAAATCATCATACGTATTTAGTACTTGCGTATAATCATCCTTTACAAATGCTTGG
TATCCTTTTTCAATGCGCTCATTATGCTTCATTACTGAAAAATATAAAAAAGCTAAAAATGCAATTAATAACA
CACTTAACGTTGTCATACCGATAGCGACCCATTTAAAAACGGTATGCCCTACTTTACGAACATATGCATAATT
TTCACTATAATCTTGTTCTTGTTTCTGATATTGTTCATCTAAAAACGAAGTTAATAAGTCTAACGTTGCCGCT
TCAATAACTTTAGTTTCAAATGGTGTTCCTTTATGTAGTTCTAAGTTTCCTTCAACTAATGCGTCAAATGATT
GTTTCTCATTGAATGCACATATAACCAGTGCTTTATATCTTGTTAAAAATTCAGCTTCCGACATTGGCAAAGG
GTCAACAACATTTTGTAATCCTCTCGTTTTGGCAATCGGCAATCCATCTCTTGTGAAAAATAATTCATCTGGT
GCCAACACAAATGTGTAGCGTGTACGATTTACTTCTTCTAAATTTTTGATATTAAGTAAGTAACGTAATTTTT
CATTTTTAGTAAATGATTTAATATTATCAAAAGGTGTATGATTGTCATTAATGTCATAATGTATTTGGAAACT
ATCACGTAGTTCAGTTAGTTCAGCATCTATAAAATATGGAGAATGTTGTTCTAATAAGTACATTAAATGAAAA
TGTTCTGGTTTAATTGAAGACTTAGGAATCTCTCTCATATCTAAACGTAATTTTGTTTTAGCTGCTTCTTCGG
CATCTAAAGGTGTTAACATATCTTGCATTTCATTTTTAGGGTTATGATTTTTAACCATCTATTTTTCCTCCTA
TAGTAACTTCAAAATATCTCCATCAGCGATTTGATAATCAATAAGTCGGTCGTTTTCAACAAGTAATTGACCT
TTCGTCATCACTTTAATTTGTGTATTCACATCGAAAATCGAAATATCCAAACTGTCTAACACTAATGCTATTA
GATTCTTAATCGGTAAATATGCTGGTACTGCTAAGTCATATGTGCCGTAATTATAATTAGTAAAATCAAATGT
TACTTTTACGTGCTGATTCATTTTTCGTTCGTCTCCCTTTATGAATTGAAAAAATGACAAATCCTAAAGTCAA
AAAAGCCCCTACAGACATCAAAATGTACGGGAATATTTTTTTCTCCTGCTTGTTTTGAATATAGGGGCTTTTA
TCACTCTCCGAAGTCTTTTCTAAGTTAGATTTGGAAAATAACACTTTTTTAGTTTCATTCAAGCGAGTCGAGT
GACTCGCTTGATTTTGAAACATATCATTTTTTATTTGTTGTTGTCTTTCTTTTTTCTGCTTAGCAATCGCATC
ATTAACCGCTTTGCTGTCTTTATTAAATAGCGTTGTATCATATTGATTTAAATCATTATTGATTCGCTTTTCC
TCTTCTTGTTGCACGTCAATATTAAGTCCGCCATTATTGCTAGATGCTGTTAAAAAAGTTAAAGCAATCACGC
TATTCATCAACATTAGATTAATCTCTCTTTCTTAAAGTGTTTTACAAACATGTTCAGTACGAAGCCAATAATC
ACAAGTACTGTTAATATTACTAGCGCCAAGCCTATAGGATGCTCTGCATTTAAATAATTGAAGAACATGTTAT
CGATATAAGATAATGGTGAAATTTTATTAGTCACACCTTGTCCAGCTGCTTTCAAATTATTCATAGCTACAAA
GTATAGACCCAATGCAGCAATCATTAAGAACATACCGATAGATTTTACCTGTCTTAGTAAATACGTATTAATA
AGTACGAAGACCATCATAGTTAAAATCACCATTAAGATGAATTTCGCTCTATAGCCAGCTAATACATGGAACT
TATTCATTGCAATTAAACCGACAATTAATCCTTCTATCAAACCAGTTGTACCAATAACACCTGACGTAATGAC
ATTATTCCAAAGATGATTTTTACTACTATAATCATCTTTAATGAAATTCATTTGTCCTTTGGCACGTTCATAA
CTATAGAAAATATATGCTGTAATCATTGATAGTAAGTACATCAATAATACGAATAATGTCGGTGAAATCACGT
CTGTATTACCATTATTAGCTAAAACATTTTCTAAGTTTTTCTTTTGAACCGGATTACTCATAAATGCTTTTAA
TGCTTGGTTTTGTCTGTCGCCATCTTTAGAATTTTGTAATACTTTTTTAAACTCTTTAGCGAATAAATCATTT
TCTTTATCGTTTTTAGCCATTTGACGGTTCAAGTCATTCGCTCTTACGCCTAATGCTCGACCTGTCGCATGTA
GCTTATTAACATTATTGTCGACTTGATTTAATTGGCCACTAACAGAATCTGCAATTGATTTTGATTCTTGTGT
ATCTGATAGCAATTGCGTACTCTTTTCAGAAATTCTACTAATTTCTTTATCTAAATTTGAAGACATCGTATTA
AATTCATCATTTTTGCCTTTATCAATTTTTGGTTCTTGTGGCTCTTCAGCAAATGTCTTTTTAACGTTTTCTA
ACTGATCAATCAGCTTAGAAATATCTTCTTTATTTTTGGTTGTATTCTTTTTGTTATTTAAAATGTCATCAAT
CAGTTTGTCTGAGTTTTCTTCCATTGAATCAATTTGATGTAACACAGCTACTTTATCGTCTTTGAAACTTTCC
ATGTCATTGATAACTTGGTCAACCATCATATCAATTAAACGTTTGTTATCGAATGGTTTATCTTCTCTGCCTT
TTGTATCTGTGTACATTTTATAATGTGCATCAAATCTAGATAATGCACTCAATTGCTGGCTTAATGCATCTTT
CGATAAGCGACCATCAAGGTTATGATTCAATGTTACATCCACAACACTCGTTGCTTTCTTATCATTTGGTTCA
TCTTGACGATTTGCTTGTCCAAATAACAATTGTAAATGCATTGTTTTATCTTTTAAGAAATCTTTCTCAGCAT
CCTTTTTCAATTTAGCAACGCCATTGACTTCAACTTTATATTCCTTGTTAGATGTATCGAGTTGAACACTTTG
ATCCTTAATGTCATATTTTTTACCATTAATTTTAATGTCGCCTTCAAAATTAAAATGAGGATCTGTTGCAACA
GTTAATTGATTAATATCATTACTTTTAATCGTTTCAGTACGTTGCACTTTGACACCATCATTAATCAAACTAC
TTGTGTCTTGCGCAACTTGGTTATCGTAATCTGTTAAATTAATGTGTTCTGCTAACGGTTTTTTCAAATTATA
TTCGTTTTTATAACGTTTTGCTTCTTTGACAATTGCTTCGTATTTATTAGCTTCATCCTCATTTAAACCTGCA
GCTATAAAGTCTTGTTTAGACATGTTATAGATAAATGTTGTATCTGTATCAGGTTCTTTGACAATATCATCAT
GAAGTTGTTTTTCTAAGTTTTCAGCGAATTGAGCATTGTTCATTTTAATGCTATTTAGCGCATCTTGTAAGTC
TTTGTTATTTGCAAGCTCATCTTGCAGTGATTCTGTTAATTGCTTACGATAGTCTTCAATCATACCTTTTGAA
AATGGTGACTCTTGTGATTGAATGATTTCTCTTAATTTATCTAAGTTTTCTTTAACAGTTTGTTTATATTCTT
CTTTACCTGTATCTTGCATACTTGATTGTTGATCAATTTGGCTGTCCATCTGTTTTAATGCATTGATATAGTT
ATCAAGTTCCACGCTATCTTTTTGCGATTTATAATCTTGTAGCATTTTATCCATCGCTGTATTGTGCTCGTCA

AATAATGAATTTTGTTTTTCAATTAAAGTCGAAACATTATAATCTGTGTTCACTCTGAACGTATCTGAATTCG
CACTCAATAATGATTTATTGTATGTTTGGAACCATTTTGTAATATCTTTGTTTGCAGAAATTGAATTTACAAG
CGTATCTGTAAATAATTCCGGGAAGTCGTTAATTGGATTTAATAAGTAATTCGAGAATTTACTATTCACACCA
TGTTCACGCGTCATAATAGCGCCAACATTTTTTTGTGCATTATGTAAATTATCAATGATGCTTGTTAAATAAA
TTTCGACCAAGTTTTTGTTAAAGTCATTAAGTACATTACTTACAACTTTTTCTGTGTTTTTAGCTACTTCTTC
TTTTTGTCCTACAGCTGTTTTATACTGTAGTGATATTTTCGATGGTGTTTTAGCGTCTAATTGCATTGCCAAT
TTTGAAAAGTTTTCTGGGATAACAATCATGACTTGGTATCCACCATTTTTCAAACCAGACTCAGCAACGTTTC
GTGTTACTGTTTCAAATTTATAGTTTTTCTCATTTGCTAACCTTTTAATAAATGCTTGACCCAGCTCAACCTT
TTTACCGTTATATGTCGTTGGTTGATCCTCGTTAACAATTGCGATATGTATTTTATTATTTTTATTACTTACA
CTTTGGGATCCTTTTTCTGATTGATCTCCATATTTTGTTTGAACAAAAAATATCATACTAACTATGGCAATTA
TAATAATTAAAGTGACAATTAATGCATAAATCCAATTTTTCTTTTTCATGCTTATTTCCTTTCAGTTGTTTTC
TTAAAAAATGATAAGCAAAGCCACATTAGAAAATGTGACTTTGCCAATTTCAGAATGCTTATTGCAAACCGAA
ATTATTAGAAAGTTGTTGGTCTTGCTCTTGAACGGCATCAGCAGTGCTATTCAATTGTTGTTTAATTTCTTCT
AATAATTGTGCAAATTTTTCTACTTTAGGACTAAGTTGTTGGAATTGCTCTTCGAAACGGCTGAAAGCTTGAC
CTTCCCAGTTCGCTGAAATTTCACCTTGTGCACGTGTTAAATCAGATAAAATTTGACGGATTTGGTCTGAACC
TTGCCCGTAAGATTGCGATTTTGCTCTGATTTCCTCTGGACTCATCTTAATCATTGCCATAACTAGAAACCTC
CTGAATATTTTAAGTTTATCAAAACTTTTTAGGGACACTATTTTTTGAAAAAGTGCTCCTTACTCAAATAATA
TATAAATTATTAGTATATGTATATAGTTTTTTAAGTATTTTTAGCTTTTTTTAAATAAATATATTGAATATAC
CCATATATTTTTAATTAACCATTCATTTTTGTAATATAAATGTGTATACTAAAATTAAATTAAATACATAAAG
GATTAAATGGTTATTATGAAGAAAACAATTTTACTGACGATGACAACTCTTACTTTATTTAGTATGTCGCCTA
ACTCGGCTCAAGCATACACGAATGATAGCAAAACATTAGAAGAAGCAAAGAAAGCACACCCAAACGCACAGTT
CAAAGTGAATAAAGACACCGGCGCGTATACTTATACTTATGACAAAAACAACGCGCCAAACAACAATCATCAA
AATCAGTCACGTACAAACGACAATCATCAACACGCAAATCAACGTGATTTTAACAACAATCAGTACCATTCTT
CATTAAGTGGTCAGTATACGCACATTAATGACGCAATTGATTCACACACACCGCCTCAAACGTCACCAAGCAA
TCCTTTGACACCAGCCATACCGAATGTCGAAGACAATGACGATGAATTAAATAACGCTTTTTCAAAAGATAAC
AAAGGGCTTATTACAGGCATCGATTTAGACGAATTGTATGACGAATTACAAATCGCCGAATTTAATGACAAAG
CAAAGACTGCTGACGGTAAACCTTTAGCATTAGGTAACGGTAAAATCATTGATCAGCCTCTTATCACAAGTAA
GAACAACTTATATACTGCTGGACAATGTACATGGTATGTCTTTGATAAACGTGCCAAAGATGGACACACGATT
AGTACATTTTGGGGAGATGCTAAAAACTGGGCAGGCCAAGCTTCAAGCAATGGCTTCAAAGTAGATAGACACC
CAACACGAGGATCAATTTTGCAAACAGTAAACGGTCCATTTGGTCATGTAGCCTACGTTGAGAAAGTTAATAT
TGATGGAAGTATTCTAATTTCAGAAATGAATTGGATTGGTGAATATATCGTTTCATCAAGAACCATCTCTGCT
TCAGAAGTTTCATCATATAGTTACATCCATTAAATTAATCATGACATCAATAAAAAGCGACCAGTTCGCAGTT
TACAATTCGTAACACTGCAAAATTGGTCGCTTTATTTTGTATGTTATTCGATTATAAAATATGATGTTTACCT
TCAAAATTGCAATAACTTGTCACCTTAAAGTTAACCGACATTTTTCAAGAATCATTATGTAATCAAACTATTT
TTTGTAGTTTATTAGATTTTCAATAACTTATTAAACATTATTATAACTAAAATTCTATTAATTTAAACAAAAT
TTAAAATAAACCATTTAATAAAATGTATTGTTCTGATATGATTAACTCATAAATAATTGGGAGGAAGCAAAAT
GTCTAAAAAGTTAAAAATTATAATTCCTATTATTATTGTCTTATTATTAATAGGTGGAATCGCATGGGGAGTT
TATGCATTTTTTGCAAACACACCGAAAAATACATACTTAAAAAGTGAACAACAAACTGCAAAAATGTATAAAG
ATTATTTTAATGACCGTTTTGAAAACGAAGTGAAGTTCCAAGAAAAGATGAAAGATAATTCATTTTTATCTTC
ATTAGAATTAAGCGCAGATGCATCAGATGAAATTGTTAAGGGGCTTGGTATTCCTAAATCTGTTGTTAATGCT
TCGAAAATTAAAATGTCATATGGACATGATCCTAAAAAAGAGAAATCAATGATTAATCTTGAACCAACAATAG
CAGACTCTGAATTAGGGAAATTCCAGTTAGCTGCAGATAAAGATAAGCATTATTTCGAATCACCATTATTTAA
AGGGAAATATAGTGTTAATAATTCTGATTTATTATCAACTTATTCAAAACTTACAGGTGAAGATGAAGAAACA
GCAAAAGAAAATGGTATTACAAACCAACAACTAAATTTAAATACTCTTTTTAGTAATGCTCAAGCACAACAAA
GTGACTACAGCAAAATTGCCGAAAAATATTCCGAACTTATTGTCGACAAATTAGATGACGATAATTTTGATAA
AGGTAAAAAAGAAGAAATTAAGGTTAATGGTGAAAAGTACAAAGTTAGACCTGTCACGTTAACACTTAGCAGA
GCTGACACTAAAAAAATTACATTAGCTGTATTAGAAGAAGCTAAAAAGGATAAAGACCTTAAAAAATTAATGG
AAGAACAAGGTGCTACAAAAGACTTTGAAAAAGACATCAAAAAAGCAATTGACGATGTCAAAGAAACTAAAAA
AGATGAATTTGCTAAAATTCAATCTAAAATTTATACCGAAAAACATACGATTGTAAAACGAGAAATTACTATT
ACAGACAAAGAAAATAATAAAACTAAAATCAAAGGTACTAATACTTTAGAAGACAATAAGTTAAAACTAGATT
ACGCACTTGATTTCGATCAAGATAAATACACGTATGCTGAAGCGAAATATACAATTAAAGGCGTATCTTCTAA
GGAAAAAGACAATAAATACAGTGATAAATACGAATTTGGTAAAAAGACAGAATATGATGAATCAAAAATCAAA
TTAGATAACCAAGAGAAAGTAGATGGCACAAAACGTCAAGATAAAGGTAAAATTACTGTCGCATTAGATAAAT
ATAGCGACGAAAATGAATTCACTTTTGAAAATAATATAGATTCTGACGTAAAAAATAACACTCAGAAATCTAC
GTTAAATATCGGCATCAAATATGCTGAAGAACCAATTAATTTCATTTTAAAATCTAGCACAAAATTGAAAGCA
GATATTGATTTTGATGATAGTGGTGCGAAAGATTTCAATAGTCTATCTTCAAAAGATCGTGAAAAACTTGAAA
AAGAAATCGAAAAAAATGGCGGCAAAATGTTTGAATCAATTTTAAAAAAGGCATCTAAATAACTAAAATATAA
AAATAAGATTGGAAGGTCATCTTGCCTTTCAATCTTATCATTGGAGGAGTGGTTAAATGAAAACAATACATTT

GTTTCGCATCTACCACTCTTTTTTATTGAAAAAGTGGTATTTGATTATCTATTTACTATTTATATTAGCGGCA
CTCGTTATTACATTAACGACAATCCAACATGTAACAGAAGATGACAATCATTTTAATATAGGTGTCGTAGATA
AAGATCAATCAAGTGAAACGAAATTAATCTTAAACTCTATTGGTAAAGGGAGTAACCTAGGAAAAAATGTGAG
CATTAAAGCATATGATGATAAGCAAGCACATACTTTGTTAAAAAAACATAAACTTCAAGGTTATTTTGTTTTT
GATAAAGGTATGACCAAGGCATTTTATAAACAAGGCGAACTACCAATTTCAGTATATACATATGATCAACAAT
CCATGAAAAGTGTCGTGCTATCTCAGCTAACAGATTCTGTTTACCAACGTCTTATGCGATCAATGGGTGGCAT
CTTAGCTTTTCAAGACTTAGCACCGAAAGCATCACATTCTGACAGTATCAATGTTATGACTGATTTGCTGATT
ACAGGATTAAACCGTTCAGGTGCATTTAACTTAGAACCGATTCATTTATACGATACGGGCAGTTATTATGCAA
TTACAGGATTTTTAACAACGGTATTCATCTTTGCACTATCTTTATTTACAGTTTTGAAAATGAATCAAGATAC
TGTATTGAAAGCGCGATTGAAAATGTTTCATTTTTCTAAAGAGCGTTTGTTAATCATTCGTGCGTTGATTACA
TGGTTTTATACTATGTTATGGAGTATCGTTGGTGTAGTTTGGATTGTGTTCAGTATTCCGAATACCTTTGAAT
TATATAATTGGCCAACGTTAGCCATTCATTTAAGCTATTATGTTACCTTTTTAATACTGTGGTTATTACTCAT
CGAACTTTTAACTACAGGCTTGTTGAATAGTATTAGTAAAGTCATATTAGCTATTGTCATCCTGGTGTTATCT
GGTTTAACAATACCTACGATTTTTTTACAACATATAGCAAATGGCGTTTTCAATATTCAACCGTTCGCAGTTG
TAACAAATCAATTGTTAGAAATTATTTTAAACAACTACATTTTAGAACTGCACCCTAGTTTCTATCTTAGCTT
CATCGCACTTTTGATAATTAATTTAGCTGTATTGGTTTGGAGGTATCGTCAATGAAGCCTTATATACAACTTG
TTGTGTTCAAGCAATGGTTACAATACATCTTGCTCGTAACAACCATTGTCATCGCACTCGTACTTATTGGTAT
CGGTTACCGTGTAGCACATGACAACTTCAAAATACCGATTACCATTCAAGATTTAGACCAAACCACTGCATCA
AAATCATTCGTCAATAAAATTAAACAATCTGACTATGTAACTATTAAAAAAGTCGATGAAGATGAAAGCTATA
TTGAAGATGATGTTACTAAAAAGGAAGCTATTTTAAGTATGCAAATTCCTAAAGGTTTCTCTCAAAAATTAAA
AGAGAACCGTTTAAAAGAAACGATACAGTTATATGGTAGAGATGACTTTATAGGTGGTATTGCTGTAGAAATT
GTTAGTAGTTCATTATATGAGCAGCAAATTCCTAACATTATTTATGAACACCTTGAGGATATGAAACAGCATC
AATCCATCGATGCTATCAACAAGTCCTATCATAAACATACACCTGAATCTAAAATCAAATTTGTGTCGCTTAC
TAAACAAGCACAACACTCTATTTCAATTAGCTTAATCTTTGCGGTGATTTTATTTGTTAGCGCTGTTCAAGTA
GTACTTCATTATCGTTTAAACCAACAAGCAGCATTGCAACGATTATCACAATATCATTTAAGCCGTTTCAAAC
TATATAGTACTTATGTAATGACACATACGATTTTGTTATTGTTGGTACTATTGGCAGTTAGTCTATATTTGTC
TCAACCACTCAGCTTAATATTTTACTTAAAATCACTGTTACTTATATTGATTTATGAGATAGGTATCGTCTTT
ATCTTATTCCATATTCAAACAATAAGTCATCGATTATTCATGACATTTATATATGCACTTGCTATGGGTATCG
TATACTTGACCATTTTCATGTAAAGGAGCGTAACTGATGATAGAAATTAATAACCTTTCAAAGCGTTACCGTA
ACAAACAGATTTTCAATCATTTAACCATGTCCTTTGATAGTAATCGATTAACCGTATTACTTGGTGATAATGG
CGCTGGAAAATCAACATTACTTCGTATGATTGCTGGTATTGAAAAAGCTAATGATGGAACTATCAACTATTTC
GGCGAAAAATGGAATCAAAGACAAATACAAAATCACATCGGTTATGTGCCACAAGACATTGCGTTATTTGAAC
ACATGACAGTGGCTGAAAACATTAAATTTTTTAAATCACTTTGTAAAAATCCAATTAACGATACAACTATCAA
CGAATATTTACAGCAATTAAATTTTGATGATACGTCTGCCAAAGTATCTACATTGTCCGGTGGGAATAAACGT
AAAATTAATATATTAGTAGGTTTACTAGGTAAACCTCGAATTCTCATTTTAGATGAACCGACAGTTGGTATTG
ATTTAAAATCTAGACATGACATCCACCAACTACTTAACATTATGAAATCTAAATGTTTAATTATATTAACTAC
CCATCATTTAGATGAAGTTGAAGCACTTGCAGATGATATCAAGTTAATTGGCCAAGATCCTTTTTATCAACAT
GTTTTAGAGGACAAACAATGGACTTATACCTATTATTAAAACGAAAAAATCCCAAGCTGCGTATGATATCGCA
ACTTGGGATTTTCTGTATTATCTACTTTGCAAGTATGACATTGGGTCTACTGCATATTGATTACCGATGCCAC
CAGACATACGTTGGAAGTGTACGTGAGGCGCTGTTGAATTACCCGTACTACCTGAATATGCAATTTGGTCACC
AGCTTTGACTTTATCACCAGCTGAAACAGTTAAACGATTATTATGCATATACCATTGGTAGTTATTACTGTTC
GCTTCTTTAATCGTTACTTGATTACCACCACCATAGTTACTCCAACCTGCTTGTACTACTGTACCATCAGTTA
ATGAGTAAACTGGTGAATTTTCAGGCATTGCATAGTCGACACCGTAATGCGCACCACCACCGTGATATTGTCC
ATATGGTTGTAGTTGTTTACGACTTGTTAACCAGCTTGCGTCTTTCGCATGACCACTAGCTGTCGCTTTACTT
GCTGATCCGCCATTTTGATTAGACGTGCCATTAGGGTAATTGACCTTTCCATTACCATCATGGCTGTTATACG
CTTGGTTGTTACTATGTGAATAATAGCTCGCGTCTGGACCTACATTTGATTGATAACCATATTGATTAATGTG
CTGTTGGCTTTGACTCGCTGTGTAGTCATTGTTATCTCCTGTTGTTGCTGGATTCACATATGTTTGACCGCTT
CCATTGGCATTTGCATTTTTTGGATAACAGTTATAAAAATAATGCGTATGTCCTTGAGCATCTACGAATGTAT
AGCTATATTCTTTATTATCAAACATTGCTTGATTCCAGTTACCATCAGGTGTGTGATGATAATCCCCATTAGA
ATCAATTGTATAATAAGTACCATAAGATACGTCTTGTGATTGTGCTGACATTTGTGTATGTGCTTGTTGGGTG
TTTGTCGTTTCTGCTGCATCTGCTTGATGCGCCATTGTAAATGTAGCGAAGCCCATCGTTGCAATCGCTGCTG
CTGTTAATTTTTTCATGTATAAAACATCCTCCATTAAAGTTAAAGTTAGTTTTCAATTAAACTGTACTGCACA
TACTAAAAGAATTAGACAACTGAGTAAAGGATTTAATTCTCATTTTCCAACTATTTAATATTCCCGAAATGTT
TTACTAAACTCATTACATTGTCATTACAAAATAGCCATACATTGATATTAAAATGACATCTCTCACTGCATTC
GTTTAACCTTTTATAAATTTTCAAAACTAACAACTAATCATTCGTCCATGTTCGCGGTTCAACGCTAATGTAT
GATAGTAATCATCCATCAAATCATATCAACCAAATTCCATTATCAATCGCTATTGATTGTCATTCAACTTTCT
AATAGTGATATGCTTCTCAGGCTTAAAAATCGTCATATCTTTTCTATTAATTAAATCATCTGTGAGCTTTAAT
GCTACTATTTCATTGCTACCATAATACTTAATATATAACGTTCTTGTAGTTAAATTTATTACGGTCTGATACA

TCGTATAGTGATTGTCAGCATCATGCGGACGTACAATTCCAATCGGTATATTTACCGCATCTAATAAATAAAA
TGCATTCATTAAATCCATTTCTTTATCATTGTTTTGAGCAATGTTTGCTTTCATAAATGCCATTCTCACAAAG
CGCTCAGTTGAAGTAAATCCACCTGGCAATCCAAATGTACCTGCTTCATTGCCTAAAGGTTCAATCGTTACAC
CTTCCAATAAATTTGCTGTTGCTGGATAAGGAGAAATATTGATATATTGCCTTAAATTACTATAATGCCAATC
TAAGTCTGGATGATTTGTTAAGACACCAATAGGGTTATCTTTTATAACCACTTCACCCTCTTTAAATGAAACT
TCGACTGATTGTCCAGTTGCATCGGAAACATGATAATGCAATGGCGGAACTTCACCGATATCATTTAAATATA
CAGCTACAACATGTATTTGGGATGCTTGTTGTTTCATATCTTCAATGCTTGTTGTATATCCCAAAATCCATGT
TACAATTTCATTTTGCGTAATATTCATCGCGTCCGCTTTGTGTGTTGATCCATATGAACTATAACCTCGGAAA
TATTGTGTTGAAATGGCAACGCCATGTTCATTAACACCATCACCATAAATAAAACCTTCCATATCTGTTCCTG
TGCCAATAAAGCCATATTGCGTTTGGCCTGTCGTGCCAGTGCGAGATGTCCAACGATAATTTCTAGGCGTCAC
TGCTGGCGAACCATCTAATGGATAATCATAATCCATCGTGCGTCCAAGAAGTACTTGATTATTTAAAGTTTGT
ATTGTGAATCCTGTGCACATTGTTCTCACTCCTCTGTACCTTCATTTACTTTAATCACTTTCAAATAAAGCTG
TTTCACTTAAACATACTATAAAAAATCAATCATACAAGCAATTAATTGATATTCATTCTCAATAACTGTGGTA
TGATATGTAAGGAAATCATGACTTATGTGTGAGTGAACGATCATCTATACATCCGTTCACTTCATCTCATGAC
TTTCTATATTTAATTTTTACAAGGAGTGACATCTGTGAATAACACACAATCTTCACCACGCAGTAATATTATT
ATTGCGATTATGTTGTCTGCATTAACATATTGGTTGTTTGCACAATCATTTATTAATATAGGACCTCTCGTTG
GTCAAACGTATCAAACCTCTCCTGCCGTGTTAAATTTATCTATTAGTTTAACTTCCTTCGCCACAGGTATCTT
CATGGTGGCTGCAGGTGATATTGCTGATAAAATAGGACAACTGAGAATGACATACATGGGTCTCATAATCAGT
ATGTTTGCATCTCTTCTATTAATTATATCGGACATCACTGCACTGCTCATCATCGGTAGAATTTTACAAGGTC
TATCAGCAGCTATCTTGTTACCTTCAACAGTTGGCGTTTTAAATAATCAATTTAAAGGAGAACATTTAAGACG
AGCGATTAGTTATCTAATGATTAGTACTGTTGGTGGCATCGGCCTAGCTGGTGTTATCGGCGGTTTAATCGCC
ACAAATTTCGGATGGCAAACGAATTTCATCATTAGTATAGTCATTGCTTTCATTGCCATATTGCTTCTAAAAG
GCACACCTGAAAAAGTAAGTCAACATAGCCATCGTCATCCATTCGATTACAAAGGTATGTCGATTTTCGCTGT
TATGATTGGTAGCTTTACATTATTGTTAACACAAGGATTCGAACAAGGTTGGTTTAGTACATTTTCATTCATT
TGTCTGAGCATTTTTATCATCACTACGTTGATATTCATCATCATCGAACGTCGACATGAAGTACCTTTTATTG
ATTTTTCAGTATTACGCAACCGTCCGTTCATTGGTGCATTTTTAAATAACTTTGTTTTAAATAGCGGTCTAGG
CGTAACAGTGGTCTTTTTCATATATGCTCAAACACACCTTGGTTTATCAGCTGCGCAATCTGGACTTGTTACA
TTGCCATATGCCATTGTGGCAGTTGCGATGATTCGTTTAGGTGAAAAAGCAACATTACGTTTCGGTGGCAAAT
TGATGCTCATCATTGGTCCGTTGTTCCCTGTCATCGGCATCACTATTATTAGCATGACTCAATTATCGGCATC
ACAATATGTCATTGCAGTTATCATTGGTTTCGTCATTTGCGCAATTGGAAATGGTTTAGTCGCAACACCCGGC
CTTACAATTGCAATTTTCAGTATGCCAAATGAAAAAGTTGGTTTAGCTACAGGATTATATAAAATGAGTGGTA
CATTAGGTGGCGCCTTTGGTATAGCACTAAGTACTACAGTTTTCAGTATGTTACAACTAAACTATGCACCAAG
TGTAGCTGCAACCGTAACATTTATCGTCAGCATTGTATTGATGATCCTTGGCTCATTGTCTGCATACATGATC
ATTCCAAAAACAGTTAAATCTTAAATATAATAGAAGAATTATGTTTCGAAATATCTTTATCACTTTAAAATGA
TATACAAGAAATTCAAGAAAAATAAGCGAAGAACGTTTTTCTCTGTTCTTCGCTTACTCTAATTATATTCACT
ATCAATTAGACAAAATGGCCATTTTCAAATATCACGCGTTGTTTCTGACCTTGAATATATTTATTATAATTCT
CTTTTTGAAAATCAGTTAACATTAATTTAGATGTACCGTATTTTAACACTTTTTGCATTGTTTCTATTCTCAT
TTTTCTAAATAACCATCCATCTTTTAACACAATACGATTAACAGCATCATATGATAATTCTACTGTTTCTTTA
ATTTCAAATGTCTTGAATGAAATAATCGTGCACATTAAAAACGTATCACCAAAGTAATAAACATCTAAATCAT
CACGTTTATGTTGTCCAACTAACAAACGACCATATTCGAACTCTTTTTCTGGATATTTCAATTCTAAAAAACT
AATAATCTCTTCTTCTTTTAATTTGAATTGCATTTAAAAACATCCTCTCTTAAGTTTTAACAAGCCTTAATTA
AAAATTTTTTCAATCACATAGTTCAATATACATCATTTCGTTATATTTTTTAATACTTTTTTTCAAAAACAAA
TATTTTATTCTTTAAAATAATGACTTTTGTATTCCTCATCACAATAAACATTTTAAAATTCTAGTTATCATAA
TCATTCAAAGGTATTAACCTTAATAATATATTCTCTCGCCTCAACCTTAATCGCATACTATAGGCGTCTGTTT
GTAGACAATAAAAGTCATTCACGTCTTCATATGTCATCAAATGTTTATCATGATATGATGAATATAATAATCG
GGTATATAACTGTATGATTAATTACACAATAAAGAGAAGGAATATAATATGAAAAAAGTGTCAATTAAAGATG
TTGCTAGAGAAGCTGGTGTATCAGTTACAACTGTGTCACATATTTTAAATCATAATGATAGTCGTTTTTCCGC
AACAACGATAAAAAAACGTACATGCTGTTTCAGAACGTTTAGGCTATGCCCCTAATAAACATGCAAAACAATTG
CGCGGCAGTAAAATTCAAACTATTGGCGTCATTTTACCTAGCTTAACAAATCCGTTTTTCTCAGCACTAATGC
AAAGTATTCATGACCATAAACCATCTGATGTTGATTTATGCTTTTTAACATCTACAGCAACTGATTTGTATGA
CAATATTAAACATTTAATTGATCGAGGTATTGACGGATTAATTATCGCACAATACATATCATCCCCGGACGCC
CTAAATAACTATCTAAAGAAACATCATGTACCTTATGTCGTACTGGATCAAAATGACCATCAAGGCTATACAG
ATTTTGTTCGGACAAATGAATATCAAGGTGGACAACTTGCAGCACAACATTTAATAGAACTCGGTCACAACAA
TATGATAGTTGTTGCACCATATGACATGATGGCGAATATGTCGACTCGTGTCGCTGGATTTGTCGATACTTTG
CGCGCGAATCAATTGCCAGAACCACAAATCGTCCATACTGAATTATCTAAGCGCGGTGGGCTAACCATTGTTG
ATGACATCATGGTTCAATCTGCCACTGCAATCTTCGCTATTAACGATGAACTCGCTATTGGCATTTTACGAGG
ACTAATTGAACATGGCATCAGTATCCCGAAAGATATCTCATTAATAGGTTATGACGACATTGATTATGCAGCG
TACGTCTCGCCACCTTTAACTACTGTGGCACAACCTATAACTGATATTGGCAAAACATCTTTAACCTTATTAC

TTCAACGATTACAGCACTTAGATAAATCCATTGATATGATTGAATTACCAACGACTTTAAAAATTCGTGCAAC
AACTGGCTATCATCTTTCAAACTAACTACGTATCTTCCGAAATATACTCATCATTGTTAGGCCCTTAGCGTTG
CTTTAATGCTGAGGGTTTTTAATCATAATTATTTCACTAAGAAATTAAAATAATAATGTATGAATTTTTAAAT
ATGATTTAAACGTTTTCAGCTTTTATGAAAACGCATGCATTTTACAAATAAAAATGGTACGATGGCACTGGTA
AAACGTTTTACTAAAAACAAATCGTGAGGTGTATAACATGAGCATTGTTGCATTACTTATCGGGTTAGGCCCC
TTAATTGGCTGGGGATTCTTCCCAACAGTCGCTTCAAAGTTTGGTGGTAAACCTGTACATCAAATTATCGGTG
CTACTGTAGGTACGTTAATCTTCGCTATTATATTAGCCGTAGTCACATCAAGCGGCTTCCCTACTGGAACCAA
TTTGCTATTCGCCTTATTATCAGGTGCTGGTTGGGGATTCGGACAAATCATTACATTTAAAGCGTTCGAATTA
GTCGGTTCATCTCGTGCCATGCCAGTCACAACAGCATTCCAATTATTAGGCGCATCTTTATGGGGTGTCTTTG
CATTAGGAAATTGGCCAGGCATTGGTCATAAAATCATTGGATTTACAGCTTTAGTCGTTATTCTAATTGGTGC
GCGTATGACAGTTTGGAGTGAACGCAAAGAAGCAAGTAACGCCAAAAATTTACGTCGTGCAGTGGTACTTCTG
TTAATTGGTGAATTTGGATACTGGTTATATTCAGCTGCACCGCAAGCAACTTCTATTGATGGCCTAACTGCCT
TTTTACCTCAAGCAATGGGTATGGTAATTGTTGCAGTCATTTATGGCTTTATGAATATGAAAGCAGAGAATCC
ATTCCGTAATAAAATTACGTGGTTACAAATTATTTCAGGTTTCTTCTTTGCATTTGGTGCCTTAACATATCTT
ATTTCAGCACAACCTAATATGAATGGTTTAGCAACTGGATTTATTCTTTCTCAAACATCCGTTGTGCTTGCTA
CATTAACTGGTATTTATTTCTTAAAACAACATAAAACGTCAAAAGAAATGGTTATTACAATCATCGGCTTAGT
ACTCATTTTAGTAGCCGCTTCTGTTACAGTATTTATAAAATAAGGAGTGTAGATGTCATGAAAAAAACAGCTG
TTTTAAATGAACATATTTCAAAAGCAATCGCGACAATTGGTCATTTTGATCTATTAACGATTAATGACGCTGG
CATGCCAATTCCAAATGATCATCGTCGTATCGACCTAGCTGTAACTAAAAACTTACCACGCTTTATTGATGTC
TTAGCTACAGTGTTAGAAGAAATGGAAATCCAAAAAATATACTTAGCAGAAGAAATAAAAGAACATAACCCTA
CACAATTGCAACAAATTAAACAATTGATTTCATCGGAAATCGAAATCATTTTCATTCCTCACGAAGAAATGAA
AAGTAACTTAGCTCACCCATTAAATAAAGGTAATATTCGTACTGGTGAAACAACGCCCTACTCTAATATTGCA
TTAGAATCGAATGTTACTTTTTAAAAGTTATAACTTGAAAGGAGCGTACACATGACCAACAAAGTTGTTATTT
TAGGTTCAACGAATGTCGATCAATTTTTAACAGTTGAAAGATATGCACAACCAGGCGAAACATTACATGTTGA
AGAAGCACAAAAAGCATTCGGCGGAGGTAAAGGTGCCAACCAGGCTATTGCCACTGCACGCATGCAAGCAGAC
ACAACATTTATTACTAAAATTGGCACTGATGGCGTTGCTGATTTCATCTTAGAAGATTTTAAAGCGGCTCATA
TTGATACATCATATATTATCAAAACAGCTGAGGCAAAAACGGGCCAAGCCTTTATCACTGTGAATGCAGAAGG
ACAAAACACCATCTATGTTTATGGTGGTGCGAATATGACGATGACACCTGAAGATGTTATTAACGCAAAAGAC
GCTATAATCAATGCAGACTTTGTCGTTGCACAATTAGAAGTACCCATCCCGGCTATTATATCTGCATTTGAAA
TTGCCAAGTCACATGGTGTGACGACAGTATTAAATCCTGCACCAGCGAAAGCATTACCTAATGAATTATTGTC
ACTAATCGATATTATTGTGCCAAACGAAACAGAAGCCGAATTGTTATCTGGGATTAAAGTAACTAATGAACAA
TCTATGAAAGACAATGCTAATTACTTTTTATCTTTAGGCATTAAGACTGTTTTGATTACGTTAGGTAAGCAAG
GTACATATTTTGCTACTAAAAATCAAAGCCAACACATCGAAGCTTATAAAGTAAATGCGATTGATACAACTGC
TGCAGGCGACACATTTATTGGTGCATTTGTCAGTCGCTTAAACAAGTCGCAAGATAACTTAGCAGATGCTATT
GATTTTGGTAATAAAGCGAGCTCACTCACTGTACAAAAACACGGCGCGCAAGCATCTATTCCTCTACTAGAAG
AAGTAAATCAAGTTTAAATGAATCAAACACAGCTATGATTTGAAGGTTTAGCATATAACATGCAACATTCGTA
TATCATGGCTGTGCTTTTTATCTTTATAAAACATCATCTACTAGAAATAATTTTCCACTAAACCTATGCTTGA
TGACTCATGTTTAGTTATAAATGAAGTGACAATTTTTTGTCATCTGTTTAACTTTCAAATTATCGCATATAAA
TATGCTATATTAATGATAATGATTATCAATTAATAAGGAGGTTATGTTATGTCTAAAGAAGCCGGTCATACAT
TTTTAGCTAAATTAGGAAAAACTCGTCTACGCCCCGGTGGTAAAGAAGCAACAGATTGGTTAATACAACAAGG
GGCATTTTCACAAGATAAACAAGTGTTAGAAGTGGCATGTAATATGTGTACGACATCTATTTATCTAGCTCAT
ACATATGGCTGTCACATTCAAGGCGTTGATATAAATAAGAAAGCATTAGAAAAAGCACAAGAAAATATCTCAG
CAGCAGGTCTTGAATCATATATTCAAGTTCAACAAGCGAATGCTGTTAAATTGCCCTTTGATGACAATCAATT
CGATATCGTTTTAAATGAAGCAATGTTAACAATGTTACCCATCGCCATAAAGGAAAAAGCATTACGCGAGTAC
TACCGAGTCTTAAAGCCTGGGGGTATCTTGTTAACACATGATATTGTCATCGTTAATGAATCACATGCCACAC
ATGTAGTTAAATCATTATCTGCAGCAATTAATGTCAATGTCTCACCGCAGACGAAACTTGGCTGGTTAGATTT
ATATCATCAAGCTGGTTTTAATCATGTACATTATCATACTGGTCCAATGAGTTTAATGACACCTAAAGGTTTA
ATTTATGATGAAGGTATTGTCGGAACTTTAAAGATTATCAACAATGCTTTAAAAAAAGAAAATCGACCAATGT
TTTGTAAAATGTTTAAAACGATGACTAAACTGCGAAAAGATATGAATTATATTACTTTTGTCGCTAAAAAAGA
GCACTAAATATAATGCCACTAACTGTACTTTATATCTATGTTTGACTGTTACTTTAATTTCTTTGTGACACTA
ATCATCTACTTAACAATATCGTTATCGTTGATTAGTAGGTCATCAATTTTGGTTAAAGACTTTTATAAACACT
CAAACATTAACACTATACATACTTAGTTAGTGGCATTATTTTTTTCTTAAAATTTTAACATCACGGGATTGGA
ACAGAAATGATATTTTCGCAAAATTTATTTATGTCCCTCCCCAACTTGCACATTATTGTAAGCTGACTTTCCG
CCAGCTTCTATGTTGGGGCCCCGCCAACTTGCATTGCCTGTAGAATTTCTTTTCGAAATTCTCTATGTTGGGG
CCCCGCCAACTTGCACATTATTGTAAGCTGACTTTCCGCCAGCTTTTGCGTTGGGGGCCCGCCAATACAACAC
TTTAGGTCCTACTATATTAATTTATGTCTCGGGCTCTAACTCTAAAATACTTATAATATACCTTTATAATTTA
TCTCCATTTGATGCTATCACTTGTTTATACCAATAGAACGAATCTTTTTTCAAGCGTTCCATCGTGCCATGAC
CATCATTATCTCGATCAACATATATTAAACCATAGCGTTTCTTCATTTCACCAGTTGTAAATGAAACAATATC

AATGATTCCCCACGGCGTATAACCGATTAAATCTACACCATCTTGATCAACTGCTTCTATCGCTGCTGTAATA
TGTACTTTTAAATATTCAATGCGATAATCATCATGAATATGTCCATCTACCACTTCATCAACTGCACCAAAAC
CATTTTCCACAATAAAAAGTGGCAACTGATAACGATCATATAACACATTCAATGTATATCTTAAGCCATCTGG
ATCAATCGCCCAACCCCAATCACTCGTTGCGATATGCGGATTCTCCACAGAATGATTCAAACCACCGTTGACG
ATGTTGTTTTCAACTGTAGTATCAACATCATGTTTTACAGCCGTAGACATGTAATAACTAAAGCCAATATAAT
CAACCGTGCCCTGCTGTAAAATACTGTCGTCCCCTTCTTGCCATCCAAAATCATATCCTTTTCGTGCCAACAT
TTTTTTAGCATAGCTTGGATAATAACCTCTTACTTGAACATCCGGGAAAAAGAAACGTAAGCGATTCGCAATT
TGTGCTTCCATCATATCTTTCGGATGACACGAATATGGATAAATGGGTACATGTGAAATCATTGTTCCAATCT
TAAATTTCGGATTAATCTCTTTACCAAGACGAACCGCTAAAGCACTGGCTAAAAGTTCATGATGTGCTACTTG
ATACAAGACTTCTTCGGGATTATCATTTTCTGTTAATGCTACCCCAGAATTTGTCCATAAAAAGATAGGATTT
GATGTGTCCATCTGATTATTAATTTCATTAAACGTCATCCAATATGTCACTTTATCTTTATATCTTTCAAATA
CAACACGCGCAAAATGCACAAAATAATCGACAACTTCTCTATTTCTAAATCCACCATAATGTTTCGCTAAATG
AAGTGGCATCTCAAAATGTGATAACGTCACAACAGGTTCAATACCTTGTGCAATTAATTCATCAAAGATACGA
TCATAAAAGGCAAGTCCTTCTTCATTTGGCACATCTTCATCCCCATTCGGAAAGATACGTGTCCACGCAATCG
ACGTACGTAAACATTTCAATCCCATTTCTTTAAACAAGGCAATATCTTCCTTATAACGATGATAAAAATCAAT
ACCTTCATGATTTGGATAATAGTGATTTGGATCTATAGATTCTGTAATCTGACGTGCTTTGCCATGTGCACCA
CTCGTCATAACATCAATTACACTTAATCCTTTGCCACCTTTATCATATCCACCTTCAAATTGATTTGCGGCAA
GAGCGCCACCCCACATAAAATTTTGCGGTAATTTTGTCATTATTACAACACACTCCTAGTTTTTAAATATTTT
AAAAATAACATCTTCAGTCGTAATGATTTCATTCATTGTCAGTTCTACTTTTCCTAAATCGGCAGAGTTGCTA
ATAACGACAATAACGTCTGAGTTATAACCTTGTTCCTGAATATATTGTTGATCGAATTGTAACAGTGTTTGCC
CAGCTTCAACACGATCATTTTGCTTTACAAAGCACTTGAATCCTTTACCTTCCAAGTCAACTGTATTCACGCC
AATATGAATGACTATGTCCACACCTTCTTCTGAACGAATACCGATAGCATGTTTAGTAGGTACAATCATAGAT
ATTAAACCATTGAACGGTGCGATTACTTTGGATTCTTCATGAGCTTTGATTGCCAAGCCTTCACCTACCATTT
TCTCTCTAAAAATTGAATCTCTTACTTCTTTAAGTAAAATACGACGACCAGCGATTGGTGCTTTCACTAAAAT
CGGTGTTGTCACTTTTGCTTGAGGTGTATAACTTGTATCTTGGCTATTAATATTGTCAGTATTCGCACCCTCT
TCAACATTAGCATCCTTAATTAATCGCTGTAATTGCTGATACACTTCATCGACATTTCCGTTTAATTTTATGA
CTAACTGCGCATCATTTTCTGCTACAGATGTCACATCAACTATTTGTTTCACTTCATTTGCTGTAGAAGGAAT
TGTATGTTGCATATGAATTGTAATGCCCTTCGTGTCATACGTTACATCAACAATGTTTTCGACACCACCCACA
GCTGTAAGAATGTCTTGGGCTTGGTCTTTATATTTATTACTCATAAATATTACCCCCTCAACTTGATAGTAGA
ATGAAAATGTATTTACTTACCTCCAATTATAATTGTACCGGTTCATTTTGTAAATGACGATACAATTATAATA
TTTTGTGCTATAATAATTACAGACAAAGTGAAATGAGGATAAAATATTGTTAAAGTACGAACATATTGCTAAG
CAACTTAATGCGTTTATACATCATTCTAATTTCAAACCTGGTGATAAGTTGCCAAGTGTAACGCAATTAAAAG
AACGTTATCAAGTAAGTAAGAGTACTATCATTAAAGCATTAGGCTTATTGGAACAAGATGGTTTGATCTATCA
GGCACAAGGTAGCGGTATATATGTGAGAAATATTGCAGATGCAAATCGTATCAATGTTTTTAAGACCAATGGT
TTTTCTAAAAGTTTAGGCGAACATCGAATGACAAGTAAGGTGCTTGTTTTTAAGGAGATTGCAACGCCACCTA
AATCTGTACAAGATGAACTCCAATTAAATGCAGATGATACCGTCTACTATTTAGAGCGATTAAGATTCGTGGA
CGATGATGTTTTATGTATCGAATATTCTTATTATCATAAAGAAATCGTGAAATATTTAAATGATGATATTGCT
AAGGGCTCTATCTTCGACTATTTAGAATCAAACATGAAACTTCGTATTGGTTTTTCAGATATTTTCTTTAATG
TAGATCAACTCAATTCAAGTGAAGCGGCATTACTACAATTATCTAAGGGCGAACCATGTTTACGTTATCACCA
AACGTTTTATACAATGACTGGCAAACCTTTTGATTCGTCTGACATCGTATTTCATTATCGTCATGCACAGTTT
TATATTCCTAGTAAAAAGTAATACATACATCAAAACGTCTATATTCCAGTTATAAACCGGAGTATAGACGTTT
TATAAGACATAATTTCTTTAAAAACAGTGAGAAGTACATTTTTAATCAGAAAATTACTTTATACTGTTCTTTA
TGTTTACATGATCTAAGAACTTATAGTTTGAATCCTATTGTATAATATCTCTTCCCCTCAAACGTTAAAACAC
ACTAAAATCACTCGGTAATATGATGATCTATACTCTGTATTTTGGTTTTAAGTGTCTGTATCCCCTTATAAAA
ATAACTACTAATATAATGACACCTACTGTGAATATAATGTCCCAAGGCATTCTCCCCCACATGATTGCTTTTA
CAAGTGGTTGTTCATAAAATGACGTTAGTCTTGAATGCCAATAACCATGTTCTAATGCATCTTTAAGTTGGAT
ATAACCTACTGGCATTAATGTAACGAACACCATACCTGCCAAGCCAATATTTAAAGCCCAACATGATATTTTA
ATCCATTTTTCAACTTTCGGTGTCCAAAATTCTTTTTTAGTAATATTTTTAAAAACATACAACGCAATTGCAA
TTGAGAACATACCATACACGCCAGCCATAGAACCATGGGCATGTGCTGCAGTCCATTGCGTACCATGTTCAAA
GTAGTTAACTGCTGGTGTGTTAATTAAAAAGCCTAATGCTCCAGCACCAATTGCATTCCACAATCCTGTTGAA
GCAAGAAAAATAAATGTACCTTTGTAAGGAAAAGCGATGTCACTATTCTTATATAAGCGATAGTGTGTGTATG
CTTCCCAAATTAATAAACAAAGCGGCACGACTTCTAATGCTGAAAAACAAGAACCCAGTGCTAGCCATATGGA
ATGATCACCTTGCCAATAGTAATGATGACCCATACCAACAATACCTGTACCTAGTAATAAGATTAATTGAAAA
TATAATGCACGAATCGTCGTTTCAATGGTAGTTAATTTCATATTAACCATTAAGAAACCAATCAACACTACGG
CAAATGCTTCAAATATACCTTCAACCCATAAGTGAACAATCCACCATCGCCAGTAATCGGCAAACGTCACATG
TGAATTCGGCATTATAAATAATGATGCAAGATAAAATAACGGAATTGCAATTGCTCCAACAAACAATAGCGTC
AATAAGTGACGTTTATCATGATTCACTTGTACTTTGTTGCGAATAGCTGGTAAAAAACCTCGACAAAGAATGA
TAACCCATAAGACCATACCTACTATAAATAATATTTGCCAAAATTTACCTAATTCTATATATTCCCAACCAAA

GTGCCCGAATAGCCACCAATGAGAATCAATCCAACCTAAAATATGACCCCATTCTCCAATCATGCTGCCCATC
ACAACTATGATTAAGGCCCAAAACAAGATATCTACTAATACGCCTTGTTTCTTTGGTTCTTTGCCCAATACTT
TAGGTACAATGTATATTCCTGCTGCTAACCAAGTCGTAGCAACCCAGAAAATAACTAATTGCACATGCCATGT
CTTCGCAATATTAAATGGAAAGAGCCTTTGTATTTCTATACCAAAAAATTTATTCTCTACATAATAATGTGCC
AGTAATTCGCCTAATAATATTTGAACCAAAAATAGTAACATGACAACGACTAAATATTTCCCAACTTTTCGTT
GACTATCAGTGATTTTACGATCCACTTCAATCTTTGGTAGTTCCCGTTCCGACTCATACGTCGGCTTCATGTC
AAATTGATAACGCTTTTGAATATATATAATGAATGCCAAGCCTGCAATCAGTAATGTTACAGATATAGCAGTC
CATAAAATCCCTGCACTAGGCAACGTATTTCCTGCATCCACATCGTATGGCCAATTATTTGTATATGAAAATG
TTTTTCCAGGTCTATCTGTTGACGATAGCCAAGCACCCCAGAAAAAGAATTGACTTAAATGTTCAACTTTATT
TCCTGCGACCATATAATCATCATTTTGAAACTGTTTAATCATATTTTCATTTAACCCTACTTGTTTAGGGTTA
TTAACAAACTCTTTTTTATAATAATCTTGCAAATATTTTAGACCGTATACTTGTCCTGGCGTTAATACAAGTT
GATCTTCTTTTTCAGAATAACGATTAATCCGTATTTCTTTCATCACTTTATCTTTAACTACGGCTAACTCTGC
ATGATTTAATTTATGAAGTTGCTTATGATAAATATCTTGTGCATAATATTTACGCATGCCAATCAGGTAATGA
TGTAATGCCTCTGCTGTATAATCTGGACCTAAATAGGAACCATTGCCTAGGTAGGAGCCATAATCTGTTAACC
CATATTTCTCATATATCGCTTGCCCACTGATCAGCTCGCCTTTTGTAACAAGCGTATGACCTTGTTGATCTAC
TATCTTTGTTGGACGAGGTGCTTCATTTTTAAAAATATACCAACCACCTACTAGTAAAATTGAAAACACAATC
ACTAAAATCGTTAGTAGCACTTTAACTAAACGTTTATATGAACTTTGCATACTCATTTATAAAATCTCCTCTT
AATTGCATTCTTACATATAAGCATACGCCGTTTTTTTATAAAGTGTTGATGACATTAAGTAATTTCAAGAATT
TGTCGAAAAATATTTAATTGAAATTTCACAAATGATATAACTTACATTTTAATTCTTGTTTAAATTAATTCGT
TCTACCATTTTTCAAAAGTGGCAATACATAAAACGAACGCCTAAATACCATAATTCCTAGTAAAAAGTAATAC
ATACATAAAAACGTCTATATCCCAGTTATAAACCGGAGTATAGACGTTTTTTTACGATAATAACAATGGCTCA
AATTGCTATTATCTTGCTTAGGTTTTTCGTTTTAGAAGAATATTGCTACAAAGACAGGCACAACTGCTACAAC
AACTACACCAACTAACACTAAAGCTATACTTGCCATTGATTCTTCTACAGGTCCTAATTCTTTGGCTGGTGCT
ACACCTAATGTGTGACCACTTGTTCCAAGTGCTAATCCTCGGGCAATAGGGTTAGTAATTCGGAAAAGCTTCA
AGAATTTATTACCTAGGGCATAAATAATGACACCATTTAAAATAACTGCTAATGATGTTAATTCTTTTATACC
ACCGATACCAGCTGATACTGGTAACGCAATCGCTGTAGTTGCTGCTTGAGGTAACATTGATAAAATAACATCA
TTGGCAAATTGTGCTAACTTCGCAAAAGTTAAAATAATTAATAACGCTACAACTGTACCGATACCAATACCTC
CGATGATACGATGCCAATGTTTAACAAGCACTTCACGCTTTTTATATAACGGAATCGCAAAACAGATTGTTGC
CGGTTCTAAGAAGAAGTAAATAATATCTCCACCTATTTTGTAAGTCTTATACGGAATGCCTGTTAAATAGAGG
AAGGCCACACCAAATACCATACTGACAAATAGCGGTGCGAATAAGAAGAAACGATTAGTTTTTTCAAATAATA
TGGTCGCTAAGAAAAATGGTATAACGGATAACAGTATTCCGAAGTAAGGTGTGTTTAGTGCTAAGTGGTTAAT
CATGAGCTTGTGCCTCCTCTATTTTGATCTTTTTTGTGACTTTGTCACCTTTAGATCTCGAAGTAACTTTCAT
AATAATTTGTGTGACATAGCCAGTACAAATAAGTAATAGTATTGTTGAGACGATTATTAGTCCAATGATTAAA
AATGGTGCTTGGCTAATGACACCTAAAGAGTTAACAACTGAGATACCGGCTGGTACGAAGAGTAAGCCAATGT
TATTTGTTAGTGTCGTTCCTACTTTTTCGACTTCGCCTAACTTAACAGCACCAGTACATAATAATACAAATAA
TAATACTAAACCGATTACTGATGCAGGCATAGGAATTGGCATAAATGATTCAATTATTTTCGATACAAAGAGT
ACTAAAGCAATTACAATGACTTGGTGAAAAAAGTGTGCTGGTTTTGATGCGTCTTTTTGTTGTTTCACGACCA
TTGCCTCCTACGTTTGATTTAACTAAAGTATAGATGGCTCACTTCGATTTGCGTGATTTTTAGTCCGAAATAC
AAAATATCATAGGTAAAATGCATAAAAAAAAGGATTACTGTTAAAGTAATCCTATCGACGCTTTAAAATCTTT
CATAAATGAACGTCCAACTTGCATCTTGACACCATTTGTCAATATTACCATATAAGTGTAGTTAAACCATTGT
TGCACTTCTTTAATGTGTTTCGTGTTAATAATATATGAACGATGAATACGTATGAAATAAGCGGGATTCAATC
GTTTTTCATAACGATTCAATGGCTCTGTTGTTTCGTATTTATGATTTGTTGTATGTATGGTTGTAATACCATT
ATGTGTGCCAATCCCAATAATATTTTGTTGCTTTAACATGTGAATTTTATCGTCAATTTCAACAGGTAAACTT
TGATCAAAATTCGCCGACATATCATTCGCAATTGCACTTGCGCTATTATCATCTTTGGCTTTAGTCGCACGAA
CTTTATTGACTGCTTGTTCAATACGTTTTTGACCAAACGGTTTCAAAATATAATCTGTCGCATTTAATTCAAA
TGCCTGTACTGCGTATTGGTCATGTGCAGTTGCAAAAATAATCGCAGGTGGCTCTTTCATCTTTTGAATTTTA
GCTCCTAATTCGATACCATTTTCATCCATTAAATTGACATCTAAAAATATAATGTCATATTGATTTATCAGTA
GTGCTTCCAATGTTTCTTTTACATTTTCTGCCTCATTAATTTCTTCAAAACCACCAATTTCATTTAATAAATA
TGTTAATTCATTACGTGCTAATGGCTCATCATCTATGATTAATGCTTTCATATTTATTCCTCCTCTTGTCTTT
CATAAGGAAGTACACACCAAAAAGTGGTACCGCTCGATGTCGATTCAAATTGTAATGCTGCGGATTTTCCAAA
TAATCCTTTTAGGCGTAAGTTTAAATTTTCTAAAGCACTACCAGTTCCAGACTCTGATTCTACAGATGTTTCT
CCCAACAAATGCATTTTATCTTTAGAAATACCCTGACCATTATCTTGTACAATAATACGTACATGTGTTGCAG
TTTCTTTAATCACTGACACGTCAATATCGTTACCTTGCTTTCGATTCGTAAACGCATGTTTGATGGCATTTTC
AACTAAAATTTGAATTAAAAATGGTGGTACAAGCACATCGCGATACTTGTCTTCAACATTAATATTGATATTA
AATCTTCCTGGATAACGTGCTTGTTCGAGTGATAAGTATGCACGCACTTGACTTAACTCTTTATCTAAAGTAA
TCGTATGTTGCTTTGAGCCTTGTAAATTCGCTCTGAAAAAATAACTCAATTCTAATAGTAACTCTCGTGCCTT
TTCGCTATTTATTCTAACTAAAGCTGAAATCGTGTTAATTGAATTGAAGAAAAAAATGCGGACTCACTTGTGCC
TGCAATGACTTAATTTCAGCATCTTTCAATAACTTACTTTGCGTTTCGGCTTCACCAAGTTCAATTTGGCTAC

TAAAAATATTTGCCAATCCTTCTGCAAGTTGACGTTCCACAAAAGTTAAATCATTAGGGTTTGTAAAATACAT
CTTCAATGTACCGACGATAGAACCATGCATCTCAAGTGGTATCACGATAGCTGCTCTAAGCGGGCAATTCGGA
TGACTACAACCAATCTCTTCTTTAGTATGAACTTCTTTCAACTTTCCTGATTTCAATACATCTTTAGACAGAC
TTGTTAATATTTCATTTGTTGGTATGTGATGATCACTACCTGCACCTACATGCGATAAGATTTCATTTTTGCT
TGTAATTGCTACGGCAGATACTTTCATTAAATTTTTAATAATCATCGCAATTTGCTGTGCCGATTCTCTATTC
AATCCTTCTTTAAAATACGGCAATGTCTGGTTCATCAATTGCAGTACATCATGTGTTTGAACAGCCTTCATTT
GCTCCTCTTGCTTTAATGTTGAAATGATAATAGACATAAAAATCGCCGTACCAACGCTATTAACAATAATCAT
TGGTAGTGCAATTAATGATATGAGGTCAACCGCATATGCTTTGTCGTGGGAAAATGTTAAAATGCTCAACATT
TGAATCATTTCCATAACAATTCCAATCATGGCACTTTTCGCAATACTCGGGTAACGCTTGCGTCTTTGAGCTT
GTAAACCAAAATAACCAGCAATTATACCGATAAATATAGATGAGATAAGATAAACTTGTGCATCCGCCCCACC
CATATACACTCTGAAAATACCTGAAATAACGCCAACAAATAGACCTACAAAAGGGCCACCAACTAATCCTGCG
ACACCTATTGTCAATACACGTGTGTTAGCTAAAGATACATCATCATCTAAACGGAAGTACACACTTCCTGACA
AACTATGTTGATGATCGATGACGATACCAGTTAAATTAGACATTAAGGCAAACAAACTGAAAATAATACATAA
TTGCCAACGTGCTTTCCATGTACGTCGACGATTCATTAAGTTTTAAAATATGGAATATTCATCAACACATAG
GCCAAAATAATAATTAAACCTACACGCTCAAGTAATAACATTGTTAGCGATAGCACGATAGCACCTCAGTGAA
TTATAATTATGTTTATTTTATCATAATAGTGCATGTAAAATTCATGAATAAATTATTAAAAAAACACGATACT
ATTTAATTACCTATACCATTAAAATTTCATATCAATTCTTATGCTAAGTTGACAATTTTCCTTTTCAAAATAA
TGACTATCATGTCACTAAATTTCTGTTAAGTATAGCGAAAAACTGCTGACATTACTTTGCCAACAGTTTCTAA
TCGCGTTATTTATGATACTTTTTAAATAAAACATGATTCTCTAAATGAACATGCTCATGTGTTAACACTTCA
AGTGCTTTTAATCTCTGATAAACGAGTCGCCAAGTACCACATGCCTCTATCGGTGGTTCATAGCTTGATGTTA
AATCGCTCATTTTCACTAACAATTGTCCCGTTGCAATGTGATCAGATACTAAATCATCAATCACTGTATTAAT
ATCGTCTACTACTTCACCTTGTTCATATTTAATGAGTTTTGGAAAATCAACATCATCTTCTTTTTGCATATGC
TCTAACATGCCATTTTTAAATGTATCGTATGTTGCTTTTAACTCAACTAAATATGGATGATTAGGTCCATGTA
CTTTCGATAACTTTGTCACATAAGGTGTTAAATTTTTAAATTCTTCTCTTAGAGGTTCATGATATGCTGCTTG
AATATATTGAATAAGTGATGAAACATTTAAAAATTTAGGATTTAACGAACCTGGTGTATTCGTTTGTTCAACT
TCATTGAGACGTTGTAATAATTCGTTCAAATCTACATTTTTCTTTTCTAAGACTGCTGCTTCTATACTTACTT
GTCCGCCACAACAAAAATCTATTCCCACACTTCTAAAAATATCTGCTGCTTTCGGATAATCAGTTACTACATC
TGCTACTATGTCATTTTTATTTATCATTATAGATAACCTCCTCATTTTCAGTGTATACAATTTAATGTATTTG
AAAATGATAAACGAGGAAATGTCATAATCATGTCATAAACAGCAAAATAAAACCCTCAAATCATGTTAGTTCA
CATAGATTTGAGGGCATTTATTATTTATTTTAGCGAGTAAGTCATATGTGCAGTGTTTAACTTAACAATATTT
TTGAAGTCTCGCTTCTTAATATCTTTACTAAGTAAGATTTCCACTTCGTTTTCTGACGTTTGGCTTAACATTC
CCATCACCATGTCGTCTTTTAGCACGACACTAGCCATACCTTCTTCAAATACATAACCACGATCCAGACCGGT
TAAAATGATTTTGTTTTTACCTTTTAACTTCATTGAGATTGAATCCGTTAATGTAATAGCTGTAAAGTATAAC
GAAATGTTATTAAAGTCTTTTGTGAAATACGGTGTTAATCTATAAAAATCATTATTCAAATGAATTAATGCAC
TTGTTTCTGCAGTATATGCATATTTATTACGTTGATTACCAACACGCACTTCAAATACTTCCGTACCTATTTG
GACTTCTAAGAAAACATCCCAAATTTTTTCTTTAGGTAAATCCGCTTTAATATCTTCTAACATAATGTTAAAA
CGATATTGTTTATTCGCATTGACAGTTAAAGGTACATACATTGTGTGCTTCTTGTTTCGTGAATGTAATTTAA
GTCTGACATCATAATGTCCTTCGCCAATCATAAATTCATTTAACGTTAGCTCTGTAACAATTTTATGATTATC
AAATTTAAATGCTGTCATTTTCACTTTTGGCTTAGCGATATCAATGCCTTCAAAGTATGGTTCGCCTTCATTG
AATTGAATGTTTAATTTACCATCTACAATATCAAAACGGTAGTATTGACCTTGACGGTAACTTTCTTCTACAG
TTCTATAGTTGTCTATATCTTTCGCTCTCGCGTAGTGCAACAATGGTCGTAATTTACTCATCACCAATGCATC
TACACGTTCTGGAACTGCTTGTATAAAGTCTCCTAGAGCATTAATCCATTGCGGTTGATCTTCTAGTTTAACT
TTAAGTGAGAAGCCATTCGTACGAGAAAAACTAAAATGACGATTTAAAAATTCTGCTAAGATTTGATCTTTAT
GAGCTTCTTCTAAATCTGCATTTAATATTTCTACAGCAATCAATCTCATGACTTCGTAAAAGTCTTCAGGTGA
AACATACGCACTACTCATATGTTCCCCTTCACGCTTTGTAGCATAATAATACGCTTTATCACTTAACACACTG
ATTCGATTTGCATTCAAATATGCTTTCATTGTAAATAATTGATCTTCTGCACTCTTTAATTCTTCTGGAAAAT
AAATGTCATTATCTTTTAGTAATGCTGTTCTATAGATTTTAGTCGGGCTTAAAGTATAGATAATTCTTGAATT
TAACAATGTAACTTCAGGTGCTGTTTCTTTAAACATAGATTGTGGTACACCACGACCATTAACACCTTTCATT
TTAATCAATAAGACATCTGAGTGATGTTCATCAATAAATGCTGCTGCATCCTTTAAAGTCTCTTTGTTTATAT
AGTCATCGGAGTCCACAAATAATACAAATTCACCTTCTGCTTGTTTTAACGCCACATTTCTAGGTTTACCTGG
ACCACCAGAATTTGTTTCGAGTTGACTTACTTTCAAGTTCAATTTATTGCGATACTTTTCAACTATTTGTAAC
GTTTGATCTGTTGAACAGTCATCAACTACAACCACTTCAAATTCAGTTTTCGGAAAATCTTGTTTCGCAAGGC
TATTAAGTAATTCTGTTATATACTTTTCTGAATTGTATGTTGGAACTATTACTGAAAATTTCATCATTATACC
TCTCCCACTTTGAATACTATATAAACTTAGCTACCAAATAAATTTCTGACTAAACGCTCACTTGATCGGCCAT
CTTGATATTTAAAATGTTTATCTAAGAATGGAATGACTTTTTCTCCTTCATAATCTTCATTGTCCAAGGCGTC
CATTAATGCGTCAAATGATTGCACAATTTTACCTGGAACAAATGATTCATATGGTTCATAAAAATCACGCGTC
GTAATATAATCTTCTAAATCAAATGCATAGAAAATCATTGGCTTTTTAAATACTGCATATTCATATATTAAAG
ATGAATAGTCACTAATTAATAAATCTGTTATGAACAGTATATCATTAACTTCTCTAAAGTCAGAAACGTCAAC

AAAATATTGTTTATGTTTGTCTGCAATATTAAGTCTATTTTTCACAAATGGATGCATTTTAAATAATACAACC
GCGTTATTTTTTTCGCAATATCTTGCTAAACGTTCGAAATCAATTTTGAAAAATGGGTAATGTGCTGTACCAT
GACCACTACCTCTAAATGTTGGTGCGAAAAGAATGACTTTCTTACCTTTAATAATTGGTAATTCATCTTCCAT
CTCTTGTTTGATCTGTGTCGCATAAGCTTCATCAAATAGTACATCAGTACGTGGAACACCTGTAGGCACTACA
TTTTTCTCTTTAATACCAAATGCTTCAGCGTAGAATGGAATATCGGTTTCAGATGATACATAAGCTTTTGTAT
AGCTACGATGATTTAATGAATCAATAAATGGTCCACCCTTTTTACCAGTACGACTAAAGCCAACTGTTTTAAA
GGCACCAACGGCATGCCATACTTGAATAACTTCTTGAGAACGTCTAAAACGCACTGTATAAATCAATGGGTGA
AAGTCATCAACAAAGATGTAGTCTGCCTTCCCAAGTAAATATGGCAATCTAAACTTGTCGATGATGCCACGTC
TATCTGTAATATTCGCTTTAAAAACAGTGTGAATATCATACTTTTTATCTAAATTTTGACGTAACATTTCGTT
ATAGATGTATTCAAAGTTTCCAGACATCGTTGGTCTAGAGTCTGATGTGAACAACACCGTATTCCCTTTTTTC
AAGTGGAAAAATTTCGTCGTATTAAATATCGCTTTAAAAATAAATTGTCTTGTATTAAATGATTGTTTGCGGA
AATACTTACGTAATTCTTTATATTTACGAACGATATAAATACTTTTAACTTCCGGAGTCGTTACAACAACATC
AAGGACAAATTCATTAACATCGCTAGAAATTTCAGGTGTAACAGTATAAACCGTTTTCTTCGAAATGCCGCCT
TTTCTAAATTCTTTTAGGTAAGTCTGCAATAAGAAATTGATTTTACCATTTTGTGTTTCTAATTCGTTGTATT
CTTCTTCTTGTTCTGGCTTTAGATTTTGATATACATCATTAATCACATCTGGGTTTAACTGTGCAATATAATC
AAGTTCTTGCTCATTCACTAATAAGTACTTATCTTCAGGTAAGTAATAACCATTATCTAAGATAGCTACATTG
AAACGACAAACGAATTGATTCCCATCTATTTTGACATCATTCGCCTTCATTGTACGTGTCTCAGTTAAATTTC
TTAATACAAAATTACTATCTTCTAAATCTAGGTTTTCACTATGTCCTTCAACGAATAACTGAACACGTTCCCA
ATAGATTTTATCTATATATATCTTACTTTTAACCAACGTTAATTCATCCTTTTCTATTTACATAATCCATTTT
AATACTGTTTTACCCCAAGATGTAGACAGGTCTGCTTCAAAAGCTTCTGTAAGATCATTAATTGTTGCAATTT
CAAATTCTTGGCCTTTTAACAACGCTAATTTATCTACAATATCTGGGTATTGAATGTATAAGTCTACAACATC
TTGGAAATCTTTTGAACCACTTCGACTACTACCAATCAACGTTAGTCCTTTTTCCAATACTAGACGTGTATTA
ACTTCTACTGGGAACTCACTTACACCTAACAGTGCAATGCTTCCTTCTGGTGAAATGTAATCGATCATTTGAT
TTATGGCTGATTGACTACCGCGACCACCCACACACTCAAATGCATGATCAAATGTTAAGCCTTCAGGTATTTT
ATTAATAAAGAAGACATCATCAACAAATGAGAAGTGACTCAATTTATAATCTGTTTTACCAAAGACATATATT
TTAGACTCTGGATATAATTTACGTAATAAAATGGCTGTAATGTAACCTAAGTTACCATCACCCCAAATACCAA
ATGTATTTTTATTTGAAATAGATTTCTTTTCAAAACGACGAATAGCATGCAAACTTACTGTTACAAGCTCTGT
ATATGAAATAATACTTAAATCAATATCATCAGGTAGTGGTACAGCTCTATCATGATTTAGCAACACAAAATCT
TGCATAAACCCATCATGTCCACTTGATCTGAAGTAGCTCGATTTTAAATAGTTTTCAGCAATGACATCGTCTT
TTTCTGTCGGCGTATTCGGTACCATAACTACTTTTGTACCTTTATTAAACACACCTTTACTGTCAAATACGAC
CTCACCAACACCTTCATGAATTAAAGACATTGGCAATTTCTGAGATAAGACATTCTCATCACGGCTACCAGTA
TAATATCTTTGATCGGCAGCACAAATTGACATATATAAAGGACGTACAATGACATAGTCACTGTAAATATCTA
CGTTGTTATACGTAACTTCAAATTGTCTAGGTGCAACTAATTGATATACTTGATTAATCATCGGCAATACCAC
CTCGAATAATAGCATTCGCTACTTTTAAATCGTAAGGTGTTGTTACTTTAATGTTATATAACTCACCTTTTAC
AAGTCGAACCGGTTTGTTTGTTTCTACAATAATCTTACAAGCATCAGATAAAATACTCTTTTGCTCATCACTC
AACTGTGCATAGCTTTCTTTTAATAAATTTATATTAAACGATTGAGGTGTTTGACCTTGGTACATTTCATTAC
GCACTGGAATTGCATCAATCGTTTGATTATCTTTAGATGTAACAATCGTATCTATAGCATCAATCACTGTGTC
TACTGCACCGTACTCTAAAGCAGCTTGAATATTTTCTTTAATAATACGATGCGTTAAAAATGGTCTAACTGCA
TCATGTGTCACAATAACATCGTCATCGTTAATACCATTTGTTGATTCAATATGTTTAACGATATTCATAATTG
TATCGTTACGATCGCTACCACCTTGAATGACTTCAATTCTTTCATCAGAAATTTTGAATTTTCTAAGTGTATC
TTTCGTATGCGTCATCCATTGTTGTGGCGTCGCGATAATAATTTTTTCAAAATCATTAATTAAAATAAATTTT
TCTAATGTATGGATTAAAATCGGTTTGTTGTCTAAATCTAAAAATTGTTTAGGTAAAGGTACGTTACCCATTC
TTGAGCCTATACCTCCAGCTAGAATACCAGCGTATTTCATAAAATACTTCCTCCATTCAACTATATCTATATT
TAATTATTTAAATTTCGTTGCATTTTCCAATTGAAAACTCATTTTAAAATCAAAACTCTAAATGTCTATGTAT
TACTTAAAATTATACATATTTTGCTTATATTTTAGCATATTTTGTTTAAACCTATATTACATTATATCAGACG
TTTTCATACACAAATAATAACATACAAGCAAACATTTCGTTTATTATTTATATCACTTAACTAATTAATTTAT
AATTTTTTATTGTTTTTAAGTTATCACTTAAAAATCGTTTGGCAAATTCGTTGTGACGCCTTTCCATCTTCTA
ATGAACAGAATTTTTGATAAAATACCGTTCGTGCTTCAATATACTCATTTGCAGTCTCATCGATTTGTTTTAA
TGCATCAATGAGTGCTGTTTGATTTTCAACAATTGGACCTGGCAACTCTTTTTTATAATCCATGTAAAAACCT
CTAAGCTCATCGCCATATTTATCTAAGTCATACGCATAGAAAATTTGCGGACGCTTTAATACACCGAAGTCGA
ACATGACAGATGAGTAGTCGGTAACTAACGCATCGCTGATTAAGTATAAATCCGAAATGTCTTCATAATCTGA
AACGTCTTTCACAAAATCATCATGTTCATCAATACGTGTCACAACTAAATAATGCATGCGTAATAAAATAACA
TAATCATCATCCAGCGCTTGACGCAAAGCTTCTATATCAAAGTTAACATTAAATTGATATGAACCTTCTCGAA
TCGCTTCATCGTCACGCCAAGTTGGCGCGTACATAATCACTTTTTTATCTAATGGAATATTTAATCTTGTCTT
AATACCATTAATATATTCAGTATCATTGCGTTTATGTGATAATTTATCATTTCTTGGATAACCTGTTTCCAAA
ATCTTATCTCGACTAACATGAAATGCATGTTGAAATATCGATGTCGAATATGGATTAGGTGACACTAGATAAT
CCCAACGCTGGCTTTCTTTTGTAAAGCCATCTTGGTAATTTTGAGTATTTGTTCCTAGCATTTTAACGTTACT
AATATCCAAACCAATCTTTTTCAATGGCGTGCCATGCCATGTTTGTAAGTACGTCGTTCGCGGTGATTTATAT

AACCAATCTGGTGTACGTGTGTTAATCATCCACGCTTTCGCTCTTGGCATCGCTAAAAACCATTTCATTGAAA
ACTTTTTAACATATGGTACATTGTGCTGTTGGAATATGTGTTCATATCCTTTTTTCACACCCCATATTAATTG
GGCATCACTATGTTCAGTTAAGTATTCATATAATGCTTTGGGGTTGTCGCTGTATTGTTTACCATGAAAGCTT
TCAAAATAAATTAGATTCTTGTTTGGCAATTTTGATAGTAATTTAAATGTCGTATATATACTATGTTCTATCA
ATTTTTTAATTGTAGTTTTAATCATGTCGTACCTCCGACGTGTTTTTGTAATTATATTAATATGTATGAGCAA
GCTCATTGTAACCATGCTTATTATAGCATTTCATCATAAAATACATTTAACCATTACACTTGTCGTTAATTAT
CATACGAAATACATGATTAATGTACCACTTTAACATAACAAAAAATCGTTATCCATTCATAACGTATGTGTTT
ACACGTTTATGAATTAGATAACGATTGGATTGATTATTTTATTTTACAAAATGACAATTCAGTTGGAAGGGTG
ATTACTTTTGATTGAATCGCCTTATGCATGAAAAATCAAAAGGTTATTCTCATTGTATAGTCCTGCTTCTCAT
CATGACATGTTGCTCACTTCATTGTCAGAACCCTTCTTGAAAACTATGCCTTATGACTCATTTGCATGGCAAG
TAATATATGCCAACATTAGCGTCTAAACAAATCTTTGACTAAACGTTCACTTGAGCGACCATCTTGATATTTA
AAATGTTTATCTAAGAATGGCACAACTTTTTCAACCTCATAATCTTCATTGTCCAAAGCATCCATTAATGCAT
CAAAGGACTGTACAATTTTACCTGGAACAAATGATTCATATGGTTCATAGAAATCACGCGTCGTAATGTAATC
TTCTAAGTCAAATGCATAGAAAATCATCGGCTTTTTAAATACTGCATATTCATATATTAAAGATGAATAATCA
CTAATCAACAAGTCTGTAACAAAGAGAATATCGTTAACTTCACGATGATCTGACACATCGATAAAGTATTGTC
TATGTTCACGTGAAATATTAAGTCTATTTTTTACGAACGGATGCATTTTGAATAACACAACTGCATTATGCTT
CTCGCAGTATCTTGCTAAACGTTCAAAATCAATTTTAAAAAATGGATAATGTGCCGTACCGTGACCATTACCT
CTAAATGTCGGTGCGAATAGAATAACTTTCTTACCTTTTATAATTGGCAATTCATCTTCCATCTCTTGTTTAA
TTTGTGTTGCATAAGCTTCATCAAATAGTACATCAGTACGTGGTACACCTGTTGGTACAACATTTTCTTCTCT
AATTCCAAATGCTTCAGCATAAAATGGAATATCGGTTTCTGATGAAACATATGCTTTCGTGTAACTACGATGG
TTTAATGAATCGATAAACGGACCACCTTTTTTACCTGTACGACTAAAGCCAACTGTTTTAAAAGCACCAACTG
CATGCCACACTTGAATAATTTCTTGTGATGGTCTAAAGCGAACCGTATAAATTAATGGATGGAAATCATCGAC
AAAAATATAATCGGCCTTACCAAGTAAATATGGCAATCTAAACTTGTCTCTCCATTTACGTCTATCCGTAATA
TTCGCCTTAAATACCGTTTTAATATCATAATCAAAATCTACTTTTTGGCGTAGTAACTCATCATATACATATT
TAAAGTTACCTGATAAATTCGGACGTGAATCTGATGTGAATAATATTGTTTTGCTTCTTTTAATATGTAGTAA
CTTTGTAATATTAAAAATAGCTTTAAATAAGAAACTTCTACTTTCAAATGAAGCTTTATGGCCTTGTTTATGA
AGCCAGTGTGCGCTTGTCGCAATGACCCCTGATTTCTCTTGAGGTAAGGTGATTTCAATATCAAATACAAATT
CGTTAACGTCACTTGCTATTTCTGGAGTAATGGTATAAACCGTATGTTGTGATACGCCACCTTTGCGGAAAAC
TTTAGCTTCGTTGGCTAACACATAGTTTGCTTTTCCATTTTGTGTAGTCAGCTCATTGTATTCTTCAATTTGC
TCTTCATTTAATGCCGCATAAGCTTGATCAATAATATTTGGATTAAGTTGTCCAATATACTCTAACTGGTCAT
GATAAACAAACAAATATTTATCCATTGGTAAATAATACCCATCGTCTAATATTGCGACGTTGAATCTACATAT
AAATGTATTACCGTCTATTTTCATTTCATTTGCTTCTAATGTTTTTGTTTCGGTTAAATTCCTCAGAACAAAT
TGTCCTGATGTTAAATCTACACCTTCACTATGTCCTTCAATATATAACTGAACACGTTCCCAGTATATGTTAT
CAATATGTATTGCTTGTTTCGTTTTTGTCATTGTTATTCCCCCATTGGTTTATTAAATCGTCCATTTCAATAC
TGTTTTTCCCCAAGATGTCGATAAATCCATTTCAAACGCTTGGACGATATCTTGCATCGTACATACATTAATT
TCATGTCCTTTTAATAAGGCTAACTTTTCAACAATGTCTGGGTACTTATGATATAAATCAACAACTTGCTCAA
AATCTTTAGAGCCGCTTCGACTACTACCAATCAACGTTAATCCTTTTTCAAGTACTAATCGTGTATTCACTTC
CACGGGTAATTCACTTACGCCTAACAAAGCAATACTGCCTTCTGGTGAAATATGTTCAACTATTTGTTGAAGT
GCAACTTGACTTCCTTTACCTCCAACACATTCAAATGCATGATCAATTTTAAGATCATCTGGTATTTGATTTA
CTGTAAAGATGTCATCTACAAATGAAAAATGACTTAATTTATAGTCTGTCTTACCAAATACATAAATTTTAGC
TTCTGGGTACAACTTACGTAGCAAAATAGCAGTAATATAACCTAAGTTACCATCACCCCAAATACCAAAGCTG
GTTTTCAAAGGTATAGATTTACGTTCAAATCGTTGTATAGCATGATAACTTACTGACACTAATTCTGTGTATG
AAATCGTACTCAAATCAATGTCATTAGGCAGCGGAACGATACGATCATGTGCCATCACAACGTAGTCTTGCAT
AAAACCATCATAACCACTAGATCTAAAATAACTAGAGGCTAAGTAATTCTCCGCAATAATATGATGTTGCTCT
GTAGGTGTATTCGGCACCATTACTACTTTCGTACCTTTTTCAAATACCCCTGTACTATCGAATACAACTTCAC
CAACAGCTTCATGAACTAATGACATTGGTAATTTTTGGCGCAGTACATTTTCATCTCTTCGACCTGTGTAATA
CCTTTGATCAGCTGCACAAATAGACAAGTATAAAGGTCTTACGATGACATGATTACCATAAATATCAACATTA
TTATATGTGACGTCGAACTGTCTCGGTGCAACGAGTTGATATACTTGATTAATCATCGGCAATATCACCTTGA
ATAATGGCATTTGCTACTTTTAAATCATACGGTGTTGTCACTTTAATGTTGTATAGTTCTCCACGTACCAATT
TAACTGGATGTCCAGATTCGACAATGATTTTACATGCATCTGATAAGATTTCTTTTTGTTCACTACTTAAGGC
GCGATAACTATCTTGTAATAATTTAATATTAAATGATTGTGGTGTTTGGCCTTGATACATTTCATTCCTTACA
GGGATACTGTGTATGTTCTGTTTATCTTTAGACATTACAATCGTATCAATTGCTTCAATGACTGTATCTACTG
CACCATATTTTGCTGCTACTTCAATATTCTCTTTAATAATACGTTGAGTTAAAAATGGTCTTACGGCATCATG
AGTTACAATCACATCATCATTATTAATTCCATTTATATTGCGAATGTGGTCGATAATGTTCATAATTGTTTCG
TTTCGATCCGTGCCACCTGCAACTACTTTGACACGTTGATCTGTAATGTTATATTTTTTTAAAATATCCTGTG
TATGGGAAATCCACTGTGCTGGCGTTGCGATAATAATCTCATTAAATTCACTCACTAAAATAAACTTCTCAAT
TGTATGGATTAAAATCGGTTTATTATCAATATCTAAAAATTGTTTTGGTAATGGCACGTTCCCCATTCTCGAA
CCAATACCTCCTGCTAAAATACCTGCATAAATCATGTTGTCCTCCATTCTGTCATTACATCATTTCCATTTAT

ACATTACTGACCTATGCCCGCACATAAGCCTAACCTATTGCTCACTTGCCTCTATTATTAAGCCAAAGGTAGT
TGTCACAATAGTGTGATAATTTTTTATAAAAATGTATTTTTGTAACTGACCATTCTAAGTTGTTTTGCCATGC
AGTTAATCATTAACACTGACGATATTAAATTGTTAAAGGTATTAATGTTTACTCTTTTTCAAATTCATTATTA
CTGCCATCATTTTACCATATATTATAATAAATTTATCTTATTAAGTGGCTGTACTTGATTTTCACTTTAAAAA
TTATCAAATATTGCCATCTCATTTTAAGTATACAAAATGCAAAACAACCGATTCACAAGCATATTTCACACAA
GTAAACCAGCTATTTAGCAACGTATATTCGAAGATGAATTATTTCGATAGTATCTGTAGACCAGACGGCATTC
GCACTTTCATAGCTATAACTATACCAGCGTTTTCGTCCTCGAAGGTGCATACTAATAAATCGTAAACATGACT
TTATCAAATCGTTCTTTCTTGTTAACTAATTTATCAAATGTCTCTGGGCCTTTTTCTAACGGTAAAAAATGAG
AAATAATAGGCTTTACATTAATATCTTTCGTCTTCATATAATGTAAGGTTGCCGTCCACTCTTTGCCCGGAAA
ATCACTGGACAAACAGTTCCAAGAGCCACATACTGTCAACTCGTTACGCAGAATTTTTTCAAAATGAACGCGA
TCAATCTCAATATCATCATATGGTATTCCGAGTAATACCACCTCGCCACCTTTTTTAGGTAGCGTCAATATTT
GACCAATCGTAACTTTAGCACCTGATGATTCTATAGCTAAATCGATTTGATTGGCGTAATGATTTTCGATGAA
TTTCTCAAGATCTTCTTCTTTTGAATTGATTGTTTGATGTGCGCCCAATGATGTAGCAATATCTAGTTTATGC
GCATCTATATCTATAGCGATGATATGTGCAGCACCAAATATTCGTGCCCATTGAATAGCTAACAATCCTATAC
TGCCACACCCCATTACTGCAACAGTCATACCAGGTTGTATATTCGATTTATAAAATCCATGCGCAACAACGGC
TGATGGCTCAACCATTGCTGCTTCAATGTAATCAACATTGTCTGGAACCTTTAAAACATTTTGCGCTGGCAAT
TTGACATATTCCGCGAACGATCCAGGTTCATATGAGCCAATGACGAACAACTTTTCACATCGTGCATATTCAC
CTTTCAAACAGTACTCACATTGATAACAAGGTATGGCTGGACAACCTGTCACTTTGTCGCCGACCTTAACATG
CGTAACATCACTTCCAATGGCATCTACAACACCTGAAAATTCATGACCAAATGGCATACCTTGAATGTATGGG
CCCATTTTTTTGTATCGTGACGTGACTGAACCACATATACCCGTTGCGCGCACTTTAACAATAACGTCATTCG
CACTTTCAATGACTGGCTTATCATTATCCTCATACCGTAAATCTTCCACGCCATATAATTTCAAAGCTTTCAC
TTGTAAATCACCTCAACTCTGATTTAATTCACAACTTTTTTCTTTTTAAAAATACCTGTCGCAAAATAACCTG
CAATGACAATGGAATTACTTACGAGTAAATGTTCCATATAAAAATCAGTGATTTGTCTTAATGGCCCAAGCAT
AAAAGTTAGCATTGATTCTACCATATTAATTCTCTCCTTCATTTTCATCTAAATCGAGCGGCTTAAGTAATGC
TTTAATACCTTCGCCTGATTTTAAATGTTGATACGCCTCGTCCCATTTCGAAATATCATATATTTTTGTCACC
AAAGCTTCAGCATTTACTAAACCATCCGCCATAAGTCGCAATGAAGGTTCCCAATCTGCTGGCTTTTGACTTC
TACTACCAACAACTGTTATTTCTTTTTGAATCACTTTTTCCATATCAAATGGAATTTCAGCATCCTTAAAAAT
ACCTATTTGACTGTAGAAACCTTTTTTGCGTAAAATATCCAAACCTTGTCGTGCTGCTGGAACTGCACCTGAA
CATTCAACAACAACATCTGCACCGTAACCGTCTGTAATGCCATTGATATACGTTTTTAAGTCTGTTTGTTGTA
AATTGACTACATAATCCATGTGCAATGCTTCTGCTTTATCTAATCTGACTTTGTCATTGTCCAATCCAGTTAC
CACAACAGTTGCGCCTTTACTTTTCAACACTTGTGCTACAAGTAATCCGATTGGCCCAGGTCCCATTACAACT
GCTACATCGCCTGAATTGACTTGAATCTTAGAAACACCATGATGTGCACATGCTAATGGTTCTGTCATAGCTG
CAGACTGATACGATACTTCGTCTGGAATATGATGCAAACTTTCTTCACGTGCAATGACATAATTAGTAAATGC
GCCATCAACTTGTGTTCCAATACCTTTTCGATGGTTGCATAAATTATAGTCTTTTGATTCACAGTATTCACAC
TCATTACAAATATAGAATGTCGTTTCAGAAGTGACACGGTCACCAACTTTAAAATCTTTAACGTCTGCTCCAA
CTTCAACGATTTCACCAGAAAATTCATGACCTAATGTCACTGGAAAATTAACTTTATAATGACCTTCATAAGT
ATGAATATCTGTGCCACAAATTCCTGCATAATGTACTTTAATCTTTACTTTATCATCTAGCGGTGTTGCAACT
TCTTTATCAAGAAGTTCTAAGTTGCCATGTCCTTCTCTTGTTTTTACTAAAGCTTTCACCACAAACACCTCGA
TTTTTAATTGAATAGACTAAATAGTTTAAAGATAAGATAGTTAACGATATTACCACCTTGATCAATACTTGAA
ATTTCAGATGAACCTTTTGGCATTTGTACATTCGTACCTTTCGCCATATCTGTGAAAATGGGTGCTACGTCTG
TTGCAATATATAGTGAAATTGCAATCATAATCGTACCCACAATGACAGAATGAATAATGTTTCCTCTTGCTGC
ACCAACAATAAACGCGACAACAAATGGTATAGTTGCTAAGTCACCAAAAGGTAGTACTTGGTTTCCTGGTAAA
ATAACGGCTAATAAAACAGTGATAGGTACTAAAATTAAGGCTGTCGAAATAACCGCTGGATGACCTAATGCTA
CAGCCGCATCCAATCCAATATAAATTTCACGTTCGCCAAAACGTTTGTTTAGCCATGTTCTTGCAGACTCTGA
AACTGGCATTAAACCTTCCATTAAGATTTTTACCATTCTAGGCATTAATACCATTACTGCAGCCATTGACATT
CCTAAATTAATGATGTCTCCAGGTTTGTAACCTGCTAACACACCAATACCTAAACCTAAAATTAAGCCGACAA
AGATAGATTCTCCAAATGCGCCAAAACGTTTTTGAATTGTTTCAGGATCAGCATCTAACTTATTCAGACCGGG
TACTTTTTGTAACAATTTAACTAAGTAAATACCTGGTGCATAAGAAATTGTACTTCCTGTAGCAATCGATACA
CCTGGTAAATCAAAGAACTCACTCATCATCGGTGCTGTCCAATCTGCTACTTTCAAACAGATAACTTGGAAAA
TAACTGCTGCTAATAACGCTTGCCAAATACTGCCTGATACGGCATAAACCATTGCTGCTGTAAACGTATAATG
CCAAAAATTCCAAATATCTACATTCATCGTCTTTGTCACTTTAGTTACTAGCAATACAACGTTAACTATGATT
CCGAGTGGAATAATAAATGCTGCGACAGATGATGCCCAAGCGATAGATGATGTTGCTGGCCAACCTACATCAA
TCACATTCAGACTGACGCCTAAATTTTTAACCATCGCTTGTGCTGCTGGCCCTAAATTTTTAACTAATAAATC
GATGACTAAGAAAATCCCTACAAAAGCCACACCTATTGTTAAACCAGACCTAAATGCCGCTCCAATTTTCTGC
CTAAAGAATAGGCCAAGCAAGAATATGACAACCGGTAAAATAACAGTTGCACCTAAATCTAAAAATCCCCTTA
CAAAATCAGTGAAGTAACTCATATTTAAACCCTCCCTGTTATATATGCATTGTCACGATACTTTCCGATTGTG
ATTACATTTGACGTTACAGTCATTTCAACGACAACCCTTGCTACATTCGACTGCAGTCCCTTTGAATTACAGT
CACTGCGTTTCTATGTCATCAACAATCATTTGTCGTGATAGTCATTTATATGCAATTTGCATATATTTATATG

TTATTGAACCACGTTACATATCAATTCCGTTATTTTTGTAACTCTGTTAAGATTTGTTGTTTTGTTTCTTCAA
TACCAATACCAGTTAAGAAATTACGTGCGTTGATAACTGGGAATTTATATTCTTTTTTTGTCATTGCAGTTGT
AACTAATAAATCTGCAGTGTCTTCATAAGGTCCAACTTCTGTAATTTTGATTTGTTTAATATCTACTTTAATA
TTGTGTTCCTTTGCTATTTCCTCAATTGCATTATTTACTACTGTTGACGTTGCAATACCTGCACCACATGCTA
CTAATACTTGTTTCATTTTCAATTCCTCCAATTAATTTTTAGTTATATTCCAAATAATCATTGATTAGTGTTG
CTAAAATTGTTTCATCTTTCGTTCGCAGAATCTGCTCCAATTTTTCTTCACTTTGAAAAATTTGCATCAACTG
TTGTAACAGCTTAAGTTGATCATCTACTTTATCCATTGCTAACATAAAAACGATTTTCACTTCTGTCTGTTGA
TCAAGTGTTCCCATTTCAATAAACGGCACTTCTTTTTCTAGAACAGCCACACCTATCGTTCTATGGTTAATAT
GTTCGACATCTGTATGCGGTATAGCGACCGAACATAGATGCGTTGGTAAACCAGTAGCAAATTCTTTTTCTCT
GTCGATGACTGCATCTTTAAACGTTGACTTCACGAACCCATTTTGAAATAACACATCTGACATTTGTGACAAT
ACGGATTCTTTATCAGTTGCCGACAAATTGAGCATTATATTTTCTTTATGCACTAATTGCTGTCCCATCCATT
TTCCCTCGCTTCTTTATTTGAATAATTTTTTAAAATCTCATTTACATCAGAATTTTTGCGACTTTGTATGATG
CGCTTAATTGCATCATTGTCTTGCGCCACATCTCTCAATTGTAGTAACGCTCTTAAGTGTGTCACTTTATCAA
CAGCAGCAATAGGTACAATAATATGGATTGCTGTGCCATCTGACATGTATATTGGTTCTTGTAATATCAACAT
ACTCATCGCCGTTTTATGTACATGCTTTTCAGAGTATGCATGTGGAATAGCAATATTTTGCATGATAACCATA
TACGAATCATCAAATACCTGTTGCATCTCATGAATATATTTAATATCAATAAAATGATTAGCAACTAACACAT
CACTTGCTTTAGCAATAGCTTCATCAATATTTTCAACATGATGTATTCTTTTCACGTGCCTTGCTGGTATCAA
GTCAGCTAAATCTAATGTCTTATTTTGTGTGACAATCGATCCATTAATGGTTGAAATTGAATTATAATTCGCA
ATAAAATCTTCTAAACCATCACGTAGTTCTGTAATGTCATTAACTGTCGTTGTGCGTTCAATTAATGCCATTA
ACTTGTTTATTTCCTTATCAATGTCAGCCGATTCCTTATTAATGTACTTCATCACTTCTTTACGTAACTTTCG
TTGCTCATTTTCAGATAAAGCTACTTTTGTGATAAATAATTTTTTATGTGTTAGGACAAACATTGGTGAAAAG
ACGATGTCATAATCTAATGTGTAATTTTCAAATGTTCTAAGTGAAATCGCATCTAAGAAAATAATTTCTGGAA
ATAAGTTTCGCAACTCGTATAACATCATTTGTGATACTGACGTGCCTTGTGTACACACGATAATAGCTTTTAT
CTTGCCATCGAAGTTTTCATCTTGACGTCTCAAACTACCTCCGAACAACATGGTTAAATATGCTATTTCATTA
TCAGGCAACGATTTTCCGAAATATTCAGTTAACGATTGGCATGATTGTTTCACCATATGAAATAAGGATTGAT
AATTTCCTTGTAAAGGATTGATTAATTCATCACGATCCGTTAAGTTATATTTAATCCTATAAAAAGCAGGCGT
TAAATGTAACAAGAGTTGCTGTGATAATTTCTCCTTATCTTCAATGTTAATAAAAGTGATTTGTTCAAAATGG
TGAATCATTTGAGCGATGGCCATCGTTAAATTCGATATGCTATCTGATTCTTGCAAATCAGTCCATTGCACAC
TTGTTGAAAGTAAGTGTAATGTCAAATATAACTTTTCCGCTTCTGGCAAATCCGGCTCATGTTGCGTCATAAT
CTCCGTTGCTTGATATTCTTTCGTATCCCTCAAATACTGATAATTAATATTTAATGGATTCATCACATGACCA
CTTTGAATTCGTCTACGAATCACACAAAGGACATAAGGCAGTGAACTAAGTGATTTGTCTATAAAGCGACTCT
TCAAAAATTGTTCTACCTGTTTGATTTTGTCTTTTTGATATGCGATATCTTCGAATGTTAAGTTGAGCGCCTT
TAAAACTTCACTTTTAGTAATATCATGATTCAACCTTTGATCAATCAATTTAATGAAGAAACGGCGAACTTCA
AATTCATCACCAACAATTTCATAACCATGTTTTCGAGAATACTTAAGTGACAAACCATGATTTTCCAATTGCT
CTTTCACATGATTTATATCGTGAATGACAGTATTTTTACTGACTTGTAAATCAATTGAAAAATGGTTTAGAGA
CATTGCGTTTTCCTTACTAAAAAGCATGAGCATTAAATAATAACGACGTGTTTCTATGCTAAAAATGACATTG
TTACCGTTTAACATTTGCTGCTCCGATACATCTCGCTTGAATAACGTCATGATTTCAGAACTTACAATAAAAT
TTCCTTGGCTTGTTCTTTCAAGTTTTGGATAACCCTCTTGTTCAAGCCACAAATTGATTTTTTGAATGCGATA
TCCTAGTTGTCTACGAGATAAACCAAATATCGATTCAAGTTCTTTACCATGAATAGTAGGATTCAATACAATT
TCTCTGAGTATTTGCTGATTATCCCCGTTCATGTTCGATCCTCCTTTGAGCATCTGAGCTCAATTTATCATCA
ATCGTCACACCGATGTACACACTTTGAACACATATTTTCAAAATGAGTATGTACATCATTGTGATGTTTCGAC
AACATTCCAATTATATCGGTATTCTTTGTGATTTTAATCTTTTAAAATAAAGCAATTGAAATTTTTACATATA
TTTTTGTGTTTTTTTGAAGCATTTTTAACATACATATCTCAATCATTATCAAATTGTCATGACCATTGTAATC
CAATACAAAAACCCTAAGGACGCTTATATCAGGCGCCTTAGGGCTAACTGTATCTATTTAATTAAGTATTATT
ATTCGTAAGTACGTAACTTATAATCTATCAAGTTCCACACTTCTTCAACATCAACTGCTGTAGCAAAATAAGC
ATTGGCAGGCTTACCTGTAACATGATTTAAATCGACAGCCATAGTACCATAAGTTAGTGGACTTTGATGTTCA
ATGTCGATATTAACGGGTACCATTGTAAACAATTCTGGTTGTAACAAATACAAAATTGTACAAGCATCATGTA
TTGGACCACCGTCCATATTAAAGTGAGTCTTGTATGTCTTCTTAAAGAATTGCAATAATTCTACGACGAACTG
TGCAACAGGATTATTGATACTTTCAAAGCGTTCAATCACGAGATCGTCGGCTAAAACTTGATGTGTTACATCT
AAACCAAACACATTTATAGTAATCCCACTTTCAAAAACGCGCTTCGCTGCTTCAGCATCAACCCAAATATTGA
ATTCTGCTGTAGGCGTCCAATTTCCAAATGTACCACCACCCATCAAAGTAATAGATTCAATATGCTCAGCGAT
TCTTGGCTCACGAATCAATGCCGTTGCTACATTCGTAAGAGGACCTGTCGCTACAATTGTTACAGGTGTATCA
CTCGTCATCACTTTGTTTATAATCATATCTGATGCTGGCATTGCAACTGCTTGACGTGATGGTGTCGACGGTA
GTTTCGGGCCATCTAATCCAGATTCCCCATGTATTTCAGAAGCAAAGGCAGCTGATTTAATTAACGGCCTATC
CGCACCTTTCGCTACTGCTATATCTTGACGTCCCATAATATCCAATACGTTCAAGGCGTTTGTCGTATTCTTG
TCAACTGATTGATTACCTGCGACTGTTGTTACAGCTAATATCTCTAGTGGACTGTCAATTGCCCCCGCTAAAA
TTAATGCTATTGCATCATCGTGTCCTGGATCACAATCCATAATAATCTTTCTTTTCATTTATATATCCACCTT
TCTTAAGTTGTTATCGATAGCTTATGTATATTTATTTATGTGGTGAATCATGTTTATTTTGAAAAATAGTTTT

AACTTTCTCATATTTTTGGATACAAACACTATTTATCTATTTTATGGCTTATAAATTTATCCGATATGCCTTA
TCAACCTACCTCGCTAAAAATAGGATGTCTACATATCTATACCGACTTTTGTCAACTCATTTTCACAACAATA
TAAACAGCAATTTATATGATTGTTACATGATTCAAACAATTTTTATGAAAAATATTTTCATACACAGAATATA
TATTGATATTAAATTTCTCAAAAGCTATATTGAGAATAATTAGGAGGGATGTTGATGAAATCTTTATTTGAAA
AAGCACAGCAGTTCGGCAAGTCCTTTATGCTACCTATCGCAATCTTACCAGCTGCAGGTCTATTGTTGGGTAT
CGGTGGTGCATTAAGTAATCCAAACACCGTTAAAGCATACCCTATTTTAGATATTACCTTATTACAAAATATT
TTTACATTAATGTCAGCTGCAGGTAGTATTGTTTTCCAAAATTTACCGGTCATCTTTGCAATTGGTGTCGCAA
TCGGATTATCTAGAAGCGATAAAGGTACTGCAGGTTTAGCTGCGCTGCTCGGTTTCTTAATTATGAACGCAAC
TATGAATGGCTTATTAACTATCACGGGCACATTGGCAAAAGATCAGCTTGCACAAAATGGACAAGGCATGGTG
CTCGGTATACAAACGGTTGAAACCGGTGTTTTTGGCGGTATTACCACAGGTATTATGACCGCAATACTTCACA
ACAAATATCACAAAGTGGTATTACCACCGTATTTAGGTTTCTTTGGTGGCTCTAGATTTGTCCCTATTGTCAC
AGCATTTGCCGCAATCTTTTTAGGTGTATTGATGTTTTTCATTTGGCCAAGCATACAAGCCGGCATTTATCAT
GTTGGTGGATTTGTAACGAAAACAGGTGCCATCGGTACTTTTGTTTATGGCTTCATCTTAAGATTGTTAGGTC
CACTCGGTTTACATCATATTTTTTACTTACCGTTTTGGCAGACGGCACTTGGTGGTACTTTAGAAGTCAAAGG
GCACTTAGTTCAAGGTACGCAGAACATCTTCTTTGCTCAACTCGGTGATCCAGATGTGACGAAGTATTATTCA
GGTGTGTCACGCTTTATGTCAGGCCGTTTTATTACGATGATGTTCGGCTTATGTGGTGCCGCACTTGCAATTT
ATCACACAGCTAAACCTGAACATAAAAAAGTTGTCGGCGGTTTAATGTTATCCGCTGCACTCACTTCATTTTT
AACAGGTATTACCGAACCTTTAGAGTTTAGTTTCTTGTTTGTCGCACCTATTCTTTATGTAATCCATGCCTTC
TTTGATGGATTAGCATTTATGATGGCAGACATTTTCAACATTACAATTGGTCAAACCTTCAGTGGAGGCTTTA
TCGATTTCTTACTTTTTGGTGTGCTACAAGGTAATAGTAAAACAAACTACCTATACGTCATACCTATTGGAAT
TGTGTGGTTCTGTTTGTATTACATCGTTTTCAGATTCTTAATTACGAAATTTAATTTCAAAACACCTGGTCGA
GAAGATAAAGCTGCAGCACAACAAGTTGAGGCTACTGAAAGAGCACAAACTATTGTTGCTGGTTTGGGAGGCC
AAGATAACATTGAAATCGTTGACTGTTGTGCAACGAGACTACGCGTCACACTTCATCAAAATGACAAAGTCGA
TAAAGTATTACTCGAAAGTACTGGTGCCAAAGGTGTAATCCAGCAAGGCACTGGTGTGCAAGTAATTTATGGG
CCTCACGTTACAGTTATCAAAAATGAAATTGAAGAATTGCTCGGGGATTAAGACTAACCGAAATATCAACAGA
ACTAATGGCAACGATGTACGAAGTAAGAAGTGACATCGTTGCTTTTATTTTTAATGTTACATTTGAAGCATTA
AGTTCATCATGCACTGTAGTGAGCCCGCAAATCGCCTCTGCTAGACAATCATCTTAATGCTATGATTAACGCT
TAAGTGCCAGATTTGAATTTAATTTCAACAACGACTTTCACTACATTAAAAATAGGGCTACTCGACACATATA
GTTGTATCAAATAGCCCTTTATACAATTTTTTGGGGTAAGGTTTTACAATTTTTGGAATGGTATAGATTTTAT
AAAAAGTTATTTAAGTTCTTCTGATTCAGCCATAATATCTTTTAATGTTTTAGCTGAATGTGCGAACTTGCTT
TGTTCTTCGTCGTTTAATGGGATTTCTACGACGTTGCGAATACCGTTTCTATTGATGACTGCTGGAACACCAA
TGTAAACATCTTCTTCATCATATTCGCCTTCTAATAATGCTGATACAGTCAATACGGCATCTTCATTTCTGAA
AATCGCTTCAGTAATTCTAGCTAATCCCATTGCAACACCATAATAAGTGGCACCTTTAGCTTGAATAATGTCA
TATGCTGCATCACGTGTTTGAACAAAAATTTGTTCAATTTGCGCTTTGCCCTCAGGACGTTGTTCAAGTAATG
TCTTCAAAGGTTGACCCGCAATATTAGCGTGTGACCATACTGGTAATTCAGTGTCACCATGTTCACCAATAAT
TTGAGCATCGACACTACGTGGCGCAACATCGAACGCTTCGCTTAACAATAATCTAAAGCGTGCAGAGTCTAAA
ATTGTACCAGAACCTATAACACGTTCTTTAGGTAAACCAGAGAATTTCCATGTTGCATACGCTAAAATATCAA
CAGGATTTGTAGCTACCAAGAAAATACCATCAAATTTTGATGCCATTACTTCACCAACAATTGATTTAAATAT
TTTCAAGTTTTTAGATACTAAATCTAAACGTGTTTCTCCAGGTTTTTGTGCAGCACCAGCACAGATGACAACT
AGATCCGCATCATGACAATCACTGTATTCGCCAGCTTTCACACGAACTGTTGTTGGAGAATATGGTGTGGCAT
GTTTTAAATCCATAACATCTCCTCGAACTTTTTCAGTGTCTAAATCAATGATGACTAATTCATCAACAATGCT
TTGGTTCACTAATGAAAATGCGTAGCTTGAACCTACTGCACCATTACCTATTAATACAACTTTGTTCCCTTTA
AATTTGTTCATTACAAAAACTCCCTTATGATTAATTCACTAACATACGTGTAGCTTCAAATATGTTAGTTTAA
TGCTGCTTATTGACGATACAAAAGCAAATAAACATCTCTTTTATTTTCAACGCATAACTTAAAAGGTCATGTG
TCATCCGCTTTTAAGTTTGTGATTTATTTCACATATAAAATGTAACACGCATTAAGTACTGGGTCAATATTAA
ATTGTGATTTATTTCACATTTTATTTTAATTTTTACACCTTTTTAATTTGTATACGATTACATCTTAGATGTC
TTTAGTCTTCGTACTTCGCCAGTGATTTTTTACACTTTCACATTTTTATTATCATGTTTAATTTTTTCTAAGA
AAACAACAATGTTTTTTGAATTAGTCAAATAAGTGCACGCAATCGTTGGGGTTCAAATACACAATTGTACACA
ATGCTTATTGATAAGTATTTAAAAAATTAAAAATGTCATACAATTATCAAATTTGCCATTTTATTTATATTTT
CTCAAACCAATTAATTGAATATCGAAATTTTTAGTAGAATAATCAAAGTAGACAGATTAAAGGAGGAGTATCA
TGCTTACAGAACAAGAGAAAGACATTATCAAACAAACGGTGCCTTTACTTAAAGAGAAAGGAACAGAAATTAC
GTCAATCTTTTATCCAAAAATGTTTAAAGCGCATCCTGAACTTTTAAACATGTTTAATCAAACGAACCAAAAA
CGAGGCATGCAATCTTCAGCATTAGCACAAGCTGTAATGGCCGCAGCGGTTAATATCGATAACTTAAGTGTTA
TTAAGCCAGTCATTATGCCAGTCGCATATAAACACTGTGCATTACAAGTTTATGCTGAGCATTATCCAATTGT
GGGGGAAAATTTATTAAAAGCCATTCAAGACGTGACAGGATTAGAAGAAAACGACCCTGTCATTCAAGCTTGG
GCGAAAGCATATGGCGTCATTGCGGATGTGTTCATCCAAATTGAAAAAGAAATTTATGATCAAATGATGTGGA
TTGGTTTTAAACCTTTCAAAATTACCAATATTAAACAAGAATCTGAAGACATTAAATCATTTACAGTTGAAAC
TGAAGAATATGACTTTAGTGAATTTACACCAGGCCAATACATCACAGTTGATGTTTCTAGTGATAAACTTCCA

TATAGAGCTAAACGTCACTATTCTATCGTATCAGGTGATAAAAACCATTTAACTTTTGGCGTTAAACGTGATG
TCACAACAGAACATGAAGGCGAAGTTTCAACAATATTACACGACGAGATTAAAGAAGGCGATATGATTAATCT
AGCTGCGCCTGTAGGTGGATTCGTATTAGAGAATACGACTGAACCACAACTTTTCTTAGGTTCAGGAATTGGT
GTTACACCTTTAGTAGCTATGTATGAAGCTGCCTCTGCCAAAGGTTTAGATACACAGATGGTTCAAGTTGCTG
AAAATGAACAACATTTACCTTTCAAAGACAACTTCAACAGTATCGCAAGCCATCATGACAACGCTAAATTATA
TACACACTTAAAAGATAAACAAGGCTATATTGGCGCTGAAGAATTAAAAGCATTTTTAGCAAATAAACCTGAA
ATTTATATCTGTGGTGGTACAAAATTCTTACAATCTATGATTGAAGCACTTAAATCTTTAAATTACGATATGG
ATCGCGTACACTACGAAACATTTATTCCAAGACTAAGCGTTGCAGTATAACTGAGAAAAGAGGTTTAGTACTC
ATATGAAACTTAATCAACGTTACGTTAAAGTATTTGCATTATATTTCGTAAGTATTGTTACTGCAAATATTGT
TGTCAAAAATAACAATTTAATCAAAACATTGATTCAAACACTTGCCGGTTACACCGTCTTTGCAGTTGGTTTG
AAGTATTTAACTAAACGTAAAAATAAATAACATATATACAAAAACAGCTATGACTTTACTTTGTCATGGCTGT
TTTTTAATTGGGAGTGGGACAGTAATGATATTTTCGCAAAATTTATTTCGTCGTCCCACCACAACATGCATTG
ATGTATGCTCACTGAATTTCATAAGAAAGGAGTTCACAAGATGACCGTAGATATTGGACGGATTTATGACAAT
AAAGATAATACCGACGCTATTCGTATCCTAGTCGATAGAGTCTGGCCGAGAGGTATTTCGAAAAGAACTGCTA
ACCTAGATTATTGGTTAAAAGACATTGCCCCTTCTACTGAGTTGCGACAATGGTTCCAACATGATCCTAAACT
TTTTGGAGCTTTTAAAGAAAAATATGAAAAAGAATTACGTGATCAGGATGTGCAAAAAGAGGCTTTTGAAAAA
TTAAAGGATATTGTAAATCAGCATAATCATGTTCTATTGTTATATTCAGCAAAAGATACTAAACATAACCAAG
CTGTAGTACTACAGCAGTTGCTCAATACTTAGGTATATATGTTTGTCTTGAATCACTATACATGTCGCTAACT
TCATTTCTAATATTTATTTTTAAAAAGCATAATTACTACAATTAATTGAACTTTAATAATCACTAAACTTGAA
CAACATTTTACTCTAACAAAATAAAGTTTTAAAATATTAATTATTGGGTTTTACATAGTAATTAGAAAGAGAG
TGTTAATGAAATGAAGTTTAAAAGACTAGCAACTATATTTTCAGCAGTATTAGTGTTATCAGGATGTGGCTCA
ATGCATTCATCTGGCAAAGACTTGAATATTTCGTTACCGTTAAAAACAAAGTCTATTGCACCTTATGAAACAG
ATGTACCAGTTAAGATTGGTGCTGCAGAATCATTATTTAAAACAAATGATCAAGGAAAAATAGAAAAAGTATT
AGTGAAATCGTATCATCAACCAAATGATAAAACGTTAGATATAGAATTAAAAGATAATATTAAATTTCAAAAC
GGTCAAAAATTGACTGCAGAAAAAGTGAAATCTAGCCTTGAAAATAGCATGAAAAAAAGCGACTTGGTCAAAT
ATTCATTACCAATATCATCAATTACCGCTAAAGGTCAAAAACTGACAATTAAAACCAACTACGCTTACCCTGA
ACTTGTATCTGAATTAGCTAATCCTTTTATGGCAATTTATGATACAGATGCTAAATCAGATGTTAATCAAACT
CCTGTTGGTACAGGCCCTTACCAAATAAAAGATTATAAGCAATCTCGAAAAATATCATTGTCGAATTTTAAGG
ACTATTGGCAAGGTAAACCGAAACTTGATCATATTACTGTGACGTACCAAGAAGACGGCAATAATCGCGTCAG
AAATTTAGAATCTCAAAAAGATGATTTAATAACTGATGTCCCAGTTAATAAAGTTCAAGACATAGAAAATAAT
CAAAATTTAAAAGTGTCAAAAGAATCTGGATTTAGAACTTCTTTACTTATGTATAATCATACTAATAAAAAAA
TGACTAAATCCGTTCGTGAAGCATTAGATCATATCATTGATAGACAAGGTATTGCAGATCATATTTATCAAGG
TTACGCGAAACCTGCAACGAGTCCATTTAATGATAAAATTCCATATATTAAAGAACCTAAGTTAACCAAACAA
AATATCGAACAAGCAAAAGCGTTATTAGCTAAAGATGGTTATACAAAAGAACACCCTTTAAAAATTAAGTTAA
TTACGTATGACGGTCGTCCAGAGCTATCGAAAATTGCGCAAGTATTACAATCAGATGCTAAAAAAGCTAATAT
TGAAATCGACATTAAAAGTGTTGATGACATAGAAGGTTACTTAAAAGACCGTTCTGCCTGGGATGCAACGATG
TATAGTTTCGGAACGATTCCTCGTGGAGATACAGGCTATTTCTTTAATCAAGCATATAAAAAAGATGGTGCTA
TTAATAAAGGAGACTACAATAATAGCAACGTTGATGATTTAATTAATCAATTGAATCATACTGTAGATATTAA
GGAACGTCACAATATTTCTAATGATATTATTAAATTATCAAGTCGAGATGTACCAAACAGTTATATTGCTTAC
AACGATCAAATAGTCGCTGCTAACTCTAAGGTTAAAAATTATAAAGTAACACCTGAAGGTATCTATTTAATCG
ATTATCGAACAACGATTGAACGATAAGGGAGTGTGGGACATTGATTTATGTCTCAGCCCCGCTTCAACTATAA
AAACCACGCCGAGGTCATTCAATATGATTATGGCGTGGTTTAATTTGCTTATATGATTGTATTACTTTCTGTT
TGATGAATTTCAATAAAATGATTATTACATATTTTTAGTGAATCATGTTCATTTTATATAAATCTAATTGTCA
CTATTTGTTTAACGTGAAACACTTTTTAACTCCAAAAAGTATTCCACTTCCACTCTTTTAGAGACAAATTATA
AAAATAGCTTATGTCACAAACATATGAGCAACATAAGCTTTAATTATTATTCGTCTACTTTTTTCTGTTGTTC
TTTAAATTCTTTCTGCAGTAAATTAACTTGTTTTAAACCTGCCCCCATTAAAATGAATACAAGTATCCACACA
ATAACGATTAAAATAAATATTTTCAACGTATCACTGCCAAGTACAGTTAAAGATGTATCCCAAATGCCATGTA
AACCAACGGCTGATAAAAAGAATATTAAAAAGCGTTTATCAAAAATATCTTTGAATTCAAAGCCATGCTGTTC
TTTCGCAATAACTATTGCAGCACCAACAATCGCTGACCAAACTAAATGGCCACCAATCGCAGTCCACGCACGT
GTGAAGACAATATCTAATAATGGGACATTTTCCCCTAAAGCGAAATTCAAAATATAACCTGCTGATTCAAAAA
CTGCGAACCCTGCACCAATAGCAGCACCGATTAATAATCCATTCAAAATCTTATTTGTTTTCAATTTATTGAC
GAAATAAACAATAATGAGTGCTTTTCCAGTTTCTTCAACTAATCCTACTAAAAATGCATCGAAAAATGTTAAA
GAACCAAACCTTTCGAATTGATCACTAAAAACGACAAATCTATATAATACCATCGTACTTAGTAATGAAAATA
CTCCGCCAATAAAGAACATGATAATAACTTCAAAAATGCTAATATTTTTAAACGCATTTGATTCATAAAAGAA
GAACAAACCCGATAATGGTACTGTTAAAGCCCCAATAAAAATGAGACCCGGAATCGCGTTAGTGTTATTAAAA
ATCGCTGCCATGACCCATAATCCAATAAATGTTACTGTGAAAGCGATGAATACTCGAGAAAAGAGCCATGGCT
TCCCCCATTCTTCTGATATGTCACAAATTGCTGGCGTAGTTTTAGCAGTACCCGCTATAAATATTTCATCTGC
TTCGTTTTTAGTATGTGGCTTAAATACTTCTGAAAACATATCTTTTAAATTAAGATTTAAGCTTTCATCATTG

CCGGCTAGTCTACCGATTGATTTGGTTGCATTATCAAATAAACTTCTTGCTTCGTTTTTTAAATCTGTATTTT
GCGCTGTTTCATGCGCTTTGGCTTCCATTTGTTTATCGATAGATTCATTATGTATGTCGGACGTCTTAGTTGA
TTGTGTACTTGCTGATGACATCCCCTCTTCACCTGTATTAGGTGCAACTGATTGCGTTTGTGTTGTCTCTTCT
TTTTCTGTATTTCTTCGATGATTAAGACCTTCCATAGACTTACCTACTTTCACTTTTCATAATAAAACTATTT
TAATTTAATATTGATGGATTTTCCATATATTTATATAACTACTTTGTTCGATTGTGTACATTCGTTTTTTTAC
TCATTTTAAAATACAACTCACATCTTACGCTTGAGTAATAAAATCAAGTATTCGGAAAATGGCCTGCCTATTC
TGTTTAGGCACCTCAAAGGCTAATATGTCCTCCGCCGATTGCTCTAAAATAGAATGATTGATGTATTGGGCAT
CATTTAGTACTATTTCTCTGACATCGTCTGGACCAGGCTCAAAATGAAATTGTAATCCAATCACATGATGATC
GATGATAAATCCTTGGTTTCTAATATGATTCCCTGAAAATAATAATTCTGCTTGTTGCGGTATTTGAAACATA
TCCTCATGCCAATGTAGTGCCTTCAATCGACTTGGTATATTCGGAATGATTTCTGATTCTAAGTAAACATCAT
CCCAACCTACTTCCTTTACTGGTGATTTTAACACCGCATAACCTAATGCTTTCGAAATTTGTTGTGCACCAAA
GCAAGCACCATAAATTGGTTTGTTTTGTGCCATCAATACTAAAATTAATTCGCGCTCTGATTGAATCCAAGGC
ATCTCATCGTCTGGATTCATTGGCCCACCAAGTATCACTAACATATCTGTTTCTTCCGCAAGAGGTAGCACGC
CATCATTCATATATGGGTGATAAATATAGACTTCGTGCCCGCTTTCCTTCGCCCACTCACCTATGTATCCAAC
ACCTTCATTTGGTGTATGTTGTAAAACATTGACTCTCATATGTTCAACATCTCCTTCAACTCTCCAATGATTT
CATCAGTTACATTATACACCACTGATTGTACGCTGATCATCCTCACCTTACTGATGCATCAAAATGATAATTA
GTCTTTGCATTTTTTAAATAAATATGGGTTGTTTTTTATATTCCGAGCAATATTTTTATATAAGTATTTTAAT
GATATTCAATTCATTACAAACCCTTGATATTACAATTGTTATCAACCATCAAAATGTGTCTATACTGTGTCTA
TTATCAGAATAAAAAGATATTTCTAATTGATTTTAAATGTCGTTATGTTATATTTCTTGTAAGGGTTTACACA
AAGTGTAAAAACGCTATATTTTATCAAATGTGATGGAGGTGACTTGCTTGAAACAAATCACATGGCACGACTT
ACAACATATCATTAAAGATGGTGATGTGATTGGTTTACCAGCATTAGCTGTAGCCAACTTACCCGCCGAAGTT
CTACGTGCTGTGTTAGCGCAACATGACACATATCATACGCCCAATGATTTAACGTTTATATTAGCGAATGATA
TCCATAGTTTAGGTGCCGCACCGGATTTAGATGATTTTATAGAACGTCGCATGATTAAACGTGTCATTATGAG
TATTTTAACGGCTTCTTCCAAAACGGCACAAGCAATGAAAAATAATGACATTGAAGCTTATTTTTTACCACAA
GGTATCATTGCAACTCATTATCGTCAGAGTAATCAATTATTACCTGGAGTTATTACTAAAATCGGATTAAACA
CAGCTGTTGATCCTAGATACGGTGGCGGTAAAGTAAATACACGAACAACTGATGATTTAGTTTCATTAGTAAC
CATCAACGATGAAACATACTTACACTACACATTCCCTAGCGTTGATGTGGCACTACTGAGAGGAACATACGCA
GATCAACAAGGTAACATTTATTTAACTCAAGAAGCGTACTTGAGCGAGTGTTATCATGTCGCATTAAACACGA
AAGCCAATCATGGGAAAGTTATTGTACAAGTTAAAGCTTTAGTTGACGACTATCAACTAAAGCCGAATGAAGT
TGTTATCCCAGGAAATCTTGTCGATTATGTATACGTCACAGAAGATGAAAAGAATCACCGTCAAGTAATTCAG
AGTCATTATTTACCAGCCTTGTCTGGAGAAGAACGAATTGATGGAATACCTGAACCCGCATTACCTTTTAATA
GTCGCAAATTGATTCTCCGACGTGCTGCTCAGTTTTTAACTTATGGCGATACAATTAGCATCGGTTATGGCAT
CAATAATGAACTCTCTAATTTATTGCATGAAGAATGTGTTGAACATGATGTGCAACCGATTTTAGATGTTGGC
ATTTTCGGTGGATTCGTTGGGAGTCGTGAACATTTTGGTATGAATTACAATGCAGATGTGCGCATGCCTCATG
ATCGAGCATGGGATTTTATTTATAACAATGGTGTATCAGTTGCCTATCTTAGCTTTGCTGAAGTTGATCAACA
CGGCAATGTCAACGTGTCATACTTCAATGACCGACTAAATGGATGTGGCGGCTTTATAGACATTACGCAATCT
GTAAATAAAATTATCTTTTCAGGTACTTTTGTAGCCGGTAGTCATGTCTCATGTCATAATCAACGATTAAACA
TTGAAACTGAAGGACAAAACCAGAAATTTGTATCAGATGTGAGCCATATCGACTTTAATGCACAATATTCACA
ATCACTCGAGCAAGAAGTCTATTTTGTTACTGATCGTGCAGTATTCGAGCTCACCAATCAAGGCTTGAAACTA
ATTGAAATTGCTCCAGGTCTTGATTTGCATAAAGATATTTTGAATCAAATGGCTTTTAAACCAATTATTGCTG
ATCATTTAAAATTAATTGATACCAGCATTTACAAAGAAAAATGGGGACAACTTAAACAATCAATTCATAAAGT
ATGAAAGTGAAGCTAAAGGGGGTATACTAATGAATTTCGATTGGATAAAAACACGTTCAGACTTCGATGATGA
CAAGCCTGCCGTTATTGATCACGCAAAACAAACATCTTGGACATACCAACAACTCAATGCACGCGCTGATAAT
ATGGCACATTATTTAACATCTCAAGGTGTTAAAAAAGGCGATGTTATCGGTATTTTTGCGCCAAATGATATTG
CAATATTAGATTTACTATTTGCTTGTTTTAAAACAGGTGCGGTTTTTTTACCATTGAATTGGCGGCTTAACCC
AAAAGAAATTGCAGCCATTGTGGAAGATGCACAATTAAAACTGCTCTTCTATGCTGAAAAACATTTAAGTTCA
CTCACCGATATTGACCAAAACTTATTGCATATGGATATTGATGTAGCGCAATATGATGAAATCGTAAATCCAG
ATTATCACCAACCTTTTCAAGCAACACCTGTTGAACCACAAGATCTTGCAGCATTAATTTATACAAGTGGTAC
AACCGGATCACCTAAAGGTGTGATGTTCTCATATGAATCATTTGTTCATAACGGCGCAAACTTAGAGCTGACG
TATAAGTTCAATTCAAACTATATTACGATTGTATCAACACCAATGTTCCATGTTTTAGGGTTTAACGATACTG
TATTACCAGTATTAATGTCAGGTGGCACACTTATTCTTCAACGTTACTTTAACGGCGAAGAATTGAATGACAT
GATTGCACAATATCACCCTACATTTATTATCATGATTCCGACAATGTATTATAGTACGCTACGTGCTAGTAAT
TTTAATCCTGAAAATTTTAAAGCTATGGATTATATCATCCAAGGTGGTTCACAACCATTACCAAGTATCCAAG
CAGCTTTTAAACAATATGGCATAAACATTATTAACGGCTATGGTTTAACTGAAGCACCTCTTGTACTTGTTAA
TACACCGGAAAATTCAAAACGTAAGCCAATGAGTATTGGTAAAGCAGTCATGTTCGTTGATGCACGTATCCTT
GATGATAACGGTGAGGAAGTACCTACTGGTGAGATTGGCGAACTTGCAATTAAGGCTAAAAATGTCACGCCAG
GATATTGGAATAAACCAGCAGAGACTGCCAAAGCATTTCATGGTCGTTATTTATTAACTGGTGACTTAGCGAA
GATGGACGACGATGGCGATATATTTATTATTGACCGCAAAAAAGAATTAATCATAACTGGTGGCGAAAATGTC

TTACCATCCGAAGTCGAAAATGCTTTAGCTGAGCATCCACTAGTAGACCGGTGTGTGGTCGTTGGCTATGATC
ACCCAAAATATGGTGAATCAATTGCTGCAGCCATTATACTTCGCGAAGATGAACCTCATTACGCTGAAATTTT
AGATCAACATATGCGAAGTCGTTTAGCAGGTTATAAAGTCCCAAGAATGTATGTACCAGTGACACATATGCCG
TTAAACAGTACGCAGAAACCAGATAAACTTGCGATTCGACAAATGATGAATGACAAAGTCTCACAAACACTTT
AAAGGTGATAAAAATTTTTAGTATTTAGTGTAAGCGTTTACAAATAAAGCGTGTTGTTTTTGAATTAAATGCA
TTTCACATTAGTATTCATATTATTTTTAGGAGGAATTTATATGACATTTGAAAAAGAAACAGTCTTAAAAACA
TTATTTCCTGAAGATGTACTTAGTATTGCTAAAGGTTTAACAGACGGTGAAGTCGAATTTTTACAACAAGTAG
ATTCATTGCTAGAAAGTAAGTACCGTGAAAATATTAATCAACATTGGATAGACGCTACTGTACCCGAGGACTT
TTTTAAAAATCTGGGAGAATTAAATTATTTTAACAATCCATTACTTTACAAGGATCGTCCAAACGCCAAAATG
CCTAGTCAACTATTTCAGTTTTTCATGTCTTACCTACTCGCGCGATTTGATATTTCCTTAGCTACCCTACTCG
GTGTTCACCAAGGTTTAGGGCATAACACTTTCTATTTCGGAGGTAGCAAAGAACAAATTGCGAAATATGTACC
TAAATTACAGTCACATGAACTGCGTACATGCTTTGCTTTAACTGAACCAGAACACGGTTCGGACGTTGCGGGA
GGTCTTGAAACAGTCGCTGAACGCCAGGGCGATACTTGGGTTATCAATGGTGAAAAGAAATGGATTGGTGGTG
CACATGTATCTGATGTCATTCCAGTATTCGCAGTAAATAAAGAAACTGGCAAACCCCATTGCTTTGTAGTCAG
ACCAGAACAAGATGGCGTCGATATTGAAGTCATTGATAATAAAATCGCACTTCGCATTGTTCCTAACGCACTA
ATTAAATTAACTAATGTCAAAGTAGATGAAGCGGATCGCTTACAAAACATAACAAGCTTTAAAGATATTGCCA
AAATTCTTTATTCAACGAGAGCAGGCGTTGCTTATATGGCTACAGGTGGTATGGCTGGCGCTTTACGTGCCAC
ATTAGATTATGTCACTGAGCGTAAGCAATTCGGCAAACCAATTAGTAAATATCAGTTAATACAAGAAAAGCTA
GCAATGATGCAAGGTAATTTAGCTCAAGCAATGGCAACATGTGCTCAATTAGCTAATATGCAAGCACATGGTG
AATATGACGAGGTTGCAACTTCAACGGCGAAGATGATGAATGCCTTACGTTTGCGTGAGACAGTAGCTATGGG
CCGCGGTATTACAGGTGGTAATGGCATACTAGCTGACGATTATGATATTGCACGTTTCTTCTCTGATGCAGAA
GCGATTTACACGTACGAAGGTACACATGAAATTAATGCCTTAGTAATTGGACGCGCTTTGACTGGAGATTCTG
CTTTCGTATAAATAGCAAAGAATTATATAAGATACAGTAATTTCACTAAAAACAGACTTATTTTAAGCATAAA
GCTTTTTCCTTAAATAAGAGGCTAAGATGACTGTCAAAGATACCTAATTAATTTTATAAAATAGCAACGTTAT
TCCAATTATCTTAATGGTTATCTTATCCTCAACTAAATTGGAGGAATCACTATGACAATTAATAAAGTAACCG
TTCTTGGCGCAGGCACAATGGGCGCTCAACTGGCAGCACTCTTTGTGAATGCTGGACTTAAAGTAAAACTATT
AGATATTGTAGTAGACAAAAACGATCCAAATCTCATTGCGAAAAAATCTTACGATAAAATTACAGATAAGAAA
CGGCCGCTACTATTCGACTTAAATCTAGCGAGTCATTTAACATATGGTAATTTTGATGATGACTTGGTAAATG
ATGATGCTGATTTATATATCGAAGCAGTCAAAGAAGATATTGAAATTAAGCATGCTGTTTGGCAACAAGTTCT
ACAACATGCTAAAGAAGATGCTTTATTCGCTACAAATACATCAGGTATTCCAATTAATGCGATTGCTCAAGTA
TTTAACGAGAAGGATCAAGAACGATTCTTTGGTCTACATTTCTTTAACCCACCACGTATTATGAAATTAGTGG
AGTTAATACCTACGTCACACACGAAGGAATCTATCATATTAGATGTAAAAAATTTCGCGCAAAATGTGTTAGG
TAAAGGTGTCATTGTCGTCAATGATGTACCTGGCTTTGTCGCAAATAGAGTCGGCACGCAAACAATGAATGAT
ATTATGTATCGCGCCGAGCAACACAAGTTAAGCATTGTAGATGTGGATGCTTTAACTGGACAAGCGATTGGTC
GTCCTAAAACAGGTACATATGCGCTATCTGACCTAGTCGGTTTAGATATTGCAGTGTCTGTAATTAAAGGCAT
GCAACAAGTACCTGAAGAAACACCTTATTTTCATGATGTCAAAATTGTAAATACGTTGTTTGACAATGGCGCA
CTCGGACGTAAAACGAAACAAGGATTTTACAAAAAGGATAAAGAAACTAAAGCTCGACTTGTTTACGATGTTG
AAAAACAAGATTATGTACCTGTATCGCAACCACAATTACCAATTTTAAATGAATTTAATAAAGACTTAGCGCA
TAACCTTGATGTCATATTCAATGCGCAAGACGAAGCGGGACTATTTTTATGGGAGACATTACGTAATAATTTC
TATTACTCTGCTATCAATGTACCTAAAGCTACCGATGATTTCCGAGACATAGACCGTGCGCTTGTCTGGGGGT
TCAACTGGAAACTTGGTCCATTCCAATTATGGGATGCAATGGGATATGAACGTGTTAAAAAACGTATGGAAGA
CGAATTTGGAGACTTACCACAATGGATTAGTGATTTAGATGGTGGCTTTTATAAAAAAGATGAGACCATCGAA
TATGCAACACCTGTTTCTCACTTCGTAAAAGATGAACTTTGGGATAAAGGTGATGCCAAACTTTCCGTAACTC
ATGATGATCAACTGTTACTGAAATTACAAAGTAAAAATAATGTCATTACCGATGAATTCAACGATGCGTTAGT
TGATGCGATTGATTTACTGGAAAATGACCATTACACAAGTATGGTTATTTATGCAGATGGTAACAATTTCAGT
GTGGGTGCTAACCTTTTCTTAATGAAAAAGGCGCATGAAGACGGTCTTGTAGATGATGTCGTTGCACAATCAA
TTGATAAATTACATTATAGCTTTAATCGTTTGAAGTATAGTTTGAAACCAGTAGTCACAGCTGTTCAAGGTCG
TGCCTTAGGCGGTGGCTGTGAGCTTGTACTTTACTCACCTATTGTTGTCGCTGCAAGTGAAACGTATATCGGT
CTTGTTGAAGCAGGCGTTGGCTTATTACCGAGTGGCGGTGGCCTTGCAGAAATGGCTGATCGCATATTACGCA
CATCGCATAAGTTTGATGACAAACAAGCTTCCATGACAAAAGTACTGACGAATATCGCATTTGCGAAAGTCTC
TACAAATGCCTTTGAGGCACGTCGTTATGGTTATTTACGTGATACAGATACGATTATTTTCAATACAGCACAA
CGTGTCGAAGTTGCGCTCAAACGTGCGAAATATGAAGCAGAAACAAACTATATTCCGAATCCTAGACATCAAT
ATATCGCTTTAGGTGAAGACTTCAAAGCATTTATCCAAGGACAATTAGATGCGCAAAGACGGGGTCATTTTAT
TAGCGACCATGATTATCATATCGCCTTAAATATTGCCACAATTTTAGCGGGTGGTGATTTACCAAGAAATACA
TTTATCAATCAACGTTACATTCAATCGTTGGAGAAAATTGGCTTTATTGACTTACTAAAATCTAAAAAATCAT
ATGAAAGAATTGCACATATGTTAAAAACTGGTAAGCCATTACGTAATTAAAAGATAGTCATTAAGAGAGGATG
ATAACCATGCAAGAAGCATACATTGTAGCTTATGGGCGTTCAGCCGCAGCGAAAGCAAAGCAAGGCGCATTAT
TCCACGAAAGACCTGATGATGTCGCAGCCAAAGTATTACAAGGCGTATTGAAACGTATTGACGGAAAATTCAA

TAAGAATATGATTGAAGATGTCATTGTTGGTACGGCTTTTCCAGAAGGATTACAAGGACAAAACATTGCACGA
ACGATTGCATTGCGTACGGGATTATCTGACACGGTACCGGGTCAAACAGTGAATCGCTACTGCTCATCAGGAT
TACAAACCATCGCGATTGCAGCCAATCAAATTATGGCTGGTCAAGGAGATATACTTGTAGCTGGTGGCGTTGA
ATTGATGAGTGCCGTACCAATGGGTGGCAACGAGCCCACAAACAATCCCACCTTACAATATGATGATATAGGT
GCGTCATATCCTATGGGTTTAACTGCTGAAAATGTAGCATCCCAATTTGACGTATCACGCGAAGATCAAGATG
CTTATGCTGTCAGAAGTCATCAACGTGCCTATGACGCACAACGTGATGGTCGGTTCAAAAATGAAATTATTCC
AATACAAGTAAACTCAGTTGAATATACAAACGCAGGACCAAAAGTACACACAAATATCTTTGACCAAGATGAA
TTTATACGACCTGACACCACGATGGAGGCATTAGCCAAATTACGTACAGTATTTAAAGCTGACGGCACTGTGA
CTGCGGGAACATCTGCCCCACTTTCTGATGGTGCAGGATTTGTAGTTTTAATGTCTGGAGATAAAGTGAAAGA
ACTCGGCGTGACACCTATTGCACGATTCGTTGGTTATAAGGCAGTAGGCGTTGACCCGAAAATTATGGGTATT
GGGCCTGCATATGCGATTCCTGAAGTATTGTCACTCAGCAATCTATCTGTTGAAGATATTGATTTGATCGAAT
TGAACGAAGCATTTGCTTCTCAAACGATTGCATCTATTAAAGAAGTAGGTCTAGATATATCACGTACGAATGT
GAATGGTGGCGCTATTGCTTTAGGTCATCCATTAGGTGCTACAGGCGCAATGTTAACCGCGCGTTTACTTAAT
GAAATGGGTAGACGTCCCGATAGCCGTTACGGCATGGTTACGATGTGTATTGGTGTCGGTATGGGTGCAGCTG
CTATATTTGAGTATGTGCGTTAGAATGGTTGATTTTGGATGAAGCGGATTCGTTTTGTTATTAAATGAAGTAG
GCTGAAGTTGAAGCAGGTTGAAGTTGAAGCGGGTTGAAGCAATTTCGTTTTATTAATGAAGCTATGTGAAACA
TAGTGATTGAACAAAAAAGTGGTTTAATGGGATGGTGGGTATTTCCGTTTTAGAATTTAACATTTACACGTCT
AATTTTAATCATTGTTTTAAATTTTATGAATCGAAGCCCTTTGATTTAATAATATTTGCTAATGCTAGTAACT
TATCTGATTGTTCATGTTTAAAATAAAGAAAACCACTCACATCAGTGTGTGTTCGAACTAGACTTGTAAGTTC
CAGTTCGGTACGACTTTCTAAAGCAATTATTATTGCTGTGATTGTCGTATATCACTTAGATGTGCGTGGTTTA
TTTTAATAGGTTAGTAATATATTAGGTCATGTTATGTTTAAGACTATAATGAATAAATAATTTAGAAATATGC
TTCCGATTGTTCGATGCTTTAATTCAGTTAGAAGCATCATAGAATGCATGATTACTGTTGTAAAGATACGTAA
TGTTTTGTATTGACTGTATATCTTTGGATAGAGTTACAAACTTATTTTGTTACTCTAGGCCCATATGTCGCAG
TACCATCTGCATGTGTTGTTACATTGTATGCATTTGTTTCGCTTGGCTTCTTGTATGTCGGGCGAGCTCCGTA
TGATACTTGGCCATTTCCGTGTGTTGTTACGTTATATGCGTTTGTTTTGCTTGGCTTGTTTTGTGTCGGACGA
GCGCCATATGATACTTGACCATTTGCATGTGTTGTTACGTTGTATGCATTTGTTTCGCTTGCCTTGTTTTGTG
TCGGACGAGCGCCATATGATACTTGACCGTTTGCATGTGTTGTTACGTTATATGCATTCGTTTCGCTTGGCTT
CTTGTATGTCGGACGAGCTCCGTATGATACTTGACCGTTTGCATGTGTTGTTACGTTATATGCGTTTGTTTTG
CTTGGCTTGTTTTGTGTTGGACGAGCGCCATATGTTACTGTGCCATCTTGATTTGTCGTTACGTTGTATGCGT
TTGTTTCTGATGGCTTATTGAATCTTGGTCTCGCTTCATATCCAAATGTTCCATCGTTGTATTCACGGATACC
TGTACCAGCATCTCTATATTTAACATATTTAGGTGTTTTGTTAAATTGCGGTCTCGGACCATATTGAGAAGCT
TCTGTTGTTTCAGTTGCTTGAGGTTTAACTTCAATATCACTTGATTCTCCTTGAGTACCTTGTAACGTTGATT
CAGTACCTTGTGGTTTTATTTCAAGTTTAGATGAGCTACCTTCAAGACCTTCTAAAATAGGGTTCGTTGTCGT
CGGTTGAGTATAATTATCGCTTAAAGATGGTCTGTTTTGTTCCATTGTTGGGAAATCTGGACCTTGAACGATT
TCACCTTGTAACGTTTTATTTTCCATAGTTGGATAGTCTGGACCTTTTACAATTTCACCTGTAATTGTGCCCT
GTGGAATTTTAACTAATGGTTGTGTTGTTTCTTCAGCTTTACCAGCCGTAGTTTTAACCTCTTGCTGGTTCCC
AACTTTAGGTAATTGAGGTTCTTCAACTTTCTTCTCTTCTTTTACAAAAGGAGATTTTGTTACAGTTTCCTCG
TATTTTTTGACAGTTTTATTTTTCCAAGATTCGTCTGCTTTTTCAACTGCTTCTCTTGTTTCTTTAACTAACT
TATCAAAATTTTCTTTATTCTCTTTTTTCTTTGTATAATCATGAATATTGGGGTCATATTTCTTTATTGTTGT
AGGTCTATTCTGATTTGTTTCCATAAAGAAATCATCGACTATTGAATTTAAGTCTTCCATCATTTCTTTTTTT
ATACGTTCATTAGTGATTCTATTCGGGTTATGCACGTCACCTAGTATTATGTCTAATTTTGCTCGTAATTCTT
TAGCATGTATACCAAATTTTTCATGACTATAATACGCTGCAAAAACTGTATCTATTTCACTAACAAATTCATA
TATTTTATCAGTTTCTTGTTTTTCAGTATTTTCGTCATAAGGCTTTAAGTCTTCATTTTTAGATTGAATATCT
CTTACTTCATTATTAAATTTTTCCACAGCGTCTTTTAATGAATTTGTGATTTCATTATATTCATTCATTGTTA
AATAGTAAACATTATACTTATGAAATGAATACATTTTTGAATTATCGTTTGGGTAAGTTGCTTTATACCATTT
TTTATAATAATCGTATTCATCTATTTTTTTCTTTAATAAATATTGATCTTCTCCTAATATCCTTTCCATTAAT
TTATCTTTTGCCTCTTTATATTCATTACCACCATATTGATACTCATCTGTTAATGCCAATGCACTTGAAAACT
GCTTTTCTAGAGCTTCTATTTTTTCCCAATAATATCTGCTATCTATTAATGTCCCATTTTTACTCTCTTTTTT
AACTTGTGATTTCCCATTATAATCCTTTGTTACTATCGCATCTGCTTTGTTATCCCATGTAAATAAGCTAGAT
GCAACTGCTAATGCGCCTAGCGAAATTATTTGCTTTTTCATAATTTTTTAATTCCTCCAAAATGTAATTGCCC
AATCTACATTAAAGAAACAAAATATTAAAAGACATTAACTATATATTAACTAGAATAAACAAAGCATTAACTA
TCTTTTTGTTATAGTTAATTAGCTTTGCAATATACAATAGTGTATAATTTTTTTGATTAATGTACATCAAAGG
AGTAACAAAGCATGACAACACAAATGAAAATCAAAACATATTTAGTTGCTGGTATTAAAGCGGCGCTCCTTGA
TACGACTGGTATTAAATTAGCAAGCAAATCTGAAACTACATCACATACGTATCAACATCAAGCGCTTGTAGAT
CAATTATATGAATTAATAGCAAACACTGACTTAAATAAATTATCGTACCTAAATTTAGATGCGTTTCAAAAAC
GCGATATTTTAGCTGCGCACTATATTGCAAAATCAGCTATACGCACTAAAAATTTGGATCAAATGACTAAAGC
GAAACATAGATTAGAAAGTATTTACGATTCAATTTCTAACCCTTTGCATTCACAAAAAAATTAATAATTCACC
AATAAATCATGTAAGTGTTTGTGACGCCAAATTTCCTTATAAATCCTTGTCATATGATTATAAACGAATGAAA

CGATTGCCTATCCCATAGGTGACAATGTTAAAAAGACCTCTGAAGGTATCATCTTGATAACTTTAGAGGTCTT
TGTTTATATTATTCAAACAAAATTCTTATAGAACGATCGAAGTATGTTTCGTCTTTCTTTTCTTCTTTAATCT
GATCAGCTAATGCTGGGTCATCTGTGATAATACCATCAACATTGGTTTGTAAGTATTTCGTTAAATCTTCTTC
GCCGTTAATGGTCCAAGTATAGACTTCTTTATTTTCCATGTGTGCTTGATTAACAAGTCTTGGCGAATAAGAA
AAATCTTCGATGACAAAGAAATCTAATGATGTTTCTTTAAAATGACCAAACTGTAACGGGATGATATAACCAC
ACTTGAGATATGGCGCTTCTTTTTTTAACTTAGTCATCACATCATAATCCAATGACATCACACGATATTGATG
TTCAACACCATGCTTTTTCAAAATATCAATAACACGTTGTGTATAATCTGCTGGTTCTTTACCATGTGGCTTT
AACTCTACTAGTAGCTTCACATTTGATTGTTTAGCCGTTTCAATAAATTCGTCTAAGGATACCAGTTTTGCTT
CATGTCCATTTTGACGCATTTTCAAACCGACGACATCTTTGAAATTAGATTCAGAAATGTTTTTATTAACACC
TGTTAAACGTTTCAAATTGTTATCATGACTAACAACAAATTGTTTATCTTTCGTCATAATTGTATCTAACTCA
ATATATTCGACATTCGCTTTTGCAGCAGCTTTCAATGACGGAATAGAATTTTCAACGCCTTTATCTTCGAAAC
CACGATGGCCAATAATGGATATATTTGTATTGATAGTATTATTGTAAAGTAAGTACATGTTATAACCGATAAA
ACATGTCACTGCAAGCACCATTGAAATTATAAAGAACCTAGATTTCCGTTTCGGTTTTGGGTATTTAAATTCT
AAGCCCGGTTGGTCTAATACATTCTCTTGTTTTAAGTGCAGTACTAACACACTGATTAATGATAATTTCGTAA
ATAAATAATAGAAGAACAATGCGCTTTTCAATACAACAAATAAAATTGATGAGACTAAAAACTTATCTCCTTC
TTCATCTACACAAATAGCAAGATATGTTGCTCCTGAAATAATTAATGTTAAAATCGCACCAATGATGAGTTCC
AATATAACTATTTCTATAACAAGTCGAAACTTATTTCGCTTCGTAATTTGCCAACTTAGTCTCATATTTTTAA
ATAACGACTGGCGGTTTAAAATCGTTAACGGTAGAGTAAATATTAATTTAAAGTTTAATATAAATACAGCAAT
CATAAAGGTACCGTAAATGATTATACCTTTTGTCGTTTTCATAAGTTCTTCCGTTAAAAATTTAGGTATGTAA
ATATTTTTTGTTAATACTGAACTTAGTCCTAGGTTAGCAATGGGTATCATTAACATTAAATAAATGACAAAGA
AAATAACTGGTACACCTATGAGTTTACGCACATTTACAAAGGCATTTTTAAAAATGGATTTAAATGTAATAAT
CTGTCGATCAAAGCCGGCATAAACCATATAAACTAACAATGAAAACTCTACATAAATCAGAAAGGCAACACTT
AATATGAATATAATAAGAAGTATCACACTGGCAGGGTGACTTACGATTTCCATCCAATTGTTAATCGTAAGTT
GGCTTTGCCCAGCTACTTTTAACATCATATTAAATAGTAAAATTAAGTATGTACTACTAATAAAAATCATGAT
TAACTGCAATAGTAAGGCGTTAATGCTAAAACGCCCTTTATTTTGATACAGTAATTTAAATACTGCCCATATA
TCTTTACTAATTCTCTTCATAATCACGCTCCGCATTGCTTTAATATTAAGTTTCATCTTAATATTTTTCATTA
CTCAGGGTCAATAAAAATTTGAAAAGACTCATATTCATATGCAAGTAGCAAATAATAACCCATTCAACATCAG
CTAAATGATGATATTGGAACCCCACCTTTAACAAGGCTTCACATTCTTTATCAGCATAGCTACTTACAAAAAT
GTCTTCCTTCAACCATTGTAGAAGTTGTCTCATATACCTACTACGTCTTGCTATTACTCTATACTCAATTGAA
AAAGAAGCATATGCCCCTTCACTCTTGAAGTTACATATGCTTTCTTTTCGGTCTGAATTGTATTTATAATTCA
ACGGGAATTTTCCCTTTGAAGTTAACATAACGGTAGGCTGCTTTAACAGCTTCATCATCGGGCGCTTCGACAT
CTTCTAATTCATATGCAATGCCCAATGTTTTCCACTTATGAACACCTAACTGATGATATGGCAGAATTTCAAA
CTTTTCGACGTTATCAAGAGAATTAATAAATTCCCCTAGTTTAATTAAATCGTCTTTATCATCAGAATAACCA
GGCACAAGGACATGTCGAATCCATACAGGTTGTTTCATATCTGACAGTTTGCGCGCGAAGTTAAGGATGTGTG
TATTAGGCTTTCCCGTCAATCTAATATGTTTGTCATTATCAATATGTTTTATATCTAATAATATCAAGTCTGT
ATGTTTTTGTAATTCTTCAAAATGCCTTTGAAATGCTTTTGTATCATTAGCACATCCAGCCGATGTGTCTAAG
CAAGTGTGCACACCATTTTCTTTTAATTCTGCAAATAATTTTTCTAAGAATGGCATTTGTAACAATGGTTCGC
CACCACTGACTGTTACACCGCCACCCGATGCATCAAAGTATGGTTTGTATGGTAATATTTCATTCACCATTTC
ATCAACTGTGACTTCTCTTGATGGCTCACTAATTTTCCAAGTATCTGGATTGTGGCAATACAAGCATCTAAGT
AAGCATCCTTGTGTAAATAATATATATCTTAATCCCGGTCCATCGACAGTACCTAAACTTTCGACAGAATGTA
AGTGTCCCTTAAGCATAGTGCTCCCACCTTAAATTTTGTTACATACTTTCATGGAATGTACGAGAAATTACAT
CTAATTGTTGTTCACGTGTTAATTTAATGAAGTTAACAGCGTAACCAGATACACGGATTGTTAACTGTGGATA
TTCTTCTGGATGTTCCATTGCATCTATTAATGTTTCACGGTTAAATACGTTAATATTTAAGTGGTGACCACAT
TGCATTGCGTAACCATCTAACATACTAGTTAAGTTACGGTTTTGATCTTCTGGTTCTTTACCTAATGATTTTG
GTACGATACTGAATGTATTTGAAATACCATCTTTACAGCAATCGTAAGGGATCTTAGCTACAGAACTTAATGA
AGATAATGCACCTTTTTGGTCACGGCCATGCATTGGGTTTGCACCTGGTGCAAATGGTTCGCCAGCTTTACGT
CCGTCTGGTGTGTTACCAGTTTTCTTACCGTATACAACGTTTGAAGTAATTGTTAATACACTCATTGTATGTT
CTGAATCACGATATGTTTTATGACTACGTAATTTAGTCATAAAGCGTTCTACTAAATCAACTGCGATATCATC
TACACGGTCGTCATTGTTACCGTATTTAGGGAAGTCGCCTTCGATTTCAAAGTCTACTACAAGACCTTCTTCG
TTACGAATTGGTTTAACTTGTGCATATTTAATTGCAGATAATGAGTCAGCTGCTACTGATAAACCAGCGATAC
CTGTTGCCATTGTACGTACAATTTCTGTATCATGTAATGCCATTTCAATACGTTCATAGCTGTATTTATCGTG
CATGTAGTGAATAACATTTAATGAGTTAATGTAAACACCTGCTAGCCAATCCATCATTTGATCAAATTTCTTG
AATACTTCGTCATATTCTAATACTTCGCTGTTAATACCTTCGAAGTTTGGACCAACTTGTGCACCAGATTTTT
CATCTTTACCACCATTGATAGCGTAAAGTAATGTTTTAGCTAAGTTCGCACGTGCACCGAAGAATTGCATTTG
TTTACCAATTGTCATCGCTGATACACAACATGCGATACCATAGTCATCGCCATAGCTTTCACGCATAATGTCA
TCATTTTCATATTGGATAGAACTTGTTTTAATACTCATTTTTGCACAGTATGTTTTGAAGTTGTCAGGTAAAC
GTACTGACCATAATACTGTTAAGTTTGGTTCTGGTGCTGGACCTAAGTTATCTAATGAGTGTAAGAAACGGAA
TGAGTTTTTCGTAACAAGTGGACGTCCGTCAATACCTACACCACCGATAGATTCAGTTACCCAAGTTGGGTCT

CCAGAGAATAATTCATTGTAATCAGGTGTACGAGCAAATTTAACAATACGTAATTTCATGATGAAGTGGTCAA
TAATTTCTTGAACTTCGCTTTCAGTAATAACGCCTGCTTTAAGGTCACGTTCAGCATAGATATCTAAGAATGT
TGATGTACGACCTAAACTCATTGCTGCACCGTTTTGTTCTTTAATTGCAGCAAGGTATGCTAAGTATAACCAT
TGAACTGCTTCTTTGAAGTTTTCTGCTGGACGGCTTAAATCGAAACCATATTTTTGTCCAAGTTCTTTTAATT
CTTTTAATGCACGATATTGTTCTGATAATTCTTCACGTAAACGAATTACATCTTCTGACATTTCTGTAGACAT
CGTGTTGAAGTCGTGCATTTTTTCTTCCATTAAGAAATCTACACCATATAAAGCTACACGACGATAGTCACCG
ATAATACGTCCACGTCCGTATGCATCAGGTAAACCAGTGATTACACCTGCTTTACGGCAGTTCAACATTTCTC
TAGAATATGCATCGAATACACCTTGGTTATGTGTTTTACGATAATCTGTAAAGATTTTTTCAGTTTCTTCGTC
TAATTCGTAACCGTAAGCTTCACAAGCTGCTTTCGCCATACGAATACCACCGAATGGTTGCATTGAACGTTTG
AATGGCTTTTCAGTTTGTACACCTACAATTGTTTCTAAATCTTTGTCTAAATAACCAGCATCATGAGATGTGA
TTGTTGAAGCTACTTTCGTGTCCATATCCCACATGCCGCCACGTTCACGTTCTTCTTTCGATAACTGCATTAC
TTGTTCCCAAAGTTTAGAAGTTGCTTCTGTTGGTCCTGCTAAAAATGAATCATTACCTTCATAAAGAGTGTAG
TTTAATTGGATAAACTCTCTTACATCTACGTGTCTGTTCCATCTTCCATTTTTAAATCCTTGCCAAGCTGTTG
CATGATTTTTATTTGTTTCTAACATACTAAACGCCTCCATCTGTATAATGTTGTGAATTTGAAATGCTATGTT
AACGTTTTCATAGTCATTGTAGCATGTTTGTTGTGATTTTTTTCACATAAATTATGTACAATTTTCATCAAAT
GTATGTCAAAACATAGAAACTGTACTAATTTGTGTAACAGTAATATGCTAATTGCATTCTGTTACACAACAGT
TATAATGTTCACTATTTTCATTGCTTTAAAAGTTAATTTAGGAGTATATATTTGTTTATAAAAAATGATTGTT
GATAAGCGTTAAATCAACACTTAGAAAGATTTACTTTTATATATGAAGAGACTGGATTAAATACTTTTATTGA
CGTAAAAATTCACTTTTGAACCGTTCAATATCTTGCCGATTTTTATATAACAGCTACAAATAAAATATAACAG
TTTGATTTTACAGCCTCGGTAAATCGTCTTGACAAACAAAAATTTTTTGCTATCACAACATTTGCAACGTCTT
AACAAGTCATCTATAAACATTTCTAAATATTTAACATTACTTATGCGTCATTTATTGCTAAAATTATTGTATT
AAAATATACATAGAATTGATGGGATATCATGAAATCAAAAATTTATATCTTGCTATTATTTCTCATTTTTTTA
TCAGCATGCGCTAATACGCGTCACTCTGAATCCGATAAAAATGTATTAACAGTTTATTCTCCGTATCAATCAA
ACTTGATTCGTCCAATTTTAAATGATTTTGAAAAACAAGAGCATGTCAAAATTGAAATCAAACATGGATCTAC
TCAAGTACTGCTTTCAAACTTGCATAATGAAGATCTTTCGGAGCGTGGTGATGTCTTTATGGGTGGTGTGTTG
TCAGAAACAATTGATCATCCAGAAGATTTTGTTCCCTATCAAGATACATCTGTAACACAGCAATTAGAGGATT
ATCGCTCGAACAATAAATATGTTACTAGTTTTCTATTAATGCCAACAGTTATAGTAGTTAATTCAGATTTACA
AGGAGATATTACGATTCGAGGTTATCAAGATTTATTAAAACCTATACTTAAAGGTAAAATTGCGTACTCAAAT
CCAAATACAACAACGACAGGCTATCAACATATGCGTGCTATTTATAGCATGCATCATCGAGTAAGTGATGTGC
ATCAATTCCAAGACCATGCGATGCAACTGTCAAAGACGTCTAAAGTCATTGAAGATGTTGCTAAAGGTAAATA
TTACGCAGGTCTAAGCTACGAACAAGATGCACGCACATGGAAAAACAAAGGTTATCCTGTATCAATCGTTTAT
CCTATTGAAGGAACAATGTTAAATGTCGATGGTATTGCTTTAGTTAAAAATGCACACCCTCATCCTAAGCGTA
AAAAGTTAGTGCAATATTTAACAAGCCGCTCAGTACAACAACGATTAGTTGCAGAGTTCGATGCCAAGTCAAT
TCGAAAGGATGTTAGTGAACAAAGCGATCAGTCTATCGAAAATCTTAAGAACATACCTTTAATACCGAAATCT
AAATTACCGGACATTCCACATCATAAATTTTTGGAGATGATTCAATGACGGCATACAAACCTTATAGACATCA
ATTGAGACGATCGTTATTTGCGTCTACTATTTTTCCAGTATTTTTAGTCCTTATTATTGGACTAGTAAGCTTC
TATGCCATCTATATTTGGATAGAGCATCGTACGATTCATCAACATGTTGATGAAAGTCAGTCATCTTTACATC
ACACTGAAAAGCAGATTCAGACATTTATTACACAGCATAATAATTCGTTTCAAGAGCTCGATTTAACAAATCA
TCATGATGTTACCGCTACAAAACGTGAATTATTAAAATTAATACACCAACAGCCCGCAACACTTTATTATGAA
TTATCTGGACCAAATCAGTTTATAACCAATAATTATGAACATTTAAACACTAAAAATATGTATTTATTTTCAA
CGCATCAGCTTAAATTCAAAAATAGTACATATATGCTTAAAATTTATATGGCTAACACCCCACGTTTAATTGA
AATTAAGAAAGATAGCCGACAATTCGCACTTATCGTTGATCAATATGACAATATTTTATACGCCAATGATGAC
CGTTTTACTATAGGTGAAAAGTATAGACCACAACAATTCGGATTTATGAATGAATCTGTAAAATTAAACCATG
CTGACCATCGTCTCATTATTTATAAAGATATACATGAAAACATTGAAGATGGTATCACGTTGTTAATTGTTAT
GGCCGTAGTTCTTGTTTTACTAGTAATATTTGGTTTCATTAGCGCAGATAATATGGCTAAACGACAAACAAAA
GATATCGAAACGATTATTCAAAAAATTTACTATGCCAAAAATCGTCATCTAGGTACATATACGCCTTTAAAAA
ACAACAGTGAACTAGAAGAAATTAATAATTATATTTACGATTTATTTGAATCAAATGAGCAACTGATCCATTC
TATCGAGCATACAGAACGTCGCTTGCGTGATATTCAGTTAAAGGAAATAGAACGCCAATTCCAACCTCATTTC
CTATTCAATACGATGCAAACAATACAATATTTAATTACGTTATCTCCGAAATTAGCACAAACTGTGGTCCAAC
AATTATCGCAAATGTTACGTTACTCATTACGTACAAACTCACATACAGTGGAATTAAACGAAGAGTTGAACTA
CATTGAACAGTATGTTGCGATACAAAACATCCGTTTTGATGACATGATTAAGCTCCATATCGAAAGTTCTGAA
GAAGCGCGTCACCAGACAATTGGTAAAATGATGCTTCAGCCACTCATAGAAAATGCTATTAAACATGGTAGAG
ATACTGAGTCATTGGACATTACTATACGCCTGACATTGGCACGTCAGAACTTACATGTTTTAGTTTGCGACAA
TGGTATCGGGATGTCGTCATCTCGCCTGCAATATGTACGCCAATCTCTTAATAATGATGTATTTGATACTAAG
CATTTGGGATTAAATCATTTACACAATAAAGCAATGATTCAATATGGTTCACATGCACGACTCCATATTTTTT
CAAAGCGTAATCAAGGTACGTTAATTTGTTATAAAATACCACTTTCAAGGGGGAATGTAGATGTTTAAGGTAG
TTATTTGTGATGATGAACGAATTATTCGAGAAGGTTTAAAGCAAATCATTCCTTGGGGAGACTATCATTTCAA
TACAATATACACTGCTAAAGATGGTGTTGAAGCATTATCACTAATTCAACAACATCAGCCAGAATTAGTAATT

ACTGATATTCGTATGCCACGTAAAAATGGCGTCGATTTACTCAATGATATTGCGCATCTAGATTGTAATGTCA
TCATATTATCGAGTTACGATGACTTTGAATATATGAAAGCCGGCATTCAACATCATGTTCTTGATTATTTATT
AAAACCAGTAGACCATGCACAATTAGAAGTTATTTAGGAAGACTTGTCCGTACCTTATTAGAGCAACAATCA
CAGAACGGCCGTAGCTTAGCATCTTGTCATGATGCCTTCCAACCACTATTAAAAGTAGAATACGATGATTATT
ATGTAAATCAGATTGTTGATCAAATTAAGCAATCTTATCAAACGAAAGTGACTGTTTCAGATTTAATTCAACA
TATCGATGTTAGTGAATCGTATGCGATGCGAACATTTAAAGATCATGTTGGTATTACAATTGTCGATTATTTA
AATCGCTATCGTATTTTACAATCTCTGCAATTGTTAGATCGACATTACAAACACTATGAAATTGCAGACAAAG
TAGGCTTTTCAGAGTATAAAATGTTTAGCTATCATTTTAAAAAATATTTACAAATGTCGCCAAGTGATTATTG
TAAGCAAGCTAAATAGATTAGCAAATAACTAATGATTATGGCGCCTACATTTTGTTCTATGAAAAAGCGGACT
GATAGAGAAGTTTTCACTTTTCTTTCAGTCCGCTTTTTAGAGAGTTTAAATATAAAACAATACTCCCTACAAT
ATCGACACCGCTATATGTCTTTATTGTAAGGAGTAAATTCATTGAGTCACCCTAAGTCGGGCAAATAAGGATA
CTTCATAACAGGGAATTATGATCTGCCCCGAGACATAGAGATGATATATTTGCAAGGGTAGAAGCAGTATCTT
TCATCTCTTCACGCACATGCCGTCGGGGCAGGATTGCCAACTATATTGCAATAGATTGGCGATACTTTTAATC
CGATTGATATTAGATTTTTAAACTTCTAATTTTCTTTTCTTCATAGAAAGCAACGATTCCTAATAGAATCATG
CCTAGGAATAATGCAACATAGAAGACGATGAAAACATCTGTCCATCCACTTAATGTATATCCAAAGATGTTTA
AACCGTTACGTGTTGGATCAGCAATAGCCGCCAAACCAACTTTCGCCATTGAGTCACCGAATAGATACGCGAA
TGAACCTGTCATTCCGTTTGCTACACTGATGGCATTTTTAGGAACAAAACCAGTCAATGATACACCAATTAAT
AATTGCGGACCAAAGATTAACGCACCTAATGCAAACAATGAAATGTTAACCATCATTACACTTGTAGCATTTG
TGTAGAATAAGACAACAAATGTAATCATAAACATACAGCCAATAGCTACAATTGCACGACGACCTTTTAATAA
GTCTGATACGTAGCCCCATAATAAACTTGCAACTAATGCACCAATTTCAAAGTAGAATATCGTATTAACTGCA
TCGCCTTTACTAAAGTGTAAATGCTCTGACACATATAACGGTGCCCAGTTATCAATACCGATTCGTACAATGT
ATACAAAGACGTTTGAAACACATAGAATCCATATAACAGGATTTCCCAGGATATATTTTTTAAAGATCTCCCA
TTTCGTCATACCTTGAGAATCAATATTTTCTTTATCGACCGGCTCTTCCCAAATTTCTTCAGCACGATTCCAT
CCTAATTCTTCTGGATCATCTTTTCCGATAAATAATGTTGCGATACCAATAAGTAATGCAATCACCGATGGGA
AAATGAACATCCCTATAACATTTCCATGGAAGAATACATTAGCACCCCAAAGTGCAACACCACCTGCAATGGC
ACCACCGATATTATGTGATGTATTCCAAAATCCTAAGTATCGGCCACGTTTCGTTCTTGGCGCCCATCTTGAA
ATCGTTGAATAACTTGCAGGTCCACCAACTGATTGGAACACCCCGTTAAGTCCCCAAAGTACAATTAATAATC
CCATTACAGAACCAAAGTAACTTAAAACAAATCCCATAATTAAAACTGTAATCGCAGATAAGATAAGTAAGAA
CGAGATAATACGTTTTGTGTTACGTCCATCGACAAAATATCCAAGTAATGTTTTCCCTAAACCGTACGTGATA
CTAAATGCTAATCCGATATAACCAAGTTCTAATGTAGATAATCCAATTTCCTCTTTTAAAAACGGTTGTGCCG
CCTTAAAGTTGTTTCGAATTAAATACATAGCCATATAAACAAAGAACACTACAAAGAAAGCTTGCATAAAGTT
TCTAAGCCATAATTTACGTTGTACCGATAATGGAATGCCTTTGTTCGGAATCTTATGGATATCAAAAAAGTTC
ATCTCTGTCACCTCAATCATTTTCGTTAAGCCAAATATATCACGTCGGTGTAAGCGCTTAATATCCTTAAAAT
TATTAAATTATCCAAAATACTGAACATAAACACATACTTAGAAACATTTTTATGTACTAAAATTAATAAATAG
TTAATAGTATTTGACAAGTTTTATCTATAAAATACTTTATGTTGTCAAAATCAACGTTTATACATATGACATA
ATGACGGAGGACATCATGATTCTATCTATATTACTTATCTTTTTCTGTATCAGACTTGTCAGCTTAAAGATAT
CTATTAATCACTCAAAACAATTAAAAGCAGACGGTGCAGTTGAATATGGCGTTAAAAATTCGAAGTTTTTAGC
AATAACACACGTTTTAATTTATGTATTGGCTGGTGTAGAGGCATTTATTAATAAAGATACATTTAGCTTTGCA
AATGGTATTGGCTTAGTTATATTAATCTTTGCTTATATCATGTTATTTATGGTTATTAAAACTTTAGGTGGTA
TTTGGACATTAAAATTATTCATTTTACCTAACCATCCTATTATTAAATCTGGATTATATAAAATTACAAAACA
CCCAAATTACTTCTTAAACATCATTCCTGAATTAATCGGTGTATTATTATTAACACACGCAACATATACAACT
ATTTTATTAGTACCGTATGCGTATTTCTTATATGTACGTATTAAACAAGAAGAGAAATTAATGAATATTTAAA
AAGATGAAAGCCTGTCATCACATTAGTCGACATCGACGTCCATCTTTAAAGTGCGTTCTGAAATATTATATTC
AGAAACCTTTAAAGTATGTCGTGTCTATGACCATTGTGACAACAGGCTTTTTTATTATCATATACCTCACTA
TCGAAAAGGTTATATACTCTTTTCAATGGTGAGGATTGTTACTGTGAATGGGTATGACCATCGCTAAACACTT
ATATAAAGTACACCTTCTTTACAAACAATGACATGATTTAAGGTGTGAAATTTTTGGCAATGGCTTGGCGACA
TATGCATTCATTCAACACTGCCATCAGTCTTACTTCAGACAAACTTCAGACTAACCTCGAGTTCGTATTATGC
CCACCACATGTCTGCTGGCTTGTCGTAAATATTAACAGATTTCAAAGTTTGACAAGCTTTTTGGAAACCTTCT
TCTACTGACATAATAGGATCTTCATGTTCAATACTTAATACATAATCATATCCATTAATAATAAGTTGACTTA
TGATATCTGCCCATACATATGGACTATGTCCATAACCAACTGTACGGAATGTCCATGCTCTTGTCGCAACGTT
ACCATATGGTTGCATATCAGTTAGACCATACATATTTACATTTTCTTGATTAATATACGTATCTTTAGCATGG
AAGTGATGAATTGCATTTGCTTGGCCTAATATGCGAATCGCAGCAATTGGGTCAATACCTTGCCACCATAAAT
GACTAGGATCTAAGTTAGCACCGATATATTCATTTGTAGCCTCACGTAACTTCAACATTGTATATGGTGTATG
CACTAAAAATCCTGCATGCAACTCTATGGCAATTTTTACATCTTGCTCTTTTGCAAACTCAGCTAAATCTTGC
CAATATGGTATCAACTTTTCATTCCACTGATAATCATAAATTTCAGAGTAGGCTGTTGGCCAAGGTGTAACAG
GCCAATTAGGCTTTTTAGCGGTATCATCTGACCCTGCAATGCCAGAAAATGTATTAACAACTGGCACGTCTAA
TAGATTTGCTAAACGGATTGTTTTACGTAACGTTTCATCGGCTTCTTTCGCTTCTATCGGATCTGGAGAAATT
GGATTGTTATGACAACTGAAACCACTTATTTGTAAGCCTCTGTCTGTGATTGACTTCATAAATGCTTGTCGCT

TGTCTTCATTTTCTAACAACTCATCTAACTTACAAAATTTATCTCCTGGGTTACCACCTGTTCCAACTTCAAT
CATATCCAATCCAGATTCTGCGACATAATCTAACATATCTTCAAAATTTTTATCGTAAAATAATACTGAAAAT
ACACCTATTTTCATTGAAAAGCACTCCTTCTTTTTATATTGAGAATTTTGTATCATATATGATAATCATTAAA
GTTGTATACTCTTATTATCAAGATCACTACGATAAATCGCTTCTATAAGGGCATTTACATTGCGTGCTTCTTC
CGGTTTCACCACAATCTCTTCTATACCTAAACACGCATTGACAAAGTTACGCGCCTGTCTCTCACCAGCAATG
TCTTCGTTATGCTCAACATTAGCTTTGCTTTCAAAAATAGTTCCAAAGCGGGGCTCATATATTTCAAATGGAA
ATAAATTGATACCGCCATCTTCTCCTGATAAACTAACGTGTACCTTATCTTCCTTTATATTTGCAGACCACGA
ACATTCAAACTGCATGCTTGCTCGATTGGCAAATGTCATATAACTAGTAACATGATCATCGACATCAAATTTA
GTGTGATCAAATGTTCCCCAATCATTAATTTGATTCGGTTGTTTGCTCAATTGATTATATGTTTTTCCTAGCA
CTTCATGCGGCACCATATCTTTACCTAATAGCCACAAAGATAAGTCTAACAAGTGGCAACCATAATCGATTAA
ACTACCGCCACCTTGCAACGCTTTATTGGTAAAAACGCCCCACCCAGGTACTTTACGCCTACGCATCGCTTGT
ACACGTGCTACTAATGGTTTACCAACCACACCTGCTTCAATTGCTTTTTTAGCAGTCATAGCGACATCTGTGT
GACGATAATGATAAGCGACAGTTAATAATTTGTGATTTTTATTAGCCGCTTCAATCATGCGATCACACTCTTC
CGTCGTCATTGCCATTGGCTTTTCGCATAAGACATGGACACCATGGTTCAATGCTTCTATAGAAAGATCAGCA
TGGAATTTATTGGGTGTACAAATGACCACCGCATCAACAAGTTTAAACAGCTCGCTAGGTGTCTCAACTGCCT
GAGGTATATTAAAGCGCTTCGCAACATCAATCATCTGCACTGTATTAATATCTTGTACTGCAACTAATGAGAC
TGTGTCTTTGAGTTTCAGCAATGCTGGAATATGACGGTCTTGTGCAATACCACCAACACCTATCACACCAACT
TTTAATTTTGTCATGATGTGCCTCCTTACCGTATGATGTTATTCAAAGTAAATTGCTTTGCCTGATTTTGCAG
ACTGATAAATCGCTTCAAGAATTTTTGTAACTACCATTGCTTGTTCCGGTTTCACAACTGGTTCAGTATCATT
TACAACTGCATCAATCCAAGCTTTTGCTTCTTCTTCAGCTTCATCCACTTCATTACCTTCATAAAAGTCGACG
CCTTTGCTTTCCAATTCAACGTGTTTGGTATAAAGTGTGCCCATGTCTTCACCATGAATACGTAGACCATCTT
TCATATCAGCACCTGCTTTCGTTCCTGATAATGAACATTTTGCCTCATCCACTTCTAAAGAATTAATCGCCCA
AGCGGATTCTAAAATGATCGTCGCTCCATTCTTCATTTTAATAAATCCAAACGCAGAATCTTCAACTGTAAAT
TCATCTGGATTCCATGAACCCCAAGCGTTTGCCGCATGATGCTGTTTATTTAATTTATGGAATGTTGAACCCA
TCACTGATTCTGGCTCATAATTATCCATCATCCATAACGTTAAATCTAAAGCGTGTGTACCGATATCGATTAA
TGGTCCTCCACCTTGAGCTTCTTCGTCTAGAAAGACACCCCATGTTGGTACTGCTCGACGACGAATGGCATGT
GCCTTTCCGAAGTAAATGTCTCCTAAGTCGCCACGTTGCGCTGCTTGATGTAAAAATTGACTATCTGGTCTGA
AGCGATTTTGATAACCTATTGTTAATTTTTTACCTGTTGATTTAGCTGTATCTATCATTTTTTGAGCTTCTGC
TGTCGTTTTAGCCATTGGTTTTTCACACATCACATGTTTACCGGCATGCAACCCTGCTACAGTAATTTCACAA
TGCGAGTCATTTGGCGTACAAACATGGATAACATCAATCGTGTCATCTTTTAACAATGCTTTGTAATCATCAT
AAACCTTTGCATTGTCAGTTCCGTATGCTTCTGCCGCACTCGCTGCTTTCGAAATGTCTACGTCACAAAATGC
GATCATTTCAACATTTTCAACTTTTTGTAAACTTGGCATGTGCTTGCCATTCGCAATACCACCACACCCAATG
ATTCCAACTTTAATCGTCATCATAACGTCCTCCTTTTAATCTTTATCGTCATTAGTGGTTCTTTATCAATTCT
GCCCCACTCCTAATTTCAAACTAACCTTTTGTCGCACCTGTTGTTAAACCTGATACTAAATAGCGTTGCAAGA
ACAAGAATACGATTGCTATAGGTACTGCAATCATAATTGCCCCTGCTGCAAACACTGTGAAATTATTTGCATA
CTTATCATTAATAAAGTTGAACAATCCAACTGCTAATGTGAATTTTTCAGGACTTCTTAATAGTATTTTAGGT
AATATAAAGTCCATAAATGGCCCCATAAAATTGAACAAAGCAACAACTGCTAAAATCGGCTTAGCTAATGGAA
GCATAATTTGTAAGAAAATACGCATATGCCCTGCACCATCAATTTTGGCAGATTCATCAAGTTCTTTTGGAAT
CGTATCGAAGTAACCTTTCACTAAAAAGGCATTCATCGGTATTGATCCACCAATATATACCAGTGTTAGTCCA
AATAAAGAATCTAATAATCCAATTGTATTTAGCAAAATATAGATTGCGACCATTGCCATTAATACAGGGAACA
TTTGTAAAATCAAAAATGTAATCAGCCCGTATTTACGACCAACAAAGCGATATCTAGAAAAAGCATATGCTGT
TAACGTGACAAATATCACACTAAACAGTGCATTTGCAGATGCTACGATAAGCGTATTTTTATACCAAGTCAGG
TATTGACTACTGTCATCGAATAGTAAGAATGCATAATTTTTAAATGTTGCATTGTCTGGTATCATTTTGGCAC
CATACAAGTTCGTACCTGGATTAAGGGAAATGCCAAATGTCCACAGTAGTGGATATAAAATGATGACAAACAT
CATCGCTATAAAACTGTAAATACCGATTGCTTTTAATATGTTTTTCTTCTTTGTCATCTTATAAACCTCCCTC
ATCTTTAAACGTACTTGTACGTCTGAATTGAATAAATGCGACAATAGCAACAATAAATCCAATAATTAATGAC
ACAACTGCACCCATGTTAAAGTTGTTAAACTCAAATGTCAGATTATACACCCAAGATATCAAGATATCTGTAC
TTCCAGCATTCTGCCCTGACACTGGTGGACCACCTTTATTAAATAGATAAATAAGATTAAAATTATTGAAATT
ACCTGCATATTGCATAATTAACAATGGCGCTGTGGCGTAAATGACATGTGGAAATGTGATATTTCTAAACTTT
TGCCAACTAGACGCACCATCCATATCTGCTGCTTCGTACCAATCTGATGAAATACTTTGCAGTACACCAGTGA
ACAGTGCAAAGACAAATGGGAAACCAAGCCATACTTGAATGCCGATTAATGCCACTTTTGCCCAAAACGGATC
ACTTAACCATGCTGGTGCTACACCTAATAAAGGTTGCAAAATATCATTATTTATCGCACCAAATTCATCATTA
AATAACGCTACAAATATTAAAATTGTCACAAATGATGGTACAGCCCAAGGTAGGATTAACACAGTACGGATAA
ATTTCTTACCTTTGACAACAGGGTGATTTACAATAATTGCCAAAAACAGCCCTAATGCAATTTGAAGTGTCGT
TGCAACAAGCGTCCATACTAATGTCCAAGTAATAACACTGAAAAATGTTTTACGCCAAACGCCAATTGTGAAT
AACGTTTTGAAGTTATCTAAACCGACCCATTCTAATGTGTGTCTCGGAGGCGCGTTGTATAAATTGTAATTTG
TAAATGCTACTCCAAACATAAATATTAATGGAAATACAACTACAAATATCAATAAGAATGTACCTGGTGAGAT
TAGTAAGTATGGGAAAGTCTTATCCCATGTTGCAACCATACGTGCCTTCGGATCTTTTATTTCCTCATTGCGA

TTAAATCGTTCAGCATTACGATATGCATCTAAAATATTAATGACATATAGCATGATTGCAAAAGCAACGAGTA
AGATAGAAATAATACCTTGTGCAAGTAAGACACGAGAATCGTCTAACTTAGGTACTGTCCCTAATGTGAACAA
TCCCCAAAAACCAATATTTAAAAAGCTATAAAATACAGAAATAAAACTGATGAAAAAGATAAAAAAATATCGTC
CCTTTAATGGGTCTTTTATTATAAAACTGTCCCAAACCAGGTATAACAGATAATAATGCTGCTAATTTAGGGT
TACGTTTCGTCATAACTAATCTCCTTTCTTATCATTTTGTGATGGATGAAGAATCTTAATATTTTGCGTTATA
TCATTCGTCGCCTCATCTAACGCTTGTTTAGGATTCTTACCATTTGAGATAAATATGCTTGCATTGCCCATCG
GTTCCCAAACTTGTCGCATTTCAGGAATATTAGGCATCGGTTCAGCATGACGTGCTTGCTTTTCAAACACTTT
TAAATTTGGATTAGATGATTTCACGTCAACACGTCCAGTAATTTCGCTCATTTCATCTGTATATTTTTGTAAT
GTATCTTTACTAGTGATATACAACATTAAATCTTTAGCCCAATACTTATGTTTACTATATTCAGATAAATACC
AACCACGTACACCGAGAAATGGTTTCATAGGTTTGCCACCATCTGTAGGTAATGTTGTTACTCCTAAATCTTT
ACCAAACGTTTCTTGATATTCATTAATGTTCCACGGTCCAGTGACAAATTGTCCTACTTTTCCTTCTTTAAAA
AGACCAATCATGACATCATGTGTTGCTGCCTTAGGAAGATACCCTTTGTCGTACCATTTTTGTAATCGTTCAG
CATTTTTGACGACATGTTTTGAATTTAGTCCTAGCTGATGAATATCATATTCACTGCCATTTTTCTTGAAAAT
ATAATCATCATTGCCGAATAAAAACGGATAATTAAAATAGAAATTTTTAGCATCAAATAACATACCGTATTGT
TTCTTTTTACTATCAGTTAGTTTGGCAGCATTAGCTTCAACTTCTTCTAACGTTTGCGGTGCATTTTTCACTA
ATTTTTTATTATAGAAAAGTGCGGTTGTTTCTACGATAGCTGGCAATGCTAGTTGCTTATTGTCATAATTCAT
CGCTTTAAGTGCTTGCTTATTGAAACCTTTCAACTCATCTTTTGATAATTTGATTTCAGCAGCTAAGCCTTGT
AGATAGGCACTTCCAGTATTATCATGTGCTAAGAAAAAGATATCTGGACCTTTTCCTACAGGAGCGTCTAGCG
AAATATTTTCTAGTTGATCATTTTGACCAATATTTACAAGCTTTACTTTGATGCCAGTTTTTTAGTATATTG
ATCCGTAATTTTTTTATAAAACGCCATTTGCTTGTCGCCATCCACCCACATCGTAAGTTGGTTAGGCTTGTCT
TTAGAATTATCTTTATTCAATGCTTTATCAATATCTTCTTTCGAACGATTAGGGCCACATGCAGTTACGATTA
ATAACATTACCACGGCTAACGTGATACATTTTAAAATTTTAGACATGAATACTCCCCCTTAGACGATACGATT
TCCTGTTTTTTCATCAAAAAAGTGACACTTATTCATATCAAATGCTAGTGTAATCTTGTCGTTCGCCATCACT
TGTGTTCTTGAATCTAATTTAGAAATCAATTCCATTCCTTGGAATGTGCTATGTACCATAATTTCTGAACCTA
ACAGTTCGGATACAACTACTTCAGATTCAAATTGTGTCTCTGGAGAGGTTTGAATAAATATTGGTTCTTCATG
AATGTCTTCAGCTCGAATACCTAAAATAATTTCCTTATCTAAGTAGCCAGCAGCTTTTAACTTTTCAAATTTT
TTATTATGTAATTTAAAATGGTGTGTTCCTACCTTCAATCCGTCCATTTCAACTGTAGCATTCAACATATTCA
TTGCTGGTGAGCCGATAAATTGCGCCACAAATATGCAATTAGGGGCATCATATATTTCTCTAGGTGTGCCGAC
TTGCATAATGTCGCCATCTTTCAAAACAACAATTCGACTAGCCATCGTCAATGCTTCAGTTTGATCATGTGTA
ACATAAATTGTCGTAGTATTAAGTCGCTTATGTAATTTCAATATTTCTGTGCGCATTTGTACTCGAAGCTTCG
CATCAAGATTCGATAATGGTTCATCCATTAAAAAGACTTTCGCATCCCTAACAATAGCTCTACCTAAAGCAAC
ACGTTGACGCTGTCCACCAGATAACGCTTTTGGTTTACGACCAAGATACTCAGTTAATCCTAATATTTCAGCT
GCTTCATTAACTTTTTGTTCAATCTCTTTTTTATTTACTTTACGTAGCTTTAGCCCAAATGCCATATTTTCAA
AAACAGTCATATGTGGATATAATGCATAGTTTTGAAATACCATCGCAATATCTCTATTCTTTGGTTCAACATC
GTTCATGCGTTCCCCATCAATATAAAAATCTCCAGATGTGATAGACTCTAGTCCAGCAACCATTCGTAATGTT
GTTGATTTACCACATCCCGATGGTCCAACAAATACAATGAATTCTTTGTCAGTAATATGTAGATTGAAATCTT
TCACTACAGTATTGTTGTTATCATACGTCTTTTTAATATGCTCTAACTTTAGTTCTGCCATGTTATGCCTCCC
ATACTTTGTTTACAGTTTGATTGTGCAATACACTTTGTGAAAACGTTTGCATAAATACTTACATAAATCATAT
AGTCACTTATTATTAATGTCAATCATAAATTGTAAACGTTTACATTACTGTCAGAATTAAACTTTTATACAGA
AAATTGATATAGCGCACAACTATTTTTGGTGTGGCAATAATGATTTTTTACGTGCGATATTTCACTGCCAATG
CTAACAACCTTGTCTTACATTTTAAGTAATATCTAAATCAAACAATTATGCCTTAGCATTTCGATTTTCATTG
AAAAAAGCAATCATTGTGTAAGTATCATACTCACACAATGATTGCTTAATTTTTTAGACTGCGGGCACTCTTC
TATAGCTACAAATCATATAGGCTACGTTCCCCATCAGGTAACTGATCAATAAACGGTTTAGGATCTTCTGCAT
ATGCATTACCTACGCCACCCTTCATTCTTTGAAAATGTAAATGTGCGCCTGTCGTTTGTTTACCTGTATTGCC
TGAATATGCAATAATATCACCTGCTTTGACTCGATCACCTACTTTGACATTATATTTGTCTAAGTGTAGATAC
CACTGGTGATATTCTCCATTGTCTTCGGCAATCTGTAATACCTTGCCGCCTAATTCATTATTAAAGAAACGTG
TTACTTTACCATTCGTCGGCGCTTTAATTGGTGTACCTTTTGGCAATGCAAAATCAATGCCATAATGCTTACC
ATCAAATGGTGAATGTTGATATTTACCAAAAGTCTCAGTAATCCGACTCCCTCTAAAAAAGTCTTCCCATTTT
GAGTGTCCTTGTCCTTTATGATTTTGTGTCAATTTATCGGTATATTGGTTAAACCATTTTTGAATGTCATCTA
TATAAATAATCGTAAATAATAGTGAAAGAAAAATGATAATTGTTGCCAAAATACGTTTTGGTCGCTTTGTCAT
AGTGGCATCCATCCTTCATATATTTTGGGTATTAAGTATATATTCTAAATCAACTATTTCATAATTAAATGCA
CTCATACTTTAGTAGTACTGCCAAATATAGATGAAGAAAATGATGGTAATTTGAATATTTTTATTGATATCAA
ATTTTATATCTAGTATAATAATTATACTTAAGAAAATAACAGAACCAGAGGAGAATTTTCAAATGGCAAAAGT
ATTATATATTACAGCACACCCTTTTAATGAACTGGTATCAAATTCTATGGCGGCTGGTAAAGCATTTATCGAA
ACATATCAACAACAACATCCTGATGATGAAGTGAAACATATTGATTTATTTGAAACTTATATTCCAGTTATTG
ACAAAGATGTATTAACTGGTTGGGGAAAAATGAGCAATGGCGAAACGTTAACTGATGATGAACAAATGAAAGT
TTCAAGATTGAGTGACATTTTAGAAGAATTTTTAAGCGCAGATAAATATGTATTTGTAACGCCGATGTGGAAC
CTTTCTTTCCCACCTGTAGTCAAAGCATATATTGATGCCATTTCAATTGCAGGTAAAACTTTTAAATATTCTG

CTGAAGGACCTCAAGGTTTGTTAACTGACAAAAAAGTATTACACATTCAATCACGTGGTGGATATTATACTGA
AGGACCTGCTGCTGATTTTGAAATGGGTGACCGTTATTTAAGAACAATTATGACATTCTTAGGTGTGCCATCA
TATGAAACAATCATTATTGAAGGACACAATGCTGAACCTCATAAGACTGAAGAAATTAAAGCAACTAGTATTA
ATAATGCTGAAAAATTAGCAACAACTTTTTAAATTTTTAATTACACTGTGTGACTCCTCAAACGTCACACAGT
TTTTAATTGTATGCATTTCCTACTTTTTTATAAATACCTATTATTTCCACACATTTAAAGTTTGATATTCACT
TTAATACTGATGTAAATATTTTTAAAATATATTATTATAGTGTTAATTAAGTATTTTAAGAAAAGGTGTGAAT
GTATGAAAGCACATAAAATTTTCTGGTTAAACTTAGCAGCAATTATTATTATTTCAATTGTAGTCAGTGGCGG
TATGTTTTTGGCAATGAAGTGGGAACAAATTCATTTGAAAGATGGTCTTAAAAAAGTACTAAGTACATATCCT
ATTAAAAACTTAGAAACGCTTTATGAAATTGATGGTCACGATAATCCCCATTATGAAAATAATGATCAAGACA
CATGGTACATAGAATCCTCTTATTCAGTTGTTGGATCAGATGAGCTTTTTAAAGAAGACCGTATGTTGTTAAA
GGTAGATAAGAACACACATAAAATAACTGGTGAATATGACACAACTACAAACGATAGAAAAAATGCTACTGAC
TCGACATACAAAAGTTATCCAGTAAAAGTAGTTAATAATAAAATCGTTTTCACAAAAGACGTAAAAGATCCCA
CGCTGAAACAAAAAATCGAAAACAATCAATTTTTGATTCAAAATGGTGATTTAACAAGCATTTTAAATAGCAA
TGATTTGAAAGTCACACATGATCCTACCACTGATTATTATAATTTATCTGGTAAGTTGTCGAATGATAATCCA
AACGTTAAACAATTAAAACGTAGATATAATATTCCCAGAAATGCATCAACAAAGGTGGAATTAAAGGGAATGA
GTGATTTAAAAGGCAATAATCATCAAGATCAGAAACTTTATTTTTATTTTTCAAGTCCTGGAAAAAACCAAAT
TATATACACAGAAAGCCTTACTTATAATAAATTAAGTGAACATTAATACTTATGATATAATTATAGAAATAAC
CAAATCATTTATTATCGTTTAACATACTAAAATCACACATTATAAAAACGCCTCACATCATTCATTTAATTGT
ACCGAATCACATCAAGTTAGATTTTAGACTAACTATTGTGGTGTACATCAAAAACGAATTTGTGGGGCGTTTT
ATATTTTACAAAGGCGACTAGCGTTTCTTATAACAACTTCAACATTACTACACTCAAGCATCACTCTAAAGTT
TGAAATCGAACCCTTTCGTTCAACAAAAATCAAAATCCACTTCAAACTATATATCAAATACATAAATTATCTT
GTGATACTATCTCGATCATATCTATAGTATGCATTTGTGTTTGCTTTCACTGAATGATACGTTTCATCAGTTA
AGTATAAACTGTCATCCTTTAAGGTAACTTGATAACCTTTCTTCCGTGCCAACCAGGCAATATCTATATAATT
TTTTCCGGCGATTTCATAACTTCTTAAATCTTCAATATGCACACTAACTTCAGGGAAAATGATTCTAATAACA
CTTTCATCAACCCAATATTTGTCATACATCCACCGCACTTGTTCAGCATATAATGGCAACTGCATATCATTGA
AATATACTCGATGGGTGTCATTATCGTATATTTTCGGACTAGGTAATGGTTCTTTTCTAATTGATAATACATC
GCCACCTATCACTGTGCCTTCTCTCGTATCATCAGCACCTCCCGACACGTCACGTGTATTCAAGTCAACCTGT
AGTCCATGTACATCTCCAATATAGGCATCTGGTTTATGTTCCATTGCATGTCCACGTGCAATCAATGCTAATA
TTGTAGATTGTGAAAATTGAGGCTCCCATTCAATGCGATTAGGATGGCTACTATAAACTCTAGGTTCATCTAT
AGCCTGCTGAATATCCATGCCAAACACTAATACATTGATTAATGTTTGCGCAACACTAGCAATGATACTTATG
GCACCAGGTGCACCTACTGTTAATATCGGCTTCCCGTGATACATCACAATCGTTGGAGCCATGTTACTTAGTG
GTCGTTTATATGGTGCAATTTCATTAATACCACCATCTACTACATCAAAGCCATCCATTGTCGTATTCAATAA
CACACCGTAGCCTGGAATCGTAATACCTGAACCATAAATCATACCAATTGATGTCGTAAATGAAGCAATATTA
CCTTCCTTATCAATCACACAAAAATGAGTTGTTTCGGTATGATTTTCTTCAACATCTGTATGACTAATGCAAT
CAGACACAACACCATGCTCTATATCAATATTTGCTTTATTGCTATCAATGAGCGTACTGCGTGCTTTTAAATA
ATCGTCATCAATTAATGACTGTACAGGCACCTCATGAAAATTATCATCCGCCAAGTATTGCGCACGATCACTA
TATGCTAAATGCATCGCTTGTATCAAATGATGCAAGTAATCAACAGATCTTGGACCCATAGATGGTAAATCGA
CATGTTCTAATAACTTCAATATTTGAATTACCGTGATACCGCCAGAACTAGATGGTCCCATTGAATAAATGTC
ATAGTCTTTAAATGTTGCACTGATTGGCGCTTTAATCTGAATGTCATATTTGGCTAGATCCTCTAAAGTGATT
GTCCCACCACATGCTTTGACAACATTGACTAATTGTTTCGCAATGTCACCTTTATAAAATGCATTAAACCCTT
GTTCTCTTAATATTTGAAATGTCTTACCTAATTCGGGTTGTACAATCCAATCACCTTCACGCCAATATTGATT
TTCATGCGTAAATACTTGCGCCGTTTCGTGATACTTTGTCAATCGTGCGTGTTGCTGGCGCGAATATTTTTCA
GTAGCCCAATTGGCTGAATGACCTTCAATGGCTAGTTCAATTGCAGGATTAATTAAATCTTCCAATGACAATT
TAGCATAACGCTTGTGAATATAATCAAACAGCTTTGGAATTGCTGGCACAGCGACAGTTTTACCATGTGTAGT
CATATCAAAAAATGATTTATATTCGCCTGAATCATCTAGATAAAATTGTTTGTCTACATGTGCAGGGGCTGTC
TCACGCGCATCAAACGCAGTTATACTGCCAGTACTTTGCTCATAATATAACAAGTACCCGCCACCACCAATAC
CTGATGCAAATGGTTCTACCACATTCAATGCCAGTTGAATTGCAATCACTGCATCCATGGCGTTGCCACCTTG
ATCTAATACATCCTTACCAATTTTAGCCGCAAGAGGATGTGATACGGAAATTAACCCTTCTTTAGATGTTTTT
GTCTGTTTGTCATTTAAGTTAATGACCATTCTATATCCTCCTACTTTCTGTTAAATATTTAAAACATTATTGA
TTAATGGCTTTTTCTACTTTTTCTAAATCTTGACGTTGCTCGTTACCAGTATCGACAAGTGGTGTAATCGGTG
ATGCAATTTTAAATTTATCGCCACGATAAAACTTAATAAATTGATCCTGATTTATCGCATTAACTACTGCTTG
TCTCAAGTTTGGATGCGTCTTAAATATACCTTTTTTAATATTTAGCATTAAAAAGACTGATTTGCGTCCATTT
TTGCGAATAATGCTTAAATTTTTATCCGACTTAATTAAATCAAAATGTTTTTGATTCACATCTGCCAACATAT
CAATTGAATGATTTCTAAGTTCTGACAATGCATTATTCGGGTCACCATTAAACTTCAATGTAATATTTTTAAT
TTTAGCTGGTCCATAACTACCTTTTTCTGTTTCGTTGAATCCTGGATTACGTTGAAACGTTGCTTGATATGCA
TTTTTCTGTGTCATAATGTATGCACCACTTGCATAAAGCGCATTTTTTCCATCTGAATTTGCAGGAATTGTAC
TGCTATCCCCATATCCTTTTGGATATTCTTGATTTACTTGATTAACAAATTTTTTAGATAAAATGCCTGCCGA
AGAGTGTGTTAAGTAATTTACCTCTCGAGGCATCGATTGATCTGTCGTAATTTTAACAATTTGATAAATACCG

TCTTTATTATTTACTTTTTGACCATCTGTCGTTAACGATTTGACGTTATACGCTTTAATCAACTTATCATAGA
TTGATTTATCGTCCTTGTCTTTCTCTTTACGCAACTGATCGATGTCCTCATCTTTTAATATCTTGATGTCATT
TATATGTTTGTGCATATTGTAAGTATTATTGTTAGGCACAGACTTTTTATCACGTGCTCTATCTAAAGAAAAC
TTAACATCTTCAGCCGATACACGCTCTCCAGTATTACGTGCTTGTCCATTGACCACTTTCGCAAAATAATCAT
CATCTCTTAACAAGAAATAAAACGCTTTATTGTCCTTATTCACAGCATAATCACGACTTAACGAACCTTTCGT
TGTTAAGTGATCATTTTCATCTAATAATAATAACCTTGTGTACATATTCATATTAATTGAATATACTGACGGC
GCAATTGAACGTATTGGATCCAATGTAGGAATTTCACCATCTTGTTGTGTCATCACAAGTGGCCGCGTATCTC
GTTCTCTACTATTGTTGTAATCAAATTGTTGCCATATTAATGCACGTGAATTTGGCAATCCAACACTATTTTT
ATCTAACACTTTATTGTCATATACTAAATTCTTTTTTGATCCATATAATGGCGCCATATACCCTTTATCAAAT
ACAACTTCATCTTCAATTTGCTTATATGTTTGTTTAACATCTGCTTCATTTTGAGTAGAAGCTTTATTTAACA
ACTGGTCTACATGTTTATCTTTCAATAAACTATTTGATCCTGTAGAACTAAATAATGCCGTCATAGCATAGTT
CGGGTCACCAAAAACTGTCATCCAGTCATCGATTTGGATATCATAATTGCCGGCTTGACGTTGTGTACGATAG
CTACCATAATCTGGTTGGATATTCATCTTCACGTTAAATCCTGCATTTTCCAATTGATCTTTGACGATATTCA
TATCATTTTCATAACTTGCTTGTCCTAGGAAATGTATTGTTGGTCGCTCGCCTTTCACTTCAACTTTCGATGA
CTTTTGAGCCACTTCTGATTTCGTAGGGACACCACAACCACTTAATACCAACGCTAAAACTATAATTGCGATA
CTAATGATTTTCTTCACATCTATCCCTACCTTTTTAATGAATTCTTGGATCTAGTGCATCACGCACTGCATCA
CCTATAAAATTAAATGCTAAAACGACGAACATAATACAAACACCAGGTACAATAGCTAAATTACTGTGCGTTT
CCAAGTAGTTACTACCGGTACGTAAAATGTTGCCCCATTCAGCTACATCAGGTGCAACACCAAGTCCTAGGAA
ACTTAAACTACTTGTTGTTAATACAACCACACCTATATTTAATGAAAAACGTACAATCATAGGCGCAATCGCA
TTCGGTAAAATATAACGCCATATGATATTCCAAGTGTTTTCACCAGTGATACGCGCTGCATCTACATATTCCA
TGCGTTTAATTTCTAAAACACTGGCACGCATTGTCCGTGCAAATGATGGTATATTACCGATACTTAAAGCAAT
AATTAAATTGGGAATACTTGCTCCAAATGATGCAATAATTGCCACCGCTAACAATAATGATGGAATTGCAAAC
ACTACATCTAAAATTCGCATTATTAAATTATCAATATGATTAAAATAACCTGCGATAGTGCCTAGTAACACAC
CAAAAATAACTGCAATAACTACTGAAATAATTGAAATTGAAAATGTCAGCTTCGTTCCGACAACTACGCGTGT
AAATAAGTCTCTACCGAAATCATCAGTACCAAACGGATAGGCTAGACTCGGTCCATGTAACAGTGCATTGAAC
TGATTTTTAGTAGCCAATGTCGTATCAAATGTAAATTGTGACACAATTGATAATGTCAGCATGTAGACTAAAA
TAAGTAACCCGATAATCGCAATACGATGTCTAGTAGTTTTTCGTATAAACGATTCCCAGCCGTTATAACTATG
TATTTGCGATGTACGTTCATAACGTCTAATACTTACAAACATTAATAATGTAAATACGTTGCCTGTTAATGTC
ATCAATAATAACAACACTTCGACAATACGTCGCCATAGGTCATGATGCTTCCATGTTTGTTCCGTTGTTAAGA
TGATAATTAAAATGATAGTTAAAACGATTAGCAATGTTTCAGCAATATAGAACGTATCGGCCACATAACCTTT
AAAAAGATTTAATGCACTCGTTAATATAACTAAAATATAAGTTGCAATAGCATAACTTGCGAATAGTTTTAAG
GAAGCTATCTTTGTTGTAAGTTTTACCATATGCCTCACTTCCTTTCGTTAATTTCACTACGTAATTTTGGATC
GATTAAAGCATAAAATATATCAATAATTAAGTTTGCTAAAGATATTACAATTGATATATATACGACCCCACCC
ATGACTGCTGGGATATCAGGTATTAGTTGTTTTTGGACGATATAACGACCGATACCATTAATGTTAAATACTT
GTTCCGTCACTGCTGAACCGCCTAGTAACTCTGCCACTAGAAGACCAACTAGCGTTACAATTGGAATAATGGC
ATTTTTCAAAATATGTTTAATAACTACTTGTGTCGTCGATAATCCTTTTGCATAAGCAGTTAAAACATAATCA
CTGCGCATTACTTCAAGTACAGAAGACCTTGTCATACGCGTGATAGAAGCAGCAATACTTGTTCCAATGACAA
GTACAGGTAAAATCAACGATATTGGATGTTCTGGCATATAAGATGGTGGCAAAATATCCAATTTCAATGAGAA
CGCTAAAATGAATAATAGCCCTTGCCAGAAACTTGGAATAGATAAACCAATTAATGCAATTATCATTAACGTG
ATATCAAGCCAACTATTTCGCTTCATCGCACTGATAATTCCAATTGGTATCGCAATAATTAATGCCACAATTA
GCGCTAATACTGCGACAATTATTGTAATTGGAATTCTTTCGCCAACTGCTTTAGTCACAACCTCATTCCCTTT
GTAAGTCGTACCTAAGTCAAAAGTAAAAACACCCTTGATGGTATCCCACAATTGAATAAAATAAGGTTCGTTA
AGATGATGTAATACATTGAATTGATGTATCTGTGCCTTTGTTGCATTTTGTCCCAGTATGCTATAAGCCGCAT
CAAGCGGTGAAAAATACAGAATGGTAAACACACTGACAATAACACCAATGATGACAATCACAGCCATGACAAT
TCGCTCAAAAATATATCTAACTAATGGCTGTAAATAAATAGTCAATAAGATGAACATCGGCAAGGCCAATATC
ACTTTGATAATGATGAACTTATGAAATAATACATTTTCAAAGTATGTTGAAAAATGTGCTTGTTCAATATTCT
TTGAATTCGTATTAGAACTTTGTGCCTTGAATATTTTTAATGCTTCTTTATGTATTTGTGTGGATAACTTTTG
CTGCGATAAATATTTATATTTTTGATGTAACGCCTGTTCGATTTCTGAAATTTCAGAATTATTAGCGTAAAAA
TTTTTCCTCTTAGCAGAAAAGAAAAACTTTATCACTGCATATAAAAATATTGGCAAGCTTAATACCGATAATA
CAAACTTGTAAAATGCCTTGTGATACAACGACTTAAAAGATTCTAAATAAGTAATTATGATAATCACTCCTTG
TAAAATAATCTTGAGTAGATTACTATGATATACAAAAGTATAGAATAAATTTACACATTTGTGAATAGGGAGG
CACAACATCATGTCAAATTTATTAGAAGTCAACAGTCTGAATGTACAATTCAATTATGATGAAACTACAGTTC
AAGCGGTAAAAAACGTCTCTTTCGAATTACGAAAAAAACATATCCTAGGTATTGTTGGTGAATCAGGATCAGG
AAAAAGTATTACTGCTAAATCTATTTTAGGGCTACTACCAGATTATCCAGATCACACATTAACAGGAGAAATT
ATTTTTAATGGGCAATCGTTAACTAATTTATCAACTTCAGCGTTACAACAAATTCGAGGCAAGGATATTTCAA
TGATTTTTCAAGATCCACTCTCTTCGTTGAATCCAAGATTAACGATTGGCAAACAAATTACAGAAGTACTATT
TCAACATAAACGTGTATCTAAATCTGAAGCAAAGTCGATGACAATAGACATTTTAGAAAAAGTAGGTATAAAA
CATGCAACTCGACAATTTGATGCTTATCCACATGAACTTTCTGGTGGTATGCGTCAACGTGTCATGATAGCAA

TGGCATTGATTTTAAAGCCACAAATTTTAATCGCAGATGAACCAACAACGGCATTAGATGCCAGTACACAAAA
TCAATTACTGCAGTTAATGAAGTCCCTTTATGAGTACACAGAAACATCTATTATTTTTATCACTCACGATTTA
GGCGCTGTGTATCAATTTTGCGACGATGTGATTGTAATGAAAGATGGAAGTGTCGTTGAAAGTGGCACGGTTG
AAAGTATTTTTAAATCGCCACAACATACCTATACGAAACGCTTAATAGATGCGATTCCAGATATTCATCAAAC
GCGTCCGCCAAGACCGTTAAACAATGATATTTTATTAAAATTCGATCGCGTGAGCGTGGATTACAAGTTACCA
AGTGGCAGCCTATACCGAGCAGTTAATGATATTAACTTGGCTATTAGAAAAGGCGAAACATTAGGCATTGTCG
GTGAGTCAGGGTCAGGGAAATCGACATTAGCTAAGACGGTCGTCGGTCTAAAGGAAGTGTCAGAAGGCTTTAT
TTGGTATAACGAATTACCATTAAGTTTATTTAAAGATGATGAATTGAAAGCTTTACGACAAGAGATACAAATG
ATTTTCCAAGATCCATTCGCATCTATTAATCCAAGATTTAAAGTCATTGATGTGATTAAACGACCACTAATCA
TTCACGGGAAAGTCAAAGATAATGATGATATTATTGAAATTATCGTATCGTTGTTAGAAAAAGTTGGCCTAGA
TCAAAGTTTCTTATATCGCTATCCACACGAATTATCTGGTGGGCAACGCCAGCGTGTAAGTATCGCGAGAGCA
CTTGCTGTAGAATCTAAAGTGATTGTTTGCGACGAGGCAGTGTCCGCTTTAGACGTTTCAATTCAAAAAGATA
TCATCGAGTTATTAAAACAATTACAGTTAGACTTCGGCATTACTTATTTATTCATCACACATGACATGGGTGT
TATCAATGAAATATGTGATCGCGTTGCAGTTATGAAAAATGGCGAAATCGTTGAACTTAATAACACAGAAGAT
ATTATCAAACATCCGCAGTCAGACTATGCAAAGCAACTTATTTCAGAAGTAGCAGTTATTGCTAAATAAAAGT
CATGCGTTGTGTAACTTTATCACTGTATGGTCTGGAATAAATCAGGCAATCTCTGATGTGTTATGTTAGATGA
CTTTGTCATACTAACTATATTGTGTTAAGTTAGGGCTCGGCACCATATCAAGTTTTAGGTGCTTTGCCATGAT
TTAAGAGTCACCCCCATACTTTGGGCATTTTGACGCCAGAATAAATCCCCCGCTACTATGTGAAGTGCGGGGG
ATTATTTATATTTTATTAGAATATTCAGATTTTTGAGTGTGTCAACTTAGCTTAGTCAATGTATATTTAACAT
CACTTACTCTTTTTCTTTCATAATTAACACATTCAAATAAACTTTGATCAAAAAACACAAAGTTAAAAGTACC
ATCTTGCAATATGCTCTCATACATTATCCCGTCATATTTAAGGCTTCGAATATAATCAGCTAAATATTGAAAT
GGCAAATAATCTATTCCTTGTTCATCGCTTGGATTTGTTATTCCTTTATGAATCTTTTTTAATGTTTGGTAAT
TTACAAAATACTTTCTAAATCCATCATCGCCAGCTTTGATTGCATTACTAGTTAAATTAGTTAAATTCGCAAT
TTTCAATTTCTCTTTTGTAACGTTTTTTTGTAACTTAACCTTACCTATATAAATAATGTCATTATGCTTAGGT
TTAACTTCTTCTATACTGACCTGTTCTTTTGTACTAAGGTATAATACGCTTATCCATTTAGAATTCAATCTTC
CTGCCGTTGCAAATCCCTTTGGTGGTGACATTAGTTCACTTTTCTCTGTAATGAACTTAACTATTCTAGATCT
ATATAATGGTTCAAATCTTTCTCTAAATTCCTCAATACTATAGTAATTAGTAGTGATATCGAGAAAGAACGCT
AAATTCTCTAAATTAATCATATTATTATGAAATCTATTGTTATACTTCAAGCTCTCACAAAATCCATCCCAGT
CATTATTTGCTACAATTAGATTTTCATTTGTATATTTTTTATCGTTTATGATTTTAGCGCCTACTAAATCTTC
CAACACTCGTCTATCTAAATTTTCATCATCTTTAAAAAGTTCATTTAAAATACAACTTATTTGAGCTTCCTCA
ACTTTAAATATACTCCAGTCGTCTTTTAATGCTATTTCAATCTTTTTACCTTCTTTTGGGCTAAAAGTATCTG
GTAAATTTATACTAATATCATATAATTCTAATGCTGGTCTTAAATAATCTCTAATAAGTTCTAATTTATCTTT
GTCCTTAGTCGTATCAAATATTTTAACACCAAGATGATTGTTATCAATATCACAATTGTCAATTTTGCTATTA
TCATTTGCAATGATTTCTACGATTTCAGTATTATTAAAACATTTTTCACATATTTTCATTTTGAGACTCCAAG
TATCTATTCATAATTTCTAGATGATGCATGATAGATAACCTTTTAATTAAACCTAAGCCTGGATACTTATTAT
TTTCATTTAATTCTTCAAATTGTCCAAGCGCATAAGATCTATTTTTAATATCTAAGTTTTTTGACCATGTTAC
TAATTTATCTACAGCCTCAAAAAACTTTTTACCTGGATTTGATCTATCATTATTAGAATCAGAGACAAAATGC
TTAACTCTTAGCTCATTAGCCTCATCAAAATAGACAATATGTATCGCAATAGCTAATGGCGAAAAGCCACCGT
CTACATATTCTCCACCTATAACAGAATAGTCTGAAAATGCTACGTATCCATCTTCCTTATAATAAAGGTGATC
GTCGCTATAAAACTCGTCATCATTATCTAAATAATCAACATTACGACTTAGTTTATTAAAAGGATCCATAATG
ATGCCTTTGTTTGGAATATTACGCAATTTACGTTTAATGTGTCTATCATCTGGTATAAAAACCATTTCAAGTT
TATTTTTATCTCCAAACATTTCCCAGTCATCTACACTTGTTAAAATAGCTAAACTATTACTTGAAAATTGCTG
TTTCAACCTATCATTGCCTTCATCAGAAGGACCCAAGTAAGCTTTTTGAATAACACTTTTGTTTTTTGTTATT
ATTTCAGTTAAATGTTCAACAGTTTCGTTACTAATCTCTTCTTCAGTTAACTTTGAATTTACTACTAAATTTA
TAGAGAATGCTTTTTCACCACAGTATTGCAAAGTATTTTTAAATGTGGTTGTATATTTAATTGGTTCAATTAT
AGGTTGAATACAATCACCAATCAAACCTTTCTCTAACAATTCTTTAATTGCAAATAGTTCATTTTGTTTCCCA
CGCAAATAAGGATAATACATATTAATCCTCCTTAAGAAAATGATATAGTTTATTTTTATCTTTCTTTGTCATT
TTTGCGAAATATAACAAGAACTTAATTTCAGTCGGAGCTGCTAAAAATTCATCTTTGTATTCTTTCATGTTGC
CTCTTTTTTTCAACTCAGAAATCATATTGTTATGCAGTTTCGTCATTGGTATTTCTTTAAACACGTCAAATAA
CACATCATACTCTGTGAAATCATTATAAGTTGGTACATCCATATGATTTGTGCAATAACTCTTTTTCTTTCT
TCTTTTCGTAAAAAGTTGTACATCGCTTTCGTGTTTAAGAGACTATTGTTTTCTTTAGGTTTTTGAACTTCAC
TCAGTGTATTTCTAGTTGTTAACACTAAAATTCCAACTGTTGTATCTTTGTATCTAGCCATAACTTTATTCAG
ATGTTTGTTATTTGTAATGACTACGACATAATTAAACACTTCATAATAATCATTAATTTGATTATCTAATCTA
TCCAACTTATCTAATTCTGTTTTAATCTCATAGACAACGCCTTTGCCGTTTAACAATATAAAATCAGCAATAC
TTTTCCCTATGGGCATCTCAGAAAGTGCAGTAGTTGTATTAACAGAATGTCGTCCTAGAAGGAGTTTATTAAG
CATTGTGTTTTTGTAAAAATATTCGTTACGATAAGACTTTTTAAGATAGTTATATAAAGACTTCATTTTCTGT
ATTACATTGATATTTTTAGGTCCCTGTATATAAAAATCATATGCCTCATCAACAACATCACTTTTATTTTTGA
TTGCAATTTGATGTAATATCTTTTGACTAAAAACACGATTAATCATATTTCTATTATCCATAGCTACGCACCT

CCTTCAACATACATTTAATACTTATCCTGTTAATATTATAAACTATTTAAATGCCTATAGACATTATTTCTA
TACACATACGATGATAAAATTTTATTGAAACCAAGTGATGATTGCAAAATAAATGAGCCTTTTCTAATGAAAT
AATTATGCCCCCTACACTAATCTAGCGAGGGGGCTGAATCATTACACACCGTATTTTTCAGTTGTTTCTGCCA
CGAAAGATTCAATGGCTTTTCTTGCTTTACGTTTTTCTTTTAATGGCAAATCACCAATCATTTTTTTAAGGTG
ATGTGGGTTTACAACACCACTATTCTGATAGTCATTTGAAATTGTTTTTAGGGCTTGTTCATCGATAGATCTC
TCTCCAGCAAATCCTTTGAACTCCGCTTCTTTTTTAATACTTTCGAAATTAACATATTCTTGATCGATATCAT
CATCCTTATTTAAAGAAGGTACAACATTGTCGATGAATTCTCTAATTAGATCTCGTTTTAACCTCAATGTCGG
ATCATCTGCATGATCTAAAATGCGTCTAATTTGTTCTTGGTTACGACGTTGTTCCGCTTTGTCTTCAAGATCA
ATTTGTCTCAATATATTCATAATATAATTCACATTAATCGTATCATTACGCATCATTTCTATTTCGAAATCAA
TATCATTTAAAATGGATACTTTATTTTTCTCAGCCGTCGCTCTTTTTACTTGATCGTACACAGCTAAATATTT
ACTTTTATAGTCTTCATTCTCTTGTTCATCCATTCCAATTTCATCAATTGTAAACTCAAACTCGTCAAATGCT
TTTAAACGTAATATTATTTTAGCTAATAAACGATAAGCTTCAACAAAGCGCTTTAGCTCTTCTTCATCTTGAA
TGTCATCAACCATGTGTGGTGTCGGCACAATCATTTTAAGCTCACGATAAGCGTCCATAAATTCTTTTTTATA
CTCTTCATAACTACGCATTAAAATTGTATCCGTATCATTTGTTTGTGAGAATACTCTCAGTGCATCGTCTGTC
TCTTTTTTCAAGTCACGATAGTTTACAATTTTACCAAATGCCTTTGATTCTTTTTCAACCCTATTTGTACGTG
AATACGCTTGAATTAAATCATGATACATTAAATTCTTATCAACATATAAAGTGTTCAGTACTTTACTATCAAA
ACCAGTTAAGAACATATTAACAACGATTAAGATATCAATTTTACTATCTTTAACGCCCTTTTTAACGTTTTTT
GAAATATGATTAAAATACTCATTAGTTGTGTCTGTTGAAAAATTCGTCTCGAACTTTTTATTATAATCGCTAA
TCATTATCTCTAATTTTTCACGTGAATGATATGGCACTTCACCATCACGATCATCTTCATTAGGTTTAAACGT
AAATATACCAGCTACCGTTAATGGTTGTTCCAACTTTTTGTTAAGTCGCTTAAATGTCTCATAATATTTAATA
AGCGCGTGAATACTTTGGACTGTAAATATACTTGAATATTGACGATTACGTGTATATTTATCATGATTATTGA
TGATATGTCGTGTTACTAATTCCACACGTTTATCCGCTAACCATACTTCTTCCGTATCAATTGCTTCAACCAT
GCTGTTATCTTCTGCTTTTAAAGCTTTATTTTTAAAAGTATTAATATAGTCAACTGAGAAACCAAGTACATTA
CCATCATGAATGGCATCTCTAATTAAATACGTATGTAAGCATCTACCGAAAATATCTGCAGTTGTTCTACCAT
CTTGACTACTATTTTCTGGAAAACGTGGCGTACCAGTGAACCCAAAGTATTGGGCATTTTTGAAATGTTGTTT
AACTAGACGATGCATGTCACCAAATTGACTGCGATGACACTCATCAATAATAAATACAACTTTATTCGTTTTA
TACTGTTCTAATAAATGGGCATTACCTTGAATCGCTTTAGCCATTTTTTGAATCGTCGTTACAATAAGTGGCA
AACTTTTATCATTTAGTTGGCGTACCAGTTGCGAGGTATTAAAAGTTTTGTCTACAGCACCCTTAGCAAATTT
ATTAAATTCCTCTTCTGTTTGACTATCCAAGTCTTTGCGGTCAACCAAAAAGATAACTTTCTTAATGTCATCT
TGCTGTGATAAAATCTGACTCGCTTTAAAAGAAGTCAACGTCTTACCACTTCCAGTTGTATGCCAAATATATC
CATTATTCCCTGTCTCAGTCGCTTGTTGAATTAGTGCTTCTACCGCATACACTTGATACGGGCGCATTGCCAT
CAGTATTCTATCTGTTTCATTAATAATCATATAGCGCGATATCATCTTAGCTAATTGACAAGGTCTCATAAAT
GACTCAGCAAACGATTGTAATGTATTGATTCGGTTATTCTGTTTATCACTCCAATAAAACATGTGACTCTTCA
ATAGTTCGCTATCATTATTAGAAAAGTATCGCGTTTCAACACCATTACTAATGATAAACATTTGTATGTAGCG
GAATAAGCCTGTGTAATTTTGTTTACGGTAACGTTTTACTTGGTTAAACGCCTCATTAATATCAATGCCTCGA
CGTTTCAATTCAACTTGGACAAGGGGTAGTCCATTGATTAATATCGTTACATCATAACGTGCTTTATATGTAT
CCTCGACAGATACTTGATTCGTCACTTGAAACTTATTTTTACACCAACTTTTCGTATCTAAAAACGACAAATA
AATCTCAGACTCATCATCACGTCTAAGTGGTAATTTATCACGTAAAATACGGGCACTCTCGAAAATACTTTTC
CCATCAATCATCGTTAACAGACGTTGAAATTCTTTATCTGTTAAGGGATTGCCTTCTAATTTGTCCGCATGAC
GCTCATTTAAAATCGTTCTAAAATTATCAAGCAATTGCTTATTATCACGTATCGTTACTCTTTCGTAACCCAA
TTGTTCAAGTTGATTCATCATTTCATTTTCTAATGCGTATTCACTTTGGTATGCCATTCATATCCCCTTCCAT
ACACTTTCTATTGCTCTAAATATATCATAAACTTTAATGAAAAATGTTTGTTTTTTATCTTCAAACGTAAATT
TATTCTAATTTTATTGTCTTATCTTTTAATATTTGTCTTTGAGGTAAGTCGTATACTAAAAACACCAACACAC
ACTATCCTTTATATTTTTATACTTCTATGCCAGAGTTCAACTTTTGAATATATTGTGTCCTCAATTGTTTGTT
CTCTGTATATATTTTATTGAATATGATTGATTGAAAAACAAAAGTGAAAATGTACATCACAATTGGAATAAAA
GCATTATAAATGCCATTATCAAAATAGGATAAAGAAAAAATAAATAAAGTGAACACTATCGGCATTGTAAAAC
GACATAAAATAATAGACCAATTATTTATAAAAAGCCAACTTCTTATTAAGTTTTTTCTATTCAGCGTAATGAT
ATGGAATATTTTGTCGTTTTTATTTTGATAAAAACTTTCAACTTTTTCATAGTGTCCATATATGGTTTGATTT
AGGTTAATTATTTTGTAGTTTGATCCAGGTTCGTAAAGGTCATTATTGCCAACTTTAAAACAATCTTTAATTA
ATGTACTTTCTTTGTTTACAACAAACCTATAGGGATGTATGTAATCACAATTTTCACACATTTTATATAATTG
AAATCTATTAACAATCGCTAAATAATTTCTGTAAGTTGAAATAGCTATAAATAAGATTAATCCATAACTTATG
CCTATTAATGAAAACCATATTGTATTAATAAATTCACTATGAGGTCTTTCTAGCAGTTTAATATATATTTTAT
AATTCAAAAATCCTAAAAGAAAATACATTGGTAACACTGTGCCAATCATAATAATTGCCCTATCGAATATTCT
ATATTTATTTTCAGTATTAATGATTTTTGAATACCAACTTTTTAACTCAGGAAATATTACAGACGTGAAAATT
GTTAGCGATAAAGTAACCATTATCTTATTGTTTTCTTTTACAAAATTAATCATCTCGTTAAGTTCCAAATTAA
TTCCCCCTTAATAACAGCATTTAATTTCCATCTATGAAAAAAGCAAAGCTCAAATCTGAACTTTGCTTTAATT
TGTCACGCCTTTATCATTTTCAAAATAGCCTCTATGCCAGTTTTACAAACTTGTAGCAACAATTTTTCATCAA
GCAACTGAATCACATCAAAAACTTCAATTGTTTACGTTGCAACTAACTTTATATTAGAACGCGCACAATATCT

AATGTTTAAAATCTATCGTCGCAAGATGCTTCCTGTAATTATCAAGTGCCATTTTCGATTGGGTTATACAATC
TAGAATCGCATGATAATTTAATGCTACAAATCGATAGTACAATATATCTACCGTGAATAACTGTGCAAATAGT
GACGTTGTAGCCGCCATACGCATTTCATTTTCATCAGTTCTGCCATAAATCAATGCATAGTCTGCAATTTGAG
CCACTGGATTATTAGCTGTACTAGATATAGTTATGATGGGAATACTGTAATGTGTGGCCACCTGTGCAATTGA
CTGCAATTCACTATGACTACCTTGATTCGTCACAAAAATCATGCAATCTCTATCATCATGCGTCGCAAATGTT
GACACAAGTAAATGCGTTTCATGTAATAACCTGACATTTAAGCCAATACGAGATAACTTTTGAAAAAGATCAC
CAATAGTCAAACTCGATGCGCCAAATCCAAATAAAAATATTGTCCTGGCATTTTTCAACACATCACAAATTGC
ATCAATTTGCGCATCCATAATATTAGTAGCTACAAATCGCATCGTATTCGTTGCTCTAGCAATCATTTTATTT
TTCAAAGTTTCTACAGGTTCATTTTCAATCAATTCCAAATGTGGATTGGTTGCAATATCTTCGGGTAAGTATC
GAGATATCGCAATCTTTAGCTCTTGAAAACCTTGATGTGTCATTTTCCGACTAAATCTAACAATTGATGCTGT
ACTAACATTCGTAACATCTGCCAAATCATTCACAGTCATATCAATGATTTTATGTGGATTCTTTAAAATGTAA
TCAGCGATTATCTTTTCTGTCTTCGTAAAATCACTCAACTGCTTATCAATGCGATATAAAATATTTGTCATCA
TTAATCACCCAACAAATCTGTCTGTCGCATCGCCTTTGTCGTTCCAAATAAATATGTACAAACGAATCCACCA
GCATACGCAGCAAGTAACCCTGCAATATAACCTAAATACATATTATCTGAGATTAATGGTAATAGTGACACAC
CACTTGGGCCTATTGCTTTGGCACCAATATGTCCAATTCCACCTATTACAGCACCACCAATACCACCGCCAAT
ACAAGCAGTTAAGAAAGGTCGACCTAATGGCAAAGTCACACCATAGATTAATGGTTCTCCAATACCTAGGAAA
CCAACTGGCAATGCACCTTTTAAAGTATTACGTAATGTTGTGTTGCGTTTACATCTTACCCAAAGTGCTAATG
CGGCACCTACTTGTCCAGCACCAGCCATCGCTGCTATTGGCAATAAGTAAGTAGCACCAGATTGGTTAATCAT
TTCTATATGAATTGGCGTAAAAATATGATGAAGCCCTAACATAACTAACGGTAGGAAGCTTGCACCAATGATA
AATCCACTAAATACGCCACCAATACTAATAATTCCGTTAACTACTGAAACTAAACTGTCTGAAACAAAACCTG
CTAATGGCATAAAGATAAAGATAGTTAATAGTCCTACGATCAACAATGCAATAGTCGGCGTTACAATAATATC
AATCGCATTTGGCACAATTTTATGCAATCTCTTTTCGACAATACTTAAAATCCAAACGGCAAAAATAACGCCA
ATAATTCCACCTTGTCCAGGTTGCAATGGTTCTCCAGTGAAGACATTCATTAAAATATTTTTACCAGCAATAC
CCGTTAATAACGTTGTACCACCAATCACGCCACCAAGTCCTGGTGTCGCACCAAATTCTTTAGCCGCATTAAT
ACCAGTGAAAATAGCTAAGTATGCTAACATACCGTCTTTAATGACATTAAATACTGTTATAAGTTGCGTAATC
CAAGCACCTGAAATATAACCTGCCACCATTAAGTTACTCAGTACTGCTGCAATACCACCAATTAATCCAGCTC
CAATAAATGCAGGAATCAACGGTATAAAGATATTGGCAATTGATTTCAATACTTTATTCGACTTACCATTCTT
TTGTTTTGCTTTATGTGCTTCTTTATTCGCCTTTGCTTTATCAGCTGCATATGATTTATAGTCCATTTTTTCA
CTATCATTGTGATGGTGTGGTATTGGGTCACCTAGTTTAACACCACTTAATTCCGCCATATGATTAGCCACTT
TATTGACTGTACCAGGTCCAACCACAACTTGAATGCGTTCATCGTGTATAACACCCATGACACCATCAATATG
CCTTAGTTCTTGATCATCTACTTTATTTTCATCTAATACTTTAATACGCACACGTGTCATACAGTTCATGACA
CTATCTATATTATCCATACCACCTACTGCAGCAATAATTCGTTCTGCAAGTTGTTGTTCTTTGGTCATTTAAA
TCCCTCCTAAGGTTGTCTATCTCTGATTGCTCGTTTAACAATGTCACCATTGTTTAGTAACCGTCTTGTTGCT
TCTTCCTTAGAAATGCCACACATACCCATAACTGTCGCAACTTTCACATCATGCTCAGATACCTGATATAACG
CCATTGCTTCATCATATGTGATAGCACATATTTCTTGAATAATACGCACTGAACGGTCGATCAGTTTTTGATT
GGTTGCTTTAACATCAATCATGAGGTTATCGTAAACTTTTCCGACACCAACCATTGTGATGGTTGAAATCATA
TTTAAAATTAATTTTTGTGCTGTACCAGACTTTAAACGCGTTGAACCAGTTAATACTTCTGGACCAACTTTAA
CTTCTACTGGATACTGCGCAATTTCACTTATAACTGCATGTTCATTGCATGAAATAGATACTGTTGTAGCACC
GATTGTGTTAGCAAATGTTAAACCGCCTATAACATATGGCGTTTTGCCACTCGCGGCAATTCCTATAACGACA
TCTTTTGATGTTAAATCTATATTTTTCAAATCTTCTTCCGCTAATTTTTTGTGATCTTCCGCACCTTCTACAG
CCATCGTCATAGCATGTTGTCCACCAGCAATAATACCTATAATTTCATGAGGGTCAGTATTGAATGTAGGTAC
ACACTCCGCTGCATCTAAGACACCCAATCTTCCACTTGTACCTGCACCGATATAGATCAATCGTCCACCCTTT
TTATACTGTGCAATTGTTTTTTTAATTACTTTTGTCAATTGTGGTATTGCCTTTCGAACTGCTAACGGGACTT
GCTGATCTTCTTTATTCATCGTAATTAAAGCCTCTTCCACAGTCATTTCATCAAGATGCATCGTCGCTTCATT
ACGCGCTTCGGTCGTACTATTTTCCATCACTTCTTACACTCCCTAGTTTTTTGAAAATCAAATGTATCATTCG
GCTCGATACAACTTAACAGTGGTAAGTCTTCTTTAATAATTTGTGCAACAACATTCACATTGTCATGTGCACT
AAGCGTTTGTCTCACAATTTGCATTTCACCTTGATAACGTCCGTTATTCAAATTATCAACGGTTACTGAACCA
ATGCGTCGTTGCGTCGTAAACTGTGGTTGAATCGAATGTGGACATATTTGTCTTGACGTTTCCGAACGAATGA
CATTTTCCGGATTATCCGGGCGTACTTTATGACACATATCGAAAAGGTAAGTCACCGTCGTATCAAACACTTC
TTCAATACATAACGTGAAATGCCTATGCTTGCAAAAATCTATAAGTTGTTTTGCCTGCCTCATTTCAATCAAT
GAGTCTCCAACTAACACTTCAGATACACCAGTTTCTTGTAGTAATTTAGCTGCAACGACAGGATGACTATGTC
TCGTTGCTTCAATTGTTGGCAAGCCTTTATGCAAAGGACCTCGCAAACCACTCCCTACAATAAAACCATATAT
TTGTGCCTTTGGATTAAATTGATAAATGAGTTCATTTTTCTTATTGACCAAGTCAACAGATAATCCCGTATCT
GGTCTTGGATAATAGTTATGACAAAATGAAAGTAATGTAAAATCATTCAATTGTTGATGTAAGCTTGTTAACA
ATTCCCGGGAAATAATACTTGCATTCAAACAGCACTTTAAACCCTGTGCCATTATCGCTTCGATTGCCTCAAT
TGATGTACTATGATCGATACGAATCATAAATTGTGCATCATATTGCCGAAGATGCTCATAAAAAGATGGTGTT
AAAATAGATGGATTAGCATCTATGAGGTAAGTCACTTGTTCATGTTTTAATAAATTGAGTAGTTTTGTGAAAT
AATGATATTTTGTCTCGTCATCTTCTTCTGGTATTTGTACAGATGTAAAAATCATTTGGTAACCTTGTTTAAT

CATTCGCTTAATATACGCTTCATCTAAAGGTTGTCCTAAATACACTGAAAAGCCTGTCAAAGTAGCCCTCCTT
AACAATATAATTATTAGGAAAATATAGTTGAGTTGTGTAATCGCTTACATTTTACTATAAGAGAAAACACATT
ACAATATTAATCAGTTAAAGCCTGTTCATTGTAATAATCTTACATATTTTTGTCACAAGTTAATTATTACACC
ATCAAAGATTATCCTTTCTTTTAAGTGCTGATAATAGCTGCTATTGCTGAATTGTTACAACAACTTTTATACA
TTTTATTCAAGATTATCTTATATTATGTTTTAATAATAATCTGTGAACAATTAAGAGATTTGAAATTGAATTT
AATAATTGTATTGAAAACGCATACTTCACCATGCTAAAATAGGAGTCGCAAACAAATAAGATTCAATAAGATG
TGATGGTAAGCTAGATAAACTAACGTATCCGTTTTATATACTACTTAGTCAATTGCTTCTTTTACATTAATTA
CATACACCAACGTGTTACTAAGTAAGATTAGGCATGAGTTAAATTAAGCTGTGATGGTTACCAACACAGTCTA
TTTGCTCGTGCCTTTTTTATTGAATCTTAAATAATAAATACAACTTTGGAGGTTGGACAAGTGAGGAAGAAAC
TTTTCGGTCAATTGCAACGTATTGGTAAAGCGCTAATGTTACCTGTTGCGATTTTACCAGCAGCTGGTCTGTT
ATTAGCTATCGGTACAGCTATGCAAGGTGAATCATTACAACACTACTTGCCGTTTATACAAAATGGTGGCGTA
CAAACTGTCGCTAAATTAATGACGGGTGCTGGTGGTATCATTTTTGATAACTTGCCTATGATTTTCGCATTAG
GTGTCGCAATCGGATTAGCTGGCGGTGATGGTGTAGCAGCTATCGCAGCATTCGTCGGTTACATAATCATGAA
CAAAACAATGGGTGACTTTTTACAAGTAACACCTAAGAACATTGGTGATCCAGCGAGTGGTTACGCTAGCATT
TTAGGTATCCCAACATTACAAACAGGTGTGTTCGGCGGTATTATAATCGGGGCCCTGGCAGCTTGGTGTTATA
ACAAGTTCTATAACATTAACTTACCATCTTATTTAGGTTTCTTCGCTGGTAAGCGTTTCGTACCTATTATGAT
GGCTACAACATCATTTATTTTAGCATTCCCAATGGCATTAATTTGGCCAACGATTCAAACAGGATTAAATGCA
TTCAGTACAGGATTATTAGACTCAAATACTGGTGTTGCCGTATTCTTATTTGGTTTCATCAAGCGTTTATTAA
TTCCATTCGGTCTACATCACATTTTCCACGCACCGTTCTGGTTCGAGTTTGGTTCATGGAAAAATGCAGCTGG
TGAAATTATTCACGGTGACCAACGTATCTTTATCGAACAAATTCGTGAAGGCGCACATTTGACAGCTGGTAAA
TTCATGCAAGGTGAATTCCCTGTTATGATGTTCGGTTTACCTGCAGCAGCTTTAGCAATTTATCACACAGCTA
AACCTGAAAACAAGAAAGTAGTAGCAGGTTTAATGGGTTCTGCTGCTTTAACATCATTCTTAACTGGTATTAC
AGAACCTTTAGAATTCTCATTCTTATTTGTAGCACCATTATTATTCTTTATTCACGCAGTACTTGATGGTTTA
TCATTCTTAACATTGTACTTATTAGATCTTCATCTAGGTTATACATTCTCAGGTGGTTTCATCGACTACTTCT
TACTCGGTATACTACCTAATAAGACACAATGGTGGTTAGTTATTCCTGTAGGTCTTGTATACGCAGTTATTTA
CTACTTCGTATTCCGATTCTTAATTGTAAAATTAAAATACAAAACACCAGGTCGTGAAGATAAACAATCACAA
GCGGCTACTGCTTCAGCAACTGAATTACCATATGCAGTATTAGAAGCTATGGGTGGCAAAGCAAACATTAAAC
ATTTAGACGCTTGTATCACACGTCTACGTGTTGAAGTCAACGATAAATCTAAAGTTGATGTTCCTGGTTTGAA
AAATTTAGGTGCATCCGGTGTATTAGAAGTCGGTAATAATATGCAAGCAATTTTTGGTCCAAAATCTGACCAA
ATCAAACATGAAATGCAACAGATTATGAATGGTCAAGTAGTAGAAAATCCTACTACTATGGAAGACGATAAAG
ACGAAACTGTTGTTGTTGCAGAAGATAAATCTGCAACAAGCGAATTGAGCCATATCGTGCATGCACCATTAAC
TGGTGAAGTAACACCATTATCAGAAGTGCCTGATCAAGTGTTCAGCGAAAAAATGATGGGTGACGGTATCGCT
ATCAAACCTTCACAAGGTGAAGTTCGTGCACCATTCAACGGTAAAGTACAAATGATTTTCCCAACAAAACATG
CAATTGGTCTTGTATCAGATAGTGGTTTAGAACTATTAATCCACATCGGTTTAGACACTGTTAAATTAAACGG
AGAAGGCTTTACTTTACATGTTGAGGAAGGTCAAGAAGTTAAACAAGGTGATTTATTAATCAACTTTGATTTA
GACTACATCCGCAATCATGCAAAGAGTGATATTACGCCTATTATCGTGACACAAGGAAACATTACAAACCTTG
ATTTTAAACAAGGTGAACATGGCAACATTTCATTTGGCAATCAATTATTTGAAGCTAAATAATGCTTACTATA
AACAGGTGCGTATACCTTCATAAGGTGACGCGCCTGTTTTTTCTTTGCTATTGTATTTTGCGGTATCATTGAT
AGTTCGCTCTCCCCTTAAATTTTGAATTTTAAGATCATCAATTAAAGCCCCCCTTCATACTCATTTCCTAAAA
AATATTAATTGTTCACTATCGTTAGCGTTTTCACAACAAAGTCAACTTACTTGACCTTACACTGTATTCGAGG
CTATCATTTTAAGTGTAAATATAGAGAAAAGGTGGTTTTTTTATGAAACAACGCATTGGAGCTTACTTAATTG
ACGCTATTCATCGAGCAGGCGTCGATAAAATTTTTGGTGTTCCTGGTGATTTTAATCTCGCTTTTCTAGACGA
TATTATCAGCAATCCCAATGTAGATTGGGTAGGAAATACAAATGAATTAAACGCAAGTTACGCAGCGGACGGT
TATGCCCGTCTTAATGGACTCGCTGCATTAGTTACTACATTTGGTGTTGGCGAATTAAGTGCCGTCAACGGTA
TCGCAGGTTCATATGCTGAACGCATACCTGTCATTGCGATTACAGGTGCGCCGACACGTGCTGTTGAACAAGC
CGGTAAATATGTACATCACTCACTTGGTGAAGGTACATTTGACGACTACCGAAAAATGTTTGCACATATAACC
GTTGCACAAGGTTATATCACACCTGAAAATGCAACAACCGAAATACCACGTTTAATTAATACAGCAATCGCCG
AAAGACGCCCAGTTCATTTACATTTACCAATCGATGTCGCAATCTCTGAAATTGAGATACCGACACCATTTGA
AGTGACGGCAACTAAAGATACGGATGCATCAACATATATAGAGTTATTAGCAACTAAACTGCATCAAGCGAAG
CAGCCTATCATCATTACTGGACATGAAATTAACAGTTTTCACCTCCATCAAGAATTAGAAGATTTTGTAAATC
AAACACAGATACCAGTAGCACAACTTTCATTAGGAAAAGGTGCTTTTAATGAGGAAAATCCATATTATATGGG
TATTTACGATGGGAAAATTGCCGAAGATAAAATACGAGATTATGTGGACAACAGCGATTTAATTTTAAATATT
GGAGCCAAATTAACAGATTCAGCAACAGCAGGATTTTCATACCAATTCAATATCGATGATGTCGTTATGTTAA
ATCATCATAATATCAAAATTGACGATTTTACAAATGACGAAATATCTCTACCATCGTTGTTAAAACAGTTATC
CAATATTTCATATACGAATAACGCATCGTTCCCTGCTTATCATCGTCCAACATCACCCGATTATACTGTTGGC
ACAGAACCATTAACACAACAAACTTATTTAAAATGATGCAAAATTTCTTAAAACCAAATGATGTCATTATTG
CTGATCAAGGTACATCATTCTTTGGTGCTTATGATTTAGCATTATACAAAAACAATACTTTTATAGGGCAACC
GTTATGGGGTTCTATCGGCTATACATTACCTGCAACATTAGGTTCACAATTAGCAGACAAAGATCGTCGTAAC

TTATTATTAATTGGTGATGGCTCATTGCAACTAACTGTTCAAGCTATTTCAACTATGATTAGACAGCATATTA
AACCGGTATTATTTGTGATTAATAATGACGGCTATACGGTAGAACGACTTATTCACGGCATGTATGAACCTTA
TAATGAAATTCACATGTGGGATTATAAAGCTTTACCAGCTGTATTTGGTGGTAAAAATGTTGAAATTCATGAC
GTTGAATCATCAAAAGATTTACAAGACACGTTTAATGCAATTAATGGTCATCCCGATGTGATGCATTTTGTCG
AAGTCAAAATGTCTGTCGAAGACGCACCGAAGAAACTGATCGATATCGCTAAAGCTTTTTCACAACAAAATAA
ATAATTTCATCGTATACAGGGTATAAGTTTAAGCGAATACTTTATTAAACGAAAAGGACTCTGATATAAGATG
ATTAATTTTAATAAAACCGCTTTAGTGTTAATCGACCTGCAAGAAGGTATACTTAAGATGGATTATGCGCCAC
ATACAGCTGAAAATGTCGTTCAAAACGCTAATAAATTAATAGATGTTTTTAGAAAAAACAATGGCTTTATCGC
TTTTGTTCGCGTGAATTTCTATGATGGTAAAGATGTATTGCAACCAAATGCAATGATCTCATTACCACCAAAA
GAAGGCGACGACTACAGTCGTTTCCATCATTTATTAGACAAGAGAGATGACGATTTTGTCATAGACAAACGAC
ATTTTAGTGCATTTGTAGGAACAGATTTGGACTTACAATTGCGACGTCGAGGAATTGATACGATTGTTCTTGG
TGGTGTCGCAACGCATATTGGCGTAGATACGACAGCGCGAGATGCCTATCAATTAAACTACAATCAGTTTTTT
GTTACAGATATGATGAGTGCACAAAACGAAACGCTACATCAATTTCCAATAGATAATGTATTCCCATTGATGG
GACAAACAATAACTACAAACGACTTTCTAAATATATTGAACTAAACATATACTTCCCCCCTTCGATCATGTTG
AGGGGGATCTTTATTTCACAAAGTATTAATACGTCGGGTTGTCTAACCTTCTATATTTAACATATTCTATATC
TGTTAAATCGTTCTTAACTTACGCCCCTACTACATAAGAAACAGTATTTATTCCGGAATTTTCAAAAAATTTA
GTATTTATTGCAAAATTATGTATCACTTTATGTTTAATTTTTGATATTATCTTAATTAAGTAGATTTTTATAA
GTTCTAAAAAGGAGAACAAATACATATATGAAGAAGAAACTAACATTTAAAGAAAACATGTTTATAGGTTCTA
TGTTATTTGGTTTATTCTTTGGTGCCGGCAATCTTATCTTCCCAATACACTTGGGTCAAGCTGCTGGTTCTAA
CGTTTTTATCGCTAACTTAGGATTTTTAATTACAGCAATTGGCTTACCATTTCTAGGTATCATTGCTATTGGC
ATTTCAAAGACATCTGGTTTATTTGAAATTGCATCGCGTGTTAATAAAACATATGCTTACATTTTCACGATTG
CCTTATATCTAGTTATCGGACCATTTTTCGCCTTACCTAGACTGGCAACGACATCATTTGAAATTGCATTTTC
GCCATTTTTATCACCAAAGCAAATCACTTTATATTTATTTATTTTTAGCTTCGTCTTCTTTGTGATTGCATGG
TTTTTTGCGAGAAAGCCATCAAGAATTTTAGAATATATCGGTAAATTTTTAAATCCGGTATTCTTAGTATTAT
TAGCAATTATTTTATTATTTGCTTTTATCCATCCATTAGGTGGCATATCTGATGCACCTATTAGTAAACAATA
TCAATCACATGCCTTATTTAACGGCTTTTTAGATGGATACAATACCTTAGATGCGCTAGCGTCATTGGCATTT
GGTATCATCATTGTTGCAACGATTAAAAAGTTAGGTATCGAAAATCCAACTGATATCGCTAAAGAAACAATTA
AGTCTGGTACTATCAGTATCATTATGATGGGGATCATTTATACCCTACTAGCAATCATGGGTACATTAAGTAT
TGGTCATTTCAAACTTAGTGAAAATGGTGGTATTGCCTTAGCGCAAATTACGCAATACTACTTAGGTAACTAC
GGTATTGTCCTGTTGTCACTTATCGTTATGGTTGCTTGTTTAAAAACAGCCATCGGTTTGATTACGGCATTTT
CAGAAACATTCGAACACCTTTTCCCTAAAATGAATTACCTAGCGATTGCAACAGTTGTAAGCTTTATTTCGTT
CTTATTCGCGAATGTTGGTTTAACTAAGATTATTATGTACTCAGTCCCAGTGTTAATGTTCTTATATCCATTA
GCAATTGCCTTGATTGTACTAACATTATTTAGTAGCAAATTCCATCATTCAAAAATTATTTATCAATGTACCA
TTTTCTTTACAATGATTGCTGCATTAGTAGATGGATTAAAAGCTAGTCCAGAGTTCATTTCAAGCACATCATT
CTCACAAACTTTGATTAATTTCAGCCAAAAATATTTACCATTATCAGACATTGGTATGGGCTGGGTTGTTCTC
AGTTTGATTGGTTTCATTATCGGCTTCATTATTTTTAAAATTAAGCATCGTAAAATTCCACAAGCATAATACT
ATGCCACAGTCATATGTTAAACATATGCTTGTGGCATTTTTTATTCATACTACATTAAACTGCAATCGTATAC
ATACATATCAATGATTATCCACAAAAAATATTAGTACTTTCATTTTACAAATCACATTAATACAAACACAACC
TTATCTTTATATTATTAAATTTACATTTGACACTTATATTGAACAACTGTAATATATTAATATTAATTCTTTA
AATTGTATAAATATAAAGGAGGGAGACCGATGAATTCAATCATTGAATTAACTGATTATTATAGCTCTAATAA
TTATGCACCACTTAAGCTTGTCATTTCTAAAGGTAAAGGTGTCAAAGTTTGGGATACTGATGGCAAACAATAT
ATAGATTGCATCTCAGGTTTTTCAGTTGCAAACCAAGGCCATTGTCATCCAACAATTGTTAAAGCGATGACTG
AACAAGCTTCAAAGTTGTCTATCATTTCACGTGTCCTTTATAGTGACAATCTCGGGAAATGGGAAGAAAAAAT
TTGTCATCTTGCTAAGAAAGACAAAGTACTCCCCCTTAACTCTGGTACTGAAGCTGTTGAAGCAGCCATTAAA
ATTGCTAGAAAATGGGGCTCTGAAGTTAAAGGCATTACTGACGGACAAGTTGAAATCATCGCTATGAATAACA
ATTTTCACGGTCGTACACTTGGCTCATTATCACTATCTAACCACGACGCATATAAAGCAGGATTTCACCCCCT
ACTTCAAGGCACTACAACAGTAGATTTTGGAGACATTGAACAATTAACACAAGCTATTTCACCGAATACAGCA
GCAATTATTTTGGAACCAATTCAAGGTGAAGGTGGCGTTAATATACCACCGAAAGGATATATTCAAGCTGTGC
GTCAACTATGTGATAAACATCAAATATTATTGATTGCAGATGAAATTCAAGTTGGTCTTGGTAGAACTGGGAA
ATGGTTTGCTATGGAATGGGAGCAAGTCGTTCCAGACATTTATATTTTAGGTAAGGCATTGGGTGGCGGCTTA
TACCCTGTATCTGCTGTACTTGCAAATAATGATGTCATGCGTGTTCTAACACCAGGTACACATGGTTCAACAT
TTGGTGGTAACCCTTTAGCCATTGCAATATCGACGGCAGCGCTTGATGTACTTAAAGATGAACAACTGGTTGA
ACGATCAGAACGCTTAGGTTCATTTTTATTAAAAGCGTTGCTACAACTTAAACATCCTAGTATTAAAGAAATT
AGAGGTCGTGGTTTATTTATAGGCATAGAGCTTAACACAGATGCTGCACCTTTTGTGGATCAACTGATTCAAC
GTGGAATCTTATGCAAAGACACGCATCGTACTATCATTCGATTGTCTCCACCTCTAGTCATTGATAGAGAGGA
AATCAATCAAATTGTTGCAGCTTTTCAAGACGTTTTTAAAAATTAACAATTAATCATTTATATATGACATAGG
AGGGATTCATGATGATTAAAGTAGGTATCGTTGGCGGTAGCGGTTATGGCGCAATTGAATTAATTCGATTATT
ACAAACACATCCTCATATAACGATTGCACACATCTACTCACATTCAAAAGTAGATGAACCGTTGAAATTAACA

TTTCCACATTTACAACATATTATGCAACATTTCGAAGCACTTACAGTGGACAATAATGACTGTGATGTAATTT
TCTTTGCGACACCAGCACCCGTAAGTAAAACATGTATCCCTCCCTTAGTAGAAAAAGGTATTCATGTTATCGA
TTTATCTGGCGCATTTAGAATTAAGAATCGTGAAATATATGAAGCATATTACAAAGAAACTGCTGCAGCACAA
GATGATTTGAATCATGCTATTTACAGCATTTCAGAATGGCAATCGTTTGATAACAATGGAACGAAGCTCATTT
CTAATCCTGGCTGTTTCCCTACAGCAACATTATTAGCATTACATCCACTTATTAGCGAAAAAATAGTAGATTT
GTCATCTATTATTATTGATGCTAAGACCGGCGTGTCAGGTGCTGGTCGTTCATTATCACAACGTGTTCATTTT
TCAGAAATGAATGAAAATCTAAGCGCTTATGCAATCGGAAACCATAAACACAAACCGGAAATCGAGCAATATT
TATCTATCGTTGCGGGTCAAGATGTATCAGTCATATTTACACCACATCTCGTACCAATGACACGAGGTATTTT
ATCAACAATTTATGTCAAATTATCATCTGAATATACAACTGAATCATTACATAAATTAATGACCTCTTATTAT
GCTAATCAGCCATTTGTCAGAATTAGAGATATTGGGACTTTTCCAACCACAAAAGAAGTACTCGGTAGTAACT
ACTGCGATATCGGTATCTATGTAGATGAAACAACGCAAACAGCAATTTTAGTATCAGTGATTGATAACCTTGT
CAAAGGGGCAAGTGGTCAAGCCATTCAAAACTTAAATATATTATATAATTTTGAAGTTACGACTGGCCTAAAA
CAATCACCAGTTTATCCATAAGGGGTGTTAGAATGAAACATCAAGAAACGACATCACAACAATATAACTTTTC
AATTATTAAACATGGCGATATTAGTACACCTCAAGGCTTCACGGCTGGTGGTATGCACATCGGTTTACGCGCT
AACAAAAAAGACTTTGGCTGGATTTACTCATCGTCTTTGGCAAGTGCAGCTGCCGTATATACTTTAAATCAGT
TTAAAGCTGCACCACTTATTGTCACTGAAGACACTTTACAAAAGTCTAAAGGAAAATTACAAGCACTTGTTGT
TAATTCAGCTAATGCAAATTCTTGTACCGGTCAACAAGGCATAGATGATGCACGACAAACACAAACATGGGTT
GCTCAACAACTTCAAATACCATCTGAGCATGTTGCTGTTGCTTCAACTGGGGTTATTGGTGAATATTTGCCTA
TGGATAAAATTAAGAATGGGACCGATCATATTAAGGATACTAAGTACACAACGCCAGGTGCGTTTAACGAGGC
AATTTTAACAACTGATACATGTACAAAACATATCGCTGTATCACTAAAAATCGATGGTAAAACCGTTACAATT
GGTGGTAGCGCCAAAGGTTCAGGTATGATTCACCCAAATATGGCTACCATGCTTGCTTTTATAACAACCGATG
CAGCGATTGAATCGAATACACTTCATCAATTATTAAAATCTTCGACTGACCATACATTTAATATGATTACTGT
TGATGGCGATACAAGTACAAATGACATGGTATTAGTCATGGCAAATCACCAAGTTGAACACCAAATACTTAAT
CAAGACCATCCACAATGGGAAACATTTGTTGATGCATTCAATTTTGTCTGTACATTTTTAGCTAAAGCTATAG
CTAGAGATGGCGAAGGCGCAACAAAGTTAATCAGCGTCAATGTGTCAGGTGCTAAGTCAATATCAGATGCAAG
AAAAATCGGTAAAACTATCGTAAGTTCAAATCTAGTAAAATCAGCTATTTTTGGCGAAGATGCCAATTTTGGT
CGAATCATTACAGCTATTGGCTATAGCGGATGTGAAATTGATCCTAACTGCACATATGTTCAACTGAACCAAA
TACCTGTCGTTGATAAAGGTATGGCTGTACTATTTGATGAGCAAGCTATGTCGAATACATTAACTCATGAACA
TGTCACAATTGACGTTCAGCTTGGTTTAGGTAACGCTACAGCGACTGCATACGGTTGTGATTTATCCTATGAT
TATGTGCGTATTAACGCATCATATCGAACATAAGGTGGTGTTGGTTAGATGAAATTTATTGTCATTAAAATTG
GTGGCAGTACACTTAGTGACATGCATCCATCAATTATTAACAATATTAAGCATTTACGATCAAACAACATCTA
CCCTATTATCGTTCATGGCGGTGGCCCTTTTATTAATGATGCATTATCAAACCAGCAAATCGAGCCACACTTT
GTTAATGGCCTAAGAGTGACTGATAAAGCAACCATGACGATTACTAAACGCACGCTCATTGCAGACGTTAACA
CGGCATTAGTAGCTCAATTTAACCAGCACCAATGTTCTGCAATAGGCTTATGTGGTGTAGATGCACAGCTGTT
TGAAATTACATCTTTTGATCAACAATATGGATATGTCGGAGTTCCGACCGCTTTAAATAAGGATGCTTTACAG
TATATATGTGATAAATTTGTACCTATCATTAATTCGATTGGTTTCAATAACCATGATGGAGAATTTTACAATA
TTAATGCTGACACGCTTGCCTATTTTATTGCATCATCATTAAAAGCACCTATTTATGTATTAAGTAATATTGC
AGGTGTACTCATCAATGATGTTGTTATACCTCATCTACCATTAGGTGATATTAATCAGTATATTGAGCATGGT
GATATTTATGGAGGTATGATTCCCAAAGTGCTTGATGCTAAAAATGCGATTGAAAATGGCTGTCCTAAAGTTA
TCATTGCATCAGGAAACAAGCCAAATATTATCGAATCTATTTACAATAATGATTTTGTTGGCACAACAATCCT
TAATTCATAACCATGAAATTAAGGACTAACAAGTTTTGACACGCGATGATTCCAGTTCGTTTATCCATTGCGC
TGAAACATTTATTTACCGTTCATCTCGTCAATAATTCCGAATACAGTATGATACAATATGAGATGTAAAAAAC
TAATAACCTTTTACAAATTTGTTTATCAAAATATTTTAAGTTTTGCAAAGCTTTTCATTGTGATTATTTTCAC
AAAATACTATAATGAGGATAGTAAATAGAGAGGAGTCCTTAAGTTGACGAAACGACAAATGGGTATATTCATT
TATGCTGGAATTATCGGTGGCTTGTTATCTGGAATTGTAAAATTAGGTTGGGAGGTCATGTTTCCACCTCGCA
CACCAGAACGTAATGCAACAAACCCACCTCAAGAGTTATTGCAACAATTAGGATTTAGTAGTGAGTTTACGCA
TCAAACATATACATTTTCAAATATGGAATTGCCTTGGGTAAGCTTTATTGTCCACTTTAGTTTTTCTATCGTC
ATTGCAATTATTTACTGCATATTAGTTAAAAAAATACGCTTACTTAGCGATGGGACAAGGTGCTGTATTTGGTA
TTGCTATTTGGGTATTATTCCACCTTATCATTATGCCAATCATGCATACTGTACCTGCTGTGTGGGATCAACC
ATTCCAAGAGCATCTGTCAGAATTCTTTGGCCATATCGTCTGGATGATGACAATTGAATTAGTGCGACAACAT
TTTGTCTATCGCTATAAATTAAATTAATACACTGACTAACATTAACGTGAGTTTTAAATCATCGTTTGAGTAT
GATGATTGATGCTCACGTTATTTTATTAACTGACATGATATGATTCCAGCCAACTTACGTGAGCATTAAAGTC
TCAAATGCGTCGTAACAAACTATTATTTTCGGTAATTTCAATATTGCTCAGTATATTTTCACCTTATCACTTA
CTTTAATCTCGTCATGATTTTGAATGATGCCATCGTGTATTCACCTTTCATTTTTCCAATAAAAAAACATCTA
ACAGTAAACATTTAGGCAGTATAGTTTAAAATCACTGCGCAATGATACTGTCAGACGTCATATTAACTACTCA
ATAACTGAAATACAGACACTTTTTTATAACCCCAGGGTGCCTGTCCTAAGAAACATACCTGTACCATAAACTG
ATCAAAAATAAATTGTTTGAACTTCACTTCACGTGATTGATAAAAGTGTGATTGTGTCATATCATAAATGTCC
AATCCTTTGATTAAACCTTCACCAATCAATTTTGTAAAACTTTCTTTTTGTGTCCATATTTGATAAAAATCAT

TTAAACTACATATTTGATGTGCTTCGTTTGTAGAGAAACACGTCACTAACGTACGCCAGTCTAAACGTTGTGA
TATCTTTTCGATATCTATACCAACTGGTTCTTTATCGACAACACACACGATATAAGGATAACTATATGATAAG
CTCACATAGATGGGCTGTCCATCACGATTGTGTTGAACAATATCTGCCTTACCTCGTGGCGAAATGTGATAAT
GCCATTCATGTGGTAATAAACCTGTGTCATGTTGAATTCCATATTGCACTAAAATATCTCCCAATCTGTGCAT
GAGTTTATCTTGATTGTATCTATAGTTGACTGTACGTGGTTTTTATATGACCAACGACTTTGTGATATTAAT
TCTTCAATACTTTTCAAGTTACTCTGTAATTGCATTACAAATACTGTCATAACTTTCCCTACTTACTTATTGA
ATATTGTTTTGATATATTGCGCCCAATGATGCAGCCAATTGTTAGTTATCGTTGGCCATTTTTCACTGATGTG
ATTCATTATTTTTAATGTTAATGTTGTATCTATCATTGCTAGTTGTTGTTCACGGTCAACACTAGTTAATCCA
ATCGTTTCGTACATGTCTTGTTTCTGTAAAATTTCATTAAATGATTCATCGCTGACGAGTTCAATTTCTTTGC
GCTTAACGCATTCTAACAAAGATTTCACCGGCATTTTATTAGGTGATAGCACATGGTAAATGATTTGTGGTGT
GTTGACCTGTGCTAATGCGACAATTTGTCTTGCAGTCGTATCCACAAAAGAAAAATCTACAGGCATTTCAGCC
AGGCTAACCCCGATACAATCCAGTTGTAACAAATCATTCATTACCATTGAAAAACGGTTAGTCTTTATATTTC
TCATATGCCATCTTCCATTGTAAGGACTCGTCAAATTACCAACACGTACAATCCGACCATCTAAGCCATTATT
TACAGCTTCTAATACTTTTAATTCACTATAAAATTTACTCCGTGTATATGGTGATGTTAGTAGTTGTCCTTTA
TAGACATCCGCTTCTGAAAATGTCACATCTTCTGTGTCTATATCAAAATAAGTTCCCACACTTATCGTAGACA
CATATATTAACCTTGCATGATGTTGTTGTGCCAAACGTATGGCATCAACAGTACCTTGAACATTTACTTTTTC
AAATTCATCATCATCACCAAAATGATCTGTACGAGCACCTGCATGAATAATCGTATCCATGTTTTCTGGTAAA
ACAACATCATCCATACACTCGAAATCACCAACAATGACTTCAATGTTTGATAACATCATTTCAACCGTCTCTT
CTGAAAAATAATCATTTAAATTCGTCATCAACTTATACCATGCTATTTCCTCATTATCAGCACGTATGAAACA
ATAAATGCGATGACTGTATCCTTGTAGTGCTTCAATCAGATAAGCACCTAAAAAACCTGTCACGCCAGTCAAT
AGTGTATTTCCTAGAGGTCGATGACTTAGACTATCCTCTAAAATACCCAAGTTATAACGAGACATAACAATCT
TTTGTAATTCCGAAAGATTATCCGGTAATGCAACTAATGATTGTTGATTTTGGTACATATAATTAACAATCTG
TCGCACGGTTTTATATTGGTATAATGTCTGCATTGAAATATGATGGCCAAATCGTTTTAAATGCGAGACAACT
AACATCGCCTCTAATGAGTTACCACCAAGTTCAAAGAAATCATCGTCAACACCGACATCATTTTGTTTCAATA
CCTCTCCAAATACATCAACAAATGTCTGCTCAATTTCATTAGAGGGTTCGCTATACACTTTATTAGACTGTTG
TATAGGTGATGGATTTGGCAAACGCGTAGTATCCACCTTGTCATTCGTGGTTAATGGCATACAATCGATATGC
GTTATAGTCTTAGGAATCATATACTTAGGCAGATGATCATTTAAATATTGCTTCAAATCCTGCTCCACTTGTT
GCTCTCCGACATAATAAGCATTCAATATATCATGCGTATCAAAGTGACTTACTGTTACAACACAATCAGATAT
ACCACGAATAGCTAATATTGCATTTTCAATTTCATCAAGTTCAATACGGTACCCGTTAACTTTCACTTGTTTA
TCTATTCTTCCTAAAAATTCAATTTGACCATCAGATGTATAACGTGCTAAATCACCACTATGATACAACTTTC
CTTTACCAAATGGATTATTTTGCCATTTATCAGCCATTAATTCTGGACGGTTAATATATCCTATCGCTAAACT
ATCACCTGCAATACACAACTCGCCTGGCATACCAATACCGCATAACAAACCATCTGACATAATATACACTTGG
ATGTTAGATAAGGGTTTGCCAATTGGAATCGTCTCAGGTATCAAATCACCACAATGATGTGACCAATACGATG
TGATGACTGTTGACTCAGATGGTCCATAGGCATTGAAATACGTGCCACAATGCTTCTCAATATATTTAACAAA
GGATGCCGTACTAGTTGCCCCGCCTGTAATCAACTTTTCAATATAAAAGTCTTCCATAACACTACACATCTGT
AACGGAATCGACGCCACCGTCACACGATGCTTATTAATGAGTTGTTGTAACTGTGCTGGATTAACACGTTCCT
CTCTATCTGGAATCACAAGCGTATGACCATTTAACAAACAACAATAAATCTCCATAACTGATGCATCAAAAAC
AATATTTGCATGTTGCAAAAATACTTCATTGTTGCCTAATTGCAATTCAGTTGACCATGCATGCACTAAATTC
AACAAATTTCGTTGTCGTATGGCAACCCCTTTAGGCATCCCGGTCGTACCAGATGTGTAAATAGCATACATCT
CATTATCTAACATCGCTGTGTTTTCAAGTTGATTGCCATGTAAATCATCATATTGTTCATTTTCCTTTGATTC
AACAAAGCCTTTAGCATTTTCCAATTGAATGACTGGTAATGTCGTTTCAATTTCAACGCCATAAGACATGACT
GCAGTTACTTTAGCATCCTCCAAAATTGCACCTTGTCGTTTATTCGGAAAATCAATATCGATAGGTATATAAG
ATGCACCTACTTTAACTGTCGCCAACATCGCCGCAATCATTTCAAAACTACGTTCTGTAAACAAGGCAACCCG
TTGACCATTGCCCACACCATTTGATAGGAGCATGTGCGCAATGGCATCCACATAGTTGTGTAATGTTTCATAC
GTCATTGTCAAATCATTCATGACTAGCGCAACATGATTACCTTGTCGTGAGACAGCTTCATTAAAGTAACTTA
TGATAGATTTATTTCCCGGGACATTAAGCATTCGATCGTTAACATGCGTATTGACCCAATTTAGAAGTTCCTC
CGTGCCGTTTGGTATATCACAAATTTGTAGTGTATCTTGATGCTTCAAAATATAATCAATCATAATCATACAT
TGATTACCCATGTGACGAACTGTTTCTGAGTGATATAAATCGGTATTATACTCGATATTGATTATATAGTCAT
CGCGATCTTCTTCAATGATGAAAGATAAATCAAATTTCGCCGTCACTGATTTGGGTTGAATGTGTGTTAATTT
ACTATGCCCAAAATGAGCATGATTCGTTTCATTGTTTTGTAGTACTAACATGACATCAAATAATGGATTCCGT
GAGGCATCATGTGATTGATCTAAGTCATTTACTAAACATTCGAATGGGTATTCTTGATGCTCGTATGCCTCCA
AACTCATTTCCTTAACCTCTTGTAAAAACTGTGTCCACATTTTATCAGGTGACGGTTGCCCTCTATATACCAA
CGTATTAGCAAACATGCCTAGCATTTGCTCCGTGCCTTTATGCATACGCGCACTCATCACACTACCGACAACA
ACATCATCTTTTCGAGCATATCTGCTTAACAACGTCATGACCACACTCATAAAGAACATAAAATCAGTAATTT
GATGCTTTTCTACATACTTTTGAAGTAGCTGTCTCATTTGTTGATTCATTGTAAATGACATCATTGCTCCATT
TGTCGTTTTAATATTTGGTCTAACATAGTCTGTCGGTAAGCTTAAAATAGGTACTTCATCTTTGAATTGAGAT
AACCAATATTGTCTATGTTTCGTCATATCACGATGCGACATCCACTCACTATAGTCTTTATATTGCAATTTAA
GTGGTAACAATAATTTATGTTGATAAAGTGCGTTAAGATCATTCATTAATTGTATATTACTCATACCGTCATT

AATGATATGATGTGTATCTATAAACAGGTATGCATGTAAGGGACTTCTAATGTATCTCACTCTAATTTGACTT
GGCTTTTCCAAATTAAAAGGTGCCACAAATTGGCGCATGATTTCTTGTTCATCCGTAAAATGCGTGTTAACTT
CTTCAAAGTCAGCTACAACATCTGCCACAATACGTTGTCGAACCTCATCATCTACAACAATATATTGTGTTCG
TAGAATCTCATGTCGCGCTATCAAATGCTGAACTGCTTGTTGCAATTGAGCTACATTAAGTCCTGATGATAAC
CGCCATAAAAAAGGTACGTTATACACCGTATCTTTATGGTTTGATTTCCATAATAAATACATACGCTTTTGTG
CAGAGCTCAGCACATAATCATCTTTAACTATAGCTTCTGGAATCACTTCATAGTTTTGTTCTTGAACCTTAGC
AATCGCTTGTGCTAGTTCAAATACAGTTGGCTTTTGTAATAAATCACCAATTTGTAATCGTTTCCCAGTAGAT
GCCTCTATCCGATTCACCACTAACGTTGCTTTTAATGAATGGCCACCTAATTCAAAGAAATTATCATGAATAC
CTACTTGATTCACATGTAAAATATCTGCAAAAATTTGGCATAGCAAGTGTTCGGTATCTGTACTCGGTGCTAC
ATAGGCATCCGTATCGACATAGTCCATGATAGGCAATGCCTTCTTATCTAATTTCCCATTAATAGTAATAGGA
ATTTGCTCAATATGCATGAAATTAACTGGTATCATGTACTCCGGTAAGGTCATACGTAATTGTGATTTAATCT
TATTATGTGATAATGTATGCATCGCTTCATAATAAGCAACGATATACTGATCTTGATCATGATTTTGAACAAT
AACAACTGCTTTATTAATACCTTGTATACGCTCGAGCGCATGCTCAACCTCTGACAACTCAATCCTAAACCCT
CGAATCTTAACTTGTTTGTCCTTTCGATATAAATAATCTATGTTGCCATCGGGTAATAGACGAACGATATCAC
CACTTCTATACATCAGCTGATTTATATTTGAATCTTTGATAAATTTATCTGCTGTCAATTCTGGCTGATTTAA
ATAACCTGCAGCTAACCCAAAGCCACTTGTACATAATTCTCCAGGAATACCAACGCCACATCGACACTCGCCT
TGCATGATATAAACATGAGTACCAAGAATCGGTTTACCAATAGGAATACGATTTGGAACTTTGTTAGGTATAT
TATACGTCGTTGTAAATGTTGTATTTTCAGTTGGTCCATAACCATTAATAATTTGAGGATGCTTCGGTTTTTG
ATTAAGCAAATCCACCCACTTAGCATTCAATACTTCTCCACCAATTAATAAGTACTTTAACGGTACCAATACT
TCTATTCGTTCACTAGCAATCTGATTAAATAATGAGGAGGTTAACCACATAGTATTAACGTCATTTTCATTGA
TTAATTGTTCTAACGCTATTGGATTTAATAATTGTTCTTTTTTAGCAACAATCAGCTTTCCACCATTGAGCAA
TGCACCATATATTTCAAATGTTGCAGCATCAAAGGCTATAGTTCCTGATAACAAAATCGTCGTCTCTTCATTT
AATGGTACATAATGATTTTGATGGACCAAGCGAACAATACCTCGGTGCGGAATTAGTGTACCTTTTGGGTTAC
CAGTTGTCCCCGACGTGTAAATAACATAAGCATGATCTTCTAACGTGTTACATTTAGAAAGATTATCAATATT
TTTCCACGCTATCTTATTCAAATCAATGTGATTAATATTTTGTTTACCATTTTCATATAAAGCTTGGTACGTT
ATTACAACTTTAGGCGTTGCATCTTTTAAAATGTACTCCTGACGATCACTTGGATAGTTCGGATCAATTGGCA
CGTAAGCCCCACCAGCTTTCAACACACCTATCATCGCTATTATCATCTCAATACTTTTTTCAGCTATGACAGC
GACACGATCATTAGGTTCAACACCATACTGGTTTCTCAAACGGTGTGCTAAATCATTCGCGCGTGCATTCAAT
GTTTGATATGTTATAAACACTCCGTCAAATTGCACAGCGACATGATTCGGCGTTGCTTCAACTTGTTGCTCAA
ATAAGGTAACAACTGTTTGCGCATCATCTATCTCAGGCAAACTTACATTGATATCGTCATATAATTGAATATC
ACGTTCTGTCATCAAATTAAGTTCATCTACAGTTGTTCGTTTATTTCCATTTTCTTCAGTAATTTGCAAATAA
ATATTTCGAACTAAGTCACTCAGCGTCTCGATTGAGAGCAAATCATATGCTGCACTGTTATATATAATTTTGA
ACCCGTGTTGATGTGGAAAAATTTCAATATCCGCTAAAGATGTATGTGCATCATGTATTTGATGTACATCCTC
AATAACATCATTACAACAAGACATCATATGATGATAACAATGAAAAATAGTCTCTAGTGAAAGCGAAGAATTC
GCGCACTGTAATTGCGACATATTTTGCAACACACATTTATTAAAATCTGTTGTAAAACGTTGACATACATCTT
TTGCATCGATTGTTAACGTTAACGGCACAATATTTCCGTGTAAATCATTTGGTAAATGTGATGGTACATGTAT
ACCTAATGTGACATCATGTTGTTGACTCATTATATGATTAGCTAAATACACACTAACAGCCAACGATGCCATA
TCTATTGATGTCATATCATCAATCAAATACGTTTGATATAAAGCTTGCTCAAATGGATGCTTAATTGGTAAAT
AACTATCAATATGGATGTCAGAGTTATTCTCTAACCGAAAATAGTTTGAGTCTAATGCTATATGCGATGCATC
TTGATTGTGCTTATCATCATTTCTATTTAAATGTGCATGCTGTCGAGTATTGTTAATAACAGTATTGCCACGA
TATGCATTGCATAAATCATCAAGAAAAATATCAATTTGACTATCATCGAAAATGGACACATGAAAATCTAATA
GTATATATGCAGCATCAGCGAACTGAAACAATTTAAATTTGAATAAAGGTGAATCATTAAAATGGTAAGTACT
TAATTCTTGCTTAAAAAAAGCTTCTAAATCATAGTTTGCGGAAGAAGTTGGAACTTGTTTTATCTCAATAAAA
GGCAGAAATTCATGAAGTATCATTGTTAAATTGTCATCGGTAGTAACATCAAAAAAATGTCTTATAGATGCAT
GTTGTTGCACAATTGTCGATAATGCATACATCATTTTAGTAGCTTCAACATTTTTAGCGAGTTTAACCCAATA
CGCATTACGGTGTGTCGTTGATTCTGTATTATTTTTGTATATACGAAAATATTCCTGCTGAAATCTCAAATTA
CCCATAATCATAAAAAGTCCTTCTTTCATATCATAATACTCATTACTTACTGAAATTGCATGATGATATGATA
ACCGACGAAATGTTAATTAACTCGTTATGTAATGATTAATATAAAACACCATTCGCAACATATGAGCGATATA
TTCTACCCTAAAATACATCTTGTATCATCGTTACAATTGGTATATTTTTCAATGTAAATTACATACATCTTCG
ATAAATAGTACACTACAACTCGTTAATCACTTTCTGTTGTTCACATCTCATTGCAAATTCAATATTGTTGTTA
CAAAATATCCATGAAGCAAGTTTATATTAAACAAACAACTCGCATAAAACAATTGTTATCCTTAAATTTTAAC
AAATTCTTAATAAATTTATCTCTATTTTAATTACGACCGAATTAATAGGTTTTCAATATAAAAAGATGCATAA
AATAAATATTTAAATAAATTCAATTTGTATTCACTTGTTTTTGTCCCCCGAATACACCAGCAACAAGCATGCT
AGCACCAATTGTTAAAACGATAAACATATACAGTCCCATTTGTAATGACGTTAAGAAAGCACCCAACACAATC
CCTAACCTAGCTAGTGTTTCTGAAAAATGAATACCTAATGCATTAACTGCACTATATGTTCCTCTTTTAGCTT
TAGGAATAATTTTAAAGCGTTGTTCTGAAACTATAGGCGAATAAATAATTTCACCTACAGTCGCAATTATCAT
AAAAACAACTAATAAGCCAAACTGATTAAGATAGGTTAGACCACTATAGCCAACAATATAAATCAGCAAACCA
GTGATTAAAGCTTTTTTAAAATCTATTTTCAATACAACTTTCGAGATTGAATACGTGAGTAAAATGACGACGA

CCGTATTAATCATTAGCAAGATTGCTAACATCTTAGCACCTGTAATATCATATGAACCTATACTTATTGTTTC
AAACTGATTCTTTAGTCTAATAGCAATATATGAGGAGATTGAAAATTCACCCATCATGATGATACTGAACCCC
GAAATCAATAACATATAATTACGGTCTTTCAAAACTAATTTATAACTGCGAAATATATTCATTATTTGTAATT
TTTGATAACGACTTGCATGCCTCTTGTCATCACTTTGCTTTACTTGATTTCGGTCTTGAGGTAACCAAATATA
TAAAATAAAGAGTACAATTAAAAATATACAAGCTGCTATTAAGAAAAGTAGTAACATACTGTAGCCATACATC
AAGCCGCCTAACAATGCCCCAATAGCTACCGATAAGTTCGTCATCCAATAGCTAATCTTGTAAATATAATGTT
CCACGTCTTCGGTAATTGCATCCATAATTAATGTGTCCATAACTGGAAATTGTAATCCCCAAACGATTGTAAA
TATGGCATATGCAACACAAAAACCAATAATTTGCCACAATTGATGTGACCCAAATATGCCCATGAACACAAGC
ATTATCACCATCGTCGCTTGATAAATAAGTACTAGCAACTTTTTCGGAAATATCTCAATAAGGTAACCAGATA
TAATGGACAATGGAAATTTTAGAACCACTAAACCAACAAGATATATACCGACAATTGATTGACTTAACATATC
TGTTAAATATAGTGCTATAAACGGTATAAATGCTGTCGTAATAATTAGCTGTAAAAAATTGCTAATCAATCGT
ACTTTCAAAGTCGTACTTAAGCGTTTCATTAATAACTACACCCCTATCCACATATATACACACTACCATTTAT
ATAAAATCTATTATTAGATTATACGTTGAAACAAAGAATTTTTAGTTATACAAAGTTATTTAACAAACAAATG
CATCGACTTTACAATGCATCTTGCATATAATGCCTCAAAAAAATTAGCACTATATGACTATATATTATTCCAA
ATGAATGAAATTTATCAATAATTGCGTAAGTGAACACATATGTGCGTAAATGTTAAATCATTGTCCTAACATA
GCCGTGACTATACTTAGATGTAATCATAAAAAAACTACTGCCATCTTTACAACAGCAGTAGTGCTTATATAAT
TCATTTATATAAAATTGAAAGCGTTAATCGCTAGCCCAGCATCCTTATTCTATTTAGCTGTATAACTTTTACT
AGCAATACGACCACTTGCAACAATAATATCTTTATCTTGGAAAGGTTCATGATTGAAGAAACGCTCTAAAATA
TCTTTAACTCCATCTTCAATACGTTTTTGTGCTTCTAAAGTCATACCTGAATAGTGAACTGTCATAGCATTTC
TAGGCATTGTTCTCCATGGATGATCAGCAGGTGCAGGTTGTGGATACCAAACATCACCAGCATATCCTTGTAA
ATGCTCGGATGCTAACGCTTCAACTAACGCATCGCGATTTACAATTTTACCACGTGCAGTATTCACTAAATAA
CTGTGTTTTTTCATACGACTTAAAACATCTTTATCAAATAAGTTATCAGTTTCTGGTGTTAATGGTGCATGAA
TTGTAATCGCATCACTTGTTGAAACAAGTTCATCAAAGCTTACAAATTTAGACAATTTATGGTCTTGTTGATT
GATTGGATCATAGTGTTGTAATGTTACATTAAATGGCGCTAATCTTTCAGCAACAAGTTGTCCAATTCGACCA
AATCCAAAAATACCAATTGTTTTGTGTTGTAATTCATGCGCATGATTACCTACTTGAGACAAGTTCCATTCAC
CTTCTACTGATTGACGATGACCTTCTTCATAGTTTCTAAGAAGTATTAATAAATCCATAACCGCATGTTCTGC
CACACTAACTGTATTACTTCCTGTAACTTCAACGACACCAATATTGTGTTCACTTGCTGCCGCTAAATCTACA
TGGTCAGATCCTACACCTGCTGTAATTGCTAATTTCAAGTTCGGTGCTTTTTCAATACGTTCACGAGTCATAT
ATGCAGGATAAAATGGCGCACTAATAATCACATCCATATCTGGTAAATGTTTATCTAAGTCTTCACCATTATC
TGCTAATATAATGAACTCATGTCCTCTTTCCTCTAAAAATGTTTTTAATCCTAATGCTTTTTTAGTATTAAGT
AATTGATTTTCTTGACCTTCTACTGCTTCTGGAAATAACGCTACGATTTTCATATCCATCTCTCCTAAATGAT
AAATTTTGACTTCCTTAAAGTACGTGTTGCATGTTGCTTGTTTTAAAAATATGACAAAAAAACGGCACCGTTT
GACATACCACCCCCTTGAATTATTGTTAAATTCAAAGCGAAGTTAGCAAACAGCACCGTGTACCTTACACCAG
CAACACTTATTCTATTGACTTATCGAAAACAAATATTGAAATGACTTCATCATCATTCAAATCAATATCTGTA
AATAATTTTACGAACTTAGCGCCTACAAGACTTTCCATCTCATTTACATCTATTTCTTTATACATCTGTTTAA
TCTTCTCTGTGCGTGTATAATGGAGCATCGATTCATTGCGTTTGTCGTTTAGGTAAAAACTCTCAACTTTACT
CAAAACACCTGTCATATGTGCAATCGCCCAATTATCTTTAAACGACACTCGAATACTATTGGGTCCTTTACCC
ACGTATTCTTTACGATAAGATCTTACTAGATTCGAGAATTGTTGTTCTTTAGTATGCGTATCTATAGTATTCA
CCTTCTTTAAAATAAGAAGTTCATTAATTGTTCACATTTTCATTATACACCTTATAAACACTTTTTCAATGAT
AACGATTATTATTATTACGAAATGTATATTTTCCTAGTTAAAAGGATATAACAAATAAACAGAAAATGTTTAT
AAACTAAGATTTATATTATTTTAAAAAATGTTTCAACAAACGCATCTAAACAACAGATCAGCATATCATACTT
ACCCCTTCAACTCTGCCTCAAGTTTACCTAAATGTCTTAATGTTGGTGTCAATGCTGCGAAGAAGAATCCTTC
TTTTAACTTGCGAACTTGTGGCGAATATGGTGTGTACGCTCTTGAACCACCATTGACAACAGAAGCTTGATTT
ACATCTAACAATAAATAGCCAATGTTCTTCTTCAATGATATTAATTCACTTAAATGTGAAGTTAAGTTACCGT
CATCTAATATTGCATAATATTCCTCTCTAAGTTGACGATAACGTTTTTTAAAAGCTTCAACATCATACTCTAA
ATATTGATTTATTCCGTTTTGCGCATTTGAAAATGCATCAATTAACTCTAAAGAACTTTTAATTGAGCCTAAT
CCTATTGGAATTTGGTAAGCAATAAATTGCGGGCGAATAGTTGCCGCAAACTGCTTCGCATCATGCGTGATGA
TTTGTGATTGTGGCACTACGACTTAATTCAATGTGATTTGATACGTTGCTGACCCGTTGACCCCTAAAAAATT
TGTTTTTTCAACGAGAGTGATACCATCTTGATTGGCACGTAGAATGAACATGACAAATTCATCTGATGATTCA
TGTTTCGAAATCGCACCAAAATAATGGTCTTCTTGAATATTACTTACAGCTGGCATACGTCCACTGACAACCA
ATTGTCCATCAACATAAGTGTGTTCAAGGTTCAACTTTTCTAAATCATTAAATGACTTCATCGGATTAGACAA
TCCGGTAGCACCTAATATTTCTCCAGATAACAATTGCTGTTGTAAGTCATTATTTAAATGTGGCTGCGTGGCA
TTTTCTAAATACGTTGAAAAAGCTAATTGGCACCATAAACAAAATCCTGTTGTCAAACAAGACTGCGATACAG
CTTCGATTACTTCAGCATTTTTTCTCAATGCCGCCTCACCGAAATAACCATCTACAAATAATTGTTGAATAAA
TGTTCTCGGATAATACGTCCCTTCATCAACTTCTACTAAATGAGGATCTAATTGTTCTTTGATAAGCGTTTCT
AAAGTCATCATCATCTCTCCTTATTCACCAAATCTTCTAAGTATAAACTGCTCAATATAACTAATGAATCGAT
CAATAATAAAACCAAATAATCCGATAAAGAATATTGCTGCTAAAACATCTTCTAAGTTCAACATATTTCGTGC
ATCAACGATTAAAAAACCTAATCCCGATTGTGCACCAATCATTTCACCAGAAACTAAAAATATCCAACTTGTT

CCTACCGCCATATGTATCCCAGCCATGATTTGTTTAAATGCCCCGGGAAATAATATATTGCGATACAATGACC
ACCCAGTTAAATTTAAATTTGCCGCTATTTTTAAATATTGAGGTTCAATGTCTCTAACGCCTTTAATAGTATT
GAACACAATTGGGAAAAAAGCAGCGATAAAAATAATCGCAATCGCTGGCAAACTACCAATACCAAACCATAGA
ACAACAAATGGTGCCCATGCTATCGGAGATATCGGCCTAATCAATTGAAATAGCGGTTCGATAGCGTTGTATA
GCCAACGATTCCTTCCAAGCAAGAAGCCCAATGGAATAGCAACCAACAATGCGACAACAAAGCCCGCTACAAA
TCTCCATAAACTAATTGCTAAATGTTGGAAAATTTCTCCAGTAACAATGAAAGTCCATATACTTTTTCCTACA
AGAACAGGACCAGGTAACAATACAGGTTGGTAATGCCCAATAATAATGACCATTTCCCAAATGCCTAAGAAAA
TAATAAATGTGATAATAGGTAATATAAATTTGTTATTTGTGGGACGTGTCATGAACGCGATGCCTCCTTATAC
AATGACGGTTCAACAAAGTCATCATATGCAGGTGGATTAAACAAATGATGTTGTTTTACCAATGTCGTAATTT
CTTGATAGCCGGATGGCTTAATTGTTAAATCACCATAGGATGTCCATGCCGCTGACTGTGTTAAAACGTCACG
ACTTTGTTTAAAATGATGCGTCATAATGTCTACACTTTGCTTGCGATCATTCATTTTAAAGCCAGACTTTTTA
TAATCTTGTACAAATGCTTGCGCTACATCCTTGTGTTGATCAAGCAATTCCCCTCTCAGTACTAGCACACAGC
AATACGCATCAGGTATAACGTCATCACCATGTTTCAAAGTCTTACCTTTGCCTAACTTTTCACCCAGTGCACC
GAATGGTTCGGCTACAGAATACCCTGTAATTCTGTGTTCACTCAATGCGGCTGGCATTTCTGCTGGCGACATT
TCATGATAGCTAAAATGCCCCGGTTTAATCTTTAATTGTTTACGTAATTCCTCAAGTAAAAGATAATGTGTTG
AATAACGATGTGGTATACCAAAATGGTAATCATCGCCATTATTATTAAATTCATTTAAGTGCATACCTTTTTG
TCCCATAATGACATTGCCTTCATGATGGCCCAATGCCACAGCCTTTATATTTGAGCCCTTCTGTTTTGATTTC
ATCGCTAGCTCTATTAAAGTTGATGCACCATCAATACGACCACTGTTTAATGCGTCCATTAAATCTGGCCAAT
TATTGAATTTAACTAATTCTAGTTTATATTTCGGATGATTGTATTGTGATAATAATTTTTTAGTCATCATCAA
ATTAGCTGAATGTGTAATCGGCAAATATCCAATTTTAATCACTTGCTGATTTTGGGCATTTTTAGACGGTTCT
TTAGACGTCCTTTGCCAATCGCATCCTGTAATTATAAAGATTCCAATGATGACGATTATGCTTAACCTTTTCA
TCGTCACTCACTCCTTATAAATAATATTCAGGTTCAACTTGATGATGATTCAATGCAAATGTTTCCATAATTT
CATTACGAATCTTAAGTAGGTGGCTATCATTACGACTGCGTGGATGTGATGCTGTAATTTCATATTGAGAAAT
AATATTGCACCCTTCACCTAACAGAACAATGCGGTCGGAAAGATAAATAGCTTCATCAATGTCATGCGTCACT
AAAATAATAGTTGATTGCGTTTTATGTTTTAGTTGCACTAGTTGATCCTGAAGTTTATAACGTGTAAATGCAT
CTAATGCACCTAATGGCTCATCCATCAATATAACGTTAGGCTTATGCACATGCGCTCGACATAGTGCCACACG
TTGTTTCATACCCCCGGACAGTTGCTCGGGAAAATGCTTTCCCCTGCCTTCTAAATCAACTAATTTAAGCTGT
GCGTTAATCTCTTCATCACTAATTTTCTGTTGGAATCCAATCCTAATGTTGTCATTAATCGTTTTCCATGGCA
GCAGATTATGATGTTGAAATAGCATTAAACAATCTGGAGACGGCTGTTGTTTAATTTCGTTATCAATAATGAC
ACGACCAGATGATGGATGAATAAATCCACCGATGATATTGAGTAAAGTAGACTTTCCGCAACCACTTTTTCCT
ATGAAAGTGACTATTTCTCCCTTGCTAATGTCCAAATTAAAGTTATGAATTACTTTATGTGATCCAAAGTGAT
GTTGTAATTGTTGAATTTTAATCATGACAAGCCCCCTTTTAATCCCTACTATTTTAATAGGAATTAAGTGTAT
ATCTAAAGGGCTATGCCAAACACAGCCCTTATATCGTGAATTGTTATATCTATAGATGATTATGGTTTGGTAT
GAGTGCTCTTATAACATAAGCGTTATTCAATTAACGACGTTATATTAATTTCAGCTCTACTAAATTTCAATAA
AATGATTGTTTTAGACAATACTTGCATTTAGTTCATTAGTTTCTTCATATCAACTACTTACATTTATAGCATT
GAGCTATTCTAATTAACTGCAATCAAAATCCACGTCAAACCTATATACATATATCTCATCAACTTGTTGTCTT
ATTAAAGTTTTTCGATGCCATCAATCGGTGTATCAACTGCTTTGCGCGCGCGACGTACCGCTTCTTCATCAGG
TGCGTTGTATAAACAAATACATTTAGACATATCTTCACATACATATGTGCGTTTAAATTCAACTTCTGGCACT
TCTTCATAATGAACAGAATTTTTCTTTTTACGTGCTAAATATTGATCCATCGTAATGCCTTCCGGAATGTTCC
AAGTTACAAGGTAATCAACATGTGCATCACCATTTTTAACTTCATCTAAATCTTTACCAACTAAGCGTACTTC
TTTGACTAATTGAATACTAATATCTGCTTCTGTTAAAAATTGTTTAGCTTCTGCTTCGTCATTTGCTTCCACA
ATAAAATAACCATGAGTTAAATTTTCAGTAGCTTGTACTTCAATTAATGTTGGAATTGTCTTCGTAGATAGTG
ATGCTGCCTTTGCTTCTAGTTCTTCTTTCGTTGATGCAAAATCAAGATTGTTAGCTTCTAATAAAAATAATGT
CATTAATAAGACCTCCTATAACAATGGTTGAAAAAATATTACTATAATTATTATCAGATTACAAGGATATTAA
GCTATTTTTTTAAATATTTAGATAAAATTCTGTAAAATATATTTTCAAAGAGTATAAAAATAATGTTAGGGTA
TAAGTAAATAAACATATATATAGGGGGCTATCATTATGGATAAAGAATCTGTCGTAGCAAGTTTAGCAAGAAA
CAAAAAGATTGCTGTAGAAACAATGACCGGTCAAAGGTACATCATAGAACGCATTTTACATACAAATGATGAA
AAACATATTCATATTTTGAAACCTAAAGATGTCGTGTTAGATGTAGATACCATCAAAGACATCGACGAAAATC
ATTTAGATGATGCAACATAAGTATAAAACGAAACGTGCGCCTATGACATAGATTAATGTTTTAGGCGCTACAA
TGTTTTATTTATGTTAATTTGTCGAAGTATGGCTCATCTAATGGATTCAATTGCAAGTTTTCAATTTGCAACG
TTGAATGTGCAATGCCATATTTTTCTTTCAACAATGATGATACTTGATCAATCGCTTGATAATCATCACCCTC
ATATTTTTTATCTAACACAACATGGGCGCTTAATGAATAATGCTCTGTTGTAATACTCCACAAATGAAATTCA
TGTACATCTAATATGCCATCTATGTTTTTAATATCTGCCATAATTTGATCAGTATCCAAATGTTGAGGCACAC
TTTCCATTAAAATTAACCACGCATTACGCGTAATTTTATAACCACCACGTAAAATGATGAGTGAAATTACAAT
ACTAATGATTGGGTCGATGATGCGCCATCCTGTAAAGTAAATCAATACAACTGCAACGATGACACCAATAGAG
TTCAATAAGTCTCCCATGAAATGCCATAATGCACTTTGAATATTGATATTGTCTTCTTGTTTTAAAGACCTTA
CAAGGATAACAGTCAAAATAATATTGACGAGTAAACCAATACTAGCAATCATAAACATAATGCCACTTTCAAT
GGGTTGCGGATAAATAATACGTACAATAGCTTCATATAAAATCCAAATTGAAATCACAATTAATGCTAATCCA

TTTAAAAATGCCGCTAATATCTCAAATCTTAAATATCCGAATGTGTAGCGTGCAGTCGGCTTCTTACTAGCAA
AATAAATTGCCAACATTGACAAACCGAGTGCTAATACATCACTAAGCATATGAAATGAGTCTGACAGCAATGC
TAATGAATTAGATACTAAACCTCCAACAAATTCAATCACTGTAAATAATAGTGTGATGATAAGTGATGCCCAT
AGCGTCTTTTTAGAACTTTGTTGAATTTTTTGATGATCGACATGATGAAAATATTTGTATCTGTTTATCGTAT
TCGCCTTTTTCCCATTCATACGTTTGCCACTCCTAAATAAATGCGTCGTATTTAATATTGTCGCTTTTAAAAA
TAATTCTTTATAGCTTCTCAGATTAATATTAATGCTTCCGGAATTAACCATAATTTATACACCATCGTTCTTC
TCATGACAACTCATTAATCTTTACCCTTTATACCTTTATGTAATATCACCTTGGAAAAAATTTTGCAGCACAC
TCGCTCTTTGCTTTAACCCTAGCATATAGAAATTTTTAATAATGTACGAGATTATTGTGTGAGTTACATAAAA
ATGACAAAAAAGTATCTCAAATTTATTGTTTAGTTTCATTAAGTTCAAAAAATCGCAATAACTTTTATGATTG
TTCCACATATTAAAACAAACGATGACTTTGCATATTGAACTTCATATTTCTAAATCACCATAATTCATAAACA
ACTCTGTCCTTATAATAGCCGCATCGCAAATTAGCATAATTAAAAACAGCAGTAAGATGATTTGCAATTAAAA
ACCATCCTACTGCTATTATTTTAGTCATTATATCCTAATATCATGATACGTGTTCCTAAATATTTTGTTTCGC
TTAAAATCTAAACAACTCCGTAAACATGCTAAGACTTACCGCTAGACTGCCTGCAAACACGCTTCAGAAACGA
TATTTATTCTAGTACAAACCTTTTAAAGCATTGCTTGTATCAATATAAATATTTTTAACTTGTTGATAGTTAC
TTAACGCACCTTTATAAGTTTCTCGACCGATACCTGATTTTTTATAACCACCAAATGGTGCGCCTTCTGGTAC
TTGGTTGTAAGTGTTAATCCAAATACGTCCTGTACGAACAGCTTTAGCAATATTTAATGCACGTGTGATATTT
TGAGAAAATACGCCGCCTGCTAAACCATACTCTGAATCATTAGCTATATCAATTGCTTCTTGATCGTCCTTCA
CTTTAATCACTGTTAACACTGGTCCAAATATTTCTTCTTGCGCTAATTTGTGATGATTATCTGGCACAGCAAT
TAATGTCGGTTCAAAGAAGAATCCTTTATCTAACCCATTTTCAGTTAAACGATGACCGCCTGCTAAAATTTGT
GCATCTGATTCTTTTGCTGCATCAATATATGATTGAATTTTATCTAATTGATCCTTACCAGTTTGACTACCCA
TTTGTGTAGCTTCATCTTGTGGATCTCCAACTTTAATATTTGAAAATGCCTCTTGTAAACGTGGCACCAATTG
ATCATAAATTTTTTCATGAACTAATAATCGAGAACCTGCACTACATACTTCACCTTGGTTGAATAAAATACCT
AACTGAATGCCTTCAACTGCAAGGTCTAAATTAGCATCATCTAATATGATATTGGCGCTTTTACCACCAAGTT
CTAATGTAGCGGGTACTAGATGTTTTGCTGCAGCTTCGGCAACTTGATAACCTACATCAGTTGAGCCCGTAAA
TGATAATTTATCTACACCATCATGATTGAAAATTGCATTACCTGATTCTGAACCTTTACCCGTTAGTATATTG
ACAACACCTTTAGGTAATACCTCTTGGAAAATTTTAGCAACTTCCAATAAACTTAATGGTGTTGAAGACGAAG
GTTGAATCACAATTGTATTACCTGCAGCAATGGCTGGCGCAATCTTCCATGCAGCTAATAGCATTGGGAAGTT
CCAAGCAACAACAGCACCTACGACGCCAATCGGCTCATGTCGTACGATACTCATTGTGTCTTTATCGATATCA
TTCACTGTACCTTCTTCTGTTTCAATAACACTTGCGAAATAATGGAAATGTCTTGCAGCAAATGGAATATCAA
TTGCTGTTGTCTCACGAATCGGTTTACCATTATTTAATGTTTCAATCATTGCAATTTTATCTTTTTGTGCCAT
TAATTTATCACAAATATCACGCAACATTTGTGCACGTTCTGATTTAGAAGTTAATGACCATGATTCAAATGCC
TCTTGCGCCACTTCGACTGCATAATCGACATCTTTATCTTTTGCTCTTGTAATATGTGATAGTGTTTCTCCAG
TTGCTGGATTAGTCACTTCGATTGTTTCGTCACTGCTACCTTTAACAAATTCCCCATTGATAAATAAACCATA
ATTCTCTGCTATATAATCTCGAACGTTTACTGCCATATTATCTCTCCTTTGTTTATTGGCTATAATATAAATC
TCTTAGCATCATATTTAATAATGCTTTTAAACGATATGATTCACATTATATAGTAAGCGTTTACATTTTATAA
AATAATCAGCTCACAGAAGTCTCATTTTTAAAACTTTTGCGTCGTTTTTGCACATAAAAATAATCTTATTTTC
GAAAACATATTGCACTTTTCAAAAATAAAACACATAATATAACTATAATACTTGAGAATAATTCTCATTAAAT
AGTTTAGTTGTTATGAAAGCGCGCTTAAAACGCGTTAATGGGACAAGGATAATTCAACAACGTTTCATTTTAC
TGAGTCATTGCACTTATCATACACATTATATTTAGCATGAGTTATATTACTAAAATAATATTACATGCGCAAT
AGTACTAGAATGGAGTTTTTTCAATGCGACTTATTTTAATCGTCATTGGCTTAATATTTACAGCGCTTGGTAT
TGCAGGTGCCGTATTACCTTTACTGCCAACGACCCCTTTTTTACTCGTAGCAGTTTTTTGTTTTGCTCGAAGT
TCAGATCGCTTTTACAATTGGCTTATTAATCAAAAAATTTATAAAGAATATGTAGAAAACTTTTATTTACATC
GAGGCTACACGCTACAACAGAAAATTAAAATTTTAATTAGCTTATACATTGTAATAGGTTTTTCAATTTATAT
GGTGGATGTTCTTGCAGTCCGTGTAGGATTAATCATCATGGTTATCATACAGACCGCTGTACTCTTTACATTT
GTAAAAACATTACCCAAATCAAATCATAAAATAGAGGAGTGATTGCCCATGTTTATGGCAGAAAATAGATTAC
AATTACAAAAAGGCAGTGCGGAAGAAACGATTGAACGTTTTTACAATAGACAAGGTATTGAAACTATTGAAGG
CTTCCAACAAATGTTTGTCACTAAAACATTAAATACCGAGGATACAGACGAAGTTAAAATCTTAACTATTTGG
GAATCTGAAGATAGCTTTAATAATTGGTTGAATTCCGATGTATTTAAAGAAGCTCATAAAAATGTACGTTTAA
AAAGTGATGACGATGGACAGCAAAGTCCAATATTATCAAATAAAGTATTCAAATATGATATTGGCTACCACTA
TCAAAAATAAATATATTAAAAACCCCTAGTCACCGCTGGTTGTCTAAACCAACTTCATGATTAGGGGGTTTTT
ATTATTTGTGACGTAAAGGTATGAATTCATCCGGCTTGTCTGAGCGCAATCCAAAATAATATTCTATTGCTTC
ACAAATACGTTGTGATGCTTGTCCATCACCATATGGATTATTCGCTTGACTCATACGTTGATACGCTTCATCA
TCCTCAATCAATTGTTTCACATTTCGAACAATATTGTCATAATCTGTGCCAATTACTCTCGATGTTCCCGCCT
CAACGCCTTCGGGACGCTCTGTATGATTCCTTAATACCAACACAGGTTTTCCAAATGTAGGAGCCTCCTCTTG
AATACCACCAGAATCTGTCAGCACGAGGTACGATTGATTTGTAAAATTATGGAACTCAATCGCATCTAATGGC
TCAATTAATTCAATCCGATTTCTCCCAGATAAATATTTTTCGGCAATCGCTCTTACCTTTGGATTACGATGCA
TTGGATAAATGAAGACAACATCTTTATATTCATCTGCCAAATCTCTTACTGCTTTAAAAATCTGATGCATCGG
TTCCCCAATATTTTCACGACGATGCGCTGTTAGTAAAATAACTTTCTTGCCTTTATGTTTATTAATAATCGTT

GAAACAAAATCATTTTGAACTGTTGTTGACAATGCGTCAATAACTGTATTTCCAGTAATAAAGATACTCTCTT
TGTCTTTGTTTTCAAAAAGTAAATTTTTAGCTGCAATTACTGTTGGTGCAAAATTCAATTCAGCAATATTACT
TACCATGACTCGATTTAACTCTTCAGGAAATGGCGAGTATTTCTGATGTGTTCGAAGTCCAGCTTCTACATGT
CCGACCGGAATTTGATGATAAAATGCTGCCAAACTTCCTACAAAAGTCGTTGTAGTATCACCATGTACTAAAA
TCATATCCGGCTGTTCCTCATTAATGATGCTATCAAGTTTAGCAAGCGTATTCGCGGTAAGGTCCGCTAACGT
TTGTTGATCTTGCATAATATTTAAATCATGATCAGCTTGAATATCAAATATACTTAACACACTATCTAACATA
TCTCTATGTTGTGCTGTAATCACAATGTTCGCTTCAAAGTTCCCATTATGATCAATTTCTTTTACTAATGGTG
CCATTTTTATTGCTTCGGGTCTCGTACCGAAAATAACCATAATTTTTTTCATAACATTATCCTCTCTGAAGTT
CAAACACATAACAGCTTATCTTTCCTTAATCATGTATGCCCTAGTTTGACACATTTTATTTGTCGATAAAATT
AAATATATTGCCATAATTATAATACGATACATCTTCAAATGATGATTTCACAACATTTTTTGTATCAAAAATC
ACTTTATGCTTCATTTTATCAAAATGACTGTCCGATAAATTTTTAAATTCTGAGTGGTCACTTAAAATCAATA
CTAGCGATGCGTCTTTGACAGCATGTGACATATCATGTTCTACAAAATCTAATGCAACATGTGGATCATAAGC
ACATACTTCTATGTCTGGTTCTTGATTTAATAGCTCATAAATATCAAATGCTGGTGATTCTCTTATATCATCA
ACATCACCTTTATAAGTTAAACCAAATACTGTGACTTTATTCCCGCTCAACGCTTTAATGATTTGTTTCGTTG
TATCAACAACATAGGCCGGCATTGAATTATTAATTTCACGTCCAGTTTGAATTAACTTTGCATTTTCAGGGTC
TTTAGCAATAATAAAGTACGGATCAACAGCTAAACAATGACCGCCTACACCTGGACCAGGTTGATGGATATTA
ACACGCGGATGTTTGTTTGCCATTTCAATCACATCTAATACATTAATATTTAAGTTATTGCAAATTTTTGTTA
ATTCATTAGCTAAAGAAATGTTCACGTCTCTATATGTGTTTTCCATTAGCTTACTCATTTCAGCAGTACGTGC
ATCTGTTTCAATCATTTCTCCCTGAACGAATGTGCGATAGACACGTTTACCCGCTTCAATACAAGCTTTAGTC
ACACCGCCAATGATGCGATTGTTATGAACTAATTCTTCTAAAATTTTTCCTGGCAGTACACGTTCTGGACAAT
GTACTAAATAAATATCTTCACCTATTGTAAACCCTAAATTTTCAATGACCGGTTTTACAAAATCATCCATCGT
TTTAGGCGCAATTGTCGACTCTACAATAATGGTATTTCCTTTTTCTAAAAATGGTAAAATACTATCTAATGCA
TGCATAACTAGCGAAATGTCACATGACCGGTACTGATCATCATTATTCGGCGTCGGAACGGCAATGATAAAAA
CATCAGATGCTTCTGGCGTTGTAGATACCTTCAATTTTCCCGATGACAGTACCTCTTCATAAACCTCTTGTAA
TCCAGGTTCTTCAATACTAATTTGACCACTTTGTAACTTATCAATCGTTTGCTGATTAATATCAACACCAAGC
ACATCGACGCCATGCTTTGCAAACATAATTGATGTTGGTAAACCAATATAACCTAAGCCAACTACTGTTAACT
TCATACTATAACCTCCAAATAAAACGATTTGTATGTTGATTTTATTTAATAGATGACTTATGCCTTATCTTTG
GTTGTCGTTTCATCCATGATGTCCGCAATAACAAGTGACATTTTTCCAGGAATAACTTCAAGTGCATTATTAT
TTTCATATAACGTATTGCTGTATATTAAATTGCCGAATAATTTTCTAAAGACCAACAACTTATTAAAATACTT
ATTTAGCATTGAAGGCATGTTGATCAATACCGTTTTATGATGTGATTGGCGACGTATTTCATACATTACTGAC
GATGTATCAAAGTAAAAACTATCTTGAGGATGATACACGCCTGTCACTTCTAATGATATTAATTGATCAATAA
ATGCTGTCAGATGTTTAATATATAATGCACTGCGCTGATTGTTAATATTTGGAATGATTGGCAATCGCTTTGA
CAATTGCATTAACCGTTGGAAATTTCCTGGGCAATGTGCACCATAAATCATTGGTGGTCTCACAATTGCTACT
TTAAACGAATCACTAATCAACTCTTGTAATGCTTGTTCAGCGAACTTTTTGGAAATACCATAATTGGTCGTAG
GGTTCATTGGTGTTTGTGTATCAATTTGATCTGATTTACCAACCTGACCTTCTTTTCCATAAACTGCCATAGT
ACTCATAAAAATAAATTGTTTAACGTCTTCAGCTTTAGCCTTTTGTGCCAATTGTTTCGTAAGCAACATATTC
ACTTGCATATAATCAGATAGCCTTGCTTGAGGTGAATTGTTGTGAACCAAAGCTGCTGTATGAATTAAAACAT
CATAATCTTTGAACGAGGTCGACTTCCATAATTGATTCCTAACATTAATTTGATCTACTTGATGTCCTTGTTC
AATAAGCTTATCTTTTAAAGCATTACCGATATATCCATGTACGCCTGTAATTAAAATATTTTTTCTCATTAGT
GATGCACACCTTCTGAAGTAACGATATTTTTAATTGTTTTATATATGATATACATATCAAGCATCATAGATTG
ATGTGTTAAGTAATAATGATCATACGCTACTTTTTGATCATCAGTAATATCATCTCTCCCCATCACTTGAGCT
AGTCCTGTCACACCTGGTCTAATCGTATGCACGTTCGCTTTTGTACGTTTTTCGATTAATTCGTATTGATTAT
AAAGCGCTGGTCTAGGACCTACAATTGACATTTCTCCTTTTAAAACATTCAATAATTGTGGCAATTCATCAAT
AGAGGTCTTACGAATGACCTTCCCTGTCTTTGTTATATACGATGTTGAATCCATTAAATCAGTTGCAACATTA
GGTGTGTCTATTTTCATTGATCTAAACTTATAAATATTAAACAATTCATTATTAATCGTCGGTCTTTTTTGTT
TGAAAATGGCTGGTCCAGGTGATTCCATTTTAATTAGTAATGCTGTAATTAACAGAATCGGACTTAAAACTAC
TAAACCATATATTGAACTCACTACATCGAATAATCGCTTCATAATTATCTCCTCACTTCAACAGATTGTAATA
CTTCATAGTATTAATTTCTCTCGTATATTTATCTTTAAACAGTTGATGTGCGCGCTCACCCATCTCGTGCAAT
TCCTTACTCGAGTGAGTGTTGATGAAGTTATAAATGGCATGTGCATCGCCATTATCAATTTGGATACCCGCAT
CATATTGTTCAACATGTTGAACGATATCAGATTGCTTATCCATGATGAGTACCAACGGTTTCTTAGCTGCAAG
GTAGCCATAATTCTTGCTCGGCACGCCTAAACCGACGCCTTCTTTAATCAGCGATGCAATACATACATCCGCA
ATTTTTAATACGTCAGCATAGTCTGTACCTGTTAAAAACTCAAACATTTTAACATTTTCAATACGATGGTCTT
CTATTGCCGTTTTGACATCTGCAAATTTCTTACCATGACCACAAAGTATTGTTAACGTTTGAGGCTGATCCTT
ATTTAATTTTAAAAATGAGATAAGTGTCTCCATATCCTGTAACTGCCCCATATTACCGCTATACAATAAAATT
TTGTCGTATTGCTCACGGTAAGCTTTAAATGTATCATTATAGATACGATTGTCTTGTAATTGACGCATGTCAT
ACCAGTTAGGAATCACATGGATATTGTCAGCATTTTTAGAAATTTGATGATTTAGTAAGTAGTTTTTCATTTC
CGTACCAAGGACAATGACATTTTCAGCATTCTTGTAGACATGTCTATTAATGTAACGCATCAGCTTATCAATC
ATGCTACCTGGACGAGTTGCACCTGTCTTAATCGCATTATCAGGTGCTATATCATACACCACAAAAGAATATT

TTTTCTTAAGCAGTCTGTGTAAAACGTCTGGTATTAATGGCAAGATTGGTGGATTAGAGTAAACAAGAATCTG
ATCATATTTCAACATTTTAGGTATATTAATCACGAATTTTGAAAATAAACTAAAGAAATTGATGATCCTTCCA
ACCTTACTTTTGTTATTAAACCTCGAATACTTGAGACGTCGAATGCGAATACCACGATGCATCTCGGTTTTAG
AAACCTGTTTATGATTACTATATTCATATGGCCATCCACACATGACATCGACATTAATGTGATTCGCAATTAA
ATCTTCCGCCAATTGAGTTGGTAACGTCGCAGAAGATACATATTCCGGATAAAAATACTGACATAAAATCAAA
ATCTTTTTTTCACTCATCTAATCGACGTCCTTTTTATTAATGAATTTAGACTTTAAACCTATAATATTTAAAA
TCACATACTTAATAATGTTGCTGAACAGCGTTGTCCAAAAGAATCCATTTAGACCAAACGCAATTGTCATTAA
AATAGTTATGAATATAAATGTAATCGTGTGAAACGTCATATAATTCGCTTGTAATGTTATTGATGCGTGTTTT
GTATTTAAAGTTTGGATCATAATACTCACTGCATTGAATAAAACACCTATATTACCTAAAATAATAAACTTCG
AATATAAACTTGAATCAATGTTATACAGTAAAGAGACAATAATTATTGTAATTGGATAACATATAATCATGAC
TAAACATAGGGCAGCTATAGCAAATAGATTAGTTTTCAAATATTGTTTCTTTATATTGTCACTTTCATTTACA
GAAATATGTGAAAGCACTACATTATTAATCGGATACAGAAATGTAGCTAACATTTTCCCAATAAATGTTGAAA
GAAATGATATAGTTACAGCTGTTCCACCTATAATTGGTAATAAGATTAATCTATCTAAGTAGAGATTCAAATT
ATTAAGGCTATTTGTACTCAGTAGCATCACATAATCTTTTACCACATTATTATCTTCACTTTGATACGCGCCT
ATAGTTAATCCCCGTAATTTAACCAATGTATATATCGTTGCAAACAATTCACTGGTAATAAAACAAACAATCC
AGTTTTGGGTTAAATAATATAGAAGTAGTCCTATCAACAAACCTAAAAATTGAATAAGAGCAATATACAAAAT
CTGATTATATTTTAAAGTCATCCTAAAAAATACATTCAGATAAATCCTTAAGCACATTAAAATATTAAGTAGA
ATTAAAAAGATAATATCGATGGTGTTCAAATTAAAAAAGTAAAGAAATACAATAATTAAAGCTATACTCTCAA
TCAGAATTGAAATTAAAAGTATCAACACAAATTTCCAGTAGTAATGATTGGATTTGTATAGATTCATATTAAT
CAATCGTATATTGTTAAGCGTATTGCCTAATACAACACTCGTGATTGTTATTATTGTATAAATCGTTAAAATA
GAACCAAACGCTTCATTACCTACTCGTTGATTAATAATTGGATAAGCTAAAAATTGTAATCCTAAAGCTATAA
GCAACGTACCAATTATTGTCTTTACACTATCCATAATAAAAACTTTCTTAACCATGATGACGCCGTCCTTTTG
TAATAAAATAGAACAACACAAACCAAAATTCACTTACAACTAAATAATTTGAACTAAACATTAATAATGTGAT
TGGATAGATTGCTATAAACATAACGAGTAAATCTATAGTGTTTGGATCATAGTTCCTAATCATTTTATAAACT
AGTAGCAAAATGCAAATCATTATGATAAAAAAACCCTAATAAGCCAAATGATAGAATCAACTCAATAATGATGT
TATGTGGTATATTTCCGATTAGTTTATAATAGTTAAATGGCCCATAGCCTAATAACGGACTTTGTTGAATAAA
GTAAATACCTTTTTCATAAATCGGTCCTCTTCCAGAAGTACCTTCTAAATTAAGTGTTCCACCTTGTAGATAT
GAAAATGTTCTAGTATTCGAACCTTTTGTAAAAAGAAAGTAAATCAATACACTAGATATGCTTAATGCAGAAA
TATACATAATGCTTTTTACTGCAATAGGTATTCCTCTTTTAAACGTAATAAGTATAAATGCAAATAAGCCGTA
AAGAATTAATAAAATAGCACCTCCACGCCCTCCTGGTATAAACACAATAGGGATATCAATTATTGTAAATAGA
ACATATATCCACTTATGTTTCACTGAACCTTTCATAATGAAATAAATGCCTAATCCGGCAGTAAATGCTGAAA
GGTACGAAGCGTTTTGATAGTTCATAAGTCCAAAATTGATATAGCTAGGTATCTCACCTGTAAGTTTTGGTAT
TAAAATTACAAAAATAAATGAAACAGAAAATATGAAAAATACTAATTTAAAAAATCTTTCTACCGTAGCCTTG
TTTATATATTTAATATAAATACCACTAATTGCCGCTGGAACTGCCCATGTTAAAAAGAATAGAATATTATTTT
TAGCTAGTTTCTCTTCCTTATCTGGTGAAAAATAATAAAAAGCTAGATAAAGCAAACATATAGCAATTAATAA
TATTAACCCTCGCGGAATTTCTTGCGTGACAATTATCTTATAAATAGCAAACACCGTAGTAATTAATGCTATA
CCAACCATGGTTGAGTAATACACCGGCTCTATAGGGAACCCTAATACTTCTTTAGTAAATGTAGAGATTACTA
TAAATATGTTCATGCTGATAATTGCACAAAGTACAAAAAATTTCATAGTTTACCTCTCTTTAAAATTTATATA
ATTTTACTAATCGTTCACTTTCTATTTGCCAATTCAAAATTTTAGACGCCTTAATTGCATTTTGACGTAAATG
ATTAAACAAATCGTGATTATCTCTTAATTTTCTAACCGCCTTTTCAATTTCTAACGGCGTAACTTCCTTTAGA
ACAATGCCAAATTTATATTTTTCATTGAGATAAATATGCTCTTTGACAGGAGATAAAATTACTGGTAAACCAG
CATGTATACATTCAAAAATTTTATTAGATACTGTATATTCAAAATTAATAGATACAGGTTTCGTCAAGACAAC
ACCAACATTACTTTCTGCTAACTTATCAACCAATTCTTTTACTTCAACTGGTTTATCCAACCTAATATTTTCC
GGGTTATAACTAATCAGTTCTTTTATCACTTCTTCATGCGGACCAAACCCTCGAATTATGAATGAAGGGGCAT
TTTGTTTAAAAGCTGATGAAGCTATAATAAACTCTTCATATCCTCTGTCCATTACAATTTGACCTTGATATAC
GATTGATTTAAAGTTTTCGATTTCTTTAAATTCTCTGCTATCATTTAAAATAGGTGCATTCGTAATAACATTC
GCTTCCTTCTTATATCCTTTAGATTGATAATATTCTTTTGCTGCATGACTTACTGTTACGAAGGCATTAACAC
GATGTTTTACAATGTGTTTTTCTATACTTTCTACAAACTTTGAAATAAGTGGAACTTTATTAATAAAGGCATT
TTTCGCATATATTTCATGCGCATCATAAACAATATTAGCTTTTTTATAATTGCTTAAATAGACCATTAATAAT
ACGTCGAAATCATTTGCATGTATCACGTCAGGTTTATAAGCTTTAATTTCTCGGATAACACCTGTTGCAAATC
TTATACGCTTAATTAATTTAGAAAGAATATTTTTTGGATCTACCTTGCTACCTAACAAACGATAATTACAATC
TAAATTTTCCAATCGCTTATTAGTAGCTTGTGAATTATTCATTCCAACAATTTTATAATCATTCGTAACGCCT
TTAATTGTTTCTATTTGTTTAAGTACCCTTGGGTCTTGAACAATATTACTCGATACAATATTTAAAATTCTCA
TATCATTAAGTATCCTTTTTATTTAAATATTCAACCGAAAATCCTTTTAGTTTGTCAGGCGTTTTCTCCCACC
ACTTGCTTTCCAAAAGTTTTTCAATTGTTTTATTGTCAAATCGCTTCTTAATCACTTTTGCAGGAACCCCACC
AACAACCTCATATGCTCCTACATTTTTAGTAACAACTGAGCCGGCTGCTATGACTGCACCAGTATTTATTGTT
AATCCATCCATAATAATTACATTTGCACCAATCCACACATCATTTTTAATTGTTGTACGGCTTGGTTGGTCAT
TAAAGTCTATAAACTTTTGCTTTATGTTAAATGGATTATTATTAGAATAAAAAATCGGTGATGAGCTAAAAAA

GTGTGTAGGATGTTTTCCTAACCCAATTTTTACATCCGAAGATATCGAACAATATCTTCCTACTTCTACATTA
TTAAAATCACTACCAAATCCAATATAACTGTATTCACCAATGTGAGAATTCCTGATTTTACACCATCTATCTA
TATAGTTATTGCCATCAAATTTTGAGTTTGTAATATACGCCAAGCGATGAATCTTAACATTCGATTCTTTAGA
GGACTGGTTTTTCAGCAAACCAATTATCTTTTCAATCGCTATCCTCATCGTCATTTCCTCCAAGTATTTCGAT
TGATAATATCTTTATAGCTTTGAATAATTTTAACTACCTTTGTCGAAACATTAGTGTCTTTATAATCAATAGC
ATCAATCATCGGTTCGTTATTGTTTTGCATCTCTCTTGCTAGTTCAACGGATTGGATTAGATTGTTATAGGTA
ATACCACCTACAATAACCGTACCTTTATCTAGTACTTCCGGTCTTTCTGTGGAAGTTCGAATAAGGACACCAG
GGAACTTCAAAATAGACGACTCTTCTGACAATGTTCCACTATCTGATAGCACAACAAATGCATCTTTTTGCAA
TGCATTATAATCAAAGAAACCAAATGGCTTTAACTGTTTAACTAGTGGATCAAATTCAAATTTACTTTCTTCA
ATTTTCTTCCAACTTCTTGGATGCGTTGAATAAATCACAGGCATTTTATACTTTTTGGCAATATCATTTATCG
CATTCATTAATGATTTAAAATTCTTTTCATTATCGATATTCTCTTCTCTATGCGCAGATACTAAAATGTATTG
TTGCGGTTCTAATCCTAGTTTATTTAAAACGTCACTGTGATTAATTTTATCTCGATGCGCTTCTATCACTTCT
GTCATCGGTGATCCTGTCACAAAGATATTCGCTTTATTGAAGCCTTCATCTAATAAATAACGTCTGCTATGTT
CCGTATAAGGTAGATTCACATCACTGACATGGTCAACAATTTTACGATTGATTTCTTCAGGTACATTCTGATC
AAAGCATCTATTACCCGCTTCCATGTGGAATACAGGAATTTTTAATCGTTTAGCAGATACTGCTGCTAAACAA
CTATTTGTATCACCAAGAATTAAAAGTGCATCTGGTTGTTCGCGTAATAAAACGTCATATGTCTTCGCAATAA
TATTCCCCATCGTTTCTCCAAGGTTACTTCCAACTGCCTCTAAGTAGTGGTCCGGTTGTCTTAATTCCAAATC
ATCAAAGAAAATTTGATTCAATGTATAATCATAATTTTGACCAGTGTGTACTAATATCTGATTAAAATATTGA
TCACATGCTTTAATCGTTGATGATAAACGAATGATTTCAGGCCTTGTACCAACTATTGTCATTAATTTCAGTT
TTTCCATGCGCTATACCTCCAAGAAATACGTATCTGGCTGATTTGGATCAAACATTTCATTCACCCACATAAT
AGTTACCATATCTGTGTCGCCTAAATTTTCAATATTATGTGTGTATCCTACTGGTATGTCTACAACTTCTAAT
TTGTCGCCAGAAACGTAATATTCAATGATTTCATCATCATTAACATGTCTAAAACGAATTACCCCTTTACCTG
ATACGACTAGAAATTTTTCGTTTTTAGTATGATGCCAGTGATTACCTTTAGTAATACCTGGTTTAGAAATATT
TACAGAAACTTGACCACGATCCGGTGTTTTTATAAATTCTGTAAAAGAACCCCTATCATCCACATTCATAAGT
AAGGGATAACTAAAGTCTGTACTAGGTAGATAGCTTAAATACGTACTATACAAATCTTTTTCAAACAAGTTAT
CTAATTTCGGCAATGTTCGATCGAGACGTGACTGTTTGAACTTGTATAATAAATCTACAATTTCTCCCAATGT
CACTTTAAATACGTTTGGTACTGTAGGTACACCATTTTCAATCGTTGGAGTTCCTTCAATAGCACGTTTTATT
TCAGCGACGATATCATCCACGTAGTTTAGCGTCAGTTCAACATTCCGATCATTAACTTGAATCTCTTCGTTAC
GTGCAATTTTGTAACAAAATGTTGCAATCACTGAGTTATAATTCGGCTTACACCACTTACCGAATAAATTTGG
CCATCGATAAATATAAACCGTATTGCCATACTCTTCGGAATACTCTCTTAATAGCTGTTCCCCTTGCAACTTA
CTCTCACCATAAGGATTATCTTGTGTTGCTTGTATTGAAGACGATAATAATATCGCCGGCTTTTTCTTATTTC
TAGTTAATATATCAAGTACATGATCTAAATAACTCACGTTTCCTAAGCTGAATTCTTTGTCGTGTTCAGGTCG
ATTAACACCTGCTAAATGCACGACAAAGTCTGCTTTCAACAACGCTGACTCTAATTCTTCCTCTTTAGTTTGT
CGATGTACTTCGAAAATATGATAATCTGTCGTTGATGTTAAATCTGCTTTCAAGTTTTTTCCTACAAAACCCT
TTGCTCCTGTAATTACAATATTCAACGTCAATCTCTCCTATCTCATTGAAGCTTTATAATCGTTCAATTCGTT
TCTAACATATTCTAGTGTTAAAAGTTTTTCTTTTATCTCTTCCACCGTTAAAATATGTGTATTATCGGAGTTA
TATTCATAAGATTGCGTAATCTTTTCGTTACCGGTTTCAACATAATTACTATAATTTAAATCTCTGGAGTCTG
CCGGCACTCTAAAATAATCACCCATATCTTCACATTGTGCGTATTCTTCTCTCGTCAACAATGTTTCTGCTTT
TTTCTCTCCATGTCGCGTACCAATGATTTCAATTGCATTATCAGCTTCAAACAATTCTAATAATGCGGTCGCA
AGATCCCCTACTGTTGAGCTTGGTGCTTTTTGAACCATGATATCGCCTGTCTCTGCATGCTTAAATGCATGAA
CAACTAGTTCCACGGCATCTTCTAAACTCATTAAAAATCTTGTCATATCAGGATCTGTAATCGTTAAAGGTTC
TCCAGCTTTGATTTTGTCGATAAACAAAGGAATTACTGATCCTCTTGAAGCCATCACATTACCGTATCTTGTA
CCACAAATAAGCGTTTGTTCACTACGAATATTTCTTGATTTGGCTATGAATACTTTTTCCATCATTGCTTTCG
AAATACCCATAGCATTAATAGGATACGCTGCCTTATCTGTAGATAAACATATGACTTTTTTAACATTTTGATG
AATAGCACTTTGTAAGACATTTTCTGTACCAATAATATTTGTCTTCACTGCCTCAACTGGAAAGAATTCACAT
GACGGCACTTGTTTTAAAGCTGCTGCATGGAATACGTAATCAACATCTCGCATTGCTGTTTCTACACTTTGAC
TATCACGCACATCACCAATGTAGAACTTTAATTTTGAATTATTATATTTTTTTCGAATGTCATCTTGTTTTTT
CTCATCGCGTGAAAAAATACGAATTTCTTTAATATTAGAATCTAAAAACCGTTTCATAACAGCATTACCGAAT
GATCCTGTGCCCCCAGTAATTAATAAAATTTTGTCATCGAACATAATTATCTCCCTTTTTGCCATTGGCATAG
TTAATAATTTTTTCTTTACTAAAGTCATTGACGATGTCTTGAATAATGGCTTCAACTTCATTACATTTCATAT
GTTGTACTTTGCCACGATAAATTTTTTCAAATACTTGTTCAGGATGAACTTCATCTTTATTCATAAGCTCTTC
AAACATTTTTTCGCCGGATCTAATCCCTGTATAAGTAATGCGTATGTCGTCTTCTTTTTTACCACTTAGCTTA
ATTAAATTACGTGCCAAATCTACAATTTTCACTGGTTCTCCCATATCTAGCACAAATACTTCGCCACCTTCTG
CTAATGCCCCTGCCTGCAAAACTAGTCTAGAAGCTTCAGGAATTGTCATAAAGTAACGTGTCATTTCAGGATG
TGTCACAGTAACTGGCCCACCTTCTTCAATTTGACTTTTGAAAAGTGGAATCACAGACCCTCTCGATCCAAGT
ACATTACCAAATCTCACTGCAACAAAATTTGTTTGATGTGTTTCATCATTTAAACTTTGAATAATCATTTCTG
CAATTCGCTTTGAAGCTCCCATGACATTAGGCGGATTAACGGCTTTATCCGTCGAAATCATAACGAATTTCTT
TACCTCTGCATTTTTAGCAGCTTCAGCAGTATTTTTCGTACCTAAAATATTATTACGTACTGCTTCTTCAGGG

TTGTCTTCCATTAACGGCACGTGCTTGTGTGCTGCTGCATGATAAACTGCGTATGGTTTATACATTTCCAAAA
TTTCAAACATACGCGCTCTATTTTGCACATCCGCTATAATAGGAACGATATCAACATTTTTTCCGAAGCGATT
TCGCAATTCACGATTGATTAAATAAATACTGTTTTCACCATGGCCAAGTAGAATAATACGTTCTGGATAGAAA
TTACAAACTTGTCTACAAATTTCTGATCCTATTGAACCACCTGCACCCGTAACTAAAATAGTTTTATTCGTCA
ATTCATTTGATATCATATCCATATCTAATTCAACAGGATCTCTGCCTAGTAAATCTTCTACTTCAACTTTTTT
AAGTTGGTTCACTTCTAACTCACCAGACATGACGTCTTCTATATTTGGCATTTTCAATAACTCAACGCCATCC
ATATGGCAAATATTATTAATTTCTTTCAAACGCTCTTGACCAATAGTTGGAATTGCAATGATGATTTTTTTAA
TCTTATATTTCCTCACTAGTTCTGGAATATCCGCAATTTCACCTTGGACTTTTACACCCTCAGTAATTGTGAT
ATTGCGTTTATGTTCGTCATCATCGACTGCTAATACCGGTTCAAGTTTCATTTCGTCACTTTTCAACATTTGT
CTAATCAGCATTGAACCTGCTTGGCCAGCACCAACAACTAAAGTTGGCTTCTTATTAAATGACTTACCTCCAA
GGTATTTCCGATAAATACGCCAAAATAACCTTGAGCCACCTATTAAAATCAAGTGCATCATCCAAGTAATTAA
ATACAATCTAAAAAACGGTCTATTGCCTGTAACAATTGTCACGACCACCATCGTAATAACGATAGATGTCGTC
ACAGCTTTAACAATTAAAATCAATTCACTCACACTGGCATATTCCCACGCTCGATGATACATATTAAAAATAA
ATGCTGAAATATGATGCGATATGAATAGTGATATAGCTGCCAATATTAATAATTTGACAGAATATGTTTTGAA
ATACGGTTCTAAAATGTAATAACTTACGAATACTGAAAATGTCACTATCAGTGAATCGATTAATGCTAGTATT
AAAAGCCGCAATTTCACAGATAAATGTGCCATAAAACCCCTCATTTGCTGTTTATAACCCAAACCATCTTTTC
TGTTTATAATCTTGTTGTGGCATTCGTTTAGGAATTTTTTTATCATCAACAACTAACTTCGCATTACTAATAA
ATCCGTTCATATCTTCATAATAATCACGTAATTTCTTATCATTAAATAAGTCTTTCATTAAGAACGGTCTGAT
TTCTGTGTTATGCGCATCTGAACCGATGAAATGTGTCAGATTGTTTTCAATCATTTGAATTGCTAATTTTCTA
ATTTTTTTACCGGAAATACCCGCTAATGACGCCGTTGTCACTTGACTTAAAGCACCTTTGTTAATTAAATCGT
ATAGTATGTCAAGGTTTTGACTTATTGCTTTATTCCGCTCTGGATGTGCAATAATCGGTACAAAGCCTTTACT
TTGTAATTCGAAAAATAATTGATCAGTATAGTGTGGAACTTCATTTGATGGAAATTCTATTAGTAAATAGCGT
GAATCATTAATACCGGTAATAACTTTTCGATCAATATCATTAAGGATTTGATCGGTAATTCTTATTTCCTGAC
CATAATAAAACTTTAGATTCAGTGCTTGTACTTCCTCTAAGCTTTCAATATGGTTTAAACATGATTTCACTTT
TTCTATAGGTGTGGTATATCGAGGATGTAAGTGATGTGATGTTACAATGATTTCTGTAACACCTTGTGTTGTC
GCTTGTTTTAAAAGATCTAGCATCTCTGTTTCATTTGTCGGACCGTCATCGATATTAGGCAATATATGATTAT
GAATATCAATCATACTTATTCATCTCCATAATAGTGATAATAACTAGAAGATTTATCTACTTTTGCCTTATTT
AAAATGACGCCCAGAATGTTACTGCCTGCTTTTTCCATAAGTATTTTAGCTTTTTTAACTTCATTTTTATCAT
TTTTTTCACTATCAATGACTAACAGACTATCTTTAATAGCACGCGCATATAGTTGTGCATCAGTCACGGTATT
AACTGGCGGTGTATCGACAATAATAATGTCGTAACGTTTATTGAAAAGATCAACTAACTCTTTAAATCTTTCA
GAACCAATTAATTCTGTTGGATTAGGAGGTACAGGACCAGCTGTTAGTAAATCTAAATTATCAATTTCTGTCG
TCGTAATAGCTTCTGACATAGTCGTTCTACCAATGATTAAATTCGATAATCCATTATTATTTTGTTCATTAAA
AATATAGTTTTGTGTTGGCTTACGCATATCCCCATCGATAATTAACGTTTTATAACCTGCTTGTGCATACGTA
ATCGCTACATTGGATACGACTGTACTTTTACCTGCACCAGGTTTTTCAGAAGTAACTAATAAGCGCTTTACTT
CGCCATTTGCTTTTGAAAACATGATGTTTGAACGTATACCCCGAAACTTTTCACTAATTGTTGATTTTGGTTT
TTCATACACAAATAGTGTTGTTGTCGTATTTTCCTTTTTTGACATAAGTAGCAACCATCCTTAATTAAATTTT
TGAATTGAACCCAATACAGGCAATCCTAATTCTTTCTCTACATCTTCTTCATCTTTAATTCGCTTATCGAAAA
TTACTTTGAAGAAGATATATATAAGCGCGACAACTAATCCTAAAAAGAATGCACCGATTAGATTCACTACAGT
TTTTGGTGCGACTTTAACTGCTGTACCGTCTGCTTTAGATAAAATTGATACGTTATCTACACTCATAATCTTC
GGAATTTGTTTACTTGTCACTTTGGCAAAGCTATTCGCAATTTTTTCCGAATCTTCCTTATGACCACTTTTAA
CTTGGATGTTGATAAGTTGTATATTTTCTTGATTCGTAATTGTCAACATGCTCGACAATTTAGATGGTGAATA
CTTATCATTTAAGTCCTTTGACACTTCATCTAAAATTCTAGGACTTTTAACAATTTCTTTATACGTATTTACA
AGTTGAATATTACTTTGAACCTCTTGTTCCATAAACTGAGGATTGTCACCCTTAGTTTGATTCACTAAGATTT
GAGTATTAGCTTGATATTTAGGTGATAAGACGAAAAATGTAACAATAGCGCTAATTATTAAAAATAATAGCGG
TAAAATAATTAAAATTTTCAAGTTTTTTTGTAATACTTCTTTAATTTTTGTTAATTCTAATGTACTTTCCATT
ATTTACCTCCCTTAAAAATTTTCATTAAAATTGGTATTATATATATAGTATTTACATATTACATATCATTTAA
ACAATTAATTACTTTTAAATCGCATTATTTACGCATATTCATTTATATGAAATTAAAAATAATTATACTCTAA
AGTAGTAATAGTTTGTGAAATGAGTATCAATCTTAATATTTTCTTTATTTAATCAAAACCACCCGTGTGAACG
GGTGGTTTGTTCTGCGGCTATAAGCCTTCCTTACTGGCCAGCCCTAAAAGGGCACTGACAAGTCAGCCAACTG
CACTACTATTCCAGCAACCCTAAAGGGTTACTCTTTTTTCTTTCTTTTTTTA
>DSM104437_scaffold_4
ACTCCTTCATAAATGTCATGTCTCAATCAAATAACCTCCAATCGAAGATTGAGTTAAGACATGATTATATAAT
TTAAAAAAGAAAAAAATGTAGGAAAAACAGCATTTTCATTTCGCCATTTTCCTACATTTTATAACCGCCATTTA
CAATGGTTTAGAAAGCGGATCTAAATTTTAATAATGATTAATGAAAGGTAGATGAACATGAGTAACTCAAACG
GTTTGTTAAATTTGTCCCTTCATTTATTTGCGTTGAACATCACGAGGCTGTTTCCATTTTCGAAAAATGGAAA
CGAGTATTGTAAGTAAAAACTCTGAAGAATCTAAGGAGATTATATAGTTTCAAGATGATGAACCAATTTAGGA
TGAAAATTTAAATGGATGGATATATTTAGATCTTACTGAATTGTAAAAATAGAATTTCAAGTTATTTGAATGT
ATTTATAAATATATCGTTATTATTAAAGTAGAGATTAGGGGCGATAACATGACTTTCGAAGAAAAATTAAGTG

AAATGTACAATGAAATTGCGAATGAGATTAGCAGTATGATACCAGTAGAGTGGGAAAAGGTATATGCAATTGC
CTATGTAGATGATCAAGGTGGAGAGGTCGTTTTTAATTATACAAAACCAGGTAGTGATGAATTGAATTATTAC
ACGAATATATCTAGAGGTTATAATGTTTCGGAAGAAATATTTGATGATTTATGGATGAATCTGTATTACTTGT
TTAAGAATTTAAGGAATTTATTTAAAGAAGAAGGACTCGAACCATGGACATCATGCGAATTTGACTTTACAAG
AGACGGCAAATTGAAAGTATCATTTGATTATATAGATTGGATTAATACAGAGTTTGATCAATTGGGCCGTGAA
AATTACTATATGTACAAAAAGTTTGGTGTTTTACCAGAAATGGAATACGAGATGGAAGAAATTAAAGAAATCG
AGCAATATATTAAAGAGCAAGATGAAGCTGAACTATAGGGGAGATAATATGACTTTCGAAGAAAAACTAAGTC
AAATGTACAACGAGATTGCGAATGAGATTAGCAGCATGATACCAGTAGAGTGGGAAAAGGTATATGTAATTGC
CTATGTAGATGATGGAGGTGGAGAGGTCATTTTTAATTATACTAAACCAGGTAGCGATGAATTGAATTATTAC
ACATATATCCCTAGAGAGTATAATGTCTCTGAAAAAGTATTTTATGATTTGTGGACGGATTTATATAGATTGT
TTAAGAAGTTAAGAAATGCATTTAAAGAAGAAGGACTTGAACCATGGACATCATGTGAATTTGACTTTGCAAG
AGATGGCAAATTAAATGTATCTTTTGATTATATTGATTGGGTAAATACAGAGTTTGATCAATTGGGCCGTGAA
AACTATTATATGTATAAAAAGTTTGGTGTTTTACCAGAAACGGAATATGAAATGGAAGAAATTCGAGCAGTAG
AGAAGTATGTTAAAGAGCAAGAGTAGCAGACATGTTATAAAAGGCTGTGCAAAACCAACCTTGTTTTACATTT
GACTCAAAGAAGAAGGTAAAAGATAAGATTATTTGCAACTTAAAAAGTCAATTAGTTTATCGGTATCCATACA
TCATGGATAAATGAGTTCAACTAATTAACAAATTACGATATAAATTTAAAATTGTCATTAATTGAAAAGGTTA
TTGTAGTGTATTTTATAAAGTTGATGTACATCGACTTATTTTTTAAAACTTATATCAAAAAAATATGAGAAAA
GTATATACTATTAAAATGTTTTTTACTATAATTATAAATAGTTAAATATTTGGTTATGAAATAAATGTGTGGG
AGGCAATTTTTAGAGATACATAGCAATGTAAAGGTATCAAATAGTTATTGCATAATTTATTATGATGACATAA
TTTTCAGTGAATAGTTGTAAATTAACACTGAAATAGATGTTTATATTAAGAAGACTTTAATATCATAATTAAT
CAAAATCTTGCGTCAAACATTTAAACGAATTTTAAGTTGATAACAATTGGTTAAACATTGTTCAAACATCACA
ATGATAAAGCATATTATCAGTATTGTAGTGTGTGGAAAATGACAGCCATCTAAGGAGAAAAATGATGAAAAGA
ATATTGGTAGTATTTTTAATGTTAGCAATTGTATTAGCAGGTTGTTCTAATAAAGGAGAAAAGTATCAAAAAG
ATATTGATAAAGTGTACAAAAAACAGAATCAAATGAATAAAATTGCCTCGAAAGTGCAAAACACTATTAAAAC
AGACATTAAACAAGAAGACAGTAATACACATGTTTATAAAGATGGTAAAGTCATTGTTATTGGTATTCAATTA
TATAAAGATCGTGAAAAAATGTATTATTTCGCATATGAAATAAAAGATGGTAAGGCAGAGATTAATAGAGAAA
TAGACCCAATTAAGTATATGAAAGACCATAAAGCAGATTATGAAGATGAAAATGTAGAAGTGGAAAAAGATTA
ACGTAGGCTTTCTTTAAAACTTTTAACCAGTTTCAAAAAGTAAATGAGCTTTAGCGACAATAATTTTTAAAAT
AAATACGATATAAATTTAAACATTTATATATAAGAACACAAACGTAAGTTTGTGAATAATAATAAAGGAGTAA
GACAATGGAAAAATCGATCAAAATAATGACAATAATAGGAATTGTTGTTCAAGGTTTAGCAACGGTATTTAGT
TTACTATTTATGGTTTTAGCAGCATCAGGTGTAATGACTACAGATGTGTCAACAACAGTTAATGGTGAGGTTG
ACCCAGTTGATGCAGAAACAGCAGCAGCAATTTTCACTGTATTATTCTTATTCCTATTTATATTTGGAATCAT
TTCAATTATTTTAGGTGCAATCGGTATGTTTAAAGCATCTAAAAACAAAAAAATGAGTGGTATATTGTTGATT
ATTGGAGCTGTAATAAGTGGTAACATAATTACATTTGCTTTATGGTTAATCAGTGGTATTAAACTACTTACTA
ATAACAAGCCTAAAGATGAAATAAGCGACTTATCATAAACATCGTATATTGAAATTTCAAGATTTCTTAAGTA
ATTATATTTCTGTTAGTTTTTAACAAATTAACCGATTAAATAAAGTGCTCTCTTAGAAGTAGATTTTCAGTCA
ATAACTGCTTTTAAGAGAGCATTTTGTATTTGATAATGCTCATAGCATCGGACAAAAAGCATTGACGAACTTA
AAAAAATACGTTAGACAATGCACTTCTAAATATTAAAGGCATCGACTAACGCATTGTTCAAATATAGTTAATT
ATTTTTATAAAATTGATGATGATCATTCAAGTAAGCATAGAATAAACCTATAATGAGTCCGCCTCCAATATAG
TTACCGATAAAAGCCGCAGCGATATTCGAAATAGCTGGTATGAAGTGCAATGTATCAACTTGATAAATTAAAC
CACCCATAAATAAGCAACTGTTGTAAACGACATGTTCATAACCCATAAAGGCGAATATGGTAACACCGAACAT
CATGACAAACATTTTTGCAAGTACATCGTCAATTTGCATGGCGATAACTAATGAAATATTAATAAAGAAGTTG
GCGAATATCGCTTTCATTAATATACTTACAAACCCAGTAGACAACGTTTTATGTTCTATAACTGCTGATAATT
GATTTAACATATCTGGCGTCATTACATTTGAAAAACGCATGAAACTAAATAAAATAGCAGCACCTAAAATATT
TCCTGCAAAGCACAATAAAAATATTTTCAATACTCTAGTTGGTTTAATTACTTTATAATACAGGCCTACAGTA
AAGTACATGAAGTTACTGGTTAGTAGTTCGGAGTTTGTAAATAAAATGAGTACTAACGCAAAGCTGAATGTAA
TGGCACTGGCCATATTCACAATGCCTGGCGGTAAATCTGGTTCGTGTGTTGCTTTAACTGATAATACGAAGAC
CGTAATAATTCCGATAATAAATCCTGCCATCATCGCGCGTAATAAATAACGTTTTAAATAAACGCTTTGTAAT
ATATCTTTTGTTCTTATTGTTTCGACTACGTTATTTACCCAGTCATCCCCATAAAATATTTTATCCCATTTAA
TATGTTTCTCCTTCACAATGACATCCTCACTTTTCATTTGTTAATCAGAGTATACTATGAATCACTTTATTTG
TGAATAATTTCACAAAATTAATTTTAAAAACTAATATTTTTGAATAACAGCTAGGATTTTTTAACAAATCATT
TATTTTAAAATAATTAATCGTCAGTTAGTATCAATGAATAAATACAGATTTTCATGCAAAAAAACCTAGTAAT
GATGCGACGATCATTACTAGGAGATTATGCGTCTATTAAGTTGTCGCTTGTTTCGGACGGCGTATAACTACAT
CGATGATGAAACCGATAATAGCAAAGAGCATGAATGGTACAAGCCAAGCTAAATCGATATCTGCTAAAGGTAA
CATCATAAACCATTTCAAAATAACACCGTGTAATAAGTTGAAACTATTTAGTATTTGTAAAATTGAAATAATC
AATGTAATAACAGTTGCGAGTCGATAGGCCCAACTGAATCTGAATGTGCTAAACATGTTAGCAAATGATATCA
GTACAAGTGCAATAGACACGGGATATATTAAAGTCAATAATGGTACAGCAATTTTTAAAATCATTTCTAAACC
AAGTGTTGTAAATAAGAACCCTATGATAGAGAAAATAAGTGCAAATATTTTATAAGAAAACTTAGGTACGTGT

TTCTTAGTAAATGTGGCGCAAGCATTGACGAGTCCTATACATGTTGTTAAACATGCAAGTATCACTGTCATAC
CAAATACTATGTTGCCAAATGAACCAAATACTCGTAATGAGTTGTACGTCAATATATCTGTACCATCTTTAAA
GTTTCCTGGAGCTGTTGATGCCCCAACGTATGCAAGTGCAAAGTAAATCATTCCAAGTAATATGGCTGCAATA
AGACCTGAAAAGCAGACATATTTTAAAATTTTCATGCGATCTGTGAGGCCTTTAAACTTATAGCCATTGACAA
TGACTACGGAAAAAGCTAACGCAGCAACAAGATCCATTGTAAAATAGCCTTCCAAACTTCCTGAAATGAAAGG
ATGTGTTATATATTTATCCTTAGGTGCACTTAGTGCAGATTCAGGGTTGAAAATGACAGCAATACTTAATAGA
GCGACCATTAATAGTAATAACGGTGTTAATAATTTACCTAAATTATCAACGATTTTCGATGGATTTAAACTAA
TCCAGTAAACGATGGCAAAAAAGATTGCTGCGAATATAATTAAAGTCCATTGGTTGTGCACAGGTAAAATGTG
TCTTGTACCAATTTCGTACGCGACATTTGCAGCACGTGGAATACCGTAAAATGCTCCGATAGACATGTAAATC
ACGACAGCAAAAATAAACCCGAACCATGGATGTATACGATTGCCTACACTTTCAACACCTTCATCATAAAATG
CAACAACAATAACAGTAATAAAGGGGAGTAATATGCCTGTAAGAGCAAAGCCTAGCATACCAATCCACATATT
TTGACCCGCTGTATGGCCAAGCATGGGCGGGAATATTAAATTTCCGGCTCCAAAAAATAGTGAAAATAACATG
AGGCCCGAAATAATAACTTGTTTTTCAATGTAATTTCCTTCCTAAAACTAGATATTCATAATAATTTAAAAA
ATCTGAAAAATAAAAACGTTCTTACTTTATCTTTAAATGACAATACTATGATTCTATTATTTTATAAAAAAGA
TTGCAATAATAACGTCTTGCTATTATTGGATAATGACAATTAAAGTGTCGTTTTTATGAATTAAAATAGGTGA
AAGTAAGTTGGAAATACGTTGAAGCGTTGTGATTTTTATGAGAAATTGTATGAGTGAGGACTGCTATTTATAA
AAGGTAAACTATGGATGAGTTTATGGTTGTTCCAATAGGTAAATAAGAGATAGCACACTGTACACATAATGAT
ACGTGGATGAGTGAATATGTCTGATAGGAAGGCCTAGTCACTCAAATGAATTTAAGTTTATTACGTGATAAAT
CACAAATCTCTCTCATGTGATAGGTCTCCCATTAAATCATGCTTATATGAAATGTTCACATATTTGTTAGCTT
TTCAAGAAATAATATTAAATATCTTTTCATAGTATAGCAATATTAAATAAATGCATTTATAATTTTAACTGTA
TTTTAAATATTAATCATGAGGTGATAAGATGAATAAAATTTCAAAGTATATTGCAATAGCATCATTATCGGTA
GCGGTTACAGTTTCAGCGCCACAAACGACAAATTCTACAGCGTTTGCCAAAAGTTCTGCTGAAGTTCAACAAA
CGCAACAAGCTTCTATACCAGCATCGCAAAAGGTGAATCTTGGTAATCAAAATATTATGGCAGTGGCTTGGTA
TCAAAATTCAGCTGAAGCAAAAGCATTATATTTACAAGGTTATAACAGTGCAAAGACTCAGTTAGATAAAGAG
ATTAAAAAGAATAAAGGTAAACATAAGTTAGCTATTGCTTTGGATTTAGATGAAACAGTTTTAGATAATTCTC
CATATCAAGGCTATGCATCAATACATAATAAACCTTTCCCAGAAGGTTGGCATGAATGGGTACAAGCTGCTAA
AGCTAAACCTGTCTATGGCGCAAAAGAATTCTTGAAATATGCTGACAAAAAAGGTGTCGATATCTACTATATT
TCTGATAGAGATAAAGAAAAAGATTTAAAGGCAACACAAAAGAACTTAAAACAACAAGGTATCCCTCAAGCTA
AGAAGAGTCATATTTTACTAAAAGGTAAAGATGATAAGAGTAAAGAATCACGCAGACAAATGGTTCAAAAGGA
TCATAAACTTGTCATGCTATTTGGAGATAATTTATTAGACTTTACAGACCCAAAAGAAGCTACAGCTGAATCT
CGTGAAGCATTAATTGAAAAACATAAAGACGATTTCGGTAAGAAATATATCATTTTCCCTAACCCAATGTATG
GTAGTTGGGAAGCTACAATTTACAACAATAACTATAAAGCAAGTGACAAAGCAAAAGATAAATTACGTAAAAA
TGCTATTAAGCAATTCGATCCTAAAACAGGCGAAGTTAAATAATATATGAATTGGACGTCTACATGTACTTAA
AGGTATATGTAGGCGTTTTTAAATTGATCCTTAAATTTTAAATGTACCGTACAAAAATAATAGACAATTAATT
GCATTTTCCATAAATTTGCTTTTAATATAAAAAGTTCGGGTATAATGAAGGATAAGTGTATGTATAACATTAC
AGAGCGCAACACGACGTACGTGCATCTAAGAATATAAACAATGTTTAAATCATAGTAGTAGGGAGAGAAATGC
ATGTTTTTAGCTTGGAATGAAATACGGCGCAACAAATTGAAGTTTGGACTAATTATTGGTGTGTTAACGATGA
TTAGTTACTTGCTATTTTTATTATCTGGATTGGCGAATGGTCTTATCAATATGAATAAAGAAGGCATTGATAA
GTGGCAAGCAGATGCCATTGTTCTAAATAAAGATGCCAATCAAACTGTGCAACAATCTGTTTTTAACAAGAAA
GATATTGAAAATAAATACAAGAAGCAAGCTACTTTGAAGCAAACAGGGGAAATTGTGTCTAATGGCCATCAAA
AAGACAATGTTTTAGTGTTCGGTGTTGAAAAGTCATCATTTTTAGTTCCGAGTTTAATAGAAGGGCATAAAGC
GACTAAAGATAATGAAGTGTTAGCTGATGAAACACTTAAAAATAAAGGATTTAAAATTGGCGACACATTATCA
CTATCTCAATCAGATGAAAAATTGCATATCGTAGGTTTTACAGAAAGTGCAAAATATAATGCGTCACCAGTCA
TTTTCACGAATGACGCTACCATTGCCAAAATCAATCCTAGATTGACTGGAGATAAAATTAATGCAGTTGTTGT
ACGTGATACAAATTGGAAAGACAAAAAATTAAACCAAGAGCTTGAAGCGGTAAGTATTAATGACTTTATTGAA
AATTTACCAGGTTATAAACCACAGAACTTAACATTAAACTTTATGATTTCATTCTTATTTGTCATTTCAGCTA
CAGTTATAGGCATTTTCTTATATGTCATGACATTACAAAAAACAAGTTTATTTGGCATATTAAAGGCTCAAGG
ATTTACGAATGGCTATTTAGCGAACGTAGTTATTTCGCAGACGCTCATATTAGCATTATTTGGTACGGCATTT
GGCTTACTGTTAACAGGCGTTACAGGTGCATTTTTACCTGATGCAGTACCTGTCAAATTCGATGTACTAACAT
TGCTCGTATTTGCAATTGTGTTAATGATTGTCTCTGTATTAGGAAGTTTATTCTCCATTTTAACAATTAGAAA
AATAGATCCGTTAAAGGCGATTGGGTAGGAGGTGTAGCAAATGTTGAAATTTGAAAATGTAACAAAGTCATTT
AAAGATGGGAATCGTAACATTGAAGCGGTTAAAGATACAAATTTTGAGATAAATAAAGGTGATATTATAGCAT
TGGTTGGACCTTCTGGCTCTGGTAAAAGTACATTTCTAACTATGGCAGGTGCTTTACAAACGCCGACATCTGG
GCACATTTTAATCAATAACCAAGATATTACGACAATGAAGCAAAAAGCATTGGCAAAAGTTAGAATGTCTGAA
ATAGGTTTTATTTTACAAGCTACAAACCTTGTACCATTTTTAACGGTAAAGCAACAATTTACATTATTGAAAA
AGAAAAATAAGAATGTTATGTCTAATGAAGACTATCAGCAACTTATGTCACAATTAGGTCTAACTTCATTGCT
TAATAAGTTACCTTCAGAAATTTCAGGTGGTCAGAAACAACGTGTGGCGATAGCCAAAGCGTTATATACGAAT
CCGTCGATTATTTTAGCGGATGAACCTACCGCGGCGTTAGATACTGAAAATGCGATTGAAGTCATTAAAATTC

TACGTGATCAAGCCAAAAAAAGAAAGAAAGCATGTATTATTGTTACACATGATGAACGACTTAAAGCATATTG
TGATCGTTCATATCATATGAAAGATGGCGTCCTTAATCTTGAAAATGAAACAGTAGAATAGTTTTATTAAGCC
GGTACATCATGTGCCGGTATTTTTATGTTTATGTATTATTTGAATAAACTTTCACATTCAATTAATAATAATT
ATTATCGAAAATTAGAAATATTCCGTGAAATATAATATTTTTTGTAGTAAAATGGCCTCTAAGTATTCAATAT
TTAAATATGGGGATTGAATATAAAATTATCGTAATGGGGGTCAATGGTTATGGATTTATTGATAGGTACATTA
TTTTTATTTTTGGTCTTAGTGATTTTTACATTATTTACATATAAAGCACCTAATGGTATGCGTGCCATGGGAG
CATTAGCTAATGCAGCAATCGCAACATTTTTAGTAGAAGCATTTAATAAATATGTTGGTGGCGAAGTATTCGG
TATTAAATTTTTAGAAGAGCTAGGAGACGCTGCGGGAGGTCTAGGTGGTGTCGCTGCCGCTGGATTAACAGCA
TTAGCTATCGGTGTGTCACCAGTATATGCATTAGTTATAGCAGCCGCGTGCGGTGGTATGGATTTATTACCAG
GTTTCTTTGCGGGTTATATGATTGGATATGTGATGAAATATACAGAGAAATATGTGCCGGATGGTGTCGACTT
AATTGGATCGATTGTCATCTTAGCGCCATTAGCTCGTCTTATTGCAGTATTATTAACGCCAGTAGTGAATAGT
ACATTGATTCGAATTGGTGATATTATCCAAAGTAGTACGAATACGAATCCAATTATCATGGGTATCATTTTAG
GTGGTATTATCACGGTTGTCGGCACAGCGCCATTGAGTTCAATGGCATTGACAGCATTATTAGGTTTAACGGG
TGTACCTATGGCTATTGGTGCCATGGCAGCATTTAGTTCGGCATTTATGAATGGGACGCTATTCCATCGCTTA
AAATTAGGTGATCGTAAGTCTACGATTGCAGTAAGTATTGAACCTTTATCACAAGCAGATATTGTATCAGCCA
ATCCAATTCCAATCTATATTACAAATTTCTTTGGTGGTGCGATTGCTGGTTTAATTATTGCTATGTCAGGTTT
AATTAACGATGCGACAGGTACAGCTACACCGATTGCAGGATTTTTAGTTATGTTTGGATTTAATCATCCGACG
ACAATTGTGATTTATGGTGTAGTAATGGCGATTGTAGGTGCGCTTGCAGGTTATCTTGGTTCAATTGTATTTA
AAAAATATCCAATTGTTACTAAGCAAGACATGATTAATCGAGGTGCAGTAGACGCATAGCATCATCATATTGA
ATAGTAAAAACAAATAAAACATAGTAACGTGATTCAGTCGATGTAACAGTCGATAATGAGTCACGTTTTTTTA
TAGAAAAATATAAGACATAAAAATGTCATAATTTATTGTCGACAAATATCATACTGTATAAACATTTATCATT
TTCTCAAGTACCTTTTACACGATGGAATGAACTTACTTTTTACGAAATTATGCGTATTTTATAAACAAATATC
ATTGATATAACGGTAAATGTAAGCGTTTACAACAGAAATAACAGCATGCTACGATATTTTTGTAAATTCACTG
ATTCAAGTATTTTAAGTCAATATGAGGAGGGATGTTATGAGCGATTCTGAGAAAGAAATTTTAAAAAGAATTA
AAGATAATCCGTTTATTTCACAACGTGAACTTGCTGAGGCAATTGGATTATCTAGACCCAGCGTAGCAAACAT
TATTTCAGGATTAATACAAAAGGAATATGTTATGGGAAAGGCATATGTTTTAAATGAAGATTATCCTATTGTT
TGTATTGGCGCAGCGAATGTAGATCGTAAGTTTTATGTGCATAAAAATTTAGTTGCAGAAACATCAAATCCTG
TAACGTCAACACGCTCTATTGGTGGCGTAGCAAGAAATATTGCTGAGAACTTAGGTAGGCTTGGCGAAACGGT
CGCTTTTTTATCTGCTAGTGGACAAGATAGTGAATGGGAAATGATTAAACGATTGTCCACACCATTTATGAAT
TTGGATCATGTTCAACAATTTGAAAATGCGAGTACAGGTTCATATACAGCTTTAATTAGTAAAGAAGGCGACA
TGACATATGGCTTAGCAGATATGGAAGTGTTTGACTACATTACGCCTGAATTTTTAATTAAGCGTTCACACTT
ATTGAAAAAGGCTAAGTGCATTATTGTAGATTTGAATTTAGGCAAAGAGGCATTAAACTTCTTATGTGCCTAT
ACCACGAAACATCAAATCAAATTAGTTATCACCACGGTTTCTTCCCCAAAAATGAAAAATATGCCTGATTCAT
TACATGCTATTGATTGGATTATCACGAATAAAGATGAAACAGAAACATACTTAAATTTAAAAATAGAATCTAC
TGATGATTTAAAAATAGCTGCTAAACGCTGGAATGATTTAGGTGTTAAAAATGTTATTGTGACAAATGGCGTG
AAAGAACTCATTTATCGAAGTGGTGAGGAAGAAATCATTAAGTCAGTTATGCCATCAAATAGTGTGAAAGATG
TTACAGGTGCAGGCGATTCATTCTGTGCTGCAGTAGTGTATAGCTGGTTAAATGGGATGTCTACTGAAGATAT
ATTAATTGCTGGTATGGTTAACGCAAAGAAAACGATAGAAACAAAATATACAGTTAGGCAAAACCTAGATCAA
CAGCAACTTTATCACGATATGGAGGATTATAAAAATGGCAAATTTACAAAAGTATATTGAGTATTCTCGAGAA
GTTCAGCAAGCACGGGAGAACAATCAACCGATTGTAGCGTTAGAATCAACAATTATATCGCATGGTATGCCGT
ACCCACAAAATGTTGAAATGGCAACAACAGTAGAGCAAATTATCAGGAATAATGGTGCCATTCCAGCAACCAT
AGCCATTATAGATGGCAAAATTAAAATTGGTTTAGAAAGCGAAGATTTAGAAATACTGGCAACTAGTAAAGAC
GTTGCTAAAGTATCTAGAAGGGATTTAGCAGAAGTTGTTGCGATGAAGTGTGTTGGTGCTACTACTGTAGCGA
CGACGATGATATGTGCTGCAATGGCTGGTATTCAATTTTTTGTTACAGGAGGTATTGGGGGCGTCCATAAAGG
TGCAGAACATACGATGGACATTTCAGCAGACTTAGAAGAACTGTCTAAAACAAATGTCACTGTTATCTGTGCA
GGTGCCAAATCAATTTTAGACTTACCTAAGACGATGGAGTATTTAGAAACAAAAGGCGTTCCAGTTATTGGAT
ATCAAACGAATGAATTGCCAGCATTCTTCACTCGCGAAAGCGGTGTTAAGTTAACAAGTTCGGTTGAAACGCC
AGAACGACTTGCTGACATTCATTTAACAAAACAGCAGTTAAATCTTGAAGGTGGCATTGTTGTTGCTAATCCA
ATTCCATATGAGCATGCCTTATCAAAAGCATATATTGAAGCAATCATAAATGAAGCTGTTGTTGAAGCGGAAA
ATCAAGGTATTAAAGGTAAGGACGCCACACCGTTCTTGTTAGGGAAAATTGTAGAAAAAACGAATGGTAAAAG
TTTAGCAGCAAATATAAAACTTGTTGAAAACAATGCGGCGTTGGGTGCTAAAATTGCTGTCGCTGTTAATAAA
TTATTGTAGGTGATGATACATGAATATTTTATTCGCTATCACAGGGATAGCATTTGCACTATTTGTTGCGTTT
TTATTCAGTTTTGATCGTAAAAACATAGACTTCAAAAAGACGTTAATAATGATATTTATTCAAGTGTTGATTG
TGTTATTTATGATGAACACAACGATTGGTTTAACAATCTTAACTGCATTAGGCTCATTTTTTGAAGGGTTAAT
AAATGTTAGTAAAGCAGGTATAAACTTTGTTTTTGGAGATATACAAAATAAAAATGGCTTTACGTTCTTTTTA
AATGTGTTGCTGCCATTAGTGTTCATTTCTGTATTAATAGGCATCTTTAATTATATTAAGGTATTACCATTTA
TTATCAAATATGTAGGTATCGCTATTAATAAAATAACTAGAATGGGGCGCTTAGAAAGTTACTTTGCTATTTC
AACAGCAATGTTTGGACAACCAGAAGTATATTTAACAATAAAAGATATTATTCCAAGATTATCTAGAGCGAAA

TTATATACAATTGCGACGTCTGGTATGAGTGCTGTTAGTATGGCAATGTTGGGTTCATATATGCAGATGATTG
AGCCCAAGTTCGTAGTTACAGCTGTAATGTTAAATATTTTTAGTGCGCTTATCATCGCCAGTGTAATCAATCC
CTATAAATCTGATGATAGTGATGTTGAAATTGATAACTTAACTAAATCAACGGAAACGAAATCAGTGAATGGA
AAAACAGGAAAACCTAAGAAAGTTGCCTTTTTCCAAATGATTGGTGATAGTGCGATGGATGGGTTTAAAATCG
CTGTTGTAGTAGCCGTAATGTTGTTAGCATTTATTTCATTAATGGAAGCAATTAATATCATGTTTGGTAGTGT
TGGTTTGAACTTTAAACAGCTTATTGGCTATGTGTTTGCACCAATCGCATTCTTAATGGGGATTCCATGGAGT
GAAGCAGTTCCAGCTGGCTCTTTAATGGCGACCAAATTAATTACAAATGAGTTTGTAGCAATGCTTGATTTTA
AAAATGTCCTGGGTGATGTATCTGCTCGAACACAAGGTATCATTTCAGTTTACTTAGTAAGCTTCGCTAATTT
TGGTACGGTTGGTATCATCGTAGGTTCAATTAAAGGCATTAGTGATAAACAAGGAGAAAAAGTTGCATCCTTT
GCAATGAGGCTGTTACTTGGTTCAACTCTAGCTTCAATCATTTCAGGATCAATTATTGGCTTAGTATTGTAAA
TTAATCGAAGTATCTAAATTAAATTCATGGCAAAGCTAAACCCCATCACCAAGTTGGCGCAACAGCGCATCAT
AACTTGGTGATGGGATTTTATCATAACAATCTACTTTTTCGTAGCCGTTTTTGAAATGTATGTTGATGGTTTA
TCTTTTTCAAAAATTGTTAATCCCGTTATATCTTTTTTATGTTTTGAAGGGACAATGAAGCTAAGTATATAAG
CAAAGACAAAAGCAACTGTAAATGAAATGGTAGATACATAGAAAGGTGAGTTACCTTTGCCAACACCATTATA
GACATAAGCAAAGATGATACCCAATATTAATCCACAAATAACACCGAATGTATTCGTACGTTTAGTGAAAATA
CCAACTGCAAATACACCAGCTAATGGAACACCGAATAATCCAGTTACAAACAAGAATAAATCCCATAAGTCAT
TTGAATTAGAAGCAATTAAGTATAGTGACATTCCAAAACCGAAAATACCTGCAATGATGATAATGAAACGTGC
AAAGTTAACTTCGTGTCGCTCGCTACCTTTTCCGAAGAAGCGTTGCTTAATGTCGATTGAAATACAAGCAGAT
ATAGAATTTAAACTAGATGAAATGGTAGACTGTGCAGCGGCGAAAATGGCTGCAATAAGTAATCCTGCTACAA
ATGGTGGCATCTCAGTCAAAATGAAATATGGCACTACAGATGATGTATTGAAGCCTTTTGGTAAAACAGCTTC
ATGTGTATAAAATGAATACAGCATTGTACCCATACCATAAAATAAGGGTGCTGAAATTAAAGCTAGGATACCA
TTTGTCCATAACGATTTATTTGTTTCTTTTAAACTATCAGAAGCTTGATAACGCTGCACGACGTCTTGACTCG
CTGTGTATTGATACAAGTTGTTGAAAATATTTCCTAGGAAAATAATTGGAATGGCAGCTGCCGCAGTATTTAG
TTTCCAATTGTCTGCACTAATTAATTTTTTGTGCTCAATCGCATCTGCAAAGACAGTGCCAAAACCGCCTTTA
ATGTTCATAACACCTAGAATAATGATAACTAAAGCACCACCTAATAAAATGACGCCTTGAATGAAATCACTCC
AAACCACACCTTCGAATCCACCTAAAAATGTATATAAAATACATAGTAAACCAACGAGTGATGCAACGATATA
AGGGTTCATGTCTGATACAGATGTAATCGCTAATGTTGGTAAGTAGATAACGATTGCTACACGCCCTAGATGA
TAAACGACAAATAATAATGAGCCAATAACACGTATGCTAGGGCCGAATCTAGCTTCTAAATATTCATATGCAG
ATGTTACCTTTAACTTTTTAAAGAATGGAACATAGAAATAAATGAGTAATGGAATAATTGCTACGATAGCGAT
GTTACCTGCAATATATGACCAATCTGTTAAAAATGCTTTCTCTGGTGTAGACATAAACGTAATTGCACTTAAT
GTAGTAGCATAAATGGAAAAACCAACTACCCAAGATGGCAAGCGACCACTTGCAGTAAAGAAACTATTCGTAC
TTTGACTCGCGCGCTTGGTAAAATAAACGCCAATGAACAACATAGCTAGTAGATAAATGATAACGGCAACCCA
GTTTAGTGTGCCAAATCCAACTTCTTTCATGGGCAACATCCCCTTTACAATGTATTGATTCTTTGATGTCTAT
AAATCGTATTTTGCAATGAGTTGATCTAATGTTTGTCGATGTGCTTCGTTAAAAGGTTTGAAAGGTCTTTTCG
GTAATCCTGCATCAATGCCACGATGACGTAATATTTCTTTCAATGTTGGATAAATCCCCATTGATAACACTGT
TTCGATAATGTCGTTTGAATCATGTTGTAGTTGGTAAGCTTCTTGAATTTGACCTTGTCTTGCTAAGTCGAAG
ATTTTTCTTGCACGGCGACCATTAACGTTATATGTAGAACCAATTGCACCATCTACGCCAGAAATCGTAGCTT
GAACTAACATTTCATCAAAGCCAGATAAGATTAATTTGTCTGGGAATGCTTTTCTAATACGTTCGAGTAGGAA
GAAGTTCGGCGCTGTATATTTAACACCAACAATTTTTTCGTGATTAAATAGCTCGCTGAATTGTTCAATAGAA
ATATTCACACCTGTTAAATCTGGTATTGCATAAATAATCATATTGTTCTGTGTTGCTTCGATAATATCGAAAT
AGTAATCTCTAATTTCTTCAAAAGTAAATGGATAGTAGAATGGTGTTACGGCAGAAAGTGCATCATAACCGAG
TTCTGTGGCATATTTCCCAAGTTCAATGGCTTCATTTAAATCTAACGAACCTACTTGAGCAATCAATTTCACT
TTATCCCCAACTGCCTCTTTGGCAACTTTGAAAACTTGCTTCTTCTGCTCTGTATTTAATAAAAAGTTTTCGC
CTGAGCTACCATTTACATAAAGACCGTCTAATTCTTCAGTTTCAATGGCATTTTGAGCAATTTGTTTAAGTCC
TTGTTCATTTACTTGACCATTTTCATCAAAAGGAACGAGTAACGCTGCATATAAACCTTTTAAATCTTTGTTC
ATTATGAAGTCCCTCCAAAAATCATTTGATAATATAGTTTACAGTTATAATTGTAAACGCTATCATAAAATGT
AACAATATCTTTTTGAAAATTGAAGTCATATTTATGTATAATTAATGAAAATATTTTTCAAAATCAATAGAAA
TGGAGTGAGTAAGGTGTATTACATCGCAATCGATATTGGAGGCACTCAAATTAAATCGGCGGTTATTGATAAG
CAATTAAATATGTTTGACTATCAACAAATACCAACGCCGGACAACAAAAGTGAGCTTATTACTGACAAAGTAT
ATGAGATTGTAACAGGATATATGAAGCAATATCAGTTGATCCAACCTGTCATAGGTATTTCATCAGCAGGCGT
TGTTGATGAACAAAAAGGCGAAATTGTATACGCAGGGCCAACCATTCCGAATTATAAAGGTACTAATTTTAAG
CGATTATTAAAATCACTGTCTCCTTATGTCAAAGTAAAAAATGATGTAAACGCTGCATTACTAGGCGAATTGA
AATTACATCAATATCAAGCAGAACGGATCTTTTGTATGACGCTTGGTACAGGCATTGGGGGTGCGTACAAAAA
TAATCAAAGTCATATTGATAATGGTGAACTTCATAAGGCAAATGAAGTTGGGTATTTATTGTATCGTCCAACT
GAAAATACAACGTTTGAGCAACGTGCTGCAACGAGTGCATTGAAAAAGCGCATGATTGACGGAGGATTTACGA
GAAGCACACATGTGCCAGTGTTGTTTGAAGCAGCTGAAGAAGGTGATGATATTGCAAAACAAATATTGAATGA
GTGGGCAGAAGATGTAGCAGAAGGGATTGCCCAAATACAAGTCATGTATGATCCAGGGCTTATATTAATTGGT
GGCGGTATATCTGAACAAGGAGACAATCTCATTAAATATATCGAGCCGAAAGTTGCACACTATTTACCAAAAG

ACTATGTTTATGCACCAATACAAACGACTAAGAGTAAAAATGATGCAGCATTATATGGCTGTTTGCAATGATG
ATTGAAAGAAGGAGTCATTCTAAAATAGAATTTGAAACCGTTACGAGAGATGAGAGCTGTTGTTAGTTCCACA
CATCACACTCTATCTAGGACCAATCTAAACTATATCAACCAACAGTGTGCCACGGGCATATTAAATTGAAGAC
GCTTGAACGTATAGATCTCAAAAATGTAAAAAAAATATTTGGTATTGAAATTAAAAAAGCACCTAGCAACTCGT
TGGGACAATCACGATGATTATCTACAGTTGCAGGTGGATTTGAATATACAACTAGTTATTTGTTGTCTAGGAT
AATAGATTTAGTATGTTGATAAGTTTGACTCAGATTCGTATTTTCTAATAAATGATAACTTACGATATCGATT
AAAAAGAGTGTCGCAATTTGTGTGTTGATAAATTGATGGTCGGTATTACGCGATTGATCCGTTGTTAAAAGTA
CTAAATCTGCACAATCTGTAAGTTTACTACCTTCGAAATTTGTGATGGCAACGACATATGCACCATGAGATTT
GGCGACTTCCGCTGCAGAAATTAATTCCGAAGTATTACCACTATTTGACATAGCAATAAACATATCCGAATGA
GATAGTAGGGATGCCGATATTTTCATTAAATGTGAATCGGTAGTAACATTACCTTTTAGCCCCATACGAATCA
TACGATAATAAAATTCAGTCGCTGATAAACCAGAGCTACCTAGTCCAGCAAAGAGTATATGTCGACTTGATTG
AAGTTTGTCGATAAAGGTTTGGATAATGTCGTTATCAATAAATTCACCAGTTTGTTGAATGATTTGTTGATGA
TATTTATGAATTCTTTGAATAATTGGGCTATTTTCAATAACTGTCTCTGTCATTTCTTGTTGAATATTAAATT
TTAAATCTTGGAAATTCTCATAATCCAGCTTATGACTAAAGCGTGTCATCGTTGCTGGTGATGTACCAATCGC
ATGGGCTAAGGAGTTAATCGTTGAAAAGGCATCGCTATAGCCATTTTGTCTTATATAATTGACGATGCGTTTA
TCAGTTTTTGTAAATAAATGTTGATAACGTTGAACACGATTCTCAAATTTCATTGTGTCACCCCTTCATCTTA
ATGATTACTATTATATATGAAAAATATTTTCAAGATAGTAAAAAGCATTGATAAAAATTATCTTAATGATATA
TTGTAAATGACTTTACGTGAAAAAACGACTTATGGAGTGAGGAATAATGTTACCACATGGATTAATAGTATCT
TGTCAGGCACTACCAGATGAACCATTGCATTCATCTTTTATTATGTCGAAAATGGCATTAGCTGCGTATGAAG
GTGGTGCTGTTGGTATTCGCGCAAATACTAAGGAAGACATTTTAGCAATTAAAGAAACGGTAGATTTACCAGT
TATTGGCATTGTGAAACGTGACTATGATCACTCAGATGTTTTCATTACTGCAACGTCAAAAGAAGTTGATGAA
CTGATAGAAAGCCAATGTGAAGTCATTGCATTGGATGCAACGTTACAGCAACGTCCGAAAGAAACGTTAGACG
AATTAGTATCATATATTAGAACACATGCACCGAACGTTGAAATCATGGCTGATATCGCGACCGTTGAAGAAGC
TAAAAATGCCGCACGACTTGGCTTTGATTATATTGGCACGACGTTACATGGCTATACTAGTTATACGCAAGGA
CAATTACTTTATCAAAATGACTTCCAATTTCTAAAAGATGTACTACAAAGTGTTGATGCAAAAGTTATTGCGG
AAGGTAATGTCATTACACCGGATATGTATAAACGCGTGATGGACTTAGGCGTTCATTGTTCAGTCGTTGGTGG
TGCGATAACACGACCAAAAGAAATTACGAAACGTTTTGTTCAAGTTATGGAAGATTAAATGATAACGATAAAA
AAACGAGATGACCATCATTAATTAAAGGTACCTAATTATCTTAGGTGGCTGAATGAATGTAATGGGTTCATCT
CGTTTTGTTTGTTTACGATAGTGATTTTATTTTCAATTTGAATTATCCAAAAATAAGTAAAGCGACGGGGATG
GTGATTAATAGCGACAATGCCACGCGTAAAAACCAAATGATGATGAGTTTCCAGACAGGTATTTTAATTTCAG
TTGCTAGTATACATGGCACTAATGCTGAGAAAAAGATAATGGCTGATACGCTTACTACACCGACGACAAATTT
AGTACTCATTGCAGCTTTAGTTACTAACAAAGATGGTAGAAACATCTCTACAATAGAAATCGCTGACGCTTTT
GCAAGTAAAGCCTGATCAGCAATTGGGAAAATATAAATAAATGGATAGAAGATATAGCCAAGCCAATCAATGA
ATGGTGTATAGTTCGCTACAATCAGTCCTAAAAAACCAATCGATAATATAGAAGGCAAAATACCAACAGTCAT
TTCTAAACCGTCTTTCAAATTGTCCCAAACGTTCTTCACGAGGGATGGTGTTAATGCATTTTGTTTCATCGCC
TCTGCATATGCAGTTTTCAGTCTGCTTCCTTCAATAGCAACTTCTTGTTCTCCTTCTTGTCCGTTATAATATT
CTGTTGATTCATTGGTGATTGGCGGTAGCCATGCAGTAATTGCAGTCACGACAAATGTGATGACTAAAGTTAT
CCAAAAGTATAAATTCCAATGCGGCATTAATCCTAAAGTTTTAGCAACGATAATCATAAAAGTTGCTGAAACT
GTTGAAAAGCCAGTCGCAATAATCGTGGCTTCTCGTTTGTTGTACATTCCTTGCTTATAGACACGATTAGTAA
TCAATAATCCTAAGGAATAACGGCCGACAAACGAAGCTACTGCATCGACAGCGGATTTTCCTGGTGTTTTAAA
AATAGGTCTCATAATAGGCTCCATATAAACACCGACAAATTCTAATAAGCCATAGCCTACTAATAAAGAAAGC
GCAATTGCACCTACTGGAATTAAGATACTTAATGGCATCATTAATTTTTCAAACAAGAACGGACCATAGTTAG
CTTTAAATAGTATTGATGGACCGATTTTAAATACATACATTATACCAATCATTGCACCTGCAACTTTAAATAA
TGTAATGACCAAGTTTGTGATTGAAGTCATAAAAGTACGTCTCACTATTGGTAACGCTGTACCAATTAAAATC
ATAATCAGTGCAACATAGGGCATAAGTGGACCTATGATTGAGCGAATGGCTAGATGAACATGATCGACGAAAA
TAGTGTTGTTACCATTAATCGTAAAAGGAATAAAGAAACATAGTATGCCCACTAAACTATAGACAAAAAAACG
CCATGCACTTGGTTGTTGTGCATTAGAATGATATTGATTCATTAAAGCAACCCCTTTGTTTAAATGAATACAC
AAAACTGTATGATGCATCTTCCCCTTGATGAGATGAATCATTATTTTAATTTAGAAAAATCTGAAAACTTACT
ATAATTGTATAGTTTGAATTATTTTCATACCAATACAAATTAACTAATTATATATAGATTGAAACTATATTAC
TTAATAAAATATTTATCTTAAATGTTGTTGTGTTGATTCAACGCCACAACTAAAAGTGTTTATAAATTATTTG
GAAATGTACATATTTGTAAATGATTAGTATCGATTTAATATCGTATTATTAAATTTTTATTAATTTTGTAGTC
TTAATCAAAAAATAATATATGTCATGTTATATTGAAGGTGCAGTTGTTTTTCATTCTCAAGAGGGGGTCAAAA
AAATACTTTTGAGGTGATTATATGTTAAGAGGACAAGAAGAAAGAAAGTATAGTATTAGAAAGTATTCAATAG
GCGTGGTGTCAGTGTTAGCGGCTACAATGTTTATTGTGTCATCACATGAAGCGCAAGCCTCGGAAAAAAACACC
AACGTCTAATGCAGCGGCACAAAAAGAAACACTAAATCAACCGGGAGAACAAGGGAATACGATAACGTCACAT
CAAATGCAGTCAGGAAAGCAATTAGACGATATGCATAAAGAGAATGGTAAAAGTGGAACAGTGACAGAAGGTA
AAGATACGCTTCAATCATCGAAGCATCAATCAACACAAAATAGTAAAACAATCAGAACGCAAAATGATAATCA
AGTAAAGCAAGATTCTGAACGACAAGGTTCTAAACAGTCACACCAAAATAACGCGATTAACAAAACTGAACGC

CAAAATGATCAGGTTCAAAATACCCATCATGCTGAACGTAATGGATCACAATCGACAACGTCACAATCGAATG
ATGTTGATAAATCACAACCATCCATTCCGGCACAAAAGGTATTACCCCATCATGATAAAGCAGCACCAACTTC
AACAACACCCCCATCTAATGATAAAACTGCACCTAAATCAACAAAAGCACAAGATGCAACCACGGACAAACAT
CCAAATCAACAAGATACACATCAACCCGCGCATCAAATCATAGATGCAACGCAAGATGATACTGTTCGCCAAA
GTGAACAGAAACCACAAGTTGGCGATTTAAGTAAACATATCGATGGTCAAAATTCCTCAGAGAAACCGACAGA
TAAAAATACTGATAATAAACAACTAATCAAAGATGCACTTCAAGCGCCTAAAACACGTTCGACTACAAATGCA
GCAGCAGATGCTAAAAAGATTCGACCACTTAAAGCGAATCAAGTACAACCACTTAACAAATATCCAGTTGTTT
TTGTACATGGATTTTTAGGATTAGTAGGCGATAATGCACCTGCTTTATATCCAAATTATTGGGGTGGAAATAA
ATTTAAAGTTATCGAAGAATTGAGAAAGCAAGGCTATAATGTACATCAAGCAAGTGTTAGTGCATTTGGTAGC
AACTATGATCGTGCTGTAGAACTTTATTATTACATCAAAGGTGGTCGCGTAGATTATGGTGCAGCACATGCAG
CTAAATACGGACATGAGCGCTATGGTAAAACATATAAAGGAATCATGCCTAATTGGGAACCTGGTAAAAAGGT
ACATCTTGTAGGGCATAGTATGGGTGGTCAAACAATTCGTTTAATGGAAGAGTTTTTAAGAAATGGTAACAAA
GAAGAAATTGCCTATCATAAAGCGCATGGTGGAGAAATATCGCCATTATTCACTGGTGGTCATAACAATATGG
TTGCATCAATCACAACATTAGCAACACCACATAATGGTTCACAAGCAGCTGATAAGTTTGGAAATACAGAAGC
TGTTAGAAAAATCATGTTCGCTTTAAATCGATTTATGGGTAACAAGTATTCGAATATCGATTTAGGATTAACG
CAATGGGGCTTTAAACAATTACCAAATGAGAGTTATATTGACTATATAAAACGCGTTAGTAAAAGCAAAATTT
GGACATCAGACGACAATGCTGCCTATGATTTAACGTTAGATGGCTCTGCAAAATTGAACAACATGACAAGTAT
GAATCCAAATATTACGTACACGACTTATACAGGTGTATCATCTCATACTGGTCCATTAGGTTATGAAAATCCT
GATTTAGGTACATTTTTCTTAATGGATACAACGAGTAGAATTATTGGTCATGATGCAAGAGAAGAATGGCGTA
AAAATGATGGTGTCGTACCAGTGATTTCGTCATTACATCCATCCAATCAACCATTTATTAATGTTACGAATGA
TGAACCTGCCACACGAAGAGGTATCTGGCAAGTTAAACCAATCATACAAGGATGGGATCATGTCGATTTTATC
GGTGTAGATTTCCTAGATTTCAAGCGTAAAGGTGCAGAACTCGCCAACTTCTATACAGGTATTATAAATGACT
TGTTGCGTGTTGAAGCAACTGAAGGTAAAGGTACACAATTGAAAGCAAGTTAAATTCATCTTCTGAATTTAAT
ATGCTATGTATTTCGTGCTACTAACATGGCACAGCAGATATAAGTAACATCACAGTGTTGAATTTCAAAATAG
TAAAGTGAAATAAAGCGCCTGTCTCATTAGCGAAAACTAAAGGGACAGGCGTATCTTTTTATGAGCTTAATAA
ATTGTATGAATAATATGGTTGATCGAATAACTGTTTATCATGATGATAAATTGAGTTTTTTAAAATAATGATA
TATTACACCATTGTTATAGCGTTTAAGAAATCAACAACTTTACGATAAATAGTGATTGCTTCGTCATTAGGTC
TACGATCAAAATCATGCTCGTTTTTATTCACGCGTTCAGATGTTGAATGTGGAACATGATTCATGATATGTTC
GCTTTCCTCAACGGGAACATCATAATCGCCATTACAATGCGCAATGAAAACAGGTGGAAGTGTTTTAAGTTCA
TCTGGTGCAATATTATATTTTGAATCAGTATAATCAGCAATGTTAATCATATTTATCCATTTACCTGTGCCAC
GTGCATAAACGTAGATTAAAAAACGTTGTGCGATCTGATCTTGAACAACCGGTGTTGGTGAAGTGAGTTGTGC
AATCATTGTTTCGTTTACGCTTTGAGCTATTTTTGCGTAATAACTATTAGTTGTTTTAAAAGGTTCAGTGTTG
ATGCGACTATAACCATAAAAATCAATAACACCATCAATATCTCTGTCTCGTGCAATTAATAGACTTAAATATG
CACCTGATGATCTGCCAAAGGTAAAAATAGGGCAATTAGAATATTGTGATTGAATCGCATCGAATGATGCGTA
GACATCCTCAATAATGCAATCGAGACTTACTTCTGGTAATAAACGATAACTTAGTTGAATTAAATCGTAATGT
TCCGTAAGGATATCGATATACTGTGGGGATAAATCGTTAGCTTTACCGAACATTAATCCACCACCGTGGATGT
AGACAATGGCACCTTTTGTTGGTTGATGCTTCGCTTTAATAATTGTGTAGGGTAATTCAAACGCATCTTTAGT
AATTACTTTATCTTTAATTTCAGTCACGACTATACAGGCTCCTTATTTTTGATATTGATGTCATTATAACACT
GTCTTAAATTTCCATGAAAAATAGTCTTAAGACGATGAGTCATGATAATTCTGTTCCAATTGACGTAAAGCGT
CACGGGTATGCTTCTTTAGACCTTCCCCATAATCCATCATTTTAACAATATCTTTAAAAGCAGCATGTGGAAT
GGCTAAATCTTCTAAATCTGCCATAGAAAATTCAAGATTGATATCATGAGGTCGCTGTTCAGCAAGTTTATGC
ACAAAGTCAGGTTCTGTGACAAAAGGCGAAGACATGCCGACCATATCTGCATGTTGTAAAGCATCTAAAGCAG
ACTCTGGAGAATTAATCCCGCCACTTGCAATTAAAGGGATGCGACCAGCTAAATGTTCATAGACAATTTGGTT
AACTGGTCGACCGAAATGTTCACCTGGTGTACGAGACGTATTTTGATAAATATGTCGCCCCCAGCTAGCGATT
GCTAAATATTGGATGTTTGAAACGTCCATGACCCAATCGATTAATTGATTGAACTCGTCAATGGTATATCCTA
AATCACTACCTCTGGTTTCTTCTGGCGTTGCTCGAAATCCTAAAATAAAATTGTCAGGTGCTTCTTTATCAAT
CACTTCTTGTACCGCACACATAACTTCTAAACATAATCTTGCACGATTTTTTAATGAGTCGGCACCGTAATGG
TCTGTACGTTTATTCGAAAAAGTTGAGAAAAATGTTTGAATCAGCAAACGTTGTGCAATCGAAATTTCCACAC
CATCAAAACCTGCTTTAATCGCGCGTAATGTAGCATCGCGATACTGCTGAATGATGCTATTGATTTTCTCATG
AGACATGGCGATAACATCGTGTTCAATCGGTGAATGTAACGTCATAGGGCTTGGTCCATACACCTTTCCAAAA
TTTAAAATTGCTTGATTTGAAAAACGACCAGCATGCGCTAGCTGAATAATAGCGAGGCTACCATGTTGTTTCA
TCGTAGATGCCATGTTAGTTAATCCAGGGATACAAGCATCATGATCAATATTAAAGCCGTATTCAAACAATTG
ACCATATGGCTCAATGTAAGCAGCGCCGGTGACTTGCATACCAGCTGAATTAGAGCGACGTGCAGCATAAGCC
AAGTCTGCTTTTGTAATATAGCCTTCTTTTGTTGATGCGTTTACGGTCATTGGTGATAATACAAAGCGATTCG
AAATTTTGATGCCATTAGGTAAGTGGATTGATTGTAAAAGTGGTTTGTATCGGTACATACTATGATTCCTTTT
CTATTCAATATTGTTTTCAAAGTACCATGGAAAGAATGAATAATCAATGATGAACAGTCTTGATAGAATAGAA
TTGGTACATGGAAAGCATTTTTAAAATTAAAATTGAACTAATGAATGTCATTTGTAGGTCTGAAAATATGAAT
ATGAAAAAGAAAAATACAAGCGAAAAGATATAAAAGTTAATTGAAAAACGTTATCATATACGTGGGTATATGA

AGAGGGAATGGTATTAAGAACGCTAAAATGTTATGTCTGTTTGACATGACAGGATAAGTTTGGAGATGACGGA
TTGGTTAAATTAAGCGTATTAGACTATGCCTTAATAGATGAAGGTAAGGATGCACAAAAGGCGTTGCAGGATT
CAGTGACACTTGCAAAATTAGCAGATCGACTTGGCTTTAAGCGAATTTGGTTTACGGAACATCATAATGTACC
AGCGTTTGCGTGTAGTAGTCCAGAACTTTTGATGATGCATACATTGGCGCAGACAAATCACATACGAGTTGGT
TCTGGTGGTGTGATGTTGCCGCACTATCGCCCTTATAAAATTGCTGAGCATTTTAGAATGATGGCAGCGTTAT
ATCCAAATCGTATTGATTTAGGTATTGGTAATAATCCAGGTACTACTATGGTAAAGCAAGCTTTAGATGGAAT
AAATCCTACATATGATAGTTACGATGAATCGATTTCGTTATTACGTGATTATCTTACAATAAAGGATAAACCA
AGTGCGCATACGTTAGGTGTCCAACCACACATTGATCATTTTCCAGAAATGTGGTTATTAAGTAGTAGCGCAA
CATCTGCCAAAATAGCTGCTGAACTAGGTATAGGACTTTCTGTTGGAACATTTTTGCTACCGGATATAAATGC
GATACATGCAGCGAAGGATAACATTGATATTTACAAAAAACATTTCCAAGCATCAACGATTAAAATGGACGCA
AAGGTGATGGCATCTGTATTTGTCATTGTAGCTGATAACGAAGCGGAAGTAGCAGCATTACAACATGCCTTAG
ATGTTTGGTTATTAGGTAAATTACAATTTGCAGAATTTGAACATTTTCCATCGGTAGACACAGCGCAAAAGTA
TAAGCTTAATGATCGAGACAAAGAGATGATTCAAGCACATCAAGCACGCATCATTGCAGGTACACAAGAACAG
GTTAGAGCACAATTAGATGATTTCATTGCTACGTTTGAAGTTGATGAGGTGTTAGTAGCACCGCTTATTCCAG
GCATTGAACAGCGTTGTAAAACATTAAAATTACTCGCGGAAATCTATTTGTAGCATTTTAAATAGAAGAGAAA
GGATGAAGATAAGATGAAAAAGTTAGCCAATTATTTATGGGTAGAAAAAGTAGGAGATTTGTATGTGTTTAGT
ATGACACCTGAATTGCAAGATGATATTGGGACAGTAGGTTATGTTGAATTCGTAAGTCCAGATGAAGTTAAAG
TGGATGATGAAATTGTGAGTATCGAAGCATCGAAAACGGTCATTGATGTGCAAACGCCATTGTCAGGAACGAT
TATTGAGCGAAATACAAAAGCGGAAGAAGAACCGACAATTTTAAACTCTGAAAAACCAGAAGAAAATTGGTTG
TTCAAATTGGATGATGTCGATAAAGAAGCATTCCTAGCATTACCGGAGGCTTAAATGGAAACGTTAAAATCAA
ATAAAGCGAGACTTGAATATTTAATCAATGATATGCGTCGAGAGAGAAATGACAATGACGTATTGGTAATGCC
ATCTTCATTTGAAGATTTGTGGGAATTATATCGAGGCTTAGCAAATGTCAGACCGGCATTACCTGTAAGTGAT
GAATATTTAGCTGTACAAGATGCTATGTTAAGTGATTTGAATCGTCAACATGTTACGGATTTGAAGGATTTGA
AGCCGATAAAAGGTGACAATATCTTTGTTTGGCAAGGTGATATCACGACGTTAAAAATCGATGCTATTGTTAA
TGCTGCAAATAGTCGTTTTCTAGGATGTATGCAAGCTAATCATGACTGCATTGATAATATTATTCATACAAAA
GCGGGTGTTCAAGTTCGACTTGATTGTGCAGAGATCATTCGACAACAAGGGCGCAATGAAGGTGTAGGTAAAG
CCAAAATAACACGTGGATATAATTTGCCAGCAAAGTATATAATTCATACGGTTGGTCCGCAAATACGTCGATT
GCCTGTTTCAAAGATGAATCATGACTTGTTAGCTAAATGTTATCTTAGCTGTCTTAAATTGGCTGATCAACAT
AGTTTAAATCATGTCGCTTTTTGCTGTATATCTACAGGTGTATTTGCTTTTCCTCAAGATGAAGCAGCAGAAA
TTGCTGTTCGAACAGTAGAAAGCTATCTCAAAGAAACAAATTCAACATTGAAAGTCGTGTTCAATGTATTTAC
AGATAAGGATTTACAACTGTATAAGGAGGCATTGAACCGTGATGCAGAGTAGTAAATGGAATGCAATGTCTCT
GTTAATGGATGACAAGACAAAGCAGGCTGAAGTATTGCGTACTGCGATTGATGAAGCAGATGCGATAGTGATT
GGAATTGGTGCAGGCATGTCTGCATCTGACGGATTTACATATGTAGGAGAGCGTTTTACGGAAAATTTCCCGG
ATTTTATTGAAAAATATCGCTTCTTTGATATGTTGCAAGCGAGTTTACATCCTTATGGCAGTTGGCAAGAGTA
TTGGGCGTTTGAGAGTCGTTTTATTACATTAAACTATTTAGATCAACCTGTAGGTCAGTCTTACCTCGCTTTA
AAATCCTTGGTGGAAGGTAAACAGTACCACATTATAACTACGAATGCAGATAATGCTTTCGATGTAGCTGATT
ATGATATGACGCATGTATTTCATATACAAGGCGAGTATATACTGCAACAGTGTAGTCAGCATTGTCATGCTCA
AACGTATCGCAATGATGATTTAATTCGTAAAATGGTTGTTGCGCAACAAGATATGCTTATACCTTGGGAGATG
ATTCCAAGATGTCCAAAATGTGATGCTCCAATGGAAGTGAATAAACGTAAAGCGGAAGTTGGCATGGTTGAAG
ATGCTGAATTTCATGCGCAACTGCAACGTTATAATGCTTTTCTAGAGCAACATCAAGATGATAAAGTGTTGTA
TTTGGAAATTGGAATTGGTTTTACTACACCACAATTTGTGAAACATCCTTTTCAGCGTATGACACGTAAAAAT
GAAAATGCACTTTATATGACGATGAATAAAAAGGCATATCGCATTCCGAATTCAATTCAAGAACGTACCATAC
ATTTGACTGATGATATCTCAACATTGATTACAACAGCACTCCGGAACGATAGCACAACGAAAAATAACAACAT
TGGAGAGACAGAAGATGTACTTAATAGAACCGATTAGGAATGGAGAATATATTACTGATGGTGCGATTGCACT
CGCCATGCAAGTTTATGTTAATCAGAATATCTTTTTAGATGACGATATTTTATTCCCTTATTATTGTGATCCA
AAAGTGGAAATTGGACGTTTTCAAAATACTGCTATAGAAGTGAATCAAGATTATATAGATAAACACAGTATTC
AAGTAGTTCGCCGAGATACTGGTGGTGGCGCTGTGTATGTTGATAAAGGTGCCGTTAATATGTGTTGTATTTT
AGAACAAGACACTTCAATTTATGGTGATTTTCAACGATTTTATCAACCAGCTATAAAGGCATTGCATACATTA
GGTGCAAGAGATGTGATACAAAGCGGTAGAAATGATTTAACATTGAACGGCAAAAAAGTGTCAGGCGCCGCAA
TGACATTAATGAACAATCGTATTTATGGCGGTTATTCGCTATTACTTGATGTTAATTATGAAGCAATGGATAA
AGTGTTAAAGCCTAATCGCAAAAAGATTGCATCGAAAGGGATTAAATCTGTGCGCGCACGTGTTGGTCATCTT
AGAGAAGCATTGGATGAAAAGTATCGTGGTATAACCATTGAAGAATTTAAAAATTTAATGGTGACGCAGATTT
TGGGAATCGATGACATTAAAGAGGCAAAAAGGTATGAATTATCCGAAGCAGATTGGGAAGCGATTGATGAATT
AGCTGATAAAAAGTATAAAAATTGGGATTGGAATTATGGCAAGTCACCCAAATATGAATATAATCGAAGTGAA
AGATTATCATCAGGTACTGTAGATATTACCATTTCTGTTGAACAAAATCGTATTGCAGATTGTAGAATTTTTG
GTGATTTCTTTGGACAAGGTGATATAAAAGATGTGGAACAAGCGCTACAAGGAACAAAAATGACAAGAGAAGA
TTTAACGCATCAGTTAAAGCAATTAGACATCGTTTATTATTTTGGTAATGTTACGGTTGAATCATTGGTTGAG
ATGATTTTAAGTTAATTTTGTTTAGGCGTTAATGTCGCACCCTCGTTGAATATGTGTATTTGCTTTTAAAATG

GTGACTATGTAGTTATGTAATTTGAACATACGATTGAAAATGAATTTAGTTAATTAGCAAAGACATCATATTT
ATAGCGTTTGTGTTTATTAGTGATTGGGTATTTGAAAATGGTCAATGATAGTTTTTAAAGGAATAAATTCATT
TTCATTTATACCAGTAAGATTAAATACTAGCTACCAAAGTCTATTTATATACGTCAAAATATAATGACTGCTA
TAATGAGTAATCAATAGACACAAAGAGGAGATTATGTGATGAATAATAAAGTATTAGTGACGGGTGGTACAGG
GTTTGTTGACATGCGAATCATTTCACGATTATTAGAACAAGGTTATGAGGTCAAAACGACGATACGTGATTTA
AGTAAAGCTGATAAAGTAATTAAAACAATGCAAGACAATGGTATTTCCACAGAGCGATTAATGTTTGTCGAAG
CGGATTTATCACAAGATGAACATTGGGATGAAGCAATGAAAGATTGTAAATATGTGTTGAGTGTGGCATCTCC
AGTGTTTTTCGGTAAAACAGACGATGCAGAAGTGATGGCGAAGCCTGCCATTGAAGGCATACAACGTATTTTA
AGAGCTGCAGAACATGCTGGCGTGAAGCGTGTGGTGATGACTGCAAACTTTGGTGCAGTTGGTTTTAGTAATA
AAGATAAAAATTCAATCACAAATGAAAGTCATTGGACAAATCAAGATGAACCAGGCTTATCAGTATATGAAAA
ATCAAAATTGTTAGCTGAAAAGGCAGCGTGGGATTTTGTTGACAATGAAAATACGACAGTAGAATTTGCCACA
ATCAATCCAGTTGCAATTTTTGGACCATCATTAGATGCACATGTTTCAGGAAGCTTTCATTTATTAGAAAATT
TATTGAATGGTTCAATGAAACGTGTACCTCAAATTCCATTAAATGTTGTTGATGTGAGAGATGTAGCAGAACT
ACACATTTTGGCAATGACAAATGAACAGGCTAATGGCAAGCGATTTATTGCTACGGCTGATGGACAAATTAAT
TTATTGGAAATTGCAAAATTAATTAAAGAAAAACGACCTGAAATAGCTCAAAAAGTTGCTACTAAAAAATTGC
CAGACTTTATTTTGAGTCTAGGTGCTAAATTTAATCATCAAGCTAAAGAAGGTAAACTTTTATTAGATATGAA
CCGCAATGTAAGTAACGAACGTGCAAAAACATTACTTGGTTGGGAACCGATTGCGACACAAGAAGAAGCAATT
TTAGCAGCAATTGATAGTATGGTTAAATTTCATTTAATATAATACAAAAACACCGTCCATAAGTAAGTGGCTT
TCCTCTTGATAAAAGGGGCATTCACTCAATATGTGACGGTGTTTTATATTTTTACGTTAACACTATTGCTGTG
GCTTTTGACGACCTTTTAATAAAATTGCTGTCGCACCTACGATAATAATAAATAGAATCGCACCAAATAATCC
CATATATTTTACTGCGTTACCGAACACGATACCGACAGCTAAAAAGTCTGTATCTGAGAATGTTGTTGCAGCA
CCACCTAATTCGCCTAAAAATGGCAAGAATAATAATGGTAAAAACGTGATTAGGATACCATTTAGAGCGGCGC
CAGCAACAGCACCTTTAATACCGCCTCTTGCATTACCGAATACAGCAGCCGTTGCACCTAAGAAGAAGTGTGC
AACTACGCCAGGTAAAATGACGACGCCACCAAATAAGAATAAGATAAACATACCGATGACACCTGTAATAAAG
CTGACAAAGAATCCAATTAATACTGCATTTTGTGCATAAGGGAACACAATAGGGCAGTCTAATGCAGGTTTAG
AATTTGGTACAAGCTTTTCAGAAATTCCTTTAAATGCTGGGACGATTTCAGCTAAGATTAAACGAACGCCGGT
TAAAATAATAAATACACCAGCAGCAAATGTCACACCTTGAATTAATGAAAAGACAATAAAGTTTTGACCATCA
CTAATAGATTCGTGTACATAACTAACGCCCGCAAATAAGCATGCGATGAAGTAAAGTAATGCCATCGTAATCG
AGATACTAATTGTACTTTCTCGTAAGAAACTTAAGCCTTTTGGAAATTTAATCTCTTCCGTTGATTTAGACTT
ACCTTTGAATAATTGACCTACAGCACCTGCGGCAAAGTAACTGATTGAGCCAAAATGACCTAACGCTACTTGG
TCATTCCCTGTAATTTTTCGCATCGTAGGTTGGAGTAATGCAGGTAAGACTGCCATGATTAATCCTAATACGA
GTGCGCCGATAACAATCGTTAGCCAGCCTTTAATATGACTGACTGTTAAAATGATTGCTAAAAACGCAGCCAT
GTAAAATGTATGATGACCTGTTAAAAAGATATATTTTAAATTAGTGAAGCGGGCAATTAAAATATTAACAATC
ATGCCACAGACCATGATGAGTGCAGCTGTTGTTCCAAAATCTTTTAAGGCTAGTGAGACGATAGCTTCGTTGT
TAGGTACGATACCTTGCACACCGAATGCGTGTTGAAATATTTTGCCGAATGGCTCAAGAGATCGAACGACGAC
ATCAGCACCTGCACTTAAAATTAAGAAGCCTAATATCGTTTTAATGGTTCCTGAAGTGATCGTTGCGGCAGGT
TTTTTCTGAACGATTAAACCTATAAAGGCAATCAGTGCAACAAGAATGGCTGGTTGACTTAAAATATCGACTA
TAAAATTAAGGATTGCTTGCATAGGTCGTACCTCCTTTAAATCATGTTAAGTTGTTGTAATTTTTCTGAGAGC
TTTTGTTGTAATTCAGCTTTATCTAAAATATTATCAAGAACTAAGACATCCCCTAGACGTTCGGCATTTTCAG
CTAAATCTCTACCACAAATAAACAAGTCAGCCATCTCTGGACTTGCTGTCATAATGTCACTATGTTCAACTTC
GATATCAGATGGTGCATTAAGTTGCCTAAGTGCTTCTTGTGCGTTCATTTCTACCATAAAACTACTTCCTAAA
CCGTGGCCACATACTACTAAAATCTTCATATTAATCATGCTCCTTTAAAATGTTTTTAATGTCTTGTGCGTTT
GTTGCAGTTAATAGTTGCTGGACTGTTTGGTTATCGCCCAGTATGGTTGCTAAATTTTGTAATACAGATAAGT
GTGAATGATTGTCGATGGCACTCAATACAAAAATGAGTGATGCGTAGTGATTTTCATCACAGAATGCCACATG
TTGGTTCAACTTTAATAGACTTAAACCAACTTGATGTACGTCATTGTTCGGTCTTGCATGTGCAATTGCAATT
TCAGGTGCGATAACGATATAAGGTCCAAGTTCATTAACGCTATCAATCATTGCTTGAACATAGCCTTGTTCAA
TAATTTGTTCTTGTAGTAATGGCTGAGAAGCTATAGTTATAGCTTCAGTCCAATCATTTACTTGTTCTTTTAC
AATGATGCGTGTTGTTGACAAAATGTCTAATGACACGTTATTAAGCCTCCTTTGTCATAGTTAAAGCAATGTG
TTGTTTAATTTTAAAAATATTCCCATCTAAGAAATCTTGTCGATATAAGTCGCTGCTTAAGCATTCGCTTAAC
TGTCCCAATGCCTTTAAATGTGCATTGGGGTGGTCCGTTGCTAATGTAATTACAAGGTGAACGGGATCGTTAG
CTTTACTACCAAAGATAATCCCTTCAGTGAAATATGTTAGTGCGAAACCTACACCATTCTGTACATAATCAGT
ACCAGCGTGAATAAGTGCAATATGTGGACTAATGACCATATATGACCCGAATTGTTCAAATTGTTTTAAAATT
GCAGCTGTATAATTTGAATAGACAATGCCATCATTGATTAAAGGTTGCACAGCCACTGCAATTGCGGATTCAA
TTGATAATGGTTGTTTATTTATAATGATGCGATGTTCAGGCAATAAATCTGCGAGTGACTTGCCATCAGTTGC
CATTTTCATGACTCGTTGTTCTCTTGAGTCATTGATAATTTGATTCAATTTTTGACGAGATTGTTGATTGATA
AATGGATCGACATGAATAACTGGTACAGCTGATATTTCACAAGGTACTGTTGAAATGACATAATCAATGTTAT
CTTGCAATAATCGACTTTCTTCCAATTGATAAATGGAATAGGCATCCCAAATGTGAAACTCAGGATACAGATG
ATTTAGTTTTGATTTTAAAAGTTGCGACGTGCCTATACCAGAACCACATAGTAAGACAACCTTAATCATTGAT

TGCTTATGTGTTGCAACACGCTCTATACTTGATGCGAAATGAATTGTAATGTATGTTAATTCATCTTCGTTGA
AGCGAATAGCAGCATCCTGTTCAATTGGGCTAATATGCTTGCTAACGGCTTCTATGATTTGAGGATAGCGACG
CATAACTTCTTGCCTCAAAGGATTAGGTTGTAGCATATCGTATTTAATACGATGTATAGCTGGTTTGATATGT
GTGATCAGACTGGTATGCAACTTGTTGTCTTTTGACATATCAATGCCTAATTCTTGGCTAACACAAGTGATCA
ATTCATGTATATTTTGCGATAAATCATGATATTCAAAGGTAATTGAAGATGCTGTATGTTCGGTCATTTTAGA
GCCTAGTAAATGTAACGTGATAAAGATAATTTCAGACTCTGGAAATGTGACATTACAACTGCGTTCTAAGTTT
TCTATCATTTTTGAAGCAATAGCATACTGATTAGTATGTCGCCATTTATTAATTTCATTGATAGGTATATCGA
ACGAAAAATTTTCATTTAGACGCTGAATGGCAATGAGTATATGATAGATTAAGCCATCGATAGCCGACTGAAC
TAAATGATAATTTTCACTATTTAATGTCTTAATAATGGCACGGCGAACCAATGCGATTGATTCTGAATTAAAG
ATATCAGCCTCTATAAAAGGTGCAGCTTGTTTCATATATTGATGTATAAAGTGTGCATACGCTTTACGATAAT
AATCTTCCTCACCAATAATATTGAATCCTTTATTGTGGACATAATTTAACTTTAAATGGTATTGATCTAGTTG
GGCTTGAATCATTTTAATATCATCTGCAATTGTCCGACGCGAAACATTAACATCTTGCGCAAGTTGCTTTGTT
GAAACAGGGTCAGTTGTTTCGAATAACTTTAAAGCGATATGTGTGAGTCGCTCATCTTTTGAAAAATGAATTT
GATTCGTTAGATTGTGCTCTAATTCATTTAATAGTGTCGCGTGAGCTGTTGTTACTTTGATGCCTGCAGCTTT
ATTACGACTGACTTGGTAATGATAAGTTTCAGCATATTGCTCAATATATGCTATATCATATTGAATGGTACGA
GGTGATACACCAAGTTGATTAGCAATGGTATTGATTGGAATAAACGTTTGCTCATGAATTAAAAGATACAAAA
TTTCGATTTGTCTATAACTTAACAACGTAATATCCTCCTATTTGTAATTGTAAGCGATTTCTCGAAAACGTAG
ATATGCAATCTCTTTCATATTTTAATCCGAAAAATTGCATATCAAAATGTTTATGGCGCAAGATTTTATAGGA
ATTTTTAAAATAAATTAGATATTCATGTTGACAATTTAAAAATGTCGCAGTATATTTAGTTAGACATCTAACG
AAATGGTGGTGCAATAAATGGAATTCACTTATTCGTATTTATTTAGAATGATTAGTCATGAGATGAAACAAAA
GGCTGATCAAAAGTTAGAGCAATTTGATATTACAAATGAGCAAGGTCATACGTTAGGTTATCTTTATGCACAT
CAACAAGATGGACTGACACAAAATGATATTGCTAAAGCATTACAACGAACAGGTCCAACTGTCAGTAATTTAT
TAAGGAACCTTGAACGTAAAAAGCTGATCTATCGCTATGTCGATGCACAAGATACGAGAAGAAAGAATATAGG
ACTGACTACCTCTGGGATTAAACTTGTAGAAGCATTCACTTCGATATTTGATGAAATGGAGCAAACACTCGTA
TCGCAGTTATCTGAAGAAGAAAATGAACAAATGAAAGCAAACTTAACTAAAATGTTATCTAGTTTACAATAAA
TGGTAAGCGTGACTGGTAGAAATCAGTCACTTTATCTTTAATATTATAGTTAGATATCTAATTGTTAGTAAGC
TAATTATTGAAAAAGACAAGGAGTATTGAACAATGAAAGACGAACAATTATATTATTTTGAGAAATCGCCAGT
ATTTAAAGCGATGATGCATTTCTCATTGCCAATGATGATAGGGACTTTATTAAGCGTTATTTATGGCATATTA
AATATTTACGTTATAGGATTTTTAGAAGATAGCCACATGATTTCTGCTATCTCTCTAACACTGCCAGTTTTTG
CTATCTTAATGGGGTTGGGTAATTTATTTGGCGTTGGTGCAGGAACTTATATTTCACGTTTATTAGGTGCGAA
AGACTATAGTAAGAGTAAATTTGTAAGTAGTTTCTCTATTTATGGTGGTATTGCACTAGGACTTATCGTGATT
TTAGTTACTTTACCATTCAGTGATCAAATCGCAGCAATTTTAGGGGCGAGAGGTGAAACGTTAGCTTTAACAA
GTAATTATTTGAAAGTAATGTTTTTAAGTGCACCTTTTGTAATTTTGTTCTTCATATTAGAACAATTTGCACG
TGCAATTGGGGCACCAATGATTTCTATGATTGGTATGTTAGCTAGTGTAGGCTTAAATATTATTTTAGATCCA
ATTTTAATTTTTGGTTTTGATTTAAACGTTGTTGGTGCAGCTTTGGGTACTGCAATCAGTAATGTTGCTGCTG
CTCTATTCTTTATCGTTTATTTTATGAAAAATAGTGACGTTGTGTCAGTTAATATTAAACTTGCGAAACCTAA
TAAAGAAATGCTTTCTGAAATCTTTAAAATCGGTATTCCTGCATTTTTAATGAGTATCTTAATGGGATTCACA
GGATTAGTTTTAAATTTATTTTTAGCACATTATGGAAACTTCGCGATTGCAAGTTATGGTATCTCATTTAGAC
TTGTGCAATTTCCAGAACTTATTATCATGGGATTATGTGAAGGTGTTGTACCACTAATTGCATATAACTTTAT
GGCAAATAAAGGCCGTATGAAAGACGTTATCAAAACAGTTATCATGTCTATCGGCGTTATCTTTGTTGTATGT
ATGATTGCTGTATTTACAATTGGACATCATATGGTCGGACTATTTACTACTGATCAAGCCATTGTTGAGATGG
CGACATTTATTTTGAAAGTAACAATGACATCATTGTTATTAAATGGTATAGGTTTCTTGTTTACTGGTATGCT
TCAAGCAACTGGTCAAGGTCGCGGTGCTACAATTATGGCCATTTTACAAGGTGCGATTATCATTCCTGTATTA
TTTATTATGAATGCTTTGTTTGGACTAACAGGTGTCATTTGGTCATTATTAATTGCTGAGTCACTTTGTGCTT
TAGCAGCAATGTTAATCGTCTATTTATTACGTGATCGTTTGACAGTTGATACATCTGAATTAATAGAAGGTTA
AATATTTCGTACACCTCTGGCTGAGTATATTTCGGTCGGAAGTGTATTTTTCGAAAAAAATAAATATATGATA
CGATTATGAAAAATAAAGTGAGATTGGCATATGTATAAATCTAAAATACTGTTGAAATATATTTTTAGTGAAG
AATTAGACGTTAAAGATTTAACTGAAGAAAAATATAATCAAGATTACGAAGCATTAACATTTAGCTTTAAAGA
GGAAACATATCAAAGTAGGTTAGCTAAGAAAACACCGACTAAAGCAGGTTATTTTGTTGCATGTTGGACAAAA
GATGAGAACAATTGTAATCAACCATACTCGAAAGAGGCGTTTGCAGATTACTTAATGATTATAGTTATTGATG
AAGAATTAAGCGGTTATTTTTTATTTCCTAGGGAATTATTGGTAGAAAAAGGTATCTTAACTACATTTGAACA
TAAAGGTAAGATGGCTTTTAGAGTTTATCCTAAGTGGTGTAATCAATTGAATAAAACAGCAGGGCAAACACAA
AAGTGGCAATGTAAATATTTTTTATCATTTAATGATGAACGGTTTCTTGTCCTACTTTATTCCAAGTGAGGAT
AAAGCTCAACATTGCAAACACACTGATTGCTGTTAATAAAATAAAACCGACATCCCATCCGAATTTATCAACT
ACAGCACCTAAGACGATGTTGGCCATTACAGCACCAAACAGATAACCAAATAATCCTGTTAATCCAGCTGCTG
TGCCAGCTGCTTTTTTAGGTACATAATCTAATGCTTGTAAACCAATTAACATAACTGGTCCATATATTAAGAA
ACCAATGGCAATTAATGAGACATTGTCTAACCAAGCATTGCCTGGAGGATTTAACCAATAAATTAATACAAAT
ACTGTGACACCTAACATAAAGAAGAAACCTGCAGGTCCACGACGACCTTTGAATAATTTATCAGAAATGTAAC

CACATAATAATGTACCAGGAATTCCAGCCCATTCGTATAAGAAGTATGCCCAACCTGATGCTTTTAAGTCGAA
ATGTTTTTCTTCACTTAAGTAGACTGGCGCCCAATCAAGTACACCATAACGCACGAAATAAACAAATATATTT
GCAAAGGCAATTGCCCATACCCATTTATTGTTTAGTACATATTTAAATAAAATTTCTTTTGTAGTTAATTCTG
TTTCTAATGTTTTCTTATCGCTTGTAGCAAAGTCATTTTTATAAATTTCGATTGGAGGTAAACCTTGAGATTG
AGGTGTGTCTCTAATCAATACGTATGAAATTGCGGCAATGATAAGTGCTAAGAGTGCAGGGTAAATGAATACA
CCTTCGAAACCTTTTAAATAACCAAAGTTGATAAATGCTGTTGTTGTAATACCCCAAGCAGCAATAGGTGCCA
TAATACCTCCACCAACATTATGCGCAACGTTCCAAAGGGCAGTCTTACTTCCGCGTTCACTTACACTAAACCA
GTGAACGAGAACACGACCTGAAGGTGGCCAGCCCATACCTTGAAACCATCCATTTAAGAATAATAGGACAAAC
ATAATACCGATACCTGATGTAAAGAACGGTACAAATCCCATTAACAAATTGACGATAGCAGTGAGTGCTAATC
CAAGAACTAAGAATATCCGAGCATTGCTCCGATCACTTACAGTACCCATAAAGAACTTACTAAATCCATATGC
GATGGAAACAGCAGAAAGTGCAAAACCTAGTTCCGCTTTTGTAAAACCTTGCTCTTGCAATGCCGGCATCGCT
AATGAAAAGTTTTTACGTAATAAATAGTACCCAGCGTAACCGATGAAAATCCCAAGAAATACTTGGAGACGTA
ATCGTTTATAGGTATCATCTATCTGATTTTCTGGCAAAGGCTTAATATGCTTTGCAGGTTTAAGAAAATTCAT
AAAATCCTCCTTAATATGTATTTATATGCATTTTGTGCGATAAACTTATATTAGACATGTATGAAAGCGTTGT
CAATATACTTTTGTGAAATCTTGCAGATTTAGTTTTTGAAGCATTTTATAAACAGTCAGAAAAGTAAATGGTC
GAACATAAATTAATAATTATGCAATTATTATGTCGTTATACTTACTAGCTGTGTATCTACGAATGGAGTGCAA
ACATATTTTTATTTGCAGAAAAGGGGTAAATGGCATATAACTATCTTTTTTATGTAGGTTGGTATAAAATTTA
TACTAATAGGAGGGATAGTATGAATATAGTAGGGCATCATCACATATCCATGTATACAAAAGACGCAAAACGT
AATAAGGATTTTTACACAAATGTCCTTGGATTACGATTAGTTGAAAAGTCGGTTAATCAAGACAATCCGTCAA
TGTATCATTTGTTTTATGGGGACGAAGTAGGTACAGCTGGAACAATTTTAAGCTTTTTTGAAATTCCCAATGC
GGGTCATAAGCAGCCAGGTACTGAAACGATTTATCGATTTTCATTATTAGTACCAAATCAAGCGGCACTTCAT
TATTTTGAAAAACGCCTTGATAATAATGGTATTAAGTCTGAACGTTTGTACTATCTTGGACAAGAAGGTATTG
TCTTTAAAGATGAAGACGACTTAGAAATCATACTGCTTGTTAATGATAGTTTTGAAGTACCACATCAATGGCA
ACATAACGCTTATAGTGAAATACCTCAAGCATATCAAATTTTAGGAATAGGGCCAGTCGAATTAAGAGTTAGA
AATGCAGCGCGTACGGTAGAATTTTTGGAAAATGTCTTAGGTTATCGCAAAAGAGATAATAAATCATTCGATG
TGCTGACATTAGCACCACAAGGTTTATATTCGGATTTTGTAGTTATTGAGCAACAGGGACAACGTGAAAGACC
TGGACGAGGTTATATCCATCATATTGCAGTTAATACACCTCAAATGAGTGACTTAGATGCAATTTACAAGAAA
TTACAACAACAACCACAAAGTAATTCAGGTATAATTGATCGCTATTTCTTTAAATCATTATACTATCGCCATA
ATTCAATTATGTATGAATTTGCGACTGAAGCGCCTGGATTTACTATTGATACACCTCTTGAACAATTAGGAAG
TCAATTGAACTTGCCTGACTTTTTAGAAGCAGAACGTGAACAAATTGAAAGTAAGTTACACGAAATATAAAGG
AGAATGTTTAATGGCAAAATTAGAAATGAATAAAAATACGCCTCTTGAGTTTGGTTTGTATTCCTTAGGTGAT
CATTTATTGAATCCATTGAAAGGTGAAAAAGTTAGTTATGAGCAACGTATTAATGAAATTATTGAAGCAAGTA
AATTAGCAGATGAAGCAGGTATTGATGTTTTTGCAGTTGGTGAAAGTCATCAGGAGCATTTTACAACACAGGC
ACATACGGTTGTGTTAGGTGCAATTGCCCAAGCGACAAAGCATATTAAAGTTTCAAGTTCTTCAACGATTATT
AGTGCAACAGATCCTGTAAGAGTATTTGAAGACTTCGCGACATTAGATTTGATTTCTCATGGTAGAGCCGAAA
TTGTAGCTGGCAGAGCATCAAGAACAGGTATTTTTGACTTGTTTGGCTATGATTTAAAAGACTATGATGAATT
GTTTGAAGAAAAATTAGGTTTACTTTTAGAGTTAAATAAAACTGAGCGTATTACTTGGTCTGGAAAATATCGT
CCAGAACTTAGAAATATGAAAATATTCCCAAGACCAATCGATAATACATTGCCAATATGGCGTGCTGTTGGTG
GTCCACCTGCAAGTGCTATTAAAGCGGGAAAACAAGGTGTGCCAATGATGATTACAACTCTTGGTGGCCCAGC
AATGAACTTTAAAGGTTCTATAGATGCTTATCGTCAAGCAGCAACTGAAGCAGGTTTCGATGCTTCGCCTAAG
TCTTTACCAGTAAGTACAGCGAGTCTGTTTTATACAGCTGAAACAACTCAGGATGCTATGAGAGAATTTTATC
CACATTTGAATACAGGGATGTCATTTATTCGTGGCGTTGGTTATCCGAAACAGCAATTTGCTAATTCGTCAGA
TTATCGAGAAGCGCTAATGGTTGGAAGCCCGCAACAAATTATTGAAAAGATATTGTATCAACACGAGTTGTAT
GGTCATCAACGTTTTATGGCACAGCTTGATTTTGGCGGTGTGCCATTTGAAAATGTTATGAAGAATATAGAGT
TAATTGGCAACGACATTATACCGGCGATTAAAAAGCATTTATCAAAATAGGAGGGGCGTCATCATGAATATTG
TATTATTGTCAGGTTCCACAGTAGGTTCTAAAACGAGAATTGCTATGGATGATTTAAAAAATGAACTAGAAGT
CATCAATGAGGGACACCAAATAGAATTGATGGATTTACGAGACCTCGAATTAGAATTTAGTGTTGGAAAGAAT
TATCTAGATACTACAGGAGATGTATATAAATTAACGACGTCGTTAATGCAGGCTGATGTGATTTTTATTGGTT
TTCCAATTTTTCAAGCTTCCATCCCTGGTGCTTTGAAAAATGTATTTGATTTGCTTCCGGTCAATGCGTTTCG
TGACAAGGTAATAGGACTTGTAGCGACAGCAGGTTCTAGTAAACATTATTTAATTCCTGAAATGCATTTAAAA
CCAATATTGAGTTACATGAAAGCACATACGATGCAAACGTATGTATTTATTGAAGAGAAAGATTTTTCAAATC
AACAAATTGTCAATGATGATGTTGTATTTCGTTTAAAAGCGTTGGCACAATCCACAATGCGAACTGCTAAAGT
ACAACAACATGTGCTTGAAGAAGAAAACAACCAATACGACTTTTAAAGTATAAAAATAAGACGCTCGGCACAC
TAAATTTGTAAGTGTTTGAGCGTCTTTTTATTTAACTATATAGCCAGTGAACGACGATAAAGGCAAGTGATGA
CAAGCATATTGAGGTAATAATGATTGTCATAAGCGGTTTAAGTGCGCGATTTTTAAGATCTTTAAATGCCACA
TTTAACCCTAAAGCAACCATGGCCATTAATAAGCAAATTGTTGATACAGTATTTAAAATATTTAGCAATGCTG
ACGGAATAGTTACATATGTATTCACTAAGGCCATAATGACAAATCCAATTAAAAAGTAAGGAATGCTTATTCG
ACCCTTGCTAGATGATTCTGATGAACGGAAACGCATAATTAAAATAAGTACAATGGTTAATGGAATCAGTAAG

AATACTCTACCAAGTTTACCAAGAAGTGCAATTTTAAGTGCATCACTACCACCAAAGCCACCAGCTAAGACAA
CGTGTGCAATTTCATGAAGACTAACGCCGGACCAAGCGCCATAAACATTTGTCGTCATTGAAAAGATAGCATA
GATAGCTGTATATATAAGTGAAAATATCGTACCAATCAATGCGATGATACCGATACTAATAGCTGTATCCTTT
TCACGTGATTTGAATATTGGAGCTACTGCAGCAATAGCGGCAGCACCACAAACGCCTGTACCTACACCTAGTA
ATAATGCGATGTTTTTGTCACCATGCAACAGTTTGTTGACAAAGAGCATCATTACAATACTAAAAATAACGAC
ACCTACATCGATGGCTAATAGTCTACTACCTTGACCGATAATATCAAATATATTGAGTTTAAGTCCGTACAGG
ATAATTGCAAATCTTAATAAATATTTAGATGAAAACGTAATACCTGAGCTATATTGTTCAGGATATCCTCTAA
AGTGACGATATAGAATAGCGATTAATATCGCGATAGTTAATGCGCCAACCTTATCTAGGATTGGCAATTTAGC
TGCTAAAAAGCTAAATAATGCGACTATAAATGTTAGTGATAGTCCAATCATAAAATGCTTATTTTTCAATGAT
GCCATGAGCAGTGCCTCCTTTAATAGCATTTTAGCACTGTTTTGTAGTATTTTTAAATATAAATTTGGAATGG
TTAATAAAGTAGTTATTAAATTAAGTTGTGTGATAGGTATCAAGGACATCAATCAAAGTAATAGGCACTACAA
CCCTTATTGGTGGGGCCCCAACAAAGAAGCTGACGAAAAGTCAGCTTACAATAATGTGCAAGTTGGCGGGGCC
CCAACATAGAGAATTTCGAAAAGAAATTCTACAAGCAATGCAAGTTGGGGAAGGACAACAAATTTAAGATACA
AGGCGTAATATTAATATGTTATTATTATGATAATTTACAGAATTATATGAAAAATAAATGAGGGTGTGATGGT
ATGTTTGGAATGAAAGTGAATGAACAAATAAGATTAAAAATTTTAGAAGCCCATGACACAGAAGCGCTTTTCA
ATTTAATCAATCGTTCAAGAAATTCACTTAGGGAATGGTTACCTTGGGTAGATGCAACTGAGCAACCATCAGA
TACGCGTGCATTTATTAAAAGAGGACTTTTGCAATTTGCTGATGGTCATGGATTTCAGTGTGGTATTTGGTAT
GAGGGAATGCTAGTTGGTGTTATCGGTTTGCATGAAATTAATCACATGCACAGAAAAACTTCAATAGGGTATT
ATTTAGATAAACAATTTGAGGGTCATGGGATTATGACACAAGCAGTTGAGGCATTGATAAAGTATTGCTTCGA
TGAGATTGACTTAAATCGAATTGAGATCAGTGCCGCAGTTCATAATGAAAAAAGTCGAGCTATTCCTGAAAGG
CTAGGATTTACTAGAGAAGGTATGTTACGTGACAATGAATTACTAAATGGTATTTATTCATCGAGTTACATCT
ATAGTTTATTAAAATCAGAATATAATGAAGGATGATAAATCAGACTACAAAAGAGTAATGACTTACATATAAG
TATTACTCTTTTATTTATTTTCAAACTAAAAAGGTTGTTGACAGTATTATTTTATAACAATATAATGATTTTG
ATAATTATTATCAAGTAGATAATGTTCATGGGAGGATGCCTTAAAATCATCATTTTAAGAGCCACGTATTATC
TTGGCATTTAGGGAGTGTGAGATAGTACGAATGAGGGCATGTCTATGGGGATAATAGAATTTCTATAATGAGG
TGTCAAAATGAAAAAGTTAACAACGCTATTATTAGCATCAACGTTATTAATTGCTGCATGTGGGAACGACGAT
AGTAAGAAGGATGATTCAAAGACATCGAAAAAAGATGATGGTGTTAAAGCAGAATTAAAACAAGCAACAAAAG
CATATGATAAATATACTGATGAACAGTTAAATGAATTTTTAAAAGGTACAGAAAAATTTGTTAAAGCGATTGA
AAATAATGATATGGCCCAAGCAAAAGCGTTATATCCAAAAGTTCGTATGTATTATGAACGCTCTGAACCAGTT
GCAGAAGCATTTGGAGATTTAGATCCTAAAATTGATGCACGTCTTGCAGATATGAAAGAAGAGAAAAAGGAAA
AAGAATGGTCAGGATATCATAAGATTGAAAAAGCATTATACGAAGATAAGAAAATTGATGATGTGACTAAAAA
AGATGCACAACAATTATTGAAAGATGCAAAAGAATTGCATGCCAAAGCTGATACATTAGATATCACACCAAAA
TTAATGTTACAAGGTTCTGTTGACCTATTAAATGAAGTTGCAACTTCTAAAATCACAGGTGAAGAAGAAATTT
ATTCACATACAGATTTATATGATTTTAAAGCGAACGTTGAAGGCGCACAAAAAATTTATGACTTATTTAAACC
TATTTTAGAGAAAAAAGATAAAAAATTAAGTGATGATATTCAAATGAACTTCGATAAAGTGAATCAGTTATTG
GATAAATATAAAGATAACAACGGCGGTTATGAGTCGTTTGAAAAAGTATCTAAGAAAGACCGTAAAGCATTTG
CAGATGCTGTTAATGCATTAGGAGAGCCACTAAGTAAAATGGCTGTGATTACTGAATGACAAATTATGAACAA
GTTAACAATAGTACGCAATTTTCAAGACGTACATTTTTGAAAATGTTAGGTATTGGCGGTGCCGGTGTTGCAA
TTGGCGCAAGTGGTGTTGGTAGCATGTGGTCTTTCAAATCAATGTTCAATACACCAGAAGATCCGGAAAAAGA
TGCGTATAATTTTATGGTAAAGTGCAACCAGGCATTACCACACCGACGCAAAAAACATGTAAATTCGTTGCAT
TAGATTTGAAATCAAAAGATCGGGATGCGATTAAAGCAATGTTTAAGAAATGGACGGTTATGGCTGATCGTAT
GATGGATGGAGATGCAGTTGGCAAGGCCAGTAACAATCCTTTAATGCCGCCAGTAGATACCGGTGAATCGATA
GGATTAGGTGCAAGCAAGTTAACGATTACCTTTGGAATTAGTAAGTCTTTGATGAAGAAAATTGGGTTATCTA
ATAAAATTCCCGATGCCTTTAAAGATTTACCGCATTTTCCGAATGATCAGTTAATAGACGATTACAGCGATGG
CGATATTATGATTCAAGCATGCTCAAATGATTCACAAGTGTCTTTTCATGCGGTTCATAATTTAGTTCGTCCG
TTTCGAGATATTGTTAAGGTGCGTTGGGCGCAATCTGGTTTTATCTCTGCTAAAGGTAAGGAAACGCCAAGGA
ATTTAATGGCATTTAAAGATGGAACAATTAATCCTAGGAAGAATAATCAACTTAAAGATTATGTGTTTATTGA
TGATGGATGGGCGAAGCATGGAACTTATTGTGTTGTCAGACGTATCCAAATACACATTGAAACGTGGGATCGC
ACTGCATTAGAAGAACAAGAGGCGACATTTGGTCGGAAACGACATAGTGGTGCGCCGTTAACAGGCGGGAAAG
AGTTTGATGAAATTGACTTAAAAGCGAAAGATAGTCACGGTGAGTACATTATAGATAAAGATGCTCATACGAG
ACTAGCGAAAGAAGCTAATACGTCAATTTTACGTAGAGCCTTTAATTATGTTGATGGTACGGATGACCGCACA
GGTAACTTCGAGACAGGCTTGTTGTTCATTGCTTTTCAAAAAGCGACCCAACAATTTATCGATATTCAAAATA
ATTTAGGCAGTAATGATAAATTAAACGAATACATTACACATAGAGGTTCTGCTTCATTTTTAGTATTACCAGG
TGTTAGTAAGGGAGGATACCTCGGTGAAACATTATTTGACTAAAATTATAGCAATGCTAATAACTGCTGCTAT
GGTGTGTAGCTTTGGGTTACTGAAAAGTCAGGCAGCAGCACAACAAAGTATTAGTGATGTATATAGTGTGATT
ACGGATGCGAAATCTGTACTTTCTAATAATTCGATATCGAATGACAATAAGCAGAAAGCAATTGAGCAAGTGG
TAAGTGCAGTTAAGAAATTATCGCTTGAAGATAATAGTGAAGGTAATGCTGTCAAATCAGATGTGAGAAAGCT
TGAAGATGCAAAAGCGAATGATGATCAAAAAGATACACTTTCGCAATTAACGAAGTCATTAATTGCTTATGAA

GAGAAATTGGCTAGTAAAGATGCGGGTTCTAAAATTAAACTATTACAACAGCAAGTCGATGCCAAAGATGCAG
CGATGACAAAAGCGATTAAAGATAAAAATAAAGCAGAGCTAGAATCGCTGAACAATAGTTTGAATCAGATTTG
GACAAGTAATGAAACGGTGATTCGCAATTATGACGCAAATCAATATGGACAAATTGAAGTCGCATTATTACAA
CTTAGAATTGCAATTCATAAGTCGCCATTAGATACGACAAAAGTGTCACATGCTTGGACAACTTTTAAATCAA
ATATTGATCATGTCGATAAGAAAAGTGGTACGTCTGCAAATGATCAATACCATGTATCACAATTAAATGATGA
GTTAGAGAAGGCGATTAAAGCTATCGACGACAATCAATTGTCGGATGCTGATGCTGCGCTTACACATTTTATA
GAAATTTGGCCGTATGTTGAAGGTCAAATTCAAACTAAAGACGGTGCTTTGTATACGAAAATTGAAGATAAAA
TACCATACTATCAAAGTGTATTAGACGAACATAATAAAGCACATGTGAAAGATGGCTTAGTAGATTTAAATAA
CCAAATTAAAGAGGTTGTTGGCCATAGTTATAGCTTTGTTGATGTGATGATTATCTTTTTACGTGAAGGTCTA
GAAGTGTTATTAATTGTAATGACATTGACTACTATGACGCGTAATGTCAAAGATAAGAAGGGTACTGCAAGTG
TGATTGGTGGTGCAATTGCCGGACTTGTACTGAGTATTATCTTAGCAATTACGTTTGTAGAAACTTTAGGTAA
TAGTGGCATTCTTCGTGAAAGTATGGAAGCGGGATTAGGTATTGTTGCGGTCATATTAATGTTTATCGTTGGT
GTTTGGATGCACAAACGTTCAAATGCGAAACGCTGGAATGATATGATTAAAAATATGTATGCCAATGCGATTA
GTAATGGTAATTTGGTATTGTTAGCGACGATTGGTTTAATATCTGTGTTGCGTGAAGGTGTCGAGGTTATCAT
TTTCTATATGGGAATGATAGGCGAATTAGCGACCAAAGATTTTGTCATAGGTATCGTCTTGGCCATCGTGATA
TTAATTGTCTTTGCGCTATTATTTAGATTTATCGTCAGATTGATACCTATATTCTATATATTTAGGGTATTGT
CGATCTTTATTTTTATTATGGGATTCAAAATGCTTGGCGTAAGTATTCAAAAGTTACAATTATTAGGTGCGAT
GCCAAGACATGTTATTGAAGGATTCCCAACGATTAACTGGTTGGGCTTCTATCCAAGTTATGAACCATTGATA
GCACAAGCTGCTTATATTATGGTAGTTGCTATCTTAATCTTTAAATTTAAAAAATAAAAAACAGGCCGAGTGC
CTGTTTTTTTTGTTGCGATGTTGGAAATACTCGGTATTGCAGTATAACGATAATCACAGCATTAATTCTTATA
AGATTAATGTGTTGGCGGTTTGCCTCGGCATGTGAATTTAACGATGAACATACTGAACTCAAAGAGCAATATG
AGTGGCAATGTGAGTAATATATTTAATGTTATGTCTGGTGGTGCAATGATACTCGCCAGCACAAAGCAAGCGA
AATAAATATATTTTCGATAATGCTTCAATGATGTGGTATCTATAAGACCGAATTTTGCAAGTCCTATGAATAA
TATTGGCAATTGGAATAAAATGCCAAAACTGAAAAGCCAACGGATAAGTTCAACTAAATACGCTTTAAAGCCA
ATGACAGGCGAAATGTTTAAAGTTAATGATAATTTTAACGCGAATTGAATGATCATTGGAAAGCCAACATAAA
ATGCAAAAGCGACGCCAGCACAGAATAATAACACGCTGAAAAAACTATATTTATAAATAAATTGACGTTCATT
ATTATGTAATCCAGGTGCAATGAATGCCCACAATTGATAAAACATGACTGGTGAAATGAAACAAAACGCGATG
AAAAATATAATCATCACGTATATTTGGATCATTTCTGTGAATGAAAATGCATGTAAGGACACATGTGCACGGG
TAATATACGTTATGAATGGTGTCATCCACCAAAATGATGAAACATATACGACGATGACCGTAATGACGAACGA
CAATAAAATTTTTACTAACCGATGGCGTAGTTCGCTAAAGTGAACCATTAATGTATCATCAGTACTATTGCTC
TCGCTGTTGCTTCGATTCCTTACTGGGTGTATCGTGAGACTCTTTATCTAAATCTTCTGTTGCAGATTTAAAT
TCTTTTAAAGTAGAACCGATAGCACGACCAAATTGTGGTAATTTTTTCGGACCAAAAATAATTAAAGCGATAA
TGCTAATGACGACAAGACTTGTCGGGCCTGTGATGCCTAAAATAAAAGTGTTAGTTATCATGATAATCAACCT
CGATCATAAGTAGTATATAGTATTTACTTTATACCGAACGATAATTATTATCAACAATATTTTATATAATAAT
TAAAAAATTGTTATAAATACATTTTACTGAGGTATTAATTCTGAAATGATAGCTTGTTCTTTTAAGTCGATAT
AAGTACAAGTACCAGCTTGAATATCGATATGCCATTTATAAATGCCAGCTTCAGCCATTTCATCACAAAATGT
TTCGAAATCTGTTTGTCCTTGTTGATGTCTAGTTAATACATCTTGAACGATAGATTGATTTGATCTTGGAGCG
ACAGGATGATTACTTTTGATAGATGAAGTAACAATATCATCTTCTGATTGATGTACGTAAGTTGCTGTGCCAT
CTTGAATATTAACAATATTGTACGTCATACCCATATCCTTAAATGCTTTGAATAGTTTTGGAAAATCAACACC
AGTAAATTGTTGATGTGCTTGTTGAATTGCAGATAATGTAAATGCCATAATAGTCTCCTTTGAATGGTTTGAT
TTAGTGAAAATGCGTGCATCATACTAAAATTAGAATGCTATTAATTATGCAGCAAGGTGTAACGAAAGTAAAA
AAATACGACTTGTAGGCGTTGAGGTGTTTGTTTCAAAATGTCATACATAGGTTGACACTCATATTTTTTTGTG
TAGATTTGACAAATATAGTTGTCAAAAAGACAAATATAATTTACAGTGTAATTAAGGAGTTGATGCGATGTGC
GTAATCGATTGAAAGAATTACGAGCACGAGATGGCTTAAACCAAACGCAGCTTGCTAAACAAGCGGGTGTTTC
AAGACAAACCATTTCGCTAATTGAGCGAAACAACTTTATGCCATCGGTATTAACTGCAATAAAGATTGCTCGT
ATCTTCAATGAAAAGGTGGAAACTATTTTTATTATTGAGGAGGATGAGGCATGAAGATACTAAGATATATCGG
ATATCTTTTACTGGGTGGACTTGTAGGTGGTATCATAGGTGGAATTTTAGGTAACTTTGATGGATTGGGTATT
GAGAATTTGACGTTTGCGACATACAACAATGTCGTTGTAATATCAATTGTTGCGACCATTATTATCATATTGG
TAGAAATCATTGTTTTGATGAATCAAAGACGTGCATTGAAGTATAAGCGGCTTGTAGATGAAGAGGTAGATAT
CGATGCAACAGATCAATATGAATTGCTTGCGAATCGTTATGTTTTAAATGGAAGTATATTAAGCATTATACAG
ACAATTATTGCCTTTGTAGTGTTACTAATTTTTGTGGTAGGACACGCTGAAGCAAATGCAATACTATTCTTTT
TAATACCATTTTTTGCTAGTGCTATTTTCAATACACAATTTACACTGTTTAATAGAAGATTTGATGATAGAAT
GCCAAAAATTGCAGATAAGAATTACACTGAAAAGCGATTGGAAATATTGGATGAAGGTGAACGCCATATAGAA
TTAATTGCATTATTTAAAACATATGCGATCAACTTATCCATATTGATACTAGCCATTGTAGTAATTGGGCTTT
ATTCAATTACTACTGGAGTTAATCAAAGCTTTAGTTTGCTACTTATCATCGCTATTTTCATATATAACGCCTT
TAGTTATTTATTGAAGAGAAGACGTTTTTATTAAAATTAAACAAAGAGGAGCATGGATATGACAACATTGTTA
AACGTAGATAGTGTGAACAAACAATACAAAGATTCGGATTTTAAATTGCAAGATGCATCTTTAACGATTTCTA
CTAATGAGACAGTTGGATTAATTGGGAAAAAATGGCTCAGGTAAATCGACATTAATTAATATTCTAGTAGGCAA

TCGACATAAAGATAACGGTAGTATCACATTTTTTGATGAAGAACATGCTATGGATGATGTCGAATATAAAGAA
CACATAGGTATAGTGTTTGATGATTTGAGAGTACCTAATAAATTGACTATTAAAGATATTGATAAAGTATTTC
AATCTATTTATACGACTTGGAATAGTCAAAAATTCTTTGATTTAATCAAATATTTTGAGTTGCCACTACAAAC
TAAAATTAAAACTTTTTCAAGAGGGATGCGAATGAAGATAGCTTTAACAATTGCGCTTTCTCATGATGTGAAG
TTATTAATCTTAGATGAAGCAACTGCAGGTATGGATGTTTCTGGACGAGAAGAAGTAATGGAACTATTAGAAG
ATTTTGTCGCGCAAGGTGGAGGCATCTTAATATCATCGCATATTTCTGAAGATATAGAACAATTAGCGGATAA
ATTAGTGTTTATGAAAGATGGACGAATGATTTTAACTGAACAGAAAGATATACTTTTAGCACAATATGGAATT
GTTACGACAGAAGATAAAGATGTTGAAATTCCTAAGCATTTAATCATAGCTTCTAGATTGTCAAAGGGGAAAT
ATCAAATTTTAGTTAAAGATTATGCAGAAATTGAAAATGCAGAACCTTTAAAACACATTGATGACGCTACGAA
AATCATAATGCGAGGTGAAGTATAATGAAAGGTATGTTCCTAAGTAGTTTTTATGCAACGAGAATGCAAACAT
ATATTTATTTTATAGTCGCTATCATAGCTGCGGGATACTTTGCAGTATTTAATCCGTTAATGAGTTCGGCAAT
GGCTGGGGTTATGTTAATCACACCTATTACTGATAATATTAAACATGAAAAAGACTCAAGATGGATGTATTAT
GTATCTACTTTACCGGTTAAACGTAGTGATTATATTAAGTCATACTTTGCCTTTTATTTAATCTTATTCGGTG
CAAGTTTAATGATTGGATTAGTTGTGACTACGATTGTGACCCAAAGTGTGATGATTGGTATTATGTCAGGTTT
AATGAGTTTTGGTATCATAGGGGCATACTCTATCATTTTCCCATTGACATTTAAATTTGGCGCTGAAAACTCT
AATGTCATTATGATATTTGCATCTATTCTACTACTTATTTCTTTCGTGGTCTTTTTCTTTATATATGGTATGG
TTAGTGGTGCATCGGCATTAGAATTTGAAAAAATTAGCACTGAAGGATGGCTAGTTGTCATAGTATATGCGGT
CATTGGTATCGTTATAACAAGTGTTTCTTATATATTGTCTATTAAAATTTTTAACAAACAAGAACTATAATCA
ATATTCTTGGTGTGAAGCTAGTCATTGAAATAATATTTAGAGCAGGTGATAAATCAATACGATTATCATCTGC
TCTTTTTGGTGTTGTGGAATGAAATGCGAGATGATAGGTTGAGGTGACATCTCTATATCATATCATTTGTTTT
TAGAAGATTAGGTTGTGTGGGAATTGCATCCTTTTATAGTGAGGATTAACGTGTATATATTATTGAGTATGTT
GCTGAAATTTATTTGCGTGTATCAATTGATGATGGTATATGCCAATGATTTCCTCGGAAATATAAAAACTCGA
TCATGTATCATAACTGATGATTGTCTCAAAAATATACTGTGAAGCACAATATTTTTGGATGCAGTTCAAATAC
ATTGATCGAGATCAAGGTTATCTATTCATCACTTTGACGTAGACGTTGATTTTTAAAATAGATGAGCATGATG
CAGAAAGTCGCAACGGATACCGAGATAATCATTACTTGGTCGTGCCCTTTAAAGATAAGGCTGATGATATATG
ATACTACAAGTATGCCAAGTGAAGAAGAAATATATTTTGCGTTTGTCAGTTCATTATGGAAATAAGGCGTGAT
TAACCATAATCCAATATAGAATATTAAAACACTGATATACATCATATTAATTTCAAACAAGTCATTTAGTTTA
TTGTTATTACTAAAAACAATTGCGGCATTAATCACACCTAAAGCGATATTGATTAATAAATGCGTATACGATA
AACGGAAACCGATAGATGACAATTTATGATTAATGTAATTTTCAGTAATGATCCAATATACACCGAAAAGACT
AATTAAAATCATAAATTGGAATATATAAATGTAACTAAAATGATCAATGCTAAATGATGACGAAGCTAAACCA
ACCAGTACCTCGCCAAAGATAATAATTGTTAGTAATGAAAAACGTTCTACTAAATGCATCATATTAACAGGTG
ATAATACAAGATATTTCTGAAATGGAATAAGTCCTGTCGCTGCAATGAATACGCCTAAAAATCCAGGGATGTA
ATGGATACTTTGTGGTAGTACTAATGATAGAAATGATAAAAATGAAATCACAAAGGCTACGGCCGCAAAAGCT
TGACATGTACGCTTATCGCCATAATCTAACCCTGTACGTATATGTAATAAATACTGGAATCCGATACTTAAAT
ACATAATTGCCACGCATAAGAAGAATGGGAAGAATGTCTTTTCAAAGTCCGGATATAGGCTGTTAGATAGAAA
GACCATGATAAACATATTAAACATCATAAACGAGACGTCTTTGAATGTAACTTGACCAAATCGATTTGTAAAA
AATGTTTGATGAGACCACATTAACCATAAGAACAAACTCATGACGATGTATTTGAAAAATAAATCAGCTGAAA
TGGAACCGTTTTGTGTTGTTAAAATCACATGTGCGATTTTTTGAATGGCATAGACGAAAATTAAATCAAAGAA
CAACTCATGGAATCCTGCACGCTTTTCAGCTAAATGTTTTGGTGTTAATGCATTAACCATAAAATTTTAACTC
CTTTAAGATGTGTAATTAATTTACTAAGTATACTATTTATTTTTTCTAGTGAATAGAGGAAGATTTGGCGATG
AAGTGGAAGGAGGGGCGACTGCAAGGTAATTGCGGAATTAACAATCATCAGCGATTTAATATTTGACTAGAGA
CGTAATGGTAAGGAAAAGTTGATGAGAAATTGATGGTGAAACCAGCTGTGAATAGCGATGCAATGATAGATAG
CATTTAATTAGAGTCATTACGCGAAATGATTAATGATAATTTGTGGTAAATCAAAGCATAATTTTGTACTATA
GATGAGGATGATAGAGCATATTTAAGAGTGTGAAATGTTAAAGTGAAACCGTTTACGTTTCCGATTGCCCAAA
CAAATTACATCATTGTATAATATGATTTGTTAAATGCATAACAAGAATGAAAATGTAACATACGTAGCAATTG
GTTTCATAAATTGGATGTTAGTGGCGTATTGGTTCATTAGACGTATTAGTAATAAAATTGTATATATCATAAG
GAGATGAATATGACATGACGAGAGTCGTATTAGCAGCAGCATACAGGACACCTATTGGCGTTTTTGGAGGTGC
GTTTAAAGACGTGCCAGCCTATGATTTAGGTGCGACTTTAATAGAACATATTATTAAAGAGACGGGTTTGAAT
CCAAGTGAGATTGATGAAGTTATCATCGGTAACGTACTACAAGCAGGACAAGGACAAAATCCAGCACGAATTG
CTGCTATGAAAGGTGGCTTACCAGAGACAGTACCTGCATTTACAGTGAATAAAGTATGTGGTTCTGGGTTAAA
GTCGATTCAATTAGCATATCAATCTATTGTGACTGGTGAAAATGACATCGTGCTAGCTGGCGGTATGGAGAAT
ATGTCTCAATCACCAATGCTTGTCAACAACAGTCGCTTTGGTTTTAAAATGGGACATCAATCAATGGTTGATA
GCATGGTATATGATGGTTTAACAGATGTATTTAATCAATATCATATGGGTATTACTGCTGAAAATTTAGTAGA
GCAATATGGTATTTCAAGAGAAGAACAAGATACATTTGCTGTAAACTCACAACAAAAAGCAGTACGTGCACAG
CAAAATGGTGAATTTGATAGTGAAATAGTTCCAGTATCGATTCCTCAACGTAAAGGTGAACCAATCGTTGTCA
CTAAGGATGAAGGTGTACGTGAAAATGTATCAGTCGAAAAATTAAGTCGCTTAAGACCAGCTTTCAAAAAAGA
CGGTACAGTTACAGCAGGTAATGCATCAGGAATCAATGATGGTGCTGCGATGATGTTAGTCATGTCAGAAGAC
AAAGCTAAAGAATTAAATATCGAACCATTGGCAGTGCTTGATGGCTTTGGAAGTCATGGTGTAGATCCTTCTA

TTATGGGTATTGCACCAGTTGGCGCTGTTGAAAAGGCTTTGAAACGTAGTAAAAAAGAATTAAGCGATATTGA
TGTATTTGAATTAAATGAAGCATTTGCAGCACAATCATTAGCTGTAGATCGCGAATTAAAATTACCTCCTGAA
AAGGTGAATGTTAAAGGTGGCGCTATTGCATTAGGACACCCTATTGGTGCATCTGGTGCTAGAGTATTAGTGA
CATTATTGCATCAACTGAATGATGAAGTTGAAACTGGTTTAACGTCATTGTGTATTGGTGGCGGTCAAGCTAT
CGCTGCAGTTGTATCAAAGTATAAATAATAAGAAAACAGGTTATCACAACAGTATTAATTACATGTTGGTATA
ACCTGTTTTTATTTGTTTATGGATTTATTGGGTAATATTAGTCATTTGATGGTTTAATTGCAAATGCTCTAAC
AGGGAACCCAGGTGCATCTTTTGGTTTAGGGCTGATAGCGTAAATGATAGCGCCACGAGTTGGTAATTGATCT
AAATTAGTTAATAACTCGACTTGGTATTTATCCTGACCAAGAATATAACGTTCGCCAACTAAATCACCATTTT
TTACAACGTCCACAGATGCATCGGTATCGAATGTTTCATGACCAACAGCTTCAACACGGCGTTCTTCAATTAA
GTACTTCAAAGCATCTAATCCCCAACCCGGTGCATGTTGTTGTCCGTTCGCATCTTTGTTTTCAAACGTTTCA
ATATTAGGCCAACGTTTTGACCAATCGGTACGAAGTGCAACAAAAGTGCCAGGTTCAATAGTACCATGCTCTT
TTTCCCATGCTTCTATATGCGCACGTGTTACAATGAAATCATTGTTGTTCGCTACTTCTGTTGAAAAGTCTAA
TACAATTAACGGCAATACCAATTCTTTTAAATCAATGTCTTCTAAATAACGTTTATTCTCGACAAAGTGAATT
GGTGCATCAATGTGAGTACCATATTGCGTTACAATATTCCAACGTTGCACATAGAAACCATGATCTTTAACAG
TGAATAAAGTTGAAACCTCGCCTTTTTCAAACTCACTAAAACGTGGTATTTCTGGATCAAATGTATGCGTTAA
ATCAACCCAAGTTGCTTGTTTTAAAGTATTTAATTGTTGCCATAAAGGATATTGTGTCATAAAATCACCCGTT
TTTAGTTTATTATATGATAAATGCTGCGATTATTCTTGGCGTTTAGCTTTAACAGCATTCACAAGTACAGTCA
ATGCATCTTTAACTTCTTCTTCTTTTCGCGTTTTCAAACCACAGTCAGGGTTTACCCAGAATAATGAGCGGTC
GATTTGTTGTAGTGAACGATTGATTGCTGTAGTAATTTCTTCTTTTGTTGGAATACGTGGACTATGAATATCA
TATACACCTAGACCAATACCTAAATCATAATTAATATCTTCAAAGTCTTTAATTAAATCACCATGGCTACGAG
ATGTTTCAATTGAAATAACATCAGCATCTAAGTCATGAATAGCATGAATGATTTGACCGAATTGAGAATAACA
CATATGTGTATGGATTTGAGTTTCATCACGAACTGAAGACGTTGCAAGTTTAAATGATAAAACAGCATCTTTA
AGATATTGTTCGTGATATTCAGAGCGTAATGGTAAGCCTTCACGTAATGCAGGTTCGTCAACTTGGATAACTT
TGATTCCTGCAGCTTCAAGTGCTAATACTTCTTCGTTGATTGCTAAAGCAATTTGATCTTGAACGACTTTACG
TGGTAAATCAACACGTTCAAATGACCAGTTTAGAATTGTTACAGGTCCAGTTAACATACCTTTAACTGGTTTA
TCTGTTAAGCTTTGTGCATAAACTGTTTCATCAACAGTTAAAGGCGCTGTCCATTTTACATCACCATAAATGA
TTGGTGGTTTTACGGCACGTGAACCATATGATTGCACCCAACCGAATTTAGTTACTAAGAAACCTTGTAATTT
TTCTCCGAAGAATTCAACCATGTCATTACGTTCAAATTCACCGTGAACTAATACATCTAAGCCGATGTCTTCT
TGAATTTTAATCCATCGAGCAATTTCATTTTTTAAGAATGTTTCATATGCTTCGTCTGTAATGCGTTTGTTCT
TCCAATCTGCACGGTATTTTCGAACTTCTCGGCTTTGTGGGAATGATCCAATAGTTGTTGTTGGTAAATCCGG
TAAGTTCAAACGTTTTTGTTGTTGTTCAATACGTTGCGCGAATGGTGATTGTCTCGAAGTACGCACGCTTTCG
AAATCATAATCTAAGTTTTTGAATGATTGATTTTGGAAACGCTCATAACGTGCTTTTAATTTATCATATTTAA
CACTATCGTTTTGATTAAATAGGCGACGCAATGCATCTAATTCGTCTAATTTTTCAGTTGCAAAGCTTAAGCC
TTCGCCAACACTTGTATCTAATGTTTCATCATCTAAAGATACTGGAACATGTAATAATGAAGATGATGGTTGA
ATGACAAGTTCATTAGTGTGTGCTAACAATTTATCGATTAAGACTTTTTTAGCTTCAATGTCACTTGCCCATA
CATTACGACCATCAATAATTCCAGCGTATAATGTTTTTGATTTATCAAAATCTCCAGCTTCAATTTGTTTAAG
GTTATAGCCATTATCATGGACAAAGTCTAAACCTAAACCACCAACAGGTAAAGAACTTAAGAATTTAAGATGT
GCATGTTCGAAGTATGTTTGAATGACTAATTTTTTAGCAATACCAGCTTCTTCGAAATAGTCATAAGCTTCAC
GTGTAATATTTTCATAGCTTTCACTGTCGTCTGTAACTAAGATTGGCTCATCAACTTGAATGTATTCAGCACC
TGCATCTATTAATGATTCAAACACTTCTTTATAAAGTGGTAATAACGTTTTAACTTTTTCTTCAAAAGTTTGG
TGACCGCCTTTTGATAATTTAACAAAAGTAATCGGACCAACAATGACAGGGTGAGCGTTAACGTTTAAAGATT
GGGCATATTTAAAGCGATCTAATAATACGTTGCGACTCACTTTAGGCTCAACATTGTCCCATTCAGGTACGAT
GTAATGATAGTTAGTGTTAAACCATTTTATAAGTGCACTTGCAACATGGTCTTTATTACCGCGAGCAATATCA
AATAATAAGTCATCATCGATAGTTCTACCTTGGAAACGTTCAGGGATGATGTTGAATAATAATGACGTATCTA
AAATATGGTCATATAAAGAGAAATCACCAACTGGGATGCTATCTAAGTGATAGTACTTTTGTAATAATAAATT
TTCTTTATGTAGATCAGTTAATGTTTGATCTAATTCTTCTTTAGAAATCTTCTTTGCCCAATAACTTTCGATG
GCTTTTTTCCATTCTCTTTTTCTACCTAATCTTGGGAATCCTAAGTTTGATGTTTTAATTGTTGTCATAATAT
TGCCTCCTTGTGAGCAGTAATAGATTTTGAGTATGCTGCAAGTTCTAATGAATCTTCGACATTTTGAAACGGT
GTGATAATGTATAAACCATTAAAATATTCATGAACAGTATCGATTAAATCCTTTGAAAGCTTAAGACTTAGTT
CTCGTGTTTTGGCTTTATCATCTTTAACTGCTTCAAATTGTTGTAAAATTTCATCTGACATCTTGATTCCTGG
CACTTCATTATGCAAAAAGAGTGCGTTTTTGTAACTTGCGATAGGCATAATGCCTATGAAAAATGGTTTGTTC
AAGTGCTTAGTGGCATGGTAAATTTCAATGATTTTCTCTTTGCTGTACACGGGTTGTGTTATAAAATAAGACA
TTCCGCTTTCTATCTTTTTCTCTAGTCGTTTTACGGCACCATCTAATTTACGAACATTAGGGTTAAAGGCGCC
AGCGATGTTGAAGTGTGTACGTTTCTTCAGCGCATCACCGTCAGTGTTAATACCTTGATTAAATCTTAGAGCG
AGTTCAGTTAATCCTTTAGAATTAACATCATAGACATTGGTTGCACCTGGTAAGTGACCAACTTTTGAAGGAT
CACCAGTTATGGCTAATATTTCGTTAACGCCAATGAGCGATAATCCAAGTAAATGGGACTGCAAGCCGATTAA
GTTTCGGTCTCGACATGTAATATGTACGAGTGGTTCAATATTGTAATATTGCTTAATTAAGCTTGCAGCAGCA
ATGTTGCTAATTCTGACAGTTGCCAATGAATTATCTGCGAGTGTTACCGCATCTACATTTGCTTTATCAAGTT

TAGCGATATTTTCAAAAAATCTATCCGTGTCCAAATGTTTCGGTGTGTCCAATTCGATAATAACGGTTGGACG
TTCTTGAACCTTAGATGTTAATGATTGTCTAACTTTATTTGAGATGGATTGAAAAGTGCTTTCGTTGGTATC
GGAATCACTTTTTTGTCATTAACAGGTTTAAGTGTCTGAATAGATTCTTTAATAAATTTGATGTGCTCTGGCG
TTGTACCACAGCAACCACCAATTAAACGAACACCTTCGCGAATTAAATTTTGAGCAACTTGGCCGAAATATTG
TGCATTGTCACTATACTTAAATTTACTATTTTCAATATCTAATAAGCTGGCATTTGGATAACAAGATAAGTAT
GCGTGCTCTGGTAATTCAATATGTGTGAAAGACTCTTGCATATGGTGTGGGCCATGATGACAATTGAGTCCCA
CGATGTTTGCACCACATTGAACGAGTTGTTTTAATCCTTCATTGATTGCCTGACCATTAACTAAGTAATTTGT
GTTTGAAGCAGTTAATTGAGCAATGATTGGAATGTCGTATTTCTTTCTCGTTCGTGAAATGACATTTGTTAGC
TCTTCTAGGTCGTAATACGTTTCGAAAAGTAGCGCGTCAACGCCTTCTTCAATTAAGGTGTCTATTTGAATTT
CAGTATGATAAAGAATAGTTTGTAAGCTGATATCCTCTTGTTTGATACCTCTAAACCCACCAACTGTGCCTAA
TATATACGTATCTTTATTTGCTGCTTTTTTTGCGATGCGTACGGCAGCTTGATGTATAGCTTTAACTTTATCT
TCAAGACCGAATCGTTTTAACTTTTCAAAATTTGCACCATAAGTATTGGTTTGAATGACATCAGCACCAGCTT
CGATATATGAACGATGGATACGTTCAACTTTATCTGGATGGCTAAGATTATATGCTTCTGGACAGGTGTCTAA
TCCTTCAGAGTATAAAATGGTTCCAATAGCGCCATCAGCTACTAAAACATTATCTTTCAATTGTGTGAGGAAT
TGACTCATTGAATGCCTCCTTTAATGCGTATTTGATGTCTGCAATGAGTTCATCAGGATCTTCGAGACCAACA
CTTAATCGGAATAGACCGAAAGTGATACCACGTTCTTGTCTCACTTCTTCAGGTAGTGTTGCGTGAGACATTG
TTGCTGGGTGCGAAAGTATTGTTTCCACACCACCTAGACTCACTGAAACGAGTGGTAATGTCAGTGCATCGAC
AAATTGTTGTGCTTTAGACTCATCAGCTAAACGTAAGCCAATAACGGCACCGCCATTTTTAGCTTGTTCTAAA
TGAGATGCAGTAAGTCCCGGGTAATAAACTTCTGAAATTTCATCTTGCTTTATTAAAAATGACACGATTTTTT
GCGCGTTTTCGACAGATTGTTTAAATCTGATTGGAAATGTTTTTAAATGTTTAGCAAGTGTCCAGCTATCTTG
AGCAGATAACATATTGCCTGTACCATTTTGTATTAAATAAAGAGCCTCACTAATTGCCTCATTATTAGTAATG
ACAGCACCAGCAATTAAATCGCTATGTCCACTTAAAAATTTTGTAGCACTATGAATGACAATATCAGCGCCTA
GTAATAAAGGTGATTGACCTAACGGTGTCATAAATGTATTGTCCACAGCTACCAGTAGTTCATGCTTTTCGGC
TATTTTAGAAACAGCTTTGATATCAGTAATTTTAAAACAGGGATTCGATGGTGTTTCGATATAAATTAATTTT
GTGTTTGATTGAATGGCACCCTCGATTTGTTCGATCTTGGTAGTATCTACGGTTGTAAATTCAATATTAAAAC
GATTCAAAATTTGCTCAGTGAGGCGAAAAGTACCGCCATATACATCATCAGGTAAGATGACATGATCACCAGA
TTTGAAAGTCAAAAGTACTGCTGAAATAGCAGCAATACCTGATGCAAAAGCAAAAGCGAATTTTCCCTGTTCT
AATCGTGCTAACTTCTCTTCTAAAAGTTCACGGTTAGGGTTGCCACTTCGTGCATAATCATATTTAACATCGC
CACCAAGACTTGTTTGATGGAATGTTGAAGAATCATAGAGTGGTGGGTTAGCTGAATGATATTCCACACCTCT
ACGCCAATCGAATATCACTTCTGTCTCTTTTGAAAGTGTCATACAATCTCTCCAATCTGAGCTTTATCTAATG
CTTGGATGATATCGTGTTCGATGTCTTCATAATTTTCAACGCCTAGTGATAAGCGGATTAAATACTCATCAAT
GCCACGTTTATCTTTTTCAGCATCTGGCATATCAACATGTGTTTGTGTGTAAGGGAAGGTCACTAATGTTTCA
GTGCCTCCTAAACTTTCTGCAAAAATACAAATGTCTAAATTTTCTAATAATTTAGCGACGCTATAGGCCTTGT
TAAGTCTTAAACTAAGCATGCCAGTTTGCCCGCTATATAGTACTTCGTCAATCGCTTGAAGTGACTGACATTT
TTTAGCAAGTTTTCTTGCGTTTGATTGCGCGCGCTCAATGCGTAAATGCAAAGTTTTAAGTCCACGTAACAAC
AAATAACTATCTATTGGTGATAGTGTTGCGCCAGTCATGTTGTGAAAATCAAACAACTGTTGTGCGAGTGATT
CATCTTTGACGGTTACGACACCTGCTAGTACATCGTTATGTCCACCAATATATTTCGTAGCTGAATGTAAGAC
TATATCAGCACCTTCTGCTAACGGTGTTGAAAGATAAGGTGTTAAAAAAGTATTGTCGATAATTGACAATAAG
CCTTTAGCTTTACAAAGTTGATAGTACGTCTTTACATCAATAGCAATCATTTGTGGGTTAGATATTGGTTCAA
TGAATAATGCAACTGTTTTATCAGTGATTTCTTTTTCAACTTGTTCATAATCTGTAAAATCAACGTACTTAAA
TTTGATATCGTATTGTTGTTCGTAAAACTCAAACAATCTAAATGTGCCCCCATATAAATCGAATGAAACTAAA
ATTTCATCATGAGGTTTAAATAGATTACATATTAATTGAATGGCTGACATTCCACTTGATGTAGCGAATGATG
CAATACCATGCTCAAGTTTGGCAAAACAGGTTTCAAATGTTGAGCGTGTAGGATTTTTAGTACGTGTATAATC
AAATCCTGTCGATTGTCCTAGTTTTGGATGCTTGTAGGCAGTAGATAAATGGATTGGATTTGCTATAGCACCG
GTTGAATCATCAGTTAATGTGATTTGGGCTAACTGTGTATCCTTCATATTAAGACCCTCCTATAAGAAAAAAT
AAAAAGCTTCCGTCCTTCGTACCCGAATGAATCGGATAAAAAGGACGAAAGCTTATGTTTCGCGGTACCACCT
TTGTTTGTTATTCCATCGCTGAAATAACCTTATTCAGTACGCATTAAAAGTAAATATGCTTACTGAACAATTA
TCACAATTTAAGTCGTTAAGTAAGGATATAGTAATGTGCTATCTCATACTTATTAACAAAAAATCGTGCGTAA
AGAATCCAGTACGCCATTTAACATCAATGTTAATACTGTATCGCTATAACGGGCGAACCCGTAGACACCTCAT
ATTGGCATCAACACTCCAAGGCCATTTTCAAACACGCTTTCAAAATCTTCTCTCAGCTACTAAAGACTCTCTG
TATAAGCAGGGTGTGTTTTACTTTCCTCTTTATTGTGTTTACGTTTCATTAAACTGTTATAAGATATTAATTA
GCTTACAGAGTAAAAAAAGATTTGTCAACAATTATTCAGAAAATTTTGATTTAAAAGTTAATTTGTTTGTGAA
ATTGTATTTGGTATCTTGAAGTTAAAAAATGAATTATTTTTTAAATAAAGTGTGGTTAATGGTTGTCTGACTC
ATTTAGAATACATAAAATATATTTAACTGTTGTTATCAAATAAAAAGTGATGTGAGTGAATTGTCAAAAAGTG
AAGATCAACGTATTACTAAAACAAAATATGAACAAATTAAGCAAATAGATATATCGGATATCAAACCGAATCC
GTATCAGCCCCGAAAAACTTTCGATGAAAATCATTTAAATGATTTGGCAGATTCAATTAAGCAATATGGTATT
TTGCAACCAATTGTGCTTAGAAAAACAGTTCAAGGTTATTACATTGTAGTTGGTGAAAGAAGGTTTAGAGCTT
CGAAAATTGCTGGTCTAAAATACGTATCAGCGATTATCAAAGATTTAACAGATGAAGATATGATGGAACTGGC

GGTCATAGAAAATTTACAACGAGAAGACTTAAATGCGATTGAAGAAGCTGAAAGTTATCAACGTTTGATGACA
GATTTGAAAATTACACAACAAGAAGTAGCGAAGCGATTGAGTAAGTCGCGCCCGTATATAGCGAATATGTTGA
GGTTATTACATTTGCCGAAAAAGATTGCTGACATGGTAAAAGATGGGCGACTGACAAGTGCACATGGACGAAC
GTTATTGGCAATTAAAGATGAACAACAAATGCTTAGGTTAGCGAAACGGGTTGTTAAAGAAAAGTGGAGTGTC
AGATATTTAGAAAACCATGTTAATGAATTAAAAAATGTTTCGTCAAAGTCGGAAACAGACAAAGTAGATATAA
CTAAGCCTAAATTTATAAAGCAACAAGAACGACAGTTGCGAGAACAGTATGGCGCAAAAGTAGATATATCGAT
AAAAAAATCAGTCGGCAAAATCTCATTTGAGTTTGAATCACAAGATGACTTTGTGAGAATCATTGAACAATTA
AATCGTAAGTATGGTAAATAGTTACACAATTTTATATAATATCACTTTGTGAATCTATAGATATATCGATAGT
TATCTATATTCTTTGAAGATGATGCGACTTCAGTAGGTTGGAAGAATACAGCAATATTAATAAAATTATTTGA
GACTATATAGAAGGGAGTAACGCTAATTATGGATCAAGTTATGAGTATTATTTCATCGCTATTCGAACCATTA
ACTAAAATTGAAACATATGAAAGTGTCGCTACTAAATTAGCGATGATAATTATATATATTATCGTTGCATTAA
TCGTAATTAAAATACTTGATAAAATGATTGCCCAAGGATTTAAAATACAAAACAAGAGTAAAAAGGGTAATAA
AAAACGTTCGAAAACATTAATATCATTGGTGCAAAATGTTGTTAAATATATTGTGTGGTTTATAGTTGTAACT
ACCATTTTAAGTAAATTTGGCATTAGTGTTGAAGGAGTAATTGCAAGTGCTGGTGTCGTAGGCTTAGCAGTAG
GTTTTGGTGCTCAAACTATAGTTAAAGACGTAATTACAGGTTTCTTTATTATATTTGAAAACCAGTTTGATGT
AGGTGATTATGTTAAGATAAACAACGGTGGTACAACTGTAGCAGAGGGAACAGTTAAATCAATTGGGCTCCGT
TCAACACGTATTAATACAATATCTGGAGAACTTACAATCTTGCCAAATAGTAGCATGGGTGAAATAACGAATT
ACTCAATTACGAATGGAACAGCAATTGTTAAAATTCCAGTATCTGTAGATGAAAATATTGATAATGTTGAGAA
GAAATTAAACAAATTATTTACATCTCTGCGTAGTAAATATTATTTATTTGTAAGTGATCCTGTTGTGATTGGT
ATTGATGCCTTTGAAGATACTAGAGTGATATTAAGGGTTTCAGCAGAAACAATACCAGGTGAAGGTTTCTCTG
GTGCGCGTATTATTCGTAAAGAAGTACAAAAAATGTTTTTACAAGAAGGTATCAAGACACCACAACCAATAAT
GACACCTTTTAATCATAGTGAAAACGGTGTTTAGTAGTTTATAATACATGGAGGTCATATTTAATGGCGTCAA
AATATGGAATAAATGATATAGTAGAAATGAAAAAACAACATGCGTGTGGAACCAATCGTTTTAAAATTATTAG
AATGGGTGCTGACATTAGAATTAAGTGTGAAAATTGTCAACGAAGCATTATGATTCCACGTCAAACGTTTGAT
AAAAAACTAAAAAAAATCATCGTATCTCAGGATGATACACAAAGATAGGAGAATGATTCATGGCTTTAACAGC
AGGTATCGTTGGCTTACCAAACGTTGGTAAATCAACATTATTTAATGCAATTACAAAGGCGGGTGCCTTGGCA
GCAAACTATCCGTTCGCAACTATAGACCCAAACGTGGGTATTGTAGAAGTGCCAGATGCAAGACTACTTAAAT
TAGAAGAAATGGTTCAACCTAAAAAAACATTACCTACTACATTTGAATTTACAGATATTGCCGGAATTGTTAA
AGGTGCTTCTAAAGGTGAAGGTTTAGGTAATAAATTCTTATCACATATTAGAGAAGTAGATGCGATTTGTCAG
GTCGTTCGTGCATTTGATGATGATAACGTAACTCATGTTGCTGGTCGAGTAGACCCTATTGATGACATCGAAG
TTATCAATATGGAATTAGTACTTGCAGACTTAGAATCAGTTGAAAAACGCTTACCTAGAATTGAAAAATTGGC
ACGTCAAAAAGATAAGACTGCTGAGATGGAAGTGCGCATTTTAAATACTATTAAAGAAGCTTTAGAAAATGGT
AAACCTGCCCGCAGTATTGACTTTAATGAAGAAGATCAAAAATGGGTGAACCAAGCGCAATTACTGACTTCTA
AAAAAATGCTTTATATCGCTAATGTTGGTGAAGATGAAATTGGTGATGATGATAATGATAAAGTAAAAGCGAT
TCGTGAATATGCAGCGCAAGAAGACTCTGAAGTGATTGTTATTAGTGCAAAAATTGAAGAAGAAATTGCTACT
TTAGATGATGAAGATAAAGAAATGTTCTTAGAAGATTTAGGTATTGAAGAACCAGGATTAGATCGATTAATTA
GAACAACTTATGAATTATTAGGATTATCAACATATTTTACTGCTGGTGTGCAAGAAGTACGTGCTTGGACATT
TAAACAAGGTATGACTGCACCTCAATGTGCTGGTATCATTCATACTGATTTTGAACGTGGATTTATCCGTGCC
GAAGTAACAAGTTATGACGACTATGTACAATATGGTGGCGAAAGTGGCGCTAAAGAAGCGGGCAGACAACGAT
TAGAAGGTAAAGAATATATTATGCAAGATGGCGATATCGTTCATTTCAGATTTAATGTATAAACGATAGAGTG
AAGTTAATTAAATAGTATATATGTAGAAGAGGCGGAATCAATTGTTCGCCTCTTTTAATTATGCGTGTTATTT
ATTAAAAGAATGGAATAATTTTACTCGCGTTAATAATATCTTGAGTGCTGAAAAATTGTTTGCCTTCGCCAGT
ATAAGCAGGCTCTAAAACAAGATTAGCCTTTGCACAATAAAGCCATTCAGGATGAATGCCACTATTAAGTATC
TCTTGGAATTCTTGAAAATCTTTAGACCAATCAATATTTAAATTCATTCCTTACCACCTCACAACCATTATAG
AACATGTGTTCGCTTTTGTGAAGTGTATTTTTAAAAAATATCATAGAAAATTTCAAATGTATTAATGTCAAAA
ATGATATGAAGAGTAGTTTGAATTATTATTGTAAAAAGTAATGGCGCATGATATAATTCTTTATTGTGAGTAA
TGAAAATTATTCCTTGCTTATCTGTTTTAAGATTGATAAGCCGTATAGACCACAAGGAGGTGCAAATATAAAA
TGAGAACATATGAAGTTATGTACATCGTACGCCCAAACATTGAGGAAGATGCTAAAAAAGCGTTAGTTGAACG
TTTCAACGGTATCTTAGCTACTGAAGGTGCAGAAGTTTTAGAAGCAAAAGACTGGGGTAAACGTCGCCTAGCT
TATGAAATCAATGATTTCAAAGATGGTTTCTACAACATCGTACGTGTTAAATCTGATAACAATAAAGCTACTG
ACGAATTCCAACGTCTAGCTAAAATCAGTGACGATATCATTCGTTACATGGTTATTCGTGAAGACGAAGACAA
GTAATAATTAGAGGGGGCGTTTAAATGCTAAATAGAGTTGTATTAGTAGGTCGTTTAACGAAAGATCCGGAAT
ACAGAACCACTCCCTCAGGTGTGAGTGTAGCGACATTCACTCTTGCAGTAAATCGTACGTTCACGAATGCTCA
AGGGGAGCGCGAAGCAGATTTTATTAACTGTGTTGTTTTTAGAAGACAAGCAGATAATGTAAATAACTATTTA
TCTAAAGGTAGTTTAGCTGGTGTAGATGGTCGCTTACAATCCCGTAATTATGAAAATCAAGAAGGTCGTCGTG
TGTTTGTTACTGAAGTTGTGTGTGATAGCGTTCAATTCCTTGAACCTAAAAATGCGCAACAAAATGGTGGCCA
ACGTCAACAAAATGAATTCCAAGATTACGGTCAAGGATTCGGTGGTCAACAATCAGGGCAAAACAATTCGTAC
AATAATTCATCAAACACGAAACAATCTGATAATCCATTTGCAAATGCAAACGGACCGATTGATATAAGTGATG

ATGACTTACCATTCTAATAAAAATTAACGAAATTAAAGCGAAAAAATTATCAAAGGAGGCACACAATCATGGC
AGGTGGACCAAGAAGAGGCGGACGTCGTCGTAAAAAAGTATGCTATTTCACAGCAAATGGTATTACACATATC
GACTACAAAGACACTGAATTATTAAAACGTTTTATCTCAGAACGCGGTAAAATTTTACCACGTCGTGTAACTG
GTACTTCAGCTAAATATCAACGTATGTTGACTACAGCTATCAAACGTTCTCGTCATATGGCATTATTACCATA
TGTTAAAGAAGAACAATAATATTTAATATATTATAACCCCCGTAGGCATAGGCTTACGGGGGTTTTTGTGTTT
TGGGATATAGAAAAAGGGCAGAAAAAGGACGATGTGAATGTTTTGTGTTCGGGATTTGCACAAAGATATGTTT
ATATTGCAAAAATAATATGAATTTAGATGCATAAAAAAAGAACTACGCATTTTAAATAAAATGCATAGCTCTT
CTTTTTCTTGCATACGAATTAAAATAACTCGCGAGACCTATAAGTCTCTTTCCTCACTAGATAGTTTATACTT
TTGGTCTGTTGAAGTCAATAATTTTATCTAAAGCTATAAAAAATCTTTTGATAGCTAATGCATTATTATAATA
GCTTTCGTTTCTTTTATATCGCTTTTGAAGTTGGTCCAAATCGTGATATCTTGCTTGGATAATTGCATTACTA
CAAACTTGATTATGTAATTCTAGAGTAGCGAAAGTATCTATGAAATTTTTTATTCCGAACATGTTTCTAGATA
TGCCTATATTATTCCCTTTTTCAAATAAATATTGAGGAGATCTACTGTCATAATTTAGATTTGCTATGATGGG
TTGGTTATGAGCTGATTTGTTTCTTATATTTTTAACTAAAGGCATTAAAATATTAGCAACTCTCAATTCTTCG
TCATTGTACTTCTTGTAATAGAAGTTGAGAAACGAAACGAATTGACCTAGTTGCATGAATTCAATGCAAACCC
ATGCGGGTGGATTTTGATAGTATTTATTCAACTTTGCGGGTAGTTGCCCTCTTTTATTCATATGCTTAAATAT
TTCGTTTTTATTTTTGATTTTGGTTTCCATAACTTCTTCTGGTGTTCTTGAATTTGTGTCAAAATTTGAATTG
TTATATGATTTATCAATACATAAGAACTCATCTATTATTTTATAACCATCTTCTTGGTTATTTTCTGTTATTA
GTTTTAAGACTAGACACTTTAAACTATGTTCAATATCTAAAGTTAAATGCAACATTGTGTATCTTAATTTCAT
ATCTATAGTTGCTAAATCTGATAAATAAGCAAATTCTATGAAATAGCCGCCATTCTTTTTTTCGAAATTTTTT
CGGAAATAAGCTAGTTTGAAGAAGTAATTATTTTTTCTAAGAATTTCATTTGCTTTTTCGGTGTCAATAATAT
TAAAAAATATATTCATCTGTTTTAATTTCGCTATTTGCTCATCAAAATTGAGCATAGGCTTAATTTCTGCTAG
TGTATCATCTTTTTCCAATATTAACTCCCCAATCGTTCAAATTTATTCATCATATCTTTCGCCATCTGATTAG
TAACATGTGTGTATATCTCTAGAGTTTTTATAATCTGAATGACCTACATGCTCTTGCATTGCTTTTAAGTTAA
TTCCTAATTGAGCAAGTGTAGATATATGAGAATGATGTAATGTATGCGTCGTTGCAGGTTTCTTAATAGAACT
AATCTCAGTTGTCTCTTTGATAGCCCCCCCTTTAGTAATGTGGCTAATTTTGTTCGAGTCGATAGGGCTACCA
GCCGTATTTGTGAATATGTACTCTCTATCAATAAACTTATCATTCCAAGCATAAGTGTTCTTAGTAAGTCGAT
GCTTTGGGTAGTGAGCCCTATGGCCTTATAGCTATTACTTCTTTCAGTTGTCTCTTTTACTCCGGATGCTTCC
ATTTTTAATCAGTTTCCCAATTAACTTTACCGTCGATATCTAGCGTTTTATCTTGATAGTTTATATTTTCTTT
ATTTATTGCAAATAGTTCACCGATACGCATGCCATTATCAATTTAAAACTGAACTATAGATTTTACCTTTTTA
TAATTACGTTTTGTCGTTGGACGTTTTTTTATACTTAATTACATAGTCGAAACAATCCAGTAACCCCTTAACT
TAATTATCTTCATTATTTTCTAAAGTGTTATTACGTTTAGCTAGTAACGCATCACTATAGGGATATCTATTTT
ATCTATTACACATATAACTTTGAATTGCTTACTATTTTAAATTAACAAATTCTATTCTCTTAGATTTAGTCCA
ATTATGTGTAGACGATTTATAGTTATTAAATTCAGAGTGGTAGCAAATTAAAGTTAATCAAGAGTTAAGATGA
AGTTAATTCATGAACACGTCTATTATTTCTATAATTGTAGCAAATAAAGCTTTACATCAAGGAGGTAATTAAA
TATGTTCAAAAAACATCATTCTAAAAATTCAATCTTATTAAAATCTATTCTATCGCTAGGTATCATCTATTCG
GGATCATTTGGAATATATTCAAAAGCAGGCGCGTCAACACAAAATTCCTCAAGTTTACAAGATAAACAATTCC
AAAAAGTTGAAGAAGTACCAAATAATTCAGAAAAAGCTTTGGTTAAAAAACTGTACGATAGATACAGCAAAAA
TACAATAAATGGAAAATCTAATAAATCTAGGAATTGGGTTTATTCAGAGAGACCTTTAAATGAAAATCAAGTT
CGTATACATTTAGAAGGGACTTACACAGTTGCTGATAGAGTATATACACCTAAGAGGAACATTACTCTTAATA
AAGAAGTTGTCACTTTAAAAGAATTGGATCATATCATAAGATTTGCTCATATTTCTTATGGCTTATATATGGG
AGAACATTTGCCTAAAGGTAACATCGTCATAAATACAAAAGATGGTGGTAAATATACATTAGAGTCGCATAAA
GAGCTACAAAAAGATAGGGAAAATGTAAAAATTAATACAGCCGATATAAAAAATGTAACTTTCGATCTTGTGA
AAAGTGTTAATGACATTGAACAAGTTTGAAATTAAGTTAAATAAGTATATACAGTGTTTTATCGCTAATACTT
TGAAAGTTAGGTATCTAAAGGTGCCTAGCTTTCTTTGTTATGATCAGCACCATCATATAGAAAATTCTTTAAT
GACGTTTATGCCGAAATCTACAAAAATAATTTCTCTTTTATGGTTAATCAATATGTTTTTATATTCGACAACC
TTAAAATTAACTTCTTTCAATTCCATAATAAGGTCTCTATAAAATAACTTTGTTTAAAAACGATTCGTATCTT
TCAGATTCAAATACCATCATTTTCTCCTAATACTTACATTTTAATTACAATTATGTAAGTTGTTTTCAGAGAC
ATCCTATTTTATATTTTGTCAGAGGTGATCTTTTGAAAACGATTTTAAAAACAATAACATATCTCGCACTTAC
TATCGTTGGCGCTTATGCTGCTCTATTCATTTTAAAAACAATAGACTCTCATGGTATAACAGATCAATTTAAC
CCATTAGTAAAGGAAGATGATTCTTATGTTAAAACGACAGAGGTGTCTACTAGAATGGATGATCAACTCCGAA
GTTATACTCAAAGTGCTTTTAATAAAGAAGGGAAAGAGACGCAATTAATGTATACTGCTACATTTGATGTTAA
ACCGCATAGATACTTGAAAATTACACATAAAGGTCATCATGTAGAAACTTTTGAAGAAGTAGAAAAGGAAGAA
GTACCAAAGAAAGCATTAGACAAACTGAGTCGATAATAGCACCCTTATATTACATTACTCATTTTAAAAATGA
TTACGAACGAAAGTAACGCATGAAGAGTAATTTAAAAAAATTTGTAATAATTATGAATAGAATTAAAAACAAG
GGGTAATACAATCTATATAGCATATAAGCTTTTGTTATGAGTTTCTAAAAAAGGAAGAGAGAGTGATATTATG
AAATTAAAATCATTAGCAGTGTTATCAATGTCAGCGGTGGTGCTTACTGCATGTGGCAATGATACTCCAAAAG
ATGAAACAAAATCAACAGAGTCAAATACTAATCAAGACACTAATACAACAAAAGATGTTATTGCATTAAAAGA
TGTTAAAACAAGCCCAGAAGATGCTGTGAAAAAAGCTGAAGAAACTTACAAAGGCCAAAAGTTGAAAGGAATT

TCATTTGAAAATTCTAATGGTGAATGGGCTTATAAAGTGACGCAACAAAAATCTGGTGAAGAGTCAGAAGTAC
TTGTTGCTGATAAAAATAAAAAAGTGATTAACAAAAAGACTGAAAAAGAAGATACAGTGAATGAAAATGATAA
CTTTAAATATAGCGATGCTATAGATTACAAAAAAGCCATTAAAGAAGGACAAAAAGAATTTGATGGTGATATT
AAAGAATGGTCACTTGAAAAAGATGATGGTAAACTTGTTTACAATATCGATTTGAAAAAAGGTAATAAAAAAC
AAGAAGTTACTGTTGATGCTAAAAACGGTAAAGTATTAAAGAGTGAGCAAGATCACTAAAATATGTCGTAACA
CTAACGATAGCAACATGTCTTAATTCATATTAATTATTAAAAATACCCTCACAACGCAATGTGATATATCCAG
TATAGATTTGAAAGGATATATGCCTAACGTGTTGTGAGGGTTTAATTGCATCTACTTATCCATGCGAATATCG
ACTTCTTCTAAATGTTTCTGATATTCTTTAACCTTACTTTCTAAAAACATTTCATATGGTACATCAAAGAAGT
CAGCTAAATGCATGGCATCTTTAAATTTAGGTTCATGGTGATGATTCTCCCATTCCCAAATTTGGTGTGCTTC
ATATTTAGTACCATATTTCTCATTTAATTGTTGTGCTAATTCGTCAATTTCTAAATTATGTTTAGTTCGTAAG
TTATATAAAATATGCATATTCATTGTTCATTAACCTCGCTTCTTGATTTAAAATTCAAAGATGCTCTAATAAT
ATGTGTATATTCTAGTCCTATAACATAAAATAATCAAGCTATGAATTCGCCAGAAAAAATAATGTATATGATA
TTGCCGGTACAGAGACTTTAAATAACGATAGCTACATTGAATAAAATTGATATTCAATTACTACTTTGAAAAA
TATTTAGATAAAAATAATTTGAATTTGTTTTAGAAAGTCTAATAACGGGTACTACTTAGAATAACGCAAAATA
AATAGAAAAAGGAGACTGAAAATTATGTTTGGATTTATTGGAATGTTAATTGTCGGTGGCTTAATTGGATGGG
CTGCTGGTGCTATTATGGGTAAAGATATCCCAGGTGGTATTTTAGGTAATATTATCGCAGGTATTATTGGATC
ATGGTTAGGTGGAACATTATTAGGACATTGGGGTCCTGAACTAGGAAGCATTTACATCTTACCAGCTTTAATT
GGTTCAATTATCTTAATTGCACTGGTAACGCTAATTTTAAGAGCTGTGCGTAAAAAATAAAATATTCAACTTA
GGTTGAATGGAAATCAAGATGGCATTCAGTTGTCATCTTTTTTATTTGCGTGAAAATGGTCAATATGTATTAT
AATATTTGAAAATGTAGGTAAGATAGGATTAAAAAGTAATAAATTAACATGAGGAAAGGTTGGAAATAAAAAA
GCGAGTATTTGACAAATAAAAAAGGATGACGTTGGGTTGCGCCATCCAGGAGTATTATGTTCATGAATATAAA
AGACTGTGTAGTCATTTAAGAACTACACCCATATAGTAACACATCACTTGATTAAAGACAATACTATTTTTAG
AGTCATTAATTTAAAATATGAATTAATTCTTTTTTTAGAATAGAATATTAAGATTGATATTGAATTAGTAGTC
AAAGTGTTATGGTAGATATGAAATACATAATGTAAGGAGTAATATTATGACGATTTATTTAGTTAGACATGGC
GAATCACAATCGAATTATGATAATAAACATTTTCGATCTTATTTTTGTGGACAATTAGATGTGCCGTTAACGG
ATTTTGGTAAGAAAAGTGCAGACGATTTATGTGATTATTTTAAAGAGAAACAGATTAAACATGTATATGTTTC
AGACTTATTAAGGACACAGCAAACGTTTGAACATATTTTTCCATATGACATTGCATCAACGACTACGCCTCTA
TTAAGAGAACGTTCGCTTGGCGTATTTGAGGGTGAATATAAAGATGAAATCAGTGCGAATCCGAAATATGAAA
AATATTTCAATGATCCAAACTTTAAAGACTTTCGTCATAGTTTTTCACAAAAAGCGCCTGAAGGAGAAAGTTA
TGAAGATGTATATCAACGCGTCGAACATTTTATGAATCATGTTGTCAATGAAGATACACAAAAAGATGATATC
GTCATTGTTGCACATCAAGTTGTCATTCGTTGTTTAATGGTTTATTTTAATAACGTTTCACGGGAAGAAGCTG
TGGATTTAAAAGTTGAAAATTGCAAACCATATATCATTGAATAGAAGTATAGAGGTTCTGAAAAGCTGTGTTT
TATAGCGGCCTTCAGAACCTTTTTTGTATTTGGAATTTAAAGGGAATCATTCTTATCAACAGACTTACCACCA
ATAGCTAATGTGATAATTGTGCTTGCGAACAAAATAATTGAAGTTATAAATGACGCTAATGTAAAAGGTGTAA
ATTGTGTGAACGATAATATCAATGATAAAAAGAACCAAGAGATAGCCCCTAGAAATTGTAAAATAGCAGAAGA
AATTCTAAAACTCATATAATCATATATAGCTAAAACAAGTGAAGGTAATGCGACTAATACCATTATAATGATG
TTGAGGGTGAATAAATATGGCTGTTCAAAAGTTACTGTGTTTGGTCCTGTTGGATGCATTGCTGCTAAGAACA
AGCAAACAATCGTTGATAAAATTGCTAAAATAATAAAAGTAATTCGAACTTTCATCATGACCATCCTTTGTTT
ATAGAGTCAATATAATTATGAAATACGTTAGAGATATAGTCAAATGCGTCAACTAATGGGGATTTTAGGATGG
ATAGAGAATTTAAGGCAATTAAAAAAGCATCAAACAGTAATATGCTGCTTGATGCCCAAATGATGACTTTAGC
TAAATTGATTAGTCACTTTTAAGGATAAAGAATTGCCATGAGTTAAAGCACATGTAATGATGTGTTACATGTC
GCAATGATGGCATTCAGTTATTTATCGATAACATCACTCTTGATACCTTTAGATTTTAAGAAATCTTTAATTT
TATCTTGTTGCTTTTTATTAACATCACCGGCATATTTTGTTGGCACGTCGACTACATTGATTTTATTTTGCGG
TTGATAGCTAAGCTTTTCAATATCTTCATCAACATTGGCGATTGTACTATTTAAAGCTTTGAAGTAATTCATC
ATTAATTCAACGGGTTTCTTATATTCTTTAGGAATATTGTTTTCAGTGACAAATTTCTTGAAATGCAAATCAT
TTTTTACAGCTAAGTTAGATAAGTGACTAAGTGTTTCTGCTTGTTTTTCAGTCACTTTTGTTTGACTATCAAT
TTGTTTATCTAGTTTATGTTGCATTATATATTTGTTATCAAGTATATCGCTATTTACAGACAAATACTTTTCT
ATAGCTTGCTTCATTTCTGCATCACTAATATCACTATTTTTCTTATCTGAGTTAAAGATATCTTTTGTTTCTA
ATTTTTTAGCGCTTTTAGGTGCATGGATGCCAGTACTTGTGTGATGATCTTCGTTATCAGATTGATCGGACGC
GCAACCTGTAAGAATTAATGCAGATGCTAAAAATGTACTTAGTAGTAATCTCTTTTTCATAATGTAATATAAC
TCCTTGGTTTATCTTTAATTGAAAAAATGTGTATTCATGTTTAATAGAGTAACATTGAATTAGTTTGGAATGT
CACGATGACCTTGCAATGACCATAGACGTAAATGATTACGTGCATGAGTTGCTTTTTCTATCAATAATGCATC
ATTTTGGACGTTGTTAAGGATAGCTTTATCTATAAATAACTGCATAATTGGTTGTATCAATTTAGATGTAGGT
ATTGTACGTAAAAGCATAATGATTTCATTCACATATTTTTCCTTCTCAATATCATTTTTCATATTGATTTGTT
TGCGTAGATAACTTGTGAACGCTTTAATTTCTGAATCAGATTTCAAATAGTGGTGTCTATCATAATAGATTAA
TTCCTCAACAGAAGCTGGTACATAGTACCCTTCATCAGAAATATTAATGCCTGACGCCTCAGACTCGACAACA
TCAAATAACACAGGGTGAAACACGAAGTCATCTTCAATATAATATAGCTCAGTACCAGCTAAAGTATCTAAGC
TATATTGCAACTCTTCTTTACTAAAAGTAGCGTCATAAAATCTTTGTGTGAGATATAGTAGATGATTAGTTGA

TAAAATACCATACAAGTTTACAGAACCAAGTATAATACGAAGAGGTAAAATATCTTTTTTATCTATTAATCCT
TTCTTAATCTCAGAAATCAATTCAGTGATTGCATCATTTCTTAAACTATCGGTTGTACCAAAATCTTCAAAGT
CATCATCTTCATCATATAGTATATCGAAGTGAACTTCGTCATCCATCATTGATAATTCTATCGCATTTTCAAA
ATCTGCTAAACTATGCTTAATCGTTTGCTCATCAATAAATTCATTGATAGCCTCTTTTATATCGCCATCAGCG
ATTTGATTCAGGTAATTTTCAATATGTTTACGCACGTCATTTGGTAAAATGAGACAATCATACCCGAAGTCAT
AATAATAATAGAAATGAGTAATTAAAAATGTGCCAGGATTCATCAAATGAATGTTTTCAAATGTAATTAGTTC
ATTGTTTAATCGATAGTTATCATCACTTTCGAGTATGCCAGTAAGAATACTAAACTCAAATAGGGAAGCATTA
GAAAACACTTGTTCAATGATATGATTATTATTGAAAAATGCTGTCATAATCAATTTAACAAGATTTTGCTTGT
TTTTATTTGAGAATCCTTTGATACCATACTGTCTGCAAATTGCTTTTAGCTCATCATTAGTTTTTAACAGTAA
GGCCTGTTCCATTGTGACATCGTTTAAGCGCGTATGTTGTCTTACACGAAAGATTTCATCATTTAAATGCATA
TGGTCTTTTGTATCTAATGTTTCGAATTCACCATATGCACTATTTAGTACCTCTGAATTGATATAACTTAAAA
ATAAATTATTTACAAAGAACGTATCTTCGTTAATTTGAATAACAAAACCTAGATGATTTGTTAGCTTTAAAAT
ATTAACCTCATGGCTATTTATAATTGTGACAGGATTACCCTTAGATATAAGTGTTTCTATAAATTTTTGTTCT
GTCTTATTCATATCATGATAAATCTTATGTAAATTTTCAGGACGTAAAGCATGCATCGTGACTTCAAATCCTT
CTTTGTCGTTCGGTAAATTAAAATATTCGCATGCATTTTGTAACTTTCCTGGGTAGATAAAATGAGCATCAAG
CATTTTTACACACCTCATTGTATTTTTTAAGTTTATCATAACATGATTTTATGCCAGGATAAAAAAATAACAG
CATCTTAACAAATGTAAGATACTGTCAGTGAAACTGTGAAAACTTTAGTTTCTGATAATATAGTCAAAGGCAT
TTAATGCTGCATTTGCACCAGCGCCCATTGAAATGATAATTTGTTTGTTCTTCTGATCTGTGACATCGCCAGC
AGCAAATATTCCAGGTACGTTAGTATTATTGTTACGATCAATAACAATTTCACCACGTTCGTTTAATTCAACA
GCATCTTTTAACCATGATGTGTTTGGAAGTAAACCAATTTGAACAAAGATACCATCTAAGTTAAGTAGATGTT
CTTCGCCGGTGCTCATGTCTTCGTAACGTATACCTGTAACATGGTCTTCTCCGACAACTTCAGTAGTTTTGGC
ATTTGTTTTGATATCAACATTTGATAAAGAACGTAAACGATCTTGTAACACGTTGTCTGCTTTTAATTCGCTA
GCGAATTCGAATAATGTAACATGATTAACGATACCAGCAAGGTCAATTGCTGCTTCAACCCCAGAGTTACCGC
CACCGATAACTGCTACGTCTTTATTTTCAAATAGAGGTCCGTCACAGTGCGGGCAGAATGCAACACCTTTATT
AATCAATTGCTCTTCACCTGGAATGTTTAGCTTACGCCAACCTGCACCAGTAGCAATAATGACTGTTTTACTT
TCTAAGACAGCACCGTTTTCTAACGTAACTTTAATTGCTTCGTCAGTCTTTTCGATATCTGTAGCACGAATGC
CTGTCATTGCATCAATGTCATATTGATCAATGTGCGCTGCTAAGTTAGAAGAAAATTCAGAACCAGTTGTTTC
TTTAACAGTAATGAAGTTCTCAATACCAGCAGTATCATTAACTTGGCCACCGATACGATCAGCAACTATACTA
GTACGTAAACCTTTACGTGCTGTGTAAATCGCTGCACTACCACTAGCAGGACCACCACCAACGATTAAGACAT
CATAAGGTTCTTTATTTTCAAACTCAGATGCATCTGCCGTACTGCCTAGTTTCGAAAGAATATCTTGGATTGT
CATACGACCATTGCCAAATTCTTCGCCATTTAAAAAGACAGCAGGGACTGCCATGATGTTTTCAGATTCTTCA
CGGAACACTGCACCATCAATCATAGAATGCGTGATGTTAGGGTTGATCACACTCATTAAGTTAAGTGCTTGAA
CGACATCAGGACATTTTTGACACGTTAAACTAATGAATGTTTCAAAATGGAATGAACCTTCTAAGTTTTTAAT
TTGGTCAATGATTGACTGTTTTTCTTTAGGTGCACGACCACTAACCTGTAAAATTGCTAAAACAAGTGAGTTA
AACTCGTGACCTAATGGAATACCTGCAAATGTTATACCTGTTTCTTCACCAGGACGATTGACTGAGAAACTTG
GTGTACGTTTTAAAGACTTTTCAGAAAGTGAAAGTCTAGGTGACATATCAGAAATTTCTGTTAATAATTCTTT
TAGTTCTTTAGATTTATCATCTGAACCAAGGCTGGCAACGAATTCAACGTTGCCCTCCATTAGTTCTAATAGT
TGTTTAAGTTGTTGTTTTAAATCAGCATTAAGCATGGTTGTAATGCCTCCTTAGATTTTACCTACTAAATCTA
AACCAGGTTGCAATGTTTTAGCGCCTTCTTCCCATTTAGCTGGGCATACTTCGCCAGGGTTTTTACGAACATA
TTGAGCTGCTTTGATTTTGTGAGCTAATGTACTAGCGTCACGTCCAATTCCGTCAGCGTTAATTTCAGATGCT
TGTACAACACCGTCTGGGTCGATAATGAATGTACCACGTTGAGCTAAGCCAGTAGCTTCATCTAATACATCAA
AATTACGAGTGATTGTTTGTGATGGGTCACCAATCATAGTGTAAGTGATTTTGCTAATTGCATCTGAATGGTC
ATGCCATGCTTTGTGTACGAAGTGAGTATCAGTTGATACTGAGAATACATTTACGCCTAATTTTTGTAATTCT
TCATATTGGTTTTGTAAGTCTTCTAATTCAGTTGGACAAACGAATGAGAAGTCAGCAGGATAGAAGCATACTA
CGCTCCAAGAACCTTTTAAATCTTCTTGTGTAACTTCTTTAAATTGATCTTTTTTTGGATCGAAAGCTTGCGC
TGTAAATGGTAAGATTTCTTTGTTAATTAATGACATAAATATCTTCCTCCTAAGAATTTAAGTATGAATTAGA
ACTATCAATTGATTGTGCTTAATTATAATAATTCTAATCTCTTAGTTAGCATTATTACATTTTGATCCAGAAT
AGTCAACTGGATAACTTTGTAAAGTGAATGATTACTTTTAAAATAAAGAAAGATAATATAAAGTGCTTTGATA
ATGGATTTTGTAGTTGATGATTTAAAAGGTTGTGTCTATATTTAATATCTTGATTTTAATGTAAAAAATGTAA
AAAAAGAAGATTTGTATTCTCAACTAAGTCAACCTTATTGATAATGGTGTGAGAATATTTGTTCGAGATGGAT
GAAGGTAATGCGTGAGAAACTGGATTTTTCAAGTACGAGACAATATTTTTAAAAGTTCAATTATTAACTTATA
AGCAAATAATTGCTATAAAAAAGTTTGGACGTGTACAATTGCAATATGAAGATTTTTAATTAACTGTAAAGTA
TCGAGGAGTGGGTAACGTGTCAGATCATGTATATAATCTTGTGAAAAAGCATCATTCTGTTAGAAAATTTAAG
AATAAGCCTTTAAGTGAAGACGTTGTTAAGAAATTGGTAGAAGCTGGACAAAGCGCTTCGACGTCAAGTTTCC
TGCAAGCATACTCAATTATTGGTATCGACGATGAGAAGATTAAGGAAAATTTACGAGAAGTTTCTGGACAACC
TTATGTTGTAGAAAATGGCTATTTATTCGTCTTTGTTATTGATTATTATCGTCATCATTTAGTTGATCAACAT
GCTGAAACTGATATGGAAAATGCATATGGTTCAACGGAAGGTTTGCTAGTAGGTGCAATCGATGCAGCATTAG
TTGCCGAAAATATTGCGGTAACTGCTGAAGATATGGGGTATGGCATTGTCTTTTTAGGATCATTAAGAAATGA

TGTTGAACGAGTTTGAGAAATTTTAGACTTACCTGACTATGTCTTCCCGATATTTGGTATGGCAGTAGGGGAA
CCCGCAGATGACGAAAATGGTGCAGCCAAGCCACGCTTACCATTTGACCATGTCTTCCATCATAATAAGTATC
ATGCTGATAAGGAAACACAGTATGCACAAATGGCAGATTACGACCAGACAATCAGCGAGTACTATGATCAACG
TACAAACGGGAATCGCAAAGAAACATGGTCGCAGCAAATTGAGATGTTCCTAGGAAACAAAGCAAGATTAGAT
ATGTTAGAACAATTGCAAAAATCAGGCTTAATACAGCGATAGCAAGATACCAAAATAACCCGCCCCCTCTAGC
TTAAAATGATAAGTATAGCTAGAGGGGGCGGGTATTTCTTGCAATGAATTAGTGTGAAGTTAATGCAGCATTA
TCATTTGATTCGAAAGTATCTTTGTCCCAATGTTTAGTTAACTTGGCGGTACCTGTACCAGCTAGCATTGAAT
CGTTCACGTTTAATGCTGTTCTACCCATGTCAATCAATGGTTCAACGGAGATGAGCACGCCGGCTAAAGCGAC
TGGCAAGTTTAACGTTGACAACACCAATATGGATGCAAATGTAGCCCCGCCACCGACGCCAGCAACGCCGAAT
GAACTAATAATCACGACAGCGATTAACGTTACAATAAATTGTAAATCAATTTCTACATTAGCGACGGGTGCGA
CCATAATTGCAAGCATGGCAGGGTAAATGCCTGCACAACCATTTTGTCCAATCGACAATCCAAATGTCGCAGC
GAAATTGGCAATACCTTCTGGCACGCCTAGACGTCTTGTTTGTGTTTGTACATTCAGTGGTAAGGCGCCCGCA
CTTGAGCGTGATGTGAATGCAAAGATTAATACTTCCAAAGTCTTTTTAATATAGCGAATAGGGCTAATGCCTA
ACAGGCTTAAAATAATTAAGTGAATGATATACATCGTAATTAATGCAGCGTACGATGCGATTAAGAATTTTCC
TAAAGTCCAAATGGCGCCAAAGTCACTTGTCGATAACGTGTTGGCCATAATTGCTAATACACCGTATGGCGTT
AAACGTAAGACGAACGTCACAATCGCCATTACTAGTGAATAGATAGCGTCAATCGCACGCTTAAGCAATTCAC
CATGATCAGGTTGTTTGCGTGCTACGCGTAAATAAGCAAATCCTATAAACGAAGCAAATATCACGACAGCAAT
CGTGGAAGTTGCACGTTGTCCAGTGAAATCTAAGAATGGATTTTTAGGCAATAATTCCAAAATTTGTTGTGGT
AACGTATGTGCTGTTAAATCTTTCGCTTGTTTAGCAATTTCGCTTCCACGTGCTTGTTCAGCGTTACCAAGAT
TAATTGTTGATGCGTCTAAACCAAACACCAAGGCATACACAACACCAACAATTGCAGCAATGGTGACAGTGCC
AATTAAAAAGATAAAAATGAGACTACCAATTTTAGCAAACTTTTCTCCGATTTGAATTTTAGTGAATGCAGCG
ACAATAGAAATGAAAATTAAAGGCATCACAATCATTTGCAACAATGCAACGTAACCTTGTCCGACAATGTTGA
ACCAGTCACTTGTTGATGTAATAACATTCGAATGTGTTCCATAGACAAGATGTAATAACACGCCAAATACAAT
GCCGGTACCTAAAGCTGTAAACACACGTTTCGCAAAAGATATATGTTTGCGAGCCATCATGTGCAATATTACG
ATGAAAATCACCAATACAATAATATTAATCAGTGTAAGAAAAGCATTCATGAACGTCACTCCTCAAATTTTTG
AATATAATTCCGACTAGTATGCTAAGAATAATATTAATTTTAAAATATTTCAAGAGGGCATTTGCAATTAAAT
GTAAACATGATGAAATTCGACCGTTGGCATAAGGGAATTTGTAAACAAATCGTTTTGAATTTAGTAGAGAAAG
GTGAAAAGGGTTAATAGTGTTATGACCTAGTCGATTAAAGTTGATATTTGAAAATGAAGCAGGGCATTAAATG
TAATAAATTAAATAAGTTGTCATTACAGCGTTAATAATAGAAATGATTTTAACAGGAAAAAAGTGATGAATAT
TTGGAAAAGATATATATCGTGCACTGTCATAAGAGATAAGATATGAGAAAACAAATGCATCCTTGAATCGAGA
TGTGGCTATGACGGTTTGATAAAAAATTGCTAACCTCATATTTATTGAATAAAAAGATAATAAATGATCAACCT
TGAAAAGACGCTTCGAACAAACACGTTGTAAAAAATTCTGTCGAATCAAAATTAGCATCTTTTGAGTGTTCGT
GTGTGCAGGTGGGAGTTCAAAAGCTATCGTAAAAAAATATTTTTGTTTGAGCAAAAAGGCGTTTGTAGTAAAT
GTGAATATGAGTGAGCCCTTGTGCGATTCGTCATTTACTAACACAGCCAAACATTTAAATTGAATGGGTTTAT
TAAGATGAATCTATTTAGGAATACACAGGAGCCATCCCATTTAAAAGTAACATACTATAAAAAATTTATTCAAA
ATTCAATATTAATAATAATGAATAATTAAAAAACCTGAAACAAATTAGTTTGATATGCTTTCTTCAAAGTAGA
TATTGAATGGAGTATATCAGCTTGTTTCAGGTTTTATGAATTGAGTATTAAATTTGAAAGTAAATTACAGTTC
TATAGGTTTATAAAAAATCAGTTTTTAAATAATAGATATATTACTGAGTCTTACAGGCTGGGACATAAATCAA
TGTTCTATGCTCTACGAAGTTATATTGGCAGTAGTTGACTGAACGAAAATGCGCTTGTAACAAGCTTTTTTAA
ATTCTAGTCAGGGGCCCCAACACAGAGAATTTCGAAAAGAAATTCTACAGGCAATGCAAGTTGGGGTGGGACG
ACGATAAAGAAATACTTTTTCTATAGAAATTAGTATTTCTTATGCATGAGTTTTACTCATGTATTCCTATTTT
TAAGTACACATTAGCTGTGGCTAATGTGTAAGAACCACTACATAATAAATTATTAGTGGCTCTTTATCATTTC
TGTCCCACTCCCTACAGGTTGAATAAACGTGCGAAGAAACCTTTTTTCTCTTCTTGAGGTTTAGGTTCTGACA
TTTCAGACTGTGAAGCTGTGCTGTCATTTTCTGCTTTCGCTTCCAATGAATCGTTCGAAGTAGCTTGTTTAGA
AGCTTCTGCATGGGCTTCTGCTACTTGTTGTTCAGCATCATGTTGCGCTTCTGCATCTACTGTAGCTTCTTTA
TTTGAAGTCTCAGCTGCATTGTCATTTTTAGACTGCTCAACGCTGGCTTTTTCAATTGCTTCAGTTGCAGAAA
TTTGATCCTCTTTATCTACTTTAGTAGTAGTGCCATCGCCTGCATTGTCTGCTTTAGTATCCGTCTTAGCGTC
TGTTGATGTATTCACTCCAGCAACCTTCGAATCTTTACTGTCAGCCTTTTCATCAGTAGCGGACTTAGCATCA
ACTTTATTTGTTGATTCAGCCAATACTTTAGCTTGAGATGCTTGTGCTTCTTGGATAGCCTCTTGTGATGTTT
GGATGGCTTTTAAGGATTCGCTATTTGAATCGATTTTAGATGTAAGTTGATTTTGAAGTTCTTTTAATTCTTG
TTGTTGCTGATGTACTTGATTCATCATTTGACCAAGTAGGTGACGTTCTTCGCGCATTTTCTGGATTTCTAGG
CGTAAATCTTCTACTAATTGAGCGACATTTTGATTAGTAGGTAAGTTTTTGTCATCGTTTTTGACAATGACTT
GCAGGAAGTCTTTTTCTTTTTCTAATTCATCAAACGCTAGATCATAACTATTTGTTTGTTTTACTTTGTCGGC
AATGTCTTTGAAAAGCTCGATATCTTCTTCTTTGAAATCAGTTGCTTCACGACCACGATATTCAGTCTTGCTT
AGTTGGTAACCACGTTCTTCTAAATGTTGAACAATTTTACGGACTTGCTTCTCACTTAGTTCTACGCGTTGTG
CAAATTCTTTAGTTAACATACTTTAAAGTCCTTTCTGCATATACTTTATGTATACTGACGTTTGTCTACGGGC
AGTGAAACGTCAGTCTTGCTTCATACATAGTTTAAACGTTATTGTTAGTGATTTCAAGTTTGTTTAAAATGGT
TTACGTAAATCCATTTCTTCTAATAAAGCATAAATAATTTCACGTGCGATACCATTGGCAAATACATACTTCA

AAATATAATTTGATTTAACACTTGTACCGTAGATATCCTTTGTCAGTTTAATATTTTTCTTTAAAAATAAGTC
CACTTTTTTAGTGTGACCATCATTAGTTGGATGAATGTAATCAATTTCAGTTTGTCTTTCAAATTGATGTTCG
TATAAAAACTGTCTACGGTGTTCAATAATGAGGCCGTTATCTTGTGTTAAATCTGCAGGTTCTTTAGGGACTT
CCATCATTAAAAAGTTAATACTTCTAGAATGAACAAGGTTTGATAATAAGTTTAGTTCATGTTCAATTTGATG
TAATGTTTGCGTCATTAACTCATCATGGTTGGGTAGGTCTTTTGCGAATAAATAAATTAAAAATGCTGAATTG
GTTGTGGCTTCATAATGTGCAGTTGCTAGACTGACAACAACATCGTTTTCTAAGCCTACGATAAAAACATAAT
CGTTTTCGGTTTTGTTGTTTTCTAATGAGCGTTTGATGATACGTTTATCTTCAGTTAAAGATAAATTTAACTT
AGTATCGTAAAGTTTAAGTGCTTCGTTGTAATGTGGATCTTTGACAGATTGAATGGTTTTAAATTCCATAAGA
ACACCTCCCCAATTTAAATAATATTATAGCATAATCGCCTGCTGTAAAAGACTGTTCATAAACTTTTAAATGG
TATAAAAAACTGTACTATCTTAAATTAGACAGTACAGTAATCTCATTTTGAATTCAGTGTGATAACTAAGCTT
TGGGACCTTTAGATGCTTCAGCAAAATGTGTAATATCAATCTCTTCATAAGCTGAATTATTTTCATGCACTTC
TTGATGTGATGATTTGTCACGAACCGCTACAACTAACATTTTATCGTCTAAAATAAGTTGTTTATATTTTTCT
AATTCATCAGGCGCTAAGTTGTAGCGTGATAAAACTGCATGTTCACCATCTTCTCCTGTTAACAGTTTAGTCA
TTCTATCACTAAATGTTCCACTTGTTGAGATAAGGGAGATTTCAGAGTCGTGTAAGTCATTTAGGTTTAATTT
ACTTTTACTAATAATTGTTAGCTCTGATTCTAAATAACCTTCAGATTTCTTTTGATTGATTACGTTGTATAAT
TCGCCAGTGTCATTTACTACAGTAATATCTGCCATAGTTGTCGCCCCTTTAAAAATTTGTTTATTTAATCTTT
TACCCTTCTTATTATAAAGTAAAACCCTTACATTATTAAGTTATAAGTCTTCATTCGCATTAAACGTCTCTGT
ACATTTATAAAACTATTAAAAGATTACCTAAGCAGTTATTGAAAAAATGCCGAAAATTTGCTATTATCGTTAA
ATAATTTACATAAACTCATATAATCTAAAGAATATGGCTTTAGAAGTTTCTACCATGTTGCCTTGAACGACAT
GACTATGAGTAACAACACAATACTAGGAGTAGCTTCAGCCATTAAATTGTAACCATGGTGGGTGATTTATATC
ATTTTATATGATGGTCACAGTTTATTTGATGAAACTTCTTTTACATTGATTGCATGACAAATTCGTTATGCAT
GTTCGTTCACTCATAAACCCTGAAACTATTATTTAGTTTGGGGATTTTTTTGTATCTAGCACCAATTTAAGAG
CAAAATGTTTCACACAAATCTGAGGAGGTTTTAAGAGTGGAGTTACTAGGACAAAAAGTAAAGGAAGACGGCG
TTGTCATTGATGAGAAGATTTTAAAAGTCGATGGATTTTTAAATCATCAAATTGATGCAAAGTTAATGAATGA
AGTTGGTCGCACTTTTTTACGAGCAATTTAAAGATAAAGGGATTACTAAAATCTTAACCATTGAAGCTTCCGGT
ATCGCACCTGCAATCATGGCTGCACTGCATTTTGATGTGCCATGTTTATTTGCGAAAAAAGCAAAACCTAGCA
CTTTGACGGATGGTTATTATGAAACATCTATTCATTCATTTACTAAAAATAAAACAAGTACGGTCATTGTTTC
AAAAGAATTTTTATCAGAAGAAGATACTGTACTCATCATCGATGACTTTTTAGCAAATGGTGATGCTTCATTA
GGATTATACGATATCGCACAGCAAGCGAATGCTAAGACAGCTGGTATTGGTATTGTTGTTGAAAAGAGTTTCC
AAAATGGGCATCAACGTTTAGAAGAAGCAGGTTTAACAGTTTCTTCTCTCTGCAAGGTTGCTTCACTAGAAGG
AAACAAAGTGACATTGGTGGGAGAAGAATAATGAAAAATTTAATCCTAAGTGTTCAACATCTTTTAGCTATGT
ACGCAGGTGCCATCTTAGTTCCAATCATTGTTGGTACAAGTTTGAAGTTTACACCTGAACAAATCGCTTACTT
AGTTACAGTAGATATATTTATGTGTGGGGTTGCCACATTTTTACAAGCCAATAAAGTAACAGGAACAGGATTA
CCAATCGTTCTTGGATGTACATTCACGGCTGTTGCGCCCATGATTTTAATTGGTCAAACGAAAGGAATAGATG
TACTTTATGGTTCGCTATTTTTATCAGGGATATTAGTTATTATCATCGCGCCTTTCTTTTCACATCTTGTAAA
ATTCTTCCCACCAGTAGTAACGGGTAGTGTTGTTACTATCATTGGTATCAATTTAATGCCAGTAGCAATGAAT
TACTTAGCTGGAGGTCAAGGTGCAAAGGACTATGGAGATGTTAAGAACATTTTGTTAGGTTTAATGACATTAA
TCATTATTCTTGTTTTACAAAGATTCACAACTGGATTTATTAAGAGTATTGCCATATTAATTGGACTCGTTTT
AGGAACGATAGGCGCTGGCTTACTTGGGATGGTCGATATTAATCAAGTCAATCATGCCGGTTGGTTAGGCATC
CCAGTGCCGTTTAGATTCTCAGGATTTAGCTTTGATGTGACATCGACGTTAGTGTTCTTTATTGTAGCTATCG
TTAGTTTAATTGAGTCGACAGGTGTCTATCATGCGTTAAGTGAAATTACCGGTAAGAAGTTAGAAAGAAAAGA
TTTTCGTAAAGGTTATACTGCGGAAGGTCTAGCGATAGTGTTAGGTTCTATATTCAATTCATTTCCGTATACA
GCCTATTCGCAAAATGTAGGACTTGTTTCTTTATCCGGCGCTAAGAAAAACAATGTTATATACGGCATGGTCG
TGTTATTGCTTATATGTGGTTGTATACCTAAGCTTGGTGCATTAGCAAATATCATACCGCTACCTGTGTTAGG
TGGTGCGATGATAGCTATGTTTGGCATGGTAATGGCATATGGTGTTAGTATATTAGGACATATCGATTTTAAA
AATCAAAACAATTTATTAATTATCGCTGTATCAGTAGGATTAGGTACTGGTATAAGCGCTGTACCACAAGCAT
TTAAAGGTTTAGGTGAACAATTTGCATGGTTGACTCAAAACGGAATTGTTTTAGGCGCAATCTCTGCAATTAT
TCTTAATTTCTTTTTTAATGGAATAAAGTATAAACAAACGGAAGAAAATGTGAAATAATATAACTAATTAATT
TGAAAAATGGAGGCTGTTTTTAATGTGGGAAAGTAAATTTGCAAAAGAATCATTAACGTTTGATGATGTGTTA
TTAATTCCAGCACAATCTGATATTTTACCGAAAGACGTTGATTTAAGCGTACAATTATCAGACAAAGTTAAAT
TGAATATTCCAGTTATTTCTGCTGGTATGGATACAGTAACTGAATCTAAAATGGCGATTGCTATGGCTCGTCA
AGGTGGTTTAGGTGTTATTCATAAAAATATGGGCGTTGAAGAACAAGCGGACGAAGTTCAAAAAGTAAAACGC
TCAGAAAATGGTGTTATTTCAAACCCATTTTTCTTAACACCAGAAGAAAGTGTTTATGAAGCGGAAGCATTAA
TGGGTAAATACCGTATTTCAGGTGTACCAATTGTTGATAATAAAGAAGATCGCAACTTAGTAGGTATTTTAAC
AAACCGTGACTTACGTTTTATTGAAGACTTCTCGATTAAAATTGTAGATGTAATGACGCAAGAAAATTTAATT
ACAGCTCCAGTGAATACAACACTTGAAGAAGCAGAAAAAATTCTCCAGAAACATAAAATTGAAAAGTTACCAT
TAGTTAAAGACGGACGTCTAGAAGGTCTTATTACTATTAAAGATATTGAAAAAGTTATCGAATTCCCTAATGC
AGCAAAAGATGAACATGGTCGTCTACTTGTAGCCGCAGCAATTGGTATTTCAAAAGATACTGATATTCGTGCT

CAAAAATTAGTCGAAGCAGGTGTGGATGTCTTAGTTATCGATACAGCACATGGTCACTCTAAAGGTGTTATCG
ATCAAGTGAAACATATTAAGAAGACTTACCCAGAAATCACATTAGTAGCAGGTAACGTAGCAACTGCAGAAGC
AACAAAAGATTTATTTGAAGCGGGTGCAGATATTGTTAAAGTTGGTATTGGCCCAGGTTCAATTTGTACGACG
CGTGTTGTAGCAGGTGTTGGTGTACCACAAATTACAGCAATTTATGATTGTGCAACTGAAGCACGCAAACATG
GTAAAGCTATCATTGCTGATGGTGGTATTAAATTCTCAGGAGATATCATTAAAGCATTAGCTGCTGGTGGACA
TGCGGTTATGTTAGGTAGCTTATTAGCAGGTACTGAAGAAAGTCCAGGCGCAACAGAAATTTTCCAAGGTAGA
CAATATAAAGTATACCGTGGTATGGGCTCTTTAGGTGCGATGGAAAAAGGTTCAAACGATCGTTACTTCCAAG
AGGACAAAGCACCTAAGAAATTTGTTCCTGAAGGTATCGAAGGACGTACGGCTTATAAAGGTGCGTTACAAGA
TACAATTTACCAATTAATGGGCGGTGTGCGTGCTGGTATGGGTTATACTGGTTCACACGATTTAAGAGAATTA
CGCGAAGAAGCACAATTTACACGTATGGGTCCTGCTGGTTTAGCAGAAAGCCATCCACATAATATTCAAATTA
CGAAAGAATCACCGAACTACTCATTCTAATTAAGATAAAGGAGAACGACAAATATGGAAATGGCAAAAGAACA
AGAGTTAATCCTTGTCTTAGACTTTGGTAGCCAATACAACCAATTAATTACACGCCGAATTCGTGAAATGGGC
GTTTATAGTGAATTACACGATCATGAAATTTCAATTGAAGAAATTAAGAAAATGAATCCAAAAGGTATCATCT
TATCAGGTGGTCCAAATTCAGTTTATGAAGAAGGTTCATTTACAATTGATCCGGAAATATATAATTTAGGAAT
TCCAGTACTTGGTATTTGTTACGGCATGCAATTAACTACTAAATTATTAGGTGGTAAAGTTGAACGTGCCAAT
GAACGTGAATACGGTAAAGCAATCATTAATGCGAAGTCAGATGAGTTATTCGTTGGCTTACCAGCAGAACAAA
CTGTTTGGATGAGTCATTCTGATAAAGTTATTGAAATTCCAGAAGGCTTTGAAGTTATCGCTGATAGCCTAAG
CACAGACTATGCAGCAATCGAAGATAAGAAACGTCGCATTTATGGTGTTCAATTCCATCCAGAAGTACGTCAT
ACAGAATATGGTAATGATTTATTAAATAATTTTGTCCGTCGTGTTTGTGATTGTAAAGGTCAATGGACAATGG
AAAACTTTATCGAAATCGAAATTGAAAAGATTCGTCAACGCGTAGGAGACCGTCGTGTATTATGTGCGATGAG
TGGCGGCGTAGATTCATCTGTTGTAGCTGTACTATTGCATAAAGCAATTGGTGATCAACTAACATGTATCTTT
GTAGACCATGGCTTACTTCGTAAAGGTGAAGGCGACATGGTTATGGAACAATTCGGTGAAGGGTTCAACATGA
ATATTATTCGTGTTAATGCGAAAGATCGTTTTATGAATAAATTAAAAGGTGTTTCAGATCCTGAACAAAAACG
TAAAATCATTGGTAATGAATTTGTATATGTATTTGATGATGAAGCATCAAAACTCAAAGGTGTAGACTTCCTT
GCGCAAGGAACACTATATACAGACGTCATCGAATCAGGTACTAAGACAGCACAAACAATCAAATCACACCACA
ATGTTGGTGGATTACCAGAAGACATGGAATTCGAATTAATCGAACCAATCAATACATTGTTTAAAGATGAAGT
ACGTAAATTAGGTATTGAACTAGGTATTCCAGAACATTTAGTGTGGAGACAACCATTCCCAGGACCTGGTCTT
GGTATTCGTGTACTTGGAGAAATTACTGAAGATAAACTAGAAATCGTTAGAGAATCAGACGCGATTTTACGCC
AAGTGATTAGAGAAGAAGGTCTTGAAAGAGAAATTTGGCAATACTTCACAGTGTTACCAAACATTCAATCAGT
AGGTGTTATGGGAGACTACCGTACGTATGATCACACAGTAGGTATTCGTGCAGTAACATCTATCGACGGCATG
ACAAGTGATTTTGCACGCATCGATTGGGAAGTCTTACAAAAGATTTCAAGTCGTATCGTAAACGAAGTAGACC
ACGTCAACCGCGTAGTCTATGACATTACATCAAAACCACCAAGCACAATTGAGTGGGAATAATTATATATAGC
AAATGATAGGCTAGAAGTACCGTTATTACGCGGTTTCTAGCCTTTTTTGGTGCGATTGGTATGGGCAACATCA
TGTGATGAAATGATGGGAGTTATATCTGTAGAAATTGGCGTACCAATTTCTTTATGTTGGGGCTCCGCAGTCA
ACTACTACCAATATAAGATTGTAGAGCCTACGATTTGGATTTTATGTCCTGGGATCGGTTTCCTATTCTTATT
AAATAGTATTTAAAAATCTATACTATCTATATTGAAATTTTTATTTATGGTTATGCCATAACTACTTTGTATT
TCCGGTACTAAACTTTGCAATATTGGGTAATAGTTATTAGCGAAACCAATAATAATGGAATTAATCACATTGA
TACTAAGTTCTTTATCTTCAGGTAATGCATTTTCTATTATTTTGATTAAGTCTATATATTTTATTTCATCAGA
ATTTTTATGTGCTAAATACTTCATAAAATCAGTTTGCACTTTTTCGAAAACACTATATCTTAAGTCCATTTGT
ACAAATATTAAGCTGACAGTTTTAGTTTCGAATTGACTTTGACCCAAAACTGAAGCAAGTTCTTCTATTGATT
GATATAGCAATCTGATATCGCCGTTATCTTCAAGCATATGAAACACTCCTTTATCATTTTAGAATAACCATGC
AATGGCTTCTTTGACTACAGTTGAGATTATTCCAGCATCACCTTTACTTAGACCGGCATTTATTGCGCCTCGT
TTAACTGCATCACCGACAGCGTTAGCTGGTACTTCTGACCATTTTAGCAATGGTTTCAATTCTTTTACAATAC
CTGAATACCCTTTATCAAAAATAGTTGCAGCTTTTTTGCCGCCAACTTGTTGTATGACATTTTTTGCTTTGCC
CCTATTATTAATAATTAGCTTTATAGCAGTTTTAACTACTTTTGTTCCGCCAATTCGTTGTTGTGCTTCTGGA
TTCATTGGCAAGTTTGGATTGCGATTAATTTTCACACCATTTATATTTTCATAACTTTGGTGAGCAGTTTTTG
TATTGTTTGTCTGTATTTCATTTGCGTTAGTTAAAGGTGAAACAGACGTTAATAAAACGGCAGTCAAGACTAG
TGAACCAATAGTTTTTGTGATTTTCATTATACATTTATCTCCTTTAATAGTGAATTTGCAAAACCAAATGTTT
GAAATATAAGTAAATAATCATTAAAAAACAAAGTGAGTGAAAGGTCCTAATTCGTATAACTAGCATTTAATAA
TTTAATTAATCGTTTGCACCAAAATAGAAAATGCCACTTAACACAATTATTAAAAGTAATGTAACTATGAAAG
AAGTCCAAGCTAATACAAGTGAACATATTAAAAGTACTAATGAAGTGACTGTTTGTAAGAACCCAAAAAGTAA
AGATTCCATTATATAAGCAAAATAAATAGTTAGTGAAAAAATTGCTACAGCAAATAACATTAGCCCTATGATT
TTCAAAATTCCAGTTGTGTAGCCAGTAACTAATAAATTTTTCACATGAACTTGATTACCATATTGTCATCCCG
TTCATAAAATAGTTTAGAAGAGATTGCTATTAAATTTAATTGTTTTGCATAAGTGTCAGAATATTATTCTTAA
CTGTTAAGAGTCGTTAATTTATTTAAATCCATTAAGGTTGTTAGAGATTATTATGCGGTGAGAAACGCGACTG
TTACAGTGGAAGATGAATTTGTATAACTAGATATGTTAATCGTAAATGTGGCGTCGTGAAACACGACTTCAAA
CATCGAGGACCAATCTATCTTTGAGATTGGTTGAAGATTGAAACATATGTAAGAAAATGACATAGTTTATGGA
AAGGTCGATAAGTAATTGAAAAATCAATCTAGATTGAAATCATGCATATCGTTTAATAAAATTTTTCGGAGAA

ATAAAAACTTACTAAAAATGAAAAGGTTTATTTGATTTATTTACTTGTATATTGTAAAAGGTTACAATGAGTC
TATAGATTATGTAGAGAACTCTATTATGCTTTTTGAGTATATGTGGGGTTCTTTCTTTATATATAAAAGGAGT
TCGGAGTGGCAACACTAAAAGATGTAAAGACTGGTAAAAAGGAAATTTTGCCATCAACTGCAAAAGATAAAAA
TGGTAAAAAAAGTTACGTTAATTTATTTTGAAAAAGACGATAAATTAGGTTTTTATGTGCAGAAAACACAAAAA
GAACGTGGAGTAGGGAAATGCGTTTCTGGTATAGCGGGTGGCGCAGTGACAGGAGGCACTACTTTAGGTCTTG
CAGGTGCAGGAGTAGGAACAGTTACTATTCCAGTAATTGGGACAGTTAGTGGAGGCGTAGTTGGAGCTGTTGG
TGGTGCTGTCGGCGGTGGTCTAACCGGTGGAGCCACATTCTGCTAATAGTGTAGGAGTGATACCATGAAAAAA
TTTTTTAATATATGGGTATGTTCGTTCTTATTTTTTAGTAATAATATCATGGGTGATAATTAGTATAAAATTT
GGTAATATCAGCACGACGCAAATATTAATTTCAAATCTGTTTATATTTATCGCTTATATACTATATTTAGTTA
AGTTCATTCGTTTCAAAAAAAAGATGAAAATACGTTTTTTGCCCAAAACCTAATTGTGATAGCATATGAAAAGT
GAGTAGTGTCCTTTTTTTAATCTAATTTACTTATGAAGCGAAAATAACGTGCAAGTAACGTGCAAGTAAAAAA
ACGACCTAAATCCCTTTAAAAACAAGGATTAGGTCGTATTTATTTGGTAATGAATTTGTCTAGCGAAAATAAC
GTGCAAGTAACGTGCAAATAAAAAAGCATGCGTAATATGGTATATATTTTATATGTCTAGGTAAAGTTTACGA
ACCTACAGATTTGAAATATTCTTCAGCAACTCCGTCTTTTATAATTCTTGAAATAACCGAGGTTTTTATTTTT
ACGTTGTTTTGAATTGTCTAAAATAGTCATACATAATCTGTTTAAACCCTTACTTAAGCTTGTAAAATGCGGA
AATAAGTTTTCCTTTTTTAGTTATTTTTTCTTGGTATATTTTAAATCCACAATTATTGCAATAAAATTCTTTC
AAATTCTTATTATCATACCAATCAACACAAATATGTCTTAGTGCAGTACGATCATAAATTTCAGAAGCAATAG
ATAAGGCATAGTCCATTACAATTTCTCCTGGTATATGCTTTTTGTGTTTATCGCTCCTATCTAATTTAGTTAT
TAATAATGCTTGAAATTCTCCATTCTTTTTAGCATCAGACGAGATTTTTCTTTTCGTTTTTCCACTAACGTCT
TTTTCAAGCTTTATATTTATAATTTTTAAGCAGAAGTATCCCATTATTTCAAGGTCTGATTTATCTATAATTA
AATACGTACGGAAGGAATTTTGATCGTTCATTGTTATTGCTTTATGTTTTAGGGAATGTGCTATTGAAGGGTT
TAAAGATGTTTGAAATGTCTTGGTTTCTTGACTTATATAGTTATGAAAGAGGGCGTTGTCCTTTATAGCATTT
CTGAGATTAGAAATGCTATAAATAAAGTTATTAAGATTTTTTGTTTTCATGTAATTTAAGAAATGGATTTGTT
TTTAGTGGGTTATTTTTAATTCTGTTCTTAAATGGTTTGAATTTTGCGGTTGCAAATATGATAGCAGCTTCTT
TTGACAAAGTAATAGAACCTTTTTCTTCTGGTACCATGATATTCACTCCTTGTACAATTGGATTTAAAGTTTA
TAAAATAATTTTACTATGTATAATAATTTGTGTAAATAGTTTCTTATTGTTACCGAATAATTTGTTTGGAAGC
GTTATGTAATGCCAATCAACACCAAAGTCCTCATGTCCTCATTCCTTTTGCGACTACATATATTGTTAATAAT
ATTGAAATTATAAACTGATAGTGATAATTTTGTTAACTGACCCATGAAAGTAGGAACCCCACCATACGGGAAT
TTCGAAATCATGGTTTTTTTGTTTTAAAGTATTGATGCAATAGATAAAGTAGCCATTCTAATATATGGAGTAT
TTATCAGCTAAAAATTAAAACAGATATGAATTTTCTTCATTAAATCATTTTTCCATATAGAAACAGAGGCAAC
GCTACTGTCACCTCTGCTTGAAATAATTAGGTTGAAATACTCTGGGTCTAAAGTCCACACCTAAATCACCCAA
ATCGCTCAAACTCTCAACCCCAGACTAAATCTCCAAACCCCAAAACACCAATACTTACCAAAGCATTGAAATG
AGTGCCAATACTGGCAACGTACCTTGTTTGAAAAAGATACTAATATTGCTTGAAAGGCCACCATAAATAGCAA
CGCCAATGATATACACTAAAATAGCTGCGCATATTTCTTTTGGATTACTGCTGATAAACAAACCGTATATTAG
CAAAACTCCGATTAAACCATTATATACGCCTTGATTCTTCAAAAGTACGTTAATATTTTTGTCTTTCAATTTA
TCGACGCTTATATTAAATGTCTCGCTAGTCTTTTTGGAAGTTGTAGCAATCGTTTCAAGGTACATAATATAGA
AAAATTCTAATGCCACAAATATGATTAAAATTATCGAGATGATATTCACTGTAACGCTCCTTTAGTATTAAAT
ATTTTCTTGTAAAAATGATTGTAGTGTCTGTGGTTGATCATTGACTAATTGTTGGAAATCATTGGATTCTTGG
TCTAATAGTCCTCTTGCTCCTGCGTCGTACATTGATGCCAATAATGCACCAAAGCCTTTAGGTTCATCGTACA
TTTTTGCAAATGTCTCTAATGAAACGGGTTCATATTTAATTTCTGTGCCTGATGCCTCAGATAAAATAGCAGC
AAGTTCTTTCATATCATAACTGTAGCCTGATAATAAATAACATTTGCCCCAAGTATCTGGATTTTTAATAATA
GCAATGACGCCTCTAGCAATATCATTTCTAGTAATATAATTAATACGACCATCACCAGCTGGATAAATCAGTT
TATGCATATTCATCAATTCTGGTAAATATGGTTTAAGTGGATCCATGTACATTGCCATTCTTACATAGGTATA
GTCAATGCCACTTGTTGCCAATAGACGTGCTGCATAACCAAAATAAGGACTCATATGGAATGGATTATTATGC
TGATCTGCGTAATAACCTATGAAAATGATATGAGCAACGCCACATTGCTTTGCAGCATATACTAAATTTTCCA
CTTCAGGAATACGTTTGAATGATGGATGGATAATACTTGGAATAAACACAACGGTATCCATTCCTTTAAATGC
TTCTACCATGCTTTCTTGATTAAAATAATCTAATTGTCGAACAGAAACTTTTCCACGCCAATCTTCTGGAACT
TTCTCAACATTTCTAACACCAATGTGAAAATGATCTATGTGATTTGCAATGGCTTGATTTGTAATATGTGTGC
CTAAATGACCTGTAGCACCTGTTAACATAATATTCATTCACTTCATCTCCTAATCTTTATATACATAACATAA
TACTTATTCGATGGTTTTCAAAACATTTGATTTTATAAAAAAATTGTAATCTGTATTTATTGTCGTCGTGTATA
GTAAATACGTATATGTTATTAATGTTGAAAATACCGTAATGACGCGTTTTAGTTGATGTGTGTCACCATGATC
TTTGAAATTTTTGACATGGTACTGCGACGATATGGTGTCATTTTTGTGTCTAAAAGTTTTACAGTTTTTAAAA
TTAAAATGGTATAAAGTGTGAATTGTATAAAAAAAGAGTCTCGACGGATAAGAATTGATTAATAACAGTTAGCA
TTTTATTAATTACCTTAACAATGATTCAAGTTTAGTTAAATGAGGTTTAATTTGAAAGGGGATAGCGCCTCAA
TATAATGTAGGTAGATTGTTCATATTACGTAATTGAAAAATCAAATTTAAATAGATTGGGGCTAAAAATTATG
AAATTTAAAGCGATAGCAAAAGCAAGTTTAGCATTGGGAATGTTAGCAACAGGTGTAATTACATCGAATGTAC
AATCAGTACAAGCGAAAGCAGAAGTTAAACAACAAAGTGAGGCTGATTTAAAACTTTATTATAGTGGACCAAG
TTTTGAATATAAAAAAGTAACTGGATATGGATTTGTTGAAGGTAAAGATAGATTCATTGATTTTATATACAAT

GGACAATATAATAAAATATCTTTAGTTGGTTCTGATAAAGATAAATATAATGAAGAAGTTAATCCAGATATAG
ATGTGTTTGTCGTTAGAGAAGGAAACGGTAGACAAGCTGATAATCATTCGATTGGTGGCGTAACAAAAACTAA
TCGAGGAGTGTATTATGACTATATACACACACCAATCCTTGAAATCAAGAAAGGTAAAGAAGAACCACAAAGT
AGTCTATACCAAATTTATAAAGAAGACATTTCGCTAAAAGAACTTGATTATAGATTAAGAGAACGTGCAATCA
AACATCACGGCTTGTATTCAAAAGGTCTTAAACAAGGTCAAATTACAATTACAATGAATGATGGCACAACACA
TACAATCGATTTAAGTCAAAAACTTGAAAAAGAGCGTATGGGTCAGTCAGTAGACGGCAAACAAATACAAAAA
ATTCTAGTAGAAATGAAATAATACTTTCTAACAACAAAGCACTACGCTGAATAGCACTTGTTTTGGAATATAT
GGAAGTTAAGCGACGTACTGTTGCTTAGCTTCTTTTTTTGAGGGTCAAAGTTACAAAACTCACACAAACAGTC
GCACCACGCATTATCTTTTACTTAACTAGCTTAATCATATTTTATGAATAGTTAAAAAAGGGTTAATGTGAAT
ATCAGAATACAGCTCCTATAATATGGTTGTATGATTCAATTTACGTAATAAAACAATCTAATCTAATATATTG
GAGCATACAACTATGAAAATGAAATCAATTGCAAAAGTAAGTTTAGTCTTGGGTATTTTAGCTACAGGTGTAA
ATACTGTAACGGAACAACCGGTGCATGCCGAAAATAAACCTATTCAAGTAAGCCAAAATAGTAAAAATTTAAA
AGCGTACTATACTCAACCGAGTGTTGAATATAAAAATGTGACAGGTTATATCAGTAGTATTCAACCTAAAGCA
GGTACTAAATTTATGAATATTATAGAAGGTAATACAGTTAATAATCTAGCTTTAGTTGGCAAAGATAAGCAAC
ACTATCATACGGGTGTACATCGTAACCTTGATATTTTTATGTTAATGAGGATAAGAGATTTGAAGGTTCAAA
GTACTCTATTGGTGGTATCACAGAAGCAAGCGATAAAGCTGTTGACCAAATAGCAGAAGCAAGAGTTATTAAA
GAAGATCATACTGGTGAATATGATTATGACTTTTTCCCATTTAAAATAGATAAAGAAGCAATGACATTGAAAG
AGGTCGATTTTAAAATAAGAAAACATCTTATCGATCATTATGGTCTTTACGGTGAAATGAGTTCAGGAACAGT
TACTGTTAAAATGAAATACTATGGCAAGTATACATTTGAATTAGATAAAAAGTTACAAGAAGACCGAATGGCC
GATATCATTAAAGTCACAGATATTGACAGAATTGAAGTTATAGTTAAAAAAGCATAATGCTTAGACTGGTCGT
CGTAATGAATTGAAATTGCAATAGAGAGGTTAAGTGATGATAAAACGTTACTTAACTTCTTTTTTATGTCTTA
AAATCATTTCAATGATACGTAGTAACACCACATAAACCTCGAAAGTACTCATTATTTTTTGCTTAAATGACTT
AATAATACTTCAACAATTGTTAAACGAGGGTTAATGTGAATAGACCAATATGTCCTCTATAATGAAGTTGTAT
GATTCTAATTACGTAAAGAACAATCGAATATAATTACGATTGGAGTATACAACTATGAAAATAAGAACAATTG
CGAAAGCGAGTTTAGCGTTAGGGCTTGTAACAACAGGTGCAAGTATAGTAACAACGCAATCGGCCAATGCAGA
AGTAGCATCAGCACTTAAAGCAGGACAGTTAGCAAAGATAAACGTATCATCAAGTGCAGTCACAACGACAACG
CAAGCAGTGAACGCAGAACAAAATCGTACGGCTAATACTGAACAGGCATCAAAGTCTAATACAGAAAAAGTAT
CAACATCGCCTTCAATTAAAACAGAACAGACGCCAAAATCTAACACACAAAAGGCAACTCAACCAACACCAAG
CGGAACAACTCAGCCTTTACCAAATGCACAACCGCAATCACCACAATCAACACCAAACGCATCAACATCGCCT
TCATCTAGTGTAGAAACACAACAACCGCAATCGCCTACCACAAAACAAGCACAAAAGGAAATAAACCCTAAAT
ATAAGGATTTAAGAACATATTATACGAAACCGAGTTTAGAATTCGAAAAGCAATTTGGTTTTATGCTGAAACC
ATGGACGACAGTTAGGTTTATGAATGTTATACCAGATTGGTTCATTTATAAAATAGCTTTAGTTGGAAAAGAC
GATAAGAAATATAAAGATGGGCCATACGATAATATTGATGTATTTATCGTTTTAGAGGACAATAAATATCAAT
TAAAAAAATATTCTGTCGGGGGTATCACGAAGACGAATAGTAAAAAAGTTGATCACAAAGCAAGAATCAGTAT
TACTAAAGAAGATGAGAAAGGCAAAATCTCACGCGATGATTCAGAATACAAGATTACTAAAGAAGAGATTTCC
TTGAAAGAGCTTGATTTTAAATTGAGAAAGCAACTCATTGAACAACATAATCTGTACGGTAATATTGGTTCAG
GAACAATTGTTATTAAAACGAAAAATGGTGGGAAGTACACGTTTGAATTACACAAAAAATTACAAAACATCGC
ATGGCAGATGTCATAGATGGCACTAGTATCGACAGAATTGAAGTGAATCTAAAATCATCGTGATGGATACTGC
ATAGAACCTATGAACGATGTGCCGAGAAGTTGAGTGACAACTATTTACATGTTGCTCAGCTTCTTTCGTGTTG
CGCAGTGATATAAGAAGACGAATATTTGATGTTGACCACACATAACATACTTATGTCCTAAATGTGTCTAAAA
ATCCCAATCTATCAAGCGTTGAAGTACAACAAAAGTCATTTTTATTTAACGAACATTATGGATTCCTTAATTT
AATTAACGATGATTCAAATTTAGTTAAACAAGGTTTAATGTGAATAGAGTCATACGCCAACTATAATAAAGCT
GTGTGATTCAATAAACGTAATCGAACAAGTCTAATAATTACGATTGGAGCATACAACTATGAAAATAAGAACA
ATTGTGAAAGCCAGTTTAGCTCTAAGTATTTTAGCAACTGGGGTTATAACATCAACGGCTCAAACTGTAAATG
CGAGTGAACATGAAGCAAAATATGAAAATGTGACAAAAGATATCTTTGACTTAAGAGACTACTATAGTGGTGA
AAGTAAAGCGCTTAAAAATGTTATCGGTTATCACTATAGTAAAGGCGGTAGACACTACCTTGTCATTGATAAA
AATAGAAAGTTCACAAGAGTACAGATATTTGGTAAAGATATTGAAAGATTTAAAGCACGCAAAAATCCGGGAT
TAGACATATTTGTTGTTAAAGAAGCGGAAAACCGTAATGGTACAGTTTATTCGTATGGTGGTGTTACTAAGAA
AAATCAAGGCGCCTATTATGATTATATAAACGCACCAAGATTCCAAATCAAGAGAGATGAAGGTGACGGTATT
GCTACGTACGGTAGAGTACACTACATTTATAAAGAAGAAGTATCACTTAAAGAATTGGATTTTAAATTGAGAC
AGTATTTAATTCAAAATTTTGATCTGTATAAAAAGTTTCCTAAAGACAGCAAGATAAAAGTGATAATGAAAGA
TGGCGGCTATTATACGTTTGAACTCAATAAAAAGTTACAAACAAACCGCATGAGTGACGTCATTGACGGTAGA
AATATTGAGAAAATAGAAGCCAATATTAGATAATTCAATAAAACATGGATAATAGTAAAATATGGATAGTATA
GAGGAGTTAGGCAGCATAGGTTGCTTAGCTTCTTTTTTGTGTCGTTATGATCGGAAATGAAGCGGGTTGATGA
AAGATAATAACGACAATAAAACTTGTAAAAATAGTTGATACCTATAGTCGCACGGTGTCGCATTAGTGACGTG
AAACAATGTGGAAAACATAATTAAATTGAAGGAAAGTGTTAATGGTGAAAAAAATTAGCATAGTGTTATAAAA
AATGATTAATACTGTTAGGAATCCATTAATTAGCTTAACGTTGGTTCATGAATAGTTAAAAAGTGGTTAATTC
ATAGCGCAATATCCGGCTTATATAATGATAGTAGATTGTTCGTATTACGTAATTGAATTAATCATATAAGCAT

ATATTAAGTTAAATTTTAAAAATAGATTGGGAGAATAATACTATGAAATTAAAAACGTTAGCTAAAGCAACAT
TAGCATTAGGTTTATTAACTACTGGTGTCATTACATCAGAAGGTCAAGCAGTAAAAGCGGCAGAAAAGCAAGA
GAGAGTACAACATTTACATGATATTAGAGATTTACATCGATACTACTCAGCACCAAGTTTTGAATATAGTAAT
ATTAGCGGTAAGGTTGAAAACTACAATGGTTCTAACGTTGTACGCTTTAACCAAAAAGATCAAAATCACCAAT
TATTCTTATTAGGAAAAGATAAAGAAGAATATAAAGAAGGTCTTCAAGGTCAAAATGTCTTTGTAGTACAAGA
ATTAATTGATCCAAACGGCAGACTATCTACTGTTGGTGGTGTAACGAAGAACAATAACAAAACTTCTGAAACT
AAAACACAATTAGTTGTTAATAAAGTTGATGGCGGAGATTTACATGCATCAATTGACTCATTTTTAATCCAAA
AAGAAGAAATCTCATTAAAAGAACTTGATTTCAAAATTAGACAACAATTAGTTAATAATTACGGATTATATAA
AGGTACATCTAAATACGGTAAAATCATTATCAATTTGAAAGACGAAAATAAAGTAGAAATTGATTTAAGTGAT
AAATTGCAATTCGAGCGCATGGGCGATGTGTTGAATAGTAAAGACATTAGAGGTATAGCAGTAACTATTAACC
AAATTTAAAGTAAGCAATCAATGACTTTAAAGTAATAAATTTTGAAGCAGCTTAACGATGAAACGTTGAATAA
ATACGCATATCCTACAAGAGGAGCGTGTTTAAAGTAACGCCGTCGTTCGGCTGTTTTTTATATTTAATAACGC
AGGTCACGAGTATACTAAAAAAACCAAATTAGACGTGAAAGCTATGTGCTTTGAATATTACTTAGATGTTCAT
TAACAATGATTCAAAGTTATTTAAACGCACGTTAATGTCAGTTTGTTTCGATGCACTTTATAATAAAGGCAGA
TGTTTCAAATTACGTAATCATAACAATCCAATACATTAAGATTGGAGTAAATAAATATGAAATTGACAGCATT
AGCAAAAGCAACATTAGCATTAGGAATATTAACTACAGGTGTGTTTACAGCAGAAAGTAAAGCCGTTCACGCG
AAAGTAGAACTTGATGAGACACAACGCAAATACTATATCTATATGTTAAAAGATTACTATTCTGAAGAAAGCT
ATGAACCAACAAACATTAGTGTTAAAAGTGAAGATTATTATGGTTCTAACGTTTTAAACTTTAACCATCGAAA
TAAAAACTTCAAAGTATTTCTACTTGGTGATGATAAAAATAAATATAAAGAAAAAACGCATGGTCTTGATGTC
TTTGCAGTACCTGAATTAATAGATACTAAAGGTGGCATATATAGCGTTGGCGGTATAACAAAGAAAAATGTGA
GACCCGTGTTTGGCTGTGTAAGTCATCCAGGTTTACTAGTTAAAAAAATCGATCCTAAACATGGCTTTTCGGT
AAATGAGTTGTTCTTTATTCAAAAGGAAGAAGTATCGTTGAAAGAACTTGATTTTAAAATAAGAAAAATGTTA
GTCGAAAAATATAGATTGTATAAAGGCGCGTCAGATAAAGGTAGAATCGTTATTAATATGAAAGACGAAAAGA
AATATGTAATTGATTTAAGTGAAAAATTAAGTTTTGATCGTATGTTTGATGTAATGGATAGTAAGCAAATTAA
AAATATTGAAGTGAATTTGAATTAATTAAGCATAATAGCATAATAGATTAAGAAGCGACTTAACAACAAAATG
TGAATTGACATGCATGTCCTTAAATAAGGAACTGTGTTAAATACATTAATGTTGTTAAGTTGTTTTTTGCATT
TCAAAGTGCAGAACAGAGTATCAACATTTGTTGTAGTAAACAATGATCCAGAAAAACAACCACATGTAAAGAG
TGAAAGTTAATTAATCTGGCCATTAAAATATATTTGTTTTTCATTAATAATAATTCACGTATATTTAAATTGA
GGTTAATTATCGCATTAAACGATGGACGTTATAATTAGAGTGTATGATTCAAATTACGTAATAAAACAATCCA
ATACATTAAGATTGGAGTAAATAAATATGAAATTGACAGCATTAGCAAAAGCAACATTAGCATTAGGGATTTT
AACAACAGGAACTTTAACAACAGAGGCTTATTCAGGTCATGCTAAACAAAATCAAAAGTCAGTAAACAAACAT
GATAAAGAAGCATTACACCGATACTACAGTGGAAACTTTAAGGAAATGAAAAACATTAATGCTTTGAGACATG
GTAAAAATAACTTACGTTTTAAATATAGAGGTATGAAGACTCAAGTATTATTGCCTGGAGATGAGTACCGTAA
ATATCAACAGCGAAGACATACAGGCTTAGATGTGTTTTTTGTTCAAGAAAAAAGAGACAAGCACGACATATCA
TATACTGTTGGTGGCGTAACAAAGTCGAATAAAACACCTGGGTTTGTTAGTGCACCATTATTAAATGTTACAA
AAGAAAATGGTGAAGATGCTTTTGTGAAAGGTTACCCTTATGATATTAAAAAAGAAGAAATATCACTAAAAGA
ATTAGATTTTAAGTTAAGAAAACATCTAATTGAAAAATATGGTCTTTATAAAACAAAATCAAAAGATGGCAAG
ATTAAAATTAACTTGAAAGATGGCAGCTTTTATAACCTTGATTTAAGATCTAAATTAAAATTCAAACATATGG
GGGAAGTCATAGATAGTAAACAAATTAAAGATATTGAAGTGAATTTGAAGTAAATAATTACGAATAAGAAAAA
TTAATTGAAGTGGCTTAACGATGAAATGTAAATTGGTGTGCACACCTTACCAAAGGGATGCATTAATCGATAT
CGTCGTTAAGTCGTTTTTTTCGTTTTATGGATTCTACCCCAATTTCCATAAAGATAAAATTTCCACCACCAAC
ATCAAAATTCTCAACATCGTAACATCACCAAATGTTATAATAAATCTATTACACAAAGAGATAAATTACTTAT
TCAAAGGCGGAGGAATCACATGTCTATTACTGAAAAACAACGTCAGCAACAAGCTGAATTACATAAAAAATTA
TGGTCGATTGCGAATGATTTAAGAGGGAATATGGATGCGAGTGAATTCCGTAATTACATTTTAGGCTTGATTT
TCTATCGCTTCTTATCTGAAAAAGCAGAACAAGAATATGCAGATGCCTTGTCAGGTGAAGACATCACGTATCA
AGAAGCATGGGCAGACGAAGAATACCGTGAAGACTTAAAAGCAGAATTAATTGACCAAGTCGGTTACTTCATT
GAGCCACAAGATTTATTCAGTGCGATGATTCGTGAAATTGAAACGCAAGATTTCGATATCGAACATTTGGCGA
CGGCAATTCGTAAAGTTGAAACATCAACACTAGGTGAAGAAAGTGAAAATGACTTTATCGGACTGTTCAGCGA
TATGGATTTGAGTTCAACGCGTTTAGGTAACAATGTCAAAGAACGTACTGCGTTAATCTCTAAAGTCATGGTT
AACCTTGACGATTTACCATTCGTTCACAGTGACATGGAAATTGATATGTTAGGTGATGCATACGAATTTCTTA
TCGGGCGCTTTGCGGCGACAGCGGGTAAAAAAGCAGGCGAGTTCTATACACCACAACAAGTATCTAAGATACT
GGCGAAGATTGTCACAGACGGTAAAGATAAATTACGTCACGTGTATGACCCAACATGTGGTTCAGGTTCACTG
TTGTTACGTGTTGGTAAAGAAACACAAGTGTATCGTTATTTCGGTCAAGAACGTAACAATACCACATACAACT
TAGCGCGTATGAATATGTTATTACATGATGTGCGTTATGAAAATTTCGATATCCGTAATGATGATACGTTGGA
AAACCCAGCCTTTTTAGGCCATACATTTGATGCGGTTATTGCGAATCCACCGTATAGTGCGAAATGGACAGCA
GATTCAAAATTTGAAAATGACGAACGATTTAGTGGTTACGGCAAGCTTGCGCCAAAATCCAAAGCAGACTTTG
CCTTTATTCAACACATGGTACATTACCTAGACGATGAAGGTACCATGGCAGTTGTGCTACCACATGGCGTCTT
ATTCCGTGGGGCTGCAGAAGGTGTGATTCGTCGTTATTTAATAGAAGAAAAGAACTACCTAGAAGCCGTGATT

GGCTTACCAGCCAATATTTTCTATGGGACAAGTATTCCAACATGTATTTTAGTATTTAAAAAATGTCGCCAAC
AAGACGACAACGTATTATTTATCGATGCATCCAATGATTTTGAAAAAGGAAAAAATCAAAACCATTTAAGCGA
TGCCCAAGTCGATCGTATTATAGCCACATATAAGCGTAAAGAAACGATTGATAAATATAGCTACAGCGCGACA
TTACAAGAGATTGCCGATAACGATTACAACCTAAATATACCGAGATATGTCGACACATTCGAAGAAGAAGAGC
CAATTGATTTAGATCAAGTCCAACAAGATTTGAAAAATATTGACAAAGAAATCGCAGAAATCGAACAAGAAAT
CAATGCATACCTGAAAGAACTTGGGGTGTTGAAAGATGAGTAATACACAAAAGAAAAATGTGCCAGAGTTGAG
ATTCCCAGGGTTTGAAGGCGAATGGGAAGAGAAGAAGTTAGGGGATCTTACAGATAGAGTAATTAGGAAAAAT
AAAAACTTAGAATCGAAAAAGCCTTTAACAATATCCGGACAGTTAGGTTTAATTGATCAAACAGAATACTTTA
GTAAATCAGTTTCGTCGAAAAATCTAGAAAATTATACACTAATAAAGAATGGAGAATTCGCGTATAACAAAAG
TTATTCTAATGGATACCCATTAGGGGCTATTAAAAGATTAACTAGATATGATAGTGGTGTATTGTCCTCTTTG
TATATTTGTTTTTCTATTAAAAGTGAAATGTCTAAAGACTTCATGGAAGCATATTTTGATTCGACACACTGGT
ATAGAGAAGTTTCTGGAATTGCAGTTGAGGGTGCAAGAAATCACGGATTATTAAATGTTTCTGTGAATGATTT
TTTTACTATTCTAATTAAATATCCAAGTTTAGAAGAACAGCAAAAAAATAGGCAAGTTCTTCAGCAAACTCGAC
CGACAAATTGAATTAGAAGAACAAAAGCTTGAATTACTTCAACAACAGAAAAAAGGCTATATGCAGAAAATCT
TCTCACAGGAATTGCGATTTAAGAATGAGAATGGTAATGATTATCCTGATTGGGAAAGAATTAAATTTTTTGA
TGTAATTGATAAAGTAATAGATTTTAGAGGGAGAACACCAAAAAAATTAAATATGGAATGGTCTGACGAAGGG
TATTTAGCATTATCAGCAGTCAATGTAAAAAAAGGCTATATTGATTTTAATGTAGAGGCGAAATATGGAAATC
TAGATTTATATACTAGATGGATGAGAGGAAATGAATTATATAAGGGGCAAGTATTATTTACAACTGAAGCGCC
AATGGGCAATGTAGCACAGGTTCCGGATAATAAAGGATATATATTAAGTCAAAGAACTATCGCGTTTAATTCA
AATGAAAAAATCACTGATAACTTTTTAGCATCATTGTTGAGCTCTGAAAATGTTTATAATGATTTATTAAAAT
TGTGTAGTGGTGCTACAGCAAAAGGTGTGAGTCAAAAAAATTTAAATCGACTATACGTTACTATTCCACATTC
CATATCAGAGCAAGAAGAGATTGCTGAATTCTTTAGAAAAATTAATCAATTGGTTGAGTTGCAAAAATATAAA
ATTGAACATACTAAAAGTCAAAAACAAGTGTTTTTACAAAAGATGTTTATTTAAGCATTTGAGCACATCTATC
AATTAAGTATTAATAAAATAATCTTAAATTGGCCGATTTTCCTTATGTAACTTTTAGTAACTAAAAATACCCG
ATTCGCTATGATAAAAAAGCGAACATTAAATTTAGACACTGTGAAAGCGCAGTGTCTTTTTTGTGTCGAAATT
GTGTACAGAATAAGTGGTTAAATAAAGATTAAGTTGAGATAAAGTGTTATTCGTAAATAAAAGAGAGTAGATC
GATAGGAATTGAATGATATTAGTTAACTATTTATTAAATTACTTAATAATGATTAATTTTTAGTTAAAGTAAG
TTTAATGTGAAGCACGACCATTGCTCATTATAATGAATGAGGATTGTTCGTATTGCGTAATAGAATAAATCAA
ATAGACTAAAAATTGGGAGCATAGAATTATGAAATTAAAAAATATTGCTAAAGCAAGTTTAGCATTAGGGATT
TTAACAACAGGGATGATTACAACTACTGCTCAGCCAGTAAAAGCAAGTACATTAGAGGTTAGATCACAAGCGA
CTCAAGATTTAAAAGATTATTATAAAGGACAAAGTTTTGATCTGAAAAATATAGGAGGATATAAGGAAGGCAA
CAAGGTTACTTTCATATATCACAATCAACAAATTGATGTGACACTAACAGGTAATGAAAAGTTAGAATTTAAT
GACGATGGAGAAATATCTAATGTTGATGTATTTGTGGTCAGAGAAAATTCTGATAGATCAGGTATCACAAGTT
CTAATGGAGGAATAACAACTTCAAATAAACAAGATATCATAGATAATGTCAAAAGTGTGAACATGCAAATAAC
TAAAAAAACTAGTAGTAATACTGAAACTGTAACTTCATCTTATTACAGTATAAGTAAAGAGAATATCTCATTA
AAAGAACTTGATTTCAAATTAAGAAAACACTTAATCGACAAAAACGGTCTTTATAAAACAGAACCTAAAAACA
GCAAAATTAGAGTTACCATGGAAGATGGTGGGTTTTACACATTTGAATTGAATAAAAGTTACAAACAAACCG
CATGGGTGATGTAATTGATGGTACTAAAATAAAAGAAATTAATGTTGAATTAAACTAATAATATTCGAGGGAG
TAAATCATGAGGGAAAATTTTAAATTACGTAAAATGAAAGTCGGTTTAGTATCAGTTGCAATTACTATGTTAT
ATATCATGACAAACGGACAAGCAGAAGCATCAGAAACTAATCAAAAGCCAAGTACAAATCAAGAATCAACAGC
TGTTACACATACTGAACAAAATTCAAAAGAAACAAAAACGGCAGAGTCTTCTAAGAACTTTGTTAATTTAGAC
CCTATCAAACCAGGAGATCAAAAGGTAACAGGGACTACTATGCCGAATCATATTATATTACTTAACATAGATG
GAAAAAGTGTTGATTCAGTAGATGGAGAGGACAGTTCATTTATAACTTCAAAAGAAGATGGGAAGTTTGAGTA
TCCTCTAAATAATCGTAAAATAGTTCACAACCAAGAAATTGAAGTATCATCTTCATCTTTAGATGATTTAGGT
GAAGATGATGAAGAGGTAGAAGAAGATTCCACTGAAAAATCTGAAGCTGAAGAAGAAAGTACAGGTGCTAAAG
TTACTTACACAACACCGCGATATGAAAAAGCGTATGAAGTACCGGAAAAACGATTAGAAGAAAAAGATGGACA
TCACCAAGTTTTCATCGAACCTATTACTGAAGGATCAGGTATTATTAAAGGGCATACGTCTGTAAAAGGTAAA
GTTGCTTTATCTATTAATAATAAATTTATTAATTTTGAAACAAATTCTAATGGTGGCCCAAATAAAGAAGAAG
CGAAATCTGGATCAGAAGGAATCTGGATGCCTATTGATGATAAAGGATACTTTAATTTTGACTTCAAAACGAA
ACGTTTCGATAATTTAGAGTTAAAAGAAGGTAATGACATTTCACTAACATTTGCACCTGATGATGATGAAGAT
GCATTAAAACCTTTAATTTTCAAAACTAAAGTAACAAGTTTGGAAGATATTGATAAAGCAGAAACTAAATATG
ACCATACTAAAGTCAACAAAGTGAAAATTTTAGATAATGTTAAAGAAGATTTACATGTTGATGAAATATATGG
AAGCTTATATCATACAGACAAAGGTAAAGGTATTCTTGATAAAGAAGGTACTAAAGTAATTAAAGGAAAGACT
AAATTCGCGAATGCAGTAGTGAAGGTAGACTCTGAACTAGGTGAAGCACAATTATTCCCTGATTTACAAGTAA
ATGAAAAAGGTGAATTTAGCTTTGACTCACATGGTGCTGGTTTTAGATTACAAAATGGAGAAAAACTAAACTT
TACAGTAGTTGATCCTATTACAGGTGAGCTGTTGAGTAATGAGTTTGTTTCTAAAGAGATTGATATTGAAGAA
ACACCAGAAGAAAAAGCAGACCGTGAATTTGATGAAAGACTTGAAAATACGCCTGCTTACTACAAGTTATACG
GCGATAAAATAGTTGGTTTCGATACTAACGATTTCCCGATTACTTGGTTTTATCCATTAGGTGAAAAGAAAGT

TGAACGTATAACACCTAAATTAGAAAAATAATTAATAAAATAAAACAGCTTAATGATGTAATGAAATTAGTGA
GTTAATCACTGACTTCAACGTCATTGAGCTGTTTTTTGTGCTTTGTTACAAAGCATTATTGAATTTATTTTAC
GTGTTCATATTTTGAAACATCAAAGCCGTCTTGTTTAGCTTTGTTGATAATGTCTTTGATTGAATGTAGTCCT
TTATCGGCGAAGTATGATCTTAAGTTGTCTTTTGTAGCTTGGTCAGCATTCTTATCTAATAACACATCAATAT
AACTTAATTCATGTTCTAAGAAGTTTGCATCATAATGTAGTACGAGTCCATTTTGAGAATAAACTTTCGCATC
TGCTTGATTACCATATCCAACAACGCCAGTTGCTAAAACACCTACCATTGCCGTAGCTACTAAAACCTTTTTA
AATTTCATATCCATCACTCCTCTAAAAATTGTAACTCCATCATAACACTGAATATTAAGAAAATTACGTTTAT
TAAGTCGATTTAATGATTTTTAATAAATAGTTAAAGTGACAAATATTGCTAAAAAGCAATTAATCTCTAATAC
GATACTTATGAATTTTCCTACTAAAACCTTGATTTCAAAGAGGGTTTAAATCAAATGAAACAATAATAAAAAA
ATGATGCAACTTAATAATAAGTACAAATTTAATTAAGAAATTAAATTGATTGCATATGTATATTTTGGTAACG
TAAAAGAGAAATATACAAAATAATTAATTATTTATATGAAAAGAGAATATAAATGAAGTATAAAACAGAGAGA
CGTGAAGCGATGGGATATTTAAAAAGGTTTACATTGTACATAAGTATTTTTATTTTAATAGTTGTTATAGCAG
GTTGTGGTAAAAGTGATGAAACAAAAGAAGATTCCAAAGAAGAACAAATTAAAAAGAGCTTTGCGAAAACGTT
AGATATGTATCCAATCAAAAATCTCAAGGATTTATACGACAAAGAAGGCTATAGAGATGGCGAATTTAAAAAG
GGCGATAAGGGTACATGGACTTTACTCACAAGTTTTTCAAAAAGTAACAAACCAGGCGAGATAGATGATGAAG
GTATGGTGCTATATCTAAATAGGAATACCAAAAAGGCAACAGGTTATTATTTTGTAAATAAAATTTATGATGA
TATTAGCAAAAATCAGAATGAGAAAAAAATACCGTGTTGAACTTAAAAATAATAAGATTGTTCTTTTAGATAAT
GTAGAAGACGAAAAACTTAAACAAAAAAATTGAAAATTTTAAATTTTTTAGTCAGTATGCCGATTTTAAAGATT
TAAAAAATTATCAAGATGGAAGTATAACAACTAATGAAAATGTACCGAGCTATGAAGCAGAATACAAATTGAA
TAATAGTGATGAAAATGTAAAAAAACTTAGAGATATTTATCCAATTACAACGAAAAAGGCTCCAATATTAAAG
TTACATATAGATGGTGATATAAAAGGAAGTTCAGTTGGATACAAAAAAATAGAATATAAATTTTCAAAAGTTA
AAGATCAAGAGACAACATTAAGAGATTATTTAAATTTTGGGCCGTCTGATGAAGATATCTAAAGTTTATTAGA
TATAGAAATAATTCTAAAACAGAGAGACGTGAAATGATGGGATATTTAAAAAGGATTGGATTGTGCATAAGCC
TGTTGATTTTAAGCATTTTTGTAACATCTTGTGATAGTGATAATAAGATCACTGGAGATTCAAAAGAAGCACA
AATTAAAAAGAGTTTTGCAAAAACTTTAGATATGTATCCAATTAAGAATCTCGAGGATTTATACGACAAAGAA
GGATATAGAGATGGTGAATTTAAAAAGGGAGACAAAGGGACGTGGGTTGTTAGATCTGAAATGATAATTCAAC
CAAAAGGTAAAAGTTTAACTTCAAGAGGCATGATATTATACATGAATAGAAATACTAGGACCACAACTGGTTA
TTTTTCAATAGAAGAAATAGATTCAAGAAAAAAACCTTGACGAGAGAGAAACAGAAAAAAAGTATCCTGTGAAA
ATTATAAACAATAAAATTATTCCAACCGTAAAGATAAAAGATGAAAAGTTGAAAAAGGAAATTGAAAACTTTA
AGTTTTTTGTTCAATATGGGAGTTTTAAAGGAATAGAGAATTATGAAAATGGTCACATTTCCTACAATTCTGA
AGGTCCTATTTATTCCGCGAGATATCAACTGAAAAATGATGATTATAATGTTAAAGAATTACGAAAAAGATAT
GATATACCAACAGAAAAGGCGCCCAAATTATTGTTGAAAGGTTCGGGGGATTTGAAAGGGTCTTCAGTTGGAT
ATAAGGAAATTGAATTTATATTTCTAGAAAATAAAAAAGAAAATATATATTTTTCAGATGGATTAAACTTAAT
TCCAGGTGACTAAAAATAACACAAAGGTTGATGGTGCTCAACAAATATTAACATGATAGAGAGAAGTGAAAAA
ATGAGGGATTCAAAAAGAGTAGTATTGTACATAAGCATTATGGTGTTGAGTATTTTTATAATTGGTTGTAATA
GTTCAAGCGAGAATGCAAAAAAATCAAAAGAAGACTCAAAAGAAGACCAAATCAAAAAGAGTTTTGCGAAAAC
GTTAGATATGTATCCAATTAAGAATCTCGAGGATCTATATGACAAAGAAGGATATCGAGATGGCGAATTTGAA
AAAGGTGATAAGGGGACGTGGACGATATATACAGATTTTGCCAAAAGTAATAAAGCGGGTGAATTGGATAGTG
AAGGTATGGTATTACATTTGAATAGAAATACGCGGACGGCTAAAGGATATTATTTTGTAAGAAAAACTTATAA
CGAAGAGGATAAGATGCTGCAAAGGAAAGATTATCGTATTAAACTTAAAAATAATAAAATTGTCCTTTTAGAT
AAAGTAGAAGATAAAAAACTCAAACATAAAATAGAAAATTTTAAATTTTTTGGGCAATATGCAGATTTTAAAG
ACTTGAAAAATTATAAAAACGGGAAAATATCGAGCAATGAAAATGTTCCATATTATGAAGCAGAGTACAAAAT
AGAAAATAGTGATAGAAATGTAAAAAAACTTAGAAATGTTTATCCAATTACAACCAGTGAATCGCCAACTTTA
AAATTTCATATAGATGGGGATATAAAAGGTAGCTCGATAGGATATAAAAAAGTGGAATATATCTTTTCAAAGC
AAAAAGATAACCAAATTTCTGTCAGTGATTTTTTAAATTTTAACCCATCAAAAGATAATCAGTAGAGATTCAA
TAAAAAATCAGACAAGAAAGATGTGGAATAATGAGAAATTTAAAGAGAGACGTATTGTACATAAGCATTATGG
CTTTGAGTATTTTTATAATAGGCTGTGATAAATCTAGTGTTACTGCTGAAAAAGCAAAAGAAGACTCAAAAGA
AGACCAAATCAAAAAGAGCTTTGCGAAAACGTTAGATATGTACCCTATTAAGAATCTCGAGGATTTATACGAT
AAAGAAGGATACCGTGATGGCGAATTTAAAAAGGGTGATAAGGGGACGTGGGTTATTAGATCTGAAATGACAA
CAGAACTAAAAGATGAAAATATGGAATCCAAAGGTATGGTCGTACGTTTAAATAGAAATACCAGAACAACTAC
AGGTGAGTATTTTGTTAGGATAATCAAAGAAGACAATGAGGGCAAGGTATATAGTGATGAACGAAAATACCCA
GTCAAAATTGATAATAAAAAAATTATTCCATTAAAATTAATCGATGATGAAAAAGTTAAAAAAGAAATTGAAG
AATTTAAATTCTTTGTACAATATGGGAATTTTAAAGAATTGGAAAACTATAAAGATGGTAAAGTGTCATATAA
TCCAGAAGTACCAATTTACTCTGCGCAATATCAATTGAAAAACAGTGATTATAATGTAGAGCAACTACGTAAG
CGATATAATATCCCAACAAGAAAGGCGCCTAAATTGTTATTGAAAGGCTCAGGTAATTTAAAAGGATCCTCAG
TCGGATATAAAAATATTGAATTTACCTTTGTTGAAAATAAGGAAGAAAATATTTACTTCACAGATAGTATTAA
TTTCAACCCAAGTGAGGATAAATAAGTATATTTAGTATTATAAAAAACACGTTAGAACATTTAATAATAAAGT
TAAATGAATTACATGTATTAAATTGAATAAGTTAGTTTTGTTTGATAACATAAAAGTGCAATAATCACTATTT

TATTAAGTTGTATGAAATATTCAAATGTTTATAAAATTAAAGCAGAGAGATGTGAAATGATGGAGTATATAAA
AAAAATTGCTTTGTACATGAGTGTATTACTTTTAATCATTTTTATTGGGGGATGTGGAAATATGAAAGAAGAA
CAGAAAAAAGAAGCAAATACAAATAAAACAGATTCAAAAGAAGAGCAAATCAAAAAAAGCTTTGCGAAAACGT
TGGATATGTATCCTACTGAAAATCTAGAAGACTTTTATAACAAAGAGGGATATCGAGATGGAGAATTTAAAAA
AGATGATAAAGGTACTTGGCTAATTAGATCTGAAATAGTTAAACAGCCAAAGGGCAAAGTGATGAAAACAAGA
GGTATGCAATTATATATTAATAGAAATACCAAAACAGCCAAAGGTTTCTTTGTTTTGAAAGAAATAAGTGAAA
ATAATAATCGTGTAAATAAAGATAAGGAGGAAAAATACGAAGTGAAAATGGTAGGAAATAAAATTATTCCTAC
TGAACAAATTAATGACGAGAAAATAAAAAAAGAAATTGAAAACTTCAAGTTTTTTGTGCAATATGGAAAGTTT
AAAAATTTCGAAAAAATACAAAAATGGTGAGTTTTCATATAATCCAGAAGCACCAATTTATTCGGCTAAATATC
AATTACAAAACGACGATTACAATGTAAGGCAACTACGTAAAAAAATATGACATTTCAACAAAAGAAGCGCCTAA
GTTACTTTTAAAAGGTGGAGGGGATTTAAAAGGTTCCTCAGTTGGTCAAAAAGATATTGAATTTACATTTGTT
GAAAGAAAAGGTGAGAATATTTATTTTAATGATAGTGTTGAATTTATACCAAGTGAGGATAAATAAATATGAG
TAAACCAGAATATCAACTAAACCCAGGTAAAATAAATGCAAATACAGAAGAAACATCTGCTATATCCTATTTT
AGTTATCAAATTGAAAATGCAAATATTAGTGGTTTGGATAAAGTAAAAATGTATCAGCAAATTGATATACTAA
AAGATAGATACAAATTCCCTTCTAATCTTGAATATGTTGATAGTTACACTGATTCTCTTACTGGAGTAACAAC
TTCTGCTTTTTTAAATAAAGATACAGGCAAAGTAACTCTCGGGATGACTGGGACTAATTTACAAGACGAAGCC
TTTAAAAAGTTAAAAGAAGGTGAATTTTCAAGACAAAATTTTACCAATGCTTTGGAAACAGTTAAAGATGGAT
ATGCAGATCTTAAAATATTATATTCTCCTGCATCTGATCAAAACTATAGATATGCGAATACACAAGAATTTAT
AAATAAAATAAAAAGTAAGTATGACATTGATTTTATTACTGGTCATTCACTAGGTGGAAGAGATGCAGTAGTT
CTAGGAATGAGTAATGGTATTCCGAACATTGTGGTTTATAATCCAGCTCCTATTTCTATAACTAGTTTGAATC
CTAATTCCCCAGATGGAAAACGATTGTTAGAGTTATATAAAAATTATAAAGGTAATATTACTAGGTTTGTTGC
AGAAAATGATGCATTGACAGAAAATCTGAAGAAATATAAGCATTATGTTTTTTTCGGTAATGATAAAGTCTTT
AAAAATGGTAAAGGTCATGGAATGGAAGGCTTTCTGACCAAAGAAGAACAAAAAGCTATAAAAAAAGAACTAA
AAAAACTACAAGGTTATGCAGAAGAAAATAATAAGTCATTTGTAAAGCATTCAAATAATGCTATCTCTAAATT
AGCTAGTATAGAATTACTTAGAGCAAATATGATGACTACAAATGGAGGAGGATTATCTTCTTCGCAGCAAAAA
GTTTTAGAAAGTTTAACAGCTTTAACAATTGCGCAGTCATTCAGTCAACTGATAGACGATGAAATTAATCAAA
TCAAAAAAATGTATAATGAAAAGAAAAAGAAATTTGGAAAAAATTGGGAAGACGCTCAAAAAGCTGGAAAAGC
TGTAGGTGAAGATTTGAGTGTAAATGGAGTTCTCAATGCTTTAGATGAAGGTCAAGTGAATGAAAGTAGTATG
GTAAGAGAACCTGAACAAATGATATCTGCAAAAGAAAGACAACTTTCAACGATAGGGTCTTCTGTATCAAATT
ATATTATGAGAGTTAGACTCAGTATTAATGAAATCGTTGATAAAGATCAAGTGCTTGCATCACAAATAGGTGG
ATTACTATGATGTTTAATCAAATTAATAATAAAAATGAATTAGAAGAATCATATGAATCTGAGAAAAAACGTA
TAGAGAATGAACTGCAAAATTTAAATGAACTTAGGCATAGAACTCGAAAAGAAAATGAACGTAGTTATGATGT
TTTTCAATATTTGAAGCACGAAATGAATTATAGTGAAGATGCCCAAAGGAAAATGACGAGAAATATAGAAGCG
TATGAGCAAGAAATCAATGAGATAATTAGAAAGCAAGAATGGAAATTAGAAGAATATAAAGAAGACTTAAAAA
AGTCTTATGAAAAGCAGTTAGATAAACTAAGTGACTGATAATTTGGAGGAGATTAAATGACTCTAATAGAACC
AGATATGAACTTAAGAATGCCAGATATAAGTACTACAGTAGAAACACTTAATCTCATATCTAAAATGGAAGCG
CAAAAAGAAAATATTCGCACAGTTATTGCACCTGAACATAAGCATAAATATAAAGATATTGAAAATGGGTTAA
AAGGTGAAGAAAAAGTATTAATTGAACAAATGGCGCAGCATTGCGAAGCTTTTAAAGCTAATTTTAAAGGCGC
AGCTCAAGGAGATTGGGTTAAAAGTGCTATGTCTGAGATTGACAGCATTAAGGATGACCTGAAAAAAATTAAT
AGCTAAACTACATATCATATTAATAAATAAAGCTCCCTTCAAAATTTTCATTTTTTCAATGTCTACTTTGGAG
GGAGCGCTTCAGTATATTCACAACTGACACTAGGCCGCTTCTTTTTAATTTATTTTATTCCCATGTAAAGTAA
TTCGAACCGAATGGACATACCACACTCACAATTGTAAAAACACAACTTAAATTACTGTCCCGCATTAAACATA
TCCACATTCCAAACAGCAACAACAAAATCCTCAAGTACCAAAAGCTATGACCACTTTATCAAAATCAACGCAA
GTCAACATCGACCGAACTACTTGCCATTAATCATCCTCACCTTACAAACCATAAAGCGCTGCAGTTAAGTATT
GAATGGCATAGCCAATTGAAATGAAAATAGCAATCGTTATAATAGCACTGCCAATGGTATATAACGTATGTTT
CGTAGATAATCCTTCAGTCTTTGAACCAAAAACACTGAGTAGAATAAATGCAATACCAATGACATATAAAACT
AAAATGACAAATAGCATGTTTATACACCTCGATTCTCTTTCGTGAGATATATCTTTGTCTCCATTATAAAGTA
AAGCGCTCAATAATTTAATTAAGTATACTATTTTTTTATTATCGAAGATTGCGAAAGATGAAACGCTCCTATA
TAATGTAAAACGGAATTATTCCTATTTACAAATAAATTGAAGTGAGGGTATATATATGGCTAAAATTCCAGTT
ACGGTATTAAGTGGTTATTTAGGCTCGGGGAAAACAACATTGTTAAATCATATTTTACAAAATCGTGAAGGTC
GACGTATCGCGGTAATTGTAAATGATATGAGTGAAGTTAATATCGATAAAGATCTTGTCGCAGATGGTGGGGG
ACTATCGCGTACAGATGAAAAATTAGTCGAACTCTCTAATGGTTGTATCTGTTGTACACTTAGAGATGATTTA
TTAAAAGAAGTTGAGCGTTTAGTGAAAAAAGGTGGCATCGATCAAATTGTTATTGAGTCAACAGGGATTTCAG
AGCCAGTACCTGTTGCACAAACTTTCTCATATATTGATGATGAACTTGGCATTGATCTTACAGCGATTTGCCG
TTTAGATACAATGGTTACAGTTGTTGATGCTAACCGCTTCGTACATGACATCAACTCAGAAGATTTATTGATG
GATCGTGATCAAAGCGTTGATGAAACAGATGAACGTTCGATTGCTGATTTATTAATTGACCAAGTTGAATTTT
GTGATGTATTGATTATTAATAAAATTGATTTAATTAGTGAAGAAGAACTTGCAAAGTTAGAAAAAGTGTTAAG
CGCATTGCAACCTACTGCTAAAATTATTAAGACAACAAATTCTGAAGTAGATTTAAAAGAAGTCTTAAATACG

CAGCGTTTTGATTTTGAAAAAGCGAGCGAGTCAGCAGGATGGATCAAAGAACTTGAGTCTGGTGGGCATGCAT
CGCATACACCTGAAACAGAAGAATATGGTATATCATCGTTTGTATATAAACGTCGACTTCCTTTCCATGCTAA
AAGGTTCAATGATTGGTTAGAAAGCATGCCAAATAATGTCGTTCGATCAAAAGGTATCATATGGCTAGCACAA
TACAATCACGTAGCATGTTTATTATCTCAAGCAGGGTCATCTTGCAATATTCATCCAGTTACATATTGGGTGG
CTAGTATGTCTGAAGCGCAACAAACACAAATATTAGCAGAACGTCAAGATGTCGCAGCTGAATGGGATCCAGA
ATATGGCGATCGTCATACACAATTTGTCATTATTGGTACAGAATTAGATGAAGAAAAATTAACAAAAGAACTC
GACGCATGCTTAGTCAATGCGCAAGAAATTGATGCAGATTGGCAACAATTTGAAGATCCATATCAATGGCAAA
TTAGACCAGCACGATAAGTTGAATGAAGTATAATCATTTTTAAATTGTGGCTTAATTGTTTGCAATTTATGAC
AATTAATAAAAAGTTAAGGTGTTCTTAGGATCTTTTTGTTTTCAATCTATTTAAAGTGTGATAGTTTTGATAA
AGCAGAAATTTGCATTTTATCCATAGGGCTAGGACATGTATGTGTCTTAGTCCTTTTTATGTTTGCTTTGATG
AAAAATGGCAAAACCATCGTACCAACTGTTAATTGAAGGTACAATCATTGTTTAACAGTCATAAAAATGAGTG
AAGTAAATGCAGCTATCAATGTACTATTAACACTTACGGTCACACGAGTGTTTCAGTCTAAACGTGCAACGCA
ATTCCGCACGTGGCCAATATATCTTGCGCTAGATCATGAAAATCATTAAGATTTCGCACGCCAAATCATGAAT
GTCTCAGACCTCGAGTATCAAACATAAAACACGCCACAAAAATATAACGCGTCAGACTACAAAGATCACGAAT
ATTCCGTACGTCCCACGAAGAACATCCTATACAAAAAAACCAAGACCATTTATTTGCAACAAACTAACAAGAC
TAACCTCACATCAATAAATCAACACAAAGCAAAGCCACCATCCCTATTGGCATAGTGGCCTGAGAATTTTAAG
TATTCAATTCGCTTAAATTATTTTGCGAAAATGTCGATAATTGCTTTGATGATTTTAATGATAGTACCTACAA
TAGCCATCGTTTTGTCCTCCTGTATGTTGATTTAACATAGTGATGAGTTGTTGATCGTTAATGTTTGAGATTA
GTTGTTACCTAAAAATTTACCAAGTAAATCTTTAAAGAATTTGAATAATTTTGCTACGAATTCCATGTGAATG
GCCCCCTTCAAATAAGATGTTCATATCTATGAGATTTTTATAACTTACTTACCAGTGAATTTCTCAATTAATC
CTTTAATGAATTTAATGATTCCTGCAATGATACCCATGTGAAAGACCTCCTTTGTTTGTTATGAAATCTTATT
TACCAGTGAATTGTTCGATTAAGCTTTTGATAACTTTAATGATGCCAGCGATGATACCCATTAAGATTACCTC
CTTTGCTTATGAGTTAACTTCATTGTACATAGTTATCTTGTGCGTAATTGATTTTTTTCCTAACTTGAATTGT
TATGATCATAACTGCGAATTTTTTATTTTAAAAGGACGGGAAATACTGCCTAACTGTACAGGTGCATTTACAA
AATCTTTACAATCATTGGTTTAATAGAGGTTAAGCTGATGAATTTGACATGAAGCAGAAATTTAATTATATTT
TATGTTATAAGTTTAAAACGAATAACACGTTAGGTCTCGTCTAGGCAAAGCATATTTATGAAGATGAATTGAA
CAGACAGAAGTAACATTTTATATACATATGTGCATGGGAGCACGCTTAAAGAGAACACTGCAACAAGGTATAT
CAACGTACCGTGATGCAGTGTCTAGTTAAACTGAAACCTGCAATAGTAAATTTTCTGCCATTATGTACAGAAT
CTACTATTGTAGGTTTTTATTTTTATACATAAGCAATTATGCGAGAGATTAGAAATAAGGAGGTTATGCAGTG
TTAAGTTTTCAATTGCTATTTTCACTGTTTGTTATTGCGCTTATCATTGCATTGATAAGTGGCTTGTTGTTTT
TAGCACCAGTTATGCCAATGAGATATATTAAATTACATTTATACATACTAGTCATGCCAGTATTATTTGCAGT
CATTGGCTTTTTCGGTATTCATGGTCAACATGTCTTAGGTCCATTTAAAATAGATCGTTTATCTTGGTTATTA
GCTGGCTTTGTAATGGCGCTTGGCTTTATTATTCAAAAGTTTTCAATGCGATATTTACTAGGTGATCATCATT
ATAGACATTACTTTCCATTGTTCACTGCGATTACGTCGTTTGCATCTTTAGCATGGATGTCTGAAGACTTAAG
ACTGATGGCACTCTGCTGGGGTATGACATTATTATGTTTAACATTGCTGATGAACGTTAATCGTTTTTGGAAA
GTGCCACGTGAGTCTGCGAAATTATCAAGTATGACATTTTTATGTGGTTGGCTTGCATTCGTTGGAGCAATTG
TAACTATTTATATTGCGACTGGCGAGTGGCGCGTGCCTCAACATATGTCTAATTCGACATGGTCATTGTTGAC
GAATGTACTACTTGTATTAGCTGTCATGATACCGGCAGCACAATTTCCTTTTCATCGATGGTTGATTGAATCT
GTAACGGCACCAACGCCAGTATCGGCAATTATGCATGCAGGAATTGTGAATGCAGGTGGTGTTATTCTAACTC
GTTTTGCGCCGATATTTGATAATGGATTTGCGTTATCATTATTACTTATCCTTTCTAGTATTTCTGTATTGTT
AGGATCGGGTATTAGCTTAGTTCAAGTTGACTATAAACGCCAATTAGTGGGCTCTACGATGAGTCAAATGGGC
TTTATGTTAGTTCAATGTGCATTGGGTGTATATTCAGCAGCGATTATTCATTTAATATTGCACGGTATTTTTA
AAGCAACATTATTTTTACAATCAGGTTCTATCGTGAAGCGATTCAATATTCCAAAACAAGCATCTGCTAAAGA
CGCTTATGGCTGGATTGTCATGGGACGTGTATTAGCTATTATCGTGGCATTTCTATTTTGGATGAGTAGTGAC
AGAAGTGCATATGAAGTGTTAAGTGCACTCATTCTAGCATGGTCATTACTTGTATCTTGGAATCAGATGGTAG
CCTTTAGTAAAGGGCGCATGGCACGTCTTGTTGGCATGATTTTGATTGCAATTGTGACATTTATCTACATCAT
CACACATAATTATTTTTACGATGTATTGCGAAATATAACAACACATGCGACAACACCGCCTACAGTGAGTGTC
ATCATTAGTGTTGTCATTTTAATCTTTGGTAGTTTATTAAGTATTTGGGTGGCGCGTCATCGATACTCTAAGG
GTTTTGCGGTATTGTACGTGTGGTTAGTTAATCTAGGTGAAGCACGCTCGAAAGCGATAGAAAGTCATCCGAA
TTATTTGAAGAAGTATTTATAGGAGGTGAAAGGTATGACAACACAGTTAAATATCAATTCAGTCATTGAAAAT
GCGAAACGTGTTATTACACCATTATCACCAATTTCGATTTTTGCAGCACGAAATCCATGGGAAGGATTAGAAG
CGGATACGTTTGAAGATGTTGCAAAATGGTTACGTGATGTTCGAGATGTGGATATTTTTCCAAATAAAGCATT
AATAGAAAGTGCTGTGGCACGTGGTGAATTAGACGAAAGTGTCTTTAATCAACTTGTTACTGATATGTTACTT
GAACATCACTACAATATCCCGCAACACTACATCAATCTTTATATTGATAACATTAAAACATTAAAAGACGTAC
CTACATCATATATGAATCATTCAAATGTTGATGTTGTTGCTGATCTACTATTAGAAAAATCAAAACGTGATAT
GGCTGAATCATATCATCACTATGATGTACGTCCGATGAGTGATGCAATAATAGATGAACAAGGTGAGCCACTT
AGCGAACAAGTGAATCGTCAAATGATTAAATGGACGAAACTTTATATCGATCAATTTCTATCGAGTTGGACAA
TGCCGAAGCGAGAGCAAAGTTTTTACCATGCATGGTTGCATTTAGCGCAACATGACCATAGTTTTACTAAAGC

ACAGCGCCAAGTGATTAAAGGCTTACCCAATGATCCTGAAATGACGATAGAGTCAGTATTAACTCATTTTTCA
ATAGCGCAGGAAGACTACCAAGCTTGTGTTGAAGGACATCTTTTGGCGTTACCGGGTTGGGCAGGTATGTTGT
ATTACCGTTCACAACAGCATCACTTTGAACAACATTTGTTAACGGATTATTTGGCCATTCGGTTAGTTGTCGA
ACAATTGCTAGTTGGTGATGAGTTTAAGTCAGTCACTAAAGATTGTGAAAGTAGATCGGAAAATTGGTTTAAG
CAAACTGTTGCATCATGGTGTTACTACAGTGATATGCCTAGCGATGTATTACTACAACATGACGTCAATGAAA
TTCAAACGTTTATTCATTTTGCAGCAACTATGAATAAAAATGTATTTAAAAATTTATGGCTAATTGCCTGGGA
AATGACATACGAATCTCAGTTAAAACAAAAAATTAAAGCAGGTCATGAGAGTGTGGCGGGCGCATTAGATGTA
AACCAAGTAAATGTCTTAGAAAATGATAACGCTAATCAGCCACATTCAGTATTGTTAAATGACACACAAGCAG
TTGATGAAAATAATAGCGAGCTAAATCAGATGGGCACATCAACGAAAGCGCAAATTGCATTTTGTATAGATGT
TCGTTCAGAACCATTTCGTAGACATATCGAAGCGGCAGGGCCCTTTGAAACGATTGGTATTGCAGGTTTCTTT
GGATTACCTATTCAAAAAGATGCCGTAGACGAACAATTCAAACATGATTCATTACCTGTCATGGTACCGCCGG
CATATCGCATTAAAGAATTTGCAGACCGCTACGATATGAATGTTTATCGACAACAGCAACAGACAATGTCATC
GATGTTTTACACATTTAAATTGATGAAAAATAATGTCATGCCTAGTCTGTTATTGCCTGAATTAAGTGGGCCA
TTTTTAAGCTTGAGTACCATTGTCAATTCGATTATGCCTAGAAAAAGTCGCGCGTCTTTACAAAAAATAAAAC
AAAAATGGTTGAAAAAGCCTGAAACAAAGTTAACGATTGATCGTGAGTTTGACCGAACATCAGACTTACCTGT
TGGATTTACTGAGCAAGAGCAAATTGATTTCGCGTTACAAGCGTTGAAATTGATGGATTTAACCGAAGCATTT
GCGCCGTTCGTTGTGTTAGCAGGTCATGCTAGTCATTCTCATAATAATCCACATCATGCATCACTTGAATGTG
GGGCTTGTGGTGGCGCATCAAGCGGTTTTAATGCTAAGTTATTAGCGATGATATGTAATCGTCCAAATGTCAG
ACAAGGATTAAAACAAGCAGGCGTAGTAATTCCGGAGACAACTGTTTTTGCGGCAGCAGAACATCATACGTCT
ACTGATACGTTGGCATGGGTATATGTGCCAGACACATTATCAGCTTTAGCTCTAGATGCATATGAATCATTGA
ATGACGCGATGCCGATGATTTCTGAACACGCGAATCGCGAACGTTTGGACAAACTGCCAACGATTGGTCGTGT
GAATCATCCAGTGGAAGAAGCGCAGCGGTTTGCGAGTGATTGGAGTGAGGTACGTCCAGAATGGGGCTTGGCT
AAAAATGCATCATTTATAATTGGACGACGCCAATTAACAAAAGGCATTGATTTAGAAGGGCGGACATTTTTAC
ACAATTATGATTGGCGTAAAGATAAAGATGGCACATTGTTAAATACCATCATTTCTGGTCCGGCACTTGTGGC
ACAATGGATTAATTTACAATATTATGCGTCGACAGTTGCGCCGCATTTTTACGGAAGTGGGAATAAAGCGACA
CAAACCGTCACGTCAGGTGTTGGTGTCATGCAAGGTAATGCGAGTGATCTGATGTATGGCTTATCATGGCAAT
CTGTTATGGCTGCTGATCGAACGATGTATCATTCGCCAATTCGTTTGCTTGTCGTTGTTCAGGCACCCGACTA
TGTTGTAGCAAGACTACTCGCGAATAATGACCATTTCGCTAGGAAGGTGTCTAATCATTGGCTGCGTTTAATG
AGCGTTAATGAGGAAGGGCGTTTTAAAAGTTGGATTTAACGATGATATGTTGCGATGCTAAGTAACTAATATA
TCATCATATCTACAATGTTCTAGCTTCTTAAAATATACTAAAAATGATAAACTTAATGTAATAGTTGATGTCG
TGATAACGATACAAAGATGAAGTAATCAATTTTAATATTATTGGAGTTAGTGATATGAAAAGAACGAAAGGTG
AAATCGAAGCTGAAATCAGTAAAGCCATTACGCAATGGGAAAAAGATTTCCTTGGCAGAGGTTCCTTGTCAGT
TAAATCAGACATTTTAAGAGATATGGTGATTATTAGTTTACAAGGTATCTTAACGCCAGCAGAATATCGTGTG
TGTAGTACGAATGAAGGATTATTAAATATTAAACGAACACGTTCTGAATTAGTTGAATCCGGTGAGCAAGATT
TGAATGATATCATTCTTAAAATTACAGGTATCAAAGTGATGAGCTTCCACAGTGATTTAAGTACAGTTACAGG
TGAACGTATTATCGTATTCAAACTTGAGGATAATTTGGAAAAGCATATTTAAGAAGGTTAGGTATGCTTCTGC
TGGCTTATTAAAGCTATAGAAGTATGCTTGGTTAAACAGCGCAACGCCATATATTTGGGTGTGCTGTTTTTCT
ATTATATGATGTGTTTATTACGTGGATGAATGCGTGGGTCTGAATATAGCCCGTGAAAGGCGTATGTCATAAA
ATTTGCGAATGGTGACAAGTCAACGCAAGCATATTTGTTGCGAGACATGCCCACTATCCTTTTACATAAAGAG
TATATGTGTGACGTATGCATATAATCGATAAAGTATACCTAAAAAATTAGGTATAATGACCATTGTTGCAATA
AGTTTTTCGGCCTTGAAACCGATAACGCATAGTATAATAGGAATAAAATATAATACAAATATCCAGTAAATCA
AATTTGAAAACGATGGATGAATAGGGGTAAAGAATCTAACTAATGTAATGATTAACGCTATATAGACAAAAAT
AACTCTATTGATACGCCTTACCCCCTCTGTATAATAACTATAAATCGTACTAAATTGAAAATAGCAAAATATT
GTTATTTCAATTATACAATGTTTTATTTGCAATATACATAACAATATGAACTTTATAATTTTGATATTGTCGG
TAAAGTGAAATTTGTGGCACATTGATATTATGGATAACATTTGTAAAATGAAAATGATGATACGAGGAGATTG
TAAGATGATAGATAAAAAATTAACATCACCGAAAATGACAGTGCCTTTATTTTTAATCGCGCTGATTGTATTT
ATAGGTATGTTTTACAGTGTAGTGACAAATCAAGAATGGCTTAAAAATATAGATATGGGATCATTAACATGGT
TTACAGATTATTTCGGTGAGCCACAACGTCAGTATGTTAACAATTTATTTAATTACTATATGACGTTTAGTGC
GGAAATTGGAGATGTCAAAGGTGTCGTGTTGATTTCCATTATCATCACAATCATACTGTTTATTAAACAGAGG
CATTTAGCGGTTTGGTTTGTGACATATTTGGTTTCAGGCGTCATCATGAACAAATTAATTAAAGATACTGTTT
TACGTCCAAGACCATATAATCATTTAGCCGTTGATACAGGCTTTTCATTTCCAAGTGGACATTCCAACGCCAG
CACATTATTATATTTCGCCTTAATGATCATAATTATTTCACTTGCTGCTAAGACAATAACAAAAGTGTTGAGT
GCGCTAGTTATGGGAATATTATGGCTTAGCATATTATTTTGTCGCCTTTATTTTCATGCGCATTACTTTTCAG
ATGTCATTGGCGGCACGTCACTAGCAATCATTTGGGTAGCGTTATTCTTAATGGTATACCCATACTTTATTAA
TCATCGACGACAACGCGTTTAGCAAACATCTTTAGACATAGTGGAGGTATATAGAAAAATGAGAATTAAAACA
CCGAGTCCATCGTATTTAAAAGGCACAAATGGACATGCGATATTATTATTACATTCATTTACAGGTACAAATC
GGGATGTGAAGCATCTTGCAGCTGAGTTAAATGCACAAGGATTTAGTTGTTATGCACCGAATTATCCAGGTCA
TGGCTTACTGTTGAAAGATTTCATGACATATAATGTAGATGATTGGTGGGAAGAAGTTGAGAAAGCTTACCAA

TTTTTAGTCAATGAAGGTTATGAATCTATCAGTGCAACGGGTGTGTCTTTAGGTGGATTAATGACATTAAAAT
TGGCGCAACACTATCCTTTGAAACGTATCGCTGTCATGTCAGCACCAAAGGAAAAGAGTGACGATGGCTTAAT
AGAACATTTAGTTTATTATAGTCAACGCATGTCGAATATTTTAAATTTAGATCAGCAAGCATCGAGTGCGCAA
TTAGCAGCAATTGATGATTATGAAGGTGAAATTACGAAGTTTCAACATTTTATTGATGATATCATGACAAATT
TAAATGTTATTAAAATGCCAGCTAATATATTATTTGGTGGTAAAGATGCGCCATCCTATGAAACAAGTGCACA
TTTTATTTATGAACATTTAGGATCAGTAGACAAAGAATTAAATGGTCTGAAGGATTCGCATCATTTAATGACG
CATGGAGAAGGCAGAGATATTTTAGAAGAAAATGTTATTCGCTTTTTCAATGCTTTAACATAATTGAAGATTT
TAAAAATTAACACTTGAGTGACGATGTTATTATATTTGTCATTTGAGTGTTTTTTGTGGCGTGATAGTATAGG
GAACTTAGCTAAAGTTAATTTGTAGTGTGATGTCGTGGAAATTTTATGGGCATTGTACCTGAAGTTGTTATGT
GGCGAGATGTTGGGGATATAGATGGTGTTGTATGTGGCGAGATATTGGCGTACGTATACAGCGTTGAACCAGA
AAATTTAATGTAGCGTAATGACAACACATTACAAGAGTTGATTATATTATGATAAATCCAGGTGCTACCGTAC
TAATGAAACAAATTTGATGCAGGTGTATTGCATGTTATTTACATACATGTTAGAGTGTTACTTAAACACGTAG
ACGCTTTAACGCTTTAAAGGAGATGCGAATGATGAAAAGACAACAATCACAATGGAAGTCATCAACTGGATTT
ATTTTAGCTAGTGCGGGTTCTGCAATCGGTCTTGGTGCCATGTGGAAATTCCCATATATGGCAGGGATTTATG
GCGGCGGTGCCTTTCTAGCTATGTTCTTAATATTCACCATTTTTGTTGGGTTGCCATTACTCATTATGGAATT
CACTGTTGGGAAAATGGGACGGACATATACAACACAAATATATAGTAAATTAACTGGTAAAAAATGGCTCAAT
ATCATTGGCTGGAACGGTAATTTGGCAGTGTTTATTTTATTTGGCTTCTATAGTGTTATCGGTGGTTGGATTG
TCATTTACATCGGACAAGTATTATGGCAATTAGTTATATTTCAACGCATCAATCATCTCCAAGAAATGAATTT
TGAAGCGGTAATATCAAATCCTTGGTTAACTGTTCTAGGGCAAGGTATATTCATATTCGCTACGATGATTATT
GTCATGTTAGGTGTTGAAAAAGGATTAGAAAAGGCATCGAAAGTTATGATGCCATTGCTGTTTGTCTTTTTAA
TCATCATTGTGATTAAGTCTTTAACATTAGATGGTGCCTTAGAAGGTGTGAAATTTATTTTACAACCAAGAGT
ATCAGAGATTACTGCTGACGGTATCTTGTTTGCGCTAGGCCAATCATTCTTTACGTTATCATTAGGAACTACA
GGTATGATTACTTATGCGAGTTATGCCTCTAAAGACATGACGATTAAGTCATCAGCTATTTCTATCGTTGTTA
TGAATATCTTTGTATCTGTATTGGCAGGTCTAGCTATATTTCCGGCGTTACATAGTTTTGGCTATGAACCACA
AGAAGGGCCTGGATTATTATTTAAAGTACTGCCAATGGTCTTTAGTCAAATGCATCTAGGCACATTATTCTAT
TTGGGATTCTTAGTGCTGTTCTTATTTGCGGCTTTAACGTCATCTATTTCTTTATTAGAATTAAATGTTTCTA
ACTTCACGAAGAATGACAATACAAAACGTAAAAAAGTCGCAGTGATCGGTAGTATTTTAGTATTTATCATTAG
TATTCCAGCAACCTTATCTTTTGGTATCTTAAAAGATGTAAGATTCGGTGCGGGAACGATTTTTGATAATATG
GATTTCATCGTTTCGAATGTATTGATGCCATTAGGCGCATTAGGTACTACGCTTGTCGTAGGACAATTATTAG
ATAAAAAATTATTACAACAATATTTTGGTAAAGATCGATTTAGATTATTCAGTGGTTGGTATTACTTAATTAA
GTATGCGATGCCTGTCGTTATTATCTTAGTCTTTATCGTGCAATTATTTAGTTAATATATAGAAGCATCTGAC
ACACAAGTATTTGTGTTGATCAGGTGTTTTTTTGTTTGGAATTGAAAAGTTAGTACTGGTTCTGAATTAATCG
AAACTTAGATAATTGCAAAATAACAAAATGTCGTTTATAATTCTTATCTGAAAAGTAAGAATTAAAATTGAGA
TATGAAGAAAGGTTTCATTCATTTGATCGGCATGTCAGATGAGGGTGCATCTATGATTACTTATGATTTAATT
GGCAATACACCATTAGTACTGTTAGAACATTATAGTGATGATAAAGTTAAAATTTATGCCAAGCTTGAACAAT
GGAATCCTGGAGGCAGTGTTAAAGACAGACTCGGGAAATATTTAGTAGAGAAGGCAATTCAAGAAGGGCGTGT
GCGTGCAGGTCAAACTATTGTTGAAGCGACTGCTGGTAATACAGGCATAGGGTTAGCTATTGCAGCGAATAGA
CATCATTTGAAATGTAAGATCTTTGCGCCGTATGGTTTTTCAGAAGAAAAGATTAATATTATGATAGCGCTTG
GTGCAGATGTTTCAAGGACGAGTCAGTCTGAAGGTATGCATGGGGCACAATTAGCTGCACGTTCCTATGCTGA
AAAATATGGTGCCGTTTATATGAATCAATTTGAATCCGAACATAATCCGGATACATATTTTCATACATTGGGA
CCCGAATTGACTTCAGCATTACAGCAAATTGATTATTTTGTGGCTGGTATTGGCTCTGGCGGTACATTTACAG
GTACCGCACGTTATTTAAAGCAACATCACGTGCAATGTTATGCCGTTGAGCCAGAAGGGTCCGTGTTAAATGG
AGGGCCAGCTCATGCACATGACACTGAAGGTATCGGTTCTGAGAAATGGCCGATATTTTTAGAGAGACGTCTT
GTAGATGGGATATTTACGATTAAAGATCAAGATGCCTTTCGAAATGTCAAAAGTTTGGCTATAAATGAAGGGT
TGTTAGTAGGCAGTTCTTCAGGTGCAGCATTACAAGGTGCATTGAATTTAAAAGCGCAATTATCTGAAGGTAC
GATTGTTGTCGTATTTCCAGATGGTAGCGATCGATATATGTCTAAGCAAATATTTAATTATGAGGAGAATGAT
TATGAATAAGAAAACTAAATTAATTCACGGTGGACACACAACGGACGACTATACAGGTGCAGTTACTACACCA
ATTTATCAAACAAGTACATATTTACAAGATGATATTGGTGATTTACGTCAAGGATATGAATATTCTCGTACTG
CGAATCCGACAAGAAGTTCTGTAGAAAGCGTTATTGCGGCATTAGAAAATGGCAAACATGGCTTTGCATTTAG
TTCAGGTGTTGCAGCAATCAGTGCAGTTGTTATGCTGTTGGACAAAGGTGATCATATTATTTTAAATTCAGAT
GTATACGGCGGTACTTATCGCGCATTGACAAAAGTATTTACACGATTTGGCATTGAAGTGGATTTTGTAGATA
CAACGCATACAGATTCAATTGTACAAGCGATACGCCCAACAACAAAGATGTTGTTTATTGAAACACCTTCTAA
TCCATTATTACGTGTTACTGACATTAAAAAGTCTGCTGAAATTGCGAAAGAACACGGTTTGATTTCAGTCGTT
GATAACACATTTATGACACCTTATTATCAGAATCCATTAGATTTAGGTATCGATATTGTTTTACATTCTGCAA
CGAAATATTTAGGTGGACATAGTGATGTCGTTGCTGGTTTAGTTGCAACATCGGATGACAAGCTTGCAGAACG
TTTAGCATTTATTTCAAATTCAACAGGTGGCATTTTAGGACCTCAAGATAGCTATTTACTTGTGAGGGGTATT
AAAACATTAGGTTTACGTATGGAACAAATTAATCGCAGTGTTATTGAAATTATTAAAATGTTACAAGCACATC
CAGCTGTGCAACAAGTGTTCCATCCAAGTATTGAAAGTCATTTAAATCATGATGTCCATATGGCTCAAGCTGA

TGGCCATACAGGTGTGATTGCATTTGAAGTGAAAGATACAGAAAGTGCCAAACAATTGATTAAAGCAACATCG
TATTACACATTAGCTGAAAGTTTAGGTGCAGTGGAAAGTTTAATTTCAGTACCTGCACTGATGACACATGCAT
CCATTCCAGCAGATATTCGAGCTAAAGAAGGTATTACAGACGGACTTGTAAGAATTTCTGTAGGTATTGAAGA
TACTGAAGATTTAGTGGATGATTTAAAACAAGCACTAGATACGTTATAAATAATAGCAGCACTGGCATATATT
TTGAGTTCAACGTTTGTTGTTCTTGAATATGCTAGTGCTTTTTTGTGCGATGTTAATATTTCGTAATTGCGTT
GTGGAGGATTGCTGGTCATTTTTATTCATTTAATTCGATAAATGTCATAGTAGCCTCACAATTCATCATTGTC
ACATGAATTGCTTATTTTTTAATTTTTTAGAAAATTCGGCAAATTGTATTGACGATATATGCAATTTTACATA
TAATGCTCTTATTCCTAGTGGATTAATAAGAATTTGTAGGAGGGGCGATGATGATTGAGTTTCGACAGGTAAG
TAAGACCTTTAATAAAAAGAAGCAAAAAATACATGCTTTAAAAGATGTATCATTTAAGGTCAATCGCAATGAT
ATTTTTGGTGTGATTGGATACAGTGGTGCTGGAAAAAGTACGTTGGTAAGACTCGTGAATCATCTTGAAGCTG
CCTCAAGTGGTCAAGTGCTTGTAGATGGACATGATATTACGAATTATAGTGAAAAAGGCATGCGAGAAATAAA
AAAAGATATCGGTATGATATTTCAGCATTTCAATTTATTAAATTCAGCTACCGTATTTAAAAATGTAGCAATG
CCACTCATTTTAAGTAAGAAAAGCAAAACAGAAATTAAGCAACGAGTAACGGAAATGCTTGAATTTGTAGGAT
TGAGTGATAAAAAAGACCAATTTCCTGATGAATTATCTGGTGGGCAGAAGCAAAGGGTGGCTATTGCAAGAGC
GCTTGTTACTAATCCGAAAATACTCCTATGCGATGAAGCAACAAGCGCATTGGATCCAGCAACGACTGCTTCG
ATATTGACGTTATTAAAGAATGTCAATCAAACCTTTGGCATTACAATTATGATGATTACACATGAAATGCGCG
TTATTAAAGACATTTGTAATCGTGTTGCTGTAATGGAAAAGGGGCAAGTGGTTGAAACAGGAACTGTTAAAGA
GGTGTTTAGTCATCCTAAAACGACGATTGCTCAAAATTTTGTGTCTACAGTTATACAGACTGAGCCAAGTACA
TCATTGATTCGTCGATTGAATGACGAACAAGTTGGCGATTTTAAAGATTATAAAATCTTCGTCGAGGAAACTC
AGGTGACACAACCGATTATAAATGACTTGATTCAAATTTGTGGCAGAGAGGTTAAAATTTTATTTTCATCTAT
GTCAGAAATACAAGGTAACACCGTATGTTATATGTGGCTTCGATTTAATATGGATCAACAATTTGATGACACG
GCAATAAATCAATATTTCAAAGAGAAAAATATTCAATTTGAGGAGGTGCATTAACATATGTTTGGTTCTGATT
TAGACAGTGCACAGTTATTACAAGCATTGTACGAAACGCTATATATGGTATCTATTGCTTTATTTTTAGGAGC
AGTGATTGGTATTCCATTAGGTGTCTTATTGGTAATTACTCGAAAACAAGGCATATGGCCCAATATAGTGATA
CATCAAGTTTTAAATCCTTTAATCAATATTTTAAGGTCACTACCATTTATTATTTTGTTAATTGCGATTGTGC
CATTCACAAAATTAGTAGTAGGTACTTCAATTGGTACGACTGCTGCCATCGTGCCTTTAACAGTATATGTGGC
ACCTTACATTGCAAGACTTGTTGAAAACTCATTATTGGAAGTAGACGAGGGGATTATTGAAGCGGCGAAAGCG
ATGGGCGCTTCACCACTACAAATCATTAGATATTTTTTAATCCCTGAAGCGTTAGGTTCATTAGTATTAGCAA
TTACCACTGCGATTATTGGACTTATTGGAAGTACGGCGATGGCAGGAGCTGTTGGCGGTGGTGGTATAGGAGA
CTTAGCTTTAGTGTATGGTTATCAAAGATTTGATACGACGGTCATTATTATTACCGTTATTGTATTAGTCATT
ATTGTCCAAGTGATTCAAACGCTAGGGAATGTTCTAGCTAGATTCATACGTAGACATTAATGGTATATAGTGA
AGATTTTGAAAGGAATTGATAGAATGAAAAGATTGATTGGGTTAGTTATCGTAGCACTTGTATTATTAGCAGC
GTGTGGTAGTAACAATGATAAAAAAGTAACAATTGGTGTCGCATCAAATGATACTAAGGCTTGGGAGAAGGTT
AAAGAATTAGCTAAAAAAGATGATATTGATGTGGAGATTAAGCACTTTTCCGATTACAATTTACCGAATAAAG
CATTAAACGATGGTGATATTGATATGAATGCATTCCAACATTTTGCATTTTTAGATCAGTATAAGAAGGCACA
TAAAGGAACAAAGATTTCAGCATTAAGTACAACAGTTTTAGCACCGTTGGGCATTTACTCAGATAAAATTAAA
GATGTCAAAAAGGTTAAAGATGGTGCTAAAGTTGTCATTCCAAATGATGTATCAAACCAAGCACGTGCACTTA
AACTATTAGAAGCAGCTGGTTTAATAAAACTGAAAAAAGATTTCGGTTTGGCAGGCACGGTGAAAGATATAAC
GTCAAATCCAAAACATTTAAAAATTACTGCAGTAGATGCACAACAAACTGCGCGTGCTTTATCTGATGTCGAT
ATTGCAGTTATTAATAATGGTGTAGCAACTAAAGCGGGTAAAGATCCTAAAAATGATCCGATATTTTTAGAAA
AATCAAATTCAGATGCTGTAAAGCCATATATTAATATTGTTGCAGTTAATGACAAAGACTTGGATAACAAAAC
ATATGCTAAAATCGTAGAATTGTATCATTCAAAAGAAGCTCAAAAAGCGTTGCAGGAAGATGTCAAAGATGGA
GAGAAACCTGTTAATTTATCTAAAGATGAGATTAAGGCAATAGAAACGTCATTAGCAAAATAAATAATATTGC
GTCCTACAAGCAAAGTTCATGCTTATGTTTGTAGGGCGTTATTGTTTGACAATAAAATTATTTCCAATAGAGA
AAGGGATTGTAATCATTTTATAGTGAAATATTATGAAATTGTAATAATTTAGATATTGTAAAATCTAATAAGT
TGTAATAATTTTAAGGGGTAATTATAAAATTTGATGATACAGTATATGATTTTTTTGTAATCATAATGTCATC
AAACATCAACCTATTATACATAATTAAATCGTATAATGATGTAGTATTCATAAATTCGGATAAAAAGAATGTT
AGGAAAGTTAAGCAAGAGGAGGATTTTAAAGTGCAAAAAAAAGTAATTGCAGCTATTATTGGGACAAGCGCGA
TTAGCGCTGTTGCGGCAACTCAAGCAAATGCGGCTACAACTCACACAGTAAAACCGGGTGAATCAGTGTGGGC
AATTTCAAATAAGTATGGGATTTCGATTGCTAAATTAAAGTCATTAAACAATTTAACATCTAATCTAATTTTC
CCAAACCAAGTACTAAAAGTATCTGGCTCAAGTAATTCTACGAGCAATAGTAGCCGTCCATCAACAAATTCAG
GTGGCGGATCATACTATACAGTACAAGCAGGTGACTCATTGTCATTAATCGCATCTAAATATGGTACAACTTA
CCAAAACATTATGCGACTTAATGGTTTAAATAATTTCTTTATTTATCCAGGTCAAAAATTAAAAGTATCAGGT
ACTGCAAGCTCAAGTAATTCTGCGAGCAATAGTAGCCGTCCATCAACGAATTCAAGCGGTGGATCATACTACA
CAGTACAAGCAGGTGACTCATTGTCATTAATCGCTTCAAAATATGGTACAACTTACCAAAAAATTATGAGCTT
AAATGGCTTAAATAATTTCTTTATATATCCGGGTCAAAAATTAAAAGTAACTGGTAATGCAACATCTTCAAAC
TCAGCATCTGCAACAACTACGAATAGAGGTTACAATACACCAGTATTCAGTCATCAAAACTTATATACATGGG
GTCAATGTACATATCATGTATTTAATCGTCGTGCTGAAATTGGTAAAGGTATTAGTACTTATTGGTGGAATGC

TAATAACTGGGATAACGCAGCGGCAGCAGATGGTTACACTATCGACAATAGACCTACTGTAGGTTCTATCGCT
CAAACAGATGTAGGTTACTATGGTCATGTTATGTTTGTAGAACGTGTAAATAACGATGGTAGTATTTTAGTTT
CAGAAATGAACTATTCAGCTGCACCAGGTATTTTAACTTACAGAACGGTACCAGCTTACCAAGTAAATAATTA
TAGATATATTCACTAAAGTCTTACGTATATAAATATATAATGAATTCCTATTACATTTCACAAGCTGAAAAGT
TGGATTGAATCCTAAACTTTATGGCGGGGCTATCAAAGTCGTGATTGTTGTAATAGGAATTTTTGTATATGAA
AAAGGATTGGTCGAATGAACTTCATGTTCTATGTTCGACCAATCCTTTTCTGAATTAGTATTCATGCTTTACT
TTGCGAATGCTTGTAAATAATCTAGCACCGTTTGTTATTAAAGTAACAACTGCCACTGCTTTTTTGAACTTAC
GTGGTGACTTAATTGAAATGTAAAAGTCTAACACGGTTCCTACAGTGCTTAAACCTAATGAAAGATATACTAA
TTTTTTATTTTTAGCATGATATTTATAGCCATTGTAGCCGTCGACTATGAAACCTGCGACATTTAGTAAACTT
GATAAACGTTGTGATTTGGAACGTTTTGCCATAATATTAATTCCCCCTAGTATCTTCTGTTTATATGAAAAGT
TGGGTGCGCTGAATTGCTAACGTTTTGCGCTATAACTACTCATATATGATAACATAATTGTACAGTATAATTT
GAAAAATTGATTTCACAAAGTTGGGGTGTCAAAGATGATTAAATGTGTCTGTTTAGTTGAAGAAACAGCTGAT
AAAATATTGCTTGTTCAAGTAAGGAATCGCGAAAAGTATTATTTCCCAGGTGGTAAAATAGAAGAAGGGGAAT
CACAAGTACAAGCGCTGTTAAGAGAAGTAAAAGAAGAATTAAATTTAACATTAACAATGGATGAAATTGAATA
TATCGGGACAATTGTAGGTCCTGCATATCCACAACAGGATATGTTAACTGAGTTAAATGGATTTCGCGCATTA
ACCAAAATCGATTGGGAAAACGTAACTATCAATAATGAAATTACGGATATACGCTGGATGGATAAAGATAATG
ATGCGTTGATTGCGCCTGCTGTCAAAGTTTGGATTGAAACGTATGGTGGTAAACATGACAAATAATGACACCA
TCATGTTACGACATTATGTCCCACAAGATTATTCGATGTTAGAAGCTTTTCAATTAAGTGAAAGTGATTTGAA
GTTTGTTAAAACGCCAGAGGAAAATATTACAGCTGCAATGTCTGATAATGAAAGGTATCCCATCGTTGTAATG
GATGGCAGGCAATGTGTGGCCTTTTTTACATTACATCGTGGAAAAGGGGTCGCACCATTTAGCGATAACCAAG
ATGCAGTATTTTTCAGGTCATTTAGTGTTGATCAACGTTATCGTAATAGAGGAATAGGTAAAGTGGTAATGGA
AAAATTGGCGTCATTTATCACTTCAACATTTCAGGGTATTAATGAGATTGTGTTAACGGTTAATACTGACAAT
CCACATGCCATGGCACTTTATCGCCAACAAGGATATCAATATATGGGAGATAGTATGTTCGTCGGAAGACCTG
TTCATATTATGGCGTTAACTATAAAATAAATTAAATTTAAAAGCATCTTTACTCATCGTCGATCACAACAATT
AATGATGAATAAAGGTGCTTTTTGTTATAGATCACCGGACAATTTACTATAGTAAAAAGCGACCTAGTGAACA
ATTGACATATATCCACAGGTCGCTTAACTTAAATTATATTGCTAGTTGCGATTAATTGATAGACTCATCATTT
TTGCGCTGTCGAGATGGTCTTTTTATTAAAAATGCCGTAATCCAAGCCGTAATCGGAATACTGATTGCAACGG
CAATACCGCCTAAAATAATAGAAATAAATTCTTGAGCAAATATTTTCGAGTTTATAATATGACCAAATGAATA
TTTAAGTTTGAAAAACCAAATAAATAAAGCAAGTTGGCCACCAAAAAAGGCAAGGTAAATCGTGTTCGCAGAT
GTCGCTAAAATTTCTCTACCAACACGCATGCCAGATTGGAATAATTCGTATTGCGTAAGTGTTGGATTCACTT
GATGCAATTCATAAATGGGTGAACTAATGGTAATCGTTAAATCTATCACAGCTGCAATAACAGCAAGAATAAT
AGTGAACACCATAAATTGAACCATATCAATGCCAATATTCATTGAATACACATATGTTTCATCTTGTTGTTCG
GTTGAAAAGCCTTGTAGATGACCGAAGTAGACCGATAAATAAATGAGTGTAATCAACAATATTGTTGTAACGA
TAGTGCTGATAAATGCAGCTTGTGTTTTAACATTGTAACTATTGAGTACGAATAAATTACAAGCGCCAATAAT
AATGCAGAAAAAGAATGTGACGACATAAATCGGTACGCCAAAAATAATCAACACAATACTAATAATTAAAATA
GCGAAATTTAAAAATAGGGTTAAATAAGAGATGAATCCCTTTTTACCTCCGAAAATTATCATCAGAAAGAGGA
GCAATAACGCCAATATAAATACAGCATTCATTGTTTCGCCCTCCTTAATGTTTCAAATATTTCCATAAACAAT
ATTGTGATAGGAATTGTAAGTACGATACCTATACCGCCTGTTAGTGCGCGCGCAATTTCTAACGACCAATTCA
TCGAAATAGTATAAGTCACAGTATTTGCATTTTTTAAAAAGATTAAAAACATAGGTAGTGCACCAGATAAATA
TGAGAATAATAAGATGTTAGTCATTGTTCCCATAATATCTTGGCCGATGTATCGCCCAGCAAGCGCCCATCTC
CTCATTGAAATGTGTGGCGTACGCTGTAAAATTTCATGCATACCACTAGCAATTGTAATTGCAACATCCATAA
TAGCGCCAAGTGAACCTATTAACACTGAGGCTAGGAAGATATCTTTCGGTGGTAATGATAAAAAGTTCATCGT
TTCATATTTAATGCCTTTACCATCTGTCATATATATGATTAATTCTGTTAAACCTATACTCAAAAAAGTTCCG
ATAATTGTACTGGCTATGGTAATGAGTGTACGCATATGCCAGCCTGTAACGAGCAATAAAGTGAGTACTGTTG
AACAGATCATGGCAATGGTCATGAGTAAGAATAAATTAATATTGCTATGTTGAATATGAATGTAAATTGCGAT
TAATATGGCAATAGAATTCAAGATTAACGATAAAATCGATTGCAGTCCGACTTTGCGACCAACCAATAATACA
GTTAATAAGAACAAACCAGTGATGATAACCGTTAAGGTATCACGCTTCTTTTCTATAATATAAGCATCACTCG
GCTTGTTAGAAATATGTAATAATACTTTTTCGTGTGTGCGAAATGCCTCAGAATCTGCTTGTGATTTGACGTA
CTGATGATTAATCGTCGTTGTTTCTCCAGCAAATTGACCATTTAATATTTTGACTTTTAATTGATTTTTATAT
TTAATATCACGATTATTTTGTGCATCTTTTGTAGGTGTCGAAGAAACATGTTTGACATCTATAATTTGACCAA
TTGGTTTGTTGTAAAAGTTCTCATTATTGAATGTAAATAAAATAGCACCAATGAATGCGATGCAGAACAAACC
TAAAATTATATTAAATGGCTTTGTAAATAAATTTCTATATTTCAAAAACAAAACCCCAATTCTATGAATGAAT
TAATATGGTGATTATACGCCCTTAATTTTTTATTTTCAAAGATATTACTGCTAAGTGTAAAACGAAAATCATC
ATTGATAGCATCGAATTACTTAATGGAATGTAGACGTTTTAGTCATTAATTGCTGAATAAGTGTTAATAATAT
GCCAATATCACTCTTTGTATAAGGCTCCTTTGTAATAGCACATATCGTTCTTTTTAATTCAGTATGATCTAAT
TTTATATCTATCCATGATTTAGATTCTGGTAAATGTATATTTTGTGATGAAATGATGTAACCTTCTTTTTGAC
GAAGGAGATACTGCGCAAGTGGTTGGCTACTGATTGTGTATACATCTGATTTAGTAATCTTGCGCAATTGTTT
TTTTACAGTTTCGGCAAATGGTGCCAAGCAATAAATATGACTATGCTCAAACTGAATTAATGGTGGGTGTGTC

GCCATCGTAATTGGATCGTCTGAAGGCGCATATAAATGATAGTGCTCTTCGAATAAAGGTAGCATATGTAATT
GTTTGTGTTTACGTATTTCTGGTGTAAGTTCCGTGAAACCAATGTCTATATTCCCATTTAATACGCTATTTAT
AATTGTGTCATGTTCTAATAAATTAGGGATGACATGTGTATCATTTTGTAAATGAAACGTTTGGATAAGTGGT
AGTAACATGTGGGATACGTCACTCTCATCATAGCCAATGTAGATACTTTTATTTTTAGTTAATCCATGGCTTT
GAAATTGTTCAATCGTGCTATCTAAATGTTCAATAATACGCAGAGCTTCATTAAATAATAATTTTCCTTCAGA
TGTGAGCGTAATATTGCGTCCTTGCTTTTTAAATAAAGACACATTAAGTTCTTGTTCTAATAATGTAATTTGA
CGGCTTATCGCTGATTGAGCAATGTTTAGTTCAAGTGCTGTTTCGGAGATATGTTCTCTTTTAGCGACCTCGA
TAAAATATCTTAATTGTTTAATTTCCATAGCGATATAGGCACCTCCAAAAATGAGTGTTTTGTAACTATTATA
GCAATATTATTGATAAATGTTCTATTTTTTAGATGAATATCTTCTATTTTATATATTGAACAGATAAATTTTT
TAGATTATAGTAATTATCATTAATAACTAATATCAGAATATTCTAAAAAAGGGGTGTGCATCATGCACAATGA
GAAATTAATTAAAGGCTTATATGACTATCGTGAGGAACATGATGCGTGTGGTATTGGTTTTTATGCGAATATG
GATAATAAAAGGTCTCACGACATCATTGATAAATCGCTTGAAATGTTGCGACGCTTAGATCATAGGGGCGGGG
TCGGCGCAGATGGCATCACTGGTGATGGCGCAGGTATTATGACTGAAATACCTTTTGCATTTTTCAAACAACA
TGTAACGGACTTTGATATCCCAGGTGAAGGTGAATATGCCGTGGGGTTATTTTTTTCCAAAGAACGCGTTTTA
GGTTCTGAACATGAAGCAGTTTTTAAAAAATATTTTGAAGGCGAAGGGTTATCAATTCTTGGTTATCGTAATG
TACCAGTTAATAAAGATGCCATTGCTAAACATGTAGCAGATACGATGCCAGTCATTCAACAAGTGTTTATTGA
TATTAGGGACATTGAAGATGTTGAAAAGCATTTGTTTTTAGCGAGAAAACAATTAGAGTTTTATTCGACTCAG
TGCGATTTAGAATTGTATTTTACGAGCTTATCACGCAAAACAATTGTATATAAAGGTTGGTTACGATCAGACC
AAATTAAAAAACTCTATACAGATTTATCGGATGATTTATATCAATCAAAGCTAGAGTTAGTGCATTCGAGATT
TAGTACGAATACATTCCCAAGTTGGAAAAGGGCACATCCTAACCGTATGTTAATGCATAATGGTGAGATTAAC
ACGATTAAAGGTAATGTAAACTGGATGCGAGCACGCCAACATAAATTAATCGAAACATTATTTGGCGAGGATC
AACATAAAGTGTTTCAAATTGTCGATGAGGATGGTAGTGACTCTGCTATTGTAGATAATGCGCTAGAGTTCTT
ATCGTTAGCCATGGAGCCAGAAAAGGCAGCGATGTTACTCATACCTGAACCTTGGTTATATAATGAAGCGAAT
GATGCAAATGTACGTGCGTTTTATGAATTTTATAGTTATTTAATGGAACCGTGGGATGGTCCTACAATGATTT
CGTTCTGTAACGGTGACAAACTTGGCGCGCTTACAGATAGAAATGGATTACGTCCAGGTCGTTATACGATTAC
TAAAGATAACTTTATTGTCTTTTCATCTGAAGTGGGTGTTGTGGACGTACCTGAAAGTAATGTTGCTTTTAAA
GGTCAATTGAATCCTGGAAAGTTATTGCTTGTTGATTTTAAACAGAATAAAGTCATTGAAAATAATGATTTAA
AAGGTGCGATTGCTGGAGAATTACCATATAAAGCGTGGATTGATAACCATAAAGTTGACTTTGATTTTGAAAA
TATACAATATCAAGATTCGCAATGGAAAGATGAGACGTTATTTAAATTACAACGTCAGTTTGCATACACGAAA
GAAGAGATTCATAAGTATATTCAGGAACTTGTAGAAGGTAAGAAGGATCCTATCGGTGCAATGGGATATGATG
CGCCAATTGCAGTGTTGAACGAGCGACCAGAATCACTATTTAATTACTTTAAACAGCTGTTTGCACAAGTTAC
GAATCCACCAATTGATGCGTATCGTGAAAAAATCGTAACGAGTGAACTTTCTTATTTAGGTGGCGAAGGTAAC
TTACTAGCACCTGATGAAACGGTTTTAGATCGTATTCAATTGAAAAGGCCGGTATTGAATGAATCACACTTAG
CAGCGATTGATCAGGAACATTTTAAATTAACTTATTTATCAACGGTATATGAAGGGGACTTGGAAGATGCGTT
AGAAGCATTAGGCCGAGAAGCAGTGAATGCTGTAAAGCAAGGCGCTCAAATTCTAGTGTTAGATGACAGTGGA
TTAGTTGATAGCAATGGCTTTGCAATGCCGATGTTACTCGCAATAAGTCATGTGCATCAATTACTTATTAAAG
CAGATTTACGTATGTCTACAAGTTTAGTCGCTAAATCTGGTGAGACACGAGAAGTGCATCATGTTGCTTGTTT
ACTCGCATATGGCGCGAATGCAATTGTGCCATACCTAGCGCAACGTACAGTTGAACAACTGACATTGACAGAA
GGGTTACAAGGCACCGTTGTCGATAATGTTAAGACATATACGGATGTATTGTCGGAAGGTGTCATTAAAGTAA
TGGCTAAGATGGGAATTTCGACAGTGCAAAGTTATCAAGGGGCACAAATATTTGAAGCGATTGGCTTGTCTCA
TGATGTGATTGATCGTTATTTTACTGGGACACAGTCTAAGTTATCTGGTATTTCGATTGATCAAATTGATGCT
GAAAATAAAGCACGTCAACAAAGTGATGATAATTATCTTGCATCAGGTAGTACATTCCAATGGAGACAACAAG
GTCAACATCATGCTTTTAATCCGGAATCTATTTTCTTATTGCAGCACGCATGTAAAGAAAATGACTATGCGCA
ATTTAAAGCATACTCTGAAGCGGTGAACAAAAATAGAACAGATCACATTAGACATTTACTTGAATTTAAAGCA
TGTACACCGATTGACATCGACCAAGTTGAACCGGTAAGTGACATTGTCAAACGCTTTAATACAGGGGCGATGA
GTTATGGATCGATTTCAGCGGAAGCACATGAAACGTTAGCACAAGCCATGAACCAATTAGGTGGAAAGAGTAA
TAGTGGTGAAGGTGGCGAAGATGCAAAACGTTATGAAGTACAAGTTGATGGAAGCAACAAAGTAAGTGCGATT
AAACAAGTTGCTTCTGGGCGGTTTGGTGTAACTAGTGATTATTTACAACATGCCAAAGAAATTCAAATTAAAG
TTGCGCAAGGTGCAAAGCCTGGTGAAGGTGGTCAATTACCTGGTACTAAGGTATATCCGTGGATTGCGAAGAC
AAGAGGGTCAACGCCAGGTATCGGTCTGATTTCACCACCGCCACATCATGATATTTATTCAATAGAAGATTTA
GCGCAACTGATACATGATTTGAAAAATGCGAATAAAGATGCAGATATCGCGGTAAAATTAGTTTCGAAAACAG
GTGTTGGTACCATTGCATCTGGGGTGGCAAAAGCATTTGCAGATAAAATTGTCATCAGTGGTTACGATGGTGG
TACAGGGGCTTCACCTAAAACGAGTATTCAGCATGCCGGTGTTCCTTGGGAGATTGGTTTAGCAGAAACACAT
CAAACATTAAAACTAAATGACTTAAGAAGTCGTGTTAAGTTAGAAACAGACGGTAAGTTATTAACTGGTAAAG
ATGTAGCGTACGCATGTGCGCTTGGAGCGGAAGAATTTGGATTTGCAACTGCACCATTAGTGGTGTTGGGCTG
TATAATGATGCGTGTATGCCATAAAGATACATGTCCAGTAGGAGTTGCAACTCAAAACAAAGATTTACGTGCT
TTATATAGAGGTAAAGCACATCATGTTGTTAATTTTATGCATTTTATTGCACAAGAATTAAGAGAAATTTTAG
CATCTTTAGGTTTGAAACGTGTAGAAGACTTAGTTGGAAGAACTGATTTATTACAACGATCATCAACATTAAA

AGCGAATAGCAAAGCGGCTAGTATTGATGTTGAAAAACTGTTATGTCCTTTCGATGGGCCAAACACAAAAGAA
ATTCAACAAAATCATAATCTTGAGCATGGATTTGATTTAACAAATTTATATGAAGTAACGAAGCCATATATTA
CTGAAGGGCGTCGCTATACAGGTAGCTTTACAGTAAATAATGAACAACGTGATGTAGGAGTTATTACAGGTAG
TGAGATTTCGAAACAATATGGAGAAGCAGGACTTCCTGAAAATACAATTAATGTTTATACGAATGGTCATGCT
GGTCAAAGTCTTGCAGCATACGCACCGAAAGGCTTAATGATTCATCATACTGGAGATGCGAATGACTATGTTG
GTAAAGGATTATCTGGTGGTACGGTCATTGTCAAAGCACCTTTTGAAGAACGACAAAATGAAATTATTGCTGG
TAACGTCTCATTCTATGGTGCGACAGGTGGTAAGGCATTTATTAACGGTAGTGCAGGAGAAAGATTCTGTATT
AGAAATAGTGGTGTAGATGTTGTCGTTGAAGGTATCGGCGACCATGGATTAGAGTACATGACTGGTGGACATG
TCATTAATTTAGGTGATGTAGGTAAGAACTTCGGTCAAGGTATGAGTGGTGGTATTGCTTACGTTATCCCGTC
TGATGTAGAAGCTTTTGTTGAAAATAATCAACTAGATACGCTTTCGTTTACAAAGATTAAACACCAAGAAGAA
AAAGCATTCATTAAGCAAATGCTGGAAGAACATGTGTCACACACGAATAGTACGAGAGCGATTCATGTGTTAA
AACATTTTGATCGCATTGAAGATGTCGTCGTTAAAGTTATTCCTAAAGATTATCAATTAATGATGCAAAAAAT
TCATTTGCACAAATCATTACATGACAATGAAGATGAAGCGATGTTAGCTGCATTTTACGATGACAGTAAAACA
ATCGACGCTAAACATAAACCAGCCGTTGTGTATTAAGGAAAGGGGGAGATACGATGGGTGAATTTAAAGGATT
TATGAAGTATGACAAACAGTACTTAGGTGAATTATCACTGGTAGACCGTTTGAAGCATCATAAAGCATATCAA
CAACGATTTACTAAAGAAGATGCCTCTATCCAAGGTGCGCGATGTATGGATTGTGGAACGCCGTTTTGTCAAA
CCGGACAACAGTATGGTAGGGAAACAATAGGTTGTCCAATTGGAAACTACATTCCTGAATGGAACGACTTAGT
GTATCATCAAGATTTTAAAACTGCTTATGAACGCTTAAGCGAAACAAATAACTTTCCTGACTTTACAGGGCGT
GTATGTCCTGCACCATGCGAAAGTGCTTGTGTGATGAAGATTAATAGAGAATCGATTGCGATTAAAGGTATTG
AACGCACAATTATTGATGAAGCTTTTGAAAATGGTTGGGTAGCGCCGAAAGTTCCGAGTCGCCGTAGAGATGA
AAAAGTGGCAATCGTTGGAAGCGGTCCAGCAGGATTAACTGCTGCTGAAGAACTTAATCTACTAGGATATCAA
GTAACTATTTATGAACGTGCTAGAGAATCAGGCGGTTTATTAATGTATGGTATTCCGAATATGAAACTTGATA
AAGATGTGGTTCGACGTCGTATTAAGTTAATGGAAGAAGCGGGCATTACTTTCATTAATGGTGTTGAAGTAGG
CGTTGATATTGATAAAGCAACGTTAGAATCTGAGTATGATGCCATTATATTATGTACTGGTGCACAAAAAGGT
AGAGATTTACCTTTAGAAGGACGCATGGGTGATGGTATACATTTCGCTATGGATTATTTAACTGAACAAACGC
AGCTGTTAAATGGAGAAATTGATGATATTACAATAACTGCAAAAGATAAGAATGTCATTATCATTGGTGCTGG
TGATACAGGGGCAGACTGTGTAGCGACAGCATTAAGAGAAAATTGTAAATCGATTGTTCAATTTAATAAATAT
ACGAAATTGCCAGAAGCAATTACATTTACAGAAAATGCATCATGGCCTTTAGCAATGCCGGTGTTTAAAATGG
ACTATGCGCACCAAGAGTACGAAGCTAAGTTTGGTAAGGAACCACGTGCATATGGTGTTCAAACAATGCGTTA
CGATGTTGACGATAAAGGACACATACGTGGTTTGTATACTCAAATTTTAGAGCAAGGCGAAAATGGTATGGTC
ATGAAAGAAGGACCTGAAAGATTTTGGCCTGCTGACCTTGTATTATTATCAATCGGCTTCGAAGGTACAGAAC
CAACTGTACCGAATGCTTTTAACATTAAAACGGATAGAAATCGAATCGTGGCGGATGATACAAACTATCAAAC
TAATAATGAAAAAGTATTTGCTGCTGGAGATGCTAGACGTGGTCAAAGTTTAGTTGTATGGGCGATTAAAGAA
GGTAGAGGCGTAGCGAAAGCAGTAGATCAGTATTTAGCGAGTAAAGTTTGTGTATAATCTTTGTATGGAAATG
GTGGTTACGTTGACGTTGTGGCATGCTGAATCGAGTTTGAAATAATCTAGTATCTATCAACGTCACATGCTAT
CATTGTAACCTAAAAACAAAGGTTTGTAAGACAACAAATAGATTAATTATAAGTAGTGATTTTTTACATTCGT
TTATAGGTCAACTGTAGTGGAAGACAATGATTTGTGGTAATCATGTAATGCTTAAAAACAATATTGACTTTTA
CAGAACGTTCATATATGATAAATATTGTGTTTAGGAGGAATACCCAAGTCCGGCTGAAGGGATCGGTCTTGAA
AACCGACAGGGGCTTAACGGCTCGCGGGGGTTCGAATCCCTCTTCCTCCGCCATCAATATTTATATTAAATAC
TATATATAATGAAGGTAAGTGCTCAAATTTTGAGTATTTACCTTTTTTATTTGTCTTTGAATGACTCGTAATT
TTTGATAATAGAAATGATAAGGCATTGAGATTGGAAGGGCATTTGGCTTGTGCAATATACATAGCTAAATGTC
TTTTTTGTTTTGTGAAATATGATGGATGGCTTGTGTGGACAAGTTTGCTATTTATAGATATGCATTTTTCAAT
TTAGGAGTTGGCCATGCATCTACACTTTATAATGGTGAGAGCGTGGTGAGGTATTGTTAATAACGCAATTGTA
GCGAGGAGTTATTGCTACATATGTCGTTATGGCTCATTGATTTTCTGAAATGGCTATCCCAGATAATTGTGAC
AAAATAAAAATATTTCTTTGAAAGCCTTTACATAACTTGTCTAGACAAGTTATACTCATTTTAAGACATTAAG
GGAGTGAAATATATGGCTGTAAAAAGAGAAGATGTAAAAGCCATCGTAACCGCTATTGGGGGAAAAGAAAATC
TTGAAGCTGCAACGCATTGTGTAACACGATTACGTTTAGTGCTGAAGGATGAAAGTAAAGTTGATAAAGACGC
ATTAAGTAATAACGCGTTGGTCAAGGGGCAGTTTAAAGCAGACCATCAATATCAAATTGTCATTGGTCCAGGA
ACAGTCGATGAAGTGTATAAGCAGTTTATTGATGAAACAGGTGCTCAAGAAGCTTCGAAAGATGAAGCGAAAA
AAGCAGCTGCACAAAAAGGGAATCCAGTACAACGTTTGATCAAATTGTTGGGGGATATTTTTATACCAATATT
ACCTGCGATTGTGACAGCTGGTTTGTTAATGGGAATCAATAATTTACTTACAATGAAAGGTTTATTTGGTCCA
AAAGCACTTATTGAGATGTATCCACAAATTGCTGATATTTCAAACATCATTAATGTGATTGCGAGTACGGCAT
TTATTTTCTTACCAGCATTAATTGGTTGGAGTAGTATGCGTGTATTTGGTGGTAGTCCGATTCTAGGCATAGT
CTTAGGTTTGATTTTAATGCATCCGCAATTAGTATCTCAGTATGATTTGGCAAAAGGGAATATTCCGACGTGG
AACTTATTTGGCTTAGAGATTAAGCAGTTGAATTACCAAGGTCAAGTGTTGCCAGTTTTAATTGCAGCTTATG
TTCTAGCTAAAATTGAAAAAGGATTAAATAAAGTCGTTCACGATTCGATAAAAATGTTGGTCGTTGGACCCGT
AGCGCTTTTAGTTACTGGATTTTTAGCATTTATTATCATTGGACCAGTTGCGTTATTGATTGGTACAGGTATT
ACATCTGGTGTTACATTTATATTCCAACATGCAGGATGGCTTGGCGGAGCAATATATGGATTGTTATATGCAC

CACTTGTAATTACAGGACTACACCATATGTTTTTAGCAGTAGATTTCCAATTGATGGGTAGCAGCTTAGGCGG
TACGTATTTATGGCCAATTGTTGCGATTTCCAATATTTGTCAGGGCTCTGCAGCATTTGGAGCATGGTTTGTC
TATAAACGTCGTAAAATGGTTAAAGAAGAAGGCTTGGCATTAACATCTTGTATTTCTGGTATGTTAGGTGTTA
CTGAACCAGCTATGTTTGGTGTGAACTTACCTCTGAAATATCCATTTATCGCTGCGATATCAACGTCTTGTGT
ATTGGGGGCAATCGTTGGTATGAATAACGTACTTGGAAAAGTTGGTGTTGGTGGCGTGCCAGCATTCATTTCA
ATTCAAAAAGAATTTTGGCCAGTATATCTTATTGTGACAGCTATTGCTATTGTTGTACCATGTATACTAACAA
TTGTGATGTCTCATTTTAGTAAACAAAAAGCGAAAGAAATTGTTGAAGATTAATAAAATAAAAAAGGGGCGTT
CGTTATTTGGACGTCCTTTATTACGTTATAAGGTGGTAATTGTGTGTCGAAAGAAATAGATTGGAGAAAATCC
GTTGTATATCAAATTTATCCTAAGTCGTTTAATGATACGACGGGGAATGGTATAGGAGATATCAACGGAATTA
TAGAAAAAATTGGATTATATCAAGTTATTGGGTGTTGATTATATTTGGTTAACACCAGTGTATGAATCACCGAT
GAATGATAATGGCTATGATATCAGCAATTATTTAGAAATCAACGAAGACTTTGGAACGATGGATGATTTTGAA
AAGTTAATCAAAGTTGCTCATCAAAAAGACTTGAAAGTGATGTTAGATATTGTCATTAATCATACGTCGACGG
AGCATGAATGGTTTAAAGAAGCCCGTAAATCTAAAGATAACCCTTATAGAGATTATTACTTTTTCAGATCATC
TGAAGACGGGCCGCCAACAAATTGGCATTCTAAATTCGGTGGTAATGCATGGAAGTATGATTCTGAGACAGAT
GAATATTATTTACATTTATTTGATGTCAGTCAAGCTGATTTAAATTGGGATAATCCGGAAGTACGTCAATCGT
TATATCGCATAGTCAATCATTGGATAGACTTCGGCGTTGATGGTTTTCGATTTGATGTCATTAACTTAATTTC
TAAAGGTGAATTTAAGGACTCTGACAAAATAGGTAAAGAATTTTATACGGATGGTCCTAGAGTGCATGAGTTT
CTGCATGAATTAAATCGTCAAACGTTTGGTAACACTGACATGATGACTGTAGGAGAAATGTCTTCGACGACGA
TTGAAAATTGTATTAAGTATACACAGCCAGAACGCCAAGAATTGAATAGTGTTTTTAATTTTCATCATCTAAA
GGTTGATTATGTTGATGGTGAAAAGTGGACAAATGCGAAGCTTGATTTTCATAAGTTAAAGGAAATTCTGATG
CAATGGCAACGAGGTATTTATGACGGTGGCGGATGGAACGCGATTTTCTGGTGTAATCATGATCAGCCACGGG
TAGTGTCTAGATTTGGTGATGATACGTCGGAAGAGATGAGGATACAAAGTGCTAAAATGTTAGCTATCGCACT
GCATATGTTGCAAGGGACGCTATATATTTACCAAGGGGAAGAAATTGGTATGACGGACCCACATTTTACATCA
ATAGCACAATATCGCGATGTTGAATCGATTAATGCCTACCATCAGTTGTTAAGTGAAGGGCATGCTGAAGCGG
ATGTATTAGCGATTTTAGGACAGAAGTCACGAGACAATTCGAGAACGCCTATGCAATGGAGTGATGATGTTAA
TGCTGGATTTACAGCTGGTAAGCCTTGGATTGATATTTCGGAAAATTATCATCAGGTCAACGTTAGACAAGCA
CTTCAGAATAAAGATTCTATTTTCTACACGTATCAAAAATTAATACAATTAAGACATACGCATGATATTATTA
CGTATGGAGACATTGTGCCACGTTTTATGGATCATGATCATTTATTTGTTTATGAACGTCATTATAAGAATCA
ACAATGGCTAGTAATTGCGAATTTCTCAGCATCGGCTGTTGATTTGCCAGAAGGATTGGCTAGAGAAGGTCGT
GTTGTGATTCAAACAGGCACAGTGGAAAATAATACGATAAGCGGGTTTGGTGCAATTGTAATCGAAACAAACG
CGTAAAATAAATTGAGTGGATGCGTTTATATGGCGAAACAAAAAAAGTTTATGAAGATTTATGAGGCGTTGAA
AGAAGATATATTAAACGGGCAGATTCAATATGGTGAACAAATTCCGTCTGAACATGATTTGGTGCAATTGTAC
CAGTCATCTCGAGAGACCGTGCGTAAGGCATTAGATTTGTTGGCATTAGACGGCATGATTCAAAAGATTCATG
GTAAAGGGTCACTTGTCATTTATCAGGAGGTTACAGAGTTTCCATTTTCTGAACTTGTTAGTTTTAAAGAAAT
GCAAGAAGAAATGGGCGTCGCATATTTAACTGAAGTTGTTGTGAATGAGGTTGTTGAAGCGCATGAAGTTCCA
GAAGTTCAACATGCTTTAAACATCAATTCTAGTGAATCACTCATTCATATTGTTAGAACTCGTCGGCTTAACC
AACATGTGAAGATTGTTGATGAAGATTATTTTCTAAAGTCGATTGTTTCAGATATAGGTAATGATGTTGCGAG
TGATTCTATTTATGATTATTTGGAAAAGGTATTAAATCTTAATATTAGTTATTCAAGTAAGTCTATTACTTTT
GAACCGTTTGATGAACAAGCATATCAATTGTTTGGTGATGTATCGGTGGCTTATTCAGCAACAGTTCGAAGTA
TTGTGTATTTAGAAAATACAATGCCGTTTCAATATAATATTTCAAAACATCTTGCAAATGAATTTAAATTTAA
TGACTTCTCAAGACGTCGTACAAAGTAAACAATGATATAAATGATTTATACTTGCAATTAACTATTAAAATAT
AGTAATATATATCTTGCCGTGCTAGGTGGGGAGGTAGCGGTTCCCTGTACTCGAAATCCGCTTTATGCGAGGC
TTAATTCCTTTGTTGAGGCCGTATTTTTGCGAAGTCTGCCCAAAGCACGTAGTGTTTGAAGATTTCGGTCCTA
TGCAATATGAACCCATGAACCATGTCAGGTCCTGACGGAAGCAGCATTAAGTGGATCATCATATGTGCCGTAG
GGTAGCCGAGATTTAGCTAACGACTTTGGTTACGTTCGTGAATTACGTTCGATGCTTAGGTGCACGGTTTTTT
ATTTTTTAAATATTAAACCGATTATTAAGAGTTGAAAATATATATTTATTTATAGAAGCTACTTTCTTGAAGA
CAATTCAGCGTATTATACGTGGAACATGTTTGTGGGAAGTAGCTTTTTTATATGTGAAGTTTGATTCAAGTGA
ACTCGATGTACAGTTTGAATGATTTTTGTGTCAATGAAAAGTAAGAAGTTATAATTTGATGATAAAGAAATGA
TGGTGAAATGAGGGGGAGTATCTTACAATAGAATTATTAATGAGATACGTTATGATTATTGACAATCAAATGC
CTACGGAGGACATATGCAAATATATTTAAGTACTTTAACAGAGTTAGATTATGATAAATCTTTAAATAGTATT
GAAGAAAGTTTTGATGATAATCCTGAAACGAGTTGGCAAGCACGTGCGAAAGTAAAACATTTAAGAAAATCTC
CTTGCTATAATTTTGAATTAGAAGTAATAGCGAAAAATGAAAATAACGATGTCGTTGGACACGTTTTATTAAT
TGAAGTAGAAATTAATAGTGATGATAAGACGTATTATGGTTTGGCGATTGCCTCTTTATCAGTTCATCCTGAA
TTACGTGGACAAAAAATTAGGTCGTGGCTTGGTTCAAGCAGTAGAAGAGCGTGCCAAAGCACAAGAGTATAGTA
CGGTTGTTGTAGACCATTGTTTTGACTACTTTGAAAAGTTGGGTTATCAAAATGCTGCTGAGCATGACATTAA
ATTAGAATCTGGTGATGCACCGTTACTTGTAAAATATTTATGGGATAATTTGACGGATGCACCACACGGAATC
GTAAAATTTCCAGAACATTTTTATTAATTGTTCAATTAAGAAGTAAAGGTATTATCATGCTATAATGAGAGGT
AATTGTTTATGGAGGTGCTAACTTGAATTATCAAGCCTTATATCGTATGTACAGACCCCAAAGTTTCGAGGAT

GTCGTCGGACAAGAACATGTCACGAAGACATTGCGCAATGCGATTTCGAAAGAAAAACAGTCGCATGCTTATA
TTTTTAGTGGTCCGAGAGGTACGGGGAAAACGAGTATTGCCAAAGTGTTTGCTAAAGCAATCAACTGTTTAAA
TAGCACTGATGGAGAACCTTGTAATGAATGTCATATTTGTAAAGGCATTACGCAGGGGACTAATTCAGATGTG
ATAGAAATTGATGCTGCTAGTAATAATGGCGTTGATGAAATAAGAAATATTAGAGACAAAGTTAAATATGCAC
CAAGTGAATCGAAATATAAAGTTTATATTATAGATGAGGTGCACATGCTAACAACAGGTGCTTTTAATGCCCT
TTTAAAGACGTTAGAAGAACCTCCAGCACACGCTATTTTTATATTGGCAACGACAGAACCACATAAAATCCCT
CCAACAATCATTTCTAGGGCGCAACGTTTTGATTTTAAAGCAATTAGCCTAGATCAAATTGTTGAACGTTTAA
AATTTGTAGCAGATGCACAACAAATTGAATGTGAAGATGCAGCCTTGGCATTTATCGCTAAAGCGTCTGAAGG
GGGTATGCGTGATGCATTAAGTATTATGGACCAAGCTATTGCATTTGGTGATGGTACGTTAACATTGCAAGAT
GCGTTGAATGTCACAGGTAGCGTACATGATGAAGCGTTGGATCACTTGTTTGATGATATTGTACAAGGTGACG
TACAAGCATCTTTTAAAAAATACCATCAGTTTATAACAGAAGGTAAAGAAGTGAATCGCCTAATAAATGATAT
GATTTATTTTGTCAGAGATACGATTATGAATAAAACATCTGAGAAAGATACTGAGTATCGAGCACTGATGAAC
TTAGAATTAGATATGTTATATCAAATGATTGATCTTATTAATGATACATTAGTGTCGATTCGTTTTAGTGTGA
ATCAAAACGTTCATTTTGAAGTGTTGTTAGTAAAATTAGCTGAGCAGATTAAGGGTCAACCACAAGTGATTGC
GAATGTAGCTGAACCAGCACAAATTGCTTCATCGCCAAACACAGATGTATTGTTGCAACGTATGGAACAGTTA
GAGCAAGAACTAAAAACACTAAAAGCACAAGGAGTGAGTGTCGCTCCTACTCAAAAATCTTCGAAAAAGCCTG
TGAGAGGTATACAAAAATCTAAAAATGCATTTTCAATGCAACAAATTGCAAAAGTGCTAGATAAAGCGAATAA
GGCAGATATCAAATTGTTGAAAGATCATTGGCAAGAAGTGATTGATCATGCCAAAAACAATGATAAAAAATCA
CTCGTTAGTTTATTGCAAAATTCGGAACCTGTGGCGGCAAGTGAAGATCACGTACTTGTGAAATTTGAGGAAG
AGATCCATTGTGAAATCGTCAATAAAGACGACGAGAAACGTAGTAGCATAGAAAGTGTTGTATGTAATATCGT
TAATAAAAACGTTAAAGTTGTTGGTGTACCATCAGATCAATGGCAAAGAGTTCGAACGGAGTATTTACAAAAT
CGTAAAAACGAAGGCGATGATATGCCAAAGCAACAAGCGCAACAAACAGATATTGCTCAAAAAGCAAAAGATC
TTTTCGGTGAAGAAACTGTACATGTGATAGATGAAGAGTGATACATGACAAGCGATATAATCGTATGTATAAT
GAAAGAAACATCATTTTATTGATAAATATTTATTGATTTTCAAGGAGGAAATGGAATATGCGCGGTGGCGGAA
ACATGCAACAAATGATGAAACAAATGCAAAAAATGCAAAAGAAAATGGCTCAAGAACAAGAAAAACTTAAAGA
AGAGCGTATTGTAGGAACAGCTGGCGGTGGCATGGTTGCAGTTACTGTAACTGGTCATAAAGAAGTTGTCGAC
GTTGAAATCAAAGAAGAAGCTGTAGACCCAGATGATATTGAAATGCTACAAGACTTAGTGTTAGCAGCTACTA
ATGAAGCGATGAATAAAGCTGATGAGCTTACTCAAGAACGTTTAGGTAAACATACTCAAGGCTTAAACATCCC
TGGAATGTGATCATAGATGCATTATCCAGAACCTATATCAAAACTTATTGATAGCTTTATGAAATTGCCAGGC
ATTGGTCCAAAGACAGCCCAACGTCTGGCTTTTCATACCTTAGATATGAAAGAAGACGATGTTGTTCAGTTTG
CCAAAGCATTAGTAGATGTTAAAAGAGAATTAACATATTGTAGCGTATGTGGTCACATTACTGAAAATGATCC
ATGTTATATTTGTGAAGATAAGCAAAGAGATCGTTCAGTTATTTGTGTTGTGGAAGATGACAAAGATGTCATA
GCTATGGAAAAAATGAGAGAATACAAAGGTTTATATCACGTTTTACATGGGTCTATTTCGCCTATGGATGGCA
TTGGACCAGAAGATATTAATATTCCTTCATTGATTGAACGCTTAAAAAGCGATGAAGTTAGCGAATTAATCTT
AGCTATGAACCCGAACTTAGAGGGGGAATCTACAGCCATGTATATTTCTAGATTAGTTAAGCCTATAGGTATC
AAAGTGACGAGATTAGCACAAGGGTTATCGGTAGGTGGCGATTTAGAGTATGCTGACGAAGTAACTTTATCTA
AAGCAATCGCAGGTAGAACAGAAATGTAATGTCTTCTATTAAACATTTTTGAATTTAATACTATAGTAAGAAA
AGTCACAGTGTAATCATTGTGGCTTTTTTTATGGTGTGGTGTGATGTACTACTTTATTTGCGGTGTGGCGGTG
GTATGGTTTACCTAGTTTTACTGAGGGATGGGTAATCTTTAGGAAGCAAGCCGTTGGTTGTGATTTGTTACTT
CTAATAGTAATGATGTGAATTGGATTATCGAATTAGATCTACGGTTATGGTGTGTTGGTGCTATTAATTTGAT
AAATGCGGTTGATGGTTAATGACTATGCAAATGAAATTCTTTTGTAATTGAAATGATAGATGCTGGCTTAGTA
GTTGTACTTCTTTGGTCTAAAGTTTATTAAATCAGCCTGTATAGCGGTGTTTTGAGAGGTTATTTAAAACTTG
TAAATTTATTTTTAATTTCTGGTAAAAAAAATAAAGTTCTGTTTTGCGGTTTTTTTTGATTGATATGGTTAGA
GAAAAATCTGTTTCTTGTTCTAAAAAACGTACTATTTATAAGTGGGGATTTTTTAAGTTCGATTTTTAGGATA
AGGGCGTTCAGTACAGATGGCAAAGGTGTAATTTTTACTGTTGTTAAGCAGTTTGAAAGCCTGTATAGTATTT
ATTTGTTGAGGCAAACAAAACAACTCAACTTAAGAAATAACTTGAATTACTAACGAAAATTAATTTTAAAAAG
TTGTTGACTTAAATGTTAATAAAATGTATAATTAATTCTTGTCGGTAAGAAAAATGAACATTGAAAACTGAAT
GACAATATGTCAACGTTAATTCCAAAAAACGTAACTATAAGTTACAAACATTATTTAGTATTTATGAGCTAAT
CAAACATCATAATTTTTATGGAGAGTTTGATCCTGGCTCAGGATGAACGCTGGC
>DSM104437_scaffold_5
GCACATACCGTAGTAACAACGAATAATGAGAAGTCAGCAGACGTCATAGTAGGGAAAATGTACCGAAGGACGG
AACAATATTCATACAAAGTAAAGAATGGAGGTTATAGATTTACATCATGCAGAAAACAACGAACTAGTTGGCA
ACATATAGAGAGATAAGTAGTGGAACGAAAAAGGGTAAATAAGTGCGCATGGTAAATCTTAGGTGAAATGAAA
GAAATGTATCGTGAGTCTCCATCGATGATGGAGCTTGTTGTAAGAGAACACAACATTCAAAAGGCAATTAAGA
AAGTTAAAAAGAATAAAGGTGCCCCTGGCATTGATGGCATGAAAGTGAGTGAAATACAAGGTCACTTTGCGCA
ATACTTTCCAAAAATAAAACAAAAACTGCTTGAAGGTACATACAAACCTCAAGCAGTTAAGAAAGTTGAAATT
CCCAAAGCAAATGGGAAAAAGCGTGTGCTTGGTATACCTGTCGTAAGAGACAGAGTGATACAACAAGCAATTA
AACAAGTGATTGAACCTAGTATCGATCGTACCTTCTCTAAACACAGTCATGGCTTTAGACCTAATCGTAGTAC

AGGAACAGCACTGAAAGAATGTGCGTCTTACTATGAAGCAGGTTACACGATAGCCGTAGATTGTGATTTAAAA
CAATGCTTTGACAACATAAATCATGATAAACTCATGTATTTATTTGAGCGCCACATTAAAGATAAAGCGGTAT
CGACATTTATCCGTAGAAGGTTACAAGTTGGTGCCATTGACCTATCTGGCGAAGTCGCAGAAAGAAAGATAGG
TGCACCGCAAGGTGGTGTGATATCACCATTACTCTGTAACATATATCTACATGAACTGGATAAAGAGCTCGAA
AAACGTAATCATCGTTTTGTGAGATATGCAGATGACTTCGTCATCTTTGTCAAGACGAAACGTGCAGGTGAAC
GTGTGATGGATAGTATTAAAACATTCATACACAAAACCCTCAAACTTGAAGTCAATAATGATAAAAGTAAGGT
GGGTTCTCCTACCCGTTTAAAGTTCTTGAGTTGTCTAATGAGCAAAGTAAATGGCACTTATCGTTTCAGACCC
ACTACGGAAGCAAAAAGAAATTTAAAACGCAACTTAAAGTGGTTAACCAGACGAAGTAGACCAGGTTCATTCC
AAGATATTATAACAGAAATTAATCGTGTGACACGAGGATGGATTAACTATTTTGGAAAAGGATTCATAAAAAG
ATTTCTTAAAGAATTAGAACCTTGGCTAAATCGCCGCATTAGGCAACTCATACTCAAAAGATGGAAGAAGATT
AAAACAAAATATAAGATGTTGCGCAAGTATGGTCTAGACCACGATAGCGCAATGAGAATCGCGAGCTCAAGAA
AGAAATACTGGCGTTTATCATCTACGCATGAGGTTCATCGTGCACTTACAACAAAACGTCTCCGCAAGTGGGG
GTTATTATCACTAACCCAACTCGCAGAGACGGCTTACGCAAGATATTGAACCGCCGAGTACGGAACCGTACGC
TCGGTGGTGTGAGAGGACGAATAATCAAGTAATGATTATTCTCCTACTCGATTTGTCCAAAACGTGCTGATGA
CATTTTAAAAGCAAGTATGGAATATCAGTCGACAGACTATAAACGGAGGTATATCTCATGGAAAACAATGATA
GCAATGAAGTAGCTTATTTGTTAAAAGATGGTAAATTTACAAAAGTATATGTCAATCAAGATAGTGTATCTTT
TGTGCCAGGGTAACAAGCAACCGAATTATTATTTAACAGTAAACCGAATTCAATTGTTATGTTACATAACCAT
CCTGGACAGTCAGGATTTTCAGAATATGATTTATTTACATTTTTTAAGCATCCATCAATAAAATCAATGACAA
TCGTTACTAATAAGGGACAAGTAAAATTTATTACCAAGTCAAATAGGTTTCATGGTAAAATAGTGAGTAAATT
TTGTGCTAAATATTTTACGCATATTAATATCATTAATGATAGCCATATTGAAAAATTATTGAAAAAACTTTAT
AGTATAAATATGATTAAATATAAAGTGAGATGATTTTAATTATGTTAGATTCACAATTAGATGGAAATGTAAC
TGCAAAAGAATTGAGTGAATCTTTTAAAGAATTAGTTGAAGAATTTGAAAGTATAGAAAAAGCGAAAAAGAAT
TCAAATTCAAAAGATAAATAGCATCCTTTTTAGACAGATAAAGAGAAAGTGGTGCTATTTTTATACGCTTTTT
TAACTATTTACTGAAAATGCAGAAGCACAAAAGTTAACATATTTAAAAGAACTTGGTGAAGATGGCGAATATA
AATATGTTGCCAAAATAGATAGTAAAACATCTAAATTATGTCATTCACTTAACGGAAAAATATTTAAAGTTAA
AGATATGATACCAGGTGTGAATGCGCCACCTATGCATCCTTGGTGTAGAAGTACCACAGTGCCACATGTTGGC
AATTGGCGGGACAAGTTCTTTAAAGAGCGTGAAGGTAAATATCAAGTAGAAGTAAAAGAAGCATAATTACAGG
AAAAAGCTAAAAACCAGATGAAAGAAATGATTAAAAATGGTAAAATAAAAATAGAAATAAATTGCGGAAAATA
AAATAGATATATGTTAGGTCATCACCTATATAATGAAAATAAAAAAAGAGCCATTTTAAATAATAAGAAATTG
CCTAGCTATACAATACTTTCTATAGATTTATTGAATGAATTGTTAAGAGAAAAAATGTCAACAGGCAATCTAA
TATTAAGTGATGAGCGATTTGATTTGAAAGAGATTATTAATTTTAATCAAATTATTGGAAAAATACATATCGA
AAATGTGTATATTGAAACCAGAAAGGGAAAAGTGCATTATTCGAAGACAGGTGCTCATATAGTACCTTATATT
GATAAGTAGGTGAAAAGTATTGAGAATTGAAGATGCATATCGTAAAGATGTTATTATCACGCTTTTGAATAAT
GAAGAATACGAAGGGTTTGTAACTGACTATGAAAATGAATTCGAGAGCGAAACAGGAAATCTTGTTGTAGATA
TACAGACTGATTTTGCTGTTTATTCGTTTGATGAAACTGAGATAAAAAGTATTAGATTATTAAAATAATTACA
AAATAGCATCCTTTCTACAAAAAGCTAAAAAAGAAATGCTTGAAATGATAAGTAATGGTAAAATGACGATGAA
ATTGAATAATGTTAAGCAAAAGCGTCATATTTTATGCACAAATGAATATAATAATAAGAAAAATAATTCATCT
TTATTACCAAGTTATACTATAATAGATTCTAATGAAAGTGAAAAGATGACTAAAAAAGAATTTATTGATATTC
CAGTTTTGTTTGATGATGAAGGGAATTTTAGAATAAAACAAGTTATAGATTATAAAAAGATAATAGGTAAATC
TTATGTGAATGGTAAATATATTGAAACGAAATTAGGTAAGGTTCATTATTCTAAAACTGGTTTTCATGTAGTT
CCGTATATTAAAAAGGAGTGATTAAATATGAATTTACAGTCATACATAGGCAAATTAGTCAAATTAACGCTTA
CTAATAATAAAATATTAATTGGAAAAGTGATAGACTTTGATGATAAAGTTGATAATTTTGATGGTTATAATTC
GATAGAAATTGATACAGGTAGAATTACATATGATATATCAGAAAATAAAATTAAAACTATTGTTTTACAATAA
TAGCATCCTTTCTACACAGATAAAGAGAAAGCGGTGCTATTTTTTGCGCTTTTATAACAACTAAGGATACAAG
ACACATAACTTCAACAATATCCTCATCTAAACCTATTGATTTCCTAAGTTCAATTTTAAACACAAAGTTTATT
TGTAAAGATGCACACCTATAAAAATCTTAATCATCGAAATGTCACAAACATCTCCATATTAAAAACTTAAATA
GTGCATTCCATTTAAACTAAACATCGATATTCCTACCAAAATAGATTTAGTAATTCAAATGATATTACATCAC
TTTCATAAAATAACTACAACCATCATAATACTTTTCAAATCTCGTCAAATTAAAGTAAATACAGCACAAATTA
ACATTTTTAAATTTATAAAATAAAACAAGATACTCAATAATCTGTAGTATCTTGTTTTGGAATAATGTAATTT
TATATCATCAACGGCTTCAAATGAGCAGTTTCATATATAAATAAACACAACTACTCAACTGAACTTGATTAAT
TAATAAATGATTTGCCAGATGACATATAACAGCATTATTAGATTAATTCTAATAGTTTATTTAAATTTTCTTC
GGTTGTCGCCCAGCTGGTTGCGAATCTAACAACACGATGTTGATCATCGTATTTTTCCCAAACAGCAAATTTA
ACTTTTTGTTCTAACTCTGCTATTTTCTCGTTACTTAAAATAAAAAATTGTTGATTGGTTGGAGAATCAAAGT
AAAGACGATAGCCTTTATTTTTAAACCCGTCTTTCATCTTATTTGCCATTTCGATAGCATGTCTGCTTATATT
AAAATATAAATTGTCCGTAAATAATTCTAAAAATTGTATGCCTGTTAACCGTCCTTTTGCTAAAAGGGCACCG
TGATGCTTGATTCGAGTGGTAAATTGTTTCGGTTCATTATTTTTCGTAAAAACAATGGCTTCCCCGCATAATG
CACCTATCTTCGTACCACCTATATAAAATACATCACAATATTTAGCGATGTCTTTAATAGTCATATCTGATTG
GTCACTCATCAATCCATACCCTAATCGTGCACCATCCATAAATAATGGAAGCTGATATTGCTTACATACTTTG

CATAACTCTTCCAATTCTGATTTAGAGTATAATGTGCCATATTCTGTAGGATGAGAAATATATACCATTCCTG
GGAATACCATATGGTCCTTTTTAAAATCACTTTTAAATGTCTCCATGTAAGTTTCAACATCTGAAGCACTAAC
TTTTCCTTCCTTAGAGGGTATAGTAATTACTTTATGTCCACTATATTCAATTGCACCGCCCTCATGCACAGCA
ACATGACCAGTGTCTGCTGAAATAACCCCTTCGTAACTTTCTAACATTGAATTAATAACAACCTGATTGGTTT
GCGTTCCACCTACTAAAAAACGGATAGTAGCATTTGGACAGTCAATTGTATCCTTAATCTTTTCAATTGCCTG
ATTTGTGAATTGATCAAAGCCATATCCCGAAGCTTGTACAAGATTTGTATCTACTAATCTTTTTAATACTTTT
TCATGAGCGCCTTCTAAATAATCATTTTCAAATGAAATCACTACATTTCCCCCTAAAACTAATATCAACATTT
TAATAAGATAAACCAATTTCAAAACTAGTTCGATATTTAAAATGTATTATGAATGGTTAAAGTTTGTATCGCA
TTATCGCGAAGTTGAATAAATATATTATACATCAAACAATACATTAATAAAATTAATTAATTTTTAATCATAG
CGACTTTTCGTGCTGGAACACATATATTAATGATTGAATTTTACTGTTAAAAGTTCTATGATTTAGGAAATAG
AGAAAAGAGGGGATTTAAATGGAACAAATTAAACTTAAAACTTTTACAGCTGAGACTTTAGAATTATTAGAAT
CAAATATCAACGAATTTTTAGGTTCTGAAGAAGCTACAAATTTGAAATTAGTAAATATTACAATAAAAGAAAT
TGAAGAAAGAACATTTCCAAATAATGAAGAAGAGTTTAATGCAATATTAACTCTATCTGTAAATAAATAATCT
GGAAAGAAAGGTACAAAAGTTTTTTGTGCCTTTCTTTATAGGAGAAATAAACTATGCAATTTTCATTTTCAAA
TGATTTAGGAACACTTTTTACCATCATTTTAGCAATTGCCTTTATCATTAATTTAGTTCTAGCGTTTATTATT
ATTTTTTTAGAAAGAAATAGACGTACAGCCAGTTCAACATGGGCGTGGTTATTTGTACTTTTTGTCTTACCAT
TGATTGGTTTTATCCTTTATTTATTTTTTGGAAGAACGGTTTCTGCGCGTAAACTAAATAAAAATAATGGGAA
CGTGTTAACAGATTTTGATGCGCTTTTAAAGCAGCAGATTGAAAGCTTTGATAAAGGTAATTATGGTACAGAT
AATAAACAAGTTCAAAAGCACCATGATTTGGTACGTATGTTGTTAATGGATCAAGATGGCTTTTTAACTGAAA
ATAATAAAATTGATCATTTTATTGACGGAAATGATTTATATGATCAAGTTTTACAAGATATTAAAAATGCTAA
AGAATACATCCATTTAGAGTATTATACATTCGCTCTAGATGGCTTAGGCCAAAGAATTTTAAAAGCGTTAGAA
GAGAAATTGAAACAAGGTCTAGAAGTGAAAATTTTATATGATGACGTTGGTTCAAAGAAAGTTAAGATGGCTA
ATTTTGATCATTTTAAATCGTTAGGTGGAGAAGTTGAAGCATTTTTTGCTTCTAAATTACCGTTATTGAATTT
CAGAATGAATAATAGAAATCATAGAAAAATCATCGTAATCGATGGTCAACTAGGTTATGTCGGAGGATTTAAC
ATTGGTGATGAATATCTAGGTTTAGGAAAATTAGGGTATTGGAGAGATACGCATTTACGTATACAAGGCGATG
CGGTTGATGCACTGCAGTTGCGATTTATTTTAGACTGGAATTCGCAAGCACATCGACCTCAATTTGAATATGA
TGAAAAATATTTTCCTAAAAAGAATAAATCATTCGGTAATGCGCCAATCCAGATTGCTGCAAGTGGTCCTGCC
AGTGACTGGCATCAAATTGAATATGGTTATACAAAAATGATTATGAGCGCTAAGAAGTCGGTATATTTGCAAT
CGCCTTATTTCATTCCGGATAACTCATATATAAATGCCATTAAAATTGCAGCTAAATCAGGGGTAGATGTTCA
TCTGATGATTCCATGTAAGCCAGATCATCCTTTAGTTTATTGGGCGACATTTTCAAATGCCTCTGACTTATTA
TCAAGTGGTGTTAAAATTTATACGTATGAAAATGGATTTATACATTCTAAAATGTGCTTAATTGATGATGAAA
TCGTATCAGTCGGCACAGCAAATATGGACTTTAGAAGTTTTGAATTAAATTTTGAAGTAAATGCCTTTGTATA
TGATGAAAATCTTGCTAAAGATTTAAGAGCGGCTTATGAACATGATATTACAAAATCAAAACAACTTACTGAA
GAATCTTATGCTAATAGACCATTGTCTGTTAAGTTTAAAGAATCGTTAGCTAAATTAGTTTCGCCAATTTTAT
AATTTATTTGTAAGGAGTCTCGATTATAGAGGCTCTTTTTATTTCTGCAATTAATGACTATAATTGTATTATG
TAAACCGTAAAAGTGACAAATGTCATTTTGAACCAATGACAAAGTATCCTATTTTAAATCGAATAAATTTACT
AATCTTAAATTAATAAAATATTAAGAGGTGATTCATTTGATTCAAATATCTAATATTAATAAATCATTTAAGA
AAAAGCGTGTTTTAAAAAACATTTCATTTGATATTGAAAAAGGTACCTGTATCGCATTAATCGGAAAAAATGG
TGCTGGAAAATCAACGTTAATTGATATATTAATTGGTAATGTTAATGCTAATTCTGGTGAGATATTTGATAAA
GACAAGTTATTACAAAGTGAAAATCGCAGTATAATGTTCCAAAAAACGATGTTTCCAGATCAATTAAAAGTTA
TTGAGATTATCAACTTATATCAATCATTTTACGAAAATCCATTACCATTGGAAGAAATAATAGAACTGACGAA
ATTTGATTCTAGTCAACTGAACCAATTTGTAAATAAACTTTCTGGTGGTCAACAACGATTACTCGATTTTGTA
TTATCTTTAATCGGACAACCACAATTGATCTTATTAGATGAACCAACATCGACTATGGATATAGAAATTAGAG
AATATTTTTGGTCAATTATTGAAAATTTAAAAGAAGATAATCGAACGATACTCTATACATCGCACTATATTGA
AGAAGTCGAACGTATGTCAGACAAAATTATTCTCATTGAAAATGGAGAAATAATACTTAATGATTCAACGTCA
CATATTAGAACCAATCAGCAATCTCAGATTACGTTATCCGATGAATATATAAGAAAGTTAAAACTAGATAAAG
ATGATTTAGTTATTCAAAAAAATCATAATGGCACTATCAAAATTATTACTTCAAATGTAAATGATACGATTTT
ATATCTTCAACAACTTCATATTAATTTGGATGATATTGAAATACAAAAAGTCTCAATTGTTGATTCATACTTC
AACAATAAAAAGCAAAGGGGATCTAATTATGATACTAAGTTACTTGAAAATCGAATTTAAAGTTATAATGCGT
AAAAAAACAACATTAATATTATCTATTTTATTTCCTGTTATATTCTATATATTATTTACTTCGATATTGGAAT
TGCCGGAAGATGTTAAACCTAAATTTTATAAAGAGTATATGTATAGTATGACGGTTTATAGTTTGTTAAGTTT
TAGTTTACTAACTTTTCCATTAGATATTATTAATGAAAAACAAAATGAATGGCGCCAAAGATTAATGGTAACA
CCATTTACTTTTACTAGTTATTATATTTCAAAAGTAGTGAAAACTATGCTGCAATTTGCAATAGCGATATTAG
TTATTTTTATGGTTGGACATTTTTATAAAGGTGTTGCAATGAGTGCAGTTCAATGGTTAGAGTCAGGAATATT
TTTATGGTTAGGTGCGTCTCTATTAATAACTTTTGGCATATTATTTTCTTTGTTAAATGATATTCAAAAAACA
AGTGCTTTAGCTAATATCGTAACAATTGGTTTAGCAGTATTAGGTGGATTGTGGTTTCCGATAAACACATTTC
CAAATTGGCTTCAACATGTTGCTCATGTTTTACCGAGCTATCATTTGCGTAAACTAGGTGTAGATATTGCTTC
AAATCATCATATCAATTTAATATCATTTGCTATAATACTCTTGTATGCTTTAGGGAGTATATTAGCAGTATAT

TGTATTAGTCATTTTAAAAGGGCGGAATAAAATATGAAATTTTTAAAAGATACTTCAATTGCTGAAATATCGT
CTATACTTTATCTGATTTTTCCTATTGCCGGTATATTTTTTAATGAAGTATATGGTCCCAAATGGTTGTATAT
TATATCAGTCATTGTCTTTTCGTTGTCGTATCTTATATTAGTTATAGTAAATAATAGACTTAATACATTAATG
TTTTACATTTTGTTGATTATTCATTATTTTATTATTTGTTATTTTGTTTTCAGTGTACATCCAATGCTAAGTT
TGTTTTTCTTTTATAGTGCTTTTGCCGTTCCATTTACTTTTAAAAATAATGTTAAAAAAACGGCAACTAATTT
TTTCATACTAACAATGATTATATGTACAATAATAACGTACTTATTTTATAACAACTATTTTGTTGCAATGATG
GTTTATTATGTCGTTATATCGTTAATAATGCTAGATAATTTTAAAAAAATGAAAAACCGTGAATATCAAAAAG
AAATAGCAGAAAAAAATAGACATATTAATACATTAATTGCTGAACAAGAGCGACATAGAATTGGTCAAGACTT
ACATGATACGTTAGGGCATGTGTTTGCAAGTTTATCATTAAAATCAGAATTAGCTTATAAACTAATAGATGCT
GATGTAGAAAAAGTAAAAGCTGAATTATTAGCAATTAATAAATTATCTCGTGAATCATTGAACAAAGTTCGAG
AAATTATTGATGATGTAAAATTACCATCATTTATTGAAGAGATTGATAGTATACGTAAAGTTTTAAAAGATGC
TGATATTGATTTTACATTTGAAAATAAAGAATTAGCGCAAGTATTAAGTCCTACTAAACAATCTATGTTAGTT
ATGATTACGCGTGAAGCGATAAATAATGTTATTAAACATGCAAATGCTTCAAAAGTTCATGGTAAATTAAAAA
CTGTAAACAATCATAAATTACTGCTTATGATTGAAGATGATGGCAAAGGTATCGATAGTGATTGTGAGGTGAA
AAGTATTTCACAGCGTGTACAACATTTAAATGGAACTTTAGCAGTCGACTCAACAAATGGAACTAAAATAATC
ATTGAAATCTCAACAGGAGGAATAGCATGACATCTTTAATTATTGCAGAAGATCAAAATATGTTACGACAGGC
AATGGTTCAATTAATTAAACTACATGGTGATTTTGAAATTTTAGCAGATACTGATAATGGTCTCGATGCAATG
AAACTTATTGAAGAATATAATCCTAACGTTGTTATTTTAGATATAGAAATGCCAGGCATGACTGGACTTGAAG
TTTTAGCGGAAATTAGAAAAAAGCATTTGAATATTAAAGTGATTATTGTAACAACTTTTAAAAGACCGGGATA
CTTTGAAAAAGCAGTTGTGAATGATGTGGATGCATATGTTTTAAAAGAACGTTCTATAGAAGAATTGGTGGAA
ACCATTAATAAAGTAAATAACGGAGAGAAAGAATATAGCGCCACATTGATGACTTCATTTTTTGTAGATAAAA
ACCCATTAACGCCCAAAGAACAAATTGTATTAAGGGAAATTGGCAATGGTTTAAGTAGTAAAGAAATAAGTGA
AAAATTATTTTTGACAGATGGAACAGTTAGAAATTATACATCTGTTATAATTGATAAATTATTTGCAGATAAT
CGTTTTGATGCTTGGAAAAAGGCAAATGAAAAAGGCTGGATCTAAATACAAACAAAAAAGTATTGAGTGTTTT
GATTTAAGAGACTTGGAAAGTGAGTCAATAATCAAAATCTTACTCAATACTTTTATTTTGGTACTCGCAATTT
AGATTTGTTAACTAATTTTTTCGCTTATTATAAAACGAAACAATCAAGTAAACGATAAAGCCTACAAAGATAC
CCAATAAAATAGATAGTACTGCCGTCACTATTAATGGTAATTTGAAAAATATTTGTAGGAAAATACCAATGAT
AATTGCGATAATTACTGCAATTAATGTGACTGTATTTTCATTTGAAATGTTCATTTATTTTCACTCCTTAACA
ATAACATTATATCATGCTATAGCTTTCCAAAATATTGAAATATGTAGATATGGCTATTGACGATATTTCTTAA
CTTTTATATGATTAATCGGAATGAAAAAAGAGAAGTAGGTGGCAATATGAAGTCAAATAAATCGCTTGCTATG
ATTGTGGTAGCCATCATTATTGTAGGTGTATTAGCATTTCAATTTATGAATCATACGGGTCCTTTCAAAAAGG
GGACGAATCATGAAACTGTACAAGATTTAAATGGTAAAGATAAAGTACATGTTCAAAGAGTTGTGGATGGTGA
TACATTTATTGCAAATCAAAATGGTAAAGAAATTAAAGTTAGGCTTATAGGGGTTGATACGCCAGAAACGGTG
AAACCGAATACGCCTGTACAACCATTTGGCAAAGAAGCATCAAATTATAGTAAGAAGACATTAACAAATCAAG
ATGTTTATTTAGAATATGATAAAGAAAAACAAGATCGCTATGGTAGAACATTGGCGTATGTATGGATAAGTAA
AGATCGTATGTACAATAAGGAATTAGTGGAAAAGGGACTTGCTAGAGAGAAGTATTTTTCACCAAATGGCAAA
TATAGAAATGTATTTATAGAAGCACAAAATAAAGCTAAACAACAGAAATTAAATATTTGGAGTAAATAATTAA
ATAGGTGATTATATATATTAAAGTAAAATATACTTGAAGCATCGATTTATTATTACCATAAATTTATATATAG
AAATAATTTAAAGGCATAATAACCATAGTGCGTATCGAGCGTAATGGATTATTATGCCTTTGATTTTACTTGA
ATATAGCAGTGATTTGATCAACCTGTTCTTTATTTAAAGGGTGTTTAGATAGCTCTATGGCTTCGCTAATAAT
ACGTCGTAACGAAGATTTTGTTGAAACTGTATCAACATATTTATCTCTTTCTTCAGAACCTTGAATGATATTA
ACTGTACCATCACTAAAGTAAATAACACCGGTACTTTGGACTTTAAAGTTAAATTTTTGTTCAATATGATCCT
TTAATGCTTTAGCATTATTTGCTGCTAATTGATACGGATCATAATCATAAAAATCATAAATAACACGATTTGG
TTGAACTGTTTCTGTAAAAGTATAGATAGACTTTCTTGATGAATTAAACTGATCATGATATTGCTGGCTAATG
TAATGACCGACAACTTTTTCAATATTACTATTGCTCATTTCTGTATCATGCTCATCAGGTACATCAAAGTGAT
AAAATGTTTTTTCACCCCAGCTTTTTACATCAACGTTTATTAAACCGATATCTGAAACAATGATAAAATCAAA
TGAACGTGCATACTCGAAAAACGGATGTTTAGTAGCTAAATTGCTTGTAACGATAATATCGTAATATTTTAAT
TTACCATTTTCGAGATAGCTATCTAAAATCTTGCGTAATTCTCTTTTAGCATTTTTGTATGCATGGTGGCCAA
CATCATTATTAGAACTCAGCATTTGACTTCTTAATTCAGCATTTTCTGCACTTAATGCTTTGTTCTTTTTAAT
AAGTTGCTTTCTTGCATAAACTTCGGTATCTATTTTACTATTACTATACCTTTGATTTAAAACTAATATACCA
ATTAATGCTACAATGATAATGATAAGTACAACATAAAAAGACATTTTTTCACCAATCCTTTTTGACTTCTTTA
ACTTTGTATACAATAATAATTAATAAAGATTAATTGTTATTCAATTTCCCACATTTTTATTAGTTGATTTTAG
TTCATCATTGTTATAATCAAATTATAAACTGACAGATATTGATGTTCAATGAATATGACGTGAAAGATTCGTG
AATTCAAGTTTATGTCGAATTTATGTTATAACGGTCATTTAAATGACAGAATTAGGTCACTCATAGTATTTTG
AAGATTGAATTCATTAATTTTAAAATGTATAATGATATTTGTGAAAGCGCTTGCTTAGGAGGTGTATTTGAGA
GTGAATGAAATGAATGCTAAAGAACAATTAGTGGACAATTTAATGAAAACATCATCGCAATTATTTAAATTTC
ACGGTGAAGTTGCCATGCAGCTTTTCTTAAATGATGAATTAAAATTACCTTCTATTGTTGAAATATGCGTGGA
ACGTAAGCGTTTAAGTGATATTGTGAAAGTTATTCCGCAATCATATGCGTTACTATACATAGATAAGCAAGAT

CAAGCAATAGCTAAAGAAGATTTATCACTTTCAAAAATTGCAAAAGTTTATGTGCAATATGATGATACAACAA
TAATGAGTATTTTCGTTTATGATGTAGTAAACGATGAATGGATTTTTAGATTGGATCCGAATATACGTATACC
TAAGAGTAACATTTACTTCCATAGTTTAAATTGGGATGTGGATTATATTAAACCGGAAATCGTTCTAATGTAT
GATTTAATGCAACACCATCAGTATCATCATTATTCCAATTATAAACGTGTCATAGATGCATTAAGCTACTATC
AATTTTTTATTTTAAAATTTGTAGTAGGTGAGCAACGTATTAAGGATGCAATCCAGAGAACAATAAATAATTA
AGAAAAAGCAATTCATAACGCAGTTGAATACATGTGTTACGAATTGCTTTTATATTAGTTTTTATCACACAAG
TTTTTTAATGCAACCCCGTGATAGCAAAACTCATATGTAGATAATACAGCTTTTTCAGCATCATCTACATGAA
TTCCAAACATCATCGAGATTTCCGAAGCACCTTGGTTAATCATTTTTAAGTTAATTTTTGATTCAGCTAAGGC
ATGTGTAATTTTATTTGCAGTACCGATGACTTTATTCATACCTTCGCCGACAATCATTAAAATTGCTAAATCA
TGCTCAATACTTAGCTCATCAACATCACATTTTTGACGAATTTCATTTAATACTTTTGTTTCTTTATTTTGAA
TTTGTTTTGAACGCATAACGATACTGATAGTATCAATACCTGAAGGCATATGATCAAATGAAATATTATTATC
CTCTAAGACACCTAATATCTTTCTAGTAAAGCCGACTTGTCTATTCATTAAATACTTCTTGATATTAATAACA
GTAAAATCTTTATCACAACTTATACCGCTAATCACATTTTTCGCATTTATTTCTCTATCATGCACTATAAATG
TACCTTTATCTTGAGGGCGGTTCGTATTTTTAATCACCACAGGGATGCGATCTTTATAAAGTGGTTGTAAGGC
TTCATCATGGAAAACACTAAAACCAGCATAAGATAATTCACGCATTTCTCGATAAGTGATTTCTTCGATTAAT
TCAGGATCTTTGATGATATTTGGATTAGCTTTATAAATACCAGACACATCGGTGAAATTTTCATAAATTGTAG
CTCTAACACCACTTGATATGATGGCGCCAGTTATATCTGATCCGCCACGTGGAAATGTAACTATATATCCTTC
ATGAGATACGCCAAAAAATCCTGGGATAATTAGTTTCTCCTCATAATCTCTTAATTTTTTAATTTCAGAGTAA
GCACTATCTAATATTTGTGCTTCTTGTGGGACGTCAGTAACAAAAATACCCGCTTCCTTCGGTGATATATATT
TTGTTGGTATACCTTGACTATTATTATATAAAGCTATCAATTGCGCATTAAAATCTTCACCACAAGAAAGTAA
TGCATCTAATAGTCTCTTTGGTTCATTTTTTAATTGATTAATATAATGTTCCAAAGTCACATCTATCGTCCGT
AAAATACTTTCATCCATTTGCAATTCTTTTACAATATCATCATAACGCTGAATAATTTCTCTTTTTTTATCAT
GATAATCAAGATGATTAATGACCTTTTCATATAATCTGATTAACAAATCAGTTGTTTTAATATCATTATCATG
TCTTTTACCTGGAGCAGAAACGATAACAATCTTTCGCTCTGGATCAGAATTAACAATATTTAAAACCTTTTTA
ATTTGAGTAGCATTGGAGACGGAGCTACCACCGAATTTAGAAACTTTCATAGTGCATGCCAACCTTTCTAAAA
AATCAGAAAATTGTATTCAGTTTTGTAAGTAAGAATGATATTATGTATTTATATTTTGAATATTACGAACTTT
ACAAAAATAAAGAATAATTCTATACTATACTATCATCTAGTGTTTTTCAATAATACATTTGTTTTTCTCAAAA
GCACAAACGGAGGTACATAAAATGAAAAAATTAAATATAGCATTATTAGGATTAGGTACTGTCGGATCTGGTG
TTGTTAAAATCATCGAAGAGAACCGACAGCAAATTCAAGATACATTAAATAAAGATATTGTCATAAAGCATAT
TCTTGTTCGAGATAAATCTAAAAAGAGACCGCTAAATATTAGCCAATATCATTTAACTGAAGATGTTAATGAA
ATTTTAAATGATGATTCATTAGATATTATCGTTGAAGTCATGGGAGGAATTGAACCAACTGTAGATTGGTTAA
GAACAGCACTTAAAAATAAAAAACATGTTATTACCGCAAATAAAGATTTATTAGCAGTACATCTTAAACTTTT
AGAAGATTTAGCAGAAGAAAATGGTGTAGCTTTAAAGTTTGAAGCGAGTGTAGCAGGTGGTATTCCGATCGTA
AATGCCATAAATAATGGTTTGAATGCGAATAATATTTCAAAATTTATGGGAATTTTAAATGGTACCTCTAATT
TTATTTTATCTAAAATGACTAAAGAGCAAACGACATTTGAGGAAGCACTTGATGAAGCGAAAAGACTTGGTTT
TGCTGAAGCGGATCCAACTGATGATGTAGAAGGGGTAGATGCAGCGCGTAAAGTTGTCATTACATCATATTTA
TCATTTAACCAAGTCATTAAATTAAACGACGTTAAACGAAGAGGAATTAGTGGCGTAACTTTAACTGATATTA
ATGTAGCCGATCAACTGGGGTATAAAATTAAATTGATTGGTAAGGGAATATATGAAAATGGCAAAGTTAATGC
ATCGGTAGAACCAACGTTAATTGATAAAAAGCATCAATTAGCAGCTGTAGAGGATGAATATAACGCGATTTAT
GTCATTGGTGATGCCGTTGGTGACACGATGTTTTATGGAAAAGGAGCAGGCAGTTTAGCAACAGGTAGTGCCG
TTGTCAGTGATTTATTGAATGTAGCATTATTCTTTGAATCAGATTTACACACATTGCCACCACATTTTGAATT
AAAGACAGATAAAACAAGGGAAATGATGGATTCAGATGCAGAAATTAATATTAAAGAAAAATCCAATTTCTTT
GTAGTAGTGAATCATGTCAAAGGTTCAATTGAAAATTTTGAAAATGAGTTAAAGGCAATATTACCATTTCACC
GATCATTAAGAGTTGCAAATTACGATAATCAATCATATGCCGCTGTTATAGTTGGATTGGAATCATCACCGGA
AGAATTAATCACTAAGCATGGATACGAAGTTGACAAAGTATACCCAGTAGAAGGAGTTTAATTATAATGAGAA
GATGGCAAGGATTAGTAGAAGAGTTTAAAGCACATTTACCAGTAAATGAAAATACACCAAAATTAACATTGAA
CGAGGGAAATACACCACTCATTCATTGTGAAAATATGTCTAAAATACTAGGTATAGATTTATATGTGAAGTAT
GAAGGTGCCAATCCGACAGGTTCATTTAAAGATCGCGGTATGGTAATGGCTGTGACAAAAGCAAAAGAGCAAG
GTAAGAAAATTGTAATATGCGCTTCGACTGGAAATACATCAGCGTCTGCAGCAGCATATGCAGCGAGAGCAGG
TTTAAAAGCTATCGTCGTAATACCAGAAGGTAAAATTGCATTAGGTAAATTGTCGCAAGCAGTAATGTATGGT
GCAGAAATCGTTTCTATTGAAGGAAACTTTGATGAAGCTTTAGAAATTGTAAAAGAAATTGCAAAAAGTGGCG
AAATCGAGCTTGTAAACTCTGTCAATCCATTTAGAATCGAAGGACAAAAGACAGGCTCATTTGAAATTGTACA
ACAATTAGACGGTGAAGCACCTGATATTTTAGCGATTCCTGTAGGTAATGCAGGTAATATTACTGCATATTGG
AAAGGCTTTAAAGAATATCATGAAGCTAAAGGATCACAATTGCCGAAAATGTTTGGCTTCCAAGCTGAAGGCG
CATCACCAATTGTTCAAAATAAAGTCATTAAAAATCCTGAAACGATTGCAACTGCTATTCGAATTGGTAATCC
TGCTAGTTGGGATAAGGCGACTAATGCTCTTAAAGAATCAAATGGATTAATAGATAGTGTTACTGATGATGAA
ATTCTAGAAGCATATCAGTTAATGACAACTAAAGAAGGTGTCTTTAGTGAACCAGCAAGTAATGCTTCTATTG
CAGGTTTAATTAAATTGCATAGACAAGGTAAATTACCTCAAGGTAAAAAAGTAGTTGCTATTTTAACTGGTAA

TGGATTAAAAGATCCTGATACTGCTATTTCACTACTAGATAATCCGATAAAGCCATTGCCAAATGATAAAGAT
AGCATTATCGATTATATTAAAGGAGCTTTATAACATGTCGAATGTTTTGGAGTTAACAATTCCTGCATCAACA
GCCAACCTTGGAGTTGGCTTTGATTCTATAGGTATGGCTTTAGATAAATTTTTGCATCTGTCTGTAAAGGAAA
CATCAGGGACAAAATGGAAATATATTTTCCATGATGATGCATCGAAGCAATTGCCTACTGACGAAACAAACTT
TATTTATCATGTAGCACAACAAGTTGCTTCTAAATATAGTGTTGACTTGCCTAATTTATGTATCGAAATGAGA
AGTGATATTCCATTGGCAAGAGGGTTAGGTTCGTCAGCTTCTGCTTTAGTAGGAGCTATATATATCGCAAATT
ATTTTGGTGACATCCAACTGTCTAAACATGAGGTATTACAATTAGCGACTGAAATCGAAGGACATCCTGATAA
TGTTGCGCCGACCATTTATGGTGGTTTAATCGCTGGATATTATAATGATGTCTCGAAAGAAACGTCAGTTGCA
CATATCGACATACCAGACGTGGATGTGATTGTAACGATACCAACTTATGAACTTAAAACAGAAGCATCAAGAC
GTGCTTTACCACAAAAATTAACACATAGTGAAGCGGTTAAAAGTAGTGCAATTAGTAATACAATGATTTGTGC
ATTAGCACAGCACAATTATGAATTAGCAGGTAAACTCATGCAACAAGATGGCTTTCATGAACCGTATCGTCAG
CATTTAATTGCTGAATTTGATGAAGTGAAAACAATTGCTAGTCAACATAATGCCTATGCAACTGTAATTAGTG
GTGCTGGACCAACTATTTTAATATTTAGTCGCAAAGAAAATAGTGGCGAATTAGTTCGCGCTTTAAATAGAAA
TGTAGTTACATGTCATTCTGAATTAGTAGATATTAATGTTAGTGGTGTTAAAGAACGAATTGTATACCAATAG
ACACTTTATATTGTAAAATAGTATTGAATGTGAAATAGAGAGGAGATTTAATGCGATATGACAAATTATAAAG
TTGTCGTTTTAGATATGGATGACACATTGCTAAATTCAGATAATGTGATATCAGAAGAAACTGCAAATTATTT
AACAGCAATTCAAGATGAAGGTTATTATGTTGTTTTAGCATCTGGTAGACCTACTGAAGGTATGATTCCAACT
GCTAGAGATTTAAAATTACCTGAACATCATAGCTATATTATTAGTTATAACGGCAGTAAAACGATTAACATGA
CTAATGAAGAAGTAGAAGTAAGTAAATCGATTGGTAAGCAGGATTTCGATGAAATTGTAGATTATTGTCGAGA
TAGAGGATTTTTCGTTCTTACATATCATGATGGTCAAATTATATACGACAGCGAACATGAATATATGAATATT
GAAGCGGAATTAACTGGTTTACCGATGAAACGTGTTGATGATATCAAAGCATATATTCAAGGCGATGTACCCA
AGGTCATGGGTGTAGATTATGTAGCGAATATTACAGAAGCTAGAATTGATTTGAATGGTGTGTTCAATGATAA
TGTAGATGCTACGACAAGTAAGCCATTCTTCTTAGAATTTATGGCCAAAGATGTTTCAAAAGGTAATGCAATT
AAAGCGTTATGTCACAAATTGGGATATTCGGTGGATCAAGTCATTGCTTTTGGTGATAGTATGAATGATAAAT
CAATGTTTGAAGTCGCAGGTCTAGCTATTGCTATGGGGAATGCATCAGATGAACTTAAGCAATATGCAGATGA
AGTTACGTTGGATCATAATGAAAATGGTATTCCGCATGCGCTCAAAAAATTGTTATAAATTTTAAAATAAGCC
TTAACACATGATATTTGAATAAGATTGAAATGCTCCTTTCAAAGTAGACATTGAAAAAATGAAAGCTTTGAAG
GGAGCTTTATTTATGATACTATAGAGTATTTGAAATACATATTTTAGATTAGTAATGATATCAAACGAATATA
GTAGATGTATATTTTTTTAATAAATCAAGTATTAATTAGTCACGGAAGGTAGATAAATAAATTTGAATCATTC
TGAATAATGACGAATGTATTAAAAAATGCAGTGACAATCTAACATTAATTGTTAAAATCACTGCAAGCATCTT
GATTTTATTTAGCTGTTTGTGTCCAGTCATGCTTCCCTCTGAAGCGTTTTACATGAGCATATATTTGGTCAGC
AGTATAATCTAAATTTGTATAAACTGTAATACTGAACGCTGATTGGTTTTCAGCAAATGATTCTTCGGAAAAT
AATTCCTTTAAGTGCGTAAATAAATCATTCATTTGTTTATGGTTTAAACCGGGATATTCCCTTAAAGTTCTTT
TTAATAATTGTCCGTTCTTTTCTTCTAATGCTTGAACTACAATAACAAATCTCTCATCTTGCTTTAGTACATC
ATCAAATAAATTTGTTTGTCCAATCATTGAATTTCACCCCTGAAAAGTTATTGTTATCTATATTATACACGAT
ATGCAAAATATCTAAAATAAAGAATGGTAACATGGTCATAATAAAATATAAACAGAATACTAGTTAATACACT
TTATACTAGAAAAAGATATTAAAGTATATCTTGCTTTACACCATTAAAAAGCGGCAAATGCTATAGATAATCT
AAAGCATCTGCCGTGTGAGAATTTATTTATTTTTGATTGTCATAATCGTGTGGTTTTAAATTAATTGTTTCTA
GCTTTACAAATTTTGTTTTGTGAATGATTTTATGAATAAAGTAAATCGACACTAGAATGATTAAAGGTAAAAA
GTTTTTAAAAGCATTTAACCATTGATCTTTTAAAATATATTCAACTGAGCCACCAAATAGCAAGAATAATAGC
GTAGTGATGACAATGATTGGTCCTAATGGATAAAAAGGTGCTTTATATGGTAGGACCTTATTAGGATCTTGAC
CTTGTTTTTTAATAGCTTGTCGCAATCGTATTTGTGACCAAATGCTTGATCCCCAAACAACTATAATCATTGA
ACCAATAATTTCAAGTAAATTAAAAACGGCATTTGAATTAAAGTTTGCATAAATAATAACAATAACAACGACT
GCATAAGTAGTTAATAATGCTCTTAAAGGTAACTTTGTTGTCTTGTTTAATTTACTTAAAAATTGGGGTGCTT
TTTTGTCTGAACTTAAGGAATACAACATTCTTCCTGTTGTATAAACACCTGAATTTGCAGCGGATAATAATGA
AGTTAAAATAACCGCGTTGATTACTGATGCTGCAAAGGCTATGCCTACTCTATCGAATACAATTGTAAATGGG
CTTTGACTTATTGAACTACTTGCTCTTAATAATGATGGATCTGTGTACGGAATAATTGCACCAATTACTGCAA
TTGATAAGACATAGAATAAAAGAATACGCCAAAATACTTGTTTAATTGCCTTAGGCATAGACTTTTTAGGGTC
ATCTGATTCACCAGCAGTTACTGCAACTACTTCTGTACCACCAACCGAAAATCCGGCGACTAATAATACGCCT
AAGAAACCAGAGATACCACCAACAAACGGTGCTTGGCCTTTTGTATAGTTTTCAAATCCATATGCATGACCAC
CTAAGATACCGAAAATCATTAAAAAGCCAAAAATAACGAATACGATAATTGTTAACACTTTAATCAATGATAA
CCAAAACTCAGTTTCTCCAAATGATTTTACAGAAAAAATGTTTAATAATAGTAAAATTGTAATAAAGATTAAG
CTCCAAGTAATGGGGTGGAAAAATTTAAATGTGTCCCAGAAATAAAGCACATTTGACGCTACTATGACATCAA
CACTTGTAACTAATGACCACAATGCCCAATACAACCATCCCATGGTAAAGCCTAGAGATGAGTCAATAAAGCG
TGTTGAATAAGAGCTGAATGAACCTGATACTGGATAAAATGTTGCCAACTCTCCAATTGATGACATTAAGAAA
TATAGCATGACACCAATAACAAGATAAGCGAGTATAGCGCCTCCAGGACCAGCTTGAGAAATGATATTACCAG
TAGCTACAAATAGACCAGTCCCAATTGCACCGCCTATAGCAATCATGGAAATGTGTCTTGAGTTAAGACTACG
GTTCATTTTATTATCTTCCATATTTAGTCTCCCATCTATTTAAATATACCCATTATTGTAAGCTTTTTAAGTG

TACTATTCAATAACTATTTAGTGCTGTAAAGCGAAAAAATTAAAATTTTCTGATTTTTTAATCATTTTGAGCA
TGTTTAATTGTAATTTTGATGGGGTTAAATTATAATATGTATTAAATTATAATTATTATAAATTGTGGAGGGA
TGACTATGTCACAACAAGACAAAAAGTTAACTGGTGTTTTTGGGCATCCAGTATCAGATCGAGAAAATAGTAT
GACAGCAGGGCCTAGGGGACCTCTTTTAATGCAAGATATTTACTTTTTAGAGCAAATGTCCCAATTTGATAGA
GAAGTAATACCAGAACGTCGAATGCATGCCAAAGGTTCTGGTGCATTTGGGACATTTACTGTAACTAAAGATA
TAACAAAATATACGAATGCTAAAATATTCTCTGAAATAGGTAAGCAAACCGAAATGTTTGCCCGTTTCTCTAC
TGTAGCAGGAGAACGTGGTGCTGCTGATGCGGAGCGTGACATTCGAGGATTTGCGTTAAAGTTCTACACAGAA
GAAGGAAACTGGGATTTAGTAGGGAATAACACACCAGTATTCTTCTTTAGAGATCCAAAGCTATTTGTTAGTT
TAAATCGCGCGGTGAAACGAGATCCTAGAACAAATATGAGAGATGCACAAAATAACTGGGATTTCTGGACGGG
GCTTCCAGAAGCATTGCACCAAGTAACGATCTTAATGTCAGATAGAGGGATTCCTAAAGATTTACGTCACATG
CATGGGTTCGGTTCACACACATACTCTATGTATAATGATTCTGGTGAACGTGTTTGGGTTAAATTCCATTTTA
GAACGCAACAAGGTATTGAAAACTTAACTGATGAAGAAGCTGCTGAAATTATAGCAACAGATCGTGATTCATC
TCAACGCGATTTATTCGAAGCCATTGAAAAAGGTGATTATCCAAAATGGACAATGTATATTCAAGTAATGACT
GAGGAACAAGCTAAAAACCATAAAGATAATCCATTTGATTTAACAAAAGTATGGTATCACGATGAGTATCCTC
TAATTGAAGTTGGAGAGTTTGAATTAAATAGAAATCCAGATAATTACTTTATGGATGTTGAACAAGCTGCGTT
TGCACCAACTAATATTATTCCAGGATTAGATTTTTCTCCAGACAAAATGTTGCAAGGGCGTTTATTCTCATAT
GGCGATGCGCAAAGATATCGATTAGGAGTTAATCATTGGCAGATTCCTGTAAACCAACCTAAAGGTGTTGGTA
TTGAAAATATTTGTCCTTTTAGTAGAGATGGTCAAATGCGTGTAGTTGACAATAACCAAGGTGGAGGAACACA
TTATTATCCAAATAACCATGGTAAATTTGATTCTCAACCTGAATATAAAAAGCCACCATTCCCAACTGATGGA
TACGGCTATGAATATAATCAACGTCAAGATGATGATAATTATTTTGAACAACCAGGTAAATTGTTTAGATTAC
AATCAGAGGACGCTAAAGAAAGAATTTTTACAAATACAGCAAATGCAATGGAAGGCGTAACGGATGATGTTAA
ACGACGTCATATTCGTCATTGTTACAAAGCTGACCCAGAATATGGTAAAGGTGTTGCAAAAGCATTAGGTATT
GATATAAATTCTATTGATCTTGAAACTGAAAATGATGAAACATACGAAAACTTTGAAAAATAAATTTGATATG
TAGTTTCTATATTGCGTAGTTGAGCAGTTTATGATATCATAATAAATCGTAAAGATTCCTAACAAGAGAGGGT
GTTTAACGTGCGCGTAAACGTAACATTAGCATGCACAGAATGTGGCGATCGTAACTATATCACTACTAAAAAT
AAACGTAATAATCCTGAGCGTATTGAAATGAAAAAATATTGCCCAAGATTAAACAAATATACGTTACATCGTG
AAACTAAGTAATTCTTATCATTCAAATACGACGATTTGAAAATAAAGCGGGCTTACCTATTATATTGGGGAGC
TCGCTTTTTTATGAAATTTTTGTAAAGAGTGATTAATGAATTGAGTTTCATCGGTAGAACAATATATGATTGT
ATTAGTTGTTACTTTATTAAAATTTGAGAATTTTTATAGAAGGAAATAGATTGCTGATTTTATAAAGTCACTT
TGTTAGCGAATGCTTGAAAGAATATTTAATATATTAGAATTTAAAATTTCAAAGCGGAATTTAATAAGTACGA
AGTAATTGTGGATATGTTTTATAAATGTTCGATAATACACTTTAATCTTAAATATGATGGTTTAGAAAATGAT
TTAACAAAGAAATGAAACTTTACTGTTGAATTATGTGAGGATTGTGTTATTATATAAATCGTAATAATTACGA
TTTGATAAAAAGTGAGGTAACTATATATGGCTAAGAAATCTAAAATAGCAAAAGAGAGAAAAAGAGAAGAGTT
AGTAAATAAATATTACGAATTACGTAAAGAGTTAAAAGCAAAAGGTGATTACGAAGCGTTAAGAAAATTACCA
AGAGATTCATCACCTACACGTTTAACTAGAAGATGTAAAGTAACTGGAAGACCTAGAGGTGTATTACGTAAAT
TTGAAATGTCTCGTATTGCGTTTAGAGAACATGCGCACAAAGGGCAAATTCCAGGTGTTAAAAAATCAAGTTG
GTAAAATCAAATTTCGTACTTTAGCCCATTTACAATATCAATAAAACAATGTACAGTATATACGAATGCTATA
AACTGAATGTTTTCTCATATTAATAAGGAAACATTCGGTTTTTAATTTGCATTTAAAAATAATTAGTTTTTAA
AGGGGCTATTTAAAGTGAAAATATTTGATTACGAAGATATTCAATTAATACCTAATAAATGCATAGTTGAAAG
TAGGTCTGAATGTGATACAACAATCCAATTTGGTCCGAAAAAATTCAAGCTACCTGTAGTTCCTGCAAATATG
CAAACAGTTATGAATGAGAAATTAGCGAAATGGTTTGCTGAAAATGATTACTTTTATATCATGCATCGTTTTG
ATGAAGAAGCAAGAATACCTTTTATAAAACATATGCAAAATTCAGGCTTATTTGCATCTATTTCAGTTGGTGT
AAAGAAAGCGGAATTTGATTTTATTGAAAAGTTAGCTCAAGAAAAATTAATCCCCGAATATATTACAATAGAT
ATTGCGCATGGTCACTCAGATTCAGTGATAAACATGATTAAACATATAAAAAACCATATACCTGATAGTTTTG
TTATTGCTGGTAATGTTGGTACGCCAAAAGGTGTTAGAGAATTAGAAAATGCTGGTGCTGATGCTACCAAAGT
CGGTATAGGTCCTGGTAGAGTTTGTATTACAAAGATTAAAACAGGTTTTGGTACTGGTGGTTGGCAGTTAGCG
GCATTAAACATATGTAGTAAAGCAGCTCGTAAACCTTTGATTGCCGATGGTGGTATAAGAACGCATGGCGACA
TTGCTAAATCAATTAGATTTGGTGCATCAATGGTCATGATTGGTTCATTATTTGCGGCACACGAAGAATCACC
TGGTGAAACTGTAGAACTTGATGGTAAACAGTATAAAGAATATTTTGGTAGTGCATCTGAATTTCAAAAAGGC
GAACATAAAAAATGTAGAAGGTAAAAAAATGTTTGTAGAACATAAGGGTTCATTAATGGATACCTTAAAAGAAA
TGCAACAAGATTTACAAAGCTCAATTTCATATGCCGGTGGAAAAGACTTGAAATCATTACGTACTGTAGATTA
TGTTATTGTTAGAAACTCTATTTTCAACGGTGATAGAGATTAATATTTATAGTAGGTGATGTAAATTAAAAAA
TTCATAGTAACTGTTGTTGCGTTTTTATCAATTATTATTATTGCGCCAATAACAGAATTTAAACCATTCATTC
ATTTACAAAATGAAGTAAGACAATATATTGACATTCACATCAATAAAGAAACAATTTCTGCGGAAAATAAATT
GGATACGCCTAAGAAACAACAATTTGCCTTTAATAATATTCAGATGAATATGTCAAAGTCAGATGTTGAGAAA
ACATTGAATAAACCAAAAAGAGTTACATTTAATGAATATGGCACGAAGTGGTATACGTATTATGATGACGATT
ACAATAATTTTATCATGGTAAGCTATATTAAAGATAAAGTAAATGCGTTATATACAAATCAAAATATCATTAC
TTCAAAATCAAAAATTAAATACAATACACCGAAATCGGTTGTAATACAAAGATTAGGCGAACCAGAGACAGAG

ATTGTTAAAGGTAGAGTGCGTTACGAACAAAATAATAAAGAATATGATGTTTTCCATAAAAATCACATTTATA
CGACGGTATTTTATGATAAGCATCGACGTAATAATGTAACAGCTGTTTTACAAGTAAGTGATGCTATGGAAAA
TAGATTAAAAGAACAATATGGAGCACCTTCGAAATCGCTTGCAGATAGTTTTGAACTACAAAATTTTGATTTA
GTTAATGCTGAAAGAAAACAACATCAATTATCTACATTGAAGTATTCTAAACAGAATTCTGAAACTGCTCGTA
AGCATAGTAAAGATATGGCCAACAATCATTATTTTGATCATACAAATTTAAAAGGTCAATCACCATTTGATCG
ATTGAAAAAAGATGGTATTACATTTAACTCAGCCGGAGAGAATTTAGCATATGGTCAAGTTAGTAGTATCTAT
GCACATCAAGGATTAATGAATTCTATTGGTCACAGAAAAAATATTTTAAATGATACGTTTAAAATATTAGGTG
TTGGTGTTGATTTTAATGATGAAAAACAACCTTTTTGGACAGAAAATTATACTGGTTAATGTTTTTGGCATGA
GATTAAAGGGTAATGTTTGTCTGTAAGGAAGTAGATAAACATGATGACAACAGATAAACCAAAAGATGCGGAT
ATAGTTGAACGCGTCAAAGATATACTTAATAAAAAAGAAAGAAAAAAATAATAATGGCTCGCTCCTGTAAATTA
TTACGGGGGCGAGTTTTAATTTGTGTAACATTTTGCAGAAATCTGTAATTCAAAATTTATATTAAAGAATGAG
ATTATCTAACAATTCGCAATTTCTGTTTGAACTGAATTTAGATTAGAATAAATAAAACCTCTTGAAAACGTAA
CTTTTTCAAGAGGTCCAAAATTTATAAAAATGAGGGCTTAACAATTACGATAATATATAACGGTTTAAAATAT
TACATTTCGCGGTACAAACCAATTACTTTCCCAATTACAGCAACATTGTCTAGGTAAATTGGCTCCATTGTAC
TATTTTCAGGTTGTAATCGATAACGATTTTTTTCTTTATAGAAGCGTTTGACAGTTGCTTCATCTTCCTCGGT
CATAGCAACAATAATGTCTCCATTTTCTGCTATGGTTTGACTGCGAACAATTACTTTGTCTCCGTCTAATATA
CCAGCCTCAATCATACTGTCGCCTACGACGTTTAATATGAATATGTCGCTATTGTGTGTCGATGTTAAGTGTT
CAGGTAATGGAAAATATTCTTCAATATTTTCTACTGCGGTAATAGGAACACCTGCTGTGACTTTACCAATAAC
TGGCACATGAATCGTTTCTTCCATATTAATATTATCACTTGTTTGATCACTTACAATTTCTATAGCACGTGGT
TTCGTCGGATCTCTTCTTATATAGCCTTTTTCTTCAAGACGTGAAAGGTGACCATGAACAGTTGAACTGGATG
CTAAGCCAACTGCTTCACCAATTTCGCGAACACTAGGCGGATAACCTTTCATTTGAACAACTTGTTTAATATA
GTTATATATTTCGCTTTGTCGTTTTGTTAATTCTCTCATATATAGGCACTCCCTAAATTAATTTATATACAGT
ATATCATTAATTCACAACCAAAACAAACATTTGTTCGTTAAAATATTGACAGAGAACATAAGTTCTGATAAAT
TACTAATACAAACAAATGTTCTAGGAGTGAAAATGATGTTTTACAATAAATATAAAAACGTATCAACATATAT
CATCATATTTTTAGTTTCAAGTGCAGCCTTTGCAATATTCTTGTTAAGTGCGAACATTAGTGCTCACTCGGAA
CAAGTGTACGAAATGACTGACCATCAAATTAAGAACAATACGATAAATAAAGCATACGAACATAAAGACCCTA
CAAACAATAGCGAACAAAGAGATGGGAAAGTGTTCGCTTTAATAAATTGATACATTGTCACAACGTTATTTTG
CCTATTTTTGCGAAATAGCGTTTTTTATTACTTTTTTGCTGATCTTAAATTTGTTATATTTTGTTAAAGTATT
ATAATGATTGAATAAACAAATTGAAGGTAGGTTTTTTAATTGAGTAATTCTGATTTGAATATCGAAAGAATTA
ACGAGTTAGCTAAAAAGAAAAAAGAAGTAGGATTAACTCAAGAAGAAGCAAAGGAGCAAACAGCCTTAAGAAA
AGCTTATCTTGAGAGTTTTAGAAAAGGGTTTAAACAACAAATTGAAAATACTAAAGTAATTGATCCAGAAGGT
AATGATGTAACACCTGAAAAAATTAAAGAGATACAACAAAAAAGAGATAATAAAAATTAAATCACAAATCTGT
AAAGAATTTTCTGACATTATAACTTGAAATAAGTATTTTACTTATCTTTTTATTTTAAAATAAGTTATAATGT
ATTTGATAAAATTGAAGAAGGGAAGATACACAAGATGTTTAATGAAAAAGATCAATTAGCTGTTGATACGCTA
CGTGCACTAAGTATCGACACAATCGAAAAAGCGAATTCTGGTCATCCAGGATTACCTATGGGAGCTGCCCCAA
TGGCTTACACTTTGTGGACACGTCATCTGAATTTTAATCCACAATCTAAAGATTACTTCAATAGAGACCGTTT
CGTATTATCTGCAGGGCATGGTTCAGCATTATTGTATAGCTTGTTACATGTTTCTGGTAGTTTAGAATTAGAA
GAATTAAAGCAATTTAGACAATGGGGTTCTAAAACACCAGGTCATCCTGAATACAGACATACAGATGGTGTAG
AAGTTACTACCGGACCACTTGGACAAGGTTTTGCTATGTCAGTAGGATTAGCTTTAGCAGAAGATCACCTAGC
AGGGAAATTTAATAAAGAAGGATATAATGTTGTAGATCATTACACATATGTATTAGCTTCTGACGGTGATTTA
ATGGAAGGTATATCGCATGAAGCAGCTTCATTTGCTGGACATAATAAATTAAGTAAATTAGTTGTTTTATACG
ATTCAAATGATATTTCATTAGATGGCGAATTAAACAAAGCTTTTTCTGAAAACACAAAAGCTCGTTTTGAAGC
ATATGGTTGGAATTACTTACTAGTTAAAGATGGTAATGATTTAGAAGAAATTGATAAAGCGATTACTACAGCT
AAATCTCAAGAAGGACCAACGATTATTGAAGTTAAAACAACAATCGGATTTGGTTCACCGAATAAAGCAGGAA
CTAATGGTGTTCATGGGGCACCTTTAGGTGAAGTTGAAAGAAAATTAACATTCGAAAATTACGGTTTAGATCC
TGAAAAACGTTTTAATGTTTCAGAAGAGGTATACGAAATTTTCCAAAATACTATGTTAAAACGTGCTAATGAA
GATGAATCTCAATGGAATTCATTATTAGAAAAATATGCAGAAACATATCCTGAATTAGCAGAAGAATTTAAAT
TAGCGATTAGTGGTAAATTGCCTAAAAATTATAAGGATGAATTACCACGTTTTGAACTGGGTCATAATGGTGC
ATCTCGTGCTGATTCTGGTACTGTTATTCAAGCAATCAGTAAAACTGTCCCTTCATTCTTTGGTGGATCAGCA
GACCTTGCTGGTTCAAACAAATCCAATGTAAATGATGCAACTGATTATAGTTCTGAAACACCTGAAGGTAAAA
ATGTGTGGTTTGGTGTACGTGAATTTGCTATGGGTGCTGCTGTAAATGGTATGGCTGCACATGGAGGTTTACA
TCCATATGGTGCAACATTCTTCGTATTTAGTGATTATTTAAAACCAGCGTTACGTTTATCATCAATTATGGGA
TTAAATGCAACGTTCATCTTCACACATGATTCAATTGCAGTAGGTGAAGATGGTCCTACTCATGAACCAATTG
AGCAATTAGCTGGATTAAGAGCCATTCCAAATATGAATGTTATCCGTCCTGCTGATGGTAATGAAACAAGAGT
AGCATGGGAAGTTGCCTTAGAATCTGAATCTACACCTACTTCATTAGTATTGACACGTCAAAACTTACCGGTA
TTAGATGTACCAGAAGATGTAGTTGAAGAAGGCGTTCGAAAAGGTGCCTATACAGTTTATGGCTCTGAAGAGA
CACCAGAATTCCTATTATTAGCTTCAGGTTCAGAAGTTAGTCTTGCAGTTGAAGCTGCTAAAGATCTTGAAAA
ACAAGGTAAATCAGTACGTGTTGTTTCAATGCCTAACTGGAATGCATTTGAACAACAATCTGAAGAATATAAA

GAATCAGTTATTCCATCAAGCGTAACAAAACGTGTTGCGATTGAAATGGCTTCACCGCTTGGATGGCATAAAT
ATGTAGGTACTGCAGGTAAAGTTATTGCTATTGACGGCTTTGGCGCAAGTGCACCTGGCGATTTAGTAGTTGA
AAAATATGGATATACAAAAGAAAATATCTTAAACCAAGTTATGAGCTTATAAGAATAATTTATAAAGCGAGTA
TGTTTAGAAGTCTAGGATGCATAATCTTAGGCTTCTTTTAAAGTGTTGAAATTTAGAGTATAGCACTTAAACT
ACATCATAAGTGATAAGTTATGAAAGTATACTATTTCAGATTAATCTTTAAAAGCTCTGTTATAACAGCATGA
TTTTTGATATTATTTTTAGTATCGATATTAAAATACTTGAATAAACTAGTTCTTGAAATAATGTGATGAATTT
AGTAAAATTCAGTAGGATAAAGAAAGCGGGTGAAACAATGGCAACTTGGTTAGCAATTATTTTTATAGTAGCT
GCATTAATTTTAGGTTTAATTGGAGGTTTCCTTTTAGCTAGAAAATATATGATGGACTACTTGAAGAAAAACC
CACCAATCAACGAAGAAATGCTTCGTATGATGATGATGCAAATGGGTCAAAAACCTTCTCAGAAGAAAATTAA
TCAAATGATGACGATGATGAATAAAAATATGGATCAAAATATGAAGAGTGCGAAAAAGTAAATTCGCAATTGA
TAGAGGCTATTTTCCAGATATGGAAATGGCCTCTTTTTATAATCAAATTAATAAGAATAAATATGTTTATTAA
AATTAAAGTTAACAAAATGACGAATAGACTGAGAAATGCTATAATTCATTTTGTATGATTTACAGAGAGTTTA
TTTAACGAGAAGGTGTCTGCGTGCTCTATTTAATATTTTCAATCATTGTAGCTTTATTTATGGGAACTATAGT
TATAGTTATTCGTATGAAAGCTCAAAATTATCCGGTAAATGAGAAAAAAATAGTTTTGCCACCGTTTTTTATG
GCGACCGGTGCATTGATGTACGTCGTTCCATATTTTAGGCTAACAGGATCGGAAATGCTAGAAGCCTTTATAA
TTGGTTTGCTTTTTTCTACAGTTCTAATTTGGACTTCTCGATTTGAAGTCAAAGGTACAGAAATTTATATGAA
ACGATCTAAAGCATTTCCAGTTATTTTGATTTCATTACTTATCATTCGTACTGTGATGAAAATATTCATTAGT
AATGAAATAGATCCTGGAGAATTAGGCGGCATGTTCTTTTTATTAGCATTCTGTATGATTGTTCCTTGGAGAG
CAGCAATGCTATATAAATACAAAAAACTAAAGAAAACATTAATCAATTAATTACTTTTAAAACCACTTGTGAT
CGACTTCTAAATCAGTCAATGAGTGGTTTTAATTTTACTTGAAAAGGTGAAATGATTATATTAAAATAAAAGC
GAACAAATGTTCTATATGGAGGCAGTAATGAAAATTATACATACAGCAGACTGGCACTTAGGGAAAATATTAA
ATGGCAAACAGCTTTTAGAAGATCAAGCGTATATTTTAGATATGTTCGTAGAAAAAATGAAAGAAGAAGAACC
TGATATCATTGTGATAGCTGGAGATTTATATGACACAACATATCCAAGTAAAGATGCAATCATGTTATTAGAA
CAAGCGATTGGAAAGTTAAATTTAGAACTGCGTATACCAATAATTATTATTAGTGGAAATCACGATGGTAAAG
AGAGGTTAAACTATGGGGCGAGTTGGTTTGAACATAATCAGTTATTTATAAGAACAGATTTTACATCGATTAA
TTCACCAATAGAGATAAATGGGGTTAATTTTTATACACTCCCTTATGCTACTGTGAGCGAAATGAAACACTAC
TTTGAAGATGACACCATTGAAACACATCAACAGGGAATTACGCGCTGTATTGAAACAATAGCACCGGAAATTG
ATGAAGATGCCGTCAATATTTTAATTAGTCATCTGACTGTTCAAGGTGGAAAGACATCTGATTCTGAAAGACC
ATTAACTATTGGAACGGTTGAATCAGTTCAGAAAGGTGTTTTTGATATATTTGATTATGTCATGCTAGGTCAC
TTGCATCATCCATTTAGTATAGAAGACGACAAAATTAAATATAGTGGCTCCTTATTGCAGTATTCATTTTCGG
AAGCGGGTCAAGCTAAAGGGTATAGACGTGTAACAATTAATGATGGCATTATTAACGATGTATTTATTCCTCT
TAAGCCACTTAGACAATTGGAAATTATCTCAGGCGAATATAATGATGTTATTAATGAAAAAGTTTATGTGAAA
AATAAAGATAATTATTTACATTTTAAACTTAAAAATATGTCTCATATTACTGATCCAATGATGAGTTTAAAAC
AAATTTATCCTAATACTTTAGCGCTGACGAATGAAACTTTTAATTACAATGAAGAAAATAATGCTATAGAAAT
AAGTGAAAAAGATGACATGTCAATTATCGAAATGTTTTATAAACATATAACTGATAAAGAATTATCGGATATC
CAATCTAAAAAGATAAAAAATATTTTAGAAAACGAATTGAGAAAGGAGGATTAATGAATGAAACCATTACATT
TAAAGTTGAATAATTTCGGCCCCTTTTTAAAAGAAGAAATTGATTTTTCTAAAATTGATAATAATGAATTGTT
TTTAATAAGTGGTAAGACTGGATCGGGTAAAACAATGATTTTTGATGCAATGACTTATGCCTTGTTTGGTAAA
GCATCAACTGAACAAAGAGAAGAAAATGATTTGAGAAGTCATTTCGCTGATGGTAAACAGCCGATGTCAGTAA
CATTTGAATTTCAATTAAATCATCGAATTTATAAAGTGCATAGACAAGGCCCTTATATCAAAGAAGGTAATAC
AACAAAAACGAACGCTAAATTTGATGTATTTGAGATGGTGGATGGCAAGTATGAAATTAGAGAAAGTAAAGTA
ATTTCAGGTACCCAATTCATTATTGAATTATTAGGAGTAAATGCAGATCAATTCCGACAATTGTTTATTTTGC
CTCAAGGTGAATTCAAACGCTTTTTAATATCAAACAGTCGTGAAAAGCAAGGGATATTAAGAACACTGTTTGA
CAGTGAAAAATTTGAAGCTATACGAGAAATATTAAAAGAAGAAGTAAAAAAAGAAAAAGCTCAAATCGAGAAT
AGATATCAACAAATTGACCTTTTATGGCAAGAAATTGAATCATTTGATGATGACAATATAAAAGGCTTATTAG
AGGTTGCCACTCAACAGATAGACAAATTGATTGAAAATATACCACTTTTACAAGCTAGGTCGAAAGAAATACT
AGCATCTGTAAATGAAAGTAAAGAAACTGCTATTAAAGAATTTGAAATAATAGAAAAGAAAACATTAGAAAAT
AATATATTAAAAGATAATATTAATCAACTCAACAAAAATAAAATTGATTTCGTTCAATTGAAAGAACAACAAC
CTGAAATAGAGGGAATTGAAGCTAAGTTAAAGTTGTTACAAGATATTACAAACCTATTGAATTATATTGAAAA
TAGAGAAAAAATTGAAACTAAAATTGCTAATAGCAAAAAAGATATTTCTAAAACCAATAATAAAATATTGAAT
CTTGATTGTGATAAGCGAAACATAGACAAAGAGAAAAAAATGTTAGAAGAAAATGGAGATTTAATTGAAAGTA
AAATCTCTTTTATTGATAAAACTAGAGTATTATTTAACGATATTAATAAGTATCAACAAAGTTATCTCAATAT
TGAACGCTTGAGAACTGAGGGTGAACAATTAGGTGATGAATTAAATGATCTAATTAAAGGTTTAGAAACGGTC
GAAGATTCAATAGGTAATAACGAAAGTGATTACGAGAAAATTATCGAACTAAATAATACGATAACGAACATAA
ATAATGAAATTAATATAATTAAAGAAAATGAAAAAGCTAAAGCTGAATTAGATAAACTATTAGGTAGTAAGCA
AGAGTTAGAGAATCAAATTAATGAAGAAACATCTATATTGAAGAATCTCGAAATAAAATTAGATCGCTACGAT
AAAACAAAATTGGACTTAAATGATAAAGAAAGCTTTATAAGTGAAATTAAATCTGCTGTAAATATTGGAGATC
AATGTCCGATATGTGGTAATGAAATTCAGGATTTAGGGCATCATATTGATTTTGACAGTATTGCTAAACGTCA

AAATGAAATTAAAGAAATTGAAGCAAATATCCACGCTATAAAATCGAATATTGCTGTGCATAATTCTGAAATT
AAATTTGTTAATGAAAAAATATCGAATATTAATATTAAAACGCAAAGTGATTTTTCACTTGAAGTATTGAATA
AGCGTCTGCTAGAAAATGAAAATGCATTGAATAATCAAAGAGATCTTAATAAATTTATAGAACAAATGAAAGA
AGAAAAAGATAATCTAACGTTGCAAATTCATAATAAACAATTGCGTCTAAATAAAAATGAATCTGAGTTGAAA
TTATGTCGAGATCTCATCACTGAATTTGAAACACTCTCGAAATATAATAATATCACTAATTTTGAGGTGGATT
ATAAGAAGTATGTTCAAGATGTGAATCAGCATCAAGAACTCTCAAAGGAGATTGAAGATAAGTTAATGCAATT
GTCTCAAAGAAAGTCAATTGAGCAAAATAATCTAAATCATTATGAAAATCAACTAGAAACTTACAATAATGAC
TTAGAATTGAATGAACAGTCTATTGAAATGGAAATGTCGAGGCTGAATTTAACTGATGACAATGATATAGATG
AAATAATAGCCTGGAGGGGCGAGCAAGAGGAATTAGAGCAGAAAAGGGATACTTATAAAAAACGTTATCATGA
ATTTGAAATGGAAATAGCTAGGTTAGAATCATTAACTAAGGATAAAGAGTTATTGGACTCTGATAAATTAAAA
GATGAATATGAGCTAAAAAAAGGAAAGATGAATACACTGATAGATGAATACTCTGCTGTTCATTATCAATGTC
AAAATAATATTAATAAAACACAATCTATAGTTTCGCATATTAATTACTTAAATCAAGAATTAAAGGATCAACA
AGAAATATTTCAATTGGCTGAAATTGTCAGTGGTAAGAATAACAAAAATCTTACATTGGAAAACTTTGTCTTA
ATTTACTATTTAGATCAAATTATTGCTCAAGCAAATCTGAGATTAGCAACAATGTCAGATAATCGATACCAAC
TAATTAGGCGAGAAGCGGTTTCTCATGGTCTTAGTGGCCTAGAAATTGATGTATTTGATTTGCATTCAAATAA
GTCTAGACATATTAGCTCGTTATCAGGTGGAGAAACTTTCCAATCGTCGCTTGCATTAGCTTTAGGGTTAAGC
GAAATTGTACAGCAGCAATCAGGAGGTATTTCACTAGAATCAATATTTATTGATGAAGGATTCGGTACATTAG
ATCAAGAAACGCTTGAAACAGCGTTAGACACTTTATTAAATCTTAAATCAACTGGTAGAATGGTTGGGATTAT
TTCACATGTGAGCGAATTGAAAAATAGAATACCTTTAGTTTTAGAAGTGAAATCAGATCAATATCAGAGTTCA
ACAAGATTCAAAAGAAATTAAAACATAAAAAATAGAGATGGTACTTTGGAATGTATAGAACAACCTTAGCCCA
TCTCTTTTTATTTAAAAATTATTTTTTCTCACGTAATAAATCTCTGATTTCAGTTAATAACACAACATTTTCT
TCCACAACTGCTTCTTCTTCGGCTTCTTCTTTCTTCATTAATGTATTTGCAATCTTAACAAAGATGAATAAAG
CAAACGCGATGATAATAAAGTCGATAACAGATTGGATAAATAAACCGTATTTAATACCCCAGAATGACCATTC
TTTAGCAAAATCAACTGATCCGAAAATTTTACCAATTAATGGCATAATGATATTTTCTACTAATGAAGATATA
ATCTTGTTGAAAGCTGCACCCATCACAACAGCAATTGCTAAATCTAAGACGTTACCTTTTAAGGCGAACTCTT
TGAATTCTTTTAACATTACACTCAACCTCTCTTTTTTAAATGTAACCTCAATTATACAACAAATAAAAAAATT
AGAAAATAGATTATTCAAGAAAATTTGATTATCGCAAAGACCTTTGTTATCCTATAAAGGTGCGTGAGTTATT
GAATAAAATGTTTAGATAATATTTTTATTCTACTACATAAAATTAAATATAGAATTGAAGGGAGTAATTAATT
ATGAATTCTTCTTCACCAGAGAATCCAAATGGAAAGAAGTATTCACCAGTCTTCATCTATAGTGCAATTGTTG
TTGCTATAGTCGTATTACTTGGTGCATTTTTACCTGAACAATTCAACTATGTTACCAATAATATTAAAATGTG
GATTACAGAAAAGTTAGGTTGGTATTATCTTATTCTTACTACGATTATCGTTTTCTTCTGTATATTCCTTATT
TTTAGTCCTATTGGAAAACTTAAACTAGGTAAACCAAATGACAAACCTGAGTTTAATACAATTTCATGGTTTG
CTATGTTGTTTAGTGCTGGTATGGGGATAGGTTTGGTGTTTTATGGTGCAGCTGAACCGATGGCGCACTTTGC
TACGCCACCTACAGCAGATCCCAAAACTACTGAAGCTTATACTGAAGCTCTACGTTCAACATTTTTCCATTGG
GGATTCCATGCTTGGGCTGTTTATGGTGTTGTTGCGTTAGCGTTGGCATATTCGCAATTCCGTAAAGGTGAAC
CAGGTTTATTATCTAGAACTTTACGTCCTCTTTTAGGTGATAAAGTAGAAGGTCCTATTGGGATTTTTATTGA
CGTTTTATCTGTATTTGCGACAATCGTTGGGGTAGCCGTTTCGTTAGGTATGGGTGCTCTACAAATTAATGGT
GGTTTACATTACTTGTTCAATGTTCCAAACAATACGTTTGTACAAGCGATTATCATCATTGTTGTTACTATCT
TATTTATAGCAAGTGCATGGTCTGGATTAAGTAAAGGTATTCAATACTTAAGTAACTTGAACATTGGTTTAGG
TACTATTTTAATGGTAGCTGCTTTAATTGTTGGACCAACTGTTCTTATTTTAAATATGTTAACTAGCTCTACG
GGTAGTTTACTAAACACATTCTTGTTTAATAGTTTTGATACAGCAGCTTTAAATCCTCAAAAACGTGAATGGA
TGTCTTCATGGACACTTTATTACTGGGGTTGGTGGTTAAGTTGGAGCCCATTCGTTGGAGTGTTTATTGCACG
AGTTTCAAAAGGACGTTCAATTAGAGAGTTCATTTCTGGTGTCTTGCTAGTTCCAGCAATTGTTAGTTTTGTT
TGGTTTAGTGTCTTTGGTGTATTAGGCATCGAGACAGGTAAGAAACACAAAGAAATTTTTGATATGACTCCTG
AAACACAGCTATTTGGAGTGTTTAATCATGTGCCATTTGGCATTGTTTTATCGTTGATTGCATTATTATTAAT
TGCATCATTCTTTATTACATCTGCTGACTCAGCAACATTTGTATTAGGAATGCAAACAACATTTGGTTCATTA
AATCCATCTAGTATGGTAAAAGTTGTTTGGGGAATTTCACAGGCCTTAATAGCATTTGTACTTTTATTAGCTG
GTGGCGGTAACGGCGCTGAAGCTTTAAATGCGATTCAAAGTGCTGCAATTATAAGTGCATTCCCATTCTCCTT
TGTCGTCATACTCATGATGGTAAGTTTCTACAAGGATGCGAACCAGGAACGTAAATTCCTAGGTTTAACATTG
ACTCCGAATAAACATCGCTTACAAGAATATATCAAGAGTCAACAAGAAGATTATGAATCTGACATTCTTGAAA
AGCGTCAGTCACGTAGAAATATAGAGAAAAAAGATAACTAATAAAATTTAGTTAAGTATTTAAATAACAAGTA
GTATGTCATTCTAGTAGCTAGAACAGATATACTACTTGTTTGTTTTTGTGGAAAATTGAGTATATTCAAAAGG
ATAAGAACGAGAATTTCGTTATTAAACTACGAATTCTCGATTTTTTTATATTTTAAGATAGGTTTATTTCTGA
CAACTTAATAGAAAGGGGTTTGACAAAGCTAAAGTGAAGTTTGACGGTATAAACGCAAATTAACTATACTTTT
ATAAAAAATTAACTCAGGCTGGGACATAAATCAATATTCTATACTCTACGAAGTTATATTGGCAGTAGTTGAC
TGAACGAAAATGCGCTTGTAACAAGCTTTTTTCAATTCTAGTCAGGGGCCCCAACACAGAGAATTTCGAAAAG
AAATTCTACAGGCAATGCGAGTTGGGGTGAGGGCCCCAACACAGAAGCTGACGAAAAGTCAGCTTACAATAAT
GTGCAAGTTGGGGTGGGACGACGAAATAAATTTTGCGAAAATATCATTTCTGTCCCACTCCCATCAAAAGAAT

GACATTGAAAAGAAATATTAAAGTTTGGCGCTATAATTTTCTTTCTGCTCAATGAAATAATACGTTTGAAAAT
TGAATTGAATCATTCGCGAAATTGATAATTATTCTCAATTAATAAAAAATACTTATTACACCTTACTTGTATG
ATAAGTTTTGCTTATATACTCTGATTAAAAAGTCAAAACCTAAATTAAGCGTTTTCTAAGCATGATGAGTTGT
TTATCAATAAATATAATAATACAATTTAATCAAGGCATATAAATATAAAAATGTATCAAGGGGGATCATTAAA
TGGCTGCAAATTTTAAAGAGCAATCAAAAAAACATTTTGACTTGAATGGCCAAAGTTATACTTACTATGATTT
AAAAGCTGTAGAAGAGCAAGGTATTACTAAAGTTTCCAATTTACCTTATTCAATTCGTGTTTTGTTAGAATCT
TTACTTCGTCAAGAAGATGATTTTGTAATTACAGACGATCATATTAAAGCTTTAAGTCAGTTTGGAAAAGATG
GAAATGAAGGCGAGGTACCATTTAAACCTTCTCGTGTTATTTTACAAGATTTCACAGGTGTACCAGCCGTAGT
TGATTTAGCTTCTTTACGTAAAGCAATGGATGACGTTGGGGGAGATATTACTAAAATTAATCCAGAAGTACCG
GTGGATTTAGTTATTGACCACTCAGTTCAAGTGGATAGCTATGCAAATCCAGAAGCTCTTGAACGTAATATGA
AATTAGAATTTGAACGTAACTATGAACGTTATCAGTTTTTAAATTGGGCAACGAAAGCATTTGATAATTACAA
TGCAGTTCCTCCTGCAACTGGAATAGTTCACCAAGTTAACTTAGAATATTTAGCAAGTGTTGTACATGTTCGT
GATGTAGATGGTGAAAAAACTGCATTTCCAGATACATTAGTTGGTACTGATTCACATACAACAATGATAAATG
GTATTGGCGTACTAGGATGGGGTGTTGGTGGTATTGAAGCTGAAGCTGGAATGCTTGGACAACCTTCTTATTT
CCCAATTCCAGAGGTTATTGGTGTACGACTAGTAAATTCATTACCACAAGGCGCAACAGCAACTGATTTAGCG
TTAAGAGTAACTCAAGAGCTACGTAAAAAAGGTGTTGTTGGTAAATTTGTGGAGTTCTTTGGTCCAGGTGTAC
AACATTTACCACTAGCAGACCGTGCTACAATTGCAAACATGGCACCAGAGTATGGAGCAACTTGCGGATTCTT
CCCAGTTGATGATGAATCTCTTAAATATATGAAGTTAACTGGTAGATCAGACGAACATATCGCGCTAGTAAAA
GAATATTTGAAACAAAACCATATGTTCTTTGATGTTGAGAAAGAAGATCCTAATTATACAGATGTTATCGAAT
TGGATTTATCAACAGTTGAAGCATCGCTTTCAGGACCAAAACGTCCTCAAGATTTAATTTTCTTAAGTGATAT
GAAATCATCATTTGAAAATTCTGTAACAGCTCCAGCAGGCAACCAAGGACACGGTTTAGATAAAAGTGAATTT
GATAAGAAAGCTGAAATTAACTTTAAAGATGGATCAAAAGCTACAATGAAAACAGGTGATATTGCAATAGCAG
CAATTACATCATGTACAAATACATCTAACCCTTATGTAATGTTAGGTGCAGGTTTAGTTGCTAAAAAAGCAGT
TGAAAAAGGCTTGAAAGTTCCTGAATACGTTAAAACTTCTCTAGCACCAGGATCAAAAGTTGTTACCGGATAT
TTAAGAGATGCTGGCTTACAACCTTATTTAGATGATTTAGGCTTCAACTTGGTTGGTTATGGATGTACAACTT
GTATCGGTAATTCAGGTCCTTTATTACCAGAAATTGAAAAAGCGATTGCTGATGAGGACCTATTAGTGACATC
TGTATTATCTGGTAACCGTAACTTTGAAGGTCGTATCCATCCTCTTGTTAAAGCCAATTACCTAGCTTCACCA
CAGTTAGTTGTTGCTTATGCATTAGCTGGAACGGTTGATATTGATTTACAGAATGAACCTATTGGTAAAGGTA
ATGACGGTGAAGATGTATATTTGAAAGATATTTGGCCATCAATTAAAGAAGTTTCAGATACCGTTGATAGTGT
TGTAACACCTGAATTATTTATTGAAGAATATAATAACGTATACAATAACAACGAATTATGGAATGAGATTGAT
GTAACTGATCAACCTCTATATGACTTTGATCCTAATTCAACATACATTCAAAATCCATCATTCTTCCAAGGAT
TATCTAAAGAACCGGGTACGATTGTTCCATTAAATGGTTTACGTGTTATGGGTAAATTCGGTGATTCTGTGAC
AACTGACCACATCTCTCCAGCAGGTGCAATTGGTAAAGATACGCCAGCTGGTAAATATTTACAAGATCACCAA
GTGCCTATTCGTGAATTTAATTCTTATGGTTCAAGACGTGGTAATCACGAAGTAATGGTTCGAGGTACGTTTG
CTAATATACGTATTAAAAACCAATTAGCGCCAGGCACTGAAGGTGGTTTTACAACTTATTGGCCAACAAATGA
AGTAATGCCTATCTTTGATGCTGCAATGAAATATAAAGAAGATGGTACAGGTTTAGTTGTATTAGCCGGTAAC
GATTATGGTATGGGTTCATCTCGTGACTGGGCAGCAAAAGGTACAAACTTATTAGGTGTTAAAACAGTTATTG
CACAAAGTTATGAACGTATCCATCGTTCAAATTTAGTTATGATGGGTGTATTACCATTAGAGTTTAAAAAAGG
TGAATCAGCTGATTCTCTTGGTCTAGATGGTACAGAAGAAATTTCTGTTAATATTGATGAAAATGTTCAACCA
CATGACTACGTCAAAGTTACTGCTAAGAAGCATGATGGTGATTTGGTAGAATTTGACGCTATGGTTCGTTTTG
ACTCACTTGTTGAAATGGATTACTATCGTCACGGTGGTATTTTACAAATGGTTTTAAGAAATAAATTAGCGCA
ATAAAAATAGATATCACAGCAAAATTTTAATCGGTATTTGAGGCACTTTGATAGTGCTCAAAAATACTTCTAT
AAACTTTATGATATAATAGGTCGGTTCAAAACCTTTGTGAAATGCTCCCTTCAAAGTAGACATTGAAAAAATG
AAAACTTTGAAGGGAGCTATATTTATGCCAATAAAACCTAAAGTAGACATAGATGTACTGCTATATATGTTTC
AAGAATATGAAAAAGGCGTGTCGTTTCAATACTTAATCGATACATTACAATTAGATATTAATGTCAATACTTT
GTATCCTAAGTATAAATATTATAAAACCCATGGTATTGAAACACTACTTACACAAAAACAATACAATCGCTAT
TCAAAGGAACTAAAATTAAAAGTTGTAAATGAATATTTGAATTCAAATCAATCAACACAAGATCTCGCCATTA
AATACAATATTCGTAGTTCGACACAAGTCAAAAATTGGATAAAAATGTATACTGTAGGAAAAGAGATTAAGAA
TCAATCTCCGAAAACTGGGGTGTATATCATGAAAGCAAGAAAAACCACATTTGAAGAACGTGTCACAATTGTT
GAATATTACTTGAATCATAAACAATCTTATAGAGAAGTTGCAGAACACTTTAATATCTCATACGGTCAAATTT
ACCAGTGGGTCCAAAAGTATCAAGCCCATGGTAAAAATGGGTTAGTAGATGGCAGGGGCAAAGGAAAACCCAA
ATCTATGATGACGCCAGAAGAACAAAAGGAAGCTGAAATTCAAGCATTAAAAGCACAAAATCGATTATTAGAA
ATGGAAAATGACGTTTTAAAAAAGTTTCAAGCATTGGAAAGAGAAATGATTCAAAGAGAAAACAAATCACGGC
ATACAAAACGATCGAAGCGTTAAAGCATCAATACCCTATCCAACGCTTGTGTCGTATTTTAGGCATTTCTAGA
GCGAGTTATTATAAGTGGGTACATTATCAACCGAGTGAACTTGAATTAGAGAATGAACAGCTTAAGCGAGAAA
TAGAATCAATCTATCATAAGTATAATGGTATTTATGGCTATCGTCGCATTTATATCTATATTCGATTAAAACT
AGGTAAACAAGTAAACCGAAAGCGAATTTATTGTTTGATGAAAGAACTTAATTTAAAAGCGGTAATACGTCAA
AAGCGAAAACCTTATCGACGTTCAACGCCACAAATCACATCAGAAAATAAGCTTAATCGACAATTTGACATTG

ATACGCCAAATAAAGTTTGGTTAACAGATGTAACAGAATTTAAAATCAAAGATGGTAGTAAGATATATTTGAG
TGCCATTTATGACTTAGGTGCTAAACGTATTGTTAGTTATGAGCTAGGCCCTTCTAACAATAATCAATTAGTA
TTTAAAACATTCAATCAAGCGATAGAAAAAGTAGAAAATACCAAAGGTATTTTATTTCATAGTGACCGAGGTT
TTCAATACACATCGAAAACATTTAAACATATGTTGGATGAATGTGGCATGATTCAAAGTATGTCACGTGTTAG
CAAATGTATCGATAATGGTCCTATGGAAGGTGTGTGGGGTACAATAAAAAGTGAAATTTTTAGAGGAAATAAG
CACTTTAAATTTAATAGTGTAGAAGAAGCTACAAAAACAATTCATGATTTTATCCATTTCTTTAATTATGAAA
GAATTACATTAAAAATGGCTGATTCTGTATAACAACAAAACCAACCATTAATTTTAATTATTTAATTGTCTAC
TAGACTGGGTGCAGTTCATTGTGTTCTTGGCCGGCCTCTTCTTGTATTTTATGTTAGAATGAGATATAAAGTT
AAACTGAAAGAAGGCTTAAAATGATATATAGTATTACAGAAATAGAAGCGCGTTATGCTGAAACTGATAAGAT
GGGTGTAATTTATCACGGGAATTATGCAACTTGGTTTGAAGTTGCGCGGTTGGATTATATATCGAAGTTAGGT
TTTAGTTATGCTGATATGGAAAAACAAGGAATCATTTCACCCGTGACTGACCTCAATGTCAATTATAAAAAGT
CTATTTTTTATCCAGAAAAAGTTAAAGTTAAAACTTGGGTTGAAAAATATTCGAGATTACGTTCAGTGTATAA
ATATGAGATTTTTAATGAAAAAGGTGAACTTGCAACTACAGGTTCCACAGAATTGATTTGCATTAAAGAAGAT
ACTTTTAAGCCTATACGGTTGGATCGTTATTTCCCAGATTGGCATGAAGCCTATAGTAAAGTGCAAACGCTCA
ATAATGAAGGGAAAACAGTAGAGATAATGGATGATATTGATTCTTTATAAGTTACTATAAATGTTTGGAGAGG
CAATATTAACAATTAATCTATGTTAAATAGTCAATACGTTAGCATTAACGCAATTAGCGTGAAGATAAGCTAA
TATATAGCAAAAACGAACTTGTTCATACAACATCAGATGTGATAAACCAAAGTGCAACTTCCGAGGTTCAGTA
CACATCAGAATGTGTAAACAAGTTCGTTTTAATTTAGTTATTTTGTGGAATAAGAAATTTCATCTTCGTGATC
AACTACATTTACAATAATGTGGTCATCTTCAAAGTACCACAAATCTTTTTCCGCTACGACAACATTTAAATCG
TCATATTGTTGTTCATAGCCAATATCAACATCTTCCTTTGGTTCAACTGTAAAAGCAGGACTAAATCCTTGCT
TGAGTTGGAATTCGCCACCATATCTTACAAAAAACACGAGCACTTTATTATTTTCAGGTAACTCAAGTTCATT
TTTAAACCAAGTTACTGCTGCATCAGTAAGTTCTATTTGCATAACAATGACCTCCTATATTTATTACAGAATG
GTAGTGGGCCAACTCGTATTGTTTGTGGAATTTCTTTTCGAAATTCTCTGTGTTGGAGCCCACCCCAACCTGC
ACATTATTGTAAGCTGAATTTCCATAAGCTTCTATGTTGGGACCCCCGACCAGAATTGAAAAAACCTAGTTAT
AAGCGCATTTCGTTCTGTCAACTTCAGCTAATATAACATTGTGGAGCCTATGACATTGATTTATGTCTCGGTC
TCAAATGTTCTTGTAAAAAACTAAGTATAGTTGGATTTTAAGTTAAAAAAATTGTTTTTGCAAAAATAAAATA
ATCCATCAACTGCAAAAAAATATGCAATGATGGACTAATGTCAAAAAATTGTTACATCCATTTTATTTTAGGT
TCTTCGCCTCTAAAAATCCTTGCGATATTAGAGCGATGTCTAATTATCAATATGATTGAAACTAAGAAACTAA
CGACTAATAAAATATAGTCTTGAATGATAAGCGAGCCAATCACACAGCAAATTGCTGCAACGATACTTGCTAA
AGAAACATATTTAAAAATCTTCAATACAACAAAGAAGATAATTGCAAGTATTAGTAAAAGTATCGGATTGACT
CCCAAGACGACACCTGCACTAGTTGCAACAGCTTTGCCACCTTGGAATTTTAAATAAACAGGATAAACATGTC
CAAGTATAGCGCATAAGCCAACAATTAAACCATTTGTAAAAAAAGTACTAATAGGGCCATCTGCGTGAACTGG
TAACCATAAAGGGAAGAAAACAGTTATGAACCCTTTGAAAATATCTAGAAATGTTACCAAGAATCCTGCAGGA
CGACCTAATACTCTAAAGCTATTAGTAGCGCCAGTATTACCACTACCAAATTGTCTAATATCTTTTTTGAAAA
ATAATTTTCCAATTACGAATCCACTTGGGAAAGCGCCGATAAGATAACTTAGTAGTAACATGACGATTATCAT
CATAAATATTACACATCCTTTAATATCTTAGGACTATTTTATCATATTCGTTTAAATTACGGCTAAAATTTTT
AAAAACGGGGATTAATATATGGAATTAAGCTATGAAAGTTAATTGATACTTGCATTTTACGCTGATTTATATA
AGAATAACTATTGTATAGTTTTAAAAACGAACGTACGTTTGCAGGAGGCGAAATCATTGGCAATGAATAAACA
AAATAATTATTCAGATGATTCAATACAGGTTTTAGAGGGGTTAGAAGCAGTTCGTAAAAGACCTGGTATGTAT
ATTGGATCAACTGATAAACGGGGATTACATCATCTAGTATATGAAATTGTCGATAACTCCGTCGATGAAGTAT
TGAATGGTTACGGTAACGAAATAGATGTAACAATTAATAAAGATGGTAGTATTTCTATAGAAGATAATGGACG
TGGTATGCCAACAGGTATACATAAATCAGGTAAACCGACAGTCGAAGTTATCTTTACTGTTTTACATGCAGGA
GGTAAATTTGGACAAGGCGGCTATAAAACTTCAGGTGGTCTTCACGGTGTTGGTGCTTCAGTTGTAAATGCAT
TGAGTGAATGGCTTGAAGTTGAAATCCATCGAGATGGTAATATATATCATCAAAGTTTTAAAAACGGTGGTTC
GCCATCTTCTGGTTTAGTGAAAAAAGGTAAAACTAAGAAAACAGGTACCAAAGTAACATTTAAACCTGATGAC
ACAATTTTTAAAGCATCTACATCATTTAATTTTGATGTTTTAAGCGAACGACTACAAGAGTCTGCGTTCTTAT
TGAAAAATTTAAAAATAACGCTTAATGATTTACGCAGTGGTAAAGAGCGTCAAGAGCATTACCATTATGAAGA
AGGAATCAAAGAGTTTGTTAGTTATGTCAATGAAGGAAAAGAAGTTTTGCATGACGTGGCTACATTTTCAGGT
GAAGCAAATGGTATAGAGGTAGACGTAGCTTTCCAATATAATGATCAATATTCAGAAAGTATTTTAAGTTTTG
TAAATAATGTACGTACTAAAGATGGTGGTACACATGAAGTTGGTTTTAAAATAGCAATGACACGTGTATTTAA
TGATTATGCACGTCGTATTAATGAACTTAAAACAAAAGATAAAAACTTAGATGGTAATGATATTCGTGAAGGT
TTAACAGCTGTTGTGTCTGTACGTATTCCAGAAGAATTATTACAATTTGAAGGACAAACGAAATCTAAATTGG
GTACTTCTGAAGCTAGAAGTGCTGTTGATTCAGTTGTTGCAGACAAATTGCCATTCTATTTAGAAGAAAAAGG
ACAATTGTCTAAATCACTTGTGAAAAAAGCGATTAAAGCACAACAAGCAAGGGAAGCTGCACGTAAAGCTCGT
GAAGATGCTCGTTCAGGTAAGAAAAACAAGCGTAAAGACACTTTGCTATCTGGTAAATTAACACCTGCACAAA
GTAAAAACACTGAAAAAAATGAATTGTATTTAGTCGAAGGTGATTCTGCGGGAGGTTCAGCAAAACTTGGACG
AGACCGCAAATTCCAAGCGATATTACCATTACGTGGTAAGGTAATTAATACAGAGAAAGCACGTCTAGAAGAT
ATTTTTAAAAATGAAGAAATTAATACAATTATCCACACAATCGGGGCAGGCGTTGGTACTGACTTTAAAATTG

AAGATAGTAATTATAATCGTGTAATTATTATGACTGATGCTGATACTGATGGTGCGCATATTCAAGTGCTATT
GTTAACATTCTTCTTCAAATATATGAAACCGCTTGTTCAAGCAGGTCGTGTATTTATAGCTTTACCTCCACTT
TATAAATTGGAAAAAGGTAAAGGCAAAACAAAGCGAGTTGAATACGCTTGGACAGACGAAGAGCTTAATAAAT
TGCAAAAAGAACTTGGTAAGGGCTTCACGTTACAACGTTACAAAGGTTTGGGTGAGATGAACCCTGAACAATT
ATGGGAAACGACGATGAACCCAGAAACACGAACTTTAATTCGTGTACAAGTTGAAGATGAAGTGCGTTCATCT
AAACGTGTAACAACATTAATGGGTGACAAAGTACAACCTAGACGTGAATGGATTGAAAAGCACGTTGAGTTTG
GTATGCAAGAGGACCAAAGTATTTTAGATAATTCTGAAGTACAAGTACTTGAAAATGATCAATTTGATGAGGA
GGAAATCTAGTGAGTGAAATAATTCAAGATTTATCACTTGAAGATGTTTTAGGTGATCGCTTTGGAAGATATA
GTAAATATATTATTCAAGAGCGTGCATTGCCAGATGTTCGTGATGGTTTAAAACCAGTACAACGTCGTATTTT
ATATGCAATGTATTCAAGTGGTAATACACACGATAAAAATTTCCGTAAAAGTGCGAAAACAGTCGGTGATGTT
ATTGGTCAATATCATCCACATGGAGACTTCTCAGTGTACAAAGCAATGGTCCGTTTAAGTCAAGACTGGAAGT
TACGACATGTCTTAATAGAAATGCATGGTAATAATGGTAGTATCGATAATGATCCGCCAGCGGCAATGCGTTA
CACTGAAGCTAAGTTAAGCTTACTAGCTGAAGAGTTATTACGTGATATTAATAAAGAGACAGTTTCTTTCATT
CCAAACTATGATGATACGACACTCGAACCAATGGTATTGCCATCAAGATTTCCTAACTTACTAGTGAATGGTT
CTACAGGTATATCTGCAGGTTACGCGACAGATATACCACCACATAATTTAGCTGAAGTGATTCAAGCAACACT
TAAATATATTGATAATCCGGATATTACAGTCAATCAATTAATGAAATATATTAAAGGTCCTGATTTTCCAACT
GGTGGTATTATTCAAGGTATTGATGGTATTAAAAAAGCTTATGAATCAGGTAAAGGTAGAATTATAGTTCGTT
CTAAAGTTGAAGAAGAAACTTTACGCAATGGACGTAAACAGTTAATTATTACTGAAATTCCATATGAAGTGAA
CAAAAGTAGTTTAGTAAAACGTATCGATGAATTACGTGCTGACAAAAAAGTCGATGGTATCGTTGAAGTACGT
GATGAAACTGATAGAACTGGTTTACGAATAGCAATTGAATTGAAAAAAGATGTGAACAGTGAATCAATCAAAA
ATTATCTTTATAAAAACTCTGATTTACAGATTTCATATAATTTCAACATGGTCGCTATTAGTGATGGTCGTCC
AAAATTGATGGGTATTCGTCAAATTATTGATAGTTATTTGAATCATCAAATTGAGGTTGTTGCAAATAGAACG
AAGTTTGAATTAGATAATGCTGAAAAACGAATGCATATCGTTGAAGGTTTGATTAAAGCGTTGTCAATTTTAG
ATAAAGTAATCGAATTGATTCGTAGCTCTAAAAACAAGCGTGACGCTAAAGGAAACCTTATCGAAGTATTCGA
GTTCACAGAAGAACAGGCTGAAGCAATTGTAATGTTACAGTTATATCGTTTAACAAACACTGACATAGTTGCG
CTTGAAGGTGAACATAAAGAACTTGAAGCATTAATCAAACAATTACGTCATATTCTTGATAACCATGATGCAT
TATTGAATGTCATAAAAGAAGAATTGAATGAAATTAAAAAGAAATTCAAATCTGAACGACTGTCTTTAATTGA
AGCAGAAATTGAAGAAATTAAAATTGACAAAGAAGTTATGGTGCCTAGTGAAGAAGTTATTTTAAGTATGACA
CGTCATGGATATATTAAACGTACTTCTATTCGTAGCTTTAATGCTAGCGGTGTTGAAGATATTGGTTTAAAAG
ATGGTGACAGTTTACTTAAACATCAAGAAGTAAATACGCAAGATACCGTACTAGTATTTACAAATAAAGGTCG
TTATCTATTTATACCGGTTCATAAATTAGCAGATATTCGTTGGAAAGAATTGGGGCAACATGTATCACAAATA
GTTCCTATCGAAGAAGATGAAGTGGTTATTAATGTCTTTAATGAAAAGGACTTTAATACAGATGCATTTTATG
TTTTTGCGACTCAAAATGGCATGATTAAGAAAAGTACAGTGCCTCTATTTAAAACAACGCGTTTTAATAAACC
TTTAATTGCTACTAAAGTTAAAGAAAATGATGATTTGATTAGTGTTATGCGCTTTGAAAGAGATCAATTAATT
ACCGTCATTACTAATAAAGGTATGTCATTAACGTATAATACAAGTGAACTATCAGATACCGGATTAAGGGCAG
CTGGTGTTAAATCAATAAATCTTAAAGCTGAAGATTTCGTTGTTATGACAGAAGGTGTTTCTGAAAATGATAC
TATATTGATGGCCACACAACGCGGCTCGTTAAAACGTATTAGTTTTAAAATCTTACAAGTTGCTAAAAGAGCA
CAACGTGGAATAACTTTATTAAAAGAATTAAAGAAAAATCCACATCGTATTGTAGCTGCACATGTAGTGACAG
GTGAACATAGTCAATATACATTATATTCAAAATCAAATGAAGAACATGGTTTAATTAATGATATTCATAAATC
TGAACAATATACAAATGGCTCATTCATTGTAGATACAGATGATTTTGGTGAAGTAATAGACATGTATATTAGC
TAAAAACTATATGCAATCACGAAATTAAATGATAAAATACAGTAATGTTAAATTTTGACTAAATTCAAGGGAT
TTATATTAAATGCTGACCAAGTACTTATCGTTAAATTAGCGATATACTTTGACCAAACTAATATAAATCTCTT
GTATTGTATAAGAAATGATGATGTATCTCTATATTTACTGAAGTTTCAATTCGGTCTAATTTTATAGAAGGTG
CATGATTCATGTAGAGGATGCGAGGGGTTTATTTTGAAAGATTTCGATAGTTTAATTCCTGGATGGTTTAAAG
AATTTGTCCATGTTGGTACCGATTTAATATGGTCTCAATATTTAATTGGTCTATTATTGACAGCTGGATTCTT
CTTTACAATTAGTTCTAAATTCGTTCAATTACGAATGTTACCTGAAATGTTTAGAGCTTTAGTAGAACGTCCA
GAAACTTTAGAAGATGGTAAGAAGGGTATTTCGCCATTCCAAGCATTTGCTATTAGTGCTGGTTCGAGAGTTG
GTACTGGTAATATTGCTGGTGTTGCGACTGCGATTGTTTTAGGCGGTCCAGGTGCAGTGTTTTGGATGTGGGT
TATTGCATTTATAGGTGCAGCGAGTGCATTTATAGAAGCGACTTTGGCTCAGGTTTATAAAGTACATGATAAA
GATGGTGGATTCCGTGGTGGTCCAGCTTACTATATTACTAAAGGTTTAAATCAAAAATGGCTAGGTATCGTAT
TTGCGATTTTAATTACAATTACATTTGCATTTGTATTTAACACAGTGCAATCTAATACAATTGCGGAGTCGTT
AAATACGCAATATAATATTAGTCCAGTAATCACAGGTATTATTTTAGCAATCGTAACAGCTATTATTATATTT
GGTGGTGTACGTAGTATTGCTACGTTATCTTCGTTAATTGTACCGATTATGGCTATCATTTACATTGGTATGG
TTTTAGTAATATTGCTATTTAATTTAGATCAAATTGTTCCTATGATAGGTACGATTATTAAAAGTGCATTTGG
TATCGAACAAGTAACTGGTGGCGCTGTAGGTGCTGCGGTTCTTCAAGGTATCAAACGTGGTTTATTCTCTAAC
GAAGCTGGTATGGGTTCTGCGCCGAATGCAGCGGCAACTGCTGCCGTGCCACACCCTGTTAAGCAAGGTTTAA
TCCAATCATTAGGTGTGTTCTTTGATACAATGTTGGTTTGTACAGCAACTGCAATCATGATTTTACTATATTC
AGGATTGAAATTTGGTGATAACGCACCTCAAGGTGTTGCAGTTACTCAATCAGCACTTAATGAGCATTTAGGT

TCTGCTGGAGGTATTTTCTTAACAATAGCAGTTACACTGTTTGCATTTTCATCTGTTGTAGGTAATTACTATT
ACGGTCAATCTAATATTGAATTTTTATCAACAAACCGTGTAATATTATTTATCTTTAGATGTCTTGTTGTAGT
ACTTGTCTTTGTCGGTGCAGTTGTAAAAACAGAAACAGTATGGAATACGGCAGACTTATTTATGGGCTTAATG
GCAATTGTAAACATTATTTCCATTATAGGACTGTCCAATGTAGCTTTTGCATTGATGAAAGATTATCAAAAGC
AGAAAAAAGAAGGCAAGAATCCTGTCTTTAAACCTGAAAACTTAGAAATTAACTTATTTGGAATTAGTGCTTG
GGGCGCTAACAAATATAAGAACTCTGATAAATAAATATTTTTAATGTAACGATTCATTATATAAAAATCAAAT
AAACCCTGAGGTTTTCAAATTAAAGCGAAAATCTCAGGGTTTTAAATTTGTACTCAATAATCATCTGTATCAT
TACACTAGTTAATCCAAGATTTGATTATATATTAATATAGTAAGGTTGAGATTTATTTCCTCCATTGGTGTGC
AAAGACTATACCATTGATAAGACATATTGTTGGATAGATGAATATAAATTTATTTTAGCTTTGCACAATTTAA
TAGTACGTTTTAGTTTGTATTGAGTATTAATTCAGATAATATATAAATAAATAGAATTATTTGTAAATATGGG
AGTCAGATTTTTTTATTATAGTTATTCCACTTTGAGAGGCATCGAAATTAAATTAAGATGTATCATATAGGAT
AGGCGTGTAATAGAACACAAGTATTTAATTTATTATACTTTCAAGTTAGGCATTTCCGATATATAATCTTAAT
AATAGAAAAATAGGTGATGATAATGGGAGAATATATTGTTACTAAAACATTGAACAACAATGTCGTAGTATGT
ACTAATAATGATCAAGAAGTTATTTTAATCGGTAAAGGTATTGGTTTTAACAAAAAAGAGGGAATGACGTTAA
ACGACCAAACTATTACAATAGAGAAAATTTATAAATTAGAGAGTGAGCAACAAAAAGCACATTATAAAAGTTT
AGTTGAAATCGCTGATGATAATGTATTACAAGTAATTATTGATTCGTTAAATTTTATTTCTAATACTGCGATG
AATGTTGATTCAAAACAACTTGTAGTTTCATTAACGGATCATATTATATTTGCATATAAACGCTTAAAACAAA
ATCAAGTTATTAGCAATCCATTTGTTATGGAAACTATGCAGTTATATAGTGATGCATATCATATTGCTAAACA
GGTAATTGATCAGTTAAATGCAGCATTAGATGTACATTTTCCTGAAGATGAGATAGGATTTATTGCATTACAT
ATTGCATCTAACACAGAAGATTTATCTATGCATGAGATGACTTTGATCAATAATGTTATTAAAAAAGGTATAG
ATATCATTGAATCAGACCTTGTGACAACTGTTGATAAGGAATCATTACAATACCAACGTTTTATAAGACATGT
ACAATTTTTAATTCGCCGATTAAGAAGAAAAGAATATATACATGCACAAGATGATTTTGTGTCTATGATTAAA
AATCACTATCCGATTTGCTATAACACAGCATATAAAATTTTAACTATGATACAAAAACAATTTGATGTTACTA
TCAGTGAGTCTGAAATTATATATTTAACATTACACATTCATCATTTTGAAGAAAGGATTAATCATTCCTAAAT
TAAGTGCATTATACGGGCGTTTCGTTACTGTAAAGGGTATTTGTATGGTATCATTGATGTTAGTAATTTTACA
TAAGTGGACTGTTTTTATACAACATCTTATGAATATTTTAAGTGGTGGTGAATCTATAATTGAATGAAAATGA
AAAGAATATAAGAAAGAACTTTTTAAAATTTACCGAATCACGGTATATGAAGTTTGTTGGTGGGAATGATTTA
GTCTTCTCATTAATAGCTCTAGTATTGTTGGGTATTGTTATTTTTATTTTCGAAAAAGTATCATACGTTTTTG
ATCCTTTTATCATCGTTTTTAAGACGATAGCAGCACCTATCATCGTCTCTTTAATTCTATTCTATCTATTTAA
CCCAATCGTAAATATGATGGAACGTTATAGAATACCAAGAGTTGCAGGTATTTCTATTATTTATCTAGCTGTA
GTAGGTGTTATTACGTTAATTGTTAATTTATTGATACCTATTATTGGTTCGCAAGTAGATAGTTTAGTTAAAA
ATTCACCGCAATATCTAGAAAAATTAATTAATTCTATTGATAAAATAGCAAATAATACGTTTTTCTCTTCGTA
TTATAGTCAAATTAATGATTGGTTAAATTCTTTACCTAAGAAAATACCATCTATGTTAAGTGAATTTACAGAT
GGCTTTGGGTCTAAAATTGCAACGTTTGCAGAAACGATTGCTAATATTGGCGTTGTGATTGTAACAACACCAT
TTGTACTATTCTTTATGCTTAAAGATGGACATCACTTCAAAGAATTTTCAACGAATATTATGCCACCGAAATT
CCGAAAAGATTTTCATGATCTACTTGAAAAAATGAGTGTTCAAGTTGGTTCATACATTCAAGGACAAATTATT
GTTTCATTCTGTATCGGTATATTGTTGTTTATCGGTTATTCGGTTATCGGGTTGAAATATAGCTTAGTATTAG
CTAGTATTGCGGCAGTTACAAGTGTTGTACCATATTTAGGGCCTACAATAGCGATTTCACCAGCTATTGTAAT
AGCTGCTATAACTTCGCCGTGGATGCTCTTAAAATTAGCAGTAGTATGGACTTTAGTACAATTTGTTGAAGGG
CACTTCATTTCACCAAATATCATGGGTAAAACACTTAAGATTCATCCACTTACAATCATTTTCATTTTACTTT
GTGCAGGCAAATTGCTTGGTATTGTAGGCGTTATTTTAGGTATTCCGGGATATGCTATTTTAAAGTATTAGT
TACTCATTTATTCCAATTATTTAAACGTCGATACAATCGTTTCTATGGTAATGATGTAGGTGAATATGATATT
AAAGAAAGTAATAAAATAGTTGAATAAGTAATTAAAAATACCAATAGACTATACGTTTGTTGGTATTTTTTAT
GGTGCTTATAATGTTGGGCACTCACATTTATGATTATCAGCACATGTTTAGCTTATAAATTTTTACGAAAGTT
TAGCCGTTTCTAAAGTACATAATAATAAAACGAGTATTTGCCACTTGATTAGTACTTCATTATTATGTCGAAA
ATAAAAACAAGTGGTATTTTTAATATATTAAGAAGCACTCATAATCAGCTGTTAATTAATAATATTTTTCATA
AGTATTGATTCATCATTTTCTTTATGTTAAATATGAATCAGTATAGATAACCATATTGTTCTGTTTGAGATAG
AAATATTTTCTATTTACTTCTTAATACAGATGTCAATATGACAAAAGTGTTATATAAATCAAAGGTAAATGAT
ATGCTATTTTTATGGTTTATATGATATATTTTGATTTATAACAGAAATAATTAGAATTGATGTGAAAAAATGA
ATCAGGAAGTTAAAAACAAAGTATTTTCAATCTTAAAAATTACGTTTGCTACAGCTTTATTTATTTTTGTAGT
AATCACGTTGTATCGAGAGTTATCTGGAATTAACTTTAAAGATACTTTGGTTGAATTTAGTAAAATCAACCGT
ATGTCCCTTGTCTTATTATTTATTGGTGGTGGGGCATCGCTTGTTATTCTCTCAATGTATGATGTGATTTTAT
CTAGAGCTTTAAAAATGGATATTTCATTAGGAAAAGTTTTAAGAGTAAGTTATATCATTAATGCATTAAATGC
GATTGTAGGCTTTGGTGGCTTTATTGGTGCCGGTGTAAGAGCAATGGTTTACAAAAACTATACGCATGATAAA
AAGAAATTGGTTCACTTTATTTCATTAATACTGATTTCAATGTTGACAGGTTTAAGCTTATTATCATTGCTAA
TTGTATTCCATGTTTTCGATGCATCTTTAATCTTAGATAAGATTACATGGGTAAGATGGGTATTATATGTAGT
GTCATTTTTCTTACCATTATTCATTATTTATTCAATGGTTAGACCACCCGATAAAAACAATCGTTTTGTAGGA
TTGTACTGCACTTTAGTGTCGTGTGTTGAATGGTTAGCAGCTGCAGTTGTATTATATTTCTGTGGTGTAATTG

TTGACGCTCATGTATCATTCATGTCCTTTATTGCAATATTTATCATTGCTGCATTATCAGGTTTAGTCAGCTT
TATTCCTGGTGGTTTCGGCGCTTTCGATTTAGTTGTATTACTAGGATTTAAAACTTTAGGTGTCCCTGAGGAA
AAAGTATTATTAATGCTACTTCTATATCGTTTTGCGTACTATTTTGTACCGGTAATTATTGCATTAATTTTAT
CATCATTTGAATTTGGTACATCAGCTAAGAAGTACATTGAGGGATCTAAATACTTTATTCCTGCTAAAGATGT
TACGTCATTTTTAATGTCTTATCAAAAGGATATTATTGCTAAAATTCCATCATTATCATTAGCAATTTTAGTA
TTCTTTACAAGTATGATCTTTTTTGTAAATAACTTAACGATTGTTTACGATGCTTTATATGATGGAAATCACT
TAACGTATTATATTCTATTGGCAATTCATACTAGTGCTTGTTTATTACTTTTACTGAATGTAGTTGGTATTTA
TAAGCAAAGTAGACGTGCCATTATCTTTGCTATGATTTCAATTTTATTAATCACAGTGGCGACATTCTTCACT
TACGCTTCATATATTTTAATAACATGGTTAGCTATTATTTTTGTTCTGCTTATTGTAGCTTTCCGTAGAGCAC
GTAGGTTGAAACGCCCAGTAAGAATGAGAAATATAGTTGCAATGCTTTTATTCAGTTTATTTATTTTATATGT
TAACCATATATTTATTGCTGGAACGTTATATGCATTAGATATTTATACGATTGAAATGCATACATCTGTATTG
CGCTATTACTTCTGGCTTACGATTTTAATCATCGCTATCATCATAGGTATGATTGCATGGTTGTTTGATTATC
AATTTAGCAAAGTACGTATTTCTTCTAAAATTGAAGATTGCGAGGAGATTATTAATCAGTACGGCGGTAATTA
TTTGAGTCACTTGATATATAGTGGTGACAAGCAGTTTTTCACTAATGAAAATAAAACAGCATTTTTAATGTAT
CGTTATAAAGCAAGTTCATTAGTGGTTCTTGGAGATCCGTTAGGTGATGAAAATGCCTTTGATGAATTGTTAG
AAGCATTCTATAATTACGCTGAGTATTTAGGCTATGATGTTATATTCTATCAAGTTACAGATCAACACATGCC
TTTATATCATAATTTCGGTAACCAATTTTTCAAATTAGGTGAAGAAGCAATTATTGATTTAACGCAATTTTCA
ACTTCAGGTAAAAAACGCCGTGGATTTAGAGCGACTTTAAATAAATTCGATGAACTTAATATTTCGTTCGAAA
TTATTGAACCACCGTTTTCAACTGAATTTATAAATGAACTTCAACATGTAAGTGATTTATGGCTAGATAATCG
TCAGGAAATGCATTTCTCTGTTGGTCAATTTAATGAAGAATACTTATCTAAAGCGCCAATTGGTGTAATGCGA
AATGAAGAAAATGAAGTAATTGCATTCTGTAGTTTAATGCCAACATACTTTAATGATGCCATTTCAGTCGATT
TAATTAGATGGTTGCCAGAGTTAGATTTACCATTAATGGATGGATTATACTTGCACATGTTACTTTGGAGTAA
AGAACAAGGTTATACAAAATTTAATATGGGTATGGCAACGTTATCAAACGTTGGTCAATTGCATTATTCATAT
TTAAGAGAACGACTTGCAGGCCGTGTCTTTGAACATTTCAACGGTCTATATCGTTTCCAAGGATTACGTCGTT
ATAAATCTAAATATAATCCGAATTGGGAACCACGCTTTTTAGTTTATCGTAAAGATAATTCGCTTTGGGAATC
ACTTTCTAAAGTAATGCGTGTAATACGTCACAAATAATTAAAATCCAAGTGCTAAGAGGTATACAGTTATGCC
TGAAGCGCTTGGATTTTTTGGTTTCTATAAAGAATGATTTATAAAAAACGGTACAGATAAGAATATTTAAGTT
TCCTATCTGTACCGTATTTTAATATTGAAATGAGCTTAAGTTAAGTCGCCTAACTTTTATTATCAGTCCAATC
AGTTTCATCAAGTATAAATGTTATTGCTTATTTTTGTATTCTTGGCGTATTTTTTGTTCTTCTGCATAGCGCT
CTGGATTTTTCTTATAAAAATCTTGGTGATAGTCTTCGGCTTTGTAAAATTGTGACGCTGGTAATATTTTTGT
TGCAATTGCCTTATCAGCATTAATCGTATTTTTAAGCTGCTCGATATAAGTCTCAGCGAGTTCTTTTTGATGA
TCATTAGTGTAGAAAATAGCTGTTTGATATTGAGGGCCACGGTCTTGATATTGACCACCTGTATCTAATGGGT
CAATGACTGAGAAAAATATTTCTAATAACTTATTGTATGAGAATAATGCAACATCATATTGAATTTCAACAGT
TTCTAAATGACCACTCGTACCTGATTTTACTTGTTCGTAAGTAGGATTTTCAATATGTCCGCCCATATATCCA
GAAGTTACTTTTTCTATGCCGTCAAAGGTGTCAAATGGTTTCGTCATACACCAAAAGCAACCTCCGGCAAAAT
AAGCTGTATTAATATTCATTTTTGACATCCTTTCATTAGACCTTAGTACGATTTATTAAGAAATCACTTGCTT
TTTGAATTGTTTTTATATAACGTTAATATGTGATTATTATAGTACACAAAAATCTATTTTTGAAGATATAAGG
TAGGTAAATATGGATAAAGAAACTAATGACAACGAATATAGACGTCAAAGTGAACATCGCACTTCGGCGCCTA
AGCGAAAAAAGAAGAAGAAAATTAGGAAATTACCTATCATTCTTCTGATTGTTGTAATTTTACTTATCGCATT
AGTTGTATATATTGTGCATAGTTACAATAGCGGTGTAGAATATGCCAAGAAACATGCGAAAGATGTTAAAGTA
CATCAATTTAATGGACCAGTAAAAAATGATGGTAAAATTTCTATTCTTGTACTCGGTGCAGATAAAGCACAAG
GTGGACAATCAAGAACAGATTCTATCATGGTTGTTCAATATGACTTTATCAATAAAAAGATGAAAATGATGTC
TGTCATGCGTGATATTTATGCAGATATTCCAGGATATGGAAAACACAAAATTAATTCAGCATACGCTTTAGGT
GGTCCAGAGCTACTTAGAAAAACACTTGATAAAAATTTAGGAATTAATCCTGAATATTATGCAGTAGTTGATT
TTACTGGATTTGAGAAAATGATTGATGAATTAATGCCAGAAGGTGTACCAATTAATGTCGAAAAAGATATGTC
GAAAAATATTGGTGTATCTTTGAAAAAGGGTAACCATAGGTTGAATGGTAAAGAATTACTTGGTTATGCAAGA
TTCCGTCACGACCCTGAAGGTGACTTCGGACGCGTGCGACGTCAGCAACAAGTGATGCAAACATTGAAAAAAG
AAATGGTTAATTTTAGAACAGTTGTTAAATTACCAAAAGTCGCAGGTATTTTAAGAGGCTATGTGAATACAAA
CATTCCTGATTCAGGGATTTTCCAAACAGGTTTGAGTTTTGGTATCCGAGGTGAAAAAGATGTTAAGTCATTG
ACTGTGCCAATCAAGAACTCATACGAAGATGTCAATACAAATACTGATGGTAGTGCATTACAGATTAATAAAA
ACACAAATAAACAAGCTATTAAAGACTTTTTAGATGAAGATTAAAAATAAACAAGGCGATTTCTATCATACTC
AGATAGAAGTCGCCTTGTTCGTTTTCAAAGAATTATTTGGCATGCATCTTATGAAGAACTATTGATCTGACTT
TCTTACGCCAGCCACACCATAATGGTTTGGTTTCATTTCTTCTATAACAACGTGAATTGCTTGTCTATTTGCT
CCCGTTGTTTTTTCTACGGCGTCAGTAACTTCGCTAACTAAATTTTTTAATTGTTCATCCGAACGACCTTCTA
ATAATTTTACATTGACGATTGGCATCATCCAAACCTCCTAATCATTATTAATTTGAGTATATCATGTAATACT
TAAAAATTCATTTGCAAATTAACAGAACACTTGTTCTAAAATAGTTGAAATAGAACACGTGTTCGTATATAAT
AGAATTCAAGTAAGGGAGGAATAACAGTGTATAATTATCATTTATTAGAAGATAGGGATGTTCTATGTATTGA
CCAAAAAAGTTTTTTTGCGAGTGTTTCTTGTATTGAAAAAGGGCTAGATCCATTAGAAACAAAGTTAGCTGTT

GTTGCAGATACTAAGCGTCAGGGTTCTGTAGTATTGGCTGCGACACCTAAATTAAAAGAATTAGGCATCAAGA
CAGGGTCGCGATTGTTTGAAATACCACATAGAAATGATATTTACATTATCAATCCAAGTATGCGTAAATATCT
TAATGTTTCAGTTGCTATTTCTAAGATTGCATTGCGTTATATTCCACCTGAAGATTTACACCAATATAGTATT
GACGAATTTTTTATGGATGTTACTGATAGCTATCATAGATTTAGTTCTACAGTACATGCATTTTGCGAAAGAC
TTAAACGTGAAATTTATGAAGAAACAGGCATTTATTGTACTGTGGGCATTGGTTCTAATATGTTATTAAGTAA
AATTGCTATGGATGTTGAAGCGAAGCATAGTCAAAATGGTATAGCTGAATGGCGATATCAAGATGTACCAACG
AAATTATGGCCAATTCAGCCCTTGCGAGATTTTTGGGGTATTAATCGTCGAACAGAAGCCAAATTGAATAAAA
GAGGAATTTTTACTATAGGAGATTTAGCGAAATATCCATATAAATTTTTAAAAAAAGAGTTCGGTATTTTAGG
TGTTGATATGCATCTACATGCGAATGGGATAGATCAGAGTAAAGTACGTGAAAAGCACAAGATCAGCAATCCA
TCGATATGCAAAAGTCAAATATTAATGAGAGATTATCATTTTGATGAAGCAAAAGTAGTAATGCAAGAGTTAA
TTGAAGATGTTGCTAGCAGAGTTCGAGCAAGAAAAAAAGTGGCAAGAACGATACATTTTGCCTTTGGCTATAG
TGATGAAGGCGGTGTACATAAGCAATATACTTTGAAAGATCCAACAAACTTAGAAAAAGATATTTATAAAGTA
GTAATGCATTTCGCAGATAAATTATGTAATAAACAAGCACTATATCGTACGCTAAGTATATCTTTGAGTCAAT
TTATTAATGAGGATGAGCGACAGTTAAGTCTGTTTGAATATGAATACCAACGCAAACGTGACGAATGTCTAGC
TAAAACGATAGACCAATTACATTTGAAATACGGCAAAGGTATTGTGTCCAAAGCAGTATCGTTTACAGAAGCA
GGTACAAAACACGGCAGATTAGGTTTAATGGCTGGACATAAAATGTAATGATTATACGGTTTAAGGAATATAT
AACTGTGATTCGTATAAAATAAGTCTCTAAAGATAAATATTTCATATATCACAATAGATTTCACAATAATATC
TAAGAATACATGGGAATTTATCAAAAGAGACTTAATAATTATTGGATATAACAATCAAAATCACTCAATGCTT
GCATACCGCGTTCTCGGTCAGTAGGATTTTTGAAACTAATTTTTAAAGCACCGTATATATCTTCGCGTACTTC
TAAGATTCTTAAGTTGCTTATAGATATGTTATGTAAACTCAGGATATAAGTCACTTTACTTATCATACCTGAT
TCATCCGGAATATCCACATATAGATCATACGCAGTATTTAGTCCACCTAGTTGTTTAGCGGGCAGTGCGTCAC
GATACGATTTAGCTTCGGCAAAAAATGATAACAATTTTTCAGAATCATTGCTTTCAATTAGTCTTTCTAAATC
TTGAAACTGACTTTTTAGCTGTCGAATCATTTCTAAAATATACGTTTTATTACTCAAGGTGATATCTTTCCAC
ATTTGTGCATTACTACTAGCTATACGAGTGATATTACGAAAACCACCAGCTGCAAGTTTATTGACTAAATGAT
GTTCTTGACCGTTCTTTTGACTAACATGAACTAAACTAGATGCAACGATATGAGGTAAATGACTTACGACGCT
TGTTACGTAGTCGTGTTCTTCAGCAGTAGTTACAATAAATTTAGCAAGAGTAGGTGATAACAGTTCTTTTAAC
GTGTTTGCTGCTTGCTCATTTCTTGCCTCATTGTAGACTAAAATATAATAAGCGTTTTCAAATAAGTGCTTTT
TAGCATTTAGTACACCAGATTTATGACTACCAGCCATTGGATGACCACTGACTAGATGAATATTATGCTTTAA
TAAATTGCATTCGTGTTGCTGTATCATTGCTTTAGTACTACCAGTATCAGAAACAATAACACCAGGTTTAGTT
GGCATATCTATAAGCTTGCTAAGATATTTATTTGTGATAGCAACAGGTGTTGCATAAATAATTACATCGGCTT
TTTTAATAGCTTCACTATAATTTAAACATTTTTCATTAATAATACCGATTGATTTAGCTTTATCTAACTGAGA
AGTATCTGCATCGTATGCAATAATATTAGTATTAGGGTTATGGTATTTTATATTGCTAGCAAGACTTCCACCA
ATTAATCCAAGCCCAACAAATAAAACTGTTGTCATATAAATCACCTTATTTCGAAATTTTCAGAATAATAACA
TTGTAAATGAGCTGTTGACACAGTGCAATAGTAAATAAAAATCGATAATAGCATTAATAGATAAACGGAGATA
AATCATCTACAATAAAGAGTATAGTAACACAATGGCAACGGAGGGGTAAATCAATGGAACCAATATTAGAAAT
GATTAAAACATTAACAGGTATTAATAGTCCTTCAGGAGACACAGAAGAAGCAATTCAATTTGTCGAAAAATAT
GCAAAAGACTTGGGTTATCAAACAACACTAACAAATAAAGGTGCGTTATTAATAACAGTGCCAGGCAAAAATG
ATGAAGTACAACGCTGTATTACTGCTCATGTTGATACTTTAGGTGCAATGGTTAAAGAAATTAAAGAAGATGG
GCGCTTAGCAATAGAATTAATTGGAGGATTCACGTATAACGCGATTGAGGGTGAATATTGCCAAATTAAAACT
GATGCTGGTCAAATATATACAGGAACAATTTGTCTGCATGAAACAAGTGTTCATGTATATAAAAATAATCATG
AAATAACTAGAGACCAAAAGCATATGGAAATAAGAGTTGATGAAGTAACTACATCAGAAGAAGATACAAAGAG
TTTAGGTATTTCAGTAGGTGATTTTGTTAGCTTTGATCCACGTACAGTTATCACGTCATCAGGTTTCATTAAA
TCTCGCCATTTAGATGATAAAGCTAGCGTAGCGATGATACTACAATTACTAAAGAAATTAAAAGAAGAGCAAA
TAATATTACCACATACAACGCAATTTTATATTTCTAATAACGAAGAAATAGGTTACGGTGCAAATGCATCAAT
TGATTCGAAAATCAAAGAATATATTGCATTAGATATGGGCGCGTTGGGAGACGGTCAAGCATCGGATGAATAT
ACAGTTTCTATTTGTGCCAAAGATGCTTCAGGTCCATATCATAAGCAATTGAAATCGCACCTAGTTAATCTTT
GCAAAATAAATAACATTCCATATAAAGTCGACATATATCCATATTATGGTTCAGATGCTTCAGCAGCTTTACA
TGCTGGTGCGGATATCAGACATGGTTTATTTGGCGCTGGCATTGAATCATCTCATGCAATGGAACGAACACAT
ATTGATTCTATTAAAGCGACAGAGAAATTACTATATGCATATTGCTTATCACCAATTGAGTAAACAATTAGTG
TTGACAAATGTGAACGACCTATGTAATATAATGAACTATAAAAATAATTAGAATTTTCTAAAGAAATAGTAGC
AGATATGAAACGTAGCAAATAGAAAGCTAATGGGTGATGGGAATTAGCACGCCATATCTTGTGAATTGGACTT
TGGAAAACAATTGAATGAGTTTTGAAAGTGAACATGAATTATGTTAACTAAGGTGGCACCACGGTAACGCGTC
CTTACAGGTATATGCGTTATGTGGTGTCTTTTTATTTAGACAAAATGTAGTAGTTAATTAAAGGTAGCAACAG
AAAGTTAGTGGATGATGTGAACTAACACCGAGATTAATGAAATTGGGTTTTGTCTGCAACAGAAAAATTATAT
ATAGTAAAGAGTGAACTATGAATATTTCGAATATTCGGTTAATTTAGGTGGTACCACGCGTCAGCGTCCTTTA
TATTGATAAGGATGCTGGCGCTTTTTTGAAAGGAGCGTATAGAATGGATATATTTTATAAAAAAATAAAAGCA
AATGTAACGCCAGAAGTTTTAGCACAACTTCATTCCAAGAAGATCATTTTGGAAAGTACAAATCAACAACAAA
CTAAAGGTCGCTATTCAGTTGTTATTTTTGATATTTATGGCACTTTAACTTTAGATAATGATGTATTATCAGT

AAGCACTTTAAAAGAATCGTATCAAATCATTGAAAGACCGTACCATTATTTAACGACTAAAATAAATGAAGAC
TACCATAATATTCAAGATGAGCAACTTAAGTCATTACCGTTTATATCTGGATATGTTGGGACGTGTAGCTTTG
ATTTAGTAAGACATGAATTTCCTAAATTGCAATCAATACAATTAGAAGATCACAAGCAGCACGATGTAAGGTT
ATATATGGTTGAACAAGTTTATGTATTTGACCATTACAAAGATGAGTTATATATCATCGCGACGAATCAATTT
TCAAATTCAACAAAATCAGATCTTGAGAATCGAGTTAATAAGTCTATCGAAGACTTAACTAAAATCCAACCAT
TCATGCCTACACAAGATTTTGATTTTAAAACTAAAGAAATTCAATCAAATATTTCTGAAGAAAGATTTATCGA
AATGATTCAGTATTTCAAAGAGAAAATAACAGAAGGGGATATGTTCCAAGTTGTGCCATCAAGAATTTACAAA
TATGCGCATCATGCTAGTCAGCATTTAAATCAACTTTCGTTTCAACTGTATCAAAATTTAAAACGACAAAACC
CAAGTCCATATATGTATTATCTTAATATCGATAAACCCTATATTGTCGGTAGTTCTCCCGAAAGTTTCGTAAG
TGTCAAAGATCAAATTGTAACAACTAATCCTATTGCAGGTACGATTCAACGTGGTGAGACGACACAAATAGAT
AATGAGAATATGAAACAACTACTTAATGATCCAAAAGAATGCAGCGAACATCGTATGCTAGTTGATTTAGGAC
GTAATGATATTCATAGAGTAAGTAAAATCGGTACCTCAAAAATTACTAAATTAATGGTTATTGAAAAATATGA
ACATGTTATGCATATCGTAAGTGAAATCACAGGTAAAATAAATCAAAATTTATCACCAATGACAGTTATTGCG
AATTTATTACCAACAGGTACCGTTTCAGGTGCACCAAAATTACGTGCAATTGAAAGAATATATGAACAATATC
CACATAAACGAGGCGTTTATAGTGGTGGTGTTGGATACATAAATTGTAATCATAACTTAGATTTTGCTTTAGC
AATTCGAACGATGATGATAGATGAGCAGTATATCAACGTAGAAGCTGGTTGTGGCGTTGTATATGATTCTATT
CCTGAAAAAGAACTAAATGAAACGAAATTGAAAGCTAAAAGCTTATTGGAGGTGAGCCCATGATCTTAGTTGT
AGATAATTATGATTCCTTTACATATAACCTAGTGGATATTGTTGCTCAACATACTGACGTCATTGTTCAATAC
CCTGATGATGATAATGTGCTGAATCAATCGGTGGACGCTGTTATTATATCTCCTGGTCCAGGGCATCCATTAG
ACGATCAACAGTTGATGAAAATCATATCAACCTATCAACACAAACCCATTTTAGGTATATGTTTAGGGGCTCA
GGCACTGACTTGTTACTACGGTGGAGAAGTCATTAAAGGCGACAAGGTTATGCACGGCAAAGTTGATACTATA
AGTATCACTTCAAATACTCACAAAGTGTTATATCAAGATGTTCCAGAACAGTTTTCAATTATGAGATATCATT
CATTAATAAGTAACCCTGACAATTTTCCAGAAGAATTGAAAATTACTGGACGTACCAAAGATTGTATACAGTC
ATTTGAGCATAAAGAAAGACCGCATTACGGTATTCAGTACCATCCTGAATCATTTGCTACAGACTATGGTGTC
AAAATAATTACAAATTTTATTAATCTAGTGAAGGAAGGATGAAAACCATGACATTACTAACAAGAATAAAAAC
TGAAACTATATTACTTGAAAGCGACATTAAAGAGCTAATCGATATACTTATTTCTCCTAGTATTGGAACTGAT
ATTAAATATGAATTACTTAGTTCCTATTCGGAGCGAGAAATCCAACAACAAGAATTAACATATATTGTACGTA
GCTTAATTAATACAATGTATCCACATCAACCATGTTATGAAGGGGCTATGTGTGTGTGCGGCACAGGTGGTGA
CAAGTCAAATAGTTTCAACATTTCAACGACTGTTGCTTTTGTTGTAGCAAGTGCTGGCGTAAAAGTTATAAAA
CATGGTAATAAAAGTATTACCTCAAATTCAGGTAGTACGGATTTGTTAAATCAAATGAACATACAAACAACAA
CTGTTGATGATACACCTAACCAATTAAATGAAAAAGACCTTGTATTCATTGGTGCAACTGAATCATATCCAAT
CATGAAGTATATGCAACCAGTTAGAAAAATGATTGGAAAGCCTACAATATTAAACCTTGTGGGTCCATTAATT
AATCCATATCACTTAACGTATCAAATGGTAGGCGTCTTTGATCCTACAAAGTTAAAGTTAGTTGCTAAAACGA
TTAAAGATTTAGGTAGAAAACGTGCAATCGTTTTACATGGTGCAAATGGTATGGATGAAGCAACACTATCTGG
TGATAATTTGATATATGAATTGACTGAAGATGGAGAAATCAAAAATTACACATTAAATGCGACTGATTATGGT
TTGAAACATGCGCCGAATAGTGATTTTAAAGGCGGTTCACCTGAAGAAAATTTAGCAATCACACTGAATATCT
TGAATGGTAAAGATCAGTCAAGTCGACGTGATGTTGTCTTACTAAATGCGGGTTTAAGCCTTTATGTTGCAGA
GAAAGTGGATACCATCGCAGAAGGCATAGAACTTGCAACTACATTGATTGATAATGGTGAAGCATTGAAAAAA
TACCATCAAATGAGAGGTGAATAATATGACGATTTTATCAGAAATTGTTAAATATAAACAGTCACTTTTACAA
AATGGCTATTATCAAGACAAACTTAATACCTTGAAAAGTGTGAAGATTCAGAATAAAAAATCTTTTATAAACG
CAATTGAGAAAGAACCAAAGCTAGCAATTATTGCAGAAATCAAATCGAAGAGTCCTACAGTTAATGACTTACC
TGAACGAGATTTATCGCAACAAATCTCAGATTATGACCAATATGGTGCAAATGCCGTGTCTATTTTAACTGAT
GAAAAGTACTTTGATGGTAGTTTTGAAAGATTACAAGCATTGACGACAAAAACAACATTACCCGTATTATGCA
AAGACTTTATTATAGACCCGCTTCAAATTGATGTTGCTAAACAAGCTGGTGCATCTATGATTTTATTGATCGT
TAACATCTTATCTGATAAACAATTGAAAGATTTATATAACTACGCTATATCGCAAAATCTAGAAGTGTTAGTT
GAAGTACATGATCGCCATGAATTAGAACGTGCCTATAAGGTTAATGCTAAATTGATTGGTGTGAATAACAGGG
ACTTAAAACGATTTGTCACAAATGTGGAACATACAAATACTATTTTAGAAAATAAAAAACCAAATCATCATTA
TATTTCTGAAAGTGGTATTCACGATGCATCTGATGTAAGAAAAATCTTGCATAGTGGTATCGATGGCTTACTA
ATAGGTGAGGCGCTTATGCGTTGTGACAATATATCTGAATTTTTACCACAACTGAAAATGCAAAAGGTGAAGT
CATGATGAAATTGAAATTTTGTGGCTTTACATCAATAAAGGATGTTACAGCGGCCAGTCAATTACCTATTGAT
GCGATAGGTTTCATCCATTATGAAAAAAGTAAAAGGCATCAAACAATTACCCAAATAAAAAAGTTAGCGTCTG
CTGTTCCAAATCATATCGATAAAGTATGTGTCATGGTAAATCCTGATTTAACAACAATTGAACACGTATTAAG
CAATACGTCAATTAACACAATACAGTTACACGGCACAGAATCTATTGATTTTATACAGGAAATTAAAAAGAAA
TATTCAAGCATTAAAATCACTAAAGCTTTAGCTGCAGATGAAAACATAATCCAAAACATAAATAAATATAAAG
AGTTCGTAGATTTATTTATTATCGACACACCCTCAGTGTCGTATGGTGGTACCGGTCAAACATATGACTGGAC
TATTTTGAAGCACATAAAAGACATACCTTACTTGATAGCAGGAGGCATTAACTCTGAAAATATTCAAAAAGTT
AATCAACTTAAATTATCACATCAAGGTTATGATCTTGCATCAGGTATAGAAGTAAATGGGCGAAAAGATATAG
AAAAAATGACAGCAATTGTAAATATTGTGAAAGGAGATAGAGAAAATGAATAAACAAATACAAACAGAAGCAG

ATGAATTAGGTTTCTTTGGTGAATACGGAGGGCAATATGTTCCAGAAACATTAATGCCAGCAATTATTGAGTT
GAAAAAAGCTTATAAAGAGGCAAAAGCAGACCCAGAGTTTCAAAGAGAACTGGAATACTATTTATCAGAGTAT
GTAGGACGCGCGACGCCACTTACATATGCTGCATCATATACTGAAAGCCTAGGTGGCGCTAAAATATATTTGA
AACGAGAGGATCTAAATCATACAGGCGCCCATAAAATTAATAATGCGTTAGGTCAAGCGTTGCTTGCTAAAAG
AATGGGCAAGAAGAAGCTTGTTGCTGAAACTGGTGCGGGTCAACATGGTGTAGCTAGTGCTACGGTTGCTGCA
TTATTTGATATGGAACTTGTTGTCTTTATGGGAAGTGAAGATATTAAAAGACAACAACTTAATGTATTTAGAA
TGGAATTACTTGGTGCAAAGGTTGTGGCAGTTGAAGATGGTCAAGGGACTTTATCGGATGCAGTTAATAAAGC
ATTGCAATATTGGGTAAGTCATGTAGATGATACACATTATTTATTAGGTTCTGCATTAGGTCCAGACCCGTTC
CCAACGATTGTTAGAGATTTTCAGAGTGTGATTGGTAAAGAAATAAAATCACAGATATTGAAGAAAGAAGGTC
GACTTCCGGATGCAATTGTAGCATGTATCGGTGGTGGCTCAAATGCAATCGGTACATTTTATCCATTTATTAA
AGATGATGTTGCATTATACGGTGTTGAAGCCGCAGGTCAAGGCGAGGATACTGATAAACATGCACTTGCAATT
GGCAAAGGATCACCTGGCGTATTACATGGTACTAAAATGTATTTAATTCAAGATGAAGGTGGGCAAGTGCAAC
TAGCACATTCTATTTCAGCAGGACTTGATTATCCTGGTATTGGGCCAGAACATTCTTATTACCACGACATTGG
TAGAGTAACTTTTGAAAATGCAAGTGATACACAAGCAATGAATGCTTTAATCAACTTTACAAAACATGAAGGT
ATTATACCTGCAATTGAAAGTGCACATGCACTGAGTTATGTTGAAAGACTAGCGCCTACGATGTCGAAAGAAG
ATATTATTGTAGTAACTATTTCTGGACGTGGCGATAAAGATATGGAAACAATTAGACAATATATGGCAGAGCG
AGGTCTTGCAAATGACTAAATTATTTATACCTTATATTATGGGCAATAAAGATTTGATTGAAAATGCAACATT
GTTGAGTGAAAATGGTACAGATATAATTGAAATTGGTGTACCTTTCTCTGATCCGGTTGCTGATGGTCCAGTT
ATCATGGAAGCAGGTCAACAGGCGATTAAACAAGGCATCACGATAGATTATATTTTCAATCAATTAGAAAAAC
ATGGTGATCAAATTAAGTGTAACTATGTATTAATGACGTATTATAATATTATTTGTCATTATGGAGAACAAGC
GTTTTTTGAAAAATGTCGAGATACTGGTGTCTACGGCTTAATTATTCCTGATTTACCATATGAATTATCGCAG
CGTTTAAAAGAACAATTTAGTCACTATGGCGTCAAAATCATATCGTTAGTTGCGATGACTACTGATGACAAAC
GTATGAAAGATATCGTATCCCATGCGGAAGGCTTTATTTATACTGTGACGATGAATGCGACAACAGGGCAAAA
CGGTGCGTTTCATCCAGAATTAAAACGAAAAATTGAGTCAATTAAAGCGATAGCCAATGTGCCAGTTGTCGCA
GGATTTGGTATAAGAACACCTCAACATGTTGCAGATATAAAAGAGGTTGCAGATGGCATCGTCATTGGTAGCG
AAATCGTTAAGCGATTTAAATCTAACACGCGTGAAGAAATCATTAAATATTTACAATCTATCCAACAAACATT
GAATAATTAAGTTTACTTGATTTAAAAAAATTAGTCGAATACTGTTTGAAAAAGTGAAAAACGGTGAATTATA
AAATTGAATAGAAATGATACAAAAGTAATATGAGCAAACCCAAACGTTCATATTACTTTTTTTGAAATTGTAT
GCAAAAATCTAAATATTACTATAAAAGTATACGCAATTAAAGCGTTTATGTTTTAGTTTTAACATTAACTATT
GTATACTTATTTAGATTAGATTTATTATTTTTGACATTTGCAGAGGGGAAATAGAAAAACTGCAAATACGGAA
ATGAAATTAATTAACGAGAGACAAATAGGAGTAATGATAATGAAGTTTACAAATTTAACAGCTAAAGAGTTTG
GTGCCTTTACAGATAGCATGCCATACAGTCATTTCACGCAAACTGTTGGCCACTATGAGTTAAAACTTGCTGA
AGGTTATGAAACACATTTAGTGGGAATAAAAAACAATAATAACGAGGTTATTGCAGCTTGCTTACTTACTGCT
GTACCTGTTATGAAAGTGTTCAAGTATTTTTATTCAAATCGCGGTCCAGTGATCGATTATGAAAATCAAGAAC
TCGTACACTTTTTCTTTAATGAATTATCAAAATATGTTAAAAAACATCGTTGTCTATACCTACATATCGATCC
ATATTTACCATATCAATACTTGAATCATGATGGCGAGATTACAGGTAATGCTGGTAATGATTGGTTCTTTGAT
AAAATGAGTAACTTAGGATTTGAACATACTGGATTCCATAAAGGATTTGATCCTGTGCTACAAATTCGTTATC
ACTCAGTGTTAGATTTAAAAGATAAAACAGCAGATGACATCATTAAAAATATGGATGGACTTAGAAAAAGAAA
CACGAAAAAAGTTAAAAAGAATGGTGTTAAAGTAAGATATTTATCTGAAGAAGAACTGCCAATTTTTAGATCA
TTTATGGAAGATACGTCAGAATCAAAAGCTTTTGCTGATCGTGATGACAAATTTTACTACAATCGCTTAAAAT
ACTACAAAGACCGTGTGTTAGTGCCTTTAGCGTATATCAACTTTGATGAATATATTAAAGAACTAAATGAAGA
GCGTGATATTTTAAATAAAGATTTAAATAAAGCATTAAAGGATATTGAAAAACGTCCTGAAAATAAAAAAGCG
CATAACAAGCGAGATAACTTACAACAACAACTTGATGCAAATGAGCAAAAGATTGAAGAAGGTAAACGTCTAC
AAGAAGAACATGGTAATGAATTACCTATCTCTGCTGGTTTCTTCTTTATCAATCCATTTGAAGTTGTTTATTA
TGCTGGTGGTACATCAAATGCATTCCGTCATTTTGCCGGAAGTTATGCAGTGCAATGGGAAATGATTAATTAT
GCATTAAATCATGGCATTGACCGTTATAATTTCTATGGTGTTAGTGGTAAATTTACAGAAGATGCTGAAGATG
CTGGTGTAGTTAAATTCAAAAAAGGTTACAATGCTGAAATTATTGAATATGTTGGTGACTTTATTAAACCAAT
TAATAAACCTGTTTACGCAGCATATACCGCACTTAAAAAAGTTAAAGACAGAATTTTTTAGGAAGGGAATTAT
CAAAACATGAAATTTACAGAGTTAACTGTTACCGAATTTGACAACTTTGTACAAAATCCATCATTGGAAAGTC
ATTATTTCCAAGTAAAAGAAAATATAGTTACCCGTGAGAATGATGGCTTTGAAGTAGTTTTATTAGGTATTAA
AGACGACAATAACAAAGTAATTGCAGCAAGCCTTTTCTCTAAAATTCCTACTATGGGAAGTTATGTTTACTAT
TCGAATCGTGGTCCAGTAATGGATTTTTCAGATTTAGGATTAGTTGATTATTATTTAAAAGAGTTAGATAAAT
ATTTACAGCAACATCAATGTTTATATGTTAAATTAGATCCGTATTGGTTATATCATCTATATGATAAAGATAT
CGTGCCATTTGAAGGTCGCGAGAAAAATGATGCCTTAGTAAACTTGTTTAAATCACATGGTTACGAGCATCAT
GGCTTTACAACTGAGTATGATACATCGAGCCAAGTACGATGGATGGGCGTATTAAACCTTGAAGGTAAAACAC
CCGAAACATTGAAAAAGACATTTGATAGTCAACGTAAACGTAATATTAATAAAGCGATAAACTATGGTGTTAA
AGTCAGATTCCTTGAACGTGATGAGTTCAATCTTTTCTTAGATTTATATCGTGAAACTGAAGAGCGTGCTGGA
TTTGTATCAAAAACAGATGATTATTTTTATAACTTTATTGACACATATGGAGATAAAGTATTAGTACCATTAG

CATATATTGACCTTGATGAATATGTGTTAAAGTTGCAACAGGAATTGAATGACAAAGAAAATCGTCGTGATCA
AATGATGGCGAAAGAAAACAAATCAGATAAGCAAATGAAGAAAATTGCAGAATTAGATAAGCAAATTGATCAT
GATCAGCATGAATTATTGAATGCAAGTGAATTGAGCAAAACGGACGGCCCAATTCTAAACCTTGCTTCTGGCG
TTTATTTTGCAAATGCATATGAAGTGAATTATTTCTCTGGTGGTTCATCAGAAAAATATAATCAATTTATGGG
ACCATACATGATGCATTGGTTTATGATTAACTATTGCTTCGATAATGGTTATGATCGTTATAATTTCTATGGT
TTATCAGGTGATTTCACAGAAAACAGTGAAGATTATGGCGTATACCGCTTTAAACGTGGATTTAATGTACAAA
TCGAAGAATTAATAGGGGATTTCTATAAACCAATTCATAAAGTGAAATATTGGTTGTTCACAACATTGGATAA
ATTACGTAAAAAATTAAAGAAATAGATGTAAATGATGTTAGGGCTTATAGTTATTGATGCTATAGGCTCTTTT
TTATATGTTTTTTAGCGAAATTTTAATTATTTCACACTAAGAAAAACTAGTATGTATTTGTCAATCTAATGTT
TTTCATTATAATGCTGTTTTATTGATTTAATTTTATGATTAAAATATTATGCAAAACATAAAATATATAGCGT
CATATTTGAGTTTTAATGATAATATTTTTATAATATATAATATAAAATAATGGTTATCTGAGTATGAAATGCA
AGTAAGTGAGGTAGTTAAAAAAATGAATCGAATTGAAAAACATGCTAAAAACACTTTTATTATTCTGATGCTC
ATTATGTTATTTTGGATTTTTATGAGTTTTATTTTCCAGAAATTATTATTTCCACCTTCGAAAAATAATTTAA
CTACATATGAAGCATTGAAATATTATACACATTTAAAGGGATATTACGGTTTGGATCATATATCAAAAGGAAT
AGCATACATTGCCTGTGTGTTAATTCCACTCAATTTCTTTTTTAGATTCAATGACATCAAAAAAGATAATAAC
TATAATAATATTATAAGTACGTTATTCCTATTATTATACTTTTTAGTTAATGGAATTTCATTGATTATTCAAG
GATTCACTGCAGAATTTACAATTAGTTTAATTAGTAAGGCAAATATTCATAATAATCATGAATTTGCTGTGAA
CTTATTTAGATATGTCATACAAGAAGGTGGTATTTCTTTTAGTACATATCTAGTGTGTAACTTTTGCATAATA
ATGTGGTTATTATTTTCGTGTTCTTTATTAGGTGGTGTAAACTCTAGTTAACAGTTAATTCTTATGAAAGGAA
TTGATAATTATGACTGAGTTTACACCTTTTTTTGATGGTTCAAATCAATTAAATTATCTTAAATTTGTAATTT
TAAGATTTCATGGTATTTCTAAAAGAAAAATTTGTAAAATGTATAATTTTGCATATTTTCGTATATTAGAAGT
TTGTGAAATGGCAAAAAAGAATGATTATCGATTTACTTATAAGGATTATAAATTTTTAAAACAATATGGTGTG
TCTAATACATTTATTTGTAAGATGTATCATATTTCAATTGAAGACTTAGAATTTTTTGAGGTGATGAACCGAT
AGGTTTGATTAAAAATATGATAGGTCAGGAGAAGCATAGACACTCCATTATCAAAAATGCAGTGGTACGGATT
TGCGGGAGTTATTCCTATTCGTGCGACCCTTTAGAAGTCGTTTAATTTTATTAAAAATTTTGTAAAGGATGGA
TATATTATGAAACCAGTATTGGATATTAAAAAAACTTTAGGACAATTAAACTTTTTAGGTGTAGATGAAAAAT
ATAAATATGAAAATGGAGAGCGTACTGATAAAACTGTTTATGCATATAAATTAGCGAGTCAAGAGCAAGGCGA
ACAAATCACTGTTAAAACGCCTAATAAAGTAGAATTAAACTATTTACAAGAATGCGAATTAGTTGAACCAGAC
GTTAAAATGTATGTTCAAATGTCAGGCGATTTTGGAACTATCGCATATAGCTGGAATGCAGAAGATATTAAAG
TAGTTGGTTCTAATAGTGCACTTAAACAAAAATAATTAGGTGATGTAAATGGATTATTATACAGCAGATAGAT
TATATCGCTACACAAATAGTTCAAATTTGAGTGAACCTATTTTAAACTATGTTGCTAGTCGTATAAATTGGGG
CGATAAAGTTTCATTAATGACTTTAGCTAAAGAAATACAGTCTAAATTTAATGATAGTTACGTTAAAGAAAAT
ACGGTAAAAGGTCGGCCTAAAATTTACGCTGACCTTTGTCTATTGTGTATGAGCTTGAGTGAAGCAGGACATG
GCAGAATGCTACAAGTGAATTTGGAAGATTGTATTTATATTGGCGATATTGATGTTTAACATATAGTATTAGT
GATTTAACATGACCGTGTAGCTTTTAGCGTATGCAGGCGCAGAAATGCAAGCACGGAGTGCGTTAAGCATTTG
GGCGGAGCCTGCGACAAATACGCGTTAAGAAAGCAAGAACGGTCATGTATCAAATCGCTTGCGATTTGATAGG
AGGCATAAATAAATATGACTTTAAAAAGCCAGTGTACCCCCCCTTACTAATAGGGGGGTAGAGCGTACAAATA
AAAGTGCCGTGGAGGCAGTTGTTGACTGGGTTCAGGTCACTTTCCATATAGCCCCTATTTCAGCAGTGATTGA
GGACGTTATCGGTTTGCCTATAACGTTGTTCAAAAAACGAAATAGTGGCATTTACTTTTATAATCGTGGTTAT
GAATTTTCAAACATTAAATTGTATTATTCAAGTGATGATGAATCAATGGGCATTCACTTACAGTTAACTGGTA
CAGGTTGTAGAGAGTTTGAACATCATTTACAACAGTTGAATAAAACTTGGCAAGATTTTTTTGATAAATGTTT
ATCAGTTAATGCAAATTTTACAAGAATTGATATTGCAATTGATGATTATAAAACATATTTAAAAGTGCCTTTA
TTAATTAAAAAGGCTGAAAAAGCTGAGTGTGTTTCACGTTTTAGAGCTGGAAGCGCAATAAACGGTTTTAATT
TGTCAGACGGAAGAAGTAAAGGCGCTACTTTTTATATTGGTTCTAAACAAAGCAATTTATATTGTCGTTTTTA
TGAAAAGAATTATGAACAAGCGTTTAAACGTCATTGTGATGTCGAAGACATTGGGTTATGGAATCGTTATGAA
ATACAAATGCGAAAGGCATATGCAGTTAATTGTGCGAAAGTTTTGAGTAGAACAGATAATATAAGCGAAATTG
TAAAGTCTATATTGCATAATAATTTGCGTTTTATCTCTCCTCCTAAAGATGGAAATGATAAAAAATCGTAAGCG
GTGGCCATTATATAGACCTTGGGCGTTATTTATTAAAGATACTGAAAAATTGAATTTAACTACTAGACCAACA
TTAAAGTCCATTGAGGATAATCTTGATTGGTTATGTAAACAAGTTGCTACTACTTTAGATACAGTGTTAACTG
CCGAAAGTATGGCGCAATCTGAGGGGTTGCTTACAGATACTGATTTTTTAGATAAAATTTTAGCGCATTCACA
ATTTAATGATGAGCATACGAATAGAATTAATCATTATTTGGAGGCTTTAAAACAAAAAAAGCACTTATCAAAA
GATAAGTGTTAAAAAAATAACTGGGATGTGATTTTTATTAAAAATTTCGTAAAGGATTTTAGAAAATCGTTTA
TGAAAATAATAAGCAAGTACATTTTAACACGATTTTCTAAAAACGGAAAGCATTTTGATATTCCTAAAGAACA
ACGCCGAGTAATTCCAAAATTTAAAAATCGTCGTAAGATGATTGTAATTTGTTTTTACTCATTATTAGCTATT
TTGTTAATTCTTTTATTAGCTTCTTTTATTAAAGCTACAAGAGCAAATAATGACAGTAAAGAAGCAGTTAATA
AAACGAATATGATTCAAAAAAAGTATGAAGATAATGCGAATACAGTTCAGTATAGTCCTAAATTAAAGTTATA
TGCTGATAAGTTTATTGATACGTATATGAATATCTCGAAAGATTCAAAAGAATTGGAATCTAGAGAAAAAGAA
TTGTTGAAATATTTTCCATCAGATTATAAAAAACCTGATGAAAAAATTTCAGATACAGAACGAAAATTGAATA

GTAAAGAATTTTATAATATTAAACGTAAAGATAAACAAACAATTATACAGTATATTGTTAATTATGATGTGAA
TATTACTGAAAAGAAAGAAGTTAAAGTTAAGAAGAAAAAGAAAAGTGAAAAAGATAAAGATGAGTATGAAACA
AAAACAGAAGAAAAGCAACGTAAAGTAAATCAAAACATTTTAATAAATATACCTATTAAGAGTGAAAATAATA
AGTATGTTGTTGTTGAATATCCTTATTTTACGCCAATCCCTGATAGTCAATTGAATAAAGCTAAAATGGTTGA
GGATAATTTGAAAGACAATAAACGGGAAGACAATCCGAAAGCAAAAGCTTTTATTGAAGATTTCTTTAATAAA
TATGCTTCTAGTAAGTCTGATGATATGGCCTATTTAATGGATAACCCTGAGGGCTTAGAAGGAACTAGAGAAG
TTTCTCAGATAAGAGAAATTAGGTTATATCCAAAAGGCGATGATTATGTTGCTAAAGTTGAAATTTTGATGAA
AGATAAAGATTCTCCTTTGGAGAATTTGGAGCATTACACATTAGATATAACAAAAAAAGATGGTAAATATTAC
GTTAAAAATATGACTAATTCTATTGGAGGTTAATGTTATGAGTGGTTTATTTAATTTTTTTGTTTTAGGTGCT
GACCGTCCATCACTTGGTGGAGTTGGTGACTGGATAAGTAATGAAGTAGGTACGGGTATTGGTTTAGTAGTAT
TGGTTGTTGGTATTGTAAAATGGGCTAGTGGTAAATATGGGCATATGGTTATATTATTTGTTGTTGGTGGCTT
TTTATTCTTAGTTTCTAAAGGTCCTGAACAGGTATTTAATGCGATTGCAGGTGTTTGGAAAATGATTTTTGGC
GGTTGAGGTGATTCCATTTGGATAAAAAAGCTTATAATTTAAAGCGAACTTTTGAACAACCGATAGTTATGTA
TGAATTTACAGATAAGTTTCGTATTAATAAGGGTTTTCGTTTAGATTTTTGGGCTACATTTTTGATTGTGTGG
TTTATATTATTTTTGTTATTTTGGTATTTATTACAGCCTGTAATTATGTCGATTGGTGGACTTATGTTTATTT
ACTTTACGGTTGCACCTTATTATATAACGAAATATATTGTTAAGTTAAAACAAGATGGAAAGAAATTATTTTT
CTTCTTATGGGATTTTGTAATATTTGTTTTTAATGTGCAATTAAGAAAAGTTAAATTAAGTTATGATGAAGAA
GTAGAATATCATGATAAAAAAATAACATTTAAATAGCCCCTATTGGGGTTATTTTTATTGGAGGGATGACATG
AATTTAAAAGCACCGTATGCAGGTTTAAGAGATAATTTGTTATTAACGAAAACTGGCGAAGTTTGGGCGTATT
ATCGTGTTCGTTCTGAGTCTATATCAACAGCAAATTATGATGCAAAAGAAGAATCAAAAAAGCGTATGCGTTA
TTTTTTAGAGTCGATTAAAGGTTATCAAGATTTTCATTTTGAAATGTATGACCACGATTTAGATTTGCGAAAT
AAATTTAAAGAAATTAGTAAAGATTTTGATGATGAAGCTTTTAATGTAGCTGAATATTATTCAAAAGAAACTA
TTGACGTATTAGAACAAGAATTACAAATGATTACGCATAATTCTTTTGTTATGGGTGTTAAAATACGTGAATT
AGCTGACGATGCAGTTACACTTAAAGAGATTTTAAAAAGTACATTATCAGATACGGCTGAGCGTATTGTTTCG
AATTTTGGTTTTGATGTGAATTTAGACGATAAAATTTTAGATAAGTATAAAACATTTGAACGAGATTTGGCTG
ATAGTTTTCTAGGTATTGGAGGCGAGCGTTTAACTGAGAATGAATTGGCTTATATTATTAGAAAGCCTTTTAT
TTCAAATGCAACACATGACGTACAGGAAGAATCTTCAAGAAGAGCTATTACAGATATTTATAATGCAGTAATT
GACCCTACTCGCTTGAGTGTTTTAGAAGTTGAAAATGATAACGAGAAATTTTATACAACAACAGTTGTTGTTG
ATGATTTTCCGTTAGATATGGAATATACACATTTATTTGAGAAAGCTCAAAATATGCCTTTTCCGGTTGAATG
CCATGTAAAAGCAAAAATCATTAATAATGAGAAAGTTAAGAAAAAATTTGACCGTGCGAAAATAAATCATAAA
CAACAAGCAAAAGAAAAGCAAGATACTGGCGACAGTGCTTCGGAAGATGTTCAAGATAATTTGTTTGTATTGA
ATCAAATGGAACGAGAAGTGACTGGCTCAGGTGTATTTATTGAGTGGGTATTTATGTTTGTAATTAAAAGTGA
TGATTATAGAGATTGTAAACGTAAAGCAAAGCGTTTGATGAAACGATTAAAGGAAACTCAAATATATGCAGTT
AATCCATTAGCTGACCAGTTACAATTGTTTTATCAATGTTTACATGGTAATGATTTATTTATTAATAAGTATT
GGTTACAAAGAACAACGGCAACAGGTATAGCAGAGAATTTATTTGGTGTGTCTCAAAGTTTGGGAACAAAAAC
GGGTTTTTATATAGGGCGTATTGATAAATTTATACGTTCTGTTTCACGTGAGGAAGCTGTTGCAAGTTCAAGG
GATATAATTTTATTTAGTTTATTGTTGGCAGCAAAAGGTATTAAGGGGGCTGTCAGTGATAGTCCGCATGTAT
TAATTACTGGTCAAACTGGTAAAGGTAAATCATTTTTAGCAAAACTATTGTGGATTTATACATCATTCTTTAA
AGGTCAAATGTTGTACTGGGATCCAAAATCTGAGTTTGCAGAATGGTTTGATAGAGTAACAGAAAGTGAAGAA
ATGCAACAAAAGTATCCCCTATTTATTAATCATTTAAAAACATTTAAATATGTAACATTGAATTATGAAGATT
CAACCAATTATGGTTGTTTAGACCCTATTGTATTTTTAGATGGTATTGAAGCCAATGAAATGTTGAAATCGGT
GTTTGATGAAATTAAATCTTTTGAAAATTATCATCATATTGAAACGGCAATTTATCAAGCTATTTCTGATACG
GTTGAAGAACGCGAAAATGGCAAAAAAGTTGGTTCATTAAATGTAATTGAAAAATTACAGAATAATGAAGATG
AGCATGTAAAAAATGCAGGCGATTTATTATTTGAAAAAACGCAAAACAATATTTTAAAACTTGTTTTTAGTGA
TGGTACGAATCCAGCTTTAAATATTCAAGAAAAAGCAACTATTTTACAGGTTAAAGGTTTAGATATGCCTAAA
GCAGATGATGATACATCAAGTTATTCAACAAGTGAGAAAAATGGGATTACATTAATGTTATTAATTGGTAAGT
TTTTAGAGAAATTCGGTTCTCGTAGAGATGTTCAAACGACTATATTTATTGATGAGGGTTGGGCTTTTAGTGC
TTCAAGACAAGGTAAAAAAGTTGGTAAGCGTATAAAAAGAACGGGACGTTCTGAGAATAATTCACTTGTATTT
ATTACACAATCGGTAAAAGATAAAGCAGATGATGATGGTGGTAACTTTGGTTGTCATTTTGCATTTGATGAAA
AAGATGAACGTGAGGATATTTTAAAATCTTTAGGCTTAGAATATTCAAAAGAATCTCCTGAAAATATGGAGAT
GTTGAAAGATTTAAAGAAAGGTCAATGTATATTCAGTGATTTTTATGGACGTGTTGGAAAAATGGTTGTACAT
TGTCCATTTGAGGAAATGACTGAAGCATTTAGAACACAAGAAGATTCTGCAAGTTCTAAAGCAGAAGAAAAAT
TTGCGATGTAAAAAATTAAAAAGATACACTTTATTGAAGTGTATCTTTTAAACGAGGTGTTATAAATGATTGA
AATATTTAAATCATTTATATTTATATTATTTGCATGGGTAATTTTTTATTGGATTATTCCCTATGGTATGTAT
TTAACGTTAAAACATAAAAATATGGAAAAGGAATCAATTATGCGTCACGATGTTTATTTGAAAAATCGTGACT
GTTTAAATAAAGATAAGTAAAGAATAAGAATCCTGAAATACAAATAATGCAAGTGTCGGTGGCTATTTTATCA
TTAGTCATTTTTGCGATTAAAAAAATATTAAATTGTTGTGTTAGTAAACTGATTAACCATCCAAGTATTTCAC
CTATTCGAGATGGAATTATTATCTTGAAGACACAAAGCAGTATAGATAAATAAAGTAAACAAGCACAAACACC

AATTAGATAAAAATAGAACAGATATAAATTATAAATCATATCAGAATTAATATTACTAAAAATTTTAGAGATA
TGGTTATTTGTAATTGCATTTAACATATCTTTGATGCCAACTAGTATTTTAGGTTGAAATATATAAGTGATAA
ATATATAAGTGATAAATATAGCTATATTAATATACGTTAATATAAATAAAGCTGTACCAATATTTAACCAACT
TAATTTAAATTTGTTCATAAGGAGGTCATCCTTTCAAATGTTTGTTTTGAATTGTATTGCTTCGTTATTTATT
TTGTTTTTATCGATTAAACGGATTAAGTATTTACTAGATAAAGAAGAAAATAGACATAAAGTTGAAATTAATA
AAATAAAAACAAATATATTTAAAAACATAAAATAATTTAAAATGATTATACAATAAGGTATAATACTTGTTAA
GTACTTGACACATATTGTAAAAGGAGCGATTTAATATTGGAAGTTTTAAGTAATTTAACAATGAATTTTATTT
TTGTAGGCTTTATTGTTATTAGTTTATGTATAGTATGTGTTTTTGTTTTAAATTATTTTTATAAAAATGATTA
TATTGAGTATTATCAATGGAAATCTTGGAAGAGAAAGCTTTTTATTATGATGTTTTTATTTATTGTATCAATA
ATTCTTTTATTTTTTGCACATCGATGTTTTGAGAAAAGTATTACTGATTATAATAAAAATATGGAAGAACAAT
TGGAAAATTAATATTTTGTTTTAAGCATGCACATTATTTGTGTATGCTTTTTATTTTTAAAGGAGTGCATATA
ATGGCAATGAATGAAATTGCTTTATTTAAAGGTATACGTATACAGCCTTATCAGAAGTATATTGATTTTATGT
TTGCAGGTGCATTAGCATTTATTTTTTTGGTATATCGTATATACATTTTGTTTATTGATTATAAATCAGTTAA
TAAAAAATTGGATATATTGACGTTGTTTGCATATTTTAAACCGCATTTATTGTATTTATTGATTGGTACAGCA
ATTATATTTGTGATATGTAAGCTTATTGCTCAGTTTTTAATACGTTTTAAAACAAAAGATTTGGCTGAAATGA
TTGAAACACAAGGGTTTCATAATCGTACAGTAATCAAGAAAACAACTGAGTATATCCCTATTGATTATAAACA
AACTGAATATGATTATTATCCAACAATGTTTTATAAGCGTGGTAAAAAATCTTTTGTTATTCATATTAAGAAA
GACGGTTCGAGGTTTCAAGATAATTATTTAGAGTTAGAGACGATTATTGAACCAATGTTTAATTCTGAATTAG
TAGAAAAAAAGCATATTGGTCGTTATTTGAGATATGAATTTATGCCACTTAAATATAAAAAGCGTATTGTTAT
GAATGGCGAACAAGAATCAAATACAATTTTTTATGATACAAAAATTGCTATTACGCATCAAATTACTTGGGAT
TTTGTGAAAGCACCTCATGGATTGGTTACAGGTATAACCGGAGGTGGTAAAACATATTTTTTATTTTATGTGA
TTCGTGAGTTATTCCGTAGACATTCAGAAGTACGTTTATTAGACCCAAAAGTTTCAGATTTGAGCTTTATGAA
GCGCGTTATTGGTGATGATAAAGTTGCAGATACTAAAGGCCAAATATTAAAACAATTGAGAGAAGCTAATAAT
GAAATGGAAGAGCGTTTTAGATTAATGAATGATAGTTCGGATTATAAAATCGGGAATGATTTCCGTAATTTTG
ATATGCGCCCCTATTTCATTATTTTTGATGAGGTAACGGCTTTTACTTCAACTTTAGATAAAAAAGAATTACA
AGAAATGAATGATTATTTAATTAATATTATTATGAAAGGTCGTCAAGCTGGTGTATTTATGTTTTTAACAGCA
CAAAGACCAGATGCAGATGTGATTAAAGGTAATGTGCGTGACCAATTAGGGTTACGTGTGTCTTTAGGTAATT
TGTCAAATGATGGTTATCGTATGACCTTTGGTCAAACTGATAAAGAATTTCAAACGATTCATGATAGTGATAT
CGGCCGAGGTTATATTTCTATACTTGGACAATATAATGAACCAATTTTATTTGATGCACCTTTAATGGAACAG
TATGATTTTGTAGAAGATGTGAAACAAATATTAAATAAGGAGTGATTTGATGAAGCATAAAAGTGTGTATCTT
TTATGCTTTTTAATTTTGCCTATTCTGCTTTTAACATCTGTGAGTGCTTACGCTGTTTCTAATCCAGTTGGAG
AGCCCTCACATGCAGTTAAGCCAAAAATTGAAAAGTATGATTTATCACATTATCGTTCAATATATAGTGAAAA
AGGCGACTGGAATCCGTTTGGTCAAGAAGAAATTTCACGACAAATTAATAATGTGTCAGATTTTTTCTTTTCT
ATGACTAAGATTTTAGCAGGTGTTACAGATTATGCGATTGAAAATTTATTTCAACTAGATGTTATTAATGATT
TTGCGGATAAAATCGGCGATTTTGTTGGCGATATATATCAAAAGTTATTGAGTAATTTAGCATTAACATTATT
TATTATTGTTTGCTTTAACGCATTTATTATTTTTAGTGTACAAGGTAATGCAAGAGAAGCATTAAAACGAGCG
TTTTTAATTATGTGTTTAATTGGTTTTGGTGTTGGTATACTTGCGAATGCAGGTAATATTATACGTGGTACAA
ACAATATTGGTAAGGACTTAAATAATATAATTATGAATAGTACATCTTCTATTGATGGTAATATTGATTATAT
TCATGAGAATTCAGGTATGAATCATATACGTAATCAATTATTTGATATGACGATATATAGAACATATTTAGTA
ATGAATTATGGAACTGTCGACGAAAAAGAGATAAAAGCAAAAGGTAAAGACCGTATTGATAACATTTTAAAAC
TTGATTATGATAAAAAAATCAGAAAAAGAATTAGATGATATTGTTAAAGATGAGGTTGAAAAGAAAAATAATAA
ATATATGACGCAAGGTTATGTATTTCAAAAGTTGGCTATTTCAGTGATTGGTTTTATTATTACACTTTTTATG
TCGATAGTATTTTTTATCAATAAGTTTTGCGAAGTTGATTTTTTCTACATTTGCTTTATTTTTTATTCTTATTTT
TAGTATTCAGTTGGATAGTGAGTTTTATACCAGGATTTGAATTATCTGTTTTCTCTGCGTTTGCAAAAACGTT
AGGCTATATTATTTTAAGTGCTTGTATGACATTCTTATTTGTGATAGTTGGTTTGTGTATCAAGCTTGCAAAT
AGTTTTATTGAACCGGATAGTCAAAATGCTTATTTTTTAAATTCAATATTTATAATTGTAATATTGTTTGTAA
TGTATAAAAAAACGGGCTCAAATTATCAATTTTGTAAGTCGTGGTAATATTAGTTTTTCTCCTAGTGCAATTGG
TGCTGGTGTGATGAATCGAACACAAGAACGTTTTAATAAGATTCGTCATGAACAAGCGCAAAATAAGAAAGCT
AAACGAGAAAATCAAAGAGATGAACCCGCCCCACCACTTCAAAATGATAATGATTTAAGAAGAAGACAACAGG
ATAAACCAATGCCTTTATTTATAAATAAAGATAATCAAAAAAATGGTAATAAGCGCAGAGAACAACAAGAGTC
AATGAATGGTAACGATGTAAAATCTGCTTCTGTTGAATCAAATGCAAATAATTATTCTAAACAACCACAAAAA
GCATCACAGCAAGAACATCAAGTACGAGAAACAAGACAGCGAAAAGATATACAAAGGTCGCCACAAGTGGTTA
ATCAGCCATTAAATAATGAGAATCATTCAATAAATCGTAAAGAACAGAAGTCAGTTCAAACTGCTTATGATAC
TGATGTTCAAAAACGACAAATACAAAATGCGACACAGAATCAACAAAGTAGACAATCTGGAAATCGTAATCAA
CCTATTACGCGTAATTATCAGTCAAAAGACCGTTTAAAAGAGCAAAAGGATATCAATAAGCATGGTAAATAAA
AAAGATATTGCTAAAAAAGTAATAGGTAAAACGCCAATAGGCGTTAAATTGAAATTAGCGAAAGTCATCATCA
TTTTATGTGTGGTGGCTTTTTTTATATTTCCAGTAATTATAATGTTTTTGTTAGCGCCTGATTTAAATAAAGG
TAAAGATGATGCTGGTTGTACTGTTAGTGGCGGTAATGTTTCGGCAAATGGTATTGATAAGTTTAACGAAAT

GCTAAAGGTGGAAAATTAGAGGGTAAAGGAAAAGAAATACAGAAAATTGCAGAAAAAAAATAAAGTACCTGTGA
ATATTTTTATGGCCATTATTGCGAGTGAATCACAATGGGGTAAAGGAGAAAATGCGACAAGACAAAATAATCC
TTTGTCTGTTATGGGTTCAAAATCGATTCATGATTCAACATATCCAACTATTGAAGATGGTCTTAATGCGGGA
GCTAAAAATTTGTATGATGTTTATATTTCTAAAGGTTTAGATACTCCTAAAAAAATAGGTCCCAAATATGCGC
CTGTTGGTGCGTCAAATGACCCTAATAATATGAATGCCCGTTGGATACCAACAGTTGAAAAAATTATGAAAGA
TTTAGGCGGTTCAGAAGCTAAAACGTCATGTAGTAATGGTAAAGGAAAGTCTATAAAGTTTAATGGTAAATTG
CCACATTGGTCAAATGATGACCCTGGAAAAGGTAATTTATACACTGCTGGTCAATGTACTTGGTATGCATACG
GTATGCGTCAAAAAATGGGTAAACCTGTATCGACTTATTGGCATGATGCACATAAATGGAATGATAGAGCTAA
AGCTGAGGGTTATAAAGTTGATAAAAATCCGGAACCTGGTGCATTATTTATTGCTGAACAAGGTGCAGGCGGT
CATGATGGGCATTATGGTCATGTGGCTGTTGTTATTGGTGTAAGTGATGGTGGTAAAACATTTAGAATAACAG
AAATGAATTGGGAAGGTGCTTTTAAAGTTAATGAACGTACATTGAAAATGACTGACGGATATAGTTTTATTCA
TGATAAGGAGTGAAAATAAATGAAGCAAAAAATTATTATGTTTACTCTTGTAACAATAATATTATTTTGTGCT
GTTTTAATTGGTTATCAGATTCCTAAACAACAAGTTAAAATGAAACAAAATCAAATTGAAGATTTGCAGGAAG
AACAACGTATATTAAGAGATAAAAATGGAGAGTTAAATAAATTAGTAAAGAGACAATCAAAAACAGTTATTTC
TGATGAAGAAAAACAAATACGTGAAGTTTCTTCAAATTTTGTTAAACAGATGTTTGAAATGAAAAAAGATTCT
TCTTTTAAATCAAAAGCACCACAAATAAAGCCTTTAGTTACAAAGGATTATTACGATACATTATTTAAGGATT
CTAAAGATAAATATGATTTATATGATGATATAACGGTTAATGATATTCATGTTTATTTTGATACGTATGACCC
TAAAAAAGATAGTTATAAAGTTTTTGTTCAGTTCGACGAACGTATTGAAACAGATGGAGATGATAAAATTGAA
CATCGTCAAACATCTGCACAATTGGATTTAGTTCGTACAGCTGAAGGTTGGCGTATTGATAATTTAAAACGTT
TCAATTTAAAACCTCTAGGACGTTAGTTTGTTTCAAGACGATAGCAATGATATAATTTACGTAAAAAGGAGTG
AATTTATCATGAGGCGATGGTTTGTATTAATTTTAGGATTAGTTATATTATTATCTGCATGTGGTCAGAAATA
TGATAAAGAAATTGATGAAGTTACAAAGTTAGAAAAAGAAAGTATTCAAGATGTGAAAAATACAAAGAAGTAT
AAAAATGTTGAGCGGTCAAAAAGTTATTATAAGATTTATAATGATGGTGAGGTTATTATAATGACTTATATGC
CTTTTAAAGATAGTAATACAAAAGTTTCAAGAGTATATAAAATAAATCAGACTAGCGATAAATATGAAGAAGA
TTCTAATATTGATGCTGAAAAGTTTGAGAAAGATAATAAACCAGTTTATGAAGAAAATAATATGAAAAAATAA
AGAATCAACTCCCTATACGCTTTAGCGGTGGTATCGGGAGTTGATGAACATATATCCTCTTATAACTTATTTT
ATAAATAACCTTTTTAAAAGTAAAGGAGGATATACATGAAAAAAAGAAAAAAACGTAAAGAATTAGACTATAA
AACACGTCTAGCTATCGCAAAGTATTTAAATGATGGTTTAAACATCATTCAGATTTCTAAGATTTTAGGTCGT
GATGATGCAGTTCTCTATAGAGAAGTAAATAAGCGTTCTGTTGATGGTGTTTATAATCCTTTATTGGCTGAAG
AAGATGCTATAAAATCGCGTAGTATTTCAGATGAACAGGTTGCTAAACGTAGACGTTTGAATGATGAAACAAA
AGCATATATTGAAGAAAAACTTTCATTAAAATGGTCTCCTAGACAGATAGCAAGTCAAATTGAAAAAGATACA
GGGTATTATATAAGTTATCCTACTATATATAGATATATTCGCAATGGATTGGTAAAAGTTGATGTTAAACGTG
ATATGAGACAAGCAGGCAAGAAATATAATAAGTCTTCTGAAAAGCGTGGCAAATTAGACGTAGGGTCTCGTGT
TATAAAGTATCGGCTCAAAAAGTATTAATAATCGTGAAGAATTTGGACATTATGAAATTGATACTGTTTGGAG
TGTTAGACCATCAACATATTGCTTGTTAACTATTATTGAAAGAAAGACAAGATATTTATATGCTAGTAGATTA
AATACACGTAAATCCAATGTAGTTTGTAATGAGATTATAAATATTATGAAACCATTATTACCCAAAAGTATAA
CTATGGATAGAGGTAAGGAGTTTGCTCTATTTGAACAAATAGAAGATGAATTGAATTGTAGTATTTATTTTAG
TGATTTGGGTTGTCCATATCAACGTGGTTCTATTGAAAATGCAAATGGACTTTTAAGACAGTATTATCCTAAA
GGTACGGATTTTGCAAATATAACACAAAAGTCATTAGATGAAGCTGTTAAACAAATTAATGCACGGCCTCGAA
TGATATTTGACTATAAATCGAGTGAGGAAATGCTAAAGTACCATGTAAGCACACAAAATTGTGAACCTATATT
AAATGACTGTGTGAGGCACGAACCCGTCAATTAATATAGGTTAATCTAAGCGAATGGTTTACAATGAGCGCGG
AAGGACAGGACGAATAAATTTTGTATATAAAATGCATAATCTAAAAAGAAGCATACTATTTAAATATGTGGTT
TTTGATAGATATTACTAGTAAAATGCTTCTTTTTCTTCTTCTGTACTCTTTTAGTAATATTTATTAAAAAAAT
TTAAGAAATTCTCATTTTTCTATTGACACACTGCCTTAAAAGAAAGAAAACCAGTTGTCAGATGTTTACCATT
AATTATATCTTGTTTAAAATTAATATTAATTCTACTATTTTTACTTTCAATACTTTTGGTCATATATCAAACT
CAATCTGCACAAATCTTATTTGTTTTTATAGATTTTCTAAATTTTGTCGCGTTAATTTTAGTTTATTTATGTA
CTAATCCTAATAATAGAAGTATTGATAAAATAGCATGTGTTAAATAAAAAGTTAATAGTAACTACAAACATAC
TTTACTAATAATCAAAACGAGTTACACAAACATACAGTTATATAAGTCCCCTTATGGAATTTCACATTCTAGT
TTACATAATATATATTATAGGAAGTAATGTGTGTGACGCAAAAGGTACCCTACATCATAATCATTATCTAATA
TCGTCACATAACTTACTTATGCTATAATCATGGTATTATATTGTTTGGAGTGATTTGATGAGATTTGTCTTTG
ATATTGATGGTACGCTTTGTTTTGACGGCCGATTAATTGATCAGACTATTATTGATACATTGTTACAATTACA
ACATGATGGTCATGAACTTATATTTGCATCAGCACGTCCAATTCGTGGTTTGTTGCCAGTTTTACCATCAGTA
TTTCATCGGCACACATTAATTGGCGCAAATGGTGCTATGATTTCACAGCAATCAAAGATTTCTGTTATCAAAC
CAATTCATACTGATACATATCACCATATCTTAAAAATAATTCAAAAGTATGAGTTAGATTTTATTATTGATGA
TGATTGGAATTATGCTGCACAACTTGACGCTGAGAACGCGATTTTTGAGCGTTTAGATCCACATAAGCTGGCC
AGTTGTATTGATGTTGCAAATATCGACACGCCAATCAAAGTTATTTTATTAAATATAGACCCGGCACAAATTA
CAACTATATTAGACGAGCTAAATAAATACCATCAAGAATTGGAAATGATTCACCATTCAAATGAGTATAACAT
TGATATAACAGCGCAAAATATTAATAAATATACTGCATTACAATATATATTTGATGCAGATGTTAAATATATA

GCATTTGGTAATGACCACAATGATATTGTCATGTTACAACATGCTAGTAGTGGCTATATTATAGGACCATCAG
AAGCATACACACACGCAATATTGAAACTTGATAAAATCAAACACATCAATAATAATGCACAAGCTATTTGCAA
AGTCTTAAAATCATATAAATAAAAAACACCCCTATCAAATGATAATCATTATCAATCGATAGGGGCTATTTTA
ATAAAATTCGTCCTCGAACATTTCGTCCTCTTCATCTAATCCAAATAATTCTGCCATTTCTCCATGTTCAATT
AACATGTTTAAATATGCATCGCGGAGTTCTTCTTCACTCATATCATTAATCATTTCTTTAAGAGAATCAATCC
ACATATTTCTGCGTAAGTGATAGTCTTCTTCAACTTCGTTTAACATGATTATATGTTTATTTGCTGCTTCTGG
ACTAGCTGTAAAGAGTAATGCAATCATATGTTTACATATCACTCGTCTTCCATCAGCATGAGGACAATTACAT
TTGGATTTTCTAGGATGTTCCATATCAATATAACAACGATATACTTTGTTACCACTGCCTTTTACTTCAGCCT
CATGCTGCGTTTCTGAAAATGATTTTAAGTTAATGACGCATTCACTTTGATAATAATTAAAGCCTCTTTCTAT
AGAACGAATACTTGCAATATCAAGTAATCCCATTAATGATACTCCTTTTTATTATTATTTTTAAATACAGAAA
ATAAAATCGATAAGTGTCTAGATTAATATACTTGATTTATCTATATTTTATAACAAGTCTAGAATTATCGCAT
TCTTAAATAACTAATATGAAAATGCTTGCACTAATTCTTTTGTATAAGGGTGTCTATCAACATTAAATAATTC
CTCTATTGCAAAATCATCGACTATCATGCCATCCTTAAGAACGATAATTCTATTAACTAAGCGTTGTAACACG
GATAAATCATGAGAAATAACGATAAAATGATTTAAGTTCGTAATCGTTTGCGCTTTTAATATATTTATTACAT
TTTGTTCAGCTATAACATCTAAATTTGAAGTTATCTCATCACATATTAAAACGCGAGGTTGTACTAATAACGA
ACGCATGACATTAAATCTTTGTAATTGTCCACCACTCACTTCGCTTGGTAATTTAGTCAATAATTGCGCGTTC
AACTCAAAAGTAGATAAATGTTGTATTAATAATTGATCCTGAGCAGTATTATCAGTTAGGCCTCTGTAATAAT
ATAACGCTTCTTTTAATGAGGTCTCAATCGTCCAATCAGGGTTAAAGCTAGTTAAAGGGTGTTGGAAAATCGG
TAACACAGCATTATCACTTAAGTAAATTTCTCCTTTAACAGGTTTAAACAAGCCAAGAACCAATGAAGCGAGC
GTACTTTTACCACAGCCACTTTCGCCTAAAATACCAACATTTTCTCCATCAGGTATAGTAATATTGATATCTT
GTAGCACCATCTGCTTTTTATTATAACCAAAAGTCACATGTTTTAACTCAATCATAGTAATCCCTCTTTAATT
GTGTTCTATATTTAATTAGACGTTCAGTATACGGATGCAAATGCTCATACTTGAAATGATTAATATTACCTCG
TTCAATGATTTGACCTTCTTTTAAAACATTAATGTACTGACAATATTTCAATACATGACTTAAGTTATGTGTG
ATAATAAATAATGTTTGACCATGTTCTAATACAATATGCTGTAATAAATCCATCACTTGATTACCGTTCAAAG
CATCCAATGATGCAACTGGTTCGTCTGCAATGATTAATTTAGGCTCCAACATGAGAACGCTTGCTATGTATAC
GCGTTCAAGTTGGCCCCCAGAAAGTTGGAAACTATATTTATTTAATATATCTTTGCTTTGTAAATTAACCCAC
GACAAAGCCTTATCAATTTTGGACAAAGCCTCTTCTTTACTATTTTTATAATGCTTACGATAAATCGCAGTTA
ACTGTTTACCTAATTTAGTATGGTCGTTAAAACTTTCTGCATAATTTTGAGAAATATAGCCAATTGTATGACC
ATAATATTGACTCAATTTACTAACATTTTCCCCATCAAATTGGTACGAATCATACGTGCAGGTTAAATCGAAT
GGTAAATATTCAAGTAAGGCTTTAGCAATCAAACTTTTTCCAGCGCCGCTCTCTCCAATCAAGGCATTAATCC
GTTGACTAAAAATTTTCAAATCAATCCCTTTAATAAGTGATTTCTCACTAGTATTCTTTATTGTTAAATTTTG
TATATCAATGAGACTCATCATATTCACCTCGTTGTTTCAGCAATCTATCTCTTAGCGTATCACCGGTTAAATT
AAAAATTAAAATAGTTATAGCAATGACTGAAGCAGGTGCAATCAACATAATTGGATGAGACGAAATAAAATCA
CGACCTTGTTGCAACATAGCGCCCCACTCTGGTGTTGGCGGTTGTGCACCTAACCCAATAAATGATAGTGAAC
TTATATATAGAATGATTTTACCGAAATCAACGACCATCAAAACGATAATAGCCGGTATAATTTTAGGTGTTAA
ATGACGTATTAATATTGTTCTTGTAGGTACATGAAATAATTGTGCCATTTTTATATAAGGCTTATTCATTTCG
CTATTAACTATACTTCTAGTCAACCTTGTGTAATTCATCCATTTTATTACTGCAATTGAAATAACTAAATTCC
ATAACGATGGTTGGAAAAAGCTTGCTAAAGCAATCATGATGATAAATTCTGGAATACTTAGACCAACATCAAT
AAACCTTAACACTAATCGTTCAATCCACCCCTTTTTATATCCGGCAAATAGACCTAGTGTAACACCTATGACA
ACGATAGCTATTAATGTTAAAACAGTAACAAACAATGTTGAACGTGCACCGATAATAATTCGGGTAAATAAAT
CTCTCCCATAATCATCAGTTCCTAATAAATGCAACCAACTAATAGGTTCAAAAGTTTGTGATAAATTGACTTT
GGTTGTATTTTCACTACTTACAAAGAATTGCAGTACAATTACCACAAAAATAAATGCAACGAATACAAAAAAT
ATCAGGTTATTCTTTGAAAATATTTTATGCATGACGGTCACTACCTTCTGATATCAATGGTGTATTGGTTTTG
TTTTTTGGATTTCCTAATTGTAGACGCTGCTTCGGATCAAGTAATAACGTTAATAAATCAGCAATCGTATTAA
TAATAACAACGAAGAAGCCAATAAATAACACGCATCCTTGAATAACAGGATAATCTCGAGATTTAATACTATC
CATTAATAGATAACCAATACCAGGTATATCAAATAAATTTTCAATCACTACAGTACCACCTATTAGACTACCA
AGTGAAATCCCTAATAATGGGATAATCGGAAAAATTGTTGGTTTTAGTAAATCATGAATTAAAATATAACGTT
CATTCATACCGCGTAATCTTGATGCTTGTACGATATTACTTTGCAATAACATCAATAAATTAGAGCGCACTAA
ACGAATGATGTATGCACACATACCTAAAGATAGTGTGAGTACAGGTAATATAAACTGACTTAGTATAACGCTA
TCTATATTCATTAAATTTGTGACAATAAATAATAAAATAATACCGATAAAGAACGCTGGTAAACTAATTGATA
GTGTTGAGATCACTCTAATCACTTTATCCGTCCACTTATGAAATCGTTTGGCTGCTATAATGCCGAGCGGTAT
AGATATGCATAACGACACTACTAATGTTGAAAATGATATGAGTAATGTTATGGGTGCATAGTTGAATAATATC
TGTGTTACCGGTTCTTTTGATTCAAAACTTTTTCCTAAATTAAAATGTAATAAATGATTCATCCAATGCCACC
ACTGTACCAATAAAGAATCATTTAATCCCAATTTATCTTTGGTTGCATTTATTTGTTCCGTCGACACTTGTGC
TACATCAAGATGTAATATTTTATCAACAGGATTTCCTGGTGATAATTTCATTAAAATGAATGTAAGTGTAGAA
ATAACAAATAAAACAACTATCATTTGTATCAGTCTATACATCATAGACTTTATTATGAACATAATAGTCCCCC
TCCTTGTGTAAGTTACCAACACTTTCTTGTTACATGAGAATGGCGCGTGTATATGCAACTTACATATTAAGAA
CCAACGTTCATTATAGTATTATCCCAAAAGAAATTGAAGTATATTTAATTTTTTAACAAAATCATTATAAAAT

ATAATATTTTGAATCAAGTCAACCATGTAAAATATAAAAAAGTCAAAACAAAAACAACTATAGCACTGTATTC
CATCTCTTTCGAAATAATTGTTACTGCAGTGTAACTTAAAAGTCGATGATTTTGTGCATATAGTTGTCGAATA
TTATTTTTTATCTTTACGGCGAAGTTCAGCGCCCTCATAGCCGTATTTTTCAATTTGCTTTTCTAATTTACGC
GCTTTTCTTTCTTTACGCCAATTTCTAGTAAAATACCATAATAGAAAACTAATTAATAAACTCATAATCGCTA
AAAATGCAGCGTATCCTAATAATGGTTGATATTTTATATCTTGAAAATTTGGAATAAAAAATGCAAGCACACC
TAATATAACAAATGTAATTACTGCAGATACAAACCATTTATTTAAAACTAAGCAACAGAATATTGTTAATAAA
ATCATTATTAATGTTGTAATCCATAAATAATTAGGCATATCGAATAATGTCATATTCATTCTCCTTTTATTTC
ATTACTTTCCTTGTATACATTTTATTATAAATTTTTAAAAACTTAAACAATAGCAGTCAGTTTCAAGCAATAT
TCTATCTACTATTAGAAAAATCATTGTTCCTTGCGACATGGAAATCGTAACATTATCGTTTAGGAGACAAAAT
TATGTATAATGAATGTATTATACCAAAGGAGTGATTATATGTCTCAAGGTTTACTTTTAAGAGAAGATGTTCC
TGTTTCAGAAACATGGGATTTAGTAGACTTATTTAAAGATGATCAACAATATTATGAAAGTATTGACGCTCTA
GTACAACAAGCAAATCAATTTCATCATACATATGCAACAACATTAAATTCAATCGAACAAATTAATACTGCTT
TAGCTGAATTAGAAAATATTTTAATTGCCTTAGATCGCTTAAGTAATTATGCAGAACTACGTTTAAGTGTAGA
TACTAGTAATATTGAGGCGCAAGTATTGAGCGCTAAATTATCTACTACATATGGTAAAATTGTTAGCCAATTA
TCATTTGTAGAGTCAGAAATACTTGAATTACCAGAAGAAATACTTCAACAATTAGAAGAATCATGTCCATATC
AACACTATATTAAACAGTTAATAAAACAAAAGCCATTCCAATTATCTGCGTCGGTAGAACAAGTATTAGCAAC
TTTATCACCTACGCTAAACAGTCCTTACGATTTATACGGCACGACAAAAATGCTAGATATTACATTCGATTCA
TTTGAACATGATGGTACAACGTACCCTGTCGACTATGCTACGTTTGAAAATGATTATGAAGATAATAAAGATC
CTGAGTTTAGACGTAAAAGTTTCAAATCGTTTAGCGATGGGATTCGAAAATATCAGCATACTACCGCGGCTAC
ATATAATATGCAAGTACAACAAGAAAAAATTGAAGCTGATTTACGTGGATTTGAATCGGTCATCGATTATTTA
CTACATAGTCAAGAAGTAACGCGTGATATGTTTGACCGTCAAATCGATATGATTATGCGTGACTTGGCACCAG
TTATGCAGAAATATGCTAAACTTTTACAACGTATTCACGAATTAGATAACATGCGTTTTGAAGACTTGAAAAT
TTCTGTAGACCCTGATTATGAACCAGAGATTTCAATTGAAGACTCAAAAAATTATATTTTCGGTGCGTTAAGT
GTTTTAGGTGATGACTATACAAACATGTTACGTGAAGCATACGATCAGCGATGGATTGATTTTGCACAAAATA
AAGGTAAAGATACAGGCGCATTTTGTGCAAGTCCATACTTTACACATTCGTATGTGTTCATTTCTTGGACTGG
TAAAATGGCTGAAGCATTTGTCCTAGCACATGAATTAGGTCATGCAGGTCATTTTACATCAGCTCAAAAACAT
CAACCATATCTTGAATCAGAAGCATCAATGTACTTTGTTGAAGCCCCTTCTACAATGAATGAAATGTTGATGG
CCAATTATTTATTTAACACAAGTGATAATCCAAGATTTAAGCGTTGGGTTATTGGCTCAATTTTATCTAGAAC
ATATTATCATAATATGGTTACCCATTTATTAGAAGCTGCTTATCAACGTGAAGTGTATCACAAAGTAGATCAA
GGTGAATCTTTAAATGCGCCGACATTAAATGAAATAATGCTAAATGTTTATAAACAATTTTTTGGAGATGTAG
TAGACATGACTGAGGGTGCTGAATTAACATGGATGCGTCAACCTCATTACTATATGGGATTATATTCGTATAC
GTATTCTGCTGGCTTAACAATCGGAACTGTCGTTTCTCAAAAGATTAAAAATGAAGGCCAACAAGCTGTTGAT
GCTTGGTTAGAAACATTGAAAAAAGGTGGTAGTGTATCACCTGTCGAACTTGCAAACATTGCAGGTGTGGACA
TTACTACAGAACAGCCACTTAAATCTACAATTCAATATATTTCTGATTTAGTCGATGAAGTTGAAAAATTAAC
AGATGAAATTGAGCAAGCAAATAACTAAGATGCGAGACAATTTATAGTAGAACAATATAAACCAAGATTTTCA
CATTACTAATTGTGAGAATCTTGGTTTTTTTTGAGGGTTTATGAATAATAGAAATATATATTTATCGTAAGGG
ATTTTATAGTAATAACTTTAAGTTATTGTTCGTAATGTGTACCTGTTAAATAAAAATAAACACTTTCAGCGAT
GTTAATAATATGATCACCAATACGTTCTAAATGTCTTGCTGCTAAATGAGCTTGTGCAGCGACAAATGGATCG
TTATCAATAAGATACGTTGCGTTAATAATATGACTATATAAGTCATCGATATCTTCATCACGCTCAATTATTT
CTCTTATTAATACGGTATCTTTCTTTTTAAATGCTTGATCTAAGTCCTTTAACATTAACATAGCTAATTTACC
CATTGTCTTTAAACGGGTTAACACATAATCATCTGTAATCTTCGTACGCAATCGAATATTGGCAATACTCGAT
GCATTATCTCCTATTCTTTCTAAATCGGAGGCGATTTTTAATGAAGAAATCATCATACGCAAATCACTCGCAA
TGGGCTGTTGCTTTGTAATTAACATGATAACTCGCTCATTAATATTATAATTTAATTGATTGATATGTTTATC
GTTTTTAACTGTTTGTCGTGCAAAGCCTCTATCGTCAATACTTAATGATTTTATACCATTTTCAATACTCACA
TAGACATTTGCACCTAACCGACGTAATTCTTTTATTAAATCATCAAGTTGCTCCTGATATCGTTGTCTAATTA
TTGCCATTATATATCAACCAAACCTTCCTGAAATATAATCTTCTGTTTTCTTGTTTGATGGGTTAGAGAAAAT
TTTATCAGTATCATCATATTCATTGACATAACCATTTAAGAAAAATGCAGTTTTATCTGATACACGAGCTGCT
TGTTGCATATTATGTGTAACCATAATAATTGTATACTTTTCTTTTAGTTCTTGAACCAACTCTTCTACTCTTA
ATGTTGAGATTGGGTCTAATGCTGATGTCGGTTCATCCATTAAAATGACTTCAGGTTCAATTGCTAAACAACG
CGCGATACAAACACGCTGTTGTTGCCCGCCGGATAAACTATATGCATTTGTGTGCAACCTATCCTTTAGTTCA
TCCCAAATTGCCGCGCCACGTAATGATTTCTCAACGATTTCATCAAGAACTTTTTTATTTTTAATACCGTGAA
TCTTTGGACCGTAAGTAATATTATCGTATATTGATTTTGGAAATGGATTAGGTTGTTGAAAGACCATGCCCAC
ATTTGTACGTAATTGTTCTTTAGAATATTTTTGATCAAATATGTCTTGATCGCGATATAGTATTTTACCAGCT
GTTTTGACTGTGGGTACTAACTCAACCATTCGATTCAAAGTTTTAATATATGTTGATTTGCCACAACCAGATG
GACCTATAATGGCAGTTATTTCATTTTCATAAATATCTAAATTAATATTTTGCAATGCATGATTTTCGCCATA
CCATAAGTCTAAATTTTGTGTTGAATATATAACAGAATGTGAGTTTGTATCATCTGGTGTTTTATGATGACTT
TGTGAGACATCAAACGTATGACTTTGAGATATTTGTTTAGTTTGTGCAAGTGTTTGCGCCATGTTTAAAACTC
CCTTTTTAATAGATAAATGAGATTGATTGTTTAAATTAGAATTTTTTACTAAATTTGTTACGTAAAATAATCG

CAACGCCATTCATTAATATTAAGATAACTAGTAAAACGATAATGCCTGCCGATGCAACATTCTGGAATTCTTC
TTGAGGCATTTTCGCCCAAGTAAATATTTGGATAGGTAATGCTGAAAATTGATCCAATATACTCCTAGGTGTT
GCCAATAATATAGTCGGTATACCGATAAGCACAAGTGGCGCTGTTTCTCCCAGTGCTCTTGAAAGAGACAAAA
TGAATCCAGTTAAAATACCAGGTAACGCTGCTGGTAAGACAACACGTCTTATCGTTTGCCATTTATTAGCACC
TAAACCGTAAGAAGCTTCGCGTACTGAGTTAGGTACAGCTCTAATTGCTTCCTGACTTGAAACAATAATAATT
GGTAATATCAGTAAGGTCATTGTTAGCGCTGCTGCCAATATACTGTTACCCATTTTCAAGGCTTCAATCCCCG
CGCCACCAACGAACAAAGTGTAACCTAATAACCCAAATACAACTGATGGTACACCAGCTAAATTGGAAATACT
GATTTTAACAAACTGAGTAAATTTGTTGTTTTTCGCATATTCTTCTAAGTATATAGCTGTACCTATTCCTAGG
ATGATTGATAATGGAATGATACTTAACATTAACCAAAGTGAACCGATTAACGCGCCTTTAACGCCAGCCATAG
ATGGTGTTGAAGAAGAAAAAATTAGTGAAAAACTGTAAATTTAAATGACTTACCCCCTTAATCAATGTTTGAGT
TAACAACGCAATAAGTACGACAAGTCCTAATAATGTACATGCTAAAAATATGAGTTTGAACACTTTATTTTTA
ACCGTTCTGGATGATAAATGTTTTTGGACAAGTTGTTGATCGACGAGTGATTGTCTATTATTATCTGTCATTT
CCATATTAATACTCCTCCCTAAAACGCTTAGAAATCCATTGAGAAAGTAAATTCATGATTAAGGTAAAGATAA
ATAATGTGAATCCTACAGCATAAATACTGTAATAAATATTTGATCCAAATGTTGCATCACCTGTCGCTATCTC
AACAATATATCCAGTCATTGTTTGAATCGAACTTGTTAAACTTAATGAAGCTGTTGGCGAACTACCTGCCGCT
AATGATACAATCATCGTTTCTCCAATTGCTCTTGAAATCGCGAGAACGATTGAAGCTACAATACCTGATGTTG
CTGCAGGAAGTACGACTTTAGTTGCTACTTCTAATTTAGTTGCTCCAAGTCCATAGGCACCTTCTCGAATTTT
ATTTGGTACAGATGCCATTGCATCCTCACTCAAACTTGTGATGAGAGGGACAATCATAATACCGACAACTAAG
CCGGGACTTATAGCATTAAACTCTCCAAGACCTGGTATGAAAGATCTTAATACTGGTGTAACAAAGGTTAATG
CAAAGAAACCAAACACAATTGTTGGTATTCCTGCTAAAATTTCTAATATCGGTTTAATTATGCGTCGTGCACG
GTCACTTGCATATTCACTTAAATAAATTGCTGCACCAAGCCCGACTGGAACTGCAAATATAGTCGCAATAACT
GTGATTTTTAAAGTCCCTATTATCAATGCCCAGATACCAAACTTAGGGTCTGAACCGGTAGGATTCCAAGTAG
TAGAAAATAGAAATTCAGTTATTGGAATTCTGGTGAAAAAAGTGATGGTTTCTAAAAGCAATGTGATTAATAT
ACCTAGTGTTGTTAAAATGGAAATCGCTGAAATTGCGGCTAAAATAACTGGTATAATTTTGTCATTATGCTTC
CCTTTTTTATTATTATTTTTTTCGATTAAAGCTTTAACATTAGTAGATGAAGTCATAATCAAACCTCTTTCTA
TTACTCCCAAAATAATTAGATTTGTAAAATAATGATGAACCACTATTTTAAGTTGATAAGCTTGTAGACTTAC
TGAATGTCAAAATCAGCGTCTCGATAAATCAAGCTTTGTACTCTAAATAATGATTCACCATCAAAATCATGTT
TCAAGACACATTTGAAATTGCGTCTTATTATTTTTTGTCTTCAGACTTTTTATCATCAGATTTCTTGTCGTCA
GATTTTTGATTTTTATCAATAAATGCTTTTAAATCATCTAGTTGTGATTTGTATGTTTTCTCTGGTGCTGCTA
CATATCCAGCTTCTTCAGCTGCTTTACCTTTATCTTCTAAGACGAATTTGATAAATTCTGACATTACTTTATT
ATCTTTTAATGCTTTTTCATTTACATAAATGAATAATGGTCTACTTAATGCATAAGAGTTATCTTGAATTGTT
TTTTTCGTAGGCTCTGTTGCTTTACCATTTTCATCTTTGATTTTAACTTCTTTTAATTTATCTTTATTTTGAA
CGTAGAAGTTATATCCAAAGTATCCGATTCCCTCTTTGTTTTTCGTTACAGAAGAAACGATAGCATTTGTATC
AGCATTTTTTTCTGCTTTAATATCTTCTTTATTCATTACTTCATTTTCAAAGAAGTCATAAGTACCATGACTT
GAGTTTGGTGATACAGCATTTATTTTTTTATCTGGCCATTTACTATTAACATCTTTCCATGTTTTAGCTTTTC
CAGAATAAATTGCTTTTAATTGCTGTTTGTCTAATTCATCTACAAAATCATTTTCTTTATTTACAGCAACCGT
TACACCATCTTGCGCAATTTTGAATTCTTTGTATTTAATATTCTTATCTTGTAATTTTTGCTTCTCTTCATCT
TTAATTGGTCTAGAAGCATCAGCGAAGTCGATATCTCCTGCAATGAATTTTTGGAAACCAGCACCTGTACCAG
CTTGTCCTGCTGAGATTTTAGCATCCGAGTGATCTTGAGCCCATTTTTCATTTAATTTCTCCACAATTGGTGC
TACTGTTGATGAGCCATCACCTTTAGCTTCCCCTTTTAAATCACTATTACCACTGCCACCATTACCGCCACCA
CAAGCACCTAATAATAGTGTTGCACCTAAAGCTGTAGTACCAACAAATTGCCATTTTTTCATTGAAATATCCT
CCCTGTATGAACAACAAATAGTATTTGTATTGATTACATCTTTATCTTAACCTTGAATTATTAACCTCATTTT
TAGAAACTGTAAATGTTACATAAAAATTTGTTAAGAAAATGTAAATATTAATTTTTGTTAATCTAAAAAATAT
TGCTTCATTAGTAAAATTAAAATGCTTCCTGAAAAATTATCGAAATTTAAATATGTACACAAAAAAATGGACA
GATGAAATTAAAAAACTACTTCATCTATCCGATTTAATATTGAATATAAAAAACACGCATCCTACGATACGTG
TAAAAATGATTATTCTTTTGAGTCAATTCGACTCCAACCTTTTTTAGTTAAAGTGATTTTGCCTGTTTCTATA
TTAATAATCTTCTGTTTATATAAGTGACCGATTGCACGTTTGAATGAACCTTTACTCATATTGAATACTTCTT
TAATCGCTTCAGGGCTTGATTTGTCCCAGAATGGTAATTCACCATCATATTCAACTAGTAAATCAAAGATGAC
TTGGCCGTCATCGTCTAAACGTTCATGTGCAAGTGGTAAAAATGAACCATTTAACTCACCTTTATCATTATGC
CCGATAATTCTAACTTGAACAGATTCACCTAATCTTGGTTCAGCTTTACGTTCTGATTCATGTACGAAAATTT
TGTAACCTGATTCGCTTAATAAAAAGCTACCAATTCGTAATACGCGGTAAGGTTTGGCTTCAATGACTTCGTT
TTTTAAATTATCGTCGTGTACAGGTGTAAACATATTTCTACAACAGATTCACTCGCTAATCGTCCATACATA
TGATTCTCACGGTCAATTCGTAATGTGACTAGCAAATGATCACCTAGTTGTGGCCATAGTGATTTCACTTTTG
GTAAATCTTCCCATGGTACTAACACTTCACGGGGTAATCCAACATCTATACGTGCCCCATCGCGATCCGTTTT
AAGTACTTTAGCAAAGTCATATTTATCTTTCGTAATATCAGGCATATTTTGAGTTGCAAATAATTCACCTGAA
CGGTTTGGATAAATGAAGAAACTATATTCTTCACCTACTTCTAATTCATCATCATCGTTCATTTCTGATTGGT
TTAACTTTACGTTTTCACCGTTTGGTCCTTTTAAAAGGTAAGTTGAACCTTGTAACCCTACTACTTCAAGGAA
TTCTATAGAACCTACTATATCTTTGTCTAATGCCATGTTATTCTCCTTCGATTCTTGTATTCTTTATTATTAT

AACATGTGTGAGTCATTCTATCATTACAACTTATATGAGGTCGAAACTGTGACGATAATATGATATAATTCTT
CCGTTAGAATATCGAATGAAAAAAAGGAGAAAATGCATGTTACAAGTAACTGATGTGAGTTTACGTTTTGGAG
ATCGTAAACTATTTGAAGATGTAAATATTAAATTTACAGAAGGTAATTGTTATGGATTAATTGGTGCGAATGG
TGCAGGTAAATCAACATTCTTAAAAATATTATCTGGTGAATTAGATTCTCAAACAGGACATGTTTCATTAGGT
AAAAATGAACGTCTAGCTGTTTTAAAACAGGACCACTATGCTTATGAAGATGAACGTGTGCTTGATGTTGTAA
TTAAAGGTCACGAACGTCTTTATGAGGTTATGAAAGAAAAAGATGAAATCTATATGAAGCCAGATTTCAGTGA
TGAAGATGGTATCCGTGCTGCTGAACTTGAAGGTGAATTTGCAGAAATGAATGGTTGGAATGCTGAGGCTGAT
GCAGCTAACCTTTTATCTGGTTTAGGTATCGATCCAACTTTACACGATAAAAAAATGGCTGAATTAGAAAACA
ACCAAAAAATTAAAGTATTATTAGCGCAAAGTTTATTCGGTGAACCAGACGTACTATTACTGGATGAGCCTAC
TAACGGTCTGGATATTCCAGCAATCAGTTGGTTAGAAGATTTCTTAATTAACTTTGATAATACTGTTATCGTA
GTATCACATGACCGCCATTTCTTAAATAATGTATGTACTCATATCGCTGATTTAGACTTTGGTAAAATTAAAG
TTTATGTTGGTAACTATGATTTTTGGTATCAATCTAGTCAGTTAGCTCAAAAGATGGCTCAAGAACAAAACAA
GAAAAAAGAAGAAAAAAATGAAAGAGTTACAAGACTTTATTGCTCGTTTCTCAGCTAACGCTTCTAAATCTAAA
CAAGCAACAAGTCGTAAAAAACAACTTGAGAAAATTGAATTAGATGATATTCAACCATCATCAAGAAGATATC
CTTTCGTTAAATTCACGCCTGAGCGTGAGATTGGTAACGACTTATTAATCGTTCAAAATCTTTCTAAAACAAT
TGACGGCGAAAAAGTATTAGATAATGTATCATTCACAATGAATCCAAATGATAAAGCGATTTTAATTGGAGAT
AGTGAAATTGCAAAAACAACATTACTTAAAATATTAGCTGGCGAAATGGAACCAGACGAAGGTTCATTTAAAT
GGGGTGTTACTACATCATTAAGTTACTTCCCTAAAGATAACTCAGAGTTCTTTGAGGGTGTAAATATGAATCT
CGTTGATTGGTTAAGACAATATGCTCCTGAAGATGAACAAACAGAAACATTTTTACGTGGTTTCTTAGGTCGT
ATGTTATTTAGTGGTGAAGAAGTTAAGAAAAAAGCTAGTGTGCTTTCAGGTGGAGAAAAAGTACGTTGTATGT
TAAGTAAAATGATGTTATCAAGTGCGAATGTACTTTTACTTGACGAACCTACTAACCACTTAGACTTAGAAAG
TATTACTGCTGTCAATGATGGTCTTAAATCATTTAAAGGTTCTATCATCTTTACTTCTTATGACTTCGAATTT
ATCAACACGATTGCAAACCGTGTTATCGATTTAAATAAACAAGGCGGCGTTTCAAAAGAAATTCCATATGAAG
AATACTTGCAAGAAATCGGCGTTTTAAAATAAACATAATATGTAGAATTTCTTTTCGAAAATCTCTGTGTTGA
GGCCCGACCAACTTGCACATTATTGTAAGTTGGCTTACTGTCACCTTCTATATTGGGACTTATTCCGCAACTT
GTATTGTTTGTAGAAATTAGGAATCCAATTTCTCTATGTGTGGCACCGACAAGGATTGAAAAAAGCTAGCTAC
AAAGTCATTTTCATTCAGTCAACTACTGTCAATATAACATTGCAGGGCCTAGGGCATTGATTCATATCCGAGA
CTATATTCAATTTAATAGACATTAACTGGGCGTCTCGATGCTAATATTTATAGCATCGAGGTGTCCTTTTTAT
TTTATTTTTTAACACATCAGCACAGTGAACACCCCTTCTCTAAATTACAAATACAGTTATCACCGCATTAAAA
GAGTACATTTATTTAATGTGCATATTGAGTATGCTATAAGTATTTTGAATTTTATTTTTCAAAAAGTCTCATT
ATCACCACCTTTTAAAATATAAGCAACGCTATAACTTGAATTTTCAGGTTATTCCAAAAAATCTAAATTATCG
GTTTACATTCATATTTGTCATATGTATAATGAACACATACCAATTTAATAATATTTTGATGAGGCGCATCAAT
CATGAGTAAAGTTTAGATTACTGTCTGCTAACAGCTAAATTTGAAAGGGTGCGATGCCGAAGCAATTATAATA
GCAGTTATAATTTGTTGGACTTTTTGGTTAAGAGCTGAGAGTTTGTCATTATTTAAAAATAATGGAGTGCATC
ACTTGTATATAGATTAAGAGCAAGTTCGCATTCCGAACTTGCTCTTTTTTTATTATTGTGTGCCCTTCCTAAC
AATTAGGAGGATTATATGGTAACAAGAAGTGTGTTGAAATTTGGCGGATCATCCGTCAGTGATTTTACAAAAA
TAAAAAGGATCGCTGAAATGTTAAAGGAGCGAGTCAATCAAGATGAACAATTAATTGTCGTTGTAAGTGCTAT
GGGTAACACAACAGATCAATTAATGACGAATGTATCGACCTTGACTAAAGCACCAAAACAACAAGAACTGGCA
TTATTATTGACAACCGGAGAGCAACAAACTGTATCTTATTTATCAATGGTATTAAATGATATCGGTGTGAATG
CCAAAGCAATGACTGGCTATCAAGCAGGTATTAAAACCATTGGCCATCATTTAAAAAGTAAAATTGCTCAAAT
TAATCCTCAAACATTTGAACAAGCCTTTCAAGAAAACGATATTTTAGTAGTTGCTGGATTTCAAGGCATCAAT
GAACATCAGGAATTAACAACTTTAGGCAGAGGCGGTTCTGATACGACCGCTGTGGCACTTGCTGTTAGTAATC
AAATACCTTGTGAAATTTATACCGACGTTGATGGTGTGTATGCCACTGACCCAAGACTTTTACCAAAAGCTAA
ACGACTAGACATCGTCTCATATGAAGAAATGATGGAAATGAGCGCTTTAGGTGCTGGTGTACTTGAAACAAGA
AGTGTTGAATTAGCTAAAAACTATAATATCCCTTTATATTTAGGAAAAACTTTATCGAACGTGAAAGGAACAT
GGATTATGTCAAATGAAGAAATATTAGAGAAAAAAGCAGTTACTGGTGTGGCTTTGGATAAACATATGATGCA
TGTAACAATTAGTTATCCCCTACCTGACAATCAGCTACTTACCCAACTATTTACGGAACTTGAAGAAGGTGCT
GTAAATGTTGATATGATTTCACAAATCGTCAACTTGGATGGGCTACAACTATCCTTCACGATTAAAGATAGTG
ATTTTCATCAAATTTCTATGATTCTTGAAACATTAAAGAATCAATATGAAGCATTAGCTTATAAAATCAATGA
GCATTATGTCAAAATTTCATTAATTGGCTCAGGCATGCGTGATATGTCAGGTGTGGCATCAAAAGCATTTTTG
ACATTAATTGAAAATAATATACCTTTCTACCAAACAACAACATCTGAAATAAGTATTTCATACGTCATTGATG
ATTTTAATGGGCAACAAGCGGTAGAAAAACTATATGACGCATTTAACATTTAATGGTAAAATGATTGTTAAAA
TATTCTAAAAATTGGAAATTATTATAAAATGGAGTGACAAGTTATGACAAAGTTAGCAGTTGTGGGTGCAACA
GGATTAGTAGGTACAAAAAATGTTGGAGACATTAAATCGTAAAAAATATTCCTTTCGATGAATTAGTATTATTTT
CATCAGCACGTTCTGCAGGGCAAGAAGTTGAATTTCAAGAAAAAACATATACAGTACAAGAATTAACTGATGC
TCGTGCAAGTGAACATTTCGATTATGTATTAATGAGTGCTGGTGGCGGTACAAGCGAACACTTTGCCCCACTC
TTTGAAAAAGCTGGTGCAATCGTTATAGACAATTCAAGTCAATGGCGTATGGCAGAAGATATTGATTTAATCG
TTCCGGAAGTCAATGAACCTACATTTACAAGAGGTATCATTGCCAATCCAAACTGCTCTACGATTCAATCTGT

TGTGCCATTAAAAGTATTGCAAGATGCTTATGGTTTAAAACGAGTGGCATATACAACATATCAAGCTGTATCA
GGTTCAGGGATGAAAGGTAAGAAAGATTTAGCTGAAGGTGTAAATGGTAAAGCACCAGAAGCATATCCACATC
CAATTTATAATAATGTGTTACCGCATATTGATGTGTTTTTAGAAAATGGATATACAAAAGAAGAACAAAAAAT
GATTGATGAGACGAGAAAAATTTTAAATGCGCCAGACTTAAAAGTAACAGCAACATGCGCACGTGTGCCTGTT
CAAGATAGTCATAGTGTTGAAATTGATGTAACGCTTGACAAAGAAGCGACTGCAGAAGATATTAAAGCATTAT
TTGATCAAGATGACCGCGTTGTTTTAGTAGACAATCCAGAGAACAATGAATATCCAATGGCAATCAATTCTAC
TAATAAAGATGAAGTGTTTGTTGGCCGTATACGTAGAGATGATTCATTAGAAAATACTTTCCATGTATGGTGT
ACATCAGACAATTTATTAAAAGGTGCTGCATTAAATGCTGTACAAGTATTGGAACAAGTTATGCGTTTAAAAG
GAGCGAATTAAAATGACACATTTATTTGAGGGTGTTGGCGTTGCACTTACAACCCCTTTTACAAATAACAAAG
TTAATCTTGAAGCTTTGAAAGCACACGTTAATTTTTTACTAGAAAATAATGCCCAAGCAATCATCGTTAATGG
AACTACTGCTGAGAGCCCTACTTTAACAACAGATGAAAAAGAACGCATTCTAAAAGCAGTTATTGATCTTGTA
GATAAACGTGTTCCTGTCATAGCAGGAACTGGCACTAATGATACTGAAAAGTCAATCCAAACTTCAATCCAAG
CTAAAGCCTTAGGTGCTGATGCAATTATGTTAATTACGCCCTACTACAACAAAACGAACCAACGTGGTTTAGT
CAAACACTTTGAAGCGATTGCAGATGCTGTGAAATTACCAGTCGTGCTGTACAATGTGCCATCAAGAACAAAT
ATGACGATTGAACCAGAAACAGTCGAAACATTAAGTCAACATCCTTATATAGTTGCTTTAAAAGATGCTACGA
ATGATTTTGAGTATTTAGAAGAAGTGAAAAAGCGCATTGATACCAATGTATTTGCTTTATATAGTGGCAATGA
TGACAATGTTGTAGAATACTATCAACGTGGTGGTCAAGGCGTTATTTCTGTCATTGCTAATGTCATTCCTAAA
GAATTTCAAGCGTTATACGATGCTCAACAAAGCGGATTAGATATTCAAGATCAATTTAAACCAATCGGCACAC
TGTTATCAGCCCTATCAGTTGATATTAACCCAATTCCTATTAAAGCTTTAACAAGTTATTTAGGATTTGGAAA
CTATGAATTACGTTTACCATTGGTTAGCCTAGAAGATACAGATACTAAAGTGCTTCGTGAAGCATATGACACA
TTTAAGGCGGGTGAAAATGAGTGAAGATATTACTAATTGGCTATGGCGCAATGAATCAACGCGTTGCTAGATT
AGCAGAAGAAAAAGGCCATGAAATTGTTGGGGTCATTGAAAATACACCGAAAGCAACAACGCCATATCAACAA
TATCAACATATTGCAGATGTTAAAGATGCCGATGTTGCAATAGATTTTTCAAATCCGAATCTATTATTCCCAT
TATTAGATGAAGAGTTTCATTTGCCATTAGTGGTAGCAACAACTGGCGAGAAAGAAAAACTACTTAATAAGTT
AGATGAATTGAGTCAAAATATGCCTGTATTTTTCAGCGCGAACATGAGTTATGGCGTTTATGCATTGACTAAA
ATTTTAGCAGCTGCTGTTCCCCTACTTGATGATTTCGATATCGAATTGACTGAGGCACATCATAATAAAAAAG
TAGATGCACCAAGTGGTACGTTAGAAAAATTATATGACGTGATTGTATCTTTGAAAGAAAATGTAACACCTGT
GTATGATAGACATGAATTAAATGAAAAACGCCAGCCACAAGATATTGGTATACATTCTATTCGTGGAGGTACG
ATTGTCGGTGAACATGAAGTTCTATTTGCCGGCACTGATGAAACGATTCAAATCACGCATCGTGCACAATCAA
AAGATATTTTTGCGAATGGTGCAATACAAGCAGCAGAACGCTTAGTTAATAAACCAAACGGCTTATATACGTT
TGACAACCTATAAACATATTAAAGGAGATCGATTATTTTATGGTACAACATTTAACAGCTGAAGAAATTATTC
AATATATAAGTGATGCTAAAAAATCTACACCAATTAAAGTATATTTAAATGGGAATTTTGAAGGCATCACATA
TCCAGAAAGTTTTAAAGTATTTGGTTCAGAACAATCAAAAGTAATCTTTTGTGAAGCAGATGATTGGAAACCT
TTTTACGAAGCATATGGTAGTCAATTCGAAGATATAGAAATTGAAATGGATCGTCGCAATTCTGCCATTCCAT
TAAAAGACTTAACAAATACGAATGCACGAATTGAACCAGGTGCGTTTATTAGAGAACAAGCCATTATTGAAGA
TGGTGCTGTCGTTATGATGGGCGCAACAATTAATATTGGCGCAGTCGTTGGCGAAGGTACAATGATTGATATG
AATGCTACTCTCGGTGGTCGTGCTACAACTGGTAAAAATGTACATGTAGGGGCTGGCGCAGTATTAGCAGGTG
TGATTGAACCCCCTAGTGCTTCACCAGTTATAATCGAGGATGATGTTTTAATCGGTGCAAATGCAGTTATTTT
AGAAGGTGTACGTGTTGGTAAAGGTGCTATTGTTGCAGCTGGTGCGATTGTGACACAAGATGTACCAGCTGGT
GCAGTTGTTGCTGGTACACCTGCAAAAGTGATTAAGCAAGCTTCTGAAGTACAAGATACTAAAAAAGAAATTG
TAGCAGCATTAAGAAAACTAAATGACTAGTGACGTCAACGTATAATAATTTCAAGGTTGAGATACGATTATGT
CTCAACCTTATTGTTTATCATTGAATAAACTTATGATCACAAATTTCGAAATACGTTACACTATTTATATAGT
AATTTAACACAAAGGATTGATAATTATGAATGAATTAGAATTTGTTACGAAACATCGCCGTCATTTACATCAA
CATCCTGAATTAAGCTTACATGAATTTGAAACAACTGCTTATATTAAAGCGTTTTTAGATAGTTTAAATATTA
AATACGATTGCCCATTGGAAACTGGCGTCATTGCATACTTAGAAGGTAATGGCTCACATACGATAGCGTATAG
AGCTGATATTGATGCGTTACCTATTTTAGAGGAAAATGATGTGCCTTATCGCAGTCAATCTGATCATGTGATG
CATGCTTGTGGACATGATGGTCATACAACTGCATTAATGCTTTTTGTACAACGTTGCAAAGACATGCAAGATG
CAAGTCAATTACCGCAAAATGTCGTTTTCATTTTCCAACCTGCAGAAGAAACTGGTGGCGGTGCAAATCGATT
AATAAAAGCCGGTGCCTTTGATAAGTATCCAATTGAAGCGGTATTTGGTATTCATGTTAACCCATTTGCTGAT
GAAGGCATTGCAGTGATAAGAGATGAAGAAATTACGGCCAGCGCAACAGAGTATCGCTTTTTCTTAACAGGCC
TGTCAAGTCATGTTGCTGATAAAGAACAAGGTCATTCTTGTGGTGAAGCATTACAACATGTATTAACTCAAAT
ATCACAAATTCAACAATTTCACCTTAACGGTTTGAAACGAAATATTGTTCATATTGGTCATTTTAAAGCTGGT
GAAGCGATTAACACTGTACCAAGTAATGGTTATTTAGAAGGTACTATTCGTACATATGATATTGATGATTTAA
CAATCGTTAAAAATCAAATGCACAAGATAGCAGAAAGTGTCAAGCTTCTGTTTAATGTAGATTGTGAAGTTAA
ATTTGCAGAAGGTTATCCCCCTACAATCAATAGTCCGAAATTACGTACTCAAATAGAGGACGCCTTAATAAAA
GCTGATTTAAATGTCTATGACAAACCAACGCCATTCTTATTTGGGGAAGATTTTAGTTTTTATGGTCAACAAC
TAGCTCCAGCTTACTTTGTTTTTATAGGAACACGAAATGAAGATAAAGGTTTTGTAACTGGTTTGCACACATC
ACATTTAAATTTTGATGAAAAAGTGTTAATAAACGTGGTTAATTTTTACGAAAATTTATTAAATAATTACAAA

GAGGTGTAATACATTGACAGCAACATGGTCTGTAAATAAGAAAATATTTTTACAAAATGCAATCACAGTCAAA
AACAATCAGCCATTAATGGCAGTTGTTAAAAATAATGCATATCACTATGACCTAGAATTTGCTGTAACTCAGT
TTATCCATGCAGGTATAGATACATTTAGCACAACATCACTACGAGAAGCAATTCAAATTAGACAACTTGCTCC
AGATGCAACAATCTTTTTAATGAATGCAGTTTACGAGTTTGATTTAGTCCGTGAACATCAAATACACATGACT
TTGCCGTCGTTGACATATTACTATAAGCATAAAAATGATTTAGCAGGTATTCATGTTCACTTAGAATTTGAAA
ATTTATTACATCGGTCTGGATTTAAAGATTTAAACGAAATTAAAGAAGTATTGAAAGATCACCATCATAATCA
AAACGCAAAAATGATTATTAGTGGTTTATGGACCCATTTTGGATATGCTGATGAATTCGATGTGTCAGATTAT
AATGTTGAACGTTCACAATGGATGGAAATTGTTGAAGCACTTTTATCTGAAGGTTATCAGTTCGACCTAATCC
ATGCTCAAAATAGTGCGAGTTTTTATCGGGAAGGACAAATATTACTACCCCACCATACACATGCGCGTGTAGG
TATTGCGTTATACGGTTCAAGACCATATAGTTCACTGAATCAACATGATATAGTTCAGTCATTAACTGTAAAA
GCACATGTTATTCAAGTGCGCGAAGTACAAGCTGGTGATTATTGCGGTTATAGCTTTGCCTTTGAAGTGACTA
AAAACAATACAAAATTAGCTGTAGTTGATATCGGTTATGGCGATGGAATTTTAAGAACTCGTGCTAAACATGA
AGCACTTATCAATGGTAAACGCTACCCGATACGTGCATTAATGATGAGCCATATGTTTGTTGAAGTAGATGGC
AATGTACATGCACAAGATGAAGTTATTCTTTATAATAATGATATCCGCATCGATGAATATACCTTTAAAGGTG
TTGGTGCAAATTCTGAACAATTAAGTGCTATGAATCATGATTCTTTAAAAAAGGAGTACATTTCAAATGACTG
TTAAATATAATCAAAATGGCGAATTAACAATGGATGGTATTAGTTTAAAAACGATTGCGCAAAGCTTTGGTAC
ACCTACCATTGTTTATGATGAACTACAAATTAGAGAACAGATGCGCCGTTACCATCGCGCATTTAAAGATAGT
GGATTAAAATACAATATTTCATACGCCTCAAAGGCATTTACTTGCATTCAAATGGTCAAACTTGTAGCTGAGG
AAGATTTACAGTTAGATGTTGTTTCTGAAGGTGAATTATATACAGCTTTAGAAGCAGGTTTTGAACCGAATCG
CATCCATTTCCATGGTAACAATAAAACGAAACATGAAATTAGGTATGCTTTAGAAAATAACATCGGTTATTTT
GTTATAGATTCATTAGAAGAAATTGAATTAATAGACCGCTATGCTAATGATACGGTTCAAGTTGTATTACGAG
TTAATCCAGGTGTTGAAGCACATACACACGAATTTATTCAAACTGGTCAAGAAGATAGTAAGTTTGGGTTATC
AATTCAATATGGCTTAGCTAAAAAAGCAATTGACAAAGTCCAACAATCTAAACACTTAAAATTAAAAGGTGTA
CATTGTCATATTGGTTCACAGATTGAAGGTACAGAAGCATTTATTGAAACTGCTAAAATTGTTTTACGTTGGC
TTAAAGAGCAAGGCATTCAAGTTGAATTATTAAACCTTGGTGGTGGCTTTGGTATTAAATATGTTGAAGGTGA
TGAAAGTTTCCCTATCGAAAGTGGTATTAAAGATATTACAGACGCAATAAAATCCGAAATTAAAGTTCTAGGT
ATAGATGCACCAGAAATAGGTATTGAACCAGGACGATCTATTGTAGGTGAAGCTGGCGTTACTTTATATGAAG
TTGGAACCATTAAAGAAATTCCAGGGATTAATAAATATGTTTCTATCGATGGCGGTATGAGTGATCATATCAG
AACTGCACTTTATGACGCAAAGTATCAAGCATTGCTTGTTAATAGAAATGAAAAAGCAGATGACAGTGTAACT
ATAGCTGGAAAATTATGCGAGTCTGGTGATATCATTATTAAAGACGCTAAATTACCTTCATCAGTCAAACGTG
GAGACTATCTTGCTATATTATCAACTGGTGCATATCATTACTCTATGGCATCCAATTACAATCAAATGCAAAA
GCCTTCTGTGTTTTTCTTAAAAGATGGCAAAGCACGTGAAGTTATAAAGCGACAATCGTTAAGACAACTCATT
ATTAATGATATAAAATAAAAATAAACAAAGTAATCCCCGAAGCACAGAAAATTATACTGTGACTCGGGGAATT
TTTATGCATTATAGGATGACTGTAGATAACATGCTTGGACATATACTCTAAAAAAGGAAGAACAGCACTTTTT
TAGTTAGAAAGTATTTATTTCTTTTTGTCATTAATCCTTTATATTTAGTTAGCCCTATTCAACTAATTTAAAG
CATCATTTACATACCACACTTTAAACAAGCATCATGAAATTAGTTTTTATTCTTTACTTGCTTTTTCAAATAA
TATAAAACGATAAGAATTAACAACAAATATAATGTATAAAACAGAATTTCATCAAATCCAAAATCTTTACTTG
TAATTAATACATCAATATGTTCTAATTCCATCATCTCTGTTTTGATATAATTATTAAACTTTGGCAAAGTCAT
TAAATGTACATTGTAAATATAATAATAAAAGTCTATGAATATGATGTTGAAAAATATGTATGTTTTATCAATA
TTAGTTTTGAAAAATAATATCCCAGAAATCATTATCGGAATCACTAATACAATCATCATTGCTGCTAAAATAT
TAATATTTAAACCAATTGCAGATAATACACCGAAAACTAATAAATTAGCAACTAAACTAAGGATAAGATATTT
CATAAAATCACCTCGTCGTTGTACACCATAATTATATTATATAAATATAATTTTCTATTGCAAACAATGTTGG
TGATATAAAAAAAGAGTTCTCAATTTGGTTGAGAAACTCATCGCGTTAAGTATTTGTAATAAGCATATAAAAA
CACCTTACTTCTTGGTAAGGTGTTAGTTAAAATCAATGATTCAAATCTAAGAATTATAGTTTAACAACGTTTG
CGGCTTGTGGACCGCGGTCGCCTTCAACTACTTCAAACTCAACAGCTTGACCTTCTTCTAATGATTTGTAACC
ATCTTGGTTAATTGCTGAAAAATGTACGAATACGTCATTTTCTCCTTCAACTTCGATAAAGCCGAATCCTTTT
TCAGCGTTAAACCATTTAACTGTACCTTGTTTCATAATCTGAAACCTCCAAGACTAAAATTCATTCAATATGC
TTATTCGCATATTACAAAAAATACATGCAAATTTCACTCAAATATTCTTTGCAATATGGAATAATCATCTGCT
TAACTTGTATTATAGTGCAGTTATTACCAAATTGCAATACAATAATAAAAAATATTCATTTAATCAATTATTG
GTAGTTTATAATATACTTCATTATTTTGTAAAACAATGAAACTATAAAATATTTGTAAATCCTCTTCACAATC
TTCGCAATAGTTCATGTCACAATTATTATAAAATGCATAAATATTATATTTGCCTGTGACATAATTATCGAAC
TGAACTTTACATTGATTAATCAATTGTTGATAGTGCTTAAACATGTCATCAAAATCTTGATATTGATATTTTT
CAACAATGTTTTCTGGCCAGTCACTGAATAACCACCATCCCTCATAATCGGCTCTAATTTTGGTAACTGTCCA
CATTATTAAAGACCCCTTCCATACTTCAAAAACTCTATACTTATTTTTATACCTTTATCCTCTGAAAATCAAG
TTTGACGACTTGAAACAAACAAAGTAGTAACTATAGTAATTATTAGTTTTTTTAAGTAAAACTACGTATAATA
ACATTATGAGGTGGTAAAAATGAGACATATACATTTACAAGTATTCGGACGCGTTCAAGGCGTCGGATTTAGA
TATTTTACACAACGCATTGCAATGAACTATAACATTGTCGGTACTGTTCAAAATGTAGATGACTATGTAGAGA
TATATGCACAAGGGGATGACGCAGATATAGAGAGATTTATTCAAGGTGTAATTGAAGGTGCCTCACCAGCATC

AAATGTAACAAGCCATCAACTTGAAGAGTTAGAACTAAATCAAAAATTATCGGATTTTCGATCAATATAAAAT
GCTAAATAAAGGAAGATGACAGTGAGATATAATATTTCTCATATATTTGGGGTGTTAGTGGGAATTCCTGTAG
CGTTTTTAACAAGCATATTTGGGATGATTGCACTTGATGTATCTTTTTTAATTGATATGTCTATTGGTATTGT
TGGCTTTTTAATGACATACCTACCGATACAAAAACTCACTTCACGCAAATATTTAAACGAAATTGGTTTGACT
AGAAAAGACTATCGCTATATTCGAAATCAGTTAAATCATACACATCAAAAACTTAGAGGTATTTTAAAAACGT
ATGTCAATATAAGATCAATTAAAGATTTTAGGCAGATTAATGATATATACCAAATTTCACGTTCTATTTATAC
GACAGTTAGACAGAGACCTGCATCATTTTATAAAGTTGAAGGCTTTTTTTATTCTCATATTGATAATGCTTTA
AATTTGGTTGATGCATATACACGTCTAGCAAAAATGCCCAAAAAATCAATTAATGAACAGCAAAAGTTAGAAC
AAACACGAATTACTTTGGATGAGGTCAAACGAACATTAATCGCTGATTTAAAGCGTCTCAACGAAGATGATTA
TGAACGTTTAGATATTGAAATGGAATTAAATAAGTTACATCAAAAACATCATCAAGATTGATGTAAAATAGCA
ACTATTGGAGAGATGATTGATGACTGAAAATAAAAGTTTCAAAGAAAGTCATCCACTAGATGATTTTATAAGC
GATAAAGAATTATCGAATAATACTATTCAAAAAGAAAAATTAACAATTGAACAACAAAAACAGGTAGACACAA
TCAGTAAACAAATTAACCCTTTAGACAATGAAGGTTTATTGGCGTTTGGTTCTGATTTACAGAAACAAATGTC
TCAATTTTCACATCAAATGTTGGATGAAGTACAAAGTAAAGATGTTGGTCCTATTGGAGATACTTTGTCAGAT
TTAATGTCAAAACTAAAGTCAGTTAATCCAAATGAGTTAAATACTGATAAACCATCTATGTTAAAAAGAATTT
TTAGCAGAGCAAAGTCGTCTATCAATGAAATCTTTTCAAGAATGCAATCAGTTAGTGCTCAAGTCGATCGCAT
AACAATTCAACTGCAGAAACATCAAACGCATTTAACAAGAGATATTGAATTATTAGATACGCTATATGATAAA
AACAAACAATACTTTGATGACTTATCATTGCATATCATTGCTGCACAGCAAAAAAAGTTGCAATTAGAAAATG
AAAAGCTACCACAATTGCAACAGCAAGCGCAGCAATCCACTAATCAAATGGATATTCAACAAGTTGCAGATAT
GCAGCAATTTATAGATAGACTAGATAAACGCATATATGACTTACAGCTTTCAAGACAAATAGCTTTGCAAACT
GCGCCACAAATTCGTATGATTCAAAATGTTAATCAAGCACTTGCCGAGAAGATACAAAGTTCAATTTTGACAA
GTATTCCACTATGGAAAAATCAAATGGCCATTGCGCTTACATTAATGAGACAGCGTAATGCAGTTGCTACACA
ACGAGCTGTCACTGATACAACTAATGATTTATTAACAGCAAATGCTGAAATGTTGAAACAAAATGCGATTGAA
ACTGCAACAGAAAATGAGCGTGGCATTGTTGATCTTGATACATTGAAACGTACACAGCGTAACATTATTGAGA
CAATTGAAGAAACATTAATTATTCAACAACACGGTCGCGAAGAACGACAATTAGCTGAAAAAGAATTACAACA
ATTAGAACAAGATTTAAAGTCACATTTAGTGAACATCAAAGGACCGAATAAACAATCATAAATTACGAATTAG
ATATAACAAAAAGGAAGCATCCTACGATTAAATTGGAGATGCTTCCTTTTATATTATTCGTTATTCCTGTTGA
TATTTAATTGGATCTTGTTTTACAAATATGCCGACTAGATAGCCTAATATCGTTGCAATAATTGCTACTGGGA
ACCACTCTAAAGAATACGCTCTTAGAGGCAATGATTCTATAAAGTTAATTTTCAGCCAACCTAACTTACTAAT
AACACTGAAAATCGACAATATAAATACGATAATAACTGGAATTTGTTGTGTAATGCGTTTTGTCGGTATGAAT
TGGGCAATTAAAATTAATAATACAACAGTTATTGCTACTGGATATACAATGCTTAATACAGGAATTGACATTG
AGATAACGGCATTTAAACCTTGGTTAGCAATAATAAAACTCATTAAAATGAAAACTAATACAAATGCTTTGTA
TGATACTTTAGGTACGATTCTATGGAAATATTCAGAAACTGCAACAATAAGCCCACATGCTGTAGTTAGACAT
GCCAGCGCCACAATGATGCCCAATAAATATTTTCCGAATGAACCAAATCCTGTTGAAGCCATTGTCGTTAATA
AATATGTCCCAATGTTTCGATCTTTGGATTTCAATTGATCTAACGTCATGTCACTTACTGGCATATGATTACC
AATATAACCTAATGAAATATATATGAAAATTAAAGCTACGGCTGCAATTAAACCAGCAGTCAAAGTTTGTTTG
AATATTTGATTTGTTTTAGTAATACCAGTTAGTTTTACTGCATTAACAACAATCATTGAAAAAGCAATTGCTG
CAATGGCATCCATTGTTAAATAGCCTTGTGTAAAGCCTTCAGCAAAACTTGAAAAATTAGAATGATATAGTGC
TTCATTGCCCTTTCCAGCACTATTACCGCTAAAGTCTAAGTATCCTTTAATAATCATCGCTAAAATAGTAATC
AATAATAATGGTGTTAATAATGAACCAATACGATCGATTAACTTAGATGGATTTAAACAAATATACAAAACGA
CTATGAAGTAGATAATCGTAAATATAAATAAAGCGATACTACTATTGCTATGTATAATTGGTGTAATTGTCAT
TTCAAAAGATGTAGATGCAGTTCTAGGTATTGCGAAAAGTGGTCCTATAGTCAAATAAATGATGATTAAGAAC
AATATTGAAAATTTAGGTGAAATTTTATTTAATGCGCCAATATATCCTTCTTTATCAAGTGCACCTACAATCA
CACCTAATAATGGTAAACCAATCCCAGTTAGAACAAACGCTAAAATGGCGGGCCAGAAAAAATTGACCACTATC
CAATCCGAGATTAGGAGGAAAAATTAGATTGCCTGCGCCAAAAAACATAGCGAAGAGCGTAAACCCAATGACC
CATGTATTTTTATTCATAGAGTTACTCCTTTTTTAAAACACGAAATAATTTACAAGTAAATATTTTAGCACAA
AAAATAATAAAGAGTCTATATGAAATATGTTGTACATAGCTGAAACAAAACACATGTGTTGTTTAAATCAAAT
TATGTCAGAAAATAAAATTCTTCAAATCCACTTTTTAAATAGTAACTGATGACTTTTATTCACAATTTCATAT
AAACTCATATTTTAAATTTATTTATAAAGATTTAAGTAGTAATTTTTTTAATAATGGAGAGAGATGTCCAGGT
AAATGCGCAACACCTTCAACGAAAATAGCATATTGACCATAAATATTATGAATTGTTTGTTCAACATCTTCAG
TTATTGGATCTTGACTCAAAAATACATTAAATACTTCAATACCAAATTTACGTGACATTTCTACAGCTTCATA
CGTATCAATAATACCATCTTGACTATAATTAAATGCAGACGGTTCGCCGTCTGAAAATACGATTAAAAATCGT
TGATGTTGATTTCGACGCATTAATCGTTCACTTGCAACTCTAATAGCAACACCATCACGATTATCATCTTGAG
GTTCAAGTGCCATAATACGTGGGCCATCTTTTTCAAAGGTTGAGTAATCATAGTTAATAATTTCATTAATGAT
ATTTGGTTGTGCATGCTCGTCTGAATCAAAGGCATCCTCACTGAATGATAAAATTTCATGTTTAATGTTCAAT
GCTTTTAACGTCTCGTGGAATAACACTACACCTTTCTTCGTTTCAGCCATTTTATCATGCATACTTGCTGACG
CATCAATTAACAATGTAAATGTCGCATCAAATGATTTACTTAAATCTTGTTTTTTGTAAAACAATTTATATTG
ATCGTCGATAAACCAATTAATTAAATCCTTTTGTAATCGCCCTTTTGTTAAATTAAAACGTGCATCTCGTTGT

TCTCGTTCAATCGTTTTCTTTATAATTTGAATTAAGTCTTTGATTTCATATTGTACGTCTTGTTTCGATTCTT
GATATTCTAAAACATATTGTGGCTCAATTTCAGGAATTTGCCATTTTATTTCTACATTTTTGTTCACACCATC
AAGTTGAAATGCTTGGCTTTGACCAACAGCATCGCCTTCTTCACGATTTAGCGTATCGTTTGATCCTTTGCCC
TTTTTGGTCATCATATCAGTCATATCATCTGTAGCATCGCCTTCACGTGCTTCATCATTACCTAATGTTTCAC
TATTTTGTCCTTCGTGAAGCTCCATTTCTAAATAGGCGCCACCTTTTGACTCACTATCTGCAGATTTAGAGTC
AGCTTTTTCACTTTCGTTATCATCATCTTCTGAAGTATCATCTTGCCCATAGACTTGACTTGCATCAGTTCTC
TTTAAATCATCAAACTCTGGACTAGCTAATGTATTATATAGTGTTTTAGGTAAGTAATAATATTCGTTTAGCA
TATCTTCTTTTAAAATATCATCTACTTGATACATAATTCTTTGAGCCAGATACATATTATCTTCAGAATTTTG
ATTTTGGAAAAAATTAGGTAAATATAAGAACATATTTACTAAAATATCATCTAAATCTGAATGTATAGATGGT
ATATCAAAGAAATCTTGACTTAAAAATGCATGTTCTAAATACAAAAATAATAAATCTGTATATTGTGTTTTAG
TACGATACACTTTAATTTGAGATTCCGTATATGATACCCGTGTATCTAAGCGAAGATCAATTAATTTAGCAGT
ACTTGGGCGCTCAACTTTAATAGAATTTAATACACGCATATCTTCTAACAATTTAAAAAGTTGTTGATAAAAT
TTAGGGTGTTTAAAAGTTTTATCTTGTACTACTTCATTTACAATTTGTACATCCATCATATGATAACCGTAAG
CAGCTAACATAACATCTGTTTTTAAACCAGCCATTTCGATATGGCTTGGTCGATGTGACCAAAACCAACTCGT
AATCAAAACATTTTGAACAGGATTATAGTAAGGGAATTTTTGGATTTTAACTTGTGTTTGTTCATTTTTTAAT
AAGAGTCGAGCTAAGTCTTGCAACATCATTACTTGCTTTGCATCTAACTGTTCGTCGTTAAATTTTATGAAAC
GATCACTCATGTTTTATCCCTCTTTAAAAGTTTAGTTCTACAGCATTATATATTGCTTGTTGTTCACGTTCAT
CTTCCAATTTATCAATTATTGTACGTTTAATTGCACGTTCAACTGGCATTACAGTGATTAAATCACACAAGTC
TAATAATGCACGGATACTAGCGGCTTCTTCAGAAATTTGTCCCTGCTTAGACATAGTACGTAAATCTTCGTTA
AACTTAATAATTTGTTCGATTTGTTTATCATCTTGTAATAAACTTTGCTCTTTAATCACATTTTTTAAAATGT
CCCCATCAATATAGTCAACGTGAATAACAACAAAGCGATTTTTTAGTGCTTCATTCATTGGCAATGTACCAAC
ATAACCTTCATTTATCGCTGCTATAACGTTAAATCCTGGTACAGCTTTGATTACTTCACCAGTGTATGGATTC
GTAATTTGACGACGATAATCTAAGACCCCATTTAATACAGGCAATGTTTCAGGTTTAGCCATATTTATTTCAT
CAATATATAAAATATGCCCCTCTTTCATAGCTTTAATAACTGGACCATCTACAAAGACAATTTCTTGTTGTCC
TTCCGCATTTGTTTTAATTGTTTTAAAGCCTAATAAGCTTTCTGTATCTAAATCAACAGAACAGTTGACTTGA
TGCATGGGTGTATCAACAACTTCACTTAATGTTTCTGCCAACTTCGTTTTCCCTGAACCCGTTGGACCTTTAA
GTAAAATATTTTTATTTAAATCAAATAATGCCTTCGCATCATTGAAAACTGTTGAATCTGAATTCTTATAATG
TTTTAGTGCCATTTTAAAACGTTCTCCTTTAAATAAATTAAAAAACTAAGACATGGAATAAGTTCTCCAAATG
CCTTAGTTTACTATTCTATTCTGATATAAAACGCAATGCTTAGTATGCGAATTGTTTATATTTTACCAAATTT
ACTAATTGAAAATCTCATATTAGGCTTCATTTCAATAATTATGCGTTTAAACTTCTTTGAAATATGTTTTATA
GTAACCGCCAACTAATCCAGAATTATCTATTATTTGATAATATTCTTCAGTTTCGTTGATAACATCATATTGT
TTGCCAACCGTTAACATGTCAGAAACTGTAAATTTCTTTGCATCTGTATTAATGACTTCTACTTTCTTGATTG
GTGTACGTTCTTTCCAAGTTTCATGTAACATAAATTTTGTCCTCACTATCGTAAAAAAATTATTTTTGTAAAG
TGAATATAAACGATAATCCACTGTTTTCAGTAATTCTAATTGATTCAATATCATCTTGCTCAAGCGTTTGATA
TGGTATTTCCACTTCATCTAAGCGCGATTTCGCTTGATTAAAGAAATCTGCATCTTTCGTTTCAAACTCAACT
TGATCGACTGCGCCATATTCCGGCATTGTCATTTCGGTTTCTACAGGCATTAAATGTATTTCGCCACCTAGAC
CACCCGTTCCAACTTTGAATACTTGGACATGATAGTCAGCATTGTCGAAGGGTTGGTATTCTGCAAATACTTC
AAGTCCGAATATATTTGTTAAAATTTGACCTGTAATATCTACATGATTCACTTTAAGAATCACTGGTCCTAAA
CCTTGCACTTGATGTAACGGATTGACCGCACTCTCAAAAGAAGGCATACCTAAACCAACGCCATAATTATTCT
CGTTTGAATATATTGAGAAAATATGACCATTGTTATCTTCTAGACTGAAATATTTATTGCCATTTAATTCTTT
AACTGTTGTAAATGGAATATCCTTATTCGATAATATTTCCGCATACTCCTCTAAACCTGAGTCAGTTGGTGTA
CGTAATCCAATACCCACAAAGTGAGATTCTTCTAATTGCTCACTTGGAACTTGTATAAATTGAAGTCTTGTTC
CTGGGCTTATATCTGCATCGCCGAAACGAATTGAGTTTTCAAGAAGTTCTTCATAATTTAATCCTAATATGTC
AACCATGAAATGTTTTGTCTGTTCTATATTTGTTGTACCTAATGTTATACTTCTAAGTCCACACATTATAGCC
ACCTCTATCTTTATCTTTACTATTATATTATAACTTATTTTAATCATAAATTATAGAATATCGGTCAGTTGAT
TTTGATTATAGATTAATATGTGAAAAATGTATTTCTTTAAAAGAACTATTTCATTTAAATAGGTAACATAATA
TTGTTATGGTTGCTATTGAGTATGTCATTAATGAGTATCTGTATATAGCAATTGAATATTTCGTTTTATGTAG
TACATAAAAAACATATACATGTCGTATCCATAAAGTGAATTTACATCTTGTTCAGATGATTTTAAATTAACTA
AAGGCGCTTATAGAATTCATAAATTTCATCGCTTGTTATTATCTAAAATGATTTCATTAGCATATTGAAACAC
GCCATTCCACCTATATTTCTATTGTGAGTCTATTTCAATAAAAATGATATATTTTTTAAGTTGTCAAAATTAA
ACTCACATTTTAATACGAAGCATTATAAAAATAAAGTACAGCAGTAAGATATTTCGACTAGAAAATAATCTTA
CTGCTGTTTGTTATTGATTTGATTACGTTTTTAGGAATAGTAAACTATTTTGTGTTGTGGATTAAGATTCTAA
TAATAAGTCTTCTGGGTTTTCAATTAATTCTTTAATTGTTTTTAAGAATCCAACTGCTTCTTTACCGTCAATA
ATTCTATGATCATAGCTTAATGCAATATACATCATTGGACGATTTTCGATTGTATCTTGATCAATCGCAATTG
GTCTTGTAATAATTGAATGCATGCCTAAGATTGCAGCTTGATTACCATTGATAATTGGCGTACTCATCATTGA
TCCAAAAATACCGCCATTTGTAATCGTAAATGAACCATTAACCATATCATCTAAGCCAAGTTTTTTCTCTCGT
GCTTTAACAGCTAAATTAGCAATTTCTGCTTCGATTTCTGCAAAATTCTTTTTATCACAATCTCTTACAAATG
GTACTAATAATCCATCATCTGTAGAAACAGCTACACCAATATCATAATATTGTTTCGTAATCATGTCGTCGCC

GTCGATTTCTGCATTAACTTCTGGATACTTTTTCAAAGCTGCTACTGAAGCTTTAGTAAAGAATGACATAAAT
CCTAATTTAGTACCATCATGATCTTTCATAAATTGTTCTTTCTTACGTTTACGCAATTCCATAACATTTGTCA
TGTCAACTTCGTTAAATGTTGTTAACATAGCTGTATTATTAGATACCTCTAATAATTTTTTGGCAGCTGTTTT
CTTTCTGCGTGACATTTTTTCACGAATCACTGGTTTTGTAGGATATTGATTGTATTTTTTCTCTTCTTTTGCA
GGTGCTTGTTGTGTTGTTTGTGTTGATGCCGGTGCCTGTTGTTTCTTATCAATATCTTCTTTACGAACCACAT
CATTTGTTTTTGGACTTACTTCAGCAAGATTGACACCATTTTCACGAGCATATCGACGCGCAGAAGGCGTAGC
ATTAATACGTTGCTGATTGTCATCATTTGCTTGATTGACTTCAGCTTTATCTACCGAATTATTTGTTGTTTCT
TCTTTTTTATTATTTGTTTCTTCATTTTGTTGTGGAGTATTGTCGTTACTATTTTCTTTAGAAGCATTGCCAC
TACCTTCGCCGATGATAGCAATTGCTTGCCCAACTTCTACAGTGTCGCCTTCACTTGCAAGTTGTTCAGATAA
TACACCTGCTTCTTCAGATACAACTTCGACATTAACTTTATCAGTTTCTAATTCAAGAATAGCTTCACCTTTT
TCTACGCTATCCCCTACGTTTTTCAACCATTCTGCAATGGTACCTTCTGTAATAGATTCTGCTAATTCTGGAA
CTTTAACCTCTGGCATGACTTATTTCCCCCTAGTTATTTTTAATGCATTTTCTATAATTTTATTTTGAACAA
GTTTATGAATTTCTCCATCGCCTTCAGCTGGAGCAGCCCTTTGAATTCTGCCATGATAACTTAAATCATATTT
ATCTGCAACTAGCACTTTAACATATGGATAGACATATAACCATGCACCTTGATTTTTAGGTTCTTCTTGTACC
CATGACACTTCTTCAAGGTTTGGCAATTGTGCTAGTAATGCTTCAATCTCTTCCTCTGGGAATGGATACAATC
TTTCAATCGCAACGAGTAACACTGATTCGTCTGGATTTTTAGCTAATGCTTCTTTTAAATCAATGAACATTTT
ACCAGTTGCTAAAATAACTTTTGTAACCTTATCCGCTTGATATGATTCTGTCAAAATTGGCTCAAATCCACCA
GAAGTAAATTCATCAATTGGTTTTGCAACTGTTTTATTTCTCAGTAAGCTTTTTGGTGACATAACAACCAATG
GTCGCATTTGTTCAGAATCTAAACTAGCTGCTTGTGCACGCAATAAGTGGAAATAATTACTTGAACTAGATAA
GTTGACAACTGTGCAATTATTTTCAGCAGCTAATTGTAAAAATCGCTCTAATCTTGCTGATGAATGTTCAGGA
CCTTGACCCTCATATGCATGAGGTAAGAATAATGTTAATCCTGAACGTTCTCCCCATTTTGAGCGAGAACTGA
ATAAGAAGTTGTCAAAAATCATTTGTGACATATTTGCAAAATCACCATATTGCGCTTCCCAAATATTGAAGCT
TTTTTTGTTTTCCACATTATAGCCGTATTCAAAACCAACTACTGCTGCTTCTGAAAGCGGAGAATTGTGTATA
TCAAATGTCGCTTTTTGATCAGGAACATGATGTAAAGGTGTATATGTTTCACCTGTTTGCTCATCATGTAACA
CGGCATGCCTATGACTGAATGTACCACGTTCACTATCTTGACCAGTTAAGCGAATCGGTGTACCATCTTGTAA
AATTGTCGCAAATGCAAGTTGTTCTGCTTGTGCCCAATCAACTAAGCCATCCTCTTTATTAAACGGCTCATGA
CGCTTCTCAAGAACTTTGTTTAACTTTTTCAAAATGTTAAAGCCATCCGGATATGTTAACAATGCATCATTTA
TTTCTTTCAAATGATCAAACGTAAATGATTGTTCGTCTGCTTGTAACGGTAATGCAAGTTCTGCAGGTTTTTC
CATATCTGGATTATCCATTTTATCAGCTTTATTAATTTTATCATGTGCTTGTCTTAGTTCCTTTTGGACTTGT
TCTATAAATGAATGCATTTCATCTTCTGAAATGACACCTTCATTAACAAGCTTTTTACCAAACACATATTCAA
CAGAGTCATGTTTGCGAATATTCTGATAAGGAACTGGATTAGTAATTGATGGTTCATCCATTTCGTTATGACC
GAAACGACGATAACCTACTAAATCAATAACGACGTCTTTATGAAACTCTTTTCTAAATTCCATTGCAATATCA
ATTGCTTCAATAGTAGCTTCAACGTCATCAGCATTGACATGGAATATTGGCACATCATAACCTTTGGCCACAT
CTGTAGAATAAGTTGTTGAACGTGCATCAATTGGTTCTGTAGTAAATCCAATTCTATTGTTAGTAATAATATG
CAATGAACCACCCGTAGAATAGCCTTTCAAGTTTCCTAAGTTCATTGTTTCGAAGTTAATTCCTTGACCAGGA
TAAGCAGCATCGCCATGTATAATAATTGGCATTGCTTTATGATGATCAGTCGTCGGAGCCCCAGCACGTTGTG
TATCATCTTGTGCTGCTCTCGTACGCCCCTCAACAACAGGTGCAACAATTTCCAAGTGACTTGGATTGTTAGC
CAGTGCAATACGCTGCATTGTACCGTATGAATCAGTAGTTTTAATGCCACCAAGGTGATATTTCACATCACCA
GTCCATCCAGCAGTTAACTGCAAGCTACCATCTTCAGGTAAGAATTTCATTGGATCTGTATGCATAAATTCTG
AAATCATCATTTCGTACGGTTTTTCTAAGACATGCGTTAAAACGTTTAAACGTCCACGGTGAGCCATGCCTAT
TTGTATATTTTTAATACCTTCTTTCGCAGCAATCGTAATAGTACGTTGTAACATCGGTACGAGTGCGTCTACC
CCTTCAATTGAAAAACGCTTTGCACCAACGAAGTTTTTATGAAGATATTTTTCGAACCCTTCAACATACGCTA
ATTGTTTAAATAGTGCCCTTTTTTCGTTGTTATTTAACGTTACTTTATATGGCGTTTCAATTCTTCTTTTTAA
CCAACCACGTTCGGTATTGTTATTAATATGTGTATACTCAAATGCAATTGGTCCTTTGTAACGTTTTTCCATT
CTTAAAATTGCTTCATAAGCATTATCATAAATGTCGGCAAAGTGATCTGAAACAATTCCTGCTGATATACCTT
CCAAAGTCTGTTGATCTAAATCAAAGTCTTCAATCTCTAATTTAGGTACATGTTTCCTTTTCGGAGGATTTAC
AGGATAAATATCGGCTTTAAGATGCCCGTATTGGCGAATATTATCAATTAAACGCATGACACGTTTAATTGTG
CCGTCGCTATTTTGACTACTTGTACTTTTTAAAGCTGGTACAATTGAGTCATCATTCTTAATTGTGCTGAATA
AGACTTGTAAATCTTCTGGTACAGATGATGGATCTTGTAAAAAGTCATCATATAGATCTAACATTAGACCTAA
ATTCGCACCGAAGTTTACAGGAGCCTCTGAAACTTCTTTTCTTTCGTTAGTCATTTTACACCCTCCACAAAAA
TGTTGAAACGCTTACAATGTAATAATATCACTATTTAGTTATTTGTTAAAGTAGATTTACCTATTTATCTGCT
AAAAAGAAGAATCAACAAAAGCGTTGATTCTTCTAGGTTTATAGATGTATTTTATTGTAAATTTTGGGCGTAC
AACAGAAATGATAAAGAACCACTGTTTATTTGTTTTATAGTCGTTCTTGGGAGTGGGACAGAAATGATATTTT
CGCAAAATTTATTTCGTCGTCCCACCCCAACTTGCATTGCCTGTAGAATTTCTTTTCGAAATTCTCTGTGTTG
GGGCCCATCCCCAACTTGCACATTATTGTAAGCTGACTTTTCGTCAGCTTCTGTGTTGGGCCCCTGACTAGAA
TTGAAAAAAGCTTGTTACAAGCGCATTTTCGTTCAGTCAACTACTGCCAATATAACTTCGTAAAGCATAGAAC
ATTGATTTATGTCCCAGCCTCACTTAAAAAATTGATCCTATGATTTTGATTGACGGCTCAAACATAATTAAAA
TATGATTTTAAACGTTGTTCCTTTGTTAATTTCACTTTTAATTTTAATCGATCCACCGTTTAATTGAATGATT

TTTTGAGCAATAGATAATCCGAGTCCATTACCGCCTTGACTTCTTGAACGAGATTTATCCACTCGATAAAAGC
GATCAAAAATGAAATCTTGATCTTCCTCTGGAATACCAATTCCATGATCTGTAATTTCAATTATTTTTTGCTT
ATTTTTTAACCTTGTCTTAACTTTAATTTTCTTATTCTTCACATCATATTTGATTGCATTATCAATAAAGATT
AAAAATAATTGTTCAAATTGATGAGGCTTCATTTTAATTTCTAAATTTTTAGATGTCAGATCCGTATCAAATT
GATAATCAGGATGTAATTGTTTTAATGAGTGTATTCGCGAGCGAATTTCATCATTAATATGCACGGTCTGCGC
TTCAGAAGAAATGTCATTTACATCTCCTTTAGTCAATTCAAGTAATTCTTCGACTAATTTTATGATACGATTC
ATTTCTTCAATAGAAATATTTAACGATTCTTCTAATACTGCTGGATCTTTTTTTCCCCATCGCTGAATCAAAT
TTAAATGACCTTGAATAATTTGTAATGGTGTTCGTAATTCATGTGATGCATCTTCAACAAATTGTCTTTGTTG
ATTAAATGATTCTTCAATTTGGCTCATCATCTCATTAAACGTATTTGCTAGATTATCTATTTCTTCATAATTT
GTATTTAATTGCAATTTATTTTGAAATCCATCTCGTCGAATCTCAATCATTTTATTTGATAAACTGACAAGCG
GTTTAGTAATTTGAGTTGAAAATACATAACTGATTGTGGCAGTTATAATTGTTGCAATCACTCCAAATGCGAG
CGCAATGATATACAATGATTTTACGATGTTATCATAATTTTCTAGTGAATGAATTAACAAGCTATACCCTTTG
AAATCTTGCGTTGTGATTGGTTCTTTAATAATTAAATATTCAATGCCTTTATAGCGTTTTTTTATTACGCGGT
CAAAATAACGGTGTTCATAACCTGGTTCAACTCTCACTGTGTTATCATTCGATGTCTCAAATAATTTATTATT
ATGTTCATCATAAATAATAATCTCTTGAAAATTACCTAAAGATGCATTCAAGTCTAATGCAGATATATCTTTA
ACAGTCTTAGAATGAAATAAATTATTAATATCGCTTGAGCTTCGTTCTGCATCATCAAGCTCACTATTATGCA
GTGTATCTTTCAAGAAAAAGATAATAATTAAACAAAACAAAAATATCGTGACAAACGTAATCATCGTGGTAAC
AATAATCCAGTTATTGCGCAATTTACGTTTTGTCATCGTATCACATACCCAACGCCACGAACTGTTTCAATCA
TTTTGTCACGATCGTATGGTTTTAATTTGTTTCGTAAATATCTTATATAAACATCTACGACATTTGTTTCTAC
TTCACTATTATAACCCCATACATGATTTAAAATTTGTTCCCGTTGCATAACATGGTTTTTATTTTCAGCTAGA
AGATATAGTAAATCATACTCTGTTTTTGTTAATTCAATTTCTGCGCCATTTACCGTCACTTTAAAAGCATTCT
TATCAATTGTAATACCGTTAACATCGATAATATCCTTTTGTGGCTGACGACGTAAAATCGCACGAATTCTTGC
TAAAAGTTCTTCAATATCAAATGGCTTAACTATATAATCGTCTGCACCGTAATCAAGCCCAGCAACTTTGTCA
TACGTGTCACTTTTCGCTGTAATTATAATGATAGGTGTAGATTGTTGTTGTCTAATTTTGCGACAAATTTCTA
AGCCATTAATTGAAGGCAACATTAAATCTAATATGATTAAATCATAGTAATGGCTAAGCGCTTTATCTAAACC
GTCTTGTCCATCATACTCAGTGTCCACATTGTAATTTTCATGTGTGAGTTCCAATTCAAGAAATCTTGCTAAG
TTTTGTTCATCTTCTACTATTAAAATTTGCGTCATTTGTACACCTCATATTACGACTTTTTCTAATAAGGTAA
TATATTTTAAATTTTGTATTTTTCTATACAATAGTTGCTTTTTAAATTAGTTTGTTTACGTTTAAATTTTATC
ACAAATCAATAACCATGTTTTAAATTTTTCAAGCTACAATCATTAATTATTTATTAACTCAAATATTTAATGA
TCAGTTAATGGAATTCTGTTAATTCTGATTGCAGTACTTAAATATAGAATACTGTAACACAAGGCCTTACCGC
AATATTAATTGTTGTCATATACTGACTTTTCACTATTGTAAAATGAATAGTTATTATCTTTATTTTCACAAAA
TGACCGTAGTTTAAACATTTTCACACATCATTTCAATGTGCGATTTTGAACTTACAAGTAACATTTCTTAATG
TATGAATTCAAAAGGAGTATGTAAACAATATAATTTTTAAACCTCATCACTTTTAAATATTCTAATTCTAATG
AGAAATACAAATTTTTTTATTTTCTAAAATAGCGTAATCATGCGTTTTATTTACTATTCTTAAAAAATATTCA
AAAAAAGTTTTAGTTTCTAATTGACAATGATTCTCACTAATGTATAATAGTAGTTGAAAATGATTATCAATAC
CACATAGAACATCCCCCCCACAACGTTTCGTTCTTGTTGGATTGGTCATTTTCAAATATTCCCCTTTTATATG
CCCGTAAAAGACAATATACGTTATAACAACGTTTTATAAAAGCAGTAAACCCTTACGACACTTTAGGTTTACT
GCTTTTGTTTTTTTACTATTTAATTGATTAATTTATTTCTAAGTAATAACGTTGATAAAATAATGCAAAATAC
GCCACCAATAATACCGGCAATAATATCTGTTGGATAATGCACACCTAGATATACACGTGATATGGAAATCAAT
AAAATCATAGCTGCACATAACCCTATAAGAATACCTTTTGAATTACCTTGATTTAATCGTTTTAATAGATAGA
TACCACTTCCAAAATATGCAGTTGATCCCATAGCATGACCGCTAGGAAAACTAAATCCTGTTATATCAATTAA
ACGCAGCAATGTAGGTCTTTCTCTATCGAATATATTTTTTAGTACTGGATTCAAAATTCCAGATAGTGCCATT
GTTAATGCAAAAAATAATGCTTCAATTTTGTGGCGCTTTAACATGAGATATGCCACAAGAAGTAATGAAATAC
ATAACATTGCCCAGACTTCACCTACTTTAGTAGCTCCAAGCATGATAGATGTCGTAATAAAGCTCTCTGATGA
ATATATAAACTCATAAACTTCATTATCAATCCATTTCCCAAGTCTTGATTCGTGGAAAAACGCGATAATTCCA
AAAACCAATGTAAAAACGATGAGCAAAGAGATACGTTTCCATTGACTCATAAAGCATTACCCCCCATTAATTC
AATGCGTCTTTAATCATCTTATCAAATAAAGCTTCTTTCGTATAACTTTGTTCATACGATTTCATATTATTGA
TAATTCGAGTTCTTTCCTGTTCCATTTCATTTAGTTCTTGTAATAAAATTTGTGCTGTTAATTGTTCTTCATC
AATCGCTTTAGCATAACCTTTATCAGCAAAATGATTTGCATTGTCAATTTGGTCGCCTCGGGATTGATCTAAA
CCTAATGGTACTAATAACATTGGTATACGTAATGTTAAGAACTCATAAATCGCATTTGATCCAGCTCTACTTA
TTACTGTGTCCGTAATTGCTAATAAATCTGTTAATTCCTCTTTAACAAATTCATATTGTATATATCCTGATTT
TTTAACTTGAGCATCTTTTAATCCTTTACCAGTTAAATGTATCACTTGATATTGTTGTAATAATGCATCTAAG
TTTTCGCGAATAATGCTATTTAATTTTTTACTTCCTAAGCTTCCACCCATAACGAGTAACACTTTTTTATTTT
CATTAAAGCCTGTTAATTGATAACCATTATGTGCATTACCATTTTTTAAATCTTCTCGAATTGTTGCTCCAAT
AAAATCAGCTTTCTCTTTAGGTAGGTAGTTTAGCGTTTCTTCAAATGTTGTATATATTTTCTTGGCAAATTTA
AGTGCTATCTTATTCGCTAATCCTGGTGTTAAGTCAGATTCATGAATAATAGTTGGTATATTTAATGATTTGG
CTGCAATAACAACTGGCACAGATACAAATCCACCTTTTGAAAATAATAGATCAGGTTTTTCTTTTTTCAAAAC
TTTACGAGCATCAAGAATACCTTTCAATACTTTAAATACGTCTTTGGCATTTTCTAAAGAAATATATCTTCTT

AATTTACCACTTGAAATAGGATAATACTTAATTTCTGGTAGTTGTGATTCAATCATTTCTCTTTCAATACCAT
TTTTAGAACCAATATAAAGCGCTTCATAACCTTGTGATAATGCAGTTGGAATTAAACTTAAATTTACTGATAC
GTGTCCAACTGTTCCCCCTCCGGTAAATGCGATTTTCTTCATTAATATTACCTCTATTCTTTTAAATTTTTAT
AATATGCATAAAATGGTTCACCTTTATCATATGGCGGATATTCCATTAATTGTTCACCGACCTTATGAAATCC
AAATTTGGCAAATAAACCTTGTGCAGGTTTGTTTAACGCAAAGGTGTCCGTTAAAATAACTTCTGCACCACGT
GCTTTAACTACATCAATAACATAATTGAATAATTCTGTAGCAGCTCCTTTATATTCTTTCGAACCAGTTAATC
GATGAATAACAAAGGCGCCTTCTCTATTTACTGGCCAGTCAATGTCATCATACCATTCTGCTTGGTCTTGGTC
GACAACAATAAAACCATATATTTGTTATTTTCCTCTAATACGTACAAATAATCTTTAGCAATATCTTCTTCA
AAATGTTCTAAAAGTGGGTACTGATCGTCCCATTGCTCGTTGTCGTGTTCTTTCATTAATTCTTTTGCTTCTT
CTACTAGATTTAAGATTTGATCTAAATCTGACATTTTACCTAGACGGATCATGTACGTCACTCCTTATATCGT
TATGTTAAACATGTTTTTCAGTACATGTTAATCTTACACACTATTAACATGCTTTAAACCTTTATTATGAATG
AAAAATAGTAATTATCACACTATGGGATAAAAATAACCAATAAAAGTATCATATACCAATTAATCGATTGTTA
AAACTAGTGAATAATACTATTTAAAAAGTATATGCTCATACTATATTAAAGCATGAATTGTTAACAGCGATGA
TAGAAATTGCGTTATTTACATCATTTGTAAATGTCTAATTTATATCTTTTAGAGTTAGACGTCAATAAAAATA
AACACGCCTCCTAAATTGCTATCTTTGCAAATTTAGGAGGCGTGTCATTAACATAATAAAACAATTTTAGCAG
TGTGTATCACTTATTTTAAAATATTAATCAATTTATCGAGAACATCATCATGTTTATTAGCTTTTTCAACTAA
TAACTCAGTAAATTTATTATTCGTTTCTTTATTAAATTCACCAGTTACCTCGAGTTTATTCGTTTGTTGGAAA
GCTTTAACTTGATTTTCTAAAGCTTGATCAAATTGCGTTGATTCATTATCAACTTTATAACCTAAAGCTGATA
AACCAATTTTAATAGTTTTAATATTTTTATCATCGTCTCCAACTTTAAATGTTTTCGTATTAGGAATGACATT
TAAAGATTGATATTTAGGTGTGTCAATAGTAACGTCTGGTTTAATGCCTTTACCGTGAATATAATGACCATCT
GGCGTTAACCATTTCATTTCAGTATATTTTAACAATGAACCGTCCTTAAACTCTCTTGTAGTTTGTACGACAC
CTTTACCGAATGTTTTTGACCCATAAACTTTAGCTTTATTATAGTCCTTTAGCGCACCAGTAAATACTTCAGA
AGCGCTAGCTGAACCTTCATTCACTAAGATGGATATATCCATGTCTTTCGCTTCTTTTAACGAGTCATTAGAA
GTTTGAATTGCTTCAGTATCTTTACCTTTTTCTAGTTTAACAACAGTTTTTCCTTTATCGATAAAAATATTTG
CCATTTTAACAGCTTCATCTAGTAGTCCACCAGGATTATTTCTTAAATCTAAAACAATCTTTTTCAAACCATC
TTTGTGAGCTTTAAGAACTGCATCTTTCAATTCACCTGATGTATCATTCTGGAATTTATTAATAGTAATAACT
CCAACCTTATCTTTTTTCTTATACTCAACACTTTTAACATGAATTTTTTCACGTTTAATCTTAACGTCTTTTT
CTTCACTGCCTCGTTGAACAGTTAAAGTTACTTCAGTGTTTTCTTTACCACGAACATCTTTGACAACTTCATC
TAATGCTTTACCTTTAATTGATTTTCCATTTACTTTAGTAATGACATCTTTAGGACGAATGCCAGCACGTTCT
GCTGGAGATCCCTTCATAGGACTAGTAACCATAATTTGATCATTTTTCTTTTGCATTTCTGCACCAATACCTA
CAAAATCACCTGAAACACCTTCATTAAAGGATTTCGTTTGTTCTTTTGTTAAATATTCAGAATAAGGATCTTT
TAATTCTTTGACCATGCCATCAATTGCAGCTTTACTTAACTTGTCAGAGTCCTGTTTTTTGTAATAATCACTA
TTTAAGATTTTATACACATTTTCAATTTTATTTAAGTTTGCTTGATCAGTTTTGTTTAAACCACTTATTTTTT
GATTTATAAAAATATATGCAACAACTGTGATGACAGCTGTTATTAGGATTGTACCAATTAAAATTGATATGAA
TTGCCAACGTTTTAAGTGAACGCGTTTCGATGAATTAGTTTGTTGGTCTTGATTGCTTGTTGCAATTTCAGCG
CGTTCATCATCGGATGAAGATGTGTGTTGCTTATCATCCATTGTATCACTTCCTTATTATGTATTATGTACTT
TTAAATTCTGTGTAGTTAATCATATCAATTTATGCAAGATAGCGCACCATATTTCACTATAATTGATTAATTT
TTAGTCACAACTTAGTTTATTTTAAATTAAAACCAGGATAACAAATTGCATCACCCTGGTTCTAACTATTATT
AAATATTATGTTAAAATTTTAGCCATTCCGTATATTCTTCATATAAATCATCAAATACAGACATTGGCAATGT
GAAATCACCATGTGTCTCAATATAATCAGACAGTATGTTAAAATCATCATCGTGTTTTGGGAAAGCAAGATCG
TCAAATATCTCTTCTGCTAGACGACCTTTATCGTCGTGTCGACCACGAACTGTCATGACAAATTGATAAAACG
AATAGTTTTTCATTAGTTCATTGTCACATCAATCACTTTTGTTTCACCTTTAATCACAGCATTTTCATCATAA
ATATTAATTGAAGCTGCTTGATCAGTGTTTGTAATTATAATTGGTGAAATAACAGATTTAGCGTTATTATTAA
TAAATTCAAGGTTGAATCTTACTAATGGATCTCCGACGTTAACTTCGTCACCACTAGACACTAACACTTCAAA
TCCTTCACCGTCTAATTGAACTGTGTCTAAACCGATATGAACTAATAATTCTAATCCGTTATCTGCTTTTAAC
CCAATCGCATGCTTAGTTGGAAAGACATTGTCAACACGTCCTGCAATTGGAGACACAACTTCTCCTTCAGTTG
GATTAATACCAAAACCTTCGCCCATCATTTTTTGTGCGAATACAGGATCTGGAATATCTTCAATTTTCACGTA
TTCTCCAGTTAATGGTGCATAAATTGCGATATCTTTCTGAACTTCTTTGCCTTTTCCGAATAATTTTTTAAAC
ATACTTTCCACTCCTACTTATCAAAATGTGATATTAAATCGCCATAACCCAATTCTTCTAATTTTTCATATGG
AATAAATTGAATTGCAGCGGAATTGATACAGTATCTTAAGCCGCCACTTTCTTTAGGTCCATCATTAAAGACG
TGTCCTAAATGACTATTTGATTCTTCTGAACGGACTTCAGTTCTTACCATACCAAATGATTTGTCGACTAATT
CTATAATTTCATCGTCATCAAGCGCTTTGGAAAAGCTAGGCCATCCGCACTCAGAATGAAACTTTTCTTCAGA
TGTAAATAAAGGTTTACCAGAAATCTTATCTACGTAAATCCCTTTAGCAAAATGATTCCAATATTCATTCATA
AATGGTGGTTCAGTACCATTTTCTTGTGTAACAATATATTCTATATCTGTTAGTTCGCTTTTATCTTTTTTAA
GCATTTTGATTCCCCCAATGTGATTCTATAAACGCTTTTCTACCTGAACCACGTTGATATTGGTAATAATGTA
CCGGGTTCTTTTTGTAATAATCTTGATGGTAGTCTTCAGCTGGATAGAAATTTTTATATGGTTTAATAGGTGT
AATCACTGGTTTCTTGAAAATACCTTGTTCATTTAATTGTTGCTTTTTAAACTCAGCAGCCTTTTTCTGATGC
TCATCATGATAGAAAATGACTGGTTGATAGCTTTCGCCTCTATCGAAAAATTGCCCTTGATCATCGGTTGGGT

CAAATGTTTTGAAATATATGTCTAATATATTTTCAAAGGAAGTAACCTCTGGATCAAACGTAATTTGTACTGC
TTCGACATGGCCGGTTTGATTCGTACATACCTGTTCATAAGTTGGGTTGTCAACATGACCGCCACTATAACCA
GATACGACTGACTTGATGCCTGGATATGATGTAAATGGTTTAACCATGCACCAGAAACATCCTCCTGCTAATG
TTGCATATTCTTTTGTCATATGAATTACCTCCTCTATCTATCTAATTATAAATTTAGTCTATAAAGGATTGAA
ATGGAAGTAATTAACACTGAAAAGATTAAATTTACTTCTTAAGGACTACGAGGCCAATCGCACCTTGACCAGT
ATGTGCAGAAATAACTGGTGTAGTTACATTTATATCGTAATTATTCACATGAAAAGCTTCATTAAAAACTTTC
TTCAATTTATCAACATATTCAATAACGTTAGCATGTGTGACACCAACGGATTTGATTTCATGATCTCCTATAA
ATTCAGCAATTTCCTTTTTCAAGTATTGGATACTAGAATTTTGAGTTCTCGCATTGTGCACAAGCTCTAAGCG
ACCATCATCTAGTGTACCAATTGGTTTGATTTTCATAAGATTACCAATCAAACCTTTTGTTTTACTAATTCTG
CCACCTTTAATTAATTGATTCAATTGCCCTATAACTACAAATAATTTAATGTTTTCTCTTAAATGATTTAACT
TTTTAACTATTTCAGAAGTTGAGACACCTTCTTTTACAAGCTCTACTAGGTGTTGTATTTGATACCCTAAACC
AAAAGAAATAGATTTTGAATCAATAACAGTTACATTAGCATCTACCATTTGACTTGCTTGGTAAGCAGTGTTA
TATGTACCACTTAATCCTGAAGAAAGATGAATACTTATGATTTCGGAGCCATCTTTTCCTAGTTCTTCATAAG
CAGATATAAATTCACCTATGGCTGGCTGACTTGTCTTTACATCTTCATCATTTTCAATATGATTAATAAATTC
TTCTGATGTAATATCTACTTGGTCAACGTATGAAACTCCTTCAATAGTTAAACTTAAAGGAATTACATGAATG
TTGTTTGCTTCTAAATATTCTTTAGATAAATCGGATGTTGAGTCTGTTACTATAATCTGTTTTGTCATGGTCG
TTTTCCCCCTTATTTTTTACGAATTAAATGTAGAAAGGTATGTGGAATTGTATTTTTCTCATCTAGTTTACCT
TCAACTGAAGAGGTAACTTCCCAGTCTTCAAATGTATAAGGTGGGAAGAACGTATCACCACGGAATTTACCTT
CAATAACAGTAATATACATGTCGTCCACTTTATCAATCATTTCTTCAAATAATGTTTGCCCTCCAAATATGAA
AACATGTCCCGGTAGTTGGTAAATATCTTCAATAGAATGAATCACATCAACGCCCTCTACGTTGAAACTTGTA
TCTGAAGTAAGTACAACATTTCGACGATTCGGTAGTGGTTTACCAATCGATTCAAATGTCTTACGACCCATTA
CTAAAGTATGACCTGTTGATAGTTTTTAACATGCTTCAAATCATTTGGTAGGTGCCAAGGTAATTGATTTTC
AAAACCAATTACTCGTTGCAAGTCATGTGCAACTAGAATGGATAAAGTCATAATTATCCTCCTTCTTCTATCA
TTTCATTTTTTATTACTAAGTTATCTTTAATTTAACACAATTTTTATCATAAAGTGTGATAGAAATAATGATT
TTGCATAATTTATGAAAACGTTTAACACAAAAAAGTACTTTTTTGCACTTGAAAATACTATGATGTCATTTTG
ATGTCTATATGGTTAGCTAACTATGCAATGACTACACTGCTATTGGAGCTTTTATTGCTGGATGCGATTCATA
GTCAACAATTTCCAAATCTTCATAATTTATGTCGAAAATAGACTTGTCACTGTTAATTTTTAATGTTGGAGGA
TTGAAACTTTCACGCGCTAATTGAGTTTGAATTGCATCAATATGATTTGAATAAATATGTGCATCTCCAAATG
TATGCACAAATTCACCTACTTCAAGTCCACATTCTTTGGCAATAAGGTGTGTCAATAAAGCGTAGCTTGCGAT
ATTATATGGCACACCTAAAAAGATATCTGCGCTACGTTGGTATAACTGGCAACTTAACTTACCATCTTGGACA
TAAAACTGGAACATTGTATGACAAGGCGGAAGTGCCATTGTATCAATTTCTGTTGGATTCCATGCAGATACGA
TGTGTCGTCTTGAATCTGGATTATGCTTAATTTGTTCAATTACTGTTTTAAGTTGATCAAAATGATTACCATC
TTTATCAACCCAATCTCGCCATTGTTTACCATAAACATTTCCTAAATCACCGAATTGCTTCGCAAATGTATCA
TCTTCAAGAATACGTTGCTTAAATTGTTTCATTTGTTCTTTATATTGTTCGTTAAATTCAGGATCACTCAATG
CACGATGTCCGAAATCTGTCATATCTGGACCTTTATACTCGTCTGATTTAATATAATTTTCAAAAGCCCATTC
GTTCCAAATATTATTATTATATTTTAATAAGTATTGGATGTTTGTATCTCCTTTAATGAACCATAATAATTCG
GTTGCTACTAATTTAAAAGAAACTTTCTTTGTCGTTAATAGTGGAAATCCTTTAGATAAGTCAAAGCGAAGTT
GATGACCAAATTTCGAAATCGTACCTGTATTTGTGCGATCATTTCGTGTATTTCCTATTTCTAAAACTTCTTC
ACAAAGACTGTGATATGCTGCATCAAATGAATTCAACATATGCGATAACACCTCATTTTCATTATTTATAGTA
TGTATATTTAGTTTGATATAATTTAACTTTATGTAGCATTTTGTTATCACTCATTTTAGGAATGTGATATTAA
TATCATGGATTCCGTTACTTAATTTATAAAATGCTGATTAAGTACCTACCACATCGTAAAGTGATAAATGTTT
CCAATTGCTAATTGTTTACCCAAATCTATAACTTTAATGCTAAAAAATTTTAAAAAAGAGGTTAACACATGAT
TTGAATATTATGTTTGATCTCCTATTAAAACAGTTAAATTTATAGAAAATATAGTTGAAGTGTACGGACTTTA
TATAACAAATAGAATACAACTAAATTCTCTATTTTCAATGACAGACACCATTTTTAATATTATAGAATGTGTT
AACCTTTATATTTATTTATGTGTACTATTTACAATTTTCGTCAAAGGCATCCTTTAAGTCCATTGCAATGTCA
TTAATATCTCTACCTTCGATAAATTCTCTAGGCATAAAATAAACTAAATCTTGACCTTTGAATAAAGCATACG
AAGGACTAGATGGTACCTGCTGAATGAATTCTCGCATAGTAGCAGTTGCTTCTTTATCTTGCCCTGCAAAAAC
TGTAACTGTATTTGTTGGTCTATGTTCATTTTGTGTTGCAACAGCTACTGCAGCTGGTCTTGCTAATCCAGCT
GCACAGCCGCATGTAGAGTTAATAACTACAAAAGTTGTGTCATCAGCATTTACTTGGTTCATATACTCTAATA
CTGCTTCGCTCGTTTCTAAACTTGTAAAACCATTTTGAGTTAATTCGCCACGCATTTGTTGCGCAATTTCTTT
CATATAAGCATCATATGCATTCATATTTAATTCCTCCAATTAAATTGTTCTGTTTGCCATTTGTTTCCATACT
GAACCAAGTGCTTCATCTCCGTTTTCAATACGAGATATGGCCATTTCAATTTGTAATTTAACTTCAAACGCAT
TGTCATTAATATGGGCTTTAAGTGCGGGAAGCTGCTCTGCATTACCTTCATCAAAGATAAACATAGCAGCACG
CCACCTAACGATCTTCTGTGAATCATCTAAAAGTAGCACCATTTCTGGTAGTGCCTCTGGATACCCTAAATCA
CTTATGCAATCCCCTGCTGTCCTTCTTACAGCAGGACTTTTATCGCGAAGTCCCTTATATAAATACGGTAAAA
TTTCTTTACTTTCAATCATGCCTAATAATACAATCGCTTGACGTCTAACCGGTACTTTTTCATCAGATAAAGC
TAAATCAAGCAGCGGTATATCTTCAAAAGTCGGCTTTGGAAAATGGTTTAACATTCGCAATCGAGTCTTCCAA
TTATCAGTCGCATGATATTCATCCAAAGAGACATGTCGATAGAAATGATAATTATTAGCTGCGTGATTTTCTT

CTAAAGCATGTTTTACCAATACGGGCAACTGTGATTCTGGATAGGTAGCTAACACTTCTTCTAGGACACCATC
CATTACTTCTTCAATATTTCCATAGCGATTTCCTAAATCTAGCCATTTACGCATAAAAACAATATTGTCATGT
TCTGTTTGCGCTTGAGTCATATGATCAACGTATGTTTGTGGTAATTGTTCTCTTAATTCCTGGTCAGCAGAAG
TTAGCTTAATTTGATACGGTATACCCTTAAAAGTTAATAATTCAGCTTTAATTTCACCAAAATGTTTATCAAT
TTGTGGGTCTTTTCCAGATTCCAATACCTGACTTTCACCAGAAAAAGCAGCTTTAATATCAGGTAATATGACT
TCCCAATCAGCTTTTGGTGCCTTATCAACAGCTAAGAAGTTCATGACATGAAAAATGGAAGTGATACCATCTA
TAGATAACAACTGATTTATAAATCTTGGTTGATTTTCTTCTACTTTTTTATAAGTATTAGATAACTTGTCTTC
TCTTGTATATGACAAAACAACTTTCATTGTATTTGGACTTGGCGTTGGCTCTATACGTAAAATTTCCATATAC
TTTATTCCCCCCGAATACTAGTTGATGAAAAATTTATTCGAATACAGCCTCTACTTTTGGCAATACTGTTTCC
CAATTTGCGTCATTTTCTTTATCTACTGAAATAAAGTCCATAACATGGAAAATTGATTTAACGCCTTCAACCT
TTAAGATGTCATTAATAAATGCTGGCTGTGAATCATCAACTTTAGTATACGTATCTGATGTCATACCTTCTCT
GCTTTCACTAAGTGTAATCTTCATTGTGTTGTGGTTCGGTGTTTCTGATATAGATATAATTTGCATTTTACTT
CCTCCTATTGAAGTTCATTTCCTAATTGTTTTATTTCTTTCCCTATTGAACACTCATTGATACAAAAATGATG
CGACTTCGTTTTCCCCTCTAATTTACGCAGACGAGTTTTGATTGGACATTGATTGCAATAAGTATTCATTAAA
TCATCTATTTTCGCGAGTGCATTTTGCTCTGAAAAAGTTAAAATACGCGACACTTCCTTTATTACTTCAAAAT
ATTGCAACCTTGTAAATAAGTGTTACTGCTTTAAATTTGACATAAAAATATTATAACATTTTTGACCCTAAGA
TTTGAATGAAACCTTTTACAATTCTATACGTAAAATGTATTTATATTCAAATGCTATTGCATCGCAAACGATT
TTTCAGTAAAATGAGTAAGTTATTTATTGAGCGGTTTCTAAACACCCGCATGATCAAAATTTAGGAGGACATT
TTAATATGTATAATGAAATTTTAGGACTAGTTACGTTTATTGCAACGTTCGTGCTTATGGTACTTATGTATCG
CTTTTTTGGTAAACAAGGTTTAATTGCATGGGTTGCAATTGGCACAATCATTGCCAACATACAAGTAATTAAA
ACTGTAGAAATCTTTGGTATTTCAGCAACTTTAGGTAATGTCATGTTTGCTTCTATTTATTTAGCAACAGATA
TATTAAACGATATTTATGGGCGTAGAGTTGCAAAAAGAGCAGTTTGGTTAGGCTTTTCATCAACATTAATTAT
GATTATTGTTATGCAATTGTCATTACATTTTATTCCTGCACCAGAAGATATGGCACAAAAAGCTTTACACGCA
ATCTTTGATGTTGTGCCACGCATTGCTTTAGGCTCAATCGTCGCATATATTATTGGTCAACATATTGATGTAT
TTATCTTTTCACTAATTAAAAAAGTATTTAGTTCTGATAAAACGTTTTTCATCCGTGCATATGGTAGTACATT
TTTAAGCTCAATAATTGATACAGCTTTATTTGTAGCTATCGCATTTATCGGAAGTTTACCTGGTACAGTTGTA
TTTGAAATATTTATTACAACGTACGTACTAAAATTAGCTTCAACAGTTTTCAATGTACCATTTGGATATATTG
CTAAATCATTTTATCGTAAAGGCAAGATTGATAAATTAGATCAAGGTTATTAACGCACTTACTACATAATAGA
ATAAGTTTTAAGGGAGTGGGACAGAAATGATATTTTCGCAAAATTTATTTCGTCGTCCCACCCCGGCAAGGTT
GACTAGAATTGAAAAAAGCTTGTTACAAGCGCATTTTCGTTCAGTCAACTACTGCCAATATAACTTCGTAGAG
CATAGAACATTGATTTATGTCCCAGCCTGAAGATGATTTTTTTAGGAAGTATATTACAGTACTACGTATTAGA
AAATCATCTTTGAATCAGCAAGTATTTTAAACACGTGTTACTTATTTAAGTGATGCGTGTTTTTTAATAGATT
GTTTAATATATTTTGTATTACAGTAATAGTAACTTTAACTAAGTATTGAATAGAAAGTAGGTTTATTATGGCG
AAAATAAATTTTGATGCTGCGACGAAAGGAAATCCAGGCATAAGTACGTGTGCCGTTGTAATCAAAGAAGATG
ATCAGCATTATACATATACACATGAGTTAGGCGAAATGGATAATCACACTGCAGAATGGGCTGCTTGTATCTA
TGCATTGAAACACGCCCGTGAATTAAACGTTAGCAATGCGTTGTTGTACACTGACTCTAAGTTAATTGCGGAT
AGTGTTAACGCTGGTTATGTAAAAAATGCAAAATTTAAACCTTATTTTGATCAATTGGAAATATTTGAACAAG
ATTTTGATTTATTATTTGTTAAATGGATTCCGAGAGAACAAAACAAAGAAGCGAATCAAAACGCACAGCAAGC
ATTGTACAAATTAATTAAAAAGAACAAATAAAAGGAGCACCGACTGACATAATTTCAATCGGTGCTCCTCTTT
TTATCGAAGAAACAAATTATTTTTTATTTTTTTTAGATTTCTTTTTCTTCTTTTTAGCTACTAGCTTTTTAGA
AGGTTTTTTTGATGCCGACTTTTTCCCTTTGGATTGGTTATCTTTTTGATTTTTCTTTTTAGCCAATAATAAA
GGTGCGTCATCTTCATTAGATTCAATACTAGGTGTTGTTTCAACATCTACCTCTTTATCTTTGCGTCGTTTTG
CGATTAAGAGTGGTGAATGTTCGTTATCTTGCAACACTTTTTCATCTGTGTTTTCATTTGTAACTTCCACATC
TTCTTCATCTTCTTTGCGACGACGTTTAGGTAAGAAGAACGGCGTATGTTTAACTTTATCGAGTGACTCGTTA
TTGTTTAGCGAATCTTTTTCTTCAACAATAGCATCTTCTTCATCTTCTTTTCTGCGACGTTTCGCTAATAAAA
GTGGTAAATGTTTAGTACCTTCTATTGAACCTTTCTTCTTGTCTTTGCTATTATCTCTAATTTCCAATTCTTC
CTCTTCATCCTCTTTACGACGACGTTTCGCAATGAAGAACCAGAAGCTAGCAAGTAATCCGGAAATACCAACA
ACGCCAAGTGCGTTTTTGATAACATTACCAATTCGGTCACCGAAATGACGCATATGAAGTGGATCGATTTCAT
CATCTTGATCCAATTTCTTTTTATGTCCAATAGCCGAACTATTGAATGGATGGTTTACAGTTCCATATCCAAT
GCTTATATGACTATTAGATTCACCGTCCGAATCTGATGAATGCTTGCTATTATTAGATGTAACAATTTGTACT
GCCAAAATTCGTGCGATACCATTATTCAATGCAGCCTCAACTTGTTGTGGCGTTTGCGCATTATTAATATCTC
TGATTGTTTCAAGTACAATTTCGTTAATTCGATTCATTGCTGCTTGTCTTTCTTCAGCAGTTCCAGTTGAACT
ATTTTTAATGCGTTCAATTGCATCTGTCGAAATATCTTTAATTTTTGAAATCGCTACTTTTTTACGTTTTTCC
AATTCTTTAGCAAATGGATTTGCTGCTTTTATTTGCGCTTTGAATTGCTCTAATTGTTGTGTGATTTCATCGA
TACTTTGCGCACGTTGAATCGCTTGAATTGCCTGTTCTTTTAATTGATTTAACTTAGATATAGCTTCTTGTTT
CTCTTTATCAGTTAAATCCTTACTAGCATTAATTTCATCAATCATATGTTGAATAGCATCAGTAATTGATTTA
ATGGCATTTGATTTGGCTTGGTCAATTGTAAATTGCTCTGGATTAAATTGTTCAATATGCGTTTGTTCTTGTT
GTTGAACTTCTTCCACCGAATGAACATCTGTTGCATTGTTAATTTGCTCAATAGCTTGTTGTTTAAGCTTATT

AACTTCCGCAATCGCAGCTTCTTTTTGTTCTGGTGTTAATGTTGCGTGATTATTGATATCATTAATCTTTTGT
TGTGCCGCATTTTCAATTGATTCAATTGCCTGTTGTTTGACTACTGCCAATTCTTTTTCTACTACTTTCACAG
GAACATTAACAATTACTTTTGATCCATCAAGTAATGTTACTTCGACTTTTGCTGTTAAGCTATCAGAAATTGT
TGCGGTTGACGGTGTATCAATAACTTCTGCTGTAAGCTGATCAACTAAGTTTATATGTGCTAGAATATCTTGT
TCTGTAATGATGTCATCACGATGTACAATGAGTTCACCATTTACAAGGATTTGTTCAGGAGTTGCGTCAAAAA
TTTCATTTACAGCAGGTTGTGTATCAACTTCCCATACATGAGTATTTCTAATATCATCTAATCCTAAATTTAA
TCCTTGATTTAATTGATCTTTAGTTTGAGCATTTTCAATATTTTCTAGTGCTCGTTTTTCAGCTTCGTCAATT
TCTTTGAGCGCTTTTGCTTTTTGCTCAGGTGTTAAATCAGGGTTTGAATTGATTTGATCACGCTTCGCATTTG
CTAATGCTTTAATTGCATTTTTCGCATTTTCTTTAGCTTTCACTAAATCTTTAATTTCTTGTAATGCTTGTGC
AAGTTGTGCTTTGGCTTGTTCAATTTCTTCTTTAGTCATCGCATTGTTAATGCCATTATGACCTTGTTGCAAT
ATTTGATTAATTCGATCTTTAAGCGCTTGTTTTTCCTTATCTGTTAGATTTGGATTTCGATCAATTTCGTCAA
TTAATGCTTGTACTTGCTTATCAACATCTTGTTTCGCATTTTCTTTAGCTTTCACTAAATCTTTAATTTCTTG
TAACGCTTGTGCAAGTTGTGCTTTGGCTTGTTCAATTTCTTCTTTAGTCATCGCATTGTTGATGCCGTTATGA
CCTTGTTGCAATATTTGATTTATTC
>DSM104437_scaffold_6
TTCAAAAGTATTTAAAATTACTGGAGAATCATATCGATTAAAAGATTATAAAACCGAAAAATCATTAAATATA
CGACAATCTTAAACCGCTAAAAAACGACATTTTCAAACCGCCATAAAACAGCATTTTTAAACCGCTATTGACA
TATAGAACAGTTTGGTCTGTCAAACAGTTTAAAAAGAAAAGTTCATACGCTAAGTGGAGGTGAACAACAACGT
GTCGCTTTAATTAGAATGATGTTAAAAGATCCGATTGTTATGTTAGCTGATGAACCAACGGGTGCGTTAGATC
CTAAAACAGGACAGATGATTATTCAATCATTATTTGATTTGGTCGATGAAAATAAAGTGCTGATTTTAGCAAC
ACATGATATGGCTATTGCAAATCAATGTGACGAAATAATAGATTTAGAACAGTATAGAAAAGTAGCATCTATG
TGATATCCAAAAGATAAAATTAAAAGCCAATCAATGGGAAATGTTGACTTCAACACTCCGTTTGATTGGCTTT
TATAATGTGTTACTAAGTTATGAAAGTAACAACGGCAAACTGAAAAAAAGCAATATAATAATATTTAATACGA
GTAGTAATAAATGTAATTGATTTTTCGTTTTAATATAGCAAAAAACAATGACTAAAGATAACAACAATATCAT
GATTGCACCTGGGAAGAACCATAATGCTGAAGCTGTGTACCAAATCATTGGTAACGCCATAATAATAAAAGAT
ATAAGACAAGAAAGGATACCAATAATATTATAAGTTCTAGTCAAAATTACACCTCCAATTTTCATTTTAAAAT
GATATGCGCAAAACAATCATTTTAAATACATCATACAATAAAATTAAATTGTGCGGTAGAAGAATTTGAAATT
AAATTGAATTGGCTTGATTAAAAGGTGATTTTATAGAAGATAATAAAAATGGTTATTTAATAGAAAACCATAA
TATAAACGACATGGCTGATAAAATATTAAAGCTTGTTAATAATGATGTTTTAGCAGAGGAGTTTGGTTCGAAA
GCGAGAGAAAACATTATAGAAAAATATTCAACGGAATCAATATTAGAAAAATGGTTAAATCTTTTCAATAGCT
AAATGAAAAAATCCTAGGTATTCATGAGTACCTAGGATTTTTACGCATTATATATACAAAAAAAGAGAGGATA
GTCCTCTCAAATAATAGTTAATAAAATTGTAGCCATTTCTTCCAGCTGTAACTACTAATACAAGCTAAGTTCA
TTACGATATGTATGATTTCAAATTCATAACCACTTACATATCCTCTTGTAAGTTTAAGTGTGAAGATTGAGCC
TAACATAACACAAATATAAAAAACATTAACATAAGGCGTTAAAAAGCCGATAATAAGTGCTAAACCTCCAACA
ACTTCACCAATGGACATCAATACTGCGATGGTAGGTGGTAAACCTAAAGAGCCTAAGAAGTGAATTGTTCCAT
CTAAATCAACAAATTTCAAAGTACCATGCACTAAGAATGATGTTGCTAACATCCATCTAATTAAAAGCATACC
TAATTTCATCATGTCACCTCCAACATACATTTTTTTCTTAAAACATAGTCCAAATTATATTTGTAAAGAAGTG
GAAAGTAAAGATTATTTATAAAAATAATTATTAAATATTTATTAAATGTTCTATTGTATTGAACAATTGTTGT
AGAAAGATGTTTAAGTCATCAGTTACTTTTTGTATTTATAAATAAAGATTAGTACAGCATAGTGCAAAGATTA
TAGGGAAATGAATTAACTATTGCATCGCTATTTATGAATCGTAAACACTATTATGAAATTGCAAGAGTAGTAT
GGACAATTAGAATGATGGTTTGAGATAAATGCGTGTTATTAACCAGTGCGTCATCTTCAAATTAGAGGCCATT
TGCTGTTTATAATAAGAAGATTTGATAATCAACTATGATAAGAAAAAAATAATAACATCCTAAATATATCATA
AACAAATTAAATGATAAAATAATAATGTGGTTTTCTATTAAAGTTGTTTGGAGGTAAATGTGTGAATAGGAAT
ATCGTTAAATTAGTTGTGTTTATGCTAATCTTAGTTGTAGCAGTAGCGGGTTGTGGACAAAAAGATACTGAAG
AGAAAACTGAAATGACGACAATAAAAGATGAATTAGGAACTGAAAAAATTAAGAAAAATCCTAAACGTGTTAT
TGTATTAGAATATAGTTTTGCTGATTATTTAGCAGCATTAGATATGAAACCTGTTGGTATTGCAGATGATGGC
AGCACTAAAAATATAACAAAGTCAGTAAGAGATAAGATTGGAGCATATGAATCGGTTGGATCTAGACCGCAAC
CGAATATGGAAGTAATAAGTAAATTAAAACCGGATTTGATCATTGCAGATGTTAGCAGACATAAGAAAATCAA
ATCAGAATTGAGCAAAATTGCGCCGACAATCATGTTAGTTAGCGGTACGGGAGATTATAATGCAAATATTGAA
GCATTTAAAACAGTCGCTAAAGCAGTAGGCAAAGAGAAAGAAGGCGAGAAGCGTCTGGAAAAGCATGATAAAA
TAAGCGGAGATTAGAAAGAAAATTGAACAGAGTACGTTAAAATCTGCATTTGCATTCGGTATCTCAAGAGCAG
GTATGTTTATTAATAATGAAGATACATTTATGGGACAATTCTTAATTAAAATGGGTATTCAACCTGAAGTCAC
AAAAGACAAAACTGCGCATGTTGGTGAACGCAAGGGTGGCCCTTATATATATTTAAATAATGAAGAGCTTGCC
AATATCAATCCAAAAGTTATGATTTTAGCTACGAATGGAAAAACAGATAAAAATAGAACGAAATTCATTGATC
CTGCAGTTTGGAAATCATTAAAAGCTGTGAAAGATAATAAAGTTTATGACGTTGACCGAAATAAGTGGTTGCA
ATCAAGAGGTATTATCGCAAGTGAAAGTATGGCAGAAGATTTAGAGAAAATTGCAGAAAAAGCAAAATAAAAA
TACAGCGCCACTCGGAAATCATATAAGAGTGGCGCTGTATTTTTAATTATGTTTGTTATTTGTTGAATGTGAT
GATTTACCTGAAAGTTTATTTCGAATAAATTTTAATACATAACCGACAAGGATTGTTTTAACAATTCTTTTAA

TGAATTGACGCATCGTTACATACCTCATTTCTCTTTATCTTATGAACTATATACCCATTCATATATGCTTTTT
AAACGTCATTGTCACAATTTAATTTTTAGGGAATATAATATAACCGTCTCTGTCTGCTTTTTTAGTAAAAATG
ACAAAAATGGCATGTATTATTGAGATGATGGTAGGGATACCTGTCCAGAAAAATAATAAGTGAAAAAGACCTT
GTCCAAATTTATCAGCATAAAATTTATGAATACCTAAACCTCCAAGAAATAATGCAACAATAACATAAATGGC
TTTATTGACTTTCATTTGTAAATCCTCCTTAACTATAATTCTACTTAAATTCGTTGTGAAAACCAATATTTCT
AACTTTAGAATTTTCAAACTTTCTAAAATTATAAGTATATCTTTTTAAAATAAGCTAGAATTTCTATATAATA
AATGTTAATAACGTAAAAGGGAATGATGACATAGTGATACGTCAAGCACGTCCAGAGGACCGATTTGATATTG
CGAAGTTAGTTTATATGGTTTGGGATGATATGGAATTAGAATTGGTAAAGCATCTACCTAAAGACATGGTATT
AGATGCAATTGAAAAAAGCTGTGTTGATGCAACATATCGAACTTTTTATCAGCATATTTTAGTTTATGAAGTA
GAAAATAAAGTAGCAGGTTGTATTATTAGCTATAGCGGTGAAAATGAATTGAAATATGAAAAAGCATGGGCAC
TACTTGACTTACCAGAAGAAATAAAACAATATGGCACGCCATTACCTGTAAAAGAAGCTAAAGACGATGAGTA
TTATATAGAAACAATTGCGACATTTGCAGCATATAGAGGTAGAGGCATTGCGACAAAGTTATTAACGTCATTA
CTTGAATCAAATACACATGTTAAATGGAGTTTGAATTGCGATATTAATAATGAAGCAGCATTAAAGTTATATC
AAAAAGTAGGTTTTATATCTGATGGACAGATTGAATTATACAAGCACATGTATCATCATTTAATTGTTAAATA
AAAAAACTCGACAGTTCGAAGTAAGTCGATTGCCGAGTAGTATCATTTTATATTAAATGCCTGCAAATAATGC
GCTAATATAAATACCTAATGCATATAATAAACCGAAAAATGTATTTGTTTTACCAGCAGCAGCCATTGCTGGC
ATCATTGTAGGCGGTGTATCATTCTTCTTGAAACGTCTGATAACTTTAACAGGCATTGGGAATGATAACAACG
CAAGTAAGTAAAATAATGAGCCACCAGGTTTAATAATGATCGTAAGTACAATAAAGGCATAAGCGATAAAGTA
CATGATTGCCATAAATGTTAAAGAAGCATTTTTACCTAATAGAATGGGTAAAGTTTTACGACCACTTGCTTTA
TCTTTGACACGGTCGCGAATATTGTTAGCCATATTAATTAAACCGATAGTGATTACTATAGGTACACTTAACC
AAATTACATAGCTTTGAATATTGCCAGTTTGAATAAAGAATGCAATAACGATAATAAACATACCCATAAATAC
GCCTGAGAATAATTCACCGAAAGGCGTCCATGAAATAGGGAAAGGGCCACCTGTATATAGGTAACCAACAGCC
ATACATACTAATCCAACTGGTAATAACCAAAATGAAGAGTTAGCAGCTAAAAACAAACCTAATATTGCTGCTA
AGATGTAAAATGCAATGGCTAATCGTAGCACAAGCTCTGGGCTCATACCGTTGCGTACAATTGCACCACCAAT
GCCTACAGATTCATGATCATCAAGGCCTTTTTTATAATCATAGTATTCGTTAAACATATTAGTTGCTGCTTGA
ATAAGTAAGCATGCTAGTAACATGGCAATGAATAGGCTGATTTTAATATGATCTTCGCTACCAAGAAAATATA
TTTTAGATGCTGCTGTACCAACTAAAACGGGTACTACGGAAGCAGTTAATGTATGAGGACGCATTAAATGCCA
ATATTTCTTAACTGTAGAATATTGCTGATATTGATTACTCATTGAATACAAAACCTCTTTAAATCATATTTTT
TATAACAAAGATATTTTAATAGAAAGTAGCATAAAGGTCAATTGACGTGAAAAATTGCAAGATAAGTTTAAGC
AATTCTTGTAACACAATTATCCAAAAATGATACAATATAATAATGAGTGAAAAAAGACGAAGAAAGAAGTGAA
ATGGATGGCTACGGGCGTATTAGAGGACGATATTGTCAAAGAGATATATGACAGCTCAAAGGAATGGGTTTCA
GTTGAAGTGAAATTATCACAGTCACTAGACCCGAGCACATTATTTCATCTCACTGACAATGAGGCAGGAGATC
GCTTTTATATGCGTTTGAATGATAATCGAACGTCATATTTTGGCTACAAAGCAATTCAATTATTCAAAAATAA
TTCTAAAAATAAACAATCTATTTTTAAAGACTGGGAAAAATTAAAACATAACATCACATTTATACATCCGCAA
TCTGAGAAACATCATCTTCGAGTTGTTGGAGGGTTTCAATTTTCAAGTCATAAATCAGATGATGAATGGCGAG
AGTTTGGACTAAATCATTTTGTATTACCTGAAGTTTTAATTTCAACTGATAATAATGGGACATTTTTAACTTA
TACAGTTAAAAGGGAAAGTTTTACTGTTGAGGCATTGAACGATTTAATGGATTTGTTCAACAATATATCGGAC
ATAGATGTGGACGAGCAAATTGGGGAAATTACTAGAAATGAAGATATTTATAAAGATGATTGGCGTCAACTTG
TAGTAGAAGCTATAGAATCTATTAATAATGAAGAAAAAATTGTACTAGCACGTAGACGGTTAATAAAGTTCGA
TAAAGATATCAGTATTCCATATATTCTAAAGCAAGCATATTCTAAAGAAAAAAACAGTTATATATTCTTGTTA
GAATCACAAGATTCTATATTCTTTTCACAAACACCTGAACAATTAATAAAGGTCAATAATAAAATACTATCGA
CTAAAGCTGTAGCAGGTACAATTAAACGTTCACAAGATGAGGACGAAGATACAAAAAATGTTGACGCATTTTT
AAAAGATAATAAAAACTTAATTGAACATCGATTTGTTGTTGACAGTATTTTACATGATATTAAACCTTATATC
ACTGAATTACATTATGATAAGACGCCTAAAATTCTAAAAAATGATCATTTATATCACTTATACACTGAAATAA
AGGCGCCACTGAAGGATGATTCGTATATTAGTTTAATTGATCATTTACATCCAACACCTGCTTTAGGTGGCTA
TCCAAAAGAATTTGCAATGGATTTTATTGAGCAGAAAGAATTTGGTACGCGAGGATTATATGGTGCACCGGTT
GGCTATATAGATATATACGATGATTGTGAATTTATTGTTGCAATTCGGTCAATGCTGATTAAGAAAGCACAAG
CAACATTATTTGCAGGGTGTGGCATTGTTAAAGATTCTGATCCAGATAGTGAATTGGCAGAAACGAACCTTAA
GTTCACACCTATGATGAATGCATTAGGAGTCGATATGAATGGGAAATCATAAAGCAGCTTTAACGAAGCAAGT
TTTTACATTTGCATCTGAGTTATATGCGTACGGCGTAAGGGAAGTAGTTATCAGTCCGGGATCACGCTCAACG
CCACTTGCACTTGCATTTGAAGCACATCCAAATATTAAAACATGGATACACCCCGATGAGCGAAGTGCAGCTT
TTTTTGCGGTAGGATTAATTAAAGGCAGTGAAAGACCTGTCGCTATATTATGTACGTCAGGTACAGCAGCAGC
GAATTATACGCCTGCAATTGCTGAAAGCCAAATTAGTAGAATTCCTTTAATCGTTTTAACAAGTGACCGTCCG
CATGAATTAAGAAGTGTAGGCGCACCACAAGCGATTAATCAAGTAAATATGTTTAATAATTATGTAAGTTATG
AGTTCGATATGCCTATTGCAGATGATAGTAAAGAGACCATTGATGCAATTTATTATCAAATGCAAATTGCTAG
TCAATATTTATATGGACCACATAAAGGGCCAATTCATTTTAACTTGCCATTTAGAGATCCGTTAACACCTGAT
TTGAATGCAACAGAATTGTTAACTTCTGAGATGAAGATTTTACCGCACTATCAAAAAAGTATAGATGCATCGG
CATTAAGACACATTTTAAATAAGAAAAAAGGTTTAATTATTGTAGGGGATATGCAGCACCAAGAAGTTGATCA

AATACTGACGTATTCAACGATATATGATTTGCCTATTTTAGCTGATCCTTTAAGTCATTTAAGAAAATTTGAT
CATCCGAATGTTATCTGTACATATGATTTGCTGTTTAGAAGCGGCTTAGACTTAAATGTGGATTTCGTAATTC
GTGTTGGAAAACCAGTGATTTCTAAAAAGTTGAATCAATGGTTAAAGAAAACTGATGCATTTCAAATATTAGT
GCAAAACAATGATAAGATTGATGTCTTTCCGATAGCCCCAGATATTTCATATGAGATTTCTGCGAATGATTTC
TTTAGGTCATTAATGGAAGACACGACCATCAATCGAGTAAGTTGGTTAGAAAAATGGCAACGCTTAGAGAAAA
AAGGGCGTAAAGAAATTAAATGTTATTTGGAACAAGCTACAGATGAGAGTGCATTCGTTGGTGAATTGATTAA
GAAAACATCTGAAAAAGATGCATTATTTATTAGTAATAGTATGCCTATCAGAGATGTAGATAACTTGTTATTG
AATAAAAATATAGATGTCTATGCGAATCGTGGTGCGAATGGTATTGATGGTATCGTTTCAACTGCACTGGGTA
TGGCTGTGCATAAACGAATAACATTATTGATAGGTGATTTATCATTTTATCATGATATGAATGGACTATTAAT
GTCAAAATTAAATAATATTCAGATGAATATTGTATTATTGAACAACGATGGTGGCGGTATTTTTTCATATTTA
CCACAAAAAGAAAGTGCAACTGACTATTTTGAACGGTTGTTTGGCACACCGACGGGATTGGATTTTGAGTATA
CAGCTAAGTTATATCAATTCGATTTTAAACGTTTTAACAGTGTTTCAGAATTTAAAAATGCCACATTGTTATC
TGAAACTTCGACGATTTATGAATTGATAACGAATCGCGAAGATAACTTTAAACAGCATCAAATTTTATATCAG
AAATTGAGTGAAATGATTCATGACACATTATAATTACTATGAAGCAAACGTTGAGACCAATCAAGTTTTAGTA
TTACTGCATGGTTTTCTTAGCGACAGTCGTACTTATCATAATCACATCGATAAATATACTGATATCTGTCATG
TCATCACTATAGACTTACCAGGACATGGCGAAGATCAGTCTTCAATGGATGAAACGTGGAATTTTGATTATAT
TACGACGTTGTTAGACCGAATTTTAGATAAATATAAAGATAAATCAATAACATTGTTTGGATATTCAATGGGT
GGGCGTGTTGCGTTATATTATGCAATTAATGGTCACATCCCTATATCTAATTTGATATTAGAAAGTACGTCAC
CAGGTATTAAAGAAGAAGCAAATCAATTGGAACGCCGTCTTGTTGATGATGCACGTGCTAAAGTATTAGACAT
AGCAGGTATTGAATTATTTGTTAATGATTGGGAAAAGTTGCCATTATTTCAATCGCAACTAGAATTACCAGTT
GAAATACAACATCAAATAAGACAACAACGATTGTCTCAATCGCCACATAAAATGGCCAAAGCATTAAGAGATT
ATGGTACAGGTCAAATGCCAAACTTATGGCCGCGCCTGAAAGAAATTAAAGTACCAACATTAATATTAGCTGG
AGAATGTGATGAAAAATTTGTACAGATTGCGAAAAAAATGGCAAATTTAATTCCTAATAGTAAATGTAAATTA
ATTTCTGATACAGGTCATACAATTCATGTGGAAGATAGTGATGAATTTGATACAATGATATTAGGATTTTTAA
AGGAGGAGCAAAATGACTAACAGACAATGGGAAACACTTAGAGAATATGATGAAATCAAATATGAATTTTACG
AAGGGATTGCTAAGGTAACAATAAATCGCCCTGAAGTACGCAATGCGTTTACACCTAAAACAGTTGCTGAAAT
GATTGACGCATTTTCACGTGCACGTGATGATCAAAACGTTTCAGTTATCGTATTAACTGGTGAAGGTGATTTA
GCATTCTGTTCTGGTGGTGACCAGAAGAAACGTGGACATGGTGGTTATGTAGGTGAAGACCAAATCCCTCGCT
TAAATGTATTAGATTTACAACGTTTAATTCGTATTATTCCAAAACCGGTTATCGCGATGGTAAAAGGTTATGC
TGTAGGTGGCGGTAATGTACTAAATGTTGTTTGTGACTTAACGATTGCTGCTGATAATGCTATTTTTGGACAA
ACTGGTCCTAAAGTAGGTTCATTTGATGCGGGTTATGGTTCAGGATATTTAGCACGTATCGTTGGACATAAGA
AAGCACGTGAAATTTGGTACTTATGTCGTCAATACAATGCACAAGAAGCTTTAGATATGGGTCTAGTAAATAC
AGTGGTACCTTTAGAGAAAGTTGAAGATGAAACTGTGCAATGGTGTAAAGAGATTATGAAACACTCACCAACA
GCTTTACGATTCCTTAAAGCAGCTATGAATGCTGACACAGATGGTTTAGCTGGTTTACAACAAATGGCTGGGG
ATGCAACATTGCTTTATTACACAACTGATGAAGCGAAAGAAGGCCGTGATGCGTTTAAAGAAAAACGTGATCC
TGACTTCGATCAATTCCCTAAATTCCCATAAGTTATTTTGAATATGATATTAAGTCACTTGCGTCGTTTATTA
GCGACGATAGGTGGCTTTTTTATTTTATAAGAATTTAAAAGGGAATTTTAATTAATTATCATAAAGATAAGGT
AGTTTGAGTAAATTAATAGAGAAAGAATAAATGTTAAAGATTATTATTACAATGAAAAAACGAGCTGGTGAGA
AATTGAATTCTCGCCAACCCGCTTTATGAAGTGAAATCATTTAAATTAGTTATACTAAGCGCTCATAAACGAT
TGGTCGCGAAGTGCCAATACCTTGTTTGGATGACGTTTGCATGAGGATATGCTGTGTATCATCATAATCAAGA
ATATAAATGATTTCTTCATTATCAATAGATGAAAATTTAATGCGTTGATGTGCCGTATCTATAAAAAGAATAT
GATATTGATTGTGACTTGTATCATCGCCATGACGTATACAAATTGATTTTTGTAATTGATGATTACTCCAATT
ACCAATGAATAAATTGATATTGGTTTGTTCTAGATGTGTGAGTTTGGTTGTGTCGTAAACTAAATTTATAAAT
TGTAGTTGATACATAGTTTTACCTGCCTTTAATCAATATCTATATTACTTTTTAGTAACCTATCATTGAACCA
TACCAGTTATAATCACGATTGAATGATAAATGTAATAGGCTTGAATCTGCATCTTGGATTGATAATTCTGTAT
CCCAAGGATTCCAGTAAATAAGTTTTTCTTGGTCATTAATTTTAGCATTACCAACAACTGCTAGCGCATGTCC
TAAATGTGGGTCATTAGGGTTTTGAGATACACTTTGTGCAAGGATCATAATTCCTACATTATCTTTTGTAAGT
TGATCAACTTGTTCATATGATGGTACGCCTTCTTGATAATGAATATCTCTGCCTTGTGATTTACCGTATTCAA
TCATTTGATTAGGGAATGTTGAGCAATTAGGAAGGTCTTGTTCACTTACTTCTGGGTATAATGTACGCATAAT
ATCATGTGCATTATAAGTGTCTGTATTTTTAGTTGCATTTAATAATGCTGCCATACTGAAACCTGCACACCAT
GAGTTATCGAATTGTTGTTCTCTAATTTTGAAGTTTTTAATGTATTTTCATATTGAACTTGATCTTCTTGTA
TCGCTTCGTTTTCTTCAACTTTAGTAGGTGTTACTGTATTTTTTAACTCTTGTTTCAATTTTGCTGAAACTGT
TTTAGCACTTTCTTTTTCTTTTACATTACCAGGTAGTGGCGTAGCTTTTACTAATCTAACTTTGCCATCTTCT
TCAAAATAAAACCCTTTTTCATCAGTAAGAACAGTGATATTTGAATTTTTATCTTTAATTTGGTCTAAATCTT
TAGCGATGAAGTTTGAAATTTTAACACTGTAATTCATGTCTTCTTTTGATTTGTTTAAATCATCTTTATTTTT
AGGGCTAAGTGTTAAAGTATAAACAATTTTACCGTCTTTTATAACTGGATAATAGTAACTATTATCTTCTTCA
CCATTAAATTTATAAATTTTAAAAGCTTCGCCAAGTTCATATTTACCAGTTTTTGCATTACTTTGTTTATCTA
ATGCTTTAGCATAACCAGCAAATTGTTGTTGTGCTAGATCTTTTACTTTTTGAGGTACTTTGTCACTCTTAAC

ATTAATTTCTAGCTGTTTAGAGTGTGAATCGGCTTTGGCTTTATTGTTATTAGCAAATGCGCCTAGTGATAAA
ATAAGCATTGAAACCATTATGATGCTTATAATATTGAATACTCTAGATTTATATGAACTATTCATATAATACC
CTCCATTTAAATCAAAATATTTTGCGTTGCTAAGACTTTGCTTATATTTTATCTAGACGAACTTAATTGTTAA
TTAAGCTGCATCTGGATTGTCTGAATTATTGTTATCGCCATTGTCTGGATTATCAGGGTTATTAGGGTTGTCA
GGGTTATTAGGATTGTCCGGATTATTAGGATTGTCCGGATTATTAGGATTGTCCGGATTATTAGGATTGTCTG
GGTTATTTGGTTCATCTGGGTTGTTAGGGTTATCAGGATTATCTGGGTTATTAGGTTGGTCATCGTTGGCAAA
ATGGATATCTTCAATATTTTGTTTTAAGAAGTTGCGTACATTTTCATTAATAAATACCGCACCATTAAATTCA
TTTGGTACACCGCCCCAATGAATTCCGATTACTTCATTTTTTTCATTAAATACAGGTGAACCTGAGTTACCAC
CAGTTGTACTTAAATCATATTGCATAGCTTCGCCTTTGAGGTAAGTGATTTTTCCTTTACTTTCCCACATTGT
TGCTACAGGTTTATCACCAGGATATCCTGTTACAGTAATATTTTGGTTAACTTGTGTTTCATCATTATTACTC
ATTGTTGCTGGTTTAACTACTTCGCCTATATGTTTGTTTTGCTCATTAGGGGAGAATTTAACGATTGCTAAAT
CACCTTCGCCTGAATATTTAGTGATTTGTTCAGCAGTGAAACCACCATTTGGATAATTGTCTTGGTTAATTGC
AGAAGGGAATGCTTTTAAAGCATGAGGATCACCGTGCGTAGCATCTACGACGTGTTTATTTGTTAAAAGTGTA
TCTTTACCTACAACTACACCGGAAGCAATAAATGTACCAGTAGGTGCTTCAACTTGAATATAAGTAACAGGTG
CATAATGACCATTCGTTGTATCTGTGATTTGGTGACGATCGTTATTTGGTAATATAACATTAGCGCGTTCACG
TTGTTCTAATGGTTTAAGGTTACCGCCTTTTTGAATCTTAGGTGTTTGCTGTTTGTCTGTCTGCGTTTGTTGT
GGATGATTGTCCATAGCTTTTGAAGATAACGCATTTGCTGCTGGAGAACTCACAAGTGTCGCTGTTGTCAAAG
TTGCAACGAATAAAGAACTAACTTTTAAAAATTTACCTTTCATCTAAAAACCTCCAAAAAATTTATTTACAAG
TTAAATATAACACTAAAATTTTTTAAGTCAATAAGAATATATAAATAAATTAAAAAGTTTTTGTAAAGATTAG
AGTTTACAAAAACATTAAGGGAATTAAAATTGAATTGTATTATACTTTATTCGATTGTGTTAAATTGATAAGG
TTGTCAATTGTTTGCATTTATCTAAAAGAATAAATAGGTAAGCAATTGAGGACTAAGGGAAAGTATTTGATTA
TAATAATAAATAGTAACTTTTGAATAATAATTGTTTAGCCAAAGGTAGATTTATATGAATGGTGATATATTAT
TTGAGTGCTTAAGTTTCGAAGAACAATTATAAGTTAGCATATTAAAACCTTTTGATAGAATCATTTTGAGTGA
GGTGTTTAAAGGAGAGGAATGTAACTCGGTAGTATTTACAGTCTCTATAGAGTATTATTTAAAACTTATAGAA
AATAAAAAACTGAACGTACTATGAATTTAGTTCACGACGTCCAGTTCAACATATTATTTTTATTTAGTATTTA
AATATTTAGCAAGTCTCTTCATACCTTCTTTTAAAACATCCATTTCATAAGCATAGGAAATCCTTACAAATCC
TTTACCGAATTCTGTAAAGGATGAGCCTGGAACGATTGCTAAATGTGTTGATTCAAGTAAATCGACACAAAAT
TCGAAATCGTCATCGGTGATATGTTTAATACTTGGGAAAATATAAAACGCGCCTTCAGGTTGAGCGGTAATCT
CAAAACCTAATTTAGTTAATTCAGATACTAAATAATTTCTTCGTTCTACATAAGCTTCGTTCATATATTTAGG
AGCTTCTAATCCTTCGTTAAGTGCTGTTATACATGCTATTTGAGCTGGAACATTGGCACAAATACAATTATAC
GCATGCATAAATGTTAATTTTTCAATGAGATATTGAGGTCCTAATAGAAAACCAATTCTTATTCCAGTTGCTG
AGTGTGATTTACTTAAACCACCAATTAATATTAATTGATCACGAATGTCTTCAAATTCAGCGAAAGATACATG
TTTACCACTAAATGTATTTTCAGCATAAATCTCATCGCTAATAATAAATATCGGATATTTTTTTAATACATTT
ACGATATTTAAAACTTCATTTCTTTTTAAAACTACGCCAGTTGGATTAGTCGGATAATTTAACAAGACAGCTT
TTGTCTTTGGAGAAATATGACTTTCTAACGCATCAGGTGTAATTTTGAATTGTGTTGCTGTTGTATCAATATA
AATTGGTTTACCACCTAGTACTTCGATGAGTGGTATGTAGCCTGCATAAATTGGTCCCGGTATTATAATTTCA
TCTCCAGGCTCTATGATAGAACGTAACGTTGTGTCTATTGCTTCACTAGCTCCATTTGTCACAATAATTTCTT
CAGGATCATAGTAAAAATGATAACGATTTTTGAAGTATTGACTAATTGCTTCGCGAGTTTCTAATAACCCTTT
ATTGTGAGAGTATGAAGTTTGATTATTGTTTATGGCATCGATATATGCACGTTTCACTACGTCTGGCATAGGA
AAGTCAGGTTGCCCAATTGTTAAATTAACACAATCATCAAGACCTTTCATGCGGTTTGAAAATTGGCGAATAC
TTGGGGCTCTTAAATATTTAGAATTAGAATTTAAAGAAAGTTTCATTTGTGACACCTCAAATATAAATCAAAT
ATTGTCTAAAAATTTAGAAATTAATAATATCATAACATACTTTTAAAGCAAAAAAGGGTAAATTACTTAAGCT
TTATTTGAAAAACGAAAAATGTCTAAATACACTGTAGTAACTACTTTTCAAAATGAATAAAGTGGTTACAGTT
AATGTACTTAGACATTATATAATTATGAGTCTTTAAACTGTGATGGTCTTTGTAAATTAATTATTGGATTTGT
CCATTTACAAACAAAGTTTGTAGATAATACATATACGATGATTACAGATATACTTATTAAATAAAGATACGTC
ATTAATGATATAGGATTATCGAATGGGTACCATTCAAATCCTCTAACAATGCCAATAATTAAACCATGTAATA
AATATACGTATAGCGTACGACTACCAATATAAGTATATAATTTTTTCTTTGTTGATATTAAATTTAGAAACGC
AGTCATTGCGATTAATATAATTCCATATAATATAAGTCGTTTAAAAGGACTGAATACACTCTGTCCTTCATTT
TCAAGTGAAGTATATGGTGAACTTCCTAATAACCAATCTGCATTGATAGGATGAATCACGTAAATGATAAAAA
ACAAAATAAACGTAATGATAGATACTGGTATTAGTTTTTTATTTTTAAAAATAGCCGTATGTTTTTTGGTGAA
AATGTAACCTAGGTAAAATATTGGGAAAAATACGATTGTCCTTGAAATGCTTAAGTAGCTATCGATGTTTCCT
GAAAAACCTGCTCCAATAGATATAATAATTGAAACTGATAGCACTTTATATGGATTAAATCTTCTAACTATTA
CTAAAATGACATGAAAGAAAAATAGCGTGATCAAAAACCATAACGCAAATACTGGGTTAAAAGGATCAAGTTG
TAATTCGTCACTTTTACCTGTTAAGAAATAATATATTGAAAAGAATGCAAAAAATATCATATAAGGTACTATC
AAACGTTTTGAAATTTTTTCTAAATAGTATGGTTTATCAATATTTTTCGCGAAATAACCAGATATAAACAAAA
ATGTTGGCATATGGAAACTATAAATAACTAGGTATAATGCTGATAAATATTTATCGCCACTAGTGTAGGGTTG
TAACATATGTCCAAATACGACTAATAATATTAAAATTGCTCTTGCGTTGTCAAAAAAATAATCTCTCTCTTTT
AATGAAGTCATATTTTATCTCCTTTATGTCCATAAGACTCTTACTAATTTATTATATTTTGTATTAGTTATGC

AACAAATTTGTCTATTGTGTCGAATAGTATTGTAAAAATTATAAAAAAAGCATATAATATTATAAATAATATG
TAGTGGAGTAGAACAACATGTATAAACAACTTGAAAAACTTATTACACTGACTAACAATGACTTAAACTTAGT
GAATAGAAGATTTGGACAACGCACGGATATCACATCTGAACAGCTAGAACTTCTCCGTATTTTATTTAATTAC
GATCGCTTATCACAGTATGATTTGACGATGAAGATTAGCAGGGAACAATCTATAGTTTCAAGGTGGATTAAGA
AATTAGTTTTGAAAGGGTACATCACAAGTCAACAATCTAGCGAAGATTTAAGATGTAAAGAATTAATTTTGAC
TGATCAAGCACGTACATTAATTTCACAAATAAATAATGCACGTTGCGAATTGATTGAAGCAAGATGTCAATGT
TTATCGGAAGTCGAATTAGATAATTTAAATCAATTACTTGATAAGTTAAATCAACGACGCATATCGTTGTAAC
ACGACAATAGCAAACACATAATTTAGTGTCATTGTGCACACAACAAAAATTGAATCATGAATTACAAGCAAAA
GTAGCGGTGATTGTTAAAATTGATGGTAAACAATCACCGCTATTTTTGCTTGTGTATGTATAAAAAAGGGATC
AAAGGTCATCCCCCATGATTGATAGTGGGGGATGACTTTTGATCCTATGTTCATGTTGCTTATTTATATTGT
GGGATGTCGAAGTATTTGCCGACTTCGCCCATTTTATCATAGTAGCCTTTGATGATTTTAGCATTGATGTTAG
CCCAATCTACATCTGTAGCATATTGGTGTGTTCCTGGATGTGCAGGATTCCATCTCATTTTGTAAAGCGTATT
TTGACCAGCTTTTACATATGAGTTGCCGATGAATTTAGCACCACCAACGATGGCTTTTGAAACTGTGTCCCAA
CCAGCTTGTTTAGCATATTTAATACCTTCACGTAAAGGATCGTTATCATATGCAGCAATACCAAATACGTTAT
GGTATTTCGTGTTAGAGTTAGTTACAACTTTGTTGTTCACTACATCTGCACCTTTTGCTAATTGAGAAGTACC
GTTACCTGTTTCTAATAGGGCATGTGAGATAAGATAAACTTCATTAATGCCATACATTTGAGCAGCTTTGTTA
AATGCAGCACCTTGGTTTTCTAATACACCTTTACCTTTTAAGAATTGATTAATTTTATCAATAGAAATATTTT
GTGGTTGGTCTAAGCGTAAGAATTGATATTTTAATGCTGGATCTTGAGCTAAACGCTTCGTATCCATTGCATG
CTTAACATCATTAAAGTTAGCATCTGTCCACTTACCTGGTACACGTTGTACTTGTGGTTTATATTGTAAACCA
GCTTGAATTTGAGCAACTTGGTTTAATGTCATACCTGTTTGATTATACTTAATTAATTCTTTAGCTAAATCAG
TCGATTTAATCCATGCTAATTTACCGTTAGATAATTTACCATAGTACCAAGTTTGTCCATTAATGACTTGTTC
TTTAACAACTGAGAATGGTTGTTCATTAAATGCTTTTAATGAGTATTTAGCTGTATCAGAATTTGGTGTTACA
TAGTAATAACCATTACCATTTTTAATTACATAAGTGTAGTTATAATCTTTGGCAGCTGATGTAGTTGGTTTAA
CAGCAGTTGGTGCAGTTAAATCTTTTGCATTTACCCAACCAGTGCGGTTATTAATAGTACCGTATAAATAAAC
ATCTTTGCCTACAGATACTTGTTTCGCTGCATTAAATGTACCTTGAGCAATGTTATTGCCTGTTAAAATGACT
TGGTTTTTAGTACCCCAAGGAACCATTGATAAGCCGTTATTTGATTTATTAACAGTATATTTTTGAGTCGTTT
TAACTTCTTTGCCTAAGTTTTGAACATTTAAGTCTTTAACATTGAACCAACCTAATGGAATATTATGACTTGT
ATTATTTAATAATACATATGTTTCATTACCATGTGCACGTTCTTTTGTTACATAGAATGTACGATCCGCATAT
TTTGCGCCGTTTTTCGCTGTTTTTTCATAAACAGAAGCACGAATACCAGTGTTGTTTGGTTTAACTTGAGCAA
TTTTGCTAACTGTTTGAGTCGTTTGTGGTTTAGTAACAGCATAAGCTTTTACAGCTGTTTTTGGTTGTTCTAC
AGCTTTTTTAGGTGCAGCAGGTACAGCTAAATATGCTTTACTAATCCATCCGGATTTACCATTTACAGTTCCA
TATAAATAGATAGATTTATCAATTTGTTGTTGCTTAGTCGCTTTAAAAGTTTGGTTACCTGTGCCAGAAACCG
CACCAGCTTCTTGTTTATAAGTACCCCAAGGTACTGAATATAATTTAGTGCCTGGTTTTACTGTATATGTTTG
CATTACATTTACAGGTGATTTTGCATTGTTATAAATAACGTCACCTTGTTTAACCCAACCAATTAAAGTTGGA
CTATTGTAATCTTTAACTAGGTAGAATTTGTTTCCACCTAGACTTGCTTCTTTTGTTACAGAAAATGTTTTTT
GAACTTCTTTCGTTGGCTTACCAGTTTTGTCATAAACTGTAGTGAATAAACCATTGTTTTTAGCATTAATTTG
AGCAACACCATTTAATGCTGAGACTGTTAATTTATTATTTGTTGTCGGCGTTGATGGCTTAGGTGTTGGTGCA
GGTGTAGGTTTAGCAGTATCAACTAAATATGCTTTACTTACCCAACCAGATTTACCATTCACAGAGCCATATA
AATAAATTGATTTATCAATTTGTTGTTGCTTTGAAGCCTTAAATGTTTGGTTTCCAGAGCCAGACACGCTACC
AGCAACTTGTTTAGATGTACCCCAAGGTACTGTATAAAGTTTCGTACCAGGTTTGATTGAATATGATTGATTT
ACATTTACAGGTGATTTAGCTGTGTTGTAAACCACATCGCCTTCTTTAACCCAACCAAATTTATTACCAGAAT
TGTAATCTTGAACGAGATAGAATTTTTGATTACCTAATGTAGCTGTTTTAGATACAGCAAATGTTTTTTGAAC
TTCATTAGTTGCCTTACCAGTTTTGTCGTAAACAGTAGTATATAAACCACTATTTGTTGGTTTGATTTGTGCG
ACACCATTATTAGCATCAACTGTTAATTTACCAGTTGATGGTGTTGATGGTTTCGACGGTGTTGTTGGTTTTG
ATGGTGTAGTAGGGGTAGTTGTAGATTGCGTACCCCATGGCGCCACTTTACCCATTTTTATTAAATATTTTTC
ATTAATTAAGTCATATAATTGATCATAACTATAATTATGACTTCTTAAATATCCATGTGGATCGGCATGGTCC
GTACCACCTAAATATTTACTTACAGCGTAGTGAGTCCATACTGTACCATTTCCATCATACTCAGCACTGTCTG
GTTTTAAACCATAATATTGTAATTGTGTAGCTGCATAGTCAGCATAGTTATTCATTGAACGTGCAAATGAAGC
ATAATCGTGTGTGTGTACGATTTCAACATTGATGAATCTAGGGTTACCGACTGCACCTACACCCCATGATAAG
TAATCCGTTGGTGCTGTTTCAATTATACGATCCCCATCAACAAACGCATGAACGAAAGCATTTTGATAATTGT
TTTTCATATAACTAATTTCGCCATTTATCGTCGAACGATCATTAGCTGTATCATGAACTACGATTCCTTCAGG
ACGACCTACGCCGTTACGGTATGCGTATTTAGGGAAGTAAGATGTATAATCTTCTTCAATCTTAGGTGCTTTT
AAATTATTTTTACGAATGTAATCGTTAATTGATGAGTTTACTTGCGGTTTATATTTTGGCAAACTCGTTTTTG
GTGCTGCAGCGACTGATCTTGGTTGTGCTGAAGCACTAAAAGTAGTTACTTTTGGTGTAGCTTCAGTTTTAGC
TTTAGGTGCAACAGGTGTTGCTTTTGGTGCTGCGGCTTTATATTGCGTTTCAAGAGCAGCAGGTTTAGCAGCT
GATTTAATTAATTCTGGATTTATTTGATTTTCTGAATTATCATCTTCATCATCAACTAAACTATATCCAGCAC
TTGTAACATTATTGTTCGTTTTAGATGCTGTTGTGTTAGTTGTTTTTGTAACAGGTTTCGCATTATTTGATGT
AGTTGATTGAGTCTCACGAGTGTCACCATTTACTTGTGTAGTATCAACCTTTTGATTTACTTGAGCATTGCCT

GTTTTATTCGCTGCTTTTGGTTGGACAATAGCAGGGTCTTGATATACTTGAGTGCCAGAAATGTTTTGCGTTG
GATTTTTTACCTCAGCTTTTGCTTGTTCAGTAGTTGCTTTAACTTTATTACTATCTAAAACGTTTTTATTAGT
AGTTTGATCTTGTGTCGTCTCAGCTGCTTGAACTTGATGTGCAGTGACTGCTGAACCTACAAGCGTTAATGCA
ACCATTGATGGTAGTTTGTAATTAAATTTTTTCGCCATTCTATTTATTACTCCTAACATTTATTAATTATTAA
TCTCATTATAGTACCTGTTTTATACACCTGTGCGTATTTAACCAATTTGTAATATACTCTTAATTTATATTTT
CACTGTAAAAAAATGAAAAAAATTTTGAATCTCAATGGTGACGTGACAAATCAAATAGAATTGATGTCAATTT
AAGAATTGGTTTATTACAAATGTTTCTATAATAGATACAACTTTTTTTAATTAAGCGAGGTCAACTTTTTTGT
CATTTCAATATGCTCGATGCCTTCCTCAAGAAATATATTACCTCTCATTTTAAAATTTAAACTTTCATAGAAT
GGAATTGCATGACATTGGGCATTCATGGTAGCTACGTAAAAACCTTCATCTATAGCTAATGACTCTACAGCTT
GCATAAGCATTCTACCCATGCCTTGTCCGCGATGTGATTTCATCACAGCTACTCGTTCTATTTTGACAGTTGT
TTCATTAATAGGGCGTATTCGAGCAGTGGCAACTGGCTGCCCATTGTCATACCCAATCAGGTGAATAGATTCA
GATTCATATTCATCAATTTCACTTTCCTCTGGGACGCCTTGTTCTTCTACAAACACTTTCTTTCTTATATAGA
AGCAATCTTCTAACATCTTTTGATTGTTTACTTTTAAAAACATAAAATCCCTCCATATCCCAACAAAATCATT
TATATTTAATTTACTAATAATAACATAGAATAACGATTGTTATTTTTCACAAAAATAATTAGACATAAAAAAT
GCTATAAAAATGATTCATAGGAACCATCTTTATAGCTAGATAGAGTGTATTTGTTATAATGCTTCTTTTCTAA
TAATTGCTGTATATTGCCAAAAGATTCGCATTTGAGCAATATTCCAATTATTCATCCCCATTGTAAACCCAGA
AGGTTTAAAGATATTTACATCTGTCACTTCTAAAGCTGCTGCGACTAAATATTGTAATGGAATACTAAGTGAT
TTCATTGTTTCAGTAATGCCATTTTCATCATATACCCATGAAAATGGTAACTCTGATTCGAAAACATCAACAC
TATTGAAAATTTCAGGTAGGGCTAAAATATAACATGCACCATCTACAATAGGATTCTCGATACTATTTTTGTA
ATAAATTAGGAGTGCTTCATAGTTTTCTCGATGCTCATGATTGACGTAAAAAAATTCACTTCTAAAATGAGCC
ATATCTTGGCTATGAATAATTTGTTGTTCTAACATATTAAACATCCCATTTATATTTGCAATCTTTTCGTATG
TTTTTCTACTCATGTATGATGCTCCTTTCATAAATAGTAAATGTTAATCGTCATATAATATTAGTTTACAACA
CCATTTTGGTTATTTGAAGCTTGTGGCGTTTGTTGAGTGCCACCTTGATTTTGATTTAAGTTTTGATCTGTAG
CAGGTTGTTGTTGATTATTGACGCTATTGTTGCTATTACTGTTATTACTGTCGTCGCTATTATTCGAAGATTT
ATCTTTATCAGTCGTATCGTTTTGTTCTTTTCTCAATAAACTACTATCTAAAGGCGTTAATGGTATTAATGAA
CCATAATGATTGATGACACGTTGATCTAAGAAATCATTTTTATCATTAATAGGTGATAATTCTAAGTCTTTAC
GAAGTAAGTTTGCATATTTTTGAATGCTGTCTACACTTGGATGATAATAGTAAATACCATTTAACATATCATC
TTTACCTTTTAATTGCGCAGTTTTAATTTCAACATCATTTGTTAAGTACATTTTTGCTAAAGCTTTAATTTCA
GAGTTAGTTAAATTATGCTTTGCATTTTTACCTACAATTTGAATCACGTTATCAAGTTTATCAATAGAATCAA
CTTCCTGTGCTTTTTGGAATAAAATCTTAATTAATTCCATTTGACGTTGTCCACGTTTCAAGTCTGAATCATG
ATGTCTAGTTCTAGCAACTGCTAAAGCCTCATCACCATTTAATTTTTGGTACCCTTTTTTAATTTTAATCTTA
CCAGTATCATCTGTATTTGGCTCATTTAAATCATATGGTACATCATAGTATATGCCACCAAGTTCATTTACAG
CTTCTACAAAGGCCTTCATATTAACGCGTACATAGTAATCAGCTGGAACATTCATCGTTGCTTCAACAGAGTC
CATTGCTGCAATTGGACCACCATATGCATGTGCATGGGTAATTTTATCGTAGTAGCCGACTTTAGGAATGTAG
CTGATTGTATCACGAGGAATACTAAGCATTCGAATTTGGTGTTTAGATTGATTAAAAGTAGTTAAAATCATAG
CGTCTGATCTAGAGTGTTCAGCATCTTGGCCTTTTTTTCTTCTTCCGTCATTATCATCGATACCTAAGAAGAG
AATAGAGATAGGTTGTTCTTCAGGATTAACTTTATTGTCTCGTAAATTTGATTGACGATTAGCATTTTTACTA
TCTTGAGAAGATTCAAATGCATCTTGAGACGTTTTAAAAAGTAACGTAGCGAAGACAATTGGAACAACAATAA
GAACCAATGCTAGAAGGATCAAAAAGTATTTTAAAAATTTATTCATGATTGATGCTCCTATATTAAATTTTTG
TTAAATCATAATCATAATTTTAAAATATAATTAGTTGAACGATATTTATACAACTTCATTACTAATACATTAT
AGCCTTAAATTGTAGTACTTTATAGTAAAAAATACAAATGTTATGTCAAAGTTCCTTGTAATTTGAATGTTTT
GTTAAAATCAAAAAGCATTTTAAAAAATATAATGCGACTTTAGTATGATGTCTAAAAAAAATTAAAGGGGTAA
TATAAATTTGAGTGATTTATACAATGTGATAAGGTATAATTAAATTTGTTGTAATAAGAACTTATATGAAGAA
AATATTACTAAGTAGCATAACGCATAAGTGGGTGACAGATAAAATTGTAAGTGATATTATAACGAAGTTAAGG
TTATTGTTTTAATTATTTAATTTGAATACAATGAGAGGCTATTAGCGAATGAAATTTAATAAAGTGAAATTGG
TTATACATGCATGCGTGCTTTTGTTCATCATTATCTCTATAGGATTAGTTTTTCATCGATTACAAACGAAAAC
GAACTCTATAGAACCTATACATAAGGAAACAAAATTATCGGATAATGCGAAATATTTGGTTGATCGTAATAAA
GGCAAAGGTGAGCCATCGAAATTAAAGGAAGTATACAATAGTAAAGATCCAAAGTATAAGAAGATTGACCAGT
ATTTACAAAATTCATTATTTAATGGTTCAGTAGCGGTATATGAAAATGGCAAATTGAAAATGAGTAAAGGCTA
TGGATATCAAGATTTTGAAAAAGGAATTAAAAATACACCAAATACGATGTTTCTAATTGGTTCAGCTCAAAAA
TTTTCAACAGGATTACTGTTAAAACAGTTAGAAGAAGAACATAAAATTAATATTAATGATCCAGTAAGCAAAT
ATATTCCATGGTTTAAAACATCTAAGCCCATTCCTTTAAAAGACTTGATGCTGCATCAAAGTGGATTATATAA
ATATAAATCTTCAAAAGACTATAAGAACTTAGATCAAGCAGTGAGAGCAATTCAAAAACGTGGCATTGATCCT
AAGAAATACAAAAAGCATATGTATAATGATGGGAATTATTTGGTGCTAGCTAAAGTTATCGAAGAAGTTACCG
GCAAATCATATGCCGAGAATTACTATACAAAAATAGGGGATCCTCTAAAACTTCAGCATTCAGCATTTTATGA
TGAAAAATCATTTAGAAAATATTTAGCAAAAGGTTATTCTTATAATAGTACAGGACTTTCTTTCTTGAAGCCT
AATGTTTTAGAACAATATTATGGTGCAGGTAATATATATATGACACCAACAGATATGGGTAAATTAATTACTC
AAATGCAACAATATAAATTATTTAGTCCTAAAATAACCAACCCATTATTACATGAGTTTGGTACGAAACAGTA

TCCAGATGAATATCGATATGGTTTCTATGTTAAGCCAACATTGAATAGACTTAACGGGGGATTCTTTGGACAA
GTCTTTACTGTTTACTATAATGATAAGTATGTAGTTGTACTTGCATTAAATGTAAAAGGAAACAATGAAGTTC
GAATCAAACATATTTATAATGATATTTTAAAACAAAATAAACCTTACAATACGAAGGGTGTTATTGTTCAATA
ATTAATATAGAAGATATAACTTGTATATGGCATTAAGGCATTGACCTTATTTGACTAGTATACGAGTTATATC
TTCTTTTTTATAGAGGTGAAAAGTTTAAAGTATAAGGTTGAAGTAGTAGGATGATAAATTTGCGTTAAGTTAA
AAAGATATAAACAATTGATGTAAATGTCAATATAAAAATGTATATAAATGATTGTATAAGAATGTATTAATTG
AATGGATAATAATGATTGTCTTTTAAGATATAGAAGGGGACTTAACCATTTAAAAATTGAAGGAAAATAAAAA
CCAGCTAACAAAAGTGTTAGCTGGTTAAACTTTACATTATTAAAATGTTGTTTATAAGTGTGAAGAGTGACCG
CCTTGCATAACCCAATATGTTCCGACAACGAAACAAAGTGTAATTACAAGAGCAAAGATAACTTTGAATGTTT
GTAAACGTCCATCTTTACCTTCAGTTAAATGCATGAACATTAATAATTGAAGTCCAGCTTGGATGAATGCAAA
GCCAAAGATAATTGTCAACTTCGCGTGGAATGTTAATGACGTGTATAGTGTTACGTAAACTGCTAAAAGCGTT
AATACGATAGATGCGATAAATCCTACAGTATGTTTCATTATTGTACTCATCCGCTATACACCATCCCTATCAT
ATATACGGCAGTAAAGATGAAAACCCAAACAACATCTAAGAAGTGCCAGTATAAACTTACTATAAATAATTTT
GGCGCATTATATTTGTCTAATCCGCGTCGTTGGATTTGGATTAATAAACAAATGGCCCAAACGATACCTAGCG
ATACGTGACAACCATGCGTTCCTAATAGGATAAAGAAACTAGACCAGTAAGAACCAATTGTTGGGTTAACGCC
TTCTGATGCATAGTGTGCGAATTCATAAATTTCGAATCCAACAAAGACTAAACCTAAAAGTAACGTAATGATC
ATCCAAAACATCATTAACTTTTGTTTTTCTTGGCGCATGTAGTAAATAGCAATACCACATGTGTAAGAACTGA
ATAATAATGCAAACGTCATTATTAAAACAAGAGGCAATTCAAATAACTCAGTAGTCATTTTACCTGCATAATC
GCCACCATGTTGCAAAGTTAATAGTGTCGCAAATAGGGTACCGAATAACGCAAATTCGGCTGTAATGAAAATC
CAAAAGCCAAGCTTATTTAATTCGCCTTCATGTGTGCGTGAATCAATAGTGTTTGTATCATGACTCATGACTT
ACAGCCTCCCTTTCTTTAATACGCGCTTCTCTTAATCTAGCTTCAGTTTCAGCAACTTCAGCAGCAGGGATAT
GATATCCGTGATCGATTTGGAAACTGCGATAAATCATAGTACCAAAAATACCGAATAAACAAATTAATGCTGG
AATTACAGTTTCGAAAATTAAGAAGAAACCGCCGATAGTCATAAAGATACCAATCCAGAATCCAACAGGAGTA
TTGTTTGGCATATGAATGTCTTTGTAATTATGGTTGTCTAAGTAATGACGACCATGTTCTTTCATATCAACAA
ATGTGTCGTAGTCATTCCAATCTGGTGTAATGGCAAAGTTGTATTTAGGTGGAATTGCTGATGCAGTAGTCCA
CTCTAGAGTACGACCAAGGCCATCCCAGTTATCTCCAGTTGCTTCACGTGGAGATTTGAAGTGACTGTATACG
ATACTAACAACAAGGAATAAGAATCCGATTGCCATTAATAATGCACCGATAGTTGAAATTAAGTTTAATAAGA
ACCAACCATCTGATGGCATATAAGTGTATAAACGACGTGGCATACCATCTAATCCAAGAATGAATTGTGGTAA
GAAACAAACGTTAAATCCGATCATGAAGAACCAGAAGCACCATTTGTTTAATGTTTCGTTTAATTTGTAACCC
ATCATCTTTGGATACCAGAAGATTAAACCAGCTAAGCAGGCAAATACAACACCAGTAACCAATGTATAGTGGA
AGTGAGCTACTAAGAAGTACGTATTGTGATATTGATAGTCAGCTGATGCCATTGCTAACATTACACCCGTAAC
ACCACCTAAAAGGAAGTTAGGGATAAATGCTAATGAGAATAGCATTGGTGACTCAAATGTAATACGTCCTTTA
TATAATGTTAATAACCAGTTAAACAATTTCACACCAGTTGGAATACCAATCAGCATTGTTGAAATTGAGAAGA
ATGAGTTGATTAACGCACCATTACCCATTGTGAAGAAATGGTGAACCCAAACTAAGAAACTAAGGAACGCGAT
ACCGGCAGTTGCCCATACCATACTTTGATGTCCGAATAAACGCTTACGAGCGAATGTCGGGATAATTTCTGAG
TAAATACCAAATGCTGGAAGGATAACGATATAAACTTCAGGGTGCCCCCATACCCAGAAGAAGTTAGCCCAAA
GCATTGGCATACCGCCATGTGCAACTGTGAAGAATGCTGTGTCAAATATTCTATCAGTTGTCATTAATGCTAA
CGCTACTGTTAAAGGAGGGAAAGCAAGAATAACAATTAATGTAGTAATAAATGTTGTTACTGTAAACATTGGC
ATTTGCATAAACTTCATAGTTGGTGTTTTACATCTTAAAATTGTTACAAAGAAGTTGATACCTGTAGCTAAGG
TACCAAGCCCTGAAATTTGTATAGCTATTAAGTAATAGTTAACACCCGGACCAGGACTGAATTCACCTGCTAG
TGGCGCATAGTTTGTCCAACCAGCTGCTGGTGAACCACCAATAATAAATGACAGGTTGAATAAAATCATACCT
GCAAAGAATAGCCAGAAACTTACGTTGTTTAATACTGGGAATGCAACATCACGTGCTCCAATTTGTAATGGAA
CAACGATATTCCATAAACCAAAGATAAATGGCATTGCCATGAAGATAATCATGATTACACCATGTGTACTAAA
AATTTCGTTATAGTGGTTAGATTCTAAAAATTTGTTATCAGGTACTGTTAATTGCGCACGAATAAGTAACGCA
TCAATACCACCACGGACGAACATTAATACGGCACAGATTAAATACATAATACCGATTTTCTTATGGTCTACAG
ATGTGAACCATTCTTTGTAAAGATATTTCCATAATTTAAAGTAAGTAATTACTGCGATTAAACCAATAACTAA
GAATGGGGCACCAATTTGTGCCATTGTAATCATCCAGTTACCTTTAACTAGTAATTGATCCCATGGAAAATTC
ATTAATGTCCACCTCCATGATCATCATTGTCTTGATCTTGCGCATCTTTTGAAATTTTCTTCATTTCTTTCGC
ATTTTTCGATTCATCTTTCTTGAACTCATTGTTATATGGTTCGTCATTTCCAAGAATCATCAATTTCATACCA
TGTCGTTTATAGTTCGCATTTGTAATTTGAGCTTTACGAGCAGGTATTAATGGTTTGTCTGATACATCTTTAA
ACATATTTTCTTCACTAGTGAAGTTTGGATCTTTCAATTCGAAATTGAAACGTTTATATGCATAGAAGATGTA
TTCTGGATCGGCTGCTGGATCAACAAACGCCATATGTGTACCATTAAATTCTAAAGCTTTATTAGGTGTGCTT
GGTAATAATTGTTTATCAAATGTATCTTGATCTAACGTTTTCTTACCTTTAACTTCTTTCACCCATTTGTCGT
AGTCTTTTTGACTAACGGCATTTACTTTAAATGTTTGACGTGAGAATCCTTCACCATTGAAGTTAGAGTTACG
ACCTCTGAACGTACCAGTTTGAGATGCTTCTAACGTCCAATTCATTGTCATGCCAGTCATGGCATATTTTTGA
CCACCTAATTGTGGAATCCAGAAACTTGTCATTGTATCCATAGCTTGAAGCTTAAATACAACAGGACGATCTT
TAGGGATTGTTAATGTATTAACAGTCTCTATATGTTCATCTGGATAAGCAAAGAACCATTTGTATCCTGCACT
TACTGCATATACAACCATTGGATCTTTCTCACTCTTCGGTGGTTTTTCGTAATCGTATAAAGTTTTAACTGTA

GGAATAGCTAAAGCAGCAACGATTATGATAGGTATTACAAACCATATTGTTTCAATGATGGCATTATGGTGCA
TCTTACCAGATTCGGCATTCTTATTATAACTATACTTGTAAATAAAAATGGCGAACATGCCAAGTACAACGAA
ACAAATAACAAGCATGAAGACGATTGAATAAAGAATCAAGAACTTCTGACTACTTGCTACTGGCCCTTTTGCG
TTGAAAATTTCTATATTTGAACAACCACTAAGTAAAATTAGTGTGCCAAATAATAGAAGCAAAGACTTAAATT
TTGACACTTTTTTGACCTCCTAATACTACAAATGTAGGGCTTAACATTAATTTTAAGTTATTACACAATATTT
ACAAGGGCTTATGGGAAAAAAATTAATAAAATTGTATCAAAAATGTTGATAAATCAAGGTGTGACGTGGGTTC
ACACATTTGTTAAAATTATGTGTACATTTTGTGACTAATAGCGTTTTTGAAAAAATTGCTTCATATTTAAAAG
GGAAGTTGAGAATAGCTTTCAATTAAAGGTTAATTGAATGAAAATAAAAAAAGTTTGAAATTGTCTAATAAAT
TGAAAATGATGGCTAGTTCATTGAAAGGAAGCGCAAACATTTTAATAAGGAATTTGCCAAAAGTAATTTATCG
TTAAACAATGAAAAGTAGAAATTTGTTTGTTCAAAATCAAAATATTCTTTTAAAAAGAGCGGTTAAGTTTTAA
TAATTTGAAAGGTAGTCCTTTGGTATACAGAAAAAAATACAATATTTGGGTTAAAGTTGCAGATGAAGTTGGAA
TAACAAATAAAATGGTAGCAAAACATGTACGTTTGCTACCATTTTATTTGCAGTATAGTGATGAGGGCTATTT
AACCTCAATATACCTAGTGGAAATTTTAATTGCACCATCTGAATCAGTGACATGATATTTAATTTGATATTTA
CCAGATTTAGATGTATCAATTTGGCCATCGACTTTAATTTTATCGGTTAAATCTCCATCTTCTTTATCAAATG
CACTTATGCCGTTTAGAAGGTTATAATCTTGACCTTTCTTAATAACGATATCATTAGCGCCTTTAATTTGTGG
TGTAGTATTCGTCGTTGCATCTGCATTTAAATTTGGTGTTACTAGTGTAGCAGAAACACCGAGGGCTGATAAT
GACTGTAATAGTTTATTCATAATGTAACCTCCGAATGATAAGTCATTTCTGAGTTAGCTTTACTATACCTTAA
TTTATATTATTTACACATCATACTATAGTCCTAATCTTACCCTATAAAGAATGAAATTTAAATATAAATTTAT
AAATAATGAGGAAATAATTATTATTTTGAAAATCTGCCACAATTGTAAAATTAATAATTCAGGGATAATTTGA
AATCAAATATAACTTTATTCGACAGTTTGAACATAAATTTTTGTGTATCAAAACAATAAGAAAAGGGAGGCGA
AAATGATAAAGAACCACTATTGATTAATTATGTAGTAGTACTTTATCATAGTCACAGCTTCTGTGATGGGGCC
TCGTCGACTTGCATTGTCTGTAGAAATTGGAAATCCAATTCCTCTTTGTTGGGGCCCATCCCCAACTTGCACA
TTATTGTAAGCTGACTTTCCGCCAGCTTCTATGTTGGGGCCCCGGACTATAATTGAAAAATGCTTGTTACAAG
CTTATTTTCATTCAATTAACAACTGTCTATATAAAATTGTTGAGCCAAGAACAAAGTGAAATGCTCCCTTCAA
AGCCTTCATTTATTCAATGTCTACTTTGAAGGGAGCATTTGATCGATTAATGTCCCAGATTTTTCTTGTTATT
CATTCACATTACATGACTAATGTGAATTTACGAATCAATACCTCGACGCATTTTTTCTGCAAGCAAAGTATTA
TTTAATACCATAGTAATTGTTAATGGACCAACGCCACCAGGAACTGGTGTAATAGCTCCAGCAATTTCTTTAA
CCGCATCATAATCAACGTCACCTTTTAATTTCCCATTTTCATCTGGCGTATTGCCAACATCGATAATTACTGC
TCCTTCTTTGACCACATCTTTTGTTACTAAACCAGGCTTACCAACTGCACTGACAATGACATCAGCATCTTTT
AAATATGATGCCATATCCTTTGAACGAGAATGTAAGATTGTTACTGATGCATTTTTTTGAAGTAGTAACTTAG
AAACTGGTTGTCCGACAATATGACTTCGTCCAATTACAACTGCATTTTTACCTTCTAAATCAATATCAGCATG
TTTTAATATTTCCATGATGCCGAGCGGTGTGCAAGGTACAAAAGTTTGTTCATCGATATATAATTTCCCTATA
TTTATTGGATGAAAACCGTCCACATCTTTATCAGGATTGATTGCTTCTAATATTTTCTGTTCGCTAACTTGTT
TTGGTAGTGGTACTTGTACCAAAATACCACTTACAGAATCATCATTATTTAGTCTATTTAGTTCGTTTAATAC
TTCCTCTTCAGTAGCTGTTTCTTCCAAATGTACGATTTCTGAAATCATACCAATTTTTTCAGCTGCTTTCTTT
TTTGATCTAACATAACTTTGACTAGCGCCATCATTACCAACTAATATAACGGATAATTTAGGTGTAAAACCCT
TTTCTTTTAGCGCTTCAACTTGATCTTGTAACCCCTGTCTGTAGTCTTTGGCAATTTGTTTACCATCTAAAAT
TTTAGCAACCATAAAAACTTCTCCTCCTATTGTCAATATCCTAACTTTGTTAGTTTATAAAACTAATTTACAT
AGCAAAAGACGTAAATTCGAACAAAAAATCAAAATAAAGTTCGATTTTTGATTGAAAAAGCAGAAATTGCTTG
TTATGCTATATCTATAATATACAACTAAAGTAATTCACAATTCAAACTTTTGAAAGGGTGTACAAAGTGAAAG
TAGCAGTCATTATGGGCAGTTCTTCCGATTGGAAAATTATGCAAGAGAGTTGTAACATGTTGGATTATTTTGA
AATTCCGTACGAAAAACAAGTAGTATCGGCACATCGTACGCCAAAAATGATGGTTCAATTTGCTTCTGAAGCG
AGAGAAAGCGGTATAGACATTATCATTGCAGGCGCTGGCGGTGCGGCACATTTACCAGGTATGGTTGCATCAT
TAACGACGCTACCAGTTATTGGAGTGCCGATTGAAACAAAAAGTTTAAAGGGTATAGATTCTTTATTATCAAT
TGTTCAAATGCCAGGAGGTATTCCGGTTGCAACGACTGCAATTGGTGCAGCAGGTGCTAAAAACGCAGGTATA
CTTGCAGCAAGAATGTTAAGTATTCAAAATCCTTCTTTAGTTGAAAAACTAAATCATTATGAATCTTCGTTAA
TTCAAAAAGTGGAGGACATGCAAAATGAACTTCAATAAATTAAAGTTTGGTGCGACTATTGGCATTATTGGTG
GTGGTCAGCTTGGAAAGATGATGGCACAATCAGCTCAAAAAATGGGTTATAAAGTGGCTGTATTGGATCCTGC
TGAAGATTGTCCATGTAGATACGTTGCACACGAATTTATACAAGCCAAGTATGACGATGAAAAGGCACTCAAT
CAATTAGGACAAAAAATGTGATGTGATTACTTATGAATTTGAAAACATTTCAGCCCAACAATTAAAACTATTAT
GTGAAAAGTACAATATTCCGCAAGGTTACCAAGCTATACAGTTATTACAAGACCGCTTAACTGAAAAAGAAAC
ATTAAAAAGTGCTGGTACCAAAGTTGTCCCGTTCATTTCAGTAAAAGAATCTACAGATATTGACAAAGCAATT
GAAACATTAGGATATCCTTTTATTGTAAAAACTAGATTTGGTGGCTACGATGGCAAAGGTCAAGTTTTAATTA
ACAACGAAAAAGACTTACAAGAAGGTTTTAAATTAATTGAAACTAGTGAATGCGTAGCTGAAAAATATTTGAA
TATCAAGAAAGAAGTATCACTTACTGTTACAAGAGGAAACAACAATCAAATTACTTATTTCCCATTACAAGAA
AATGAGCATAGAAATCAAATACTTTTCAAAACAATTGTTCCAGCGAGAATAGATAAAACAGCTGAGGCGAAAG
AGCAAGTTAATAAAATTATCCAATCGATTCATTTCATTGGAACATTTACAGTTGAATTTTTTATAGATAGTAA
CAACCAATTGTATGTGAACGAGATTGCACCAAGGCCTCACAATTCCGGACATTATTCAATTGAAGCATGTGAT

TATTCACAATTTGATACTCATATTTTAGCAGTTACCGGACAATCATTACCAAATTCAATTGAATTATTAAAGC
CTGCAGTCATGATGAACTTACTAGGTAAAGACTTAGATTTATTGGAAAATGAATTTAATGAACATCCAGAGTG
GCACTTACATATTTATGGTAAGTCTGAGCGTAAAGATAGTAGAAAAATGGGGCATATGACTGTACTAACGAAT
GATGTAAACCAAACTGAACAAGATATGTACGCTAAATTTGAGGGGAGTAATTAAAGCATGACATTATTATATG
AAGGAAAAGCGAAGCGTATTTTCTCAACAAATCAAGAAAATGAATTAAGAGTTGAATATAAAGATGAAGTTAC
TGCTGGAAACGGGGCTAAGAAAGACACAATGGCAGGTAAGGGGCGATTAAATAATCAAATTACTTCTATTATA
TTTAAATATTTACAAGAAAATGGAATAGAAAGTCACTTTATTAAACAATTATCTGAAACAGATCAATTAGTTA
AGCCTGTGAAAATAATTCCATTAGAAGTAGTTGTCCGTAATATTGCTAGTGGATCTATTACAAAGCGTTTAGG
TTTTGAAAATGGTGAAGTTTTTAGAGAACCACTTGTGGAATTTTTCTATAAAAATGATGCATTAAATGATCCT
TTGATTACAGATGATCACATCAAATTACTGAATATTGCGATAGATAAAGATATTGAAACATTAAAAACGAAAG
CGCTAGAAATTAATCAAGTATTAAAACAATTAATGGATGCGATGAATTTAAAATTAGTAGATTTTAAAATCGA
ATTTGGAAAGACTGAGACTGGTCAAATTTTGTTAGCGGATGAAATATCTCCAGATACATGTCGAATTTGGGAT
AAAGCTACCAATGCAAACTTTGATAAAGATGTATATAGAAATAACACTGGATCACTGATTGAAACATATCAAA
TATTTTTAAACAAATTGGAGGATTTAAAATAATGAAAACAATTGAACTACATATCACATTACAACCACAAGTA
TTAGATACGCAAGGACAAACGCTTACTCGAGCTGTACATGACTTAGGTTATGCACAAGTGAATGATATTCGTG
TAGGAAAAGTATTATATATGACAGTGGATGAGGTTAGTGATGAAAAGGTACACAACATTATTACAACTCTAAG
TGAAAAATTGTTTGCAAATACAGTGATTGAAGAATATAGCTATAAAGTGTTAGATGATGAAAAGGAGAATGCA
TAAAATGAAATTTGCGGTTCTTGTTTTTCCAGGTTCGAATTGTGATAGAGACATGTTTAATGCTGCTATTAAA
AGTGGTGTTGAAGCGGAATATGTAGATTATAGAGAAACATCACTAAGTGGATTTGATGGCGTACTTATTCCTG
GTGGATTTTCATTCGGGGATTACTTAAGATCTGGGGCAATGGCTAGTGTAGCGCCGATTATTTCGGAAGTTAA
ACGTCTTGCAGCTGAAGGTAAGCCAGTATTAGGTGTTTGTAATGGGTTTCAAATTTTAACTGAAATAGGCTTA
TTACCTGGTGCATTATTGCATAACGATTCACATTTATTTATTAGTAGAAATGAAAAGTTAGAAATAGTGAATA
ATCAAACGGCATTTACAAATCTTTATGAACAAGGTGAAAAAGTTATATATCCTGTAGCTCACGGTGAAGGTCA
TTATTATTGTACTGATGAAATATATCAACAATTAAAAGCTAACAATCAAATTATTCTGAAATATGTGAATAAT
CCGAACGGTTCATATGATGATATTGCAGGAATTGTTAATGAAAAAGGCAATGTATGTGGCATGATGCCACATC
CTGAAAGAGCTTTAGAAACGTTGTTAGGTACTGATAGTGGTGTGAAATTATTTGAAGCGATGGTAAAAAGTTG
GAGGGAACAACATGTCTAAATTTATCGAACCAAGCGTTGAAGAAATTAAACTTGAAAAAGTATATCAAGATAT
GGGATTAAGTGATCAAGAATATGAAAAAGTTTGCGATATTTTAGGCAGACAACCTAACTTTACAGAAACAGGT
ATCTTTTCTGTTATGTGGAGTGAACATTGCTCTTATAAACATTCTAAACCGTTTTTAAAGCAATTTCCTACGT
CAGGTGAGCATGTGCTTATGGGGCCTGGTGAAGGTGCAGGGGTAGTCGATATAGGTGATAATCAAGCCGTAGT
ATTTAAAGTAGAGTCTCACAATCATCCATCAGCAATTGAACCATATCAAGGGGCTGCTACAGGCGTTGGTGGA
ATCATTCGTGACATTGTCTCTATTGGGGCTAGACCTATTAATTTGTTAAACAGTCTTAGATTTGGAGAATTAG
ATAATAAACAAAACCAAAGATTACTTAAAGGTGTTGTAAAGGGTATCGGAGGTTATGGTAACTGCATTGGTAT
TCCAACAACTGCTGGTGAAATCGAATTTGATGAACGTTATGATGGCAATCCACTTGTTAATGCAATGTGTGTT
GGTGTTATCAATCACGACATGATTCAAAAAGGCACAGCAAAAGGTGTAGGTAATTCGGTCATTTATGTTGGTT
TGAAAACTGGTCGAGATGGTATTCATGGTGCTACTTTTGCATCTGAAGAATTGACGGAAGAAAGCGAAAGTAA
ACGACCTTCTGTACAAATCGGTGATCCATTTGTAGGTAAAAAATTAATGGAAGCAACACTTGAAGCAATTACA
TTTGATGAATTAGTTGGTATTCAAGATATGGGTGCTGCTGGTTTAACATCTTCATCGTCTGAAATGGCGGCAA
AAGGTGGTAGTGGGTTACATTTGAGATTAGAACAAGTGCCAACACGTGAGCCAGGTATTTCTCCTTATGAAAT
GATGCTTTCAGAAACTCAAGAACGTATGTTACTAGTTGTTGAAAAAGGTACTGAACAAAAATTCTTAGATTTA
TTTGATAAGCACGAATTGGATAGTGCTGTTATAGGTGAAGTTACAGATACAAATCGTTTTGTTTTAACATATG
ATGACGAAGTTTATGCTGACATTCCAGTTGAACCACTAGCTGATGAAGCACCTGTATATATTTTAGAAGGAGA
AGAAAAAGATTATAATACTTCTAAAAATGATTATACACACATCGATGTTAAAGATACTTTCTTTAAATTACTT
AAGCATCCGACTATAGCATCTAAACACTATTTATATGATCAATACGACCAACAAGTTGGTGCCAATACGATAA
TTAAGCCAGGACTTCAAGCATCGGTAGTACGTGTGGAAGGCACAAATAAGGCGATTGCTTCAACAATTGATGG
TGAAGCGCGTTATGTATATAACAATCCATATGAAGGTGGAAAGATGGTAGTAGCTGAAGCTTATCGAAATTTA
ATTGCCGTGGGTGCAACACCATTAGCAATGACAGATTGTTTAAATTATGGTTCTCCTGAAAAGAAAGAAATCT
ATCAACAGTTGATAGATTCAACGAAAGGTATGGCAGAAGCATGCGACATTCTTAAGACACCAGTAGTTTCTGG
AAATGTATCTTTATATAACGAAACGAAAGGTACTTCTATTTTCCCAACACCAGTTGTTGGAATGGTAGGTTTG
ATTGAAAATGTAAATTATTTAAATGATTTTGAACCTCAAGTTGGAGATAAATTATATTTAATCGGTGATACTA
AGGACGACTTTGGTGGTAGTCAACTTGAAAAGTTAATTTATGGCAAAGTTAATCATGAATTTGAGTCATTAGA
TTTGAGTTCAGAAGTTGAAAAAGGTGAATCAATCAAGACCGCTATTCGTGAAGGACTATTATCACATGTTCAA
ACAGTTGGTAAAGGTGGCTTACTGATTACCTTAGCTAAACTAAGTGCGCATTACGGTTTAGGATTAAAATCTT
CAATAGATATAACAAATGCACAATTGTTTAGTGAGACACAAGGCCGATATGTTGTTTCTGTTAAATCAGGTAA
AACTTTAAATATTGATAATGCAATAGAAATTGGACTTTTAACAGATAGTGATAATTTCAAAGTAACAACACCA
TATACAGAGATTAGTGAAAATGTTTCAGATATTAAACAAATATGGGAAGGGGCAATTGCTCAATGTTTAACTA
CTCAGGATTAAACGAAGAATGTGGCGTGTTTGGTATTTGGAATCATCCTGAAGCAGCGCAACTAACATATATG
GGACTTCATAGTTTGCAACATCGTGGTCAAGAAGGTGCAGGTATAGTTGTTTCTGATCAAAATGAATTAAAAG

GCGAGCGAGGATTAGGCTTACTAACTGAAGCGATTAAAGATGATCAAATGGAACGATTAAAAGGATATCAACA
TGCAATTGGTCACGTCCGTTATGCTACTTCAGGTAATAAAGGTATTGAAAATATTCAACCGTTTCTGTATCAC
TTTTATGATATGAGTGTAGGTATTTGTCATAATGGTAACCTCATTAATGCTAAATCATTGCGTCAGAATTTAG
AAAAACAAGGTGCTATCTTCCATTCGTCTTCTGATACTGAAGTCATTATGCATTTGATTCGACGCAGTAAGGC
ACCAACTTTTGAAGAAGCATTAAAAGAAAGTCTTCGAAAAATTAAAGGTGGTTTTACATTTGCAATTTTAACA
AAGGATGCGTTATATGGCGCAGTAGATCCAAATGCTATCAGACCACTTGTTGTAGGTAAAATGAAAGATGGGA
CATACATCCTTGCAAGTGAAACATGTGCAATAGATGTGTTAGGTGCAGAATTCGTTCAAGATATTCATGCAGG
TGAATATGTCGTGATTAACGATAAAGGTATTACAGTTAAATCTTATACACATCATACGACAACTGCAATTTCT
GCGATGGAATATATTTATTTTGCTAGACCAGACTCAACAATAGCTGGTAAAAATGTCCATGCAGTACGTAAAG
CTTCTGGTAAAAAATTAGCCCAAGAAAGCCCTGTAAATGCTGATATGGTCATCGGTGTACCCAATTCATCGTT
ATCAGCTGCGAGTGGTTATGCTGAAGAAATAGGTTTGCCATATGAAATGGGACTAGTTAAAAATCAATATGTT
GCAAGAACATTTATTCAACCAACTCAAGAATTACGTGAGCAAGGTGTGAGAGTGAAGTTATCTGCGGTAAAAG
ATATAGTAGATGGGAAAAATATCATTCTTGTTGATGATTCTATTGTTCGCGGTACGACAATTCGACGCATTGT
GAAAATGTTAAAAGATTCTGGTGCAAATAAAGTGCATGTGCGTATAGCATCACCGGAATTTATGTTCCCAAGT
TTTTATGGAATCGATGTTTCAACTACGGCAGAATTAATTTCTGCAAGCAAATCACCTGAAGAAATTAAAGATT
ATATTGGCGCTGATTCATTAGCATATCTATCTGTAGATGGGTTAATTGAATCAATTGGTTTAGATTATGACGC
GCCATATAGTGGCTTATGTGTAGAAAGTTTCACTGGAGATTATCCTGCAGGGTTATACGATTATGAAGCAAAT
TATAAAGCACATTTAAGTCATCGACAAAAGCAATATATTTCTAAAAATAAACACTTTTTTGATAGTGAGGGAA
ATTTAAATGTCTAAAGCATATGAACAATCTGGTGTAAATATTCATGCTGGTTATGAAGCTGTAGAAAGAATGT
CTAGTCATGTTAAACGTACGATGCGTAAAGAAGTTATCGGTGGTTTAGGTGGATTCGGTGCTACATTTGATTT
ATCACAATTAAATATGACAGCACCAGTTTTAGTTTCTGGAACAGACGGAGTAGGAACGAAATTAAAACTAGCT
ATCGACTATGGTAAACATGATTCGATAGGTATCGATGCAGTCGCAATGTGTGTTAATGATATTTTAACGACAG
GTGCAGAACCATTATATTTTTTAGATTACATCGCTACAAATAAAGTTGTTCCTGAAGTTATTGAACAAATTGT
TAAAGGTATTAGTGATGCATGTGTTGAAACGAATACTGCACTTATCGGTGGAGAGACTGCTGAAATGGGTGAA
ATGTATCACGAAGGCGAATATGATGTAGCCGGATTTGCTGTTGGAGCAGTTGAAAAGGATGACTATGTAGATG
GTTCAGAAGTGAAAGAGGGACAAGTTGTTATAGGACTTGCGTCAAGTGGCATTCATTCAAATGGATATAGTTT
AGTGCGCAAATTAATTAATGAATCAGGCATTGATTTGGCATCAAACTTCGATAATCGTCCATTTATAGATGTC
TTTTTAGAACCAACTAAATTATATGTCAAACCTGTACTTGCTTTGAAAAAAGAAGTTTCTATTAAGGCAATGA
ATCATATTACTGGTGGAGGTTTTTATGAAAATATTCCACGTGCATTGCCAGCCGGATATGCTGCTAGAATTGA
TACTACATCATTTCCAACGCCAAAAATATTTGATTGGTTACAACAACAAGGCAATATAGATACAAATGAAATG
TATAACATTTTTAACATGGGTATTGGCTATACGGTTATCGTTGATGAAAAAGATGCATCACGCGCTTTGAAGA
TTTTAGCAGAACAAAATGTGGAAGCCTATCAAATTGGACATATTGTGAAAAATGAGTCAACTGCAATTGAATT
GTTGGGGGTATAACAATGGTTAAAATTGCGATTTTTGCATCAGGTTCAGGAAGTAACTTTGAAAATATAGTTG
AGCATGTTGAATCAGGAAAACTTGAAAATATTGAAGTTACGGCGCTATATACGGATCATCAAAATGCGTTTTG
TATAGATAGAGCAAAAAAGCACGGTATTCCTGTTTATATTAATGAACCAAAACAATTTGATTCAAAAGCAGCG
TATGAACAACATTTAGTAACATTATTAAATAAAGATAAGGTAGAGTGGATTATTTTAGCTGGTTACATGCGTC
TAATTGGTCCAGATTTATTAGCTTCATTTGCAGGTAAAATATTGAATATACATCCATCTCTATTGCCGAAATA
TAAGGGGATTGACGCAATAGGCCAAGCATATCATAGTGGCGATACTATTACTGGTTCGACAGTACATTATGTT
GATAGTGGTATGGATACGGGAGAAATTATTGAACAGAGAAAATGTGATATTAGACCGGACGATTCAAAAGAAC
AATTAGAAGAGAAAGTAAAAAAATTGGAATATGAGTTATATCCAAGTGTTATTGCTAAAATTGTAAAATAAGG
AGTATGTATGTGATGAAGAAAGCTATTTTGAGCGTATCAAATAAAACAGGTATTGTAGAGTTTGCTAAAGCGT
TAACGCAATTAAATTATGAATTATATTCAACAGGTGGTACTAAACGTATATTAGATGAAGCAAATGTACCAGT
TCGTTCTGTTTCAGACTTAACACATTTTCCAGAAATAATGGATGGCCGTGTTAAAACATTACATCCGGCTGTT
CATGGTGGCATTTTAGCTGATCGAAATAAACCGCAGCATTTAAATGAATTATCAGAACAACATATAGATTTAA
TTGATATGGTAGTAGTTAATTTATATCCATTCCAACAAACTGTTGCAAACCCTGATGTGACGATGGACGAAGC
AATTGAAAATATTGATATTGGTGGTCCAACAATGTTACGTGCTGCAGCTAAAAACTATAAACATGTAACAACA
ATTGTACATCCGGCAGATTATCATGAAGTATTGACGCGATTAAGAAACGATTCGTTAGATGAGTCATATAGAC
AATCATTAATGATTAAAGTTTTTGAGCATACTGCAGAATATGATGAAGCGATTGTACGTTTCTTTAAAGGGGA
TAAAGAAACTTTAAGATATGGAGAAAATCCACAACAATCAGCGTATTTTGTGAGAACTTCGAATGCTAAGCAC
ACGATTGCAGGCGCTAAACAATTACATGGGAAACAATTAAGCTATAACAATATTAAAGATGCAGATGCTACAC
TAGCTTTAGTTAAAAAGTTTGATACACCTGCTGCAGTTGCGGTTAAACACATGAATCCATGTGGTGTTGGTAT
CGGTGACACGATAGAGCAAGCATTTCAACATGCGTATGAAGCGGATAGTCAATCAATATTTGGTGGAATTGTT
GCATTAAACCGAGCTGTAACACCTGAGTTAGCAGAGCAATTGCATAGTATCTTTTTGGAAGTCATTATTGCAC
CAAAATTTACAGATGAAGCATTAGATATTTTAAAACAAAAGAAGAATGTAAGATTATTAGAAATTGATATGAC
TATAGACAGTAACGAAGAAGAGTTTGTTTCAGTATCTGGCGGATATTTAGTTCAAGATAAAGATAATTATGTC
GTACCAAAAGATGAAATGAAAGTTGTTACAGAAGTAGTACCTACAGATGAACAATGGGAAGCAATGTTATTAG
GATGGAAAGTTGTACCATCAGTAAAAAGTAATGCAATTATTTTAAGTAATAATAAACAAACTGTAGGTATAGG
TGCTGGACAAATGAATCGTGTCGGTGCTGCTAAAATTGCGTTAGAGAGAGCTATTGAAATCAATGATCATGTA

GCGTTAGTATCTGATGGATTTTTCCCTATGGGAGATACAGTTGAACTTGCAGCACAACATGGTATAAAGGCAA
TTATCCAACCGGGTGGTTCGATTAAAGATCAAGATTCAATTGATATGGCTAATAAACATGGTATTGCAATGGT
AGTCACAGGCACTCGACATTTTAAACACTAATTTTAAAGGGGCGTAACATAAATGAATATATTAGTAATTGGT
GCTGGTGGACGAGAACATGCACTTGCATATAAACTTAATCAATCGAATCTAGTTAAACAAGTGTTTGTCATTC
CAGGTAATGAGGCAATGACACCTATAGCTGAAGTACACACTGAAATTTCAGAATCTAATCATCAAGGGATACT
AGATTTTGCTAAACAGCAAAATGTTGATTGGGTAGTTATAGGTCCAGAACAGCCGCTAATTGATGGATTAGCA
GACATTTTACGAGCGAATGGTTTCAAAGTGTTTGGTCCAAATAAGCAAGCAGCTCAAATCGAAGGCTCAAAAT
TATTTGCTAAAAAGATAATGGAAAAATATAATATTCCAACTGCTGATTATAAAGAAGTTGAGCGAAAAAAGGA
TGCTTTAACATATATTGAAAACTGTGAATTGCCCGTTGTTGTCAAGAAAGATGGGTTAGCTGCTGGGAAAGGC
GTTATTATTGCAGATACTATTGAAGCAGCCAGAAGTGCTATTGAGATTATGTATGGTGATGAAGAAGAAGGTA
CTGTTGTATTTGAAACGTTTTTAGAAGGTGAAGAGTTCTCGCTAATGACATTTGTTAATGGTGATTTAGCAGT
TCCTTTCGACTGTATTGCACAAGATCATAAACGCGCATTTGATCATGATGAAGGACCAAATACTGGTGGTATG
GGGGCTTATTGTCCAGTACCACATATTAGTGACGATGTTTTAAAACTTACAAATGAAACAATTGCACAACCCA
TTGCAAAGGCAATGCTTAATGAAGGTTATCAATTCTTCGGTGTATTATACATTGGTGCTATTTTAACTAAAAA
TGGTCCAAAAGTAATAGAATTTAATGCCCGTTTTGGTGATCCTGAAGCTCAAGTATTATTAAGTCGCATGGAA
AGTGATTTAATGCAGCATATTATTGATTTAGATGAAGGAAAACGTACTGAATTCAAATGGAAAAATGAATCTA
TTGTAGGGGTCATGTTGGCATCAAAAGGATATCCTGATGCATATGAAAAAGGGCATAAAGTAAGTGGCTTTGA
TTTAAATGAAAACTATTTTGTTAGTGGATTAAAGAAGCAAGGTAATACCTTTGTTACTTCAGGTGGTAGAGTT
ATACTTGCCATCGGAAAAGGTGACAATGTACAAGATGCACAGCGAGACGCATACGAAAAAGTATCACAAATAC
AAAGTGACCATTTATTCTATCGTCATGACATTGCGAATAAAGCACTACAACTTAAATAAGTATATTTAAAAAA
TACTAAGATTAGCTATGAAGAAATCTATAACGATAGTTTTTTCATAGCTTTTTTAGTTGTAGAGTCTAGGACA
TTGATTTCTGTACCAAATTTGTGATTATTCATATGTAATACAAAAGAGGCGCCACAACATGTTTGGATGAACA
AAATAACATGTTTGTGGCACCTCTTTTGTTTAGTATGGAATAAATGGTTTTCTTTTTCTATACAATGAATTTC
TAATTTAGTATCTATACAATTATGGATAAAATTTAACCTACACGACCAAGACGAACATCATCTATGCCCGTGA
TGGGTAAGGTGATTGAACAATAATATGCCAAAGTAATAATGGCAATTAAAACTATAATAAAGATTATATCTTT
ATATGAGAAAGGTACGTTGTAATAGTAAGTACGAGGACCATCTCTAAATCCTTTCGACTCCATCGCAACTGAT
AATTGATGTGCCTTTCTAATATTTTGGCTTAATAGAGGTATAATTAAATGCTTAAATCGCTTTAACCCTCTAT
AATTTGCCGCGTCTATCATCTGATAGCGCATTTTTAAAGATCTGCGAAGCTGTATTAAAGAACTAATCATTAA
AGGTATCATACGAATGGCAGCCATGAATGCATAAGCAACTTTTGATTTAACCTTTAAATGCTGCATTAAACTA
TAAAATATCATGACAACCTGAGATGTAAGTGCGATTAAGATACCGAAAAATGATATAGCAATGGTTCTTAATG
ATACATGTAAACCACGAACTAAACTTTCTGTTGTAATATGGATAAATCCGAATTTCAAAATTGTATGGCTACC
ATTCCCGTATAAAATCATGAATAGGGAAGAGAGTAATGCAAAGCCAATACTTATAGTTATAAAAATTGATGTA
ATTTTAAACTGAGTACCATTAAACATCAATAAGAAAACTAACATTAAGATAGTGATATAAAGCATAAAATCGA
AATTATGCACAAATATAATAAAGAAAAATAGTATAATTCCAAGAAATAGTTTCGTTATAATGTTGACATCATC
AACAAATGATTGCCGAACTTTCCATTGCTCATACATTCGTATCACCATCACAATCTAGTAACGTACCATCTGA
AATTTTAAGTCTTCTTGATGGATAACGTTCAATTATTTCATCGTCATGTGTAACCATGACAATACTTTGTCCC
AAATTAATTCGTTTTTGGAAAAGTTTGATCAACTGGAATGTATTATGGCTATCAAGTCCAAATGTCGGTTCAT
CTAAAAAGATGATATCAGCTTTAGAACTTAGTGCGGTAGCTACGCTAAGGCGTCGTTTTTGACCAATAGACAA
CTCATAAGGATGTTGATCTTTAACATTTTCTAAATCTAAAAGTTTTAAAAGTTGTATCGTTTCATCATCACTT
TGATCTTTAGAAAGGTGATTAAAATGAATGTTAATTTCATCATAAACCGAATTTGTTATAAATTGTAATTCTG
GGTTTTGATAAACTAGATACATGTGTTTTGCTGCATGTTTAATTTTTGTTAAACGTTGATTTTCAAAATAAAC
ATCACCTTGATATTTAATCAATTGCATAATTGATTCAAGCAAGGTAGTTTTACCACTGCCATTTGCCCCTGTA
ATTGTAATCCACTCACCTAAACCAATTTCTAAATCTGAGAATGAAAGCAACGTTGATTTACCGCGAATAATAC
GTCCATTTTTAAATTGTAATAAGTGTGAGTTTGTTGTTGGAAAGTCAACACGACTTGGTGCGAATTCCCATGC
ACGTGGATGCCACACACCGTATTCACTGAGTAAATGAACATACTTCTGTAATATGATTTCAGGACATTCATCG
GCAATGATATTTCCGTTATAATCCATCAAAATGACGCGGTCGACATGATTCCAGATGTGTTTAACTTTATGTT
CAACGATTACAACAGTTTGATCTTCCCAAAGTTCAATTAGTTTAGTCCATAAATCTTCCGTTGCTTGAACATC
TAACATTGCTGTCGGTTCATCTAAAAACAATGTTTTTGATTGTTGAAGAATGGTTTCAACAATTGCCAATTTC
TGTTTCATCCCGCCACTTAAATCTTTGATATACGTTTCAGGGGTAACATTTAAATTGACCATATTTAAAGCAT
TGATAATTAACGCATCCATGTCTTCACGTGGTACTTGTCTATTTTCTAAAACGAATGCAAGTTCTTCGTATAC
TTTTGGCATACAAAACTGGCTATCAGGGTCTTGGAAAATAACGCCACTTAATGGGTCAACGATTAGTTCATCA
TATTTCATAGGTAATTCAATTAAATTAGGAACAATACCACTTAATACATTCAGAAGTGTACTTTTACCGCAAC
CAGAAGGACCGAGTAAAAGTACTTTTTCTTTGTCTTGAATAGTGATATTTAAATGATCGAAAATTTTACGCTG
ACCACTTGGATATTTTAATCGTAAATCACTTACTTTTAACACTCTGAATGCTCCTCTTATAAGTTGTCGTAAT
CTTCTTTAGCAGCTGGTCTAAATAATTTTGTTACGCCTGTCTTATCTAAAGCTTTTACTAAAAGGTAAGATAG
GACGCCGGCGACTACTGCACCACTAATTAATCTAAATACGATGAATAATGTTAAGTTCCAACCTGCAACTTCA
TTTAAATACCCATAGAAATAATCTATCGGGAAAGCTGCGATTGCTGTACAAAAACCTGCTAACATAGCTACCA
TAACTGAACGTGATTGATATTTAAAAATTGCAAAGACAAGTTCACACGCTAAACCTTGTATAAAAGCGTAAAC

GATTGTCGGAATATCGAAACGACCCATAATGATAGTTTCGCCTGCACCTGCAGCAAATTCAGCCAGTAAAGCA
ATACCTGGCTTTGGGATAATTAGATAGCAGACAATAGCTGCCATGAACCAAACCCCGTTTGTTAATTGTTCGA
GGTGAAGGCCTGTAGCTTGCACACCATTGTAAACAAACCACCATAAATTGTAAATAACTGCGAATACTACTGA
AATAAGAACGGTTACTAGTATTTCAGATAGCTTTAAACCTTTTGACATTTTTACATCCTCCTAATAAAAAAAC
GCACAACCATCCATAGGAAAGTTATGCGTTCACAATATATATTAGTAAAACATATGTATAGTAACACTTTCCT
ACGCTAGTTCAAGCTAGATCAGGTTCAAAGGGTTTGAGGGCAAGCCTCATCTCAGTATAAAACACCCCTAGTG
TGTGCGATTTATTTAATTAATTATACTGTAAGACGTTTGTAAACTTATGTCAATAGGTTGTCTTCATGAAATT
TCGTTTAATTCGATTTAGAATTTATAATATTGGCATTGGATTTAAATTGAAGATGTAGTAGGAATGTTAGTAA
TTAAAGATATAAAAATATGTGACATGTAATAATATTGAGCTGATAAATGAAGAGGGATACATGTGCCGAGCGC
AAGCAAAAAAGCCCCTAACAACTAAAGCTTGTAAGGTAGGAGAGGGATACATGCGCCGAGCACATGCAAAAAA
GCCCCTAACAACTAAAAGTTGTAAGGGGCTCTAAATTAATTTAGTGTATCTTGGATATCTTGTTTTGTTTGAT
TAATATCTTCTGTTTTTACTTCTTTTTTATCTTTTAATTTTTCTTCAACTTCTTTAGCTTTTTCTGCTGCTTT
TTTATTTTGATTTTCATTAGACATGATTAATTCCTCCCAAATTGGATAATTATTTATATATAAATCTTACCCG
GTTGTACTTTCGTTAAACTTTTCTAAGTCTATAGCACTATTTATTCATTTATCTAAAGACAACAACATTAGAT
TAATATATAATGATTTTGAGGTGAACATAATGTCTTTTCTTAGGAAACACGCCGAAATTATTTTTAGCTATTT
AATCGGTATCGTTTCACTCTTCACTGGTCTCATTATTTTAATTAACTTGCCATTAATTAAACAATTAAATGGT
GGTAAAAAAGTTGATACACATGTTCATAATGTGTGGGAATTTCTGAATGCATTTTTCAGTGAAATTATTAAAG
TAATGAGTCGATTTATAGGTAATTTCCCTATAGTTAGTGCAATTGTGATAATTATATTCGGTATTTTAGTTAT
GTTGATTGGTCATACATTACTTAGAACTATTAAGTATGACTATGATATTTCTATCTTTTTCTTAGTTATCGGT
ATCATGTATTTTATTATTACTCTTATATTAATGACTCAAGTTTATGGATTCTTTGCAGTGATTTTCATTATTC
CATTTACAATTCATATAGGATATATCGTCTATAAAGATGAATTGAATCAGGAAAATGTAAAAAATCATTTCAT
GTGGATAATTGTGAGTTATGGTATAAGTTACTTAATTACACAAATTGCATTGTATGGCAGAATTGATGCTAAT
GAAATAGAGTCAATTGATATCTTAAGTGTCAATGCTTTCTTTATAATTATGTGGTTACTTGGTCAAATGGCTA
TTTGGAATTTCTTGTTCTTGCGCCGAGCTTTACCTTTAACAAAGCAAGAATTAGGTGAAGAGGAGCCAGAATT
ATCAAGAACAAGTAAAGGGAATGTCACGAATCAAACTAAAATTCACTTGAAACAACTCCAAGATAAGACTACA
GAATATGCACGTAAGACAAGAAGAAGTGTCGATTTAGATAAAATTAGAGCTAAAAGAGATAAATTCAAAAAGA
AAGTTAATGATATTATCGATATTCAAGAAGACGATATTCCTGATTGGATGAGAAAACCGAAATGGGTTAAACC
AATGTATGTCGAACTATTTTGTGGTGTCGTCATCTTTTTATTCACATTTTTAGAATTTAATAATCGTAATGCA
TTATTTGTATATGGTGATTGGAAATTATCACAGACACAATATGTTATTGAATGGGTTACATTACTAATTCTGT
TATTCATTATTATCGCATATATCGCTACAACGTTAACTTTCCACTTGAAAGGTAAGTTTTATTATTTACAACT
ATTTATGGGGAGCATTTTATTCTTTAAATTGTTAACGGAATTTATAAATATAATGATTCACGGACTACTACTT
TCAGTGTTCATTACGCCAACATTACTATTAATGTTATTGGCAATCATCATTTCTTATTCGTTACAATTACGAG
AGCGACCATAATTAAAAGCATTATAAAAGTACTATCTATTAAACATTTTGATGTGTACGCTATAAGTTAGATA
TATCTCTAACTTACTTAGATACATGTCAATGAAGTTTATGGATAGTACTTTTTTTGTAACTAGATTTGATTGA
TTCAGGTGATGTGAATTAAGTATTGAAAATTGTATACAAAGTTTTAGTGCAAATAAAATAGTTGAAAAGTTAT
CCATTTGTAAAATCAAGAAAACTAGTAAATAGTTGAAGCGACTTATGGAATTTGCGAAACGATATATAGTATT
TCCTTTGTAGAAATTTAACATATATCATTCGAATTACTAATTTGTTAAAATCAACAGTAAGATTAGAAGTAGA
TGATATTGAAATTTGGCAAACAATTTAATCTATATAAAACTACAACGAAACACAGAAAGGAAGTTGTCAGATG
AAAATAGCAACTCTGAACAAAGGCAAAGAAACAAAATATTTTAATGGATATCCTTTAATTGAAGAAGAGGATA
TCTATTCACAAGATCATTTAAAAGAAGGAGATATTTTTCAAATTGTGACTGATAAATCACAATATGTTGCAAC
GGCTTATGTTGGTCGCCAACATAAAGGATTAGGTTGGGTTCTAACATACGATAAAGCTCAAGAAATCAACACA
GCTTTCTTTGTGAAATTGTTTAATACTGCATTAGCAGAACGTGATTATTATTTTAATATAGATGGAACAAATG
CTTTTAGATTATTTAATGCTGAAGGTGATGGTGTTGGGGGATTAACAATCGACAATTACGATGGTCATTTGTT
GATTCAATGGTACTCAAAAGGTATTTATAAATTTAAATATGCCATTCTTGAAGCGGTTAGAAAAGTATTTGAT
TATAAATCTATTTACGAAAAAGTAAGATTTAAAGACAGCGAATATAGTGGTGGTTTTGTTGAAGGCGATGCAC
CTGAGTTTCCAATTGTTATCGAAGAAAACTTCACATTTTATAATGTAGACCTTGAAGATGGTTTGATGACAGG
TATCTTTTTAGATCAAAAAGAAGTGCGCAAGAAATTAAGGGATCAATATGCCAAAGAACGCCATGTTTTAAAC
TTATTTAGTTATACAGGTGCTTTTTCTGTAATAGCAGCAAGTGAGGCATCTTCAACAACAAGTGTAGATTTGG
CTAATCGTTCTCGTAGTTTAACTGAAGAAAATTTTGGATTAAATGCTATTGATCCTAAATCTCAATATATTTA
TGTCATGGACACTTTTGATTTCTATAAATATGCTGCACGACATGGACATAGTTATGACACAATCGTGATCGAT
CCACCTAGCTTTGCACGCAACAAAAAACGTACATTTTCAGTGCAAAAAGATTATGACAAATTAATTACTGGCG
CCTTAAATATCTTATCATCTGAAGGAACATTATTATTATGTACAAACGCAAGTGTATATCCATTAAAGCAATT
TAAAAATACTATTAAAAAGACGCTTGAAGAGAGTGGCGTTGATTATGAATTAACTGAAGTTATGGGATTACCA
AAAGATTTTAAAACACATCCACATTATAAGCCATCTAAATATTTAAAAGCTGTTTTTGTAAATATTAGACATT
AATTTGATAAAAAGGGGTATAGTATCTTATTGAGAAAAAGAAGGGTGATGATACAATGGGATTCAAAAACAAT
TTAACATCAAATTTAACAAATAAAATTGGTAATTCAGTTTTTAAAATAGAAAATGTTGACGGAAAAGGTGCAA
TGCCAACGACGATTCAAGAATTGAGAGAAAGACGCGAACGTGCTGAAGCAATTGTAAAGAGAAAGTCTTTAAT
GTCATCAACAATGAGCGTTGTTCCAATTCCGGGTTTAGATTTGGTGTTGATTTAAAATTAATGAAAGATATT

ATCGAAGATGTAAATAAAATATACGGATTAGATCATAAGCAAGTTAATAGCCTTGGGGATGATGTAAAAGAAA
GAATTATGTCTGCAGCAGCAATTCAAGGTAGTCAATTTATTGGTAAAAGAATTTCAAGTGCATTTTTAAAAAT
TGTAATTAGAGATGTAGCAAAACGAACTGCAGCTAAACAAACAAAATGGTTTCCTGTTGTAGGACAAGCTGTG
TCTGCATCTATTAGTTACTATTTTATGAATAAAATTGGAAAAGATCACATTCAAAAATGCGAAAATGTTATTA
AAAATGTCATGTAGGTGCTATAATAGTTTTGCAATTTGCAAATTTTACTGAAACCGGTTTTAAACGAATTGAA
TTTAAAGCATGGTTTTGGTAAAGTTAAGGTATAAAACTAAGTTAGTATTGTAATAATATTGAAGATTCTAACT
ATACGAAGGAGAAATGTAATTATGGAACAAAATTCATATGTAATCATCGACGAGACTGGTATTCACGCTAGAC
CAGCAACAATGTTAGTACAAACAGCTTCAAAATTCGATTCTGATATTCAATTAGAATATAACGGTAAAAAGGT
TAACTTAAAATCAATCATGGGTGTTATGAGCCTTGGTGTTGGTAAAGATGCTGAAATTACAATTTATGCTGAC
GGTAGTGATGAATCTGACGCCATTCAAGCAATCAGTGACGTCTTATCAAAAGAAGGATTGACTAAATAATTAT
GTCTAAATTAATTAAAGGAATTGCTGCATCTGATGGTGTTGCAATTGCTAAAGCTTATTTGTTAGTTGAGCCA
GACTTAACATTCGACAAAAATGAAAAAGTCACTGATGTTGAAGGAGAAGTTGCTAAGTTCAATAACGCTATCG
AAGCTTCTAAAGTTGAGTTAACTAAAATTAGAAATAATGCAGAGGTTCAATTGGGTGCTGATAAAGCTGCTAT
CTTTGATGCACATTTATTAGTTTTAGATGACCCTGAATTAATTCAACCAATTCAAGATAAGATTAAAAATGAA
AACGCTAATGCTGCTTCAGCATTAACGGATGTAACAACACAATTTGTTACAATTTTTGAATCTATGGATAACG
AATACATGAAAGAACGTGCGGCTGATATTCGCGACGTTTCTAAACGTGTGTTATCACATATTTTAGGTGTAGA
ATTACCGAATCCGAGTATGATTGATGAAAGCGTTGTTATTGTAGGGAATGACTTAACGCCATCTGATACTGCT
CAATTAAATAAAGAATTCGTACAAGGTTTTGCTACAAACATTGGCGGAAGAACAAGTCACTCTGCAATTATGA
GTCGTTCTTTAGAAATTCCAGCAATTGTTGGTACAAAATCAATTACTCAAGAAGTTAAACAAGGCGACATGAT
TATCGTAGATGGATTAAATGGTGATGTAATCGTTAATCCAACTGAAGATGAGTTAATCGCTTATCAAGATAAA
CGTGAGCGTTATTTTGCTGACAAGAAAGAATTACAAAAACTACGTGATGCTGATACTGTTACAGTTGATGGTG
TTCACGCAGAGCTTGCTGCAAATATTGGTACACCTAATGATTTGCCAGGTGTTATTGAAAATGGTGCACAAGG
TATCGGCTTATATAGAACTGAGTTTTTATATATGGGTCGTGACCAAATGCCTACAGAAGAAGAACAATTTGAA
GCTTATAAAGAAGTATTAGAAGCAATGGACGGTAAACGTGTTGTTGTACGTACTTTAGATATAGGTGGAGATA
AAGAATTATCATACTTAAACTTGCCTGAAGAAATGAATCCATTCTTAGGTTACCGTGCGATTCGTTTATGCCT
TGCGCAACAAGATATTTTCAGACCACAGCTACGTGCATTATTACGTGCATCAGTTTATGGTAAGTTAAATATC
ATGTTCCCAATGGTTGCAACAATTAACGAATTTAGAGAAGCTAAAGCTATATTATTAGAAGAAAAAGAAAACC
TTAAAAATGAAGGTCATGACATTTCGGATGATATAGAATTAGGAATCATGGTAGAGATACCTGCAACAGCAGC
ATTAGCTGATGTTTTTGCTAAAGAAGTAGATTTCTTCAGTATCGGTACAAATGATTTAATTCAATACACATTA
GCTGCTGACCGTATGTCAGAGCGTGTATCATATCTGTACCAACCATATAACCCTTCAATCTTACGTTTAGTTA
AACAAGTTATTGAAGCGTCACATAAAGAAGGTAAATGGACAGGTATGTGTGGTGAAATGGCTGGAGATGAAAC
AGCTATTCCATTATTGCTTGGTTTAGGTTTAGATGAGTTCTCTATGAGTGCAACGTCTATTCTGAAAGCAAGA
AGACAAATTAATGGTTTAAGTAAAAATGAAATGACTGAACTTGCTAACCGTGCAGTCGACTGTGCAACGCAAG
AAGAAGTTATTGAATTAGTTAACAACTACGTAAAATAATTAATTCTTTCGAAAGACAGAATTATAAATAAACC
CGAAGTATTCATTGTGAGTAATTATGAATACTTCGGGTTCTTTTTTATGATTTATGTTAATTTCAATGGAATG
TTGTTGCTAACTAATTAAACAACTATGTGTCATTAACAAAATGTTGTTCTTTAATTAATGTTAGTTAATTTAA
AAATCGATATTTAATTTATGATAGCTATAAATGATGAAGTTGATTTAGATGAAAATTAATCTTGGATATTTAA
TACTTTGTTGATTTCATCAAGATCAACATGGTACATTGGATTGCCATTCAACAAAATAAACGGAGTTGAAAAA
GCATCAAAATCTATCATTTCGTTTCGATATTGTTGATTGTTGATATTTCTCTCTTCAAAATCAATTTGATGCT
CATTTAGATAATTTTTTACAAATGTACAAGGTGGACAATCATTCTGCGTATAAACGATTATTTCTGACATGTA
TCATGCTCCTTCGTGCTTTATTCTAATGTAATATACACAATTATACATTGAAATTCAAATTTGTGAACACATT
GTGGACTGACATAAATTATACACAATTTCATAGCGCATAATGTTTTAAAAATACACATTATAGCAATTATAAT
TAAGTTCATTGTGTTGTCATCAAAAAAAGAAAAAGGTGATGTTTTTAAATGGATACAGTTGAAATCAGTCGGT
TTTTGACAGCTATGACTTTAGCAGTTCATATCATTTTTGCAACGATTGGTGTTGGTATGCCTTTAATGTTCGC
AATTGCAGAATTTTTAGGTATTCGCAAAAATGATCTTCAATATATAGCTATGGCCAAAAGATGGGCTAAAGCT
TATACAATTACTGTAGCAGTGGGAGTTGTTACAGGTACAATTATAGGACTTCAATTATCATTGATTTGGCCTA
CATTTATGGAAATGGGTGGACACGTTATTGCACTTCCATTATTTATGGAAACATTTGCGTTCTTCTTTGAAGC
TATTTTCTTAAGTATATATTTATATACTTGGGATCGTTTTAAAAATAAATGGACACATTTCTTAATTAGTATA
CCAGTAATTATTGGTGGCTCTTTCTCAGCATTCTTCATTACTTCAGTGAATTCATTTATGAATACGCCTGCAG
GTTTTGAGTTGAAGAATGGAAAGATGGTCAATGTTCAACCTATAGAAGCGATGTTTAACCCATCGTTTATAGT
TCGATCATTTCACGTAATTACTACAGCTGGTATGACGATGGCATTTGTTATTGCATCAATAGCAGCTTTTAAA
TTATTGCGTAATCGTCAACCTAAAGATACTGTCTACCATAAGAAAGCTTTGAAAATATCTATGATAGTTGGAT
TCTTTTCAACATTACTTTCTATGTTGGCAGGGGATTTATCTGCAAAATTTTTGCATAAATTCCAACCTGAAAA
ATTAGCAGCTTATGAATGGCATTTCGATACATCTTCCCATGCCAAATTATTATTATTTGGTGTGTTAGATGAA
AAGACTCAGCAAGTTAAAGGTGCGATTGAATTACCTGGACTATTGAGTTTCTTAGCAGATAATAGTGTCAAAA
CTAAAGTGCAAGGGTTAAATGATTTTCCAAAAAGTTTACATCCGCCTATGATTGTCCATTATTTCTTTGATTT
AATGGTAACGATGGGAATTTTATGTTTTGTCATTTCAGGTGTTTATGTCTTAACTTTAATGTTTAAAAAGCTT
AGAAAGTTTTCTACTCATAAATGGATGCTTTACGGAATATTATTAACCGGACCAGCTTCAATGCTAGCTATAG

AATTTGGATGGTTCTTAACAGAGATGGGTAGACAGCCTTGGATTGTTCGTGGTTATATGCGCGTGGCAGAAGC
AGCAACACAAGCAGGCGGAATAACCTTCGTTACAATTTTATTTGGCATATTGTACATCATTTTAATGTATACA
TGTGCATACGTATTAATTCGTATGTTTAAAAATAAACCGGCGTATGAAGATGTAAATCGCTTAGCCAAGAAGC
AAGGAGGAGAAATAGAAAAATGATTTATGCATTTATAGGTATAACAGTGTTATGGTTGTTTTTATTTTGCTAT
ATCATTATTGCTTCTATAGATTTTGGGGCAGGTTTCTTCGCATTGCATTCAAAGTTAACTGGTGATGAAAAGA
AAATTAATCACTTAATTTCACGTTATTTGAACCCAGTTTGGGAAGTTACGAATGTATTCTTTGTTTTCTTCTT
CGTAGGATTCGTAGGTTTCTTTCCAGAGTCAATCAAATATTTAGGCACGGTATTGTTAATACCAGGTTCGATA
GCACTGATTATGATATCGTTGAGAAACAGTTTTTATGCATTTGAAAATTATGGTCAAGATACAAAATTAGCAT
GGATGATCATGTATGGGGTAAGTGGATTATTAATTCCAGCTTCATTATCTACTGCTTTAACTATTACAGAAGG
TGGCTATATTAATGTTCGAAACAATGTTATCGATCTAGATTGGGTGCAGTTACTATTAAGCCCATTTGCTTGG
TCTGTAGTATTCTTGGCAATTATTTCAGTTTTATATATTTCATCAGGATTTTTGACATATTATGCTAAAAAAG
CAAATGACGAACCAGCATATAATTTAACAAGACAATGGCACATATTTTTAGGGCCGCCGATGATTATCATTTG
TTTATTCGTATTTCTATCATTACGCATTCAAAATTCTGAACATTTTTATTCAGCTGTTTTTGACTACTGGTGG
ATGTTTGCTATAAGTTTCTTATTCTTTGCATTAGCTTCATTGTTAACATTCTTTAAGAAAAAACATGGTTTGG
CTTTTGTATTTGTTATTTTACAAATGATGTTCGCGTTCTTTGGCTATGGAATTAGTAAATTGCCTTACCTTTT
ATATCCGTTTGTAAAAATTACAGATGCATATGTTAATCCAGAAATGGGCTGGACATTAGTGATTGTCTTTATT
TTAGGTTTACTTTTATTACTTCCATCGTTAATATTATTATTAAGATTATTTGTTTTCGACAAAGAATATGTTG
AAGGAAAGAAATCATAAGTTGAGACGATACCCCAACTTTCATTTACTTGCATTACTGCATTTAGATAATTTAG
TTGGGGTGCTCATTTATAATAATTTTAAATTTATATTTTCAATGAATTGATGAAAATCATTTTATGGAGGGCG
AGTATGGGTAAAGAATATGTAGTCATCGGTCTAGGTCGTTTTGGAGGTAGTATCGTTCGTGAGTTGAATGCAT
TAGACATGGATGTAATGGCCATCGACCATGATGAAAATAGAGTGAATGAATATAGTGATATCGCTACTCATGC
GGTTGTTGCAGACACAACAGATGAAGCAGTTATGAAAAGTTTAGGTATCCGTAACTTTGATCATGTCATTGTG
GCAATTGGTGAAAATATTCAATCAAGTACGTTGACGACTTTAATTTTAAAAGAGTTAGGTGTAAAAAAAGTAA
CTGCTAAAGCACAAAATGATTATCATGCAAAGATTTTAAATAAAATTGGAGCAGATACTGTTGTGCACCCTGA
GCGTGATATGGGTAGACGTATTGCGCATAATGTTGCGAGTGCAAGTGTACTTGATTATCTTGAGTTGGCAGAC
GAGCATTCTATTGTAGAATTGAAAGCAACTGAAAAGATGGCGGGGAAGTCTATCATTGATTTAGATATAAGAG
CACAATATGGAATTAACATTATTGCAATTAAACGAGGCAAAGAGTTCATCATTTCACCAAATCCAAATATTAA
TTTAGAAATAGGTGATATTTTAATTATGATTGGACATGATAATGATTTAAATCGCTTTGAAAAAAAATATTGCG
ACGAGATAATCGGGAGTGGGACAGAAATGATATTTTCTCAAAATTTATTTCGTCGTCCCACCCCAACTTACAT
TGTCTGTAGAAATTGGGAATCCAATTTCTCTTTGTTGGGGCCCACACCCCAACTCGCATTGCCTGTAGAATTT
CTTTTCGAAATTCTCTATGTTGGGGCCCATCCCCAACTTGCACATTATTGTAAGCTGACTTTTCGTCAGCTTC
TTTGTTGGGGCCCCTGACTAGAATTGAAAAAAGCTTGTTACAAAGTGCATTTTCGTTCAGTCAACTACTGCCA
ATATAACTTTGTAGAGCATTGAACATTGATTTATGTCTCAAGCTCATCATATATAAAGAAAAAGCTACTAACA
ATGTGTGGTGAGTTTAATTAAAACTTCACTTCAAAGTTAGTAGCTTTTTGATTATTTATTGTTTGATTCTTTT
TGCTCGTTTACCTTCATAATGACTGGTAAAATCATTGGTTTTCTAGCCGTTTTTTCAAATAAATAAGGTTGTA
ATGTTTCAATGATAGAAGATTTAATCTGATGCCATTGAATATCTTTATTTTGATTTAACTTACTAATAACGTC
AGTTTTGATTTTGCGTTGTGCATCATAAATTAATTGACCTGATTCTCTCATATATACAAATCCTCGAGAAATA
ATGTCTGGACCAGAAAGTAATTTATTTGTATTAAAATCAATACTAACAACAACGATAACTAAACCTTCCTCAG
ATAATAGCTTACGGTCTCTTATTACAACATTACCGATATCACCGATACCACTACCATCAACAAGTACATTACC
AGATGGAATGCGACCAGCTTTACGTGCTGAATCGTGTGTTAAAGCTAAGACATCTCCAATATCAAAGATGAAG
ACATTATCTTCTTCAACGCCGCATTCAACACCAGTCTCACCATGTGCTTTTAACATACGGTATTCACCATGAA
TAGGTAAGAAATATTTCGGCTTGATTAATCGAAGCATTAATTGTTGATCACCTTGAGAACCATGCCCTGAAGT
ATGGATGTTAGAAATCTTGCTGTGGATAACATCTGCACCAGCTTTATACAAGGAATTAATCGTTCTGTTAATA
CTTTTTGTATTACCTGGGATAGGTGATGAACTAAATACAACGGTATCTTCAGGTATAATTTTAATTTGCTTAT
GAGTACCATTAGCAATTCTAGATAATGCTGCCATTGGTTCACCTTGAGAACCAGTACATAGTATCAATAACTC
ATGCTTCGGTACGGTATTAATTTTATTAGGTTCAATAAATGTTTCAGGTGGTGCTTTAATATAACCAAGTTCC
ATACCGATTTTAATATTGTTTTCCATCGAACGACCGAACGTAACAATTTTACGGTTATTTTTGATAGCAGCTT
CAACTGCTTGTTGAACTCGGTAAATATTAGAAGCGAAGGTAGCAAATATAATACGACCTTTACAATTACGGAA
GATCTTATCTACGTTTTGACCAACTTCACGTTCGCTTAAAGTAAAATCAGGCACAAGTGAATTTGTTGAGTCT
GAAAGTAAACATAGAACGCCTTCTTCGCCTAATTGAGCCATTTTAGCAATGTTTGCTGGTTTGCCTACAGGTG
TAAAATCAAATTTAAAGTCACCGGTATGAACTACTTTTCCTTCAGGTGTATCTACGATGACGCCATAAGTTTC
AGGAATACTATGCGTAGTTAAGTAGAAAGAAATCGTAAAGTGCTTAGATTTAATCACACTGTCCTCATTGATT
TCATTTAGTTTAGCAGTACGTAATAAATGATGTTCTTCAAGTTTATTACGGATTAAACCTAATGCTAAAGGAC
CACCATAAATAGGTATATTAAGTTGTTTTAATAGGAAGGGCACACCGCCTATATGGTCTTCGTGACCATGTGT
TATAAATAGGCCAACAATTTTATCTTGGTTTTGAACTAGATATGTGTAGTCAGGTATAACATAATCAATCCCT
AATAAGTTATCATCAGGGAATTTGATACCTGCATCGATAATGACAATTTCGTCTTTATACTCAACTGCATAAG
TATTTTTACCGATTTCACCTAGACCTCCAAGTGCATATACACCTACTTCATTTGGATGTAATTGTTTCATTAT
TTAGCAATCTCCACATTAAAGTGTTCTGAGTTTTCTTTTTCGTAATCTAAATGTGCGCCCTCTAATTTAGTGA

TAAATTCGATATTAAAATTACGATCTTTCAAGTAACGACGTACTTGTTCTTCTGTTTGAGCTTCAACATAAAG
TGATTGTGTATTTTCACGCACAATTACCTCGTCTCTGTTATGTTGATAAAAAACTTTAAATACTGCCATGTTA
AAAATTCCTCCTAAGAATGTTTGTTTAATTTATTGTTAACCCTAGTAAAATCGTATTGGAGTATATATCGATA
AATTCATTCCAATCATCTCTATAATTTAACTTAATAACGATTTGGTTAATAACTAGGTTCATCATGTCGTTCA
TTTTAAAAAATTAGTGAAATAACACTAAAATTTCAGTTAAATCTTATTTTACATGATGAATGATAATAAATAA
AGCAGTTTATCTCACTAGAAATCTAAGTATTTCGAATAAATGTGCAGTTCTATTTCTTAAAATAAATGTAGAG
ATTAAAAATTAATGTTAAGAAAGAATTAAAGGATTTTATATGAAACGGTAAAATTTAAATGAGGGGATAAAAA
GTTTGGTAGAAAGGTGAATATATTTAACTGTTGTTTATTCGAAAAAGTTGTACAAGAAAAAGCTTAGGACATC
ATATTTATCCTAAGCTTTGATAATTAGTAAAAATGTGCTTTAAACTTCTACAGCATCTGTATGTGGTTGTAAT
GGGTGATCTTTGTCAATGTGATCATAGAACATTACACCATTTAAATGGTCAATTTCATGTTGGAAAACAATTG
CTGGATATCCTTTTAGACGTAATTGTATATCATTACCTTCGATGTCTTTGGCTTTAATTGTAATTCTATTATG
ACGGTGAACTAGACCAGCAACATTATCATCGACACTAAGGCAACCTTCACCAGTTGGTAAATAAGCTTCTTGA
ACGCTATGACTTACAATTTTTGGGTTCACAAGCATATAGTCATAAGATTTGCCACTGCCATCATCTGGTATTA
AAACAGCAATCATACGTTTAGAAATATTAATTTGAGGTGCAGCCAAACCAACGCCTGAACGTAAACCATATCG
TTTCGCGATTTCCTCATCTTGACTATTTACTAAAAACTCTCTCATGGCGATTAATGTTTCTTTTTCTTCTTTA
GTTAATGGTAATTCTAACTCAGCTGCTTTTTGACGCAAAGTTGGATGACCATCTCTAATGATGTCTTTCATTG
TTAACATATATTGCACCTTCCTTATTTTAATTTGTTTTAGTTGAATGACAGTAAAAAGGTTGTTAAGATACTC
ATACATTTTTATGTGTAAATATCTACAAAGTTAACCAACTACTGCCAATGTTTATTTTAGATAGTATATGTAA
ATTTTCAGATATGCTAATTGCTTAAAAAATGATTAAAATGTTGATTTCAAGCAATGATACTTTAGAAATTTAT
TTATCATCTTGACTTTAAAAATTATATTATAAATGACGTAACTGTCAACAGATATACTTAGTAATGAAGATGT
GTAATGTAATTGTTTAAAATTGATTTCCAAGCAGATTTTATTTATCATTTAATTTAAATAGCAAGTGGAGGTA
CAAGTAATGAAATTTGGAAAAACAATCGCAGTAGTATTAGCATCTAGTGTCTTGCTTGCAGGATGTACTACGG
ATAAAAAAGAAATTAAGGCATATTTAAAGCAAGTGGATAAAATTAAAGACGATGAAGAACCAATTAAAACTGT
TGGTAAGAAAATTGCTGAATTAGATGAGAAAAAGAAAAAATTAACTGAAGATGTCAATAGTAAAGATACAACA
GTTCGCGGTAAAGCAGTAAAAGATTTAATTAAAAATGCTGATGATCGTCTAAAGGAATTTGAAAAAGAAGAAG
ACGCAATTAAGAAGTCTGAACAAGACTTTAAGAAAGCAAAAAGTCACGTTGATAACATTGATAATGATGTTAA
ACGTAAAGAAGTAAAACAATTAGATGATGTATTAAAAGAAAAATATAAGTTACACAGTGATTACGCGAAAGCA
TATAAAAAGGCTGTAAACTCAGAGAAAACATTATTTAAATATTTAAATCAAAATGACGCGACACAACAAGGTG
TTAACGAAAAATCAAAAGCAATAGAAGAGAACTATAAAAAGTTAAAAGAAGTATCAGATAAGTATACGAAAGT
ACTAAATAAGGTTGGTAAAGAAAAGCAAGACGTTGATCAATTTAAATAATTAATATAATACAGATGGTAGGAA
ACAACTAATACAGTTCCTATTATCTGTATCTTTTTTTATTAAAACAGAACTTTTTCAAATGGTTTAACAGTCC
CATTTATTTGTGGTACAATTAGTAAGGATAAAATGAATTTCTATACAATTATGGGAAAGGTATGGTGAATTGA
ATGGCTCCTAAGTTACAAGCCCAATTCGATGCAGTAAAAGTTTTAAATGATACTCAATCGAAATTTGAAATGG
TTCAAATTTTGGATGAGAATGGTAACGTCGTAAATGAAGACTTAGTACCTGATCTTACGGATGAACAATTAGT
AGAATTAATGGAAAGAATGGTATGGACTCGTATCCTTGATCAACGTTCTATCTCATTAAACAGACAAGGACGT
TTAGGTTTCTATGCACCAACTGCTGGTCAAGAAGCATCACAATTAGCGTCACAATACGCTTTAGAAAAAGAAG
ATTACATTTTACCGGGATACAGAGATGTTCCTCAAATTATTTGGCATGGTTTACCATTAACTGAAGCTTTCTT
ATTCTCAAGAGGTCACTTCAAAGGAAATCAATTCCCTGAAGGCGTTAATGCTTTGAGCCCACAAATTATTATC
GGTGCACAATACATTCAAGCTGCTGGTGTTGCATTTGCACTTAAAAAACGTGGTAAAAATGCAGTTGCAATCA
CTTACACTGGTGACGGTGGTTCTTCACAAGGTGATTTCTACGAAGGTATTAACTTTGCAGCAGCTTATAAAGC
ACCTGCAATTTTCGTTATTCAAAACAATAACTATGCAATTTCAACACCAAGAAGCAAGCAAACTGCTGCTGAA
ACATTAGCTCAAAAAGCAATTGCTGTAGGTATTCCTGGTATCCAAGTTGATGGTATGGATGCGTTAGCTGTAT
ATCAAGCAACTAAAGAAGCACGTGACCGCGCAGTTGCAGGTGAAGGTCCAACATTAATTGAAACTATGACATA
TCGTTATGGTCCTCATACAATGGCTGGTGACGATCCAACTCGTTACAGAACTTCAGACGAAGATGCTGAATGG
GAGAAAAAAGACCCATTAGTACGTTTCCGTAAATTCCTTGAAAACAAAGGTTTATGGAATGAAGACAAAGAAA
ATGAAGTTATTGAACGTGCAAAAGCTGATATTAAAGCAGCAATTAAAGAGGCTGATAACACTGAAAAACAAAC
TGTTACTTCTCTAATGGAAATTATGTATGAAGATATGCCTCAAAACTTAGCAGAACAATATGAAATTTACAAA
GAGAAGGAGTCGAAGTAAGCCATGGCACAAATGACAATGGTTCAAGCGATTAATGATGCGCTTAAAACTGAAC
TTAAAAATGACCAAGATGTTTTAATTTTTGGTGAAGACGTTGGTGTTAACGGCGGTGTTTTCCGTGTTACTGA
AGGACTACAAAAAGAATTTGGTGAAGATAGAGTATTCGATACACCTTTAGCTGAATCAGGTATTGGTGGTTTA
GCAATGGGTCTTGCAGTTGAAGGATTCCGTCCGGTTATGGAAGTACAATTCTTAGGTTTCGTATTCGAAGTAT
TTGATGCGATTGCTGGACAAATTGCACGTACTCGTTTCCGTTCAGGCGGTACTAAAACTGCACCTGTAACAAT
TCGTAGCCCATTTGGTGGTGGCGTACACACACCTGAATTGCACGCAGATAACTTAGAAGGTATTTTAGCTCAA
TCTCCAGGTCTAAAGGTTGTTATTCCTTCAGGCCCATACGATGCGAAAGGTTTATTAATTTCTTCTATTAGAA
GTAATGACCCAGTCGTATACTTAGAGCATATGAAATTGTATCGTTCATTCCGTGAAGAAGTACCTGAAGAAGA
ATATACAATTGACATTGGTAAGGCTAATGTGAAAAAAGAAGGTAATGACATTTCAATCATCACATACGGTGCA
ATGGTTCAAGAATCAATGAAAGCTGCAGAAGAACTTGAAAAAGATGGTTATTCTGTTGAAGTAATTGACTTAC
GTACTGTTCAACCAATCGATGTTGACACAATTGTAGCTTCAGTTGAAAAAACTGGTCGTGCAGTTGTAGTTCA

AGAAGCACAACGTCAAGCTGGTGTTGGTGCAGCAGTTGTAGCTGAATTAAGTGAACGTGCAATCCTTTCATTA
GAAGCACCTATTGGAAGAGTTGCAGCAGCAGATACAATTTATCCATTCACTCAAGCTGAAAATGTTTGGTTAC
CAAACAAAAATGACATCATCGAAAAAGCAAAAGAAACTTTAGAATTTTAATACATTTTAAAAGTTAACGAAGT
TAGCGTATTTTAGTCTCATTGATTAAAATGAAATGTTTAATTTACGAAATCTTAGGAGGGCAAAAACGTGGCA
TTTGAATTTAGATTACCCGATATCGGGGAAGGTATCCACGAAGGTGAAATTGTAAAATGGTTTGTTAAAGCTG
GAGATACTATTGAAGAAGACGATGTTTTAGCTGAGGTACAAAACGATAAATCAGTAGTAGAAATCCCATCACC
AGTATCTGGTACTGTTGAAGAAGTTATGGTAGAAGAAGGTACAGTAGCTGTAGTTGGTGACGTTATTGTTAAA
ATCGATGCACCTGATGCAGAAGATATGCAATTTAAAGGTCATGATGATGATTCATCATCTAAAGAAGAACCTG
CGAAAGAGGAAGCGCCAGCAGAGCAAGCACCTGTAGCTACTCAAACTGAAGAAGTAGATGAAAACAGAACTGT
TAAAGCAATGCCTTCAGTACGTAAATACGCACGTGAAAAAGGTGTTAACATTAAAGCAGTTTCAGGATCTGGT
AAAAATGGTCGTATTACAAAAGAAGATGTAGATGCATACTTAAATGGTGGTGCACCAACAGCTTCAAATGAAT
CAGCTGCTTCAACTACAAGTGAAGAAGTTGCTGAAACTCCTGCAGCACCTGCAGCAGTATCATTAGAAGGCGA
CTTCCCAGAAACAACTGAAAAAATCCCTGCTATGCGTAGAGCAATTGCGAAAGCAATGGTTAACTCTAAGCAT
ACTGCACCTCATGTAACATTAATGGATGAAATTGATGTTCAAGCATTATGGGATCACCGTAAGAAATTTAAAG
AAATCGCAGCTGAACAAGGTACTAAGTTAACATTCTTACCATATGTTGTTAAAGCGCTTGTTTCTGCATTGAA
AAAATACCCAGCACTTAATACTTCATTCAATGAAGAAGCTGGTGAAATTGTGCATAAACATTACTGGAATATC
GGTATTGCGGCGGACACTGATAGAGGTTTATTAGTACCTGTAGTGAAACATGCTGACCGTAAATCTATTTTCC
AAATTTCTGATGAAATTAATGAATTAGCTGTTAAAGCACGTGATGGTAAATTAACAGCCGATGAAATGAAAGG
TGCAACATGCACAATCAGTAATATCGGTTCAGCTGGTGGACAATGGTTCACTCCAGTTATCAATCACCCAGAA
GTAGCAATCTTAGGAATTGGCCGTATTGCTCAAAAACCTATCGTTAAAGATGGAGAAATTGTTGCAGCACCAG
TATTAGCATTATCATTAAGCTTTGACCACAGACAAATTGATGGTGCAACTGGCCAAAATGCAATGAATCACAT
TAAACGTTTATTAAATAATCCAGAATTATTATTAATGGAGGGGTAAAACATGGTAGTTGGAGATTTCCCAATT
GAAACAGATACTATAGTAATCGGAGCAGGTCCTGGTGGATACGTTGCAGCAATTCGTGCAGCTCAATTAGGAC
AAAAAGTAACAATCGTTGAGAAAGGTAATCTTGGTGGTGTTTGCTTAAACGTAGGATGTATTCCTTCAAAAGC
ATTACTACATGCTTCTCACCGTTTTGTTGAAGCACAACATTCTGAAAACTTAGGTGTTATTGCTGAAAGTGTT
TCTTTAAACTTCCAAAAAGTTCAAGAATTCAAATCATCAGTTGTTAATAAATTAACTGGTGGTGTTGAAGGCT
TACTTAAAGGTAACAAAGTAAACATCGTTAAAGGTGAAGCATATTTCGTAGATAACAATAGCTTACGTGTTAT
GGACGAAAAGAGCGCACAAACATACAACTTTAAAAATGCAATCATTGCAACAGGTTCAAGACCAATTGAAATT
CCTAATTTCAAATTCGGTAAACGTGTTATCGACTCAACAGGTGCTTTAAACTTACAAGAAGTACCAGGTAAAT
TAGTTGTAGTTGGTGGAGGATACATTGGATCAGAATTAGGTACAGCATTTGCTAACTTTGGTTCAGAAGTAAC
AATCCTTGAAGGTGCTAAAGATATCTTAGGTGGCTTCGAAAAACAAATGACACAACCTGTTAAAAAAGGTATG
AAAGAAAAAGGTGTTGAAATCGTTACTGAAGCTATGGCTAAATCTGCTGAAGAAACAGATAACGGAGTTAAAG
TTACTTATGAAGCTAAAGGCGAAGAGAAAACAATCGAAGCTGATTATGTATTAGTAACTGTAGGTCGTCGTCC
AAACACAGACGAATTAGGCCTAGAAGAATTAGGTGTTAAATTCGCTGACCGTGGATTATTAGAAGTTGATAAA
CAAAGCCGTACGTCTATCAGCAATATCTATGCAATTGGTGATATCGTTCCAGGTTTACCACTTGCTCACAAAG
CTAGCTATGAAGCTAAAGTTGCTGCTGAAGCAATTGATGGTCAAGCTGCTGAAGTTGATTACATTGGTATGCC
AGCAGTATGCTTTACTGAACCAGAATTAGCTACAGTTGGTTATTCAGAAGCGCAAGCTAAAGAAGAAGGTTTA
GCAATTAAAGCTTCTAAATTCCCATATGCAGCAAATGGTCGTGCATTATCATTAGATGATACTAACGGATTTG
TTAAACTTATTACACTTAAAGAAGATGATACTTTAATCGGTGCTCAAGTAGTTGGTACTGGTGCATCAGATAT
TATCTCTGAATTAGGTTTAGCAATTGAAGCTGGTATGAATGCTGAAGATATCGCATTAACAATCCATGCACAT
CCAACATTAGGTGAGATGACTATGGAAGCAGCAGAAAAAGCTATCGGATACCCAATCCATACAATGTAATAAC
TGATTATCTATAAAGATTCAGTCATTAAAAGCTGTAGCATATGCTACGGCTTTTTTGTTTTAGGTAAAGTAAT
GTAAGGAAATTGATTTGAGATATCGTTAACATGTGACATGCATGTTATACTAGCGATGCTAACAAAAGAATTG
AAATGGAGGGTTCAACAATGGAATATGAGTATCCAATTGATTTAGACTGGAGTAATGAAGAGATGATTTCAGT
GATAAATTTCTTTAATCATGTAGAGAAGTATTATGAATCCGGCGTGACGGCAGGCGACTTTATGGGTGCATAT
AAAAGATTTAAAGAAATTGTGCCTGCTAAAGCAGAGGAAAAACAAATTTTTAATACTTTCGAAAAAAGTAGTG
GCTATAATAGTTACAAAGCAGTTCAAGATGTAAAAACTCACTCTGAAGAACAAAGAGTAACAGCTAAAAAATA
ATTCGTTCGAAATTAACACAATTTAATAGGAATTTTTCTTTAAAACTATTGCTAATAAAGCTATATTTTGATA
CCTTTATCAAGTGTTAAACAAAATGTTTGATAAAAGTAAACTTAATATAGCTTTTTTAGGTGGAAAAATAAAT
GAACATAGGTAATAAAATTAAAAATCTTAGAAGAATTAAAAATTTAACGCAAGAAGAACTTGCTGAACGTACA
GACTTATCGAAAGGCTACATTTCACAAATAGAAAGTGAACATGCCTCACCAAGTATGGAAACTTTCTTAAATA
TTATAGAGGTGTTAGGAACGACGCCAAGTGAATTTTTTAAAGACAGTGAAAATGAAAAAGTATTATACAAGAA
GGAAGAACAAGTTATTTATGATGAGTATGATGAAGGTTATATATTAAATTGGTTAGTTTCAAAGTCAAATGAA
TATGATATGGAGCCATTAATATTAACTTTAAAGCCTGGCGCATCATATAAAAATTTTAATCCATCAGAGTCTG
ATACGTTTATTTATTGTATGTCAGGTCAGATAACACTTAATTTAGGCAAAGAGGTATATCAAGCACAAGAAGA
AGACGTTTTGTATTTTAAAGCACGAGATAATCATCGTTTGTCAAATGAATCAAACAATGAAACACGAATACTT
ATTGTAGCGACAGCTTCATATTTATAGGGGGGATCTTATTTGGAACCGTTATTATCATTAAAATCAGTTAGTA
AAAGCTATGATGATCTTAATATCTTAGATGACATAGATATTGATATTGAATCAGGATACTTTTATACATTATT

AGGTCCTTCAGGTTGTGGTAAAACAACAATTTTAAAATTAATTGCAGGGTTTGAATATCCTGACAGTGGTGAA
GTGATTTATCAAAACAAACCAATTGGTAATTTACCACCAAATAAACGTAAAGTGAATACAGTCTTTCAAGATT
ATGCATTATTTCCACACTTAAACGTCTATGATAATATCGCTTTCGGTTTGAAATTAAAAAAATTATCAAAAAC
CGAAATTGATCAAAAAGTAACTGAGGCATTAAAATTAGTTAAGCTTTCAGGTTATGAAAAAAGAAATATTAAT
GAAATGAGTGGCGGACAAAAGCAACGTGTTGCAATTGCACGTGCTATCGTAAATGAACCAGAAATATTATTAT
TAGATGAATCTTTATCCGCATTAGATTTGAAATTGCGCACTGAAATGCAATATGAATTACGGGAATTGCAATC
TAGATTAGGTATTACATTTATATTTGTAACACATGATCAAGAAGAAGCGTTAGCACTAAGTGACTTTCTTTTT
GTATTAAAAGATGGGAAAATTCAACAATTTGGTACGCCAACGGATATATATGACGAACCAGTGAATCGATTTG
TAGCTGATTTTATTGGAGAATCTAATATTGTTGAAGGACGCATGGTTAGAGATTATGTCGTGAATATTTATGG
GCAAGATTTCGAATGTGTCGATATGGGTATTCCTGAAAATAAAAAAGTAGAAGTCGTTATTCGACCAGAAGAT
ATATCATTAATCAAAGCTGAAGAAGGATTATTTAAAGCAACTGTTGATTCTATGTTATTTAGAGGGGTCCACT
ATGAAATATGTTGTATAGACAATAAAGGTTATGAATGGGTAATACAAACGACTAAAAAAGCTGAAGTAGGCAG
TGAAGTTGGTCTTTATTTTGATCCTGAAGCCATTCATATTATGGTTCCTGGAGAAACAGAAGAAGAGTTTGAT
AAACGTATTGAAAGCTATGAGGAAGTAGACAATGCGTAATACTAATAAATTTCTCTTAATCCCGTATTTATTA
TGGATGGTTATATTTATTATTGTACCAGTTGTATTACTCATTTATTTTTCATTTTTAGATATCAATGGACATT
TTAGTTTCACGAATTATCAACAAATTTTTACTACAAAATATTTAAAAATGTTTGCATATTCAATTTTATATGT
CGCTTTAATAACAATTATTACGTTGGCTATAAGTTATCCAGCTGCCTATTATATTACTCGTTCGAAATTTCAA
AATATCTTATTAATGATAATGATTATTCCAACATGGATAAATTTATTGTTAAAGACATATGCTTTTATAGGTT
TATTAAGTCATGATGGCGTGATTAATCAATTTTTCCACTTATTTAATTTACCATCATTCAATTTGTTATTTAC
AACTGGTGCGTTTTTAGTAGTGGCAAGTTACATTTATATACCATTTATGATTTTACCTATATTTAATAGCATG
AAAGCAATTCCTAATAATTTATTGCAGGCCTCAAGTGATTTAGGTGCTAGTCCTTTTTATACTTTTAGAAAAG
TAATCATGCCGTTAACAAAAGAAGGTGTTATGACTGGGATTCAAGTAACCTTTATTCCATCACTTTCATTATT
TATGATTACAAGATTAATTGCAGGTAATAAAGTCATAAATATAGGTACGGCAATAGAGGAACAATTTTTAACA
ATTCAAAATTATGGTATGGGATCAACTATAGCTATATTCTTAATTGTATTTATGGCATTCATTTTAATCATTA
CAAAATCATCTAATGGGAGAGGGTGAATCATATGAAATGGTATGGAAAGCTGTATATCGGGATACTTTTAGCG
ATTTTATACATCCCAATATTCTTTTTAATGTTCTATTCATTCAATTCGGCTGGTAATATGATTCACTTTGAAC
ATTTTACATTAGAGCATTATCAATCATTATTTCAAAATGATCATTTAATGTCGGTCATTTTTAATACGATAGC
TGTAGCACTTTTAGCAGCCTCAATTTCTACAGTTATTGGTACATTTGGTGCCATTGCTATTTATTATTTAAGA
AATAAAAAGTTTAAAGTAACTTTACTAACATTGAATAATGTCTTGATGGTATCATCCGACGTTGTCATAGGTG
CATCATTCTTAATTATGTTTACAACGATTGGCCATTTTACTGGTCTTGGTTTAGGATTTTGGACAGTTCTAAT
ATCTCATATTGCATTTTGCATACCTATAGTTGTGATTATCGTCTTACCACAACTGTATGAAATGAATAATAAT
ATGTTAAATGCTGCAAGAGATTTAGGAGCGACTGAACCACAATTATTAAGCAACATTATTATTCCTAATATTT
TACCTTCTATTATAGGAGGTTTCTTTATGGCTTTAACTTATTCACTAGACGATTTCACAGTAAGTTTCTTCGT
TACTGGTAATGGCTTTAGTGTGTTATCAGTTGAAGTTTATGCTATGGCGAGAAAAGGAATTAGTATGGAAATT
AATGCGATTTCAACATTATTGTTTGCTGTTATTGTATTAGGAATACTAGGATATTATTTGATTCAATACGTGA
TTAATAAGAAAAAACTAATCAAGCGAGGTGTAAAATAATGAAACGTTTTTTACAACTCATTATAGGTTCATTA
GTTGTGGGTATGCTTTGTCTTACTTTAAGTCATTGGTTTAAATCTAAAGAACAAGTGCATACAAATCAAAAAA
TTTACGTATACAATTGGGGCGAATATATTGATCCAGAGTTAATTAAGAAATTTGAAAAAGAAACTGGCATTCA
AGTCGTTTATGAAACTTTCGATTCAAATGAAGCGATGGAAGCCAAAATTCGTAATGGCGGTACACATTATGAT
GTTGCTTTTCCTAGTGAATATACAGTTCAAAAATTGAAAAGAGATCATTTATTGTTACCAATAGATCATAATA
AGGTACCTAATATTAAAAATTTAGATTCAGATTATATGAATATGTCATTTGATAAAGGCAATAAATATTCTTT
ACCTTATTTCTTTGGAACTGTAGGTATTTTATATAATAAAGAAAAGTATCCAAATGAATCATTTGATAGTTGG
AAGTCATTGTATAATCCTAAATTTAAAAACCAAATTTTACTAGTTGACGGTGCTAGAGAGATTATAGGCATGA
GTTTGAATAAACTTGGGTATAATCTTAATGACCGTAATTCGCACCATTTAAAAGAAGCAGAGCGAGATTTAAC
CAAACTAGCACCACAAGTAAGAGGTGTCGTAGGTGATGAAATTACCATGATGCTTCAACAAAATGAAGGTAAC
ATAGCGGTTGTTTGGAGTGGTGTTGCAGCACCTCTAGTGCAAGAAGGGGATAAATATAATTATGTTATTCCTA
AAGAAGGATCGAATTTATGGTTCGACAATATGGTAATTCCAAAAACGGCACAAAATAAAGAGGGTGCATATAA
ATTTATGAATTTTTTATTAGATGCTAAAAATAACAAGCAAAATACAGAATTCGTAGGCTATGCAACGCCAAAC
AAGGCTGCTCGACAATTGTTGCCTAAAGAGATTAAAGACGACCATCGTTTCTATCCGACTAAGAAAGAGCAAG
AACGCCTTGAAGTTTATAAAGATTTAGGACCTGAAGTTTTAAGTGAATACAATGAAAACTTTTTGAATTTCAA
AATGTCATTAAAATAAAATCATAAATAGTAGTTATTTACTGTATATTTAGTTATAATAACGGGATAAGTACTA
TAAGGAGTTGTAACGAATGACTGGAGAACAATTTACTCAAATTAAACGTCCAGTAAGTAGATTAACTGAAAAA
GTTCTAGGTTGGTTATGTTGGGTGATGTTATTAGTGCTTACTGTCATTACGATGTTTATAGCACTTGTTTCAT
TCAGTAATAACACATCGATTGCTAATCTTGAAAATACATTAAACAATAATGCATTTATCCAGCAATTATTAGC
TGGAAATGGCTATAATACAACACAATTTGTAATATGGTTACAAAATGGTATATGGGCTATTATCGTTTACTTT
ATTGTTTGTTTGTTGATTTCATTTTTAGCTCTCATATCTATGAATATAAGAATCTTGTCAGGTTTCTTATTCT
TAATATCAGCAATTGTAACGATTCCTTTAGTTTTACTTATCGTTACTTTAATCATTCCGATATTATTCTTTAT
TATTGCGATGATGCTATTTATAAGAAAAGATAAAGTTGAAATGGTTGCGCCACAATATTATGAAGAGTATAAC

GGACCGATTTATGATTATCGTGAACCTGTGTATGAGCGCCCACAACCGAAAGATGATTATTATGATGTGCCTA
AATATGAAAAAGAATTGGATAAATCAAATACTGTATATGATCAAGAACAGGAAAGAGATAAATATGATCAATT
TCCTAAACGTGCAGTTGAAAGTGAATATAATCATGATGAGCACACTGAGGAAGAACCATCAGTATTATCCAGA
CAGGCTAAATACAAACAAAAAAGTACTGAAGAACTAGGTATTGAAGATGATGGTTATTATGCAGAACCTGAAG
TTGATCCAAAAGAATTGAAAGCACAACAAAAGCGAGAAAAAGCTGAAATTAAAGCTAAGAAAAAAGAAAAACG
TAAAGCTTATAACCAACGAATGAAAGAACGTAGGAAAAATCAACCTAGCGCAGTTAGTCAACGTCGAATGAAT
TTTGAAGAGCGACGTCAAATTTACAACAATGATATTTCTGAAGAACGCAATTCAAGTGAAGTTAAGGACAAAA
AAGAGCAAGAATAAATATTGATGATACCCAGATTTAGTAAGTAGAGGTCTTTTGCTTATTAATCTGGGTTTTT
ATATGAGGTTAATTATCGATAATGTCTAATTAAAGTGTTTAGGTGTCAAAATTTTAAATGACGATTTCTCATT
ACCATACACCTAAATTACCATCAATCTGAATTCAGATATTTATTATAAAAATTAGATGAAAAATATGTTAATA
TACAAGTAAATTAATGTGCGTATATCTATAGGCTCGTAGTATACTTATTAAAATATTAAACAAAGAAAGGATT
TTAAGATGAATAGAAAACCTGAATTAATCATGGCTTGGATTGCAAATAGTATTAGTATTATTTATTTATTAGT
TATGGGGCTATCCTATTTTTCTTTAAAAAGTGGTAATGCATCACAACGCGAAGAATTAGCGAAGCAATTATCT
CAGAACGGTGGCAATGTTTCTTTAGATATGCTTCAGACAACAATTGGTGCATTAGCAATTATTTTATTAATTT
CAACACTTTATGGTATATTTGCGACAATTTGTATTAAAGGGCGTAGAAAATTATCGATTATACTTTTTGTTAT
CGCGATAATTGTAAGTTTGATGGCTCTTAATTTAATTGCAATTGTCTTATGGATTATCGTGATGATTATGTTG
ATTTCTAAAAAAGAATCAAAAGAAACTACACATAAGGACGATGAGTATATTTATCATTAATATGTTCATAGCA
AAAGAAAAACCATTAAAATGTTAAGTTGTTAATTATTAGATACAACCAACATTTTAATGGTTTTATTTTTAAC
CTTGTAGTTCTTGGAATGTTTGAACGATTAAATAGACATTTAAAATACTTAGAATAATAATAAGTGTCCATGA
AATGATATTAACCCATGTTTTATTATAGAAAGGCCCCATCAAGTCTTTATTACTAGTAGCTAATTGTAATGGT
ATTAAACAGAATGGCAGAGCAATACTTAAAAATACTTGAGAGAATACAAGTAGTTGCTCAATTTTAGCTGCAT
TACCTTTAAAAATGATTAAGCATACGATAACAGGAATGACAGCAAGGGAACGTGTAATTAAACGTCTTAACCA
ATTTGGTATGTGCAATCTTAAAAATCCTTCCATTACAATTTGTCCTGCTAAAGTACCAGTAATCGTTGAATTT
TGACCTGACGCTAATAATGCAACTGCAAATAATGTACTCATGATTGCACCCATTGTTGCACCTAGTACAGGTT
CAGTTTTTAAGGCGTGATATAAATCATAGAAACCACCTAAATCGTCAGCGTTCGAGTTGAAAAATAGTGATGC
TCCTAACACTAATAATAAGCAATTGACTACAAATGCGATTGATAACTGAATGTTCGAATCTATCGTAGCAAAT
TTAATCGCTTGCGCCTTTTCTTCATTGTTATGTCTTGAGTATGTTCTAGATTGTACAATTGATGAATGTAAGT
ACAAATTATGAGGCATAATTGTAGCGCCAATAATACCTAATGCAATATAGAGAATGCCGTTATTTGTAATGAT
TTCACTATGTGGTATAAATCCATTTAACACAGCGTTCAACTGTGGTGATGAAATATAGACTTCAAATATAAAG
ATGAATAACACTGTGAAAATTAATGTACCAACAATAGCTTCAATTTTTCTAAAACCATATTTCATTATAAATA
GTAGTAAAAATACATCAAGTACAGTTATTAGTGCACCGACGATTAAAGGTATGTTAAATAGGAGATTAAGAGC
AATAGCACTACCAATAACCTCAGCAATATCTGTGGCGATAATTGCTAGTTCTGCAATAATCCAAAAGATTATA
GCAATAGGTCTTGATAAATAATGTCGTGTCATTTGAGCTAAGTCCATACCTGTTGCTATTCCTAATCTCACTG
TCATGCTTTGAAGTAACATTGCTGATAAACTTGAAATAAGAATTACGAATAGCAAAGTATAGCCATATTGCGC
GCCACCTTGCATTGATGTTATCCAGTTTCCGGGATCCATGTAACCAACAGCGACTAATAACCCAGGCCCAAGA
AATGATAAAAATTTCTGTTTATTTGAACTGCGATGATCGAATTTAATTGTATTGTTTATTTCGTCTAAACTTA
ATTGTTCATTTGTTGAATGTCGTTTATTATTCATTTTGACTCACCTCTACTTCTTATGGACAATATAATAACC
TAATAAAATGATTAGGTCAACCTAAAATTATTTTAATTTTTTTAAAAGTATGAATTATTATGTTGAACGTTCG
ATTTAATATTAAGAAAAAAGAGCACGAAATCATGGATGTGATTTAGTGCTCTATTATTTAAACAAAGTTATTT
ATGCAGAATAAAATGGTAAGAACTGATCGAAGGTTTCTTCTAAAAATGCAATAAATGCTTTGTCACTTTTTAA
TCTTTTATCTTGTGGTGTGATTGCACGCGCTATAAAGAATTCACCTTTTTTTACATTGATGGCTCTTTGTATC
GCTTCTTTTAAATCATCATCCGTTAAATCTTTAATAAATGGTTTATCAGGTTTCATATGATCTAAGCAAACAC
GATAATCATCAGGTAATTGTTGAATAGCTTTAAATTTTCTTTCAAAAACTTTTGCACGTGTTGCTTTATCTTT
TGCTTCATGCATGATTCCAAACATAACAAACAGTTGATCTTCAAACATACCAATTTGGAAATGAGGTAACATT
TTATAGCCTCGTTTGCTTGTAGCAAAAGCAACCCATGTATCTTTAGGAGGATTCACACTTCTTCTAGCATGTT
TTGCTACGTGAGGATAAAATGTTTCACCAGTTTGACTTGTAAAGAAGTCACTAAAATATTCTCCTAATTCATG
GAGTTGTGGTCGTATGTATTCGTTTAAAGCTTCCATTCGTGCGTCTAAACCTTCTACGTTGAACGCTTTGAAA
TCTTTAGGTTTAAATGTATATTTTGTCATAGAGTACCTCCTGTATAGTGTTTAAAAATATTGTAGCATATGTT
TTATCAATTGCGGCTTAAGACGCTCTCATTAAAATTAGTATTTTCATTTTTATCATAAGAAGTTATTTTTATA
AAATGTTGATGTATAATAAAGATGTTAGGAGATGATGTTATGGCACTTTATGGATTTGCCCAAGGACTTATTC
AAGAAGCAGGAATTAGAATTAAACAATTGATGGAGCAAAATTTAACAATTGAAACAAAGTCAAATCCGAATGA
CCTTGTTACAAATGTAGATAAAGCAACAGAAGATTTTATTTTTGATACAATTTTAGAAACATATCCCAATCAT
CAAGTATTAGGTGAAGAAGGGCATGGTCATGACATCGATATTTCCAAAGGTACGGTATGGGTTGTTGACCCAA
TAGACGGTACATTGAATTTTGTTCATCAACAAGAAAATTTCGCGATTTCAATTGGTATTTATATCGATGGTAA
ACCTTATGCAGGTTTTGTATATGATGTTATGGCTGATGTCTTATATCATGCTAAAGTAGGGGAAGGTGCATAT
CGAGGTAGCCAACCCTTGAAACCATTGAATGATTCTAATCTAAGACAAAGCATTATTGGGATCAATCCGAACT
GGTTAACTAAACCAATTTTAGGAGAAATCTTTAAAGAAATTGTTAATGATTCTAGAAGTGCAAGGGCATATGG
TAGTGCAGCGCTTGAAATTGTTTCTGTTGCTACCGGTAATTTAGAAGCATATATGACACCTAGACTCCAACCG

TGGGATTTTGCTGGAGGATTGGTTATTTTATATGAAGTAAATGGACAAGCTTCAAATTTATTGGGTGAACCGC
TAACAATTGGTGGTCCAAACTCAGTTTTAGTTGGTAATCGTGGTGTTCATAGAGAGATCCTCGATGTATATTT
AGAGCCACATCGCGATGCGTTAATACAATTACATGAACGACGATTTAAAAGAAAATCAAAATAATTACCAAAA
GCGTAATATGTAGTCCTAACAATTAGGTAACATATTACGCTTTTATATCTTAAATAATAAAAACACGCGCCAA
TATAAAACCATTTAAATGTTCATCATGAACATGCGTGGTCTTTAATCAACGCGTGTTATTGTCGATATAACTT
ATAGCCAATCGTTTTCTCGATATTTTTTCTTTGTAGTAAATCCAATTCCAAAGGTTGCAACGAGTAATATAAA
TGTTAAAATCATCATTGGTACATTTGATGCACCAACAGCAAAACTAAATAATACTAAGAAAATAACAGCTAAT
ATAGAAAATACCCAAAAGATATTTTTAGATTTTTTTTGTTTCATCCTATCCCCACCTTTTGCTTATCTTTTCT
CAATTATATGATATAATAAAAAAGTTGTAATTAAAAGTGGGATTTTACTTAAGAAAGAAGGAAACTATTTATA
TGACTAATAAAAGAGAAGATGTCCGCAATATAGCAATTATTGCTCACGTTGACCATGGTAAAACAACTTTAGT
AGATGAGTTGTTAAAACAATCTGGTATATTCAGAGAAAATGAACATGTCGATGAACGTGCAATGGACTCTAAC
GATATTGAAAGAGAGCGTGGAATTACGATTCTAGCCAAAAATACGGCTGTTGATTATAAAGGTACACGTATTA
ATATTTTGGATACACCAGGACATGCAGACTTTGGTGGAGAAGTAGAACGTATTATGAAAATGGTTGATGGGGT
TGTCTTAGTAGTAGATGCGTATGAAGGTACAATGCCTCAAACACGTTTTGTACTTAAAAAAGCGCTAGAACAA
AACCTGAAACCTGTTGTTGTTGTTAATAAAATTGATAAACCATCAGCACGTCCAGAGGGTGTTGTAGATGAAG
TTTTAGATTTATTTATTGAATTAGAAGCAAACGATGAACAATTAGAATTCCCTGTTGTTTATGCTTCAGCAGT
AAATGGTACAGCTAGCTTAGATCCTGAAAAACAAGATGATAATTTACAATCATTATATGAAACAATTATTGAT
TATGTACCAGCTCCAATTGATAACAGTGATGAGCCATTACAATTCCAAGTAGCATTGTTGGACTACAATGATT
ATGTTGGACGTATTGGTATTGGTCGTGTATTCAGAGGTAAAATGCGTGTCGGAGATAATGTATCACTAATTAA
ATTAGACGGTACAGTGAAAAACTTCCGTGTAACTAAAATCTTTGGTTACTTTGGATTAAAACGTTTAGAAATT
GAAGAAGCACAAGCTGGAGATTTAATTGCTGTTTCAGGTATGGAAGACATTAATGTTGGTGAAACTGTAACAC
CACATGACCATCAAGAAGCATTGCCAGTTCTACGTATTGATGAGCCTACTCTTGAAATGACATTTAAAGTTAA
CAATTCACCATTTGCTGGCCGTGAAGGTGACTTTGTAACAGCGCGTCAAATTCAAGAACGTTTAAATCAACAA
TTAGAAACAGATGTATCTTTGAAAGTTTCTAACACAGATTCTCCAGATACATGGGTAGTTGCTGGTCGAGGTG
AATTGCATTTATCAATCCTTATTGAAAATATGCGTCGTGAAGGTTATGAATTACAAGTTTCAAAACCACAAGT
AATTATTAAAGAAATTGATGGTGTAATGTGTGAACCATTTGAACGTGTGCAATGTGAAGTGCCACAAGAAAAT
GCAGGTGCGGTTATTGAATCATTAGGTGCACGTAAAGGTGAAATGGTTGATATGACTACAACTGATAATGGAC
TTACACGTTTAATCTTTAATGTACCGGCTCGTGGTATGATTGGTTATACGACTGAATTTATGTCAATGACAAG
AGGTTACGGTATTATTAACCATACATTTGAAGAATTTAGACCACGTATTAAAGCACAAATTGGCGGTCGTCGT
AATGGTGCATTAATTTCAATGGATCAAGGTTCTGCAAGTACTTATGCCATTTTAGGACTTGAAGATAGAGGTG
TAAACTTCATGGAACCTGGTACTGAAGTTTATGAAGGTATGATTGTTGGTGAACATAATCGTGAAAATGATTT
AACTGTTAACATCACTAAAACAAAACATCAAACTAACGTACGTTCTGCAACGAAAGACCAAACACAAACAATG
AATAGACCGCGTATTCTAACATTGGAAGAAGCGTTACAATTCATTAATGATGATGAACTTGTTGAGGTTACAC
CAGAAAGCATACGTTTAAGAAAGAAAATTTTAAACAAAAATGTTCGTGAAAAAGAAGCAAAGCGTATCAAACA
AATGATGCAAGAAAACGAATAATTTAACATAATAAAAGAGAAGCTGAGCAATGAAAGTTGCTTAGCTTCTCTT
TTGGCATATAATATTTGAAAAAGTGTTTAAATGCTATTATTTCCTGTTACTTTCACATTTTCAAATTTCTAAC
TTGTTTATTCAGATATTAATCGAGCGGATAGTGTTGCGCACGTTGTTTAGGTGTGTCTAATCGACTTTTACAA
TCATCGCACATAAATGTTCGAATAGGATTGTTTTTAAGTCGTTTAGACTCAGTTGTGCGCTCATCTATAAATA
CTTTTGCATCACAGATAATGCATTGCACTTGTCTCAAATTAAATCACCTCAATATGAGTTACATGGTTGATTC
GATATGAATAACCATCTTCTTGATTGTAAATAAAACTATCTACACCATTATCGCTGTAAAGTCGTTTACCGTC
TTTTGCAAATTGGAAAAATAAATAGGGTAATAGATCTATCGGTATATCTAAATGATCATGCTCATTTGATAAT
CGAATAGTAGTTGCAGAGTCATGTGGTTCGGAATGTTTGAAAAATGGTGTCATATTAATGACAAATGAACCTT
CTAACATGGCACGTTTTTTATATTTTATTTCTGAATTTAAAGTAGGCGGATTAGTTTGTCCTTCTAGGATAGC
ACGATTCCATTCATGATTATCTTCAAAGTCGATTGGTTTGAACCATCAAATACACCTTTTTCTAAATCTTCG
ATGCTAACTTTTCTATCATCGAAAATCCAAGTCGTACTATCTAATGTTATAGGAAACTTTACGGCTCCTTTAA
TTTGTATCATTTTCCCACTCCCTATCAATGTATATAGCATTATTTTAACACAAATTGCTAACGACACATTTTA
AATCTACTTGCTTTTTAATATTTAGTAAGATAAACTTTTAGTAAGACTTGAGAATTTATATAGAGGGGGAGCG
TGTCATGGCGAAACAAACAACAATGAAAAATGCAGCTTTGAAACAATTGACTAAAGATGCTGATGAAATCTTA
CATCTGATTAAAGTTCAACTAGATAATTTAACATTACCTTCATGCCCATTATATGAAGAAGTACTAGATACAC
AAATGTTTGGACTTCAAAAAGAAGTTGATTTTGCTGTTAAATTAGGTTTAGTTGACCGCGAAGATGGCAAACA
AATAATGTTACGTCTTGAGAAAGAACTTTCAAAATTACATGAAGCTTTTACACTTGTTTAAGGTTTAATATAT
AAGCATATTAAAACGGCGTGAGATATCGACAATTGAGCTGCGCCGATTTTATATTTATAGTTTAATTACTGAT
TAAAGTGGCTGTTATGTTGTCTTCTTGATGACATGTCAGTCACTTTTATTGGGTTTTAGCAATTCACGATATT
AAGTAAACAAATATTCTATAAATTACCTCAAGATACCTGTTAATATTTGTGATTATGACGATGTCATGTATAT
AATAAGTAACATGGATTTTTAAAATTAAAGTATAAAACGAAATCATTTGAAGTATAAATTGTAGAATTGGATG
ACTGAATATATGAAGAATTTTAGAAGTATTTTACGGTATATTGGTAAAACCTCAAAGTTTATTGATTACCCGT
TATTAGTTACATATATTGTATTGAGTTTAATTGGATTAGTGATGGTATATAGTGCCAGTATGGTTCCAGCAAC
TAAAGGCACATTGACTGGTGGTATCGATGTTCCAGGAACGTATTTTTACAACCGACAATTAGCATATGTCATA

ATGAGTTTTATGATTGTATTTTTTATTGCATTTTTAATGAATGTTAAATTACTGAGTAATATTAAAGTGCAAA
AAGGTATGATTATAACTATCGTCTCGCTATTATTACTGACGTTAGTAATCGGTAAAGATATTAATGGTTCTAA
AAGTTGGATAAACTTAGGATTTATGAACTTACAGGCATCTGAGTTATTAAAAATTGCAATCATATTATATATC
CCATTTATGATCAGTAAAAAAATGCCTAGAGTATTAAGTAAACCAAAATTAATTTTAAGTCCTATTGTATTAG
CATTAGGTTGTACGTTTTTAGTTTTCCTACAAAAAGACGTAGGGCAAACATTACTAATATTAATTATTTTAGT
TGCGATCATTTTTTATTCAGGAATTGGGGTAAACAAAGTCCTAAGATTTGGTATACCAGCAGTGCTAGGATTC
TTAGTAGTATTTGTCATTGCATTAATGGCTGGTTGGTTACCAAGTTATTTAACAGCCAGATTTAGTACGCTAA
CAGATCCATTCCAATTCGAATCAGGAACTGGATACCATATTTCCAATTCATTGCTTGCGATAGGTAACGGTGG
CGTATTTGGAAAAGGATTAGGAAATAGTGCAATGAAATTGGGCTATTTACCAGAACCACATACAGATTTTATT
TTTGCAATTATTTGCGAAGAATTAGGTTTAATCGGAGGATTGCTAGTTATTACTTTAGAGTTCTTTATTGTAT
ATCGTGCCTTTCAGTTTGCAAATAAAACATCATCATATTTTTATAAACTTGTGTGTGTTGGGATTGCCACATA
CTTTGGAAGCCAAACGTTTGTAAACATTGGCGGTATTTCGGCAACAATTCCATTAACTGGTGTGCCATTGCCA
TTTATCAGTTTTGGTGGATCATCAATGATTAGTTTAAGTATTGCTATGGGATTACTTCTGATTGTAGGTAAAC
AAATCAAAGTAGACCAGCAACGAAAGAAACAACAACAAAAAGTTGATATAAGAAGACAATTTAATTAAAAAAT
ACTAGCCAATATTTAGTACGAGATGACTTCTGTCATAAATCGATGCTAAACATTGGCTATTTATATACTCTTT
GTTAAATTGGACTGATGAGTTAAATAACGGAAGTTAGGCAACCAAACAACGCTTAACTTCCTTTTTGTTTTGA
TATTACGAGATTACTCATGATTATCAAATTTTATAAACGAAACAACGTATAATTATAACTAAATTTTATGACT
AAAAATTACTAAATGTGGTGGCGAAATTAGAATCATGATGATAAGATAAAGTTATCGCTATTGATAATAAATG
TTGATGTTTTGCAATTTTATTTTTAATTTTTTAGAGTTGTAGAAATCGAAAATGTTTCACAACAAAAACGTGA
ACTACCTGTTGTTGCATAGTCATTTTTGAAATTATTATTTTTGAAGCGGAGGTTTTAATTGTCTCAAGAATGT
TAAATTTTGAAAATTTGAAACATAACTATAGCAAAACAGAGCGCTTTAAGATAAAATTTTATTATCTAATTAG
TTAGAAAATTCTAATAGTTTGAGGAGTGTATCGCTATTGAAACAAATAAAAAAGTTACTTGTTGCTAACCGTG
GAGAAATTGCAATTCGTATATTCAGAGCGGCAGCAGAATTAGACATCAGCACAGTTGCAATTTATTCGAATGA
AGACAAAAGTTCATTACATAGATATAAAGCAGATGAATCCTATTTAGTTGGAAGTGATTTAGGTCCTGCTGAA
AGTTATTTAAATATTGAGCGTATCATTGATGTAGCAAAACAAGCGAATGTGGATGCGATTCATCCTGGCTATG
GATTTTTAAGTGAAAATGAACAATTTGCGCGTCGTTGTGCAGAAGAAGGAATTAAATTTATTGGTCCTCATTT
AGAACATTTAGATATGTTTGGAGATAAAGTTAAAGCTCGTACAACGGCTATCAAGGCAGATTTACCAGTTATT
CCTGGTACAGACGGTCCAATTAAATCATATGAATTAGCAAAAGAATTTGCAGAAGAAGCTGGTTTCCCGCTAA
TGATTAAAGCCACAAGTGGTGGCGGCGGTAAAGGTATGAGAATCGTTCGTGAAGAAAGTGAATTAGAAGATGC
TTTCCATAGAGCAAAATCAGAAGCTGAAAAATCATTTGGTAATAGTGAAGTTTACATAGAAAGATACATTGAT
AGTCCAAAGCATATTGAAGTACAAGTTATAGGTGACGAACATGGAAATATCGTACACTTATTTGAACGTGATT
GTTCAGTACAACGTCGTCATCAAAAAGTTGTAGAAGTTGCACCATCAGTTGGATTATCACCAACATTACGTCA
ACGTATTTGTGATGCTGCAATTCAATTGATGGAAAATATTAAATATGTCAATGCAGGTACTGTTGAATTTCTA
GTATCTGGTGACGAATTCTTCTTTATAGAAGTTAACCCTCGTGTACAAGTAGAGCATACAATTACAGAGATGG
TAACAGGAATTGATATTGTTAAGACACAAATTTTAGTTGCAGCAGGTGCCGATTTATTTGGTGAAGAAATTAA
TATGCCGCAACAAAAAGATATTACAACATTGGGCTATGCCATCCAATGTCGTATTACAACAGAAGATCCGTTA
AATGATTTCATGCCGGATACTGGAACAATCATTGCTTATCGTTCAAGCGGTGGCTTTGGTGTACGTCTTGATG
CTGGAGATGGTTTCCAAGGTGCTGAGATATCACCTTATTATGATTCATTACTCGTAAAATTATCTACACACGC
GATATCATTTAAACAAGCAGAAGAAAAAATGGTACGTTCATTACGAGAAATGCGTATTCGTGGTGTTAAAACT
AATATTCCATTCTTAATTAATGTAATGAAGAATAAAAAGTTCACAAGTGGTGATTACACAACTAAATTTATTG
AAGAAACACCAGAACTTTTCGACATTCAGCCGTCTCTAGATAGAGGTACTAAAACATTAGAATATATAGGTAA
TGTAACAATTAATGGTTTCCCAAATGTTGAGAAACGTCCGAAACCAGACTATGAATTAGCATCAATTCCAACT
GTATCTTCAAGTAAAATCGCTTCATTTAGTGGTACGAAACAATTGCTTGATGAAGTAGGTCCAAAAGGTGTAT
CTGAATGGGTTAAAAAGCAGGATGATGTCTTACTAACAGATACAACTTTTAGAGATGCACACCAATCATTATT
AGCTACACGAGTTAGAACTAAGGATATGATTAATATCGCATCCAAAACAGCGGACGTATTTAAAGATGGTTTC
TCACTAGAAATGTGGGGCGGTGCTACATTTGATGTGGCATATAATTTCTTGAAGGAAAACCCATGGAACGAC
TTGAACGTCTACGTAAAGCTATTCCAAATGTATTATTCCAAATGTTGTTACGTGCTTCAAACGCAGTTGGTTA
TAAAAACTATCCTGATAATGTTATTCATAAATTCGTACAAGAAAGTGCTAAAGCAGGCATAGATGTCTTTAGA
ATTTTCGATTCATTAAACTGGGTAGATCAAATGAAAGTTGCCAATGAAGCAGTACAAGAAGCGGGCAAAATCT
CTGAAGGTACTATTTGTTATACAGGTGACATTTTAAATCCTGAGCGATCAAACATTTATACTTTAGAGTATTA
TGTCAAACTAGCTAAAGAGTTAGAACGTGAAGGTTTCCATATTTTAGCGATTAAAGATATGGCAGGCTTATTA
AAACCTAAAGCCGCTTACGAATTGATTGGTGAGTTAAAATCAGCTGTAGATTTACCAATTCATCTTCACACTC
ATGATACAAGTGGTAATGGTTTATTAACATACAAACAAGCAATAGATGCTGGTGTCGATATCATTGATACTGC
TGTTGCTTCAATGAGTGGTTTAACAAGTCAGCCAAGCGCCAATTCGTTATATTATGCATTGAATGGCTTCCCA
CGCCACCTTAGAACTGATATTGAAGGTATGGAGTCACTTAGTCATTATTGGTCAACTGTACGTACTTATTATT
CAGACTTTGAAAGTGATATCAAATCACCGAATACTGAAATTTATCAACATGAAATGCCTGGTGGACAGTATTC
GAATTTAAGCCAACAAGCTAAAAGTTTAGGTTTAGGCGAAAGATTTGATGAAGTTAAAGATATGTATCGCAGA
GTGAATTTCTTATTTGGTGATATCGTAAAAGTAACACCATCGTCTAAAGTAGTTGGTGATATGGCACTTTATA

TGGTACAAAATGATCTTGATGAACAATCCGTGATTACAGATGGCTATAAATTAGATTTCCCAGAATCAGTAGT
GTCGTTCTTCAAAGGCGAAATAGGACAACCTGTAAATGGTTTTAATAAAGATTTACAAGCGGTTATTTTAAAA
GGCCAAGAAGCACTAACAGCTCGTCCAGGTGAATATCTAGAGCCGGTTGATTTTGAAAAAGTCCGTGAGTTGC
TTGAAGAAGAGCAACAAGGTCCTGTTACGGAGCAAGATATTATTAGTTATGTACTATATCCAAAAGTATATGA
ACAATATATTCAAACTAGAAATCAATACGGAAACTTATCGTTACTTGATACGCCTACATTCTTCTTTGGAATG
CGTAATGGTGAAACAGTAGAAATCGAAATCGATAAAGGTAAACGATTAATTATTAAACTAGAAACGATTAGTG
AACCAGATGAAAATGGTAATAGAACGATTTACTATGCGATGAATGGTCAAGCGAGACGTATTTACATTAAAGA
TGAAAATGTACATACAAATGCGAACGTTAAACCAAAAGCAGATAAGAGTAATCCAAGTCATATAGGTGCGCAA
ATGCCAGGTTCAGTAACTGAAGTCAAGGTTAGTGTAGGTGAATCTGTGAAAGCTAACCAGCCGTTGCTAATTA
CTGAAGCTATGAAAATGGAAACAACAATTCAAGCACCATTTGACGGTGTGATTAAACAAGTAACTGTAAATAA
TGGTGACACAATAGCGACAGGCGATTTATTAATCGAAATTGAAAAAGCAACTGACTAAAATGATTAAAATAAA
ACGAGATTACACAACAATTGACGTGTAATCTCGTTTTTACATGTCTTAGGTTTAATTTTAAATATTGATATAA
AAAACCGTAATTTTAATGGGCGTGCCATTAAAATTACGGTTTGCTTTTTTGTTATTGCTTGTCACTTCTTACT
GATCGTAACATTAGCATGATAAAGTATGTTGTCATACCAAATAAATAAGTGATAAATAATGCATGGAAAAGTG
CGATTATCAGGTTAACATTTGTCATAATAGATAATGCACCTGTGATAACTTGTAATATAACAAGTATAAATGC
AGCTGTATAACCATAATGAACAGTACGGTTATTTGGATAATTTTTAACAGCGTGAATATAAGTAATCATAATA
ATCGTAAACACTATAAAAGCCATGATACGATGCGTGAGTTGAACCCAATCTTGTTCTGAATGTGGTACAAGAT
CGTGGAATGGCAATGGCCAACCACCATATGCTAAACTTGCATCCGCATGTCTCACTAGTGCACCAGTATAAAC
ACCACAATAAATGATGATTGCCATTAACCATGTTAAACGTCTTAATGGCTTTTTGATATATAATTCGTCAGCT
TCATATTTTTGATCTATAGAGAAAATAATCAATGTTATTAAAAATACAGATGAGAAACTGATTAATGATATAC
CAAAGTGCAATGCTAAAACGTAATCGTTTTGTTGCCAAATAACAGCGGCAGCTCCGATTAATGCTTGCAATAA
TAAGAATCCAACACTAATGATTGATAAAGGTTTAATTTCTTTAATATAGCCTATATGTTTCCATGCAGTGATA
ACTAACCATAAGACCATTAATAAAGACAAAGCTGAAACGGCTCTATGACTTAACTCAATAATCGTATCAATAG
GAAAGAATTCTGGAATCAACGCACCATGACATAGTGGCCAAGAAGAACCACAACCATCAGCTGATCCGGTTTT
GGTAACTAAGGCTCCACCAAGTTGTACAAATGTCATCATTAACGTTGCTACGACACCTAACCATTTTAAATTC
TTTTTGCCAAACAATTTATACACCCCATCATAAAAAAACGCATCACTGAGAATTAGAATTAATAGCCGGATTT
GAGATGATACTATAAATTGACCTGATTGTAGGATATGAAAAGATGGGATCCAACGCAGGAAATATGATTTAAT
TTTACAAGTATTGTATGTGGACGACATAAAAAGAACTTAATCGTAATGATTTTTTATTGGGATAATTATGTAA
TCCACGTTTACTCAAATTTGAAGCTTAAATATTAATCTCTTTTAGATGCATGATTCTACTTATTAAAATTTTC
TAACATATACATTATAGCAATAAATTAAATGTTATTTGTGTCACAAAATTGACATTTTCTCTTGTCGTTAATT
TGTCACATTTTAATTTTATTAAACCTAGTCTTTTTATGCAATCTAAGATATCATAATCTTATATGAACAAATT
TAAGGAGGGGGATTATGAGCAAAGAGCATACTTTGTCACAAAATATTAGCAGAGTTAACTTCAAAGAATTGCA
ACAGATAATTAAAATGGGACTTGTTCAAGGTAACTTAATTCCGGCTTTTGCGGGCGCATGGCTAGCAGTTGTA
ATGACAAATCATTCCTTCTTATCATCAATACCTCAAATTTTATTAATGCTATTAGGATCTACTTTAATTATGG
GTGGCGCATGTGCGTTAAATAATTATTACGACCAAGATATTGATCGTATTATGCCTAGTAAACAAAATAGACC
AACTGTAAATAATAGAATTACAGATCAAAATTTATTACTATTAAGTTTTGGTATGATGTTAGTTGGAGAAATT
TGTTTGTTTTTATTGAATATACCATCAGGCGTACTTGGTCTTATGGGGATTGTAGGTTATGTGTCTTATTACT
CAATATGGTCTAAAAGACATACAACATGGAACACAGTGATTGGGAGTTTTCCTGGAGCAGTACCACCACTAAT
TGGATGGGTTGCAATTGAAGGACAAATTAGTTTAACAGCGATTGCGCTGTTTTTAGTTGTATTTTGTTGGCAA
CCAATTCATTTTTATGCCTTAGCTATTAAACGTAAAGATGAATATGCACTTGCAAATATTCCAATGTTACCAT
CAGTTAAGGGCTTTAAACGTACACGTGTCAGTATGTTTATCTGGTTGATTATTTTATTGCCAGTACCTTTATT
ACTAATAAATTTAGGTGTCGTATTCGTAGTGTTAGCTACATTATTAAATTTAGGATGGATTGCATTAGGTTTA
ACAACATTTAAGAAAAATTCAGATCAAACAAAATGGGCAACACAAATGTTTATATATTCACTAAATTATTTAG
TGATCTTTTTCGTGTTAGCTGTGATTGTTTCATTACTTACTTTGATCTAGATTAATTAAGTTAGGATGAAAAA
TATGGGCGTTCCAATTTTACCAACGATTAGTACGACATGTATTGTCATTAGTGCAATTTTAATTGCCATTGGT
TGGAGACTTATTTGGAAAAGGGAAATAAATAAACACAAAAATGTTATGTTAGCTGCAGCTGTTTTTGCTTTAA
CTTTTTTCTTAATCTATGCAAGTAGAACGATTTTTATCGGTAATACAGCATTTGGCGGACCAGCATCAATTAA
GAAATATTATACGATTTTCTTATTTTTCCACATTAATTTAGCAACAATTGGTGGTATTCTAGGTCTAGTTCAA
ATTATTACTGCATTTAAAGATAAATATAATGTACACCGCAAATTCGGGCCATTCGCTTCAGTTATATGGTTCT
GTACTGCAATTACAGGTGTAGCAGTTTACTTATTATTATATGTATTATATCCAGGTGGAGAAACGACATCACT
GATTAAAGCAACATTTGGTCATTAATATGCTTATACAAATGATACTAATCTGATAAGGCAATTTGTTTTATCA
GGTTTTTTGATGAAGAATAGTCGGACAGTTGATGGTATTCATTTGATACCTTAACTGTCCATTTTTTTATACA
TATTTTAATCTTAATAATGAAATTCAACCTGAAAACATAAAGATAGGTTATGATATAGAGTGTAGGGAAAATC
TTTATGACGTTAAAATTATGCCTGAAGATGATTTTGTTTTATCGAAACGACACAGTTATGTCAGTGTTGAAAA
TTTGCTAATTAATGTGTTGTTTTTTACAATAGTATTAATACTTTTTAGGTGGTTGATTGATGAAAAAGTTAAT
TATAAGAGTCGTCGGTGTATTATTTTTAATTGGATTTTTAATATATCTTTTTTATTCACCGAGATTAAAATTT
GATGTATTAGAGAACCCGAATAAAGGTAATAAAGTAAATAGATCTGAACAGGTGAATAAATCAAATAACCATG
CTGAAAATCCAAAGCCTAAAGAAGGTGTTGGTACATGGGTAGGTAAAAATATTAAAGTGCTTACTTCTAAATT

TGGACAAGCAGATCGTGTCTACCCTTTTAGAGATGGTTACAAAAATTATGTGTTTAAAGACAAAAACAGTTAT
TACATTGTTTCAACTAAACGTGAAGAAATCGTTTCAGTGTATGCTACAGGTGAGAAAGTCAATGTTAGTCCGT
TAAAAATAGGACAACATTCTGCAGAAATTTTTAATCATACAAGTATTAATCCAGAACCGTCCTTTAAAGTTGA
TGGTAAAAAATATGAATTTGAACTTTCAGATGAAGATTTAAAAACACAAACACTGATTAAATATGGCGACATA
TATGCACAAGTGTATTCTGATCAACAATCGAAAAAGGTGCTAAGTGTACGATTTTTAACAAAAGAAATGTTAG
CAGATATTGAACCTTATCAATTAAATTCTAATTCTACGTCAGAAGAGCATAATAAGCGTCCAGTTGAGCAAAA
TCCAAATCAATTAATTTCTCTTTATGAAGTAACGAATGAAATGAGAAAATTAAAAGGATTAAAACCATTGAAA
ATCAATAGCGATTTAGCACATATTGCATCTAATAACTTATATGAAGCGACCTCTAATGGTTCTGATAGTGTTG
AATTTACAGAGGACGCATTAAGAGGGCAATTAGATAAAAATCACGTTACTTATAAAACAACTGCTCAAAATGT
TGGTTATGCGTTTAATGATGTACCAACATTAATCCATAGTTGGATGAATTCAGATATACATCGATCTCGTCTA
TTAAATTCAAAATACGATGAGATGGGTGGAGATGTAATGAGAGATTATTATTCACTAATTTTCTTAGAAAAAT
AACAATGAGGGGTATTTATGATTAATGAAGCTTCACTAGCGATATTAGATGATATTGATGAACTAGCTGATAT
GATAGTTGCATCAGATATTTATGCATCATTTGAGCAAGCTAAACAGGCGCTCGAAAACAATGACGAAGCACAT
TTACTTTATCAATCATTTTTAAAATCAAAAGAGAAATATGATGAAGTAATGCGATTCGGGAAATATCATCCTG
ATTATAAGAAAGTTATGTTAGAGACAAGACAACGCAAAAGAGCATATGAGATGCTTGACGTTGTGATGCATTA
TAAAGCTAGGGAAATGGCACTTCAACATTTAATTGATGAAGTTGTTACGAAAATTGCGTACGCTGTTTCAGAG
CATGTCAAAATAGAAACAGGTAATCCATTCTTTCAAACATCACATAGTGGTTGTACGACGGGCGGATCCTGTA
ATTGTTCATTATAAAAAACATCGAGTCAGAAAAAGATGGTTATTGAAACCACTAACTAGCATCTGACTCGATG
TTTTTATTTTTTCGGGATTGTTTGTTTGAATTGTTGTGCTAAATCTGGTCGATCTGTCACAATCGTGTGTGCA
CCTTTTTGGTATAAATCATTCATCAGATCTATACTATTTACGCCATAATAGCCTGGAATGACATTCATATCAT
TTAACCATTTGATAAAACGAGATGAAGTCAAATCAATGCCTTTAAAATGAGTAGGCATTTGGAACGTTTGTGC
TAATGGTTGGTAGTACCTGCCACCTAATAAATGATATTTTAAAAATGCCTCTGTAACTTCCTGTTGGCTAGCA
CCAATTGCAACGGATCCTTGTGCAATTTTATTAAAACGGACGATTTGTTCTTTATAAAAACTTGTCACAAGAA
CGCGGTCAAATGCTTGATTCTCTGCAATTGTATCAAACATAATTTGTGGTGCGATAGAACCTTCATAGGATTC
AGGAGCATCTTTTAAGTCTACGTTTATATACATATCAGGATATTGCTTCAGCAACTCATCGAAGGTTAGTATA
GCTGTGTGTGCATGACCACGATATGGTGTTAGTCCATTGATATCTTTGAAGTGATAAGCTGCGTCTAATTTCT
TTAATTCTGCTAATGTATGGGCACTAACTTTTCCAGAGCCATTCGTCGTTCTATCAACAGTTGCGTCATGAAA
AACGATAAGCTGTTGATCTTTTGTGAGTCTCACATCTGTTTCAAAGCCATCAACGCCTAATTGTTTAGCATAG
TCAAATGCAAGTTGCGTTTGCTCTGGTCTTAAAGCCATACCACCGCGATGCGCAAATATATATGGTGCATTGC
CTTTGAAAAAAGCAGGGATGGTTTGCTTTTTAGTAATCACTTTATTTTTATTTATCATTAATAAACTACTTAC
AAATCCAGCACCGACTAGTACCGCATTTAAAATGTTTCTGTTTACTTTTTTCATAAAAAATTCCTCCTCGTTT
ACTTTTATAGCTTAACAAATTCATAGTTCTGACTACAGCGAAATGTGTGGTTAATCATATAGTGGTGTTCTTG
AGAATTGAATTAATATAATGATGTTTAATATAATCTAAATACATTTATCGAGCAATTACATAAGACATAATTA
AGGTAGCTTAATATTTTAAAATTAATAAAGACATGATATGATTAATGCTGAAAATAAATGGAGTGAGGTTTAA
ATGAATTTAATCCCAAGAACTAGTATTGTAGTTTATTTAAAACATATGAAACATGAACGACAAATCCGAAAAT
ATGGACATATCGTTCATTCAAATAGAGATCGTAAATTTGTAATTATGTATGTGAATGAGCAAGATGTTGATCA
AATTGTACATAAACTAATGCAACTTAAATACGTTAGACACATTGATGGCTCACCATATAAATACTTAAAGAAA
ACTTACGAAAAAGAGAAACACGAAATATACAATTAATATTATTGAAGTTCTGGTATCCATTTTTGGAGCCTTA
ACACACTAATTAAATAATTATAATATAACGATAAATCATGAAATGCTTGAGGGGGCTGTACATCGATACCTAA
AATAGGTATGTCGAATTTTTCAGCACAATCATTAATCATTTGAAACTTTTTATTTGAATCTGGATATGGATAT
TTAATAGCGTTTATCATGATATAAAATGCTTGGAAATGATTGCCAGTTGTAGGATTCATAATGATTGCTACAG
ATGACTTTTTATTTTCAGCTTGTGGTGAATGAAAATAGATAATTTTATCAATACGTTGATGGTTATATTCAAT
CATCGTCATGAATTCGAACAAATCTGTTAGACCTTCACCTAGTGTAATAAATGTTTGTTTCATTGGTTTATCA
TTCTCCTTTTTCTCAATAAATTATATTAAATAACTAAATGAATTGGAAGTGTGAACAACATGCGCGTCATTGC
AGGTAAACATAAAAGTAAAGCTTTAGAAAGTATGGAAGGCCGTAATACGAGACCAACTATGGATAAAGTTAAA
GAAGGCATCTTTAATAGTTTATATGATGTGTCAGGTATAGGTTTAGATTTATTTGCAGGAAGCGGGGCGCTTG
GAATAGAAGCACTCTCTCGAGGTATGGATAAGGTTATCTTTGTTGATCAGAATTTTAAAGCTGTAAAAGTTAT
TAAATCAAATCTTGCGAATTTGGATTTAGAGGCGCAATCTGAAGTTTATAAAAATAATGCAGATAGAGCTTTA
AAAGCATTGTCAAAACGTGATATTCAATTTGATGTCATTTTCTTAGATCCACCTTATAATAAAGGTCTCATTG
ATAAAGCTTTAAAACTAATTTCAGAGTTTAATTTATTGAAAGAAAATGGTATCATCGTTTGTGAATTTAGCAA
TCATGAAGAAATAGACTATCAACCGTTTAATATGATTAAACGTTACCATTATGGGTTGACAGACACATTGTTA
TTAGAAAAGGGAGAATAGCATGGAACATACAATAGCGGTCATTCCGGGTAGTTTTGACCCCATTACTTATGGT
CATTTAGACATTATTGAGAGAAGTACAGATAGATTTGATGAAATTCATGTCTGTGTTCTTAAAAATAGTAAAA
AAGAAGGTACGTTTAGTTTAGAAGAGCGTATGGATTTAATTGAACAATCTGTTAAACATTTACCTAATGTCAA
GGTTCATCAATTTAGTGGTTTACTAGTCGATTATTGTGAACAAGTAGGAGCTAAAACAATCATACGTGGTTTA
AGAGCAGTCAGTGATTTTGAATATGAATTACGCTTAACTTCAATGAATAAAAAGTTGAACAATGAAATTGAAA
CGTTATATATGATGTCTAGTACTAATTATTCATTTATAAGTTCAAGTATTGTTAAAGAAGTTGCAGCTTATCG
AGCAGATATTTCTGAATTCGTTCCACCTTATGTTGAAAAGGCATTGAAGAAGAAATTTAAGTAATAAAAATAA

CAGTATTTTAGGTTTATCATGGTTTACAATCCTAAAATACTGTTTTCATTTGTTAACGATATTGCTGTATGAC
AGGCGTGTTGAAATCTGTTTGTTGTTGCCCGCTTATTGCATTGTATATGTGTGTTGCTTTGATTTCATTTGTG
AAGTAATGTGCATTGCTTTTGTTAATATTGGTTATATATTGTCTTTCTGGGAACGCTGTTTTTAAATGCTTTA
AATATTGTCTGCCACGGTCGTTCATCGCTAATACTTTAACTGCGTGAATGTTTCTCGTAACATCTGTAGGTTT
AATGTTTAATAATACATTCATTAACAGTCTTTGGATATGCGTATATGTATAACGCTTTGTTTTTAGTAATTTT
ATAAAATGATGAAAATCAGTTGCTTCATAAATGTTAGATTTCAAACGATTTTCAAAACCTTCAGTAACAGTAT
AAATATTTTTTAATGACTCTGTAGTCATAGCTATGATTTGATATTTCAAATATGGAAATATTTGATTTAATGT
AATATGAGGTGTTACGTACAGGTGTTGAATATCTTTAGGTACCACATGATGCCAATGATCATCTTGACTAATG
ATTGATGTTCTAATAGATGTACCACTTGCAAACTGATGGTGTTGAATTAATGAATCATGATGTTGAGCATTTT
CTCGTTTAATAGAAATTGCATTGATGTTTTTAGCATGTTTAGCAATTGCTTTCAGATAACTAATACCAAGTAT
GTTGTTAGGACTTTCTAGTGCTTCATGATGCTCTAATAATTCGCTAATGATACGAGGGTAGCTTTTACCTTCT
TTTACTTTTTGTGAAAAGGATTCGGATTGTTCAATTTCATTAATGCTGTGAGCTAATTGCTTTAATGTTTTGA
TATCATTATTTTCACTACCAAATGCAATTGTATCGACACTCATATAATCTGCGACTTTAACTGCTAGTTCGGC
AAAATGATCGCCAGATGATAAACTGGCAGTTGCTGGTAGTTCGATAACTAAATCAGCTGTTGATAATGCCATT
TTTGCACGAGTAAACTTATTATAGATTGCCGGTTCGCCACGCATGACAAAGTTACCACTCATTATTGCAATAG
TAACGTCAGCATTTGTAAGTTTTTTAGATTGATTAATATGATATTGATGCCCATTATGAAAGGGATTATATTC
TGTGATTAAGCCAACGCTTTTCATTTGCAATTCGTCCTTTCGGTATTCATTATTAATATTGTAACAGAACATG
ATATGTTAAGAAAAAATCTTGACAACTTGTTCTTAGAAAGTTAAAATAAATTTTGTGCTTGTTAGAGGTGAAG
CCATATGAAATGGTCAATTACGCAATTAAGGAAATATCAAGGTAAGCCATTTGAATTTGATCAAACGGTGAGT
TTTGACAATTTAAAAGAATCATTAGATTTAATTGATTTATCTCCAATTACAATCCAAGGTCAGTTAACCATTA
AGTCAACAGAAGTCGTTGCGGATATTCACATTACTGGAACGTATACAATGCCTTGTGCACGTACTCTTGTACC
AGTTAAAGTCCCACTAGATGTAACTACTACAGAAGTATTTGATTTAGAAGGGTACAATCAGTATAACGATGAT
CAAGATGATGTAGATGAACACTATCACATTATTAAAGATGGTATGGTTAATCTTCAGGATATTGTCGAGGATA
TAGTTATTATTGAGAAACCAATGAGAGCTTATTCAGAGCAAAGTGACCAAATGTTGACAGTAGGTAATGGTTG
GGAAGTAATCGATGAGGATCAATTAGATGAGCTTGCTAAACAGCAAGAACAAGATGATTCAGAATCACGACAA
GTTGATCCAAGGCTTCAAAAATTACAACAATTATATGATAAAGAGCAATAAGTAGTTTAATTAATGATATAAT
GTTGTTAATTAAACTAACATTTAGTATTTTTCGTTTATAATCTAAGGAGGATATATCATGGCAGTACCAAAAA
GAAGAACTTCTAAAACTAGAAAAAAACAAACGTCGTACGCATTTCAAAATTTCAGTACCAGGTATGACTGAATG
CCCAAACTGTGGCGAATACAAATTATCACACCGTGTATGTAAAAACTGTGGTTCTTACAATGGCGAAGAAGTA
GCAGCTAAATAATTTTAGTTACTCAATATAAAAAGTCCCGCTTAAAATGATTGTTTTAAGTGGGACTTTTTAT
ATTGCGAAAAATAATTGGCGAACGAGGTAACTGGATACCTCATCCGTCAATTAAAATTTGTTAATTTAATAAT
TAAATATAAAGACGATTTATTAGTTTTTACGTTTTCTAGGTAATACGAATGCAACGATGCTACTTAAAGCTAA
TAATGCCATTAATGGTAATGTCATATCTTTATTTGATTCTTCACCAGTTTGTGGTAATGATTTTGCTTTATTT
TCTTGTGTATTTTTATTGTTTTGGCTTTGAGTGTGTCCATCATTTGTGTTTTTAATGTTTGCTTTTTGTAATG
GAGCACTATCTTTTGCTTCGCTAGAACCTGCTGAAGTTTGAACAACATCTTTTGTTGTTTTTGATGAAGCAGT
TGTTGGTTTTGCAACATTTTGAGTCGTAGATACTACCTTAGTTGGAGTTGTACTACTTGATTCTACTTCACCT
TTAGCTGGTTTTGTAGCAGGCGTTTTGTCTTTACCTGACTCACTAGATGCGTCATTTTCTTTTTCAACACTTG
GTAATTGTTTATTGTCATCTTTTTGGCTGTCTTGTTTTTGTGATTCTTTTTCAACAGGTGATGGTGTTGGTTT
GCTAGGCGTAGCTGGAGTAGCTTCCTTCTTAGCTGAGTTATCTTGTTGTTCTTTTTTGTTAGATTTATCGGTA
TTGGCTTTTGTAAATGCTTCTTTATCAACGATTCTGACATGGTATTGTCCATCATAATCAATCGTTTTTACGT
GAACTTTAACGATAGCATCATATAGAGTTTTACCTTCAACATATGGGAAAATAATTGTTCTAGTATTATTTAT
AGCATCTTTGCTTATAGTTCTAACACGTTGGCCTTCAACCATGAAATCTTTCCAGTAATCGTCATTAGTAGTT
TCCATGACCATATATTTTTTGCCGTTAAGCATACCTGTTTTAATAGGGTGTTTAACAAAAGTATCCATCATAG
ATTCGTTATTCTCAACACTTTCATAAACAACATATTTTGTATCTTGTAAATCAGTCATTTTTTCATTTGTTGG
TTGTACATTTTGGAATTCAGTAATAGCTGATTTCACTTGCTCATCTAAAGCTTTCTTTGTATCCTCTAATTTC
TTCTTGTACTCAGCCTTTAATTTTTCAGGAAGTTTATCTTGAATTTTATTTAATTCATAAACTTGTCTTTCTA
GTGTTTTCGCTTTTTTATATGGTGCTAATAATTTTTCAGCTTTATAATCTTCTTCAGTTTTGAATTTATCTGC
ACTGTTATAAATTGGTTGTGCGAATTCCATTAATGTGTAATCGTATTTTTCTTCTTTGTTATTGAAGTGTGTT
GAACTAACAATTTTAACAGCTTTTGTTCCATTTGATACAGAGAAGCGAATGTAAGCATAATCTTTAACAGTAT
CGTATGATACTAATTTAATTGGCAACTTTTTGTCACCCTCATAAACTTCAAATTTTCTCCAAAATTGACCTGA
TTGTAATCCTAATTCAATTTCTGGTTTTGAATCAGTGAAAATAACTCTAGCAGGTTTAACAGAGCTGGCATAA
TGATAAAATTGTTGCTCACCATTTTCTTTTTTCATTTCAAAATCAATTGGACGAGAGTTTGGTGCGCTATGAT
CTTTATCTTTTATTGCAGGGTTTTTAATCGCTTCTCTAAGTTCCTGATTCAAAATAGGATATGTATTGTTATC
GGCTTTTGCTGCTGGTTTAACTGCTTTTGTTTCCTTAGGGGCTTTAACTTCTTTAACTTCTTTAGCTTCTTTT
GTTTCAGAAGTAGGGGCCTCAACTTCTTTATTAGATACTGAGACAGCATTAGCTACTGGTTTAGTTTCTGGAG
CTTTTTCAGTTGTTGTTGTTGGACTTGCAAGTGCTTCAGTTTTTGGTTGTGCTTCTGTATTTGTACCACCTGT
TTCTTCAGCTGCTTGTGCTTCGCCATTTGACATTAATAATAAAAGTGTACTAATTGCTACAGATGCAACGCCT
AGTGATGACTTTCTAATTGAATAAAATGATTTAAATTCTTTTTGCTGTTTGTTCATGTTGTAGAAACAACTCC

TAATTGTATATTATCAACTGATAATCATTATCAATTGATTACTTGTATTGTACATTGAAATGGTAGTTAATTG
CAACGTTATTTTATAAAAAAATAACCTATGTTATAGATTATGAAATATCTATGACATAGGTTAAATAAAATTT
AAAATAAAAAATATATTAAGTTATGAGTGTGACGATGATTTATTTAGATTCTTTTCTTTTGAAAAGTAATAAA
GATAATGAACCTAAAAGGGCAAGTGTTGCGAAGGCAACTGTGCTAATAAAGTTATCAACTGAAGTTAAACCAG
TTTTTGGTAATTCTTTAGCTTTAGATGCTTGTTTAGGCGTTTCGTTTTGTTTTGTAACTTTGTTAGTTTGTTG
TGATTTATTGTCACTTACAGCTTGACTGTTGCTTTCAGATTTCGCTGTTGCAACATCTTTAACAGGTGTTTGA
ACTTTATTTTGTTCTTGAGCTGTTTGTGTTGTTTTCACTGTACGAGCACTTTGTGTTTGACTTTGATCTTTTG
TTGTATCAGTACTTACAACTTTAGTAGAGTGATTGTCTTCAACTTTGCTTGTTGTAGTTACAGTAGGTTTAAC
TTTTTCAACTTTAGGTTGAACTGGTTTAGTTTGCTCAGTAGGTGTTTTAGGTTGAGCAGGTTTTGGTTGAACC
GGTTTAACATTGTTTGGTTTTGCTGCGTCAGCTAATGTAGGAATTGCTTTTTCAAATTCCAAATGCGTAGTAT
ATCTATGATTGTAATTAATTTGTGGCACGACAATATGTACTTTAGTAGTTAAGCTCTTATATCCAGGTTCAAC
TGCAACATTGATTGTTCTAGTATCCGCTTTTTTATTATCGTTAACAACAGTTGTTGCTAATTCTTGATTGTTT
GCATTGTAAAATTTGTATTCTTTCCAGAATGATGCATTGTTTAACACGGTTTGGAAATAATATTTATTATTTT
GTTTAATTACTTTACCAGGGTGTTGCATATAGTCATCCATGTGTGACTTCTCTGAAGAGCCATCTTTTTGCAC
TTGGAAATTAATTGGTTGTGATGTTGCTTGAGAAACTTGTGTGCTTTGATTATTAGTTGCGTTCGTAGCTTCT
GTTGCCGCATTGACTTGTTGGCTGTCTGCGCCTATGTATACAAGGGAACCTAAAATGATAGATGCTGTACCCA
TTGTAATCTTTTTCATAGCTGATGAACGTTGTTCTGATTGATACTTACTGTTTAAATAATGTTTTGTCATGTT
GTTTTCCTCCTAAGGATACAAATTATGTGTTGTTATATGAGATGATTAACCATTGCAATTGATAATGGTTATC
AATTATACTAGCACATCTATTTTCGTATAACAATAATAAATTGATTATAAAATAAATATTGACAATGATAATC
ATTATTATTTATGATTTTAGTAAAGACTTAAAAGCAATCATAAAAAGGAGGATTATGTTTTGAAAAATATTTT
AAAAGTTTTTAATACAACGATTTTAGCGTTAATTATCATCATCGCGACATTCAGTAATTCTGCAAATGCCGCA
GATAGCGGTACTTTGAATTATGAGGTTTACAAATACAATACCAATGACACGTCAATTGCTAATGACTATTTTA
ATAAACCGGCAAAGTACATTAAGAAAAATGGTAAATTGTATGTTCAAATAACTGTCAACCACAGTCATTGGAT
TACTGGAATGAGTATCGAAGGACATAAAGAAAATATTATTAGTAAAAACACTGCCAAAGATGAACGCACTTCT
GAATTTGAAGTAAGTAAGTTGAACGGTAAAATAGATGGAAAAATTGACGTTTATATCGATGAAAAAGTAAATG
GTAAGCCATTCAAATATGACCATCATTACAACATTACATATAAATTTAATGGACCAACTGATGTAGCAGGTGC
TAATGCACCAGGTAAAGATGATAAAAATTCTGCTTCAGGTAGTGACAAAGGATCTGATGGAACGACTACTGGT
CAAAGTGAATCTAACAGTTCGAATAAAGACAAAGTAGAAAATCCACAAACAAATGCTGGTACACCTGCATATA
TATATGCAATACCAGTTGCATCCTTAGCATTATTAATCGCAATCACATTGTTTGTTAGAAAAAAATCTAAAGG
CAATGTGGAATAATGAGAAATGTTAAACAAATTGCTACAAAATCTATTATAGCTATTATTAGCTTAGGTATAC
TTACATATACAACAATGATTGGTAGCGTGTTGGCTGATGAGATAAAATATCCATCAGCCAAATTTAATCAACC
TGAAGCAAAAGATAAAACAGAATTAACTACATCAATTTTTGATGAAAAGATAAAAGAGAATAAAGCGTTAGAG
TTAGTAATTTTTAATCAAGAAAATAAAAATGTAACTGAGGAACAACAACTAGTTGACGAAAAGGCGCAATTGA
TTTCAGATATGACTGGTAAAATTTACTTGCAAGTAAAGCTAAAAGGTCAAATTGATAAAGAACAACTTGTTTT
TCAAAATGACAAAAATGAAGAATTTCCTTTTGTTATAAAAGATGAAAAGGATGACACAATAGTAAGAATTTTA
ATTGAACAGCATATGGATAAAATCAATATGCATGTTAAAACGTTGGCTGAAAAGAAAAATCTAGATAACAAAG
AAATGGTGTATTCTATTCATTTTAAAGAGAAAAAAGTACAACATGATGATGCAAAAGAAGTGCCTTCAAAACA
TCAAAGTCAAGAAAATAATCAAGATCAGCTTAAAAAAGATATTGATGACAAAAAAGATAGTCAAAAATCAGAC
ATTAAGGAAAGACGTACTAGCCTTTTTACTGAAAAAGGCTTAAATGATATTCCTGTACAAAAAGATAAAGTGC
AACAAGACAGTAATAAAAAGATTGAAAATGAGCGACCTAAAGCATCAGGTACATTAAAAGTTGAAAATAATCC
TTCAACAGTAAAAAAAGGTTGAAAATAATCACAAAGAGCAACCGAAACATAAAGATGAAAAATCAAAAAAGGAA
AAGAAAAAAGTAGTTGAAAAAGAAAAAGCGTTACCAGCTTTTAATAGAGATGATGATAGCAAGAATAGTAGTC
AATTATCTAGTGATATTAAAGAACTTGATGAACCAAATCATAAAAAGCAATATATGTTATTTGCAGCTGGCAT
TGTGTTAGCAACCATTTTACTTATTTCGGCACATTTATACAGCAGAAAGAGAGGTAACCAAGTTTGAGAATCA
TAAAGTATTTAACCATTTTAGTGATAAGCGTCGTTATCTTAACCAGCTGTCAATCTTCCAGTTCTCAAGAATC
AACTAAATCCGGCGAATTCAGAATCGTACCAACAACTGTCGCATTGACAATGACATTGGACAAATTGGATTTA
CCAATTGTCGGCAAACCCACGTCATATAAGACATTGCCTAATCGCTATAAAGATGTACCGGAAATTGGTCAAC
CAATGGAGCCGAATGTTGAAGCTGTTAAAAAGTTAAAACCAACACATGTTTTGAGTGTGTCAACGATTAAAGA
TGAAATGCAACCATTTTACAAACAATTAAATATGAAAGGCTACTTTTATGATTTTGATAGTTTAAAAGGGATG
CAAAAGTCGATTACACAATTAGGTGATCAATTTGATCGTAAAGCACAAGCAAAAGAATTAAATGACCATTTAA
ATTCTGTAAAGCAAAAAATTGAAAATAAAGCAGCTAAACAAAAGAAACATCCCAAAGTATTAATATTAATGGG
TGTACCTGGTAGCTATTTAGTAGCAACTGATAAATCATATATTGGTGATTTAGTTAAAATAGCAGGTGGAGAA
AATGTTATTAAAGTGAAAGATCGTCAATATATTTCGTCTAATACTGAAAATTTGTTGAATATAAATCCAGATA
TTATTTTACGATTACCACACGGAATGCCTGAAGAAGTTAAGAAAATGTTTCAAAAAGAATTTAAACAAAATGA
TATTTGGAAACATTTTAAAGCTGTGAAAAATAATCATGTTTATGACTTAGAGGAAGTGCCATTCGGTATTACA
GCAAATGTTGATGCTGATAAGGCAATGACTCAATTATATGATTTATTTTATAAGGATAAAAAATAGTGAGTTG
ATATGATGATAAAAAAATAAAAAGAAACTACTATTTTTATGTTTGTTAGTCATTTTAATCGCAACTGCTTATAT
TTCGTTTGTAACCGGTACAATTAAATTGTCATTTAATGACCTATTTACAAAATTTACAACTGGTAGCAATGAA

GCAGTGGATTCAATCATTGATTTGCGATTGCCACGTATATTAATTGCATTAATGGTTGGCGCAATGTTAGCAG
TTTCTGGAGCATTATTACAAGCAGCACTACAAAATCCTTTGGCAGAGGCGAATATCATTGGCGTTTCTTCAGG
TGCACTTATAATGAGAGCACTTTGTATGTTGTTTATTCCACAATTGTACTTTTACTTACCATTATTAAGTTTT
ATTGGAGGTTTAATACCATTTTTAATAATTATATTGTTGCATTCTAAATTTAGATTCAATGCTGTAAGTATGA
TATTAGTAGGTGTTGCGTTATTCGTATTATTAAATGGTGTTTTAGAAATTTTAACTCAAAACCCTTTAATGAA
AATTCCTCAAGGCTTAACAATGAAAATATGGAGCGACGTATACATATTAGCAGTATCAGCATTATTGGGATTA
ATATTAACATTACTATTGTCCACTAAATTGAATTTACTAAATTTAGATGACGTACAAGCGCGAAGTATCGGTT
TCAATATTGATCGTTACAGATGGTTAACAGGTTTATTAGCAGTATTTTTAGCAAGTGCAACTGTTGCGATTGT
TGGACAACTAGCCTTTTTAGGTATTATTGTGCCACATGTGGTTAGAAAGCTAGTTGGGGGCAATTACAGAGTA
CTTATTCCATTTTCTACAGTTATTGGTGCATGGCTATTGTTAGTGGCTGATTTATTAGGACGAGTGATACAGC
CTCCTTTAGAAATTCCAGCCAATGCTATTTTAATGATTGTCGGTGGTCCAATGCTAATTTACTTAATTTGTCA
AAGTCAACGAAATCGAATCTAAAACGGTACAATAACTGGTTTAGTAGTGACTGAAGAAACGTCGTTAATAAAA
GAGGATTTTTATGAGAATGAAGCGATTTTTAACTATTGTACAAATTTTATTGGTTGTAATTATTATCATTTTT
GGTTACAAAATTGTTCAAACATATATTGAAGACAAGCAAGAACGCGCAAATTATGAGAAATTACAACAAAAAT
TTCAAATGCTGATGAGCAAACATCAAGCACATGTGAGACCACAATTTGAATCACTTGAAAAAATAAATAAAGA
CATTGTTGGATGGATAAAATTATCAGGAACATCATTAAATTATCCAGTACTACAAGGTAAGACAAATCACGAT
TATTTAAATTTAGATTTTGAGCGAGAACATCGACGTAAAGGTAGTATTTTTATGGATTTTAGAAATGAATTGA
AGAATTTAAATCATAATACTATTTTATATGGGCACCATGTCGGTGATAATACGATGTTTGATGTGTTAGAAGA
TTATTTAAAGCAATCTTTTTATGAAAAACACAAGATAATTGAATTTGACAATAAATATGGTAAATATCAATTG
CAAGTATTTAGTGCATATAAAACTACTACTAAAGATAATTACATACGTACAGATTTTGAAAATGATCAAGATT
ATCAACAATTTTTAGATGAGACAAAACGTAAATCTGTAATTAATTCAGATGTTAATGTAACGGTAAAAGATAA
AATAATAACTTTATCAACGTGCGAAGATGCATATAGTGAAACAACGAAAAGAATTGTTGTTGTCGCAAAAATA
ATTAAGGTAAGTTAAACAGAAAAGAGGATAATTATGAAATTCATGGCAGAAAATAGGCTGACGTTAACAAAAG
GAACAGCAAAAGATATTATAGAACGATTTTACACGAGACATGGGATTGAAACATTAGAAGGCTTTGATGGCAT
GTTTGTTACACAAACTTTAGAACAAGAAGATTTTGATGAAGTGAAAATTTTAACAGTTTGGAAATCAAAGCAA
GCTTTTACGGATTGGTTAAAATCTGATGTCTTTAAAGCAGCACATAAACATGTTAGAAGTAAAAATGAAGATG
AAAGTAGCCCGATTATCAATAACAAAGTAATTACATATGATATAGGCTATAGTTACATGAAATAATTGAATTT
AAAGAGGTTGCAGTACTTGTTATGACAATTGGTATTGTTAGCTTTTGTAACGACCATACATGATACCGATGAT
GGTCGTTTTTTAATGAACACAAACATGCTAACAACAAATTGCTAAAGCGTAGTTTGATTTGAATGTGCTTTGA
AAATAATCATCAATTATATCATTTTGATTTATTTTGAAGAAGAAAAGAATTAATAATGATATTTTGGGGCGTA
TGATTTATAGTAGTAGTATCGATAAAAGAAATTTATACAATTTGAAATAATATGAGTGATAAAATCGATTAAT
TAATATTAACTATACAATCAAATGTTTTCATCTTATTAATGATTAATATTTTTATTTTTAAAAATAAAGCGAG
GAGCTATCTATGGAACAAATTACTTCTGCACAAAATAATAGAATTAAACAAGCGAACAAGCTAAAAAAGAAAC
GTGAGAGGGATAAAACTGGATTAGCTTTAATTGAAGGTGTGCATTTAATTGAAGAAGCTTATCAAAGTGGGAT
TGTAATTACACAATTATTTGCAATTGAACCGGCAAGATTAGATCAGCAAATTATCGCATACGCGCAAGAAGTT
TTTGAAATAAACATGAAAGTTGCTGAATCTTTATCAGGTACAGTGACACCACAAGGGTTTTTCGCAATCATTG
AGAAGCCACAATATGATATTTCTAAAGCACAACAAGTATTGCTTATCGATCGTGTTCAAGATCCTGGAAATTT
AGGCACATTAATTAGAACTGCGGATGCTGCTGGAATGGATGCTGTAATAATGGAGAAGGGTACGACAGATCCT
TATCAAGATAAAGTATTGCGAGCGAGTCAAGGTAGTGTTTTTCATTTGCCAGTTATGACTCAAGATCTCGATA
CGTTTATTACTCAATTTAATGGTCCTGTTTATGGTACAGCACTTGAAAACGCAGTGGCATACAAAGAAGTTAC
TTCAAGTGATTCTTTTGCATTACTATTAGGTAATGAGGGAGAAGGTGTTAATCCTGAATTATTAGCACATACT
ACACAAAATTTAATCATACCTATTTATGGTAAAGCTGAAAGTTTAAATGTAGCGATTGCAGGTAGTATTTTAC
TTTATCATTTGAAAGGTTGACCGTGTTGAAAGTTTTCCGATATAATTATAATTAATTGTTTAACAGAACATTT
CTACGTGATTGCATACACAATTGAATATAAACCAATAAAAAGGCATGGACATTTATATAAATAATTGTTTTAG
GGAGAATAATCGTGACTGCAAGTTATTCCAATTATTTAAAGTCTTTTCACCTTTTTGGTTACTTAAAGAGATT
TAAGTCGGAAAGACAATCCGTTATCAATATTAAACAAGTGTATGCTTAGGCATAAATTTGGGTGGTACCACGG
AAATGACTTTCGTCCCTTATTTTTTAAGAGGATGAAAGTCTTTTTTTAGTTAAACAACAAATATGATAAATAG
AAAATGAATAGTTCGAATAGGGAGGTCAGTGACATATGTCTGAACAACAAACAATGTCAGAGTTAAAACAACA
AGCGCTTGTAGATATTAATGAAGCAAATGATGAACGTGCACTGCAAGAAGTTAAAGTGAAATACTTAGGTAAA
AAAGGGTCAGTTAGCGGACTAATGAAATTGATGAAGGATTTGCCGAATGAAGAGAAACCTGCATTTGGTCAAA
AAGTGAATGAATTGCGTCAAACAATTCAAAATGAATTAGATGAAAGACAACAGATGTTAGTTAAAGAAAAATT
AAATAAGCAATTGGCTGAAGAAACAATTGATGTATCATTACCAGGTCGTCATATTGAAATCGGTTCAAAGCAT
CCATTAACACGTACAATAGAAGAAATTGAAGACTTATTCTTAGGTTTAGGTTATGAAATTGTGAATGGATATG
AAGTTGAACAAGATCATTATAACTTCGAAATGCTGAATTTACCTAAATCACACCCTGCACGTGATATGCAAGA
TAGTTTCTATATTACGGATGAAATTTTATTACGTACGCATACATCACCAGTGCAGGCACGTACGATGGAATCA
CGTCATGGTCAAGGTCCAGTTAAAATTATTTGCCCTGGTAAAGTGTATCGTCGTGACTCTGATGATGCGACAC
ATAGTCATCAATTTACACAAATTGAAGGATTAGTTGTTGATAAAAACGTTAAAATGAGTGATTTGAAAGGTAC
TTTAGAATTGTTAGCTAAGAAATTATTTGGTGCTGATCGTGAAATTCGTTTACGTCCAAGTTACTTCCCATTC

ACTGAACCTTCTGTAGAAGTTGATGTGTCATGTTTTAAATGTAAAGGAAAAGGTTGTAATGTGTGTAAACACA
CAGGATGGATTGAGATTTTAGGTGCTGGAATGGTACATCCTAATGTATTAGAAATGGCTGGTTTTGATTCTTC
AGAGTACTCTGGATTTGCATTTGGTATGGGACCAGACCGTATTGCAATGTTAAAATATGGTATAGAAGATATT
CGTCATTTCTATACTAATGATGTGAGATTTTTAGATCAATTTAAAGCGGTAGAAGATAGAGGTGACATGTAAT
GTTGATATCAAATGAATGGTTGAAAGAATATGTAACAATCGATGATTCTGTAAGTAATTTGGCAGAACGTATT
ACGCGCACAGGTATTGAAGTGGATGATTTAATTGACTACACAAAAGATATCAAAAATTTAGTTGTCGGCTTCG
TTAAGTCAAAAGAGAAACATCCTGATGCTGATAAATTAAATGTTTGCCAAGTTGATATCGGAGAAGACGAACC
TGTACAAATCGTATGTGGTGCACCGAACGTTGATGCAGGACAATATGTCATTGTTGCTAAAGTAGGTGGAAGA
TTGCCTGGTGGTATTAAAATTAAGCGTGCCAAATTACGCGGTGAACGTTCAGAAGGTATGATTTGTTCGTTAC
AAGAAATTGGTATTTCAAGTAACTATGTACCGAAAAGTTTTGAATCAGGCATTTATGTTTTTAGTGAATCCCA
AGTTCCAGGAACAGATGCCTTACAAGCTTTATATTTAGATGATCAAGTAATGGAATTTGATTTAACGCCGAAT
CGTGCTGATGCTTTAAGTATGATAGGTACTGCTTATGAAGTTGCAGCATTATATAATACAAAAATGACTAAGC
CAGACACAACATCAAATGAGCTTGAGTTATCTGCAAATGATGAACTGACTGTGACAATTGAAAATGAAGATAA
AGTACCATATTATAGTGCACGTGTTGTTCACGACGTGACAATTGAACCTTCGCCAATTTGGATGCAAGCACGC
TTAATAAAAGCGGGTATACGTCCTATTAATAATGTTGTTGACATTTCAAATTATGTGTTATTAGAATACGGTC
AACCATTGCACATGTTTGATCAAGATGCGATTGGTTCACAACAAATTGTTGTTCGTCAAGCGAATGAAGGCGA
AAAAATGACAACATTAGATGATACAGAACGTGAATTATTAACGAGCGATATTGTCATTACTAATGGACAAACT
CCAATTGCATTAGCTGGTGTTATGGGTGGCGATTTTTCAGAAGTTAAAGAACATACATCAAATATAGTGATTG
AAGGTGCTATTTTTGATCCAGTTTCAATTCGTCATACATCAAGACGTTTAAATTTACGCAGTGAATCATCTAG
TCGTTTTGAAAAAGGAATAGCTACTGAATTTGTAGATGAAGCAGTCGACCGTGCATGTTATTTATTACAAACT
TATGCAAACGGAAAAGTGCTAAAAGATAGAGTGTCTTCAGGAGAACTTGGTGCATTTATTACACCAATCGACA
TCACTGCTGATAAAATTAATCGCACTATTGGATTTGATTTGTCACAAAATGATATTGTTACTATTTTTAATCA
ACTAGGGTTTGATACAGAAATAAATGATGATGTTATTACAGTGCAAGTACCATCACGTCGTAAAGATATTACA
ATTAAAGAAGATTTAATTGAAGAAGTTGCACGTATTTATGGGTACGACGATATTCCATCAACGTTACCTGTCT
TCGAAAAAGTTACTAGTGGTCAGCTAACTGATCGCCAATATAAAACGAGAATGGTTAAAGAAGTGTTAGAAGG
TGCTGGATTAGACCAAGCTATTACGTATTCGTTAGTTTCTAAAGAAGATGCTACTGCATTTGCGATGCAACAG
CGTCAAACAATTGATTTATTGATGCCAATGAGTGAAGCGCATGCGTCATTACGTCAAAGTTTATTACCACATT
TAATCGAAGCGGCATCATATAATGTGGCACGCAAAAATAAAGATGTAAAATTATTTGAAATCGGCAATGTCTT
CTTTGCTAATGGAGAAGGTGAACTACCAGATCAAGTTGAATATTTAAGTGGTATTTTAACTGGAGATTATGTA
GTCAATCAATGGCAAGGTAAGAAAGAAACGGTTGATTTCTATTTAGCAAAAGGTGTCGTGGATCGAGTATCTG
AAAAGTTAAACCTTGAATTTAGTTATCGTCGTGCTGATATTGATGGATTACATCCAGGTCGTACTGCTGAAAT
CTTATTAGAGAATAAAGTTGTTGGTTTTATTGGTGAATTACATCCAACATTAGCAGCTGATAATGATTTAAAA
CGTACGTATGTTTTTGAGTTGAATTTTGATGCATTAATGGCTGTGTCGGTAGGTTACATTAATTACCAGCCAA
TTCCGAGATTCCCAGGCATGTCTCGTGACATTGCATTAGAAGTAGATCAAAATATTCCAGCAGCTGATTTATT
ATCAACGATTCATGCACACGGTGGCAATATATTAAAAGATACACTTGTCTTTGATGTATATCAGGGCGAACAT
TTAGAAAAAGGTAAAAAATCAATTGCAATACGTTTAAATTATTTAGACACAGAAGAAACATTGACAGATGAGC
GCGTTTCAAAAGTACAAGCGGAAATTGAAGCAGCATTAATTGAACAAGGTGCTGTTATTAGATAATGATTTAA
ACCCCATGTATAAGGATATCTGAAGTAGATTGATATCCCTAACATGGGGTTTTATTTTTGGGTTCACCAATTA
GGTTCCAATGCATTTAAAAAGTCAAAGAGGAACAGCGGATTACAGTTGATGCTTCGCATCACTGCATATAAAG
TCATACTTATGAGCAAAGCAAATTAGTATGACTTACCTAATAGGATTTCCTATTAGGATCCAATTTATCTTGA
TTAAAAATCAAAGAGGCACAGCGGATTACAGTTGGTGCTTTGCACAACTGCATAAAAGCCTCTAATGATTATA
AATCAAAGAGGCTTTAAAATTTTTTGGGCTTTTTCACGATTTTTAAAATGCTTTTTTGAAATGGTATCTAAAC
GTGAAAGACCGTATTTTTTTATAATTTTGGCGGCGATTACATCGACTTTAGCACCGGCACCTTTAGGAATCGT
CATATTAATATTTTTTGATATTTGATCCATATATGTAACAAATGCGTATCGAGAAATTATGCTTGCCACTGCA
ATGGCTAATGACTTTGATTCTCCTTTTGTTTCAAATTTTGTTTTCTTTGGAAGTGGTATATCTGATAATGCGT
AATGGCTATACACTTCGCGTTTTGCGAACTGATCAATCACAATATAATCCAATTGAGACGAATCAATTTTTTC
AAGTACATTTTTGATGGCTTCATTATGAAGAACAGCTTTCATTTTTACTTGAGTCCAGCCTTTTGCTTGCTGA
ATATTATATTTTTCATTGTGTAGTGTTAATAGTGAATGTGGAATGAAAGTAACCAATTGCTCAGCAAGTTCTA
CGATTTTAGTATCGGTTAATTTTTTTGAATCATCTACACCCAAAGTTTTTAAAATAAGGACATGCTCTTTGGT
AACAAAAGCAGCACATACAGTCAATGGACCAAAGTAATCGCCACTTCCAGCCTCATCACTACCAATACAGTTA
AATTGATCATACATTAAAGACTGTTCAAGAAAAGAATTAGCCATATTTTTCTTTTTAGTTTTATTAGTATTCA
ATTGAGAATGCTGCGGTAGAAGTTCTTGAGACACAGCTTCTGCATGATTACCTTGAAACATGACTTTACCTGA
TTGGTAAATATTTACAGTTGTATTTTGATACTTTGCACGTGCTTTCATACCTTGAGGTAAATTCTCAGTATCA
AAAGAAATTCGTGACATTAATGTCGTTATGTCTTTATCCGACAATTTAAAAACGATATTCGCCATTTGTGTAA
ATCCTTTCATATAATTTCATTATCATTAGCAAAAATAATCATATCATAAATGATAATAACGTCAGAAACATTT
ATATATGTTGTAAAGAATTAATTGCGGATACTTACAAAATACATAGTGGTCATGTATAATGTTTATGAAAATG
TTATAGTGACACAATTTAAACATGATAAAGTATTGAATAGAAAATTCTTTACATTATTTCATTTCATTATAAA
AAGAACATTAAGTCATCTATAGAGATTGTGTTGTTGTTTTATCATAATTGCAATATAACAATATTAGTATCAA

ATTCAGACAAACTATCCAGTCATATTTTATAACGTGAATTTAGATTTTAAAAAAAGGATCAGGAGAAATTATA
AATGGCACAGTTTAAAAACAAGGTAAATGTATCAATTAATGATCAGCTTTTTACAATTGTTGGGGAAGATAAC
CCAGAGCACATACGATATGTAGCACATTTAGTTGATGATAAAATAAAAGAATTAGGGTATAAAGCAGCAGGTT
TAGATACTTCAAGAAAAGCAATATTAACTGCTGTGAATATTATGCATGAAAAAGTACTACTAGAAGAAGAAAA
TCGACGTTTGAAACAACAAATTCACAAATTGCAGCAGCGTGAGCAATAAATGGTCATTGATTTTATCATAATC
ATTTTCTTTGTGTATTTTGTCATCGTTGGATTCAGACGAGGTTTTTGGTTATCTATGATACATTTGAGTGCAA
CGATTGTATCATTGTGGATTGCCAGTCAATTTTACAAATCTATTGTAGAAAGATTAATTGTATTTATTCCATA
TCCTAAAACAACAGCATTTAATACAACTTTTGCGTTTCATTTTAATCATCTACAAAATCGATTTGAAGCGATT
GTAGCTTTTTTAATGATTACATTGTTTTGTAAGTTCATTTTGTATCTAATTATCGTAACTTTTGATAAAATAA
TAGCGTATCAAAACATTCATATTTTCAGTCGTGCAATGGGAATGATAGTTGGTGTGTTTATGACGATAATTGT
CTTACACTTTACGTTATATCTATTGGCATTATATCCTAACGAAGCATTACAACATCAGCTTAAAATATCTATT
GTGAGTCATTCATTGATTTTTCACATCCCATATTTATCGGCTTTCACCATTAATTTATAAATTATCATAAGAG
GTCAGGACAAAGGTATATATTAATGATACGTCCTGGTCTTCTTTTTATGGAGTGTTATTATGACAAAAAAAGA
TGTTATCAAACTATTAGAACAAATTGCTACTTATATGGAATTAAAAGGGGAAAATACTTTTAAAATATCAGCG
TATCGAAAAGCAGCTCAAAGTCTTGAATTAGATGAACGACCATTAGATGAAATATCTGATGTAACGGACTTAA
AAGGCATTGGTAAAGGTGTTGCAGAAGTAATCAATGATTACCGTGAGACCGGTGAATCTCAGTATTTACAGCA
ATTACAGGAAGAAGTTCCGGAAGGTCTTATTCCACTTTTGAAAATTCAAGGACTTGGAAGCAAGAAAATTGCT
AAGCTATATAAAGAGTTGAATATTGTTGATAAAGCGTCACTTCAAGTTGCTTGTGAAAATGGAAAAGTTAGTG
AATTAAGCGGATTTGCTAAGAAAACGGAACAAAACATATTAGAAGCTGTGAAACAACTTGGTGCTAAGAAAGA
TAGATATCCAATTGATCAAATGAGAAGACTTAATCAAGAAATCATTGATTATATAGATACATTAAATTATATC
GATCAATATTCATCTGCAGGAAGCTTCCGTCGTTTTAAAGAAATGAGCAAAGATTTAGATTTCATAATAAGTA
CCGATAACCCAAAAGCAGTGCAGCAGCAATTATTAAATATTCCCAATAAAGTAAAAGAAGTTGCAGTGGGGAA
CACAAAAGTTTCATTAGAATTAGCGTATGATGATGAAACGATTGGTGTCGATTTTCGATTAATTGAACCAAGT
GCTTTTTATCATACATTGCAGCATTTTACTGGGTCAAAAGAACATAATATAAGAATTCGACAACTTGCTAAAG
CACGTGATGAAAAAGTAAGTGAATATGGAATTGAACAAGCTGATGGTACATTAATTCAATATGATAGTGAAGC
CAAGATATATGAACATTTTAATGTGAATTTTATACCACCTGCTATGCGAGAAGATGGTAGCGAATTTGATAAA
GATCTAAGTAATATCATTACATTAGATGATATTAATGGTGATATTCATATGCATACAACGTATAGTGATGGTG
CGTTTTCTATTCGAGACATGGTAGAAGCAAATATCGCAAAAGGTTATAAATTCATGGTAATTACTGATCATTC
ACAAAGTTTACGTGTTGCTAATGGCTTACAAGTAGAAAGACTTTTAAGACAAAACGAAGAAATTAAGGCTTTA
GATAAAGAATATAGTGAAATTGATATTTATTCAGGTACAGAAATGGATATATTAACTGATGGCTCGCTGGATT
ATGATGATGAAATTTTAGCACAACTTGATTATGTAATTGGAGCTATTCATCAAAGCTTTAACCAATCAGAAGA
ACAAATTATGGAACGATTAGCTAATGCATGTCGCAATCCATACGTGCGACATATAGCGCATCCAACAGGGCGT
ATTATAGGTAGAAGAGATGGTTATAAACCGAATATTGAACAATTAATGGCATTAGCTGAAGAAACGAATACAG
TATTAGAAATTAATGCCAATCCACATCGATTGGATCTGAACGCTGATATCGTTCGTAAATATCCAAATGTGAA
ATTAACTATTAACACTGATGCGCATCATACAAATCATTTAGATTTTATGAATTATGGCGTAGCAACTGCGCAA
AAAGGATTTGTAACAAAAGATAGAGTGATTAACGCATTATCGCGTGAAGCTTTTAAAGACTTTATTGAAAATA
ATATAAAACTTAAGAAATAGAGGGATTTTATGAGACAAAAAACATTAGACGTCTTAGAATTTGAAAAAATAAA
ATCACTCGTTGCCAATGAAACAATTAGTGACTTAGGCTTGGAAAAGGTCAATCAAATGATGCCAGCTACTAAT
TTTGAAACGGTTGTTTTTCAAATGGAAGAAACGGATGAGATTGCTCAAATCTATAATAAGCATCGTTTACCAA
GCTTGAGTGGCTTATCTAAAGTATCAGCATTCATTCATCGCGCTGATATTGGCGGCGTTTTAAATGTATCAGA
GCTTAACTTGATAAAAAGATTAATTCAAGTACAAAATCAATTTAAGACATTTTATAATCAATTGGTTGAAGAA
GATGAAGGTGTTAAATACCCAATATTAGATGACAAGATGAATCAATTACCTGTGTTAACCGATCTTTTTCAAC
AAATAAATGAAACATGCGATACGTATGATTTATATGATAATGCGAGTTATGAATTGCAAGGGATTAGAAGTAA
AATTTCTAGCACGAATCAACGTATTAGACAAAATTTGGACCGTATTGTTAAAAGCCAAGCAAATCAGAAAAAA
TTATCAGATGCAATTGTAACAGTTAGGAATGAAAGAAACGTTATACCTGTCAAAGCTGAATATCGACAAGATT
TTAATGGGATTGTACATGATCAATCTGCTTCAGGACAAACATTGTATATTGAGCCATCATCAGTTGTTGAAAT
GAATAATCAAATTAGTCGATTACGTCATGATGAAGCAATTGAAAAAGAACGCATTTTAACGCAACTAACTGGT
TATGTGGCTGCGGACAAAGATGCACTACTTGTGGCAGAACAAGTCATGGGTCAGTTAGATTTTTTAATCGCAA
AAGCGAGATATAGTAGAAGTATTAAAGGAACAAAGCCGATATTTAAAGAGGAATGTACTGTATATTTACCTAA
AGCATACCATCCATTATTAAATCGTGAGACTGTTGTAGCTAATACCATCGAATTTATGGAAGATATTGAAACG
GTAATTATTACAGGACCGAATACAGGTGGTAAAACAGTAACATTAAAAACATTAGGTTTAATTATTGTTATGG
CTCAATCAGGATTGTTGATTCCAACACTTGATGGTAGTCAGTTAAGTATATTTAAAAATGTATATTGCGATAT
CGGAGATGAACAATCAATAGAACAATCATTATCAACTTTTTCATCTCATATGACGAATATAGTTGAAATTTTA
AAGCATGCAGACAAACATAGTTTAGTTTTATTTGATGAATTAGGTGCAGGTACAGATCCAAGTGAAGGTGCTG
CATTAGCAATGAGCATTTTAGATCATGTTAGAAAAAATTGGTTCTCTAGTAATGGCAACGACGCACTATCCTGA
ACTTAAAGCATATAGTTATAATCGAGAAGGCGTTATGAATGCGAGTGTAGAATTTGATGTAGATACTTTGAGT
CCAACGTATAAGTTATTAATGGGTGTGCCGGGTCGTTCAAATGCTTTTGACATTTCTAAAAAGTTAGGTCTTA
GTTTGAATATTATTAATAAGGCTAAGACGATGATTGGTACTGATGAAAAAGAAATAAATGAAATGATTGAATC

ATTAGAGCGTAATTACAAACGTGTAGAGACACAGAGGTTAGAACTGGACCGTCTTGTAAAAGAAGCGGAGCAA
GTGCATGATGATTTATCTAAGCAGTATCAACAATTCCAAAATTATGAAAAGTCTCTAATAGAGGAAGCGAAAG
AAAAAGCAAATCAGAAGATTAAAGCTGCAACAAAAGAAGCTGACGATATTATTAAAGACTTAAGACAATTGCG
TGAACAAAAAGGTGCAGATGTTAAAGAACATGAATTGATTGATAAGAAGAAACGATTAGATGATCATTATGAA
GCGAAATCTATAAAGCAAAATGTACAAAAGCAAAAATACGATAAAATTGTTGCTGGTGATGAAGTAAAAGTAT
TATCTTACGGTCAAAAGGGTGAAGTTTTAGAAATTGTCAATGATGAAGAAGCAATTGTTCAAATGGGAATTAT
TAAAATGAAGTTACCTATTGAAGATTTAGAGAAAAAACAAAAAGAAAAAGTTAAGCCAACGAAAATGGTTACA
CGTCAAAACCGTCAAACAATTAAAACTGAACTAGACTTACGAGGCTATCGTTATGAGGATGCTTTAATTGAAC
TAGATCAATATTTAGATCAAGCAGTTTTAAGTAATTACGAACAAGTTTATATCATTCACGGTAAAGGTACAGG
TGCACTTCAAAAAGGTGTACAACAACATTTGAAAAAGCATAAAAGTGTTAGTGACTTTAGAGGTGGTATGCCA
AGCGAAGGTGGATTTGGCGTTACCGTTGCAACACTAAAATAAATTATAATTTGATAAATTAAATAGCTGCAGT
TAAAATAATGTAAAGCAACAAGAATACATTTCAAACATGTTATTTGAAATAAGCATAAAAATTGAGCAAATAG
AAATACATGAAGCATGTTATCTGATATAATTTGAACATCATAATAATAATTAAGGAGGATTGGCATTTATGGC
AATCGTAAAAGTAACAGATGCAGATTTCGATTCAAAAGTAGAATCTGGTGTACAATTAGTAGATTTTTGGGCA
ACATGGTGTGGTCCATGTAAAATGATCGCTCCGGTATTAGAAGAATTAGCAGCTGACTATGAAGGTAAAGCTG
ACATTTTAAAATTAGATGTTGATGAAAATCCATCTACTGCAGCTAAATATGAAGTGATGAGTATTCCAACATT
AATCGTCTTTAAAGACGGTCAACCAGTTGATAAAGTTGTTGGTTTCCAACCAAAAGAAAACTTAGCTGAAGTT
TTAGATAAACATTTATAAGTTACAACCAATGACGACTGGGGCATTTCTTTAATGAATTGCTCCAGTTTTTGTT
TGTGTTTTTAATATAAAAAGTTGAATGATAAGTCATCATATTGTTTACGACTTGAGAATGGTGGGATTAATAA
ATCTATGAACGTTAAATGATAATCTAGCATGCTGATAGATTTGTAGCAGTTGGTTTGATAAAACAATGTTCAA
TTTTACATGATGTGCATGAAAAGTCATACTCGAAGATGTTGATTATGAGCTAGAATTAGTGGTGATAAATTTG
AAGCACTTTTGTAGCATCATTCATTTTAAAATTAGAAGGGGGGATATTATTGGAAGACTATAAGCAACGAATT
AAAAATAAATTAAATGTCGTACCTATGGAACCAGGCTGCTATTTAATGAAAGATCGTAATGATCAAGTGATAT
ATGTTGGCAAAGCTAAAAAGCTAAGAAATCGATTGAGATCATATTTTACGGGTGCTCATGATGCTAAAACAAC
GAGACTGGTTGGAGAAATACGGCATTTTGAATTTATTGTCACGTCAAGTGAAACAGAGTCACTTTTACTGGAA
TTGAATCTGATTAAACAATATCAACCAAGATATAATATATTATTAAAGGATGATAAAAGTTATCCATTCATTA
AAATTACGAAGGAAAAATATCCTAGACTACTAGTGACGAGAACTGTAAAACAAGGTACTGGCAAATATTTCGG
ACCGTATCCGAATGCATATTCTGCTCAAGAAACTAAAAAGTTATTAGACAGAATATATCCATATCGCAAATGT
GATAAGATGCCAGATAAATTATGTCTTTATTACCATATTGGACAATGTTTAGGACCATGTGTATATGACGTTG
ATTTGAGTAAATACGCACAAATGACGAAGGAAATTACTGATTTTCTGAATGGGGAAGACAAAACAATTTTAAA
AAGTTTAGAAGAGCGAATGTTAACTGCAAGTGAATCACTTGATTTTGAACGGGCTAAAGAATATAGAGATTTA
ATTCAACATATTCAAAATCTAACTAACAAACAAAAAATTATGTCATCAGATAAAACGATTCGTGATGTCTTTG
GTTATAGCGTTGATAAAGGATGGATGTGTATCCAAGTTTTCTTTATACGACAAGGTAATATGATAAAGCGAGA
TACAACGATGATTCCATTACAGCAAACAGAAGAAGAAGAATTTTATACATTTATTGGACAATTTTATAGCTTA
AACCAACATATTTTACCTAAGGAAGTTCATGTACCACGTAATTTGGATAAAGAAATGATTCAATCTGTTGTGG
ACACTAAAATCGTTCAACCTGCGCGAGGTCCCAAAAAAGATATGGTTGACCTAGCTGCACATAACGCTAAAGT
ATCTTTAAATAATAAATTTGAATTAATATCACGTGATGAGTCCAGAACAATTAAAGCTATTGAAGAACTTGGA
ACACAAATGGGAATTCAAACACCAATTAGAATTGAAGCATTCGATAATTCTAATATTCAAGGTGTGGATCCAG
TGTCAGCAATGGTTACATTTGTCGACGGTAAACCAGATAAGAAAAATTATAGAAAGTATAAAATCAAAACGGT
TAAAGGTCCAGATGATTACAAATCAATGAGAGAAGTAGTAAGACGCCGATATTCTCGCGTTTTAAACGAAGGA
TTACCATTACCTGATTTAATAATAGTAGATGGTGGTAAAGGACATATGAACGGGGTTATTGATGTGCTACAAA
ACGAATTAGGTCTTGATATCCCTGTTGCAGGTTTGCAGAAAAATGATAAACACCAAACATCTGAATTATTATA
TGGCGCTAGTGCAGAAATAGTACCACTGAAGAAAAATAGCCAAGCATTTTATTTGTTGCACCGTATCCAAGAT
GAGGTTCACAGATTCGCAATCACATTTCATAGGCAAACACGTCAAAAGACAGGCTTGAAATCAATCCTTGATG
ATATAGAGGGTATCGGTAGCAAACGTAAAACATTATTATTACGTTCATTCGGTTCAATCAAGAAAATGAAGGA
AGCTACACTTGAAGATTTTAAAAATATAGGTATTCCTGAAAACGTCGCAAAGAACCTACATGAACAATTGCAT
AAATAAATTACTGGGCGTGAGAATCATTCTCATGCCCCAAAATATATGAAATAATGTTACAATATGGTTAACA
AAGTTATTTTTTTGAATGTACGTGGAAGCGTTTTCTAAATGAGAAAGTTTATCATCACAAGCAATTTTAAATA
TCGAATCCAAATGTTACATTGTTCGACATGCAGTTTGGTGATCGTACAGTGTGTATTTGTCTTTAAAATGTTT
CGCTTAATGATAATGACAACGCTAGAAACGATATCACTTTAGGGGGTTAAGCGTACGTTTAATATAAATAGCT
CAATAAATTAAATATTCGTTCACAGGGGGGACTCCTTTTGGCTCAATCAAAAAATGAATTTTATCTAAGACGT
ATTCACTCGTTATTAGGTATTATCCCAATAGGTGCATTTTTGGTCGTTCATTTATTAGTGAATCACCAAGCAA
CACAAGGTGCTGAAGCGTTTAATAAGGCATCTAACTTTATGGAATCATTACCATTTCTAATTATTGTAGAATT
TTTATTTATATACATTCCGTTGTTATATCACGGTTTGTTTGGTATACACATTGCATTTACAGCAAAAGAAAAT
GTTGGACATTACTCGATTTTTAGAAACTGGATGTTCTTCTTCCAAAGAGTGAGTGGTATCTTAACATTTATCT
TTATTGGTATCCATTTATGGCAAACACGTTTACAAAAAGCATTTTACGGCAAAGAAGTGAATTACGATTTAAT
GCACGAAACATTGCAACATCCTGGATGGGCAATATTTTATATTATTTGTATTATTGCTGTTGTGTTCCACTTT
GCAAATGGCTTATGGTCATTCTTAGTTACTTGGGGAGGACTTCAATCTCCAAAATCACAACGAGTATTTACAT

GGGTTTCATTAATCGTATTCTTAGTTATTTCGTATATTGGTGTTACTGCAATTATTGCCTTTATGTAATACAT
CGCATTTAACAAATATAGATTGAAAATTTAGGGGAGTGAAATTTTTATGGCAGAGAAACATCTTATTGTTGTC
GGAGGTGGCCTAGCGGGCTTAATGTCAACAATTAAAGCGGCAGAAAAAGGTGCACATGTAGATTTGTTCTCAG
TTGTACCAGTAAAGCGTTCGCACTCTGTTTGTGCCCAAGGTGGCATTAATGGTGCGGTCAATACTAAAGGGGA
AGGCGATTCTCCTTGGATTCACTTTGATGATACAGTGTATGGTGGCGACTTCCTTGCAAACCAACCACCTGTT
AAAGCGATGACAGAGGCAGCACCTAAAATTATTCATTTATTAGACCGTATGGGCGTAATGTTCAATAGAACAA
ATGAAGGTCTATTAGATTTAGACGTTTCGGTGGTACATTACACCACAGAACAGCATATGCAGGGGCAACAAC
TGGACAACAATTATTATATGCATTGGATGAACAAGTTCGTGCATATGAAGTAGATGGATTAGTTACGAAGTAT
GAAGGATGGGAATTCCTTGGCATAGTTAAAGGTGACGATGATAGTGCAAGAGGTATCGTTGCACAAAATATGA
CAACTGCTGAGATTGAAACATTTGGTTCAGATGCAGTTATTATGGCAACGGGTGGCCCCGGTATTATTTTCGG
TAAAACAACAAACTCAATGATTAATACAGGATCAGCGGCTTCCATTGTTTACCAACAAGGCGCTATTTATGCT
AATGGTGAGTTCATTCAAATTCATCCTACTGCAATCCCTGGTGATGATAAACTTCGACTAATGAGTGAATCAG
CACGTGGTGAAGGTGGACGAATTTGGACATATAAAGATGGTAAGCCTTGGTACTTCTTAGAAGAGAAATATCC
TGATTATGGTAACTTAGTACCTCGTGATATCGCAACGCGTGAAATTTTCGATGTATGTATTAACCAAAAATTA
GGTATTAATGGCGAAAACATGGTATATCTTGATTTGTCACATAAAGATCCACATGAGTTAGATGTAAAACTAG
GTGGTATCATTGAAATTTATGAAAAATTTACTGGTGATGACCCACGCAAAGTACCAATGAAGATTTTCCCAGC
TGTCCACTATTCAATGGGTGGTCTATATGTAGATTATGATCAAATGACAAATATTAAAGGGTTATTTGCAGCT
GGAGAATGTGACTTCTCTCAACATGGTGGTAACCGCTTAGGTGCCAATTCATTATTGTCAGCGATTTACGGTG
GTACAGTTGCAGGCCCGAATGCCATTGATTATATTGCTAATATTGATCGTTCATATACTGATATGGATGAAAG
TATTTTTGAAAAACGTAAAGCTGAAGAACAAGAACGTTTTGAAAAATTAATGGCTATGCGTGGTACTGAAAAT
GCTTATAAATTACATCGTGAACTTGGAGAAATTATGACAGCTAATGTAACAGTTGTTCGTGAAAATGAAAAAC
TGTTAGAAACAGATAAAAAGATTGTTGAATTGATGAAGCGTTATGAAAATATAGATATGGAAGATACACAAAC
TTGGAGTAACCAAGCGGTATTCTTCACTCGTCAACTATGGAATATGTTAGTACTTGCACGTGTGATTACGATT
GGGGCATATAATCGTAATGAATCACGTGGTGCCCATTATAAACCAGAATTCCCAGAGCGTAATGACGAAGAGT
GGTTAAAAACAACAATGGCCTCATTCCAAGGTGCATTTGAAAAACCACAGTTTACTTATGATGACGTCGATGT
GAGTTTAATACCACCTCGTAAACGTGATTACACAAGTAAGTCTAAAGGGGGTAAAAAATAATGACTGACCAAC
CAGTAAAAGACACACCACAACATGAAACACAATCTAAACCGAAACAAAAAACAGTAAAATTAATTATTAAACG
TCAAGATACAAGTGATTCAAAACCATATGAAGAAACGTTTGAAATTCCGTATCGTGAAAATTTAAATGTCATT
GCATGTTTAATGGAAATTAGACGTAATCCAGTTAATATTAAAGGCGAAAAAACAACGCCAGTAGTATGGGACA
TGAACTGTTTAGAAGAAGTATGTGGTGCGTGTTCAATGGTTATCAATGGTCGTGCAAGACAATCATGTTCAGC
GATTGTTGATCAATTAGAACAGCCTATTCGATTAGAGCCTATGAATACATTCCCAGTTATCCGTGACTTACAA
GTTGATCGTTCTAGAATGTTCGATAACTTAAAACGTATGAAAGCATGGATCCCAATTGATGGAACGTATGATT
TAGGTCCGGGACCACGTATGCCAGAGAAAAAACGTCAAACAGCTTATGAATTATCTAAATGTATGACATGTGG
TGTATGTTTAGAGGTTTGTCCTAATGTTACTGAAAATAATAAATTCGTTGGTGCGCAAGCAATCTCGCAAGTT
CGTTTGTTTAATTTGCACCCAACAGGATCTATGACTAAAGATGAACGTTTAAATGCATTAATGGGTACTGGTG
GCTTACAGCAGTGTGGTAATTCACAAAACTGTGTTAATGCTTGCCCTAAAGGTATTCCATTAACAACATCCAT
TGCAGCAATGAACAGAGAAACAACATTCCACATGTTTAAATCTTTCTTTGGTTCAGACCATGAAGTAGAATAA
AAATTAATCCCTTTTGAGTTAAAATGTTATGTTCCTTTCAAAATTAAATTGAATGGAGATACACAATAACTTG
GAAGGGATTTTTTTAATAAGCAGATTACATGACAATATGATAGTGGGCTGATTTAAAATAAATATTATAATGA
CAAAAATGGTAAACACAGTGCATAATACTAAAATCCAATGCTTAGTTATGATAAACTAAAAATTAAATTAAAA
GTAAGGATGACCGAATATGAATAAACCAATAGGTGTAATAGACTCTGGTGTCGGAGGTTTGACAGTAGCTAAA
GAAATTATGCGTCAGTTGCCAAATGAGACGATTTATTACTTAGGTGATATTGGGCGATGCCCATATGGGCCAA
GACCAGGAGAACAAGTAAAACAATATACAGTTGAAATCGCTCGTAAATTAATGGAATTTGATATAAAAATGCT
CGTGATTGCTTGTAATACAGCAACTGCTGTAGCTTTAGAATATTTACAAAAGACCTTATCAATCCCAGTGATT
GGCGTAATTGAACCAGGTGCTAGAACAGCAATAATGACTACTAGAAATCAAAATGTATTAGTACTAGGAACGG
AAGGCACAATTAAATCTGAAGCATATCGTACGCATATTAAACGTATCAATCCACATGTAGAGGTACATGGCGT
TGCCTGTCCAGGTTTTGTGCCACTTGTAGAACAAATGAGATATAGTGATCCAACAATTACAAGCATTGTCATT
CATCAAACACTGAAACGTTGGCGTAATAGTGAGTCTGATACTGTCATTTTAGGATGTACCCACTATCCATTGC
TCTATAAACTTATCTATGATTATTTTGGTGGTAAAAAGACAGTGATTTCGTCTGGATTAGAAACGGCTCGTGA
AGTTAGTGCATTGCTAACATTTAGTAATGAACATGCAAGTTATACTGAACATCCAGATCATCGATTTTTTGCA
ACAGGTGATCCTACTCACATTACTAACATTATCAAAGAGTGGTTAAATTTATCTGTCAATGTGGAACGTATAT
CAGTGAATGACTAGGAGGATTTTTAATGAAAGAGATTGTTATTGCATCGAATAATCAAGGGAAAATAAATGAC
TTTAAAGTGATATTTCCAGATTACCACGTAATAGGTATTTCAGAACTAATACCAGATTTTGATGTGGAAGAAA
CAGGATCAACATTTGAAGAAAATGCTATATTAAAATCAGAAGCTGCAGCAAAAGCATTGAATAAAACGGTCAT
AGCTGATGACAGTGGACTTGAAGTTTTTGCATTAAATGGTGAGCCAGGTATATACTCGGCACGTTATGCTGGT
GAAAATAAAAGCGATGAAGCAAATATTGAAAAATTATTAAATAAGCTTGGTAATACAACTGATCGTCGTGCGC
AATTTGTTTGTGTCATAAGTATGAGTGGCCCTGATATGGAAACAAAAGTATTTAAAGGTACGGTTTCAGGTGA
AATTGCAGATGGAAAATATGGCGATAATGGTTTTGGTTATGATCCGATATTTTATGTGCCGAAATTAGATAAA

ACCATGGCGCAACTTTCTAAAGAAGAAAAAGGTCAAATTAGCCATAGACGAAATGCAATTAATTTATTAGAAG
CTTATCTTGCGGGTGATCAACATGTCTAAATGGATTATTGTGAGTGATAACCATACTGAATCAGGCGTTTTAT
ATCAAATTTATGAAATGCACCCAGATGCAGATGTATATTTACATTTAGGAGATTCAGAATTCGCGTATGATGA
TACGGAACTTAGCTTATTTAATAGAGTAAAAGGCAATTGTGATTTTTACCCAGAATTTGAAAATGAAGCGGTC
GCAAAGTGTAACGACGTGTATGCTTTTTATACTCACGGCCATTTATATCAAGTCAATCGAACAAGACATTTAT
TAGCTGAGCAAGCACGCGAATTAGGTTGTGTATTTGCTTTTTATGGACATACACATGTGGCAAAATATGAATA
TATTAATGGTGTTCATGTGATTAATCCTGGAAGTATATCTCAATCTAGAAGTTCAATGGAAGAAACATATGCT
GAAGTCTTGATTGATGATCAAACTTTACATGGCACTGTCAATTTCAAAAATCGACATCACGAAACAATCAGTC
ATACTACTTTTTAAATAGAAAGTAGCTATGACTTTTTTTAGTTTATAGACTGATTTAACTAGGAGGTCGTGAC
ATGAATATTTGTTTAAAAGTATTTCTGACAATTTTATAAGATAACTGGAATGAATAACACATTTATGTTATGA
AAAGAATTGTACTTTTGAGGAATGTGATTGTTGATGGTCCTATATGGTTGAATCATGTTGATTATTTGGTTAA
AAGTGTATTGTTAAAAAGTATAAAATTAAGATTCCAAGAAGTCAGGAAAATTGAAAATTTAGTTAAAAGCATG
AATCGCATACATATTTAAAAGGATTTCTAAAGGCATAAATTATATCAATAATTTGGTGGTTAAAATATGGAGT
AGCAAAATTCTTGAAATTCAGAAATGTAGTGACACAAATATTAATGTTTAGTTAATAAATAGTTAATGTAGTT
TTTTGTATATCTCTATTATTATAAATATATACAGAATAAATATTTGAGGATAGGTGTTATTAATTATGAAAAA
GAATTTTATTGGGAAATCAATTTTAAGCATAGCTGCTATTAGTTTAACGGTATCAACGTTTGCCGGTGAATCT
CATGCACAAACTAAAAACGCTGAAACTGTAAAAAAAATATAACGAGTATCAAACAAACTTTAAAAAACAAGTAA
ATAAAAAAGTTGTGGATGCACAAAAAGCTGTAAACTTGTTCAAACGTACAAGAACTGTTGCAACACACCGTAA
AGCACAAAGAGCTGTTAATTTAATTCATTTCCAACACAGCTATGAAAAGAAAAAAATTACAAAGACAAATCGAT
CTAGTTTTAAAATATAATACTTTAAAATAATTGATAGAAGCCATGCTTTAGAGTGTGGCTTTTTGCTATATTC
AAAGGTATAATTAGTTTATAATATTGTGTTCATTTGAATGGTGAGCTTATTGGGAAAAATAATTATTTAAAAG
TGAATGCAATAGTTGTTTATGTTTTGATCAAGCGAGGTGGCTATTGTGAAAAGGAAATCGAATGTATTAACAA
TCATAAAAATTGTAACGGATATAGTGGCAACCGTGTTAATCATACGTGCTTTATTAAGCAGTCGTAAAGATGC
TAAATAAATTGGAAGTTGCAGCATATAAAAAAGCACGCCAAAACGTTAGCATTTTTTTCTAACGTTTGGCGTG
CAACTATTTAATTCAGTGTGTTTTTTAATACCAAGTAATTGAATCGATTTCGCCGTTTTTAACTACGGGAATA
TTTTTTCTATGTTCTTCTAATGGACGCGTGATATCAAACGTATAGTAACCGTTTTTACCACCATCTATAATTC
TCACTACAGCTATAGTATTACCTTTACCTAATTTTTTAGCAATGACATATAAATCTTCAACCGTTTTACCTGT
ATAAGTTCTTTCTTTCTCGGCGTCTTTAATTAATCTATCAGCACGTTCTTCATCTTTTTTCTCATCATCTGCT
AATTTTTTAGCAATTTCTAAACCTTTCCATTCATAACTAAAGAAAGCTTTAGCATCATTTGAATGAGTTAGAA
GACCTGTAGCGATAACTGTAGATGCAATAATTACTTTTGTAATATTTTTTTTCATAATAAGTTCTCCCTGTAA
AATAGATTTGTACTTACTAATGCGAGTACACTGCTATTTTACAAACGGTTTCATACATTTACCAGTTAACACT
TCATTAATTAAAATTAAGTGCAAATTAATAAGGTTTAATGCAACCGAAAAAGAATAATGATGAATTTGATGTT
ATTATTTTTATTTGAGGTATTATTGAATTTGTTGATAGTATAATAATAGTAGTAAACGTCACTCAACTATGAT
TGAATGATTTTTATTGTTAAAACGATATTTATATTTCTCTACATTTTGTTTGCAATATTGGGTAGGTAAACAC
TCTAAATTGTTATACATGTAGCTGTTTTGATAATTAATGTTTATAAAACAAAGAAATGATTCATAGTAGAGAA
GTAATTAATATGTATAATTTTACTTATAAAGAAATAAGAGATATATTCTAATTAAGGTTGATATGTTATGATT
AATATATAAGTTAAAAATTATACTATTTAGTTGAATTATGCATTTTCGAAGTAAGCATAATTCAACACTTACA
TTTTTAAATAGATAAAATAGTGTAAAATCACGTGCCAAGATTACTATAGAGTAGGCATTGAGTATTATTGGCA
ATCACTTAAGTGATTGTTATTTTTTCGTGGTCAATAATAAAGATATAAATAAATATATTGAGGTCAAACGATG
ATAATTAAAATTTTAACAATTCTATTACTACTTTGTATATTGAGCTATTTGGTTACAAATAGAAAGAAGCCTT
TTCTGTTCTTAAAGACACTCTTTATGGGTGTGGTATTTATCTTTATAGGATATATTTCACTGGCAATATCTGC
CGTAATTATTTATGGTATTATTCAATTTATCACAATTGATTTTGGTAGTTTTTTCTTAATGGGTATTATATTG
ATCTTGATTTCAAGTATATTCCAATTATTTATAGTTAGATTACTTTTTAGAAAAAAGAATGTAGATTTGACAG
AGGTTGTCGTTTTAGAACATTTAATTCAATGGTTCTTAGTTTACTTTGCGATCTATCAAGCAGTAAATGAAAA
AATGGACATTAATGATATTAATATCGACAATTTCCAATCTGTCTTTTTTGACGTGTCTAATTTGAATTTAGTA
ATTCTACCAACGTTAATCATTAGCTGGGTCACAATATTTAACTATAGAATGAGAAGTTACAAATAAAATCTAT
GAAATTATACCATCAGACACCAACATTCAAATGGTGTCTTTTTTGTTGTGTGGTTTTATTTTTGAAATTCGAA
AAAGTAGAGGCATGAATTTTTTGACTAGTGTATAAGTGCTGATGAGTCACAAGATAGATAGCTATATTTTGTC
TATATTATAAAGTGTTTATAGTTAATTAATAATTAGTTAATTTCAAAAGTTGTATAAATAGGATAACTTAATA
AATGTAAGATAATGATTTGGAGGATAATTAACATGAAAAATAAATTGATAGCAAAATCTTTATTAGCAATAGC
GGCAATTGGTATTACTACAACTACAATTGCGTCAACAGCAGATGCGAGCGAAGGATACGGTGTAAATGGCGGT
TATAAAATGTAGGAAAATGGCGAAATGAAAATGCTGTTTTTCCTACATTTTTTCTTTTTTAAATTATATAATC
ATGTCTTAACTCAATCTTCGATTGGAGGTTATTTGATTGAGACATGACATTTATGAAGGAG
>DSM104437_scaffold_7
GTGTAAAGGATTTTATGTGGATACAGTAGGTAGAAATAAAAAAGTGATTGAAAATTATATTCGTAATCAATTA
CAAGAGGATATCGTTGCAGACCAAATCTCAATGGAAGAATATTTAGATCCTTTCACTGGAGAGAAAAATAAAA
AAGAAAGAAAAAAGAGTAACCCTTTAGGGTTGCTGGAATAGTAGTGCAGTTGGCTGACTTGTCAGTGCCCTTT
TAGGGCTGGCCAGTAAGGAAGGCTTATAGCCGCAGAACAAACCACCCGTTCACACGGGTGGTTTTGATTTAGC

AAGCATTGATAGAGGCAATACTTTTGCGTAAAATAATAATTGAATAAAGTATTATGACTCGTGAAAATTTAGT
GACATGAATCGAAATGCCGAGACGTCATTGAGTATAAAGGAAAGTAGTTCTGCATTAATCACGAAGTAGAGCA
TGACAACGAGGAATAACTATAGGGAGATGGTTTTGGAATGACGAATGTCTTGTATCAACATGGTACATTAGGT
ACGTTAATGGCTGGCTTACTAGAAGGAACAGCTACAATTAATGAATTGTTAGAACATGGGAATTTAGGGATTG
CAACGTTAACAGGGTCTGATGGCGAAGTAATATTTTTAGACGGGAAGGCATATCATGCTAACGAGCATAAAGA
ATTTATAGAATTAAAAGGCGATGAGAAAGTACCCTATGCATCGATTACTAATTTTAAAGCGAGTAAGACATTT
CCATTGCAACAATTATCACAAGATGATGTATTCGCACAAATTAAAAATGAAATGTTAAGTGAGAATTTATTTT
CGGCTGTTAAAATTTATGGCACATTTAAACATATGCATGTACGAATGATGTCTGCTCAGCAACCGCCATATAC
ACGTTTGATTGATTCAGCACGCAGACAACCTGAGGAAAAAAGACAAGATATTCGTGGTGCCATTGTTGGATTT
TTTACACCAGAATTATTTCATGGCGTAGGGTCTGCTGGTTTTCATATACATTTTGCGGATGATGAAAGAGCTT
ATGGTGGACATGTTCTTGACTTTGAAGTGGATGACGTTGTCGTTGAGATACAAAACTTTGAAACATTCCAACA
ACATTTCCCGGTAAATAACGAGACGTTTGTTAAAGCGAAAATAGACTATAAAGATGTGGCAGAAGAAATTAGA
GAAGCTGAATAGTCTAATATAATTAAAAGACCTTAGCGATATTAGGAACAGGTGGTTCTAAATGCATCGTTAA
GGTCTTTTTATATTATATATGTGGTTACATATTTTGTTGATACGCGCGTAAAAAGCTATAAATAGTATCAAGA
TATGGGGTGTCAAGATTGTGTTCACGTGCACGTCGATAAATAAAGCCTTGAATCGCTTCGACTTCAAGAGGTT
GCTGATGCACAATGTCGTAATACATGCTCGTTCCCATTTCGTCGGGATAACCTTGATATATAGTCATAATAGT
ATCGACAGTTTGTTCTGAAAAGTTTAACCCCTCTGCCTGTGCAACACGACAACCATCTAGTAATAGCTGTCTA
CACAGTGTACGTATTTCAGGATTATGCATGATTGCAACTGTCTGTCTACCGAGTGCTGTGATAGAATTAATGC
CCAAGTTAACTAACAATTTATACCAAATAGCTTGCTGAATATTTGCTTCTAATACAATGTCGATTTGACTGTC
TTGGACCAAATCTCTAAATTGTCGTGTTAATGCATTATCTTTTATACGTAATTGATAATCTCTGAAGTGCGTA
ACAACATCGCCTTTCTTTTGACCACTTATATAGACAACTGCTTGGCATATGTTTTTAAATGGAATATGTTCGA
GTTGACCATAACCATTTTGGGCTAAAATGATGAGCGTGTCCTCATGTGCAAGATGAGTTAAATGAGGAATAAC
TGCATCAAGTTGATGTGTTTTGACTGCAATAATAATAACATCAAATGTGTTTGTGACATCTTCATAACCTTTC
ACAACAATATCTTGTGCAGGTGCATGTGGTACAGTATAATATGTTATTGTTTTGGCGTGTCTCCCGATAAGCG
TTGTATGAGGCAATGATTGTTGTAATTCATAGGCAATAGTTGTACCGACAGCGCCTGGGCCGATAATCGCAAC
TGATAACATCTTCTTCTTTCCTTTCTATAAAAGATTCATTATACTATTTCTAATAATACTGCAAAAAGAGGTT
GAATAACATTGAAAACAGTAAGTCAATTAATAGATATGAAACAAAAGCAAACTAAAATTTCTATGGTAACAGC
TTATGATTTTCCAAGTGCTAAACAAGTGGAAGCAGCGGGTATTGATATGATTCTTGTTGGGGATTCACTTGGT
ATGACTGTATTAGGTTATGAAAGTACCGTACAAGTGACGTTAGCAGATATGATTCATCACGGTCGTGCAGTAA
GAAGAGGCGCACCAAATACATTTGTAGTTGTTGATATGCCGATTGGTGCAGTAGGCATCTCTATGACACAAGA
CTTAAATCATGCTCTAAAGCTTTATCAGGAGACGAATGCAAATGCAATTAAAGCAGAAGGAGCGCATATTACA
CCATTTATTGAAAAAGCAACGGCAATTGGTATTCCTGTTGTTGCACATTTAGGATTAACACCTCAAAGTGTTG
GCGTTATGGGGTATAAATTACAAGGTGCAACGAAAGAAGCAGCGGAGCAACTTATTTTAGATGCGAAAAATGT
AGAACAAGCTGGTGCAGTTGCGTTAGTACTTGAAGCAATTCCAAATGATTTAGCAGAAGAAATTAGCAAGCAC
TTAACAATTCCAGTCATTGGTATTGGTGCAGGAAAAGGTACAGATGGTCAAGTATTGGTTTATCACGATATGT
TAAATTATGGTGTTGAACATAAAGCGAAATTTGTGAAGCAATTTGCTGATTTTAGTGTTGGTGTGGATGGTTT
AAAACAATACGATCAAGAAGTTAAATCTGGTGCATTTCCATCAGAAGAATATACGTATAAAAAGAAAATTATG
AATGAGGTTAATAACAATGACTAAGCTGATTACTACGGTAAAAGAGATGCAACACATTGTTAAAGCAGCCAAA
CGCTCAGGAACAACGATTGGTTTTATCCCAACAATGGGTGCCTTACATGATGGACATTTAACGATGGTACGCG
AATCAGTTAGTACAAATGATATTACAGTTGTGAGTGTATTTGTTAATCCATTACAATTTGGTCCAAACGAAGA
TTTCGATGCTTATCCGAGACAAATTGATAAAGATTTAGAATTAGTATCAGAAGTAGGGGCGGACATTGTTTTT
CATCCTGCAGTAGAAGATATGTATCCAGGTGAATTAGGTATTGATGTCAAAGTAGGTCCATTAGCTGATGTGT
TAGAAGGAGCGAAGCGTCCAGGGCATTTTGATGGGGTGGTAACAGTAGTTAATAAGCTATTTAATATTGTTAT
GCCGGATTATGCTTATTTTGGTAAAAAAGATGCCCAGCAATTGGCTATTGTCGAGCAAATGGTAAAAGACTTC
AATCATGCCGTTGAAATTATTGGTATTGATATCGTTCGAGAAGCAGATGGTTTGGCGAAAAGTTCAAGAAATG
TTTATTTAACGGAGCAAGAACGACAAGAAGCGGTACATTTAAGTAAAAGTTTGCTATTAGCACAAGCGTTGTA
TCAAGACGGTGAACGTCAAAGTAAAGTAATTATAGACAGGGTTACTGAATATCTTGAATCACATATAAGTGGA
CGCATTGAAGAAGTTGCTGTTTATAGTTATCCTCAATTAGTAGAACAACACGAAATAACTGGACGCATATTTA
TCTCGTTAGCTGTCAAATTTTCAAAAGCGCGTTTAATAGATAATATAATAATTGGAGCTGAATAAAGTGATAA
GAACAATGATGAATGCTAAAATTCATAGAGCAAGAGTGACTGAGTCAAATTTAAATTATGTAGGTAGTATTAC
GATTGATTCAGATATTTTGGAAGCGGTAGACATCTTGCCAAATGAAAAAGTAGCCATCGTGAATAATAATAAT
GGTGCACGTTTTGAAACATACGTAATAGCTGGTGAAAGAGGTAGTGGTAAAATTTGTTTGAACGGTGCAGCAT
CAAGACTTGTTGAAGTAGGCGATGTAGTCATAATCATGACTTATGCACAGTTGAATGAAGAAGAAATTAAAAA
CCATGCACCTAAAGTAGCAGTAATGAATGAAGACAATGTCATTATTGAAATGATTCATGAAAAAGAAAATACG
ATAGTGTTATAAAATAGTATAAGTTAGAAACCTCTAGTAGATAACAATGTAGTTATCCATTAGGGGTTTTTAT
TTTTATGCCAATTGGGTTAGCGTAATAGAAGTAAAGGTATTATCGTTGCAATTTTATCAGTGTTAAATGATGG
ACATATTTATAAAGAAATAGTTTTGCGAATTGATTATCTAAAACAGTATTCAGAATTTATTTGGTAGAAAATA
GAATTCTTAACTTAACGAGATAAGTTAATGACATGTTGTTTAGTTTCGTGAAGTAAAAATATTGAATTGATAG

CAATTATATAATAATATTATATGAAATAGATAAAAATAATATAGATTAAAGGAGGAATGCCTATGACAAAAAT
TTTTAAAATATCATTTTTGATTTTGTTAGTAGTAATTGTTTCTAGTTATTATGGTGAAAGTTTTGTCTTAGCT
GATAATAATCCTATCGTCAATTCAGAAAATGGTAGTGATAATAATTTTGAATCAGGAACAATTGAACGTAATC
AAAATGAGCTTTATGAAGTAGGTGATTATAGTACGCCCTTAACTGAGCGAGTTTTCGGTAAAGATCAAAGAAC
GGTTGTTAATAATATTTTACAAAGACCTTATAAGCAGACTGTATTATTAAATATGACGTTTTCAAATAACCGT
GTATATAAAGGCACCGGTACTATGATTGGTAAGGACATTGTATTGACAGCGGCTCATAATGTCTATTCTAAAG
ACGATAAGGGTTGGGCTAAAAAGATTGATGTCTATGCTGGAGTAAATGGGCAAACATATACAATAGGAAAGGC
CTTTTCGCATAAGTTTTTTGTATCTAAATCTTGGATTAATAATGCTCCTACAAAAGAAGATATAGCAATTATT
AAGTTAAATAGTAATTTAGGAAATAAAACAGGTTACTTAACTTTGAACACACGCATTAGCAAAGGTGAAAATA
TTGAAATTTCTGGATTTCCAGGAGACAAGTCAGACAACAGACAATATAAAGGTAAAGGGAAATTAGAAAGTTT
TGATGAGAATGAAATGTATTATACGGTAGACACATTTTCAGGACAAAGTGGTAGTGCGATAAGAGATTCAAAA
AACAATATCATCGGAGTACATGCATACGGCAGATATAATCATAATTCAGGCGTGAGAATAAATGATTTAAAAT
TAGATTACATAAATTATTGGATAGGTAAGTACAGAAGTCATCCATATAATAAGAAAGTGAAAGTAATTAAAAG
TAAGTATATATATTGGAAAAACATTGAAATTACAAAAAAGGGTGATAATAAATTAATTAAAAAAGATAAAGCG
TACACAGCTAAACTCTATTACAATATTCCAAATGGGTATAAATATTACTCTTTATACGACGAAAAAAATAGAT
GGATGGGTTACTTTAATAGCAATGATATTAAGGTAGTAAAATAGGAAAGGATGAATAAGATGAAAAAAGTTAT
AAGTTTATTTGTTGCAATTTTATCAGTGTTGATTTTTACGGATAATGCGAACGCCGAAAATAACAAGCCTGAA
GGTGTGAATGAAAACAGCAATTTAATACCAGTTAGACAACCGGATGCAAATTATCCAGGGCCAGTTAGTGACA
TAGCACGTGTGAAAAAACTTGATAATTTAATAGATGGCACGAAAGATCCAGATGTTATCAAGCAGTTGAAAGA
TGATAATGAGGTGCCGAATAATGCGGTAAAGGCAAAAGATTCTAAAATAGTATCCGACTGCAGACATTTACTA
AAACATCCAGATAATTTAATGGATGGCACGAAAGATCCAAATGTTATTAATCAGTTGAAAAACGCAAATGGAA
CATCGAATAATAGTGCTGTAAATCCAAGTATTAAAGCAAATGATATTCACACAAAAAGTAATAAACAAATGGC
TAAACAACAAAGTATTCATTTGGATAGCAAGAGCAAGTCAGTACATAAGCAACAATTGAAAGAATTACCAAAA
ACAGGAGTAAATAACGAAAGTAATACAGCAATTATGGTTTTATTTATTGGTTTAATATTGCTTGTAGTAAGTT
TAGTGACAAGATTTATAAAATTAAATAGCCATCAATAATAAAAAGGGCCACACCTTGGTTTAAGATTCCAAGC
GTGTGGCCTGCTCTTGTTATACAGTTCTATCACTATGTCACTTTTAATCAAAAGCGACCGTAATTGCTGCATA
TCAATTAACGATAGGAATGATTAATTGTGAATCATATTTACCACCAGTATAAATCATGTGCATGCCTTTATTT
ACGGTTTCTTCATGTTCATAACGCGTTTTCATGCCAGGGGTACTATTACCAATTACAATTTCACTACCTTTTA
CAACAACTTCCAATGTTTCGCCTTGTTTAAATAGCGTGCCTGAAGGTAACAATTCGATTTCAACAGGTACAAT
CTCATCTTGTGACAACTTTAATTCTGTTTCATGTTTATGCCAAGGTTGCGCAATTGTGGATTTTTCTTGATCT
AATTCACGATGTGATACGCGTAACCAACCGGTAGCTACTTGACCATTTTCAATATGATTAAAATCAGGGAAGT
TAACTTCATTACCACGACGATCTAACTTTTTAATACCTGCAAATAAATCCATATCGTCTGAGTCTTTAGTGCT
TACCCATAGTTTTAAGTTCATGTTTCCAACTAACTCAGTATCTTTGTCAAACGTATATTTAAAACTTACATCT
TGTCGTTTATCTTCAGAGTCATATTGTGCGACATGCGCGCTACTAATCTTTGCATGATTCAATGTGTGATTTT
CAGCATTCAAATACAATGGTGTATATTCTGTGTTAGGTAAAGGGAACGCTGACGCTGATTTGAATTCGCCTTT
ATAAAATTGATCTCTAACTTCATAAATGACATGAGGCGTATCTTTCCAATCGTTATTTTCTTCTTTAAGGTAA
AAATCAAAGAATGATTTTTGGCGTTCGAGATTTCTCTAGCGTAGTAACTTTCCCACTCTTTACGTCCATGCA
CATATAGCCATTTTTCTTCAGATGCAGCTTGTTTAAATCCTTCAAAAGAGCCACGGTTGTGCAAACCTTGTGT
AGACCAACTAGCACATGTTAGTAGAGGTGTTTTAATTTGTGATAATGGCACTTGACGCTGTTTCCAAAAATCA
TCGAACAAAGGGTGTTCTTGTTGTGCTTGAATCATGTCTTCGATATTTGGATTATCTGTCCATCTCGCAAAAA
TACCTTGTATCCAGAAACGATAAAAGCCAGTATCCGGTATACCTCCGTGAAAGGCTACTTCTCTATACATATC
ATTTAATCCTTCCCAAGGAATCATTGCTTTTAAATGTGGTGGATTTAATGATGCGACCCACCATTGAGTCACC
GCAAGATAAGAAACACCATTTGTTCCGATATTTCCATTACTCCATGGCTGTTTTGCTGCCCATTCAATCACTT
CGTAATAATCTTCCGCTTCTCTTTTTGACCATGGAGATAAGACGCCTTTTGATTTGTCACTACCGCGTAATGC
AACTTTAACTACAACATAATCATTTGGCACCCAAAATCCTGGGTCTGGTGATTCTTCAGGTGTAAAACTAGAT
GTCGGAATGGCGCCTAATGTTGGCCAAAGGGCACCCATATTTGTGATTTTAGGCTTATTATCTTTACCGTAAG
TATCTGCAGACATAACTACAGGGAATTTGCCATCTTTATTTGGTCTGAAAATATTAATATATAATTTTTCGCC
ATCGCGCATTTCCACAGTGACATCTTTTTCCATAATCATTTCTTGATTTCCATATTGAACACTGTCGACAACG
ATGTGGTTAATACCGGCTTTCACTTCATTGACATGAGTTACAGTTAATTTTGGATTTCCTAGTAAATGTTGGT
TCATTGTTATCACCTTTTTCTGTTTGTTTGTATCACCTATATTAAACAGGTAAACATTGTGTGCCAATCATCA
ATGTTGGAGATTTGTGGGATAATGAACATAACGTTTATAATTGTTAATAATCATTGGGGAGGACTTGATACAA
ATGACTATAGATCGACGTATTAGAAAATCACAAGTTGCAATGAGAAATGTATTTATTGAACTTTTACATCAAC
ACCAATTAGAAGAGATTACTGTGCAACAAATTGCAGATTTAGCTGATGTTAACCGTAGTACATTTTATACGCA
TTATTATGATAAATATGATTTGCTAGAAAAAATTAGAGAATCAGCAGTTGGAGGAAATAAGAGACTTCATTCAT
GATGAGAAGTTAAATGGAGCAGTTAAGTTATCCACGGATAATATACATCAAATTATGACCGTTTTAATAGAAA
TGATAGAAAAAGACATCGCATTTTATCAATTGATGTTTCGTATGGGTAAAGATTCAAATATCCACGAAAAACT
TTATGATTTAATTATGTGTCATTTACAACGTTATAAAAATGAAAATGATCAAATTAATGGTATACCATTTTCC
TATTTCATGAGTTATGTTTCAGGTGCGGGATTGTCTTTGATAAGACATTGGGTAGAAGATCCAAACCGTATAG

ACAAAGTTGACTTAATAAGGCATTTTAATACGATTGTCAATCAAGGGCCAGCAACGATAATTAAAAATATACA
ATAAAATAAGGCAAGCCATGAATCGAGAGTGGCTTGCCTTGCTAAATAACCTAAGCTTATTAAATGATATCTT
TATCAATAACATTATTGTTGGATTGCAGTATAGTCTTGACCATGTAACATCCAACGAACACCGTATTTATCGG
TAAAGATGCCCATTTTGCCACCCCAGAATTGGTCAGCTAACGGTAATTCTATTTCAATTGAAGAATGATCTTT
AATTTGCTCATAGAATGCTTCAACTTTATCAGCATCTTCCTTATTGTTAACATCATAATCAATTAATAATGAT
ATGCCATTATTAATTTTGTCAGCGCGACCAAAAGAATCAGAACATAACACTTTTACGCCAAGCACTTCAAACT
CAGCATGCATAGTTGCTTCTTGCGCTTCTTCCTTAGTCATACCAAAATGTGACGCTTGTTCTTCGCCAACTTC
TAAACGTTTAACGTCAGTTGCACCAAATACTTCTTCGTAATATGCAAGGGCTTCTTTTGAATTTTTAAAAGCG
ATATAAGGGAATAATGCTGTCATGTAAAACACCTCAATGTATTATTTTTATTACTAACACCATGTTACATTAA
AAAATGGCAAAAGTATAACTTTTATGATGAATTGGAGTCAATCTTTTGTAAAATACACGATAAATTATAAGAT
GAAATTGATTTAAATGAAGTAAACATATATATGACATACAAAAAATGAAAATGACACAGAGACGTATGTGTTG
TTTAGTGTTTTAAAATTCAAGTTTAAAAAATGCTTTTAGATACATAAGGGACTTTCGAAATATAAATGCATTA
AAATGAATTTCTAACTTTTTTGACTGACATTTAATATGAATCATATATAATAGAAGTTAATGATTAAATGTGG
GAGGTTAAATCATGATTAATGCTGAACCTATTATCAGTAAAATGAAAAATCAAAAAATTAATTATGACAAGGT
GTTGAAGAAATTAATCGGGCAGTGGGAAAGAGAAGCAATCAGACCTAAAATCTTATTGCATAGTTGTTGTGCA
CCTTGTAGTACATATACATTAGAGTTTTTAACACAATATGCAGACATTGCAATTTATTTCGCGAATTCAAATA
TTCATCCGAAAAATGAATATTTACGACGTGCTAAAGTGCAAGAACAATTTGTAGAAGATTTTAATCGCAAGAC
TGGTGCGAATGTGAAGTATATTGAAGCACCTTATGAACCGCATAAGTTTGTGAAAATGGTGAAGGATAAAGAA
TTAGCTGATGAAAAAGAAGGCGGCTTACGTTGTACCGCTTGTTTTGAAATGCGTTTGGATATTGTAGCGAAAG
CAGCTGTAGAACATGGCTATGATTATTTTGGCAGTGCAATTACATTATCACCTAAAAAGAACGCACAATTAAT
CAATGAACTTGGTATGGATGTCCAAAAAATATACGATGTGAACTATTTGCCAAGTGATTTTAAGAAAAGTAAA
GGTTATGAGCGTTCCATAGAAATGTGTAATGACTATAATATCTTTAGGCAGTGTTATTGTGGATGTGTCTTTG
CAGCGATGCAACAAGGTATTGATTTTAAAACAGTTAATAAAGAAGCGAAAGCATTTTTAGAACAATATCCAGA
TTAATCATTGGAAATGAGATAAAAGGCAGGCGCATATACAAGCCATGCCCTATTAAAAATTATCAAATAGAAA
CCCGAGACTTTAGTGAAATGAATCATACTAAAATCTCGGGTTATTTATATCATCGTAAATATTGTAGCTATAA
TAAATAACTGGATCCTGAGACATAAAACAATGTCTCACTCTAATTACATCATTTTATGAACAAATCTTATAAT
TGCTAAAATGACAATGACAAGTGTTGAAATAAGTGAAAACACTTTAAACTTTTTGTAGTCATTTTTCGTTTCA
TTTTCTCCTGTTTTCATCATTTCACGTAGCTCTTGCTCAATTTCTAAATCAAGATCTGTTTTACGATAACCAT
TCAAACCTTGACGTTCATCTTCTTCAGGTTGATTTTGTTGCTGTGTGTCTTTGTTGTCAGAAGTCGCTACTGT
TTTTTTATTATCTGTTTCTTTAGTCATAACAAACGCCTCCGTTATAAAACGCTATATTTAATGATATGTGATA
TTTAAGCCATTATTTTGAAAAATGGTGATAAATATATCTTCTTGAGTTATATCATAATTTTAATAAGACGATT
CAGCAAGTTTTAAAGTATTATTTGACTACGTTGGATTAGGCATCTAGTCCTATAATATCACTGACATTGTCAA
AATGATGATCTTTTAAGTAACGTGCGATGCCTTTGTTCATTTTCTTAGTTAAACCTGGGCCTTCAATAACAAG
TGATGAATAAATTTGAATAAGTGACGCACCGTGACGCATCATTTTGATTGCATCTTCAGTACTGAATACGCCG
CCTGTACCTATAATTAAAAATTCACCATTTGTTTGCTGATAAGCATACTTAATCAATTTTAAATTACGTTCAA
ATAATGAACGACCACTCAAACCGCCTTCTTCGACTTTATTAGCAGAAGTTAAACCATCTCGTTGTCGCGTTGT
GTTTGCTAAGATGATACCGTCAAATGTCTCAGTAATCGCTGGTAATAGTGCTTTTAAGCCATCGAAATCCATA
TCAGACGTTAGTTTTAAATAAATTGGCACTGTTACATCATGTTGTTTTTTAAATGCTGTTAAAGCTTGGCATA
ACATTGAAAATTCATCTTTATCATGGAAGTTTTGAAGATTTTCAGTATTTGGAGAACTGATATTGACTGTGAA
AAATGAAACGTCGTGTTTAAACGTATCAATGACTTTTATATAATCTTGATAACGCGCTTCATAAGGTGTCATT
TTATTCACACCAACATTGATACCAACAGGTACTTGATAAGCATTTTTACGCAAATGACTGAGTGCTTTGTTCA
TACCAATATTATTGAAGCCCATACGATTTATCAAGGCATCATCTTCTAATAATCTGAACATGCGTGGTTGAGG
GTTACCCGGTTGAGGTTTAGGTGTGATACCACCTAATTCTAAAGCACCGAATCCAAGGTGTTCCAATGCTTTT
GGTACTTCGCAAGATTTGTCGAAACCAGCTGCTAAGCCAATTGGATTGTCGTACGTATTACCTTGTATCGTTT
GTGATAACGTTGGATTCTTATAAGTAAATAGTTTATCGACGACTGGGAATAAAACCGGAAATTTTTGTAACGT
TTTTAATGCATCGATAGTTAGTCCGTGTGCTTTTTCGGGTTCGATTTTGAATAAGAAAGGTTTAATTAATTTG
TACATGAGTATGCTCCTATTTCATTATATTTGAGGCTTACTATCCTCAACTTAATATATGTGAAATATATTCT
TTTAATAGACTAGCATTTCCCTACATAATTTCCTAGTTAAAACTAAAAAGTTTTGAAAATTGACGCAAGTACC
ACAAATAAAATTGAGATTAAATCATCTTAATTAGGCAATATAATAGTATAAAGTAAGCGTAATGGAAGGTGTT
TGTATGAATGAACAATGGTTAGAGCATTTACCTTTAAAAGATATTAAAGAGATTTCACCAGTGAGTGGTGGCG
ATGTAAACGAAGCATATCGAGTCGAAACGGATACGGATACATTTTTCTTACTTGTCCAACGTGGACGTAAAGA
ATCATTTTATGCTGCAGAAATTGCAGGTTTAAATGAATTTGAACGTGCAGGTATCACGGCACCTAGAGTAATT
GCAAGTGGCGAGGTTAACGGTGATGCGTATTTAGTGATGACGTATTTAGAAGAAGGGGCTTCAGGGAGTCAAC
GCCAATTAGGGCAACTCGTAGCTCAATTACACAGTCAGCAACAAGAAGAAGGCAAATTTGGCTTCTCATTACC
TTATGAAGGTGGCGATATTTCTTTTGATAATCATTGGCAAGACGATTGGTGTACCATTTTTGTCGACAAGCGC
ATGGATCATTTGAAAGATGAGTTGCTAAATAGAGGGCTATGGGATGCCAACGATATCAAAGTATATGACAAAG
TGCGACGTCAAATTGTGGCGGAATTAGAAAAGCATCAAAGTAAACCGTCTTTATTACATGGTGACCTATGGGG
TGGCAATTATATGTTCTTACAAGATGGTCGTCCGGCGTTATTTGATCCAGCACCATTATATGGTGACAGAGAA

TTTGATATCGGTATTACAACGGTATTTGGTGGTTTTACGAGCGAATTTTATGATGCGTATAATAAACATTATC
CACTTGCAAAAGGTGCATCCTATAGACTTGAATTTTATCGTTTATATTTATTGATGGTGCATTTATTGAAATT
TGGGGAGATGTACCGTGATAGTGTTGCGCACTCTATGGATAAGATTTTACAAGATACAACAGGTTAGTTATTA
CGTTAGATTAAGATAATAATATGCACAGATATTTTTACAATGAGAAGGCCTTCAGCTGCCTCAATAAAAATAT
TTGTGCTTTTTTATTGTTGGAAAATAAAATTTTAATCTCTATTGTTAATTTCTATAATGCAAAACAAGATTGA
GTTGCAATAAAAGTGATTTTCTAACTTTTTGTTTAATAAAATTCTAGGAATGATACATATTTATTAATACAAT
AATTTTGAATATAATCACAAAACAATATTTAAGTATTATTGAATGTTTGAATATCATATATTGATACAGTTTC
TAATAATTTTAAAATAATTTAAATGGGAAGAGGTGTAAATGATGAGTACAGTTCAAAGTGATATTTTTAAGAC
CAATAGTGCATCATCATCTATTAAAAGCGCTGTTGAAACATGTAATAATGTGTCGAAACCGGATAAAGATGAA
AGTACAACAGTAAGTGGAAATAATAATGCTCATAGTGTGATAGATGATTTGATGAGTAAGAATCAATCTGTTG
CTGAAGCAATACGAACTGCGAGCGATAATATACAAAAAGTTGGTGAGGCTTTTGACCAAACTGACGTAATGAT
TGGTAATGAAATTGGTAAAAATTAAAACGTGGTGAAATGATGTCGAATAAACTGGATGAAATCAATAAAATAA
TCACAGCGAAACATGAGCAAATGGATGACTTATATGATGAAAAGCGAGAGGTTAAAGCATTGATAGATGAAAG
TGATGCGCTTAATCATTCGATAGATCAATTATATCAACATTTAGGTGAGCGTTATTATAGTAGCAATATGGCT
AGTCGTATGGAACAGTTCCGCGATGAATTTCATTTTGCGAAACGACGTTCAACGGTAGCGTTACACGAGCAGC
AACAGCAAATTCAACATGGCATTCGTAAAGCGGAAGAAGAGATGATTGACTTGGAAATGCGAAGGAATGTTGA
AATTGAGACGGTGACAAAGGAGGAAAATAAATGGAAACAATAGGAAGCATTATTTATTTAAAAGAAGGTTCGC
AAAAGTTAATGATTATTAATAGAGGACCAATTGTAGAAATTGAAAATCAAAAGTATATGTTTGACTATTCTGG
ATGTAAATATCCGATTGGTGTTGTGGAAGATGAAATTTATTATTTTAACGAGGAAAATATAGATTCAGTTATT
TTTAAAGGTTATTCTGATCAAGATGAGGTTAGATTTCAAGAGTTGTTTGATAATATGAAACAAAATTTGGATA
GTGAAATACAACGTGGAAAAGTTACACAACAATAAAGAAATACTTTTTCTTTATTGGGGTGGACGACGAAAT
AAATTTTGTAAAAATATGATTTCTGTCTCACCGCCAAAATGTTATATCCAAGTACTGAGGTTGGATGATACCA
TTAAATGTCATTCATAATCATTTGAACTAACCGTAGCATCTTTAAATTTAATAAAAAAGACACATACCAACTT
CCGAAATGTAGATGAATTCTCTACAATAACGGAAGTTTTTCTTTTAATATTGAAATTTCTCAAGGATAGGTCT
ATACTTTATAAATCGTAATTATTACGATTTATAGTCAGGAACAATAACTTGAAATAGATCATTGAGGGAGTGT
TAATATGCAACATCATAAAGTGGCTATTATCGGTGCCGGTGCTGCAGGTATAGGTATGGCCATTACCTTAAAA
GATTTCGGTATAACAGATGTCATTATTTTAGAAAAAGGAACAGTAGGCCATTCATTTAAACATTGGCCGAAAT
CGACCCGTACGATTACGCCATCATTTACGTCTAATGGATTTGGCATGCCTGATATGAATGCAATTTCTATGGA
TACTTCACCAGCATTTACATTTAATGAAGAACATATTTCCGGAGAAACATATGCTGAATATTTACAAGTGGTT
GCCAACCATTACGAGCTGAATATCTTTGAAAATACAGTTGTTACAAATATATCTGTAGATGATGCATATTATA
CGATTGCAACGACAACAGAGACATATCACGCGGATTATATCTTTGTCGCAACAGGTGATTATAATTTCCCTAA
AAAGCCATTTAAATATGGTATTCATTATAGTGAAATTGAAGACTTTGATAACTTTAATAAGGGGCAATATGTG
GTTATCGGAGGTAATGAAAGTGGCTTTGATGCTGCATATCAACTTGCAAAAAATGGCTCTGACATCGCACTTT
ATACTAGCACAACCGGTTTAAATGATCCGGATGCTGATCCTAGTGTTAGATTGTCACCTTATACACGTCAGCG
ACTAGGTAATGTCATTAAACAAGGTGCTCGTATTGAAATGAATGTACATTATACAGTTAAAGATATTGATTTT
AACAACGGACAGTATCATATCAGTTTTGATAGCGGACAAAGTGTGCTTACGCCTCATGAACCAATACTAGCAA
CTGGCTTTGATGCAACAAAAAATCCAATCGTTCAACAATTATTTGCGACAACAAATCAAGATATTAAATTAAC
AACACATGATGAATCGACCCGTTATCCGAATATTTTTATGATTGGTGCAACAGTTGAAAATGATAATGCCAAA
TTATGCTATATCTATAAATTTAGAGCGCGATTTGCAGTACTTGCACATCTTTTAACACAGCGGGAAGGCTTAC
CAGCTAAACAAGATGTCATTGAAAGTTACCAAAAAAATCAAATGTATTTAGATGATTATTCATGTTGTGAAGT
GTCATGCACATGTTAGAAGTGAAATATGATATGAGAACTGGGCATTATACGCCCATACCTAATGAACCTCATT
ATTTGGTTATTAGTCATGCGGATAAACTTAACGCAACAGAAAAAGCGAAATTAAGATTATTAATCATAAAACA
GAAATTAGATATTTCATTGGCAGAAAGTGTAGTTTCTTCGCCTATAGCGAGCGAACATGTGATAGAACAATTG
ACACTATTTCAACATGAGCGACGACATTTAAGACCTAAAATAAGTGCGACATTTTTAGCCTGGTTGTTGATAT
TTTTAATGTTTGCATTGCCAATTGGTATTGCTTATCAATTTTCAGATTGGTTTCAAAATCAGTATGTATCAGC
ATGGATAGAATATTTAACTCAAACAACATTGCTCAATCACGATATATTACAGCATATATTATTTGGTGATTAT
GGTGTGCTATCACTTGGAACATATTCGCTCGTATGGGCATTGCCGGTTGTAATATTGATTAGTTTATCAACTG
CTATAATTGATCAAACAGGATTCAAATCATGGATGATATGGGCAATTGAACCGTCAATGTTACGGATAGGATT
ACAAGGCAATGATATCGTGCCACTATTAGAAGGATTTGGATGTAATGCAGCCGCCATTTCACAAGCAGCACAC
CAATGCCATACCTGCACGAAGACACAGTGTATGAGTTTAATAAGCTTTGGTAGTTCTTGTAGTTATCAAATAG
GTGCGACATTATCTATTTTTAGTGTAGCTGGAAAGTCATGGCTATTTATGCCGTACTTAATATTAGTACTTTT
AGGTGGCATCTTACATAATAGGATATGGTTGAAAAAGAATAATCAACAACTTAGCGTTCCGCTACCTTATGAT
AGACAATTACATATGCCAAATATACGTCAAATGTTGCTACAAATGTGGCAAAATATACAAATGTTTATCGTTC
AAGCGCTACCTATTTTTATCACAATCTGTCTTATTGTTAGTATTTTATCACTAACGCCAATTTTGAATGTTTT
ATCACAAATATTTACACCTATATTATCGTTATTAGGCATCTCGTCAGAATTGTCACCAGGGATTTTATTTTCA
ATGATTCGAAAAGACGGCATGCTCTTGTTTAATTTGCATCAAGGCGCCTTATTACAAGGAATGACAGCAACAC
AGTTACTACTACTTGTGTTTTTTAGTTCAACATTTACAGCGTGCTCGGTCACAATGACGATGCTTTTGAAACA
TTTAGGTGGTCAGTCAGCACTAAAATTAATTGGAAAGCAAATGTTGACATCATTGTCTTTAGTTATTGGTGTA

GGCATCATTGTTAAAATAGTAATGCTGATTATTTAAAAAAGAAATGAACTATAACTGAATATAGAGTCATGTC
AGTCAATAGGAAAGATATCTTGGAATATGCTATTCATATGAAGTATAAGAGGAGAGTCGCAGATGAAAATAGT
TATTATAGGTGGGTTTTTAGGTGGCGGTAAAACGACTGTCTTAAATCATTTGCTCGCTGAATCATTAAAGGAG
TCGCTGAAACCGGCAGTTATCATGAATGAATTTGGTAAAATGAGTGTTGATGGTGCCTTAGTATCTGAAGACA
TACCTTTAAGTGAACTGACAGAGGGGTGTATCTGTTGTGCAATGAAAGCAGATGTATCAGAACAATTACATCA
ATTATATTTAAAAGAGCAACCAGACATTGTATTTATTGAATGTAGTGGGATTGCAGAACCGGTCTCTGTCTTA
GATGCTTGTTTAACGCCTATTTTAGCTCCGTTTACAACCATTACACATATGATTGGTGTTGTAGATGCAAGCA
TGTATAAACATATAAAATCATTTCCAAAAGACATCCAAGGCTTATTTTATGAGCAATTAGCATATTGTTCTGT
CTTATTTGTTAATAAAATAGATTCAGCAGATGTTGAAACAACGAGCAAACTATTGAAAGATTTAGAAGTTATT
AACCCAGAGGCAGATATACAAGTCGGTATGCATGGCAGCGTCACTTTACCAATATCAGTTAGACAAATGACAG
TTACTTCCGACAATAAACATGAATCATTACACAAAACAATAAATCATCAATTTGTGCAATCACCAGTCAAATG
TACTAAAGCAGAGTTTATAAAACGTTTAGCATGCCTTCCGTCTCATATTTATAGGTTGAAAGGGTTTATGACA
TTTGAAGACACCGCACATACGTATCTCATTCAATTTACACAAGGACAATATGAATTAACACCTGTAGCATTTT
CAAAAAAAGTGCCAGAATATTTGGTGCTTATCGGAAAGGGTATTTCAAAAGAAGACTATCAATGTTTGGAACA
GTAGTGTTTTCAGTGGGAAAGAATGAACAGTATATAGATAATAAATGCCCCACTTGTTATATAAATTGTAGTG
AGAGGGGCATTTATAAATTATTTTATTAGGTATATTTCTTTATTGTTAAATACAGATCTATTAATAGTATCCA
TAAGGTCAAAGATAATAATTGATATTATTTTTAAAGCTACAAATAAATATAAGATAAATTCAGGTACTATCTA
TTTAATCAATAAATTGTGTTGTCAGAGTTATCAATACGTATTAATACCTTGCCATTTGTATGATGATTAAATG
TATAGTCAAGTGCTTTGTCAATTTCGTCTATTGTGAAAATATGGTCGTCGATGTCTGGTATGATATTGTTTTG
TTCGATGATTTTATTTAAAATTTTTAATTGCTCACCATCCGAACGTACAAATATAAAACGATAATCTATCGAC
TTTTTTTGAGCTTTTTTATCGAATTTTTTACCTGCTAAACCAAATAGCAATTTTTTAAATGGTGAAAAATTAT
TTTTCACAGCAAATGCTTTGTTAGGCGCATTAATAAGACTGACAATAGTACCACCAGGTTTCATAACTGATAA
TTCTTTATCGAATTCATTTGGTCCAAGTGTGTCTATAACATAATCAATGTCAGATAAAACTTCACAGTAATTC
TCAGTTTTGTAGTCTATATATTGATCGACACCCTTAGCTAAAAATTGATCTTTAAGTCTCGGACTTCCGGATA
CAATAACTTTAAGCCCTAATGATTTTGCTATAGGTACTGCCATTTGTCCAAAGCTGCCTGAACCACCAGGTAT
AAACAATGTTTTACCAGGTATAGCTTTTAATTCTTCAGTAATTGCTTGATAAGCAGTTAAACCTGTTAACGGC
ACTGCTGCACCTGTTTTAAGGTTTAAATTTTTAGGTAATTTTGCAATAGCATTAGATTCAATAGAAACATACT
CTGCAAAAGCACCTATACGATCGATTGGTAATCGTGAATATATTTTATCGCCTAATTTATAATCTTCGACTTT
ACTTCCTACTTTAACAATTGTTCCTGTGAGTTCATTACCAATAGTTTGAGGCATTTTATAGTTTTGAATTAAT
TTGACACTTCCATTAATAGTTAAATTTTCTAATGGATTAATTGCTGCATAAGCTACTTTTACAAGTACCTCAG
TAGATTTGATCTCTGGAATAGTAATATCTCTAATTTGTACATCTCTATTTTTATTGTATTTCTTTATTTGAAT
GGCTTTCATAGTATATATGGCTCCTCAGTGATTAAAATGCATTACAATATTTTCATTAGACCCCACAAATATT
TCACTCTATAAGTATATTATCAATTTATAAATGTAACTTTTCAGCATGGTGCAGTTGTATAAAGCTATAAAAT
CAATTAACTCCACATATTTAATAATACATCAGCAAAGCCTTCAGGTTTTTGAATATAACCTAAGTGACCACCT
GGAATATCTACAATAGGTATGCCAGTTTCGTTATTTATATAAAAGTTAACATCTTGTGGGAAGGAGCCTCTAG
AATCTGTCCCATTTAGTAGGGTGATTTTATCGTTGTATTTTGTGAAATCATCCAAAGTAATATCTGAATGCGT
ATATTGTCTAATTTCAAATTCTGACCAGAACATCGTACGTTTGTACTGTTCTATACGTCCTTCTTCAGTATCA
GCAGGTTGAGACATCATTTTTGCATCAATTGGTGCGATATTTAATGTTTCACCAAATGTTTTCATGCCTTTTT
CTAAGCCTTCTGTTAAAATTTGATGCACAATGTCATCATTTTTATCTTTCCAATAAGTACTGTCTGGTAAAAA
TGTATTAATTGGTGGTTCGTGAAATGCAATCTTTTTAACGACTTCAGGGTAATCTTTTAACACATGCATCGCA
ACGATTGAACCTGAACTTGAACCTAATATATAGACAGGTTCATCACTTAATGACTTTGCAAGTTCGGCAATGT
CCTGTGCGTCGCGTTTGACACGATAATCACTGTCAGGGTTAGAAGCGGAATCAGGAAGTGGTTCAGTTAACTC
GCTTTCTCCATAATCACGACGATCAACGGCTACAACAGTAAAATGGTCTTTTAACTGTTCTGCAAGAGGCAGA
AAAATGTCTCCGGTACCGTTTGCACCAGGAATAAAGATGAGCACGGGTCCTTGTCCGACTTGGTGGTATCGTA
ATTTAGCGCCTTGTAATTCTAAAGTTTCCATATTCAATGACCTCCATTTGTTAATTGTTAGATGATAAACCTA
ATAATTTAGCACCATTTGTATAACTTATTTTCTCTTTTTCTTCATCTGTTAAACCCAGTTCATCTAAAAATAC
ACCTAATTTTTCAGGCTCAATATATGGATAATCAGCAGCATAAAGAATTCTATCAATACCTACTTCTTTCTTG
ACTAAATCAAACTGTGGCTTCGTTAACATGCCACTCGGTGTGATATAAAAATTATTTTTAAAGTAATAGCTTA
CAGGGTGGTTCAAATGTTCAGCGAATAAAGCTTCATCCATACGTTCTAAGAAGAAGGGGATAAACTCACCCCA
ATGTCCAATAATCATATTTAACTTTGGATAACGATCAAAAATGCCAGATAATACAAGATGTATTGCATGAATA
CCGACATCAATGTGCCAACCATAACCAAAACAAGCAAATGTTGCCGCAGTTACTTCAGGATAATTTCCTTTAT
AGTATGATTGATAAATGTCACTGTTAACTGGCGCGGGATGTAGATAAATCGGTACGTCTAAATTTTCAGCTGT
TTTGAAAATAATGTCATATTTGTCTTGATCAAGAAAACCATCTTGTGCACGTCCCATAATGAGCGCACCTTTG
AATCCTAAATCATTGATGCAACGTTCGAATTCTCGCGCTGCGGCTTCAGGCTCATTGATAGGTAAAGTTGCAA
AGCCTACAAAGCGATTGGGATATTGTGCAATATAATTTGCCAATTGATCATTTGCTTTTTGACATAATTCAAT
GGCTTTCTGACCAACTAAATTTGAAGGAGACCCATTTCCATAAGATAAGACTTGAATTTGAACGTCTTGATTA
TTCATAAATTGGATACGTTCATCATGATGTGATAATTCGTCGGCATTTGTAAAACCGGTCTTTTTTTCAAGGC
CTTCTAACATTACTTTCATCGGTACACCTTTAGGATCTGCTGATATCGCATTCATCGTTTCTTTTTGAATATC

TTCAATGACATAATGTTCTTCAAACGTAATACTTTTCATTTAATTCGCCTCCATATTTTTTTGCATGTATATT
GCATCTATTGCAGAAGCATTCTTTATTTACCTCTAAATTTAATATTTGTAACATAAAATTGATCTACCAAGGC
ATCTCTCCATCGCCATTAATAAATGTACCTGTTGGACCATCTGCATCAATGGTTGCTAATTGAATGATTGGCT
TGATCCCTTCAGAAACATGTTTTGAATTATTGCTAAAATCACCAACTAAATCAGTATTTGTAGCGCCTGGATC
AGCAGCATTGATTTGCATGTTAGGTAATCCTTTAGCGTATTGTAGCGTTAGCATTGTTACTGCCGATTTAGAC
GAACAATAAGCTAATGAATTCACTTTAGATTCAGCTGTTTCGGGGTTTGTAACCATTCCAAATGAACCTAAAC
CACTTGATACGTTGACGACAACAGGTTGTTCAGATTTTTCTAAGAGAGGGACGAATGTGTTCATCATTCGAAC
GATACCAAATACATTCGTTTGATATACTTCTTCAACGTCACGAGGTGTCAATTTGGAAGGTGCTGAAAATTGA
CCAGATATACCTGCATTGTTAATGAGGATATCAAGACGGCCTTCTTTTTCAGCAATCATGTTATAAGCATTTT
TGACTGAGTAGTCACTTGTAACATCTAACTGTACATAATGAACACCTAATTTTTGTGATGCTTGTTGTCCTCT
TACGTCATTACGAGAACCTATATAAACTTTGTAACCCAATGCTTTAAGTGCCTCTGCACTTGCATAGCCTAAC
CCTTTATTGCCTCCTGTGATTAACACAATTTTAGTCATTACGTTCCACCTCATCTAAATAAATGTTTAATAAA
TAATTTCTGTACGCTTCAATTGAAATATGGCGATGCTCTATTTGGAAGGCAAATACACTAGTTGATAATGCTT
GCAACAGCATATCTGTTTTGAACTTCTTGTAAATGTCGTCATCGCTTTTAAATAAGTCATAATAAAAATTAAA
TAATTCTTGATAAAATGCGCTTTGGTAAAAACGTAATTTATTGTTGCCTGCTTCAATACATTGCAGTAGTGCC
TTATTATCGATTTTAAATTGTAAAAGATAATCTAACGACACTTGCATAACCTCATAATCAGAATGATAGTCAT
CTTTAATTTGCTTAAAATGAGTGATGAAAATATCAAGGTCTCTTTGTATGACGTAGTAGCATAAATCGCTTTT
ATCTTTGAAATGTCGATACAATGTCCCCATACCGATACCTAGTTCTTTAGCAATACGATTCATACTAATGTTT
TCAACGCCTTCTTCATCAAAAAGTTTGTGTGCTATTTCTTCAATTCGTTGCCTATTCTCTTTTGCATCTTTTC
GCATGATTACACCTACTTAAAATTCTCTAAAATTGACAAACGGATAACTCTCCGTTTATTATAAAGCGTGTTA
AGAAAGATAGCAATGAATTTGCAATAACTATTAAATATCATAAAAGAAAAGAGTGTTGCGTATGTCTTTAAAT
AAAGAGCAAAGACGTATTACAGCTGAAGAATTGCAAGCACATTTCGAAGCATCAACTTTATCGGTTCAAATGA
TTGCAGAAAAACTGAATGTTACCACAGAAGATGTGGAAAAAGCATTAGCTATGAAAGCGCCACTAGGCATTTT
TAGTCATCAATTACAACGATTTATTCATTTAGTATGGGATGTCAGAAATGTAATAAATGACAATATTAAGGAA
AATGGACAAACACCAGAACCATATACGTATTTAAAAGGTGAAAAAGAGGACTATTGGTTTTTAAGATAAAAGG
GGAGTGGATCTAAAATGAAAGATATTTTAGTAATAGGCGCAACAGGGAAGCAAGGTAATGCAGTTGTAAAACA
GTTGTTAGAGGATGGATGGTATGTAAGTGCATTAACTAGAAATAAGAATAATCGTAAACTTTCAGACATAGGA
CATCCGCATTTATCAATCGTTGAAGGTGATCTGAGTGATAGTGATAGTTTGCAATCAGCGATGAAAGGGAAGT
ATGGCTTATACAGTATTCAGCCAATCGTTAAAGATGATGTTAGCGAAGAATTAAGACAAGGTATGAAGATAAT
TGAAATAGCTGAGCAAGAAAATATTCAACATATTGTGTATAGTACTGCGGGAGGGGTTAATCGAAATCGAACT
GGCCCACACTTTGAAGTACTAGCAAAAATAGAAAATAGATTAATGGAATCTAATATAAACGCAACAGTTATCA
AACCATCATTCTTTATGGATAATTTTTTACGCATTGCTAAAGTCGAAGATGAACGTATTACATTACCAGAATT
TATTAATCCGAATATAAAATTCACAATGATTTCTTCTATTGATATAGCTAAAATTGCATCGTATATTTTTGCA
CATCCACAAAGCTTTACGCATCAATCAATAGAGATTGGTTCCGATGAAGTTACATTAAGTGAAGCAGCAACAA
TTTTTAGTGAAGTGACAGGTAAATCGACTGTTATTGAAGGAGAATTTGTTAGTAGTGTTGCAGAAAAGCAATG
GTTGGAAGAAAAAGGTTATGAAGTAGACTTTGAATTAATGGCTGAAATAAATCCAACAAGATTATCATTAAGT
GATTGGCTTAAAGTCCAAGACTATAATAAGTAATGAATGGGGTGCAATCATGATTCAATCAATGTGGTTTAAT
TTGCATGTTCAAGATTTAGAAAAGAGCGCACAGTTTTATAAAGCGTTAGGATTTAAAATAAATAGAAACCCAC
AAATGTTAGATAAAATGGTGGGCATTCAAATAGGTCAAACAACCGTAATTTTAATAGAAAACAAGCATTTTCA
AAATGTAAGTCAGCAAAGCCTTAATACTGAACCAAATGAAGTGATGATTTCTCTAGGTGTGAACACAAATGAA
GAAGTTGACCAGTTGGTGAATAAAGTGAAAGAAGCAGGTGGCACAGTCGTTCAAGAACCAACAGTAAGCCAAG
GGTTTTATGGTGCAATGTTCAAAGATCTTGATGGACACCATTTTAATTTTTTGGTCTGCTAAAATGAAATTCT
CAAAAGAATAAATAAAAAGCCCATACAGAGATGAAGTTTAATGTGGGAGTGGGACAGAAATGATATTTTCGCA
AAATTTATTTCGTCGTCCCACCCCAACTTGCATTGTCTGTAGAAATTGGGAATCCAATTTCTCTTTGTTGGGG
CCCATCCCCAACTTGCATTGCCTGTAGAATTTCTTTTCGAAATTCTCTGTGTTGGGGCCCCTGACTAGAATTG
AAAAAAGCTTGTTACAAGCGCATTTTCGTTCAGTCAACTACTGCCAATATAACTTCGTAGAGCATAGAACATT
GATTTATGTCCCAGCCTGGACACATTAGTGTCTTCAATCAGTATGGGCTTTGTTTATGAGGTCAGTTGGCACC
TTAAGTGCAATACCTCGTGAGCAAATATTAATTTTGAATATAACCGTATTGTTGGCGAAGATCATAAACTTTA
TCTGGTGTGACATCTTGACCGATTGGATCAACCACTTTTATTGTGGAAAATGTTGTTAATACTTGTCCTCGGA
TCATGCTTAAGTAGTCTTGACGCAATGCATGTTGTTCTTGTTTTTCAGCTACAGTAAGTGGTTGTACTTTTTC
TTTATTTGCAAGTTCATTAATTCTATCTAAAATTTTCATATTGTCATATCCTCCTTAGAATCAAGTGTTGATA
TATTTTGAGATTATTTCTATAAGGTAATTTATCAACACATTCAGTAATGTTTTTAGTTTCTCGGTCTTTTGAC
CAGCAGATTTTTGGAATTAAAATAAGGGGTTTGAGACACGATTTTAATAGTAGTACGATGGCAAACTGATGTT
ATTGAAGTGTCGTTTGATATACATGTCTGCAATGTTGTTTTATAAGACAGGATGACGTAGCTGAGCAAAGAAA
TAATCATTGTCGAATGACATCATGTGTCTTATTAAAATTGCAATGGGCATAGCATAGGTATATTCACAGTCGA
TTTTCTTGATTTCTACATTATCATAATACGTAGTGTAAAAGTTATACGACAGTCGATAGTCGATTCGCTTTTT
ACCACAAGCAATTATTATGTGTTGCTTCAACGTCGACTTTAATTTATTTGTAATGTATGTCACTACACCCAGA
CTACTCTGATTAATATTTAATATATTATGCGTGCGTTCAAACCCCTGAGATTCATCTGTCTCGCTCACGTCAA

TCCCTCCTATCTTAGTATTTAAACAACGTATATCAACTATACGACATGTGTTGTTTAGACATACTATACTACT
ATGTCGTACGTTTGCCAATAAACACGCATATATCATTTGTTGCCTAAACAACATATTGTCTGAAGTGTTGTTT
AAGTACGCAAAAACAAAAATGCTTGTCACTAAAATCTATATGTTTTAATGACAAGCTTATTTTCATTCAGTTA
ACGACTGCCAATATGGCTTTGTAGAGTATAGGACGTTGATTTATGTCCTAGACACTGCAAATTAATCGTAATA
CGTTAAATTTTATTGTGTGTACAAGTATGAACCTAAGTCACATAGTGATTTCAAGATGCATGACTAAATACTA
AGTTTTATTAAAAATGTGCTATATGATGAATTAAAAATGGTAGGTTGCGATCGATAAACACATCGGCTGTCTC
TTGACTCGTGTTAAACCAATGTAATGCTAAGCCTGAGATACCAGCTGCTAAAAAACTCGCACTATTTACAATG
ATATCGCGTTGATGCTCTGGGTAGCTGTTTTCTAACATAATCGCAAAAATATCTTCTAATTCATTATTAATAC
GTTGATGTGCTTTGTTGCAAAATGCTTCACTATTTAATTCGCAAGACTTTGCAGCATCTTTGATATAACTCGC
AATTGAAATGAAAATATTACGCAGTACCTTTTCATTAATGACATTAGAAATGCTCAAGTTATCATTCAAGTCT
TTTAAAATGGTTACAGACAATGTGTAGTCTAGGAGGTCTTCTTTATCAGTGAAATGAGCGTAAAATGTTGCAC
GATTTAGCGTAGCAATATCAGTGATATCTTTAACTGTAATTTGAGATAATTTCTTTTCTCTAGAAATTTTAAG
AAAAGCATCGACAAGCAATTGTTTGGTTCTAACGATTCTTGGATCTATGGATTTACTTTCCATCATTTGTTCA
CTACCTAACATCTGTTGTTTTAACACTTATTATCAGTATCTTCTATTATATTGTCAATTGTGTTGATGATATG
TAAGTGCTGATACGCTCCTAACAAATAATGTACCTTTTTGGTAAGCATTTGAATGTCTTTCTTCTATTGTATT
AATGGATTTTAAAAATGAAGATAAAATAAAAGTGTATTGTAACAATATGTTTGAAAAATTGCCAATTTCTTTA
TCTAAAAATTCAGAATTTTGTGATATACTATTTTTCATAGTCTTGAGTTATAGATGATACTGCTAGCCTAAAT
ATAACAACATCGGCATGCACATCTTAGAATTTAAGCTAGTGGCAGCTTATAACTTAAGGCTCTTTATTATTTG
CATACAGATGTATATACATAAATGGAAGGATGATATTATGACATCAATTAAAACAATTACACCCAAAGATTTT
ATATTTAGAGTTTTATCAGGTGTCGCCATAGGTATTGTTGCGGGTTTAGTTCCTAATGCGATACTTGGAGAAA
TTTTTAAATACTTTATGGATTATCATCCGATTTTTAAAACGTTATTAGGCGTTGTCGTTGCAATTCAATTTAC
AGTACCTGCTTTGATTGGTGCATTGGTTGCCATGAAATTTGACCTATCACCATTAGCAATTGCTGTTGTGGCG
AGTGCAGCATATGTAGGAAGCGGTGCAGCGCAGTTTAAAAATGGTGCTTGGATGATTACGGGTATTGGTGATT
TAATTAATACAATGATTACAGCAGCGATTGCAGTGTTGTTCATTTTACTTATTCAACATCGCGTAGGTAGTAT
GGCATTGATTGTATTCCCAACAGTTGTTGGCGGTATTTCAGGTGCAATTGGTGTTTTAATATTGCCGTATACG
AAAATGATTACAACAGCCATCGGTAATATGGTTAATGGTTTTACAGAATTACAACCGATTGTCATGAGTATTT
TGATTTCAATGGTATTTAGTCTTATTATTATTTCTCCACTATCAACTGTGGCCATTGCCTTTGCGATAGGTAT
TACAGGTTTAGCAGCTGGATCAGCGTCAATTGGTATCTCCGCGACTGAAGCAGTATTGATAATTGGTACAAGT
AAAGTGAATCGTTTAGGGGTGCCGTTATCCGTCTTCTTTGGAGGCGTTAAAATGATGATACCTAATATGGTGA
AATATCCAATTCTTATGTTACCAATATTGACAACAGCCATTGTATCAGGTTTGGTATCAGCATTAGTAGGTAT
TCATGGAACGAAAGAATCAGCTGGATTTGGTTTTATAGGGATGGTAGGACCGATTAATGCATTTAAATTTATG
GAGGTTGATTCTGCTTGGTTGAGTGTCTTACTAATTGTAGTAGCGTTCTTTGTTGTACCATTTGTAACAGCTT
GGCTTGCAGATATCATTTATCGTAAAGTGTTTCGTTTATATACTAATGATATTTTTAAATTCATGGGATAAGG
ACAGTATGTTTTTTGAAAATATGAGACCGAAAATTATTTAATGTTATTGTGAATTGCGAGAATGAAGTTGAAC
AATATAATTGAACAGCAGATAATTTGCGCTAATAAAAGATAAGTCAATCCACAAAAGATATGTGCATAGTCAC
TTTGAATATTTAATATTAATAGCAATCAATACATGTGATGGAACCTATGTATTGATTGCTTTTTAATTGTGTT
TAACTGATCAAATTAAGTTGTATTTGATGACGGTAATGTTACAAGCATGTAACAAAAATACAATCTGTAATAT
TACGAAAGCTGTAATAGTTGAGCTAAATATAGTAAAAAACTTATGCATTTATGCTTATTTCAAGGATTGATTA
GATTCAAAAAGAAACATATTTCACATTACAGTATAAAACAACCATATTTCAACATTACAAATAACACTTGATA
TTGTAATGTTTTGTAAAGAAAGTGTAATTTACTTGCTACTTTTTTATGATATAGTGAGTACCGTTGAAAGAAA
TAAAAGACAATTTAAACTTGCACTACGACATTCAAATTCAAAATATAATTACTAAAAACATAAACTTACAATT
ATTACTGGAGGATTTTTTACTATGAAAAAGACAATTATGGCATCATCATTAGCAGTGGCATTAGGTGTAACAG
GTTACGCAGCAAGCACAGGACATGAAGCACACGCTGCTGAAGTAAACGTTGATCAAGCACACTTAGTTGACTT
AGCGCATAATCACCAAGATCAATTAAATGCAGCTCCAATCAAAGATGGTGCATATGACATCCACTTTGTAAAA
GATGGTTTCCAATATAACTTCACTTCAAATGGTACTACATGGTCATGGAGCTATGAAGCAGCTAATGGTCAAA
CTGCTGGTTTCTCAAACGTTGCAGGTGCAGACTACACTACTTCATACAACCAAGGTTCAAATGTACAATCAGT
AAGCTACAATGCACAATCAAGTAACTCAAACGTTGAAGCTGTTTCAGCTCCAACTTACCATAACTACAGCACT
TCAACTACTTCAAGTTCAGTGAGATTAAGCAATGGTAATACTGCAGGTGCTACTGGTTCATCAGCAGCTCAAA
TCATGGCTCAACGTACTGGTGTTTCAGCTTCTACATGGGCTGCAATCATCGCTCGTGAATCAAATGGTCAAGT
AAATGCTTACAACCCATCAGGTGCTTCAGGTTTATTCCAAACTATGCCAGGTTGGGGTCCAACAAACACTGTT
GACCAACAAATCAACGCAGCTGTTAAAGCATACAAAGCACAAGGTTTAGGTGCTTGGGGATTCTAATTCCTTC
ATAATAAATAAAAGTAATGTTTAAACATTAAATATATAGCTCTTCCCATAGAAATTGGGGAGAGTTTTTTTAT
TAATCAAAAATACATTTCTTAAAAAGTGGATATTGAAGTTAGGGAAATTTATTGGACTTTATTGTATTACTGC
TAAAATACTTGTCTTTATTAAGTGCCAAGTGGAATGAAACTTCATTTGAGGCTGTACTGAGTACACTAGATAC
AAACTAAACGCACATTTTATGATTAAATTAAATGAAACTGGAGATAAAAATGACTATAAACATAATTGAATAT
AATAGGTCTTATAAGGAAGAGCTAATCGAGTTTATTTTATCGATTCAAAAAAATGAATTTAATATAAAAATTG
ATAGAGATGATCAACCTGATTTGGAGAACATCGAACATAACTATTTGAATAGTGGAGGGCAATTTTGGCTTGC
AATAAACAATCATCAAAATATTGTTGGTACTATAGGTTTGATTAGACTAGATAATAATATGTCTGCACTTAAA

AAAATGTTTGTTGATGAAGGTTATAGGAATTTAAAAATAGGTAAAAAACTTTTAGATAAAGTTATAATGACAT
GTAAAGAACAAAATATCGATGGTATTTATTTAGGAACAATTGATAAATTTATTAGTGCACAGTATTTTTATTC
TAATAATGGCTTCCGTGAAATTAAGCGAGGAGATTTACCAAGTAGTTTTCCTAAATTAGATGTAGATAATAGG
TTTTATTACAGAAATTTAAAAGGTTAATTCGAATCAATGAATAATTGATATGGCGACCTTAGTAATATTCATT
TTAAAAGTGGCATATAGCGTTTAAATATAAAAGGCTGACGTGATATATCGTTTGATTTAAATTAGAGGATATT
ATGCATTTCACTTTATTTTCAATACATGGTAATTTATTCATTATAGAAGTTTTGAAAAAATGAAATTTAAAGG
GTATATTTAAATAATTGTGTAAAAGTAAGATGCATAAAATATAAAACGAACCTTTTTTTGTAAAAATATAATG
AAAACAACAGTTATTTTGTTAAAAATTCAAGAATCTATGATAATCTATGTTATGTAGTAAAATCGACAAATTT
ATATGTGGTTTGCAGTAATGAAATTAACTGTAATAATTGTTTTCTACTTAATAAAAAATTTAAAACTATCTTA
ATTAAGGGTTAAATCAATTAATGAAAATCAGTGCAATATTTTATTTTCAAAAATATTATGATATGCTACAAAA
TAGAGAAAAATAAATGGGGCGTTTATTAACATGGATACAAAAGACTTTAAACGTTTAGAAAAAATGTATTCCC
CGCGATACTTACCCGGATTAGATGGATTGAGGGCATTCGCAGTTATAGGAATCATTATTTATCACTTGAATGC
ACAATGGTTAAGTGGGGGCTTTTTAGGAGTAGATACATTCTTCGTTATTTCAGGTTATTTAATAACAAGTTTG
TTGATAAGTGAGTATTATCGGACGCAAAAAATCGATTTGCTAGAGTTTTGGAAGCGACGATTGAAACGACTCA
TTCCGGCAGTGTTGTTTTTAATCTGTGTCGTGCTTACGTTCACACTGATATTTAAACCGGAATTGATTATACA
AATGAAACGAGATGCTATTGCAGCTATATTCTATGTTTCAAACTGGTGGTACATTTCACAGAATGTAGATTAT
TTTAACCAATTTGCTATTGAACCACTCAAACATTTATGGTCTTTAGCCATTGAAGAACAATTTTACTTGCTTT
TCCCATTGGTTATCACGTTCTTATTACATAGATTTAAACCGAGAAATATTATTCAAACGCTATTTATTGTATC
GTTGATTTCTTTAGGACTTATGATAGTGATTCATTTCATCACTGGAGATAATTCACGTGTGTATTTTGGGACA
GATACACGACTGCAAACTTTATTGCTTGGTTGTATATTAGCATTTATTTGGCCTCCGTTTGCTTTGAAAAAAG
ATATTTCTAAAAAGATTGTCGTATCATTAGATATTATAGGGATATCTGGTTTTGCGGTTCTAATGACTTTGTT
CTTTATAGTTGGAGACCAAGATCAATGGATCTATAATGGAGGATTTTACATTATATCATTTGCAACTTTATTC
ATTATTGCAATTGCGGTACATCCTTCTAGTTTATTTGCTAAATTTTTAAGTATGAAACCTTTGCTAATTATAG
GTAAACGATCATATAGCTTATATTTATGGCATTATCCTATCATTGTTTTTGTGAACAGTTATTACGTACAAGG
ACAAATACCTGTATACGTCTATGTGATAGAAATTGTATTAACAGCGTTAATGGCTGAAATTTCGTATCGCTTT
ATTGAAACACCTATACGTAAAAAAGGATTTAAAGCTTTTGCATTTTTACCTAAAAAGAAGGGGCAATTTGCTA
GAACAGTGTTAGTTATCCTATTATTGGTTCCGTCTATCGTTGTGCTCAGTGGACAGTTTGATGCACTTGGCAA
ACAACATGAAGCCGAGAAGAAAGAGAAGAAGACGGAATTTAAAACAACGAAGAAAAAAGTCGTTAAAAAAGAT
AAGCAAGAGGATAAGCAGACAGCGAATAGCAAAGAGGATATTAAAAAGTCATCACCACTATTAATTGGTGACT
CGGTCATGGTGGATATTGGTAATGTCTTTACTAAGAAAATACCAAATGCACAAATTGATGGTAAAGTTGGACG
GCAACTCGTTGATGCTACACCAATTGTGAAATCGCAATATAAAGACTATGCTAAAAAAGGTCAAAAAGTTGTA
GTAGAGCTTGGTACAAATGGGGCATTTACGAAAGATCAATTAAATGAACTATTGGATAGTTTTGGAAAAGCAG
ACATTTATTTAGTTTCTATTAGAGTACCTAGAGATTATGAAGGTAGAATAAATAAATTAATTTATGAGGCAGC
TGAAAAGCGCTCTAATGTACATCTAGTCGATTGGTATAAAGCTTCTGCAGGTCATCCGGAATACTTTGCATAT
GACGGCATACATTTGGAATATGCAGGTAGTAAAGCGCTGACTGATTTGATTGTAAAAACGATGGAAACACATG
CTACAAATAAAAAATAACTTGATGCACTAAACTTTTGAAATATTACATTACTTCTGATATTTATTGTCAGTTC
GTGTGTATTTCATTAAAAGTTTAGTGCTTTTTTATTTTCAAATCCCATGTTAACGGTGCAGAAAAAGTGTTGT
AAACATTCTAATTGGTATATTACATTCAATGAAGCTTTATTAGGAACAGATTACATTATGATAACAAAGCCCG
CAAGACACCTAATCTCTGTTATAGTTTATTTTGTCGCAAAACTATAAAAGTTATAATTATTTGCATACTAAAA
AAATAAAAAATATAAAATTTAAAATAATTGAGTCGCTAATGACTATATGTATAGAGTGTTTTGATTATTGGGA
GGATATTTAATTATGAAAAAAATCGTTACAGCTACAATCGCTACAGCAGGACTTGCCACTATCGCATTTGCAG
GACATGATGCACAAGCCGCAGAACAAAATAACAATGGATATAATTCTAATGACGCTCAATCATACAGCTATAC
GTATACAATTGATGCACAAGGTAATTATCATTACACTTGGACAGGAAATTGGAATCCAAGTCAATTAACGCAA
AACAACACATACTATTACAATAACTACAATACGTATAGTTATAACAATGCATCTTACAATAACTACTATAATC
ATTCATATCAATACAATAACTATACAAACAATAGCCAAACAGCAACAAATAACTATTATACTGGTGGTTCAGG
TGCAAGTTATAGCACAACAAGTAATAATGTTCATGTGACTACAACTGCAGCGCCATCTTCAAATGGTCGTTCA
ATTTCTAATGGTTATGCATCAGGAAGTAACTTATATACTTCAGGACAATGTACTTATTATGTATTTGATCGTG
TTGGTGGGAAAATTGGTTCAACATGGGGTAACGCAAGTAATTGGGCTAGCGCAGCTGCATCATCTGGCTATAC
AGTGAACAATACACCAAAAGCTGGTGCTATCATGCAAACAACACAAGGCTATTACGGTCATGTTGCTTACGTT
GAAGGCGTTAACAGCAACGGTTCTGTTCGTGTTTCAGAAATGAACTATGGACATGGTGCTGGTGTGGTTACGT
CTCGTACAATTTCAGCAAACCAAGCAGGTTCATATAATTTCATTCATTAATCAAATGTAAATCAAATGACGTC
AATATTCTCTAACATGAGAGTATTGGCGTTTTTGTTTTATATAAATATAAATGAGAGCGGTTTATTCACTAAT
CTTTAGGGAACTAAGTAATAAAATGATAATTTATACTATGTCAGTATGATTGAGAGTGATTCAATTTAGATGA
AAACCATGAAAAAATATATCAAAACAGCATTTTTTTGTAGTATGTATTGGTTAATTGTTCAACTAAATATAGC
AAATTTAGGTACAAGAATTCCTGACAAGTATTTTCGTCAGAAGCATATAATATTTAAATCATTTAACTTTGAG
AAGCATGGAAAATTTTGGAACAAATGGTTTTACGTAAGAAAATGGAAACATAAGATTTTAGATGGTCATCAGC
TTAATCAAAATATATATGATCAGCGTCATTTAATGACAATCAATACTGATGAAATTGAAAAAATGATTATAGA
GACAAAGAGGGCAGAGTTGATCCATTGGATATCGATACTTCCAGTCATCATATTTAATAAAGGCCCTCGTTTA

GTAAAGTATATAAATATTTTCTATGCAATGATAACTAATGTTCCAATCATTATTGTGCAACGTTATAATCGAC
CGAGATTAACGCAGTTACTACGCATATTAAAACGAAGAGGTGAACGTCATGACTAAACATATCATCGTTATTG
GTGGTGGCTTAGGTGGGATTTCTGCAGCAATTCGAATGGCGCAAAGTGGCTATTCGGTCTCATTATATGAACA
AAATAATCATATAGGAGGCAAAGTGAATCGTCATGAATCAGATGGCTTTGGCTTTGATTTAGGTCCATCTATT
TTAACGATGCCTTATATTTTTGAAAAAATATTCGAATATAGCAATAAGCAAATGTCAGACTACGTTACAATCA
AGCGATTGCCACATCAATGGCGTAGCTTTTTTCCAGATGGAACGACTATCGATTTGTATGAAGGTATTAAAGA
AACAGGTCAGCATAATGTGATATTGTCGAAACAGGATATAGAGGAACTGCAAAATTATTTGAATTATACAAGA
CGAATCGATCGTATTACTGAAAAAGGGTATTTTAACTATGGTTTAGATACACTATCTCAAATTATTAAATTTC
ATGGGCCATTAAATGCTCTTATTAATTATGATTATGTACATACTATGCAACAGGCCATAGACAAGCGTATCTC
GAATCCATACTTGCGACAAATGTTAGGCTATTTTATCAAATATGTAGGTTCTTCATCATACGATGCGCCAGCT
GTATTATCTATGTTATTCCATATGCAACAAGAGCAAGGCCTTTGGTATGTAGAAGGTGGAATCCATCATTTAG
CCAATGCCTTGGAAAAGCTAGCGCGTGAAGAAGGTGTCACAATTCATACAGGTGCACGTGTGGACAATATTAA
AACATATCAAAGACGTGTGACGGGTGTCAGATTAGATACAGGTGAGTTTGTAAAGGCAGATTATATTATTTCA
AATATGGAAGTCATACCTACTTATAAATATTTAATTCACCTTGATACTCAACGATTAAACAAATTAGAGAGGG
AATTTGAGCCGGCAAGCTCAGGATATGTGATGCATTTAGGTGTTGCTTGCCAATACCCGCAATTAGCACATCA
TAATTTCTTTTTTACGGAAAATGCTTATCTCAATTATCAACAAGTTTTTCATGAAAAGGTATTGCCAGATGAT
CCGACCATTTATCTAGTAAATACGAATAAAACTGATCACACACAAGCGCCAGTAGGTTATGAAAATATCAAAG
TCTTACCACATATTCCATATATTCAAGATCAGCCTTTTACCACTGAAGATTATGCGAAGTTTAGGGATAAAAT
TTTGGATAAATTAGAAAAAATGGGACTTACTGATTTAAGAAAACACATTATTTATGAAGATGTTTGGACACCG
GAGGATATTGAAAAAAATTATCGTTCTAATCGTGGTGCAATATATGGTGTTGTAGCAGATAAAAAGAAAAACA
AAGGATTTAAATTTCCTAAAGAAAGTCAGTATTTTGAAAACTTGTACTTTGTAGGTGGATCAGTAAATCCTGG
TGGTGGCATGCCAATGGTTACATTAAGTGGGCAACAAGTCGCAGACAAAATAAACGCGCGAGAAGCGAAGAAT
AGGAAGTGATATCTATGAAATGGTTATCACGAATATTAACAGTAATAGTGACCATGTCTATGGCGTGTGGTGC
ATTGATATTTAATCGTAGACATCAGCTAAAGGCGAAAACGCTGAACTTCAATCATAAATCATTAACAATTATT
ATTCCGGCTAGAAACGAAGAAAAAAGAATAGGTCATTTACTACATTCGATAATACAACAGCAAGTTCCAGTAG
ATGTCATTGTTATGAATGACGGATCGACAGATGAAACAGCACGTGTAGCGCGTTCATATGGTGCGACTGTAGT
AGATGTTGTTGATGATGCTGACGGCAAATGGTATGGGAAATCACATGCTTGTTATCAAGGTGTGACGCATGCA
TGTACGAATCGTATTGCTTTTGTAGATGCTGATGTAACTTTCTTAAGGAAAGATGCTGTTGAAACGTTGATTA
ATCAGTATCAATTACAAGATGAAAAAGGATTGTTAAGCGTACAGCCTTATCACATAACAAAGCGTTTCTACGA
AGGGTTTTCAGCGATATTTAATTTAATGACAGTCGTTGGTATGAATGTATTTTCTACCTTAGACGACGGTCGG
ACTAACCAGCATGCATTTGGACCGGTGACATTAACAAATAAAGAAGATTATTATGCAACTGGAGGTCATAAAA
GTGCGAACCGTCATATTATTGAAGGATTTGCTTTAGGAAGTGCATATACTTCACAATCATTGCCCGTAACAGT
TTATGAAGGATTTCCATTTGTTGCATTTCGCATGTATCAAGAAGGATTTCAGTCATTACAAGAAGGATGGACA
AAGCATTTGTCGACTGGGGCAGATGGCACAAAGCCTAAGATCATGGCAGCAATTGTGTTGTGGTTGTTCGGTT
CTATCGCGAGTATTTTAGGGCTATGTCTTAGTTTAAAATATCGCCAAATGTCTGTAGGAAAAATGTTAACAGT
TTACTTGAGCTATACTACACAATTTATTCATCTGCATCGAAGGGTCGGCCAATTTTCTAATTTATTAATGGTA
TGTCATCCATTGTTATTTATGTTTTTCACTAAAATTTTCATCCAATCTTGGAAACAAACGCATCGTTATGGTG
TAGTTGAATGGAAAGGTCGTCAATATTCTATATCTAAAGAACAATAAATCAAGGTAATGGCATTTCAATATAG
GAGGACTAGTATGACAATGATGGCTATGAATTTTAAATATTGTCATAAAATCATGAAGAAACATTCAAAAAGC
TTTTCTTACGCTTTTGACTTGTTACCAGAAGATCAAAGAAAAGCGGTTTGGGCAATTTATGCTGTGTGTCGTA
AAATTGATGACAGTATAGATGTTTATGGCGATATTCAATTTTTAAATCAAATAAAAGAAGATATACAATCTAT
TGAAAAATACCCATATGAACATCATCACTTTCAAAGTGATCGTAGAATCATGATGGCTCTTCAGCATGTTGCA
CAACATAAAAATATCGCCTTTCAATCTTTCTATAATCTCATTGATACTGTATATAAAGATCAACAATTTACAA
TGTTTGAAACGGACGCTGAATTATTCGGATATTGTTATGGTGTTGCTGGTACAGTAGGTGAAGTATTGACGCC
GATTTTAAGTGATCATGAAACACATCAGACATACGATGTCGCAAGAAGGCTTGGTGAATCGTTGCAATTGATT
AATATATTAAGAGATGTCGGTGAAGATTTTGAAAATGAACGGATATATTTTAGTAAGCAACGATTAAAGCAAT
ATGAAGTTGATATTGCTGAAGTGTACCAAAATGGTGTTAATAATCATTATATTGACTTATGGGAATATTATGC
AGCTATCGCAGAAAAAGATTTTCGAGATGTTATGGATCAAATCAAAGTATTTAGTATTGAAGCACAACCAATC
ATAGAATTAGCAGCACGTATATATATTGAAATACTGGACGAAGTGAGACAGGCTAACTATACATTACATGAAC
GTGTTTTTGTGGATAAGCGGAAAAAAGCAAAGTTGTTTCATGAAATAAATAGTAAATATCATAGAATATAGGT
GGTTGAATAATGAAGATTGCAGTAATTGGTGCAGGTGTCACAGGATTAGCAGCGGCAGCCCGTATTGCTTCTC
AAGGTCATGAAGTGACGATATTTGAAAAAAATACTAACGTAGGCGGGCGTATGAATCAATTAAAGAAAGACGG
CTTTACATTTGATATGGGCCCCACAATTGTCATGATGCCAGATGTTTATAAAGATGTATTTACAATGTGTGGT
AAAAATTATGAAGATTATATTGAATTGAGACAATTACGTTATATTTACGATGTGTATTTTGACCGCGATGATC
GTATAACGGTGCCTACAGATTTAGCTGAATTACAGCACATGCTAGAAAGTATAGAACCTGGTTCAACGCATGG
TTTTATGTCATTTTTAACGGATGTTTATAAAAAATATGAAATTGCACGTCGCTATTTCTTAGAAAGAACGTAT
CGCAAACCAAGTGACTTTTATAATATGACGTCACTTGTGCAAGGTGCTAAGTTAAAAACGTTAAATCATGCAG
ATCAGCTAATTGAACATTATATTGATAACGAAAAGATACAAAAGCTTTTAGCGTTTCAAACGTTATACATAGG

AATTGATCCAAAACGAGGCCCGTCACTATATTCAATTATTCCTATGATTGAAATGATGTTTGGTGTGCATTTT
ATTAAAGGCGGTATGTATGGCATGGCTCAAGGGCTAGCGCAATTAAATAAAGATTTAGGTGTTAATATTGAAC
TTAATGCTGAAATTGAGCAAATTATTATTGATCCTAAATTCAAACGAGCTGATGCGATAAAAGTGAATGGTGA
CATAAGAAAATTTGATAAAATTTTATGTACGGCTGATTTCCCTAGTGTTGCGGAATCATTAATGCCAGATTTT
GCACCTATTAAAAAGTATCCACCACATAAAATTGCAGACTTAGATTACTCTTGTTCAGCATTTTTAATGTATA
TCGGTATAGATATTGATGTGACAGATCAAGTGAGACTTCATAATGTTATTTTTTCAGATGATTTTAGAGGCAA
TATTGAAGAAATATTTGAGGGACGTTTATCATATGATCCTTCTATTTATGTGTATGTTCCAGCGGTCGCTGAT
AAATCACTTGCGCCAGAAGGCAAAACTGGTATTTATGTGTTAATGCCGACGCCGGAACTTAAAACAGGTAGCG
GAATCGATTGGTCAGATGAAGCTTTGACGCAACAAATAAAGGAAATTATTTATCGTAAATTAGCAGCGATTGA
AGTATTTGAAGATATAAAATCGCATATTGTTTCAGAAACAATCTTTACGCCAAATGATTTTGAGCAAACGTAT
CATGCGAAATTTGGTTCGGCATTCGGTTTAATGCCAACTTTAGCGCAAAGTAATTATTATCGTCCACAAAATG
TATCGAGAGATTATAAAGATTTATATTTTGCAGGTGCAAGTACGCATCCAGGTGCAGGCGTTCCTATTGTCTT
AACGAGTGCGAAAATAACTGTAGACGAAATGATTAAAGATATTGAGCGTGGCGTGTAAGGGAGTAGTCTAAGA
GAAAGATGTGAGAAAAGTATAAGGGGAACGTAAAAGGCATAGTGAAATAAGTATGTGCTAAAGAAGTTTTATT
TAACAATGAATGACGCCTGTTAAGGTTCACGTTTGAATACGTACCTTGATGGGCGTTATACTTTATATAAGCA
TATCTTACTGGTTGCGCAAGCGGATATGCATGACAGTTAAATCATAACGATTGAGCGTATATTGATCTGAAAA
TTCCTAATATTGACAATAATAATGTACCTACCTAAAATGAAAATAAATTCATTCAATACAACGTGTGTCATAA
GTTCTGTTGAACATAACGGTGAATGGCGATGTCATTGTAACCATCTAAATGAGAGCGATACAAGTTGTTAGGA
TTATGATAGTCGCCCAGGCTATGTTTAATGATAGAGGAGGAATTTGATGATCTCTAACAAATTAGCAAATATC
CCTGATAGTTATTTCGGGAAAACGATGGGACGTAAAATCGAACATGGACCATTGCCATTAATTAATATGGCAG
TTGGGATCCCTGATGGGCCCACACCACAAGGTATTATTGATCATTTTCAACAAGCACTAACTATTCCTGAAAA
TCAAAAATATGGTGCTTTTCATGGTAAAGAAGCGTTCAAGCAAGCTATTGTAGATTTTTATCAAAGACAATAT
AATGTGACGTTAGATAAAGAAGATGAAGTATGCATTTTATATGGTACAAAAAATGGATTAGTGGCAGTACCAA
CATGTGTTATCAATCCAGGAGACTATGTATTACTACCTGATCCAGGCTACACGGATTATTTAGCAGGTGTACT
TTTAGCTGATGGCAAGCCTGTCCCACTTAATTTAGAACCGCCACATTATTTGCCAGATTGGTCCAAAGTTGAT
TCACAAATAATAGATAAAACAAAACTCATTTATTTAACGTATCCAAATAATCCAACTGGATCGACAGCTACGA
AAGAAGTTTTTGATGAAGCGATAGCTAAATTTAAAGATACAGACACTAAAATCGTACATGATTTTGCTTATGG
TGCTTTCGGTTTCGACGCTAAAAATCCTAGTATACTTGCTTCGGAAAATGGGAAAGATGTTGCGATTGAAATT
TATTCCTTGTCTAAAGGTTATAACATGTCAGGTTTTAGAGTCGGTTTTGCAGTTGGTAATAAAGATATGATTC
AAGCTTTGAAAAAGTACCAAACGCACACAAATGCTGGGATGTTTGGTGCATTACAAGATGCCGCAATATATGC
TTTGAATCATTATGATGATTTTTTAGAAGAACAAAGCAATATATTTAAAGCACGTCGTGACCGATTCGAAGCC
ATGCTTGCAAAAGCTGATTTGCCATTTGTTCATGCTAAAGGTGGCATATATGTATGGTTGGAAACACCGCCTG
GTTATGACAGCGAGCAATTTGAGCAATTTTTAGTGAAAGAGAAGTCAATACTTGTTGCTCCAGGGAAACCATT
TGGAGAGAATGGTAATCGTTATGTGAGAATTTCATTGGCGTTAGATGACCAGAAATTAGACGAAGCTGCAATA
AGATTAACAGAACTAGCATATTTATATGAATAATAAAGTTAAGGGTGATGTTAAAATGACAAAAATTAAAATC
ATGAGTGTACGTGATGAAGATATGCCTTATATCAAGGCGTGGGCAGAGAAACATCATGTTGAGGTAGATATTA
CTAAAGAAGCATTAACTGATGACAATGTTGAAGGTGTCGCTGGTTATGATGGCTTATCGCTATCACAACAAAT
ACCATTATCAGAGCATGTTTATAAACGATTAAATGAACTAGGTATTAAACAAATCGCACAACGAAGTGCTGGA
TTTGATACATATGATTTGGAGTTAGCAAACAAGTATAACTTGATTGTGTCAAATGTGCCGTCATACTCTCCGA
ATTCAATTGCGGAGTTTGCTGTGAACCAGGCTATTAATGTGGTTCGTCATTTTAATCAAATTCAAACGAAAGT
GCGAGAGCATGATTTTAGATGGGAACCTACTATTTTATCGAAATCCATTAAAGATTTAAAAGTAGCTGTTATT
GGTACAGGTCGAATTGGCCGTGTAGTAGCCGATATATTTGCCAATGGTTATCAAAGTGATGTGGTCGCATACG
ACCCGTTTCCTAATGCTAAAATTGCAACGTATGTCGATTACAAAGATACGATTGAGGAAGCGGTTGAAGGTGC
TGATATCGTGACATTACATGTACCTGCAACGAAATATAATCATTATCTATTTAATGCTGAATTATTTAAACAT
TTTAAAAAGGGCGCAGTATTTGTCAATTGTGCGAGAGGTTCTTTAGTAGATACTAAGGCGTTATTAGAAGCAT
TAGACAATGGTGTGATTAAAGGTGCAGCACTTGATACGTATGAATTTGAACGCAAACTTTTCCCAAGTGATCA
AAGGGGCAAAACACTGAACGATCCATTGTTAGAATCGTTGATTGACAGGGAAGATGTCATATTAACACCACAT
ATTGCGTTTTATACTGAAGCTGCAGTTGAAAATCTAATTGTCGATGCATTAGATGCAACATTAGATGTATTGC
AGACTGGAGATACCAGGTTGCGAGTAAATTAAAAATCGAACTGATGAGATAATTTGGATTGTTGGGATTCTCA
TCAGTTCGATTTTTTAATTTGGTGTTGATGACGTTGAATATTGCCTAATTAAACGACATCGTAACCTTGATCT
TCAATTGCGTCTTTCATTTGAGATACAGCAACTTTACTGTCATCATATTGAACACTTACTTGACCATTTTCAA
GGTTAACGTCAGCTGAAGTGACACCGTCAATATTATTTAATGCAGATTCAACAGCACTTTTGCAGTGACCACA
GCTCATACCTTCAACATTTAAAATTTCTTGTGACATAGCATTCACCTCCTTTTAGAGCCACTTATTATTTATA
ATAATTAGTGGCTCTTATGCAGTTGGAGCGAAGATCCAACTGTAAACCATAGTGTACTTATTATTTATAATAA
TGTTGGAACCTAATAGGCGGAGCTATTAGGTAAGACACACTAATTCGCCAAGCGAAAAGTGTAACTATATATG
CAGTAGGAGCGAAGATCCAACTGTAAACCGCGACATTATAGAGCCAATCACTAGTCTACCTCAGAATCACCAA
CGAATCCTTCATTATTAAGGAATCTAGGCATCTTTACGGCGTGGTTCTAATCGCATCTTTTTCAATCTAAGTG
CGTTTGTGACAACACTTACTGAACTTAGTGCCATTGCAGCACCAGCAACCCATGGCGCAAGTAAGCCCAATGC

AGCTATAGGGATACCGGCAATATTATAGCCGAATGCCCAAAATAGATTTTGACGAATATTACGAATGGTTGCT
TTACTTGCATAAATGGCTTTAGGAATAAGCATCAAGTCGCCACCAAGAATAGTAATATCAGCCGCTTCAATGG
CAACTTCTGTACCTGTACCAATGGCGATACCGATATCAGCTTTAACTAATGCAGGTGCATCATTTACACCGTC
ACCAACCATTGCAACCTTCTTACCTTGTTGCTGTAGTTTCGCAATTTGTGCAGCTTTTTCTTCTGGTAAAATA
TCTGCAATAACAGTATCTATGCCTACTTGTTTTGCAATGGCTTGAGCAGTGTTTTTATTATCGCCAGTTAACA
TGGCAACTTCAATGCCCATATCATGCAATTGTTTTATAGCATCTTTGGCATGATCTTTGACAGTATCTGCCAC
TGCGATGATACCAGTTAATGAATAATTAACAGCAATGAGCATAGCTGTTTTGCCATCTCGTTCATAATGTGTT
AAATCATCAGAAATATGCTTAGGCAAGCTAATATCATTGTCAGCCATTAATTTACGGTTACCAACCAATATAT
GGTGATGATCAATCGTTGCTTCAATACCATGGCCAGGTACTGCTTTAAATGTTGTTGTCTCAGTTAATGTTAA
TTGCTTTTCTTTTGCATAATTGACAATGGCTTCTGCCAATGGGTGTTCAGAATCTTTTTCAGCAGTAGCGAGT
AGTTGTAGCGTTTGATTGTCACCATGATAATCTGTCACGACTGGACGACCATTTGTAATGGTACCCGTCTTAT
CTAAAACGATGGTATCAATTAGATGTGTGCGTTCAACAAACTCGCCACCTTTAAATAAAATACCATTTTCAGC
AGCGCGACCAGTACCTACCATAATAGAAGTTGGTGTAGCAAGTCCTAATGCGCATGGGCAAGCAATGACGAGA
ACGGAAATACTCGCAACAAGTGCAGGTTCAAATGTACCTGGTGTAACTAAAGTAATCCACACGATAAATGTTA
ATAGTGCGATACCAACAACGATAGGAACGAAATAACCAGAAATAATATCTGCCAATCGTTGAATCGGCGCTTT
AGAACTTTGAGCTTCTTCGACAACTTTAATAATATTTGCCAACGCAGTGTCCCCGCCAACTTTTGTTGCTGTC
ATAGTAATAGTACCGTTTTTGTTCATCGTTGAACCAATTACAGTATCATCAACATTCTTCTCAACAGGTATAG
ATTCACCTGTTAACATAGATTCGTCGATGGCAGTCATACCTTTAATAATTTTACCATCAACAGGTATCTTTTC
ACCTGGTTTAACGATAAGTGTATCTCCAACATGTACTTCATTTAGAGGAATCATCACTTCATTACCATCTTTT
AAAATGCGTGCTTCTTTAGCTTGCAAACTTAATAATTCGCCAAGCGCATTGGTTGTTTGAGACTTCGCTCTAG
CTTCTAAATACTTACCGAATAAGATTAAGGTAATTAGTACGGCGCTTGTTTCAAAGTATAAATGCGGTTGCGT
TGTTGAGCCATTTAGCCAACGAACCATTTCATAAATACTGTAAAAATATGCTGCACTTGTACCAACAGCAACA
AGTACATCCATATTGGCGCCACCATTTCTTAAGTTTTTATAAGCACCTACATAAAATTGCCATCCAATAATAA
ATTGTACAGGTGTAGCTAAAATAAATTGGAACCATGGATTCGTAAATAGTGCTGGTATATGCATATTGAAAAG
ATGCACAAACATTAACATTAATAGTGGTAAAGATAATATTGCTGATATGATAAGCTTAATCAATTTATGTTGT
AGCGCTTCAGCTTTGCGTGACGTTTGATCTTTATTGTTATCTTTAATAGACGCGTCATAACCTAATTTTTGAA
TGCGAGTGACAAGTTTATCAGCATCTGTTTCTTCAGGATAATAGTCAACTTTAGCTTGCTCTGTTGTTAAATT
GACAGTTGCATTTTGAACACCGTCCATTTTATTTAACACTTTTTCAATACGGCTTGAGCATGCAGCACAAGTC
ATACCTGTAATGTCTAATTCGACAGTTTCTACAGCGACACCGTAACCTAAATGTTGAATCGTATTAATAAATT
CTTGGACATCATGTTGATCAGGGTTATACTCAACAGTTGCTTTCTCTGTAGTTAAATTCACTTGGGCATTAAC
GTCATCAAGTTTATTCAGTTTCTTTTCGATACGATTTGAACATGCGGCACAAGTCATACCAGTGATATCTAAT
GTTGTTTTTTTCGTATTAGCCACATTATTGCCTCCCTGTGATTCATTGTTACACGTCTAATGTACCCCCTATA
GGTATATAAGTCAATCGTTGCGTTTTACTTTTACCAATTTTAAAAAGAAAAAGAGAGATTGCCGTGCAAACAT
TGCACAACAATCTCTCTATTTAGCATCTATATAAAAGAAGTCGTAATTAATATTAAAGGATGTATGAATGAAA
GGTAAAACTTAAATGACTTCTGGATTTGCAATTCTTAGGTGACTTAAAAGAAATCAGATGGGTTAAATTCTTT
CGCATGTAAATTTTTAAAATTATTAAGATGGATGACTTTATCATCATCTTTGTTGTCTGACATCTGACGACTT
GTGTCGTTTGTCATTTCGTAATCTTGTATCGTTTTAACTAAACCTAGAATGATTAAGATAGCAACTGTGCCTG
TAATTAATATTCTCATCGCTATCATACTCCTTTATATTTCTCTTACTCACATTGTACGTTAATAGATTAAAAA
ATGAATGCGTCTGTAGTATTGAATTTATCTACTACTTTAGCAGTATTTGATGCGAGAATTGTATAGATAAGGG
TGATAAGGTCACGATTTCATAATGTTATAGCGTATTAGTTGCATAGGCGAATGTTTATTTGATTAACTTTTAA
ACAAGTAAATAAGTTATTAGGAGTTGAAAGGTGTAATGACTGAAAAAGAAAAAATGTTAGCAGAAAAAATGGTA
CGATGCAAACTTTGATCAAGACTTAATCAATGAACGTGCACGAGCGAAAGATATTTGCTTTGAATTAAATCAT
ACAAGACCGAGTGACACAATTAAAAGAAAGGAATTAATCGATCAATTATTTCAAACAACAACAGATAATGTAA
GTATTTCGATTCCTTTTGATACAGATTATGGTTGGAACGTTAAACTAGGAAAAAATGTCTATGTAAACACCAA
TTGTTATTTTATGGATGGTGGACAGATTACAATTGGCGATAATGTTTTTATTGGACCTAATTGTGGATTCTAC
ACAGCGACACATCCACTTAATTTTCATCATAGAAATGAAGGTTTTGAAAAAGCCGGACCCATTCATATTGGCA
GTAATACTTGGTTTGGCGGTCATGTAGCCGTGCTACCAGGGGTGACGATTGGAGAAGGTAGTGTGATTGGTGC
TGGTAGTGTTGTCACTAAAGATATTCCGCCACACAGCTTAGCGGTTGGAAACCCTTGTAAAGTCGTTCGTAAA
ATTGATAATGACCTACCATCCGAAACATTAAATGACGAAACGATAAAGTAGTAGTTGCGACACATATAAATTT
TAAAAAGTAACCAATCAATTTCAAAATGCTTGAGAATGAGGGATAAAGCATAAAAATGAAAGCGATTGCAAAA
CTGACCTTAATCATAGTAAAATGAGTTTAAATGATGTTATATCATTAAATCAAAGGGTTAAACCACTTTCAAA
ATTTATAGGGGGTAAGTGTACATGGTAGTAGAATTTAAAAATGAACCTGGTTACGATTTTTCAGTCCAAGAAA
ATGTGGATATGTTTAAGAAAGCATTGAAAGACGTGGAAAAAGAATTAGGTCAAGACATACCATTAGTGATTAA
TGGAGAGAAGATTTTCAAAGATGACAAAATTAAATCAATCAATCCTGCAGACACATCACAAGTAATTGCAAAT
GCATCAAAGGCAACAAAACAAGATGTTGAAGATGCATTTAAAGCAGCAAACGAAGCTTATAAATCTTGGAAAA
CATGGTCAGCAAATGATCGTGCAGAGCTTATGTTACGTGTTTCGGCAATTATTCGTCGTCGAAAAGCGGAAAT
TGCAGCAATTATGGTTTATGAAGCAGGTAAGCCATGGGATGAAGCAGTTGGTGATGCAGCTGAAGGTATTGAT
TTTATTGAATACTATGCACGTTCAATGATGGACTTGGCTCAAGGCAAGCCAGTACTTGATCGTGAAGGTGAAC

ACAATAAATATTTCTATAAATCAATTGGTACAGGTGTTACAATTCCGCCGTGGAACTTCCCGTTTGCGATTAT
GGCTGGTACAACTTTAGCACCAGTTGTGGCAGGTAATACAGTGTTATTGAAACCTGCTGAGGATACACCTTAT
ATCGCTTATAAATTAATGGAAATTTTAGAAGAAGCAGGATTACCAAAAGGCGTAGTTAACTTTGTTCCTGGTG
ATCCGAAAGAAATTGGTGATTACTTAGTTGATCATAAAGATACGCATTTCGTTACATTTACAGGATCACGTGC
GACAGGTACTAGAATTTATGAACGTAGTGCCGTAGTTCAAGAAGGTCAAAACTTCTTGAAACGTGTTATCGCT
GAAATGGGTGGTAAAGATGCCATTGTAGTTGATGAAAATATTGATACAGATATGGCGGCAGAAGCTATTGTAA
CGTCAGCGTTTGGTTTCTCTGGTCAAAAATGTTCTGCATGTTCACGTGCAATTGTGCATAAAGACGTTTATGA
CGAAGTATTAGAGAAATCAATCAAATTAACAAAAGAATTAACGTTGGGCAATACTGTAGACAATACGTATATG
GGCCCAGTTATTAATAAAAAACAATTTGATAAGATTAAAAATTATATTGAGATTGGTAAAGAAGAAGGTAAAT
TAGAACAAGGTGGCGGCACTGATGATTCAAAAGGTTACTTTGTAGAACCAACAATCATTTCAGGACTTAAATC
TAAAGATAGAATCATGCAAGAAGAAATTTTTGGTCCAGTTGTTGGCTTTGTGAAAGTTAACGATTTTGATGAA
GCAATTGAGGTTGCAAATGATACAGATTACGGTTTAACTGGTGCAGTGATTACAAATAATCGCGAGCATTGGA
TTAAAGCGGTAAACGAATTTGATGTAGGTAATTTATACCTTAACAGAGGCTGTACTTCAGCAGTTGTTGGATA
CCATCCATTTGGTGGTTTCAAAATGTCAGGTACTGACGCTAAAACAGGTAGCCCAGACTACTTATTACACTTC
TTAGAACAAAAGGTTGTTTCAGAAATGTTCTAATGCTTCATAATGAACAAGGGCCATCCATTGCGGTTGGCTC
TTTTTATTTATAACTTTAAAAATTAAGTAATAGCAACCATATTTTTAGAAAAGGATGATAGGTATAAGAATAT
GAGATACGTTATTTCAATAATCATGGCAATTGTATTAGCAATTTGGTCATTGAAACAACTGAGTCAAAGTCAT
TTAGATAGCGGATTTATTTTTTTCTTCATAGTTTATGTGTTGTGTATCAGTTATTTTAATAGCGATAAGCACG
ATAAAAATAAAAAACGATAGTAGTACACGAATGCATTAATGTGTAAACTATCGTTTTTATTATGAATGCTGGT
GACGCTTATGAGCCTCTCATCTACTATTTAATCTTAATATTTATTATCTTATGGAAAATTTTATCAATTTCTT
CACCAGGCCAGTCAATTTTTTCATCTTTTATCCAATGCATAAAACCTTCAATCAACGAGTCATAAATATAAAA
TATGAGATTATCTGGGATGTGGCTGTCAATATCGATACGGTCTCTATTATTTTTGATATCGTTATGCATAATT
TTAATGCAGACATTTTTTAATACTTTATTAAATTCAGCGTCTTCTTCTTGAAATTCTGCAATATCTCGCAAAT
CTTCTTTATTGTTAATTGTATCGCTAATCGTTTGAAATGGGTGGTTAAGACGATCGCTAATGTCTCTGGCGAA
ATAGTCTTTAGTCAATTGATTAAATAAATATTCAAGAAGATCATATTTATCATAAAAATGTTTATAAAATGTT
GTGCGGTGTACGAGTGCGTTGTCGCAAATTTGATTGACAGTAATCGTTTGGAATAATTGCTGTTCTAACAATT
GCAGCAAGCTACTTGACAATGCTTTTTTTGTCTTTATAACTCGTAAATCAGTCTCTTTCATCTACATTTCTCC
TTTGTGTGTAGTTTAATATACAAAATTTTCCAATTGATTCTTTAAGAGGTCGGGGATAGATACTATAATCAAA
CAATAATCTACACTGTATAGTTAAACTATAACGTTGATTTTAAAAGGAGGCAATATACTTTGGCAAAATTTTT
ATATAAGATGGGGACATTCATCGCAAAACATAAATGGTCCGCGGTCATTGCATGGATAGTGATTGTAGCTGCA
ATATTGATACCACTTGCTACAAATGCACCGAAATTTGACAATGATATTAAAATGACAGGATTAGAGTCGTTAG
ATACGAATAAAAAGATTGAAAAACATTTTAATCAAGATAGTGAAAAGGCACAAATACGTGTTGTCTTTAAGAC
GACTAAAGATGATGGTATTGTGCAACCGAACATTACTAAAGATATTAAGAAAACGTTAGATGATATTAAAAAA
GATGACAAGCACATTGATAAAATATCAGATCCTTATGAAAATAAGCAAATTAGTAAAGATAAAACAACAGCAT
TTGCAGATATCACATATGACGTTAGTCAAACATCTTTAAAAGATGGCTCTAGAGATAATGTTAAAAGTCATTT
GAAAGACTTACGTGACAATCATAATGTACAAACAGAGTTAACTGGTACAGGAATGACATCTACAGAAGTAGGC
GGTAATTCTGAACTAGTTGGTATCATTGTTGCATTTGTAGTGTTATTGATTACATTTGGTTCAGTCATTGCTG
CAGGATTACCAATTATAAGTGCATTGATTGGTTTAGCATCTGGGGTTGGCATTATCAGCTTGTTAACTTATGC
ATTTGATATACCAAATGTCACACTGACATTAGCGGTAATGATTGGGCTCGCTGTTGGTATCGATTATGCATTA
TTTATTTTGTTTAGATATCGACAAGTAATGAAAACTGAAACAGACTATGTTAAAGGTATCGGACTTGCAGTGG
GAACAGCAGGTAGCGCAGTCATCTTCGCTGGTGTGACAGTTGTGATTGCAGTATGTGGTTTATCATTAGTAGG
TATTGACTTCTTAGCGGTAATGGGATTTGCTTCGGCAATTAGTGTTGTTTTTGCAGTACTTAGCGCTTTAACT
TTATTACCTGCATTAATAAGCATTTTCCATAAACGCATTAAAGTGAATAAATTACAGTCTAAATTCAAAAAAG
ATATTGATACGCCATGGTCTAAATTTATTACAGGAAATGCACTTGCGGCAGTATTACTAGGTTTAATCATATT
AGTAGCAGCTGCAATACCAGTTAGTCATATGCGACTTGGTATTCCAGATGACGGTGTTAAACCAGCAGATTCT
ACACAGAAAAAAGCTTACGATATTATCTCGGATAAATTTGGTGAAGGATTTAATGGCCAAATTCCGATGTTGA
TAAATGTGAAAGATAAAAAGGATGATCCACAAGGATTACAACAAGATTTACAATCTGTGTATAAAGATATTAA
AGATAAGAAAAATGTAGATATCGTTACGCCGCCACAAATGAGTAAAGATAATGATTATGCTTTGATGGTCGTG
ATACCTAAGCAAGGGCCTAATGCAGAGAGCACAAATGATTTAGTGCATGATTTACGAGATTATCATAGAGATG
CACAAGATAAATATGGGTTCAAAACGGAGATTTCGGGACAAAGTGTCATTAATATCGATATGTCTAAAAAGTT
AAATGAGGCGATACCTTTATTTGCAACAGTCATTGTTGTATTGGCATTTTTCTTATTAATGATTGTCTTCCGT
TCAATTCTAATACCGTTAAAAGCAGTTTTAGGATTTGTCTTGTCATTAATGGCAACGCTAGGATTTACAACAT
TAGTAATGCAAGATGGTTTTATGAAAGGATTATTCGGCATAGAAACGACAGGACCAATTCTAGCCTTCTTGCC
GGTTATTACAATTGGTATACTATTCGGCTTAGCAATGGATTACGAAGTCTTCCTTATGTCGCGAATTCATGAA
GAATATAGTAAAACAGGGGATAATGATTATTCTATCAAAGTAGGATTGAAAGAAAGTGGTCCTGTTATTGTAG
CAGCTGCATTAATCATGTTTAGTGTCTTCTTTGCGTTTGTATTCCAAGAAGATGTCATGATTAAATCAATGGG
TATGGCATTAGCGTTTGGTGTACTCTTTGATGCCTTCGTAGTCAGAATGATGTTAATTCCTGCATTAACTAAA
TTGTTTGGAAAAGGTTCGTGGTATTTACCAGCATGGTTGAATCGCATTATTCCACGAGTTGATATTGAAGGAC

ATGCACTTGAAAAATATAAAACGGTTGAATCACAGGAATCTGAAGCTAAAGATAGTAAGGAAACATACGACAC
AACCTTTAAAGTATATCCACAAGGTGCAACAAATGTATCGAAATATCAAGATGTACATGGGCAAGATGATGCA
CATTCAATAGTACTAGATGATAAAACGATGGCATTGTACCAAGAAGTGAAACAGCAATCAGCAAGTTCATTGT
TCTTATATGATGCTTTAATTGATTATCAAAACAAGCATCAACTAAATTCAAAACAACAAGTAACGAACATAGA
ACAGTTGAATAAAAATATTGAAAAGCTCAATCAATTGTTAGAAAAAAACTTAAGAAATAAATCATAAAATAAC
ATGTACATGCCTCCGCTTTAAATGTCTATCAATGATATTTGAAGTGGAGGTTTTTTGCATGTAAGAGATTATA
TAAAACATTTTTGTAATGTCACAAATTTTATATGAAAAATGAAAATTATTTAAATAAAGTATTGCGAGCTACA
ATTTTAAGGGTTATGATTTTTAAGTGATAATGATTATTATTTCTAATAGGAGAGCGAAATAGTATGTTAAACA
TTAAAAATGGTGAAATGAATAAGGCTTATAAAATAAAGCGAATGGATATTGCTAATGAGAATATGTTGTATCG
TCTAAGTGCCTTTGGGTTAACAGATGACGCTATCATAACGATTAAACAAAAATGTTTATTTAAAGGGCCATGT
ATTATTGAAGTAAACGGACAACAGTTAAGTATTAGACATTGCGATGCTTGTTCTATTGCATTAGAAGAATAGG
AGCCGGACGAATGGAAAATTATTGTATTTTAGGAAATCCTAATGTTGGTAAAACATCTTTGTTTAACGCTTTA
ACTGGTTCGTATGAATATATAGGCAACTGGAGTGGCGTAACGGTAGAGAAAAAAGTAGGAAAGCTTAAAGAAA
ATGTAGGACAATTAATTGATTTACCAGGTACATATGATTTATCGCCGATTTCTAAAGATGAAACTGTAGTGAC
AGACTATTTATTAAACGATTCATTTTCAGGAATCATTAACATTGTTGATGCGAGTCAACTAAAAAGAAATATG
CAATTAACAGTACAATTGTTAGAACTTAATCAACCAATATACATCGGATTAAATATGATTGATGTAGCAACAA
AGCGCGGGATAAAAATCGATTATCATAAATTGATGAAAAAGTTAAAAACACCTATTTTTCCAGTTGTGGCACG
TACAGGCAAAGGAACTAAACATTTACTTGGCGAAATAAAGCATCTAGGGGAAGGGTATCAACCGCATTTTAAA
ATCAATTATGGTGAGAAGATTGAAGAGACAATTAAAAATATGTGTCAAATAATAATGACAGAGACATCTCATG
ATAAATATCAAGCTAGATTTATTGCGATTCAATTCTTATTAAATAACATGCAAATTGCAAACGAACTTAATTC
AGAGGTTGTAAATAAATTGTCGAGTTTACGTGATCAAGTTGCTAAACAAGTAGAAGCTGTATCTGTTCGTCGA
GAGATGGAGCGTATACGCAATCATTATATTGAGACGTTATTGCAAGACGTGGTAACGTATCCAGATGAAGATA
AGCAATATTTCAGCTCTAGAATAGACAAGATATTAACACATAAATATATAGGGATGCCTATCTTTCTAGCGAT
TATGTGGTTGATCTTTCAAACTACATTTACATGGATTGGCACACCTTTGTCGGATCAACTCGATGCATTTATA
GGTGGTACATTTACGGATAGTGTGAAGACGATTATGAATTATCTTGGCGTTATACCATTTTTACAAGATTTAA
TTACAGATGGCATTATTGCTGGTGTAGGATCAGTATTAGTCTTTGTACCACAAATTGTTGTGCTCTTTTTCTT
TATATCTTTATTAGAAGATTCAGGTTATATGGCACGTATTGCGGTTTTAATGGATCGTATTATGGAATCGTTC
GGTTTAAGCGGGAAGTCTTTTATACCGATGATTATCGGATTTGGTTGTAACGTACCGAGTATTATGGCTGCGC
GCAGTATTGAAAATGAAAAAGAGCGTTTAACAACGATATTAATTGCACCATTCATGTCATGCTCTGCTAGACT
GCCAGTGTATGCGTTGTTCGTAGGTATCTTTTTCAAAGAGAATCAATCTTTAGTAGTATTAAGCTTGTATGTT
TTAGGTATTATTATGGCATTTTTAGTCAGTACAGTACTTACTAAAACGGTTTTGAAAAATGATAATGCAATCT
TTATTGTGGAATTGCCAACATACCGTGTGCCTTCCATTAAAACGTTGTGGCGTAGTACTTGGGAAAAGGCTAA
GGGCTTTGTTCGTAAAGCAGGTACATTTATTTTTGGCGGCTCAGTAGTCATTTGGCTATTAAGTTATGTAGGA
CCACATGGTATCAATGTGAATATCAATCAAAGTTTTTTACATATGGTTGGTAGCTTCTTTGGTATGTTAGTGC
AACCGCTCGGATTTGGAACATGGCAAGCAGGTGCTACGTTAGTGCCAGGATTTTTAGCAAAAGAAGTTATCGT
AAGTTCGATGGCGATTATCTATTCATCAGGTGATGCAGGCCTTGTTAATGTGATTCAAAATCAATTCACACCG
CTATCTGCGTATGCATTTATGATATTTATACTATTGTATATTCCTTGTGTTTCTACAGTGGCAGCAATCCGTA
AAGAGACTTATTCATGGAAATGGACGGCACTTGCAGTTGCCTATCCTCTAGTCACTGCATATGTCTTAACATT
CATTTTTTATCAAGTCGGACATTTATTTGTTTAAGGAGTGGTTAAAGTGTCAGTCATTATTAACATTTTAATT
TTTTTAGCAATTTTCGGATATGCCTTGTATACGTTAGTTAAATTTTTCAAACGTTCGAAACAAGGAAAATGTG
GTACATGTGACATTGATCGTGATTGTTGTGGAACAGAACAGCACACAGCGAATCATTTTCCTGGGAAATAATT
TAAAAAATGAGCACAGGTGAGGGCGTCCTGATGCTCATTTTTTGATACGCAATATGTTAAAGTATCATGATTT
ACCTTTGACGACTAATTCATTGTCCTCGATATCTAAATCCACATGTTTAACGTCAGTATGATCTAAATAGTAA
TCTGTAATTTTGTCACGTACTTGCTGTTCAACAATACGTCTTAATGGGCGTGCGCCTAAGTCTTCATCATAAC
CTTCTTCAATTAACCAATCTTTTGCATCTTGAGAGACATTCATCGTAATACCTTTTTTATCTAATGTTACTTG
GACATCGTCTAATAATAAGTTGACGATATCTTGTAATGCATCTTTATCTAAATGTAAGAATTCAACGATACCG
TTGAAGCGGTTAAGGAATTCAGGGCGGAAGAATTTTTTCATTTCATGCATAATGTCATCGTATTCTTGGTCTG
TACCGTTACCAAAGCCAGCATTTGATGTACAAATAATAATTGTATTTTTAAAGTTGATGACATTACCTTGACC
ATCAGTCAAATTACCATCATCCATTACTTGTAATAACAATGTTAAAATTTGTGGATTTGCTTTTTCGATTTCA
TCAAATAGAATGACTGAATATGGATTACGGCGTACTTTTTCAGTTAACGTATTTGAATTGTCATCATAACCGA
CATAACCAGCAGTTGTACCAATCATTTTTGAAACAGCTGTTGTGTCACTATATTCACTCATATCAAGTCGAAT
CAGTGCATCTTTATTACCAAATAGATCAATTGCTAATTGTTTAGCAAGCTCTGTTTTACCAACACCAGTAGGG
CCAACAAATAGGAAACTGCCAATTGGACGGTTGCCGTCATCAAACCCAGCACGATTACGGCGAATTGCACGAG
AAACCATTTCTACAGCTTGATCTTGACCTATGATTTTACTTCTTAAACGATTAGAAATATTTTTTAAACGTTC
AATATCGTTATCATCCATTTGAGAAACTGGAATACCAGTTAATCGTTGAATAGTATCTGAAATATCATGAACA
GTAGCAACGGCAGTATGTTCACCATTACTATTTTCTAATTTATCTTGTAATGATTTGATTTCATTTTGAATGT
CGTCAGCTTTTTTATATTCTTCAGCACTTACCGCTTTACGCTTATCATGTTCCAATTCAGAAATACGTTTTTC
AGTCGCGACTTTATCAATAGCAGGGCTTTGTGCAGATAAATGTGCTGCTGTAATATCTAACACATCGATTGCT

TTATCTGGTAATAATCGTTGTGGAATATATTGAATTGATAAGTCAACACATGCTTTTAATACGTCATCTGGTA
ATTTTACTTGATGGTGTTCTTCGAATTTTTCGCGAATACCTTTTAAAATTTCAACAGTATCTTTAGCACTTGG
TTCATTAACAAGTACTTCATTAAATCTGCGCGTTAATGCAGCATCTTTAAGAATATTGTTTCGATATTCATCT
TGAGTCGTTGCACCTATAATTGAAATTTCACCACGACTTAATGCTGGTTTTAAAATATCAGATAACCCTTTAC
TACCGGAATCACTTCCAGTTGCACCGGAACCGATGATTTGGTGAATTTCGTCAAAGAAGAGCACAGCATTTTG
TGATGATTTAACGCCTTCAACTAACTTTTGGATGTTTTCTTCAAAGGCGCCACGATATTGCGTTCCAGCTTCA
AGCGATGAAATATCTATAGATATAATTTCTTTATCTTTGATTGCTGCAGGTACGTTCCCTTCAACGATTGCTT
GCGCTAAACCTTCGACAATCGCAGTTTTACCAACCCCAGCTTCTCCGACTAGGATAGGATTGTTTTTAGTGCG
TCTACTTAAGACTTCTGCAGTCTCTTGGATTTCTTTATCACGACCGATGACTGGATCTAATAATCCGTCACGA
GCCTCTTGCGTAAGATTGCGACCAATTTGTTCTAAATAATTGCCATTGCCACCTTGTTGACCTTGTTGACCTT
GTTGTCTTAGTGCTGCTTGTTGAAAAGCTTGCGCACTTTGTTCGGCAGAATGATTACCACCCTGTTGCGTAAA
TTGTGCTAATTCTTCCGGTGAAACTTCTTTACCATTAATATAGTATTTCTTGTTACTAGTCTGATTTGAGCTT
TGCATGTCTTGCATCATTCTTCGTAAAATAGAATCGAAGTCACTATTGAAAAAATTGTTATTCATAAACATCA
CCTCTAAATTTTTAAACTAATAAGTAAAAGAAAATACATTGAAACTATATGCAAACTATAAACAATGGCAATT
CTATTTTTCCACCATTGTAGTTTTAAAAACGGCGCATTGTAGGCACTTTATGAACTTTTTGTGTAATTTTTGA
AGTAAGTAAGAATTTAAAAGTAAATATTAATCAATGGTTAAGAAATGTATGTGTTGATACATAGTTAATTAAG
AAATGGTATATTGTGTTGGAAGTCTGTGTGAATTTTGATTTGGAAGTGAATAGTTGATCTTGATAAAGCATAA
AGTGTTAATCATTTTCATAAAGTTAAATGAATAGTATGATGATGCAAAAGGAGTGTGTGTTTATGGTATATAA
GAGTTACTATGATTCTCCTGTTGGACGATTGGAACTTTTGAGTGATGGGGTAAGTCTGACCGCTGTTTTGTTT
GAAAATCAACAAGGTGATGGTAATAGGGAAGAAAATACGTCTTTAGCGATATTTAAAGAGGCGACACAGTGGT
TAGACGCGTATTTTAAAGGTGACAATCCTGAAATCACAATACCTTTAAAGCCAACAGGTAGTGATTTTCAACA
GTGTGTTTGGAATGAATTAAGACAGATTCCTTATGGGACGTTAACAACGTATGGTGCTATTGCCAAAAAAGTA
GGCAAGGTGCTTGATAAGCCAAAAATGTCAGCTCAAGCGGTTGGAGGTGCAGTCGGAAGTAATCCATTATCAA
TTATCGTGCCATGTCATCGTGTTGTCGGTAAAACGGGTAGCTTAACAGGATTCGGTGGAACGATTAATAATAA
AATTAAGCTGTTAGAGTTGGAGAATATCGATATGTCAAAGCTTTATGTACCAAAACATAGCACGAAGCCGTAA
CGTATAAAGGATCCAGGCATTAGGAAAACTTTAATAATATGTCAAATAGGCATAATGAATTCATTCAAGACGA
AGCATTATAAATAGAAAACAGCAGTAAGACGATTTCAATTAGAAGTGGTCTTACTGCTGTTTTTAGGTTATAT
TTAAATGTCTTTCAAATACACAGATTACTCTGGTCTGTGATATTCGCGAACGTTATTTTCAATATTTGATAAG
TAGAAAATATGGCGATCTTCATGAACAGCATCTTGTTCTTCGTCAAAGTCTACGTCATCAAAACGTTTGAAGA
ATGTTTCATATGCATCAACAGATACTTCAGTACGGTTATTTAATAATGCTTTGTGAGCAGCCTGATCTAAATG
ATCTTTGTAGCCTTCAACTAATGTCGCACTATAGAATTCACCAACTGAACCTGAGCCATAACTGAATAAACCG
ATTGTTTCACCAGCTTGTAAATCACGATTTTCAAGTAATGAAATTAGGCTTAAATATAGTGATCCAGTATAAA
TGTTACCGACATAACGGTTATAATCTACAGCATCTTCATATCCTGAACGTAAACGCTCTTGAGTTGTTTCATC
AGCGTTATCAATGATTGACTCTAATGCCTTTTTACCCATTTTTGTAAATGGAACATGGAAGCATAGAGATGCG
AAGTCAGCTAGCGACTTACCTTGACGTTTTGCGTATTCATTCCAGCTTTGTTGGAATGAGCGGATATAAGCAT
CTTTAGATAATGCACCATCAACTAATGGATATTTATGTCCAGTTGGACGCCAGAAATCATAAACGTCTTCAGT
GTAAGCAACAGCATCTTGATTTAATGCCAAAATGCTTGGATTATGTGCAATAACCATCGCAACTGCGCCAGCA
CCTTGTGTTGGCTCGCCGCCTGAATTCAATCCATAACGTGCTGTATCTGTAGCAATAACTAATACTTTTTCAT
TCGGTCTAGTTGCTAAATAATCTTTAGCTAATTGAATTGCTGGTGTTGCAGCATAACAAGCTTCTTTCATTTC
AAAGCAACGTGCAAAAGGTTGAATACCTAATAAGTTGTGAATTTGAACAGCGGCTGCTTTAGCAGCATCAACT
GCTGATTCAGTTGCCACAATTACCATACCAATTTTCTTTTTGTCTTCGTCTGTTATAATGTCCTTAGCAGCAT
TAGCGCCCATTGAAACGATGTCTTGGTTTACAGGACTAACAGCCATTTCAGTTTGACCAATACCAATTAAAAA
TTTGTTTGGATCTACTTGGCGTGCTTCTGCTAATTTAGCCATGTCTACATAGTACTTTGGAACGTAAAAGTTT
ATTTTATCGATACCTATTGTCATAAGGTTTTACATCCTTTCAAATTTATAAAAGCAACACTTGTATCATATCA
GAATATGCTAATTTTATACAAAGAAATATACTAAATTTAAAAATGATTTGTCGTTTCTTGTAAGCGTTTTAAT
TTACCTTGTTTTATTTTAAAGTCAAAACGACAATTATTCAATTGAATTTTAAGACGAATGACGTTATTTTGTG
GTAAAAATAGTAGTGATTGAAATGTTAAAAATTATGTGAAGTAAAAGGAGTACGTCCATGCAAAATTTAGATA
AGAATTTTCGACATTTATCTCGTAAAGAAAAGTTACAACAATTGGTTGATAAGCAATGGTTATCAGAAGAACA
ATTCGACATTTTACTGAATCATCCATTAATCGATGAAGAAGTAGCCAATAGTTTAATTGAAAATGTCATCGCG
CAAGGTGCATTACCCGTTGGATTATTACCGAATATCATTGTGGACGATAAGGCATATGTTGTACCTATGATGG
TGGAAGAGCCTTCAGTTGTCGCTGCAGCTAGTTATGGTGCAAAGCTAGTGAATCAGACTGGCGGATTTAAAAC
GGTATCTTCTGAACGTATTATGATAGGTCAAATCGTCTTTGATGGCGTTGACGATACTGAAAAATTATCAGCA
GACATTAAAGCTTTAGAAAAGCAAATTCATAAAATTGCGGATGAGGCATATCCTTCTATTAAAGCGCGTGGTG
GTGGTTACCAACGTATAGCGATTGATACATTTCCTGAGCAACAGTTACTATCTTTAAAAGTATTTGTTGATAC
GAAAGATGCTATGGGCGCTAATATGCTTAATACGATTTTAGAGGCCATAACTGCATTTTTAAAAAATGAATTT
CCGCAAAGCGATATTTTAATGAGTATTTTATCCAATCATGCAACAGCGTCCGTTGTTAAAGTTCAAGGCGAAA
TTGATGTTAAAGATTTAGCAAGGGGCGAGAGAACTGGGGAAGAGGTTGCCAAACGAATGGAACGTGCTTCTGT
ATTGGCACAAGTTGATATTCATCGTGCTGCAACACATAATAAAGGTGTTATGAATGGCATACATGCTGTTGT

TTAGCAACAGGAAATGATACGCGTGGTGCAGAAGCAAGTGCGCATGCATATGCGAGTCGTGACGGACAGTATC
GTGGTATTGCTACATGGCGTTACGATCAAGATCGTCAACGATTGATTGGTACAATTGAAGTGCCAATGACATT
AGCAATTGTTGGGGGTGGTACGAAAGTATTACCAATAGCTAAAGCTTCATTAGAGCTACTAAATGTAGAGTCA
GCACAAGAATTAGGTCATGTAGTTGCTGCCGTTGGTTTAGCGCAAAACTTTGCAGCATGTCGCGCGCTTGTGT
CAGAAGGTATTCAACAAGGTCATATGAGTTTACAATATAAATCATTAGCTATCGTTGTAGGAGCAAAAGGTGA
TGAAATTGCTAAAGTAGCTGAAGCATTGAAGCAAGAACCCCGTGCAAATACACAAGCAGCGGAACGTATTTTA
CAAGAAATTAGACAACAATAGTAAATTGCGAATAGCGGTTTTAAAGAAAAGTTTAAGTCAGTCCATCGTAGCG
CTTAAATAAATTAATATGAACAAAAAATAAAAACTTCTATCACACGCATACAGGAAATTGAAGCGTTCGTGAT
AGAAGTTTTTTGATTAGTAAATATAGTTAAAGTTACGTGCTGCCTTTTTGCTTAGTACACGTGTATTGTATTT
TAATGGGCGATAAAAGTTGTATTCTGAAACTTTAATACTACCGTTTTTATATACTTGTTCAACAACTGCGACA
TGTCCATAATATCCTGCAGTTGATTGCATTACAGCAAATTTTCTTGGTGTACGACCAGTCGCATATTTTGATT
TACGTGCATTGTTATACCAATTTTTGGCATTACCCCATTTGTTAGAGACAGGTTTGCCAACTTGTGCGCGACG
TTTAAATGCCCACCATGTACATTGGTTTTTACTATAATAATTAACTGCGGCATGCGATTGATGCGTTTGTTCG
ACGGATTGATAAGTGAAATCTGCTGAGAAAAGTACTGCAAAAGCAATTAATAAACGAATTAGTATCTTTTTAT
ATTCCATTTTTATCTCCCAAGTTAATTTTTTTCTTTATTCAACGTTATTTATGATAACAAAATTTTTTTAGAA
AACAATGCAAGCAATCAACAATAAAAGTAACTGTAATGAAGTTGTCATATAAAGTGTTGAATTGTAATAAAAA
TGGATTTTTTCATAAAATGTAGAAATTAATGTATTTAACAATTAATTGTCATAGTGATACAAGGATGTCAGAA
TTGATTAACAGCGGATATTAGTGGGAAAATATAAATGATTTAAAAGAAAAGGTACGTGGTTTTATAACTGAAA
GAAAATAGCAATCACAACATACTAAATGTAGCGATAACATTAAAAATAGCGCCCTGCAATCAGAGCGCTAACA
TGAGATGATGTGACTCCATGTATTCATGTATCATTGTACATTTGTGAATTGGTTGAAAACTGATAGTGCCCGT
TTAATTACAGGTACTGAGATGCTTTCAGGACATGGATTTGTTCGAAAATATGACAATATTTCACTATGGCTCT
TCACTTCTCTAGGGAAATTGCTATCTTGATTAATCCAATCAACCAATTCACCTAATGGTGTGTCATCACCAAG
AAAACTTTGCATAAATTCGTAAAAGCTCAATACCTCACCTCGAATAACAAACTTGAGCCATTAATATAATACC
ATAAAAGAATGTGTATTTTACATGAAACTTTTTATAAAGCAATTTTATAAATCTAAATTTATTGAATGTTTAA
GAAGTTGTGTATAATGAAATAAACGTGTTGAAATAGTTAGGATGATGTTAGTGGATATCAAACATATGAAATA
TTTTATTGAAGTCGTTAAGCAAGGGGGCATGACTAATGCTTCCAAATCATTATATATTGCACAACCTACAATT
AGTAAAGCAATTAAAGATATTGAAAATGAAATGGGCACGCCCTTATTTGATAGAAGTAAAAGACATTTAATTC
TTACCGATGCAGGTCAAATTTTTTATGAGAAAAGTAAAGAAATTGTTGCACTGTATGATTATTTACCATCTGA
AATGGAACGCTTGAATGGACTGGAAACAGGGCATATAAACATGGGTATGTCGGCAGTCATGAATATGAAGATT
CTTATCAATATTCTTGGTGCATTCCATCAACAATATCCAAATGTTACATATAATTTAATAGAAAATGGCGGCA
AAACGATTGAACAGCAAATTATCAATGATGAAGTAGATATAGGCGTGACCACTTTGCCAGTCGATCATCATAT
TTTCGATTATACTACCCTAGATAAGGAAGATTTGCGACTTATCGTGAGCAGAGAGCATCGACTCGCAAAATAT
GAAACTGTTAAACTCGAAGATTTAGCAGGTGAAGACTTCATTTTATTTAATAAAGACTTTTACTTGAATGATA
AAATTATTGAAAATGCTAAAAACGTTGGCTTTGTTCCGAATACTGTAGCGCAAATTTCACAATGGCATGTTAT
AGAAGATTTAGTTACGAATGAATTAGGTATTAGTATTTTACCAACATCAATTTCAGAGCAACTAAATGGAGAT
GTGAAGCTGCTACGCATTGAAGATGCTCATGTACATTGGGAATTAGGTGTTGTTTGGAAGAAGGATAAACAAT
TAAGTCATGCCACAACGAAATGGATAGAATTTTTGAAAGACCGTTTAGGCTAACATATTAATAAAGCACTCAT
TATTTAAGGCGCATCATTACGTGGGTCATTGAAATAATGAGTGTTTTTTTGTGAAAATGAAGTGAAATTTAGA
GAGCGTTTCCATAGAAAATAGTAATACAAACTATAAAAAAAGAGTATTTTTATATTGTGTACGCCATCTTTAT
AATAGTTATTGTAACAATTTAGACATATTTAGAAAGGGATGGCGCCATGCACAAAGTCCAATTAATAATCAAA
CTACTACTACAACTAGGAATCATCATTGTGATTACTTATATTGGCACAGAAATTCAAAAGATTTTTCATCTTC
CCTTAGCCGGCAGTATTGTTGGTCTATTTTTATTTTATTTACTATTACAATTTAAGATTGTACCGCTAACTTG
GGTAGAAGACGGTGCAAACTTTTTATTAAAGACGATGGTCTTTTTCTTCATACCGTCAGTTGTAGGTATTATG
GATGTTGCTTCCGAAATTACGCTAAATTATATACTCTTTTTCGCAGTCATTATCATAGGAACATGTATCGTTG
CATTATCTTCAGGTTATATTGCTGAAAAAATGTCTGTTAAACATAAACATCGTAAAGGTGTAGACGCTTATGA
ATGATTACGTGCAAGCCTTATTAATGATTTTGTTGACTGTCGTTTTATATTATTTCGCTAAAAGGTTACAACA
AAAATATCCGAACCCATTTTTGAATCCAGCATTAATTGCATCTTTAGGAATTATTTTTGTCTTACTTATCTTT
GGAATTAGTTATAACGGGTATATGAAAGGTGGCAGTTGGATCAACCATATTTTAAACGCAACGGTCGTATGTT
TAGCGTACCCACTTTATAAAAATAGAGAGAAAATTAAAGACAATGTCTCTATCATTTTTGCAAGTGTATTAAC
TGGCGTCATGCTGAATTTCATGTTAGTGTTCTTAACACTTAAAGCATTTGGCTATTCTAAAGACGTCATTGTA
ACGTTATTGCCCCGATCTATAACAGCCGCAGTAGGTATCGAAGTGTCACATGAACTAGGTGGTACAGATACGA
TGACCGTACTTTTTATTATCACAACGGGTTTAATCGGTAGTATTTTAGGTTCGATGTTATTAAGATTTGGAAG
ATTTGAATCTTCTATCGCCAAAGGTTTAACGTATGGGAATGCGTCACATGCATTTGGCACAGCTAAAGCACTA
GAAATGGATATTGAATCCGGTGCATTTAGTTCAATTGGGATGATTTTAACTGCAGTTATTAGTTCAGTGTTAA
TACCTGTTCTAATTTTATTATTCTATTAATTTAGATATTTAAAATGATAGACAGAAAGGGAGGCTATTAGTAA
TAATGGCAAAAATAAAAGCAAATGAAGCATTAGTTAAAGCATTACAAGCATGGGATATAGATCACTTGTATGG
TATTCCAGGAGACTCAATCGACGCAGTAGTCGATAGTTTACGTACAGTGAGAGATCAATTTAAATTTTATCAT
GTACGTCATGAAGAAGTAGCAAGCTTAGCGGCTGCTGGTTACACAAAATTAACTGGTAAAATCGGTGTGGCAT

TAAGTATCGGTGGCCCTGGTTTAATTCATTTATTAAATGGTATGTATGATGCCAAAATGGATAATGTACCGCA
ATTAATATTATCTGGACAAACGAATAGTACAGCACTTGGAACGAAAGCATTCCAAGAAACAAATTTACAAAAA
TTATGTGAAGATGTAGCCATTTATAATCACCAAATTGAAAAAGGTGACAATGTGTTTGAAATTGTTAACGAAG
CAATTCGTACGGCATATGAACAAAAAGGTGTAGCTGTTGTTATTTGTCCTAACGACTTATTAACTGAAAAAAT
TAAAGATACAACGAATAAACCAGTAGATACATCAAGACCAACTGTTGTATCACCAAAATATAAAGACATTAAA
AAAGCTGTTAAATTAATCAATAAGAGTAAAAAGCCGGTTATGTTGATTGGTGTTGGTGCAAAACATGCGAAAG
ATGAGCTACGTGCGTTTGTTGAAGCGGCAAAAATTCCGGTCGTTCATTCATTACCTGCTAAGACAATCTTACC
GGATGACCATCCATACAGTATTGGTAACTTAGGTAAAATAGGTACAAAAACCTCATATCAGACAATGCAGGAT
GCAGATTTACTAATTATGGTAGGTACAAACTATCCATATGTAGATTACTTACCTAAGAAAAATATTAAAGCAA
TCCAAATCGACACAAATCCTAAAAATATCGGACATCGTTTTAATATTAATGTAGGAATTGTTGGAGACAGTAA
AATTGCATTGCATCAGTTAACTGAAAATATTAAACATGTTGCGGAAAGACCATTTTTAAACAAAACGTTAGAA
CGTAAAGCGGTTTGGGATAAATGGATGGAACAAGATAAAAATAATAATAGCAAACCATTACGTCCAGAACGTT
TAATGGCATCAATTAACAAATTTATTAAAGATGATGCAGTAATTTCAGCAGATGTTGGTACTGCAACAGTTTG
GTCTACACGTTATTTAAATCTTGGTGTGAACAACAAGTTCATCATTTCAAGTTGGTTAGGTACAATGGGTTGC
GGTCTTCCAGGAGCTATGGCATCAAAAATTGCATATCCAAATAGACAAGCGATTGCCATTGCTGGTGACGGTG
CATTCCAAATGGTAATGCAAGACTTCGCTACAGCAGTACAATATGATTTACCTTTAACTGTATTTGTACTTAA
TAACAAACAGTTAGCATTTATTAAATATGAACAACAAGCAGCTGGTGAATTAGAATATGCAGTTGATTTTTCT
GATATGGATCATGCAAAATTTGCTGAGGCAGCAGGTGGTAAAGGTTATACAATTAAGAGTGCTAGCGAAGTAG
ATGCTATAGTCGAAGAGGCATTAGCACAAGATGTACCAACGATTGTAGATGTATATGTTGATCCTAATGCTGC
GCCATTACCAGGTAAAATTGTAAATGAAGAAGCGCTTGGTTATGGTAAGTGGGCATTTAGATCAATTACTGAA
GATAAGCATTTAGATTTAGATCAAATTCCACCAATTTCAGTGGCAGCAAAACGTTTCTTATAACTGATTTAAA
GGTTATCACAATTGAATTGAACTATAAAAACGGTAATTTCTATTTCAACAAAATGGGAATTGCCGTTTTGTTT
ATTTATCACAAATGATCGTACTGAATTGATGATAAAATTGTGAAAAAGTTGTTGAAAACGCTTTTACAAATAT
GTATAATAGCTATGAATTAGATATCACTTGCGTGTTACTGGTAATGCAGGCATGAGCAAACAACCGCACTATG
AGAATAGTCTTGTTTGTTCATGCCTGCTTTTTTTGTACATGGAAGCGGAAATTGAGATAGGGGATGTTTATAT
GTTTAAGAAATTGTTTGGACAATTGCAACGTATCGGTAAAGCATTAATGTTACCTGTTGCGATTTTACCAGCA
GCTGGTATTTTATTAGCGTTTGGTAACGCAATGCACAACGAACAATTAGTAGAAATTGCACCATGGTTAAAAA
ACGATATCATTGTAATGATTTCGTCGGTCATGGAAGCAGCAGGACAAGTTGTATTTGATAACTTGCCATTATT
ATTTGCAGTTGGTACAGCACTTGGATTAGCAGGAGGAGACGGTGTTGCAGCATTAGCAGCGCTAGTAGGTTAC
TTAATTATGAATGCAACAATGGGGAAAGTGTTGCACATTACAATTGATGACATTTTCTCATATGCCAAAGGGG
CAAAAGAATTAAGTCAAGCAGCGAAAGAACCGGCACATGCCTTAGTATTAGGTATTCCAACATTACAAACGGG
TGTGTTTGGCGGTATTATCATGGGTGCTTTAGCCGCATGGTGCTACAACAAATTTTATAACATTACACTACCA
CCATTTTTAGGATTCTTTGCAGGTAAACGATTTGTACCGATTGTGACATCGGTCGTAGCGATTGCAACAGGTG
TGGTTTTAAGCTTTGCGTGGCCACCAATTCAAGACGGATTAAATAGTTTATCGAATTTCTTATTAAATAAAAA
TTTAACATTAACAACGTTTATATTCGGTATTATTGAACGCTCATTAATTCCATTTGGTTTACATCATATTTTC
TATTCACCGTTCTGGTTTGAATTCGGTAGTTATACAAATCACGCAGGTGAATTAGTTCGTGGTGACCAACGTA
TTTGGATGGCACAATTGAAAGATGGCGTACCATTTACTGCTGGTGCATTTACTACTGGTAAATATCCATTTAT
GATGTTTGGTTTACCAGCGGCAGCATTTGCGATTTATAAAAATGCACGACCAGAACGTAAAAAAGTCGTGGGT
GGTTTAATGTTATCAGCAGGATTAACTGCATTTTTAACTGGTATCACTGAGCCATTAGAATTTTCATTCTTAT
TTGTAGCACCAGTACTTTATGGAATTCACGTATTATTAGCTGGTACATCATTCTTAGTAATGCATTTATTAGG
CGTTAAAATTGGTATGACATTCTCAGGTGGTTTCATAGATTATATTTTATATGGTTTATTAAACTGGGATCGT
TCACACGCATTATTAGTTATTCCAGTCGGTATTGTATATGCTATCGTGTATTACTTCTTATTCGACTTTGCAA
TTCGTAAGTTTAAATTGAAAACACCAGGTCGTGAAGATGAAGAAACTGAAATTCGTAACTCTAGTGTCGCAAA
ATTACCATTTGATGTCTTAGATGCAATGGGTGGAAAAGAAAACATTAAACATTTAGATGCATGTATTACACGT
CTGCGCGTAGAAGTGGTTGATAAATCAAAAGTAGATGTAGCAGGTATTAAAGCTTTAGGCGCATCAGGTGTAT
TAGAAGTTGGAAACAATATGCAAGCTATCTTTGGTCCAAAATCAGATCAAATTAAACATGATATGGCCAAGAT
TATGAGTGGTGAAATTACGAAACCAAGTGAAACGACAGTGACTGAAGAAATGTCAGATGAACCAGTTCACGTA
GAAGCACTTGGAACAACAGATATCTATGCACCAGGTGTCGGTCAAATCATTCCATTATCAGAAGTACCTGATC
AAGTATTCGCTGGTAAAATGATGGGTGATGGTGTTGGCTTTATCCCTGAAAAAGGTGAAATTGTAGCACCGTT
TGATGGTACAGTGAAAACAATCTTCCCTACGAAACATGCGATAGGATTAGAATCTGAAAGTGGTGTCGAAGTA
CTTATTCATATTGGTATCGATACAGTGAAACTGAATGGCGAAGGATTCGAAAGTCTGATTAACGTTGATGAAA
AAGTAACACAAGGTCAACCATTAATGAAAGTGAATTTAGCATACTTGAAATCACACGCACCAAGCATCGTTAC
ACCAATGATTATTACAAATCTTGAAAATAAAGAACTTGTCATTGAAGATGTACAAGATGCTGATCCAGGTAAG
CTAATTATGACAGTCAAATAATGATTAAAAATTAAACAGCATATCAAATGAATGAACTTTTTGTCATTCGTAG
TGCGTATGCGAAGTAGCGAGTTGAAACAGAATACGTTACAAAAGAGCTTGAGACATTAAGTTCTTAGGCAATG
TAAAAAAGCTGATTTCTATTAATTATTTGATAGAAATTAGCTTTTTTGATATGTATTTTATAATGTACAGCTC
GTTGAGCTGCTATTTTCCTTATATTAAGTGCCATTAATACAAAACCTAGCTCTCGTTTAACTTTATTTATTCC
TCGAACTGACATACGAATGAAACTCAAAATAGCTTTCATAAATCCAAAAACAGGCTCTACATCAATTTTTCTT

TGACTATAGATTTTTTTCGTTTCTGGTTCAGAACGCTTTTGATTAATTTGGGCTTTAAAGTATTCCCAATTAT
AATTCTTCATAATTTTCTTATTGGATTTCGAATTTGGCTTCATGCATTGATGTCTCAAAGAACATGATGAACA
GTCATCACATTCATATAGTTTGAAGTCTCGTTTAAAACCATATCTATCATTATGGTATGCATATCTTTTAAAA
CCTATTCTTTTGTTATTAGGACATATAAATTCATCATTAAGTTCGTCATATTTCCAATTTTGAGTGTTGAAAA
TGGAATTAATGTTCCAGTATCTGTTGGATTTTGAAATAGGTCATAGGATAAAACAAATTGAGAATTTGTCGCT
ATTTGTAAATTGTATCCTGGCTTAAGTTGGCCATTTTTCATATGGTCTTCTTTCATTCTCATAAAAGTTGCAT
CATGATCAGTTTTAGAAAAGCTATTTCTATCTTTAAGAATCGATTTTTGCTCTTCATATTTACTTTTTCTTTC
AGAATAATCATCAAATTTATTTTTGAACTTCTTAATCTTAGTTCTTTTTTACGGGTCTATTTTCTAATTTGAG
CACAATCTTCGTTCTCAATAGAATGATTTAAATCTTCGATTTCTTTATCTAAATGACTACCAATTAAATCTAT
TTCTTCTATTGTTAAATCGCTATATCCATCTTCTTTTATCTCTGGTATTATTTTTCTTCAACTAAGTCACGAT
ATAATGCTTTTGAATTTTCGTTCAATTTCGATTCGTGATTTTGAATACTTTTCTTCCACACAAATGTATATCT
ATTGGCATTAGCTTCTACTTTTGTACCATCAATAAAAATTGAATTATCATCAATAAGATTTTTCTTTAAACAT
TGACTATGGAACTGAATAAATAAAGATTCAATTAACGCATCAGTATTAGGATTCACTCTAAAACGATTAATAG
TTTTATAAGAAGGTATTTGATCTTGAGCTAACTACATCATTCGAATACTGTCATGAAGTAATTTCTCTATTCT
ACGACCAGAAAATACAGATTGAGTATATGCATATAAGATGATTTTTAATATCATTTTTGGATGATAGGATGTT
GCGCCACGATGATGTCTGAATTCATCGAATTCGCTATCAGGTATCGTTTCAAAAATTTCATTAACATATCGCG
AAATATCATTTTGAGGAATTCTAACAGAAGTTTCTATTGGTAGTGTAAGTTGGGTCATGTTATAATTTTTATA
CATAAGGCACCTCGTTAATTTAGTTTAGTGGTATTTATTAAATTATGCGAAAGTGTCTTATTTTTTTAAAGTA
TTTCAAAGTAAAATTACATGTTAATACGTAGTACTAATGGCGAGACTCCTGAGGGAGCAGTGCCAGTCGAAGA
CCGAGGCTGAGACGGCACCCTAGGAAAGCGAAGCCATTCAATACGAAGTATTGTATAAATAGAGAACAGCAGT
AAGATAATTTTCAATTAGAAAATATCTTACTGCTGTTTTTTTAGGGATTTATGTTCCAGCCTGTTTTTAGTGC
GCAATGATGTATAGCAGATGTGTTGATGATAATAAGTTAAATATTAGTGTTAGATATAGAAAACATTGCTTAT
GTTTTTGTCACATTTTAGAAAAATGCATCTTCGCGACTAGCCAAATTAATAGTCTCATTGAAAAGTTTGATTT
TCCGTAATAAAGTTCAATTGCTTGTATTTTTATCATGAAAGTAATAAAAATAAATTAACATGATTTTAAATCT
ATTTGTAAGATAAGGAGATTTGTCATTATGACAACAGAAGGTCTATTAGTTGCAGAGAAAGAAATCGAAGTGA
ATGGTTACGACATTGATGCGATGGGTGTCGTTAGTAATATCGTTTATATTAGATGGTTCGAAGATTTGAGAAC
AGCATTTATTAATCAGCACATGAATTACTCAACAATGATCAATCAAGGCATTTCACCTATACTTATGAAAACG
GAAGCAGAGTATAAAGTACCTGTCACAATACATGACAAACCAGTAGGTCGTATTTACTTAGTTAAAGCAAGCA
AGATGAAATGGGTGTTTCAGTTTGAAATTGTGTCCGCACATGGCGTGCATTGTATTGGTACACAGACAGGCGG
TTTTTACAGATTGAGCGATAAGAAGATAACCTCTGTGCCACAAGTGTTTCAAGACATTTTAGCAACAAAATAA
TGACTTCATTTTAAAATATAAAAAGTAAGAAGGTGTTCGAAATGGTTAAGCAATTAAATAGTGTCGAAGCATT
CCGTGAATTTATTCATCAATATCCGTTAGCAGTCGTACATGTCATGCGCGATCAGTGTAGCGTGTGTCATGCC
GTTTTACCACAAATTGAAGACTTGATGCAATCATATCCCAATGTGCCATTAGCTGTGATTAATCAAAGTCAGG
TGGAAGCTATTGCTGGAGAATTAAATATTTTCACTGTACCTGTGGATTTAATTTTTATGAATGGAAAAGAAAT
GCATCGTCAAGGGCGTTTTATCGATATGCAACGTTTTGAACATCATCTTAAGCAAATGAATGATAGTGTGAAT
AACGATGTCGATGAGCATGAATATCGCAAATGATTAGCATTGCTAAGATTATGTAGACATCATAACTTATTTC
CCAGTAAATATTGGTAGTAATAAGAATCAGCATGGTACATTAATACTATAGTAGAAATCATCAAAGAGGAGTG
ACGACAAATGCGTAAAAAAATGGTCTACACTTGCGTTTGGATTTTTAGTAGCAGCATACGCACATATTAGAATT
AAAGAAAAACGCAGTGTGAAAAGTTATATGTTAGAACAAGGTATACGATTATCTAGAGCTAAGCGTCGTTTTA
TGTATAAAGAAGAAGCGATGAAAGCATTAGAAAAAATGGCGCCACAGACAGCAGGTGAATATGAGGGCACCAA
TTATCAGTTTAAGATGCCAGTAACAGTGGATAAGCACTACGGTTCAACCGTTTATACCGTTAACGATAAACAA
GATAAGCATCAACGCGTTATATTATATGCACATGGAGGGGCATGGTTCCAAGACCCACTCAAAATTCATTTCG
AATTTATTGATGAACTTGCAGAAACACTCAATGCTAAAGTCATCATGCCAGTATATCCGAAGATTCCGCATCA
AGATTATCAAGCGACGTATGTACTTTTTGAAAAGTTGTACCATGATTTATTGAATCAAGTAGCAGATTCTAAA
CAAATCGTTGTAATGGGTGACTCTGCGGGCGGTCAAATTGCTTTATCATTTGCTCAATTGTTAAAAGAAAAAC
ATATTGTGCAACCAGGACATATTGTATTAATTTCACCAGTTTTAGATGCAACGATGCAGCATCCTGAAATTCC
TGACTACTTAAAGAAAGACCCAATGGTAGGTGTGGATGGCAGTGTGTTCTTAGCTGAACAATGGGCAGGGGAC
ACGCCTTTAGATAACTACAAAGTATCACCAATTAATGGTGACTTAGATGGTTTAGGTCGTATTACATTAACTG
TTGGCACAAAAGAAGTACTGTACCCAGATGCTTTGAACTTATCGCAATTGTTGAGTGCGAAAGGTATCGAACA
TGACTTTATACCTGGATATTACCAATTCCATATTTATCCAGTATTTCCGATACCAGAGCGCCGACGCTTTTTA
TACCAAGTGAAAAATATTATTAATTAAAATAAAAAACTCTGATATCACCTACAAGGTAGCTACATTGTTAAAT
AATAGTTACTTGCTAAAGTGATTATCAGAGTTTTCTTATTCATCTAATGCTTCAGGTTGTCTTTTAAGTGATA
GTAATAAAATTAAAATCATAATAAGAATGATGCCTACGATTTGAAATGGTTTGAATGCCACATGTAACCATAG
ACTGCTTGCGATAACGGCTACGACAGGTTCAACAGTTCCGAATAATGTTGTTTCTTTCGCTGATATGTATTGT
AAACTGTCGATAAAGAAATAAAATGCGAGTGCGGTACCACCGATAATACCAAAGATTAAAAATAATATAATTG
ATATGTCCCATTTAGTGATATCAATGTGCCAAATTGGATGGCGTAAATTCATCGCAACACCAGAAATAAGCAT
TGCCCACCCGACAATTAGAATCGAACCGAAGCGGTTAAGTAGGTCTGAAGGATAAATTGTGTAAAAAGCGAGT
GCTACACCAGCTAAAATACCCCAGAATAAACTTGCAGGATTGACGACTAAATTAGAAAATGAACCATTCGTTA

ATAATAAAAATGTTCCTAATAGTGTCATGATAATAGCAAGCACATCAAATAATGTTAGTTTTGCAACGCCTCT
TATGACAAACCAAATAATAATATAAACTGGCGCAATGTATTGTAGTAATGTTGCAATCGCAGCATTACCTGTA
TTAATAGATGCCATATAAGCATATTGTACAACTAACATGCCCAGTATACTGAAGATAAGTAATTTGCCTAACA
TACGTCGATCTTGGAATACGCTAAATATTGAACGTTTGGGTTGCATCATTTTGTACATAATAAGTAAGAAAAC
ACCACTGACTACAAGTCGAGCAGTGACGTACCAATCGACATTAATATTCTTATATTTGAACAAGAAATCAGAA
ACAGTACCACCTAATCCCCATAAACAAGCACCGACAATGGCAAGTATAATTCCTTTTAAACGCAAAGCTGAAT
GATTGTTCGCCAAGTATCTCACCCCTTTAAAACTTAACACCAACAACATAAAATATGTCGGTGTATGATAATG
ATTTAATTCATTATATCGACTTTTAAGAAAGAGTGAAATGAGAAAATAAAAAAATCATCTAAGATGTAGTGGT
CTAGATGATAGATAAATGAATCGATATGGGAAATGAATGTTGAAACACAGAGGATAAACTATTTAAATGAGAT
TTCAGGAAGACTCAAATGTTTTTGCGTAAGAGATCCATGATGAGAATGCTGACTATCTAAAACGAAATGTGAT
ACATCATGTCCAGTCATACTCGTATATAAAATAATGACTAAATAAGAAGTGAACGTTATAAAGATAAGTGCTA
AATAGATAGAAATTGCAACTTTAACGGAATGACTCAATTGCATCTTTATCGCCTCCACTAAGTTATACTAAAT
ATTTTCATCTTCAGTATAAACAGCCATTGTTAAGTTGATGTAAATTTAGCTTTAAAAAAAGTAAAGATTCAAC
CTATAGACTATAATCATTCCTATCAGAAAACTAAAAATAATATCCTACTTTAATTATCTTATTGCAAATATAT
GTGTAATAAGTTATTTTTGAATATAGTGTGCGGTTTTATAAACACAGCACAAAAACAAAAATAAATAGCTCGA
AAGATAAAATGTATAAATATTAAAAATTTTTAGAAATATTACTCACTTTCAGATGTAAAAAAGTTATCAAAAA
ACTTTCACACAACTGTTTGAAATATAGAGTGAAAAGTTAATTAGTAAATATTAATGACAACATAAGTAGTTTT
TGAAACAATCCATTTTAAAAATCTTTCACAAATATGAAATATTTCACTTAAATATTTGATTTGATAACAGATA
AAAGTTGAAAACGTTTTCTTAAGGGTGTACGATGTTTATGGTCTGAAAATAAAATAAACATAGAGAGAGGTTT
TTGGGAAATGGATATTCTGTTAGGTGTAGGCACATTAGTCTTGGTTTTAGTGATCATGACGCTATTTTTAAAG
TATGCACCGTACGGTAAACAAGGACTACAAGCATTATCCGGTGCAGCTTGTGCGACATTTCTACCACAAGCAT
TCTTAAGTTATGCTATTGGTGGCGTATTTGATATTAAATTTCTACAAGATATTGGGGATTTAGCAGGAAGTTT
AAGCGGTATTGCAGTAGGTATTTTAACGTGCTTAAACTTAGGCGTTGCACCAGTATTTGCGGTCATTGTTGGT
TTAGTATTACATGACTTTAAATTATTACCAGCATTTATCGCAGCTTATGGCGTAGCTTTTTTAATTAAATGGA
TTGAAAAGAAAGTTCCAGAAGGCTTGGACTTAATTGTGGTGATCTTATTTGCACCAGCAATTGCTTTCGGACT
TGCATCAATCATTACACCTGGTGTGCTAGCAACATTAAAACAAATAGGTAGTGCTGTCACTGCGGTAGGTGAT
AACAATCCATATGCATTAGCGGTCATTTTAGGACTAGTGATTCCTGTTACAGGTATGACACCGCTAAGTTCAA
TGGTATTAACAAGTTTATTAGGACTGACAGGTGTTCCTATGGCTATTGGAGCACTAACATGTACAGGTAGCTC
ATTTGTTAACCTTATTTTATTCCGAAAATTAAACATTGGTGGACCGTCAAAAGCATTTGCAGTTTGTATTGAG
CCATTGACGCAAATTGATTTAATAGCGCAATATCCAGTACAACTATATGGTACAAATGCACTGATTGGGGTCG
TCAACGCATGTATCGTGACATTTAGCGGACTAGTAATAGGTGTCAAAGGTATGGCAACACCAATTGCAGGCGC
CATCGTATTGTTTGGCTTTAATAATGCAGTGACATCAATTGTAACAATCGCAACAGTGATTATTGTCAGCATT
GTCTTAGCCTATATTATTGGAACACTAATTAATAAGTTTAATTTAATGAATATCAACTTTAAAATGCCGAGCA
AAAAGAATCATATTAAGGAGAGTGTTTAATTATGGCAAAGAGCTATGATTATCAAAGTGCTTTCGATATTATC
GGACCAGTTATGATGGGCCCTTCAAGTTCTCATACGGCTGGTGCAGTAAAGATTGGGAATTCAGCACGCGCAA
TATTGGGAGATATTCCTAAAAATATTGAAATACATTATTATGAATCATTTGCTCAAACACACCAAGGACATGG
TACAGATGTCGCTATTGTGGGTGGAGCAATGGGATATTCAACATTTGATAACCGAATTAAATCTTCATTAGAA
ATTGCAGCTGATGATGGTATTAAAATAGATATCATCGAAGAAGAAGGCGATAGTATTGGTCAACATCCAAACT
GTGCTTATATTAAATCATCTCGTAATGATGGTCGCTACATTGAAATTATCGGTATTTCAATCGGTGGTGGTAC
AATTAAAATTAAAGGTATTCATATAAATGGACTAGAAGTAGATTTAAATCATGGATTACCAATTTTAGTTGTC
GACGGAAATATGACTAAAGCACAAGTAAATCATTTCATTAATGATATCAACGATATGAAATTAGACTGTAAAG
ATGAATTTATTAAGATAGATGAAGATAAATGCTTGGTTGTAATACCATTAAATAAAGCAATCTCAGAATCGAC
ATTAAATACAATTAAAGAGAAATACAGTGACGTAAACGTTTCCTATATAAATTAGAGGGGGAATAAACATGTT
TGATTCAATTAGAGAGACTATAGATTATGCCGTAGAAAATAATATATCATTTGCGGATATCATGGTTAAAGAA
GAAATGGAATTAAGCGGTAAATCACGTGATGAAGTGCGAGCGCAAATGAAACAAAATTTAGATGTCATGCGAG
ACGCAGTAATCAAAGGGACGACAGGTGATGGCGTTGAAAGTGTAACGGGCTACACTGGTCATGACGCTGCTAA
ACTACGTGATTATAATGAAACACATCATGCTTTGTCTGGATATGAAATGATTGACGCAGTCAAAGGTGCCATT
GCAACAAATGAAGTCAATGCTGCGATGGGTATTATTTGTGCAACGCCAACAGCTGGTTCCTCGGGTACCATTC
CCGGTGCACTTTTTAAATTAGAAAAAACACATGATTTAACAGAAGAGCAAATGATTGATTTCTTATTCACTTC
AGCGTTGTTTGGGCGTGTCGTAGCAAACAATGCAAGTGTAGCTGGTGCAACAGGTGGCTGCCAAGCTGAAGTT
GGTTCAGCATCTGCAATGGCGGCAGCTGCAGCAGTAGCTATATTCGGAGGATCACCAGAAGCATCCGGGCACG
CTATGGCATTAGCGATAAGCAATTTATTAGGATTAGTTTGTGACCCAGTAGCCGGACTTGTTGAAATTCCTTG
TGTTATGAGAAATGCAATTGGTTCGGGTAACGCTTTAATTTCAGCAGATTTAGCATTAGCAGGTATTGAAAGT
AGAATACCTGTTGATGAAGTTATTGAAGCAATGGATAAGGTTGGTCGTAACCTTCCTGCATCATTACGTGAAA
CTGGATTGGGTGGACTAGCAGGCACACCAACTGGCGAAGCAATTAAACGTAAAATCTTTGGCACAGCTGAAGA
TATGGTTAAAAATAATTAATGAGATAACAATAAGATATTGATAAAAAAGAAGCATCTGTCTGGATCATTAGTA
CCAGATAGATGCTTCTTAATTATCTCTGAAGAAGTTGAAAAATTTATGGGAAATACATGCTAATCAAAATAGT
GATTGCAAAAGCGCCCCCGTATATCTAAAATAACTTAAAAGGAGTGATGATATGCTAAGACTATCAATCATCT

TTATAGCATTCATCATTAACACAACGATTACATATGGATATACGACTGAGGGAACGTGGGTCAATCTATTATT
TAAATCTTTATCGCTCAGCATGATTATCGTTTTCATGTTCTACTATATTCGTTTTGTTATTGAGAAAAAGCGT
TAAATCAAAGCTATGGGGATAGTATTTGAAAAGAAAAAATAAAAGGGACGATGTAAATTGTCCCTGAGAAGTT
TAAAATTTTATATGTTGGCTTGTTATGTTAAGTGAATTGACATGGTCGTCTTGTTTATATTATGTGATTCAAA
CATTACTAGTCTTGTAAATCTAATTCGTAAAATGCTAAATCTAACCATTGATTAAATTTAAAACCTACATTGG
TCAGTGTGCCGGCATGCTTAAAAGCAAACTTTTGATGCAACTGAATACTCGCTTTGTTGGAAGCATCAATGCC
TGCAACTAGCGCACGATAACCTTTAGCTTTTGCTTCCACAATTAAATGGTGTAGTAATTGACTAGCAATACCT
TTTCCTCTAGCTGAAGCATCGACATAAATAGAATGTTCGATTGTATATAGGTATGCTGGCCAAGGTCTAAACG
AACCGAACGTCGCAAACCCTAAGACACTTCCATTTTCCTCAAATACAAAGATAGGCTCATGCTTACGTTGTTT
CGTTTCAAACCATGCGACACGTTCGTCTATGGTTTGTGGTTCATAAGTATAAACAGCTGTAGTATTGATAATG
GCATCATTGTATATCACTAATATAGCGTTTAAATCCTCTTTTTTAGCGCATCTAATCATAACAATTCCCCCTT
AGTAATTATTAAAAGCGTTTCGTTATTTGAATGCAAATATGTGTAATGAAATCTAACGTAAAAGCATACATGT
AAATTTTATAGTATAAAATGAATTGCTATGAGTCACTTTGAAATTAATGGTATACTATATGAAATGTTAATAG
GCATTGTGAAATGTATAAAAGGAGCCTTAACGTATGAAAAAATGGACAAATCGATTAATGACAATCGCTGGTG
TGGTACTTATCCTAGTGGCAGCATATTTGTTTGCTAAACCACATATCGATAATTATCTTCACGATAAAGATAA
AGATGAAAAGATTGAACAATATGATAAAAATGTAAAAGAACAGGCGAGTAAGGATAAAAAGCAGCAAGCTAAA
CCTCAGATTCCGAAAGATAAATCGAAAGTGGCAGGCTATATTGAAATTCCAGATGCTGATATTAAAGAACCAG
TATATCCAGGACCAGCAACACCTGAACAATTAAATAGAGGTGTAAGCTTTGCAGAAGAAAACGAATCACTAGA
TGATCAAAATATTTCAATTGCAGGGCACACTTTCATTGACCGTCCGAACTATCAATTTACAAATCTTAAAGCA
GCCAAAAAAGGTAGTATGGTGTACTTTAAAGTTGGTAATGAAACACGTAAGTATAAAATGACAAGTATAAGAG
ATGTTAAGCCTACAGATGTAGAAGTTCTAGATGAACAAAAAGGTAAAGATAAACAATTAACATTAATTACTTG
TGATGATTACAATGAAAAGACAGGCGTTTGGGAAACACGTAAAATCTTTGTAGCTACAGAAGTTAAATAATCC
ATTACGCTAATGGATGAATATATTGAGTGGAAAACAGTCTTGATTGCGAGACTGTTTTTTTGTTTGGTCTGAG
GTAACAATGACGACGTGTCATTGGTGGAGATTGTAAAAATACATAATAAAAAGGAGCGACAATGGATGCCGCT
CCTTTTTATGTACTACATACCGATTTTCAACCATCTCTTTCTACTTAGTAATAAGACAATAGTATTAACTATA
AATAGAAGAACGAAGAATGATACTGTATTTATAATTTCAGTAGGACATATAAATGTTGACTCGTTATTCAATA
TTTTTTCTACGGCACGATACATCGTGTTGCTCGCCTCAAATGGAGCAACGATACCAAATATATTTTTATTAAT
GGCAACTAAGATGACTGAACCAATCCAATATACAATACTGATACCTAAGCTGATTAAAATGTTAGGTGAAACC
ATACTAATTGTTCCAACAACTAAGATATATTGTAAGATAACGAGTGAAAATAAGATTATTAATAGTAAGTAAT
GTGAGAAATCCGAATATATAATTGAAATAATAGTGATACTTAGAATTATGAACACTAAACATTCAAAAAATAA
CACTGCTACCTTTTTATAGAAGAAGGTAAAGATATTATCGCCAATCAGCTTGTAAAATAATATATTTTTATTC
GAATACTCTTTATTAATAAAATATGCAATAACAAATGAAAATAGTAAGAACCCTAATTGCGTTGCAACAGTAT
ATGAACTGAAGAAAAACTGGCTATAGCTTAAACTTTTAACTTTGTCTATTCCTATTGGTAAAAAATACCCAAG
TAAGAAAAGGAATATGAATAGCACACCAAGCGTGTAAATAATTTTATTGGAAATACTTTTTTTAAATTCTAAT
TTCAAAGTGGACACCTCAATTATAAATTAATGTAATCATTTATGACTTCTTCTTTTGATTGGTACTCTTCTAT
TTGAAGGTCTTTAAAAATAAAGTATTTACCCGGCAAAGCACTTAAATCGGATAAATTATGTGTAATATTGATA
ATAGTTTTAGTTTGATGGCTTTGAATAAAATCATTTAAAAATTCATAAATTTCATTAACTGTTTTCTTGTCTA
AAGCGTTTGTAACTTCATCTAATATGATTAAATCATGATCTTCCAATAAGAAACTTAATAATTTAAGCTTTTG
TTTTTGACCATCACTCAAGTTTTTTAGTAGTACGTTAGACGGTATTGCTTCGATGTTTAGTATGTTATAAATC
TTATCGAATGTTTGTCGATTGTTTTCAAATTTTGATTTTAACAATGATAATAAAAAATCTTTTGTTATATCAT
TAGGAATATTTGATGAACTTGATATTAAGGTTACATTTTGAGAAATGGACTTAGGGATATTTCCACTATTATT
TAGTAAGAAATCTTTAGCTAATAAAGATTTTCCTACACCATTTTTACCAACAATGTGATTGATCTGACCAAGA
TAGAAATTTAAATCACAATTGTCAACTAGTTGTTTGTTTTTAACTTTTAAAGAATAGTTATTTAGTTTCATGT
ATACAACTCCTATGTATAAAGGGATTTATTACACCGATATTTAATTGTATTTTTGAAAATTCTTTCACATTAT
GTATAGATGTTATAAATTAGTATATCACACTATATTTTGTCCAAATGATAAATATATCGTTATATTTTTACAA
TATTCTGAAATTTATGTTCGCCTTCGAATGCTATATCCAGTGTAATGTGTTTTGCATATATGAAAGCAATTTC
AAAATGTGAATATAGGTTCATTGTGGTATGACAAACTTCATTGCTTGTCATGAGATGGATATAATGAAGACGA
CTAAAAGAGCAATGAGGAGGATAATCATGGTTAAGGCTATTGCGGTAGATATGGACGGGACGTTTTTAGATTC
GAAAAAGACATACGATAAACCAAGATTTGAAGCGATTTTTACTGAACTTAGAAATAGAGATATTACATTTATT
GCTGCGAGTGGCAATCAATATGCGAAGTTGAAGTCTATTTTCGGGGATAGAGATATGTATTTTATTTCTGAAA
ATGGTGCAGTTATTTATAAAGGCAATGAGTTATATAATTATAAAAGCTTTAATCGTCAGGTGTTTCAACAGGT
TGTCGATTACTTAAATATGAACCAAGGTATTGATCAACTCGTCATCTGTGGTTTGAAAAGCGCGTATATTTTG
AAACATACTTCTGAAGCATTTAAAGAAGATACGAGATTTTATTATCATCAGTTAAAAGAAATTGATAGTCTAC
AACAATTACCTGAGGATGATTATGTCAAAATAGCATTTAATATTAATCGTGAGACGCATCCGAATGTTGACGA
AGAAGTAGCAACGCAATTCAGCAATGATATTAAACTTGTCTCAAGTGGGCACGATAGTATTGATATTATTATG
CCAAACATGACTAAAGGGCAGGCATTAAAACGATTGTTAGATAAATGGGAAATGTCTCCTTCAGAGCTCATGG
CCTTTGGAGATGCGAATAATGATAAAGATATGCTAGCGTTCGCTAAGCATAGTTATGTGATGGAAAATAGTCA
TGATGAAGAATTGTTCAATATTGCCTCTGCTGTCGCTCCAAGTAACGATAAGCAAGGTGTTTTAACAATTATC

GAACAAGAAGTATTGAACAAGTAGGTAGTACATCATCAATGAAGTAGCTATTATGATACTAAATACATGTTAA
ATGAAAACTCTGAATACGAATCATCAAATATTAATATATCTCAAACAGGTTAGACAATAATGCCATAGTAAAA
TAAGCGCACTAAAATCATTTGAATAGATGATAAAATAAAGCGGATTTTAATCAAATCGATTTTGATACAGATA
TACAAAATAAAACAATTCATGATTTTGAATACTTTTCTTCCACGCAAATGTATACCTATTGGCATTAGCTTCT
ACTTTTGTACATCAATAAAAATCGAATTATCATCAATAAGGTTTTGCTTTAAACATTGACTATGAAACTGGAT
AAATAAAGATTCAATTAATGCATCAGTATTAGGATTTACTCTAAAACGATTAATAGTTTTATAAGAAGGTGTT
TGATTTTGAGCTAACCACATCATTCGAATACTGTCATGAAGTAGTTTCTCTATTCTACGACCAGAAAATACAG
ATTGAGTGTATGCATATAAGATAATTTTTACATCATTTTTGGATGATAAGATGTTGCGCCACGATGGTGTCTG
AATTCATCGAATTCACTATTAGGTATCGTTTCAACAATTTCATTAACATATCGCGAAATATCAATTTGAGGAA
TTCTAACAGAAGTTTCTATTGGTAGTGTAAGTTGGATCAAGTTATAATTTTTATACATAAGGCACTTCATTAA
TTTAGTTTAGTGGTATTTATTAAATTATACGAAGGGACCCAACACAGAAAATTCAAATTATTGAATTAAACAT
TTATGTGCAAGTTTGGCAAAGTGTCTTATTTTTTAAATTATTTAAAAGTAAAATTACATGTTAATACGTAGTA
TTAATGGCGAGACTCCTGAGGGAGCAGTGCCAGTCGAAGACCGAGGCTGAGACGGCACCCTAGGAAGGGACCC
ATCATCAAAAATTCTATTTATAGAATTTTACAGTAATGTGCCAGATGGGCATAGCGAAGCCATTCAATACGAA
GTATTGTATAAATATAGAACAGCAGTAAGATATCTTCTAATTGAAAATTATCTTACTGCTGTTTTTTAGGGAT
TTATGTCCCAGCCTGTTTAAATATATATTAATTTAAACGTGTTTCACATGTACCAGTGTTAATGACAGATAAT
GCTGCGTTTAAACCACCTTCAACAAGGTTTTGTACTGCTTCATCAGAGAAGAAAGCAATATGTGGTGTTACTA
AAATTCTTTCATGTTCGATTAACTCTAATAATGTTTTATCGTCAATGTCTTTATTAGTCCAGTCATTTGTGAA
GTATGCTGCTTCATTTTCATAAGTATCAATCGCAGCACCTAACAAAGTACCATCGTTCACTGCAGCGATTAAA
TCAGGTGTATTGATGACTGCACCACGTGCTGCGTTAACTAAGATTGCACCTTTTTTCACATGATTAAACATTG
CTTTATCGAATAAATGATAGCTTTCTTTGTTCGCTGGAACATGTAAAGAAATAATATCGGCATCTTTAATAGC
TTCTTTAACACTATCTTTATAAGTTAAAAAGTCTAAATCTTTATTAGGATAGGCGTCATAAGCTGTAATTGTA
GCACCAAATCCTGCATATATTTTAGCTGTAGCAGCACCGATACGACCCGTACCAATAATTGCAACAGTCATAT
TTTTAACTGGTTTAGACATGATTTCTGCTTGCCAAGTAAAATCATGTGCTTGTACACGGCGTTCAATATCTGG
GAAGCGACGCACTAATTGTAGGGCGATAGAAACAGAATACTCTGCAATTGTTTCAGGTGAATAACTAGGAACG
TTAGATATCACAATGTTGTGTTTTTTAGCTAAATCTAAATCATACATATCAAATCCAGCAGTACGTTGTGCAA
TTTGTTTAATACCGTAAGATTCTAATTTAGGATAAACGTCATTTTCTAACTTACCAAATTGCATTGTAGTTAC
GCCATCGTAATCTTTTAATTGATCGACTGTAGCACTTGATAATAGCTCTTTAGAAGTAGTTACTTCGACATTA
TTCTTTTTTCCCCAATTTAATGCCATCTCTTTCTCATAATCACGCGTACCAAAGAACATAATTTTTGTCATTA
TTAAAAACCTCGCTTTTAAAAGATTGAAAAGTAAATGAGTGAAATTAAAGATTATGTACATTAAAATCACGCC
ACAATTTAATTGTGAAAAATATCACAAATATATTATAACACTAAATTTTCCAAAATTCAAAAGTGTGTTTTAT
TGCAGAAAACTTATAACATGTACACAAGTTATAGTGAATTGCAAACGGATTACTTTAGTCTTTTTAAAACATG
AAGTATAATTTGTATAGTAATAAATATAAAAATTGGAGGCTATGTTCAATGAGCAATATGAATCAAACAATTA
TGGATGCATTTCATTTCAGACATGCGACTAAGCAATTCGATCCACAAAAGAAAGTTTCGAAAGAAGATTTTGA
AACAATATTAGAGTCAGGTAGATTGTCTCCAAGTTCTCTTGGGTTAGAACCTTGGAAGTTTGTCGTGATTCAA
GATCAAGCGTTACGTGATGAATTAAAAGCGCACAGTTGGGGCGCAGCAAAGCAATTAGATACAGCGAGCCATT
TTGTGCTAATTTTTGCGCGTAAAAATGTAACGTCAAGATCACCGTATGTACAACATATGTTAAGAGATATTAA
AAAATATGAGGCACAAACGATTCCAGCTGTTGAACAAAAAATTCGATGCATTCCAAGCAGATTTTCATATTTCC
GATAATGATCAAGCCTTGTATGATTGGTCAAGTAAACAAACGTATATCGCATTAGGCAATATGATGACGACAG
CTGCATTATTAGGTATTGATTCATGCCCGATGGAAGGTTTTAGTCTGGATACAGTGACAGACATTTTAGCAAA
TAAAGGTATCTTAGATACTGAGCAATTTGGTTTATCAGTGATGGTTGCATTTGGCTACAGACAACAAGATCCA
CCGAAAAATAAAACACGCCAAGCTTATGAAGATGTTATTGAATGGGTTGGACCAAAAGAATAAATAGAATACC
GTATGTCTAAATATATAAAATTAAAAAGTTAGCAATAAAAAAGCCTGCGATTACATAAATGAATCGCAGGCTT
TTGCGTGAAAAAATTGTATTAATAAAGTATGGATGTTTATTTTTCTGGAACAAGGTCAGTATTTGAATGAACT
GTGATGCCAAACCCTTCTGGTGCCGTAAATGTATGTGTTGAGGCGTCGGGTTGATAAATATCAACATGTGTTA
ATCCATAACTTTGTGCATTGTTTTGTCTTGCTTGATTAGATTGCCAAGTGTTAGCGGCAATATGATGGTGATA
GTGATTCGTTGACATAAATAGCGCACGTGGAAAATCAGACACATGTTGGAATCCTAATTGTTCAATGTAACAT
TGATATGCTGCGTCTAAATCATGTGTTTTTAAATGTAAGTGTCCAATCATGCCTTTTGCTGGCATTCCTTGCC
AACCTTCATCAGTACGATGTGTTAATAAAGTTTGGCTATCAACTTCTAAAGTATCCATTTTAACTTTGCCATT
TTGCCATTCCCATGAAGATGAAGGTCTATCGCGATAGACTTCAATACCATTACCTTCGGGGTCGTTGAAATAT
AAAGCTTCACTGACTAAATGATCACCAGCGCCAATGCCCATATTTTTTTGTGCCACGAAATATAAGAAGTTAG
CTAGATCCTCAGTAGTTGGTAATAAAAATGCTATATGAAAAAGTCCTGCTTCACGTGGGGAAGTCTGACGGCC
GTCTTCTAATAAATGTAACGTTAGAGTATGGCCACCAGTCCCAACAGATAATACGGTTGTGTTATCGTCAGAA
CTTTTAACGGATAGTCCTAAAATGTTTTTGTAAAATGTTGTCATTAAGTCTAAGTCTCTTACGTTCAGTTCAA
TGTTTGTCACTTGTGTTGCTGTTTTATCGTGAAATGCCATTATGCATCGCCTCTTTTTCTATTTTTCTATAAG
TTAGTATAAAAAGTATACCAGAAAAGAAAATGAATTGACAGCATAAAGTTTGAAATGCAAAATAACTAGTCGT
TTTGCAATTTTATATTGATGCGAACAAATAAAAAGCGATGGCACAGTTGCACCATCGCAAAATTTATTTAACC
AAGATATACATCTTGATATGAATCTTCTTTTTCTAACATATGTTTGGCAAATGAACATGAGGCAATGATTTTT

AAATGATTTTCGCGAGCATGTTCAACAACTGCTTTAACTAGTTTTTTGCCAACACCTTGACCACCGAGTTCAT
CAGATACGCCTGTATGATCAATGTTAATTTCATTATTATCCACAAAACGGTATGTGATTTCAGCTAAAGCATG
ATTTTCATCATCATCAATATAGAATTTGTTCTCGCCTTGTTTAATTTCAAGGTTACTCATACATATCAACTCC
TATCATGATTGATTATAGTATTTCCCTATTCTATTTTAACTTAAACGAAGTCAAAGGTGCATGACAGTCATGT
GACGACATTGCCACATTTATGTAGTCGTTTTTATTAAGCGCAGTTTGAAATAAAGATGAAAACACGTATCTTG
ACATTAAATCTATTCAGCTATATAATTTATCTCGAAATCGAAATAAAATGAAAAAGTTGGTGATGATATGGAT
CGAACGAAACAATCGCTCAATGTCTTTGTCGGAATGAATAGAGCATTAGACACATTAGAGCAAATTACAAAAG
AAGACGTAAAACGTTATGGATTAAATATTACTGAATTTGCAGTGCTTGAGTTGCTTTATAATAAAGGTCCGCA
ACCAATTCAACGTATTAGAGACCGCGTATTAATTGCAAGTAGCAGCATTTCATATGTTGTAAGTCAATTAGAG
GACAAAGGTTGGATTACACGTGAAAAGGATAAAGATGATAAACGTGTATATATGGCTTGTTTAACTGAAAAAG
GTCAAAGTCAAATGGCAGATATTTTCCCTAAGCATGCTGAGACATTAACAAAAGCGTTTGATGTGTTAACAAA
GGATGAATTAACAATCTTACAACAAGCGTTTAAGAAACTAAGTGCACAATCTACAGAAGTGTAAGGCGTGCAC
TAAAAATTTACATTAAAATATCTCGATTTCGAGATAAATACACTAAAAAGACAAAGAGGGTATATAAAATGGC
AAATAATCATGAATTATTAGGTATTCACCATGTTACTGCAATAACAGATAATGCAGAACGTAATTATAAATTT
TTTACAGAAGTACTAGGCATGCGCTTAGTTAAAAAGACAGTCAATCAAGATGATATTTATACGTATCATACAT
TTTTTGCAGATGATGTAGGTTCAGCAGGTACAGATATGACATTCTTCGATTTCCCAGATATTCCTAAAGGGCA
GGCTGGAACAAATTCTATTACAAGACCGTCTTTTAGAGTGCCTAACGATGACGCATTAACATATTATGAACAG
CGCTTTGATGAGTTTGGTGTCAAACACGAAGGTATTCAAGAACTATTTGGTAAAAAAGTGTTGCCATTTGAAG
AAGTCGATGGCCAAGTGTATCAATTAATTTCAGATGAGTTAAATGAAGGTGTAGCACCTGGTGTACCTTGGAA
GAATGGACCGGTTCCAGTAGATAAAGCGATTTATGGATTAGGACCCATTGAAATTAAAGTAAGTTATTTTGAC
GACTTTAAAAATATTTTAGAGACTGTTTACGGTATGACAACTATTGCGCATGAAGATAATGTCGCATTACTTG
AAGTTGGCGAAGGAGGCAATGGTGGCCAGGTAATCTTAATAAAAGATGATAAAGGGCCAGCAGCACGTCAAGG
TTATGGTGAGGTACATCATGTGTCATTTCGTGTGAAAGATCATGATGCAATAGAAGCGTGGGCAACGAAATAT
AAAGAGGTAGGTATTAACAACTCAGGCATCATTAATCGTTTCTATTTTGAAGCATTATATGCACGTGTGGGGC
ATATTTTAATAGAAATTTCAACAGATGGACCAGGATTTATGGAAGATGAACCTTATGAAACATTAGGCGAAGG
GTTATCATTACCACCATTTTTAGAAAATAAAAGAGAATATATAGAATCGGAAGTTAGACCTTTTAATACGAAG
CGTCAACATGATTAATTGGAATGAGGAGGATTTGTGATGGAACATATTTTTCGTGAAGGACAAAAAGGTGCGC
CAACGCTAATATTGTTGCATGGTACAGGTGGTGATGAGTTCGATTTATTGCCATTAGGCGAAGCATTGAATGA
AAATTATCACTTGTTAAGTATTAGAGGACAAGTTTCAGAAAATGGGATGAACCGTTATTTCAAACGTCTTGGT
GAAGGTGTTTATGATGAAGAAGATTTGGCATTTCGTGGACAAGAATTGTTAACGTTCATTAAAGAAGCTGCTG
AACGTTATGAATTTGATATTGAAAAGGCTGTGCTTGTTGGATTTTCAAATGGATCAAATATAGCAATTAACTT
AATGTTGCGTTCAGAAGCACCATTTAAAAAAGCATTGTTATATGCACCGTTATATCCAATTGAAGTAACGTCA
ACGAAGAATTTATCAGATGTCAGTGTGTTGCTTTCTATGGGGAAACATGATCCAATTGTACCATTAGCTGCAA
GTGAACAAGTCATTAACTTGTTTAAGACACGTGGGGCACAAGTCGAAGAAGTTTGGGTGAATGGTCATGAAAT
TACAGAAACTGGATTAACGGCTGGTCAACAAATACTTGGGAAATAACAGTTCTATTAAGAAGCGGACAGATGG
AAAAGATTTTTACTTTTCATCTGCCCGCTTTTTTGATTTTGAAGTGTTGTACTAAATTTTACAATAGTATAGA
TATTTTAATCGATATGAGATTTGCCGGTAATACGCTTAATTAAACCTTTATAGAGTACAGGTATGAGTAAGAT
GAAACCGAACAATCCCATAATAGGGAATACTTTTCCAATTAATGAAATGAAACCGATAAATGTACTAATATAA
GTGATGACAGCCATTGTAATAATAATGATGAAGTAACGTCTGCTAAATGGAACGCTGAAACGTGACGCAAATG
CATACATTAATCCAACAACAGTATTGTAGATGACAAGTATCATAATGACAGACATAATAATACCGATTGACGG
AGACATTTGTGTCGCTAATTTTAATGTAGGTAAGTCGACGTGTTTAATTTTATCGAATTGAGAAATTAAACCT
AGGTTAATCATCATGAGTAAAAATGTAATGATTAAACCGCCAATCAAGCCCCCGTATAACGTTGAGTCACGAT
ACTTAACTTTACTACCCATCACTGATAAGAAGCTGAAGGCAGCAGCAATTTGCAAGCTTGCATAGTTAATTGC
ATCAAACCACCATCCAGGTGATAATGATTTCTGCTTATGAATCTGAGCATCATTATTAGCGGCAGTAAAATCA
AGATGACTCGTTGTGAAATAGTAGACCGCAATCATAATGACAATCGCAATTAAAAATGGGGTAACACCGCCAA
GCACAGCAATTAAACGATCGAATTTTAGAAACAGTGTTGCTAAAATAAAGGCGACTAATATGAGTGCGCTCAG
CCAATACGGTAAGTTGAAACTTTGATGAATGGTTGAAGCGCCACCTGCAGTCATAATAATAGCTAAAGACAAC
ATAAACATTGTCAAAATAATATCAAAACCTCTTGCAATAGAGGGATATAAGAAATAGTTAATTGAATCAGAAT
GATTTCTAGACTTTAGATGATGACCTGTATGCATGACAATCATTCCACCTAAAGTAATCAATAGTCCTGTCAC
AATAATGCCTGAAATGCTATACACGCCATGACTTGTGAAAAACTGGAAAATTTCTTGACCAGTAGCAAAGCCG
GCACCAACGACAACACCAACAAAGGCAAATGCCACAATAATGGACTCTTTTAAGATACGCATGATTTAAAAAT
GTCCCTTCGTAATTTTAAGTAATATAGAAAATGTAACATACATGTTAATGAAAAATATAGTACTAATATAGTA
TTTTGTTAAATTGGAGTAGAAGCGAGGGCGTCGGTCATTTCATTAATTTATTAGTTGATTTTGCATTTTTTTG
CTGTGAAGTTGTTATAATACAGTTAACAGGAACTAGCATAGATACACCAATCCCCTCACTACTCGCAATAGTG
AGGGGATTTTTCGGTGTAGTTAGGTCGACTATTTACCATCGCGTTTGCGTAGCCAATGCGTAAACAAAGTACC
ATTAAGTAAGTGCACGATGCATGCACCAAATGTCGTCTTTAGTCTAAGTAACGATCATGCATTAACATTTTCA
AAATATCTATTTGAGCTTGAAGCTCTTTACCTTTATTTGTATTACGGATTTTTTTACGTTGCAATTCTTTATC
AACAATCCGTTTAATTGACAGTTCGTCGATACCTTCGGAAAGAATTTTTTCTTTTGCGTTAAATTGTTGATGT

GCAACGAGTTGCATTCCGAATGAATTATACAACAATGTATAACCTGCAATGCCAGTTGTTGACTGATAAGCTT
TTGAAAAGCCACCATCAATGACAAGCATTTTTCCATCAGCCTTGATAGGATCTTCGCCATTAATTTCTTTCAC
TGGTGTGTGACCATTAATAATGCGTCCTTCATCTGGATTTAATCCAAAATCACTGAGCATTTTACGAACCATA
TTCACATCTTCACGTAGATGATAGTACGGGTTTTTTTCTTCTTTATGAGAAGCTTTATCTGCAATAAAATATC
GCTCAAACGTAGTCATCGCACGTTTACCAAATAGTGAAGAATATTTCCCAGTCCATAAATACCAAACTAAATC
AGTGGATAAGTCATCAGTATTTTCTTTTTCATCAAATGATTTACGGACATGATATTCAAACACATCTAATAAC
TCTTGGCCACTGTATGTCTGACCATCAATTTCGAATGATTCCATCTCACCATTTTCATCAACTGGAATACAAC
CATGAATGAGTAAATTGCCATTATAAGGTAAGTAAAGTGAGCCTTTACGCATCAAGAAAGACATATGACGACG
TAATTTTTCAGATTGCTGGAATGACAATAATAGTTTATTCATGACTTCTTCTTCTTCAGGTAGTAATTCTGCT
GGGTTATCACGGTTAACAGTTTGGAAGCATGTATCTTTTAATGGATATGTTTTTCCGTATACTGTTATTTCAT
TAGTATCGTAATTAACTTTTTCAAGTACAAGTCGTTCGTCCATTTCAAAGTTTGGACGGCGCTTAATAACCGG
TATTTCCAGTTTGAATTGAATCATCGCAATAGCTTGATGAATTTTAGTAATCTGACTTTCTTCGCGTTGTGTT
AAACGTTCATGTTTATCAGGTCTTTTTTTAGGTTTAAAAGCAGGATTATCTGCATCATAGTATTTTTCGGCTA
ATGTTAGCAAAGGTCTTAAATTAATACCGTAAGCATCTTCAACGATATCTAGATTGTCATAACGTGCACAAAT
ACGTAGTAAATTAGCTAAACATACTTTAGACCCTGCATAAGCACCAACCCATAGCACATCATGATTACCCCAT
TGAATATCCAGAGAATGATAATTAATCAGCGTGTCCATGATTTTATCTGGTTGCGGGCCACGATCATAAATAT
CGCCGACAACGTGTAAATGATCGACGACTAAGCGTTGTACAGAATACGCAAGCCCTATAATTAAATCATCCGC
TTGCTTAAGCTCAATCACTTGATTTACAAGTGTTTCGTAGTAAGATTTTTTATTTTTGAATTCGTTGTTTTTA
TATAATAACTCTTCTACGATGAAAACGTATTGCTCCGGCAAGGCACGTCGAAGTTTTGAACGCGTATATTTTG
AAGAACAATATTTAATTAGCTGAATGAGATGTTCAATCGTCGTAATATACCAAACATTTAATTGTCCGTAATT
TTGAAAATCGCATTTAATAAGCTGCAATTTGTCTTCTGGATAGTAAACAAGGGCAGTCAAATCGTTAAGTTCT
TTCGTTGATAACTTGTCTTTGAAAATATCATTGATTTTCGCTCGCACGTTCCCAGAACCGTTGCGTAATACGT
GTTGGAAAGCTTCATATTCACCATGTAAATCGCTGACGAAATGCTCCGTACCTTTAGGCAATTCTAGAATAGA
TTCTAAATTAATAATTTCAGTCGCAAGCTTTTCTGGAGTATCGAAATGTTGAGATAATAAATCCAAATATTTC
TTTTTTAATTCTCTTTCAGTAATTTGAGTCATTGTGTGTCACTCCTGTAGTATATTTCAATCATTGGTTTGTA
AAATTAATACGCTTACAATGATTATCAAGAAAACGTTAACATTTTTCAATAAGTATGCTTGAATTGATAAAAA
TAGAGTAAACATATTGTGAATTTTAGTGTTGAGAATCTATGGTTGTATCATTCATATTAGTAGCGTTGAAGTG
TACCCGCATAAATGTGAAGTGATAAAGTTGTTGAAGCAGAGTTGAAATAGCTTGGCAGAGATACGATTATCGT
ATAAGTGAGCATATTTTGTACATTACAAGTGGGGGGATTCTGGAATATATAAAAGATAGAATGAGTGGTATGC
TGACATATCAAAAATCCCCGCACCATTATGGTGAAGGGATTTGAAAATGATTTTGAATTACGATGGAATCTGT
GTGTATTTATTCATCTAATACTTTAAGTTACTAAATATTTTTCTTCTTGAAAATAAATACTACAAGTATTAAG
AAAATAGAAATATAAGCAATATTACCAATAAACAATTCATGAAGTTCTAATTTTGTCGACTTACTAAAGCCTT
TTTCAACTGTTTGAAGCATAATATTCATCATATTAATAGGATTCCACTTTAGCCAGTCTATTTTTTCTAAAAG
TGCCGTTTGAATAACTGCTAAAATAGAACTTGCAAAATAAAAAACAATACCTACAGCAATGGCTACACCCGTT
GAATTTGTTGCAGATGATAATAGCAACGTTAAGCTTAAAACTAACCAAACGCCAACAAATGTGCCTAAACTAA
CTAATAATAATTGATTCAATAAAGATAATTGATTACCGCTACTTTCAAATATATTTAAATCATTAAAGAATAA
AGACCCAATAACAATTGAAGCAATAATTGTAATAACAAAATAAATTAAAGAAATAATAAATAATGTGATGATT
TTGCTAACAATCATAGTTGTTCTTGAATATTCACGATAGAGTAAATTTTTAATCGTACCGTAATGAAATTCCA
TTGAAATGATTGTACTTGCTTGAATAATTAATAAAAATGCAAACCATGAAAAACCATTATAAGCAGATGTAAA
AGATTCCTGTGGCGTAAAAATTTCATTGTATTTTGTAGCGATATAACCTTGAGCAACCATTAGTAGAATAAAG
ACAATAGGTGCGATAAAAGTTGATTTCTTTTTAAATAATTTGAAACATTCTTGTTTAATTAAAGTTCCCATTT
TATGCTTGATCCTCCCTTTCTATGATTTCTAGTAATACATTTTCTAAATCATTATCTTTAAATTGTTTAAGTT
CTTCTTCCGATGTTTCTGTAACAATTTTACCTTTGTTAATAATAAGGATAGAGTTTGTGATTTTAACTAACTC
ACTTAAAATATGACTCGAAATTAAGAAAGTGACACCTTCTTGCGCTTTTTGTACAATCAATTCACGTACATCT
CGCACAGCTTTTGGATCTAAGCCGTTCATTGGTTCATCTAAGATAATGAATTGAGGTTTATTTAAAAATGCTA
TAGCAATTCCTAATTTTTGTTTCATACCAAGAGAATACGTTTTAGCTTTTTTATGAATGTATTCATCCATATG
AAGTTGTGAGACAATTTTATCGATATCTTGAGTGTTTTTTGATTCGTTCAATAACTTCAAGTTTTCATATCCA
GACATAAAAGGATATATTCCTGGATTTTCAATCAATGCACCGATATTGCTTTTGCTGTCCTTGCTGTTAATAA
CATTAAAATTCCCGCTTTGGAAACTAGAGTAACCTAATATAACTTTCATTAAAGTTGTTTTACCTGCACCATT
CGCTCCAACAAGACCAACTATTTGTCCGCGTTTCAGCTTAAATGATACATCTTCGAGAATCGTTTTGTTGCCT
ATCTTCTTTGTTAAATGTTCTATTGTTAAAACATCCATATCACTATCTCCTTTGCTATTAAATAATCTGAATC
TTAAGATTTTATATATAAAATTAATTTTATCACATAGCAAGTGTTATAAAAATATGGATTTAAAAAACTTAAA
TAATATAAAATATAAATATTAATATGAGTATTTTTGCAGCATGCAAATGGCATTGCTACATTTATTAAAATGC
GCAATTTGTTATGAGTGACTTTTTTGAATAGAAGGCATATTACAGTGACGAAAACAGTTTGTGTGGAAGACAG
GGAAAATTCTGCTTATTTAAAATGATAGTTTGTATTATAGATGGATTTGTGAGGTGGGTTTGCGGAATAAATG
GTGCATAACTAAGTAAATATATGTTAATTACAACAATATTTGACAAAAATGGGAGTGACAACAATAATATAGT
TAACCGGTTAACTAATGATAAAGTGAATAATAAATTTTTGTCTGAAATGTTAAATGTATCAAAACCAGCAATT
ACTAAATCGATAAAAAAACTTTTAGCGAAAGGCTTAGTAGTTGAATCGCACAATGAATTTAACAAAAGAGAAG

TTAATTATTCATTAACACAAGAAGGAAAAAAATTATCTTATATACATGATGAATTACATGAAAAAGCGGTAAA
AAAATATGAAGAAGTACTTAAAGTCTTTGATAATGATGAAATGGCAGTTATTGTAGAGTTTTTAAACCGTAGT
ATAGAAGAATTAAAAAAAGAAGAAGATGGTTTAAATGACTAATAGTAATATCAGATTATATATTCTAGGATTT
TTAGCATTTTTCGCATCATTGATTCAAAACATATACACGCCTATTATTCCGAGACTATATGACGATTTTCAAG
TACCACTATTTTGGATAAATGCGACAGTTGGCGGTTTTATATTTATCGTTGCAGTTATGCAAATTGTACTTGG
TAGAAGTATAGATTCTCGTGATTCGAAAAAAGTACTTTTAACGGGATTGGGAATAGTAATCATATCTAGCTTT
ATTTGTGCAATGACACATAACTTTATATTATTTGCTATATCAAGGTTATTTCAAGCAATTGGTTGCGGAATTA
TTCCTCTCGTGACTTTAACTTTATTAGCCAAACTAAGTACAGATAATGGTAGAGCTCAAGCTATGGCGAACTA
TCAAATATTTTTATCATGTGCGCCAGCTTTGGCACCAATCCTAGGGAGTACTTTAGGTGCAAGATGGGATTAT
ATTGGTATTTTTAGTTTTTTGCTTGTTATATCTATCGTATTATTTCTGATTATCTTTTTTATTGATATTCCAA
ATGTTGAAAAAGGGATTGTAAAATTAACTGAAAAAATTGAAGAAAAATATTTGATCGATAAAGTGTTTATTAC
TTTAGTGACATTGGGATTTTTAATATTTTTGACGTACTTTTCAATATTAGTTTATTTACCGACATTATTAAAT
AATACCTATGATATAGGCGTTGGAATATCAGGAGTTTTGTTTTTACCTATTACTGTTAGTGTTATATTAGGAA
GTTTATTTTATAAACGATTGTCAAAAAAATATAATGAAATAATGATACTTCGTGTTACTATAACTGGATTTGC
TATATTTACATTATTGTTTGGATGGTTAAACGAATCAAATGTAATTATATTATCAGTAATTATATTTATATTA
GGTACTTGTGTTGGTATTGTGCCAGCTTTGCTGTCTACTATAATTTCTAAAAGGTTTGAACATATAAAAGGAA
AAGTGTTAGGTGTATTTAACTTTGTGAGATATATTGGAATGACTGTCGGCGCATTATTAATTGGTATCATTTC
TCACTCATTGGTACCATTTTATTTCACAACTGTAACTATCATGTTAATAATAATATTTCTTTATATAAAGATA
GGTGACTTTCAGCTAAAGTTTGCCAAATAAACTTAAAAAAGCAGTGAATCATTTCAATAGATGGAATGATTTT
ACTGCTTTTTGTGTATAGAAATTTAAAATTCATGCCTTTATACTAACAACAATATTATTTTACTCTTTTACGT
TTGCCGATGTAACGTATAGCAAAGTAAATGACTGCGATAATGATGATAACATACATAATACGTGAATATGTAT
GAAGACCAGTCATCAACATACCAAAGCTATCGCTCAAAGTGCGTCCTAGTAAAATTAAACCGAAATTCCAAAT
TGTTGTACCTATTAATGAAATAACGGTAAATGTCACAACATTCATGCGGTTTACACCGGCTGGAATGGTAATC
AATACACGTAATACAGGTATGAAACGACAGATAAATACAGCCCATACGCCATACTTTTTAAACCAATCATTTG
CTCTCTTTAAATCCTTACTTTTCAATTTAATCCACTTACCGTGTCGATCAATAAAACGATATAGACGTTCTTC
TGAAATCAAACGACAGATATAATATAAAATTAACAGCCCTATAAACGATGCGATGGTTGCAATAATAAATAAG
GTTAAAATTGATAAATGTGATTTAACAGACATAAGGCCAGCAAATGTCAGAATAATTTCCGATGGTACGATAG
GTAAAACATTTTCTAATAAAATTAATATAAAAATGGCTGCATACCCAAAACGACTAATAAATTCAGTGATAAT
TTGTTCCATTATTAAAAATAAGCTCCTTTAATTTTCAGCAATTGCAGACAAATAGAAACTGGATAAATTCAAA
TGAATAATGAATGATGTCGTATGCGATGTTGAAACAAGCATTTGTCTTAAAATGTATTATAAGCAATAAATCA
AAAAATTGCTTGCTATAAGTACGAAAAGTCAACAAATGATCATCTAAATAACAGGCGCTGCACAATGAATGAG
CAATGTAGTTTTATGCAATTTAAAATGAATGTCAAATATGTAAGAAAGCCTGCAATATACGTTTTAAAACACT
AGCATTGCAGTATTTTTAATTAACAAATGATAGGCTACTATGTAACTAAAAGTTCCTTTATATCGTTTTAATA
GCAAAAGTTAAGCGGAATTTAATAAAATTTTTATAGATTCTATTGTAAACGCTTTCCATGTCTGATAATATTA
TAAAAAATAACTTTGGTACAATTTTGTGACAAAGTAGGGGTGGACATGCCAGATGTCCGAAAATTTTAACGTT
TCAGGGTAAGTTTTAAAAAATTAATTAGGGAAGTAGGAATAGTTATGAAGGAAAAACGAACTAACGTAAGGTG
GATGTTCGCGCTTGCGTTCTTCTTTATTGGGGTTATTGCGTATATGGATAGAGCAAACATTTCATATATCGCT
AAGCAAATGATGGATGATTTAGGGATGACAAAGCCACAATTTGGTTTATTGGCATCATTCTTCTCTCTAGGTT
ATGCATTAATGCAAGTACCATCGGGGATGTTGGCTGAAAAATTCGGTCCACGTAAGATGATTACAATTGCATT
AGTTTGGTGGAGTGCATTTACAATCTTAACGGGTATGATTAAGAACCACGGTTTAATTTATTTAGTGAGATTC
TTATTTGGTGTAGGTGAGGCGCCAATGTACCCTTCTAATGCTGTGTTTAACTCATTTTGGTTCTCTAAAAATG
AAAAAGGTAGAGCATCAAGTGCATTATTAGCAGGATCATATTTCGGACCTGTATTAGCACCAATAGTTACAAT
TGCTATTGTTAACGCATTTAACTGGCAAGCAGTATTTTACATTTTTGGTGCAGTAGGTATTTTAATGGCTGTA
TTATGGGCGATTATTGCCAAAGACTTACCTGAGCAACATAGAATGGTTAATGAAGCGGAGAAACGTTTCATTA
TGGAAAATCGTGATATCGTAGCTACTGAAAAGTCATCACCACCATGGAATGATTTCTTTAAGCGTTTTAGCTT
CTATGCAATTGCAATTCAATACTTTGTTGTACAATTTATCATTACATTATTCTTAATTTGGTTACCGACGTAT
TTAACAGAAGTATTCCACGTTAACTTTAAAGAAATGAGCATTAGTTCATTACCTTGGTTATTAATGTTCTTCT
TAATCTTATCAGCAGGTGCAATTTCTGACCGTGTATTAGGATTAGGTCGTTCAAAATTCGTAGCTAGAGGTGT
AATTGCAATCGCAGGATTTATTGTGTTTGCAGTTTCAATTATCTTTGCTGTACGTACAGGAAATTTATATGTA
AGTATTTTCTGGTTATCACTAGGTCTTGGTGGTATCGGTATTTCAATGGGTATGAGTTGGGCTGCAGCAACTG
ACTTAGGACGTAACTTCTCTGGTACAGTATCAGGGTGGATGAACTTATGGGGTAATATAGGTGCATTAATCAG
TCCGCTATTAGCAGGTCTATTCGTAGAACATTTGGGTTGGACAATGACATTCCAATTGTTAATCGTTCCAGCA
GTAATCGCTGTGATTATGTGGTTCTATGTGAAACCAGATCAACCTTTAATTGTTAGTGATGATAAAGCAATAG
AAAAATAATTTAAACAAGCAGTAAGCTTTCACATTGTTGGGGCTTATTGCTTTTTTTACGTTGAAATTGAAAC
TTTTTAAAACAGATATGGTTTAAGATGAAAGAGAAGTTATTAAAATGATGTATGTAAAGAAATAAAGTTTTAA
AACATTAGTCAGGTAACGCTTATAAAAGTACATATAAATTTAGGAGGTTATAAATATGAAGGAAAACTTAGA
GAAATATATTAGAAGTTTGCCTCTTATAGGTCTAATTATAAGTATTTTTTTAATTAGTTTATTCTTCTTAATT
TATCGAGTAGAAGGAAATTTTTGTGTAATCATTTTGTACTGCTTACTGCCATTATTTGTAAATACGTCACTAT

ATATATTATACGTAAGCATCTTTCGATATTTTAAAAAATGAACTGACATCGAATGTCATTTTAAATAACGAAT
ATAGGAACTATTCAAGCTTATGAAGCAAATATGTTTTATAAACTTTCTTAAATTTACTATAGTTTGGATCTTG
AACCCTAGGAATAATATGAAGTAAATAGACACTAAGTCTATGTGAATGTTTTTCTAACCCTAAATGTATAATT
CGCTAAAATAATTTCAACATGTCACAAATAAATTTTAACTAGCGCAAAGGTGGGCGACCAAAGGTTCAACGAT
GTTAAATAACATTAGAAATTAATTTTAATTGGACTTTAAAAGTTTTTAAATTTAGATAATTGAGCATAAGGTG
TTATAATGACATATGTTGCGTAATTAAAATTTATAGCAACAAATTCATTTTAACTATGCTAATAAAAAGATTA
TGGAAATATTTTGACAAGGAAAGGAGAAGTCGAAATGACATCTTTTTGACATCACTCATAAAAATCAATCGAC
TTAACTTAGACTTTTATAAAGGTGTAAGACAGGGACTGTTAATGATTATTCCCGCAATAATCGGTTACTTATG
TGGTAATTTCCAATTTGGATTATTAGTTGCAACCGGAACACTAGCCCATATTTATGTTTTTAAAGGTCCGTCG
CGATCTAAGCTGCGAACTGTAATAATTTGTAATTTAGCGTTTGCAATATGTATGATGCTTGGTACGCTAACAG
CCAAAACGCCACTCGTTTTTGGAATGACATTATTAATTGTTACGGTTATACCATTTTATATATTTACTGCCTT
AAAAATAGCTGGACCGTCATCGACATTCTTCATTGTGACATTCAGTCTACCCATTAACTTACCTATAGCTCCC
GAAGAAGCATTATATAGAGGCTTTGCGATTTTAGTAGGCGGTATACTTGCCACTATGATGGTGTTAATCACGA
TCGTATTTTCTAAAAACAAAGCTGAAGAACAAGCAATTCAAAATGATTTTAAACTCATATCTAAGTTGTTACA
CACTTATAATGATAAATCTGCATTTTTACAAGTAGCAAAAACAGCGGTGGATAATTTTAAAGCATCTGATAAA
TTATTAATCACTTCTACTTCAAGTAACGATAAATTAAGTAGACGTTTCCAAAAATTATTATTATTACACACAT
CTGCCCAAGGGATTTATTCTGAACTGTTAGAGTTGAACGCTAAACAAATTCGACCATTGCCAGATGAGTTAAT
TGAAATGATGGATCATATCATTGCACAACTAGATAATAGTGAGGAAAATGTAAGATATTGGCGAAAAGAAGTG
ACAGTAACAGAGGAATTTCAAAATTTATTCAACCATATATTGAAAATTGATGAAATGGTGCATGCAAATGAAG
CGCGTATTGCGTATGAAGCAGACATGCGAAAACCTTTATATAGTAAACGCATTTATCAAAATTTAACATTAGA
CTCTATTGTTTTTAGAAATACATTGAGATATACAGCGATTATGATGATAGCGATATTTATTGCGTTAATGTTT
GATTTTGAAAAAGCATACTGGATACCGTTATCTGCACATACAATATTACTAGGAACATCAACTATACATGCAA
TCGAGAGAGGTATGGCACGAGGTTTAGGTACTATTTTAGGTGTGTTAGTACTTTCAGTCATATTGTTGTTTTC
AATACCAACACCTGTTGCAGTAATTTTAATGGGCATTGCAGCATTGTTTACTGAAGCATTGGTGGGAGCAAAT
TATGCGATTGCAGTAGTTTTTATTACAATACAAGTTATTTTAATGAATGGATTAGCATCACAGAATTTAACAA
TTAACATTGCGTTTCCAAGAGTTATTGACGTTGCAATGGGTATTGTGATTGCAATCATAGGTTTATTTGTCCT
TGGACAACGTACCGCATCCGCATTGCTTCCTAATGTAATGGCTGAAGTTGTTCGTAAAGAAGCAACGCTCTTC
CATTATTTGTTTTCTGAAAACCAATATAAAGATGATGTGTATCAGAAAAACACGGCTATGAACCTATCTGTGA
AATTAAATAACATGACGCAAGTTTATAATGCAGCTAATGGTGAATTATTTAGTGATAAGACATTGATTCAAAA
TTATTATCCTAGCTTATTCGCATTAGAAGAAATTAGCTTTATGTTAAATAGAGCCATGGCTAATGAAGATAGA
CTTACAATAAATGAACAATTAATGGGCGAGTATTTAGCAACATTTGAAAACATCGCTAAGCATTTAGAATTAA
ATACAGAATTAGAAATAAAGATATTACCAGATTTACCACAGTATAATTATATTCAATCAGCAATGATGAATAT
TCAACACAATAGTTTTTGTGAGAGAGACAAGAACGTTTAAAATAAAATATTTATCAGGTCAGACTAACGTTTG
TCTGAAGTTATTATGACGGGAGTCTAAAAAATTGTGTTTAATATGTTTCATTTTATAATTATGGTGGTATATA
ACATGAATTATAACATATTGGAGGTTAAGGAAATGCCCTTTACAATGAAAGGTTACCCCGAAAAATGGAAAGA
TTTTGAAGTAATTAAACGGCAAAAAGCGATAGAAATTGCGAATGCAATGTTAAAAGAAGGATATCTTGAAAAA
GATGTTGTTCCTATTGCGGCGAAAAAAGCGAAAGATTGGTATCGAGCGTTATCGAAAGAAGAAATCAAAGCAT
TAGAAGAAGAGAACTTCGTACAATTCCATACAGCAACAATGGAAAAAACAGAGGTGGATAGTGAATATGCAAC
AACTGATAATGAAGAACATATTCAAGAACAATCAAATCAAGATAAATCGTACTTTGATAGCGAATCAATTGGC
AACCAAAGTGGTGGCGTTCGACAAAGAGAACCGCTTATGGATGGTCCACATCCAAAAGTTGTGGAATCATTTA
AAGACGTATAACATCTCTATCAATTAAACACTTGTACCTATATAACGGTAACAGGTGTTTTTTTGCGTTAAAT
CATTTTAAAATTACTTCCTATAATTGAAGGTTTTGATACATAATTCGAATTGTTTGAGTTTCTTTGTGCTGTT
TCAGTATCAGCCCAAAGTTCGATAGTGTATTAGTATTATCTTAATAAAATGTTAGGTACAATAAAGATGATTA
TATATCGGAGGTTAGTATAAAAATGTATGTAGATCGAAAACCATCACTATATTTAGAGGATTTGCGACATGAT
TTTAAAAATAGTTTAAGTAAATTTGAAAATGGTGATGAAGCATTTGATACGTTATTAGGTTTCGTAGAGTTAG
ATCATATTTATTCGTCAGCACTAAAGGAAATAAGCACTAAACTGAGTATTTTAGACGATAATTTCAATCACAT
TTATAAACACAACCCTATACATCATATGGAGCGACGTGTGAAAGAAATGCGCAGTTTAATAGAAAAGCTTAAT
CGTAAAGGATTACAGATTAGCGCAGAAACTGCCAAAGAACATATACTGGATATTGCCGGAATTCGCGTAGTAT
GTAATTACTTAGATGATATTTATTTGATTGAAGAGATGTTGCTTAAACAAGAAGACGTACAATTGATAAAACG
TAAAGATTATATTCAGCACCCTAAAGAAAATGGTTACCGCAGTTTACATATCGTTGTATCCATTCCAGTCTTT
TTAGCAGAACGCGTTGAGGTATTGCCTGTTGAAATTCAAATTAGAACGATAGGTATGGATATGTGGGCAAGTT
TAGAACATAAAATACGTTATAAAAACAATGCAGAGACGGAAAAGTATCGAGATTTACTGAAAGAATGTGCGAC
AGAGATTACTGAAGTTGAAGATAAATTACAACAAATTCATTCTGAAATAACAGAGTAGACGTTTAGTAGTAGT
GTTGCTTAATTTGAGTGATTTTGTAAATCAACATGGTACGAAATCACAAAAATAAGACGCCTAGTATAATAAA
TTTCTTTAAAATGTAGAAAAAGTACTTGCTCGTGACGTCGGGTCATCAACTATGGTGATTGTAGGAGGTGGAA
TCTATGTCTAACTATTCGACTGGAGAACTCGCGAAATTATGCAATGTGACAACGCGAACGATTCAATATTATG
ATCGCAAAGGTATTTTGAAACCACAAGGATTTACAGAAGGAAAGCGTCGTGTGTATACAGAACAACAGCGACA
AACATTAGAGTTAATCTTATTGCTTAAAGATTTAGGTTGTGCGTTAAGCGATATAGATATGTTGCTAAAAGGT

GAAGGTACTTTGAAGACACTCAATACTTTACTAACTATGAAACAACAAGAAATTAATCAACAAGTCAAACAGC
AACAAGCGGTATTAAACAAAATTAAAAATGTTCAATATTACGTAAATAAAGCGTCGACGTCTCCAATCACACA
TTTAAAAGACATAGAGCATGTCATGAGTAAATCAGCTGAAATGAAAAGTATTCGTCGTAACATTTGGATTAGT
GCGGGTATTATAGGAATAATTCAATATTCTAGCATTATAAGTGCAATTTTGATGAAAAATAAATGGCCGTTTC
TAATTGCGTTGCCGTTTATGATTGGATACGGCATTGGTGTTACTTTTTATTACCAACAAAAGGTTGCCTATTT
ATGTCCTAACTGCCAGCATATATTCTCACCATCTTTGTGGGCAGTTATCAAAGCGAAACATACAGCGACAACA
CGTCGATTCGAATGTCCGAACTGTCATGAAACGCATTATTGCATTGAAGTACCTAAAGCGCATATGAGTACAG
AACAATTAGAAATATCCCGCATACAACATAACAATTAAATTATTCGTGTGATAGTGACCATTCCCTTTAAACT
TACATCGAAAAGGGGTGGTCGCTATTTTAGTACGCGTAAAGAAAGCGCTTGCAAACGATTAAGAACTATATTA
ACATACTTGTATACAAGTATGTCGTGTAATGAAGGAGTGGCTTTGATGATGTATGGATATCCAGAGAAATGGT
TGGAAGGTATGACAACTGGAGAAGGTATCGCGGCAGAATTACGCTTAGGTATTGTGAATGGTCACATAGCTGA
AGGTACGTTACTCACTGAAAATCAAATGGCAAAGCAATTTAATGTGAGTCGTTCGCCAATTCGAGATGCATTT
AAATTATTGCAACAAAATCAACTCATCCAATTAGAAAGAATGGGTGCACATGTGTTGCCGTTTGGGGAACAAG
AAAAGAAAGAAATGTACGATTTGCGACTGATGCTAGAGTCTTTTGCATTTTCAAGAGTTAAAAATCAAGAGCG
ACTACCTATCGTAAAAGAAATGAAGAAACAACTTGAAATGATGAAAGTGGCAGTAAAATTTGAGGATGCAGAA
TCATTTACGAAGCATGACTTTGAATTTCATGAAACATTAATCAAAGCATCTAATCATCAATATTTAAACTCAT
TTTGGAGTCATTTAAAACCAGTAATGATGGCACTCGTTTTAACATCCATGCGACAACGTATGCAACAGAACCC
GCAAGATTTTGAACGCATACATCATAATCATCAAGTGTTTATTGATGCAGTGGAACAATACGACAGTCAAATT
TTGAAAGAAGCGTTTCACTTAAATTTCGACGATGTTGGTAAAGATATTGAAGGATTTTGGTTGAATTAAAATG
AAAGAAAGAAGGATTTGTCATGAAATATATGATTGGTGTCGACATTGGAACGACGAGTACCAAGTCAGTCTTA
TATGATGACAATGGAACATTCATCATGAAACATCAAATCGGCTATGATTTACACACACCAAACGTTGATGTCT
CAGAAGAAAACCCAGATGAATTATTTGATGCGGTATTAATGACTATTAAATATGTTATGAGAGAATCCAAAGT
TAATAAAGATGATATTAAATTTGTGTCATTTAGTGCGCAAATGCATAGCTTGATTGCGATGGATCAGAAACAT
CAAAGATTAACAAATAATATTACTTGGGCAGATAACCGCGCTGCAAAATATGCAACAGTAATAAATGAAGTGC
ATGATGGCAATGCGATTTATCAGCGAACAGGTACGCCTATTCATCCTATGTCGCCTTTAGTGAAAATTTTTTG
GATGAAACATGAATGGCAAGATGTATTTCAACGTACTGCTAAGTTTGCAGATATTAAAACATACATTTTCTAT
CATTTATTTGATACATATATCATTGATTATTCAATGGCTTCCGCAACAGGGATGTTTAATTTAGAAACATTAG
ATTGGGATGTTGGGGCATTAGAATTGCTTGGTATTTCCAAGGAAATGTTGCCGGAATTAGTGCCAACAACGTA
CGTAATGAAAGGCATGAAAGAACGTTATGCAACATTAATGGGGCTTAATGAAGATACACCGTTTGTTATTGGT
GCGAGTGATGGGGTCCTTTCTAATTTGGGTGTCAATAGCGTTGGTAAAGGAGAAGTTGCTGTCACAATCGGTA
CATCTGGGGCGATTCGTACTGTGATAGATAAACCGCGTACTGATTACAAAGGTAGAATATTTTGTTATGTCTT
AACAGAGGACCACTACGTCATCGGAGGTCCTATAAATAATGGTGGTGTCGTATTGAGATGGTTGCGCGACGAG
TTGCTAGCGAGTGAAGTCGAAACTGCGAAACGTCTCGGTGTTGATCCTTATGATGTCTTAACTCAAATTGCAA
AGCGTGTTAAACCAGGTGCAGATGGTTTAATATTCCATCCCTATTTAGCTGGAGAACGTGCACCGCTTTGGAA
CGCAAATGCAAGAGGTTCATTCTTCGGTTTAACTTTATCTCATAAAAAAGAACATATGATTCGCGCTGCATTA
GAAGGTGTTCTTTACAATTTGTATACCGTCTACCTTGCACTCATTGAAGTAATGAATGAAACGCCTAAGATGA
TAAAAGCAACAGGTGGTTTTGCGAAAAGTGAAGTATGGCGTCAAATGATGTCAGATATATTTGACACAGAGTT
AGTGGTTCCTGAAAGCTATGAAAGTTCATGCTTAGGTGCCTGCGTGCTTGGACTTAAAGCTGTAGGTGACATT
GAAGATTTTTCAATCGTTTCATCGATGGTCGGTGCTACAAATAATCATATGCCGTTTGAAGAAAATGTCGCTG
TTTACCAAGAGCTCGTATCCATTTTTATCAATTTAAGTCGTTCTTTAACAGAGAATTATGAACAAATTGCAGA
TTTTCAACGCCAACATATGGCTGAAAATAAAACACAATAAATACGTCACTTAAGCATCTTAGATAAAGTTGTT
GTGCCATGACTACTATATATTATGTGGAATACGTGCGCGACGACGGTGTTACATTCTATTGAAAAGAGAAAGA
GGGGAATGTCATGTTTAACGAAATATGGCCGTTAATCAGTGTTGTTTTAGGTATCGTTATTTTATTAGTATTA
ATTATTGGGTTTAAATTAAATACATTTATTTCATTAATTATCACATCGATGATTACAGCGTTAATGCTAGGTA
TACCATTGACTAAAATTATGGAGACGATTGAGAAAGGGATGGGCAGTACGCTCGGCCACATTGCACTAATATT
CGGTTTAGGTGCCATACTTGGGAAATTACTTGCTGATGGTGGTGGTGCAACTCGAATTGCAGACACGTTGATT
CAAAAATTTGGTCAAAAACATGTACAATGGGCAATGCTTGTTGCAGCATTTATCGTCGGTATTGCATTGTTCT
TTGAAGTAGGTTTAGTCTTACTGATTCCATTAGTATTTACAGTAGCAAAACGCGCAAATGTTTCAGTGTTAAA
ACTAGGACTACCTATGGTAACAGCTTTATCTGTGACACATGGCTTTTTACCACCACATCCAGGACCGGTAGTC
ATCGCAAAAGAATTAAAGGCAAATGTTGGAGATGTATTACTGTACGGTATGATTATTGCCATTCCAGTTACAC
TCATTGCAGGACCTATATTTAACAAAGTTGCACAAAAAATGATTCCGTCTGCGTATACACGAGAAGGCGATAT
TTCAGCGTTAGGTGCACAAAAAGAATTTACGGATCAAGAGATGCCAGGATTTGGTATGAGTTTATTAACTGCA
ACATTACCAGTCATATTAATGTTAGTGTCTACGATAACGCAACTTGTAACTGGGCACGACAAACCTACAAATC
TATTTGAATCTATCATTTATATGATAGGAACAGCAGGGACAGCCATGCTAATAGCAGTATTATTTGCAATCGT
TACGATGGGATTAATGAGAAAACGTAAGATGAATCATATTATGGAATCAGTGACGAATGCGATTTATCCAATC
GGAATGATGTTATTGATTATTGGCGGTGGCGGTACATTTAAACAAGTATTAATTGACGGTGGTGTCGGAAATA
CAATTGCTAAAATGTTTGAAGGTACAGAGATGTCTCCAATTTTACTAGCATGGATTGTTGCAGCTGTGCTACG
TATCGCATTAGGTTCGGCTACAGTAGCTGCGATTTCAACTACAGGTATTGTCTTACCATTATTACAATCATCA

GATGTAAATGTTGCATTAGTTGTACTTGCGATAGGTGCAGGTAGTGTGATTTTGTCTCATGTAAATGACGCAG
GATTTTGGATGTTTAAAGAATATTTTGGTTTAACTGTAAAAGAAACATTCTTAACATGGTCATTATTAGAAAC
GATTATTTCCGTTTCAGGTATCATCTTCATTCTATTCATCAGCTTATTTGTTTAACTAAATATTAAGTAAACG
TGTTTTAAGGTAATTGCAATATATTGCCTTGAAACACGTTTATTTTTATGCAGAAAATCGTCTGAAATAGTCA
GAATCCGAACGATATAAAAAGTATATTGGGGATTTTAGTGTAATGAAAGGCGTTTCAAGACATGTTTTTGTGC
GTATTGTACAGGCGATAATTATGAAACGCTTAGTATATTGTTTTAAATTAGATAATGATGAATTTAATTTGAA
AAATAAGTATAAAAAAATACAAGCCTTGTGTGACAAGGGTTTATGATGACTTGAATACAATTTATAGGTATATT
TCAAATAATAAAATTATCAATTAACATAAAATTAATGACAATCTTAACTTTTCATTAACTCGCTTTTTTGTAT
TGCTTTTAAAAACCGAACAATATAGACTTGCATTTATTAAGTTTAAAAAAATTAATGAATTTTGCATTTAAAG
GGAGATATTATAGTGAAAAACAATCTTAGGTACGGCATTAGAAAACATAAATTGGGAGCAGCATCAGTATTCT
TAGGAACAATGATCGTTGTTGGGATGGGACAAGATAAAGAAGCTGCAGCATCAGAACAAAAGACAACTACAGT
AGAAGAAAATGGGAATTCAGCTACTGAAAATAAAGTAAACGAAACACAAACAACTACAACTAACGTTAATACT
ATAGATGAAACACAATCATACAGCGCAACAGCAACAGAACAACCGTCAAACGCAACACAAGTAACAACTGAAG
AAGCACCAAAAGCAGTACAAGCACCACAAACTGCACAACCAGCAAATTTAGAAACAGTTAAAGAAGAGGTAGT
TAAGGAAGAAGCGAAACCTCAAGTTAAGGAAACAACACAATCTCAAGACAATAGCGGAGATCAAAGACAAGTA
GATTTAACACCTAAAAAGGCCACACAAAATCAAGTCGCAGAAACACAAGTTGAAGTGGCACAGCCAAGAACGG
CATCAGAAAGTAAACCACGTGTGACAAGATCAGCAGACGTAGTTGAAGCTAAGGAAGCTAGTGACGAGAAAGT
GGAAACGGGTACAGATGTGACAAGTAAAGTTACAGTAGAAAGTGGTTCTATTGAGGCACCTCAAGGAAATAAA
GTAGAGCCACATGCTGGTCAACGTGTCGTATTGAAATACAAATTGAAATTCGCAGATGGATTAAAAAGAGGAG
ATTATTTTGATTTTACATTATCAAATAATGTAAATACTTATGGGGTTTCAACAGCTAGAAAGGTACCAGAGAT
TAAAAATGGCTCAGTTGTAATGGCTACAGGTGAGATCTTAGGGAATGGTAACATAAGATATACATTTACTAAC
GAAATTGAACACAAGGTAGAGGTAACAGCTAATTTAGAAATCAACTTATTTATTGACCCTAAAACTGTACAAA
GCAATGGAGAACAAAAGATTACTTCTAAATTAAATGGTGAAGAAACAGAAAAAACAATACCAGTTGTTTATAA
TCCAGGTGTTAGCAATAGTTATACAAATGTAAATGGATCAATTGAAACATTTAATAAAGAATCTAATAAATTT
ACACATATAGCTTATATTAAGCCAATGAATGGAAACCAGTCAAACACTGTATCAGTAACAGGGACGTTGACTG
AAGGTAGTAATTTAGCTGGTGGACAACCTACTGTTAAAGTATATGAATATCTAGGGAAAAAAGATGAATTGCC
ACAAAGTGTTTATGCAAATACATCAGATACTAACAAATTCAAAGATGTAACAAAGGAAATGAATGGAAAATTG
AGTGTGCAAGACAATGGTAGTTACTCATTGAATTTAGATAAGTTGGATAAAACGTATGTCATTCATTATACAG
GTGAATATTTGCAAGGGTCAGATCAGGTTAATTTTAGAACTGAATTATATGGGTATCCAGAACGAGCATATAA
ATCTTACTATGTTTATGGGGGATATCGTTTAACTTGGGATAATGGTTTAGTTTTATATAGCAATAAAGCTGAC
GGCAATGGTAAAAATGGACAAATTATTCAAAATAATGATTTTGAATATAAAGAAGATACTGCAAAAGGAACTA
TGAGCGGGCAGTACGATGCCAAGCAAATTATTGAAACAGAAGAAAATCAAGACAATACACCGCTTGACATTGA
TTACCACACAGCTATAGATGGTGAGGGTGGTTATGTTGATGGGTATATTGAAACAATAGAAGAAACGGATTCA
TCAGCTATTGATATCGATTACCATACTGCTGTGGATAGTGAAGCGGGTCACGTTGGAGGATACACTGAGTCCT
CTGAGGAATCAAATCCAATTGACTTTGAAGAATCGACACATGAAAATTCAAAACATCACGCTGATGTTGTTGA
ATATGAAGAGGATACAAACCCAGGTGGTGGCCAAGTAATAACTGAGTCTAACTTAGTTGAATTTGACGAAGAG
TCTACAAAAGGTATTCTAACTGGCGCAGTGAGCGACCATACAACAGTTGAAGATACGAAAGAATATACGACTG
AAAGTAATCTGATTGAACTAGTAGATGAATTACCTGAAGAGCATGGTCAAGCACAAGGACCAATCGAGGAAAT
TACTGAAAACAATCATCATATTTCTCATTCTGGTTTAGGAACTGAAAATGGTCACGGGAATTATGGTGTGATT
GAAGAAATCGAAGAAAATAGCCACGTTGATATTAAGAGTGAATTAGGTTATGAAGGTGGCCAAAATAGCGGTA
ACCAGTCATTCGAGGAAGACACAGAAGAAGATAAACCTAAATATGAACAAGGTGGCAATATCGTAGATATCGA
TTTCGATAGTGTACCTCAAATTCATGGTCAAAATAAAGGTGATCAGTCATTCGAAGAAGATACAGAGAAAGAC
AAGCCTAAATATGAACATGGTGGTAATATCATTGATATCGACTTCGACAGCGTGCCACATATTCATGGATTCA
ATAAGCACACTGAAATTATTGAAGAAGATACAAACAAAGATAAACCAAATTATCAATTCGGTGGACACAATAG
TGTTGAATATGAAGAAGATACACTTCCACAAGTAAGTGGTCATAATGAAGGTCAACAAACGATTGAAGAAGAT
ACAACGCCGCCAACACCACCAACACCAGAAGTACCAAGTGAGCCGGAAACACCAACACCACCGACACCAGAAG
TACCAAGTGAGCCGGAAACACCAACACCGCCAACACCAGAGGTACCAAGTGAGCCGGAAACACCAACACCACC
AACGCCAGAGGTACCAGCTGAACCTGGTAAACCAGTACCACCTGCCAAAGAAGAACCTAAAAAGCCTTCTAAA
CCAGTGGAACAAGGTAAGGTAGTAACACCTGTCATTGAAATCAATGAAAAGGTTAAAGCAGTGGCACCAACTA
AAAAAGCACAATCTAAGAAATCTGAACTACCTGAAACAGGTGGAGAAGAATCAACAAACAAAGGTATACTGTT
CGGAGGATTGTTTAGTATTTTAGGATTTGCATTATTACGCAGAAATAAAAAGAATCACAAAGCATAATCAATC
TAAAATTGACAGGTTTATTTCATAAATTATATGAAGTAAGCCTGTTTTTTAAAATTAAATCAAATTTCCCAAG
AAATAATTACATACTTAATGACACTATGAAGGCGTTCTAATTAGTGTTAAAATGACGTTGATACATAGATTTA
ATACTTAGGAAAAGGAGCACATTAACTTTGAAAAAAATAAAAAAGGCAATCATTCCCGCTGCTGGTTTAGGGA
CTAGATTTTTACCAGCAACTAAAGCGATGCCAAAGGAAATGCTTCCTATCTTAGATAAACCCACAATACAATA
TATCGTTGAAGAAGCTGCAAGAGCTGGAATTGAAGATATTATTATAGTGACAGGTCGCCACAAACGCGCGATT
GAAGATCATTTTGATAGTCAAAAAGAATTAGAAATGGTGTTAAAAGAAAAAGGTAAATCTGAGTTACTAGAAA
AAGTTCAGTATTCAACAGAACTTGCGAATATTTTTTATGTAAGGCAGAAAGAACAAAAAGGTTTAGGGCATGC

GATTAGTTCTGCGCGTCAATTTATCGGCAATGAACCATTTGCGGTATTATTAGGTGACGATATTGTTGAATCT
GAAGTGCCAGCAGTGAAACAGTTGATTGACGTTTATGAAGAAACAGGGCACTCAGTTATAGGTGTCCAAGAAG
TTCCGGAAGCAGATACTCATCGTTATGGGATTATTGACCCATTAACTAAAAACGGACGTCAGTATGAAGTGAA
AAAGTTTGTTGAAAAGCCAGCACAAGGTACGGCACCATCAAACCTAGCTATTATGGGACGTTATGTATTAACG
CCTGAAATTTTCGATTATTTAAAAACGCAAAAAGAAGGCGCAGGTAACGAAATTCAGTTAACAGATGCGATTG
AACGTATGAATAATGATAACCAAGTGTATGCATATGACTTCGAAGGCGAACGTTATGACGTTGGAGAAAAATT
AGGCTTCGTTAAAACAACGATAGAATATGCATTGAAAGATGACAGTATGCGAGAAGAATTAACACGATTTATT
AAAGAATTAGGGTTATAACATTAAAAAGCATGATACTCAATAAAATTGGAACACGAAATTGTGAACTAAATTG
AATGAGTGTCATGCTTAATTTATGTGTTGTGATATATAGTAGGGTATTGAAATAATATAAAAACAAAAGAAAG
TTAATTATGTATTTTTATTTATACTAAATTGAGAAGTATTATGATTGCTTATTTATTTTATCAAGAGGTAAAT
CTGTTTAAATTTAAAAGAAAAATTTTCTTGGTTTGAGAATCTTGGAAGTATCTGTGAATATGCTTTTAATAGT
GTTATATTGTATTTTATTTATTTTGATTAACACAATCCTTTCATCTGCAAGGGATCGTTCTTTGATTAGCATA
CCTTTATTATTTAATCTATTAACTGACCTAACAATTTGAGGATACATAAATCTAATACTTTCAAACAAATCTT
TTAAATTTAGTATCTCATTTTTTCGAGATGATATTACATATAAGATAGATAAATCAAGAAATGTTAAATTAAA
TCGATATTTTAAAAAATCTTTTGAGTACATAATGTTCATAAAAAGTATAAAAAATTCTTTGCTATATTTTGGA
GCCCATTGATATTTTATGTAGTCTCTTTCCTTATTAAATCCTTTAATTATTTTATCTAACTCATTAATAACAC
AAGCAATCTTTTTACGTTGTATTGGAGTAACTGAAACAAAAATACAGCGTTCATCTGCTTCGTTTCTAGACTT
ATTTATAAATCCTTTTTTAGATAGTGACTTAATGTTCTTTACAACATCAGATTGTTTGTAAAGTATGTCACCA
ATGATTTCTTTTAGTGAAATACTGTGTTCATTTTTGTAATAAAAATATGCTAATATCAACAATTCTTTTAATG
ATAATTTATATTGTTGCTTTAACTCTTGAGTGAGAAAAAATATGTACGCTTCTACATTTATGAATTTATTGAC
CTTTTCGAAAGTTTGATAGTCCACCTTACATTCTCCTACTATTTTCTGATGTCCCAATACTTTATATTATTTA
AAGTAATATAAAAGCAATCTCTTTTGAAGACATGTTAACCTAAATATTTTTATATATAATATATATAAAATAT
AAATAACTAAAAATCACGGAATAACAAATGACAAATTCATTTATTATCCAAAAATAAAAACAATTGAACAAAA
TCAAAATATAGTGTTATGATTCATTTGTGTTTATAGCTAGCACCACAAACTAAATATATTTGGAGTTATTACT
AGTTAAAGACAAATATTATTTGACGTGACAATTAAAAGTAAGGGATGAAGACTTGATGAATGATTTGAAAAGC
AAGAGCAATATTAAATTAATGAAAAGAGTATTAACTACATATGAGCTGCGTAAATATTTAAAAAAATATTTTT
GTCTTACATTAGATAATTATTTAGTATTAGCATATTTAGATGTATTTAAAAATGATGAAGGTAAATATTTTAT
GAGAGACATTATAAGTTATATCGGTATAGACCAATCTAGAATTGTTAAAAGCGTAAAAGAATTATCAAAAAAG
GGTTACTTGAATAAATGTAGAGACCCACATGATAGTAGAAATGTAATCATTGTTGTTTCTGTAAAACAACACA
ATTATATTAAAAATCTTTTGAGTGAAATAAATATTAATGAAACATAAAGTATGTTTCGAGATTTTAATATCTT
GGTGCAGTTAAATGTATTTTTAAATGCGTTAAGGTATTTTTTATTTTAGAAATTCAATGCTATTGAGTTGATG
GGTTTTTCCAACAGATAAGAATGAATTATTCATTGAAAACTAAAAATAATAATTTTATTTGTAGATTGTTTTA
AGTGTTTCAATATATAAAATAATTTTATAAAGATAAGTGTTATATTCCATTGTGCAACTCCTATTGTATTTGT
AAAATTACATGTAAATTCACTGTAAGTAAAGTGGAAAATATGGAAAAAGGAGTATGCAAATGAGAGATAAGAA
AGGACCGGTAAATAAAAGAGTAGATTTTCTATCAAATAAATTGAATAAATATTCAATAAGAAAATTTACAGTT
GGAACAGCATCTATTTTAATTGGCTCACTAATGTATTTGGGAACTCAACAAGAGGCAGAAGCAGCTGAAAACA
ATATTGAGAATCCAACTACATTAAAAGATAATGTCCAATCAAAAGAAGTGAAGATTGAAGAAGTAACAAACAA
AGACACTGCACCACAGGGTGTAGAAGCTAAATCTGAAGTAATTTCAAACAAAGACACAATCGAACATGAACCA
TCAGTAAAAGCTGAAGATATATCAAAAAAGGAGGATACACCAAAAGAAGTAGCTGATGTTGCTGAAGTTCAGC
CGAAATCGTCAGTCACTCATAACGCAGAGACACCTAAGGTTAGAAAAGCTCGTTCTGTTGATGAAGGCTCTTT
TGATATTACAAGAGATTCTAAAAATGTAGTTGAATCTACCCCAATTACAATTCAAGGTAAAGAACATTTTGAA
GGTTACGGAAGTGTTGATATACAAAGCAATCCAGAGGATCTAAAGGTATCTGAAGTCACTCGTTTCAATAATA
AAAGTATTGGAAAAAAACGAATTGACAGGAGCATTACAATTAAAGAATAAAGTTTCTTTTAAAAATGACTTTGA
GTTTAACATTAGAGTAGCTAATAATCATCAATCGGTAACAACTGGCGCTGATGGATGGGGGTTCTTATTTAGT
AAAGGTGACGGTAACGAATATTTACAAAAGGGAGGTATATTAGGTCCCAAAGGAATGGAAAATTCAGCAGGAT
TTAAAATTGATACAGGGTATAATTTTAAAGATCCCATGGATAAAGAAGAGAAACAGGCAGGACAAGGATTTAA
AGGGTATGGTACGTTTGTAAAAACAGGTGCTGATGGAACTACAGCAAAGGTTGGAACAAACATACCAACAAGA
GGTAAAGCGGATAATTCATTCCAATATGCAGATAATTCTATTGATACAACAGATGGAAAATTCCATGGTCAAT
TACTTAATAATTTAAAATTAGCGTACAATGAAAAATCGGGGATAATGAGAGCCGAATATGCTGGTAAAATTTG
GGAAGCTAATATATCAGATTTGGGATTGGATAAATCTGAAGCATATAACTTTTTAATTACTTCTAGTCAAAGA
CAGGGAACATCTCAAGGTGTATATGCAAATGGTTGGATGAGAACAGATTTAAATAACTCAACATTTAAACTAA
CGCCTAATCTAGTTAATAAAACAGAAACAAAAACAGTAGAGATACCGTTCGAGACAAAACGTGAATTTAATCC
GAAGTTACAACCTGGTGAAGAGCGAGTGAAACAAGAAGGACAACCAGGAAGTAAGACAATCACAACACCAATC
ACAGTGAACCCATTAACAGGTGAAAAAGTTGGTGAGGGTCAACCAACAGAAGAGATCACAAAACAACCAGTAG
ATAAGATTGTAGAGTTTGGTGGCGAGAAAATACCGCAAGGTCATAAAGATATCTTCGATCCAAACTTACCAAC
AGATCAAACGGAAAAAGTACCAGGTAAACCAGGAATTAAGAATCCAGACACAGGAAAAGTGATCGAAGAGCCA
GTGGATGATGTGATTAAAC
>DSM104437_scaffold_8

TTCAAAAGTATTTAAAATTACTGGAGAATCATATCGATTAAAAGATTATAAAACCGAAAAATCATTAAATATA
CGACAATCTTAAACCGCTAAAAAACGACATTTTCAAACCGCCATAAAACAGCATTTTTAAACCGCTATTGACA
TACGGTCCAAGAGAAAAGAAACCAGTGAGTATTAATCACAATATCGTAGAGTACAATGATGGTACTTTTAAAT
ATCAATCTAGACCAAAATTTAACTCAACACCTAAATATATTAAGTTTAGACATGATTATAACATTGTGGAATA
TAATGACGGTACATTTGGATATGGACCACGTCCGCATTTTAATAAACCAGCAGCGAAAACTGATGCAACTATA
AAAAAAGAACAAAAATTGATTCAAGCTCAAAATCTTGTGAGAGAATTTGAAAAGACCCATACTGTGAGCGCAC
ATAGAAAAGCACAAAAGGCAGTCAACTTAGTATCATTTGAATACAATGTTAAGAAAATGATATTGCAAGAGCG
AATTGATGAAGTATTAAAGCAAGGATTAGTGAGATAATACTTCTGTCATTATTATAAGTTCAAAATAATTTAA
TCTAATATTAATTTCTATTAATAAAAAGACTATGCTATTTAATAGTAGGTTAATGTAACTTTCCTAAAATTGA
CTATATAATCATTAAGTATCAATTTTTAAAGGAGAATTTACAATGAAAATTAAAAAATATATAGTAGCAGGAA
CATTAGCAGTACTATTATCAACAACAGCAGTATCAACGTTAAACGGGAACAAAGCAGAAGCAAGTAGTTATGA
CAATTATTTAACTCAAAGTAAATATCACGATAAAAAAATTGCTGAAGAGTTGAAATCACTACTTAGACAATCG
AACGTATATGACTTGGCTGCAGGTAGCTTAAATCGATATTACAAACGTACGATTATGATGAATGAATATAGAG
CAAAAGCAGCACTAAAGAAAAATGATTTCAAATCAATGGCTGAAGCTAAAGTTGGATTAGAAAACATTTACAA
AGAAATTGATGAAATTATAAATAGATAATAAAGAAAACAGGTTGAGACAATAAATATCATTGTCTTCGCCTGT
TTTTTAATTTGTATAGTTAAAAATTCTACAATTTAATATTTATGTATGAGCAATGAATATACAATAATAGGGG
AGACTAATACAAATATAATAGTTAGAAATGTTATGCACAAAATTGCTTTGTACAATCTAAAAAATATTTATAA
TGCTCCCATAGATAAAATCAAATGCTGTAAGAAACGTAGTGAATATTACTCAATCAGCATATTATTGGAAGAA
AAAGTTAACGTGTGTAGAACATGCCACAATACCAAATATAATGAATAAAAATAAGACGATAAGCATGGCGACA
CTTTTTAAACAACCTCTATTTGTTTCTTCCGAATTTCTTTGTTGAACCTTTTTATAATCTTCAAGATGCTTTG
TGGTCTTTTTATTTATATGTTTATTCATGATGTTTACTCCTTAATCTAAATGTAAATTGCATTAAAATAGTTG
AAAACATACCTTGAAATAAAGATATAAAGCTAAAGTTGAGAAGAGTGTTATTTTAAATTTAAGCAAAATTAAC
TGTAAATAAGTTAATTTGATGATAACATGTTTCGCTTATTTTTAAAATTGATAGTTTTTAAACTGACGCATTA
AAATATTAATGAAACACTAGAACACATAAATGAATAATAAAGGTGCGCAGTATAGAATTATTAATGCAACACT
AAGTATAAATATTGTTAACTGTAAACTATCTTTAGTTTTAATATAAACAATTAAGATAAGCAGTGTTAAAAGA
ACCATATATATTAATGACGAAGATGTCCATACAAAATCTGCGTCTGTCGTAGTTAACAGCGGTACTATAATTA
GTCCGAAGTTAATCATGCATGCTATATATACAATGATGTTATACACAATGTTAATTTTTGGTCACCACCTTAG
CTGTTATATTTTAAATGCTTGGAAATCGTGCATTTTTAAGTGTAATGATTATCTGAATCTTTGATTACCGTTT
GTTTTTCGTTTAAAAGACTATATATAGTATGCCTTTATATTTCTAAATGTTTTTTAATATTTAATTCTCGTCA
ATATTTGGATTATGAATCATCAATTCAAGTACTTTGTTAGATGAGTCAATTGTAAATTCAGATCCATAGTTAA
CATGACTGACCTTTAATTTATTGTCTATATAATTGATTGCGATTGCGACATCATAATCTTCATCTATGACAAA
TAAATTATTTTCATTTGTAACAACTTCATGTTTCCCAGTGTAAACAACATTAACTTTCCAATCTTTAAAAACC
ATTTGTCATCCTCCTTGAACATTTAAACTGATTCAATTTAAGTTTAACTTATTCATACAACTTCTTACAATAT
CTAGATTAGAGATAAATAGATTTCTAGAAGTTATTTTCAATTATATGTACTTATTATACTTTTTATCTTCTTA
TTTCAGTATAGTTTTAAAACGATTTTAAAATAATTCTGCAAATATATTAACACATAATGTGTTCGATAAGTTT
TGAACAATTTCAAAACTTTTAAATAAAGGGTTTGACAATATGAATTAAAAATGTTTGAAACTGAAAAATTAAA
TCTTATATCGTGTTTATAAGCATGAATATATATTGAAAAGCATAATATATATATTACTATTCATCGATGTTTA
ATTTTATACTGCAACTAAAATAGCCGACACACATCATTTCTGATGTTATCGGCTAATGTTAAAATTTGAATAT
TTGAGTGTGCATGACCATTAATTTGTCATTTCTTCTTTTTCCCAATCGATTTTATATCTTTCTGAAGAACGAT
CCGTCCATTTATCTTTAGTATTAGTACCTTTCCAATTTGTTGAAGTCCAATGTAATTGATAGTCATCACGAAC
TCGTTCGTATATTACATCTATATTTGATTGTTGTTTGGTTGCTTTCCTATCCATAGTAATAACTGTAGCGAAG
TCTGGTGAAAACCCTGAAGATAATAGAGAACTTGCTTTGTTAGGATCAAGGAAGTTATCTGCTGCTTTCATTG
AACCGTTTCTTGTTTTCATGAAAAGTTGATTGCCGTATACTGGGTTCCAAGAATCTCTGTCGTATGGTCCCCA
ATTTTGATTCACCATATTGTTAAATATAACTTTCCAACCTACTTTTTTATCAGTTGGACTTTCTAAAATTGTT
TTGAAATCTGGTTGAACATATTTGAGTGTATGACCAATTGAAACATTTGCACCAATCAAACCGCCAATTTTTC
CAGAATCATCACCAGTCACATTACCGTTGAATCCATAAGTTAATGTACTCATATATTCTTTAGTATCAATTGA
ATTTCTAGGATAGTAATCAGAAATTTGTGCTACTTCATTATCAGGTAGTTGCAACTGTACCTTAAAGGCTGAA
GGCCAGGCTAAACCACTTTTATTAGCGCCTTCTTCGCTATAAACTCTATACTGACCAGCAATGGTACCTTTCG
TTCTGATGACTAATATTTTTTTATTGTGATTTTTATCATCGATAAAACTATAAAATACTTTTTTGTGCATGCC
ATTTTCTTTATCATAAGTGACTAAATCACCTGTTTTTACTATAGTATTGCTTCCTATATCTGTAGTACCGGTT
TTAATATTAATATCAGAATCTGCGGCATTAGCGACAGGATTCATTAATATGGAACCTAGCAATAGTGTTGTTG
TTACTGAGCTGACTATACGTGTTTTCATTTTCATCATCCTTCTATTTTTTAAAACGATTTGAGGAAACAATAA
TCAATATGTCAATTTAGAATAATGCAGTTGAGACATATCAAATATTTATCATCGTTTATATTAGTATATTTAA
TGTAGCAACTGTTAAATGATTGAGTGATGATGAGTGAATATTTTAAGAATATGTTTTTAACTTTTAATTAATA
TTTGAAAGGAAGCATTTCAATTTCGAGGCTTAGTCAAAGTTGAATGAACTCTTTATGAACCAAGGAAAAGACA
AAGCTAATTTTATTGATTAATTTCTTTAAAACTAGGTATTTATTTAACTTTAAAATGTAATCGATTACAATAT
AAAAATACAAATATCTTAGAATTAAATCAATTAATTAACTATTAAATAAAAGTTAACTGTATATTAACTAGTA
TGATCAATCAATTATGAATAGAGAAAAATTAATTGTGTTGGAATATGGGTGAGTTTAAATATAATAATTCGTG

AAAACTTCTTGTGAAAAAATATAAAAAGGTGTAGTGTAACATTATAAAATGTTATATACAAGATAAAATGCAGA
CTTCTAGTTATAGGCGTTATAAGATAATCTTAAACCTAGGTTAGCTTTTAATGTTGAAACTAAATGGAAAGTG
ATAAATATAAAAATGATAGGGGATTATAATGAAACTATTTTATATTGCATTTCTAATTATTATATGGCTGAAT
ATATTTTTAGGAAATGAAATCATCCATACACTGACTGTTTTAATAACAACATTGTATATTGTTAATAGAAGAA
AGAGGATTAAAAATGAAAGAGTTGAATGAAATAATAAACGCTATTCAATTTTTGTTTGAGTCTGAATCAGGCT
ACAAAATATCGAAAAATTCAGGGGTGCCGTATCAGACCGTGCAAGATTTGAGGAATGGTAAAACGAAAATAGA
AGATGCTAGATTTAGAACGATTATAAAACTATATAGTTATTATGCTACATTAAAAGAGCAGTAATAATTGAAT
TAAGAGCCTAAAACGTAACCACGTCTTAGGCTCTACAATATTATATAGACAAATATTGACCAAACAAAAATGC
CCATGTAACAAACATTTTTCAATTATGGGGTGGGGCCCCAACACAGAAGCTGACGAAAAGTCAGCTTACAATA
ATGTGCAAGTTGGGGATGGGCCCCAACACAGAGAATTTCAAAAAGAAATTCTACAGACAATGCAAGTTGGAGC
GGCCCCCAACACAGAGAATGATTTTGCATTTCTATTAGACTCCAAATCCCATTTAATATTCTGTGTACTAATT
TAAATATTGTGTAAATATTATTGATACATTTTGTAGTATTACTCAATTATTGTTTATCCTTAGTATTTTCGAT
ATAAATCATACTACCTTATTTAATCAATGAGGACTTCGACTGTTATTTTTTGAGGATTTTTAACATAAACATT
TGCAAGATCAGAATGTAATCTTTTACTTAAATCAATTGTAAAGTTATTACCACCACCGGTAATATGAATATTA
CCTTTATTGCGATTTTCATTATATAGTATTTTATTTTTTATTAATGATTCACGTACTCGGAAATCAATTTCTT
TTAACGTTAAAACTGGTTTGTTACCTTGATATATCTTATGTGCGCCGATAAATGTTTGTAGTTTATCTTTATA
TTGTACATAAATATTATAAGGCTTATCAGAAGACGATGCTGCAGGTGTAACACCACCTGTAAATGTATCAGTA
TATACCCACCATAATCGTCTAGTGTCTTTGTCTTTTAAACCAAACACGTCTACATAGCGGTCTCTTAATTGAT
TAATGTTGCCCCAACTTTCAGCACCCCATAAGCGTATATGTGCAGACCAAGAATAACGCTTAAATTCAACATA
AATGTTGCCGTCATCATATTGGTATAACCATCTATTTGAAAATGTGAGAGGCTTTTGTGTGTAATATTTGATT
AATTCATTGATGTTCGTTTCGTCTTGGCCAATATGATAAGCTTTAGCTTCAGATGAAAAACCGATTGGTGTGT
TAGGAAGTTGAGCTGAAGAAGTACCTAATATTAACAATGTTGCTGTTAATATTATTTTGTTCGTGATGTTCTT
TTTCATTTGAACATCTCCTTTCTGTGGATTTATCATTGCGAGGAATATCAAATATAAAAGTGAGCGGCGTCAA
TATAATGAATGAGATTATTATTTGTTGACTCCGATTTCGATACTAGTTAGATTTTTCGAGGGTAACTTCAATT
TTGGCATTTTGAGGATTTTTAACATATTTATTTGCGTCAGTTGACGGTAATCTCTTACTTAAATCAATTGTGT
AATTATTATCGTCACCAGTAATCGTGAGTTGACCTTTATTATATTTATCTTGATATAATTTTTTGTTTTTAAT
TAATGTTTGTCGGATACGGAAATCAACTTCTTTTAAAGTTAAAACTGGCTTATTACCTGTATAAAATGGATAT
CCACCAATAAATGTTTCATCTTTATCTTTATATGTTACATTCAATTTATAGTGTTTATCGTTAGGTTTTGCTG
CAGGTGTAACGCCACCAGTAAATGTTTCTTGAGATAGTGCATATGAGTCAATAGTTTCTTGGTCTTTTATGCC
GAAAATATCTACATATTTATCTCTTAATTGATTAATATTACCCCAACTTTCAGGGCCAAATACTTGAATATGG
CTATACCATGATATCTGAAGTAACGTTGCATGAATTGTGCCATTATCTTTTTGCCATAACCATTTATTTGAAA
AGGTTAAATGTTGCTGAGTGTAATACTTGATTAACTCACTTACATTTGTTTCCTCTTGATTAATATAATAAGC
TTTAGCTTCAGATTTAGAACTGATTGGTGTGTTAGGAAGTTGTGTTGATACAGCGCCTAATAGTAACAACGCT
GATGATAAAATAATTTTTTTCGTAATGTTTTTTTTCATTTAAAATGCTCCTTGTATTTTTTATAAATAAGGGT
TACTTAAATAATTTTAGTATTTACTCCGCAATTAAGGTTAATTAGATTTTTCAAGAATGACTTCAATTTCTGC
ATGTCGAGGATTTTTAACATAACGGTTTGTGTCAGTTAATTTTAACTTCTTACTTAAATCAATCGTATAATTA
TTACCGCCACCCGTAATCTTAATTTGACCTTTATTAAATTCTCCGTTATATAGCTTTTTATTATTTATTAATG
TTTGCCGGATACGGAAATCTAGTTCTTTTAAAGTTAATACTGGTTTATTTCCTTTGTAAAATTCGAGACCACC
GATGATAGTCTGCTGTTTATCGCTATATTTTAAAAATAATTTATAAGACTTATCGGATGATGTAGCTGCAGGT
GTTACACCACCTGTAAATGTTTCATCATAAGTCCAGTAACCTTCAACTGTATCCTCATCTTTAGTTCCGAATA
TATCAACGTATTTATCTCTTAACTGATTAATGTTTCCCCAACTTTCAGCACCAAATACTTGAATATGACTATA
CCAAACCCACGTTTGCAATGTTGCATGAATGCTACCATTGGGCTTTTGCCATAGCCATTTTCCAGATAATGAA
AAATGCGGCTGAGTGTAATATTTGATTAGTTCATTGATATTAGTCTCGTTTTCACTGATATTATAGGCTTTTG
CTTCAGATGAAAAACTGATTGGCGTTTTAGGAAGTTGTGTTGATGTGGTTCCTAAAAGTAACAATGCTGTTGA
TAAAACTAATTTATTCATGATGTTCTTTTTCATATGAAAATCTCCTTTGCGTGAATTACCCAAAGTATATAAG
CTTTTACACCGATTCGGAATTAAATAAAAGCTAAAACTATGTTAAATAAACTTAAACAGTTAGTAGTGTTATT
TAAGCAAAACTTATCATTTTTAATTTTTGGACAGAAACAGTACCTAATAAAGTAGGCGAGAGTTATATATTAA
AAACGACACGTTAAAATTATTCTCTAATCAATTGCATTAAATTGTTTGATAATTGAATTTTCTAACTACTTGA
AAATTAGTTATACTTTAAATGTAGTACTTATTTTAATAATTTCCTACTACTTAAATTTAATATTAATAAAATG
TTCATTTAATTATTGATTAAATATTACAAATTTTAATAGTAGGTTGTGTTTATTTTGTATGCGCTTACAATTT
AGGTGTAACTAATATAAAAGGAGTTGTTATTAATGAAAAATTTACGAAACAGAAGTTTTTTAACTTTATTAGA
CTTTTCACGACAAGAGGTAGAATTCTTATTAACACTCTCCGAGGATTTAAAACGTGCTAAATATATTGGCACT
GAAAAGCCTATGTTAAAAAATAAAAATATTGCACTGTTATTTGAAAAAGATTCTACAAGAACACGATGTGCAT
TTGAAGTTGCAGCACATGATCAAGGTGCAAATGTAACTTATTTAGGCCCAACTGGATCACAAATGGGTAAAAA
AGAAACAACTAAAGATACTGCACGTGTGCTTGGTGGAATGTATGATGGCATTGAATACCGTGGTTTTTCACAA
AGAACAGTAGAAACTTTAGCTGAATATTCAGGCGTACCAGTGTGGAATGGTTTAACTGATGAAGATCATCCTA
CTCAAGTTCTTGCTGATTTCTTAACAGCAAAAGAAGTCTTAAAAAAAGATTATGCAGATATTAACTTTACATA
TGTTGGAGATGGTCGTAATAACGTTGCAAATGCATTAATGCAAGGTGCTGCCATTATGGGTATGAACTTCCAT

TTAGTTTGTCCAAAAGAATTAAATCCAACAGATGAATTATTAAATCGCTGTAAAAATATTGCCGCTGAAAATG
GTGGCAACATATTAATCACAGATGATATTGACCAAGGTGTAAAAGGTTCGGATGTAATTTACACTGATGTTTG
GGTATCAATGGGTGAACCTGATGAAGTATGGAAAGAACGACTTGAATTATTGAAACCATATCAAGTAAATAAA
GAAATGATGGATAAAACTGGTAATCCAAATGTTATTTTTGAGCATTGCTTACCATCTTTCCATAATGCTGATA
CGAAAATTGGTCAACAAATTTTTGAAAAATATGGAATTCGAGAAATGGAAGTTACAGATGAAGTATTCGAAAG
TAAAGCTTCAGTTGTATTCCAAGAAGCTGAGAACAGAATGCATACAATCAAAGCAGTCATGGTTGCTACATTG
GGTGAATTTTAAATGATATAAGGAAGTGAATATGATGGCGAAAATAGTAGTAGCATTAGGTGGTAATGCTTTA
GGAAAATCACCTCAAGAACAACTCGAGCTTGTTAAAAATACTGCGAAATCATTAGTAGGATTAATAACAAAAG
GACATGAGATTGTTATTAGTCATGGTAATGGGCCACAGGTTGGAAGCATTAATTTGGGACTTAACTATGCTGC
AGAACACAACCAAGGTCCGGCATTTCCATTTGCTGAATGTGGCGCAATGAGTCAAGCTTACATAGGCTATCAA
TTACAAGAAAGCTTACAAAACGAATTGCATTCTATTGGAATGGATAAACAAGTGGTAACACTAGTGACACAAG
TTGAAGTTAATGAAAATGATCCGGCATTTAACAATCCTTCAAAACCAATTGGGTTATTTTACAACAAAGAAGA
AGCTGAACAAATTCAAAAAGAAAAAGGATTTATATTTGTTGAAGATGCTGGAAGAGGATATAGACGTGTTGTT
CCTTCACCACAACCCATCTCTATTATTGAATTAGAGAGTATTAAAACACTTATTAAAAATGATACACTCGTTA
TTGCTGCTGGTGGTGGAGGGATACCAGTAATTAGAGAGCAACATGATGGTTTAAAGGTATTGATGCAGTTAT
AGACAAAGATAAAACAAGTGCGTTGTTGGGTGCTAATATTCAATGCGATCAATTGATTATTTTAACAGCAATT
GATTATGTATATATTAATTTTAACACTGAAAACCAACAGCCTTTGGAAACAACAAATGTTGATGAATTAAAAC
GATATATAGACGAAAATCAATTTGCAAAAGGAAGTATGTTACCAAAAATTGAAGCAGCCATATCATTTATTGA
AAACAATCCAAAAGGAAGTGTGCTTATAACATCATTAAATGAATTAGATGCTGCCTTAGAGGGTAAAGTAGGT
ACTGTGATTAAAAAGTAATTGAATTGAAACGCTTTTCAATTACTATATGTCAAATGCATGATTTTTATTATTT
ATGTGCACCCCTGAAAATAATGCCTCTATTTTGATGCGGGGTGCACTTTCTTAATTTATATTTATAAAATCTT
TAAGGTAGAAATGCTAGGTTAAATGTCGAAGGAGATGAAACCGTGGAAAATACAATTAATGAAAGTGAAAAGA
AAAAACGATTTAAATTAAAAATGCCAGGTGCATTTATGATTTTATTCATTTTAACGGTTGTTGCAGTTATAGC
AACATGGGTTATTCCTGCTGGTGCATATTCTAAACTTTCTTACGAACCTTCATCCCAAGAACTAAAGATAGTT
AACCCTCATAACCAAGTGAAAAAGGTTCCGGGTACGCAACAGGAACTAGACAAAATGGGGGTTAAAATTAAGA
TTGAACAATTTAAATCAGGTGCAATTAATAAGCCGGTATCAATTCCGAATACTTATGAAAGATTAAAGCAACA
TCCAGCTGGACCAGAACAAATAACAAGTAGCATGGTTGAAGGTACGATAGAAGCGGTCGATATCATGGTATTC
ATTCTTGTACTAGGGGGACTTATTGGCGTAGTTCAAGCCAGTGGTTCTTTTGAATCGGGATTGTTAGCTTTAA
CGAAGAAAACAAAAGGGCATGAATTTATGCTAATTGTGTTTGTATCAATACTAATGATTATCGGCGGGACATT
ATGTGGTATTGAAGAAGAAGCTGTAGCATTCTATCCGATTTTAGTCCCTATATTTATAGCGTTAGGATACGAT
TCTATCGTTTCAGTTGGTGCCATATTCCTTGCCAGCTCTGTCGGTAGTACATTTTCAACTATTAACCCGTTCT
CGGTTGTAATTGCCTCTAATGCCGCTGGTACAACTTTTACGGATGGCTTGTATTGGAGAATAGGCGCTTGTAT
TGTCGGTGCGATTTTTGTTATTAGTTATTTATATTGGTACTGTAAAAAAATTAAAAAAGATCCTAAAGCGTCA
TATTCTTATGAAGACAAAGATGCTTTTGAACAGCAATGGTCTGTATTAAAAGATGACGATAGTGCCCATTTTA
CTTTGCGTAAGAAGATAATCCTTACATTATTTGTACTACCATTTCCAATTATGGTATGGGGAGTTATGACGCA
AGGTTGGTGGTTCCCAGTTATGGCTTCAGCATTTTTAATATTTGCAATTATAATAATGTTTATTGCTGGGACA
GGTAAATCTGGATTGGGAGAAAAAGGAACTGTAGATGCATTTGTCAATGGCGCATCAAGTTTAGTAGGTGTAT
CTTTAATTATTGGCTTAGCTCGAGGTATTAATTTAGTGTTGAATGAAGGTATGATTTCAGATACAATCTTACA
CTTTTCATCATCTTTAGTTCAACATATGAGTGGACCATTATTTATCATCGTATTACTATTTATTTTCTTCTGT
TTAGGTTTTATCGTGCCATCATCTTCTGGATTAGCGGTATTATCAATGCCTATCTTTGCACCACTAGCTGATA
CAGTAGGTATACCAAGATTCGTCATCGTTACGACATATCAATTTGGTCAATATGCGATGTTATTCTTAGCGCC
GACTGGACTTGTTATGGCCACACTACAAATGTTAAACATGCGATATTCACATTGGTTCCGATTTGTATGGCCG
GTAGTTGCTTTTGTATTGATTTTCGGTGGCGGAGTACTAATTACGCAAGTACTAATTTATTCATAATTTGAAA
TGCTATATTATAAAAATACTAATTGGGTTTTATGCATCTCGTAGGTTTGTAGAAATACTAAACTAAGCGAGGT
GCATTATTATTTTTGATTAAGAAAATAATGACGGTAATCATAACACTAGTAAGTAATTGATACAATGCTCTAT
TTAATAATGATATTTTTAAAATTTGTTTTTAATGTAATGTTAGATCTATGGTATATTATATTTATCGTGGTAA
ATATGTATTTGCTGTAAACAGTTTTTGTAACACATGACACATATGGTTGTGAAAATAGCATATATAAAGGATG
GCTATAAATGACACATTTGACAAAGGTTTAGATACACTAACTGGAATATGCGTAGTATTATTATTTAGTAAA
TATTTTGTGGCGTATGCAAATATGGTGTTTGATTGGAAGTTAAGATGGTATTTGCTAGAAAACATACCACATT
TGCCAATTATATTATTTATTCTGATGTTTATTTTCGGAGTACCTTCTGAAATGATAAAAGATAGGCAAAGGAA
AAATAACGGCGTTTAATTTATCGATATTTAGAGGTGATAAAATTTGTCAACTATTACAAAGACTAAAATAAAA
ATCTTTATTAATTATTTTATTGGTATATTAGCCTTGGCGTTTGCTGCTTATATATTATTTAAATAAAGGTTAG
AGTGAAACGTGTTTATGAACTAGACGTTCTAGTATAGTTACTACAGCTTTAGTCAGAAGGTATCATTGATATG
ATCATATTAAATCAAAGAGGCATTGATATACACTAAAAAGAGGCAAGATTACCTGCCTCTTTTTTAGTTATTA
AATATGCGTGTTAATTCTTGGTAATAGTGTTACTGAGACGCATTACGAAGAAATTTATCTTGATTTTCCTTTT
TTAAAAAGAAGTGAAGATATCCTATAAAGACTCTAAGTACTATTACAGTAGCTAATAACACTTCGATAAACAA
AAGACCTTTCCAAATGTCTGGAAACATAAGTACAGGCAAACTGTTCTTTAAAGCAGTTGCTGAGATTACTAAA
GGGAATGTGAAAGCTGAAAATACGGGCGAAAAAGGTTCTTTTAGTAATTTAGGCAATTGTATAATGATATAAA

AATAAAATATTTGAGCTAATATTAAAAATATAATTACGATAAAAGCATTCGCCTTAGGAAATGCTATAACATA
TGCTGCAGCGACTAAAGAAAATGGTGCACAAATTGTCGATGTGTTCGGTTTGATTGACGTCTGCAATGGAAAT
GCTTTTAATCGCTTGAAAACAATAGGTAAGACAATACAAGTAGCTACAAAGCCATATATTACTGTTAATTGAC
CTATGAAAAAATATCCGCTAACGGGTGCCGTCAATCCTGCGATAGCAATACCAATAAAAAGTACAGTCCACGA
AGGATAAACATTTTCAAGTGAAAAATCTTTTAAATATTTTATTGAAAAAATAATCATATGCGTCATAATTCCC
ACAAGGCATAAAATCCAAATAGGCGTTATTAAGCTATTGATAAAAGTTATGTTACTAAAAAATGTATTTAAAT
AAGTAGTACCTAAAAAGCCAGACATGAAAAATGTTGTGAACACTGATGAAACTAGAGGACTGTTTAATTGTTC
TTTCACATTATTAGAATATTTGAGCATAGTACATAAAAGGTGAATCCAAATCAAGAAAGCAAAGATTCCGCAA
ACAGCGTTTAAAGTAAGAGATAAGTCTTTTAATAGATTACCCAGGCCTAATAATCCTAAGACTAGTCCTGACG
TTACTAAAGGTGCTTTTTGAAGTCTCATGATTTACAACCTTTCTTATGTGATTTTCTTCACTAATTATATCAT
GATAGCTATGGCCAATTAATAAGAAGAGTGTGTACTATTACGTTATTAGAGTATGTATATTGGATTGATAGAC
ACATAATTGACATTTAAATCTCAAAATTAATGATATTTGAGGTATGCTTTGCAACCTAAATATTGGAATATGT
GGAAAAGTAATTATTTAATTTAAATGATGATTGAAGATTTACAAGGGGGTGTACAAAATGAGAAATCAAATTC
AAAAACTATTAGACAGTGATTTGAGCAGTTTACATATATCGAAACAAACAGGAGTTCCACAAAGCACAATACA
CAGAATGAGAAAAAAAGAAAGATCATTAGACAATATGTCATTGAAAAACGCTGAACTACTTTATAAATTTGCC
AATAGTATATTTAGCAATGAAAATTAAGAAGAATAATTAGTGAATTAAATAATTAAAAAACCACGTCTTGATG
GACGTGGTTATTTTTTTAAGATGTATATAATTATAGGATTTCGGGTCCCTGAAAAGTCCCTAAAATTTTGAAT
AGTATCAAAATCTATAAGAATAATATAATAATAAAAATCTCTACGCATGCAAATGGTGAGTGTTAAAAATCTT
GGTGTAGTGTTGGTGTAGTTCAGGTGCAGTATAGATGAAATTTGAGCACAAAATTACTTAATTAAAAATGTAT
AAACAAGTGTCAGACGCGTGTCAATTGTGTGTCAAAAAATTCTATAACGAATAAGCATGCTTGTTTTAATTAC
TGTATTTTCGGGGCTTATGCGGGGGACGTTTTGGTGACGCATCATACTATAATACTGAAATTCAAAACAAAAA
GAGCCCCGTAATCACGGAACTCTTTTGTTTGGTAATGCGTATAAAAATACCTATAAACGTCCTGGGAGGGATT
CGAACCCCCGACCGATGGCTTAGAAGGCCATTGCTCTATCCAGCTGAGCTACCAGGACACGTTTAACAACACA
AGAATTATTATATCTAAATGAACTTAAATTAGCAATACCTTGTAAATAAAAAATGTTTATATTTTTCACTATT
ATAGAGCTATTTATCTAAAAAGGTTCAATAAGACTTAAATACGAATTCAGGCAACTTAATTGTGTTAAATACA
GTTTTGAATGCCTAACTGTATTTCTTTTCTCTTTAAAATACAGTTAAGTACATTATAAGATGTTGTGCGGATA
AACAAACTAATTGCATCAAATTTATTTTAAAATAACAACAACAAAACGTTAAGCGAATAACATTTCGGTGATT
TAAAAGCTACGCACGTTTTTGTTATCTTCAAATTTAAATTTTAAGGAGTGTTTTCAATGGAAGGTTTATTTAA
CGCAATTAAAGATACCGTAACTGCAGCAATTAATAATGACGGCGCAAAATTAGGCACAAGCATTGTGAGCATC
GTTGAAAATGGCGTAGGTTTATTAGGTAAATTATTCGGATTCTAATATCAATATGTTATGTAAGTAATAAGTA
TTATTTCAAAGGTGAGGGAGAGATTTAAATGACTGGACTAGCAGAAGCAATCGCAAATACTGTGCAAGCTGCA
CAACAACATGATAGTGTGAAATTAGGCACAAGTATCGTAGACATCGTTGCTAACGGTGTGGGTTTACTAGGTA
AATTATTTGGATTCTAATATAATAACTAATATTCTTTAAAATAAACTGGGTGAGCATACTTTAATGTTATGCA
CTCAGTTTATTTTATTTGCAGAAATTTGAGCCTCTGTTAAGATTTAGATACATAGACAATATAGGAGATGGGG
AAATTGGGATATAAAAATATTTTGATAGACTTTGATGATACAATTGTTGATTTTTATGATGCAGAAGAATGGG
CGTTTCACTATATGGCGAATGTTTTTAATCATAAAGCAACAAAAGATGATTTTTTAACATTTAAAAAAATCAA
TCACCAACATTGGGAAGCTTTTCAACAAAATAAATTAACGAAGTCTGAAGTATTATCAGAACGATTTGTGAAT
TACTTCAAACATCATCAAATGGAAGTTGATGGGCATCGTGCAGATGTGTTATTTAGAAATGGATTAGCAGAAG
CTAAAGTTAAATACTTTGATCAAACATTAGAAACAATTGTCGAATTATCGAAAAGACATGATTTATATATTGT
TACTAATGGTGTAACCGAAACGCAAAAGAGAAGGTTAAATCAGACGCCGTTGCATAAATATATTAAAAAGATA
TTTATATCTGAGGAAACAGGATATCAAAAACCTAATCCGGAATTTTTTAATTATGTTTTTAATGATATTGGTG
AGGATGAAAGACAGCACTCGATTATAGTTGGAGATTCTTTAACATCTGACATTCTAGGTGGAATCAATGCGGG
TATAGCTACTTGCTGGTTTAATTTTAGAGGATTTGATCATAATCCAGGAATTATACCTGATTATGAAATTAAT
TCATGGAAACAACTAAATGATATTGTACGTTAAGACAAATAATTTGAAAATGATTTACTATTAGCTTAAGAAA
GTAATTGTAAAAAAGACACTACGAAAAGATGCATTTTCATGCTCGTAGTGTCGAATATTATTGTCTTATGGTT
TCCACAATAAGACATCATGTCCCTCTATATTTTTAGCTTCCACATCTGTAAATCCATGATGTAAAAAGAAATC
CTTAGAATCATTTCTGCCAATGGCTTTAATTGGCATGTTGAAACTCTTTGCAAAATCAATCAATTCTGAAGCG
TACCCTCTGTTTTGATATTTTGGTAATACTTCTAACTTCCATAATAATATATAATCTTCAAAATCTGGGAAGT
AGATTTCTTCGACATCACCTTTTTTTAGTAATGCCATTCTAGCTCCTAATTGATCTCCGACAAATATGCCATA
AAATGGTGAATCTGAACTTGCATCAATCATTTGACCGTTTAACTCATTGACCATGTATAAGTCTTTGTTGCCA
AACGCTCTAAAGTTTTCGAATAATTCGTCAGTTTTGTAATTAATTTCAAGACGTTTGATTTCACTCATATTTA
TCTACCCCTTATTTGTTTACTACTATTATTATACATTAAACCACTTGTGTTTTTCATTGATTTGAAAATGTAA
AACAATGAATATTATAAATTTTCCATGATTAAATTGATTTTCAAGCCGTAAAATTGTCAATCACCGGTATAGA
CTTTATACTATAGTTTGTAATCGCGTACTTAAGGAGAAAAAACATGGACTGTAAAGTAGTTAGTTTAAATGAA
AAAGATCAGTTTATACCAAAAATAAAGAGCAGTGACCCTGTAATAACAGGATTATTTCAATATGACGCAGCTC
AACAAACTAGTTTTGAAAAAAGGATGTCTAAAGAAAATAATGGAAGAGAAGCGGCATTAGCGAATGTTATTCG
TGAATATATGAGTGATTTAAAGCTTTCTAGTGAACAAGAATTAAACATACAACATTTAGCTAATGGTTCAAAA
GTTGTGATTGGTGGACAACAAGCAGGGCTTTTCGGGGGACCATTGTATACATTCCATAAAATATTTTCAATCA

TTACTTTATCTAAGGAATTAACGGATACACATAAGCAACAAGTAGTACCAGTTTTTTGGATTGCAGGAGAAGA
TCATGATTTCGATGAAGTGAATCATACATTTGTTTATAACGAAAATCATGGGTCGCTGCATAAGGTTAAATAT
CATACAATGGAGATGCCAGAGACGACTGTCTCTAGATATTATCCTGATAAGGCTGAGTTGAAACAAACTTTAA
AAACGATGTTCATTCTTATGAAAGAAACTGTTCATACACAAGGTCTACTGGAGATTTGTGACAGAATTATTGA
CCAATATGACTCGTGGACTGATATGTTTAAAGCAATACTGCATGAAACATTTAAAGCATATGGCGTTCTATTT
ATAGATGCGCAGTTTGAGCCGTTAAGAAAAATGGAAGCGCCTATGTTTAAAAAGATTTTGAAAAAACATCAGT
TGCTTGATGATGCTTTTAGAGCAACACAACAACGTACTCAAAATCAAGGCTTGAATGCGATGATACAAACAGA
TACAAATGTTCATTTATTCTTACATGATGAAAATATGCGTCAATTAGTTTCGTATGATGGTAAGCATTTTAAA
TTAAATAAAACAGATAAGACATATATAAAGGAAGAAATTATAAATATTGCGGAAAATCAACCTGAATTATTTT
CTAATAATGTAGTGACAAGACCATTAATGGAAGAATGGTTATTTAACACGGTGGCATTTGTTGGAGGACCGAG
TGAAATTAAGTACTGGGCTGAACTAAAAGATGTATTTGAACTATTTGATGTTGAAATGCCTATCGTGATGCCA
AGGCTTAGAATTACTTATTTAAATGACCGTATAGAAAAATTACTTTCGAAATACAATATTCCATTAGAAAAAG
TGTTAGTCGATGGTGTTGAAGGAGAAAGAAGTAAGTTTATTAGAGAACAAGCATCACATCAATTTATTGAAAA
GGTAGAAGGTATGATTGAACAACAGCGTCGTCTAAACCAAGACTTATTAGATGAAGTGGCGGGGAATCAAAAT
AATATTAACCTTGTGAATAAAAATAATGAAATTCATATACAACAGTATGATTATTTGTTAAAACGTTATCTTT
TAAACATTGAAAGAGAAAACGAGATCAGTATGAAGCAATTTAGAGAAATTCAAGAAACACTCCATCCAATGGG
AGGATTACAAGAAAGAATATGGAATCCACTTCAAATTTTGAATGATTTTGGGACAGATGTGTTCAAGCCCTCC
ACCTATCCACCACTTTCTTACACTTTTGATCATATTATTATAAAACCTTAATATACCAAGGGTTTAGCCCGAT
TTATCTTAATGATAAATCGGGCATTTTTTTGTTTTTTAAAATAAATTTCACAAATTTTGTATAAATAGTGGTG
GATAGTGGGGAGATGTGGTAAATTATATATAAGGTGAGGTGATAAAAAATGTTCATGGGAGAATACGATCATC
AATTAGATACAAAAGGACGTATGATTATACCGTCCAAGTTTCGTTATGACTTAAATGAGCGTTTTATTATCAC
AAGAGGCCTTGATAAATGTTTATTCGGTTACACTCTAGACGAATGGCAACAGATTGAAGAGAAAATGAAAACC
TTACCTATGACAAAAAAAGACGCACGTAAGTTTATGCGTATGTTCTTCTCTGGTGCTGTTGAAGTAGAACTTG
ATAAGCAAGGGCGTATTAACATCCCTCAAAACTTGAGGAAATACGCTAATTTAACTAAAGAATGTACAGTAAT
CGGTGTTTCAAATCGTATTGAGATTTGGGATAGAGAAACTTGGAATGATTTCTATGAAGAATCTGAAGAAAGT
TTCGAAGATATTGCTGAAGATTTAATAGATTTTGATTTTTAAAATGGAGGAATTGAAGTGTTTCATCATATCA
GCGTTATGTTAAACGAAACCATTGATTATTTAAATGTAAAAGAAAATGGTGTGTACATTGACTGTACGCTAGG
TGGAGCGGGACATGCCCTTTATTTACTAAATCAATTAAATGACGACGGAAGATTAATAGCAATCGATCAAGAC
CAAACTGCAATTGATAATGCTAAAGAGGTATTGAAGGATCATTTGCATAAGGTGACTTTTGTTCATAGCAACT
TCCGTGAATTAACTCAAATATTAAAAGACTTAAACATTGAAAAAGTAGATGGAATTTATTACGACTTGGGTGT
TTCAAGCCCACAACTCGACATTCCAGAACGAGGATTCAGTTATCACCATGACGCAACATTAGACATGCGTATG
GACCAAACACAAGAACTAACAGCATATGAAATTGTTAACAATTGGTCATATGAAGCGTTAGTGAAGATTTTTT
ATCGTTATGGCGAGGAGAAATTTTCAAAACAGATAGCTCGAAGAATCGAAGCACATCGTGAACAACAACCAAT
AACAACAACATTAGAATTAGTTGACATTATAAAAGAAGGTATTCCTGCAAAAGCAAGAAGAAAAGGCGGACAT
CCTGCAAAACGAGTATTTCAAGCACTACGAATTGCAGTAAACGATGAATTGTCAGCTTTTGAAGATTCAATAG
AACAAGCGATTGAATTAGTGAAAGTAGACGGCAGGATATCGGTAATCACTTTCCATTCTTTAGAAGATCGTTT
ATGTAAACAGGTGTTCCAAGAATATGAAAAAGGTCCAGAGGTACCAAGAGGATTACCAGTTATACCAGAAGCA
TATACACCTAAGTTAAAGCGTGTTAATCGTAAACCGATTACCGCTACAGAAGAAGATTTAGATGACAATAACA
GAGCACGAAGCGCGAAATTACGTGTAGCTGAAATACTTAAATAAGGAGCAATTTATAATGGCTGTAGAAAAAG
TGTACCAACCATATGACGAACAAGTTTATAATAGTATACCGAAGCAACAACCACAAACTAAGCCCGAAAAGAA
GACTGTTTCGAGAAAAGTGGTTGTACAATTAACTAAATTTGAAAAAGTTTTATACATAACTTTGATTACTGTA
ATTGCTATGTTAAGTATTTATATGCTATCTTTAAAAATGGATGCGTATGATACGCGAGGAAAGATTGCAGATT
TAGATTATAAAATAGATAAACAATCAAGTGAAAACAGTGCTTTACAATCTGAAATCAAAAAGAATTCTTCTTA
TGAACGCATATACGAAAAGGCTAAGAAACAGGGGATGAGCCTTGAGAACGATAATGTAAAGGTAGTGCGTAGT
AATGGCGAAGCAAAAAATTAAAATTAAAAAAAATAAAATAGGGGCAGTCCTACTTGTTGGTTTATTCGGACTG
CTCTTTTTTATATTGGTTTTAAGAATTTCATATATCATGATTACTGGACATTCTAATGGTCAAGATTTAGTCA
TGAAGGCAAATGAAAAGTATTTAGTTAAGAATGCACAACAACCAGAACGAGGGAAGATATATGATCGTAATGG
TAAAGTGCTAGCAGAAGATGTAGAAAGATATAAACTTGTTGCAGTAATAGATAAAAAGGCGAGTGCCAATTCT
AAAAAACCTAGGCATGTAGTTGATAAAAAAGAGACTGCAAAGAAATTATCTACAGTCATTGATATGAAGCCAG
AGGAAATTGAAAAGAGACTTAGTCAAAAGAAAGCTTTCCAAATTGAATTTGGACGCAAAGGAACAAATTTAAC
GTATCAGGACAAATTGAAAATAGAGAAAATGAATTTGCCTGGTATTTCTTTATTGCCTGAAACAGAACGCTTT
TATCCAAATGGCAATTTTGCATCACACTTAATTGGTAGAGCTCAGAAAAATCCAGATACTGGTGAACTTAAAG
GTGCGCTTGGAGTTGAAAAGATTTTTGATAGTTATTTAAGTGGATCTAAAGGATCATTGAGATATATTCATGA
TATTTGGGGATATATCGCACCAAATACTAAAAAAGAGAAGCAGCCTAAACGTGGTGATGATGTCCATTTAACA
ATCGATTCAAATATTCAAGTATTTGTTGAAGAAGCTTTAGATGGCATGGTTGAAAGATACCAGCCGAAAGATT
TATTTGCGGTTGTCATGGATGCCAAAACTGGAGAAATTTTAGCATACAGTCAGCGACCAACATTTAATCCTGA
AACTGGTAAAGACTTTGGTAAAAAGTGGGCAAATGACCTATATCAAAATACATACGAGCCTGGATCAACATTT
AAATCATATGGGTTAGCAGCTGCTATTCAAGAAGGTGCTTTTGATCCTGATAAGAATATAAATCTGGACATA

GAGATATTATGGGTTCACGTATTTCAGACTGGAATAGAGTCGGTTGGGGTGAAATCCCAATGTCACTCGGATT
TACTTATTCATCTAATACATTGATGATGCATTTACAAGATTTAGTTGGTGCAGACAAAATGAAATCTTGGTAT
GAACGATTTGGATTTGGAAAATCAACTAAAGGTATGTTTGATGGAGAAGCACCTGGTCAAATTGGATGGAGTA
ATGAGTTGCAACAAAAAACGTCATCATTTGGTCAATCGACAACTGTAACACCTGTTCAAATGTTACAAGCGCA
ATCAGCGTTCTTTAATGATGGTAATATGTTAAAACCATGGTTTGTGAATAGCGTTGAAAATCCTGTTAGTAAA
AGACAATTTTATAAAGGTCAAAAACAAATCGCAGGCAAACCAATAACAAAAGATACTGCTGAAAAAGTTGAAA
AGCAATTGGATTTAGTTGTGAATAGTAAGAAGAGTCACGCTGCAAACTATCGTATTGATGGTTATGAGGTCGA
AGGTAAGACTGGTACAGCACAAGTCGCTGCACCTAATGGTGGTGGATACGTTAAAGGTCCAAACCCATATTTT
GTAAGTTTTATGGGTGACGCGCCGAAGAAAAATCCTAAAGTTATTGTATACGCTGGTATGAGCTTGGCACAAA
AAAATGACCAAGAAGCTTATGAATTAGGTGTTAGTAAAGCGTTTAAACCAATAATGGAAAATACTTTGAAATA
TTTAAATGTAGGTAAATCAAAAGATGACACATCTAATGCAGAGTATAGTAAAGTGCCAGATGTTGAAGGTCAA
GACAAACAAAAAGCTATTGATAATGTGAGTGCAAAATCATTAGAACCAGTTACTATCGGTACTGGCACACAAA
TAAAAGCACAATCTATAAAAGCAGGGAATAAAGTCTTACCTCATAGTAAAGTACTGTTATTAACAGATGGAGA
CTTAACTATGCCTGACATGACAGGATGGACGAAAGAAGATGTCATTGCTTTTGAAAACCTAACAAATATTAAA
GTAAATTTAAAAGGTAGCGGTTTTGTGTCCCACCAATCAATTAGTAAGGGACAAAAACTTACTGAAAAAGATA
AAATAGACGTAGAATTTTCATCAGAGAATGTAGACAGCAATTCGACGAATAATTCTGATTCGAATTCAGATGA
TAAGAAGAAATCTGACAGTAAAACTGACAAGGATAAGTCGGACTAACAAGAATGGCTACATTAAATAAGGGTG
TATTGTCACTATTTGTTTTTACTAAAGTAATGCTGTGATAGAAAACAACATCTTCATTTAATCTTTATAAATA
AGTGTTGATAGAGAATTTTAAGGTAGACGAGGTCAGATTAACTTGTTTGTTATAGGCAAACTTTTTATACTGG
CCTCTTATGCCGTAAAAAAGAAAAATTATAAATTTTGATTACAACTTTGTTTATTTAATTGAAAAATTGATAA
TATATTAGTGGTTAAAAAAGTCTGGATTAAGAAGAGGAGATAGTTATGATTTTTGTATATGCGTTATTAGCGC
TAGTGATTACATTTGTTTTGGTACCTGTTTTAATACCTACATTAAAAAGGATGAAATTTGGTCAAAGTATTCG
AGAAGAAGGTCCACAAAGCCATATGAAGAAGACTGGTACACCAACGATGGGTGGACTAACATTTCTATTAAGT
ATTGTGATAACGTCTTTGGTGGCTATTATATTTGTAGATCAAGCTAATCCAATCATACTGTTATTATTTGTGA
CGATTGGTTTTGGGTTAATTGGTTTTATAGATGATTATATTATTGTTGTTAAAAAGAATAACCAAGGTTTAAC
AAGTAAACAGAAGTTTTTGGCGCAAATTGGTATTGCGATTATTTTCTTTGTTTTAAGTAATGTGTTTCATTTG
GTGAATTTTTCTACGAGCATACATATTCCATTTACGAATGTAGCAATCCCACTATCATTTGCATATGTTATTT
TCATTGTTTTTTGGCAAGTAGGTTTTTCTAATGCGGTAAATTTAACAGATGGTTTAGATGGATTAGCAACTGG
ACTGTCAATTATCGGATTTACAATGTATGCCATCATGAGCTTTGTGTTAGGAGAAACGGCAATTGGTATTTTC
TGTATCATTATGTTGTTTGCACTTTTAGGATTTTTACCATATAACATTAACCCTGCTAAAGTGTTTATGGGAG
ATACAGGTAGCTTAGCTTTAGGTGGTATATTTGCTACGATTTCAATCATGCTTAATCAGGAATTATCATTAAT
TTTTATAGGTTTAGTATTCGTAATTGAAACATTATCCGTTATGTTACAAGTCGCTAGCTTTAAATTGACTGGA
AAGCGTATATTTAAAATGAGTCCGATTCATCATCATTTTGAATTGATAGGATGGAGCGAATGGAAAGTAGTTA
CAGTATTTTGGGCCGTTGGTCTGATTTCAGGTTTAATCGGTTTATGGATTGGAGTGCATTAAGATGCTTAATT
ATACAGGGTTAGAAAATAAAAATGTATTAGTTGTCGGTTTGGCAAAAAGTGGTTATGAAGCAGCTAAATTATT
AAGTAAATTAGGTGCGAATGTAACTGTCAATGATGGAAAAGACTTATCACAAGATGCTCATGCAAAAGATTTA
GAATCTATGGGCATTTCTGTTGTAAGTGGAAGTCATCCATTAACGTTGCTTGATAATAATCCAATAATTGTTA
AAAATCCTGGAATACCTTATACAGTATCTATTATTGATGAAGCAGTGAAACGAGGTTTGAAAATTTTAACAGA
AGTTGAGTTAAGTTATCTAATCTCTGAAGCACCAATCATAGCTGTAACGGGTACAAATGGTAAAACGACAGTT
ACTTCTCTAATTGGAGATATGTTTAAAAAAAGTCGCTTAACTGGAAGATTATCCGGCAATATTGGTTATGTTG
CATCTAAAGTAGCACAAGAAGTAAAGCCTACAGATTATTTAGTTACAGAGTTGTCGTCATTCCAGTTACTTGG
AATCGAAAAGTATAAACCACACATTGCTATAATTACTAACATTTATTCGGCGCATCTTGATTACCATGAAAAT
TTAGAAAACTATCAAAATGCTAAAAAGCAAATATATAAAAATCAAACGGAAGAAGATTATTTGATTTGTAATT
ATCATCAAAGACAAGTGATAGAGTCGGAAGAATTAAAAGCTAAGACATTGTATTTCTCAACTCAACAAGAAGT
TGATGGTATTTATATTAAAGATAGTTTTATCGTTTATAAAGGTGTTCGTATTATTAACACTGAAGATCTAGTA
TTGCCTGGTGAACATAATTTAGAAAATATATTAGCAGCTGTGCTTGCTTGTATTTTAGCTGGTGTACCTATTA
AAGCAATTATTGATAGTTTAACTACATTTTCAGGAATAGAGCATAGATTGCAATATGTTGGTACTAATAGAAC
TAATAAATATTATAATGATTCCAAAGCAACAAACACGCTAGCAACACAGTTTGCCTTAAATTCATTTAATCAA
CCAATCATTTGGTTATGTGGTGGTTTGGATCGAGGGAATGAATTTGACGAACTCATTCCTTATATGGAAAATG
TTCGCGCGATGGTTGTATTCGGACAAACGAAAGCTAAGTTTGCTAAACTAGGTAATAGTCAAGGGAAATCGGT
CATTGAAGCGAACAATGTCGAAGACGCTGTTGATAAAGTACAAGATATTATAGAACCAAATGATGTTGTATTA
TTGTCACCTGCTTGTGCGAGTTGGGATCAATATAGTACTTTTGAAGAGCGTGGAGAGAAATTTATTGAAAGAT
TCCGTGCCCATTTACCATCTTATTAAAGGGTGTGAGTATTGATGGATGATAAAACGAAGAACGATCAACAAGA
ATCAAATGAAGATAAAGATGAATTAGAATTATTTACGAGGAATACATCTAAGAAAAGACGGCAAAGAAAAAGA
TCAAAGGCTACACATTTTTCTAATCAAAATAAAGATGATACATCTCAACAAACTGATTTTGATGAAGAAATTT
ACTTGATAAATAAAGACTTCAAAAAAGAAGAAAGCAATGATGAAAATAATGATTCTGCTTCTAGTCATGCGAA
TAATAATAATATCGATGATTCTACAGACTCTAATATTGAAAATGAGGATTATAGATATAATCAAGAAATTGAC
GACCAAAATGAATCGAATGGAATTGCAGTCGACAACGAACAACCTCAATCAGCTCCTAAAGAACAAAATAGCG

ACTCGAATGATGAGGAAACAGTAACGAAAAAAGAACGAAAAAGTAAAGTAACACAATTAAAGCCATTAACACT
TGAAGAAAAGCGGAAGTTAAGACGTAAGCGACAAAAACGAATCCAATACAGTGTTATTACAATATTGGTATTG
TTGATTGCTGTTATATTAATTTACATGTTTTCACCACTTAGTAAAATTGCGCATGTAAATATAAATGGAAATA
ATCACGTTAGTACTTCAAAGATAAACAAAGTTTTAGGTGTTAAAAATGATTCAAGGATGTATACGTTTAGTAA
AAAAAATGCTATTAATGATCTCGAAGAGGATCCATTAATCAAAAGTGTTGAGATACACAAGCAATTACCAAAC
ACATTAAACGTAGATATCACAGAAAATGAAATTATTGCTTTAGTGAAATATAAAGGTAAATATTTACCTTTAT
TAGAAAATGGTAAATTGCTTAAAGGTTCAAATGATGTCAAAATTAATGATGCACCTGTCATGGATGGTTTCAA
AGGTACAAAAGAAGATGATATGATTAAGGCGTTATCTGAAATGACACCTGAAGTTAGACGATATATTGCCGAA
GTGACATACGCCCCAAGTAAAAACAAACAAAGCAGAATTGAATTGTTTACGACAGATGGACTTCAAGTAATCG
GTGATATTTCGACGATATCTAAGAAAATGAAATATTATCCGCAGATGTCACAATCATTATCAAGGGATAGTTC
GGGTAAACTAAAAACACGAGGCTATATTGATTTATCAGTCGGTGCTTCATTTATCCCATACCGTGGAAACACG
TCTAGTCAATCAGAAAGCGATAAAAATGTGACTAAATCATCTCAAGAGGAAAATCAAGCAAAAGAAGAATTAC
AAAGCGTTTTAAACAAAATTAACAAACAATCAAGTAAGAATAATTAAAAAATTTTGATATTGTCTATGTTTAT
AGTTCACAAGCCATTCAACGTATTGTAAACTAAGGATAGTGTATTTTTTTAATAGTAATTTGTCAGGAGGTGC
CTATCTATGGAAGAACATTACTACGTAAGTATTGATATTGGATCATCAAGCGTAAAAACAATAGTAGGCGAGA
AATTTCACAATGGTATAAATGTGATAGGTACAGGACAAACCTACACGAGCGGTATAAAAAATGGTTTAATTGA
TGATTTTGATATTGCGCGACAAGCAATCAAAGACACAATTAAAAAGGCATCAATCGCTTCGGGTGTTGATATT
AAAGAAGTTTTCCTGAAATTACCTATCATTGGAACGGAAGTTTATGATGAATCAAATGAAATCGACTTTTATG
AGGATACAGAAATCAACGGTTCACATATCGAAAAAGTATTAGAAGGTATTAGAGAAAAAAATGATGTGCAAGA
AACAGAAGTAATTAATGTGTTCCCGATTCGTTTTATAGTCGATAAAGAAAATGAGGTTTCAGACCCTAAAGAA
TTAATTGCCAGACATTCATTAAAGGTTGAAGCAGGCGTAATTGCTATTCAAAAATCGATTTTAATTAATATGA
TTAAATGCGTTGAAGCATGTGGTGTTGATGTATTAGATGTTTACTCTGATGCATATAACTATGGTTCAATCCT
AACAGCTACTGAAAAAGAGTTAGGTGCATGTGTCATTGATATTGGTGAAGACGTTACGCAAGTTGCTTTTTAT
GAACGCGGTGAATTAGTAGATGCTGATTCTATCGAAATGGCAGGGCGTGATATTACAGACGATATTGCACAAG
GATTAAACACTTCTTATGAAACTGCTGAAAAAGTTAAACACCAATATGGTCATGCATTCTATGATTCTGCTTC
AGATCAAGATATCTTCACTGTTGAACAGGTTGATAGTGATGAAACAGTACAGTATACTCAAAAAGATTTGAGT
GACTTTATTGAAGCGCGTGTAGAAGAAATATTCTTCGAAGTATTTGATGTTTTACAAGATTTAGGATTAACAA
AAGTAAATGGTGGGTTTATTGTAACTGGTGGATCTGCAAACTTACTTGGCGTAAAAGAATTATTATCAGATAT
GGTAAGTGAAAAAGTTAGAATTCACACGCCATCGCAAATGGGAATTAGAAAACCTGAATTTTCTTCAGCAATT
TCTACAATTTCTAGTAGTATCGCTTTTGATGAGTTATTAGATTATGTTACAATTAATTATCATGATAATGAAG
AAACTGAAGAAGATGTTATTGATGTGAAAGACAAAGATAACGAATCTAAATTAGGCGGATTTGATTGGTTTAA
ACGTAAAACAAACAAAAAAGATACTCATGAAAATGAAGTAGAGTCAACAGATGAAGAAATTTATCAATCAGAA
GATAATCATCAGGAACATAAACAGAATCATGAACATGTTCAAGACAAAGATAAAGAAGAAAGTAAATTCAAAA
AACTAATGAAATCTCTATTTGAATGATTATTGGCCAATAAAACTAGGAGGAAATTTAAATGTTAGAATTTGAA
CAAGGATTTAATCATTTAGCGACTTTAAAGGTCATTGGTGTAGGTGGTGGCGGTAACAACGCCGTAAACCGAA
TGATTGACCATGGAATGAATAATGTTGAATTTATCGCTATCAACACAGACGGTCAAGCTTTAAACTTATCTAA
AGCTGAATCTAAAATCCAAATCGGTGAAAAATTAACACGTGGTTTAGGAGCAGGAGCTAATCCTGAAATCGGT
AAAAAAGCTGCAGAGGAATCTCGTGAACAAATTGAAGATGCAATCCAAGGTGCAGACATGGTATTTGTTACTT
CTGGTATGGGTGGCGGAACTGGTACTGGTGCAGCACCAGTCGTTGCTAAAATTGCAAAAGAAATGGGCGCATT
AACTGTTGGTGTTGTAACTCGTCCATTTAGTTTTGAAGGACGTAAACGTCAAACTCAAGCTGCTGCTGGAGTA
GAAGCTATGAAAGCTGCAGTAGATACATTAATCGTTATACCAAATGACCGTTTATTAGATATCGTTGACAAAT
CTACGCCAATGATGGAAGCATTTAAAGAAGCTGATAACGTGTTACGCCAAGGTGTACAAGGTATCTCAGACTT
AATCGCTGTTTCTGGTGAAGTAAACTTAGACTTTGCAGACGTTAAGACAATTATGTCTAACCAAGGTTCTGCA
TTAATGGGTATTGGTGTTTCTTCTGGTGAAAATAGAGCGGTAGAAGCTGCTAAAAAAGCAATCTCTTCTCCAT
TACTTGAAACATCTATCGTTGGTGCACAAGGTGTGCTTATGAATATTACTGGTGGCGAGTCATTGTCATTATT
TGAAGCACAAGAGGCTGCTGATATTGTCCAAGATGCTGCAGATGAAGACGTTAATATGATTTTCGGTACAGTT
ATTAATCCTGAATTACAAGATGAGATTGTTGTAACAGTTATTGCAACTGGTTTTGATGACAAACCAACATCAC
ATGGTCGTAAATCTGGTAGCACTGGATTCGGAACAAGCGTAAATACTTCTAGCAATGCAACTTCTAAAGATGA
ATCATTCACTTCAAATTCATCAAATGCACAAGCAACTGATAGTGTAAGTGAAAGAACACATACAACTAAAGAA
GATGATATTCCTAGCTTCATTAGAAATAGAGAAGAAAGACGTTCAAGAAGAACAAGACGTTAATCGGTTAATA
TATATACACAAATAATTCAACACAAATCATCAGATAACATATCTGATGATTTTTTTACTAATTTTTAGAACAT
GTAGAAGGACATTTAAGTTTTTCAAAGTTAATAAAAGTGTTTAAGTATCGTGTCAAAATTAAGTCAAAAATTA
TTTGCGCAACATTTTAACTTTAAACATAAATGTTATATTATATAATTATTAACTTTGTACAGTTAGACGAAGA
TAATTTAAATGAAATGATGGTGACGATCGAGTGAATGATAATTTTAAAAAGCAACCGCATCATTTAATATATG
AAGAATTATTACAACAAGGTATTACTCTAGGTATTACAACTAGAGGCGATGGTTTAAGTGACTATCCTAAAAA
TGCTTTTAATATGGCGAGATATATTGATGATCGCCCATATAATATTACTCAACATCAATTGCAATTAGCTGAA
GAAATTGCGTTTGATAGAAAAAAATTGGGTGTTTCCCATCCAAACACATGAAAATAAAGTCGCTTGTATTACAA
AGGATGATATAGGCACAAATATAGACACTTTAACTGATGCGCTTCATGGTATTGATGCGATGTACACATATGA

TAGTAATGTCTTATTAACGATGTGTTATGCAGACTGTGTACCAGTATATTTTTATAGTACAAAACATCATTTT
ATTGCATTGGCGCATGCAGGTTGGCGTGGTACCTATACTGAAATTGTAAAAGAAGTGCTAAAACATGTGAACT
TTGATTTGAAAGACTTACATGTCGTTATTGGACCATCTACATCATCAAGTTATGAAATTAATGATGATATTAA
AAATAAATTTGAAACATTGCCAATTGATAGTGCCAACTATATTGAAACTAGAGGACGAGATCGTCATGGTATT
GATTTGAAAAAAGCCAATGCTGAATTATTAAATTATTATGGTGTTCCTAAAGAAAATATTTATACGACAGCGT
ATGCTACATCTGAACATTTAGAATTATTTTTCTCTTATCGATTAGAAAAAGGTCAAACAGGACGCATGTTAGC
ATTCATTGGTCAACAGTAAACAAGGAGGAGATATGTTTGCGTGTGAAAGATAATTTACAACAAATCTCAACAC
AAATTAATGACAAAAGTGAAAAAAATAATTTTTCAACAAAACCAAACGTGATTGCAGTTACAAAATATGTTAC
AATAGAGCGAGCTAAAGAAGCGTATGAGGCTGGAATAAGACATTTTGGTGAGAATAGATTGGAAGGCTTTTTT
CAAAAGAAAGAAGCATTACCATCAGATGCGGTGATTCATTTTATAGGATCATTACAATCTCGAAAAGTTAAGG
ACGTTATAAACGACGTAGATTATTTCCATGCTTTAGATCGATTGAGCTTAGCCAAAGAAATTAACAAACGTGC
AGAACATAAAATTAAATGTTTCTTGCAAGTGAACGTTTCGGGAGAAGCTTCTAAACATGGTATTGCTTTAGAA
GATGTTGATCAGTTTATAGATGATCTTAAAAAATATGACAAAATCGAAATTGTAGGTTTAATGACGATGGCAC
CATTGACAGATGATGAAGCATATATTAGATCGTTATTTAAACAGTTACGTTTGAAAAAAGAAGAAATACAACG
ACTCAATTTAGAATATGCGCCTTGTGATGAATTATCAATGGGAATGAGTAATGACTATCTTATTGCAGTTGAA
GAAGGTGCGACGTTTGTTAGAATTGGGACTAAACTTGTAGGAGAAGAGGAGTGAGCCACTTGGCTTTAAAAGA
TTTATTTAGTGGATTTTTTTGTAATAGATGATGAAGAGGAAGTAGAAGTACCTGACAAACAACAACAGGTCAAT
GAAGCGCCAGCAAAAGAGCAGTCACAACAAACAACAAAACAAAACGCAATCAAATCAGTCCCTCAAAAATCTG
CATCAAGATATACAACAACGTCAGAAGAAAGGAATAACCGTATGTCTAATTATTCAAAAAATAATTCACGTAA
TGTTGTAACTATGAACAATGCTACACCAAACAATGCATCACAAGAAAGTTCAAAAATGTGTTTATTCGAACCA
CGTGTTTTCTCAGATACACAAGATATTGCTGATGAGCTTAAAAACCGCCGTGCGACACTTGTCAATTTACAAC
GTATTGATAAAGTATCAGCGAAAAGAATTATTGATTTTTTAAGCGGTACTGTTTATGCAATCGGTGGAGATAT
CCAACGTGTAGGTACTGATATTTTCTTATGTACGCCTGATAATGTGGAAGTAGCTGGAAGCATTACAGACCAT
ATTGAAAATATGGAACATTCATTCGACTAAGGAGTTAACATATGGATATAAATGTGCTAGCTACAATATTTAA
ATTTATCCTTTTTGTTGTTGAAATTTATTATTTCGGCATGATTATATATTTCTTTACATCTTGGGTACCAAGT
ATTAGAGAAACTAAGGTAGGTTATTTATTAGCGAAAATATATGAACCTTTCTTACAACCATTTAGAAAAGTAA
TTCCACCTATTGGAATTATCGACATATCATCAATCGCTGCAATTATCGTTTTAGTATTATTCCAAAAAGGGTT
ACTCCAAATCTTTAATTGGATTTTAATTCAATTACAATAAATTAAGCGACATAACGAGGCAAGGGTACATGAT
ACTTTAGCCTCGTTTTTGATATGTATTTTTCTGAATATAAGGGCAATAGATGGTATTTTATAAATTTTTTAAG
GTAGTGATTAACATAGATATTTATCAACACTTTAGGCAGGAAGAATACGAATTAATTGATCAGCTAACGGATA
AATGTGATCAAGCGGAACACCATTATGCACCAGTATTAACGCATTTTTTAGATCCAAGAGGGCAATATATATT
GGAAGTGATTTGTGGCAGTTATGAAGATTTAAACGTATCTTTTTATGGCGGGCCTAATGCTGAAAGAAAAAGA
GCAATCATTTCGCCGAACTATTATGAACCTAAAGAAAGTGACTTTGAATTAACTTTAATGGAAATAGAGTATC
CTGAAAAAATTCGTCACTTTAAAACATCAACATATTTTAGGGACATTAATGTCTTTAGGTATCGAACGCGAACA
AGTTGGAGATATAATTGTGAATGAACGAATTCAATTTGTTTTGACAAGTAGATTGGAATCATTTATTATGTTA
GAATTACAACGTATTAAAGGCGCATCAGTTAAACTTTATACTATTCCAGTAACAGATATGATACAATCTAATG
AGAATTGGAAAAATGAAAGTGCAACAGTTAGTTCTTTAAGGTTAGATGTTGTTATTAAAGAAATGATACGTAA
ATCACGTACGATTGCGAAACAACTAATCGAAAAAAAACGTGTTAAAGTGAATCACACTATTGTTGATTCAGCA
GATTTTCAATTACAAGCAAATGATTTAATATCCATCCAAGGTTTTGGTAGAGCACACATTACTGACTTAGGTG
GTAAAACTAAAAAAGATAAAACGCACATTACCTATAGAACATTATTCAAATAGTAATGATTTAAGGAGGATAA
CAAATGCCTTTTACACCAAATGAAATTAAGAATAAAGAGTTTTCACGTGTAAAGAATGGTTTAGAACCTACTG
AAGTTGCTAATTTTTTTGGAGCAACTAAGCACTGAAATTGAACGTCTTAAAGAAGATAAAAAACAACTTGAAAA
AGTAATCGAAGAGAGAGATACTAATATTAAGTCTTATCAAGACGTGCATCAATCTGTAAGTGATGCTTTGATA
CAAGCCCAAAAAGCTGGTGAAGAAACTAAACAAGCTGCAGAGAAACAAGCTGAAGCGATTATAGCTAAGGCAG
AAGCGCAAGCTAATCAAATGGTTGGTGACGCGGTAGAAAAAGCACGCCGTTTAGCATTCCAGACTGAAGATAT
GAAACGTCAATCAAAAGTATTTAGATCGCGTTTCCGTATGTTAGTTGAAGCGCAATTAGACTTATTAAAAAAC
GAAGATTGGGATTACTTGTTGAATTATGATTTAGACGCTGAACAAGTGACGCTTGAAAATATTCATCATTTGC
ATGAAAATGATTTAAAGCCAGATGAAGTTGCAGCAAATGCACAAAATAATGCATCAAATACACCAGACAATAA
TCAACAATCCAATGATTCAGAAACAACTAAGAAGTAAGAATTAAATAAAGACAGACGCGTAATATACATTTAA
CTTTTCACAGCGAATTAGGTAATGGTGAGAGCCTAGTAAAAGCATGTATGTTATATCACTGGCTTTTTAATAT
TTAAATAATGTAATGAGAGAACTCTAAGTTGAGTTAATAAGGGTGGTACCGCGAGCAATCGTCCCTTTTAATT
TAACTTAGAGTTTTTTAAATTTTTAAGGAGTGAAAAAAATGGATTACAAAGAAACGTTATTAATGCCTAAAAC
AGATTTCCCAATGCGAGGTGGTTTACCAAACAAGGAGCCGCAAATTCAAGAAAAATGGGATGCAGAAGATCAA
TACCATAAAGCGTTAGAAAAAAAATAAAGGTAACGAAACATTCATTTTACATGATGGCCCACCATACGCGAATG
GTAACTTACATATGGGACATGCCTTGAACAAAATTTTAAAAGACTTTATTGTACGTTATAAAACTATGCAAGG
GTTCTATGCACCATACGTACCAGGTTGGGATACACATGGTTTACCAATTGAACAAGCATTAACGAAAAAAGGT
GTTGACCGAAAGAAAATGTCAACAGCTGAATTCCGTGAGAAATGTAAAGAATTTGCTTTAGAACAAATTGAAT
TACAGAAAAAAGATTTTAGACGTTTAGGTGTTCGTGGTAACTTTAATAATCCATATATCACATTAAAACCTGA

ATACGAAGCTGCACAAATTCGTATTTTTGGAGAAATGGCAGATAAAGGTTTAATTTATAAAGGTAAAAAGCCA
GTTTATTGGTCTCCTTCAAGTGAGTCTTCATTAGCAGAAGCAGAAATTGAATATCACGATAAACGTTCAGCAT
CAATTTACGTTGCATTTGACGTTAAAGATGACAAAGGTGTCGTTGATGCAGATGCTAAATTTATTATCTGGAC
AACAACGCCATGGACAATTCCATCAAATGTTGCGATTACCGTTCATCCTGAATTAAAATATGGTCAATACAAT
GTAAATGGCGAAAAATATATTATTGCAGAAGCCTTATCTGACGCTGTAGCAGAAGCACTGGATTGGGATAAAG
CATCAATCAAATTAGAAAAAGAATACACAGGTAAAGAATTAGAGTATGTTGTAGCACAACATCCATTCTTAGA
CAGAGAATCGTTAGTGATTAATGGTGATCATGTTACTACAGATGCTGGTACAGGTTGTGTACATACAGCACCA
GGCCACGGGGAAGATGACTATATTGTTGGTCAAAAATATGAATTACCAGTAATTAGTCCAATCGATGATAAAG
GTGTATTTACTGAAGAAGGCGGCCAATTTGAAGGAATGTTCTATGACAAAGCTAATAAAGCCGTTACTGATTT
ATTAACAGAAAAAGGTGCACTATTAAAATTAGACTTTATTACACATAGCTATCCACACGACTGGAGAACAAAA
AAACCTGTAATCTTCCGTGCTACACCACAATGGTTTGCTTCAATCAGTAAAGTAAGACAAGATATTTTAGATG
CAATCGAAAATACAAACTTCAAAGTAAATTGGGGTAAAACACGTATTTACAATATGGTTCGTGACCGTGGCGA
ATGGGTTATTTCTCGTCAACGTGTTTGGGGTGTACCGTTACCAGTATTTTATGCTGAAAATGGCGAAATTATC
ATGACGAAAGAAACAGTGAATCATGTTGCTGATTTATTTGCAGAACACGGTTCAAATATTTGGTTTGAAAGAG
AAGCGAAAGACTTACTACCAGAAGGATTTACACATCCAGGCAGCCCTAACGGTACATTTACTAAAGAAACAGA
CATTATGGACGTTTGGTTTGATTCTGGTTCATCACACCGTGGCGTGTTGGAAACAAGACCGGAATTAAGTTTC
CCAGCAGATATGTATTTAGAAGGTAGTGACCAATATCGTGGTTGGTTCAACTCTTCTATCACAACTTCAGTTG
CTACAAGAGGAGTATCACCTTATAAATTCTTACTTTCTCATGGTTTTGTTATGGATGGTGAAGGTAAGAAAAT
GAGTAAATCTTTAGGTAATGTGATTGTACCTGACCAAGTGGTTAAACAAAAAGGTGCTGATATTGCGAGACTT
TGGGTAAGTAGTACGGACTATTTAGCTGATGTTAGAATTTCTGATGAAATTTTAAAACAAACATCTGATGTTT
ATCGTAAAATCAGAAATACATTAAGATTTATGTTAGGTAACATTAACGATTTCAATCCTGACACAGATAGCAT
TCCTGAATCAGAGTTATTAGAAGTGGATCGTTACTTGCTAAATCGTTTACGTGAATTTACTGCAAGTACGATT
AACAACTATGAAAACTTTGACTACTTAAATATTTATCAAGAAGTTCAAAACTTTATCAATGTTGAGTTAAGTA
ATTTCTATTTGGATTACGGTAAAGACATTTTATATATTGAACAACGTGATTCTCATATCCGTCGCAGTATGCA
AACAGTGTTATATCAAATTTTAGTTGATATGACGAAGTTGTTAGCACCAATCTTAGTGCATACAGCTGAAGAA
GTTTGGTCTCATACACCACATGTTAAAGAAGAAAGTGTTCACTTAGCAGACATGCCTAAAGTTGTAGAAGTAG
ATCAAGCTTTATTGGATAAATGGCGTACATTTATGAATTTACGTGATGATGTGAATCGTGCATTAGAAACTGC
TCGTAATGAAAAAGTTATTGGTAAATCATTAGAAGCTAAAGTTACGATTGCTAGTAACGATAAATTTAATGCA
TCTGAATTCTTAACTTCATTTGATGCATTACATCAATTATTTATCGTGTCACAAGTTAAAGTTGTAGATAAGT
TAGATGATCAGGCAACAGCTTATGAACATGGTGATATTGTCATCGAACATGCAGATGGTGAAAAATGTGAAAG
ATGTTGGAACTATTCAGAGGATCTTGGTGCTGTTGATGAATTGACGCATCTATGTCCACGATGCCAACAAGTT
GTAAAATCACTTGTATAATTGAAATTGTATAAAGTACTCATACAGATGATATAAATTAAAGCTCTCTTCATAA
TCATGTTGTAGTTTTTGTTGACATGATGAAGGGAGTTTTTTACGAATAAAAAAATGACCAAGTTACTGGTTA
TATGTGTTAAAAAATGTGTAATTTACTAAAATAAAAATTATTCAGGAATGGTACAAATTCTCTAAGGCTTATA
AATGCGTTATAGTTGCTATTCTCAATTATGTTCGCGATAATTTTAAGTAAAAGTAAGCACAGATATTGAATTT
GATAGGAGTTAATTGAATGTATCATAACAGTAACGCAAACTTTGTAAATGGTATCACTTTAAATGTAAGAGAT
AAGAATGAATTAAAGCCATTTTATGAGGACGTATTAGGATTAAATATTATAAATGAGACATTAACATCGATAC
AATATGAAGTAGGCCAAAATAATCATGTCATTACACTTGTTGAATTACAAAATGGACGTGAACCTTTAATGTC
CGAAGCGGGACTGTTTCATATCGCAATTAAACTACCTCAAATTAGTGATTTAGCTAATTTACTAATTCATTTA
AGCGAATATGATATTCCAGTTAACGGTGGTATACAGCCTGCTTCGTTATCATTATTTTTTGAAGACCCGGAAG
GAAACGGTTTTAAATTTTATGTTGATAAAGACGAAGCGCAATGGACGAGGCAAAATGATTTAGTAAAAATTGA
TATTAGACCATTAAATGTACCGAGATTAGTGAGTCATGCAACAAAATTGTTATGGTTAGGTATTCCAGATGAC
GCTATTATAGGTGCATTGCATATTAAGACAATTCATTTATCAGAGGTAAAAGAGTACTACCTCGATTATTTTG
GATTAGAGCAATCGGCATATATGGATGATTATTCAATATTTTTAGCATCGAATGGCTATTATCAACATTTGGC
CATGAATGATTGGGTATCAGCAACGAAACGTGTAGAAAATTTTGATACGTATGGATTAGCAATTGTTGACTTT
CATTATCTTGAAACAACACATTTAAATTTACAAGGTCCGGATGGTATCTATTATCGCTTTAATCATATCGAAG
TTGAAGGTTAGTATATGCTTTGAATGGACGAACCATATAATGAATCGTTTTTAATGATCTTTTTATACAAGCT
ATGAAGGAGGCTGGGACATAAATCCCTAAAAAACAGCAGTAAGATAATTTTCAATTAGAAAATATCTTACTGC
TGTTCTCTATTTATACAATACTTCGTATTGAATGGCTTCGCTTTGCCCGTCTGGCACATTACTGTAAAATTCT
ATAAATAGAATTTTTGATGACGGGTCCCTTCCTAGGGTGCCGTCTCAGCCACCCCAACCGGCACATTGTTGTA
AGCTGACTATATTACTACGTATTAACATGTAATTTTACTTTGAAATACTTTAAAGCCCAAATTAATCAAAAGC
TTTCTGAACCAGAAACGAAAAAAATCTATAGTTAAAGAAAAATTGATGTAGAGCCTGTTTTTGGATTTATGAA
GGCTATTTTGGGTTTCACTAGAATGTCAGTTCGAGGAATAAATAAAGTTAAACGAGAGCTAGGTTTTGTATTA
ATGGTACTTAATATAAGGAAAATAGCAGCTCAACGAGCTGTACATTATAAAATACATATCAAAAAAGCTGATT
TCTATCAAATAATTAATAGAAAACAGCTTTTTTAAATTGCCTAAGAACTTAATGTCCCAGCCTGTTTCCTAAT
TTTTAATGTCATCTTAAATATGATAAATGAATAATTAAGTTCATATTTAATGTCAAAACATAGTAGTTTATCA
AGTATTGAGTTAGTAACATTAGATTTAATGTAATATGCTTACTTTTTTTATTAGCAGGTGTAAGCTATAATAT
AAAGAGTTGTCTCATGGACGATTGATTGGAGGAACGAAAATGCACAAAAAATATTTTATTGGCACTTCCATTT

TAATAGCAGTATTTGTCGTTATATTTGACCAAGTTACTAAATATATTATAGCTACTACAATGAAAATTGGAGA
TTCATTTGAAGTGATACCGCACTTTTTAAACATAACATCACATCGAAATAATGGTGCTGCATGGGGAATATTG
AGTGGAAAAAATGACATTTTTCTTTATTATTACCATTATTATATTAATAGCCTTAGTATATTTCTTTATTAAAG
ATGCTCAATATAATTTGTTTATGCAAGTTGCTATTAGTTTACTTTTTGCAGGTGCACTTGGAAACTTTATTGA
TAGAATTTTAACAGGAGAAGTTGTTGACTTTATTGATACAAATATTTTTGGTTATGATTTTCCAATATTTAAT
ATCGCAGATTCAAGTTTAACAATTGGTGTAATATTAATTATTATTGCCTTATTAAAGGATACTTCCAATAAAA
AGGAGAAGGAGGTTAAGTAATGGAGACTTATGAATTTAACATTACAGATAAAGAACAAACAGGTATGCGTGTA
GATAAGTTGCTGCCTGAATTAAATAGTGATTGGTCTCGTAACCAGATACAAGATTGGATTAAAGCAGGTTTAG
TCGTTGCAAACGATAAAGTTGTTAAATCTAATTATAAAGTGAAACTTAATGATCATATAGTTGTCACTGAAAA
AGAAGTGGTTGAAGCTGATATTCTACCTGAAAATTTAAATTTAGATATTTATTATGAAGATGACGATGTTGCA
GTTGTATATAAACCGAAAGGCATGGTAGTTCATCCATCACCAGGGCATTATACCAATACATTAGTTAATGGTT
TAATGTATCAAATTAAAAATTTGTCAGGTATTAATGGAGAAATTCGTCCAGGTATTGTTCACCGTATAGATAT
GGATACTTCTGGTTTATTAATGGTTGCTAAAAATGATATTGCTCATCGTGGGCTTGTAGAACAATTAATGGAT
AAATCTGTTAAAAGAAAATATATCGCTTTAGTTCACGGGAATATTCCTCATGATTACGGTACAATCGATGCGC
CAATTGGTAGAAACAAAAATGATCGTCAATCTATGGCTGTTGTTGATGATGGTAAGGAAGCAGTGACACATTT
TAACGTACTAGAACATTTTAAAGATTATACGCTTGTTGAATGTCAACTTGAAACAGGACGTACGCATCAAATC
CGTGTGCACATGAAATATATTGGCTTCCCATTAGTTGGTGATCCAAAGTATGGACCGAAAAAGACATTGGATA
TTGGTGGTCAAGCTCTACATGCTGGACTTATTGGATTCGAACATCCAGTAACAGGTGAATATATTGAAAGACA
TGCTGAATTACCACAAGACTTTGAAGATTTATTAGATACAATTCGAAAAAGAGATGCATAATTGTGTTGTGCT
ATAATTACGATAACATTATTATGTAAACTATAAGTATTTATCGTTTTCGTTTTTTTAAAATGAAAACACATTA
TAAAATAGATTAAGGTTAACATTTTTACTTAGATCGGTTAATTTTTCAATATAGATTGACAAAGCGCCTGATT
AGTATTATCCTTATAACAGTTTAAGTACAACATAAAGAGTCTTTAATTGAGCGTCCAGAGAGACGTCAAAGAC
ATGAAAATATAGATATATACTAAAATGATCATAACACTTTCACATATGCAAGAATAAGTAGTAATACTACGAT
TCTTCTATGTATATAGGCTGATTGTATATACTAAACCTCATGTCGATATTAAAGACATGAGGTTTTTTGTTTC
TAAATTTTGAAAGGGTGAGAATATGTCTGAACGTATCATAATGGATGATGCCGCAATACAACGTACAGTGACG
AGAATCGCTCATGAAATTTTGGAGTATAACAAAGGTACTGATAATTTAATTCTTTTAGGTATCAAAACAAGAG
GTGAATATTTAGCGAATCGTATACAAGATAAAATTCATCAAATTGAGCAACAACGTATACCTACTGGAACAAT
TGATATTACATACTTTAGAGATGATATAGAGCACATGTCATCACTTACGACAAAAGACGCAATAGACATCGAC
ACAGATATTACAGATAAAGTAGTCATCATTATTGACGATGTGCTGTATACTGGTCGAACGGTTCGTGCTTCAC
TTGATGCTATTTTGCTAAATGCTAGACCTATTAAAATTGGTTTAGCTGCTTTGGTTGATCGAGGACATCGTGA
GTTACCAATTCGAGCAGATTTTGTTGGTAAAAATATACCTACTTCTAAAGAGGAAACGGTAAGTGTCTATTTA
GAAGAAATGGATCAAAGAAATGCAGTTATAATTAAATAATACCCCTTTTAATTCAGTACGAGAGACTGAAAAA
GGTGGGGGAGTTGAATGATAAATAAGTTTTCATATGCTTGTTTATCTAATAATACCCTCATGTTCGACAACTA
TAAGTCAATTTCTATGGACGAAAATGACTTGTAGCAACTTGTCACACTTTTTGTCTCTTTACATATACTGATG
TAAAGAGATTTTTTTATTATCGGAGGAAATGAATATTATGCAAAATGATGAAATGTTTGAACGAACAGTAAAA
CCCGTACTAGATGTAAATGAAAAACCACAACCAGCGCAATGGGCATTTTTAAGCTTACAACATTTATTTGCGA
TGTTTGGCGCAACAGTACTAGTACCATTCTTAACAGGACTACCAATATCAGCAGCGTTACTAGCTTCGGGAAT
CGGTACATTACTTTATATCTTAATAACGAAGGCGCAAATACCAGCATACTTGGGATCTAGCTTTGCATTTATC
ACACCAATTATCACGGGATTAAGTACGCATAGCTTAGGAGACATGCTTGTAGCATTATTCATGAGTGGTGTTA
TGTACGTCATCATCGGAATTCTAATCAAATTAAGTGGGACAGCATGGTTAATGAAATTATTACCACCAGTTGT
TGTTGGACCAGTCATCATGGTAATTGGTTTAAGCTTAGCGCCTACTGCAGTCAATATGGCAATGTATGAAAAT
CCTGGAGATATGAAAGGTTACAATATCAGTTTCTTAATTGTTGCAATGATAACCTTGCTTGTAACAATAGTCG
TTCAAGGTTTTTTTAAAGGATTCCTATCTTTAATTCCAGTTCTTGTAGGTATTATCGTAGGTTATGTGGTAGC
GATTTTTATGGGGATTGTGAAATTTGATGCAATTATGTCAGCAAAATGGATAGATTTCCCTCATATTTATCTG
CCATTTAAAGATTATGTACCTTCATTTCACTTAGGACTTGTACTTGTAATGATTCCGATTGTGTTCGTAACAG
TAAGTGAACATATTGGGCACCAAATGGTACTGAATAAAATCGTAGGTAGAAACTTCTTTGAAAAGCCAGGACT
TGATAAATCAATCATTGGTGATGGTGTTTCTACAATGTTTGCCAGTATTATTGGTGGACCACCAAGTACAACA
TACGGTGAAAATATCGGTGTATTAGCGATTACCAGAATATACAGTATTTACGTCATTGGTGGTGCAGCAGTTA
TAGCAATTGTTTTAGCATTCATTGGTAAGTTCACTGCATTAATTTCTTCTATACCTACACCAGTTATGGGAGG
AGTATCTATATTACTTTTCGGTATTATTGCAGCAAGTGGCTTAAGAATGTTAGTTGAAAGCAAAGTAGATTTT
GCGAACAATCGAAATTTAGTTATAGCTTCAGTAATTTTAGTTGTAGGTATCGGTAATTTAGTATTTAACTTAA
AAGAAATTGGTATCAACCTTCAAATTGAGGGGATGGCATTAGCTGCACTTTCAGGAATTATTTTGAACTTAAT
CTTACCTAAAGAGAAAAAACAAAACAATTAAGATTTACAAATTAAGGAGGGCGCTTTTATGAATCATTTATTA
TCAATGGAACATTTATCTACAGATCAAATATACAAACTTATCCAAAAGGCAAGTCAATTTAAATCTGGTGAAC
GTCAACTACCAAACTTTGAAGGGAAATATGTCGCAAATTTATTCTTTGAAAATTCTACTCGAACAAAATGTAG
TTTTGAAATGGCAGAACTTAAGCTAGGGTTAAAAACGATTAGCTTTGAAACATCAACATCATCTGTTTCAAAA
GGTGAATCTTTATATGACACATGTAAAACTTTAGAAAGTATTGGCTGTGATTTATTAGTCATTAGACATCCGT
TTAATAACTACTATGAAAAATTAGCGAATATTAACATCCCAATTGCGAATGCTGGTGATGGTAGTGGACAACA

TCCAACACAAAGTTTACTTGATTTAATGACGATATATGAAGAATATGGATATTTTGAAGGCTTGAATGTATTG
ATTTGTGGAGACATTAAAAATTCACGTGTCGCACGTAGTAATTACCATAGTTTAAAAGCATTAGGTGCAAACG
TAATGTTTAATAGCCCAAATGCTTGGATTGATGATTCTCTAGAAGCACCTTATGTAAATATAGATGATGTTAT
AGAAACAGTGGATATAGTTATGTTATTAAGAATTCAACATGAAAGACATGGGCTTGCAGAAGAAACTAGATTT
GCAGCAGATGATTATCATCAAAAGCATGGCTTAAATGAAGTGCGCTATAACAAATTACAAGAACATGCTATTG
TTATGCATCCGGCACCTGTGAATAGAGGAGTAGAAATACAAAGCGATTTAGTAGAAGCTTCAAAATCAAGAAT
TTTTAAGCAAATGGAAAATGGCGTTTACTTAAGAATGGCAGTCATTGATGAATTATTAAAATAGGTAAGGGGA
CGAAAATGATGAAATTAATTAAAAACGGTAAAGTATTACAAAATGGCGAATTACAACAAGCAGATATTTTAAT
TGATGGTAAGGTAATTAAACAAATTGCACCTGCAATTGAACCAAGCAATGGTGTTGACATCATAGATGCGAAA
GGTCACTTTGTGTCACCTGGATTTGTCGATGTTCATGTTCATTTACGCGAACCTGGTGGTGAATATAAAGAGA
CAATTGAAACTGGTACTAAAGCTGCTGCTAGAGGCGGATTTACAACTGTATGTCCAATGCCTAACACAAGACC
GGTACCAGATTCTGTAGAACATTTTGAAGCTTTACAAAAATTAATCGATGACAATGCTCAAGTACGTGTATTA
CCTTATGCTTCAATTACAACACGTCAATTAGGTAAAGAATTGGTTGATTTCCCAGCACTAGTAAAAGAAGGTG
CCTTTGCGTTTACAGATGACGGTGTAGGAGTACAAACTGCAAGCATGATGTATGAAGGCATGATTGAAGCTGC
AAAAGTAAACAAAGCCATCGTAGCACACTGTGAAGATAATTCATTAATCTATGGTGGTGCAATGCATGAAGGG
AAACGCAGTAAAGAGTTAGGTATACCAGGTATTCCAAACATTTGTGAATCTGTTCAAATCGCAAGAGATGTAC
TATTAGCTGAAGCAGCAGGTTGTCATTATCATGTATGTCATGTTTCTACTAAAGAAAGTGTTAGAGTCATTCG
TGACGCTAAACGCGCAGGTATTCATGTTACAGCTGAAGTTACACCACACCATTTATTATTAACAGAAGATGAT
ATTCCTGGTAACAATGCCATTTATAAAATGAATCCACCATTGAGAAGTACTGAAGATAGAGAGGCTTTGTTAG
AAGGGTTACTAGACGGTACAATTGACTGTATCGCAACAGACCATGCACCACATGCACGTGATGAAAAAGCGCA
ACCAATGGAAAAAGCACCATTCGGAATAGTTGGTAGTGAAACAGCATTCCCATTATTATATACGCATTTTGTA
AAAAATGGTGATTGGACATTACAACAATTAGTAGATTACTTAACAATTAAACCATGTGAGACATTTAATTTAG
AATACGGCACATTAAAAGAAAATGGTTATGCAGATTTAACAATCATTGATTTAGATAGTGAACAAGAAATTAA
AGGAGAAGATTTCTTATCAAAAGCAGATAATACACCATTTATCGGCTATAAAGTTTATGGAAATCCGATCTTA
ACAATGGTTGAAGGCGAAGTTAAATTTGAGGGGGATAAATAATATGCAAAGCAAACGTTATCTAGTGTTAGAA
GACGGTTCTTTTTACGAAGGCTACCGTTTAGGATCTGATAACTTAACTGTAGGAGAAATTGTATTTAATACAG
CGATGACAGGTTATCAAGAAACTATTTCTGATCCATCATATACAGGTCAGATCATTACTTTTACGTATCCATT
AATCGGTAATTATGGTATCAATAGAGACGATTTTGAATCATTAGTACCTACATTAAACGGTATTGTAGTGAAA
GAAGCGAGTGCGCATCCAAGTAATTTTAGACAGCAAAAGACACTTCATGACGTTTTAGAATTGCATCAAATTC
CAGGGATTGCAGGTGTTGATACAAGAAGTATTACGCGTAAAATTCGACAACACGGTGTGTTAAAAGCTGGTTT
TACTGATCGAAAAGAAGATATTGATCAACTTGTCAAACATTTACAACAAGTAGAATTACCTAAAAACGAGGTA
GAAATCGTTTCGACTAAAACACCGTATGTTTCGACAGGTAAGGATCTAAGTGTCGTACTTGTAGACTTTGGTA
AGAAGCAAAATATTGTTCGAGAATTAAACGTCAGAGGTTGTAACGTCACAGTTGTACCATATACAACTACTGC
CGAAGAAATTTTAGCAATGGCTCCAGATGGCGTTATGCTATCAAACGGACCAGGTAATCCTGAAGTTGTAGAA
TGTGCGATTCCAATGATTCAAGGAATTTTAGGGAAAATTCCGTTCTTTGGTATCTGTCTAGGACATCAACTTT
TTGCATTATCTCAAGGAGCAAGCTCATTTAAAATGAAGTTTGGTCATCGTGGTGCGAACCATCCAGTTAAAAA
TTTAGAGACTGGAAAAGTTGATATTACGAGTCAAAACCATGGATATGCAATAGATATAGATTCGTTAAAAAGT
ACTGATTTAGAAGTTACTCATCTTGCATTAAATGATGGTACTGTAGAAGGTTTAAAACATAAAACATTACCAG
CATTTTCTGTTCAATACCATCCTGAAGCAAATCCAGGACCGTCAGATTCAAACTATCTATTTGATGATTTTGT
AGCAATGATGACTAATTTTAAGGAAAAGGAGCGTCATATCAATGCCTAAACGTAATGATATCAAAACAATTTT
AGTAATAGGGTCTGGGCCAATTATCATAGGTCAAGCAGCTGAATTTGATTATGCTGGAACACAAGCATGTCTA
GCTTTAAAAGAAGAGGGATACCGAGTTATTCTTGTAAATTCAAATCCAGCGACAATCATGACTGATAAGGAAA
TTGCGGATAAAGTATATATCGAACCGTTAACTCATGATTTTATAGCGCGAATTATACGTAAAGAGCAACCTGA
CGCTTTACTTCCAACTTTAGGTGGTCAAACAGGTTTAAACATGGCGATTCAACTACACGAAAGTGGTGTGCTT
CAAGATAATAACGTCCAATTATTAGGAACTGAGCTAACATCAATTCAACAAGCAGAAGACCGTGAAATGTTTA
GAACATTAATGAATGATTTAAACATTCCTGTACCAGAGAGTGACATTGTAAATACAGTAGAGCAAGCCTTTAA
ATTCAAAGAGCAAGTGGGATACCCGCTAATTGTTAGACCGGCATTTACGATGGGTGGTACCGGAGGCGGTATT
TGTCATAATGATGAAGAATTACATGAAATCGTCTCAAATGGTCTTCATTATAGTCCAGCAACGCAATGTTTAT
TAGAAAAATCTATCGCAGGTTTTAAAGAAATCGAATACGAAGTAATGCGTGATAAAAACGATAATGCCATCGT
TGTATGTAACATGGAAAATATTGATCCAGTTGGTATTCATACAGGCGATTCAATTGTTGTGGCTCCTAGCCAA
ACATTATCAGATGTTGAGTATCAAATGTTACGTGATGTTTCATTAAAAGTTATTCGAGCTTTAGGTATCGAAG
GTGGTTGTAATGTTCAATTAGCATTAGATCCCCATTCATTCGATTATTATATTATAGAAGTAAATCCACGTGT
ATCACGTTCATCAGCGTTAGCTTCAAAAGCAACAGGATATCCTATTGCAAAATTAGCTGCTAAAATCGCGGTT
GGTCTAACATTAGATGAAATGTTAAATCCAATTACAGGAACATCTTATGCAGCGTTTGAACCAACTTTAGACT
ATGTGATTTCAAAAATACCAAGATTTCCTTTTGATAAATTTGAAAAAGGAGAACGAGAGCTTGGCACACAAAT
GAAAGCAACAGGTGAAGTTATGGCCATTGGTCGAACTTACGAAGAATCATTGTTAAAAGCAATTCGATCACTT
GAGTATGGTGTGCATCACTTAGGATTACCAAATGGTGAAAGCTTCGATCTTGATTATATTAAAGAACGTATTT
CACACCAAGATGATGAACGATTATTTTTCATCGGCGAAGCAATTAGAAGAGGCACAACATTAGAAGAAATTCA

TAATATGACTCAGATTGATTACTTCTTCTTACACAAGTTCCAAAACATTATTGATATTGAGCATCAATTAAAA
GAACATCAAGGTGATTTAGAATATCTTAAATATGCAAAAGATTATGGATTTAGTGATAAAACAATAGCGCATC
GCTTTAATATGACAGAAGAAGAAGTATATCAATTGCGTATGGAAAATGATATTAAACCTGTTTACAAGATGGT
TGATACTTGCGCAGCTGAATTTGAATCTTCAACACCATATTATTATGGTACATACGAAACTGAAAATGAATCC
ATAGTTACTGACAAAGAAAAAATCTTAGTATTAGGCTCTGGACCAATTCGAATCGGCCAAGGTGTAGAATTTG
ACTATGCGACAGTTCACGCCGTTTGGGCAATTCAAAAAGCAGGGTACGAAGCGATAATTGTGAATAACAATCC
AGAAACAGTTTCAACAGACTTCTCAATTTCTGACAAATTATACTTTGAACCTTTAACTGAAGAAGATGTGATG
AATATCATTAATTTAGAAAAACCTAAAGGTGTCGTTGTACAATTTGGAGGACAAACAGCGATTAATTTAGCAG
ACAAATTGGCTAAACATGGTGTTAAAATACTTGGTACTTCACTAGAAAATCTAAATCGTGCTGAAGATAGAAA
AGAATTTGAAGCACTATTAAGAAAAATTAACGTGCCACAGCCACAAGGGAAAACAGCTACATCACCTGAGGAA
GCATTAGCGAATGCTGCAGAAATCGGATATCCGGTTGTAGTAAGACCTTCTTATGTATTAGGTGGTCGCGCAA
TGGAAATTGTAGACAATGACAAAGAGTTAGAAAACTATATGACCCAGGCTGTAAAAGCGAGTCCGGAACATCC
GGTACTAGTCGATAGATATTTAACTGGTAAAGAAATTGAAGTTGATGCGATTTGTGATGGAGAAACGGTCATT
ATTCCAGGAATCATGGAACATATTGAACGTGCTGGTGTGCATAGTGGTGACTCAATCGCTGTATATCCGCCAC
AAACTTTGACAGAAGACGAGTTAGCAACACTTGAGGACTATACTATAAAATTAGCTAAAGGTTTAAACATCAT
TGGCTTAATCAACATTCAATTCGTTATAGCTCACGATGGTGTGTATGTTTTAGAAGTAAATCCACGTTCTAGT
AGAACGGTACCATTCTTAAGTAAAATTACTGATATTCCAATGGCACAATTAGCTATGCGAGCAATCATTGGGG
AAAAACTAACAGATATGGGGTATCAAGAAGGGGTTCAACCATATGCTGAGGGTGTATTTGTGAAAGCACCGGT
ATTTAGTTTTAATAAATTGAAAAATGTTGATATTACTTTAGGACCTGAAATGAAGTCTACAGGTGAAGTAATG
GGGAAAGATACTACATTAGAAAAGGCGTTATTCAAAGGGTTAACAGGTAGTGGTGTTGAAGTTAAAGATCACG
GTACAGTATTAATGACCGTCAGTGACAAAGATAAAGAGGAAGTTGTTAAATTGGCACAACGCTTAAATGAAGT
TGGCTATAAAATTTTAGCAACGTCTGGAACAGCTAATAAATTAGCTGAGTATGACATACCTGCAGAAGTAGTA
GGCAAAATTGGTGGCGAAAACGATTTATTAACACGTATTCAAAATGGTGATGTTCAAATCGTTATAAATACAA
TGACTAAAGGTAAAGAAGTAGAAAGAGATGGCTTCCAAATTAGACGTACTACAGTTGAAAATGGTATTCCATG
TTTGACATCTTTAGATACAGCTAATGCCTTAACGAATGTAATTGAAAGTATGACATTTACAATGCGTCAAATG
TAAATCAATCAAACTGTATCGGTGGGGCTGTAATTAACCATTTACTTAAAGAAGTTTATATTACAGCCTCATT
ATTTTAATGAATTGCTTAACGTAAAGGGAGACATATATGATGAAAGATTTACCAATTATTGCATTAGATTTTG
AATCAAAAGAAAAAGTAAATCAATTTTTAGATTTATTTGATGAATCATTATTCGTAAAAGTAGGTATGGAACT
TTTTTATCAAGAAGGCCCTCAATTAATTAATGAGATAAAAGAAAGAGGCCATGATGTATTTTTAGATTTAAAG
CTGCATGATATTCCTAATACAGTTGGTAAGGCGATGGAAAGACTAGCTAAATTGAATGTTGATCTGGTAAATG
TTCATGCTGCTGGTGGTGTAAAAATGATGTCTGAGGCCATTAAAGGATTAAGAAAACATAATCAACATACAAA
AATTATTGCAGTAACACAGCTTACGTCAACAACAGAAGATATGTTACGACACGAACAAAATATACAAACATCG
ATTGAAGAGGCCGTTTTAAATTATGCCAAGTTAGCAAATGCAGCTGGTTTAGATGGCGTTGTTTGTTCACCTC
TTGAAAGTCGTATGTTGACTGAAAAGTTAGGTACATCATTTTTAAAAGTAACACCAGGTATTAGACCTAAAGG
TGCATCTCAAGATGACCAACACCGTATTACGACACCGGAAGAAGCAAGACAGCTTGGTTCGACGCATATTGTA
GTCGGTAGACCGATTACACAAAGTGACAATCCAGTCGAAAGTTATCATAAAATTAAAGAAAGTTGGTTAGTAT
AATGGCTAAAGAAATTGCAAAATCATTATTAGATATTGAAGCTGTAACATTATCACCAAATGATTTATATACA
TGGAGTTCAGGTATTAAATCACCGATTTACTGTGATAACCGTGTTACGTTAGGTTATCCTTTAGTTCGAGGCG
CAATCCGCGATGGTTTAATTAACTTAATTAAAGAACACTTTCCTGAAGTAGAAGTTATTTCTGGTACTGCAAC
AGCTGGTATTCCACATGCAGCTTTTATTGCTGAAAAATTAAAATTACCAATGAATTATGTTCGTTCATCAAAT
AAGAGTCATGGTAAGCAAAATCAAATTGAAGGTGCTAAAAGTGAAGGTAAAAAAGTAGTTGTGATAGAAGATT
TAATTTCGACAGGGGGATCTTCAGTCACAGCAGTTGAAGCCTTAAAACAAGCAGGTGCAGAAGTATTAGGTGT
TGTAGCTATCTTTACTTATGGTTTGAAAAAAGCAGATGATACATTTAGCAATATTCAACTACCTTTTTACACT
TTAAGTGATTACAATGAATTAATTGAAGTAGCTGAAAATGAAGGTAAAATTTCTAGTGAAGATATCCAAACAT
TAGTTGAATGGAGAGACAACTTAGCATAATATAGACACTAGAAGGAGGAATTCAACATATGAATGACAAAACA
TCTAATGATTTATATGGGAAGATAAAACATTGTAACGAATTTATCAATCATTCTAATGATTCCAATCTATCTA
GTAGTCACGATGTCGACGAAAGTTCAACGAAGCAAAAACATATAAAAAATAAAACAACTATAGATCATAATGA
TGATTTATTTAAACATGTAAAGGATATATTACGTAAACAAGGACAAATTTAAAAACAAAAAAGCTATAGTGTT
TAATATCATGGGCTGCTTAACCGCATCCGAATATTACAAACACTATAGCTTTTATTATATAAAAAATTTATTA
GCGGATAATTAACTTTATTAAAATCCAACCAATTAAGAATACGAGTAATAGGACTAATACTGGAATCACATAA
TGTAACATAACGTCCCTCCTTTAACTTAATTTTAATTGTAATCAAATTTGACAATAAGTCAAAACATTAATAC
CTATGATAAGTATCATTTATTAACATATGTATCATATTTTTAATCTTGCGTAATTTTTATCGTTAACTATGTG
ATTTAATCAACAGGCCAATAAGACATTTGTCATTCGTGCATATAACGGTACGACGCGCTTTTGTCATTTTAAT
TATGTTAAGATAATAGTAGTTATAGAAGTTCAATCTATAGGAGGCATAGCATGGATATTCCAAAAATCACGAC
ATTTTTAATGTTTAATAACCAAGCTGAAGAAGCTGTTAAACTATACACAAGCTTATTTGAAGATAGTGAGATT
ATAACAATGGCTAAGTATGGTGAAAATGGACCTGGTGATCCTGGGACTGTACAACACTCAATATTTACATTAA
ATGGACAAGTATTCATGGCAATTGATGCTAATAGTGGCACAGAATTACCAATGAATCCAGCGATTTCATTATT
TGTTACAGTAAAAGATACTATTGAAATGGAACGACTATTTAATGGATTAAAAGATGAAGGTGCCATTTTAATG

CCAAAAACGAATATGCCACCATACAGAGAGTTTGCTTGGGTTCAAGATAAGTTTGGAGTAAGTTTTCAATTAG
CATTACCTGAGTAAAAGGATTCGCACAGCGTTGAATGATAAAGAAACACTTTTTCTTATGCATGCGTTTTACC
TATGTATTTCTATTTTAAGTACACATTAGCATTTTAGTTTCACTCATTTTTAAAATCACAAAGATAATTTGTA
ACTTAAATGATGATACGTTATTTAAAAACACGATACTTCGTTTCAATGAACGCATTAAATAATAAATAAACAC
CTCGCAAAAGAAAGTAGTCCTTTCTTATGGTGAGGTGTTTATTTGTCGACAACTTGATTATGATTGTTTCATT
TTTTGAATAAGTTCATAATCAGGTGTAGCATACAAAGTTTTTTGATTGTCATATGTTACAAACCCAGGCTTTG
CACCTGATGGTTTGTGCACATTTTTAATTAACGTGTAATCAACAGGTATTTGTCCAGAATTACCAGCTTTTGA
AAAATATCCTGCTAACATAGCCGCTTCCTTGATTGTCGTATCACTTGGTGCGTCATTAAATATAACGACATGT
GAACCAGGAATATCTTTTGTGTGTAACCATGTGTGAGTTTTTTTAGCTTTTTTATTTGTTAAATAATCATTTT
GCTTGTTATTCTTGCCAACATATATATCGTCGCCATCAGTTGATACATAATGTTGTAATTGAATCTGCGCTTT
CTTTTTCTTAGTTTGATTTTTACGCTGTTTCATAAAGCCTTGTTCTGCTAATTCATCTCTAATTTCATCAATG
TCATGGACAGAAATATGATGTAATTGTTGTTCGATTGTTGAAAAATAATCTATATTGTCTTTCGTCAATTGAA
TTTGATGTTGTAATTCACGTTCTCTCGTTTTCATACGATTATATTGTTTATAATAATATTGAGCATTTGCTGA
TGGGGATTTTGTAGGATTTAAAGGAATGACAACTTCTTCATTCGTATAATAATTCAACGCCGTCACTTCTTTA
TCACCTTGCTTAATTCGATATATATTAGCAGTGATCAATTCACCATATAACTGTTCAGTATCTTTATTTTTAG
ACTGCTCATATTCTTCAATCAACTTCGCTAATTTATTTTGATATTTGTGCAACTGCTGTTGAACAAATCGAAC
TAAATCATTCGCACGTTGTTTAACGCGTTCACGTTCGCCACGCGCATCATAAAAACGATCAAGTAAATCATTT
AATGAATCGTACGTAACTGTATCATCATTAAATTGATTTAACTTTATAAAATAGAAATCCTCTTTACCTGTTT
CATGATTTTTATGAAAAATAGGAGTAGGTGGTAACTTGGTTTCTGCCATAACTTCGTCGAATGCTTCTGGTAA
TGTTGATGAAGTCATAAATTGACGACGATTAACGATTTCATTCGTAATTAAAGGGCTAAATCCTTCAAACTGA
TTCAATAATTGTTTAGCAATATTACCTGCGTTAAAATCGATATATTTCAACACCTCTGCACCTGTAATATCAT
ACGGATTCATTTTGTGCTGAGTAGGTGGTGCTTCATAATTAAATCCTGGCATTACTGTACGATAGTGATTCGT
ATTTGGTGTTAAGTGTTTAAATCCTTCAATTATTTTGCGATTTTCATCTACTAAAATTAAGTTACTATGTTTA
CCCATAATCTCTAGGATAACAGTGCGGTAAATAGTATCGCCAATTTCATCTTTACTCTTTATATCGATTTCAA
TGCGACGATCATTACCAATTTGCTTAATCGATTCGATAATACCACCTTCTAAGTGTTTTCTAAAAACACGCGC
AAACATGGGTGGATTAAATGGATTATCATATTTTTTAGTAGTCAATTGTAATCTTGAAAAGTTTGGATGGATT
GACAATAACAATTGATGGTTTTGTCTATTTTGACGTACAACCATTAGTATCGTGTCATTATCAGGTTGATTGA
TTTTGTGAACGCGTCCTGTTGTTAAAAATTGTAGAGACTCAACCATTTTCTTTGTAAATAAGCCATCATAAGC
CATAATTTATCTTCCACCTTCTTCATATCATTCGTTTCATTTTAACATGATTGTAACTTTTCGGTATAGTTAT
TGAACCATACATTATGCCACACATTTTACATTATCTAAAAGTTTGCAATTCAATTTATTTAATTAAAATTAAT
CGTGCAATATAACGATATTGTTAGACTTAATAGAAATTATGGCATGCAATTTCAAATATGCTATACAATATAA
AGAACAATGTGATATCATATTTAAATAATAGAAGATTAGCTTAGAGAGGTCGTAAGGCATGGATAATGAAAAA
GGATTGTTAATCGTTTTATCAGGACCATCTGGAGTAGGTAAAGGTACTGTTAGAAAACGAATATTTGAAGATC
CAAGTACATCATATAAGTATTCTATTTCAATGACAACACGTCAAATGCGTGAAGGTGAAGTTGATGGCGTAGA
TTACTTTTTTAAAACTAGGGATGCGTTTGAAGCTTTAATCAAAGATGACCAATTTATAGAATATGCTGAATAT
GTAGGCAACTATTATGGTACACCAGTTCAATATGTTAAAGATACAATGGACGAAGGTCATGATGTATTTTTAG
AAATTGAAGTAGAAGGTGCAAAGCAAGTTAGAAAGAAATTTCCAGATGCATTATTTATTTTCTTAGCACCTCC
AAGTTTAGATCACTTGAGAGAGCGATTAGTAGGTAGAGGAACAGAATCCGATGAGAAAATACAAAGTCGTATT
AACGAAGCACGTAAAGAAGTCGAAATGATGAATTTATACGATTACGTTGTAGTTAATGATGAAGTAGAACTTG
CGAAGAATAGAATTCAATGTATTGTAGAAGCTGAGCACTTAAAAAGAGAGCGCGTAGAAGCTAAGTATAGAAA
AATGATTTTGGAGGCTAAAAAATAATGTTAAATCCACCATTAAACCAATTAACGTCACAAATTAAATCAAAGT
ATTTAATTGCAACAACTGCAGCGAAAAGAGCGCGTGAAATTGATGAACAACCTGAAACTGAATTATTAAGTGA
ATATCATTCATTTAAACCAGTTGGTAGAGCGTTAGAAGAAATTGCTGACGGTAAAATTCGCCCTGTTATTTCA
AGCGATTATTATGGTAAAGAATAGTTTCGACCATTAAAAATATGTGAAGCTAGACGTACATCAAATACGTCAA
GGCATATCCAAATCAAACGCACCTATGTAGGTGTGTTTTTTTAGTTTATCCAATGATTGATGTTATAATAATA
CTAAATTTGTATCTATAAAAAAGTAATGAGCATTTGTGCGCATATGATGATGTAAAGCGTAAATGTCATAGTG
ATATTTAAGGGAGATTAAATATGAAGAAAATATTATTAGCCGTTACAGGTGGCATTGCGGCATATAAAGCAAT
TGATTTGACAAGTAAGTTAACACAATCTGGGTATGAAGTTCGCGTTATGTTAACGAATCACGCACAAAAATTT
GTGACACCATTAGCATTTCAAGCAATAAGTCGAAATGCTGTTTATACAGATACTTTTATAGAAGAAAATCCTT
CAGAAATACAGCATATTGCATTAGGTGATTGGGCAGATGCAATCATTGTTGCACCTGCAACGGCAAATACAAT
TGCAAAATTGAGTGTAGGTATTGCTGATGATTTGGTGACATCAACGTTGCTAGCAACAGAGACACCGAAATTT
ATTGCGCCTGCTATGAATGTGCATATGTATGAAAATAAACGTACGCAGCAAAATATTAATATTTTAAAAGAAG
ATGGGTATCATTTTATCGAACCAGGAAGCGGATTTCTAGCATGTGGTTATGTTGCTAAAGGACGTATGGAAGA
ACCGCTTCAAATCGTTTCTGTTATTGATGCTCATTTTCAAAATAGTAATCGTTTAGCTAATAGTTCATTTCAA
GATAAACGCGCATTGGTTACAGCAGGACCAACTATTGAAGTTATCGATCCAGTCAGATTTGTATCCAATCGTT
CTTCTGGAAAAATGGGCTATGCAATAGCTGAAGCATTGCGAAATCGAGGAGCTATCGTGACGTTAGTTGCTGG
TCCTACAACACTAGAGGATCCAAAAGATATTGAAGTTATTCATGTTCAAAGTGCTGAAGAAATGTTTGAACAA
GTGACAAGCCGATTTGACGAACAAGATATTGTTGTAAAAGCAGCAGCCGTATCTGACTATACACCAGTTGATG

TATTAGAACATAAGATGAAAAAGCAGGATGGTGATTTATCAGTATCTTTTAAACGTACTAAAGACATTCTTAA
ATATTTAGGTGAACATAAAACATCACAGTATTTAATAGGCTTTGCAGCAGAGACTGAAGATATTGAAAATTAT
GCACAACAAAAATTACGCAAGAAAAATGCAGATGTGATTATTTCAAATAATGTTGGGGATATGTCTATCGGAT
TTAGTTCTGATGATAATGAATTGACAATGCATTTTAAAAATAATGAAAAGGTAAATATCAAGAAAGGAAAAAA
AGTAGTATTAGCTGCACAAATTTTAGATGAACTAGAAACTAGGTGGCAATAATGATAGCGAAAGTCATAGTCG
ATGTCGCGTCGAAGAGCGTTGACTTTAAATTTGATTATATAATTCCTGAACAACTCGAATCTGTCATCCAACC
TGGTGTGCGTGTGATTGTACCTTTTGGACCAAGAACGATTCAAGGTTATGTAATGGAAGTAACAGCAGAACCT
GATGCACAACTTGACGTTTCGAAGTTAAAAAAAATCATAGAAGTGAAAGATATACAACCTGAATTAACATCAG
AATTAATAGCTTTAAGTGAGTGGATGGGTTCAACTCATGTCATTAAACGTATTTCTATGCTAGAAGTGATGCT
TCCGAGTGCTATTAAAGCGAAGTATAAAAAAGCATTTAAGATGAAAGATGACATAGAGCTACCTTCAGCTTTA
TTACAAAAATTTGATAAGCATGGTTACTATTATTATAAAGATGCGCAAAAAAATAATGATATTCAATTGCTTA
TGAAGTTGTTAAAAGATGATATCGTTGAAGAAAAAACGATTCTCACACAAAATATAACTAAAAAAACCAAGCG
TGCTGTTCGTGTCATTGAAGGGTATCATCCTGATGAAGTATTAGCTAAGTTGGAGAAAGTTATTAAACAATAC
GATTTGTATGCTTACTTGTCTGAAGAACAACATAAAACAATATTTTTAACTGATATTGAGGATATGGGCTTTT
CAAAATCCAGTTTAGATGGACTTATCAAAAAAGGTTATGTTGAAAAATATGACGCGGTTGTTGATAGAGACCC
ATTTAAAGATCGTGTTTTCGAACAAGAATCAAAACAGCAATTAACAGAAGATCAATATAAAGCATATGAAGCG
ATTAAAGCTAAAATTGTAAGCCAAGAGCAAGAAACATTTTTACTTCATGGTGTGACGGGATCAGGTAAAACAG
AAGTATATTTACAAACGATAGAAGATGTTTTAAGCCAAGGAAAACAGGCGATGATGTTAGTTCCTGAAATCGC
TCTAACACCGCAAATGGTTTTACGCTTCAAACGTCGATTTGGTGATGACGTTGCTGTATTACATTCTGGCTTA
TCTAATGGGGAACGTTATGATGAGTGGCAAAAAATTAGGGATGGTCGTGCGAGAGTAAGTGTTGGTGCAAGGT
CAAGTGTGTTCGCACCTTTCAAAAATTTAGGGTTAATCATCATTGATGAAGAACATGAATCTACATATAAACA
AGAAGATTATCCGAGATATCACGCTAGAGAAATTGCCCAATGGCGAAGTGAATATCATCACTGTCCAGTCATT
TTAGGAAGTGCAACACCATGTCTTGAAAGTTATGCACGAGCTGAAAAAGGCGTTTATCATTTGCTATCATTAC
CAAACAGAGTGAACCAACAAGCTTTACCTGAAATTGATATAGTAGACATGCGTGAAGAATTGAGTGAAGGTAA
TCGGTCAATGTTTTCAAAAGATTTACGTGAAGCCATACAATTAAGATTAGATCGACAGGAACAAGTTGTTTTA
TTTTTAAATCGACGTGGTTATGCATCGTTTATGTTATGTCGGGATTGTGGATATGTACCGCAATGTCCAAACT
GTGATATTTCATTAACGTATCATAAAACGACAGACTTATTAAAATGTCACTATTGTGGTTACCAAGAGACGCC
ACCGAATCAATGTCCAAATTGTGAGAGTGAACACATTCGACAAGTAGGTACTGGTACTCAGAAAGTTGAAGAA
CTATTGCAACAAGAATTTGAAGATGCGCGCATAATTAGGATGGATGTAGATACAACCTCAAAGAAAGGTGCAC
ATGAAAAGTTATTGACTGAATTCGAAAAAGGTAACGGTGACATTTTACTAGGTACTCAGATGATTGCGAAAGG
ATTAGATTATCCAAATATTACTTTAGTTGGTGTGCTGAATGCAGATACAATGTTAAATTTACCTGATTTTCGG
GCGAGCGAACGTACTTATCAACTATTAACGCAAGTGGCTGGTAGAGCTGGTCGTCATGAAAAGGCAGGTCAAG
TCATCATTCAAACGTATAATCCAGATCATTATTCAATATTGGATGTTCAAAAAAATGATTATTTAACATTTTA
TCGTCAGGAAATGGAATATCGTAAATTAGGAAAGTATCCACCGTATTATTATTTGATTAATTTCACAATCTCA
CATAAAGAAATGAAGAAGGTTATGGAAGCATCGCAGCATGTTCATAAAATTTTATTACAGCATTTAACAGAAA
AAGCGCTTGTGCTAGGTCCATCTCCGGCAGCACTTGCGAGAATCAACAATGAATTTAGATTCCAAATTTTAGT
GAAATATAAAAGTGAACCTGGATTATTACAAGCCATTCAGTTTTTAGATGACTATTACCATGAAAAATTTATA
AAAGAAAAATTAGCATTGAAGATTGATATTGATCCGCAGATGATGATGTAACATTACTAATTATTAGAAACAA
GTTCAGTATGTACGAGTATTTGAACCAGTGTTTGAATATTTACTTTAAGTACAGAATAAGGGCAGAATATACA
ACTGTTAACTATTTAAATTAGCAGTTTATATTCTGTCTTTTTATATGGCTTTATAACTTACGTGATTTTGGTT
TGATAAGGAATTTATTAGTATTTTCATTTACGAATTCTGATTTTAATGCAAGCGCCATACTTACTGATGAATA
AAAGTACTAATGATTAAGCAAACTATTTTTAAATGGCAAATTGAAATAGTGCAAATTTTAAACTTATAAATTA
ATGGCTATAAACTATTTTATATCTATCTATGTTTTAACTACTTAAATTATTAGACAAAACAATTTTCTTAAAT
TATCAATTAAAGTATTTAAAAATATTGATGGTTAATGTAAAATATCAATATAAATAAGTTTTTTAAATTTATG
TATGTTTATTTGATTCAAACAAAATAACTTAAGAGGAGAAAGTTTATGAAAAAGACACTGGGATGTTTACTTT
TAATTATGCTTTTAGTCGTAGCAGGTTGTTCTTTTGGTGGTAATCATAAATTATCATCAAAGAAATCAGAAGA
ATCAAAACAAGAAACTGTAAAAAAAGAATCGGAAGAAGAGAAAGACCCAGATTTAGAGAAATTTGAAGAAATA
GAGAAGAAAATGAAAGGAATTAAAGATGCGCCATCTCTAGATAAGTTGGATCCATTAATGACAGAAAAGTCGT
TTACGAATAGTAAAGGGATTCAAGGATGGAAAGATTACAAAGAATTAATGGGTAAAGTGGAACTTGCAGATTA
TAGATTTACAAAAGATTCAAAAGGATCTTCAATAAAAGATGTTGATGCATTCTTTAAAGGTAAGAAAGGTATA
AAAAGGAAAGTGATTGAAACACACGATGATGTAAAACAAGTTGATTATTGGTATGTAGATCCAGATGGAAAGA
AAATTGGCAATTCAAACACACCTGTTTTTTACGCAGAAATTATGACAAAATATAAAGATGGAAAGTTAGTTTA
TGCATCAGTCGAACCAGGTTCTTACGTAATACATAAAGATGATGCAATTAAATATGACGATTATTCTAAGTTA
AAAAAATTAAGTCAACTAACTAAACTTGATCATCCAAAACCAGTTCCATATAGCGTAGCTCAAATCAAATCTT
TCGGAGTACCTTTAACAAGCGTTTCATTTATGACACATGGATCAAAGGATACTAAAGATGAAGTGTTGCCGGC
ATTGGCCTATTTCACTTTTTCACCAAAAAATTATGAAGACAAGTCTAATCCAGATCCAAAAGTTTTAAATTTA
GTACATATGGATTTCTTAAATGCATCTAGTGATTTTGGTAACGCACATTTTGTTGTTTTAAGTAAATATATTA
AAGAGTATGAATCAAACTATGAAACAGCGTCAGATGATTCTTTAAAATAGTATTTACTGTGTGAAAAATAAAT

AGTGTACTACATTAAATAATCGCAATAATAATCCCGATAAACAATCAGCATTACTGCTTATCATATAGAGTTC
GTAATATCTATAACTCTATGATTCGCAATTAATAAATGATTGTCATCGGGATTTATTTTTATCAATTTATAAT
GTAACATTACCATGTTCGTCAGCTGGTTTGAAAACAGTAATTATCGCACTAATAATTGCTAATATATGTGGGA
TGACTGTCCAAAAGAATATTAAGTGTAGGAAACCTTGCATGTTTTTGCCGGCATAAAATTTATGAATACCAAA
ACTACCTAAGAACAATGCTAATAAAATATAAATAACTTTGTTTACTTGCATTTCTTTCCCTCCAGTTGAATTG
CTTATAATGACATTAACTTCTCTTTTTATTATACCCACTTTTAGTTCAAACATTCTAGTTTAAGCATTTCCAA
TCATCTAGATTTCAGTTATTCAATCCTTACAATAAATTTAGGATTACACTTCAGTTGCATTGTATTATTTTAC
GTGTGAAATATACGTAATGAATCACATGACAATCTTTAAATTGAAAAATATACATTCTATGATGTAAGGTCGC
ATTTTTAATATATTTACGTTATAATGGTTTGATGAATTTTATTGAGGAGTAAGAGCTATGGCGATTAAAAAGT
TAGTACCAGCATCGCATCCTATTTTAACGAAAAAAGCGCAAGCAGTTATAAAATTTGATGATTCGTTAAAAAG
ATTATTACAAGATTTAGAAGATACAATGTATGCACAAGAAGCAGCTGGCTTATGTGCACCTCAAATTAATCAG
TCATTGCAAGTGGCAATCATTGATATGGAAATGGAAGGATTATTACAACTTGTTAATCCGAAAATTATTAGTC
AATCAAATGAAACGATAACAGACTTAGAAGGTTCAATTACATTGCCAGATGTTTACGGCGAAGTGACAAGAAG
TAAAATGATCGTTGTCGAAAGTTATGACGTCAATGGGAACAAAGTTGAACTAACTGCACATGAAGATGTAGCA
AGAATGATTTTGCATATTATAGATCAAATGAACGGTATCCCTTTTACAGAACGTGCGGACCGTATTTTAACAG
ATAAAGAAGTGGAGGCATATTTTATAAATGACTAAAATAATATTTATGGGTACACCAGACTTTTCAACAACTG
TTTTAGAAATGCTTATTGCAGAACATGATGTCATTGCAGTCGTAACGCAACCAGATCGACCTGTTGGACGTAA
ACGTGTTATGACACCACCACCAGTTAAAAAAGTTGCAATGAAATATGATTTACCTGTATATCAGCCTGAAAAA
CTAAGTGGATCAGAAGAATTAGAACAATTGCTTCAATTAGATGTAGATTTAATTGTAACTGCTGCTTTTGGAC
AATTATTACCTGAATCATTGTTGGCATTACCAAAACTTGGCGCAATTAATGTACATGCATCATTGTTACCGAA
GTATAGAGGTGGTGCACCAATTCATCAGGCAATTATCGATGGTGAACAAGAAACCGGCATAACAATTATGTAT
ATGGTTAAAAAATTAGATGCGGGTAATATTATTTCGCAACAAGCAATTAAAATAGAAGAAAATGATAATGTCG
GTACGATGCATGATAAATTAAGTGTATTAGGGGCAGATTTATTAAAAGAAACTTTACCATCTATTATAGAGGG
CACAAATGAAAGTGTACCTCAAGATGATGCGCAAGCAACATTTGCTTCCAATATTCGACGCGAAGATGAGCGA
ATTAGCTGGAATAAACCAGGAAGACAAGTGTTTAATCAAATTCGAGGATTATCACCATGGCCAGTTGCTTATA
CAACTATGGATGACACTAACTTGAAAATATACGATGCTGAACTCGTTGAGACTAATAAGATAAACGAGCCTGG
AACAATTATAGAAACGACTAAAAAAGCCATTATTGTTGCTACAAATGATAATGAAGCTGTTGCAATTAAAGAT
ATGCAATTGGCTGCGAAAAAGAGAATGTTAGCTGCCAATTATTTAAGTGGTGCGCAAAACACACTAGTAGGGA
AGAAACTTATATGATAGAAAACGTGAGAAGTCTTGCTTTTGACACGATTCAAGATATATTAAATGAAGGTGCG
TATAGTAACTTGCGTATCAATGAAGTGTTGTCAGAAAATGAATTAAATGCAATGGATAAAGCTTTATTTACAG
AAATTGTCTACGGAACCGTTAAAAGAAAATATACGTTAGATTTTTATTTAAAGCCTTTTGTGAAAACAAAGAT
TAAGGCATGGGTTAGGCAATTATTATGGATGAGTATTTATCAATATGTTTATTTAGATAAAGTTCCAAATCAT
GCCATTATTAATGAAGCAGTTGAAATAGCAAAAGAACGCGGTGGCTATCATAATGGTAATGTCGTAAATGGTA
TTTTACGTACAATGATGCGTAGTGACTTACCTGATTTTAATGAAATTGCAGATCCTAAAAAAAGAATGGCAAT
CGAGTATAGTATGCCGAAGTGGATTATAGATCATTGGGCAACACATTATGGTCTCGAAGAAACTGAAACAATC
TTACAGTCATTTTTAGAAACGACATCTACAACTGTGCGTGCCAACCTGACGCGAGCATCATTAGATGATATTA
TTGAAAAGTTGCAAGACGAAGGTTATGACGTTGAAAAAGATCATGACTTACCTTATTGTCTCCATATAGGAGG
ACAACCAATTATTCATTCTCGTTCATTTAAGGATGGATTCGTTTCAATTCAAGATAAAAGCTCAATGTTTGTT
GCGCACATTATGAATGTAGACCGACATGATCACGTATTAGATGCATGTAGTGCACCTGGCGGTAAGGCTTGTC
ACATTGCTGAAGTTTTAATGCCAGAAGGGCAAGTTGACGCTTCAGATATACATGATCACAAAATAGACTTAAT
TAATTTTAATATAAAAAAATTACGATTAACAAATATTAAAGCCTTTCAACATGATGCGACAAAACCTTATGAT
AAAACATACGATAAGATACTTGTTGATGCACCATGTAGCGGATTAGGTGTAATGAGACATAAGCCGGAGATTA
AGTATACTCAAAGCAAACAACATATTGAGTCACTAGTTGATTTACAGCTTGAAATATTGGAAAATGTAAAAAA
CAATGTAAAAATAGGTGGCGAAATCATCTATTCAACATGTACAATAGAGCAACTAGAAAATGAAAACGTGATT
TATACGTTTTTGAAAAATAATAAAAACTTCGAATTTGAACCGTTTCAACATCCGATAACTGGAGAGTTGGTCA
AAACGTTACAAATCATGCCGCAAGACTTTAATTCAGATGGATTCTTTATCACTAAGATAAAAAGAAAGGACAA
TTAGGAATGATAACTGCTGAAAAGAAAAAGAAGAATAAATTTCTTCCAAATTTTGACAAGCAATCAATATATT
CATTGCGATTTGACGAAATGCAAAACTGGCTCGTTGAACAAGGGCAACAAAAATTTCGAGCGAAACAGATTTT
TGAATGGTTATATCAAAAAAGAGTAGATTCGATTGATGAAATGACGAACTTATCGAAAGACTTACGACAGCTC
TTAAAAGATAACTTTACTGTTACAACTTTAACAACTGTAGTAAAACAAGAAAGTAAAGACGGTACAATTAAAT
TCTTATTTGAATTACAAGATGGCTATACAATTGAAACTGTTTTAATGAGACATGATTATGGAAATTCAGTATG
TGTAACAACACAAGTAGGTTGTCGTATCGGATGTACGTTTTGTGCTTCTACACTTGGCGGCTTAAAAAGAAAC
CTTGAAGCTGGCGAAATTGTTTCACAAGTTTTAACAGTTCAAAAAGCCCTTGATGCTACAGAAGAGCGCGTAT
CTCAAATTGTCATAATGGGTATCGGTGAACCATTTGAAAATTATGATGAAATGATGGACTTTTTAAGAATCGT
CAATGATGATAACAGTTTAAATATTGGTGCACGTCACATTACAGTATCAACATCAGGTATCATTCCTAGAATA
TATGAATTTGCGGATGAAGATATTCAAATTAATTTTGCTGTAAGCTTACACGCCGCAAAAGATGAAGTGCGAT
CACGCTTGATGCCAATTAACCGTGCATATAATGTTGAGAAGTTAATCGAAGCAATTCAATATTATCAAGAAAA
AACAAATCGTCGTGTTACTTTTGAATATGGTCTGTTTGGTGGTGTAAATGACCAACTAGAACATGCAAGAGAA

TTAGCACATTTAATAAAAGGCTTAAATTGCCATGTTAACTTAATTCCTGTCAACCATGTTCCAGAAAGAAATT
ATGTAAAAACGGCTAAAAATGATATCTTTAAATTTGAAAAAGAATTAAAGAGACTAGGAATTAATGCCACAAT
ACGTCGTGAACAAGGTTCGGATATTGACGCAGCTTGTGGTCAATTAAGAGCAAAGGAACGACAAGTAGAAACG
AGGTAAAGACAAATGCTAGAGGCACAATTTTTTACTGATACTGGACAACATAGAGATAAGAATGAAGATGCGG
GTGGTATTTTTTATAATCAAACTAATCAACAACTTTTAGTTCTGTGTGATGGTATGGGCGGCCATAAAGCAGG
AGAAGTTGCAAGTAAATTTGTTACAGATGAGTTGAAATCCCGTTTTGAAGCGGAAAATCTTATAGAACAACAT
CAAGCTGAAAATTGGTTGCGTAATAATATAAAAGATATAAATTTTCAGTTATATCACTATGCACAAGAAAATG
CAGAATATAAAGGTATGGGTACAACATGTGTTTGTGCACTTGTTTTTGAAAAATCAGTTGTGATAGCAAATGT
CGGTGATTCTAGAGCCTATGTTATTAATAGTCGACAAATTGAACAAATTACTAGTGATCACTCATTTGTTAAT
CATCTTGTTTTAACGGGTCAAATTACGCCGGAAGAAGCATTTACACATCCACAACGTAATATTATTACAAAGG
TGATGGGCACAGATAAACGTGTGAGTCCAGATTTGTTTATTAAGCGATTAAATTTTTATGATTATTTATTATT
AAATTCAGATGGATTAACTGATTATGTTAAAGACAATGAAATTAAGCGTTTGTTAGTAAAAGAAGGTACAATA
GAAGATCATGGTAATCAATTAATGCAATTGGCATTAGATAACCATTCGAAAGATAACGTTACTTTCATACTCG
CGGCTATTGAAGGTGATAAAGTATGATAGGTAAAATAATAAATGAACGATATAAAATTGTAGATAAGCTTGGC
GGCGGTGGCATGAGTACCGTTTATCTTGCAGAAGATACGATACTTAACATTAAAGTTGCAATTAAGGCGATTT
TTATACCACCTAGAGAAAAAGAAGAAACATTAAAACGTTTTGAACGAGAAGTACATAACTCATCACAGCTATC
ACATCAAAATATAGTAAGTATGATCGATGTTGATGAAGAAGATGACTGTTACTACTTAGTAATGGAATATATT
GAAGGTCCGACTTTGTCTGAGTATATTGAAAGTCATGGCCATTAAGTGTTGACACGGCGATTAATTTTACGA
ATCAAATATTGGATGGCATTAAACATGCGCATGATATGCGTATTGTACATAGAGATATTAAGCCACAAAATAT
ATTAATTGACAGCAATAAAACGTTGAAAATATTTGATTTTGGAATTGCTAAAGCTTTAAGTGAGACGTCTTTA
ACTCAGACTAATCATGTGTTAGGTACTGTGCAGTACTTTTCGCCAGAACAAGCAAAAGGTGAGGCAACGGATG
AATGTACAGATATTTATTCTATAGGTATTGTGTTATATGAAATGCTTGTTGGTGAGCCGCCATTTAATGGAGA
AACTGCAGTTAGCATTGCGATTAAACATATTCAGGATTCTGTTCCAAATGTGACAACAGATGTACGTAAGGAT
ATTCCGCAATCTTTAAGTAATGTCATTTTACGCGCTACAGAAAAAGACAAAGCGAATCGTTACAAAACAATTC
AAGAAATGAAAGATGATTTGAGTAGTGTTTTACATGAAAATCGAGCGAATGAAGATGTCTATGAACTCGATAA
AATGAAAACGATAGCGGTACCTTTGAAAAAAGAAGATCTAGCAAAGCATATTAGTGAACATAAGTCGAATCAA
CCTAAACGTGAAACGACGCAAGTACCTATTGTAAATGGGCCTGCTCATCATCAGCAATTCCAAAAGCCAGAAG
GTACGGTGTACGAACCAAAACCTAAAAAGAAATCAACACGAAAGATTGTGCTCTTATCATTAATCTTTTCGTT
GTTAATGATTGCACTTGTTTCTTTTGTGGCAATGGCAATGTTTGGTAATAAATACGAAGAGACACCTGATGTA
ATCGGGAAATCTGTAAAAGAAGCAGAGCAAATATTCAATAAAAACAACCTGAAATTGGGTAAAATTTCTAGAA
GTTATAGTGATAAATATCCTGAAAATGAAATTATTAAGACAACTCCCAATACAGGTGAACGTGTTGAACGTGG
TGACAGTGTTGATGTTGTTATATCAAAAGGCCCTGAAAAGGTTAAAATGCCAAATGTCATTGGTTTACCTAAG
GAGGAAGCCTTGCAGAAATTAAAATCGTTAGGTCTTAAAGATGTTACGATTGAAAAAGTATATAATAATCAAG
CGCCAAAAGAATACATTGCAAATCAAAGTGTAACCGCAAATACTGAAATCGCTATTCATGATTCTAATATTAA
ACTATATGAATCTTTAGGCATTAAGCAAGTTTATGTAGAAGACTTTGAACATAAATCCTTTAGCAAAGCTAAA
AAAGCCTTAGAAGAAAAAGGGTTTAAAGTTGAAAGTAAGGAAGAGTATAGTGACGATATTGATGAGGGTGATG
TGATTTCTCAATCTCCTAAAGGAAAATCAGTAGATGAGGGGTCAACGATTTCATTTGTTGTTTCTAAAGGTAA
AAAAAGCGACTCATCAGATGTCAAAACGACAACTGAATCGGTAGATGTACCATACACTGGTAAAAATGATAAG
TCACAAAAAGTTAAAGTTTATATTAAAGATAAAGATAATGACGGTTCAACTGAAAAAGGTAGTTTCGACATTA
CTAGTGATCAACGTATAGACATTCCTTTAAGAATTGAAAAAGGAAAAACAGCAAGTTATATTGTTAAAGTTGA
CGGTAAAACTGTAGCTGAAAAAGAAGTCAGCTATGATGAAGTTTAAATATAATTGTAATAAGTGTACCGAGGT
TTCTATTTGGAAGTCTCGGTATTTTTTGTTGAGATTGCGGTAGTTTTAAAATGCTTTATGACATCATATACAG
GTGAGCATTGTCTATCAATTCTGTTTCACTCCCACAATATTTATATCATTTGAAATGATCATTTATATATTAA
TTTTCCAGTCTTTGATATAATAAATGCAGTAAAATTAATCGAGAGGTGCCATTTTGAAGACAGGTCGAATAGT
GAAATCAATTAGTGGGGTATATCAAGTAGACGTTAATGGCGAACGTTTCAATACAAAACCACGAGGATTATTT
AGAAAGAAAAAATTTTCACCAGTAGTTGGAGATATAGTGGAATTTGATGTACAAAACATTAATGAAGGCTATA
TTCATCAAGTGTATGAGCGAAAAAATGAGTTGAAAAGACCACCTGTAAGTAATATAGATACACTAGTAATTGT
AATGAGTGCTGTCGAGCCAAATTTTTCAACGCAATTATTAGATCGATTTTTAGTTATTGCACATTCGTATCAG
TTAGATGCGAGAGTTTTGGTGACTAAAAAAGATAAAACACCAATTGAAAAGCAGCTTGAAATTAATGAGTTGT
TGAAAATATATGAAAATATTGGCTATGAGACTGAATTTATTGGAAATGATGATGATCGAAAAAAAATTGTAGA
AGCTTGGCCAGCAGGACTTATAGTACTTAGTGGTCAATCAGGTGTCGGTAAGTCCACTTTCTTAAATCATTAT
CGTCCAGAACTTAATCTTGAGACAAATGATATATCAAAATCATTAAATCGAGGAAAGCATACTACAAGACATG
TCGAACTATTCGAACGTCAAAACGGTTATATTGCAGACACACCTGGATTCAGTGCTTTAGATTTTGATCATAT
AGATAAAGATGAAATAAAAGATTATTTTCTTGAATTAAATCGATATGGTGAAACATGTAAGTTTAGAAATTGT
AATCATATCAAAGAACCTAATTGTAATGTTAAGCATCAATTAGAGATAGGGAATATTGCGCAATTTAGATACG
ACCATTATTTACAACTATTTAATGAAATTTCAAATAGAAAGGTTAGATATTAAATGACAAAACTATATCCATC
ATTATTATCTGTTGATTTTTTGGATTTACAACATGAATTAAAACGACTTGAAGAAGCAGGTGTCGACGGAGTT
CATTTTGATGTTATGGATGGTCAATTTGTGCCTAATATATCTATTGGTTTACCAATATTAGATGCAGTGAGAA

AAGGCACAACATTACCTATAGACGTACATTTGATGATTGAAAATCCAGAAAAGTATATTGCATCATTTGCAGA
ACATGGTGCCGATATGATTTCAATTCATGTCGAATCAACGCCTCATATCCATCGTGCTATTCAAATGATTAAA
CATTTAGATAAAAAAGCTGGTGTAGTAATTAATCCTGGTACACCAATATCACAAATTGAACCTATTTTAGACA
TTGTTGATTATGTACTAGTGATGACAGTTAACCCAGGGTTTGGTGGTCAATCATTTATTGATCAATGCGTAGA
AAAAATAGCGGGTCTTAATGCTATTAAAATGGAACGTCAATTAAACTTTGATATTGAAGTTGATGGAGGCGTA
AATACCGATACAGCGAAAGTTTGTGTTGAAAATGGTGCTACAATGTTAGTAACAGGTTCATTTTTCTTTAAAC
AAGAGGATTATAAAAAAGTCACACAAGCATTGAAAGGTTGAGTGTATATGCATATAAATTTATTATGTTCTGA
TCGACACTTGCCGCAAGATATTTGGGCCAAAAGTAATGAAGGTAAATGGGGCGGCGTTGATAGAGGTGCTTTG
ATTTTATTGAAGCATCAAATTATCCCTTTTTTCTCAGTGGGAGACTTTGATTCAGTCAGTAAAGAAGAACGCC
AACTTCTAACAGAACAGTTACAAATCAAACCAGTTCAAGCTGAAAAAGCTGATACGGATTTAGCTTTAGCGGT
TGATAAAGCTGTTGCACTTGGGTTTGATAGTATTACAATTTATGGTGCAACAGGCGGACGATTAGATCACTTT
TTTGGGGCAATTCAGTTATTATTGAAAAAAGCATATTATAAACATGATGTTCATATAGAAGTTATCGATCAAC
AAAATAAAATTGAATTATTACCTAAAGGTCAACATACAGTTGAAAAAGATAAGAGTTATCCGTACATTTCATT
TATACCGATGACTGATGATGTAGAACTTTCTCTAGCAGGTTTTAAATATAATTTAGCTAGACAAATGCTTAAT
ATAGGTTCTACTTTAACTATTTCAAATGAAATTGAGTCTTTGCAAGCGAAAGTAACTGTACATGATGGGTTGA
TTTTGCAAATTAGAAGTACAGATTTAAATTAAGTATATTCAAATTTGTGAGGATTTTTAATAAAGTGACGTTT
ATAGATAGAATTTATAACATAATAATAGATGAATCACGCTGTAATTAATGAAGATTATGGCGTGATTTATTAT
CGAAAGGAAAATTGTAGGTCCTAGTACATTGATTTATGTGCCAAAATCGATATTGAACCCATAAGTCCAATTT
GACAGAGAACCATTATTAAACGTATTAACCAAATTATATTGATAGTTCGAATTTCACTATGAACCATTATGTA
TTGTTTGGTAACAAAAAAAGAACTAAGAGGTTGAGCTCTTAGTTCTTAAGTAAATGTGTCCTCCACATAAGGT
CACATTTTTTATGTGCAATATATTTATTAAACTCTAGTTACTTTACCAGATTTTAAAGCACGTGCAGAAACCC
AAACTTTTTTAGGTTTACCGTCAACTAGGATTCTAACTTTTTGAAGGTTAGCGTTCCATCTACGTTTAGTAGA
GTTTAAAGCGTGTGAACGTCTGTTACCAGTCGAAGCTTTACGACCTGTTACGAAACATTGTTTACCCATATGA
GTACCTCCTTTAATAAATATAAATACACATAACTACTTATATACTTAACTAAGATAGCATAGTTTCATTTGAA
AAACAATGAATAATTTTCACATAAAAGTCAAAAATACTGAGTTTTGTGATATAATTGTAGACTGTGAAGTTAT
GTAGTATGATATTTTAGAGAAATAAGTGAATGAAACATTTAAACTTTTTATTTAATGATACTACATCTATTAA
AAGAAAACGCCTATAGATAATCGTCATGAGTGTAAATTCAGTTTACTCAAAATATAATAGGTGATTTAAGCGT
AAGTCATTCTGATAAGTTGTACTATTGATATTTTATAAAAATAATCAATGAAACTTAAAGCGTTTATGACTAC
ACTAAGTGGTAAAATTTTAAAAGTGATTAGGGAAAACGAATAATAAACTTTGAAATTTAGGAGGGACAAAGAT
ATGACATTAGAGATTTCAAATGATTACGGTAAAATTGATATTTCAAACGAAGTGATTGCTTCGGTTGTAGGTG
GAAAAGCCGTTGAATGTTATGGTATTGTAGGTATGGCATCTAGACAACAAGTTAGAGATGGTATTGCGGAAAT
ACTAGGACATGAAAATTATGCTAAAGGCATCAAAGTAACTGAAAATAATGGCGTAGTGAATATAGATATGTAC
ATTATTGTAAGTTACGGTGTGAAAATATCTGAAGTTGCCAATAATGTACAATCAACAGTGAAATATACTTTGG
AAAAATCACTTAATGTATCAGTAAATTCAATCAATATATATGTACAAGGTGTACGTGTGAATAATACAGGCAA
GAAAGCTTAGGAGGACAACTTGAAATGATTAGCAAAATTAATGGTAAATTATTTGCCGATATGATTATACAAG
GGGCACAAAATTTATCTAACAATGCAGATTTGGTAGATTCTTTGAATGTGTATCCAGTGCCAGATGGTGATAC
AGGAACAAATATGAATCTTACTATGACTTCAGGTCGCGAAGAAGTAGAGAATAATTTGTCGAAAAATATCGGC
GAATTAGGTAAAACATTCTCGAAAGGTTTACTAATGGGTGCAAGAGGTAACTCTGGTGTCATCTTGTCACAAT
TATTCAGAGGATTTTGTAAAAATATTGAAAGTGAATCTGAAATTAACTCAAAATTGTTAGCTGAAAGTTTTCA
AGCTGGTGTTGAAACGGCATATAAAGCTGTTATGAAACCAGTTGAAGGTACAATACTTACAGTTGCAAAAGAT
GCTGCGCAAGCCGCAGTAGAAAAAGCAAATAATACTGAAGATTGTATAGAATTAATGGAGTACATTATTGTAA
AAGCCAATGAATCACTTGAAAACACACCAAACTTATTAGCTGTACTTAAAGAAGTTGGTGTTGTTGATAGTGG
TGGTAAAGGTTTGTTATGCGTTTACGAAGGATTCTTAAAAGCGCTTAAAGGTGAAAAAGTTGAAGCCAAAGTT
GCAAAGATAGATAAAGATGAATTTGTACATGATGAACATGATTTCCATGGTGTAATTAATACTGAAGATATTA
TTTATGGCTATTGTACCGAAATGATGGTTCGTTTTGGAAAGAATAAAAAAGCCTTTGATGAACAAGAATTCAG
GCAAGATATGAGTCAATTTGGTGATTCTTTATTAGTCATTAATGATGAAGAAATTGTGAAAGTTCACGTGCAT
ACCGAACACCCAGGTAAAGTGTTTAATTATGGTCAACAATATGGTGAATTAATTAAACTTAAGGTTGAAAATA
TGAGAGAACAGCACCGTGAAGTGATTCGAAAAGAACAGCACACGGCTAAACCGAAAATGGAAACGGTTGAAAC
AGCAATTATTACTATTTCTATGGGTGAAGGTATTTCAGAGATATTTAAATCAATGGGTGCCACACATATCATT
AGTGGTGGACAAACGATGAATCCTTCTACAGAAGATATCGTTAAAGTCATTGAACAATCAAAATGTAAACGTG
CAATTATTTTACCAAATAATAAAAATATCTTAATGGCAAGTGAACAAGCAGCGAGTATTGTTGATGCAGAAGC
AGTTGTTATTCCAACGAAATCTATTCCTCAAGGTATAAGCGCACTATTCCAATATGATGTGGACGCAACACTT
GAAGAAAATAAAGCGCAAATGGCTGATTCAGTAAATAACGTTAAATCTGGTTCATTAACGTACGCTGTTCGTG
ATACGAAAATTGATGGCGTTGAGATAAAAAAAGACGCGTTTATGGGCTTGATTGAAGATAAGATTGTAAGCAG
CCAAAGTGATCAATTAACAACGGTTACTGAGTTGTTAAATGAGATGTTAGCAGAAGATAGTGAAATATTGACT
GTGATTATTGGTCAAGATGCAGAGCAAGAAGTTACAGATAACATGATAAACTGGATCGAAGAGCAATATCCAG
ATGTAGAAGTGGAAGTTCATGAAGGTGGACAACCAATTTATCAATATTTCTTTTCAGTAGAATAAAAATTTAA
AATAAAAAACTACCAATGATAAATCATCAGTTGGTAGTTTTTTATTTTGCTATTTTAGTGTTATTGCGGGTTA

AAAGTATCATTCTCGAGTTGCTAACAATGTCATGTTCAACTTAGTCATGATAAAATAAATAACATACAAAATG
ATACGTAAAATCAAATAAAACATAGGTGATTTATTTTGGCTAAAGTAAACTTAATAGAAAGTCCATATTCTCT
TTTACAATTAAAAGGTATAGGTCCTAAGAAAATAGAAGCATTGCAACAACTAAATATTCATACAGTGGAAGAT
CTTGTTCTTTATTTGCCAACTAGATATGAAGATAATACAGTGATTGATTTGAATCAAGCAGAAGATCAATCTA
ACGTTACGATAGAAGGACAAGTATATACAGCTCCAGTTGTTGCATTTTTTGGAAGAAATAAATCAAAATTAAC
CGTTCATTTAATGGTAAATAATATTGCTGTCAAATGTATTTTTTTCAATCAACCGTATTTAAAAAAGAAAATC
GAATTAAATCAAACTATAACTGTTAAAGGTAAGTGGAATAGGGTTAAACAGGAAATTACTGGTAATAGGGTTT
TCTTTAATTCACAAGGGACACAAACTCAAGAAAACGCAGATGTTCAATTAGAACCAGTCTATCGTATTAAGGA
AGGTATTAAACAAAAGCAAATACGAGACCAAATTAGACAAGCGTTAAATGATGTGACAATTCATGAATGGTTA
ACTGATGAACTAAGAGAAAAATATAAATTAGAGACCTTGGACTTTACTTTGAACACGTTACATCATCCAAAAA
GTAAAGAGGATTTATTACGTGCTCGTAGAACCTATGCATTTACTGAACTGTTTTTATTCGAATTACGTATGCA
ATGGCTAAATAGATTAGAAAAGTCATCTGACGAAGCAATTGAAATTGATTATGACTTAGACCAAGTTAAATCA
TTTATTGATCGTTTACCTTTTGAACTAACTGAAGCACAGAAATCCAGTGTTAATGAAATTTTTAGAGATTTAA
AAGCACCAATACGTATGCATCGATTACTTCAAGGTGATGTAGGTTCAGGAAAAACAGTTGTTGCTGCAATTTG
TATGTATGCATTAAAAACGGCTGGTTATCAATCAGCATTGATGGTACCAACTGAAATTTTAGCAGAGCAACAT
GCTGAAAGTTTAATGGCTTTATTTGGAGATTCTATGAACGTTGCATTGTTAACTGGGTCAGTAAAAGGTAAGA
AACGAAAAATACTTTTAGAACAACTTGAAAATGGTACGATTGATTGTTTAATTGGAACTCATGCATTGATTCA
AGATGATGTGATTTTCCATAATGTTGGTTTAGTAATTACAGATGAACAACATCGATTTGGTGTGAATCAACGC
CAGCTTTTAAGAGAAAAAGGTGCAATGACAAATGTGTTATTTATGACAGCAACCCCGATACCAAGAACACTAG
CAATATCAGTTTTTGGTGAGATGGATGTGTCTTCAATTAAACAATTACCAAAAGGTCGTAAACCTATCATTAC
TACTTGGGCAAAGCATGAGCAATACGATAAAGTTTTGATGCAAATGACTTCAGAGTTGAAAAAAGGTCGTCAA
GCATATGTCATTTGCCCGCTTATAGAAAGTTCTGAGCATCTCGAAGATGTTCAAAATGTTGTCGCATTGTACG
AGTCTTTACAACAGTATTATGGTGTTTCCCGTGTAGGGTTATTGCATGGCAAGTTGTCTGCCGATGAAAAAGA
TGAGGTCATGCAAAAGTTTAGCAATCATGAGATAGATGTTTTAGTTTCTACTACTGTTGTTGAAGTAGGTGTT
AATGTACCGAATGCAACTTTTATGATGATTTATGATGCGGATCGCTTTGGATTATCAACTTTACATCAGTTAC
GCGGTCGTGTAGGTAGAAGTGACCAGCAAAGTTACTGTGTTTTAATTGCATCCCCTAAAACAGAAACAGGAAT
TGAAAGAATGACAATTATGACACAAACAACGGATGGATTTGAATTGAGTGAACGAGACTTAGAAATGCGTGGT
CCTGGAGATTTCTTTGGTGTTAAACAAAGTGGATTGCCAGATTTCTTAGTTGCCAATTTAGTTGAAGATTATC
GTATGTTAGAAGTTGCTCGTGATGAAGCAGCTGAACTTATTCAATCTGGCGTATTCTTTGAAAATACTTATCA
ACATTTACGTCATTTTGTTGAAGAAAATTTATTACATCGTAGTTTTGACTAATTGCCATGCTGATTTGTCAAT
TTGAGTGCAACACTTCGTTAATTGAGTGATATGACACTTGAACTATTTAAATGTAAAGTGGTATTTTAACAAT
TTATAAATTTTCGACTAAATAATAGCTAAATAATACAGTTATTTGTTGAGTCGGTTAAATAGAAAGTGTTATG
ATATGTGAGGAATGTTTAAGACTAGGTACTAAAAAAATGAGGGGTGAGACGTTGAAACTAAAGAAAGATAAACG
TAGAGAAGCAATCAGACAACAAATTGATAGCAATCCCTTCATCACAGACCATGAACTAAGCGACTTATTTCAA
GTGAGTATACAAACAATTCGTTTAGATCGCACTTATTTAAACATACCAGAATTAAGGAAGCGTATTAAATTAG
TTGCTGAAAAGAATTATGACCAAATAAGTTCTATTGAAGAACAAGAATTTATTGGTGATTTGATTCAAGTCAA
TCCAAATGTTAAAGCGCAATCAATTTTAGATATTACATCGGATTCTGTTTTTCATAAAACTGGAATTGCGCGT
GGTCATGTGCTGTTTGCTCAGGCAAATTCATTATGTGTTGCGCTAATTAAGCAACCAACAGTTTTAACTCATG
AGAGTAGCATTCAATTTATTGAAAAAGTAAAATTAAATGATACGGTAAGAGCAGAAGCACGAGTTGTAAATCA
AACTGCAAAACATTATTACGTCGAAGTAAAGTCATATGTTAAACATACATTAGTTTTCAAAGGAAATTTTAAA
ATGTTTTATGATAAGCGAGGATAAAATTATGGTTAAATTAGCAATTGATATGATGGGTGGCGACAATGCGCCT
GATATCGTATTAGAAGCCGTACAAAAGGCTGTTGAAGACTTTAAAGATCTAGAAATTATACTTTTCGGTGACG
AAAAAAAGTATAATCTGAACCATGAACGAATCGAATTTAGACATTGTTCTGAAAAGATTGAAATGGAAGATGA
GCCTGTTAGAGCGATTAAACGTAAAAAAGATAGCTCAATGGTAAAAATGGCTGAAGCTGTGAAATCTGGTGAA
GCAGATGGATGTGTGTCAGCAGGTAATACTGGTGCTTTAATGTCAGCTGGTTTATTCATTGTTGGACGTATTA
AAGGTGTAGCTAGACCGGCTTTAGTAGTAACATTGCCAACGATTGATGGAAAAGGTTTTGTCTTTTTAGACGT
TGGTGCAAATGCTGATGCTAAACCTGAACACTTATTACAGTATGCGCAACTAGGGGATATTTATGCTCAAAAA
ATTAGAGGTATTGATAATCCGAAAATCTCATTATTAAATATAGGAACCGAGCCAGCTAAAGGTAATAGTTTAA
CGAAAAAATCATATGAGTTATTAAATCATGATCATTCATTGAATTTTGTTGGGAATATTGAAGCGAAGACATT
AATGGATGGCGATACAGATGTTGTAGTTACCGATGGCTATACAGGGAACATGGTCCTTAAAAATTTAGAAGGT
ACTGCAAAATCAATCGGTAAAATGTTAAAAGATACGATTATGAGTAGTACTAAAAATAAATTAGCAGGTGCAA
TATTGAAGAAAGATTTAGCTGAATTCGCTAAAAAGATGGACTACTCAGAATACGGTGGTTCCGTATTATTAGG
ATTGGAAGGTACTGTAGTTAAAGCACACGGTAGTTCAAATGCTAAAGCTTTTTATTCTGCAATTAGACAAGCG
AAAATCGCAGGAGAACAAAATATTGTACAAACAATGAAAGAGACTGTAGGTGAATCAAATGAGTAAAACAGCA
ATTATTTTTCCGGGACAAGGTGCCCAAAAAGTTGGTATGGCACAAGATTTGTTTAACAACAATGATCAAGCAA
CTGAAATTTTAACTTCAGCAGCGAACACATTAGACTTTGATATTTTAGAGACAATGTTTACTGATGAAGAAGG
TAAATTGGGTGAAACTGAAAACACGCAACCAGCTTTATTGACGCATAGTTCGGCATTATTAGCAGCGCTAAAA
AATTTGAATCCTGATTTTACTATGGGGCATAGTTTAGGTGAATATTCAAGTTTAGTTGCAGCTGACGTATTAT

CATTTGAAGATGCAGTTAAAATTGTTAGAAAACGTGGTCAATTAATGGCGCAAGCATTTCCTACTGGTGTAGG
AAGCATGGCTGCAGTATTGGGATTAGATTTTGATAAAGTCGATGAAATTTGTAAGTCATTATCATCTGATGAC
AAAATAATTGAACCAGCAAACATTAATTGCCCAGGTCAAATTGTTGTTTCAGGTCACAAAGCTTTAATTGAAG
AGCTAGTAGAAAAAGGTAAATCATTAGGTGCAAAACGTGTCATGCCTTTAGCAGTATCTGGACCATTCCATTC
ATCGCTAATGAAAGTGATTGAAGAAGATTTTGCAAGTTACATTAATCAATTTGAATGGCGTGATGCTAAGTTT
CCTGTAGTTCAAAATGTAAATGCGCAAGGTGAAACCGACAAAGAAGTAATTAAATCTAATATGGTCAAGCAAT
TATATTCACCAGTACAATTCATTAACTCAACAGAATGGCTAATAGACCAAGGTGTTGATCATTTTATTGAAAT
TGGTCCTGGAAAAGTTTTATCTGGCTTAATTAAAAAAATAAATAGAGATGTTAAGTTAACATCAATTCAAACT
TTAGAAGATGTGAAAGGATGGAATGAAAATGACTAAGAGTGCTTTAGTAACAGGTGCATCAAGAGGAATTGGA
CGTAGTATTGCGTTACAATTAGCAGAAGAAGGATATAATGTAGCAGTAAACTATGCAGGCAGCAAAGAGAAAG
CTGAAGCAGTAGTCGAAGAAATCAAAGCTAAAGGTGTTGACAGTTTTGCGATTCAAGCAAATGTTGCCGATGC
TGATGAAGTTAAAGCAATGATTAAAGAAGTAGTTAGCCAATTTGGTTCTTTAGATGTTTTAGTAAATAATGCA
GGTATTACTCGCGATAATTTATTAATGCGTATGAAAGAACAAGAGTGGGATGATGTTATTGACACAAACTTAA
AAGGTGTATTTAACTGTATCCAAAAAGCAACACCACAAATGTTAAGACAACGTAGTGGTGCTATCATCAATTT
ATCAAGTGTTGTTGGAGCAGTAGGTAATCCGGGACAAGCAAACTATGTTGCAACAAAAGCAGGTGTTATTGGT
TTAACTAAATCTGCGGCTCGTGAATTAGCATCTCGTGGTATCACTGTAAATGCAGTTGCACCTGGTTTTATTG
TTTCTGATATGACAGATGCCTTAAGTGATGAGCTTAAAGAACAAATGTTGACTCAAATTCCGTTAGCACGTTT
TGGTCAAGACACAGATATTGCTAATACAGTAGCGTTCTTAGCATCAGACAAAGCAAAATATATTACAGGTCAA
ACAATCCATGTAAATGGTGGAATGTACATGTAATATATTTGAGCTAAAGCTCAAGATAAAGACGTAGTAATCA
ATAAATTGATAACAGGGATAGAGGTACGCCTGAGCTAAAGCTCATTGACGCAGTGGTTGACTGGTCATCCGAT
GGAGAATTGCCTGACCTAGTCAACTTTGCGGGGGGAATTCTAAGTAACCTAGATAAGGTTCCAGAATTTCTCC
CTAAGAAACACTAATCAATAAATTGATAAGAAGATTAGAGGAACGCTTGAGCTAAAGCTCATGCATAAGAAAC
AGTAATCAATAAATTGATAACTGGGATAGAGGTACGCCTGAGCTAAAGCTCATGCATAAGAAACAGTAATCAA
TAAATTGATAACTGGGATAGAGGTACGCCTGAGCTAAAGCTCATGCATAAGAAACAGTAATCAATAAATTGAT
AACTGTTTCTAAAATTTCTACTTGTTTTTTAGAATTTAAAATGGGAAAATATAATAGTCTATGTATAGGCATT
TTTAAAGGAGGTGAATCGACGTGGAAAATTTCGATAAAGTAAAAGATATCATCGTTGACCGTTTAGGTGTAGA
CGCTGATAAAGTAACTGAAGATGCATCTTTCAAAGATGATTTAGGCGCTGACTCACTTGATATCGCTGAATTA
GTAATGGAATTAGAAGACGAGTTTGGTACTGAAATTCCTGATGAAGAAGCTGAAAAAATCAACACTGTTGGTG
ATGCTGTTAAATTTATTAACAGTCTTGAAAAATAATAAATCTTACATCTGGGTCGTCAGTATTGTCGACTCAG
TTTTTTTCTTTAATTATCAATAGTTTTAACGTAAAATTAAAGATGATTCAAGAGCAACACATAAAGGAGATAA
AATAATGTCTAAACAAAAGAAAAGTGAGATAGTTAATCGTTTTAGAAAGCGCTTTGATACTAAAATGACAGAG
TTAGGCTTTACTTATCAAAATATTGATTTATACCAACAAGCATTTTCGCATTCGAGTTTTATTAATGATTTTA
ATATGAATCGTTTAGACCATAATGAGCGTTTAGAGTTTTTGGGTGATGCGGTATTAGAATTGACGGTTTCACG
ATATTTATTTGATAAACATCCCAACTTGCCAGAAGGGAATTTAACAAAAATGCGTGCCACTATTGTATGTGAG
CCCTCACTTGTAATATTTGCGAATAAAATTGGATTGAACGAAATGATTTTACTTGGTAAAGGTGAAGAGAAAA
CAGGGGGACGTACAAGACCATCATTAATATCAGATGCATTCGAAGCATTTATTGGGGCATTGTATTTGGATCA
AGGACTAGATATAGTTTGGAAATTTGCTGAGAAAGTTATTTTCCCACATGTAGAACAAAATGAGTTATTAGGA
GTGGTAGATTTTAAAACACAATTCCAAGAATATGTGCACCAGCAAAATAAAGGTGATGTAACCTATAATTTAA
TAAAAGAAGAGGGACCGGCACATCATCGTCTATTCACTTCAGAAGTTATTCTGCAAGGGGAAGCAATAGCTGA
AGGTAAAGGGAAAACGAAAAAAGAATCAGAACAACGTGCTGCTGAAAGTGCCTATAAGCAATTAAAACAAATT
AAATAGAAGTTTATATGATACGACATATAGGTGTTCGATTTAGGATTTAAGGAAGAATTTTTAATATTTTATG
GAACAACTTCGAAATTTAATATACAAATATTAAAATATCGTATACATGTGTTCTTTAAAATTGTGATAAGGAG
TTTAGGATGGTTTATTTAAAATCAATAGATGCCATTGGATTTAAGTCTTTTGCAGATCAAACCAATGTTCAAT
TCGATAAAGGTGTAACTGCAATTGTTGGTCCAAATGGAAGCGGTAAAAGTAATATTACAGATGCTATTAAATG
GGTGTTGGGTGAACAATCGGCTAAATCATTACGTGGCTCAAAAATGGAAGATATTATCTTCTCAGGTGCAGAA
CATCGCAAAGCTCAAAATTATGCTGAAGTACAGTTAAGATTAGATAATCATTCTAAAAAGCTCAGTGTTGATG
AAAACGAAGTTATTGTAACAAGAAGATTGTATCGAAGTGGTGAAAGTGAGTACTACATAAATAATGACCGTGC
AAGATTAAAAGATATTGCCGATTTATTTTTAGATTCTGGATTGGGAAAAGAAGCATATAGCATTATCTCGCAA
GGTAGAGTTGATGAAATACTAAATGCTAAACCAATTGATAGACGTCAAATTATTGAAGAATCGGCTGGTGTAC
TTAAATATAAAAAACGTAAAGCTGAATCATTAAATAAACTTGACCAAACAGAAGATAATTTAACGAGAGTAGA
AGACATTTTATATGATTTGGAAGGTCGTGTAGAACCTCTAAAAGAGGAGGCAGCTATAGCTAAAGAATATAAG
ACACTTTCACACCAAATGAAACATAGTGACATTGTAGTTACAGTGCACGATATTGATCAATATACAAATGACA
ATAGACAATTAGATCAACGTTTAAATGATTTACAAGGTCAACAAGCAAATAAAGAAGCTGACAAGCAACGTTT
AAGCCAACAAATTCAACAATATAAAGGTAAACGTCATCAACTTGATAATGATGTTGAATCGCTTAATTATCAA
TTAGTAAAAGCTACGGAAGCCTTTGAAAAATATACGGGACAATTAAATGTTTTAGAAGAACGTAAGAAAAATC
AATCTGAAACAAATGCACGATATGAAGAAGAACAAGAAAATTTAATGGAGCTTTTAGAAAATATATCAAATGA
GATTTCTGAAGCTCAAGATACTTATAAGTCTCTGAAAAGTAAACAAAAAGAACTCAATGCTGTCATTCGTGAA
CTTGAAGAACAACTATATGTTTCAGACGAAGCACATGATGAAAAATTGGAAGAAATTAAAAACGAATACTATA

CATTAATGTCAGAGCAATCAGATGTTAACAATGATATTCGTTTTTAAAGCATACTATAGAAGAGAATGAGGC
TAAAAAATCAAGACTAGATTCTCGATTAGTTGAAGTTTTTGAGCAATTGAAAGATATTCAGGGTCAAATAAAA
ACGACAAAAAAAGAATATCAACAGACCAACAAAGAACTTTCTGCTGTAGATAAAGAAATTAAAAATATAGAAA
AAGATCTCACTGATACAAAAAAAGCACAAAATGAATACGAAGAGAAATTGTATCAAGCATATCGATATACCGA
AAAAATGAAAACACGTATTGATAGTTTGGCAACGCAAGAGGAAGAATATACTTATTTTTTCAATGGCGTCAAA
CATATTTTGAAAGCTAAAAATAAAGAATTAAAGGGTATTCATGGTGCAGTTGCGGAAATTATTGATGTGCCAT
CTAAATTAACTCAGGCAATTGAAACAGCATTAGGTGCTTCATTACAACATGTCATTGTAGATTCAGAAAAAGA
TGGACGCCAGGCTATTCAATTTTTAAAAGAACGTAATTTAGGTCGTGCGACGTTTTTACCATTAAATGTTATA
CAGAGTAGAGTGGTAGCGACTGATATTAAATCTATTGCTAAAGAGGCAAACGGATTTATTAGTATCGCTTCGG
AAGCAGTTAAAGTAGCACCAGAATATCAAAATATTATCGGGAATTTATTAGGTAATACGATTATCGTTGATCA
TTTAAAGCATGCAAATGAATTGGCACGTGCGATTAAATATCGAACTCGTATTGTTACTTTGGAAGGTGATATT
GTAAATCCTGGTGGTTCTATGACTGGTGGTGGCGCTCGTAAGTCAAAAAGTATTCTGTCTCAAAAAGACGAGT
TGACAACAATGAGACACCAATTAGAAGATTACTTGCGTCAAACAGAATCATTTGAACAACAATTTAAAGAGTT
GAAGATAAAAAGTGATCAATTAAGTGAACTGTATTTTGAAAAAAGTCAAAAGCATAATACACTTAAAGAGCAA
GTGCATCATTTTGAAATGGAGCTCGATAGATTAACTACACAAGAAACACAAATAAAAAATGATCATGAAGAAT
TCGAATTTGAAAAAAAATGATGGTTATACGAGTGACAAAAGTCGACAAACTTTGAGTGAAAAAGAAACTCATCT
AGAAAGTATTAAAGCATCTTTAAAACGACTAGAAGATGAAATTGAACGCTACACAAAACTTTCTAAAGAAGGT
AAGGAAAGCGTTACTAAAACACAACAAACCTTACATCAGAAACAATCTGATCTTGCTGTGGTTAAAGAGCGTA
TTAAAACACAACAACAGACAATAGATCGATTAAATAATCAAAATCAACAAACTAAACATCAATTAAAAGATGT
TAAAGAAAAAATTGCATTCTTTAATTCGGATGAAGTGATGGGCGAACAAGCTTTTCAAAATATTAAAGATCAA
ATTAATGGTCAACAAGAAACGAGAACACGCTTATCTGATGAATTAGATAAATTGAAACAACAACGTATTGAGT
TGAATGAACAAATCGATGCGCAAGAAGCTAAACTACAAGTTTGTCACCAAGATATTTTAGCTATCGAAAATCA
CTACCAAGATATTAAAGCTGAACAATCAAAGCTAGATGTATTAATTCATCATGCGATAGATCATTTAAATGAT
GAATATCAATTGACTGTTGAACGTGCGAAATCTGAATATACGAGTGATGAATCGATTGACGCATTACGTAAAA
AAGTTAAGTTAATGAAGATGTCGATTGATGAACTAGGTCCTGTAAACTTAAATGCAATTGAACAATTTGAAGA
GTTAAATGAACGTTATACATTTTTAAGTGAACAACGTACAGATCTTCGTAAAGCTAAAGAAACATTAGAGCAA
ATTATAAGTGAAATGGATCAAGAGGTTACTGAAAGATTTAAAGAAACTTTCCATGCTATTCAAGGACATTTTA
CAGCTGTGTTCAAACAATTGTTTGGTGGAGGAGATGCAGAATTGCAATTAACTGAAGCCGATTATTTAACAGC
TGGTATTGATATTGTGGTACAACCACCGGGTAAAAAGTTGCAACATTTATCGTTACTAAGTGGTGGTGAGCGT
GCATTAACTGCTATTGCTTTACTATTTGCAATTTTAAAAGTAAGATCTGCACCTTTTGTTATATTAGATGAGG
TTGAAGCTGCATTAGATGAAGCGAATGTTATTAGATACGCAAAATATTTAAATGAGTTATCAGACGAAACACA
ATTCATTGTTATTACACACCGTAAAGGAACAATGGAATTTGCTGATAGGTTATACGGTGTGACAATGCAAGAA
TCAGGTGTTACTAAACTTGTGAGTGTGAATTTAAATACAATAGATGATGTGTTGAAGGAGGAGCAATAATGAG
CTTTTTTAAACGCTTAAAAGATAAATTTGCAACAAATAAAGAAAATGAAGAAGTTAAATCCTTAACAGAAGAA
CAAGGTCAAGACAAATTAGAAGATACACATTCTGAAGGTTCAACGCAGGACGTAAATGATTTAGCAGAAAATG
CTGAAGTGAAAAAGAAGCCACGCAAGTTAAGTGAAGCGGATTTTGATGACGATGGCTTAATATCAATTGAAGA
TTTTGAAGAAATTGAAGCTCAAAAAATGGGTGCTAAATTTAAGGCAGGACTCGAAAAATCTCGTCAAAATTTC
CAAGAACAATTAAATAATTTGATAGCGAGATATCGTAAAGTAGATGAAGACTTTTTTGAAGCTTTAGAAGAAA
TGTTAATCACTGCAGACGTCGGTTTTAATACTGTGATGACGTTAACTGAAGAATTACGTATGGAAGCACAACG
ACGTAATATTCAAGATACTGAAGATTTGCGTGAAGTCATTGTTGAAAAAAATCGTAGAGATTTACCATCAAGAA
GATGATAATTCAGAAGCTATGAACTTAGAAGATGGTCGTTTAAATGTCATTTTAATGGTTGGTGTGAATGGTG
TTGGTAAAACAACAACAATTGGAAAATTAGCTTACCGATATAAAATGGAAGGTAAAAAAGTAATGTTAGCTGC
GGGCGATACTTTTAGAGCGGGTGCTATTGATCAATTGAAAGTTTGGGGCGAACGTGTTGGTGTAGATGTAATT
AGCCAAAGTGAAGGTTCTGATCCAGCTGCTGTTATGTATGATGCGATTAATGCCGCTAAAAACAAAGGTGTTG
ATATTTTAATCTGTGATACCGCTGGACGTTTACAAAATAAAACAAATCTAATGCAAGAATTAGAAAAAGTTAA
GCGTGTAATTAATCGAGCAGTGCCAGATGCGCCTCATGAAGCATTACTATGTTTAGATGCTACAACTGGTCAG
AATGCGTTGTCACAAGCTAGAAACTTTAAAGAAGTAACAAATGTTACAGGTATTGTATTAACGAAATTAGATG
GTACAGCCAAAGGTGGTATCGTATTAGCCATTCGTAATGAATTGCACATTCCAGTTAAATATGTAGGTTTAGG
TGAGCAATTAGATGACTTACAACCATTTAACCCTGAAAGTTATGTCTACGGCTTATTCGCTGATATGATTGAA
CAAAATGAAGAAATAACAACAGTTGAAAATGATCAAATTGTAACAGAAGAAAAGGACGATAATCATGGGGCAA
AATGATTTAGTTAAAACGTTACGAATGAATTATTTGTTTGATTTTTATCAATCCTTATTGACGAATAAACAAC
GTAATTATTTGGAATTATTTTATCTCGAAGATTATTCTTTGAGTGAAATCGCAGATACTTTTAATGTGAGTAG
ACAAGCAGTTTATGATAATATAAGAAGAACTGGCGATTTAGTTGAAGATTATGAAAAGAAATTGGAATTATAC
CAGAAATTTGAGCAACGCCGAGAAATATATGATGAAATGAAACAACATTTAAGTAATCCAGAACAAATACAAC
GTTATATTCAACAATTAGAAGACTTAGAATAGATATAAATTGAGGGAGGACATCGATATGGCATTTGAAGGCT
TATCAGAACGCCTGCAAGCGACGATGCAAAAAAATGCGTGGTAAGGGTAAACTTACTGAAGCTGATATAAAGAT
AATGATGCGTGAAGTAAGATTAGCGTTACTTGAGGCTGACGTAAACTTTAAAGTGGTAAAAGAATTTATTAAA
ACAGTATCAGAACGCGCATTAGGTTCCGATGTAATGCAATCATTAACACCTGGGCAACAAGTTATTAAAATAG

TTCAAGATGAATTAACGAAGTTGATGGGTGGAGAAAATTCATCGATTAATATGTCAAATAAACCACCTACTGT
TGTTATGATGGTTGGTTTACAAGGTGCTGGTAAAACAACAACTGCAGGTAAATTAGCATTATTGATGCGTAAA
AAATACAACAAAAAACCTATGTTAGTTGCAGCAGATATTTATCGTCCAGCAGCGATAAATCAATTACAAACAG
TAGGGAAACAAATTGATATTCCTGTATACAGTGAAGGGGATCAAGTAAAGCCACAACAAATTGTAACTAATGC
ATTAAAACATGCTAAAGAAGAACATTTAGACTTTGTAATCATTGATACAGCAGGTCGATTACACATCGATGAA
GCATTGATGAACGAATTAAAAGAAGTAAAAGACATTGCTAAACCAAACGAAATTATGTTAGTTGTCGATTCAA
TGACGGGTCAAGATGCTGTCAATGTTGCAGAATCTTTTGACGATCAACTTGATGTCACAGGTGTTACCTTAAC
TAAATTAGATGGTGATACACGTGGTGGTGCAGCTTTATCTATTCGTTCGGTGACACAAAAACCAATTAAATTT
GTTGGTATGAGTGAAAAGTTAGATGGTTTAGAGCTATTCCATCCTGAACGTATGGCATCACGTATTTTAGGTA
TGGGTGATGTGTTAAGTTTAATTGAAAAAGCGCAACAAGATGTGGATCAAGAAAAAGCAAAAGATTTAGAGAA
AAAGATGCGTGAATCATCATTTACTTTAGATGATTTTTTAGAACAGCTTGATCAGGTGAAAAATTTAGGACCA
CTGGATGATATTATGAAAATGATTCCAGGTATGAATAAAATGAAAGGTCTAGATAAGCTTAATATGAGTGAAA
AGCAAATTGATCATATTAAAGCGATTATCCAGTCAATGACGCCGGCTGAAAGAAACAATCCAGACACATTGAA
TGTATCACGTAAAAAGCGTATTGCTAAAGGTTCTGGTCGTTCATTACAAGAAGTCAATCGTTTGATGAAACAA
TTTAACGATATGAAGAAAATGATGAAACAATTCACTGGTGGCGGTAAAGGTAAAAAAGGTAAACGCAATCAAA
TGCAAAATATGTTAAAAGGTATGAACTTACCATTTTAATGTTTAATTTAACACGCTGATTTTTGTTGCAATGA
GATGTGCATCCCGAAAGTTATACTAAAGAATATAACTTTATGGGGTGCATATTTTTAGATACACAATAATAAT
TAAAAACTGAAATTGCTGATTTGCTAGAACATGAGGTTATTGTTGCTAATATTAATAAGTTATATTGGTGGAA
CATGTGCGTTAGAAGTGGTACCATTCATGTCCTTACGAGATAAATTAACTTAACACGTATGTGGTGAAGGTTA
ATATGAATTTTTATAAACTATTAACAGTCTAATTTAGTAATCACATAGTTATTTTTGTAAGAAAAAGGTTCAA
TAATTTTCCTGGTAAAGAAAAAACTCTTTACAAACATTCACACACCTGTTAATATTATTTCTTGTAGAAAATA
AAAATTAAAACATGACTTAAAGGAGATTTTATAAATGGCAGTTAAAATTCGTTTAACACGTTTAGGTTCAAAA
AGAAATCCATTCTATCGTATCGTAGTAGCAGATGCTCGTTCTCCACGTGACGGACGTATCATCGAACAAATCG
GTACTTATAACCCAACGAGCGCTAATGCTCCAGAAATTAAAGTTGACGAAGCGTTAGCTTTAAAATGGTTAAA
TGATGGTGCGAAACCAACTGATACAGTTCACAATATCTTATCAAAAGAAGGTATTATGAAAAAATTTGACGAA
CAAAAGAAAGCTAAGTAATTTAGCGTAAAATTGTTCTAACAATAAGAATAACTCGTTTACACTGACAGTTATT
ACTCAATGATACGTTGGGAATATCACATGTTAGTAATATAGAACGTATTGGGTACCATAATGGTGCCCTTTTT
CTTTGAATAATTTTCAATTAAAATAGAAGTGTTCAAAGCATAGAGTTGGAGGTAATAGAATGAGAGTTGAAGT
TGGTCAAATTGTTAACACACATGGTATTAAAGGTGAAATTAAAGTAAAATCCAATTCAGACTTTACAGACGTT
CGTTTTCAACCCGGTCAAGTGCTGACAGTTGTGCATAACAATAACGACCTTGAATATACTGTTAAGTCACATA
GAGTGCATAAAGGGCTTCATATGCTTACATTTGAAGGTATTAATAATATTAATGATATTGAGCATTTAAAAGG
GAGTTCTATTTATCAAGAGCGTGATCATGAAGATATCGTACTTGAGGAAAATGAATTTTATTATTCAGATATT
ATAGGATGTACAGTTTTTGATGATCAAGAAACACCAATAGGTCGTGTAATTAATATCTTTGAAACAGGTGCAA
ATGATGTGTGGGTGATTAAAGGATCTAAAGAATATTTGATTCCTTATATTGCTGATGTTGTAAAAGAAGTGGA
TGTTGAAAATAAAAAAATTATCATCACGCCAATGGAAGGATTGTTGGATTAATGAAAATTGATTATTTAACTT
TATTTCCTGAAATGTTTGATGGTGTTTTAAATCATTCAATTATGAAACGTGCCCAAGAAAACAATAAATTACA
AATCAATACGGTTAATTTTAGAGATTATGCAATTAACAAGCACAACCAGGTAGATGATTATCCGTATGGTGGC
GGACAAGGTATGGTGTTAAAGCCTGAACCTGTTTTTAATGCGATGGAAGACTTAGATGTCACAGAACAAACAC
GCGTTATTTTAATGTGTCCACAAGGCGAGCCATTTTCACATCAGAAAGCTGTTGAATTAAGCAAGGCTGACCA
CATCGTTTTCATATGCGGACATTATGAAGGTTACGATGAACGTATCCGAACACATCTTGTCACAGATGAAATA
TCAATGGGTGACTATGTTTTAACAGGTGGAGAATTGCCAGCGATGACCATGACTGATGCTATTGTTAGACTGA
TTCCAGGTGTTTTAGGTAATGAACAGTCACATCAAGACGATTCATTTTCAGATGGGTTATTAGAGTTTCCGCA
ATATACACGTCCGCGTGAATTTAAAGGTCTAACAGTTCCGGATGTTTTATTGTCTGGAAATCATGCCAATATT
GATGCATGGAGACATGAGCAAAAGTTGATCCGCACATATAATAAAAGACCTGACTTAATTGAAAAATATCCAT
TAACTAATGCAGATAAGCAAATATTAGAAAGATATAAAATAGGATTGAAAAAAGGTTAGTATTGTGCTAACAT
AATCTGAGTGTGGAAACACAAAAATCACTATGATCCGCTGCTATATATTTGTCGAGGCAAGAACATAGGTTGA
AGAGGAGAATTTATAATGACAAATCACAAATTAATCGAAGCAGTAACTAAATCACAATTACGTACAGACTTAC
CAAGTTTCCGTCCTGGTGATACTTTACGTGTACACGTACGTATCATTGAGGGTACTCGTGAGCGTATCCAAGT
ATTCGAAGGCGTTGTAATTAAACGTCGTGGCGGTGGCGTTTCTGAAACGTTTACAGTTCGTAAAATTTCATCA
GGTGTTGGCGTGGAACGTACATTCCCATTACACACACCAAAAATTGAAAAAATCGAAGTTAAACGTCGTGGTA
AAGTACGTCGTGCTAAATTATATTACTTACGTAGTTTACGTGGTAAAGCTGCTAGAATCCAAGAAATTCGTTA
ATCAGCATTTAAACAAAGCTATGTATGAGTCATATTCGACTCAAACAACAAAGCCATCTAGGTCACTTTTATG
GAGTGATATAGATGGCTTTTTTTGCTGTTTAGTTAATGTAAAGTTGATAATGTAATTGTTTTTTTATTTATTC
ATAACGTGTTTGCTCAAAATGGTTATATAAACAAAAATCTACTAAGTGCCATTGAAAAGGACTTAGTAGATTT
ATTAAATATATGCGTAGCCACATTATTTTTGTCTTACCCATCTCGTGAAAATAATACTACATATAATGCCAAA
TATAGTAATTGTTATAAGTAAATAATAGTATGGTGGGGTATAGCTCAATTGAATCTTTGTTATATTTTTAGGT
GCTTTAATGCCGGTCATAACACCATTTACTTGTTCAACTTTAAGACTTTGATCACCTGATGTCGCTTTCATAC
CTTGATTATATGCTGTTGGCAAAACAATATACCCAGATGAATTTTTATTTTTAGTAATAGTATAACCTTGCTT

TGTCTTACTAACTTTGACAGCCTCTAATGAATTTGAAGTGTCTTTAAGCGTGGTATAATCTTCGCCGTATATC
CCTTTTAAATTTACACGATACTTACCTTTAGGCAACGATAATTTAATTGCATCTGAAGATTTAATACGTATCG
TTACAGGTGTTACAAAACGACGGTATTTATATGTGAGTTTGTTTCTTTCTAGTGTATATTCATTAACTTTAAC
ATAATGAGCTTTATCCGGCGAAAGTAATTCTAAATCCATTTCAAAATACAAATCTTTAAATTGATTAGAAACT
GATTTTGGCAACTGTACAGTTAGACCACCATTATTTTGTTTAACTTGTAATAAATGTTTTGAAGGAGATTGCC
AGGCTGCACTATTTAATTTAATTGTTGAATCTGATAGTAAATTTTTATTGGCTTTAAAATGTGTATTTACATC
TTTAGTATTGTTAGAAACAATCCCTTGCAACATTGCTTGTTCTTTATCTAATGGAGATTTTAATTCTTTATTG
GAAAAGACCTTATTTGTAATATGTGCACTTGGATAATGGATGGTATTTTTAGAATGAATCCAACGAACTTTAT
TGTCTTTGTGTTCAGAGTTAATTTTAAATCCATATGGTAAGTTGTAATCATGATTTACTCTAATTCGATCATT
TACATTCCAAAGCGATAGTAAATTTTGACGATTGCCAAGTAATCTATAAGTGCTGTTTTTATCGATTGGCATA
TTAATTTGGAGTGTCTTGTCATAATATTTTAAAATGTCTCCATTAAAAATACTAGAATATAATGAAATGCCAT
TATAATGATATATAAATGGTGAATTTAATGCATAGTCTGACATATAATCAATGCGATTAAATGGGCCTGTTGC
ATTTTGATTTATCTTTTTTATAAGCTGGTTTACATAGTTACTATGATAATCATGTTGTTTCAACGTTGATATT
GATTGTTGATAAGGTTTGATTGCCATATTTTTGTTGTTATCTAAAATGACGATTTGTTGAATCATAACGATTA
ATACTATTAATACAACGGTTAATTTTTTATAACGCCATAAACTAAATTTTAAAATAACGGCAAGCACCATTAG
CAGGATAATACCTACTATAAGTACGAGTGGGTGTGTCGGTGATAGTAATACATAAAGTAATGCGATGATGCTT
ACTGGTATTGTTCTGATTAAATAATATTTCATATTTAATGTTGATAAATGCTGAATAAACAATCCGCAAAGAG
CACTTGATGATAGTGCTAAGATATACACCCAACGCCTTTCTGGAAATGAAAAACCATTAAAAGCACTGTCGAA
ATACTGTGATAATGAACCAATAAATAATATCCATGTTACTATTGCGAAAAGTCTATAAAAGTAAAAACGATAC
AGTTTGAATGACAATAATGCAACGATAGTAAGAATTGAAATCGTAATATAAAATCCATCGCTAAAGAAAAAAT
AATGATAATCAAGTGGTGTCAAAAACGGTATATCAACATTGGGATTTTGCTTTCTGTCATTTTCCAAAAACGC
AGAAATGCCAGTGAATAAACCAAATACACTTGATAACACACTCAAAACTGCAGCAGATATGACGCAAATTAAT
TTTTGTGTTCTAGAGACAATGTCATATTTGTAAGTGAAAATGAGTCGATATAAATAGTAGCAACCTATAATAA
TAGCTTGATAATAACTGAAATAAAAATTGCTAAATAGTGTTAAGGCTATCGCAACAATGAATATACCGATTTT
GCGTTGTTGAAAATATCTTTCCAAACCAAGAATCGATAATGGTAATAAATATAATAAATTTCCATAAAATGAC
CAAGTAAAATTAAAGTATATAACGACAGTTGACATGCCGTATAAAATCGTAGCGATCATATTTGTTGAGCGTT
TAAAGTGTAATATTTTAAATAAGTAGAAGGTCACGACAAATGTTATGATAGCTCGTATCATGGCCATAATAAG
TTGGTTTGTCGGCCAAAAATGTATTGTTGTCGGATTAAATATACCAACCGTTTCTCCTATTTTAATGAATAGA
AAATTTAGCCACATTAAAGGTGACAGCGAATAATAATATGATAGTCCTTTCATATAATCGCCACCTAATCCAA
ACGATGCATCATATAAACTAGAAAAACTACTTAGATGTTCATACAAATACATTTGAAATGGCATCATTTGACG
GAATCCATCTCCAGCCCCACTAAAAACAGTACCATTCACAATATAATCATAGATATGAGTAGAAAATAAAATA
AGCGTTAATATTACACTAATGAAAGTTATAACAAAGAATTGTTTGACGTTTGAATTTAGCCACTTTTTTAACA
CAACATTATCCTCAACTTTCAAATTTAAAATTAAGTTTAACTGAAACTAAAGTTAATGAGGTTCTTGATAGGT
AAAGACGAAGATGACTGTGGAACAGATACCTTATCATAGTTACTTAAACTTTGGATCATTTTCAGTTTATCAT
TAAACAAATATATTGAATAATAAAAATGTCATACTGATAAAGATGAATGTCACTTAATAAGTAACTTAGATTT
ATTAAATGAAAATTTTTAATTAGAGAAAACTTGGAATAATCACTTATACCTGAATCTAAAGCATTGTTAAGAA
GTGTGACAATGTTAAAATAAATATAGTTGAATTAATGAATTTGTTCTATAATTAACAGGTTATAGATTTTAAT
AATGAGAAAAGAATTGACGAAAGTAAGGTGAATTGAATGGTTATTCAATGGTATCCAGGACATATGGCGAAAG
CCAAAAGAGAAGTAAGTGAACAATTAAAAAAAGTAGATGTAGTGTTTGAACTAGTAGATGCAAGAATTCCATA
TAGTTCAAGAAACCCTATGATAGATGAAGTTATTAACCAAAAACCACGTGTTGTTATATTAAATAAAAAAGAT
ATGTCTAATTTAAATGAGATGTCAAAATGGGAACAATTTTTTATTGATAAAGGATACTATCCTGTATCAGTGG
ATGCTAAGCACGGTAAAAATTTAAAGAAAGTGGAAGCTGCAGCAATTAAGGCGACTGCTGAAAAATTTGAACG
CGAAAAAGCAAAAGGACTTAAACCAAGAGCGATTAGAGCAATGATTGTTGGAATTCCAAATGTTGGTAAGTCC
ACATTAATAAATAAACTGGCAAAGCGTAGTATTGCACAGACTGGTAATAAACCAGGTGTGACCAAACAACAAC
AATGGATTAAAGTTGGTAATGCATTACAACTATTAGATACACCAGGGATTCTTTGGCCTAAATTTGAAGATGA
AGAAGTCGGTAAGAAGTTGAGTTTAACAGGCGCGATTAAAGATAGTATTGTGCACTTAGATGAAGTTGCCATC
TATGGTCTGAACTTTTTAATTCAAAATGATTTAGCGCGCTTAAAAGCACATTATAAAATCGAAGTTCCTGAAG
ATGCAGAAATCATAGCGTGGTTTGATGCGATAGGGAAAAAACGTGGCTTAATTCGACGTGGTAATGAAATTGA
TTACGAAGCAGTCATTGAACTGATTATTTATGATATTCGAAATGCTAAAATAGGAAATTATTGTTTTGATATT
TTTAAAGATATGACTGAGGAATTAGCAAATGACGCTAACAATTAAAGAAGTAACGCAGTTGATTAATGCGGTT
AATACAATAGAAGAATTAGAAAATCATGAATGCTTTTTAGATGAGCGAAAAGGTGTACAAAATGCCATAGCCA
GACGCAGAAAAGCGTTAGAACAAGAACAAGCTTTAAAAGAAAAGTATGCTGAAATGACTTACTTTGAAAATGA
AATATTAAAAGAGCATCCTAATGCTATTATTTGTGGTATTGACGAAGTTGGAAGAGGACCTTTAGCAGGTCCA
GTCGTTGCATGCGCAACAATTTTAAATTCAAATCACAATTATTTGGGCCTTGATGACTCGAAAAAAGTACCTG
TTACGAAACGTCTAGAATTAAATGAAGCACTAAAAAATGAAGTTACTGCTTTTGCATATGGTATCGCTACAGC
TGAAGAAATAGATGAATTTAATATTTATAAAGCTACTCAAATCGCCATGCAGCGAGCTATTGATGGATTATCA
GTACAACCAACGCATTTATTGATAGACGCGATGACGCTTGATAATGCACTGACTCAAGTATCTTTAATCAAGG
GTGATGCAAGAAGTGTATCTATTGCAGCGGCAAGTATCATGGCAAAGGTTTTTCGTGATGATTATATGACACA

GTTATCTAAAGATTATCCTGAATATGGTTTTGAAAAAAACGCGGGTTACGGTACCAAACAACATTTACTAGCA
ATCGATGATATTGGCATTATGAAAGAGCATAGAAAAAGCTTTGAACCTATAAAATCGTTACTGTAAATCGTAA
AATTCAATTAAATGATAAAAAAGTAGCATTTAAGTTTACAATAGCGCTTACATTTTTTATAATTGTAAGGGAA
CTTAACCTAAGTAACAGGAGGATGGAAGATGAATATCCACGAGTATCAAGGTAAAGAAATATTTCGTTCAATG
GGCGTTGCAGTTCCAGAAGGACGAGTAGCATTTACTGCTGAAGAAGCGGTGGAGAAAGCAAAAGAATTAAATT
CAGATGTTTATGTTGTAAAAGCACAAATTCATGCTGGAGGTAGAGGTAAAGCAGGCGGAGTAAAAATTGCTAA
ATCTTTATCAGAGGTAGAAACATATGCAAAAGAATTATTAGGGAAAACTTTGGTGACACATCAAACTGGTCCA
GAAGGTAAAGAAATTAAGCGTTTATATATCGAAGAAGGTTGTGCTATTCAAAAAGAATATTACGTTGGATTCG
TTATTGATCGTGCGACTGACCAAGTAACATTGATGGCGTCTGAAGAAGGGGGCACTGAGATTGAAGAAGTTGC
TGCGAAGACTCCTGAAAAGATCTTCAAAGAAACTATTGATCCAGTAATCGGACTTTCACCATTCCAAGCAAGA
CGAATTGCGTTTAATATTAATATTCCTAAAAAATCTGTTAACAAAGCTGCTAAATTCTTATTAGCACTTTATA
ATGTATTCATTGAAAAAGATTGTTCAATCGTAGAAATCAACCCATTAGTTACAACAGCTGATGGTGATGTATT
GGCATTAGATGCTAAAATTAATTTTGATGATAATGCATTATTCAGACATAAAGATGTTGTAGAATTACGTGAT
TTAGAAGAAGAAGATCCGAAAGAGATTGAAGCGTCTAAACATGATTTATCATACATTGCATTAGATGGTGACA
TCGGATGTATGGTTAATGGTGCAGGTTTAGCCATGGCAACAATGGATACGATTAATCATTTCGGTGGAAACCC
AGCCAATTTCTTAGATGCAGGCGGAAGCGCTACTAGAGAAAAAGTAACTGAAGCATTTAAAATCATTTTAGGT
GATGAAAATGTTAAAGGTATTTTTGTAAACATTTTCGGTGGCATTATGAAATGTGATGTTATCGCAGAAGGTA
TCGTTGAAGCTGTAAAAGAAGTAGATTTAACTTTACCACTAGTTGTACGCTTAGAAGGTACAAATGTTGAGTT
AGGTAAAAAAATCTTAAAAGACTCAGGATTAGCAATTGAACCAGCAGCAACAATGGCTGAAGGTGCACAAAAA
ATTGTTAAACTAGTCAAAGAAGCATAAGGAAAGGATGGGAGCACTAAGATGAGTGTATTTATAGATAAGAATA
CTAAAGTAATGGTACAAGGTATTACAGGGTCTACTGCCCTTTTCCATACAAAACAAATGCTTGATTATGGTAC
GAAAATAGTAGCAGGTGTGACGCCTGGTAAAGGTGGTCAAGTTGTTGAAGGCGTTCCTGTTTTCAACACTGTT
GAAGAAGCTAAAAATGAAACCGGGGCAACGGTTTCAGTCATTTACGTTCCAGCACCATTTGCTGCAGACTCAA
TTTTAGAAGCAGCTGATGCAGACTTAGATATGGTTATTTGTATCACTGAACATATTCCTGTATTAGACATGGT
TAAAGTTAAACGCTACTTACAAGGTAGAAAAACACGTTTAGTTGGTCCGAACTGTCCAGGTGTGATTACAGCA
GATGAATGTAAAATTGGTATTATGCCTGGCTATATTCACAAAAAAGGTCATGTTGGTGTAGTATCACGTTCAG
GTACATTAACATATGAAGCAGTGCACCAATTGACTGAAGAAGGTATTGGTCAAACTACAGCTGTTGGTATTGG
TGGAGACCCAGTAAATGGCACAAACTTTATTGATGTTTTAAAAGCATTCAATGAAGATGACGAAACGAAAGCA
GTTGTTATGATTGGTGAAATCGGTGGTACGGCTGAAGAAGAAGCTGCTGAATGGATTAAAGCGAATATGACAA
AACCAGTTGTAGGCTTTATCGGTGGACAAACAGCACCTCCTGGAAAACGTATGGGACATGCTGGTGCAATCAT
TTCAGGTGGTAAAGGTACTGCTGAAGAGAAAATTAAAACATTAAATAGTTGTGGTGTGAAAACAGCGGCAACA
CCTTCAGAAATTGGTTCAACATTAATTGAAGCTGCTAAAGAAGCAGGAATTTATGAATCATTATTAACTGTTA
ATAAATAAAGTTAAAAGATGATATAAATGGCTATAGCCCATTCCATACTTTATTAAAGTATCGGAAATGGGCT
ATTTGTCGTGCGATGAATTTTAGTACACTTTTCATATTTTGGAAAGGCAAGTTAATTATTTTACCTCCTTCAA
TAGCTTTCTAAAACAAAGTGAATTTTTATTATAATATATAGTGCGCTATGTTATAATATTTATATATTATTGC
AAGGAGTGTTTCATCATGAATAAACAACAAAGTAAAGTACGCTATTCAATTAGAAAAGTTAGTATTGGAATTT
TGTCAATTTCAATTGGTATGTTTTTGGCATTGGGTATGTCGAACAAAGCATATGCAGATGAAATTGATAAATC
TAAAGATTTTACAAGAGGGTATGAGCAAAATGTATTCGCGAAATCAGAGTTAAATGCTAATAAAAATACGACA
AAAGACAAAATAAAAAATGAAGGTGTTGTTAAAACATCGGACACAAGTTTAAAGTTAGACAACAAATCAGCAA
TTTCAAATGGAAATGAAATTAATCAAGATATAAAGATTTCAAATACTCCGAAAAACTCAAGCCAAGGTAACAA
TCTAGTTGTTAATAACAATGAACCTACTAAAGAAATTAAAATTGCAAACTTGGAAGCTCAAAATTCTAATCAG
AAGAAAACGAATAAAGTTACTAATAATTACTTTGGTTACTACAGTTTTAGAGAAGCTCCAAAAACACAAATCT
ATACTGTAAAAAAAGGAGACACACTTAGTGCTATAGCGTTAAAATACAAAACTACAGTTTCAAATATTCAAAA
TACAAATAATATAGCAAATCCTAATTTAATATTTATTGGTCAAAAATTAAAAGTGCCAATGACACCATTAGTA
GAACCAAAACCAAATACAGTGTCTTCAAATAATAAAAGTAATAGTAATAGCAGTACATTAAATTATTTGAAAA
CATTAGAGAATAGAGGATGGGATTTCGACGGTAGTTATGGATGGCAATGTTTCGATTTAGTCAATGTATATTG
GAATCATCTTTATGGTCATGGATTAAAAGGATATGGAGCTAAAGATATACCATATGCAAATAATTTTAATAGT
GAAGCTAAAATTTATCACAACACACCAACTTTCAAAGCTGAACCTGGGGACTTAGTGGTTTTTAGTGGAAGAT
TTGGTGGAGGATATGGTCATACAGCTATTGTTTTAAATGGTGATTATGATGGAAAATTAATGAAGTTCCAAAG
TTTAGATCAAAACTGGAATAATGGTGGATGGCGTAAATCAGAGGTTGCACATAAAGTTGTTCATAATTATGAA
AATGATATGATTTTTATTAGACCATTTAAAAAAGCATAATTTAAATCAAAGGCAGGACATTGAAATATGAAAT
TTACAACTTTAAGTGAAGAAGAATTTACCAACTACACCAAAAAACACTTCAAACATTATACACAGTCTATAGA
ATTATATAATTATAGAAATAAAATAAATTATGAAGCACATATTGTGGGAGTGAAGAATGATAAAAATGAAGTT
ATAGCTGCATGTTTATTAACAGAAGCACGTATTTTTAAATTCTACAAATATTTCTACTCTCATAGAGGCCCTT
TACTTGATTATTTCGATGCTAAATTAGTTTGTTACTTTTTTAAAGAATTATCTAAATTCATTTATAAAAATAG
AGGAGTATTTATTCTTGTTGATCCATATTTAATAGAGAATTTAAGAGATGCAAATGGTAGGGTAATAAAGAAT
TATAATAATTCAGTGATAGTAAAGATGCTAGGGAAAATTGGGTATCTCCATCAAGGTTATACAACAGGATATT
CAAATAAAAGTCAAATTAGGTGGATTTCTGTATTGGATTTAAAAGATAAAGATGAGAATCAACTTTTAAAAGA

AATGGAATACCAAACTAGAAGAAATATAAAAAAGACTATTGAGATTGGTGTTAAGGTTGAAGATTTATCTATT
GAAGAAACAAATCGATTTTATAAATTGTTTCAAATGGCTGAAGAAAAACATGGTTTTCATTTCATGAATGAAG
ATTATTTTAAACAAATGCAAGAAATATATAAAGGTAAGGCAATGTTAAAGATAGCTTGTATAGATCTTAATGA
ATATCAAGATAAATTAAAAATACAATTATTGAAAATCGAAAATGAAATGATGACTGTGAACAGATCATTAAAT
GAAAATCCAAATTCTAAAAAGAATAAATCAAAATTAAATCAGTTAAATATGCAATTATCTAGTATTAATAATA
GAATTAGTAAAACCGAAGAACTAATATTAGAAGATGGACCTGTTTTGGATTTAGCTGCTGCTTTATTTATATG
TACTGATGATGAAGTTTATTATCTATCAAGTGGATCAAATCCTAAATATAATCAGTATATGGGTGCATATCAT
CTACAATGGCATATGATAAAATATGCAAAATCACATAATATTAATAGGTATAATTTTTATGGAATAACAGGCG
TCTTTAGTAATGAGGCGGATGATTTTGGTGTACAACAATTTAAAAAAGGTTTTAATGCACATGTTGAAGAATT
AATTGGTGATTTCATCAAACCAGTAAGACCGATTCTATTTAAATTTGCAAAACTTATTTATAAGGTTTGATTA
TAAAGTATGTTGGAAATTGAAATTTTAAATTCTTTCCAACATACTTTTCACTTTTAAAGTCAGTATTTTTACT
CCTTAAAATAACATTACATCGACAATGTTTTTGTATTAGAAATGTAAAACAAAAATAGAATATGATTTTGTAT
GATTACAAAAAGGAGTTGATTTATTGATTAGACTATTTTTGCTTAAGTTATACTGGTCACACTTTTCGACTAA
ACAAATTCATCAATTTTTAATGGCATATCCTAATGTAATTAAAGAGGAGGGAAGAAAAAAAGATAGTTATTTA
TGTGAATGGGTGAATAGGGAAGAAAATGTTCATTTATTACGTAAATACTATGCTTTTATAAAACTTGATCATA
ACGATATTATTAAAGAACTGCAGAAATTAAAAGTAAGTTACATTACATATATGGATTCTGAATACCCAGTGCT
ATTAAAAGAAATATATCAATTTCCATTACTTCTTTTCTATAAAGGGAACATCAAATTAATAAATAATATGCAT
CATTTGGCAGTAGTAGGTGCAAGAGATTCTACAAGTTATACCCAACAGTCTTTAGAATTTTTATTATCAAATG
ATAAAAGCAAATATTTAACAATTGTTTCCGGCCTTGCTCAAGGAGCTGATGCAATGGCACATCAAATAGCTTT
AAAATACAATCTCCCTACAATTGCAGTTTTAGCCTTTGGCCATCAAACACATTATCCCAAAAGTACATTAGCA
TTAAGAAATAAAATAGAAGAAAAAGGTTTAGTTATATCCGAATATCCACCACATACACCAATTGCTAAATATA
GATTTCCTGAGCGCAATAGAATTATCAGCGGTTTGTCAAAAGGGGTTTTAATTACTGAGGCTAAGGAACAAAG
TGGCAGTCACATCACGATAGATTTTGCATTAGAGCAAAATAGAAATGTTTATGTTTTACCTGGATCTATGTTT
AATCCTATGACAAAAGGTAATTTATTACGTATCCAAGAAGGTGCTAAGGTAGTATTAAACGCTAATGATATAT
TTGAAGACTACTATATTTAAAACTAAAAATAACGAGTTGCTTCATATTTCTTTTGTTACCCTTTGTAATTTAT
TTTTTAATAAAATCTATAAAAAAATAGACAGGGAAAATGATTTGTTTAGATATAAAACGTTGACAAAAGCAAAA
TTAAGCGTTTATCATTTATCTTTAGTAATTAGATTAGCGAGGGGGAAATGACATTGGCAGATAATTTAGTCAT
TGTTGAATCGCCTGCAAAAGCAAAAACCATTGAAAAGTATTTAGGTAAGAAATATAAAGTTATAGCTTCAATG
GGACACGTCAGAGACTTACCAAGAAGTCAAATGGGTGTCGACACTGAAGATAATTACGAACCAAAATATATAA
CAATACGCGGAAAAGGTCCTGTTGTAAAAGAATTGAAAAAGCATGCAAAAAAAGCGAAAAACGTCTTTCTCGC
AAGTGACCCCGACCGTGAAGGTGAAGCAATTGCTTGGCATTTATCAAAAATTTTAGAGCTTGAAGATTCTAAA
GAAAATCGCGTTGTTTTCAACGAAATAACTAAAGACGCTGTTAAAGAAAGTTTTAAAAATCCTAGAGAAATTG
AAATGAACTTAGTCGATGCACAACAAGCGCGTCGAATATTAGATAGATTGGTTGGCTATAACATCTCGCCAGT
TCTATGGAAAAAAGTAAAAAAAGGGTTGTCAGCGGGTCGAGTTCAATCTGTTGCACTTCGTTTAGTCATTGAC
CGTGAAAATGAAATTCGAAACTTTAAACCAGAAGAATATTGGACTATTGAAGGAGAATTTAGATACAAAAAAT
CAAAATTCAATGCTAAATTCCTTCATTATAAAAATAAACCTTTTAAATTAAAAACGAAAAAAGATGTTGAGAA
AATTACAGCTGCATTAGATGGAGATCAATTCGAAATTACAAACGTGACTAAAAAAGAAAAAACGCGTAATCCA
GCAAACCCATTTACAACTTCTACATTACAACAAGAGGCGGCACGTAAATTAAACTTTAAAGCTAGAAAAACAA
TGATGGTCGCACAACAATTATATGAAGGTATAGATTTGAAAAAACAAGGTACGATTGGTTTAATAACATATAT
GAGAACCGATTCTACACGTATTTCAGATACTGCCAAAGCTGAAGCAAAACAGTATATAACTGATAAATACGGT
GAATCTTACACTTCTAAACGTAAAGCATCAGGGAAACAAGGTGACCAAGATGCCCATGAGGCTATTAGACCTT
CAAGTACTATGCGTACGCCAGATGATATGAAGTCATTTTTAACGAAAGACCAATACCGATTATACAAATTAAT
TTGGGAACGATTTGTTGCTAGTCAAATGGCTCCAGCAATACTTGATACAGTCTCATTAGACATAACACAAGGT
GACATTAAATTTAGAGCGAATGGTCAAACAATCAAGTTTAAAGGATTTATGACACTCTATGTAGAAACTAAAG
ATGATAGTGATAGCGAAAAGGAAAATAAACTGCCTAAATTAGAGCAAGGTGATAAAGTCACAGCAACTCAAAT
TGAACCAGCTCAACACTATACACAACCACCTCCAAGATATACTGAGGCGAGATTAGTAAAAACACTTGAAGAA
TTGAAAATTGGGCGACCATCAACATATGCACCGACAATAGATACGATTCAAAAGCGTAACTATGTCAAATTAG
AAAGTAAGCGTTTTGTTCCTACTGAGTTGGGAGAAATAGTTCATGAACAAGTGAAAGAATACTTCCCAGAGAT
TATTGATGTGGAATTCACAGTGAATATGGAAACGTTACTTGATAAGATTGCAGAAGGCGACATTACATGGAGA
AAAGTAATCGACGGTTTCTTTAGTAGCTTTAAACAAGATGTTGAACGTGCTGAAGAAGAGATGGAAAAGATTG
AAATCAAAGATGAGCCAGCCGGTGAAGACTGTGAAGTTTGTGGTTCTCCTATGGTTATAAAAATGGGACGCTA
TGGTAAGTTCATGGCTTGCTCAAACTTCCCGGATTGTCGTAATACAAAAGCGATAGTTAAGTCTATTGGTGTT
AAATGTCCAAAATGTAATGATGGTGACGTCGTAGAAAGAAAATCTAAAAAGAATCGTGTCTTTTATGGATGTT
CGAAATATCCTGAATGCGACTTTATCTCTTGGGATAAGCCGATTGGAAGAGATTGTCCAAAATGTAACCAATA
TCTTGTTGAAAATAAAAAAGGCAAGACAACACAAGTAATATGTTCAAATTGCGATTATAAAGAGGCAGCGCAG
AAATAATATTTTTATTTCCTAGAGACAATTTAAGAATGTTAAATAGAATCATTAGTGAATCTTATTTTAAAGA
TAGTAATGGATTAATCTAAATAAGTGCGGATAATATAAACATAACAACATAATTAAAAGACATAAATGACAAT
AAAAGGAGTATAGAAATGACTCAAACTGTAAATGTAATAGGTGCTGGTCTTGCCGGTTCAGAAGCGGCATATC

AATTAGCTGAAAGAGGAATTAAAGTTAATCTAATAGAGATGAGACCTGTTAAACAAACACCAGCGCACCATAC
TGATAAATTTGCGGAACTTGTATGTTCCAATTCATTACGCGGAAATGCTTTAACTAATGGTGTGGGTGTTTTA
AAAGAAGAAATGAGAAGATTGAATTCTATAATTATTGAAGCGGCTGATAAGGCACGAGTTCCAGCTGGTGGTG
CATTAGCAGTTGATAGACACGATTTTTCAGGTTATATTACTGAAACACTCAAAAATCATGAAAATATCACAGT
TATTAATGAAGAAATTAATGCCATTCCAGATGGATACACAATTATCGCAACAGGACCACTTACTACAGAAACG
CTTGCTCAAGAAATAGTGGACATTACTGGTAAAGATCAACTTTATTTCTATGATGCAGCTGCTCCAATTATTG
AAAAAGAATCTATTGATATGGATAAAGTTTACTTAAAGTCCCGTTATGATAAAGGTGAAGCTGCATATTTAAA
CTGTCCTATGACTGAGGATGAATTTAATCGTTTTTATGATGCAGTATTAGAAGCTGAAGTTGCGCCTGTAAAT
TCATTTGAAAAAGAAAAATATTTCGAGGGTTGTATGCCTTTTGAAGTAATGGCAGAACGCGGACGCAAGACAT
TACTATTTGGACCAATGAAACCAGTAGGATTAGAAGATCCAAAGACTGGGAAACGTCCTTATGCAGTGGTTCA
ATTAAGACAAGATGACGCTGCTGGCACACTCTACAATATTGTCGGCTTCCAAACGCATTTAAAATGGGGAGCT
CAAAAAGAAGTTATTAAATTAATTCCAGGCTTAGAAAATGTTGATATTGTTAGATATGGTGTGATGCATAGAA
ATACCTTCATTAACTCACCGGACGTATTAAACGAGAAATATGAATTGATTTCACAACCAAACATACAGTTTGC
GGGACAAATGACTGGTGTTGAAGGTTATGTGGAAAGCGCAGCTAGTGGCTTAGTTGCAGGCATCAATCTTGCG
CATAAAATATTAGGCAAAGGTGAGGTAGTATTTCCGAGAGAAACTATGATTGGAAGTATGGCTTACTATATTT
CTCATGCTAAAAACAATAAGAATTTCCAACCTATGAATGCTAACTTTGGGTTATTACCATCTTTAGAAACTAG
AATTAAAGATAAAAAAGAACGCTATGAAGCACAAGCTAATAGAGCTTTGGATTACTTAGAAAATTTCAAAAAA
ACTTTATAAAATAGTTAGAAAGACTAGATATGCTATTCATTCTTAAGTCATCAACGAGTAAGTAATGACTTTC
TAAATGGAAAATACTTATCCTAGTCTTTTTAATTTTGGAATTGTTACGTATTTCTGACAATTGAGAATTCGCA
TTCAAAAAATATCTAAATAAATAACACGCAATAAGTTGATTGATGTAACATGTAAGAGAATGTTTTAAATAAA
CTTTATTTAAAAGACAATGAAATAATAAATGGCAAGGCTATTAATAAAGACTTTTAGTAATTAATTTAAAAAA
GAGGTATTCTAATTAACAGGTTTTCCGATTAGTTACAATTATTTAATTCTCAAAAGATTTAGAATTGATTATC
AAATTACTGTAAGCCCTTTGCTGTATATGCTACAATTCTTATTGATGGAGGGTAAATGTATTGAATCATATTC
AAGAAGCGTTTTTAAATACATTGAAAGTTGAACGGAATTTTTCGGAACATACATTGAAATCATATCAAGATGA
CTTAATTCAGTTTAATCAATTTTTAGAACAAGAACATTTACAGTTGAATACTTTTGAATACAGAGATGCTAGA
AATTATTTGAGCTATTTATATTCAAATCATTTGAAAAGAACATCTGTTTCTCGTAAAATCTCAACGTTAAGAA
CTTTCTATGAATATTGGATGACGCTTGATGAGAACATTATTAATCCATTTGTTCAATTAGTACATCCGAAAAA
AGAAAAATATCTTCCGCAATTCTTTTACGAAGAAGAAATGGAAGCGTTATTTAAAACTGTAGAAGAGGACACT
TCAAAAAGTTTACGGGATCGAGTTATTCTTGAATTGTTATATGCTACAGGTATCCGTGTTTCGGAATTAGTTA
ATATTAAAAAACAAGATATAGATTTTTACGCGAATGGTGTTACCGTGTTAGGAAAAGGGAGCAAAGAGCGCTT
TGTACCGTTTGGTGCTTATTGTAGACAAAGCATCGAAAATTATTTAGAACATTTCAAACCTATTCAGTCATGC
AATCATGATTTTCTTATTGTAAATATGAAGGGTGAAGCAATCACTGAACGCGGTGTACGATATGTTTTAAATG
ATATTGTTAAACGAACAGCAGGCGTAAGTGAGATTCATCCCCACAAGCTCAGACATACATTTGCAACGCATTT
ATTGAATCAAGGTGCAGACCTAAGAACAGTACAATCATTATTAGGTCATGTTAATTTGTCAACAACTGGTAAA
TATACACACGTATCTAACCAACAATTAAGAAAAGTGTATCTAAATGCACATCCTCGAGCGAAAAAGGAGAATG
AAACATGAGTAATACAACATTACATGCAACAACAATTTATGCTGTAAGACATAATGGGAAAGCAGCTATGGCT
GGAGATGGGCAAGTAACGCTTGGTCAACAAGTCATCATGAAACAAACGGCAAGAAAAGTGCGACGTTTATATG
AAGATAAAGTGTTAGCTGGTTTCGCAGGTAGTGTAGCAGATGCGTTTACGTTATTTGAAAAATTCGAAACAAA
ATTACAACAGTTTAGTGGTAACTTAGAAAGAGCTGCTGTTGAATTGGCACAAGAATGGCGAGGCGATAAACAA
TTACGTCAATTAGAAGCTATGCTAATTGTAATGGATAAAGATGCTATTTTAGTTGTCAGTGGAACTGGCGAAG
TTATTGCCCCAGATGATGACCTTATCGCTATTGGATCAGGAGGCAACTACGCATTAAGCGCAGGACGTGCATT
GAAACGCCATGCATCGCATTTGTCTGCTGAAGAAATGGCATATGAGAGCTTGAAAGTAGCGGCTGATATTTGT
GTCTTTACCAACGATAATATTGTTGTCGAAACACTATAATAATCAGAGCACGATAAATAATTACGAGCAATTA
ATTTTAGTTAAAAGACGGAGGAATGAAATTAATGGATACAGCTGGAATAAGATTAACTCCAAAAGAAATCGTA
TCTAAATTAAATGAATACATCGTTGGACAAAATGATGCTAAACGTAAAGTGGCAATTGCCCTACGTAATCGAT
ACAGAAGAAGTTTATTAGATGAGGAATCAAAGCAAGAAATTTCACCTAAAAATATTTTGATGATTGGACCAAC
CGGCGTTGGTAAAACTGAAATTGCAAGAAGAATGGCCAAAGTTGTCGGCGCACCATTTATAAAAGTAGAAGCT
ACTAAATTTACTGAGGTAGGTTATGTAGGACGAGATGTTGAAAGTATGGTTAGAGATCTTGTTGATGTTTCAG
TAAGGTTAGTCAAGGCGCAGAAAAAAATCATTGGTACAAGATGAAGCAACAGCTAAGGCTAATGAAAAACTTGT
TAAGTTATTAGTTCCAAGTATGAAAAAGAAAGCATCTCAAACGAATAATCCTTTAGAGTCACTTTTCGGAGGT
GCAATTCCAAATTTCGGACAAAATAACGAAGATGAAGAAGAACCACCTACTGAGGAAATTAAAACAAAACGTT
CTGAAATTAAGAGACAATTAGAAGAAGGCAAACTTGAAAAAGAAAAGGTAAGAATTAAAGTCGAACAAGATCC
TGGTGCTTTAGGTATGCTAGGTACAAATCAAAATCAGCAAATGCAAGAGATGATGAATCAATTAATGCCTAAA
AAGAAAGTTGAGCGAGAAGTTGCTGTTGAGACGGCAAGGAAAATACTAGCTGATAGTTATGCGGATGAACTAA
TTGATCAAGAAAGCGCTAACCAAGAAGCACTTGAATTAGCAGAACAAATGGGTATCATCTTTATAGATGAAAT
CGACAAAGTTGCGACGAATAATCATAATAGTGGTCAAGATGTCTCAAGACAAGGTGTTCAAAGAGATATTTTA
CCTATACTTGAAGGTAGCGTTATTCAAACCAAATATGGTACTGTGAATACTGAACATATGCTGTTTATAGGTG
CTGGAGCTTTCCATGTATCTAAGCCGAGTGACTTGATACCAGAATTGCAAGGTCGTTTTCCGATTAGAGTTGA

ACTTGATAGTTTATCGGTAGAAGATTTTGTAAGAATTTTGACAGAACCAAAATTGTCATTAATTAAACAATAT
GAAGCATTGCTTCAAACAGAAGAAGTTACTGTAAACTTTACCGATGAAGCAATTACTCGCTTAGCTGAGATTG
CCTATCAAGTAAATCAAGATACAGACAATATTGGTGCACGTCGACTTCATACAATTTTAGAAAAGATGCTAGA
AGATTTATCATTCGAAGCACCAAGTATGCCGAATGCAGTTGTAGATATTACCCCACAATATGTTGATGATAAA
TTAAAATCAATTTCAACAAATAAAGATTTAAGTGCATTTATTCTATAAAAATATACAAAAGGAGAAAAATTCA
TGAGCTTATTATCTAAAACGAGAGAGTTAAACACGTTACTTCAAAAACACAAAGGTATTGCGGTTGATTTTAA
AGATGTAGCACAAACGATTAGTAGCGTAACTGTAACAAATGTATTTATTGTATCGCGTCGAGGTAAAATTTTA
GGATCGAGTCTAAATGAATTATTAAAAAGTCAAAGAATTATTCAAATGTTGGAAGAAAGACATATTCCAAGTG
AATATACAGAACGATTAATGGAAGTTAAACAAACAGAATCAAATATTGATATCGACAATGTATTAACAGTTTT
CCCACCTGAAAACAGAGAATTATTCATAGATAGTCGTACAACTATCTTCCCAATTTTAGGTGGAGGAGAAAGA
TTAGGTACATTAGTACTTGGTCGAGTACATGATGATTTTAATGAAAATGATTTGGTACTAGGTGAATATGCTG
CTACAGTTATTGGTATGGAAATCTTACGTGAGAAGCATAGTGAAGTAGAAAAAGAAGCGCGCGATAAAGCTGC
TATTACAATGGCAATTAATTCATTATCTTATTCTGAAAAAGAAGCAATTGAACATATCTTTGAAGAACTTGGC
GGTACGGAAGGCCTATTAATCGCATCAAAAGTTGCAGATAGAGTTGGTATTACTAGATCTGTAATTGTAAATG
CACTACGTAAATTAGAAAGTGCTGGTGTAATTGAATCACGTTCTTTAGGAATGAAAGGTACTTTCATTAAAGT
TAAAAAAGAAAAAATTCTTAGATGAATTAGAAAAAAGTAAGTAAAACGATGAGTCTGGGGCATAATTCAATGTC
CTAGTCGTGACGATGTTATCTTGGCAGTAGTCAACGAAATGACTTTGATGATTATGTTGAAGTCATTTCTTTA
TTTTTGTCTAATTTTCAATCAAATTGATATTCCACATTAACATGATAGAATTACCTTTAAATTTATGAAAAAA
TGCTTGAAAAACATTTGCTTTAGACACGGCATTATGTTATATTTATTTTCGGCTATAAAGCCAATACACACAT
ATCAAACGTGATTGTGTAAAGGGTGCAATATTTGTATATTGTCTTTACAATAGTTTGCTATGGAGGTAATTAA
CCAATAGGAGGAATTTATAATGGCAGTAATTTCAATGAAACAATTACTAGAAGCGGGTGTTCACTTCGGTCAC
CAAACACGTCGTTGGAACCCAAAAATGAAAAAATATATCTTCACTGAGAGAAATGGTATTTATATCATCGACT
TACAAAAAACAGTGAAAAAAGTAGACGAGGCATACAACTTCTTGAAACAAGTTTCAGAAGATGGTGGACAAGT
CTTATTCGTAGGAACTAAAAAACAAGCACAAGAATCAGTTAAATCTGAAGCAGAACGTGCTGGTCAATTCTAC
ATTAACCAAAGATGGTTAGGTGGATTATTAACTAACTATAAAACGATCTCAAAACGAATCAAACGTATTTCTG
AAATTGAAAAAATGGAAGAAGATGGTTTATTCGAAGTATTACCTAAAAAAGAAGTAGTAGAACTTAAAAAAGA
ATATGACCGTTTAATCAAATTCTTAGGCGGAATTCGTGATATGAAATCAATGCCTCAAGCATTATTCGTAGTT
GACCCACGTAAAGAGCGTAATGCAATTGCTGAAGCTCGTAAATTAAATATTCCTATCGTAGGTATCGTTGACA
CTAACTGTGATCCTGACGAAATTGACTACGTTATCCCAGCAAACGACGATGCTATCCGTGCGGTTAAATTATT
AACTGCTAAAATGGCAGATGCAATCTTAGAAGGTCAACAAGGCGTTTCTAATGAAGAAGTAGCTGCAGAACAA
AACATCGATTTAGATGAAAAAGAAAAATCAGAAGAAACAGAAGCAACTGAAGAATAATCAACTGTTGAATCTG
ACTTAGATATAGTTTAAATGGGTGATAAGATATTAATGCTTATCACCTTTTTTAAAAAGAAAATCGAGGCAAA
TTACAAATATTCAATTAGAGTATTGGCAATCTTGCCTATAATAATGCTAAAATCATAATATATAAAATGATAA
CTTATTGGAGGAATAATGAATGGCAACTATTTCAGCAAAACTTGTTAAAGAATTACGTGAAAAAACTGGCGCG
GGTATGATGGATTGTAAAAAAGCGCTAACTGAAACTGATGGTGACATCGATAAAGCGATTGACTACCTACGTG
AAAAAGGTATTGCTAAAGCAGCTAAAAAAGCAGACCGTATTGCGGCTGAAGGTTTAGTACATGTAGAAACTAA
AGGTAACGACGCAGTTATCGTTGAAATCAACTCTGAAACAGACTTTGTTGCTCGTAACGAAGGTTTCCAAGAG
TTAGTTAAAGAAATCGCTAATCAAGTATTAGATACAAAAGCTGAAACTGTTGAAGCTTTAATGGAAACAACTT
TACCAAATGGTAAATCAGTTGATGAAAGAATTAAAGAAGCAATTTCAACAATCGGTGAAAAATTAAGTGTTCG
TCGTTTTGCTATCAGAACTAAAACTGATAACGATGCTTTCGGCGCTTACTTACACATGGGTGGACGCATTGGT
GTATTAACAGTTGTTGAAGGTTCAACTGACGAAGAAGCAGCAAGAGACGTTGCTATGCATATCGCTGCAATCA
ACCCTAAATATGTTTCTTCTGAACAAGTTAGCGAAGAAGAAATCAACCACGAAAGAGAAGTTTTAAAACAACA
AGCATTAAATGAAGGTAAACCAGAAAACATCGTTGAAAAAATGGTGGAAGGACGTTTACGTAAATACTTACAA
GAAATTTGTGCTGTAGATCAAGACTTCGTTAAAAACCCTGATGTAACAGTTGAAGCTTTCTTAAAAACAAAAG
GTGGAAAACTTGTTGACTTCGTACGCTATGAAGTAGGCGAAGGTATGGAAAAACGCGAAGAAAACTTTGCGGA
TGAAGTTAAAGGACAAATGAAATAATCTGTCATAAAGTAAAACAAGGAAGAAGACACCTTTAATGTTGCTTTA
TTAAAATGTAAATCATTCTAATAAAACGACAACTGTGTCTTCTTTACTTGTATATGTTACATATATTCACGAT
AGAGAGGATAAGAAAATGGCTCAAATTTCTAAATATAAACGTGTAGTTTTGAAACTAAGTGGTGAAGCGTTAG
CTGGAGAAAAAGGATTTGGCATAAATCCAGTAATTATTAAAAGTGTTGCTGAGCAAGTGGCTGAAGTTGCTAA
AATGGACTGTGAAATCGCAGTAATCGTTGGTGGCGGAAACATTTGGAGAGGTAAAACAGGTAGTGACTTAGGT
ATGGACCGTGGAACTGCTGATTACATGGGTATGCTTGCAACTGTAATGAATGCCTTAGCATTACAAGATAGTT
TAGAACAATTGGATTGTGATACACGAGTATTAACATCTATTGAAATGAAGCAAGTGGCTGAACCTTATATTCG
TCGTCGTGCAATTAGACACTTAGAAAAGAAACGTGTAGTTATTTTTGCTGCAGGTATTGGAAACCCATACTTC
TCTACAGATACTACAGCGGCATTACGTGCTGCAGAAGTTGAAGCAGATGTTATTTTAATGGGCAAAAATAATG
TAGATGGTGTATATTCTGCAGATCCTAAAGTAAACAAAGATGCGGTAAAATATGAACATTTAACGCATATTCA
AATGCTTCAAGAAGGTTTACAAGTAATGGATTCAACAGCATCCTCATTCTGTATGGATAATAACATTCCGTTA
ACTGTTTTCTCTATTATGGAAGAAGGAAATATTAAACGTGCTGTTATGGGTGAAAAGATAGGTACGTTAATTA
CAAAATAAATTTAGAGGTGTAAAATAATGAGTGACATTATTAATGAAACTAAATCAAGAATGCAAAAATCAAT

CGAAAGCTTATCACGTGAATTAGCTAATATCAGTGCAGGAAGAGCTAATTCAAATTTATTAAACGGCGTAACA
GTTGATTACTATGGTGCACCAACACCTGTACAACAATTAGCAAGCATCAATGTTCCAGAAGCACGTTTACTTG
TTATTTCTCCATACGACAAAACTTCTGTAGCTGACATCGAAAAAGCGATAATAGCGGCTAACTTAGGTGTCAA
CCCAACAAGTGATGGTGAAGTGATACGTATTGCAGTACCTGCCTTAACAGAAGAGCGTAGAAAAGAGCGCGTT
AAAGATGTTAAGAAAATTGGTGAAGAAGCTAAAGTATCTGTTCGAAATATTCGTCGTGATATGAATGATCAGT
TGAAAAAAGATGAAAAAAATGGCGACATTACTGAAGATGAGTTGAGAAGTGGTACTGAAGATGTTCAGGAAGC
AACAGACAATTCAATAAAAGAAATTGATCAAATGATTGCTGATAAAGAAAAAGATATTATGTCAGTATAAAAC
TAATATACAATGACATATTAAAATGCCAGTATTAAACGATAATGTAACATTTAAAATGGGCATGTTTAATTAA
ATCAAAGATGCATGTGATAATTTAAATTCACAATGAGCATAAAAATGGTGTTTAAACAAGTTAATTAAACATA
TACTTTATAAATAATAGGCATTAGGTATATTGCTATAATAAAGTTATGTAATTTTTAACCTCAGTATGTATGT
CACATTTCTGGTGTAAACTGTACCGAGTCAGACTTTGGTACAGTTTTTTATTTGCTTATTCAATGCATTAAA
TGAGTATGATAAAATGATAATGATTGTTTAGTAACTTATACTATATGACAGAGATGATCAGGCTCGGAGGAAA
GACCATGTTTAAAAAGCTAATAAATAAAAAGAACACTATAAATAATTATAATGAAGAATTAGACTCGTCTAAT
ATACCTGAACATATCGCTATTATTATGGATGGTAATGGGCGATGGGCTAAGAAGCGAAAAATGCCTAGAATTA
AAGGCCATTACGAAGGTATGCAAACAATAAAAAAAAATTACTAGGATAGCTAGTGATATTGGTGTTAAGTACTT
AACTTTATATGCCTTTTCCACTGAAAATTGGTCAAGACCTGAAAGTGAAGTAAATTATATTATGAATTTGCCT
GTCAATTTCTTAAAGACATTCTTACCGGAACTAATTGAAAAAAATGTCAAAGTTGAAACAATTGGATTTACTG
ATAAGTTGCCAAAATCAACGATAGAAGCAATTAATAATGCTAAAGAAAAGACAGCTAATAATACCGGCTTAAA
ATTAATATTTGCAATTAATTATGGTGGCAGAGCAGAACTTGTTCATAGTATTAAAAATATGTTTGACGAGCTT
CATCAACAAGGTTTAAATAGTGATATCATAGATGAAACATATATAAACAATCATTTAATGACAAAAGACTATC
CTGATCCAGAGTTGTTAATTCGTACTTCAGGAGAACAAAGAATAAGTAATTTCTTGATTTGGCAAGTTTCGTA
TAGTGAATTTATCTTTAATCAAAAATTATGGCCTGACTTTGACGAAGATGAATTAATTAAATGTATAAAAATT
TATCAGTCACGTCAAAGACGCTTTGGCGGATTGAGTGAGGAGTAGTATAGTATGAAAGTTAGAACGCTGACAG
CTATTATTGCCTTAATCGTATTCTTGCCTATCTTGTTAAAAGGCGGCCTTGTGTTAATGATATTTGCTAATAT
ATTAGCATTGATTGCATTAAAAGAATTGTTGAATATGAATATGATTAAATTTGTTTCAGTTCCTGGTTTAATT
AGTGCAGTTGGTCTTATCATCATTATGTTGCCACAACATGCAGGGCCATGGGTACAAGTAATTCAATTAAAAA
GTTTAATTGCAATGAGCTTTATTGTATTAAGTTATACTGTCTTATCTAAAAACAGATTTAGTTTTATGGATGC
TGCATTTTGCTTAATGTCTGTGGCTTATGTAGGCATTGGTTTTATGTTCTTTTATGAAACGAGATCAGAAGGA
TTACATTACATATTATATGCCTTTTTAATTGTTTGGCTTACAGATACAGGGGCTTACTTGTTTGGTAAAATGA
TGGGTAAACATAAGCTTTGGCCAGTAATAAGTCCGAATAAAACAATCGAAGGATTCATAGGTGGCTTGTTCTG
TAGTTTGATAGTACCACTTGCAATGTTATATTTTGTAGATTTCAATATGAATGTATGGATATTACTTGGAGTG
ACATTGATTTTAAGTTTATTTGGTCAATTAGGTGATTTAGTGGAATCAGGATTTAAGCGTCATTTCGGCGTTA
AAGACTCAGGTCGTATACTACCTGGACACGGTGGTATTTTAGACCGATTTGACAGCTTTATGTTTGTGTTACC
ATTATTAAATATTTTATTAATACAAGCTTAATGCTGAGAACAAATCAATAAACGTAAAGAGGAGTTGCTGAGA
TAATTTAATGAATCTCAGAACTCCTTTTGAAAATTATACGCAATATTAACTTTGAAAATTAGACGTTATATTT
TGTGATTTGTCAGTATCATATTATAATGACTTATGTTACGTATACAGCAATCAATTTTAAAATAAAAGAAATT
TATAAACAATCGAGGTGTAGCGAGTGAGCTATTTAGTTACAATAATTGCATTTATTATTGTTTTTGGTGTACT
AGTAACTGTTCATGAATATGGCCATATGTTTTTTGCGAAAAGAGCAGGCATTATGTGTCCAGAATTTGCGATC
GGTATGGGGCCAAAAATTTTTAGTTTTAGAAAAAATGAAACACTTTACACTATTAGGTTATTGCCTGTTGGTG
GATATGTTCGTATGGCAGGAGATGGCTTAGAAGAGCCACCAGTCGAGCCCGGTATGAACGTTAAAATTAAACT
GAATGAAGAAAATGAAATAACACATATCATATTAGATGATCATCATAAGTTTCAACAAATTGAAGCGATCGAA
GTTAAAAAATGTGATTTTAAGGATGACTTATTCATAGAAGGTATCACTGCTTATGATAATGAAAGACATCATT
TTAAAATTGCTAGAAAGTCTTTCTTTGTTGAAAATGGTAGCTTAGTTCAAATTGCTCCGAGAGACAGACAATT
TGCACATAAAAAGCCATGGCCGAAATTTTTAACATTATTTGCGGGACCGTTATTTAACTTTATATTAGCTTTA
GTCCTATTTATTGGTCTTGCATATTATCAAGGTACACCTACGTCTACTGTAGAACAAGTCGCAGATAAGTATC
CAGCACAACAAGCAGGATTACAAAAAGGTGATAAAATTGTTCAAATTGGCAAATATAAAATATCTGAATTTGA
TGATGTTGATAAGGCGTTAGATAAAGTTAAAGATAATAAGACGACTGTTAAATTTGAACGTGATGGTAAAACG
AAGTCAGTTGAATTAACACCTAAAAAGACTGAAAGAAAACTGACTAAAGTAAGTTCAGAGACGAAGTATGTTC
TCGGATTCCAACCAGCGAGTGAACATACACTTTTTAAACCAATTGTATTCGGATTTAAAAGCTTTTTAATCGG
TAGTACTTATATTTTTACAGCTGTAGTAGGTATGTTGGCTAGTATATTTACGGGCGGATTCTCATTTGATATG
TTAAATGGTCCGGTTGGTATTTATCATAACGTAGACTCAGTTGTTAAAGCGGGTATCATTAGCTTAATTGGTT
ACACTGCGTTATTAAGTGTAAACTTAGGTATTATGAATTTAATTCCTATTCCTGCACTAGACGGTGGTCGTAT
TTTATTTGTTATATATGAAGCGATTTTCAGAAAACCAGTTAATAAAAAAGCGGAAACAACGATTATTGCTATT
GGTGCCATTTTCATGGTCGTTATTATGATATTAGTAACGTGGAATGATATTCGACGATATTTCTTATAATTTA
GGAGGATAAATAATTATGAAGCAATCCAAAGTTTTTATACCAACGATGCGTGACGTGCCATCAGAAGCAGAAG
CACAAAGTCATCGTTTATTATTGAAATCGGGTTTGATAAAACAAAGTACAAGTGGGATTTATAGTTATTTACC
GCTAGCAACACGTGTGTTAAATAATATTACTGCAATTGTGCGACAAGAAATGGAACGTATCGATTCTGTTGAA
ATTTTAATGCCAGCGTTACAACAAGCTGAATTATGGGAAGAATCAGGACGTTGGGGTGCATATGGCCCAGAAT

TAATGCGTTTACAAGATAGACATGGAAGACAATTTGCATTAGGTCCAACACATGAAGAATTAGTTACATCAAT
AGTAAGAAATGAATTGAAATCATACAAACAATTACCGATGACATTATTCCAAATTCAATCTAAATTCCGTGAT
GAAAAGAGACCACGTTTTGGTTTACTTCGTGGTCGTGAATTTATTATGAAAGATGCGTATTCATTCCATGCTG
ACGAGGCATCATTAGATCAAACGTATCAAGATATGTATCAAGCGTATAGCCGTATTTTTGAGAGAGTTGGCAT
TAACGCAAGACCAGTAGTTGCAGATTCAGGTGCTATTGGCGGTAGCCATACACATGAATTTATGGCATTAAGT
GCTATCGGTGAGGATACAATCGTTTACAGTAAAGAAAGCGATTATGCTGCTAACATCGAAAAAGCAGAAGTCG
TTTACGAACCAAATCATAAGCATACTACTGTGCAACCTATAGAAAAAATTGAAACACCAAATGTTAAGACTGC
GCAAGAATTGGCAGACTTTTTAGGTAGACCAGTAGATGAAATCGTTAAAACGATGATTTTCAAAGTTGATGGC
GAATATATTATGGTTTTAGTGCGTGGACATCATGAAATTAATGACATTAAATTAAAATCTTATTTCGGCACAG
ATAATATTGAATTAGCAACACAAGACGAAATTGTTAATTTAGTTGGTGCAAATCCTGGTTCACTAGGTCCTGT
AATTGATAAAGAAATCAAAATTTATGCAGATAATTTTGTGCAAGATTTAAATAATTTAGTTGTCGGTGCTAAC
GAAGATGGTTATCACTTAATTAATGTAAATGTAGGTAGAGACTTCAACGTTGATGCATATGGCGATTTCCGTT
TTATTTTAGAAGGCGAAAAGTTAAGTGATGGTTCAGGCGTTGCACATTTTGCTGAAGGTATTGAAGTTGGTCA
AGTATTTAAATTGGGTACTAAGTATTCAGAATCAATGAATGCTACATTCTTAGATAATCAAGGAAAAGCTCAA
CCGTTAATTATGGGTTGTTATGGTATTGGAATTTCTAGAACGCTAAGTGCAATTGTTGAACAAAATCACGATG
ATAATGGTATTGTTTGGCCTAAATCAGTTACTCCATTTGATTTACATTTAATTTCTATTAATCCTAAGAAAGA
TGATCAACGAGAACTAGCGGATGCACTATATGCTGAATTTAATACTAAATTTGATGTGTTGTACGATGATCGT
CAGGAACGTGCGGGTGTCAAATTTAATGATGCCGATTTAATTGGTTTACCACTGCGAATTGTTGTTGGTAAAC
GTGCATCGGAAGGTATTGTAGAAGTTAAAGAACGTTTAACAGGTGATAGCGAAGAAGTCCACATTGATGACTT
AATCACTGTCATTACAAATAAATATGATAACTTAAAATAATTAAGATCGAATGAATTATAAGAGTAGGAAAAA
GCTGAAAGAAATCAGATGCTTATGTCCTGCTCTTATTATTTTTGATATAATGATTATTCGATGAAAAATGACT
GAAGACATAGTATAATTAAAGATAAATTTGTTTTAACAATATTATGATTAGCCAAATATAAAGCATTTAATTT
TCTATCATTACTATGCTCACATAATCTAAATATTGTTCGAACACGTAAAAGTAATTTCTATTTAAGGTGGTAA
TTGTCTTGGCAATGACAGAGCAACAAAAATTTAAAGTGCTTGCTGATCAAATTAAAATTTCAAATCAATTAGA
TGCTGAAATTTTAAATTCAGGTGAACTGACACGTATAGATGTTTCTAACAAAAACAGAACATGGGAATTTCAT
ATTACATTACCACAATTCTTAGCTCATGAAGATTATTTATTATTTATAAATGCAATAGAGCAAGAGTTTAAAG
ATATCGCCAACGTTACATGTCGTTTTACGGTAACAAATGGCACGAATCAAGATGAACATGTAATTAAATACTT
TGGACACTGTATTGACCAAACAGCTTTATCTCCAAAAGTTAAAGGTCAATTGAAACAGAAAAAGCTTATTATG
TCTGGAAAAGTATTAAAAGTAATGGTATCAAATGACATTGAACGTAATCATTTTGATAAGGCATGCAATGGAA
GTCTTATCAAAGCGTTTAGAAATTGTGGTTTTGATATCGATAAAATCATATTCGAAACAAATGATAATGATCA
AGAACAAAACTTAGCTTCTTTAGAAGCACATATACAAGAAGAAGACGAACAAAGTGCACGATTGGCAACAGAG
AAACTTGAAAAAATGAAAGCTGAAAAAGCGAAACAACAAGATAACAACGAAAGTGCTGTCGATAAGTGTCAAA
TTGGTAAGCCGATTCAAATTGAAAATATTAAACCAATTGAATCTATTATTGAGGAAGAGTATAAAGTTGCAAT
AGAGGGTGTCATTTTTGATATTAACTTAAAAGAACTTAAAAGTGGTCGTCATATCGTAGAAATTAAAGTGACT
GACTATACGGACTCTTTAGTTTTAAAAATGTTTACTCGTAAAAACAAAGATGATTTAGAACATTTTAAAGCGC
TAAGTGTTGGTAAATGGGTTAGGGCTCAAGGTCGTATTGAAGAAGATACATTTATTAGAGATTTAGTTATGAT
GATGTCTGATATTGAAGAGATTAAAAAAGCGACAAAAAAAGATAAGGCTGAAGAAAAGCGTGTAGAATTCCAC
TTGCATACTGCAATGAGTCAAATGGATGGTATACCAAATATTAGTGCGTATGTTAAACAGGCAGCAGACTGGG
GACATCCAGCCATTGCGGTTACAGACCATAATGTTGTGCAAGCATTTCCAGATGCTCACGCAGCAGCGGAAAA
ACATGGTATTAAAATGATATACGGTATGGAAGGTATGTTAGTTGATGATGGTGTTCCGATTGCATACAAACCA
CAAGATGTCGTATTAAAAGATGCTACTTATGTTGTATTCGACGTTGAGACAACTGGTTTATCAAATCAGTATG
ATAAAATCATCGAGCTTGCAGCTGTGAAAGTTCATAACGGCGAAATCATCGATAAGTTTGAAAGGTTTAGTAA
TCCGCATGAACGATTATCGGAAACGATTATCAATTTGACGCATATTACTGATGATATGTTAGTAGATGCCCCT
GAGATTGAAGAAGTACTTACAGAATTTAAAGAATGGGTTGGCGATGCGATATTCGTAGCACATAATGCTTCGT
TTGATATGGGCTTCATCGACACGGGATATGAACGTCTTGGATTTGGACCATCTACGAATGGTGTTATCGATAC
ATTAGAATTATCTCGTACGATTAATACTGAATATGGTAAACATGGTTTAAATTTCTTAGCTAAAAAATACGGT
GTAGAATTAACGCAACATCACCGTGCCATTTATGATACAGAGGCAACAGCTTACATTTTCATAAAAATGGTTC
AACAAATGAAAGAATTAGGCGTATTAAATCATAACGAAATCAACAAAAAACTCAGTAATGAAGATGCATATAA
ACGTGCAAGACCTAGTCATGTCACATTAATTGTACAAAACCAACAAGGTCTTAAAAATCTATTTAAAATTGTA
AGTGCATCATTAGTGAAGTATTTCTACCGTACGCCTCGAATTCCACGTTCATTGTTAGATGAATATCGTGAGG
GATTATTGGTAGGTACAGCGTGTGATGAAGGTGAATTATTTACGGCAGTTATGCAGAAGGACCAGAGCCAAGT
TGAAAAAATTGCCAAATATTATGATTTTATTGAAATTCAACCACCGGCACTTTATCAAGATTTAATTGATAGA
GAGCTTATTAGAGATACTGAAACATTACATGAAATTTATCAACGTTTAATACATGCAGGTGACACAGCGGGTA
TTCCTGTTATTGCGACAGGAAATGCACACTATTTGTTTGAACATGATGGTATCGCACGTAAAATTTTAATAGC
ATCACAACCCGGCAATCCACTTAATCGCTCAACTTTACCGGAAGCACATTTTAGAACTACAGATGAAATGTTA
AACGAGTTTCATTTTTTAGGTGAAGAAAAAGCGCATGAAATTGTTGTGAAAAATACAAACGAATTAGCAGATC
GAATTGAACGTGTTGTTCCTATTAAAGATGAATTATACACACCGCGTATGGAAGGTGCTAACGAAGAAATTAG
AGAACTAAGTTATGCAAATGCGCGTAAATTGTATGGTGAAGACCTGCCTCAAATCGTAATTGATCGATTAGAA

AAAGAATTAAAAAGTATTATCGGTAATGGATTTGCGGTAATTTACTTAATTTCGCAACGTTTAGTTAAAAAAT
CATTAGATGATGGATACTTAGTTGGTTCCCGTGGTTCAGTAGGTTCTAGTTTTGTAGCGACAATGACTGAGAT
TACTGAAGTAAACCCGTTACCGCCACACTATATTTGTCCGAACTGTAAAAGGAGTGAATTTTTCAATGATGGT
TCAGTAGGATCAGGATTCGATTTACCTGATAAGACGTGTGAAACTTGTGGAGCGCCACTTATTAAAGAAGGAC
AAGATATTCCGTTTGAAACATTTTTAGGATTTAAAGGAGATAAAGTTCCTGATATCGACTTAAACTTTAGTGG
TGAATATCAACCGAATGCCCATAACTACACAAAAGTATTATTTGGTGAGGATAAAGTATTCCGTGCAGGTACA
ATTGGTACCGTTGCTGAAAAGACTGCTTTTGGTTATGTTAAAGGTTATTTGAATGATCAAGGCATCCACAAAA
GAGGCGCTGAAATAGATCGACTCGTTAAAGGATGTACAGGTGTTAAACGTACAACCGGACAGCATCCAGGGGG
TATTATTGTAGTACCTGATTACATGGATATTTATGATTTTACGCCGATACAATATCCTGCCGATGATCAAAAT
TCAGCATGGATGACGACACATTTTGATTTCCATTCTATTCATGATAATGTATTAAAACTTGATATACTTGGAC
ATGATGATCCAACAATGATTCGTATGCTTCAAGATTTATCAGGAATTGATCCAAAAACAATACCTGTAGATGA
TAAAGAAGTTATGCAGATTTTTAGTACACCTGAAAGTTTGGGTGTTACTGAAAATGAAATTTTATGTAAAACA
GGTACATTTGGAGTACCAGAATTCGGTACAGGATTCGTGCGTCAAATGTTAGAAGATACAAAGCCAACAACAT
TTTCTGAATTAGTTCAAATCTCAGGATTATCTCATGGGACAGATGTGTGGTTAGGCAATGCTCAAGAATTAAT
TAAAACCGGTATATGTGATTTATCAAGTGTAATTGGTTGTCGTGATGATATCATGGTTTATTTAATGTATGCT
GGTTTAGAACCATCAATGGCTTTTAAAATAATGGAGTCAGTACGTAAAGGTAAAGGTTTAACTGAAGAAATGA
TTGAAACGATGAAAGAAAATGAAGTGCCGGATTGGTATTTAGATTCATGTCTTAAAATTAAGTACATGTTCCC
TAAAGCCCATGCAGCAGCATACGTTTTAATGGCAGTACGTATCGCATATTTCAAAGTACATCATCCACTTTAT
TACTATGCATCTTACTTTACAATTCGTGCGTCAGACTTTGATTTAATAACGATGATTAAAGATAAAACAAGCA
TTCGAAATACTGTAAAAGACATGTATTCTCGCTATATGGATCTAGGTAAAAAAGAAAAAGACGTATTAACTGT
CTTGGAAATTATGAATGAAATGGCGCATCGAGGTTATCGAATGCAACCGATTAGTTTAGAAAAGAGTCAGGCG
TTCGAATTTATCATTGAAGGCGATACACTTATTCCGCCGTTCATATCAGTGCCTGGGCTTGGCGAAAACGTTG
CGAAACGAATTGTTGAAGCTCGTGACGATGGACCATTTTTATCAAAAGAAGATTTAAACAAAAAAGCTGGATT
ATCTCAGAAAATTATTGAGTATTTAGATGAGTTAGGCTCATTACCGAATTTACCAGATAAAGCTCAACTTTCG
ATATTCGATATGTAAAATGAAATAATTAAGCTATTTATTTAATGCGTATGGCATAGTCGAAGAAATACAAAAT
TGTTGCTGGACACAAAGTTATGCCTGTATTTTTTTAGCGTCTTACGAGTCTATTCGAATGTAATGGTGAAATA
AATGAACAAACTTTTACAAGAATCTCTGATTAATAGTGAAGTCATTTGTTTCAAGCATAAACTTATGCTATAA
TTAAGTTGCTTAAAAATTAGTGAACTCAGGCAGAAGAGTGGGAGATTCCCGCTCTTTTCTATTTGCCAAAAAG
GGAGGCCTGTATGAGTAAAATTACAGAACAAGTAGAAGTGATCGTTCAACCAATAATGGAAGACTTGAATTTT
GAACTTGTAGACGTTGAATATGTCAAAGAGGGTAGAGATCATTTTCTTAGAATCTCTATTGATAAAGAAGGTG
GCGTAGATTTAAATGATTGTACGCTAGCTTCTGAAAAAATAAGTGAAGCTATGGATGCAAATGATCCTATTCC
TGAAATGTATTATTTAGACGTAGCGTCACCTGGTGCAGAACGTCCAATTAAAAAAGAACAAGATTTCCAAAAT
GCAATAACTAAACCTGTATTTGTTTCTTTATATGTACCAATTGAAGGTGAAAAGGAATGGTTAGGCATTTTAC
AAGAAGTCAATAATGAAACAATTGTAGTACAAGTTAAAATCAAAGCAAGAACGAAAGATATAGAGATACCGAG
AGACAAAATAGCAAAAGCACGTCACGCAGTTATGATTTAACGTGATGAGGAGGAAAAAACGTGTCAAGTAATG
AATTATTATTAGCTACTGAGTATTTAGAAAAAGAAAAGAAGATTCCTAGAGCAGTATTAATTGATGCTATTGA
AGCAGCTTTAATTACTGCATACAAAAAGAATTATGATAGTGCAAGAAATGTCCGTGTGGAATTAAATATGGAT
CAAGGTACTTTCAAAGTTATCGCTCGTAAAGATGTTGTTGAAGAAGTATTTGACGACAGAGATGAAGTGGATT
TAAGTACAGCGCTTGTTAAAAACCCTGCATATGAAATTGGTGATATATACGAAGAAGATGTAACACCTAAAGA
TTTTGGTCGTGTAGGTGCTCAAGCAGCGAAACAAGCAGTAATGCAACGTCTTCGTGATGCTGAACGTGAAATT
TTATTTGAAGAATTTATAGACAAAGAAGAAGACATACTTACTGGAATTATTGACCGTGTTGACCATCGTTATG
TATATGTGAATTTAGGTCGTATCGAAGCTGTTTTATCTGAAGCAGAAAGAAGTCCTAACGAAAAATATATTCC
TAACGAACGTATCAAAGTATATGTTAACAAAGTGGAACAAACGACAAAAGGTCCTCAAATCTATGTTTCTCGT
AGCCATCCAGGTTTATTAAAACGTTTATTTGAACAAGAAGTTCCAGAAATTTACGATGGTACTGTAATTGTTA
AATCAGTAGCACGTGAAGCTGGCGATCGCTCTAAAATTAGTGTCTTCTCTGAAAACAATGATATAGATGCTGT
TGGTGCATGTGTTGGTGCCAAAGGCGCGCGTGTTGAAGCTGTTGTTGAAGAGCTAGGTGGCGAAAAAATCGAC
ATCGTTCAATGGAATGAAGATCCAAAAGTATTTGTAAAAAATGCTTTAAGCCCTTCTCAAGTTTTAGAAGTTA
TTGTTGATGAAACAAATCAATCTACGGTAGTTGTTGTTCCAGATTATCAATTGTCATTAGCGATTGGTAAAAG
AGGACAAAACGCACGTCTAGCTGCTAAATTAACCGGCTGGAAAATTGATATTAAATCAGAAACAGATGCGCGT
GAAGCGGGTATCTATCCAGTAGTTGAAGCTGAAAAAGTAACTGAAGAAGATATTGCTTTAGAAGATGCTGACA
CAACAGAATCAACCGAAGAGGTAAATGATGTTTCAGTTGAAACAAATGTAGAGAAAGAATCTGAATAATAGGT
TGGAGTGAAGTATCTATGAAAAAGAAAAAAATTCCGATGCGAAAATGTATTCTTTCAAATGAAATGCATCCCA
AAAAAGATATGATTCGTGTTGTTGTTAATAAAGAAGGCGAAATCTTTGCGGATGTTACTGGAAAGAAACAAGG
CCGTGGCGCATATGTTTCTAAAGATGTTGCTATGGTTGAAAAAGCACAACAAAAAGAAATTTTAGAAAAATAT
TTTAAAGCATCTAAAGAGCAATTGGATCCTGTTTACAAAGAAATTATTAGATTAATTTATAGAGAAGAAATCC
CAAAATGAGTATAGATCAAATATTAAACTTTTTAGGATTAGCAATGAGAGCTGGTAAAGTAAAAACAGGTGAA
TCAGTCATTGTTAATGAGATTAAAAAAGGAAATTTGAAGCTCGTTATTGTTGCAAATGATGCGTCTGATAATA
CAGCTAAATTAATTACAGATAAATGTAAGAGTTACAAAGTTCCATTCAGAAAGTTTGGAAATCGAAATGAATT

GGGAATAGCACTTGGAAAAGGTGAGCGTGTTAATGTAGGGATTACTGACCCAGGCTTTGCTAAAAAGTTGCTA
TCAATGATAGATGAATATCATAAGGAGTGATTATATGAGTAAACAAAGAATTTACGAATATGCGAAAGAATTA
AATCTAAAGAGTAAAGAGATTATAGATGAGTTAAAAAGCATGAATATTGAGGTTTCAAATCATATGCAAGCTT
TGGAAGATGACCAAATTAAAGCATTAGATAAAAAGTTCAAAAAAGAACAAAAGAACGACAATAAACAAAGCAC
TCAAAATAATCACCAAAAATCAAACAATCAAAACCAAAATAAAGGGCAACAAAAAGATAACAAAAAGAATCAA
CAACAAAATAATAAAGGCAACAAAGGCAATAAAAAGAATAATAGAAATAATAAGAAAAATAACAAGAATAATA
AACCACAAAATCAACCAGCTGCTCCAAAAGAAATACCATCAAAAGTGACATATCAAGAAGGTATTACAGTAGG
CGAATTTGCGGATAAATTAAATGTTGAATCATCCGAAATTATCAAAAAATTATTCTTACTTGGTATTGTTGCT
AATATCAATCAATCATTAAATCAAGAAACAATCGAATTAATTGCCGATGATTATGGCGTTGAGGTTGAAGAAG
AAGTTGTGATTAATGAAGAAGATTTATCAATCTACTTTGAAGACGAAAAAGATGATCCAGAAGCAATTGAGAG
ACCAGCAGTTGTAACAATTATGGGACATGTTGACCATGGTAAAACGACTTTATTAGATTCAATTCGTCATACA
AAAGTTACAGCAGGTGAAGCAGGCGGAATCACTCAACATATTGGTGCATATCAAATTGAAAACGATGGCAAAA
AAATCACTTTCTTAGATACACCGGGACATGCTGCATTTACAACGATGCGTGCGCGTGGTGCACAAGTAACAGA
TATTACAATTTTAGTAGTTGCAGCTGACGATGGTGTTATGCCACAAACAATTGAAGCAATTAACCATGCTAAA
GAAGCAGAAGTACCAATTATTGTTGCAGTAAATAAAATTGATAAACCAACTTCAAATCCTGATCGAGTTATGC
AAGAATTAACTGAATATGGTTTAATTCCTGAAGATTGGGGCGGCGAAACAATTTTCGTTCCACTTTCTGCATT
AAGTGGTGATGGTATCGACGATTTATTAGAAATGATAGGATTAGTTGCAGAAGTTCAAGAACTTAAAGCAAAT
CCTAAAAACCGTGCTGTTGGTACAGTTATCGAAGCTGAATTAGATAAATCACGTGGTCCTTCTGCATCATTAT
TAGTACAAAATGGTACATTAAATGTTGGTGATGCGATTGTAGTTGGTAATACTTACGGCCGTATCCGTGCAAT
GGTTAATGACTTAGGTCAAAGAATCAAAACGGCTGGTCCATCAACGCCTGTTGAAATTACAGGTATTAATGAT
GTGCCACAAGCTGGGGATCGCTTTGTTGTATTTAGTGATGAAAAACAAGCTCGTCGTATTGGTGAATCAAGAC
ACGAAGCTAGCATTGTACAACAACGTCAAGAAAGTAAAAATGTTTCATTAGATAACCTGTTTGAACAAATGAA
ACAAGGTGAAATGAAAGATTTAAACGTTATTATTAAAGGTGATGTTCAAGGTTCTGTTGAAGCTTTAGCTGCA
TCATTAATGAAAATTGATGTTGAAGGCGTAAATGTTCGTATCATTCATACAGCGGTTGGTGCAATTAATGAGT
CAGACGTGACACTTGCTAATGCCTCAAATGGTATTATCATTGGTTTCAATGTTCGTCCAGACAGTGGTGCAAA
ACGTGCTGCAGAAGCTGAAAATGTTGATATGCGTTTACACAGAGTTATTTATAATGTTATCGAAGAAATTGAA
TCAGCGATGAAAGGTTTACTTGATCCAGAATTTGAAGAACAAGTTATCGGACAAGCTGAAGTTCGTCAAACAT
TCAAAGTTTCTAAAGTTGGTACTATTGCTGGATGTTATGTTACTGAAGGTAAAATTACGCGAAATGCTGGTGT
ACGTATTATTCGTGATGGTATTGTTCAATATGAAGGCGAATTAGATACACTTAAACGTTTCAAAGATGATGCT
AAGGAAGTTGCAAAAGGTTATGAATGTGGTATTACAATTGAAAACTACAATGACCTTAAAGAAGGCGATGTTA
TCGAAGCATTTGAAATGGTTGAAATTAAGCGTTAATTAAATAAATTACAAGCTAAAAGTATAGTTAAGATTGA
TATGCTCCCTATAAATATTGCACTTTTTAAGTGTCTACTTTATAGGGAGCATATTTGATACTAGCTTTTGGTT
TTTTATTAGAATAGATTACCTATTAAAAGTTACGTTATATGGACATGATTTTGTATAAAATTTTGTGGTGGCC
TAGAATGATTTTTAATGACAAAATATAATGTCGACTATTATTGGAAAATTTTCTGTTGAAATGCCTATCTTAC
GGCAAACTTTATTTGATTTTATAGGCTTAATTTATTAAAATAACGTGTGAGCTAAAATAATTGTTTAAGCATT
GTTACACTAAAAAATGCAAATAACAATTGAACTTAAAGATAAAGAGGTGACAAGAATGAGCAGTATGAGAGCA
GAGCGTGTTGGTGAACAAATGAAGAAGGAATTAATGGATATCATCAACAATAAAGTCAAAGATCCTCGAGTTG
GTTTTATTACAATTACAGATGTTGTTTTAACAAATGATTTATCGCAGGCTAAAGTATTTTTAACTGTATTAGG
TAACGATAAAGAAGTAGAAAATACATTTAAAGCACTTGATAAAGCAAAAGGCTTCATTAAGTCTGAATTAGGT
TCTAGAATGCGATTACGTATTATGCCGGAATTAATGTATGAATATGATCAATCAATCGAATATGGTAATAAAA
TTGAACGAATGATTCAAGATTTACACAAACAAGATAGATAATTTAGTGTTAGGTATCTGGAAAATGTTTGATA
ATTTCTTAATATCGGTATATTAACATTAAACAGTTAATACATAGATGTGTAGAAATAGTTAACATTTTCCAGT
TTTTTTATGAATAAATTCAGTTGAAACGCTATTAAAATATATTTTAAAAAAGAAGGTGACTATATGTATAATG
GGATATTACCAGTATATAAAGAGCGCGGTTTAACAAGTCATGACGTTGTATTCAAATTGCGTAAAATATTAAA
AACTAAAAAAATAGGTCACACGGGTACGCTTGATCCCGAAGTTGCAGGCGTGTTACCGGTATGTATAGGTAAT
GCAACGAGAGTTAGTGATTATGTTATGGATATGGGCAAAGCTTATGAAGCAACTGTATCGATAGGAAGAAGTA
CAACGACTGAAGATCAAACGGGTGATACATTGGAAACAAAAGGTGTACACTCAGCAGATTTTAATAAGGACGA
TATTGACCGATTGTTAGAAAGTTTTAAAGGTATCATTGAACAAATTCCGCCGATGTACTCATCCGTCAAAGTA
AATGGTAGAAAATTATATGAATATGCGCGTAATAATGAAACAGTTGAAAGACCAAAGCGTAAAGTTAATATTA
AAGACATTGGGCGTATATCTGAATTAGATTTTAAAGAAAATGAGTGTCATTTTAAAATACGCGTCATCTGTGG
TAAAGGTACATATATTAGAACGCTAGCAACTGATATTGGTGTGAAATTAGGCTTTCCGGCACATATGTCGAAA
TTAACACGAATCGAGTCTGGTGGATTTGTGTTGAAAGATAGCCTTACATTAGAACAAATAAAAGAACTTCATG
AGCAGGATTCATTGCAAAATAAATTGTTTCCTTTAGAATATGGATTAAAGGGTTTGCCAAGCATTAAAATTAA
AGATTCGCACATAAAAAAACGTATTTTAAATGGGCAGAAATTTAATAAAAATGAATTTGATAACAAAATTAAA
GACCAAATTGTATTTATTGATGATGATTCAGAAAAAGTATTAGCAATTTATATGGTACACCCTACAAAAGAAT
CAGAAATTAAACCTAAAAAAGTCTTTAATTAAAGGAGATAGAATTTATGAAAGTCATAGAAGTGACACATCCT
ATACAATCTAAACAGTATATTACAGAGGATGTTGCAATGGCATTCGGATTTTTCGATGGCATGCATAAAGGTC
ATGACAAAGTCTTTGATATATTAAACGAAATAGCTGAGGCACGCAGTTTAAAAAAAGCGGTGATGACATTTGA

TCCGCATCCATCTGTCGTGTTGAATCCTAAAAGAAAACGAACAACGTATTTAACGCCACTTTCAGATAAAATC
GAAAAAATTAGCCAACATGATATTGATTATTGTATAGTGGTTAATTTTTCATCTAGGTTTGCTAATGTGAGCG
TTGAAGATTTTGTTGAAAATTATATAATTAAAAATAATGTAAAAGAAGTCATTGCTGGTTTTGATTTTACTTT
TGGTAAATTTGGAAAAGGTAATATGACTGTTCTTCAAGAATATGATGCGTTTAATACGACAATTGTGAGTAAA
CAAGAAATTGAAAATGAAAAAATTTCTACAACTTCTATTCGTCAAGATTTAATCAATGGTGAGTTGCAAAAAG
CGAATGATGCTTTAGGCTATATATATTCTATTAAAGGCACTGTAGTGCAAGGTGAAAAAAGGGGAAGAACTAT
TGGCTTCCCAACAGCTAACATTCAACCTAGTGATGATTATTTGTTGCCTCGTAAAGGTGTTTATGCTGTTAGT
ATTGAAATCGGCACTGAAAATAAATTATATCGAGGGGTAGCTAACATAGGTGTAAAGCCAACATTTCATGATC
CTAACAAAGCAGAAGTTGTCATCGAAGTGAATATCTTTGACTTTGAGGATAATATTTATGGTGAACGAGTGAC
CGTGAATTGGCATCATTTCTTACGTCCTGAGATTAAATTTGATGGTATCGACCCATTAGTTAAACAAATGAAT
GATGATAAATCGCGTGCTAAATATTTATTAGCAGTTGATTTTGGTGATGAAGTAGCTTATAATATCTAGAGTT
GCGTATAGTTATATAAACAATCTATACCACACCTTTTTCTTAGTAGGTCGAATCTCCAACGCCTAACTCGGAT
TAAGGAGTATTCAAACATTTTAAGGAGGAAATTGATTATGGCAATTTCACAAGAACGTAAAAACGAAATCATT
AAAGAATACCGTGTACACGAAACTGATACTGGTTCACCAGAAGTACAAATCGCTGTACTTACTGCAGAAATCA
ACGCAGTAAACGAACACTTACGTACACACAAAAAAGACCACCATTCACGTCGTGGATTATTAAAAATGGTAGG
TCGTCGTAGACATTTATTAAACTACTTACGTAGTAAAGATATTCAACGTTACCGTGAATTAATTAAATCACTT
GGTATCCGTCGTTAATCTTAATATAACGTCTTTGAGGTTGGGGCATATTTATGTTCCAACCTTAATTTATATT
AAAAAAGCTTTTTACAAATATTAACATTTATTATATGTTAAGCTAATATTGAGTGAATAATAAGGTTACAATG
AGATAAAGATGATATAAGTACACCTAGAGTAATAATCAAGATATTAAAAATAAAGTATGTTTTTTTAAAAAAT
ATAACTTATATTTATACTGATAAGGGTGGGACGATAAGTCTATTTTGTAAATAATAGATGGATATCCCGCTCT
CTTTTTTTCCAATTCAATATTTTATAACTAATATTAAAATACGATAATAAATGATATGATATAACTATTAGAT
TCAAGAGAGGAGATTTATAATGTCTCAAGAAAAGAAAGTTTTTAAAACTGAATGGGCAGGAAGATCTTTAACG
ATTGAAACAGGGCAATTAGCTAAACAAGCAAATGGCGCTGTATTGGTTCGTTATGGAGATACAGTCGTGTTAT
CGACGGCAACTGCATCAAAAGAACCTCGTGATGGAGATTTCTTCCCATTAACAGTGAACTATGAAGAAAAAAT
GTACGCTGCGGGTAAAATTCCTGGTGGATTTAAAAAGAGAGAAGGACGTCCTGGTGACGATGCAACATTAACT
GCGCGATTAATTGATAGACCAATTAGACCTTTATTCCCTAAAGGATATAAGCATGATGTTCAAATTATGAACA
TGGTATTAAGTGCAGATCCTGATTGTTCACCACAAATGGCTGCAATGATTGGTTCATCTATGGCGCTTAGTGT
GTCGGATATTCCATTCCAAGGGCCAATCGCCGGTGTAAATGTGGGTTATATTGACGGTAAATATATCATTAAC
CCAACAGTAGAAGAAAAAGAAGTTTCTCGTTTAGACCTTGAAGTAGCTGGTCATAAAGATGCAGTAAACATGG
TAGAGGCAGGCGCTAGTGAGATTACTGAACAAGAAATGTTAGAGGCGATTTTCTTTGGTCATGAAGAGATTCA
ACGTTTAGTTGAGTTCCAACAACAAATCGTCGACCACATTCAACCTGTTAAACAAGAATTTATTCCAGCAGAG
CGTGATGAAGCGCTAGTTGAACGTGTAAAATCTTTAACCGAAGAAAAAGGACTTAAAGAAACAGTTTTAACAT
TTGATAAACAACAACGAGATGAAAATCTTGATAACTTAAAAGAAGAAATCGTCAATGAATTTATCGATGAAGA
AGATCCAGAGAATGAATTACTTATTAAAGAAGTTTATGCAATTTTAAATGAATTAGTGAAAGAAGAAGTTCGA
CGTTTAATTGCAGATGAAAAAATTAGACCAGACGGCCGTAAACCTGATGAAATCCGTCCATTAGATTCTGAAG
TTGGTATTTTACCTAGAACGCATGGTTCAGGTCTATTTACACGTGGTCAGACTCAAGCACTTTCAGTTTTAAC
ATTAGGTGCTTTAGGCGATTATCAATTAATTGATGGTTTAGGACCTGAAGAAGAAAAAAGATTCATGCATCAT
TATAACTTCCCGAATTTTTCAGTAGGTGAAACTGGTCCAGTACGTGCGCCAGGTCGTCGTGAAATTGGACATG
GTGCGTTAGGTGAAAGAGCATTAAAATATATTATTCCTGATACTGCTGATTTCCCATATACAATTCGTATTGT
AAGTGAGGTACTTGAATCAAACGGTTCATCATCTCAAGCGTCAATTTGTGGATCAACATTAGCATTAATGGAT
GCGGGCGTACCGATTAAAGCACCAGTTGCTGGTATTGCTATGGGCCTTGTTACACGTGAAGATAGCTATACGA
TTTTAACTGATATCCAAGGTATGGAAGATGCATTAGGTGATATGGACTTTAAAGTCGCTGGTACTAAAGAAGG
TATTACAGCAATCCAAATGGATATTAAAATTGATGGTTTAACGCGTGAAATTATCGAAGAGGCTCTAGAACAA
GCGAGACGTGGTCGTTTAGAAATAATGAATCATATGTTACAAACAATTGATCAACCACGTACTGAATTAAGTG
CTTACGCGCCAAAAGTTGTAACTATGACAATTAAACCAGATAAGATTAGAGATGTTATCGGACCTGGTGGTAA
AAAAATTAACGAAATTATTGATGAAACAGGCGTTAAATTAGATATTGAACAAGATGGTACTATCTTTATTGGG
GCCGTTGATCAAGCTATGATAAATCGTGCTCGTGAAATCATTGAGGAAATTACACGTGAAGCGGAAGTAGGTC
AAACTTATCAAGCCACTGTTAAACGTATTGAAAAATACGGTGCGTTTGTAGGCCTATTCCCGGGTAAAGATGC
GTTGCTTCACATTTCACAAATTTCAAAAAATAGAATTGAAAAAGTGGAAGATGTATTAAAAATCGGTGACACA
ATTGAAGTTAAGATTACTGAAATTGATAAACAAGGTCGAGTAAATGCTTCACACAGAGCATTAGAAGAATAAT
ATTTAAAGTCATATGACGACAATGTATCGTCATGTGATTTTTTTATGCAATTTTTTACGAAGTGATCCATTTT
GAATTTGTTGTATTGAACATTTTAAAACGCTTTATTATTTTGTGTGTAACTGTTAATTATCCTGTATGTATAG
TGATTAATAGTGTACATCAAGTGTTTTTTAACTTATAATGAATAGTGAGTTTATATATGGACGGGTAACAAAT
TTAGGAGGTAAGATTTTGAGTTTAATAAAGAAAAAGAATAAAGATATTCGCATTATACCATTAGGCGGTGTTG
GCGAAATTGCTAAAAATATGTATATCGTTGAAGTAGACGATGAAATGTTTATGTTAGATGCTGGACTTATGTT
TCCAGAAGACGAAATGCTAGGTATTGATATTGTTATACCAGACATTTCATACGTACTTGAAAATAAAGATAAA
TTGAAGGGTATATTCCTTACACACGGACATGAGCACGCGATTGGTGCAGTGAGTTATGTTTTAGAACAATTAG
ATGCACCAGTATATGGATCTAAATTGACAATAGCGTTAATTAAAGAAAATATGAAAGCCCGTAATATTGATAA

AAAAGTTCGCTACTACACAGTTAATAATGATTCAATTATGAGATTCAAAAACGTGAATATTAGTTTCTTTAAT
ACGACACACAGTATTCCTGATAGTTTAGGTGTTTGTATTCATACTTCATATGGTGCCATTGTGTATACAGGTG
AATTTAAGTTTGACCAAAGTTTACATGGACATTATGCACCAGATATTAAACGTATGGCAGAGATTGGTGAAGA
AGGCGTATTTGTCTTAATCAGTGATTCTACTGAGGCAGAGAAACCTGGATATAATACTCCGGAAAATGTGATT
GAACATCATATGTATGACGCTTTTGCAAAAGTGCGAGGTCGCTTGATTGTTTCATGTTATGCTTCGAACTTTA
TACGTATACAGCAAGTTTTAAATATTGCTAGCAAGCTAAATCGTAAAGTGTCATTTTTAGGAAGATCACTTGA
AAGTTCATTTAATATTGCTCGTAAAATGGGGTATTTCGACATTCCTAAAGATTTGCTAATTCCTATAACAGAA
GTTGATAATTATCCTAAAAATGAAGTGATAATTATAGCTACTGGTATGCAAGGAGAACCTGTAGAAGCCTTAA
GTCAAATGGCGCAACATAAGCATAAAATTATGAATATCGAAGAAGGCGATTCTGTATTTTTAGCAATTACGGC
TTCTGCTAATATGGAAGTTATCATTGCGAATACATTAAATGAGCTTGTACGTGCTGGCGCACATATTATTCCA
AATAACAAGAAGATTCATGCTTCAAGTCATGGTTGCATGGAAGAATTAAAAATGATGATTAATATTATGAAAC
CTGAATACTTTATTCCTGTACAAGGTGAATTTAAAATGCAGATAGCACATGCGAAGCTAGCAGCTGAAGCAGG
TGTTGCACCAGAAAAGATTTTCCTTGTGGAAAAAGGAGATGTCATTAATTACAACGGTAAAGATATGATATTA
AATGAAAAGGTAAATTCAGGAAATATTTTAATAGATGGTATTGGTATTGGGGATGTAGGAAATATCGTGTTGA
GAGACCGTCATCTTTTAGCAGAAGATGGTATCTTTATTGCTGTAGTAACGTTAGATCCTAAAAATAGACGTAT
AGCTGCGGGACCTGAAATTCAATCTCGTGGGTTTGTATATGTACGTGAAAGTGAAGACTTATTACGTGAAGCA
GAAGAGAAAGTACGTGAAATAGTAGAGGCTGGTTTACAAGAAAAACGCATAGAATGGTCTGAAATTAAGCAAA
ATATGCGTGATCAAATTAGTAAACTATTATTCGAAAGTACAAAACGTCGTCCTATGATTATTCCAGTAATTTC
TGAAATTTAATCAAAAAGTCATTAACATAAAAGAGGTCAGAACAAGTCACTGAAATATAATGGTTGTCATGGA
CAATTTACTTATATTTTATGATGGTCAATTGAAAGGGGTAACGATTAATCTGTTATCTTAAGTAAATTGATAC
ATAGATGATATTGTTCTAACCTCTTTCATCGTCTGTTTGGACTACATATTCTAAACATCAAATAGGAAATTAT
ATATAATAACGTCGTTTTAACTAAGGCAACATAAGGAGGTGCGTCAATTGGCACAAGCAAAAAAGAAATCGAC
AGCTAAGAAAAAAACAACATCAAAAAAAAGAACAAATTCGAGGAAAAAGAAGAATGATAATCCGATACGTTAT
GTCATAGCTATTTTAGTAGTTGTATTAATGGTGTTGGGTGTTTTCCAATTAGGAATAATAGGTCGTCTAATTG
ACAGCTTCTTTAATTATTTATTTGGGTACAGTAGATATTTAACATATATTTTAGTACTCTTAGCAACTGGTTT
TATTACATACTCTAAACGTATTCCTAAAACTAGACGAACGGCTGGTTCGATTGTATTGCAAATTGCATTGCTA
TTTGTATCACAGTTAGTTTTTCATTTTAATAGTGGTATCAAAGCTGAAAGAGAACCTGTACTTTCTTATGTGT
ATCAGTCATACCAACACAGTCATTTCCCAAATTTTGGTGGCGGTGTATTAGGCTTTTATTTATTAGAGTTAAG
CGTACCTTTAATTTCATTATTTGGTGTATGTATTATTACTATTTTATTATTATGCTCAAGTGTTATTTTTATTA
ACAAACCATCAACATCGTGAAGTAGCAAAAGTTGTACTGGAAAATATAAAAGCTTGGTTTGGTTCATTTAATG
AAAAAATGTCGGAAAAAAACCAAGAAAAACAATTGAAGCGTGAAGAAAAAGCGAGACTTAAAGAAGAACAAAA
GGCACGTCAAAATGAACAGCCACAAATAAAAGATGTGAGTGATTTTACGGAAGTGCCTCAAGAAAGAGATATT
CCAATTTATGGGCATACTGAAAATGAAAGTAAAAGTCAGAGTCAACCAAGTCGGAAAAAGCGAGTGTTCGATG
CAGAGAATAGTTCGAATAACATCGTAAATCATCATCAAGCAGATCAGCAAGAACAATTAACAGAACAAACTCA
TAACAGTGTTGAAAGTGAAAACACTATTGAAGAAGCTGGTGAAGTTACGAATGTATCGTATGTTGTTCCACCG
TTAACTTTACTTAATCAACCTGCAAAACAAAAAGCAACATCTAAAGCTGAAGTGCAACGTAAAGGACAAGTAC
TAGAGAATACATTAAAAGATTTTGGGGTAAATGCAAAAGTGACACAAATTAAAATTGGTCCTGCAGTAACTCA
ATATGAAATTCAACCAGCTCAAGGGGTTAAAGTGAGTAAAATTGTAAACCTGCACAATGATATCGCATTAGCT
TTAGCAGCAAAAGATGTTAGAATCGAAGCGCCAATACCTGGTCGCTCTGCAGTAGGTATTGAAGTGCCAAATG
AGAAAATTTCATTAGTTTCACTAAAAGAAGTTTTAGATGAAAAATTCCCGTCTAATAATAAACTAGAAGTTGG
ATTAGGAAGAGATATATCAGGTGATCCAATTACTGTTCCACTAAATGAAATGCCACACTTATTGGTGGCAGGA
TCGACGGGTAGTGGTAAATCTGTTTGTATAAATGGTATTATTACAAGTATTTTATTAAATGCTAAACCGCATG
AAGTTAAACTTATGTTAATCGATCCGAAAATGGTTGAACTAAATGTTTATAACGGAATTCCACATTTATTAAT
TCCGGTTGTTACAAATCCTCATAAAGCTGCTCAAGCTTTAGAAAAAATTGTAGCTGAGATGGAAAGACGTTAT
GATTTATTCCAACATTCATCAACTAGAAATATTAAAGGTTATAACGAATTAATCCGTAAGCAAAATCAAGAAT
TAGATGAGAAGCAACCAGAATTACCTTATATCGTTGTTATTGTAGATGAGCTTGCAGATTTAATGATGGTAGC
TGGTAAAGAAGTTGAAAATGCGATTCAACGTGTCACACAAATGGCACGTGCAGCAGGTATACATTTGATTGTA
GCGACACAAAGACCTTCTGTGGATGTAATTACAGGTATCATTAAAAATAATATTCCATCTAGAATAGCTTTTG
CTGTGAGTTCTCAAACAGATTCAAGAACTATTATTGGTACTGGCGGCGCAGAAAAGTTACTTGGTAAAGGTGA
CATGTTATACGTTGGAAATGGTGACTCATCACAAACACGTATTCAAGGGGCGTTTTTAAGTGACCAAGAGGTG
CAAGATGTTGTAAATTATGTAGTAGAACAACAACAGGCAAATTATGTAAAAGAAATGGAACCAGATGCACCAG
TGGATAAATCGGAAATGAAAAGTGAAGATGCTTTATATGATGAAGCGTATTTGTTTGTTGTTGAACAACAAAA
GGCAAGTACATCATTGTTACAACGCCAATTTAGAATTGGTTATAATAGAGCATCTAGGTTGATGGATGATTTA
GAACGCAATCAGGTAATCGGTCCACAAAAAGGAAGCAAGCCTAGACAAGTTTTAATAGATCTTAATAATGACG
AGGTGTAAAAAAATGTCAGAAATGAATGCGGTATATAACGTTAAACAATACATTTTAAATTTGATTAAGCAAA
ATAAATTGGAATATGGTGACCAACTTCCAAGTAATTTATCAATTGCCAGAGAATTAAATGTAAAAACCGACGA
TGTTTATGAAGCAATTCAGGCATTGATTACTGAACAAGTCATTAAAGATAATTTTGAAGAGGGCACAAGTGTT
AAGTCACTGCCCCCTTTCTTTTATCCATTGAATGAACTTATAAGTATTGGGCAAATGATTAAAAATGCAGGAT

TTGAATGCGGAACTGAATACTTAAATTTTGATGAGCAACCAGCAACTATGTTAGATGCAAATTTGTTGAGCGT
TGAAGAAGGATATCCAGTAACCATTATAGAACGATTACGAACTGCCGATGGAGAACCGGTCGTCTATTGTTTA
GATAAAATTGCTAAAAAAGAATTAACATGTACAGAGTATCAAATGAGCAATGGATCGATTCTAAGTGCGATAA
AAGAACAAAGTAATCATAATATTTGTTATGCTGATACAGAAATTGAAGCGGTAAATTATGAACCTCGAATATC
CGAAGTACTGAATGCTTCGCCACACGAAGGTTTGATTTTATTAAAAATTACGCACTATAATGAATCAGATGAA
CCTATTTTGTATTCATTAAATTATATGAAAAATAGCTTAGTTCAATTTAAAATCACTAGAAAAATATAGATGT
AAGAAGATATAGAGGAGGCTATATATTTGAGTAGACAATCTCAACCAAATATACATATCAAAGTTTCACCAAC
AACTAAATTTAAAACAACTACTATAGTTTTTAAATTTATGGCACCTTTAGAATATGACACAATAACAGCTAGA
TCATTATTAAGCAAATTATTAGTTCGAGCAACTAAGAAATGGCCAACCGATAAGTCGTTTAATAATCACTTAG
CCGATTTATATGGTGCGTATGTGAATAGTACAATTTCAAAATTCAAAGATCAGCATGTCATTACATTTTCATT
AGAAATTGTTAATGAACGTTATTTAAGAAACGGTGAATCATTATTTAATCAAGGATTAGATTTATTACACGAA
ATCATTTGGAATCCATTAATTGAAAATAAAGCATTCAATGATAATTTTGTTAACCAAGAGAAAACATTATTAG
CCAAAAAAATAGAAGCAATGGTAGATAATAAAGCACAATATTCGTTTTTAAAATTACTCGACCATATGTTTGA
AAATGAAGCATATAAATACTTATCTACAGGACAACTAGAACAAATCCCACATATTACTGCTGAAACACTATAT
CATACATATCAATCAATGATTAATAATGATCAATGTTCTGTTTATGTTGTCGGCAATGTAGAACCTGAAAGTG
TTGAGAAACAAATACGTGAAAAATTTGCACTTAAACCATTCGATAAACATCAATTCCAACATTCTACTCATCA
TTTACACGATGAAGAAGTTGATTATATTGTTGAATATGATGACGTGGATCAAGCTAAATTAAATATGGGATAC
CGTTTTCCAACACAATATGGACAAAGTGGATATGCTGCCTTTGTTGTATTTAACATGATGTTTGGAGGAGATC
CTTCATCTGTTTTATTTAATGAAGTGCGAGAAAAGCAAAGTTTAGCGTACTCTATACATTCACAAATTGATGG
CAAAAATGGCTATTTATTTGTTTTGAGTGGGGTTTCAAGTGATAAGTACGAAACTGCAAAAGATACTATTATA
AGTGAATTTGAAAAAAATAAAAGCAGGAGATTTCACTGAAGAAAAATTAGAGTTAGCTAAAAAAGTAATCATTT
CTCATCGATATGAATCTGAAGATCGTCCGAAAAGTATTATAGAGATTATGCATAACCAAATATTATTAGAGCA
ACCACAAAGCAAAGAAACATTTATAAATGATATACAGAAGGTAAGTCGCGAAGATATTGTTACTGTTGCTGAA
AAAGCATTTTTAGATACAATCTATGTGTTGACAAAAGGAGGGGATAAATAATGAAAGAGCGTTATTATGAATT
AATAGACGAAAGAGTATTCGAACAAGAATTAGAAAATGGTTTACGATTATTTATTATCCCCAAACCAGGTTTT
CAAAAGACATTTGTCACTTACACTACACAATTTGGTTCATTAGATAATCAATTCAAACCCCTCGGACAAGACC
AATTTGTTACTGTACCTGATGGAGTTGCTCACTTTTTAGAACATAAATTATTTGAAAAAGAAGAAGAAGACTT
ATTTACTGCGTTTGCTGAAGATAATGCACAAGCAAATGCGTTTACAAGCTTTGATCGTACAAGCTACTTGTTC
AGTGCAACTGATAATATTGAAAACAACATTAAACGTTTACTTACAATGGTTGAAACGCCTTATTTTACAAAAG
AAACTGTTGATAAAGAAAAAGGTATTATTGCAGAAGAAATAAAAATGTATCAAGAACAACCTGGATATAAATT
AATGTTTAATACATTGCGTGCAATGTATCAACAACATCCAATACGTGTTGATATTGCCGGTAGTGTAGAAAGT
ATATACGATATTACAAAAGATGATTTGTATCTATGTTATGAAACGTTTTATCATCCATCAAATATGGTTTTAT
TTGTTGTTGGTGATGTGAATCCAGAAGAAATATGTCGAATAGTAAAACAGCACGAGGATGCTCGTAATAAAGT
AAACCAACCTAAAATCGAACGAGGACTTGTTGATGAACCGGAGGATGTTAAAGAAGCATTTGTTACTGAATCT
ATGAAAATTCAATCACCAAGACTAATGCTTGGTTTTAAAAATAAACCATTACAAGAAGCGCCTCAAAAATATG
TACAACGTGATTTAGAAATGTCATTATTCTTTGAGTTAATTTTTGGGGAAGAAACAGATTTTTATCAGAATTT
ATTAAACGAAGGACTTATCGATGATACATTTGGTTATCAATTTGTACTAGAGCCGACGTATAGTTTTTCAATC
GTGACAAGCGCTACTGAAGAACCAGATAAATTAAAAAAATTATTATTAGATGAGTTGCGTGATAAAAAAGGCA
ATTTCCAAGATGCACAAGCATTTGAACTTTTGAAAAAGCAATTTATAGGTGAATTCATATCAAGTTTAAACTC
ACCTGAATATATTGCTAATCAATATACTAAATTGTATTTTGAAGGTGTTAGCGTGTTTGACATGTTAGACATT
GTTGAAAATATCACGTTAGATAGTATTAACGAAACGTCATCGTTATATTTAAATTTAGATCAGCAAGTCGATA
GTCGTTTGGAGATTAAAAAGTAATGAAAGCATTAGTATTAGGTGGTTCTGGTTCAATTGGTTCTGAGATAGTC
AAACAATTATTAACTGATGGATTTGAAGTTTATGTGCAATATTATCGTACTGATATAAATGAATTAACTAGCA
AATTTAATGATGATAAAGTTCATTTTATACAAACGGATTTATCTCAAACAACTGATATTGACAAAACATTTGG
TGACATTAAATCATTAGACTGTTTAATATATGCAAGTGGTCAGTCTTTATATGGTGTTTTACAAGATATGAAA
GACCATGATATTGATGCATGTTATCAGTTAAATGTCTTGCAATTAATTCGATTATGTAGATATTTCGTTGATG
TTTTACGTCAAAGTGACAATGGAAGAATTATTGTAATTTCATCAATTTGGGGTGAGACAGGAGCTAGTATGGA
AACTATTTATTCGACGATGAAAAGTGCACAATTAGGTTTCGTTAAGGCGCTTAGTCAAGAGCTTGCACTAACA
TCAGTGACAGTAAATGCTATCGCACCTGGATTTGTAGCCGGTAATATGGCAAGTGAGTGGCAAGAAGATGAAC
TTCAAGCAATGATAACTGAATTACCACAACAGCGATTGGTTTACCGAGTGAGGTTGCTCATACATGCGCCTA
TTTATATCACCCAAATGCTAGAAGTGTTACTGGAACTATACAGAAAGTTAATGGTGCTTGGTATATATAAAAT
ATATAACTATTTTTATGAGCAAATGTTTTTATGATTGATGTTATAATATGAATATTACAATGAATCATAATTT
GTTAATTAAACAAGGGGTGAGGTTCACCATGACAGTTGCAGAGAAAAAAGAATGGTACCTAGAATACGAAATT
GAAATTAATAGACCGGGTCTTTTAGGTGATGTATCTAGTTTATTAGGTATGTTAGGTATAAGTATTGTTACAA
TTAATGGTGTTGATCAAGGTAAACGAGGCCTTTTAATTAAAACTGACAATCTTGAAAAAGTTGAACGTTTTGA
GCAAATAGCTCGTGGTATAAATGAAATTGAAATAACAAAGCTTAAAAAACCAGAATTAAGAGACCGTCTTGCA
GTTAGACATGGTAGATATATTGAGCAAGATGCAAAAGATAAGAAAACCTTTCGATTTGAGCGTGAAGATTTAG
GTTTGTTAGTAGACTTTTTAGCTGAATTGTTCAAAGAAGAAGGTCATAAGTTGATTGGCATTAGAGGTATGCC

ACGGGTTGGTAAAACTGAATCAATTGTTGCGGGAAGTGTTTGTGCACATAAGAGATGGTTATTTATTAGTTCT
ACTTTAATAAAACAAACTGTACGTAGCTCTCTAATTAAAGGGGAATATGATGCCAATCATGTATACATTATTG
ATGGTGCAGTTACTGCCAGAGAATCTAATCCAAAACATCAAGAGCTTGTTAACGAAGTTATGACGTTACCATC
AATCAAAGTCGTTGAACATCCAGATTTATTTGTTGAAACAAGTACTTGTACAATGGAAGATTTTGACTATATT
ATCGAATTGAGAGAAAATGAAAATCAAGAAATACATTACGAAGAAATGAAGAAACAGACAGTCCAAAGTAAGA
ATAATTTAGATTTTGGAGATCCATTTGGTGGTGGTTTTGGTTTCTTCGAGTAAGTGTAAGGAGGCTATGAATT
GAAAACGGTCGGTGAAGCGCTAAAAGGTAGACGTGAAAGGTTAGGAATGACTTTAACAGAATTAGAGCAACGT
ACTGGAATTAAACGTGAAATGCTAGTGCATATTGAAAATAATGAATTCGATCAACTACCGAATAAAAATTACA
GCGAAGGATTTATTAGAAAATATGCAAGCGTAGTAAATATTGAACCTAACCAATTAATTCAAGCTCATCAAGA
TGAAATTCCATCGAACCAAGCCGAATGGGACGAAGTAATTACAGTTTTCAATAATAATAAAGATTTAGATTAT
AAGAGTAAATCAAAAGAGCCAATACAATTATTAGTAATCATGGGTATTACAGTTTTAATAACTTTATTGTTAT
GGATCATGTTAGTTTTAATATTTTAACAGAAATAAATTAGTGAGAAATGAGGATGTATAATGAATATTCCGAA
CCAGATTACGGTTTTTAGAGTAGTGTTAATACCAGTTTTTATATTGTTTGCGTTAGTTGATTTTGGATTTGGC
AATGTGTCATTTCTAGGAGGATATGAAATAAGAATTGAGTTATTAATCAGTGGTTTTATTTTTATATTGGCTT
CCCTTAGCGATTTTGTTGATGGTTATTTAGCTAGAAAATGGAATTTAGTTACAAATATGGGGAAATTTTTGGA
TCCATTAGCGGATAAATTATTAGTTGCAAGTGCTTTAATTGTACTTGTGCAACTAGGACTAACAAATTCTGTA
GTAGCAATCATTATTATTGCCAGAGAATTTGCCGTAACTGGTTTACGTTTACTACAAATTGAACAAGGATTCG
TAAGTGCAGCTGGTCAATTAGGTAAAATTAAAACAGCAGTTACTATGGTAGCAATTACTTGGTTGTTATTAGG
TGATCCATTAGCAACATTGATTGGTTTGTCATTAGGACAAATTTTATTATACATTGGCGTTATTTTTACTATC
TTATCTGGTATTGAATACTTTTATAAAGGTAGAGATGTTTTTAAACAAAAATAAATATTTGTTTATACTAGAT
TTCATTTTCATATGGAATCTAGTTTTTTTAATCCCAATTTTAGAAATTAGCCACGCAATTGTTTATAATGATA
TATTGTAAAACGATATTTGTTCATTTTTTAGGCAAATGTGTTGTAGCATCTGATACATTGAATCTAAAATTGA
TGTGAATTTTTAAATGAAATACATGAAAAAATGAATTAAACGATACAAGGGGGATATAAATGTCAATTGCCAT
TATTGCTGTAGGCTCAGAACTATTGCTAGGTCAAATCGCTAATACCAACGGACAATTTCTATCTAAAGTATTT
AATGAAATTGGACAAAATGTATTAGAACATAAAGTTATTGGAGATAATAAAAAACGTTTAGAATCAAGTGTAC
GTCATGCGTTAGAAAAATATGATACTGTTATTTTAACAGGTGGCTTAGGTCCTACGAAAGATGACTTAACGAA
GCATACAGTGGCCCAGATTGTTGGTAAAGATTTAGTTATTGATGAGCCTTCTTTAAAATATATTGAAAGCTAT
TTTGAGGAACAAGGACAAGAAATGACACCTAATAATAAACAACAGGCTTTAGTAATTGAAGGTTCAACTGTAT
TAGCAAATCATCATGGCATGGCTCCAGGAATGATGGTGAATTTTGAAAACAAACAAATTATTTTATTACCAGG
TCCACCGAAAGAAATGCAACCAATGGTGAAAAATGAATTGTTGTCACATTTTATAAACCATAATCGAATTATA
CATTCTGAACTATTAAGATTTGCGGGAATAGGTGAATCTAAAGTAGAAACAATATTAATAGATCTTATCGATA
AACAGACTAATCCTACGATTGCGCCTTTGGCGGGAAGTCATGAAGTATATATTAGATTGACTGCAAATGCCGA
CTCAAAAGAACAAGCACAATCATTGATTCAACCTGTTAAACAAGAAATTCTTGATCGTATTGGAGAATATTAT
TATGGTTCAGATGACACATTAATTGAGCAAGCTGTAATAAAGAAAATTCATGAACCTTTTGTAATATATGATG
GTATTACTAATGGTGCTTTATATCATCGATTGAAAGAAGTGGATTTAAACGATGTTCTAAAGGGTATGATTAA
TCACAATGAAAACTTTGTTGATATTAATAAACCTATTGAGCAGCAATTAAAAGATGCAGTGCAATTTGTTAAT
AAATTGTTTAATGTGTCATCAGCAATTATTCTATTAGAGTATGATGGTGTAGTCCATATAGGCTATGATAATA
ACTTTGAATTTAAAACTGAGCAATTTAAAATGTCTAAATCTAGAAATTTATTAAAGAACAGAAGTCAAAATTA
TGCGCTCATAAGATTATTAAATTGGCTTAGAACAACAAATTAATTGTATTATCGATAAAAATATAAGCACGTT
TGTTCGTTTTTGCGTTTTGATTTCAAGATTTTATACGAACAAATATTCGCAAAACACTTGTATTTTATTTTGA
ATCCTTGTATAGTATTGGTAAGATAATTAAAAGATAGCAATTTCAATTAGGAGGTCTCGCTTTGGATAACGAT
CGTCAAAAAGCTTTAGATACAGTAATTAAAAATATGGAGAAATCTTTCGGTAAAGGTGCCGTAATGAAGTTGG
GTGACAATATAGGTCGCCGAGTTTCAACTACATCAACTGGTTCAGTTACATTAGATAATGCGCTAGGTGTAGG
TGGCTATCCTAAAGGACGAATTATTGAAATTTATGGTCCTGAAAGTTCTGGTAAGACAACAGTAGCGCTTCAC
GCTATTGCTGAAGTACAAAGTAATGGCGGGGTGGCAGCATTTATCGATGCTGAACATGCTTTAGATCCAGAAT
ATGCTCAAGCATTAGGCGTGGATATCGATAATTTATATTTATCGCAACCGGATCATGGTGAACAAGGTCTTGA
AATCGCCGAAGCATTTGTTAGAAGTGGTGCAGTTGATATTGTAGTTGTAGACTCAGTTGCTGCTTTAACACCT
AAAGCTGAAATTGAAGGAGAAATGGGAGACACTCACGTTGGTTTACAAGCTCGTTTAATGTCACAAGCGTTAC
GTAAACTTTCAGGTGCTATTTCTAAATCAAATACAACTGCTATTTTCATCAACCAAATTCGTGAAAAAGTTGG
TGTTATGTTCGGTAATCCAGAGACTACACCAGGTGGACGTGCATTAAAATTCTATAGTTCAGTAAGACTAGAA
GTACGTCGTGCAGAACAGCTTAAACAAGGACAAGAAATTGTAGGTAATAGAACTAAAATTAAAGTCGTTAAAA
ATAAAGTGGCACCACCATTTAGAGTAGCTGAAGTTGATATTATGTATGGACAAGGTATTTCTAAAGAGGGTGA
ACTTATTGATTTAGGTGTTGAAAACGACATCGTTGATAAATCAGGAGCATGGTATTCTTACAATGGCGAACGA
ATGGGTCAAGGTAAGGAAAATGTTAAAATGTACTTGAAAGAAAATCCACAAATTAAAGAAGAAATTGATCGTA
AATTGAGAGAAAAATTAGGTATATCTGATGGTGATGTTGAAGAAACAGAAGATACACCAAAGTCATTATTTGA
CGAAGAATAGTACACAAATTTATATCTATAGTTAAACTTAGCAAATATCCTTATAGGATTGATTGAAAGTGAT
ATTCATCTCATAAAGCTAGAATAATATCTAACTTTATGGGATGCACTACAAATCGAGACTATAAGGTTTTTTA
TTTTATTTATTATTACATTATCAATAGTTTTATAATCGAGCTTCAAAACTTTAGAAAATAGTAGAAATAGCAT

TCAATATAGTGCAAAAGTGCAAATTGATAACTTGACACTTATCTCCTATAAACCGTACAATTAATTTGTATGA
TTTATATATAATTTCATAAAGTCATATTGAATTTCATATAAAGAGCAAACCCTAGAAAAGGAGGTGTTTGTGT
GAATTTATTAAGCCTCCTACTCATTTTGCTGGGGATCATTCTAGGAGTTGTTGGAGGGTATGTTGTTGCCCGA
AATTTGTTGCTTCAAAAGCAATCACAAGCTAGACAAACTGCCGAAGATATTGTAAATCAAGCACATAAAGAAG
CTGACAATATCAAAAAAGAGAAATTACTTGAGGCAAAAGAAGAAAACCAAATCCTAAGAGAACAAACTGAAGC
AGAACTACGAGAAAGACGTAGCGAACTTCAAAGACAAGAAACCCGACTTCTTCAAAAAGAAGAAAACTTAGAG
CGTAAATCTGATCTATTAGATAAAAAAGATGAGATTTTAGAGCAAAAAGAATCAAAAATTGAAGAAAAACAAC
AACAAGTAGATGCAAAAGAGAGTAGTGTTCAAACGTTAATAATGAAGCATGAACAAGAATTAGAACGCATCTC
CGGTCTCACTCAAGAAGAAGCTATTAATGAGCAACTTCAAAGAGTAGAGGAAGAACTGTCACAAGATATTGCA
GTACTTGTTAAAGAAAAAGAAAAAGAAGCTAAAGAAAAAGTTGATAAAACAGCAAAAGAATTATTAGCTACAG
CAGTACAAAGATTAGCAGCAGATCACACAAGTGAATCAACGGTATCAGTAGTTAACTTACCTAATGATGAGAT
GAAAGGTCGAATCATTGGACGAGAAGGACGAAACATCCGCACACTTGAAACTTTAACTGGCATTGATTTAATT
ATTGATGACACACCAGAAGCGGTTATATTATCTGGTTTTGATCCAATAAGAAGAGAAATTGCTAGAACAGCAC
TTGTTAACTTAGTATCTGATGGACGTATTCATCCAGGTAGAATTGAAGATATGGTCGAAAAAGCTAGAAAAGA
AGTAGACGATATTATTAGAGAAGCAGGTGAACAAGCTACATTTGAAGTAAACGCACATAATATGCATCCTGAC
TTAGTAAAAATTGTAGGGCGTTTAAACTATCGTACAAGTTACGGTCAAAATGTACTTAAACATTCAATTGAAG
TTGCGCATCTTGCTAGTATGTTAGCTGCTGAGCTAGGCGAAGATGAGACATTAGCGAAACGAGCTGGACTTTT
ACATGATGTTGGTAAAGCAATTGATCATGAAGTAGAAGGTAGTCATGTTGAAATCGGTGTAGAATTAGCGAAA
AAATATGGTGAAAATGAAACAGTTATTAATGCAATCCATTCTCATCATGGTGATGTTGAACCTACATCTATTA
TATCTATCCTTGTTGCTGCTGCAGATGCATTGTCTGCGGCTCGTCCAGGTGCAAGAAAAGAAACATTAGAGAA
TTATATTCGTCGATTAGAACGTTTAGAAACGTTATCAGAAAGTTATGATGGTGTAGAAAAAGCATTTGCGATT
CAGGCAGGTAGAGAAATCCGAGTGATTGTATCTCCTGAAGAAATTGATGATTTAAAATCTTATCGATTGGCTA
GAGATATTAAAAATCAGATTGAAGATGAATTACAATATCCTGGTCATATCAAGGTGACAGTTGTTCGAGAGAC
TAGAGCAGTAGAATATGCGAAATAATTTTTGTCTCCCTCACAAATTAGTGAGGGAGCTTTTTTAAGTTGTAGT
CTCAATCTAGTTAGACAGCACTTTATTGGTAATAACTATATTAAACAGTAGTTATTTGAAAGTAAGTCGGACC
GTATATAAAATAAGAAGTTATTGCTTTTAATAAAAATGTTTTAGGCTTCATAATTACTATATTTATATTATGT
AAACCTATAAAGATGATTGCTTTTCTATCCAATAAAAAAGAGGCTGGAACATTAAGTTCTTAGGCAATGTAAA
AAAGCTGATTTCTATTAATTATTTGATAGAAATCAGCTTTTCTGATATGTATTTTATAATGTACAGCTCGTTG
AGCTGCTATTTTTCTTATATTAAGTGCCGTTAATACAAAACCTAGGTCTCGTTTAACTTTATTTATTCCTCGA
ACTGACATTCGAGTGAAACCCAAAATAGCCTTCATAAATCCAACAACAGACTCTACAACAATTTTTCTTTGAC
TATAGATTTTTTTCGTTTCTGGTTCAGAAAGCTTTTGATTAACTTGGGCTTTAAAGTATTCACAATTATAATT
CTTCATAATTTTCTTATTGGATTTTGAATTTGGCTTCATGCATTGATGTCTCAAAGAACATGCTGAACAGTCA
TCACATTCATATAGTTTGAAGTCACGTTTAAAACCATATCTATCATTACGGTATGCATATCTTTTAAAACCTA
TTCTTTTGTTATTAGGACATATAAATTCATCATTAAGTTCGTCATATTTCCAATTTTGAGTGTTAAAAATGTC
ACTTTTAAACTTTCTAG
>DSM104437_scaffold_9
CGACGGCTTAGGTGCTACTTCAGCCACTAGATACGGTGATAATCGTACTAACTATAGATTCGATGTTGGACAG
GCTGTATACGCGCCTGGAACATTAATATATGTGTTTGAAATTATAGATGGTTGGTGTCGCATTTATTGGAACA
ATCATAATGAGTGGATATGGCATGAGAGATTGATTGTGAAAGAAGTGTTTTAATATAATGTTTGCTTAAATGT
TGTATTGTGATATAATAACATTATTCTTTAGATAACATTACTCTCAAGATTTAAATGTGCATAACAGGCAGGT
ACTTCGGTACTTGCCTATTTTTTTTATGTTATACTTACTTTTATATAGTAGGAGTGAACTATATAGCCGGGCA
GAGGCCATGTATCTGACTGTTGGTCCCACAGGAGACATCTTCCTTGTCATCACTCGATACATATATCTTGATA
ACATAGAGTTGTTACAGTCGCTACACCACTCATACTAGTTACTGGGTGGTTTTTTATATGTTTAAAAACCGTT
ATAAATGCTATAATAATGCTATACATGTGATAAAATGAGGTAATATATATGAACGAAAAAACTGGTATAGACC
AATTTATGAGAAAAGAAATCGAAAGTTTAGGCGTAAGTTACGATGAACAACAAAGCAGCAATGTTGAAATAGC
GGAAGCGCTAAAAACAGCAAGTAAAAGTTTAAGCGGAAAAGTAGGTAAACCAGAATTTTTATTTTTTAGCGAT
GAATTTTTAGTCGTTGTTGAGGATAAAAAAGATATACATAAGCATGAAATGAAGTCTGAACGAGGTGAACTAA
TACTAGATTCACCAGAAATATTGAAAGATTATGCTGTAAATGGCGCAGTGCATTATGCGCGGCACATTATTAA
ACATACAAATATAGTTGAAAAGGTTTTTGCGGTTGGCGCATCAGGTGATGGGCATTCGAATAAAATTAGCATA
CATTATGTAGATAGCAAAAGCTATAAATATATTTCAGATATAAACAACTTAGAGGATTTGAAAGAAGAAAACA
TTGAAGAATTTTATCGTGTTTCTGTGTTAGGAGAGTTGCCTAAAGAAGAAAGAGAGTTAATAGAAGTTAATAA
AATTGCTGCCGATTTGCACGAGGACTTAAGGAACTATGGTTCGCTTGAAGGTGAAAAGAAAGCTTCTGTAGTG
TCCGCAATTTTACTAGCTTTAGAAAATGAAGAAGTGATTTTTAATGTGGATAAACTACAAGGACTTCAAGGTG
AGGGTGTTAAAGATGGCGAAATCCTCTTTGATGCTATTGATAAATATTTAAGAAATAAAAGTTTAATGCCTCA
TGCTAAAATTGGTGAATTAAAAGATAACTTCACTTTTATACAGAACGATTTGACGTTAAATAGATCAAGAGAA
GACTTAAAGATGACACCATTAAAATATTTTACTATCAAGCTAAGTAATAAATTAAAGAAAAATATCAAACATT
CTGATATGGATATACTAGGTAATTTTTATGGTGAGTTTGTAAAATATGGAGGAAATGATGGTAATTCATTAGG
CATAGTCTTAACACCTAGACATATTACAAACCTGATGTGCGAATTAATTGATATTGATGAAAATGATTATGTA

TTAGATCCATGCTGTGGTTCAGGTGGGTTTTTAATAGCTGCCATGAATAGAATGTTAGGAAAAACAAATGATG
AAATCAAAAAAGCTGAAATAAAACAAAATCAATTACATGGAATTGAATTACAACAAAAGTTATTCACTATAGC
AACTACAAATATGATTTTACGTGGTGATGGTAAAAGTAATCTAAAACGTGATGACATATTCCATGTTGAAACA
GGTCTATATAAAAATCAAATTACTAAGGCATTAATTAACCCCCCATATTCACAAGCTAAAACGAAAAACTTAA
GTCACTTATCAGAAATTAGTTTTATCAATGAAACGTTGTCACTAATGAAAAAGGACGCTAAGTTAGCCGCTAT
CGTTCCACAGAGTACAATGATTGGTAAAACTAATAATGATAAGAATTACAAGCGCGAAATACTAGAAAAGCAT
TCGTTAGAAACAGTAATTACGCTTAATAAAGATACCTTTTATGGCGTGGGAGTAAATCCGTGTATAGCAATTT
TCACTGCAGGTGTTCCACAAGACAACAAAAAACGTGTTAACTTTGTAAACTTTTCTGATGATGGTTATATAGT
GCGTAAACATGTGGGATTAGTTGGAGATGGTACCGAGAAATCAAAAAAAGAATATCTTTTAAACGTGTTAAAT
GATTATGAAGATGCGGATACTAACTTTTTAGTTAAATCCCCTATTACTTGGAAGGATGAATGGTTGCATAGCT
TCTTTTACTACAATGAAGAAATTCCTACAGATGAAGATTTCGAAAAGACAATAGCTGATTACTTATCATTTGA
ATTTGATATGAAACTGCACGGCAGGGGGGATTTATTCGATAATGAAACTGAGTGATAGAGAATGGAAGGCGTT
TACAACTGATGAGTTATTCAGTATTAAATCTGGGACTACGTCTGACTTTTCAAAATTGAATTTAAAAAACGAT
TCAAAGTTACCGTGTATAGGGGCAAAATATAAAAATAATGGTGTGGTAGGTTTCGCTAATAATGAAGAACTGA
AAGTAGAAGGAAACGCTTTAGTTTTTATTAAAACTGGAGAAGGTTCTGTTGGACTAACTCTTTATAAAGCAGA
AGATTTTATACCGCACAAAAATGTTTACATAGGCTATAACGTAAGCTTAAATAAATATATAGGCTTGTTTATT
GCAACTATGTACAATAAACAGAAATTTATATATAACTATGGCTATGGTCTAAACCAATTAAGAGTAAAAAGAC
GTAAAATATTGCTTCCTTCTAAAAATAATAGTCCTGATTACGAATTCATGGAACGATACATCAAAGAAAAATA
TTTTAATCTAAAATCACATATAAAAGAGAAACAAAAACACAAAATTTCTGATTGGCGAGAGTTAGATGAGGTT
GAGTGGCTACCCTTCTCAACTTCAGAAGTTTTTAATACTATTCAACGAGGAAAAAGGTTAACTAAATCTAATC
AAATAAAAGGATTAACACCTTACATATCCTCTAGTGGTATTAATAACGGATTAGATAACTTTATTTCAAATAG
CGAAAAAATTAGAGTTTTTGATAATTGTTTAACAATAGCAAACAGCGGAAGTGTAGGAAGTACTTTTTATCAT
ACGTATAGATTCATTGCTAGTGATCACGTTACTCACTTAAAAAATAATAATTTAAATAAGTTTACTTATTTAT
TTATAGCTAATATGCTACGTAGACTAGAAGGAAAGTATAGTTTTAATCGTGAAATTAATGATTTTCGTATTAA
AAGAGAACGCATATTACTCCCCACTAAAAACAATCAGCCCGACTATGAATTTATGGAGCAATACATGAAGCGA
AAAGAAAATAAGATATTAGATAGATTATAAATGATCAAACCACACCACCTATTAATTTAGGAGTGTGGTTATT
TTTTTGTGTTTTTTTCGGGGCGAAAAAAGGGCAAATTATTTAAATAAGGGCAAACATGCGTGGAAAACACAGA
GGTTTAAAAATGCCAAAACCGTTGATATGACAAGGTTTTTATACGTTTGTATACAACGACGAATTATCTATTC
GCCATCACATTATGACGATATGTTTATTTTAAACACACAAGCTCATGCTCGTCTTGATCAAATGGCACAACAG
TTTGAAGTTGTTTGTAATGGCTTGAACGAAAATGAAGGACAAGCTATTCAAACAATGGATCAATCTGCCTCGT
TAATTCGTTCAAACTTAATTCAAGTTAAAGAACAATTAGAAAAACTAGCAGTTTACTAATTAATTTATTAAAA
GCTACTTGTTTTCATTGAGAATAAGTAGCTTTTTCAAACACAAAAGTTTTATAAACACATAAATGGGTGATGA
GCTATGTTTAAAAGGACAAAACTAATCTTAATAGCAACAATACTGCTATCAGGATGTTCAGCGATGGAAAGTG
AATCAAAAAAAGACACGAATACAGAAACAAAATCAGTACCAGAAGAAATGGAAGCTTCAAAATATGTAGGACA
AGGCTTTCAACCACCTGCAGAAAAAGATGCGATTGAATTTGCGAAGAAGCATCGTAAAGAATTTGAAAAAGTA
GGTGAACAATTCTTTAAAGATAACTTTGGACTAAAAGTTAAAGCTACAAATGTTGTAGGTAAAGATGATGGTG
TAGAAGTTTATGTGCATTGTGAAGATCATGGTATTGTATTTAATGCAAGTCTACCTTTGTACAAAGATGCCAT
CCATCAAAAAGGATCAATGCGTAGTAATGACAACGGTGATGATATGAGTATGATGGTGGGTACAGTGCTGAGT
GGCTTTGAATATCGAGCGCAAAAAGAAAAGTATGATAATTTATATAAATTTTTTAAAGAAAATGAAAAGAAAT
ATCAATATACAGGTTTTACTAAAGAAGCAATAAACAAGACGCAAAACGTCGGGTATCAAAATGAATACTTTTA
TATTACATATTTATCAAGAAACTTAAAAGAATATCGTAAATATTACGAACCATTGATTCATAAAAATGATAAA
GAATTTAAAGAAGGTATGCAAAAAGCTAGAAAAGAGCTAAACTATACTGCTAATACAAATACTGTAGCAACGT
TATTTAGTACGAATGATGAAAGGAATAGAAAAGAAAAGATAAATAATGTAATAGATTTATCCGAGAAAATTGA
AAGAACAAAAGATATGCCAATCAAGAATACTATAACTACTCAATTAGGAAATAAACTTATTGGCACAAAAAAA
GCTCGTTTTGATGATAAGAAAGTAGTGTCGTTTGGAGCATTTGAAGATGAATATAATAAATGATAGAGATTTA
ACAGAATTGAGTAGTTATATGGTTTATCAAGACATCAATAAAGATAATAACTTTACAGTTAACGAAAAACGAT
TTAAGCAGGCAGATGAATTTGAAGATTTATATAGAGAGAAACTATAAGACGCAAATAAATTAAGAGAGTATAA
TTATTTACAAAATGAAACTTTTAAAAGCGCATAAATGGGTGATGAGATATGCTTAAAAAGGCGAAATTAATCT
TAATAGCAACGCTACTGTTATCAGGATGTTCAGCGATGGAAAGTGAATCAAAAAAAGACACAAAAGCAGAAAC
AAAATCTGTGCCAGAAGAAATGGAAGCTTCAAAATATGTAGGTCAAGGTTTCCAACCACCTGCAGAAAAAGAT
GCGATTGAATTTGCGAAGAAGCATCGTAAAGAATTTGAAAAAGTAGGTGAACAATTTTTTATGGACAACTTCG
GTCTAAAAGTTAAAGCTACCAATGTTGTAGGTAAAGATGATGGTGTAGAAGTTTATGTGCATTGCGATGATCA
TGGCATTGTATTTAATGCAAGTTTGCCCTTGTACAAAGATGCCATCCATCAAAAAGGATCAATGCGTAGTAAT
GACAATGGTAATGATATGAGTATGGTGGTGGGTACAGTGCTTAGTGGCTTTGAATATCGAGCGCAAAAAGAAA
AGTATGACAATTTATATAAATTTTTCAAAGAGAATGAAAAGAAATATCAATATACAGGATTTACAAACGAGGC
AATTAACAAGACACAAAACGTCGGGTATCAAAATGAATACTTTTATATTACATATTTATCAAGAAACTTAAAA
GAATATCGTAAATATTATGAACCATTGATTCGTAAAAATGATAAAGAATTTAAAGAAGGTATGCAACGAGCCA
GAAAAGAGCTAAATTATACTGCAAATACTGATGTAGTTTCTACGCTTTTTTCTACTAAGAAAAACTTTACTAA

AGATAATACAGTAGATGATGTGATTGAATTGAGTAATAAATTAAAAGATAAACCCAATATGCCTCAAAAATCT
CAAGTTACTATTCAATTAGGAAAATCTAGTATAAATACTAAAAAACCATTTTATGACGATATAAATCCAATTG
AAGGGTAAGTGAATATAAAGTGAATAAAATAAATGATAGGGACATAGCAGAATTGAACGGTTATTGGGTTTAT
CAAGATATAAATGAATGATAGATTTTAAAGTAAATGAAAAACGGTTCAAACAAATAGAAAATCATAATATGAA
AGGCATACCTTAATATAGAAATATACGAGTTTCTTGATGATCAGGGGAATCTAACTTGTCAACAGATAATAAT
CTATCAAGGAACTTCAAATGAGGCAGTAAATCCTAATAACCCATTGAAATCTTTAGGTAATGGAGATGATTGG
CTTCAAAATGCTAAATCAACGGATAATTAAATAAAAATCAGACATTGTTTAACGCAAGTAGATGTATTTGAAG
ATTTATATAGAGAGAAACTATAAGACGTAAATAAATTAAGAGAGTATAATTATTTGCAAAATGAGAGTTTTAA
AAGCGCATAAATGGGTGATGATATATGCTTAAAAAAGCAAAATTAATATTAATAGCAACACTTCTACTATCAG
GATGTTCCATGATGAAAAATGAATCAAGTAAAGACACGAATACAGAAACTAAATCTGTACCAGAAGAAATGGA
TGCTTCAAAATATGTAGGACAAGGATTCCAACCATCTGCAGAAAAAGATGCGATTGAATTTTCGAAGAAGCAT
AAAGATAAAATTGCTAAGCGAGGCGAACAATTTTTTATGGATAACTTCGGTCTAAAAGTTAAAGCTACAAATG
TTATAGGTAGTGGCGACGGTGTAGAAGTATTCGTGCATTGTGATGACCACGACATCGTATTTAATGCAAGTAT
TCCATTTGATAAATCAATTATTGACAGTGATAGTTCATTAAGAAGTAAGGATAAAGGCGATGATATGAGTACT
TTAGTTGGTACAGTGTTGAGTGGCTTTGAATATCGAGCACATAAGGAAGAGTTGGATAATTTAACAGAAGTTT
TAAAAGAATATAAAAGTAAATATAAATATACTGGTTATACTGAAAATGCAATTATGAAAACGCAGAATAGTGG
TTTTAGAAATGAGTATTATTATTTGACTGCAATTCCATATACTTTAGATGAATATAAAAAGTATTTTCAACCC
TTAATCAAAGAGGATGATAAGTCTTTTAGGGACGGTATGAAAAACTCAAAAAAACAACTTAAAGATAAATCTC
GTCCATATATTGTTACAACTTTATTTAGTACTAAAGATAATTTTACTAAAGATAATACAATAGACGAGATGAT
TGATTTTTCTGAAGTACTAAAAAAGAAAAAGAATATTCCTCGTGATTTGAATGTTTCTCTTCAAATTTCTAAT
AAATATATAAATACAAAAAGACCTAATTATAGTAAGAAGGATGTAATAGAAATAGGTGTATTTAATCATGAAA
AAGCTAACACGAATGATTAAACTAAAAACAATAAAAAGATAGGTTCAAAAAAGCCATCCTAACAACAGACAAA
ACCACAAACCAATCAAACCTACCCAACCAATGAACTCAAATCACCAATGCATCACTCTCCGATAACGTAACTA
TGATCTTATTTAAGCATAGTTATTGAGGTTTTGTGATATATAGTATAAAATTAATGAGAATTAAATTTAATAA
TGTAAAATTCATCTTCGGGGTCGGGTGTAATTCCCAACCGGCAGTAAAAAAAGCCTGCGACCTGCTAGTATGT
ATCATATTAGTGGCTGATCTAGTGAGATTCTAGAGCCGACAGTATAGTCTGGATGGGAGAAGATGGAGGTTTT
TTGTTGTGCAATAATCCTCCTATTCTTACGAGATGAATGGAAGGAGAAAATTGAATATGCAACAAAATAAACG
TCTTATCACAATAAGTATGTTGAGCGCGATTGCGTTTGTGTTAACTTTTATCAAGTTTCCTATACCATTTTTG
CCACCATACTTAACTTTAGATTTTAGTGATGTACCGTCACTACTAGCTACATTTACGTTTGGACCAGTTGCCG
GTATCGTAGTTGCACTGGTTAAAAATTTATTGAACTACTTATTTAGTATGGGCGATCCAGTTGGACCATTTGC
TAACTTTTTAGCAGGCGCAAGTTTCTTATTAACTGCTTACGCCATCTATAAAAATAAACGTTCAACAAAATCT
TTGATTACTGGATTAATCATTGCAACAATCGTTATGACTATCGTGTTGAGTATTTTGAACTATTTCGTTCTAT
TACCTTTGTACGGTATGATATTTAACTTAGCTGATATCGCAAATAATCTTAAAGTAATCATTGTTTCAGGAAT
TATACCATTCAATATTATTAAAGGTATCGTTATTTCTATTGTATTTATTTTACTATATAGAAGGCTTGCGAAT
TTCTTGAAAAGAATTTAATCAAATTAAAGCAAAATAATATACACATAATAATAAAAAGCAGGTGACTATCAAT
AAACGATAGCTTGCCTGCTTTTTCTATGGAACATTTGTCTAAAAAATCAATTATTCAAATTTTAAAGCGTCCC
CATCAAATGATTCGTCTGCAATTTTAATAGAATCTGTAGGGCATCCATCAATTGCATCTTCCATATCTTCATA
TAATTCCTCAGGCACTTCTGCAGTACCTTGGTTATCGTCAAGGATTACGAAAGCAATACCTTCGTCGTCGTAA
TCATATATATCTGGCGCTGCTGCACCGCATGCACCACATGCAATACAAGTATCCATATCAACGATTGTATATT
TTGCCAATGTCTTCGCCTCCTTTGATAAAAATGCTAAAATAGTAATGTGACTAAAATTTTAGACAGCATCATT
TTTATTTTCAAATTATCCGTTTTACAGAGTGAGGGTTAAACTTGCAACACATTATAAAAACAGCATTACAACA
AACATTTAACTATAAAACAAATAAAAGTATTTACAATATCTTAGTTGGTAAGAAATCTCACCAAACCTTTTTT
GACGCTTGTAGTCAACAACAGTTGTCATTATATCACAGTTTACCACTATTAAAATATCCGTCTTTTGAGCTAT
TTTTAGAAAATATAACTGAATTTAATGCTGAAATGGAAATCATGTTGCATCCTAGATATACATTTGAAAGTAT
GGGGCAAACATTTCAAGCAATTCAACTATTAGTGCAAACCATGTCTAATACTATGCAGCAAGATTTTCGTTTC
GTACCAATCTCTCAAAATAATAAGATACAAGAAACTGTAAAGATTGTCTATAACTATATTAAAGAAAACCAAT
TACAAATTGATTTTGAAAATGAACTACATAACTTGTTTAAAGCAATAACTTTAAAAGGGCCATGTTACTTACA
TTATTATTTGCAAGGCTATGATGAACCAATGTATACGAGACAGCAAGTTAGTTTAATAGAAAAGCTATCTCAA
CAGCAATTGTTTGAATACGAAATGAATAATTTAGTGACAATGATGTTTGAATTAGAAAGTGGAGAATATACTA
TTTTATCAAAAATAATAATGAAACCTACATTATTAAACCAAACTTATATTACTTATACAAGATTGCTTGAACA
ATTCACGATGGAAGATATAGCGGCTCAACAACAAGTTAAAATCAATACTATCGAAGATCATGTACTTGAAATC
TTAATCAAAGGTTATATGTCTAATTACGATGATTATGTTGAACAAGAAGATCAACTGCAATTTTTAAATTTTT
ATCAACAACATCGTGGTGAACGATTAAAATTTTACAAAGAACAATTTGACACGTTATCATATTTTCAATTAAA
AGTATTAATCGTTGGATTTGAAAGAGGTGATCTGAATGTTGCATGATATTTTACGAAACAAATTTGGCTTCGA
GAATTTTAAACCGGGACAACAGGAAATTATAGAAAGTATAATGTCTCAACAACACACTCTAGGTATTCTTCCA
ACTGGAAGTGGAAAGAGTTTGTGTTATCAAATACCTACGTATTTATCAGGTAAGCCGACATTAATTATCTCAC
CGTTAATATCTTTAATGGATGACCAAGTTATGCAGTTGAAAATAAATGGAGAAAAACGTGTAACATGTATTCA
CTCTGGTATGGATGAAATTGAGAAAAAGCATAATATTAAATGTTTACGACATAGCCGCTTCATCTTTCTAAGT

CCAGAATTTCTCCTGCAACCGTCAAATTTTAAATTAATATCTATGATAGACTTTGGCATGATTGTTCTAGATG
AAGCACATTGCCTATCTGAATGGGGATATGATTTCAGACCACATTATGCTCTAATAGGAAAAGTAACAAAGCA
TTTTAAAGAAGCGGTTGTCTTAGCATTGACAGCAACTGCACCACCGCATTTACAAGATGATTTGACGGAAATG
TTAGCGATTCAATTCAAAGTTATTAAAACTACAATGAATCGCCCAAACATAAGCTTTAAGCATCTTAATTTTC
ATGATGATGAAGATAAAATTGAATGGTTGCTGCCGTTTCTACAACAGTCGGGACCAACGATTATTTATGTCTC
ATCGAAAAAGATGTGTCTGAATTTAGCGCACCTTATTTATGATTCAGGTTTTCTTACAGGCATTTATCATGGT
GATATGAATTATCAAGAGCGACACACAGTTCAACAACAATTTTTAAATAATGATATTCCGATTATAGTCGCAA
CGAGTGCTTTTGGTATGGGAATTAATAAAAAAGATATTCGCACAATCATTCACTTTCATCTTTCAACAAGTCC
TTCTAACTACATTCAAGAAATTGGCCGTGCGGGTCGCGATGGTGAACTAAGTCAGGCAATTAGTTTATTTCAA
CCGGACGATAAATACATTTTAGAAACATTATTATTTGCAGATATGATAACAGAAGAAGATGTACAAAATTTCG
AAATAGGAGAATTTTTAGCACCCGATAAACAAGACGTTTTGACAACGTTGCATTCATTCTATAGTATCGGCGC
CTTGAAACAGATATTTAAGCAATCATTTAAACGAAAGCAATTAGGATTCTTTCGCATGATTGGCTATTGCAAA
TTAGATCAATGTAGACGAAAGTATTTATTAGAATTTTTCGGTGAATATCCACCGGCACAAGATCGATGTTGTG
ACAATGATTCTAATATAACTGATATCGTAATTTTAAATAAGAAGAAGGTAATTAGAAGTATTGGATTTGATGA
AAAGTTGCAAAATTTATTTCTCAGATAGTATTACTTTACTAAAAGAAAATTGACAAGCTATAATTAGTGTATA
CACAATTGAAAAATGATTGAAATAATTTTGAAAAATATACATAAACATATGTCATGTGGGTATATTTTATGTA
AAATCATTGTAATAGAATAGAAAGGAAGATGGCTATGTCTAATAATTTTAAAGATGACTTTGAAAAAAATCGT
CAATCGATAGACACAAATTCACATCAAGACCATACGGAAGATGTTGAAAAAGACCAATCAGAATTAGAACATC
AGGATACAATAGAGAATACGGAGCAACAGTTTCCGCCAAGAAATGCCCAAAGAAGAAAAAGACGCCGTGATTT
AGCAACGAATCATAATAAACAAGTTCACAATGAATCACAAACATCTGAAGACAATGTTCAAAATGAGGCTGGC
ACAATAGATGATCGTCAAGTTGAATCATCACACAGTACTGAAAGTCAAGAACCTAGCCATCAAGACAGTACAC
CTCAACATGAAGAGGAATATTATAATAAGAATGCTTTTGCAATGGATAAATCACATCCAGAACCAATCGAAGA
CAATGATAAACACGATACTATTAAAAATGCAGAAAATAACACTGAGCATTCAACAGTTTCTGATAAGAGTGAA
GTTGAACAATCTCAGCAACCTAAACCATATTTTACAACAGGTGCTAACCAATCAGAAACATCAAAAAATGAAC
ATGATAATGATTCTGTAAAACAAGATCAAGATGAACCTAAAGAACATCATAATAGTAAAAAAGCAGCAGCTAT
TGGTGCTGGAACAGCAGGTGTAGCAGGTGCAATGGCTGCTTCTAAAGCTAAGAAACATTCAAATGACGCTCAA
AACAAAAGTAATTCTGGCAAGGCGAATAACTCGACTGAGGATAAAGCGTCTCAAGATAAGTCTAAAGATCATC
ATAATGGCAAAAAAGGTGCAGCGATCGGTGCTGGAACAGCAGGTTTGGCTGGAGGCGCAGCAAGTAAAAGTGC
TTCTGCCGCTTCAAAACCACATGCCTCTAATAATGCAAGCCAAAACCATGATGAACATGACCATCATGACAGA
GATAAAGAACGTAAAAAAGGTGGCATGGCCAAAGTATTGTTACCATTAATTGCAGCTGTACTAATTATCGGTG
CATTAGCGATATTTGGAGGCATGGCATTAAACAATCATAATAATGGTACAAAAGAAAATAAAATCGCGAATAC
AAATAAAAATAATGCTGATGAAAGTAAAGACAAAGACACATCTAAAGACGCTTCTAAAGATAAATCAAAATCT
ACAGACAGTGATAAATCAAAAGATGATCAAGACAAAGCGACTAAAGATGAAACTGATAATGATCAAAACAACG
CTAATCAAGCGAACAACCAAGCACAAAATAATCAAAATCAACAACAAGCTAATCAAAATCAACAACAGCAACA
ACAACGTCAAGGTGGTGGCCAAAGACATACAGTGAATGGTCAAGAAAACTTATACCGTATCGCAATTCAATAC
TACGGTTCAGGTTCACCGGAAAATGTTGAAAAAATTAGACGTGCCAATGGTTTAAGTGGTAACAATATTAGAA
ACGGTCAACAAATCGTTATTCCATAATATAACTATATAAATTGTAACTGAACTGCTATATACAAACGTGCATA
ATATAAAGTAACTCTAAATATTTTAGAGAACTTAAATTGCGCTTATAAGTATGTAGCGGTTTTTTCCTTTTTC
AAAGTTTGTTATTTAACAAGGTGTTGTCTCGGATATTGACATATAAATTAAACTTTTCAAATAGTCATCAAAA
AGATAAAACACTACAATCAACAAATTTAACGAGGAAGAATAAAAAATAAATCAACATATTAAATTGTAGTGTT
ATTCAACTCCGTAGCTAACAATTCTCTATGCACATTAAACAAATTGTCAAAAATATATCATAAATCTTCAAGC
ACAGACTTAGCGTATCAATCACTGAACTGTTATAATAGTTTGGGATTAAAGGAGGCCGAAACAATGCAAAAAG
TTGAAAGTATCATAATTGGTGGAGGGCCATGCGGATTAAGTGCGGCTATTGAACAAAAAAGAAAAGGTATTGA
TACCTTAATTATTGAAAAGGGTAATGTCGTTGAATCAATCTACAATTATCCTACTCACCAAACATTTTTCTCA
TCAAGTGATAAATTAAGTATTGGGGACGTACCGTTTATCGTTGAAGAAAGTAAACCAAGACGTAATCAAGCGC
TAGTTTATTACCGAGAAGTTGTAAAACATCATCAATTAAAAGTAAATGCATTTGAAGAAGTATTAACTGTTAA
AAAAATGAATAATAAATTTACAATTACTACGACGAAAGATGTTTATGAATGTCGATTTTTAACAATCGCGACA
GGCTATTATGGTCAGCATAATACATTAGAAGTTGAAGGTGCGGATTTACCTAAAGTGTTCCATTATTTTAAAG
AGGCACATCCGTATTTTGATCAAGATGTTGTTATTATTGGTGGTAAGAATTCGGCTATCGATGCTGCTTTGGA
GTTGGAAAAAGCTGGTGCTAACGTGACGGTTCTATATCGTGGTGGAGATTATTCACCTTCAATTAAACCGTGG
ATACTTCCAAATTTCACAGCATTAGTAAATCATGAAAAAATTGACATGGAATTTAATGCTAATGTTACCCAAA
TAACTGAAGATACTGTGACTTATGAAGTAAATGGTGAAAGTAAAACGATACACAATGATTATGTATTTGCGAT
GATTGGTTATCATCCCGATTATGAATTTTTAAAATCTGTAGGCATTCAAATTAATACAAATGAATTTGGAACA
GCGCCTATGTATAATAAAGAAACATACGAAACAAATATCGAAAATTGCTACATTGCAGGTGTGATTGCTGCAG
GGAATGATGCGAATACCATTTTTATTGAAAATGGTAAATTCCACGGTGGCATTATTGCTCAAAGCATGCTAGC
TAAGAAATAAACGCCGTTAGAATCATAAAAACAAAGGTCTATGTACTAGATAACTTAGTTTTACAACGACTGA
CATTCATGATATGTCAAATGAGGTTGATGACTAATTATTGTACATAGACCTTTTTATATTACGTAATCATTAT
AATTCAAAATATGATTTGATTTCAGCTTTATCTAAATTGTTGCTTAACGCAACTAATAATTTTAATCTTGCTT

TTGGACCATTCAAACCGTTAGAAAAAATAAAACCTTGTTGTGCGAGTTGGTAACCACCACCATCGTATGCGTA
AGTTGGACTCACAATACCATTAAAGGAACGTGAAACTAGCACAATAGGTATATTTAAAGATACTAATTGTTGA
ATGCCTTCTAATGCGCTTGGAGGTATGTTGCCTTGTCCTAACGCTTCAATAACCATACCATCGATACCTTCAC
GACTATAAAAACTAAAAATGTCACCTGGCATACCCATATATGCTTTTACTAATGGTACATTTAGTTTCTCATT
GACATTTTCCAATGCTTGTTGGCGATATGGCATATGATGGAATTGCACACGATCCTTTGTCAATACACCTAGC
GGACCATGATTTGGACTTTGAAATGTGTTTGTATTAGACGTATGTGTTTTGGTAACATTACGCGCCGTATGAA
TTTCATCATTAAATACAACCATCACGCCTTTATGACGAGCCTTTTCATCAGAGGCAACGCGAATAGCGGAAAT
ATAATTATATAATCCGTCAGAACCAATTTCATTAGACGAGCGCATTGCGCCAGTAATAACAACAGGTTGCTCG
ATACCTAATATTAAATCAAGTAAAAAGGCAGTTTCTTCTAACGTATCGGTACCATGCGTGATAACGAAACCAT
CATAATATTTATTTGATACTGCTTCTAAAATAATATCCTTTAATTGTTTAACATGTTGGATTGTCATATGAGG
TGATGGTACATTAAAAGGATTTAATTCATCTATTTGTGCATATTGATTTATGACATCTTGATGCATTGAAATA
GGGTTGATATCATTTGTTACTACTTTATTAGATTGGTCTTGTGACATACTAATGGTGCCACCAGTATGAATAA
CCAGTAGATGTTTCATATATTTCCTCCTATATTTCATTTACCTAATTATGATAAAATATTATTCATAAAACGA
CAAGGAAGGGAAATGACGCATGAAAGCCATTAATATTGCATTAGATGGTCCAGCTGCTGCCGGAAAAAGTACA
ATTGCGAAACGTGTAGCCAGCGAACTATCAATGATTTATGTCGATACAGGAGCAATGTATCGTGCATTAACAT
ACAAATATTTAAAATTAAACAAAACTGAGGACTTTGCAAAACTAGTTGACCAAACAACATTAGATTTAACTTA
TAAAGCAGATAAAGGTCAATGTGTCATTTTAGATAACGAAGATGTAACAGACTTTTTAAGAAATAATGATGTG
ACGCAACATGTTTCATACGTTGCATCTAAAGAGCCAGTACGTTCATTCGCCGTTAAAAAACAAAAAGAGTTAG
CTGCAGAAAAAGGTATCGTAATGGATGGTCGCGATATCGGAACTGTAGTGCTACCAGATGCAGATTTAAAAGT
ATATATGATTGCATCAGTTGAAGAGCGAGCAGAAAGAAGATATAAAGATAATCAATTAAGAGGTATCGAATCA
AATTTTGAAGATTTAAAACGTGATATTGAAGCTCGTGATCAATATGACATGAACCGTGAAATATCACCATTAA
GAAAAGCAGATGATGCAGTGACATTAGATACGACAGGCAAGTCGATTGAAGAAGTTACTGACGAAATTTTAGC
GATGGTGAGTAAAATTAAATAATCTAATAAGTCATAACTATCTATTTGTAATGTGCAACGCTTAACGCATATA
CAAAATGAATGTGCTGAGAATGATTTACTCAAATTAAAAGGTGATTTTTATTCAATGATGAATGAAAGTTGCC
TTTTTATTTTTGGTAAAAGTTAATGCGTCAGTGAATTGTGTAAGTTTTTCAAAAAGTAAAAAGAAATAATAAA
GGTGAATTATTAGAATTCCAAAAATAATTCATTACATTCATAAAGCATTTTACAAATGGTAAGAAAATGAGTG
TTACAAATCTAAATATTGCAAAAGAAGCTGATTTAGTCACAAAAAATGTCCTATGTAATAATTCGAGAAAGAT
GCACTATATACAGTCTTCTTACTATTCAAATGTAAAAGTTGCTTATTTGCGTGGCTTTTTGTTTTATAAAAGT
ATAAAATTTTACTATAATATATCTTGTAGAGAACAATGAAATGACAGACTTATAAAACAAAATAGTAAGCTAT
GCTAATTTTTATACAAAAAATATGTATAAACATAGCGGACTTTATTACTTGTTTGAATATATGGTTGTAATAA
ATATATGTCCAACTAATTCACAGAATGAGATTAAGTGACATATTTATGGATACAACTAATTTACTAAATGTAA
TAAAGGTAGAATTTCTTGACAATTCTGTCAGTTTATAAGATGTTATAAATATGTAGTGTATAAGGAGGCAAAC
AAGATGACTGAAGAATTCAATGAATCAATGATTAACGATATTAAAGAAGGTGACAAAGTCACTGGCGAGGTAC
AGCAAGTTGAAGACAAGCAAGTTGTTGTTCATATCAACGGTGGTAAATTTAATGGGATTATTCCTATTAGTCA
ACTATCTACGCATCATATTGATAGCCCAAGTGAAGTTGTAAAAGAGGGCGACGAAGTTGAAGCATATGTCACT
AAAGTTGAGTTTGATGAAGAAAATGAAACTGGAGCTTACATCTTATCTAGAAGACAACTTGAAACTGAGAAGT
CTTATAGTTATTTACAAGAAAAATTAGATAATAATGAAATCATCGAAGCGAAAGTAACAGAAGTAGTTAAAGG
TGGTTTGGTTGTTGATGTAGGACAAAGAGGTTTTGTTCCAGCTTCACTAATTTCAACAGACTTCATTGAGGAT
TTCTCTGTATTTGATGGACAAACAATTCATATTAAAGTTGAAGAATTGGATCCTGAAAATAATAGAGTCATTT
TAAGCCGTAAAGCAGTTGAACAAGAAGAAAATGATGCTAAAAAAGATCAATTATTACAATCTTTAAATGAAGG
CGATGTTATTGATGGTAAAGTAGCACGTTTAACTCAATTTGGTGCATTTATAGACATTGGCGGTGTTGATGGT
TTAGTGCATGTATCTGAACTTTCTCACGAACATGTTCAAACACCAGAAGAAGTAGTTTCAATTGGTCAAGATG
TTAAAGTTAAAATTAAATCTATTGATAGAGATACAGAACGTATTTCATTATCAATCAAAGATACGTTACCAAC
ACCTTTCGAAAATATTAAAGGTCAATTCCACGAAAATGATGTCATTGAAGGTGTCGTAGTAAGATTGGCAAAC
TTTGGTGCATTTGTAGAAATTGCACCAGGTGTACAAGGACTTGTACATATTTCTGAAATTGCACACAAACACA
TTGGTACGCCAGGTGAAGTGTTAGAACCTGGTCAACAAGTAAATGTTAAAATATTAGGTATTGATGAAGAGAA
TGAAAGAGTATCACTATCTATTAAAGCAACATTACCAAACGAAGATGTTGTTGAAAGTGATCCTTCTACGACT
AAGGCGTACTTAGAAAACGAAGAAGAAGATAATCCAACAATTGGCGATATGATTGGTGATAAACTTAAAAATC
TTAAACTATAATTTAATATTTAATAGTCAACTCCACATGTTTATGATTGCATGTGGAGTATTTTTTTGTACCA
AAATATACTCGGAATGATAACGTGGACAAATTTAACTAAGTGTTTAAAGAGATAAAGTTTTAAGTGCTGATTT
TTATCATTACAGTAATAAACTCATTTTGAATACACAGTCTCATGTGATATTATTAAAAAGATATAAGAAAGAG
AGGAAGTTAGCTTATGACTAAACCTATAGTAGCTATTGTAGGTAGGCCTAATGTAGGTAAATCTACAATTTTT
AATAGAATAGTTGGAGAACGTGTTTCGATTGTGGAAGACACGCCAGGTGTAACACGAGATCGTATTTATTCTT
CAGGTGAATGGTTAACACATGATTTCAATATTATTGATACAGGTGGTATTGAAATTGGTGATGCACCATTCCA
AACACAAATTAGAGCGCAGGCAGAAATCGCCATAGATGAAGCGGATGTTATTATATTTTTATGGTTAACGTGCGT
GAAGGATTGACACAAAGCGATGAAATGGTCGCTCAAATTTTATACAAATCTAAAAAACCGGTCGTATTAGCGG
TTAACAAAGTAGATAATATGGAAATGCGTACAGACGTGTATGATTTCTATTCATTAGGATTTGGTGAACCGTA
TCCGATATCAGGGTCACATGGTTTAGGTCTTGGTGACTTGTTAGATGCAGTTGTTTCTCATTTTGGTGAAGAG

GAAGAAGATCCTTATGATGAAGATACAATTCGACTATCCATTATTGGACGACCAAACGTAGGTAAATCAAGTT
TAGTAAATGCTATTTTAGGTGAAGATCGCGTTATCGTTTCTAATGTTGCAGGGACAACGAGAGACGCTATTGA
TACAGAGTATAGTTATGATGGACAAGATTATGTTTTAATCGATACTGCTGGTATGCGTAAAAAAGGAAAAGTA
TATGAATCAACTGAGAAATATTCAGTACTAAGAGCTTTAAAAGCGATTGAACGTTCAAATGTTGTTTTAGTGG
TCATTGATGCAGAACAAGGCATCATTGAACAAGATAAACGCGTTGCAGGATATGCACATGAACAAGGTAAAGC
AGTCGTGATTGTCGTAAATAAATGGGATACTGTGGAAAAAGATAGTAAAACGATGAAGAAATTTGAAGATGAA
GTACGTAAAGAATTCCAATTTTTAGATTATGCACAAATTGCTTTTGTGTCTGCTAAAGAACGCACAAGATTAC
GTACATTATTCCCTTACATTAATGAAGCAAGTGAAAACCATAAAAAACGTGTTCAAAGTTCAACTTTAAATGA
AGTCGTTACTGATGCAATTTCCATGAACCCTACACCAACAGACAAAGGTAGACGTTTGAATGTCTTTTATGCA
ACACAAGTTGCTATAGAACCACCGACATTTGTTGTATTTGTTAATGATGTAGAATTAATGCATTTTTCTTATA
AACGCTATTTAGAGAATCAAATCCGTGCCGCTTTTGGTTTTGAAGGTACACCAATTCATATTATAGCTCGAAA
GAGAAATTAACGATTGGGGGATAACAATGACTAAAATTACCGTTTTTGGTATGGGAAGTTTTGGGACAGCCCT
TGCCAATGTTCTTGCAGAAAATGGACATGATGTTTTGATGTGGGGTAAAAATCAAGATGCTGTTGATGAATTA
AATACATGTCATACAAATAAAAAGTATTTAAAATACGCGAAATTAGATGTTAACATCATCGCTACTTCAGATA
TGACCAAAGCAATTCAATTTGCAGATATTTACTTAATGGCTTTACCTACTAAAGCAATGCGAGAAGTTGCTAC
TCAAATTAATGATAAGCTGACCTCTAAAAAGACTTTTATACATGTTGCTAAAGGTATTGAAAATGGGACGTTT
AAACGTGTGTCAGAAATGATTGAAGATTCTATTTCACCCGAATATAATGCAGGTATTGGCGTGTTGTCAGGGC
CAAGTCATGCGGAAGAAGTTGTAGTCAAGCAACCAACTACAGTTGCTGCATCATCAAAAGATAAAAGTGTAAG
TAAATTAACGCAAGATTTATTTATGAATGATTATTTGCGTGTGTACACGAATGATGACTTGATTGGTGTTGAA
CTTGGTGGTGCATTGAAAAATATCATCGCAGTAGCAAGTGGTATCGTAGCTGGAATTGGCTACGGTGATAATG
CAAAAGCTGCGTTAATGACTCGTGGTTTAGCGGAAATTAGTAGATTAGGTGAAAAGTTAGGTGCCGATCCTAT
GACATTTCTAGGTTTAGGTGGTATCGGTGACTTAATCGTTACTTGCACATCAACACATTCTCGAAATTTCACA
TTAGGATATAAACTTGGACAAGGTGAATCAATGGATCAAGCATTATCTGAAATGAATATGGTTGTTGAAGGTA
TTTATACAACTAAATCAGTTTATCATTTAGCTAAAGAAAAAAATGTGGATATGCCAATTACAAATGCATTATA
TAGAGTATTATTTGAAAATATCTCAGTAAAAGAATGCGTAAAAGATTTAATGGAGCGCGATAAAAAATCTGAA
TAAAATGTGTATTATTACACATATACGGCTAATAATTACGTAAAATGAATAGAAATGGCTTAAATTCAACGTT
TTTGCCACATAAATGATTGCGATTTATGTAATGCTTGTGTTAAAGTTTTACCATAATGATATAAACATATCAT
TATCAAACCTCAGGAGGTGAATGTCTAATGAACAAAACAGATTTAATCAATGCAGTTGCAGAGCAAGCTGATT
TAACTAAAAAAGAAGCTGGTTCAGCAGTAGATGCTGTATTCGAATCAATCCAAAACTCACTTGCTAAAGGTGA
AAAAGTACAATTAATTGGTTTCGGTAACTTTGAGGTACGTGAACGTGCTGCACGTAAAGGTCGTAACCCTCAA
ACTGGTAAAGAAATTGATATCCCAGCAAGTAAAGTTCCAGCATTCAAAGCTGGTAAAGCATTAAAAGATGCTG
TAAAATAATTTTACTTAAAAAGCCCTGAATAAGGGCTTTTTATTTTGCTTTTAATACTTACAACCTGTACATA
ATTTGTAATGTTCTTCTAAGTTTTTAATCTTTGGAACATTCATTGTTTAAGCGTTCAAAATATTACATGACAT
AATAGTTTTGATAAAAATAGCAACTACAAAATATACATATGAATAATGAGATTAATTGTTAGCGTTGAATTTA
CTTAAAAGGTAACCATGTCTACTATAGTATTTTACGTTATTTAAAAAGATGAATAATGTAAATGAAGTAAAGG
TTATTATGAGAATTACAAAAGCTACATAAATTACTGTTAGTTTAAATTGAAATTTAAAAATGATAAGTAATCT
TAATGCTTCAAAAGATTTAATTTCTTACATATAATGTTAATATGAATAGTGATAATATCGTGAGGTGTAGAC
ATGGAAACAACTGTTAGCAAATTGGAAAGACAAATAGAAGAAAGATTAAAGGGTGTATCAGAATATGAATCCA
TTAATATTAATCATCGGTTAGGTAAGTTACTTGATTCATATGATATTCCAGATGTTGCCAAAGTAGCATGCCT
CACAATTGACACATCAATGAGACACCTTGATGACATTACATATAATCATTTGTCTAAACATTCCATTTTGATT
GGAGATTTAATTAGTGCGCATTTTTATACGCTACTAGCAGAAATCAATGATTTATCATTTCAAAATGAAATTA
GTAAAGCAATTGTTGAAATTAATGAATTGAAATCTTCATTACATCATCAAGCTTTAAATGATTATGAAATTTC
TCAAGCAATAGTAAAAATTGAAACACTTTTCCCATATATTACGTTATCTCATTTCGGTATCAATATTGATGAG
TCGGAAATATATAATTATTTATTTGAAGATATGTCTGATTACTATCCATCATACTTCAAGAAATATAATCAAA
GTGAAGTAAAGCATTACTTACACGATATCCAAAAAAGTTATTTAAAAAGTAGAGGTAATTAAGCATGGCTGAC
AATAAAGCAAATAAAGAGCAAGTACATCGCGTTTTTCAAAATATTTCAAAAAAATATGACAAATTAAATAATA
TTATTAGTTTTGAGCAGCATAAAGTATGGAGAAAACGCGTCATGAAAGACATGGGTGTTAGAAAAGGGACGAA
AGCATTAGATGTTTGTTGTGGTACTGGTGATTGGACAATCGCATTAAGCAAAGCTGTAGGACCAACAGGTGAA
GTTACTGGTATTGACTTTAGTGAGAATATGTTAGAAGTAGGAAAAGAAAAAACTGCTTCAATGGAAAATGTTA
AACTTGTTCATGGTGATGCAATGGAATTGCCGTTTGAAGACAATTCTTTTGATTATGTAACAATTGGGTTTGG
ATTAAGGAATGTGCCAGACTATTTAGTCGCGTTAAAAGAAATGAATAGAGTACTTAAACCAGGTGGTATGGTG
GTATGTCTTGAAACGAGCCAACCAACTTTGCCAGTCTTTAAACAAATGTATGCATTATACTTTAAATTTGTTA
TGCCTATTTTTGGGAAATTATTTGCAAAATCAAAAGAAGAATATGAATGGTTACAGCAATCTACGTTTAATTT
TCCTGGAAAAGAAGAGTTGAAGCGCATGTTTGAAGAAGCGGGTTTCATAAATGTAAGAGTACGTAGTTTTACA
GGGGGCGTTGCTGCAATGCACCTTGGCTATAAAGAAAAAGATAATACCAAAGGTGATTAACGTGGCAAAGTTA
AACATGAACAATGAAATTAAGAAAGTGGAACAACGACTTGAAAAGGCAATAAAAAGTAAAGATTCTGTATTAG
AACAGGCATCATTACACTTATTATCTTCTGGTGGTAAAAGAGTACGCCCAGCATTTGTAATTCTGAGTAGCCA
ATTTGGCAAAGATGAACAAACGTCTGAACAAACGTATCAAGTTGCAGTCGCATTAGAGTTAATTCATATGGCA

ACACTTGTTCATGATGACGTTATTGATAAAAGCGACAAGCGTCGAGGCAAGTTAACCATATCAAAGAAATGGG
ATCAGACAACTGCTATTTTAACTGGGAATTTTTTATTGGCATTAGGACTTGAACACTTAATGGCCGTTAAAGA
TAATCGTGTACATCAATTAATATCTGAATCTATAGTTGATGTTTGTAGAGGGGAACTTTTCCAATTTCAAGAC
CAATTTAACAGTCAACAGACAATTATCAATTACTTACGACGTATCAATCGCAAAACAGCACTGTTAATTCAAT
TATCAACTGAAGTTGGTGCAATTACATCTCAATCTGATAAAGAGACTGTACGAAAATTGAAAATGATTGGTCA
TTATATAGGTATGAGCTTCCAAATCATTGATGATGTATTAGACTTCACAAGTACCGAAAAGAAATTAGGTAAG
CCGGTCGGAAGTGATTTGTTGAATGGTCATATTACTTTGCCTATTTTATTGGAAATGAGAAAAAATCCAGAAT
TCAAAGTGAAAATTGAGCAATTACGACGTGATAGTGAACGTAAAGAATTTGAAGAATGTATCCAAATCATTAG
AAAATCTGACAGCATCGATGAGGCTAAGGCAGTAAGTTCGAAGTATTTAAGTAAAGCTTTGGATTTGATTTCT
GAGTTACCAGATGGACATCCGAAATCACTACTTTTAAGTTTGACGAAAAAAATGGGTTCAAGAAACACGTAGT
ATTTATGAAAAGTATTGAAAGCGCTTTACCAACCTGTTAATATATAATAGTAATATACATAAACCTATTAAAC
ACAGGGGGATACATAAAGTGGAACGTACATTTTTAATGATTAAACCAGATGCAGTACAAAGAAATCTAATTGG
TGAAGTAATTTCAAGAATTGAAAGAAAAGGACTAAAACTTGTCGGTGGTAAATTAATGCAAGTACCAATGGAA
CTTGCTGAAACACATTATGGTGAACACCAAGGTAAACCATTTTATAATGATTTAATTTCATTTATTACATCAG
CACCAGTGTTCGCAATGGTAGTTGAAGGTGAAGATGCAGTTAATGTATCTAGACATATTATTGGCAGCACAAA
TCCTTCAGAAGCTTCACCAGGATCAATTAGAGGTGATTTAGGTTTAACTGTTGGTAGAAATATCATTCACGGT
TCAGATTCATTAGAGTCTGCTGAACGTGAAATTAATCTATGGTTTAATGAAAATGAAATTACTAGCTATGCTT
CACCACGTGATGCATGGTTATATGAATAAAATTTAAACTGTAAACCTTTACGATTTATTTATAAAGGTAGAAA
GGGTTTTGTTATGTGGTTAGTCATTATGATTATACATAACAAGGCCCGTTTTTTATGTTGTAGTAAATTACTT
GAAAAATTTTATAGTTTTTTTGGTAACACGTATTAAAAAGAGAGGAATATTCTTTATCAAATGAAACTAAACT
AAAAGAAGGGGTTGATAGAATGAAGAATATTATTTCAATTATTTTAGGGATTTTAATGTTCTTAAAATCAATG
GAATTATTATATGGTGCTATATTTTTAGATAAACCACTTAATCCTATAACAAAAATTATTTTTATACTGACTC
TCATTTATATTTTTTATGTATTAGTAAAAGAATTGATTATATTTTTGAAGTCAAAGTATAACGAAAGCGCTTA
ACATATGTATATTTTTAATATCATAATTTTTTTAAACGGACTGATTAACTTTATTAATAATTAACAGTTCGTT
CTTTTGTATTAAGAAATGTAGTCAGTATATTATTTGCTAAAGTTGCGATACGATTATATTAAAACGGCTAATC
ATTTTTAATTAATGATTATATGATGCAACTGTTTAGAAATTCATGATACTTTTCTACAGACGAATATATTATA
ATTAATTTTAGTTCGTTTAATATTAAGATAATTCTGACATTTAAAATGAGATGTCATCCATTTTCTTAATTAA
GCTTGAAAACAAACATTTATGAATCCACAATGAATGTGATAAGATTAACAACATATTATAATGTTATTGCGGA
AGTATGAAAGGAGCGAGTGTGTATGAGATATCTAACTTCAGGAGAATCACATGGACCTCAATTAACAGTTATT
GTCGAAGGTATACCAGCAAATTTAGAAATTAAAGTTGAAGATATTAATAAAGAAATGTTCAAGCGTCAAGGTG
GTTATGGTCGTGGCCGACGCATGCAAATTGAGAAAGATACAGTAGAGATTGTTTCGGGTGTGAGAAATGGTTA
TACATTAGGTAGCCCTATTACAATGGTTGTTACTAATGATGATTTTACACATTGGCGAAAAATTATGGGTGCT
GCGCCAATAAGCGACGAAGAACGAGAAAATATGAAACGTACAATTACGAAGCCAAGACCGGGACATGCAGATT
TAGTTGGCGGTATGAAATATAATCATCGTGACTTACGAAATGTATTAGAACGTTCATCTGCCAGAGAAACAGC
AGCACGTGTAGCTGTCGGTGCATTATGCAAAGTTTTATTAGAACAATTAGATATCGAAATATACAGTCGTGTT
GTTGAGATAGGTGGCATTAAAGATAAAGATTTTTATGATTCAGAAACATTTAAAGCAAACCTTGATCGAAATG
ACGTCCGTGTAATTGATGATGGCATCGCACAAGCAATGCGCGATAAAATTGATGAAGCGAAAAACGATGGTGA
TTCAATAGGGGGCGTAGTTCAAGTTGTAGTTGAAAATATGCCTGTTGGTGTAGGTAGTTATGTACATTATGAT
CGTAAATTAGATGGAAGAATAGCACAGGGTGTCGTTAGTATTAATGCATTTAAAGGTGTAAGTTTTGGAGAAG
GATTTAAAGCAGCTGAAAAGCCTGGTAGCGAAATTCAAGACGAAATTCTCTACAATACTGAATTGGGCTATTA
TCGTGGGTCAAATCACTTAGGTGGTTTAGAAGGCGGTATGTCAAATGGAATGCCAATTATCGTTAATGGTGTA
ATGAAACCTATTCCTACGTTATATAAACCATTAAATTCAGTTGATATAAATACTAAAGAAGACTTTAAGGCAA
CAATTGAACGTTCTGATAGTTGTGCTGTTCCTGCAGCAAGTATCGTCTGCGAACATGTCGTAGCATTTGAAAT
TGCGAAAGCATTATTGGAAGAATTCCAATCAAATCATATTGAGCAACTTAAACAACAAATTATTGAGCGCAGA
CAATTAAATATTGAGTTTTAACAACAAGAACAATTGAGGTGTGATCATGAAATTACAAACAACCTATCCTTCA
AACAATTATCCAATATTTGTTGAACATGGTGCAATTGACCATATTAGCACGTATATTGATCAGTTTGATCAAA
GTTTTATATTAATTGACGAGCATGTAAATCAATATTTTGCTGATAAATTTAATGATATTTTATCTTTTGGAAA
TGTACATAAAGTTATTATTCCAGCTGGTGAAAAGACGAAAACATTTGAGCAATATCAAGAAACATTAGAATAC
ATTTTGTCACATCATGTAACGCGTAATACAGCGATTATAGCTGTTGGTGGTGGTGCGACAGGAGATTTTGCAG
GATTTATTGCGGCGACACTTTTACGAGGTGTTCACTTTATACAAGTGCCAACGACTATACTAGCGCATGATTC
TAGCGTTGGCGGTAAAGTGGGTATTAACTCAAAGCAAGGTAAAAACCTTATCGGTGCATTTTATCGTCCAGAT
GCTGTAATTTATGACTTAGACTTTTTAAATACTTTGCCATTTGATGAAATATTGAGTGGCTATGCGGAAGTAT
ATAAGCATGCCTTATTAAATGGTAAATCTGCTACACATGACATCGAGCAACATTTTAAAGATCGAGCGTCATT
ACAATCATTAAATGGAATTGATAAATATATAACTAAAGGCATTGAAACAAAATTAAATATCGTTGTTGCTGAT
GAAAAAGAGCAAGGTGTTCGAAAGTATTTAAATTTAGGACATACATTTGGTCATGCCGTGGAGTATAACCACA
AAATTGCACACGGTCATGCCGTAATGATAGGCATAATATATCAATTTATTGTTGCGAATATATTGTTCAATTC
TAATCACGATATCCAACATTATATTAATTATTTAACGAAATTAGGTTATCCTTTAGAAACAATAACCGACATA
GATTTCGAAACGATATATCAATACATGCTAAGTGATAAAAAGAATGATAAGCAAGGTGTACAAATGGTCTTGA

TTAAACATTTTGGAGATATCGTTGTACAACATGTTGATCAACTAACATTACAACATGCATGTGAACAATTAAA
AACATATTTTAAGTAGGTGAATGAAATGGTAAATGAACAAATCATTGATATTTCAGGTCCGTTAAAGGGCGAA
ATAGAAGTGCCGGGCGATAAATCAATGACACACCGTGCAATCATGTTGGCGTCGCTAGCTGAAGGTGTATCTA
CTATATATAAGCCACTACTTGGCGAAGATTGTCGTCGTACGATGGACATTTTCCGACTGTTAGGTGTAGAAAT
CAAAGAAGATGATGAAAAATTAGTTGTGACTTCCCCAGGATATCAATCTTTTAACACGCCACATCAAGTATTG
TATACAGGTAATTCTGGTACGACAACACGATTATTGGCAGGTTTGTTAAGTGGTTTAGGTATTGAAAGTGTCT
TGTCTGGAGATGTTTCCATAGGTAAAAGGCCGATGGATCGTGTTTTAAGACCGTTAAAAAGTATGAATGCAAA
TATTGAAGGAATTGAAGACAATTACACCCCATTAATCATTAAACCGTCTGTAATTAAAGGTATTAATTACAAG
ATGGAAGTGGCAAGTGCACAAGTGAAAAGTGCCATCTTATTCGCAAGTCTATTTTCTAAGGAACCGACCATCA
TTAAAGAATTAGATGTAAGTCGAAATCATACTGAGACGATGTTCAGACATTTTAATATTCCAATTGAAGCAGA
AGGGTTATCAATTACTACAACCCCTGAAGCAATTCGATACATTAAACCTGCAGATTTTCATGTTCCTGGCGAT
ATTTCATCTGCAGCGTTCTTTATTGTTGCAGCACTTATCACACCAGGAAGTGATGTAACAATTCATAATGTTG
GAATCAATCCAACACGTTCAGGTATTATTGATATTGTTGAAAAAATGGGCGGTAATATTCAACTTTTCAATCA
AACAACTGGTGCTGAACCTACTGCTTCTATTCGTATACAATACACACCAATGCTTCAACCAATAACAATCGAA
GGAGAATTAGTGCCAAAAGCAATTGATGAACTGCCTGTAATAGCATTACTTTGTACACAAGCAGTTGGCACGA
GTACAATTAAAGATGCCGAGGAATTAAAAGTAAAAGAAACAAATAGAATTGATACAACGGCTGATATGTTAAA
CTTGTTAGGGTTTGAATTACAACCAACTAATGATGGATTGATTATTCATCCGTCAGAATTTAAAACAAATGCA
ACAGTTGATAGTTTAACTGATCATCGAATAGGAATGATGCTTGCAGTTGCTTCTCTACTTTCAAGCGAGCCAG
TCAAAATCAAACAATTTGATGCCGTAAATGTATCATTTCCAGGATTTTTACCAAAACTAAAGCTTTTAGAAAA
TGAGGGATAATATAAAATGGAAGATATCTATAAATTAATAGACGATATCAATCTACAAAAACTAGAAAATTTA
GACTCTCGTGTTAATGAAGCAATAACTACTGACAACGATGACGCATTATTTATTCTAGGAGAGACACTTTACA
ATTTTGGATTAATGCCACAAGGTTTGGAAGTATTCCGCGTGTTATATCACAAATATCCAGACGAAAGTGAATT
GCTGATTTATTTTATTGAAGGTTTAATGTCTGAAAATCAAACTGACGAAGCGTTAGAATATTTAAGTTATGTT
GAACCATCACCTGAAAAGTTGATGTTAGAAGCAGATTTATATCAACAAATTAATATGATGGAAGTTGCTATTG
ATAAATTACAAGAAGCACTTGAACTAGAGCCAAATGATCCAATAATCCATTTTGCATTGGCTGAAATGTTATA
TTATGATGGTCAATATTTACGTGCTACCTCTGAATACGAAACCGTTTTAGAAACTGGTGAATATCAAGTTAAT
GGTGTAAACTTATTCTCTCGTATGGCAGATTGTAGTTTACAAAGTGGTAACTATAGTGATGCGATTCGCTTAT
ACGATGAAATTAATGAAGATGAAATGACTTCAGAAGATTATCTCAAAAAAGCCATTTCTTACGATAAAAATGA
CATCACTCAAGAAGCAATTAAAATAATGACTACATTACTTTCTAAAGATCCTGATTATATTCAAGGCTACTTG
TATTTACAATCATTATATGAAAATGAAAAAAATTATCCAGATGCTATTGAAACGGGTAAAGAAGGATTACGAC
TAAGTCAATTTTATAAAGAGTTAATGTATACAACTGGATGTTTGGAAATAGAACACGGCGATGCTAATGAAGG
TGTATTATTGTTAAAACAAGCATTAGAGGTTGATAATGCTTACCAAGAACCTTTATTGATTTTAAGCGATTTA
TATCGTAATGAAGAAGATTATGAAGCAATCATTGAATTATTAACATATGTAGATGAAGAAGATTTGGATCCTA
CATTCATGTGGCATTTAGCGTTCGCCTATGGTCAAGAAGAGCGAGATAAAGAAGCTCAACATTTCTTTGAATT
AGCATATCCAACGATGAAAACAAATATTGATTTTATGAGTGATTATTATTTCTATTTAATTGAAATTGGTCAA
AAGGAACAAGCAATTACTATTTTAAATCAATTGTTGGAACTAGAGCCAAGTAACGAAAATTGGCATGACGAAT
TATCAAGATTGCAATCTTAAAATGGGGTATTTGATGTCAGAGACACTCAATCAAATTAAAGAAAGTTTTATTG
AATATTTACTTTTTCAATATCGATTTAAGTCGCGTATTGCAGTTTGGGTGCTTAACTATATAAAAGTGAATGA
GGCGAAATTGGCAAACACTCATTTTGTAGATACAAAAATTAATCATCATGAAACATTAGAAATTGCAGAAGTG
GGAAGTCACGCTTCTGCAATTCAATTTACTAAGCGTAATATAAAGTTAATGAATACAAATGAAATATTTGATT
ATATCGCTAATCATAACTGTGCTTTTGATATTCAAATTCACTTCGCAAACGTGTCAAAGCGTGAGCAAAGGCT
AGATGATTTAATAGTTGCTCAACTAACGGAATCGCCCAGTTATCAAACTTATCTTCATGACTTAAATAGTATG
GCTATAGATCGGCATAAACATGCGTTATTAATAGATTACTTGTTACACAATATTGATTTAAGTTTACAGATGA
ATGAAAAGCAACGTTTTTATCAACTAACGCAAATTTTAAATACACTCAAGCTAGTAAATAAACATAATCAATT
TGAGGACTTAGCAGATGACGATTAATACATTTTGGCAATATACACTTTATCAACGTTCTTGGTTAATGATGCT
ACTCATTTGTAATATATTAGGGATGATTTATGGTTATATATGGTATGGTGAACAATTAAGTCATACGCCGTGG
CATTTTAAGATTTTTGTTCCAGATAGCCCAACTGCAATATTATACTTAGTTATTTCTATCTCATTGATATTAA
TTCAAAAACAAAATAGTATTATCGATGCACTGGCATTTGTAACATTATTCAAATATGGGATTTGGGCAGTTAT
AATGAATATATTATTTATTATTGAACAAGGTGATATTACAGTAAATGGTTTGGTATTAATGTTTTCCCACAGT
ATAATGGCTGTTCAGGCAATTTACTTCTATCCTAGGTTTAAACGTTCTATGATTGGAATCAGTGTAGCGATGA
TTTGGGTATTTTTGAATGATTATATTGACTATTTTCACCTTCAATTTCCATATTATGATTTTATAACTACACA
TGTGTGGCAAATTGGTGTATTATCATGTTGTCTAAGTGTTTTTGGACTTTTGCTTTATATTGAATTAAACAAA
TTATTAAAGTGTAAATGATTTGATAAAAACGGCAAATGACAGTAAAATATTTATAAAGGAGAGTGAGTTTATT
GTCTTCAATTTTTACTATGATAATTTATTTCGTAATATTAATGGTGCTTCCTTTATGGGCACAACACAAAGTT
AAATCTAACTATGAGAAATATTCACAAGTTAGATCAACTAGTGGTAAAACGGGTCGTGAAGTTGCATTAGAAA
TTTTACATGCAAACGGTATTTATGATGTCGAAGTTGTTAAAGGTGAAGGATTCTTAACAGATCATTACGATCC
TAAAAAGAAAGTTGTTTCACTATCTCCTGCAAACTATGACAGACCATCAGTTGCTGGAACTGCAATTGCAGCC
CACGAAGTTGGTCATGCCATTCAAGATCACCAAGGATATTGGTTCTTAAGATTCAGAGCAGCATTAGTACCTG

TTGCAAACCTAGGGAGTTCATTAAGTTATATGATCATCATGTTAGGTATTATCTTAACAGCGATAGGTAGTGC
ATTTGGTTCAACTGCTTTATGGATTGGTGCTGGTTTAATGTCATTAGCGGTATTATTCTCAATTGTTACGTTA
CCAGTTGAGTTTGATGCGAGTTCAAGAGCGATGAAACAAATTACGGCATTGAACATTGTAAATGAAAAAGAAT
ACAAGCATGCGCGTAAAGTATTATCTGCAGCAGCAATGACTTATGTTGCAGCAACTGCTGTCGCTGTAGCAGA
GCTTGTACGTATTATTTTGATTGCGCGTTCAAGTGATTAATCTTATAGATTAACAATTAACTTAAATTAGACA
CTTATTAACTAGCTAATCACATTTTTGTGTGGTTGCTAGTTTTTTTGTGCTTATTTTTCTCATGGTAGATTAT
TCACTATATTATTTTATTGTAGTAGTACGTATCATAAAGTTATAATCCCAATTTCAATTGATTTAATTTAGTT
AGGAATGCATTACATTTATATAAACTGAATTATTAAACTTCTAAAATAAACAACTTGTCATCTAGTTTAGCTA
GAGTCAGTAGATTGCACTAAACATGCATGAGACTTCAAATTGATATATACTATATTAGAAATGCAATATAAGG
AGAACGTAGAAATGAAATCAATGGTAGAAATGCAACGTGAAGTTGATGAATATATTGGACAATTTAAAACAGG
ATATTTTTCACCATTAGCTAACTTAGCTAGATTGACTGAAGAAGTGGGCGAACTTGCACGTGAAATAAATCAT
ACCTATGGTGAAAAAAAGAAGAAAGATTCAGAGGAAGCAAATACGATTAAAGCAGAATTAGGTGATAATTTAT
TTGTGTTGTTATGTTTAGCGAATTCAATGGGAATAGATATGACAGAGAGCTTTAATGAAACTATGGAAAAGTT
TAATACAAGAGATAAAAATCGATTCGAAAGAAAGTGAATATAAAAATATATGCTTTATTTGGATTTGATTAAG
ATTAAAAACATCCATCAATGAATTGCATCGCATGAAATCATATTATTTTTAAAGTAAGTATAACTAGATGAAT
GATTATATATACAACACCTGTTTTTTAGATATTTGAAATTATAAACTCAACATTACTATAAACTTAAATTTAA
ACAATTATCTACGCATCGAGAATAATAGAAATAGAAAGAAAGGATGGGCAAGCGAATGAAGATAGGTATAACA
TGTTACCCGTCCATGGGTGGTTCTGGAATTATTGCCACAGAATTAGGAATTAAATTAGCAGAGCGAGGTCACG
AAGTACATTTTATCACTTCAAATATCCCGTTTAGAATAAGAAAACCATTACCGAATATGATTTTTCATCAAGT
TGAAGTGAATCAATATGCAGTATTTCAATATCCACCGTATGATATTACGTTAAGTACTAAAATCGCTGAAGTT
ATTAAAGAATATGATCTTGATTTGTTGCATATGCACTATGCTGTACCTCATGCAATTTGTGGTATTTTAGCTC
GTGAGATGTCAGGTAAAGATATAAAAATTATGACAACGCTACACGGTACTGATATTACTGTTTTGGGCTATGA
TCATTCACTCCAAGGTGCAATTAAATTTGGCATTGAAAAAAGTGATATTGTGACAAGTGTGAGTAAATCTTTA
GCGCAAGAAACACATGAAATTATCGAAACTAACAAAGAAATTATACCGATTTATAATTTTGTACGTGAAAATG
AGTTTCCAACTAAACATAACACAGCACTAAAATCACAATTTGGTATAGCACCAGATGAAAAAGTGTTAATACA
TGTATCGAATTTTAGACAAGTAAAACGCATAGATACTATTATAGAAACATTTGCAAAAGTACGTGAAAAGATA
CCTAGTAAATTAATTTTACTTGGAGATGGTCCTGAATTAGTGCCAATGCGTCAATTGACGAAAGAGTTAAACG
TAGAAGAGGATGTTTTATTTTTAGGGAAGCAAGATTGCGTAAGTGAATTTTATCAATTATCTGACTTAGTATT
ATTATTAAGTGAAAAAGAAAGCTTTGGACTAACTTTACTTGAGGCTATGAAAACAGGCGTCGTTCCAATTGGT
TCAAATGCCGGTGGAATTAAAGAGGTTATTAAACATGGTGAAACTGGATTTGTTGTAGATGTGGGTGATTGTG
ATTCAGCGAGTGACTATGCTATCCGATTACTTGAAGATAAGGCGTTATACAACAAACTTCAGAAAAACATGCT
AGCAGATATTGCTGAACGTTTTGGATCAGAACTCATTACAGATCAATATGAGTATTATTATCAAAAAATGCTA
AATGAACATAACAAAAGTAAAGGCGAGTAATAATATGGATAAATCATTATTTGAACAGGCAAGGCCTATATTA
GAACAAATTCAAGACAATGGTTTTGAAGCATATTATGTAGGTGGCTCTGTAAGAGATTATGTCATGGGAAGAA
ATATTCATGATATAGATATCACAACAAGTGCAACGCCGGATGAAATAGAATCAATCTTTAGTCATACGATACC
TGTAGGTAAAGAACATGGCACGATAAATGTAGTTTTTAATGATGAAAATTATGAAGTGACAACATTCCGGGCT
GAAGAAGATTATGTCGATCACCGTAGACCAAGTGGTGTTACATTTGTTCGTGATTTATACGAAGATTTGCAAC
GAAGAGATTTCACGATGAATGCGATAGCAATGGATACAGCATACAAATTGTATGATTATTTTGATGGTCAACA
AGATATTAATAATCGAATAATAAGAACTGTAGGTATAGCTGAGGAACGATTCCAAGAAGATGCTTTACGTATG
ATTCGATGTTTAAGATTCCAGTCACAATTATCATTTGATATTGCAACGGAAACATTCGAAGCGATGCGTACAC
AAATGGCAGATATTAAATTTTTATCAATTGAGCGTATAGTGATTGAACTAACTAAATTAATGCGAGGTATTAA
TGTTGAAGAGAGTTTTAATCATTTAAAATCGCTGAAAGCATTTAATTATATGCCATATTTCGAACATCTTGAT
ATGAATCAAATTAATGTGACTGAAGCAATTGATTTAGAATTATTGATTGCTATAGCATCAGTTAAATTTGATA
TTAATTACTCATTGAAGCCTTTAAAGCTAAGTAACCGACAAGTAAAAGATATCAATCAATATATTCAAATTAT
GAATGCATTACCAAGTATTATTACAAAAGAACAATTAAAAATGTTTGTTTATGATTATGATACGAATCTCATT
AAAAATGTAATGGTTGCAGCAGACGTGTTAAAGGCAAATGATATTCAAGGACATGAACCATTAATCGTTAACC
TTCAAACGATTGATGAAACATTACATCGTTTACCTATGCATAATAGAAAAGACATGATGGTTAATGGCGGTGT
ACTTATGGCACATTTAAATGCCAAAAGTGGTCCATGGTTAAAAGATGTGCTAAGACAAATTGAGATAGCGATT
GTAACAGGTAAAGTAAGCAACGAAGAAACTGAAATTTTGAAATGGGTGGATAATCATGTCAAAATATAGTCAA
GATGTACTTCAATTACTCTATAAAAATAAACCGAATTATATATCTGGACAAAGCATTGCGGAATCACTTAATA
TATCGCGCACTGCAGTAAAAAAAGTGATTGACCAATTAAAATTAGAGGGATGTAAAATAGATTCAGTAAATCA
TAAAGGGCATTTATTACAACAGCTCCCAGATATTTGGTATCAAGGTATAATAGACCAATATACAAAAAGTTCT
GCTTTGTTTGATTTTAGTGAAGTATACGATTCAATAGATTCTACACAACTTGCTGCGAAAAAGTCACTTGTTG
GAAATCAATCTTCATTTTTTATCTTGAGTGATGAACAAACGAAAGGTCGTGGGCGATTTAATAGACATTGGAG
TTCTTCAAAAGGGCAAGGACTTTGGATGTCTGTCGTGTTAAGACCTAACGTTGCATTCTCAATGATATCTAAA
TTTAATTTATTTATTGCATTAGGGATAAGAGATGCGATTCAACATTTTAGTCAAGATGAAGTCAAAGTGAAAT
GGCCGAATGATATATATATTGATAATGGTAAAGTGTGTGGTTTCTTAACTGAAATGGTTGCTAATAATGATGG
TATAGAAGCAATAATATGTGGTATAGGTATTAATTTGACGCAACAACTAGATGACTTTGATGAAAGTATTAGA

CATAGAGCAACAAGTATACAATTAAATGATAAAAATAAATTAGATAGATATCAATTTTTAGAGAGATTACTTC
AAGAAATTGAAAAAAGATATAATCAATTTTTAACGTTACCTTTTTCTGAAATTCGTGAAGAATATATTGCAGC
TTCTAATATTTGGAATAGAACGTTGCTATTTACAGAAAATGATAAACAGTTTAAAGGACAAGCAATTGATTTA
GATTACGATGGCTATCTAATTGTTAGAGATGAAGCGGGTGAATCACACCGTTTAATTAGTGCAGATATAGATT
TTTAACACTAAAGCAAGGAGAGATAGCTATGGGTATGGCAACCTATGCCGTTGTGGATTTGGAAACAACAGGC
AACCAATTAGATTTTGACGATATCATTCAAATTGGTATAACGTTTGTGCGCAATAACCAAATTATTGATACAT
ATCATTCTATGATTAGAACAAATTTAGAGATTCCGCCATTCATTCAAGCGTTAACATCCATTGAAGAAAATAT
GCTACAACAAGCGCCATATTTTAATCAAGTTGCACAAGAAATCTACGATAAGATAAAAGATTGCATTTTTGTC
GCTCATAATGTAGATTTTGATTTAAATTTTATTAAAAAGGCATTTAAAGATTGTAATATTCAATATCGTCCCA
AAAAAGTTATAGATACACTAGAAATCTTTAAAATAGCTTTTCCTACAGATAAAAGTTATCAATTAAGTGAATT
AGCAGAAGCACATGGTATTAACTTAGCAAATGCCCATCGAGCTGACGAGGATGCCGCTACTACTGCTAAATTG
ATGATATTAGCTTTTGAAAAATTTGAAAAGTTACCGTTAGATACTTTAAAGCAATTATATTATTTAAGTAAAC
AGTTGAAATACGATTTGTATGATATTTTCTTTGAGATGGTTAGGCAATATGATGCAAAGCCATTGGATAAATC
GTACGAAAAGTTCGAACAGATTATCTATCGTAAGCAAGTTGATTTTAAAAAGCCGACAACAAATTATAATGGC
AGCTTGAAGTCTTTATATAGCAAAGCTGTAGATCAACTTGGCTTAACATATCGACCGCAGCAATTATATTTAG
CGGAAACTATATTAGATCAGCTCATGCATAGTGAAAAAGCAATGATTGAAGCATCACTAGGCAGTGGTAAATC
ATTAGCATATTTACTTGCGGCCTTGATGTATAATATTGAAACTGGAAAGCATGTTATGATTTCTACCAATACC
AAATTACTGCAAAGTCAATTGTTAGAAAAAGATATTCCAGCAATGAATGAAGCGTTAAATTTTAAAATTAATG
CACTATTGATTAAAAGTAAAAGTGATTACATTTCATTGGGGCTTATTAGTCAAATTTTAAAAGATGACACAAG
TAACTACGAAGTGAATATCTTGAAAATGCAATTGCTGATTTGGATTACAGAGACGCCGTCCGGAGATATACAG
GAATTAAATTTAAAAGGTGGTCAAAAGATGTATTTTGACCAGAAAATTGAAACATATGTACCGGCACGTCACG
ATGTCCATTATTATAATTTTATTAAGCGAAATGCACAGAACATTCAAATTGGTATTACAAATCATGCACATTT
AATTCATTCTGATGTTGAAAATTCAATATATCAATTGTTTGATGATTGTATAGTGGATGAAGCACATCGCTTA
CCAGACTATGCCTTAAATCAAGTGACAAATGAGTTGAGTTATGCTGATATTAAATATCAGCTTGGTTTGATTG
GTAAAAACGAAAATGAAAAATTGTTAAAGGCAATTGATCAACTGGAAAAGCAGCGAATTTTGGAAAAGTTAGA
TATTGCACCAATTGATATTTTTGGCTTGAAGGCTAGTATGAATGAGATTCATGAGTTAAATGAGCAATTGTTT
TCAACGATATTTACGATTATCAATGATTCTGATGTTTATGATGATGATATTCATCGCTTCCATAATGTATTTA
CTTTTGAAACAAAAGACATTTTGAAGGATTTACATGCGATTATCGATAAGTTAAATAAAACGCTAGAAATATT
CAATGGCATTAGTCATAAAACAGTTAAATCATTACGGAAACAGTTGTTGTATTTAAAAGATAAATTTAAAAAT
ATCGAACAAAGTTTAAAAGCTGGACATACTAGTTTCATTTCAATTAAAAATTTATCTCAAAAATCAACAATAC
GTTTATATGTGAAAGATTACGCTGTAAAAGATGTATTAACGAAACAAGTATTGGAAAAATTTAAATCACTCAT
TTTTATATCTGGTACTTTAAAATTTAATCATTCGTTTGAAGCTTTTAAACAGTTATTCAACAAAGATGTTCAC
TTTAATACATTTGAAGTTAACACGTCGTTACAAAGTGCAAAAAATACAAGTGTATTTATACCGAGTGATGTAG
CATCTTATCAATATAAAAATATAGATGAGTATGTAGCATCGATTGTAAGCTATATTATTGAATATACGACTAT
AACGTCATCAAAATGTTTAGTCTTATTTACGAGTTATAAAATGATGCATATGGTACAAGATATGCTAAATGAA
TTGCCAGAATTTGAAGATTATGTTGTATTAACACAACAGCAGAACCAAAATTATAAAATAGTACAACAGTTTA
ATAATTTTGATAAGGCCATACTGCTTGGTACGTCAACATTTTTTGAAGGTTTTGATTTTCAAGCAAATGGTAT
TAAATGTGTGATGATAGCGAAGTTACCGTTTATGAACAAGCATAATGCAAAATATTGGCTTATGGATTCTGAA
TTCACTTCAACTTTCAAGGAATATGTATTACCTGATGCAGTTACACGTTTTAGACAAGGACTTGGAAGATTAA
TTAGAAATGAAAATGATCGCGGCATAATAGTTTCATTTGATGATCGACTCATAAACAGTAATTATAAAAACTT
TTTTGAACAAACACTTGAAAATTATCGTCAGAAAAAAGGCGACATTCAACAGTTTGGAAAATTATTAAGACAA
ATTCAAAAAAAGAAAAAGTGAAATACAAAGTAAAATTCTTGTGACATGACATGTGCTATGTTCTGTTACAAGA
ATTTTTGTTTGGTCGTCAAAATTTGAGTTTTATAATTAAACTTGAATGTTAGCACAAAAAACGACCTTCATAC
TAATAATATTGAGGATTTATTATATATTTTGTCTGAAACTGATGATTGTATTGTCTTAATATAAATTAATGTT
CAAATCTGTGCTTAAATCATTACCATTTACACGTTATCTTTTAGTCTAGTTCTAGAACTTGAAAGCGGATTTT
GTTAAAATGAATGAGTATTGTATAACGAAAAAATAAATAGGAGAATGGTTATGAAAACAACGATTAAACAAGC
GAAAGATCATTTAAACCAAGACGTTACAATTGGTGCTTGGCTAACAAATAAACGTTCAAGTGGTAAAATCGCC
TTTTTACAATTACGTGATGGAACAGGCTTTATGCAAGGCGTAGTAGTTAAATCAGAAGTTGATGAAGAGGTAT
TCAAACTTGCGAAAGAAATTACTCAAGAATCATCTCTATATGTTACAGGCACAATTACAGAAGATAATCGTTC
TGACTTAGGATACGAAATGCAAGTGAAATCAATTGAAGTTATTTCAGAAGCGCATGACTATCCGATTACACCT
AAAAATCATGGTACAGAATTCTTAATGGATCACCGTCATTTATGGTTACGTTCTAAAAAACAACATGCTGTAA
TGAAAATTAGAAATGAAGTTATTCGTGCAACGTATGAATTTTTCAACAAAGATGGATTTACAAAGGTTGATCC
ACCAATTTTGACAGCAAGTGCGCCAGAAGGTACAAGTGAATTATTCCATACTAAATACTTTGATCAAGATGCG
TTTTTATCTCAAAGTGGTCAGTTATACTTAGAAGCTGCAGCTATGGCACACGGAAAAGTATTTTCATTTGGTC
CAACTTTCAGAGCTGAAAAATCAAAAACACGTAGACACTTGATCGAGTTCTGGATGATTGAAGGGGAAATGGC
TTTCACAAATCATGCTGAAAGTTTAGAAATTCAAGAACAATATGTAACACATGTTGTAAAATCAGTTTTAGAA
AATTGTAAACTAGAGTTGAAAATGTTAGAGCGTGATACATCAAAACTTGAAAAAGTTGCGACACCATTCCCTA
GAATTTCATATGATGATGCAATTGAATTCTTAAAAGCAGAAGGCTTTGATGATATTGAATGGGGCGAAGATTT

TGGTGCGCCACATGAAACAGCCATTGCTAATCATTATGATTTACCGGTGTTTATTACTAATTATCCAACTAAA
ATTAAGCCTTTCTATATGCAACCAAATCCTGAAAATGAAGAAACTGTATTATGTGCAGACTTAATTGCACCTG
AAGGATACGGTGAAATTATAGGTGGATCTGAACGTGTGGATGACTTAGAATTGTTAGAACAACGCGTTAAAGA
ACATGGATTAGACGAAGAAGCATATAGTTACTACTTAGACTTACGTCGTTATGGTAGTGTGCCACACTGTGGA
TTTGGTTTAGGTTTAGAGCGTACAGTAGCATGGATTTCTGGTGTTGAACACGTTCGTGAAACTGCGCCATTCC
CAAGATTATTAAACCGTTTATATCCATAAGTTTTAGGATCGTTACTTAATATTTATATTATGATAATAAAAAG
AATCGTCCAGCCTATATTAATAGCTGGGCGATTTATGCTTGGAAAATGAAGATTGATAACGGCTTATATTAAT
CATGAACAAACTTTTGTTTAGTAACTATATTTGGAAAATAAATAGTTCATCTGATTATCCATAAAGTTCATTG
TTAAAATCTAGTTTTAAAAAATACTGTTATTTAAAATTTGTTTGCTACTAGTTAAATATATTAAGCATTTTAG
TCGAAAATTAAAGAAAATGATTTATACTATTAAGTAATGGTTAAATTTAAAAGGGGGCACGAACATGGATAAA
TATCAATTAAAAGCAAGACCTGTAGTGATACGAAGAGAATTATTAGACCATTACAGTGACTTAGGTTTAGATG
AGCAAGATTTAGTCATTTTGCTTAAATTGATTTATGCTTCCGAAACATCAAATAAACAGCCTTCAATTGAATT
ACTTCAGAAGGGCTCAACTATGCAACCACGTGATATTACAATGGTTATTCAAAATTTGATACAACGTGAATTG
CTAGAATTACAAGTTCAAAAAGATGAAGAAGGTAGATTTACCGAATATATGAATCTAGATCCTTTTTTCGAAA
AATTAAGTCATATATTGAAGCAACAAAGTATGGAAACGAAAGAGCAAAATAGTAAAGAAAAAATTTAAACAATT
ATTTAGAGTTTTAGAGGATACATTTGCAAGGCCGTTATCTCCATATGAAATAGAAACGTTAAATCAATGGATT
GATGTTGATAAACACGATACTGCAATTATACAAGCGGCATTAGACGAGGCGAATAGTTTGAATAAACTTAGCT
TTAAATATATGGATCGTATTTTACTTAATTGGAAGAAAAACAATGTTAAAACAATTGACGATTCTAGAAAAAT
AAGAGAAAAATTCAATAAACCGAAAATGACGCACACAGTTAAAACGGTACCTAAATTTGATTGGTTAAACGGG
GAGAACCTTGATGGTAAGTAAGAAAAAAGCATTAGAAATGATTGATGTTATAGCAAATATGTTTCCGGATGCA
GAATGTGAATTAAAACATGATAATCCGTTCGAATTAACTATTGCTGTATTATTGTCAGCGCAATGTACAGACG
TTCTAGTAAATAGAGTAACAACTGAGCTGTTTAAAAAATACAAAACGCCTGAAGATTATTTGGCAGTCAGTGA
TGAAGAACTTATGAATGATATTCGTTCTATCGGTCTGTATCGTAACAAAGCTAAGAATATTAAGAAACTTTGT
CAATCTTTGATTGATCAATTTAATGGAGAAATACCACAAACACATAAGGAATTAGAAAGTTTAGCAGGTGTAG
GACGTAAAACTGCTAATGTAGTCATGAGTGTAGCATTTGATGAACCTTCGTTAGCTGTTGATACGCATGTAGA
ACGTGTTTCTAAACGCTTAGGTATTAATCGTTGGAAAGATAATGTGAGACAGGTAGAAGACCGTTTATGTTCT
GTCATACCTAGAGATAGATGGAATAGGAGCCATCATCAACTCATTTTCTTTGGAAGATACCACTGTTTAGCCC
GAAAACCTAAATGCGATATTTGTCCACTATTAGAAGATTGTAGAGAAGGACAAAAACGTTATAAAGCTAGTTT
GAAAGAAGCGTGATAATATGACAAATATTACAAAAGAGGTATTTGATAATTTAGAACAAGAGATTGATGTATT
TGCCAAAAATAAGACATTAGGTTCTAGTGAAGCAAAACCTTACTTGGATGAATACCATAGTAAAATTATTGAT
TATTTTAAGCAGGTTAATGACATCACTGGCAATATTGATTTTGATAATTTAAATCAATATCCTGTTGTGCCAA
TGAATTTTAAAGAAAGATATGATTATATGATTGAACGTAAATATCATTTTATGGGCTATCGACAAATGAAGAC
CTTTAAAACTGAATTGATTAAAATGAATGCTAGTTATCAAACAAGGTTAAAGAATAAGCAGGTATAGACAAGT
TTGATCATTTTCTAATGTTAAAAAGATATAAGACTAATTCATGCACACATATTTGTATTTATGTTAGATGTCG
TGTCATTACAACGTCTTAATATAAAAACTGTCCATCTACATCATAATGGATATCTTGAGGTATGCTTTAAGAT
ATCTATTTCGAATCGTAGGTGGATTTCTTATATCTATGATTGTGAAAATATGTTGCAAACATGTTGATATTTC
ACTGATTAACGTACTCAGGGAATTGATAAACATATCAATTTAAAATAACGTCTAAGATGATTATGAGAAAATA
TAATCATAATTTTAAAAATGGGAACAACCACTTATTGAAATTCAATAATTTTACATAGCTAAAGATGTGAATA
TGAAAGACTATTTAGATGTTTCAAAATGTATGTGAATGTCTGATTTTACGGTTTCTACTAATATAGCAATAGC
GTATGAATACTTATAAAAAAATCCTCAGCTACACCATAAATATGGTATAACTGAGGATTTTATGTTGAGTGGA
TTAGTTGAATATACCTGTTAATCCACCGCTGTTTCTAGATTGTTGAGATCTAGTATTGTTATTTGATTGTGCA
GTACCACTGCTGTTTGCTGATGTGTCACTACCACCGTGTGCACTACGATTTGTAGTGTTGTTATCTTGACTAC
CAGAAACATTGATTGATGGGATACTACCACTTACTGAGCTAGGACGTTTAAAGTCTTCGCCATCTCTAGATGA
AATTTTTGACATCACATTTTCATATAAGAACTGTGGATATTCTTGTTGGCTATGTCCCACAAATGAGTTTTCA
CCATATTGTTTAACTTTGCTGAAGCCCATCCACACTGACATAGTGTATTGAGGTGTAAAGCCGTTAATCCACA
CGTCTTTCGCTGCATTATCAGGTAAATTATATTGTGAATAAGTTTCAGCACCGTAAGTACCAGTACCTGTCTT
AGCACCCATATTTACTCCAGATACACCATGGCCATATGCAGAACCATATGGTTTAAATGTACCTTTTAGCATC
TCAGCTAACATGTATGCAGTGTAATCACTCATCGCTTTATGGCTAGTATGATCGTATTCGATTGTTTCACCAT
CACGAGTAACTACTTTTTGAATTGAATGCGCGTTGTTATAAGTACCACCGTTAGCGATTGCAGCAAATGCTGA
TGCTAATTGTGTTGGTGAGAATTCTGAAGCAGAACCACCAAGTACTTCAGATGGACCAATATCGCCTTCGTAG
TTTAAGCCAAGTTTGGCAGCGAATTTCTTAGGTGCATCATTACCAGCATTTTGCTTAACTGATTGCCAAGCTT
TTAAAGCTGGGATATTGAAACTTTGTCGTAAAGCATCATAAATTGATACAGTACCGTGACTCTTCACATCGTA
GTTTCTAAATGTAGAGCCATCAACTTGATATGAAGATTCATCTTGAATCGCATGGTTTGTTGCCCATTTCATA
TTTTCAATGGCAGGTCCATACGCTAAGAAAGGTTTTAAAGATGAACCAGTAGGGTGTGGATCTGTTGCTTGGT
TTCTGTTAACGACGTCTTTGAAATCACGTCCACCAGATATAGCAACTAAACCACCAGTTTTACTATCAAGAAT
CGTTGCACCAACTTGTTGGTCTTTATTCTTGTAGAAGCTACCATTATCAACATCATTTTGTAATGTTTTTTGA
ACATCTTTATCCATGTTTGTATAAATTTTAATACCACTTTGTAATACATTACCTAAATTTTCATCTTTGAATG
CTTTATTATTCATTAATTCAGATTTTACAAAGTTAACGTATGAATTATACTCAGAATCTTGATTTGTATCAAT

GTTTTGACGTTCTTCAGCAGTACGATTTACTAAGTTCGCTTTTAAATCGATTTTCTTAGCATCTTCCCACTGT
TTATCTGTAATGCGTTTATGATAATGCATTAAGTATAAAACAGTGTTTTTACGATCTTCAGCAGCTTTTGGAT
GATCATAAATATTATAGTTGTTTGGAACCTGAGGTAAACCGGCTAAATAAGCTTCTTCCGCTAAGTTTAAATC
TTTTAAATCTTTATTAAAGTAATACTTAGCAGCAGCTTTAATACCTGTTACGCCATCAGAGTAGTAAATTTTG
TTTAGATATACTTGGAAGATATCATCTTTACTATACTCTTGTTCTAAACGATATGATAAGTATGCTTCTTGAG
CTTTACGTCCAATAGATTTATGTTGTGATAAAAATGCATCTTTAACAACTTGTTGTGTTAATGTTGAGGCACC
TTCAGAACCAAATCCACCAGTCAAGTTCTTACCAATTGCACCGAATAAACGTTTATAATCAAGTGCGCCGTGT
TCGTAGAAACGATTGTCTTCAGTTGCAAGTACTGCGTCTTTCATTGATTTCGGCACGTCTTTTAAATTTACAT
GCTCATGTCTTTGGCCATTATCTAATGTTTTAACAAGTTCTCCGTTCTTGTCATATATCTTTGCAGGAATCGG
ATCTTGTAATTTAGCTTCGGTAAAAGCAGGTGCTTTCCAAGCATAATAAGCAAACAATAAGATACCTAGTAAA
AGAACAACGAAAAATGCAATAATCATGAAGCCAATAATCTTAATAATCGTTCTCTTCACATTTCTATTCTTTT
TTGAGTTGGATTTCCCACCATTATTACCATTTTTCTTAGGCTGAGAAGATCCTTTGTTTTCCGTCATACGCGG
TCCTCACTTTCATCTAATATCAACTTATCAACTGCTTTTAGATAGTCTAATCTTGGTTGATACTGATAAGGAA
TATGGTAACCATTTTTTCGTATTTCATCAACTGTTATAGACTTTTTAATATTATCTTTATATCTCTTCCAAAA
TATTTCGAATTTTGAATAGGGTAAAAGATAAACTTCATCTAGCGTTTTAAAACGAATCATTAAAAACACAATA
CCTTTTTGTTGATATGCATTTTTCATATGTTCGACTTGATGATCATGAATATTATTTAAAGGAAAGGACGTCT
TGTTTTTAGTTTCCTTTGCTTCAAAATCAATATAATAACCTTGATAAACGCCGTTGTAATCAGTTGTTGAAGG
TGTACGAAAATAAGCTTCGTTAATCACAGCTTTACTCCGTTTGGGATAGTTGACATTAACTATTTGTACCGGC
GTAGGCTTTTTGTGAATAACTGCAATGTCGCTTTTAAGATAAAACGCATTTGAATGTTCGATATCTTTTTCAA
GTGACATACCACGTCCACCATACTCAATATTACTAAAGGCAGCGGTCTTTTTCCCTCCGTCTATAGCACTATT
TTTACGATATGGTTTACCATTTGGATAATTCATAAATTTCACCACACAAGTTTGGTAGTAAACGTTGTTTATT
ATACACAACATTATAGTGAAACACCAAACTTAAATTAGTATAAATGCGAAAGACACAATACACCAAAGCTTAT
TTTAACGTGATTTCAGCATTAACACAATAATATTATAATACATTTACCGTTAATGTTGGAATTTTACACACAA
TTTTAATTAATTGAAAAGTTTTGCTTATAAAAATGAATACTCATTACAATATTTTTGAATTTTGTTATAAAAG
TCGAATCATGATCAGTAATTATTATTTTTGAGGCAAGTACAAGTATGTGTTGTTAATCAGCAGGGGTGTATTT
GATTTAAATGATTTTATTAGATTATAAGTATTTTCATCGTATAACAACATACACGATAAAATTTTGTATTTAT
ATAGGAAGTTTTTAGATTTACTTGATTTAACAGATAACACATTTAAAAAATAGTTAAATCAAATTATATAGAG
TGTTAAAACATGACGAATACAGTATATTGTGATAAATTAAAAAATGTAGGAGGCGTCCTCATGAATGATGTAG
TCGAATCACTAATTTATGAAGTTAACAACATGCAACAAAATTTTGAAAATGTGAAATCACAACAACAAGATCA
TGATTTTTACCAAACTGTAAAGCCATATACTGAACATATTGATAGCATGCTCAATGAGATCAAATTACATCGT
GAATTTATTATAGAAGTACCTTATATGAATTCAAGGAAATTTGAGCTACTGATTGCTAACATTGAACAACTTT
CTGTCGAATGTCATTTTAAGCGAACAAGTCGAAAGTTATTTATTGAAAAGCTTAAAAGTGTTCAATATGATTT
ACAAAATATATTAGATGGCGTAACAAAAGAGGGTACTCATGGTTAAAACAGTTTATGTAACAGGTTACAAATC
ATTCGAATTAAACATTTTTAAAGATGACGCACCTGAAGTACATTATTTAAAACAATTTATAAAACATAAAATT
GAACAACTGTTGGATGAAGGATTAGAATGGGTGTTAATACAAGGGCAAATGGGTATAGAATTATGGACTGCAG
AAGTTGTTATTGAATTACAACGAACATATGATTCGTTAAAGTTTGCTGTAATTACACCGTTCCAAGGACATAC
AGAAAAGTGGAATGAACATAATCAAAGTAAGTATGCCAACATAATTAAGCATGCAGATTATGTTGATAGTATT
TTTCATACGCCGTATCAAGGTCCTTTTCAATTTAAACAAGCAGATCAATTTATGCTTGAACATTCGGACCAAA
CATTGCTCATTTATGATGAGGAACAAGAGGCGAGTCCTAAGTTCTTCAAGCAGATGTTAGTTGATTTTATGGA
TAAAACAAACTATACTTGTGATATTGTGACGTTCGATGAATTAACAGCATTCATCAATGACTTACAGTGGTCT
GAAGATCAAAGTTTCTAATGAGGTGGAAAAAATGTCAGATGTTTCATTGAAATTATCAGCAAAAGATATTTAT
GAAAAAGATTTTGAAAAAACGATGGCTCGTGGCTATAGAAGAGAAGAAGTAGATGCATTTTTAGATGACATTA
TTGCTGATTATCAAAAAATGGCCGATATGAATAATGAAGTTGTAAAATTATCAGAAGAGAATCATAAACTTAA
AAAAGAATTAGAAGAATTAAGACTACGTGTAGCAACATCTAGACCTCAGGACAATAAAAGTTTTTCTTCGAAT
AATACAACAACAAATACATCTTCAAATAATGTAGATATTTTAAAACGTATTTCAAACTTAGAAAAAGCTGTAT
TTGGTAAATAATTACTAAATATAAAAGTTTAACTGTCTTACTAATAATGACTATGTTATAATTTTAAAAGTGA
TATTTTGGGTAATCGCTATATTATATAGAGGAAAGTCCATGCTCACACAGTCTGAGATGATTGTAGTGTTCGT
GCTTGATGAAACAATAAATCAAGGCATTAATTTGACGGCAATGAAATATCCTAAGTCTTTCGATATGGATAGA
GTAATTTGAAAGTGCCACAGTGACGTAGCTTTTATAGAAATATAAAAGGTGGAACGCGGTAAACCCCTCGAGT
GAGCAATCCAAATTTGGTAGGAGCACTTGTTTAACGGAATTCAACGTATAAACGAGACACACTTCGCGAAATG
AAGTGGTGTAGACAGATGGTTATCACCTGAGTACCAGTGTGACTAGTGCACGTGATGAGTACGATGGAACAGA
ACATGGCTTATAGAAATATCACTACTAGTTTAGCTCTCCTAGATGATGGAGAGCTTTTTTCATGAAAAGAACA
CTTAAAATTAACGCCTTGTCTTGATATAATGACACTGCCTTGTTTTAAAATAATAAGCGAATGCGTTAATGTA
TCAGCGATTAAATTTGTTGGAAATGTATAAAAAACACAAGCTAAGAATAAAATACCTGTATAAAAGGAGAATC
ATATATGTTTCAATTACTTGCAGTTTGTCCGATGGGATTAGAAGCTGTTGTTGCTAGGGAAATTCAAGAATTA
GGCTATGAAACAAATGTTGAAAATGGTCGTATATTTTTTGAAGGAGACGCAAGTGCAATTGTAAAGGCAAATT
TATGGTTGCGCACAGCAGACCGAATCAAAATTGTTGTTGGACGTTTTAACGCAACAACGTTTGACGAATTATT
CGAACAAACCAAAGCGCTCCCTTGGGAATCTATTATTGATAAAGAAGGTAACTTCCCAGTTCAAGGTAGAAGC

GTTAAATCAACACTACATAGTGTGCCAGATTGTCAGGCGATAACTAAGAAAGCTATTGTTGAACGATTACGTC
GTGCATATAACGAAAAGGGTTGGTTAAATGAATCAGGTGCCAAATACCCTGTTGAAGTTGCCATTTTAAAAGA
TAATGTATTATTGACCATCGACACATCAGGTTCTGGTTTGAACAGACGTGGATATAGATTAGCACAAGGTGAA
GCACCAATTAAAGAAACGTTGGCAGCAAGTTTAATCCGTCTCGCAAACTGGAAAGGTGATACACCTTTAATTG
ATCCATTTTGCGGTTCAGGTACTATAGCTATAGAAGCTTGTTTAATTGCTCAAAATATCGCACCTGGTTTTAA
TCGCGAATTCGTATCAGAGCAATGGAACATCATGCCAGCAAATATTTATGATGATTACCGTGATGAAGCGGAT
AAGATGGCTGATTATGATAAGGAAATCGAAGTATATGCTTCTGATATTGATCCAGAAATGGTAGAGATTGCTA
AGCGTAATGCTGAAGAAGTTGGGTTGTCTGATATTATTAAATTTAGTGTAAAAGATGTCAATACATTAACAAT
TGATACAGAAGAACCGGTGGCGTTAATTGGAAATCCTCCATATGGTGAACGTATTGGTGATCGTGAAGAAGTT
GAAGAAATGTACCGTTATATTGGTAAACTAATGAAACAACATCCATTTTTATCTACATACATTTTAACAAGTA
ATAAAGAATTTGAATATTTAGTAGATCGTAAAGCAACGAAGCGACGTAAGTTATTTAATGGATATATAGAATG
TACTTATTATCAATACTGGGGTAAAAAGACAGAACGTAAAACTATCGAAAACTAAAGTGTTTATAATGCGTGA
CTAAAATTAGTAATAATTATGTTCTCATGATAATAGGTATTTTTGAAAAATGGAGGAGTCTATAAATGGGTAA
AAAAATGGGTCTAGGTTTATCTATAGCATTGGTTGTTATTGGTATTGCCGTTGTATGTTTAATGATTTTTTCT
AGTCAAAAAACGACTTATTTTGGTTATATGAATAGTAATACAAATGCAGAAAAAGTTGTCAGTGAAAAAGATG
GATTAGTCAAACATAATATCAAAGTAGAACCATCTAATGATTTCAAGCCGAAAAAAGGAGACTTTGTAAAATT
AGTTTCTAAAGATGGTGGGAAGACATTTTATAAACAAGAGATTGTTAAACATGATGACGTCCCACACGGTTTA
ATGATGAAAATTCACGACATGCATATGAATTAATAAAAAAGCATCTATAACGTAATTTGGAAGAAGTAGAGTT
ATCTTCTTATGCGTTTTAGATGCTTTTTTATCTTCAAATTCAAAAAATCGTACTGATGGAAAAGTAAAAAACT
TTTTCATCAGTACGATTTATTATAGAACCCGTTTTAAGTAATTATAGTGTCGAGAATCATTTTGTAAATTTCG
TCAGTTCAAAACTTTAAGTGCATTATTGTTTAGCACGACGATCTTTAATATATAATATTAATTCAATTATAAA
TACAAGGATAAGTGATAATCCAAATAATGGCATTAATAATCCTAATACAATTAAACATATTATGAGTGATATA
GATGCTGGTTTTTTTACGCGATGTGGTACTTTTACTGCAGTATTTTTTGTACGCTTTATCCAAGAGACAAACC
CCATGCCAATGGCTACTAATAAAGCGATACATACAAATAAATTAATGATTTTATTTAAAATGCCGAATAAATG
ACCTTCGTGAAGCGGAATGCCCCATGTAAACCATTTAGCAATAATACCGTAGTCATCATATTTAATCGTACCT
AACTTTTTACCGCTATATTGATCGAAATAAAGTGTTTGTTCCTTGTAAGGTGATACATCTAGCCCAGTAACAC
CTGAATTACTAGTATTAGATACAATAAAGGCACCATTTCTATCACTTGGATATACGATTGAAAACGGCTTTTT
AATACCAGATTTTTGCGCCTGTTCAACGACCTTATCAATAGATATTTGATAATCTAATTTGGTTTGAGGCATT
GCTGCATTACCATGATGACCACTTGAACCTTTTTCGGATGAAGCTGGCTGTTTATTTTTACGTGTAGCCCAAG
GTAATTCGTTCAAATCTGATTTAGGTGGATTAATCGCTAATTCACCTTGACCAAGGTCACCGTTTGTGTCCAT
CATACCGGCAATTTTACTACCCATAAAACCAGACCATGGCAATCCAGTTATGACTAATACAAATACTGGAATT
GCAATGATAACTCCAATCATTGCGTGCCACTTTTGCCAACGAAGTGCCTTACTTTTGTTAGAAATTAAATGCT
TCTTAATGAGTAAATAAGTACCAGATAAAATCATGAATATCGTCCAACATGCGGTTAACTCTACTAAATAATT
AATAATAGTATTTTCAGTAAACAAAGAACTATGTATACTTCGCATCACATTAGAATACGTATGTTTCGCATTT
TGATCTGCAACAAATTGATTGTTACGATCTAGAAAGACGTAACGTTGATTTCCTGCCATATCACTCAGTGTAA
TTCGTTTGTTATATGGTTCATCAAGTATGCTAACTTTACTTACAAAGAATCCTTCATATTGTTGTTCAACTTG
ATGTACTGCATCATTTAATGTTTGATGCGTTTTTACATCACTGTCACCAAAAAACTTATTCTTATAAATATTA
TTTTCAACTTCTGGAAAGAACAAGTAACCAATGCCCGAAATGGTTAAAGTGATTAACAGTGGAGCAATAAATA
TTGCTGCATAGAAATGTAATCTTTGTAATGGATTAAATGTATTTTTCATATTTCCCTCCCAATTGGCTATTAT
ACGGTGTCAATTCTGTGACGTGTGTGAACAAACTGTGACAATATTTATTTTCTAGAAAAATTTAACGATGATT
TGTGATATTTAGAAAAATGAACTTTTAAGTTGGAATGTTTGAAGAAAATTGATTATTCGTATGTTTTATCAAG
CAGCTATGATAAAATTTAAACATAATACAATGCGAGCCATTCAACGATCTTTGTTTAAATGGATATCGATATT
GTATGAATTCGTTTTAACAAGCAAGCATTTCTATGTGAACGAACCAAAGGGGAAAGTAACATGATTAATAAAG
AACAATTAGATCTTTTATATAAATTAAAAAAAGAAGTTGAAAAGTCGCGAAATGAAGCACTTTTACATACAAT
TAACCAAGTAATTAAGAAAGTATATTTGCAGCAATATACATGTTCGTTCGTTGGACATTTTTCTGCAGGTAAA
TCGACACTGATAAATTTATTAATTGAACAAGATATCTTACCAAGTTCACCTGTACCAACGACAAGTAATACTG
CTATCGTGTCAGTTTCAGACAATCACGATATTATTGCTAATTTGCCGAATCAAACGTATGCCAAATTATCTAA
TTATGATGAAGTAAGGGAAATGAATCGCCAAAATGTCGACGTTGAATCTGTAGAAATTAATTTTCAATCAACT
AAATTTGAAAATGGGTTTACGTTGCAAGATACACCAGGTGTTGATTCAAATGTTGCATCACATCAGTCAATAA
CAGAACAATATATGTATACAAGCAATATGATATTTTATACGGTTGACTATAACCACGTTCAATCTGAACTTAA
CTTTAAGTTTATGAAGCATATAAATGATGTTGGAATACCTGTTGTGTTTATCATTAATCAAATTGACAAGCAT
CAAGACGATGAATTGTCATTCTCTACGTTTAAATCTCGAGTTGAAAAATCAATTGCTGATTGGGGCATTAAAT
TAGAACGCATCTTTTATGTATCTAAATTTGATCACCCTGAAAATGAACTTGAAGTATTATCAACTTATCTAGT
TTCATTAGATCAACATAGAGAAACAATAGAGGATTATACATTAAGAACGGTTGAATATATTACCGAAGCTCAG
CTAGATTATATTCAGTCTGAAATTCAGGAAGTATTAGAAGATTTGGGTATCGAAGAAGCAGAGTTTGAACAAG
CATTTTTAAATAGTCAACAGCATCAAGCAATTAGTGAAGAGGCACAACTTTTAAATAATCCAGATGAATTAAT
GGCATTTTTAAAGAATAAGCGTAAAAATATTTTAGAAAATGCATACATTATGCCGCATAATATGAGAGAAATG
TTACGAAGTTATTTGGAAAGTATGTCTCAAGACTTTAATGTTGGCGGATTTTTTAATAAAAAGAAGAAAAAGC

TACAAATTCAACAACAGCGATTATTAACAGCGACAGATGCGTTACAAGAACATGTTAATCAACAAATTCGTCA
ACCAATGCGAGAAGATATGTCATTTGTTACACGTTTTATCAATAAAAAAGAAGCTTCAGATAAAGTATTAAAT
CAGCATTATGACGTTAAGTCGGAAATGATTGAAGCTTTATATCAACCACAAACATCAATCAGCAATACTTATG
TACTTACATTTTCAGACGAAGTGGTTAAAGCCATTAAGAAATACGTTGAACAACAGTCAACACCGATTTTTAA
AGAAATTATAGAAAATGTGCAGGCAGATGAATTACCAACAGAAGAAAGTGATGATTTAAAAGAATATCAACGT
TATACAGAATTAAATGAGCTGCGTCAGTCATTGACGACTAAGAATTATCGTCACTACTATATTCATTTAGATG
AATCTCTAGATAAATTAGTAGATCGACAAGAGACAACATATCAAGTGGCTACTGATAATGCTCAGGGTAATCG
AGATAATCAACAGCTAAATCAGAATACCGCTACAACAAATATGTCTATAGATATTCAAAAAGCACTTGATATA
ATTTCGGATGTGCCTTTGTTCAAGCGTACAAAGCAGGATATCCACGAAACATTAACACGTATAGATAATAAAT
TAATAAAAATAGGCGTATTTGGAACATTTAGTGCGGGTAAAAGTAGTTTGATAAATGCATTATTAGGCGAGCA
TATTTTAGTCAGTTCTCCAAATCCTACTACAGCAGCTACTACAGAAATATCTTATGGAGACGAGAGTTATATA
ACGCTAAAATCACAATCGCAATTATTAGATGAAATTAATGCAGTAGTTGAATACCAAGATATATCTTTTTCAA
CTATAGAAGACTTTATTAATTCAGATTTAGAAAAGTTAAAATCACATTTAAATAAAAATCAACTCGCTTTTGT
TCATGCAGTTGAAAAACATTATAAATTGTATGTCAGTATGTTGGAAAACGGAGAAAAACATGCCATTAATCAA
CAAGAATTGAAAAAGTGGAGTGCAGAAGATGAATATGCAACATTTGTTAAAACAGTACACATTGCATTAATGC
ATGATTGGTTAAAAGGTAAAATAATTGTTGATTCCTTAGGGCTACACTCAAATAACCAAAGGCATACAAATGA
AACAGAGCAAATTTTAACTTCTTCAGACTTAATATTGTATGTGAGTTATTTTAATCATTCATTTACTGATAAT
GACAAGGCGTTTATAGAACACATGAAAGATATGAACCAGTTGAATGAAAACCAAGCATTTAAAATGGTAATTA
ATGCTGCTGATTTAGCAGAAAGTCAAGATGATCTTGAAGCGGTTGAAACATATGTATCAGATGCATTAAGACA
AGTACACTTACAATCAGACATTTTTGCTGTATCAAGTCGAAATGCATTGCAAGCTGAAGATAAGGGCATTGAT
CAATTAAAACAAAGCATACAACAATTTGTTGATGTTGATTCTAAATCAATTTTAGAACAACAAATGATTCATC
AGCTTCAGCAAATGGATCGTTCTTATGTAGAGATGATTACAGAATTCGAAACAAATAAAGCTGATATTTCACG
TCGCCAACAAAGGTTAACAGACTATAAAGATAAACAGCGTTTACAACATCAATTAATTGATGCGACGCTACAA
CATACAGACAACGAAGTTGAAGAACAAGTTTATCATTTAAATGCACGTTTAAAATTACAACTACTTGACGATG
TTAAATCAGTGTTTAATTCTCAAATGACGCAAAATAGTGATTTTAATGAAGAAAAGAAAGTGTCTACGAAAGT
ATACTTAGATCAAATTCATCAACGATTGTTTTTAGAGCAATCTTTAATTACAGAACGTATAAAAAAATACTTT
AATAAGCAACTCACTGAGCAAATTGCCCCAATCGTTCAACAATTAGCAGATTTACATGTCATTGTTAATCCTC
AGTTTAACTTTGAATCAGCTAATATAGAGCAACCATTATTGCACATCGATTTCAATGATATGCTAAATGCATT
GCCTAAACAATTAACAAAACGTAAAATTTTGAATCCAAATGGGCAAAGAGATATACATGAATCAATTTGTCAA
AGTACGTTAGGATTATTACAACCACAAATGGGATTATTGAGGCAACAGCTTGAATTATATGTAAAGCAAATGG
CTGTAGAAGCTGAATCGCAATTTGAAAGTTTTGAAGCTAATATTCAAACGCAAATAAACGATTTATTAGCATT
TGATTTAGATACAACACTTATCAATCAATTGAAAGATAAACATCAACAACTGAAAACTATTTTATATTAAGAA
AGAAGGAACGTTTTAAATGCCAAATAAAATATTACTTGTAGATGGTATGGCGCTATTATTTAGACATTTCTAC
GCTACAAGTCTTCATAAACAATTTATGTACAATTCACAAGGTGTACCTACAAATGGAATACAAGGATTTGTGC
GTCATATCTTTTCGGCAATACATGAAATACGCCCTACACATGTAGCTGTATGTTGGGATATGGGACAATCAAC
TTTTAGAAATGATATGTTTGATGGTTATAAGCAAAATCGTTCTGCACCACCAGAAGAATTGATACCACAATTT
GATTATGTTAAAGAAATTTCAGAGCAATTTGGCTTTGTAAATATTGGCGTTAAAAACTATGAAGCGGATGATG
TTATAGGTACATTAGCACAACAATATTCAACTGATAACGATGTCTATATTATTACGGGCGACAAAGATTTACT
GCAATGTATTAATGACAATGTTGAAGTTTGGCTAATTAAAAAAGGTTTTAACATTTATAATAGATATACATTA
CATCGTTTTAACGAAGAATATGACCTTGAACCACAACAACTAATCGATATTAAAGCGTTTATGGGTGATACAG
CAGATGGATATGCAGGTGTTAAAGGAATTGGTGAGAAAACGGCTATTAAGTTAATTCAGCAATATCAAAGCGT
TGAAAATGTGGTTGAAAACATTGATGCATTATCAGCTGGACAACGTAATAAGATTAATGACAATTTAGATGAA
CTATATTTATCGAAGCGACTGGCTGAAATTCACACACAAGTACCAATTGATAGTGAAGCGCTTTTTGAGAAAA
TGTCATTTGCAACAACGCTTAATCATATTTTATCAATTTGCAACGAACATGAATTACATGTTTCAGGAAAATA
TATTTCATCTCATTTTTAAAAAGTATAAGATTTTATGAAAAATTAAACCAATTAAGTATAAAAAGACTATTTC
TATCGATACAAGGTAGAAATAGTCTTTTTGCGTATTTGGATATGTCAAAGTGAATAAATAAGTCGAATTTTGA
TAGATACATATTTGATTAATCAATAGAGTTAAAGAATTATATTTCATCCAAATAAATTACCATGCACTACAAA
ATAGTACAGTTACAACTTCATTCATTTAAAAAAGAGATTTAATTATTTAGTTTTTGAGTCAATGTATTAATGC
AACTATAAATCAAATGGAGTATAAGAAATAAATATAATGGTATAATTGCACATAATATTTAAGAATAAGAATA
ATATATTAAATATTTATTAACAAATTATAGATAAAATATGAATAATTAATTAATGGTATTTACATATGCATAC
CCACAAGATAAAATGATTGTGAAATAATTCACAAAATCAAAAGGAGTGTTGCAAATGTTAGTTGCAGTAGTCA
AAGAATTAAAACAAGGTGAAGGACGTGTAGCTTGCACACCCGAAAATGTGCGTAAGTTAACGGATGCAGGACA
TAAAGTAATTGTAGAAAAAAAATGCTGGCATTGGTTCAGGATTTTCTGACGATATGTATGAAAAAGAAGGCGCT
AAGATTGTAACTCACGAACAAGCATGGGAAGCAGATCTGGTTATCAAAGTAAAAGAACCACATACAAGTGAAT
ATCAATATTTTAAAAAAGATCAAATCATCTGGGGATTCTTACATTTAGCATCTTCAAAGGAAATAGTAGAAAA
AATGCAAGAAGTTGGTGTTACTGCTATTAGTGGTGAAACCATTATAAAAAATGGAAAAGCAGAATTATTAGCG
CCAATGAGTGCTATAGCAGGTCAACGCTCAGCAATTATGGGAGCTTACTACTCTGAAGCACAACATGGTGGTC
AAGGTACTTTAGTGACTGGTGTACATGAAAATGTGGATATACCTGGTAGTACATATGTGATTTTCGGTGGTGG

CGTAGCAGCAACAAATGCAGCAAATGTTGCCTTGGGACTAAATGCTAAAGTAATCATTATCGAGTTAAACGAT
GATCGCATTAAATATTTACAAGACATGTATGCAGAAAAAGATGTCACAGTAGTCAAATCAACACCAGAAAATT
TAGCAGAACAAATAAAAAAAGCAGATGTATTTATTTCTACAATTTTAATTCCAGGTGCGAAACCGCCAAAATT
GGTTACTCGTGAGATGGTTAAATCAATGAAAAAAGGTTCAGTATTAATCGATATAGCTATTGACCAAGGTGGA
ACTATTGAAACAATTAGACCAACTACAATTTCTGATCCAGTGTATGAAGAAGAAGGTGTGATTCATTACGGTG
TACCAAACCAACCAGGAGCAGTCCCAAGAACTTCAACAATGGCATTAGCACAAGGAAATATTGATTATATATT
AGAAATTTGTGACAAAGGCTTAGAACAAGCAATTAAAGATAATGAAGCCTTAAGTACTGGTGTAAACATTTAC
CAAGGACAAGTGACAAATCAAGGATTAGCTACATCACATGACCTAGATTATAAAGAAATATTAAATGTTATCG
AATAGATAGTAATTTAAATGAAATTGAGTGAAATGAATATTTTAAATATAGCATTATAGTTTGGACTAAAAAT
TTACAAAACGGAAGGATGTAATTATTATGACAACCAACACAGTTACATTACAAACAGCACATATTGTAAGTTT
AGGAGATATCGAAGAAGCTAAAGCAAGCATTAAACCATTTATTCGTCGAACACCTCTAATTAAATCAATGTAT
TTAAGCCAAAGTATAACTAAAGGGAATGTATTTCTAAAATTAGAAAATATGCAATTCACAGGATCTTTTAAAT
TTAGAGGCGCTAGCAATAAAATTAATCACTTAACAGATGAACAAAAAGAAAAATGCATTATCGCAGCATCTGC
TGGGAACCATGCACAAGGTGTTGCTTTAACAGCTAAATTATTAGGCATTGATGCAACTATCGTAATGCCTGAA
ACAGCACCACAAGCAAAACAACAAGCAACAAAAGGCTATGGGGCAAAGGTTATTTTAAAAGGTAAAAACTTTA
ACGAAACTAGACTTTATATGGAAGAATTAGCGAAAGAAAATGGCATGACAATCGTTCATCCATATGACGATAA
GTTTGTAATGGCAGGCCAAGGAACAATTGGTTTAGAAATTTTAGATGATATTTGGAATGTGAATACAGTCATC
GTACCAGTTGGCGGTGGAGGATTAATTGCAGGTATTGCCACCGCATTAAAATCATTTAACCCTTCAATTCATA
TTATCGGTGTTCAATCTGAGAATGTTCATGGTATGGCTGAGTCTTTCTATAAGAGAGATTTAACTGAACATCG
AGTGGATAGCACAATAGCAGATGGTTGTGATGTAAAAGTTCCTGGTGAACAAACATATGAAGTAGTTAAACAT
TTAGTAGATGAATTTATTCTTGTTACTGAAGAAGAAATTGAACATGCTATGAAAGATTTAATGCAGCGTGCCA
AAATTATTACTGAAGGTGCAGGCGCATTACCAACAGCTGCAATTTTAAGTGGAAAAATAAACAATAAATGGCT
TGAAGATAAAAATGTTGTTGCATTAGTTTCAGGCGGGAATGTTGACTTAACTAGAGTTTCAGGTGTCATTGAA
CATGGACTGAATATTGCAGATACAAGCAAGGGTGTGGTAGGTTAAAACATTTAATCTTAAAAATGAGGTGTAA
TTATGTCAAATGGTAAAGAATTACAAAAAAATATAGGTTTCTTCTCAGCGTTTGCTATTGTTATGGGGACAGT
TATTGGTTCAGGAGTATTCTTTAAAATATCAAACGTAACAGAAGTAACAGGAACAGCAGGAATGGCCTTGTTT
GTATGGTTCCTAGGCGGCATCATTACCATTTGTGCGGGGTTAACAGCAGCAGAACTTGCTGCTGCAATCCCTG
AAACAGGTGGCTTAACGAAGTATATAGAATATACATACGGTGATTTCTGGGGCTTCTTATCAGGCTGGGCACA
ATCATTTATTTATTTTCCAGCAAACGTAGCAGCATTGTCTATCGTATTTGCGACACAGTTAATTAATTTATTC
CATTTATCTATAGGTTCATTAATACCAATAGCAATCGCATCTGCGTTATCTATTGTGTTGATAAATTTCCTAG
GTTCAAAAGCAGGTGGAATTTTACAATCAGTTACATTAGTAATTAAACTGATTCCAATCATCGTTATCGTAAT
TTTTGGTATTTTTCAATCTGGAGATATCACTTTTTCATTAATTCCAACTACAGGTAATTCAGGAAATGGTTTC
TTTACAGCAATTGGTAGTGGTTTATTAGCAACTATGTTTGCATATGATGGTTGGATTCATGTAGGAAATGTTG
CGGGGGAACTTAAAAATCCTAAACGCGATTTACCTTTAGCGATTTCAGTTGGTATCGGTTGTATTATGGCTGT
GTATTTATTAATTAACGCTACATTCTTATTAACGTTGCCAATTGAACTACTTGCTGGTAATTTAAATGCAGCT
TCAGATACATCAAAAATATTATTTGGTGAAAATGGCGGTAAGATTATTACAATTGGTATATTAATTTCTGTTT
ATGGTACGATCAATGGCTATACTATGACTGGTATGCGCGTACCATATGCAATGGCTGAAAGAAAATTATTGCC
ATTTAGCCACTTATTCGCAAAATTAACAAAATCTGGCGCACCATGGTTTGGCGCAATTATACAACTTATAATC
GCTATCATCATGATGTCAATGGGAGCATTTGATACAATTACAAACATGTTAATCTTTGTTATTTGGTTGTTCT
ATTGTATGTCATTTGTTGCGGTAATAATTTTAAGAAAACGTGAACCAAATATGGAGCGACCATATAAAGTACC
GTTATATCCAATCATACCTTTAATTGCTATTTTGGCAGGATCATTTGTATTAATTAATACACTGTTTACACAA
TTTATACTAGCAATTATTGGGATTCTAATCACAGCACTTGGTATACCAGTTTATTACTATAAGAAGAAACAAA
AAGCAGCATAAGGTAAGATAACTAGCATTGAGAATAAATGGATGGACTACTAATAAATTTAAAGTTTTACACA
TTAAAATCAAAAACCATTCAATTATTCTATCGAACAGACAAATTTCTGTTATGGAATTTGTCTGTTTTCAAAA
GTATAGGGAGGCAAATAGAGATGGAAAAGCCGTCAAGAGAGGCATTTGAAGGCAATAATAAGTTGTTAATAGG
AATTGTTTTAAGTGTAATAACGTTTTGGCTATTTGCACAATCATTGGTTAATGTTGTACCAATACTTGAAGAT
AGTTTCAATACAGATATTGGAACGGTAAATATCGCCGTTAGTATAACTGCTTTATTTTCAGGAATGTTTGTAG
TAGGAGCAGGTGGTCTTGCTGATAAATATGGCAGAATTAAACTCACGAACATTGGTATTATCTTAAATATATT
GGGTTCATTATTAATCATTATTTCAAATATTCCTTTATTACTTATTTTAGGAAGATTAATTCAAGGACTTTCA
GCAGCATGTATTATGCCTGCAACTTTGTCTATTATTAAGTCATATTACATTGGGAAAGATAGACAACGCGCTT
TAAGTTATTGGTCAATTGGCTCATGGGGCGGCTCAGGTGTTTGTTCATTTTTTGGAGGTGCAGTTGCAACGCT
TTTAGGTTGGCGTTGGATTTTCATCCTATCAATTATAATTTCATTAATTGCGCTATTTCTTATTAAAGGCACA
CCTGAAACTAAATCTAAATCGATTTCTCTAAATAAATTTGACATTAAAGGTCTGGTTCTTTTAGTCATTATGC
TCCTCAGTTTAAACATTTTAATTACTAAAGGATCAGAATTAGGTGTAACCTCACTTCTTTTTATCACTTTATT
AGCAATTGCAATTGGATCTTTTAGTTTATTTATAGTTCTTGAAAAGCGTGCTACAAATCCTTTAATCGATTTT
AAATTATTTAAAAATAAAGCTTATACAGGTGCAACAGCTTCAAACTTTTTGTTAAATGGTGTTGCGGGAACAT
TAATAGTAGCCAACACATTTGTTCAAAGAGGTTTAGGATATTCTTCATTGCAAGCAGGAAGTTTATCAATCAC
TTATTTAGTAATGGTACTAATTATGATTCGTGTTGGTGAAAAGTTACTTCAAACACTCGGATGCAAGAAACCA

ATGTTAATTGGAACAGGAGTTCTTATTGTCGGAGAATGTCTCATTTCATTAACATTTTTGCCAGAAATATTGT
ATGTCATTTGTTGTGTTATAGGTTATTTATTCTTTGGTTTAGGACTAGGAATATATGCTACACCATCAACAGA
TACAGCAATTGCAAATGCACCGTTAGAAAAAGTAGGCGTTGCTGCAGGTATCTATAAAATGGCTTCTGCATTA
GGTGGAGCATTTGGCGTCGCATTAAGTGGTGCAGTTTATGCAATCGTATCAAATATGACAAACATTTATACAG
GTGCAATGATTGCATTATGGTTAAACGCAGGTATGGGAATTTTATCATTCGTTATCATTTTGTTACTTGTGCC
TAAACAAAACGACACTCAATTATGATAATTGAGAATTAAATTGAAATCATATAAGTCGCTACAATATTAAACA
AAAATATAAACCGATTCTTATGTGTCATTATTTTAAATGAACATAGGGATTGGTTTTTTATTACTCTTTTACG
CTACTTTATTTATAATTATTATAAATTGTCACAAATTCAATTTACCTTACAATATATTTTGTGTTATTATATT
CTGTAGCATAAATAAATTGTTCAACACATAGTTGTAATGTGTTTCAATACTTTTTGGATAGATTGCGAAATTG
TATTGAATCGTCATCGTTTTAAATTTTTAAATGAGAATGGAATGAGCATTACAATACACAAGCAATCAAAAGT
AAATACATTCACAACACAACAGAGACATAACAACAAGATAAGGAGTGAACAATAGCTGTGAATTATCGTGATA
AAATTCAAAAGTTTAGTATTCGTAAATATACCGTTGGTACATTTTCAACGGTCATTGCGACATTGGTATTTTT
AGGATTCAATACATCACAAGCACATGCTGCTGAAACAAATCAACCAGCAAGCGTGGTTAAACAGAAACAACAA
AGTAATAATGAACAGACTGAGAATCGAGAATCTCAAGTACAAAATTCTCAAAATTCACAAAATGGTCAATCAT
TATCTGCTACTCATGAAAATGAGCAACCAAATATTAATCAAACAAACCATGTAGATGTAAGTGCAGAACAATC
ATCTACAACTGAAAATGAACAAGCAGTATCTCAAAATAAGCAAACAAAGAAAGATACAGCAGATGCTACGCCA
ACACAATCGGCTAAAGCGGAAAGCAAGCATGAACAAAATGAAAGTCGTTCTGCTAACAAAAAAGAAAACGACA
ATAAAGCGGCTCATGTAGAAAATCATGAAGCAAATGTAGTCACAGCTTCAGATTCACCTGATAATGGTAACGT
ACAACATGACCGAAATGAACTACAAGCATTTTTTGATGCAAATTATCATGATTATCGCTTTATTGACCGCGAA
AATGCAGATTCTGGGACATTTAACTATGTAAAAGGCATTTTTGACAAGATTAATACGTTATTAGGAAGCAATG
ATCCAATAAACAATAAAGACTTGCAACTTGCATATAAAGAATTGGAACAAGCTGTTGCATTGATCCGCACTAT
GCCTCAACGTCAACAAACTAGCCGACGCTCAAGCAGAATTCAAACGCGTTCAGTTGAGTCAAGAGCTGCAGAA
CCTAGATCAGTATCAAGTTATCAAAATGCAGACTCTTCATACTACGTTGAAAATGCTAATGACGGTTCAGGGT
ATCCTGTAGGTACATATATTAATGCATCAAACAAAGGTGCGCCATTCTATCTTCCTACAGCCCCATGGAATAC
TTTGAAAGCATCAGCTGCAAAAGAAATAGTACTGATCACAGCGAAACAAACTGGAGACGGATATCAATGGGTA
ATTAAGTTTAACAAAGGTCATGCACCACATGAAAATATGATTTTCTGGTTTGCTTTACCGTCAGATCAAGTGC
CAGTTGGACGTACTGACTTTGTAACAGTGAATGCAGACGGGACGAATGTACAATGGAGTAATGGTGCGGGAGC
TGGTGCTTACAAACCACTTCCACAAATGTGGCCATATGGTGTAAATGACTCACGTTCGCGAGACTTTAAAATT
AGAAATAGAAGCGGACAAGTTATTTATGATTGGACTGATGTTCATGTAAATACATTGAGAGACTTAGCGCGAG
CAGGAGATTATTTCAGTGAAGCTGGAGCACCCGCTGCGACTAAAGCTATAGGGGAACAAACGTTTAGATATAT
TAATGGTGAAAGACCAACAGAATCCCCTGGTGCATCAAAAGTTTATACTTTTATTGGTGCTGGCGATGCAAGT
TATACGATTTCATTTAAAACACAAGGACCAACAACGAACAAATTGTATTATGCAGCCGGTGGTCGTGCTCTAG
AATACAATCAATTATTTATGTATAGCCAACTATACGTAGAATCAACGCAAGACCACCAACAACGACTTGATGG
TTTTAGACAAGTAGTTAATCGTTCTTATCGTTTAGATACAACAAAAAGAGTAGAAGTAAGTCAGGGTAACATT
CAAACTAAAAAAGTACTTGAAAGTACAAACCTAAATATCGATGACTTTATGGATGATCCATTAAGTTATGTTA
AGACACCGAGTAATAAAGTGTTAGGTTTTTATCCAACAAATGCGAATCCCAATGCATATAGACATGGTGGAAC
TAATCCATTAAATGAATATCAATTAAGTCAGTTATTTACTGATGATAAACTACAACAGGCAGCTAGAACTGGC
AATCCAATACGATTAATGATTGGATTTGACTATCCAGATGGTCATGGCAACGCAGAAACTTTAGTACCAGTTA
ACTTGACGGTATTACCTGAAATTCAACATAATATTAAATTTTATAAAAATGATGACACACAAAATATTGCCGA
TAAACCAGCTTCAAGACAAGCAGGACATCCAATCTTCTATGTATATGCAGGTAATCAAGGGAATGCTTCAGTG
AATTTAGGTGGAAGCGTAACATCTATTCAACCGTTACGTATTAATTTAACAAGTAGTGAAAATTTTACAGATA
GAGATTGGCAAATAACAGGTATTCCACGTACGTTACACATTGAAAACTCGACAAATAGACCTAATAATGCCAG
AGAACGTAACATTGAATTGGTTGGTAATTTATTACCGGGTGACTATTTTGGAACAATACGTTTTGGCCGTAAA
GAACAATTATTCGAAATACGTGTTAAGCCACATACACCAACAATTACTACAACTGCTGAACAACTAAGAGGTA
CAGCATTACAAAAAGTGCCAATTACAGTTACGAATATTCCGTTGGACCCATCGTCATTAGTTTATATCGTTTA
TCCAACAGATCAAACAAGAGATGGTGGATCTGAAGCAGACCAAATGCCTTCAAACTATTCAATACTGGCGACT
GGTACACCTGATGGTGTGCATAATACCATAACAATACAACCAAAAGACTATACTGTGTTTATTCCTCCGGTAG
GTAAACAAATTAGAGCATTGATTTATTACAATAAAGTTGTTGCTTCTAATATGAGTAATGCGGTTACTATTTT
GCCGGATGATATTCCGCCGACAATTAATAATCCAGTTGGCTTAAATGCAAAGTATTATCGTGGTGATGAAGTC
AACTTTACAATGGGAGTTTCAGATAGACATTCTGGAATAAAAAATACAACTATTACTACTTTACCGAATGGTT
GGACATCAAATTTAACTAAAGCGGATAAGAATAATGGCTCATTATCTATTACTGGTAGAGTGAGTATGAATCA
GGCATTTAACAGTGATATTACATTTAAAGTGTCAGCGACAGATAATGTCAATAATACGACAAATGATAGTCAA
TCTAAACATGTGACAGTACATGTAGGTAAAATTAGTGAAGATGCGCATCCAATTGTATTAGCAAATTCTGAAA
AAGTTGTCGTTGTAAATCCGACATCTGTATCTAATGATGAAAAACAACGAATAATTACTGCATTTATGAATAA
AAACCAAAATATAAGAGGTTATTTAGCTTCATCAAATCCAGTTACAGTTGATAATAATGGTAATGTCACATTA
CATTACCGTGATGGCTCATCAACAACGCTTGATGCTACAAATGTGATGACATACGAACCAGTTGTGAAACCTG
AATATCAAACTGCCAATGCACCTAAAACGGCAACGGTAACAATTGCTAAAGGACAATCATTTAGTATAGGTGA
TATTAAACAATATTTCACTTTAAGTAATGGACAAGCGATTCCAAATGGAACATTTACAAATATTACTTCGGAC

AGAACGATTCCAACAGCACAAGAAGTTAGTCAAATGAATGCAGGTACACAGTTATACCATATCGTCGCTTCAA
ACGCGTATCATAAAGACACTGAAGACTTATATATTAGTTTGAAAATCATCGATGTGAAACAACCAGAGGGTGA
TCAACGTGTATATCGTACATCAACATATGATTTAACTACTGATGAAATTTCAAAAGTAAAACAAGCATTTATT
AATGCCAATAGGGATGCGATTACGTTTGCAGAAGGAGATATATCGGTTACAAATACACCAAATGGTGCCAATG
TGAGTACCGTTACTGTCAATATTAATAAAGGTCGATTAACGAAATCGTTTACATCAAACCTTGCTAATATGAA
TTTCTTACGTTGGGTTAATTTCCCACAAGATTATACTGTATCTTGGACAAATGCGAAAATCGCTAGTAGACCA
ACTGATGGTGGTTTATCATGGTCTGATGATCATAAATCATTAATTTATCGCTATGATGCAACATTAGGTACAC
CAATTACGACGAATGATATTTTAACGATGTTAAAAGCAACAACGACAGTGCCTGGATTGCGTAATAACATTGC
CGGTAATGAGAAAACTCAAGCTGAAGCTGGTGGCAGACCAAACTATAGACCAACTGGATATTCACAAGCAAAT
GCTTCATCAGATGGGCAACGTCAATATACGTTAAATGGACAAGTAATCCAAGTTCTAGATATTATTAACCCGA
CGAATGGTTTCGGTGGTCAAACGATTACAAACTCAAATGTTCGTGCAAACCATAGTAATTCAACTGTTGTTAG
TGTCAATGAACCGGCAGCTAATGGTGCGGGTGCATTTACAATTGATCATGTGGTAAAAAATAATACTACGCAT
AATGCAGCTGATGCGGTATACAAAGCGCAGTTATACTTATCTCCATATGGACCAAAACAATATGTTGAACATT
TAAATCAAAATACGGGAAATACTACCGATGCTATTAACATTTATTTTGTACCTAGTGATCTAGTAAATCCAAC
TATTTCAGTAGGTAATTACGCAAATCATCAAGTATTCTCTGGTGAAACATTTACCAATACGATTACAGCGAAT
GATAACTTTGGTGTGCAATCTGTAACTGTACCAAATACATCACAAATTACAGGTACTGTTGACAATAATCATC
AGCACGTTTCTGCAACAGCGCCAAATGTGACATCTGCTACAAATAAAACGATCAATTTAGTGGCAACTGATAC
AAGCGGCAATACTGCAACAACGTCATACAATGTCACAATTAAACCTTTACGTGATAAATATCGAGTTGGAACA
TCATCAACGGCTGCTAATCCAGTGAGAATTGCTAATATTTCGAATAATGCGATAGTATCACAAGCTGATCAAA
CAGCAATTATTAATTCATTGACGTTTACTGAAACAGTACCAAATAGAAGTTATGCAAGAGCAAGTGCAAATGA
AATCACTAGTAAAACAGTTAGTAATGTGAGTCGTACTGGAAATAATGCCAATGTCACAGTAACTGTTACTTAT
CAAGATGGAACAACATCAACAGTGACTGTACCTGTAAAGCATGTCATTCCAGAAATCGTTGCACATTCGCATT
ACACTGTACAAGGCCAAGACTTCCCAGCAGGTAATGGTTCTAGTGCATCAGATTACTTTAAGTTATCTAATGG
TAGTGCCATTCCAGATGCAACTATTACATGGGTAAGTGGACAAGCGCCAAATAAAGATAATACGCGTATTGGT
GAAGATATAACAGTAACTGCACATATCTTAATTGATGGCGAAACAACGCCGATTACGAAAACAGCAACATATA
AAGTAGTAAGAACTGTACCGAAACATGTCTTTGAAACTGACAGAGGTGTTTTATACCCAGGTGTTTCAGATAT
GTATGATGCAAAACAATATGTTAAGCCAGTGAATAATTCTTGGTCGACAAATGCGCAAAATATGAATTTCCAA
TTTGTGGGAACTTATGGACCTAACAAAGATGTTGTAGGTATATCAACGCGTCTTATTAGAATCACATATGATA
ATAGACAAACTGAAGAATTAACTATTTTATCTAAAGTTAAACCAGACCCACCGAGAATTGATGGCAACTCTGT
AACATATAAAGCAGGTCTTACAAACCAAGAAGTTAAAGTTAATAACGTATTAAGTAACTCGACGGTTAAATTG
TTTAAAGCTGACAATACGCCATTAAATGTTACAAATATTATCCATGGCAGTGGCTATAGTTCGGTTGTGACAG
TAAGTGATGCGTTACCAAATGGTGGAATTAAAGCGAAATCTTCAATTTCAATGAACAATGTGACGTATACGAC
GCAAGACGAACATGGTCAAGTTGTTACAGTAACAAGAAATGAATCTGTTGATTCAAATGATAGTGCTTCTGTT
ACAGTAACACCACAATTACAAGCAACTACTGAAGGCGCTGTATTTATTAAAGGTGGCGACGGTTTTGATTTCG
GACACGTAGAAAGATTTATTCAAAACCCACCACATGGCGCAACGGTCGCATGGCACGATAATCCAGATACATG
GAAGAATACAGTCGGTAACACTCATAAAACTGCGGTTGTAACATTACCAAGTGGTCAAGGTACGCGTAATGTT
GAAGTTCCAGTCAAAGTTTATCCAGTTGCTAATGCTAAGGCGCTATCACGTGATGTGAAAGGTCAAAATTTGA
CTAATGGAACGGATGCGATGAGCTACATTACATTTGATCCAAATACAAACACAAATGGTATCACTGCAGCATG
GGCAAATAGACAACAACCAAATAACCAACAAGCAGGCGTTCAACATTTAAATGTCGATGTCACATATCCGGGT
ATTTCAGCTGCTAAACGAGTTCCTGTAACTGTGAACGTATATCAATTTGAATTCCCTCAAACTACTTATACCA
CAACAGTTGGTGGCACTTTAGCAAGTGGTACGCAAGCATCGGGATATGCACATATGCAAAACGCTACTGGTTT
ACCAACTGATGGATTTACGTATAAATGGAATAATGCAGCAACGGGTACAAACGATGCAAACTGGGCAGCAATG
AATAAACCGAATGCGGCTAAAGTCGTTAATGCGAAATATGATGTTATCTATAATGGACATACATTTGCAACAT
CTCTACCAGCGAAGTTTGTAGTAAAAGATGTACAACCAGCGAAACCAACTGTGACTGAAACAGCGGCAGGAGC
GATTACAATTGCACCTGGAGCAAACCAAACAGTGAATACACATGCCGGTAACGTAACAACATACGCTGATAAA
TTAGTTATTAAACGTAACGGCAGTGTTGTGACGACATTTACACGTCATAATAATACGAGTCCATGGGTTAAAG
AAGCATCAGCAACTAATGTAACAGGTATTGTTGGAACGAATAATGGTATTACTGTTGCAGCAGGTACTTTCAA
TCCTGCTGATACAATTCAAGTTGTTGCAACACAAGGTAGTGGTGAAACAATCAGTGACGAACAACGTAGTGAT
GATTTCACAGTTGTCGCACCTCAACCGAACCAAGCGACTACTAAGATTTGGCAAAATGGTCATATTGATATCA
CGCCTAATAATCCATCAGGGCATTTAATTAATCCAACTCAAGCAATGGATATTGCTTACACTGAAAAAGTGGG
TAATGGTGCAGAACATAGTAAGACAATTAATGTTGTTCGTGGTCAAAATAATCAATGGACAATTGCGAATAAG
CCTGACTATGTAACGTTAGATGCACAAACTGGTAAAGTGACATTCAATGCCAATACTATCAAGCCAAATTCAG
CAATTACCATTACACCGAAAGCAGGTACAGGTCACTCAGCAAGCAGTAATCCAAGTACATTAACTGCACCGGC
AGCACATACTGTCAACACAACAGAAATTGTGAAAGATTATGGTTCAAATGTAACAGCATCTGAAATTAACAAT
GCAGTTCAAGTTGCTAATAAACGTACTGCAACGATTAAAAATGGCACAGCAATGCCTACTAATTTAGCTGGTG
GTAGCACAACGACGATTCCTGTGACAGTAACATACAATGATGGCAGTACTGAAGAAGTACAAGAGTCCATTTT
CACAAAAGCGGATAAACGTGAGTTAATCACAGCTAAAAATCATTTAGATGATCCAGTGAGTACAGATGGTAAA
AAGCCAGGTACAATTACGCAGTACAATAATGCAATGCATAATGCGCAACAACAAATCAATGCTGCGAAAACAG

AAGCGCAACAAGTGATTAATAATGAGCGTGCAACACCACAACAAGTGAACGCAGCATTATCTAAAGTCCAAGC
AGCACAAACTAAGATAAATGAAGCTAAAGCTTTACTTCAAAATAAAGAAGATAATAGCCAATTAGTAACGTCT
AAAAATAACTTACAAAGTTCTGTGAACCAAGTACCATCAACTGCGGGTATGACGCAACAAAGTATTGATAACT
ATAATGCGAAGAAACGTGAAGCAGAATCGGAAATTACAGCTGCACAACGTATCATTGACAATGGAGATGCAAC
TGCACAACAAATTTCAGATGAAAAACATCGTGTCGATAACGCATTAACAGCATTAAACCAAGCGAAACATGAT
TTAACTGCAGATACACATGCCTTAGAGCAAGCAGTGCAACAATTGAATCGCACAGGTACAACGACTGGTAAGA
AGCCAGCAAGTATCACTGCGTATAATAATTCAATTCGTGCACTTCAAAGTGACTTAACAAGTGCTAAAAATAG
CGCTAATGCTATCATTCAGAAGCCAATAAGAACAGTGCAAGAGGTACAATCTGCATTAACAAATGTAAATCGT
GTCAATGAGCGATTAACGCAAGCAATTAATCAATTAGTACCTTTAGCTGATAATAGTACTTTAAGAACTGCTA
AGACGAAACTTGATGAAGAAATTAATAAATCAGTAACTACTGATGGTATGACACAATCATCAATCCAAGCATA
TGAAAATGCTAAACGTGCGGGTCAAACAGAATCAACAAATGCACAAAATGTTATTAACAATGGTGACGCGACA
GACCAACAAATTGCCGCAGAAAAAGCTAAAGTAGAAGAAAAATATAATAGCTTAAAACAAGCAATTGCTGGAT
TAACACCAGACTTGGCACCATTACAAACTGCAAAAACGCAGTTGCAAAATGATATTGATCAGCCAACGAGTAC
GACTGGTATGACAAGCGCATCTGTTGCTACATTTAATGAAAAACTTTCAGCAGCTAGAACTAAAATTCAAGAA
ATTAATCGCGTACTGGCATCTCATCCAGATGTTGCAACGATTCGTCAAAACGTGACAGCAGCGAATGCTGCTA
AATCAGCACTTGATCAAGCACGTAATGGCTTAACAGTCGATAAAGCGCCTTTAGAAAATGCGAAAAATCAACT
ACAACATAGTATTGATACGCAAACAAGTACAACTGGTATGACGCAAGATTCTATTAATGCATACAATGCGAAG
TTAACAGCTGCACGTAATAAGATTCAACAAATCAATCAAGTATTAGCTGGTTCACCGACAGTTGATCAAATTA
ATACAAATACGTCTGCAGCAAACCAAGCTAAATCTGATTTGGATCATGCGCGTCAAGCATTAACACCAGATAA
AACACCGCTTCAAAATGCGAAAACGCAATTAGAACAAAGTATTAATCAACCAACAGATACAACAGGTATGACG
ACCGCTTCGTTAAATGCGTACAACCAAAAATTACAAGCAGCGCGTCAAAAGTTAACTGAAATTAATCAAGTGT
TGAATGGCAACCCAACTGTCCAAAATATCAATGATAAAGTGGCAGAGGCAAACCAAGCTAAGGATCAACTAAA
TACAGCACGTCAAGGTTTAACATTAGATAGACAGCCAGCGTTAACAACATTACATGGTGCATCTAACTTAAAC
CAAGCACAACAAAATAATTTCACGCAACAAATTAATGCTGCTCAAAATCATGCTGCGCTTGAAACAATTAAGT
CTAACATTACGGCTTTAAATACTGCGATGACAAAATTAAAAGAAAGTGTTGCGGATAATAATTCTATTAAATC
AGGTCAAAACTACACTGACGCAACTCAAGCTAATAAACAAGCGTATGATAATGCAGTTAATGCGGCTAAAGGT
GTCATTGGGGAAACGAATAATCCAACGATGGATGTTAATACAGTGAACCAAAAAGCAGCATCTGTTAAATCGA
CGAAAGATGCTTTAGATGGTCAACAAAACTTACAACGTGCGAAAACAGAAGCAACAAATGCGATTACACATGC
AAGTGATTTAAACCAAGCACAAAAGAATGCATTAACACAACAAGTGAATAGTGCGCAAAATGTTCAAGCAGTA
AATAATATTAAACAAACAACTCAAAGCTTAAACACTGCAATGACAGGTCTAAAACGTGGCGTTGCTAATCATA
ACCAAGTCGTACAAAGTGATAATTATGTCAACGCAGATACTAATAAGAAAAATGATTACAACAATGCATACAA
CCATGCGAATGACATTATTAATGGTAATCCACAACATCCAGTTATAACACCAAGTGATGTTAACAATGCTTTA
TCAAATGTCACAAGTAAAGAACATGCATTGAATGGTGAAGCTAAGTTAAATACTGCGAAACAAGAAGCGAACA
CTGCATTAGGTCAATTAAATAACTTGAATAATGCACAACGTCAAAACTTACAATCGCAAATTAATGGTGCGCA
TCAAATCGAGACAGTTAATACGATTAAGCAAAATGCAACTAACTTAAATAGTGCAATGGGTAATTTAAGACAT
GCTGTTGCAGATAAAGAGCAAGTCAAACGTACAGAAGATTATGCGGATGCAGATACAGCTAAACAAAATGCAT
ATAATAGTGCCGTTTCAAGTGCTGAAACAATCATTAATCAATCAACAAATCCGACAATGTCTGTTGATGATGT
TAATCGTGCAACTTCAGCTGTTACTACTAATAAAACTGCATTAAATGGTGATGAAAAATTAGCACAATCTAAA
ACAGATGCTGCAAGAGCAATTGATGCATTACCACATTTAAATAATGCACAAAAAGCAGATGTTAAATCTAAAA
TTAATGCTGCATCAAATATTGCTGGCGTAAATACTGTTAAACAACAAGGTACAGATTTAAATACAGCAATGGG
TAACTTGCAAGGTGCGATCAATGATGAACAAACGACACTTAATAGTCAAAATTATCAAGATGCGACACCTAGT
AAGAAAACAGCATACACAAATGCGGTACAAGCTGCGAAAGATATTTTAAATAAATCAAATGGTCAAAATAAAA
CGAAAGATCAAGTTACTGAAGCGATGAATCAAGTGAATTCGGCTAAAAATAACTTAGATGGTACGCGTTTATT
AGATCAAGCGAAGCAAACAGCGAAACAGCAGTTAAATAATATGACGCATTTAACAACTGCACAAAAAACGAAT
TTAACAAACCAAATTAATAGTGGTACTACTGTCGCTGGTGTTCATACGGTTCAATCAAATGCCAATACATTAG
ATCAAGCCATGAATACGTTAAGACAAAGTATTGCCAACAAAGATGCGACTAAAGCAAGTGAAGATTACGTAGA
TGCTAATAATGATAAGCAAACAGCATATAACAACGCAGTAGCCGCTGCTGAAACGATTATTAATGCAAATAGT
AATCCAGAAATGAATCCAAGTACGATTACACAAAAAGCAGAGCAAGTGAATAGTTCTAAAACGGCACTTAACG
GTGATGAAAACTTAGCTACGGCAAAACAAAATGCGAAAACGTACTTAAACACATTAACGAGTATTACAGATGC
TCAAAAGAACAATTTGATTAGTCAAATTAGTAGTGCGACAAGAGTGAGTGGTGTTGATACTGTAAAACAAAAT
GCGCAACATCTAGACCAAGCTATGGCTAGCTTACAAAATGGTATTAATAACGAATCTCAAGTTAAATCTTCTG
AGAAATATCGTGATGCTGATACAAATAAACAACAAGAGTATGATAATGCTATTACTGCAGCGAAAGCGATTTT
AAATAAATCGACAGGTCCAAACACTGCGCAAAATGCAGTTGAAGCAGCATTGCAACGTGTGAATACTGCGAAA
GATGCATTGAATGGTGATGCAAAATTAATTGCAGCTCAAAACGCAGCAAAACAACATCTAGGTACTTTAACAC
ATATCACTACAGCACAACGTAATGATTTAACAAATCAAATTTCACAAGCTGCAAACTTAGCTGGTGTTGAATC
AGTTAAACAAAGTGCAAATAGTTTAGATGGCGCAATGGGTAACTTACAAACTGCTATCAACGATAAATCTGGA
ACATTAGCGAGCCAAAACTTCTTGGATGCTGATGAGCAAAAACGTAATGCTTACAATCAAGCTGTATCAAATG
CCGAAACAATTTTAAATAAACAAACTGGACCGAATACAGCGAAAACTGCAGTTGAACAAGCACTTAATAATGT

TAATAGTGCGAAACATGCATTAAATGGTACGCAAAACTTAAATAATGCGAAACAAGCAGCGATTACAGCAATC
AATGGCGCATCTGATTTAAATCAAAAACAAAAAGATGTATTAAAAGCACAAGCTAATGGTGCTCAACGCGTTT
CTAATGCACAAGATGTACAGCGCAATGCGACTGAACTGAACACGGCAATGGGTACATTAAAACATGCCATCGC
AGATAAGACGAATACGTTAGCGAGCAGTAAATATGTTAACGCCGATAGCACTAAACAAAATGCATACACAACT
AAAGTTACCAATGCTGAACATATTATTAGCGGTACGCCAACGGTTGTTACGACACCTTCAGAAGTAACAGCTG
CAGCTAATCAAGTAAACAGCGCGAAACAAGAATTAAATGGTGACGAAAGATTACGTGTAGCAAAACAAAACGC
CAATACTGCTATTGATGCATTAACGCAATTAAATACTCCTCAAAAAGCTAAATTAAAAGAACAAGTGGGACAA
GCCAATAGATTGGAAGACGTACAAACTGTTCAAACAAATGGACAATCATTGAACAATGCAATGAAAGGCTTAA
GAGATAGTATTGCTAACGAAACAACAGTCAAAGCAAGTCAAAACTATACAGACGCAAGTCCGAATAACCAATC
AACATATAATAGCGCTGTGTCAAATGCGAAAGGTATCATTAATCAAACTAACAATCCAACTATGGATACTAGT
GCGATTACCCAAGCTACAACACAAGTGAATAATGCTAAAAATGGTTTAAACGGTGCTGAAAACTTAAGAAATG
CACAAAACACTGCTAAGCAAAACTTAAATACGTTATCACACTTAACAAATAACCAAAAATCTGCAATCTCATC
ACAAATTGATCGTGCAGGTCATGTGAGTGAGGTAACAGCTGCTAAAAATGCAGCAACTGAGTTAAACACGCAA
ATGGGTAACTTGGAACAAGCTATCCATGATCAAAACACAGTTAAGCAAAGTGTTAACTTTACTGATGCAGATA
AAGCTAAACGTGATGCGTATACAAATGCGGTAAGCAGAGCAGAAACAATTCTGAATAAAACGCAAGGTGCAAA
TACTTCAAAACAAGATGTTGAAGCAGCTATTCAAAGTGTTACTAGTGCTAAAAATGCATTGAATGGTGATCAA
AATGTCACAAATGCGAAGAATACAGCTAAACATGCATTAAATAACTTAACGTCTATTAATAATGCACAAAAAC
GTAACTTAACAACTAAAATTGATCAAGCAACAACTGTAGCTGGTGTAGAAGCGGTTTCTAATACAGGTACGCA
ATTGAATACAGCGATGGCTAACTTGCAAAATGGTATTAATGATAAAACAAATACACTAGCGAGCGAAAACTAT
CATGATGCTGATTCAGATAAGAAAACTGCTTATACTCAAGCCGTTACGAACGCAGAAAATATTTTAAATAAAA
ATAGTGGATCAAATTTAGACAAAGTTGCCGTTGAAAACGCGTTGTCACAAGTAACAAATGCAAAAGGTGCCCT
AAATGGTAACCATAATTTAGAGCAAGCTAAATCAAATGCAAACACTACTATAAACGGACTTCAACATTTAACA
ACTGCACAAAAAGATAAATTGAAACAACAAGTGCAACAAGCACAAAATGTTGCAGGTGTAGATACTGTTAAAT
CAAGTGCCAACACATTAAATGGTGCTATGGGTACGTTAAGAAATAGCATTCAAGATAACACAGGTACGAAAAA
TGGCCAAAACTATCTTGATGCTACAGAAAGTAACAAAACAAACTATAACAATGCTGTTGATCGTGCTAATGGT
GTCATTAATGCAACAAGCAATCCAAATATGGATGCTAATGCAATTAACCAAATCGCTACACAAGTGACATCAA
CGAAAAATGCATTAGATGGTACACATAATTTAACACAAGCGAAACAAACAGCAACAAATGCCATCGATGGTGC
TACTAACTTAAATAAAGCGCAAAAAGATGCGTTGAAAGCACAAGTGACAAGTGCGCAACGTGTTGCAAATGTA
ACAAGTATCCAACAAACTGCAAATGAACTTAATACAGCTATGGGTCAATTACAACATGGTATTGATGATGAAA
ATGCAACAAAACAAACTCAAAAATATCGTGACGCTGAACAAAATAAGAAAACTGCTTATGATCAAGCTGTAGC
TGCTGCGAAAGCAATTTTAAATAAACAAACTGGATCAAATTCAGATAAAGCAGCAGTTGACCATGCATTACAA
CAAGTAACAAGTACGAAAGATGCATTGAATGGTGATGCTAAACTGGCAGAAGCGAAAGCGGCAGCTAAACAAA
ACTTAGGCACTTTAAACCATATTACGAATGCACAACGTACTGCGTTAGAAGGTCAAATCAATCAAGCGACGAC
TGTTGATGGCGTTAATACTGTGAAAACAAATGCAAATACATTAGACGGCGCAATGAATAGCTTGCAAGGTTCA
ATCAATGATAAAGATGCGACATTAAGAAATCAAAATTATCTTGATGCAGATGAATCAAAACGAAATGCATATA
CGCAAGCTGTCACAGCGGCTGAAGGCATTTTAAATAAACAAACTGGTGGTAACACATCTAAAGCAGACGTTGA
TAATGCATTAAATGCAGTTACAAGAGCGAAAGCGGCTTTAAATGGTGCTGACAACTTAAGAAATGCGAAAACT
TCAGCAACAAATACGATTAATGGGTTACCACACTTAACACAATTACAAAAAGACAACTTGAAGCATCAAGTTG
AACAAGCGCAAAATGTAGCTGGTGTAAATGGTGTTAAAGATAAAGGTAATACGTTAAATACTGCCATGGGTGC
ATTACGTACAAGTATCCAAAATGATAATACGACGAAAACAAGTCAAAATTATCTTGATGCATCTGATAGCAAC
AAAAATAATTACAATACTGCTGTAAATAATGCAAATGGTGTTATTAATGCAACGAACAATCCAAATATGGATG
CTAATGCGATTAATGGCATGGCAAATCAAGTCAATACAACAAAAGCAGCGTTAAATGGTGCACAAAACTTAGC
TCAAGCTAAAACAAATGCGACGAACACAATTAACAACGCACATGACTTAAACCAAAAACAAAAAGATGCATTA
AAAACACAAGTTAACAATGCACAACGTGTATCTGATGCAAATAACGTTCAACGTACAGCTACTGAATTGAACA
GTGCGATGACAGCACTTAAAGCAGCTATTGCTGATAAAGAAAGAACAAAAGCAAGCGGTAATTATGTCAATGC
TGATCAAGAAAAACGTCAAGCGTATGATTCAAAAGTGACTAACGCTGAAAATATTATTAGTGGTACACCGAAT
GCGACATTAACAGTCAATGACGTAAATAGTGCGGCATCACAAGTCAATGCGGCTAAAACAGCATTAAATGGTG
ATAACAACTTACGTGTAGCGAAAGAGCATGCCAACAATACAATTGATGGCTTAGCGCAATTGAATAATGCACA
AAAAGCAAAATTAAAAGAACAAGTTCAAAGTGCAACTACATTAGATGGCGTTCAAACTGTTAAAAATAGTTCT
CAAACGTTGAATACAGCGATGAAAGGCTTAAGAGATAGTATTGCGAATGAAGCAACAATTAAAGCAGGTCAAA
ACTACACTGACGCAAGTCCAAATAATCGTAACGAGTACGACAGTGCAGTTACTGCAGCAAAAGCAATCATTAA
TCAAACATCGAACCCAACGATGGAACCAAATACTATTACGCAAGCAACATCACAAGTGACAACTAAAGAACAT
GCATTAAATGGTGCGCAAAACTTAGCTCAAGCTAAGACAACTGCGAAAAACAACTTAAATAACTTAACATCAA
TTAACAATGCACAAAAAGATGCGTTAACGCGTAGCATTGATGGTGCAACAACAGTAGCTGGTGTAAATCAAGA
AACTGCCAAAGCAACAGAATTAAATAACGCAATGCACAGTTTACAAAATGGTATCAATGATGAGACACAAACA
AAACAAACTCAGAAATACCTAGATGCTGAGCCAAGTAAGAAATCAGCTTATGATCAAGCTGTAAATGCAGCGA
AAGCAATTTTAACAAAAGCTAGTGGTCAAAATGTAGACAAAGCAGCAGTTGAACAAGCATTACAAAATGTGAA
CAGTACGAAGACGGCGTTGAACGGTGATGCGAAATTAAATGAAGCTAAAGCCGCTGCGAAACAAACGTTAGGT

ACATTAACACACATTAATAATGCACAACGTACAGCGTTAGATAATGAAATTACACAAGCAACAAATGTTGAAG
GTGTTAATACAGTTAAAGCCAAAGCGCAACAATTAGATGGTGCTATGGGTCAATTAGAAACATCAATACGTGA
TAAAGACACGACGTTACAAAGTCAAAATTATCAAGATGCTGATGATGCGAAACGAACTGCATATTCTCAAGCA
GTAAATGCAGCAGCAACTATTTTAAATAAAACAGCTGGCGGTAATACACCTAAAGCAGATGTTGAAAGAGCAA
TGCAAGCTGTTACACAAGCAAATACTGCATTAAACGGTATTCAAAACTTAGAACGTGCGAAACAGGCTGCTAA
CACAGCGATTACAAATGCTTCGGACTTAAATACAAAACAAAAAGAAGCATTAAAAGCACAAGTAACAAGTGCA
GGACGAGTATCTGCAGCAAATGGTGTTGAACATACTGCGACTGAATTAAATACTGCGATGACAGCTTTAAAAC
GTGCCATTGCTGATAAAGCTGAGACAAAAGCTAGTGGTAACTATGTCAATGTTGATGCGAATAAACGCCAAGC
ATATGATGAAAAAGTGACAGCTGCAGAAAATATCGTTAGTGGTACACCAACACCAACGTTAACACCATCAGAT
GTTACAAATGCAGCAACACAAGTAACGAATGCGAAGACGCAGTTAAACGGTAATCATAATTTAGAAGTAGCGA
AACAAAATGCTAACACAGCAATTGATGGTTTAACTTCTTTAAATGGTCCGCAAAAAGCTAAACTTAAAGAACA
AGTGGGTCAAGCGACGACGTTGCCAAATGTTCAAACTGTTCGTGATAATGCACAAACATTAAACACTGCAATG
AAAGGTCTACGAGAAAGCATTGCGAATGAAGCAACGATTAAAGCAGATCAAAACTACACGGATGCAAGTCAAA
GTAAACAAACTGACTACAACAATGCAGTCACTGCAGCAAAAGCAATCATTGGTCAAACAACTAGTCCAACAAT
GAATGCGCAAGAAATCAATCAAGCGAAAGACCAAGTGACAGCTAAACAACAAGCGTTAAACGGTCAAGAAAAC
TTAAGAACTGCGCAAACAAATGCGAAGCAACATTTGAATGGCTTAAGTGACTTAACTAATGCTCAAAAAGAAG
CAGCCAAACGCCAAATCGAAGGTGCAACGCATGTTAATGAAGTAACACAAGCGCAAAATAATGCGGACGCATT
AAATACAGCTATGACGAACTTGAAAAATGGTATTCAAGATCAAAATACGATTAAGCGAGGTGTTAACTTCACT
GATGCAGATGAAGCGAAACGTAATGCATATACAAATGCAGTGACGCAAGCTGAACAAATTTTAAATAAAGCAC
AAGGTCCAAATACTGCAAAAGACGGTGTCGAAACTGCGTTACAAAATGTACAACGTGCTAAAAACGATTTGAA
CGGTAATCAAAATGTTGCGAATGCTAAGACAACTGCGAAAAATGCATTAAATAATTTAACATCAATTAATAAT
GCACAAAAAGAAGCATTGAAATCACAAATTGAAGGTGCGACAACAGTTGCAGGTGTAAATCAAGTGTCTACAA
CGGCATCTGAATTAAATACTGCAATGAGCAACTTACAACGTGGTATCAACGATGAAGCAGCTACAAAAGCAGC
TCAAAAATATACTGATGCAGATAGAGAAAAACAAACAGCATACAATGATGCCGTAACAGCAGCTAAAACGTTA
TTAAATAAAACAGCTGGTTCAAATGATAATAAAGCAGCCGTTGAACAAGCATTACAACGTGTGAATACTGCGA
AAAATGCATTAAATGGTGATGCGCGCTTAAATGAAGCGAAGAAAACAGCTAAACAACAATTAGCGACAATGTC
ACATTTAACTGATGCTCAAAAAGCAAACTTAACATCGCAAATTGAACGCGGTACGACTGTTGCAGGTGTTCAA
GGTATTCAAGCTAATGCTGGTACTTTAAATCAAGCAATGAATCAATTAAGACAAAGTATTGCTTCTAAAGATG
CGACTAAATCAAGTGAAGATTATCAAGACGCGAATACAGATTTACAAAATGCATACAACCGTGCAGTATCAGA
TGCTGAAGGTATTATTAGTGCAACGAATAACCCTGAAATGAATCCAGATACGATTAATCAAAAAGCGAGCCAA
GTGAATAGTGCGAAGTCAGCATTGAACGGTGACGAAAAATTAGCAGCCGCAAAACAAACTGCGAAAACAGATA
TCGGTCGTTTAACAGACTTAAACAATGCCCAACGAACTGCGGCAAATGCAGAAGTGGATCAAGCACCAAACCT
TGCAGCGGTAACAGCAGCTAAAAATAAAGCGACATCGTTAAATACAGCGATGGGTAATTTGAAACATGCACTT
GCTGAAAAGGATAATACGAAGCGAAGTGTCAATTACACAGATGCGGATCGACCAAAACAACAAGCGTATGACA
CTGCAGTTACACAAGCCGAAGCAATTACTAATGCCAATGGCAGCAACGCGAATGAAACACAAGTTCAAGCAGC
GCTTAACCAATTGAATCAAGCTAAAAATGACTTGAATGGTGATAATAAAGTTGCTCAAGCTAAAGAAGCAGCG
AAACGTGCATTAGCTTCATATAATAACTTGAATAATGCGCAATCAATTGCAGCAACTAGTCAAATTGACAATG
CAACGACAGTAGCAGGCGTAACTGCTGCACAAAATACTGCTAATGAATTAAATACAGCTATGGGTCAACTTCA
AAATAGTATTAATGACCAAAACACTGTTAAACAACAAGTGAACTTTACAGATGCTGACCAAGGTAAGAAAGAT
GCTTACACAAATGCTGTTACGAATGCTCAAGGTATTTTAGATAAAGCACACGGTCAAAATATGACAAAAGCAC
AAGTTGAAGCTGCATTAAATCAAGTAACGACTGCTAAGAATGCTTTAAACGGTGACGCAAATGTAAGACAAGC
AAAATCAGATGCGAAAGCAAACTTAGGTACATTAACACACTTGAATAATGCACAAAAACAAGATTTAACATCA
CAAATCGAAGGCGCAACAACAGTCAACGGTGTAAATGGTGTTAAAACGAAAGCACAAGACTTAGATGGTGCAA
TGCAACGATTAGAATCAGCAATCGCAAATAAAGATCAAACTAGAGCGAGCGAAAACTACATCGACGCAGATCC
AACTAAGAAAACAGCATTTGATAATGCCATCACACAAGCTGAATCTTACTTAAATAAAGATCATGGTGCGAAT
AAAGATAAGCAAGCTGTTGAACAAGCAATTCAAAGTGTAACGACTGCTAAGAATGCTTTAAACGGTGACGCGA
ACTTACAACGCGCTAAAACTGAAGCTACACAAGCTATCGATAACTTGACACATTTGAATACACCACAAAAAAC
AGCGTTAAAACAACAAGTGAACGCTGCGCAACGTGTATCAGGTGTAACTGATCTGAAAAATAGTGCTACATCA
CTTAATAATGCGATGGATCAATTAAAACAAGCAATTGCTGATCATGACACAATTGTTGCTGGTGGTAATTACA
CTAATGCAAGTCCTGATAAGCAAGGTGCTTATACTGATGCATATAATGCTGCGAAAAACATTGTAAATGGTTC
ACCTAATGTGGTTACAAATGCAGCAGATGTTACAGCAGCAACACAACGTGTTAATAATGCTGAAACAGGTTTA
AATGGTGATTCAAACTTAGCAACTGCGAAGCAACAAGCTAAAGATGCATTACGTCAAATGACGCATTTATCTG
CTGCACAAAAACAAAGTATTACTGGTCAAATTGATAGTGCGACACAAGTAACTGGCGTTCAAAGTGTAAAAGA
CAATGCAACAAATCTTGACAATGCAATGAATCAACTTCGAAATAGTATTGCGAATAAAGATGAAGTTAAAGCG
AGTCAGCCATATGTTGATGCAGATACAGATAAACAAAATGCATACAACACAGCTGTTACAAGTGCTGAAAATA
TCATTAATGCAACGAGTCAGCCGACACTTGATCCATCTGCAGTAACACAAGCAGCTAATCAAGTGAACACTAA
CAAAACTGCGCTTAATGGTGTACAAAACTTAGCGAATAAAAAGCAAGAAACGACTGCTAACATCAACCAATTA
AGTCATTTAAATAATGCTCAAAAGCAAGATTTAAATACGCAAGTGACAAATGCACCAAATATTAGCACAGTAA

ATCAAGTGAAAACTAAAGCTGAACAATTAGATCAAGCAATGGAACGTTTAATCAACGGAATCCAAGACAAAGA
TCAAGTGAAACAAAGTGTTAACTTTACAGATGCAGATCCAGAAAAACAAACAGCATACAACAATGCGGTAACG
GCTGCTGAAAATATTATTAATCAAGCAAATGGTACAAATGCGAACCAATCACAAGTTGAAGCAGCACTTTCAA
CTGTAACAACTACTAAACAAGCGTTGAATGGTAATAGAAAAGTAACAGATGCTAAAAACAATGCAAACCAAAC
ATTATCTACATTAGATAACTTAAACAATGCACAAAAAGGTGCTGTTACTGGAAACATCAATCAAGCGCACACT
GTAGCTGAAGTAACGCAAGCCATTCAAACAGCTCAGGAACTGAATACAGCGATGGGTAACTTGAAAAATAGCT
TGAATGATAAAGACACTACACTTGGCAGTCAAAACTTTGCAGATGCAGATCCAGAGAAGAAAAATGCATACAA
TGAAGCGGTTCATAATGCTGAAAATATTTTAAATAAATCTACAGGTACAAACGTGCCTAAAGATCAAGTTGAA
GCAGCTATGAATCAAGTGAATACTACAAAAGCAGCGCTTAATGGTACTCAAAACCTTGAAAAAGCTAAACAAC
ACGCAAATACAGCAATTGACGGTTTAAGCCATTTAACAAATGCACAAAAAGATGCATTAAAACAATTGGTACA
ACAATCGACTACTGTTGCAGAAGCACAAGGTAATGAACAAAAAGCAAACAATGTTGATGCAGCAATGGACAAA
TTACGTCAAAGTATTGCAGATAATGCGACAACAAAACAAAACCAAAATTATACTGATGCAAGTCCGAATAAAA
AGGATGCGTACAATAATGCTGTCACAACTGCACAAGGTATTATTGATCAAACTACAAGTCCAACTTTAGATCC
GACTGTTATCAATCAAGCTGCTGGACAAGTAAGCACAACTAAAAATGCATTAAATGGTAATGAAAACCTAGAG
GCGGCGAAACAACAAGCGACACAATCATTAGGTTCATTAGACAACTTAAATAATGCGCAAAAACAAACAGTTA
CTGATCAAATTAATGGCGCGCATACTGTTGATGAAGCAAATCAAATTAAGCAAAATGCGCAAAACTTAAATAC
AGCGATGGGTAACTTGAAACAAGCGATTGCTGACAAAGATGCTACGAAAGCAACAGTAAACTTCACTGATGCA
GATCAAGCAAAACAACAAGCATATAACACTGCTGTTACAAATGCTGAAAACATTATTTCAAAAGCTAATGGTG
GCAATGCAACACAAACTGAAGTTGAACAAGCAATCCAACAAGTAAATGCAGCAAAACAAGCATTAAATGGTAA
TGCCAACGTTCAACATGCAAAAGACGAAGCAACAGCATTAATTAATAGCTCTAATGACCTTAACCAAGCACAA
AAAGACGCATTAAAACAACAAGTTCAAAATGCAACTACTGTAGCTGGTGTAAACAATGTTAAACAAACAGCAC
AAGAGTTAAACAATGCGATGACACAATTAAAACAAGGCATTGCAGATAAAGAACAAACAAAAGCTGATGGTAA
CTTTGTCAATGCAGATCCTGACAAGCAAAATGCATATAATCAAGCAGTAGCAAAAGCTGAAGCATTAATTAGT
GGTACGCCTGATGTAGTCGTTACACCTAGCGAAATTACTGCAGCTTTAAATAAAGTTACGCAAGCTAAAAATG
ATTTGAATGGTAATACGAACTTAGCAACGGCGAAACAAAATGTTCAACAAGCTATTGATCAATTGCCAAACTT
GAATCAAGCGCAACGTGATGAATACAACAAACAAATTACGCAAGCAACTCTTGTACCAAACGTCAATGCTATT
CAACAAGCGGCGACAACGCTTAATGACGCGATGACACAATTGAAACAAGGTATTGCGAATAAAGCACAAATTA
AAGGTAGCGAGAACTATCACGATGCTGATACTGACAAGCAAACAGCATATGATAATGCAGTAACAAAAGCAGA
AGAATTGTTAAAACAAACAACAAATCCAACAATGGATCCAAATACAATTCAACAAGCATTAACTAAAGTGAAT
GACACAAATCAAGCACTTAACGGTAATCAAAAATTAGCTGATGCTAAACAAGCAGCTAAGACAAATCTTGGCA
CATTAGATCATTTAAATGATGCTCAAAAACAAGCGTTAACAACACAAGTTGAACAAGCACCAGATATTGCAAC
AGTTAATAATGTTAAGCAAAATGCTCAAAATCTGAATAATGCTATGACTAACTTAAACAATGCATTACAAGAT
AAAACTGAGACATTAAATAGCATTAACTTTACTGATGCAGATCAAGCTAAGAAAGATGCTTATACTAATGCGG
TAGCACATGCAGAAGGTATTTTATCTAAAGCAAATGGCAGCAATGCAAGTCAAACTGAAGTGGAACAAGCGAT
ACAACGTGTGAACGAAGCGAAACAAGCATTGAATGGTAATGACAATGTACAACGTGCAAAAGATGCAGCGAAA
CAAGTAATTACAAATGCAAATGATTTAAATCAAGCGCAAAAAGATGCATTAAAACAACAAGTCGATGCTGCGC
AAACTGTTGCAAATGTAAACACAATTAAGCAAACAGCACAAGATTTAAATCAAGCAATGACACAATTGAAGCA
AGGTATTGCAGATAAAGACCAAACTAAAGCAAATGGTAACTTTGTCAATGCAGATACTGATAAGCAAAATGCA
TATAACAATGCGGTAGCACATGCTGAACAAATCATTAGTGGTACACCAAATGCAAACGTGGATCCACAACAAG
TGGCTCAAGCGTTACAACAAGTGACTCAAGCTAAGGGTGATTTAAACGGTAACCATAACTTACAAGTTGCTAA
AGACAATGCGAATACTGCCATTGATCAGTTACCAAACTTAAATCAACCACAAAAAACAGCGTTAAAAGACCAA
GTGTCACATGCAGAACTTGTTACAGGTGTTAATGCTATTAAGCAAAATGCTGATGCATTAAATAATGCAATGG
GTACGTTGAAACAACAAATTCAAGCAAACAGTCAAGTACCGCAATCAGTTGACTTTACACAAGCGGATCAAGA
CAAACAACAAGCATATAACAATGCAGCTAACCAAGCGCAACAAATTGCAAATGGAACACCAACACCTGTATTG
TCGCCTGATACAGTAACACAAGCAGTTACAACTATGAATCAAGCGAAAGATGCATTAAACGGTGATGAAAAAT
TAGCGCAAGCGAAACAGGATGCTTTAGCAAATCTGGATACGTTACGTGACTTAAATCAACCACAACGTGATGC
GTTACGTAATCAAATCAACCAAGCACAAGCGTTAGCTACAGTCGAACAAACTAAACAAAATGCACAAAATGTG
AATGCAACAATGGGTAACTTGAAACAAGGTATTGCAAATAAAGATACTGTCAAAGCAAGTGAGAACTATCATG
ATGCTGATGCCGATAAGCAAACAGCATATACAAATGCAGTGTCTCAAGCGGAAGGTATTATCAATCAAACGAC
AAATCCAACGCTTAATCCAGATGAAATAACACGTGCATTAACTCAAGTAACTGATGCTAAAAATGGCTTAAAC
GGTGAAGCTAAATTAGCAACTGAAAAGCAAAATGCTAAAGATGCCGTAAGTGGAATGACGCATTTAAACGATG
CTCAAAAACAAGCATTAAAAGGTCAAATCGATCAATCGCCTGAAATTGCTACAGTGAACCAAGTTAAACAAAC
AGCAACGAGCCTAGATCATGCAATGGATCAATTATCACAAGCTATTAATGATAAAGCTCAAACATTAGCGGAC
GGTAATTACTTAAATGCAGATCCTGACAAACAAAATGCGTATAAACAGGCAGTAGCAAAAGCTGAAGCATTAT
TGAATAAACAAAGTGGTACTAATGAAGTACAAGCCCAAGTTGAAAGTATCACTAATGAAGTGAAAACAGCGAA
ACAAGCATTAAATGGTAATGATAATTTAGCGAATGCTAAGCAACAAGCGAAACAACAATTGGCGAATTTAACA
CACTTAAATGATGCTCAAAAACAATCATTTGAAAATCAAATAACACAAGCGCCACTTGTTACAGATGTCACTA
CAATTAATCAAAAAGCGCAAACGTTAGATCATGCGATGGAATTATTAAGAAATAGTATTGCAGATAACCAAGC

GACATTAGCATCTGAAGATTATCATGATGCAACTGCGCAAAGACAAAATGACTATAACCAAGCTGTAACAGCT
GCTAATAATATCATTAATCAAACAACATCACCTACGATGAATCCAGATGATGTTAATCGTGCAACAACTCAAG
TGAATAACACGAAAGTTGCATTAGATGGTGATGAAAACCTTGTAGCAGCTAAGCAACAAGCGAACAATAGACT
TGATCAATTAGATCATTTAAATAATGCGCAAAAGCAACAGTTACAATCACAAATTACGCAATCATCTGATATT
GCAGCAGTTAATGGTCACAAGCAAACTGCAGAATCTTTAAATACCGCAATGGGTAACTTAATCAATGCGATTG
CAGATCATCAAGCCGTTGAACAACGTGGTAACTTCATCAATGCTGATACTGATAAACAAACTGCTTATACTAC
AGCAGTAAATGAAGCAGAAGCAATGATTAACAAACAAACTGGTCAAAATGCGAACCAAACAGAAGTAGAACAA
GCTATTACTAAAGTTCAAACAACACTTCAAGCGTTAAATGGAGATCATAATTTACAAGTTGCTAAAACAAATG
CGACGCAAGCAATTGATGCTTTAACAAGCTTAAATGATCCTCAAAAAACAGCATTAAAAGACCAAGTTACTGC
TGCAACATTAGTAACTGCAGTTCATCAAATTGAACAAACTGCGAATACGCTTAACCAAGCAATGCATGGCTTA
AGAGAAAGTATTCAAGATAACGCAACAACTCAAGCAAATAGCAAATATATCAACGAAGATCAACCAGAGCAAC
AAAACTACGATCAAGCAGTGCAAGCTGCAAATAACATTATCAATGAGCAAACAGCGACATTAGATAATAATGC
GATTAATCAAGCAGCAACAACTGTGAATACAACGAAAGCAGCATTACATGGTGATGTGAAGTTACAAAATGAT
AAAGATCGTGCGAAACAAACGGTTAGTCAATTAGCACATCTAAACAATGCTCAAAAACATATGGAAGATACGT
TAATTGATAGTGAAACAACTAGAACAGCCGTTAACCATGATTTGACTGAAGCACAAGCATTAGATCAACTTAT
GGATACATTACAACAAAGTATTGCTGACAAAGATGCAACACGTGCGAGCAGTGCCTATGTCAATGCAGAACCG
AATAAAAAACAAGCATATGATGAAGCAGTTCAAAATGCTGAGTCTATCATTGCAGGATTAACTAATCCAACTA
TCAATAAAGGCAATGTTTCAAGTGCGACACAAGCAGTAACATCATCTAAAAATGCATTAGACGGTGTTGAACG
ATTAGCGCAAGATAAGCAAACTGCTGGAAATTCTCTAAATCATTTAGATCAATTAACGCCAGCACAACAACAA
GCGCTAGAAAATCAAATTAATAATGCAACAACTCGTGATAAAGTGGCTGAAATCATTGCACAAGCGCAAGCAT
TAAATGAAGCGATGAAAGCATTAAAAGAAAGTATTAAAGATCAACCACAAACTGAAGCAAGTAGTAAATTTAT
TAATGAAGATCAAGCACAAAAAGATGCTTATACGCAAGCTGTACAACACGCGAAAGATTTGATTAACAAAACA
ACTGATCCTACATTAGCTAAATCAATCATTGATCAAGCGACACAGGCAGTAACTGATGCTAAAAACAACTTAC
ATGGTGATCAAAAATTAGCTCAAGATAAGCAACGTGCAACAGAAACGTTAAATAACTTGTCTAACTTGAATAC
ACCACAACGTCAAGCACTTGAAAATCAAATCAATAATGCAGCAACTCGTGACGAAGTAGCACAAAAATTAACT
GAAGCACAAGCACTTAACCAAGCAATGGAAGCTTTACGTAATAGCATTCAAGATCAACAGCAAACAGAAGCGG
GTAGCAAGTTTATCAATGAAGATAAGCCGCAAAAAGATGCATACCAAGCAGCAGTTCAACATACCAAAGATTT
GATTAATCAAACTAGCAATCCAACGCTTGATAAAGCACAAGTTGAACAATTGACACAAGCTGTTAACCAAGCT
AAAGATAACCTACATGGTGATCAAAAACTTGCAGACGATAAACAACATGCAGTTACTGATTTAAATCAATTAA
ATGGTTTGAACAATCCGCAACGTCAAGCGCTTGAAAGTCAAATAAACAATGCAGCAACTCGTGGTGAAGTAGC
ACAAAAATTAACTGAAGCACAAGCACTTAACCAAGCAATGGAAGCTTTACGTAATAGCATTCAAGATCAACAG
CAAACAGAATCTGGTAGCAAGTTTATTAATGAAGATAAACCGCAAAAAGATGCTTACCAAGCAGCAGTTCAAA
ATGCAAAAGATTTAATCAACCAAACAGGTAATCCAACACTCGACAAATCACAAGTAGAACAATTAACACAAGC
AGTAACAACTGCAAAAGATAATCTACATGGTGATCAAAAACTAGCTCGTGATCAACAACAAGCGGTAACGACT
GTTAATGCATTACCAAACTTAAATCATGCACAACAACAAGCATTAACTGATGCAATTAATGCAGCGCCTACAA
GAACAGAGGTTGCACAACATGTTCAAACTGCTACTGAACTTGATCACGCGATGGAAACATTGAAAAATAAAGT
TGATCAAGTGAAAACAGATAAGGCCCAACCAAATTACACTGAAGCATCGACGGATAAAAAAGAAGCAGTGGAT
CAAGCGTTACAAGCTGCACAAAGCATTACAGAACCTAATAATGGTTCAAATGCAAATAAAGATGCTGTAGAAC
AAGCATTAACTAAGCTTCAAGAAAAAGAAAATGAGTTAAATGGTAATGAGAGAGTCGCTGAAGCTAAAACACA
AGCGAAACAAACAGTTGACCAATTAACACATTTAAATGCTGATCAAATTGCAACTGCAAAACAAAACATTGAT
CAAGCGATGAAACTTCAACCAATCGCTTAATTAGTAGATCAAGCAACGCAATTGAACAAATCTATGGATCAAT
TACAACAAGCGGTCAATGATCATACAAACGTTGAGCAAACTGTAGATTACACGCAAGCAGATTCAGATAAGCA
AAATGCTTATAAACAAGCTATTGCAGATGCTGAAAATGTATTAAAACAAAATGCGAATAAGGAACAAGTGGAT
CAAGCACTTCAAAACATTTTAAATGCAAAACAAGCATTAAATGGTGATGAACGCGTAGCACTTGCTAAAACAA
ATGGTAAACATGATATTGACCAATTAAATACATTAAACAATGCACAACAAGATGGATTTAAAGGCAGAATTGA
TCAATCTAATGACTTAAATGAGATCAAACAAATTGTAGATGAAGCTAAAGCTCTAAATCGTGCAATGAATCAA
TTGTCTCAAGAAATCACTGGCAATGAAGGACGTACGAAAGGTAGTACGAACTATGTCAATGCAGATACACAAG
TTAAACGCGTGTACGATGATGCAGTTGATAAAGCGAAGGAAGCACTAAATAAAGCGACAGGTCAAAACTTAAC
TGCAGAAGAAGTTATCAAGTTGAACGATGCGGTAACAGCAGCGAAACAGGCATTAAATGGTGAAGAAAGACTT
AACAAGCGTAAGTCTGAAGCATTACAAAGATTGGATCAATTAACACATCTAAACAATGCTCAAAGACAATTAG
CAACACAACAAATTAATAATGCTGAAACATTAAATAAAGCGTTTCGAGCAATTAATAGAGCTGTTCAATTAGA
TGATGCAATGGGTGCAGTACAACAGTATATTGATGATCATCATTTTGATGTCATTAGCAGTACAAATTACATC
AATGCAGACGATAACTTGAAAGCAAATTATGATAATGCCATTACAAATGCAGCGCATGAGTTAGATAAAGTTC
AAGGTAGTGCAATCACGAAAGCTGAGGCAGAACAATTAAAACAAAATATTATAGATGCGCAACAAGCCTTAAA
TGGTGATCAAAATCTTGCAACTGCTAAAGATAAAGCCAATGCCTTTGTTGATACATTAAATGGTTTAAATCAA
CAACAACAAGATCTTGCACATCAAGCAATTAATAATGCCGATACTGTAACAGGTATAGCGAATATTATTAATG
ATCAAATCGACTTGAATAACGCTATGGAAACATTGAAACACTTAGTTGACAATGAAATTCCAACTGCAGAACA
AACTATCAATTACCAAAACGCTGACGACGTTGCTAAATCAATCTTTGATGATGCAAAACGTCTGGCAAATACA

TTGCTAAATAGTGATAACACAAATGTGAATGATATTAACGGCGCAATTCAAGCAGTCAATGATGCAATCCAAA
ATCTTAATGGTGATCAACGTCTACAAGAAGCTAAGGACAAAGCAATTCAAAATGTTAACAAAGTCTTAGCAGA
TAAATTGAAAGAAATAGAAGCTTCAAATGCGACGGATCAAGACAAGCTTATTGCGAAAAATAAAGCAGAAGAA
TTGGCAAACAGTATCATTAACAACATTAATAAAGCAACAAGTAATCAGGATGTATCTCAAGTTCAAACAGCTG
GCAACCATGCGATTGAACAAGTGCATGCCAATGAAATACCGAAAGCTAAAATTGATGCAAATAAAGATGCTGA
TAAGCAAGTACAAGCATTAATTGACGAAATTGATCGAAATCCAAATCTAACAGATAAGGAAAAACAAGCACTT
AAAGATCGAATAAATCAAATATTGCAACAAGGTCATAACGGCATCAACAATGCGATGACTAAAGAAGAAATTG
AACAAGCCAAAGCACAACTTGCACAAGCGTTACAAGAAATTAAAGATTTAGTGAAAGCTAAAGAAAATGCGA
>DSM104437_scaffold_10
GTTTGTGTCAGATGACATTAAATAGCATCTCCTCGTGTTGATTATTTTGGTTGGCTGACCAATATTTATTCTA
GCACGTAGAGATGCATTTTTTGTGACAATGGTAGAACCTTTTCTGAACCATACGCATAGCGTATGGTTTTCTT
TTCAAGAAAAAAAAGACAAAGCTGTTATGATCTTAGCTTTGTCTTTTATGTGTTAGTCATCATGTATACGACA
TCCTACTACTAACGTTTATATATTCGAATTTTTATTTCATAATAATCTTCATGATCTTTGTCTTTATGCTCAA
CATGTAGTCCTGTTTGTTGAATCGCTTGGATACTTTTGCCTACCTCATCTCGTGCTTGAGTGACATCTTGTGC
AAAGCGAAGGTTTTGTGCTTTGACTTTTTCGGGCCCCGTTTTTTGGCGTACTCTATCTTCAGTCTGTTTCACA
TTTAGCTTTTGTGCAATGACTTGCTCAATCAACGCTTCTTGTTCGCTATCAGACAATGATAATACCGCTCTCG
CATGGCGTTCAGTAATTTTACCTTCTCTTAAGCGAAGTAGTACTTTCGGCGCCAACTTCAATAAACGCAACTT
ATTTGCAATAAAGCTTTGACTTTTACCTAAACTTTTTGCCAATTCACTTTGCGTTGTATCACCAATTTCCAAT
AATTTCTTATAGGCTTCCGCTTCTTCAACAACAGACAAATTTTCTCTTTGAATATTCTCAATTAATGCAACAA
CAGCCGTCTCTTCATCATCCATATCACGAATAATAACGTCTGCTTGAGGTAAATTTAGTGATTGTATTGCTCT
AAATCGGCGCTCTCCAGCAATAATTTCAAACATATCTTCTTCAATCGGTCTTACAACAATAGGTTGTAGTAAA
CCATGTTCATGTATTGATTCAGCAAGTTCTTTAATTTTATTTGGTTCAAACACCTGTCTTGGTTGATAACGGT
TGGGAACGATACGTTCAATTTGAATGGATTCAACATTACTATTGCGATCTTCTTCAATATGTCCAATGATGTC
ATCTTTGTTTTTCAAACCAAATAATTTTGAAAAAGGTTTTTTCATTATCCATTCGCTCCTTTTATGTTAGTTG
TAAAGGATTATGCATTATTTTTCAAGTAAAGGAGTCTTATTAGGCGTCCCTGGTTTTCTTGGATACTTTTTCG
GCGTCTGTCTTTTTTTATCAATAATGAACATCTGGCGCTCTCCAGCATCTTCTGGCAATTCAAAGGTATGTGT
TTCTGTAACATTACCACCTAACACACTAATTGCAAATTTTGCTTCTTCTAATTCTTCTTCACCTTTTGAAGAT
TTTAATGCAACAAACTGTCCACCTTTTTTAACTAGCGGTAAGCACAATTCACTTAACACGGATAATCTAGCTA
CTGCTCTTGCAGTAACAACATCATAAGACTCCCTGTAGACACCCCTTACCAAATGTTTCTGCTCTATTGTGTAT
AAAGCTGACATCCTGTAATTGTAATTCTGACGCTAAATGGTTTAAAAATTGAATACGCTTATTTAATGAATCA
ACAATCGTCACTTTTAACTGCGGAAACATTATTTTTAACGGAATACTTGGAAAACCAGCTCCAGCGCCTACAT
CACATATACTTATAGGCTGATTAAAATCAAAATAAAAACTAGGTGCAACGGAATCATAAAAATGTTTCAAATA
TACATCGTGTTCATCTGTAATACTTGTCAAATTCATCTTTTCATTCCATTCAACAAGTAAACGATAATATGTT
TGAAACTGTTGTTTTTGAGTCTCAGTTAATTCAATATTATGTTCTTTTAATTGTTCTGCTAACCATTCTACAG
TCATTAGTCACTCACCCTTTGGAGTTTACCTTGTTCTAAGTAAATCAATAATATAGATATGTCTGCTGGATTT
ACCCCTGATATTCTAGAAGCTTGTGCAATATTTAAAGGTTTTACTTCTGATAATTTTTCTCGCGCTTCAGTCG
CCAAACTATCAATCTTACTATAATCTAAGTCTTCTGGAATTTTCTTCTCTTCCATACGCTTAACTTTCTCAAC
TTGTTGTAGTGATTTATTGATATAACCTTCATATTTTGTTTGTATTTCTACTTGTTCTTCAACATCTGCATTC
AATTGATGTTCTTCTTCTAAAATTTCTAAAATTATATCGTAAGTCATTTCAGGTCTGCGTAATAAATCGATAG
CTAAAATACCATCTTTTAAGCGAGAACCACCATGTTGTTCAATAATCGCTTGCGTATGTTCGTTTGGTTTAAT
ACGAATATCTGATAAACGCTTAATTTCCGCATCAATTTGCTGACGTTTTTCATTAAAACGTGCATATCGTTCT
TCAGAAATCATACCAAGTTCATATCCCATATCCGTCAATCTCAAATCAGCATTATCATGACGTAGTAACAAAC
GATATTCTGCACGTGATGTTAGTAAACGATAAGGTTCATTAGTACCTTTAGTTACAAGATCATCGATTAAGAC
ACCAATATATGCATCTGAACGACTTAATATCTTTTCGCCTGTGTTTAACACTTTACCTGCAGCGTTAATACCT
GCCATCAATCCTTGTCCTGCTGCTTCTTCATAACCAGATGTACCATTAATTTGACCTGCAGTGTATAAGTTTT
TAATCATTTTCGTTTCAAGTGTAGGCCATAACTGCGTTGGCACAATCGCATCATATTCAATTGCGTAGCCGGC
ACGCATCATATCTGCTTTTTCAAGACCTGGTATCGTCTCTAACATTTGACGTTGCACATGTTCAGGAAGACTT
GTAGATAATCCTTGCACATATACTTCATTTGTATTACGACCTTCAGGCTCTAAGAAAAGTTGATGTCGCGGCT
TATCATTAAATCGAACAAATTTATCTTCAATTGAAGGGCAATAACGTGGCCCGGTTCCTTTAATCATCCCTGA
ATACATTGCAGATAGATGTAAATTATCATCGATAACTTTGTGTGTTTCAGCATTAGTATACGTTAGCCAACAT
GGCAATTGATCTAATATATATTCTGTTGTTTCAAAGCTGAATGCACGACCTACATCGTCACCTGGTTGTATTT
CAGTCTTCGAATAGTCAATTGTTTTTGAATTTACACGCGGTGGTGTACCTGTTTTAAAACGAACAATATCAAA
ACCAAGTTCTCTTAAATTGTCTGATAATGTGATTGATGGTAATTGGTGATTTGGTCCACTTGAATACTTCATA
TTACCTAAAATGATTTCACCACGTAAAAATGTTCCCGTTGTAATAATTACTGCTTTAGATAAATACTCTGTAC
CAATATTTGTACGTACACCTTTAACTTCATTATCTTCTATAATAAGTTCGTCTACCATACCTTGCATTATATG
CAAATTTTCTTCATCTTCAATCACGCGTTTCATTTCTTGTTGATAAAGTACTTTATCTGCTTGCGCTCTTAGT
GCTCTTACAGCAGGTCCTTTACCTGTATTTAACATTCTCATTTGAATGTGTGTTTTATCGATTGTTTTTGCCA
TTTGTCCACCTAAAGCATCAATTTCACGAACAACGATACCTTTAGCTGGTCCACCTACAGATGGGTTACATGG

CATAAATGCAATATTATCTAAATTTATTGTTAGCATTAATGTTTTAGCACCACGTCTTGCAGATGCTAAACCT
GCTTCTACACCTGCATGTCCCGCACCTATAACGATTACATCATATTCTTGAACCACAATATAAACCTCCTTAT
TTGATATCTTACTAGCCTTCTTAAGACGGTATTCCGTCTATTTCAATTACTATTTACCTAAGCAGAATTGACT
GAATAACTGATCGATGAGTTCATCACTTGCAGTCTCACCAATAATTTCTCCTAATATTTCCCAAGTTCTAGTT
AAATCAATTTGTACCATATCCATAGGCACACCAGATTCTGCTGCATCAATCGCATCTTGTATCGTTTGTCTTG
CTTGTTTTAATAATGAAATATGTCTTGAATTAGAAACATAAGTCATATCCTGATTTTGTACTTCTCCACCAAA
GAACAAATCTCGAATTTGTATTTCTAATTCATCAATACCTTCTTGTTTTAACATTGAAGTTTGAATTAATGGC
GTATCACCTATCATATCTTTAACTTCATTAATATCTATGTTTTGCTCTAAATCCATTTTATTAACAATTACGA
TTACATCTTCATTTTTAACCACTTCATATAATGTGTAATCTTCTTGAGTCAATGCTTCGTTATTGTTTAATAC
AAATAAAATTAAGTCTGCTTGGCTAAGAGCCTTTCTAGAGCGTTCAACACCAATCTTCTCTACTATATCTTCT
GTCTCGCGTATACCAGCAGTATCAACTAATCTTAATGGCACGCCACGAACATTGACGTACTCTTCTAAGACAT
CTCTTGTAGTACCTGCTACCTCAGTTACAATCGCTTTATTATCTTGTATTAAATTATTTAACATCGATGATTT
ACCTACGTTTGGTTTACCAACAATAACTGTAGATAAACCTTCACGCATAATTTTACCCTGCGCACCGGTATCT
AATAAACGATTAATTTCCTGTTTGATTTCTTTAGACTGCTCTAAAAGAAATTCAGTAGTCGCATCTTCAACAT
CATCGTATTCAGGATAATCAATATTCACTTCTACTTGAGCGAGTATCTCTAATATAGATTGACGTTGTTTTTT
GATTAAGTCACTTAGACGACCTTCAATTTGATTCATCGCAACCTTAGAAGCTCTATCTGTCTTCGAGCGAATA
AAGTCCATAACTGCTTCAGCTTGAGATAAATCAATACGACCATTTAAAAAGGCTCGTTTTGTAAATTCACCTG
GCTCAGCCATTCGAGCGCCATATGTCATAGTAAGTTCCAGCACTCTATTAATCGTTAATATACCACCATGACA
ATTAATTTCTATAATATCTTCGCGTGTAAATGTTTTTGGCGCTCTTAACACAGACACCATAACTTCTTCAACC
ACTTCTTTAGACTCTGGATCAATAATATGACCGTAATTAATCGTATGTGATGGAACATCATTTAAAAGATGTT
TTCCTTTATATAATATGTCTGCAATTTCAACGGCTTGCGGTCCAGACAATCGAACAATTCCAATTGCCCCTTC
ACCCATTGGTGTTGAAATACTCGTAATTGTATCTAAATCCATATTGCTACTCGCCTCCTTCAACGATGTGAAT
ACATTTTAAAGTAAGTTATTATAACCCTAAGGTAAGTCTTACGTTTGTCTGAGGTAAGACTTCGGGAGGTGTT
GAGTGGTTAATGTTTACCTTCCCCTACCCTATCATTACTTAATTTTTTTATTAAAAACTTTGGCAATTTTAAG
TACGTGCTCAAGACTATTCTGTATTTGTAAAGTCGTCATATCTTTAGCTGGCTGTCTTGCTATTACAATAATA
TCTTTGGCCAATATATGCGACTTATGTACTTTGAAATTTTCACGTATTGCTCTTTTAATCTTGTTTCTTAACA
CTGCATTACCTAGTTTTTTAGAAACACTAATACCTAAGCGAAAATGGTCTATTTCTTTATTATTACAAGTGTA
TACAACAAATTGTCTGTTGGCTACAGAATGACCTTTTTTATATATTCTCTGAAAATCTGCATTCTTTTTAATT
CGGTAAGCTTTTTCCAATAACATCACTCGCTTATTTATCGTTTTTATTTGAAGCTATATTTAAACTTCTATTG
AGCTTATAACATAAATTTCTATTTATTCTTAATTTAAACGAAAAAAAAGATCACTGATGAGTCAGTGATCTTA
TGCAGATAAAACTTTACGGCCTTTACGACGACGGCGCGCTAAAACTTTACGGCCATTTTTTGTGCTCATGCGT
TTTCTGAAACCATGAACTTTACTATGTTTACGTTTATTTGGTTGATAAGTACGTTTTACCATGCAAAACACCT
CCATCTTTATACAGTTATCTAATATACCAGATTATGCAGACTCTGATCACTTTAATAGATAAGATTTTGCGTT
TCTTAGATTAAAATTCTCGTATTAGCTCATTGATTATCTAGTCATAATTCAAGCAACTACTACAATATAACAA
AATCCTTTTTATAACGCAAGTTCATTTTATGCTACTGCTCAATTTTTTTACTTTTATCGATTAAAGATAGAAA
TACACGATGCGAGCAATCAAATTTCATAACATCACCATGAGTTTGGTCCGAAGCATGAGTGTTTACAATGTTT
GAATACCTTATACAGTTCTTATACATACTTTATAAATTATTTCCCAAGCTGTTTGATACACTCACTAACAGA
TACTCTATAGAAGGAAAAGTTATCCACTTATGCACATTTACAGTTTTCAGAATTGTGGATAATTAGAAATTAC
ACACAAAGTTATACTATTTTTAGCAACATATTCACAGGTATTTGACATATAGAGAACTGAAAAAGTATAATTG
TGTGGATAAGTCGTCCAACTCATGATTTTATAAGGATTTATTTATTGATATTTACATAAAAATACTGTGCATA
ACTAATAAGCAGGATAAAGTTATCCACTGATTGTTATTAACTTGTGGATAATTATTAACATGGTGTGTTTAGA
AGTTATCCACGGCTGTTATTTTTGTGTATAACTTAAAAATTTAAGAAAGATGGAGTAAATTTATGTCGGAAAA
AGAAATTTGGGAAAAAGTGCTTGAAATTGCTCAAGAAAAATTATCAGCTGTAAGTTACTCAACTTTCCTAAAA
GATACTGAACTTTACACGATCAAAGATGGTGAAGCTATCGTATTATCGAGTATTCCTTTTAATGCAAATTGGT
TAAATCAACAATATGCTGAAATTATCCAAGCAATCTTATTTGATGTTGTAGGCTATGAAGTAAAACCTCACTT
TATTACTACTGAAGAATTAGCAAATTATAGTAATAATGAAACTGCTACTCCAAAAGAAACAACAAAACCTTCT
ACTGAAACAACTGAGGATAATCATGTGCTTGGTAGAGAGCAATTCAATGCCCATAACACATTTGACACTTTTG
TAATAGGACCTGGTAACCGCTTTCCACATGCAGCGAGTTTAGCTGTGGCCGAAGCACCAGCCAAAGCGTACAA
TCCATTATTTATCTATGGAGGTGTTGGTTTAGGAAAAACCCATTTAATGCATGCCATTGGTCATCATGTTTTA
GATAATAATCCAGATGCCAAAGTGATTTACACATCAAGTGAAAAATTCACAAATGAATTTATTAAATCAATAC
GTGATAACGAAGGTGAAGCTTTCAGAGAAAGATATCGTAATATCGACGTCTTATTAATCGATGATATTCAGTT
CATACAAAATAAAGTACAAACACAAGAAGAATTTTTCTATACTTTTAATGAATTGCATCAGAATAACAAGCAA
ATAGTTATTTCGAGTGATCGACCACCAAAGGAAATTGCACAATTAGAAGATCGATTACGTTCACGCTTTGAAT
GGGGGCTAATTGTTGATATTACGCCACCAGATTATGAAACTCGAATGGCAATTTTGCAGAAGAAAATTGAAGA
AGAAAAATTAGATATTCCACCAGAAGCTTTAAATTATATAGCAAATCAAATTCAATCTAATATTCGTGAATTA
GAAGGTGCATTAACACGTTTACTTGCATATTCACAATTATTAGGAAAACCAATTACAACTGAATTAACTGCTG
AAGCTTTAAAAGATATCATTCAAGCACCAAAATCTAAAAAGATTACCATCCAAGATATTCAAAAAATTGTAGG
CCAGTACTATAATGTTAGAATTGAAGATTTCAGTGCAAAAAAACGTACAAAGTCAATTGCATATCCGCGTCAA

ATAGCTATGTACTTGTCTAGAGAGCTTACAGATTTCTCATTACCTAAAATTGGTGAAGAATTTGGTGGGCGTG
ATCATACGACCGTCATTCATGCTCATGAAAAAATATCTAAAGATTTAAAAGAAGATCCTATTTTTAAACAAGA
AGTAGAGAATCTTGAAAAAGAAATAAGAAATGTATAAGTAGGAAACTTTGGGAAATGTAATCTGTTATATAAC
AGCACTAATAATAACTGTCATTTTTTACATTTCTATATACTAATGTGGCAAGATGAGCAAAACTCATTTTGTG
GATAATGTTTAAAAGTCATACACATCATACACAAGTTATCAACATGTGTATAACTTCGCCAAATCTATGTTTT
TAAGACTTATCCACCAATCCACAGCACCTACTACTATTACTAAGAACTTAAAACCTATATAATTATATATAAA
CGACTGGAAGGAGTTTTAATTAATGATGGAATTCACTATTAAAAGAGATTATTTTATTACACAATTAAATGAC
ACATTAAAAGCTATTTCACCAAGAACAACATTACCTATATTAACTGGTATCAAAATCGATGCGAAAGAACATG
AAGTTATACTAACTGGTTCAGACTCTGAAATTTCAATAGAAATCACTATTCCTAAAACTGTAGATGGCGAAGA
TATTGTCAATATTTCAGAAACAGGCTCAGTAGTACTTCCTGGACGATTCTTTGTTGATATTATAAAAAAATTA
CCTGGTAAAGATGTTAAATTATCTACAAATGAACAATTCCAGACATTAATTACATCAGGTCATTCTGAATTTA
ATTTGAGTGGCTTAGATCCAGATCAATATCCTTTATTACCTCAAGTTTCTAGAGATGACGCAATTCAATTGTC
GGTAAAAGTGCTTAAAAACGTAATTGCACAAACGAATTTTGCAGTGTCCACCTCAGAAACACGCCCAGTACTA
ACTGGTGTGAACTGGCTTATACAAGAAAATGAATTAATATGCACAGCGACTGACTCGCACCGCTTGGCTGTAA
GAAAGTTGCAGTTAGAAGATGTTTCTGAAAACAAAAATGTCATCATTCCAGGTAAGGCTTTAGCTGAATTAAA
TAAAATTATGTCTGACAATGAAGAAGACATTGATATCTTCTTTGCTTCAAACCAAGTTTTATTTAAAGTTGGA
AATGTGAACTTTATTTCTCGATTATTAGAAGGACATTATCCTGATACAACACGTTTATTCCCTGAAAACTATG
AAATTAAATTAAGTATAGACAATGGGGAGTTTTATCATGCGATTGATCGTGCATCTTTATTAGCACGTGAAGG
TGGTAATAACGTTATTAAATTAAGTACAGGTGATGACGTTGTTGAATTGTCTTCTACATCACCAGAAATTGGT
ACTGTAAAAGAAGAAGTCGATGCAAACGATGTTGAAGGTGGTAACCTGAAAATTTCATTCAACTCTAAATATA
TGATGGATGCTTTAAAAGCAATCGATAATGATGAGGTTGAAGTTGAATTCTTCGGTACAATGAAACCATTTAT
TCTAAAACCAAAAGGTGACGACTCAGTAACGCAATTAATTTTACCAATCAGAACTTACTAAAAATAAATATAA
ATAAAGGATGACGTGATTAATTAAAACGTCATCCTTTATTTTTTGGCAAAAATAATTCTAGATGCGTATGTAA
AATAAATTTGACAGCATTTTAAACAGCAAATAAAAGACGCCAATTAAATTTATGACAAATGTATCCAAAATTT
AATAAGTGTGCTTATATGCCCTTTAAATTTAAAATTTTAATAGTCAATAACAAGTTGAATATAAAAGTTAAAC
GCCGTTAAATAGCGTTAAAAAATTGAAAATGACAGTATTGCCCAAAAATAAGAATTAATTATTTATATGTAAA
CGGTTTCTACCTCTATTTTAAATGAAATTTGTGACAAAAAAAGGTATAATATATTAATGACATACAAAGAAAT
GGAGTGATTATTTTGGTTCAAGAAGTTGTAGTAGAAGGAGACATTAATTTAGGTCAATTTCTAAAAACAGAAG
GGATTATTGAATCTGGTGGTCAAGCAAAATGGTTCTTGCAAGACGTTGAAGTATTAATTAATGGAGTGCGTGA
AACACGTCGCGGTAAAAAGTTAGAACATCAAGATCGTATAGATATCCCAGAATTACCTGAAGATGCTGGTTCT
TTCTTAATCATTCATCAAGGTGAACAATGAAGTTAAATACACTCCAATTAGAAAATTATCGTAACTATGATGA
GGTTACGTTGAAATGTCATCCTGACGTGAATATCCTCATTGGAGAAAATGCACAAGGAAAGACAAATTTACTT
GAATCAATTTATACCTTAGCTTTAGCAAAAAGTCATAGAACGAGTAATGATAAGGAACTCATACGTTTTAATG
CTGATTATGCTAAAATAGAAGGTGAGCTTAGTTATAGACACGGCACGATGCCATTAACAATGTTTATAACTAA
AAAAGGTAAACAAGTCAAAGTGAATCACTTAGAGCAAAGTCGACTAACTCAATATATTGGACACCTCAATGTG
GTTCTATTTGCGCCAGAAGATTTGAATATTGTAAAAGGCTCTCCTCAAATAAGACGACGCTTTATAGATATGG
AGTTGGGCCAAATTTCTGCTGTTTACTTAAATGATTTAGCTCAATACCAACGTATTTTAAAGCAAAAGAATAA
TTACTTAAAGCAGTTACAATTAGGCCAAAAAAAGGACTTAACAATGTTGGAAGTATTAAATCAGCAGTTTGCT
GAATATGCAATGAAAGTAACTGATAAACGTGCACATTTTATTCAAGAGCTAGAGTCGTTAGCTAAACCGATTC
ATGCTGGTATCACAAATGATAAAGAAGCGTTGTCGCTAAATTATTTACCTAGTCTTAAATTTGATTATGCTCA
AAATGAAGCGGCACGACTTGAAGAAATTATGTCTATTCTTAGCGATAATATGCAAAGAGAAAAAGAACGAGGC
ATTAGCTTATTCGGACCACATCGAGATGATATAAGTTTTGATGTGAATGGCATGGATGCTCAAACATATGGTT
CTCAAGGACAGCAACGTACAACGGCTTTGTCCATTAAATTAGCTGAAATTGAGTTAATGAATATCGAAGTTGG
GGAATATCCCATCTTATTATTAGACGATGTACTCAGTGAATTAGATGATTCGCGTCAAACGCATTTATTAAGT
ACGATTCAGCATAAAGTACAAACATTTGTCACTACGACATCTGTAGATGGTATTGATCATGAAATCATGAATA
ACGCTAAATTGTATCGTATTAATCAAGGTGAAATTATAAAGTAACAGAAAGCGATGGTGACTGCATTGTCAGA
TGTAAACAACACGGATAATTATGGTGCTGGGCAAATACAAGTATTAGAAGGTTTAGAAGCAGTACGTAAAAGA
CCAGGTATGTATATAGGATCGACTTCAGAGAGAGGTTTGCACCATTTAGTGTGGGAAATTGTCGATAATAGTA
TCGATGAAGCATTAGCTGGTTATGCAAATCAAATTGAAGTTGTTATTGAAAAAGATAACTGGATTAAAGTAAC
GGATAACGGACGTGGTATCCCAGTTGATATTCAAGAAAAAATGGGACGTCCAGCTGTCGAAGTTATTTTAACT
GTTTTACATGCTGGTGGTAAATTCGGCGGTGGCGGATACAAAGTATCTGGTGGTTTACATGGTGTTGGTTCAT
CAGTTGTAAACGCATTGTCACAAGACTTAGAAGTATATGTACACAGAAATGAGACTATATATCATCAAGCATA
TAAAAAAGGTGTACCTCAATTTGACTTAAAAGAAGTTGGCACAACTGATAAGACAGGTACTGTCATTCGTTTT
AAAGCAGATGGAGAAATCTTCACAGAGACAACTGTCTACAACTATGAAACATTACAGCAACGTATTAGAGAGC
TTGCTTTCTTAAACAAAGGAATTCAAATCACATTAAGAGATGAACGTGATGAAGAAAACGTTAGAGAAGACTC
CTATCATTATGAGGGCGGTATTAAATCTTATGTTGAGTTATTGAACGAAAATAAAGAACCTATTCATGATGAG
CCAATTTATATTCATCAATCTAAAGATGATATTGAAGTAGAAATTGCGATTCAATATAACTCAGGATATGCCA
CAAATCTTTTAACTTACGCAAATAACATTCATACGTACGAAGGTGGTACGCATGAAGACGGATTCAAACGTGC

ATTAACGCGTGTCTTAAATAGTTATGGTTTAAGTAGCAAGATTATGAAAGAAGAAAAAGATAGACTTTCTGGT
GAAGATACACGTGAAGGCATGACAGCAATTATATCTATCAAACATGGTGATCCTCAATTCGAAGGTCAAACGA
AGACAAAATTAGGTAATTCTGAAGTGCGTCAAGTTGTAGATAAATTATTCTCAGAGCACTTTGAACGATTTTT
ATATGAAAATCCACAAGTCGCACGTACAGTGGTTGAAAAAGGTATTATGGCGGCACGTGCACGTGTTGCTGCG
AAAAAAGCGCGTGAAGTTACACGTCGTAAATCAGCGTTAGATGTAGCAAGTCTTCCAGGTAAATTAGCCGATT
GCTCTAGTAAAAGTCCTGAAGAATGTGAGATTTTCTTAGTCGAAGGGGACTCTGCCGGGGGGTCTACAAAATC
TGGTCGTGACTCTAGAACACAGGCGATTTTACCATTACGAGGTAAGATATTAAATGTTGAAAAAGCACGATTA
GATAGAATTTTGAATAACAATGAAATTCGTCAAATGATTACAGCATTTGGTACAGGAATCGGTGGCGACTTTG
ATCTAGCGAAAGCAAGATATCACAAAATCGTCATTATGACTGATGCCGATGTGGATGGAGCGCATATTAGAAC
ATTGTTATTAACATTCTTCTATCGATTTATGAGACCGTTAATTGAAGCAGGCTATGTGTATATTGCACAGCCA
CCGTTGTATAAACTGACACAAGGTAAACAAAAGTATTATGTATACAATGATAGGGAACTTGATAAACTTAAAT
CTGAATTGAATCCAACACCAAAATGGTCTATTGCACGATACAAAGGTCTTGGAGAAATGAATGCAGATCAATT
ATGGGAAACAACAATGAACCCTGAGCACCGCGCTCTTTTACAAGTAAAACTTGAAGATGCGATTGAAGCGGAC
CAAACATTTGAAATGTTAATGGGTGATGTTGTAGAAAACCGTAGACAATTTATAGAAGATAATGCAGTTTATG
CAAACTTAGACTTCTAAGCGCTGTGAACTGAACTTTTGAAGGAGGAACTCTTGATGGCTGAATTACCTCAATC
AAGAATAAATGAACGAAATATTACCAGTGAAATGCGTGAATCATTTTTAGATTATGCGATGAGTGTTATCGTT
GCTCGTGCATTGCCAGATGTTCGTGACGGTTTAAAACCAGTACATCGTCGTATACTATATGGATTAAATGAAC
AAGGTATGACACCGGATAAATCATATAAAAAATCAGCACGTATCGTTGGTGACGTAATGGGTAAATATCACCC
TCATGGTGACTTATCTATTTATGAAGCAATGGTACGTATGGCTCAAGATTTCAGTTATCGTTATCCGCTTGTT
GATGGCCAAGGTAACTTTGGTTCAATGGATGGAGATGGCGCAGCAGCAATGCGTTATACTGAAGCACGTATGA
CTAAAATCACACTTGAACTGTTACGAGATATTAATAAAGATACAATAGATTTTATCGATAACTATGATGGTAA
TGAAAGAGAGCCGTCAGTCTTACCTGCTCGATTCCCTAACTTGTTAGCCAATGGTGCATCAGGTATCGCGGTA
GGTATGGCAACGAATATTCCACCACATAACTTAACAGAATTAATCAATGGTGTACTTAGCTTAAGTAAAAACC
CTGATATTTCAATTGCTGAGTTAATGGAGGATATTGAAGGTCCTGATTTCCCAACTGCTGGACTTATTTTAGG
TAAGAGTGGTATTAGACGTGCATATGAAACAGGTCGTGGTTCAATTCAAATGCGTTCTCGTGCAGTTATTGAA
GAACGTGGAGGCGGACGTCAACGTATTGTTGTCACTGAAATTCCTTTCCAAGTGAATAAGGCTCGTATGATTG
AAAAAATTGCAGAGCTCGTTCGTGACAAGAAAATTGACGGTATCACTGATTTACGTGATGAAACAAGTTTACG
TACTGGTGTGCGTGTCGTTATTGATGTGCGTAAGGATGCAAATGCTAGTGTCATTTTAAATAACTTATACAAA
CAAACACCTCTTCAAACATCATTTGGTGTGAATATGATTGCACTTGTAAATGGTAGACCGAAGCTTATTAATT
TAAAAGAAGCGTTGGTACATTATTTAGAGCATCAAAAGACAGTTGTTAGAAGACGTACGCAATACAACTTACG
TAAAGCTAAAGATCGTGCCCACATTTTAGAAGGATTACGTATCGCACTTGACCATATCGATGAAATTATTTCA
ACGATTCGTGAGTCAGATACAGATAAAGTTGCAATGGAAAGCTTGCAACAACGCTTCAAACTTTCTGAAAAAC
AAGCTCAAGCTATTTTAGACATGCGTTTAAGACGTCTAACAGGTTTAGAGAGAGACAAAATTGAAGCTGAATA
TAATGAGTTATTAAATTATATTAGTGAATTAGAAGCAATCTTAGCTGATGAAGAAGTGTTATTACAGTTAGTT
AGAGATGAATTGACTGAAATTAGAGATCGTTTCGGTGATGATCGTCGTACAGAAATTCAATTAGGTGGATTTG
AAGACTTAGAAGATGAAGACTTAATTCCAGAAGAACAAATAGTAATTACTTTGAGCCATAATAACTACATTAA
ACGTTTGCCGGTATCTACATATCGTGCTCAAAACCGTGGTGGTCGTGGTGTTCAAGGTATGAACACATTGGAA
GAAGATTTTGTCAGTCAATTGGTAACTTTAAGTACACATGACCATGTATTGTTCTTTACTAACAAAGGTCGTG
TATACAAACTTAAAGGTTATGAAGTGCCTGAGTTATCAAGACAGTCTAAAGGTATTCCTGTAGTGAATGCTAT
TGAACTTGAAAATGATGAAGTCATTAGTACAATGATTGCTGTTAAAGACCTTGAAAGTGAAGACAACTTCTTA
GTGTTTGCAACTAAACGTGGTGTCGTTAAACGTTCAGCATTAAGTAACTTCTCAAGAATAAATAGAAATGGTA
AGATTGCGATTTCGTTCAGAGAAGATGATGAGTTAATTGCAGTTCGCTTAACAAGTGGTCAAGAAGATATCTT
GATTGGTACATCACATGCATCATTAATTCGATTCCCTGAATCAACATTACGTCCTTTAGGCCGTACAGCAACG
GGTGTGAAAGGTATTACACTTCGTGAAGGTGACGAAGTTGTAGGGCTTGATGTAGCTCATGCAAACAGTGTTG
ATGAAGTATTAGTAGTTACTGAAAATGGTTATGGTAAACGTACGCCAGTTAATGACTATCGTTTATCAAATCG
TGGTGGTAAAGGTATTAAAACAGCTACGATTACTGAGCGTAATGGTAATGTTGTATGTATCACTACAGTAACT
GGTGAAGAAGATTTAATGATTGTTACTAATGCAGGTGTCATTATTCGACTAGATGTTGCAGATATTTCTCAAA
ATGGTCGTGCAGCACAAGGTGTTCGCTTAATTCGCTTAGGTGATGATCAATTTGTTTCAACGGTTGCTAAAGT
AAAAGAAGATGCAGAAGATGAAACGAATGAAGATGAGCAATCTACTTCAACTGTATCTGAAGATGGTACTGAA
CAACAACGTGAAGCGGTTGTAAATGATGAAACACCAGGAAATGCAATTCATACTGAAGTGATTGATTCAGAAG
AAAATGATGAAGATGGACGTATTGAAGTAAGACAAGATTTCATGGATCGTGTTGAAGAAGATATACAACAATC
ATCAGATGAAGATGAAGAATAATAAAAAAATAAGACTTCCCTATATGTAGGGAAGTCTTATTTTTGTGCTAGA
AAGTAATGCTTTACTATATTCAATGATTAGTAATGACTAACTTTCTAATTGTTTCATTGCGTAAGGTATTTCA
TTGATAAGTCTTGATGGTGGTACCACATACATATCTTTTGCAAGGTTTTCGCCAATAAAACTATGTGTATATG
TGGCACTCATAACCGCTTCTTTTAAGTTATCAAATTGACCGACAAAACTTGTAATCATACCAGCAAGTGTATC
GCCCATACCACCAGTCGCCATTGCTGGACTACCGATTGTCAATTTAAAGTCTTCATCTTTAAAGAAAATTTCA
GTACCATGTTTTTTAAGTACAACAGTTGCGCCTAAACGATCAACTGCTTCACGATTACGCTCATATGTCTGTT
CCTCAATAGGAATACCACTTAATCGCTCCCATTCTTTGAGATGCGGAGTAAAAATCACGCGACATGTAGGTAA

TTGCGGTTTCAGTTTACTAAAGATTGTAATCGCATCGCCGTCTACGATTAAATTTTGATGCGGTTGTATATTT
TGTAGTAGGAATGTAATGGCATTATTTCCTTTGAAATCAACGCCAAGACCTGGACCAATTAGTATACTGTCAG
TCATTTCAATCATTTTCGTCAACATTTTCGTATCATTAATATCAATAACCATCGCTTCTGGGCAACGAGAATG
TAATGCTGAATGATTTGTTGGATGTGTAGCTACAGTGATTAAACCACTACCGCTAAATACACATGCACGAGCC
GCTAACATAATGGCACCACCTAAGTTAGCAGATCCACCAATTAATAAAATTTTGCCATAATCACCTTTATGTG
AATTTTCTTTACGCTTAGGAATGTTAATAGAATTTAACGTTTCCATAGTGATATAACCTCCCATGTAAAAGCC
TTTTCCGAATTTATTCAATTTTAAAAATATATAGTAACTTTTAACAAAATGTATTATAAATTTCTGAATTCAT
TATTATTTGTCGTTAAATACAATAGAAAATACTATACCTGTATATGCAATTCGTCAATAGATAAATTATTAAA
TATGCTTACAACAATCTTAATATCCTTTAACGCACTACAATAGTGCTCTGATAATAGGTTATAAATGTACGTA
AAACCATTGTTTCAATAAAAATGAAAACGTATACTTCAAGAAGGATGGGTTACTTAATATAAACAAGGGGGTA
ACATATATGACTTTATATTTAGATGGTGAGACACTAACAATTGAGGATATTAAATCATTTTTACAACAACAAT
CAAAGATTGAAATTATTGATGATGCGTTAGAACGTGTCAAAAAAAGTAGAGCGGTAGTTGAACGTATTATTGA
AAATGAGGAAACGGTTTACGGTATTACTACAGGTTTTGGGTTATTTAGTGATGTACGTATAGACCCGACGCAA
TATAATGAATTACAAGTGAATCTGATACGCTCACATGCCTGTGGACTAGGTGAGCCATTTTCAAAAGAAGTAG
CATTAGTCATGATGATTTTACGATTGAATACATTATTAAAAGGTCATTCAGGTGCCACTTTAGAATTAGTGAG
ACAATTACAATTTTTTATAAATGAACGTATTATACCGATAATCCCACAGCAAGGCTCTCTCGGTGCATCAGGA
GATTTAGCGCCATTATCACACTTAGCATTAGCATTAATTGGTGAAGGGAAAGTATTGTACAGAGGGGAAGAAA
AGGATAGTGACGATGTATTAAGAGAATTAAATAGACAACCTTTGAACCTTCAGGCTAAAGAAGGTTTAGCATT
GATTAATGGTACGCAAGCTATGACAGCTCAAGGTGTCATTAGTTATATAGAAGCAGAAGATTTAGGTTACCAA
TCTGAATGGATTGCTGCATTAACGCATCAGTCTCTTAATGGCATTATAGATGCATATCGACATGATGTGCACG
CTGTTCGTAATTTTCAAGAACAGATTAATGTGGCAGCGCGTATGCGTGATTGGTTAGAAGGATCAGCATTAAC
AACGCGACAAGCAGAAATACGTGTACAAGATGCATATACGTTGCGTTGTATACCACAAATCCATGGCGCGAGT
TTTCAAGTATTCAATTATGTTAAACAGCAATTAGAATTTGAAATGAATGCGGCTAATGATAATCCACTTATAT
TTGAGGAAGCAAATGAAACGTTTGTTATTTCAGGTGGTAACTTCCATGGACAACCTATTGCTTTTGCATTAGA
TCATCTTAAATTAGGTGTAAGTGAATTAGCAAACGTATCGGAACGTCGTCTAGAGCGACTAGTAAATCCTCAA
TTAAATGGTGATTTACCAGCATTTCTTAGTCCAGAGCCAGGATTGCAAAGTGGCGCGATGATTATGCAATATG
CTGCTGCAAGTCTCGTTTCTGAAAATAAAACTTTAGCGCATCCAGCGAGTGTTGATTCTATCACTTCATCTGC
GAACCAAGAAGATCACGTATCTATGGGAACTACAGCTGCTAGACATGGTTATCAAATTATTGAAAATGCAAGA
CGTGTGCTGGCAATCGAATGTGTTATTGCATTACAAGCAGCAGAGTTAAAAGGTGTTGAAGGTTTATCACCAA
AAACACGTCGTAAATATGAGGAGTTTCGAAGTATCGTGCCATCCATTACACATGACCGTCAATTTCATAAAGA
TATTGAAGCGGTTGCACAGTATTTAAAGCAATCAATTTATCAAACGACTGCATGTCACTAAATCGACATGAGT
GATGTTTGAAATCGTTACTTGAAAAAGCAAATATAGTTTGCTATATTAAAAATAACTTAATAAGACATTGTTC
GTAGGACAAGTAATATATAGTGTTCGATATCAGAGAGCTTGTGGTTAGTGTGAACAAGAATCAACATATATAT
GAATCTACCTACTTAATTTAAAAGAACAATCGGTGATAACCGTTATTTTAGTGAAGTGCAATTTAGGTTTAGT
GTATCTTTATAACTTAAATTGTTAAATAGGGTGGCAACGCGTAGACCACGTCCCTTGTAGGGATGTGGTCTTT
TTTTATTTTCTAAAAAATAAAGACGTACGTCATTCCATAATAAATGATAATTATTTGGGAAAGGATGAAGGTT
AATGTTAGACATTAGATTATTCAGAAATGAGCCTGACACAGTTAAGAGCAAAATTGAATTACGTGGAGATGAT
CCAAAAGTTGTAGATGAAATTTTAGAATTGGATGAGCAACGACGTAAATTAATTAGTGCAACAGAAGAAATGA
AAGCACGTCGTAATAAAGTAAGCGAAGAAATCGCATTAAAAAAACGTAATAAAGAAAATGCTGATGATGTGAT
TGCTGAAATGCGCACATTAGGTGACGATATTAAAGAAAAAGATAGTCAATTAAATGAAATTGATAATAAAATG
ACAGGTATCCTTTGTCGTATTCCAAATTTAATAAGTGATGATGTACCTCAAGGTGAATCTGATGAAGATAACG
TTGAAGTTAAAAAGTGGGGTACACCACGTGAGTTTTCATTTGAACCCAAAGCACATTGGGATATTGTAGAAGA
ATTGAAAATGGCTGATTTTGATCGTGCAGCAAAAGTTTCAGGTGCGCGTTTTGTATATTTAACAAATGAAGGT
GCGCAATTAGAGCGTGCTTTAATGAACTATATGATTACAAAACATACAACACAACATGGTTATACAGAAATGA
TGGTACCACAGCTTGTGAACGCAGATACAATGTATGGTACAGGTCAATTACCTAAATTTGAAGAAGATTTATT
TAAAGTAGAAAAAGAAGGATTATATACAATTCCAACTGCTGAAGTACCATTAACGAATTTCTACCGTAATGAA
ATTATTCAACCAGGTGTACTTCCTGAAAAATTCACTGGTCAATCTGCATGTTTCCGTAGTGAAGCAGGATCAG
CAGGTAGAGATACAAGAGGATTAATTCGTTTACATCAATTCGATAAAGTGGAAATGGTACGTTTTGAACAACC
TGAAGATTCATGGAATGCTTTAGAAGAAATGACAACAAACGCAGAAGCAATTCTAGAAGAGTTAGGTTTACCA
TACCGTCGTGTTATTTTATGTACAGGTGATATTGGATTTAGTGCAAGCAAAACATATGATTTAGAAGTTTGGT
TACCAAGCTACAATGATTATAAAGAAATTAGTTCATGCTCAAACTGTACGGATTTCCAAGCGCGTCGTGCTAA
CATCCGCTTCAAGCGTGACAAAGCAGCTAAACCAGAATTAGCACATACATTAAATGGTAGTGGTTTAGCAGTT
GGACGTACATTTGCTGCTATTGTTGAAAATTACCAAAATGAAGATGGAACAGTAACAATTCCAGAAGCATTAG
TACCATTTATGGGTGGTAAAACACAAATTTCAAAACCAGTTAAATAAAGGCTTTAGCTACAAGCTTTAAAAAG
TATATATCTACGTATACTTAAAGCAAGGGCAAGATACTTTAAATAATATTTTAAAAAGTGGTGACGAAGCTGT
CGCCACTTTTTTTGTGCTGTAAAAGTATAATAGTGAGCCAATGCACATAACAACAATAAATTAAGTTTGTGGT
TTAATGGGGTGAACGCATTTCATTATAGCAACAATACGGGATAATTATGATGAACTAAAACAATCTAAAACGT
AACAATTTTGAGCATCACTATTATAGGAAATTAAGTGATAAAAAACTGATTTCGTTGATGTAGTATGAGTAAT

ATCGATGGAGTAGGGTAGGGGGAGAGGATGATGATTATAAGGGAGTGTTACATGAATCAATATCCCAGACTCA
TCATTAGATATAAAAAATTATAAAATTAGTACTTAAAAACAACTACAAATCCGTAGAAAATATGGAGGTAGTC
TTAAATAAAAAAATGAAAATTCTCAAAAATAAAAAGTTGATATAAAGCTGATTGAAGACTCCAGTATGTCTAA
CCTCAGACAAACTGGAGTCTAATGTTATTGCTTAGGGTATAGAACTGTATTAGACTAGGTATATTATTTTTTC
GTAATTATATAAATATAAAGTGGCAAAGGAGGTAATTGAGATGGCAACACATTTAAGTTTTAGACAAGGCGTA
CAAGAGTGTATCCCAACATTATTGGGTTATGCCGGTGTTGGTATTTCATTTGGTATTGTGGCTTCGTCTCAAA
ACTTTAGTATTTTAGAAATTATCTTGTTATGTCTTGTTATATATGCCGGTGCTGCGCAATTTATTATGTGCGC
GTTGTTTATAGCAGGTACACCGATATCAGCGATTGTACTAACTGTATTTATCGTAAATTCAAGAATGTTCCTT
TTAAGTATGTCGCTTGCACCAAACTTCAAGACATATGGGTTTTGGAACCGTGTTGGATTAGGTTCATTAGTAA
CTGACGAAACGTTTGGCGTCGCCATTACACCTTATTTAAAAGGAGAAGCTATCAATGATCGTTGGATGCATGG
TCTTAATATCACAGCATATTTATTTTGGGCAATTTCATGTGTAGCTGGGGCTTTATTTGGCGAATATATCTCA
AATCCGCAAACGCTAGGGTTAGATTTTGCTATCACGGCTATGTTTATCTTTTTGGCCATTGCGCAATTTGAAT
CAATTACTAAATCGCGATTAAGAATTTACATAGTACTCATTATTGCCGTCATAGTAATGATGTTATCGCTAAG
TATGTTTATGCCTTCATATCTAGCAATATTAATTGCAGCCACAATTTCAGCAGCGTTAGGAGTGATGATGGAA
CGATGATAACTCATATGAACATGTTAATACTTATTTTATTGTGTGGTATCGTAACGCTATTAATTCGAATTAT
ACCTTTTATCATGATTTCAAAAGTGCAATTGCCTGATGTCGTGGTTCGATGGCTATCATTTATCCCAATCACA
CTATTTACGGCACTTGTCATAGACAGCATTATTCAACAGACGCCTCATGGTGAGGGGTATACATTAAACATCC
CTTACATTATCGCGCTCATTCCGACGGTTATTTTATCTATAATCACGCGTAGTTTAACTATTACAATTATTAG
TGGGATTGTTATCATGGCAGCATTACGATTTTTCTTTTAAAAATAACTGAAAATCATTGAACAGATATTTAAA
CTTAGGTAAACTGTTAGTAATCAGAAAATTCTGATATACAAATCGTCTATGTTAAGATTGGGAATCGTGTATA
CGTAAGCGATTTGGCTAACGCATGACAATGATAACAACATTTTAAATGATAAAAGTAATTCATCACTGAATCT
CAACTAACACATAACAATTTCATATTTCTTATTGTGAGAAGTTGAGGGACTTGGCCCTGTGATACTTCAGCAA
CCGACTTTATAGCACGGTGCTAAAACCAACGAGTTACTCGAATGATAAGTATAAAGACTTCTTACTTTTCAAT
AGGGTGAGAAGTTTTTTTTGTTTAAGGAGGAAAGAACAATGACAAATTACACAGTAGATACTTTAAATTTAGG
GAAATTTATTACAGAATCTGGGGAAGTCATAGATAACTTGCGTTTGAGATATGAGCATGTCGGTTATCATGGA
CAACCATTAGTTGTAGTTTGTCATGCATTAACTGGCAATCATTTAACATATGGAACAGATGATTATCCGGGTT
GGTGGCGAGAAATTATTGATGGGGGATATATACCCATTCACGATTATCAATTTTTAACATTTGATGTTATTGG
TAGTCCTTTCGGTTCAAGTTCACCTTTAAACGACCCTCATTTTCCTAAAAAATTAACATTAAGAGATATAGTC
AGAGCGAATGAACGAGGTATACAAGCCCTTGGTTATGATAAGATTAATATTTTAATAGGGGGAAGTCTTGGAG
GTATGCAAGCAATGGAACTACTTTACAATCAACAGTTTGAAGTAGATAAAGCCATTATCCTTGCTGCAACAAG
TCGAACATCATCTTATAGTAGAGCTTTCAATGAAATTGCAAGGCAAGCCATTCATCTTGGTGGTAAGGAAGGT
CTAAGTATTGCACGCCAATTAGGTTTTTTGACATATCGATCATCAAAAAGTTATGATGAACGTTTCACGCCGG
ATGAAGTAGTCGCATACCAACAACATCAAGGTAATAAATTTAAAGAACATTTTGATTTGAATTGTTATCTGAC
ACTGCTAGATGTATTGGATAGTCACAACATTGACCGAGGTCGCACAGACGTAACGCATGTTTTTAAAAATTTA
GAAACAAAAGTGTTAACGATGGGGTTCATAGATGATTTGCTATATCCGGATGATCAAGTTCGTGCATTAGGTG
AACGTTTTAAATATCATCGTCATTTCTTCGTGCCTGATAATGTTGGGCATGATGGATTCCTACTAAATTTTAG
TACCTGGGCACCTAACTTATATCATTTCTTAAATTTAAAGCATTTTAAGCGTAAGTAATAATATGTATATATT
TTCAAAAAATGAAGCCACTAAATCACAGTTATCATATTCAAAGACATCTAGAGTACATGAGAGTGGAAATGAA
TTGTAAATCTAAATCAAGCTTGATTGCTATGTAGTGGGATTAAATAATCATGTTGAAAATGAAAGCACACAAA
AGCGATTCAAATGAGTATGTTAATTACTTATAAAACATGAAAGTTATTATTCAATTAAATGAAATAGAAGCAT
ACAATCTATAAATTGTTAAATTCCATTAAAGAGGTTAAAATAATAGCTATAGTTAAAAATATGGGAGTGGCTT
ATGTGTTTTCAAAAATACAACCTAAAGCAACAATAATTGCAACGATTGCGTTGGTATTTGTCGCTTTAGCTTT
ATATCTAGTGCCTGGTTTAGGACTAATATTTGCATTATTTGCAACCATACCAGGTATCGTTTTATGGAATAAA
TCAATACAATCTTTCGGGATTAGTGCACTTATTACAGTAATTATAACAACTGTTTTAGGTAATACTTTCGTTT
TAAGTGCCATCATATTAGTCTTAATTGCAAGTTTAATTATTGGTCAATTGCTCAAAGAAAGAACGTCTAAAGA
AAGAATATTATACGTAACAACAGTAGCGATGAGCTTAATTTCATTAATCGCTTTTATGTTACTACAAACATTC
GGAAGAATTCCACCATCAGCGAGCATAGTAAAACCTTTCAAGCAAACATTACATGAAGCGATTACGATGAGCG
GTGCCGATGCGAATATGACCCAAATATTAGAAGAAGGGTTTAGACAAGCGACCGTTCAATTACCAGGTTTCAT
CATTATCATTACATTTTTAATCGTCTTAATTAACTTAATCGTTACATTTCCGATTTTACGAAAATTTAAAATC
GCTACACCTGTATTTAAGCCACTTTTCGCGTGGCAAATGAGCGGTATTTTATTATGGATATACATTATTGTTA
TCATATGTTTATTATTTACAGGTCAACCGAGTGTGTTCCAGAGCATTCTTTTAAACTTCCAACTTGTGTTATC
ATTAGTAATGTATATTCAAGGTTTAAGTGTTATTCATTTCTTTGGTAAAGCGAAAGGTTTGCCGAATGCAATA
ACGATTTTACTATTGATTATCGGTACAATACTGACACCTACGACACATATTGTAGGACTACTTGGTGTTATCG
ATTTAAGTTTGAATTTGAAGCGAATCATGAAAAATAATTCTAAAAAGTGAATAGAGGTGGAATAATGAATCGG
CAGTCCACTAAGAAAGCTTTACTAATACCATTTGTCATCATGATCATCACAGCAATTGTTTTAATGGGTGTAT
GGTTTATCTTTAATAGTCTTATAGCACTAATTGCATCTATCGTTCTTGTCGTGATGATTATTGTTAGCATCGT
TTTATTCAGACAAGCTTTAATGAAAATGGATAGTTATGTAGATGGTTTGAGTGCTCAAATTTCAACAACAAAT
AATAAAGCAATCAAACATTTACCAATTGGTATCATTGTTTTAGATGAAAATGATCACATCGAATGGGTTAACC

AATTTATGACAGATCATATGGAAGCAAATGTCATTTCTGAATCTGTAAATGAAGTATTTCCAAACATTTTAAA
GCAATTAGATAGAGTGAAATCCGTTGAAATAGAATATAATCAGTATCATTTCCAAGTACGTTATTCTGAGAAT
GATCACTGCCTCTATTTCTTTGATATAACTGAACAAGTACAAACAAATGAACTATATGAAAATTCTAAACCAA
TCATTGCGACATTATTTTTAGATAACTACGATGAGATTACTCAAAATATGAATGATACGCAGCGTTCGGAAAT
CAATTCAATGGTAACGCGTGTTATTAGTCGATGGGCAACTGAGTATAATATATTCTTTAAAAGGTATAGTTCT
GATCAATTCGTAGCCTATTTAAATCAAAAAATATTAGCTGACTTAGAAGAATCTAAATTTGATATCTTGAGTC
AATTACGTGAAAAAAGTGTTGGTTATCGTGCCCAATTAACATTAAGTATTGGTGTTGGTGAAGGTACTGAAAA
TTTAATCGACTTAGGTGAATTATCACAATCAGGCCTAGACTTAGCATTAGGACGCGGTGGCGACCAAGTTGCA
ATTAAAAGTATTAATGGTAATGTGCGTTTCTATGGCGGTAAGACTGACCCGATGGAGAAACGTACTCGTGTAA
GAGCACGTGTGATCTCACATGCGTTAAAAGATATCCTTGCAGAGGGTGACAAAGTCATTATCATGGGACATAA
ACGTCCTGACTTAGATGCAATTGGTGCAGCAATCGGTGTGTCTAGATTTGCAATGATGAATAATTTAGAAGCA
TACATCGTATTAAATGAGACTGACATTGATCCAACATTACGACGCGTGATGAACGAAATTGATAAAAAGCCAG
AGTTAAGAGAACGATTTATAACATCAGATGATGCTTGGGATATGATGACATCTAAGACAACCGTAGTGATTGT
TGATACGCATAAACCGGAACTGGTTTTAGATGAAAATGTTTTAAATAAAGCAAACCGTAAAGTTGTTATCGAT
CATCATAGACGTGGTGAAAGCTTCATCTCTAATCCATTGTTGATATATATGGAACCATACGCAAGTTCAACAG
CAGAACTTGTAACAGAATTACTGGAATATCAACCAACAGAACAACGTTTAACACGTCTTGAATCAACAGTGAT
GTATGCAGGTATTATTGTAGATACAAGAAACTTTACATTACGAACAGGATCAAGAACATTCGATGCAGCGAGT
TATTTACGTGCACATGGTGCAGATACGATTCTAACGCAACATTTCTTAAAAGATGATGTGGATACTTATATTA
ATCGATCTGAATTAATTCGAACTGTAAAAGTCGAAGATAATGGCATAGCCATTGCGCATGGTTCAGACGATAA
AATTTATCATCCAGTAACAGTTGCACAAGCAGCAGATGAACTGTTAAGTTTAGAAGGTATTGAAGCATCATAT
GTTGTTGCGAGACGTGAAGATAATCTAATTGGTATATCTGCACGTTCACTCGGTTCAGTAAATGTCCAGTTAA
CAATGGAAGCACTTGGTGGCGGTGGACATTTAACCAATGCGGCAACACAACTTAAAGGTGTGACAGTCGAAGA
GGCGATAGCACAATTACAACAAGCAATTACAGAACAATTAAGTAGGAGTGAAGATGCATGAAAGTAATTTTTA
CACAAGATGTTAAAGGTAAAGGTAAAAAAGGTGAAGTTAAAGAAGTACCAGTAGGTTATGCAAATAACTTCTT
ATTGAAAAAGAATTATGCTGTAGAAGCAACACCAGGTAACCTTAAACAATTAGAGTTACAGAAAAAACGTGCA
AAACAAGAACGCCAACAAGAAATTGAAGATGCTAAAGCATTAAAAGAAACGTTATCAAACATTGAAGTTCAAG
TATCAGCAAAAACTGGTGAAGGTGGTAAATTGTTTGGGTCAGTAAGTACAAAACAAATTGCCGAAGCACTAAA
AGCACAACATGATATTAAAATTGATAAACGTAAAATGGATTTACCAAATGGAATTCATTCCCTAGGATATACG
AATGTACCTGTTAAATTAGATAAAGAAGTTGAAGGTACGATTCGCGTACACACAGTTGAACAATAAAGTTGGA
TTGAAATAAGAGGTGTAACCATTCATGGATAGAATGTATGAGCAAAATCAAATGCCGCATAACAATGAAGCTG
AACAGTCTGTCTTAGGTTCAATTATTATAGATCCAGAATTGATTAATACTACTCAGGAAGTTTTGCTTCCTGA
GTCGTTTTATAGGGGTGCCCATCAACATATTTTCCGTGCAATGATGCACTTAAATGAAGATAACAAAGAAATT
GATGTAGTAACATTGATGGATCAGTTATCAAGTGAAGGTACGTTGAATGAAGCGGGTGGTCCGCAATATCTTG
CAGAGTTATCTACAAATGTACCAACGACACGAAATGTTCAGTATTATACGGACATTGTTTCTAAGCATGCATT
AAAACGTAGATTGATTCAAACTGCAGATAGTATTGCCAATGATGGATATAATGATGAACTTGAACTAGATGCG
ATTTTAAGTGATGCAGAACGTCGAATTTTAGAGCTATCATCTTCTCGTGAAAGCGATGGCTTTAAAGACATTC
GAGACGTCTTAGGACAAGTATATGAAACAGCTGAAGAGCTTGATCAAAATAGTGGTCAAACACCAGGTATTCC
TACAGGATATCGAGATTTAGACCAAATGACAGCAGGGTTCAACCGAAATGATTTAATTATCCTTGCAGCGCGT
CCATCTGTAGGTAAGACTGCGTTCGCACTTAATATTGCACAAAAAGTTGCAACGCATGAAGATATGTATACAG
TTGGTATTTTCTCACTAGAGATGGGTGCTGATCAGTTAGCCACACGTATGATTTGTAGTTCTGGTAATGTTGA
CTCAAACCGCTTAAGAACGGGTACTATGACTGAGGAAGATTGGAGTCGTTTTACTATAGCGGTAGGTAAATTA
TCACGTACGAAGATTTTTATTGATGATACACCGGGTATTCGAATTAATGATCTACGTTCTAAATGTCGTCGAT
TAAAGCAAGAACATGGCTTAGACATGATTGTGATTGACTACTTACAGTTGATTCAAGGTAGTGGTTCACGTGC
GTCTGATAACAGACAACAGGAAGTTTCTGAAATCTCTCGTACATTAAAAGCATTAGCCCGTGAATTAGAATGT
CCAGTTATCGCGTTGAGTCAGTTATCTCGTGGTGTTGAACAACGACAAGATAAACGTCCAATGATGAGTGATA
TTCGTGAATCTGGTTCGATTGAGCAAGATGCCGATATCGTTGCATTCTTATACCGTGATGATTACTATAACCG
TGGCGGCGATGAAGATGATGACGATGATGGTGGTTTTGAGCCACAAACGAATGATGAAAACGGTGAAATTGAA
ATCATCATTGCTAAGCAACGTAATGGTCCAACAGGCACAGTTAAGTTACACTTTATGAAACAATATAATAAAT
TTACAGATATCGATTATGCACATGCTGATATGATGTAAAAAAGTTTTTCCGTACAATAATCATTTTAATGATA
AAATTGTACGGTTTTTATTTTGTTCTGAACGGGTTGATATATGTTAAGTTTGTGTATTGAAAGTGATGAATTA
GTACTGGCAACGCCTCTGTTAAAGGGTTTTTAGGACGTTGAAGACGATTTGTTAAAATGGTTTTCTTTTAAAA
AAGGCCGAAAATCAATGTTCGATTTTTATTTGCATTATGGTCTCGATATTGGTAGAATATCAAATGGTTAAAT
GAGAAAAACTTGGAGGTGCTCACATGTCATCAATCGTAGTAGTTGGGACACAATGGGGAGACGAAGGAAAAGG
AAAAATAACGGATTTCTTGGCAGAACAGTCAGATGTTATCGCGCGTTTTTCAGGTGGTAATAATGCAGGCCAT
ACCATTCAATTTGGCGGAGAAACATATAAATTACATTTAGTACCATCTGGTATCTTTTACAAAGACAAATTAG
CGGTAATCGGTAACGGAGTCGTTGTTGATCCAGTTGCACTATTGAAAGAATTAGACGGATTAAATGAACGTGG
CATTCCTACAAGTAATTTACGTATATCTAATCGTGCGCAAGTGATTCTACCATATCACTTAGTACAAGATGAA
TATGAAGAACGTTTACGTGGTGATAATAAGATTGGTACAACTAAAAAAGGTATCGGTCCAGCATATGTAGATA

AAGTTCAACGTATCGGTATTCGTATGGCAGATTTACTTGAAAAAGAAACATTCGAAAGATTATTAAAATCAAA
CATTGAATATAAACAAGCATATTTCAAAGGTATGTTTAACGAAACATGTCCATCATTTGATGATATCTTTGAA
GAATATTATGCTGCAGGTCAACGTTTAAAAGAATTTGTAACAGACACATCAAAAATCTTAGACGATGCATTTG
TAGCAGATGAAAAGGTACTTTTCGAAGGTGCGCAAGGTGTAATGTTAGATATCGACCATGGTACATATCCATT
CGTTACATCAAGTAATCCAATTGCAGGTAACGTTACTGTTGGTACAGGTGTAGGTCCTACATTCGTTTCGAAG
GTAATTGGTGTATGTAAAGCTTATACATCACGTGTTGGTGATGGTCCATTCCCTACTGAATTATTCGATGAAG
ATGGACATCATATTAGAGAGGTTGGTCGTGAATACGGTACAACAACAGGACGTCCACGTCGTGTAGGTTGGTT
TGATTCAGTTGTATTACGTCACTCTCGTCGTGTAAGTGGTATTACAGATTTATCTATTAACTCAATCGATGTT
TTAACAGGCCTAGACACAGTGAAAATCTGTACAGCTTATGAATTAGACGGTAAAGAAATTACTGAGTACCCAG
CAAACTTAGATCAATTAAAACGTTGTAAACCAATCTTTGAAGAGTTACCAGGTTGGACAGAAGACGTAACAAG
TGTGCGTACTTTAGAAGAATTACCTGAAAATGCACGTAAATATTTAGAGCGTATTTCAGAATTATGTAATGTA
CAAATTTCTATCTTCTCAGTTGGTCCAGATAGAGAACAAACAAACCTATTAAAAGAATTGTGGTAGAACTTTA
TATAAGTCATACACAATGATTATAAATACATGAGCCTTCTATCTTTAATGGTAGGAGGCTTTTGTTATGTTTG
CTTCTGTATCGATTCGATTATTTAGATAAAAATTACTAACGTAAAGGTGATATTTGCTAGTCATAATTTAAAA
GATTAGATGATATTTAACGAAATTAAGAAGAAATACTTGAATGTAATAAGTCTGATGTCGAAAATAGCTATTA
AAATAGAGTAGACGTAAGTGTAAATGAAAGCACCTAAAATAGAAAAATTTCAAAAATAGCGTAATTATTATAA
TAAATAGACAGCCAATAAAATGCAATTTTTCACTTATAACATTCTTCAAAAAATAATAGCAAAATTATGTAAA
AAATATCTTGTCATGGCAAGATTGGCTGTGCTATAATCTATCTTGTGCTTAAGAACGGCTCCTTGGTCAAGCG
GTTAAGACACCGCCCTTTCACGGCGGTAACACGGGTTCGAGTCCCGTAGGAGTCACCATTTTTTAGGTCTCGT
AGTGTAGCGGTTAACACGCCTGCCTGTCACGCAGGAGATCGCGGGTTCGATTCCCGTCGAGACCGTACAAATG
CCTATCCAAGAGGATAGGCATTTTTTTGCGTTTAATATTATATTAATAAAAGATATATGGACGAATGATAATC
ATATTGATTTATCTGTTCGTCCATTTTCTTTATAATGTATGAACCTCAAGTAACTTAGTGGTTGGATATGAAA
GATAAACGTAGCCAATAAAATCTTAATTAGACGTACAAACATATGCTACTGTCAACATATTTCTTCGTTGTGA
TATGCCACCAATCCTCCATAACATCAATTGTTAAAGTAACGAATAATGAATAATGATATTTATTTTCTGAGCA
ATGACGTGCAACTAGAAGTTGCCATTACCCTAATTTTATTATTGGAATAGAGACCTCATCATTGTGTTAAATA
TCATTGTCACAATCCGCCGTGAGAAACTAATAAAAAATATTAATATATAAGTTTATATTGGAAAATAGAATTA
ATAGCTTATAAATGGTAAATTATATAATAGGTTACTATACGTTATAAGATGGAAAATGCGCACAATAACAAAA
ATAGTAAGCGACATCCTGTGATTTTTTACACAAACATAAACGATAAAGAACAAAAAATGATAAAATAATATTA
ATGATTTAAGAAAAGAGGTTTATGCAAATGGCTAGAAAAGTTGTTGTAGTTGATGATGAAAAACCGATTGCTG
ATATTTTAGAATTTAACTTAAAAAAAGAAGGATACGATGTTTACTGTGCATACGATGGTAATGATGCAGTCGA
CTTAATTTATGAAGAAGAACCAGACATCGTATTATTAGATATCATGTTACCTGGTCGTGATGGTATGGAAGTA
TGTCGCGAAGTGCGCAAAAAATACGAAATGCCAATTATTATGCTAACTGCTAAAGACTCAGAAATTGATAAAG
TGCTTGGTTTAGAACTAGGTGCAGATGACTATGTAACAAAACCGTTTAGTACACGTGAATTAATTGCACGTGT
GAAAGCGAATTTACGCCGTCATTACTCACAACCAGCGCAAGACACTGGGAACGTGACGAATGAAATCACAATT
AAAGATATTGTGATTTATCCAGACGCATATTCTATTAAAAAACGTGGCGAAGATATTGAATTAACACATCGTG
AATTTGAATTGTTCCATTATTTATCAAAACATATGGGACAAGTAATGACACGTGAACATTTATTACAAACAGT
ATGGGGCTATGATTACTTTGGCGATGTACGTACGGTCGATGTAACGATTCGTCGTTTACGTGAAAAGATTGAA
GATGATCCGTCACATCCTGAATACATTGTGACGCGTAGAGGCGTTGGATATTTCCTCCAACAACATGAGTAGA
GGTCGAAACGAATGAAGTGGCTAAAACAACTACAATCCCTTCATACTAAACTTGTAATTGTTTATGTATTACT
GATTATCATTGGTATGCAAATTATCGGGCTGTATTTTACAAATAATCTTGAAAAAGAGCTGCTTGATAATTTT
AAGAAGAATATTACGCAGTACGCTAAACAATTAGAAATTAGTATTGAAAAAGTATATGACGAAAAGGGCTCCG
TAAATGCACAAAAAGATATTCAAAATTTATTAAGTGAGTATGCCAACCGTCAAGAAATTGGAGAAATTCGTTT
TATAGATAAAGACCAAATTATTATTGCGACGACGAAGCAGTCTAACCGTAGTCTAATCAATCAAAAAGCGAAT
GATAGTTCTGTCCAAAAAGCACTATCACTAGGACAATCAAACGATCATTTAATTTTAAAAGATTATGGCGGTG
GTAAGGACCGTGTCTGGGTATATAATATCCCCGTTAAAGTCGATAAAAAGGTAATTGGTAATATTTATATCGA
ATCAAAAATTAATGACGTTTATAACCAATTAAATAATATAAATCAAATATTCATTGTTGGTACAGCTATTTCA
TTATTAATCACAGTCATCCTAGGATTCTTTATAGCTCGAACGATTACCAAACCAATCACCGATATGCGTAACC
AGACGGTCGAAATGTCCAGAGGTAACTATACGCAACGTGTGAAGATTTATGGTAATGATGAAATTGGCGAATT
AGCTTTAGCATTTAATAACTTGTCTAAACGTGTACAAGAAGCGCAGGCTAATACTGAAAGTGAGAAACGTAGA
CTGGACTCAGTTATCACCCATATGAGTGATGGTATTATTGCAACAGACCGTCGTGGACGTATTCGTATCGTCA
ATGATATGGCACTTAAGATGCTTGGTATGGCAAAAGAAGACATCATCGGATATTACATGTTAAGTGTATTAAG
TCTTGAAGATGAATTTAAACTGGAAGAAATTCAAGAGAATAATGATAGTTTCTTATTAGATTTAAATGAAGAA
GAAGGTCTAATCGCACGTGTTAACTTTAGTACGATTGTGCAGGAAACAGGATTTGTAACTGGTTATATCGCTG
TGTTACATGACGTTACTGAACAACAACAAGTTGAACGTGAGCGTCGTGAATTTGTTGCTAATGTATCACATGA
GTTACGTACACCTTTAACTTCTATGAATAGTTACATTGAAGCACTTGAAGAAGGTGCATGGAAAGATGAGGAA
CTTGCGCCACAATTTTTATCTGTTACCCGTGAAGAAACAGAACGAATGATTCGACTGGTCAATGACTTGCTAC
AGTTATCTAAAATGGATAATGAGTCTGATCAAATCAACAAAGAAATTATCGACTTTAACATGTTCATTAATAA
AATTATTAATCGACATGAAATGTCTGCGAAAGATACAACATTTATTCGAGATATTCCGAAAAAGACGATTTTC

ACAGAATTTGATCCTGATAAAATGACGCAAGTATTTGATAATGTCATTACAAATGCGATGAAATATTCTAGAG
GCGATAAACGTGTCGAGTTCCACGTGAAACAAAATCCACTTTATAATCGAATGACGATTCGTATTAAAGATAA
TGGCATCGGTATTCCTATCAATAAAGTCGATAAGATATTCGACCGATTCTATCGTGTAGATAAGGCACGTACG
CGTAAAATGGGTGGTACTGGATTAGGACTAGCCATTTCGAAAGAGATTGTGGAAGCGCACAATGGTCGTATTT
GGGCAAACAGTGTAGAAGGTCAAGGTACATCTATCTTTATCACACTTCCATGTGAAGTCATTGAAGACGGTGA
TTGGGATGAATAATAAGGAGCATATTAAATCTGTCATTTTAGCGCTACTTGTCTTGATGAGTGTCGTATTGAC
ATATATGGTATGGAACTTTTCTCCTGATATTGCAAATGTCGACAATACAGATAGTAAGAAGAGTGAAACGAAA
CCTTTAACGACACCTATGACAGCCAAAATGGATACAACTATTACGCCATTTCAGATTATTCATTCGAAAAATG
ATCATCCAGAAGGTACGATTGCGACGGTTTCTAATGTGAATAAGCTGACGAAACCTTTAAAAAATAAAGAAGT
GAAGTCCGTGGAACATGTTCGTCGTGATCATAACTTGATGATTCCTGATTTGAGCAGTGATTTTACATTATTC
GATTTTACGTATGATTTACCGTTATCAACATATCTTGGCCAAGTACTGAACATGAATGCGAAAGTACCAAATC
ATTTCAATTTCAATCGTTTGGTCATAGATCATGATGCTGATGATAATATCGTGCTTTATGCTATAAGCAAAGA
TCGCCACGATTACGTAAAATTAACAACTACAACGAAAAATGATCATTTTTTAGATGCATTAGCAGCAGTGAAA
AAAGACATGCAACCATACACAGATATCATCACAAACAAAGATACAATTGATCGTACGACGCATGTTTTTGCAC
CAAGTAAACCTGAAAAGTTAAAAACATATCGCATGGTATTTAACACGATTAGTGTTGAGAAAATGAATGCTAT
ACTATTTGACGATTCAACCATCGTTCGTAGTTCAAAGAGTGGTGTTACAACCTACAACAATAATACAGGTGTC
GCAAACTATAACGATAAAAATGAAAAATATCATTATAAAAACTTGTCCGAAGATGAAGCAAGTTCCAGCAAAA
TGGAAGAAACGATTCCAGGAACCTTTGATTTTATTAATGGTCATGGTGGTTTCTTAAACGAAGACTTCAGATT
GTTTAGTACGAATAATCAGTCAGGCGAGTTAACATATCAACGTTTCCTTAACGGTTATCCAACGTTTAATAAA
GAAGGCTCTAATCAAATTCAAGTCACTTGGGGTGAAAAAGGCGTCTTTGATTATCGTCGTTCGTTATTACGCA
CCGACGTTGTTTTAAACAGTGAAGATAATAAAACGTTGCCGAAATTAGAGTCTGTACGTTCAAGCTTAGCGAA
CAATAGTGATATTAATTTTGAAAAAGTAACAAACATCGCTATCGGTTACGAAATGCAGGATAACTCAGATCAT
AATCACATTGAAGTGCAGATTAACAGTGAACTCGTACCGCGTTGGTATGTAGAATATGATGGCGAATGGTATG
TTTATAACGATGGGAGGCTTGAATAAATGAACTGGAAACTGACAAAGACACTTTTCATTTTCGTGTTTATTCT
TGTCAACATCGTGTTAGTATCGATTTATGTTAATAAAGTCAATCGCTCACACATTAATGAAGTCGAGAGTAAC
AATGAAGTTAATTTTCAGCAAGAAGAAATTAGGGTGCCAGCCAGTATTTTAAATAAATCTGTTAAAGGCATAC
AATTAGAACAAATTACGGGACGATCAAAAGACTTTAGTTCAAAAGCTAAAGGTGATTCTGATTTATCCACATC
AGATAGTGGAAAATTATTAAATGCGAACATCAGCCAATCGGTAAAAGTTAGTGATAATAACTTAAAAGATTTA
AAAGATTACGTTAACAAGCGCGTATTTAAAGGTGCTGAATATCAATTAAGCGAGATTAGTTCAGATTCTGTAA
AATATGAACAAACGTATGATGATTTTCCGATTTTAAATAACAGCAAAGCGATGTTGAACTTTAAAATAGAAGA
TAACAAAGCGACTAGTTATAAACAATCAATGATGGATGACATTAAGCCCACAGATGGTGCAGATAAGAAGCAT
CAAGTGATTAGTGTGAGAAAAGCAATCGAGGCATTATATTATAATCGTTACTTGAAAAAAGGTGATGAAGTCA
TTAATGCTAGACTTGGTTACTATTCAGTCGTGAATGAAACGAATGTTCAATTGTTACAACCAAACTGGGAAAT
TAAAGTGAAGCATGACGGTAAGGACAAAACGAATACTTACTATGTCGAAGCGACAAATAATAACCCTAAAATT
ATTAATCATTAATATGAATCGTAATAAGCTAGCATTGCAAGCTCATCATATGTGAGAAGCAGTGCTAGCTTTT
TGCTGGTACGGTTTATTATGGCTGACAGGCGACATTTCTGTGTCTCCAACGTGCGCATTTATTCATATTTTAA
GTAGTACCGCATTGTAAAATTAGTGTAACTGCTATTTTAAAAACTTTAGTATTTGTCTAATCATTGTTATAAT
AATTAAGAAATTCATTGCACGTGATTATCAAAATTTAAATATAAGAAACCGGTCGATGAACTAAAGTTACATA
ATAGGAAAGGTATACAAAACAGCTAATATACTGATAGTTTCTGTAGGGAAAATCGTATATTTGTACTGATGTA
TATTGTAGTCATATAGAGAGATTGACTGCTTAAATAGAAAGGATGAGCCGCTTGATACGCATGAGTGTATTAG
CAAGTGGTAGTACAGGTAACGCCACTTTTGTAGAAAATGAAAAAGGTAGTCTATTAGTTGATGTTGGTTTGAC
TGGCAAGAAAATGGAAGAATTGTTTAGTCAAATTGACCGTAATATTCAAGATTTAAATGGTATTTTAGTAACC
CATGAACATATTGATCATATTAAAGGATTAGGTGTTTTGGCGCGTAAATATCAATTGCCAATTTATGCGAATG
AAAAGACTTGGCAGGCAATTGAAAAGAAAGATAGTCGCATCCCTATGGATCAGAAATTCATTTTTAATCCTTA
TGAAACAAAATCTATTGCAGGTTTCGATGTTGAATCGTTTAACGTGTCACATGATGCGATAGATCCACAATTT
TATATTTTCCATAATAACTATAAGAAGTTTACGATTTTAACGGATACGGGATACGTGTCTGATCGTATGAAAG
GTATGATACGTGGCAGCGATGCGTTTATTTTTGAGAGTAATCATGACGTCGATATGTTGAGAATGTGTCGTTA
TCCATGGAAGACGAAACAACGTATTTTAGGCGATATGGGTCATGTATCTAATGAGGATGCGGGTCATGCGATG
ACAGACGTGATTACAGGTAACACGAAACGTATTTACTTATCGCATTTATCACAAGATAATAATATGAAAGATT
TGGCGCGTATGAGTGTTGGTCAAGTATTGAACGAACACGATATTGATACGGAAAAAGAAGTATTGCTATGTGA
TACGGATAAAGCTATTCCAACGCCAATATATACAATATAAATGAGAGTACCCTATAAAGTTCGGCACTGCTGT
GAGACGACTTTATCGGGTGCTTTTTTATGTTGTTGGTGGGAAATGGCTGTTGTTGAAATTAAGGTTCTAGTTG
AAATGTGAAAAATAATTCGATATTAAATGTAATTTATAAATAATTTACATAAAATCAATCATTTTAATATAAG
GATTATGATAATATATTGGTGTATGACAGTTAATGGAGGGAACGAAATGAAAGCTTTATTACTTAAAACAAGT
GTATGGCTCGTTTTGCTTTTTAGTGCAATGGGATTATGGCAAGTCTCGAGCGCGGCTGAGCAACATACACCAA
TGAAAGCACATGCAGTAACAACGATAGACAAAGCAACAACAGATAGGCAACTAGTACTGCCAACAAAGGAAGC
GGCTCATCAATCTGGTGAAGAAGCGGCAACCAACGTATCAGCATCAGCACAGGGAACAGCTGATGATACAAAC
AACAAAGTAACATCCAACGCACCATCTAACAAACCATCTACAGCAGTTTCAACAACAGTAAACGAAACGCACG

ATGTAGATGCACAACAAGCCTCAACACAAAAACCAACTCAATCAGCAACATTCAAATTATCAAATGCTAAAAC
AGCATCACTTTCACCACGAATGTTTGCTGCTAATGCACCACAAACAACAACACATAAAATATTACATACAAAT
GATATCCATGGCCGACTAGCCGAAGAAAAAGGGCGTGTCATAGGTATGGCTAAATTAAAAACAGTAAAAGAAC
AAGAAAAGCCTGATTTAATTTTAGACGCAGGAGACGCTTTCCAAGGTTTACCACTTTCAAACCAGTCTAAAGG
TGAAGAAATGGCTAAAGCGATGAATGCAGTAGGTTATGATGCTATGGCAGTGGGTAACCACGAATTTGACTTT
GGATACGACCAGTTGAAAAAGTTAGAGGGTATGTTAGACTTCCCGATGTTAAGTACTAACGTTTATAAAGATG
GAAAACGCGCATTTAAGCCTTCAACGATTGTAACGAAAAATGGTATTCGTTATGGAATTATTGGCGTAACGAC
ACCAGAAACAAAGACGAAAACAAGACCTGAAGGCATTAAAGGTGTTGAATTTAGAGATCCATTACAAAGTGTG
ACAGCGGAAATGATGCGTATTTATAAAGACGTAGATACATTTGTTGTTATATCACATTTAGGTATTGATCCTT
CAACACAAGAAACATGGCGTGGCGATTACTTAGTGAAACAATTAAGTCAAAATCCACAATTGAAGAAACGTAT
TACAGTCATTGATGGTCATTCACATACCGTACTTCAAAATGGTCAAATTTATAACAATGATGCATTAGCACAA
ACAGGTACAGCACTTGCGAATATCGGTAAGGTTACATTTAATTACCGCAATGGAGAGGTATCAAATATTAAAC
CGTCATTGATTAATGTTAAAGACGTTGAAAATGTAACACCGAACAAAGCATTAGCTGAACAAATTAATCAAGC
TGATCAAACATTTAGAGCACAAACTGCAGAGGTAATTATTCCAAACAATACCATTGATTTCAAAGGAGAAAGA
GATGACGTTAGAACGCGTGAAACAAATTTAGGAAACGCGATTACAGATGCTATGGAAGCATATGGCGTTAAGA
ATTTCTCTAAAAAGACTGACTTTGCCGTGACAAATGGTGGAGGTATTCGCGCCTCTATCGCCAAAGGTAAGGT
GACACGCTATGATTTAATCTCAGTATTACCATTTGGAAATACGATTGCGCAAATTGATGTAAAAGGTTCAGAC
GTCTGGACTGCTTTCGAACATAGTTTAGGCGCACCAACAACACAAAAAGATGGTAAGACAGTGTTAACAGCGA
ATGGTGGTTTACTACATATCTCTGATTCAATCCGTGTTTACTATGATATGAATAAACCATCTGGCAAACGAAT
TAACGCTATTCAAATTTTAAATAAAGAGACAGGTAAGTTTGAAAATATTGATTTAAAACGTGTATATCACGTA
ACGATGAATGACTTCACAGCATCAGGTGGCGACGGATATAGTATGTTCGGTGGCCCTAGAGAAGAAGGTATTT
CATTAGATCAAGTACTAGCAAGTTATTTAAAAACAGCTAACTTAGCTAAGTATGATACGACAGAACCACAACG
TATGTTATTAGGTAAACCAGCAGTAAGTGAACAACCAGCTAAAGGACAACAAGGTAGCAAAGGTAGTGAGTCT
GGTAAAGATACACAACCAATTGGTAAAGACAAAGTGATGAATCCAGCGAAACAACCAGCGACAGGTAAAGTTG
TGTTGTTACCAACGCATAGAGGAACTGTTAGTAGCGGTGCAGAAGGTTCTGATTGCGCATTAGAAGGAACTGC
TGTATCAAGTAAGAGTGGGAAACAATTGACTAAAATGTCAGCGTCTAAAGGTAGCGGGCATGAGAAACAGTTA
CCAAAAACTGGAACTAATCAAAGTTCAAGCCCAGCAGCGATATTTGTATTAGTAGCAGGTATAGGTTTAATCG
CAACTGTACGACGTAGAAAAGCTAGCTAAAATATATTGAAAACAATACTACTGTATTTCTTAAATAAGAGGTA
CGGTAGTGTTTTTTTATGGAAAAAAGCTATAACCGTTGATAAATATGGGAAATAAAAACGGGGATAAGTAATA
AGACATCAAGGTATTTATCCACAGAAATGGGGATAGTTATCCAGAATTGTGTACAATTTAAAGAGAAATACCC
ACAATGCCCACAGAGTTATCCACAAATACACAGGTTATACACTAAAAACCGGGCATAAATGTCAGGAAAATAT
CAAAAACTGTAAAAAAACTTGGTATAATAAGAGGGAACAGTGTGAACAAGTTAATAACTTGTGGATAACTGGA
AAGTTGATAACAATTTGGAGGACCAAACGACATGAAAATCACCATTTTAGCTGTAGGGAAACTAAAAGAGAAA
TATTGGAAGCAAGCCATAGCAGAATATGAAAAACGTTTAGGCCCATACACCAAGATAGACATCATAGAAGTTC
CAGACGAAAAAGCACCAGAAAATATGAGCGACAAAGAAATTGAGCAAGTAAAAGAAAAAGAAGGCCAACGAAT
ACTAGCCAAAATCAAACCACAATCAACAGTCATTACATTAGAAATACAAGGAAAGATGCTATCTTCCGAAGGA
TTGGCCCAAGAATTGAACCAACGCATGACCCAAGGGCAAAGCGACTTTGTATTCGTCATTGGCGGATCAAACG
GCCTGCACAAGGACGTCTTACAACGTAGTAACTACGCACTATCATTCAGCAAAATGACATTCCCACATCAAAT
GATGCGGGTTGTGTTAATTGAGCAAGTGTATAGAGCATTTAAGATTATGCGTGGAGAAGCATATCATAAATGA
TGCGGTTTTTTCAGCCGCTTCATAAAGGGATTTTGAATGTATCAGAACATATGAGGTTTATGTGAATTGCTGT
TATGTTTTTAAGAAGCTTATCATAAGTAATGAGGTTCATGATTTTTGACATAGTTAGCCTCCGCAGTCTTTCA
TTTCAAGTAAATAATAGCGAAATATTCTTTATACTGAATACTTATAGTGAAGCAAAGTTCTAGCTTTGAGAAA
ATTCTTTCTGCAACTAAATATAGTAAATTACGGTAAAATATAAATAAGTACATATTGAAGAAAATGAGACATA
ATATATTTTATAATAGGAGGGAATTTCAAATGATAGACAACTTTATGCAGGTCCTTAAATTAATTAAAGAGAA
ACGTACCAATAATGTAGTTAAAAAATCTGATTGGGATAAAGGTGATCTATATAAAACTTTAGTCCATGATAAG
TTACCCAAGCAGTTAAAAGTGCATATAAAAGAAGATAAATATTCAGTTGTAGGGAAGGTTGCTACTGGGAACT
ATAGTAAAGTTCCTTGGATTTCAATATATGATGAGAATATAACAAAAGAAACAAAGGATGGATATTATTTGGT
ATATCTTTTTCATCCGGAAGGAGAAGGCATATACTTATCTTTGAATCAAGGATGGTCAAAGATAAGTGATATG
TTTCCGCGGGATAAAAATGCTGCAAAACAAAGAGCATTAACTTTATCTTCCGAACTCAATAAATATATTACAT
CAAATGAATTTAATACTGGAAGATTTTATTACGCAGAAAATAAAGATTCATCTTATGATTTAAAAAATGATTA
TCCATCAGGATATTCTCATGGATCAATAAGATTCAAATATTATGATTTGAATGAAGGATTCACAGAAGAAGAT
ATGCTAGAGGATTTAAAGAAATTTTTAGAACTATTTAATGAATTAGCTTCAAAAGTTACAAAAACATCCTATG
ATAGCTTGGTCAATAGCATAGACGAAATACAGGAAGACAGCGAAATTGAAGAAATTAGAACAGCACAAAAAGA
TAAGACACTCAAGGAAGTGGAAGCACCTAAAGGAATAATTCCAAAATATAAAAAAGGTGTATCAAAGACTACT
AAAAATGATTCAGAAATTGAAAAATCAAATAAAGAGAATAAATTAACCGGTAAAGTTGGAGAAAAATTAGCGC
TAAATTACTTTAATGAGCTAATTGATAATAAAATAGACGAAGATAAGAAAGAACAGTTTAGGAATATTTTAAA
TGATAATCCAGGCTCTCAACACGGTCATGGCTATGATTTAGTAGCTTTTGATCCAACAAATACAGATAAAGCT
GTAGAAAAATTTATTGAAATTAAAACATCTACATCTTCTAGTATTGAGGAACCATTTTTTATGTCGCTAAATG

AAATGTTTGCTATGAAAGAATATAAGCAGAAATATTTAATATTAAGAATATTTAATGTTTCCGGTAAAGAACC
ACAATTTTATTTTATAGATCCATATGCAAATTATTCTGAATTTAAAGATGTAGATGATCTCATTGACAAAGTA
TTTAATGTAGAAGCTATTCAGTATAAAGTTTTTGGCGAAAAATGATTACTTGAACAAGAGCTAAAATAAAATT
GTGATCTAATAAAAATAGAAACTGTAATTTAAATAAAACTTTCTAAATAAGCTAACTGATAAAAAATCAGTTT
GTCCACAGTCTGAAACAAGATTCCTATATTCTTTAGGAATCTTGTTTTTTCTATTTTTATGGTGATAAAGAGC
AGATAAGATAATGTGTAATAATCACAAAAAAGTTAAATATTTTAAGGCTTGTTTAATTATTAATGATTTTATA
TATAAAGAGCAGTATAATAAAGTTGTTAATATATTATGAATAATATTCAAGTAATTTTATTGTTTTTTAATTT
GTCGATATTTAAGTTGAGTTAAATTTAAAGGGTGTAATTTGTTTTACAATGATGAAGATAATTAGTCTATCAA
AATAAAGGGGTTGGGACTGTTATGAGTGATAATTTGTCATTATTCATTGACTATATCAATGATAATATAATCT
ATGGTAGTGAAATCAAACGGGAGAAATTAGAGAATTTATTTAATCAATTTGCTATAAAAAATGTTGAAAAGAA
CATTGTCTATGATGAACTGAAATCTTTAGATATTACAATCATTGAGTCACAGGATTCATATAAAAATAAATTG
AAGAGATTATTTTCGGTTCTGTTGCAAAGTAAAAAAATATAGCTAACCACTAATTTATCATGTCAGTGTTCGC
TTAACTTGCTAGCATGATGCTAATTTCGTGGCATGGCGAAAATCCGTAGATCTGAAGAGACCTGCGGTTCTTT
TTATATAGAGCGTAAATACATTCAATACCTTTTAAAGTATTCTTTGCTGTATTGATACTTTGATACCTTGTCT
TTCTTACTTTAATATGACGGTGATCTTGCTCAATGAGGTTATTCAGATATTTCGATGTACAATGACAGTCAGG
TTTAAGTTTAAAAGCTTTAATTACTTTAGCCATTGCTACCTTCGTTGAAGGTGCCTGATCTGTAATTACCTTT
TGAGGTTTACCAAATTGTTTAATGAGACGTTTGATAAACGCATATGCTGAATGATTATCTCGTTGCTTACGCA
ACCAAATATCTAATGTATGTCCCTCTGCATCAATGGCACGATATAAATAGCTCCATTTTCCTTTTATTTTGAT
GTACGTCTCATCAATACGCCATTTGTAATAAGCTTTTTTATGCTTTTTCTTCCAAATTTGATACAAAATTGGG
GCATATTCTTGAACCCAACGGTAGACCGTTGAATGATGAACGTTTACACCACGTTCCCTTAATATTTCAGATA
TATCACGATAACTCAATGTATATCTTAGATAGTAGCCAACGGCTACAGTGATAACATCCTTGTTAAATTGTTT
ATATCTGAAATAGTTCATACAGAAGACTCCTTTTTGTTAAAATTATACTATAAATTCAACTTTGCAACAGAAC
CGTATTATGGAATAGAGATGTTGGTAACATTTATACAGGATCATTATACTTAAGTTTAATTTCGTTATTACAG
AACCACACATTCCAACCAGAAGAGAAAGTATGTCTATTTAGTTATGGTTCAGGAGCAGTAGGAGAAATCTTTA
GTGGTTCAATCGTTAAAGGATATGACAAAGCATTAGATAAAGAGAAACACTTAAATATGCTAGAATCTAGAGA
GCAATTATCAGTCGAAGAATACGAAACATTCTTTAACAGATTTGATAATCAAGAATTTGATTTCGAACGTGAA
TTGACACAAGATCCATATTCAAAAGTATACTTATACAGTATAGAAGACCATATCAGAACATATAAGATAGAGA
AATAAACTAGTGGCCGATTGTGCTTGATGAGCTTGGGACATAAATCCTAACTCGAAATAAATAAGCATATCAC
TAAACTGATTTTTTAAAGTTTACAGTGATATGCTTATTTTTTTATCTTACGATTTTGTACGTGCATGCTTGCC
TAGGGGTATGGCTCGAGCCATTAGTCTCTCGCACATACTATTCCCTCAGGCGTCAGCACTTACAAAATCGGTT
GTAATTTTCATTTTTATACGCATTCTTACTGAGATTATACTAATAAGAGGAATAGTAAAAGCAATTCTAAGTA
AAATTGCAGATAAGAGGTTTGTTAAAAGCAGTTCTAAGTAAAATTGCAGATAAGAGGTTTGTTAAAAGCAGTT
CTCAGTAAAATTACAGATAAGAGGTACGTTAAAAGCAGTTCTAAGTAAAATTGCAGATAAGAGGTTTGTTAAA
AGCAGTTCTAAGTAAAATTGCAGATAAGAGGTACGTTAAAAGCAATTCCATGCAAAATTGCTGATAAGGGGTA
AGTTAAAAGCAGTTCTCAGTAAAATTGCAGATAAGAGGTACGTTAAAAGCAGTTCTAGGCAAAATTGCAGATA
AGAGGTGCGTTAAAAGCAGTTCTCAGTAAAATTGCTGATAAGGGGTAAGTTAAAAGCAATCCTAAGTAAAATT
GCAGATAAGGGGTACAGAAAAACTAGACTTGATTACAAAATGGAGCTTGGGACATAAATGATTTTTTAAAAAT
GAGATGAGACGTAGATTAACTCCATAATCAATACGAATCTATCGACTTCTTTATTTATGATATTCATCTCTTT
TTAATGGAAATAAAAGTGCGATTAATGTGATAATACAGTTACGTTAATTAAAAAAATAAAAATGCAAGGAGAG
GTAATATGCTAACTGTATATGGACATAGAGGATTACCTAGTAAAGCTCCGGAAAATACAATTGCATCATTTAA
AGCTGCTTCAGAAGTAGAAGGTATAAACTGGTTGGAGTTAGATGTTGCAATTACAAAAGATGAACAACTGATT
ATCATTCATGATGATTATTTAGAACGGACTACAAATATGTCCGGGGAAATAACTGAATTGAATTATGATGAAA
TTAAAGATGCTTCTGCAGGATCTTGGTTTGGTGAAAAATTCAAAGATGAACATTTGCCAACTTTCGATGATGT
AGTAAAAATAGCAAATGAATATAATATGAATTTAAATGTAGAATTAAAAGGTATTACTGGACCGAATGGACTA
GCACTTTCTAAAAGTATGGTTAAGCAAGTGGAAGAACAATTAACAAACTTAAATCAGAATCAAGAAGTGCTCA
TTTCAAGCTTTAATGTTGTGCTTGTTAAACTTGCAGAAGAAATCATGCCACAATATAACAGAGCAGTTATATT
CCATACAACTTCGTTTCGTGAAGACTGGAGAACACTTTTAGATTACTGTAATGCTAAAATAGTAAACACTGAA
GATGCCAAACTTACTAAAGCAAAAGTAAAAATGGTAAAAGAAGCGGGTTATGAATTGAACGTATGGACTGTAA
ACAAACCAGCACGTGCAAACCAACTTGCTAATTGGGGAGTTGATGGTATCTTTACAGACAATGCAGATAAAAT
GGTGCATTTGTCTCAATAGAAAGTTAGAGGTGAGTCTTACGTTTCAGTGACGGTAGACTTACCTTTAACATGT
TACATACTAAAAAATTAATTTGAATAAGAAAGAGAGACATATATGAAATACGATGATTTTATAGTAGGAGAAA
CATTCAAAACAAAAAGCCTTCATATTACAGAAGAAGAAATTATCCAATTTGCAACAACTTTTGATCCTCAATA
TATGCATATAGATAAAGAAAAAGCAGAACAAAGTAGATTTAAAGGTATCATTGCATCTGGCATGCATACACTT
TCAATATCATTTAAATTATGGGTAGAAGAAGGTAAATACGGAGAAGAAGTTGTAGCAGGAACACAAATGAATA
ACGTTAAATTTATTAAACCTGTATACCCAGGTAATACATTGTACGTTATCGCTGAAATTACAAATAAGAAATC
CATAAAAAAAGAAAATGGACTCGTTACAGTGTCACTTTCAACATACAATGAAAATGAAGAAATTGTATTTAAG
GGAGAAGTAACAGCACTTATTAATAATTCATAATAAAACAGTGAAGCAACCATCGTTACGGATTGCTTCACTG
TTTTGTTATTCATCTATATCGTATTTTTTATTACCGTTCTCATATAGCTCATCATACACTTTACCTGAGATTT

TGGCATTGTAGCTAGCCATTCCTTTATCTTGTACATCTTTAACATTAATAGCCATCATCATGTTTGGATTATC
TTTATCATATGATATAAACCACCCAATTTGTCTGCCAGTTTCTCCTTGTTTCATTTTGAGTTCTGCAGTACCG
GATTTGCCAATTAAGTTTGCATAAGATCTATAAATATCTTCTTTATGTGTTTTATTTACGACTTGTTGCATAC
CATCAGTTAATAGATTGATATTTTCTTTGGAAATAATATTTTTCTTCCAAACTTTGTTTTTCGTGTCTTTTAA
TAAGTGAGGTGCGTTAATATTGCCATTATTTTCTAATGCGCTATAGATTGAAAGGATCTGTACTGGGTTAATC
AGTATTTCACCTTGTCCGTAACCTGAATCAGCTAATAATATTTCATTATCTAAATTTTTGTTTGAAATTTGAG
CATTATAAAATGGATAATCACTTGGTATATCTTCACCAACACCTAGTTTTTTCATGCCTTTTTCAAATTTCTT
ACTGCCTAATTCGAGTGCTACTCTAGCAAAGAAAATGTTATCTGATGATTCTATTGCTTGTTTAAGTCGATA
TTACCATTTACCACTTCATATCTTGTAACGTTGTAACCACCCCAAGATTTATCTTTTTGCCAACCTTTACCAT
CGATTTTATAACTTGTTTTATCGTCTAATGTTTTGTTATTTAACCCAATCATTGCTGTTAATATTTTTTGAGT
TGAACCTGGTGAAGTTGTAATCTGGAACTTGTTGAGCAGAGGTTCTTTTTTATCTTCGGTTAATTTATTATAT
TCTTCGTTACTCATGCCATACATAAATGGATAGACGTCATATGAAGGTGTGCTTACAAGTGCTAATAATTCAC
CTGTTTGAGGGTGGATAGCAGTACCTGAGCCATAATCATTTTTCATGTTGTTATAAATACTCTTTTGAACTTT
AGCATCAATAGTTAGTTGAATATCTTTGCCATCTTTTTTCTTTTTCTCTATTAATGTATGTGCGATTGTATTG
CTATTATCGTCAACGATTGTGACACGATAGCCATCTTCATGTTGGAGCTTTTTATCGTAAAGTTTTTCGAGTC
CCTTTTTACCAATAACTGCATCATCTTTATAGCCTTTATATTCTTTTTGTTTTAATTCTTCAGAGTTAATGGG
ACCAACATAACCTAATAGATGTGAAGTCGCTTTTTCTAGAGGATAGTTACGACTTTCTGTTTCATTAGTTGTA
AGATGAAATTTTTTTGCGAAATCACTTAAATATTCATCCATTTTTTTAACGGTTTTAAGTGGAACGAAGGTAT
CATCTTGTACCCAATTTTGATCCATTTGTTGTTTGATATAGTCTTCAGAAATACTTAGTTCTTTAGCGATTGC
TTTATAATCTTTTTTAGATACATTCTTTGGAACGATGCCTATCTCATATGCTGTTCCTGTATTGGCCAATTCC
ACATTGTTTCGGTCTAAAATTTTACCACGTTCTGATTTTAAATTTTCAATATGTATGCTTTGGTCTTTCTGCA
TTCCTGGAATAATGACGCTATGATCCCAATCTAACTTCCACATACCATCTTCTTTAACAAAATTAAATTGAAC
GTTGCGATCAATGTTACCGTAGTTTGTTTTAATTTTATATTGAGCATCTACTCGTTTTTTATTTTTAGATACT
TTTTTTATTTTACGATCCTGAATGTTTATATCTTTAACGCCTAAACTATTATATATTTTTATCGGACGTTCAG
TCATTTCTACTTCACCATTATCGCTTTTAGAAATATAACTGCTATCTTTATAAACTTGTTTGAAATTTTTATC
TTCAATTGCATCAATAGTATTATTAATTTCTTTATCTTTTGAAGCATAAAAATATATACCAAACCCGACAACT
ACAACTATTAAAATAAGTGGAACAATTTTTATCTTTTTCATCAATATCCTCCTTATATAAGACTACATTTGTA
GTATATTACAAATGTAGTATTTATGTCAAAATAATGTTATAATTTTTGTGATATGGAGGTGTAGAAGGTGTTA
TCATCTTTTTTAATGTTAAGTATAATCAGTTCATTGCTCACGATATGTGTAATTTTTTTAGTGAGAATGCTCT
ATATAAAATATACTCAAAATATTATGTCACATAAGATTTGGTTATTAGTGCTCGTCTCCACGTTAATTCCATT
AATACCATTTTACAAAATATCGAATTTTACATTTTCAAAAGATATGATGAATCGAAATGTATCTGACACGACT
TCTTCGGTTAGTCATATGTTAGATGGTCAACAATCATCTGTTACGAAAGACTTAGCAATTAATGTTAATCAGT
TTGAGACCTCAAATATAACGTATATGATTCTTTTGATATGGGTATTTGGTAGTTTGTTGTGCTTATTTTATAT
GATTAAGGCATTCCGACAAATTGATGTTATTAAAAGTTCGTCATTGGAATCGTCATATCTTAATGAACGACTT
AAAGTATGTCAAAGTAAGATGCAGTTCTACAAAAAGCATATAACAATTAGTTATAGTTCAAACATTGATAATC
CGATGGTATTTGGTTTAGTGAAATCCCAAATTGTACTACCAACTGTCGTAGTCGAAACCATGAATGACAAAGA
AATTGAATATATTATTCTACATGAACTATCACATGTGAAAAGTCATGACTTAATATTCAACCAGCTTTATGTT
GTTTTTAAAATGATATTCTGGTTTAATCCTGCACTATATATAAGTAAAACAATGATGGACAATGACTGTGAAA
AAGTATGTGATAGAAACGTTTTAAAAATTTTGAATCGCCATGAACATATACGTTATGGTGAATCGATATTAAA
ATGCTCTATTTTAAAATCTCAGCACATAAATAATGTGGCAGCACAATATTTACTAGGTTTTAATTCAAATATT
AAAGAACGTGTTAAGTATATTGCACTTTATGATTCAATGCCTAAACCTAATCGAAACAAGCGTATTGTTGCGT
ATATTGTATGTAGTATATCGAGCTTCACATGAAACAGCTAAAGAAGCTTTGGGCGATAAAGAGTTAAGAGCCA
TTGCACATGAGTTAACTAAAACAGTTAAGGATAACATGAGTGTTGATTGGTCTAAACGAGACAGTGCTAAAGC
TAAAATGAGAGTTCAAGTTAGACGCCTATTAAAGAAATATGGCTATCCACCAGATCTTCAAAAAAATGGCTGTG
GAACAAGTTGTAGAGCAAGCAGAATTAATGGCAAGTCAGCAATAAAAAAATAAATCATAATGAGTCCGGGACA
TAAAGTTCTTGGATAAGTGAAAAAAGACAATTTCTATTGAAATAATATAGAAATTGTCTTTTTTATAAATTTT
TTGATTATTTTCAGCTCGTTGAGCTACTACTTTTCTTATATTAAGTGCCATTAATACAAAACCAAGTTCTCTT
TTGACTTTATTGAGTCCTCGGACAGACATCCGAGTGAAACCCAAAATAGCCTTCATAAATCCAAAAACAGGTT
CCACATCAATTTTTCTTTGACTGTAGATATTTTTTGTTTCTGGTTCTGAAAGCTTTTTGTTAATTTGGGATTT
AAAATATTCCCAGTTATAATTCTTCATTATTTTTTTGTTTGTTTTTGAATTGAAGTTCATACATTGATTTTTC
AGAGGACATTCTGAACAATCATCACATTCATATAATTTGAAGTCTCGCTTATAACCATACTTATCATGACGAT
AGGCATATCTTTTAAAACCTAGCCGTTTATTATTCGGACAAATGAATTCGTCATTAATTTCGTCATAGTTCCA
ATTTTGAGTATTAAAGATGTCACTTTTATATTTTTTAGTTTTATCTTTTATAAACATTCCATATGTTATGAGT
GGCGTTCGATTAAAGTCATCTATAATTGCCTTATAATTTGATTCACTACCATAACCTGCATCAGCTACAATAT
ATTCAGGTAAATGACCGTAGGTCTCTTGAATTGAATTTAAAAATGGAATCATCGTTCTAGTATCCGTTGGATT
TTGATACACATTATAAGATAAAACAAATTGGGAATTTGTTGCTATTTGTAAATTATACCCTGGCTTAAGTTGT
CCATTTTTCATGTGATCTTCTTTCATTCTCATAAATGTCGCATCATAATCTGTCTTAGAATAACTATTTCTAT
CCTTTAAAATAGATTTTTGAAATTCGTATCGATACTTTCGCTCAAAATAATCATTGATTTGCTTTTTGTATTT

TTTGATTTTAGTTCTTTTGAGACGTATTTGTTTTCTTGTTTTAGTACATTTTTCATTGTTGATATGTTGGTTT
AAATCTTCGATTTCTTTATCTAAGTGACTACCAATCAAATCTATTTCTTCTTTTGTTAATTCATTATCATGAT
CTTCTTTAATTTCCGGTATGATTTTATTGGTTACCAATTCATGGTAGAGGGCTTTAGAATCCTCATTCATCTT
TGATTCATGGTTTTGAATACTCTTTTTCCATACAAATGTATATCGATTGGCATTTGCTTCAATTTTTGTACCA
TCAATAAAAATAGCTTTATCATCTATAAGATTTTGTTTTACACACTGACTGTAAAATTGAATAAATAAAGATT
CTAATAAAGCATCTACTTTTGGATTTACTCTAAATTGATTAATTGTTTTATAAGAAGGTTTTTGATTTTGTGA
TAGCCACATCATTCGGATGCTATCATTAAGCATTTTTTCTATTTTACGACCTGAGAATACAGATTGTGTGTAG
GCATATAGAATCACTTTTAACATCATTTTAGGATGGTACGAAGTTGCACCACGGTGATGTCTGAATTCGTCGA
ATTCATTGTCAGGAATTGTTTCAACAATATCATTTACAGTAAAACGATGTTGATTTGTTTTGTTTCCATATTG
ACCTCCATGTATTTGCTATGATTTCAAAATCCATTTTTGACGTGCCTTAGGGTTGAGTGGATGCATAATTTCA
TTTGTTACTGGATTGATGAGCTTTTTTACTTTCTTTTTATGAGGTTTTAACATTTCCATCACTTGTTCGACAC
GGTCGATAACAACTGGTCGCTTCGCATAGGCACCATAAGCAAGAATCACTGTGTCACTTTCACTAATCGCTTT
CATCAAATGAATATCTGTGTGCTCATCGTATGGCTCTTTGATATGTTTAAGATTCTCTGGAGTTTTAATATTA
GAGAATAGATTTACAAGATATACAGCACCGTATCGTTCTGAATTGGCTAATTGGTTGAGGATAAGAACAGTTG
TGAGATCGAGTGATAATACACCGTCTAAATGAGGATACATCGTTATCACTGTGCATGCAGCTTTCTTTTCATC
CCATGTTTTCTTGAGTAAATAGCGGTGCTGTTCATCATCGCTAAATATGGCTTCTGTGTGTATCGTACTTTTG
ATTGTATTCATCATCGTCACTTCCTTTAGTATTCTTCTGGTAAAAGCATCACATAATAAAAAGCGTCCACGTC
ATCTTCACGAATGACGTAGACTTTCTTAGGTAATGCATTTTGATTTTTTTCATAGTTTGTATAGTGATATTCC
AATTTGTATGTGGGTTGTTCTTGTTCATGTGTGATTGAGAGTATATTCTCATCTTCTTGTAATTTAAAAATGT
GTAGGTAATCTGTATGAGGCTGGTTATCTTTTTTTTCTACCATGTGCCAAAGTAAGATTTGAAGGTCTAGTGG
AATACTTTCATTAATTCCTCTTGTGATGTATCGATTGATTTTCATGCTATTTCCCTCCCTTCTGCTTTTCTTT
CATGATGTCGATGATTTCGTTGATAACTGTGACGGATAATTGAGCAACTATGATCCAATTATTCATGGTCCTG
ACCTCCTTGTTTTAGTAAATGACGTTCATCAATAATGATATTTTGAGTATCTGTAAGGTACAGAAAGTCCATG
TCAAAATGGTCTAAGTATCCGACACTGATGAGTTGGTTATTGGCATACATTAGAAATGGATAGATACTTAGCT
CATGTAGTTCATCATTATAGTAGGTATAAGTCTCGAGTGTGAGATGTACCAGTGGAGAATCATTAATAAAACG
TTCCGGTAGAATATTTTCTGCTGCTTCCTCCAGCGCTTCACATTCCCAAGCTTCGTTATTAGATAGTTGGAAT
AGGTGGGTTATATATTGTTTGAGTTCTTGAGTGATGGTTTTCATATTATTGCCTCCTAGATAGTGTAATAGTG
ATGTAGTTCATATACATCATTGAGATAATATATATTTGATTTGTCATTTATTACGAATCCCGGTGGGAATAAG
AGAAAATTCCATATAAAAACCCGCTACAAACGTTGGTATGCCAAGGAAATCCTGAAATTCCGCCTATTTTGAC
AAACAATCAACTCATTATTTATAAGTATTGATGATAGGGTTGTGTCTCTGCTTCCTTATATATATTATTTATT
TATAAAAAGTAACGGGATTTTGGGATTGTGCTTGCACAATCCTTCTGTTTCTTCGAATCTGCAAATCCCAATC
ATTTCCCGATAAAAAATCATTGTGGGATGTTCTTTAGCAATTTCAATATAAGCATTGTATAGTTATGAAAAAA
TTACGACAATAACTGTTTCATTAGATAAGTGTTATTGAAATTGATAAAGAGCAATTCTTGAAAATAGTTAGAT
AAAATAAGCGAAAGAATATAGTGAAAATTATTGTTATAACAATGATTCTATTAGCTAAATAGTAAGATATAGT
GTTTGGGGCAAAAATAAAGACGAAGTGCTGAGATGCACTTCGTCGAGTTGTTTATTATTGAAAAGTTGTTTAA
TGATTTCGTTATTAAGTTTGAGTGTGACATAGAATTGTTTTTTATGATTACCATCTTTTTTAATATCAATGCG
ATCAATCACTGATAGATACAATGCTTTGAGTCGAGATTTTTCTATGTGTTTAATATCATGAAAGATGTGTTGT
AATAGTTTACTGATTTCTTTGGCATCAAATAAAGTCTTATCTTCATTTTGTTGATTTTTGAGTTGGTTGATTT
GATTCGTAATGTCATTGAGTTGCTTTTCATATTTTTGAATACTTGGTCTGATTACTGATGTTAAGTCCGGATT
ATCCTCGATGGTTTTAATCAAGTTATTTATTTTGATTTGTACTTCATCATATTGTTGTTGCTTATAAGCAATA
TCGTGATGAAGTGCAGCGCCATCAACTTGATTTTCTTGATTGACGTGTGTTACTACGCGTTGAATGACTTTAT
CACTTTTGACTATTTCAAGTATTTGCTTCATCACATAATCTTCAATCACATCAGCTCTTACACTGTTTGCCGA
ACATACTTTGGAACCCTTGTTCCGAAAATTACTACATGAATAGTAACGAATACGTTTCTTAGTCCCGTCTTTA
AGAGTATTCGTGGTATTGCTTGCTGCCATAGGTGCGCCACATTGGGGACAGTGAATAATGCCTGTAAGCAGAT
TCGTTCCTTTGCCATGGACTTGGGGTTTTTGACTGACTTGTTTCTTACGCATTTGTACTTTATCCCATAAATC
TTGATTAATAATGGGGGAATGCTTACCTTCAGCTATCACTGGTTTATCATTCAGCCCTTTACGACGTTTTTCA
CTCCAATCTTTGTATTTCGCAAATTGAATTTTGCCGATATAGAAAGGGTTAGCTAAGATGTATGTGATTGAAC
TAATACTGAAAGGTTTCCCCTTTTTAGTGACATATCCTTTGTGATTCAATGCATTGGCAATTTTACGATAGCC
ATGTCCTTTGGCATAGCACTCGAATATATATTTTACAATATTAGCTTCATGTTGGTTAATCATTAGCTCGTGT
TTACTATCTGGTATTTTGTCATAACCTAGTGGTAAATTGCCTTGATAATAGCCTTCTTGGGCACGTCTCGTTT
GACCCATAAATACGTTCTCGACAATGTTATTACGTTCGAATTCTGAGAAACTCGCAAGTATCTGTAACATGAG
TTTACCAGAAGAAGTATTGACTTCCATACGCTCTGACAAACTGAAAAATTCGACATTTTGTTTGTGTAAATCT
TCGACAATTTTGAGAAGATCAGATGTATTACGAGCTAATCGGTTTGTTTTGTATACCATAACACAGTCGATAT
TGCCTTCTTTTGCATCTTTCAACATACGTTGGAGCTCAGGTCTGTTCATAGATTTACCTGAAATACCACGGTC
AGCGTATATATCTTTAACTTCAAAATGATGGAAGTCACAGTATTCTTTGATTTGATTGATTTGTCCGTCGATA
CTATAACCTTCTGTGCTTTGCATTTCTGTTGATACACGTACATAGATACCGACACGTTTTGTTTTAAGTTGTT
GCATTATGTTACATCCTTTCTTCATTTATGCAATCGATGATTGCATGGTTTGATTGACAATATTGAGTGGTTC
ATTTTTGAAATAGATTCCTATAAGATTTTTATCTTTCGTAATGTGAATGGTTTCAATATAGGGGTACAATATG

TTTAACGTGAAACGTTTTTGAATAATATTTTGAATGATGTGTTGTATTTGATGCCCATTGATAGATGTAGTGC
GTTGCGGTTGTTGACGTAATGATTGTGTTTGCTCTCTGAACGTTTCTGCATCAATGATGCCTTGTGCCAACTT
TTCTATCAGTTGTTCTTGAGTCAATGTGTGATGTTTTTCTATGTTTCTTTGTCTTTTGATGCGTTTGTCAATC
GCACTTTTAATTTTTGTGTAGATGCGTTGATTTTGATAAAAGTCTCGGCACACTTCTAATACTTTATCTTCAA
GTGTTTGTGCATTGATGCCTTTAAAATCACAGACAAAGCGTGAAGCATTCATGTTTTTAGGACAGACGTAGTA
ACGTAATGTATGATTCTTTTTTCTAATGGTCATATTTGTAAGTGTTGCATTACAACATGGGCATTTGATTTTT
TGTTTGAGTTGATTATCCGAAGATGTCTGTTTGGTTTGTTTTTGCGATCGAAGTCTCTGCGCTTGCTCATATA
TACTTGTGGAAACAATAGAAGGAAACATATTGTCGAATTGGCCATATTGATTGTTGACACGACCACAATAATT
AGGATTGATGATAATGTTACGAACTTGATAGGGTTGTCGATTGATATACGTGTTATCTTCTTCTAATAACTGT
GCAATTTTCTTATAACCATGGCCTTTAATGTAATAATTGAATACAGCCTTTACCGTTGGTGACTCATTTTGAT
TGATGATGAATGTTCCGTGGTGATAATCGTAACCAAAAGGTGCATGTGTTGTAATCAATCGACCTTGCTTTGC
TTTTTCTTGAAGCCCATTTCTGACTTGTTCTCCAATGTTATCCGATTCAAGTTCGGCTAAACTGATGAAGATA
TTAAGCTTGAGTCGGTCGAAAGCTTGATCCATATCAAAGTAACCATCATGTACGCTTAAGATATGAACATGGT
ACGTTTGACATAATTTGATGAGTTTTAATGCATTTTTCAGATTACGATGCAACCTATTAAGACGATAACAGCA
TAATATGTCACACTGTCCTTGTTGAATTAATTGTGTTATTTGTCGATACCCACTACGATTATCTTTGCGACCT
GATTGTTTGTCGCTATAAAAGTTGATATGTTGAATATGATGTTTTTCGGCTATTGCTTCGATAGCTTGTTTCT
GTGCTGCAAGAGATTGTTGTTTCATCGTACTTTGACGTAAATAGCCTATGACTTGTTTCATATCGGCTCCTCC
TTTCACAGTGATAATATATATTTATGGATGAATTGATATATAAGCCCAACATCAATGAGATGTTGGGCGTCCA
TATTAGTCATTTGTTTGATTGATTTCTTCAATTACCAAATCGGCTAATATCTCGATAAGTTCATCCATGTTTT
TCACTCCGTTATTTGTTCTATCTTCAATACGTCGATTATTCAGTTTGATGCTTCACAGTTGTATGATAAAGAC
AATTAGAAATCTTCGTGAACTCCTGAAGGGCCTATCCCTTCATTAGCGGATTTAAAAAGTTCTTTCGCAGCTT
TGTTATCATTTGACGGTGTCCAATTTTGAAGTAACGACTTATCTTTAGTTAATCCGAGGATAGATGCAAACTC
TACATCTAATTTTAGATGGTAAAATACAAGTGATTGTTTTTTACCGCTATTATCTTTGACACTTCTTTTAGTT
GTTTGGCGTCCACGGTCAGCTAATATGAAACCTTTATCTCTTAAGGCGTTGACAACATTATTAACATCTTGAA
ATTGATGATTGTTTAGCATCTGTTTAAAAACGTTCGCAATTATTTTTACTTCGATATGGTCATCTTTTAATGA
GATTAATCCATAGTTCTCAAACATATTTTTCAAAGCACCTTCATCTGAAAACTTACCTCTGTTTTGTGCTACA
AATTGAATGATGACATCGATAGCTTTATCAGCTAATGAGCGTTCAGAGATTGTATGACCATGATAATCAATAA
AGTAGTCTCTTATTTTAGCGATATCAATATCTGTAGCTAAAACACGACCTAATATTTTTGCAGATGTTGTAAT
GACTGCATAACGCTTAAACATACGATTGCCTGTGTTGCTTTTATCATCTTTCAATTTAGCTTCAAACCAATCT
ACTTCCTTGTAAAACCATTGAATAACTTCATCTTCACGATTTATAAGATATTTAGCTACTAACGGTAAAACAT
GACCATAGTTTAGTGCTACAGCTTTTTTAATATTGTCAGCATTGGTCGCATTTGTAGTGAATTGTTCATTAAT
CTCGATGGTTCTTACACGTAATCCATCGTTTTGAGCTGAATCATTAAAAATACTGTGTTCTGACGTTGAAATG
ACAGAAGTACCCCAATTCTTAGGCGTTTTAACTTCTCCATGAACGTTTGAACGTTGACGACCTTGACCTTCAG
TGATGGAGTACAATAACCCCGTTGTATCTCTAAAAGTTGCTGATGAGAGTTCATCAAATACAATAGGTATACC
AAAATTGTTACTCAAGTAACCTTCAAGTGCATTACGTGTGGCATTCCAATTTCTAAAGAGAGTTTCATTACCT
TTGGTAGGGTTACCAGCGACTGATACAGCTAAAGAAGCTGCAGTTGACTTACCGGTTGAGGATTGACCTGTAA
AACTAAAAATGATTCCGGCAAATTCGGTTTCATGTTTGTGCTTCAGGAAACTCGTCACTAAGGCAGAAATACC
AAATACGACTGCTAATTCTAAAAGAAGAGAACCTTTAACCTCTTTTAGATACATGTTAAACCAATTATCAAAT
GTACCTTTAGGAGTTAAGTCATAAGCATTATCACAAATGGCGTCAGATGGAGATTTATTATCAAATTCTTTAG
TAGTATAGATTTCACTTAACGATACAATAGGACCAAACGGTGTTTCCAGTATACCTACCCCTTCATATAAGTA
GGAAATGGGTAATTGATTGCGCATTTGTTGCAACGCATAACCTAAATCTTTTGTATATTTTTCATTAATACTA
AATCCATACTTCATTAAAGATGGCAGTTTTTGTGTTGTTAAAATATCACTAGATTCAACAATTTCTTTTTGAT
CCTCGTCTGTAATAATTACTTTTTCAGTGTTAGTTTTAGGGTCAATAAACTTATTTTCGATAACGATAGGACC
TGCGATTTCAACTTCAGTAGGCATTCCTCCTTTTTCTTTGGGAGGCTTGTCTTTATACCAACCTTTTTTTGAT
TTGTATCGTGGTGAAGGATTAAATGAAGGGTTAGTTTGAGTCATTAGCGAACACCTCCTTTCGAAGGGTTGCT
GTTATTATATGGATTTGGCCCTGTTTTAAGATAAACCAAGTGTCCATGAGTATTTTTACCAATAATAACAAAT
GGAATACGTGGCGCATGTTTTACAAAGTACGCGAACCAACGTCCGACATTTTGTTGTACAGCTTTTGAGCATT
GTACATTTGCACGACTGTTCAGATCATGAAATGTAAACGTTTGCCCCTCAGGTAAATCGAACACAATTCCTAA
AATTCTTCTTTTCAACTCTTTAAAATCATCTTTTTGATTGTAAGTCATATGAGTTCCTCCCAAATATTATTAA
TTAAGTCTATCTTCTGGTTTTTTTCAGAAGATTACATTGCTTATAATAGAGATGTACTGTCTTTATTGCTCGA
ATAGTATTGAGCAAAAGACGAACATATAATTGCAAGACTTTTCGCTTGACAAATTAGAAAAGAGGTAACCAAA
GTGTATGATTTTTTAGAATATTTACATCGTTATTGTGTAAAATCGATTAATAATTGGGAGGCTGTTGCAGATA
TATCATGCAAAGTTGTACGTGAAAAAGTTAGTAAAGGTTATTATGTTAAACAAGATAGCGTTGTAATTGATGA
TAATGATATACCTTCAATTATTAAAATAAGTGGAAATTATGTAGCAGCAGATATATTAGTTACTAGATATTTA
GATGGAATGGGTGAATTTATAAAGGTATCAGAATTAGATCAGTCGAGCTATTACGAATGGTTTATGTTTGTTG
AAGAAACTAGAAGAATATTTAGAGGTACAGAAATAGTTTATCGATTTTTAAAATATCTATTTACGCATACCTA
TGAACATAAAAACGAAGAACAAAAAATAGAAAAATATATGAAGAATATTATCATAGATAATCATTTTAGAGGG
GGGCATAGTAATGATATAGTTCGTTTAGATATGAAAGATGATAATGTGTTATTTTATAATTTTGATATATACG

ATACAAATATTCTTTATGAAAAAATAGACGAGGAAAAATTACTTAGAATTTTAGATGAAATAAGTAATACTGA
CAATTACAAAGCGCTATTTAACATTGTAAGTGAGAACCAAAAGAAATTTTGGAAATCAAATATTAAGCCAATA
GTAAGTGAATGTTTAGAAGATATAAATACGATACCATTTGATTTATATGTTTATGTAAATAAGTATATAAGTA
ATAAAATATTTAACTGTGATAATTATTTAATTACTAAAAATGAGGTTGTGGATTTTTATTTTAATCATGGTAG
TGACTTTAATAATTTCAAGATGGGACAGAAATGGACAAAGTCTAAATTTAAGCAGGAAAATAAACTTAAAATA
CACGAATATATTATTGCAGTAACTTATGTGAATTTATTAAATCATTCATATATGAAATATATCAATGCTGAAA
ATTACTATGACCCTAGCAAAAAAATAACAAAAGGAATGAAAAATAATTATCATAAATTTACAGATTCATTCAG
TGATTTTGTAGAAATGGTGGACGATATAACATGGAGAATCCGTGATTCATTTGATTATATTGGAGGATTAAAA
AACAAAGAATTTAACGAAGATGAGAATGAATATATCGAACAATTAATTCAATTTGTTATATTATCTAAATTCG
GCTATATGAATGTAGAAAATCACTTTGATTATATTGAGAACTTAAATACGCTTAATAATGAATTAACGACTAT
ATTAAAAAGTGAGTTCGCGCACATTTCAAATGATATGTATAATAATTTATTCAGTAATTTGTTTGATGTTGTA
AATACATGGAAAAAGGATTATTCAAATAACTTTTATAATAATACTCAGAATACTATAACTACAGAAAAGTTAG
ACATAATATCTCGATTTATAAAACAATATGATAAACCAGTTGACCGAGACAAACATGGTAAACGAGTTAAAGC
AGAAGTGTTTGAAGGTATAAGTGATTTTCATAGGCGTGTAAAAGAAGGATTTGATAATAATGATGTGCTATTA
CAATTAAGATTAGAAATGAGCGTACATGAAAATGATAAATTTACTAGTTAAGTAATGGGGATTTTTTGTAAAA
TCTAATTCAAAAGCAATATAAATATTTAAATATAAAAAAGGGGTAAGTTATGGACTTAAAAAAATTTGAAGAA
CTATATAAAGATTATAATGATGATGATGTAATAGAACAATTTGCCATATATAAAATAAAAGATGAAAAGCAAG
TAATGGAAATTACTGATAAAAAGCTAACCAGTGAAGATAAACAAGTTTTTCAGAGAATAGCTAATTTAAGTTA
TCAATTTAATGTAGTAAAGGAAATGTATGATTACTATCTTTATGATTTTCAAGTAATTACTGATTATATCAAA
GATAAAAGAAACCTAAATAATAAGTTTAAATCTGTTGACTTTGTCAACCTATATTTTTTACATTTCTTATCTA
CTGCAAGGACTTTTTATCAATATACTGAAAATTTATTTAAGTCTATAGGTAAGAAAGAATTTGAGAAATGGAA
AAATAATATATCAAATATTTATGATAATAATTTTTCTTATAGATTATGTTATCAAATGAGGAATTTTACACAG
CATCATGGTTTGGCAATTACGAATATTAAAGAGAAATTAAATTTAGATTCTAATGGTAAAGAAATAAATGACT
ATCAATTGTTCATTGATCCATCTATATTACTAAATACTAACTATAATTGGAAGAATTCAATTAAGAGCGATTT
AGAAAATCATAATGGATTAATTGATTTTTATCAATTAATAATAGATTATGACAAGTGTATAAAAGATATACTT
TTGTATTCATATATAGGGTTAATAGATAAGTATAGCACTGAATTAAACCAATTAATGACAAAAGCTATGGCAA
TAGAAGGAGAGGCAGGTATACCTTTTTTAAGAAAAGCTAAAAAGAAAGAACTTAGAGAATATTATAAAAATAT
TGAATTTACGGAATTACAAGGAAGTAAAGCAATAAATGATATATTTAATATGATGTGTGATTTATATACTCAA
CTCAATACATCTAAAATTTGTTTCAATAGATGAATATATTTAAACTGAGTAAAAGATGAATAGATAAATGGAC
CAATTAGCAAAATAACAGATTACAAGTCTAATAATATTAAAATATTGTGAAGATATTTATGTTTTTTTTATAG
AATTACTTTAGAGTTAATCCAATATACAGGGTATTTAACTATAGAAGAGATTACTATGTTTGTTTTTTATCAG
AGATCCTTCTGAAATTTCTTTGAAAATAAAGCAAACTGAAAATTTTAGAAAACTTAAAAGGGTTTAAAGCGAA
GATTTAGTTAATACTTTTAAAAAAGTCCAAAAATAGGTAATACAACTTTAAATAAAGTTCCAAGTGCTGTATA
TTTTATGTAATTTTGTGTAGGTTTAGATATTAAAAAAGTATAGTTAAAATAGGGAATTCATCAAAAAAAGTAA
GTGCATTATTTAATAAAGAAGTGAAAATAGAATGGGTAAAAGAAACACTGATTCAATATAAAAGTATTGAACC
ATGTAATTTTAATAATGATTTAAATTTTTGGATTGAATATATTGAATATGTTGACAAAAAGCTTCCTCCTTTC
GATACAAATACTGTTAATGACGAAAATTTTGATGTGTTTTATGAGTTGTTTGATCAATCAAAATCAATAAAAG
ATTTAAATATAATTTCCTCTGCTTCTAATCATATTAGGTTATATAAAAATAAAATTAATAATAAAATAGTTGT
TAAAGAGATATTTTGTGTACCTATTGTAAATGAAAGGATATACAACACAAAGAAAAGCATACTTAAAGAATCA
AACACTGATATTGTGTCGCAAAAAATCCTATAATTGCTAGATTTACTAGAAGAAAGTACAAAGAAGAATTTAA
ATAATTCATTAGATGAAGTATAAAAAAAGCTTTTTATTAGTGATTAATAATTTTTATAATTTAAATTGCTGCA
AAAATGTGAAAGATGCTGCACCTTTTAAAAGTAATAAAAAATAAATATTTAAACTTCTAGAGGAGTTAGGAAA
AGTGCAATTTTTACAATTAATGCATATCCTACAAAATTCATTGAGAACAATTTTTCGAAAAAGAAGAACTATA
TGAAATGATTATAAGAAAAATTACATTATTATGGTATTAATGAATTATTATTTATTGACCTAGATAGTCATAT
ATATTGGATAATAGAAGGAAATACGTATGGTTATAATAATATAACAAATGGGTTAAATTTAATTAACGAATAA
TAGCTAAAATCTGATAAGTAAATGGTATAATATTTGTAAATATAAATTGTTTGGAGGTATCATATGTCTTTAT
TTGATAGACTAAGTGTTCTAAAAAATAAATCAGAAATAAAACTTATAGAAAGTAAGCAAGTAGTTGAAGATAA
AGTATTTGAAATACGACATGGTAAATTAGATGATATACCAATGACTAAAGAAAGAAAGCGTTCAGAAAAAACG
AGGAAAAAACTTGAAAAACATGAATCAAAGGTAAAAGAAAAAGAAAAAAGAAAGCAACAAAGAGAAGAAGAAA
TAGAAAAAAGGAGAATAGCAAAAGAAGAGCAAGAAAAGCGTGATGTTTGGATTAAAGAGAATGTATTTAAAAT
TATAGTAAATGAGGAAGGTAAACGAATAATTTTTTGTCCTAAATGTGGTAGTGAGAATAATGTAACTGCTACT
ACTTGGAACGTAGATGAAGCTGAAAATAGTGAGGATATTGGAACATATTTTAAGAGAACTTGTAATTTATGCG
GACATTCTCAAAAATTTAATAAAGATAACCCTAAAAAAATTTATGAATAGGAATAAAGAATTGATACATTTTC
TGGATAAGTGAAAAAGACAAATTCTATTGAAATAATATAGAATTTGTCTTTTTTTATAATTTTTAGTTGTTTA
AATGTTAGAATTTATAAAACCTCATTATCAACCGATACGCAGAGGCGTATCATAAGTAGGGGTAGTATTAGCC
AATTTAATAAATAAAGAGGTGACAGAATGGACAATCACATAAGAATTTTTGATAATTTATTTCAAAGTAATAT
TTCAAAATTCCAAAACCTTACTAGTAAATCATACATTATAAGAAATGATAATGAAAAGAATAGTTATCTTCCT
ATGGTTCAAGAGATAAGAGAGTTGTTTGGAAAAGAGGGAGAAATTTTTTCTAAAAGTATAGGAACACATCCTG

ACTTTTTTGGAATAGAAAATACTAATGAATACCAGTACATTTGTTCCCTGTTTGTAGATATATCTGGGTCTAC
AAAGCTAGCTTTGAAATATAGTTTGGATAAAGTTAAACTTTATAAAAATGCGATAATTTCTAGTGCTATAGAA
ATATTTCGTGCATTTGACGGTCATATTCATAGAATACAAGGAGATGCGGTGCTTGTTTATTTTGGACATAAAG
AACTGGAAAAAAGTGATGCAATAATTAATGCAATCAACGCTGCGTCATTAATGCAGTATTTTAACGCCACAAC
ATTAAAAAAGTTTTTTGAAAGTGAAAATTTAGAGCCGTTAAAGATTAGAATAGGAATTGATTTTGGTGATGAT
TCTAGTGTATTGTGGTCAAAATATGGAATTGATGGAATTAATGAAATAACTTCTACCAGTATACATACTGACT
TAGCATCAAAATTCCAAAACAAAGCCCCTTCAAATAAGATTATGATTGGAGAAAATATAAATAAATATCTTGA
TATTCCTAAAAAGTTTAGAAGTATAAAAATTGAGAAGAACAATGGTGTTGATGTTGAGAAAAGATATATTTTA
AATACAAATAATCTGGGAAGATATTCTATGGAAGTGTTTGAATGGGAAAAATACTTGAATAGTTTTTCAATGT
TGCCTCCTTTTTCTACAGAAAATGAACAATTTTACTCACCTAGAGATTTGAAAATAAGATGTTGGATTATTGA
TGAAAAAAATCAGGATAAATATGAATATATTGAAAGAGGTAGTGCTTTAAAAAAAGAAATGAATTTGTTATTT
AAATTAGAAATTTATAATCAATGTCTAGAATTTAAAAATATTAAATGGAGAGTGGTAAACTACGGAGAGGAGG
CAAAAAAGGACAAGGAATTAGAATTTGAAATGAATCAGTATGAAGGGTATCAGTATTGTAATCAAAAGACAGC
TTATACAGGATTACATTTTATGGAATGTTATTTGTACGACATCAATGATAAAATAATTTGTCATGATAGTTTT
GGTCTTTTTATAAATGATAATAATAGAGAAGTGAGAAAATTAGGAATAGAGGATTGAGTAATGTGAAAAAAGA
TGAGTACATAAAGTTACATCAAAGCTATCTAAATGAATATATTAAATTTGCTGATGCCAAGGCATTAGCTATC
ATAAGTATTAATGGCTTTATTTTAAATTTTAACTTTTCAAAACGTAATATTAAATTTCATAATACTGAAGACA
TTTTCAATTTTACAGCTTTTATATTATTGATAATTACAATTATACTAGCAGCATTTGCAGTATATCCAAGAAC
AAATAATAGAAGCGAAAAAGGAATTATTTTTTGGGATAATATAAATAGTATGGGAGAAAAAGAATTTATAGAG
AAAGTGAAATTTGAAAAAGAGGAAGAATTACTTGAAAAGACGATACAACAAAATTATTTTTTAGCAAAAACTG
CATCTATGAAATATTCAATCATTAGAAAGGTTTTTATAATTTCAGCATTAGGTTATTCCTGTTTATTATTTTC
AAGTATCTTTCAGATTATATGCTCTTGAATTTTCAGTTTTAACATGAAAAGGAGAAAAATAAAAATATGGCAA
CAATTGGATTTGAAGAAAAGTTATGGCAAGCGGCGGATAAGTTACGTGGAAGTATGGATGCAGCAGAATATAA
AAATGTAGCATTAGGTATTATATTTTTAAAATATGTTTCTGATTCTTTTGAGGAAAAGTATGAAGAATTATTA
AATGATGAATATGCCGATGAAGAAGATAAAGATGAATACTTAGCAGAAAATATTTTCTGGGTTCCTAAAGAAT
CAAGATGGCAATACATAAATGATAATTCTAAGAAACCAGAAATTGGTCAAATTATCGATAAAGCAATGATAGC
AATTGAAAGAGAAAATGAGTCTTTAAAAGGCGTATTACCTAAAGATTACGCAAGACCTGCATTAGATAAAGAA
AAATTAGGCGATATTATTGACCTATTTACATTCAAAGTTGGAGATTCTGAAAGTAAGAAGCAAGATATTTTAG
GTCGTGTTTATGAATACTTTATCGCAAAATTTGCAAGCGCTGAGGGTAAGAATGCTGGGGAATTCTATACACC
AGCTTCAATAGTTAAATTGCTTGTTGAAATGATTGAACCATATCAAGGTAGAATTTATGACCCTTGTTGTGGG
TCGGGCGGAATGTTTGTACAGAGTGAACATTTTATCGAACGTCATCAAGGTCGTATAGATGATATTGCTGTTT
ATGGTCAAGAATCTAATCCGACTACTTGGAAATTAGCGAAAATGAATTTGGCTATTCGAGGTATAGATAATAA
TTTAGGTGATCATCATGCAGACACATTTCATAATGATTTACATAAAGATTTGAAAGCTGATTTTATATTGGCT
AATCCACCATTCAATGCAAGTGATTGGGGACGAGAAAAATTACAAGATGATTACCGCTGGAAATTTGGTATTC
CACCTAAAGGCAATGCCAACTATGCATGGATTGAACATATGATTTCTAAACTTGCCCCAAGTGGTACAGCAGG
ATTTGTTTTAGCGAATGGTTCGATGTCTACAAGTGGTAAAGATGAATTAGAAATACGTAAAAACTTAATTGAA
CAAGACATGGTTGAGTGTATTGTCACATTACCAGGGCAACTATTCTATTCAACTCAAATTCCAGTATGTTTGT
GGTTCATGACAAAAAATAAAGCTAAAAATGGCAAGAATGAACGACGTGGAGAAGTACTATTTATAGATGCTAG
AAATATAGGAAGCATGGTATCAAGAACATTAAAAGAATTCTCAGATGATGAAATAAAAGATATAGCAAATGTT
TATCACTCATGGCGTGGAACAAACAATAACCAATATGAAGATAAAGCAGGTTTCTGTAAAGTAGCAAAGACAG
AAGAAATAAAAAACAATGAATATATCTTAACGCCGGGACGTTATGTAGGATTAGCAGAAGTAGAAGAAGACTC
AGAACCATTTGAACAGAAAATGGAAAGAATCACAGCAGATTTAAGTGAACAATTTGCGAAATCAAAAGAACTC
GAGGACCAAATCCGTAAGTCATTGGAGGGATTAGGTTATGGAGTTTGAAACATTTAATTTAACTGATTTATAT
ACTATTAGTTCAGGTTTATCAAAAAATAGAAAATATTTTGGTACTGGTACACCTTTTTTAACATTTAAAGATG
TATTCGATAATTTAATATTACCTAATGAGTTTTCTGGGCAAGTTATAACTGAAGAAAAAGAAAGAGAAAAGTA
TTCAGTTAAAAAAGGAGACCTTTTTTTAACAAGAACTAGTGAGAAACAAAATGAGTTAGGTATAAGTGCAGTA
GCTTTAAAAGACTATAAAAATGCGACTTTTAATGGATTTACCAAAAGATTAAGACCAAACAAATATTGTGAGA
ATAAATTGTTACCAGTATTTGCTGCATTTTATTTTAGAAGTAATAATTTTAGAAATCAAGTAAATTCTATGTC
TATCATGTCTACAAGAGCAAGTTTAAATAACGAAATGATTTCAAAACTTAAAATTACTATACCTAGTCTTCAA
AATCAGATGAAAATTAGTCATATACTATTAGCACTTTTAAAAAAAGAAAAAATCAATCAAAAAATCATAGCAA
ATCTTGAAGAACTTTCTCAAACACTATTCAAACGTTGGTTTGTTGATTTTGAATTTCCTGATGAAAATGGAAA
TCCATACAAATCAAGCGGTGGAGAGATGGTTGATAGTGAATTGGGTGAAATACCTAGGAGTTGGAAAGTTGAT
GAATTAGGAAATTATATAAAAATAAAAAGTGGTAAAAGACCTAAAAATAAAGTTGATAAAGAAGATATAGAAA
ATGTGGTGCCAATAATTGGTGCTAGTAAAATAGTGGGTTATACGAATGATTATTTATATAATGAAAAAATTAT
TATAATTGGTAGGGTAGGAACTCATGGTGTAATCCAAAGGTTTTCTACAAGAACATGGCCATCAGATAATACT
TTTGTAATAACAAGTGATTTTGAAAGCATTATATATCAAGTACTTAAAAGTATAGACTATATATCATTGAATA
GAGGTTCTACACAACCTCTATTATCTCAAAAAGATATTAAGAATACTAAAGTAGTAATGCCCACCAATGCAAC
TTTGCTTTCAAAATATCAAAAGAAAAACAATCATATTTTAAAAATGATGGATCAAAAAATCATAGAAAACAAG

AAATTAACCCAACTCCGTGACACATTATTACCAAAATTAATGTCAGGTGAAATTGAGATACCTGATGACATCG
AGGTGAATGAAGATGATCTTTCAATTTAGTGAAGATGATTTAGAACAGGTTGCACTGAAGTGGTTTGAGGGTC
TTGGCTATTCTGTTAAGAATGGTCGAGATAGTAGTGACAATGGGATTATGAATGAACGGGAGAGCGACAAAGA
TGTTGTGCTTGATGATCGTTTGGAAGCAGCTTTGAGACGCATTAATCCTGAACTAAACAATAGTGCAATTGAA
CAGGCAATTCATGAAATTTCTATTGAGAAGTCTCCTAGTTTATTAGAGAATAATCGTTCTTTCCATGAAATGA
TTACTAATGGTATCGAGGTTGAACACTATAATGATGATGGTGAGACGATTAATGATTTGGTCTATGTGCTTGA
CTTTGATAACCCAGGAAATAATGATTTCTTAGCGGTTAATCAATTAACTGTTGTAAATGGAGATTATAAAAAA
AGACCTGATATTGTGTTATTCATAAATGGATTACCAGTAGTGGTAATTGAACTGAAAAATTCTACAAATGAAT
CTGTTGGTATTGAAGACGGGTATCATCAGTTAGAAACTTATAAAATGAGAATTCCACAATTATTTAATCATAA
TGCAGTATTAGTAACGAGTGATGGTGTCAATACAAAAGCTGGTTCTTTAACTGCTGATTATGACCGTTTTATG
ACTTGGCGTACTAAAGATGGAAAAACTGAAGATTCATCAACGTTTCGTAGTTTAGATATATTGATTCACGGTA
TGTTAAATAAAGAGGTACTATTAGATTTAATTCGTCATTTTGTTTTGTTCCAAGATGATGGTAAAGGAAATAT
AGTTAAAATATTAGCTGCATATCATCAATATTATGCGGTTAATAAAGCTGTTGATCGTGCTATGGAAGCAACA
TCTGAACATGGTGATGGTAAGGGCGGCGTTATTTGGCACACACAAGGTTCTGGTAAGAGTTTGACAATGGTCT
TCTTCTCAGGAAAGTTAATTCAAAAATTAAATAATCCAACATTAGTGGTCGTAACAGACCGTAATGACTTAGA
CAATCAATTATATGGTACTTTTGTTAAGTCCAAAGGACGCTCAGGTAAAGGATTACTAAGACAAACACCTAAA
CAAGCAGAATCACGAAAAGAATTGAAAGAACTATTATCTGTAGAATCAGGTGGTATTGTATTTACAACTATGC
AGAAATTCGAACCTGAAGAAGGTCAAGTGACAATGGATGCTTTAACAGAACGTAAAAATGTAGTAGTTATGGC
AGACGAAGCACACCGCACACAATATGGTTTTAAAGCAACTTACAATGAAAAAGGTGAAGGTATAAAATATGGT
TATGCAAAATATTTAAGAGACGCTTTACCTAACGCTTCGTTCGTTGGTTTTACTGGAACACCTGTATCTTCGA
CAGATAAGAATACGCAAATGGTATTTGGTAATTATATTGATGTATATGATATGACACAGTCTGTAGAAGACGG
TAGTACGGTTAAAATATACTATGAAAGTCGTATTATTCCATTAAATTTACCAACTGATTTAGATATTGATGAT
GCATATGATGTCATTACTTTTGGCCAAGAAGAAGATACTAAATCTCGATTGAAATCTAAATGGTCACGTATAG
AAGCTCTATCTGGTGCAGAAAAACGAGTACATGCTTTAGCGACTGAAATTATTAATCACTTTGAAACAAGACA
GAAGGCAATGAACGGTAAAGGTATGATTGTCACAATGAGTCGTCGTATTGCAGTTGATTTATATGATGAAATT
ATAAGACTTAAACCTGAATGGCATTCTGATGATGACGACAAAGGTGTAATTAAAGTCGTTATGACAGGTTCAT
CAAGTGATCCGCAAAACTTCCAAAAACATATAGGTCCGAAAAAGAGAAGAAACCTTTTAGAGAATCGTATGAA
AGATATTAATGATGAACTGCAACTCGTAATTGTGCGAGACATGTGGTTAACAGGATTTGATGTACCTTCAATG
CATACAATGTACATAGATAAACCGATGAAAGGTCATAATTTAATGCAAGCTATTGCACGTGTTAATAGAGTAT
TTAAAGATAAACCAGGTGGCTTAATTGTTGACTATGTAGGTATTGCTGAAAGCCTGAAGGAAGCACTAAAAGA
GTATACGGACTCTGATAGAGAACAAACAGGTATAGATACAGATAAAGCGCTAGAACTAATGTTAGTGAAATAC
GATATCATTCAAGATATGCTATATAACCATGACTATTCTGACTTTGAGTCAGAAGATCAATTAAATCGCTATA
ATGCTATCTCAAATACGATGGATTATATCATTGGATTAGGTGAGAAAGAAAGAGAGCGATTTGTGACAACAGT
TACAGAACTTTCTAAGGCTTTTGCATTATGTGCAACAGAGGAAGCTGCTCAAGAACTAAATAGTGAAATTGCG
TTTTTAAAAGCAGTAAAATCAGGACTTGTTAAATTATTAGCTCCACCTAATGAAGGAAGCAAGCTTAAAAAGA
CACCTGCTGAAATAGAAGCAGAAATAAATCAATTGATTTCAAAGTCAGTTGTAACTGAAGAAGTTGTAGATAT
TTATCAAACACTCGGAATTGAAAATCCAGATATCTCAATATTGTCGGATGATTTCTTAAAAGATGTAGAAGGA
CTACAGCAAAAAAATGTTGCAGTTGAATTGTTGAACAAATTACTCAAAGGACAAGTTAAGTCATTAATGAAAA
CTAATACTACTGTATCTAAAAGGTTTTCTGAGATGCTAAGTAAATCAATACAAAAATATAATAACCGTTCTAT
CGAAGCATCAAAAGTCATAGAAGAATTAATTCAAATGGCGAAAGAAATAAATCAAGAAAAAGAAAGAGGAAAA
GAATTAGGTTTAAGTACTGAAGAAATTGCATTTTATGACGCATTAGCTTCACATGAAACAGCAAAAGAAGCGA
TGGATGATAAAGAATTAAGGGCTATTGCACATGAATTAACAAAAACGGTTAAGAATAATATGAGCGTAGACTG
GTCAAAACGTGAGAGCGCAAAGGCTAAAATGAGAGTCCAAGTAAGACGACTATTAAAGAAATATGGTTATCCA
CCAGACATACAAAAAATGGCAGTAGAACAAGTAGTGGAACAAGCAGAATTAATGGCAAGTCAACAATATAAAT
AAATACTGTTTATATTGTTGACCTGTTAGATACTTTTTTAAAATTAAACATTTAAGACAGAGTATATAAAAAA
GTGTCAATATACCGGTAGACTATAGAGAAAAGGAGATTGATATGAAATTCGACTTAATTATAAGTAATATTAT
GATAGGGATATTAGTGATTTGTTCTGCATTTGCCTTAATGTTTGTCTTTTATATTCAAAGAAAGTTAACGCCG
GAACAATACTTTTCTTTTTCAGAATTCTTTTTAATAGGAAGAGGTTTAACTATCAAGAAAATTTTGAATAGAA
TGATATTGATTGCTAGTTTTAACTGTATTTTGTTTTTAATACTTAAGAATTTTTTTGAAATTGAATTAGTATG
TAGTGTGTGTATACTAAGTACATTGTTGGGTTCGATTTTAATTATTTATCCGGCGTTTAAAATTAGGCATGAT
TTACCTAACAATGAGATGAAAAGTTTGTTGAACTATATGTATGTATCTTTTGTTTTTTTAGCGCTTATTATTT
CTACTCTTACTGTATTTACTTTAGTAGTATTATTTGATGATATTAGTATAGTAACACTTTGGCAAGAGCATAA
AAGTGGAATAATTTTATATCTTCTATTGGTGATACCTAGTCTATTTTTTAATCCTACAAATTATGAAAAGAAA
ATGGAATATGCCATTGAAAAACATCGTGAGAGTAATAGAGAGATAAATGAGACGATAATAAAAAATAATAATG
AAACAATTATACCAGACGAGTCGAAAAGTTCTTCACCATTCAAAATTAAAAAAGTTTTTAGATTGTTGAATAG
GATTATGAAAGATGATTGAATATTATTTGAAGAAAATTATACATTTATTTGAAAATAATAAATGCGAAATTTT
ACATTTAAAAATGAATTTTAATGATAACTTTGATATGTTAAGTTATATTTATTGTATTGAGAACATGCATAGA
GGTTCAAATATTATAAAGATAGCGGAGTATATATTAGTAAAGTATTTTCAAAAATATTGTATAAAAAAAGACT

TTTCTATAGGTCCATTTCAAGTTAAAAAAAGTTTTTGTGTGTCTAATAATTTATATTTAGAGTCACTAGATAA
ATTATTGGAGTTGCACAGTAGTGCTCATGTAATTAATGAATTTATTGAAAATAAAAAATATTATTTGAATAAT
AATGAGATTTTATCCTTATATCATTCAGGGAAGGTTATGGATACTAGCTTTTCAACTTTAATGTATATAGGAT
TATTTAAACATTTTTCTAGTTATTTAAGAAAACACGAATAAAAAACAACTGATGATAACAAGTTAACAAATTA
TTAAATTTAAATCACTCTAGTTCATACCAAAAAGTCTTTAACACAAACAAAAAAGGAGGAAACTTAAATTCCT
CCTCAACCTTTATTACACCTATAATTCTATTTAACGTCTTCGTCCATTTGGGCTTCAAATTCATCTAGTAGT
GCTCGTGCTTCTGCTTAATTTGAATCATACAAGGTGTTTTTTGTTATTCCTAAAATTTGGCATAGCTAAATAA
ATCTGTATTTTCTATTTGTCTTTTTTGGCATTTATGATAACCCAAGTATCTTTTGGAACGGTAACTGTAGTTG
TTGATTGAATTAATTCGTTTTCACTTTCATCGAAGCTTAGTAATGATTTATATAAATCTGATTTCCAAACCCA
AACTTCTTTTTGGGTTTGCTGTGTTGCCTTATCAGTATTCTTTACTTCATATTTAGCTTGATGCTTAACGGAC
TCAGATATATTATTTTTATCCATTTTAGGCTTGAAATGAGTTGTAGGTTTTTTATCCTTAGGATGATCTCTAT
AGACCATGACATAGTTATTTGTATTTTTTCCTATTTCATTGATTATTAATATATTAGCTGGATTATTTTTAGG
ACCACCAGTAAAAATTTCTTTTTTATTTGATGTGATTACTTTCTTTTCCATACCCCAATGGTTAGTCATATTA
GCTGTAATACCAATGATTGTTAGTGTCAACGCAAGAGTAAATAATGTTCCTAATATATAGCGTGTGATTTTAT
GAGGAATTAATAACCAACTAACAAAAGTACATAAAGTTAAAACAATCGTTGTTATCATATTATTTATCCTCAC
TTTCAATAATATTCATTTTTAAAAATGGAACTAAAATGATACCTATAATTGCAAAGAAAATGCCAATTATAAA
TGCAGTATTAAAGCCATCGATTGATGCTTGTTGAGCAAGGTGTGCATATTTGAGTGGATTGCTTTCTTTTAAA
CTATCTGCAGGCATATGGTTGTTAATAATATTTTGAGTAACAGATGTCAGTAATGCTACTGCTATAGATGATG
CTACTTGTCTAGCAGTATTATTTACTGCTGTAGCATGTGTGCCTTTTTCAACTGGTAAAGCATCCATCGCATT
TGTTGTGAGTGGCATCATTAAAATTGCCACACCAAACATTCTTACTGCATATAGAACAAGAATAAGTGTTGGT
GATGTGTTAACTGTTAAAAATCCAAATGGAACTGAAGCTAAAGTTAAAATAGTGAAACCAACGAACGCTAATC
TTTTAATGCCAATTTTATTGTACAGTATTCCTGAAATTGGAGAGATAAATCCCAACATCAGTGCTCCAGCTAA
AAGTGTTAAACCAGATTCAAATGGTGAGAAACCGTGTATATTCTGTAAGTAAGTGGGTAACATCATTTCAACA
CCATACATTGCCATAGTTACAACGATTAAACCAATTGTCGCTATCATGAAACCTTTAGATTTAAAAACGCGTA
AATCCAAGAAAGGATCTTTAGATTTTAATTGTTTGATAACGAACAAGGTAAGAACAATGATACTTAAAAAAAT
TGGTAAAATAACGTAATTTATATTTCCCCATCCTTCGCTTGCAACGTTTGAAAATCCCCATAAAAATAATCCG
AAACCGATACAAGAAAGTATTAGAGAATAGAGATCTAATTTAAGTTTACGATTAGGTATAACATCTTTCATGA
AGAAGAAGCTTAATATAAATGCTATCGCTATAATAATGATTGGAAGAACAAAAATATTACGCCATGAGTCTGA
AATTGTAATACCTAGTACCTCGTGGTTTTTGTTTAATATCCAACCAGCAAAAGTTGGACCCATTGCAGGTGCG
AGACCTACTGCTAAACCACTCAGACCCATTGCAGTACCACGTTGTTTTATTGGAAACATATAAATAATGATGA
TTTGTAACAATGGCATCATTAAACCAACAGCAATTGCTGTAACAATACGTCCAGCTAGAAATATTAACCAGCC
ATCTTGATTTGATGGTGCGATTGCAGTAAGAATTAATCCGAATAATAGGGCGCCGTAAGAGTATACATGTAAC
AATCTAGTAGATATGCGCGTCGCTAAAAATGCCGAAAGTGGTACCATAATACCATTTGCGAGTAAAAACCAAG
TTGTTGCTTGTTGAGCAGTACTAAAATCGATATCAAAATCGTTCATCAAAGTAGGTAGAGCGGTACCTAATGA
AGTCTGCATCAATGCACCAGAGAATGTAGCTAATAATATAATGCTCATTATAATTTTGGGTTTATATTGATTG
CCATGAATATCTAAATTCATTGCATTACTTTTTTGAGTAGACATAAAGCAAGCCTCCTATAGGTTTTTAAAAT
TAATACTTTGAAAATATAACACCTACTTTATAAACAGAACAGAACAAAATCATATACTTTGTACAATTTGTGA
CACTTTGTTTTATTTTGTGTTATATTCATTTATAGGAGTGATCATATGGGGTATAAAAGGAAAACGGTCTTAA
CTCAGCGTAATATAAAAAATGCTTTAGTGAGTTTATTAGAGAACAATAAGTTTGATTTCATTACAATCAATCA
AATAGTAGAAGAAGCGGAAATAACAAGAAGTACTTTTTACAGGTATTATGATGATAAATATGAATTAATATCA
GAAATAGAAGAAGAAATTTTAGAACATATTCATAAAGAAAGGCAAAAGATAGACGAGAACGCTTCAGAGAAGA
ATTTATTAGATATTGAGATGTTTAAATATCTGTTTGAAACATTAGAGCCATATTCGAACACGATATATCGATT
GTTAGGATATAATGGTGATTCCAGTTTTGAGATGAAACTCAAGAATGAAATATCAAGAAGATTTATAGATATA
GAACAACGTAATCATATTTCTCAAGTACGAGCAGACTTAGTAAAGGAATATGTATTTGTTATTATGATAAAAA
CATTACAATATTGGTCAGCTCATAAAGACGAAGTAGATATTGATGAAATAGCTTTAACAATTAGGGATATCCA
GTTAAAAGGATTAAGAAAAGCAATAGGGTTATAATTTTAATGAATAACAAAAAAGGAGGAACATTATATTCCT
CCTTGACCTTCATTACATCCATTACATCTATAATTCAATTTTAACGTCTTCGTCCATTTGGGCTTCAAATTCA
TCTAGAAGTGCTCGTGCTTCTGCAATTGATTGTGTGTTCATCAATTGATGGCGAAGTTCGCTAGCGCCTCTTA
TGCCACGCACATAGATTTTAAAGAATCTACGCAAGCTCTTGAATTGTCGTATTTCATCTTTCTCATATTTGTT
AAACAATGATAAATGTAATCTCAACAGATCTAATAGTTCTTTGCTTGTGTGTTCGCGTGGTTCTTTTTCAAAA
GCGAATGGATTATGGAAAATGCCTCTACCAATCATGACGCCATCAATGCCATATTTTTCTGCAAGTTCAAGTC
CTGTTTTTCTATCGGGAATATCACCGTTAATTGTTAACAATGTATTTGGTGCAATTTCGTCACGTAAATTTCT
AATAGCTTCGATTAATTCCCAATGTGCATCTACTTTACTCATTTCTTTACGTGTACGAAGATGAATAGATAAA
TTGGCAATATCTTGTTCGAAGACGTGCTTCAACCAATCTTTCCATTCATCGATTTCATAGTAGCCAAGGCGTG
TTTTAACACTTACCGGAAGTCCACCTGCTTTAGTTGCTTGAATAATTTCGGCAGCGACGTCAGGTCTTAAGAT
TAAGCCGGAACCCTTACCCTTTTTAGCAACATTTGCTACAGGACATCCCATATTTAAGTCTATTCCTTTAAAG
CCCATTTTAGCTAATTGAATACTCGTTTCACGGAACTGTTCTGGCTTATCTCCCCATATATGAGCGACCATCG
GCTGTTCATCTTCGCTAAAAGTTAAGCGTCCACGCACACTATGTATGCCTTCAGGGTGGCAAAAGCTCTCGGT

ATTTGTAAATTCAGTGAAAAACACATCCGGTCTAGCTGCTTCACTTACAACGTGTCGAAAGACGATATCTGTA
ACGTCTTCCATTGGCGCCAAAATAAAAAATGGACGTGGTAATTCACTCCAAAAATTTTCTTTCATAATATATT
TATACCCTCTTTATAATTAGTATCTCGATTTTTTATGCATGATGATATTACCACAAAAGACGAACTTATACAA
AAGGAATTTTAGTTGATACAACCATTTGAAAGGGAAGTCTAAGAGTAGTCTAAAATGAATGTTGTGGTAAGTT
GATCAGTATACAGATCAAGGATTATCGTATTAGATTGTTCATTATTAATGATACACTACTTATGAAAATGATT
CAGAATTTTCTTTGGCTACTTTTACAGTAAAGCGATTTTTTAGTTATCTTATAACAAAGACAAATTTATAAAG
GTGATATTATGGAAGATTTAAAGCATTCTTTAAAAAGTTTAGGTTGGTGGGATTTATTTTTTGCGATACCTAT
GTTTCTGCTATTCGCATACCTTCCAAACTATAATTTTATAACGATATTTCTTAACATTGTTATCATTATTTTC
TTTTCCATAGGTTTGATTTTAACTACGCATATAATTATAGATAAAATTAAGAGCAGCACGAAATGATTCATTA
ATACGAATGTGATTAAACATAAAACTGAAGGAGCGATTACAATGGCGACTAAGAAAGATGTACATGATTTATT
TTTAAATCATGTGAATTCAAACGCGGTTAAGACAAGAAAGATGATGGGAGAATATATTATTTATTATGATGGC
GTGGTTATAGGTGGTTTGTATGATAATAGATTATTGGTCAAGGCGACTAAAAGTGCTCAGCAGAAATTGCAAG
ATAATACATTAGTTTCCCCGTACCCAGGTGCTAAAGAAATGATATTAATTCCAGACTTTGACGAAGCAACAAA
TCTCACTGATTTATTTAAGACCATAAAAAATGATTTGAAAAAGTGAAGTGTGGGTGCAGAGAGAACTAAGCCC
ATCGATAAATGGTCGCTTGTTAAAGAAGAGTGACCGTCACTCTTCTTTATGTGCATATTTTATTTTGTCTGTT
TTGTTAACGATCAGCAGTGTAACAAATATGAGTAAGGATAAAATGAGTATAATATAGAAACCGAATTTATCAT
TAATTTCATTAATCCATCTTCCTAAAAATGGAGCAATGAAACTCTGCAGTAACAATGAAATTGACGTCCATAT
CGTAAATGAGCGACCGACATATTTATCTGAAACAGTGTTCATTATAGCTGTATTCATATAAATTCTGATTGAT
GAAATTGAGTAGCCTAGTATAAATGATCCTATGAATAAGTAAAATGCTGAGTTTATCCAAATAAATAGTGCTG
AATTTATGACTAATATGAAATATAACAAAAATATCAATACTTTAGTTGAGATTTTCTTCGAAAGAATAGCTGA
AATTAAACCTGCACATAATCCTCCAATGCCATATAACATATCTGAAAAACCAAATTGTACAGACGAAATTTTT
AAAACATTATATACATATCCTGGTAATGATATGTTAAAGATCATTGTAAACACCATTGGTATGATTGAAATAA
CTCCAAAAATAAATATCATCATGTTGTCTTTTAAAAATTTCCATCCTAATAAATATTCTTGCAATAAGCTATT
TGTTGATTCTTCCTCTGAATGAGTTGGTTTATCTACATGCAATCTAAATAACATAAAAATGCTGATTAGAAAC
ATCATTATAGTCATCGCTATAATTAGAGTGAATCCATTTATCTCATATAATATTCCTGATAATCCACCTGCAA
TAAACATACCTGTTTGCAAACTAATCTCTAATAGAGAATTTGCATCTGTATATTGATCTGGTTTTAAAATCTG
TTTAACTAAACTTCTAGATGTTGCCATATACGTAGTCCAACCTATCCCATTAACAATCGCAAATCCAATCACT
AAATAGGTCTCAAATCCTATCATTACGAGTGCTATGACAATGAGTAAATATAATATTACCTGAAGAAGATAGG
TGATTAAAATAATATTTCGTCTATTATATTTATCGGCTAGTCCACCTATTATAGGAGAAGCTAAGAAACCGGA
TAATACATTTAAAGCTAACATAATACCTAATAGTTGGGAGTCGTTAGTTTTGTCGATTAAATACCAATTAGCC
CCAACTGTACTAATTCCAACTCCGAATGCTGAAATTATTTCTGCTAAAAAGAACAATCTGAAGTTTTTAATTT
TCAACAAATCAGTCATAAATATCACCTTTTTTCAATGCAAGATTTATATTTGCGAGCTCTTCTATAAATTGAG
AGTCGTTTAAAATATCGAATGGTTTATTGCCTTCGCCGATCAAAAATCTATAGTTAGTAATATTCATAAACTC
AAATATTAATTTGAATTGGTTGACTAAAGGTTGTGCTTTGGTATGTGGATTGTCGCCACCTATGATTAAAATC
AAATACTTTTTTTGTGACATGATTTCTTTGAAGTTGTCTATTTGCGTATCTCGCAATGACTCGGTCCATCTAT
CAATGAAGAGTTTTAAAGACGCACTCATGGAGTACCAATAGAGTGGTGTTGAAAAAATAATAATATCTGATTC
TAAAACTTTGATTAAAATTTGTTCGTAATCATCATTATGAAAGTTGGCACCTTTGCTATGACGATTATCTGTA
ACCTTTTCAATGTTGCTTTGATATAAATTCACAAAGTTGACATTTAAATTCTTCAATAGATTCTCTACTGCGA
TAGCTGAATTGCCATCTTTTCTACTACTTCCAAATAAACAAGTAATCATAGTCATAACTCCTTTCGATTTACC
TTTATAATAATATTTAATATATTATTATTAAATCAGAATTCTTAGAATTCAGGATTCGATAAAAGTGAATCCT
AAAGGAGGTACTACTTTGAACTTAAACATTTTAAAATCATTTTTAGTAACTAGTGAAACAAAGAACCTTACTA
AAGCATCAGAGTTACTTAACTATTCACAGTCAACTGTATCTACACATATTGAAAAATTAGAAAGGCAATTAGA
TGTTAAATTATTTTATAGAAAAAAATATGGTATGGAACTAACGGAAGAAGGCTTAGCATACGTTAAATACGCT
AAAGTGATTTTAGATAGTAATAGCGAATACGAGAGAGAAATAAAAGGACTTTACAATAAGAAGGTAAATATAA
GTATTAACATGCAAGAAAGTCAGTATTTGTATCGCTACTATAATAAGATTAGTGAATGGTTAGCTGAACACCC
ATATGTAAACTTGAAGTTTAAATCCGCACATTCTAATTTCTATATTAAAGAAGAAATTGCTAATTTCAAATCG
GATATTAGCCTTATCACAGACGAAAAGATTATTAATAGTAACTTAACTGCTATTCCTATAACTGAAGAACGTT
TAATATTTGTTACTAACAAAGAGATGAAAGATTTCAAATTAAACGATATTCAAAACCATACATTACTTGTTAC
TGAAAAAGGGTGTAGTTACAGAGAACAGTTAGAAAAGATTCTCTATAAATATAGCTTTTCAACAAAACAAATG
ATTGAATTTCTAGGAATTGAGTCATTGAAAAAACACTTGAAAAATTCAGATGGGATTGCGCTATTGCCGGAGT
TTATTGTTGCAGATGAATTACAAAATAAAAGTTTGTTTCAAATAGATGTCGATGTCGATATTCCGAATTTAGA
AACAACTTTGATAATTAATCCTGAATCCAGTAAGCAAGTACTAGAATCTTTTGTAAAAGATGTCTTTTTATAA
ATATTGGTGAAAACGTGTAGTTATGGTGAAACTCAAAGATAATAATTTAAATGAGATGTTAATGAAAAAGTAA
TTCAATATAAAAACAGGTGATTTATATCTTTAATAAGGATTATTTCTAGTTGAAATTTCAATTGCGGGCATTC
ATTTTCAAAATAGTAAAGAATTTAATAGATGAAAACCTTTGAATCATACTAGTTAGTATGGATTGATTAATAT
TTTAACCTGCTAAATGAGTAGCTTATGTATTTTAAATAGCTGTCATCTAGCAGGTTCTTTATTATTAATAAGT
TCAGATAAATTATTTAGTCTGTTTTCTAATGTTGATAATAATAACTTTAAATCTGTACTTTCATTGTGAGAT
ATATAGTACATCATAAATTTTTATATTTGGATTGAGACTCTTTGGAAGATTTTCAATAAATGAATATTTCACA

AACTTCCACTTATCAGGATTATAAATTATAATTTCTGCAACTGCATTATCGAAGATTCTAAACAATTTTTTGA
TTTCTATTTCTTTTAGAGATAAAGTAGGGAAAATGTCGATATATTCTTCAAGCGCATAAAGACTATGTTTAAC
AGAATCGGTTATGTTCATTGAATATATCACACCAATATTTATATTTTGCCATCCAAAAGAAGAAATAACATTA
TCATAAAATCCATTGAATATAAATTCAGTCATCCCCTCTGAACTTCTCCATAAATATAATGGTGAATAAGTAT
TTGTAATGCAATTACTAGTTGTTTGACTTATTAAATAAGCTTTTATAATTAAATTTTTAAATCCATCAGTCTT
ATAACCATTTAATCGAACTCTATCTCTAATTATTTCCATATTGTAATCACTAGGCAACTTAACATGATATTGC
ATTGCATGCATTTAGCATACCCCCTTTTATAAAAAGGATAGCAATAATAAGTAAAATCTCATATTATCCAATT
GTGATATAGTTATCATAAAAAGTGATAGGTGATTAAATTGAACTTTAATGATTTGGAAATTTTTATAACTGTA
TGTGAAGAAGCATCTATCAATAAAGCTGCAATTAAACTTAGATATGCACAATCTAATATATCTCAAAGAATTA
GCAAGCTTGAAAATGAATTAGGTGTAGTTTTGCTTTTTAGAAATCAAAAAGGTGCTAAGGCAACTAAAGCAGG
CGAAGAATTTTTAGCGTATAGCAAAAAAGTATTAAGAGATACAGAGACTATAAAAAATAAAATGAAATATAAC
ACTATGTCTATTTTATGCTCAGAACTGTTATTTAATTATTTATCTGAGAGCGAAGAAATTATGATGTCGAATA
ACTCAATTAATTTTATTTCTAGTGGAAATATTAGAAAAGCTATAGAAAAAAATAATTATGATAAGGTTATTTC
ATTCATAAAAATTAATGACTCAAATTATAGACTTAGTAATGTTGATACTATGAAAGTAACGCTTTACAGTAAT
GGAAGTAATTATGATAAAGAGCCTTTACTAATAAATAAAGATGAGTTTTGTCCTTTAAGGAAAATAACTTTAG
ATATTAAGCTTGATTCACAACGGGTAATGGAATTTGATTCATTGGCAGCAATAATAAACTTAGTTGAACAAGG
AAAAGGAAAGGCTTTATTACCTATGACTTTTGAGAATAAAAGAGATATAGTACAGGACATTTCTAAGATATTT
GAAGTTAGCTACTATACTTATAATCATATTATGCATCATTAATTGAAAGTTGATTGAGTTTAAATTACAAGAA
CGATATTAAGATTCTAGTAGTTAGAATGAGCGATGTGATAGAAAAGAATTTTTGCTTTTTAATTGGTAAATTC
GTTATTTTAAAGTAGTGGTCTTTGTAGATAGGAAAAAGCGGATTCCCTTATCAGCCTTTTCCACTCATAAAAT
TTATTATTGTAACTTTTATGAGGTGAGGAGCTAAATATTGTTCGAACTAAATTTTACTTAGACCTTTTCTCTT
TCAATTCTTTTTTAATTTGATCAGGAGATTTCCTATCCTTTGATGGTTTTAGTAATTTCTGTACCGTTTCGTC
TCTAAGCATATCACCACCAAACTGATGATTATATGCAGGTACCATGTAAGCAACAAAACCGTCGTCCTTATTA
TTATTGATATAGCCTTCAATGATTCTTTCATCAAGAGGACCTTTGTCCAAACTTGTAAAATGTACCGACTTAA
TCGTATTAGGCTCTTTTGTATTATGTTTAAGATACAATGTAATACGTTCTTGTTGCTGCTTGTAGTAAAATTC
TTTTTGTTTCATAAGCTCGTTATACTTCATTATAAGAATGACGCCTACAATAATGAAGGCTATAGTGAATATA
GTACCGATAATAATTAAAATGTATTTTTTTGACATGATATCAACTCCGATATTAGTAAGGGCATAGGAGCAAT
TGAAATTTCCAAACAAGGTACACGAGTGCTTCTGAATTTATCGGCACGCTTTTACATAGATCAAAAGAACGCG
CACTAAAATAATCATTTTATCTATGTAACTTACATCTTTGATACTACAGAAGTTCGTGCATTTGTTCAATCGT
CTCATAACCATTAAATAGCGTTAATTATGAATGAAGACATCTATAACATTTATTTATCTTATAACATAGTACA
ATTACAAAGCATCACAACGGCGACTGTACTCACCTTAAATCATAACGACAACCGCTAAAAAATGATTTGAAAA
ACTAATCATTAATTGCCATACTCAACTATTTTGAAAAGTATCAATTTCACATTATCAAATTATTGAGTATCTC
AATTTATTGTTATACACAAACAGTATTTTTGTTATCATAGAGTTAATAAATATAATAAATTGAATAAGTAACT
TCACAAAAATTAGAATGATTCAATTCAATCACAATTATTTGAAAATAGAGGTGAGCAGGTGAACGATATGTTA
ATTAGTCTTGTAATCCCAGTTTTTGCTTTGTTAGTTATTGGTGGTATTATTTGGATGATTATAGAAGGTATAG
TACATATTTCAAAAAAGAATAAAGCAATTGATAACTTTTTTAACCAAGTTAATAAAGTAAGTGAGACATATAA
ATTCGCTACTACTTTTTTATTTCTAATCTTGGCTACGGCTGGTATTTCTCAATTTTATCTATATTATATAGTA
TCAGCGTTTCTTTTTTGGCTTGTCATACTTACCTTTGGTATTGCAGGTATCATTTTTTTAATGCCATATGGAT
TATGTTTTCTACCGTTTTATAAGCAAAAAAAGAAAAAACAGACATTTAAAAAATACATGGTTTACACTACGAT
TGGTTTGTCAATTTGTCTAGGCTTATCTCTAGTTTTGGTTCACACTACGAAAATTTATATGGACGAAGGTGGC
GTAAGATACTATTACGGTAGTTTTGTAATGAAACAAGCGGGCGGTTATGCTTATTTAGCTTTAGCAGTACTTT
CAACGTTGTTAATTGTTGCGAAAAAAGCTACAAATAAAAATAAAGAAATCGAAACCGTCGACAATACAAATAT
AACGGAAAGATAATTAAGGGAGTGCTCATTCAGGAGTGCTCTTTTTTGATGTCCAAATTTAGTTGCAAATGAA
GGCATAGAAGAAATTTTTATAGTCATTTATTGGATAATGAATTATGGTCATATCGATTAATTTTATTAGTGAG
GATTTTACAAACATTTGTTGAATAAATATGGTAATGATAATATATGAATATTTTTGAAAAAACACTAATATGA
TTAAATTATTTGTTAAAATGGCATTAAGTAAAAAACAAACAGGGAACAAGGTGGGATTTCATGAGTCAATTGC
TAAATGATACGTTATCGGCTTGGTTGTTAATTGAATCTTTAAGTCCAGGAGAAGTAAATTTTACAGCGGAAGA
TATACTCTCAGCTGAACATTTTAAAAATGGTGCAAAGCAAGCACAACTTCAAAGTTTTGATGAATATTTTGAA
ATATGGAATGCTGAACGCTTTATTATATCAGAAGAAAAATCAGAGACTGGGGAACTTATATTTAAATTTTATA
GACATTGCTTCCGCTATAATGAAATTAATTTGAAAATTCAGGATATTTTTGATGATTATTCTGAGATTCATAA
TCCAAATGGGACACACTGTTATGGTTACACATTTAACACAGATAAACACGGCAAAGTGATAGTTGATTCTATA
CATATTCCGATGATTATGAGTGCATTAAAAGAAATTGAAAAGAACAAAAATGCCAATATAGAAGAAAAATTTA
ATGATTCTGTTGAAAAATTTGTTCAAAAAGTAAAAGAAATTTTAGCAGATGAACCAATTAATGAATTTAAATT
GAAGAAGATGGACAAAGCTTATGATGAGTACTTTTCTGTATTAAATTCAAAGAAAGATGGATTATTTGGACAT
TATGTAGCAATAGAATATGTGAAAGATAGTGATTTACCACAGCCGGAATTTAACAGCTTCTTCATAAGTGATA
TTGAGAAAGCAAGAAAATCTCCCAACCAAACTTTAATTGATTACATTGAAGGTGTAGAAGAAAGTCAGCGCAT
AGAAGTAGATGAAAATAAAGAAATGTTTGACAAATTTTTACATCCTTCACGTTTGCCTGATGGACGATGGCCA
TCACAGACTGAGTTTAGATTGTCTTTAATGCAACAACTTGCTGTAAACCAAATTACGAGTGGTAATGAAAGAA

TAAGTTCAGTTAATGGGCCACCAGGTACAGGTAAGACTACTTTATTAAAAGATATATTTGCTCATCTAGTAGT
TGAAAGAGGTAAAGAGTTAGCTAAACTAAATAATCCTAAAGATGCATTTGTCAAAACAAAAATTCATGAAACG
GATGATAAATACGTATACTTACTAAAGGAATCTATTGCCAAATATAAGATGGTAGTCGCATCTAGTAATAATG
GAGCTGTTGAAAATATATCTAAAGATTTACCGAAAATTGAAGAAATTATAAGAAATCCCGAAAAATGTAAATT
CCCTAAATATGAACAGAATTATGCAAATTTAGCACATGAATTAAAAGATTTTGCTGAAATAGCTGAAGATTTG
ATTGGTGAAAGTGCCTGGGGCTTATTTTCTGGAGTTTTTGGTAAAAGTACTAATATTAACCAAGTATTGAGTC
ATATGTTAAAACAAGATGCGAATGATATTGGCTTTGCTAAATTACTACAAAATGAGAATAATCGTATGAGTTA
TAACGAGTTAATGAGTGAATGGCAATCACATCAATGTGCATTTTTAGAAGAGTTGAGGCATGTTGAAATGTTA
AAAGAAGAATCTATTAGAGCATATGATGTTTATAAAAATTGTGAGTCTTTCTCTAAGATTGAACAGGTTATTA
ATAGTGAAAAAACAAGTATTGAAGAACAGATATATCATTTAGATAATGAAACGTTACGAGACAATAAAGAAAT
TGAAGATTTGGGTGATCGAATTAATTATAATACAAAGCAACTCGAAACTTTAAATGAGTTAATTAAATCCATC
AAAGAAAGCAACAAAGGTTTTATTAACAAACTGAAAGCGATGTTTAATTCAGAAGAAGATGAAAGCTATAAAG
ATCATAATAAAGAGAAGCAACAATTATTAACACAACAGTTAGAGTTAGAGAAATGTAAAAAAAACAAACATGA
AGACCTTGTTAGCAAACTAAAAGAAAAAGAGAAATTAATTAAACAATTAACTAAAGTACAGTTGCAATTAGAC
GAGTTAAATTCACAATTACAAGAGTTAGAAGCATATCGTATTGAGTCAAAAATTACAATTCCAGAAAAAGATT
TTTGGAGTGACAACAATTATGATGAGCGCCAAGTTACTAATCTGTGGACGAGTGACGAACTTCAATACAGACG
TGCCATGCTTTTTTTAAGAGCAATGATATTGCATAAATTATTATTGATTGCTAATAATACAACTATTTATTAT
GCGATTAATGATTTTAAAGATAGAAGGAAATTAATTGATGCAAATCCAGATAAAGTACACAACGCATGGAATG
TGATGCATTTAATATTTCCAGTAGTTAGTACGACGTTTGCAAGCTTTAAATCTATGTATGGGGGCATACCAAA
AGATTTCATAGACTACTTATTTATTGATGAAGCAGGACAAGCAATACCTCAAGCAGCTGTGGGAGCATTATAT
CGTTCAAAAAAAGTTGTAGCTGTAGGTGATCCGATTCAAATAGAACCGGTTGTGACTTTAGAAAGTCATTTAA
TTGATAACATTCGTAAAAATTATCATGTTCCGGAATATCTAGTTTCTAAAGAAGCTTCTGTGCAGTCTGTTGC
AGACAACGCCAATCAATATGGCTTTTGGAAATCTGATGCTACTGATAGTAATCAAAAAACATGGATAGGCATA
CCTTTATGGGTGCACAGACGATGTTTAAAACCTATGTTCACGATAGCTAACCAAATCGCTTATAATAATAAAA
TGGTGTTGCCAAGTAATATTACAAAAGTAGGTAAAACAGGTTGGTATGACGTTAAAGGAAACGCAGTTCAAAA
ACAATTTGTGAAAGAGCATGGTGAAAAAGTAGTGGGATTATTAGCTGATGATTGGATTGAAGCAATTAAGGAA
GGTAAAAATGAACCGAGCTCATTTGTAATATCGCCTTTTTCAGCAGTACAGCAACAGATTAAACGTATGTTAA
AGCAACAACTACCGACTAGAATTGATATTGAACGTACAAAAATTAATCAATGGGTCGATAAATCCATTGGTAC
TGTTCATACTTTTCAAGGTAAAGAGGCTCAGAAGGTGTATTTTGTAATAGGTACTGATAATACCCAAGATGGT
GCTGTGAACTGGTCATGCGAAAAACCAAACTTGTTAAACGTTGCAGTGACAAGAGCTAAGAAAGAGTTTTATG
TAATTGGCGACATGCAAAGAATACAGATGAAACCATTTTATGAGACGATTTTTAAAGAAAGAAATGTAAAATG
AAATTGAAATAACAGTATTACTTGATACTTAATTATCGAGCAATTTATACATTTTAAAAGGAGCAAAATTGAA
TAATGAAGAAATTTAACAACTGGATATTAAATGCAATAAGTGGATCTCAAACAGACAAGAATGAAACAACTGA
AGAATTAAAAGGGGCAAAATTTATCATTTTATATGCATATTCAATGCTCGTTTTGCTTGCGTTAGTAATTTCT
AACATATTCATTCACATTTTGGAGCCTAAACTATCAATTACCACTCAAATCATCATCGTTTTGATTTTAATTG
AAACACTAATTGGACTGCGTTTCTTGAAAGCGTACGATGTTAAGCGTGGCAAAGATAAAGAAAATAAGAAAAA
TAGTAAGGATTTCGTTAAACTAAAATCAATTTTAGTAGCAATTTTATTTACATCATTGGCGCTGACAGCAGGC
ACTGTAGCTGATATATACGGTTTCACTGACTTAGGAAATACTAGAAGTGATTTAATCGTTTGGAGCATAGGTG
GTATTATATTTGGCCTCGTATGTTACACAATGGAAGATAAAAAATAACGATAAGGAGCTGGCGATTATAAAGC
TAGCTCCTTTTTTAACTTATGTAAATGGCGGTTATAAAATGTAGGAAAATGGCGAAATGAAAATGCTGTTTTT
CCTACATTTTTTCTTTTTTAAATTATATAATCATGTCTTAACTCAATCTTCGATTGGAGGTTATTTGATTGAG
ACATGACATTTATGAAGGAGT
>DSM104437_scaffold_11
TTTTTTCAATTCTAGTCAGGGGCCCCAACAAAGAGAAATTGGATTCCCAATTTCTACAGACAATGCAAGTTGG
GGTGTGGGCCCCAACACAGAGAATTTCGAAAAGAAATTCTACAGGCAATGCGAGTTGGGGTGGGACGACGAAA
TATATTTTGCGAAAATATCATTTCTGTCCCACTCCCTATGCAGCTATCCACTTCATATGTATGGCGCATACTT
GATAAATGAATATCATCAAGATTTTAAAAGTATCGCTAATTTTAAAGTCTTAAAACGCATGATTAATGTCATG
ATGTGTTATTGTGTTTTGTTTTTAGAGCCACGTATTTCCTAGGAAATATAGAGGAACATTCAACGTAAAATTC
TCGAAAATTAATTTTAATCATTCTATAAGTGAATGGTTATTTTCAGAAGCGATAACATTTTTACTTTACATGA
GTATTTAACTTTCTATTTCAAATTACCTAGAATTATAGCAGCTATCACGATAATAATAATACCTGCCCAAATA
CCCGTCATCTGACGACGATCTTTTCTTTCTCCTAAAATGAAAATACCGCCTAAGGTTGAAACGATAACAAGTA
ACTGTGATAATGAGAAACTTGTAGCTACACCAACTTTTGGTTGAGAATAGAACATGAACAAGTTACCAATTCC
CCAAATCACACCTGGCAATAGATTAAGTGCTGTTGATTTAAGTGATGTTTTATGATTCATGGATAGGATAAAG
CCACCAATTGCCATACCGACAGATTGGAAGAACAATGCATCAGTTCCACCAACACCAAAGATGTCACCAAGTA
CAACGAAACCTACATATCCAACTGTAGATACAATTAAAATACCCATTGCTTTTTTAAATTCAGGATTATCTGA
TTGACGTTCATTTTTTAGCTTTAAGTGAAGTAAGTGCTACACCAGTAACTAATAATATCATGGCGATTAAACCA
AAGATAATTTGAGTCATTGAAGACCATTCACCTAAAAAGATAACGCTGAATAATGTTGTACCAACTAATTGCA
TACCTGTAGAAACTGGCATAGTATTTGATACACCGATTAAACTAATAGATTTAAGCTGATTCGCTTGTCCAAA

CGCCCATAATGCACCAGAAATAAGACCGACAATAATTACAGTAGGATTATTGAATTTTGCGAAACCAGTAATA
AGTAAACCTAATCCAACAATGAGTGCGCCTAGCGTCGTACCACGAATTTGGTTGTAAGGTCCACCGCCGACGA
ACACATTAATAAGAACGACACTTCCCCAGAATAAAGCAGGTAAAAGTGCGATTAAGAAATCAAGAAATTGCAA
GACTACTCACTCTCCTATACTATTTATGCAAGTGCTCCCCTTAATTGACACTTGCATGTTATTGTAAAGCATT
CTGCCCTAATAGTAAAAAACAACTACTTATAATTTTCAATATTTACAAAAAGTAGAAAAACCCACAATACATG
CTAATGATGTACTGTTGGGTATTTGCTTGGTATTTTATTCAGGGATTGTTTGATCTTTTACGTTACGTGATTC
GAATTTGAATTTAATGTACTCATAGTTGATATGGAAGCCTAGACCATTAAATCGTTCGATGAGTTTATTATTT
TTATTACGAATTTTAAAATAAACTTTTCTAATATCAAAGTTATCAAAAGCATATTGCAATGCATACGCAAGCA
ACTCGAAAGCGATACCTTTCAAACGGTAATCTACATGTGAACTGAAGTATTTAATCTCGGCGATTGATTGTTG
TGAATCAATTTCAAGGTATAAATAACCTTTAAGTAAACCTTCGCTAACAAATAAAAACAAATGATGATGGTCG
TCTAAACTATCAATGATATCTTGTGGTGACTGTGCCTGATATTTAAATGTAGTTGTGTGTAATTTGCTGAAAG
CACGATAAAACCCTTTGTGATAAGGAATGATATTTGGTTGGTGCATATCTTCTTCTAATCTTGTACGGGCTTC
TATGTAATAGTCAGTGAAGTTATAACTTGCTTGAATAACTTTCATTAATGGTTTGTATTGTTGAATGCCTTCT
TCAAAAGAAAAGTTAAAGACGGCATCGTCAGGTTGGTTCGAAGTCATTGCTTTAAACAGCTTTTCAAACGTAT
CGACAGATAATACATAATTGTCAGCTACGAAAGGGCCTATGACTTTGAATTTATTTTTATCGTATGCAAAAGA
CAATATGATTGCTTTGATTGAATCGTTTTCTTTAATCGAAAATACACCAGGTGATTCAATCGCATCGGTTATT
AATACATCTATTTCATTGTACTGTTGAGGAAGTTTATATAAGTATGATGCTGATTCATAATTCGAATGTTCAA
TGAACGATTTTATTTGTGTAATATCATATAACTGAACTATGCTCATGTCATTACCTCCGTACTTTTTGTTACT
TTTATTATATAGTATTTCAACTGAAATGAAAGTTAATAGTGATATTAACATGTTACAATACATTTAACACATC
ATTTGCAATCATTGAATTTAAATCAAAGATTAGTGGAATAGATGAAGCACGTTCGAAATAAAAGAACGTATGA
GAAAGGATAATTTATGAAATCTTTAATATTAGCTGAAAAACCATCAGTCGCAAGAGATATTGCTGATGCTTTA
CAAATAAATCAGAAGCGTAATGGTTACTTTGAAAATAACCAATATATTGTCACGTGGGCGTTAGGTCATCTAG
TGACAAATGCGACACCTGAACAATACGATAAAAATTTAAAGGAATGGCGATTAGAAGACCTTCCAATTATACC
TAAATATATGAAAACTGTTGTTATTGGTAAAACAAGCAAACAATTTAAAACAGTAAAAGCGTTAATTTTAGAT
AATAAAGTGAAAGATATTATTATTGCAACAGATGCTGGACGAGAAGGTGAACTAGTTGCAAGATTGATTTTGG
ATAAAGTTGGTAACAAAAAGCCAATCCGTCGATTATGGATTAGCTCAGTTACTAAAAAAGCTATTCAACAAGG
TTTTAAAAATTTAAAAGACGGTCGTCAATATAACGATTTGTATTATGCAGCGTTAGCGAGAAGTGAGGCGGAT
TGGATTGTTGGGATTAATGCAACGCGTGCACTAACAACAAAGTATGATGCACAGCTATCCCTGGGACGTGTTC
AGACACCAACGATTCAATTAGTAAATACACGACAACAAGAGATTAATCAGTTCAAACCACAACAATACTATAC
ATTATCATTAACGGTAAAAGGGTTTGATTTTCAGCTAGAATCAAATCAGCGATATACCAATAAAGAAACTTTA
GAACAGATGGTTAATAATTTGAAAAATGTCGATGGTAAGATTAAATCTGTTGCTACTAAACATAAGAAGTCGT
ATCCGCAATCACTGTACAATTTAACAGATTTACAACAAGATATGTATAGACGTTATAAAATTGGACCTAAAGA
AACATTGAATACACTTCAAAGTTTATATGAGAGACATAAAGTCGTAACCTATCCAAGAACAGATTCAAACTAT
TTAACAACTGATATGGTAGATACTATGAAAGAACGTATTCAGGCGACGATGGCAACAACATATAAAGACCAAG
CACGCCCATTAATGTCTAAAACATTTTCATCAAAAATGTCGATATTTAATAATCAAAAAGTATCTGATCACCA
TGCAATTATTCCTACAGAAGTGAGACCTGTCATGTCAGACTTAAGTAATAGAGAACTAAAGTTATACGATATG
ATTGTCGAGCGTTTTTTAGAAGCTTTAATGCCTCCGCACGAGTATGACGCGATAACTGTAACTTTAGAGGTTG
CAGGGCACACATTTGTTTTGAAAGAGAATGTAACAACTGTTTTAGGTTTTAAATCTATTAGACAAGGTGAATC
TATTACAGAGATGCAACAGCCTTTTTCAGAAGGCGATGAAGTGAAGATTTCAAAAACAAACATTAGAGAACAT
GAAACAACACCTCCAGAATATTTTAATGAAGGTTCGTTATTAAAAGCGATGGAGAACCCTCAGAACTTTATTC
AATTGAAGGATAAAAAATATGCGCAAACTTTAAAGCAAACAGGTGGTATCGGCACAGTTGCAACAAGGGCCGA
CATTATCGATAAATTATTTAATATGAATGCTATTGAATCAAGAGACGGTAAAATTAAAGTAACGTCAAAAGGT
AAACAAATATTAGAATTAGCACCAGAAGAATTAACGTCGCCACTTTTAACTGCACAATGGGAAGAAAAACTAC
TTTTAATTGAACGTGGTAAATATCAGGCGAAAACATTCATTAATGAAATGAAAGATTTTACGAAAGATGTTGT
AAATGGGATTAAAAATAGTGATCGTAAATATAAACACGATAATTTAACAACCACTGAATGCCCAACGTGTGGT
AAATTCATGATTAAAGTTAAAACTAAAAATGGTCAGATGCTTGTGTGCCAAGATCCATCTTGTAAGACGAAAA
AGAATGTACAGCGCAAAACAAATGCAAGATGTCCAAACTGTAAAAAGAAATTAACGTTGTTTGGTAAAGGGAA
AGAAGCGGTATATCGTTGTGTTTGTGGACATTCTGAAACGCAAGCACATATGGATCAGCGTATGAAGTCTAAA
TCCTCTGGTAAAGTATCTCGTAAAGAAATGAAAAAGTATATGAATAAAAATGAAGGTTTAGACAATAATCCGT
TTAAAGATGCATTAAAGAACTTGAATTTATAGATAAAATCGAACAAAGTTGAATCAGAAAAACGAAAAGTTCG
CTTTTGGTATTGTTTTTTATTAAGAATGATATTAAACTATTAAGGTATTTTAAAAAAAGGAGCATCCATTCGT
GAAAAACTATTTCCAGTTCGATAAATATGGAACAAACTTTAAAAGAGAAATCTTAGGCGGTATCACAACTTTC
TTATCTATGGCCTATATTTTAGCAGTTAACCCGCAAGTTTTAAGTTTAGCAGGTGTTAAAGGCGTATCAGAAG
ATATGAAAATGGACCAAGGTGCCATTTTTGTAGCGACTGCATTAGCAGCATTTGTAGGCTCGCTATTCATGGG
ACTAATAGCTAAATATCCAATTGCGTTAGCACCAGGTATGGGATTAAATGCATTCTTTGCATTTACTGTTGTG
TTAACCATGGGTATTCCTTGGCAAGTTGGTTTAACAGGCGTTTTATTCTCAGGTATATTCTTTGCGATATTAA
CCGTCACAGGGTTTAGGGAAGTTATTATTAATGCAATTCCTTATCAAATGAAGATGGCTGTTTCAGCAGGTAT
AGGATTATTCATTACATTTGTAGGTTTACAAAGTTCGGGTATTATAGTTAAAAATGAATCTACATTAGTAACA

TTGGGACATTTAACTGATCCACCAGTACTTTTAGCGATATTCGGTATCGTTATTACAGTTATTTTATATGCAA
TTAAATTGCCGGGTTCTATCTTTATCGGTATGATCATCACAGCAATCGTTGGTATGTTCACTGGATTGATTCA
AATGCCATCTGGTATTGTAGGTAAGATTCCAAGTATTGAACCAACGTTCGGTGCTGCATTTGAAGCGTTTAAA
GACCCAAGTCAATTATTAACGATACAGTTCTTAATTGTTATTTTAACGTTCTTATTTATTGATTTCTTTGATA
CAGCTGGTACATTAGTAGCCGTTGCTACTCAAGCAGGTATTATGAAAGATAATAAATTACCAAGAGCTGGTAG
AGCATTGTTCTCAGATTCTTTAGCTACGATTGTAGGATCAGTTTTTGGAACAACTACAACAACTTCTTATATC
GAATCAACTTCAGGTGTTGCTGTAGGTGCTAGAACAGGATTTGCTAGTATCGTAACAGGTTGTTGTTTCCTAT
TAGCGCTGTTCTTTAGTCCGTTAATCGCTGTTGTAACAAGTGCAGTTACGACACCAGCTTTAGTGGTGGTAGG
TGTATTAATGGCTGCAAACTTTGCTGAGATAAACTGGAAAAGTTTTGAAGTTGCAGTGCCAGCATTCATTACA
ATCATTATGATGCCTTTATCATACTCAATTGCAACTGGTATTGCGTGTGGATTTATTTTCTATCCAATTACAA
TGCTTATCTCTAAAAAACATAAAGAAGTACATCCGATAATGTATGTATTAATGGTACTATTCATTCTGTACTT
CATATTTGTTCATGGTTAAATCCATACACAAAAAACGACTGCCAATGATTGGTAGTCGTTTTTTCTTTGAAGT
AAGGATAAAATAAGAAGCGAAATAACATAAAGCAATATTATGAATGTCTTTAAATATCCGATATAAATATACT
TCTTTTAAAATTGTTGGACGTGTTTCTTCTATATTATATACCTAATTAAAAAGGTTTTAAGAATTATTCTCTA
TGTCCATCTTTGTATTGTGTGTAAATGACACGAGCATTTTTATTAGGTATATAGGTTGGCGCTGATAATACAA
CTAGGACAAATAGAATCATTAGGAAAAATAAAATCCAATGAATCAAAATTCCTAAGAAGGGGATAATAGCTAA
AAAACTACCTACTATACCAAATATTGGAATAATCGCTATAGGTCTAATCGCATTTTGTCTATTCACTAATAAT
ATAATAGTAAGAATTAGATAAAGTAATGCATTAAAGAGTAAAGGCTGCCATCCAAAACTTAAAATAAACGTTG
CACCTAAAAAAGGAATGCCATAGAAAAACTCACAAAGTATTAAAAAGATACCTATAAGTGCTAAAGATAATTT
TGGTCCATTTTTCATATTCATTCACCACCGTTCTTATGACTAATTATATAGAATAATTTTCAATGATAATAAT
AAAATGATTCAAGGTACGAATTTACAATAAAGAAGGAAATAAAAAATATCTCGAAAATAGTTGAACTGACTAA
GTATATAAAGTAAAATATAAAAGTATGTGTTAGACAGATAGAAAAATTATGTGAAAAGCCTTGATTTATCGAT
GTTTTTCGTGTAATATAACTAACGTTGGACTTTAAGAAAAGCGATGAAGCGAAAGGTTACTGACACACCCGGC
CGCTTTGCCATGGCGCTGTGTAAGATAGTTTTCGTGGAGAAGTCTATCACTAAATGTAGACGAATAAGGAGGG
AAAATTATGGCAAAACAAAAAATCAGAATCAGATTAAAAGCTTATGATCACCGCGTGATTGATCAATCAGCAG
AGAAGATTGTAGAAACAGCGAAACGTTCTGGTGCAGATGTTTCTGGACCAATTCCGTTACCAACTGAGAAATC
AGTTTACACAATCATCCGTGCCGTGCATAAGTATAAAGATTCACGTGAACAATTCGAACAACGTACACACAAA
CGTTTAATCGATATTGTAAACCCAACACCAAAAACAGTTGACGCTTTAATGGGCTTAAACTTACCATCTGGTG
TAGACATCGAAATCAAATTATAATAGACAATTTTAGGAGGTGGACTTTCGATGACCAAAGGAATCTTAGGAAG
AAAAATTGGGATGACACAAGTATTCGGAGAAAACGGTGAATTAATCCCTGTAACAGTAGTAGAAGCTAAAGAA
AATGTTGTATTACAAAAGAAAACTGTAGAAGTTGATGGATACAACGCAATCCAAGTTGGATTTGAAGACAAAA
AAGCATACAAAAAAGATGCAAAATCTAATAAATATGCTAATAAACCAGCTGAAGGTCACGCTAAAAAAGCTGA
CGCAGCACCTAAGCGCTTCATTCGTGAATTCCGCAATGTAGACGTGGATGCTTACGAAGTAGGTCAAGAAGTC
TCAGTAGATACTTTTGTAGCTGGCGACGTTATTGACGTAACAGGCGTATCAAAAGGTAAAGGTTTCCAAGGTG
CAATTAAACGCCACGGACAATCTCGTGGACCTATGTCACATGGTTCTCATTTCCACAGAGCACCAGGTTCTGT
AGGTATGGCTTCAGATGCTTCTAGAGTATTTAAAGGCCAAAAAATGCCAGGACGTATGGGTGGAAACACAGTA
ACTGTTCAAAACTTAGAAGTAGTTCAAGTTGACACAGAAAACAAAGTTATCTTAGTAAAAGGTAACGTACCTG
GACCTAAAAAAGGTTTAGTAGAAATCAGAACTTCAATTAAAAAAGGTAATAAATAATAAGAAGTGAAAGGAGG
AAATGCATAATGGCTAATTATGATGTTTTAAAATTAGACGGAACTAAATCAGGTTCAATCGAATTAAGCGATG
CAGTATTCGGTATTGAGCCAAATAATAGCGTTTTATTCGAAGCTATTAATTTACAACGTGCTTCATTACGTCA
AGGTACGCATGCTGTTAAGAATCGTTCAGCAGTAAGCGGTGGCGGACGTAAACCATGGAAGCAAAAAGGAACA
GGTCGTGCTCGTCAAGGTACAATCCGTGCTCCACAATGGCGTGGCGGTGGTATCGTATTCGGACCAACTCCAA
GAAGTTATGCATACAAAATGCCTAAGAAAATGCGTCGTTTAGCTTTACGCTCAGCATTATCTTTCAAAGCTCA
AGAGAATGGCTTAACTGTAGTTGACGCATTTAACTTCGAAGCTCCAAAAACTAAAGAATTCAAAAATGTATTA
TCTACATTAGAACAACCTAAAAAAGTATTAGTAGTTACTGAAAACGAAGATGTAAATGTTGAATTATCAGCAC
GCAACATCCCTGGCGTTCAAGTGACAACTGCTCAAGGTTTAAATGTTTTAGATATCACTAATGCTGACAGCTT
AGTAATTACTGAAGCTGCTGCTAAAAAAGTTGAGGAGGTGCTCGGATAATGGAAGCAAGAGATATTCTTAAGC
GCCCCGTAATCACTGAGAAATCTTCTGAAGCAATGGCTGAAGACAAATACACTTTCGACGTTGATACTCGTGT
TAACAAAACACAAGTAAAAATGGCAGTTGAAGAAATCTTCAACGTAAAAGTTGCAAGTGTTAATATCATGAAT
TACAAACCTAAGAAAAAACGTATGGGCCGTTACCAAGGCTATACAAACAAAAGAAGAAAAGCGATTGTAACTC
TTAAAGAAGGATCAATCGACTTATTTAACTAATAAACCAATTACCATTAAGGAGGTAAGCGACAATGGCTATT
AAAAAGTATAAGCCAATAACAAATGGTCGTCGTAATATGACTTCGTTAGATTTCGCAGAAATCACGAAAACTA
CACCTGAAAAGTCATTATTAAAACCGCTACCGAAAAAAGCGGGACGTAACAACCAAGGTAAATTGACTGTAAG
ACACCATGGTGGTGGACACAAACGTCAATACCGTGTTATCGATTTCAAACGTAACAAAGATGGTATCAATGCA
AAAGTTGATTCTATTCAATATGATCCAAACCGCTCAGCAAACATCGCTTTAGTTGTATATGCAGACGGTGAAA
AACGATATATCATTGCTCCTAAAGGATTAGAAGTAGGTCAAATCGTTGAAAGTGGTGCTGAAGCTGACATCAA
AGTTGGTAACGCATTACCATTACAAAACATTCCAGTTGGTACAGTAGTACACAACATCGAGCTTAAACCTGGT
AAAGGTGGACAAATCGCTCGTTCAGCTGGTGCAAGTGCTCAAGTACTTGGTAAAGAAGGTAAGTACGTATTAA

TCAGATTAAGATCTGGTGAAGTTCGTATGATCTTATCTACTTGCCGTGCTACAATCGGTCAAGTTGGTAACCT
ACAACACGAATTAGTTAACGTTGGTAAAGCCGGACGTTCAAGATGGAAAGGTATTCGTCCAACAGTTCGTGGT
TCTGTAATGAACCCTAACGATCACCCACACGGTGGTGGTGAAGGTCGTGCTCCTATCGGTAGACCATCTCCAA
TGTCACCATGGGGTAAACCTACGCTTGGTAAGAAAACTCGTCGTGGTAAAAAATCATCAGACAAACTTATCGT
TCGTGGACGTAAGAAAAAATAATATAATCAACTTATTTGGGTGTGCGGCTTAAAGCTGCACGCACATAATAAG
AAGGGAGGCGCCCAAATGGCTCGTAGTATTAAAAAAGGACCTTTCGTCGATGAGCATTTAATGAAAAAAGTTG
AAGCTCAAGAAGGAAGCGAAAAGAAACAAGTAATCAAAACATGGTCACGTCGTTCTACAATTTTCCCTAATTT
CATCGGACATACATTTGCAGTATACGACGGACGTAAACACGTACCTGTATATGTAACTGAAGATATGGTAGGT
CATAAATTAGGTGAGTTTGCTCCTACTCGTACATTCAAAGGACACGTTGCAGACGACAAGAAAACAAGAAGAT
AATATCTATTAAGTAGAGGAGGACATCCTAATGGAAGCAAAAGCGGTTGCTAGAACAATAAGAATCGCACCTC
GTAAAGTAAGACTAGTTCTTGACTTAATCAGAGGTAAAAATGTTGCTGAAGCTATTGCAATTTTAAAATTAAC
AAACAAAGCTTCATCACCAGTAATTGAAAAAGTATTAATGTCCGCTTTAGCTAATGCTGAACATAACTATGAC
ATGAACACAGATGAATTAGTAGTTAAAGAAGCATATGCTAACGAAGGACCAACATTAAAACGTTTCCGTCCAC
GTGCGCAAGGTCGTGCAAGTGCGATTAACAAACGTACAAGCCACATTACAATCGTCGTAAGTGACGGTAAAGA
AGAAGCTAAAGAAGCTTAATTAACTTTTAAGGAGGGAATACTGTGGGTCAAAAAATTAATCCAATCGGACTTC
GTGTTGGTATTATCCGTGATTGGGAAGCTAAATGGTATGCTGAAAAAGACTTCGCTTCACTTTTACACGAAGA
TTTAAAAATCCGTAAATTTATTGATAATGAATTAAAAGAAGCATCAGTTTCTCACGTAGAGATTGAACGTGCT
GCAAACCGTATCAACATTGCAATTCATACTGGTAAACCTGGTATGGTAATTGGTAAAGGCGGTTCAGAAATCG
AAAAATTACGCAACAAATTAAATGCGTTAACTGATAAAAAAGTACACATCAACGTAATTGAAATCAAAAAAGT
TGATCTTGACGCTCGTTTAGTAGCTGAAAACATCGCACGTCAATTAGAAAACCGTGCTTCATTCCGTCGTGTA
CAAAAACAAGCAATCACTAGAGCTATGAAACTTGGTGCTAAAGGTATCAAAACTCAAGTATCTGGTCGTTTAG
GCGGAGCTGACATCGCTCGTGCTGAACAATATTCAGAAGGAACTGTTCCACTTCATACGTTACGTGCTGACAT
CGATTATGCACACGCTGAAGCTGACACTACTTACGGTAAATTAGGCGTTAAAGTATGGATCTATCGTGGAGAA
GTTCTTCCTACTAAGAACACTAGTGGAGGAGGAAAATAATAATGTTACTACCAAAACGTGTAAAATATCGTCG
TCAACATCGTCCTAAAACAACTGGTCGTTCTAAAGGCGGTAACTACGTAACATTTGGTGAGTTTGGTTTACAA
GCTACAACAACGTCTTGGATCACATCTCGTCAAATCGAATCTGCTCGTATAGCAATGACACGTTACATGAAAC
GTGGCGGGAAAGTTTGGATTAAAATCTTCCCACATACACCATATACTAAAAAACCTTTAGAAGTACGTATGGG
TGCTGGTAAAGGTGCGGTTGAAGGCTGGATCGCAGTTGTTAAACCAGGTAGAATTTTATTCGAAGTTGCTGGC
GTTTCTGAAGAAGTTGCGCGTGAAGCACTACGTTTAGCAAGTCACAAACTTCCAGTAAAAACTAAGTTTGTAA
AACGTGAGGAATTGGGTGGTGAAACAAATGAAAGCTAAGGAAATTAGAGACTTAACCACTTCAGAAATCGAAG
AACAAATCAAATCTTCAAAAGAAGAGCTTTTTAACCTACGCTTTCAGTTAGCTACAGGTCAATTAGAAGAAAC
TGCACGTATTCGTACAGTAAGAAAAACGATTGCACGTCTAAAAACTGTTGCTCGTGAAAGAGAAATTGAACAA
AGTAAGGCTAATCAATAATTAAGTAAGAGGAGGTTACAAAAGTGAGCGAAAGAAACGATCGTAAAGTTTATGT
AGGTAAAGTTGTTTCAGACAAAATGGACAAGACTATTACAGTACTTGTTGAAACTTACAAAACACACAAATTA
TACGGTAAACGAGTAAAATACTCTAAAAAATACAAAACTCATGATGAAAACAATTCAGCTAAATTAGGAGACA
TTGTTAAAATTCAAGAAACTCGTCCTTTATCAGCAACAAAACGTTTTCGTTTAGTAGAGATTGTTGAAGAGTC
AGTAATTATTTAATACAAGTTTAGAGATAAGGGAGGTTTAACTAATGATCCAACAAGAAACACGCTTGAAAGT
AGCAGACAACTCTGGTGCTCGTGAAGTTCTTACAATCAAAGTATTAGGTGGATCTGGTCGTAAAACAGCAAAC
ATCGGCGATGTTATCGTATGTACTGTTAAAAATGCAACACCAGGTGGCGTTGTTAAAAAAGGTGACGTTGTCA
AAGCTGTAATCGTACGTACTAAGTCAGGTGTTCGTCGTAATGACGGTTCATACATCAAATTTGATGAAAATGC
ATGTGTTATCATCCGTGATGACAAAGGCCCACGTGGTACTCGTATCTTCGGACCTGTTGCTCGTGAATTACGT
GAAGGTAACTTCATGAAAATCGTATCATTAGCACCAGAAGTACTTTAATTAATAACAACCAATACAAATAAGG
AGGTGCCCACATGCATATCAAAAAAGGTGACAACGTTAAAGTTATCGCAGGTAAAGACAAAGGTAAAGAAGGT
AAAGTAATTGCTACTCTACCTAAAAAAGACCGTGTCGTTGTGGAAGGTGTTAACATTATGAAAAAACACCAAA
AACCAACTCAATTAAATCCTGAAGGTGGAATCTTAGAAACAGAGGCAGCAATCCATGTTTCTAATGTACAATT
ATTGGACCCTAAAACAAACGAACCAACTCGTGTAGGTTACAAATTTGTTGATGGTAAAAAAGTTCGTATCGCT
AAAAAATCTGGCGAAGAAATTAAATCTAATAATTAATAACAAGTTGAAAGGAGGATCCCACTTTGAACCGTTT
AAAAGAAAAGTTTAACACTGAAGTTACTGAAAACTTAATGAAAAAATTCAATTATAGTTCAGTAATGGAAGTA
CCAAAAATAGATAAAATCGTTGTGAACATGGGTGTAGGTGACGCAGTACAAAATTCTAAAGTATTAGACAATG
CTGTTGAAGAATTAGAATTGATCACTGGTCAAAAACCATTAGTAACTAAAGCTAAAAAATCAATCGCGACTTT
CCGTTTACGTGAAGGTATGCCAATCGGTGCGAAAGTAACACTTCGCGGTGAAAGAATGTATGAATTCTTAGAC
AAATTAATTTCAGTATCATTACCACGTGTACGTGACTTCCAAGGTGTTTCTAAAAAAGCATTTGACGGACGCG
GTAACTACACTTTAGGTGTTAAAGAACAATTAATTTTCCCAGAAATCGACTATGATAAAGTAAGTAAAGTTAG
AGGAATGGATATTGTTATCGTAACGACTGCTAACACTGATGAAGAAGCTCGTGAATTGTTAGCTAACTTCGGT
ATGCCATTCCGTAAATAATTATTTAAAGGAGGCTAATTAAGTGGCTAAAACTTCAATGGTTGCTAAGCAACAA
AAAAAACAAAAATATGCAGTTCGTGAATACACTCGTTGTGAACGTTGTGGCCGTCCACATTCTGTATATCGTA
AATTTAAATTATGCCGTATTTGTTTCCGTGAATTAGCTTACAAAGGCCAAATCCCTGGCGTTCGTAAAGCTAG
CTGGTAATAAAAAAGAGTCTGAAAGGAGGCAACAATCAATGACAATGACAGATCCAATCGCAGATATGCTTAC

TCGTGTAAGAAACGCAAACATGGTGCGTCACGAGAAGTTAGAATTACCTGCATCAAATATTAAAAAAGAAATT
GCTGAAATCTTAAAGAGTGAAGGTTTCATTAAAAATGTTGAATACGTAGAAGATGATAAACAAGGTGTACTTC
GTTTATTCTTAAAATATGGTCAAAACGATGAGCGTGTTATCACAGGATTAAAACGTATTTCAAAACCAGGTTT
ACGTGTTTATGCAAAAGCTAGCGAAATGCCTAAAGTATTAAATGGTTTAGGTATTGCATTAGTATCAACTTCT
GAAGGTGTAATCACTGACAAAGAAGCAAGAAAACGTAATGTTGGTGGAGAAATTATCGCATACGTTTGGTAAT
AAAAAATAAGGAGGTGCCATAACATGAGTCGTGTTGGTAAGAAAATTATTGACATCCCTAGTGACGTAACAGT
AACTTTTGATGGAAATCATGTAACTGTTAAAGGTCCTAAAGGTGAATTATCAAGAACTTTAAATGAAAGAATG
ACATTCAAACAAGAAGAAAACACAATTGAAGTTGTAAGACCATCTGATTCTAAAGAAGATAGAACAAACCATG
GTACAACTCGTGCTTTATTAAACAATATGGTACAAGGTGTTTCTCAAGGATACGTAAAAGTACTTGAACTTGT
TGGTGTAGGTTACCGTGCTCAAATGCAAGGTAAAGACTTAATCCTTAACGTTGGTTATTCTCACCCAGTAGAA
ATTAAAGCTGAAGAAAACATTACTTTCTCAGTTGAGAAAAACACAGTCGTTAAAGTTGAAGGTATTTCAAAAG
AACAAGTTGGAGCATTAGCATCTAACATCCGTTCAGTAAGACCTCCAGAACCTTACAAAGGTAAAGGTATTCG
TTACCAAGGTGAATACGTTCGCCGTAAAGAAGGTAAAACTGGTAAATAATAGATAAACTCTAAAGAAAGGAGT
ATTGAAATGATCAGTAAAATTGATAAAAATAAAGTGCGTTTAAAAAGACATGCTCGTGTTCGTACTAACTTAT
CAGGTACAGCTGAAAAGCCACGTTTAAACGTATATCGTTCAAACAAGCATATCTACGCTCAAATTATTGATGA
TAATAAAGGCGTAACATTAGCTCAAGCTTCTTCAAAAGACAGCGACATTGCTACTACAGCAACTAAAGTTGAA
TTAGCAACTAAAGTCGGTGAAGCAATTGCTAAAAAAGCTGCTGACAAAGGCATTAAAGAAATCGTATTTGACC
GTGGAGGATATTTATATCACGGACGTGTTAAAGCATTAGCTGAAGCAGCAAGAGAAAGCGGATTAGAATTTTA
ATTTAAAGGAGGGACAAATACATGGCTCGTAGAGAAGAAGAGACGAAAGAATTTGAAGAACGCGTTGTTACAA
TCAACCGTGTAGCAAAAGTTGTAAAAGGTGGTCGTCGTTTCCGTTTCACTGCATTAGTTGTAGTTGGAGACAA
AAATGGTCGTGTAGGTTTCGGTACTGGTAAAGCTCAAGAGGTACCAGAAGCAATCAAAAAAGCTGTTGAAGCA
GCTAAAAAAGATTTAGTAGTTGTTCCACGTGTTGAAGGTACAACTCCACACACAATTACTGGCCGTTACGGTT
CAGGAAGCGTATTTATGAAACCGGCTGCACCTGGTACAGGAGTTATCGCTGGTGGTCCTGTTCGTGCCGTACT
TGAATTAGCAGGTATTACTGATATCTTAAGTAAATCATTAGGATCAAACACACCAATCAACATGGTTCGTGCT
ACAATCGATGGTTTACAAAACCTTAAAAATGCTGAAGATGTTGCGAAATTACGTGGCAAAACAGTAGAAGAAT
TATACAATTAAGGAGGGAAAACTAGTTATGGCTAAATTACAAATTACCCTCACTCGTAGTGTTATTGGTCGTC
CTGAAACACAACGTAAAACTGTTGAAGCTTTAGGTCTTAAAAAGACTAACAGTTCAGTAGTTGTTGAAGATAA
CCCTGCTATTCGTGGGCAAATCAACAAAGTTAAGCACTTAGTAACAGTAGAAGAAAAATAATCAAGGAGGTGC
CGAAATGAAATTACATGAGTTAAAACCGGCAGAAGGTTCACGTAAAGAACGCAATCGTGTTGGACGTGGTGTT
GCGACAGGTAACGGTAAAACAAGTGGTCGCGGACACAAAGGTCAAAAAGCTCGTTCAGGCGGTGGTGTAAGAC
CAGGATTTGAAGGTGGTCAATTACCATTATTCCGTCGTTTACCAAAACGTGGTTTTACTAACATAAATCGTAA
AGAATATGCTATTGTTAACTTAGACCAACTTAATAAATTTGAAGATGGTACTGAAGTAACTCCAGCTTTATTA
GTAGAATCTGGTGTTGTTAAGAATGAAAAATCTGGTATCAAAATACTAGGTAATGGTTCACTTGATAAGAAAT
TGACAGTGAAAGCTCATAAATTCTCAGCTTCAGCAGCAGAAGCTATTGATGCTAAAGGTGGAGCACACGAGGT
GATCTAATGATTCAAACCCTTGTGAACTTCTTTAGAACAAAAGAGGTTCGTAACAAGATTTTCTTCACACTAG
CAATGTTAGTAATTTTTAAAATAGGGACTTATATACCAGCTCCAGGAGTAAATCCTGCAGCTTTTGATAATCC
CCAAGGTTCTCAAGGTGCCACTGAGTTATTAAATACTTTTGGTGGCGGAGCCTTGAAACGATTTTCTATTTTT
GCAATGGGTATTGTACCCTACATCACTGCATCAATCGTAATGCAATTATTACAAATGGATATTGTCCCTAAAT
TCTCAGAATGGGCAAAACAAGGTGAAGTAGGTAGAAGAAAGTTAAATAACGTTACTCGTTATTTAGCAATTTC
TTTAGCATTTATCCAATCTATAGGTATGGCATTCCAATTTAATAATTATCTCAAAGGTGCGCTGATTATCAAT
CAGTCAATTATGAGTTATTTATTAATAGCACTAGTTTTGACAGCAGGAACTGCTTTCTTAATATGGCTTGGTG
ATCAAATCACTCAGTTCGGTGTTGGTAATGGTATTTCTATTATCATATTTGCGGGTATTTTATCAACGTTGCC
AGCCAGTCTAATTCAATTTGGACAAACTGCGTTCGTTGGGCAAGAAGATACATCATTAGCATGGTTGAAAGTA
TTAGGACTTTTAGTGTCACTGATACTATTAACTGTAGGTGCTATTTATGTACTTGAAGCCGTTCGTAAAATTC
CGATTCAATATGCTAAGAAACAAACTGCTCAAAGATTAGGTTCACAGGCAACTTATCTACCTTTAAAAGTGAA
CTCAGCCGGTGTTATTCCTGTAATCTTTGCAATGGCGTTCTTCTTACTGCCTAGAACATTAACGTTGTTCTAT
CCAGATAAAGAATGGGCACAAAACATTGCGAATGCTGCAAATCCTTCAAGTAATGTTGGTATGGTTGTATATA
TTGTACTAATTATACTATTCACATACTTCTATGCATTTGTACAAGTTAATCCGGAAAAAATGGCTGATAACCT
TAAGAAACAAGGTAGCTATGTTCCAGGCATTAGACCTGGTGAACAAACCAAAAAATATATTACTAAAGTTTTA
TATCGTTTAACTTTTGTAGGTTCTATCTTCTTAGCCGTTATTTCAATTCTACCGATATTAGCAACTAAGTTTA
TGGGATTACCACAATCAATTCAGATTGGTGGTACAAGTTTACTTATCGTTATTGGTGTAGCGATTGAAACAAT
GAAATCTTTAGAAGCTCAAGTATCTCAAAAAGAATATAAAGGCTTTGGTGGTAGATAATTTTAGGAGGGCACT
TATGAATATCATTTTGATGGGTTTACCTGGCGCAGGTAAAGGAACTCAAGCAAGTGAAATTGTCAAGAAATTC
CCAATACCCCACATTTCAACTGGTGACATGTTCAGAAAAGCTATAAAAGAAGAAACTGAATTAGGTAAAGAAG
CTAAGTCTTATATGGACCGTGGCGAATTAGTTCCTGATGAAGTGACTGTAGGTATCGTTAAGGAAAGAATTTC
TGAAGACGATGCAAAAAAAGGCTTTTTATTAGATGGCTTCCCAAGAACAATCGAGCAAGCTGAGGCATTAAAT
AATATTATGTCTGAGCTTGACAGAAACATTGATGCTGTCATCAATATCGAAGTTCCGGAAGAAGAATTAATGA
ACCGTCTTACAGGTCGTCGAATCTGTGAGTCATGTGGTACAACGTATCATCTTGTATTTAATCCTCCGAAGGT

CGAAGGTATTTGTGATATCGATGGTGGTAAATTGTATCAACGAGAAGATGATAATCCTGAAACGGTAGCTAAT
CGTTTGAGTGTTAATATTAAACAATCTAAGCCTATTTTAGATTTCTATGATCAAAAAGGTGTATTGAAAAATA
TTGATGGTTCAAAAGATATTAGCGACGTTACCAAAGATGTCATTGATATTTTAGATCATTTGTAATAGATCAA
CTTTTAGCGGTCTATTTTAAGTGAGGCATGATTTAATCATATTAGATAGATGAGAGTGGCAATTAACGATAAT
TAATTCCCGTAATTTTTGTTTAACTTATAAAATTAATCACTGATAGATTCGTAACAATAAAGTTGTCTATTGA
CGATTACCTTACTATTGCAAAAAGGGGGAAGTTAATCAATGGCTAAACAAGATGTAATTGAATTAGAAGGTAC
TGTATTAGATACTTTACCGAACGCAATGTTTAAAGTAGAATTAGAAAATGGTCATGAGATTTTAGCTCACGTA
AGTGGTAAAATCAGAATGAATTACATTCGTATTCTACCTGGCGACAAAGTAACTGTTGAGATGTCTCCGTACG
ATTTAACACGCGGAAGAATTACTTATCGTTATAAATAATCGTCACTCCATAATATAGGGAGGTATAAAAATGA
AAGTAAGACCATCAGTAAAACCTATTTGCGAAAAATGTAAAGTCATTAAACGTAAAGGTAAAGTAATGGTAAT
TTGTGAAAATCCAAAACACAAACAAAGACAAGGTTAATAAAAGAGAGGTGTAAATTAATATGGCACGTATTGC
AGGAGTAGATATTCCACGTGAAAAACGCGTAGTTATCTCATTAACTTATATATACGGTATCGGTACGTCAACT
GCTCAAAAAATTCTTGAAGAAGCTAACGTATCAGCTGATACTCGTGTGAAAGATTTAACTGATGACGAATTAG
GTCGCATCCGTGAAGTTGTAGACGGTTATAAAGTCGAAGGTGACTTACGTCGTGAAACTAACTTAAATATCAA
ACGTTTAATGGAAATTTCATCATACCGTGGTATCCGTCACCGTCGTGGTTTACCAGTTCGTGGTCAAAAAACG
AAAAACAACGCGCGTACTCGTAAAGGACCAGTTAAAACGGTAGCTAACAAGAAAAAATAATAGGTAAAGGAGG
CAAATTTTAAATGGCACGTAAACAAGTATCTCGTAAACGTAGAGTGAAAAAGAATATTGAAAATGGTGTAGCA
CACATCCGTTCAACATTCAACAACACTATTGTAACTATCACTGATGAGTTCGGTAATGCTTTATCATGGTCAT
CAGCTGGTGCATTAGGATTCAAAGGATCTAAAAAATCAACACCATTTGCAGCACAAATGGCTTCTGAAACTGC
ATCTAAATCAGCTATGGAGCATGGTTTAAAAACAGTTGAAGTAACAGTTAAAGGACCTGGTCCAGGTCGCGAA
TCAGCTATTCGTGCATTACAATCTGCAGGTTTAGAAGTAACTGCGATCAGAGACGTTACTCCAGTACCTCATA
ACGGTTGTCGTCCACCAAAACGTCGTCGTGTATAATTTATGATGGTATTGTTACAGGTCACTGAGCAAACATT
TTAAATTAAGTCGACGTATATAAGGAGGATATTTAAATGATAGAAATCGAAAAACCTAGAATTGAGACAATTG
AAATTAGTGAAGATGCTAAATTCGGTAAGTTCGTTGTTGAACCACTAGAACGTGGCTACGGTACTACACTAGG
AAACTCCTTACGTCGTATCCTACTATCTTCATTACCAGGTGCAGCCGTTAAGTATATTGAAATTGAGGGAGTT
TTACATGAATTCTCAGCAGTAGACAATGTAGTTGAAGATGTTTCTACAATTATTATGAACATTAAACAATTAG
CATTGAAAATTTACTCTGAAGAAGATAAAACTTTAGAAATTGATGTACGTGATGAAGGCGAAGTAACAGCAAG
CGACATTACACATGACAGTGATGTTGAAATTTTAAACCCAGAGCTTAAAATTGCAACAGTATCTAAAGGTGGT
CACTTAAAAATTCGTCTAGTTGCTAACAAGGGTAGAGGTTACGCATTAGCAGAACAAAATAATACTAGTGATT
TACCAATTGGTGTAATCCCTGTTGATTCATTGTATTCACCTGTTGAACGTGTGAACTATACTGTTGAAAATAC
ACGTGTAGGTCAAAGCAGTGATTTTGATAAATTAACATTGGATGTTTGGACTAATGGTTCAATCACACCACAA
GAATCAGTTTCATTAGCAGCAAAAATAATGACTGAACACTTGAATATCTTCGTTGGTCTTACTGATGAAGCGC
AAAACGCTGAAATCATGATTGAAAAAGAAGAAGATCAAAAAGAAAAAGTATTAGAAATGTCTATTGAAGAATT
AGACTTATCTGTACGTTCATATAACTGCTTAAAACGCGCAGGAATCAATTCTGTTCAAGAGTTAGCTGACAAA
TCTGAAGCTGACATGATGAAAGTGCGTAATTTAGGTCGTAAATCTTTAGAAGAAGTTAAATACAAATTAGAAG
ATTTAGGATTAGGATTAAGAAAAGAAGATTGATAAAGGAGGTTAACTCATGGGTTACAGAAAATTAGGTCGTA
CTTCTGATCAACGTAAAGCTATGTTACGTGACTTAGCTACATCACTTATTATTAGTGAACGTATTGAAACTAC
AGAAGCTCGTGCAAAAGAAGTTCGCAGTGTTGTTGAGAAATTAATCACTTTAGGTAAAAAAGGAGATTTAGCT
TCTCGTCGTAATGCAGCTAAAACTTTACGTAATGTTGAAATCTTAAACGAAGATGAAACTACACAAACTGCAC
TTCAAAAATTATTTGGTGAAATCGCAGAGCGTTACACAGAACGTCAAGGTGGTTACACTCGTATCCTTAAACA
AGGCCCTCGTCGCGGTGACGGTGCTGAATCAGTAATTATCGAATTAGTATAAGAATTTGTATAAATACACTAA
CTACTTTTATATAAAGCAAATGAGTGCAACGATAATGTTTGCCACATACAAATATTGTCTAGCTCAGAGTACC
CCATCAACTAAATAAAGATTTAAAGCGTGAACTCAACAAACTTATGATGGGGTGCGCGCTTTTTTATTGTATA
AGCTATCGGACTTTTGAACAGAAATGTTTACAAGTCCGATTTCTTTATATCTACTTTTTTGAGTGTGAATCTA
AAAATATGATCAAGCTAGTTTGATCAATTTCTCTAGAACTGAATGAAGTGAAAGTACTATCGTTGAATCAAAC
GCGGCCAACCCTATGTTAGTAAATCATTTATTTTATTAACGTTTATTCATAATTGTTAGAGAGGAATAAATGT
CCTTATCATTAGTTGTTAAACAACCCTTTAATGATTAGAAATGAGTCAGAAAATACTAGTAATATATAGCAAA
CCCAGTCAATGTCATATACAGCGATTTATAAATATTGTAAAATATGATAGATACACGAATCGTAAAGGAGAGA
GACAAGTGGAGGATAAGAATTCAGTTATTGTATTTAAAAATGTTTCATTTCAATATCAAAGTGATGCATCCTT
CACATTGAAAGATGTTTCTTTTAATATACCTAAAGGTCAGTGGACATCTATTGTTGGTCATAACGGTTCTGGA
AAATCTACAATTGCCAAGTTAATGATTGGCATAGAGAAAGTTAAATCTGGAGAAATTTTTTATAATAATCAAG
CTATAACAGATGATAATTTTGAAAAGTTAAGAAAAGACATAGGAATTGTATTTCAGAATCCGGATAATCAATT
TGTTGGTTCAATTGTAAAATACGATGTGGCATTTGGACTCGAAAATCATGCGGTTCCACATGACGAAATGCAT
AGAAGAGTCAGCGAAGCACTTAAACAAGTTGATATGTTAGAACGTGCAGATTATGAACCTAATGCATTATCTG
GGGGCCAGAAGCAGCGTGTGGCTATAGCAAGTGTATTAGCACTTAACCCCTCTGTCATTATATTAGATGAGGC
GACTTCTATGTTAGATCCTGATGCACGTCAAAATTTATTGGATTTAGTGAGAAAAGTTAAATCAGAACATAAT
ATTACAATCATTTCTATTACGCATGATTTATCTGAGGCGATGGAAGCAGATCATGTTATCGTTATGAATAAAG
GGACTGTATATAAAGAAGGTACAGCGATTGAAATTTTCGACCATGCAGAAGAGTTAACAACAATAGGTTTAGA

TTTGCCATTCCCAATCAAAATAAATCAAATGCTGGGATACCAAACATCATTCTTAACTTATGAAGGGCTGGTG
GATCAACTATGACTATACGCTTTGACAATGTAAGTTATACCTATCAAAAAGGGACACCATACCAGCATCAAGC
TATTCATGATGTTAATACAGAATTTGAACAAGGTAAATATTACGCCATCGTTGGACAAACGGGTAGTGGTAAA
TCAACATTGATACAAAATATTAATGCACTGTTAAAGCCAACTACTGGGACAGTTACAGTTGATGACATTACTA
TTACACATAAGACCAAAGATAAATATATTAGACCTGTAAGAAAAAGAATTGGAATGGTATTTCAATTTCCCGA
ATCTCAATTATTTGAGGACACAGTAGAGCGTGAAATGATATTTGGACCTAAAAACTTTAAAATGAATTTAGAT
GAAGCCAAAAACTATGCCCATCGTCTGTTGATGGATTTAGGTTTTTCAAGAGATGTAATGTCACAATCACCAT
TTCAAATGTCAGGTGGACAAATGCGTAAAATAGCGATTGTATCGATATTGGCAATGAATCCTGATATTATCGT
GGTTGATGAACCCACAGCAGGACTTGATCCACAAAGTAAACGACAAGTAATGCGATTACTAAAGTCACTACAA
ACAGATGAAAATAAGGCAATTATCCTAATTTCACATGATATGAATGAAGTCGCGCGTTATGCAGATGAAGTCA
TCGTTATGAAAGAAGGTAGTATAGTGTCGCAAACATCACCAAAAGAGCTCTTCAAAGATAAAGAAAAATTGGC
GGATTGGCATATTGGTTTGCCAGAAATTGTTCAGTTACAATATGACTTTGAACAAAAACATCAAACAAAATTA
AAAGATATTGCCTTAACTGAAGAAGCATTTGTAAGCTTGTATAAGGAGTGGCAACATGAAAAATAAATTGATT
ATAGGGCGATATTTACCAATTAATTCTTTTGTTCATCATCTTGATCCAAGAGCAAAGTTAATGTTTGTTTTTC
TTTTTATTATATTAATATTTTTCTGTCATTCACCATTAACATATCTTTGGGTGTTTGCACTTATATTATTCTT
TATGAGATTAGCAAAAATTCAATTATGGTTCCTAATCAAAGGTTTAACGCCAATATTTTTCTTTTTAATCTTC
ACATTAATGATGCATATATTTTTAACTAAAGGTGGATATGTATTAGTTGAATGGCATGGCATCACGATTGAAA
CTAATGGTATTTTAGAAGGGCTATATATATCACTGCGTTTAATTGGTATTGTGATGATTGCAACAATTATGAC
ACTATCTACAAGTCCAATTGATTTAACAGATGCGTTTGAAAGATTACTTGCACCACTAAAGATGTTTAAATTA
CCAGTTCATCAATTAAGTATGATAATGTCCATTGCGTTACGATTCATCCCGACGTTAATGGATGAGTTAGATA
AAATCATTTTGGCGCAAAAGTCGCGTGGCTCTGAAATAAGTTCAGGGAACATTGCAACACGTATTAAATCATT
TATTCCATTACTAGTGCCACTATTCATCTCAGCTTTCCAACGTGCAGAAGAATTAGCGGTTGCAATGGAAGTT
AGGGGTTATGATGCCAATGTTCAAAGAACGAGCTACAGACAGCTTAAATGGCAATTGAGAGATACATTATCTT
TAATCATGATTATCCCATTTGCAATTATTTTATTCGTATTAAAATATTCAGGAGTGTAAATTCATGCGTATAT
TAGTAGAAATTGCGTATCAAGGAAATAATTTTCTAGGTTTTCAAATTCAACAAAATGGTCGAACGGTACAGCA
ACAATTTGAAAAGCTATTACAACGCATGCATAAAAGACATGTGAGAATCCATCCTTCAAGTAGGACTGATAGA
GGTGTACATGCGATACAGCAATACTTTCATTTCGACACAGAGCTGAATATACCTATGTCACAATGGCAATATG
CAATGAACCGAACGTTACCTGATGATATTTATGTCAACAATGTAGTTACAGTCGATGACGATTTTCATTGTCG
TTACGATTGTGTTGGTAAACGCTATCGATATAAAGTATATCAAGCACAACACCGCGATCCATTTCAAAGTGGT
TTGAAAACATTCATTCCTGAAACGTTGGATTTAGATAAAATGAACAGAGCGGCACAACAATTCATAGGTACAC
ATGATTTTACGGGTTTTTGTTCGCAAAAAACTGAAGTAGAAAGCAAAGTTAGAACATTATACCAAAGTGAAAT
TGTGAAAACCGATGATGGCTTTGATTATATTGTCACTGGATCTGGATTTTTATATAACATGGTGCGTGTCTTA
GTTGCATTTTTAATAGAAGTCGGAAAAGGGCGTCACGAAGTATCTGATGTACCAAAACTTCTTGAATCAAAGA
ATCGTAAGAATGTACCTTTTACTGCACCGGCTGAAGGATTATATTTAGAGAAAATTTATTTAGATGAAAATGA
GTTATTAAAAGACTTTGGTAACGATATCAAAATACATCGTAAAAAATCATTACAAAATGACTGATTGCCATTG
ACAAAAAGCTTGTGAAATTATAAGATTATTAACGGTATTGTTTTATATCCACCCCACGATAAGCCCCGGAAAC
TTATTGTGTTACAAGATATATAAGCAGAAACGAACAACAGTTAACAAAATAAATGAAATTAAACGTTTTAAAA
ATGAAACAAATGAAATCATCTATTAGGTTATGAAACTGTTTATAGCTTGAATAGAAGCATTTATTTTTTAGGA
GGACAATTATTATGCGTCAAACATTTATGGCAAATGAATCAAACATTGAGCGCAAATGGTATGTTATCGATGC
TGAAGGCCAAACATTAGGTCGTTTATCATCAGAAGTAGCATCTATCTTACGCGGTAAAAATAAAGCAACTTAC
ACACCACACGTTGATACTGGTGATTATGTAATCGTTATTAATGCATCAAAAATCGAATTTACTGGTAACAAAG
AAACTGACAAAGTTTACTACCGTCACTCAAATCACCCAGGTGGTATCAAATCAATCACTGCTGGTGAATTAAG
AAGAACTAATCCAGAACGTTTAATTGAAAACTCAATTAAAGGTATGTTACCAAGCACTCGTTTAGGCGAAAAA
CAAGGTAAAAAATTATTTGTATATGGTGGCGCTGAACATCCACACGCTGCACAACAACCAGAAAACTACGAAT
TACGTGGTTAATTAGAAGGAGGAAATGACTTTGGCACAAGTTGAATATAGAGGCACAGGCCGTCGTAAAAACT
CAGTAGCACGTGTACGTTTAGTACCAGGTGAAGGTAACATCACAGTTAATAACCGTGACGTACGCGAATACTT
ACCATTCGAATCATTAATTTTAGACTTAAACCAACCATTTGATGTAACTGAAACTAAAGGTAACTATGATGTT
TTAGTTAACGTTCATGGTGGTGGTTTCACTGGACAAGCTCAAGCTATCCGTCACGGAATCGCTCGTGCATTAT
TAGAAGCAGATCCTGAATACAGAGGTTCTTTAAAACGCGCTGGATTACTTACTCGTGACCCACGTATGAAAGA
ACGTAAAAAACCAGGTCTTAAAGCAGCTCGTCGTTCACCTCAATTCTCAAAACGTTAAGCTGCAACTGTTGCG
AACTTGTTCGTTTACAGATGGAGTTCGCAAATTGCGTCAGCAATTTGTGTTCAACTGAAGAAACGTTTACAAC
ACTCTTTGGATTATTCCAAAGAGTGTTTTTTGTATTTTTATAGAGTCATTTAGACACCATTTAGTTACCCCTA
GGGAACTAATTTGTTTCAATTCTGCTTAAAATTCAATGATATGAAACTTTAGGAAACCATATGAAATGACCTT
GTTTTTCTTTGGGATTCGTACGACAAGTAGATAGAGGTTGTAATGATATTCGATAGATATCGTATTGAATGTT
TGCAAACTGAGGTTGAAGGACAGCTTTCGAGTAAAAAGGAAAATTTTCGAGTAACTCAAAAGAAAAATATAAA
AAAATCATGAGAAACCAATGATATCAAGCGTTTTGTGAGCTTTCGAGTAAAGAGGATAATTTTCGAGTAACTC
AAAAGAAAATACTAAAAAATCATGGGAAATCATTGATATCAAGGGTTTTGTGAGCTTTCGAGTAAAGAGGCGG
ATTTTCGAGTAGTATTATTTAATAAGAATTACAGGTTAAATAAAATACCTACTAATCAGAAGGTGACAGAGAT

TGTAGAATTTCTATCAGATGGAAAGTTAGCAAGTAGGCTAGAAATTCAAGAAACAATGGGACTTCAAAAAAGT
TATACATCTGAGTTAATTGCTGAACTGAAGAAGTTAGAGATTATCGGATCTGAAGGTAGAGGTCTGGGAACTA
GATATTATTTAATTAGAGACTAATAAATTCATCAAGTGATAACCGGACGATAACCTATTGATATACATAAAAA
CATTGATAAATCAATATATTTACGACCGGACGATAACCGGACGATACGAGAGTATATACCACCGGACAGCAGT
TTATTTAGTTACCCTTTAGTTACCCCGGAGTGATATTTGGTGCAATACTATGCAATCGAAAAAATCAGAAATC
CTATAATATCATGCCTTAAGACAATATATGCAACTATACGAAACCCTAAAGTAAATCAGGTCTTAAAGCAGCT
CGTCGTTCACCTCAATTCTCAAAACGTTAATTGTCGGACGATATATACACAAAACACCTCGATGTTATGTCGA
GGTGTTTTTTTGCGTTTGTGTGGGGAATATGAAAAGTATGTAGTATAAATGAACGTTCCTTTATTTAATGTCT
CAGGAATAGAATAAATTCAACTATGAATTATTGATTTACACGTATAATTTGTACATGCTTACACAATCAAAAG
TGTACATTATTAAATTGTCATTTAGCTTGCGGAGGACGGACGATAACCGGTCGATAATGTTCTAATCATAGTG
GTTCAAGGGAAAGGGCTATCGGATGAAAACCGGACGATAACTCAAGAACCATGATTTAAGAGTGTTATCAAAA
TAAGGAGTGACTTAATTGAAAGATTCTATATTTTGGAAGAAAGCTTTTATTCCTGTTTATTTTATTGTTGCGA
TGCTGGTGTTTCTACTTTTTAGGTTTTATATTAAAACAGACAACTTTTCTATGTATTTAATGATTATCTTCTT
AATTTGTTTAGGAACTGCATCTATCATTTATAACTATAAAAACAATCGATAAATATAAGTATGAAATGAATAA
GTCACAATTAGATCTAAGTCTTGCTGTGGCTTTTTTTATCGAAATGACAATTTCATGTTGCTATAAAACAGCA
TTTTCAAACCGTCATAAAACAACATTTTCAGCCCGTCATAAAACAACATTTTCAGCCCGCCATAAAACGACAA
TTTCAAACCGCCATTGACTAAACACCTCATTCCCAAATATGCTAACAATCCTCCACATCAATCAATCCGACAT
TCTTCATTATCACACTGCACAAAATCTACTCATGCATTTTTGGAATACTTAGTATTACAAATAACGATTTTTA
TTCATCTTACAAAGGATATATAATGTACTGAAGGCAATTTTTATGTATTACAAATCTAATCGTACATGTAAAA
TTTTGAAAAACATCATTAATTTTGCGTAACTATCATTAGATTACAAATCACAATGCAATTACATGTAATACAC
ATCTACACATCACATTTGAAGGGAAATGAATATAAATGACTGATAAAAAGTACACTGCAGCCGATATGGTTAT
TGATACTTTAAAAAATAATGGGGTAGAATATGTTTTTGGTATTCCGGGTGCAAAGATAGACTATCTATTTAAT
GCTTTAATTGATGATGGTCCTGAACTTATTGTCACGCGTCATGAACAAAATGCCGCAATGATGGCGCAAGGTA
TTGGAAGATTAACAGGCAAACCTGGTGTAGTACTTGTTACAAGTGGCCCTGGTGTAAGTAATTTAACGACTGG
ACTATTAACAGCTACATCTGAAGGGGATCCTGTATTAGCGTTAGGTGGCCAAGTGAAACGTAATGATTTATTA
CGATTAACGCATCAAAGTATTGATAATGCTGCGCTATTAAAATATTCATCAAAATACAGTGAAGAAGTACAAG
ATCCTGAATCATTATCAGAAGTTATGACAAATGCAATTCGAATTGCTACTTCAGGAAAAAATGGCGCAAGTTT
TATTAGTATTCCGCAAGACGTTATTTCTTCACCAGCTGAATCTAAAGCGATATCACTTTGCCAAAAACCAAAT
TTAGGAGTACCGAGTGAACAAGATATTAATGATGTCATTGAAGCGATTAAAAATGCATCATTTCCTGTTTTAT
TAGCTGGTATGAGAAGTTCAAGTGCAGACGAAACAAATGCCATTCGCAAATTAGTTGAGCGCACGAATTTACC
AGTTGTAGAAACATTCCAAGGTGCAGGTTTAATTAGTCGTGAATTAGAAAATCATTTCTTTGGGCGAGTGGGT
TTATTCCGTAATCAAGTTGGTGATGAATTATTACGTAAAAGTGATTTAGTTGTTACAATCGGTTATGATCCTA
TAGAATATGAGGCTAGTAACTGGAATAAAGAATTAGATACGCAAATAATCAATATTGACGAAGTTCAAGCTGA
AATTACTAATTATATGCAACCGAAAAAAGAGTTGATTGGTAATATTGCTAAAACGATTGAAATGATTTCTGAC
AAAGTAGATGAACCATTTATAAATCAACAACATTTAGATGAATTAGAACAGTTAAGAGCGCATATTGATCAAG
AAACTGGTATTAAAGCGACACATGAAGAAGGCATCTTACACCCAGTTGAAATTATAGAATCTATGCAAAAGGT
ATTAACTGATGAAACAACAGTGACAGTTGATGTTGGTAGTCACTATATTTGGATGGCACGTAATTTCAGAAGT
TACAATCCAAGACATTTATTATTTAGTAACGGTATGCAAACACTTGGTGTAGCATTACCGTGGGCAATTTCAG
CTGCACTTGTACGCCCTAATACGCAAGTTGTATCCGTTGCTGGCGATGGAGGCTTTTTATTTTCATCACAAGA
TTTAGAAACGGCCGTACGTAAAAATCTAAACATTATTCAACTTATTTGGAACGATGGAAAATATAACATGGTT
GAATTCCAAGAAGAAATGAAATATAAACGTTCATCAGGTGTCGACTTCGGTCCTGTAGATTTCGTAAAATATG
CTGAATCATTTGGCGCAAAAGGTTTACGAGTTACTAATCAAGAAGAATTAGAAGCGGCAATTAAAGAGGGGTA
TGAAACAGATGGCCCAGTATTAATTGATATACCTGTAAATTACAAAGATAATATCAAACTTTCAACAAATATG
TTACCTGACGTATTTAACTAAAATACAGATAAATGTTAATGAGGAGTGGGAAATTTTATGGCTAGTGTTTTAT
ATCAACATGGTACATTAGGCACATTAATGGCAGGTTTATTAAAAGGAACTGCATCAATAAATGAATTATTGCA
GCATGGTGATTTAGGTATCGCTACACTAACAGGTTCAAATGGTGAGGTCATCTTTTTAGATGGAAAAGCCTAT
CATGCAAATGAACACAAAGAATTTGTGGAATTAAAAGGTGATGAGTTAACACCATATGCAACTGTAACTAAAT
TCGTAGCAGATACAAGCTATGAAACGAAAGATAAATCTTCTGAAGCAGTTTTTGCAGAAATTAAGGAAAAGAT
GTTGAGTGAAAATTTATTTTCTGCAGTAAAAATTTCAGGCTTATTTAAAAAAATGCATGTACGTATGATGCCG
GCTCAAGAGCCACCATATACACGTTTAATTGATTCAGCTAGAAGACAACCTGAACAAACTGAAACGTACGTCA
AAGGTTCAGTTGTTGGTTTCTTTACACCAGAATTATTCCATGGTATTGGATCAGCAGGTTTCCATGTACACTT
TGCCAATGATGATCGTAACTTTGGTGGGCATGTCTTAGACTTTGAAGTAGAAGATGTTAAAGTAGAAATCCAA
AATATTGAAACATTTGAACAGCATTTTCCAATTCATGATGAAGATTTCACTAATGCAAATATTGACTATAAAG
ATATTTCAGATGAAATTAGAGAAGCTGAATAACATTAACTGTATAATGAATGCGTAAATGTATTATCAGTTTA
TGAAAATTGACTTCATAATGCGCGATTTAGAAATGGTAGTTTGTTAATATGATTAATCATGACTACAATAGAA
CAAATATATTTATGATTACGTCTATGTAATAAAATAAATCCCTTCACTATTAGCTGTAGTGAGGGGATTTATT
TAGGTTTCAGATATTTGAGATTTGCTGTTATGTTTAGATTATAAATTGAGGAAGACAATAATATAGAATTTAC
TTTAGTATAGGCCAATCTATTGTCTGGAGAAGCAAACAACAATAATTTGCAAGTTCAATAACTAAAAAGACAA

ACGCCAATTTTTCAGCGCTTGCCCTATAAAACTATTTTCAAATTATTATTTAACTACAATTTGTATTTGTTTG
ATTGTACTTGGATCAATTGAATTTTTAGCATCGTCATTTTTCTTTAGATTCACTACTTTTTTATTACCATCAT
TCAAAGTAAGTGTATAAGTTGCTGTTTGTGCATTATTAATTTTTTCTGTTGTAACACCACGTTGAGAAGCTAA
TTCATTTTTTACTTTACTGTCAATTTCTTGATAAAGTACCTTTTTATTTTCTGGAAAAACAAAGTAAGTTCGA
TGTAATGCAGTAATACCATCTACTGAAATCGTGTAAGGAACAGTGTGATAACCATCCATTGTCATTTCTTTAT
AGCCGTTATTACTATCTGCAGATGCTTCGTGACTAGGTAATGCAGCTCCGACCGTTGATAATAATCCCAATGC
TAAAGTGGCAGTAACAAATGATTTTAGTTTCATATCTCAATCTCCTTTTAAAATTGTGGTATATTACACTATT
TATATAACACGTTTCATAAAGTTTAAAACATACTTTTTGATTTTTTTATATTTTTGATTTTTAGTAATAGTTT
TTAACGAGGGATATGACCCGTGTATTTTGAAAAGTGGTAAGCAATGTTTTTAGTTTATAGGGATTCATTAGTT
GTATATAATAAGCATGAAAAGCATATAGTCGATCTTATACTTTTAATTGAGACTTATAGTGTATTTAAGTTAT
AGATAAATTCATTAAAATATTATGGAGGAATGAGTATGTCAAAGCGTCTATTATTGTTTGATTTTGATGAAAC
ATATTTTAAACATAATACAAATGAAGAAGATTTAAGTCATTTAAGAGAAATGGAAAAATTGTTGGAAAAACTA
ACTAATAATAATGAAGTGATCACTGCTGTATTGACAGGAAGTACATTTCAAAGTGTCATGGATAAAATGGATC
AAGTTAATATGACGTTTAAACCATTACATATCTTTTCTGATTTGAGTTCTAAAATGTTCACTTGGAATAATGG
CGAATATGTTGAATCAGAAACATATAAAAAGAAAGTCTTGAGCGAACCATTTTTATTTGAAGATATTGAGGAT
ATATTACGTCATATTTCTGCGCAATATAATGTCGAATTTATTCCACAAAGAGCATTTGAAGGTAATGAAACAC
ATTATAATTTTTATTTTCATTCAACAGGTAATCACAACAATGATAGGCGTATTCTAGAAGCACTTGTCAGATA
CGCAAATGACCAAAACTATACAGCGAGATTTAGTCGAAGCAATCCATTAGCTGGTGATCCTGAAAATGCGTAT
GATATTGACTTTACACCGTCAAATGCTGGAAAATTATATGCTACTCAATTTCTGATGAAGAAATATAATATAC
CCGTAAAATCAATATTAGGTTTTGGAGATAGCGGCAATGACGAAGCATATTTAAGCTATCTTGAACATGCCTA
TTTGATGTCGAATAGTAGAGATGAAGCATTAAAGCAGAAATTTAGATTAACAAAATATCCATATTATCAAGGG
ATAACACTGCATGTTAAAGAATTTGTTGAAGGTAAGTATGATTATTGATTGGTTGAGTGATATGTATACTTTT
TAAGTGCAAATATTAATATTTAAAATTTGAGGAATGATTAGATAATATGGTGAATCGACTACACTTTAGTTTT
AAAAAATTTATTTGTAAGTTGATTTGTCTAACTAAGTGTAGACGATTCATAGTTTAATCAATATATTTGAATA
CTTTGTAAATTAAATAAACATTTAATATTGATACTATTGAAGATATAGAGGGCTTTGATCTCTATTGGCTTTT
TTCATCGATTAATTCAATCCATTTTTTGGATACTGCTTCGTAAGAAAAGTTTGTCTTTATTGTTGTTCTAGCA
TTTTCAGAAAACTGAGATATCAATGTTTCTTTTTTTAATACATGAATTATTTTTTTTGCCAATTCATTAATAT
TATCCTTCTTTTTTGTTATTATTCCATTTATATTATTTTGGATTATAGATTTAGGACCATAATTAATAGGATA
TGAAATAACTGGAACACCGTAACTCATGGCCTCTAGTAAACTCATTGGGAGACCTTCATAATCTGATGTCAGT
ACACTTAGAGAGGTTTTTGTATAAAGAGCACGTATGTCAAATGCGTAATCATGGATGAACACATTATCTTGCA
ATTTAAGTTTTTTAATAAGTTTTGTGTAATTTTCTTTTTGCGTACCGGTACCGTATATGTGTAACTTAACATC
CTTTACTTTTTTTCTTACAATATTTATAGCGGAAATGATATGTGTGATATTTTTCATAGCTACAAATCTTTGT
AATACAATAATAGAATTTTTTTCTCGAATATTTTGTGTTAAATTTTCTTCAAAAGATATAGCATTAGGAATAA
CCTTTATATTATTGTAATCACCAAATTGTTTTATAATGTCATCTTTTTGTTCTTTAGTTAATACAACTAAAGT
ATCTAACTTTTTAAGGTTTAGTAACATGTTTCTGAACTCTGGTTTAATCTCAGCGCCATAGGAGTAAGGGGAC
ATGAAATGATTCGTATGTATAGTAAATATTTTTTTGATATCATTATTCTGCATGTTCTCTACTTTATGATAAG
TACCTATACCGTCTAGTATTAATACGTCGTTTGAAGTTAAGTTTTTATCCAACCAATATGAATGGAACGCTTT
ATTATTTTTAAAGTTTAATACTTCTTTGCTGTTTTTATCAAAATAAAATATATTGACTATATTTTCGTTATCC
CCATACCAATATGATAAGTAACAAAAGCCGTCATTTGATAAATATTTAACTTCACTCATTTTATTTTTATCGT
TGAAAGTTATTTCTTTTCGAAGTATTTTATTTTGAAAAACATGTCTTTTAAAGCGTTTTCGATTTTGAAAATA
ATCAATGTGTGATAATTGACCATTTATCCATTTTTTATACATTATGTAATGGTTATCTTTAAAGTATCTAGCA
TATTTTTTTTCTTCATATTCGTCTTTTTGAATATAAAAGCCTTCTTGATTTAAAAGATTTAATTTTTCAAATG
TTTGCATATTTTTATTTTCGATTTCTGTATTTTCAACATCATACAGCATCTTCAATTCTTCGTATAAGTTAAC
AATTTTTACTTGTTTATGTAACCGTCCTGAATTTGTTAGCTTAGCTCTTAATGGATCATAGTTTTTGTCATCC
AAAGTTAATAAGGCACATGAATAGCCTTTTTCAGCTAAAAGTTTAGATCTATTTAACATAACTGATGTTATAC
CACCTGCTTTTTCTGGTATTGAATACATTAACATATAAGTAGTCATAATTTCCTCCTATTTCTTGCACTTGAT
AATTAATTCTCCGTAATTATAGTAGTAATCTTCATCTACATATATTTTGGAGAACGTTTCTAAAGAACTGTAA
TTTTCTATTTCAGCAACTATAATATGATTTAATGCTAAATAAGGATTAGAGCCATTAAAAACAATTTTGGGCT
TTAATATTTCATAATCCTTAAAAACCTCAGAAATTTTTTCGTTATTTTTTTGTACTTCAATCTTGTTTAACTT
TAATATTAATTCATTGTTGTAATTATACTCATTTAATGTCATGTTGATAAATCCCTGTTTTCTCCATCATTAA
AATTTTATTCGATGCCCATCTATTTATCTAAGTAAATAGATAATATCATTTTATTATTTATAGCGTTTTAGGT
TATGTACAATTATATTTTCTTATTTATTAGGTTGGTTGTCAATGTTGTTGGAACTTTTCAGCTGATATGCTGT
AAAAGCTACTAAAGATAAATAATGTAATGAAGTATTGACAGGATATTATGAGGAACTAGATTCTTTCTATTTA
AAAATTTTTAGTTTGAAATACTTATTGATGGTAAACTGATTATCGAACACAGCGAATTGACAGTGACTAAGTT
GCAATAAAAAATTTCAAATTGTAGGACTGACCGTTATGACTAAGAGAAAAAATGTGAATATACTTTAAAATAGAA
GTAATACAACTCGTTGTATCAAGTATAAATGTTACTGATGTCTTAATGCGTCTTTGAATTCCTATTCAAAAAA
TATCAAAGTGGTTAACTAAATGTTATAAAAATAGAGGAAATGCTTTTGCAGATAATTGGAAAATGATGTGAAT
CTATCAAAAAGACTTAAAGTTTTAGAAGAACATATAATAAAAAAAGGTTATGCACATCTTTAATAAAGAATAA

AAAGCAATTTTGATTTCATAGACAAAAACTTCCACAAGATTCAGGCAACGTATATGTGTGAGGTATTTTGTGT
CTTTGAAAGCGGATAGGATGATGTAAAAAAACGTGATTATTCATTCCAACATATTCGACGAGAAATGTAAAAA
AGCAAATTCATAAAATTTCCAGGGGAAGCAAAAAATATAAGGTAGCGTAAAAATGACGCAGGAACTGGAGAAA
GAATTGATACCAAAATAAACATAAGAGGTTAAGAATATATGAATAAAACAAACTTTGGGGATTATTTAAATAA
AAGTAAGAGCAGGAAAATATATAAGATTTTTGATCAAAATTTGAAGCGAGAGTTTAGTTATTGGCACTTTAGG
AAACTTTTCAAATCTTATACAAGGCAGTCTGATGTACATGAGTTAAAAGTGTGTACTCAAAATGATGGTACTA
TGGAGCAAATATGGATAGATAAAGCAAATCAATTTGGAGTGGCAGTGACTTGGAAAGATAATAGTATTATTGG
ATTTGTTATGCGTCCGTTAATCAATTATGATGCTATTAAGAATGAAACGACTTTAAAATACGCAGTGCCGACA
ACAGATTGCTATGAGGTTTTTTGGGGTGGAGATAATGAATTATATAATTATCACTATTTATACCCACATCAAA
GGTATGCTATTGATTTAGTTATTAAAGAAAATCATAAAACATATAAGAACAAAGGAAAAGTAAATACTGATTA
TTTTTATTTTGGAAAAGATATCATTGCGCCAGCAAATGGTACAGTTGAAAAAGTAGCGAATGGGGTTCAAGAC
AACAGTATTGGAAGCACGAATGAATCACAGTTTTTAGGAAATTATATTGTAATCAAGCACGCAGAAAATGAGT
ATAGCCTAATAGCTCATTTACAACAATATTCAATCATTGTGAATGAGGGGCAAAATGTTAAATATGGTGACAT
CATTGGGAAGGTTGGGAATTCTGGCAATTCTACTGAACCTCATATACATTTTCAAGTAATGAATGATAAGAAT
ATTGAAGCATGTACATCTTTGAAAATTCGATTTATAAATAATCGAGAACTTATCAAGGGTGATGTGGTCTGCG
GATTACAAGGTGAATGATGGCGATATTTATAAAATCTCGACACTATAAAAATGGTATAGTGCCGAGATTTTCT
TGCTTATTTAGTTAATTCAAAGCGTACGCCGGATTCATTTGTAGTCGATGTATCTTTGTTTGTAATAGAGTAT
CCAGTCATTGAAATTTTAGATTCAATATCTGAAGCGGGATTTGTAGATTCGGGATTATAGAAGCTACATTCAT
AAGTTTTATCATTTTTCTTTTTAAGAATAAACATTCCTTTGGCTTTAACTTCGACTTTAGTACCATTAATTTC
AAAAGTTTTAGCGCTATCACTATAATTGACACCAGCCCAAACCGATTCATTATCTTTCACAACGTGGAAGTCA
TCATCTTGTTTAACGACAGTTACTTGGCTTGCTTTGGATTTAAAATTATCTTTTGTTATACCTGGATACAACA
CATATCCATATTTATTGTCTTTATCAGAATGCTTTTGAGTTACTTCATAATACTCATCAGTTTTGTCTTCTGA
TTTTTGACTTTTATTTATATCACTCCACTTACCAGTATGACTTTCTTTTTTTACAGTTATTTTCGATTTGTTT
AAAAAATGATAGCCTATATTGTTTTTAGGTTTATTGGTGGACTCTAAAAAGACGGAATTGGTATCCTGATTAT
CGGAAGCGGTTGTTTGTTTATCGTCTGTATATAACGTATACCCATTCGTTTTGCGATTTTCAATCGTTGTAAC
TGGATTCTTTGATGAATCAGTACTTTTAATGCCAGTTCCTAAGAAGACAATTTTATCGTTTAATATGAAATAT
GATTTTTTGGCAGTTAAAGTTTTGTCCTGATTTTCAAAATCCATTCCGATACTAGCATGTTGGTCATCGAATT
TTGTTCCGCCTACAAAAGTTTTACTCGACTTTTTAACATCCGTACCTTTAGGTTCTTCATTTTCTATAGTTGT
AGTACCTGCTAAACGTTTCATATCGGCAGTCGCCCAGAAGTTATCACGGTAGTGTTTCACATCGCTATTGTAT
AAATAAGACATTCCAGCACCAGTATGCCAACCTTTTAGGTTTTCATTATTGATACTTTCGTAACGTGCGACGT
TTTTCGACGTCATACTTAAACCAAATGCAAAGTCTAAGCCTTTATTGTGATAGGTGACACGATCCATGTCATT
ATATATTTTAAGTTGTTGTGTTAAATCGTTAGTAGAAATAGTGCTGTCTTCCATTAAAGACTTCATTTTGCTT
ATATCCGAATAAGAGCTTAAATAATCGTTTTGTTTATAACTTGAATCAGATTTAACTGAAGTTTTAACGATTT
GTTTATATTTAGCTTTTGTTGAATCATCCATGGCATCACTTAATCTCAACAATGATTTCATTACAGTTGCAGA
TACTGAGTGACTCGTTTCATTTTCACGGCTAATGGCTCTACCACGTGATAAATCCATCATTTCACCTTTATAA
ATGAGTGGCATAAATCCTTCATCAATCCACGACTTTAATGTTGTATCATTTTGATTACTATCTTTAAAAGATG
TTTCTTTTATCATCGGCATCATTTGAGAAATACCCTCTAAGAGTACAACGCCATAAGCACCAGTGTATGGGAC
GTCTTGATGATCAATATAAGAGCCGTCTTTATAGAATCCATTACGTTCTTTACCAGTTGCATTACTTTGAACG
TAAGTGAAGACTTTATTAAATGCATCTATTGATTCTTTCATCATAGTTGTATCTTCTTCGATAATACTTTCTA
AAAGTTTTACTTTAGCAATGTCTACTAAATTTCCCCCTTTAGCTTGTTCAGGTTGTCCTACTGATGATAATAT
TTTGTCACTTTCTGGGGCGAAAGTTTTGATAGGGGCAGTGTATTTTTTCTTTTCATCACTTGAAATATCACCA
TTCAGTAATATTAGTGTATTTGTTAATGCTCTAGGTGTTCCAATTTCATAATCCCACCAATTTAAGTTGGTAT
TTCTAGAAGTTTTATTTTTAAAATTCTCAGTCAGTTCTTTTACTTTATCAGTCGGTTCTTTTCCATATGCATT
TTTATGCAGCCACTCAAGGGCATCTTTAACTTTGTTTTTATTATCCGGAGTATTCAACTTAGTATTTTTATGC
TTCATCGCTTCTGCGATTTTCTCAATATTACGATAGGTACGAGTCATATCCGCAGACTTCGTATCTAAATCTT
TTGAGTTTTCCCACAAGTATTTCCTATCACTTTCAGTTTTCATGCTACTTAGTAAATTCGTCGCCTCTTTCTC
CGTAGCCTCAAACTTCTTTTTCATTGCATCATTATTCTCATCATACTGATTATAACCGTAGTTAACGTTCAGC
CATGTGTTCCTCAACTTTTCATAATCTGTCGTTTGAACATTCGTATCAGCCACAGCGATTTGATGTTTATCAA
CACTTCTGAATTCACTATCATTAAAAGTAATCACACCAGCCATTAATAACGTAATGGTAGATATTTTTTGCCA
TTTCTTCATTCTATATGTCATTGACATGTCTCCTTTTTGTGTTGCGCGTGCGCAATGAATATTATGATTAAAT
AATGATTCAATTTTTCAAAATTCGTTAACGTATACAAATGACTGTTTACTGTCAAACAATCCACAAAGAATGT
TGATGCATATAAACAATCGATCACCCAAATTTTCCGCATCACCCAAAAATTGCAACGAAAATAATTTTTAAAA
AATTAAATATTATCAATATTAGTGATTATATCATTTAAATATTTAAGTTATACAATTTTTAAAGTAATTAAA
AACGATGAAATGTAAAATGTTAAAAAAGAGACAAATTTAAATTTGAGTAATAAAATAAGAAGTATTCAAACGA
AGATGGCAATATATAAACCAACATAACTTTAGACGACCAAACATAACTGAAAGGATAGTCGCAATGAAAACTA
AAGAAGTCGTAGCGCTCATGAATATATCTCAAGACACTTTAAGATATTATGAAAAGGTTGGTGTGATTCCACC
TGTTAATCGAGATGAAAATGGATATAGAATATATAATGATAGTGATTTAAATTGGATTTATTTAGTGAAAAAT
TTGCGAAATGCAGGCGTCAGTATTGAATCGCTTATCGAATTTTGTAGGTTAGCGCAGCTTCCTAAAAATGAAA

ATATTCAAGCACAGCAAAAGCAAATTTTAAATAAGCAACTCGAAGAATTAAATGAAAACTTAAAGACAATTCA
TGATGCGAGAGACTTACTACAATATAAAATTGATAATTATGATAATCATATTGCTAAAATCAATGCTAGTGAT
AATCATGATGCCAATGTTGAACGTCTTTGGGAGAGAAAGTAAACCTATGGCTTGCTATAGAGTTTGCTCCATG
GTTTATAGTGAGCTTACAAGAAAAAGGAGGTTCATTTTATGAATCATATTGAAATAAGTAAAGATGTAAAGAT
ACCAGTATTAGGTTTCGGAGTATTTCAAATTCCTCAAGAACAAACAGCAGAAGCGGTTAAAGAAGCAATTAAA
GCGGGTTATAGACATATTGATACAGCGCAAAGTTATTTAAATGAAACTGAAGTAGGTCAAGGTATTGAAGCTT
CAGGTATTGATAGAAGTGAACTATTCATTACGACTAAAGTATGGATTGAAAATGTTAACTATGAAGATACTAT
CAAATCAATCGAACGATCATTACAACGATTAAATTTAGACTATCTTGATCTAGTGCTTATTCACCAACCTTAT
AATGATGTATATGGCTCATGGCGTGCATTAGAAGAGTTAAAAGAAAATGGTAAAATTAAAGCAATCGGTGTAT
CTAACTTCGGCGTTGATCGAATTGTAGATTTAGGTATTCACAATCAAATTCAACCACAAGTGAACCAAATAGA
AATTAATCCATTCCATCAACAAGAAGAACAAGTTGCAGCACTTCAACAAGAAAATGTTGTGGTAGAAGCTTGG
GCACCATTTGCAGAAGGTAAAAATCAATTATTCCAAAATCAATTGTTGCAGGCCATTGCAGATAAATATAACA
AATCAATTGCACAAGTTATTTTACGCTGGTTAGTAGAACGTGACATCGTTGTACTCGCAAAATCAGTGAATCC
AGAACGTATGGCTCAAAACTTAGATATATTTGATTTTGAACTTACAGAAGAAGATAAACAACAAATTGCGACA
TTAGAAGAAAGTAATAGCCAATTTTTCTCACATGCTGATCCAGAGATGATTAAAGCACTTACAAGCAGAGAAT
TAGATGTTTAATCATAATATAACAAAGTAAATAATTTACATTACACTAACCTGAAACTTGGATGTTAAAGTCT
ATGTTTCAGGTTATTTTTTACTCTTAAAAAGTGAAAGGAGCAAGCAAATAAGTTATTATATATTTTTATATTA
TGCAATAACTTACCTAAGGTTATAATTAGATAATTAATATGTCTTGGAATTAATTATCTAATAAGTTGTATAG
CAGGTGGCGATATGACATATATACCCTTTTCTATTATTACATTAATTACAGGTATCATTATGCATATGACAAT
GTACTTTGTAAAATTTGAATGTTGGAAAATGCCATTATTCATGAAGATTATTTTTACAATCTAAAAGCAGTAA
AGACTCATAATATAGCCAAAAACGTTAATAAAAGTTTGTTAAATGATAAGGGGGTGAGTATTGGTAAATTTAT
TCAAAAGGTTAAAGGGAAAAATCCTACCTAGAGAGATCCAAAAACTAAATGGACTATTTCCAAAAATAAAGGG
CAACCACATGGTGATTCTTATTGGAAGTTAATAAATAACAAAGGAAACAGAATAGATTCTTTAACTAAAGAAG
GAAAAAATCTTAAGGGAATAAGGTGTTGTAAATTATGTGCTTTGATATAAATAATTTGAATATAAAAAAGTTG
AATTTTAGAAAGGTAAAAAACGCAATTCATTTAAGTTTAAGTGGCGATAAATACCAGTTTTTGGAGGACGGTA
AAACTATCAATAATACTTATTTTTTAGCTGTATATGATAATGCAATAAACATTTTTGAAGATTTGTTTTCGCC
TTCTGATTCTATAAATTTGGTTCATGTAGTATATGTTTATAATGAACCTTATAAGAAAACAAATGTTATAAGA
AAATTTACATCGTTAACAATAGATGAATTGTATTACTTTAAAGAATATAAAAAGGAGAAGGATGATAATATTT
TGTGCGAGGAGTTTTCATATAAGTGTTCCATAAAAGATATAAAATATAAAAAATTATTAAAAGCTATATCCAA
TAAAGACTTTAAAGGGTTAGTTCCAAATATCAATCAGAATGATAATTATTCTGAAATATACATAATTAACAAT
ACTAAAAACGTAATATATCATTTATATGATGATAGAGGTCTATGGCTAGCTTTTAATAATAATGAAGATTATG
CAAGATATTCTGAAAAATATAATGATTTAATATCAGAATTTGATAACGAAGATATCTAATTTTTTAATATTCG
CAGCTATTATTTTTTTACTTGTTTTATGTTTATTTTTTAATCTAACAAATGACAAACCATTAAAAGTAACATC
CCAACATCAAACAAAAAACAATTCATCAAATGAAAATCGTTATAAAACCAAGTCATTAAACACGCAATAATTA
AAATTTTCCACTCATTTTAATTTGGAACTTCAAACGTCGAATTCCAAAAACGAAACTCCAAATTCCAAAAACG
CAACTCCAAAATTAAAAGCATCTCCCTGTTACTCGGGAAATGCTTTTTACCTACTGATTACCCTGTTATTAAT
GATTTTACAACGGAAACCATGTCGTCATGTATGACCAAAGTAGCGTCGCTATCATAAGGTGTTCGATCTTTAT
TGATAATAATTAAATTGTCGCCTTTAAAATTTGATATTAATCCTGCGGCAGGTTGTACAACGAGTGATGAACC
TAGTACAACAAGGGTGTCGGCATGTTCAATTTTATTTAATGCCCGTATGATGGTAGGTTGATCTAACATTTCA
CCGTATAATACGATGTCCGGTCGAATGGCGCCACCACAATTATCACAATGTTTCAAAGTTCTATCAATGACAT
CTGACTTCGTATAAGATTTATGACATGCATTACAATAAAAACGATTTAACGTGCCATGTAATTCATCAACATG
TTGACTTCCAGCGTCTGAGTGCAAACCATCGATATTTTGCGTGATGACACCTAAAGATTGTTGATTACGTTCT
AATTTTGCAATCCAATCATGAACGATATTAGGCTTCGTATCGACAAATAGTAAACGCTTATGGCAGAAATTGA
TAAAACCTTCAGGATCATCTTCTAAATAATCACGGCTTAACAAGTATTCTGGTGAAAGCCCATCTTTTGAAAT
TTCATCAAATAAGCCACCCATTGAACGGAAATCTGGAACACCACTTGCGACAGATACACCAGCACCTGTGAAA
AATGTAATACGATTCGAACTATCTATAATATGTTTTAATGTCTCTAAATCATTTTCCAAATTAAACACCCCAT
CAAAATAATGAATGATTTATAACGTCTTTTGCTTTTATTATAGTATATTCATTTTACGATTATCAAAAGCGCA
GTTTAAACAGTTCTTTTTAAAAATGAATTATACATATAATCGAACAGTTAAATTATATTAGTTTTTACGCAAT
TTTTATTATAGAATAAGCATGTATATTAAAATTGATGTCTAAACATAAAGGATAATGACCCATCGCCTAAATA
AAGGAGGCAATTTTACATTGAATAAACATGAACGTTTGGACGAAATTGCTAAACTAGTGAATAAAAAGGGCAC
GATAAGAACGAATGAAATCGTCGAAGGTTTAAATGTGTCTGATATGACAGTTCGAAGAGATTTAATTGAATTG
GAAAATAAAGGGATTTTAACGAAGATTCATGGTGGTGCACGCAGTAATTCAACATTTCAGTATAAAGAAATAT
CACATAAAGAAAAACATACCCGTCAAATTGCAGAAAAACGATTTATAGCTAGAAAAGCTGCATCATTAATTGA
AGATGGGGATACTTTGTTTTTCGGACCAGGAACAACAGTGGAACTATTAGCAGAAGAAGTCAATCATCATACG
CTCACAATTATTACGAATTGTTTGCCGGTGTATAAAATTTTGTTAGAAAAACAAACAGCACATTTTCGTGTCT
ATTTAATTGGTGGTGAAATGCGCCATATTACAGAAGCATTTGTAGGTGAAATGGCGAATGCCATGTTGGAAAA
ACTAAGATTTAGCAAGATGTTCTTTAGTAGTAACGCAGTAAATAAAGGTGCTGTGATGACATCTACATTAGAT
GAAGCCTATACGCAACAACTGGCACTCAGTAATTCAATTGAAAAATACTTGTTAATCGATCATACGAAAGTTG

GCAAAGAAGATTTTACATCATTTTGTCAGTTAAACGAATTGACTGCAGTGGTCATGGATTATGAAGATGAAGA
AAAGGTAGAAACGATTAAAACATATATTGAAGTAGTCGACTAAATTAAAAATATAACTGCATTGTTAAAATAT
GTGTCTATATGCTCGTAAGTGAATGTGAATTTGCATTCATTTATGGGCTATTTTTTATGCAAATTTATGTAAA
CGGGAACCTCGACAAAGTTTTACTACAATTTCAGACGAATACCTAATTAAAACAACACCACAGTATGAATGTT
CACTTATTGTTTGAAAATATGTCATAAACAAACAGATTATAAACATATTATGTTGATTTATTGTTTGTTTATG
TTTATAATTTAAACGTAATCAAATTGAAAGCCTTTTCTCAAGATTTTCATAACCTACAAATAAACATGTCATA
TTTTAAGAAAGGTATTTCAAAATTAAAGTAAAAAGGAGTCTTATTATGGCGATTATTATTGGTTCAGATGAAG
CTGGCAAACGATTAAAAGAAGTCATCAAATCATACTTATTAGACAATAAATATGATGTTGTTGACGTAACAGA
AGGACAGGAAGTTGACTTTGTTGATGCAACTTTGGCTGTAGCAAAAGATGTTCAAAGTCAAGAAGGTAACTTA
GGTATTGTTATCGATGCATTTGGTGCAGGTAGCTTTATGGTTGCTACAAAAATCAAAGGCATGATTGCTGCTG
AAGTATCAGATGAACGCTCTGGATATATGACTAGAGGCCATAACAATTCAAGAATGATCACTATGGGGTCTGA
AATTGTTGGAGACACATTAGCGAAAAATGTCGTTAAAGGTTTTGTTGAAGGTAAATACGATGGTGGTAGACAC
CAAATCCGTGTAGATATGCTTAATAAAATGTGCTAGGGAGGAAAAATTACTATGAAGATTGCATTAGGATGCG
ACCATATTGTTACAGATACAAAAATGCGTGTATCTGAATTTTTAAAATCAAAAGGACATGAAGTCATTGACGT
AGGAACATACGATTTCACAAGAACACATTATCCAATTTTTGGTAAAAAAGTTGGCGAACAAGTTGTTAGCGGT
AATGCAGACTTAGGTGTTTGTATTTGTGGAACAGGTGTTGGTATTAACAATGCTGTAAATAAAGTACCTGGCG
TTCGTTCAGCACTAGTACGTGATATGACATCAGCTTTATATGCTAAAGAAGAATTAAATGCGAACGTTATTGG
CTTTGGTGGACGTATTATAGGTGAGTTATTAATGTGCGATATTATCGATGCATTTATTAATGCTGAATATAAA
CCAACTGAAGAGAACAAAAAATTAATCGCTAAAATTAAACATTTAGAAACAAGCAATGCAGATCAAGCTGATC
CACATTTCTTTGATGAATTCTTAGAAAAATGGGACAGAGGCGAATACCACGATTAAGGGACGACACATATGAT
TTTAACTTTAACATTAAATCCATCCGTTGATATTTCATATCCATTAACAGCATTGAAATTAGATGATGTTAAT
CGTGTTCAAGAAGTAAGTAAAACAGCTGGTGGTAAAGGTTTAAATGTAACAAGAGTACTTGCGCAAGTAGGTG
AGCCAGTCCTTGCGAGTGGTTTTATTGGCGGTGAATTAGGTCAATTTATTGCTAAAAAATTAGATCATGCCGG
TATCAAGCATGCATTTTATAACATTAAGGGTGAAACACGAAACTGCATTGCAATTTTACATGAAGGGCAACAA
ACAGAAATTTTAGAGCAAGGACCGGAGATTGATAATCAAGAGGCTGCAGGATTTATTAAACATTTTGAACAAT
TATTAGAAAAAGTTGAAGCAGTTGCTATTTCAGGTAGCTTACCTAAAGGATTAAATCAAGATTATTATGCACA
AATCATTGAACGATGCCAAAACAAAGGTGTGCCTGTAATATTAGACTGCTCGGGTGCAACTTTGCAAACAGTT
TTGGAAAATCCATACAAACCAACAGTCATTAAACCAAACATTTCTGAACTATATCAATTGCTTAACCAGCCAC
TTGATGAAAGTTTAGAAAGTTTGAAACAGGCAGTGTCACAACCCTTATTCGAAGGGATTGAGTGGATTATTGT
GTCTTTAGGTGAACAAGGTGCATTCGCTAAGCACAATCATACATTCTATAGAGTGAATATTCCGATAATAAGT
GTATTAAATCCGGTTGGTTCAGGTGATTCTACGGTTGCTGGAATTACTTCAGCAATTCTCAATCATGAAAACG
ACCACGATTTACTAAAAAAAGCCAATACTTTAGGCATGCTGAATGCGCAAGAAGCACAAACAGGTTATGTCAA
TTTAAATAATTATGATGATTTATTCAATCAAATTGAAGTTTTAGAGGTGTAAAAAATGTCAAAATCGAATCAA
AAAATCGCATCAATTGAACAATTAAGTAACAACGAAGGTATCATTTCTGCATTAGCATTTGACCAACGTGGTG
CTTTAAAACGCATGATGGCAAAACATCAAACTGAAGAACCAACTGTAGCTCAAATTGAACAATTAAAAGTATT
AGTTGCGGAAGAATTAACACAATATGCATCTTCAATTTTATTAGATCCTGAATACGGTTTACCAGCTTCTGAT
GCACGTAACAAAGATTGTGGATTATTACTTGCTTATGAAAAAACTGGTTATGACGTAAATGCTAAAGGTCGTT
TACCTGACTGTTTAGTTGAATGGTCTGCAAAACGATTAAAAGAACAAGGCGCAAATGCTGTTAAATTCTTACT
TTACTATGATGTAGATGATACAGAAGAAATCAATATTCAAAAGAAAGCATATATCGAAAGAATCGGTTCAGAA
TGTGTAGCTGAAGATATTCCATTCTTTTTAGAAGTATTAACATACGATGACAACATTCCTGATAACGGTAGTG
TTGAATTCGCAAAAGTTAAGCCTAGAAAGGTTAACGAAGCAATGAAATTATTCTCAGAACCACGCTTTAACGT
TGATGTATTGAAAGTTGAAGTACCAGTAAACATGAAATATGTAGAAGGCTTTGCTGAAGGTGAAGTGGTTTAT
ACAAAAGAGGAAGCGGCACAACACTTTAAAGATCAAGATGCAGCAACTCATTTACCATACATTTACTTAAGTG
CTGGTGTTTCTGCGGAATTATTCCAAGAAACTTTAAAATTTGCTCACGAAGCTGGTGCTAAATTCAACGGTGT
ACTTTGTGGCCGTGCTACATGGTCTGGTGCTGTTCAAGTATACATTGAACAAGGTGAAGATGCTGCTCGTGAA
TGGTTACGTACAACTGGTTTCAAAAATATTGATGACTTAAACAAAGTATTAAAAGATACTGCGACATCTTGGA
AACAAAGAAAATAAGTATAAAAGGGGATTAATGCTATGAATAGAGAAGAAGTCCAATTATTAGGTTTTGAAAT
TGTTGCATTTGCAGGGGATGCACGTTCTAAGTTTTTAGAAGCATTAACAGCAGCTCAAGCTGGAGATTTTGCA
AAAGCAGATGCACTGATAGAAGAAGGAAACAATTGCATTGCGGAAGCGCATAGAGCACAAACAAGCCTGTTGG
CTAAAGAAGCGCAAGGTGATGATATTGCATATAGTGTGACGATGATGCATGGTCAAGACCATTTAATGACAAC
AATTTTACTGAAAGATTTAATGAAGCATTTATTAGAATTTTATAAAAGAGGGTGATGACAATGATGCAAAAAT
TGATCGCACAAATTGAAAAAGGGAAGCCATTTTTCGAGAAATTATCACGTAACATATATTTAAGAGCCATCCG
TGATGGATTTATTTCTGCAATGCCTGTCATCTTATTCTCAAGTATCTTTCTATTAATTGCATACGTGCCAAAT
ATATTTGGTTTTAAATGGGATAAAGGCATGGAAGCCATTTTAATGAAACCATATAACTATACGATGGGACTTG
TAGCATTTTTAGTAGCAGGAACGACAGCGAAATCTTTAACAGATTCATTTAATCGTAAGCTAGAAAGTACAAA
TCAAATTAACTTTATTTCTACGATGCTAGCAGCGATGTGTGGATTCTTGTTCTTAGCTTCTGATCCAGCCAAA
GATGGTGGATTCTTAAGTGCATTTATGGGAACTAAAGGTTTATTAACAGCATTCTTATCAGCATTTGTAACTG
TTATTGTTTATAACTTCTGTGTGAAGCGCAATATTACAATTAAAATGCCGAAAGAAGTACCGCCGAATATTTC

ACAAGTATTTAAGGACTTAATTCCATTTTCAGCGGTAATCATCATTCTTTATGCATTAGATTTAGTCATTCGC
AACAGCTTTAAATCAAATGTAGCGGAAGGTATTTTAAAATTATTCGAACCATTATTTACAGCAGCAGATGGAT
GGATTGGTGTCACAATTATCTTTGGTGCCTTTGCATTATTCTGGTTTGTAGGAATTCATGGTCCATCAATAGT
AGAACCAGCAATTGCAGCCATTACATATGCGAATATCGAAGCGAACTTCAAGTTGCTTCAAGCTGGAGAACAC
GCAGATAAAATTATTACATCAGGTACACAAATGTTTATCGTTACTTTTGGGGGTACGGGTGCGACATTGGTCG
TCCCATTCATGTTTATGTGGATGACGAAATCTAAACGAAACAAAGCGATTGGACGTGCATCAGTTGTACCAAC
ATTCTTTGGTGTAAACGAACCAATTTTATTTGGTGCACCACTAGTATTGAATCCTGTGTTCTTTATTCCATTT
GTATTAGCACCAATTGTTAACGTATGGATTTTCAAACTTTTCGTTGAAGTGTTAGGAATGAATAGTTTTAGTG
TGAATTTACCTTGGACAACACCAGGTCCATTGGGCATTATCATGGGTACAGGTTTTGGTTTATGGTCATTCGT
ACTAGCTATTACTTTGATTGTTGTAGATATCATTATTTACTACCCATTCCTAAAAGTTTATGATAGTGAAATT
CTTGATGAAGAAGAAGGACGTAAAGAAAGTAATTCAGATTTAAAAGAAAAAGTTGCAGCAAACTTTGATACGA
AAAAAGCTGATTCAATTTTAGCGGCAAGTGGTGTATCAGACGATGCAGCTAAAGCTTCAAATATCACAGAACA
AACAAATGTCTTAGTACTTTGTGCCGGTGGTGGTACAAGTGGATTATTAGCCAACGCACTTAATAAAGCAGCT
GAAGAATATCATGTGCCAGTGAAAGCAGCAGCTGGCGGATATGGCGCACATATGGACATTATGAAAGAGTACC
AATTAATCATTTTAGCGCCACAAGTTGCGTCTAACTATGAAGATATTAAGCAAGATACAGATAGATTAGGTAT
TAAATTAGCGAAGACTCAAGGTGCTGAATATATCAAATTGACACGAGATGGCCAAGCAGCCCTTGATTTCGTT
CAACAACAATTTGAAAATTAAGAATAGGAGTTTTTCATATGACTAAAACATTACCTGAAGATTTTATTTTTGG
TGGAGCAACAGCAGCATATCAAGCAGAAGGTGCGACAAATACGGATGGTAAAGGTCGTGTAGCATGGGATACG
TATTTAGAAGAAAACTATTGGTACACTGCAGAACCAGCGAGTGATTTTTATAACAGATATCCAGTGGATTTAG
AATTAAGTGAAAAATTCGGTGTTAATGGTATCCGTATTTCAATTGCATGGTCTCGTATCTTCCCAAATGGATA
TGGCGAAGTTAACCCTAAAGGTGTTGAGTATTACCATAAGTTATTTGCAGAGTGTCATAAACGTCATGTCGAA
CCATTTGTAACATTGCATCATTTTGATACACCAGAAGTGTTACACAAAGATGGCGATTTTCTAAATCGTAAAA
CGATTGACTACTTTGTAGATTATGCTGAATTTTGTTTTAAAGAATTCCCAGAAGTAAAATATTGGACTACATT
CAATGAAATTGGACCTATTGGTGATGGTCAATATTTAGTAGGTAAATTCCCTCCAGGTATTAAATATGATTTT
GAAAAAGTTTTCCAATCTCATCACAATATGATGGTTGCTCATGCAAGAGCAGTAAAATTATTTAAAGATGGCG
GCTATCAAGGAGAAATTGGTGTTGTACACGCATTGCCAACAAAATATCCGTTCGATCCATCAAATCCTGAAGA
TGTTCGTGCAGCAGAATTAGAAGATATCATCCATAATAAATTCATCTTAGATGCAACATATTTAGGTAAGTAT
TCTCGTGAAACGATGGAAGGTGTGCAACATATCTTATCTGTTAATGGCGGTAAATTAAACATTACAGATGAAG
ATTATGCCATTTTAGATGCAGCCAAAGATTTAAACGACTTCTTAGGTATCAATTACTACATGAGTGATTGGAT
GAGAGGTTACGATGGAGAATCTGAAATCACGCATAATGCGACAGGTGATAAAGGCGGTTCTAAATACCAACTT
AAAGGTGTCGGACAACGTGAATTTGATGTCGACGTACCTCGCACAGACTGGGACTGGATGATCTATCCTCAAG
GTTTATATGATCAAATCATGCGTGTTGTTAAAGATTATCCTAACTATCATAAGATTTATATCACTGAAAATGG
TTTAGGATATAAAGATGAATTTATTGAATCTGAAAAAACAGTTCATGATGATGCACGTATTGATTATGTAAGA
CAACATTTAAACGTTATTGCAGATGCTATTAAAGATGGTGCAAATGTTAAAGGTTACTTTATTTGGTCATTAA
TGGATGTGTTCTCTTGGTCAAATGGTTATGAAAAACGATATGGTTTATTCTATGTCGATTTTGAGACTCAAGA
ACGTTATCCTAAGAAAAGTGCTTATTGGTACAAAGAGTTAGCCGAAACAAAAGAAATTAAATAATTATAGATA
TTAAAAGGCCTATCAGTCGAGTGCTGGTAGGTCTTTATTTATAGAAAGAGATAAATTAAACATGCATATACAT
ATTTATATATTAATCAATGGGTGACACACCATTGAAAGCTAAAGAAGCAATCAATTAACAAACGTTAACGGAC
AACTTTCCAAAGTCATCTTAAAAGTTAATGATGTATTCAAAATCAACTAAAGGACATTTTTAATCACATCCTT
TATACTTATATAAAAGACATGAATGGGAGTTAAAAGTGTGAAGAAAATTTTTGTAATTATTACGACATTGTTA
GCGGTCGCTATTATTATAGGCTCTATTATTACGGTGGTGTTTAGTCAACGACAGGCACAAACATTTAAAATAC
AACAACAGCAATTTGTTAAAAAGCCAATTCCAACATTGTTCTTACATGGATTTGGTGGTAGTGCCAATTCTGA
AAAGTTTATGGTGAAGCAAGCAGAAAAAAGAGGGGTAACTAAAGATATCATCACTGCTTACGTTTCAAAGGAT
GGTGCAGTGACATTTAAAGGGAAACTAAGAAAAGATGCAGTGAATCCTATTGTGAAAATTGAACTTGAAAATA
ACAGACAAGGCTATTTGGATAAGAATGCTGCGTGGTTTAAAAATGTACTGACTAAGTTACAATCTGAATACAA
TTTCGATAAATTCAACTTTGTCGGTCACTCAATGGGGAACTTAACATTTGCACAATATATGATGACATATGGT
AACGACAAGTCACTGCCACAGTTAAACAAACAAGTGAATATTGCAGGGACATTTAATGGTGTTTTAAATATGA
ATGAAGATGTCAATGAAATTACAGTTGATAAAGATGGTAAACCAAGTCGAATGAATCAACCTTATCAGCAATT
ACGCGTGCTAAAAGATATTTATAAAGGTAAAGGTATCGAAGTGCTAAATATTTATGGAGATTTGAAAGATGGT
ACACATTCAGACGGCAGAGTGTCTAATAGTTCTTCGAAATCATTAAAATATTTACTGGGAAATAGTCCGAAAA
GTTATCGAGAATCTAAATATGAAGGTGAGCCTGCACAACATAGTCAGCTTCATGAGAATGAAAATGTTGCTAA
TGAGTTAATCGACTTCTTATGGAAAAAATAAAGCAACTGCTAGTTTTTCTGGGGCAAAAAAGCTTGTAATATT
AGAGCTTTTCAAAAATTATCTATTGTTTGAGAGTTTCCATAAAATGTCTAAGTCGTAACATTTTAAATTTATG
ATTATAGCAAATATAATGTGGTTGAAGTTACATGAATGAATCAGTGCTTAAGTCAAAATATAACAACTAAGAA
TGTATTTGCATACAGACATCACGAAGACACAATTCAAACTTAACGCTTAAGTAACAAATAAGTAAAAGTTTCA
AATAAAGAAAGGTGATTAATGATGCAAAATAAACAAATTCTTTTTAACAAAATACCAGAAGGCATGCCTCAAG
AGGATACATTCAAAATTGAAGAGATTGACACGCCTAAACTTGAGAGCGATGGTGTACTTGTACAAACGCTGTA
TATTTCAGTGGATCCATATATGAGAGGACGTATGACTAAGGCAGATTCGTATGTACAGCCTTTTGAAATTGGT

AAACCGATTGTTAGCCATGTCGTTGCAAAAGTAATTGATTCGACACTCGCAGATTATAAAAAAGGAGATGTCG
TTGTAGGTATGTTGCCTTGGCGAATCATTAATCATGTCCAAGCGGATCAAATTACTAAAGTTCCAACGACAGA
TGTACCACTTGATTTATATTTAAGTGTTTTAGGAATGCCAGGACAAACAGCATATCATGGTCTGCTTGATATT
GGACAACCTAAAGCCGGTGATACTGTTGTGGTTTCAGCTGCATCTGGTGCTGTAGGTTCAGTTGTAGGTCAAA
TTGCTAAGATTAAAGGTTGCCGTGTTGTAGGTATTGCGGGTGGCGACAAAAAAGTAAATTATTTAACAGAGAC
ACTGGGCTTTGACGCTGGTATTGATTATAAGAAAGAGGATTTTGCAGAAGCGTTAGCACAAGCAGTACCAGAT
GGTATTGATGTTTATTTTGAAAATGTTGGTGGTACAGTTGGCGATGAAGTATTTAAGCATTTAAATCGTTTTG
CACGTGTACCAGTATGTGGCGCAATTTCGTCATATAATCATCCTGAAGCCGATATCGGACCACGTATTCAAGG
TACGCTTATTAAGAAGCAAGTAATGATGAGAGGCTTCTTAGTAAGTGAATTCGCCAATGCTTTTAAAGAAGCT
AGTGAACAGCTCGCAACATGGGTTCAAGAAGGTAAAATTCAGTCACAAGTAACCATTGAAGATGGTTTTGAAA
ATGCGCCACATGCATTTAAAAATTTATTTACTGGCGATAACTTTGGTAAACAAGTTATTAAAGTAACGGAATA
ATAAAGTATACGTTTTAACAAACTAACTTTGTTAATTGACCAAAATTTAATAATTTTTTATCTAAAGCAAATT
TGTTCAGTAACATGGTAGAAATAAGTCTAAAATGTAATACGAAAGTCGTCTGTAATTATTTATATATTCAGAT
GAAAAAGGAGTCATGATGCTGCGATAAAAGCACACATGGCTTCTATTTTGTATAATAAGAAATAAGTTCATAA
ATGAGGTGAAACGATATGAAAATGATAGGATTTGAAAAGCCCTTTAAATTGGAAGAAGGAAATTTATTTAAAG
TATACGAACAAAGAAAGCCAACGCCAGAAAACGATGATATCTTAGTCAAAGTAAATAGCATTAGTGTAAATCC
CGTAGATACAAAGCAACGACAAATGGAAGTCACTCAAGCACCTCGTGTACTTGGGTTTGATGCTATTGGTACT
GTTGAAGCTATCGGACCAGACGTTACATTATTTAGTCCGGGTGACGTTGTATTTTATGCTGGATCGCCAAATA
GACAAGGATCAAATGCGACATATCAATTGGTTTCAGAAGCTATAGTTGCAAAAGCGCCACACAACATAAGCGC
TAATGAAGCGGTTAGTCTACCGCTAACTGGTATTACGGCATATGAAACGTTTTTTGATACATTTAAAATTTCA
CATAATCCTTCTGAAAATGTAGGTAAATCCGTGTTAATCATTAATGGCGCTGGGGGTGTAGGAAGTATTGCCA
CTCAAATTGCTAAACGGTATGGTTTAACTGTCATTACAACAGCATCACGACAAGAAACAACAGAGTGGTGTGA
AAAGATGGGAGCAGATATCGTATTGAATCATAAAGAGGATTTAGTGCGTCAATTTAAAGAAAAAGAAATTCCA
TTAGTAGATTATATATTTTGTACCTATAATACAGACTTGTATTACAATACTATGATTGAACTGATAAAACCAT
TAGGGCATATTACAACTATCGTAGCTTTTAATGAAGATCAAGATTTAAATGCATTAAAATTAAAAAGTATAAC
ATTTACACATGAATTTATGTTTGCGCGTCCAATTCATCGAACGCCTGATATGATTAAACAACATGAGTATTTA
GAAGATATTACGAAAAATATAGAATCAGGACATTACCAACCAACAACGACACAAGTTTTTGAAGGTTTATCAC
CTGAAAACTTATATCAAGCACATCTATTGTTGGAAAAACAGTCAATGATTGGGAAATTAGTAATTAATATATA
GTTTAGTAAGTAAAGTCGCCTTATGCAGAAAATACTGTATGGGGCGACTTATTTTTGTGTCTAGCGACTATTG
ATTCTAAAGGGAAATATGACAACTTAGAAGGTATCAATTCAATAATGCATTAGAAAGGCATTACTTAAATACA
ATAAAATACTTTGTTGTTCAATGATAAATAATTTGAGTATGTTTTATATATCATTTTTAAAAGATGAAGCATT
ATTAATTGTGTCTATATAAAATTACAAATGATTAAAATGTTGCAGTTGTGGGTATGAATTTACCATTACACAG
AAAGGGTTTGTATTTATGAAAAATAAATCAGTAAGTCTAGTGTTTTGGGTATCGTTAGTGATTTGTACGATAT
TTGTTGCTTTTGGTGCAATATTTCCGAAACAGTTAGAAAAACTCACGCAAAACATTACTACATTTATTGCGCT
ACATTTTTCTTGGTATTATTTATTGCTCGTTTTAGTTATTTTATTTGTCTGTGTGTATATTTTATTTTCAAGA
TACGCAAACATTACATTAGGTGAAGAAGGCGAAGATCCTGAATTCTCATTGCCGTCTTGGTTTGCTATGTTAT
TTAGTGCTGGTATGGGAATAGGACTTGTGTTCTGGACAACAGCAGAACCAATCAGTCATGCCTTCAAATTAAC
GCCTATTCATAAAGCTGGCACGCAATCTGCAATTAATGATGCAATGCAGTTTTCATTTTTCCATTGGGGGATT
CATGCATGGGCAGTCTATGGTATTGTCGCGCTTGTGTTTGCATATTTTAGCTTTCATAAAGGATATCCTGGCT
TAGTCAGCGCTACATTGACGCCTTTATTAGGTGAAAAGGCAATGCGTGGACCTTTAGGTGGTGCCATTGATGT
ATTGGCAGTCATAGCTACAGTAACAGGCGTTGCTGCAACATTAGGTTTCGGTGCATTGCAAATAAACGAAGGT
TTACATTTTTTATTTAATGTACCGAGTAACTTCACGATGCAAGTAATCTTGATTGTCATTACAACCATATTAT
TTACTTGGTCAGCATGGTCAGGTATCGATAAAGGTATCAAAACGTTGAGTAATATCAACATGTTACTTGCTTT
TGTAGTACTTATTGGTCTTTTTATTGTTGGGCCAACGCTATACATTTTAAATACATTTACGAATGGTCTAGGT
AATTATATTGCAAATTTCTTTAGTATGAGTTTACGCATTCCGTCAGGCGGTCAAAAATTCCAATGGTTACAAA
ATTGGACAATCTTTTACTGGGCATGGTGGATATCTTGGGCACCATTTGTAGGTATATTTATTGCACGTGTGTC
AAAAGGACGTACGATTAAAGAATTTATTCTAGGTGTACTTTTTGTCCCGGCATTAGTTTGCTTTATATTCTTT
GCGGTATTCGGCGCGTCAGCAATTTACTTACAAGATAATCATATTGCCGATATTGCTAAAGCTGCGACAGAAA
CAGCAACCTTTGCAACGCTACAACATTACCCACTAGGTTTTGTATTAAGCTTAATTACTTTACTTGTTATTAT
GATTTTCTTTGTTACATCAGCGGATTCGGCAACTTATGTTTTAGGTATGTTGAGTTCAAGAGGTGATATTAAT
CCGAAGTCATTTGTCAAAGTGAGTTGGGGTATCATTATGGCATTATTTGCAATTATCATGATCTACACAGGTG
GTACTCAAGCGATTCAAAATTTATTAATCATTGCTGCGTTACCATTTTCCGTGGTCATTATAGCGATGATATG
GTCGCTTTTAAAATCTTTGAGTGAGGAGAAACCGAGAAATAGTAATAAGGTTTTAATTAAACATCGTGACCCA
GATATTTTAGAATATCGTTCTTCAAAACATATTGACGAAAATTAATTAATAATGAGGTTTTATTTACAAAAAC
TAGGGAATATATTACTAAATGGTACTTCATCTTATATATTAAAAATAAATTTATAGTTATTAATTTATTATGT
TTAAGAAAAAAATCTGATGGGTACATATTTAATATGTTTTTAAATTAACTTTAACTAAGGAGGTGTCGTTAGGG
GTGAAACGACTTAAAAACTTTATCCTTGGCTTATTAATAGTAGTAATCGTGGGGTTCCTGTTATTCATGTATA
TCCAAGATGGACGTATAACTGAATATCAAGACTACTTCCTTCAATTTGAATGGTTCCAACCATTACTTATTTC

ACTTGCTGCACTATTGATATTAATAGGTCTTATATTAGTATTTAGTATCTTCAAACCTACGCATCGAAAACCT
GGATTATACAAAGACTTCGATGATGGTCATGTTTACGTATCTCGTAAAGCTGTTGAAAAGACGGCATTCGATA
CGGTTGCAAAATATGATCAAGTAAGACAACCAAATGTAGTTGCAAAACTATATAACAAAAAAAATAAATCTTA
TATCGACATTAAAACTGACTTTTTCGTACCAAATAATGTTCAAGTTCAATCTTTAACTGAAGCAATTCGTTCA
GATATTAAAAAGAATGTAGAATATTTCACAGAAATGCCAGTACGTAAGTTAGAAGTTAATGTGAGAGATCAGA
AAACTTCTGGTCCACGAGTGTTGTAAGGGAGGTATATCATGGCTAACAATCATAACCAAAACGGACAAGACTC
TACACAACAAGTGATTAATTTTTTAAAAGTATTTAAATGGAGAATCGTTGGTTTCTTAGCGTTTCTATTAATC
GCGATATTATTCTTAACGTTAGGTTTTTGGAAAACAATACTTATCATCGTTTTATGTTTAATTGGTGTAGGTA
TTGGGTATATGAAAGACCGTAAGCAAGATTTTATGAATTTTTTAAATCGATGGAGTTAATTCATCAATTTAGA
ATTTAATTAAAAAAATATAAAAAACAATAAGTTAAAGGAGAATCTATTATGACTGTAGATAACAATAAAGCAA
AACAAGCATACGACAATCAAACTGGTGTTAACGAAAAAGAAAGAGAAGAGCGTCAAAAACAACAAGAACAAAA
TCAAGAACCTCAATTCAAAAATAAATTAACTTTCTCTGATGAAGTTGTTGAAAAAATTGCTGGTATCGCTGCA
CGTGAAGTTAAAGGTATCTTAGACATGAAAGGTGGCTTAACTGATACATTCACTAATGCATTCTCAAGTGGCA
ACAACGTTACACAAGGTGTATCTGTTGAAGTTGGTGAAAAACAAGCTGCTGTAGACTTAAAAGTAATTTTAGA
ATATGGTGAATCAGCACCTAAAATTTTCCGTAAAGTAACTGAATTAGTAAAAGAACAAGTTAAATATATTACT
GGTTTAGATGTTGTTGAAGTTAACATGCAAGTTGACGATGTAATGACTCAAAAAGAATGGAAACAAAAACACG
AAAAAAATAATGAAAACAATAACCAAGAAAGACAAGGTTTACAATAAGTTATTCGATAAAAAAGCTCGGCACA
CCATTTTAGTGCCGAGCTTTTTTGTGATTTTTAATAATTTGAATATGAGAAATGTATCTTTCTCTCATTTGTT
TAGATTGTATTTAATAAGTTAATGTAATCCTTGAGCTCACGATTAATAAAATTCTATAACCTTAATTATTTTC
TCGATACAAAGGGTTATTAACTTTAATATAAGTATAATGATGTGCCTCATCTTCAAGACGCATTGTTGTAACA
CACTTATAATCTATAAATGGCGCGAACATGGTATCTTTAATTTCATTTATGCGATTCTCATTTACTTGATTAG
ATTTGTGTGTCGAAAGCACAAGTTGATCAAAGATGTTATCTAGTACATCACGAACGATATACCACATATGTCT
TTCCAAGTTTGAATCATTTGATGCTTTAGAGATTGTAAGAATTAATTCGCCTAAATGGTTTTGAACGGTAGAA
TAAAAGGCTTTGTTAAACACAGACGTTTTTGAATCAGTAAGAATTCTTGATTTTTCATGGAAATGAGATGTAC
TGTATCCCATTTCATTTAGTTGTGCTTTATCAATTCTTAAACCTTCAAAGTCTCTAATATACATCGTGTCCAA
TAAACCATCTTTCCGAAATGTTGCAATAGCATTTTGCAAGTGAGCCTCTAATGCAATGCCATATTTAGTAACT
AGTGGGATTACGAGACCAAGCAATGCTTTACTATAAGTTTCAATCCACGATTTCGCCGATGATTCAAAATCAG
ACAATGATGCAGCTGATTGATAACGTTTAATCAATGTCACGACAGGTGATTCATTGTTAAATGGGTAGGTTGC
AACTAAACTTGAAGGAATCATTGGTGTGACTTCTTGTGGAATCATTTGGTATATGTTTTTTCTAAATAATGTA
CCTAATTGTTCACTTCGTTCAGTTTGATAGTCTGCTTCGTCTTGCTCATTATAAAAATGAATACCAGCAACTT
CATCAATAATTGTTGATGCATAGGACTTAAATATGCCATCTTTCTCCAAAATATCATTTAAAATACGTGTCAT
TAGTGGACCATTGTGCGTTGTTTGTTCTGATAATGTACGAATTTCACCTGTAATATGAACGTTTGTCGACAAT
TTGATGTGTGGCGACATAGCTGGGTATTTAGGAACTAATGTTCTGAAAGATAAACCAGCATAATAATCCAACG
TATGTTTTGCTTCAATGATTAATTCTTTATCTACTTCTGCTTGATAATCAGAATGTAATACGTCATCTAATTG
CCATGGATGTACAATCATAATGTGATAATCATTAAAGTTAAACTTTGGCGTAAATTCATTTTCTAATTGTTTA
ATTAAGTCCGGAAATAGTTGATGAACAGTTGTATCATAATCTTTAGACAGTGACATAGTACGGCTTAATTTAC
TGTGAATCAGTACTATTTTCAACTTAATAGGTTGATTGAATTCTGAAGAATATAGGAATGTTTGTAATGCATT
TAAACCTTTACGTAATTTAGCCCCAGGATGTAGCGGATGACCTTCAATAACGGCTTGCTCTGAACGCAAGTAA
CTATCTTCGCTATTTTCGATAATATTAAATAAAGGTGTAGAATCATGTTGCATTGACAGTGCTTGATAGCTAA
TTGCAAATGTCATATTAGTTGCACTGTTTATTAAATCTTGCTGAAATTGATCACTAGCAGCATTTTTTAAATC
TGGTGCTTCAATTAAAATACACTCAAGAATTTCATTTGGATGGTGTACTCGTGTAATCGTATTTGTAATGTCA
TCTTTAATGTAGAAAGGGCCTTCAACATCAATTCGATCAAAGGCATGTTCTCCAGTGATAGGAGCATATAATG
TTTGCTTAGCTTGTGGGAACTGGATTTCTAGTATATGAGTCGTTGAGATATCTAACATAATCAAATCACGACT
CAATATTTTCTTACTTTGAGTGCGCGCTTTAACTAAGTTTTCGCGATGCATTGATGTGACTAATCTCTGAGTG
ACTTTATCTCTTCCTTGTAAAATCATCTCTTTAAAAATATTAGCCCAATCGTTATTATGTTGTTGTAAAAATA
AATATGTTTCTTGTTCTTCTTTGTTAAATTTTAATGTCTGTTCTTTAAAAATTAAGTTCAAGTTCATAATTCA
CCTCTATGAAATATTTTACAAAAGCAAGATAGATTTGTATAATCCATATTAATGATAATGATTCTTATTATCA
ACAGAATGCGGGTGTAAGTTTTATGACAAAATATTTTTTTAGCAGTTCTTTTCTACTATTTCTAGGTAATTGG
ATTGGACAAATAGGGCTAAATTGGTTTGTACTTACCACTTATCATAACGCAGTTTATCTGGGGATTGTCAATT
TTTGCAGACTTGTACCAATATTATTACTAAGTGTGTGGGCAGGGGCAATTGCCGATAAATATGATAAAGGGCG
ATTGCTGAGAATTACAATTTCATCATCATTTTTAGTAACTGCAATTTTATGTGTGCTTACGTATAGTTTCACT
GCAATTCCAATTAGCATCATTATTATATATGCGACATTAAGAGGGATTTTAAGTGCGGTTGAAACACCTTTAA
GACAAGCAATCTTACCAGATTTATCAGATAAAATATCTACTACACAAGCTGTATCATTTCATTCATTCATCAT
TAATATTTGTCGTTCAATAGGGCCTGCCATTGCTGGTGTCATATTAGCAGTCTATCATGCGCCAACAACATTT
CTTGCACAGGCAATTTGTTATTTTATCGCAGTTTTATTATGTTTACCATTACATTTTAAAGTAACTAAAATGC
CTGAAGATGCAACAAGATATATGCCTTTAAAAGTCATTATAGATTACTTCAAATTACATATGGAAGGTCGACA
AATATTTATAACATCATTATTGATTATGGCGACAGGTTTTTCATATACGACACTTTTACCAGTTTTGACAAAC
AAAGTATTTCCAGGGAAATCTGAAATATTTGGTATCGCTATGACGATGTGTGCCATTGGTGGTATTATTGCAA

CGCTAGTTTTACCTAAAGTACTTAAATATATTGGTATGGTAAATATGTATTATTTAAGTTCATTTTTATTTGG
CATTGCTTTGTTAGGTGTGGTATTTCACAATATTGTCATCATGTTCATTTGTATTACATTGATTGGGTTATTT
AGTCAATGGGCACGTACGACAAATCGCGTTTATTTTCAAAATAATGTTAAAGATTATGAACGTGGTAAAGTAC
TGAGTATTATTATGATGGATAGAGGTATGATTCCATTGGGAAGTCTGTTAATGAGTATATGTGCAGATGTGTT
TGGCATTGTTAGAACTTTTTCAATAATGGGAATAAGTACTATATGCATTACAATGGTATTCTATTTTATAAAT
AGAAAGTTGAAGTTAAAGTTGGAGGAAAGTAATCATGGTATATCTTGAATGGGCAAAGGCAGATAGAAATATT
CAATATCGTGTAATTAACGCCATTATTAAAGAACGTATTTACCCCGAGCAAACATTTATTTCGCAAAAAGGAT
CTTTAATTGAAATTCAGTATCATATGCATGTGTTGACTATTGAAGTTGTTAGAAAAAGTGCATTAGAACGCTA
TGAGTTTACAGGGGATATTACTTATTTAAATAAAGGTGAAACGTCATTAATTATAACTTTAGAAGGTTTGTTA
GATGTGTTGAATCATGACTTTGATATCCCTATTTCAGAGCGACTACGCGAAGAGTTAATACACAGTCGAGATA
GTTTAGTTGAAACATATAAGCAAATGTCTCATAGACAAACATTAATAAGTCAAAGTTTTAAATTTTCAAGATT
ACCACAAGATATTAACTTTTTTTCATGGTTACAACATGTAAAAGATAGTGATAAGACAGATGATTTAACTTAT
TCTGAGAGTTTGGTACCAGAGGGGCATCCAACACACCCTTTAACCAAAACGAAATTGCCCTTAACTATGGAAG
AAGTACGAGCATATGCACCTGAGTTTGAAAAAGAAATCCCTTTGCAAATTATGATGATTGAAAAAGACCATGT
TGTGTGCACAGCTATGGATGGTAATGATCAATTTATTATTGATGAAATAATTCCCGAATACTACAATCAGATT
CGTGTGTTTTTGAAGAGTTTAGGTTTGAAAAATGAAGACTATAGAGCGATTTTAGTACATCCTTGGCAATATG
ATCATACGATAGGGAAATATTTTGAAGCATGGATTGCTAAAAAAATATTAATTCCAACGCCGTTTACAATACT
TTCAAAAGCAACTTTATCATTTAGGACGATGTCTTTAATTGATAAACCATACCATGTTAAGTTGCCCGTCGAT
GTACAAGCAACAAGTGCCGTTAGAACAGTCTCAACTGTGACTACTGTAGATGGACCAAAGTTAAGTTATGCTT
TACAAAACATGTTGAATCAATATCCAGGATTTAAAGTTGCTATGGAACCGTTCGGTGAATATGCAAATGTTGA
TAAAGATATGGCACGTCAGCTTGCTTGTATTATAAGACAAAAACCTGAAATTGATGGAAAAGGTGCAACAGTT
GTTAGTGCAAGTCTAGTTAATAAAAATCCAATAGATCAAAAAGTTATCGTGGATAGTTACTTAGAGTGGTTAA
ATCAAGGAATTACTAAAGAAAGTATTACGACATTTATTGAACGATACTCTCAAGCATTAATCCCGCCTTTAAT
TGCTTTTATTCAAAATTATGGAATTGCATTAGAAGCACACATGCAAAATACAGTAGTGAACTTGGGGCCACAT
TTTGACATTCAATTTTTAGTGAGAGATTTAGGTGGTTCTAGAATTGATTTAGAAACATTACAACATCGTGTAT
CAGATATTAAAATTACAAATGATAGTTTAATAGCTGATTCTATAGATGCAGTGATTGCAAAATTCCAACATGC
TGTTATTCAAAATCAAATGGCAGAATTAATCCATCATTTTAATCAGTATGATTGTGTTGAAGAAACCGAATTA
TTTAACATAGTACAGCAAGTAGTAGCGCATGCCATTAACCCAACACTACCACATGCAAATGAGTTAAAAGATA
TTTTGTTTGGACCAACAATTACTGTCAAAGCGTTGTTAAATATGAGAATGGAAAATAAAGTAAAGCAATATTT
AAATATTGAGTTAGATAATCCGATAAAAAAAGAGGTGTAGTACTACATGGCACACGTTAACATAAATATATCG
AAGATTAAATATAACGCCAAAGTACTTCAAACAGTTTTTCAAAGTAAAAATATGCAATTCACACCAGTAATTA
AGTGCATAGCTGGTGACCGTACAATTGTAGAAAGCTTAAAAGCGTTAGGTATCAATCATGTTGCAGAATCCAG
ATTGGATAACATAATTAGTATTGCAGATCAGGATTTAACATATACGTTATTGCGTACGCCTGCCAAAAAAGAG
ATTTCAGATATGATAGAAAAAGTTGATATGAGTATCCAAACAGAATTATCTACCATACATCAAATAAATGAAG
TAGCAGAAGTTTTAGGAAAAAAACATAAGATTTTATTAATGATAGATTGGAAAGATGGCCGTGAAGGTGTTCT
GACTTATGATGTTTTAGATTATATTAAAGAGATTATTCACTTGAAAAATATCCATTTTGTAGGTTTAGCATTC
AATTTTATGTGCTTTAAATCTGATGCGCCAAGTGATGATGATATCTTTATGATTAATCGATTCGTCTCAGCTG
TTGAAAGAGAAATAGGATATCGATTGAAAATTATTTCAGGTGGTAATTCGAGCATGTTACCACAACTTTTATA
TAATGATTTGGGTAAAATAAATGAATTACGAATAGGTGAAACGTTGTTTAGAGGTGTTGATACAACGACAAAT
CAAGCGATAGCTATGTTATATCAAGATGCGATTACATTGGAGGCTGAGATTTTAGAAATTAAACCTCGAGTTA
ATACACAAACGCATGAATCATTCTTACAAGCTATTGTAGATATTGGCTATTTAGACACTAAAGTCGATAATAT
TTCACCTATGGATCAGCATATCAATATTTTAGGCGCTTCGAGTGATCATTTAATGCTTGATTTAAATGGTCAA
GGACACTATCAAGTTGGAGATCATATATCTTTTTCATTAAATTATGAAGCACTTTCTCATTCGATGTATATGA
AAAATTTACATAAGGTATATATTGATGATTCAAAGATTGATACGCTACTTCAAAATTTTGATGTGAAATCTCC
CGCTATGGTAAACCAATATTAATATATTTATCAATAAGTCTAAGTTGACAAGTTATACTACATATCGTTTACA
CGGTTGTATCATAATTGTTCAACTTAGATTTTTTGTATTTGTTGATTTATCAAATAAAGTGCAACAGTTCGTC
ACATAAAATTGCAACAGATAATATCAGCTGAATTACAGGGATAACGGTCATGCTAAATGGTGTCAATTGCATT
AATGCAAAGATGATATAGCCATGATACATTATCAGTATTTTGTCTAAGGAAATGTGCTAATTGTAGTCATAAT
TATTAGAGGAAATATATATAGTCATACATTTTAGTAATATAAAAAGATTGAACGCTACTTGACAATGATAATT
GTTATCAATAAAATAATAAATGAAGTTATACATATTAAGGAGTGGAACGATGAGAGGTCTAAAAACTTTTAGT
ATATTGGGATTAATAGTTGCCTTATTTTTAGTTGCAGCTTGTGGTAATACGGATAATTCAAGTAAAAAAGAAT
CATCAACTAAAGATACTATTTCGGTAAAAGATGAAAATGGTACAGTAAAAGTACCTAAAGATGCAAAACGTAT
CGTTGTATTAGAGTACTCATTTGCAGATGCATTAGCAGCATTAGACGTTAAACCAGTTGGTATTGCTGATGAT
GGTAAGAAAAAACGTATCATTAAACCAGTTAGAGAAAAAATTGGGGATTATACTTCTGTAGGTACACGTAAAC
AGCCAAACTTAGAGGAAATTAGTAAATTAAAACCGGATTTAATTATCGCTGATAGCAGTAGACATAAAGGTAT
TAATAAAGAATTAAACAAAATTGCACCAACATTATCATTAAAGAGTTTTGATGGTGACTACAAGCAAAATATC
GATGCTTTCAAGACAATTGCGAAAGCTTTAGATAAAGAAAAAGAAGGCGAAAAACGTCTTGCTGAGCATGATA
AATTAATCAATAAGTATAAAGATGAAATTAAATTTGATAGAAATCAAAAAGTGCTTCCAGCAGTTGTTGCTAA

AGCTGGTTTATTAGCACATCCAAACTATTCATATGTTGGACAATTTTTAAACGAATTAGGATTTAAAAATGCA
TTAAGTGACGATGTAACAAAAGGTTTAAGTAAATATTTGAAAGGACCTTACTTACAATTAGACACTGAACATT
TAGCTGATTTAAATCCAGAGCGTATGATCATTATGACTGATCACGCTAAAAAAGATTCTGCTGAATTCAAGAA
ATTACAAGAAGATGCAACATGGAAAAAATTGAATGCAGTTAAAAATAATCGCGTGGATATTGTTGACCGTGAT
GTTTGGGCAAGATCTCGTGGCTTAATTTCTTCTGAAGAAATGGCTAAAGAACTTGTTGAATTATCAAAAAAAG
[truncated: 828,943 more chars]
